# Evaluation method for the potential functionome harbored in the genome and metagenome

**DOI:** 10.1186/1471-2164-13-699

**Published:** 2012-12-12

**Authors:** Hideto Takami, Takeaki Taniguchi, Yuki Moriya, Tomomi Kuwahara, Minoru Kanehisa, Susumu Goto

**Affiliations:** 1 Microbial Genome Research Group, Japan Agency for Marine-Earth Science & Technology (JAMSTEC), 2-15 Natsushima, Yokosuka, 237-0061, Japan; 2 Advanced Science & Innovation Group, Mitsubishi Research Institute Inc. (MRI), 2-10-3, Nagata-cho, Chiyoda-ku, Tokyo, 100-8141, Japan; 3 Bioinformatics Center, Institute for Chemical Research, Kyoto University, Gokasho, Uji, Kyoto, 611-0011, Japan; 4 Department of Microbiology, Faculty of Medicine, Kagawa University, 1750-1 Miki, Kagawa, 761-0793, Japan

## Abstract

**Background:**

One of the main goals of genomic analysis is to elucidate the comprehensive functions (functionome) in individual organisms or a whole community in various environments. However, a standard evaluation method for discerning the functional potentials harbored within the genome or metagenome has not yet been established. We have developed a new evaluation method for the potential functionome, based on the completion ratio of Kyoto Encyclopedia of Genes and Genomes (KEGG) functional modules.

**Results:**

Distribution of the completion ratio of the KEGG functional modules in 768 prokaryotic species varied greatly with the kind of module, and all modules primarily fell into 4 patterns (universal, restricted, diversified and non-prokaryotic modules), indicating the universal and unique nature of each module, and also the versatility of the KEGG Orthology (KO) identifiers mapped to each one. The module completion ratio in 8 phenotypically different bacilli revealed that some modules were shared only in phenotypically similar species. Metagenomes of human gut microbiomes from 13 healthy individuals previously determined by the Sanger method were analyzed based on the module completion ratio. Results led to new discoveries in the nutritional preferences of gut microbes, believed to be one of the mutualistic representations of gut microbiomes to avoid nutritional competition with the host.

**Conclusions:**

The method developed in this study could characterize the functionome harbored in genomes and metagenomes. As this method also provided taxonomical information from KEGG modules as well as the gene hosts constructing the modules, interpretation of completion profiles was simplified and we could identify the complementarity between biochemical functions in human hosts and the nutritional preferences in human gut microbiomes. Thus, our method has the potential to be a powerful tool for comparative functional analysis in genomics and metagenomics, able to target unknown environments containing various uncultivable microbes within unidentified phyla.

## Background

One of the main goals of genomic and metagenomic analyses is to extract the comprehensive functions (functionome) harbored in an individual organism or a whole community in various environments. However, evaluating the potential functionome is still difficult when compared with the functional annotation of individual genes or proteins; i.e. based on a similarity search against a reference database such as the NCBI-NR database of non-redundant protein sequences [1], usually employing a variant of the BLAST program [2], or on the protein domain search against a protein family database such as PFAM [3]. This is mainly because a standard methodology for extracting functional category information, such as individual metabolism, energy generation and transportation systems, has not yet been fully established. Traditionally, clusters of orthologous groups (COGs) have been used for functional classification of proteins, particularly in microbial genome sequencing projects. The COGs database provides 17 functional categories for orthologous groups in order to facilitate functional studies and serves as a platform for functional annotation of newly sequenced genomes and studies on genome evolution [4]. Although the COG functional categories are often used within Standards in Genomic Sciences (http://standardsingenomics.org/index.php/sigen) as a standard analysis, through combination with the Integrated Microbial Genomes (IMG) system [5], no large functional differences are usually observed in such broad categories; even between phenotypically different organisms (http://img.jgi.doe.gov/cgi-bin/w/main.cgi?section) and also whole microbial communities in different environments [6-8]. Thus, it is difficult to differentiate the functional potentials between different genomes and metagenomes by analysis based on COG classification.

**Figure 1:**
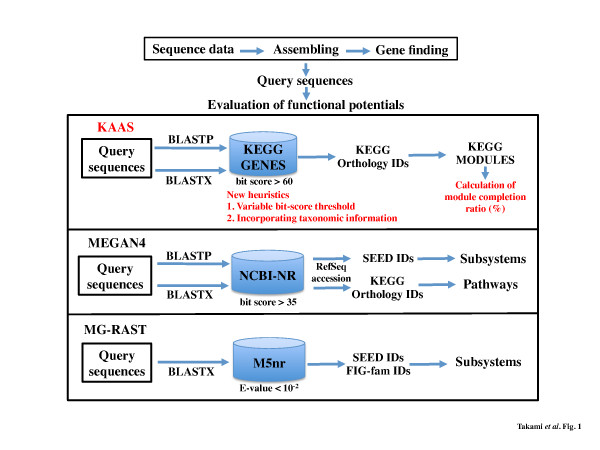
**Workflow of evaluation of the potential functionomes.** Detailed workflow of the three annotation servers, KAAS, MEGAN4, and MG-RAST using query sequences after gene finding process of sequenced data; KAAS and MEGAN4 use BLASTP and BLASTX for amino acid and nucleotide query sequences respectively and the MG-RAST uses only BLASTX. All use different databases, i.e. KEGG GENES for KAAS, NCBI-NR for MEGAN4, and M5nr [15] for MG-RAST (M5nr includes the SEED as a subset.), and different default threshold values for the BLAST hits. Each server converts the hit entries to the corresponding orthology IDs for functional annotation and pathway/module/subsystem mapping. Red colored texts of KAAS indicate its improvements in the current study (see Assignment of the query sequences to KO identifiers in the Methods section).

Recently, more detailed and comprehensive functional categories facilitated in KEGG [9] and SEED [10] have been used for comparative genomics and as metagenomics tools to highlight functional features represented by KAAS (KEGG Automatic Annotation Server) [11], MG-RAST (Rapid Annotations using Subsystems Technology server for metagenomic project) [12] and MEGAN [13,14] (Figure 1). They all employ a similarity-based method for functional annotations, but utilize different databases for protein sequences, default threshold values and orthology IDs for mapping annotated sequences to functional categories depending on their desired outputs, namely pathways in KEGG or subsystems in SEED. Notably, KAAS has been applied to protein coding sequences from several metagenomic samples, and their annotated KEGG pathways and other classifications are already available (http://www.genome.jp/kegg/catalog/org_list3.html).

**Figure 2:**
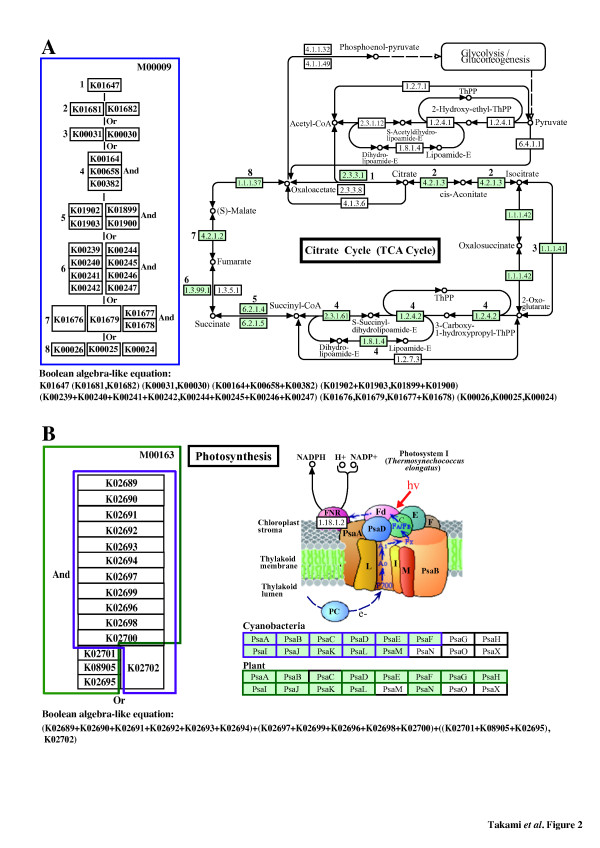
**KEGG functional modules.A**: A pathway module. The module M00009 comprising 8 reactions is defined for the citrate cycle (TCA cycle) core module and represented as a Boolean algebra-like equation of KO identifiers or K numbers for computational applications. The relationship between this module and the corresponding KEGG pathway map is also shown by indicating corresponding K number sets in the module and EC numbers in the pathway map using the same index. In each K number set, vertically connected K numbers indicate a complex and therefore represent “And” or “+” in the Boolean algebra-like equation, whereas horizontally located K numbers indicate alternatives and represent “Or” or “,” in the equation. **B**: A structural complex module. The structural complex module M00163 comprising 12 (cyanobacteria) or 14 (plant) components is defined for the type I photosystem. The Boolean algebra-like equation and the corresponding KEGG pathway map are also shown. The KEGG pathway map shows the *Thermosynechococcus elongatus* (cyanobacteria) photosystem. Green and purple boxes indicate plant and cyanobacteria components, respectively.

The outputs of these systems include functional distributions of each sample by hierarchical classification using KEGG and/or SEED and comparisons between several samples when necessary. However, it is still difficult to evaluate the functional potentials via the current classification systems (such as pathway map-based analysis) because the functional information from different organisms such as microbes, plants, and animals has been mixed up. On the other hand, KEGG MODULE, a database that collects pathway modules and other functional units, presents a promising tool for functional classification [16]. Pathway modules in KEGG MODULE are smaller pieces of subpathways, manually defined as consecutive reaction steps, operon or other regulatory units, and phylogenetic units obtained by genome comparisons (Figure 2A). This database also contains molecular complex modules, comprising multiple molecules such as the subunits of transporters and receptors, functional sets, and signature modules (Figure 2B). As of December 2011, 179 pathway modules have been defined for energy, carbohydrate, lipid, nucleotide, and amino acid metabolism, including genetic and environmental information processing pathways. In total, 434 KEGG modules (179 pathways, 248 structure complexes, 4 functional sets, and 3 signatures) can be accessed through the website (http://www.genome.jp/kegg-bin/get_ htext?ko00002.keg).

This background motivated us to develop a new evaluation method using the KEGG MODULE database to differentiate the comprehensive and detailed functional potentials between different genomes and metagenomes. In this study, we first calculated the completion ratio of each KEGG module in reference species whose genomic sequences have been completely determined. Then we characterized the functional potentials between phenotypically different bacilli and human gut microbiomes from 13 healthy individuals. Finally, we validated the effect of database dependency on the accuracy of KO assignment.

## Results and discussion

### Distribution patterns of the module completion ratio in 768 prokaryotic species

KEGG modules are modular functional units derived from the KEGG pathways, and are categorized into pathway modules, structural complexes, functional sets and genotypic signatures. Each KEGG module is designed for automatic functional annotation by a Boolean algebra-like equation of KEGG Orthology IDs (see Methods for more details). However, it remains un-catalogued as to which species possess common modules or if certain modules demonstrate universality or rareness between specific species, phyla etc. Specific information regarding the phylogenetic profiles of each module holder would be especially useful for annotating metagenomes.

**Figure 3:**
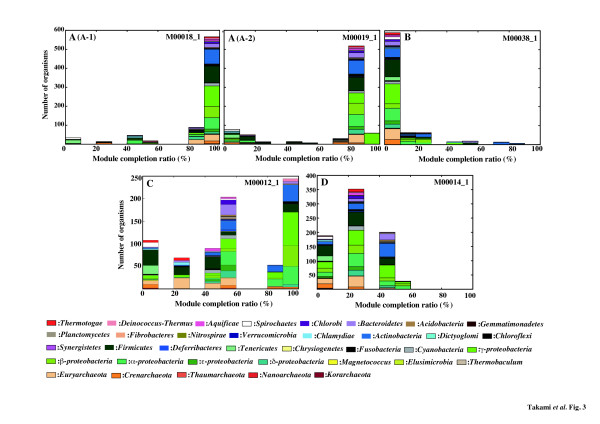
**Typical completion patterns to the KEGG modules by 768 prokaryotic species.A**: universal modules. (A-1) The modules completed by more than 70% of 768 prokaryotic species. M00018_1, which is threonine biosynthesis (aspartate homoserine threonine) is one of examples of the pattern A-1. (A-2) The modules for which more than 70% of 768 prokaryotic species show a module completion ratio of >80%. M00019_1, which is leucine biosynthesis (pyruvate 2-oxoisovalerate leucine) is one of examples of the pattern A-2. **B**: Restricted modules completed by less than 30% of 768 prokaryotic species. M00038_1, which is tryptophan metabolism (tryptophan kynurenine 2-aminomuconate) is one of examples of the pattern B. **C**: Diversified modules. These are the modules that vary in the module completion ratio among 768 prokaryotic species. M00012_1, which is glyoxylate cycle is one of examples of the pattern C. **D**: Non-prokaryotic modules completed by no prokaryotic species. M00014_1, which is glucuronate pathway (uronate pathway) is one of examples of the pattern D. Breakdown of taxonomic variations that complete each KEGG module is summarized in Table 1 and shown in Supplementary Table S2 in detail.

Thus, we first examined distribution patterns of the completion ratios of the KEGG modules in the 768 prokaryotic species whose genomic sequences have been completed (Additional file 1: Table S1). Although distribution of the module completion ratios in the 768 species varied greatly depending on the kind of module (Additional file 2: Figure S1-S3 and Additional file 1: Table S2), we found that it could be categorized into 4 patterns (universal, restricted, diversified and non-prokaryotic) regardless of the module type (pathway, structural complex, signature, or functional set), when considering 70% of all species to represent a majority measurement for the patters (Table 1 and Figure 3).

**Table 1:** Classification of the KEGG modules based on the module completion ratio of 768 prokaryotes

**Completion pattern**	**Definition of module type**	**Subtype**	**Pathways [203]**		**Structural complexes [263]**		**Functional sets**[4]		**Signatures**[3]	
			**No. of modules (%)**		**No. of modules (%)**		**No. of modules (%)**		**No. of modules (%)**	
			**Total**	**Rare**	**Total**	**Rare**	**Total**	**Rare**	**Total**	**Rare**
A	Universal	A-1	15 (7.4)	0 (0)	9 (3.4)	0 (0)	1 (25)	0 (0)	0 (0)	0 (0)
		A-2	8 (3.9)	1 (1.9)	0 (0)	0 (0)	1 (25)	0 (0)	0 (0)	0 (0)
B	Restricted	-	22 (10.8)	17 (31.5)	119 (45.2)	95 (89.6)	0 (0)	0 (0)	3 (100)	3 (100)
C	Diversified	-	79 (38.9)	36 (66.7)	54 (20.5)	11 (10.4)	1 (25)	1 (100)	0 (0)	0 (0)
D	Non-prokaryotic	-	79 (38.9)	-	81 (30.8)	-	1 (25)	-	0 (0)	-

[ ] shows total number of the KEGG modules containing branched modules. “Rare” indicates the modules completed by less than 10% of 768 prokaryotic species. Universal: the modules completed by more than 70% of 768 prokaryotic species, Restricted: the modules completed by less than 30% of 768 prokaryotic species. Diversified: the modules that varies in the module completion ratio among 768 prokaryotic species, Non-prokaryotic: the modules not to be completed by any prokaryotic species.

Pattern A defined as “universal” comprised modules completed by more than 70% of the 768 species (Figure 3A-1), and more than 70% of the 768 species possessed a module completion ratio of >80% (Figure 3A-2). Of 205 pathway modules containing submodules, modules grouped into pattern A account for only 11.3% (Table 1) and mainly belong to the categories of central carbohydrate metabolism and cofactor and vitamin biosynthesis. Although there are many species, more than 70% of the 768 prokaryotes possessed a module completion ratio of 80%, species with 100% completion ratio is very limited in the pattern A-2. M00019_1, shown as a representative of pattern A-2 (Figure 3), is a pathway module for leucine biosynthesis comprising 7 reaction steps. The 1st reaction in this module, from pyruvate and thiamine diphosphate to 2-(α-hydroxyethyl) thiamine diphosphate plus CO_2_ is catalyzed by acetolactate synthase (EC 2.2.1.6) comprising 3 (K01652, K01653, and K11258) subunits. However, since most of the species, except for 58 species within *Gammaproteobacteria*, do not have the genes assigned to K11258 of the acetolactate synthase II small subunit, the module completion ratio in the remaining 518 species becomes 85.7%. Thus, this small subunit may not necessarily be crucial for the pyruvate and thiamine reaction to occur in these species. Pattern B defined as “restricted” comprised modules completed by less than 30% of the species (Figure 3B), and accounted for 10.8% of all the pathway modules, and 17 modules were rare modules completed by less than 10% of the 768 species (Table 1). Pattern C defined as “diversified” accounted for 38.9% of all the pathway modules, and comprised modules ranging widely in completion ratios. M00012_1 (the glyoxylate cycle comprising 5 reactions) is one of representatives of pattern C (Figure 3C). As shown in Figure S4 (Additional file 2). 1 or several KO identifiers were assigned to each reaction in this module; however, KO identifiers, except for K01637 and K01638 assigned to the 3rd and 4th reactions, were also assigned to other pathway modules such as the TCA (Krebs) cycle (M00009_1), 1st carbon oxidation (M00010_1), 2nd carbon oxidation (M00011_1), reductive TCA cycle (M00173_1) and C4-dicarboxylate cycle (nicotinamide adenine dinucleotide (NAD)^+^-malic enzyme type) (M00171_1). Some KO identifiers assigned to many of the modules, categorized into pattern C, were also assigned to several other independent modules. Thus, when the module completion ratio is low, the relationship between the module completion ratio of the targeted module and others to which the same KO identifiers are assigned should be considered. Pattern D, which accounted for 38.9% of all pathway modules, comprised nonprokaryotic modules that are not completed by prokaryotic species (Figure 3D).

Of the 263 structural complex modules containing submodules redefined from modules with various complex patterns, 119 modules were categorized into pattern B (45.6%) and 95 were rare modules (Table 1). Pattern C accounted for only 20.5% in the structural modules compared with 38.9% in the pathway modules. Thus, it was hypothesized that most of the structural complex modules, except for pattern D, are shared only in limited prokaryotic species.

Non-prokaryotic modules account for 38.9% of pathway and 30.8% of structural complex modules respectively, and other modules were classified into various taxonomic patterns such as prokaryotic, Bacteria-specific and Archaea-specific based on the module completion profiles as shown in Table 2. These 4 patterns indicate the universal and unique nature of each module and also the versatility of the KO identifiers mapped to each module. Thus, the 4 criteria and taxonomic classification for each module should be helpful for interpretation of results based on module completion profile. A breakdown of all the modules grouped into the 4 patterns is summarized in Table S3-S5 (Additional file 1).

**Table 2:** Breakdown of taxonomic patterns of the KEGG modules

**Pathway [203]**		**Structural complex [263]**	
Major taxonomic pattern	Number (%)	Major taxonomic pattern	Number (%)
Non-prokaryote	79 (38.9)	Non-prokaryote	81 (30.8)
Prokaryote	52 (25.6)	Bacteria-specific	45 (17.1)
Bacteria-specific	25 (12.3)	Prokaryote	42 (16)
*Gammaproteobacteria*-specific	8 (3.9)	*Proteobacteria*-specific	24 (9.1)
*Euryarchaeota*-specific	6 (3)	Archaea-specific	10 (3.8)
*Cyanobacteria*-specific	4 (2)	*Cyanobacteria*-specific	10 (3.8)
*Proteobacteria*-specific	4 (2)	*Firmicutes*-specific	10 (3.8)
*Alphaproteobacteria*-specific	3 (1.5)	*Gammaproteobacteria*-specific	8 (3)
*Proteobacteria/Firmicutes/Actinobacteria*	3 (1.5)	*Proteobacteria/Firmicutes*	4 (1.5)
Archaea-specific	2 (1)	*Actinobacteria*-specific	3 (1.1)
*Chloroflexi*-specific	2 (1)	*Alphaproteobacteria*-specific	3 (1.1)
*Crenarchaeota*-specific	2 (1)	*Proteobacteria/Actinobacteria*	3 (1.1)
*Firmicutes*-specific	2 (1)	*Gammaproteobacteria/Firmicutes/Fusobacteria*	2 (0.8)
*Actinobacteria*-specific	1 (0.5)	*Actinobacteria/Verrucomicrobia/Nitrospirae*	1 (0.4)
*Betaproteobacteria*-specific	1 (0.5)	*Betaproteobacteria*-specific	1 (0.4)
*Betaproteobacteria/Actinobascteria/ Cyanobacteria*	1 (0.5)	*Euryarchaeota*-specific	1 (0.4)
*Betaproteobacteria/Chloroflexi*	1 (0.5)	*Firmicutes/Actinobacteria*	1 (0.4)
*Cyanobacteria/Euryarchaeota*	1 (0.5)	*Firmicutes/Fusobacteria*	1 (0.4)
*Cyanobacteria/Chlorobi*	1 (0.5)	*Frmicutes*-specific	1 (0.4)
*Gammaproteobacteria/ Firmicutes/Cyanobacteria*	1 (0.5)	*Gammaproteobacteria/Firmicutes*	1 (0.4)
*Proteobacteria/Actinobacteria*	1 (0.5)	*Proteobacteria/Actinobacteria/Deonococcus-Thermus*	1 (0.4)
*Proteobacteria/Bacteroidetes*	1 (0.5)	*Proteobacteria/Actinobacteria/Verrcomicrobia*	1 (0.4)
*Proteobacteria/Fusobacteria/ Gemmatimonadetes*	1 (0.5)	*Proteobacteria/Chloroflexi/Deonococcus-Thermus*	1 (0.4)
*Proteobacteria/Firmicutes*	1 (0.5)	*Proteobacteria/Chlamydiae/Chlorobi*	1 (0.4)
**Functional set [**[4]**]**		*Proteobacteria/Chlamydiae*	1 (0.4)
Major taxonomic pattern	Number (%)	*Proteobacteria/Chrysiogenetes/Spirochaetes*	1 (0.4)
Prokaryote	3 (75)	*Proteobacteria/Cyanobacteria*	1 (0.4)
Non-prokaryote	1 (25)	*Proteobacteria/Cyanobacteria/Chlorobi*	1 (0.4)
**Signature [**[3]**]**		*Proteobacteria/Firmicutes/Actinobacteria*	1 (0.4)
Major taxonomic pattern	Number (%)	*Proteobacteria/Magnetococcus*	1 (0.4)
*Proteobacteria*-specific	1 (33.3)	*Proteobacteria/Magnetococcus/Aquificae*	1 (0.4)
*Betaproteobacteria*-specific	1 (33.3)		
*Gammaproteobacteria*-specific	1 (33.3)		

[ ] shows total number of the KEGG modules containing branched modules.

### Comparative functionome analysis of bacilli based on the KEGG modules

*Bacillus* and its related species in genera such as *Oceanobacillus* and *Geobacillus* reclassified from genus *Bacillus* (*Bacillus*-related species) are known to thrive in a wide range of environmental conditions: pH 2–12, temperatures between 5–78°C, salinity from 0 to 30% NaCl, and pressures from 0.1 Mpa (atmospheric pressure) to at least 30 MPa (pressure at a depth of 3000 m) [17]. The genome structure of these species within family *Bacillaceae* is comparatively similar, and the core structure comprising more than 1,400 orthologous groups is well conserved among *Bacillaceae*[18]. Therefore, moderately related bacillar genomes from 8 species with different phenotypic properties were selected to test our evaluation method for potential functionome using KEGG modules, in order to differentiate the functional potentials harbored in their genomes.

**Figure 4:**
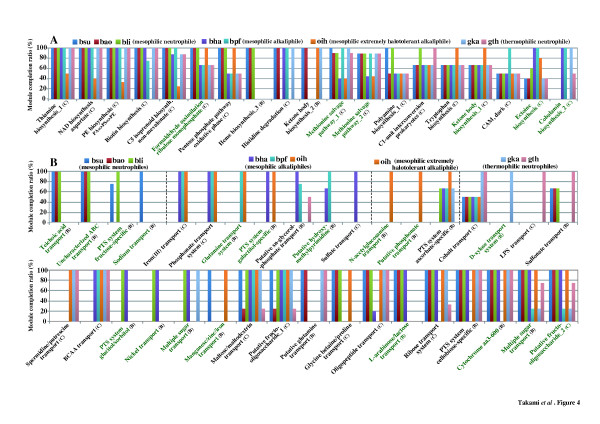
**Comparison of module completion patterns in 8 phenotypically different *Bacillus*-related species.A**: Pathway modules showing remarkable differences appeared among the 8 species. **B**: Structural complex modules showing remarkable differences appeared among the 8 species. Upper histogram indicates common or specific modules in the species possessing each phenotype (from left to right; mesophilic neutrophile; mesophilic alkaliphile; mesophilic, extremely halotolerant alkaliphile; and thermophilic neutrophile). Green letters show rare modules completed by less than 10% of 768 prokaryotic species described in Figure 3. Alphabet in parentheses shows the patterns of completion profile based on the module completion ratio as shown in Table 1 and Figure 3. **A**: Universal module, **B**: Restricted module, **C**: Diversified module, **D**: Non-prokryotic module. bsu, *B. subtilis*; bao, *B. amyloliquefaciens*; bli, *B. licheniformis*; bha, *B. halodurans*; bpf, *B. pseudofirmus*; oih, *O. iheyensis*; gka, *G. kaustophilus*; and gth, *G. thermoglucosidasius* .

The gene products from 8 bacillar genomes were assigned to KO identifiers constructing each module in 111 pathway and 84 structural complex modules as shown in Figure S5 (Additional file 2). There was significant difference in the module completion ratio by 8 bacilli in terms of at least 19 pathway and 35 complex modules (Figure 4A and B). In particular, the completion ratio in *Oceanobacillus iheyensis*, a mesophilic, extremely halotolerant alkaliphile [19], was very low in 4 modules for thiamine biosynthesis (M00127_1), NAD biosynthesis (M00115_1), phosphatidylethanolamine biosynthesis (M00092_1) and biotin biosynthesis (M00123_1). These 4 modules were completed by all bacilli except for *O. iheyensis* although they are categorized into one of the diversified modules (pattern C). In addition, all bacilli almost completed the module for C5 isoprenoid biosynthesis (M00096_1), categorized into one of the universal modules (pattern A-2) in spite of very low module completion ratio by *O. iheyensis*. Conversely, 2 modules belonging to pattern C for tryptophan biosynthesis (M00023_1) and ketone body biosynthesis (M00088_1) were completed by only *O. iheyensis*, although other species partially completed them. Through these results it was evident that *O. iheyensis* differs from other bacilli in its metabolic potentials.

Some of the completed structural complex modules were found to be shared in bacilli with the same phenotypic properties, or to be independently species specific (Figure 4B). For example, the *Firmicutes*-specific modules for the teichoic acid transport system (M00251_1) were shared only among 3 mesophilic neutrophiles (*Bacillus subtilis*[20], *Bacillus amyloliquefaciens*[21], and *Bacillus licheniformis*[22]), although this module is widely shared in other genera such as *Staphylococcus*, *Clostridium*, and *Listeria* within phylum *Firmicutes*. Similarly, *Bacillus*-specific uncharacterized ATP-binding cassette (ABC) transport system (M00315_1) was also found to be shared among 3 mesophilic neutrophiles. On the other hand, 2 other modules, the iron (III) transport system (M00190_1) and phosphonate transport system (M00223_1) which are shared in many prokaryotic species within various phyla and belonged to pattern C, were shared only among 3 mesophilic alkaliphiles (*Bacillus halodurans*[23], *Bacillus pseudofirmus*[24], and *O. iheyensis*). Although it has been previously reported that the orthologous genes for the phosphonate transport system were shared between *O. iheyensis* and *B. halodurans*[19], it could be easily visualized using our new evaluation method that this system was also shared in other mesophilic and alkaliphilic *B. pseudofirmus*, whose genome sequence has been completed recently. In addition, another putative phosphonate transport system (M00224_1) and the N-acetylglucosamine transport system (M00205_1) categorized into one of the restricted modules (pattern B) were found to be conserved only in *O. iheyensis*. Although how the differentiated functional modules confer phenotypic properties directly or indirectly is still unclear, a series of the above results should be helpful in better understanding of the physiological properties.

### Comparative functionome analysis of humans and human gut microbiomes

**Figure 5:**
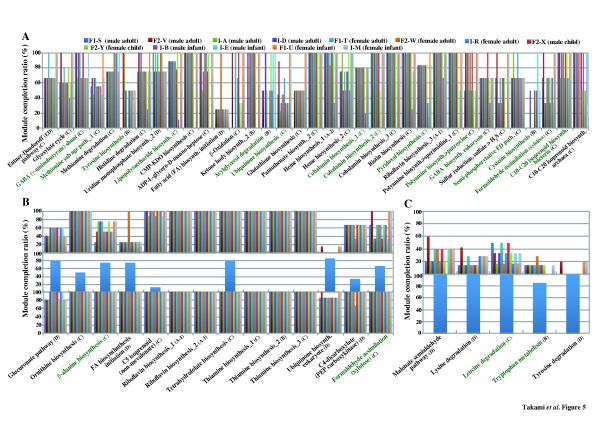
**Comparison of module completion patterns in humans and human gut microbiomes from 13 healthy individuals.A**: Typical pathway modules showing remarkable differences in the module completion ratio appeared among human gut microbiomes from 13 healthy individuals. **B**: Typical pathway modules possessing complementary relationships between humans and human gut microbiomes in the module completion ratio. **C**: Typical pathway modules for which the completion ratio in the human gut microbiome is very low in contrast to that in humans. Green letters show rare modules completed by less than 10% of 768 prokaryotic species described in Figure 3. Detailed information of the 13 individuals has been previously described [6]. Alphabet in parentheses shows the patterns of completion profile based on the module completion ratio as shown in Table 1 and Figure 3. **A**: Universal module, **B**: Restricted module, **C**: Diversified module, **D**: Non-prokryotic module.

The completion ratio of each KEGG module was compared between humans and human gut microbiomes to illustrate their metabolic linkage. The metagenomic data of gut microbiomes from 13 healthy Japanese individuals, previously reported on, was used [6]. Detailed information for all metagenomic samples are summarized in Table S7 (Additional file 1). The gene products from metagenomes of the microbiomes from the 13 individuals were assigned to KO identifiers constructing each module in 158 pathway and 150 structural complex modules as shown in Figure S6 (Additional file 2). Similarly, the gene products from the human genome were completely or partially assigned to KO identifiers in 144 pathway and 84 structural complex modules. There was a significant difference in the module completion ratios of 13 individuals in terms of at least 35 pathway modules (Figure 5A).

The most complete 16S rRNA gene sequence-based enumerations available in human gut microbiomes indicate that more than 90% of phylotypes belong to just two of the 70 known divisions of Bacteria, the *Bacteroidetes* and the *Firmicutes*, with the remaining phylotypes distributed among eight other phyla [25]. Pairwise comparison of the completion ratio of the KEGG module clearly demonstrated the well-recognized functional complementation of the gut microbiome to the human host, which includes essential amino acid and vitamin biosynthesis (Additional file 2: Figure S6). The contributors completing the modules for vitamin production are *Firmicutes*, *Bacteroidetes*, *Actinobacteria*, and *Gammaproteobacteria*. Completion patterns of the KEGG module for these amino acids and vitamins mainly fall into patterns C (diversified module) and D (non-prokaryotic module) except for riboflavin biosynthesis (M00125_1~_3) belonging to one of the universal modules (A-1), indicating that these modules are involved in the nutritional supply for the gut microbiome as well as for the host (Figure 5B). Inter-individual variation was also evident in the completion ratio of the module for vitamins. For example, the module (M00124_1) belonging to pattern C for pyridoxal (vitamin B6) biosynthesis was mainly attributable to *Bacteroidetes* in adults and *Gammaproteobacteria* in infants; however, its completion ratio in 2 male infants (In-B and In-E) was extremely low (33.33%) (Figure 5A). Inter-individual variations in completion ratios were also observed in modules (M00133_1 and M00134_1) for polyamine biosynthesis, for example, putrescine, spermidine, and spermine. Similarly, the completion ratio of the KEGG modules (M00135_1 and M00136_1) for γ-aminobutyric acid (GABA) varied among individuals, and *Gammaproteobacteria* mainly contributed to GABA production. Because these polyamines and GABA are essential biological substances that act as cell growth promoters and inhibitory neurotransmitters respectively, in humans, these variations may be linked to susceptibilities to certain diseases. Indeed, a recent report on metabolic changes in gut microbiomes after bariatric surgery for obese patients demonstrated their potential for polyamine production in the gut; elevated protein putrefaction because of the bypassed food passage promoted putrescine and GABA production from gut microbiota [26].

**Figure 6:**
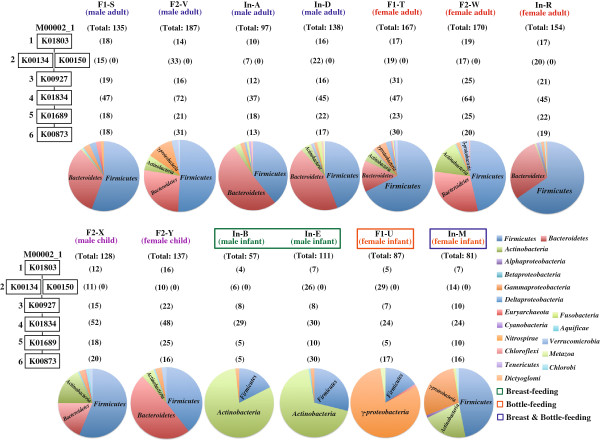
**Taxonomic variation in genes assigned to KOs associated with glycolysis in human gut microbiomes from 13 healthy individuals.** The pathway module M00002_1 comprising 6 steps shows glycolysis (core module involving 3-carbon compounds), and K number in each box indicates KO assigned for every individual reaction. Because K00134 and K00150 have the relationship of “Or” as explained in Figure 2, if the gene from human gut microbiomes is assigned to either K00134 or K00150, the reaction at the 2nd step can be executed. Pie charts show taxonomic breakdown of the genes assigned to KOs in all 6 steps. Numbers in parentheses indicate the number of the genes assigned to KO in each reaction step.

Interestingly, gut microbiomes showed preference for amino acid catabolism. As shown in Figure 5C, the gut microbiome did not seem to utilize exogenous lysine (M00032_1), leucine (M00036_1), and aromatic amino acids such as tryptophan (M00038_1) and tyrosine (M00044_1). To our knowledge, this is a novel finding on the nutritional preference of gut microbes. This may be one of the mutualistic representations of gut microbiomes to avoid nutritional competition with the host because these aromatic amino acids are precursors of various biological substances such as catecholamines, melatonin, serotonin, thyroid hormones, and NAD. To assess the taxonomic composition of gut microbiomes, the module for glycolysis (M00002_1) was analyzed (Figure 6). Each gene product mapped to this module was taxonomically assigned, and distribution at the phylum level was calculated. Consistent with a previous report [6], adult and child gut microbiomes are constituted by 2 major phyla, *Firmicutes* and *Bacteroidetes*. Analysis of the module for glycolysis clearly differentiated the gut microbial composition between adults and infants as well as among infants. In particular, it was highlighted that *Actinobacteria* was a major phylum in breast-fed infants, whereas *Gammaproteobacteria* was predominant in a bottle-fed infant. It was also evident that the microbial composition in an infant with mixed feeding (breast and bottle) showed a pattern intermediate between those in breast- and bottle-fed infants. Thus, the new evaluation method based on the KEGG modules is expected not only to highlight the metabolic linkage between host and commensal microbes but also to identify microbiome-based biomarkers for particular diseases.

## Conclusions

We developed a new evaluation method for potential functionomes based on the KEGG modules. Modules with branching or different component structures in a complex were redefined depending on the number of branching or types of component structures. The module completion ratio was calculated by counting the number of genes assigned to KO identifiers constructing each module defined by a Boolean algebra-like equation.

Using this new method, we found significant difference in module completion ratio by 8 bacilli in terms of at least 19 pathway and 35 complex modules, although how the differentiated functional modules confer phenotypic properties directly or indirectly is unclear thus far. Because the coverage of KEGG modules over whole metabolic and signaling networks is continuously increasing, differences in module completion ratio will provide some important clues to the understanding of phenotypic properties. Furthermore, variations in the functional potential of human gut microbiomes from 13 healthy individuals could be characterized by the pathway and structural complex module units, and the complementarity between biochemical functions in human hosts and nutritional preferences in human gut microbiomes identified. In addition, taxonomic variation of the contributors to each module clarified by this method will prove informative when considering ecological dynamics.

Functional annotations to metagenomic sequences remain difficult because metagenomic data targeting various environments still contains incomplete genes from various unidentified species, absent in a reference database. In this study, we used the KAAS system for functional annotation to the human metagenomes and also attempted to estimate database dependency on the accuracy of the KO assignment using the *E. coli* draft genome. As a result, the KAAS system could correctly assign to KO groups with an accuracy rate of approximately 80%, even if the gene hosts were not classified into known phyla within the reference database (see the Methods section). Thus, our method will work well for comparative functional analysis in metagenomics, able to target unknown environments containing various uncultivable microbes within unidentified phyla, although further verification studies on database dependency for metagenomics should be performed.

## Methods

### KEGG MODULE

KEGG MODULE [16] is a collection of pathway modules and other functional units designed for automatic functional annotation or pathway enrichment analysis. Pathway modules such as the TCA cycle core module (Figure 2A) are tighter functional units than KEGG pathway maps and are defined as consecutive reaction steps, operon or other regulatory units, and phylogenetic units obtained by genome comparisons. Other functional units include (1) structural complexes representing sets of protein subunits for molecular machineries such as photosystems (Figure 2B), (2) functional sets representing other types of essential sets such as aminoacyl-tRNA synthases, and (3) signature modules representing markers of phenotypes such as enterohemorrhagic *E. coli* pathogenicity signature for Shiga toxin. The latest KEGG MODULE is available from the KEGG FTP site (http://www.kegg.jp/kegg/download). Each module is defined by the combination of KO identifiers so that it can be used for annotation and interpretation purposes in individual genomes or metagenomes. Notations of the Boolean algebra-like equation (Figure 2 and Additional file 1: Table S6) for this definition include space-delimited items for pathway elements, comma-separated items in parentheses for alternatives, a plus sign to define a complex, and a minus sign for an optional item. Some modules have branching points in their reaction cascades (Additional file 2: Figure S7), leading to different products or alternative reaction pathways. These modules are divided into several parts depending on the branching patterns and are redefined as submodules for accurate calculation of the completion ratio. In the example shown in Figure S7 (Additional file 2), there are 2 pathways for the module for heme biosynthesis (M00121_1): 1 for protoheme (C00032) and the other for siroheme (C00748). Consequently, 3 submodules were defined: M00121_1 for the original, M00121_2 for protoheme production, and M00121_3 for siroheme production. By redefinition of the modules based on the above mentioned policy the number of pathway and structural complex modules increased from 179 to 205 and from 248 to 263, respectively. The module completion ratio (see the section below) was calculated for each submodule in this study to examine fine-grained functional categories. A breakdown of the functional categories of all KEGG modules containing newly reidentified modules is summarized in Table 3.

**Table 3:** Breakdown of small functional categories of the KEGG modules

**Pathway modules [203]**		**Structural complex modules [263]**	
Small functional category	Number (%)	Small functional category	Number (%)
Cofactor & vitamin biosynthesis	30 (14.6)	Saccharide and polyol transport system	29 (11.0)
Carbon fixation	14 (6.8)	ATP synthesis	27 (10.3)
Central carbohydrate metabolism	14 (6.8)	Phosphotransferase system (PTS)	24 (9.1)
Lipid metabolism	14 (6.8)	Mineral and organic ion transport system	23 (8.7)
Glycan metabolism	13 (6.3)	Phosphate and amino acid transport system	19 (7.2)
Aromatic amino acid metabolism	11 (5.4)	ABC-2 type and other transport systems	16 (6.1)
Methane metabolism	11 (5.4)	Bacterial secretion system	14 (5.3)
Fatty acid metabolism	10 (4.9)	RNA processing	13 (4.9)
Sterol biosynthesis	10 (4.9)	Ubiquitin	13 (4.9)
Cystein & methionine metabolism	7 (3.4)	Metallic cation, iron-siderophore and vitamin B12 transport system	12 (4.6)
Glycosaminoglycan metabolism	7 (3.4)	Protein processing	9 (3.4)
Other carbohydrate metabolism	7 (3.4)	Spliceosome	9 (3.4)
Polyamine biosynthesis	6 (2.9)	Repair system	8 (3.0)
Telpenoid backbone biosynthesis	6 (2.9)	DNA polymerase	7 (2.7)
Lysine metabolism	5 (2.4)	Photosynthesis	7 (2.7)
Pyrimidine metabolism	5 (2.4)	RNA polymerase	7 (2.7)
Akaloid & other secondardy metabolite	4 (1.9)	Peptide and nickel transport system	6 (2.3)
LPS metabolism	4 (1.9)	Replication system	6 (2.3)
Other terpenoid biosynthesis	4 (1.9)	Carbohydrate metabolism	5 (1.9)
Arginine & proline metabolism	3 (1.5)	Proteasome	5 (1.9)
BCAA metabolism	3 (1.5)	Ribosome	3 (1.1)
Other amino acid metabolism	3 (1.5)	Glycan metabolism	1 (0.4)
Phenylpropanoid & flavonoid biosyntesis	3 (1.5)	**Functional set modules [**[4]**]**	
Purine metabolism	3 (1.5)	Small functional category	Number (%)
Histidine metabolism	2 (1.0)	Aminoacyl-tRNA	2 (50)
Metabolic capacity	2 (1.0)	Nucleotide sugar	2 (50)
Serin & threonine metabolism	2 (1.0)	**Signature modules [**[3]**]**	
Nitrogen fixation	1 (0.5)	Small functional category	Number (%)
Sulfur metabolism	1 (0.5)	Genotypic signature	3 (100)

[ ] shows total number of the KEGG modules containing branched modules.

### Calculation of the module completion ratio based on a Boolean algebra-like equation

The completion ratio of all KEGG functional modules in each organism was calculated based on a Boolean algebra-like equation (Additional file 1: Table S6). For this analysis, 1 genome was selected from each of the 768 available prokaryotic species shown in Table S1 (Additional file 1) and a reference genome set was constructed to cover all completely sequenced prokaryotes, excluding draft genomes as of December 2011.

As one of examples, M00009_1 is a core pathway module for the TCA cycle comprising 8 reactions as shown in Figure 2A. In each KO number set, vertically connected KO identifiers indicate a complex and therefore represent “And” or “+” in the Boolean algebra-like equation, whereas horizontally located K numbers indicate alternatives and represent “Or” or “,” in the equation. When genes are assigned to all KO identifiers in each reaction according to the Boolean algebra-like equation, the module completion ratio becomes 100%. If genes are not assigned to KO identifiers in 2 reactions, the module completion ratio is calculated as 75% (6/8 x 100 = 75). On the other hand, M00163_1 and M00163_2 comprising 14 components in plants and 12 in cyanobacteria represent a complex module for photosystem I. If genes assigned to KO identifiers in 2 of those components are missing in plants, the module completion ratio is calculated as 85.7% and 83.3% in the case of cyanobacteria (Figure 2B). A stand-alone calculation system of module completion ratio for Linux and Mac OS X is available from the download site of ExtremoBase (http://www.jamstec.go.jp/gbrowser/cgi-bin/top.cgi).

### Assignment of the query sequences to KO identifiers

Efficient and accurate computational methods are required for the functional annotation of rapidly growing sequence data from complete genomes and metagenomes. KAAS is an efficient tool for assigning KO identifiers to genes from complete genomes based on a BLAST search of the KEGG GENES database combined with a bidirectional best-hit method [11]. Because of the efficiency, KAAS is used to assign KO identifiers to protein sequences from metagenome projects (http://www.genome.jp/kegg/catalog/org_list3.html) and to users’ own data from other genome and metagenome projects.

We applied a slightly modified version of the KAAS system that has improved the accuracy of KO assignments by (i) using a variable bit-score threshold instead of a fixed one (60 in the original KAAS system) to avoid missed annotations when there are sufficient high-scoring hits for KO assignment, and (ii) considering taxonomic information of each KO when more than 1 candidate KO is obtained (Figure 1). This modification resulted in improved positive predictive value (#true positives / #all positives) by 2–5% in the KO reassignment tests for 30 selected species (Additional file 2: Figure S8). The latest stand-alone KAAS system for Linux and Mac OS X is available from the web site of KAAS HELP (http://www.genome.jp/tools/kaas/help.html). We used this new KAAS to estimate database dependency on accuracy of the KO assignment. We selected *Escherichia coli* as a representative of prokaryotic species and constructed 4 different types of datasets: without *E. coli* and closely related species (1,239 species), without all species within family *Enterobacteriales* (1,200 species), without all species within class *Gammaproteobacteria* (1,040 species), and without all species within phylum *Proteobacteria* (755 species).

In addition, we created artificially fragmented protein sequences from *E. coli* to confirm the accuracy of KO assignment to the truncated proteins because the gene products predicted from the end of assembled contigs and singletons were often truncated in the sequences produced by 2^nd^ generation DNA sequencer. The draft genome of *E. coli* isolated from infants in Trondheim, Norway (accession: ERX127960), which appears to contain several sequencing errors, was used for this analysis. The short read sequences of *E. coli* produced by a 454 GS FLX Titanium sequencer (92.4 Mb in total) were assembled by Newbler ver. 2.6 and the contigs longer than 500 bp were used in this analysis. By using MetaGeneAnnotator [27], 4,410 complete and 178 partial CDSs were identified from the contigs. The amino acid sequences of complete CDSs were randomly fragmented to 50, 60, 80, 100, 120, 150, and 200 residues in length, and each fragment was subjected to verification of database dependency based on the accuracy of KO identifier assignment (Additional file 2: Figure S9).

In general, because most microbes thriving in natural environments are uncultivable, many genes in environmental metagenomes do not show significant similarity to those from known species in the public genome database. Especially when microbial genomes belonging to the same phylum as the query microbe are missing in the genome database, the accuracy rate of KO assignment to proteins phylogenetically distant from known phyla is expected to be low. In fact, when all species within phylum *Proteobacteria* were not included in the data set, the accuracy rate of KO assignment to full proteins of *E. coli* decreased to 80%, but the accuracy rate of approximately 70% was maintained even in the proteins fragmented to about 100 residues (Additional file 2: Figure S9). Considering these results, even if the genes from unidentified phyla of the so-called Candidate division are included in the metagenomes, the KAAS system can presumably assign KO identifiers to genes longer than 300 bp (100 amino acids) with an accuracy rate of approximately 70%.

### Application of the evaluation method for functionome to genomic analysis

The completed genomic sequences of 8 moderately related species within *Bacillaceae* were selected and obtained from the KEGG FTP site (ftp://ftp.bioinformatics.jp/). A breakdown of the selected species is as follows: *B. subtilis*[20], *B. amyloliquefaciens*[21], and *B. licheniformis*[22] (mesophilic neutrophile); *B. halodurans*[23] and *B. pseudofirmus*[24] (mesophilic alkaliphile); *O. iheyensis*[19] (mesophilic, extremely halotolerant alkaliphile); and *Geobacillus kaustophilus*[28] and *G. thermoglucosidasius* (thermophilic neutrophile). Amino acid sequences were used for assignment of KO identifiers to the gene products from each species by the KAAS system (Figure 1) and the KO assigned ones were mapped to the KEGG modules. The completion ratio of each module in the 8 species was calculated based on the Boolean algebra-like equation, and the ratios were compared with each other to differentiate the potential functionome among the 8 species.

### Application of the evaluation method for functionome to metagenomic analysis

The metagenomic Sanger sequences of human gut microbiomes were selected from 13 healthy individuals [6] to apply our evaluation method for potential functionome using the KEGG modules to comparative metagenomic analysis focusing on differentiation of functional potentials between individuals. Amino acid sequences of the gene products predicted by MetaGeneAnnotaor [27] from the assembled contigs and also singlets in metagenomic sequences were used in this study and obtained from KEGG Metagenomes (http://www.genome.jp/kegg/catalog/org_list3.html). Sample ID and number of coding sequences (CDSs) used in this study are as follows: Male adult: F1-S (54,151), F2-V (65,156), and In-A (35,260); Female adult: F1-T (65,156), F2-W (57,213), and In-R (63,356); Female child: F2-X (57,446); F2-Y (64,942); Male infant: In-B (20,063) and In-E (37,652); and Female infant: F1-U (35,260) and In-M (34,330). Complete CDSs account for 40-55% of all CDSs in each sample and their average length was about 180 to 217 a.a. Average length of partial CDSs in each sample was 146 to 175 a.a. and the CDSs longer than 100 a.a. account for more than 80% of all CDSs in all 13 samples. The detailed information for metagenomic samples of human gut microbiomes are summarized in Table S7 (Additional file 1). KO identifiers were assigned to 35-55% of the complete and partial CDSs identified in the metagenomic sequences from the 13 individuals using the KAAS system and 31-39% of KO assigned CDSs were then mapped to the KEGG functional modules (Additional file 1: Table S7). The completion ratio of each module by human gut microbiomes from 13 healthy individuals was calculated based on the Boolean algebra-like equation and compared with each other to differentiate the potential functionome between the 13 individuals. Human genome sequence was also obtained from the KEGG FTP site and KO identifiers were assigned to 10,508 CDSs. The KO assigned 1,321 CDSs were mapped to the KEGG functional modules similar to human gut microbiomes.

Alternatively, we also employed assembled contig sequences of short reads produced (>500 bp) by an Illumina GA to compare their functionome with those by the Sanger sequences. Short reads from the metagenomics of human intestinal tract (MetaHIT) project [29] were subjected to CDS identification, KO assignment and KEGG module mapping (of the KO assignment CDSs). We obtained two sections of the MetaHit sequence data (MH_0001: Danish female and MH_0005: Danish male) from the MetaHIT database (http://www.bork.embl.de/~arumugam/Qin_et_al_2010/) and approximately 28,000 and 27,000 CDSs containing partial sequences were identified by MetaGeneAnnotator [27], respectively. KO identifiers were assigned to 40.5-41.5% to the CDSs from the 2 Danish individuals and 33-34% of KO assigned CDSs were mapped to the KEGG modules, in a similar manner as the Sanger sequences (Additional file 1: Table S7). Ultimately, it was found that there were no discernible differences in KO ID assignment and mapping ratios of KO assigned CDSs to the KEGG modules when comparing between Sanger and Illumina reads using contigs longer than 500 bp for analysis.

## Abbreviations

KEGG: Kyoto Encyclopedia of Genes and Genomes; KO: KEGG Orthology; KAAS: KEGG Automatic Annotation Server; COGs: Clusters of orthologous groups; GABA: γ-aminobutyric acid; NAD: Nicotinamide adenine dinucleotide; ABC: ATP-binding cassette.

## Competing interests

The authors declare that they have no competing interests.

## Authors’ contribution

HT conceived the study and performed data analysis throughout the study. TT redefined the KEGG modules and analyzed the data. SG, YM, and MK confirmed the KEGG modules. TK performed data analysis of the human microbiome. HT, TK, and SG wrote the manuscript. All authors have read and approved the final manuscript.

## Supplementary Material

Additional file 1 **Tables S1–S7. Table S1.** List of 768 prokaryotic species used in this study. The additional data are available with the online version of this paper. **Table S2.** Taxonomic patterns of the prokaryotes which complete the KEGG modules (205 pathways, 263 structural complexes, 4 functional sets, and 3 signatures). Functional annotation of each module is listed in Table S3-S5. Figures S1-S3 were drawn based on this table. **Table S3.** Characterization of the 205 KEGG pathway modules containing submodules based on the module completion patterns in 768 prokaryotic species. **Table S4.** Characterization of the 263 KEGG structural complex modules containing submodules based on the module completion patterns in 768 prokaryotic species. **Table S5.** Characterization of the 7 KEGG modules (4 functional sets and 3 signatures) based on the module completion patterns in 768 prokaryotic species. Table S6. Notations of Boolean algebra-like equations for all KEGG modules containing redefined ones. Table S7. Summary of metagenomic sequences of human gut microbiome. Click here for file

Additional file 2 **Figures S1–S9. Figure S1.** Distribution patterns of the completion ratio of the KEGG pathway modules in 768 prokaryotic species. The completion ratio of 205 pathway modules containing submodules were evaluated in this study. **Figure S2.** Distribution patterns of the completion ratio of the KEGG structural complex modules in 768 prokaryotic species. The module completion ratio of 263 structural complex modules containing submodules was evaluated in this study. **Figure S3.** Distribution patterns of the completion ratio of the KEGG functional set and signature modules in 768 prokaryotic species. The module completion ratio of 7 functional set and signature modules was evaluated in this study. **Figure S4.** Distribution of KO identifiers mapped to the module for glyoxylate cycle (M00012) in other pathway modules. KO identifiers, except for K01637 and K01638 colored light green, are also shared in several other modules. **Figure S5.** Module completion patterns in 8 phenotypically different *Bacillus*-related species. (A) Pathway module. (B) Structural complex module. bsu, *Bacillus subtilis* ; bao, *Bacillus amyloliquefaciens* ; bli, *Bacillus licheniformis* ; bha, *Bacillus halodurans* ; bpf, *Bacillus pseudofirmus* ; oih, *Oceanobacillus iheyensis* ; gka, *Geobacillus kaustophilus* ; and gth, *Geobacillus thermoglucosidasius*. Green characters show rare modules, which are completed by less than 10% of 768 prokaryotic species. **Figure S6.** Module completion patterns in human and human gut microbiomes. (A)-1–3, Pathway module. (B)-1–3, Structural complex module. Upper histogram shows the module completion pattern in gut microbiomes from 13 healthy individuals [18]. Middle histogram shows module completion patterns in humans. Lower histogram shows module completion patterns in human gut microbiomes plus humans. Green characters show rare modules, which are completed by less than 10% of 768 prokaryotic species. **Figure S7.** Definition of submodules for the KEGG module with branching. The heme biosynthesis pathway (glutamate => protoheme => siroheme) module (M00212) has branching at the intermediate compound uroporphyrinogen III (C01051), where this module was divided into 2 parts. Submodules are defined as M00121_1 (original), M00121_2 (left-side branching), and M00121_3 (right-side branching). Ovals with C numbers, rectangles with R numbers, and K numbers represent metabolites, enzymatic reactions, and KO, respectively. KO is used for mapping functional annotation of genes to the modules. Black K numbers indicate KO common to all 3 newly redefined submodules (M00121_1, M00121_2, and M00121_3), and blue and red K numbers correspond to reactions specific to M00121_2 and M00121_3, respectively. **Figure S8.** Positive predictive values (ppv) of the KO reassignment tests by KAAS. We performed KO reassignment tests for 30 species (7 eukaryotes, 20 bacteria, 3 archaea) by original (old) and improved (new) KAAS and found that new KAAS showed 2-5% improvements compared with the old KAAS. Three letter codes in X axis indicate species. abbreviations as follows: hsa: *Homo sapiens*, dre: *Danio rerio*, dme: *Drosophila melanogaster*, cel: *Caenorhabditis elegans*, ath: *Arabidopsis thaliana*, sce: *Saccharomyces cerevisiae,* cho: *Cryptosporidium hominis*, eco: *Escherichia coli*, nme: *Neisseria meningitidis*, hpy:*Helicobacter pylori*, rpr: *Ricketsia prowazekii*, bsu:*Bacillus subtilis*, sau: *Staphylococcus aureus*, lmo: *Listeria monocytogenes*, lla: *Lactococcus lactis*, lpl: *Lactobacillus plantarum*, cau: *Chloroflexus aurantiacus*, mge: *Mycoplasma genitalium*, mtu: *Mycobacterium tuberculosis*, blo: *Bifidobacterium longum*, ctr:*Chlamydia trachomatis*, pcu:*Protochlamydia amoebophila*, bbu:*Borrelia burgdorferi*, syn: *Synechocystis* sp., bth: *Bacteroides thetaiotaomicron*, dra: *Deinococcus radiodurans*, aae:*Aquifex aeolicus*, mja: *Methanocaldococcus jannaschii*, ape:*Aeropyrum pernix*, neq:*Nanoarchaeum equitans.*Blue bar: old KAAS, Red bar: new KAAS. **Figure S9.** Effect of database dependency on accuracy of the KO assignment. *Escherichia coli* isolated from Norwegian infant (Draft genome sequenced by 454 GS FLX Titanium). Blue diamonds show the results using the data set without proteins from the genera *Escherichia, Salmonella, Shigella,* and *Yersinia* (1,239 species). Similarly, red squares, green triangles, and purple dots show the results without proteins from the order *Enterobacteriales* (1,200 species), class *Gammaproteobacteria* (1,040 species), and phylum *Proteobacteria* (755 species), respectively. KO identifiers specific to the genera *Escherichia, Salmonella, Shigella*, and *Yersinia* (16 KO identifiers), order *Enterobacteriales* (90), class *Gammaproteobacteria* (203), or phylum *Proteobacteria* (370) were removed in advance from the protein data set. Here, the accuracy is defined by the sensitivity TP/(TP+FN), where TP and FN are the numbers of true positives and false negatives, respectively. We also used truncated proteins to confirm effect of amino acid (a.a.) sequence lengths on the accuracy of KO assignments. The 4,410 proteins from *E. coli* isolate were randomly fragmented into 50, 60, 80, 100, 120, 150, and 200 a.a. in length, and each length of a.a. sequences was used for verification of the accuracy of KO assignment. Click here for file
