# Supplementary material for: Evaluation method for the potential functionome harbored in the genome and metagenome
Source: BMC Genomics. 2012 Dec 12;13:699. doi: 10.1186/1471-2164-13-699 (PMC3541978; doi:10.1186/1471-2164-13-699)
Supplement: Additional file 1 — Tables S1–S7. Table S1. List of 768 prokaryotic species used in this study. The additional data are available with the online version of this paper. Table S2. Taxonomic patterns of the prokaryotes which complete the KEGG modules (205 pathways, 263 structural complexes, 4 functional sets, and 3 signatures). Functional annotation of each module is listed in Table S3-S5. Figures S1-S3 were drawn based on this table. Table S3. Characterization of the 205 KEGG pathway modules containing submodules based on the module completion patterns in 768 prokaryotic species. Table S4. Characterization of the 263 KEGG structural complex modules containing submodules based on the module completion patterns in 768 prokaryotic species. Table S5. Characterization of the 7 KEGG modules (4 functional sets and 3 signatures) based on the module completion patterns in 768 prokaryotic species. Table S6. Notations of Boolean algebra-like equations for all KEGG modules containing redefined ones. Table S7. Summary of metagenomic sequences of human gut microbiome. [file 1471-2164-13-699-S1.pdf]

Table S1. List of 768 prokaryotic species used in this study

ID	Speceis	Sequencing status	KEGG abbreviation	Phylum/Class	Total nos. of Phylum/Class
1	Escherichia coli K-12 MG1655	Complete	eco	Gammaproteobacteria	126
2	Salmonella enterica subsp. enterica serovar Typhi CT18	Complete	sty	Gammaproteobacteria	
3	Yersinia pestis CO92 (biovar Orientalis)	Complete	ype	Gammaproteobacteria	
4	Yersinia pseudotuberculosis IP32953 (serotype I)	Complete	yps	Gammaproteobacteria	
5	Yersinia enterocolitica subsp. enterocolitica 8081	Complete	yen	Gammaproteobacteria	
6	Shigella flexneri 301 (serotype 2a)	Complete	sfl	Gammaproteobacteria	
7	Shigella sonnei	Complete	ssn	Gammaproteobacteria	
8	Shigella boydii Sb227	Complete	sbo	Gammaproteobacteria	
9	Shigella dysenteriae	Complete	sdy	Gammaproteobacteria	
10	Pectobacterium atrosepticum	Complete	eca	Gammaproteobacteria	
11	Erwinia tasmaniensis	Complete	eta	Gammaproteobacteria	
12	Photorhabdus luminescens	Complete	plu	Gammaproteobacteria	
13	Photorhabdus asymbiotica	Complete	pay	Gammaproteobacteria	
14	Buchnera aphidicola APS	Complete	buc	Gammaproteobacteria	
15	Wigglesworthia glossinidia	Complete	wbr	Gammaproteobacteria	
16	Sodalis glossinidius	Complete	sgl	Gammaproteobacteria	
17	Enterobacter sp. 638	Complete	ent	Gammaproteobacteria	
18	Enterobacter cloacae SCF1	Complete	esc	Gammaproteobacteria	
19	Enterobacter sakazakii	Complete	esa	Gammaproteobacteria	
20	Cronobacter turicensis	Complete	ctu	Gammaproteobacteria	
21	Klebsiella pneumoniae	Complete	kpn	Gammaproteobacteria	
22	Citrobacter koseri ATCC BAA-895	Complete	cko	Gammaproteobacteria	
23	Citrobacter rodentium	Complete	cro	Gammaproteobacteria	
24	Serratia proteamaculans	Complete	spe	Gammaproteobacteria	
25	Proteus mirabilis	Complete	pmr	Gammaproteobacteria	
26	Edwardsiella ictaluri	Complete	eic	Gammaproteobacteria	
27	Edwardsiella tarda	Complete	etr	Gammaproteobacteria	
28	Candidatus Blochmannia floridanus	Complete	bfl	Gammaproteobacteria	
29	Candidatus Hamiltonella defensa	Complete	hde	Gammaproteobacteria	
30	Dickeya dadantii Ech703	Complete	dda	Gammaproteobacteria	
31	Xenorhabdus bovienii	Complete	xbo	Gammaproteobacteria	
32	Pantoea ananatis	Complete	pam	Gammaproteobacteria	
33	Candidatus Riesia pediculicola	Complete	rip	Gammaproteobacteria	
34	Rahnella sp. Y9602	Complete	rah	Gammaproteobacteria	
35	Haemophilus influenzae Rd KW20 (serotype d)	Complete	hin	Gammaproteobacteria	
36	Haemophilus ducreyi	Complete	hdu	Gammaproteobacteria	
37	Haemophilus somnus 129PT	Complete	hso	Gammaproteobacteria	
38	Pasteurella multocida	Complete	pmu	Gammaproteobacteria	
39	Mannheimia succiniciproducens	Complete	msu	Gammaproteobacteria	
40	Actinobacillus pleuropneumoniae L20 (serotype 5b)	Complete	apl	Gammaproteobacteria	
41	Actinobacillus succinogenes	Complete	asu	Gammaproteobacteria	
42	Aggregatibacter aphrophilus	Complete	aap	Gammaproteobacteria	
43	Aggregatibacter actinomycetemcomitans	Complete	aat	Gammaproteobacteria	
44	Xylella fastidiosa 9a5c	Complete	xfa	Gammaproteobacteria	
45	Xanthomonas campestris pv. campestris ATCC 33913	Complete	xcc	Gammaproteobacteria	
46	Xanthomonas axonopodis	Complete	xac	Gammaproteobacteria	
47	Xanthomonas oryzae KACC10331	Complete	xoo	Gammaproteobacteria	
48	Stenotrophomonas maltophilia K279a	Complete	sml	Gammaproteobacteria	
49	Pseudoxanthomonas suwonensis	Complete	psu	Gammaproteobacteria	
50	Vibrio cholerae O1	Complete	vch	Gammaproteobacteria	

51	Vibrio vulnificus CMCP6	Complete	vvu	Gammaproteobacteria
52	Vibrio parahaemolyticus	Complete	vpa	Gammaproteobacteria
53	Vibrio harveyi	Complete	vha	Gammaproteobacteria
54	Vibrio splendidus	Complete	vsp	Gammaproteobacteria
55	Vibrio sp. Ex25	Complete	vex	Gammaproteobacteria
56	Vibrio fischeri	Complete	vfi	Gammaproteobacteria
57	Aliivibrio salmonicida LFI1238	Complete	vsa	Gammaproteobacteria
58	Photobacterium profundum	Complete	ppr	Gammaproteobacteria
59	Pseudomonas aeruginosa PAO1	Complete	pae	Gammaproteobacteria
60	Pseudomonas putida KT2440	Complete	ppu	Gammaproteobacteria
61	Pseudomonas syringae pv. tomato DC3000	Complete	pst	Gammaproteobacteria
62	Pseudomonas fluorescens Pf-5	Complete	pfl	Gammaproteobacteria
63	Pseudomonas entomophila	Complete	pen	Gammaproteobacteria
64	Pseudomonas mendocina ymp	Complete	pmy	Gammaproteobacteria
65	Pseudomonas stutzeri	Complete	psa	Gammaproteobacteria
66	Cellvibrio japonicus	Complete	cja	Gammaproteobacteria
67	Azotobacter vinelandii	Complete	avn	Gammaproteobacteria
68	Psychrobacter arcticum	Complete	par	Gammaproteobacteria
69	Psychrobacter cryohalolentis	Complete	pcr	Gammaproteobacteria
70	Psychrobacter sp. PRwf-1	Complete	prw	Gammaproteobacteria
71	Acinetobacter sp. ADP1	Complete	aci	Gammaproteobacteria
72	Acinetobacter baumannii ATCC 17978	Complete	acb	Gammaproteobacteria
73	Moraxella catarrhalis	Complete	mct	Gammaproteobacteria
74	Shewanella oneidensis	Complete	son	Gammaproteobacteria
75	Shewanella denitrificans	Complete	sdn	Gammaproteobacteria
76	Shewanella frigidimarina	Complete	sfr	Gammaproteobacteria
77	Shewanella amazonensis	Complete	saz	Gammaproteobacteria
78	Shewanella baltica OS155	Complete	sbl	Gammaproteobacteria
79	Shewanella loihica	Complete	slo	Gammaproteobacteria
80	Shewanella putrefaciens	Complete	spc	Gammaproteobacteria
81	Shewanella sediminis	Complete	sse	Gammaproteobacteria
82	Shewanella pealeana	Complete	spl	Gammaproteobacteria
83	Shewanella sp. MR-4	Complete	she	Gammaproteobacteria
84	Shewanella halifaxensis	Complete	shl	Gammaproteobacteria
85	Shewanella woodyi ATCC 51908	Complete	swd	Gammaproteobacteria
86	Shewanella piezotolerans WP3	Complete	swp	Gammaproteobacteria
87	Idiomarina loihiensis	Complete	ilo	Gammaproteobacteria
88	Colwellia psychrerythraea	Complete	cps	Gammaproteobacteria
89	Pseudoalteromonas haloplanktis	Complete	pha	Gammaproteobacteria
90	Pseudoalteromonas atlantica	Complete	pat	Gammaproteobacteria
91	Saccharophagus degradans	Complete	sde	Gammaproteobacteria
92	Marinobacter aquaeolei	Complete	maq	Gammaproteobacteria
93	Alteromonas macleodii	Complete	amc	Gammaproteobacteria
94	Psychromonas ingrahamii	Complete	pin	Gammaproteobacteria
95	Teredinibacter turnerae	Complete	ttu	Gammaproteobacteria
96	Ferrimonas balearica	Complete	fbl	Gammaproteobacteria
97	Coxiella burnetii RSA 493	Complete	cbu	Gammaproteobacteria
98	Legionella pneumophila Philadelphia 1	Complete	lpn	Gammaproteobacteria
99	Methylococcus capsulatus	Complete	mca	Gammaproteobacteria
100	Francisella tularensis subsp. tularensis SCHU S4	Complete	ftu	Gammaproteobacteria
101	Francisella novicida U112	Complete	ftn	Gammaproteobacteria
102	Francisella philomiragia	Complete	fph	Gammaproteobacteria

103	Thiomicrospira crunogena	Complete	tcx	Gammaproteobacteria	
104	Nitrosococcus oceani	Complete	noc	Gammaproteobacteria	
105	Nitrosococcus halophilus	Complete	nhl	Gammaproteobacteria	
106	Allochromatium vinosum	Complete	alv	Gammaproteobacteria	
107	Alkalilimnicola ehrlichei	Complete	aeh	Gammaproteobacteria	
108	Halorhodospira halophila	Complete	hha	Gammaproteobacteria	
109	Thioalkalivibrio sp. HL-EbGR7	Complete	tgr	Gammaproteobacteria	
110	Halothiobacillus neapolitanus	Complete	hna	Gammaproteobacteria	
111	Hahella chejuensis	Complete	hch	Gammaproteobacteria	
112	Chromohalobacter salexigens	Complete	csa	Gammaproteobacteria	
113	Halomonas elongata	Complete	hel	Gammaproteobacteria	
114	Alcanivorax borkumensis	Complete	abo	Gammaproteobacteria	
115	Kangiella koreensis	Complete	kko	Gammaproteobacteria	
116	Marinomonas sp. MWYL1	Complete	mmw	Gammaproteobacteria	
117	Aeromonas hydrophila	Complete	aha	Gammaproteobacteria	
118	Aeromonas salmonicida	Complete	asa	Gammaproteobacteria	
119	Tolumonas auensis	Complete	tau	Gammaproteobacteria	
120	Dichelobacter nodosus	Complete	dno	Gammaproteobacteria	
121	Acidithiobacillus ferrooxidans ATCC 53993	Complete	afe	Gammaproteobacteria	
122	Baumannia cicadellinicola	Complete	bci	Gammaproteobacteria	
123	Candidatus Carsonella ruddii	Complete	crp	Gammaproteobacteria	
124	Candidatus Ruthia magnifica	Complete	rma	Gammaproteobacteria	
125	Candidatus Vesicomyosocius okutanii	Complete	vok	Gammaproteobacteria	
126	Gamma proteobacterium HdN1	Complete	gpb	Gammaproteobacteria	
127	Neisseria meningitidis Z2491 (serogroup A)	Complete	nma	Betaproteobacteria	61
128	Neisseria gonorrhoeae FA 1090	Complete	ngo	Betaproteobacteria	
129	Chromobacterium violaceum	Complete	cvi	Betaproteobacteria	
130	Laribacter hongkongensis	Complete	lhk	Betaproteobacteria	
131	Ralstonia solanacearum GMI1000	Complete	rso	Betaproteobacteria	
132	Ralstonia pickettii 12J	Complete	rpi	Betaproteobacteria	
133	Ralstonia eutropha JMP134	Complete	reu	Betaproteobacteria	
134	Cupriavidus metallidurans	Complete	rme	Betaproteobacteria	
135	Cupriavidus taiwanensis	Complete	cti	Betaproteobacteria	
136	Burkholderia mallei ATCC 23344	Complete	bma	Betaproteobacteria	
137	Burkholderia pseudomallei K96243	Complete	bps	Betaproteobacteria	
138	Burkholderia thailandensis	Complete	bte	Betaproteobacteria	
139	Burkholderia vietnamiensis	Complete	bvi	Betaproteobacteria	
140	Burkholderia sp. 383	Complete	bur	Betaproteobacteria	
141	Burkholderia cenocepacia AU1054	Complete	bcn	Betaproteobacteria	
142	Burkholderia cepacia	Complete	bam	Betaproteobacteria	
143	Burkholderia ambifaria MC40-6	Complete	bac	Betaproteobacteria	
144	Burkholderia multivorans ATCC 17616 (JGI)	Complete	bmu	Betaproteobacteria	
145	Burkholderia xenovorans	Complete	bxe	Betaproteobacteria	
146	Burkholderia phymatum	Complete	bph	Betaproteobacteria	
147	Burkholderia phytofirmans	Complete	bpy	Betaproteobacteria	
148	Polynucleobacter sp. QLW-P1DMWA-1	Complete	pnu	Betaproteobacteria	
149	Polynucleobacter necessarius	Complete	pne	Betaproteobacteria	
150	Bordetella pertussis	Complete	bpe	Betaproteobacteria	
151	Bordetella parapertussis	Complete	bpa	Betaproteobacteria	
152	Bordetella bronchiseptica	Complete	bbr	Betaproteobacteria	
153	Bordetella petrii	Complete	bpt	Betaproteobacteria	
154	Bordetella avium	Complete	bav	Betaproteobacteria	

155	Achromobacter xylosoxidans	Complete	axy	Betaproteobacteria	
156	Taylorella equigenitalis	Complete	teq	Betaproteobacteria	
157	Rhodoferax ferrireducens	Complete	rfr	Betaproteobacteria	
158	Polaromonas sp. JS666	Complete	pol	Betaproteobacteria	
159	Polaromonas naphthalenivorans	Complete	pna	Betaproteobacteria	
160	Acidovorax avenae	Complete	aav	Betaproteobacteria	
161	Acidovorax sp. JS42	Complete	ajs	Betaproteobacteria	
162	Verminephrobacter eiseniae	Complete	vei	Betaproteobacteria	
163	Delftia acidovorans	Complete	dac	Betaproteobacteria	
164	Variovorax paradoxus S110	Complete	vap	Betaproteobacteria	
165	Comamonas testosteroni	Complete	ctt	Betaproteobacteria	
166	Alicycliphilus denitrificans BC	Complete	adn	Betaproteobacteria	
167	Methylibium petroleiphilum	Complete	mpt	Betaproteobacteria	
168	Herminiimonas arsenicoxydans	Complete	har	Betaproteobacteria	
169	Minibacterium massiliensis	Complete	mms	Betaproteobacteria	
170	Herbaspirillum seropedicae	Complete	hse	Betaproteobacteria	
171	Candidatus Zinderia insecticola CARI	Complete	zin	Betaproteobacteria	
172	Leptothrix cholodnii	Complete	lch	Betaproteobacteria	
173	Thiomonas intermedia	Complete	tin	Betaproteobacteria	
174	Nitrosomonas europaea	Complete	neu	Betaproteobacteria	
175	Nitrosomonas eutropha	Complete	net	Betaproteobacteria	
176	Nitrosospira multiformis	Complete	nmu	Betaproteobacteria	
177	Aromatoleum aromaticum EbN1	Complete	eba	Betaproteobacteria	
178	Azoarcus sp. BH72	Complete	azo	Betaproteobacteria	
179	Dechloromonas aromatica	Complete	dar	Betaproteobacteria	
180	Thauera sp. MZ1T	Complete	tmz	Betaproteobacteria	
181	Thiobacillus denitrificans	Complete	tbd	Betaproteobacteria	
182	Methylobacillus flagellatus	Complete	mfa	Betaproteobacteria	
183	Methylotenera mobilis	Complete	mmb	Betaproteobacteria	
184	Methylovorus sp. SIP3-4	Complete	mei	Betaproteobacteria	
185	Accumulibacter phosphatis	Complete	app	Betaproteobacteria	
186	Sideroxydans lithotrophicus	Complete	slt	Betaproteobacteria	
187	Gallionella capsiferriformans	Complete	gca	Betaproteobacteria	
188	Helicobacter pylori 26695	Complete	hpy	Epsilonproteobacteria	17
189	Helicobacter hepaticus	Complete	hhe	Epsilonproteobacteria	
190	Helicobacter acinonychis	Complete	hac	Epsilonproteobacteria	
191	Wolinella succinogenes	Complete	wsu	Epsilonproteobacteria	
192	Sulfurimonas denitrificans	Complete	tdn	Epsilonproteobacteria	
193	Sulfuricurvum kujiense	Complete	sku	Epsilonproteobacteria	
194	Campylobacter jejuni NCTC11168	Complete	cje	Epsilonproteobacteria	
195	Campylobacter fetus	Complete	cff	Epsilonproteobacteria	
196	Campylobacter curvus	Complete	ccv	Epsilonproteobacteria	
197	Campylobacter hominis ATCC BAA-381	Complete	cha	Epsilonproteobacteria	
198	Campylobacter concisus 13826	Complete	cco	Epsilonproteobacteria	
199	Arcobacter butzleri	Complete	abu	Epsilonproteobacteria	
200	Sulfurospirillum deleyianum	Complete	sdl	Epsilonproteobacteria	
201	Nitratiruptor sp. SB155-2	Complete	nis	Epsilonproteobacteria	
202	Sulfurovum sp. NBC37-1	Complete	sun	Epsilonproteobacteria	
203	Nitratifractor salsuginis	Complete	nsa	Epsilonproteobacteria	
204	Nautilia profundicola	Complete	nam	Epsilonproteobacteria	
205	Geobacter sulfurreducens	Complete	gsu	Deltaproteobacteria	28
206	Geobacter metallireducens	Complete	gme	Deltaproteobacteria	

207	Geobacter uraniumreducens	Complete	gur	Deltaproteobacteria	
208	Geobacter lovleyi	Complete	glo	Deltaproteobacteria	
209	Geobacter bemidjiensis	Complete	gbm	Deltaproteobacteria	
210	Pelobacter carbinolicus	Complete	pca	Deltaproteobacteria	
211	Pelobacter propionicus	Complete	ppd	Deltaproteobacteria	
212	Desulfovibrio vulgaris Hildenborough	Complete	dvu	Deltaproteobacteria	
213	Desulfovibrio desulfuricans G20	Complete	dde	Deltaproteobacteria	
214	Lawsonia intracellularis	Complete	lip	Deltaproteobacteria	
215	Desulfomicrobium baculatum	Complete	dba	Deltaproteobacteria	
216	Desulfohalobium retbaense	Complete	drt	Deltaproteobacteria	
217	Bdellovibrio bacteriovorus	Complete	bba	Deltaproteobacteria	
218	Desulfotalea psychrophila	Complete	dps	Deltaproteobacteria	
219	Desulfurivibrio alkaliphilus	Complete	dak	Deltaproteobacteria	
220	Desulfobulbus propionicus	Complete	dpr	Deltaproteobacteria	
221	Candidatus Desulfococcus oleovorans	Complete	dol	Deltaproteobacteria	
222	Desulfatibacillum alkenivorans	Complete	dal	Deltaproteobacteria	
223	Desulfobacterium autotrophicum	Complete	dat	Deltaproteobacteria	
224	Anaeromyxobacter dehalogenans 2CP-C	Complete	ade	Deltaproteobacteria	
225	Anaeromyxobacter sp. Fw109-5	Complete	afw	Deltaproteobacteria	
226	Myxococcus xanthus	Complete	mxa	Deltaproteobacteria	
227	Stigmatella aurantiaca	Complete	sur	Deltaproteobacteria	
228	Sorangium cellulosum	Complete	scl	Deltaproteobacteria	
229	Haliangium ochraceum	Complete	hoh	Deltaproteobacteria	
230	Syntrophus aciditrophicus	Complete	sat	Deltaproteobacteria	
231	Syntrophobacter fumaroxidans	Complete	sfu	Deltaproteobacteria	
232	Desulfarculus baarsii	Complete	dbr	Deltaproteobacteria	
233	Rickettsia prowazekii	Complete	rpr	Alphaproteobacteria	91
234	Rickettsia typhi	Complete	rty	Alphaproteobacteria	
235	Rickettsia canadensis	Complete	rcm	Alphaproteobacteria	
236	Rickettsia conorii	Complete	rco	Alphaproteobacteria	
237	Rickettsia felis	Complete	rfe	Alphaproteobacteria	
238	Rickettsia akari	Complete	rak	Alphaproteobacteria	
239	Rickettsia rickettsii Sheila Smith	Complete	rri	Alphaproteobacteria	
240	Rickettsia massiliae	Complete	rms	Alphaproteobacteria	
241	Rickettsia bellii RML369-C	Complete	rbe	Alphaproteobacteria	
242	Orientia tsutsugamushi Boryong	Complete	ots	Alphaproteobacteria	
243	Wolbachia wMel	Complete	wol	Alphaproteobacteria	
244	Wolbachia wBm	Complete	wbm	Alphaproteobacteria	
245	Wolbachia pipientis	Complete	wpi	Alphaproteobacteria	
246	Anaplasma marginale St. Maries	Complete	ama	Alphaproteobacteria	
247	Anaplasma phagocytophilum	Complete	aph	Alphaproteobacteria	
248	Ehrlichia ruminantium Welgevonden (South Africa)	Complete	eru	Alphaproteobacteria	
249	Ehrlichia canis	Complete	ecn	Alphaproteobacteria	
250	Ehrlichia chaffeensis	Complete	ech	Alphaproteobacteria	
251	Neorickettsia sennetsu	Complete	nse	Alphaproteobacteria	
252	Candidatus Pelagibacter ubique	Complete	pub	Alphaproteobacteria	
253	Mesorhizobium loti	Complete	mlo	Alphaproteobacteria	
254	Mesorhizobium sp. BNC1	Complete	mes	Alphaproteobacteria	
255	Parvibaculum lavamentivorans	Complete	pla	Alphaproteobacteria	
256	Sinorhizobium meliloti 1021	Complete	sme	Alphaproteobacteria	
257	Sinorhizobium medicae	Complete	smd	Alphaproteobacteria	
258	Agrobacterium tumefaciens C58	Complete	atu	Alphaproteobacteria	

259	Rhizobium etli CFN 42	Complete	ret	Alphaproteobacteria
260	Rhizobium leguminosarum	Complete	rle	Alphaproteobacteria
261	Candidatus Liberibacter asiaticus	Complete	las	Alphaproteobacteria
262	Brucella melitensis bv. 1 16M	Complete	bme	Alphaproteobacteria
263	Brucella abortus 9-941	Complete	bmb	Alphaproteobacteria
264	Brucella suis 1330	Complete	bms	Alphaproteobacteria
265	Brucella ovis	Complete	bov	Alphaproteobacteria
266	Ochrobactrum anthropi	Complete	oan	Alphaproteobacteria
267	Bradyrhizobium japonicum	Complete	bja	Alphaproteobacteria
268	Bradyrhizobium sp. ORS278	Complete	bra	Alphaproteobacteria
269	Rhodopseudomonas palustris CGA009	Complete	rpa	Alphaproteobacteria
270	Nitrobacter winogradskyi	Complete	nwi	Alphaproteobacteria
271	Nitrobacter hamburgensis	Complete	nha	Alphaproteobacteria
272	Oligotropha carboxidovorans	Complete	oca	Alphaproteobacteria
273	Bartonella henselae	Complete	bhe	Alphaproteobacteria
274	Bartonella quintana	Complete	bqu	Alphaproteobacteria
275	Bartonella bacilliformis	Complete	bbk	Alphaproteobacteria
276	Bartonella tribocorum	Complete	btr	Alphaproteobacteria
277	Xanthobacter autotrophicus	Complete	xau	Alphaproteobacteria
278	Azorhizobium caulinodans	Complete	azc	Alphaproteobacteria
279	Starkeya novella	Complete	sno	Alphaproteobacteria
280	Methylobacterium extorquens	Complete	mex	Alphaproteobacteria
281	Methylobacterium radiotolerans	Complete	mrd	Alphaproteobacteria
282	Methylobacterium sp. 4-46	Complete	met	Alphaproteobacteria
283	Methylobacterium populi	Complete	mpo	Alphaproteobacteria
284	Methylobacterium chloromethanicum	Complete	mch	Alphaproteobacteria
285	Methylobacterium nodulans	Complete	mno	Alphaproteobacteria
286	Beijerinckia indica	Complete	bid	Alphaproteobacteria
287	Methylocella silvestris	Complete	msl	Alphaproteobacteria
288	Hyphomicrobium denitrificans	Complete	hdn	Alphaproteobacteria
289	Rhodomicrobium vannielii	Complete	rva	Alphaproteobacteria
290	Candidatus Hodgkinia cicadicola	Complete	hci	Alphaproteobacteria
291	Caulobacter crescentus CB15	Complete	ccr	Alphaproteobacteria
292	Caulobacter sp. K31	Complete	cak	Alphaproteobacteria
293	Phenylobacterium zucineum	Complete	pzu	Alphaproteobacteria
294	Brevundimonas subvibrioides	Complete	bsb	Alphaproteobacteria
295	Asticcacaulis excentricus	Complete	aex	Alphaproteobacteria
296	Silicibacter pomeroyi	Complete	sil	Alphaproteobacteria
297	Ruegeria sp. TM1040	Complete	sit	Alphaproteobacteria
298	Rhodobacter sphaeroides 2.4.1	Complete	rsp	Alphaproteobacteria
299	Jannaschia sp. CCS1	Complete	jan	Alphaproteobacteria
300	Roseobacter denitrificans	Complete	rde	Alphaproteobacteria
301	Paracoccus denitrificans	Complete	pde	Alphaproteobacteria
302	Dinoroseobacter shibae	Complete	dsh	Alphaproteobacteria
303	Ketogulonicigenium vulgare	Complete	kvu	Alphaproteobacteria
304	Maricaulis maris	Complete	mmr	Alphaproteobacteria
305	Hyphomonas neptunium	Complete	hne	Alphaproteobacteria
306	Hirschia baltica	Complete	hba	Alphaproteobacteria
307	Zymomonas mobilis	Complete	zmo	Alphaproteobacteria
308	Novosphingobium aromaticivorans	Complete	nar	Alphaproteobacteria
309	Sphingopyxis alaskensis	Complete	sal	Alphaproteobacteria
310	Sphingomonas wittichii	Complete	swi	Alphaproteobacteria

311	Sphingobium japonicum	Complete	sjp	Alphaproteobacteria	
312	Erythrobacter litoralis	Complete	eli	Alphaproteobacteria	
313	Gluconobacter oxydans	Complete	gox	Alphaproteobacteria	
314	Granulibacter bethesdensis	Complete	gbe	Alphaproteobacteria	
315	Acidiphilium cryptum JF-5	Complete	acr	Alphaproteobacteria	
316	Gluconacetobacter diazotrophicus PAl 5 (Brazil)	Complete	gdi	Alphaproteobacteria	
317	Acetobacter pasteurianus	Complete	apt	Alphaproteobacteria	
318	Rhodospirillum rubrum	Complete	rru	Alphaproteobacteria	
319	Rhodospirillum centenum	Complete	rce	Alphaproteobacteria	
320	Magnetospirillum magneticum	Complete	mag	Alphaproteobacteria	
321	Azospirillum sp. B510	Complete	azl	Alphaproteobacteria	
322	Parvularcula bermudensis	Complete	pbr	Alphaproteobacteria	
323	Candidatus Puniceispirillum marinum	Complete	apb	Alphaproteobacteria	
324	Magnetococcus sp. MC-1	Complete	mgm	Magnetococcus	1
325	Desulfurispirillum indicum	Complete	din	Chrysiogenetes	1
326	Bacillus subtilis	Complete	bsu	Firmicutes	104
327	Bacillus halodurans	Complete	bha	Firmicutes	
328	Bacillus anthracis Ames	Complete	ban	Firmicutes	
329	Bacillus cereus ATCC 14579	Complete	bce	Firmicutes	
330	Bacillus cytotoxis NVH 391-98	Complete	bcy	Firmicutes	
331	Bacillus thuringiensis 97-27	Complete	btk	Firmicutes	
332	Bacillus weihenstephanensis	Complete	bwe	Firmicutes	
333	Bacillus licheniformis ATCC 14580	Complete	bli	Firmicutes	
334	Bacillus amyloliquefaciens FZB42	Complete	bay	Firmicutes	
335	Bacillus clausii	Complete	bcl	Firmicutes	
336	Bacillus pumilus	Complete	bpu	Firmicutes	
337	Oceanobacillus iheyensis	Complete	oih	Firmicutes	
338	Geobacillus kaustophilus	Complete	gka	Firmicutes	
339	Geobacillus thermodenitrificans	Complete	gtn	Firmicutes	
340	Anoxybacillus flavithermus	Complete	afl	Firmicutes	
341	Staphylococcus aureus N315 (MRSA/VSSA)	Complete	sau	Firmicutes	
342	Staphylococcus epidermidis ATCC 12228	Complete	sep	Firmicutes	
343	Staphylococcus haemolyticus	Complete	sha	Firmicutes	
344	Staphylococcus saprophyticus	Complete	ssp	Firmicutes	
345	Listeria monocytogenes EGD-e	Complete	lmo	Firmicutes	
346	Listeria innocua	Complete	lin	Firmicutes	
347	Listeria welshimeri SLCC5334	Complete	lwe	Firmicutes	
348	Lysinibacillus sphaericus	Complete	lsp	Firmicutes	
349	Exiguobacterium sibiricum	Complete	esi	Firmicutes	
350	Macrococcus caseolyticus	Complete	mcl	Firmicutes	
351	Brevibacillus brevis	Complete	bbe	Firmicutes	
352	Paenibacillus sp. JDR-2	Complete	pjd	Firmicutes	
353	Alicyclobacillus acidocaldarius	Complete	aac	Firmicutes	
354	Bacillus tusciae	Complete	bts	Firmicutes	
355	Lactococcus lactis subsp. lactis IL1403	Complete	lla	Firmicutes	
356	Streptococcus pyogenes SF370 (serotype M1)	Complete	spy	Firmicutes	
357	Streptococcus pneumoniae TIGR4 (virulent serotype 4)	Complete	spn	Firmicutes	
358	Streptococcus agalactiae 2603 (serotype V)	Complete	sag	Firmicutes	
359	Streptococcus mutans UA159	Complete	smu	Firmicutes	
360	Streptococcus thermophilus CNRZ1066	Complete	stc	Firmicutes	
361	Streptococcus sanguinis	Complete	ssa	Firmicutes	
362	Streptococcus suis 05ZYH33	Complete	ssu	Firmicutes	

363	Streptococcus gordonii	Complete	sgo	Firmicutes
364	Streptococcus equi subsp. zooepidemicus MGCS10565	Complete	sez	Firmicutes
365	Lactobacillus plantarum WCFS1	Complete	lpl	Firmicutes
366	Lactobacillus johnsonii NCC 533	Complete	ljo	Firmicutes
367	Lactobacillus acidophilus NCFM	Complete	lac	Firmicutes
368	Lactobacillus sakei	Complete	lsa	Firmicutes
369	Lactobacillus salivarius	Complete	lsl	Firmicutes
370	Lactobacillus delbrueckii ATCC 11842	Complete	ldb	Firmicutes
371	Lactobacillus brevis	Complete	lbr	Firmicutes
372	Lactobacillus casei ATCC 334	Complete	lca	Firmicutes
373	Lactobacillus gasseri	Complete	lga	Firmicutes
374	Lactobacillus reuteri DSM 20016	Complete	lre	Firmicutes
375	Lactobacillus helveticus	Complete	lhe	Firmicutes
376	Lactobacillus fermentum	Complete	lfe	Firmicutes
377	Pediococcus pentosaceus	Complete	ppe	Firmicutes
378	Enterococcus faecalis	Complete	efa	Firmicutes
379	Oenococcus oeni	Complete	ooe	Firmicutes
380	Leuconostoc mesenteroides	Complete	lme	Firmicutes
381	Leuconostoc citreum	Complete	lci	Firmicutes
382	Clostridium acetobutylicum	Complete	cac	Firmicutes
383	Clostridium perfringens 13	Complete	cpe	Firmicutes
384	Clostridium tetani E88	Complete	ctc	Firmicutes
385	Clostridium novyi	Complete	cno	Firmicutes
386	Clostridium thermocellum	Complete	cth	Firmicutes
387	Clostridium difficile 630	Complete	cdf	Firmicutes
388	Clostridium botulinum A ATCC 3502	Complete	cbo	Firmicutes
389	Clostridium beijerinckii	Complete	cbe	Firmicutes
390	Clostridium kluyveri DSM 555	Complete	ckl	Firmicutes
391	Clostridium phytofermentans	Complete	cpy	Firmicutes
392	Alkaliphilus metalliredigens	Complete	amt	Firmicutes
393	Alkaliphilus oremlandii	Complete	aoe	Firmicutes
394	Symbiobacterium thermophilum	Complete	sth	Firmicutes
395	Syntrophomonas wolfei	Complete	swo	Firmicutes
396	Syntrophothermus lipocalidus	Complete	slp	Firmicutes
397	Veillonella parvula	Complete	vpr	Firmicutes
398	Acidaminococcus fermentans	Complete	afn	Firmicutes
399	Desulfitobacterium hafniense Y51	Complete	dsy	Firmicutes
400	Desulfotomaculum reducens	Complete	drm	Firmicutes
401	Pelotomaculum thermopropionicum	Complete	pth	Firmicutes
402	Candidatus Desulforudis audaxviator	Complete	dau	Firmicutes
403	Thermincola potens JR	Complete	tjr	Firmicutes
404	Syntrophobotulus glycolicus	Complete	sgy	Firmicutes
405	Heliobacterium modesticaldum	Complete	hmo	Firmicutes
406	Finegoldia magna	Complete	fma	Firmicutes
407	Anaerococcus prevotii	Complete	apr	Firmicutes
408	Eubacterium eligens	Complete	eel	Firmicutes
409	Butyrivibrio proteoclasticus	Complete	bpb	Firmicutes
410	Ethanoligenens harbinense	Complete	eha	Firmicutes
411	Ruminococcus albus	Complete	ral	Firmicutes
412	Thermaerobacter marianensis	Complete	tmr	Firmicutes
413	Clostridiales genomosp. BVAB3	Complete	clo	Firmicutes
414	Thermoanaerobacter tengcongensis	Complete	tte	Firmicutes

415	Thermoanaerobacter sp. X514	Complete	tex	Firmicutes	
416	Thermoanaerobacter pseudethanolicus	Complete	tpd	Firmicutes	
417	Thermoanaerobacter italicus	Complete	tit	Firmicutes	
418	Carboxydothermus hydrogenoformans	Complete	chy	Firmicutes	
419	Moorella thermoacetica	Complete	mta	Firmicutes	
420	Ammonifex degensii	Complete	adg	Firmicutes	
421	Caldicellulosiruptor saccharolyticus	Complete	csc	Firmicutes	
422	Caldicellulosiruptor bescii	Complete	ate	Firmicutes	
423	Thermosediminibacter oceani	Complete	toc	Firmicutes	
424	Thermoanaerobacterium thermosaccharolyticum	Complete	ttm	Firmicutes	
425	Coprothermobacter proteolyticus	Complete	cpo	Firmicutes	
426	Natranaerobius thermophilus	Complete	nth	Firmicutes	
427	Halothermothrix orenii	Complete	hor	Firmicutes	
428	Halanaerobium sp. sapolanicus	Complete	has	Firmicutes	
429	Acetohalobium arabaticum	Complete	aar	Firmicutes	
430	Mycoplasma genitalium	Complete	mge	Tenericutes	19
431	Mycoplasma pneumoniae	Complete	mpn	Tenericutes	
432	Mycoplasma pulmonis	Complete	mpu	Tenericutes	
433	Mycoplasma penetrans	Complete	mpe	Tenericutes	
434	Mycoplasma gallisepticum	Complete	mga	Tenericutes	
435	Mycoplasma mycoides subsp. mycoides SC PG1	Complete	mmy	Tenericutes	
436	Mycoplasma mobile	Complete	mmo	Tenericutes	
437	Mycoplasma hyopneumoniae 232	Complete	mhy	Tenericutes	
438	Mycoplasma synoviae	Complete	msy	Tenericutes	
439	Mycoplasma capricolum	Complete	mcp	Tenericutes	
440	Mycoplasma agalactiae PG2	Complete	maa	Tenericutes	
441	Mycoplasma arthritidis	Complete	mat	Tenericutes	
442	Ureaplasma parvum serovar 3 ATCC 700970	Complete	uur	Tenericutes	
443	Ureaplasma urealyticum serovar 10 ATCC 33699	Complete	uue	Tenericutes	
444	Phytoplasma OY	Complete	poy	Tenericutes	
445	Phytoplasma AYWB	Complete	ayw	Tenericutes	
446	Candidatus Phytoplasma mali	Complete	pml	Tenericutes	
447	Acholeplasma laidlawii	Complete	acl	Tenericutes	
448	Mesoplasma florum	Complete	mfl	Tenericutes	
449	Mycobacterium tuberculosis H37Rv	Complete	mtu	Actinobacteria	80
450	Mycobacterium bovis AF2122/97	Complete	mbo	Actinobacteria	
451	Mycobacterium leprae TN	Complete	mle	Actinobacteria	
452	Mycobacterium avium paratuberculosis	Complete	mpa	Actinobacteria	
453	Mycobacterium smegmatis	Complete	msm	Actinobacteria	
454	Mycobacterium ulcerans	Complete	mul	Actinobacteria	
455	Mycobacterium vanbaalenii	Complete	mva	Actinobacteria	
456	Mycobacterium gilvum	Complete	mgi	Actinobacteria	
457	Mycobacterium abscessus ATCC 19977	Complete	mab	Actinobacteria	
458	Mycobacterium sp. MCS	Complete	mmc	Actinobacteria	
459	Mycobacterium marinum M	Complete	mmi	Actinobacteria	
460	Corynebacterium glutamicum ATCC 13032	Complete	cgl	Actinobacteria	
461	Corynebacterium efficiens	Complete	cef	Actinobacteria	
462	Corynebacterium diphtheriae	Complete	cdi	Actinobacteria	
463	Corynebacterium jeikeium	Complete	cjk	Actinobacteria	
464	Corynebacterium urealyticum	Complete	cur	Actinobacteria	
465	Corynebacterium aurimucosum	Complete	car	Actinobacteria	
466	Nocardia farcinica	Complete	nfa	Actinobacteria	

467	Rhodococcus sp. RHA1	Complete	rha	Actinobacteria
468	Gordonia bronchialis	Complete	gbr	Actinobacteria
469	Tsukamurella paurometabola	Complete	tpr	Actinobacteria
470	Segniliparus rotundus	Complete	srt	Actinobacteria
471	Streptomyces coelicolor	Complete	sco	Actinobacteria
472	Streptomyces avermitilis	Complete	sma	Actinobacteria
473	Streptomyces griseus	Complete	sgr	Actinobacteria
474	Streptomyces scabiei	Complete	scb	Actinobacteria
475	Tropheryma whipplei Twist	Complete	twh	Actinobacteria
476	Leifsonia xyli xyli CTCB07	Complete	lxx	Actinobacteria
477	Clavibacter michiganensis subsp. michiganensis	Complete	cmi	Actinobacteria
478	Microbacterium testaceum	Complete	mts	Actinobacteria
479	Arthrobacter sp. FB24	Complete	art	Actinobacteria
480	Arthrobacter aurescens	Complete	aau	Actinobacteria
481	Renibacterium salmoninarum	Complete	rsa	Actinobacteria
482	Kocuria rhizophila	Complete	krh	Actinobacteria
483	Micrococcus luteus	Complete	mlu	Actinobacteria
484	Rothia mucilaginosa	Complete	rmu	Actinobacteria
485	Beutenbergia cavernae	Complete	bcv	Actinobacteria
486	Brachybacterium faecium	Complete	bfa	Actinobacteria
487	Jonesia denitrificans	Complete	jde	Actinobacteria
488	Kytococcus sedentarius	Complete	kse	Actinobacteria
489	Xylanimonas cellulosilytica	Complete	xce	Actinobacteria
490	Sanguibacter keddieii	Complete	ske	Actinobacteria
491	Cellulomonas flavigena	Complete	cfl	Actinobacteria
492	Intrasporangium calvum	Complete	ica	Actinobacteria
493	Propionibacterium acnes KPA171202	Complete	pac	Actinobacteria
494	Nocardioides sp. JS614	Complete	nca	Actinobacteria
495	Kribbella flavida	Complete	kfl	Actinobacteria
496	Thermobifida fusca	Complete	tfu	Actinobacteria
497	Nocardiopsis dassonvillei	Complete	nda	Actinobacteria
498	Thermomonospora curvata	Complete	tcu	Actinobacteria
499	Streptosporangium roseum	Complete	sro	Actinobacteria
500	Frankia sp. CcI3	Complete	fra	Actinobacteria
501	Frankia alni	Complete	fal	Actinobacteria
502	Acidothermus cellulolyticus	Complete	ace	Actinobacteria
503	Nakamurella multipartita	Complete	nml	Actinobacteria
504	Geodermatophilus obscurus	Complete	gob	Actinobacteria
505	Kineococcus radiotolerans	Complete	kra	Actinobacteria
506	Saccharopolyspora erythraea	Complete	sen	Actinobacteria
507	Saccharomonospora viridis	Complete	svi	Actinobacteria
508	Thermobispora bispora	Complete	tbi	Actinobacteria
509	Amycolatopsis mediterranei	Complete	amd	Actinobacteria
510	Actinosynnema mirum	Complete	ami	Actinobacteria
511	Salinispora tropica	Complete	stp	Actinobacteria
512	Salinispora arenicola	Complete	saq	Actinobacteria
513	Micromonospora aurantiaca	Complete	mau	Actinobacteria
514	Catenulispora acidiphila	Complete	cai	Actinobacteria
515	Stackebrandtia nassauensis	Complete	sna	Actinobacteria
516	Arcanobacterium haemolyticum	Complete	ahe	Actinobacteria
517	Mobiluncus curtisii	Complete	mcu	Actinobacteria
518	Bifidobacterium longum NCC2705	Complete	blo	Actinobacteria

519	Bifidobacterium adolescentis	Complete	bad	Actinobacteria	
520	Gardnerella vaginalis	Complete	gva	Actinobacteria	
521	Rubrobacter xylanophilus	Complete	rxy	Actinobacteria	
522	Conexibacter woesei	Complete	cwo	Actinobacteria	
523	Acidimicrobium ferrooxidans DSM 10331	Complete	afo	Actinobacteria	
524	Cryptobacterium curtum	Complete	ccu	Actinobacteria	
525	Slackia heliotrinireducens	Complete	shi	Actinobacteria	
526	Atopobium parvulum	Complete	apv	Actinobacteria	
527	Eggerthella lenta	Complete	ele	Actinobacteria	
528	Olsenella uli	Complete	ols	Actinobacteria	
529	Chlamydia trachomatis D/UW-3/CX (serovar D)	Complete	ctr	Chlamydiae	8
530	Chlamydia muridarum	Complete	cmu	Chlamydiae	
531	Chlamydophila pneumoniae CWL029	Complete	cpn	Chlamydiae	
532	Chlamydophila caviae	Complete	cca	Chlamydiae	
533	Chlamydophila abortus	Complete	cab	Chlamydiae	
534	Chlamydophila felis	Complete	cfe	Chlamydiae	
535	Candidatus Protochlamydia amoebophila	Complete	pcu	Chlamydiae	
536	Waddlia chondrophila	Complete	wch	Chlamydiae	
537	Borrelia burgdorferi B31	Complete	bbu	Spirochaetes	14
538	Borrelia garinii	Complete	bga	Spirochaetes	
539	Borrelia afzelii	Complete	baf	Spirochaetes	
540	Borrelia turicatae	Complete	btu	Spirochaetes	
541	Borrelia hermsii	Complete	bhr	Spirochaetes	
542	Borrelia duttonii	Complete	bdu	Spirochaetes	
543	Borrelia recurrentis	Complete	bre	Spirochaetes	
544	Treponema pallidum subsp. pallidum Nichols	Complete	tpa	Spirochaetes	
545	Treponema denticola	Complete	tde	Spirochaetes	
546	Spirochaeta smaragdinae	Complete	ssm	Spirochaetes	
547	Leptospira interrogans serovar lai	Complete	lil	Spirochaetes	
548	Leptospira borgpetersenii JB197	Complete	lbj	Spirochaetes	
549	Leptospira biflexa serovar Patoc Patoc 1 (Paris)	Complete	lbi	Spirochaetes	
550	Brachyspira hyodysenteriae	Complete	bhy	Spirochaetes	
551	Candidatus Koribacter versatilis	Complete	aba	Acidobacteria	5
552	Acidobacterium capsulatum	Complete	aca	Acidobacteria	
553	Acidobacterium sp. MP5ACTX9	Complete	acm	Acidobacteria	
554	Terriglobus saanensis	Complete	tsa	Acidobacteria	
555	Solibacter usitatus	Complete	sus	Acidobacteria	
556	Bacteroides thetaiotaomicron	Complete	bth	Bacteroidetes	35
557	Bacteroides fragilis YCH46	Complete	bfr	Bacteroidetes	
558	Bacteroides vulgatus	Complete	bvu	Bacteroidetes	
559	Porphyromonas gingivalis W83	Complete	pgi	Bacteroidetes	
560	Parabacteroides distasonis	Complete	pdi	Bacteroidetes	
561	Paludibacter propionicigenes	Complete	ppn	Bacteroidetes	
562	Odoribacter splanchnicus	Complete	osp	Bacteroidetes	
563	Candidatus Azobacteroides pseudotrichonymphae	Complete	aps	Bacteroidetes	
564	Prevotella ruminicola	Complete	pru	Bacteroidetes	
565	Salinibacter ruber	Complete	sru	Bacteroidetes	
566	Rhodothermus marinus	Complete	rmr	Bacteroidetes	
567	Chitinophaga pinensis	Complete	cpi	Bacteroidetes	
568	Pedobacter heparinus	Complete	phe	Bacteroidetes	
569	Pedobacter saltans	Complete	psn	Bacteroidetes	
570	Sphingobacterium sp. 21	Complete	shg	Bacteroidetes	

571	Cytophaga hutchinsonii	Complete	chu	Bacteroidetes	
572	Dyadobacter fermentans	Complete	dfe	Bacteroidetes	
573	Spirosoma linguale	Complete	sli	Bacteroidetes	
574	Leadbetterella byssophila	Complete	lby	Bacteroidetes	
575	Marivirga tractuosa	Complete	mtt	Bacteroidetes	
576	Gramella forsetii	Complete	gfo	Bacteroidetes	
577	Flavobacterium johnsoniae	Complete	fjo	Bacteroidetes	
578	Flavobacterium psychrophilum	Complete	fps	Bacteroidetes	
579	Capnocytophaga ochracea	Complete	coc	Bacteroidetes	
580	Robiginitalea biformata	Complete	rbi	Bacteroidetes	
581	Zunongwangia profunda	Complete	zpr	Bacteroidetes	
582	Croceibacter atlanticus	Complete	cat	Bacteroidetes	
583	Riemerella anatipestifer	Complete	ran	Bacteroidetes	
584	Maribacter sp. HTCC2170	Complete	fbc	Bacteroidetes	
585	Cellulophaga algicola	Complete	cao	Bacteroidetes	
586	Weeksella virosa	Complete	wvi	Bacteroidetes	
587	Flavobacteriaceae bacterium	Complete	fba	Bacteroidetes	
588	Candidatus Sulcia muelleri GWSS	Complete	smg	Bacteroidetes	
589	Blattabacterium sp. (Blattella germanica)	Complete	bbl	Bacteroidetes	
590	Candidatus Amoebophilus asiaticus	Complete	aas	Bacteroidetes	
591	Fibrobacter succinogenes	Complete	fsu	Fibrobacteres	1
592	Fusobacterium nucleatum	Complete	fnu	Fusobacteria	5
593	Leptotrichia buccalis	Complete	lba	Fusobacteria	
594	Sebaldella termitidis	Complete	str	Fusobacteria	
595	Streptobacillus moniliformis	Complete	smf	Fusobacteria	
596	Ilyobacter polytropus	Complete	ipo	Fusobacteria	
597	Opitutus terrae	Complete	ote	Verrucomicrobia	4
598	Coraliomargarita akajimensis	Complete	caa	Verrucomicrobia	
599	Methylacidiphilum infernorum	Complete	min	Verrucomicrobia	
600	Akkermansia muciniphila	Complete	amu	Verrucomicrobia	
601	Gemmatimonas aurantiaca	Complete	gau	Gemmatimonadetes	1
602	Rhodopirellula baltica	Complete	rba	Planctomycetes	4
603	Pirellula staleyi	Complete	psl	Planctomycetes	
604	Planctomyces limnophilus	Complete	plm	Planctomycetes	
605	Isosphaera pallida	Complete	ipa	Planctomycetes	
606	Elusimicrobium minutum	Complete	emi	Elusimicrobia	2
607	Uncultured Termite group 1 bacterium phylotype Rs-D17	Complete	rsd	Elusimicrobia	
608	Thermanaerovibrio acidaminovorans	Complete	tai	Synergistetes	2
609	Aminobacterium colombiense	Complete	aco	Synergistetes	
610	Synechocystis sp. PCC6803	Complete	syn	Cyanobacteria	16
611	Synechococcus sp. WH8102	Complete	syw	Cyanobacteria	
612	Synechococcus elongatus PCC6301	Complete	syc	Cyanobacteria	
613	Cyanobacteria Yellowstone A-Prime	Complete	cya	Cyanobacteria	
614	Thermosynechococcus elongatus	Complete	tel	Cyanobacteria	
615	Microcystis aeruginosa	Complete	mar	Cyanobacteria	
616	Cyanothece sp. ATCC 51142	Complete	cyt	Cyanobacteria	
617	Cyanobacterium UCYN-A	Complete	cyu	Cyanobacteria	
618	Gloeobacter violaceus	Complete	gvi	Cyanobacteria	
619	Anabaena sp. PCC7120	Complete	ana	Cyanobacteria	
620	Nostoc punctiforme	Complete	npu	Cyanobacteria	
621	Anabaena variabilis	Complete	ava	Cyanobacteria	
622	Anabaena azollae 0708	Complete	naz	Cyanobacteria	

623	Prochlorococcus marinus SS120	Complete	pma	Cyanobacteria	
624	Trichodesmium erythraeum	Complete	ter	Cyanobacteria	
625	Acaryochloris marina	Complete	amr	Cyanobacteria	
626	Chlorobaculum tepidum	Complete	cte	Chlorobi	10
627	Chlorobaculum parvum NCIB 8327	Complete	cpc	Chlorobi	
628	Chlorobium chlorochromatii	Complete	cch	Chlorobi	
629	Chlorobium phaeobacteroides DSM 266	Complete	cph	Chlorobi	
630	Chlorobium limicola	Complete	cli	Chlorobi	
631	Chlorobium vibrioformis	Complete	pvi	Chlorobi	
632	Pelodictyon luteolum	Complete	plt	Chlorobi	
633	Pelodictyon phaeoclathratiforme	Complete	pph	Chlorobi	
634	Prosthecochloris aestuarii	Complete	paa	Chlorobi	
635	Chloroherpeton thalassium	Complete	cts	Chlorobi	
636	Dehalococcoides ethenogenes	Complete	det	Chloroflexi	11
637	Dehalococcoides sp. CBDB1	Complete	deh	Chloroflexi	
638	Dehalogenimonas lykanthroporepellens	Complete	dly	Chloroflexi	
639	Roseiflexus sp. RS-1	Complete	rrs	Chloroflexi	
640	Roseiflexus castenholzii DSM13941	Complete	rca	Chloroflexi	
641	Chloroflexus aurantiacus	Complete	cau	Chloroflexi	
642	Chloroflexus sp. Y-400-fl	Complete	chl	Chloroflexi	
643	Herpetosiphon aurantiacus	Complete	hau	Chloroflexi	
644	Thermomicrobium roseum	Complete	tro	Chloroflexi	
645	Sphaerobacter thermophilus	Complete	sti	Chloroflexi	
646	Anaerolinea thermophila	Complete	atm	Chloroflexi	
647	Deinococcus radiodurans	Complete	dra	Deinococcus-Thermus	7
648	Deinococcus geothermalis	Complete	dge	Deinococcus-Thermus	
649	Deinococcus deserti	Complete	ddr	Deinococcus-Thermus	
650	Truepera radiovictrix	Complete	tra	Deinococcus-Thermus	
651	Thermus thermophilus HB27	Complete	tth	Deinococcus-Thermus	
652	Meiothermus ruber	Complete	mrb	Deinococcus-Thermus	
653	Oceanithermus profundus	Complete	opr	Deinococcus-Thermus	
654	Aquifex aeolicus	Complete	aae	Aquificae	9
655	Hydrogenobaculum sp. Y04AAS1	Complete	hya	Aquificae	
656	Hydrogenobacter thermophilus	Complete	hth	Aquificae	
657	Thermocrinis albus	Complete	tal	Aquificae	
658	Sulfurihydrogenibium sp. YO3AOP1	Complete	sul	Aquificae	
659	Sulfurihydrogenibium azorense	Complete	saf	Aquificae	
660	Persephonella marina	Complete	pmx	Aquificae	
661	Thermovibrio ammonificans	Complete	tam	Aquificae	
662	Desulfurobacterium thermolithotrophum	Complete	dte	Aquificae	
663	Thermotoga maritima	Complete	tma	Thermotogae	11
664	Thermotoga petrophila	Complete	tpt	Thermotogae	
665	Thermotoga lettingae	Complete	tle	Thermotogae	
666	Thermotoga sp. RQ2	Complete	trq	Thermotogae	
667	Thermotoga neapolitana	Complete	tna	Thermotogae	
668	Thermotoga naphthophila	Complete	tnp	Thermotogae	
669	Thermosipho melanesiensis	Complete	tme	Thermotogae	
670	Thermosipho africanus	Complete	taf	Thermotogae	
671	Fervidobacterium nodosum	Complete	fno	Thermotogae	
672	Petrotoga mobilis	Complete	pmo	Thermotogae	
673	Kosmotoga olearia	Complete	kol	Thermotogae	
674	Dictyoglomus thermophilum	Complete	dth	Dictyoglomi	2

675	Dictyoglomus turgidum	Complete	dtu	Dictyoglomi	
676	Thermodesulfovibrio yellowstonii	Complete	tye	Nitrospirae	2
677	Candidatus Nitrospira defluvii	Complete	nde	Nitrospirae	
678	Thermobaculum terrenum	Complete	ttr	Thermobaculum	1
679	Deferribacter desulfuricans SSM1	Complete	ddf	Deferribacteres	3
680	Denitrovibrio acetiphilus	Complete	dap	Deferribacteres	
681	Calditerrivibrio nitroreducens	Complete	cni	Deferribacteres	
682	Methanocaldococcus jannaschii	Complete	mja	Euryarchaeota	60
683	Methanocaldococcus fervens	Complete	mfe	Euryarchaeota	
684	Methanocaldococcus vulcanius	Complete	mvu	Euryarchaeota	
685	Methanocaldococcus sp. FS406-22	Complete	mfs	Euryarchaeota	
686	Methanocaldococcus infernus	Complete	mif	Euryarchaeota	
687	Methanococcus maripaludis S2	Complete	mmp	Euryarchaeota	
688	Methanococcus aeolicus	Complete	mae	Euryarchaeota	
689	Methanococcus vannielii	Complete	mvn	Euryarchaeota	
690	Methanococcus voltae	Complete	mvo	Euryarchaeota	
691	Methanosarcina acetivorans	Complete	mac	Euryarchaeota	
692	Methanosarcina barkeri	Complete	mba	Euryarchaeota	
693	Methanosarcina mazei	Complete	mma	Euryarchaeota	
694	Methanococcoides burtonii	Complete	mbu	Euryarchaeota	
695	Methanohalophilus mahii	Complete	mmh	Euryarchaeota	
696	Methanohalobium evestigatum	Complete	mev	Euryarchaeota	
697	Methanosaeta thermophila	Complete	mtp	Euryarchaeota	
698	Methanospirillum hungatei	Complete	mhu	Euryarchaeota	
699	Methanocorpusculum labreanum	Complete	mla	Euryarchaeota	
700	Methanoculleus marisnigri	Complete	mem	Euryarchaeota	
701	Methanoplanus petrolearius	Complete	mpi	Euryarchaeota	
702	Candidatus Methanoregula boonei	Complete	mbn	Euryarchaeota	
703	Candidatus Methanosphaerula palustris	Complete	mpl	Euryarchaeota	
704	Methanocella paludicola	Complete	mpd	Euryarchaeota	
705	Methanothermobacter thermautotrophicus	Complete	mth	Euryarchaeota	
706	Methanothermobacter marburgensis	Complete	mmg	Euryarchaeota	
707	Methanosphaera stadtmanae	Complete	mst	Euryarchaeota	
708	Methanobrevibacter smithii ATCC 35061	Complete	msi	Euryarchaeota	
709	Methanobrevibacter ruminantium	Complete	mru	Euryarchaeota	
710	Methanobacterium sp. AL-21	Complete	mel	Euryarchaeota	
711	Methanothermus fervidus	Complete	mfv	Euryarchaeota	
712	Methanopyrus kandleri	Complete	mka	Euryarchaeota	
713	Archaeoglobus fulgidus	Complete	afu	Euryarchaeota	
714	Archaeoglobus profundus	Complete	apo	Euryarchaeota	
715	Ferroglobus placidus	Complete	fpl	Euryarchaeota	
716	Halobacterium sp. NRC-1	Complete	hal	Euryarchaeota	
717	Halobacterium salinarum R1	Complete	hsl	Euryarchaeota	
718	Haloarcula marismortui	Complete	hma	Euryarchaeota	
719	Haloquadratum walsbyi	Complete	hwa	Euryarchaeota	
720	Natronomonas pharaonis	Complete	nph	Euryarchaeota	
721	Halorubrum lacusprofundi	Complete	hla	Euryarchaeota	
722	Halorhabdus utahensis	Complete	hut	Euryarchaeota	
723	Halomicrobium mukohataei	Complete	hmu	Euryarchaeota	
724	Haloterrigena turkmenica	Complete	htu	Euryarchaeota	
725	Natrialba magadii	Complete	nmg	Euryarchaeota	
726	Haloferax volcanii	Complete	hvo	Euryarchaeota	

727	Halalkalicoccus jeotgali	Complete	hje	Euryarchaeota	
728	Halogeometricum borinquense	Complete	hbo	Euryarchaeota	
729	Thermoplasma acidophilum	Complete	tac	Euryarchaeota	
730	Thermoplasma volcanium	Complete	tvo	Euryarchaeota	
731	Picrophilus torridus	Complete	pto	Euryarchaeota	
732	Pyrococcus horikoshii	Complete	pho	Euryarchaeota	
733	Pyrococcus abyssi	Complete	pab	Euryarchaeota	
734	Pyrococcus furiosus	Complete	pfu	Euryarchaeota	
735	Thermococcus kodakaraensis	Complete	tko	Euryarchaeota	
736	Thermococcus onnurineus	Complete	ton	Euryarchaeota	
737	Thermococcus gammatolerans	Complete	tga	Euryarchaeota	
738	Thermococcus sibiricus	Complete	tsi	Euryarchaeota	
739	Thermococcus barophilus	Complete	tba	Euryarchaeota	
740	Aciduliprofundum boonei	Complete	abi	Euryarchaeota	
741	Uncultured methanogenic archaeon RC-I	Complete	rci	Euryarchaeota	
742	Aeropyrum pernix	Complete	ape	Crenarchaeota	23
743	Staphylothermus marinus	Complete	smr	Crenarchaeota	
744	Staphylothermus hellenicus	Complete	shc	Crenarchaeota	
745	Ignicoccus hospitalis	Complete	iho	Crenarchaeota	
746	Desulfurococcus kamchatkensis	Complete	dka	Crenarchaeota	
747	Desulfurococcus mucosus	Complete	dmu	Crenarchaeota	
748	Thermosphaera aggregans	Complete	tag	Crenarchaeota	
749	Ignisphaera aggregans	Complete	iag	Crenarchaeota	
750	Hyperthermus butylicus	Complete	hbu	Crenarchaeota	
751	Sulfolobus solfataricus	Complete	sso	Crenarchaeota	
752	Sulfolobus tokodaii	Complete	sto	Crenarchaeota	
753	Sulfolobus acidocaldarius	Complete	sai	Crenarchaeota	
754	Sulfolobus islandicus L.S.2.15	Complete	sis	Crenarchaeota	
755	Metallosphaera sedula	Complete	mse	Crenarchaeota	
756	Pyrobaculum aerophilum	Complete	pai	Crenarchaeota	
757	Pyrobaculum islandicum	Complete	pis	Crenarchaeota	
758	Pyrobaculum calidifontis	Complete	pcl	Crenarchaeota	
759	Pyrobaculum arsenaticum	Complete	pas	Crenarchaeota	
760	Caldivirga maquilingensis	Complete	cma	Crenarchaeota	
761	Thermoproteus neutrophilus	Complete	tne	Crenarchaeota	
762	Vulcanisaeta distributa	Complete	vdi	Crenarchaeota	
763	Thermofilum pendens	Complete	tpe	Crenarchaeota	
764	Acidilobus saccharovorans	Complete	asc	Crenarchaeota	
765	Nitrosopumilus maritimus	Complete	nmr	Thaumarchaeota	2
766	Cenarchaeum symbiosum A	Complete	csy	Thaumarchaeota	
767	Nanoarchaeum equitans	Complete	neq	Nanoarchaeota	1
768	Candidatus Korarchaeum cryptofilum	Complete	kcr	Korarchaeota	1

Table S2. Taxonomic patterns of the prokaryotes which complete the KEGG modules (205 pathways, 263 structural complexes, 4 functional sets, and 3 signatures). Functional annotation of each module is listed in Table S2-S4. Figures S1-S3 were drawn based on this table.

(M00001_1)

Phyla	Module completion ratio (%)									
	0--10	10--20	20--30	30--40	40--50	50--60	60--70	70--80	80--90	90--100
Gammaproteobacteria	1	0	0	0	0	4	15	0	32	74
Betaproteobacteria	1	0	0	0	0	1	2	0	14	43
Epsilonproteobacteria	0	0	0	0	0	1	11	0	0	5
Deltaproteobacteria	0	0	0	0	0	0	1	0	6	21
Alphaproteobacteria	10	0	1	0	0	12	7	0	30	31
Magnetococcus	0	0	0	0	0	0	0	0	1	0
Chrysiogenetes	0	0	0	0	0	0	0	0	0	1
Firmicutes	0	0	0	0	0	2	3	0	10	89
Tenericutes	0	0	0	0	1	3	2	0	8	5
Actinobacteria	0	0	0	0	0	0	2	0	4	74
Chlamydiae	0	0	0	0	0	0	0	0	6	2
Spirochaetes	0	0	0	0	0	0	1	0	10	3
Acidobacteria	0	0	0	0	0	0	1	0	2	2
Bacteroidetes	0	1	0	0	0	1	3	0	5	25
Fibrobacteres	0	0	0	0	0	0	0	0	1	0
Fusobacteria	0	0	0	0	0	0	0	0	0	5
Verrucomicrobia	0	0	0	0	0	0	0	0	0	4
Gemmatimonadetes	0	0	0	0	0	0	0	0	1	0
Planctomycetes	0	0	0	0	0	0	0	0	0	4
Elusimicrobia	0	0	0	0	0	0	0	0	0	2
Synergistetes	0	0	0	0	0	0	0	0	0	2
Cyanobacteria	0	0	0	0	0	0	0	0	6	10
Chlorobi	0	0	0	0	0	0	0	0	0	10
Chloroflexi	0	0	0	0	0	2	0	0	3	6
Deinococcus-Thermus	0	0	0	0	0	0	0	0	0	7
Aquificae	0	0	0	0	0	1	2	0	3	3
Thermotogae	0	0	0	0	0	0	2	0	1	8
Dictyoglomi	0	0	0	0	0	0	0	0	0	2
Nitrospirae	0	0	0	0	0	0	0	0	1	1
Thermobaculum	0	0	0	0	0	0	0	0	0	1
Deferribacteres	0	0	0	0	0	0	0	0	0	3
Euryarchaeota	0	0	0	0	1	10	22	0	22	5
Crenarchaeota	0	0	0	0	0	5	8	0	8	2
Thaumarchaeota	0	0	0	0	0	2	0	0	0	0
Nanoarchaeota	1	0	0	0	0	0	0	0	0	0
Korarchaeota	0	0	0	0	0	0	1	0	0	0
Total	13	1	1	0	2	44	83	0	174	450

(M00002_1)

Phyla	Module completion ratio (%)									
	0--10	10--20	20--30	30--40	40--50	50--60	60--70	70--80	80--90	90--100
Gammaproteobacteria	1	0	0	0	0	0	4	0	62	59
Betaproteobacteria	1	0	0	0	0	0	1	0	10	49
Epsilonproteobacteria	0	0	0	0	0	0	5	0	8	4
Deltaproteobacteria	0	0	0	0	0	0	1	0	18	9
Alphaproteobacteria	10	0	0	0	0	1	11	0	46	23
Magnetococcus	0	0	0	0	0	0	0	0	1	0
Chrysiogenetes	0	0	0	0	0	0	0	0	1	0
Firmicutes	0	0	0	0	0	4	2	0	38	60
Tenericutes	0	1	0	0	0	0	3	0	15	0
Actinobacteria	0	0	0	0	0	0	2	0	3	75
Chlamydiae	0	0	0	0	0	0	0	0	0	8
Spirochaetes	0	0	0	0	0	0	1	0	3	10
Acidobacteria	0	0	0	0	0	0	0	0	4	1
Bacteroidetes	0	1	0	0	0	0	2	0	24	8
Fibrobacteres	0	0	0	0	0	0	1	0	0	0
Fusobacteria	0	0	0	0	0	0	0	0	1	4
Verrucomicrobia	0	0	0	0	0	0	0	0	2	2
Gemmatimonadetes	0	0	0	0	0	0	0	0	1	0
Planctomycetes	0	0	0	0	0	0	0	0	0	4
Elusimicrobia	0	0	0	0	0	0	0	0	0	2
Synergistetes	0	0	0	0	0	0	0	0	0	2
Cyanobacteria	0	0	0	0	0	0	0	0	11	5
Chlorobi	0	0	0	0	0	0	0	0	9	1
Chloroflexi	0	0	0	0	0	0	2	0	3	6
Deinococcus-Thermus	0	0	0	0	0	0	0	0	2	5
Aquificae	0	0	0	0	0	0	4	0	3	2
Thermotogae	0	0	0	0	0	2	1	0	7	1
Dictyoglomi	0	0	0	0	0	0	0	0	2	0
Nitrospirae	0	0	0	0	0	0	0	0	1	1
Thermobaculum	0	0	0	0	0	0	0	0	0	1
Deferribacteres	0	0	0	0	0	0	0	0	3	0
Euryarchaeota	0	0	0	0	0	1	10	0	38	11
Crenarchaeota	0	0	0	0	0	1	2	0	20	0
Thaumarchaeota	0	0	0	0	0	0	2	0	0	0
Nanoarchaeota	1	0	0	0	0	0	0	0	0	0
Korarchaeota	0	0	0	0	0	0	0	0	1	0
Total	13	2	0	0	0	9	54	0	337	353

(M00003_1)

Phyla	Module completion ratio (%)									
	0--10	10--20	20--30	30--40	40--50	50--60	60--70	70--80	80--90	90--100
Gammaproteobacteria	1	0	0	0	0	0	4	18	58	45
Betaproteobacteria	1	0	0	0	0	1	0	9	11	39
Epsilonproteobacteria	0	0	0	0	0	0	0	4	9	4
Deltaproteobacteria	0	0	0	0	0	0	1	8	17	2
Alphaproteobacteria	10	0	0	1	0	0	1	22	39	18
Magnetococcus	0	0	0	0	0	0	0	1	0	0
Chrysiogenetes	0	0	0	0	0	0	0	0	1	0
Firmicutes	0	0	0	1	0	2	10	29	42	20
Tenericutes	0	0	1	0	0	4	14	0	0	0
Actinobacteria	0	0	0	0	0	0	2	8	9	61
Chlamydiae	0	0	0	0	0	0	0	0	8	0
Spirochaetes	0	0	0	0	0	0	0	9	4	1
Acidobacteria	0	0	0	0	0	0	1	1	2	1
Bacteroidetes	0	1	0	0	0	0	0	6	19	9
Fibrobacteres	0	0	0	0	0	0	0	1	0	0
Fusobacteria	0	0	0	0	0	0	0	0	4	1
Verrucomicrobia	0	0	0	0	0	0	0	2	1	1
Gemmatimonadetes	0	0	0	0	0	0	0	1	0	0
Planctomycetes	0	0	0	0	0	0	0	1	2	1
Elusimicrobia	0	0	0	0	0	0	0	0	2	0
Synergistetes	0	0	0	0	0	0	0	0	0	2
Cyanobacteria	0	0	0	0	0	0	2	8	5	1
Chlorobi	0	0	0	0	0	0	0	0	0	10
Chloroflexi	0	0	0	0	0	2	2	2	3	2
Deinococcus-Thermus	0	0	0	0	0	0	0	0	2	5
Aquificae	0	0	0	0	0	0	2	6	1	0
Thermotogae	0	0	0	1	0	1	4	2	2	1
Dictyoglomi	0	0	0	0	0	0	2	0	0	0
Nitrospirae	0	0	0	0	0	0	1	0	1	0
Thermobaculum	0	0	0	0	0	0	0	1	0	0
Deferribacteres	0	0	0	0	0	0	0	0	3	0
Euryarchaeota	0	0	0	1	0	23	21	15	0	0
Crenarchaeota	0	0	0	7	0	5	11	0	0	0
Thaumarchaeota	0	0	0	0	0	0	2	0	0	0
Nanoarchaeota	1	0	0	0	0	0	0	0	0	0
Korarchaeota	0	0	0	0	0	0	1	0	0	0
Total	13	1	1	11	0	38	81	154	245	224

(M00004_1)

Phyla	Module completion ratio (%)									
	0--10	10--20	20--30	30--40	40--50	50--60	60--70	70--80	80--90	90--100
Gammaproteobacteria	0	0	0	0	1	5	0	20	12	88
Betaproteobacteria	1	0	0	0	0	2	0	22	10	26
Epsilonproteobacteria	0	0	0	0	0	2	0	13	2	0
Deltaproteobacteria	0	0	0	0	0	1	0	12	1	14
Alphaproteobacteria	1	10	0	0	3	7	0	10	42	18
Magnetococcus	0	0	0	0	0	1	0	0	0	0
Chrysiogenetes	0	0	0	0	0	0	0	1	0	0
Firmicutes	0	0	0	0	0	6	0	45	35	18
Tenericutes	0	3	0	0	2	12	0	1	1	0
Actinobacteria	0	0	0	0	0	0	0	7	3	70
Chlamydiae	0	0	0	0	0	0	0	0	0	8
Spirochaetes	0	0	0	0	0	8	0	4	1	1
Acidobacteria	0	0	0	0	0	0	0	0	0	5
Bacteroidetes	1	1	0	0	0	3	0	10	2	18
Fibrobacteres	0	0	0	0	0	1	0	0	0	0
Fusobacteria	0	0	0	0	0	3	0	1	1	0
Verrucomicrobia	0	0	0	0	1	0	0	0	0	3
Gemmatimonadetes	0	0	0	0	0	0	0	1	0	0
Planctomycetes	0	0	0	0	0	0	0	0	1	3
Elusimicrobia	0	0	0	0	0	2	0	0	0	0
Synergistetes	0	0	0	0	0	2	0	0	0	0
Cyanobacteria	0	0	0	0	0	0	0	0	1	15
Chlorobi	0	0	0	0	0	0	0	1	0	9
Chloroflexi	0	0	0	0	0	0	0	2	6	3
Deinococcus-Thermus	0	0	0	0	0	0	0	3	3	1
Aquificae	0	0	0	0	0	0	0	5	1	3
Thermotogae	0	0	0	0	0	0	0	4	1	6
Dictyoglomi	0	0	0	0	0	0	0	1	1	0
Nitrospirae	0	0	0	0	0	0	0	1	0	1
Thermobaculum	0	0	0	0	0	0	0	1	0	0
Deferribacteres	0	0	0	0	0	0	0	3	0	0
Euryarchaeota	0	24	9	0	13	1	0	13	0	0
Crenarchaeota	0	0	9	0	14	0	0	0	0	0
Thaumarchaeota	0	0	0	0	0	2	0	0	0	0
Nanoarchaeota	1	0	0	0	0	0	0	0	0	0
Korarchaeota	0	1	0	0	0	0	0	0	0	0
Total	4	39	18	0	34	58	0	181	124	310

(M00005_1)

Phyla	Module completion ratio (%)									
	0--10	10--20	20--30	30--40	40--50	50--60	60--70	70--80	80--90	90--100
Gammaproteobacteria	1	0	0	0	0	0	0	0	0	125
Betaproteobacteria	0	0	0	0	0	0	0	0	0	61
Epsilonproteobacteria	0	0	0	0	0	0	0	0	0	17
Deltaproteobacteria	0	0	0	0	0	0	0	0	0	28
Alphaproteobacteria	4	0	0	0	0	0	0	0	0	87
Magnetococcus	0	0	0	0	0	0	0	0	0	1
Chrysiogenetes	0	0	0	0	0	0	0	0	0	1
Firmicutes	0	0	0	0	0	0	0	0	0	104
Tenericutes	3	0	0	0	0	0	0	0	0	16
Actinobacteria	0	0	0	0	0	0	0	0	0	80
Chlamydiae	4	0	0	0	0	0	0	0	0	4
Spirochaetes	1	0	0	0	0	0	0	0	0	13
Acidobacteria	0	0	0	0	0	0	0	0	0	5
Bacteroidetes	0	0	0	0	0	0	0	0	0	35
Fibrobacteres	0	0	0	0	0	0	0	0	0	1
Fusobacteria	0	0	0	0	0	0	0	0	0	5
Verrucomicrobia	0	0	0	0	0	0	0	0	0	4
Gemmatimonadetes	0	0	0	0	0	0	0	0	0	1
Planctomycetes	0	0	0	0	0	0	0	0	0	4
Elusimicrobia	0	0	0	0	0	0	0	0	0	2
Synergistetes	0	0	0	0	0	0	0	0	0	2
Cyanobacteria	0	0	0	0	0	0	0	0	0	16
Chlorobi	0	0	0	0	0	0	0	0	0	10
Chloroflexi	0	0	0	0	0	0	0	0	0	11
Deinococcus-Thermus	0	0	0	0	0	0	0	0	0	7
Aquificae	0	0	0	0	0	0	0	0	0	9
Thermotogae	0	0	0	0	0	0	0	0	0	11
Dictyoglomi	0	0	0	0	0	0	0	0	0	2
Nitrospirae	0	0	0	0	0	0	0	0	0	2
Thermobaculum	0	0	0	0	0	0	0	0	0	1
Deferribacteres	0	0	0	0	0	0	0	0	0	3
Euryarchaeota	0	0	0	0	0	0	0	0	0	60
Crenarchaeota	0	0	0	0	0	0	0	0	0	23
Thaumarchaeota	0	0	0	0	0	0	0	0	0	2
Nanoarchaeota	1	0	0	0	0	0	0	0	0	0
Korarchaeota	0	0	0	0	0	0	0	0	0	1
Total	14	0	0	0	0	0	0	0	0	754

(M00006_1)

Phyla	Module completion ratio (%)									
	0--10	10--20	20--30	30--40	40--50	50--60	60--70	70--80	80--90	90--100
Gammaproteobacteria	23	0	0	0	0	13	0	0	0	90
Betaproteobacteria	24	0	0	0	0	11	0	0	0	26
Epsilonproteobacteria	15	0	0	0	0	2	0	0	0	0
Deltaproteobacteria	12	0	0	0	0	2	0	0	0	14
Alphaproteobacteria	29	0	0	0	0	25	0	0	0	37
Magnetococcus	1	0	0	0	0	0	0	0	0	0
Chrysiogenetes	1	0	0	0	0	0	0	0	0	0
Firmicutes	42	0	0	0	0	26	0	0	0	36
Tenericutes	17	0	0	0	0	1	0	0	0	1
Actinobacteria	6	0	0	0	0	4	0	0	0	70
Chlamydiae	0	0	0	0	0	0	0	0	0	8
Spirochaetes	5	0	0	0	0	0	0	0	0	9
Acidobacteria	0	0	0	0	0	0	0	0	0	5
Bacteroidetes	15	0	0	0	0	2	0	0	0	18
Fibrobacteres	1	0	0	0	0	0	0	0	0	0
Fusobacteria	4	0	0	0	0	0	0	0	0	1
Verrucomicrobia	1	0	0	0	0	0	0	0	0	3
Gemmatimonadetes	1	0	0	0	0	0	0	0	0	0
Planctomycetes	0	0	0	0	0	1	0	0	0	3
Elusimicrobia	2	0	0	0	0	0	0	0	0	0
Synergistetes	2	0	0	0	0	0	0	0	0	0
Cyanobacteria	0	0	0	0	0	1	0	0	0	15
Chlorobi	1	0	0	0	0	0	0	0	0	9
Chloroflexi	2	0	0	0	0	3	0	0	0	6
Deinococcus-Thermus	3	0	0	0	0	3	0	0	0	1
Aquificae	5	0	0	0	0	1	0	0	0	3
Thermotogae	4	0	0	0	0	1	0	0	0	6
Dictyoglomi	0	0	0	0	0	2	0	0	0	0
Nitrospirae	1	0	0	0	0	0	0	0	0	1
Thermobaculum	0	0	0	0	0	0	0	0	0	1
Deferribacteres	3	0	0	0	0	0	0	0	0	0
Euryarchaeota	46	0	0	0	0	14	0	0	0	0
Crenarchaeota	23	0	0	0	0	0	0	0	0	0
Thaumarchaeota	2	0	0	0	0	0	0	0	0	0
Nanoarchaeota	1	0	0	0	0	0	0	0	0	0
Korarchaeota	1	0	0	0	0	0	0	0	0	0
Total	293	0	0	0	0	112	0	0	0	363

(M00007_1)

Phyla	Module completion ratio (%)									
	0--10	10--20	20--30	30--40	40--50	50--60	60--70	70--80	80--90	90--100
Gammaproteobacteria	0	0	0	0	0	0	0	10	0	116
Betaproteobacteria	1	0	0	0	0	0	0	3	0	57
Epsilonproteobacteria	0	0	0	0	0	0	0	2	0	15
Deltaproteobacteria	0	0	0	0	0	0	0	2	0	26
Alphaproteobacteria	1	0	10	0	0	0	0	6	0	74
Magnetococcus	0	0	0	0	0	0	0	1	0	0
Chrysiogenetes	0	0	0	0	0	0	0	0	0	1
Firmicutes	0	0	0	0	0	8	0	15	0	81
Tenericutes	3	0	0	0	0	0	0	16	0	0
Actinobacteria	0	0	0	0	0	0	0	1	0	79
Chlamydiae	0	0	0	0	0	0	0	0	0	8
Spirochaetes	0	0	7	0	0	0	0	2	0	5
Acidobacteria	0	0	0	0	0	0	0	0	0	5
Bacteroidetes	2	0	0	0	0	0	0	2	0	31
Fibrobacteres	0	0	0	0	0	0	0	1	0	0
Fusobacteria	0	0	0	0	0	0	0	3	0	2
Verrucomicrobia	0	0	0	0	0	1	0	0	0	3
Gemmatimonadetes	0	0	0	0	0	0	0	0	0	1
Planctomycetes	0	0	0	0	0	0	0	0	0	4
Elusimicrobia	0	0	0	0	0	0	0	2	0	0
Synergistetes	0	0	0	0	0	0	0	2	0	0
Cyanobacteria	0	0	0	0	0	0	0	0	0	16
Chlorobi	0	0	0	0	0	0	0	0	0	10
Chloroflexi	0	0	0	0	0	0	0	0	0	11
Deinococcus-Thermus	0	0	0	0	0	0	0	0	0	7
Aquificae	0	0	0	0	0	0	0	0	0	9
Thermotogae	0	0	0	0	0	0	0	0	0	11
Dictyoglomi	0	0	0	0	0	0	0	1	0	1
Nitrospirae	0	0	0	0	0	0	0	0	0	2
Thermobaculum	0	0	0	0	0	0	0	1	0	0
Deferribacteres	0	0	0	0	0	0	0	0	0	3
Euryarchaeota	0	0	38	0	0	7	0	1	0	14
Crenarchaeota	0	0	8	0	0	15	0	0	0	0
Thaumarchaeota	0	0	0	0	0	0	0	2	0	0
Nanoarchaeota	1	0	0	0	0	0	0	0	0	0
Korarchaeota	0	0	1	0	0	0	0	0	0	0
Total	8	0	64	0	0	31	0	73	0	592

(M00008_1)

Phyla	Module completion ratio (%)									
	0--10	10--20	20--30	30--40	40--50	50--60	60--70	70--80	80--90	90--100
Gammaproteobacteria	18	0	0	18	0	0	22	0	0	68
Betaproteobacteria	13	0	0	9	0	0	13	0	0	26
Epsilonproteobacteria	15	0	0	0	0	0	0	0	0	2
Deltaproteobacteria	13	0	0	13	0	0	1	0	0	1
Alphaproteobacteria	28	0	0	9	0	0	6	0	0	48
Magnetococcus	1	0	0	0	0	0	0	0	0	0
Chrysiogenetes	1	0	0	0	0	0	0	0	0	0
Firmicutes	38	0	0	51	0	0	15	0	0	0
Tenericutes	17	0	0	2	0	0	0	0	0	0
Actinobacteria	5	0	0	38	0	0	27	0	0	10
Chlamydiae	0	0	0	8	0	0	0	0	0	0
Spirochaetes	4	0	0	8	0	0	2	0	0	0
Acidobacteria	0	0	0	1	0	0	4	0	0	0
Bacteroidetes	10	0	0	9	0	0	16	0	0	0
Fibrobacteres	0	0	0	1	0	0	0	0	0	0
Fusobacteria	2	0	0	2	0	0	1	0	0	0
Verrucomicrobia	1	0	0	1	0	0	2	0	0	0
Gemmatimonadetes	1	0	0	0	0	0	0	0	0	0
Planctomycetes	0	0	0	2	0	0	2	0	0	0
Elusimicrobia	1	0	0	1	0	0	0	0	0	0
Synergistetes	1	0	0	1	0	0	0	0	0	0
Cyanobacteria	1	0	0	1	0	0	14	0	0	0
Chlorobi	1	0	0	9	0	0	0	0	0	0
Chloroflexi	5	0	0	4	0	0	2	0	0	0
Deinococcus-Thermus	3	0	0	3	0	0	1	0	0	0
Aquificae	6	0	0	3	0	0	0	0	0	0
Thermotogae	4	0	0	2	0	0	5	0	0	0
Dictyoglomi	0	0	0	2	0	0	0	0	0	0
Nitrospirae	1	0	0	1	0	0	0	0	0	0
Thermobaculum	0	0	0	0	0	0	1	0	0	0
Deferribacteres	3	0	0	0	0	0	0	0	0	0
Euryarchaeota	51	0	0	9	0	0	0	0	0	0
Crenarchaeota	23	0	0	0	0	0	0	0	0	0
Thaumarchaeota	2	0	0	0	0	0	0	0	0	0
Nanoarchaeota	1	0	0	0	0	0	0	0	0	0
Korarchaeota	1	0	0	0	0	0	0	0	0	0
Total	271	0	0	208	0	0	134	0	0	155

(M00009_1)

Phyla	Module completion ratio (%)									
	0--10	10--20	20--30	30--40	40--50	50--60	60--70	70--80	80--90	90--100
Gammaproteobacteria	0	2	1	0	0	5	6	4	13	95
Betaproteobacteria	0	1	0	0	0	0	2	2	3	53
Epsilonproteobacteria	0	0	0	0	0	2	6	9	0	0
Deltaproteobacteria	0	1	0	3	0	2	2	5	15	0
Alphaproteobacteria	1	0	0	0	0	0	2	2	5	81
Magnetococcus	0	0	0	0	0	0	0	0	1	0
Chrysiogenetes	0	0	0	0	0	0	0	1	0	0
Firmicutes	8	22	3	22	0	16	5	4	24	0
Tenericutes	19	0	0	0	0	0	0	0	0	0
Actinobacteria	2	1	0	1	0	4	3	7	45	17
Chlamydiae	0	0	0	0	0	6	0	1	1	0
Spirochaetes	8	2	0	0	0	1	0	0	3	0
Acidobacteria	0	0	0	0	0	0	0	2	3	0
Bacteroidetes	1	0	5	0	0	0	2	4	21	2
Fibrobacteres	0	0	0	0	0	0	1	0	0	0
Fusobacteria	2	0	0	1	0	2	0	0	0	0
Verrucomicrobia	0	0	0	0	0	0	0	1	3	0
Gemmatimonadetes	0	0	0	0	0	0	0	0	1	0
Planctomycetes	0	0	0	0	0	0	0	1	3	0
Elusimicrobia	0	1	1	0	0	0	0	0	0	0
Synergistetes	0	0	0	1	0	1	0	0	0	0
Cyanobacteria	1	0	0	0	0	7	3	5	0	0
Chlorobi	0	0	0	0	0	0	0	6	3	1
Chloroflexi	0	0	0	3	0	0	0	3	4	1
Deinococcus-Thermus	0	0	0	0	0	0	0	0	2	5
Aquificae	0	0	0	0	0	0	6	3	0	0
Thermotogae	1	1	3	6	0	0	0	0	0	0
Dictyoglomi	0	0	2	0	0	0	0	0	0	0
Nitrospirae	0	0	0	0	0	0	1	1	0	0
Thermobaculum	0	0	0	0	0	0	0	0	1	0
Deferribacteres	0	0	0	0	0	0	0	0	3	0
Euryarchaeota	1	6	2	21	0	9	4	4	13	0
Crenarchaeota	0	7	1	0	0	1	0	13	1	0
Thaumarchaeota	0	0	0	0	0	0	2	0	0	0
Nanoarchaeota	1	0	0	0	0	0	0	0	0	0
Korarchaeota	0	0	0	1	0	0	0	0	0	0
Total	45	44	18	59	0	56	45	78	168	255

(M00010_1)

Phyla	Module completion ratio (%)									
	0--10	10--20	20--30	30--40	40--50	50--60	60--70	70--80	80--90	90--100
Gammaproteobacteria	12	0	0	1	0	0	0	0	0	113
Betaproteobacteria	1	0	0	0	0	0	0	0	0	60
Epsilonproteobacteria	0	0	0	0	0	0	3	0	0	14
Deltaproteobacteria	1	0	0	0	0	0	4	0	0	23
Alphaproteobacteria	1	0	0	1	0	0	2	0	0	87
Magnetococcus	0	0	0	0	0	0	0	0	0	1
Chrysiogenetes	0	0	0	0	0	0	0	0	0	1
Firmicutes	29	0	0	8	0	0	16	0	0	51
Tenericutes	19	0	0	0	0	0	0	0	0	0
Actinobacteria	4	0	0	0	0	0	0	0	0	76
Chlamydiae	6	0	0	0	0	0	0	0	0	2
Spirochaetes	10	0	0	0	0	0	0	0	0	4
Acidobacteria	0	0	0	0	0	0	0	0	0	5
Bacteroidetes	6	0	0	0	0	0	0	0	0	29
Fibrobacteres	0	0	0	0	0	0	0	0	0	1
Fusobacteria	2	0	0	0	0	0	0	0	0	3
Verrucomicrobia	0	0	0	0	0	0	1	0	0	3
Gemmatimonadetes	0	0	0	0	0	0	0	0	0	1
Planctomycetes	0	0	0	0	0	0	0	0	0	4
Elusimicrobia	1	0	0	1	0	0	0	0	0	0
Synergistetes	0	0	0	1	0	0	0	0	0	1
Cyanobacteria	1	0	0	0	0	0	0	0	0	15
Chlorobi	0	0	0	0	0	0	0	0	0	10
Chloroflexi	0	0	0	3	0	0	0	0	0	8
Deinococcus-Thermus	0	0	0	0	0	0	0	0	0	7
Aquificae	0	0	0	0	0	0	6	0	0	3
Thermotogae	4	0	0	1	0	0	6	0	0	0
Dictyoglomi	0	0	0	2	0	0	0	0	0	0
Nitrospirae	0	0	0	0	0	0	1	0	0	1
Thermobaculum	0	0	0	0	0	0	0	0	0	1
Deferribacteres	0	0	0	0	0	0	0	0	0	3
Euryarchaeota	24	0	0	8	0	0	8	0	0	20
Crenarchaeota	7	0	0	2	0	0	0	0	0	14
Thaumarchaeota	0	0	0	0	0	0	0	0	0	2
Nanoarchaeota	1	0	0	0	0	0	0	0	0	0
Korarchaeota	0	0	0	1	0	0	0	0	0	0
Total	129	0	0	29	0	0	47	0	0	563

(M00011_1)

Phyla	Module completion ratio (%)									
	0--10	10--20	20--30	30--40	40--50	50--60	60--70	70--80	80--90	90--100
Gammaproteobacteria	0	0	2	0	3	3	0	0	18	100
Betaproteobacteria	0	0	1	0	2	2	0	0	3	53
Epsilonproteobacteria	0	0	0	0	2	3	0	0	12	0
Deltaproteobacteria	0	0	1	0	5	2	0	0	19	1
Alphaproteobacteria	1	0	0	0	2	1	0	0	3	84
Magnetococcus	0	0	0	0	0	0	0	0	1	0
Chrysiogenetes	0	0	0	0	0	1	0	0	0	0
Firmicutes	15	0	30	0	22	11	0	0	25	1
Tenericutes	19	0	0	0	0	0	0	0	0	0
Actinobacteria	2	0	5	0	2	8	0	0	45	18
Chlamydiae	0	0	0	0	0	1	0	0	7	0
Spirochaetes	8	0	1	0	2	0	0	0	3	0
Acidobacteria	0	0	0	0	0	2	0	0	3	0
Bacteroidetes	1	0	0	0	2	6	0	0	24	2
Fibrobacteres	0	0	0	0	1	0	0	0	0	0
Fusobacteria	3	0	1	0	1	0	0	0	0	0
Verrucomicrobia	0	0	0	0	0	0	0	0	4	0
Gemmatimonadetes	0	0	0	0	0	0	0	0	1	0
Planctomycetes	0	0	0	0	0	1	0	0	3	0
Elusimicrobia	0	0	0	0	2	0	0	0	0	0
Synergistetes	0	0	0	0	1	1	0	0	0	0
Cyanobacteria	1	0	7	0	3	5	0	0	0	0
Chlorobi	0	0	0	0	0	6	0	0	3	1
Chloroflexi	0	0	0	0	3	2	0	0	5	1
Deinococcus-Thermus	0	0	0	0	0	0	0	0	2	5
Aquificae	0	0	0	0	0	7	0	0	2	0
Thermotogae	0	0	1	0	8	2	0	0	0	0
Dictyoglomi	0	0	2	0	0	0	0	0	0	0
Nitrospirae	0	0	0	0	0	1	0	0	1	0
Thermobaculum	0	0	0	0	0	0	0	0	1	0
Deferribacteres	0	0	0	0	0	0	0	0	0	3
Euryarchaeota	0	0	5	0	13	7	0	0	34	1
Crenarchaeota	1	0	3	0	4	12	0	0	3	0
Thaumarchaeota	0	0	0	0	2	0	0	0	0	0
Nanoarchaeota	1	0	0	0	0	0	0	0	0	0
Korarchaeota	0	0	0	0	0	1	0	0	0	0
Total	52	0	59	0	80	85	0	0	222	270

(M00012_1)

Phyla	Module completion ratio (%)									
	0--10	10--20	20--30	30--40	40--50	50--60	60--70	70--80	80--90	90--100
Gammaproteobacteria	7	0	5	0	5	22	0	0	13	74
Betaproteobacteria	1	0	0	0	6	6	0	0	2	46
Epsilonproteobacteria	0	0	0	0	4	13	0	0	0	0
Deltaproteobacteria	1	0	1	0	4	15	0	0	1	6
Alphaproteobacteria	1	0	1	0	3	30	0	0	16	40
Magnetococcus	0	0	0	0	0	1	0	0	0	0
Chrysiogenetes	0	0	0	0	0	1	0	0	0	0
Firmicutes	34	0	17	0	27	8	0	0	0	18
Tenericutes	19	0	0	0	0	0	0	0	0	0
Actinobacteria	2	0	2	0	7	19	0	0	13	37
Chlamydiae	0	0	6	0	0	1	0	0	1	0
Spirochaetes	10	0	0	0	1	3	0	0	0	0
Acidobacteria	0	0	0	0	0	3	0	0	0	2
Bacteroidetes	2	0	4	0	1	23	0	0	0	5
Fibrobacteres	0	0	0	0	0	1	0	0	0	0
Fusobacteria	2	0	0	0	3	0	0	0	0	0
Verrucomicrobia	0	0	0	0	0	3	0	0	1	0
Gemmatimonadetes	0	0	0	0	0	1	0	0	0	0
Planctomycetes	0	0	0	0	0	4	0	0	0	0
Elusimicrobia	2	0	0	0	0	0	0	0	0	0
Synergistetes	1	0	0	0	1	0	0	0	0	0
Cyanobacteria	1	0	0	0	7	8	0	0	0	0
Chlorobi	0	0	0	0	0	10	0	0	0	0
Chloroflexi	0	0	3	0	1	0	0	0	1	6
Deinococcus-Thermus	0	0	0	0	0	1	0	0	0	6
Aquificae	0	0	0	0	6	3	0	0	0	0
Thermotogae	5	0	6	0	0	0	0	0	0	0
Dictyoglomi	2	0	0	0	0	0	0	0	0	0
Nitrospirae	0	0	0	0	1	1	0	0	0	0
Thermobaculum	0	0	0	0	1	0	0	0	0	0
Deferribacteres	0	0	0	0	0	3	0	0	0	0
Euryarchaeota	8	0	23	0	12	16	0	0	1	0
Crenarchaeota	8	0	1	0	0	6	0	0	4	4
Thaumarchaeota	0	0	0	0	0	2	0	0	0	0
Nanoarchaeota	1	0	0	0	0	0	0	0	0	0
Korarchaeota	1	0	0	0	0	0	0	0	0	0
Total	108	0	69	0	90	204	0	0	53	244

(M00013_1)

Phyla	Module completion ratio (%)									
	0--10	10--20	20--30	30--40	40--50	50--60	60--70	70--80	80--90	90--100
Gammaproteobacteria	40	0	24	0	40	22	0	0	0	0
Betaproteobacteria	10	0	3	0	4	27	0	0	17	0
Epsilonproteobacteria	17	0	0	0	0	0	0	0	0	0
Deltaproteobacteria	9	0	6	0	10	3	0	0	0	0
Alphaproteobacteria	17	0	13	0	9	35	0	0	17	0
Magnetococcus	1	0	0	0	0	0	0	0	0	0
Chrysiogenetes	1	0	0	0	0	0	0	0	0	0
Firmicutes	59	0	25	0	13	7	0	0	0	0
Tenericutes	18	0	1	0	0	0	0	0	0	0
Actinobacteria	13	0	8	0	19	40	0	0	0	0
Chlamydiae	7	0	0	0	1	0	0	0	0	0
Spirochaetes	11	0	0	0	3	0	0	0	0	0
Acidobacteria	1	0	2	0	2	0	0	0	0	0
Bacteroidetes	20	0	12	0	3	0	0	0	0	0
Fibrobacteres	1	0	0	0	0	0	0	0	0	0
Fusobacteria	3	0	2	0	0	0	0	0	0	0
Verrucomicrobia	3	0	1	0	0	0	0	0	0	0
Gemmatimonadetes	0	0	0	0	0	1	0	0	0	0
Planctomycetes	1	0	3	0	0	0	0	0	0	0
Elusimicrobia	2	0	0	0	0	0	0	0	0	0
Synergistetes	2	0	0	0	0	0	0	0	0	0
Cyanobacteria	12	0	4	0	0	0	0	0	0	0
Chlorobi	10	0	0	0	0	0	0	0	0	0
Chloroflexi	4	0	3	0	4	0	0	0	0	0
Deinococcus-Thermus	0	0	1	0	5	1	0	0	0	0
Aquificae	9	0	0	0	0	0	0	0	0	0
Thermotogae	11	0	0	0	0	0	0	0	0	0
Dictyoglomi	2	0	0	0	0	0	0	0	0	0
Nitrospirae	2	0	0	0	0	0	0	0	0	0
Thermobaculum	0	0	1	0	0	0	0	0	0	0
Deferribacteres	0	0	3	0	0	0	0	0	0	0
Euryarchaeota	42	0	3	0	13	2	0	0	0	0
Crenarchaeota	9	0	5	0	9	0	0	0	0	0
Thaumarchaeota	2	0	0	0	0	0	0	0	0	0
Nanoarchaeota	1	0	0	0	0	0	0	0	0	0
Korarchaeota	0	0	0	0	1	0	0	0	0	0
Total	340	0	120	0	136	138	0	0	34	0

(M00014_1)

Phyla	Module completion ratio (%)									
	0--10	10--20	20--30	30--40	40--50	50--60	60--70	70--80	80--90	90--100
Gammaproteobacteria	22	0	52	0	45	7	0	0	0	0
Betaproteobacteria	14	0	29	0	11	7	0	0	0	0
Epsilonproteobacteria	5	0	12	0	0	0	0	0	0	0
Deltaproteobacteria	1	0	20	0	7	0	0	0	0	0
Alphaproteobacteria	15	0	47	0	17	12	0	0	0	0
Magnetococcus	0	0	1	0	0	0	0	0	0	0
Chrysiogenetes	0	0	1	0	0	0	0	0	0	0
Firmicutes	36	0	47	0	21	0	0	0	0	0
Tenericutes	18	0	1	0	0	0	0	0	0	0
Actinobacteria	10	0	21	0	47	2	0	0	0	0
Chlamydiae	6	0	2	0	0	0	0	0	0	0
Spirochaetes	9	0	2	0	3	0	0	0	0	0
Acidobacteria	0	0	0	0	5	0	0	0	0	0
Bacteroidetes	3	0	10	0	22	0	0	0	0	0
Fibrobacteres	0	0	0	0	1	0	0	0	0	0
Fusobacteria	3	0	1	0	1	0	0	0	0	0
Verrucomicrobia	0	0	2	0	2	0	0	0	0	0
Gemmatimonadetes	0	0	1	0	0	0	0	0	0	0
Planctomycetes	0	0	0	0	4	0	0	0	0	0
Elusimicrobia	2	0	0	0	0	0	0	0	0	0
Synergistetes	1	0	1	0	0	0	0	0	0	0
Cyanobacteria	0	0	15	0	1	0	0	0	0	0
Chlorobi	0	0	10	0	0	0	0	0	0	0
Chloroflexi	3	0	4	0	4	0	0	0	0	0
Deinococcus-Thermus	2	0	3	0	2	0	0	0	0	0
Aquificae	0	0	9	0	0	0	0	0	0	0
Thermotogae	1	0	10	0	0	0	0	0	0	0
Dictyoglomi	0	0	0	0	2	0	0	0	0	0
Nitrospirae	0	0	2	0	0	0	0	0	0	0
Thermobaculum	0	0	0	0	1	0	0	0	0	0
Deferribacteres	0	0	3	0	0	0	0	0	0	0
Euryarchaeota	18	0	38	0	4	0	0	0	0	0
Crenarchaeota	15	0	8	0	0	0	0	0	0	0
Thaumarchaeota	2	0	0	0	0	0	0	0	0	0
Nanoarchaeota	1	0	0	0	0	0	0	0	0	0
Korarchaeota	1	0	0	0	0	0	0	0	0	0
Total	188	0	352	0	200	28	0	0	0	0

(M00015_1)

Phyla	Module completion ratio (%)									
	0--10	10--20	20--30	30--40	40--50	50--60	60--70	70--80	80--90	90--100
Gammaproteobacteria	6	0	0	0	0	9	0	0	0	111
Betaproteobacteria	1	0	0	0	0	2	0	0	0	58
Epsilonproteobacteria	1	0	0	0	0	2	0	0	0	14
Deltaproteobacteria	1	0	0	0	0	2	0	0	0	25
Alphaproteobacteria	18	0	0	0	0	5	0	0	0	68
Magnetococcus	0	0	0	0	0	0	0	0	0	1
Chrysiogenetes	0	0	0	0	0	0	0	0	0	1
Firmicutes	10	0	0	0	0	15	0	0	0	79
Tenericutes	18	0	0	0	0	1	0	0	0	0
Actinobacteria	4	0	0	0	0	5	0	0	0	71
Chlamydiae	6	0	0	0	0	1	0	0	0	1
Spirochaetes	8	0	0	0	0	1	0	0	0	5
Acidobacteria	1	0	0	0	0	3	0	0	0	1
Bacteroidetes	9	0	0	0	0	3	0	0	0	23
Fibrobacteres	0	0	0	0	0	0	0	0	0	1
Fusobacteria	2	0	0	0	0	0	0	0	0	3
Verrucomicrobia	0	0	0	0	0	0	0	0	0	4
Gemmatimonadetes	0	0	0	0	0	0	0	0	0	1
Planctomycetes	0	0	0	0	0	0	0	0	0	4
Elusimicrobia	1	0	0	0	0	0	0	0	0	1
Synergistetes	0	0	0	0	0	1	0	0	0	1
Cyanobacteria	1	0	0	0	0	0	0	0	0	15
Chlorobi	0	0	0	0	0	0	0	0	0	10
Chloroflexi	0	0	0	0	0	2	0	0	0	9
Deinococcus-Thermus	0	0	0	0	0	0	0	0	0	7
Aquificae	0	0	0	0	0	0	0	0	0	9
Thermotogae	3	0	0	0	0	1	0	0	0	7
Dictyoglomi	0	0	0	0	0	0	0	0	0	2
Nitrospirae	0	0	0	0	0	0	0	0	0	2
Thermobaculum	0	0	0	0	0	1	0	0	0	0
Deferribacteres	0	0	0	0	0	0	0	0	0	3
Euryarchaeota	30	0	0	0	0	18	0	0	0	12
Crenarchaeota	5	0	0	0	0	18	0	0	0	0
Thaumarchaeota	2	0	0	0	0	0	0	0	0	0
Nanoarchaeota	1	0	0	0	0	0	0	0	0	0
Korarchaeota	0	0	0	0	0	1	0	0	0	0
Total	128	0	0	0	0	91	0	0	0	549

(M00016_1)

Phyla	Module completion ratio (%)									
	0--10	10--20	20--30	30--40	40--50	50--60	60--70	70--80	80--90	90--100
Gammaproteobacteria	0	1	1	0	0	0	3	5	16	100
Betaproteobacteria	1	0	0	0	0	0	0	0	1	59
Epsilonproteobacteria	0	0	0	0	0	0	0	1	16	0
Deltaproteobacteria	0	0	0	0	0	1	10	8	2	7
Alphaproteobacteria	1	0	2	0	0	0	1	10	7	70
Magnetococcus	0	0	0	0	0	0	0	0	0	1
Chrysiogenetes	0	0	0	0	0	0	0	0	1	0
Firmicutes	3	2	4	2	1	1	20	45	18	8
Tenericutes	16	2	0	0	0	0	1	0	0	0
Actinobacteria	0	2	1	1	0	2	4	3	13	54
Chlamydiae	0	0	0	0	0	8	0	0	0	0
Spirochaetes	8	1	0	0	0	0	3	0	2	0
Acidobacteria	0	0	0	0	0	0	0	3	2	0
Bacteroidetes	0	0	0	0	0	1	10	24	0	0
Fibrobacteres	0	0	0	0	0	0	1	0	0	0
Fusobacteria	0	1	0	0	0	1	0	0	3	0
Verrucomicrobia	0	0	0	0	0	0	1	1	2	0
Gemmatimonadetes	0	0	0	0	0	0	0	0	0	1
Planctomycetes	0	0	0	0	0	0	4	0	0	0
Elusimicrobia	0	0	0	0	0	0	0	2	0	0
Synergistetes	0	0	0	0	0	1	0	0	1	0
Cyanobacteria	0	0	0	0	0	0	14	2	0	0
Chlorobi	0	0	0	0	0	0	0	10	0	0
Chloroflexi	0	0	0	0	1	0	3	7	0	0
Deinococcus-Thermus	0	0	0	0	4	1	2	0	0	0
Aquificae	0	0	0	0	0	0	2	2	3	2
Thermotogae	0	0	0	0	0	0	0	0	7	4
Dictyoglomi	0	0	0	0	0	0	1	1	0	0
Nitrospirae	0	0	0	0	0	0	1	1	0	0
Thermobaculum	0	0	0	0	0	1	0	0	0	0
Deferribacteres	0	0	0	0	0	0	0	3	0	0
Euryarchaeota	0	1	3	1	7	2	7	13	23	3
Crenarchaeota	0	2	5	2	12	0	2	0	0	0
Thaumarchaeota	0	0	0	2	0	0	0	0	0	0
Nanoarchaeota	0	1	0	0	0	0	0	0	0	0
Korarchaeota	0	0	0	0	0	1	0	0	0	0
Total	29	13	16	8	25	20	90	141	117	309

(M00017_1)

Phyla	Module completion ratio (%)									
	0--10	10--20	20--30	30--40	40--50	50--60	60--70	70--80	80--90	90--100
Gammaproteobacteria	0	1	2	0	5	24	0	23	19	52
Betaproteobacteria	0	1	0	0	0	16	0	39	5	0
Epsilonproteobacteria	0	0	0	0	1	12	0	1	3	0
Deltaproteobacteria	0	0	2	0	0	11	0	11	4	0
Alphaproteobacteria	0	2	16	0	7	1	0	24	31	10
Magnetococcus	0	0	0	0	0	1	0	0	0	0
Chrysiogenetes	0	0	0	0	0	0	0	1	0	0
Firmicutes	3	2	2	0	7	18	0	18	29	25
Tenericutes	18	0	1	0	0	0	0	0	0	0
Actinobacteria	1	0	0	0	1	13	0	60	3	2
Chlamydiae	0	0	7	0	0	1	0	0	0	0
Spirochaetes	9	0	0	0	0	2	0	3	0	0
Acidobacteria	0	0	0	0	0	1	0	3	1	0
Bacteroidetes	0	0	2	0	4	6	0	21	2	0
Fibrobacteres	0	0	0	0	0	0	0	1	0	0
Fusobacteria	0	0	1	0	1	0	0	1	2	0
Verrucomicrobia	0	0	0	0	0	2	0	2	0	0
Gemmatimonadetes	0	0	0	0	0	0	0	1	0	0
Planctomycetes	0	0	0	0	0	1	0	3	0	0
Elusimicrobia	0	0	0	0	0	2	0	0	0	0
Synergistetes	0	0	0	0	1	1	0	0	0	0
Cyanobacteria	0	0	0	0	1	12	0	1	2	0
Chlorobi	0	0	0	0	0	3	0	7	0	0
Chloroflexi	0	0	0	0	3	2	0	6	0	0
Deinococcus-Thermus	0	0	0	0	0	4	0	3	0	0
Aquificae	0	0	0	0	0	9	0	0	0	0
Thermotogae	0	0	1	0	3	1	0	5	1	0
Dictyoglomi	0	0	0	0	0	2	0	0	0	0
Nitrospirae	0	0	0	0	0	2	0	0	0	0
Thermobaculum	0	0	0	0	0	0	0	1	0	0
Deferribacteres	0	0	0	0	0	3	0	0	0	0
Euryarchaeota	1	1	0	0	4	44	0	9	1	0
Crenarchaeota	3	3	1	0	1	1	0	14	0	0
Thaumarchaeota	0	0	0	0	0	2	0	0	0	0
Nanoarchaeota	1	0	0	0	0	0	0	0	0	0
Korarchaeota	0	0	0	0	0	0	0	1	0	0
Total	36	10	35	0	39	197	0	259	103	89

(M00018_1)

Phyla	Module completion ratio (%)									
	0--10	10--20	20--30	30--40	40--50	50--60	60--70	70--80	80--90	90--100
Gammaproteobacteria	1	0	0	0	4	5	0	0	8	108
Betaproteobacteria	1	0	0	0	0	0	0	0	1	59
Epsilonproteobacteria	0	0	0	0	0	0	0	0	2	15
Deltaproteobacteria	0	0	0	0	1	1	0	0	16	10
Alphaproteobacteria	1	0	2	0	19	0	0	0	9	60
Magnetococcus	0	0	0	0	0	0	0	0	0	1
Chrysiogenetes	0	0	0	0	0	0	0	0	0	1
Firmicutes	4	0	1	0	7	1	0	0	5	86
Tenericutes	18	0	0	0	1	0	0	0	0	0
Actinobacteria	0	0	1	0	0	0	0	0	2	77
Chlamydiae	0	0	0	0	8	0	0	0	0	0
Spirochaetes	8	0	1	0	0	0	0	0	1	4
Acidobacteria	0	0	0	0	0	0	0	0	0	5
Bacteroidetes	0	0	0	0	5	3	0	0	7	20
Fibrobacteres	0	0	0	0	0	0	0	0	0	1
Fusobacteria	0	0	1	0	1	0	0	0	0	3
Verrucomicrobia	0	0	0	0	0	1	0	0	0	3
Gemmatimonadetes	0	0	0	0	0	0	0	0	1	0
Planctomycetes	0	0	0	0	0	0	0	0	4	0
Elusimicrobia	0	0	0	0	0	0	0	0	0	2
Synergistetes	0	0	0	0	0	0	0	0	0	2
Cyanobacteria	0	0	0	0	0	0	0	0	1	15
Chlorobi	0	0	0	0	0	0	0	0	0	10
Chloroflexi	0	0	0	0	0	0	0	0	4	7
Deinococcus-Thermus	0	0	0	0	0	0	0	0	0	7
Aquificae	0	0	0	0	0	0	0	0	0	9
Thermotogae	0	0	0	0	1	4	0	0	0	6
Dictyoglomi	0	0	0	0	0	0	0	0	0	2
Nitrospirae	0	0	0	0	0	0	0	0	1	1
Thermobaculum	0	0	0	0	0	0	0	0	0	1
Deferribacteres	0	0	0	0	0	0	0	0	3	0
Euryarchaeota	0	0	1	0	1	1	0	0	23	34
Crenarchaeota	0	0	7	0	0	1	0	0	1	14
Thaumarchaeota	0	0	0	0	0	0	0	0	0	2
Nanoarchaeota	1	0	0	0	0	0	0	0	0	0
Korarchaeota	0	0	0	0	0	0	0	0	0	1
Total	34	0	14	0	48	17	0	0	89	566

(M00019_1)

Phyla	Module completion ratio (%)									
	0--10	10--20	20--30	30--40	40--50	50--60	60--70	70--80	80--90	90--100
Gammaproteobacteria	7	2	1	0	2	0	0	2	54	58
Betaproteobacteria	1	0	0	0	0	0	0	1	59	0
Epsilonproteobacteria	0	0	2	0	0	0	0	0	15	0
Deltaproteobacteria	0	2	0	0	1	1	0	0	24	0
Alphaproteobacteria	10	11	2	0	2	0	0	2	64	0
Magnetococcus	0	0	0	0	0	0	0	0	1	0
Chrysiogenetes	0	0	0	0	0	0	0	0	1	0
Firmicutes	8	17	2	0	4	2	0	3	68	0
Tenericutes	18	1	0	0	0	0	0	0	0	0
Actinobacteria	1	2	2	0	1	2	0	1	71	0
Chlamydiae	8	0	0	0	0	0	0	0	0	0
Spirochaetes	9	0	0	0	0	0	0	1	4	0
Acidobacteria	0	0	0	0	0	0	0	1	4	0
Bacteroidetes	1	5	0	0	2	0	0	1	26	0
Fibrobacteres	0	0	0	0	0	0	0	0	1	0
Fusobacteria	2	0	0	0	0	0	0	0	3	0
Verrucomicrobia	0	0	0	0	0	0	0	0	4	0
Gemmatimonadetes	0	0	1	0	0	0	0	0	0	0
Planctomycetes	0	0	0	0	0	0	0	0	4	0
Elusimicrobia	0	1	0	0	0	0	0	0	1	0
Synergistetes	0	0	0	0	0	1	0	0	1	0
Cyanobacteria	1	0	0	0	0	0	0	4	11	0
Chlorobi	0	0	0	0	0	0	0	0	10	0
Chloroflexi	0	0	0	0	0	0	0	0	11	0
Deinococcus-Thermus	0	0	0	0	0	0	0	0	7	0
Aquificae	0	0	0	0	0	0	0	0	9	0
Thermotogae	0	3	2	0	0	0	0	0	6	0
Dictyoglomi	0	0	0	0	0	0	0	0	2	0
Nitrospirae	0	0	0	0	0	0	0	0	2	0
Thermobaculum	0	0	0	0	0	0	0	0	1	0
Deferribacteres	0	0	0	0	0	0	0	0	3	0
Euryarchaeota	5	3	1	0	2	1	0	4	44	0
Crenarchaeota	7	2	0	0	0	0	0	9	5	0
Thaumarchaeota	0	0	0	0	0	0	0	0	2	0
Nanoarchaeota	0	1	0	0	0	0	0	0	0	0
Korarchaeota	0	0	0	0	0	0	0	1	0	0
Total	78	50	13	0	14	7	0	30	518	58

(M00020_1)

Phyla	Module completion ratio (%)									
	0--10	10--20	20--30	30--40	40--50	50--60	60--70	70--80	80--90	90--100
Gammaproteobacteria	1	0	0	7	0	0	13	0	0	105
Betaproteobacteria	1	0	0	0	0	0	10	0	0	50
Epsilonproteobacteria	0	0	0	0	0	0	12	0	0	5
Deltaproteobacteria	0	0	0	12	0	0	10	0	0	6
Alphaproteobacteria	20	0	0	7	0	0	3	0	0	61
Magnetococcus	0	0	0	0	0	0	1	0	0	0
Chrysiogenetes	0	0	0	1	0	0	0	0	0	0
Firmicutes	20	0	0	33	0	0	44	0	0	7
Tenericutes	19	0	0	0	0	0	0	0	0	0
Actinobacteria	5	0	0	3	0	0	5	0	0	67
Chlamydiae	7	0	0	0	0	0	0	0	0	1
Spirochaetes	9	0	0	0	0	0	2	0	0	3
Acidobacteria	0	0	0	2	0	0	3	0	0	0
Bacteroidetes	2	0	0	1	0	0	24	0	0	8
Fibrobacteres	0	0	0	0	0	0	1	0	0	0
Fusobacteria	1	0	0	2	0	0	2	0	0	0
Verrucomicrobia	0	0	0	3	0	0	1	0	0	0
Gemmatimonadetes	0	0	0	0	0	0	1	0	0	0
Planctomycetes	0	0	0	1	0	0	2	0	0	1
Elusimicrobia	1	0	0	1	0	0	0	0	0	0
Synergistetes	1	0	0	1	0	0	0	0	0	0
Cyanobacteria	0	0	0	13	0	0	2	0	0	1
Chlorobi	0	0	0	5	0	0	5	0	0	0
Chloroflexi	0	0	0	5	0	0	6	0	0	0
Deinococcus-Thermus	0	0	0	6	0	0	1	0	0	0
Aquificae	0	0	0	9	0	0	0	0	0	0
Thermotogae	0	0	0	11	0	0	0	0	0	0
Dictyoglomi	0	0	0	2	0	0	0	0	0	0
Nitrospirae	0	0	0	2	0	0	0	0	0	0
Thermobaculum	0	0	0	1	0	0	0	0	0	0
Deferribacteres	0	0	0	3	0	0	0	0	0	0
Euryarchaeota	0	0	0	3	0	0	54	0	0	3
Crenarchaeota	1	0	0	10	0	0	12	0	0	0
Thaumarchaeota	0	0	0	0	0	0	2	0	0	0
Nanoarchaeota	1	0	0	0	0	0	0	0	0	0
Korarchaeota	0	0	0	1	0	0	0	0	0	0
Total	89	0	0	145	0	0	216	0	0	318

(M00021_1)

Phyla	Module completion ratio (%)									
	0--10	10--20	20--30	30--40	40--50	50--60	60--70	70--80	80--90	90--100
Gammaproteobacteria	7	0	0	0	0	8	0	0	0	111
Betaproteobacteria	1	0	0	0	0	1	0	0	0	59
Epsilonproteobacteria	0	0	0	0	0	0	0	0	0	17
Deltaproteobacteria	2	0	0	0	0	0	0	0	0	26
Alphaproteobacteria	24	0	0	0	0	0	0	0	0	67
Magnetococcus	0	0	0	0	0	0	0	0	0	1
Chrysiogenetes	0	0	0	0	0	0	0	0	0	1
Firmicutes	8	0	0	0	0	6	0	0	0	90
Tenericutes	19	0	0	0	0	0	0	0	0	0
Actinobacteria	4	0	0	0	0	29	0	0	0	47
Chlamydiae	8	0	0	0	0	0	0	0	0	0
Spirochaetes	9	0	0	0	0	0	0	0	0	5
Acidobacteria	0	0	0	0	0	1	0	0	0	4
Bacteroidetes	4	0	0	0	0	5	0	0	0	26
Fibrobacteres	0	0	0	0	0	0	0	0	0	1
Fusobacteria	1	0	0	0	0	1	0	0	0	3
Verrucomicrobia	0	0	0	0	0	0	0	0	0	4
Gemmatimonadetes	0	0	0	0	0	0	0	0	0	1
Planctomycetes	0	0	0	0	0	0	0	0	0	4
Elusimicrobia	1	0	0	0	0	0	0	0	0	1
Synergistetes	0	0	0	0	0	0	0	0	0	2
Cyanobacteria	0	0	0	0	0	0	0	0	0	16
Chlorobi	0	0	0	0	0	0	0	0	0	10
Chloroflexi	0	0	0	0	0	2	0	0	0	9
Deinococcus-Thermus	0	0	0	0	0	3	0	0	0	4
Aquificae	0	0	0	0	0	7	0	0	0	2
Thermotogae	0	0	0	0	0	0	0	0	0	11
Dictyoglomi	0	0	0	0	0	0	0	0	0	2
Nitrospirae	0	0	0	0	0	1	0	0	0	1
Thermobaculum	0	0	0	0	0	1	0	0	0	0
Deferribacteres	0	0	0	0	0	0	0	0	0	3
Euryarchaeota	23	0	0	0	0	12	0	0	0	25
Crenarchaeota	0	0	0	0	0	23	0	0	0	0
Thaumarchaeota	1	0	0	0	0	1	0	0	0	0
Nanoarchaeota	1	0	0	0	0	0	0	0	0	0
Korarchaeota	0	0	0	0	0	1	0	0	0	0
Total	113	0	0	0	0	102	0	0	0	553

(M00022_1)

Phyla	Module completion ratio (%)									
	0--10	10--20	20--30	30--40	40--50	50--60	60--70	70--80	80--90	90--100
Gammaproteobacteria	0	0	1	0	0	0	0	1	7	117
Betaproteobacteria	1	0	0	0	1	0	0	0	0	59
Epsilonproteobacteria	0	0	0	0	0	0	0	1	1	15
Deltaproteobacteria	0	0	1	0	0	0	0	6	2	19
Alphaproteobacteria	14	7	0	0	0	0	0	0	0	70
Magnetococcus	0	0	0	0	0	0	0	0	0	1
Chrysiogenetes	0	0	0	0	0	0	0	0	0	1
Firmicutes	10	2	2	0	1	0	0	2	6	81
Tenericutes	18	0	0	0	0	0	0	0	0	1
Actinobacteria	0	0	0	0	0	0	0	1	3	76
Chlamydiae	0	0	0	0	0	1	0	0	1	6
Spirochaetes	8	0	0	0	0	0	0	1	0	5
Acidobacteria	0	0	0	0	0	0	0	0	0	5
Bacteroidetes	1	0	0	0	0	0	0	0	31	3
Fibrobacteres	0	0	0	0	0	0	0	0	1	0
Fusobacteria	0	1	0	0	0	0	0	0	2	2
Verrucomicrobia	0	0	0	0	0	0	0	0	0	4
Gemmatimonadetes	0	0	0	0	0	0	0	0	0	1
Planctomycetes	0	0	0	0	0	0	0	0	0	4
Elusimicrobia	1	0	0	0	0	0	0	0	0	1
Synergistetes	1	0	0	0	0	0	0	0	0	1
Cyanobacteria	0	0	0	0	0	0	0	0	1	15
Chlorobi	0	0	0	0	0	0	0	0	0	10
Chloroflexi	0	0	0	0	0	0	0	0	0	11
Deinococcus-Thermus	0	0	0	0	0	0	0	0	0	7
Aquificae	0	0	0	0	0	0	0	9	0	0
Thermotogae	2	3	0	0	0	0	0	0	0	6
Dictyoglomi	0	0	0	0	0	0	0	0	0	2
Nitrospirae	0	0	0	0	0	0	0	0	0	2
Thermobaculum	0	0	0	0	0	0	0	0	0	1
Deferribacteres	0	0	0	0	0	0	0	0	0	3
Euryarchaeota	3	0	0	0	2	4	0	42	0	9
Crenarchaeota	8	0	0	0	0	0	0	0	0	15
Thaumarchaeota	0	0	0	0	0	0	0	2	0	0
Nanoarchaeota	1	0	0	0	0	0	0	0	0	0
Korarchaeota	0	0	0	0	0	0	0	1	0	0
Total	68	13	4	0	4	5	0	66	55	553

(M00023_1)

Phyla	Module completion ratio (%)									
	0--10	10--20	20--30	30--40	40--50	50--60	60--70	70--80	80--90	90--100
Gammaproteobacteria	8	0	0	1	0	0	2	0	0	115
Betaproteobacteria	1	0	0	0	0	0	0	0	0	60
Epsilonproteobacteria	0	0	0	0	0	0	3	0	0	14
Deltaproteobacteria	1	0	0	0	0	0	1	0	0	26
Alphaproteobacteria	25	0	0	0	0	0	1	0	0	65
Magnetococcus	0	0	0	0	0	0	0	0	0	1
Chrysiogenetes	0	0	0	0	0	0	0	0	0	1
Firmicutes	32	0	0	1	0	0	16	0	0	55
Tenericutes	19	0	0	0	0	0	0	0	0	0
Actinobacteria	9	0	0	15	0	0	41	0	0	15
Chlamydiae	5	0	0	1	0	0	2	0	0	0
Spirochaetes	10	0	0	0	0	0	0	0	0	4
Acidobacteria	0	0	0	0	0	0	0	0	0	5
Bacteroidetes	3	0	0	0	0	0	3	0	0	29
Fibrobacteres	0	0	0	0	0	0	1	0	0	0
Fusobacteria	2	0	0	0	0	0	0	0	0	3
Verrucomicrobia	0	0	0	0	0	0	0	0	0	4
Gemmatimonadetes	0	0	0	0	0	0	0	0	0	1
Planctomycetes	0	0	0	0	0	0	0	0	0	4
Elusimicrobia	1	0	0	0	0	0	0	0	0	1
Synergistetes	2	0	0	0	0	0	0	0	0	0
Cyanobacteria	1	0	0	0	0	0	2	0	0	13
Chlorobi	0	0	0	0	0	0	0	0	0	10
Chloroflexi	0	0	0	0	0	0	0	0	0	11
Deinococcus-Thermus	0	0	0	0	0	0	1	0	0	6
Aquificae	0	0	0	0	0	0	0	0	0	9
Thermotogae	5	0	0	0	0	0	0	0	0	6
Dictyoglomi	0	0	0	0	0	0	0	0	0	2
Nitrospirae	0	0	0	0	0	0	0	0	0	2
Thermobaculum	0	0	0	0	0	0	0	0	0	1
Deferribacteres	0	0	0	0	0	0	0	0	0	3
Euryarchaeota	6	0	0	0	0	0	4	0	0	50
Crenarchaeota	8	0	0	0	0	0	0	0	0	15
Thaumarchaeota	0	0	0	0	0	0	2	0	0	0
Nanoarchaeota	1	0	0	0	0	0	0	0	0	0
Korarchaeota	1	0	0	0	0	0	0	0	0	0
Total	140	0	0	18	0	0	79	0	0	531

(M00024_1)

Phyla	Module completion ratio (%)									
	0--10	10--20	20--30	30--40	40--50	50--60	60--70	70--80	80--90	90--100
Gammaproteobacteria	8	0	0	3	0	0	38	0	0	77
Betaproteobacteria	1	0	0	0	0	0	10	0	0	50
Epsilonproteobacteria	0	0	0	2	0	0	15	0	0	0
Deltaproteobacteria	1	0	0	1	0	0	25	0	0	1
Alphaproteobacteria	22	0	0	22	0	0	26	0	0	21
Magnetococcus	0	0	0	0	0	0	1	0	0	0
Chrysiogenetes	0	0	0	0	0	0	1	0	0	0
Firmicutes	15	0	0	21	0	0	67	0	0	1
Tenericutes	18	0	0	1	0	0	0	0	0	0
Actinobacteria	1	0	0	28	0	0	51	0	0	0
Chlamydiae	7	0	0	1	0	0	0	0	0	0
Spirochaetes	8	0	0	2	0	0	4	0	0	0
Acidobacteria	0	0	0	4	0	0	1	0	0	0
Bacteroidetes	1	0	0	4	0	0	30	0	0	0
Fibrobacteres	0	0	0	1	0	0	0	0	0	0
Fusobacteria	1	0	0	1	0	0	2	0	0	1
Verrucomicrobia	1	0	0	0	0	0	3	0	0	0
Gemmatimonadetes	0	0	0	0	0	0	0	0	0	1
Planctomycetes	0	0	0	0	0	0	4	0	0	0
Elusimicrobia	1	0	0	0	0	0	1	0	0	0
Synergistetes	2	0	0	0	0	0	0	0	0	0
Cyanobacteria	1	0	0	0	0	0	15	0	0	0
Chlorobi	0	0	0	8	0	0	2	0	0	0
Chloroflexi	0	0	0	1	0	0	10	0	0	0
Deinococcus-Thermus	0	0	0	2	0	0	5	0	0	0
Aquificae	0	0	0	0	0	0	9	0	0	0
Thermotogae	5	0	0	0	0	0	6	0	0	0
Dictyoglomi	0	0	0	0	0	0	2	0	0	0
Nitrospirae	0	0	0	0	0	0	2	0	0	0
Thermobaculum	0	0	0	1	0	0	0	0	0	0
Deferribacteres	0	0	0	0	0	0	3	0	0	0
Euryarchaeota	5	0	0	7	0	0	48	0	0	0
Crenarchaeota	8	0	0	10	0	0	5	0	0	0
Thaumarchaeota	0	0	0	1	0	0	1	0	0	0
Nanoarchaeota	0	0	0	0	0	0	1	0	0	0
Korarchaeota	0	0	0	0	0	0	1	0	0	0
Total	106	0	0	121	0	0	389	0	0	152

(M00025_1)

Phyla	Module completion ratio (%)									
	0--10	10--20	20--30	30--40	40--50	50--60	60--70	70--80	80--90	90--100
Gammaproteobacteria	4	0	7	0	0	10	0	37	0	68
Betaproteobacteria	0	0	1	0	0	0	0	14	0	46
Epsilonproteobacteria	0	0	0	0	0	2	0	15	0	0
Deltaproteobacteria	1	0	0	0	0	7	0	20	0	0
Alphaproteobacteria	21	0	22	0	0	19	0	27	0	2
Magnetococcus	0	0	0	0	0	0	0	1	0	0
Chrysiogenetes	0	0	0	0	0	0	0	1	0	0
Firmicutes	9	0	11	0	0	16	0	68	0	0
Tenericutes	18	0	0	0	0	1	0	0	0	0
Actinobacteria	1	0	3	0	0	24	0	52	0	0
Chlamydiae	7	0	1	0	0	0	0	0	0	0
Spirochaetes	8	0	1	0	0	2	0	3	0	0
Acidobacteria	0	0	1	0	0	3	0	1	0	0
Bacteroidetes	1	0	3	0	0	13	0	18	0	0
Fibrobacteres	0	0	0	0	0	1	0	0	0	0
Fusobacteria	1	0	1	0	0	1	0	1	0	1
Verrucomicrobia	0	0	0	0	0	3	0	1	0	0
Gemmatimonadetes	0	0	0	0	0	0	0	0	0	1
Planctomycetes	0	0	0	0	0	2	0	2	0	0
Elusimicrobia	0	0	1	0	0	1	0	0	0	0
Synergistetes	0	0	2	0	0	0	0	0	0	0
Cyanobacteria	1	0	0	0	0	14	0	1	0	0
Chlorobi	0	0	0	0	0	8	0	2	0	0
Chloroflexi	0	0	0	0	0	0	0	11	0	0
Deinococcus-Thermus	0	0	0	0	0	1	0	6	0	0
Aquificae	0	0	0	0	0	3	0	6	0	0
Thermotogae	1	0	4	0	0	0	0	6	0	0
Dictyoglomi	0	0	0	0	0	0	0	2	0	0
Nitrospirae	0	0	0	0	0	0	0	2	0	0
Thermobaculum	0	0	1	0	0	0	0	0	0	0
Deferribacteres	0	0	0	0	0	0	0	3	0	0
Euryarchaeota	1	0	3	0	0	6	0	50	0	0
Crenarchaeota	7	0	3	0	0	6	0	7	0	0
Thaumarchaeota	0	0	1	0	0	0	0	1	0	0
Nanoarchaeota	0	0	1	0	0	0	0	0	0	0
Korarchaeota	0	0	1	0	0	0	0	0	0	0
Total	81	0	68	0	0	143	0	358	0	118

(M00026_1)

Phyla	Module completion ratio (%)									
	0--10	10--20	20--30	30--40	40--50	50--60	60--70	70--80	80--90	90--100
Gammaproteobacteria	10	1	0	0	0	1	2	0	34	78
Betaproteobacteria	0	1	0	0	0	0	1	0	59	0
Epsilonproteobacteria	2	0	0	0	0	0	0	0	0	15
Deltaproteobacteria	1	1	0	0	0	0	7	0	19	0
Alphaproteobacteria	24	0	0	0	0	0	2	0	63	2
Magnetococcus	0	0	0	0	0	0	0	0	1	0
Chrysiogenetes	0	0	0	0	0	0	0	0	0	1
Firmicutes	29	0	0	0	0	0	1	0	15	59
Tenericutes	19	0	0	0	0	0	0	0	0	0
Actinobacteria	5	1	0	0	0	0	2	0	12	60
Chlamydiae	8	0	0	0	0	0	0	0	0	0
Spirochaetes	9	0	0	0	0	0	0	0	4	1
Acidobacteria	0	0	0	0	0	0	1	0	4	0
Bacteroidetes	5	1	0	0	0	0	1	0	1	27
Fibrobacteres	0	0	0	0	0	0	0	0	1	0
Fusobacteria	2	0	0	0	0	0	0	0	0	3
Verrucomicrobia	0	0	0	0	0	0	1	0	3	0
Gemmatimonadetes	0	0	0	0	0	0	0	0	1	0
Planctomycetes	0	0	0	0	0	0	1	0	3	0
Elusimicrobia	0	0	0	0	0	0	1	0	0	1
Synergistetes	0	0	0	0	0	0	0	0	2	0
Cyanobacteria	1	0	0	0	0	0	0	0	15	0
Chlorobi	0	0	0	0	0	0	10	0	0	0
Chloroflexi	0	0	0	0	0	1	1	0	7	2
Deinococcus-Thermus	0	0	0	0	0	0	0	0	1	6
Aquificae	0	0	0	0	0	0	0	0	9	0
Thermotogae	5	0	0	0	0	0	0	0	1	5
Dictyoglomi	0	0	0	0	0	0	0	0	0	2
Nitrospirae	0	0	0	0	0	0	1	0	1	0
Thermobaculum	0	0	0	0	0	0	0	0	1	0
Deferribacteres	0	0	0	0	0	0	0	0	3	0
Euryarchaeota	6	0	0	0	0	0	3	0	46	5
Crenarchaeota	9	0	0	0	0	0	0	0	14	0
Thaumarchaeota	0	0	0	0	0	0	1	0	1	0
Nanoarchaeota	1	0	0	0	0	0	0	0	0	0
Korarchaeota	1	0	0	0	0	0	0	0	0	0
Total	137	5	0	0	0	2	36	0	321	267

(M00027_1)

Phyla	Module completion ratio (%)									
	0--10	10--20	20--30	30--40	40--50	50--60	60--70	70--80	80--90	90--100
Gammaproteobacteria	23	0	0	28	0	0	57	0	0	18
Betaproteobacteria	7	0	0	19	0	0	30	0	0	5
Epsilonproteobacteria	15	0	0	2	0	0	0	0	0	0
Deltaproteobacteria	3	0	0	15	0	0	8	0	0	2
Alphaproteobacteria	20	0	0	45	0	0	26	0	0	0
Magnetococcus	1	0	0	0	0	0	0	0	0	0
Chrysiogenetes	1	0	0	0	0	0	0	0	0	0
Firmicutes	43	0	0	32	0	0	28	0	0	1
Tenericutes	19	0	0	0	0	0	0	0	0	0
Actinobacteria	9	0	0	16	0	0	28	0	0	27
Chlamydiae	7	0	0	1	0	0	0	0	0	0
Spirochaetes	12	0	0	1	0	0	1	0	0	0
Acidobacteria	0	0	0	2	0	0	3	0	0	0
Bacteroidetes	10	0	0	24	0	0	1	0	0	0
Fibrobacteres	1	0	0	0	0	0	0	0	0	0
Fusobacteria	3	0	0	2	0	0	0	0	0	0
Verrucomicrobia	1	0	0	2	0	0	1	0	0	0
Gemmatimonadetes	0	0	0	1	0	0	0	0	0	0
Planctomycetes	1	0	0	2	0	0	1	0	0	0
Elusimicrobia	2	0	0	0	0	0	0	0	0	0
Synergistetes	1	0	0	1	0	0	0	0	0	0
Cyanobacteria	5	0	0	5	0	0	6	0	0	0
Chlorobi	0	0	0	10	0	0	0	0	0	0
Chloroflexi	3	0	0	6	0	0	2	0	0	0
Deinococcus-Thermus	0	0	0	1	0	0	6	0	0	0
Aquificae	9	0	0	0	0	0	0	0	0	0
Thermotogae	9	0	0	2	0	0	0	0	0	0
Dictyoglomi	2	0	0	0	0	0	0	0	0	0
Nitrospirae	2	0	0	0	0	0	0	0	0	0
Thermobaculum	1	0	0	0	0	0	0	0	0	0
Deferribacteres	2	0	0	1	0	0	0	0	0	0
Euryarchaeota	25	0	0	20	0	0	13	0	0	2
Crenarchaeota	2	0	0	19	0	0	2	0	0	0
Thaumarchaeota	1	0	0	1	0	0	0	0	0	0
Nanoarchaeota	1	0	0	0	0	0	0	0	0	0
Korarchaeota	0	0	0	1	0	0	0	0	0	0
Total	241	0	0	259	0	0	213	0	0	55

(M00028_1)

Phyla	Module completion ratio (%)									
	0--10	10--20	20--30	30--40	40--50	50--60	60--70	70--80	80--90	90--100
Gammaproteobacteria	14	0	2	0	0	1	0	16	0	93
Betaproteobacteria	1	0	0	0	0	1	0	0	0	59
Epsilonproteobacteria	3	0	0	0	0	1	0	0	0	13
Deltaproteobacteria	0	0	1	0	0	0	0	3	0	24
Alphaproteobacteria	11	0	6	0	0	1	0	3	0	70
Magnetococcus	0	0	0	0	0	0	0	0	0	1
Chrysiogenetes	0	0	0	0	0	0	0	0	0	1
Firmicutes	27	0	3	0	0	6	0	3	0	65
Tenericutes	19	0	0	0	0	0	0	0	0	0
Actinobacteria	5	0	3	0	0	1	0	2	0	69
Chlamydiae	8	0	0	0	0	0	0	0	0	0
Spirochaetes	9	0	0	0	0	0	0	1	0	4
Acidobacteria	0	0	0	0	0	0	0	1	0	4
Bacteroidetes	6	0	1	0	0	6	0	21	0	1
Fibrobacteres	0	0	0	0	0	0	0	1	0	0
Fusobacteria	2	0	0	0	0	0	0	0	0	3
Verrucomicrobia	0	0	0	0	0	0	0	0	0	4
Gemmatimonadetes	0	0	0	0	0	0	0	0	0	1
Planctomycetes	0	0	0	0	0	0	0	3	0	1
Elusimicrobia	1	0	0	0	0	0	0	0	0	1
Synergistetes	1	0	1	0	0	0	0	0	0	0
Cyanobacteria	1	0	0	0	0	0	0	0	0	15
Chlorobi	0	0	0	0	0	0	0	0	0	10
Chloroflexi	0	0	0	0	0	2	0	1	0	8
Deinococcus-Thermus	0	0	0	0	0	0	0	1	0	6
Aquificae	0	0	0	0	0	0	0	2	0	7
Thermotogae	3	0	0	0	0	0	0	0	0	8
Dictyoglomi	0	0	0	0	0	0	0	0	0	2
Nitrospirae	0	0	0	0	0	0	0	0	0	2
Thermobaculum	0	0	0	0	0	1	0	0	0	0
Deferribacteres	0	0	0	0	0	0	0	0	0	3
Euryarchaeota	5	0	4	0	0	5	0	10	0	36
Crenarchaeota	8	0	0	0	0	14	0	1	0	0
Thaumarchaeota	0	0	0	0	0	2	0	0	0	0
Nanoarchaeota	1	0	0	0	0	0	0	0	0	0
Korarchaeota	0	0	0	0	0	0	0	1	0	0
Total	125	0	21	0	0	41	0	70	0	511

(M00029_1)

Phyla	Module completion ratio (%)									
	0--10	10--20	20--30	30--40	40--50	50--60	60--70	70--80	80--90	90--100
Gammaproteobacteria	7	0	2	0	26	86	0	0	5	0
Betaproteobacteria	1	0	0	0	21	38	0	0	1	0
Epsilonproteobacteria	0	0	2	0	0	15	0	0	0	0
Deltaproteobacteria	1	0	0	0	8	16	0	0	3	0
Alphaproteobacteria	19	0	0	0	39	27	0	0	6	0
Magnetococcus	0	0	0	0	1	0	0	0	0	0
Chrysiogenetes	0	0	0	0	0	1	0	0	0	0
Firmicutes	4	0	14	0	4	54	0	0	28	0
Tenericutes	15	0	4	0	0	0	0	0	0	0
Actinobacteria	3	0	1	0	2	56	0	0	18	0
Chlamydiae	7	0	1	0	0	0	0	0	0	0
Spirochaetes	1	0	8	0	3	1	0	0	1	0
Acidobacteria	0	0	0	0	0	2	0	0	3	0
Bacteroidetes	6	0	2	0	20	7	0	0	0	0
Fibrobacteres	0	0	0	0	0	1	0	0	0	0
Fusobacteria	0	0	2	0	0	3	0	0	0	0
Verrucomicrobia	0	0	0	0	1	3	0	0	0	0
Gemmatimonadetes	0	0	0	0	0	1	0	0	0	0
Planctomycetes	0	0	0	0	0	4	0	0	0	0
Elusimicrobia	1	0	0	0	0	1	0	0	0	0
Synergistetes	0	0	0	0	0	2	0	0	0	0
Cyanobacteria	1	0	0	0	0	15	0	0	0	0
Chlorobi	0	0	0	0	0	10	0	0	0	0
Chloroflexi	0	0	0	0	0	5	0	0	6	0
Deinococcus-Thermus	0	0	0	0	0	0	0	0	7	0
Aquificae	0	0	0	0	0	9	0	0	0	0
Thermotogae	0	0	3	0	1	7	0	0	0	0
Dictyoglomi	0	0	0	0	0	2	0	0	0	0
Nitrospirae	0	0	0	0	0	2	0	0	0	0
Thermobaculum	0	0	0	0	1	0	0	0	0	0
Deferribacteres	0	0	0	0	0	3	0	0	0	0
Euryarchaeota	0	0	8	0	0	41	0	0	11	0
Crenarchaeota	0	0	8	0	0	15	0	0	0	0
Thaumarchaeota	0	0	0	0	0	2	0	0	0	0
Nanoarchaeota	1	0	0	0	0	0	0	0	0	0
Korarchaeota	0	0	0	0	0	1	0	0	0	0
Total	67	0	55	0	127	430	0	0	89	0

(M00030_1)

Phyla	Module completion ratio (%)									
	0--10	10--20	20--30	30--40	40--50	50--60	60--70	70--80	80--90	90--100
Gammaproteobacteria	105	20	1	0	0	0	0	0	0	0
Betaproteobacteria	50	10	1	0	0	0	0	0	0	0
Epsilonproteobacteria	0	15	2	0	0	0	0	0	0	0
Deltaproteobacteria	9	8	11	0	0	0	0	0	0	0
Alphaproteobacteria	61	28	2	0	0	0	0	0	0	0
Magnetococcus	0	0	1	0	0	0	0	0	0	0
Chrysiogenetes	0	0	1	0	0	0	0	0	0	0
Firmicutes	71	29	4	0	0	0	0	0	0	0
Tenericutes	19	0	0	0	0	0	0	0	0	0
Actinobacteria	71	9	0	0	0	0	0	0	0	0
Chlamydiae	8	0	0	0	0	0	0	0	0	0
Spirochaetes	13	0	1	0	0	0	0	0	0	0
Acidobacteria	4	1	0	0	0	0	0	0	0	0
Bacteroidetes	20	13	2	0	0	0	0	0	0	0
Fibrobacteres	0	1	0	0	0	0	0	0	0	0
Fusobacteria	3	2	0	0	0	0	0	0	0	0
Verrucomicrobia	1	2	1	0	0	0	0	0	0	0
Gemmatimonadetes	1	0	0	0	0	0	0	0	0	0
Planctomycetes	4	0	0	0	0	0	0	0	0	0
Elusimicrobia	1	1	0	0	0	0	0	0	0	0
Synergistetes	2	0	0	0	0	0	0	0	0	0
Cyanobacteria	8	8	0	0	0	0	0	0	0	0
Chlorobi	0	10	0	0	0	0	0	0	0	0
Chloroflexi	5	6	0	0	0	0	0	0	0	0
Deinococcus-Thermus	0	4	3	0	0	0	0	0	0	0
Aquificae	7	2	0	0	0	0	0	0	0	0
Thermotogae	10	1	0	0	0	0	0	0	0	0
Dictyoglomi	0	2	0	0	0	0	0	0	0	0
Nitrospirae	1	1	0	0	0	0	0	0	0	0
Thermobaculum	0	1	0	0	0	0	0	0	0	0
Deferribacteres	0	1	2	0	0	0	0	0	0	0
Euryarchaeota	48	11	1	0	0	0	0	0	0	0
Crenarchaeota	23	0	0	0	0	0	0	0	0	0
Thaumarchaeota	2	0	0	0	0	0	0	0	0	0
Nanoarchaeota	1	0	0	0	0	0	0	0	0	0
Korarchaeota	1	0	0	0	0	0	0	0	0	0
Total	549	186	33	0	0	0	0	0	0	0

(M00031_1)

Phyla	Module completion ratio (%)									
	0--10	10--20	20--30	30--40	40--50	50--60	60--70	70--80	80--90	90--100
Gammaproteobacteria	15	0	2	0	109	0	0	0	0	0
Betaproteobacteria	1	0	0	0	60	0	0	0	0	0
Epsilonproteobacteria	3	0	0	0	14	0	0	0	0	0
Deltaproteobacteria	1	0	0	0	27	0	0	0	0	0
Alphaproteobacteria	5	0	14	0	72	0	0	0	0	0
Magnetococcus	0	0	0	0	1	0	0	0	0	0
Chrysiogenetes	0	0	0	0	1	0	0	0	0	0
Firmicutes	30	0	1	0	73	0	0	0	0	0
Tenericutes	19	0	0	0	0	0	0	0	0	0
Actinobacteria	8	0	0	0	70	1	0	0	1	0
Chlamydiae	8	0	0	0	0	0	0	0	0	0
Spirochaetes	9	0	0	0	5	0	0	0	0	0
Acidobacteria	0	0	0	0	5	0	0	0	0	0
Bacteroidetes	6	0	1	0	28	0	0	0	0	0
Fibrobacteres	0	0	0	0	1	0	0	0	0	0
Fusobacteria	2	0	0	0	3	0	0	0	0	0
Verrucomicrobia	0	0	0	0	4	0	0	0	0	0
Gemmatimonadetes	0	0	0	0	1	0	0	0	0	0
Planctomycetes	0	0	0	0	4	0	0	0	0	0
Elusimicrobia	1	0	0	0	1	0	0	0	0	0
Synergistetes	1	0	0	0	0	0	0	0	1	0
Cyanobacteria	0	0	1	0	15	0	0	0	0	0
Chlorobi	0	0	0	0	10	0	0	0	0	0
Chloroflexi	0	0	0	0	3	0	0	0	8	0
Deinococcus-Thermus	0	0	0	0	0	2	0	0	5	0
Aquificae	0	0	0	0	9	0	0	0	0	0
Thermotogae	3	0	0	0	8	0	0	0	0	0
Dictyoglomi	0	0	0	0	2	0	0	0	0	0
Nitrospirae	0	0	0	0	2	0	0	0	0	0
Thermobaculum	0	0	0	0	0	0	0	0	1	0
Deferribacteres	0	0	0	0	3	0	0	0	0	0
Euryarchaeota	9	0	0	0	35	0	0	0	12	4
Crenarchaeota	8	0	0	0	0	0	0	0	1	14
Thaumarchaeota	0	0	0	0	0	0	0	0	2	0
Nanoarchaeota	1	0	0	0	0	0	0	0	0	0
Korarchaeota	0	0	0	0	0	0	0	0	0	1
Total	130	0	19	0	566	3	0	0	31	19

(M00032_1)

Phyla	Module completion ratio (%)									
	0--10	10--20	20--30	30--40	40--50	50--60	60--70	70--80	80--90	90--100
Gammaproteobacteria	7	0	84	0	35	0	0	0	0	0
Betaproteobacteria	3	0	13	0	45	0	0	0	0	0
Epsilonproteobacteria	17	0	0	0	0	0	0	0	0	0
Deltaproteobacteria	13	1	10	0	4	0	0	0	0	0
Alphaproteobacteria	2	0	42	0	46	1	0	0	0	0
Magnetococcus	0	0	1	0	0	0	0	0	0	0
Chrysiogenetes	1	0	0	0	0	0	0	0	0	0
Firmicutes	79	1	22	0	2	0	0	0	0	0
Tenericutes	19	0	0	0	0	0	0	0	0	0
Actinobacteria	8	5	35	0	32	0	0	0	0	0
Chlamydiae	0	0	8	0	0	0	0	0	0	0
Spirochaetes	11	0	3	0	0	0	0	0	0	0
Acidobacteria	0	0	2	0	3	0	0	0	0	0
Bacteroidetes	6	4	3	0	22	0	0	0	0	0
Fibrobacteres	1	0	0	0	0	0	0	0	0	0
Fusobacteria	5	0	0	0	0	0	0	0	0	0
Verrucomicrobia	0	0	4	0	0	0	0	0	0	0
Gemmatimonadetes	0	0	0	0	1	0	0	0	0	0
Planctomycetes	1	0	3	0	0	0	0	0	0	0
Elusimicrobia	2	0	0	0	0	0	0	0	0	0
Synergistetes	2	0	0	0	0	0	0	0	0	0
Cyanobacteria	15	1	0	0	0	0	0	0	0	0
Chlorobi	7	0	3	0	0	0	0	0	0	0
Chloroflexi	4	0	5	0	2	0	0	0	0	0
Deinococcus-Thermus	0	1	0	0	6	0	0	0	0	0
Aquificae	9	0	0	0	0	0	0	0	0	0
Thermotogae	11	0	0	0	0	0	0	0	0	0
Dictyoglomi	2	0	0	0	0	0	0	0	0	0
Nitrospirae	1	1	0	0	0	0	0	0	0	0
Thermobaculum	0	0	1	0	0	0	0	0	0	0
Deferribacteres	3	0	0	0	0	0	0	0	0	0
Euryarchaeota	48	11	1	0	0	0	0	0	0	0
Crenarchaeota	23	0	0	0	0	0	0	0	0	0
Thaumarchaeota	2	0	0	0	0	0	0	0	0	0
Nanoarchaeota	1	0	0	0	0	0	0	0	0	0
Korarchaeota	1	0	0	0	0	0	0	0	0	0
Total	304	25	240	0	198	1	0	0	0	0

(M00033_1)

Phyla	Module completion ratio (%)									
	0--10	10--20	20--30	30--40	40--50	50--60	60--70	70--80	80--90	90--100
Gammaproteobacteria	1	0	1	0	71	30	0	0	1	22
Betaproteobacteria	1	0	0	0	42	10	0	0	1	7
Epsilonproteobacteria	0	0	1	0	15	0	0	0	0	1
Deltaproteobacteria	0	0	0	0	22	2	0	0	1	3
Alphaproteobacteria	1	0	2	0	76	7	0	0	0	5
Magnetococcus	0	0	0	0	1	0	0	0	0	0
Chrysiogenetes	0	0	0	0	0	0	0	0	0	1
Firmicutes	5	0	0	0	93	3	0	0	1	2
Tenericutes	18	0	0	0	1	0	0	0	0	0
Actinobacteria	1	0	1	0	47	10	0	0	0	21
Chlamydiae	0	0	0	0	8	0	0	0	0	0
Spirochaetes	9	0	0	0	4	1	0	0	0	0
Acidobacteria	0	0	0	0	5	0	0	0	0	0
Bacteroidetes	0	0	0	0	35	0	0	0	0	0
Fibrobacteres	0	0	0	0	1	0	0	0	0	0
Fusobacteria	1	0	0	0	4	0	0	0	0	0
Verrucomicrobia	0	0	0	0	3	1	0	0	0	0
Gemmatimonadetes	0	0	0	0	1	0	0	0	0	0
Planctomycetes	0	0	0	0	3	1	0	0	0	0
Elusimicrobia	0	0	0	0	2	0	0	0	0	0
Synergistetes	0	0	0	0	2	0	0	0	0	0
Cyanobacteria	0	0	0	0	12	4	0	0	0	0
Chlorobi	0	0	0	0	10	0	0	0	0	0
Chloroflexi	0	0	0	0	9	1	0	0	0	1
Deinococcus-Thermus	0	0	0	0	7	0	0	0	0	0
Aquificae	0	0	0	0	9	0	0	0	0	0
Thermotogae	0	0	0	0	11	0	0	0	0	0
Dictyoglomi	0	0	0	0	2	0	0	0	0	0
Nitrospirae	0	0	0	0	1	1	0	0	0	0
Thermobaculum	0	0	0	0	1	0	0	0	0	0
Deferribacteres	0	0	0	0	3	0	0	0	0	0
Euryarchaeota	3	0	0	0	47	10	0	0	0	0
Crenarchaeota	7	0	1	0	13	2	0	0	0	0
Thaumarchaeota	0	0	0	0	1	0	0	0	0	1
Nanoarchaeota	1	0	0	0	0	0	0	0	0	0
Korarchaeota	0	0	0	0	1	0	0	0	0	0
Total	48	0	6	0	563	83	0	0	4	64

(M00034_1)

Phyla	Module completion ratio (%)									
	0--10	10--20	20--30	30--40	40--50	50--60	60--70	70--80	80--90	90--100
Gammaproteobacteria	1	25	19	12	20	17	2	1	6	23
Betaproteobacteria	1	0	11	23	20	5	1	0	0	0
Epsilonproteobacteria	0	7	7	2	1	0	0	0	0	0
Deltaproteobacteria	0	1	4	11	8	1	0	1	2	0
Alphaproteobacteria	7	32	14	14	11	7	0	0	6	0
Magnetococcus	0	0	0	0	1	0	0	0	0	0
Chrysiogenetes	0	0	0	0	1	0	0	0	0	0
Firmicutes	0	22	19	14	7	24	2	1	15	0
Tenericutes	2	17	0	0	0	0	0	0	0	0
Actinobacteria	0	30	25	12	6	1	1	5	0	0
Chlamydiae	6	2	0	0	0	0	0	0	0	0
Spirochaetes	0	8	0	3	0	0	0	0	3	0
Acidobacteria	0	0	0	4	0	1	0	0	0	0
Bacteroidetes	2	28	3	1	1	0	0	0	0	0
Fibrobacteres	0	1	0	0	0	0	0	0	0	0
Fusobacteria	0	2	0	2	0	1	0	0	0	0
Verrucomicrobia	0	3	0	0	1	0	0	0	0	0
Gemmatimonadetes	0	0	0	0	0	1	0	0	0	0
Planctomycetes	0	0	1	0	2	1	0	0	0	0
Elusimicrobia	0	1	1	0	0	0	0	0	0	0
Synergistetes	0	0	0	0	2	0	0	0	0	0
Cyanobacteria	0	0	1	5	6	0	1	1	2	0
Chlorobi	0	0	9	1	0	0	0	0	0	0
Chloroflexi	0	0	0	6	2	3	0	0	0	0
Deinococcus-Thermus	0	3	0	1	2	1	0	0	0	0
Aquificae	0	0	0	0	0	3	0	0	6	0
Thermotogae	0	0	0	0	10	1	0	0	0	0
Dictyoglomi	0	0	0	0	0	2	0	0	0	0
Nitrospirae	0	0	0	1	0	1	0	0	0	0
Thermobaculum	0	0	1	0	0	0	0	0	0	0
Deferribacteres	0	0	0	2	0	1	0	0	0	0
Euryarchaeota	0	6	8	20	11	15	0	0	0	0
Crenarchaeota	0	0	0	0	12	11	0	0	0	0
Thaumarchaeota	0	0	0	2	0	0	0	0	0	0
Nanoarchaeota	1	0	0	0	0	0	0	0	0	0
Korarchaeota	0	0	0	0	0	1	0	0	0	0
Total	20	188	123	136	124	98	7	9	40	23

(M00034_2)

Phyla	Module completion ratio (%)									
	0--10	10--20	20--30	30--40	40--50	50--60	60--70	70--80	80--90	90--100
Gammaproteobacteria	1	25	32	0	32	6	6	0	23	1
Betaproteobacteria	1	0	34	0	20	6	0	0	0	0
Epsilonproteobacteria	0	7	9	0	1	0	0	0	0	0
Deltaproteobacteria	0	1	14	0	7	3	2	0	1	0
Alphaproteobacteria	7	32	28	0	11	7	6	0	0	0
Magnetococcus	0	0	0	0	1	0	0	0	0	0
Chrysiogenetes	0	0	0	0	1	0	0	0	0	0
Firmicutes	0	22	32	0	7	27	0	0	0	16
Tenericutes	2	17	0	0	0	0	0	0	0	0
Actinobacteria	0	29	38	0	7	6	0	0	0	0
Chlamydiae	6	2	0	0	0	0	0	0	0	0
Spirochaetes	0	8	3	0	0	0	3	0	0	0
Acidobacteria	0	0	4	0	0	1	0	0	0	0
Bacteroidetes	2	28	4	0	1	0	0	0	0	0
Fibrobacteres	0	1	0	0	0	0	0	0	0	0
Fusobacteria	0	2	2	0	0	1	0	0	0	0
Verrucomicrobia	0	3	0	0	1	0	0	0	0	0
Gemmatimonadetes	0	0	0	0	0	1	0	0	0	0
Planctomycetes	0	0	1	0	3	0	0	0	0	0
Elusimicrobia	0	1	1	0	0	0	0	0	0	0
Synergistetes	0	0	0	0	2	0	0	0	0	0
Cyanobacteria	0	0	6	0	6	1	1	0	1	1
Chlorobi	0	0	10	0	0	0	0	0	0	0
Chloroflexi	0	0	5	0	3	3	0	0	0	0
Deinococcus-Thermus	0	3	1	0	2	1	0	0	0	0
Aquificae	0	0	0	0	0	3	5	0	1	0
Thermotogae	0	0	0	0	10	1	0	0	0	0
Dictyoglomi	0	0	0	0	0	2	0	0	0	0
Nitrospirae	0	0	1	0	0	1	0	0	0	0
Thermobaculum	0	0	1	0	0	0	0	0	0	0
Deferribacteres	0	0	2	0	0	1	0	0	0	0
Euryarchaeota	0	6	28	0	11	15	0	0	0	0
Crenarchaeota	0	0	0	0	12	11	0	0	0	0
Thaumarchaeota	0	0	2	0	0	0	0	0	0	0
Nanoarchaeota	1	0	0	0	0	0	0	0	0	0
Korarchaeota	0	0	0	0	0	1	0	0	0	0
Total	20	187	258	0	138	98	23	0	26	18

(M00035_1)

Phyla	Module completion ratio (%)									
	0--10	10--20	20--30	30--40	40--50	50--60	60--70	70--80	80--90	90--100
Gammaproteobacteria	1	0	29	0	0	54	0	33	0	9
Betaproteobacteria	1	0	0	0	0	26	0	33	0	1
Epsilonproteobacteria	0	0	6	0	0	11	0	0	0	0
Deltaproteobacteria	0	0	0	0	0	8	0	17	0	3
Alphaproteobacteria	6	0	12	0	0	36	0	30	0	7
Magnetococcus	0	0	0	0	0	1	0	0	0	0
Chrysiogenetes	0	0	0	0	0	0	0	1	0	0
Firmicutes	0	0	44	0	0	50	0	10	0	0
Tenericutes	2	0	7	0	0	10	0	0	0	0
Actinobacteria	0	0	7	0	0	18	0	20	0	35
Chlamydiae	7	0	0	0	0	0	0	0	0	1
Spirochaetes	0	0	10	0	0	4	0	0	0	0
Acidobacteria	0	0	0	0	0	2	0	3	0	0
Bacteroidetes	2	0	4	0	0	9	0	12	0	8
Fibrobacteres	0	0	0	0	0	0	0	1	0	0
Fusobacteria	0	0	3	0	0	2	0	0	0	0
Verrucomicrobia	0	0	0	0	0	3	0	1	0	0
Gemmatimonadetes	0	0	0	0	0	0	0	0	0	1
Planctomycetes	0	0	0	0	0	4	0	0	0	0
Elusimicrobia	0	0	1	0	0	1	0	0	0	0
Synergistetes	0	0	0	0	0	1	0	1	0	0
Cyanobacteria	0	0	0	0	0	6	0	9	0	1
Chlorobi	0	0	0	0	0	1	0	6	0	3
Chloroflexi	0	0	0	0	0	5	0	5	0	1
Deinococcus-Thermus	0	0	6	0	0	1	0	0	0	0
Aquificae	0	0	0	0	0	7	0	2	0	0
Thermotogae	0	0	1	0	0	10	0	0	0	0
Dictyoglomi	0	0	0	0	0	2	0	0	0	0
Nitrospirae	0	0	0	0	0	2	0	0	0	0
Thermobaculum	0	0	0	0	0	1	0	0	0	0
Deferribacteres	0	0	0	0	0	3	0	0	0	0
Euryarchaeota	0	0	1	0	0	29	0	29	0	1
Crenarchaeota	0	0	0	0	0	0	0	17	0	6
Thaumarchaeota	0	0	0	0	0	0	0	2	0	0
Nanoarchaeota	1	0	0	0	0	0	0	0	0	0
Korarchaeota	0	0	0	0	0	1	0	0	0	0
Total	20	0	131	0	0	308	0	232	0	77

(M00036_1)

Phyla	Module completion ratio (%)									
	0--10	10--20	20--30	30--40	40--50	50--60	60--70	70--80	80--90	90--100
Gammaproteobacteria	8	54	0	10	0	5	5	0	18	26
Betaproteobacteria	1	10	0	5	0	1	2	0	30	12
Epsilonproteobacteria	0	17	0	0	0	0	0	0	0	0
Deltaproteobacteria	0	13	0	7	0	7	0	0	1	0
Alphaproteobacteria	11	25	0	6	0	8	11	0	23	7
Magnetococcus	0	1	0	0	0	0	0	0	0	0
Chrysiogenetes	0	1	0	0	0	0	0	0	0	0
Firmicutes	10	60	0	15	0	14	5	0	0	0
Tenericutes	18	1	0	0	0	0	0	0	0	0
Actinobacteria	1	29	0	15	0	31	3	0	1	0
Chlamydiae	7	1	0	0	0	0	0	0	0	0
Spirochaetes	10	1	0	1	0	2	0	0	0	0
Acidobacteria	0	2	0	1	0	2	0	0	0	0
Bacteroidetes	1	13	0	8	0	12	1	0	0	0
Fibrobacteres	0	1	0	0	0	0	0	0	0	0
Fusobacteria	2	3	0	0	0	0	0	0	0	0
Verrucomicrobia	0	4	0	0	0	0	0	0	0	0
Gemmatimonadetes	0	0	0	1	0	0	0	0	0	0
Planctomycetes	0	2	0	2	0	0	0	0	0	0
Elusimicrobia	1	1	0	0	0	0	0	0	0	0
Synergistetes	0	1	0	1	0	0	0	0	0	0
Cyanobacteria	5	11	0	0	0	0	0	0	0	0
Chlorobi	0	10	0	0	0	0	0	0	0	0
Chloroflexi	0	4	0	0	0	5	2	0	0	0
Deinococcus-Thermus	0	0	0	4	0	2	1	0	0	0
Aquificae	0	9	0	0	0	0	0	0	0	0
Thermotogae	0	11	0	0	0	0	0	0	0	0
Dictyoglomi	0	2	0	0	0	0	0	0	0	0
Nitrospirae	0	1	0	0	0	1	0	0	0	0
Thermobaculum	0	0	0	1	0	0	0	0	0	0
Deferribacteres	0	3	0	0	0	0	0	0	0	0
Euryarchaeota	9	40	0	10	0	1	0	0	0	0
Crenarchaeota	12	7	0	4	0	0	0	0	0	0
Thaumarchaeota	0	2	0	0	0	0	0	0	0	0
Nanoarchaeota	0	1	0	0	0	0	0	0	0	0
Korarchaeota	0	1	0	0	0	0	0	0	0	0
Total	96	342	0	91	0	91	30	0	73	45

(M00037_1)

Phyla	Module completion ratio (%)									
	0--10	10--20	20--30	30--40	40--50	50--60	60--70	70--80	80--90	90--100
Gammaproteobacteria	121	0	5	0	0	0	0	0	0	0
Betaproteobacteria	57	0	4	0	0	0	0	0	0	0
Epsilonproteobacteria	17	0	0	0	0	0	0	0	0	0
Deltaproteobacteria	24	0	4	0	0	0	0	0	0	0
Alphaproteobacteria	81	0	10	0	0	0	0	0	0	0
Magnetococcus	1	0	0	0	0	0	0	0	0	0
Chrysiogenetes	1	0	0	0	0	0	0	0	0	0
Firmicutes	104	0	0	0	0	0	0	0	0	0
Tenericutes	19	0	0	0	0	0	0	0	0	0
Actinobacteria	75	0	5	0	0	0	0	0	0	0
Chlamydiae	8	0	0	0	0	0	0	0	0	0
Spirochaetes	14	0	0	0	0	0	0	0	0	0
Acidobacteria	3	0	2	0	0	0	0	0	0	0
Bacteroidetes	34	0	1	0	0	0	0	0	0	0
Fibrobacteres	1	0	0	0	0	0	0	0	0	0
Fusobacteria	5	0	0	0	0	0	0	0	0	0
Verrucomicrobia	4	0	0	0	0	0	0	0	0	0
Gemmatimonadetes	0	0	1	0	0	0	0	0	0	0
Planctomycetes	4	0	0	0	0	0	0	0	0	0
Elusimicrobia	2	0	0	0	0	0	0	0	0	0
Synergistetes	2	0	0	0	0	0	0	0	0	0
Cyanobacteria	16	0	0	0	0	0	0	0	0	0
Chlorobi	10	0	0	0	0	0	0	0	0	0
Chloroflexi	9	0	2	0	0	0	0	0	0	0
Deinococcus-Thermus	6	0	1	0	0	0	0	0	0	0
Aquificae	9	0	0	0	0	0	0	0	0	0
Thermotogae	11	0	0	0	0	0	0	0	0	0
Dictyoglomi	2	0	0	0	0	0	0	0	0	0
Nitrospirae	2	0	0	0	0	0	0	0	0	0
Thermobaculum	1	0	0	0	0	0	0	0	0	0
Deferribacteres	3	0	0	0	0	0	0	0	0	0
Euryarchaeota	60	0	0	0	0	0	0	0	0	0
Crenarchaeota	23	0	0	0	0	0	0	0	0	0
Thaumarchaeota	2	0	0	0	0	0	0	0	0	0
Nanoarchaeota	1	0	0	0	0	0	0	0	0	0
Korarchaeota	1	0	0	0	0	0	0	0	0	0
Total	733	0	35	0	0	0	0	0	0	0

(M00038_1)

Phyla	Module completion ratio (%)									
	0--10	10--20	20--30	30--40	40--50	50--60	60--70	70--80	80--90	90--100
Gammaproteobacteria	104	9	3	0	3	5	0	0	1	1
Betaproteobacteria	21	9	1	0	16	6	0	2	6	0
Epsilonproteobacteria	17	0	0	0	0	0	0	0	0	0
Deltaproteobacteria	15	9	1	0	1	0	0	0	2	0
Alphaproteobacteria	62	16	4	0	7	2	0	0	0	0
Magnetococcus	1	0	0	0	0	0	0	0	0	0
Chrysiogenetes	1	0	0	0	0	0	0	0	0	0
Firmicutes	73	19	1	0	9	0	0	2	0	0
Tenericutes	19	0	0	0	0	0	0	0	0	0
Actinobacteria	45	7	17	0	5	0	0	1	5	0
Chlamydiae	6	2	0	0	0	0	0	0	0	0
Spirochaetes	14	0	0	0	0	0	0	0	0	0
Acidobacteria	1	2	1	0	0	0	0	1	0	0
Bacteroidetes	21	3	1	0	0	8	0	0	2	0
Fibrobacteres	1	0	0	0	0	0	0	0	0	0
Fusobacteria	5	0	0	0	0	0	0	0	0	0
Verrucomicrobia	4	0	0	0	0	0	0	0	0	0
Gemmatimonadetes	0	0	0	0	1	0	0	0	0	0
Planctomycetes	3	1	0	0	0	0	0	0	0	0
Elusimicrobia	2	0	0	0	0	0	0	0	0	0
Synergistetes	2	0	0	0	0	0	0	0	0	0
Cyanobacteria	12	3	1	0	0	0	0	0	0	0
Chlorobi	10	0	0	0	0	0	0	0	0	0
Chloroflexi	1	5	5	0	0	0	0	0	0	0
Deinococcus-Thermus	2	0	2	0	3	0	0	0	0	0
Aquificae	9	0	0	0	0	0	0	0	0	0
Thermotogae	11	0	0	0	0	0	0	0	0	0
Dictyoglomi	0	2	0	0	0	0	0	0	0	0
Nitrospirae	1	1	0	0	0	0	0	0	0	0
Thermobaculum	0	1	0	0	0	0	0	0	0	0
Deferribacteres	3	0	0	0	0	0	0	0	0	0
Euryarchaeota	45	15	0	0	0	0	0	0	0	0
Crenarchaeota	23	0	0	0	0	0	0	0	0	0
Thaumarchaeota	2	0	0	0	0	0	0	0	0	0
Nanoarchaeota	1	0	0	0	0	0	0	0	0	0
Korarchaeota	1	0	0	0	0	0	0	0	0	0
Total	538	104	37	0	45	21	0	6	16	1

(M00039_1)

Phyla	Module completion ratio (%)									
	0--10	10--20	20--30	30--40	40--50	50--60	60--70	70--80	80--90	90--100
Gammaproteobacteria	51	0	74	0	1	0	0	0	0	0
Betaproteobacteria	29	0	32	0	0	0	0	0	0	0
Epsilonproteobacteria	15	0	2	0	0	0	0	0	0	0
Deltaproteobacteria	19	0	9	0	0	0	0	0	0	0
Alphaproteobacteria	50	0	38	0	3	0	0	0	0	0
Magnetococcus	1	0	0	0	0	0	0	0	0	0
Chrysiogenetes	0	0	1	0	0	0	0	0	0	0
Firmicutes	94	0	10	0	0	0	0	0	0	0
Tenericutes	19	0	0	0	0	0	0	0	0	0
Actinobacteria	45	0	34	0	1	0	0	0	0	0
Chlamydiae	8	0	0	0	0	0	0	0	0	0
Spirochaetes	13	0	1	0	0	0	0	0	0	0
Acidobacteria	0	0	5	0	0	0	0	0	0	0
Bacteroidetes	17	0	18	0	0	0	0	0	0	0
Fibrobacteres	1	0	0	0	0	0	0	0	0	0
Fusobacteria	5	0	0	0	0	0	0	0	0	0
Verrucomicrobia	2	0	2	0	0	0	0	0	0	0
Gemmatimonadetes	0	0	1	0	0	0	0	0	0	0
Planctomycetes	1	0	3	0	0	0	0	0	0	0
Elusimicrobia	2	0	0	0	0	0	0	0	0	0
Synergistetes	2	0	0	0	0	0	0	0	0	0
Cyanobacteria	11	0	5	0	0	0	0	0	0	0
Chlorobi	5	0	5	0	0	0	0	0	0	0
Chloroflexi	11	0	0	0	0	0	0	0	0	0
Deinococcus-Thermus	6	0	1	0	0	0	0	0	0	0
Aquificae	7	0	2	0	0	0	0	0	0	0
Thermotogae	10	0	1	0	0	0	0	0	0	0
Dictyoglomi	2	0	0	0	0	0	0	0	0	0
Nitrospirae	2	0	0	0	0	0	0	0	0	0
Thermobaculum	1	0	0	0	0	0	0	0	0	0
Deferribacteres	3	0	0	0	0	0	0	0	0	0
Euryarchaeota	41	0	19	0	0	0	0	0	0	0
Crenarchaeota	23	0	0	0	0	0	0	0	0	0
Thaumarchaeota	2	0	0	0	0	0	0	0	0	0
Nanoarchaeota	1	0	0	0	0	0	0	0	0	0
Korarchaeota	1	0	0	0	0	0	0	0	0	0
Total	500	0	263	0	5	0	0	0	0	0

(M00040_1)

Phyla	Module completion ratio (%)									
	0--10	10--20	20--30	30--40	40--50	50--60	60--70	70--80	80--90	90--100
Gammaproteobacteria	48	0	0	0	0	76	0	0	0	2
Betaproteobacteria	11	0	0	0	0	50	0	0	0	0
Epsilonproteobacteria	17	0	0	0	0	0	0	0	0	0
Deltaproteobacteria	27	0	0	0	0	1	0	0	0	0
Alphaproteobacteria	26	0	0	0	0	40	0	0	0	25
Magnetococcus	1	0	0	0	0	0	0	0	0	0
Chrysiogenetes	1	0	0	0	0	0	0	0	0	0
Firmicutes	99	0	0	0	0	5	0	0	0	0
Tenericutes	19	0	0	0	0	0	0	0	0	0
Actinobacteria	80	0	0	0	0	0	0	0	0	0
Chlamydiae	7	0	0	0	0	1	0	0	0	0
Spirochaetes	14	0	0	0	0	0	0	0	0	0
Acidobacteria	5	0	0	0	0	0	0	0	0	0
Bacteroidetes	35	0	0	0	0	0	0	0	0	0
Fibrobacteres	1	0	0	0	0	0	0	0	0	0
Fusobacteria	4	0	0	0	0	1	0	0	0	0
Verrucomicrobia	4	0	0	0	0	0	0	0	0	0
Gemmatimonadetes	0	0	0	0	0	1	0	0	0	0
Planctomycetes	4	0	0	0	0	0	0	0	0	0
Elusimicrobia	2	0	0	0	0	0	0	0	0	0
Synergistetes	2	0	0	0	0	0	0	0	0	0
Cyanobacteria	16	0	0	0	0	0	0	0	0	0
Chlorobi	10	0	0	0	0	0	0	0	0	0
Chloroflexi	11	0	0	0	0	0	0	0	0	0
Deinococcus-Thermus	7	0	0	0	0	0	0	0	0	0
Aquificae	9	0	0	0	0	0	0	0	0	0
Thermotogae	11	0	0	0	0	0	0	0	0	0
Dictyoglomi	2	0	0	0	0	0	0	0	0	0
Nitrospirae	2	0	0	0	0	0	0	0	0	0
Thermobaculum	1	0	0	0	0	0	0	0	0	0
Deferribacteres	3	0	0	0	0	0	0	0	0	0
Euryarchaeota	60	0	0	0	0	0	0	0	0	0
Crenarchaeota	23	0	0	0	0	0	0	0	0	0
Thaumarchaeota	2	0	0	0	0	0	0	0	0	0
Nanoarchaeota	1	0	0	0	0	0	0	0	0	0
Korarchaeota	1	0	0	0	0	0	0	0	0	0
Total	566	0	0	0	0	175	0	0	0	27

(M00042_1)

Phyla	Module completion ratio (%)									
	0--10	10--20	20--30	30--40	40--50	50--60	60--70	70--80	80--90	90--100
Gammaproteobacteria	120	0	6	0	0	0	0	0	0	0
Betaproteobacteria	54	0	6	0	0	1	0	0	0	0
Epsilonproteobacteria	17	0	0	0	0	0	0	0	0	0
Deltaproteobacteria	24	0	4	0	0	0	0	0	0	0
Alphaproteobacteria	78	0	11	0	0	2	0	0	0	0
Magnetococcus	1	0	0	0	0	0	0	0	0	0
Chrysiogenetes	1	0	0	0	0	0	0	0	0	0
Firmicutes	104	0	0	0	0	0	0	0	0	0
Tenericutes	19	0	0	0	0	0	0	0	0	0
Actinobacteria	69	0	9	0	0	2	0	0	0	0
Chlamydiae	8	0	0	0	0	0	0	0	0	0
Spirochaetes	14	0	0	0	0	0	0	0	0	0
Acidobacteria	3	0	1	0	0	1	0	0	0	0
Bacteroidetes	32	0	3	0	0	0	0	0	0	0
Fibrobacteres	1	0	0	0	0	0	0	0	0	0
Fusobacteria	5	0	0	0	0	0	0	0	0	0
Verrucomicrobia	4	0	0	0	0	0	0	0	0	0
Gemmatimonadetes	0	0	1	0	0	0	0	0	0	0
Planctomycetes	4	0	0	0	0	0	0	0	0	0
Elusimicrobia	2	0	0	0	0	0	0	0	0	0
Synergistetes	2	0	0	0	0	0	0	0	0	0
Cyanobacteria	14	0	2	0	0	0	0	0	0	0
Chlorobi	10	0	0	0	0	0	0	0	0	0
Chloroflexi	9	0	2	0	0	0	0	0	0	0
Deinococcus-Thermus	6	0	1	0	0	0	0	0	0	0
Aquificae	9	0	0	0	0	0	0	0	0	0
Thermotogae	11	0	0	0	0	0	0	0	0	0
Dictyoglomi	2	0	0	0	0	0	0	0	0	0
Nitrospirae	2	0	0	0	0	0	0	0	0	0
Thermobaculum	1	0	0	0	0	0	0	0	0	0
Deferribacteres	3	0	0	0	0	0	0	0	0	0
Euryarchaeota	3	0	57	0	0	0	0	0	0	0
Crenarchaeota	15	0	8	0	0	0	0	0	0	0
Thaumarchaeota	2	0	0	0	0	0	0	0	0	0
Nanoarchaeota	1	0	0	0	0	0	0	0	0	0
Korarchaeota	1	0	0	0	0	0	0	0	0	0
Total	651	0	111	0	0	6	0	0	0	0

(M00043_1)

Phyla	Module completion ratio (%)									
	0--10	10--20	20--30	30--40	40--50	50--60	60--70	70--80	80--90	90--100
Gammaproteobacteria	126	0	0	0	0	0	0	0	0	0
Betaproteobacteria	61	0	0	0	0	0	0	0	0	0
Epsilonproteobacteria	17	0	0	0	0	0	0	0	0	0
Deltaproteobacteria	28	0	0	0	0	0	0	0	0	0
Alphaproteobacteria	91	0	0	0	0	0	0	0	0	0
Magnetococcus	1	0	0	0	0	0	0	0	0	0
Chrysiogenetes	1	0	0	0	0	0	0	0	0	0
Firmicutes	104	0	0	0	0	0	0	0	0	0
Tenericutes	19	0	0	0	0	0	0	0	0	0
Actinobacteria	80	0	0	0	0	0	0	0	0	0
Chlamydiae	8	0	0	0	0	0	0	0	0	0
Spirochaetes	14	0	0	0	0	0	0	0	0	0
Acidobacteria	5	0	0	0	0	0	0	0	0	0
Bacteroidetes	35	0	0	0	0	0	0	0	0	0
Fibrobacteres	1	0	0	0	0	0	0	0	0	0
Fusobacteria	5	0	0	0	0	0	0	0	0	0
Verrucomicrobia	4	0	0	0	0	0	0	0	0	0
Gemmatimonadetes	1	0	0	0	0	0	0	0	0	0
Planctomycetes	4	0	0	0	0	0	0	0	0	0
Elusimicrobia	2	0	0	0	0	0	0	0	0	0
Synergistetes	2	0	0	0	0	0	0	0	0	0
Cyanobacteria	16	0	0	0	0	0	0	0	0	0
Chlorobi	10	0	0	0	0	0	0	0	0	0
Chloroflexi	11	0	0	0	0	0	0	0	0	0
Deinococcus-Thermus	7	0	0	0	0	0	0	0	0	0
Aquificae	9	0	0	0	0	0	0	0	0	0
Thermotogae	11	0	0	0	0	0	0	0	0	0
Dictyoglomi	2	0	0	0	0	0	0	0	0	0
Nitrospirae	2	0	0	0	0	0	0	0	0	0
Thermobaculum	1	0	0	0	0	0	0	0	0	0
Deferribacteres	3	0	0	0	0	0	0	0	0	0
Euryarchaeota	60	0	0	0	0	0	0	0	0	0
Crenarchaeota	23	0	0	0	0	0	0	0	0	0
Thaumarchaeota	2	0	0	0	0	0	0	0	0	0
Nanoarchaeota	1	0	0	0	0	0	0	0	0	0
Korarchaeota	1	0	0	0	0	0	0	0	0	0
Total	768	0	0	0	0	0	0	0	0	0

(M00044_1)

Phyla	Module completion ratio (%)									
	0--10	10--20	20--30	30--40	40--50	50--60	60--70	70--80	80--90	90--100
Gammaproteobacteria	65	0	9	0	4	39	0	0	9	0
Betaproteobacteria	19	0	5	0	4	6	0	0	27	0
Epsilonproteobacteria	17	0	0	0	0	0	0	0	0	0
Deltaproteobacteria	21	0	0	0	0	7	0	0	0	0
Alphaproteobacteria	45	0	12	0	6	15	0	0	13	0
Magnetococcus	1	0	0	0	0	0	0	0	0	0
Chrysiogenetes	1	0	0	0	0	0	0	0	0	0
Firmicutes	97	0	1	0	6	0	0	0	0	0
Tenericutes	19	0	0	0	0	0	0	0	0	0
Actinobacteria	40	0	13	0	2	25	0	0	0	0
Chlamydiae	8	0	0	0	0	0	0	0	0	0
Spirochaetes	14	0	0	0	0	0	0	0	0	0
Acidobacteria	2	0	0	0	0	3	0	0	0	0
Bacteroidetes	17	0	0	0	3	15	0	0	0	0
Fibrobacteres	1	0	0	0	0	0	0	0	0	0
Fusobacteria	5	0	0	0	0	0	0	0	0	0
Verrucomicrobia	4	0	0	0	0	0	0	0	0	0
Gemmatimonadetes	0	0	0	0	0	1	0	0	0	0
Planctomycetes	2	0	2	0	0	0	0	0	0	0
Elusimicrobia	2	0	0	0	0	0	0	0	0	0
Synergistetes	2	0	0	0	0	0	0	0	0	0
Cyanobacteria	5	0	11	0	0	0	0	0	0	0
Chlorobi	10	0	0	0	0	0	0	0	0	0
Chloroflexi	8	0	0	0	0	3	0	0	0	0
Deinococcus-Thermus	4	0	3	0	0	0	0	0	0	0
Aquificae	9	0	0	0	0	0	0	0	0	0
Thermotogae	11	0	0	0	0	0	0	0	0	0
Dictyoglomi	2	0	0	0	0	0	0	0	0	0
Nitrospirae	1	0	1	0	0	0	0	0	0	0
Thermobaculum	1	0	0	0	0	0	0	0	0	0
Deferribacteres	3	0	0	0	0	0	0	0	0	0
Euryarchaeota	59	0	0	0	1	0	0	0	0	0
Crenarchaeota	21	0	2	0	0	0	0	0	0	0
Thaumarchaeota	2	0	0	0	0	0	0	0	0	0
Nanoarchaeota	1	0	0	0	0	0	0	0	0	0
Korarchaeota	1	0	0	0	0	0	0	0	0	0
Total	520	0	59	0	26	114	0	0	49	0

(M00045_1)

Phyla	Module completion ratio (%)									
	0--10	10--20	20--30	30--40	40--50	50--60	60--70	70--80	80--90	90--100
Gammaproteobacteria	55	0	1	0	0	1	0	17	0	52
Betaproteobacteria	28	0	5	0	0	0	0	17	0	11
Epsilonproteobacteria	15	0	2	0	0	0	0	0	0	0
Deltaproteobacteria	18	0	0	0	0	0	0	9	0	1
Alphaproteobacteria	47	0	9	0	0	0	0	12	0	23
Magnetococcus	1	0	0	0	0	0	0	0	0	0
Chrysiogenetes	1	0	0	0	0	0	0	0	0	0
Firmicutes	67	0	4	0	0	3	0	10	0	20
Tenericutes	19	0	0	0	0	0	0	0	0	0
Actinobacteria	36	0	5	0	0	0	0	29	0	10
Chlamydiae	7	0	0	0	0	0	0	0	0	1
Spirochaetes	11	0	2	0	0	0	0	1	0	0
Acidobacteria	1	0	2	0	0	0	0	2	0	0
Bacteroidetes	9	0	3	0	0	1	0	9	0	13
Fibrobacteres	0	0	1	0	0	0	0	0	0	0
Fusobacteria	4	0	0	0	0	0	0	0	0	1
Verrucomicrobia	4	0	0	0	0	0	0	0	0	0
Gemmatimonadetes	0	0	0	0	0	0	0	1	0	0
Planctomycetes	4	0	0	0	0	0	0	0	0	0
Elusimicrobia	2	0	0	0	0	0	0	0	0	0
Synergistetes	0	0	0	0	0	0	0	1	0	1
Cyanobacteria	15	0	1	0	0	0	0	0	0	0
Chlorobi	10	0	0	0	0	0	0	0	0	0
Chloroflexi	6	0	0	0	0	1	0	4	0	0
Deinococcus-Thermus	1	0	1	0	0	0	0	5	0	0
Aquificae	8	0	1	0	0	0	0	0	0	0
Thermotogae	5	0	0	0	0	0	0	6	0	0
Dictyoglomi	2	0	0	0	0	0	0	0	0	0
Nitrospirae	2	0	0	0	0	0	0	0	0	0
Thermobaculum	1	0	0	0	0	0	0	0	0	0
Deferribacteres	2	0	0	0	0	0	0	0	0	1
Euryarchaeota	49	0	0	0	0	0	0	5	0	6
Crenarchaeota	19	0	0	0	0	0	0	4	0	0
Thaumarchaeota	1	0	1	0	0	0	0	0	0	0
Nanoarchaeota	1	0	0	0	0	0	0	0	0	0
Korarchaeota	1	0	0	0	0	0	0	0	0	0
Total	452	0	38	0	0	6	0	132	0	140

(M00046_1)

Phyla	Module completion ratio (%)									
	0--10	10--20	20--30	30--40	40--50	50--60	60--70	70--80	80--90	90--100
Gammaproteobacteria	64	0	47	0	0	12	0	3	0	0
Betaproteobacteria	25	0	13	0	0	17	0	5	0	1
Epsilonproteobacteria	13	0	4	0	0	0	0	0	0	0
Deltaproteobacteria	15	0	13	0	0	0	0	0	0	0
Alphaproteobacteria	44	0	24	0	0	13	0	7	0	3
Magnetococcus	1	0	0	0	0	0	0	0	0	0
Chrysiogenetes	1	0	0	0	0	0	0	0	0	0
Firmicutes	69	0	29	0	0	6	0	0	0	0
Tenericutes	17	0	2	0	0	0	0	0	0	0
Actinobacteria	42	0	19	0	0	18	0	1	0	0
Chlamydiae	8	0	0	0	0	0	0	0	0	0
Spirochaetes	11	0	2	0	0	1	0	0	0	0
Acidobacteria	3	0	1	0	0	1	0	0	0	0
Bacteroidetes	32	0	3	0	0	0	0	0	0	0
Fibrobacteres	1	0	0	0	0	0	0	0	0	0
Fusobacteria	3	0	1	0	0	1	0	0	0	0
Verrucomicrobia	3	0	0	0	0	1	0	0	0	0
Gemmatimonadetes	1	0	0	0	0	0	0	0	0	0
Planctomycetes	3	0	1	0	0	0	0	0	0	0
Elusimicrobia	2	0	0	0	0	0	0	0	0	0
Synergistetes	0	0	2	0	0	0	0	0	0	0
Cyanobacteria	1	0	15	0	0	0	0	0	0	0
Chlorobi	7	0	3	0	0	0	0	0	0	0
Chloroflexi	8	0	3	0	0	0	0	0	0	0
Deinococcus-Thermus	4	0	3	0	0	0	0	0	0	0
Aquificae	9	0	0	0	0	0	0	0	0	0
Thermotogae	7	0	4	0	0	0	0	0	0	0
Dictyoglomi	2	0	0	0	0	0	0	0	0	0
Nitrospirae	2	0	0	0	0	0	0	0	0	0
Thermobaculum	1	0	0	0	0	0	0	0	0	0
Deferribacteres	3	0	0	0	0	0	0	0	0	0
Euryarchaeota	36	0	22	0	0	2	0	0	0	0
Crenarchaeota	15	0	8	0	0	0	0	0	0	0
Thaumarchaeota	0	0	2	0	0	0	0	0	0	0
Nanoarchaeota	1	0	0	0	0	0	0	0	0	0
Korarchaeota	1	0	0	0	0	0	0	0	0	0
Total	455	0	221	0	0	72	0	16	0	4

(M00047_1)

Phyla	Module completion ratio (%)									
	0--10	10--20	20--30	30--40	40--50	50--60	60--70	70--80	80--90	90--100
Gammaproteobacteria	121	0	0	5	0	0	0	0	0	0
Betaproteobacteria	60	0	0	1	0	0	0	0	0	0
Epsilonproteobacteria	16	0	0	1	0	0	0	0	0	0
Deltaproteobacteria	27	0	0	1	0	0	0	0	0	0
Alphaproteobacteria	83	0	0	8	0	0	0	0	0	0
Magnetococcus	1	0	0	0	0	0	0	0	0	0
Chrysiogenetes	1	0	0	0	0	0	0	0	0	0
Firmicutes	104	0	0	0	0	0	0	0	0	0
Tenericutes	19	0	0	0	0	0	0	0	0	0
Actinobacteria	70	0	0	10	0	0	0	0	0	0
Chlamydiae	8	0	0	0	0	0	0	0	0	0
Spirochaetes	14	0	0	0	0	0	0	0	0	0
Acidobacteria	5	0	0	0	0	0	0	0	0	0
Bacteroidetes	35	0	0	0	0	0	0	0	0	0
Fibrobacteres	1	0	0	0	0	0	0	0	0	0
Fusobacteria	5	0	0	0	0	0	0	0	0	0
Verrucomicrobia	4	0	0	0	0	0	0	0	0	0
Gemmatimonadetes	1	0	0	0	0	0	0	0	0	0
Planctomycetes	4	0	0	0	0	0	0	0	0	0
Elusimicrobia	2	0	0	0	0	0	0	0	0	0
Synergistetes	2	0	0	0	0	0	0	0	0	0
Cyanobacteria	13	0	0	3	0	0	0	0	0	0
Chlorobi	10	0	0	0	0	0	0	0	0	0
Chloroflexi	11	0	0	0	0	0	0	0	0	0
Deinococcus-Thermus	7	0	0	0	0	0	0	0	0	0
Aquificae	9	0	0	0	0	0	0	0	0	0
Thermotogae	11	0	0	0	0	0	0	0	0	0
Dictyoglomi	2	0	0	0	0	0	0	0	0	0
Nitrospirae	2	0	0	0	0	0	0	0	0	0
Thermobaculum	1	0	0	0	0	0	0	0	0	0
Deferribacteres	3	0	0	0	0	0	0	0	0	0
Euryarchaeota	60	0	0	0	0	0	0	0	0	0
Crenarchaeota	23	0	0	0	0	0	0	0	0	0
Thaumarchaeota	2	0	0	0	0	0	0	0	0	0
Nanoarchaeota	1	0	0	0	0	0	0	0	0	0
Korarchaeota	1	0	0	0	0	0	0	0	0	0
Total	739	0	0	29	0	0	0	0	0	0

(M00048_1)

Phyla	Module completion ratio (%)									
	0--10	10--20	20--30	30--40	40--50	50--60	60--70	70--80	80--90	90--100
Gammaproteobacteria	1	1	3	0	0	1	0	0	11	109
Betaproteobacteria	1	0	0	0	0	0	0	0	4	56
Epsilonproteobacteria	0	0	2	0	0	0	0	0	15	0
Deltaproteobacteria	0	0	0	0	0	0	0	0	23	5
Alphaproteobacteria	1	10	0	0	0	0	0	0	2	78
Magnetococcus	0	0	0	0	0	0	0	0	1	0
Chrysiogenetes	0	0	0	0	0	0	0	0	1	0
Firmicutes	1	3	0	0	0	0	1	5	37	57
Tenericutes	14	5	0	0	0	0	0	0	0	0
Actinobacteria	0	0	1	0	0	0	0	1	8	70
Chlamydiae	7	0	1	0	0	0	0	0	0	0
Spirochaetes	4	4	0	0	0	0	1	0	2	3
Acidobacteria	0	0	0	0	0	0	0	0	1	4
Bacteroidetes	2	0	0	0	0	0	0	1	11	21
Fibrobacteres	0	0	0	0	0	0	0	0	1	0
Fusobacteria	0	0	0	0	0	0	0	0	4	1
Verrucomicrobia	0	0	0	0	0	0	0	0	2	2
Gemmatimonadetes	0	0	0	0	0	0	0	0	0	1
Planctomycetes	0	0	0	0	0	0	0	0	0	4
Elusimicrobia	0	0	0	0	0	0	0	0	2	0
Synergistetes	0	0	0	0	0	0	0	0	2	0
Cyanobacteria	1	0	0	0	0	0	0	0	0	15
Chlorobi	0	0	0	0	0	0	0	0	9	1
Chloroflexi	0	0	1	0	0	0	1	2	1	6
Deinococcus-Thermus	0	0	0	0	0	0	0	0	1	6
Aquificae	0	0	0	0	0	0	0	0	3	6
Thermotogae	0	1	0	0	0	0	0	0	4	6
Dictyoglomi	0	0	0	0	0	0	0	0	2	0
Nitrospirae	0	0	0	0	0	0	0	0	1	1
Thermobaculum	0	0	0	0	0	0	0	0	1	0
Deferribacteres	0	0	0	0	0	0	0	0	3	0
Euryarchaeota	0	1	0	0	0	0	1	17	36	5
Crenarchaeota	7	1	0	0	0	0	0	3	12	0
Thaumarchaeota	0	0	0	0	0	0	0	0	2	0
Nanoarchaeota	1	0	0	0	0	0	0	0	0	0
Korarchaeota	0	1	0	0	0	0	0	0	0	0
Total	40	27	8	0	0	1	4	29	202	457

(M00049_1)

Phyla	Module completion ratio (%)									
	0--10	10--20	20--30	30--40	40--50	50--60	60--70	70--80	80--90	90--100
Gammaproteobacteria	1	0	0	0	0	0	4	0	50	71
Betaproteobacteria	0	1	0	0	0	0	6	0	39	15
Epsilonproteobacteria	0	0	0	0	0	0	0	0	5	12
Deltaproteobacteria	0	0	0	0	0	0	12	0	15	1
Alphaproteobacteria	1	0	0	0	0	10	23	0	52	5
Magnetococcus	0	0	0	0	0	0	1	0	0	0
Chrysiogenetes	0	0	0	0	0	0	0	0	1	0
Firmicutes	0	0	0	2	0	2	14	0	55	31
Tenericutes	0	5	0	9	0	0	4	0	1	0
Actinobacteria	0	0	0	0	0	0	14	0	55	11
Chlamydiae	0	0	0	0	0	6	1	0	0	1
Spirochaetes	0	1	0	2	0	1	8	0	2	0
Acidobacteria	0	0	0	0	0	0	3	0	2	0
Bacteroidetes	1	0	0	0	0	1	7	0	22	4
Fibrobacteres	0	0	0	0	0	0	0	0	1	0
Fusobacteria	0	0	0	0	0	0	0	0	4	1
Verrucomicrobia	0	0	0	0	0	0	1	0	2	1
Gemmatimonadetes	0	0	0	0	0	0	0	0	1	0
Planctomycetes	0	0	0	0	0	0	3	0	1	0
Elusimicrobia	0	0	0	0	0	0	0	0	2	0
Synergistetes	0	0	0	0	0	0	2	0	0	0
Cyanobacteria	0	0	0	0	0	1	3	0	12	0
Chlorobi	0	0	0	0	0	0	5	0	5	0
Chloroflexi	0	0	0	0	0	0	5	0	6	0
Deinococcus-Thermus	0	0	0	0	0	0	3	0	4	0
Aquificae	0	0	0	0	0	0	0	0	4	5
Thermotogae	0	0	0	0	0	1	6	0	4	0
Dictyoglomi	0	0	0	0	0	0	2	0	0	0
Nitrospirae	0	0	0	0	0	0	1	0	1	0
Thermobaculum	0	0	0	0	0	0	0	0	1	0
Deferribacteres	0	0	0	0	0	0	3	0	0	0
Euryarchaeota	0	0	0	0	0	0	12	0	47	1
Crenarchaeota	0	0	0	5	0	2	9	0	7	0
Thaumarchaeota	0	0	0	0	0	0	0	0	2	0
Nanoarchaeota	0	0	0	0	0	1	0	0	0	0
Korarchaeota	0	0	0	0	0	0	0	0	1	0
Total	3	7	0	18	0	25	152	0	404	159

(M00050_1)

Phyla	Module completion ratio (%)									
	0--10	10--20	20--30	30--40	40--50	50--60	60--70	70--80	80--90	90--100
Gammaproteobacteria	1	0	0	1	0	0	3	0	49	72
Betaproteobacteria	0	1	0	0	0	0	5	0	40	15
Epsilonproteobacteria	0	0	0	0	0	0	0	0	5	12
Deltaproteobacteria	0	0	0	0	0	0	12	0	15	1
Alphaproteobacteria	1	0	0	0	0	10	23	0	52	5
Magnetococcus	0	0	0	0	0	0	1	0	0	0
Chrysiogenetes	0	0	0	0	0	0	0	0	1	0
Firmicutes	0	0	0	0	0	0	18	0	57	29
Tenericutes	0	5	0	10	0	2	1	0	1	0
Actinobacteria	0	0	0	0	0	0	14	0	55	11
Chlamydiae	0	0	0	0	0	2	1	0	4	1
Spirochaetes	0	2	0	1	0	4	7	0	0	0
Acidobacteria	0	0	0	0	0	0	3	0	2	0
Bacteroidetes	1	0	0	1	0	0	7	0	22	4
Fibrobacteres	0	0	0	0	0	0	0	0	1	0
Fusobacteria	0	0	0	0	0	0	0	0	4	1
Verrucomicrobia	0	0	0	0	0	0	1	0	2	1
Gemmatimonadetes	0	0	0	0	0	0	0	0	1	0
Planctomycetes	0	0	0	0	0	0	3	0	1	0
Elusimicrobia	0	0	0	0	0	0	0	0	2	0
Synergistetes	0	0	0	0	0	0	2	0	0	0
Cyanobacteria	0	0	0	0	0	0	3	0	13	0
Chlorobi	0	0	0	0	0	0	5	0	5	0
Chloroflexi	0	0	0	0	0	0	5	0	6	0
Deinococcus-Thermus	0	0	0	0	0	0	3	0	4	0
Aquificae	0	0	0	0	0	0	0	0	4	5
Thermotogae	0	0	0	0	0	0	7	0	4	0
Dictyoglomi	0	0	0	0	0	0	2	0	0	0
Nitrospirae	0	0	0	0	0	0	1	0	1	0
Thermobaculum	0	0	0	0	0	0	0	0	1	0
Deferribacteres	0	0	0	0	0	0	3	0	0	0
Euryarchaeota	0	0	0	0	0	19	40	0	1	0
Crenarchaeota	0	1	0	11	0	11	0	0	0	0
Thaumarchaeota	0	0	0	0	0	0	2	0	0	0
Nanoarchaeota	0	0	0	1	0	0	0	0	0	0
Korarchaeota	0	0	0	0	0	0	1	0	0	0
Total	3	9	0	25	0	48	173	0	353	157

(M00051_1)

Phyla	Module completion ratio (%)									
	0--10	10--20	20--30	30--40	40--50	50--60	60--70	70--80	80--90	90--100
Gammaproteobacteria	3	0	0	2	0	0	121	0	0	0
Betaproteobacteria	1	0	0	0	0	0	60	0	0	0
Epsilonproteobacteria	0	0	0	1	0	0	16	0	0	0
Deltaproteobacteria	0	0	0	0	0	0	28	0	0	0
Alphaproteobacteria	11	0	0	1	0	0	79	0	0	0
Magnetococcus	0	0	0	0	0	0	1	0	0	0
Chrysiogenetes	0	0	0	0	0	0	1	0	0	0
Firmicutes	1	0	0	3	0	0	100	0	0	0
Tenericutes	18	0	0	0	0	0	1	0	0	0
Actinobacteria	0	0	0	2	0	0	78	0	0	0
Chlamydiae	7	0	0	1	0	0	0	0	0	0
Spirochaetes	8	0	0	1	0	0	5	0	0	0
Acidobacteria	0	0	0	0	0	0	5	0	0	0
Bacteroidetes	2	0	0	0	0	0	33	0	0	0
Fibrobacteres	0	0	0	0	0	0	1	0	0	0
Fusobacteria	1	0	0	0	0	0	4	0	0	0
Verrucomicrobia	0	0	0	0	0	0	4	0	0	0
Gemmatimonadetes	0	0	0	0	0	0	1	0	0	0
Planctomycetes	0	0	0	0	0	0	4	0	0	0
Elusimicrobia	0	0	0	0	0	0	2	0	0	0
Synergistetes	0	0	0	0	0	0	2	0	0	0
Cyanobacteria	0	0	0	1	0	0	15	0	0	0
Chlorobi	0	0	0	0	0	0	10	0	0	0
Chloroflexi	0	0	0	1	0	0	10	0	0	0
Deinococcus-Thermus	0	0	0	0	0	0	7	0	0	0
Aquificae	0	0	0	0	0	0	9	0	0	0
Thermotogae	1	0	0	0	0	0	10	0	0	0
Dictyoglomi	0	0	0	0	0	0	2	0	0	0
Nitrospirae	0	0	0	0	0	0	2	0	0	0
Thermobaculum	0	0	0	0	0	0	1	0	0	0
Deferribacteres	0	0	0	0	0	0	3	0	0	0
Euryarchaeota	0	0	0	0	0	0	60	0	0	0
Crenarchaeota	1	0	0	0	0	0	22	0	0	0
Thaumarchaeota	0	0	0	0	0	0	2	0	0	0
Nanoarchaeota	1	0	0	0	0	0	0	0	0	0
Korarchaeota	0	0	0	0	0	0	1	0	0	0
Total	55	0	0	13	0	0	700	0	0	0

(M00051_2)

Phyla	Module completion ratio (%)									
	0--10	10--20	20--30	30--40	40--50	50--60	60--70	70--80	80--90	90--100
Gammaproteobacteria	3	0	2	0	0	8	0	113	0	0
Betaproteobacteria	1	0	0	0	0	0	0	60	0	0
Epsilonproteobacteria	0	0	0	0	0	1	0	16	0	0
Deltaproteobacteria	0	0	0	0	0	0	0	28	0	0
Alphaproteobacteria	11	0	0	0	0	3	0	77	0	0
Magnetococcus	0	0	0	0	0	0	0	1	0	0
Chrysiogenetes	0	0	0	0	0	0	0	1	0	0
Firmicutes	1	0	1	0	0	2	0	100	0	0
Tenericutes	18	0	0	0	0	0	0	1	0	0
Actinobacteria	0	0	0	0	0	2	0	78	0	0
Chlamydiae	7	0	0	0	0	1	0	0	0	0
Spirochaetes	8	0	0	0	0	1	0	5	0	0
Acidobacteria	0	0	0	0	0	0	0	5	0	0
Bacteroidetes	2	0	0	0	0	2	0	31	0	0
Fibrobacteres	0	0	0	0	0	0	0	1	0	0
Fusobacteria	1	0	0	0	0	0	0	4	0	0
Verrucomicrobia	0	0	0	0	0	0	0	4	0	0
Gemmatimonadetes	0	0	0	0	0	0	0	1	0	0
Planctomycetes	0	0	0	0	0	0	0	4	0	0
Elusimicrobia	0	0	0	0	0	0	0	2	0	0
Synergistetes	0	0	0	0	0	0	0	2	0	0
Cyanobacteria	0	0	0	0	0	1	0	15	0	0
Chlorobi	0	0	0	0	0	0	0	10	0	0
Chloroflexi	0	0	1	0	0	0	0	10	0	0
Deinococcus-Thermus	0	0	0	0	0	0	0	7	0	0
Aquificae	0	0	0	0	0	0	0	9	0	0
Thermotogae	1	0	0	0	0	0	0	10	0	0
Dictyoglomi	0	0	0	0	0	0	0	2	0	0
Nitrospirae	0	0	0	0	0	0	0	2	0	0
Thermobaculum	0	0	0	0	0	0	0	1	0	0
Deferribacteres	0	0	0	0	0	0	0	3	0	0
Euryarchaeota	0	0	0	0	0	0	0	60	0	0
Crenarchaeota	1	0	0	0	0	0	0	22	0	0
Thaumarchaeota	0	0	0	0	0	2	0	0	0	0
Nanoarchaeota	1	0	0	0	0	0	0	0	0	0
Korarchaeota	0	0	0	0	0	0	0	1	0	0
Total	55	0	4	0	0	23	0	686	0	0

(M00051_3)

Phyla	Module completion ratio (%)									
	0--10	10--20	20--30	30--40	40--50	50--60	60--70	70--80	80--90	90--100
Gammaproteobacteria	1	0	4	0	3	6	0	0	65	47
Betaproteobacteria	1	0	0	0	0	0	0	0	56	4
Epsilonproteobacteria	0	0	0	0	1	0	0	0	16	0
Deltaproteobacteria	0	0	0	0	0	0	0	0	28	0
Alphaproteobacteria	11	0	0	0	1	2	0	0	77	0
Magnetococcus	0	0	0	0	0	0	0	0	1	0
Chrysiogenetes	0	0	0	0	0	0	0	0	1	0
Firmicutes	1	0	0	0	1	4	0	0	91	7
Tenericutes	18	0	0	0	0	1	0	0	0	0
Actinobacteria	0	0	0	0	0	5	0	0	73	2
Chlamydiae	7	0	0	0	0	1	0	0	0	0
Spirochaetes	8	0	0	0	0	1	0	0	4	1
Acidobacteria	0	0	0	0	0	0	0	0	5	0
Bacteroidetes	1	0	1	0	0	2	0	0	25	6
Fibrobacteres	0	0	0	0	0	0	0	0	1	0
Fusobacteria	1	0	0	0	0	0	0	0	1	3
Verrucomicrobia	0	0	0	0	0	0	0	0	4	0
Gemmatimonadetes	0	0	0	0	0	0	0	0	1	0
Planctomycetes	0	0	0	0	0	0	0	0	4	0
Elusimicrobia	0	0	0	0	0	0	0	0	2	0
Synergistetes	0	0	0	0	0	2	0	0	0	0
Cyanobacteria	0	0	0	0	0	1	0	0	15	0
Chlorobi	0	0	0	0	0	0	0	0	10	0
Chloroflexi	0	0	1	0	0	0	0	0	10	0
Deinococcus-Thermus	0	0	0	0	0	0	0	0	7	0
Aquificae	0	0	0	0	0	0	0	0	9	0
Thermotogae	1	0	0	0	0	0	0	0	3	7
Dictyoglomi	0	0	0	0	0	0	0	0	2	0
Nitrospirae	0	0	0	0	0	0	0	0	2	0
Thermobaculum	0	0	0	0	0	0	0	0	1	0
Deferribacteres	0	0	0	0	0	0	0	0	3	0
Euryarchaeota	0	0	0	0	0	0	0	0	9	51
Crenarchaeota	1	0	0	0	0	2	0	0	5	15
Thaumarchaeota	0	0	0	0	0	0	0	0	2	0
Nanoarchaeota	1	0	0	0	0	0	0	0	0	0
Korarchaeota	0	0	0	0	0	0	0	0	1	0
Total	52	0	6	0	6	27	0	0	534	143

(M00052_1)

Phyla	Module completion ratio (%)									
	0--10	10--20	20--30	30--40	40--50	50--60	60--70	70--80	80--90	90--100
Gammaproteobacteria	1	0	0	2	0	0	2	0	0	121
Betaproteobacteria	0	0	0	1	0	0	20	0	0	40
Epsilonproteobacteria	0	0	0	0	0	0	17	0	0	0
Deltaproteobacteria	0	0	0	0	0	0	2	0	0	26
Alphaproteobacteria	1	0	0	1	0	0	7	0	0	82
Magnetococcus	0	0	0	0	0	0	0	0	0	1
Chrysiogenetes	0	0	0	0	0	0	0	0	0	1
Firmicutes	0	0	0	6	0	0	26	0	0	72
Tenericutes	1	0	0	2	0	0	16	0	0	0
Actinobacteria	0	0	0	6	0	0	9	0	0	65
Chlamydiae	0	0	0	0	0	0	0	0	0	8
Spirochaetes	0	0	0	0	0	0	3	0	0	11
Acidobacteria	0	0	0	0	0	0	0	0	0	5
Bacteroidetes	1	0	0	2	0	0	4	0	0	28
Fibrobacteres	0	0	0	0	0	0	0	0	0	1
Fusobacteria	0	0	0	1	0	0	2	0	0	2
Verrucomicrobia	0	0	0	1	0	0	0	0	0	3
Gemmatimonadetes	0	0	0	0	0	0	1	0	0	0
Planctomycetes	0	0	0	0	0	0	0	0	0	4
Elusimicrobia	0	0	0	0	0	0	1	0	0	1
Synergistetes	0	0	0	0	0	0	2	0	0	0
Cyanobacteria	0	0	0	0	0	0	0	0	0	16
Chlorobi	0	0	0	0	0	0	1	0	0	9
Chloroflexi	0	0	0	0	0	0	3	0	0	8
Deinococcus-Thermus	0	0	0	0	0	0	0	0	0	7
Aquificae	0	0	0	0	0	0	0	0	0	9
Thermotogae	0	0	0	0	0	0	6	0	0	5
Dictyoglomi	0	0	0	2	0	0	0	0	0	0
Nitrospirae	0	0	0	0	0	0	0	0	0	2
Thermobaculum	0	0	0	0	0	0	0	0	0	1
Deferribacteres	0	0	0	0	0	0	0	0	0	3
Euryarchaeota	0	0	0	0	0	0	0	0	0	60
Crenarchaeota	0	0	0	0	0	0	5	0	0	18
Thaumarchaeota	0	0	0	0	0	0	0	0	0	2
Nanoarchaeota	0	0	0	1	0	0	0	0	0	0
Korarchaeota	0	0	0	0	0	0	0	0	0	1
Total	4	0	0	25	0	0	127	0	0	612

(M00053_1)

Phyla	Module completion ratio (%)									
	0--10	10--20	20--30	30--40	40--50	50--60	60--70	70--80	80--90	90--100
Gammaproteobacteria	1	0	0	0	0	0	6	17	48	54
Betaproteobacteria	0	0	1	0	0	0	0	5	42	13
Epsilonproteobacteria	0	0	0	0	0	0	4	11	2	0
Deltaproteobacteria	0	0	1	1	0	9	12	4	0	1
Alphaproteobacteria	1	0	0	0	0	0	5	45	38	2
Magnetococcus	0	0	0	0	0	1	0	0	0	0
Chrysiogenetes	0	0	0	0	0	0	1	0	0	0
Firmicutes	0	0	2	7	0	12	40	20	22	1
Tenericutes	0	4	4	5	0	4	2	0	0	0
Actinobacteria	0	0	0	0	0	3	11	22	37	7
Chlamydiae	0	0	0	0	0	0	0	7	1	0
Spirochaetes	0	0	1	4	0	5	3	1	0	0
Acidobacteria	0	0	0	0	0	0	3	2	0	0
Bacteroidetes	1	0	0	2	0	11	11	8	2	0
Fibrobacteres	0	0	0	0	0	0	0	1	0	0
Fusobacteria	0	0	0	0	0	2	2	0	1	0
Verrucomicrobia	0	0	0	0	0	0	3	1	0	0
Gemmatimonadetes	0	0	0	0	0	0	1	0	0	0
Planctomycetes	0	0	0	0	0	2	1	1	0	0
Elusimicrobia	0	0	0	0	0	0	2	0	0	0
Synergistetes	0	0	0	2	0	0	0	0	0	0
Cyanobacteria	0	0	0	0	0	2	12	2	0	0
Chlorobi	0	0	0	0	0	4	6	0	0	0
Chloroflexi	0	0	0	0	0	0	10	1	0	0
Deinococcus-Thermus	0	0	0	0	0	1	6	0	0	0
Aquificae	0	0	0	0	0	0	2	5	2	0
Thermotogae	0	0	6	0	0	5	0	0	0	0
Dictyoglomi	0	0	0	0	0	2	0	0	0	0
Nitrospirae	0	0	0	0	0	1	1	0	0	0
Thermobaculum	0	0	0	0	0	0	0	1	0	0
Deferribacteres	0	0	0	0	0	2	1	0	0	0
Euryarchaeota	0	0	0	0	0	5	14	23	18	0
Crenarchaeota	0	0	0	3	0	5	11	4	0	0
Thaumarchaeota	0	0	0	0	0	2	0	0	0	0
Nanoarchaeota	0	0	0	0	0	1	0	0	0	0
Korarchaeota	0	0	0	0	0	0	1	0	0	0
Total	3	4	15	24	0	79	171	181	213	78

(M00055_1)

Phyla	Module completion ratio (%)									
	0--10	10--20	20--30	30--40	40--50	50--60	60--70	70--80	80--90	90--100
Gammaproteobacteria	126	0	0	0	0	0	0	0	0	0
Betaproteobacteria	61	0	0	0	0	0	0	0	0	0
Epsilonproteobacteria	17	0	0	0	0	0	0	0	0	0
Deltaproteobacteria	28	0	0	0	0	0	0	0	0	0
Alphaproteobacteria	91	0	0	0	0	0	0	0	0	0
Magnetococcus	1	0	0	0	0	0	0	0	0	0
Chrysiogenetes	1	0	0	0	0	0	0	0	0	0
Firmicutes	104	0	0	0	0	0	0	0	0	0
Tenericutes	19	0	0	0	0	0	0	0	0	0
Actinobacteria	80	0	0	0	0	0	0	0	0	0
Chlamydiae	8	0	0	0	0	0	0	0	0	0
Spirochaetes	14	0	0	0	0	0	0	0	0	0
Acidobacteria	5	0	0	0	0	0	0	0	0	0
Bacteroidetes	35	0	0	0	0	0	0	0	0	0
Fibrobacteres	1	0	0	0	0	0	0	0	0	0
Fusobacteria	5	0	0	0	0	0	0	0	0	0
Verrucomicrobia	4	0	0	0	0	0	0	0	0	0
Gemmatimonadetes	1	0	0	0	0	0	0	0	0	0
Planctomycetes	4	0	0	0	0	0	0	0	0	0
Elusimicrobia	2	0	0	0	0	0	0	0	0	0
Synergistetes	2	0	0	0	0	0	0	0	0	0
Cyanobacteria	16	0	0	0	0	0	0	0	0	0
Chlorobi	10	0	0	0	0	0	0	0	0	0
Chloroflexi	11	0	0	0	0	0	0	0	0	0
Deinococcus-Thermus	7	0	0	0	0	0	0	0	0	0
Aquificae	9	0	0	0	0	0	0	0	0	0
Thermotogae	11	0	0	0	0	0	0	0	0	0
Dictyoglomi	2	0	0	0	0	0	0	0	0	0
Nitrospirae	2	0	0	0	0	0	0	0	0	0
Thermobaculum	1	0	0	0	0	0	0	0	0	0
Deferribacteres	3	0	0	0	0	0	0	0	0	0
Euryarchaeota	60	0	0	0	0	0	0	0	0	0
Crenarchaeota	23	0	0	0	0	0	0	0	0	0
Thaumarchaeota	2	0	0	0	0	0	0	0	0	0
Nanoarchaeota	1	0	0	0	0	0	0	0	0	0
Korarchaeota	1	0	0	0	0	0	0	0	0	0
Total	768	0	0	0	0	0	0	0	0	0

(M00056_1)

Phyla	Module completion ratio (%)									
	0--10	10--20	20--30	30--40	40--50	50--60	60--70	70--80	80--90	90--100
Gammaproteobacteria	126	0	0	0	0	0	0	0	0	0
Betaproteobacteria	61	0	0	0	0	0	0	0	0	0
Epsilonproteobacteria	17	0	0	0	0	0	0	0	0	0
Deltaproteobacteria	28	0	0	0	0	0	0	0	0	0
Alphaproteobacteria	91	0	0	0	0	0	0	0	0	0
Magnetococcus	1	0	0	0	0	0	0	0	0	0
Chrysiogenetes	1	0	0	0	0	0	0	0	0	0
Firmicutes	104	0	0	0	0	0	0	0	0	0
Tenericutes	19	0	0	0	0	0	0	0	0	0
Actinobacteria	80	0	0	0	0	0	0	0	0	0
Chlamydiae	8	0	0	0	0	0	0	0	0	0
Spirochaetes	14	0	0	0	0	0	0	0	0	0
Acidobacteria	5	0	0	0	0	0	0	0	0	0
Bacteroidetes	35	0	0	0	0	0	0	0	0	0
Fibrobacteres	1	0	0	0	0	0	0	0	0	0
Fusobacteria	5	0	0	0	0	0	0	0	0	0
Verrucomicrobia	4	0	0	0	0	0	0	0	0	0
Gemmatimonadetes	1	0	0	0	0	0	0	0	0	0
Planctomycetes	4	0	0	0	0	0	0	0	0	0
Elusimicrobia	2	0	0	0	0	0	0	0	0	0
Synergistetes	2	0	0	0	0	0	0	0	0	0
Cyanobacteria	16	0	0	0	0	0	0	0	0	0
Chlorobi	10	0	0	0	0	0	0	0	0	0
Chloroflexi	11	0	0	0	0	0	0	0	0	0
Deinococcus-Thermus	7	0	0	0	0	0	0	0	0	0
Aquificae	9	0	0	0	0	0	0	0	0	0
Thermotogae	11	0	0	0	0	0	0	0	0	0
Dictyoglomi	2	0	0	0	0	0	0	0	0	0
Nitrospirae	2	0	0	0	0	0	0	0	0	0
Thermobaculum	1	0	0	0	0	0	0	0	0	0
Deferribacteres	3	0	0	0	0	0	0	0	0	0
Euryarchaeota	60	0	0	0	0	0	0	0	0	0
Crenarchaeota	23	0	0	0	0	0	0	0	0	0
Thaumarchaeota	2	0	0	0	0	0	0	0	0	0
Nanoarchaeota	1	0	0	0	0	0	0	0	0	0
Korarchaeota	1	0	0	0	0	0	0	0	0	0
Total	768	0	0	0	0	0	0	0	0	0

(M00057_1)

Phyla	Module completion ratio (%)									
	0--10	10--20	20--30	30--40	40--50	50--60	60--70	70--80	80--90	90--100
Gammaproteobacteria	126	0	0	0	0	0	0	0	0	0
Betaproteobacteria	61	0	0	0	0	0	0	0	0	0
Epsilonproteobacteria	17	0	0	0	0	0	0	0	0	0
Deltaproteobacteria	28	0	0	0	0	0	0	0	0	0
Alphaproteobacteria	91	0	0	0	0	0	0	0	0	0
Magnetococcus	1	0	0	0	0	0	0	0	0	0
Chrysiogenetes	1	0	0	0	0	0	0	0	0	0
Firmicutes	104	0	0	0	0	0	0	0	0	0
Tenericutes	19	0	0	0	0	0	0	0	0	0
Actinobacteria	80	0	0	0	0	0	0	0	0	0
Chlamydiae	8	0	0	0	0	0	0	0	0	0
Spirochaetes	14	0	0	0	0	0	0	0	0	0
Acidobacteria	5	0	0	0	0	0	0	0	0	0
Bacteroidetes	35	0	0	0	0	0	0	0	0	0
Fibrobacteres	1	0	0	0	0	0	0	0	0	0
Fusobacteria	5	0	0	0	0	0	0	0	0	0
Verrucomicrobia	4	0	0	0	0	0	0	0	0	0
Gemmatimonadetes	1	0	0	0	0	0	0	0	0	0
Planctomycetes	4	0	0	0	0	0	0	0	0	0
Elusimicrobia	2	0	0	0	0	0	0	0	0	0
Synergistetes	2	0	0	0	0	0	0	0	0	0
Cyanobacteria	16	0	0	0	0	0	0	0	0	0
Chlorobi	10	0	0	0	0	0	0	0	0	0
Chloroflexi	11	0	0	0	0	0	0	0	0	0
Deinococcus-Thermus	7	0	0	0	0	0	0	0	0	0
Aquificae	9	0	0	0	0	0	0	0	0	0
Thermotogae	11	0	0	0	0	0	0	0	0	0
Dictyoglomi	2	0	0	0	0	0	0	0	0	0
Nitrospirae	2	0	0	0	0	0	0	0	0	0
Thermobaculum	1	0	0	0	0	0	0	0	0	0
Deferribacteres	3	0	0	0	0	0	0	0	0	0
Euryarchaeota	60	0	0	0	0	0	0	0	0	0
Crenarchaeota	23	0	0	0	0	0	0	0	0	0
Thaumarchaeota	2	0	0	0	0	0	0	0	0	0
Nanoarchaeota	1	0	0	0	0	0	0	0	0	0
Korarchaeota	1	0	0	0	0	0	0	0	0	0
Total	768	0	0	0	0	0	0	0	0	0

(M00058_1)

Phyla	Module completion ratio (%)									
	0--10	10--20	20--30	30--40	40--50	50--60	60--70	70--80	80--90	90--100
Gammaproteobacteria	126	0	0	0	0	0	0	0	0	0
Betaproteobacteria	61	0	0	0	0	0	0	0	0	0
Epsilonproteobacteria	17	0	0	0	0	0	0	0	0	0
Deltaproteobacteria	28	0	0	0	0	0	0	0	0	0
Alphaproteobacteria	91	0	0	0	0	0	0	0	0	0
Magnetococcus	1	0	0	0	0	0	0	0	0	0
Chrysiogenetes	1	0	0	0	0	0	0	0	0	0
Firmicutes	104	0	0	0	0	0	0	0	0	0
Tenericutes	19	0	0	0	0	0	0	0	0	0
Actinobacteria	80	0	0	0	0	0	0	0	0	0
Chlamydiae	8	0	0	0	0	0	0	0	0	0
Spirochaetes	14	0	0	0	0	0	0	0	0	0
Acidobacteria	5	0	0	0	0	0	0	0	0	0
Bacteroidetes	35	0	0	0	0	0	0	0	0	0
Fibrobacteres	1	0	0	0	0	0	0	0	0	0
Fusobacteria	5	0	0	0	0	0	0	0	0	0
Verrucomicrobia	4	0	0	0	0	0	0	0	0	0
Gemmatimonadetes	1	0	0	0	0	0	0	0	0	0
Planctomycetes	4	0	0	0	0	0	0	0	0	0
Elusimicrobia	2	0	0	0	0	0	0	0	0	0
Synergistetes	2	0	0	0	0	0	0	0	0	0
Cyanobacteria	16	0	0	0	0	0	0	0	0	0
Chlorobi	10	0	0	0	0	0	0	0	0	0
Chloroflexi	11	0	0	0	0	0	0	0	0	0
Deinococcus-Thermus	7	0	0	0	0	0	0	0	0	0
Aquificae	9	0	0	0	0	0	0	0	0	0
Thermotogae	11	0	0	0	0	0	0	0	0	0
Dictyoglomi	2	0	0	0	0	0	0	0	0	0
Nitrospirae	2	0	0	0	0	0	0	0	0	0
Thermobaculum	1	0	0	0	0	0	0	0	0	0
Deferribacteres	3	0	0	0	0	0	0	0	0	0
Euryarchaeota	60	0	0	0	0	0	0	0	0	0
Crenarchaeota	23	0	0	0	0	0	0	0	0	0
Thaumarchaeota	2	0	0	0	0	0	0	0	0	0
Nanoarchaeota	1	0	0	0	0	0	0	0	0	0
Korarchaeota	1	0	0	0	0	0	0	0	0	0
Total	768	0	0	0	0	0	0	0	0	0

(M00059_1)

Phyla	Module completion ratio (%)									
	0--10	10--20	20--30	30--40	40--50	50--60	60--70	70--80	80--90	90--100
Gammaproteobacteria	126	0	0	0	0	0	0	0	0	0
Betaproteobacteria	61	0	0	0	0	0	0	0	0	0
Epsilonproteobacteria	17	0	0	0	0	0	0	0	0	0
Deltaproteobacteria	28	0	0	0	0	0	0	0	0	0
Alphaproteobacteria	91	0	0	0	0	0	0	0	0	0
Magnetococcus	1	0	0	0	0	0	0	0	0	0
Chrysiogenetes	1	0	0	0	0	0	0	0	0	0
Firmicutes	104	0	0	0	0	0	0	0	0	0
Tenericutes	19	0	0	0	0	0	0	0	0	0
Actinobacteria	80	0	0	0	0	0	0	0	0	0
Chlamydiae	8	0	0	0	0	0	0	0	0	0
Spirochaetes	14	0	0	0	0	0	0	0	0	0
Acidobacteria	5	0	0	0	0	0	0	0	0	0
Bacteroidetes	35	0	0	0	0	0	0	0	0	0
Fibrobacteres	1	0	0	0	0	0	0	0	0	0
Fusobacteria	5	0	0	0	0	0	0	0	0	0
Verrucomicrobia	4	0	0	0	0	0	0	0	0	0
Gemmatimonadetes	1	0	0	0	0	0	0	0	0	0
Planctomycetes	4	0	0	0	0	0	0	0	0	0
Elusimicrobia	2	0	0	0	0	0	0	0	0	0
Synergistetes	2	0	0	0	0	0	0	0	0	0
Cyanobacteria	16	0	0	0	0	0	0	0	0	0
Chlorobi	10	0	0	0	0	0	0	0	0	0
Chloroflexi	11	0	0	0	0	0	0	0	0	0
Deinococcus-Thermus	7	0	0	0	0	0	0	0	0	0
Aquificae	9	0	0	0	0	0	0	0	0	0
Thermotogae	11	0	0	0	0	0	0	0	0	0
Dictyoglomi	2	0	0	0	0	0	0	0	0	0
Nitrospirae	2	0	0	0	0	0	0	0	0	0
Thermobaculum	1	0	0	0	0	0	0	0	0	0
Deferribacteres	3	0	0	0	0	0	0	0	0	0
Euryarchaeota	60	0	0	0	0	0	0	0	0	0
Crenarchaeota	23	0	0	0	0	0	0	0	0	0
Thaumarchaeota	2	0	0	0	0	0	0	0	0	0
Nanoarchaeota	1	0	0	0	0	0	0	0	0	0
Korarchaeota	1	0	0	0	0	0	0	0	0	0
Total	768	0	0	0	0	0	0	0	0	0

(M00060_1)

Phyla	Module completion ratio (%)									
	0--10	10--20	20--30	30--40	40--50	50--60	60--70	70--80	80--90	90--100
Gammaproteobacteria	4	0	0	0	0	0	0	5	50	67
Betaproteobacteria	1	3	0	0	0	0	0	0	57	0
Epsilonproteobacteria	0	0	0	0	0	0	1	0	16	0
Deltaproteobacteria	1	1	0	0	0	0	2	17	7	0
Alphaproteobacteria	15	3	1	0	0	0	4	68	0	0
Magnetococcus	0	0	0	0	0	0	0	1	0	0
Chrysiogenetes	0	0	0	0	0	0	0	1	0	0
Firmicutes	98	1	0	0	0	1	0	4	0	0
Tenericutes	19	0	0	0	0	0	0	0	0	0
Actinobacteria	27	49	4	0	0	0	0	0	0	0
Chlamydiae	0	0	0	0	0	0	2	6	0	0
Spirochaetes	10	0	0	0	0	0	1	3	0	0
Acidobacteria	0	0	0	0	0	1	0	4	0	0
Bacteroidetes	1	4	0	0	0	0	1	0	29	0
Fibrobacteres	0	0	0	0	0	0	0	1	0	0
Fusobacteria	1	0	0	0	0	0	0	4	0	0
Verrucomicrobia	0	0	0	0	0	0	1	3	0	0
Gemmatimonadetes	0	0	0	0	0	0	0	0	1	0
Planctomycetes	0	0	0	0	0	0	0	2	2	0
Elusimicrobia	1	0	0	0	0	0	0	1	0	0
Synergistetes	0	0	0	0	0	0	0	2	0	0
Cyanobacteria	0	0	0	0	16	0	0	0	0	0
Chlorobi	0	0	0	0	0	0	0	3	7	0
Chloroflexi	7	4	0	0	0	0	0	0	0	0
Deinococcus-Thermus	5	0	0	0	2	0	0	0	0	0
Aquificae	0	0	0	0	0	0	4	5	0	0
Thermotogae	11	0	0	0	0	0	0	0	0	0
Dictyoglomi	0	0	0	0	2	0	0	0	0	0
Nitrospirae	0	0	0	0	0	0	0	2	0	0
Thermobaculum	0	1	0	0	0	0	0	0	0	0
Deferribacteres	0	0	0	0	0	0	0	3	0	0
Euryarchaeota	58	2	0	0	0	0	0	0	0	0
Crenarchaeota	23	0	0	0	0	0	0	0	0	0
Thaumarchaeota	2	0	0	0	0	0	0	0	0	0
Nanoarchaeota	1	0	0	0	0	0	0	0	0	0
Korarchaeota	1	0	0	0	0	0	0	0	0	0
Total	286	68	5	0	20	2	16	135	169	67

(M00061_1)

Phyla	Module completion ratio (%)									
	0--10	10--20	20--30	30--40	40--50	50--60	60--70	70--80	80--90	90--100
Gammaproteobacteria	73	8	0	2	0	7	8	0	7	21
Betaproteobacteria	33	15	0	9	0	3	1	0	0	0
Epsilonproteobacteria	15	2	0	0	0	0	0	0	0	0
Deltaproteobacteria	22	4	0	1	0	1	0	0	0	0
Alphaproteobacteria	46	9	0	6	0	11	12	0	5	2
Magnetococcus	1	0	0	0	0	0	0	0	0	0
Chrysiogenetes	1	0	0	0	0	0	0	0	0	0
Firmicutes	61	10	0	2	0	8	7	0	8	8
Tenericutes	19	0	0	0	0	0	0	0	0	0
Actinobacteria	50	9	0	3	0	11	5	0	1	1
Chlamydiae	8	0	0	0	0	0	0	0	0	0
Spirochaetes	12	1	0	0	0	0	1	0	0	0
Acidobacteria	0	0	0	3	0	1	1	0	0	0
Bacteroidetes	13	0	0	3	0	5	0	0	12	2
Fibrobacteres	1	0	0	0	0	0	0	0	0	0
Fusobacteria	2	1	0	0	0	1	0	0	1	0
Verrucomicrobia	2	0	0	0	0	1	0	0	1	0
Gemmatimonadetes	1	0	0	0	0	0	0	0	0	0
Planctomycetes	0	0	0	2	0	2	0	0	0	0
Elusimicrobia	1	0	0	0	0	1	0	0	0	0
Synergistetes	1	1	0	0	0	0	0	0	0	0
Cyanobacteria	15	0	0	1	0	0	0	0	0	0
Chlorobi	10	0	0	0	0	0	0	0	0	0
Chloroflexi	10	1	0	0	0	0	0	0	0	0
Deinococcus-Thermus	4	2	0	1	0	0	0	0	0	0
Aquificae	9	0	0	0	0	0	0	0	0	0
Thermotogae	4	0	0	2	0	0	4	0	1	0
Dictyoglomi	1	1	0	0	0	0	0	0	0	0
Nitrospirae	2	0	0	0	0	0	0	0	0	0
Thermobaculum	0	1	0	0	0	0	0	0	0	0
Deferribacteres	3	0	0	0	0	0	0	0	0	0
Euryarchaeota	51	4	0	3	0	1	1	0	0	0
Crenarchaeota	16	5	0	1	0	0	1	0	0	0
Thaumarchaeota	2	0	0	0	0	0	0	0	0	0
Nanoarchaeota	1	0	0	0	0	0	0	0	0	0
Korarchaeota	1	0	0	0	0	0	0	0	0	0
Total	491	74	0	39	0	53	41	0	36	34

(M00063_1)

Phyla	Module completion ratio (%)									
	0--10	10--20	20--30	30--40	40--50	50--60	60--70	70--80	80--90	90--100
Gammaproteobacteria	3	0	0	1	0	0	7	0	0	115
Betaproteobacteria	1	0	0	2	0	0	5	0	0	53
Epsilonproteobacteria	0	0	0	0	0	0	0	0	0	17
Deltaproteobacteria	1	0	0	0	0	0	1	0	0	26
Alphaproteobacteria	15	0	0	0	0	0	76	0	0	0
Magnetococcus	0	0	0	0	0	0	0	0	0	1
Chrysiogenetes	0	0	0	0	0	0	0	0	0	1
Firmicutes	97	0	0	2	0	0	0	0	0	5
Tenericutes	19	0	0	0	0	0	0	0	0	0
Actinobacteria	80	0	0	0	0	0	0	0	0	0
Chlamydiae	0	0	0	0	0	0	8	0	0	0
Spirochaetes	10	0	0	0	0	0	3	0	0	1
Acidobacteria	0	0	0	0	0	0	0	0	0	5
Bacteroidetes	4	0	0	2	0	0	4	0	0	25
Fibrobacteres	0	0	0	1	0	0	0	0	0	0
Fusobacteria	1	0	0	0	0	0	0	0	0	4
Verrucomicrobia	0	0	0	0	0	0	3	0	0	1
Gemmatimonadetes	0	0	0	0	0	0	0	0	0	1
Planctomycetes	0	0	0	0	0	0	1	0	0	3
Elusimicrobia	0	0	0	1	0	0	0	0	0	1
Synergistetes	0	0	0	0	0	0	0	0	0	2
Cyanobacteria	13	0	0	0	0	0	0	0	0	3
Chlorobi	0	0	0	0	0	0	0	0	0	10
Chloroflexi	11	0	0	0	0	0	0	0	0	0
Deinococcus-Thermus	7	0	0	0	0	0	0	0	0	0
Aquificae	0	0	0	0	0	0	0	0	0	9
Thermotogae	11	0	0	0	0	0	0	0	0	0
Dictyoglomi	2	0	0	0	0	0	0	0	0	0
Nitrospirae	0	0	0	0	0	0	1	0	0	1
Thermobaculum	1	0	0	0	0	0	0	0	0	0
Deferribacteres	0	0	0	0	0	0	0	0	0	3
Euryarchaeota	60	0	0	0	0	0	0	0	0	0
Crenarchaeota	23	0	0	0	0	0	0	0	0	0
Thaumarchaeota	2	0	0	0	0	0	0	0	0	0
Nanoarchaeota	1	0	0	0	0	0	0	0	0	0
Korarchaeota	1	0	0	0	0	0	0	0	0	0
Total	363	0	0	9	0	0	109	0	0	287

(M00064_1)

Phyla	Module completion ratio (%)									
	0--10	10--20	20--30	30--40	40--50	50--60	60--70	70--80	80--90	90--100
Gammaproteobacteria	22	0	8	0	0	16	0	22	0	58
Betaproteobacteria	1	0	1	0	0	19	0	34	0	6
Epsilonproteobacteria	1	0	1	0	0	0	0	1	0	14
Deltaproteobacteria	6	0	4	0	0	5	0	10	0	3
Alphaproteobacteria	58	0	3	0	0	10	0	11	0	9
Magnetococcus	0	0	0	0	0	0	0	0	0	1
Chrysiogenetes	0	0	0	0	0	0	0	0	0	1
Firmicutes	93	0	6	0	0	3	0	0	0	2
Tenericutes	19	0	0	0	0	0	0	0	0	0
Actinobacteria	55	0	20	0	0	4	0	1	0	0
Chlamydiae	6	0	0	0	0	0	0	2	0	0
Spirochaetes	9	0	1	0	0	3	0	1	0	0
Acidobacteria	0	0	0	0	0	1	0	1	0	3
Bacteroidetes	25	0	2	0	0	4	0	4	0	0
Fibrobacteres	1	0	0	0	0	0	0	0	0	0
Fusobacteria	1	0	0	0	0	2	0	2	0	0
Verrucomicrobia	1	0	0	0	0	1	0	1	0	1
Gemmatimonadetes	0	0	0	0	0	0	0	0	0	1
Planctomycetes	1	0	1	0	0	1	0	1	0	0
Elusimicrobia	1	0	0	0	0	0	0	1	0	0
Synergistetes	0	0	0	0	0	0	0	2	0	0
Cyanobacteria	7	0	5	0	0	4	0	0	0	0
Chlorobi	0	0	0	0	0	0	0	10	0	0
Chloroflexi	2	0	2	0	0	6	0	1	0	0
Deinococcus-Thermus	7	0	0	0	0	0	0	0	0	0
Aquificae	0	0	0	0	0	4	0	5	0	0
Thermotogae	11	0	0	0	0	0	0	0	0	0
Dictyoglomi	0	0	2	0	0	0	0	0	0	0
Nitrospirae	0	0	1	0	0	1	0	0	0	0
Thermobaculum	0	0	0	0	0	0	0	1	0	0
Deferribacteres	0	0	0	0	0	1	0	1	0	1
Euryarchaeota	51	0	4	0	0	2	0	3	0	0
Crenarchaeota	23	0	0	0	0	0	0	0	0	0
Thaumarchaeota	2	0	0	0	0	0	0	0	0	0
Nanoarchaeota	1	0	0	0	0	0	0	0	0	0
Korarchaeota	1	0	0	0	0	0	0	0	0	0
Total	405	0	61	0	0	87	0	115	0	100

(M00065_1)

Phyla	Module completion ratio (%)									
	0--10	10--20	20--30	30--40	40--50	50--60	60--70	70--80	80--90	90--100
Gammaproteobacteria	126	0	0	0	0	0	0	0	0	0
Betaproteobacteria	61	0	0	0	0	0	0	0	0	0
Epsilonproteobacteria	17	0	0	0	0	0	0	0	0	0
Deltaproteobacteria	28	0	0	0	0	0	0	0	0	0
Alphaproteobacteria	91	0	0	0	0	0	0	0	0	0
Magnetococcus	1	0	0	0	0	0	0	0	0	0
Chrysiogenetes	1	0	0	0	0	0	0	0	0	0
Firmicutes	104	0	0	0	0	0	0	0	0	0
Tenericutes	19	0	0	0	0	0	0	0	0	0
Actinobacteria	80	0	0	0	0	0	0	0	0	0
Chlamydiae	8	0	0	0	0	0	0	0	0	0
Spirochaetes	14	0	0	0	0	0	0	0	0	0
Acidobacteria	5	0	0	0	0	0	0	0	0	0
Bacteroidetes	35	0	0	0	0	0	0	0	0	0
Fibrobacteres	1	0	0	0	0	0	0	0	0	0
Fusobacteria	5	0	0	0	0	0	0	0	0	0
Verrucomicrobia	4	0	0	0	0	0	0	0	0	0
Gemmatimonadetes	1	0	0	0	0	0	0	0	0	0
Planctomycetes	4	0	0	0	0	0	0	0	0	0
Elusimicrobia	2	0	0	0	0	0	0	0	0	0
Synergistetes	2	0	0	0	0	0	0	0	0	0
Cyanobacteria	16	0	0	0	0	0	0	0	0	0
Chlorobi	10	0	0	0	0	0	0	0	0	0
Chloroflexi	11	0	0	0	0	0	0	0	0	0
Deinococcus-Thermus	7	0	0	0	0	0	0	0	0	0
Aquificae	9	0	0	0	0	0	0	0	0	0
Thermotogae	11	0	0	0	0	0	0	0	0	0
Dictyoglomi	2	0	0	0	0	0	0	0	0	0
Nitrospirae	2	0	0	0	0	0	0	0	0	0
Thermobaculum	1	0	0	0	0	0	0	0	0	0
Deferribacteres	3	0	0	0	0	0	0	0	0	0
Euryarchaeota	60	0	0	0	0	0	0	0	0	0
Crenarchaeota	23	0	0	0	0	0	0	0	0	0
Thaumarchaeota	2	0	0	0	0	0	0	0	0	0
Nanoarchaeota	1	0	0	0	0	0	0	0	0	0
Korarchaeota	1	0	0	0	0	0	0	0	0	0
Total	768	0	0	0	0	0	0	0	0	0

(M00066_1)

Phyla	Module completion ratio (%)									
	0--10	10--20	20--30	30--40	40--50	50--60	60--70	70--80	80--90	90--100
Gammaproteobacteria	118	0	0	0	0	8	0	0	0	0
Betaproteobacteria	46	0	0	0	0	15	0	0	0	0
Epsilonproteobacteria	17	0	0	0	0	0	0	0	0	0
Deltaproteobacteria	17	0	0	0	0	11	0	0	0	0
Alphaproteobacteria	70	0	0	0	0	21	0	0	0	0
Magnetococcus	1	0	0	0	0	0	0	0	0	0
Chrysiogenetes	1	0	0	0	0	0	0	0	0	0
Firmicutes	103	0	0	0	0	1	0	0	0	0
Tenericutes	19	0	0	0	0	0	0	0	0	0
Actinobacteria	80	0	0	0	0	0	0	0	0	0
Chlamydiae	8	0	0	0	0	0	0	0	0	0
Spirochaetes	14	0	0	0	0	0	0	0	0	0
Acidobacteria	0	0	0	0	0	5	0	0	0	0
Bacteroidetes	35	0	0	0	0	0	0	0	0	0
Fibrobacteres	1	0	0	0	0	0	0	0	0	0
Fusobacteria	2	0	0	0	0	3	0	0	0	0
Verrucomicrobia	4	0	0	0	0	0	0	0	0	0
Gemmatimonadetes	1	0	0	0	0	0	0	0	0	0
Planctomycetes	3	0	0	0	0	1	0	0	0	0
Elusimicrobia	2	0	0	0	0	0	0	0	0	0
Synergistetes	2	0	0	0	0	0	0	0	0	0
Cyanobacteria	11	0	0	0	0	5	0	0	0	0
Chlorobi	10	0	0	0	0	0	0	0	0	0
Chloroflexi	10	0	0	0	0	1	0	0	0	0
Deinococcus-Thermus	7	0	0	0	0	0	0	0	0	0
Aquificae	9	0	0	0	0	0	0	0	0	0
Thermotogae	11	0	0	0	0	0	0	0	0	0
Dictyoglomi	2	0	0	0	0	0	0	0	0	0
Nitrospirae	1	0	0	0	0	1	0	0	0	0
Thermobaculum	1	0	0	0	0	0	0	0	0	0
Deferribacteres	3	0	0	0	0	0	0	0	0	0
Euryarchaeota	60	0	0	0	0	0	0	0	0	0
Crenarchaeota	23	0	0	0	0	0	0	0	0	0
Thaumarchaeota	2	0	0	0	0	0	0	0	0	0
Nanoarchaeota	1	0	0	0	0	0	0	0	0	0
Korarchaeota	1	0	0	0	0	0	0	0	0	0
Total	696	0	0	0	0	72	0	0	0	0

(M00067_1)

Phyla	Module completion ratio (%)									
	0--10	10--20	20--30	30--40	40--50	50--60	60--70	70--80	80--90	90--100
Gammaproteobacteria	126	0	0	0	0	0	0	0	0	0
Betaproteobacteria	61	0	0	0	0	0	0	0	0	0
Epsilonproteobacteria	17	0	0	0	0	0	0	0	0	0
Deltaproteobacteria	28	0	0	0	0	0	0	0	0	0
Alphaproteobacteria	91	0	0	0	0	0	0	0	0	0
Magnetococcus	1	0	0	0	0	0	0	0	0	0
Chrysiogenetes	1	0	0	0	0	0	0	0	0	0
Firmicutes	104	0	0	0	0	0	0	0	0	0
Tenericutes	19	0	0	0	0	0	0	0	0	0
Actinobacteria	80	0	0	0	0	0	0	0	0	0
Chlamydiae	8	0	0	0	0	0	0	0	0	0
Spirochaetes	14	0	0	0	0	0	0	0	0	0
Acidobacteria	5	0	0	0	0	0	0	0	0	0
Bacteroidetes	35	0	0	0	0	0	0	0	0	0
Fibrobacteres	1	0	0	0	0	0	0	0	0	0
Fusobacteria	5	0	0	0	0	0	0	0	0	0
Verrucomicrobia	4	0	0	0	0	0	0	0	0	0
Gemmatimonadetes	1	0	0	0	0	0	0	0	0	0
Planctomycetes	4	0	0	0	0	0	0	0	0	0
Elusimicrobia	2	0	0	0	0	0	0	0	0	0
Synergistetes	2	0	0	0	0	0	0	0	0	0
Cyanobacteria	16	0	0	0	0	0	0	0	0	0
Chlorobi	10	0	0	0	0	0	0	0	0	0
Chloroflexi	11	0	0	0	0	0	0	0	0	0
Deinococcus-Thermus	7	0	0	0	0	0	0	0	0	0
Aquificae	9	0	0	0	0	0	0	0	0	0
Thermotogae	11	0	0	0	0	0	0	0	0	0
Dictyoglomi	2	0	0	0	0	0	0	0	0	0
Nitrospirae	2	0	0	0	0	0	0	0	0	0
Thermobaculum	1	0	0	0	0	0	0	0	0	0
Deferribacteres	3	0	0	0	0	0	0	0	0	0
Euryarchaeota	60	0	0	0	0	0	0	0	0	0
Crenarchaeota	23	0	0	0	0	0	0	0	0	0
Thaumarchaeota	2	0	0	0	0	0	0	0	0	0
Nanoarchaeota	1	0	0	0	0	0	0	0	0	0
Korarchaeota	1	0	0	0	0	0	0	0	0	0
Total	768	0	0	0	0	0	0	0	0	0

(M00068_1)

Phyla	Module completion ratio (%)									
	0--10	10--20	20--30	30--40	40--50	50--60	60--70	70--80	80--90	90--100
Gammaproteobacteria	126	0	0	0	0	0	0	0	0	0
Betaproteobacteria	61	0	0	0	0	0	0	0	0	0
Epsilonproteobacteria	17	0	0	0	0	0	0	0	0	0
Deltaproteobacteria	28	0	0	0	0	0	0	0	0	0
Alphaproteobacteria	91	0	0	0	0	0	0	0	0	0
Magnetococcus	1	0	0	0	0	0	0	0	0	0
Chrysiogenetes	1	0	0	0	0	0	0	0	0	0
Firmicutes	104	0	0	0	0	0	0	0	0	0
Tenericutes	19	0	0	0	0	0	0	0	0	0
Actinobacteria	80	0	0	0	0	0	0	0	0	0
Chlamydiae	8	0	0	0	0	0	0	0	0	0
Spirochaetes	14	0	0	0	0	0	0	0	0	0
Acidobacteria	5	0	0	0	0	0	0	0	0	0
Bacteroidetes	35	0	0	0	0	0	0	0	0	0
Fibrobacteres	1	0	0	0	0	0	0	0	0	0
Fusobacteria	5	0	0	0	0	0	0	0	0	0
Verrucomicrobia	4	0	0	0	0	0	0	0	0	0
Gemmatimonadetes	1	0	0	0	0	0	0	0	0	0
Planctomycetes	4	0	0	0	0	0	0	0	0	0
Elusimicrobia	2	0	0	0	0	0	0	0	0	0
Synergistetes	2	0	0	0	0	0	0	0	0	0
Cyanobacteria	16	0	0	0	0	0	0	0	0	0
Chlorobi	10	0	0	0	0	0	0	0	0	0
Chloroflexi	11	0	0	0	0	0	0	0	0	0
Deinococcus-Thermus	7	0	0	0	0	0	0	0	0	0
Aquificae	9	0	0	0	0	0	0	0	0	0
Thermotogae	11	0	0	0	0	0	0	0	0	0
Dictyoglomi	2	0	0	0	0	0	0	0	0	0
Nitrospirae	2	0	0	0	0	0	0	0	0	0
Thermobaculum	1	0	0	0	0	0	0	0	0	0
Deferribacteres	3	0	0	0	0	0	0	0	0	0
Euryarchaeota	60	0	0	0	0	0	0	0	0	0
Crenarchaeota	23	0	0	0	0	0	0	0	0	0
Thaumarchaeota	2	0	0	0	0	0	0	0	0	0
Nanoarchaeota	1	0	0	0	0	0	0	0	0	0
Korarchaeota	1	0	0	0	0	0	0	0	0	0
Total	768	0	0	0	0	0	0	0	0	0

(M00069_1)

Phyla	Module completion ratio (%)									
	0--10	10--20	20--30	30--40	40--50	50--60	60--70	70--80	80--90	90--100
Gammaproteobacteria	126	0	0	0	0	0	0	0	0	0
Betaproteobacteria	61	0	0	0	0	0	0	0	0	0
Epsilonproteobacteria	17	0	0	0	0	0	0	0	0	0
Deltaproteobacteria	28	0	0	0	0	0	0	0	0	0
Alphaproteobacteria	91	0	0	0	0	0	0	0	0	0
Magnetococcus	1	0	0	0	0	0	0	0	0	0
Chrysiogenetes	1	0	0	0	0	0	0	0	0	0
Firmicutes	104	0	0	0	0	0	0	0	0	0
Tenericutes	19	0	0	0	0	0	0	0	0	0
Actinobacteria	80	0	0	0	0	0	0	0	0	0
Chlamydiae	8	0	0	0	0	0	0	0	0	0
Spirochaetes	14	0	0	0	0	0	0	0	0	0
Acidobacteria	5	0	0	0	0	0	0	0	0	0
Bacteroidetes	35	0	0	0	0	0	0	0	0	0
Fibrobacteres	1	0	0	0	0	0	0	0	0	0
Fusobacteria	5	0	0	0	0	0	0	0	0	0
Verrucomicrobia	4	0	0	0	0	0	0	0	0	0
Gemmatimonadetes	1	0	0	0	0	0	0	0	0	0
Planctomycetes	4	0	0	0	0	0	0	0	0	0
Elusimicrobia	2	0	0	0	0	0	0	0	0	0
Synergistetes	2	0	0	0	0	0	0	0	0	0
Cyanobacteria	16	0	0	0	0	0	0	0	0	0
Chlorobi	10	0	0	0	0	0	0	0	0	0
Chloroflexi	11	0	0	0	0	0	0	0	0	0
Deinococcus-Thermus	7	0	0	0	0	0	0	0	0	0
Aquificae	9	0	0	0	0	0	0	0	0	0
Thermotogae	11	0	0	0	0	0	0	0	0	0
Dictyoglomi	2	0	0	0	0	0	0	0	0	0
Nitrospirae	2	0	0	0	0	0	0	0	0	0
Thermobaculum	1	0	0	0	0	0	0	0	0	0
Deferribacteres	3	0	0	0	0	0	0	0	0	0
Euryarchaeota	60	0	0	0	0	0	0	0	0	0
Crenarchaeota	23	0	0	0	0	0	0	0	0	0
Thaumarchaeota	2	0	0	0	0	0	0	0	0	0
Nanoarchaeota	1	0	0	0	0	0	0	0	0	0
Korarchaeota	1	0	0	0	0	0	0	0	0	0
Total	768	0	0	0	0	0	0	0	0	0

(M00070_1)

Phyla	Module completion ratio (%)									
	0--10	10--20	20--30	30--40	40--50	50--60	60--70	70--80	80--90	90--100
Gammaproteobacteria	126	0	0	0	0	0	0	0	0	0
Betaproteobacteria	61	0	0	0	0	0	0	0	0	0
Epsilonproteobacteria	17	0	0	0	0	0	0	0	0	0
Deltaproteobacteria	28	0	0	0	0	0	0	0	0	0
Alphaproteobacteria	91	0	0	0	0	0	0	0	0	0
Magnetococcus	1	0	0	0	0	0	0	0	0	0
Chrysiogenetes	1	0	0	0	0	0	0	0	0	0
Firmicutes	104	0	0	0	0	0	0	0	0	0
Tenericutes	19	0	0	0	0	0	0	0	0	0
Actinobacteria	80	0	0	0	0	0	0	0	0	0
Chlamydiae	8	0	0	0	0	0	0	0	0	0
Spirochaetes	14	0	0	0	0	0	0	0	0	0
Acidobacteria	5	0	0	0	0	0	0	0	0	0
Bacteroidetes	35	0	0	0	0	0	0	0	0	0
Fibrobacteres	1	0	0	0	0	0	0	0	0	0
Fusobacteria	5	0	0	0	0	0	0	0	0	0
Verrucomicrobia	4	0	0	0	0	0	0	0	0	0
Gemmatimonadetes	1	0	0	0	0	0	0	0	0	0
Planctomycetes	4	0	0	0	0	0	0	0	0	0
Elusimicrobia	2	0	0	0	0	0	0	0	0	0
Synergistetes	2	0	0	0	0	0	0	0	0	0
Cyanobacteria	16	0	0	0	0	0	0	0	0	0
Chlorobi	10	0	0	0	0	0	0	0	0	0
Chloroflexi	11	0	0	0	0	0	0	0	0	0
Deinococcus-Thermus	7	0	0	0	0	0	0	0	0	0
Aquificae	9	0	0	0	0	0	0	0	0	0
Thermotogae	11	0	0	0	0	0	0	0	0	0
Dictyoglomi	2	0	0	0	0	0	0	0	0	0
Nitrospirae	2	0	0	0	0	0	0	0	0	0
Thermobaculum	1	0	0	0	0	0	0	0	0	0
Deferribacteres	3	0	0	0	0	0	0	0	0	0
Euryarchaeota	60	0	0	0	0	0	0	0	0	0
Crenarchaeota	23	0	0	0	0	0	0	0	0	0
Thaumarchaeota	2	0	0	0	0	0	0	0	0	0
Nanoarchaeota	1	0	0	0	0	0	0	0	0	0
Korarchaeota	1	0	0	0	0	0	0	0	0	0
Total	768	0	0	0	0	0	0	0	0	0

(M00071_1)

Phyla	Module completion ratio (%)									
	0--10	10--20	20--30	30--40	40--50	50--60	60--70	70--80	80--90	90--100
Gammaproteobacteria	126	0	0	0	0	0	0	0	0	0
Betaproteobacteria	61	0	0	0	0	0	0	0	0	0
Epsilonproteobacteria	17	0	0	0	0	0	0	0	0	0
Deltaproteobacteria	28	0	0	0	0	0	0	0	0	0
Alphaproteobacteria	91	0	0	0	0	0	0	0	0	0
Magnetococcus	1	0	0	0	0	0	0	0	0	0
Chrysiogenetes	1	0	0	0	0	0	0	0	0	0
Firmicutes	104	0	0	0	0	0	0	0	0	0
Tenericutes	19	0	0	0	0	0	0	0	0	0
Actinobacteria	80	0	0	0	0	0	0	0	0	0
Chlamydiae	8	0	0	0	0	0	0	0	0	0
Spirochaetes	14	0	0	0	0	0	0	0	0	0
Acidobacteria	5	0	0	0	0	0	0	0	0	0
Bacteroidetes	35	0	0	0	0	0	0	0	0	0
Fibrobacteres	1	0	0	0	0	0	0	0	0	0
Fusobacteria	5	0	0	0	0	0	0	0	0	0
Verrucomicrobia	4	0	0	0	0	0	0	0	0	0
Gemmatimonadetes	1	0	0	0	0	0	0	0	0	0
Planctomycetes	4	0	0	0	0	0	0	0	0	0
Elusimicrobia	2	0	0	0	0	0	0	0	0	0
Synergistetes	2	0	0	0	0	0	0	0	0	0
Cyanobacteria	16	0	0	0	0	0	0	0	0	0
Chlorobi	10	0	0	0	0	0	0	0	0	0
Chloroflexi	11	0	0	0	0	0	0	0	0	0
Deinococcus-Thermus	7	0	0	0	0	0	0	0	0	0
Aquificae	9	0	0	0	0	0	0	0	0	0
Thermotogae	11	0	0	0	0	0	0	0	0	0
Dictyoglomi	2	0	0	0	0	0	0	0	0	0
Nitrospirae	2	0	0	0	0	0	0	0	0	0
Thermobaculum	1	0	0	0	0	0	0	0	0	0
Deferribacteres	3	0	0	0	0	0	0	0	0	0
Euryarchaeota	60	0	0	0	0	0	0	0	0	0
Crenarchaeota	23	0	0	0	0	0	0	0	0	0
Thaumarchaeota	2	0	0	0	0	0	0	0	0	0
Nanoarchaeota	1	0	0	0	0	0	0	0	0	0
Korarchaeota	1	0	0	0	0	0	0	0	0	0
Total	768	0	0	0	0	0	0	0	0	0

(M00072_1)

Phyla	Module completion ratio (%)									
	0--10	10--20	20--30	30--40	40--50	50--60	60--70	70--80	80--90	90--100
Gammaproteobacteria	125	1	0	0	0	0	0	0	0	0
Betaproteobacteria	61	0	0	0	0	0	0	0	0	0
Epsilonproteobacteria	4	13	0	0	0	0	0	0	0	0
Deltaproteobacteria	21	7	0	0	0	0	0	0	0	0
Alphaproteobacteria	90	1	0	0	0	0	0	0	0	0
Magnetococcus	1	0	0	0	0	0	0	0	0	0
Chrysiogenetes	1	0	0	0	0	0	0	0	0	0
Firmicutes	104	0	0	0	0	0	0	0	0	0
Tenericutes	19	0	0	0	0	0	0	0	0	0
Actinobacteria	80	0	0	0	0	0	0	0	0	0
Chlamydiae	8	0	0	0	0	0	0	0	0	0
Spirochaetes	14	0	0	0	0	0	0	0	0	0
Acidobacteria	5	0	0	0	0	0	0	0	0	0
Bacteroidetes	35	0	0	0	0	0	0	0	0	0
Fibrobacteres	1	0	0	0	0	0	0	0	0	0
Fusobacteria	5	0	0	0	0	0	0	0	0	0
Verrucomicrobia	4	0	0	0	0	0	0	0	0	0
Gemmatimonadetes	1	0	0	0	0	0	0	0	0	0
Planctomycetes	4	0	0	0	0	0	0	0	0	0
Elusimicrobia	2	0	0	0	0	0	0	0	0	0
Synergistetes	2	0	0	0	0	0	0	0	0	0
Cyanobacteria	16	0	0	0	0	0	0	0	0	0
Chlorobi	10	0	0	0	0	0	0	0	0	0
Chloroflexi	11	0	0	0	0	0	0	0	0	0
Deinococcus-Thermus	7	0	0	0	0	0	0	0	0	0
Aquificae	7	2	0	0	0	0	0	0	0	0
Thermotogae	11	0	0	0	0	0	0	0	0	0
Dictyoglomi	2	0	0	0	0	0	0	0	0	0
Nitrospirae	2	0	0	0	0	0	0	0	0	0
Thermobaculum	1	0	0	0	0	0	0	0	0	0
Deferribacteres	0	3	0	0	0	0	0	0	0	0
Euryarchaeota	1	59	0	0	0	0	0	0	0	0
Crenarchaeota	1	22	0	0	0	0	0	0	0	0
Thaumarchaeota	0	2	0	0	0	0	0	0	0	0
Nanoarchaeota	0	1	0	0	0	0	0	0	0	0
Korarchaeota	0	1	0	0	0	0	0	0	0	0
Total	656	112	0	0	0	0	0	0	0	0

(M00073_1)

Phyla	Module completion ratio (%)									
	0--10	10--20	20--30	30--40	40--50	50--60	60--70	70--80	80--90	90--100
Gammaproteobacteria	126	0	0	0	0	0	0	0	0	0
Betaproteobacteria	61	0	0	0	0	0	0	0	0	0
Epsilonproteobacteria	17	0	0	0	0	0	0	0	0	0
Deltaproteobacteria	28	0	0	0	0	0	0	0	0	0
Alphaproteobacteria	90	0	0	1	0	0	0	0	0	0
Magnetococcus	1	0	0	0	0	0	0	0	0	0
Chrysiogenetes	1	0	0	0	0	0	0	0	0	0
Firmicutes	104	0	0	0	0	0	0	0	0	0
Tenericutes	19	0	0	0	0	0	0	0	0	0
Actinobacteria	80	0	0	0	0	0	0	0	0	0
Chlamydiae	8	0	0	0	0	0	0	0	0	0
Spirochaetes	14	0	0	0	0	0	0	0	0	0
Acidobacteria	5	0	0	0	0	0	0	0	0	0
Bacteroidetes	35	0	0	0	0	0	0	0	0	0
Fibrobacteres	1	0	0	0	0	0	0	0	0	0
Fusobacteria	5	0	0	0	0	0	0	0	0	0
Verrucomicrobia	4	0	0	0	0	0	0	0	0	0
Gemmatimonadetes	1	0	0	0	0	0	0	0	0	0
Planctomycetes	4	0	0	0	0	0	0	0	0	0
Elusimicrobia	2	0	0	0	0	0	0	0	0	0
Synergistetes	2	0	0	0	0	0	0	0	0	0
Cyanobacteria	16	0	0	0	0	0	0	0	0	0
Chlorobi	10	0	0	0	0	0	0	0	0	0
Chloroflexi	11	0	0	0	0	0	0	0	0	0
Deinococcus-Thermus	7	0	0	0	0	0	0	0	0	0
Aquificae	9	0	0	0	0	0	0	0	0	0
Thermotogae	11	0	0	0	0	0	0	0	0	0
Dictyoglomi	2	0	0	0	0	0	0	0	0	0
Nitrospirae	2	0	0	0	0	0	0	0	0	0
Thermobaculum	1	0	0	0	0	0	0	0	0	0
Deferribacteres	3	0	0	0	0	0	0	0	0	0
Euryarchaeota	60	0	0	0	0	0	0	0	0	0
Crenarchaeota	23	0	0	0	0	0	0	0	0	0
Thaumarchaeota	2	0	0	0	0	0	0	0	0	0
Nanoarchaeota	1	0	0	0	0	0	0	0	0	0
Korarchaeota	1	0	0	0	0	0	0	0	0	0
Total	767	0	0	1	0	0	0	0	0	0

(M00074_1)

Phyla	Module completion ratio (%)									
	0--10	10--20	20--30	30--40	40--50	50--60	60--70	70--80	80--90	90--100
Gammaproteobacteria	126	0	0	0	0	0	0	0	0	0
Betaproteobacteria	61	0	0	0	0	0	0	0	0	0
Epsilonproteobacteria	17	0	0	0	0	0	0	0	0	0
Deltaproteobacteria	28	0	0	0	0	0	0	0	0	0
Alphaproteobacteria	90	1	0	0	0	0	0	0	0	0
Magnetococcus	1	0	0	0	0	0	0	0	0	0
Chrysiogenetes	1	0	0	0	0	0	0	0	0	0
Firmicutes	104	0	0	0	0	0	0	0	0	0
Tenericutes	19	0	0	0	0	0	0	0	0	0
Actinobacteria	80	0	0	0	0	0	0	0	0	0
Chlamydiae	8	0	0	0	0	0	0	0	0	0
Spirochaetes	14	0	0	0	0	0	0	0	0	0
Acidobacteria	5	0	0	0	0	0	0	0	0	0
Bacteroidetes	35	0	0	0	0	0	0	0	0	0
Fibrobacteres	1	0	0	0	0	0	0	0	0	0
Fusobacteria	5	0	0	0	0	0	0	0	0	0
Verrucomicrobia	4	0	0	0	0	0	0	0	0	0
Gemmatimonadetes	1	0	0	0	0	0	0	0	0	0
Planctomycetes	4	0	0	0	0	0	0	0	0	0
Elusimicrobia	2	0	0	0	0	0	0	0	0	0
Synergistetes	2	0	0	0	0	0	0	0	0	0
Cyanobacteria	16	0	0	0	0	0	0	0	0	0
Chlorobi	10	0	0	0	0	0	0	0	0	0
Chloroflexi	11	0	0	0	0	0	0	0	0	0
Deinococcus-Thermus	7	0	0	0	0	0	0	0	0	0
Aquificae	9	0	0	0	0	0	0	0	0	0
Thermotogae	11	0	0	0	0	0	0	0	0	0
Dictyoglomi	2	0	0	0	0	0	0	0	0	0
Nitrospirae	2	0	0	0	0	0	0	0	0	0
Thermobaculum	1	0	0	0	0	0	0	0	0	0
Deferribacteres	3	0	0	0	0	0	0	0	0	0
Euryarchaeota	60	0	0	0	0	0	0	0	0	0
Crenarchaeota	23	0	0	0	0	0	0	0	0	0
Thaumarchaeota	2	0	0	0	0	0	0	0	0	0
Nanoarchaeota	1	0	0	0	0	0	0	0	0	0
Korarchaeota	1	0	0	0	0	0	0	0	0	0
Total	767	1	0	0	0	0	0	0	0	0

(M00075_1)

Phyla	Module completion ratio (%)									
	0--10	10--20	20--30	30--40	40--50	50--60	60--70	70--80	80--90	90--100
Gammaproteobacteria	126	0	0	0	0	0	0	0	0	0
Betaproteobacteria	61	0	0	0	0	0	0	0	0	0
Epsilonproteobacteria	17	0	0	0	0	0	0	0	0	0
Deltaproteobacteria	28	0	0	0	0	0	0	0	0	0
Alphaproteobacteria	91	0	0	0	0	0	0	0	0	0
Magnetococcus	1	0	0	0	0	0	0	0	0	0
Chrysiogenetes	1	0	0	0	0	0	0	0	0	0
Firmicutes	104	0	0	0	0	0	0	0	0	0
Tenericutes	19	0	0	0	0	0	0	0	0	0
Actinobacteria	80	0	0	0	0	0	0	0	0	0
Chlamydiae	8	0	0	0	0	0	0	0	0	0
Spirochaetes	14	0	0	0	0	0	0	0	0	0
Acidobacteria	5	0	0	0	0	0	0	0	0	0
Bacteroidetes	35	0	0	0	0	0	0	0	0	0
Fibrobacteres	1	0	0	0	0	0	0	0	0	0
Fusobacteria	5	0	0	0	0	0	0	0	0	0
Verrucomicrobia	4	0	0	0	0	0	0	0	0	0
Gemmatimonadetes	1	0	0	0	0	0	0	0	0	0
Planctomycetes	4	0	0	0	0	0	0	0	0	0
Elusimicrobia	2	0	0	0	0	0	0	0	0	0
Synergistetes	2	0	0	0	0	0	0	0	0	0
Cyanobacteria	16	0	0	0	0	0	0	0	0	0
Chlorobi	10	0	0	0	0	0	0	0	0	0
Chloroflexi	11	0	0	0	0	0	0	0	0	0
Deinococcus-Thermus	7	0	0	0	0	0	0	0	0	0
Aquificae	9	0	0	0	0	0	0	0	0	0
Thermotogae	11	0	0	0	0	0	0	0	0	0
Dictyoglomi	2	0	0	0	0	0	0	0	0	0
Nitrospirae	2	0	0	0	0	0	0	0	0	0
Thermobaculum	1	0	0	0	0	0	0	0	0	0
Deferribacteres	3	0	0	0	0	0	0	0	0	0
Euryarchaeota	60	0	0	0	0	0	0	0	0	0
Crenarchaeota	23	0	0	0	0	0	0	0	0	0
Thaumarchaeota	2	0	0	0	0	0	0	0	0	0
Nanoarchaeota	1	0	0	0	0	0	0	0	0	0
Korarchaeota	1	0	0	0	0	0	0	0	0	0
Total	768	0	0	0	0	0	0	0	0	0

(M00075_2)

Phyla	Module completion ratio (%)									
	0--10	10--20	20--30	30--40	40--50	50--60	60--70	70--80	80--90	90--100
Gammaproteobacteria	126	0	0	0	0	0	0	0	0	0
Betaproteobacteria	58	0	0	0	0	0	0	0	0	3
Epsilonproteobacteria	17	0	0	0	0	0	0	0	0	0
Deltaproteobacteria	27	0	0	0	0	0	0	0	0	1
Alphaproteobacteria	90	0	0	0	0	0	0	0	0	1
Magnetococcus	1	0	0	0	0	0	0	0	0	0
Chrysiogenetes	1	0	0	0	0	0	0	0	0	0
Firmicutes	103	0	0	0	0	0	0	0	0	1
Tenericutes	19	0	0	0	0	0	0	0	0	0
Actinobacteria	80	0	0	0	0	0	0	0	0	0
Chlamydiae	8	0	0	0	0	0	0	0	0	0
Spirochaetes	14	0	0	0	0	0	0	0	0	0
Acidobacteria	5	0	0	0	0	0	0	0	0	0
Bacteroidetes	35	0	0	0	0	0	0	0	0	0
Fibrobacteres	1	0	0	0	0	0	0	0	0	0
Fusobacteria	5	0	0	0	0	0	0	0	0	0
Verrucomicrobia	4	0	0	0	0	0	0	0	0	0
Gemmatimonadetes	1	0	0	0	0	0	0	0	0	0
Planctomycetes	4	0	0	0	0	0	0	0	0	0
Elusimicrobia	2	0	0	0	0	0	0	0	0	0
Synergistetes	2	0	0	0	0	0	0	0	0	0
Cyanobacteria	16	0	0	0	0	0	0	0	0	0
Chlorobi	10	0	0	0	0	0	0	0	0	0
Chloroflexi	11	0	0	0	0	0	0	0	0	0
Deinococcus-Thermus	7	0	0	0	0	0	0	0	0	0
Aquificae	9	0	0	0	0	0	0	0	0	0
Thermotogae	11	0	0	0	0	0	0	0	0	0
Dictyoglomi	2	0	0	0	0	0	0	0	0	0
Nitrospirae	2	0	0	0	0	0	0	0	0	0
Thermobaculum	1	0	0	0	0	0	0	0	0	0
Deferribacteres	3	0	0	0	0	0	0	0	0	0
Euryarchaeota	60	0	0	0	0	0	0	0	0	0
Crenarchaeota	23	0	0	0	0	0	0	0	0	0
Thaumarchaeota	2	0	0	0	0	0	0	0	0	0
Nanoarchaeota	1	0	0	0	0	0	0	0	0	0
Korarchaeota	1	0	0	0	0	0	0	0	0	0
Total	762	0	0	0	0	0	0	0	0	6

(M00075_3)

Phyla	Module completion ratio (%)									
	0--10	10--20	20--30	30--40	40--50	50--60	60--70	70--80	80--90	90--100
Gammaproteobacteria	126	0	0	0	0	0	0	0	0	0
Betaproteobacteria	61	0	0	0	0	0	0	0	0	0
Epsilonproteobacteria	17	0	0	0	0	0	0	0	0	0
Deltaproteobacteria	28	0	0	0	0	0	0	0	0	0
Alphaproteobacteria	91	0	0	0	0	0	0	0	0	0
Magnetococcus	1	0	0	0	0	0	0	0	0	0
Chrysiogenetes	1	0	0	0	0	0	0	0	0	0
Firmicutes	104	0	0	0	0	0	0	0	0	0
Tenericutes	19	0	0	0	0	0	0	0	0	0
Actinobacteria	80	0	0	0	0	0	0	0	0	0
Chlamydiae	8	0	0	0	0	0	0	0	0	0
Spirochaetes	14	0	0	0	0	0	0	0	0	0
Acidobacteria	5	0	0	0	0	0	0	0	0	0
Bacteroidetes	35	0	0	0	0	0	0	0	0	0
Fibrobacteres	1	0	0	0	0	0	0	0	0	0
Fusobacteria	5	0	0	0	0	0	0	0	0	0
Verrucomicrobia	4	0	0	0	0	0	0	0	0	0
Gemmatimonadetes	1	0	0	0	0	0	0	0	0	0
Planctomycetes	4	0	0	0	0	0	0	0	0	0
Elusimicrobia	2	0	0	0	0	0	0	0	0	0
Synergistetes	2	0	0	0	0	0	0	0	0	0
Cyanobacteria	16	0	0	0	0	0	0	0	0	0
Chlorobi	10	0	0	0	0	0	0	0	0	0
Chloroflexi	11	0	0	0	0	0	0	0	0	0
Deinococcus-Thermus	7	0	0	0	0	0	0	0	0	0
Aquificae	9	0	0	0	0	0	0	0	0	0
Thermotogae	11	0	0	0	0	0	0	0	0	0
Dictyoglomi	2	0	0	0	0	0	0	0	0	0
Nitrospirae	2	0	0	0	0	0	0	0	0	0
Thermobaculum	1	0	0	0	0	0	0	0	0	0
Deferribacteres	3	0	0	0	0	0	0	0	0	0
Euryarchaeota	60	0	0	0	0	0	0	0	0	0
Crenarchaeota	23	0	0	0	0	0	0	0	0	0
Thaumarchaeota	2	0	0	0	0	0	0	0	0	0
Nanoarchaeota	1	0	0	0	0	0	0	0	0	0
Korarchaeota	1	0	0	0	0	0	0	0	0	0
Total	768	0	0	0	0	0	0	0	0	0

(M00075_4)

Phyla	Module completion ratio (%)									
	0--10	10--20	20--30	30--40	40--50	50--60	60--70	70--80	80--90	90--100
Gammaproteobacteria	126	0	0	0	0	0	0	0	0	0
Betaproteobacteria	61	0	0	0	0	0	0	0	0	0
Epsilonproteobacteria	17	0	0	0	0	0	0	0	0	0
Deltaproteobacteria	28	0	0	0	0	0	0	0	0	0
Alphaproteobacteria	91	0	0	0	0	0	0	0	0	0
Magnetococcus	1	0	0	0	0	0	0	0	0	0
Chrysiogenetes	1	0	0	0	0	0	0	0	0	0
Firmicutes	104	0	0	0	0	0	0	0	0	0
Tenericutes	19	0	0	0	0	0	0	0	0	0
Actinobacteria	80	0	0	0	0	0	0	0	0	0
Chlamydiae	8	0	0	0	0	0	0	0	0	0
Spirochaetes	14	0	0	0	0	0	0	0	0	0
Acidobacteria	5	0	0	0	0	0	0	0	0	0
Bacteroidetes	35	0	0	0	0	0	0	0	0	0
Fibrobacteres	1	0	0	0	0	0	0	0	0	0
Fusobacteria	5	0	0	0	0	0	0	0	0	0
Verrucomicrobia	4	0	0	0	0	0	0	0	0	0
Gemmatimonadetes	1	0	0	0	0	0	0	0	0	0
Planctomycetes	4	0	0	0	0	0	0	0	0	0
Elusimicrobia	2	0	0	0	0	0	0	0	0	0
Synergistetes	2	0	0	0	0	0	0	0	0	0
Cyanobacteria	16	0	0	0	0	0	0	0	0	0
Chlorobi	10	0	0	0	0	0	0	0	0	0
Chloroflexi	11	0	0	0	0	0	0	0	0	0
Deinococcus-Thermus	7	0	0	0	0	0	0	0	0	0
Aquificae	9	0	0	0	0	0	0	0	0	0
Thermotogae	11	0	0	0	0	0	0	0	0	0
Dictyoglomi	2	0	0	0	0	0	0	0	0	0
Nitrospirae	2	0	0	0	0	0	0	0	0	0
Thermobaculum	1	0	0	0	0	0	0	0	0	0
Deferribacteres	3	0	0	0	0	0	0	0	0	0
Euryarchaeota	60	0	0	0	0	0	0	0	0	0
Crenarchaeota	23	0	0	0	0	0	0	0	0	0
Thaumarchaeota	2	0	0	0	0	0	0	0	0	0
Nanoarchaeota	1	0	0	0	0	0	0	0	0	0
Korarchaeota	1	0	0	0	0	0	0	0	0	0
Total	768	0	0	0	0	0	0	0	0	0

(M00076_1)

Phyla	Module completion ratio (%)									
	0--10	10--20	20--30	30--40	40--50	50--60	60--70	70--80	80--90	90--100
Gammaproteobacteria	115	11	0	0	0	0	0	0	0	0
Betaproteobacteria	61	0	0	0	0	0	0	0	0	0
Epsilonproteobacteria	17	0	0	0	0	0	0	0	0	0
Deltaproteobacteria	28	0	0	0	0	0	0	0	0	0
Alphaproteobacteria	88	2	0	1	0	0	0	0	0	0
Magnetococcus	1	0	0	0	0	0	0	0	0	0
Chrysiogenetes	1	0	0	0	0	0	0	0	0	0
Firmicutes	90	12	0	2	0	0	0	0	0	0
Tenericutes	19	0	0	0	0	0	0	0	0	0
Actinobacteria	71	9	0	0	0	0	0	0	0	0
Chlamydiae	8	0	0	0	0	0	0	0	0	0
Spirochaetes	13	1	0	0	0	0	0	0	0	0
Acidobacteria	1	4	0	0	0	0	0	0	0	0
Bacteroidetes	21	13	0	1	0	0	0	0	0	0
Fibrobacteres	1	0	0	0	0	0	0	0	0	0
Fusobacteria	4	1	0	0	0	0	0	0	0	0
Verrucomicrobia	2	2	0	0	0	0	0	0	0	0
Gemmatimonadetes	1	0	0	0	0	0	0	0	0	0
Planctomycetes	3	0	0	1	0	0	0	0	0	0
Elusimicrobia	2	0	0	0	0	0	0	0	0	0
Synergistetes	2	0	0	0	0	0	0	0	0	0
Cyanobacteria	16	0	0	0	0	0	0	0	0	0
Chlorobi	10	0	0	0	0	0	0	0	0	0
Chloroflexi	11	0	0	0	0	0	0	0	0	0
Deinococcus-Thermus	7	0	0	0	0	0	0	0	0	0
Aquificae	9	0	0	0	0	0	0	0	0	0
Thermotogae	5	6	0	0	0	0	0	0	0	0
Dictyoglomi	0	2	0	0	0	0	0	0	0	0
Nitrospirae	2	0	0	0	0	0	0	0	0	0
Thermobaculum	0	0	0	1	0	0	0	0	0	0
Deferribacteres	3	0	0	0	0	0	0	0	0	0
Euryarchaeota	58	2	0	0	0	0	0	0	0	0
Crenarchaeota	19	4	0	0	0	0	0	0	0	0
Thaumarchaeota	2	0	0	0	0	0	0	0	0	0
Nanoarchaeota	1	0	0	0	0	0	0	0	0	0
Korarchaeota	1	0	0	0	0	0	0	0	0	0
Total	693	69	0	6	0	0	0	0	0	0

(M00077_1)

Phyla	Module completion ratio (%)									
	0--10	10--20	20--30	30--40	40--50	50--60	60--70	70--80	80--90	90--100
Gammaproteobacteria	115	0	11	0	0	0	0	0	0	0
Betaproteobacteria	61	0	0	0	0	0	0	0	0	0
Epsilonproteobacteria	17	0	0	0	0	0	0	0	0	0
Deltaproteobacteria	28	0	0	0	0	0	0	0	0	0
Alphaproteobacteria	89	0	1	0	0	1	0	0	0	0
Magnetococcus	1	0	0	0	0	0	0	0	0	0
Chrysiogenetes	1	0	0	0	0	0	0	0	0	0
Firmicutes	90	0	12	0	0	2	0	0	0	0
Tenericutes	19	0	0	0	0	0	0	0	0	0
Actinobacteria	71	0	9	0	0	0	0	0	0	0
Chlamydiae	8	0	0	0	0	0	0	0	0	0
Spirochaetes	13	0	1	0	0	0	0	0	0	0
Acidobacteria	1	0	4	0	0	0	0	0	0	0
Bacteroidetes	21	0	13	0	0	1	0	0	0	0
Fibrobacteres	1	0	0	0	0	0	0	0	0	0
Fusobacteria	4	0	1	0	0	0	0	0	0	0
Verrucomicrobia	2	0	2	0	0	0	0	0	0	0
Gemmatimonadetes	1	0	0	0	0	0	0	0	0	0
Planctomycetes	3	0	0	0	0	1	0	0	0	0
Elusimicrobia	2	0	0	0	0	0	0	0	0	0
Synergistetes	2	0	0	0	0	0	0	0	0	0
Cyanobacteria	16	0	0	0	0	0	0	0	0	0
Chlorobi	10	0	0	0	0	0	0	0	0	0
Chloroflexi	11	0	0	0	0	0	0	0	0	0
Deinococcus-Thermus	7	0	0	0	0	0	0	0	0	0
Aquificae	9	0	0	0	0	0	0	0	0	0
Thermotogae	5	0	6	0	0	0	0	0	0	0
Dictyoglomi	0	0	2	0	0	0	0	0	0	0
Nitrospirae	2	0	0	0	0	0	0	0	0	0
Thermobaculum	0	0	0	0	0	1	0	0	0	0
Deferribacteres	3	0	0	0	0	0	0	0	0	0
Euryarchaeota	58	0	2	0	0	0	0	0	0	0
Crenarchaeota	19	0	4	0	0	0	0	0	0	0
Thaumarchaeota	2	0	0	0	0	0	0	0	0	0
Nanoarchaeota	1	0	0	0	0	0	0	0	0	0
Korarchaeota	1	0	0	0	0	0	0	0	0	0
Total	694	0	68	0	0	6	0	0	0	0

(M00078_1)

Phyla	Module completion ratio (%)									
	0--10	10--20	20--30	30--40	40--50	50--60	60--70	70--80	80--90	90--100
Gammaproteobacteria	117	9	0	0	0	0	0	0	0	0
Betaproteobacteria	61	0	0	0	0	0	0	0	0	0
Epsilonproteobacteria	17	0	0	0	0	0	0	0	0	0
Deltaproteobacteria	28	0	0	0	0	0	0	0	0	0
Alphaproteobacteria	88	2	1	0	0	0	0	0	0	0
Magnetococcus	1	0	0	0	0	0	0	0	0	0
Chrysiogenetes	1	0	0	0	0	0	0	0	0	0
Firmicutes	93	11	0	0	0	0	0	0	0	0
Tenericutes	19	0	0	0	0	0	0	0	0	0
Actinobacteria	72	8	0	0	0	0	0	0	0	0
Chlamydiae	8	0	0	0	0	0	0	0	0	0
Spirochaetes	13	1	0	0	0	0	0	0	0	0
Acidobacteria	1	3	1	0	0	0	0	0	0	0
Bacteroidetes	23	8	4	0	0	0	0	0	0	0
Fibrobacteres	1	0	0	0	0	0	0	0	0	0
Fusobacteria	4	1	0	0	0	0	0	0	0	0
Verrucomicrobia	3	1	0	0	0	0	0	0	0	0
Gemmatimonadetes	1	0	0	0	0	0	0	0	0	0
Planctomycetes	2	1	1	0	0	0	0	0	0	0
Elusimicrobia	2	0	0	0	0	0	0	0	0	0
Synergistetes	2	0	0	0	0	0	0	0	0	0
Cyanobacteria	16	0	0	0	0	0	0	0	0	0
Chlorobi	10	0	0	0	0	0	0	0	0	0
Chloroflexi	11	0	0	0	0	0	0	0	0	0
Deinococcus-Thermus	7	0	0	0	0	0	0	0	0	0
Aquificae	9	0	0	0	0	0	0	0	0	0
Thermotogae	7	4	0	0	0	0	0	0	0	0
Dictyoglomi	0	2	0	0	0	0	0	0	0	0
Nitrospirae	2	0	0	0	0	0	0	0	0	0
Thermobaculum	0	1	0	0	0	0	0	0	0	0
Deferribacteres	3	0	0	0	0	0	0	0	0	0
Euryarchaeota	58	2	0	0	0	0	0	0	0	0
Crenarchaeota	19	4	0	0	0	0	0	0	0	0
Thaumarchaeota	2	0	0	0	0	0	0	0	0	0
Nanoarchaeota	1	0	0	0	0	0	0	0	0	0
Korarchaeota	1	0	0	0	0	0	0	0	0	0
Total	703	58	7	0	0	0	0	0	0	0

(M00079_1)

Phyla	Module completion ratio (%)									
	0--10	10--20	20--30	30--40	40--50	50--60	60--70	70--80	80--90	90--100
Gammaproteobacteria	73	0	52	0	1	0	0	0	0	0
Betaproteobacteria	52	0	9	0	0	0	0	0	0	0
Epsilonproteobacteria	16	0	1	0	0	0	0	0	0	0
Deltaproteobacteria	26	0	2	0	0	0	0	0	0	0
Alphaproteobacteria	78	0	13	0	0	0	0	0	0	0
Magnetococcus	1	0	0	0	0	0	0	0	0	0
Chrysiogenetes	1	0	0	0	0	0	0	0	0	0
Firmicutes	93	0	11	0	0	0	0	0	0	0
Tenericutes	19	0	0	0	0	0	0	0	0	0
Actinobacteria	48	0	32	0	0	0	0	0	0	0
Chlamydiae	8	0	0	0	0	0	0	0	0	0
Spirochaetes	13	0	1	0	0	0	0	0	0	0
Acidobacteria	1	0	4	0	0	0	0	0	0	0
Bacteroidetes	12	0	23	0	0	0	0	0	0	0
Fibrobacteres	1	0	0	0	0	0	0	0	0	0
Fusobacteria	5	0	0	0	0	0	0	0	0	0
Verrucomicrobia	2	0	2	0	0	0	0	0	0	0
Gemmatimonadetes	0	0	1	0	0	0	0	0	0	0
Planctomycetes	3	0	0	0	0	1	0	0	0	0
Elusimicrobia	2	0	0	0	0	0	0	0	0	0
Synergistetes	2	0	0	0	0	0	0	0	0	0
Cyanobacteria	16	0	0	0	0	0	0	0	0	0
Chlorobi	10	0	0	0	0	0	0	0	0	0
Chloroflexi	10	0	1	0	0	0	0	0	0	0
Deinococcus-Thermus	7	0	0	0	0	0	0	0	0	0
Aquificae	9	0	0	0	0	0	0	0	0	0
Thermotogae	11	0	0	0	0	0	0	0	0	0
Dictyoglomi	2	0	0	0	0	0	0	0	0	0
Nitrospirae	2	0	0	0	0	0	0	0	0	0
Thermobaculum	1	0	0	0	0	0	0	0	0	0
Deferribacteres	3	0	0	0	0	0	0	0	0	0
Euryarchaeota	60	0	0	0	0	0	0	0	0	0
Crenarchaeota	23	0	0	0	0	0	0	0	0	0
Thaumarchaeota	2	0	0	0	0	0	0	0	0	0
Nanoarchaeota	1	0	0	0	0	0	0	0	0	0
Korarchaeota	1	0	0	0	0	0	0	0	0	0
Total	614	0	152	0	1	1	0	0	0	0

(M00080_1)

Phyla	Module completion ratio (%)									
	0--10	10--20	20--30	30--40	40--50	50--60	60--70	70--80	80--90	90--100
Gammaproteobacteria	50	24	10	6	18	8	5	2	1	2
Betaproteobacteria	14	3	16	20	8	0	0	0	0	0
Epsilonproteobacteria	3	0	6	2	6	0	0	0	0	0
Deltaproteobacteria	10	3	9	3	3	0	0	0	0	0
Alphaproteobacteria	79	4	6	2	0	0	0	0	0	0
Magnetococcus	0	0	0	0	1	0	0	0	0	0
Chrysiogenetes	0	1	0	0	0	0	0	0	0	0
Firmicutes	100	2	2	0	0	0	0	0	0	0
Tenericutes	19	0	0	0	0	0	0	0	0	0
Actinobacteria	80	0	0	0	0	0	0	0	0	0
Chlamydiae	6	0	2	0	0	0	0	0	0	0
Spirochaetes	11	2	1	0	0	0	0	0	0	0
Acidobacteria	2	1	2	0	0	0	0	0	0	0
Bacteroidetes	26	9	0	0	0	0	0	0	0	0
Fibrobacteres	1	0	0	0	0	0	0	0	0	0
Fusobacteria	1	2	2	0	0	0	0	0	0	0
Verrucomicrobia	2	1	0	1	0	0	0	0	0	0
Gemmatimonadetes	0	1	0	0	0	0	0	0	0	0
Planctomycetes	1	0	1	2	0	0	0	0	0	0
Elusimicrobia	2	0	0	0	0	0	0	0	0	0
Synergistetes	0	2	0	0	0	0	0	0	0	0
Cyanobacteria	16	0	0	0	0	0	0	0	0	0
Chlorobi	8	2	0	0	0	0	0	0	0	0
Chloroflexi	10	1	0	0	0	0	0	0	0	0
Deinococcus-Thermus	7	0	0	0	0	0	0	0	0	0
Aquificae	1	5	2	1	0	0	0	0	0	0
Thermotogae	11	0	0	0	0	0	0	0	0	0
Dictyoglomi	2	0	0	0	0	0	0	0	0	0
Nitrospirae	0	0	1	1	0	0	0	0	0	0
Thermobaculum	1	0	0	0	0	0	0	0	0	0
Deferribacteres	0	1	1	1	0	0	0	0	0	0
Euryarchaeota	59	1	0	0	0	0	0	0	0	0
Crenarchaeota	23	0	0	0	0	0	0	0	0	0
Thaumarchaeota	2	0	0	0	0	0	0	0	0	0
Nanoarchaeota	1	0	0	0	0	0	0	0	0	0
Korarchaeota	1	0	0	0	0	0	0	0	0	0
Total	549	65	61	39	36	8	5	2	1	2

(M00081_1)

Phyla	Module completion ratio (%)									
	0--10	10--20	20--30	30--40	40--50	50--60	60--70	70--80	80--90	90--100
Gammaproteobacteria	98	0	0	25	0	0	3	0	0	0
Betaproteobacteria	60	0	0	0	0	0	1	0	0	0
Epsilonproteobacteria	17	0	0	0	0	0	0	0	0	0
Deltaproteobacteria	28	0	0	0	0	0	0	0	0	0
Alphaproteobacteria	87	0	0	3	0	0	1	0	0	0
Magnetococcus	1	0	0	0	0	0	0	0	0	0
Chrysiogenetes	1	0	0	0	0	0	0	0	0	0
Firmicutes	97	0	0	7	0	0	0	0	0	0
Tenericutes	19	0	0	0	0	0	0	0	0	0
Actinobacteria	70	0	0	10	0	0	0	0	0	0
Chlamydiae	8	0	0	0	0	0	0	0	0	0
Spirochaetes	14	0	0	0	0	0	0	0	0	0
Acidobacteria	1	0	0	1	0	0	3	0	0	0
Bacteroidetes	22	0	0	13	0	0	0	0	0	0
Fibrobacteres	1	0	0	0	0	0	0	0	0	0
Fusobacteria	5	0	0	0	0	0	0	0	0	0
Verrucomicrobia	3	0	0	1	0	0	0	0	0	0
Gemmatimonadetes	1	0	0	0	0	0	0	0	0	0
Planctomycetes	4	0	0	0	0	0	0	0	0	0
Elusimicrobia	2	0	0	0	0	0	0	0	0	0
Synergistetes	2	0	0	0	0	0	0	0	0	0
Cyanobacteria	16	0	0	0	0	0	0	0	0	0
Chlorobi	10	0	0	0	0	0	0	0	0	0
Chloroflexi	11	0	0	0	0	0	0	0	0	0
Deinococcus-Thermus	7	0	0	0	0	0	0	0	0	0
Aquificae	9	0	0	0	0	0	0	0	0	0
Thermotogae	10	0	0	1	0	0	0	0	0	0
Dictyoglomi	1	0	0	1	0	0	0	0	0	0
Nitrospirae	2	0	0	0	0	0	0	0	0	0
Thermobaculum	1	0	0	0	0	0	0	0	0	0
Deferribacteres	3	0	0	0	0	0	0	0	0	0
Euryarchaeota	58	0	0	2	0	0	0	0	0	0
Crenarchaeota	23	0	0	0	0	0	0	0	0	0
Thaumarchaeota	2	0	0	0	0	0	0	0	0	0
Nanoarchaeota	1	0	0	0	0	0	0	0	0	0
Korarchaeota	1	0	0	0	0	0	0	0	0	0
Total	696	0	0	64	0	0	8	0	0	0

(M00082_1)

Phyla	Module completion ratio (%)									
	0--10	10--20	20--30	30--40	40--50	50--60	60--70	70--80	80--90	90--100
Gammaproteobacteria	12	0	114	0	0	0	0	0	0	0
Betaproteobacteria	1	0	60	0	0	0	0	0	0	0
Epsilonproteobacteria	3	0	14	0	0	0	0	0	0	0
Deltaproteobacteria	12	0	16	0	0	0	0	0	0	0
Alphaproteobacteria	20	0	71	0	0	0	0	0	0	0
Magnetococcus	0	0	1	0	0	0	0	0	0	0
Chrysiogenetes	0	0	1	0	0	0	0	0	0	0
Firmicutes	21	0	83	0	0	0	0	0	0	0
Tenericutes	18	0	1	0	0	0	0	0	0	0
Actinobacteria	61	0	19	0	0	0	0	0	0	0
Chlamydiae	0	0	8	0	0	0	0	0	0	0
Spirochaetes	12	0	2	0	0	0	0	0	0	0
Acidobacteria	0	0	5	0	0	0	0	0	0	0
Bacteroidetes	4	0	31	0	0	0	0	0	0	0
Fibrobacteres	1	0	0	0	0	0	0	0	0	0
Fusobacteria	2	0	3	0	0	0	0	0	0	0
Verrucomicrobia	1	0	3	0	0	0	0	0	0	0
Gemmatimonadetes	0	0	1	0	0	0	0	0	0	0
Planctomycetes	0	0	4	0	0	0	0	0	0	0
Elusimicrobia	0	0	2	0	0	0	0	0	0	0
Synergistetes	2	0	0	0	0	0	0	0	0	0
Cyanobacteria	0	0	16	0	0	0	0	0	0	0
Chlorobi	0	0	10	0	0	0	0	0	0	0
Chloroflexi	4	0	7	0	0	0	0	0	0	0
Deinococcus-Thermus	0	0	7	0	0	0	0	0	0	0
Aquificae	0	0	9	0	0	0	0	0	0	0
Thermotogae	11	0	0	0	0	0	0	0	0	0
Dictyoglomi	0	0	2	0	0	0	0	0	0	0
Nitrospirae	0	0	2	0	0	0	0	0	0	0
Thermobaculum	0	0	1	0	0	0	0	0	0	0
Deferribacteres	0	0	3	0	0	0	0	0	0	0
Euryarchaeota	60	0	0	0	0	0	0	0	0	0
Crenarchaeota	22	0	1	0	0	0	0	0	0	0
Thaumarchaeota	1	0	1	0	0	0	0	0	0	0
Nanoarchaeota	1	0	0	0	0	0	0	0	0	0
Korarchaeota	1	0	0	0	0	0	0	0	0	0
Total	270	0	498	0	0	0	0	0	0	0

(M00083_1)

Phyla	Module completion ratio (%)									
	0--10	10--20	20--30	30--40	40--50	50--60	60--70	70--80	80--90	90--100
Gammaproteobacteria	59	0	0	0	0	0	0	0	0	67
Betaproteobacteria	5	0	0	0	0	0	0	0	0	56
Epsilonproteobacteria	0	0	0	0	0	0	0	0	0	17
Deltaproteobacteria	6	0	0	0	0	0	0	0	0	22
Alphaproteobacteria	3	0	0	0	0	0	0	0	0	88
Magnetococcus	0	0	0	0	0	0	0	0	0	1
Chrysiogenetes	1	0	0	0	0	0	0	0	0	0
Firmicutes	14	0	0	0	0	0	0	0	0	90
Tenericutes	18	0	0	0	0	0	0	0	0	1
Actinobacteria	70	0	0	0	0	0	0	0	0	10
Chlamydiae	0	0	0	0	0	0	0	0	0	8
Spirochaetes	14	0	0	0	0	0	0	0	0	0
Acidobacteria	0	0	0	0	0	0	0	0	0	5
Bacteroidetes	5	0	0	0	0	0	0	0	0	30
Fibrobacteres	1	0	0	0	0	0	0	0	0	0
Fusobacteria	3	0	0	0	0	0	0	0	0	2
Verrucomicrobia	0	0	0	0	0	0	0	0	0	4
Gemmatimonadetes	0	0	0	0	0	0	0	0	0	1
Planctomycetes	0	0	0	0	0	0	0	0	0	4
Elusimicrobia	2	0	0	0	0	0	0	0	0	0
Synergistetes	0	0	0	0	0	0	0	0	0	2
Cyanobacteria	0	0	0	0	0	0	0	0	0	16
Chlorobi	0	0	0	0	0	0	0	0	0	10
Chloroflexi	5	0	0	0	0	0	0	0	0	6
Deinococcus-Thermus	0	0	0	0	0	0	0	0	0	7
Aquificae	0	0	0	0	0	0	0	0	0	9
Thermotogae	0	0	0	0	0	0	0	0	0	11
Dictyoglomi	0	0	0	0	0	0	0	0	0	2
Nitrospirae	1	0	0	0	0	0	0	0	0	1
Thermobaculum	0	0	0	0	0	0	0	0	0	1
Deferribacteres	0	0	0	0	0	0	0	0	0	3
Euryarchaeota	60	0	0	0	0	0	0	0	0	0
Crenarchaeota	23	0	0	0	0	0	0	0	0	0
Thaumarchaeota	2	0	0	0	0	0	0	0	0	0
Nanoarchaeota	1	0	0	0	0	0	0	0	0	0
Korarchaeota	1	0	0	0	0	0	0	0	0	0
Total	294	0	0	0	0	0	0	0	0	474

(M00085_1)

Phyla	Module completion ratio (%)									
	0--10	10--20	20--30	30--40	40--50	50--60	60--70	70--80	80--90	90--100
Gammaproteobacteria	126	0	0	0	0	0	0	0	0	0
Betaproteobacteria	61	0	0	0	0	0	0	0	0	0
Epsilonproteobacteria	17	0	0	0	0	0	0	0	0	0
Deltaproteobacteria	27	0	0	1	0	0	0	0	0	0
Alphaproteobacteria	91	0	0	0	0	0	0	0	0	0
Magnetococcus	1	0	0	0	0	0	0	0	0	0
Chrysiogenetes	1	0	0	0	0	0	0	0	0	0
Firmicutes	104	0	0	0	0	0	0	0	0	0
Tenericutes	19	0	0	0	0	0	0	0	0	0
Actinobacteria	80	0	0	0	0	0	0	0	0	0
Chlamydiae	8	0	0	0	0	0	0	0	0	0
Spirochaetes	14	0	0	0	0	0	0	0	0	0
Acidobacteria	5	0	0	0	0	0	0	0	0	0
Bacteroidetes	35	0	0	0	0	0	0	0	0	0
Fibrobacteres	1	0	0	0	0	0	0	0	0	0
Fusobacteria	5	0	0	0	0	0	0	0	0	0
Verrucomicrobia	4	0	0	0	0	0	0	0	0	0
Gemmatimonadetes	1	0	0	0	0	0	0	0	0	0
Planctomycetes	4	0	0	0	0	0	0	0	0	0
Elusimicrobia	2	0	0	0	0	0	0	0	0	0
Synergistetes	2	0	0	0	0	0	0	0	0	0
Cyanobacteria	16	0	0	0	0	0	0	0	0	0
Chlorobi	10	0	0	0	0	0	0	0	0	0
Chloroflexi	11	0	0	0	0	0	0	0	0	0
Deinococcus-Thermus	7	0	0	0	0	0	0	0	0	0
Aquificae	9	0	0	0	0	0	0	0	0	0
Thermotogae	11	0	0	0	0	0	0	0	0	0
Dictyoglomi	2	0	0	0	0	0	0	0	0	0
Nitrospirae	2	0	0	0	0	0	0	0	0	0
Thermobaculum	1	0	0	0	0	0	0	0	0	0
Deferribacteres	3	0	0	0	0	0	0	0	0	0
Euryarchaeota	60	0	0	0	0	0	0	0	0	0
Crenarchaeota	23	0	0	0	0	0	0	0	0	0
Thaumarchaeota	2	0	0	0	0	0	0	0	0	0
Nanoarchaeota	1	0	0	0	0	0	0	0	0	0
Korarchaeota	1	0	0	0	0	0	0	0	0	0
Total	767	0	0	1	0	0	0	0	0	0

(M00086_1)

Phyla	Module completion ratio (%)									
	0--10	10--20	20--30	30--40	40--50	50--60	60--70	70--80	80--90	90--100
Gammaproteobacteria	17	0	0	0	0	0	0	0	0	109
Betaproteobacteria	5	0	0	0	0	0	0	0	0	56
Epsilonproteobacteria	6	0	0	0	0	0	0	0	0	11
Deltaproteobacteria	2	0	0	0	0	0	0	0	0	26
Alphaproteobacteria	36	0	0	0	0	0	0	0	0	55
Magnetococcus	0	0	0	0	0	0	0	0	0	1
Chrysiogenetes	0	0	0	0	0	0	0	0	0	1
Firmicutes	61	0	0	0	0	0	0	0	0	43
Tenericutes	19	0	0	0	0	0	0	0	0	0
Actinobacteria	7	0	0	0	0	0	0	0	0	73
Chlamydiae	7	0	0	0	0	0	0	0	0	1
Spirochaetes	0	0	0	0	0	0	0	0	0	14
Acidobacteria	0	0	0	0	0	0	0	0	0	5
Bacteroidetes	3	0	0	0	0	0	0	0	0	32
Fibrobacteres	0	0	0	0	0	0	0	0	0	1
Fusobacteria	1	0	0	0	0	0	0	0	0	4
Verrucomicrobia	4	0	0	0	0	0	0	0	0	0
Gemmatimonadetes	0	0	0	0	0	0	0	0	0	1
Planctomycetes	2	0	0	0	0	0	0	0	0	2
Elusimicrobia	1	0	0	0	0	0	0	0	0	1
Synergistetes	1	0	0	0	0	0	0	0	0	1
Cyanobacteria	0	0	0	0	0	0	0	0	0	16
Chlorobi	0	0	0	0	0	0	0	0	0	10
Chloroflexi	0	0	0	0	0	0	0	0	0	11
Deinococcus-Thermus	0	0	0	0	0	0	0	0	0	7
Aquificae	0	0	0	0	0	0	0	0	0	9
Thermotogae	11	0	0	0	0	0	0	0	0	0
Dictyoglomi	2	0	0	0	0	0	0	0	0	0
Nitrospirae	1	0	0	0	0	0	0	0	0	1
Thermobaculum	1	0	0	0	0	0	0	0	0	0
Deferribacteres	0	0	0	0	0	0	0	0	0	3
Euryarchaeota	45	0	0	0	0	0	0	0	0	15
Crenarchaeota	11	0	0	0	0	0	0	0	0	12
Thaumarchaeota	2	0	0	0	0	0	0	0	0	0
Nanoarchaeota	1	0	0	0	0	0	0	0	0	0
Korarchaeota	1	0	0	0	0	0	0	0	0	0
Total	247	0	0	0	0	0	0	0	0	521

(M00087_1)

Phyla	Module completion ratio (%)									
	0--10	10--20	20--30	30--40	40--50	50--60	60--70	70--80	80--90	90--100
Gammaproteobacteria	29	0	0	1	0	0	13	0	0	83
Betaproteobacteria	7	0	0	2	0	0	9	0	0	43
Epsilonproteobacteria	17	0	0	0	0	0	0	0	0	0
Deltaproteobacteria	12	0	0	2	0	0	5	0	0	9
Alphaproteobacteria	28	0	0	8	0	0	33	0	0	22
Magnetococcus	0	0	0	0	0	0	1	0	0	0
Chrysiogenetes	0	0	0	1	0	0	0	0	0	0
Firmicutes	74	0	0	6	0	0	16	0	0	8
Tenericutes	19	0	0	0	0	0	0	0	0	0
Actinobacteria	16	0	0	5	0	0	28	0	0	31
Chlamydiae	7	0	0	0	0	0	1	0	0	0
Spirochaetes	11	0	0	2	0	0	1	0	0	0
Acidobacteria	1	0	0	1	0	0	3	0	0	0
Bacteroidetes	13	0	0	6	0	0	12	0	0	4
Fibrobacteres	1	0	0	0	0	0	0	0	0	0
Fusobacteria	5	0	0	0	0	0	0	0	0	0
Verrucomicrobia	4	0	0	0	0	0	0	0	0	0
Gemmatimonadetes	0	0	0	0	0	0	0	0	0	1
Planctomycetes	2	0	0	1	0	0	1	0	0	0
Elusimicrobia	2	0	0	0	0	0	0	0	0	0
Synergistetes	2	0	0	0	0	0	0	0	0	0
Cyanobacteria	15	0	0	0	0	0	0	0	0	1
Chlorobi	10	0	0	0	0	0	0	0	0	0
Chloroflexi	4	0	0	0	0	0	4	0	0	3
Deinococcus-Thermus	0	0	0	0	0	0	2	0	0	5
Aquificae	9	0	0	0	0	0	0	0	0	0
Thermotogae	11	0	0	0	0	0	0	0	0	0
Dictyoglomi	2	0	0	0	0	0	0	0	0	0
Nitrospirae	1	0	0	1	0	0	0	0	0	0
Thermobaculum	1	0	0	0	0	0	0	0	0	0
Deferribacteres	1	0	0	0	0	0	2	0	0	0
Euryarchaeota	43	0	0	8	0	0	9	0	0	0
Crenarchaeota	8	0	0	9	0	0	6	0	0	0
Thaumarchaeota	2	0	0	0	0	0	0	0	0	0
Nanoarchaeota	1	0	0	0	0	0	0	0	0	0
Korarchaeota	0	0	0	1	0	0	0	0	0	0
Total	358	0	0	54	0	0	146	0	0	210

(M00088_1)

Phyla	Module completion ratio (%)									
	0--10	10--20	20--30	30--40	40--50	50--60	60--70	70--80	80--90	90--100
Gammaproteobacteria	34	0	0	34	0	0	58	0	0	0
Betaproteobacteria	11	0	0	4	0	0	46	0	0	0
Epsilonproteobacteria	15	0	0	2	0	0	0	0	0	0
Deltaproteobacteria	11	0	0	5	0	0	8	0	0	4
Alphaproteobacteria	22	0	0	30	0	0	38	0	0	1
Magnetococcus	0	0	0	1	0	0	0	0	0	0
Chrysiogenetes	0	0	0	1	0	0	0	0	0	0
Firmicutes	20	0	0	39	0	0	44	0	0	1
Tenericutes	18	0	0	0	0	0	1	0	0	0
Actinobacteria	14	0	0	26	0	0	38	0	0	2
Chlamydiae	7	0	0	0	0	0	1	0	0	0
Spirochaetes	3	0	0	3	0	0	8	0	0	0
Acidobacteria	2	0	0	0	0	0	3	0	0	0
Bacteroidetes	13	0	0	1	0	0	20	0	0	1
Fibrobacteres	1	0	0	0	0	0	0	0	0	0
Fusobacteria	1	0	0	3	0	0	1	0	0	0
Verrucomicrobia	4	0	0	0	0	0	0	0	0	0
Gemmatimonadetes	0	0	0	1	0	0	0	0	0	0
Planctomycetes	2	0	0	2	0	0	0	0	0	0
Elusimicrobia	2	0	0	0	0	0	0	0	0	0
Synergistetes	1	0	0	0	0	0	1	0	0	0
Cyanobacteria	13	0	0	3	0	0	0	0	0	0
Chlorobi	10	0	0	0	0	0	0	0	0	0
Chloroflexi	3	0	0	1	0	0	7	0	0	0
Deinococcus-Thermus	0	0	0	3	0	0	4	0	0	0
Aquificae	9	0	0	0	0	0	0	0	0	0
Thermotogae	5	0	0	6	0	0	0	0	0	0
Dictyoglomi	2	0	0	0	0	0	0	0	0	0
Nitrospirae	1	0	0	0	0	0	1	0	0	0
Thermobaculum	0	0	0	1	0	0	0	0	0	0
Deferribacteres	0	0	0	3	0	0	0	0	0	0
Euryarchaeota	0	0	0	50	0	0	8	0	0	2
Crenarchaeota	2	0	0	21	0	0	0	0	0	0
Thaumarchaeota	0	0	0	0	0	0	2	0	0	0
Nanoarchaeota	1	0	0	0	0	0	0	0	0	0
Korarchaeota	0	0	0	1	0	0	0	0	0	0
Total	227	0	0	241	0	0	289	0	0	11

(M00088_2)

Phyla	Module completion ratio (%)									
	0--10	10--20	20--30	30--40	40--50	50--60	60--70	70--80	80--90	90--100
Gammaproteobacteria	92	0	0	0	0	0	0	0	0	34
Betaproteobacteria	18	0	0	0	0	0	0	0	0	43
Epsilonproteobacteria	17	0	0	0	0	0	0	0	0	0
Deltaproteobacteria	23	0	0	0	0	0	0	0	0	5
Alphaproteobacteria	30	0	0	0	0	0	0	0	0	61
Magnetococcus	1	0	0	0	0	0	0	0	0	0
Chrysiogenetes	1	0	0	0	0	0	0	0	0	0
Firmicutes	85	0	0	0	0	0	0	0	0	19
Tenericutes	19	0	0	0	0	0	0	0	0	0
Actinobacteria	46	0	0	0	0	0	0	0	0	34
Chlamydiae	8	0	0	0	0	0	0	0	0	0
Spirochaetes	14	0	0	0	0	0	0	0	0	0
Acidobacteria	5	0	0	0	0	0	0	0	0	0
Bacteroidetes	35	0	0	0	0	0	0	0	0	0
Fibrobacteres	1	0	0	0	0	0	0	0	0	0
Fusobacteria	5	0	0	0	0	0	0	0	0	0
Verrucomicrobia	4	0	0	0	0	0	0	0	0	0
Gemmatimonadetes	1	0	0	0	0	0	0	0	0	0
Planctomycetes	4	0	0	0	0	0	0	0	0	0
Elusimicrobia	2	0	0	0	0	0	0	0	0	0
Synergistetes	2	0	0	0	0	0	0	0	0	0
Cyanobacteria	16	0	0	0	0	0	0	0	0	0
Chlorobi	10	0	0	0	0	0	0	0	0	0
Chloroflexi	9	0	0	0	0	0	0	0	0	2
Deinococcus-Thermus	1	0	0	0	0	0	0	0	0	6
Aquificae	9	0	0	0	0	0	0	0	0	0
Thermotogae	11	0	0	0	0	0	0	0	0	0
Dictyoglomi	2	0	0	0	0	0	0	0	0	0
Nitrospirae	2	0	0	0	0	0	0	0	0	0
Thermobaculum	1	0	0	0	0	0	0	0	0	0
Deferribacteres	2	0	0	0	0	0	0	0	0	1
Euryarchaeota	53	0	0	0	0	0	0	0	0	7
Crenarchaeota	23	0	0	0	0	0	0	0	0	0
Thaumarchaeota	2	0	0	0	0	0	0	0	0	0
Nanoarchaeota	1	0	0	0	0	0	0	0	0	0
Korarchaeota	1	0	0	0	0	0	0	0	0	0
Total	556	0	0	0	0	0	0	0	0	212

(M00088_3)

Phyla	Module completion ratio (%)									
	0--10	10--20	20--30	30--40	40--50	50--60	60--70	70--80	80--90	90--100
Gammaproteobacteria	122	0	0	0	0	0	0	0	0	4
Betaproteobacteria	44	0	0	0	0	0	0	0	0	17
Epsilonproteobacteria	17	0	0	0	0	0	0	0	0	0
Deltaproteobacteria	28	0	0	0	0	0	0	0	0	0
Alphaproteobacteria	79	0	0	0	0	0	0	0	0	12
Magnetococcus	1	0	0	0	0	0	0	0	0	0
Chrysiogenetes	1	0	0	0	0	0	0	0	0	0
Firmicutes	99	0	0	0	0	0	0	0	0	5
Tenericutes	19	0	0	0	0	0	0	0	0	0
Actinobacteria	67	0	0	0	0	0	0	0	0	13
Chlamydiae	8	0	0	0	0	0	0	0	0	0
Spirochaetes	14	0	0	0	0	0	0	0	0	0
Acidobacteria	5	0	0	0	0	0	0	0	0	0
Bacteroidetes	35	0	0	0	0	0	0	0	0	0
Fibrobacteres	1	0	0	0	0	0	0	0	0	0
Fusobacteria	5	0	0	0	0	0	0	0	0	0
Verrucomicrobia	4	0	0	0	0	0	0	0	0	0
Gemmatimonadetes	1	0	0	0	0	0	0	0	0	0
Planctomycetes	4	0	0	0	0	0	0	0	0	0
Elusimicrobia	2	0	0	0	0	0	0	0	0	0
Synergistetes	2	0	0	0	0	0	0	0	0	0
Cyanobacteria	16	0	0	0	0	0	0	0	0	0
Chlorobi	9	0	0	0	0	0	0	0	0	1
Chloroflexi	11	0	0	0	0	0	0	0	0	0
Deinococcus-Thermus	7	0	0	0	0	0	0	0	0	0
Aquificae	9	0	0	0	0	0	0	0	0	0
Thermotogae	11	0	0	0	0	0	0	0	0	0
Dictyoglomi	2	0	0	0	0	0	0	0	0	0
Nitrospirae	2	0	0	0	0	0	0	0	0	0
Thermobaculum	1	0	0	0	0	0	0	0	0	0
Deferribacteres	3	0	0	0	0	0	0	0	0	0
Euryarchaeota	60	0	0	0	0	0	0	0	0	0
Crenarchaeota	22	0	0	0	0	0	0	0	0	1
Thaumarchaeota	2	0	0	0	0	0	0	0	0	0
Nanoarchaeota	1	0	0	0	0	0	0	0	0	0
Korarchaeota	1	0	0	0	0	0	0	0	0	0
Total	715	0	0	0	0	0	0	0	0	53

(M00089_1)

Phyla	Module completion ratio (%)									
	0--10	10--20	20--30	30--40	40--50	50--60	60--70	70--80	80--90	90--100
Gammaproteobacteria	2	0	1	0	0	119	0	4	0	0
Betaproteobacteria	1	0	0	0	0	60	0	0	0	0
Epsilonproteobacteria	0	0	0	0	0	17	0	0	0	0
Deltaproteobacteria	0	0	2	0	0	25	0	1	0	0
Alphaproteobacteria	1	0	11	0	0	79	0	0	0	0
Magnetococcus	0	0	0	0	0	1	0	0	0	0
Chrysiogenetes	0	0	0	0	0	1	0	0	0	0
Firmicutes	0	0	8	0	0	96	0	0	0	0
Tenericutes	0	0	1	0	0	18	0	0	0	0
Actinobacteria	0	0	48	0	0	30	0	2	0	0
Chlamydiae	0	0	0	0	0	8	0	0	0	0
Spirochaetes	0	0	3	0	0	11	0	0	0	0
Acidobacteria	0	0	0	0	0	4	0	1	0	0
Bacteroidetes	2	0	21	0	0	12	0	0	0	0
Fibrobacteres	0	0	0	0	0	1	0	0	0	0
Fusobacteria	0	0	2	0	0	3	0	0	0	0
Verrucomicrobia	0	0	0	0	0	4	0	0	0	0
Gemmatimonadetes	0	0	0	0	0	1	0	0	0	0
Planctomycetes	1	0	1	0	0	2	0	0	0	0
Elusimicrobia	0	0	0	0	0	2	0	0	0	0
Synergistetes	0	0	0	0	0	2	0	0	0	0
Cyanobacteria	0	0	0	0	0	16	0	0	0	0
Chlorobi	0	0	0	0	0	10	0	0	0	0
Chloroflexi	0	0	4	0	0	7	0	0	0	0
Deinococcus-Thermus	0	0	3	0	0	4	0	0	0	0
Aquificae	0	0	1	0	0	8	0	0	0	0
Thermotogae	0	0	0	0	0	11	0	0	0	0
Dictyoglomi	0	0	0	0	0	2	0	0	0	0
Nitrospirae	0	0	0	0	0	2	0	0	0	0
Thermobaculum	0	0	0	0	0	1	0	0	0	0
Deferribacteres	0	0	0	0	0	3	0	0	0	0
Euryarchaeota	60	0	0	0	0	0	0	0	0	0
Crenarchaeota	23	0	0	0	0	0	0	0	0	0
Thaumarchaeota	2	0	0	0	0	0	0	0	0	0
Nanoarchaeota	1	0	0	0	0	0	0	0	0	0
Korarchaeota	1	0	0	0	0	0	0	0	0	0
Total	94	0	106	0	0	560	0	8	0	0

(M00090_1)

Phyla	Module completion ratio (%)									
	0--10	10--20	20--30	30--40	40--50	50--60	60--70	70--80	80--90	90--100
Gammaproteobacteria	123	0	0	3	0	0	0	0	0	0
Betaproteobacteria	60	0	0	1	0	0	0	0	0	0
Epsilonproteobacteria	17	0	0	0	0	0	0	0	0	0
Deltaproteobacteria	27	0	0	1	0	0	0	0	0	0
Alphaproteobacteria	91	0	0	0	0	0	0	0	0	0
Magnetococcus	1	0	0	0	0	0	0	0	0	0
Chrysiogenetes	1	0	0	0	0	0	0	0	0	0
Firmicutes	100	0	0	4	0	0	0	0	0	0
Tenericutes	19	0	0	0	0	0	0	0	0	0
Actinobacteria	77	0	0	3	0	0	0	0	0	0
Chlamydiae	8	0	0	0	0	0	0	0	0	0
Spirochaetes	14	0	0	0	0	0	0	0	0	0
Acidobacteria	5	0	0	0	0	0	0	0	0	0
Bacteroidetes	34	0	0	1	0	0	0	0	0	0
Fibrobacteres	1	0	0	0	0	0	0	0	0	0
Fusobacteria	5	0	0	0	0	0	0	0	0	0
Verrucomicrobia	3	0	0	1	0	0	0	0	0	0
Gemmatimonadetes	1	0	0	0	0	0	0	0	0	0
Planctomycetes	4	0	0	0	0	0	0	0	0	0
Elusimicrobia	2	0	0	0	0	0	0	0	0	0
Synergistetes	2	0	0	0	0	0	0	0	0	0
Cyanobacteria	16	0	0	0	0	0	0	0	0	0
Chlorobi	10	0	0	0	0	0	0	0	0	0
Chloroflexi	11	0	0	0	0	0	0	0	0	0
Deinococcus-Thermus	7	0	0	0	0	0	0	0	0	0
Aquificae	9	0	0	0	0	0	0	0	0	0
Thermotogae	11	0	0	0	0	0	0	0	0	0
Dictyoglomi	2	0	0	0	0	0	0	0	0	0
Nitrospirae	2	0	0	0	0	0	0	0	0	0
Thermobaculum	1	0	0	0	0	0	0	0	0	0
Deferribacteres	3	0	0	0	0	0	0	0	0	0
Euryarchaeota	60	0	0	0	0	0	0	0	0	0
Crenarchaeota	23	0	0	0	0	0	0	0	0	0
Thaumarchaeota	2	0	0	0	0	0	0	0	0	0
Nanoarchaeota	1	0	0	0	0	0	0	0	0	0
Korarchaeota	1	0	0	0	0	0	0	0	0	0
Total	754	0	0	14	0	0	0	0	0	0

(M00091_1)

Phyla	Module completion ratio (%)									
	0--10	10--20	20--30	30--40	40--50	50--60	60--70	70--80	80--90	90--100
Gammaproteobacteria	114	0	0	0	0	11	0	0	0	1
Betaproteobacteria	53	0	0	0	0	8	0	0	0	0
Epsilonproteobacteria	17	0	0	0	0	0	0	0	0	0
Deltaproteobacteria	28	0	0	0	0	0	0	0	0	0
Alphaproteobacteria	55	0	0	0	0	30	0	0	0	6
Magnetococcus	1	0	0	0	0	0	0	0	0	0
Chrysiogenetes	1	0	0	0	0	0	0	0	0	0
Firmicutes	103	0	0	0	0	0	0	0	0	1
Tenericutes	18	0	0	0	0	1	0	0	0	0
Actinobacteria	77	0	0	0	0	1	0	0	0	2
Chlamydiae	8	0	0	0	0	0	0	0	0	0
Spirochaetes	14	0	0	0	0	0	0	0	0	0
Acidobacteria	5	0	0	0	0	0	0	0	0	0
Bacteroidetes	34	0	0	0	0	1	0	0	0	0
Fibrobacteres	1	0	0	0	0	0	0	0	0	0
Fusobacteria	5	0	0	0	0	0	0	0	0	0
Verrucomicrobia	4	0	0	0	0	0	0	0	0	0
Gemmatimonadetes	1	0	0	0	0	0	0	0	0	0
Planctomycetes	3	0	0	0	0	1	0	0	0	0
Elusimicrobia	2	0	0	0	0	0	0	0	0	0
Synergistetes	2	0	0	0	0	0	0	0	0	0
Cyanobacteria	14	0	0	0	0	2	0	0	0	0
Chlorobi	10	0	0	0	0	0	0	0	0	0
Chloroflexi	11	0	0	0	0	0	0	0	0	0
Deinococcus-Thermus	6	0	0	0	0	1	0	0	0	0
Aquificae	9	0	0	0	0	0	0	0	0	0
Thermotogae	11	0	0	0	0	0	0	0	0	0
Dictyoglomi	2	0	0	0	0	0	0	0	0	0
Nitrospirae	1	0	0	0	0	1	0	0	0	0
Thermobaculum	1	0	0	0	0	0	0	0	0	0
Deferribacteres	3	0	0	0	0	0	0	0	0	0
Euryarchaeota	60	0	0	0	0	0	0	0	0	0
Crenarchaeota	23	0	0	0	0	0	0	0	0	0
Thaumarchaeota	2	0	0	0	0	0	0	0	0	0
Nanoarchaeota	1	0	0	0	0	0	0	0	0	0
Korarchaeota	1	0	0	0	0	0	0	0	0	0
Total	701	0	0	0	0	57	0	0	0	10

(M00092_1)

Phyla	Module completion ratio (%)									
	0--10	10--20	20--30	30--40	40--50	50--60	60--70	70--80	80--90	90--100
Gammaproteobacteria	126	0	0	0	0	0	0	0	0	0
Betaproteobacteria	61	0	0	0	0	0	0	0	0	0
Epsilonproteobacteria	17	0	0	0	0	0	0	0	0	0
Deltaproteobacteria	28	0	0	0	0	0	0	0	0	0
Alphaproteobacteria	91	0	0	0	0	0	0	0	0	0
Magnetococcus	1	0	0	0	0	0	0	0	0	0
Chrysiogenetes	1	0	0	0	0	0	0	0	0	0
Firmicutes	104	0	0	0	0	0	0	0	0	0
Tenericutes	19	0	0	0	0	0	0	0	0	0
Actinobacteria	80	0	0	0	0	0	0	0	0	0
Chlamydiae	8	0	0	0	0	0	0	0	0	0
Spirochaetes	14	0	0	0	0	0	0	0	0	0
Acidobacteria	5	0	0	0	0	0	0	0	0	0
Bacteroidetes	35	0	0	0	0	0	0	0	0	0
Fibrobacteres	1	0	0	0	0	0	0	0	0	0
Fusobacteria	5	0	0	0	0	0	0	0	0	0
Verrucomicrobia	4	0	0	0	0	0	0	0	0	0
Gemmatimonadetes	1	0	0	0	0	0	0	0	0	0
Planctomycetes	4	0	0	0	0	0	0	0	0	0
Elusimicrobia	2	0	0	0	0	0	0	0	0	0
Synergistetes	2	0	0	0	0	0	0	0	0	0
Cyanobacteria	16	0	0	0	0	0	0	0	0	0
Chlorobi	10	0	0	0	0	0	0	0	0	0
Chloroflexi	11	0	0	0	0	0	0	0	0	0
Deinococcus-Thermus	7	0	0	0	0	0	0	0	0	0
Aquificae	9	0	0	0	0	0	0	0	0	0
Thermotogae	11	0	0	0	0	0	0	0	0	0
Dictyoglomi	2	0	0	0	0	0	0	0	0	0
Nitrospirae	2	0	0	0	0	0	0	0	0	0
Thermobaculum	1	0	0	0	0	0	0	0	0	0
Deferribacteres	3	0	0	0	0	0	0	0	0	0
Euryarchaeota	60	0	0	0	0	0	0	0	0	0
Crenarchaeota	23	0	0	0	0	0	0	0	0	0
Thaumarchaeota	2	0	0	0	0	0	0	0	0	0
Nanoarchaeota	1	0	0	0	0	0	0	0	0	0
Korarchaeota	1	0	0	0	0	0	0	0	0	0
Total	768	0	0	0	0	0	0	0	0	0

(M00093_1)

Phyla	Module completion ratio (%)									
	0--10	10--20	20--30	30--40	40--50	50--60	60--70	70--80	80--90	90--100
Gammaproteobacteria	2	0	0	0	0	0	3	0	0	121
Betaproteobacteria	1	0	0	1	0	0	0	0	0	59
Epsilonproteobacteria	0	0	0	0	0	0	0	0	0	17
Deltaproteobacteria	0	0	0	0	0	0	0	0	0	28
Alphaproteobacteria	1	0	0	11	0	0	2	0	0	77
Magnetococcus	0	0	0	0	0	0	0	0	0	1
Chrysiogenetes	0	0	0	0	0	0	0	0	0	1
Firmicutes	0	0	0	56	0	0	5	0	0	43
Tenericutes	2	0	0	14	0	0	0	0	0	3
Actinobacteria	0	0	0	44	0	0	0	0	0	36
Chlamydiae	0	0	0	0	0	0	0	0	0	8
Spirochaetes	0	0	0	11	0	0	2	0	0	1
Acidobacteria	0	0	0	0	0	0	0	0	0	5
Bacteroidetes	1	0	0	0	0	0	2	0	0	32
Fibrobacteres	0	0	0	0	0	0	0	0	0	1
Fusobacteria	0	0	0	3	0	0	0	0	0	2
Verrucomicrobia	0	0	0	0	0	0	0	0	0	4
Gemmatimonadetes	0	0	0	0	0	0	0	0	0	1
Planctomycetes	0	0	0	3	0	0	0	0	0	1
Elusimicrobia	0	0	0	0	0	0	0	0	0	2
Synergistetes	0	0	0	0	0	0	0	0	0	2
Cyanobacteria	0	0	0	16	0	0	0	0	0	0
Chlorobi	0	0	0	0	0	0	0	0	0	10
Chloroflexi	0	0	0	5	0	0	5	0	0	1
Deinococcus-Thermus	0	0	0	5	0	0	2	0	0	0
Aquificae	0	0	0	9	0	0	0	0	0	0
Thermotogae	0	0	0	11	0	0	0	0	0	0
Dictyoglomi	0	0	0	2	0	0	0	0	0	0
Nitrospirae	0	0	0	0	0	0	0	0	0	2
Thermobaculum	0	0	0	1	0	0	0	0	0	0
Deferribacteres	0	0	0	0	0	0	0	0	0	3
Euryarchaeota	21	0	0	10	0	0	29	0	0	0
Crenarchaeota	23	0	0	0	0	0	0	0	0	0
Thaumarchaeota	2	0	0	0	0	0	0	0	0	0
Nanoarchaeota	1	0	0	0	0	0	0	0	0	0
Korarchaeota	1	0	0	0	0	0	0	0	0	0
Total	55	0	0	202	0	0	50	0	0	461

(M00094_1)

Phyla	Module completion ratio (%)									
	0--10	10--20	20--30	30--40	40--50	50--60	60--70	70--80	80--90	90--100
Gammaproteobacteria	126	0	0	0	0	0	0	0	0	0
Betaproteobacteria	61	0	0	0	0	0	0	0	0	0
Epsilonproteobacteria	17	0	0	0	0	0	0	0	0	0
Deltaproteobacteria	26	0	2	0	0	0	0	0	0	0
Alphaproteobacteria	88	0	3	0	0	0	0	0	0	0
Magnetococcus	1	0	0	0	0	0	0	0	0	0
Chrysiogenetes	1	0	0	0	0	0	0	0	0	0
Firmicutes	104	0	0	0	0	0	0	0	0	0
Tenericutes	19	0	0	0	0	0	0	0	0	0
Actinobacteria	80	0	0	0	0	0	0	0	0	0
Chlamydiae	8	0	0	0	0	0	0	0	0	0
Spirochaetes	14	0	0	0	0	0	0	0	0	0
Acidobacteria	5	0	0	0	0	0	0	0	0	0
Bacteroidetes	32	0	3	0	0	0	0	0	0	0
Fibrobacteres	1	0	0	0	0	0	0	0	0	0
Fusobacteria	5	0	0	0	0	0	0	0	0	0
Verrucomicrobia	4	0	0	0	0	0	0	0	0	0
Gemmatimonadetes	1	0	0	0	0	0	0	0	0	0
Planctomycetes	4	0	0	0	0	0	0	0	0	0
Elusimicrobia	2	0	0	0	0	0	0	0	0	0
Synergistetes	2	0	0	0	0	0	0	0	0	0
Cyanobacteria	16	0	0	0	0	0	0	0	0	0
Chlorobi	10	0	0	0	0	0	0	0	0	0
Chloroflexi	11	0	0	0	0	0	0	0	0	0
Deinococcus-Thermus	7	0	0	0	0	0	0	0	0	0
Aquificae	9	0	0	0	0	0	0	0	0	0
Thermotogae	11	0	0	0	0	0	0	0	0	0
Dictyoglomi	2	0	0	0	0	0	0	0	0	0
Nitrospirae	2	0	0	0	0	0	0	0	0	0
Thermobaculum	1	0	0	0	0	0	0	0	0	0
Deferribacteres	3	0	0	0	0	0	0	0	0	0
Euryarchaeota	59	0	1	0	0	0	0	0	0	0
Crenarchaeota	23	0	0	0	0	0	0	0	0	0
Thaumarchaeota	2	0	0	0	0	0	0	0	0	0
Nanoarchaeota	1	0	0	0	0	0	0	0	0	0
Korarchaeota	1	0	0	0	0	0	0	0	0	0
Total	759	0	9	0	0	0	0	0	0	0

(M00095_1)

Phyla	Module completion ratio (%)									
	0--10	10--20	20--30	30--40	40--50	50--60	60--70	70--80	80--90	90--100
Gammaproteobacteria	28	60	29	0	4	3	0	1	1	0
Betaproteobacteria	11	47	3	0	0	0	0	0	0	0
Epsilonproteobacteria	15	2	0	0	0	0	0	0	0	0
Deltaproteobacteria	7	15	1	0	0	1	0	0	4	0
Alphaproteobacteria	18	50	21	0	1	0	0	1	0	0
Magnetococcus	0	1	0	0	0	0	0	0	0	0
Chrysiogenetes	0	1	0	0	0	0	0	0	0	0
Firmicutes	18	28	23	0	0	0	0	12	23	0
Tenericutes	18	0	0	0	0	0	0	0	1	0
Actinobacteria	9	18	49	0	0	0	0	1	1	2
Chlamydiae	7	0	0	0	0	0	0	0	1	0
Spirochaetes	3	4	0	0	0	1	0	0	6	0
Acidobacteria	1	4	0	0	0	0	0	0	0	0
Bacteroidetes	11	2	8	0	3	10	0	1	0	0
Fibrobacteres	1	0	0	0	0	0	0	0	0	0
Fusobacteria	0	3	0	0	0	0	0	1	1	0
Verrucomicrobia	4	0	0	0	0	0	0	0	0	0
Gemmatimonadetes	0	1	0	0	0	0	0	0	0	0
Planctomycetes	2	2	0	0	0	0	0	0	0	0
Elusimicrobia	2	0	0	0	0	0	0	0	0	0
Synergistetes	1	1	0	0	0	0	0	0	0	0
Cyanobacteria	4	9	3	0	0	0	0	0	0	0
Chlorobi	0	10	0	0	0	0	0	0	0	0
Chloroflexi	3	2	0	0	0	6	0	0	0	0
Deinococcus-Thermus	0	1	6	0	0	0	0	0	0	0
Aquificae	9	0	0	0	0	0	0	0	0	0
Thermotogae	5	6	0	0	0	0	0	0	0	0
Dictyoglomi	2	0	0	0	0	0	0	0	0	0
Nitrospirae	1	1	0	0	0	0	0	0	0	0
Thermobaculum	0	0	0	0	1	0	0	0	0	0
Deferribacteres	0	3	0	0	0	0	0	0	0	0
Euryarchaeota	0	0	2	0	6	39	0	3	10	0
Crenarchaeota	0	0	0	0	7	11	0	0	5	0
Thaumarchaeota	0	0	0	0	0	2	0	0	0	0
Nanoarchaeota	1	0	0	0	0	0	0	0	0	0
Korarchaeota	0	0	0	0	1	0	0	0	0	0
Total	181	271	145	0	23	73	0	20	53	2

(M00096_1)

Phyla	Module completion ratio (%)									
	0--10	10--20	20--30	30--40	40--50	50--60	60--70	70--80	80--90	90--100
Gammaproteobacteria	2	3	0	2	0	0	0	2	85	32
Betaproteobacteria	1	0	0	0	0	0	0	0	58	2
Epsilonproteobacteria	0	0	0	0	0	0	0	0	17	0
Deltaproteobacteria	0	3	2	0	0	0	0	0	18	5
Alphaproteobacteria	2	10	0	0	0	0	0	19	47	13
Magnetococcus	0	0	0	0	0	0	0	0	1	0
Chrysiogenetes	0	0	0	0	0	0	0	0	1	0
Firmicutes	0	8	14	8	0	2	1	6	43	22
Tenericutes	14	3	0	0	0	1	0	0	1	0
Actinobacteria	0	1	0	0	0	0	0	2	27	50
Chlamydiae	0	0	1	0	0	0	0	1	6	0
Spirochaetes	0	7	0	0	0	0	0	0	7	0
Acidobacteria	0	0	0	0	0	0	0	1	4	0
Bacteroidetes	2	3	10	0	0	0	0	2	11	7
Fibrobacteres	0	0	0	0	0	0	0	0	1	0
Fusobacteria	0	1	1	0	0	0	0	0	3	0
Verrucomicrobia	0	0	0	0	0	0	0	0	4	0
Gemmatimonadetes	0	0	0	0	0	0	0	0	1	0
Planctomycetes	0	0	0	0	0	0	0	0	4	0
Elusimicrobia	0	0	0	0	0	0	0	0	2	0
Synergistetes	0	0	0	0	0	0	0	0	2	0
Cyanobacteria	0	0	0	0	0	0	0	1	3	12
Chlorobi	0	0	0	0	0	0	0	0	2	8
Chloroflexi	0	6	0	0	0	0	0	0	5	0
Deinococcus-Thermus	0	0	0	0	0	0	0	0	1	6
Aquificae	0	0	0	0	0	0	0	1	8	0
Thermotogae	0	0	0	0	0	0	0	0	11	0
Dictyoglomi	0	0	0	0	0	0	0	0	2	0
Nitrospirae	0	0	0	0	0	0	0	1	1	0
Thermobaculum	0	0	0	0	0	0	0	0	0	1
Deferribacteres	0	0	0	0	0	0	0	0	3	0
Euryarchaeota	0	56	4	0	0	0	0	0	0	0
Crenarchaeota	0	23	0	0	0	0	0	0	0	0
Thaumarchaeota	0	2	0	0	0	0	0	0	0	0
Nanoarchaeota	1	0	0	0	0	0	0	0	0	0
Korarchaeota	0	1	0	0	0	0	0	0	0	0
Total	22	127	32	10	0	3	1	36	379	158

(M00097_1)

Phyla	Module completion ratio (%)									
	0--10	10--20	20--30	30--40	40--50	50--60	60--70	70--80	80--90	90--100
Gammaproteobacteria	113	0	0	5	0	0	2	0	0	6
Betaproteobacteria	5	0	0	56	0	0	0	0	0	0
Epsilonproteobacteria	17	0	0	0	0	0	0	0	0	0
Deltaproteobacteria	22	0	0	1	0	0	3	0	0	2
Alphaproteobacteria	27	0	0	38	0	0	14	0	0	12
Magnetococcus	0	0	0	1	0	0	0	0	0	0
Chrysiogenetes	1	0	0	0	0	0	0	0	0	0
Firmicutes	89	0	0	11	0	0	4	0	0	0
Tenericutes	18	0	0	1	0	0	0	0	0	0
Actinobacteria	39	0	0	22	0	0	18	0	0	1
Chlamydiae	8	0	0	0	0	0	0	0	0	0
Spirochaetes	14	0	0	0	0	0	0	0	0	0
Acidobacteria	0	0	0	2	0	0	3	0	0	0
Bacteroidetes	17	0	0	6	0	0	12	0	0	0
Fibrobacteres	1	0	0	0	0	0	0	0	0	0
Fusobacteria	4	0	0	1	0	0	0	0	0	0
Verrucomicrobia	3	0	0	1	0	0	0	0	0	0
Gemmatimonadetes	0	0	0	0	0	0	1	0	0	0
Planctomycetes	0	0	0	1	0	0	2	0	0	1
Elusimicrobia	1	0	0	1	0	0	0	0	0	0
Synergistetes	2	0	0	0	0	0	0	0	0	0
Cyanobacteria	0	0	0	1	0	0	13	0	0	2
Chlorobi	0	0	0	0	0	0	9	0	0	1
Chloroflexi	4	0	0	0	0	0	3	0	0	4
Deinococcus-Thermus	1	0	0	0	0	0	2	0	0	4
Aquificae	9	0	0	0	0	0	0	0	0	0
Thermotogae	11	0	0	0	0	0	0	0	0	0
Dictyoglomi	2	0	0	0	0	0	0	0	0	0
Nitrospirae	1	0	0	1	0	0	0	0	0	0
Thermobaculum	0	0	0	0	0	0	1	0	0	0
Deferribacteres	3	0	0	0	0	0	0	0	0	0
Euryarchaeota	43	0	0	0	0	0	17	0	0	0
Crenarchaeota	19	0	0	0	0	0	4	0	0	0
Thaumarchaeota	2	0	0	0	0	0	0	0	0	0
Nanoarchaeota	1	0	0	0	0	0	0	0	0	0
Korarchaeota	1	0	0	0	0	0	0	0	0	0
Total	478	0	0	149	0	0	108	0	0	33

(M00097_2)

Phyla	Module completion ratio (%)									
	0--10	10--20	20--30	30--40	40--50	50--60	60--70	70--80	80--90	90--100
Gammaproteobacteria	112	0	8	0	0	6	0	0	0	0
Betaproteobacteria	5	0	56	0	0	0	0	0	0	0
Epsilonproteobacteria	17	0	0	0	0	0	0	0	0	0
Deltaproteobacteria	22	0	4	0	0	2	0	0	0	0
Alphaproteobacteria	27	0	51	0	0	13	0	0	0	0
Magnetococcus	0	0	1	0	0	0	0	0	0	0
Chrysiogenetes	1	0	0	0	0	0	0	0	0	0
Firmicutes	92	0	12	0	0	0	0	0	0	0
Tenericutes	18	0	1	0	0	0	0	0	0	0
Actinobacteria	41	0	22	0	0	17	0	0	0	0
Chlamydiae	8	0	0	0	0	0	0	0	0	0
Spirochaetes	14	0	0	0	0	0	0	0	0	0
Acidobacteria	0	0	4	0	0	1	0	0	0	0
Bacteroidetes	22	0	13	0	0	0	0	0	0	0
Fibrobacteres	1	0	0	0	0	0	0	0	0	0
Fusobacteria	5	0	0	0	0	0	0	0	0	0
Verrucomicrobia	2	0	2	0	0	0	0	0	0	0
Gemmatimonadetes	0	0	1	0	0	0	0	0	0	0
Planctomycetes	0	0	3	0	0	1	0	0	0	0
Elusimicrobia	1	0	1	0	0	0	0	0	0	0
Synergistetes	2	0	0	0	0	0	0	0	0	0
Cyanobacteria	0	0	0	0	0	2	0	0	0	14
Chlorobi	0	0	0	0	0	0	0	5	0	5
Chloroflexi	4	0	3	0	0	2	0	2	0	0
Deinococcus-Thermus	1	0	2	0	0	4	0	0	0	0
Aquificae	9	0	0	0	0	0	0	0	0	0
Thermotogae	11	0	0	0	0	0	0	0	0	0
Dictyoglomi	2	0	0	0	0	0	0	0	0	0
Nitrospirae	1	0	1	0	0	0	0	0	0	0
Thermobaculum	0	0	1	0	0	0	0	0	0	0
Deferribacteres	3	0	0	0	0	0	0	0	0	0
Euryarchaeota	43	0	17	0	0	0	0	0	0	0
Crenarchaeota	19	0	4	0	0	0	0	0	0	0
Thaumarchaeota	2	0	0	0	0	0	0	0	0	0
Nanoarchaeota	1	0	0	0	0	0	0	0	0	0
Korarchaeota	1	0	0	0	0	0	0	0	0	0
Total	487	0	207	0	0	48	0	7	0	19

(M00098_1)

Phyla	Module completion ratio (%)									
	0--10	10--20	20--30	30--40	40--50	50--60	60--70	70--80	80--90	90--100
Gammaproteobacteria	91	0	0	0	0	35	0	0	0	0
Betaproteobacteria	43	0	0	0	0	16	0	0	0	2
Epsilonproteobacteria	17	0	0	0	0	0	0	0	0	0
Deltaproteobacteria	22	0	0	0	0	6	0	0	0	0
Alphaproteobacteria	87	0	0	0	0	4	0	0	0	0
Magnetococcus	1	0	0	0	0	0	0	0	0	0
Chrysiogenetes	1	0	0	0	0	0	0	0	0	0
Firmicutes	80	0	0	0	0	24	0	0	0	0
Tenericutes	11	0	0	0	0	8	0	0	0	0
Actinobacteria	64	0	0	0	0	12	0	0	0	4
Chlamydiae	8	0	0	0	0	0	0	0	0	0
Spirochaetes	11	0	0	0	0	3	0	0	0	0
Acidobacteria	5	0	0	0	0	0	0	0	0	0
Bacteroidetes	33	0	0	0	0	2	0	0	0	0
Fibrobacteres	1	0	0	0	0	0	0	0	0	0
Fusobacteria	4	0	0	0	0	1	0	0	0	0
Verrucomicrobia	3	0	0	0	0	1	0	0	0	0
Gemmatimonadetes	1	0	0	0	0	0	0	0	0	0
Planctomycetes	3	0	0	0	0	1	0	0	0	0
Elusimicrobia	2	0	0	0	0	0	0	0	0	0
Synergistetes	2	0	0	0	0	0	0	0	0	0
Cyanobacteria	7	0	0	0	0	8	0	0	0	1
Chlorobi	10	0	0	0	0	0	0	0	0	0
Chloroflexi	11	0	0	0	0	0	0	0	0	0
Deinococcus-Thermus	5	0	0	0	0	2	0	0	0	0
Aquificae	9	0	0	0	0	0	0	0	0	0
Thermotogae	11	0	0	0	0	0	0	0	0	0
Dictyoglomi	2	0	0	0	0	0	0	0	0	0
Nitrospirae	2	0	0	0	0	0	0	0	0	0
Thermobaculum	1	0	0	0	0	0	0	0	0	0
Deferribacteres	3	0	0	0	0	0	0	0	0	0
Euryarchaeota	60	0	0	0	0	0	0	0	0	0
Crenarchaeota	23	0	0	0	0	0	0	0	0	0
Thaumarchaeota	2	0	0	0	0	0	0	0	0	0
Nanoarchaeota	1	0	0	0	0	0	0	0	0	0
Korarchaeota	1	0	0	0	0	0	0	0	0	0
Total	638	0	0	0	0	123	0	0	0	7

(M00099_1)

Phyla	Module completion ratio (%)									
	0--10	10--20	20--30	30--40	40--50	50--60	60--70	70--80	80--90	90--100
Gammaproteobacteria	126	0	0	0	0	0	0	0	0	0
Betaproteobacteria	61	0	0	0	0	0	0	0	0	0
Epsilonproteobacteria	17	0	0	0	0	0	0	0	0	0
Deltaproteobacteria	26	0	2	0	0	0	0	0	0	0
Alphaproteobacteria	88	0	3	0	0	0	0	0	0	0
Magnetococcus	1	0	0	0	0	0	0	0	0	0
Chrysiogenetes	1	0	0	0	0	0	0	0	0	0
Firmicutes	104	0	0	0	0	0	0	0	0	0
Tenericutes	19	0	0	0	0	0	0	0	0	0
Actinobacteria	80	0	0	0	0	0	0	0	0	0
Chlamydiae	8	0	0	0	0	0	0	0	0	0
Spirochaetes	14	0	0	0	0	0	0	0	0	0
Acidobacteria	5	0	0	0	0	0	0	0	0	0
Bacteroidetes	32	0	3	0	0	0	0	0	0	0
Fibrobacteres	1	0	0	0	0	0	0	0	0	0
Fusobacteria	5	0	0	0	0	0	0	0	0	0
Verrucomicrobia	4	0	0	0	0	0	0	0	0	0
Gemmatimonadetes	1	0	0	0	0	0	0	0	0	0
Planctomycetes	4	0	0	0	0	0	0	0	0	0
Elusimicrobia	2	0	0	0	0	0	0	0	0	0
Synergistetes	2	0	0	0	0	0	0	0	0	0
Cyanobacteria	16	0	0	0	0	0	0	0	0	0
Chlorobi	10	0	0	0	0	0	0	0	0	0
Chloroflexi	11	0	0	0	0	0	0	0	0	0
Deinococcus-Thermus	7	0	0	0	0	0	0	0	0	0
Aquificae	9	0	0	0	0	0	0	0	0	0
Thermotogae	11	0	0	0	0	0	0	0	0	0
Dictyoglomi	2	0	0	0	0	0	0	0	0	0
Nitrospirae	2	0	0	0	0	0	0	0	0	0
Thermobaculum	1	0	0	0	0	0	0	0	0	0
Deferribacteres	3	0	0	0	0	0	0	0	0	0
Euryarchaeota	59	0	1	0	0	0	0	0	0	0
Crenarchaeota	23	0	0	0	0	0	0	0	0	0
Thaumarchaeota	2	0	0	0	0	0	0	0	0	0
Nanoarchaeota	1	0	0	0	0	0	0	0	0	0
Korarchaeota	1	0	0	0	0	0	0	0	0	0
Total	759	0	9	0	0	0	0	0	0	0

(M00100_1)

Phyla	Module completion ratio (%)									
	0--10	10--20	20--30	30--40	40--50	50--60	60--70	70--80	80--90	90--100
Gammaproteobacteria	125	0	0	0	0	1	0	0	0	0
Betaproteobacteria	58	0	0	0	0	3	0	0	0	0
Epsilonproteobacteria	17	0	0	0	0	0	0	0	0	0
Deltaproteobacteria	23	0	0	0	0	5	0	0	0	0
Alphaproteobacteria	89	0	0	0	0	2	0	0	0	0
Magnetococcus	1	0	0	0	0	0	0	0	0	0
Chrysiogenetes	1	0	0	0	0	0	0	0	0	0
Firmicutes	103	0	0	0	0	1	0	0	0	0
Tenericutes	19	0	0	0	0	0	0	0	0	0
Actinobacteria	68	0	0	0	0	12	0	0	0	0
Chlamydiae	8	0	0	0	0	0	0	0	0	0
Spirochaetes	14	0	0	0	0	0	0	0	0	0
Acidobacteria	5	0	0	0	0	0	0	0	0	0
Bacteroidetes	35	0	0	0	0	0	0	0	0	0
Fibrobacteres	1	0	0	0	0	0	0	0	0	0
Fusobacteria	5	0	0	0	0	0	0	0	0	0
Verrucomicrobia	4	0	0	0	0	0	0	0	0	0
Gemmatimonadetes	1	0	0	0	0	0	0	0	0	0
Planctomycetes	4	0	0	0	0	0	0	0	0	0
Elusimicrobia	2	0	0	0	0	0	0	0	0	0
Synergistetes	2	0	0	0	0	0	0	0	0	0
Cyanobacteria	15	0	0	0	0	1	0	0	0	0
Chlorobi	10	0	0	0	0	0	0	0	0	0
Chloroflexi	7	0	0	0	0	4	0	0	0	0
Deinococcus-Thermus	7	0	0	0	0	0	0	0	0	0
Aquificae	9	0	0	0	0	0	0	0	0	0
Thermotogae	11	0	0	0	0	0	0	0	0	0
Dictyoglomi	2	0	0	0	0	0	0	0	0	0
Nitrospirae	2	0	0	0	0	0	0	0	0	0
Thermobaculum	1	0	0	0	0	0	0	0	0	0
Deferribacteres	3	0	0	0	0	0	0	0	0	0
Euryarchaeota	60	0	0	0	0	0	0	0	0	0
Crenarchaeota	23	0	0	0	0	0	0	0	0	0
Thaumarchaeota	2	0	0	0	0	0	0	0	0	0
Nanoarchaeota	1	0	0	0	0	0	0	0	0	0
Korarchaeota	1	0	0	0	0	0	0	0	0	0
Total	739	0	0	0	0	29	0	0	0	0

(M00101_1)

Phyla	Module completion ratio (%)									
	0--10	10--20	20--30	30--40	40--50	50--60	60--70	70--80	80--90	90--100
Gammaproteobacteria	125	0	1	0	0	0	0	0	0	0
Betaproteobacteria	61	0	0	0	0	0	0	0	0	0
Epsilonproteobacteria	17	0	0	0	0	0	0	0	0	0
Deltaproteobacteria	27	1	0	0	0	0	0	0	0	0
Alphaproteobacteria	91	0	0	0	0	0	0	0	0	0
Magnetococcus	1	0	0	0	0	0	0	0	0	0
Chrysiogenetes	1	0	0	0	0	0	0	0	0	0
Firmicutes	104	0	0	0	0	0	0	0	0	0
Tenericutes	19	0	0	0	0	0	0	0	0	0
Actinobacteria	80	0	0	0	0	0	0	0	0	0
Chlamydiae	8	0	0	0	0	0	0	0	0	0
Spirochaetes	14	0	0	0	0	0	0	0	0	0
Acidobacteria	5	0	0	0	0	0	0	0	0	0
Bacteroidetes	35	0	0	0	0	0	0	0	0	0
Fibrobacteres	1	0	0	0	0	0	0	0	0	0
Fusobacteria	5	0	0	0	0	0	0	0	0	0
Verrucomicrobia	4	0	0	0	0	0	0	0	0	0
Gemmatimonadetes	1	0	0	0	0	0	0	0	0	0
Planctomycetes	4	0	0	0	0	0	0	0	0	0
Elusimicrobia	2	0	0	0	0	0	0	0	0	0
Synergistetes	2	0	0	0	0	0	0	0	0	0
Cyanobacteria	16	0	0	0	0	0	0	0	0	0
Chlorobi	10	0	0	0	0	0	0	0	0	0
Chloroflexi	11	0	0	0	0	0	0	0	0	0
Deinococcus-Thermus	7	0	0	0	0	0	0	0	0	0
Aquificae	9	0	0	0	0	0	0	0	0	0
Thermotogae	11	0	0	0	0	0	0	0	0	0
Dictyoglomi	2	0	0	0	0	0	0	0	0	0
Nitrospirae	2	0	0	0	0	0	0	0	0	0
Thermobaculum	1	0	0	0	0	0	0	0	0	0
Deferribacteres	3	0	0	0	0	0	0	0	0	0
Euryarchaeota	60	0	0	0	0	0	0	0	0	0
Crenarchaeota	23	0	0	0	0	0	0	0	0	0
Thaumarchaeota	2	0	0	0	0	0	0	0	0	0
Nanoarchaeota	1	0	0	0	0	0	0	0	0	0
Korarchaeota	1	0	0	0	0	0	0	0	0	0
Total	766	1	1	0	0	0	0	0	0	0

(M00102_1)

Phyla	Module completion ratio (%)									
	0--10	10--20	20--30	30--40	40--50	50--60	60--70	70--80	80--90	90--100
Gammaproteobacteria	125	0	1	0	0	0	0	0	0	0
Betaproteobacteria	61	0	0	0	0	0	0	0	0	0
Epsilonproteobacteria	17	0	0	0	0	0	0	0	0	0
Deltaproteobacteria	28	0	0	0	0	0	0	0	0	0
Alphaproteobacteria	91	0	0	0	0	0	0	0	0	0
Magnetococcus	1	0	0	0	0	0	0	0	0	0
Chrysiogenetes	1	0	0	0	0	0	0	0	0	0
Firmicutes	104	0	0	0	0	0	0	0	0	0
Tenericutes	19	0	0	0	0	0	0	0	0	0
Actinobacteria	80	0	0	0	0	0	0	0	0	0
Chlamydiae	8	0	0	0	0	0	0	0	0	0
Spirochaetes	14	0	0	0	0	0	0	0	0	0
Acidobacteria	5	0	0	0	0	0	0	0	0	0
Bacteroidetes	35	0	0	0	0	0	0	0	0	0
Fibrobacteres	1	0	0	0	0	0	0	0	0	0
Fusobacteria	5	0	0	0	0	0	0	0	0	0
Verrucomicrobia	4	0	0	0	0	0	0	0	0	0
Gemmatimonadetes	1	0	0	0	0	0	0	0	0	0
Planctomycetes	4	0	0	0	0	0	0	0	0	0
Elusimicrobia	2	0	0	0	0	0	0	0	0	0
Synergistetes	2	0	0	0	0	0	0	0	0	0
Cyanobacteria	16	0	0	0	0	0	0	0	0	0
Chlorobi	10	0	0	0	0	0	0	0	0	0
Chloroflexi	11	0	0	0	0	0	0	0	0	0
Deinococcus-Thermus	7	0	0	0	0	0	0	0	0	0
Aquificae	9	0	0	0	0	0	0	0	0	0
Thermotogae	11	0	0	0	0	0	0	0	0	0
Dictyoglomi	2	0	0	0	0	0	0	0	0	0
Nitrospirae	2	0	0	0	0	0	0	0	0	0
Thermobaculum	1	0	0	0	0	0	0	0	0	0
Deferribacteres	3	0	0	0	0	0	0	0	0	0
Euryarchaeota	60	0	0	0	0	0	0	0	0	0
Crenarchaeota	23	0	0	0	0	0	0	0	0	0
Thaumarchaeota	2	0	0	0	0	0	0	0	0	0
Nanoarchaeota	1	0	0	0	0	0	0	0	0	0
Korarchaeota	1	0	0	0	0	0	0	0	0	0
Total	767	0	1	0	0	0	0	0	0	0

(M00103_1)

Phyla	Module completion ratio (%)									
	0--10	10--20	20--30	30--40	40--50	50--60	60--70	70--80	80--90	90--100
Gammaproteobacteria	126	0	0	0	0	0	0	0	0	0
Betaproteobacteria	61	0	0	0	0	0	0	0	0	0
Epsilonproteobacteria	17	0	0	0	0	0	0	0	0	0
Deltaproteobacteria	28	0	0	0	0	0	0	0	0	0
Alphaproteobacteria	91	0	0	0	0	0	0	0	0	0
Magnetococcus	1	0	0	0	0	0	0	0	0	0
Chrysiogenetes	1	0	0	0	0	0	0	0	0	0
Firmicutes	104	0	0	0	0	0	0	0	0	0
Tenericutes	19	0	0	0	0	0	0	0	0	0
Actinobacteria	80	0	0	0	0	0	0	0	0	0
Chlamydiae	8	0	0	0	0	0	0	0	0	0
Spirochaetes	14	0	0	0	0	0	0	0	0	0
Acidobacteria	5	0	0	0	0	0	0	0	0	0
Bacteroidetes	35	0	0	0	0	0	0	0	0	0
Fibrobacteres	1	0	0	0	0	0	0	0	0	0
Fusobacteria	5	0	0	0	0	0	0	0	0	0
Verrucomicrobia	4	0	0	0	0	0	0	0	0	0
Gemmatimonadetes	1	0	0	0	0	0	0	0	0	0
Planctomycetes	4	0	0	0	0	0	0	0	0	0
Elusimicrobia	2	0	0	0	0	0	0	0	0	0
Synergistetes	2	0	0	0	0	0	0	0	0	0
Cyanobacteria	16	0	0	0	0	0	0	0	0	0
Chlorobi	10	0	0	0	0	0	0	0	0	0
Chloroflexi	11	0	0	0	0	0	0	0	0	0
Deinococcus-Thermus	7	0	0	0	0	0	0	0	0	0
Aquificae	9	0	0	0	0	0	0	0	0	0
Thermotogae	11	0	0	0	0	0	0	0	0	0
Dictyoglomi	2	0	0	0	0	0	0	0	0	0
Nitrospirae	2	0	0	0	0	0	0	0	0	0
Thermobaculum	1	0	0	0	0	0	0	0	0	0
Deferribacteres	3	0	0	0	0	0	0	0	0	0
Euryarchaeota	60	0	0	0	0	0	0	0	0	0
Crenarchaeota	23	0	0	0	0	0	0	0	0	0
Thaumarchaeota	2	0	0	0	0	0	0	0	0	0
Nanoarchaeota	1	0	0	0	0	0	0	0	0	0
Korarchaeota	1	0	0	0	0	0	0	0	0	0
Total	768	0	0	0	0	0	0	0	0	0

(M00104_1)

Phyla	Module completion ratio (%)									
	0--10	10--20	20--30	30--40	40--50	50--60	60--70	70--80	80--90	90--100
Gammaproteobacteria	126	0	0	0	0	0	0	0	0	0
Betaproteobacteria	61	0	0	0	0	0	0	0	0	0
Epsilonproteobacteria	17	0	0	0	0	0	0	0	0	0
Deltaproteobacteria	28	0	0	0	0	0	0	0	0	0
Alphaproteobacteria	91	0	0	0	0	0	0	0	0	0
Magnetococcus	1	0	0	0	0	0	0	0	0	0
Chrysiogenetes	1	0	0	0	0	0	0	0	0	0
Firmicutes	104	0	0	0	0	0	0	0	0	0
Tenericutes	19	0	0	0	0	0	0	0	0	0
Actinobacteria	80	0	0	0	0	0	0	0	0	0
Chlamydiae	8	0	0	0	0	0	0	0	0	0
Spirochaetes	14	0	0	0	0	0	0	0	0	0
Acidobacteria	5	0	0	0	0	0	0	0	0	0
Bacteroidetes	35	0	0	0	0	0	0	0	0	0
Fibrobacteres	1	0	0	0	0	0	0	0	0	0
Fusobacteria	5	0	0	0	0	0	0	0	0	0
Verrucomicrobia	4	0	0	0	0	0	0	0	0	0
Gemmatimonadetes	1	0	0	0	0	0	0	0	0	0
Planctomycetes	4	0	0	0	0	0	0	0	0	0
Elusimicrobia	2	0	0	0	0	0	0	0	0	0
Synergistetes	2	0	0	0	0	0	0	0	0	0
Cyanobacteria	16	0	0	0	0	0	0	0	0	0
Chlorobi	10	0	0	0	0	0	0	0	0	0
Chloroflexi	11	0	0	0	0	0	0	0	0	0
Deinococcus-Thermus	7	0	0	0	0	0	0	0	0	0
Aquificae	9	0	0	0	0	0	0	0	0	0
Thermotogae	11	0	0	0	0	0	0	0	0	0
Dictyoglomi	2	0	0	0	0	0	0	0	0	0
Nitrospirae	2	0	0	0	0	0	0	0	0	0
Thermobaculum	1	0	0	0	0	0	0	0	0	0
Deferribacteres	3	0	0	0	0	0	0	0	0	0
Euryarchaeota	60	0	0	0	0	0	0	0	0	0
Crenarchaeota	23	0	0	0	0	0	0	0	0	0
Thaumarchaeota	2	0	0	0	0	0	0	0	0	0
Nanoarchaeota	1	0	0	0	0	0	0	0	0	0
Korarchaeota	1	0	0	0	0	0	0	0	0	0
Total	768	0	0	0	0	0	0	0	0	0

(M00105_1)

Phyla	Module completion ratio (%)									
	0--10	10--20	20--30	30--40	40--50	50--60	60--70	70--80	80--90	90--100
Gammaproteobacteria	126	0	0	0	0	0	0	0	0	0
Betaproteobacteria	61	0	0	0	0	0	0	0	0	0
Epsilonproteobacteria	17	0	0	0	0	0	0	0	0	0
Deltaproteobacteria	28	0	0	0	0	0	0	0	0	0
Alphaproteobacteria	91	0	0	0	0	0	0	0	0	0
Magnetococcus	1	0	0	0	0	0	0	0	0	0
Chrysiogenetes	1	0	0	0	0	0	0	0	0	0
Firmicutes	104	0	0	0	0	0	0	0	0	0
Tenericutes	19	0	0	0	0	0	0	0	0	0
Actinobacteria	80	0	0	0	0	0	0	0	0	0
Chlamydiae	8	0	0	0	0	0	0	0	0	0
Spirochaetes	14	0	0	0	0	0	0	0	0	0
Acidobacteria	5	0	0	0	0	0	0	0	0	0
Bacteroidetes	35	0	0	0	0	0	0	0	0	0
Fibrobacteres	1	0	0	0	0	0	0	0	0	0
Fusobacteria	5	0	0	0	0	0	0	0	0	0
Verrucomicrobia	4	0	0	0	0	0	0	0	0	0
Gemmatimonadetes	1	0	0	0	0	0	0	0	0	0
Planctomycetes	4	0	0	0	0	0	0	0	0	0
Elusimicrobia	2	0	0	0	0	0	0	0	0	0
Synergistetes	2	0	0	0	0	0	0	0	0	0
Cyanobacteria	16	0	0	0	0	0	0	0	0	0
Chlorobi	10	0	0	0	0	0	0	0	0	0
Chloroflexi	11	0	0	0	0	0	0	0	0	0
Deinococcus-Thermus	7	0	0	0	0	0	0	0	0	0
Aquificae	9	0	0	0	0	0	0	0	0	0
Thermotogae	11	0	0	0	0	0	0	0	0	0
Dictyoglomi	2	0	0	0	0	0	0	0	0	0
Nitrospirae	2	0	0	0	0	0	0	0	0	0
Thermobaculum	1	0	0	0	0	0	0	0	0	0
Deferribacteres	3	0	0	0	0	0	0	0	0	0
Euryarchaeota	60	0	0	0	0	0	0	0	0	0
Crenarchaeota	23	0	0	0	0	0	0	0	0	0
Thaumarchaeota	2	0	0	0	0	0	0	0	0	0
Nanoarchaeota	1	0	0	0	0	0	0	0	0	0
Korarchaeota	1	0	0	0	0	0	0	0	0	0
Total	768	0	0	0	0	0	0	0	0	0

(M00106_1)

Phyla	Module completion ratio (%)									
	0--10	10--20	20--30	30--40	40--50	50--60	60--70	70--80	80--90	90--100
Gammaproteobacteria	126	0	0	0	0	0	0	0	0	0
Betaproteobacteria	61	0	0	0	0	0	0	0	0	0
Epsilonproteobacteria	17	0	0	0	0	0	0	0	0	0
Deltaproteobacteria	28	0	0	0	0	0	0	0	0	0
Alphaproteobacteria	91	0	0	0	0	0	0	0	0	0
Magnetococcus	1	0	0	0	0	0	0	0	0	0
Chrysiogenetes	1	0	0	0	0	0	0	0	0	0
Firmicutes	104	0	0	0	0	0	0	0	0	0
Tenericutes	19	0	0	0	0	0	0	0	0	0
Actinobacteria	80	0	0	0	0	0	0	0	0	0
Chlamydiae	8	0	0	0	0	0	0	0	0	0
Spirochaetes	14	0	0	0	0	0	0	0	0	0
Acidobacteria	5	0	0	0	0	0	0	0	0	0
Bacteroidetes	35	0	0	0	0	0	0	0	0	0
Fibrobacteres	1	0	0	0	0	0	0	0	0	0
Fusobacteria	5	0	0	0	0	0	0	0	0	0
Verrucomicrobia	4	0	0	0	0	0	0	0	0	0
Gemmatimonadetes	1	0	0	0	0	0	0	0	0	0
Planctomycetes	4	0	0	0	0	0	0	0	0	0
Elusimicrobia	2	0	0	0	0	0	0	0	0	0
Synergistetes	2	0	0	0	0	0	0	0	0	0
Cyanobacteria	16	0	0	0	0	0	0	0	0	0
Chlorobi	10	0	0	0	0	0	0	0	0	0
Chloroflexi	11	0	0	0	0	0	0	0	0	0
Deinococcus-Thermus	7	0	0	0	0	0	0	0	0	0
Aquificae	9	0	0	0	0	0	0	0	0	0
Thermotogae	11	0	0	0	0	0	0	0	0	0
Dictyoglomi	2	0	0	0	0	0	0	0	0	0
Nitrospirae	2	0	0	0	0	0	0	0	0	0
Thermobaculum	1	0	0	0	0	0	0	0	0	0
Deferribacteres	3	0	0	0	0	0	0	0	0	0
Euryarchaeota	60	0	0	0	0	0	0	0	0	0
Crenarchaeota	23	0	0	0	0	0	0	0	0	0
Thaumarchaeota	2	0	0	0	0	0	0	0	0	0
Nanoarchaeota	1	0	0	0	0	0	0	0	0	0
Korarchaeota	1	0	0	0	0	0	0	0	0	0
Total	768	0	0	0	0	0	0	0	0	0

(M00107_1)

Phyla	Module completion ratio (%)									
	0--10	10--20	20--30	30--40	40--50	50--60	60--70	70--80	80--90	90--100
Gammaproteobacteria	123	0	0	0	0	3	0	0	0	0
Betaproteobacteria	55	0	0	0	0	6	0	0	0	0
Epsilonproteobacteria	17	0	0	0	0	0	0	0	0	0
Deltaproteobacteria	28	0	0	0	0	0	0	0	0	0
Alphaproteobacteria	86	0	0	0	0	5	0	0	0	0
Magnetococcus	1	0	0	0	0	0	0	0	0	0
Chrysiogenetes	1	0	0	0	0	0	0	0	0	0
Firmicutes	103	0	0	0	0	1	0	0	0	0
Tenericutes	19	0	0	0	0	0	0	0	0	0
Actinobacteria	62	0	0	0	0	18	0	0	0	0
Chlamydiae	8	0	0	0	0	0	0	0	0	0
Spirochaetes	14	0	0	0	0	0	0	0	0	0
Acidobacteria	5	0	0	0	0	0	0	0	0	0
Bacteroidetes	35	0	0	0	0	0	0	0	0	0
Fibrobacteres	1	0	0	0	0	0	0	0	0	0
Fusobacteria	5	0	0	0	0	0	0	0	0	0
Verrucomicrobia	4	0	0	0	0	0	0	0	0	0
Gemmatimonadetes	0	0	0	0	0	1	0	0	0	0
Planctomycetes	4	0	0	0	0	0	0	0	0	0
Elusimicrobia	2	0	0	0	0	0	0	0	0	0
Synergistetes	2	0	0	0	0	0	0	0	0	0
Cyanobacteria	12	0	0	0	0	4	0	0	0	0
Chlorobi	9	0	0	0	0	1	0	0	0	0
Chloroflexi	11	0	0	0	0	0	0	0	0	0
Deinococcus-Thermus	7	0	0	0	0	0	0	0	0	0
Aquificae	9	0	0	0	0	0	0	0	0	0
Thermotogae	11	0	0	0	0	0	0	0	0	0
Dictyoglomi	2	0	0	0	0	0	0	0	0	0
Nitrospirae	2	0	0	0	0	0	0	0	0	0
Thermobaculum	1	0	0	0	0	0	0	0	0	0
Deferribacteres	3	0	0	0	0	0	0	0	0	0
Euryarchaeota	60	0	0	0	0	0	0	0	0	0
Crenarchaeota	23	0	0	0	0	0	0	0	0	0
Thaumarchaeota	2	0	0	0	0	0	0	0	0	0
Nanoarchaeota	1	0	0	0	0	0	0	0	0	0
Korarchaeota	1	0	0	0	0	0	0	0	0	0
Total	729	0	0	0	0	39	0	0	0	0

(M00108_1)

Phyla	Module completion ratio (%)									
	0--10	10--20	20--30	30--40	40--50	50--60	60--70	70--80	80--90	90--100
Gammaproteobacteria	126	0	0	0	0	0	0	0	0	0
Betaproteobacteria	61	0	0	0	0	0	0	0	0	0
Epsilonproteobacteria	17	0	0	0	0	0	0	0	0	0
Deltaproteobacteria	28	0	0	0	0	0	0	0	0	0
Alphaproteobacteria	91	0	0	0	0	0	0	0	0	0
Magnetococcus	1	0	0	0	0	0	0	0	0	0
Chrysiogenetes	1	0	0	0	0	0	0	0	0	0
Firmicutes	104	0	0	0	0	0	0	0	0	0
Tenericutes	19	0	0	0	0	0	0	0	0	0
Actinobacteria	80	0	0	0	0	0	0	0	0	0
Chlamydiae	8	0	0	0	0	0	0	0	0	0
Spirochaetes	14	0	0	0	0	0	0	0	0	0
Acidobacteria	5	0	0	0	0	0	0	0	0	0
Bacteroidetes	35	0	0	0	0	0	0	0	0	0
Fibrobacteres	1	0	0	0	0	0	0	0	0	0
Fusobacteria	5	0	0	0	0	0	0	0	0	0
Verrucomicrobia	4	0	0	0	0	0	0	0	0	0
Gemmatimonadetes	1	0	0	0	0	0	0	0	0	0
Planctomycetes	4	0	0	0	0	0	0	0	0	0
Elusimicrobia	2	0	0	0	0	0	0	0	0	0
Synergistetes	2	0	0	0	0	0	0	0	0	0
Cyanobacteria	16	0	0	0	0	0	0	0	0	0
Chlorobi	10	0	0	0	0	0	0	0	0	0
Chloroflexi	11	0	0	0	0	0	0	0	0	0
Deinococcus-Thermus	7	0	0	0	0	0	0	0	0	0
Aquificae	9	0	0	0	0	0	0	0	0	0
Thermotogae	11	0	0	0	0	0	0	0	0	0
Dictyoglomi	2	0	0	0	0	0	0	0	0	0
Nitrospirae	2	0	0	0	0	0	0	0	0	0
Thermobaculum	1	0	0	0	0	0	0	0	0	0
Deferribacteres	3	0	0	0	0	0	0	0	0	0
Euryarchaeota	60	0	0	0	0	0	0	0	0	0
Crenarchaeota	23	0	0	0	0	0	0	0	0	0
Thaumarchaeota	2	0	0	0	0	0	0	0	0	0
Nanoarchaeota	1	0	0	0	0	0	0	0	0	0
Korarchaeota	1	0	0	0	0	0	0	0	0	0
Total	768	0	0	0	0	0	0	0	0	0

(M00109_1)

Phyla	Module completion ratio (%)									
	0--10	10--20	20--30	30--40	40--50	50--60	60--70	70--80	80--90	90--100
Gammaproteobacteria	126	0	0	0	0	0	0	0	0	0
Betaproteobacteria	61	0	0	0	0	0	0	0	0	0
Epsilonproteobacteria	17	0	0	0	0	0	0	0	0	0
Deltaproteobacteria	28	0	0	0	0	0	0	0	0	0
Alphaproteobacteria	91	0	0	0	0	0	0	0	0	0
Magnetococcus	1	0	0	0	0	0	0	0	0	0
Chrysiogenetes	1	0	0	0	0	0	0	0	0	0
Firmicutes	104	0	0	0	0	0	0	0	0	0
Tenericutes	19	0	0	0	0	0	0	0	0	0
Actinobacteria	80	0	0	0	0	0	0	0	0	0
Chlamydiae	8	0	0	0	0	0	0	0	0	0
Spirochaetes	14	0	0	0	0	0	0	0	0	0
Acidobacteria	5	0	0	0	0	0	0	0	0	0
Bacteroidetes	35	0	0	0	0	0	0	0	0	0
Fibrobacteres	1	0	0	0	0	0	0	0	0	0
Fusobacteria	5	0	0	0	0	0	0	0	0	0
Verrucomicrobia	4	0	0	0	0	0	0	0	0	0
Gemmatimonadetes	1	0	0	0	0	0	0	0	0	0
Planctomycetes	4	0	0	0	0	0	0	0	0	0
Elusimicrobia	2	0	0	0	0	0	0	0	0	0
Synergistetes	2	0	0	0	0	0	0	0	0	0
Cyanobacteria	16	0	0	0	0	0	0	0	0	0
Chlorobi	10	0	0	0	0	0	0	0	0	0
Chloroflexi	11	0	0	0	0	0	0	0	0	0
Deinococcus-Thermus	7	0	0	0	0	0	0	0	0	0
Aquificae	9	0	0	0	0	0	0	0	0	0
Thermotogae	11	0	0	0	0	0	0	0	0	0
Dictyoglomi	2	0	0	0	0	0	0	0	0	0
Nitrospirae	2	0	0	0	0	0	0	0	0	0
Thermobaculum	1	0	0	0	0	0	0	0	0	0
Deferribacteres	3	0	0	0	0	0	0	0	0	0
Euryarchaeota	60	0	0	0	0	0	0	0	0	0
Crenarchaeota	23	0	0	0	0	0	0	0	0	0
Thaumarchaeota	2	0	0	0	0	0	0	0	0	0
Nanoarchaeota	1	0	0	0	0	0	0	0	0	0
Korarchaeota	1	0	0	0	0	0	0	0	0	0
Total	768	0	0	0	0	0	0	0	0	0

(M00110_1)

Phyla	Module completion ratio (%)									
	0--10	10--20	20--30	30--40	40--50	50--60	60--70	70--80	80--90	90--100
Gammaproteobacteria	123	0	0	3	0	0	0	0	0	0
Betaproteobacteria	55	0	0	6	0	0	0	0	0	0
Epsilonproteobacteria	17	0	0	0	0	0	0	0	0	0
Deltaproteobacteria	28	0	0	0	0	0	0	0	0	0
Alphaproteobacteria	86	0	0	5	0	0	0	0	0	0
Magnetococcus	1	0	0	0	0	0	0	0	0	0
Chrysiogenetes	1	0	0	0	0	0	0	0	0	0
Firmicutes	103	0	0	1	0	0	0	0	0	0
Tenericutes	19	0	0	0	0	0	0	0	0	0
Actinobacteria	62	0	0	18	0	0	0	0	0	0
Chlamydiae	8	0	0	0	0	0	0	0	0	0
Spirochaetes	14	0	0	0	0	0	0	0	0	0
Acidobacteria	5	0	0	0	0	0	0	0	0	0
Bacteroidetes	35	0	0	0	0	0	0	0	0	0
Fibrobacteres	1	0	0	0	0	0	0	0	0	0
Fusobacteria	5	0	0	0	0	0	0	0	0	0
Verrucomicrobia	4	0	0	0	0	0	0	0	0	0
Gemmatimonadetes	0	0	0	1	0	0	0	0	0	0
Planctomycetes	4	0	0	0	0	0	0	0	0	0
Elusimicrobia	2	0	0	0	0	0	0	0	0	0
Synergistetes	2	0	0	0	0	0	0	0	0	0
Cyanobacteria	12	0	0	4	0	0	0	0	0	0
Chlorobi	9	0	0	1	0	0	0	0	0	0
Chloroflexi	11	0	0	0	0	0	0	0	0	0
Deinococcus-Thermus	7	0	0	0	0	0	0	0	0	0
Aquificae	9	0	0	0	0	0	0	0	0	0
Thermotogae	11	0	0	0	0	0	0	0	0	0
Dictyoglomi	2	0	0	0	0	0	0	0	0	0
Nitrospirae	2	0	0	0	0	0	0	0	0	0
Thermobaculum	1	0	0	0	0	0	0	0	0	0
Deferribacteres	3	0	0	0	0	0	0	0	0	0
Euryarchaeota	60	0	0	0	0	0	0	0	0	0
Crenarchaeota	23	0	0	0	0	0	0	0	0	0
Thaumarchaeota	2	0	0	0	0	0	0	0	0	0
Nanoarchaeota	1	0	0	0	0	0	0	0	0	0
Korarchaeota	1	0	0	0	0	0	0	0	0	0
Total	729	0	0	39	0	0	0	0	0	0

(M00112_1)

Phyla	Module completion ratio (%)									
	0--10	10--20	20--30	30--40	40--50	50--60	60--70	70--80	80--90	90--100
Gammaproteobacteria	126	0	0	0	0	0	0	0	0	0
Betaproteobacteria	61	0	0	0	0	0	0	0	0	0
Epsilonproteobacteria	17	0	0	0	0	0	0	0	0	0
Deltaproteobacteria	28	0	0	0	0	0	0	0	0	0
Alphaproteobacteria	90	0	1	0	0	0	0	0	0	0
Magnetococcus	1	0	0	0	0	0	0	0	0	0
Chrysiogenetes	1	0	0	0	0	0	0	0	0	0
Firmicutes	104	0	0	0	0	0	0	0	0	0
Tenericutes	19	0	0	0	0	0	0	0	0	0
Actinobacteria	78	0	2	0	0	0	0	0	0	0
Chlamydiae	8	0	0	0	0	0	0	0	0	0
Spirochaetes	14	0	0	0	0	0	0	0	0	0
Acidobacteria	5	0	0	0	0	0	0	0	0	0
Bacteroidetes	35	0	0	0	0	0	0	0	0	0
Fibrobacteres	1	0	0	0	0	0	0	0	0	0
Fusobacteria	5	0	0	0	0	0	0	0	0	0
Verrucomicrobia	4	0	0	0	0	0	0	0	0	0
Gemmatimonadetes	1	0	0	0	0	0	0	0	0	0
Planctomycetes	4	0	0	0	0	0	0	0	0	0
Elusimicrobia	2	0	0	0	0	0	0	0	0	0
Synergistetes	2	0	0	0	0	0	0	0	0	0
Cyanobacteria	5	0	0	0	0	0	0	11	0	0
Chlorobi	10	0	0	0	0	0	0	0	0	0
Chloroflexi	9	0	2	0	0	0	0	0	0	0
Deinococcus-Thermus	6	0	1	0	0	0	0	0	0	0
Aquificae	9	0	0	0	0	0	0	0	0	0
Thermotogae	11	0	0	0	0	0	0	0	0	0
Dictyoglomi	2	0	0	0	0	0	0	0	0	0
Nitrospirae	2	0	0	0	0	0	0	0	0	0
Thermobaculum	1	0	0	0	0	0	0	0	0	0
Deferribacteres	3	0	0	0	0	0	0	0	0	0
Euryarchaeota	60	0	0	0	0	0	0	0	0	0
Crenarchaeota	23	0	0	0	0	0	0	0	0	0
Thaumarchaeota	2	0	0	0	0	0	0	0	0	0
Nanoarchaeota	1	0	0	0	0	0	0	0	0	0
Korarchaeota	1	0	0	0	0	0	0	0	0	0
Total	751	0	6	0	0	0	0	11	0	0

(M00113_1)

Phyla	Module completion ratio (%)									
	0--10	10--20	20--30	30--40	40--50	50--60	60--70	70--80	80--90	90--100
Gammaproteobacteria	31	93	2	0	0	0	0	0	0	0
Betaproteobacteria	9	52	0	0	0	0	0	0	0	0
Epsilonproteobacteria	17	0	0	0	0	0	0	0	0	0
Deltaproteobacteria	14	12	2	0	0	0	0	0	0	0
Alphaproteobacteria	64	27	0	0	0	0	0	0	0	0
Magnetococcus	1	0	0	0	0	0	0	0	0	0
Chrysiogenetes	0	1	0	0	0	0	0	0	0	0
Firmicutes	75	29	0	0	0	0	0	0	0	0
Tenericutes	19	0	0	0	0	0	0	0	0	0
Actinobacteria	18	28	34	0	0	0	0	0	0	0
Chlamydiae	7	1	0	0	0	0	0	0	0	0
Spirochaetes	13	1	0	0	0	0	0	0	0	0
Acidobacteria	1	4	0	0	0	0	0	0	0	0
Bacteroidetes	13	17	5	0	0	0	0	0	0	0
Fibrobacteres	1	0	0	0	0	0	0	0	0	0
Fusobacteria	5	0	0	0	0	0	0	0	0	0
Verrucomicrobia	4	0	0	0	0	0	0	0	0	0
Gemmatimonadetes	0	1	0	0	0	0	0	0	0	0
Planctomycetes	3	1	0	0	0	0	0	0	0	0
Elusimicrobia	2	0	0	0	0	0	0	0	0	0
Synergistetes	2	0	0	0	0	0	0	0	0	0
Cyanobacteria	15	1	0	0	0	0	0	0	0	0
Chlorobi	10	0	0	0	0	0	0	0	0	0
Chloroflexi	5	6	0	0	0	0	0	0	0	0
Deinococcus-Thermus	0	7	0	0	0	0	0	0	0	0
Aquificae	9	0	0	0	0	0	0	0	0	0
Thermotogae	11	0	0	0	0	0	0	0	0	0
Dictyoglomi	2	0	0	0	0	0	0	0	0	0
Nitrospirae	2	0	0	0	0	0	0	0	0	0
Thermobaculum	1	0	0	0	0	0	0	0	0	0
Deferribacteres	1	2	0	0	0	0	0	0	0	0
Euryarchaeota	47	13	0	0	0	0	0	0	0	0
Crenarchaeota	12	11	0	0	0	0	0	0	0	0
Thaumarchaeota	2	0	0	0	0	0	0	0	0	0
Nanoarchaeota	1	0	0	0	0	0	0	0	0	0
Korarchaeota	1	0	0	0	0	0	0	0	0	0
Total	418	307	43	0	0	0	0	0	0	0

(M00114_1)

Phyla	Module completion ratio (%)									
	0--10	10--20	20--30	30--40	40--50	50--60	60--70	70--80	80--90	90--100
Gammaproteobacteria	1	10	28	30	47	10	0	0	0	0
Betaproteobacteria	1	2	10	10	31	7	0	0	0	0
Epsilonproteobacteria	0	2	4	11	0	0	0	0	0	0
Deltaproteobacteria	0	0	2	8	18	0	0	0	0	0
Alphaproteobacteria	9	12	17	16	30	7	0	0	0	0
Magnetococcus	0	0	0	0	1	0	0	0	0	0
Chrysiogenetes	0	0	0	1	0	0	0	0	0	0
Firmicutes	2	6	28	32	35	1	0	0	0	0
Tenericutes	0	7	7	5	0	0	0	0	0	0
Actinobacteria	0	6	1	7	47	19	0	0	0	0
Chlamydiae	0	0	8	0	0	0	0	0	0	0
Spirochaetes	0	1	1	8	4	0	0	0	0	0
Acidobacteria	0	0	2	1	2	0	0	0	0	0
Bacteroidetes	1	1	0	6	24	3	0	0	0	0
Fibrobacteres	0	0	0	1	0	0	0	0	0	0
Fusobacteria	0	0	3	1	1	0	0	0	0	0
Verrucomicrobia	0	0	0	2	1	1	0	0	0	0
Gemmatimonadetes	0	0	0	1	0	0	0	0	0	0
Planctomycetes	0	0	0	0	3	1	0	0	0	0
Elusimicrobia	0	1	1	0	0	0	0	0	0	0
Synergistetes	0	0	1	0	1	0	0	0	0	0
Cyanobacteria	0	0	0	2	14	0	0	0	0	0
Chlorobi	0	0	0	0	10	0	0	0	0	0
Chloroflexi	0	0	1	0	9	1	0	0	0	0
Deinococcus-Thermus	0	0	3	1	3	0	0	0	0	0
Aquificae	0	0	0	5	4	0	0	0	0	0
Thermotogae	0	0	0	4	7	0	0	0	0	0
Dictyoglomi	0	0	0	0	2	0	0	0	0	0
Nitrospirae	0	0	0	0	2	0	0	0	0	0
Thermobaculum	0	0	0	0	0	1	0	0	0	0
Deferribacteres	0	0	0	2	1	0	0	0	0	0
Euryarchaeota	1	10	13	11	25	0	0	0	0	0
Crenarchaeota	0	0	1	8	14	0	0	0	0	0
Thaumarchaeota	0	0	0	1	1	0	0	0	0	0
Nanoarchaeota	1	0	0	0	0	0	0	0	0	0
Korarchaeota	0	0	0	0	1	0	0	0	0	0
Total	16	58	131	174	338	51	0	0	0	0

(M00115_1)

Phyla	Module completion ratio (%)									
	0--10	10--20	20--30	30--40	40--50	50--60	60--70	70--80	80--90	90--100
Gammaproteobacteria	9	0	3	0	7	6	0	0	11	90
Betaproteobacteria	1	0	0	0	1	5	0	0	8	46
Epsilonproteobacteria	0	0	1	0	3	0	0	0	3	10
Deltaproteobacteria	0	0	0	0	1	0	0	0	1	26
Alphaproteobacteria	14	0	0	0	15	1	0	0	10	51
Magnetococcus	0	0	0	0	0	0	0	0	0	1
Chrysiogenetes	0	0	0	0	0	0	0	0	0	1
Firmicutes	0	0	0	0	47	5	0	0	0	52
Tenericutes	0	0	5	0	14	0	0	0	0	0
Actinobacteria	0	0	0	0	12	3	0	0	8	57
Chlamydiae	7	0	0	0	0	1	0	0	0	0
Spirochaetes	0	0	1	0	9	1	0	0	0	3
Acidobacteria	0	0	0	0	0	0	0	0	0	5
Bacteroidetes	2	0	0	0	2	9	0	0	0	22
Fibrobacteres	0	0	0	0	0	0	0	0	0	1
Fusobacteria	1	0	1	0	1	0	0	0	0	2
Verrucomicrobia	0	0	0	0	0	0	0	0	0	4
Gemmatimonadetes	0	0	0	0	0	0	0	0	0	1
Planctomycetes	0	0	0	0	0	0	0	0	0	4
Elusimicrobia	0	0	0	0	0	0	0	0	0	2
Synergistetes	0	0	0	0	1	0	0	0	0	1
Cyanobacteria	0	0	0	0	1	0	0	0	0	15
Chlorobi	0	0	0	0	0	0	0	0	0	10
Chloroflexi	0	0	0	0	1	0	0	0	5	5
Deinococcus-Thermus	1	0	1	0	2	0	0	0	2	1
Aquificae	0	0	0	0	0	0	0	0	0	9
Thermotogae	0	0	0	0	4	0	0	0	7	0
Dictyoglomi	0	0	0	0	0	0	0	0	0	2
Nitrospirae	0	0	0	0	0	0	0	0	0	2
Thermobaculum	0	0	0	0	0	0	0	0	0	1
Deferribacteres	0	0	0	0	0	0	0	0	0	3
Euryarchaeota	0	0	7	0	0	36	0	0	17	0
Crenarchaeota	0	0	12	0	1	4	0	0	6	0
Thaumarchaeota	0	0	0	0	0	2	0	0	0	0
Nanoarchaeota	1	0	0	0	0	0	0	0	0	0
Korarchaeota	0	0	1	0	0	0	0	0	0	0
Total	36	0	32	0	122	73	0	0	78	427

(M00116_1)

Phyla	Module completion ratio (%)									
	0--10	10--20	20--30	30--40	40--50	50--60	60--70	70--80	80--90	90--100
Gammaproteobacteria	3	39	19	1	0	0	0	4	60	0
Betaproteobacteria	1	39	18	1	0	0	1	1	0	0
Epsilonproteobacteria	1	16	0	0	0	0	0	0	0	0
Deltaproteobacteria	0	15	4	0	1	0	1	4	3	0
Alphaproteobacteria	1	76	14	0	0	0	0	0	0	0
Magnetococcus	0	1	0	0	0	0	0	0	0	0
Chrysiogenetes	0	1	0	0	0	0	0	0	0	0
Firmicutes	34	23	11	2	2	1	2	2	27	0
Tenericutes	19	0	0	0	0	0	0	0	0	0
Actinobacteria	2	10	4	0	2	0	2	58	2	0
Chlamydiae	0	6	0	0	0	0	0	0	2	0
Spirochaetes	11	3	0	0	0	0	0	0	0	0
Acidobacteria	0	1	4	0	0	0	0	0	0	0
Bacteroidetes	0	4	3	0	0	0	23	5	0	0
Fibrobacteres	0	1	0	0	0	0	0	0	0	0
Fusobacteria	2	2	1	0	0	0	0	0	0	0
Verrucomicrobia	0	1	0	0	0	0	0	0	3	0
Gemmatimonadetes	0	0	0	1	0	0	0	0	0	0
Planctomycetes	0	3	1	0	0	0	0	0	0	0
Elusimicrobia	2	0	0	0	0	0	0	0	0	0
Synergistetes	1	1	0	0	0	0	0	0	0	0
Cyanobacteria	0	0	1	0	0	0	0	2	5	8
Chlorobi	0	0	0	0	0	0	0	0	10	0
Chloroflexi	0	0	5	0	0	0	1	1	4	0
Deinococcus-Thermus	0	0	7	0	0	0	0	0	0	0
Aquificae	0	9	0	0	0	0	0	0	0	0
Thermotogae	5	0	6	0	0	0	0	0	0	0
Dictyoglomi	2	0	0	0	0	0	0	0	0	0
Nitrospirae	0	2	0	0	0	0	0	0	0	0
Thermobaculum	0	0	0	0	0	0	0	0	1	0
Deferribacteres	0	3	0	0	0	0	0	0	0	0
Euryarchaeota	27	15	4	1	0	0	12	1	0	0
Crenarchaeota	5	12	4	2	0	0	0	0	0	0
Thaumarchaeota	0	0	2	0	0	0	0	0	0	0
Nanoarchaeota	1	0	0	0	0	0	0	0	0	0
Korarchaeota	0	0	1	0	0	0	0	0	0	0
Total	117	283	109	8	5	1	42	78	117	8

(M00117_1)

Phyla	Module completion ratio (%)									
	0--10	10--20	20--30	30--40	40--50	50--60	60--70	70--80	80--90	90--100
Gammaproteobacteria	6	1	0	0	0	2	2	31	17	67
Betaproteobacteria	1	0	0	0	0	6	4	11	39	0
Epsilonproteobacteria	0	0	1	14	1	1	0	0	0	0
Deltaproteobacteria	0	4	2	6	11	0	5	0	0	0
Alphaproteobacteria	1	0	0	0	1	6	30	53	0	0
Magnetococcus	0	0	0	0	0	0	0	1	0	0
Chrysiogenetes	0	0	0	1	0	0	0	0	0	0
Firmicutes	22	41	19	12	10	0	0	0	0	0
Tenericutes	19	0	0	0	0	0	0	0	0	0
Actinobacteria	2	42	16	12	3	2	3	0	0	0
Chlamydiae	0	2	0	6	0	0	0	0	0	0
Spirochaetes	9	1	1	2	1	0	0	0	0	0
Acidobacteria	0	0	0	4	0	1	0	0	0	0
Bacteroidetes	2	8	16	9	0	0	0	0	0	0
Fibrobacteres	0	0	0	1	0	0	0	0	0	0
Fusobacteria	1	3	0	1	0	0	0	0	0	0
Verrucomicrobia	0	1	2	1	0	0	0	0	0	0
Gemmatimonadetes	0	0	0	1	0	0	0	0	0	0
Planctomycetes	0	0	0	0	1	0	3	0	0	0
Elusimicrobia	1	1	0	0	0	0	0	0	0	0
Synergistetes	1	1	0	0	0	0	0	0	0	0
Cyanobacteria	0	0	1	2	12	0	1	0	0	0
Chlorobi	0	8	2	0	0	0	0	0	0	0
Chloroflexi	0	3	4	4	0	0	0	0	0	0
Deinococcus-Thermus	0	0	0	6	0	1	0	0	0	0
Aquificae	0	0	0	4	5	0	0	0	0	0
Thermotogae	5	6	0	0	0	0	0	0	0	0
Dictyoglomi	2	0	0	0	0	0	0	0	0	0
Nitrospirae	0	0	1	0	1	0	0	0	0	0
Thermobaculum	0	1	0	0	0	0	0	0	0	0
Deferribacteres	0	0	0	1	2	0	0	0	0	0
Euryarchaeota	0	1	29	25	5	0	0	0	0	0
Crenarchaeota	0	0	8	15	0	0	0	0	0	0
Thaumarchaeota	0	0	2	0	0	0	0	0	0	0
Nanoarchaeota	1	0	0	0	0	0	0	0	0	0
Korarchaeota	0	0	0	1	0	0	0	0	0	0
Total	73	124	104	128	53	19	48	96	56	67

(M00118_1)

Phyla	Module completion ratio (%)									
	0--10	10--20	20--30	30--40	40--50	50--60	60--70	70--80	80--90	90--100
Gammaproteobacteria	4	0	0	0	0	7	0	0	0	115
Betaproteobacteria	2	0	0	0	0	4	0	0	0	55
Epsilonproteobacteria	17	0	0	0	0	0	0	0	0	0
Deltaproteobacteria	18	0	0	0	0	6	0	0	0	4
Alphaproteobacteria	11	0	0	0	0	2	0	0	0	78
Magnetococcus	0	0	0	0	0	0	0	0	0	1
Chrysiogenetes	0	0	0	0	0	0	0	0	0	1
Firmicutes	73	0	0	0	0	30	0	0	0	1
Tenericutes	19	0	0	0	0	0	0	0	0	0
Actinobacteria	40	0	0	0	0	36	0	0	0	4
Chlamydiae	8	0	0	0	0	0	0	0	0	0
Spirochaetes	11	0	0	0	0	3	0	0	0	0
Acidobacteria	5	0	0	0	0	0	0	0	0	0
Bacteroidetes	28	0	0	0	0	7	0	0	0	0
Fibrobacteres	1	0	0	0	0	0	0	0	0	0
Fusobacteria	4	0	0	0	0	1	0	0	0	0
Verrucomicrobia	4	0	0	0	0	0	0	0	0	0
Gemmatimonadetes	1	0	0	0	0	0	0	0	0	0
Planctomycetes	3	0	0	0	0	0	0	0	0	1
Elusimicrobia	2	0	0	0	0	0	0	0	0	0
Synergistetes	2	0	0	0	0	0	0	0	0	0
Cyanobacteria	0	0	0	0	0	16	0	0	0	0
Chlorobi	9	0	0	0	0	1	0	0	0	0
Chloroflexi	11	0	0	0	0	0	0	0	0	0
Deinococcus-Thermus	7	0	0	0	0	0	0	0	0	0
Aquificae	9	0	0	0	0	0	0	0	0	0
Thermotogae	11	0	0	0	0	0	0	0	0	0
Dictyoglomi	2	0	0	0	0	0	0	0	0	0
Nitrospirae	2	0	0	0	0	0	0	0	0	0
Thermobaculum	1	0	0	0	0	0	0	0	0	0
Deferribacteres	3	0	0	0	0	0	0	0	0	0
Euryarchaeota	57	0	0	0	0	3	0	0	0	0
Crenarchaeota	23	0	0	0	0	0	0	0	0	0
Thaumarchaeota	2	0	0	0	0	0	0	0	0	0
Nanoarchaeota	1	0	0	0	0	0	0	0	0	0
Korarchaeota	1	0	0	0	0	0	0	0	0	0
Total	392	0	0	0	0	116	0	0	0	260

(M00119_1)

Phyla	Module completion ratio (%)									
	0--10	10--20	20--30	30--40	40--50	50--60	60--70	70--80	80--90	90--100
Gammaproteobacteria	3	0	0	13	0	0	29	0	0	81
Betaproteobacteria	2	0	0	0	0	0	16	0	0	43
Epsilonproteobacteria	0	0	0	0	0	0	13	0	0	4
Deltaproteobacteria	0	0	0	1	0	0	6	0	0	21
Alphaproteobacteria	10	0	0	14	0	0	27	0	0	40
Magnetococcus	0	0	0	0	0	0	0	0	0	1
Chrysiogenetes	0	0	0	0	0	0	0	0	0	1
Firmicutes	4	0	0	24	0	0	35	0	0	41
Tenericutes	18	0	0	1	0	0	0	0	0	0
Actinobacteria	0	0	0	6	0	0	34	0	0	40
Chlamydiae	8	0	0	0	0	0	0	0	0	0
Spirochaetes	10	0	0	0	0	0	1	0	0	3
Acidobacteria	0	0	0	0	0	0	3	0	0	2
Bacteroidetes	1	0	0	2	0	0	18	0	0	14
Fibrobacteres	0	0	0	0	0	0	0	0	0	1
Fusobacteria	1	0	0	3	0	0	0	0	0	1
Verrucomicrobia	0	0	0	0	0	0	0	0	0	4
Gemmatimonadetes	0	0	0	0	0	0	1	0	0	0
Planctomycetes	0	0	0	0	0	0	2	0	0	2
Elusimicrobia	1	0	0	0	0	0	1	0	0	0
Synergistetes	0	0	0	0	0	0	0	0	0	2
Cyanobacteria	1	0	0	0	0	0	14	0	0	1
Chlorobi	0	0	0	0	0	0	10	0	0	0
Chloroflexi	0	0	0	0	0	0	6	0	0	5
Deinococcus-Thermus	0	0	0	2	0	0	3	0	0	2
Aquificae	0	0	0	0	0	0	2	0	0	7
Thermotogae	0	0	0	1	0	0	10	0	0	0
Dictyoglomi	0	0	0	2	0	0	0	0	0	0
Nitrospirae	0	0	0	0	0	0	0	0	0	2
Thermobaculum	0	0	0	0	0	0	1	0	0	0
Deferribacteres	0	0	0	0	0	0	2	0	0	1
Euryarchaeota	3	0	0	22	0	0	23	0	0	12
Crenarchaeota	1	0	0	7	0	0	11	0	0	4
Thaumarchaeota	0	0	0	0	0	0	2	0	0	0
Nanoarchaeota	0	0	0	1	0	0	0	0	0	0
Korarchaeota	1	0	0	0	0	0	0	0	0	0
Total	64	0	0	99	0	0	270	0	0	335

(M00119_2)

Phyla	Module completion ratio (%)									
	0--10	10--20	20--30	30--40	40--50	50--60	60--70	70--80	80--90	90--100
Gammaproteobacteria	56	0	0	0	0	0	0	0	0	70
Betaproteobacteria	16	0	0	0	0	0	0	0	0	45
Epsilonproteobacteria	0	0	0	0	0	0	0	0	0	17
Deltaproteobacteria	12	0	0	0	0	0	0	0	0	16
Alphaproteobacteria	72	0	0	0	0	0	0	0	0	19
Magnetococcus	1	0	0	0	0	0	0	0	0	0
Chrysiogenetes	0	0	0	0	0	0	0	0	0	1
Firmicutes	44	0	0	0	0	0	0	0	0	60
Tenericutes	19	0	0	0	0	0	0	0	0	0
Actinobacteria	21	0	0	0	0	0	0	0	0	59
Chlamydiae	8	0	0	0	0	0	0	0	0	0
Spirochaetes	10	0	0	0	0	0	0	0	0	4
Acidobacteria	4	0	0	0	0	0	0	0	0	1
Bacteroidetes	3	0	0	0	0	0	0	0	0	32
Fibrobacteres	0	0	0	0	0	0	0	0	0	1
Fusobacteria	4	0	0	0	0	0	0	0	0	1
Verrucomicrobia	2	0	0	0	0	0	0	0	0	2
Gemmatimonadetes	1	0	0	0	0	0	0	0	0	0
Planctomycetes	0	0	0	0	0	0	0	0	0	4
Elusimicrobia	1	0	0	0	0	0	0	0	0	1
Synergistetes	1	0	0	0	0	0	0	0	0	1
Cyanobacteria	6	0	0	0	0	0	0	0	0	10
Chlorobi	0	0	0	0	0	0	0	0	0	10
Chloroflexi	7	0	0	0	0	0	0	0	0	4
Deinococcus-Thermus	2	0	0	0	0	0	0	0	0	5
Aquificae	0	0	0	0	0	0	0	0	0	9
Thermotogae	1	0	0	0	0	0	0	0	0	10
Dictyoglomi	2	0	0	0	0	0	0	0	0	0
Nitrospirae	0	0	0	0	0	0	0	0	0	2
Thermobaculum	0	0	0	0	0	0	0	0	0	1
Deferribacteres	0	0	0	0	0	0	0	0	0	3
Euryarchaeota	59	0	0	0	0	0	0	0	0	1
Crenarchaeota	20	0	0	0	0	0	0	0	0	3
Thaumarchaeota	2	0	0	0	0	0	0	0	0	0
Nanoarchaeota	1	0	0	0	0	0	0	0	0	0
Korarchaeota	1	0	0	0	0	0	0	0	0	0
Total	376	0	0	0	0	0	0	0	0	392

(M00119_3)

Phyla	Module completion ratio (%)									
	0--10	10--20	20--30	30--40	40--50	50--60	60--70	70--80	80--90	90--100
Gammaproteobacteria	13	0	0	0	0	0	0	0	0	113
Betaproteobacteria	1	0	0	0	0	0	0	0	0	60
Epsilonproteobacteria	0	0	0	0	0	0	0	0	0	17
Deltaproteobacteria	1	0	0	0	0	0	0	0	0	27
Alphaproteobacteria	22	0	0	0	0	0	0	0	0	69
Magnetococcus	0	0	0	0	0	0	0	0	0	1
Chrysiogenetes	0	0	0	0	0	0	0	0	0	1
Firmicutes	42	0	0	0	0	0	0	0	0	62
Tenericutes	19	0	0	0	0	0	0	0	0	0
Actinobacteria	10	0	0	0	0	0	0	0	0	70
Chlamydiae	8	0	0	0	0	0	0	0	0	0
Spirochaetes	10	0	0	0	0	0	0	0	0	4
Acidobacteria	1	0	0	0	0	0	0	0	0	4
Bacteroidetes	3	0	0	0	0	0	0	0	0	32
Fibrobacteres	0	0	0	0	0	0	0	0	0	1
Fusobacteria	4	0	0	0	0	0	0	0	0	1
Verrucomicrobia	0	0	0	0	0	0	0	0	0	4
Gemmatimonadetes	1	0	0	0	0	0	0	0	0	0
Planctomycetes	0	0	0	0	0	0	0	0	0	4
Elusimicrobia	1	0	0	0	0	0	0	0	0	1
Synergistetes	0	0	0	0	0	0	0	0	0	2
Cyanobacteria	0	0	0	0	0	0	0	0	0	16
Chlorobi	0	0	0	0	0	0	0	0	0	10
Chloroflexi	1	0	0	0	0	0	0	0	0	10
Deinococcus-Thermus	2	0	0	0	0	0	0	0	0	5
Aquificae	0	0	0	0	0	0	0	0	0	9
Thermotogae	1	0	0	0	0	0	0	0	0	10
Dictyoglomi	2	0	0	0	0	0	0	0	0	0
Nitrospirae	0	0	0	0	0	0	0	0	0	2
Thermobaculum	0	0	0	0	0	0	0	0	0	1
Deferribacteres	0	0	0	0	0	0	0	0	0	3
Euryarchaeota	60	0	0	0	0	0	0	0	0	0
Crenarchaeota	23	0	0	0	0	0	0	0	0	0
Thaumarchaeota	2	0	0	0	0	0	0	0	0	0
Nanoarchaeota	1	0	0	0	0	0	0	0	0	0
Korarchaeota	1	0	0	0	0	0	0	0	0	0
Total	229	0	0	0	0	0	0	0	0	539

(M00120_1)

Phyla	Module completion ratio (%)									
	0--10	10--20	20--30	30--40	40--50	50--60	60--70	70--80	80--90	90--100
Gammaproteobacteria	2	0	0	1	0	0	1	0	0	122
Betaproteobacteria	1	0	0	0	0	0	2	0	0	58
Epsilonproteobacteria	0	0	0	0	0	0	1	0	0	16
Deltaproteobacteria	0	0	0	0	0	0	7	0	0	21
Alphaproteobacteria	11	0	0	4	0	0	2	0	0	74
Magnetococcus	0	0	0	0	0	0	0	0	0	1
Chrysiogenetes	0	0	0	0	0	0	0	0	0	1
Firmicutes	0	0	0	0	0	0	3	0	0	101
Tenericutes	11	0	0	5	0	0	2	0	0	1
Actinobacteria	0	0	0	0	0	0	5	0	0	75
Chlamydiae	8	0	0	0	0	0	0	0	0	0
Spirochaetes	0	0	0	1	0	0	5	0	0	8
Acidobacteria	0	0	0	0	0	0	0	0	0	5
Bacteroidetes	2	0	0	0	0	0	2	0	0	31
Fibrobacteres	0	0	0	0	0	0	0	0	0	1
Fusobacteria	0	0	0	0	0	0	0	0	0	5
Verrucomicrobia	0	0	0	0	0	0	2	0	0	2
Gemmatimonadetes	0	0	0	0	0	0	0	0	0	1
Planctomycetes	0	0	0	0	0	0	2	0	0	2
Elusimicrobia	0	0	0	0	0	0	1	0	0	1
Synergistetes	0	0	0	0	0	0	0	0	0	2
Cyanobacteria	0	0	0	0	0	0	2	0	0	14
Chlorobi	0	0	0	0	0	0	0	0	0	10
Chloroflexi	0	0	0	0	0	0	0	0	0	11
Deinococcus-Thermus	0	0	0	0	0	0	0	0	0	7
Aquificae	0	0	0	0	0	0	0	0	0	9
Thermotogae	0	0	0	0	0	0	0	0	0	11
Dictyoglomi	0	0	0	0	0	0	2	0	0	0
Nitrospirae	0	0	0	0	0	0	0	0	0	2
Thermobaculum	0	0	0	0	0	0	0	0	0	1
Deferribacteres	0	0	0	0	0	0	0	0	0	3
Euryarchaeota	1	0	0	50	0	0	9	0	0	0
Crenarchaeota	1	0	0	22	0	0	0	0	0	0
Thaumarchaeota	0	0	0	2	0	0	0	0	0	0
Nanoarchaeota	1	0	0	0	0	0	0	0	0	0
Korarchaeota	0	0	0	1	0	0	0	0	0	0
Total	38	0	0	86	0	0	48	0	0	596

(M00121_1)

Phyla	Module completion ratio (%)									
	0--10	10--20	20--30	30--40	40--50	50--60	60--70	70--80	80--90	90--100
Gammaproteobacteria	0	3	0	2	0	2	0	0	3	116
Betaproteobacteria	0	1	0	0	0	0	0	0	2	58
Epsilonproteobacteria	0	0	0	0	0	0	0	0	0	17
Deltaproteobacteria	0	0	0	0	0	0	0	0	1	27
Alphaproteobacteria	0	5	0	0	0	13	45	0	26	2
Magnetococcus	0	0	0	0	0	0	0	0	1	0
Chrysiogenetes	0	0	0	0	0	0	1	0	0	0
Firmicutes	28	5	0	3	0	2	1	0	40	25
Tenericutes	0	19	0	0	0	0	0	0	0	0
Actinobacteria	0	6	0	0	0	0	0	0	0	74
Chlamydiae	0	0	0	0	0	0	1	0	0	7
Spirochaetes	1	10	0	0	0	0	1	0	2	0
Acidobacteria	0	0	0	0	0	0	0	0	0	5
Bacteroidetes	1	4	0	4	0	2	0	0	5	19
Fibrobacteres	0	0	0	0	0	0	0	0	0	1
Fusobacteria	0	1	0	0	0	0	0	0	0	4
Verrucomicrobia	0	0	0	0	0	0	0	0	0	4
Gemmatimonadetes	0	0	0	0	0	0	0	0	0	1
Planctomycetes	0	0	0	0	0	0	0	0	0	4
Elusimicrobia	0	2	0	0	0	0	0	0	0	0
Synergistetes	0	1	0	0	0	0	0	0	0	1
Cyanobacteria	0	0	0	0	0	0	0	0	1	15
Chlorobi	0	0	0	0	0	0	0	0	0	10
Chloroflexi	0	3	0	0	0	0	0	0	1	7
Deinococcus-Thermus	0	0	0	0	0	0	0	0	0	7
Aquificae	0	0	0	0	0	0	0	0	5	4
Thermotogae	0	9	0	0	0	0	0	0	0	2
Dictyoglomi	1	1	0	0	0	0	0	0	0	0
Nitrospirae	0	0	0	0	0	0	0	0	0	2
Thermobaculum	0	0	0	0	0	0	0	0	0	1
Deferribacteres	0	0	0	0	0	0	0	0	2	1
Euryarchaeota	0	9	0	0	0	0	0	0	2	49
Crenarchaeota	0	6	0	1	0	0	0	0	1	15
Thaumarchaeota	0	0	0	0	0	0	0	0	0	2
Nanoarchaeota	0	1	0	0	0	0	0	0	0	0
Korarchaeota	0	0	0	1	0	0	0	0	0	0
Total	31	86	0	11	0	19	49	0	92	480

(M00121_2)

Phyla	Module completion ratio (%)									
	0--10	10--20	20--30	30--40	40--50	50--60	60--70	70--80	80--90	90--100
Gammaproteobacteria	3	0	4	0	0	1	0	48	0	70
Betaproteobacteria	1	0	0	0	0	0	0	52	0	8
Epsilonproteobacteria	0	0	0	0	0	0	0	16	0	1
Deltaproteobacteria	0	0	7	0	0	4	0	4	0	13
Alphaproteobacteria	1	0	4	0	0	0	0	82	0	4
Magnetococcus	0	0	0	0	0	0	0	0	0	1
Chrysiogenetes	0	0	0	0	0	0	0	0	0	1
Firmicutes	11	0	34	0	0	27	0	1	0	31
Tenericutes	14	0	5	0	0	0	0	0	0	0
Actinobacteria	1	0	6	0	0	4	0	2	0	67
Chlamydiae	0	0	0	0	0	0	0	0	0	8
Spirochaetes	0	0	9	0	0	2	0	0	0	3
Acidobacteria	0	0	0	0	0	0	0	1	0	4
Bacteroidetes	2	0	5	0	0	2	0	19	0	7
Fibrobacteres	0	0	1	0	0	0	0	0	0	0
Fusobacteria	0	0	5	0	0	0	0	0	0	0
Verrucomicrobia	0	0	2	0	0	0	0	0	0	2
Gemmatimonadetes	0	0	0	0	0	0	0	1	0	0
Planctomycetes	0	0	0	0	0	0	0	1	0	3
Elusimicrobia	0	0	2	0	0	0	0	0	0	0
Synergistetes	0	0	1	0	0	1	0	0	0	0
Cyanobacteria	0	0	0	0	0	0	0	11	0	5
Chlorobi	0	0	0	0	0	2	0	5	0	3
Chloroflexi	0	0	2	0	0	1	0	3	0	5
Deinococcus-Thermus	0	0	1	0	0	0	0	0	0	6
Aquificae	0	0	0	0	0	0	0	3	0	6
Thermotogae	0	0	9	0	0	2	0	0	0	0
Dictyoglomi	0	0	0	0	0	2	0	0	0	0
Nitrospirae	0	0	0	0	0	0	0	1	0	1
Thermobaculum	0	0	0	0	0	0	0	0	0	1
Deferribacteres	0	0	0	0	0	0	0	0	0	3
Euryarchaeota	35	0	19	0	0	4	0	2	0	0
Crenarchaeota	22	0	1	0	0	0	0	0	0	0
Thaumarchaeota	2	0	0	0	0	0	0	0	0	0
Nanoarchaeota	1	0	0	0	0	0	0	0	0	0
Korarchaeota	1	0	0	0	0	0	0	0	0	0
Total	94	0	117	0	0	52	0	252	0	253

(M00121_3)

Phyla	Module completion ratio (%)									
	0--10	10--20	20--30	30--40	40--50	50--60	60--70	70--80	80--90	90--100
Gammaproteobacteria	126	0	0	0	0	0	0	0	0	0
Betaproteobacteria	61	0	0	0	0	0	0	0	0	0
Epsilonproteobacteria	17	0	0	0	0	0	0	0	0	0
Deltaproteobacteria	28	0	0	0	0	0	0	0	0	0
Alphaproteobacteria	91	0	0	0	0	0	0	0	0	0
Magnetococcus	1	0	0	0	0	0	0	0	0	0
Chrysiogenetes	1	0	0	0	0	0	0	0	0	0
Firmicutes	100	0	0	0	0	0	0	0	0	4
Tenericutes	19	0	0	0	0	0	0	0	0	0
Actinobacteria	80	0	0	0	0	0	0	0	0	0
Chlamydiae	8	0	0	0	0	0	0	0	0	0
Spirochaetes	14	0	0	0	0	0	0	0	0	0
Acidobacteria	5	0	0	0	0	0	0	0	0	0
Bacteroidetes	35	0	0	0	0	0	0	0	0	0
Fibrobacteres	1	0	0	0	0	0	0	0	0	0
Fusobacteria	5	0	0	0	0	0	0	0	0	0
Verrucomicrobia	4	0	0	0	0	0	0	0	0	0
Gemmatimonadetes	1	0	0	0	0	0	0	0	0	0
Planctomycetes	4	0	0	0	0	0	0	0	0	0
Elusimicrobia	2	0	0	0	0	0	0	0	0	0
Synergistetes	2	0	0	0	0	0	0	0	0	0
Cyanobacteria	16	0	0	0	0	0	0	0	0	0
Chlorobi	10	0	0	0	0	0	0	0	0	0
Chloroflexi	11	0	0	0	0	0	0	0	0	0
Deinococcus-Thermus	7	0	0	0	0	0	0	0	0	0
Aquificae	9	0	0	0	0	0	0	0	0	0
Thermotogae	11	0	0	0	0	0	0	0	0	0
Dictyoglomi	2	0	0	0	0	0	0	0	0	0
Nitrospirae	2	0	0	0	0	0	0	0	0	0
Thermobaculum	1	0	0	0	0	0	0	0	0	0
Deferribacteres	3	0	0	0	0	0	0	0	0	0
Euryarchaeota	60	0	0	0	0	0	0	0	0	0
Crenarchaeota	23	0	0	0	0	0	0	0	0	0
Thaumarchaeota	2	0	0	0	0	0	0	0	0	0
Nanoarchaeota	1	0	0	0	0	0	0	0	0	0
Korarchaeota	1	0	0	0	0	0	0	0	0	0
Total	764	0	0	0	0	0	0	0	0	4

(M00122_1)

Phyla	Module completion ratio (%)									
	0--10	10--20	20--30	30--40	40--50	50--60	60--70	70--80	80--90	90--100
Gammaproteobacteria	29	0	26	0	12	5	0	0	54	0
Betaproteobacteria	4	0	8	0	6	2	0	0	41	0
Epsilonproteobacteria	13	0	3	0	0	1	0	0	0	0
Deltaproteobacteria	8	0	1	0	0	4	0	0	15	0
Alphaproteobacteria	34	0	9	0	0	3	0	0	37	8
Magnetococcus	0	0	0	0	0	0	0	0	1	0
Chrysiogenetes	0	0	0	0	0	0	0	0	1	0
Firmicutes	50	0	6	0	0	16	0	0	32	0
Tenericutes	18	0	1	0	0	0	0	0	0	0
Actinobacteria	32	0	4	0	1	9	0	0	34	0
Chlamydiae	8	0	0	0	0	0	0	0	0	0
Spirochaetes	9	0	1	0	0	2	0	0	2	0
Acidobacteria	1	0	3	0	0	1	0	0	0	0
Bacteroidetes	23	0	3	0	1	6	0	0	2	0
Fibrobacteres	1	0	0	0	0	0	0	0	0	0
Fusobacteria	1	0	0	0	0	2	0	0	2	0
Verrucomicrobia	1	0	2	0	1	0	0	0	0	0
Gemmatimonadetes	0	0	0	0	0	0	0	0	1	0
Planctomycetes	3	0	1	0	0	0	0	0	0	0
Elusimicrobia	1	0	1	0	0	0	0	0	0	0
Synergistetes	0	0	1	0	0	0	0	0	1	0
Cyanobacteria	0	0	0	0	0	0	0	0	16	0
Chlorobi	0	0	0	0	0	1	0	0	9	0
Chloroflexi	1	0	2	0	0	1	0	0	7	0
Deinococcus-Thermus	0	0	0	0	1	3	0	0	3	0
Aquificae	5	0	4	0	0	0	0	0	0	0
Thermotogae	0	0	6	0	1	1	0	0	3	0
Dictyoglomi	0	0	2	0	0	0	0	0	0	0
Nitrospirae	0	0	0	0	0	0	0	0	2	0
Thermobaculum	1	0	0	0	0	0	0	0	0	0
Deferribacteres	0	0	0	0	0	2	0	0	1	0
Euryarchaeota	0	0	5	0	30	25	0	0	0	0
Crenarchaeota	9	0	2	0	9	3	0	0	0	0
Thaumarchaeota	0	0	0	0	0	2	0	0	0	0
Nanoarchaeota	1	0	0	0	0	0	0	0	0	0
Korarchaeota	0	0	1	0	0	0	0	0	0	0
Total	253	0	92	0	62	89	0	0	264	8

(M00122_2)

Phyla	Module completion ratio (%)									
	0--10	10--20	20--30	30--40	40--50	50--60	60--70	70--80	80--90	90--100
Gammaproteobacteria	59	0	0	0	0	53	0	0	0	14
Betaproteobacteria	19	0	0	0	0	42	0	0	0	0
Epsilonproteobacteria	16	0	0	0	0	1	0	0	0	0
Deltaproteobacteria	8	0	0	0	0	20	0	0	0	0
Alphaproteobacteria	38	0	0	0	0	53	0	0	0	0
Magnetococcus	0	0	0	0	0	1	0	0	0	0
Chrysiogenetes	0	0	0	0	0	1	0	0	0	0
Firmicutes	57	0	0	0	0	34	0	0	0	13
Tenericutes	19	0	0	0	0	0	0	0	0	0
Actinobacteria	29	0	0	0	0	51	0	0	0	0
Chlamydiae	8	0	0	0	0	0	0	0	0	0
Spirochaetes	9	0	0	0	0	5	0	0	0	0
Acidobacteria	4	0	0	0	0	1	0	0	0	0
Bacteroidetes	20	0	0	0	0	15	0	0	0	0
Fibrobacteres	1	0	0	0	0	0	0	0	0	0
Fusobacteria	1	0	0	0	0	0	0	0	0	4
Verrucomicrobia	3	0	0	0	0	1	0	0	0	0
Gemmatimonadetes	0	0	0	0	0	1	0	0	0	0
Planctomycetes	2	0	0	0	0	2	0	0	0	0
Elusimicrobia	2	0	0	0	0	0	0	0	0	0
Synergistetes	2	0	0	0	0	0	0	0	0	0
Cyanobacteria	16	0	0	0	0	0	0	0	0	0
Chlorobi	0	0	0	0	0	10	0	0	0	0
Chloroflexi	3	0	0	0	0	6	0	0	0	2
Deinococcus-Thermus	0	0	0	0	0	7	0	0	0	0
Aquificae	6	0	0	0	0	3	0	0	0	0
Thermotogae	6	0	0	0	0	5	0	0	0	0
Dictyoglomi	2	0	0	0	0	0	0	0	0	0
Nitrospirae	0	0	0	0	0	1	0	0	0	1
Thermobaculum	1	0	0	0	0	0	0	0	0	0
Deferribacteres	0	0	0	0	0	3	0	0	0	0
Euryarchaeota	57	0	0	0	0	3	0	0	0	0
Crenarchaeota	23	0	0	0	0	0	0	0	0	0
Thaumarchaeota	2	0	0	0	0	0	0	0	0	0
Nanoarchaeota	1	0	0	0	0	0	0	0	0	0
Korarchaeota	1	0	0	0	0	0	0	0	0	0
Total	415	0	0	0	0	319	0	0	0	34

(M00122_3)

Phyla	Module completion ratio (%)									
	0--10	10--20	20--30	30--40	40--50	50--60	60--70	70--80	80--90	90--100
Gammaproteobacteria	58	0	0	0	0	0	0	0	0	68
Betaproteobacteria	19	0	0	0	0	0	0	0	0	42
Epsilonproteobacteria	14	0	0	0	0	0	0	0	0	3
Deltaproteobacteria	8	0	0	0	0	0	0	0	0	20
Alphaproteobacteria	44	0	0	0	0	0	0	0	0	47
Magnetococcus	0	0	0	0	0	0	0	0	0	1
Chrysiogenetes	0	0	0	0	0	0	0	0	0	1
Firmicutes	57	0	0	0	0	0	0	0	0	47
Tenericutes	19	0	0	0	0	0	0	0	0	0
Actinobacteria	34	0	0	0	0	0	0	0	0	46
Chlamydiae	8	0	0	0	0	0	0	0	0	0
Spirochaetes	9	0	0	0	0	0	0	0	0	5
Acidobacteria	4	0	0	0	0	0	0	0	0	1
Bacteroidetes	23	0	0	0	0	0	0	0	0	12
Fibrobacteres	1	0	0	0	0	0	0	0	0	0
Fusobacteria	1	0	0	0	0	0	0	0	0	4
Verrucomicrobia	3	0	0	0	0	0	0	0	0	1
Gemmatimonadetes	0	0	0	0	0	0	0	0	0	1
Planctomycetes	2	0	0	0	0	0	0	0	0	2
Elusimicrobia	2	0	0	0	0	0	0	0	0	0
Synergistetes	1	0	0	0	0	0	0	0	0	1
Cyanobacteria	0	0	0	0	0	0	0	0	0	16
Chlorobi	0	0	0	0	0	0	0	0	0	10
Chloroflexi	3	0	0	0	0	0	0	0	0	8
Deinococcus-Thermus	0	0	0	0	0	0	0	0	0	7
Aquificae	9	0	0	0	0	0	0	0	0	0
Thermotogae	6	0	0	0	0	0	0	0	0	5
Dictyoglomi	2	0	0	0	0	0	0	0	0	0
Nitrospirae	0	0	0	0	0	0	0	0	0	2
Thermobaculum	1	0	0	0	0	0	0	0	0	0
Deferribacteres	0	0	0	0	0	0	0	0	0	3
Euryarchaeota	2	0	0	0	0	0	0	0	0	58
Crenarchaeota	9	0	0	0	0	0	0	0	0	14
Thaumarchaeota	0	0	0	0	0	0	0	0	0	2
Nanoarchaeota	1	0	0	0	0	0	0	0	0	0
Korarchaeota	1	0	0	0	0	0	0	0	0	0
Total	341	0	0	0	0	0	0	0	0	427

(M00123_1)

Phyla	Module completion ratio (%)									
	0--10	10--20	20--30	30--40	40--50	50--60	60--70	70--80	80--90	90--100
Gammaproteobacteria	3	0	1	0	0	2	0	1	0	119
Betaproteobacteria	1	0	6	0	0	1	0	6	0	47
Epsilonproteobacteria	0	0	2	0	0	1	0	0	0	14
Deltaproteobacteria	1	0	1	0	0	1	0	4	0	21
Alphaproteobacteria	33	0	8	0	0	0	0	1	0	49
Magnetococcus	0	0	0	0	0	0	0	0	0	1
Chrysiogenetes	0	0	0	0	0	0	0	0	0	1
Firmicutes	36	0	33	0	0	5	0	12	0	18
Tenericutes	19	0	0	0	0	0	0	0	0	0
Actinobacteria	26	0	8	0	0	0	0	3	0	43
Chlamydiae	2	0	3	0	0	0	0	0	0	3
Spirochaetes	9	0	1	0	0	0	0	1	0	3
Acidobacteria	3	0	2	0	0	0	0	0	0	0
Bacteroidetes	10	0	1	0	0	1	0	2	0	21
Fibrobacteres	0	0	0	0	0	0	0	1	0	0
Fusobacteria	1	0	2	0	0	0	0	1	0	1
Verrucomicrobia	1	0	0	0	0	1	0	0	0	2
Gemmatimonadetes	0	0	0	0	0	0	0	0	0	1
Planctomycetes	0	0	0	0	0	0	0	0	0	4
Elusimicrobia	0	0	0	0	0	1	0	0	0	1
Synergistetes	1	0	0	0	0	0	0	1	0	0
Cyanobacteria	0	0	2	0	0	0	0	10	0	4
Chlorobi	0	0	1	0	0	0	0	0	0	9
Chloroflexi	11	0	0	0	0	0	0	0	0	0
Deinococcus-Thermus	6	0	1	0	0	0	0	0	0	0
Aquificae	0	0	0	0	0	0	0	1	0	8
Thermotogae	0	0	11	0	0	0	0	0	0	0
Dictyoglomi	0	0	2	0	0	0	0	0	0	0
Nitrospirae	0	0	0	0	0	0	0	2	0	0
Thermobaculum	1	0	0	0	0	0	0	0	0	0
Deferribacteres	0	0	0	0	0	0	0	0	0	3
Euryarchaeota	22	0	24	0	0	1	0	5	0	8
Crenarchaeota	12	0	11	0	0	0	0	0	0	0
Thaumarchaeota	0	0	0	0	0	0	0	2	0	0
Nanoarchaeota	1	0	0	0	0	0	0	0	0	0
Korarchaeota	0	0	1	0	0	0	0	0	0	0
Total	199	0	121	0	0	14	0	53	0	381

(M00124_1)

Phyla	Module completion ratio (%)									
	0--10	10--20	20--30	30--40	40--50	50--60	60--70	70--80	80--90	90--100
Gammaproteobacteria	1	4	0	8	0	6	13	0	38	56
Betaproteobacteria	1	0	0	1	0	9	49	0	1	0
Epsilonproteobacteria	0	2	0	11	0	4	0	0	0	0
Deltaproteobacteria	2	0	0	11	0	5	10	0	0	0
Alphaproteobacteria	12	0	0	8	0	1	70	0	0	0
Magnetococcus	0	0	0	0	0	1	0	0	0	0
Chrysiogenetes	0	0	0	1	0	0	0	0	0	0
Firmicutes	47	48	0	9	0	0	0	0	0	0
Tenericutes	19	0	0	0	0	0	0	0	0	0
Actinobacteria	6	19	0	48	0	7	0	0	0	0
Chlamydiae	7	1	0	0	0	0	0	0	0	0
Spirochaetes	10	0	0	0	0	1	3	0	0	0
Acidobacteria	2	2	0	1	0	0	0	0	0	0
Bacteroidetes	2	0	0	3	0	2	21	0	7	0
Fibrobacteres	0	0	0	1	0	0	0	0	0	0
Fusobacteria	3	1	0	0	0	1	0	0	0	0
Verrucomicrobia	0	0	0	0	0	3	1	0	0	0
Gemmatimonadetes	0	0	0	0	0	1	0	0	0	0
Planctomycetes	0	0	0	0	0	3	1	0	0	0
Elusimicrobia	0	0	0	1	0	1	0	0	0	0
Synergistetes	2	0	0	0	0	0	0	0	0	0
Cyanobacteria	0	0	0	7	0	6	3	0	0	0
Chlorobi	0	0	0	9	0	1	0	0	0	0
Chloroflexi	3	6	0	2	0	0	0	0	0	0
Deinococcus-Thermus	2	5	0	0	0	0	0	0	0	0
Aquificae	0	1	0	8	0	0	0	0	0	0
Thermotogae	11	0	0	0	0	0	0	0	0	0
Dictyoglomi	2	0	0	0	0	0	0	0	0	0
Nitrospirae	0	0	0	2	0	0	0	0	0	0
Thermobaculum	0	0	0	1	0	0	0	0	0	0
Deferribacteres	0	0	0	3	0	0	0	0	0	0
Euryarchaeota	55	5	0	0	0	0	0	0	0	0
Crenarchaeota	21	2	0	0	0	0	0	0	0	0
Thaumarchaeota	2	0	0	0	0	0	0	0	0	0
Nanoarchaeota	1	0	0	0	0	0	0	0	0	0
Korarchaeota	0	1	0	0	0	0	0	0	0	0
Total	211	97	0	135	0	52	171	0	46	56

(M00125_1)

Phyla	Module completion ratio (%)									
	0--10	10--20	20--30	30--40	40--50	50--60	60--70	70--80	80--90	90--100
Gammaproteobacteria	1	0	0	0	0	1	0	0	0	124
Betaproteobacteria	0	0	0	0	0	0	0	0	0	61
Epsilonproteobacteria	0	0	0	0	0	0	0	0	0	17
Deltaproteobacteria	0	0	0	0	0	0	0	0	0	28
Alphaproteobacteria	11	0	0	0	0	3	0	0	0	77
Magnetococcus	0	0	0	0	0	0	0	0	0	1
Chrysiogenetes	0	0	0	0	0	0	0	0	0	1
Firmicutes	28	0	0	0	0	1	0	0	0	75
Tenericutes	17	0	0	0	0	1	0	0	0	1
Actinobacteria	9	0	0	0	0	10	0	0	0	61
Chlamydiae	0	0	0	0	0	0	0	0	0	8
Spirochaetes	9	0	0	0	0	1	0	0	0	4
Acidobacteria	0	0	0	0	0	0	0	0	0	5
Bacteroidetes	3	0	0	0	0	0	0	0	0	32
Fibrobacteres	0	0	0	0	0	0	0	0	0	1
Fusobacteria	1	0	0	0	0	0	0	0	0	4
Verrucomicrobia	0	0	0	0	0	0	0	0	0	4
Gemmatimonadetes	0	0	0	0	0	0	0	0	0	1
Planctomycetes	0	0	0	0	0	0	0	0	0	4
Elusimicrobia	1	0	0	0	0	0	0	0	0	1
Synergistetes	0	0	0	0	0	0	0	0	0	2
Cyanobacteria	0	0	0	0	0	1	0	0	0	15
Chlorobi	0	0	0	0	0	0	0	0	0	10
Chloroflexi	3	0	0	0	0	0	0	0	0	8
Deinococcus-Thermus	0	0	0	0	0	0	0	0	0	7
Aquificae	0	0	0	0	0	0	0	0	0	9
Thermotogae	2	0	0	0	0	0	0	0	0	9
Dictyoglomi	2	0	0	0	0	0	0	0	0	0
Nitrospirae	0	0	0	0	0	0	0	0	0	2
Thermobaculum	0	0	0	0	0	0	0	0	0	1
Deferribacteres	0	0	0	0	0	0	0	0	0	3
Euryarchaeota	48	0	0	0	0	8	0	0	0	4
Crenarchaeota	23	0	0	0	0	0	0	0	0	0
Thaumarchaeota	2	0	0	0	0	0	0	0	0	0
Nanoarchaeota	1	0	0	0	0	0	0	0	0	0
Korarchaeota	1	0	0	0	0	0	0	0	0	0
Total	162	0	0	0	0	26	0	0	0	580

(M00125_2)

Phyla	Module completion ratio (%)									
	0--10	10--20	20--30	30--40	40--50	50--60	60--70	70--80	80--90	90--100
Gammaproteobacteria	2	0	0	0	0	0	0	0	0	124
Betaproteobacteria	1	0	0	0	0	0	0	0	0	60
Epsilonproteobacteria	0	0	0	0	0	0	0	0	0	17
Deltaproteobacteria	0	0	0	0	0	0	0	0	0	28
Alphaproteobacteria	13	0	0	0	0	0	0	0	0	78
Magnetococcus	0	0	0	0	0	0	0	0	0	1
Chrysiogenetes	0	0	0	0	0	0	0	0	0	1
Firmicutes	28	0	0	0	0	0	0	0	0	76
Tenericutes	18	0	0	0	0	0	0	0	0	1
Actinobacteria	11	0	0	0	0	0	0	0	0	69
Chlamydiae	0	0	0	0	0	0	0	0	0	8
Spirochaetes	9	0	0	0	0	0	0	0	0	5
Acidobacteria	0	0	0	0	0	0	0	0	0	5
Bacteroidetes	3	0	0	0	0	0	0	0	0	32
Fibrobacteres	0	0	0	0	0	0	0	0	0	1
Fusobacteria	1	0	0	0	0	0	0	0	0	4
Verrucomicrobia	0	0	0	0	0	0	0	0	0	4
Gemmatimonadetes	0	0	0	0	0	0	0	0	0	1
Planctomycetes	0	0	0	0	0	0	0	0	0	4
Elusimicrobia	1	0	0	0	0	0	0	0	0	1
Synergistetes	0	0	0	0	0	0	0	0	0	2
Cyanobacteria	0	0	0	0	0	0	0	0	0	16
Chlorobi	0	0	0	0	0	0	0	0	0	10
Chloroflexi	3	0	0	0	0	0	0	0	0	8
Deinococcus-Thermus	0	0	0	0	0	0	0	0	0	7
Aquificae	0	0	0	0	0	0	0	0	0	9
Thermotogae	2	0	0	0	0	0	0	0	0	9
Dictyoglomi	2	0	0	0	0	0	0	0	0	0
Nitrospirae	0	0	0	0	0	0	0	0	0	2
Thermobaculum	0	0	0	0	0	0	0	0	0	1
Deferribacteres	0	0	0	0	0	0	0	0	0	3
Euryarchaeota	7	0	0	0	0	0	0	0	0	53
Crenarchaeota	6	0	0	0	0	0	0	0	0	17
Thaumarchaeota	0	0	0	0	0	0	0	0	0	2
Nanoarchaeota	1	0	0	0	0	0	0	0	0	0
Korarchaeota	1	0	0	0	0	0	0	0	0	0
Total	109	0	0	0	0	0	0	0	0	659

(M00125_3)

Phyla	Module completion ratio (%)									
	0--10	10--20	20--30	30--40	40--50	50--60	60--70	70--80	80--90	90--100
Gammaproteobacteria	1	0	0	0	0	0	1	0	0	124
Betaproteobacteria	0	0	0	0	0	0	2	0	0	59
Epsilonproteobacteria	0	0	0	0	0	0	0	0	0	17
Deltaproteobacteria	0	0	0	0	0	0	0	0	0	28
Alphaproteobacteria	10	0	0	0	0	0	2	0	0	79
Magnetococcus	0	0	0	0	0	0	0	0	0	1
Chrysiogenetes	0	0	0	0	0	0	0	0	0	1
Firmicutes	0	0	0	28	0	0	2	0	0	74
Tenericutes	4	0	0	15	0	0	0	0	0	0
Actinobacteria	0	0	0	9	0	0	3	0	0	68
Chlamydiae	0	0	0	0	0	0	0	0	0	8
Spirochaetes	7	0	0	2	0	0	0	0	0	5
Acidobacteria	0	0	0	0	0	0	0	0	0	5
Bacteroidetes	2	0	0	1	0	0	1	0	0	31
Fibrobacteres	0	0	0	0	0	0	0	0	0	1
Fusobacteria	0	0	0	1	0	0	0	0	0	4
Verrucomicrobia	0	0	0	0	0	0	0	0	0	4
Gemmatimonadetes	0	0	0	0	0	0	0	0	0	1
Planctomycetes	0	0	0	0	0	0	0	0	0	4
Elusimicrobia	0	0	0	1	0	0	0	0	0	1
Synergistetes	0	0	0	0	0	0	0	0	0	2
Cyanobacteria	0	0	0	0	0	0	0	0	0	16
Chlorobi	0	0	0	0	0	0	1	0	0	9
Chloroflexi	0	0	0	2	0	0	1	0	0	8
Deinococcus-Thermus	0	0	0	0	0	0	0	0	0	7
Aquificae	0	0	0	0	0	0	0	0	0	9
Thermotogae	0	0	0	2	0	0	0	0	0	9
Dictyoglomi	0	0	0	2	0	0	0	0	0	0
Nitrospirae	0	0	0	0	0	0	0	0	0	2
Thermobaculum	0	0	0	0	0	0	0	0	0	1
Deferribacteres	0	0	0	0	0	0	0	0	0	3
Euryarchaeota	6	0	0	0	0	0	54	0	0	0
Crenarchaeota	5	0	0	1	0	0	17	0	0	0
Thaumarchaeota	0	0	0	0	0	0	2	0	0	0
Nanoarchaeota	1	0	0	0	0	0	0	0	0	0
Korarchaeota	0	0	0	1	0	0	0	0	0	0
Total	36	0	0	65	0	0	86	0	0	581

(M00126_1)

Phyla	Module completion ratio (%)									
	0--10	10--20	20--30	30--40	40--50	50--60	60--70	70--80	80--90	90--100
Gammaproteobacteria	1	0	0	0	2	6	0	0	47	70
Betaproteobacteria	1	0	0	0	1	11	0	0	20	28
Epsilonproteobacteria	0	0	0	0	0	16	0	0	1	0
Deltaproteobacteria	0	0	9	0	9	6	0	0	1	3
Alphaproteobacteria	1	0	2	0	10	24	0	0	21	33
Magnetococcus	0	0	0	0	1	0	0	0	0	0
Chrysiogenetes	0	0	0	0	1	0	0	0	0	0
Firmicutes	0	0	4	0	24	17	0	0	42	17
Tenericutes	4	0	12	0	3	0	0	0	0	0
Actinobacteria	0	0	0	0	5	10	0	0	45	20
Chlamydiae	0	0	1	0	6	1	0	0	0	0
Spirochaetes	7	0	2	0	2	3	0	0	0	0
Acidobacteria	0	0	0	0	3	2	0	0	0	0
Bacteroidetes	1	0	1	0	0	3	0	0	14	16
Fibrobacteres	0	0	0	0	0	0	0	0	1	0
Fusobacteria	0	0	1	0	0	1	0	0	2	1
Verrucomicrobia	0	0	0	0	1	1	0	0	2	0
Gemmatimonadetes	0	0	0	0	0	1	0	0	0	0
Planctomycetes	0	0	0	0	0	1	0	0	3	0
Elusimicrobia	0	0	0	0	1	1	0	0	0	0
Synergistetes	0	0	0	0	1	1	0	0	0	0
Cyanobacteria	0	0	0	0	0	7	0	0	8	1
Chlorobi	0	0	0	0	0	2	0	0	8	0
Chloroflexi	0	0	1	0	2	8	0	0	0	0
Deinococcus-Thermus	0	0	0	0	0	2	0	0	5	0
Aquificae	0	0	1	0	5	3	0	0	0	0
Thermotogae	0	0	0	0	1	10	0	0	0	0
Dictyoglomi	0	0	0	0	2	0	0	0	0	0
Nitrospirae	0	0	0	0	1	1	0	0	0	0
Thermobaculum	0	0	0	0	1	0	0	0	0	0
Deferribacteres	0	0	1	0	1	1	0	0	0	0
Euryarchaeota	38	0	18	0	3	1	0	0	0	0
Crenarchaeota	9	0	14	0	0	0	0	0	0	0
Thaumarchaeota	0	0	2	0	0	0	0	0	0	0
Nanoarchaeota	1	0	0	0	0	0	0	0	0	0
Korarchaeota	1	0	0	0	0	0	0	0	0	0
Total	64	0	69	0	86	140	0	0	220	189

(M00127_1)

Phyla	Module completion ratio (%)									
	0--10	10--20	20--30	30--40	40--50	50--60	60--70	70--80	80--90	90--100
Gammaproteobacteria	10	0	0	0	0	9	0	0	0	107
Betaproteobacteria	1	0	0	0	0	0	0	0	0	60
Epsilonproteobacteria	1	0	0	0	0	8	0	0	0	8
Deltaproteobacteria	1	0	0	0	0	2	0	0	0	25
Alphaproteobacteria	17	0	0	0	0	11	0	0	0	63
Magnetococcus	0	0	0	0	0	0	0	0	0	1
Chrysiogenetes	0	0	0	0	0	0	0	0	0	1
Firmicutes	9	0	0	0	0	42	0	0	0	53
Tenericutes	19	0	0	0	0	0	0	0	0	0
Actinobacteria	8	0	0	0	0	20	0	0	0	52
Chlamydiae	8	0	0	0	0	0	0	0	0	0
Spirochaetes	9	0	0	0	0	2	0	0	0	3
Acidobacteria	0	0	0	0	0	0	0	0	0	5
Bacteroidetes	11	0	0	0	0	7	0	0	0	17
Fibrobacteres	0	0	0	0	0	0	0	0	0	1
Fusobacteria	2	0	0	0	0	2	0	0	0	1
Verrucomicrobia	1	0	0	0	0	0	0	0	0	3
Gemmatimonadetes	0	0	0	0	0	1	0	0	0	0
Planctomycetes	0	0	0	0	0	2	0	0	0	2
Elusimicrobia	1	0	0	0	0	1	0	0	0	0
Synergistetes	0	0	0	0	0	1	0	0	0	1
Cyanobacteria	1	0	0	0	0	4	0	0	0	11
Chlorobi	0	0	0	0	0	0	0	0	0	10
Chloroflexi	1	0	0	0	0	10	0	0	0	0
Deinococcus-Thermus	2	0	0	0	0	0	0	0	0	5
Aquificae	0	0	0	0	0	0	0	0	0	9
Thermotogae	2	0	0	0	0	0	0	0	0	9
Dictyoglomi	0	0	0	0	0	0	0	0	0	2
Nitrospirae	0	0	0	0	0	0	0	0	0	2
Thermobaculum	0	0	0	0	0	1	0	0	0	0
Deferribacteres	0	0	0	0	0	0	0	0	0	3
Euryarchaeota	1	0	0	0	0	10	0	0	0	49
Crenarchaeota	1	0	0	0	0	9	0	0	0	13
Thaumarchaeota	0	0	0	0	0	0	0	0	0	2
Nanoarchaeota	1	0	0	0	0	0	0	0	0	0
Korarchaeota	0	0	0	0	0	1	0	0	0	0
Total	107	0	0	0	0	143	0	0	0	518

(M00127_2)

Phyla	Module completion ratio (%)									
	0--10	10--20	20--30	30--40	40--50	50--60	60--70	70--80	80--90	90--100
Gammaproteobacteria	86	0	0	0	0	0	0	0	0	40
Betaproteobacteria	53	0	0	0	0	0	0	0	0	8
Epsilonproteobacteria	15	0	0	0	0	0	0	0	0	2
Deltaproteobacteria	15	0	0	0	0	0	0	0	0	13
Alphaproteobacteria	85	0	0	0	0	0	0	0	0	6
Magnetococcus	1	0	0	0	0	0	0	0	0	0
Chrysiogenetes	1	0	0	0	0	0	0	0	0	0
Firmicutes	29	0	0	0	0	0	0	0	0	75
Tenericutes	19	0	0	0	0	0	0	0	0	0
Actinobacteria	62	0	0	0	0	0	0	0	0	18
Chlamydiae	5	0	0	0	0	0	0	0	0	3
Spirochaetes	9	0	0	0	0	0	0	0	0	5
Acidobacteria	5	0	0	0	0	0	0	0	0	0
Bacteroidetes	29	0	0	0	0	0	0	0	0	6
Fibrobacteres	1	0	0	0	0	0	0	0	0	0
Fusobacteria	4	0	0	0	0	0	0	0	0	1
Verrucomicrobia	3	0	0	0	0	0	0	0	0	1
Gemmatimonadetes	0	0	0	0	0	0	0	0	0	1
Planctomycetes	4	0	0	0	0	0	0	0	0	0
Elusimicrobia	2	0	0	0	0	0	0	0	0	0
Synergistetes	0	0	0	0	0	0	0	0	0	2
Cyanobacteria	16	0	0	0	0	0	0	0	0	0
Chlorobi	9	0	0	0	0	0	0	0	0	1
Chloroflexi	6	0	0	0	0	0	0	0	0	5
Deinococcus-Thermus	7	0	0	0	0	0	0	0	0	0
Aquificae	9	0	0	0	0	0	0	0	0	0
Thermotogae	11	0	0	0	0	0	0	0	0	0
Dictyoglomi	2	0	0	0	0	0	0	0	0	0
Nitrospirae	2	0	0	0	0	0	0	0	0	0
Thermobaculum	0	0	0	0	0	0	0	0	0	1
Deferribacteres	3	0	0	0	0	0	0	0	0	0
Euryarchaeota	30	0	0	0	0	0	0	0	0	30
Crenarchaeota	20	0	0	0	0	0	0	0	0	3
Thaumarchaeota	2	0	0	0	0	0	0	0	0	0
Nanoarchaeota	1	0	0	0	0	0	0	0	0	0
Korarchaeota	1	0	0	0	0	0	0	0	0	0
Total	547	0	0	0	0	0	0	0	0	221

(M00127_3)

Phyla	Module completion ratio (%)									
	0--10	10--20	20--30	30--40	40--50	50--60	60--70	70--80	80--90	90--100
Gammaproteobacteria	6	0	0	0	0	6	0	0	0	114
Betaproteobacteria	1	0	0	0	0	1	0	0	0	59
Epsilonproteobacteria	1	0	0	0	0	1	0	0	0	15
Deltaproteobacteria	1	0	0	0	0	6	0	0	0	21
Alphaproteobacteria	15	0	0	0	0	25	0	0	0	51
Magnetococcus	0	0	0	0	0	0	0	0	0	1
Chrysiogenetes	0	0	0	0	0	0	0	0	0	1
Firmicutes	16	0	0	0	0	69	0	0	0	19
Tenericutes	19	0	0	0	0	0	0	0	0	0
Actinobacteria	8	0	0	0	0	5	0	0	0	67
Chlamydiae	5	0	0	0	0	3	0	0	0	0
Spirochaetes	9	0	0	0	0	1	0	0	0	4
Acidobacteria	0	0	0	0	0	0	0	0	0	5
Bacteroidetes	2	0	0	0	0	9	0	0	0	24
Fibrobacteres	0	0	0	0	0	0	0	0	0	1
Fusobacteria	2	0	0	0	0	3	0	0	0	0
Verrucomicrobia	0	0	0	0	0	1	0	0	0	3
Gemmatimonadetes	0	0	0	0	0	0	0	0	0	1
Planctomycetes	0	0	0	0	0	0	0	0	0	4
Elusimicrobia	1	0	0	0	0	0	0	0	0	1
Synergistetes	0	0	0	0	0	2	0	0	0	0
Cyanobacteria	0	0	0	0	0	0	0	0	0	16
Chlorobi	0	0	0	0	0	0	0	0	0	10
Chloroflexi	1	0	0	0	0	5	0	0	0	5
Deinococcus-Thermus	2	0	0	0	0	4	0	0	0	1
Aquificae	0	0	0	0	0	0	0	0	0	9
Thermotogae	11	0	0	0	0	0	0	0	0	0
Dictyoglomi	0	0	0	0	0	2	0	0	0	0
Nitrospirae	0	0	0	0	0	0	0	0	0	2
Thermobaculum	0	0	0	0	0	0	0	0	0	1
Deferribacteres	0	0	0	0	0	0	0	0	0	3
Euryarchaeota	0	0	0	0	0	21	0	0	0	39
Crenarchaeota	0	0	0	0	0	20	0	0	0	3
Thaumarchaeota	0	0	0	0	0	2	0	0	0	0
Nanoarchaeota	1	0	0	0	0	0	0	0	0	0
Korarchaeota	0	0	0	0	0	1	0	0	0	0
Total	101	0	0	0	0	187	0	0	0	480

(M00128_1)

Phyla	Module completion ratio (%)									
	0--10	10--20	20--30	30--40	40--50	50--60	60--70	70--80	80--90	90--100
Gammaproteobacteria	14	88	24	0	0	0	0	0	0	0
Betaproteobacteria	2	17	42	0	0	0	0	0	0	0
Epsilonproteobacteria	17	0	0	0	0	0	0	0	0	0
Deltaproteobacteria	28	0	0	0	0	0	0	0	0	0
Alphaproteobacteria	45	46	0	0	0	0	0	0	0	0
Magnetococcus	0	1	0	0	0	0	0	0	0	0
Chrysiogenetes	1	0	0	0	0	0	0	0	0	0
Firmicutes	103	1	0	0	0	0	0	0	0	0
Tenericutes	19	0	0	0	0	0	0	0	0	0
Actinobacteria	80	0	0	0	0	0	0	0	0	0
Chlamydiae	8	0	0	0	0	0	0	0	0	0
Spirochaetes	14	0	0	0	0	0	0	0	0	0
Acidobacteria	5	0	0	0	0	0	0	0	0	0
Bacteroidetes	35	0	0	0	0	0	0	0	0	0
Fibrobacteres	1	0	0	0	0	0	0	0	0	0
Fusobacteria	5	0	0	0	0	0	0	0	0	0
Verrucomicrobia	4	0	0	0	0	0	0	0	0	0
Gemmatimonadetes	1	0	0	0	0	0	0	0	0	0
Planctomycetes	4	0	0	0	0	0	0	0	0	0
Elusimicrobia	2	0	0	0	0	0	0	0	0	0
Synergistetes	2	0	0	0	0	0	0	0	0	0
Cyanobacteria	16	0	0	0	0	0	0	0	0	0
Chlorobi	10	0	0	0	0	0	0	0	0	0
Chloroflexi	11	0	0	0	0	0	0	0	0	0
Deinococcus-Thermus	7	0	0	0	0	0	0	0	0	0
Aquificae	9	0	0	0	0	0	0	0	0	0
Thermotogae	11	0	0	0	0	0	0	0	0	0
Dictyoglomi	2	0	0	0	0	0	0	0	0	0
Nitrospirae	2	0	0	0	0	0	0	0	0	0
Thermobaculum	1	0	0	0	0	0	0	0	0	0
Deferribacteres	3	0	0	0	0	0	0	0	0	0
Euryarchaeota	60	0	0	0	0	0	0	0	0	0
Crenarchaeota	23	0	0	0	0	0	0	0	0	0
Thaumarchaeota	2	0	0	0	0	0	0	0	0	0
Nanoarchaeota	1	0	0	0	0	0	0	0	0	0
Korarchaeota	1	0	0	0	0	0	0	0	0	0
Total	549	153	66	0	0	0	0	0	0	0

(M00129_1)

Phyla	Module completion ratio (%)									
	0--10	10--20	20--30	30--40	40--50	50--60	60--70	70--80	80--90	90--100
Gammaproteobacteria	6	18	0	76	0	26	0	0	0	0
Betaproteobacteria	2	8	0	31	0	20	0	0	0	0
Epsilonproteobacteria	0	5	0	12	0	0	0	0	0	0
Deltaproteobacteria	0	7	0	19	0	2	0	0	0	0
Alphaproteobacteria	10	18	0	22	0	40	1	0	0	0
Magnetococcus	0	0	0	1	0	0	0	0	0	0
Chrysiogenetes	0	0	0	1	0	0	0	0	0	0
Firmicutes	4	54	0	41	0	5	0	0	0	0
Tenericutes	11	7	0	1	0	0	0	0	0	0
Actinobacteria	3	13	0	39	0	19	6	0	0	0
Chlamydiae	6	2	0	0	0	0	0	0	0	0
Spirochaetes	2	8	0	4	0	0	0	0	0	0
Acidobacteria	0	0	0	0	0	3	2	0	0	0
Bacteroidetes	3	15	0	12	0	3	2	0	0	0
Fibrobacteres	0	1	0	0	0	0	0	0	0	0
Fusobacteria	0	2	0	3	0	0	0	0	0	0
Verrucomicrobia	0	1	0	2	0	0	1	0	0	0
Gemmatimonadetes	0	1	0	0	0	0	0	0	0	0
Planctomycetes	0	0	0	3	0	1	0	0	0	0
Elusimicrobia	2	0	0	0	0	0	0	0	0	0
Synergistetes	0	1	0	1	0	0	0	0	0	0
Cyanobacteria	0	12	0	3	0	0	1	0	0	0
Chlorobi	0	10	0	0	0	0	0	0	0	0
Chloroflexi	2	7	0	2	0	0	0	0	0	0
Deinococcus-Thermus	3	4	0	0	0	0	0	0	0	0
Aquificae	0	0	0	9	0	0	0	0	0	0
Thermotogae	5	6	0	0	0	0	0	0	0	0
Dictyoglomi	0	0	0	0	0	2	0	0	0	0
Nitrospirae	0	0	0	2	0	0	0	0	0	0
Thermobaculum	0	0	0	0	0	1	0	0	0	0
Deferribacteres	0	0	0	3	0	0	0	0	0	0
Euryarchaeota	12	22	0	26	0	0	0	0	0	0
Crenarchaeota	16	2	0	3	0	2	0	0	0	0
Thaumarchaeota	2	0	0	0	0	0	0	0	0	0
Nanoarchaeota	1	0	0	0	0	0	0	0	0	0
Korarchaeota	1	0	0	0	0	0	0	0	0	0
Total	91	224	0	316	0	124	13	0	0	0

(M00130_1)

Phyla	Module completion ratio (%)									
	0--10	10--20	20--30	30--40	40--50	50--60	60--70	70--80	80--90	90--100
Gammaproteobacteria	126	0	0	0	0	0	0	0	0	0
Betaproteobacteria	61	0	0	0	0	0	0	0	0	0
Epsilonproteobacteria	17	0	0	0	0	0	0	0	0	0
Deltaproteobacteria	28	0	0	0	0	0	0	0	0	0
Alphaproteobacteria	91	0	0	0	0	0	0	0	0	0
Magnetococcus	1	0	0	0	0	0	0	0	0	0
Chrysiogenetes	1	0	0	0	0	0	0	0	0	0
Firmicutes	104	0	0	0	0	0	0	0	0	0
Tenericutes	19	0	0	0	0	0	0	0	0	0
Actinobacteria	80	0	0	0	0	0	0	0	0	0
Chlamydiae	8	0	0	0	0	0	0	0	0	0
Spirochaetes	14	0	0	0	0	0	0	0	0	0
Acidobacteria	5	0	0	0	0	0	0	0	0	0
Bacteroidetes	35	0	0	0	0	0	0	0	0	0
Fibrobacteres	1	0	0	0	0	0	0	0	0	0
Fusobacteria	5	0	0	0	0	0	0	0	0	0
Verrucomicrobia	4	0	0	0	0	0	0	0	0	0
Gemmatimonadetes	1	0	0	0	0	0	0	0	0	0
Planctomycetes	4	0	0	0	0	0	0	0	0	0
Elusimicrobia	2	0	0	0	0	0	0	0	0	0
Synergistetes	2	0	0	0	0	0	0	0	0	0
Cyanobacteria	16	0	0	0	0	0	0	0	0	0
Chlorobi	10	0	0	0	0	0	0	0	0	0
Chloroflexi	11	0	0	0	0	0	0	0	0	0
Deinococcus-Thermus	7	0	0	0	0	0	0	0	0	0
Aquificae	9	0	0	0	0	0	0	0	0	0
Thermotogae	11	0	0	0	0	0	0	0	0	0
Dictyoglomi	2	0	0	0	0	0	0	0	0	0
Nitrospirae	2	0	0	0	0	0	0	0	0	0
Thermobaculum	1	0	0	0	0	0	0	0	0	0
Deferribacteres	3	0	0	0	0	0	0	0	0	0
Euryarchaeota	60	0	0	0	0	0	0	0	0	0
Crenarchaeota	23	0	0	0	0	0	0	0	0	0
Thaumarchaeota	2	0	0	0	0	0	0	0	0	0
Nanoarchaeota	1	0	0	0	0	0	0	0	0	0
Korarchaeota	1	0	0	0	0	0	0	0	0	0
Total	768	0	0	0	0	0	0	0	0	0

(M00131_1)

Phyla	Module completion ratio (%)									
	0--10	10--20	20--30	30--40	40--50	50--60	60--70	70--80	80--90	90--100
Gammaproteobacteria	2	0	124	0	0	0	0	0	0	0
Betaproteobacteria	1	0	60	0	0	0	0	0	0	0
Epsilonproteobacteria	7	0	10	0	0	0	0	0	0	0
Deltaproteobacteria	4	0	24	0	0	0	0	0	0	0
Alphaproteobacteria	1	0	90	0	0	0	0	0	0	0
Magnetococcus	0	0	1	0	0	0	0	0	0	0
Chrysiogenetes	0	0	1	0	0	0	0	0	0	0
Firmicutes	48	0	56	0	0	0	0	0	0	0
Tenericutes	19	0	0	0	0	0	0	0	0	0
Actinobacteria	9	0	71	0	0	0	0	0	0	0
Chlamydiae	6	0	2	0	0	0	0	0	0	0
Spirochaetes	7	0	7	0	0	0	0	0	0	0
Acidobacteria	0	0	5	0	0	0	0	0	0	0
Bacteroidetes	17	0	18	0	0	0	0	0	0	0
Fibrobacteres	0	0	1	0	0	0	0	0	0	0
Fusobacteria	4	0	1	0	0	0	0	0	0	0
Verrucomicrobia	0	0	4	0	0	0	0	0	0	0
Gemmatimonadetes	0	0	1	0	0	0	0	0	0	0
Planctomycetes	0	0	4	0	0	0	0	0	0	0
Elusimicrobia	1	0	1	0	0	0	0	0	0	0
Synergistetes	1	0	1	0	0	0	0	0	0	0
Cyanobacteria	1	0	15	0	0	0	0	0	0	0
Chlorobi	0	0	10	0	0	0	0	0	0	0
Chloroflexi	3	0	8	0	0	0	0	0	0	0
Deinococcus-Thermus	0	0	7	0	0	0	0	0	0	0
Aquificae	0	0	9	0	0	0	0	0	0	0
Thermotogae	1	0	10	0	0	0	0	0	0	0
Dictyoglomi	0	0	2	0	0	0	0	0	0	0
Nitrospirae	0	0	2	0	0	0	0	0	0	0
Thermobaculum	0	0	1	0	0	0	0	0	0	0
Deferribacteres	0	0	3	0	0	0	0	0	0	0
Euryarchaeota	10	0	50	0	0	0	0	0	0	0
Crenarchaeota	0	0	23	0	0	0	0	0	0	0
Thaumarchaeota	0	0	2	0	0	0	0	0	0	0
Nanoarchaeota	1	0	0	0	0	0	0	0	0	0
Korarchaeota	0	0	1	0	0	0	0	0	0	0
Total	143	0	625	0	0	0	0	0	0	0

(M00132_1)

Phyla	Module completion ratio (%)									
	0--10	10--20	20--30	30--40	40--50	50--60	60--70	70--80	80--90	90--100
Gammaproteobacteria	126	0	0	0	0	0	0	0	0	0
Betaproteobacteria	61	0	0	0	0	0	0	0	0	0
Epsilonproteobacteria	17	0	0	0	0	0	0	0	0	0
Deltaproteobacteria	28	0	0	0	0	0	0	0	0	0
Alphaproteobacteria	91	0	0	0	0	0	0	0	0	0
Magnetococcus	1	0	0	0	0	0	0	0	0	0
Chrysiogenetes	1	0	0	0	0	0	0	0	0	0
Firmicutes	104	0	0	0	0	0	0	0	0	0
Tenericutes	19	0	0	0	0	0	0	0	0	0
Actinobacteria	80	0	0	0	0	0	0	0	0	0
Chlamydiae	8	0	0	0	0	0	0	0	0	0
Spirochaetes	14	0	0	0	0	0	0	0	0	0
Acidobacteria	5	0	0	0	0	0	0	0	0	0
Bacteroidetes	35	0	0	0	0	0	0	0	0	0
Fibrobacteres	1	0	0	0	0	0	0	0	0	0
Fusobacteria	5	0	0	0	0	0	0	0	0	0
Verrucomicrobia	4	0	0	0	0	0	0	0	0	0
Gemmatimonadetes	1	0	0	0	0	0	0	0	0	0
Planctomycetes	4	0	0	0	0	0	0	0	0	0
Elusimicrobia	2	0	0	0	0	0	0	0	0	0
Synergistetes	2	0	0	0	0	0	0	0	0	0
Cyanobacteria	16	0	0	0	0	0	0	0	0	0
Chlorobi	10	0	0	0	0	0	0	0	0	0
Chloroflexi	11	0	0	0	0	0	0	0	0	0
Deinococcus-Thermus	7	0	0	0	0	0	0	0	0	0
Aquificae	9	0	0	0	0	0	0	0	0	0
Thermotogae	11	0	0	0	0	0	0	0	0	0
Dictyoglomi	2	0	0	0	0	0	0	0	0	0
Nitrospirae	2	0	0	0	0	0	0	0	0	0
Thermobaculum	1	0	0	0	0	0	0	0	0	0
Deferribacteres	3	0	0	0	0	0	0	0	0	0
Euryarchaeota	60	0	0	0	0	0	0	0	0	0
Crenarchaeota	23	0	0	0	0	0	0	0	0	0
Thaumarchaeota	2	0	0	0	0	0	0	0	0	0
Nanoarchaeota	1	0	0	0	0	0	0	0	0	0
Korarchaeota	1	0	0	0	0	0	0	0	0	0
Total	768	0	0	0	0	0	0	0	0	0

(M00133_1)

Phyla	Module completion ratio (%)									
	0--10	10--20	20--30	30--40	40--50	50--60	60--70	70--80	80--90	90--100
Gammaproteobacteria	25	0	0	0	0	50	0	0	0	51
Betaproteobacteria	7	0	0	0	0	31	0	0	0	23
Epsilonproteobacteria	1	0	0	0	0	15	0	0	0	1
Deltaproteobacteria	2	0	0	0	0	7	0	0	0	19
Alphaproteobacteria	54	0	0	0	0	32	0	0	0	5
Magnetococcus	0	0	0	0	0	0	0	0	0	1
Chrysiogenetes	0	0	0	0	0	1	0	0	0	0
Firmicutes	47	0	0	0	0	38	0	0	0	19
Tenericutes	18	0	0	0	0	1	0	0	0	0
Actinobacteria	40	0	0	0	0	36	0	0	0	4
Chlamydiae	8	0	0	0	0	0	0	0	0	0
Spirochaetes	12	0	0	0	0	1	0	0	0	1
Acidobacteria	0	0	0	0	0	5	0	0	0	0
Bacteroidetes	4	0	0	0	0	18	0	0	0	13
Fibrobacteres	0	0	0	0	0	1	0	0	0	0
Fusobacteria	4	0	0	0	0	1	0	0	0	0
Verrucomicrobia	0	0	0	0	0	4	0	0	0	0
Gemmatimonadetes	0	0	0	0	0	1	0	0	0	0
Planctomycetes	0	0	0	0	0	3	0	0	0	1
Elusimicrobia	1	0	0	0	0	0	0	0	0	1
Synergistetes	2	0	0	0	0	0	0	0	0	0
Cyanobacteria	1	0	0	0	0	2	0	0	0	13
Chlorobi	0	0	0	0	0	10	0	0	0	0
Chloroflexi	4	0	0	0	0	4	0	0	0	3
Deinococcus-Thermus	1	0	0	0	0	0	0	0	0	6
Aquificae	6	0	0	0	0	3	0	0	0	0
Thermotogae	10	0	0	0	0	1	0	0	0	0
Dictyoglomi	0	0	0	0	0	2	0	0	0	0
Nitrospirae	1	0	0	0	0	0	0	0	0	1
Thermobaculum	1	0	0	0	0	0	0	0	0	0
Deferribacteres	0	0	0	0	0	1	0	0	0	2
Euryarchaeota	0	0	0	0	0	3	0	0	0	57
Crenarchaeota	0	0	0	0	0	17	0	0	0	6
Thaumarchaeota	0	0	0	0	0	0	0	0	0	2
Nanoarchaeota	0	0	0	0	0	1	0	0	0	0
Korarchaeota	0	0	0	0	0	1	0	0	0	0
Total	249	0	0	0	0	290	0	0	0	229

(M00133_2)

Phyla	Module completion ratio (%)									
	0--10	10--20	20--30	30--40	40--50	50--60	60--70	70--80	80--90	90--100
Gammaproteobacteria	56	0	0	0	0	0	0	0	0	70
Betaproteobacteria	46	0	0	0	0	0	0	0	0	15
Epsilonproteobacteria	16	0	0	0	0	0	0	0	0	1
Deltaproteobacteria	22	0	0	0	0	0	0	0	0	6
Alphaproteobacteria	78	0	0	0	0	0	0	0	0	13
Magnetococcus	0	0	0	0	0	0	0	0	0	1
Chrysiogenetes	0	0	0	0	0	0	0	0	0	1
Firmicutes	45	0	0	0	0	0	0	0	0	59
Tenericutes	19	0	0	0	0	0	0	0	0	0
Actinobacteria	74	0	0	0	0	0	0	0	0	6
Chlamydiae	8	0	0	0	0	0	0	0	0	0
Spirochaetes	11	0	0	0	0	0	0	0	0	3
Acidobacteria	4	0	0	0	0	0	0	0	0	1
Bacteroidetes	33	0	0	0	0	0	0	0	0	2
Fibrobacteres	1	0	0	0	0	0	0	0	0	0
Fusobacteria	3	0	0	0	0	0	0	0	0	2
Verrucomicrobia	4	0	0	0	0	0	0	0	0	0
Gemmatimonadetes	0	0	0	0	0	0	0	0	0	1
Planctomycetes	2	0	0	0	0	0	0	0	0	2
Elusimicrobia	2	0	0	0	0	0	0	0	0	0
Synergistetes	0	0	0	0	0	0	0	0	0	2
Cyanobacteria	10	0	0	0	0	0	0	0	0	6
Chlorobi	10	0	0	0	0	0	0	0	0	0
Chloroflexi	8	0	0	0	0	0	0	0	0	3
Deinococcus-Thermus	3	0	0	0	0	0	0	0	0	4
Aquificae	0	0	0	0	0	0	0	0	0	9
Thermotogae	0	0	0	0	0	0	0	0	0	11
Dictyoglomi	0	0	0	0	0	0	0	0	0	2
Nitrospirae	1	0	0	0	0	0	0	0	0	1
Thermobaculum	0	0	0	0	0	0	0	0	0	1
Deferribacteres	2	0	0	0	0	0	0	0	0	1
Euryarchaeota	35	0	0	0	0	0	0	0	0	25
Crenarchaeota	0	0	0	0	0	0	0	0	0	23
Thaumarchaeota	2	0	0	0	0	0	0	0	0	0
Nanoarchaeota	1	0	0	0	0	0	0	0	0	0
Korarchaeota	0	0	0	0	0	0	0	0	0	1
Total	496	0	0	0	0	0	0	0	0	272

(M00133_3)

Phyla	Module completion ratio (%)									
	0--10	10--20	20--30	30--40	40--50	50--60	60--70	70--80	80--90	90--100
Gammaproteobacteria	53	0	0	0	0	0	0	0	0	73
Betaproteobacteria	13	0	0	0	0	0	0	0	0	48
Epsilonproteobacteria	7	0	0	0	0	0	0	0	0	10
Deltaproteobacteria	10	0	0	0	0	0	0	0	0	18
Alphaproteobacteria	76	0	0	0	0	0	0	0	0	15
Magnetococcus	0	0	0	0	0	0	0	0	0	1
Chrysiogenetes	1	0	0	0	0	0	0	0	0	0
Firmicutes	41	0	0	0	0	0	0	0	0	63
Tenericutes	19	0	0	0	0	0	0	0	0	0
Actinobacteria	51	0	0	0	0	0	0	0	0	29
Chlamydiae	8	0	0	0	0	0	0	0	0	0
Spirochaetes	11	0	0	0	0	0	0	0	0	3
Acidobacteria	4	0	0	0	0	0	0	0	0	1
Bacteroidetes	33	0	0	0	0	0	0	0	0	2
Fibrobacteres	1	0	0	0	0	0	0	0	0	0
Fusobacteria	3	0	0	0	0	0	0	0	0	2
Verrucomicrobia	3	0	0	0	0	0	0	0	0	1
Gemmatimonadetes	0	0	0	0	0	0	0	0	0	1
Planctomycetes	2	0	0	0	0	0	0	0	0	2
Elusimicrobia	2	0	0	0	0	0	0	0	0	0
Synergistetes	0	0	0	0	0	0	0	0	0	2
Cyanobacteria	8	0	0	0	0	0	0	0	0	8
Chlorobi	9	0	0	0	0	0	0	0	0	1
Chloroflexi	6	0	0	0	0	0	0	0	0	5
Deinococcus-Thermus	3	0	0	0	0	0	0	0	0	4
Aquificae	0	0	0	0	0	0	0	0	0	9
Thermotogae	0	0	0	0	0	0	0	0	0	11
Dictyoglomi	0	0	0	0	0	0	0	0	0	2
Nitrospirae	1	0	0	0	0	0	0	0	0	1
Thermobaculum	1	0	0	0	0	0	0	0	0	0
Deferribacteres	2	0	0	0	0	0	0	0	0	1
Euryarchaeota	30	0	0	0	0	0	0	0	0	30
Crenarchaeota	0	0	0	0	0	0	0	0	0	23
Thaumarchaeota	2	0	0	0	0	0	0	0	0	0
Nanoarchaeota	1	0	0	0	0	0	0	0	0	0
Korarchaeota	0	0	0	0	0	0	0	0	0	1
Total	401	0	0	0	0	0	0	0	0	367

(M00134_1)

Phyla	Module completion ratio (%)									
	0--10	10--20	20--30	30--40	40--50	50--60	60--70	70--80	80--90	90--100
Gammaproteobacteria	54	0	0	0	0	67	0	0	0	5
Betaproteobacteria	24	0	0	0	0	34	0	0	0	3
Epsilonproteobacteria	14	0	0	0	0	3	0	0	0	0
Deltaproteobacteria	24	0	0	0	0	3	0	0	0	1
Alphaproteobacteria	24	0	0	0	0	34	0	0	0	33
Magnetococcus	1	0	0	0	0	0	0	0	0	0
Chrysiogenetes	1	0	0	0	0	0	0	0	0	0
Firmicutes	63	0	0	0	0	40	0	0	0	1
Tenericutes	19	0	0	0	0	0	0	0	0	0
Actinobacteria	57	0	0	0	0	20	0	0	0	3
Chlamydiae	7	0	0	0	0	1	0	0	0	0
Spirochaetes	12	0	0	0	0	2	0	0	0	0
Acidobacteria	2	0	0	0	0	3	0	0	0	0
Bacteroidetes	29	0	0	0	0	6	0	0	0	0
Fibrobacteres	1	0	0	0	0	0	0	0	0	0
Fusobacteria	4	0	0	0	0	0	0	0	0	1
Verrucomicrobia	3	0	0	0	0	1	0	0	0	0
Gemmatimonadetes	0	0	0	0	0	1	0	0	0	0
Planctomycetes	2	0	0	0	0	2	0	0	0	0
Elusimicrobia	2	0	0	0	0	0	0	0	0	0
Synergistetes	0	0	0	0	0	2	0	0	0	0
Cyanobacteria	16	0	0	0	0	0	0	0	0	0
Chlorobi	9	0	0	0	0	1	0	0	0	0
Chloroflexi	5	0	0	0	0	6	0	0	0	0
Deinococcus-Thermus	0	0	0	0	0	7	0	0	0	0
Aquificae	5	0	0	0	0	4	0	0	0	0
Thermotogae	0	0	0	0	0	11	0	0	0	0
Dictyoglomi	2	0	0	0	0	0	0	0	0	0
Nitrospirae	1	0	0	0	0	1	0	0	0	0
Thermobaculum	1	0	0	0	0	0	0	0	0	0
Deferribacteres	3	0	0	0	0	0	0	0	0	0
Euryarchaeota	43	0	0	0	0	16	0	0	0	1
Crenarchaeota	23	0	0	0	0	0	0	0	0	0
Thaumarchaeota	2	0	0	0	0	0	0	0	0	0
Nanoarchaeota	1	0	0	0	0	0	0	0	0	0
Korarchaeota	0	0	0	0	0	1	0	0	0	0
Total	454	0	0	0	0	266	0	0	0	48

(M00135_1)

Phyla	Module completion ratio (%)									
	0--10	10--20	20--30	30--40	40--50	50--60	60--70	70--80	80--90	90--100
Gammaproteobacteria	31	0	0	58	0	0	35	0	0	2
Betaproteobacteria	15	0	0	25	0	0	16	0	0	5
Epsilonproteobacteria	15	0	0	2	0	0	0	0	0	0
Deltaproteobacteria	14	0	0	9	0	0	5	0	0	0
Alphaproteobacteria	24	0	0	32	0	0	29	0	0	6
Magnetococcus	1	0	0	0	0	0	0	0	0	0
Chrysiogenetes	1	0	0	0	0	0	0	0	0	0
Firmicutes	48	0	0	32	0	0	15	0	0	9
Tenericutes	16	0	0	3	0	0	0	0	0	0
Actinobacteria	16	0	0	32	0	0	24	0	0	8
Chlamydiae	8	0	0	0	0	0	0	0	0	0
Spirochaetes	8	0	0	4	0	0	2	0	0	0
Acidobacteria	0	0	0	4	0	0	1	0	0	0
Bacteroidetes	5	0	0	18	0	0	8	0	0	4
Fibrobacteres	0	0	0	1	0	0	0	0	0	0
Fusobacteria	4	0	0	1	0	0	0	0	0	0
Verrucomicrobia	3	0	0	0	0	0	1	0	0	0
Gemmatimonadetes	0	0	0	1	0	0	0	0	0	0
Planctomycetes	0	0	0	3	0	0	1	0	0	0
Elusimicrobia	1	0	0	1	0	0	0	0	0	0
Synergistetes	2	0	0	0	0	0	0	0	0	0
Cyanobacteria	2	0	0	9	0	0	5	0	0	0
Chlorobi	6	0	0	4	0	0	0	0	0	0
Chloroflexi	4	0	0	2	0	0	5	0	0	0
Deinococcus-Thermus	0	0	0	4	0	0	3	0	0	0
Aquificae	8	0	0	1	0	0	0	0	0	0
Thermotogae	10	0	0	1	0	0	0	0	0	0
Dictyoglomi	2	0	0	0	0	0	0	0	0	0
Nitrospirae	1	0	0	1	0	0	0	0	0	0
Thermobaculum	0	0	0	1	0	0	0	0	0	0
Deferribacteres	3	0	0	0	0	0	0	0	0	0
Euryarchaeota	42	0	0	16	0	0	2	0	0	0
Crenarchaeota	17	0	0	6	0	0	0	0	0	0
Thaumarchaeota	2	0	0	0	0	0	0	0	0	0
Nanoarchaeota	1	0	0	0	0	0	0	0	0	0
Korarchaeota	1	0	0	0	0	0	0	0	0	0
Total	311	0	0	271	0	0	152	0	0	34

(M00136_1)

Phyla	Module completion ratio (%)									
	0--10	10--20	20--30	30--40	40--50	50--60	60--70	70--80	80--90	90--100
Gammaproteobacteria	67	0	20	0	0	27	0	3	0	9
Betaproteobacteria	30	0	14	0	0	17	0	0	0	0
Epsilonproteobacteria	17	0	0	0	0	0	0	0	0	0
Deltaproteobacteria	28	0	0	0	0	0	0	0	0	0
Alphaproteobacteria	51	0	22	0	0	18	0	0	0	0
Magnetococcus	1	0	0	0	0	0	0	0	0	0
Chrysiogenetes	1	0	0	0	0	0	0	0	0	0
Firmicutes	103	0	1	0	0	0	0	0	0	0
Tenericutes	19	0	0	0	0	0	0	0	0	0
Actinobacteria	73	0	7	0	0	0	0	0	0	0
Chlamydiae	8	0	0	0	0	0	0	0	0	0
Spirochaetes	14	0	0	0	0	0	0	0	0	0
Acidobacteria	5	0	0	0	0	0	0	0	0	0
Bacteroidetes	35	0	0	0	0	0	0	0	0	0
Fibrobacteres	1	0	0	0	0	0	0	0	0	0
Fusobacteria	5	0	0	0	0	0	0	0	0	0
Verrucomicrobia	4	0	0	0	0	0	0	0	0	0
Gemmatimonadetes	1	0	0	0	0	0	0	0	0	0
Planctomycetes	4	0	0	0	0	0	0	0	0	0
Elusimicrobia	2	0	0	0	0	0	0	0	0	0
Synergistetes	2	0	0	0	0	0	0	0	0	0
Cyanobacteria	16	0	0	0	0	0	0	0	0	0
Chlorobi	10	0	0	0	0	0	0	0	0	0
Chloroflexi	10	0	1	0	0	0	0	0	0	0
Deinococcus-Thermus	7	0	0	0	0	0	0	0	0	0
Aquificae	9	0	0	0	0	0	0	0	0	0
Thermotogae	11	0	0	0	0	0	0	0	0	0
Dictyoglomi	2	0	0	0	0	0	0	0	0	0
Nitrospirae	2	0	0	0	0	0	0	0	0	0
Thermobaculum	1	0	0	0	0	0	0	0	0	0
Deferribacteres	3	0	0	0	0	0	0	0	0	0
Euryarchaeota	60	0	0	0	0	0	0	0	0	0
Crenarchaeota	23	0	0	0	0	0	0	0	0	0
Thaumarchaeota	2	0	0	0	0	0	0	0	0	0
Nanoarchaeota	1	0	0	0	0	0	0	0	0	0
Korarchaeota	1	0	0	0	0	0	0	0	0	0
Total	629	0	65	0	0	62	0	3	0	9

(M00137_1)

Phyla	Module completion ratio (%)									
	0--10	10--20	20--30	30--40	40--50	50--60	60--70	70--80	80--90	90--100
Gammaproteobacteria	123	0	0	3	0	0	0	0	0	0
Betaproteobacteria	61	0	0	0	0	0	0	0	0	0
Epsilonproteobacteria	17	0	0	0	0	0	0	0	0	0
Deltaproteobacteria	22	0	0	6	0	0	0	0	0	0
Alphaproteobacteria	76	0	0	15	0	0	0	0	0	0
Magnetococcus	1	0	0	0	0	0	0	0	0	0
Chrysiogenetes	1	0	0	0	0	0	0	0	0	0
Firmicutes	91	0	0	13	0	0	0	0	0	0
Tenericutes	19	0	0	0	0	0	0	0	0	0
Actinobacteria	41	0	0	39	0	0	0	0	0	0
Chlamydiae	8	0	0	0	0	0	0	0	0	0
Spirochaetes	14	0	0	0	0	0	0	0	0	0
Acidobacteria	1	0	0	4	0	0	0	0	0	0
Bacteroidetes	23	0	0	12	0	0	0	0	0	0
Fibrobacteres	1	0	0	0	0	0	0	0	0	0
Fusobacteria	5	0	0	0	0	0	0	0	0	0
Verrucomicrobia	1	0	0	3	0	0	0	0	0	0
Gemmatimonadetes	1	0	0	0	0	0	0	0	0	0
Planctomycetes	1	0	0	3	0	0	0	0	0	0
Elusimicrobia	2	0	0	0	0	0	0	0	0	0
Synergistetes	2	0	0	0	0	0	0	0	0	0
Cyanobacteria	15	0	0	1	0	0	0	0	0	0
Chlorobi	9	0	0	1	0	0	0	0	0	0
Chloroflexi	10	0	0	1	0	0	0	0	0	0
Deinococcus-Thermus	2	0	0	5	0	0	0	0	0	0
Aquificae	8	0	0	1	0	0	0	0	0	0
Thermotogae	11	0	0	0	0	0	0	0	0	0
Dictyoglomi	2	0	0	0	0	0	0	0	0	0
Nitrospirae	2	0	0	0	0	0	0	0	0	0
Thermobaculum	1	0	0	0	0	0	0	0	0	0
Deferribacteres	3	0	0	0	0	0	0	0	0	0
Euryarchaeota	60	0	0	0	0	0	0	0	0	0
Crenarchaeota	23	0	0	0	0	0	0	0	0	0
Thaumarchaeota	2	0	0	0	0	0	0	0	0	0
Nanoarchaeota	1	0	0	0	0	0	0	0	0	0
Korarchaeota	1	0	0	0	0	0	0	0	0	0
Total	661	0	0	107	0	0	0	0	0	0

(M00138_1)

Phyla	Module completion ratio (%)									
	0--10	10--20	20--30	30--40	40--50	50--60	60--70	70--80	80--90	90--100
Gammaproteobacteria	123	0	0	3	0	0	0	0	0	0
Betaproteobacteria	61	0	0	0	0	0	0	0	0	0
Epsilonproteobacteria	17	0	0	0	0	0	0	0	0	0
Deltaproteobacteria	22	0	0	6	0	0	0	0	0	0
Alphaproteobacteria	76	0	0	15	0	0	0	0	0	0
Magnetococcus	1	0	0	0	0	0	0	0	0	0
Chrysiogenetes	1	0	0	0	0	0	0	0	0	0
Firmicutes	91	0	0	13	0	0	0	0	0	0
Tenericutes	19	0	0	0	0	0	0	0	0	0
Actinobacteria	41	0	0	39	0	0	0	0	0	0
Chlamydiae	8	0	0	0	0	0	0	0	0	0
Spirochaetes	14	0	0	0	0	0	0	0	0	0
Acidobacteria	1	0	0	4	0	0	0	0	0	0
Bacteroidetes	23	0	0	12	0	0	0	0	0	0
Fibrobacteres	1	0	0	0	0	0	0	0	0	0
Fusobacteria	5	0	0	0	0	0	0	0	0	0
Verrucomicrobia	1	0	0	3	0	0	0	0	0	0
Gemmatimonadetes	1	0	0	0	0	0	0	0	0	0
Planctomycetes	1	0	0	3	0	0	0	0	0	0
Elusimicrobia	2	0	0	0	0	0	0	0	0	0
Synergistetes	2	0	0	0	0	0	0	0	0	0
Cyanobacteria	15	0	0	1	0	0	0	0	0	0
Chlorobi	9	0	0	1	0	0	0	0	0	0
Chloroflexi	10	0	0	1	0	0	0	0	0	0
Deinococcus-Thermus	2	0	0	5	0	0	0	0	0	0
Aquificae	8	0	0	1	0	0	0	0	0	0
Thermotogae	11	0	0	0	0	0	0	0	0	0
Dictyoglomi	2	0	0	0	0	0	0	0	0	0
Nitrospirae	2	0	0	0	0	0	0	0	0	0
Thermobaculum	1	0	0	0	0	0	0	0	0	0
Deferribacteres	3	0	0	0	0	0	0	0	0	0
Euryarchaeota	60	0	0	0	0	0	0	0	0	0
Crenarchaeota	23	0	0	0	0	0	0	0	0	0
Thaumarchaeota	2	0	0	0	0	0	0	0	0	0
Nanoarchaeota	1	0	0	0	0	0	0	0	0	0
Korarchaeota	1	0	0	0	0	0	0	0	0	0
Total	661	0	0	107	0	0	0	0	0	0

(M00139_1)

Phyla	Module completion ratio (%)									
	0--10	10--20	20--30	30--40	40--50	50--60	60--70	70--80	80--90	90--100
Gammaproteobacteria	126	0	0	0	0	0	0	0	0	0
Betaproteobacteria	61	0	0	0	0	0	0	0	0	0
Epsilonproteobacteria	17	0	0	0	0	0	0	0	0	0
Deltaproteobacteria	28	0	0	0	0	0	0	0	0	0
Alphaproteobacteria	91	0	0	0	0	0	0	0	0	0
Magnetococcus	1	0	0	0	0	0	0	0	0	0
Chrysiogenetes	1	0	0	0	0	0	0	0	0	0
Firmicutes	104	0	0	0	0	0	0	0	0	0
Tenericutes	19	0	0	0	0	0	0	0	0	0
Actinobacteria	80	0	0	0	0	0	0	0	0	0
Chlamydiae	8	0	0	0	0	0	0	0	0	0
Spirochaetes	14	0	0	0	0	0	0	0	0	0
Acidobacteria	5	0	0	0	0	0	0	0	0	0
Bacteroidetes	35	0	0	0	0	0	0	0	0	0
Fibrobacteres	1	0	0	0	0	0	0	0	0	0
Fusobacteria	5	0	0	0	0	0	0	0	0	0
Verrucomicrobia	4	0	0	0	0	0	0	0	0	0
Gemmatimonadetes	1	0	0	0	0	0	0	0	0	0
Planctomycetes	4	0	0	0	0	0	0	0	0	0
Elusimicrobia	2	0	0	0	0	0	0	0	0	0
Synergistetes	2	0	0	0	0	0	0	0	0	0
Cyanobacteria	16	0	0	0	0	0	0	0	0	0
Chlorobi	10	0	0	0	0	0	0	0	0	0
Chloroflexi	11	0	0	0	0	0	0	0	0	0
Deinococcus-Thermus	7	0	0	0	0	0	0	0	0	0
Aquificae	9	0	0	0	0	0	0	0	0	0
Thermotogae	11	0	0	0	0	0	0	0	0	0
Dictyoglomi	2	0	0	0	0	0	0	0	0	0
Nitrospirae	2	0	0	0	0	0	0	0	0	0
Thermobaculum	1	0	0	0	0	0	0	0	0	0
Deferribacteres	3	0	0	0	0	0	0	0	0	0
Euryarchaeota	60	0	0	0	0	0	0	0	0	0
Crenarchaeota	23	0	0	0	0	0	0	0	0	0
Thaumarchaeota	2	0	0	0	0	0	0	0	0	0
Nanoarchaeota	1	0	0	0	0	0	0	0	0	0
Korarchaeota	1	0	0	0	0	0	0	0	0	0
Total	768	0	0	0	0	0	0	0	0	0

(M00140_1)

Phyla	Module completion ratio (%)									
	0--10	10--20	20--30	30--40	40--50	50--60	60--70	70--80	80--90	90--100
Gammaproteobacteria	0	0	0	2	0	0	94	0	0	30
Betaproteobacteria	1	0	0	0	0	0	54	0	0	6
Epsilonproteobacteria	0	0	0	0	0	0	16	0	0	1
Deltaproteobacteria	0	0	0	0	0	0	16	0	0	12
Alphaproteobacteria	0	0	0	1	0	0	62	0	0	28
Magnetococcus	0	0	0	0	0	0	1	0	0	0
Chrysiogenetes	0	0	0	0	0	0	1	0	0	0
Firmicutes	0	0	0	0	0	0	15	0	0	89
Tenericutes	0	0	0	4	0	0	13	0	0	2
Actinobacteria	0	0	0	0	0	0	59	0	0	21
Chlamydiae	0	0	0	0	0	0	8	0	0	0
Spirochaetes	0	0	0	0	0	0	11	0	0	3
Acidobacteria	0	0	0	0	0	0	3	0	0	2
Bacteroidetes	1	0	0	1	0	0	24	0	0	9
Fibrobacteres	0	0	0	0	0	0	1	0	0	0
Fusobacteria	0	0	0	0	0	0	2	0	0	3
Verrucomicrobia	0	0	0	0	0	0	3	0	0	1
Gemmatimonadetes	0	0	0	0	0	0	0	0	0	1
Planctomycetes	0	0	0	0	0	0	3	0	0	1
Elusimicrobia	0	0	0	0	0	0	1	0	0	1
Synergistetes	0	0	0	0	0	0	0	0	0	2
Cyanobacteria	0	0	0	1	0	0	15	0	0	0
Chlorobi	0	0	0	0	0	0	10	0	0	0
Chloroflexi	0	0	0	1	0	0	0	0	0	10
Deinococcus-Thermus	0	0	0	0	0	0	5	0	0	2
Aquificae	0	0	0	0	0	0	9	0	0	0
Thermotogae	0	0	0	0	0	0	0	0	0	11
Dictyoglomi	0	0	0	0	0	0	0	0	0	2
Nitrospirae	0	0	0	0	0	0	0	0	0	2
Thermobaculum	0	0	0	0	0	0	1	0	0	0
Deferribacteres	0	0	0	0	0	0	3	0	0	0
Euryarchaeota	4	0	0	26	0	0	22	0	0	8
Crenarchaeota	1	0	0	17	0	0	5	0	0	0
Thaumarchaeota	0	0	0	0	0	0	2	0	0	0
Nanoarchaeota	1	0	0	0	0	0	0	0	0	0
Korarchaeota	0	0	0	1	0	0	0	0	0	0
Total	8	0	0	54	0	0	459	0	0	247

(M00141_1)

Phyla	Module completion ratio (%)									
	0--10	10--20	20--30	30--40	40--50	50--60	60--70	70--80	80--90	90--100
Gammaproteobacteria	1	0	0	0	0	125	0	0	0	0
Betaproteobacteria	1	0	0	0	0	60	0	0	0	0
Epsilonproteobacteria	0	0	0	0	0	17	0	0	0	0
Deltaproteobacteria	0	0	0	0	0	28	0	0	0	0
Alphaproteobacteria	0	0	0	0	0	91	0	0	0	0
Magnetococcus	0	0	0	0	0	1	0	0	0	0
Chrysiogenetes	0	0	0	0	0	1	0	0	0	0
Firmicutes	4	0	0	0	0	100	0	0	0	0
Tenericutes	2	0	0	0	0	17	0	0	0	0
Actinobacteria	3	0	0	0	0	77	0	0	0	0
Chlamydiae	0	0	0	0	0	8	0	0	0	0
Spirochaetes	0	0	0	0	0	14	0	0	0	0
Acidobacteria	0	0	0	0	0	5	0	0	0	0
Bacteroidetes	2	0	0	0	0	33	0	0	0	0
Fibrobacteres	0	0	0	0	0	1	0	0	0	0
Fusobacteria	2	0	0	0	0	3	0	0	0	0
Verrucomicrobia	2	0	0	0	0	2	0	0	0	0
Gemmatimonadetes	0	0	0	0	0	1	0	0	0	0
Planctomycetes	0	0	0	0	0	4	0	0	0	0
Elusimicrobia	0	0	0	0	0	2	0	0	0	0
Synergistetes	0	0	0	0	0	2	0	0	0	0
Cyanobacteria	0	0	0	0	0	16	0	0	0	0
Chlorobi	0	0	0	0	0	10	0	0	0	0
Chloroflexi	0	0	0	0	0	10	0	0	0	1
Deinococcus-Thermus	0	0	0	0	0	7	0	0	0	0
Aquificae	0	0	0	0	0	9	0	0	0	0
Thermotogae	0	0	0	0	0	11	0	0	0	0
Dictyoglomi	0	0	0	0	0	2	0	0	0	0
Nitrospirae	0	0	0	0	0	2	0	0	0	0
Thermobaculum	0	0	0	0	0	1	0	0	0	0
Deferribacteres	0	0	0	0	0	3	0	0	0	0
Euryarchaeota	4	0	0	0	0	56	0	0	0	0
Crenarchaeota	1	0	0	0	0	22	0	0	0	0
Thaumarchaeota	0	0	0	0	0	2	0	0	0	0
Nanoarchaeota	1	0	0	0	0	0	0	0	0	0
Korarchaeota	0	0	0	0	0	1	0	0	0	0
Total	23	0	0	0	0	744	0	0	0	1

(M00141_2)

Phyla	Module completion ratio (%)									
	0--10	10--20	20--30	30--40	40--50	50--60	60--70	70--80	80--90	90--100
Gammaproteobacteria	1	0	0	125	0	0	0	0	0	0
Betaproteobacteria	1	0	0	60	0	0	0	0	0	0
Epsilonproteobacteria	0	0	0	17	0	0	0	0	0	0
Deltaproteobacteria	0	0	0	28	0	0	0	0	0	0
Alphaproteobacteria	0	0	0	91	0	0	0	0	0	0
Magnetococcus	0	0	0	1	0	0	0	0	0	0
Chrysiogenetes	0	0	0	1	0	0	0	0	0	0
Firmicutes	4	0	0	100	0	0	0	0	0	0
Tenericutes	2	0	0	17	0	0	0	0	0	0
Actinobacteria	3	0	0	77	0	0	0	0	0	0
Chlamydiae	0	0	0	8	0	0	0	0	0	0
Spirochaetes	0	0	0	14	0	0	0	0	0	0
Acidobacteria	0	0	0	5	0	0	0	0	0	0
Bacteroidetes	2	0	0	33	0	0	0	0	0	0
Fibrobacteres	0	0	0	1	0	0	0	0	0	0
Fusobacteria	2	0	0	3	0	0	0	0	0	0
Verrucomicrobia	2	0	0	2	0	0	0	0	0	0
Gemmatimonadetes	0	0	0	1	0	0	0	0	0	0
Planctomycetes	0	0	0	4	0	0	0	0	0	0
Elusimicrobia	0	0	0	2	0	0	0	0	0	0
Synergistetes	0	0	0	2	0	0	0	0	0	0
Cyanobacteria	0	0	0	16	0	0	0	0	0	0
Chlorobi	0	0	0	10	0	0	0	0	0	0
Chloroflexi	0	0	0	11	0	0	0	0	0	0
Deinococcus-Thermus	0	0	0	7	0	0	0	0	0	0
Aquificae	0	0	0	9	0	0	0	0	0	0
Thermotogae	0	0	0	11	0	0	0	0	0	0
Dictyoglomi	0	0	0	2	0	0	0	0	0	0
Nitrospirae	0	0	0	2	0	0	0	0	0	0
Thermobaculum	0	0	0	1	0	0	0	0	0	0
Deferribacteres	0	0	0	3	0	0	0	0	0	0
Euryarchaeota	4	0	0	56	0	0	0	0	0	0
Crenarchaeota	1	0	0	22	0	0	0	0	0	0
Thaumarchaeota	0	0	0	2	0	0	0	0	0	0
Nanoarchaeota	1	0	0	0	0	0	0	0	0	0
Korarchaeota	0	0	0	1	0	0	0	0	0	0
Total	23	0	0	745	0	0	0	0	0	0

(M00142_1)

Phyla	Module completion ratio (%)									
	0--10	10--20	20--30	30--40	40--50	50--60	60--70	70--80	80--90	90--100
Gammaproteobacteria	126	0	0	0	0	0	0	0	0	0
Betaproteobacteria	61	0	0	0	0	0	0	0	0	0
Epsilonproteobacteria	17	0	0	0	0	0	0	0	0	0
Deltaproteobacteria	28	0	0	0	0	0	0	0	0	0
Alphaproteobacteria	91	0	0	0	0	0	0	0	0	0
Magnetococcus	1	0	0	0	0	0	0	0	0	0
Chrysiogenetes	1	0	0	0	0	0	0	0	0	0
Firmicutes	104	0	0	0	0	0	0	0	0	0
Tenericutes	19	0	0	0	0	0	0	0	0	0
Actinobacteria	80	0	0	0	0	0	0	0	0	0
Chlamydiae	8	0	0	0	0	0	0	0	0	0
Spirochaetes	14	0	0	0	0	0	0	0	0	0
Acidobacteria	5	0	0	0	0	0	0	0	0	0
Bacteroidetes	35	0	0	0	0	0	0	0	0	0
Fibrobacteres	1	0	0	0	0	0	0	0	0	0
Fusobacteria	5	0	0	0	0	0	0	0	0	0
Verrucomicrobia	4	0	0	0	0	0	0	0	0	0
Gemmatimonadetes	1	0	0	0	0	0	0	0	0	0
Planctomycetes	4	0	0	0	0	0	0	0	0	0
Elusimicrobia	2	0	0	0	0	0	0	0	0	0
Synergistetes	2	0	0	0	0	0	0	0	0	0
Cyanobacteria	16	0	0	0	0	0	0	0	0	0
Chlorobi	10	0	0	0	0	0	0	0	0	0
Chloroflexi	11	0	0	0	0	0	0	0	0	0
Deinococcus-Thermus	7	0	0	0	0	0	0	0	0	0
Aquificae	9	0	0	0	0	0	0	0	0	0
Thermotogae	11	0	0	0	0	0	0	0	0	0
Dictyoglomi	2	0	0	0	0	0	0	0	0	0
Nitrospirae	2	0	0	0	0	0	0	0	0	0
Thermobaculum	1	0	0	0	0	0	0	0	0	0
Deferribacteres	3	0	0	0	0	0	0	0	0	0
Euryarchaeota	60	0	0	0	0	0	0	0	0	0
Crenarchaeota	23	0	0	0	0	0	0	0	0	0
Thaumarchaeota	2	0	0	0	0	0	0	0	0	0
Nanoarchaeota	1	0	0	0	0	0	0	0	0	0
Korarchaeota	1	0	0	0	0	0	0	0	0	0
Total	768	0	0	0	0	0	0	0	0	0

(M00143_1)

Phyla	Module completion ratio (%)									
	0--10	10--20	20--30	30--40	40--50	50--60	60--70	70--80	80--90	90--100
Gammaproteobacteria	126	0	0	0	0	0	0	0	0	0
Betaproteobacteria	61	0	0	0	0	0	0	0	0	0
Epsilonproteobacteria	17	0	0	0	0	0	0	0	0	0
Deltaproteobacteria	27	1	0	0	0	0	0	0	0	0
Alphaproteobacteria	91	0	0	0	0	0	0	0	0	0
Magnetococcus	1	0	0	0	0	0	0	0	0	0
Chrysiogenetes	1	0	0	0	0	0	0	0	0	0
Firmicutes	104	0	0	0	0	0	0	0	0	0
Tenericutes	19	0	0	0	0	0	0	0	0	0
Actinobacteria	80	0	0	0	0	0	0	0	0	0
Chlamydiae	8	0	0	0	0	0	0	0	0	0
Spirochaetes	14	0	0	0	0	0	0	0	0	0
Acidobacteria	5	0	0	0	0	0	0	0	0	0
Bacteroidetes	35	0	0	0	0	0	0	0	0	0
Fibrobacteres	1	0	0	0	0	0	0	0	0	0
Fusobacteria	5	0	0	0	0	0	0	0	0	0
Verrucomicrobia	4	0	0	0	0	0	0	0	0	0
Gemmatimonadetes	1	0	0	0	0	0	0	0	0	0
Planctomycetes	4	0	0	0	0	0	0	0	0	0
Elusimicrobia	2	0	0	0	0	0	0	0	0	0
Synergistetes	2	0	0	0	0	0	0	0	0	0
Cyanobacteria	16	0	0	0	0	0	0	0	0	0
Chlorobi	10	0	0	0	0	0	0	0	0	0
Chloroflexi	11	0	0	0	0	0	0	0	0	0
Deinococcus-Thermus	7	0	0	0	0	0	0	0	0	0
Aquificae	9	0	0	0	0	0	0	0	0	0
Thermotogae	11	0	0	0	0	0	0	0	0	0
Dictyoglomi	2	0	0	0	0	0	0	0	0	0
Nitrospirae	2	0	0	0	0	0	0	0	0	0
Thermobaculum	1	0	0	0	0	0	0	0	0	0
Deferribacteres	2	1	0	0	0	0	0	0	0	0
Euryarchaeota	60	0	0	0	0	0	0	0	0	0
Crenarchaeota	23	0	0	0	0	0	0	0	0	0
Thaumarchaeota	2	0	0	0	0	0	0	0	0	0
Nanoarchaeota	1	0	0	0	0	0	0	0	0	0
Korarchaeota	1	0	0	0	0	0	0	0	0	0
Total	766	2	0	0	0	0	0	0	0	0

(M00144_1)

Phyla	Module completion ratio (%)									
	0--10	10--20	20--30	30--40	40--50	50--60	60--70	70--80	80--90	90--100
Gammaproteobacteria	49	0	0	0	0	0	0	0	54	23
Betaproteobacteria	0	0	0	0	0	0	0	0	0	61
Epsilonproteobacteria	0	0	0	0	0	0	1	1	3	12
Deltaproteobacteria	5	2	3	0	0	0	0	1	4	13
Alphaproteobacteria	3	0	0	0	0	0	0	0	0	88
Magnetococcus	0	0	0	0	0	0	0	0	0	1
Chrysiogenetes	0	0	0	0	0	0	0	0	0	1
Firmicutes	53	7	22	0	0	1	2	0	11	8
Tenericutes	19	0	0	0	0	0	0	0	0	0
Actinobacteria	27	1	1	0	0	0	1	0	1	49
Chlamydiae	7	0	0	0	0	0	0	0	0	1
Spirochaetes	9	0	2	0	0	0	0	0	0	3
Acidobacteria	0	0	0	0	0	0	0	0	0	5
Bacteroidetes	10	0	1	0	0	1	3	2	3	15
Fibrobacteres	0	0	0	0	0	0	0	0	1	0
Fusobacteria	3	0	2	0	0	0	0	0	0	0
Verrucomicrobia	0	0	0	0	0	0	0	0	0	4
Gemmatimonadetes	0	0	0	0	0	0	0	0	0	1
Planctomycetes	1	0	0	0	0	0	0	0	0	3
Elusimicrobia	0	0	1	0	0	1	0	0	0	0
Synergistetes	0	1	1	0	0	0	0	0	0	0
Cyanobacteria	15	1	0	0	0	0	0	0	0	0
Chlorobi	0	0	0	0	0	0	0	0	9	1
Chloroflexi	0	0	0	0	0	0	0	0	3	8
Deinococcus-Thermus	0	0	0	0	0	0	0	0	0	7
Aquificae	0	0	0	0	0	0	0	0	7	2
Thermotogae	0	0	3	0	0	7	1	0	0	0
Dictyoglomi	0	0	2	0	0	0	0	0	0	0
Nitrospirae	0	0	0	0	0	0	0	0	1	1
Thermobaculum	0	0	0	0	0	0	0	0	0	1
Deferribacteres	0	0	0	0	0	0	0	0	0	3
Euryarchaeota	20	4	1	0	3	7	7	8	10	0
Crenarchaeota	0	0	1	0	5	0	1	8	8	0
Thaumarchaeota	0	0	0	0	0	0	0	2	0	0
Nanoarchaeota	1	0	0	0	0	0	0	0	0	0
Korarchaeota	0	0	0	0	0	0	0	0	1	0
Total	222	16	40	0	8	17	16	22	116	311

(M00144_2)

Phyla	Module completion ratio (%)									
	0--10	10--20	20--30	30--40	40--50	50--60	60--70	70--80	80--90	90--100
Gammaproteobacteria	49	0	0	0	0	0	0	0	0	77
Betaproteobacteria	0	0	0	0	0	0	0	0	0	61
Epsilonproteobacteria	0	0	0	0	0	0	0	3	6	8
Deltaproteobacteria	5	2	3	0	0	0	0	4	1	13
Alphaproteobacteria	3	0	0	0	0	0	0	0	0	88
Magnetococcus	0	0	0	0	0	0	0	0	0	1
Chrysiogenetes	0	0	0	0	0	0	0	0	0	1
Firmicutes	53	7	22	0	0	2	0	12	3	5
Tenericutes	19	0	0	0	0	0	0	0	0	0
Actinobacteria	27	1	1	0	0	1	0	1	2	47
Chlamydiae	7	0	0	0	0	0	0	0	0	1
Spirochaetes	9	0	2	0	0	0	0	0	0	3
Acidobacteria	0	0	0	0	0	0	0	0	0	5
Bacteroidetes	10	0	1	0	0	0	1	5	1	17
Fibrobacteres	0	0	0	0	0	0	0	0	1	0
Fusobacteria	3	0	2	0	0	0	0	0	0	0
Verrucomicrobia	0	0	0	0	0	0	0	0	0	4
Gemmatimonadetes	0	0	0	0	0	0	0	0	0	1
Planctomycetes	1	0	0	0	0	0	0	0	1	2
Elusimicrobia	0	0	1	0	0	1	0	0	0	0
Synergistetes	0	1	1	0	0	0	0	0	0	0
Cyanobacteria	15	1	0	0	0	0	0	0	0	0
Chlorobi	0	0	0	0	0	0	0	9	0	1
Chloroflexi	0	0	0	0	0	0	0	3	2	6
Deinococcus-Thermus	0	0	0	0	0	0	0	0	2	5
Aquificae	0	0	0	0	0	0	0	0	0	9
Thermotogae	0	0	3	0	0	8	0	0	0	0
Dictyoglomi	0	0	2	0	0	0	0	0	0	0
Nitrospirae	0	0	0	0	0	0	0	1	0	1
Thermobaculum	0	0	0	0	0	0	0	0	1	0
Deferribacteres	0	0	0	0	0	0	0	0	1	2
Euryarchaeota	20	4	1	3	3	4	2	17	5	1
Crenarchaeota	0	1	4	1	0	0	6	11	0	0
Thaumarchaeota	0	0	0	0	0	0	2	0	0	0
Nanoarchaeota	1	0	0	0	0	0	0	0	0	0
Korarchaeota	0	0	0	0	0	0	0	1	0	0
Total	222	17	43	4	3	16	11	67	26	359

(M00144_3)

Phyla	Module completion ratio (%)									
	0--10	10--20	20--30	30--40	40--50	50--60	60--70	70--80	80--90	90--100
Gammaproteobacteria	49	0	0	0	0	0	0	0	1	76
Betaproteobacteria	0	0	0	0	0	0	0	0	0	61
Epsilonproteobacteria	0	0	0	0	0	0	1	2	6	8
Deltaproteobacteria	5	2	3	0	0	0	1	2	1	14
Alphaproteobacteria	3	0	0	0	0	0	0	0	0	88
Magnetococcus	0	0	0	0	0	0	0	0	0	1
Chrysiogenetes	0	0	0	0	0	0	0	0	0	1
Firmicutes	53	7	20	2	0	2	11	1	3	5
Tenericutes	19	0	0	0	0	0	0	0	0	0
Actinobacteria	27	1	1	0	0	1	0	1	2	47
Chlamydiae	7	0	0	0	0	0	0	0	0	1
Spirochaetes	9	0	2	0	0	0	0	0	0	3
Acidobacteria	0	0	0	0	0	0	0	0	0	5
Bacteroidetes	10	0	1	0	0	0	6	1	0	17
Fibrobacteres	0	0	0	0	0	0	0	0	1	0
Fusobacteria	3	0	2	0	0	0	0	0	0	0
Verrucomicrobia	0	0	0	0	0	0	0	0	0	4
Gemmatimonadetes	0	0	0	0	0	0	0	0	0	1
Planctomycetes	1	0	0	0	0	0	0	0	1	2
Elusimicrobia	0	0	1	0	0	1	0	0	0	0
Synergistetes	0	1	1	0	0	0	0	0	0	0
Cyanobacteria	15	1	0	0	0	0	0	0	0	0
Chlorobi	0	0	0	0	0	0	9	0	0	1
Chloroflexi	0	0	0	0	0	0	3	0	2	6
Deinococcus-Thermus	0	0	0	0	0	0	0	0	2	5
Aquificae	0	0	0	0	0	0	0	0	0	9
Thermotogae	0	0	3	0	7	1	0	0	0	0
Dictyoglomi	0	0	2	0	0	0	0	0	0	0
Nitrospirae	0	0	0	0	0	0	0	1	1	0
Thermobaculum	0	0	0	0	0	0	0	0	1	0
Deferribacteres	0	0	0	0	0	0	0	0	1	2
Euryarchaeota	20	4	1	6	3	0	19	6	1	0
Crenarchaeota	0	1	4	1	0	0	16	1	0	0
Thaumarchaeota	0	0	0	0	0	0	2	0	0	0
Nanoarchaeota	1	0	0	0	0	0	0	0	0	0
Korarchaeota	0	0	0	0	0	0	1	0	0	0
Total	222	17	41	9	10	5	69	15	23	357

(M00145_1)

Phyla	Module completion ratio (%)									
	0--10	10--20	20--30	30--40	40--50	50--60	60--70	70--80	80--90	90--100
Gammaproteobacteria	126	0	0	0	0	0	0	0	0	0
Betaproteobacteria	61	0	0	0	0	0	0	0	0	0
Epsilonproteobacteria	17	0	0	0	0	0	0	0	0	0
Deltaproteobacteria	28	0	0	0	0	0	0	0	0	0
Alphaproteobacteria	91	0	0	0	0	0	0	0	0	0
Magnetococcus	1	0	0	0	0	0	0	0	0	0
Chrysiogenetes	0	0	0	1	0	0	0	0	0	0
Firmicutes	103	0	1	0	0	0	0	0	0	0
Tenericutes	19	0	0	0	0	0	0	0	0	0
Actinobacteria	78	2	0	0	0	0	0	0	0	0
Chlamydiae	8	0	0	0	0	0	0	0	0	0
Spirochaetes	14	0	0	0	0	0	0	0	0	0
Acidobacteria	5	0	0	0	0	0	0	0	0	0
Bacteroidetes	35	0	0	0	0	0	0	0	0	0
Fibrobacteres	1	0	0	0	0	0	0	0	0	0
Fusobacteria	5	0	0	0	0	0	0	0	0	0
Verrucomicrobia	4	0	0	0	0	0	0	0	0	0
Gemmatimonadetes	1	0	0	0	0	0	0	0	0	0
Planctomycetes	4	0	0	0	0	0	0	0	0	0
Elusimicrobia	2	0	0	0	0	0	0	0	0	0
Synergistetes	2	0	0	0	0	0	0	0	0	0
Cyanobacteria	0	0	0	0	0	0	0	0	0	16
Chlorobi	10	0	0	0	0	0	0	0	0	0
Chloroflexi	3	5	3	0	0	0	0	0	0	0
Deinococcus-Thermus	7	0	0	0	0	0	0	0	0	0
Aquificae	9	0	0	0	0	0	0	0	0	0
Thermotogae	11	0	0	0	0	0	0	0	0	0
Dictyoglomi	2	0	0	0	0	0	0	0	0	0
Nitrospirae	2	0	0	0	0	0	0	0	0	0
Thermobaculum	1	0	0	0	0	0	0	0	0	0
Deferribacteres	3	0	0	0	0	0	0	0	0	0
Euryarchaeota	60	0	0	0	0	0	0	0	0	0
Crenarchaeota	23	0	0	0	0	0	0	0	0	0
Thaumarchaeota	2	0	0	0	0	0	0	0	0	0
Nanoarchaeota	1	0	0	0	0	0	0	0	0	0
Korarchaeota	1	0	0	0	0	0	0	0	0	0
Total	740	7	4	1	0	0	0	0	0	16

(M00146_1)

Phyla	Module completion ratio (%)									
	0--10	10--20	20--30	30--40	40--50	50--60	60--70	70--80	80--90	90--100
Gammaproteobacteria	126	0	0	0	0	0	0	0	0	0
Betaproteobacteria	61	0	0	0	0	0	0	0	0	0
Epsilonproteobacteria	17	0	0	0	0	0	0	0	0	0
Deltaproteobacteria	28	0	0	0	0	0	0	0	0	0
Alphaproteobacteria	91	0	0	0	0	0	0	0	0	0
Magnetococcus	1	0	0	0	0	0	0	0	0	0
Chrysiogenetes	1	0	0	0	0	0	0	0	0	0
Firmicutes	104	0	0	0	0	0	0	0	0	0
Tenericutes	19	0	0	0	0	0	0	0	0	0
Actinobacteria	80	0	0	0	0	0	0	0	0	0
Chlamydiae	8	0	0	0	0	0	0	0	0	0
Spirochaetes	14	0	0	0	0	0	0	0	0	0
Acidobacteria	5	0	0	0	0	0	0	0	0	0
Bacteroidetes	35	0	0	0	0	0	0	0	0	0
Fibrobacteres	1	0	0	0	0	0	0	0	0	0
Fusobacteria	5	0	0	0	0	0	0	0	0	0
Verrucomicrobia	4	0	0	0	0	0	0	0	0	0
Gemmatimonadetes	1	0	0	0	0	0	0	0	0	0
Planctomycetes	4	0	0	0	0	0	0	0	0	0
Elusimicrobia	2	0	0	0	0	0	0	0	0	0
Synergistetes	2	0	0	0	0	0	0	0	0	0
Cyanobacteria	16	0	0	0	0	0	0	0	0	0
Chlorobi	10	0	0	0	0	0	0	0	0	0
Chloroflexi	11	0	0	0	0	0	0	0	0	0
Deinococcus-Thermus	7	0	0	0	0	0	0	0	0	0
Aquificae	9	0	0	0	0	0	0	0	0	0
Thermotogae	11	0	0	0	0	0	0	0	0	0
Dictyoglomi	2	0	0	0	0	0	0	0	0	0
Nitrospirae	2	0	0	0	0	0	0	0	0	0
Thermobaculum	1	0	0	0	0	0	0	0	0	0
Deferribacteres	3	0	0	0	0	0	0	0	0	0
Euryarchaeota	60	0	0	0	0	0	0	0	0	0
Crenarchaeota	23	0	0	0	0	0	0	0	0	0
Thaumarchaeota	2	0	0	0	0	0	0	0	0	0
Nanoarchaeota	1	0	0	0	0	0	0	0	0	0
Korarchaeota	1	0	0	0	0	0	0	0	0	0
Total	768	0	0	0	0	0	0	0	0	0

(M00147_1)

Phyla	Module completion ratio (%)									
	0--10	10--20	20--30	30--40	40--50	50--60	60--70	70--80	80--90	90--100
Gammaproteobacteria	126	0	0	0	0	0	0	0	0	0
Betaproteobacteria	61	0	0	0	0	0	0	0	0	0
Epsilonproteobacteria	17	0	0	0	0	0	0	0	0	0
Deltaproteobacteria	28	0	0	0	0	0	0	0	0	0
Alphaproteobacteria	91	0	0	0	0	0	0	0	0	0
Magnetococcus	1	0	0	0	0	0	0	0	0	0
Chrysiogenetes	1	0	0	0	0	0	0	0	0	0
Firmicutes	104	0	0	0	0	0	0	0	0	0
Tenericutes	19	0	0	0	0	0	0	0	0	0
Actinobacteria	80	0	0	0	0	0	0	0	0	0
Chlamydiae	8	0	0	0	0	0	0	0	0	0
Spirochaetes	14	0	0	0	0	0	0	0	0	0
Acidobacteria	5	0	0	0	0	0	0	0	0	0
Bacteroidetes	35	0	0	0	0	0	0	0	0	0
Fibrobacteres	1	0	0	0	0	0	0	0	0	0
Fusobacteria	5	0	0	0	0	0	0	0	0	0
Verrucomicrobia	4	0	0	0	0	0	0	0	0	0
Gemmatimonadetes	1	0	0	0	0	0	0	0	0	0
Planctomycetes	4	0	0	0	0	0	0	0	0	0
Elusimicrobia	2	0	0	0	0	0	0	0	0	0
Synergistetes	2	0	0	0	0	0	0	0	0	0
Cyanobacteria	16	0	0	0	0	0	0	0	0	0
Chlorobi	10	0	0	0	0	0	0	0	0	0
Chloroflexi	11	0	0	0	0	0	0	0	0	0
Deinococcus-Thermus	7	0	0	0	0	0	0	0	0	0
Aquificae	9	0	0	0	0	0	0	0	0	0
Thermotogae	11	0	0	0	0	0	0	0	0	0
Dictyoglomi	2	0	0	0	0	0	0	0	0	0
Nitrospirae	2	0	0	0	0	0	0	0	0	0
Thermobaculum	1	0	0	0	0	0	0	0	0	0
Deferribacteres	3	0	0	0	0	0	0	0	0	0
Euryarchaeota	60	0	0	0	0	0	0	0	0	0
Crenarchaeota	23	0	0	0	0	0	0	0	0	0
Thaumarchaeota	2	0	0	0	0	0	0	0	0	0
Nanoarchaeota	1	0	0	0	0	0	0	0	0	0
Korarchaeota	1	0	0	0	0	0	0	0	0	0
Total	768	0	0	0	0	0	0	0	0	0

(M00148_1)

Phyla	Module completion ratio (%)									
	0--10	10--20	20--30	30--40	40--50	50--60	60--70	70--80	80--90	90--100
Gammaproteobacteria	126	0	0	0	0	0	0	0	0	0
Betaproteobacteria	61	0	0	0	0	0	0	0	0	0
Epsilonproteobacteria	17	0	0	0	0	0	0	0	0	0
Deltaproteobacteria	28	0	0	0	0	0	0	0	0	0
Alphaproteobacteria	91	0	0	0	0	0	0	0	0	0
Magnetococcus	1	0	0	0	0	0	0	0	0	0
Chrysiogenetes	1	0	0	0	0	0	0	0	0	0
Firmicutes	104	0	0	0	0	0	0	0	0	0
Tenericutes	19	0	0	0	0	0	0	0	0	0
Actinobacteria	80	0	0	0	0	0	0	0	0	0
Chlamydiae	8	0	0	0	0	0	0	0	0	0
Spirochaetes	14	0	0	0	0	0	0	0	0	0
Acidobacteria	5	0	0	0	0	0	0	0	0	0
Bacteroidetes	35	0	0	0	0	0	0	0	0	0
Fibrobacteres	1	0	0	0	0	0	0	0	0	0
Fusobacteria	5	0	0	0	0	0	0	0	0	0
Verrucomicrobia	4	0	0	0	0	0	0	0	0	0
Gemmatimonadetes	1	0	0	0	0	0	0	0	0	0
Planctomycetes	4	0	0	0	0	0	0	0	0	0
Elusimicrobia	2	0	0	0	0	0	0	0	0	0
Synergistetes	2	0	0	0	0	0	0	0	0	0
Cyanobacteria	16	0	0	0	0	0	0	0	0	0
Chlorobi	10	0	0	0	0	0	0	0	0	0
Chloroflexi	11	0	0	0	0	0	0	0	0	0
Deinococcus-Thermus	7	0	0	0	0	0	0	0	0	0
Aquificae	9	0	0	0	0	0	0	0	0	0
Thermotogae	11	0	0	0	0	0	0	0	0	0
Dictyoglomi	2	0	0	0	0	0	0	0	0	0
Nitrospirae	2	0	0	0	0	0	0	0	0	0
Thermobaculum	1	0	0	0	0	0	0	0	0	0
Deferribacteres	3	0	0	0	0	0	0	0	0	0
Euryarchaeota	60	0	0	0	0	0	0	0	0	0
Crenarchaeota	23	0	0	0	0	0	0	0	0	0
Thaumarchaeota	2	0	0	0	0	0	0	0	0	0
Nanoarchaeota	1	0	0	0	0	0	0	0	0	0
Korarchaeota	1	0	0	0	0	0	0	0	0	0
Total	768	0	0	0	0	0	0	0	0	0

(M00149_1)

Phyla	Module completion ratio (%)									
	0--10	10--20	20--30	30--40	40--50	50--60	60--70	70--80	80--90	90--100
Gammaproteobacteria	17	0	0	0	0	0	0	0	0	109
Betaproteobacteria	3	0	0	0	0	0	0	1	0	57
Epsilonproteobacteria	7	0	0	0	0	5	0	5	0	0
Deltaproteobacteria	7	0	1	0	0	1	0	18	0	1
Alphaproteobacteria	2	0	1	0	0	0	0	0	0	88
Magnetococcus	0	0	0	0	0	0	0	1	0	0
Chrysiogenetes	0	0	0	0	0	0	0	1	0	0
Firmicutes	67	0	3	0	0	2	0	30	0	2
Tenericutes	19	0	0	0	0	0	0	0	0	0
Actinobacteria	3	0	1	0	0	8	0	22	0	46
Chlamydiae	0	0	0	0	0	0	0	8	0	0
Spirochaetes	10	0	1	0	0	0	0	3	0	0
Acidobacteria	0	0	0	0	0	0	0	5	0	0
Bacteroidetes	2	0	0	0	0	0	0	31	0	2
Fibrobacteres	0	0	0	0	0	0	0	1	0	0
Fusobacteria	4	0	1	0	0	0	0	0	0	0
Verrucomicrobia	0	0	0	0	0	0	0	4	0	0
Gemmatimonadetes	0	0	0	0	0	0	0	1	0	0
Planctomycetes	0	0	0	0	0	1	0	3	0	0
Elusimicrobia	2	0	0	0	0	0	0	0	0	0
Synergistetes	2	0	0	0	0	0	0	0	0	0
Cyanobacteria	1	0	1	0	0	8	0	6	0	0
Chlorobi	0	0	0	0	0	0	0	8	0	2
Chloroflexi	3	0	0	0	0	0	0	4	0	4
Deinococcus-Thermus	0	0	0	0	0	1	0	0	0	6
Aquificae	0	0	1	0	0	8	0	0	0	0
Thermotogae	10	0	1	0	0	0	0	0	0	0
Dictyoglomi	0	0	2	0	0	0	0	0	0	0
Nitrospirae	0	0	0	0	0	1	0	0	0	1
Thermobaculum	0	0	0	0	0	0	0	0	0	1
Deferribacteres	0	0	0	0	0	0	0	0	0	3
Euryarchaeota	14	0	24	0	0	3	0	3	0	16
Crenarchaeota	6	0	0	0	0	2	0	14	0	1
Thaumarchaeota	0	0	0	0	0	0	0	2	0	0
Nanoarchaeota	1	0	0	0	0	0	0	0	0	0
Korarchaeota	0	0	0	0	0	0	0	1	0	0
Total	180	0	37	0	0	40	0	172	0	339

(M00150_1)

Phyla	Module completion ratio (%)									
	0--10	10--20	20--30	30--40	40--50	50--60	60--70	70--80	80--90	90--100
Gammaproteobacteria	59	0	2	0	0	1	0	13	0	51
Betaproteobacteria	48	0	3	0	0	0	0	5	0	5
Epsilonproteobacteria	3	0	1	0	0	0	0	13	0	0
Deltaproteobacteria	17	0	4	0	0	1	0	6	0	0
Alphaproteobacteria	78	0	7	0	0	0	0	1	0	5
Magnetococcus	1	0	0	0	0	0	0	0	0	0
Chrysiogenetes	1	0	0	0	0	0	0	0	0	0
Firmicutes	68	0	33	0	0	0	0	3	0	0
Tenericutes	19	0	0	0	0	0	0	0	0	0
Actinobacteria	68	0	8	0	0	2	0	0	0	2
Chlamydiae	8	0	0	0	0	0	0	0	0	0
Spirochaetes	13	0	1	0	0	0	0	0	0	0
Acidobacteria	5	0	0	0	0	0	0	0	0	0
Bacteroidetes	35	0	0	0	0	0	0	0	0	0
Fibrobacteres	1	0	0	0	0	0	0	0	0	0
Fusobacteria	3	0	2	0	0	0	0	0	0	0
Verrucomicrobia	4	0	0	0	0	0	0	0	0	0
Gemmatimonadetes	1	0	0	0	0	0	0	0	0	0
Planctomycetes	4	0	0	0	0	0	0	0	0	0
Elusimicrobia	2	0	0	0	0	0	0	0	0	0
Synergistetes	2	0	0	0	0	0	0	0	0	0
Cyanobacteria	16	0	0	0	0	0	0	0	0	0
Chlorobi	5	0	0	0	0	0	0	5	0	0
Chloroflexi	11	0	0	0	0	0	0	0	0	0
Deinococcus-Thermus	7	0	0	0	0	0	0	0	0	0
Aquificae	5	0	4	0	0	0	0	0	0	0
Thermotogae	11	0	0	0	0	0	0	0	0	0
Dictyoglomi	2	0	0	0	0	0	0	0	0	0
Nitrospirae	2	0	0	0	0	0	0	0	0	0
Thermobaculum	1	0	0	0	0	0	0	0	0	0
Deferribacteres	2	0	0	0	0	1	0	0	0	0
Euryarchaeota	34	0	26	0	0	0	0	0	0	0
Crenarchaeota	23	0	0	0	0	0	0	0	0	0
Thaumarchaeota	1	0	1	0	0	0	0	0	0	0
Nanoarchaeota	1	0	0	0	0	0	0	0	0	0
Korarchaeota	1	0	0	0	0	0	0	0	0	0
Total	562	0	92	0	0	5	0	46	0	63

(M00151_1)

Phyla	Module completion ratio (%)									
	0--10	10--20	20--30	30--40	40--50	50--60	60--70	70--80	80--90	90--100
Gammaproteobacteria	55	0	0	1	0	0	4	0	0	66
Betaproteobacteria	3	0	0	0	0	0	0	0	0	58
Epsilonproteobacteria	1	0	0	0	0	0	0	0	0	16
Deltaproteobacteria	21	0	0	7	0	0	0	0	0	0
Alphaproteobacteria	5	0	0	1	0	0	14	0	0	71
Magnetococcus	0	0	0	0	0	0	0	0	0	1
Chrysiogenetes	1	0	0	0	0	0	0	0	0	0
Firmicutes	104	0	0	0	0	0	0	0	0	0
Tenericutes	19	0	0	0	0	0	0	0	0	0
Actinobacteria	79	0	0	1	0	0	0	0	0	0
Chlamydiae	8	0	0	0	0	0	0	0	0	0
Spirochaetes	14	0	0	0	0	0	0	0	0	0
Acidobacteria	1	0	0	4	0	0	0	0	0	0
Bacteroidetes	34	0	0	1	0	0	0	0	0	0
Fibrobacteres	1	0	0	0	0	0	0	0	0	0
Fusobacteria	5	0	0	0	0	0	0	0	0	0
Verrucomicrobia	3	0	0	1	0	0	0	0	0	0
Gemmatimonadetes	0	0	0	1	0	0	0	0	0	0
Planctomycetes	2	0	0	2	0	0	0	0	0	0
Elusimicrobia	2	0	0	0	0	0	0	0	0	0
Synergistetes	2	0	0	0	0	0	0	0	0	0
Cyanobacteria	16	0	0	0	0	0	0	0	0	0
Chlorobi	10	0	0	0	0	0	0	0	0	0
Chloroflexi	11	0	0	0	0	0	0	0	0	0
Deinococcus-Thermus	4	0	0	2	0	0	1	0	0	0
Aquificae	2	0	0	0	0	0	0	0	0	7
Thermotogae	11	0	0	0	0	0	0	0	0	0
Dictyoglomi	2	0	0	0	0	0	0	0	0	0
Nitrospirae	1	0	0	1	0	0	0	0	0	0
Thermobaculum	1	0	0	0	0	0	0	0	0	0
Deferribacteres	3	0	0	0	0	0	0	0	0	0
Euryarchaeota	60	0	0	0	0	0	0	0	0	0
Crenarchaeota	23	0	0	0	0	0	0	0	0	0
Thaumarchaeota	0	0	0	1	0	0	1	0	0	0
Nanoarchaeota	1	0	0	0	0	0	0	0	0	0
Korarchaeota	1	0	0	0	0	0	0	0	0	0
Total	506	0	0	23	0	0	20	0	0	219

(M00151_2)

Phyla	Module completion ratio (%)									
	0--10	10--20	20--30	30--40	40--50	50--60	60--70	70--80	80--90	90--100
Gammaproteobacteria	126	0	0	0	0	0	0	0	0	0
Betaproteobacteria	61	0	0	0	0	0	0	0	0	0
Epsilonproteobacteria	17	0	0	0	0	0	0	0	0	0
Deltaproteobacteria	27	0	0	1	0	0	0	0	0	0
Alphaproteobacteria	91	0	0	0	0	0	0	0	0	0
Magnetococcus	1	0	0	0	0	0	0	0	0	0
Chrysiogenetes	1	0	0	0	0	0	0	0	0	0
Firmicutes	83	0	0	0	0	0	1	0	0	20
Tenericutes	19	0	0	0	0	0	0	0	0	0
Actinobacteria	78	0	0	2	0	0	0	0	0	0
Chlamydiae	8	0	0	0	0	0	0	0	0	0
Spirochaetes	14	0	0	0	0	0	0	0	0	0
Acidobacteria	4	0	0	1	0	0	0	0	0	0
Bacteroidetes	34	0	0	1	0	0	0	0	0	0
Fibrobacteres	1	0	0	0	0	0	0	0	0	0
Fusobacteria	5	0	0	0	0	0	0	0	0	0
Verrucomicrobia	4	0	0	0	0	0	0	0	0	0
Gemmatimonadetes	0	0	0	1	0	0	0	0	0	0
Planctomycetes	3	0	0	1	0	0	0	0	0	0
Elusimicrobia	2	0	0	0	0	0	0	0	0	0
Synergistetes	2	0	0	0	0	0	0	0	0	0
Cyanobacteria	16	0	0	0	0	0	0	0	0	0
Chlorobi	10	0	0	0	0	0	0	0	0	0
Chloroflexi	11	0	0	0	0	0	0	0	0	0
Deinococcus-Thermus	7	0	0	0	0	0	0	0	0	0
Aquificae	9	0	0	0	0	0	0	0	0	0
Thermotogae	11	0	0	0	0	0	0	0	0	0
Dictyoglomi	2	0	0	0	0	0	0	0	0	0
Nitrospirae	1	0	0	1	0	0	0	0	0	0
Thermobaculum	1	0	0	0	0	0	0	0	0	0
Deferribacteres	3	0	0	0	0	0	0	0	0	0
Euryarchaeota	60	0	0	0	0	0	0	0	0	0
Crenarchaeota	23	0	0	0	0	0	0	0	0	0
Thaumarchaeota	2	0	0	0	0	0	0	0	0	0
Nanoarchaeota	1	0	0	0	0	0	0	0	0	0
Korarchaeota	1	0	0	0	0	0	0	0	0	0
Total	739	0	0	8	0	0	1	0	0	20

(M00151_3)

Phyla	Module completion ratio (%)									
	0--10	10--20	20--30	30--40	40--50	50--60	60--70	70--80	80--90	90--100
Gammaproteobacteria	126	0	0	0	0	0	0	0	0	0
Betaproteobacteria	61	0	0	0	0	0	0	0	0	0
Epsilonproteobacteria	17	0	0	0	0	0	0	0	0	0
Deltaproteobacteria	28	0	0	0	0	0	0	0	0	0
Alphaproteobacteria	91	0	0	0	0	0	0	0	0	0
Magnetococcus	1	0	0	0	0	0	0	0	0	0
Chrysiogenetes	1	0	0	0	0	0	0	0	0	0
Firmicutes	104	0	0	0	0	0	0	0	0	0
Tenericutes	19	0	0	0	0	0	0	0	0	0
Actinobacteria	13	0	0	0	0	0	2	0	0	65
Chlamydiae	8	0	0	0	0	0	0	0	0	0
Spirochaetes	14	0	0	0	0	0	0	0	0	0
Acidobacteria	4	0	0	1	0	0	0	0	0	0
Bacteroidetes	35	0	0	0	0	0	0	0	0	0
Fibrobacteres	1	0	0	0	0	0	0	0	0	0
Fusobacteria	5	0	0	0	0	0	0	0	0	0
Verrucomicrobia	4	0	0	0	0	0	0	0	0	0
Gemmatimonadetes	1	0	0	0	0	0	0	0	0	0
Planctomycetes	4	0	0	0	0	0	0	0	0	0
Elusimicrobia	2	0	0	0	0	0	0	0	0	0
Synergistetes	2	0	0	0	0	0	0	0	0	0
Cyanobacteria	16	0	0	0	0	0	0	0	0	0
Chlorobi	10	0	0	0	0	0	0	0	0	0
Chloroflexi	11	0	0	0	0	0	0	0	0	0
Deinococcus-Thermus	4	0	0	3	0	0	0	0	0	0
Aquificae	9	0	0	0	0	0	0	0	0	0
Thermotogae	11	0	0	0	0	0	0	0	0	0
Dictyoglomi	2	0	0	0	0	0	0	0	0	0
Nitrospirae	2	0	0	0	0	0	0	0	0	0
Thermobaculum	1	0	0	0	0	0	0	0	0	0
Deferribacteres	3	0	0	0	0	0	0	0	0	0
Euryarchaeota	55	0	0	1	0	0	4	0	0	0
Crenarchaeota	23	0	0	0	0	0	0	0	0	0
Thaumarchaeota	2	0	0	0	0	0	0	0	0	0
Nanoarchaeota	1	0	0	0	0	0	0	0	0	0
Korarchaeota	1	0	0	0	0	0	0	0	0	0
Total	692	0	0	5	0	0	6	0	0	65

(M00152_1)

Phyla	Module completion ratio (%)									
	0--10	10--20	20--30	30--40	40--50	50--60	60--70	70--80	80--90	90--100
Gammaproteobacteria	55	1	70	0	0	0	0	0	0	0
Betaproteobacteria	3	0	58	0	0	0	0	0	0	0
Epsilonproteobacteria	1	0	16	0	0	0	0	0	0	0
Deltaproteobacteria	21	7	0	0	0	0	0	0	0	0
Alphaproteobacteria	5	1	85	0	0	0	0	0	0	0
Magnetococcus	0	0	1	0	0	0	0	0	0	0
Chrysiogenetes	1	0	0	0	0	0	0	0	0	0
Firmicutes	104	0	0	0	0	0	0	0	0	0
Tenericutes	19	0	0	0	0	0	0	0	0	0
Actinobacteria	79	1	0	0	0	0	0	0	0	0
Chlamydiae	8	0	0	0	0	0	0	0	0	0
Spirochaetes	14	0	0	0	0	0	0	0	0	0
Acidobacteria	1	4	0	0	0	0	0	0	0	0
Bacteroidetes	34	1	0	0	0	0	0	0	0	0
Fibrobacteres	1	0	0	0	0	0	0	0	0	0
Fusobacteria	5	0	0	0	0	0	0	0	0	0
Verrucomicrobia	3	1	0	0	0	0	0	0	0	0
Gemmatimonadetes	0	1	0	0	0	0	0	0	0	0
Planctomycetes	2	2	0	0	0	0	0	0	0	0
Elusimicrobia	2	0	0	0	0	0	0	0	0	0
Synergistetes	2	0	0	0	0	0	0	0	0	0
Cyanobacteria	16	0	0	0	0	0	0	0	0	0
Chlorobi	10	0	0	0	0	0	0	0	0	0
Chloroflexi	11	0	0	0	0	0	0	0	0	0
Deinococcus-Thermus	4	2	1	0	0	0	0	0	0	0
Aquificae	2	0	7	0	0	0	0	0	0	0
Thermotogae	11	0	0	0	0	0	0	0	0	0
Dictyoglomi	2	0	0	0	0	0	0	0	0	0
Nitrospirae	1	1	0	0	0	0	0	0	0	0
Thermobaculum	1	0	0	0	0	0	0	0	0	0
Deferribacteres	3	0	0	0	0	0	0	0	0	0
Euryarchaeota	60	0	0	0	0	0	0	0	0	0
Crenarchaeota	23	0	0	0	0	0	0	0	0	0
Thaumarchaeota	0	1	1	0	0	0	0	0	0	0
Nanoarchaeota	1	0	0	0	0	0	0	0	0	0
Korarchaeota	1	0	0	0	0	0	0	0	0	0
Total	506	23	239	0	0	0	0	0	0	0

(M00153_1)

Phyla	Module completion ratio (%)									
	0--10	10--20	20--30	30--40	40--50	50--60	60--70	70--80	80--90	90--100
Gammaproteobacteria	15	0	0	0	0	0	0	0	0	111
Betaproteobacteria	17	0	0	0	0	0	0	0	0	44
Epsilonproteobacteria	5	0	0	0	0	0	0	0	0	12
Deltaproteobacteria	3	0	0	0	0	0	0	0	0	25
Alphaproteobacteria	27	0	0	0	0	0	0	0	0	64
Magnetococcus	1	0	0	0	0	0	0	0	0	0
Chrysiogenetes	0	0	0	0	0	0	0	0	0	1
Firmicutes	53	0	0	0	0	0	0	0	0	51
Tenericutes	19	0	0	0	0	0	0	0	0	0
Actinobacteria	10	0	0	0	0	3	0	0	0	67
Chlamydiae	0	0	0	0	0	0	0	0	0	8
Spirochaetes	14	0	0	0	0	0	0	0	0	0
Acidobacteria	2	0	0	0	0	0	0	0	0	3
Bacteroidetes	16	0	0	0	0	0	0	0	0	19
Fibrobacteres	1	0	0	0	0	0	0	0	0	0
Fusobacteria	5	0	0	0	0	0	0	0	0	0
Verrucomicrobia	1	0	0	0	0	0	0	0	0	3
Gemmatimonadetes	1	0	0	0	0	0	0	0	0	0
Planctomycetes	1	0	0	0	0	0	0	0	0	3
Elusimicrobia	2	0	0	0	0	0	0	0	0	0
Synergistetes	2	0	0	0	0	0	0	0	0	0
Cyanobacteria	6	0	0	0	0	0	0	0	0	10
Chlorobi	2	0	0	0	0	0	0	0	0	8
Chloroflexi	9	0	0	0	0	0	0	0	0	2
Deinococcus-Thermus	6	0	0	0	0	0	0	0	0	1
Aquificae	3	0	0	0	0	0	0	0	0	6
Thermotogae	11	0	0	0	0	0	0	0	0	0
Dictyoglomi	2	0	0	0	0	0	0	0	0	0
Nitrospirae	0	0	0	0	0	0	0	0	0	2
Thermobaculum	0	0	0	0	0	0	0	0	0	1
Deferribacteres	1	0	0	0	0	0	0	0	0	2
Euryarchaeota	44	0	0	0	0	2	0	0	0	14
Crenarchaeota	15	0	0	0	0	8	0	0	0	0
Thaumarchaeota	2	0	0	0	0	0	0	0	0	0
Nanoarchaeota	1	0	0	0	0	0	0	0	0	0
Korarchaeota	0	0	0	0	0	1	0	0	0	0
Total	297	0	0	0	0	14	0	0	0	457

(M00154_1)

Phyla	Module completion ratio (%)									
	0--10	10--20	20--30	30--40	40--50	50--60	60--70	70--80	80--90	90--100
Gammaproteobacteria	73	53	0	0	0	0	0	0	0	0
Betaproteobacteria	14	47	0	0	0	0	0	0	0	0
Epsilonproteobacteria	17	0	0	0	0	0	0	0	0	0
Deltaproteobacteria	28	0	0	0	0	0	0	0	0	0
Alphaproteobacteria	17	74	0	0	0	0	0	0	0	0
Magnetococcus	1	0	0	0	0	0	0	0	0	0
Chrysiogenetes	1	0	0	0	0	0	0	0	0	0
Firmicutes	104	0	0	0	0	0	0	0	0	0
Tenericutes	19	0	0	0	0	0	0	0	0	0
Actinobacteria	80	0	0	0	0	0	0	0	0	0
Chlamydiae	8	0	0	0	0	0	0	0	0	0
Spirochaetes	14	0	0	0	0	0	0	0	0	0
Acidobacteria	5	0	0	0	0	0	0	0	0	0
Bacteroidetes	35	0	0	0	0	0	0	0	0	0
Fibrobacteres	1	0	0	0	0	0	0	0	0	0
Fusobacteria	5	0	0	0	0	0	0	0	0	0
Verrucomicrobia	4	0	0	0	0	0	0	0	0	0
Gemmatimonadetes	1	0	0	0	0	0	0	0	0	0
Planctomycetes	4	0	0	0	0	0	0	0	0	0
Elusimicrobia	2	0	0	0	0	0	0	0	0	0
Synergistetes	2	0	0	0	0	0	0	0	0	0
Cyanobacteria	16	0	0	0	0	0	0	0	0	0
Chlorobi	10	0	0	0	0	0	0	0	0	0
Chloroflexi	11	0	0	0	0	0	0	0	0	0
Deinococcus-Thermus	7	0	0	0	0	0	0	0	0	0
Aquificae	9	0	0	0	0	0	0	0	0	0
Thermotogae	11	0	0	0	0	0	0	0	0	0
Dictyoglomi	2	0	0	0	0	0	0	0	0	0
Nitrospirae	2	0	0	0	0	0	0	0	0	0
Thermobaculum	1	0	0	0	0	0	0	0	0	0
Deferribacteres	3	0	0	0	0	0	0	0	0	0
Euryarchaeota	60	0	0	0	0	0	0	0	0	0
Crenarchaeota	23	0	0	0	0	0	0	0	0	0
Thaumarchaeota	2	0	0	0	0	0	0	0	0	0
Nanoarchaeota	1	0	0	0	0	0	0	0	0	0
Korarchaeota	1	0	0	0	0	0	0	0	0	0
Total	594	174	0	0	0	0	0	0	0	0

(M00154_2)

Phyla	Module completion ratio (%)									
	0--10	10--20	20--30	30--40	40--50	50--60	60--70	70--80	80--90	90--100
Gammaproteobacteria	73	53	0	0	0	0	0	0	0	0
Betaproteobacteria	14	47	0	0	0	0	0	0	0	0
Epsilonproteobacteria	17	0	0	0	0	0	0	0	0	0
Deltaproteobacteria	28	0	0	0	0	0	0	0	0	0
Alphaproteobacteria	17	74	0	0	0	0	0	0	0	0
Magnetococcus	1	0	0	0	0	0	0	0	0	0
Chrysiogenetes	1	0	0	0	0	0	0	0	0	0
Firmicutes	104	0	0	0	0	0	0	0	0	0
Tenericutes	19	0	0	0	0	0	0	0	0	0
Actinobacteria	80	0	0	0	0	0	0	0	0	0
Chlamydiae	8	0	0	0	0	0	0	0	0	0
Spirochaetes	14	0	0	0	0	0	0	0	0	0
Acidobacteria	5	0	0	0	0	0	0	0	0	0
Bacteroidetes	35	0	0	0	0	0	0	0	0	0
Fibrobacteres	1	0	0	0	0	0	0	0	0	0
Fusobacteria	5	0	0	0	0	0	0	0	0	0
Verrucomicrobia	4	0	0	0	0	0	0	0	0	0
Gemmatimonadetes	1	0	0	0	0	0	0	0	0	0
Planctomycetes	4	0	0	0	0	0	0	0	0	0
Elusimicrobia	2	0	0	0	0	0	0	0	0	0
Synergistetes	2	0	0	0	0	0	0	0	0	0
Cyanobacteria	16	0	0	0	0	0	0	0	0	0
Chlorobi	10	0	0	0	0	0	0	0	0	0
Chloroflexi	11	0	0	0	0	0	0	0	0	0
Deinococcus-Thermus	7	0	0	0	0	0	0	0	0	0
Aquificae	9	0	0	0	0	0	0	0	0	0
Thermotogae	11	0	0	0	0	0	0	0	0	0
Dictyoglomi	2	0	0	0	0	0	0	0	0	0
Nitrospirae	2	0	0	0	0	0	0	0	0	0
Thermobaculum	1	0	0	0	0	0	0	0	0	0
Deferribacteres	3	0	0	0	0	0	0	0	0	0
Euryarchaeota	60	0	0	0	0	0	0	0	0	0
Crenarchaeota	23	0	0	0	0	0	0	0	0	0
Thaumarchaeota	2	0	0	0	0	0	0	0	0	0
Nanoarchaeota	1	0	0	0	0	0	0	0	0	0
Korarchaeota	1	0	0	0	0	0	0	0	0	0
Total	594	174	0	0	0	0	0	0	0	0

(M00155_1)

Phyla	Module completion ratio (%)									
	0--10	10--20	20--30	30--40	40--50	50--60	60--70	70--80	80--90	90--100
Gammaproteobacteria	69	0	0	1	0	0	1	0	0	55
Betaproteobacteria	6	0	0	0	0	0	2	0	0	53
Epsilonproteobacteria	15	0	0	0	0	0	2	0	0	0
Deltaproteobacteria	13	0	0	0	0	0	0	0	0	15
Alphaproteobacteria	11	0	0	0	0	0	2	0	0	78
Magnetococcus	1	0	0	0	0	0	0	0	0	0
Chrysiogenetes	0	0	0	1	0	0	0	0	0	0
Firmicutes	81	0	0	0	0	0	0	0	0	23
Tenericutes	19	0	0	0	0	0	0	0	0	0
Actinobacteria	12	0	0	0	0	0	0	0	0	68
Chlamydiae	8	0	0	0	0	0	0	0	0	0
Spirochaetes	11	0	0	0	0	0	0	0	0	3
Acidobacteria	0	0	0	0	0	0	0	0	0	5
Bacteroidetes	15	0	0	0	0	0	0	0	0	20
Fibrobacteres	1	0	0	0	0	0	0	0	0	0
Fusobacteria	4	0	0	1	0	0	0	0	0	0
Verrucomicrobia	1	0	0	0	0	0	2	0	0	1
Gemmatimonadetes	0	0	0	0	0	0	1	0	0	0
Planctomycetes	0	0	0	0	0	0	0	0	0	4
Elusimicrobia	2	0	0	0	0	0	0	0	0	0
Synergistetes	2	0	0	0	0	0	0	0	0	0
Cyanobacteria	0	0	0	0	0	0	0	0	0	16
Chlorobi	10	0	0	0	0	0	0	0	0	0
Chloroflexi	3	0	0	0	0	0	1	0	0	7
Deinococcus-Thermus	0	0	0	2	0	0	5	0	0	0
Aquificae	5	0	0	0	0	0	1	0	0	3
Thermotogae	11	0	0	0	0	0	0	0	0	0
Dictyoglomi	2	0	0	0	0	0	0	0	0	0
Nitrospirae	2	0	0	0	0	0	0	0	0	0
Thermobaculum	0	0	0	0	0	0	0	0	0	1
Deferribacteres	2	0	0	0	0	0	0	0	0	1
Euryarchaeota	44	0	0	3	0	0	1	0	0	12
Crenarchaeota	15	0	0	2	0	0	6	0	0	0
Thaumarchaeota	0	0	0	0	0	0	2	0	0	0
Nanoarchaeota	1	0	0	0	0	0	0	0	0	0
Korarchaeota	1	0	0	0	0	0	0	0	0	0
Total	367	0	0	10	0	0	26	0	0	365

(M00155_2)

Phyla	Module completion ratio (%)									
	0--10	10--20	20--30	30--40	40--50	50--60	60--70	70--80	80--90	90--100
Gammaproteobacteria	69	0	0	0	0	50	0	0	0	7
Betaproteobacteria	6	0	0	0	0	43	0	0	0	12
Epsilonproteobacteria	15	0	0	0	0	2	0	0	0	0
Deltaproteobacteria	13	0	0	0	0	15	0	0	0	0
Alphaproteobacteria	12	0	0	0	0	68	0	0	0	11
Magnetococcus	1	0	0	0	0	0	0	0	0	0
Chrysiogenetes	0	0	0	0	0	1	0	0	0	0
Firmicutes	81	0	0	0	0	23	0	0	0	0
Tenericutes	19	0	0	0	0	0	0	0	0	0
Actinobacteria	12	0	0	0	0	68	0	0	0	0
Chlamydiae	8	0	0	0	0	0	0	0	0	0
Spirochaetes	11	0	0	0	0	3	0	0	0	0
Acidobacteria	0	0	0	0	0	5	0	0	0	0
Bacteroidetes	15	0	0	0	0	20	0	0	0	0
Fibrobacteres	1	0	0	0	0	0	0	0	0	0
Fusobacteria	4	0	0	0	0	1	0	0	0	0
Verrucomicrobia	1	0	0	0	0	2	0	0	0	1
Gemmatimonadetes	0	0	0	0	0	1	0	0	0	0
Planctomycetes	0	0	0	0	0	3	0	0	0	1
Elusimicrobia	2	0	0	0	0	0	0	0	0	0
Synergistetes	2	0	0	0	0	0	0	0	0	0
Cyanobacteria	0	0	0	0	0	16	0	0	0	0
Chlorobi	10	0	0	0	0	0	0	0	0	0
Chloroflexi	3	0	0	0	0	8	0	0	0	0
Deinococcus-Thermus	0	0	0	0	0	0	0	0	0	7
Aquificae	5	0	0	0	0	4	0	0	0	0
Thermotogae	11	0	0	0	0	0	0	0	0	0
Dictyoglomi	2	0	0	0	0	0	0	0	0	0
Nitrospirae	2	0	0	0	0	0	0	0	0	0
Thermobaculum	0	0	0	0	0	1	0	0	0	0
Deferribacteres	2	0	0	0	0	1	0	0	0	0
Euryarchaeota	44	0	0	0	0	9	0	0	0	7
Crenarchaeota	17	0	0	0	0	5	0	0	0	1
Thaumarchaeota	0	0	0	0	0	2	0	0	0	0
Nanoarchaeota	1	0	0	0	0	0	0	0	0	0
Korarchaeota	1	0	0	0	0	0	0	0	0	0
Total	370	0	0	0	0	351	0	0	0	47

(M00156_1)

Phyla	Module completion ratio (%)									
	0--10	10--20	20--30	30--40	40--50	50--60	60--70	70--80	80--90	90--100
Gammaproteobacteria	66	0	0	0	0	0	0	6	0	54
Betaproteobacteria	13	0	1	0	0	4	0	9	0	34
Epsilonproteobacteria	1	0	0	0	0	1	0	0	0	15
Deltaproteobacteria	19	0	2	0	0	4	0	3	0	0
Alphaproteobacteria	42	0	1	0	0	1	0	2	0	45
Magnetococcus	0	0	0	0	0	0	0	1	0	0
Chrysiogenetes	0	0	0	0	0	0	0	1	0	0
Firmicutes	102	0	1	0	0	1	0	0	0	0
Tenericutes	19	0	0	0	0	0	0	0	0	0
Actinobacteria	80	0	0	0	0	0	0	0	0	0
Chlamydiae	6	0	0	0	0	2	0	0	0	0
Spirochaetes	12	0	0	0	0	0	0	2	0	0
Acidobacteria	2	0	1	0	0	2	0	0	0	0
Bacteroidetes	13	0	0	0	0	3	0	19	0	0
Fibrobacteres	1	0	0	0	0	0	0	0	0	0
Fusobacteria	5	0	0	0	0	0	0	0	0	0
Verrucomicrobia	1	0	0	0	0	1	0	2	0	0
Gemmatimonadetes	0	0	0	0	0	0	0	1	0	0
Planctomycetes	1	0	1	0	0	1	0	1	0	0
Elusimicrobia	2	0	0	0	0	0	0	0	0	0
Synergistetes	2	0	0	0	0	0	0	0	0	0
Cyanobacteria	15	0	1	0	0	0	0	0	0	0
Chlorobi	5	0	1	0	0	0	0	4	0	0
Chloroflexi	10	0	1	0	0	0	0	0	0	0
Deinococcus-Thermus	7	0	0	0	0	0	0	0	0	0
Aquificae	5	0	4	0	0	0	0	0	0	0
Thermotogae	11	0	0	0	0	0	0	0	0	0
Dictyoglomi	2	0	0	0	0	0	0	0	0	0
Nitrospirae	1	0	1	0	0	0	0	0	0	0
Thermobaculum	1	0	0	0	0	0	0	0	0	0
Deferribacteres	1	0	0	0	0	2	0	0	0	0
Euryarchaeota	60	0	0	0	0	0	0	0	0	0
Crenarchaeota	23	0	0	0	0	0	0	0	0	0
Thaumarchaeota	2	0	0	0	0	0	0	0	0	0
Nanoarchaeota	1	0	0	0	0	0	0	0	0	0
Korarchaeota	1	0	0	0	0	0	0	0	0	0
Total	532	0	15	0	0	22	0	51	0	148

(M00157_1)

Phyla	Module completion ratio (%)									
	0--10	10--20	20--30	30--40	40--50	50--60	60--70	70--80	80--90	90--100
Gammaproteobacteria	1	0	0	0	0	0	1	0	1	123
Betaproteobacteria	1	0	0	0	0	0	0	0	0	60
Epsilonproteobacteria	0	0	0	0	0	0	0	0	2	15
Deltaproteobacteria	0	0	0	0	0	0	0	0	0	28
Alphaproteobacteria	1	0	0	0	0	0	0	0	0	90
Magnetococcus	0	0	0	0	0	0	0	0	0	1
Chrysiogenetes	0	0	0	0	0	0	0	0	0	1
Firmicutes	5	1	0	0	0	0	0	0	4	94
Tenericutes	3	0	0	0	0	0	0	0	1	15
Actinobacteria	0	0	0	0	0	0	0	1	2	77
Chlamydiae	6	0	0	0	0	0	0	0	0	2
Spirochaetes	10	0	0	0	0	0	0	0	0	4
Acidobacteria	0	0	0	0	0	0	0	0	0	5
Bacteroidetes	1	0	0	0	0	0	0	0	1	33
Fibrobacteres	1	0	0	0	0	0	0	0	0	0
Fusobacteria	0	0	0	0	0	0	0	0	0	5
Verrucomicrobia	0	0	0	0	0	0	0	0	0	4
Gemmatimonadetes	0	0	0	0	0	0	0	0	0	1
Planctomycetes	0	0	0	0	0	0	0	1	0	3
Elusimicrobia	0	0	0	0	0	0	0	0	1	1
Synergistetes	1	1	0	0	0	0	0	0	0	0
Cyanobacteria	0	0	0	0	0	0	0	0	0	16
Chlorobi	0	0	0	0	0	0	0	0	1	9
Chloroflexi	0	0	0	0	0	0	0	0	1	10
Deinococcus-Thermus	7	0	0	0	0	0	0	0	0	0
Aquificae	0	0	0	0	0	0	0	0	0	9
Thermotogae	0	0	0	0	0	0	0	0	0	11
Dictyoglomi	0	0	0	0	0	0	0	0	2	0
Nitrospirae	0	0	0	0	0	0	0	0	0	2
Thermobaculum	0	0	0	0	0	0	0	0	0	1
Deferribacteres	0	0	0	0	0	0	0	0	0	3
Euryarchaeota	58	0	0	0	0	0	0	0	2	0
Crenarchaeota	23	0	0	0	0	0	0	0	0	0
Thaumarchaeota	2	0	0	0	0	0	0	0	0	0
Nanoarchaeota	1	0	0	0	0	0	0	0	0	0
Korarchaeota	1	0	0	0	0	0	0	0	0	0
Total	122	2	0	0	0	0	1	2	18	623

(M00158_1)

Phyla	Module completion ratio (%)									
	0--10	10--20	20--30	30--40	40--50	50--60	60--70	70--80	80--90	90--100
Gammaproteobacteria	126	0	0	0	0	0	0	0	0	0
Betaproteobacteria	61	0	0	0	0	0	0	0	0	0
Epsilonproteobacteria	17	0	0	0	0	0	0	0	0	0
Deltaproteobacteria	28	0	0	0	0	0	0	0	0	0
Alphaproteobacteria	91	0	0	0	0	0	0	0	0	0
Magnetococcus	1	0	0	0	0	0	0	0	0	0
Chrysiogenetes	1	0	0	0	0	0	0	0	0	0
Firmicutes	104	0	0	0	0	0	0	0	0	0
Tenericutes	19	0	0	0	0	0	0	0	0	0
Actinobacteria	80	0	0	0	0	0	0	0	0	0
Chlamydiae	8	0	0	0	0	0	0	0	0	0
Spirochaetes	14	0	0	0	0	0	0	0	0	0
Acidobacteria	5	0	0	0	0	0	0	0	0	0
Bacteroidetes	35	0	0	0	0	0	0	0	0	0
Fibrobacteres	1	0	0	0	0	0	0	0	0	0
Fusobacteria	5	0	0	0	0	0	0	0	0	0
Verrucomicrobia	4	0	0	0	0	0	0	0	0	0
Gemmatimonadetes	1	0	0	0	0	0	0	0	0	0
Planctomycetes	4	0	0	0	0	0	0	0	0	0
Elusimicrobia	2	0	0	0	0	0	0	0	0	0
Synergistetes	2	0	0	0	0	0	0	0	0	0
Cyanobacteria	16	0	0	0	0	0	0	0	0	0
Chlorobi	10	0	0	0	0	0	0	0	0	0
Chloroflexi	11	0	0	0	0	0	0	0	0	0
Deinococcus-Thermus	7	0	0	0	0	0	0	0	0	0
Aquificae	9	0	0	0	0	0	0	0	0	0
Thermotogae	11	0	0	0	0	0	0	0	0	0
Dictyoglomi	2	0	0	0	0	0	0	0	0	0
Nitrospirae	2	0	0	0	0	0	0	0	0	0
Thermobaculum	1	0	0	0	0	0	0	0	0	0
Deferribacteres	3	0	0	0	0	0	0	0	0	0
Euryarchaeota	60	0	0	0	0	0	0	0	0	0
Crenarchaeota	23	0	0	0	0	0	0	0	0	0
Thaumarchaeota	2	0	0	0	0	0	0	0	0	0
Nanoarchaeota	1	0	0	0	0	0	0	0	0	0
Korarchaeota	1	0	0	0	0	0	0	0	0	0
Total	768	0	0	0	0	0	0	0	0	0

(M00159_1)

Phyla	Module completion ratio (%)									
	0--10	10--20	20--30	30--40	40--50	50--60	60--70	70--80	80--90	90--100
Gammaproteobacteria	122	0	0	0	0	0	0	2	2	0
Betaproteobacteria	60	0	0	0	0	0	0	0	1	0
Epsilonproteobacteria	16	0	0	0	0	0	0	1	0	0
Deltaproteobacteria	23	0	0	0	0	0	0	2	2	1
Alphaproteobacteria	91	0	0	0	0	0	0	0	0	0
Magnetococcus	1	0	0	0	0	0	0	0	0	0
Chrysiogenetes	1	0	0	0	0	0	0	0	0	0
Firmicutes	80	0	0	0	0	0	1	0	2	21
Tenericutes	18	0	0	0	0	0	0	0	0	1
Actinobacteria	79	0	0	0	0	0	1	0	0	0
Chlamydiae	0	0	0	0	0	0	0	8	0	0
Spirochaetes	3	0	0	0	0	0	1	8	1	1
Acidobacteria	5	0	0	0	0	0	0	0	0	0
Bacteroidetes	29	0	0	0	0	0	0	6	0	0
Fibrobacteres	0	0	0	0	0	0	0	1	0	0
Fusobacteria	2	0	0	0	0	0	0	0	0	3
Verrucomicrobia	4	0	0	0	0	0	0	0	0	0
Gemmatimonadetes	1	0	0	0	0	0	0	0	0	0
Planctomycetes	4	0	0	0	0	0	0	0	0	0
Elusimicrobia	2	0	0	0	0	0	0	0	0	0
Synergistetes	0	0	0	0	0	0	0	0	0	2
Cyanobacteria	16	0	0	0	0	0	0	0	0	0
Chlorobi	10	0	0	0	0	0	0	0	0	0
Chloroflexi	11	0	0	0	0	0	0	0	0	0
Deinococcus-Thermus	0	0	0	0	0	0	0	0	0	7
Aquificae	8	0	0	0	0	0	0	0	1	0
Thermotogae	9	0	0	0	0	0	0	0	0	2
Dictyoglomi	0	0	0	0	0	0	0	0	2	0
Nitrospirae	2	0	0	0	0	0	0	0	0	0
Thermobaculum	1	0	0	0	0	0	0	0	0	0
Deferribacteres	3	0	0	0	0	0	0	0	0	0
Euryarchaeota	0	0	0	0	0	0	0	0	1	59
Crenarchaeota	0	0	0	0	0	0	1	5	10	7
Thaumarchaeota	0	0	0	0	0	0	0	0	0	2
Nanoarchaeota	0	0	0	0	0	0	1	0	0	0
Korarchaeota	0	0	0	0	0	0	0	0	1	0
Total	601	0	0	0	0	0	5	33	23	106

(M00160_1)

Phyla	Module completion ratio (%)									
	0--10	10--20	20--30	30--40	40--50	50--60	60--70	70--80	80--90	90--100
Gammaproteobacteria	126	0	0	0	0	0	0	0	0	0
Betaproteobacteria	61	0	0	0	0	0	0	0	0	0
Epsilonproteobacteria	17	0	0	0	0	0	0	0	0	0
Deltaproteobacteria	28	0	0	0	0	0	0	0	0	0
Alphaproteobacteria	91	0	0	0	0	0	0	0	0	0
Magnetococcus	1	0	0	0	0	0	0	0	0	0
Chrysiogenetes	1	0	0	0	0	0	0	0	0	0
Firmicutes	104	0	0	0	0	0	0	0	0	0
Tenericutes	19	0	0	0	0	0	0	0	0	0
Actinobacteria	80	0	0	0	0	0	0	0	0	0
Chlamydiae	8	0	0	0	0	0	0	0	0	0
Spirochaetes	14	0	0	0	0	0	0	0	0	0
Acidobacteria	5	0	0	0	0	0	0	0	0	0
Bacteroidetes	35	0	0	0	0	0	0	0	0	0
Fibrobacteres	1	0	0	0	0	0	0	0	0	0
Fusobacteria	5	0	0	0	0	0	0	0	0	0
Verrucomicrobia	4	0	0	0	0	0	0	0	0	0
Gemmatimonadetes	1	0	0	0	0	0	0	0	0	0
Planctomycetes	4	0	0	0	0	0	0	0	0	0
Elusimicrobia	2	0	0	0	0	0	0	0	0	0
Synergistetes	2	0	0	0	0	0	0	0	0	0
Cyanobacteria	16	0	0	0	0	0	0	0	0	0
Chlorobi	10	0	0	0	0	0	0	0	0	0
Chloroflexi	11	0	0	0	0	0	0	0	0	0
Deinococcus-Thermus	7	0	0	0	0	0	0	0	0	0
Aquificae	9	0	0	0	0	0	0	0	0	0
Thermotogae	11	0	0	0	0	0	0	0	0	0
Dictyoglomi	2	0	0	0	0	0	0	0	0	0
Nitrospirae	2	0	0	0	0	0	0	0	0	0
Thermobaculum	1	0	0	0	0	0	0	0	0	0
Deferribacteres	3	0	0	0	0	0	0	0	0	0
Euryarchaeota	60	0	0	0	0	0	0	0	0	0
Crenarchaeota	23	0	0	0	0	0	0	0	0	0
Thaumarchaeota	2	0	0	0	0	0	0	0	0	0
Nanoarchaeota	1	0	0	0	0	0	0	0	0	0
Korarchaeota	1	0	0	0	0	0	0	0	0	0
Total	768	0	0	0	0	0	0	0	0	0

(M00161_1)

Phyla	Module completion ratio (%)									
	0--10	10--20	20--30	30--40	40--50	50--60	60--70	70--80	80--90	90--100
Gammaproteobacteria	126	0	0	0	0	0	0	0	0	0
Betaproteobacteria	61	0	0	0	0	0	0	0	0	0
Epsilonproteobacteria	17	0	0	0	0	0	0	0	0	0
Deltaproteobacteria	28	0	0	0	0	0	0	0	0	0
Alphaproteobacteria	91	0	0	0	0	0	0	0	0	0
Magnetococcus	1	0	0	0	0	0	0	0	0	0
Chrysiogenetes	1	0	0	0	0	0	0	0	0	0
Firmicutes	104	0	0	0	0	0	0	0	0	0
Tenericutes	19	0	0	0	0	0	0	0	0	0
Actinobacteria	80	0	0	0	0	0	0	0	0	0
Chlamydiae	8	0	0	0	0	0	0	0	0	0
Spirochaetes	14	0	0	0	0	0	0	0	0	0
Acidobacteria	5	0	0	0	0	0	0	0	0	0
Bacteroidetes	35	0	0	0	0	0	0	0	0	0
Fibrobacteres	1	0	0	0	0	0	0	0	0	0
Fusobacteria	5	0	0	0	0	0	0	0	0	0
Verrucomicrobia	4	0	0	0	0	0	0	0	0	0
Gemmatimonadetes	1	0	0	0	0	0	0	0	0	0
Planctomycetes	4	0	0	0	0	0	0	0	0	0
Elusimicrobia	2	0	0	0	0	0	0	0	0	0
Synergistetes	2	0	0	0	0	0	0	0	0	0
Cyanobacteria	1	0	0	0	0	0	0	0	1	14
Chlorobi	10	0	0	0	0	0	0	0	0	0
Chloroflexi	11	0	0	0	0	0	0	0	0	0
Deinococcus-Thermus	7	0	0	0	0	0	0	0	0	0
Aquificae	9	0	0	0	0	0	0	0	0	0
Thermotogae	11	0	0	0	0	0	0	0	0	0
Dictyoglomi	2	0	0	0	0	0	0	0	0	0
Nitrospirae	2	0	0	0	0	0	0	0	0	0
Thermobaculum	1	0	0	0	0	0	0	0	0	0
Deferribacteres	3	0	0	0	0	0	0	0	0	0
Euryarchaeota	60	0	0	0	0	0	0	0	0	0
Crenarchaeota	23	0	0	0	0	0	0	0	0	0
Thaumarchaeota	2	0	0	0	0	0	0	0	0	0
Nanoarchaeota	1	0	0	0	0	0	0	0	0	0
Korarchaeota	1	0	0	0	0	0	0	0	0	0
Total	753	0	0	0	0	0	0	0	1	14

(M00161_2)

Phyla	Module completion ratio (%)									
	0--10	10--20	20--30	30--40	40--50	50--60	60--70	70--80	80--90	90--100
Gammaproteobacteria	126	0	0	0	0	0	0	0	0	0
Betaproteobacteria	61	0	0	0	0	0	0	0	0	0
Epsilonproteobacteria	17	0	0	0	0	0	0	0	0	0
Deltaproteobacteria	28	0	0	0	0	0	0	0	0	0
Alphaproteobacteria	91	0	0	0	0	0	0	0	0	0
Magnetococcus	1	0	0	0	0	0	0	0	0	0
Chrysiogenetes	1	0	0	0	0	0	0	0	0	0
Firmicutes	104	0	0	0	0	0	0	0	0	0
Tenericutes	19	0	0	0	0	0	0	0	0	0
Actinobacteria	80	0	0	0	0	0	0	0	0	0
Chlamydiae	8	0	0	0	0	0	0	0	0	0
Spirochaetes	14	0	0	0	0	0	0	0	0	0
Acidobacteria	5	0	0	0	0	0	0	0	0	0
Bacteroidetes	35	0	0	0	0	0	0	0	0	0
Fibrobacteres	1	0	0	0	0	0	0	0	0	0
Fusobacteria	5	0	0	0	0	0	0	0	0	0
Verrucomicrobia	4	0	0	0	0	0	0	0	0	0
Gemmatimonadetes	1	0	0	0	0	0	0	0	0	0
Planctomycetes	4	0	0	0	0	0	0	0	0	0
Elusimicrobia	2	0	0	0	0	0	0	0	0	0
Synergistetes	2	0	0	0	0	0	0	0	0	0
Cyanobacteria	16	0	0	0	0	0	0	0	0	0
Chlorobi	10	0	0	0	0	0	0	0	0	0
Chloroflexi	11	0	0	0	0	0	0	0	0	0
Deinococcus-Thermus	7	0	0	0	0	0	0	0	0	0
Aquificae	9	0	0	0	0	0	0	0	0	0
Thermotogae	11	0	0	0	0	0	0	0	0	0
Dictyoglomi	2	0	0	0	0	0	0	0	0	0
Nitrospirae	2	0	0	0	0	0	0	0	0	0
Thermobaculum	1	0	0	0	0	0	0	0	0	0
Deferribacteres	3	0	0	0	0	0	0	0	0	0
Euryarchaeota	60	0	0	0	0	0	0	0	0	0
Crenarchaeota	23	0	0	0	0	0	0	0	0	0
Thaumarchaeota	2	0	0	0	0	0	0	0	0	0
Nanoarchaeota	1	0	0	0	0	0	0	0	0	0
Korarchaeota	1	0	0	0	0	0	0	0	0	0
Total	768	0	0	0	0	0	0	0	0	0

(M00161_3)

Phyla	Module completion ratio (%)									
	0--10	10--20	20--30	30--40	40--50	50--60	60--70	70--80	80--90	90--100
Gammaproteobacteria	126	0	0	0	0	0	0	0	0	0
Betaproteobacteria	61	0	0	0	0	0	0	0	0	0
Epsilonproteobacteria	17	0	0	0	0	0	0	0	0	0
Deltaproteobacteria	28	0	0	0	0	0	0	0	0	0
Alphaproteobacteria	91	0	0	0	0	0	0	0	0	0
Magnetococcus	1	0	0	0	0	0	0	0	0	0
Chrysiogenetes	1	0	0	0	0	0	0	0	0	0
Firmicutes	104	0	0	0	0	0	0	0	0	0
Tenericutes	19	0	0	0	0	0	0	0	0	0
Actinobacteria	80	0	0	0	0	0	0	0	0	0
Chlamydiae	8	0	0	0	0	0	0	0	0	0
Spirochaetes	14	0	0	0	0	0	0	0	0	0
Acidobacteria	5	0	0	0	0	0	0	0	0	0
Bacteroidetes	35	0	0	0	0	0	0	0	0	0
Fibrobacteres	1	0	0	0	0	0	0	0	0	0
Fusobacteria	5	0	0	0	0	0	0	0	0	0
Verrucomicrobia	4	0	0	0	0	0	0	0	0	0
Gemmatimonadetes	1	0	0	0	0	0	0	0	0	0
Planctomycetes	4	0	0	0	0	0	0	0	0	0
Elusimicrobia	2	0	0	0	0	0	0	0	0	0
Synergistetes	2	0	0	0	0	0	0	0	0	0
Cyanobacteria	1	0	0	1	0	0	3	0	0	11
Chlorobi	10	0	0	0	0	0	0	0	0	0
Chloroflexi	11	0	0	0	0	0	0	0	0	0
Deinococcus-Thermus	7	0	0	0	0	0	0	0	0	0
Aquificae	9	0	0	0	0	0	0	0	0	0
Thermotogae	11	0	0	0	0	0	0	0	0	0
Dictyoglomi	2	0	0	0	0	0	0	0	0	0
Nitrospirae	2	0	0	0	0	0	0	0	0	0
Thermobaculum	1	0	0	0	0	0	0	0	0	0
Deferribacteres	3	0	0	0	0	0	0	0	0	0
Euryarchaeota	60	0	0	0	0	0	0	0	0	0
Crenarchaeota	23	0	0	0	0	0	0	0	0	0
Thaumarchaeota	2	0	0	0	0	0	0	0	0	0
Nanoarchaeota	1	0	0	0	0	0	0	0	0	0
Korarchaeota	1	0	0	0	0	0	0	0	0	0
Total	753	0	0	1	0	0	3	0	0	11

(M00162_1)

Phyla	Module completion ratio (%)									
	0--10	10--20	20--30	30--40	40--50	50--60	60--70	70--80	80--90	90--100
Gammaproteobacteria	126	0	0	0	0	0	0	0	0	0
Betaproteobacteria	61	0	0	0	0	0	0	0	0	0
Epsilonproteobacteria	17	0	0	0	0	0	0	0	0	0
Deltaproteobacteria	26	2	0	0	0	0	0	0	0	0
Alphaproteobacteria	91	0	0	0	0	0	0	0	0	0
Magnetococcus	1	0	0	0	0	0	0	0	0	0
Chrysiogenetes	1	0	0	0	0	0	0	0	0	0
Firmicutes	102	0	1	1	0	0	0	0	0	0
Tenericutes	19	0	0	0	0	0	0	0	0	0
Actinobacteria	80	0	0	0	0	0	0	0	0	0
Chlamydiae	7	1	0	0	0	0	0	0	0	0
Spirochaetes	14	0	0	0	0	0	0	0	0	0
Acidobacteria	5	0	0	0	0	0	0	0	0	0
Bacteroidetes	33	2	0	0	0	0	0	0	0	0
Fibrobacteres	1	0	0	0	0	0	0	0	0	0
Fusobacteria	5	0	0	0	0	0	0	0	0	0
Verrucomicrobia	4	0	0	0	0	0	0	0	0	0
Gemmatimonadetes	1	0	0	0	0	0	0	0	0	0
Planctomycetes	4	0	0	0	0	0	0	0	0	0
Elusimicrobia	2	0	0	0	0	0	0	0	0	0
Synergistetes	2	0	0	0	0	0	0	0	0	0
Cyanobacteria	0	0	0	0	0	0	1	4	6	5
Chlorobi	0	0	10	0	0	0	0	0	0	0
Chloroflexi	10	1	0	0	0	0	0	0	0	0
Deinococcus-Thermus	7	0	0	0	0	0	0	0	0	0
Aquificae	9	0	0	0	0	0	0	0	0	0
Thermotogae	11	0	0	0	0	0	0	0	0	0
Dictyoglomi	2	0	0	0	0	0	0	0	0	0
Nitrospirae	1	1	0	0	0	0	0	0	0	0
Thermobaculum	1	0	0	0	0	0	0	0	0	0
Deferribacteres	3	0	0	0	0	0	0	0	0	0
Euryarchaeota	59	1	0	0	0	0	0	0	0	0
Crenarchaeota	23	0	0	0	0	0	0	0	0	0
Thaumarchaeota	2	0	0	0	0	0	0	0	0	0
Nanoarchaeota	1	0	0	0	0	0	0	0	0	0
Korarchaeota	1	0	0	0	0	0	0	0	0	0
Total	732	8	11	1	0	0	1	4	6	5

(M00163_1)

Phyla	Module completion ratio (%)									
	0--10	10--20	20--30	30--40	40--50	50--60	60--70	70--80	80--90	90--100
Gammaproteobacteria	126	0	0	0	0	0	0	0	0	0
Betaproteobacteria	61	0	0	0	0	0	0	0	0	0
Epsilonproteobacteria	17	0	0	0	0	0	0	0	0	0
Deltaproteobacteria	28	0	0	0	0	0	0	0	0	0
Alphaproteobacteria	91	0	0	0	0	0	0	0	0	0
Magnetococcus	1	0	0	0	0	0	0	0	0	0
Chrysiogenetes	1	0	0	0	0	0	0	0	0	0
Firmicutes	104	0	0	0	0	0	0	0	0	0
Tenericutes	19	0	0	0	0	0	0	0	0	0
Actinobacteria	80	0	0	0	0	0	0	0	0	0
Chlamydiae	8	0	0	0	0	0	0	0	0	0
Spirochaetes	14	0	0	0	0	0	0	0	0	0
Acidobacteria	5	0	0	0	0	0	0	0	0	0
Bacteroidetes	35	0	0	0	0	0	0	0	0	0
Fibrobacteres	1	0	0	0	0	0	0	0	0	0
Fusobacteria	5	0	0	0	0	0	0	0	0	0
Verrucomicrobia	4	0	0	0	0	0	0	0	0	0
Gemmatimonadetes	1	0	0	0	0	0	0	0	0	0
Planctomycetes	4	0	0	0	0	0	0	0	0	0
Elusimicrobia	2	0	0	0	0	0	0	0	0	0
Synergistetes	2	0	0	0	0	0	0	0	0	0
Cyanobacteria	0	0	0	0	0	1	0	15	0	0
Chlorobi	10	0	0	0	0	0	0	0	0	0
Chloroflexi	11	0	0	0	0	0	0	0	0	0
Deinococcus-Thermus	7	0	0	0	0	0	0	0	0	0
Aquificae	9	0	0	0	0	0	0	0	0	0
Thermotogae	11	0	0	0	0	0	0	0	0	0
Dictyoglomi	2	0	0	0	0	0	0	0	0	0
Nitrospirae	2	0	0	0	0	0	0	0	0	0
Thermobaculum	1	0	0	0	0	0	0	0	0	0
Deferribacteres	3	0	0	0	0	0	0	0	0	0
Euryarchaeota	60	0	0	0	0	0	0	0	0	0
Crenarchaeota	23	0	0	0	0	0	0	0	0	0
Thaumarchaeota	2	0	0	0	0	0	0	0	0	0
Nanoarchaeota	1	0	0	0	0	0	0	0	0	0
Korarchaeota	1	0	0	0	0	0	0	0	0	0
Total	752	0	0	0	0	1	0	15	0	0

(M00163_2)

Phyla	Module completion ratio (%)									
	0--10	10--20	20--30	30--40	40--50	50--60	60--70	70--80	80--90	90--100
Gammaproteobacteria	126	0	0	0	0	0	0	0	0	0
Betaproteobacteria	61	0	0	0	0	0	0	0	0	0
Epsilonproteobacteria	17	0	0	0	0	0	0	0	0	0
Deltaproteobacteria	28	0	0	0	0	0	0	0	0	0
Alphaproteobacteria	91	0	0	0	0	0	0	0	0	0
Magnetococcus	1	0	0	0	0	0	0	0	0	0
Chrysiogenetes	1	0	0	0	0	0	0	0	0	0
Firmicutes	104	0	0	0	0	0	0	0	0	0
Tenericutes	19	0	0	0	0	0	0	0	0	0
Actinobacteria	80	0	0	0	0	0	0	0	0	0
Chlamydiae	8	0	0	0	0	0	0	0	0	0
Spirochaetes	14	0	0	0	0	0	0	0	0	0
Acidobacteria	5	0	0	0	0	0	0	0	0	0
Bacteroidetes	35	0	0	0	0	0	0	0	0	0
Fibrobacteres	1	0	0	0	0	0	0	0	0	0
Fusobacteria	5	0	0	0	0	0	0	0	0	0
Verrucomicrobia	4	0	0	0	0	0	0	0	0	0
Gemmatimonadetes	1	0	0	0	0	0	0	0	0	0
Planctomycetes	4	0	0	0	0	0	0	0	0	0
Elusimicrobia	2	0	0	0	0	0	0	0	0	0
Synergistetes	2	0	0	0	0	0	0	0	0	0
Cyanobacteria	0	0	0	0	0	0	1	0	3	12
Chlorobi	10	0	0	0	0	0	0	0	0	0
Chloroflexi	11	0	0	0	0	0	0	0	0	0
Deinococcus-Thermus	7	0	0	0	0	0	0	0	0	0
Aquificae	9	0	0	0	0	0	0	0	0	0
Thermotogae	11	0	0	0	0	0	0	0	0	0
Dictyoglomi	2	0	0	0	0	0	0	0	0	0
Nitrospirae	2	0	0	0	0	0	0	0	0	0
Thermobaculum	1	0	0	0	0	0	0	0	0	0
Deferribacteres	3	0	0	0	0	0	0	0	0	0
Euryarchaeota	60	0	0	0	0	0	0	0	0	0
Crenarchaeota	23	0	0	0	0	0	0	0	0	0
Thaumarchaeota	2	0	0	0	0	0	0	0	0	0
Nanoarchaeota	1	0	0	0	0	0	0	0	0	0
Korarchaeota	1	0	0	0	0	0	0	0	0	0
Total	752	0	0	0	0	0	1	0	3	12

(M00164_1)

Phyla	Module completion ratio (%)									
	0--10	10--20	20--30	30--40	40--50	50--60	60--70	70--80	80--90	90--100
Gammaproteobacteria	1	0	0	0	0	0	1	0	1	123
Betaproteobacteria	1	0	0	0	0	0	0	0	0	60
Epsilonproteobacteria	0	0	0	0	0	0	0	0	2	15
Deltaproteobacteria	0	0	0	0	0	0	0	0	0	28
Alphaproteobacteria	1	0	0	0	0	0	0	0	0	90
Magnetococcus	0	0	0	0	0	0	0	0	0	1
Chrysiogenetes	0	0	0	0	0	0	0	0	0	1
Firmicutes	5	1	0	0	0	0	0	0	4	94
Tenericutes	3	0	0	0	0	0	0	0	1	15
Actinobacteria	0	0	0	0	0	0	0	1	2	77
Chlamydiae	6	0	0	0	0	0	0	0	0	2
Spirochaetes	10	0	0	0	0	0	0	0	0	4
Acidobacteria	0	0	0	0	0	0	0	0	0	5
Bacteroidetes	1	0	0	0	0	0	0	0	1	33
Fibrobacteres	1	0	0	0	0	0	0	0	0	0
Fusobacteria	0	0	0	0	0	0	0	0	0	5
Verrucomicrobia	0	0	0	0	0	0	0	0	0	4
Gemmatimonadetes	0	0	0	0	0	0	0	0	0	1
Planctomycetes	0	0	0	0	0	0	0	1	0	3
Elusimicrobia	0	0	0	0	0	0	0	0	1	1
Synergistetes	1	1	0	0	0	0	0	0	0	0
Cyanobacteria	0	0	0	0	0	0	0	0	0	16
Chlorobi	0	0	0	0	0	0	0	0	1	9
Chloroflexi	0	0	0	0	0	0	0	0	1	10
Deinococcus-Thermus	7	0	0	0	0	0	0	0	0	0
Aquificae	0	0	0	0	0	0	0	0	0	9
Thermotogae	0	0	0	0	0	0	0	0	0	11
Dictyoglomi	0	0	0	0	0	0	0	0	2	0
Nitrospirae	0	0	0	0	0	0	0	0	0	2
Thermobaculum	0	0	0	0	0	0	0	0	0	1
Deferribacteres	0	0	0	0	0	0	0	0	0	3
Euryarchaeota	58	0	0	0	0	0	0	0	2	0
Crenarchaeota	23	0	0	0	0	0	0	0	0	0
Thaumarchaeota	2	0	0	0	0	0	0	0	0	0
Nanoarchaeota	1	0	0	0	0	0	0	0	0	0
Korarchaeota	1	0	0	0	0	0	0	0	0	0
Total	122	2	0	0	0	0	1	2	18	623

(M00165_1)

Phyla	Module completion ratio (%)									
	0--10	10--20	20--30	30--40	40--50	50--60	60--70	70--80	80--90	90--100
Gammaproteobacteria	0	1	0	0	0	11	47	58	9	0
Betaproteobacteria	1	0	0	1	0	0	38	10	11	0
Epsilonproteobacteria	0	0	0	0	0	0	17	0	0	0
Deltaproteobacteria	0	0	0	1	6	3	18	0	0	0
Alphaproteobacteria	10	1	0	0	1	7	46	11	15	0
Magnetococcus	0	0	0	0	0	1	0	0	0	0
Chrysiogenetes	0	0	0	0	0	0	1	0	0	0
Firmicutes	0	0	1	8	7	25	62	0	1	0
Tenericutes	0	1	2	1	0	15	0	0	0	0
Actinobacteria	0	0	0	1	0	13	63	1	2	0
Chlamydiae	0	0	0	0	0	8	0	0	0	0
Spirochaetes	0	0	0	7	0	2	5	0	0	0
Acidobacteria	0	0	0	1	1	0	3	0	0	0
Bacteroidetes	1	0	0	1	0	2	29	2	0	0
Fibrobacteres	0	0	0	0	0	1	0	0	0	0
Fusobacteria	0	0	0	0	0	1	4	0	0	0
Verrucomicrobia	0	0	0	0	1	2	0	0	1	0
Gemmatimonadetes	0	0	0	0	1	0	0	0	0	0
Planctomycetes	0	0	0	0	1	2	1	0	0	0
Elusimicrobia	0	0	0	0	0	2	0	0	0	0
Synergistetes	0	0	0	0	0	2	0	0	0	0
Cyanobacteria	0	0	0	0	0	1	0	0	0	15
Chlorobi	0	0	0	0	0	0	10	0	0	0
Chloroflexi	0	0	0	2	5	2	2	0	0	0
Deinococcus-Thermus	0	0	0	0	0	0	7	0	0	0
Aquificae	0	0	0	0	0	4	5	0	0	0
Thermotogae	0	0	0	0	1	6	3	1	0	0
Dictyoglomi	0	0	0	0	0	2	0	0	0	0
Nitrospirae	0	0	0	0	1	0	1	0	0	0
Thermobaculum	0	0	0	0	0	1	0	0	0	0
Deferribacteres	0	0	0	0	0	0	3	0	0	0
Euryarchaeota	0	0	0	2	13	24	20	0	1	0
Crenarchaeota	0	0	1	0	15	0	7	0	0	0
Thaumarchaeota	0	0	0	0	0	0	2	0	0	0
Nanoarchaeota	1	0	0	0	0	0	0	0	0	0
Korarchaeota	0	0	0	0	1	0	0	0	0	0
Total	13	3	4	25	54	137	394	83	40	15

(M00166_1)

Phyla	Module completion ratio (%)									
	0--10	10--20	20--30	30--40	40--50	50--60	60--70	70--80	80--90	90--100
Gammaproteobacteria	1	0	0	115	0	0	10	0	0	0
Betaproteobacteria	1	0	0	48	0	0	12	0	0	0
Epsilonproteobacteria	0	0	0	17	0	0	0	0	0	0
Deltaproteobacteria	1	0	0	27	0	0	0	0	0	0
Alphaproteobacteria	10	0	0	65	0	0	16	0	0	0
Magnetococcus	0	0	0	1	0	0	0	0	0	0
Chrysiogenetes	0	0	0	1	0	0	0	0	0	0
Firmicutes	5	0	0	97	0	0	2	0	0	0
Tenericutes	1	0	0	18	0	0	0	0	0	0
Actinobacteria	1	0	0	76	0	0	3	0	0	0
Chlamydiae	0	0	0	8	0	0	0	0	0	0
Spirochaetes	0	0	0	13	0	0	1	0	0	0
Acidobacteria	0	0	0	5	0	0	0	0	0	0
Bacteroidetes	1	0	0	32	0	0	2	0	0	0
Fibrobacteres	0	0	0	1	0	0	0	0	0	0
Fusobacteria	0	0	0	5	0	0	0	0	0	0
Verrucomicrobia	0	0	0	3	0	0	1	0	0	0
Gemmatimonadetes	0	0	0	1	0	0	0	0	0	0
Planctomycetes	0	0	0	4	0	0	0	0	0	0
Elusimicrobia	0	0	0	2	0	0	0	0	0	0
Synergistetes	0	0	0	2	0	0	0	0	0	0
Cyanobacteria	0	0	0	1	0	0	1	0	0	14
Chlorobi	0	0	0	10	0	0	0	0	0	0
Chloroflexi	0	0	0	10	0	0	1	0	0	0
Deinococcus-Thermus	0	0	0	7	0	0	0	0	0	0
Aquificae	0	0	0	9	0	0	0	0	0	0
Thermotogae	2	0	0	8	0	0	1	0	0	0
Dictyoglomi	0	0	0	2	0	0	0	0	0	0
Nitrospirae	0	0	0	2	0	0	0	0	0	0
Thermobaculum	0	0	0	1	0	0	0	0	0	0
Deferribacteres	0	0	0	3	0	0	0	0	0	0
Euryarchaeota	0	0	0	2	0	0	58	0	0	0
Crenarchaeota	1	0	0	0	0	0	22	0	0	0
Thaumarchaeota	0	0	0	0	0	0	2	0	0	0
Nanoarchaeota	1	0	0	0	0	0	0	0	0	0
Korarchaeota	0	0	0	0	0	0	1	0	0	0
Total	25	0	0	596	0	0	133	0	0	14

(M00167_1)

Phyla	Module completion ratio (%)									
	0--10	10--20	20--30	30--40	40--50	50--60	60--70	70--80	80--90	90--100
Gammaproteobacteria	0	0	1	0	0	0	11	48	66	0
Betaproteobacteria	1	0	0	1	0	0	0	39	20	0
Epsilonproteobacteria	0	0	0	0	0	0	0	17	0	0
Deltaproteobacteria	0	0	0	0	0	7	3	18	0	0
Alphaproteobacteria	1	10	0	0	0	1	7	47	25	0
Magnetococcus	0	0	0	0	0	0	1	0	0	0
Chrysiogenetes	0	0	0	0	0	0	0	1	0	0
Firmicutes	0	0	0	9	0	5	26	63	1	0
Tenericutes	0	0	3	1	0	0	15	0	0	0
Actinobacteria	0	0	0	1	0	0	12	65	2	0
Chlamydiae	0	0	0	0	0	0	8	0	0	0
Spirochaetes	0	0	0	7	0	0	3	4	0	0
Acidobacteria	0	0	0	1	0	1	0	3	0	0
Bacteroidetes	1	0	0	1	0	0	2	31	0	0
Fibrobacteres	0	0	0	0	0	0	1	0	0	0
Fusobacteria	0	0	0	0	0	0	1	4	0	0
Verrucomicrobia	0	0	0	0	0	1	2	0	1	0
Gemmatimonadetes	0	0	0	0	0	1	0	0	0	0
Planctomycetes	0	0	0	0	0	1	2	1	0	0
Elusimicrobia	0	0	0	0	0	0	2	0	0	0
Synergistetes	0	0	0	0	0	0	2	0	0	0
Cyanobacteria	0	0	0	0	0	0	1	0	0	15
Chlorobi	0	0	0	0	0	0	0	10	0	0
Chloroflexi	0	0	0	3	0	4	2	2	0	0
Deinococcus-Thermus	0	0	0	0	0	0	0	7	0	0
Aquificae	0	0	0	0	0	0	4	5	0	0
Thermotogae	0	0	0	0	0	0	6	5	0	0
Dictyoglomi	0	0	0	0	0	0	2	0	0	0
Nitrospirae	0	0	0	0	0	1	0	1	0	0
Thermobaculum	0	0	0	0	0	0	1	0	0	0
Deferribacteres	0	0	0	0	0	0	0	3	0	0
Euryarchaeota	0	0	0	15	0	24	20	0	1	0
Crenarchaeota	0	1	0	14	0	0	8	0	0	0
Thaumarchaeota	0	0	0	0	0	0	2	0	0	0
Nanoarchaeota	1	0	0	0	0	0	0	0	0	0
Korarchaeota	0	0	0	1	0	0	0	0	0	0
Total	4	11	4	54	0	46	144	374	116	15

(M00168_1)

Phyla	Module completion ratio (%)									
	0--10	10--20	20--30	30--40	40--50	50--60	60--70	70--80	80--90	90--100
Gammaproteobacteria	11	0	0	0	0	26	0	0	0	89
Betaproteobacteria	3	0	0	0	0	7	0	0	0	51
Epsilonproteobacteria	2	0	0	0	0	13	0	0	0	2
Deltaproteobacteria	6	0	0	0	0	13	0	0	0	9
Alphaproteobacteria	1	0	0	0	0	52	0	0	0	38
Magnetococcus	0	0	0	0	0	1	0	0	0	0
Chrysiogenetes	0	0	0	0	0	1	0	0	0	0
Firmicutes	55	0	0	0	0	45	0	0	0	4
Tenericutes	18	0	0	0	0	1	0	0	0	0
Actinobacteria	6	0	0	0	0	31	0	0	0	43
Chlamydiae	1	0	0	0	0	7	0	0	0	0
Spirochaetes	11	0	0	0	0	2	0	0	0	1
Acidobacteria	0	0	0	0	0	2	0	0	0	3
Bacteroidetes	3	0	0	0	0	14	0	0	0	18
Fibrobacteres	0	0	0	0	0	1	0	0	0	0
Fusobacteria	3	0	0	0	0	2	0	0	0	0
Verrucomicrobia	0	0	0	0	0	2	0	0	0	2
Gemmatimonadetes	0	0	0	0	0	0	0	0	0	1
Planctomycetes	0	0	0	0	0	1	0	0	0	3
Elusimicrobia	2	0	0	0	0	0	0	0	0	0
Synergistetes	2	0	0	0	0	0	0	0	0	0
Cyanobacteria	1	0	0	0	0	7	0	0	0	8
Chlorobi	0	0	0	0	0	3	0	0	0	7
Chloroflexi	1	0	0	0	0	4	0	0	0	6
Deinococcus-Thermus	0	0	0	0	0	0	0	0	0	7
Aquificae	0	0	0	0	0	9	0	0	0	0
Thermotogae	11	0	0	0	0	0	0	0	0	0
Dictyoglomi	1	0	0	0	0	1	0	0	0	0
Nitrospirae	0	0	0	0	0	2	0	0	0	0
Thermobaculum	0	0	0	0	0	1	0	0	0	0
Deferribacteres	0	0	0	0	0	3	0	0	0	0
Euryarchaeota	9	0	0	0	0	28	0	0	0	23
Crenarchaeota	6	0	0	0	0	4	0	0	0	13
Thaumarchaeota	0	0	0	0	0	2	0	0	0	0
Nanoarchaeota	1	0	0	0	0	0	0	0	0	0
Korarchaeota	1	0	0	0	0	0	0	0	0	0
Total	155	0	0	0	0	285	0	0	0	328

(M00169_1)

Phyla	Module completion ratio (%)									
	0--10	10--20	20--30	30--40	40--50	50--60	60--70	70--80	80--90	90--100
Gammaproteobacteria	17	0	0	0	0	106	0	0	0	3
Betaproteobacteria	5	0	0	0	0	48	0	0	0	8
Epsilonproteobacteria	8	0	0	0	0	9	0	0	0	0
Deltaproteobacteria	2	0	0	0	0	12	0	0	0	14
Alphaproteobacteria	3	0	0	0	0	5	0	0	0	83
Magnetococcus	0	0	0	0	0	0	0	0	0	1
Chrysiogenetes	0	0	0	0	0	0	0	0	0	1
Firmicutes	47	0	0	0	0	57	0	0	0	0
Tenericutes	19	0	0	0	0	0	0	0	0	0
Actinobacteria	39	0	0	0	0	41	0	0	0	0
Chlamydiae	8	0	0	0	0	0	0	0	0	0
Spirochaetes	8	0	0	0	0	4	0	0	0	2
Acidobacteria	2	0	0	0	0	2	0	0	0	1
Bacteroidetes	4	0	0	0	0	22	0	0	0	9
Fibrobacteres	0	0	0	0	0	1	0	0	0	0
Fusobacteria	2	0	0	0	0	3	0	0	0	0
Verrucomicrobia	1	0	0	0	0	1	0	0	0	2
Gemmatimonadetes	0	0	0	0	0	1	0	0	0	0
Planctomycetes	0	0	0	0	0	4	0	0	0	0
Elusimicrobia	1	0	0	0	0	1	0	0	0	0
Synergistetes	0	0	0	0	0	2	0	0	0	0
Cyanobacteria	15	0	0	0	0	1	0	0	0	0
Chlorobi	1	0	0	0	0	9	0	0	0	0
Chloroflexi	4	0	0	0	0	7	0	0	0	0
Deinococcus-Thermus	4	0	0	0	0	3	0	0	0	0
Aquificae	6	0	0	0	0	3	0	0	0	0
Thermotogae	0	0	0	0	0	11	0	0	0	0
Dictyoglomi	0	0	0	0	0	2	0	0	0	0
Nitrospirae	0	0	0	0	0	2	0	0	0	0
Thermobaculum	1	0	0	0	0	0	0	0	0	0
Deferribacteres	0	0	0	0	0	0	0	0	0	3
Euryarchaeota	37	0	0	0	0	23	0	0	0	0
Crenarchaeota	16	0	0	0	0	7	0	0	0	0
Thaumarchaeota	0	0	0	0	0	2	0	0	0	0
Nanoarchaeota	1	0	0	0	0	0	0	0	0	0
Korarchaeota	0	0	0	0	0	1	0	0	0	0
Total	251	0	0	0	0	390	0	0	0	127

(M00170_1)

Phyla	Module completion ratio (%)									
	0--10	10--20	20--30	30--40	40--50	50--60	60--70	70--80	80--90	90--100
Gammaproteobacteria	10	0	0	35	0	0	81	0	0	0
Betaproteobacteria	6	0	0	55	0	0	0	0	0	0
Epsilonproteobacteria	2	0	0	15	0	0	0	0	0	0
Deltaproteobacteria	15	0	0	9	0	0	4	0	0	0
Alphaproteobacteria	29	0	0	34	0	0	28	0	0	0
Magnetococcus	0	0	0	1	0	0	0	0	0	0
Chrysiogenetes	0	0	0	1	0	0	0	0	0	0
Firmicutes	38	0	0	59	0	0	7	0	0	0
Tenericutes	18	0	0	1	0	0	0	0	0	0
Actinobacteria	23	0	0	56	0	0	1	0	0	0
Chlamydiae	8	0	0	0	0	0	0	0	0	0
Spirochaetes	11	0	0	2	0	0	1	0	0	0
Acidobacteria	0	0	0	4	0	0	1	0	0	0
Bacteroidetes	2	0	0	18	0	0	15	0	0	0
Fibrobacteres	1	0	0	0	0	0	0	0	0	0
Fusobacteria	1	0	0	3	0	0	1	0	0	0
Verrucomicrobia	2	0	0	1	0	0	1	0	0	0
Gemmatimonadetes	0	0	0	0	0	0	1	0	0	0
Planctomycetes	0	0	0	2	0	0	2	0	0	0
Elusimicrobia	1	0	0	1	0	0	0	0	0	0
Synergistetes	0	0	0	2	0	0	0	0	0	0
Cyanobacteria	1	0	0	13	0	0	2	0	0	0
Chlorobi	3	0	0	7	0	0	0	0	0	0
Chloroflexi	3	0	0	7	0	0	1	0	0	0
Deinococcus-Thermus	0	0	0	1	0	0	6	0	0	0
Aquificae	9	0	0	0	0	0	0	0	0	0
Thermotogae	7	0	0	4	0	0	0	0	0	0
Dictyoglomi	1	0	0	1	0	0	0	0	0	0
Nitrospirae	2	0	0	0	0	0	0	0	0	0
Thermobaculum	0	0	0	1	0	0	0	0	0	0
Deferribacteres	0	0	0	3	0	0	0	0	0	0
Euryarchaeota	30	0	0	26	0	0	4	0	0	0
Crenarchaeota	7	0	0	16	0	0	0	0	0	0
Thaumarchaeota	0	0	0	2	0	0	0	0	0	0
Nanoarchaeota	1	0	0	0	0	0	0	0	0	0
Korarchaeota	0	0	0	1	0	0	0	0	0	0
Total	231	0	0	381	0	0	156	0	0	0

(M00171_1)

Phyla	Module completion ratio (%)									
	0--10	10--20	20--30	30--40	40--50	50--60	60--70	70--80	80--90	90--100
Gammaproteobacteria	11	21	92	0	2	0	0	0	0	0
Betaproteobacteria	3	6	45	0	7	0	0	0	0	0
Epsilonproteobacteria	2	13	2	0	0	0	0	0	0	0
Deltaproteobacteria	6	0	14	0	8	0	0	0	0	0
Alphaproteobacteria	1	3	39	0	47	1	0	0	0	0
Magnetococcus	0	0	1	0	0	0	0	0	0	0
Chrysiogenetes	0	0	1	0	0	0	0	0	0	0
Firmicutes	14	70	17	0	3	0	0	0	0	0
Tenericutes	18	1	0	0	0	0	0	0	0	0
Actinobacteria	4	22	26	0	28	0	0	0	0	0
Chlamydiae	1	7	0	0	0	0	0	0	0	0
Spirochaetes	7	6	1	0	0	0	0	0	0	0
Acidobacteria	0	1	3	0	1	0	0	0	0	0
Bacteroidetes	3	5	24	0	3	0	0	0	0	0
Fibrobacteres	0	1	0	0	0	0	0	0	0	0
Fusobacteria	0	5	0	0	0	0	0	0	0	0
Verrucomicrobia	0	0	3	0	1	0	0	0	0	0
Gemmatimonadetes	0	0	1	0	0	0	0	0	0	0
Planctomycetes	0	0	1	0	3	0	0	0	0	0
Elusimicrobia	1	1	0	0	0	0	0	0	0	0
Synergistetes	0	2	0	0	0	0	0	0	0	0
Cyanobacteria	1	7	8	0	0	0	0	0	0	0
Chlorobi	0	1	2	0	7	0	0	0	0	0
Chloroflexi	0	4	2	0	5	0	0	0	0	0
Deinococcus-Thermus	0	0	5	0	2	0	0	0	0	0
Aquificae	0	7	2	0	0	0	0	0	0	0
Thermotogae	0	11	0	0	0	0	0	0	0	0
Dictyoglomi	0	1	1	0	0	0	0	0	0	0
Nitrospirae	0	0	2	0	0	0	0	0	0	0
Thermobaculum	0	1	0	0	0	0	0	0	0	0
Deferribacteres	0	0	3	0	0	0	0	0	0	0
Euryarchaeota	7	26	22	0	5	0	0	0	0	0
Crenarchaeota	6	2	11	0	4	0	0	0	0	0
Thaumarchaeota	0	0	2	0	0	0	0	0	0	0
Nanoarchaeota	1	0	0	0	0	0	0	0	0	0
Korarchaeota	0	1	0	0	0	0	0	0	0	0
Total	86	225	330	0	126	1	0	0	0	0

(M00172_1)

Phyla	Module completion ratio (%)									
	0--10	10--20	20--30	30--40	40--50	50--60	60--70	70--80	80--90	90--100
Gammaproteobacteria	10	0	22	0	0	91	0	3	0	0
Betaproteobacteria	2	0	7	0	0	44	0	8	0	0
Epsilonproteobacteria	6	0	11	0	0	0	0	0	0	0
Deltaproteobacteria	1	0	9	0	0	13	0	5	0	0
Alphaproteobacteria	1	0	5	0	0	47	0	38	0	0
Magnetococcus	0	0	0	0	0	1	0	0	0	0
Chrysiogenetes	0	0	0	0	0	1	0	0	0	0
Firmicutes	32	0	65	0	0	7	0	0	0	0
Tenericutes	18	0	1	0	0	0	0	0	0	0
Actinobacteria	14	0	36	0	0	30	0	0	0	0
Chlamydiae	8	0	0	0	0	0	0	0	0	0
Spirochaetes	8	0	3	0	0	3	0	0	0	0
Acidobacteria	0	0	2	0	0	3	0	0	0	0
Bacteroidetes	2	0	10	0	0	21	0	2	0	0
Fibrobacteres	0	0	1	0	0	0	0	0	0	0
Fusobacteria	0	0	5	0	0	0	0	0	0	0
Verrucomicrobia	0	0	1	0	0	3	0	0	0	0
Gemmatimonadetes	0	0	0	0	0	1	0	0	0	0
Planctomycetes	0	0	1	0	0	3	0	0	0	0
Elusimicrobia	1	0	1	0	0	0	0	0	0	0
Synergistetes	0	0	2	0	0	0	0	0	0	0
Cyanobacteria	1	0	14	0	0	1	0	0	0	0
Chlorobi	1	0	2	0	0	7	0	0	0	0
Chloroflexi	3	0	3	0	0	5	0	0	0	0
Deinococcus-Thermus	0	0	4	0	0	3	0	0	0	0
Aquificae	6	0	3	0	0	0	0	0	0	0
Thermotogae	0	0	11	0	0	0	0	0	0	0
Dictyoglomi	0	0	1	0	0	1	0	0	0	0
Nitrospirae	0	0	2	0	0	0	0	0	0	0
Thermobaculum	0	0	1	0	0	0	0	0	0	0
Deferribacteres	0	0	0	0	0	3	0	0	0	0
Euryarchaeota	24	0	19	0	0	17	0	0	0	0
Crenarchaeota	7	0	10	0	0	6	0	0	0	0
Thaumarchaeota	0	0	2	0	0	0	0	0	0	0
Nanoarchaeota	1	0	0	0	0	0	0	0	0	0
Korarchaeota	0	0	1	0	0	0	0	0	0	0
Total	146	0	255	0	0	311	0	56	0	0

(M00173_1)

Phyla	Module completion ratio (%)									
	0--10	10--20	20--30	30--40	40--50	50--60	60--70	70--80	80--90	90--100
Gammaproteobacteria	1	1	6	0	7	28	49	0	33	1
Betaproteobacteria	1	0	0	0	0	9	43	0	5	3
Epsilonproteobacteria	0	0	0	0	0	3	1	0	5	8
Deltaproteobacteria	0	0	1	0	0	5	5	0	7	10
Alphaproteobacteria	1	0	1	0	1	30	46	0	8	4
Magnetococcus	0	0	0	0	0	0	0	0	1	0
Chrysiogenetes	0	0	0	0	0	0	0	0	1	0
Firmicutes	5	11	21	0	13	24	11	0	18	1
Tenericutes	19	0	0	0	0	0	0	0	0	0
Actinobacteria	0	1	2	0	4	16	19	0	33	5
Chlamydiae	0	0	0	0	6	2	0	0	0	0
Spirochaetes	7	1	2	0	0	4	0	0	0	0
Acidobacteria	0	0	0	0	0	2	1	0	0	2
Bacteroidetes	1	0	1	0	0	17	9	0	6	1
Fibrobacteres	0	0	0	0	0	1	0	0	0	0
Fusobacteria	0	2	1	0	1	1	0	0	0	0
Verrucomicrobia	0	0	0	0	0	0	1	0	2	1
Gemmatimonadetes	0	0	0	0	0	0	1	0	0	0
Planctomycetes	0	0	0	0	0	1	2	0	1	0
Elusimicrobia	0	0	2	0	0	0	0	0	0	0
Synergistetes	0	0	0	0	1	1	0	0	0	0
Cyanobacteria	1	0	1	0	3	4	2	0	5	0
Chlorobi	0	0	0	0	0	0	0	0	0	10
Chloroflexi	0	0	2	0	1	2	2	0	2	2
Deinococcus-Thermus	0	0	0	0	0	0	4	0	2	1
Aquificae	0	0	0	0	0	0	1	0	0	8
Thermotogae	0	0	3	0	8	0	0	0	0	0
Dictyoglomi	0	0	0	0	1	1	0	0	0	0
Nitrospirae	0	0	0	0	0	0	1	0	1	0
Thermobaculum	0	0	0	0	0	1	0	0	0	0
Deferribacteres	0	0	0	0	0	0	0	0	0	3
Euryarchaeota	0	1	4	0	6	25	7	0	17	0
Crenarchaeota	0	0	3	0	5	0	1	0	3	11
Thaumarchaeota	0	0	0	0	0	2	0	0	0	0
Nanoarchaeota	1	0	0	0	0	0	0	0	0	0
Korarchaeota	0	0	0	0	0	1	0	0	0	0
Total	37	17	50	0	57	180	206	0	150	71

(M00174_1)

Phyla	Module completion ratio (%)									
	0--10	10--20	20--30	30--40	40--50	50--60	60--70	70--80	80--90	90--100
Gammaproteobacteria	19	0	82	0	0	18	0	7	0	0
Betaproteobacteria	3	0	23	0	0	17	0	14	0	4
Epsilonproteobacteria	8	0	9	0	0	0	0	0	0	0
Deltaproteobacteria	4	0	21	0	0	3	0	0	0	0
Alphaproteobacteria	27	0	37	0	0	17	0	10	0	0
Magnetococcus	1	0	0	0	0	0	0	0	0	0
Chrysiogenetes	0	0	1	0	0	0	0	0	0	0
Firmicutes	64	0	36	0	0	4	0	0	0	0
Tenericutes	19	0	0	0	0	0	0	0	0	0
Actinobacteria	13	0	39	0	0	23	0	5	0	0
Chlamydiae	7	0	1	0	0	0	0	0	0	0
Spirochaetes	10	0	4	0	0	0	0	0	0	0
Acidobacteria	0	0	3	0	0	2	0	0	0	0
Bacteroidetes	10	0	23	0	0	2	0	0	0	0
Fibrobacteres	1	0	0	0	0	0	0	0	0	0
Fusobacteria	5	0	0	0	0	0	0	0	0	0
Verrucomicrobia	1	0	2	0	0	1	0	0	0	0
Gemmatimonadetes	0	0	0	0	0	1	0	0	0	0
Planctomycetes	1	0	2	0	0	1	0	0	0	0
Elusimicrobia	2	0	0	0	0	0	0	0	0	0
Synergistetes	2	0	0	0	0	0	0	0	0	0
Cyanobacteria	10	0	6	0	0	0	0	0	0	0
Chlorobi	5	0	4	0	0	1	0	0	0	0
Chloroflexi	9	0	2	0	0	0	0	0	0	0
Deinococcus-Thermus	3	0	4	0	0	0	0	0	0	0
Aquificae	6	0	3	0	0	0	0	0	0	0
Thermotogae	10	0	1	0	0	0	0	0	0	0
Dictyoglomi	2	0	0	0	0	0	0	0	0	0
Nitrospirae	2	0	0	0	0	0	0	0	0	0
Thermobaculum	1	0	0	0	0	0	0	0	0	0
Deferribacteres	2	0	1	0	0	0	0	0	0	0
Euryarchaeota	34	0	24	0	0	2	0	0	0	0
Crenarchaeota	23	0	0	0	0	0	0	0	0	0
Thaumarchaeota	2	0	0	0	0	0	0	0	0	0
Nanoarchaeota	1	0	0	0	0	0	0	0	0	0
Korarchaeota	1	0	0	0	0	0	0	0	0	0
Total	308	0	328	0	0	92	0	36	0	4

(M00175_1)

Phyla	Module completion ratio (%)									
	0--10	10--20	20--30	30--40	40--50	50--60	60--70	70--80	80--90	90--100
Gammaproteobacteria	116	0	0	0	0	0	0	0	0	10
Betaproteobacteria	50	0	0	0	0	0	0	0	0	11
Epsilonproteobacteria	15	0	0	0	0	0	0	0	0	2
Deltaproteobacteria	12	0	0	0	0	0	0	0	0	16
Alphaproteobacteria	69	0	0	0	0	0	0	0	0	22
Magnetococcus	0	0	0	0	0	0	0	0	0	1
Chrysiogenetes	0	0	0	0	0	0	0	0	0	1
Firmicutes	83	0	0	0	0	0	0	0	0	21
Tenericutes	19	0	0	0	0	0	0	0	0	0
Actinobacteria	78	0	0	0	0	0	0	0	0	2
Chlamydiae	8	0	0	0	0	0	0	0	0	0
Spirochaetes	13	0	0	0	0	0	0	0	0	1
Acidobacteria	5	0	0	0	0	0	0	0	0	0
Bacteroidetes	33	0	0	0	0	0	0	0	0	2
Fibrobacteres	0	0	0	0	0	0	0	0	0	1
Fusobacteria	3	0	0	0	0	0	0	0	0	2
Verrucomicrobia	2	0	0	0	0	0	0	0	0	2
Gemmatimonadetes	1	0	0	0	0	0	0	0	0	0
Planctomycetes	4	0	0	0	0	0	0	0	0	0
Elusimicrobia	2	0	0	0	0	0	0	0	0	0
Synergistetes	2	0	0	0	0	0	0	0	0	0
Cyanobacteria	8	0	0	0	0	0	0	0	0	8
Chlorobi	0	0	0	0	0	0	0	0	0	10
Chloroflexi	8	0	0	0	0	0	0	0	0	3
Deinococcus-Thermus	7	0	0	0	0	0	0	0	0	0
Aquificae	7	0	0	0	0	0	0	0	0	2
Thermotogae	11	0	0	0	0	0	0	0	0	0
Dictyoglomi	2	0	0	0	0	0	0	0	0	0
Nitrospirae	1	0	0	0	0	0	0	0	0	1
Thermobaculum	1	0	0	0	0	0	0	0	0	0
Deferribacteres	1	0	0	0	0	0	0	0	0	2
Euryarchaeota	27	0	0	0	0	0	0	0	0	33
Crenarchaeota	23	0	0	0	0	0	0	0	0	0
Thaumarchaeota	2	0	0	0	0	0	0	0	0	0
Nanoarchaeota	1	0	0	0	0	0	0	0	0	0
Korarchaeota	1	0	0	0	0	0	0	0	0	0
Total	615	0	0	0	0	0	0	0	0	153

(M00176_1)

Phyla	Module completion ratio (%)									
	0--10	10--20	20--30	30--40	40--50	50--60	60--70	70--80	80--90	90--100
Gammaproteobacteria	23	0	0	7	0	0	15	0	0	81
Betaproteobacteria	4	0	0	17	0	0	27	0	0	13
Epsilonproteobacteria	10	0	0	6	0	0	1	0	0	0
Deltaproteobacteria	7	0	0	13	0	0	6	0	0	2
Alphaproteobacteria	25	0	0	11	0	0	29	0	0	26
Magnetococcus	0	0	0	1	0	0	0	0	0	0
Chrysiogenetes	0	0	0	1	0	0	0	0	0	0
Firmicutes	63	0	0	15	0	0	9	0	0	17
Tenericutes	19	0	0	0	0	0	0	0	0	0
Actinobacteria	24	0	0	20	0	0	36	0	0	0
Chlamydiae	8	0	0	0	0	0	0	0	0	0
Spirochaetes	10	0	0	2	0	0	2	0	0	0
Acidobacteria	0	0	0	2	0	0	3	0	0	0
Bacteroidetes	8	0	0	10	0	0	15	0	0	2
Fibrobacteres	0	0	0	1	0	0	0	0	0	0
Fusobacteria	3	0	0	2	0	0	0	0	0	0
Verrucomicrobia	0	0	0	0	0	0	0	0	0	4
Gemmatimonadetes	1	0	0	0	0	0	0	0	0	0
Planctomycetes	0	0	0	0	0	0	1	0	0	3
Elusimicrobia	2	0	0	0	0	0	0	0	0	0
Synergistetes	2	0	0	0	0	0	0	0	0	0
Cyanobacteria	0	0	0	0	0	0	3	0	0	13
Chlorobi	6	0	0	3	0	0	1	0	0	0
Chloroflexi	6	0	0	2	0	0	1	0	0	2
Deinococcus-Thermus	1	0	0	1	0	0	3	0	0	2
Aquificae	6	0	0	2	0	0	1	0	0	0
Thermotogae	10	0	0	1	0	0	0	0	0	0
Dictyoglomi	2	0	0	0	0	0	0	0	0	0
Nitrospirae	0	0	0	1	0	0	1	0	0	0
Thermobaculum	0	0	0	0	0	0	1	0	0	0
Deferribacteres	3	0	0	0	0	0	0	0	0	0
Euryarchaeota	13	0	0	38	0	0	9	0	0	0
Crenarchaeota	1	0	0	16	0	0	6	0	0	0
Thaumarchaeota	0	0	0	0	0	0	2	0	0	0
Nanoarchaeota	1	0	0	0	0	0	0	0	0	0
Korarchaeota	0	0	0	1	0	0	0	0	0	0
Total	258	0	0	173	0	0	172	0	0	165

(M00177_1)

Phyla	Module completion ratio (%)									
	0--10	10--20	20--30	30--40	40--50	50--60	60--70	70--80	80--90	90--100
Gammaproteobacteria	126	0	0	0	0	0	0	0	0	0
Betaproteobacteria	61	0	0	0	0	0	0	0	0	0
Epsilonproteobacteria	17	0	0	0	0	0	0	0	0	0
Deltaproteobacteria	28	0	0	0	0	0	0	0	0	0
Alphaproteobacteria	91	0	0	0	0	0	0	0	0	0
Magnetococcus	1	0	0	0	0	0	0	0	0	0
Chrysiogenetes	1	0	0	0	0	0	0	0	0	0
Firmicutes	104	0	0	0	0	0	0	0	0	0
Tenericutes	19	0	0	0	0	0	0	0	0	0
Actinobacteria	80	0	0	0	0	0	0	0	0	0
Chlamydiae	8	0	0	0	0	0	0	0	0	0
Spirochaetes	14	0	0	0	0	0	0	0	0	0
Acidobacteria	5	0	0	0	0	0	0	0	0	0
Bacteroidetes	35	0	0	0	0	0	0	0	0	0
Fibrobacteres	1	0	0	0	0	0	0	0	0	0
Fusobacteria	5	0	0	0	0	0	0	0	0	0
Verrucomicrobia	4	0	0	0	0	0	0	0	0	0
Gemmatimonadetes	1	0	0	0	0	0	0	0	0	0
Planctomycetes	4	0	0	0	0	0	0	0	0	0
Elusimicrobia	2	0	0	0	0	0	0	0	0	0
Synergistetes	2	0	0	0	0	0	0	0	0	0
Cyanobacteria	16	0	0	0	0	0	0	0	0	0
Chlorobi	10	0	0	0	0	0	0	0	0	0
Chloroflexi	11	0	0	0	0	0	0	0	0	0
Deinococcus-Thermus	7	0	0	0	0	0	0	0	0	0
Aquificae	9	0	0	0	0	0	0	0	0	0
Thermotogae	11	0	0	0	0	0	0	0	0	0
Dictyoglomi	2	0	0	0	0	0	0	0	0	0
Nitrospirae	2	0	0	0	0	0	0	0	0	0
Thermobaculum	1	0	0	0	0	0	0	0	0	0
Deferribacteres	3	0	0	0	0	0	0	0	0	0
Euryarchaeota	0	0	2	58	0	0	0	0	0	0
Crenarchaeota	0	0	0	9	14	0	0	0	0	0
Thaumarchaeota	0	0	0	2	0	0	0	0	0	0
Nanoarchaeota	0	0	0	1	0	0	0	0	0	0
Korarchaeota	0	0	0	1	0	0	0	0	0	0
Total	681	0	2	71	14	0	0	0	0	0

(M00178_1)

Phyla	Module completion ratio (%)									
	0--10	10--20	20--30	30--40	40--50	50--60	60--70	70--80	80--90	90--100
Gammaproteobacteria	0	0	0	0	0	0	1	0	4	121
Betaproteobacteria	0	0	0	0	0	0	0	0	0	61
Epsilonproteobacteria	0	0	0	0	0	0	0	0	2	15
Deltaproteobacteria	0	0	0	0	0	0	0	1	1	26
Alphaproteobacteria	0	0	0	0	0	0	0	1	0	90
Magnetococcus	0	0	0	0	0	0	0	0	0	1
Chrysiogenetes	0	0	0	0	0	0	0	0	0	1
Firmicutes	0	0	0	0	0	0	1	0	2	101
Tenericutes	0	0	0	0	0	0	0	0	1	18
Actinobacteria	0	0	0	0	0	0	0	0	0	80
Chlamydiae	0	0	0	0	0	0	0	0	0	8
Spirochaetes	0	0	0	0	0	0	0	0	0	14
Acidobacteria	0	0	0	0	0	0	0	0	0	5
Bacteroidetes	0	0	0	0	0	0	0	0	0	35
Fibrobacteres	0	0	0	0	0	0	0	0	0	1
Fusobacteria	0	0	0	0	0	0	0	0	0	5
Verrucomicrobia	0	0	0	0	0	0	0	0	0	4
Gemmatimonadetes	0	0	0	0	0	0	0	0	0	1
Planctomycetes	0	0	0	0	0	0	0	0	0	4
Elusimicrobia	0	0	0	0	0	0	0	0	0	2
Synergistetes	0	0	0	0	0	0	0	0	0	2
Cyanobacteria	0	0	0	0	0	0	0	0	0	16
Chlorobi	0	0	0	0	0	0	0	0	0	10
Chloroflexi	0	0	0	0	0	0	0	0	0	11
Deinococcus-Thermus	0	0	0	0	0	0	0	0	0	7
Aquificae	0	0	0	0	0	0	0	0	0	9
Thermotogae	0	0	0	0	0	0	0	0	0	11
Dictyoglomi	0	0	0	0	0	0	0	0	0	2
Nitrospirae	0	0	0	0	0	0	0	0	0	2
Thermobaculum	0	0	0	0	0	0	0	0	0	1
Deferribacteres	0	0	0	0	0	0	0	0	0	3
Euryarchaeota	0	0	0	0	0	60	0	0	0	0
Crenarchaeota	0	0	0	0	0	23	0	0	0	0
Thaumarchaeota	0	0	0	0	0	2	0	0	0	0
Nanoarchaeota	0	0	0	0	0	1	0	0	0	0
Korarchaeota	0	0	0	0	0	1	0	0	0	0
Total	0	0	0	0	0	87	2	2	10	667

(M00179_1)

Phyla	Module completion ratio (%)									
	0--10	10--20	20--30	30--40	40--50	50--60	60--70	70--80	80--90	90--100
Gammaproteobacteria	0	0	0	0	7	119	0	0	0	0
Betaproteobacteria	0	0	0	0	1	60	0	0	0	0
Epsilonproteobacteria	0	0	0	0	3	14	0	0	0	0
Deltaproteobacteria	0	0	0	0	3	25	0	0	0	0
Alphaproteobacteria	0	0	0	0	3	88	0	0	0	0
Magnetococcus	0	0	0	0	0	1	0	0	0	0
Chrysiogenetes	0	0	0	0	0	1	0	0	0	0
Firmicutes	0	0	0	1	2	101	0	0	0	0
Tenericutes	0	0	0	0	2	17	0	0	0	0
Actinobacteria	0	0	0	0	1	79	0	0	0	0
Chlamydiae	0	0	0	0	0	8	0	0	0	0
Spirochaetes	0	0	0	0	0	14	0	0	0	0
Acidobacteria	0	0	0	0	0	5	0	0	0	0
Bacteroidetes	0	0	0	0	1	34	0	0	0	0
Fibrobacteres	0	0	0	0	0	1	0	0	0	0
Fusobacteria	0	0	0	0	0	5	0	0	0	0
Verrucomicrobia	0	0	0	0	1	3	0	0	0	0
Gemmatimonadetes	0	0	0	0	0	1	0	0	0	0
Planctomycetes	0	0	0	0	0	4	0	0	0	0
Elusimicrobia	0	0	0	0	0	2	0	0	0	0
Synergistetes	0	0	0	0	0	2	0	0	0	0
Cyanobacteria	0	0	0	0	0	16	0	0	0	0
Chlorobi	0	0	0	0	0	10	0	0	0	0
Chloroflexi	0	0	0	0	0	11	0	0	0	0
Deinococcus-Thermus	0	0	0	0	0	7	0	0	0	0
Aquificae	0	0	0	0	0	9	0	0	0	0
Thermotogae	0	0	0	0	0	11	0	0	0	0
Dictyoglomi	0	0	0	0	0	2	0	0	0	0
Nitrospirae	0	0	0	0	0	2	0	0	0	0
Thermobaculum	0	0	0	0	0	1	0	0	0	0
Deferribacteres	0	0	0	0	0	3	0	0	0	0
Euryarchaeota	0	0	0	0	0	0	0	0	0	60
Crenarchaeota	0	0	0	0	0	0	0	0	0	23
Thaumarchaeota	0	0	0	0	0	0	0	0	0	2
Nanoarchaeota	0	0	0	0	0	0	0	0	0	1
Korarchaeota	0	0	0	0	0	0	0	0	0	1
Total	0	0	0	1	24	656	0	0	0	87

(M00180_1)

Phyla	Module completion ratio (%)									
	0--10	10--20	20--30	30--40	40--50	50--60	60--70	70--80	80--90	90--100
Gammaproteobacteria	126	0	0	0	0	0	0	0	0	0
Betaproteobacteria	61	0	0	0	0	0	0	0	0	0
Epsilonproteobacteria	17	0	0	0	0	0	0	0	0	0
Deltaproteobacteria	28	0	0	0	0	0	0	0	0	0
Alphaproteobacteria	91	0	0	0	0	0	0	0	0	0
Magnetococcus	1	0	0	0	0	0	0	0	0	0
Chrysiogenetes	1	0	0	0	0	0	0	0	0	0
Firmicutes	104	0	0	0	0	0	0	0	0	0
Tenericutes	19	0	0	0	0	0	0	0	0	0
Actinobacteria	80	0	0	0	0	0	0	0	0	0
Chlamydiae	8	0	0	0	0	0	0	0	0	0
Spirochaetes	14	0	0	0	0	0	0	0	0	0
Acidobacteria	5	0	0	0	0	0	0	0	0	0
Bacteroidetes	35	0	0	0	0	0	0	0	0	0
Fibrobacteres	1	0	0	0	0	0	0	0	0	0
Fusobacteria	5	0	0	0	0	0	0	0	0	0
Verrucomicrobia	4	0	0	0	0	0	0	0	0	0
Gemmatimonadetes	1	0	0	0	0	0	0	0	0	0
Planctomycetes	4	0	0	0	0	0	0	0	0	0
Elusimicrobia	2	0	0	0	0	0	0	0	0	0
Synergistetes	2	0	0	0	0	0	0	0	0	0
Cyanobacteria	16	0	0	0	0	0	0	0	0	0
Chlorobi	10	0	0	0	0	0	0	0	0	0
Chloroflexi	11	0	0	0	0	0	0	0	0	0
Deinococcus-Thermus	7	0	0	0	0	0	0	0	0	0
Aquificae	9	0	0	0	0	0	0	0	0	0
Thermotogae	11	0	0	0	0	0	0	0	0	0
Dictyoglomi	2	0	0	0	0	0	0	0	0	0
Nitrospirae	2	0	0	0	0	0	0	0	0	0
Thermobaculum	1	0	0	0	0	0	0	0	0	0
Deferribacteres	3	0	0	0	0	0	0	0	0	0
Euryarchaeota	60	0	0	0	0	0	0	0	0	0
Crenarchaeota	23	0	0	0	0	0	0	0	0	0
Thaumarchaeota	2	0	0	0	0	0	0	0	0	0
Nanoarchaeota	1	0	0	0	0	0	0	0	0	0
Korarchaeota	1	0	0	0	0	0	0	0	0	0
Total	768	0	0	0	0	0	0	0	0	0

(M00181_1)

Phyla	Module completion ratio (%)									
	0--10	10--20	20--30	30--40	40--50	50--60	60--70	70--80	80--90	90--100
Gammaproteobacteria	126	0	0	0	0	0	0	0	0	0
Betaproteobacteria	61	0	0	0	0	0	0	0	0	0
Epsilonproteobacteria	17	0	0	0	0	0	0	0	0	0
Deltaproteobacteria	28	0	0	0	0	0	0	0	0	0
Alphaproteobacteria	91	0	0	0	0	0	0	0	0	0
Magnetococcus	1	0	0	0	0	0	0	0	0	0
Chrysiogenetes	1	0	0	0	0	0	0	0	0	0
Firmicutes	104	0	0	0	0	0	0	0	0	0
Tenericutes	19	0	0	0	0	0	0	0	0	0
Actinobacteria	80	0	0	0	0	0	0	0	0	0
Chlamydiae	8	0	0	0	0	0	0	0	0	0
Spirochaetes	14	0	0	0	0	0	0	0	0	0
Acidobacteria	5	0	0	0	0	0	0	0	0	0
Bacteroidetes	35	0	0	0	0	0	0	0	0	0
Fibrobacteres	1	0	0	0	0	0	0	0	0	0
Fusobacteria	5	0	0	0	0	0	0	0	0	0
Verrucomicrobia	4	0	0	0	0	0	0	0	0	0
Gemmatimonadetes	1	0	0	0	0	0	0	0	0	0
Planctomycetes	4	0	0	0	0	0	0	0	0	0
Elusimicrobia	2	0	0	0	0	0	0	0	0	0
Synergistetes	2	0	0	0	0	0	0	0	0	0
Cyanobacteria	16	0	0	0	0	0	0	0	0	0
Chlorobi	10	0	0	0	0	0	0	0	0	0
Chloroflexi	11	0	0	0	0	0	0	0	0	0
Deinococcus-Thermus	7	0	0	0	0	0	0	0	0	0
Aquificae	9	0	0	0	0	0	0	0	0	0
Thermotogae	11	0	0	0	0	0	0	0	0	0
Dictyoglomi	2	0	0	0	0	0	0	0	0	0
Nitrospirae	2	0	0	0	0	0	0	0	0	0
Thermobaculum	1	0	0	0	0	0	0	0	0	0
Deferribacteres	3	0	0	0	0	0	0	0	0	0
Euryarchaeota	60	0	0	0	0	0	0	0	0	0
Crenarchaeota	23	0	0	0	0	0	0	0	0	0
Thaumarchaeota	2	0	0	0	0	0	0	0	0	0
Nanoarchaeota	1	0	0	0	0	0	0	0	0	0
Korarchaeota	1	0	0	0	0	0	0	0	0	0
Total	768	0	0	0	0	0	0	0	0	0

(M00182_1)

Phyla	Module completion ratio (%)									
	0--10	10--20	20--30	30--40	40--50	50--60	60--70	70--80	80--90	90--100
Gammaproteobacteria	126	0	0	0	0	0	0	0	0	0
Betaproteobacteria	61	0	0	0	0	0	0	0	0	0
Epsilonproteobacteria	17	0	0	0	0	0	0	0	0	0
Deltaproteobacteria	28	0	0	0	0	0	0	0	0	0
Alphaproteobacteria	91	0	0	0	0	0	0	0	0	0
Magnetococcus	1	0	0	0	0	0	0	0	0	0
Chrysiogenetes	1	0	0	0	0	0	0	0	0	0
Firmicutes	104	0	0	0	0	0	0	0	0	0
Tenericutes	19	0	0	0	0	0	0	0	0	0
Actinobacteria	80	0	0	0	0	0	0	0	0	0
Chlamydiae	8	0	0	0	0	0	0	0	0	0
Spirochaetes	14	0	0	0	0	0	0	0	0	0
Acidobacteria	5	0	0	0	0	0	0	0	0	0
Bacteroidetes	35	0	0	0	0	0	0	0	0	0
Fibrobacteres	1	0	0	0	0	0	0	0	0	0
Fusobacteria	5	0	0	0	0	0	0	0	0	0
Verrucomicrobia	4	0	0	0	0	0	0	0	0	0
Gemmatimonadetes	1	0	0	0	0	0	0	0	0	0
Planctomycetes	4	0	0	0	0	0	0	0	0	0
Elusimicrobia	2	0	0	0	0	0	0	0	0	0
Synergistetes	2	0	0	0	0	0	0	0	0	0
Cyanobacteria	16	0	0	0	0	0	0	0	0	0
Chlorobi	10	0	0	0	0	0	0	0	0	0
Chloroflexi	11	0	0	0	0	0	0	0	0	0
Deinococcus-Thermus	7	0	0	0	0	0	0	0	0	0
Aquificae	9	0	0	0	0	0	0	0	0	0
Thermotogae	11	0	0	0	0	0	0	0	0	0
Dictyoglomi	2	0	0	0	0	0	0	0	0	0
Nitrospirae	2	0	0	0	0	0	0	0	0	0
Thermobaculum	1	0	0	0	0	0	0	0	0	0
Deferribacteres	3	0	0	0	0	0	0	0	0	0
Euryarchaeota	60	0	0	0	0	0	0	0	0	0
Crenarchaeota	23	0	0	0	0	0	0	0	0	0
Thaumarchaeota	2	0	0	0	0	0	0	0	0	0
Nanoarchaeota	1	0	0	0	0	0	0	0	0	0
Korarchaeota	1	0	0	0	0	0	0	0	0	0
Total	768	0	0	0	0	0	0	0	0	0

(M00183_1)

Phyla	Module completion ratio (%)									
	0--10	10--20	20--30	30--40	40--50	50--60	60--70	70--80	80--90	90--100
Gammaproteobacteria	0	0	0	0	0	1	0	4	0	121
Betaproteobacteria	0	0	0	0	0	0	0	2	0	59
Epsilonproteobacteria	0	0	0	0	0	6	0	1	0	10
Deltaproteobacteria	0	0	0	0	0	0	0	2	0	26
Alphaproteobacteria	0	0	0	0	0	3	0	2	0	86
Magnetococcus	0	0	0	0	0	0	0	0	0	1
Chrysiogenetes	0	0	0	0	0	0	0	0	0	1
Firmicutes	0	0	0	0	0	0	0	5	0	99
Tenericutes	0	0	0	0	0	0	0	15	0	4
Actinobacteria	0	0	0	0	0	0	0	1	0	79
Chlamydiae	0	0	0	0	0	0	0	7	0	1
Spirochaetes	0	0	0	0	0	0	0	11	0	3
Acidobacteria	0	0	0	0	0	0	0	0	0	5
Bacteroidetes	0	0	0	0	0	0	0	35	0	0
Fibrobacteres	0	0	0	0	0	0	0	1	0	0
Fusobacteria	0	0	0	0	0	0	0	2	0	3
Verrucomicrobia	0	0	0	0	0	0	0	0	0	4
Gemmatimonadetes	0	0	0	0	0	0	0	1	0	0
Planctomycetes	0	0	0	0	0	0	0	2	0	2
Elusimicrobia	0	0	0	0	0	0	0	2	0	0
Synergistetes	0	0	0	0	0	0	0	2	0	0
Cyanobacteria	0	0	0	0	0	0	0	0	0	16
Chlorobi	0	0	0	0	0	0	0	10	0	0
Chloroflexi	0	0	0	0	0	0	0	11	0	0
Deinococcus-Thermus	0	0	0	0	0	0	0	0	0	7
Aquificae	0	0	0	0	0	0	0	0	0	9
Thermotogae	0	0	0	0	0	0	0	0	0	11
Dictyoglomi	0	0	0	0	0	0	0	0	0	2
Nitrospirae	0	0	0	0	0	0	0	0	0	2
Thermobaculum	0	0	0	0	0	0	0	1	0	0
Deferribacteres	0	0	0	0	0	0	0	0	0	3
Euryarchaeota	60	0	0	0	0	0	0	0	0	0
Crenarchaeota	23	0	0	0	0	0	0	0	0	0
Thaumarchaeota	2	0	0	0	0	0	0	0	0	0
Nanoarchaeota	1	0	0	0	0	0	0	0	0	0
Korarchaeota	1	0	0	0	0	0	0	0	0	0
Total	87	0	0	0	0	10	0	117	0	554

(M00183_2)

Phyla	Module completion ratio (%)									
	0--10	10--20	20--30	30--40	40--50	50--60	60--70	70--80	80--90	90--100
Gammaproteobacteria	0	0	0	4	0	0	122	0	0	0
Betaproteobacteria	0	0	0	1	0	0	60	0	0	0
Epsilonproteobacteria	0	0	0	0	0	0	13	0	0	4
Deltaproteobacteria	0	0	0	1	0	0	27	0	0	0
Alphaproteobacteria	0	0	0	2	0	0	86	0	0	3
Magnetococcus	0	0	0	0	0	0	1	0	0	0
Chrysiogenetes	0	0	0	0	0	0	1	0	0	0
Firmicutes	0	0	0	4	0	0	100	0	0	0
Tenericutes	0	0	0	15	0	0	4	0	0	0
Actinobacteria	0	0	0	0	0	0	80	0	0	0
Chlamydiae	0	0	0	7	0	0	1	0	0	0
Spirochaetes	0	0	0	11	0	0	3	0	0	0
Acidobacteria	0	0	0	0	0	0	5	0	0	0
Bacteroidetes	0	0	0	35	0	0	0	0	0	0
Fibrobacteres	0	0	0	1	0	0	0	0	0	0
Fusobacteria	0	0	0	2	0	0	3	0	0	0
Verrucomicrobia	0	0	0	0	0	0	4	0	0	0
Gemmatimonadetes	0	0	0	1	0	0	0	0	0	0
Planctomycetes	0	0	0	2	0	0	2	0	0	0
Elusimicrobia	0	0	0	2	0	0	0	0	0	0
Synergistetes	0	0	0	2	0	0	0	0	0	0
Cyanobacteria	0	0	0	0	0	0	16	0	0	0
Chlorobi	0	0	0	10	0	0	0	0	0	0
Chloroflexi	0	0	0	11	0	0	0	0	0	0
Deinococcus-Thermus	0	0	0	0	0	0	7	0	0	0
Aquificae	0	0	0	0	0	0	9	0	0	0
Thermotogae	0	0	0	0	0	0	11	0	0	0
Dictyoglomi	0	0	0	0	0	0	2	0	0	0
Nitrospirae	0	0	0	0	0	0	2	0	0	0
Thermobaculum	0	0	0	1	0	0	0	0	0	0
Deferribacteres	0	0	0	0	0	0	3	0	0	0
Euryarchaeota	60	0	0	0	0	0	0	0	0	0
Crenarchaeota	23	0	0	0	0	0	0	0	0	0
Thaumarchaeota	2	0	0	0	0	0	0	0	0	0
Nanoarchaeota	1	0	0	0	0	0	0	0	0	0
Korarchaeota	1	0	0	0	0	0	0	0	0	0
Total	87	0	0	112	0	0	562	0	0	7

(M00184_1)

Phyla	Module completion ratio (%)									
	0--10	10--20	20--30	30--40	40--50	50--60	60--70	70--80	80--90	90--100
Gammaproteobacteria	126	0	0	0	0	0	0	0	0	0
Betaproteobacteria	61	0	0	0	0	0	0	0	0	0
Epsilonproteobacteria	17	0	0	0	0	0	0	0	0	0
Deltaproteobacteria	28	0	0	0	0	0	0	0	0	0
Alphaproteobacteria	91	0	0	0	0	0	0	0	0	0
Magnetococcus	1	0	0	0	0	0	0	0	0	0
Chrysiogenetes	1	0	0	0	0	0	0	0	0	0
Firmicutes	104	0	0	0	0	0	0	0	0	0
Tenericutes	19	0	0	0	0	0	0	0	0	0
Actinobacteria	80	0	0	0	0	0	0	0	0	0
Chlamydiae	8	0	0	0	0	0	0	0	0	0
Spirochaetes	14	0	0	0	0	0	0	0	0	0
Acidobacteria	5	0	0	0	0	0	0	0	0	0
Bacteroidetes	35	0	0	0	0	0	0	0	0	0
Fibrobacteres	1	0	0	0	0	0	0	0	0	0
Fusobacteria	5	0	0	0	0	0	0	0	0	0
Verrucomicrobia	4	0	0	0	0	0	0	0	0	0
Gemmatimonadetes	1	0	0	0	0	0	0	0	0	0
Planctomycetes	4	0	0	0	0	0	0	0	0	0
Elusimicrobia	2	0	0	0	0	0	0	0	0	0
Synergistetes	2	0	0	0	0	0	0	0	0	0
Cyanobacteria	16	0	0	0	0	0	0	0	0	0
Chlorobi	10	0	0	0	0	0	0	0	0	0
Chloroflexi	11	0	0	0	0	0	0	0	0	0
Deinococcus-Thermus	7	0	0	0	0	0	0	0	0	0
Aquificae	9	0	0	0	0	0	0	0	0	0
Thermotogae	11	0	0	0	0	0	0	0	0	0
Dictyoglomi	2	0	0	0	0	0	0	0	0	0
Nitrospirae	2	0	0	0	0	0	0	0	0	0
Thermobaculum	1	0	0	0	0	0	0	0	0	0
Deferribacteres	3	0	0	0	0	0	0	0	0	0
Euryarchaeota	0	0	0	0	0	0	0	0	12	48
Crenarchaeota	0	0	0	0	0	0	0	1	21	1
Thaumarchaeota	0	0	0	0	0	0	2	0	0	0
Nanoarchaeota	0	0	0	0	0	0	0	0	0	1
Korarchaeota	0	0	0	0	0	0	1	0	0	0
Total	681	0	0	0	0	0	3	1	33	50

(M00184_2)

Phyla	Module completion ratio (%)									
	0--10	10--20	20--30	30--40	40--50	50--60	60--70	70--80	80--90	90--100
Gammaproteobacteria	126	0	0	0	0	0	0	0	0	0
Betaproteobacteria	61	0	0	0	0	0	0	0	0	0
Epsilonproteobacteria	17	0	0	0	0	0	0	0	0	0
Deltaproteobacteria	28	0	0	0	0	0	0	0	0	0
Alphaproteobacteria	91	0	0	0	0	0	0	0	0	0
Magnetococcus	1	0	0	0	0	0	0	0	0	0
Chrysiogenetes	1	0	0	0	0	0	0	0	0	0
Firmicutes	104	0	0	0	0	0	0	0	0	0
Tenericutes	19	0	0	0	0	0	0	0	0	0
Actinobacteria	80	0	0	0	0	0	0	0	0	0
Chlamydiae	8	0	0	0	0	0	0	0	0	0
Spirochaetes	14	0	0	0	0	0	0	0	0	0
Acidobacteria	5	0	0	0	0	0	0	0	0	0
Bacteroidetes	35	0	0	0	0	0	0	0	0	0
Fibrobacteres	1	0	0	0	0	0	0	0	0	0
Fusobacteria	5	0	0	0	0	0	0	0	0	0
Verrucomicrobia	4	0	0	0	0	0	0	0	0	0
Gemmatimonadetes	1	0	0	0	0	0	0	0	0	0
Planctomycetes	4	0	0	0	0	0	0	0	0	0
Elusimicrobia	2	0	0	0	0	0	0	0	0	0
Synergistetes	2	0	0	0	0	0	0	0	0	0
Cyanobacteria	16	0	0	0	0	0	0	0	0	0
Chlorobi	10	0	0	0	0	0	0	0	0	0
Chloroflexi	11	0	0	0	0	0	0	0	0	0
Deinococcus-Thermus	7	0	0	0	0	0	0	0	0	0
Aquificae	9	0	0	0	0	0	0	0	0	0
Thermotogae	11	0	0	0	0	0	0	0	0	0
Dictyoglomi	2	0	0	0	0	0	0	0	0	0
Nitrospirae	2	0	0	0	0	0	0	0	0	0
Thermobaculum	1	0	0	0	0	0	0	0	0	0
Deferribacteres	3	0	0	0	0	0	0	0	0	0
Euryarchaeota	0	0	0	0	0	0	0	0	2	58
Crenarchaeota	0	0	0	0	0	0	0	0	0	23
Thaumarchaeota	0	0	0	0	0	0	0	0	2	0
Nanoarchaeota	0	0	0	0	0	0	0	0	0	1
Korarchaeota	0	0	0	0	0	0	0	0	1	0
Total	681	0	0	0	0	0	0	0	5	82

(M00185_1)

Phyla	Module completion ratio (%)									
	0--10	10--20	20--30	30--40	40--50	50--60	60--70	70--80	80--90	90--100
Gammaproteobacteria	60	0	0	0	0	1	0	0	0	65
Betaproteobacteria	8	0	0	0	0	2	0	1	0	50
Epsilonproteobacteria	14	0	2	0	0	0	0	0	0	1
Deltaproteobacteria	20	0	0	0	0	0	0	0	0	8
Alphaproteobacteria	45	0	0	0	0	0	0	2	0	44
Magnetococcus	1	0	0	0	0	0	0	0	0	0
Chrysiogenetes	0	0	0	0	0	0	0	0	0	1
Firmicutes	88	0	0	0	0	0	0	0	0	16
Tenericutes	19	0	0	0	0	0	0	0	0	0
Actinobacteria	60	0	1	0	0	0	0	0	0	19
Chlamydiae	8	0	0	0	0	0	0	0	0	0
Spirochaetes	11	0	0	0	0	0	0	0	0	3
Acidobacteria	5	0	0	0	0	0	0	0	0	0
Bacteroidetes	35	0	0	0	0	0	0	0	0	0
Fibrobacteres	0	0	0	0	0	0	0	0	0	1
Fusobacteria	4	0	0	0	0	0	0	0	0	1
Verrucomicrobia	3	0	0	0	0	0	0	0	0	1
Gemmatimonadetes	0	0	1	0	0	0	0	0	0	0
Planctomycetes	1	0	0	0	0	0	0	0	0	3
Elusimicrobia	2	0	0	0	0	0	0	0	0	0
Synergistetes	2	0	0	0	0	0	0	0	0	0
Cyanobacteria	5	0	0	0	0	0	0	0	0	11
Chlorobi	7	0	2	0	0	0	0	0	0	1
Chloroflexi	10	0	0	0	0	0	0	1	0	0
Deinococcus-Thermus	7	0	0	0	0	0	0	0	0	0
Aquificae	9	0	0	0	0	0	0	0	0	0
Thermotogae	11	0	0	0	0	0	0	0	0	0
Dictyoglomi	2	0	0	0	0	0	0	0	0	0
Nitrospirae	1	0	0	0	0	0	0	0	0	1
Thermobaculum	0	0	0	0	0	0	0	0	0	1
Deferribacteres	3	0	0	0	0	0	0	0	0	0
Euryarchaeota	60	0	0	0	0	0	0	0	0	0
Crenarchaeota	23	0	0	0	0	0	0	0	0	0
Thaumarchaeota	2	0	0	0	0	0	0	0	0	0
Nanoarchaeota	1	0	0	0	0	0	0	0	0	0
Korarchaeota	1	0	0	0	0	0	0	0	0	0
Total	528	0	6	0	0	3	0	4	0	227

(M00186_1)

Phyla	Module completion ratio (%)									
	0--10	10--20	20--30	30--40	40--50	50--60	60--70	70--80	80--90	90--100
Gammaproteobacteria	97	0	0	2	0	0	1	0	0	26
Betaproteobacteria	33	0	0	2	0	0	2	0	0	24
Epsilonproteobacteria	14	0	0	0	0	0	1	0	0	2
Deltaproteobacteria	10	0	0	0	0	0	0	0	0	18
Alphaproteobacteria	74	0	0	0	0	0	2	0	0	15
Magnetococcus	1	0	0	0	0	0	0	0	0	0
Chrysiogenetes	1	0	0	0	0	0	0	0	0	0
Firmicutes	82	0	0	1	0	0	2	0	0	19
Tenericutes	19	0	0	0	0	0	0	0	0	0
Actinobacteria	78	0	0	0	0	0	0	0	0	2
Chlamydiae	8	0	0	0	0	0	0	0	0	0
Spirochaetes	14	0	0	0	0	0	0	0	0	0
Acidobacteria	5	0	0	0	0	0	0	0	0	0
Bacteroidetes	35	0	0	0	0	0	0	0	0	0
Fibrobacteres	1	0	0	0	0	0	0	0	0	0
Fusobacteria	3	0	0	0	0	0	0	0	0	2
Verrucomicrobia	4	0	0	0	0	0	0	0	0	0
Gemmatimonadetes	1	0	0	0	0	0	0	0	0	0
Planctomycetes	3	0	0	0	0	0	0	0	0	1
Elusimicrobia	2	0	0	0	0	0	0	0	0	0
Synergistetes	0	0	0	0	0	0	0	0	0	2
Cyanobacteria	15	0	0	0	0	0	0	0	0	1
Chlorobi	10	0	0	0	0	0	0	0	0	0
Chloroflexi	8	0	0	0	0	0	0	0	0	3
Deinococcus-Thermus	5	0	0	0	0	0	0	0	0	2
Aquificae	9	0	0	0	0	0	0	0	0	0
Thermotogae	10	0	0	0	0	0	0	0	0	1
Dictyoglomi	2	0	0	0	0	0	0	0	0	0
Nitrospirae	1	0	0	0	0	0	0	0	0	1
Thermobaculum	0	0	0	0	0	0	1	0	0	0
Deferribacteres	3	0	0	0	0	0	0	0	0	0
Euryarchaeota	38	0	0	0	0	0	1	0	0	21
Crenarchaeota	15	0	0	0	0	0	0	0	0	8
Thaumarchaeota	2	0	0	0	0	0	0	0	0	0
Nanoarchaeota	1	0	0	0	0	0	0	0	0	0
Korarchaeota	1	0	0	0	0	0	0	0	0	0
Total	605	0	0	5	0	0	10	0	0	148

(M00187_1)

Phyla	Module completion ratio (%)									
	0--10	10--20	20--30	30--40	40--50	50--60	60--70	70--80	80--90	90--100
Gammaproteobacteria	124	0	0	2	0	0	0	0	0	0
Betaproteobacteria	56	0	0	5	0	0	0	0	0	0
Epsilonproteobacteria	17	0	0	0	0	0	0	0	0	0
Deltaproteobacteria	28	0	0	0	0	0	0	0	0	0
Alphaproteobacteria	90	0	0	1	0	0	0	0	0	0
Magnetococcus	1	0	0	0	0	0	0	0	0	0
Chrysiogenetes	1	0	0	0	0	0	0	0	0	0
Firmicutes	101	0	0	3	0	0	0	0	0	0
Tenericutes	19	0	0	0	0	0	0	0	0	0
Actinobacteria	78	0	0	2	0	0	0	0	0	0
Chlamydiae	8	0	0	0	0	0	0	0	0	0
Spirochaetes	14	0	0	0	0	0	0	0	0	0
Acidobacteria	5	0	0	0	0	0	0	0	0	0
Bacteroidetes	35	0	0	0	0	0	0	0	0	0
Fibrobacteres	1	0	0	0	0	0	0	0	0	0
Fusobacteria	5	0	0	0	0	0	0	0	0	0
Verrucomicrobia	4	0	0	0	0	0	0	0	0	0
Gemmatimonadetes	1	0	0	0	0	0	0	0	0	0
Planctomycetes	4	0	0	0	0	0	0	0	0	0
Elusimicrobia	2	0	0	0	0	0	0	0	0	0
Synergistetes	2	0	0	0	0	0	0	0	0	0
Cyanobacteria	16	0	0	0	0	0	0	0	0	0
Chlorobi	10	0	0	0	0	0	0	0	0	0
Chloroflexi	11	0	0	0	0	0	0	0	0	0
Deinococcus-Thermus	7	0	0	0	0	0	0	0	0	0
Aquificae	9	0	0	0	0	0	0	0	0	0
Thermotogae	11	0	0	0	0	0	0	0	0	0
Dictyoglomi	2	0	0	0	0	0	0	0	0	0
Nitrospirae	2	0	0	0	0	0	0	0	0	0
Thermobaculum	1	0	0	0	0	0	0	0	0	0
Deferribacteres	3	0	0	0	0	0	0	0	0	0
Euryarchaeota	37	0	0	9	0	0	11	0	0	3
Crenarchaeota	12	0	0	0	0	0	6	0	0	5
Thaumarchaeota	2	0	0	0	0	0	0	0	0	0
Nanoarchaeota	1	0	0	0	0	0	0	0	0	0
Korarchaeota	1	0	0	0	0	0	0	0	0	0
Total	721	0	0	22	0	0	17	0	0	8

(M00188_1)

Phyla	Module completion ratio (%)									
	0--10	10--20	20--30	30--40	40--50	50--60	60--70	70--80	80--90	90--100
Gammaproteobacteria	70	0	0	9	0	0	10	0	0	37
Betaproteobacteria	6	0	0	0	0	0	4	0	0	51
Epsilonproteobacteria	6	0	0	1	0	0	0	0	0	10
Deltaproteobacteria	4	0	0	3	0	0	3	0	0	18
Alphaproteobacteria	27	0	0	4	0	0	5	0	0	55
Magnetococcus	0	0	0	1	0	0	0	0	0	0
Chrysiogenetes	1	0	0	0	0	0	0	0	0	0
Firmicutes	32	0	0	4	0	0	10	0	0	58
Tenericutes	19	0	0	0	0	0	0	0	0	0
Actinobacteria	20	0	0	4	0	0	10	0	0	46
Chlamydiae	0	0	0	0	0	0	0	0	0	8
Spirochaetes	11	0	0	0	0	0	1	0	0	2
Acidobacteria	0	0	0	0	0	0	2	0	0	3
Bacteroidetes	28	0	0	6	0	0	0	0	0	1
Fibrobacteres	0	0	0	0	0	0	0	0	0	1
Fusobacteria	3	0	0	0	0	0	0	0	0	2
Verrucomicrobia	0	0	0	1	0	0	0	0	0	3
Gemmatimonadetes	0	0	0	1	0	0	0	0	0	0
Planctomycetes	0	0	0	1	0	0	0	0	0	3
Elusimicrobia	2	0	0	0	0	0	0	0	0	0
Synergistetes	0	0	0	1	0	0	1	0	0	0
Cyanobacteria	3	0	0	1	0	0	4	0	0	8
Chlorobi	7	0	0	0	0	0	1	0	0	2
Chloroflexi	2	0	0	0	0	0	1	0	0	8
Deinococcus-Thermus	1	0	0	0	0	0	0	0	0	6
Aquificae	7	0	0	1	0	0	1	0	0	0
Thermotogae	0	0	0	0	0	0	0	0	0	11
Dictyoglomi	0	0	0	0	0	0	0	0	0	2
Nitrospirae	1	0	0	0	0	0	1	0	0	0
Thermobaculum	0	0	0	0	0	0	0	0	0	1
Deferribacteres	1	0	0	0	0	0	1	0	0	1
Euryarchaeota	19	0	0	3	0	0	5	0	0	33
Crenarchaeota	5	0	0	2	0	0	14	0	0	2
Thaumarchaeota	0	0	0	0	0	0	0	0	0	2
Nanoarchaeota	1	0	0	0	0	0	0	0	0	0
Korarchaeota	0	0	0	0	0	0	0	0	0	1
Total	276	0	0	43	0	0	74	0	0	375

(M00189_1)

Phyla	Module completion ratio (%)									
	0--10	10--20	20--30	30--40	40--50	50--60	60--70	70--80	80--90	90--100
Gammaproteobacteria	20	0	0	2	0	0	9	0	0	95
Betaproteobacteria	8	0	0	1	0	0	4	0	0	48
Epsilonproteobacteria	1	0	0	0	0	0	2	0	0	14
Deltaproteobacteria	2	0	0	0	0	0	2	0	0	24
Alphaproteobacteria	35	0	0	0	0	0	9	0	0	47
Magnetococcus	0	0	0	0	0	0	0	0	0	1
Chrysiogenetes	0	0	0	0	0	0	0	0	0	1
Firmicutes	50	0	0	1	0	0	32	0	0	21
Tenericutes	19	0	0	0	0	0	0	0	0	0
Actinobacteria	9	0	0	4	0	0	12	0	0	55
Chlamydiae	8	0	0	0	0	0	0	0	0	0
Spirochaetes	12	0	0	0	0	0	1	0	0	1
Acidobacteria	0	0	0	0	0	0	2	0	0	3
Bacteroidetes	28	0	0	0	0	0	0	0	0	7
Fibrobacteres	1	0	0	0	0	0	0	0	0	0
Fusobacteria	3	0	0	0	0	0	1	0	0	1
Verrucomicrobia	1	0	0	0	0	0	2	0	0	1
Gemmatimonadetes	0	0	0	0	0	0	1	0	0	0
Planctomycetes	3	0	0	0	0	0	0	0	0	1
Elusimicrobia	2	0	0	0	0	0	0	0	0	0
Synergistetes	1	0	0	0	0	0	0	0	0	1
Cyanobacteria	3	0	0	0	0	0	12	0	0	1
Chlorobi	0	0	0	0	0	0	0	0	0	10
Chloroflexi	2	0	0	0	0	0	6	0	0	3
Deinococcus-Thermus	1	0	0	0	0	0	3	0	0	3
Aquificae	1	0	0	0	0	0	1	0	0	7
Thermotogae	10	0	0	0	0	0	1	0	0	0
Dictyoglomi	2	0	0	0	0	0	0	0	0	0
Nitrospirae	1	0	0	0	0	0	0	0	0	1
Thermobaculum	0	0	0	0	0	0	1	0	0	0
Deferribacteres	0	0	0	0	0	0	1	0	0	2
Euryarchaeota	27	0	0	5	0	0	9	0	0	19
Crenarchaeota	9	0	0	8	0	0	6	0	0	0
Thaumarchaeota	2	0	0	0	0	0	0	0	0	0
Nanoarchaeota	1	0	0	0	0	0	0	0	0	0
Korarchaeota	1	0	0	0	0	0	0	0	0	0
Total	263	0	0	21	0	0	117	0	0	367

(M00190_1)

Phyla	Module completion ratio (%)									
	0--10	10--20	20--30	30--40	40--50	50--60	60--70	70--80	80--90	90--100
Gammaproteobacteria	36	0	0	5	0	0	1	0	0	84
Betaproteobacteria	10	0	0	1	0	0	0	0	0	50
Epsilonproteobacteria	13	0	0	0	0	0	1	0	0	3
Deltaproteobacteria	15	0	0	3	0	0	0	0	0	10
Alphaproteobacteria	39	0	0	1	0	0	3	0	0	48
Magnetococcus	1	0	0	0	0	0	0	0	0	0
Chrysiogenetes	0	0	0	0	0	0	0	0	0	1
Firmicutes	63	0	0	8	0	0	3	0	0	30
Tenericutes	19	0	0	0	0	0	0	0	0	0
Actinobacteria	40	0	0	3	0	0	4	0	0	33
Chlamydiae	8	0	0	0	0	0	0	0	0	0
Spirochaetes	11	0	0	0	0	0	0	0	0	3
Acidobacteria	5	0	0	0	0	0	0	0	0	0
Bacteroidetes	33	0	0	0	0	0	0	0	0	2
Fibrobacteres	1	0	0	0	0	0	0	0	0	0
Fusobacteria	2	0	0	0	0	0	0	0	0	3
Verrucomicrobia	3	0	0	1	0	0	0	0	0	0
Gemmatimonadetes	1	0	0	0	0	0	0	0	0	0
Planctomycetes	0	0	0	0	0	0	4	0	0	0
Elusimicrobia	2	0	0	0	0	0	0	0	0	0
Synergistetes	1	0	0	0	0	0	0	0	0	1
Cyanobacteria	3	0	0	0	0	0	0	0	0	13
Chlorobi	9	0	0	0	0	0	1	0	0	0
Chloroflexi	3	0	0	0	0	0	0	0	0	8
Deinococcus-Thermus	2	0	0	0	0	0	0	0	0	5
Aquificae	9	0	0	0	0	0	0	0	0	0
Thermotogae	8	0	0	1	0	0	1	0	0	1
Dictyoglomi	2	0	0	0	0	0	0	0	0	0
Nitrospirae	1	0	0	0	0	0	0	0	0	1
Thermobaculum	1	0	0	0	0	0	0	0	0	0
Deferribacteres	3	0	0	0	0	0	0	0	0	0
Euryarchaeota	43	0	0	1	0	0	3	0	0	13
Crenarchaeota	22	0	0	0	0	0	0	0	0	1
Thaumarchaeota	2	0	0	0	0	0	0	0	0	0
Nanoarchaeota	1	0	0	0	0	0	0	0	0	0
Korarchaeota	1	0	0	0	0	0	0	0	0	0
Total	413	0	0	24	0	0	21	0	0	310

(M00191_1)

Phyla	Module completion ratio (%)									
	0--10	10--20	20--30	30--40	40--50	50--60	60--70	70--80	80--90	90--100
Gammaproteobacteria	70	0	0	0	0	0	0	0	0	56
Betaproteobacteria	57	0	0	0	0	0	4	0	0	0
Epsilonproteobacteria	17	0	0	0	0	0	0	0	0	0
Deltaproteobacteria	25	0	0	1	0	0	2	0	0	0
Alphaproteobacteria	77	0	0	0	0	0	1	0	0	13
Magnetococcus	1	0	0	0	0	0	0	0	0	0
Chrysiogenetes	1	0	0	0	0	0	0	0	0	0
Firmicutes	104	0	0	0	0	0	0	0	0	0
Tenericutes	19	0	0	0	0	0	0	0	0	0
Actinobacteria	61	0	0	0	0	0	19	0	0	0
Chlamydiae	8	0	0	0	0	0	0	0	0	0
Spirochaetes	11	0	0	0	0	0	1	0	0	2
Acidobacteria	5	0	0	0	0	0	0	0	0	0
Bacteroidetes	35	0	0	0	0	0	0	0	0	0
Fibrobacteres	1	0	0	0	0	0	0	0	0	0
Fusobacteria	4	0	0	0	0	0	1	0	0	0
Verrucomicrobia	4	0	0	0	0	0	0	0	0	0
Gemmatimonadetes	1	0	0	0	0	0	0	0	0	0
Planctomycetes	4	0	0	0	0	0	0	0	0	0
Elusimicrobia	2	0	0	0	0	0	0	0	0	0
Synergistetes	2	0	0	0	0	0	0	0	0	0
Cyanobacteria	16	0	0	0	0	0	0	0	0	0
Chlorobi	10	0	0	0	0	0	0	0	0	0
Chloroflexi	9	0	0	0	0	0	2	0	0	0
Deinococcus-Thermus	1	0	0	0	0	0	6	0	0	0
Aquificae	9	0	0	0	0	0	0	0	0	0
Thermotogae	5	0	0	0	0	0	6	0	0	0
Dictyoglomi	2	0	0	0	0	0	0	0	0	0
Nitrospirae	2	0	0	0	0	0	0	0	0	0
Thermobaculum	1	0	0	0	0	0	0	0	0	0
Deferribacteres	3	0	0	0	0	0	0	0	0	0
Euryarchaeota	40	0	0	1	0	0	14	0	0	5
Crenarchaeota	17	0	0	0	0	0	1	0	0	5
Thaumarchaeota	2	0	0	0	0	0	0	0	0	0
Nanoarchaeota	1	0	0	0	0	0	0	0	0	0
Korarchaeota	1	0	0	0	0	0	0	0	0	0
Total	628	0	0	2	0	0	57	0	0	81

(M00192_1)

Phyla	Module completion ratio (%)									
	0--10	10--20	20--30	30--40	40--50	50--60	60--70	70--80	80--90	90--100
Gammaproteobacteria	85	0	0	10	0	0	2	0	0	29
Betaproteobacteria	60	0	0	0	0	0	0	0	0	1
Epsilonproteobacteria	17	0	0	0	0	0	0	0	0	0
Deltaproteobacteria	22	0	0	5	0	0	1	0	0	0
Alphaproteobacteria	83	0	0	2	0	0	0	0	0	6
Magnetococcus	1	0	0	0	0	0	0	0	0	0
Chrysiogenetes	1	0	0	0	0	0	0	0	0	0
Firmicutes	99	0	0	5	0	0	0	0	0	0
Tenericutes	19	0	0	0	0	0	0	0	0	0
Actinobacteria	78	0	0	2	0	0	0	0	0	0
Chlamydiae	8	0	0	0	0	0	0	0	0	0
Spirochaetes	13	0	0	1	0	0	0	0	0	0
Acidobacteria	5	0	0	0	0	0	0	0	0	0
Bacteroidetes	34	0	0	1	0	0	0	0	0	0
Fibrobacteres	1	0	0	0	0	0	0	0	0	0
Fusobacteria	4	0	0	1	0	0	0	0	0	0
Verrucomicrobia	4	0	0	0	0	0	0	0	0	0
Gemmatimonadetes	1	0	0	0	0	0	0	0	0	0
Planctomycetes	4	0	0	0	0	0	0	0	0	0
Elusimicrobia	2	0	0	0	0	0	0	0	0	0
Synergistetes	2	0	0	0	0	0	0	0	0	0
Cyanobacteria	16	0	0	0	0	0	0	0	0	0
Chlorobi	10	0	0	0	0	0	0	0	0	0
Chloroflexi	10	0	0	1	0	0	0	0	0	0
Deinococcus-Thermus	7	0	0	0	0	0	0	0	0	0
Aquificae	9	0	0	0	0	0	0	0	0	0
Thermotogae	11	0	0	0	0	0	0	0	0	0
Dictyoglomi	2	0	0	0	0	0	0	0	0	0
Nitrospirae	2	0	0	0	0	0	0	0	0	0
Thermobaculum	1	0	0	0	0	0	0	0	0	0
Deferribacteres	3	0	0	0	0	0	0	0	0	0
Euryarchaeota	60	0	0	0	0	0	0	0	0	0
Crenarchaeota	23	0	0	0	0	0	0	0	0	0
Thaumarchaeota	2	0	0	0	0	0	0	0	0	0
Nanoarchaeota	1	0	0	0	0	0	0	0	0	0
Korarchaeota	1	0	0	0	0	0	0	0	0	0
Total	701	0	0	28	0	0	3	0	0	36

(M00193_1)

Phyla	Module completion ratio (%)									
	0--10	10--20	20--30	30--40	40--50	50--60	60--70	70--80	80--90	90--100
Gammaproteobacteria	73	0	11	0	0	7	0	1	0	34
Betaproteobacteria	20	0	5	0	0	2	0	5	0	29
Epsilonproteobacteria	15	0	0	0	0	0	0	0	0	2
Deltaproteobacteria	20	0	4	0	0	0	0	2	0	2
Alphaproteobacteria	52	0	2	0	0	1	0	3	0	33
Magnetococcus	0	0	1	0	0	0	0	0	0	0
Chrysiogenetes	1	0	0	0	0	0	0	0	0	0
Firmicutes	88	0	1	0	0	1	0	4	0	10
Tenericutes	19	0	0	0	0	0	0	0	0	0
Actinobacteria	51	0	8	0	0	2	0	4	0	15
Chlamydiae	8	0	0	0	0	0	0	0	0	0
Spirochaetes	13	0	0	0	0	0	0	0	0	1
Acidobacteria	4	0	0	0	0	0	0	1	0	0
Bacteroidetes	34	0	1	0	0	0	0	0	0	0
Fibrobacteres	1	0	0	0	0	0	0	0	0	0
Fusobacteria	2	0	1	0	0	0	0	0	0	2
Verrucomicrobia	4	0	0	0	0	0	0	0	0	0
Gemmatimonadetes	1	0	0	0	0	0	0	0	0	0
Planctomycetes	3	0	1	0	0	0	0	0	0	0
Elusimicrobia	2	0	0	0	0	0	0	0	0	0
Synergistetes	1	0	0	0	0	1	0	0	0	0
Cyanobacteria	7	0	8	0	0	1	0	0	0	0
Chlorobi	10	0	0	0	0	0	0	0	0	0
Chloroflexi	5	0	0	0	0	0	0	0	0	6
Deinococcus-Thermus	5	0	0	0	0	1	0	0	0	1
Aquificae	9	0	0	0	0	0	0	0	0	0
Thermotogae	10	0	0	0	0	0	0	0	0	1
Dictyoglomi	2	0	0	0	0	0	0	0	0	0
Nitrospirae	2	0	0	0	0	0	0	0	0	0
Thermobaculum	1	0	0	0	0	0	0	0	0	0
Deferribacteres	2	0	0	0	0	1	0	0	0	0
Euryarchaeota	50	0	7	0	0	0	0	0	0	3
Crenarchaeota	11	0	12	0	0	0	0	0	0	0
Thaumarchaeota	2	0	0	0	0	0	0	0	0	0
Nanoarchaeota	1	0	0	0	0	0	0	0	0	0
Korarchaeota	1	0	0	0	0	0	0	0	0	0
Total	530	0	62	0	0	17	0	20	0	139

(M00194_1)

Phyla	Module completion ratio (%)									
	0--10	10--20	20--30	30--40	40--50	50--60	60--70	70--80	80--90	90--100
Gammaproteobacteria	75	0	8	0	0	0	0	0	0	43
Betaproteobacteria	50	0	11	0	0	0	0	0	0	0
Epsilonproteobacteria	16	0	1	0	0	0	0	0	0	0
Deltaproteobacteria	21	0	1	0	0	0	0	1	0	5
Alphaproteobacteria	76	0	15	0	0	0	0	0	0	0
Magnetococcus	1	0	0	0	0	0	0	0	0	0
Chrysiogenetes	1	0	0	0	0	0	0	0	0	0
Firmicutes	31	0	22	0	0	0	0	2	0	49
Tenericutes	15	0	1	0	0	0	0	2	0	1
Actinobacteria	41	0	9	0	0	3	0	23	0	4
Chlamydiae	8	0	0	0	0	0	0	0	0	0
Spirochaetes	12	0	2	0	0	0	0	0	0	0
Acidobacteria	5	0	0	0	0	0	0	0	0	0
Bacteroidetes	34	0	1	0	0	0	0	0	0	0
Fibrobacteres	1	0	0	0	0	0	0	0	0	0
Fusobacteria	1	0	3	0	0	0	0	0	0	1
Verrucomicrobia	2	0	1	0	0	0	0	0	0	1
Gemmatimonadetes	1	0	0	0	0	0	0	0	0	0
Planctomycetes	4	0	0	0	0	0	0	0	0	0
Elusimicrobia	2	0	0	0	0	0	0	0	0	0
Synergistetes	2	0	0	0	0	0	0	0	0	0
Cyanobacteria	9	0	6	0	0	1	0	0	0	0
Chlorobi	10	0	0	0	0	0	0	0	0	0
Chloroflexi	3	0	3	0	0	2	0	0	0	3
Deinococcus-Thermus	0	0	1	0	0	0	0	0	0	6
Aquificae	9	0	0	0	0	0	0	0	0	0
Thermotogae	0	0	0	0	0	0	0	0	0	11
Dictyoglomi	0	0	0	0	0	0	0	0	0	2
Nitrospirae	2	0	0	0	0	0	0	0	0	0
Thermobaculum	0	0	1	0	0	0	0	0	0	0
Deferribacteres	3	0	0	0	0	0	0	0	0	0
Euryarchaeota	52	0	8	0	0	0	0	0	0	0
Crenarchaeota	22	0	0	0	0	0	0	1	0	0
Thaumarchaeota	2	0	0	0	0	0	0	0	0	0
Nanoarchaeota	1	0	0	0	0	0	0	0	0	0
Korarchaeota	1	0	0	0	0	0	0	0	0	0
Total	513	0	94	0	0	6	0	29	0	126

(M00195_1)

Phyla	Module completion ratio (%)									
	0--10	10--20	20--30	30--40	40--50	50--60	60--70	70--80	80--90	90--100
Gammaproteobacteria	126	0	0	0	0	0	0	0	0	0
Betaproteobacteria	61	0	0	0	0	0	0	0	0	0
Epsilonproteobacteria	17	0	0	0	0	0	0	0	0	0
Deltaproteobacteria	28	0	0	0	0	0	0	0	0	0
Alphaproteobacteria	91	0	0	0	0	0	0	0	0	0
Magnetococcus	1	0	0	0	0	0	0	0	0	0
Chrysiogenetes	1	0	0	0	0	0	0	0	0	0
Firmicutes	104	0	0	0	0	0	0	0	0	0
Tenericutes	19	0	0	0	0	0	0	0	0	0
Actinobacteria	80	0	0	0	0	0	0	0	0	0
Chlamydiae	8	0	0	0	0	0	0	0	0	0
Spirochaetes	14	0	0	0	0	0	0	0	0	0
Acidobacteria	5	0	0	0	0	0	0	0	0	0
Bacteroidetes	35	0	0	0	0	0	0	0	0	0
Fibrobacteres	1	0	0	0	0	0	0	0	0	0
Fusobacteria	5	0	0	0	0	0	0	0	0	0
Verrucomicrobia	4	0	0	0	0	0	0	0	0	0
Gemmatimonadetes	1	0	0	0	0	0	0	0	0	0
Planctomycetes	4	0	0	0	0	0	0	0	0	0
Elusimicrobia	2	0	0	0	0	0	0	0	0	0
Synergistetes	2	0	0	0	0	0	0	0	0	0
Cyanobacteria	16	0	0	0	0	0	0	0	0	0
Chlorobi	10	0	0	0	0	0	0	0	0	0
Chloroflexi	11	0	0	0	0	0	0	0	0	0
Deinococcus-Thermus	7	0	0	0	0	0	0	0	0	0
Aquificae	9	0	0	0	0	0	0	0	0	0
Thermotogae	11	0	0	0	0	0	0	0	0	0
Dictyoglomi	2	0	0	0	0	0	0	0	0	0
Nitrospirae	2	0	0	0	0	0	0	0	0	0
Thermobaculum	1	0	0	0	0	0	0	0	0	0
Deferribacteres	3	0	0	0	0	0	0	0	0	0
Euryarchaeota	44	0	3	0	0	0	0	0	0	13
Crenarchaeota	14	0	0	0	0	0	0	5	0	4
Thaumarchaeota	2	0	0	0	0	0	0	0	0	0
Nanoarchaeota	1	0	0	0	0	0	0	0	0	0
Korarchaeota	1	0	0	0	0	0	0	0	0	0
Total	743	0	3	0	0	0	0	5	0	17

(M00196_1)

Phyla	Module completion ratio (%)									
	0--10	10--20	20--30	30--40	40--50	50--60	60--70	70--80	80--90	90--100
Gammaproteobacteria	114	0	12	0	0	0	0	0	0	0
Betaproteobacteria	58	0	3	0	0	0	0	0	0	0
Epsilonproteobacteria	17	0	0	0	0	0	0	0	0	0
Deltaproteobacteria	24	0	4	0	0	0	0	0	0	0
Alphaproteobacteria	81	0	7	0	0	0	0	1	0	2
Magnetococcus	1	0	0	0	0	0	0	0	0	0
Chrysiogenetes	1	0	0	0	0	0	0	0	0	0
Firmicutes	32	0	45	0	0	1	0	2	0	24
Tenericutes	15	0	4	0	0	0	0	0	0	0
Actinobacteria	56	0	11	0	0	0	0	12	0	1
Chlamydiae	8	0	0	0	0	0	0	0	0	0
Spirochaetes	12	0	2	0	0	0	0	0	0	0
Acidobacteria	5	0	0	0	0	0	0	0	0	0
Bacteroidetes	34	0	1	0	0	0	0	0	0	0
Fibrobacteres	1	0	0	0	0	0	0	0	0	0
Fusobacteria	1	0	4	0	0	0	0	0	0	0
Verrucomicrobia	2	0	2	0	0	0	0	0	0	0
Gemmatimonadetes	1	0	0	0	0	0	0	0	0	0
Planctomycetes	4	0	0	0	0	0	0	0	0	0
Elusimicrobia	2	0	0	0	0	0	0	0	0	0
Synergistetes	2	0	0	0	0	0	0	0	0	0
Cyanobacteria	11	0	5	0	0	0	0	0	0	0
Chlorobi	10	0	0	0	0	0	0	0	0	0
Chloroflexi	3	0	8	0	0	0	0	0	0	0
Deinococcus-Thermus	1	0	6	0	0	0	0	0	0	0
Aquificae	9	0	0	0	0	0	0	0	0	0
Thermotogae	0	0	11	0	0	0	0	0	0	0
Dictyoglomi	0	0	1	0	0	0	0	0	0	1
Nitrospirae	2	0	0	0	0	0	0	0	0	0
Thermobaculum	0	0	0	0	0	1	0	0	0	0
Deferribacteres	3	0	0	0	0	0	0	0	0	0
Euryarchaeota	52	0	8	0	0	0	0	0	0	0
Crenarchaeota	23	0	0	0	0	0	0	0	0	0
Thaumarchaeota	2	0	0	0	0	0	0	0	0	0
Nanoarchaeota	1	0	0	0	0	0	0	0	0	0
Korarchaeota	1	0	0	0	0	0	0	0	0	0
Total	589	0	134	0	0	2	0	15	0	28

(M00197_1)

Phyla	Module completion ratio (%)									
	0--10	10--20	20--30	30--40	40--50	50--60	60--70	70--80	80--90	90--100
Gammaproteobacteria	75	0	8	0	0	0	0	0	0	43
Betaproteobacteria	50	0	11	0	0	0	0	0	0	0
Epsilonproteobacteria	16	0	1	0	0	0	0	0	0	0
Deltaproteobacteria	21	0	1	0	0	0	0	1	0	5
Alphaproteobacteria	76	0	15	0	0	0	0	0	0	0
Magnetococcus	1	0	0	0	0	0	0	0	0	0
Chrysiogenetes	1	0	0	0	0	0	0	0	0	0
Firmicutes	31	0	22	0	0	0	0	2	0	49
Tenericutes	15	0	1	0	0	0	0	2	0	1
Actinobacteria	41	0	9	0	0	3	0	23	0	4
Chlamydiae	8	0	0	0	0	0	0	0	0	0
Spirochaetes	12	0	2	0	0	0	0	0	0	0
Acidobacteria	5	0	0	0	0	0	0	0	0	0
Bacteroidetes	34	0	1	0	0	0	0	0	0	0
Fibrobacteres	1	0	0	0	0	0	0	0	0	0
Fusobacteria	1	0	3	0	0	0	0	0	0	1
Verrucomicrobia	2	0	1	0	0	0	0	0	0	1
Gemmatimonadetes	1	0	0	0	0	0	0	0	0	0
Planctomycetes	4	0	0	0	0	0	0	0	0	0
Elusimicrobia	2	0	0	0	0	0	0	0	0	0
Synergistetes	2	0	0	0	0	0	0	0	0	0
Cyanobacteria	9	0	6	0	0	1	0	0	0	0
Chlorobi	10	0	0	0	0	0	0	0	0	0
Chloroflexi	3	0	3	0	0	2	0	0	0	3
Deinococcus-Thermus	0	0	1	0	0	0	0	0	0	6
Aquificae	9	0	0	0	0	0	0	0	0	0
Thermotogae	0	0	0	0	0	0	0	0	0	11
Dictyoglomi	0	0	0	0	0	0	0	0	0	2
Nitrospirae	2	0	0	0	0	0	0	0	0	0
Thermobaculum	0	0	1	0	0	0	0	0	0	0
Deferribacteres	3	0	0	0	0	0	0	0	0	0
Euryarchaeota	43	0	17	0	0	0	0	0	0	0
Crenarchaeota	18	0	4	0	0	0	0	0	0	1
Thaumarchaeota	2	0	0	0	0	0	0	0	0	0
Nanoarchaeota	1	0	0	0	0	0	0	0	0	0
Korarchaeota	1	0	0	0	0	0	0	0	0	0
Total	500	0	107	0	0	6	0	28	0	127

(M00197_2)

Phyla	Module completion ratio (%)									
	0--10	10--20	20--30	30--40	40--50	50--60	60--70	70--80	80--90	90--100
Gammaproteobacteria	77	0	49	0	0	0	0	0	0	0
Betaproteobacteria	50	0	11	0	0	0	0	0	0	0
Epsilonproteobacteria	16	0	1	0	0	0	0	0	0	0
Deltaproteobacteria	22	0	6	0	0	0	0	0	0	0
Alphaproteobacteria	75	0	13	0	0	0	0	1	0	2
Magnetococcus	1	0	0	0	0	0	0	0	0	0
Chrysiogenetes	1	0	0	0	0	0	0	0	0	0
Firmicutes	32	0	45	0	0	1	0	2	0	24
Tenericutes	15	0	4	0	0	0	0	0	0	0
Actinobacteria	56	0	11	0	0	0	0	12	0	1
Chlamydiae	8	0	0	0	0	0	0	0	0	0
Spirochaetes	12	0	2	0	0	0	0	0	0	0
Acidobacteria	5	0	0	0	0	0	0	0	0	0
Bacteroidetes	34	0	1	0	0	0	0	0	0	0
Fibrobacteres	1	0	0	0	0	0	0	0	0	0
Fusobacteria	1	0	4	0	0	0	0	0	0	0
Verrucomicrobia	2	0	2	0	0	0	0	0	0	0
Gemmatimonadetes	1	0	0	0	0	0	0	0	0	0
Planctomycetes	4	0	0	0	0	0	0	0	0	0
Elusimicrobia	2	0	0	0	0	0	0	0	0	0
Synergistetes	2	0	0	0	0	0	0	0	0	0
Cyanobacteria	10	0	6	0	0	0	0	0	0	0
Chlorobi	10	0	0	0	0	0	0	0	0	0
Chloroflexi	3	0	8	0	0	0	0	0	0	0
Deinococcus-Thermus	1	0	6	0	0	0	0	0	0	0
Aquificae	9	0	0	0	0	0	0	0	0	0
Thermotogae	0	0	11	0	0	0	0	0	0	0
Dictyoglomi	0	0	1	0	0	0	0	0	0	1
Nitrospirae	2	0	0	0	0	0	0	0	0	0
Thermobaculum	0	0	0	0	0	1	0	0	0	0
Deferribacteres	3	0	0	0	0	0	0	0	0	0
Euryarchaeota	43	0	17	0	0	0	0	0	0	0
Crenarchaeota	18	0	5	0	0	0	0	0	0	0
Thaumarchaeota	2	0	0	0	0	0	0	0	0	0
Nanoarchaeota	1	0	0	0	0	0	0	0	0	0
Korarchaeota	1	0	0	0	0	0	0	0	0	0
Total	520	0	203	0	0	2	0	15	0	28

(M00197_3)

Phyla	Module completion ratio (%)									
	0--10	10--20	20--30	30--40	40--50	50--60	60--70	70--80	80--90	90--100
Gammaproteobacteria	77	0	49	0	0	0	0	0	0	0
Betaproteobacteria	50	0	11	0	0	0	0	0	0	0
Epsilonproteobacteria	16	0	1	0	0	0	0	0	0	0
Deltaproteobacteria	22	0	6	0	0	0	0	0	0	0
Alphaproteobacteria	76	0	15	0	0	0	0	0	0	0
Magnetococcus	1	0	0	0	0	0	0	0	0	0
Chrysiogenetes	1	0	0	0	0	0	0	0	0	0
Firmicutes	33	0	71	0	0	0	0	0	0	0
Tenericutes	15	0	4	0	0	0	0	0	0	0
Actinobacteria	70	0	10	0	0	0	0	0	0	0
Chlamydiae	8	0	0	0	0	0	0	0	0	0
Spirochaetes	12	0	2	0	0	0	0	0	0	0
Acidobacteria	5	0	0	0	0	0	0	0	0	0
Bacteroidetes	34	0	1	0	0	0	0	0	0	0
Fibrobacteres	1	0	0	0	0	0	0	0	0	0
Fusobacteria	1	0	4	0	0	0	0	0	0	0
Verrucomicrobia	2	0	2	0	0	0	0	0	0	0
Gemmatimonadetes	1	0	0	0	0	0	0	0	0	0
Planctomycetes	4	0	0	0	0	0	0	0	0	0
Elusimicrobia	2	0	0	0	0	0	0	0	0	0
Synergistetes	2	0	0	0	0	0	0	0	0	0
Cyanobacteria	10	0	6	0	0	0	0	0	0	0
Chlorobi	10	0	0	0	0	0	0	0	0	0
Chloroflexi	3	0	8	0	0	0	0	0	0	0
Deinococcus-Thermus	1	0	6	0	0	0	0	0	0	0
Aquificae	9	0	0	0	0	0	0	0	0	0
Thermotogae	0	0	11	0	0	0	0	0	0	0
Dictyoglomi	0	0	2	0	0	0	0	0	0	0
Nitrospirae	2	0	0	0	0	0	0	0	0	0
Thermobaculum	0	0	1	0	0	0	0	0	0	0
Deferribacteres	3	0	0	0	0	0	0	0	0	0
Euryarchaeota	43	0	4	0	0	0	0	0	0	13
Crenarchaeota	14	0	0	0	0	0	0	5	0	4
Thaumarchaeota	2	0	0	0	0	0	0	0	0	0
Nanoarchaeota	1	0	0	0	0	0	0	0	0	0
Korarchaeota	1	0	0	0	0	0	0	0	0	0
Total	532	0	214	0	0	0	0	5	0	17

(M00197_4)

Phyla	Module completion ratio (%)									
	0--10	10--20	20--30	30--40	40--50	50--60	60--70	70--80	80--90	90--100
Gammaproteobacteria	77	0	49	0	0	0	0	0	0	0
Betaproteobacteria	50	0	11	0	0	0	0	0	0	0
Epsilonproteobacteria	16	0	1	0	0	0	0	0	0	0
Deltaproteobacteria	22	0	6	0	0	0	0	0	0	0
Alphaproteobacteria	76	0	15	0	0	0	0	0	0	0
Magnetococcus	1	0	0	0	0	0	0	0	0	0
Chrysiogenetes	1	0	0	0	0	0	0	0	0	0
Firmicutes	33	0	67	0	0	0	0	0	0	4
Tenericutes	15	0	4	0	0	0	0	0	0	0
Actinobacteria	70	0	10	0	0	0	0	0	0	0
Chlamydiae	8	0	0	0	0	0	0	0	0	0
Spirochaetes	12	0	2	0	0	0	0	0	0	0
Acidobacteria	5	0	0	0	0	0	0	0	0	0
Bacteroidetes	34	0	1	0	0	0	0	0	0	0
Fibrobacteres	1	0	0	0	0	0	0	0	0	0
Fusobacteria	1	0	4	0	0	0	0	0	0	0
Verrucomicrobia	2	0	2	0	0	0	0	0	0	0
Gemmatimonadetes	1	0	0	0	0	0	0	0	0	0
Planctomycetes	4	0	0	0	0	0	0	0	0	0
Elusimicrobia	2	0	0	0	0	0	0	0	0	0
Synergistetes	2	0	0	0	0	0	0	0	0	0
Cyanobacteria	10	0	6	0	0	0	0	0	0	0
Chlorobi	10	0	0	0	0	0	0	0	0	0
Chloroflexi	3	0	8	0	0	0	0	0	0	0
Deinococcus-Thermus	1	0	6	0	0	0	0	0	0	0
Aquificae	9	0	0	0	0	0	0	0	0	0
Thermotogae	0	0	11	0	0	0	0	0	0	0
Dictyoglomi	0	0	2	0	0	0	0	0	0	0
Nitrospirae	2	0	0	0	0	0	0	0	0	0
Thermobaculum	0	0	1	0	0	0	0	0	0	0
Deferribacteres	3	0	0	0	0	0	0	0	0	0
Euryarchaeota	43	0	17	0	0	0	0	0	0	0
Crenarchaeota	18	0	5	0	0	0	0	0	0	0
Thaumarchaeota	2	0	0	0	0	0	0	0	0	0
Nanoarchaeota	1	0	0	0	0	0	0	0	0	0
Korarchaeota	1	0	0	0	0	0	0	0	0	0
Total	536	0	228	0	0	0	0	0	0	4

(M00198_1)

Phyla	Module completion ratio (%)									
	0--10	10--20	20--30	30--40	40--50	50--60	60--70	70--80	80--90	90--100
Gammaproteobacteria	85	0	8	0	0	0	0	3	0	30
Betaproteobacteria	24	0	5	0	0	0	0	1	0	31
Epsilonproteobacteria	17	0	0	0	0	0	0	0	0	0
Deltaproteobacteria	24	0	0	0	0	1	0	1	0	2
Alphaproteobacteria	58	0	1	0	0	0	0	2	0	30
Magnetococcus	1	0	0	0	0	0	0	0	0	0
Chrysiogenetes	1	0	0	0	0	0	0	0	0	0
Firmicutes	80	0	6	0	0	0	0	6	0	12
Tenericutes	18	0	1	0	0	0	0	0	0	0
Actinobacteria	55	0	6	0	0	0	0	11	0	8
Chlamydiae	8	0	0	0	0	0	0	0	0	0
Spirochaetes	13	0	0	0	0	0	0	1	0	0
Acidobacteria	5	0	0	0	0	0	0	0	0	0
Bacteroidetes	35	0	0	0	0	0	0	0	0	0
Fibrobacteres	1	0	0	0	0	0	0	0	0	0
Fusobacteria	5	0	0	0	0	0	0	0	0	0
Verrucomicrobia	4	0	0	0	0	0	0	0	0	0
Gemmatimonadetes	1	0	0	0	0	0	0	0	0	0
Planctomycetes	4	0	0	0	0	0	0	0	0	0
Elusimicrobia	2	0	0	0	0	0	0	0	0	0
Synergistetes	2	0	0	0	0	0	0	0	0	0
Cyanobacteria	15	0	0	0	0	0	0	1	0	0
Chlorobi	10	0	0	0	0	0	0	0	0	0
Chloroflexi	6	0	0	0	0	0	0	5	0	0
Deinococcus-Thermus	1	0	1	0	0	2	0	3	0	0
Aquificae	9	0	0	0	0	0	0	0	0	0
Thermotogae	3	0	0	0	0	0	0	8	0	0
Dictyoglomi	0	0	2	0	0	0	0	0	0	0
Nitrospirae	1	0	1	0	0	0	0	0	0	0
Thermobaculum	0	0	0	0	0	0	0	1	0	0
Deferribacteres	3	0	0	0	0	0	0	0	0	0
Euryarchaeota	54	0	0	0	0	0	0	6	0	0
Crenarchaeota	23	0	0	0	0	0	0	0	0	0
Thaumarchaeota	2	0	0	0	0	0	0	0	0	0
Nanoarchaeota	1	0	0	0	0	0	0	0	0	0
Korarchaeota	1	0	0	0	0	0	0	0	0	0
Total	572	0	31	0	0	3	0	49	0	113

(M00199_1)

Phyla	Module completion ratio (%)									
	0--10	10--20	20--30	30--40	40--50	50--60	60--70	70--80	80--90	90--100
Gammaproteobacteria	113	0	0	0	0	0	0	0	0	13
Betaproteobacteria	60	0	0	0	0	0	0	0	0	1
Epsilonproteobacteria	17	0	0	0	0	0	0	0	0	0
Deltaproteobacteria	28	0	0	0	0	0	0	0	0	0
Alphaproteobacteria	83	0	0	0	0	0	0	0	0	8
Magnetococcus	1	0	0	0	0	0	0	0	0	0
Chrysiogenetes	1	0	0	0	0	0	0	0	0	0
Firmicutes	83	0	0	5	0	0	0	0	0	16
Tenericutes	19	0	0	0	0	0	0	0	0	0
Actinobacteria	63	0	0	2	0	0	0	0	0	15
Chlamydiae	8	0	0	0	0	0	0	0	0	0
Spirochaetes	13	0	0	0	0	0	0	0	0	1
Acidobacteria	5	0	0	0	0	0	0	0	0	0
Bacteroidetes	35	0	0	0	0	0	0	0	0	0
Fibrobacteres	1	0	0	0	0	0	0	0	0	0
Fusobacteria	5	0	0	0	0	0	0	0	0	0
Verrucomicrobia	4	0	0	0	0	0	0	0	0	0
Gemmatimonadetes	1	0	0	0	0	0	0	0	0	0
Planctomycetes	4	0	0	0	0	0	0	0	0	0
Elusimicrobia	2	0	0	0	0	0	0	0	0	0
Synergistetes	2	0	0	0	0	0	0	0	0	0
Cyanobacteria	11	0	0	1	0	0	4	0	0	0
Chlorobi	10	0	0	0	0	0	0	0	0	0
Chloroflexi	8	0	0	0	0	0	2	0	0	1
Deinococcus-Thermus	3	0	0	0	0	0	0	0	0	4
Aquificae	9	0	0	0	0	0	0	0	0	0
Thermotogae	9	0	0	0	0	0	0	0	0	2
Dictyoglomi	1	0	0	0	0	0	0	0	0	1
Nitrospirae	2	0	0	0	0	0	0	0	0	0
Thermobaculum	0	0	0	1	0	0	0	0	0	0
Deferribacteres	3	0	0	0	0	0	0	0	0	0
Euryarchaeota	58	0	0	0	0	0	2	0	0	0
Crenarchaeota	23	0	0	0	0	0	0	0	0	0
Thaumarchaeota	2	0	0	0	0	0	0	0	0	0
Nanoarchaeota	1	0	0	0	0	0	0	0	0	0
Korarchaeota	1	0	0	0	0	0	0	0	0	0
Total	689	0	0	9	0	0	8	0	0	62

(M00200_1)

Phyla	Module completion ratio (%)									
	0--10	10--20	20--30	30--40	40--50	50--60	60--70	70--80	80--90	90--100
Gammaproteobacteria	114	0	0	0	0	0	0	1	0	11
Betaproteobacteria	43	0	0	0	0	0	0	2	0	16
Epsilonproteobacteria	17	0	0	0	0	0	0	0	0	0
Deltaproteobacteria	25	0	1	0	0	0	0	0	0	2
Alphaproteobacteria	66	0	2	0	0	0	0	4	0	19
Magnetococcus	1	0	0	0	0	0	0	0	0	0
Chrysiogenetes	1	0	0	0	0	0	0	0	0	0
Firmicutes	104	0	0	0	0	0	0	0	0	0
Tenericutes	19	0	0	0	0	0	0	0	0	0
Actinobacteria	24	0	40	0	0	1	0	1	0	14
Chlamydiae	8	0	0	0	0	0	0	0	0	0
Spirochaetes	14	0	0	0	0	0	0	0	0	0
Acidobacteria	5	0	0	0	0	0	0	0	0	0
Bacteroidetes	35	0	0	0	0	0	0	0	0	0
Fibrobacteres	1	0	0	0	0	0	0	0	0	0
Fusobacteria	5	0	0	0	0	0	0	0	0	0
Verrucomicrobia	4	0	0	0	0	0	0	0	0	0
Gemmatimonadetes	1	0	0	0	0	0	0	0	0	0
Planctomycetes	4	0	0	0	0	0	0	0	0	0
Elusimicrobia	2	0	0	0	0	0	0	0	0	0
Synergistetes	2	0	0	0	0	0	0	0	0	0
Cyanobacteria	16	0	0	0	0	0	0	0	0	0
Chlorobi	10	0	0	0	0	0	0	0	0	0
Chloroflexi	11	0	0	0	0	0	0	0	0	0
Deinococcus-Thermus	3	0	0	0	0	0	0	4	0	0
Aquificae	9	0	0	0	0	0	0	0	0	0
Thermotogae	11	0	0	0	0	0	0	0	0	0
Dictyoglomi	2	0	0	0	0	0	0	0	0	0
Nitrospirae	2	0	0	0	0	0	0	0	0	0
Thermobaculum	1	0	0	0	0	0	0	0	0	0
Deferribacteres	3	0	0	0	0	0	0	0	0	0
Euryarchaeota	60	0	0	0	0	0	0	0	0	0
Crenarchaeota	23	0	0	0	0	0	0	0	0	0
Thaumarchaeota	2	0	0	0	0	0	0	0	0	0
Nanoarchaeota	1	0	0	0	0	0	0	0	0	0
Korarchaeota	1	0	0	0	0	0	0	0	0	0
Total	650	0	43	0	0	1	0	12	0	62

(M00201_1)

Phyla	Module completion ratio (%)									
	0--10	10--20	20--30	30--40	40--50	50--60	60--70	70--80	80--90	90--100
Gammaproteobacteria	124	0	0	0	0	0	0	0	0	2
Betaproteobacteria	61	0	0	0	0	0	0	0	0	0
Epsilonproteobacteria	17	0	0	0	0	0	0	0	0	0
Deltaproteobacteria	28	0	0	0	0	0	0	0	0	0
Alphaproteobacteria	78	0	0	0	0	0	0	1	0	12
Magnetococcus	1	0	0	0	0	0	0	0	0	0
Chrysiogenetes	1	0	0	0	0	0	0	0	0	0
Firmicutes	103	0	0	0	0	0	0	1	0	0
Tenericutes	19	0	0	0	0	0	0	0	0	0
Actinobacteria	52	0	3	0	0	0	0	25	0	0
Chlamydiae	8	0	0	0	0	0	0	0	0	0
Spirochaetes	14	0	0	0	0	0	0	0	0	0
Acidobacteria	5	0	0	0	0	0	0	0	0	0
Bacteroidetes	35	0	0	0	0	0	0	0	0	0
Fibrobacteres	1	0	0	0	0	0	0	0	0	0
Fusobacteria	5	0	0	0	0	0	0	0	0	0
Verrucomicrobia	4	0	0	0	0	0	0	0	0	0
Gemmatimonadetes	1	0	0	0	0	0	0	0	0	0
Planctomycetes	4	0	0	0	0	0	0	0	0	0
Elusimicrobia	2	0	0	0	0	0	0	0	0	0
Synergistetes	2	0	0	0	0	0	0	0	0	0
Cyanobacteria	15	0	0	0	0	0	0	1	0	0
Chlorobi	10	0	0	0	0	0	0	0	0	0
Chloroflexi	8	0	0	0	0	0	0	3	0	0
Deinococcus-Thermus	7	0	0	0	0	0	0	0	0	0
Aquificae	9	0	0	0	0	0	0	0	0	0
Thermotogae	11	0	0	0	0	0	0	0	0	0
Dictyoglomi	2	0	0	0	0	0	0	0	0	0
Nitrospirae	2	0	0	0	0	0	0	0	0	0
Thermobaculum	1	0	0	0	0	0	0	0	0	0
Deferribacteres	3	0	0	0	0	0	0	0	0	0
Euryarchaeota	60	0	0	0	0	0	0	0	0	0
Crenarchaeota	23	0	0	0	0	0	0	0	0	0
Thaumarchaeota	2	0	0	0	0	0	0	0	0	0
Nanoarchaeota	1	0	0	0	0	0	0	0	0	0
Korarchaeota	1	0	0	0	0	0	0	0	0	0
Total	720	0	3	0	0	0	0	31	0	14

(M00202_1)

Phyla	Module completion ratio (%)									
	0--10	10--20	20--30	30--40	40--50	50--60	60--70	70--80	80--90	90--100
Gammaproteobacteria	115	0	0	0	0	0	0	0	0	11
Betaproteobacteria	61	0	0	0	0	0	0	0	0	0
Epsilonproteobacteria	17	0	0	0	0	0	0	0	0	0
Deltaproteobacteria	28	0	0	0	0	0	0	0	0	0
Alphaproteobacteria	89	0	0	0	0	0	0	1	0	1
Magnetococcus	1	0	0	0	0	0	0	0	0	0
Chrysiogenetes	1	0	0	0	0	0	0	0	0	0
Firmicutes	98	0	1	0	0	1	0	4	0	0
Tenericutes	19	0	0	0	0	0	0	0	0	0
Actinobacteria	80	0	0	0	0	0	0	0	0	0
Chlamydiae	8	0	0	0	0	0	0	0	0	0
Spirochaetes	14	0	0	0	0	0	0	0	0	0
Acidobacteria	5	0	0	0	0	0	0	0	0	0
Bacteroidetes	35	0	0	0	0	0	0	0	0	0
Fibrobacteres	1	0	0	0	0	0	0	0	0	0
Fusobacteria	4	0	0	0	0	0	0	1	0	0
Verrucomicrobia	4	0	0	0	0	0	0	0	0	0
Gemmatimonadetes	1	0	0	0	0	0	0	0	0	0
Planctomycetes	4	0	0	0	0	0	0	0	0	0
Elusimicrobia	2	0	0	0	0	0	0	0	0	0
Synergistetes	2	0	0	0	0	0	0	0	0	0
Cyanobacteria	16	0	0	0	0	0	0	0	0	0
Chlorobi	10	0	0	0	0	0	0	0	0	0
Chloroflexi	11	0	0	0	0	0	0	0	0	0
Deinococcus-Thermus	7	0	0	0	0	0	0	0	0	0
Aquificae	9	0	0	0	0	0	0	0	0	0
Thermotogae	11	0	0	0	0	0	0	0	0	0
Dictyoglomi	2	0	0	0	0	0	0	0	0	0
Nitrospirae	2	0	0	0	0	0	0	0	0	0
Thermobaculum	1	0	0	0	0	0	0	0	0	0
Deferribacteres	3	0	0	0	0	0	0	0	0	0
Euryarchaeota	60	0	0	0	0	0	0	0	0	0
Crenarchaeota	23	0	0	0	0	0	0	0	0	0
Thaumarchaeota	2	0	0	0	0	0	0	0	0	0
Nanoarchaeota	1	0	0	0	0	0	0	0	0	0
Korarchaeota	1	0	0	0	0	0	0	0	0	0
Total	748	0	1	0	0	1	0	6	0	12

(M00203_1)

Phyla	Module completion ratio (%)									
	0--10	10--20	20--30	30--40	40--50	50--60	60--70	70--80	80--90	90--100
Gammaproteobacteria	126	0	0	0	0	0	0	0	0	0
Betaproteobacteria	61	0	0	0	0	0	0	0	0	0
Epsilonproteobacteria	17	0	0	0	0	0	0	0	0	0
Deltaproteobacteria	28	0	0	0	0	0	0	0	0	0
Alphaproteobacteria	91	0	0	0	0	0	0	0	0	0
Magnetococcus	1	0	0	0	0	0	0	0	0	0
Chrysiogenetes	1	0	0	0	0	0	0	0	0	0
Firmicutes	104	0	0	0	0	0	0	0	0	0
Tenericutes	19	0	0	0	0	0	0	0	0	0
Actinobacteria	80	0	0	0	0	0	0	0	0	0
Chlamydiae	8	0	0	0	0	0	0	0	0	0
Spirochaetes	14	0	0	0	0	0	0	0	0	0
Acidobacteria	5	0	0	0	0	0	0	0	0	0
Bacteroidetes	35	0	0	0	0	0	0	0	0	0
Fibrobacteres	1	0	0	0	0	0	0	0	0	0
Fusobacteria	5	0	0	0	0	0	0	0	0	0
Verrucomicrobia	4	0	0	0	0	0	0	0	0	0
Gemmatimonadetes	1	0	0	0	0	0	0	0	0	0
Planctomycetes	4	0	0	0	0	0	0	0	0	0
Elusimicrobia	2	0	0	0	0	0	0	0	0	0
Synergistetes	2	0	0	0	0	0	0	0	0	0
Cyanobacteria	16	0	0	0	0	0	0	0	0	0
Chlorobi	10	0	0	0	0	0	0	0	0	0
Chloroflexi	11	0	0	0	0	0	0	0	0	0
Deinococcus-Thermus	7	0	0	0	0	0	0	0	0	0
Aquificae	9	0	0	0	0	0	0	0	0	0
Thermotogae	11	0	0	0	0	0	0	0	0	0
Dictyoglomi	2	0	0	0	0	0	0	0	0	0
Nitrospirae	2	0	0	0	0	0	0	0	0	0
Thermobaculum	1	0	0	0	0	0	0	0	0	0
Deferribacteres	3	0	0	0	0	0	0	0	0	0
Euryarchaeota	59	0	0	0	0	0	0	0	0	1
Crenarchaeota	13	0	2	0	0	0	0	1	0	7
Thaumarchaeota	2	0	0	0	0	0	0	0	0	0
Nanoarchaeota	1	0	0	0	0	0	0	0	0	0
Korarchaeota	1	0	0	0	0	0	0	0	0	0
Total	757	0	2	0	0	0	0	1	0	8

(M00204_1)

Phyla	Module completion ratio (%)									
	0--10	10--20	20--30	30--40	40--50	50--60	60--70	70--80	80--90	90--100
Gammaproteobacteria	125	0	0	0	0	0	0	0	0	1
Betaproteobacteria	61	0	0	0	0	0	0	0	0	0
Epsilonproteobacteria	17	0	0	0	0	0	0	0	0	0
Deltaproteobacteria	28	0	0	0	0	0	0	0	0	0
Alphaproteobacteria	80	0	0	0	0	2	0	0	0	9
Magnetococcus	1	0	0	0	0	0	0	0	0	0
Chrysiogenetes	1	0	0	0	0	0	0	0	0	0
Firmicutes	104	0	0	0	0	0	0	0	0	0
Tenericutes	19	0	0	0	0	0	0	0	0	0
Actinobacteria	80	0	0	0	0	0	0	0	0	0
Chlamydiae	8	0	0	0	0	0	0	0	0	0
Spirochaetes	14	0	0	0	0	0	0	0	0	0
Acidobacteria	5	0	0	0	0	0	0	0	0	0
Bacteroidetes	35	0	0	0	0	0	0	0	0	0
Fibrobacteres	1	0	0	0	0	0	0	0	0	0
Fusobacteria	5	0	0	0	0	0	0	0	0	0
Verrucomicrobia	4	0	0	0	0	0	0	0	0	0
Gemmatimonadetes	1	0	0	0	0	0	0	0	0	0
Planctomycetes	4	0	0	0	0	0	0	0	0	0
Elusimicrobia	2	0	0	0	0	0	0	0	0	0
Synergistetes	2	0	0	0	0	0	0	0	0	0
Cyanobacteria	16	0	0	0	0	0	0	0	0	0
Chlorobi	10	0	0	0	0	0	0	0	0	0
Chloroflexi	11	0	0	0	0	0	0	0	0	0
Deinococcus-Thermus	7	0	0	0	0	0	0	0	0	0
Aquificae	9	0	0	0	0	0	0	0	0	0
Thermotogae	11	0	0	0	0	0	0	0	0	0
Dictyoglomi	2	0	0	0	0	0	0	0	0	0
Nitrospirae	2	0	0	0	0	0	0	0	0	0
Thermobaculum	1	0	0	0	0	0	0	0	0	0
Deferribacteres	3	0	0	0	0	0	0	0	0	0
Euryarchaeota	60	0	0	0	0	0	0	0	0	0
Crenarchaeota	23	0	0	0	0	0	0	0	0	0
Thaumarchaeota	2	0	0	0	0	0	0	0	0	0
Nanoarchaeota	1	0	0	0	0	0	0	0	0	0
Korarchaeota	1	0	0	0	0	0	0	0	0	0
Total	756	0	0	0	0	2	0	0	0	10

(M00205_1)

Phyla	Module completion ratio (%)									
	0--10	10--20	20--30	30--40	40--50	50--60	60--70	70--80	80--90	90--100
Gammaproteobacteria	126	0	0	0	0	0	0	0	0	0
Betaproteobacteria	61	0	0	0	0	0	0	0	0	0
Epsilonproteobacteria	17	0	0	0	0	0	0	0	0	0
Deltaproteobacteria	28	0	0	0	0	0	0	0	0	0
Alphaproteobacteria	91	0	0	0	0	0	0	0	0	0
Magnetococcus	1	0	0	0	0	0	0	0	0	0
Chrysiogenetes	1	0	0	0	0	0	0	0	0	0
Firmicutes	100	0	0	2	0	0	0	0	0	2
Tenericutes	18	0	0	1	0	0	0	0	0	0
Actinobacteria	63	0	0	1	0	0	1	0	0	15
Chlamydiae	8	0	0	0	0	0	0	0	0	0
Spirochaetes	14	0	0	0	0	0	0	0	0	0
Acidobacteria	5	0	0	0	0	0	0	0	0	0
Bacteroidetes	35	0	0	0	0	0	0	0	0	0
Fibrobacteres	1	0	0	0	0	0	0	0	0	0
Fusobacteria	4	0	0	1	0	0	0	0	0	0
Verrucomicrobia	4	0	0	0	0	0	0	0	0	0
Gemmatimonadetes	1	0	0	0	0	0	0	0	0	0
Planctomycetes	4	0	0	0	0	0	0	0	0	0
Elusimicrobia	2	0	0	0	0	0	0	0	0	0
Synergistetes	2	0	0	0	0	0	0	0	0	0
Cyanobacteria	16	0	0	0	0	0	0	0	0	0
Chlorobi	10	0	0	0	0	0	0	0	0	0
Chloroflexi	11	0	0	0	0	0	0	0	0	0
Deinococcus-Thermus	7	0	0	0	0	0	0	0	0	0
Aquificae	9	0	0	0	0	0	0	0	0	0
Thermotogae	11	0	0	0	0	0	0	0	0	0
Dictyoglomi	2	0	0	0	0	0	0	0	0	0
Nitrospirae	2	0	0	0	0	0	0	0	0	0
Thermobaculum	1	0	0	0	0	0	0	0	0	0
Deferribacteres	3	0	0	0	0	0	0	0	0	0
Euryarchaeota	60	0	0	0	0	0	0	0	0	0
Crenarchaeota	23	0	0	0	0	0	0	0	0	0
Thaumarchaeota	2	0	0	0	0	0	0	0	0	0
Nanoarchaeota	1	0	0	0	0	0	0	0	0	0
Korarchaeota	1	0	0	0	0	0	0	0	0	0
Total	745	0	0	5	0	0	1	0	0	17

(M00206_1)

Phyla	Module completion ratio (%)									
	0--10	10--20	20--30	30--40	40--50	50--60	60--70	70--80	80--90	90--100
Gammaproteobacteria	126	0	0	0	0	0	0	0	0	0
Betaproteobacteria	61	0	0	0	0	0	0	0	0	0
Epsilonproteobacteria	17	0	0	0	0	0	0	0	0	0
Deltaproteobacteria	27	0	1	0	0	0	0	0	0	0
Alphaproteobacteria	90	0	1	0	0	0	0	0	0	0
Magnetococcus	1	0	0	0	0	0	0	0	0	0
Chrysiogenetes	1	0	0	0	0	0	0	0	0	0
Firmicutes	100	0	3	0	0	0	0	1	0	0
Tenericutes	19	0	0	0	0	0	0	0	0	0
Actinobacteria	22	0	30	0	0	0	0	3	0	25
Chlamydiae	8	0	0	0	0	0	0	0	0	0
Spirochaetes	14	0	0	0	0	0	0	0	0	0
Acidobacteria	5	0	0	0	0	0	0	0	0	0
Bacteroidetes	35	0	0	0	0	0	0	0	0	0
Fibrobacteres	1	0	0	0	0	0	0	0	0	0
Fusobacteria	5	0	0	0	0	0	0	0	0	0
Verrucomicrobia	4	0	0	0	0	0	0	0	0	0
Gemmatimonadetes	1	0	0	0	0	0	0	0	0	0
Planctomycetes	4	0	0	0	0	0	0	0	0	0
Elusimicrobia	2	0	0	0	0	0	0	0	0	0
Synergistetes	2	0	0	0	0	0	0	0	0	0
Cyanobacteria	16	0	0	0	0	0	0	0	0	0
Chlorobi	10	0	0	0	0	0	0	0	0	0
Chloroflexi	11	0	0	0	0	0	0	0	0	0
Deinococcus-Thermus	7	0	0	0	0	0	0	0	0	0
Aquificae	9	0	0	0	0	0	0	0	0	0
Thermotogae	11	0	0	0	0	0	0	0	0	0
Dictyoglomi	2	0	0	0	0	0	0	0	0	0
Nitrospirae	2	0	0	0	0	0	0	0	0	0
Thermobaculum	1	0	0	0	0	0	0	0	0	0
Deferribacteres	3	0	0	0	0	0	0	0	0	0
Euryarchaeota	60	0	0	0	0	0	0	0	0	0
Crenarchaeota	23	0	0	0	0	0	0	0	0	0
Thaumarchaeota	2	0	0	0	0	0	0	0	0	0
Nanoarchaeota	1	0	0	0	0	0	0	0	0	0
Korarchaeota	1	0	0	0	0	0	0	0	0	0
Total	704	0	35	0	0	0	0	4	0	25

(M00207_1)

Phyla	Module completion ratio (%)									
	0--10	10--20	20--30	30--40	40--50	50--60	60--70	70--80	80--90	90--100
Gammaproteobacteria	80	0	12	0	0	2	0	7	0	25
Betaproteobacteria	26	0	1	0	0	0	0	0	0	34
Epsilonproteobacteria	16	0	1	0	0	0	0	0	0	0
Deltaproteobacteria	19	0	0	0	0	0	0	2	0	7
Alphaproteobacteria	52	0	2	0	0	0	0	2	0	35
Magnetococcus	1	0	0	0	0	0	0	0	0	0
Chrysiogenetes	1	0	0	0	0	0	0	0	0	0
Firmicutes	33	0	7	0	0	5	0	43	0	16
Tenericutes	4	0	0	0	0	0	0	11	0	4
Actinobacteria	8	0	1	0	0	2	0	38	0	31
Chlamydiae	8	0	0	0	0	0	0	0	0	0
Spirochaetes	11	0	1	0	0	0	0	1	0	1
Acidobacteria	5	0	0	0	0	0	0	0	0	0
Bacteroidetes	30	0	3	0	0	0	0	1	0	1
Fibrobacteres	0	0	1	0	0	0	0	0	0	0
Fusobacteria	1	0	1	0	0	0	0	3	0	0
Verrucomicrobia	2	0	1	0	0	1	0	0	0	0
Gemmatimonadetes	1	0	0	0	0	0	0	0	0	0
Planctomycetes	1	0	3	0	0	0	0	0	0	0
Elusimicrobia	2	0	0	0	0	0	0	0	0	0
Synergistetes	2	0	0	0	0	0	0	0	0	0
Cyanobacteria	3	0	1	0	0	1	0	5	0	6
Chlorobi	10	0	0	0	0	0	0	0	0	0
Chloroflexi	3	0	0	0	0	1	0	7	0	0
Deinococcus-Thermus	0	0	0	0	0	0	0	6	0	1
Aquificae	9	0	0	0	0	0	0	0	0	0
Thermotogae	0	0	0	0	0	0	0	5	0	6
Dictyoglomi	0	0	0	0	0	0	0	2	0	0
Nitrospirae	1	0	0	0	0	0	0	1	0	0
Thermobaculum	0	0	0	0	0	0	0	1	0	0
Deferribacteres	3	0	0	0	0	0	0	0	0	0
Euryarchaeota	38	0	3	0	0	0	0	8	0	11
Crenarchaeota	6	0	0	0	0	0	0	3	0	14
Thaumarchaeota	2	0	0	0	0	0	0	0	0	0
Nanoarchaeota	1	0	0	0	0	0	0	0	0	0
Korarchaeota	1	0	0	0	0	0	0	0	0	0
Total	380	0	38	0	0	12	0	146	0	192

(M00208_1)

Phyla	Module completion ratio (%)									
	0--10	10--20	20--30	30--40	40--50	50--60	60--70	70--80	80--90	90--100
Gammaproteobacteria	64	0	0	5	0	0	9	0	0	48
Betaproteobacteria	33	0	0	1	0	0	3	0	0	24
Epsilonproteobacteria	15	0	0	2	0	0	0	0	0	0
Deltaproteobacteria	17	0	0	1	0	0	0	0	0	10
Alphaproteobacteria	54	0	0	5	0	0	3	0	0	29
Magnetococcus	1	0	0	0	0	0	0	0	0	0
Chrysiogenetes	1	0	0	0	0	0	0	0	0	0
Firmicutes	60	0	0	3	0	0	2	0	0	39
Tenericutes	19	0	0	0	0	0	0	0	0	0
Actinobacteria	49	0	0	7	0	0	2	0	0	22
Chlamydiae	8	0	0	0	0	0	0	0	0	0
Spirochaetes	6	0	0	0	0	0	0	0	0	8
Acidobacteria	5	0	0	0	0	0	0	0	0	0
Bacteroidetes	29	0	0	0	0	0	1	0	0	5
Fibrobacteres	1	0	0	0	0	0	0	0	0	0
Fusobacteria	5	0	0	0	0	0	0	0	0	0
Verrucomicrobia	4	0	0	0	0	0	0	0	0	0
Gemmatimonadetes	1	0	0	0	0	0	0	0	0	0
Planctomycetes	4	0	0	0	0	0	0	0	0	0
Elusimicrobia	2	0	0	0	0	0	0	0	0	0
Synergistetes	1	0	0	0	0	0	0	0	0	1
Cyanobacteria	14	0	0	0	0	0	0	0	0	2
Chlorobi	10	0	0	0	0	0	0	0	0	0
Chloroflexi	9	0	0	0	0	0	0	0	0	2
Deinococcus-Thermus	5	0	0	0	0	0	0	0	0	2
Aquificae	9	0	0	0	0	0	0	0	0	0
Thermotogae	11	0	0	0	0	0	0	0	0	0
Dictyoglomi	2	0	0	0	0	0	0	0	0	0
Nitrospirae	2	0	0	0	0	0	0	0	0	0
Thermobaculum	1	0	0	0	0	0	0	0	0	0
Deferribacteres	2	0	0	0	0	0	0	0	0	1
Euryarchaeota	48	0	0	0	0	0	2	0	0	10
Crenarchaeota	23	0	0	0	0	0	0	0	0	0
Thaumarchaeota	2	0	0	0	0	0	0	0	0	0
Nanoarchaeota	1	0	0	0	0	0	0	0	0	0
Korarchaeota	1	0	0	0	0	0	0	0	0	0
Total	519	0	0	24	0	0	22	0	0	203

(M00209_1)

Phyla	Module completion ratio (%)									
	0--10	10--20	20--30	30--40	40--50	50--60	60--70	70--80	80--90	90--100
Gammaproteobacteria	85	0	0	4	0	0	9	0	0	28
Betaproteobacteria	38	0	0	6	0	0	0	0	0	17
Epsilonproteobacteria	15	0	0	0	0	0	2	0	0	0
Deltaproteobacteria	20	0	0	0	0	0	4	0	0	4
Alphaproteobacteria	60	0	0	0	0	0	5	0	0	26
Magnetococcus	1	0	0	0	0	0	0	0	0	0
Chrysiogenetes	1	0	0	0	0	0	0	0	0	0
Firmicutes	40	0	0	1	0	0	7	0	0	56
Tenericutes	19	0	0	0	0	0	0	0	0	0
Actinobacteria	24	0	0	4	0	0	11	0	0	41
Chlamydiae	7	0	0	0	0	0	0	0	0	1
Spirochaetes	13	0	0	0	0	0	0	0	0	1
Acidobacteria	1	0	0	1	0	0	1	0	0	2
Bacteroidetes	27	0	0	2	0	0	1	0	0	5
Fibrobacteres	1	0	0	0	0	0	0	0	0	0
Fusobacteria	0	0	0	0	0	0	0	0	0	5
Verrucomicrobia	4	0	0	0	0	0	0	0	0	0
Gemmatimonadetes	1	0	0	0	0	0	0	0	0	0
Planctomycetes	2	0	0	0	0	0	1	0	0	1
Elusimicrobia	2	0	0	0	0	0	0	0	0	0
Synergistetes	2	0	0	0	0	0	0	0	0	0
Cyanobacteria	8	0	0	0	0	0	3	0	0	5
Chlorobi	10	0	0	0	0	0	0	0	0	0
Chloroflexi	7	0	0	0	0	0	1	0	0	3
Deinococcus-Thermus	3	0	0	0	0	0	0	0	0	4
Aquificae	9	0	0	0	0	0	0	0	0	0
Thermotogae	10	0	0	0	0	0	0	0	0	1
Dictyoglomi	2	0	0	0	0	0	0	0	0	0
Nitrospirae	2	0	0	0	0	0	0	0	0	0
Thermobaculum	1	0	0	0	0	0	0	0	0	0
Deferribacteres	2	0	0	0	0	0	0	0	0	1
Euryarchaeota	48	0	0	4	0	0	2	0	0	6
Crenarchaeota	23	0	0	0	0	0	0	0	0	0
Thaumarchaeota	2	0	0	0	0	0	0	0	0	0
Nanoarchaeota	1	0	0	0	0	0	0	0	0	0
Korarchaeota	1	0	0	0	0	0	0	0	0	0
Total	492	0	0	22	0	0	47	0	0	207

(M00210_1)

Phyla	Module completion ratio (%)									
	0--10	10--20	20--30	30--40	40--50	50--60	60--70	70--80	80--90	90--100
Gammaproteobacteria	16	0	0	1	0	0	2	0	0	107
Betaproteobacteria	1	0	0	0	0	0	0	0	0	60
Epsilonproteobacteria	0	0	0	0	0	0	3	0	0	14
Deltaproteobacteria	0	0	0	0	0	0	5	0	0	23
Alphaproteobacteria	10	0	0	1	0	0	4	0	0	76
Magnetococcus	1	0	0	0	0	0	0	0	0	0
Chrysiogenetes	0	0	0	0	0	0	1	0	0	0
Firmicutes	102	0	0	0	0	0	0	0	0	2
Tenericutes	19	0	0	0	0	0	0	0	0	0
Actinobacteria	50	0	0	3	0	0	2	0	0	25
Chlamydiae	3	0	0	0	0	0	5	0	0	0
Spirochaetes	9	0	0	0	0	0	3	0	0	2
Acidobacteria	1	0	0	0	0	0	4	0	0	0
Bacteroidetes	4	0	0	0	0	0	20	0	0	11
Fibrobacteres	0	0	0	0	0	0	0	0	0	1
Fusobacteria	5	0	0	0	0	0	0	0	0	0
Verrucomicrobia	0	0	0	0	0	0	1	0	0	3
Gemmatimonadetes	0	0	0	0	0	0	0	0	0	1
Planctomycetes	0	0	0	0	0	0	1	0	0	3
Elusimicrobia	1	0	0	0	0	0	0	0	0	1
Synergistetes	2	0	0	0	0	0	0	0	0	0
Cyanobacteria	0	0	0	0	0	0	1	0	0	15
Chlorobi	0	0	0	0	0	0	8	0	0	2
Chloroflexi	11	0	0	0	0	0	0	0	0	0
Deinococcus-Thermus	7	0	0	0	0	0	0	0	0	0
Aquificae	0	0	0	0	0	0	5	0	0	4
Thermotogae	11	0	0	0	0	0	0	0	0	0
Dictyoglomi	2	0	0	0	0	0	0	0	0	0
Nitrospirae	0	0	0	0	0	0	0	0	0	2
Thermobaculum	1	0	0	0	0	0	0	0	0	0
Deferribacteres	0	0	0	0	0	0	3	0	0	0
Euryarchaeota	60	0	0	0	0	0	0	0	0	0
Crenarchaeota	23	0	0	0	0	0	0	0	0	0
Thaumarchaeota	2	0	0	0	0	0	0	0	0	0
Nanoarchaeota	1	0	0	0	0	0	0	0	0	0
Korarchaeota	1	0	0	0	0	0	0	0	0	0
Total	343	0	0	5	0	0	68	0	0	352

(M00211_1)

Phyla	Module completion ratio (%)									
	0--10	10--20	20--30	30--40	40--50	50--60	60--70	70--80	80--90	90--100
Gammaproteobacteria	70	0	0	0	0	39	0	0	0	17
Betaproteobacteria	42	0	0	0	0	6	0	0	0	13
Epsilonproteobacteria	12	0	0	0	0	5	0	0	0	0
Deltaproteobacteria	16	0	0	0	0	6	0	0	0	6
Alphaproteobacteria	84	0	0	0	0	7	0	0	0	0
Magnetococcus	0	0	0	0	0	1	0	0	0	0
Chrysiogenetes	1	0	0	0	0	0	0	0	0	0
Firmicutes	49	0	0	0	0	10	0	0	0	45
Tenericutes	19	0	0	0	0	0	0	0	0	0
Actinobacteria	60	0	0	0	0	15	0	0	0	5
Chlamydiae	8	0	0	0	0	0	0	0	0	0
Spirochaetes	14	0	0	0	0	0	0	0	0	0
Acidobacteria	4	0	0	0	0	1	0	0	0	0
Bacteroidetes	30	0	0	0	0	2	0	0	0	3
Fibrobacteres	1	0	0	0	0	0	0	0	0	0
Fusobacteria	4	0	0	0	0	0	0	0	0	1
Verrucomicrobia	4	0	0	0	0	0	0	0	0	0
Gemmatimonadetes	1	0	0	0	0	0	0	0	0	0
Planctomycetes	4	0	0	0	0	0	0	0	0	0
Elusimicrobia	2	0	0	0	0	0	0	0	0	0
Synergistetes	2	0	0	0	0	0	0	0	0	0
Cyanobacteria	6	0	0	0	0	9	0	0	0	1
Chlorobi	1	0	0	0	0	3	0	0	0	6
Chloroflexi	10	0	0	0	0	0	0	0	0	1
Deinococcus-Thermus	5	0	0	0	0	1	0	0	0	1
Aquificae	2	0	0	0	0	7	0	0	0	0
Thermotogae	5	0	0	0	0	0	0	0	0	6
Dictyoglomi	2	0	0	0	0	0	0	0	0	0
Nitrospirae	2	0	0	0	0	0	0	0	0	0
Thermobaculum	1	0	0	0	0	0	0	0	0	0
Deferribacteres	0	0	0	0	0	1	0	0	0	2
Euryarchaeota	47	0	0	0	0	9	0	0	0	4
Crenarchaeota	23	0	0	0	0	0	0	0	0	0
Thaumarchaeota	2	0	0	0	0	0	0	0	0	0
Nanoarchaeota	1	0	0	0	0	0	0	0	0	0
Korarchaeota	1	0	0	0	0	0	0	0	0	0
Total	535	0	0	0	0	122	0	0	0	111

(M00212_1)

Phyla	Module completion ratio (%)									
	0--10	10--20	20--30	30--40	40--50	50--60	60--70	70--80	80--90	90--100
Gammaproteobacteria	62	0	0	3	0	0	2	0	0	59
Betaproteobacteria	33	0	0	3	0	0	1	0	0	24
Epsilonproteobacteria	16	0	0	1	0	0	0	0	0	0
Deltaproteobacteria	23	0	0	2	0	0	1	0	0	2
Alphaproteobacteria	61	0	0	0	0	0	2	0	0	28
Magnetococcus	0	0	0	1	0	0	0	0	0	0
Chrysiogenetes	1	0	0	0	0	0	0	0	0	0
Firmicutes	40	0	0	12	0	0	3	0	0	49
Tenericutes	18	0	0	0	0	0	0	0	0	1
Actinobacteria	40	0	0	2	0	0	5	0	0	33
Chlamydiae	8	0	0	0	0	0	0	0	0	0
Spirochaetes	13	0	0	0	0	0	0	0	0	1
Acidobacteria	1	0	0	4	0	0	0	0	0	0
Bacteroidetes	28	0	0	2	0	0	0	0	0	5
Fibrobacteres	1	0	0	0	0	0	0	0	0	0
Fusobacteria	3	0	0	0	0	0	0	0	0	2
Verrucomicrobia	2	0	0	1	0	0	0	0	0	1
Gemmatimonadetes	1	0	0	0	0	0	0	0	0	0
Planctomycetes	0	0	0	0	0	0	0	0	0	4
Elusimicrobia	2	0	0	0	0	0	0	0	0	0
Synergistetes	0	0	0	0	0	0	2	0	0	0
Cyanobacteria	13	0	0	0	0	0	1	0	0	2
Chlorobi	10	0	0	0	0	0	0	0	0	0
Chloroflexi	3	0	0	2	0	0	2	0	0	4
Deinococcus-Thermus	3	0	0	0	0	0	1	0	0	3
Aquificae	9	0	0	0	0	0	0	0	0	0
Thermotogae	1	0	0	1	0	0	0	0	0	9
Dictyoglomi	0	0	0	0	0	0	0	0	0	2
Nitrospirae	2	0	0	0	0	0	0	0	0	0
Thermobaculum	1	0	0	0	0	0	0	0	0	0
Deferribacteres	2	0	0	0	0	0	0	0	0	1
Euryarchaeota	59	0	0	0	0	0	1	0	0	0
Crenarchaeota	21	0	0	1	0	0	0	0	0	1
Thaumarchaeota	2	0	0	0	0	0	0	0	0	0
Nanoarchaeota	1	0	0	0	0	0	0	0	0	0
Korarchaeota	1	0	0	0	0	0	0	0	0	0
Total	481	0	0	35	0	0	21	0	0	231

(M00213_1)

Phyla	Module completion ratio (%)									
	0--10	10--20	20--30	30--40	40--50	50--60	60--70	70--80	80--90	90--100
Gammaproteobacteria	96	0	0	0	0	0	4	0	0	26
Betaproteobacteria	46	0	0	0	0	0	0	0	0	15
Epsilonproteobacteria	17	0	0	0	0	0	0	0	0	0
Deltaproteobacteria	27	0	0	0	0	0	0	0	0	1
Alphaproteobacteria	86	0	0	0	0	0	0	0	0	5
Magnetococcus	1	0	0	0	0	0	0	0	0	0
Chrysiogenetes	1	0	0	0	0	0	0	0	0	0
Firmicutes	103	0	0	1	0	0	0	0	0	0
Tenericutes	19	0	0	0	0	0	0	0	0	0
Actinobacteria	78	0	0	1	0	0	1	0	0	0
Chlamydiae	8	0	0	0	0	0	0	0	0	0
Spirochaetes	13	0	0	0	0	0	1	0	0	0
Acidobacteria	5	0	0	0	0	0	0	0	0	0
Bacteroidetes	35	0	0	0	0	0	0	0	0	0
Fibrobacteres	1	0	0	0	0	0	0	0	0	0
Fusobacteria	5	0	0	0	0	0	0	0	0	0
Verrucomicrobia	4	0	0	0	0	0	0	0	0	0
Gemmatimonadetes	1	0	0	0	0	0	0	0	0	0
Planctomycetes	4	0	0	0	0	0	0	0	0	0
Elusimicrobia	2	0	0	0	0	0	0	0	0	0
Synergistetes	2	0	0	0	0	0	0	0	0	0
Cyanobacteria	16	0	0	0	0	0	0	0	0	0
Chlorobi	10	0	0	0	0	0	0	0	0	0
Chloroflexi	11	0	0	0	0	0	0	0	0	0
Deinococcus-Thermus	7	0	0	0	0	0	0	0	0	0
Aquificae	9	0	0	0	0	0	0	0	0	0
Thermotogae	11	0	0	0	0	0	0	0	0	0
Dictyoglomi	2	0	0	0	0	0	0	0	0	0
Nitrospirae	2	0	0	0	0	0	0	0	0	0
Thermobaculum	1	0	0	0	0	0	0	0	0	0
Deferribacteres	3	0	0	0	0	0	0	0	0	0
Euryarchaeota	60	0	0	0	0	0	0	0	0	0
Crenarchaeota	23	0	0	0	0	0	0	0	0	0
Thaumarchaeota	2	0	0	0	0	0	0	0	0	0
Nanoarchaeota	1	0	0	0	0	0	0	0	0	0
Korarchaeota	1	0	0	0	0	0	0	0	0	0
Total	713	0	0	2	0	0	6	0	0	47

(M00214_1)

Phyla	Module completion ratio (%)									
	0--10	10--20	20--30	30--40	40--50	50--60	60--70	70--80	80--90	90--100
Gammaproteobacteria	91	0	0	0	0	0	2	0	0	33
Betaproteobacteria	61	0	0	0	0	0	0	0	0	0
Epsilonproteobacteria	17	0	0	0	0	0	0	0	0	0
Deltaproteobacteria	28	0	0	0	0	0	0	0	0	0
Alphaproteobacteria	91	0	0	0	0	0	0	0	0	0
Magnetococcus	1	0	0	0	0	0	0	0	0	0
Chrysiogenetes	1	0	0	0	0	0	0	0	0	0
Firmicutes	96	0	0	1	0	0	0	0	0	7
Tenericutes	19	0	0	0	0	0	0	0	0	0
Actinobacteria	78	0	0	0	0	0	0	0	0	2
Chlamydiae	8	0	0	0	0	0	0	0	0	0
Spirochaetes	10	0	0	0	0	0	0	0	0	4
Acidobacteria	5	0	0	0	0	0	0	0	0	0
Bacteroidetes	35	0	0	0	0	0	0	0	0	0
Fibrobacteres	1	0	0	0	0	0	0	0	0	0
Fusobacteria	2	0	0	0	0	0	0	0	0	3
Verrucomicrobia	4	0	0	0	0	0	0	0	0	0
Gemmatimonadetes	1	0	0	0	0	0	0	0	0	0
Planctomycetes	4	0	0	0	0	0	0	0	0	0
Elusimicrobia	2	0	0	0	0	0	0	0	0	0
Synergistetes	1	0	0	1	0	0	0	0	0	0
Cyanobacteria	15	0	0	0	0	0	0	0	0	1
Chlorobi	10	0	0	0	0	0	0	0	0	0
Chloroflexi	11	0	0	0	0	0	0	0	0	0
Deinococcus-Thermus	7	0	0	0	0	0	0	0	0	0
Aquificae	9	0	0	0	0	0	0	0	0	0
Thermotogae	11	0	0	0	0	0	0	0	0	0
Dictyoglomi	2	0	0	0	0	0	0	0	0	0
Nitrospirae	2	0	0	0	0	0	0	0	0	0
Thermobaculum	1	0	0	0	0	0	0	0	0	0
Deferribacteres	3	0	0	0	0	0	0	0	0	0
Euryarchaeota	60	0	0	0	0	0	0	0	0	0
Crenarchaeota	23	0	0	0	0	0	0	0	0	0
Thaumarchaeota	2	0	0	0	0	0	0	0	0	0
Nanoarchaeota	1	0	0	0	0	0	0	0	0	0
Korarchaeota	1	0	0	0	0	0	0	0	0	0
Total	714	0	0	2	0	0	2	0	0	50

(M00215_1)

Phyla	Module completion ratio (%)									
	0--10	10--20	20--30	30--40	40--50	50--60	60--70	70--80	80--90	90--100
Gammaproteobacteria	100	0	0	1	0	0	0	0	0	25
Betaproteobacteria	47	0	0	0	0	0	1	0	0	13
Epsilonproteobacteria	17	0	0	0	0	0	0	0	0	0
Deltaproteobacteria	26	0	0	0	0	0	1	0	0	1
Alphaproteobacteria	71	0	0	1	0	0	1	0	0	18
Magnetococcus	1	0	0	0	0	0	0	0	0	0
Chrysiogenetes	1	0	0	0	0	0	0	0	0	0
Firmicutes	88	0	0	4	0	0	2	0	0	10
Tenericutes	19	0	0	0	0	0	0	0	0	0
Actinobacteria	60	0	0	4	0	0	15	0	0	1
Chlamydiae	8	0	0	0	0	0	0	0	0	0
Spirochaetes	13	0	0	0	0	0	0	0	0	1
Acidobacteria	4	0	0	0	0	0	0	0	0	1
Bacteroidetes	35	0	0	0	0	0	0	0	0	0
Fibrobacteres	1	0	0	0	0	0	0	0	0	0
Fusobacteria	4	0	0	0	0	0	0	0	0	1
Verrucomicrobia	4	0	0	0	0	0	0	0	0	0
Gemmatimonadetes	1	0	0	0	0	0	0	0	0	0
Planctomycetes	4	0	0	0	0	0	0	0	0	0
Elusimicrobia	2	0	0	0	0	0	0	0	0	0
Synergistetes	2	0	0	0	0	0	0	0	0	0
Cyanobacteria	16	0	0	0	0	0	0	0	0	0
Chlorobi	10	0	0	0	0	0	0	0	0	0
Chloroflexi	9	0	0	0	0	0	1	0	0	1
Deinococcus-Thermus	4	0	0	0	0	0	0	0	0	3
Aquificae	9	0	0	0	0	0	0	0	0	0
Thermotogae	11	0	0	0	0	0	0	0	0	0
Dictyoglomi	2	0	0	0	0	0	0	0	0	0
Nitrospirae	2	0	0	0	0	0	0	0	0	0
Thermobaculum	1	0	0	0	0	0	0	0	0	0
Deferribacteres	3	0	0	0	0	0	0	0	0	0
Euryarchaeota	60	0	0	0	0	0	0	0	0	0
Crenarchaeota	23	0	0	0	0	0	0	0	0	0
Thaumarchaeota	2	0	0	0	0	0	0	0	0	0
Nanoarchaeota	1	0	0	0	0	0	0	0	0	0
Korarchaeota	1	0	0	0	0	0	0	0	0	0
Total	662	0	0	10	0	0	21	0	0	75

(M00216_1)

Phyla	Module completion ratio (%)									
	0--10	10--20	20--30	30--40	40--50	50--60	60--70	70--80	80--90	90--100
Gammaproteobacteria	125	0	0	0	0	0	0	0	0	1
Betaproteobacteria	56	0	0	0	0	0	0	0	0	5
Epsilonproteobacteria	17	0	0	0	0	0	0	0	0	0
Deltaproteobacteria	28	0	0	0	0	0	0	0	0	0
Alphaproteobacteria	72	0	0	0	0	0	1	0	0	18
Magnetococcus	1	0	0	0	0	0	0	0	0	0
Chrysiogenetes	1	0	0	0	0	0	0	0	0	0
Firmicutes	91	0	0	0	0	0	1	0	0	12
Tenericutes	19	0	0	0	0	0	0	0	0	0
Actinobacteria	52	0	0	0	0	0	0	0	0	28
Chlamydiae	8	0	0	0	0	0	0	0	0	0
Spirochaetes	13	0	0	0	0	0	0	0	0	1
Acidobacteria	5	0	0	0	0	0	0	0	0	0
Bacteroidetes	35	0	0	0	0	0	0	0	0	0
Fibrobacteres	1	0	0	0	0	0	0	0	0	0
Fusobacteria	5	0	0	0	0	0	0	0	0	0
Verrucomicrobia	4	0	0	0	0	0	0	0	0	0
Gemmatimonadetes	1	0	0	0	0	0	0	0	0	0
Planctomycetes	4	0	0	0	0	0	0	0	0	0
Elusimicrobia	2	0	0	0	0	0	0	0	0	0
Synergistetes	2	0	0	0	0	0	0	0	0	0
Cyanobacteria	16	0	0	0	0	0	0	0	0	0
Chlorobi	10	0	0	0	0	0	0	0	0	0
Chloroflexi	8	0	0	0	0	0	1	0	0	2
Deinococcus-Thermus	6	0	0	0	0	0	0	0	0	1
Aquificae	9	0	0	0	0	0	0	0	0	0
Thermotogae	10	0	0	0	0	0	0	0	0	1
Dictyoglomi	2	0	0	0	0	0	0	0	0	0
Nitrospirae	2	0	0	0	0	0	0	0	0	0
Thermobaculum	1	0	0	0	0	0	0	0	0	0
Deferribacteres	3	0	0	0	0	0	0	0	0	0
Euryarchaeota	60	0	0	0	0	0	0	0	0	0
Crenarchaeota	23	0	0	0	0	0	0	0	0	0
Thaumarchaeota	2	0	0	0	0	0	0	0	0	0
Nanoarchaeota	1	0	0	0	0	0	0	0	0	0
Korarchaeota	1	0	0	0	0	0	0	0	0	0
Total	696	0	0	0	0	0	3	0	0	69

(M00217_1)

Phyla	Module completion ratio (%)									
	0--10	10--20	20--30	30--40	40--50	50--60	60--70	70--80	80--90	90--100
Gammaproteobacteria	122	0	0	0	0	0	0	0	0	4
Betaproteobacteria	61	0	0	0	0	0	0	0	0	0
Epsilonproteobacteria	17	0	0	0	0	0	0	0	0	0
Deltaproteobacteria	28	0	0	0	0	0	0	0	0	0
Alphaproteobacteria	90	0	0	1	0	0	0	0	0	0
Magnetococcus	1	0	0	0	0	0	0	0	0	0
Chrysiogenetes	1	0	0	0	0	0	0	0	0	0
Firmicutes	102	0	0	1	0	0	0	0	0	1
Tenericutes	19	0	0	0	0	0	0	0	0	0
Actinobacteria	79	0	0	1	0	0	0	0	0	0
Chlamydiae	8	0	0	0	0	0	0	0	0	0
Spirochaetes	14	0	0	0	0	0	0	0	0	0
Acidobacteria	5	0	0	0	0	0	0	0	0	0
Bacteroidetes	35	0	0	0	0	0	0	0	0	0
Fibrobacteres	1	0	0	0	0	0	0	0	0	0
Fusobacteria	4	0	0	0	0	0	1	0	0	0
Verrucomicrobia	4	0	0	0	0	0	0	0	0	0
Gemmatimonadetes	1	0	0	0	0	0	0	0	0	0
Planctomycetes	4	0	0	0	0	0	0	0	0	0
Elusimicrobia	2	0	0	0	0	0	0	0	0	0
Synergistetes	2	0	0	0	0	0	0	0	0	0
Cyanobacteria	16	0	0	0	0	0	0	0	0	0
Chlorobi	10	0	0	0	0	0	0	0	0	0
Chloroflexi	11	0	0	0	0	0	0	0	0	0
Deinococcus-Thermus	7	0	0	0	0	0	0	0	0	0
Aquificae	9	0	0	0	0	0	0	0	0	0
Thermotogae	11	0	0	0	0	0	0	0	0	0
Dictyoglomi	2	0	0	0	0	0	0	0	0	0
Nitrospirae	2	0	0	0	0	0	0	0	0	0
Thermobaculum	1	0	0	0	0	0	0	0	0	0
Deferribacteres	3	0	0	0	0	0	0	0	0	0
Euryarchaeota	60	0	0	0	0	0	0	0	0	0
Crenarchaeota	23	0	0	0	0	0	0	0	0	0
Thaumarchaeota	2	0	0	0	0	0	0	0	0	0
Nanoarchaeota	1	0	0	0	0	0	0	0	0	0
Korarchaeota	1	0	0	0	0	0	0	0	0	0
Total	759	0	0	3	0	0	1	0	0	5

(M00218_1)

Phyla	Module completion ratio (%)									
	0--10	10--20	20--30	30--40	40--50	50--60	60--70	70--80	80--90	90--100
Gammaproteobacteria	124	0	0	1	0	0	0	0	0	1
Betaproteobacteria	52	0	0	0	0	0	0	0	0	9
Epsilonproteobacteria	17	0	0	0	0	0	0	0	0	0
Deltaproteobacteria	28	0	0	0	0	0	0	0	0	0
Alphaproteobacteria	76	0	0	0	0	0	0	0	0	15
Magnetococcus	1	0	0	0	0	0	0	0	0	0
Chrysiogenetes	1	0	0	0	0	0	0	0	0	0
Firmicutes	104	0	0	0	0	0	0	0	0	0
Tenericutes	19	0	0	0	0	0	0	0	0	0
Actinobacteria	67	0	0	4	0	0	0	0	0	9
Chlamydiae	8	0	0	0	0	0	0	0	0	0
Spirochaetes	14	0	0	0	0	0	0	0	0	0
Acidobacteria	5	0	0	0	0	0	0	0	0	0
Bacteroidetes	35	0	0	0	0	0	0	0	0	0
Fibrobacteres	1	0	0	0	0	0	0	0	0	0
Fusobacteria	5	0	0	0	0	0	0	0	0	0
Verrucomicrobia	4	0	0	0	0	0	0	0	0	0
Gemmatimonadetes	1	0	0	0	0	0	0	0	0	0
Planctomycetes	4	0	0	0	0	0	0	0	0	0
Elusimicrobia	2	0	0	0	0	0	0	0	0	0
Synergistetes	2	0	0	0	0	0	0	0	0	0
Cyanobacteria	16	0	0	0	0	0	0	0	0	0
Chlorobi	10	0	0	0	0	0	0	0	0	0
Chloroflexi	11	0	0	0	0	0	0	0	0	0
Deinococcus-Thermus	5	0	0	0	0	0	1	0	0	1
Aquificae	9	0	0	0	0	0	0	0	0	0
Thermotogae	11	0	0	0	0	0	0	0	0	0
Dictyoglomi	2	0	0	0	0	0	0	0	0	0
Nitrospirae	2	0	0	0	0	0	0	0	0	0
Thermobaculum	1	0	0	0	0	0	0	0	0	0
Deferribacteres	3	0	0	0	0	0	0	0	0	0
Euryarchaeota	59	0	0	1	0	0	0	0	0	0
Crenarchaeota	23	0	0	0	0	0	0	0	0	0
Thaumarchaeota	2	0	0	0	0	0	0	0	0	0
Nanoarchaeota	1	0	0	0	0	0	0	0	0	0
Korarchaeota	1	0	0	0	0	0	0	0	0	0
Total	726	0	0	6	0	0	1	0	0	35

(M00219_1)

Phyla	Module completion ratio (%)									
	0--10	10--20	20--30	30--40	40--50	50--60	60--70	70--80	80--90	90--100
Gammaproteobacteria	110	0	1	0	0	0	0	1	0	14
Betaproteobacteria	60	0	1	0	0	0	0	0	0	0
Epsilonproteobacteria	17	0	0	0	0	0	0	0	0	0
Deltaproteobacteria	28	0	0	0	0	0	0	0	0	0
Alphaproteobacteria	88	0	1	0	0	0	0	0	0	2
Magnetococcus	1	0	0	0	0	0	0	0	0	0
Chrysiogenetes	1	0	0	0	0	0	0	0	0	0
Firmicutes	99	0	0	0	0	1	0	2	0	2
Tenericutes	19	0	0	0	0	0	0	0	0	0
Actinobacteria	75	0	5	0	0	0	0	0	0	0
Chlamydiae	8	0	0	0	0	0	0	0	0	0
Spirochaetes	13	0	0	0	0	0	0	0	0	1
Acidobacteria	5	0	0	0	0	0	0	0	0	0
Bacteroidetes	35	0	0	0	0	0	0	0	0	0
Fibrobacteres	1	0	0	0	0	0	0	0	0	0
Fusobacteria	5	0	0	0	0	0	0	0	0	0
Verrucomicrobia	4	0	0	0	0	0	0	0	0	0
Gemmatimonadetes	1	0	0	0	0	0	0	0	0	0
Planctomycetes	4	0	0	0	0	0	0	0	0	0
Elusimicrobia	2	0	0	0	0	0	0	0	0	0
Synergistetes	2	0	0	0	0	0	0	0	0	0
Cyanobacteria	16	0	0	0	0	0	0	0	0	0
Chlorobi	10	0	0	0	0	0	0	0	0	0
Chloroflexi	9	0	2	0	0	0	0	0	0	0
Deinococcus-Thermus	4	0	2	0	0	0	0	0	0	1
Aquificae	9	0	0	0	0	0	0	0	0	0
Thermotogae	10	0	0	0	0	1	0	0	0	0
Dictyoglomi	2	0	0	0	0	0	0	0	0	0
Nitrospirae	2	0	0	0	0	0	0	0	0	0
Thermobaculum	1	0	0	0	0	0	0	0	0	0
Deferribacteres	3	0	0	0	0	0	0	0	0	0
Euryarchaeota	60	0	0	0	0	0	0	0	0	0
Crenarchaeota	23	0	0	0	0	0	0	0	0	0
Thaumarchaeota	2	0	0	0	0	0	0	0	0	0
Nanoarchaeota	1	0	0	0	0	0	0	0	0	0
Korarchaeota	1	0	0	0	0	0	0	0	0	0
Total	731	0	12	0	0	2	0	3	0	20

(M00220_1)

Phyla	Module completion ratio (%)									
	0--10	10--20	20--30	30--40	40--50	50--60	60--70	70--80	80--90	90--100
Gammaproteobacteria	123	0	0	0	0	0	0	0	0	3
Betaproteobacteria	58	0	0	0	0	0	0	0	0	3
Epsilonproteobacteria	17	0	0	0	0	0	0	0	0	0
Deltaproteobacteria	28	0	0	0	0	0	0	0	0	0
Alphaproteobacteria	81	0	0	0	0	0	0	0	0	10
Magnetococcus	1	0	0	0	0	0	0	0	0	0
Chrysiogenetes	1	0	0	0	0	0	0	0	0	0
Firmicutes	103	0	0	0	0	0	0	1	0	0
Tenericutes	19	0	0	0	0	0	0	0	0	0
Actinobacteria	80	0	0	0	0	0	0	0	0	0
Chlamydiae	8	0	0	0	0	0	0	0	0	0
Spirochaetes	14	0	0	0	0	0	0	0	0	0
Acidobacteria	5	0	0	0	0	0	0	0	0	0
Bacteroidetes	35	0	0	0	0	0	0	0	0	0
Fibrobacteres	1	0	0	0	0	0	0	0	0	0
Fusobacteria	5	0	0	0	0	0	0	0	0	0
Verrucomicrobia	4	0	0	0	0	0	0	0	0	0
Gemmatimonadetes	1	0	0	0	0	0	0	0	0	0
Planctomycetes	4	0	0	0	0	0	0	0	0	0
Elusimicrobia	2	0	0	0	0	0	0	0	0	0
Synergistetes	2	0	0	0	0	0	0	0	0	0
Cyanobacteria	16	0	0	0	0	0	0	0	0	0
Chlorobi	10	0	0	0	0	0	0	0	0	0
Chloroflexi	11	0	0	0	0	0	0	0	0	0
Deinococcus-Thermus	7	0	0	0	0	0	0	0	0	0
Aquificae	9	0	0	0	0	0	0	0	0	0
Thermotogae	11	0	0	0	0	0	0	0	0	0
Dictyoglomi	0	0	0	0	0	0	0	2	0	0
Nitrospirae	2	0	0	0	0	0	0	0	0	0
Thermobaculum	1	0	0	0	0	0	0	0	0	0
Deferribacteres	3	0	0	0	0	0	0	0	0	0
Euryarchaeota	60	0	0	0	0	0	0	0	0	0
Crenarchaeota	23	0	0	0	0	0	0	0	0	0
Thaumarchaeota	2	0	0	0	0	0	0	0	0	0
Nanoarchaeota	1	0	0	0	0	0	0	0	0	0
Korarchaeota	1	0	0	0	0	0	0	0	0	0
Total	749	0	0	0	0	0	0	3	0	16

(M00221_1)

Phyla	Module completion ratio (%)									
	0--10	10--20	20--30	30--40	40--50	50--60	60--70	70--80	80--90	90--100
Gammaproteobacteria	70	0	0	7	0	0	4	0	0	45
Betaproteobacteria	22	0	0	0	0	0	3	0	0	36
Epsilonproteobacteria	17	0	0	0	0	0	0	0	0	0
Deltaproteobacteria	14	0	0	1	0	0	5	0	0	8
Alphaproteobacteria	52	0	0	1	0	0	1	0	0	37
Magnetococcus	1	0	0	0	0	0	0	0	0	0
Chrysiogenetes	1	0	0	0	0	0	0	0	0	0
Firmicutes	25	0	0	5	0	0	53	0	0	21
Tenericutes	3	0	0	1	0	0	13	0	0	2
Actinobacteria	19	0	0	1	0	0	16	0	0	44
Chlamydiae	8	0	0	0	0	0	0	0	0	0
Spirochaetes	3	0	0	0	0	0	1	0	0	10
Acidobacteria	4	0	0	0	0	0	0	0	0	1
Bacteroidetes	35	0	0	0	0	0	0	0	0	0
Fibrobacteres	1	0	0	0	0	0	0	0	0	0
Fusobacteria	0	0	0	0	0	0	4	0	0	1
Verrucomicrobia	3	0	0	1	0	0	0	0	0	0
Gemmatimonadetes	0	0	0	0	0	0	1	0	0	0
Planctomycetes	3	0	0	0	0	0	0	0	0	1
Elusimicrobia	2	0	0	0	0	0	0	0	0	0
Synergistetes	0	0	0	0	0	0	2	0	0	0
Cyanobacteria	8	0	0	2	0	0	5	0	0	1
Chlorobi	0	0	0	1	0	0	9	0	0	0
Chloroflexi	4	0	0	0	0	0	0	0	0	7
Deinococcus-Thermus	0	0	0	0	0	0	2	0	0	5
Aquificae	9	0	0	0	0	0	0	0	0	0
Thermotogae	0	0	0	0	0	0	4	0	0	7
Dictyoglomi	0	0	0	0	0	0	0	0	0	2
Nitrospirae	2	0	0	0	0	0	0	0	0	0
Thermobaculum	0	0	0	0	0	0	0	0	0	1
Deferribacteres	0	0	0	0	0	0	2	0	0	1
Euryarchaeota	42	0	0	0	0	0	17	0	0	1
Crenarchaeota	9	0	0	1	0	0	0	0	0	13
Thaumarchaeota	2	0	0	0	0	0	0	0	0	0
Nanoarchaeota	1	0	0	0	0	0	0	0	0	0
Korarchaeota	0	0	0	0	0	0	0	0	0	1
Total	360	0	0	21	0	0	142	0	0	245

(M00222_1)

Phyla	Module completion ratio (%)									
	0--10	10--20	20--30	30--40	40--50	50--60	60--70	70--80	80--90	90--100
Gammaproteobacteria	19	0	0	0	0	0	0	2	0	105
Betaproteobacteria	3	0	1	0	0	0	0	1	0	56
Epsilonproteobacteria	6	0	0	0	0	0	0	0	0	11
Deltaproteobacteria	0	0	0	0	0	0	0	0	0	28
Alphaproteobacteria	12	0	1	0	0	0	0	7	0	71
Magnetococcus	0	0	0	0	0	0	0	0	0	1
Chrysiogenetes	0	0	0	0	0	0	0	0	0	1
Firmicutes	5	0	0	0	0	0	0	4	0	95
Tenericutes	7	0	0	0	0	2	0	3	0	7
Actinobacteria	4	0	1	0	0	2	0	3	0	70
Chlamydiae	8	0	0	0	0	0	0	0	0	0
Spirochaetes	2	0	3	0	0	1	0	0	0	8
Acidobacteria	1	0	1	0	0	0	0	1	0	2
Bacteroidetes	12	0	9	0	0	0	0	0	0	14
Fibrobacteres	0	0	0	0	0	0	0	0	0	1
Fusobacteria	3	0	0	0	0	0	0	0	0	2
Verrucomicrobia	0	0	0	0	0	0	0	0	0	4
Gemmatimonadetes	0	0	0	0	0	0	0	0	0	1
Planctomycetes	0	0	0	0	0	0	0	0	0	4
Elusimicrobia	1	0	0	0	0	0	0	0	0	1
Synergistetes	0	0	0	0	0	0	0	0	0	2
Cyanobacteria	0	0	0	0	0	0	0	0	0	16
Chlorobi	0	0	0	0	0	0	0	0	0	10
Chloroflexi	0	0	0	0	0	0	0	0	0	11
Deinococcus-Thermus	0	0	0	0	0	0	0	0	0	7
Aquificae	0	0	0	0	0	0	0	0	0	9
Thermotogae	0	0	0	0	0	0	0	0	0	11
Dictyoglomi	0	0	0	0	0	0	0	0	0	2
Nitrospirae	0	0	0	0	0	0	0	0	0	2
Thermobaculum	0	0	0	0	0	0	0	0	0	1
Deferribacteres	0	0	0	0	0	0	0	0	0	3
Euryarchaeota	8	0	1	0	0	2	0	2	0	47
Crenarchaeota	7	0	0	0	0	0	0	1	0	15
Thaumarchaeota	0	0	0	0	0	0	0	0	0	2
Nanoarchaeota	1	0	0	0	0	0	0	0	0	0
Korarchaeota	0	0	0	0	0	0	0	0	0	1
Total	99	0	17	0	0	7	0	24	0	621

(M00223_1)

Phyla	Module completion ratio (%)									
	0--10	10--20	20--30	30--40	40--50	50--60	60--70	70--80	80--90	90--100
Gammaproteobacteria	65	0	0	16	0	0	2	0	0	43
Betaproteobacteria	25	0	0	6	0	0	3	0	0	27
Epsilonproteobacteria	15	0	0	1	0	0	0	0	0	1
Deltaproteobacteria	12	0	0	9	0	0	0	0	0	7
Alphaproteobacteria	56	0	0	4	0	0	0	0	0	31
Magnetococcus	0	0	0	1	0	0	0	0	0	0
Chrysiogenetes	0	0	0	0	0	0	0	0	0	1
Firmicutes	71	0	0	4	0	0	0	0	0	29
Tenericutes	7	0	0	0	0	0	1	0	0	11
Actinobacteria	64	0	0	0	0	0	1	0	0	15
Chlamydiae	8	0	0	0	0	0	0	0	0	0
Spirochaetes	13	0	0	0	0	0	0	0	0	1
Acidobacteria	5	0	0	0	0	0	0	0	0	0
Bacteroidetes	35	0	0	0	0	0	0	0	0	0
Fibrobacteres	1	0	0	0	0	0	0	0	0	0
Fusobacteria	3	0	0	0	0	0	0	0	0	2
Verrucomicrobia	3	0	0	1	0	0	0	0	0	0
Gemmatimonadetes	1	0	0	0	0	0	0	0	0	0
Planctomycetes	3	0	0	1	0	0	0	0	0	0
Elusimicrobia	2	0	0	0	0	0	0	0	0	0
Synergistetes	1	0	0	1	0	0	0	0	0	0
Cyanobacteria	8	0	0	0	0	0	0	0	0	8
Chlorobi	10	0	0	0	0	0	0	0	0	0
Chloroflexi	7	0	0	1	0	0	0	0	0	3
Deinococcus-Thermus	4	0	0	0	0	0	2	0	0	1
Aquificae	6	0	0	3	0	0	0	0	0	0
Thermotogae	11	0	0	0	0	0	0	0	0	0
Dictyoglomi	2	0	0	0	0	0	0	0	0	0
Nitrospirae	2	0	0	0	0	0	0	0	0	0
Thermobaculum	1	0	0	0	0	0	0	0	0	0
Deferribacteres	0	0	0	3	0	0	0	0	0	0
Euryarchaeota	49	0	0	0	0	0	0	0	0	11
Crenarchaeota	22	0	0	0	0	0	1	0	0	0
Thaumarchaeota	0	0	0	0	0	0	0	0	0	2
Nanoarchaeota	1	0	0	0	0	0	0	0	0	0
Korarchaeota	1	0	0	0	0	0	0	0	0	0
Total	514	0	0	51	0	0	10	0	0	193

(M00224_1)

Phyla	Module completion ratio (%)									
	0--10	10--20	20--30	30--40	40--50	50--60	60--70	70--80	80--90	90--100
Gammaproteobacteria	103	0	0	0	0	0	0	0	0	23
Betaproteobacteria	44	0	0	0	0	1	0	0	0	16
Epsilonproteobacteria	17	0	0	0	0	0	0	0	0	0
Deltaproteobacteria	26	0	0	0	0	0	0	0	0	2
Alphaproteobacteria	69	0	0	0	0	0	0	0	0	22
Magnetococcus	1	0	0	0	0	0	0	0	0	0
Chrysiogenetes	1	0	0	0	0	0	0	0	0	0
Firmicutes	100	0	0	0	0	0	0	0	0	4
Tenericutes	19	0	0	0	0	0	0	0	0	0
Actinobacteria	77	0	0	0	0	1	0	0	0	2
Chlamydiae	8	0	0	0	0	0	0	0	0	0
Spirochaetes	14	0	0	0	0	0	0	0	0	0
Acidobacteria	5	0	0	0	0	0	0	0	0	0
Bacteroidetes	35	0	0	0	0	0	0	0	0	0
Fibrobacteres	1	0	0	0	0	0	0	0	0	0
Fusobacteria	5	0	0	0	0	0	0	0	0	0
Verrucomicrobia	4	0	0	0	0	0	0	0	0	0
Gemmatimonadetes	1	0	0	0	0	0	0	0	0	0
Planctomycetes	4	0	0	0	0	0	0	0	0	0
Elusimicrobia	2	0	0	0	0	0	0	0	0	0
Synergistetes	2	0	0	0	0	0	0	0	0	0
Cyanobacteria	14	0	0	0	0	0	0	0	0	2
Chlorobi	10	0	0	0	0	0	0	0	0	0
Chloroflexi	8	0	0	0	0	0	0	0	0	3
Deinococcus-Thermus	7	0	0	0	0	0	0	0	0	0
Aquificae	9	0	0	0	0	0	0	0	0	0
Thermotogae	11	0	0	0	0	0	0	0	0	0
Dictyoglomi	2	0	0	0	0	0	0	0	0	0
Nitrospirae	2	0	0	0	0	0	0	0	0	0
Thermobaculum	1	0	0	0	0	0	0	0	0	0
Deferribacteres	3	0	0	0	0	0	0	0	0	0
Euryarchaeota	59	0	0	0	0	0	0	0	0	1
Crenarchaeota	23	0	0	0	0	0	0	0	0	0
Thaumarchaeota	2	0	0	0	0	0	0	0	0	0
Nanoarchaeota	1	0	0	0	0	0	0	0	0	0
Korarchaeota	1	0	0	0	0	0	0	0	0	0
Total	691	0	0	0	0	2	0	0	0	75

(M00225_1)

Phyla	Module completion ratio (%)									
	0--10	10--20	20--30	30--40	40--50	50--60	60--70	70--80	80--90	90--100
Gammaproteobacteria	90	0	1	0	0	1	0	11	0	23
Betaproteobacteria	45	0	1	0	0	0	0	0	0	15
Epsilonproteobacteria	17	0	0	0	0	0	0	0	0	0
Deltaproteobacteria	27	0	0	0	0	0	0	1	0	0
Alphaproteobacteria	89	0	2	0	0	0	0	0	0	0
Magnetococcus	1	0	0	0	0	0	0	0	0	0
Chrysiogenetes	1	0	0	0	0	0	0	0	0	0
Firmicutes	104	0	0	0	0	0	0	0	0	0
Tenericutes	19	0	0	0	0	0	0	0	0	0
Actinobacteria	80	0	0	0	0	0	0	0	0	0
Chlamydiae	8	0	0	0	0	0	0	0	0	0
Spirochaetes	14	0	0	0	0	0	0	0	0	0
Acidobacteria	5	0	0	0	0	0	0	0	0	0
Bacteroidetes	35	0	0	0	0	0	0	0	0	0
Fibrobacteres	1	0	0	0	0	0	0	0	0	0
Fusobacteria	5	0	0	0	0	0	0	0	0	0
Verrucomicrobia	4	0	0	0	0	0	0	0	0	0
Gemmatimonadetes	1	0	0	0	0	0	0	0	0	0
Planctomycetes	4	0	0	0	0	0	0	0	0	0
Elusimicrobia	2	0	0	0	0	0	0	0	0	0
Synergistetes	2	0	0	0	0	0	0	0	0	0
Cyanobacteria	16	0	0	0	0	0	0	0	0	0
Chlorobi	10	0	0	0	0	0	0	0	0	0
Chloroflexi	11	0	0	0	0	0	0	0	0	0
Deinococcus-Thermus	7	0	0	0	0	0	0	0	0	0
Aquificae	9	0	0	0	0	0	0	0	0	0
Thermotogae	11	0	0	0	0	0	0	0	0	0
Dictyoglomi	2	0	0	0	0	0	0	0	0	0
Nitrospirae	2	0	0	0	0	0	0	0	0	0
Thermobaculum	1	0	0	0	0	0	0	0	0	0
Deferribacteres	3	0	0	0	0	0	0	0	0	0
Euryarchaeota	60	0	0	0	0	0	0	0	0	0
Crenarchaeota	23	0	0	0	0	0	0	0	0	0
Thaumarchaeota	2	0	0	0	0	0	0	0	0	0
Nanoarchaeota	1	0	0	0	0	0	0	0	0	0
Korarchaeota	1	0	0	0	0	0	0	0	0	0
Total	713	0	4	0	0	1	0	12	0	38

(M00226_1)

Phyla	Module completion ratio (%)									
	0--10	10--20	20--30	30--40	40--50	50--60	60--70	70--80	80--90	90--100
Gammaproteobacteria	90	0	1	0	0	1	0	11	0	23
Betaproteobacteria	45	0	1	0	0	0	0	4	0	11
Epsilonproteobacteria	17	0	0	0	0	0	0	0	0	0
Deltaproteobacteria	27	0	0	0	0	0	0	0	0	1
Alphaproteobacteria	89	0	2	0	0	0	0	0	0	0
Magnetococcus	1	0	0	0	0	0	0	0	0	0
Chrysiogenetes	1	0	0	0	0	0	0	0	0	0
Firmicutes	104	0	0	0	0	0	0	0	0	0
Tenericutes	19	0	0	0	0	0	0	0	0	0
Actinobacteria	80	0	0	0	0	0	0	0	0	0
Chlamydiae	8	0	0	0	0	0	0	0	0	0
Spirochaetes	14	0	0	0	0	0	0	0	0	0
Acidobacteria	5	0	0	0	0	0	0	0	0	0
Bacteroidetes	35	0	0	0	0	0	0	0	0	0
Fibrobacteres	1	0	0	0	0	0	0	0	0	0
Fusobacteria	5	0	0	0	0	0	0	0	0	0
Verrucomicrobia	4	0	0	0	0	0	0	0	0	0
Gemmatimonadetes	1	0	0	0	0	0	0	0	0	0
Planctomycetes	4	0	0	0	0	0	0	0	0	0
Elusimicrobia	2	0	0	0	0	0	0	0	0	0
Synergistetes	2	0	0	0	0	0	0	0	0	0
Cyanobacteria	16	0	0	0	0	0	0	0	0	0
Chlorobi	10	0	0	0	0	0	0	0	0	0
Chloroflexi	11	0	0	0	0	0	0	0	0	0
Deinococcus-Thermus	7	0	0	0	0	0	0	0	0	0
Aquificae	9	0	0	0	0	0	0	0	0	0
Thermotogae	11	0	0	0	0	0	0	0	0	0
Dictyoglomi	2	0	0	0	0	0	0	0	0	0
Nitrospirae	2	0	0	0	0	0	0	0	0	0
Thermobaculum	1	0	0	0	0	0	0	0	0	0
Deferribacteres	3	0	0	0	0	0	0	0	0	0
Euryarchaeota	60	0	0	0	0	0	0	0	0	0
Crenarchaeota	23	0	0	0	0	0	0	0	0	0
Thaumarchaeota	2	0	0	0	0	0	0	0	0	0
Nanoarchaeota	1	0	0	0	0	0	0	0	0	0
Korarchaeota	1	0	0	0	0	0	0	0	0	0
Total	713	0	4	0	0	1	0	15	0	35

(M00227_1)

Phyla	Module completion ratio (%)									
	0--10	10--20	20--30	30--40	40--50	50--60	60--70	70--80	80--90	90--100
Gammaproteobacteria	98	0	0	0	0	0	3	0	0	25
Betaproteobacteria	44	0	0	1	0	0	0	0	0	16
Epsilonproteobacteria	17	0	0	0	0	0	0	0	0	0
Deltaproteobacteria	21	0	0	0	0	0	2	0	0	5
Alphaproteobacteria	90	0	0	1	0	0	0	0	0	0
Magnetococcus	1	0	0	0	0	0	0	0	0	0
Chrysiogenetes	1	0	0	0	0	0	0	0	0	0
Firmicutes	96	0	0	5	0	0	1	0	0	2
Tenericutes	19	0	0	0	0	0	0	0	0	0
Actinobacteria	80	0	0	0	0	0	0	0	0	0
Chlamydiae	8	0	0	0	0	0	0	0	0	0
Spirochaetes	14	0	0	0	0	0	0	0	0	0
Acidobacteria	5	0	0	0	0	0	0	0	0	0
Bacteroidetes	35	0	0	0	0	0	0	0	0	0
Fibrobacteres	1	0	0	0	0	0	0	0	0	0
Fusobacteria	5	0	0	0	0	0	0	0	0	0
Verrucomicrobia	4	0	0	0	0	0	0	0	0	0
Gemmatimonadetes	1	0	0	0	0	0	0	0	0	0
Planctomycetes	4	0	0	0	0	0	0	0	0	0
Elusimicrobia	2	0	0	0	0	0	0	0	0	0
Synergistetes	2	0	0	0	0	0	0	0	0	0
Cyanobacteria	16	0	0	0	0	0	0	0	0	0
Chlorobi	10	0	0	0	0	0	0	0	0	0
Chloroflexi	11	0	0	0	0	0	0	0	0	0
Deinococcus-Thermus	7	0	0	0	0	0	0	0	0	0
Aquificae	9	0	0	0	0	0	0	0	0	0
Thermotogae	11	0	0	0	0	0	0	0	0	0
Dictyoglomi	2	0	0	0	0	0	0	0	0	0
Nitrospirae	2	0	0	0	0	0	0	0	0	0
Thermobaculum	1	0	0	0	0	0	0	0	0	0
Deferribacteres	3	0	0	0	0	0	0	0	0	0
Euryarchaeota	60	0	0	0	0	0	0	0	0	0
Crenarchaeota	23	0	0	0	0	0	0	0	0	0
Thaumarchaeota	2	0	0	0	0	0	0	0	0	0
Nanoarchaeota	1	0	0	0	0	0	0	0	0	0
Korarchaeota	1	0	0	0	0	0	0	0	0	0
Total	707	0	0	7	0	0	6	0	0	48

(M00228_1)

Phyla	Module completion ratio (%)									
	0--10	10--20	20--30	30--40	40--50	50--60	60--70	70--80	80--90	90--100
Gammaproteobacteria	107	0	0	3	0	0	3	0	0	13
Betaproteobacteria	45	0	0	4	0	0	0	0	0	12
Epsilonproteobacteria	6	0	0	2	0	0	3	0	0	6
Deltaproteobacteria	15	0	0	6	0	0	1	0	0	6
Alphaproteobacteria	81	0	0	0	0	0	1	0	0	9
Magnetococcus	1	0	0	0	0	0	0	0	0	0
Chrysiogenetes	0	0	0	1	0	0	0	0	0	0
Firmicutes	47	0	0	13	0	0	7	0	0	37
Tenericutes	19	0	0	0	0	0	0	0	0	0
Actinobacteria	76	0	0	3	0	0	1	0	0	0
Chlamydiae	8	0	0	0	0	0	0	0	0	0
Spirochaetes	14	0	0	0	0	0	0	0	0	0
Acidobacteria	5	0	0	0	0	0	0	0	0	0
Bacteroidetes	35	0	0	0	0	0	0	0	0	0
Fibrobacteres	1	0	0	0	0	0	0	0	0	0
Fusobacteria	2	0	0	1	0	0	0	0	0	2
Verrucomicrobia	4	0	0	0	0	0	0	0	0	0
Gemmatimonadetes	1	0	0	0	0	0	0	0	0	0
Planctomycetes	4	0	0	0	0	0	0	0	0	0
Elusimicrobia	2	0	0	0	0	0	0	0	0	0
Synergistetes	2	0	0	0	0	0	0	0	0	0
Cyanobacteria	6	0	0	7	0	0	3	0	0	0
Chlorobi	10	0	0	0	0	0	0	0	0	0
Chloroflexi	11	0	0	0	0	0	0	0	0	0
Deinococcus-Thermus	7	0	0	0	0	0	0	0	0	0
Aquificae	9	0	0	0	0	0	0	0	0	0
Thermotogae	8	0	0	3	0	0	0	0	0	0
Dictyoglomi	2	0	0	0	0	0	0	0	0	0
Nitrospirae	2	0	0	0	0	0	0	0	0	0
Thermobaculum	1	0	0	0	0	0	0	0	0	0
Deferribacteres	0	0	0	0	0	0	2	0	0	1
Euryarchaeota	59	0	0	1	0	0	0	0	0	0
Crenarchaeota	23	0	0	0	0	0	0	0	0	0
Thaumarchaeota	2	0	0	0	0	0	0	0	0	0
Nanoarchaeota	1	0	0	0	0	0	0	0	0	0
Korarchaeota	1	0	0	0	0	0	0	0	0	0
Total	617	0	0	44	0	0	21	0	0	86

(M00229_1)

Phyla	Module completion ratio (%)									
	0--10	10--20	20--30	30--40	40--50	50--60	60--70	70--80	80--90	90--100
Gammaproteobacteria	75	0	2	0	0	0	0	0	0	49
Betaproteobacteria	61	0	0	0	0	0	0	0	0	0
Epsilonproteobacteria	17	0	0	0	0	0	0	0	0	0
Deltaproteobacteria	28	0	0	0	0	0	0	0	0	0
Alphaproteobacteria	91	0	0	0	0	0	0	0	0	0
Magnetococcus	1	0	0	0	0	0	0	0	0	0
Chrysiogenetes	1	0	0	0	0	0	0	0	0	0
Firmicutes	104	0	0	0	0	0	0	0	0	0
Tenericutes	19	0	0	0	0	0	0	0	0	0
Actinobacteria	80	0	0	0	0	0	0	0	0	0
Chlamydiae	2	0	6	0	0	0	0	0	0	0
Spirochaetes	14	0	0	0	0	0	0	0	0	0
Acidobacteria	5	0	0	0	0	0	0	0	0	0
Bacteroidetes	35	0	0	0	0	0	0	0	0	0
Fibrobacteres	1	0	0	0	0	0	0	0	0	0
Fusobacteria	5	0	0	0	0	0	0	0	0	0
Verrucomicrobia	4	0	0	0	0	0	0	0	0	0
Gemmatimonadetes	1	0	0	0	0	0	0	0	0	0
Planctomycetes	4	0	0	0	0	0	0	0	0	0
Elusimicrobia	2	0	0	0	0	0	0	0	0	0
Synergistetes	2	0	0	0	0	0	0	0	0	0
Cyanobacteria	16	0	0	0	0	0	0	0	0	0
Chlorobi	10	0	0	0	0	0	0	0	0	0
Chloroflexi	11	0	0	0	0	0	0	0	0	0
Deinococcus-Thermus	7	0	0	0	0	0	0	0	0	0
Aquificae	9	0	0	0	0	0	0	0	0	0
Thermotogae	11	0	0	0	0	0	0	0	0	0
Dictyoglomi	2	0	0	0	0	0	0	0	0	0
Nitrospirae	2	0	0	0	0	0	0	0	0	0
Thermobaculum	1	0	0	0	0	0	0	0	0	0
Deferribacteres	3	0	0	0	0	0	0	0	0	0
Euryarchaeota	60	0	0	0	0	0	0	0	0	0
Crenarchaeota	23	0	0	0	0	0	0	0	0	0
Thaumarchaeota	2	0	0	0	0	0	0	0	0	0
Nanoarchaeota	1	0	0	0	0	0	0	0	0	0
Korarchaeota	1	0	0	0	0	0	0	0	0	0
Total	711	0	8	0	0	0	0	0	0	49

(M00230_1)

Phyla	Module completion ratio (%)									
	0--10	10--20	20--30	30--40	40--50	50--60	60--70	70--80	80--90	90--100
Gammaproteobacteria	85	0	1	0	0	2	0	0	0	38
Betaproteobacteria	18	0	1	0	0	0	0	1	0	41
Epsilonproteobacteria	16	0	1	0	0	0	0	0	0	0
Deltaproteobacteria	26	0	0	0	0	0	0	1	0	1
Alphaproteobacteria	66	0	6	0	0	1	0	1	0	17
Magnetococcus	1	0	0	0	0	0	0	0	0	0
Chrysiogenetes	1	0	0	0	0	0	0	0	0	0
Firmicutes	104	0	0	0	0	0	0	0	0	0
Tenericutes	19	0	0	0	0	0	0	0	0	0
Actinobacteria	80	0	0	0	0	0	0	0	0	0
Chlamydiae	8	0	0	0	0	0	0	0	0	0
Spirochaetes	14	0	0	0	0	0	0	0	0	0
Acidobacteria	5	0	0	0	0	0	0	0	0	0
Bacteroidetes	35	0	0	0	0	0	0	0	0	0
Fibrobacteres	1	0	0	0	0	0	0	0	0	0
Fusobacteria	5	0	0	0	0	0	0	0	0	0
Verrucomicrobia	4	0	0	0	0	0	0	0	0	0
Gemmatimonadetes	1	0	0	0	0	0	0	0	0	0
Planctomycetes	4	0	0	0	0	0	0	0	0	0
Elusimicrobia	2	0	0	0	0	0	0	0	0	0
Synergistetes	2	0	0	0	0	0	0	0	0	0
Cyanobacteria	12	0	4	0	0	0	0	0	0	0
Chlorobi	10	0	0	0	0	0	0	0	0	0
Chloroflexi	11	0	0	0	0	0	0	0	0	0
Deinococcus-Thermus	7	0	0	0	0	0	0	0	0	0
Aquificae	9	0	0	0	0	0	0	0	0	0
Thermotogae	11	0	0	0	0	0	0	0	0	0
Dictyoglomi	2	0	0	0	0	0	0	0	0	0
Nitrospirae	2	0	0	0	0	0	0	0	0	0
Thermobaculum	1	0	0	0	0	0	0	0	0	0
Deferribacteres	3	0	0	0	0	0	0	0	0	0
Euryarchaeota	60	0	0	0	0	0	0	0	0	0
Crenarchaeota	23	0	0	0	0	0	0	0	0	0
Thaumarchaeota	2	0	0	0	0	0	0	0	0	0
Nanoarchaeota	1	0	0	0	0	0	0	0	0	0
Korarchaeota	1	0	0	0	0	0	0	0	0	0
Total	652	0	13	0	0	3	0	3	0	97

(M00231_1)

Phyla	Module completion ratio (%)									
	0--10	10--20	20--30	30--40	40--50	50--60	60--70	70--80	80--90	90--100
Gammaproteobacteria	122	0	0	0	0	0	0	1	0	3
Betaproteobacteria	54	0	0	0	0	0	0	0	0	7
Epsilonproteobacteria	17	0	0	0	0	0	0	0	0	0
Deltaproteobacteria	28	0	0	0	0	0	0	0	0	0
Alphaproteobacteria	81	0	0	0	0	0	0	4	0	6
Magnetococcus	1	0	0	0	0	0	0	0	0	0
Chrysiogenetes	1	0	0	0	0	0	0	0	0	0
Firmicutes	104	0	0	0	0	0	0	0	0	0
Tenericutes	19	0	0	0	0	0	0	0	0	0
Actinobacteria	80	0	0	0	0	0	0	0	0	0
Chlamydiae	8	0	0	0	0	0	0	0	0	0
Spirochaetes	14	0	0	0	0	0	0	0	0	0
Acidobacteria	5	0	0	0	0	0	0	0	0	0
Bacteroidetes	35	0	0	0	0	0	0	0	0	0
Fibrobacteres	1	0	0	0	0	0	0	0	0	0
Fusobacteria	5	0	0	0	0	0	0	0	0	0
Verrucomicrobia	4	0	0	0	0	0	0	0	0	0
Gemmatimonadetes	1	0	0	0	0	0	0	0	0	0
Planctomycetes	4	0	0	0	0	0	0	0	0	0
Elusimicrobia	2	0	0	0	0	0	0	0	0	0
Synergistetes	2	0	0	0	0	0	0	0	0	0
Cyanobacteria	16	0	0	0	0	0	0	0	0	0
Chlorobi	10	0	0	0	0	0	0	0	0	0
Chloroflexi	11	0	0	0	0	0	0	0	0	0
Deinococcus-Thermus	7	0	0	0	0	0	0	0	0	0
Aquificae	9	0	0	0	0	0	0	0	0	0
Thermotogae	11	0	0	0	0	0	0	0	0	0
Dictyoglomi	2	0	0	0	0	0	0	0	0	0
Nitrospirae	2	0	0	0	0	0	0	0	0	0
Thermobaculum	1	0	0	0	0	0	0	0	0	0
Deferribacteres	3	0	0	0	0	0	0	0	0	0
Euryarchaeota	60	0	0	0	0	0	0	0	0	0
Crenarchaeota	23	0	0	0	0	0	0	0	0	0
Thaumarchaeota	2	0	0	0	0	0	0	0	0	0
Nanoarchaeota	1	0	0	0	0	0	0	0	0	0
Korarchaeota	1	0	0	0	0	0	0	0	0	0
Total	747	0	0	0	0	0	0	5	0	16

(M00232_1)

Phyla	Module completion ratio (%)									
	0--10	10--20	20--30	30--40	40--50	50--60	60--70	70--80	80--90	90--100
Gammaproteobacteria	88	0	1	0	0	1	0	3	0	33
Betaproteobacteria	47	0	2	0	0	0	0	3	0	9
Epsilonproteobacteria	15	0	1	0	0	0	0	0	0	1
Deltaproteobacteria	24	0	2	0	0	0	0	0	0	2
Alphaproteobacteria	43	0	4	0	0	2	0	4	0	38
Magnetococcus	0	0	1	0	0	0	0	0	0	0
Chrysiogenetes	1	0	0	0	0	0	0	0	0	0
Firmicutes	103	0	0	0	0	0	0	0	0	1
Tenericutes	19	0	0	0	0	0	0	0	0	0
Actinobacteria	78	0	2	0	0	0	0	0	0	0
Chlamydiae	8	0	0	0	0	0	0	0	0	0
Spirochaetes	13	0	0	0	0	0	0	0	0	1
Acidobacteria	3	0	2	0	0	0	0	0	0	0
Bacteroidetes	35	0	0	0	0	0	0	0	0	0
Fibrobacteres	1	0	0	0	0	0	0	0	0	0
Fusobacteria	5	0	0	0	0	0	0	0	0	0
Verrucomicrobia	4	0	0	0	0	0	0	0	0	0
Gemmatimonadetes	1	0	0	0	0	0	0	0	0	0
Planctomycetes	4	0	0	0	0	0	0	0	0	0
Elusimicrobia	2	0	0	0	0	0	0	0	0	0
Synergistetes	2	0	0	0	0	0	0	0	0	0
Cyanobacteria	5	0	0	0	0	0	0	2	0	9
Chlorobi	10	0	0	0	0	0	0	0	0	0
Chloroflexi	5	0	2	0	0	0	0	0	0	4
Deinococcus-Thermus	3	0	2	0	0	0	0	0	0	2
Aquificae	9	0	0	0	0	0	0	0	0	0
Thermotogae	10	0	1	0	0	0	0	0	0	0
Dictyoglomi	2	0	0	0	0	0	0	0	0	0
Nitrospirae	2	0	0	0	0	0	0	0	0	0
Thermobaculum	1	0	0	0	0	0	0	0	0	0
Deferribacteres	3	0	0	0	0	0	0	0	0	0
Euryarchaeota	60	0	0	0	0	0	0	0	0	0
Crenarchaeota	23	0	0	0	0	0	0	0	0	0
Thaumarchaeota	2	0	0	0	0	0	0	0	0	0
Nanoarchaeota	1	0	0	0	0	0	0	0	0	0
Korarchaeota	1	0	0	0	0	0	0	0	0	0
Total	633	0	20	0	0	3	0	12	0	100

(M00233_1)

Phyla	Module completion ratio (%)									
	0--10	10--20	20--30	30--40	40--50	50--60	60--70	70--80	80--90	90--100
Gammaproteobacteria	125	0	1	0	0	0	0	0	0	0
Betaproteobacteria	57	0	3	0	0	1	0	0	0	0
Epsilonproteobacteria	17	0	0	0	0	0	0	0	0	0
Deltaproteobacteria	27	0	1	0	0	0	0	0	0	0
Alphaproteobacteria	91	0	0	0	0	0	0	0	0	0
Magnetococcus	1	0	0	0	0	0	0	0	0	0
Chrysiogenetes	0	0	1	0	0	0	0	0	0	0
Firmicutes	104	0	0	0	0	0	0	0	0	0
Tenericutes	19	0	0	0	0	0	0	0	0	0
Actinobacteria	26	0	0	0	0	0	0	4	0	50
Chlamydiae	8	0	0	0	0	0	0	0	0	0
Spirochaetes	14	0	0	0	0	0	0	0	0	0
Acidobacteria	5	0	0	0	0	0	0	0	0	0
Bacteroidetes	35	0	0	0	0	0	0	0	0	0
Fibrobacteres	1	0	0	0	0	0	0	0	0	0
Fusobacteria	5	0	0	0	0	0	0	0	0	0
Verrucomicrobia	4	0	0	0	0	0	0	0	0	0
Gemmatimonadetes	1	0	0	0	0	0	0	0	0	0
Planctomycetes	4	0	0	0	0	0	0	0	0	0
Elusimicrobia	2	0	0	0	0	0	0	0	0	0
Synergistetes	2	0	0	0	0	0	0	0	0	0
Cyanobacteria	16	0	0	0	0	0	0	0	0	0
Chlorobi	10	0	0	0	0	0	0	0	0	0
Chloroflexi	11	0	0	0	0	0	0	0	0	0
Deinococcus-Thermus	7	0	0	0	0	0	0	0	0	0
Aquificae	9	0	0	0	0	0	0	0	0	0
Thermotogae	11	0	0	0	0	0	0	0	0	0
Dictyoglomi	2	0	0	0	0	0	0	0	0	0
Nitrospirae	2	0	0	0	0	0	0	0	0	0
Thermobaculum	1	0	0	0	0	0	0	0	0	0
Deferribacteres	3	0	0	0	0	0	0	0	0	0
Euryarchaeota	60	0	0	0	0	0	0	0	0	0
Crenarchaeota	23	0	0	0	0	0	0	0	0	0
Thaumarchaeota	2	0	0	0	0	0	0	0	0	0
Nanoarchaeota	1	0	0	0	0	0	0	0	0	0
Korarchaeota	1	0	0	0	0	0	0	0	0	0
Total	707	0	6	0	0	1	0	4	0	50

(M00234_1)

Phyla	Module completion ratio (%)									
	0--10	10--20	20--30	30--40	40--50	50--60	60--70	70--80	80--90	90--100
Gammaproteobacteria	97	0	0	3	0	0	0	0	0	26
Betaproteobacteria	60	0	0	1	0	0	0	0	0	0
Epsilonproteobacteria	17	0	0	0	0	0	0	0	0	0
Deltaproteobacteria	27	0	0	1	0	0	0	0	0	0
Alphaproteobacteria	90	0	0	0	0	0	0	0	0	1
Magnetococcus	1	0	0	0	0	0	0	0	0	0
Chrysiogenetes	1	0	0	0	0	0	0	0	0	0
Firmicutes	102	0	0	2	0	0	0	0	0	0
Tenericutes	19	0	0	0	0	0	0	0	0	0
Actinobacteria	78	0	0	2	0	0	0	0	0	0
Chlamydiae	8	0	0	0	0	0	0	0	0	0
Spirochaetes	14	0	0	0	0	0	0	0	0	0
Acidobacteria	5	0	0	0	0	0	0	0	0	0
Bacteroidetes	35	0	0	0	0	0	0	0	0	0
Fibrobacteres	1	0	0	0	0	0	0	0	0	0
Fusobacteria	5	0	0	0	0	0	0	0	0	0
Verrucomicrobia	4	0	0	0	0	0	0	0	0	0
Gemmatimonadetes	1	0	0	0	0	0	0	0	0	0
Planctomycetes	4	0	0	0	0	0	0	0	0	0
Elusimicrobia	2	0	0	0	0	0	0	0	0	0
Synergistetes	2	0	0	0	0	0	0	0	0	0
Cyanobacteria	16	0	0	0	0	0	0	0	0	0
Chlorobi	10	0	0	0	0	0	0	0	0	0
Chloroflexi	11	0	0	0	0	0	0	0	0	0
Deinococcus-Thermus	7	0	0	0	0	0	0	0	0	0
Aquificae	9	0	0	0	0	0	0	0	0	0
Thermotogae	8	0	0	3	0	0	0	0	0	0
Dictyoglomi	1	0	0	1	0	0	0	0	0	0
Nitrospirae	2	0	0	0	0	0	0	0	0	0
Thermobaculum	1	0	0	0	0	0	0	0	0	0
Deferribacteres	3	0	0	0	0	0	0	0	0	0
Euryarchaeota	59	0	0	1	0	0	0	0	0	0
Crenarchaeota	23	0	0	0	0	0	0	0	0	0
Thaumarchaeota	2	0	0	0	0	0	0	0	0	0
Nanoarchaeota	1	0	0	0	0	0	0	0	0	0
Korarchaeota	1	0	0	0	0	0	0	0	0	0
Total	727	0	0	14	0	0	0	0	0	27

(M00235_1)

Phyla	Module completion ratio (%)									
	0--10	10--20	20--30	30--40	40--50	50--60	60--70	70--80	80--90	90--100
Gammaproteobacteria	119	0	1	0	0	0	0	1	0	5
Betaproteobacteria	57	0	0	0	0	1	0	2	0	1
Epsilonproteobacteria	17	0	0	0	0	0	0	0	0	0
Deltaproteobacteria	28	0	0	0	0	0	0	0	0	0
Alphaproteobacteria	91	0	0	0	0	0	0	0	0	0
Magnetococcus	1	0	0	0	0	0	0	0	0	0
Chrysiogenetes	1	0	0	0	0	0	0	0	0	0
Firmicutes	104	0	0	0	0	0	0	0	0	0
Tenericutes	19	0	0	0	0	0	0	0	0	0
Actinobacteria	80	0	0	0	0	0	0	0	0	0
Chlamydiae	8	0	0	0	0	0	0	0	0	0
Spirochaetes	14	0	0	0	0	0	0	0	0	0
Acidobacteria	5	0	0	0	0	0	0	0	0	0
Bacteroidetes	35	0	0	0	0	0	0	0	0	0
Fibrobacteres	1	0	0	0	0	0	0	0	0	0
Fusobacteria	5	0	0	0	0	0	0	0	0	0
Verrucomicrobia	4	0	0	0	0	0	0	0	0	0
Gemmatimonadetes	1	0	0	0	0	0	0	0	0	0
Planctomycetes	4	0	0	0	0	0	0	0	0	0
Elusimicrobia	2	0	0	0	0	0	0	0	0	0
Synergistetes	2	0	0	0	0	0	0	0	0	0
Cyanobacteria	16	0	0	0	0	0	0	0	0	0
Chlorobi	10	0	0	0	0	0	0	0	0	0
Chloroflexi	11	0	0	0	0	0	0	0	0	0
Deinococcus-Thermus	7	0	0	0	0	0	0	0	0	0
Aquificae	9	0	0	0	0	0	0	0	0	0
Thermotogae	11	0	0	0	0	0	0	0	0	0
Dictyoglomi	2	0	0	0	0	0	0	0	0	0
Nitrospirae	2	0	0	0	0	0	0	0	0	0
Thermobaculum	1	0	0	0	0	0	0	0	0	0
Deferribacteres	3	0	0	0	0	0	0	0	0	0
Euryarchaeota	60	0	0	0	0	0	0	0	0	0
Crenarchaeota	23	0	0	0	0	0	0	0	0	0
Thaumarchaeota	2	0	0	0	0	0	0	0	0	0
Nanoarchaeota	1	0	0	0	0	0	0	0	0	0
Korarchaeota	1	0	0	0	0	0	0	0	0	0
Total	757	0	1	0	0	1	0	3	0	6

(M00236_1)

Phyla	Module completion ratio (%)									
	0--10	10--20	20--30	30--40	40--50	50--60	60--70	70--80	80--90	90--100
Gammaproteobacteria	38	0	0	15	0	0	8	0	0	65
Betaproteobacteria	11	0	0	6	0	0	1	0	0	43
Epsilonproteobacteria	8	0	0	1	0	0	3	0	0	5
Deltaproteobacteria	12	0	0	3	0	0	0	0	0	13
Alphaproteobacteria	25	0	0	11	0	0	6	0	0	49
Magnetococcus	1	0	0	0	0	0	0	0	0	0
Chrysiogenetes	0	0	0	1	0	0	0	0	0	0
Firmicutes	5	0	0	1	0	0	17	0	0	81
Tenericutes	16	0	0	0	0	0	0	0	0	3
Actinobacteria	8	0	0	9	0	0	5	0	0	58
Chlamydiae	0	0	0	2	0	0	0	0	0	6
Spirochaetes	8	0	0	4	0	0	0	0	0	2
Acidobacteria	5	0	0	0	0	0	0	0	0	0
Bacteroidetes	32	0	0	3	0	0	0	0	0	0
Fibrobacteres	0	0	0	0	0	0	0	0	0	1
Fusobacteria	0	0	0	0	0	0	1	0	0	4
Verrucomicrobia	3	0	0	0	0	0	0	0	0	1
Gemmatimonadetes	0	0	0	0	0	0	0	0	0	1
Planctomycetes	3	0	0	0	0	0	0	0	0	1
Elusimicrobia	2	0	0	0	0	0	0	0	0	0
Synergistetes	0	0	0	0	0	0	1	0	0	1
Cyanobacteria	5	0	0	3	0	0	7	0	0	1
Chlorobi	8	0	0	2	0	0	0	0	0	0
Chloroflexi	4	0	0	1	0	0	2	0	0	4
Deinococcus-Thermus	0	0	0	1	0	0	4	0	0	2
Aquificae	9	0	0	0	0	0	0	0	0	0
Thermotogae	1	0	0	1	0	0	3	0	0	6
Dictyoglomi	0	0	0	1	0	0	0	0	0	1
Nitrospirae	2	0	0	0	0	0	0	0	0	0
Thermobaculum	0	0	0	0	0	0	1	0	0	0
Deferribacteres	0	0	0	0	0	0	2	0	0	1
Euryarchaeota	36	0	0	3	0	0	5	0	0	16
Crenarchaeota	13	0	0	0	0	0	1	0	0	9
Thaumarchaeota	2	0	0	0	0	0	0	0	0	0
Nanoarchaeota	1	0	0	0	0	0	0	0	0	0
Korarchaeota	0	0	0	0	0	0	0	0	0	1
Total	258	0	0	68	0	0	67	0	0	375

(M00237_1)

Phyla	Module completion ratio (%)									
	0--10	10--20	20--30	30--40	40--50	50--60	60--70	70--80	80--90	90--100
Gammaproteobacteria	61	0	5	0	1	1	0	0	0	58
Betaproteobacteria	7	0	1	0	0	0	0	0	0	53
Epsilonproteobacteria	10	0	0	0	0	0	0	0	0	7
Deltaproteobacteria	1	0	0	0	0	0	0	0	0	27
Alphaproteobacteria	36	0	2	0	1	0	0	0	1	51
Magnetococcus	0	0	0	0	0	0	0	0	0	1
Chrysiogenetes	1	0	0	0	0	0	0	0	0	0
Firmicutes	47	0	3	0	0	1	0	0	2	51
Tenericutes	18	0	0	0	0	0	0	0	1	0
Actinobacteria	24	0	1	0	1	0	0	0	1	53
Chlamydiae	8	0	0	0	0	0	0	0	0	0
Spirochaetes	12	0	0	0	0	0	0	0	0	2
Acidobacteria	5	0	0	0	0	0	0	0	0	0
Bacteroidetes	30	0	2	0	0	0	0	0	0	3
Fibrobacteres	1	0	0	0	0	0	0	0	0	0
Fusobacteria	0	0	0	0	0	0	0	0	0	5
Verrucomicrobia	2	0	0	0	0	0	0	0	0	2
Gemmatimonadetes	0	0	1	0	0	0	0	0	0	0
Planctomycetes	2	0	0	0	0	0	0	0	1	1
Elusimicrobia	2	0	0	0	0	0	0	0	0	0
Synergistetes	0	0	0	0	0	0	0	0	0	2
Cyanobacteria	5	0	2	0	0	2	0	0	1	6
Chlorobi	7	0	0	0	0	0	0	0	1	2
Chloroflexi	0	0	0	0	0	0	0	0	1	10
Deinococcus-Thermus	0	0	0	0	0	0	0	0	0	7
Aquificae	7	0	0	0	0	0	0	0	0	2
Thermotogae	2	0	0	0	0	0	0	0	0	9
Dictyoglomi	0	0	0	0	0	0	0	0	0	2
Nitrospirae	0	0	1	0	0	0	0	0	0	1
Thermobaculum	0	0	0	0	0	0	0	0	0	1
Deferribacteres	0	0	0	0	0	0	0	0	0	3
Euryarchaeota	35	0	5	0	0	1	0	0	2	17
Crenarchaeota	10	0	0	0	0	1	0	0	1	11
Thaumarchaeota	1	0	0	0	0	0	0	0	0	1
Nanoarchaeota	1	0	0	0	0	0	0	0	0	0
Korarchaeota	0	0	0	0	0	0	0	0	0	1
Total	335	0	23	0	3	6	0	0	12	389

(M00238_1)

Phyla	Module completion ratio (%)									
	0--10	10--20	20--30	30--40	40--50	50--60	60--70	70--80	80--90	90--100
Gammaproteobacteria	45	0	0	4	0	0	1	0	0	76
Betaproteobacteria	23	0	0	0	0	0	0	0	0	38
Epsilonproteobacteria	7	0	0	0	0	0	0	0	0	10
Deltaproteobacteria	26	0	0	0	0	0	0	0	0	2
Alphaproteobacteria	56	0	0	0	0	0	2	0	0	33
Magnetococcus	1	0	0	0	0	0	0	0	0	0
Chrysiogenetes	1	0	0	0	0	0	0	0	0	0
Firmicutes	14	0	0	2	0	0	3	0	0	85
Tenericutes	15	0	0	0	0	0	2	0	0	2
Actinobacteria	27	0	0	1	0	0	0	0	0	52
Chlamydiae	3	0	0	0	0	0	0	0	0	5
Spirochaetes	12	0	0	0	0	0	0	0	0	2
Acidobacteria	5	0	0	0	0	0	0	0	0	0
Bacteroidetes	32	0	0	1	0	0	0	0	0	2
Fibrobacteres	0	0	0	0	0	0	0	0	0	1
Fusobacteria	0	0	0	0	0	0	0	0	0	5
Verrucomicrobia	4	0	0	0	0	0	0	0	0	0
Gemmatimonadetes	1	0	0	0	0	0	0	0	0	0
Planctomycetes	4	0	0	0	0	0	0	0	0	0
Elusimicrobia	2	0	0	0	0	0	0	0	0	0
Synergistetes	1	0	0	0	0	0	0	0	0	1
Cyanobacteria	10	0	0	6	0	0	0	0	0	0
Chlorobi	9	0	0	1	0	0	0	0	0	0
Chloroflexi	10	0	0	0	0	0	0	0	0	1
Deinococcus-Thermus	5	0	0	0	0	0	0	0	0	2
Aquificae	9	0	0	0	0	0	0	0	0	0
Thermotogae	10	0	0	0	0	0	0	0	0	1
Dictyoglomi	2	0	0	0	0	0	0	0	0	0
Nitrospirae	2	0	0	0	0	0	0	0	0	0
Thermobaculum	1	0	0	0	0	0	0	0	0	0
Deferribacteres	2	0	0	0	0	0	0	0	0	1
Euryarchaeota	60	0	0	0	0	0	0	0	0	0
Crenarchaeota	23	0	0	0	0	0	0	0	0	0
Thaumarchaeota	2	0	0	0	0	0	0	0	0	0
Nanoarchaeota	1	0	0	0	0	0	0	0	0	0
Korarchaeota	1	0	0	0	0	0	0	0	0	0
Total	426	0	0	15	0	0	8	0	0	319

(M00239_1)

Phyla	Module completion ratio (%)									
	0--10	10--20	20--30	30--40	40--50	50--60	60--70	70--80	80--90	90--100
Gammaproteobacteria	27	0	7	0	2	5	0	0	9	76
Betaproteobacteria	6	0	3	0	2	1	0	0	5	44
Epsilonproteobacteria	3	0	0	0	1	2	0	0	4	7
Deltaproteobacteria	2	0	1	0	0	2	0	0	0	23
Alphaproteobacteria	32	0	7	0	3	3	0	0	0	46
Magnetococcus	1	0	0	0	0	0	0	0	0	0
Chrysiogenetes	1	0	0	0	0	0	0	0	0	0
Firmicutes	12	0	12	0	4	5	0	0	4	67
Tenericutes	3	0	9	0	3	0	0	0	1	3
Actinobacteria	1	0	1	0	0	3	0	0	2	73
Chlamydiae	0	0	0	0	0	0	0	0	0	8
Spirochaetes	7	0	1	0	0	0	0	0	0	6
Acidobacteria	0	0	1	0	0	0	0	0	1	3
Bacteroidetes	29	0	0	0	0	3	0	0	1	2
Fibrobacteres	0	0	0	0	0	0	0	0	1	0
Fusobacteria	0	0	1	0	0	0	0	0	0	4
Verrucomicrobia	0	0	0	0	0	1	0	0	1	2
Gemmatimonadetes	0	0	0	0	0	0	0	0	0	1
Planctomycetes	1	0	0	0	1	0	0	0	1	1
Elusimicrobia	1	0	0	0	0	0	0	0	0	1
Synergistetes	0	0	0	0	0	0	0	0	0	2
Cyanobacteria	0	0	0	0	0	1	0	0	1	14
Chlorobi	0	0	0	0	0	0	0	0	0	10
Chloroflexi	0	0	1	0	0	0	0	0	0	10
Deinococcus-Thermus	0	0	0	0	0	0	0	0	0	7
Aquificae	0	0	0	0	0	0	0	0	0	9
Thermotogae	0	0	0	0	0	0	0	0	0	11
Dictyoglomi	0	0	0	0	0	0	0	0	0	2
Nitrospirae	1	0	0	0	0	0	0	0	0	1
Thermobaculum	0	0	0	0	0	0	0	0	0	1
Deferribacteres	0	0	0	0	0	0	0	0	2	1
Euryarchaeota	15	0	1	0	1	1	0	0	0	42
Crenarchaeota	0	0	0	0	0	0	0	0	1	22
Thaumarchaeota	0	0	0	0	0	0	0	0	0	2
Nanoarchaeota	1	0	0	0	0	0	0	0	0	0
Korarchaeota	0	0	0	0	0	0	0	0	0	1
Total	143	0	45	0	17	27	0	0	34	502

(M00240_1)

Phyla	Module completion ratio (%)									
	0--10	10--20	20--30	30--40	40--50	50--60	60--70	70--80	80--90	90--100
Gammaproteobacteria	22	0	0	3	0	0	7	0	0	94
Betaproteobacteria	8	0	0	1	0	0	0	0	0	52
Epsilonproteobacteria	5	0	0	0	0	0	0	0	0	12
Deltaproteobacteria	6	0	0	0	0	0	0	0	0	22
Alphaproteobacteria	28	0	0	0	0	0	1	0	0	62
Magnetococcus	1	0	0	0	0	0	0	0	0	0
Chrysiogenetes	0	0	0	0	0	0	0	0	0	1
Firmicutes	16	0	0	1	0	0	3	0	0	84
Tenericutes	16	0	0	0	0	0	2	0	0	1
Actinobacteria	3	0	0	6	0	0	8	0	0	63
Chlamydiae	7	0	0	0	0	0	1	0	0	0
Spirochaetes	8	0	0	1	0	0	1	0	0	4
Acidobacteria	0	0	0	2	0	0	3	0	0	0
Bacteroidetes	6	0	0	0	0	0	1	0	0	28
Fibrobacteres	0	0	0	0	0	0	0	0	0	1
Fusobacteria	1	0	0	0	0	0	0	0	0	4
Verrucomicrobia	1	0	0	0	0	0	0	0	0	3
Gemmatimonadetes	0	0	0	0	0	0	0	0	0	1
Planctomycetes	0	0	0	4	0	0	0	0	0	0
Elusimicrobia	1	0	0	0	0	0	0	0	0	1
Synergistetes	0	0	0	0	0	0	0	0	0	2
Cyanobacteria	3	0	0	6	0	0	0	0	0	7
Chlorobi	0	0	0	0	0	0	1	0	0	9
Chloroflexi	0	0	0	1	0	0	0	0	0	10
Deinococcus-Thermus	0	0	0	0	0	0	0	0	0	7
Aquificae	7	0	0	0	0	0	0	0	0	2
Thermotogae	0	0	0	0	0	0	0	0	0	11
Dictyoglomi	0	0	0	0	0	0	0	0	0	2
Nitrospirae	0	0	0	0	0	0	0	0	0	2
Thermobaculum	0	0	0	0	0	0	0	0	0	1
Deferribacteres	0	0	0	0	0	0	0	0	0	3
Euryarchaeota	6	0	0	0	0	0	1	0	0	53
Crenarchaeota	0	0	0	0	0	0	4	0	0	19
Thaumarchaeota	0	0	0	2	0	0	0	0	0	0
Nanoarchaeota	1	0	0	0	0	0	0	0	0	0
Korarchaeota	1	0	0	0	0	0	0	0	0	0
Total	147	0	0	27	0	0	33	0	0	561

(M00241_1)

Phyla	Module completion ratio (%)									
	0--10	10--20	20--30	30--40	40--50	50--60	60--70	70--80	80--90	90--100
Gammaproteobacteria	72	0	0	16	0	0	0	0	0	38
Betaproteobacteria	56	0	0	5	0	0	0	0	0	0
Epsilonproteobacteria	17	0	0	0	0	0	0	0	0	0
Deltaproteobacteria	28	0	0	0	0	0	0	0	0	0
Alphaproteobacteria	91	0	0	0	0	0	0	0	0	0
Magnetococcus	1	0	0	0	0	0	0	0	0	0
Chrysiogenetes	1	0	0	0	0	0	0	0	0	0
Firmicutes	104	0	0	0	0	0	0	0	0	0
Tenericutes	19	0	0	0	0	0	0	0	0	0
Actinobacteria	80	0	0	0	0	0	0	0	0	0
Chlamydiae	8	0	0	0	0	0	0	0	0	0
Spirochaetes	14	0	0	0	0	0	0	0	0	0
Acidobacteria	5	0	0	0	0	0	0	0	0	0
Bacteroidetes	35	0	0	0	0	0	0	0	0	0
Fibrobacteres	1	0	0	0	0	0	0	0	0	0
Fusobacteria	5	0	0	0	0	0	0	0	0	0
Verrucomicrobia	4	0	0	0	0	0	0	0	0	0
Gemmatimonadetes	1	0	0	0	0	0	0	0	0	0
Planctomycetes	4	0	0	0	0	0	0	0	0	0
Elusimicrobia	2	0	0	0	0	0	0	0	0	0
Synergistetes	2	0	0	0	0	0	0	0	0	0
Cyanobacteria	16	0	0	0	0	0	0	0	0	0
Chlorobi	10	0	0	0	0	0	0	0	0	0
Chloroflexi	11	0	0	0	0	0	0	0	0	0
Deinococcus-Thermus	7	0	0	0	0	0	0	0	0	0
Aquificae	9	0	0	0	0	0	0	0	0	0
Thermotogae	11	0	0	0	0	0	0	0	0	0
Dictyoglomi	2	0	0	0	0	0	0	0	0	0
Nitrospirae	2	0	0	0	0	0	0	0	0	0
Thermobaculum	1	0	0	0	0	0	0	0	0	0
Deferribacteres	3	0	0	0	0	0	0	0	0	0
Euryarchaeota	60	0	0	0	0	0	0	0	0	0
Crenarchaeota	23	0	0	0	0	0	0	0	0	0
Thaumarchaeota	2	0	0	0	0	0	0	0	0	0
Nanoarchaeota	1	0	0	0	0	0	0	0	0	0
Korarchaeota	1	0	0	0	0	0	0	0	0	0
Total	709	0	0	21	0	0	0	0	0	38

(M00242_1)

Phyla	Module completion ratio (%)									
	0--10	10--20	20--30	30--40	40--50	50--60	60--70	70--80	80--90	90--100
Gammaproteobacteria	39	0	0	1	0	0	3	0	0	83
Betaproteobacteria	59	0	0	0	0	0	0	0	0	2
Epsilonproteobacteria	3	0	0	0	0	0	0	0	0	14
Deltaproteobacteria	5	0	0	1	0	0	3	0	0	19
Alphaproteobacteria	46	0	0	1	0	0	1	0	0	43
Magnetococcus	0	0	0	0	0	0	0	0	0	1
Chrysiogenetes	0	0	0	0	0	0	0	0	0	1
Firmicutes	23	0	0	0	0	0	5	0	0	76
Tenericutes	18	0	0	0	0	0	0	0	0	1
Actinobacteria	51	0	0	1	0	0	0	0	0	28
Chlamydiae	0	0	0	0	0	0	0	0	0	8
Spirochaetes	10	0	0	0	0	0	0	0	0	4
Acidobacteria	5	0	0	0	0	0	0	0	0	0
Bacteroidetes	31	0	0	0	0	0	0	0	0	4
Fibrobacteres	0	0	0	0	0	0	0	0	0	1
Fusobacteria	0	0	0	0	0	0	0	0	0	5
Verrucomicrobia	2	0	0	1	0	0	0	0	0	1
Gemmatimonadetes	1	0	0	0	0	0	0	0	0	0
Planctomycetes	4	0	0	0	0	0	0	0	0	0
Elusimicrobia	2	0	0	0	0	0	0	0	0	0
Synergistetes	0	0	0	0	0	0	0	0	0	2
Cyanobacteria	12	0	0	1	0	0	0	0	0	3
Chlorobi	1	0	0	0	0	0	0	0	0	9
Chloroflexi	8	0	0	0	0	0	0	0	0	3
Deinococcus-Thermus	3	0	0	0	0	0	1	0	0	3
Aquificae	1	0	0	0	0	0	1	0	0	7
Thermotogae	0	0	0	0	0	0	0	0	0	11
Dictyoglomi	0	0	0	0	0	0	0	0	0	2
Nitrospirae	0	0	0	1	0	0	0	0	0	1
Thermobaculum	0	0	0	0	0	0	0	0	0	1
Deferribacteres	0	0	0	0	0	0	0	0	0	3
Euryarchaeota	33	0	0	1	0	0	0	0	0	26
Crenarchaeota	23	0	0	0	0	0	0	0	0	0
Thaumarchaeota	0	0	0	0	0	0	1	0	0	1
Nanoarchaeota	1	0	0	0	0	0	0	0	0	0
Korarchaeota	1	0	0	0	0	0	0	0	0	0
Total	382	0	0	8	0	0	15	0	0	363

(M00243_1)

Phyla	Module completion ratio (%)									
	0--10	10--20	20--30	30--40	40--50	50--60	60--70	70--80	80--90	90--100
Gammaproteobacteria	124	0	0	0	0	0	0	0	0	2
Betaproteobacteria	61	0	0	0	0	0	0	0	0	0
Epsilonproteobacteria	17	0	0	0	0	0	0	0	0	0
Deltaproteobacteria	25	0	0	0	0	0	1	0	0	2
Alphaproteobacteria	91	0	0	0	0	0	0	0	0	0
Magnetococcus	1	0	0	0	0	0	0	0	0	0
Chrysiogenetes	1	0	0	0	0	0	0	0	0	0
Firmicutes	70	0	0	2	0	0	4	0	0	28
Tenericutes	19	0	0	0	0	0	0	0	0	0
Actinobacteria	39	0	0	6	0	0	11	0	0	24
Chlamydiae	0	0	0	0	0	0	2	0	0	6
Spirochaetes	14	0	0	0	0	0	0	0	0	0
Acidobacteria	5	0	0	0	0	0	0	0	0	0
Bacteroidetes	35	0	0	0	0	0	0	0	0	0
Fibrobacteres	1	0	0	0	0	0	0	0	0	0
Fusobacteria	5	0	0	0	0	0	0	0	0	0
Verrucomicrobia	3	0	0	0	0	0	1	0	0	0
Gemmatimonadetes	1	0	0	0	0	0	0	0	0	0
Planctomycetes	4	0	0	0	0	0	0	0	0	0
Elusimicrobia	2	0	0	0	0	0	0	0	0	0
Synergistetes	2	0	0	0	0	0	0	0	0	0
Cyanobacteria	2	0	0	1	0	0	0	0	0	13
Chlorobi	10	0	0	0	0	0	0	0	0	0
Chloroflexi	2	0	0	2	0	0	1	0	0	6
Deinococcus-Thermus	3	0	0	0	0	0	0	0	0	4
Aquificae	9	0	0	0	0	0	0	0	0	0
Thermotogae	11	0	0	0	0	0	0	0	0	0
Dictyoglomi	2	0	0	0	0	0	0	0	0	0
Nitrospirae	1	0	0	0	0	0	1	0	0	0
Thermobaculum	1	0	0	0	0	0	0	0	0	0
Deferribacteres	2	0	0	0	0	0	0	0	0	1
Euryarchaeota	59	0	0	1	0	0	0	0	0	0
Crenarchaeota	23	0	0	0	0	0	0	0	0	0
Thaumarchaeota	2	0	0	0	0	0	0	0	0	0
Nanoarchaeota	1	0	0	0	0	0	0	0	0	0
Korarchaeota	1	0	0	0	0	0	0	0	0	0
Total	649	0	0	12	0	0	21	0	0	86

(M00244_1)

Phyla	Module completion ratio (%)									
	0--10	10--20	20--30	30--40	40--50	50--60	60--70	70--80	80--90	90--100
Gammaproteobacteria	76	0	0	4	0	0	23	0	0	23
Betaproteobacteria	22	0	0	0	0	0	5	0	0	34
Epsilonproteobacteria	12	0	0	0	0	0	5	0	0	0
Deltaproteobacteria	21	0	0	5	0	0	0	0	0	2
Alphaproteobacteria	56	0	0	0	0	0	2	0	0	33
Magnetococcus	1	0	0	0	0	0	0	0	0	0
Chrysiogenetes	1	0	0	0	0	0	0	0	0	0
Firmicutes	83	0	0	4	0	0	0	0	0	17
Tenericutes	19	0	0	0	0	0	0	0	0	0
Actinobacteria	30	0	0	5	0	0	3	0	0	42
Chlamydiae	8	0	0	0	0	0	0	0	0	0
Spirochaetes	13	0	0	1	0	0	0	0	0	0
Acidobacteria	4	0	0	0	0	0	1	0	0	0
Bacteroidetes	33	0	0	0	0	0	0	0	0	2
Fibrobacteres	1	0	0	0	0	0	0	0	0	0
Fusobacteria	4	0	0	1	0	0	0	0	0	0
Verrucomicrobia	3	0	0	1	0	0	0	0	0	0
Gemmatimonadetes	1	0	0	0	0	0	0	0	0	0
Planctomycetes	4	0	0	0	0	0	0	0	0	0
Elusimicrobia	2	0	0	0	0	0	0	0	0	0
Synergistetes	2	0	0	0	0	0	0	0	0	0
Cyanobacteria	4	0	0	0	0	0	2	0	0	10
Chlorobi	10	0	0	0	0	0	0	0	0	0
Chloroflexi	9	0	0	2	0	0	0	0	0	0
Deinococcus-Thermus	6	0	0	1	0	0	0	0	0	0
Aquificae	4	0	0	0	0	0	5	0	0	0
Thermotogae	11	0	0	0	0	0	0	0	0	0
Dictyoglomi	2	0	0	0	0	0	0	0	0	0
Nitrospirae	2	0	0	0	0	0	0	0	0	0
Thermobaculum	1	0	0	0	0	0	0	0	0	0
Deferribacteres	3	0	0	0	0	0	0	0	0	0
Euryarchaeota	42	0	0	0	0	0	1	0	0	17
Crenarchaeota	8	0	0	0	0	0	0	0	0	15
Thaumarchaeota	2	0	0	0	0	0	0	0	0	0
Nanoarchaeota	1	0	0	0	0	0	0	0	0	0
Korarchaeota	0	0	0	0	0	0	0	0	0	1
Total	501	0	0	24	0	0	47	0	0	196

(M00245_1)

Phyla	Module completion ratio (%)									
	0--10	10--20	20--30	30--40	40--50	50--60	60--70	70--80	80--90	90--100
Gammaproteobacteria	80	0	6	0	0	23	0	11	0	6
Betaproteobacteria	42	0	4	0	0	11	0	2	0	2
Epsilonproteobacteria	8	0	1	0	0	2	0	6	0	0
Deltaproteobacteria	4	0	0	0	0	2	0	20	0	2
Alphaproteobacteria	66	0	0	0	0	17	0	7	0	1
Magnetococcus	0	0	0	0	0	0	0	1	0	0
Chrysiogenetes	0	0	0	0	0	0	0	1	0	0
Firmicutes	0	0	1	0	0	55	0	12	0	36
Tenericutes	0	0	0	0	0	19	0	0	0	0
Actinobacteria	7	0	1	0	0	44	0	19	0	9
Chlamydiae	8	0	0	0	0	0	0	0	0	0
Spirochaetes	11	0	1	0	0	1	0	1	0	0
Acidobacteria	4	0	0	0	0	0	0	1	0	0
Bacteroidetes	34	0	0	0	0	0	0	0	0	1
Fibrobacteres	0	0	0	0	0	1	0	0	0	0
Fusobacteria	0	0	0	0	0	3	0	0	0	2
Verrucomicrobia	3	0	0	0	0	0	0	1	0	0
Gemmatimonadetes	1	0	0	0	0	0	0	0	0	0
Planctomycetes	1	0	2	0	0	1	0	0	0	0
Elusimicrobia	1	0	1	0	0	0	0	0	0	0
Synergistetes	0	0	0	0	0	1	0	1	0	0
Cyanobacteria	0	0	0	0	0	5	0	7	0	4
Chlorobi	1	0	1	0	0	0	0	4	0	4
Chloroflexi	2	0	0	0	0	2	0	4	0	3
Deinococcus-Thermus	6	0	0	0	0	1	0	0	0	0
Aquificae	3	0	0	0	0	2	0	4	0	0
Thermotogae	0	0	0	0	0	9	0	0	0	2
Dictyoglomi	0	0	0	0	0	0	0	2	0	0
Nitrospirae	1	0	0	0	0	0	0	1	0	0
Thermobaculum	0	0	0	0	0	0	0	1	0	0
Deferribacteres	0	0	0	0	0	0	0	3	0	0
Euryarchaeota	3	0	0	0	0	8	0	14	0	35
Crenarchaeota	4	0	4	0	0	10	0	5	0	0
Thaumarchaeota	2	0	0	0	0	0	0	0	0	0
Nanoarchaeota	1	0	0	0	0	0	0	0	0	0
Korarchaeota	0	0	0	0	0	0	0	1	0	0
Total	293	0	22	0	0	217	0	129	0	107

(M00246_1)

Phyla	Module completion ratio (%)									
	0--10	10--20	20--30	30--40	40--50	50--60	60--70	70--80	80--90	90--100
Gammaproteobacteria	77	0	9	0	0	24	0	11	0	5
Betaproteobacteria	41	0	5	0	0	8	0	6	0	1
Epsilonproteobacteria	8	0	1	0	0	2	0	6	0	0
Deltaproteobacteria	4	0	0	0	0	2	0	21	0	1
Alphaproteobacteria	61	0	5	0	0	17	0	8	0	0
Magnetococcus	0	0	0	0	0	0	0	1	0	0
Chrysiogenetes	0	0	0	0	0	0	0	1	0	0
Firmicutes	0	0	1	0	0	55	0	48	0	0
Tenericutes	0	0	0	0	0	19	0	0	0	0
Actinobacteria	7	0	1	0	0	44	0	28	0	0
Chlamydiae	8	0	0	0	0	0	0	0	0	0
Spirochaetes	11	0	1	0	0	1	0	1	0	0
Acidobacteria	4	0	0	0	0	0	0	1	0	0
Bacteroidetes	34	0	0	0	0	0	0	1	0	0
Fibrobacteres	0	0	0	0	0	1	0	0	0	0
Fusobacteria	0	0	0	0	0	3	0	2	0	0
Verrucomicrobia	3	0	0	0	0	0	0	1	0	0
Gemmatimonadetes	1	0	0	0	0	0	0	0	0	0
Planctomycetes	1	0	2	0	0	1	0	0	0	0
Elusimicrobia	1	0	1	0	0	0	0	0	0	0
Synergistetes	0	0	0	0	0	1	0	1	0	0
Cyanobacteria	0	0	0	0	0	5	0	10	0	1
Chlorobi	1	0	1	0	0	0	0	8	0	0
Chloroflexi	2	0	0	0	0	2	0	7	0	0
Deinococcus-Thermus	6	0	0	0	0	1	0	0	0	0
Aquificae	3	0	0	0	0	2	0	4	0	0
Thermotogae	0	0	0	0	0	9	0	2	0	0
Dictyoglomi	0	0	0	0	0	0	0	2	0	0
Nitrospirae	1	0	0	0	0	0	0	1	0	0
Thermobaculum	0	0	0	0	0	0	0	1	0	0
Deferribacteres	0	0	0	0	0	0	0	3	0	0
Euryarchaeota	3	0	0	0	0	8	0	49	0	0
Crenarchaeota	4	0	4	0	0	10	0	5	0	0
Thaumarchaeota	2	0	0	0	0	0	0	0	0	0
Nanoarchaeota	1	0	0	0	0	0	0	0	0	0
Korarchaeota	0	0	0	0	0	0	0	1	0	0
Total	284	0	31	0	0	215	0	230	0	8

(M00247_1)

Phyla	Module completion ratio (%)									
	0--10	10--20	20--30	30--40	40--50	50--60	60--70	70--80	80--90	90--100
Gammaproteobacteria	109	0	0	3	0	0	0	0	0	14
Betaproteobacteria	28	0	0	3	0	0	1	0	0	29
Epsilonproteobacteria	17	0	0	0	0	0	0	0	0	0
Deltaproteobacteria	14	0	0	6	0	0	1	0	0	7
Alphaproteobacteria	59	0	0	9	0	0	2	0	0	21
Magnetococcus	1	0	0	0	0	0	0	0	0	0
Chrysiogenetes	1	0	0	0	0	0	0	0	0	0
Firmicutes	53	0	0	0	0	0	0	0	0	51
Tenericutes	19	0	0	0	0	0	0	0	0	0
Actinobacteria	72	0	0	0	0	0	0	0	0	8
Chlamydiae	8	0	0	0	0	0	0	0	0	0
Spirochaetes	12	0	0	0	0	0	0	0	0	2
Acidobacteria	5	0	0	0	0	0	0	0	0	0
Bacteroidetes	33	0	0	1	0	0	0	0	0	1
Fibrobacteres	1	0	0	0	0	0	0	0	0	0
Fusobacteria	0	0	0	0	0	0	0	0	0	5
Verrucomicrobia	3	0	0	0	0	0	0	0	0	1
Gemmatimonadetes	1	0	0	0	0	0	0	0	0	0
Planctomycetes	3	0	0	0	0	0	0	0	0	1
Elusimicrobia	2	0	0	0	0	0	0	0	0	0
Synergistetes	2	0	0	0	0	0	0	0	0	0
Cyanobacteria	16	0	0	0	0	0	0	0	0	0
Chlorobi	9	0	0	0	0	0	0	0	0	1
Chloroflexi	11	0	0	0	0	0	0	0	0	0
Deinococcus-Thermus	6	0	0	1	0	0	0	0	0	0
Aquificae	7	0	0	2	0	0	0	0	0	0
Thermotogae	8	0	0	0	0	0	0	0	0	3
Dictyoglomi	2	0	0	0	0	0	0	0	0	0
Nitrospirae	0	0	0	2	0	0	0	0	0	0
Thermobaculum	1	0	0	0	0	0	0	0	0	0
Deferribacteres	0	0	0	0	0	0	0	0	0	3
Euryarchaeota	60	0	0	0	0	0	0	0	0	0
Crenarchaeota	23	0	0	0	0	0	0	0	0	0
Thaumarchaeota	2	0	0	0	0	0	0	0	0	0
Nanoarchaeota	1	0	0	0	0	0	0	0	0	0
Korarchaeota	1	0	0	0	0	0	0	0	0	0
Total	590	0	0	27	0	0	4	0	0	147

(M00248_1)

Phyla	Module completion ratio (%)									
	0--10	10--20	20--30	30--40	40--50	50--60	60--70	70--80	80--90	90--100
Gammaproteobacteria	6	0	0	0	0	3	0	0	0	117
Betaproteobacteria	5	0	0	0	0	1	0	0	0	55
Epsilonproteobacteria	3	0	0	0	0	11	0	0	0	3
Deltaproteobacteria	3	0	0	0	0	4	0	0	0	21
Alphaproteobacteria	26	0	0	0	0	12	0	0	0	53
Magnetococcus	0	0	0	0	0	0	0	0	0	1
Chrysiogenetes	0	0	0	0	0	0	0	0	0	1
Firmicutes	2	0	0	0	0	18	0	0	0	84
Tenericutes	13	0	0	0	0	5	0	0	0	1
Actinobacteria	1	0	0	0	0	6	0	0	0	73
Chlamydiae	6	0	0	0	0	2	0	0	0	0
Spirochaetes	4	0	0	0	0	5	0	0	0	5
Acidobacteria	0	0	0	0	0	0	0	0	0	5
Bacteroidetes	4	0	0	0	0	16	0	0	0	15
Fibrobacteres	0	0	0	0	0	1	0	0	0	0
Fusobacteria	3	0	0	0	0	0	0	0	0	2
Verrucomicrobia	0	0	0	0	0	1	0	0	0	3
Gemmatimonadetes	0	0	0	0	0	0	0	0	0	1
Planctomycetes	0	0	0	0	0	0	0	0	0	4
Elusimicrobia	1	0	0	0	0	1	0	0	0	0
Synergistetes	2	0	0	0	0	0	0	0	0	0
Cyanobacteria	0	0	0	0	0	0	0	0	0	16
Chlorobi	5	0	0	0	0	0	0	0	0	5
Chloroflexi	0	0	0	0	0	0	0	0	0	11
Deinococcus-Thermus	0	0	0	0	0	2	0	0	0	5
Aquificae	5	0	0	0	0	2	0	0	0	2
Thermotogae	0	0	0	0	0	0	0	0	0	11
Dictyoglomi	0	0	0	0	0	0	0	0	0	2
Nitrospirae	0	0	0	0	0	0	0	0	0	2
Thermobaculum	0	0	0	0	0	0	0	0	0	1
Deferribacteres	0	0	0	0	0	0	0	0	0	3
Euryarchaeota	9	0	0	0	0	1	0	0	0	50
Crenarchaeota	2	0	0	0	0	1	0	0	0	20
Thaumarchaeota	0	0	0	0	0	0	0	0	0	2
Nanoarchaeota	1	0	0	0	0	0	0	0	0	0
Korarchaeota	0	0	0	0	0	0	0	0	0	1
Total	101	0	0	0	0	92	0	0	0	575

(M00249_1)

Phyla	Module completion ratio (%)									
	0--10	10--20	20--30	30--40	40--50	50--60	60--70	70--80	80--90	90--100
Gammaproteobacteria	109	0	0	0	0	0	1	0	0	16
Betaproteobacteria	42	0	0	0	0	0	3	0	0	16
Epsilonproteobacteria	15	0	0	0	0	0	0	0	0	2
Deltaproteobacteria	26	0	0	0	0	0	0	0	0	2
Alphaproteobacteria	62	0	0	0	0	0	4	0	0	25
Magnetococcus	1	0	0	0	0	0	0	0	0	0
Chrysiogenetes	1	0	0	0	0	0	0	0	0	0
Firmicutes	104	0	0	0	0	0	0	0	0	0
Tenericutes	19	0	0	0	0	0	0	0	0	0
Actinobacteria	80	0	0	0	0	0	0	0	0	0
Chlamydiae	8	0	0	0	0	0	0	0	0	0
Spirochaetes	14	0	0	0	0	0	0	0	0	0
Acidobacteria	5	0	0	0	0	0	0	0	0	0
Bacteroidetes	35	0	0	0	0	0	0	0	0	0
Fibrobacteres	1	0	0	0	0	0	0	0	0	0
Fusobacteria	5	0	0	0	0	0	0	0	0	0
Verrucomicrobia	4	0	0	0	0	0	0	0	0	0
Gemmatimonadetes	1	0	0	0	0	0	0	0	0	0
Planctomycetes	4	0	0	0	0	0	0	0	0	0
Elusimicrobia	2	0	0	0	0	0	0	0	0	0
Synergistetes	2	0	0	0	0	0	0	0	0	0
Cyanobacteria	15	0	0	0	0	0	0	0	0	1
Chlorobi	9	0	0	0	0	0	0	0	0	1
Chloroflexi	11	0	0	0	0	0	0	0	0	0
Deinococcus-Thermus	7	0	0	0	0	0	0	0	0	0
Aquificae	9	0	0	0	0	0	0	0	0	0
Thermotogae	11	0	0	0	0	0	0	0	0	0
Dictyoglomi	2	0	0	0	0	0	0	0	0	0
Nitrospirae	2	0	0	0	0	0	0	0	0	0
Thermobaculum	1	0	0	0	0	0	0	0	0	0
Deferribacteres	3	0	0	0	0	0	0	0	0	0
Euryarchaeota	60	0	0	0	0	0	0	0	0	0
Crenarchaeota	23	0	0	0	0	0	0	0	0	0
Thaumarchaeota	2	0	0	0	0	0	0	0	0	0
Nanoarchaeota	1	0	0	0	0	0	0	0	0	0
Korarchaeota	1	0	0	0	0	0	0	0	0	0
Total	697	0	0	0	0	0	8	0	0	63

(M00250_1)

Phyla	Module completion ratio (%)									
	0--10	10--20	20--30	30--40	40--50	50--60	60--70	70--80	80--90	90--100
Gammaproteobacteria	87	0	0	0	0	5	0	0	0	34
Betaproteobacteria	25	0	0	0	0	3	0	0	0	33
Epsilonproteobacteria	12	0	0	0	0	0	0	0	0	5
Deltaproteobacteria	9	0	0	0	0	1	0	0	0	18
Alphaproteobacteria	55	0	0	0	0	2	0	0	0	34
Magnetococcus	0	0	0	0	0	0	0	0	0	1
Chrysiogenetes	0	0	0	0	0	0	0	0	0	1
Firmicutes	90	0	0	0	0	2	0	0	0	12
Tenericutes	19	0	0	0	0	0	0	0	0	0
Actinobacteria	8	0	0	0	0	6	0	0	0	66
Chlamydiae	7	0	0	0	0	0	0	0	0	1
Spirochaetes	10	0	0	0	0	1	0	0	0	3
Acidobacteria	4	0	0	0	0	0	0	0	0	1
Bacteroidetes	21	0	0	0	0	0	0	0	0	14
Fibrobacteres	1	0	0	0	0	0	0	0	0	0
Fusobacteria	3	0	0	0	0	1	0	0	0	1
Verrucomicrobia	3	0	0	0	0	0	0	0	0	1
Gemmatimonadetes	1	0	0	0	0	0	0	0	0	0
Planctomycetes	3	0	0	0	0	0	0	0	0	1
Elusimicrobia	2	0	0	0	0	0	0	0	0	0
Synergistetes	2	0	0	0	0	0	0	0	0	0
Cyanobacteria	8	0	0	0	0	0	0	0	0	8
Chlorobi	9	0	0	0	0	0	0	0	0	1
Chloroflexi	4	0	0	0	0	0	0	0	0	7
Deinococcus-Thermus	7	0	0	0	0	0	0	0	0	0
Aquificae	6	0	0	0	0	0	0	0	0	3
Thermotogae	11	0	0	0	0	0	0	0	0	0
Dictyoglomi	2	0	0	0	0	0	0	0	0	0
Nitrospirae	1	0	0	0	0	0	0	0	0	1
Thermobaculum	0	0	0	0	0	0	0	0	0	1
Deferribacteres	2	0	0	0	0	0	0	0	0	1
Euryarchaeota	50	0	0	0	0	0	0	0	0	10
Crenarchaeota	23	0	0	0	0	0	0	0	0	0
Thaumarchaeota	2	0	0	0	0	0	0	0	0	0
Nanoarchaeota	1	0	0	0	0	0	0	0	0	0
Korarchaeota	1	0	0	0	0	0	0	0	0	0
Total	489	0	0	0	0	21	0	0	0	258

(mod_M00251_1)

Phyla	Module completion ratio (%)									
	0--10	10--20	20--30	30--40	40--50	50--60	60--70	70--80	80--90	90--100
Gammaproteobacteria	125	0	0	0	0	1	0	0	0	0
Betaproteobacteria	61	0	0	0	0	0	0	0	0	0
Epsilonproteobacteria	17	0	0	0	0	0	0	0	0	0
Deltaproteobacteria	27	0	0	0	0	1	0	0	0	0
Alphaproteobacteria	91	0	0	0	0	0	0	0	0	0
Magnetococcus	1	0	0	0	0	0	0	0	0	0
Chrysiogenetes	1	0	0	0	0	0	0	0	0	0
Firmicutes	79	0	0	0	0	3	0	0	0	22
Tenericutes	19	0	0	0	0	0	0	0	0	0
Actinobacteria	79	0	0	0	0	1	0	0	0	0
Chlamydiae	8	0	0	0	0	0	0	0	0	0
Spirochaetes	14	0	0	0	0	0	0	0	0	0
Acidobacteria	5	0	0	0	0	0	0	0	0	0
Bacteroidetes	35	0	0	0	0	0	0	0	0	0
Fibrobacteres	1	0	0	0	0	0	0	0	0	0
Fusobacteria	4	0	0	0	0	1	0	0	0	0
Verrucomicrobia	4	0	0	0	0	0	0	0	0	0
Gemmatimonadetes	1	0	0	0	0	0	0	0	0	0
Planctomycetes	4	0	0	0	0	0	0	0	0	0
Elusimicrobia	2	0	0	0	0	0	0	0	0	0
Synergistetes	2	0	0	0	0	0	0	0	0	0
Cyanobacteria	16	0	0	0	0	0	0	0	0	0
Chlorobi	10	0	0	0	0	0	0	0	0	0
Chloroflexi	11	0	0	0	0	0	0	0	0	0
Deinococcus-Thermus	7	0	0	0	0	0	0	0	0	0
Aquificae	9	0	0	0	0	0	0	0	0	0
Thermotogae	11	0	0	0	0	0	0	0	0	0
Dictyoglomi	2	0	0	0	0	0	0	0	0	0
Nitrospirae	2	0	0	0	0	0	0	0	0	0
Thermobaculum	1	0	0	0	0	0	0	0	0	0
Deferribacteres	3	0	0	0	0	0	0	0	0	0
Euryarchaeota	60	0	0	0	0	0	0	0	0	0
Crenarchaeota	23	0	0	0	0	0	0	0	0	0
Thaumarchaeota	2	0	0	0	0	0	0	0	0	0
Nanoarchaeota	1	0	0	0	0	0	0	0	0	0
Korarchaeota	1	0	0	0	0	0	0	0	0	0
Total	739	0	0	0	0	7	0	0	0	22

(mod_M00252_1)

Phyla	Module completion ratio (%)									
	0--10	10--20	20--30	30--40	40--50	50--60	60--70	70--80	80--90	90--100
Gammaproteobacteria	120	0	0	0	0	0	0	0	0	6
Betaproteobacteria	24	0	0	0	0	1	0	0	0	36
Epsilonproteobacteria	17	0	0	0	0	0	0	0	0	0
Deltaproteobacteria	23	0	0	0	0	1	0	0	0	4
Alphaproteobacteria	82	0	0	0	0	1	0	0	0	8
Magnetococcus	0	0	0	0	0	0	0	0	0	1
Chrysiogenetes	1	0	0	0	0	0	0	0	0	0
Firmicutes	99	0	0	0	0	0	0	0	0	5
Tenericutes	19	0	0	0	0	0	0	0	0	0
Actinobacteria	60	0	0	0	0	3	0	0	0	17
Chlamydiae	8	0	0	0	0	0	0	0	0	0
Spirochaetes	14	0	0	0	0	0	0	0	0	0
Acidobacteria	5	0	0	0	0	0	0	0	0	0
Bacteroidetes	35	0	0	0	0	0	0	0	0	0
Fibrobacteres	1	0	0	0	0	0	0	0	0	0
Fusobacteria	5	0	0	0	0	0	0	0	0	0
Verrucomicrobia	4	0	0	0	0	0	0	0	0	0
Gemmatimonadetes	1	0	0	0	0	0	0	0	0	0
Planctomycetes	4	0	0	0	0	0	0	0	0	0
Elusimicrobia	2	0	0	0	0	0	0	0	0	0
Synergistetes	2	0	0	0	0	0	0	0	0	0
Cyanobacteria	15	0	0	0	0	0	0	0	0	1
Chlorobi	10	0	0	0	0	0	0	0	0	0
Chloroflexi	9	0	0	0	0	0	0	0	0	2
Deinococcus-Thermus	7	0	0	0	0	0	0	0	0	0
Aquificae	9	0	0	0	0	0	0	0	0	0
Thermotogae	11	0	0	0	0	0	0	0	0	0
Dictyoglomi	2	0	0	0	0	0	0	0	0	0
Nitrospirae	1	0	0	0	0	0	0	0	0	1
Thermobaculum	1	0	0	0	0	0	0	0	0	0
Deferribacteres	3	0	0	0	0	0	0	0	0	0
Euryarchaeota	51	0	0	0	0	0	0	0	0	9
Crenarchaeota	23	0	0	0	0	0	0	0	0	0
Thaumarchaeota	2	0	0	0	0	0	0	0	0	0
Nanoarchaeota	1	0	0	0	0	0	0	0	0	0
Korarchaeota	1	0	0	0	0	0	0	0	0	0
Total	672	0	0	0	0	6	0	0	0	90

(mod_M00253_1)

Phyla	Module completion ratio (%)									
	0--10	10--20	20--30	30--40	40--50	50--60	60--70	70--80	80--90	90--100
Gammaproteobacteria	97	0	0	0	0	0	0	0	0	29
Betaproteobacteria	60	0	0	0	0	0	0	0	0	1
Epsilonproteobacteria	17	0	0	0	0	0	0	0	0	0
Deltaproteobacteria	24	0	0	0	0	0	0	0	0	4
Alphaproteobacteria	91	0	0	0	0	0	0	0	0	0
Magnetococcus	1	0	0	0	0	0	0	0	0	0
Chrysiogenetes	1	0	0	0	0	0	0	0	0	0
Firmicutes	85	0	0	0	0	2	0	0	0	17
Tenericutes	19	0	0	0	0	0	0	0	0	0
Actinobacteria	79	0	0	0	0	0	0	0	0	1
Chlamydiae	8	0	0	0	0	0	0	0	0	0
Spirochaetes	14	0	0	0	0	0	0	0	0	0
Acidobacteria	5	0	0	0	0	0	0	0	0	0
Bacteroidetes	34	0	0	0	0	0	0	0	0	1
Fibrobacteres	1	0	0	0	0	0	0	0	0	0
Fusobacteria	5	0	0	0	0	0	0	0	0	0
Verrucomicrobia	3	0	0	0	0	0	0	0	0	1
Gemmatimonadetes	1	0	0	0	0	0	0	0	0	0
Planctomycetes	0	0	0	0	0	0	0	0	0	4
Elusimicrobia	2	0	0	0	0	0	0	0	0	0
Synergistetes	2	0	0	0	0	0	0	0	0	0
Cyanobacteria	15	0	0	0	0	0	0	0	0	1
Chlorobi	10	0	0	0	0	0	0	0	0	0
Chloroflexi	10	0	0	0	0	0	0	0	0	1
Deinococcus-Thermus	0	0	0	0	0	0	0	0	0	7
Aquificae	9	0	0	0	0	0	0	0	0	0
Thermotogae	10	0	0	0	0	1	0	0	0	0
Dictyoglomi	2	0	0	0	0	0	0	0	0	0
Nitrospirae	2	0	0	0	0	0	0	0	0	0
Thermobaculum	1	0	0	0	0	0	0	0	0	0
Deferribacteres	3	0	0	0	0	0	0	0	0	0
Euryarchaeota	60	0	0	0	0	0	0	0	0	0
Crenarchaeota	23	0	0	0	0	0	0	0	0	0
Thaumarchaeota	2	0	0	0	0	0	0	0	0	0
Nanoarchaeota	1	0	0	0	0	0	0	0	0	0
Korarchaeota	1	0	0	0	0	0	0	0	0	0
Total	698	0	0	0	0	3	0	0	0	67

(mod_M00254_1)

Phyla	Module completion ratio (%)									
	0--10	10--20	20--30	30--40	40--50	50--60	60--70	70--80	80--90	90--100
Gammaproteobacteria	40	0	0	0	0	35	0	0	0	51
Betaproteobacteria	7	0	0	0	0	16	0	0	0	38
Epsilonproteobacteria	2	0	0	0	0	8	0	0	0	7
Deltaproteobacteria	4	0	0	0	0	4	0	0	0	20
Alphaproteobacteria	33	0	0	0	0	10	0	0	0	48
Magnetococcus	0	0	0	0	0	1	0	0	0	0
Chrysiogenetes	1	0	0	0	0	0	0	0	0	0
Firmicutes	5	0	0	0	0	8	0	0	0	91
Tenericutes	5	0	0	0	0	8	0	0	0	6
Actinobacteria	5	0	0	0	0	8	0	0	0	67
Chlamydiae	6	0	0	0	0	2	0	0	0	0
Spirochaetes	0	0	0	0	0	2	0	0	0	12
Acidobacteria	0	0	0	0	0	0	0	0	0	5
Bacteroidetes	3	0	0	0	0	2	0	0	0	30
Fibrobacteres	0	0	0	0	0	1	0	0	0	0
Fusobacteria	2	0	0	0	0	0	0	0	0	3
Verrucomicrobia	0	0	0	0	0	0	0	0	0	4
Gemmatimonadetes	0	0	0	0	0	0	0	0	0	1
Planctomycetes	0	0	0	0	0	0	0	0	0	4
Elusimicrobia	2	0	0	0	0	0	0	0	0	0
Synergistetes	2	0	0	0	0	0	0	0	0	0
Cyanobacteria	2	0	0	0	0	0	0	0	0	14
Chlorobi	4	0	0	0	0	3	0	0	0	3
Chloroflexi	0	0	0	0	0	0	0	0	0	11
Deinococcus-Thermus	0	0	0	0	0	1	0	0	0	6
Aquificae	4	0	0	0	0	4	0	0	0	1
Thermotogae	0	0	0	0	0	0	0	0	0	11
Dictyoglomi	0	0	0	0	0	0	0	0	0	2
Nitrospirae	0	0	0	0	0	1	0	0	0	1
Thermobaculum	0	0	0	0	0	0	0	0	0	1
Deferribacteres	1	0	0	0	0	0	0	0	0	2
Euryarchaeota	10	0	0	0	0	4	0	0	0	46
Crenarchaeota	0	0	0	0	0	0	0	0	0	23
Thaumarchaeota	1	0	0	0	0	1	0	0	0	0
Nanoarchaeota	1	0	0	0	0	0	0	0	0	0
Korarchaeota	0	0	0	0	0	0	0	0	0	1
Total	140	0	0	0	0	119	0	0	0	509

(mod_M00255_1)

Phyla	Module completion ratio (%)									
	0--10	10--20	20--30	30--40	40--50	50--60	60--70	70--80	80--90	90--100
Gammaproteobacteria	2	0	0	0	0	1	0	0	0	123
Betaproteobacteria	1	0	0	0	0	0	0	0	0	60
Epsilonproteobacteria	0	0	0	0	0	17	0	0	0	0
Deltaproteobacteria	0	0	0	0	0	0	0	0	0	28
Alphaproteobacteria	2	0	0	0	0	2	0	0	0	87
Magnetococcus	0	0	0	0	0	0	0	0	0	1
Chrysiogenetes	0	0	0	0	0	0	0	0	0	1
Firmicutes	81	0	0	0	0	18	0	0	0	5
Tenericutes	19	0	0	0	0	0	0	0	0	0
Actinobacteria	76	0	0	0	0	4	0	0	0	0
Chlamydiae	0	0	0	0	0	0	0	0	0	8
Spirochaetes	0	0	0	0	0	0	0	0	0	14
Acidobacteria	0	0	0	0	0	0	0	0	0	5
Bacteroidetes	2	0	0	0	0	7	0	0	0	26
Fibrobacteres	0	0	0	0	0	0	0	0	0	1
Fusobacteria	0	0	0	0	0	0	0	0	0	5
Verrucomicrobia	0	0	0	0	0	2	0	0	0	2
Gemmatimonadetes	0	0	0	0	0	0	0	0	0	1
Planctomycetes	0	0	0	0	0	0	0	0	0	4
Elusimicrobia	0	0	0	0	0	1	0	0	0	1
Synergistetes	1	0	0	0	0	1	0	0	0	0
Cyanobacteria	15	0	0	0	0	1	0	0	0	0
Chlorobi	0	0	0	0	0	0	0	0	0	10
Chloroflexi	10	0	0	0	0	1	0	0	0	0
Deinococcus-Thermus	0	0	0	0	0	3	0	0	0	4
Aquificae	0	0	0	0	0	1	0	0	0	8
Thermotogae	8	0	0	0	0	3	0	0	0	0
Dictyoglomi	2	0	0	0	0	0	0	0	0	0
Nitrospirae	0	0	0	0	0	0	0	0	0	2
Thermobaculum	1	0	0	0	0	0	0	0	0	0
Deferribacteres	0	0	0	0	0	0	0	0	0	3
Euryarchaeota	49	0	0	0	0	5	0	0	0	6
Crenarchaeota	22	0	0	0	0	1	0	0	0	0
Thaumarchaeota	0	0	0	0	0	1	0	0	0	1
Nanoarchaeota	1	0	0	0	0	0	0	0	0	0
Korarchaeota	1	0	0	0	0	0	0	0	0	0
Total	293	0	0	0	0	69	0	0	0	406

(mod_M00256_1)

Phyla	Module completion ratio (%)									
	0--10	10--20	20--30	30--40	40--50	50--60	60--70	70--80	80--90	90--100
Gammaproteobacteria	20	0	0	0	0	1	0	0	0	105
Betaproteobacteria	33	0	0	0	0	0	0	0	0	28
Epsilonproteobacteria	0	0	0	0	0	0	0	0	0	17
Deltaproteobacteria	2	0	0	0	0	1	0	0	0	25
Alphaproteobacteria	23	0	0	0	0	1	0	0	0	67
Magnetococcus	0	0	0	0	0	0	0	0	0	1
Chrysiogenetes	0	0	0	0	0	0	0	0	0	1
Firmicutes	24	0	0	0	0	0	0	0	0	80
Tenericutes	19	0	0	0	0	0	0	0	0	0
Actinobacteria	4	0	0	0	0	0	0	0	0	76
Chlamydiae	8	0	0	0	0	0	0	0	0	0
Spirochaetes	14	0	0	0	0	0	0	0	0	0
Acidobacteria	5	0	0	0	0	0	0	0	0	0
Bacteroidetes	2	0	0	0	0	0	0	0	0	33
Fibrobacteres	0	0	0	0	0	0	0	0	0	1
Fusobacteria	1	0	0	0	0	4	0	0	0	0
Verrucomicrobia	4	0	0	0	0	0	0	0	0	0
Gemmatimonadetes	0	0	0	0	0	0	0	0	0	1
Planctomycetes	4	0	0	0	0	0	0	0	0	0
Elusimicrobia	1	0	0	0	0	1	0	0	0	0
Synergistetes	0	0	0	0	0	0	0	0	0	2
Cyanobacteria	8	0	0	0	0	0	0	0	0	8
Chlorobi	0	0	0	0	0	0	0	0	0	10
Chloroflexi	11	0	0	0	0	0	0	0	0	0
Deinococcus-Thermus	0	0	0	0	0	0	0	0	0	7
Aquificae	9	0	0	0	0	0	0	0	0	0
Thermotogae	11	0	0	0	0	0	0	0	0	0
Dictyoglomi	0	0	0	0	0	2	0	0	0	0
Nitrospirae	0	0	0	0	0	0	0	0	0	2
Thermobaculum	1	0	0	0	0	0	0	0	0	0
Deferribacteres	0	0	0	0	0	0	0	0	0	3
Euryarchaeota	60	0	0	0	0	0	0	0	0	0
Crenarchaeota	23	0	0	0	0	0	0	0	0	0
Thaumarchaeota	2	0	0	0	0	0	0	0	0	0
Nanoarchaeota	1	0	0	0	0	0	0	0	0	0
Korarchaeota	1	0	0	0	0	0	0	0	0	0
Total	291	0	0	0	0	10	0	0	0	467

(mod_M00257_1)

Phyla	Module completion ratio (%)									
	0--10	10--20	20--30	30--40	40--50	50--60	60--70	70--80	80--90	90--100
Gammaproteobacteria	126	0	0	0	0	0	0	0	0	0
Betaproteobacteria	61	0	0	0	0	0	0	0	0	0
Epsilonproteobacteria	17	0	0	0	0	0	0	0	0	0
Deltaproteobacteria	28	0	0	0	0	0	0	0	0	0
Alphaproteobacteria	91	0	0	0	0	0	0	0	0	0
Magnetococcus	1	0	0	0	0	0	0	0	0	0
Chrysiogenetes	1	0	0	0	0	0	0	0	0	0
Firmicutes	100	0	0	0	0	0	0	0	0	4
Tenericutes	19	0	0	0	0	0	0	0	0	0
Actinobacteria	80	0	0	0	0	0	0	0	0	0
Chlamydiae	8	0	0	0	0	0	0	0	0	0
Spirochaetes	14	0	0	0	0	0	0	0	0	0
Acidobacteria	5	0	0	0	0	0	0	0	0	0
Bacteroidetes	35	0	0	0	0	0	0	0	0	0
Fibrobacteres	1	0	0	0	0	0	0	0	0	0
Fusobacteria	5	0	0	0	0	0	0	0	0	0
Verrucomicrobia	4	0	0	0	0	0	0	0	0	0
Gemmatimonadetes	1	0	0	0	0	0	0	0	0	0
Planctomycetes	4	0	0	0	0	0	0	0	0	0
Elusimicrobia	2	0	0	0	0	0	0	0	0	0
Synergistetes	2	0	0	0	0	0	0	0	0	0
Cyanobacteria	16	0	0	0	0	0	0	0	0	0
Chlorobi	10	0	0	0	0	0	0	0	0	0
Chloroflexi	11	0	0	0	0	0	0	0	0	0
Deinococcus-Thermus	7	0	0	0	0	0	0	0	0	0
Aquificae	9	0	0	0	0	0	0	0	0	0
Thermotogae	11	0	0	0	0	0	0	0	0	0
Dictyoglomi	2	0	0	0	0	0	0	0	0	0
Nitrospirae	2	0	0	0	0	0	0	0	0	0
Thermobaculum	1	0	0	0	0	0	0	0	0	0
Deferribacteres	3	0	0	0	0	0	0	0	0	0
Euryarchaeota	60	0	0	0	0	0	0	0	0	0
Crenarchaeota	23	0	0	0	0	0	0	0	0	0
Thaumarchaeota	2	0	0	0	0	0	0	0	0	0
Nanoarchaeota	1	0	0	0	0	0	0	0	0	0
Korarchaeota	1	0	0	0	0	0	0	0	0	0
Total	764	0	0	0	0	0	0	0	0	4

(mod_M00258_1)

Phyla	Module completion ratio (%)									
	0--10	10--20	20--30	30--40	40--50	50--60	60--70	70--80	80--90	90--100
Gammaproteobacteria	21	0	0	0	0	7	0	0	0	98
Betaproteobacteria	3	0	0	0	0	3	0	0	0	55
Epsilonproteobacteria	0	0	0	0	0	3	0	0	0	14
Deltaproteobacteria	2	0	0	0	0	2	0	0	0	24
Alphaproteobacteria	34	0	0	0	0	4	0	0	0	53
Magnetococcus	1	0	0	0	0	0	0	0	0	0
Chrysiogenetes	0	0	0	0	0	0	0	0	0	1
Firmicutes	5	0	0	0	0	2	0	0	0	97
Tenericutes	1	0	0	0	0	10	0	0	0	8
Actinobacteria	5	0	0	0	0	2	0	0	0	73
Chlamydiae	7	0	0	0	0	1	0	0	0	0
Spirochaetes	6	0	0	0	0	1	0	0	0	7
Acidobacteria	0	0	0	0	0	0	0	0	0	5
Bacteroidetes	7	0	0	0	0	1	0	0	0	27
Fibrobacteres	0	0	0	0	0	0	0	0	0	1
Fusobacteria	0	0	0	0	0	0	0	0	0	5
Verrucomicrobia	0	0	0	0	0	0	0	0	0	4
Gemmatimonadetes	0	0	0	0	0	0	0	0	0	1
Planctomycetes	0	0	0	0	0	0	0	0	0	4
Elusimicrobia	1	0	0	0	0	1	0	0	0	0
Synergistetes	1	0	0	0	0	0	0	0	0	1
Cyanobacteria	1	0	0	0	0	0	0	0	0	15
Chlorobi	0	0	0	0	0	0	0	0	0	10
Chloroflexi	0	0	0	0	0	0	0	0	0	11
Deinococcus-Thermus	0	0	0	0	0	0	0	0	0	7
Aquificae	2	0	0	0	0	1	0	0	0	6
Thermotogae	0	0	0	0	0	2	0	0	0	9
Dictyoglomi	0	0	0	0	0	0	0	0	0	2
Nitrospirae	0	0	0	0	0	0	0	0	0	2
Thermobaculum	0	0	0	0	0	0	0	0	0	1
Deferribacteres	0	0	0	0	0	0	0	0	0	3
Euryarchaeota	11	0	0	0	0	6	0	0	0	43
Crenarchaeota	2	0	0	0	0	4	0	0	0	17
Thaumarchaeota	1	0	0	0	0	1	0	0	0	0
Nanoarchaeota	0	0	0	0	0	0	0	0	0	1
Korarchaeota	0	0	0	0	0	0	0	0	0	1
Total	111	0	0	0	0	51	0	0	0	606

(mod_M00259_1)

Phyla	Module completion ratio (%)									
	0--10	10--20	20--30	30--40	40--50	50--60	60--70	70--80	80--90	90--100
Gammaproteobacteria	27	0	0	0	0	0	1	0	0	98
Betaproteobacteria	33	0	0	0	0	0	0	0	0	28
Epsilonproteobacteria	14	0	0	3	0	0	0	0	0	0
Deltaproteobacteria	19	0	0	0	0	0	4	0	0	5
Alphaproteobacteria	2	0	0	1	0	0	1	0	0	87
Magnetococcus	0	0	0	0	0	0	1	0	0	0
Chrysiogenetes	1	0	0	0	0	0	0	0	0	0
Firmicutes	98	0	0	2	0	0	4	0	0	0
Tenericutes	19	0	0	0	0	0	0	0	0	0
Actinobacteria	74	0	0	0	0	0	6	0	0	0
Chlamydiae	8	0	0	0	0	0	0	0	0	0
Spirochaetes	11	0	0	0	0	0	3	0	0	0
Acidobacteria	0	0	0	0	0	0	5	0	0	0
Bacteroidetes	24	0	0	0	0	0	11	0	0	0
Fibrobacteres	1	0	0	0	0	0	0	0	0	0
Fusobacteria	5	0	0	0	0	0	0	0	0	0
Verrucomicrobia	4	0	0	0	0	0	0	0	0	0
Gemmatimonadetes	0	0	0	0	0	0	1	0	0	0
Planctomycetes	4	0	0	0	0	0	0	0	0	0
Elusimicrobia	2	0	0	0	0	0	0	0	0	0
Synergistetes	2	0	0	0	0	0	0	0	0	0
Cyanobacteria	16	0	0	0	0	0	0	0	0	0
Chlorobi	9	0	0	0	0	0	1	0	0	0
Chloroflexi	2	0	0	1	0	0	7	0	0	1
Deinococcus-Thermus	0	0	0	0	0	0	5	0	0	2
Aquificae	9	0	0	0	0	0	0	0	0	0
Thermotogae	11	0	0	0	0	0	0	0	0	0
Dictyoglomi	2	0	0	0	0	0	0	0	0	0
Nitrospirae	2	0	0	0	0	0	0	0	0	0
Thermobaculum	0	0	0	0	0	0	1	0	0	0
Deferribacteres	0	0	0	0	0	0	3	0	0	0
Euryarchaeota	49	0	0	1	0	0	10	0	0	0
Crenarchaeota	23	0	0	0	0	0	0	0	0	0
Thaumarchaeota	2	0	0	0	0	0	0	0	0	0
Nanoarchaeota	1	0	0	0	0	0	0	0	0	0
Korarchaeota	1	0	0	0	0	0	0	0	0	0
Total	475	0	0	8	0	0	64	0	0	221

(mod_M00260_1)

Phyla	Module completion ratio (%)									
	0--10	10--20	20--30	30--40	40--50	50--60	60--70	70--80	80--90	90--100
Gammaproteobacteria	0	0	0	1	0	0	0	0	2	123
Betaproteobacteria	0	0	0	0	0	0	0	0	1	60
Epsilonproteobacteria	0	0	0	0	0	0	0	0	0	17
Deltaproteobacteria	0	0	0	0	0	0	0	0	6	22
Alphaproteobacteria	0	0	0	1	0	0	1	0	1	88
Magnetococcus	0	0	0	0	0	0	0	0	0	1
Chrysiogenetes	0	0	0	0	0	0	0	0	0	1
Firmicutes	0	0	0	0	0	0	0	0	4	100
Tenericutes	0	0	0	0	0	0	0	0	1	18
Actinobacteria	0	0	0	0	0	0	0	0	8	72
Chlamydiae	0	0	0	0	0	0	0	0	6	2
Spirochaetes	0	0	0	0	0	0	11	0	3	0
Acidobacteria	0	0	0	0	0	0	0	0	4	1
Bacteroidetes	0	0	0	1	0	0	1	0	1	32
Fibrobacteres	0	0	0	0	0	0	0	0	0	1
Fusobacteria	0	0	0	0	0	0	0	0	1	4
Verrucomicrobia	0	0	0	0	0	0	0	0	1	3
Gemmatimonadetes	0	0	0	0	0	0	0	0	0	1
Planctomycetes	0	0	0	0	0	0	0	0	3	1
Elusimicrobia	0	0	0	0	0	0	0	0	0	2
Synergistetes	0	0	0	0	0	0	1	0	1	0
Cyanobacteria	0	0	0	0	0	0	0	0	11	5
Chlorobi	0	0	0	0	0	0	0	0	2	8
Chloroflexi	0	0	0	0	0	0	0	0	4	7
Deinococcus-Thermus	0	0	0	0	0	0	0	0	4	3
Aquificae	0	0	0	0	0	0	0	0	2	7
Thermotogae	0	0	0	0	0	0	0	0	7	4
Dictyoglomi	0	0	0	0	0	0	0	0	2	0
Nitrospirae	0	0	0	0	0	0	0	0	1	1
Thermobaculum	0	0	0	0	0	0	0	0	1	0
Deferribacteres	0	0	0	0	0	0	0	0	2	1
Euryarchaeota	52	8	0	0	0	0	0	0	0	0
Crenarchaeota	23	0	0	0	0	0	0	0	0	0
Thaumarchaeota	2	0	0	0	0	0	0	0	0	0
Nanoarchaeota	1	0	0	0	0	0	0	0	0	0
Korarchaeota	1	0	0	0	0	0	0	0	0	0
Total	79	8	0	3	0	0	14	0	79	585

(mod_M00261_1)

Phyla	Module completion ratio (%)									
	0--10	10--20	20--30	30--40	40--50	50--60	60--70	70--80	80--90	90--100
Gammaproteobacteria	126	0	0	0	0	0	0	0	0	0
Betaproteobacteria	61	0	0	0	0	0	0	0	0	0
Epsilonproteobacteria	17	0	0	0	0	0	0	0	0	0
Deltaproteobacteria	28	0	0	0	0	0	0	0	0	0
Alphaproteobacteria	91	0	0	0	0	0	0	0	0	0
Magnetococcus	1	0	0	0	0	0	0	0	0	0
Chrysiogenetes	1	0	0	0	0	0	0	0	0	0
Firmicutes	104	0	0	0	0	0	0	0	0	0
Tenericutes	19	0	0	0	0	0	0	0	0	0
Actinobacteria	80	0	0	0	0	0	0	0	0	0
Chlamydiae	8	0	0	0	0	0	0	0	0	0
Spirochaetes	14	0	0	0	0	0	0	0	0	0
Acidobacteria	5	0	0	0	0	0	0	0	0	0
Bacteroidetes	35	0	0	0	0	0	0	0	0	0
Fibrobacteres	1	0	0	0	0	0	0	0	0	0
Fusobacteria	5	0	0	0	0	0	0	0	0	0
Verrucomicrobia	4	0	0	0	0	0	0	0	0	0
Gemmatimonadetes	1	0	0	0	0	0	0	0	0	0
Planctomycetes	4	0	0	0	0	0	0	0	0	0
Elusimicrobia	2	0	0	0	0	0	0	0	0	0
Synergistetes	2	0	0	0	0	0	0	0	0	0
Cyanobacteria	16	0	0	0	0	0	0	0	0	0
Chlorobi	10	0	0	0	0	0	0	0	0	0
Chloroflexi	11	0	0	0	0	0	0	0	0	0
Deinococcus-Thermus	7	0	0	0	0	0	0	0	0	0
Aquificae	9	0	0	0	0	0	0	0	0	0
Thermotogae	11	0	0	0	0	0	0	0	0	0
Dictyoglomi	2	0	0	0	0	0	0	0	0	0
Nitrospirae	2	0	0	0	0	0	0	0	0	0
Thermobaculum	1	0	0	0	0	0	0	0	0	0
Deferribacteres	3	0	0	0	0	0	0	0	0	0
Euryarchaeota	0	0	60	0	0	0	0	0	0	0
Crenarchaeota	0	0	23	0	0	0	0	0	0	0
Thaumarchaeota	0	0	2	0	0	0	0	0	0	0
Nanoarchaeota	1	0	0	0	0	0	0	0	0	0
Korarchaeota	0	0	1	0	0	0	0	0	0	0
Total	682	0	86	0	0	0	0	0	0	0

(mod_M00262_1)

Phyla	Module completion ratio (%)									
	0--10	10--20	20--30	30--40	40--50	50--60	60--70	70--80	80--90	90--100
Gammaproteobacteria	126	0	0	0	0	0	0	0	0	0
Betaproteobacteria	61	0	0	0	0	0	0	0	0	0
Epsilonproteobacteria	17	0	0	0	0	0	0	0	0	0
Deltaproteobacteria	28	0	0	0	0	0	0	0	0	0
Alphaproteobacteria	91	0	0	0	0	0	0	0	0	0
Magnetococcus	1	0	0	0	0	0	0	0	0	0
Chrysiogenetes	1	0	0	0	0	0	0	0	0	0
Firmicutes	104	0	0	0	0	0	0	0	0	0
Tenericutes	19	0	0	0	0	0	0	0	0	0
Actinobacteria	80	0	0	0	0	0	0	0	0	0
Chlamydiae	8	0	0	0	0	0	0	0	0	0
Spirochaetes	14	0	0	0	0	0	0	0	0	0
Acidobacteria	5	0	0	0	0	0	0	0	0	0
Bacteroidetes	35	0	0	0	0	0	0	0	0	0
Fibrobacteres	1	0	0	0	0	0	0	0	0	0
Fusobacteria	5	0	0	0	0	0	0	0	0	0
Verrucomicrobia	4	0	0	0	0	0	0	0	0	0
Gemmatimonadetes	1	0	0	0	0	0	0	0	0	0
Planctomycetes	4	0	0	0	0	0	0	0	0	0
Elusimicrobia	2	0	0	0	0	0	0	0	0	0
Synergistetes	2	0	0	0	0	0	0	0	0	0
Cyanobacteria	16	0	0	0	0	0	0	0	0	0
Chlorobi	10	0	0	0	0	0	0	0	0	0
Chloroflexi	11	0	0	0	0	0	0	0	0	0
Deinococcus-Thermus	7	0	0	0	0	0	0	0	0	0
Aquificae	9	0	0	0	0	0	0	0	0	0
Thermotogae	11	0	0	0	0	0	0	0	0	0
Dictyoglomi	2	0	0	0	0	0	0	0	0	0
Nitrospirae	2	0	0	0	0	0	0	0	0	0
Thermobaculum	1	0	0	0	0	0	0	0	0	0
Deferribacteres	3	0	0	0	0	0	0	0	0	0
Euryarchaeota	60	0	0	0	0	0	0	0	0	0
Crenarchaeota	23	0	0	0	0	0	0	0	0	0
Thaumarchaeota	2	0	0	0	0	0	0	0	0	0
Nanoarchaeota	1	0	0	0	0	0	0	0	0	0
Korarchaeota	1	0	0	0	0	0	0	0	0	0
Total	768	0	0	0	0	0	0	0	0	0

(mod_M00263_1)

Phyla	Module completion ratio (%)									
	0--10	10--20	20--30	30--40	40--50	50--60	60--70	70--80	80--90	90--100
Gammaproteobacteria	126	0	0	0	0	0	0	0	0	0
Betaproteobacteria	61	0	0	0	0	0	0	0	0	0
Epsilonproteobacteria	17	0	0	0	0	0	0	0	0	0
Deltaproteobacteria	28	0	0	0	0	0	0	0	0	0
Alphaproteobacteria	91	0	0	0	0	0	0	0	0	0
Magnetococcus	1	0	0	0	0	0	0	0	0	0
Chrysiogenetes	1	0	0	0	0	0	0	0	0	0
Firmicutes	104	0	0	0	0	0	0	0	0	0
Tenericutes	19	0	0	0	0	0	0	0	0	0
Actinobacteria	80	0	0	0	0	0	0	0	0	0
Chlamydiae	8	0	0	0	0	0	0	0	0	0
Spirochaetes	14	0	0	0	0	0	0	0	0	0
Acidobacteria	5	0	0	0	0	0	0	0	0	0
Bacteroidetes	35	0	0	0	0	0	0	0	0	0
Fibrobacteres	1	0	0	0	0	0	0	0	0	0
Fusobacteria	5	0	0	0	0	0	0	0	0	0
Verrucomicrobia	4	0	0	0	0	0	0	0	0	0
Gemmatimonadetes	1	0	0	0	0	0	0	0	0	0
Planctomycetes	4	0	0	0	0	0	0	0	0	0
Elusimicrobia	2	0	0	0	0	0	0	0	0	0
Synergistetes	2	0	0	0	0	0	0	0	0	0
Cyanobacteria	16	0	0	0	0	0	0	0	0	0
Chlorobi	10	0	0	0	0	0	0	0	0	0
Chloroflexi	11	0	0	0	0	0	0	0	0	0
Deinococcus-Thermus	7	0	0	0	0	0	0	0	0	0
Aquificae	9	0	0	0	0	0	0	0	0	0
Thermotogae	11	0	0	0	0	0	0	0	0	0
Dictyoglomi	2	0	0	0	0	0	0	0	0	0
Nitrospirae	2	0	0	0	0	0	0	0	0	0
Thermobaculum	1	0	0	0	0	0	0	0	0	0
Deferribacteres	3	0	0	0	0	0	0	0	0	0
Euryarchaeota	60	0	0	0	0	0	0	0	0	0
Crenarchaeota	23	0	0	0	0	0	0	0	0	0
Thaumarchaeota	2	0	0	0	0	0	0	0	0	0
Nanoarchaeota	1	0	0	0	0	0	0	0	0	0
Korarchaeota	1	0	0	0	0	0	0	0	0	0
Total	768	0	0	0	0	0	0	0	0	0

(mod_M00264_1)

Phyla	Module completion ratio (%)									
	0--10	10--20	20--30	30--40	40--50	50--60	60--70	70--80	80--90	90--100
Gammaproteobacteria	126	0	0	0	0	0	0	0	0	0
Betaproteobacteria	61	0	0	0	0	0	0	0	0	0
Epsilonproteobacteria	17	0	0	0	0	0	0	0	0	0
Deltaproteobacteria	28	0	0	0	0	0	0	0	0	0
Alphaproteobacteria	91	0	0	0	0	0	0	0	0	0
Magnetococcus	1	0	0	0	0	0	0	0	0	0
Chrysiogenetes	1	0	0	0	0	0	0	0	0	0
Firmicutes	104	0	0	0	0	0	0	0	0	0
Tenericutes	19	0	0	0	0	0	0	0	0	0
Actinobacteria	80	0	0	0	0	0	0	0	0	0
Chlamydiae	8	0	0	0	0	0	0	0	0	0
Spirochaetes	14	0	0	0	0	0	0	0	0	0
Acidobacteria	5	0	0	0	0	0	0	0	0	0
Bacteroidetes	35	0	0	0	0	0	0	0	0	0
Fibrobacteres	1	0	0	0	0	0	0	0	0	0
Fusobacteria	5	0	0	0	0	0	0	0	0	0
Verrucomicrobia	4	0	0	0	0	0	0	0	0	0
Gemmatimonadetes	1	0	0	0	0	0	0	0	0	0
Planctomycetes	4	0	0	0	0	0	0	0	0	0
Elusimicrobia	2	0	0	0	0	0	0	0	0	0
Synergistetes	2	0	0	0	0	0	0	0	0	0
Cyanobacteria	16	0	0	0	0	0	0	0	0	0
Chlorobi	10	0	0	0	0	0	0	0	0	0
Chloroflexi	11	0	0	0	0	0	0	0	0	0
Deinococcus-Thermus	7	0	0	0	0	0	0	0	0	0
Aquificae	9	0	0	0	0	0	0	0	0	0
Thermotogae	11	0	0	0	0	0	0	0	0	0
Dictyoglomi	2	0	0	0	0	0	0	0	0	0
Nitrospirae	2	0	0	0	0	0	0	0	0	0
Thermobaculum	1	0	0	0	0	0	0	0	0	0
Deferribacteres	3	0	0	0	0	0	0	0	0	0
Euryarchaeota	0	0	0	0	0	1	0	0	0	59
Crenarchaeota	23	0	0	0	0	0	0	0	0	0
Thaumarchaeota	0	0	0	0	0	0	0	0	0	2
Nanoarchaeota	0	0	0	0	0	0	0	0	0	1
Korarchaeota	0	0	0	0	0	0	0	0	0	1
Total	704	0	0	0	0	1	0	0	0	63

(mod_M00265_1)

Phyla	Module completion ratio (%)									
	0--10	10--20	20--30	30--40	40--50	50--60	60--70	70--80	80--90	90--100
Gammaproteobacteria	59	0	0	13	0	0	0	0	0	54
Betaproteobacteria	60	0	0	0	0	0	1	0	0	0
Epsilonproteobacteria	16	0	0	1	0	0	0	0	0	0
Deltaproteobacteria	28	0	0	0	0	0	0	0	0	0
Alphaproteobacteria	90	0	0	0	0	0	1	0	0	0
Magnetococcus	1	0	0	0	0	0	0	0	0	0
Chrysiogenetes	1	0	0	0	0	0	0	0	0	0
Firmicutes	39	0	0	40	0	0	0	0	0	25
Tenericutes	8	0	0	2	0	0	0	0	0	9
Actinobacteria	57	0	0	21	0	0	1	0	0	1
Chlamydiae	8	0	0	0	0	0	0	0	0	0
Spirochaetes	7	0	0	4	0	0	0	0	0	3
Acidobacteria	5	0	0	0	0	0	0	0	0	0
Bacteroidetes	35	0	0	0	0	0	0	0	0	0
Fibrobacteres	1	0	0	0	0	0	0	0	0	0
Fusobacteria	0	0	0	5	0	0	0	0	0	0
Verrucomicrobia	4	0	0	0	0	0	0	0	0	0
Gemmatimonadetes	1	0	0	0	0	0	0	0	0	0
Planctomycetes	4	0	0	0	0	0	0	0	0	0
Elusimicrobia	2	0	0	0	0	0	0	0	0	0
Synergistetes	1	0	0	1	0	0	0	0	0	0
Cyanobacteria	16	0	0	0	0	0	0	0	0	0
Chlorobi	10	0	0	0	0	0	0	0	0	0
Chloroflexi	11	0	0	0	0	0	0	0	0	0
Deinococcus-Thermus	7	0	0	0	0	0	0	0	0	0
Aquificae	9	0	0	0	0	0	0	0	0	0
Thermotogae	11	0	0	0	0	0	0	0	0	0
Dictyoglomi	2	0	0	0	0	0	0	0	0	0
Nitrospirae	2	0	0	0	0	0	0	0	0	0
Thermobaculum	1	0	0	0	0	0	0	0	0	0
Deferribacteres	3	0	0	0	0	0	0	0	0	0
Euryarchaeota	60	0	0	0	0	0	0	0	0	0
Crenarchaeota	23	0	0	0	0	0	0	0	0	0
Thaumarchaeota	2	0	0	0	0	0	0	0	0	0
Nanoarchaeota	1	0	0	0	0	0	0	0	0	0
Korarchaeota	1	0	0	0	0	0	0	0	0	0
Total	586	0	0	87	0	0	3	0	0	92

(mod_M00266_1)

Phyla	Module completion ratio (%)									
	0--10	10--20	20--30	30--40	40--50	50--60	60--70	70--80	80--90	90--100
Gammaproteobacteria	59	0	0	42	0	0	0	0	0	25
Betaproteobacteria	61	0	0	0	0	0	0	0	0	0
Epsilonproteobacteria	16	0	0	1	0	0	0	0	0	0
Deltaproteobacteria	28	0	0	0	0	0	0	0	0	0
Alphaproteobacteria	91	0	0	0	0	0	0	0	0	0
Magnetococcus	1	0	0	0	0	0	0	0	0	0
Chrysiogenetes	1	0	0	0	0	0	0	0	0	0
Firmicutes	39	0	0	57	0	0	0	0	0	8
Tenericutes	8	0	0	10	0	0	0	0	0	1
Actinobacteria	57	0	0	22	0	0	1	0	0	0
Chlamydiae	8	0	0	0	0	0	0	0	0	0
Spirochaetes	7	0	0	0	0	0	0	0	0	7
Acidobacteria	5	0	0	0	0	0	0	0	0	0
Bacteroidetes	35	0	0	0	0	0	0	0	0	0
Fibrobacteres	1	0	0	0	0	0	0	0	0	0
Fusobacteria	0	0	0	2	0	0	0	0	0	3
Verrucomicrobia	4	0	0	0	0	0	0	0	0	0
Gemmatimonadetes	1	0	0	0	0	0	0	0	0	0
Planctomycetes	4	0	0	0	0	0	0	0	0	0
Elusimicrobia	1	0	0	0	0	0	1	0	0	0
Synergistetes	1	0	0	1	0	0	0	0	0	0
Cyanobacteria	16	0	0	0	0	0	0	0	0	0
Chlorobi	10	0	0	0	0	0	0	0	0	0
Chloroflexi	11	0	0	0	0	0	0	0	0	0
Deinococcus-Thermus	7	0	0	0	0	0	0	0	0	0
Aquificae	9	0	0	0	0	0	0	0	0	0
Thermotogae	11	0	0	0	0	0	0	0	0	0
Dictyoglomi	2	0	0	0	0	0	0	0	0	0
Nitrospirae	2	0	0	0	0	0	0	0	0	0
Thermobaculum	1	0	0	0	0	0	0	0	0	0
Deferribacteres	3	0	0	0	0	0	0	0	0	0
Euryarchaeota	60	0	0	0	0	0	0	0	0	0
Crenarchaeota	23	0	0	0	0	0	0	0	0	0
Thaumarchaeota	2	0	0	0	0	0	0	0	0	0
Nanoarchaeota	1	0	0	0	0	0	0	0	0	0
Korarchaeota	1	0	0	0	0	0	0	0	0	0
Total	587	0	0	135	0	0	2	0	0	44

(mod_M00267_1)

Phyla	Module completion ratio (%)									
	0--10	10--20	20--30	30--40	40--50	50--60	60--70	70--80	80--90	90--100
Gammaproteobacteria	76	0	0	1	0	0	22	0	0	27
Betaproteobacteria	48	0	0	0	0	0	13	0	0	0
Epsilonproteobacteria	16	0	0	0	0	0	1	0	0	0
Deltaproteobacteria	26	0	0	0	0	0	2	0	0	0
Alphaproteobacteria	87	0	0	0	0	0	4	0	0	0
Magnetococcus	1	0	0	0	0	0	0	0	0	0
Chrysiogenetes	1	0	0	0	0	0	0	0	0	0
Firmicutes	66	0	0	0	0	0	33	0	0	5
Tenericutes	15	0	0	0	0	0	4	0	0	0
Actinobacteria	54	0	0	5	0	0	15	0	0	6
Chlamydiae	8	0	0	0	0	0	0	0	0	0
Spirochaetes	9	0	0	0	0	0	1	0	0	4
Acidobacteria	5	0	0	0	0	0	0	0	0	0
Bacteroidetes	35	0	0	0	0	0	0	0	0	0
Fibrobacteres	1	0	0	0	0	0	0	0	0	0
Fusobacteria	3	0	0	0	0	0	2	0	0	0
Verrucomicrobia	4	0	0	0	0	0	0	0	0	0
Gemmatimonadetes	1	0	0	0	0	0	0	0	0	0
Planctomycetes	4	0	0	0	0	0	0	0	0	0
Elusimicrobia	1	0	0	0	0	0	1	0	0	0
Synergistetes	2	0	0	0	0	0	0	0	0	0
Cyanobacteria	16	0	0	0	0	0	0	0	0	0
Chlorobi	10	0	0	0	0	0	0	0	0	0
Chloroflexi	11	0	0	0	0	0	0	0	0	0
Deinococcus-Thermus	7	0	0	0	0	0	0	0	0	0
Aquificae	9	0	0	0	0	0	0	0	0	0
Thermotogae	11	0	0	0	0	0	0	0	0	0
Dictyoglomi	2	0	0	0	0	0	0	0	0	0
Nitrospirae	2	0	0	0	0	0	0	0	0	0
Thermobaculum	1	0	0	0	0	0	0	0	0	0
Deferribacteres	3	0	0	0	0	0	0	0	0	0
Euryarchaeota	60	0	0	0	0	0	0	0	0	0
Crenarchaeota	23	0	0	0	0	0	0	0	0	0
Thaumarchaeota	2	0	0	0	0	0	0	0	0	0
Nanoarchaeota	1	0	0	0	0	0	0	0	0	0
Korarchaeota	1	0	0	0	0	0	0	0	0	0
Total	622	0	0	6	0	0	98	0	0	42

(mod_M00268_1)

Phyla	Module completion ratio (%)									
	0--10	10--20	20--30	30--40	40--50	50--60	60--70	70--80	80--90	90--100
Gammaproteobacteria	59	0	0	49	0	0	1	0	0	17
Betaproteobacteria	61	0	0	0	0	0	0	0	0	0
Epsilonproteobacteria	16	0	0	1	0	0	0	0	0	0
Deltaproteobacteria	28	0	0	0	0	0	0	0	0	0
Alphaproteobacteria	91	0	0	0	0	0	0	0	0	0
Magnetococcus	1	0	0	0	0	0	0	0	0	0
Chrysiogenetes	1	0	0	0	0	0	0	0	0	0
Firmicutes	38	0	0	49	0	0	1	0	0	16
Tenericutes	8	0	0	11	0	0	0	0	0	0
Actinobacteria	57	0	0	21	0	0	1	0	0	1
Chlamydiae	8	0	0	0	0	0	0	0	0	0
Spirochaetes	7	0	0	7	0	0	0	0	0	0
Acidobacteria	5	0	0	0	0	0	0	0	0	0
Bacteroidetes	35	0	0	0	0	0	0	0	0	0
Fibrobacteres	1	0	0	0	0	0	0	0	0	0
Fusobacteria	0	0	0	3	0	0	0	0	0	2
Verrucomicrobia	4	0	0	0	0	0	0	0	0	0
Gemmatimonadetes	1	0	0	0	0	0	0	0	0	0
Planctomycetes	4	0	0	0	0	0	0	0	0	0
Elusimicrobia	2	0	0	0	0	0	0	0	0	0
Synergistetes	1	0	0	1	0	0	0	0	0	0
Cyanobacteria	16	0	0	0	0	0	0	0	0	0
Chlorobi	10	0	0	0	0	0	0	0	0	0
Chloroflexi	11	0	0	0	0	0	0	0	0	0
Deinococcus-Thermus	7	0	0	0	0	0	0	0	0	0
Aquificae	9	0	0	0	0	0	0	0	0	0
Thermotogae	11	0	0	0	0	0	0	0	0	0
Dictyoglomi	2	0	0	0	0	0	0	0	0	0
Nitrospirae	2	0	0	0	0	0	0	0	0	0
Thermobaculum	1	0	0	0	0	0	0	0	0	0
Deferribacteres	3	0	0	0	0	0	0	0	0	0
Euryarchaeota	60	0	0	0	0	0	0	0	0	0
Crenarchaeota	23	0	0	0	0	0	0	0	0	0
Thaumarchaeota	2	0	0	0	0	0	0	0	0	0
Nanoarchaeota	1	0	0	0	0	0	0	0	0	0
Korarchaeota	1	0	0	0	0	0	0	0	0	0
Total	587	0	0	142	0	0	3	0	0	36

(mod_M00269_1)

Phyla	Module completion ratio (%)									
	0--10	10--20	20--30	30--40	40--50	50--60	60--70	70--80	80--90	90--100
Gammaproteobacteria	99	0	0	0	0	0	27	0	0	0
Betaproteobacteria	59	0	0	0	0	0	2	0	0	0
Epsilonproteobacteria	17	0	0	0	0	0	0	0	0	0
Deltaproteobacteria	28	0	0	0	0	0	0	0	0	0
Alphaproteobacteria	90	0	0	0	0	0	1	0	0	0
Magnetococcus	1	0	0	0	0	0	0	0	0	0
Chrysiogenetes	1	0	0	0	0	0	0	0	0	0
Firmicutes	50	0	0	1	0	0	31	0	0	22
Tenericutes	14	0	0	0	0	0	4	0	0	1
Actinobacteria	72	0	0	0	0	0	4	0	0	4
Chlamydiae	8	0	0	0	0	0	0	0	0	0
Spirochaetes	13	0	0	0	0	0	0	0	0	1
Acidobacteria	5	0	0	0	0	0	0	0	0	0
Bacteroidetes	35	0	0	0	0	0	0	0	0	0
Fibrobacteres	1	0	0	0	0	0	0	0	0	0
Fusobacteria	1	0	0	0	0	0	4	0	0	0
Verrucomicrobia	4	0	0	0	0	0	0	0	0	0
Gemmatimonadetes	1	0	0	0	0	0	0	0	0	0
Planctomycetes	4	0	0	0	0	0	0	0	0	0
Elusimicrobia	1	0	0	0	0	0	1	0	0	0
Synergistetes	2	0	0	0	0	0	0	0	0	0
Cyanobacteria	16	0	0	0	0	0	0	0	0	0
Chlorobi	10	0	0	0	0	0	0	0	0	0
Chloroflexi	11	0	0	0	0	0	0	0	0	0
Deinococcus-Thermus	7	0	0	0	0	0	0	0	0	0
Aquificae	9	0	0	0	0	0	0	0	0	0
Thermotogae	11	0	0	0	0	0	0	0	0	0
Dictyoglomi	2	0	0	0	0	0	0	0	0	0
Nitrospirae	2	0	0	0	0	0	0	0	0	0
Thermobaculum	1	0	0	0	0	0	0	0	0	0
Deferribacteres	3	0	0	0	0	0	0	0	0	0
Euryarchaeota	60	0	0	0	0	0	0	0	0	0
Crenarchaeota	23	0	0	0	0	0	0	0	0	0
Thaumarchaeota	2	0	0	0	0	0	0	0	0	0
Nanoarchaeota	1	0	0	0	0	0	0	0	0	0
Korarchaeota	1	0	0	0	0	0	0	0	0	0
Total	665	0	0	1	0	0	74	0	0	28

(mod_M00270_1)

Phyla	Module completion ratio (%)									
	0--10	10--20	20--30	30--40	40--50	50--60	60--70	70--80	80--90	90--100
Gammaproteobacteria	58	0	0	30	0	0	1	0	0	37
Betaproteobacteria	60	0	0	0	0	0	1	0	0	0
Epsilonproteobacteria	16	0	0	1	0	0	0	0	0	0
Deltaproteobacteria	28	0	0	0	0	0	0	0	0	0
Alphaproteobacteria	91	0	0	0	0	0	0	0	0	0
Magnetococcus	1	0	0	0	0	0	0	0	0	0
Chrysiogenetes	1	0	0	0	0	0	0	0	0	0
Firmicutes	38	0	0	32	0	0	2	0	0	32
Tenericutes	8	0	0	8	0	0	0	0	0	3
Actinobacteria	57	0	0	22	0	0	1	0	0	0
Chlamydiae	8	0	0	0	0	0	0	0	0	0
Spirochaetes	6	0	0	7	0	0	0	0	0	1
Acidobacteria	5	0	0	0	0	0	0	0	0	0
Bacteroidetes	35	0	0	0	0	0	0	0	0	0
Fibrobacteres	1	0	0	0	0	0	0	0	0	0
Fusobacteria	0	0	0	4	0	0	0	0	0	1
Verrucomicrobia	4	0	0	0	0	0	0	0	0	0
Gemmatimonadetes	1	0	0	0	0	0	0	0	0	0
Planctomycetes	4	0	0	0	0	0	0	0	0	0
Elusimicrobia	2	0	0	0	0	0	0	0	0	0
Synergistetes	1	0	0	1	0	0	0	0	0	0
Cyanobacteria	16	0	0	0	0	0	0	0	0	0
Chlorobi	10	0	0	0	0	0	0	0	0	0
Chloroflexi	11	0	0	0	0	0	0	0	0	0
Deinococcus-Thermus	7	0	0	0	0	0	0	0	0	0
Aquificae	9	0	0	0	0	0	0	0	0	0
Thermotogae	11	0	0	0	0	0	0	0	0	0
Dictyoglomi	2	0	0	0	0	0	0	0	0	0
Nitrospirae	2	0	0	0	0	0	0	0	0	0
Thermobaculum	1	0	0	0	0	0	0	0	0	0
Deferribacteres	3	0	0	0	0	0	0	0	0	0
Euryarchaeota	60	0	0	0	0	0	0	0	0	0
Crenarchaeota	23	0	0	0	0	0	0	0	0	0
Thaumarchaeota	2	0	0	0	0	0	0	0	0	0
Nanoarchaeota	1	0	0	0	0	0	0	0	0	0
Korarchaeota	1	0	0	0	0	0	0	0	0	0
Total	584	0	0	105	0	0	5	0	0	74

(mod_M00271_1)

Phyla	Module completion ratio (%)									
	0--10	10--20	20--30	30--40	40--50	50--60	60--70	70--80	80--90	90--100
Gammaproteobacteria	106	0	0	1	0	0	0	0	0	19
Betaproteobacteria	61	0	0	0	0	0	0	0	0	0
Epsilonproteobacteria	17	0	0	0	0	0	0	0	0	0
Deltaproteobacteria	28	0	0	0	0	0	0	0	0	0
Alphaproteobacteria	91	0	0	0	0	0	0	0	0	0
Magnetococcus	1	0	0	0	0	0	0	0	0	0
Chrysiogenetes	1	0	0	0	0	0	0	0	0	0
Firmicutes	61	0	0	0	0	0	2	0	0	41
Tenericutes	19	0	0	0	0	0	0	0	0	0
Actinobacteria	66	0	0	1	0	0	0	0	0	13
Chlamydiae	8	0	0	0	0	0	0	0	0	0
Spirochaetes	13	0	0	0	0	0	0	0	0	1
Acidobacteria	5	0	0	0	0	0	0	0	0	0
Bacteroidetes	35	0	0	0	0	0	0	0	0	0
Fibrobacteres	1	0	0	0	0	0	0	0	0	0
Fusobacteria	2	0	0	0	0	0	0	0	0	3
Verrucomicrobia	4	0	0	0	0	0	0	0	0	0
Gemmatimonadetes	1	0	0	0	0	0	0	0	0	0
Planctomycetes	4	0	0	0	0	0	0	0	0	0
Elusimicrobia	2	0	0	0	0	0	0	0	0	0
Synergistetes	2	0	0	0	0	0	0	0	0	0
Cyanobacteria	16	0	0	0	0	0	0	0	0	0
Chlorobi	10	0	0	0	0	0	0	0	0	0
Chloroflexi	11	0	0	0	0	0	0	0	0	0
Deinococcus-Thermus	7	0	0	0	0	0	0	0	0	0
Aquificae	9	0	0	0	0	0	0	0	0	0
Thermotogae	11	0	0	0	0	0	0	0	0	0
Dictyoglomi	2	0	0	0	0	0	0	0	0	0
Nitrospirae	2	0	0	0	0	0	0	0	0	0
Thermobaculum	1	0	0	0	0	0	0	0	0	0
Deferribacteres	3	0	0	0	0	0	0	0	0	0
Euryarchaeota	60	0	0	0	0	0	0	0	0	0
Crenarchaeota	23	0	0	0	0	0	0	0	0	0
Thaumarchaeota	2	0	0	0	0	0	0	0	0	0
Nanoarchaeota	1	0	0	0	0	0	0	0	0	0
Korarchaeota	1	0	0	0	0	0	0	0	0	0
Total	687	0	0	2	0	0	2	0	0	77

(mod_M00272_1)

Phyla	Module completion ratio (%)									
	0--10	10--20	20--30	30--40	40--50	50--60	60--70	70--80	80--90	90--100
Gammaproteobacteria	59	0	0	51	0	0	0	0	0	16
Betaproteobacteria	61	0	0	0	0	0	0	0	0	0
Epsilonproteobacteria	16	0	0	1	0	0	0	0	0	0
Deltaproteobacteria	28	0	0	0	0	0	0	0	0	0
Alphaproteobacteria	91	0	0	0	0	0	0	0	0	0
Magnetococcus	1	0	0	0	0	0	0	0	0	0
Chrysiogenetes	1	0	0	0	0	0	0	0	0	0
Firmicutes	39	0	0	64	0	0	0	0	0	1
Tenericutes	8	0	0	11	0	0	0	0	0	0
Actinobacteria	58	0	0	22	0	0	0	0	0	0
Chlamydiae	8	0	0	0	0	0	0	0	0	0
Spirochaetes	7	0	0	7	0	0	0	0	0	0
Acidobacteria	5	0	0	0	0	0	0	0	0	0
Bacteroidetes	35	0	0	0	0	0	0	0	0	0
Fibrobacteres	1	0	0	0	0	0	0	0	0	0
Fusobacteria	0	0	0	5	0	0	0	0	0	0
Verrucomicrobia	4	0	0	0	0	0	0	0	0	0
Gemmatimonadetes	1	0	0	0	0	0	0	0	0	0
Planctomycetes	4	0	0	0	0	0	0	0	0	0
Elusimicrobia	2	0	0	0	0	0	0	0	0	0
Synergistetes	1	0	0	1	0	0	0	0	0	0
Cyanobacteria	16	0	0	0	0	0	0	0	0	0
Chlorobi	10	0	0	0	0	0	0	0	0	0
Chloroflexi	11	0	0	0	0	0	0	0	0	0
Deinococcus-Thermus	7	0	0	0	0	0	0	0	0	0
Aquificae	9	0	0	0	0	0	0	0	0	0
Thermotogae	11	0	0	0	0	0	0	0	0	0
Dictyoglomi	2	0	0	0	0	0	0	0	0	0
Nitrospirae	2	0	0	0	0	0	0	0	0	0
Thermobaculum	1	0	0	0	0	0	0	0	0	0
Deferribacteres	3	0	0	0	0	0	0	0	0	0
Euryarchaeota	60	0	0	0	0	0	0	0	0	0
Crenarchaeota	23	0	0	0	0	0	0	0	0	0
Thaumarchaeota	2	0	0	0	0	0	0	0	0	0
Nanoarchaeota	1	0	0	0	0	0	0	0	0	0
Korarchaeota	1	0	0	0	0	0	0	0	0	0
Total	589	0	0	162	0	0	0	0	0	17

(mod_M00273_1)

Phyla	Module completion ratio (%)									
	0--10	10--20	20--30	30--40	40--50	50--60	60--70	70--80	80--90	90--100
Gammaproteobacteria	62	0	0	1	0	0	2	0	0	61
Betaproteobacteria	44	0	0	10	0	0	0	0	0	7
Epsilonproteobacteria	17	0	0	0	0	0	0	0	0	0
Deltaproteobacteria	23	0	0	4	0	0	0	0	0	1
Alphaproteobacteria	83	0	0	2	0	0	1	0	0	5
Magnetococcus	1	0	0	0	0	0	0	0	0	0
Chrysiogenetes	1	0	0	0	0	0	0	0	0	0
Firmicutes	31	0	0	1	0	0	8	0	0	64
Tenericutes	8	0	0	0	0	0	0	0	0	11
Actinobacteria	42	0	0	5	0	0	0	0	0	33
Chlamydiae	8	0	0	0	0	0	0	0	0	0
Spirochaetes	3	0	0	2	0	0	0	0	0	9
Acidobacteria	5	0	0	0	0	0	0	0	0	0
Bacteroidetes	33	0	0	1	0	0	0	0	0	1
Fibrobacteres	0	0	0	0	0	0	0	0	0	1
Fusobacteria	0	0	0	0	0	0	0	0	0	5
Verrucomicrobia	3	0	0	1	0	0	0	0	0	0
Gemmatimonadetes	1	0	0	0	0	0	0	0	0	0
Planctomycetes	3	0	0	1	0	0	0	0	0	0
Elusimicrobia	1	0	0	1	0	0	0	0	0	0
Synergistetes	1	0	0	0	0	0	1	0	0	0
Cyanobacteria	14	0	0	2	0	0	0	0	0	0
Chlorobi	2	0	0	7	0	0	0	0	0	1
Chloroflexi	6	0	0	2	0	0	0	0	0	3
Deinococcus-Thermus	4	0	0	0	0	0	1	0	0	2
Aquificae	9	0	0	0	0	0	0	0	0	0
Thermotogae	9	0	0	0	0	0	2	0	0	0
Dictyoglomi	2	0	0	0	0	0	0	0	0	0
Nitrospirae	2	0	0	0	0	0	0	0	0	0
Thermobaculum	1	0	0	0	0	0	0	0	0	0
Deferribacteres	3	0	0	0	0	0	0	0	0	0
Euryarchaeota	56	0	0	0	0	0	0	0	0	4
Crenarchaeota	23	0	0	0	0	0	0	0	0	0
Thaumarchaeota	2	0	0	0	0	0	0	0	0	0
Nanoarchaeota	1	0	0	0	0	0	0	0	0	0
Korarchaeota	1	0	0	0	0	0	0	0	0	0
Total	505	0	0	40	0	0	15	0	0	208

(mod_M00274_1)

Phyla	Module completion ratio (%)									
	0--10	10--20	20--30	30--40	40--50	50--60	60--70	70--80	80--90	90--100
Gammaproteobacteria	85	0	0	0	0	0	0	0	0	41
Betaproteobacteria	61	0	0	0	0	0	0	0	0	0
Epsilonproteobacteria	17	0	0	0	0	0	0	0	0	0
Deltaproteobacteria	28	0	0	0	0	0	0	0	0	0
Alphaproteobacteria	91	0	0	0	0	0	0	0	0	0
Magnetococcus	1	0	0	0	0	0	0	0	0	0
Chrysiogenetes	1	0	0	0	0	0	0	0	0	0
Firmicutes	68	0	0	4	0	0	0	0	0	32
Tenericutes	13	0	0	0	0	0	2	0	0	4
Actinobacteria	65	0	0	0	0	0	0	0	0	15
Chlamydiae	8	0	0	0	0	0	0	0	0	0
Spirochaetes	13	0	0	0	0	0	1	0	0	0
Acidobacteria	5	0	0	0	0	0	0	0	0	0
Bacteroidetes	35	0	0	0	0	0	0	0	0	0
Fibrobacteres	1	0	0	0	0	0	0	0	0	0
Fusobacteria	3	0	0	0	0	0	0	0	0	2
Verrucomicrobia	4	0	0	0	0	0	0	0	0	0
Gemmatimonadetes	1	0	0	0	0	0	0	0	0	0
Planctomycetes	4	0	0	0	0	0	0	0	0	0
Elusimicrobia	2	0	0	0	0	0	0	0	0	0
Synergistetes	2	0	0	0	0	0	0	0	0	0
Cyanobacteria	16	0	0	0	0	0	0	0	0	0
Chlorobi	10	0	0	0	0	0	0	0	0	0
Chloroflexi	10	0	0	0	0	0	1	0	0	0
Deinococcus-Thermus	7	0	0	0	0	0	0	0	0	0
Aquificae	9	0	0	0	0	0	0	0	0	0
Thermotogae	11	0	0	0	0	0	0	0	0	0
Dictyoglomi	2	0	0	0	0	0	0	0	0	0
Nitrospirae	2	0	0	0	0	0	0	0	0	0
Thermobaculum	1	0	0	0	0	0	0	0	0	0
Deferribacteres	3	0	0	0	0	0	0	0	0	0
Euryarchaeota	60	0	0	0	0	0	0	0	0	0
Crenarchaeota	23	0	0	0	0	0	0	0	0	0
Thaumarchaeota	2	0	0	0	0	0	0	0	0	0
Nanoarchaeota	1	0	0	0	0	0	0	0	0	0
Korarchaeota	1	0	0	0	0	0	0	0	0	0
Total	666	0	0	4	0	0	4	0	0	94

(mod_M00275_1)

Phyla	Module completion ratio (%)									
	0--10	10--20	20--30	30--40	40--50	50--60	60--70	70--80	80--90	90--100
Gammaproteobacteria	89	0	0	3	0	0	1	0	0	33
Betaproteobacteria	61	0	0	0	0	0	0	0	0	0
Epsilonproteobacteria	17	0	0	0	0	0	0	0	0	0
Deltaproteobacteria	28	0	0	0	0	0	0	0	0	0
Alphaproteobacteria	91	0	0	0	0	0	0	0	0	0
Magnetococcus	1	0	0	0	0	0	0	0	0	0
Chrysiogenetes	1	0	0	0	0	0	0	0	0	0
Firmicutes	39	0	0	8	0	0	1	0	0	56
Tenericutes	19	0	0	0	0	0	0	0	0	0
Actinobacteria	75	0	0	3	0	0	0	0	0	2
Chlamydiae	8	0	0	0	0	0	0	0	0	0
Spirochaetes	10	0	0	0	0	0	0	0	0	4
Acidobacteria	5	0	0	0	0	0	0	0	0	0
Bacteroidetes	35	0	0	0	0	0	0	0	0	0
Fibrobacteres	1	0	0	0	0	0	0	0	0	0
Fusobacteria	2	0	0	0	0	0	0	0	0	3
Verrucomicrobia	4	0	0	0	0	0	0	0	0	0
Gemmatimonadetes	1	0	0	0	0	0	0	0	0	0
Planctomycetes	4	0	0	0	0	0	0	0	0	0
Elusimicrobia	2	0	0	0	0	0	0	0	0	0
Synergistetes	1	0	0	1	0	0	0	0	0	0
Cyanobacteria	16	0	0	0	0	0	0	0	0	0
Chlorobi	10	0	0	0	0	0	0	0	0	0
Chloroflexi	11	0	0	0	0	0	0	0	0	0
Deinococcus-Thermus	7	0	0	0	0	0	0	0	0	0
Aquificae	9	0	0	0	0	0	0	0	0	0
Thermotogae	11	0	0	0	0	0	0	0	0	0
Dictyoglomi	2	0	0	0	0	0	0	0	0	0
Nitrospirae	2	0	0	0	0	0	0	0	0	0
Thermobaculum	1	0	0	0	0	0	0	0	0	0
Deferribacteres	3	0	0	0	0	0	0	0	0	0
Euryarchaeota	60	0	0	0	0	0	0	0	0	0
Crenarchaeota	23	0	0	0	0	0	0	0	0	0
Thaumarchaeota	2	0	0	0	0	0	0	0	0	0
Nanoarchaeota	1	0	0	0	0	0	0	0	0	0
Korarchaeota	1	0	0	0	0	0	0	0	0	0
Total	653	0	0	15	0	0	2	0	0	98

(mod_M00276_1)

Phyla	Module completion ratio (%)									
	0--10	10--20	20--30	30--40	40--50	50--60	60--70	70--80	80--90	90--100
Gammaproteobacteria	85	0	1	0	0	0	0	2	0	38
Betaproteobacteria	61	0	0	0	0	0	0	0	0	0
Epsilonproteobacteria	17	0	0	0	0	0	0	0	0	0
Deltaproteobacteria	7	0	11	0	0	0	0	2	0	8
Alphaproteobacteria	25	0	66	0	0	0	0	0	0	0
Magnetococcus	1	0	0	0	0	0	0	0	0	0
Chrysiogenetes	0	0	0	0	0	0	0	0	0	1
Firmicutes	51	0	2	0	0	1	0	7	0	43
Tenericutes	18	0	0	0	0	0	0	1	0	0
Actinobacteria	74	0	0	0	0	1	0	2	0	3
Chlamydiae	8	0	0	0	0	0	0	0	0	0
Spirochaetes	13	0	0	0	0	0	0	0	0	1
Acidobacteria	5	0	0	0	0	0	0	0	0	0
Bacteroidetes	35	0	0	0	0	0	0	0	0	0
Fibrobacteres	1	0	0	0	0	0	0	0	0	0
Fusobacteria	1	0	0	0	0	1	0	0	0	3
Verrucomicrobia	4	0	0	0	0	0	0	0	0	0
Gemmatimonadetes	0	0	0	0	0	1	0	0	0	0
Planctomycetes	4	0	0	0	0	0	0	0	0	0
Elusimicrobia	0	0	0	0	0	0	0	0	0	2
Synergistetes	1	0	0	0	0	0	0	1	0	0
Cyanobacteria	16	0	0	0	0	0	0	0	0	0
Chlorobi	10	0	0	0	0	0	0	0	0	0
Chloroflexi	11	0	0	0	0	0	0	0	0	0
Deinococcus-Thermus	7	0	0	0	0	0	0	0	0	0
Aquificae	9	0	0	0	0	0	0	0	0	0
Thermotogae	11	0	0	0	0	0	0	0	0	0
Dictyoglomi	2	0	0	0	0	0	0	0	0	0
Nitrospirae	2	0	0	0	0	0	0	0	0	0
Thermobaculum	1	0	0	0	0	0	0	0	0	0
Deferribacteres	0	0	0	0	0	0	0	1	0	2
Euryarchaeota	60	0	0	0	0	0	0	0	0	0
Crenarchaeota	22	0	0	0	0	1	0	0	0	0
Thaumarchaeota	2	0	0	0	0	0	0	0	0	0
Nanoarchaeota	1	0	0	0	0	0	0	0	0	0
Korarchaeota	1	0	0	0	0	0	0	0	0	0
Total	566	0	80	0	0	5	0	16	0	101

(mod_M00277_1)

Phyla	Module completion ratio (%)									
	0--10	10--20	20--30	30--40	40--50	50--60	60--70	70--80	80--90	90--100
Gammaproteobacteria	107	0	3	0	0	0	0	0	0	16
Betaproteobacteria	61	0	0	0	0	0	0	0	0	0
Epsilonproteobacteria	17	0	0	0	0	0	0	0	0	0
Deltaproteobacteria	27	0	1	0	0	0	0	0	0	0
Alphaproteobacteria	91	0	0	0	0	0	0	0	0	0
Magnetococcus	1	0	0	0	0	0	0	0	0	0
Chrysiogenetes	1	0	0	0	0	0	0	0	0	0
Firmicutes	84	0	12	0	0	0	0	0	0	8
Tenericutes	19	0	0	0	0	0	0	0	0	0
Actinobacteria	78	0	1	0	0	0	0	0	0	1
Chlamydiae	8	0	0	0	0	0	0	0	0	0
Spirochaetes	14	0	0	0	0	0	0	0	0	0
Acidobacteria	5	0	0	0	0	0	0	0	0	0
Bacteroidetes	35	0	0	0	0	0	0	0	0	0
Fibrobacteres	1	0	0	0	0	0	0	0	0	0
Fusobacteria	3	0	0	0	0	0	0	1	0	1
Verrucomicrobia	4	0	0	0	0	0	0	0	0	0
Gemmatimonadetes	0	0	1	0	0	0	0	0	0	0
Planctomycetes	4	0	0	0	0	0	0	0	0	0
Elusimicrobia	2	0	0	0	0	0	0	0	0	0
Synergistetes	2	0	0	0	0	0	0	0	0	0
Cyanobacteria	16	0	0	0	0	0	0	0	0	0
Chlorobi	10	0	0	0	0	0	0	0	0	0
Chloroflexi	11	0	0	0	0	0	0	0	0	0
Deinococcus-Thermus	7	0	0	0	0	0	0	0	0	0
Aquificae	9	0	0	0	0	0	0	0	0	0
Thermotogae	11	0	0	0	0	0	0	0	0	0
Dictyoglomi	2	0	0	0	0	0	0	0	0	0
Nitrospirae	2	0	0	0	0	0	0	0	0	0
Thermobaculum	1	0	0	0	0	0	0	0	0	0
Deferribacteres	3	0	0	0	0	0	0	0	0	0
Euryarchaeota	60	0	0	0	0	0	0	0	0	0
Crenarchaeota	23	0	0	0	0	0	0	0	0	0
Thaumarchaeota	2	0	0	0	0	0	0	0	0	0
Nanoarchaeota	1	0	0	0	0	0	0	0	0	0
Korarchaeota	1	0	0	0	0	0	0	0	0	0
Total	723	0	18	0	0	0	0	1	0	26

(mod_M00278_1)

Phyla	Module completion ratio (%)									
	0--10	10--20	20--30	30--40	40--50	50--60	60--70	70--80	80--90	90--100
Gammaproteobacteria	120	0	0	0	0	1	0	1	0	4
Betaproteobacteria	61	0	0	0	0	0	0	0	0	0
Epsilonproteobacteria	17	0	0	0	0	0	0	0	0	0
Deltaproteobacteria	28	0	0	0	0	0	0	0	0	0
Alphaproteobacteria	91	0	0	0	0	0	0	0	0	0
Magnetococcus	1	0	0	0	0	0	0	0	0	0
Chrysiogenetes	1	0	0	0	0	0	0	0	0	0
Firmicutes	104	0	0	0	0	0	0	0	0	0
Tenericutes	19	0	0	0	0	0	0	0	0	0
Actinobacteria	80	0	0	0	0	0	0	0	0	0
Chlamydiae	8	0	0	0	0	0	0	0	0	0
Spirochaetes	14	0	0	0	0	0	0	0	0	0
Acidobacteria	5	0	0	0	0	0	0	0	0	0
Bacteroidetes	35	0	0	0	0	0	0	0	0	0
Fibrobacteres	1	0	0	0	0	0	0	0	0	0
Fusobacteria	4	0	0	0	0	0	0	1	0	0
Verrucomicrobia	4	0	0	0	0	0	0	0	0	0
Gemmatimonadetes	1	0	0	0	0	0	0	0	0	0
Planctomycetes	4	0	0	0	0	0	0	0	0	0
Elusimicrobia	2	0	0	0	0	0	0	0	0	0
Synergistetes	2	0	0	0	0	0	0	0	0	0
Cyanobacteria	16	0	0	0	0	0	0	0	0	0
Chlorobi	10	0	0	0	0	0	0	0	0	0
Chloroflexi	11	0	0	0	0	0	0	0	0	0
Deinococcus-Thermus	7	0	0	0	0	0	0	0	0	0
Aquificae	9	0	0	0	0	0	0	0	0	0
Thermotogae	11	0	0	0	0	0	0	0	0	0
Dictyoglomi	2	0	0	0	0	0	0	0	0	0
Nitrospirae	2	0	0	0	0	0	0	0	0	0
Thermobaculum	1	0	0	0	0	0	0	0	0	0
Deferribacteres	3	0	0	0	0	0	0	0	0	0
Euryarchaeota	60	0	0	0	0	0	0	0	0	0
Crenarchaeota	23	0	0	0	0	0	0	0	0	0
Thaumarchaeota	2	0	0	0	0	0	0	0	0	0
Nanoarchaeota	1	0	0	0	0	0	0	0	0	0
Korarchaeota	1	0	0	0	0	0	0	0	0	0
Total	761	0	0	0	0	1	0	2	0	4

(mod_M00279_1)

Phyla	Module completion ratio (%)									
	0--10	10--20	20--30	30--40	40--50	50--60	60--70	70--80	80--90	90--100
Gammaproteobacteria	113	0	0	0	0	0	2	0	0	11
Betaproteobacteria	60	0	0	0	0	0	0	0	0	1
Epsilonproteobacteria	17	0	0	0	0	0	0	0	0	0
Deltaproteobacteria	28	0	0	0	0	0	0	0	0	0
Alphaproteobacteria	90	0	0	0	0	0	0	0	0	1
Magnetococcus	1	0	0	0	0	0	0	0	0	0
Chrysiogenetes	1	0	0	0	0	0	0	0	0	0
Firmicutes	68	0	0	8	0	0	2	0	0	26
Tenericutes	19	0	0	0	0	0	0	0	0	0
Actinobacteria	76	0	0	0	0	0	1	0	0	3
Chlamydiae	8	0	0	0	0	0	0	0	0	0
Spirochaetes	14	0	0	0	0	0	0	0	0	0
Acidobacteria	5	0	0	0	0	0	0	0	0	0
Bacteroidetes	32	0	0	3	0	0	0	0	0	0
Fibrobacteres	1	0	0	0	0	0	0	0	0	0
Fusobacteria	3	0	0	1	0	0	0	0	0	1
Verrucomicrobia	4	0	0	0	0	0	0	0	0	0
Gemmatimonadetes	1	0	0	0	0	0	0	0	0	0
Planctomycetes	4	0	0	0	0	0	0	0	0	0
Elusimicrobia	2	0	0	0	0	0	0	0	0	0
Synergistetes	2	0	0	0	0	0	0	0	0	0
Cyanobacteria	16	0	0	0	0	0	0	0	0	0
Chlorobi	10	0	0	0	0	0	0	0	0	0
Chloroflexi	11	0	0	0	0	0	0	0	0	0
Deinococcus-Thermus	7	0	0	0	0	0	0	0	0	0
Aquificae	9	0	0	0	0	0	0	0	0	0
Thermotogae	11	0	0	0	0	0	0	0	0	0
Dictyoglomi	2	0	0	0	0	0	0	0	0	0
Nitrospirae	2	0	0	0	0	0	0	0	0	0
Thermobaculum	1	0	0	0	0	0	0	0	0	0
Deferribacteres	3	0	0	0	0	0	0	0	0	0
Euryarchaeota	59	0	0	0	0	0	0	0	0	1
Crenarchaeota	23	0	0	0	0	0	0	0	0	0
Thaumarchaeota	2	0	0	0	0	0	0	0	0	0
Nanoarchaeota	1	0	0	0	0	0	0	0	0	0
Korarchaeota	1	0	0	0	0	0	0	0	0	0
Total	707	0	0	12	0	0	5	0	0	44

(mod_M00280_1)

Phyla	Module completion ratio (%)									
	0--10	10--20	20--30	30--40	40--50	50--60	60--70	70--80	80--90	90--100
Gammaproteobacteria	106	0	0	1	0	0	1	0	0	18
Betaproteobacteria	60	0	0	0	0	0	0	0	0	1
Epsilonproteobacteria	17	0	0	0	0	0	0	0	0	0
Deltaproteobacteria	28	0	0	0	0	0	0	0	0	0
Alphaproteobacteria	89	0	0	0	0	0	0	0	0	2
Magnetococcus	1	0	0	0	0	0	0	0	0	0
Chrysiogenetes	1	0	0	0	0	0	0	0	0	0
Firmicutes	79	0	0	7	0	0	0	0	0	18
Tenericutes	19	0	0	0	0	0	0	0	0	0
Actinobacteria	78	0	0	0	0	0	1	0	0	1
Chlamydiae	8	0	0	0	0	0	0	0	0	0
Spirochaetes	14	0	0	0	0	0	0	0	0	0
Acidobacteria	5	0	0	0	0	0	0	0	0	0
Bacteroidetes	35	0	0	0	0	0	0	0	0	0
Fibrobacteres	1	0	0	0	0	0	0	0	0	0
Fusobacteria	4	0	0	0	0	0	0	0	0	1
Verrucomicrobia	4	0	0	0	0	0	0	0	0	0
Gemmatimonadetes	1	0	0	0	0	0	0	0	0	0
Planctomycetes	4	0	0	0	0	0	0	0	0	0
Elusimicrobia	2	0	0	0	0	0	0	0	0	0
Synergistetes	2	0	0	0	0	0	0	0	0	0
Cyanobacteria	16	0	0	0	0	0	0	0	0	0
Chlorobi	10	0	0	0	0	0	0	0	0	0
Chloroflexi	9	0	0	0	0	0	0	0	0	2
Deinococcus-Thermus	7	0	0	0	0	0	0	0	0	0
Aquificae	9	0	0	0	0	0	0	0	0	0
Thermotogae	11	0	0	0	0	0	0	0	0	0
Dictyoglomi	2	0	0	0	0	0	0	0	0	0
Nitrospirae	2	0	0	0	0	0	0	0	0	0
Thermobaculum	1	0	0	0	0	0	0	0	0	0
Deferribacteres	3	0	0	0	0	0	0	0	0	0
Euryarchaeota	60	0	0	0	0	0	0	0	0	0
Crenarchaeota	23	0	0	0	0	0	0	0	0	0
Thaumarchaeota	2	0	0	0	0	0	0	0	0	0
Nanoarchaeota	1	0	0	0	0	0	0	0	0	0
Korarchaeota	1	0	0	0	0	0	0	0	0	0
Total	715	0	0	8	0	0	2	0	0	43

(mod_M00281_1)

Phyla	Module completion ratio (%)									
	0--10	10--20	20--30	30--40	40--50	50--60	60--70	70--80	80--90	90--100
Gammaproteobacteria	126	0	0	0	0	0	0	0	0	0
Betaproteobacteria	61	0	0	0	0	0	0	0	0	0
Epsilonproteobacteria	17	0	0	0	0	0	0	0	0	0
Deltaproteobacteria	28	0	0	0	0	0	0	0	0	0
Alphaproteobacteria	91	0	0	0	0	0	0	0	0	0
Magnetococcus	1	0	0	0	0	0	0	0	0	0
Chrysiogenetes	1	0	0	0	0	0	0	0	0	0
Firmicutes	90	0	0	0	0	0	0	0	0	14
Tenericutes	19	0	0	0	0	0	0	0	0	0
Actinobacteria	80	0	0	0	0	0	0	0	0	0
Chlamydiae	8	0	0	0	0	0	0	0	0	0
Spirochaetes	14	0	0	0	0	0	0	0	0	0
Acidobacteria	5	0	0	0	0	0	0	0	0	0
Bacteroidetes	35	0	0	0	0	0	0	0	0	0
Fibrobacteres	1	0	0	0	0	0	0	0	0	0
Fusobacteria	3	0	0	0	0	0	0	0	0	2
Verrucomicrobia	4	0	0	0	0	0	0	0	0	0
Gemmatimonadetes	1	0	0	0	0	0	0	0	0	0
Planctomycetes	4	0	0	0	0	0	0	0	0	0
Elusimicrobia	2	0	0	0	0	0	0	0	0	0
Synergistetes	2	0	0	0	0	0	0	0	0	0
Cyanobacteria	16	0	0	0	0	0	0	0	0	0
Chlorobi	10	0	0	0	0	0	0	0	0	0
Chloroflexi	11	0	0	0	0	0	0	0	0	0
Deinococcus-Thermus	7	0	0	0	0	0	0	0	0	0
Aquificae	9	0	0	0	0	0	0	0	0	0
Thermotogae	11	0	0	0	0	0	0	0	0	0
Dictyoglomi	2	0	0	0	0	0	0	0	0	0
Nitrospirae	2	0	0	0	0	0	0	0	0	0
Thermobaculum	1	0	0	0	0	0	0	0	0	0
Deferribacteres	3	0	0	0	0	0	0	0	0	0
Euryarchaeota	60	0	0	0	0	0	0	0	0	0
Crenarchaeota	23	0	0	0	0	0	0	0	0	0
Thaumarchaeota	2	0	0	0	0	0	0	0	0	0
Nanoarchaeota	1	0	0	0	0	0	0	0	0	0
Korarchaeota	1	0	0	0	0	0	0	0	0	0
Total	752	0	0	0	0	0	0	0	0	16

(mod_M00282_1)

Phyla	Module completion ratio (%)									
	0--10	10--20	20--30	30--40	40--50	50--60	60--70	70--80	80--90	90--100
Gammaproteobacteria	119	0	0	0	0	0	0	0	0	7
Betaproteobacteria	61	0	0	0	0	0	0	0	0	0
Epsilonproteobacteria	17	0	0	0	0	0	0	0	0	0
Deltaproteobacteria	28	0	0	0	0	0	0	0	0	0
Alphaproteobacteria	91	0	0	0	0	0	0	0	0	0
Magnetococcus	1	0	0	0	0	0	0	0	0	0
Chrysiogenetes	1	0	0	0	0	0	0	0	0	0
Firmicutes	76	0	0	0	0	0	0	0	0	28
Tenericutes	19	0	0	0	0	0	0	0	0	0
Actinobacteria	80	0	0	0	0	0	0	0	0	0
Chlamydiae	8	0	0	0	0	0	0	0	0	0
Spirochaetes	13	0	0	0	0	0	0	0	0	1
Acidobacteria	5	0	0	0	0	0	0	0	0	0
Bacteroidetes	35	0	0	0	0	0	0	0	0	0
Fibrobacteres	1	0	0	0	0	0	0	0	0	0
Fusobacteria	2	0	0	0	0	0	0	0	0	3
Verrucomicrobia	4	0	0	0	0	0	0	0	0	0
Gemmatimonadetes	1	0	0	0	0	0	0	0	0	0
Planctomycetes	4	0	0	0	0	0	0	0	0	0
Elusimicrobia	2	0	0	0	0	0	0	0	0	0
Synergistetes	1	0	0	0	0	0	0	0	0	1
Cyanobacteria	16	0	0	0	0	0	0	0	0	0
Chlorobi	10	0	0	0	0	0	0	0	0	0
Chloroflexi	11	0	0	0	0	0	0	0	0	0
Deinococcus-Thermus	6	0	0	0	0	0	0	0	0	1
Aquificae	9	0	0	0	0	0	0	0	0	0
Thermotogae	11	0	0	0	0	0	0	0	0	0
Dictyoglomi	2	0	0	0	0	0	0	0	0	0
Nitrospirae	2	0	0	0	0	0	0	0	0	0
Thermobaculum	1	0	0	0	0	0	0	0	0	0
Deferribacteres	3	0	0	0	0	0	0	0	0	0
Euryarchaeota	60	0	0	0	0	0	0	0	0	0
Crenarchaeota	23	0	0	0	0	0	0	0	0	0
Thaumarchaeota	2	0	0	0	0	0	0	0	0	0
Nanoarchaeota	1	0	0	0	0	0	0	0	0	0
Korarchaeota	1	0	0	0	0	0	0	0	0	0
Total	727	0	0	0	0	0	0	0	0	41

(mod_M00283_1)

Phyla	Module completion ratio (%)									
	0--10	10--20	20--30	30--40	40--50	50--60	60--70	70--80	80--90	90--100
Gammaproteobacteria	68	0	0	14	0	0	3	0	0	41
Betaproteobacteria	1	0	0	60	0	0	0	0	0	0
Epsilonproteobacteria	17	0	0	0	0	0	0	0	0	0
Deltaproteobacteria	28	0	0	0	0	0	0	0	0	0
Alphaproteobacteria	91	0	0	0	0	0	0	0	0	0
Magnetococcus	1	0	0	0	0	0	0	0	0	0
Chrysiogenetes	1	0	0	0	0	0	0	0	0	0
Firmicutes	64	0	0	1	0	0	16	0	0	23
Tenericutes	10	0	0	1	0	0	0	0	0	8
Actinobacteria	70	0	0	4	0	0	3	0	0	3
Chlamydiae	8	0	0	0	0	0	0	0	0	0
Spirochaetes	12	0	0	1	0	0	1	0	0	0
Acidobacteria	5	0	0	0	0	0	0	0	0	0
Bacteroidetes	35	0	0	0	0	0	0	0	0	0
Fibrobacteres	1	0	0	0	0	0	0	0	0	0
Fusobacteria	2	0	0	0	0	0	0	0	0	3
Verrucomicrobia	4	0	0	0	0	0	0	0	0	0
Gemmatimonadetes	1	0	0	0	0	0	0	0	0	0
Planctomycetes	4	0	0	0	0	0	0	0	0	0
Elusimicrobia	2	0	0	0	0	0	0	0	0	0
Synergistetes	2	0	0	0	0	0	0	0	0	0
Cyanobacteria	16	0	0	0	0	0	0	0	0	0
Chlorobi	10	0	0	0	0	0	0	0	0	0
Chloroflexi	11	0	0	0	0	0	0	0	0	0
Deinococcus-Thermus	7	0	0	0	0	0	0	0	0	0
Aquificae	9	0	0	0	0	0	0	0	0	0
Thermotogae	11	0	0	0	0	0	0	0	0	0
Dictyoglomi	1	0	0	0	0	0	1	0	0	0
Nitrospirae	2	0	0	0	0	0	0	0	0	0
Thermobaculum	1	0	0	0	0	0	0	0	0	0
Deferribacteres	3	0	0	0	0	0	0	0	0	0
Euryarchaeota	60	0	0	0	0	0	0	0	0	0
Crenarchaeota	23	0	0	0	0	0	0	0	0	0
Thaumarchaeota	2	0	0	0	0	0	0	0	0	0
Nanoarchaeota	1	0	0	0	0	0	0	0	0	0
Korarchaeota	1	0	0	0	0	0	0	0	0	0
Total	585	0	0	81	0	0	24	0	0	78

(mod_M00284_1)

Phyla	Module completion ratio (%)									
	0--10	10--20	20--30	30--40	40--50	50--60	60--70	70--80	80--90	90--100
Gammaproteobacteria	126	0	0	0	0	0	0	0	0	0
Betaproteobacteria	61	0	0	0	0	0	0	0	0	0
Epsilonproteobacteria	17	0	0	0	0	0	0	0	0	0
Deltaproteobacteria	28	0	0	0	0	0	0	0	0	0
Alphaproteobacteria	91	0	0	0	0	0	0	0	0	0
Magnetococcus	1	0	0	0	0	0	0	0	0	0
Chrysiogenetes	1	0	0	0	0	0	0	0	0	0
Firmicutes	104	0	0	0	0	0	0	0	0	0
Tenericutes	19	0	0	0	0	0	0	0	0	0
Actinobacteria	80	0	0	0	0	0	0	0	0	0
Chlamydiae	8	0	0	0	0	0	0	0	0	0
Spirochaetes	14	0	0	0	0	0	0	0	0	0
Acidobacteria	5	0	0	0	0	0	0	0	0	0
Bacteroidetes	35	0	0	0	0	0	0	0	0	0
Fibrobacteres	1	0	0	0	0	0	0	0	0	0
Fusobacteria	5	0	0	0	0	0	0	0	0	0
Verrucomicrobia	4	0	0	0	0	0	0	0	0	0
Gemmatimonadetes	1	0	0	0	0	0	0	0	0	0
Planctomycetes	4	0	0	0	0	0	0	0	0	0
Elusimicrobia	2	0	0	0	0	0	0	0	0	0
Synergistetes	2	0	0	0	0	0	0	0	0	0
Cyanobacteria	16	0	0	0	0	0	0	0	0	0
Chlorobi	10	0	0	0	0	0	0	0	0	0
Chloroflexi	11	0	0	0	0	0	0	0	0	0
Deinococcus-Thermus	7	0	0	0	0	0	0	0	0	0
Aquificae	9	0	0	0	0	0	0	0	0	0
Thermotogae	11	0	0	0	0	0	0	0	0	0
Dictyoglomi	2	0	0	0	0	0	0	0	0	0
Nitrospirae	2	0	0	0	0	0	0	0	0	0
Thermobaculum	1	0	0	0	0	0	0	0	0	0
Deferribacteres	3	0	0	0	0	0	0	0	0	0
Euryarchaeota	60	0	0	0	0	0	0	0	0	0
Crenarchaeota	23	0	0	0	0	0	0	0	0	0
Thaumarchaeota	2	0	0	0	0	0	0	0	0	0
Nanoarchaeota	1	0	0	0	0	0	0	0	0	0
Korarchaeota	1	0	0	0	0	0	0	0	0	0
Total	768	0	0	0	0	0	0	0	0	0

(mod_M00285_1)

Phyla	Module completion ratio (%)									
	0--10	10--20	20--30	30--40	40--50	50--60	60--70	70--80	80--90	90--100
Gammaproteobacteria	126	0	0	0	0	0	0	0	0	0
Betaproteobacteria	61	0	0	0	0	0	0	0	0	0
Epsilonproteobacteria	17	0	0	0	0	0	0	0	0	0
Deltaproteobacteria	28	0	0	0	0	0	0	0	0	0
Alphaproteobacteria	91	0	0	0	0	0	0	0	0	0
Magnetococcus	1	0	0	0	0	0	0	0	0	0
Chrysiogenetes	1	0	0	0	0	0	0	0	0	0
Firmicutes	104	0	0	0	0	0	0	0	0	0
Tenericutes	19	0	0	0	0	0	0	0	0	0
Actinobacteria	80	0	0	0	0	0	0	0	0	0
Chlamydiae	8	0	0	0	0	0	0	0	0	0
Spirochaetes	14	0	0	0	0	0	0	0	0	0
Acidobacteria	5	0	0	0	0	0	0	0	0	0
Bacteroidetes	35	0	0	0	0	0	0	0	0	0
Fibrobacteres	1	0	0	0	0	0	0	0	0	0
Fusobacteria	5	0	0	0	0	0	0	0	0	0
Verrucomicrobia	4	0	0	0	0	0	0	0	0	0
Gemmatimonadetes	1	0	0	0	0	0	0	0	0	0
Planctomycetes	4	0	0	0	0	0	0	0	0	0
Elusimicrobia	2	0	0	0	0	0	0	0	0	0
Synergistetes	2	0	0	0	0	0	0	0	0	0
Cyanobacteria	16	0	0	0	0	0	0	0	0	0
Chlorobi	10	0	0	0	0	0	0	0	0	0
Chloroflexi	11	0	0	0	0	0	0	0	0	0
Deinococcus-Thermus	7	0	0	0	0	0	0	0	0	0
Aquificae	9	0	0	0	0	0	0	0	0	0
Thermotogae	11	0	0	0	0	0	0	0	0	0
Dictyoglomi	2	0	0	0	0	0	0	0	0	0
Nitrospirae	2	0	0	0	0	0	0	0	0	0
Thermobaculum	1	0	0	0	0	0	0	0	0	0
Deferribacteres	3	0	0	0	0	0	0	0	0	0
Euryarchaeota	60	0	0	0	0	0	0	0	0	0
Crenarchaeota	23	0	0	0	0	0	0	0	0	0
Thaumarchaeota	2	0	0	0	0	0	0	0	0	0
Nanoarchaeota	1	0	0	0	0	0	0	0	0	0
Korarchaeota	1	0	0	0	0	0	0	0	0	0
Total	768	0	0	0	0	0	0	0	0	0

(mod_M00286_1)

Phyla	Module completion ratio (%)									
	0--10	10--20	20--30	30--40	40--50	50--60	60--70	70--80	80--90	90--100
Gammaproteobacteria	126	0	0	0	0	0	0	0	0	0
Betaproteobacteria	61	0	0	0	0	0	0	0	0	0
Epsilonproteobacteria	17	0	0	0	0	0	0	0	0	0
Deltaproteobacteria	28	0	0	0	0	0	0	0	0	0
Alphaproteobacteria	91	0	0	0	0	0	0	0	0	0
Magnetococcus	1	0	0	0	0	0	0	0	0	0
Chrysiogenetes	1	0	0	0	0	0	0	0	0	0
Firmicutes	104	0	0	0	0	0	0	0	0	0
Tenericutes	19	0	0	0	0	0	0	0	0	0
Actinobacteria	80	0	0	0	0	0	0	0	0	0
Chlamydiae	8	0	0	0	0	0	0	0	0	0
Spirochaetes	14	0	0	0	0	0	0	0	0	0
Acidobacteria	5	0	0	0	0	0	0	0	0	0
Bacteroidetes	35	0	0	0	0	0	0	0	0	0
Fibrobacteres	1	0	0	0	0	0	0	0	0	0
Fusobacteria	5	0	0	0	0	0	0	0	0	0
Verrucomicrobia	4	0	0	0	0	0	0	0	0	0
Gemmatimonadetes	1	0	0	0	0	0	0	0	0	0
Planctomycetes	4	0	0	0	0	0	0	0	0	0
Elusimicrobia	2	0	0	0	0	0	0	0	0	0
Synergistetes	2	0	0	0	0	0	0	0	0	0
Cyanobacteria	16	0	0	0	0	0	0	0	0	0
Chlorobi	10	0	0	0	0	0	0	0	0	0
Chloroflexi	11	0	0	0	0	0	0	0	0	0
Deinococcus-Thermus	7	0	0	0	0	0	0	0	0	0
Aquificae	9	0	0	0	0	0	0	0	0	0
Thermotogae	11	0	0	0	0	0	0	0	0	0
Dictyoglomi	2	0	0	0	0	0	0	0	0	0
Nitrospirae	2	0	0	0	0	0	0	0	0	0
Thermobaculum	1	0	0	0	0	0	0	0	0	0
Deferribacteres	3	0	0	0	0	0	0	0	0	0
Euryarchaeota	60	0	0	0	0	0	0	0	0	0
Crenarchaeota	23	0	0	0	0	0	0	0	0	0
Thaumarchaeota	2	0	0	0	0	0	0	0	0	0
Nanoarchaeota	1	0	0	0	0	0	0	0	0	0
Korarchaeota	1	0	0	0	0	0	0	0	0	0
Total	768	0	0	0	0	0	0	0	0	0

(mod_M00287_1)

Phyla	Module completion ratio (%)									
	0--10	10--20	20--30	30--40	40--50	50--60	60--70	70--80	80--90	90--100
Gammaproteobacteria	108	0	15	0	0	0	0	2	0	1
Betaproteobacteria	61	0	0	0	0	0	0	0	0	0
Epsilonproteobacteria	17	0	0	0	0	0	0	0	0	0
Deltaproteobacteria	28	0	0	0	0	0	0	0	0	0
Alphaproteobacteria	91	0	0	0	0	0	0	0	0	0
Magnetococcus	1	0	0	0	0	0	0	0	0	0
Chrysiogenetes	1	0	0	0	0	0	0	0	0	0
Firmicutes	85	0	16	0	0	0	0	0	0	3
Tenericutes	19	0	0	0	0	0	0	0	0	0
Actinobacteria	78	0	2	0	0	0	0	0	0	0
Chlamydiae	8	0	0	0	0	0	0	0	0	0
Spirochaetes	14	0	0	0	0	0	0	0	0	0
Acidobacteria	5	0	0	0	0	0	0	0	0	0
Bacteroidetes	35	0	0	0	0	0	0	0	0	0
Fibrobacteres	1	0	0	0	0	0	0	0	0	0
Fusobacteria	3	0	1	0	0	0	0	0	0	1
Verrucomicrobia	4	0	0	0	0	0	0	0	0	0
Gemmatimonadetes	0	0	1	0	0	0	0	0	0	0
Planctomycetes	4	0	0	0	0	0	0	0	0	0
Elusimicrobia	2	0	0	0	0	0	0	0	0	0
Synergistetes	2	0	0	0	0	0	0	0	0	0
Cyanobacteria	16	0	0	0	0	0	0	0	0	0
Chlorobi	10	0	0	0	0	0	0	0	0	0
Chloroflexi	11	0	0	0	0	0	0	0	0	0
Deinococcus-Thermus	7	0	0	0	0	0	0	0	0	0
Aquificae	9	0	0	0	0	0	0	0	0	0
Thermotogae	11	0	0	0	0	0	0	0	0	0
Dictyoglomi	2	0	0	0	0	0	0	0	0	0
Nitrospirae	2	0	0	0	0	0	0	0	0	0
Thermobaculum	1	0	0	0	0	0	0	0	0	0
Deferribacteres	3	0	0	0	0	0	0	0	0	0
Euryarchaeota	60	0	0	0	0	0	0	0	0	0
Crenarchaeota	23	0	0	0	0	0	0	0	0	0
Thaumarchaeota	2	0	0	0	0	0	0	0	0	0
Nanoarchaeota	1	0	0	0	0	0	0	0	0	0
Korarchaeota	1	0	0	0	0	0	0	0	0	0
Total	726	0	35	0	0	0	0	2	0	5

(mod_M00288_1)

Phyla	Module completion ratio (%)									
	0--10	10--20	20--30	30--40	40--50	50--60	60--70	70--80	80--90	90--100
Gammaproteobacteria	126	0	0	0	0	0	0	0	0	0
Betaproteobacteria	61	0	0	0	0	0	0	0	0	0
Epsilonproteobacteria	17	0	0	0	0	0	0	0	0	0
Deltaproteobacteria	28	0	0	0	0	0	0	0	0	0
Alphaproteobacteria	91	0	0	0	0	0	0	0	0	0
Magnetococcus	1	0	0	0	0	0	0	0	0	0
Chrysiogenetes	1	0	0	0	0	0	0	0	0	0
Firmicutes	104	0	0	0	0	0	0	0	0	0
Tenericutes	19	0	0	0	0	0	0	0	0	0
Actinobacteria	80	0	0	0	0	0	0	0	0	0
Chlamydiae	8	0	0	0	0	0	0	0	0	0
Spirochaetes	14	0	0	0	0	0	0	0	0	0
Acidobacteria	5	0	0	0	0	0	0	0	0	0
Bacteroidetes	35	0	0	0	0	0	0	0	0	0
Fibrobacteres	1	0	0	0	0	0	0	0	0	0
Fusobacteria	5	0	0	0	0	0	0	0	0	0
Verrucomicrobia	4	0	0	0	0	0	0	0	0	0
Gemmatimonadetes	1	0	0	0	0	0	0	0	0	0
Planctomycetes	4	0	0	0	0	0	0	0	0	0
Elusimicrobia	2	0	0	0	0	0	0	0	0	0
Synergistetes	2	0	0	0	0	0	0	0	0	0
Cyanobacteria	16	0	0	0	0	0	0	0	0	0
Chlorobi	10	0	0	0	0	0	0	0	0	0
Chloroflexi	11	0	0	0	0	0	0	0	0	0
Deinococcus-Thermus	7	0	0	0	0	0	0	0	0	0
Aquificae	9	0	0	0	0	0	0	0	0	0
Thermotogae	11	0	0	0	0	0	0	0	0	0
Dictyoglomi	2	0	0	0	0	0	0	0	0	0
Nitrospirae	2	0	0	0	0	0	0	0	0	0
Thermobaculum	1	0	0	0	0	0	0	0	0	0
Deferribacteres	3	0	0	0	0	0	0	0	0	0
Euryarchaeota	0	0	0	60	0	0	0	0	0	0
Crenarchaeota	7	0	0	16	0	0	0	0	0	0
Thaumarchaeota	0	0	0	2	0	0	0	0	0	0
Nanoarchaeota	0	0	0	1	0	0	0	0	0	0
Korarchaeota	0	0	0	1	0	0	0	0	0	0
Total	688	0	0	80	0	0	0	0	0	0

(mod_M00289_1)

Phyla	Module completion ratio (%)									
	0--10	10--20	20--30	30--40	40--50	50--60	60--70	70--80	80--90	90--100
Gammaproteobacteria	126	0	0	0	0	0	0	0	0	0
Betaproteobacteria	61	0	0	0	0	0	0	0	0	0
Epsilonproteobacteria	17	0	0	0	0	0	0	0	0	0
Deltaproteobacteria	28	0	0	0	0	0	0	0	0	0
Alphaproteobacteria	91	0	0	0	0	0	0	0	0	0
Magnetococcus	1	0	0	0	0	0	0	0	0	0
Chrysiogenetes	1	0	0	0	0	0	0	0	0	0
Firmicutes	104	0	0	0	0	0	0	0	0	0
Tenericutes	19	0	0	0	0	0	0	0	0	0
Actinobacteria	80	0	0	0	0	0	0	0	0	0
Chlamydiae	8	0	0	0	0	0	0	0	0	0
Spirochaetes	14	0	0	0	0	0	0	0	0	0
Acidobacteria	5	0	0	0	0	0	0	0	0	0
Bacteroidetes	35	0	0	0	0	0	0	0	0	0
Fibrobacteres	1	0	0	0	0	0	0	0	0	0
Fusobacteria	5	0	0	0	0	0	0	0	0	0
Verrucomicrobia	4	0	0	0	0	0	0	0	0	0
Gemmatimonadetes	1	0	0	0	0	0	0	0	0	0
Planctomycetes	4	0	0	0	0	0	0	0	0	0
Elusimicrobia	2	0	0	0	0	0	0	0	0	0
Synergistetes	2	0	0	0	0	0	0	0	0	0
Cyanobacteria	16	0	0	0	0	0	0	0	0	0
Chlorobi	9	0	0	1	0	0	0	0	0	0
Chloroflexi	11	0	0	0	0	0	0	0	0	0
Deinococcus-Thermus	7	0	0	0	0	0	0	0	0	0
Aquificae	9	0	0	0	0	0	0	0	0	0
Thermotogae	11	0	0	0	0	0	0	0	0	0
Dictyoglomi	2	0	0	0	0	0	0	0	0	0
Nitrospirae	2	0	0	0	0	0	0	0	0	0
Thermobaculum	1	0	0	0	0	0	0	0	0	0
Deferribacteres	3	0	0	0	0	0	0	0	0	0
Euryarchaeota	60	0	0	0	0	0	0	0	0	0
Crenarchaeota	23	0	0	0	0	0	0	0	0	0
Thaumarchaeota	2	0	0	0	0	0	0	0	0	0
Nanoarchaeota	1	0	0	0	0	0	0	0	0	0
Korarchaeota	1	0	0	0	0	0	0	0	0	0
Total	767	0	0	1	0	0	0	0	0	0

(mod_M00290_1)

Phyla	Module completion ratio (%)									
	0--10	10--20	20--30	30--40	40--50	50--60	60--70	70--80	80--90	90--100
Gammaproteobacteria	125	1	0	0	0	0	0	0	0	0
Betaproteobacteria	57	4	0	0	0	0	0	0	0	0
Epsilonproteobacteria	17	0	0	0	0	0	0	0	0	0
Deltaproteobacteria	26	2	0	0	0	0	0	0	0	0
Alphaproteobacteria	91	0	0	0	0	0	0	0	0	0
Magnetococcus	1	0	0	0	0	0	0	0	0	0
Chrysiogenetes	1	0	0	0	0	0	0	0	0	0
Firmicutes	98	6	0	0	0	0	0	0	0	0
Tenericutes	19	0	0	0	0	0	0	0	0	0
Actinobacteria	21	59	0	0	0	0	0	0	0	0
Chlamydiae	8	0	0	0	0	0	0	0	0	0
Spirochaetes	7	7	0	0	0	0	0	0	0	0
Acidobacteria	5	0	0	0	0	0	0	0	0	0
Bacteroidetes	35	0	0	0	0	0	0	0	0	0
Fibrobacteres	0	1	0	0	0	0	0	0	0	0
Fusobacteria	5	0	0	0	0	0	0	0	0	0
Verrucomicrobia	4	0	0	0	0	0	0	0	0	0
Gemmatimonadetes	1	0	0	0	0	0	0	0	0	0
Planctomycetes	3	1	0	0	0	0	0	0	0	0
Elusimicrobia	2	0	0	0	0	0	0	0	0	0
Synergistetes	2	0	0	0	0	0	0	0	0	0
Cyanobacteria	15	1	0	0	0	0	0	0	0	0
Chlorobi	10	0	0	0	0	0	0	0	0	0
Chloroflexi	10	1	0	0	0	0	0	0	0	0
Deinococcus-Thermus	7	0	0	0	0	0	0	0	0	0
Aquificae	9	0	0	0	0	0	0	0	0	0
Thermotogae	11	0	0	0	0	0	0	0	0	0
Dictyoglomi	2	0	0	0	0	0	0	0	0	0
Nitrospirae	2	0	0	0	0	0	0	0	0	0
Thermobaculum	1	0	0	0	0	0	0	0	0	0
Deferribacteres	3	0	0	0	0	0	0	0	0	0
Euryarchaeota	19	33	8	0	0	0	0	0	0	0
Crenarchaeota	6	17	0	0	0	0	0	0	0	0
Thaumarchaeota	2	0	0	0	0	0	0	0	0	0
Nanoarchaeota	1	0	0	0	0	0	0	0	0	0
Korarchaeota	0	1	0	0	0	0	0	0	0	0
Total	626	134	8	0	0	0	0	0	0	0

(mod_M00291_1)

Phyla	Module completion ratio (%)									
	0--10	10--20	20--30	30--40	40--50	50--60	60--70	70--80	80--90	90--100
Gammaproteobacteria	126	0	0	0	0	0	0	0	0	0
Betaproteobacteria	61	0	0	0	0	0	0	0	0	0
Epsilonproteobacteria	17	0	0	0	0	0	0	0	0	0
Deltaproteobacteria	28	0	0	0	0	0	0	0	0	0
Alphaproteobacteria	91	0	0	0	0	0	0	0	0	0
Magnetococcus	1	0	0	0	0	0	0	0	0	0
Chrysiogenetes	1	0	0	0	0	0	0	0	0	0
Firmicutes	104	0	0	0	0	0	0	0	0	0
Tenericutes	19	0	0	0	0	0	0	0	0	0
Actinobacteria	80	0	0	0	0	0	0	0	0	0
Chlamydiae	8	0	0	0	0	0	0	0	0	0
Spirochaetes	14	0	0	0	0	0	0	0	0	0
Acidobacteria	5	0	0	0	0	0	0	0	0	0
Bacteroidetes	35	0	0	0	0	0	0	0	0	0
Fibrobacteres	1	0	0	0	0	0	0	0	0	0
Fusobacteria	5	0	0	0	0	0	0	0	0	0
Verrucomicrobia	4	0	0	0	0	0	0	0	0	0
Gemmatimonadetes	1	0	0	0	0	0	0	0	0	0
Planctomycetes	4	0	0	0	0	0	0	0	0	0
Elusimicrobia	2	0	0	0	0	0	0	0	0	0
Synergistetes	2	0	0	0	0	0	0	0	0	0
Cyanobacteria	16	0	0	0	0	0	0	0	0	0
Chlorobi	10	0	0	0	0	0	0	0	0	0
Chloroflexi	11	0	0	0	0	0	0	0	0	0
Deinococcus-Thermus	7	0	0	0	0	0	0	0	0	0
Aquificae	9	0	0	0	0	0	0	0	0	0
Thermotogae	11	0	0	0	0	0	0	0	0	0
Dictyoglomi	2	0	0	0	0	0	0	0	0	0
Nitrospirae	2	0	0	0	0	0	0	0	0	0
Thermobaculum	1	0	0	0	0	0	0	0	0	0
Deferribacteres	3	0	0	0	0	0	0	0	0	0
Euryarchaeota	60	0	0	0	0	0	0	0	0	0
Crenarchaeota	23	0	0	0	0	0	0	0	0	0
Thaumarchaeota	2	0	0	0	0	0	0	0	0	0
Nanoarchaeota	1	0	0	0	0	0	0	0	0	0
Korarchaeota	1	0	0	0	0	0	0	0	0	0
Total	768	0	0	0	0	0	0	0	0	0

(mod_M00292_1)

Phyla	Module completion ratio (%)									
	0--10	10--20	20--30	30--40	40--50	50--60	60--70	70--80	80--90	90--100
Gammaproteobacteria	126	0	0	0	0	0	0	0	0	0
Betaproteobacteria	61	0	0	0	0	0	0	0	0	0
Epsilonproteobacteria	17	0	0	0	0	0	0	0	0	0
Deltaproteobacteria	28	0	0	0	0	0	0	0	0	0
Alphaproteobacteria	91	0	0	0	0	0	0	0	0	0
Magnetococcus	1	0	0	0	0	0	0	0	0	0
Chrysiogenetes	1	0	0	0	0	0	0	0	0	0
Firmicutes	104	0	0	0	0	0	0	0	0	0
Tenericutes	19	0	0	0	0	0	0	0	0	0
Actinobacteria	80	0	0	0	0	0	0	0	0	0
Chlamydiae	8	0	0	0	0	0	0	0	0	0
Spirochaetes	14	0	0	0	0	0	0	0	0	0
Acidobacteria	5	0	0	0	0	0	0	0	0	0
Bacteroidetes	35	0	0	0	0	0	0	0	0	0
Fibrobacteres	1	0	0	0	0	0	0	0	0	0
Fusobacteria	5	0	0	0	0	0	0	0	0	0
Verrucomicrobia	4	0	0	0	0	0	0	0	0	0
Gemmatimonadetes	1	0	0	0	0	0	0	0	0	0
Planctomycetes	4	0	0	0	0	0	0	0	0	0
Elusimicrobia	2	0	0	0	0	0	0	0	0	0
Synergistetes	2	0	0	0	0	0	0	0	0	0
Cyanobacteria	16	0	0	0	0	0	0	0	0	0
Chlorobi	10	0	0	0	0	0	0	0	0	0
Chloroflexi	11	0	0	0	0	0	0	0	0	0
Deinococcus-Thermus	7	0	0	0	0	0	0	0	0	0
Aquificae	9	0	0	0	0	0	0	0	0	0
Thermotogae	11	0	0	0	0	0	0	0	0	0
Dictyoglomi	2	0	0	0	0	0	0	0	0	0
Nitrospirae	2	0	0	0	0	0	0	0	0	0
Thermobaculum	1	0	0	0	0	0	0	0	0	0
Deferribacteres	3	0	0	0	0	0	0	0	0	0
Euryarchaeota	60	0	0	0	0	0	0	0	0	0
Crenarchaeota	23	0	0	0	0	0	0	0	0	0
Thaumarchaeota	2	0	0	0	0	0	0	0	0	0
Nanoarchaeota	1	0	0	0	0	0	0	0	0	0
Korarchaeota	1	0	0	0	0	0	0	0	0	0
Total	768	0	0	0	0	0	0	0	0	0

(mod_M00293_1)

Phyla	Module completion ratio (%)									
	0--10	10--20	20--30	30--40	40--50	50--60	60--70	70--80	80--90	90--100
Gammaproteobacteria	126	0	0	0	0	0	0	0	0	0
Betaproteobacteria	61	0	0	0	0	0	0	0	0	0
Epsilonproteobacteria	17	0	0	0	0	0	0	0	0	0
Deltaproteobacteria	28	0	0	0	0	0	0	0	0	0
Alphaproteobacteria	91	0	0	0	0	0	0	0	0	0
Magnetococcus	1	0	0	0	0	0	0	0	0	0
Chrysiogenetes	1	0	0	0	0	0	0	0	0	0
Firmicutes	104	0	0	0	0	0	0	0	0	0
Tenericutes	19	0	0	0	0	0	0	0	0	0
Actinobacteria	80	0	0	0	0	0	0	0	0	0
Chlamydiae	8	0	0	0	0	0	0	0	0	0
Spirochaetes	14	0	0	0	0	0	0	0	0	0
Acidobacteria	5	0	0	0	0	0	0	0	0	0
Bacteroidetes	35	0	0	0	0	0	0	0	0	0
Fibrobacteres	1	0	0	0	0	0	0	0	0	0
Fusobacteria	5	0	0	0	0	0	0	0	0	0
Verrucomicrobia	4	0	0	0	0	0	0	0	0	0
Gemmatimonadetes	1	0	0	0	0	0	0	0	0	0
Planctomycetes	4	0	0	0	0	0	0	0	0	0
Elusimicrobia	2	0	0	0	0	0	0	0	0	0
Synergistetes	2	0	0	0	0	0	0	0	0	0
Cyanobacteria	16	0	0	0	0	0	0	0	0	0
Chlorobi	10	0	0	0	0	0	0	0	0	0
Chloroflexi	11	0	0	0	0	0	0	0	0	0
Deinococcus-Thermus	7	0	0	0	0	0	0	0	0	0
Aquificae	9	0	0	0	0	0	0	0	0	0
Thermotogae	11	0	0	0	0	0	0	0	0	0
Dictyoglomi	2	0	0	0	0	0	0	0	0	0
Nitrospirae	2	0	0	0	0	0	0	0	0	0
Thermobaculum	1	0	0	0	0	0	0	0	0	0
Deferribacteres	3	0	0	0	0	0	0	0	0	0
Euryarchaeota	60	0	0	0	0	0	0	0	0	0
Crenarchaeota	23	0	0	0	0	0	0	0	0	0
Thaumarchaeota	2	0	0	0	0	0	0	0	0	0
Nanoarchaeota	1	0	0	0	0	0	0	0	0	0
Korarchaeota	1	0	0	0	0	0	0	0	0	0
Total	768	0	0	0	0	0	0	0	0	0

(mod_M00294_1)

Phyla	Module completion ratio (%)									
	0--10	10--20	20--30	30--40	40--50	50--60	60--70	70--80	80--90	90--100
Gammaproteobacteria	126	0	0	0	0	0	0	0	0	0
Betaproteobacteria	61	0	0	0	0	0	0	0	0	0
Epsilonproteobacteria	17	0	0	0	0	0	0	0	0	0
Deltaproteobacteria	28	0	0	0	0	0	0	0	0	0
Alphaproteobacteria	91	0	0	0	0	0	0	0	0	0
Magnetococcus	1	0	0	0	0	0	0	0	0	0
Chrysiogenetes	1	0	0	0	0	0	0	0	0	0
Firmicutes	104	0	0	0	0	0	0	0	0	0
Tenericutes	19	0	0	0	0	0	0	0	0	0
Actinobacteria	80	0	0	0	0	0	0	0	0	0
Chlamydiae	8	0	0	0	0	0	0	0	0	0
Spirochaetes	14	0	0	0	0	0	0	0	0	0
Acidobacteria	5	0	0	0	0	0	0	0	0	0
Bacteroidetes	35	0	0	0	0	0	0	0	0	0
Fibrobacteres	1	0	0	0	0	0	0	0	0	0
Fusobacteria	5	0	0	0	0	0	0	0	0	0
Verrucomicrobia	4	0	0	0	0	0	0	0	0	0
Gemmatimonadetes	1	0	0	0	0	0	0	0	0	0
Planctomycetes	4	0	0	0	0	0	0	0	0	0
Elusimicrobia	2	0	0	0	0	0	0	0	0	0
Synergistetes	2	0	0	0	0	0	0	0	0	0
Cyanobacteria	16	0	0	0	0	0	0	0	0	0
Chlorobi	10	0	0	0	0	0	0	0	0	0
Chloroflexi	11	0	0	0	0	0	0	0	0	0
Deinococcus-Thermus	7	0	0	0	0	0	0	0	0	0
Aquificae	9	0	0	0	0	0	0	0	0	0
Thermotogae	11	0	0	0	0	0	0	0	0	0
Dictyoglomi	2	0	0	0	0	0	0	0	0	0
Nitrospirae	2	0	0	0	0	0	0	0	0	0
Thermobaculum	1	0	0	0	0	0	0	0	0	0
Deferribacteres	3	0	0	0	0	0	0	0	0	0
Euryarchaeota	60	0	0	0	0	0	0	0	0	0
Crenarchaeota	23	0	0	0	0	0	0	0	0	0
Thaumarchaeota	2	0	0	0	0	0	0	0	0	0
Nanoarchaeota	1	0	0	0	0	0	0	0	0	0
Korarchaeota	1	0	0	0	0	0	0	0	0	0
Total	768	0	0	0	0	0	0	0	0	0

(mod_M00295_1)

Phyla	Module completion ratio (%)									
	0--10	10--20	20--30	30--40	40--50	50--60	60--70	70--80	80--90	90--100
Gammaproteobacteria	126	0	0	0	0	0	0	0	0	0
Betaproteobacteria	61	0	0	0	0	0	0	0	0	0
Epsilonproteobacteria	17	0	0	0	0	0	0	0	0	0
Deltaproteobacteria	28	0	0	0	0	0	0	0	0	0
Alphaproteobacteria	91	0	0	0	0	0	0	0	0	0
Magnetococcus	1	0	0	0	0	0	0	0	0	0
Chrysiogenetes	1	0	0	0	0	0	0	0	0	0
Firmicutes	104	0	0	0	0	0	0	0	0	0
Tenericutes	19	0	0	0	0	0	0	0	0	0
Actinobacteria	80	0	0	0	0	0	0	0	0	0
Chlamydiae	8	0	0	0	0	0	0	0	0	0
Spirochaetes	14	0	0	0	0	0	0	0	0	0
Acidobacteria	5	0	0	0	0	0	0	0	0	0
Bacteroidetes	35	0	0	0	0	0	0	0	0	0
Fibrobacteres	1	0	0	0	0	0	0	0	0	0
Fusobacteria	5	0	0	0	0	0	0	0	0	0
Verrucomicrobia	4	0	0	0	0	0	0	0	0	0
Gemmatimonadetes	1	0	0	0	0	0	0	0	0	0
Planctomycetes	4	0	0	0	0	0	0	0	0	0
Elusimicrobia	2	0	0	0	0	0	0	0	0	0
Synergistetes	2	0	0	0	0	0	0	0	0	0
Cyanobacteria	16	0	0	0	0	0	0	0	0	0
Chlorobi	10	0	0	0	0	0	0	0	0	0
Chloroflexi	11	0	0	0	0	0	0	0	0	0
Deinococcus-Thermus	7	0	0	0	0	0	0	0	0	0
Aquificae	9	0	0	0	0	0	0	0	0	0
Thermotogae	11	0	0	0	0	0	0	0	0	0
Dictyoglomi	2	0	0	0	0	0	0	0	0	0
Nitrospirae	2	0	0	0	0	0	0	0	0	0
Thermobaculum	1	0	0	0	0	0	0	0	0	0
Deferribacteres	3	0	0	0	0	0	0	0	0	0
Euryarchaeota	60	0	0	0	0	0	0	0	0	0
Crenarchaeota	23	0	0	0	0	0	0	0	0	0
Thaumarchaeota	2	0	0	0	0	0	0	0	0	0
Nanoarchaeota	1	0	0	0	0	0	0	0	0	0
Korarchaeota	1	0	0	0	0	0	0	0	0	0
Total	768	0	0	0	0	0	0	0	0	0

(mod_M00296_1)

Phyla	Module completion ratio (%)									
	0--10	10--20	20--30	30--40	40--50	50--60	60--70	70--80	80--90	90--100
Gammaproteobacteria	126	0	0	0	0	0	0	0	0	0
Betaproteobacteria	61	0	0	0	0	0	0	0	0	0
Epsilonproteobacteria	17	0	0	0	0	0	0	0	0	0
Deltaproteobacteria	28	0	0	0	0	0	0	0	0	0
Alphaproteobacteria	91	0	0	0	0	0	0	0	0	0
Magnetococcus	1	0	0	0	0	0	0	0	0	0
Chrysiogenetes	1	0	0	0	0	0	0	0	0	0
Firmicutes	103	0	1	0	0	0	0	0	0	0
Tenericutes	19	0	0	0	0	0	0	0	0	0
Actinobacteria	80	0	0	0	0	0	0	0	0	0
Chlamydiae	8	0	0	0	0	0	0	0	0	0
Spirochaetes	14	0	0	0	0	0	0	0	0	0
Acidobacteria	5	0	0	0	0	0	0	0	0	0
Bacteroidetes	34	0	1	0	0	0	0	0	0	0
Fibrobacteres	1	0	0	0	0	0	0	0	0	0
Fusobacteria	5	0	0	0	0	0	0	0	0	0
Verrucomicrobia	4	0	0	0	0	0	0	0	0	0
Gemmatimonadetes	1	0	0	0	0	0	0	0	0	0
Planctomycetes	4	0	0	0	0	0	0	0	0	0
Elusimicrobia	2	0	0	0	0	0	0	0	0	0
Synergistetes	2	0	0	0	0	0	0	0	0	0
Cyanobacteria	16	0	0	0	0	0	0	0	0	0
Chlorobi	10	0	0	0	0	0	0	0	0	0
Chloroflexi	10	0	1	0	0	0	0	0	0	0
Deinococcus-Thermus	7	0	0	0	0	0	0	0	0	0
Aquificae	9	0	0	0	0	0	0	0	0	0
Thermotogae	11	0	0	0	0	0	0	0	0	0
Dictyoglomi	2	0	0	0	0	0	0	0	0	0
Nitrospirae	2	0	0	0	0	0	0	0	0	0
Thermobaculum	1	0	0	0	0	0	0	0	0	0
Deferribacteres	3	0	0	0	0	0	0	0	0	0
Euryarchaeota	60	0	0	0	0	0	0	0	0	0
Crenarchaeota	23	0	0	0	0	0	0	0	0	0
Thaumarchaeota	2	0	0	0	0	0	0	0	0	0
Nanoarchaeota	1	0	0	0	0	0	0	0	0	0
Korarchaeota	1	0	0	0	0	0	0	0	0	0
Total	765	0	3	0	0	0	0	0	0	0

(mod_M00297_1)

Phyla	Module completion ratio (%)									
	0--10	10--20	20--30	30--40	40--50	50--60	60--70	70--80	80--90	90--100
Gammaproteobacteria	126	0	0	0	0	0	0	0	0	0
Betaproteobacteria	61	0	0	0	0	0	0	0	0	0
Epsilonproteobacteria	17	0	0	0	0	0	0	0	0	0
Deltaproteobacteria	28	0	0	0	0	0	0	0	0	0
Alphaproteobacteria	91	0	0	0	0	0	0	0	0	0
Magnetococcus	1	0	0	0	0	0	0	0	0	0
Chrysiogenetes	1	0	0	0	0	0	0	0	0	0
Firmicutes	104	0	0	0	0	0	0	0	0	0
Tenericutes	19	0	0	0	0	0	0	0	0	0
Actinobacteria	80	0	0	0	0	0	0	0	0	0
Chlamydiae	8	0	0	0	0	0	0	0	0	0
Spirochaetes	14	0	0	0	0	0	0	0	0	0
Acidobacteria	5	0	0	0	0	0	0	0	0	0
Bacteroidetes	35	0	0	0	0	0	0	0	0	0
Fibrobacteres	1	0	0	0	0	0	0	0	0	0
Fusobacteria	5	0	0	0	0	0	0	0	0	0
Verrucomicrobia	4	0	0	0	0	0	0	0	0	0
Gemmatimonadetes	1	0	0	0	0	0	0	0	0	0
Planctomycetes	4	0	0	0	0	0	0	0	0	0
Elusimicrobia	2	0	0	0	0	0	0	0	0	0
Synergistetes	2	0	0	0	0	0	0	0	0	0
Cyanobacteria	16	0	0	0	0	0	0	0	0	0
Chlorobi	10	0	0	0	0	0	0	0	0	0
Chloroflexi	11	0	0	0	0	0	0	0	0	0
Deinococcus-Thermus	7	0	0	0	0	0	0	0	0	0
Aquificae	9	0	0	0	0	0	0	0	0	0
Thermotogae	11	0	0	0	0	0	0	0	0	0
Dictyoglomi	2	0	0	0	0	0	0	0	0	0
Nitrospirae	2	0	0	0	0	0	0	0	0	0
Thermobaculum	1	0	0	0	0	0	0	0	0	0
Deferribacteres	3	0	0	0	0	0	0	0	0	0
Euryarchaeota	60	0	0	0	0	0	0	0	0	0
Crenarchaeota	23	0	0	0	0	0	0	0	0	0
Thaumarchaeota	2	0	0	0	0	0	0	0	0	0
Nanoarchaeota	1	0	0	0	0	0	0	0	0	0
Korarchaeota	1	0	0	0	0	0	0	0	0	0
Total	768	0	0	0	0	0	0	0	0	0

(mod_M00298_1)

Phyla	Module completion ratio (%)									
	0--10	10--20	20--30	30--40	40--50	50--60	60--70	70--80	80--90	90--100
Gammaproteobacteria	126	0	0	0	0	0	0	0	0	0
Betaproteobacteria	61	0	0	0	0	0	0	0	0	0
Epsilonproteobacteria	17	0	0	0	0	0	0	0	0	0
Deltaproteobacteria	28	0	0	0	0	0	0	0	0	0
Alphaproteobacteria	91	0	0	0	0	0	0	0	0	0
Magnetococcus	1	0	0	0	0	0	0	0	0	0
Chrysiogenetes	1	0	0	0	0	0	0	0	0	0
Firmicutes	102	0	0	0	0	0	0	0	0	2
Tenericutes	19	0	0	0	0	0	0	0	0	0
Actinobacteria	80	0	0	0	0	0	0	0	0	0
Chlamydiae	8	0	0	0	0	0	0	0	0	0
Spirochaetes	14	0	0	0	0	0	0	0	0	0
Acidobacteria	5	0	0	0	0	0	0	0	0	0
Bacteroidetes	35	0	0	0	0	0	0	0	0	0
Fibrobacteres	1	0	0	0	0	0	0	0	0	0
Fusobacteria	5	0	0	0	0	0	0	0	0	0
Verrucomicrobia	4	0	0	0	0	0	0	0	0	0
Gemmatimonadetes	1	0	0	0	0	0	0	0	0	0
Planctomycetes	4	0	0	0	0	0	0	0	0	0
Elusimicrobia	2	0	0	0	0	0	0	0	0	0
Synergistetes	2	0	0	0	0	0	0	0	0	0
Cyanobacteria	16	0	0	0	0	0	0	0	0	0
Chlorobi	10	0	0	0	0	0	0	0	0	0
Chloroflexi	11	0	0	0	0	0	0	0	0	0
Deinococcus-Thermus	7	0	0	0	0	0	0	0	0	0
Aquificae	9	0	0	0	0	0	0	0	0	0
Thermotogae	11	0	0	0	0	0	0	0	0	0
Dictyoglomi	2	0	0	0	0	0	0	0	0	0
Nitrospirae	2	0	0	0	0	0	0	0	0	0
Thermobaculum	1	0	0	0	0	0	0	0	0	0
Deferribacteres	3	0	0	0	0	0	0	0	0	0
Euryarchaeota	60	0	0	0	0	0	0	0	0	0
Crenarchaeota	23	0	0	0	0	0	0	0	0	0
Thaumarchaeota	2	0	0	0	0	0	0	0	0	0
Nanoarchaeota	1	0	0	0	0	0	0	0	0	0
Korarchaeota	1	0	0	0	0	0	0	0	0	0
Total	766	0	0	0	0	0	0	0	0	2

(mod_M00299_1)

Phyla	Module completion ratio (%)									
	0--10	10--20	20--30	30--40	40--50	50--60	60--70	70--80	80--90	90--100
Gammaproteobacteria	33	0	45	0	0	1	0	0	0	47
Betaproteobacteria	22	0	29	0	0	0	0	0	0	10
Epsilonproteobacteria	17	0	0	0	0	0	0	0	0	0
Deltaproteobacteria	19	0	1	0	0	0	0	0	0	8
Alphaproteobacteria	47	0	31	0	0	2	0	2	0	9
Magnetococcus	1	0	0	0	0	0	0	0	0	0
Chrysiogenetes	1	0	0	0	0	0	0	0	0	0
Firmicutes	40	0	0	0	0	0	0	0	0	64
Tenericutes	0	0	0	0	0	0	0	3	0	16
Actinobacteria	57	0	1	0	0	1	0	1	0	20
Chlamydiae	6	0	0	0	0	0	0	0	0	2
Spirochaetes	4	0	0	0	0	0	0	0	0	10
Acidobacteria	4	0	0	0	0	0	0	0	0	1
Bacteroidetes	29	0	2	0	0	0	0	0	0	4
Fibrobacteres	0	0	1	0	0	0	0	0	0	0
Fusobacteria	2	0	0	0	0	0	0	1	0	2
Verrucomicrobia	2	0	1	0	0	0	0	0	0	1
Gemmatimonadetes	1	0	0	0	0	0	0	0	0	0
Planctomycetes	3	0	0	0	0	0	0	1	0	0
Elusimicrobia	2	0	0	0	0	0	0	0	0	0
Synergistetes	2	0	0	0	0	0	0	0	0	0
Cyanobacteria	9	0	0	0	0	0	0	2	0	5
Chlorobi	10	0	0	0	0	0	0	0	0	0
Chloroflexi	3	0	0	0	0	1	0	0	0	7
Deinococcus-Thermus	2	0	0	0	0	0	0	0	0	5
Aquificae	9	0	0	0	0	0	0	0	0	0
Thermotogae	3	0	0	0	0	0	0	0	0	8
Dictyoglomi	2	0	0	0	0	0	0	0	0	0
Nitrospirae	1	0	0	0	0	0	0	0	0	1
Thermobaculum	1	0	0	0	0	0	0	0	0	0
Deferribacteres	3	0	0	0	0	0	0	0	0	0
Euryarchaeota	51	0	2	0	0	1	0	0	0	6
Crenarchaeota	16	0	0	0	0	0	0	0	0	7
Thaumarchaeota	2	0	0	0	0	0	0	0	0	0
Nanoarchaeota	1	0	0	0	0	0	0	0	0	0
Korarchaeota	1	0	0	0	0	0	0	0	0	0
Total	406	0	113	0	0	6	0	10	0	233

(mod_M00300_1)

Phyla	Module completion ratio (%)									
	0--10	10--20	20--30	30--40	40--50	50--60	60--70	70--80	80--90	90--100
Gammaproteobacteria	63	0	0	0	0	1	0	1	0	61
Betaproteobacteria	31	0	0	0	0	0	0	1	0	29
Epsilonproteobacteria	17	0	0	0	0	0	0	0	0	0
Deltaproteobacteria	28	0	0	0	0	0	0	0	0	0
Alphaproteobacteria	52	0	3	0	0	0	0	3	0	33
Magnetococcus	1	0	0	0	0	0	0	0	0	0
Chrysiogenetes	1	0	0	0	0	0	0	0	0	0
Firmicutes	104	0	0	0	0	0	0	0	0	0
Tenericutes	19	0	0	0	0	0	0	0	0	0
Actinobacteria	78	0	1	0	0	1	0	0	0	0
Chlamydiae	8	0	0	0	0	0	0	0	0	0
Spirochaetes	14	0	0	0	0	0	0	0	0	0
Acidobacteria	5	0	0	0	0	0	0	0	0	0
Bacteroidetes	35	0	0	0	0	0	0	0	0	0
Fibrobacteres	1	0	0	0	0	0	0	0	0	0
Fusobacteria	5	0	0	0	0	0	0	0	0	0
Verrucomicrobia	4	0	0	0	0	0	0	0	0	0
Gemmatimonadetes	1	0	0	0	0	0	0	0	0	0
Planctomycetes	4	0	0	0	0	0	0	0	0	0
Elusimicrobia	2	0	0	0	0	0	0	0	0	0
Synergistetes	2	0	0	0	0	0	0	0	0	0
Cyanobacteria	16	0	0	0	0	0	0	0	0	0
Chlorobi	10	0	0	0	0	0	0	0	0	0
Chloroflexi	11	0	0	0	0	0	0	0	0	0
Deinococcus-Thermus	7	0	0	0	0	0	0	0	0	0
Aquificae	9	0	0	0	0	0	0	0	0	0
Thermotogae	11	0	0	0	0	0	0	0	0	0
Dictyoglomi	2	0	0	0	0	0	0	0	0	0
Nitrospirae	2	0	0	0	0	0	0	0	0	0
Thermobaculum	1	0	0	0	0	0	0	0	0	0
Deferribacteres	3	0	0	0	0	0	0	0	0	0
Euryarchaeota	60	0	0	0	0	0	0	0	0	0
Crenarchaeota	23	0	0	0	0	0	0	0	0	0
Thaumarchaeota	2	0	0	0	0	0	0	0	0	0
Nanoarchaeota	1	0	0	0	0	0	0	0	0	0
Korarchaeota	1	0	0	0	0	0	0	0	0	0
Total	634	0	4	0	0	2	0	5	0	123

(mod_M00301_1)

Phyla	Module completion ratio (%)									
	0--10	10--20	20--30	30--40	40--50	50--60	60--70	70--80	80--90	90--100
Gammaproteobacteria	126	0	0	0	0	0	0	0	0	0
Betaproteobacteria	61	0	0	0	0	0	0	0	0	0
Epsilonproteobacteria	17	0	0	0	0	0	0	0	0	0
Deltaproteobacteria	28	0	0	0	0	0	0	0	0	0
Alphaproteobacteria	85	0	0	0	0	1	0	0	0	5
Magnetococcus	1	0	0	0	0	0	0	0	0	0
Chrysiogenetes	1	0	0	0	0	0	0	0	0	0
Firmicutes	104	0	0	0	0	0	0	0	0	0
Tenericutes	19	0	0	0	0	0	0	0	0	0
Actinobacteria	80	0	0	0	0	0	0	0	0	0
Chlamydiae	8	0	0	0	0	0	0	0	0	0
Spirochaetes	14	0	0	0	0	0	0	0	0	0
Acidobacteria	5	0	0	0	0	0	0	0	0	0
Bacteroidetes	35	0	0	0	0	0	0	0	0	0
Fibrobacteres	1	0	0	0	0	0	0	0	0	0
Fusobacteria	5	0	0	0	0	0	0	0	0	0
Verrucomicrobia	4	0	0	0	0	0	0	0	0	0
Gemmatimonadetes	1	0	0	0	0	0	0	0	0	0
Planctomycetes	4	0	0	0	0	0	0	0	0	0
Elusimicrobia	2	0	0	0	0	0	0	0	0	0
Synergistetes	2	0	0	0	0	0	0	0	0	0
Cyanobacteria	16	0	0	0	0	0	0	0	0	0
Chlorobi	10	0	0	0	0	0	0	0	0	0
Chloroflexi	11	0	0	0	0	0	0	0	0	0
Deinococcus-Thermus	7	0	0	0	0	0	0	0	0	0
Aquificae	9	0	0	0	0	0	0	0	0	0
Thermotogae	11	0	0	0	0	0	0	0	0	0
Dictyoglomi	2	0	0	0	0	0	0	0	0	0
Nitrospirae	2	0	0	0	0	0	0	0	0	0
Thermobaculum	1	0	0	0	0	0	0	0	0	0
Deferribacteres	3	0	0	0	0	0	0	0	0	0
Euryarchaeota	60	0	0	0	0	0	0	0	0	0
Crenarchaeota	23	0	0	0	0	0	0	0	0	0
Thaumarchaeota	2	0	0	0	0	0	0	0	0	0
Nanoarchaeota	1	0	0	0	0	0	0	0	0	0
Korarchaeota	1	0	0	0	0	0	0	0	0	0
Total	762	0	0	0	0	1	0	0	0	5

(mod_M00302_1)

Phyla	Module completion ratio (%)									
	0--10	10--20	20--30	30--40	40--50	50--60	60--70	70--80	80--90	90--100
Gammaproteobacteria	123	0	0	0	0	0	0	0	0	3
Betaproteobacteria	49	0	0	0	0	0	0	0	0	12
Epsilonproteobacteria	17	0	0	0	0	0	0	0	0	0
Deltaproteobacteria	28	0	0	0	0	0	0	0	0	0
Alphaproteobacteria	91	0	0	0	0	0	0	0	0	0
Magnetococcus	1	0	0	0	0	0	0	0	0	0
Chrysiogenetes	1	0	0	0	0	0	0	0	0	0
Firmicutes	104	0	0	0	0	0	0	0	0	0
Tenericutes	19	0	0	0	0	0	0	0	0	0
Actinobacteria	71	0	0	0	0	0	0	1	0	8
Chlamydiae	8	0	0	0	0	0	0	0	0	0
Spirochaetes	14	0	0	0	0	0	0	0	0	0
Acidobacteria	5	0	0	0	0	0	0	0	0	0
Bacteroidetes	35	0	0	0	0	0	0	0	0	0
Fibrobacteres	1	0	0	0	0	0	0	0	0	0
Fusobacteria	5	0	0	0	0	0	0	0	0	0
Verrucomicrobia	4	0	0	0	0	0	0	0	0	0
Gemmatimonadetes	1	0	0	0	0	0	0	0	0	0
Planctomycetes	4	0	0	0	0	0	0	0	0	0
Elusimicrobia	2	0	0	0	0	0	0	0	0	0
Synergistetes	2	0	0	0	0	0	0	0	0	0
Cyanobacteria	16	0	0	0	0	0	0	0	0	0
Chlorobi	10	0	0	0	0	0	0	0	0	0
Chloroflexi	11	0	0	0	0	0	0	0	0	0
Deinococcus-Thermus	7	0	0	0	0	0	0	0	0	0
Aquificae	9	0	0	0	0	0	0	0	0	0
Thermotogae	11	0	0	0	0	0	0	0	0	0
Dictyoglomi	2	0	0	0	0	0	0	0	0	0
Nitrospirae	2	0	0	0	0	0	0	0	0	0
Thermobaculum	1	0	0	0	0	0	0	0	0	0
Deferribacteres	3	0	0	0	0	0	0	0	0	0
Euryarchaeota	60	0	0	0	0	0	0	0	0	0
Crenarchaeota	23	0	0	0	0	0	0	0	0	0
Thaumarchaeota	2	0	0	0	0	0	0	0	0	0
Nanoarchaeota	1	0	0	0	0	0	0	0	0	0
Korarchaeota	1	0	0	0	0	0	0	0	0	0
Total	744	0	0	0	0	0	0	1	0	23

(mod_M00303_1)

Phyla	Module completion ratio (%)									
	0--10	10--20	20--30	30--40	40--50	50--60	60--70	70--80	80--90	90--100
Gammaproteobacteria	59	0	0	48	0	0	0	0	0	19
Betaproteobacteria	61	0	0	0	0	0	0	0	0	0
Epsilonproteobacteria	16	0	0	1	0	0	0	0	0	0
Deltaproteobacteria	28	0	0	0	0	0	0	0	0	0
Alphaproteobacteria	91	0	0	0	0	0	0	0	0	0
Magnetococcus	1	0	0	0	0	0	0	0	0	0
Chrysiogenetes	1	0	0	0	0	0	0	0	0	0
Firmicutes	39	0	0	65	0	0	0	0	0	0
Tenericutes	8	0	0	11	0	0	0	0	0	0
Actinobacteria	58	0	0	22	0	0	0	0	0	0
Chlamydiae	8	0	0	0	0	0	0	0	0	0
Spirochaetes	7	0	0	7	0	0	0	0	0	0
Acidobacteria	5	0	0	0	0	0	0	0	0	0
Bacteroidetes	35	0	0	0	0	0	0	0	0	0
Fibrobacteres	1	0	0	0	0	0	0	0	0	0
Fusobacteria	0	0	0	5	0	0	0	0	0	0
Verrucomicrobia	4	0	0	0	0	0	0	0	0	0
Gemmatimonadetes	1	0	0	0	0	0	0	0	0	0
Planctomycetes	4	0	0	0	0	0	0	0	0	0
Elusimicrobia	2	0	0	0	0	0	0	0	0	0
Synergistetes	1	0	0	1	0	0	0	0	0	0
Cyanobacteria	16	0	0	0	0	0	0	0	0	0
Chlorobi	10	0	0	0	0	0	0	0	0	0
Chloroflexi	11	0	0	0	0	0	0	0	0	0
Deinococcus-Thermus	7	0	0	0	0	0	0	0	0	0
Aquificae	9	0	0	0	0	0	0	0	0	0
Thermotogae	11	0	0	0	0	0	0	0	0	0
Dictyoglomi	2	0	0	0	0	0	0	0	0	0
Nitrospirae	2	0	0	0	0	0	0	0	0	0
Thermobaculum	1	0	0	0	0	0	0	0	0	0
Deferribacteres	3	0	0	0	0	0	0	0	0	0
Euryarchaeota	60	0	0	0	0	0	0	0	0	0
Crenarchaeota	23	0	0	0	0	0	0	0	0	0
Thaumarchaeota	2	0	0	0	0	0	0	0	0	0
Nanoarchaeota	1	0	0	0	0	0	0	0	0	0
Korarchaeota	1	0	0	0	0	0	0	0	0	0
Total	589	0	0	160	0	0	0	0	0	19

(mod_M00304_1)

Phyla	Module completion ratio (%)									
	0--10	10--20	20--30	30--40	40--50	50--60	60--70	70--80	80--90	90--100
Gammaproteobacteria	124	0	2	0	0	0	0	0	0	0
Betaproteobacteria	61	0	0	0	0	0	0	0	0	0
Epsilonproteobacteria	17	0	0	0	0	0	0	0	0	0
Deltaproteobacteria	28	0	0	0	0	0	0	0	0	0
Alphaproteobacteria	91	0	0	0	0	0	0	0	0	0
Magnetococcus	1	0	0	0	0	0	0	0	0	0
Chrysiogenetes	1	0	0	0	0	0	0	0	0	0
Firmicutes	100	0	2	0	0	0	0	1	0	1
Tenericutes	19	0	0	0	0	0	0	0	0	0
Actinobacteria	80	0	0	0	0	0	0	0	0	0
Chlamydiae	8	0	0	0	0	0	0	0	0	0
Spirochaetes	14	0	0	0	0	0	0	0	0	0
Acidobacteria	5	0	0	0	0	0	0	0	0	0
Bacteroidetes	35	0	0	0	0	0	0	0	0	0
Fibrobacteres	1	0	0	0	0	0	0	0	0	0
Fusobacteria	5	0	0	0	0	0	0	0	0	0
Verrucomicrobia	4	0	0	0	0	0	0	0	0	0
Gemmatimonadetes	1	0	0	0	0	0	0	0	0	0
Planctomycetes	4	0	0	0	0	0	0	0	0	0
Elusimicrobia	2	0	0	0	0	0	0	0	0	0
Synergistetes	2	0	0	0	0	0	0	0	0	0
Cyanobacteria	16	0	0	0	0	0	0	0	0	0
Chlorobi	10	0	0	0	0	0	0	0	0	0
Chloroflexi	11	0	0	0	0	0	0	0	0	0
Deinococcus-Thermus	7	0	0	0	0	0	0	0	0	0
Aquificae	9	0	0	0	0	0	0	0	0	0
Thermotogae	11	0	0	0	0	0	0	0	0	0
Dictyoglomi	2	0	0	0	0	0	0	0	0	0
Nitrospirae	2	0	0	0	0	0	0	0	0	0
Thermobaculum	1	0	0	0	0	0	0	0	0	0
Deferribacteres	3	0	0	0	0	0	0	0	0	0
Euryarchaeota	60	0	0	0	0	0	0	0	0	0
Crenarchaeota	23	0	0	0	0	0	0	0	0	0
Thaumarchaeota	2	0	0	0	0	0	0	0	0	0
Nanoarchaeota	1	0	0	0	0	0	0	0	0	0
Korarchaeota	1	0	0	0	0	0	0	0	0	0
Total	762	0	4	0	0	0	0	1	0	1

(mod_M00305_1)

Phyla	Module completion ratio (%)									
	0--10	10--20	20--30	30--40	40--50	50--60	60--70	70--80	80--90	90--100
Gammaproteobacteria	117	0	0	0	0	0	0	0	0	9
Betaproteobacteria	61	0	0	0	0	0	0	0	0	0
Epsilonproteobacteria	17	0	0	0	0	0	0	0	0	0
Deltaproteobacteria	28	0	0	0	0	0	0	0	0	0
Alphaproteobacteria	91	0	0	0	0	0	0	0	0	0
Magnetococcus	1	0	0	0	0	0	0	0	0	0
Chrysiogenetes	1	0	0	0	0	0	0	0	0	0
Firmicutes	103	0	0	0	0	0	0	0	0	1
Tenericutes	19	0	0	0	0	0	0	0	0	0
Actinobacteria	80	0	0	0	0	0	0	0	0	0
Chlamydiae	8	0	0	0	0	0	0	0	0	0
Spirochaetes	14	0	0	0	0	0	0	0	0	0
Acidobacteria	5	0	0	0	0	0	0	0	0	0
Bacteroidetes	35	0	0	0	0	0	0	0	0	0
Fibrobacteres	1	0	0	0	0	0	0	0	0	0
Fusobacteria	3	0	0	0	0	0	0	0	0	2
Verrucomicrobia	4	0	0	0	0	0	0	0	0	0
Gemmatimonadetes	1	0	0	0	0	0	0	0	0	0
Planctomycetes	4	0	0	0	0	0	0	0	0	0
Elusimicrobia	2	0	0	0	0	0	0	0	0	0
Synergistetes	2	0	0	0	0	0	0	0	0	0
Cyanobacteria	16	0	0	0	0	0	0	0	0	0
Chlorobi	10	0	0	0	0	0	0	0	0	0
Chloroflexi	11	0	0	0	0	0	0	0	0	0
Deinococcus-Thermus	7	0	0	0	0	0	0	0	0	0
Aquificae	9	0	0	0	0	0	0	0	0	0
Thermotogae	11	0	0	0	0	0	0	0	0	0
Dictyoglomi	2	0	0	0	0	0	0	0	0	0
Nitrospirae	2	0	0	0	0	0	0	0	0	0
Thermobaculum	1	0	0	0	0	0	0	0	0	0
Deferribacteres	3	0	0	0	0	0	0	0	0	0
Euryarchaeota	60	0	0	0	0	0	0	0	0	0
Crenarchaeota	23	0	0	0	0	0	0	0	0	0
Thaumarchaeota	2	0	0	0	0	0	0	0	0	0
Nanoarchaeota	1	0	0	0	0	0	0	0	0	0
Korarchaeota	1	0	0	0	0	0	0	0	0	0
Total	756	0	0	0	0	0	0	0	0	12

(mod_M00306_1)

Phyla	Module completion ratio (%)									
	0--10	10--20	20--30	30--40	40--50	50--60	60--70	70--80	80--90	90--100
Gammaproteobacteria	103	0	0	1	0	0	1	0	0	21
Betaproteobacteria	60	0	0	0	0	0	0	0	0	1
Epsilonproteobacteria	17	0	0	0	0	0	0	0	0	0
Deltaproteobacteria	28	0	0	0	0	0	0	0	0	0
Alphaproteobacteria	91	0	0	0	0	0	0	0	0	0
Magnetococcus	1	0	0	0	0	0	0	0	0	0
Chrysiogenetes	1	0	0	0	0	0	0	0	0	0
Firmicutes	97	0	0	0	0	0	6	0	0	1
Tenericutes	19	0	0	0	0	0	0	0	0	0
Actinobacteria	80	0	0	0	0	0	0	0	0	0
Chlamydiae	8	0	0	0	0	0	0	0	0	0
Spirochaetes	14	0	0	0	0	0	0	0	0	0
Acidobacteria	5	0	0	0	0	0	0	0	0	0
Bacteroidetes	35	0	0	0	0	0	0	0	0	0
Fibrobacteres	1	0	0	0	0	0	0	0	0	0
Fusobacteria	3	0	0	0	0	0	2	0	0	0
Verrucomicrobia	4	0	0	0	0	0	0	0	0	0
Gemmatimonadetes	1	0	0	0	0	0	0	0	0	0
Planctomycetes	4	0	0	0	0	0	0	0	0	0
Elusimicrobia	2	0	0	0	0	0	0	0	0	0
Synergistetes	2	0	0	0	0	0	0	0	0	0
Cyanobacteria	16	0	0	0	0	0	0	0	0	0
Chlorobi	10	0	0	0	0	0	0	0	0	0
Chloroflexi	11	0	0	0	0	0	0	0	0	0
Deinococcus-Thermus	7	0	0	0	0	0	0	0	0	0
Aquificae	9	0	0	0	0	0	0	0	0	0
Thermotogae	11	0	0	0	0	0	0	0	0	0
Dictyoglomi	2	0	0	0	0	0	0	0	0	0
Nitrospirae	2	0	0	0	0	0	0	0	0	0
Thermobaculum	1	0	0	0	0	0	0	0	0	0
Deferribacteres	3	0	0	0	0	0	0	0	0	0
Euryarchaeota	60	0	0	0	0	0	0	0	0	0
Crenarchaeota	23	0	0	0	0	0	0	0	0	0
Thaumarchaeota	2	0	0	0	0	0	0	0	0	0
Nanoarchaeota	1	0	0	0	0	0	0	0	0	0
Korarchaeota	1	0	0	0	0	0	0	0	0	0
Total	735	0	0	1	0	0	9	0	0	23

(mod_M00307_1)

Phyla	Module completion ratio (%)									
	0--10	10--20	20--30	30--40	40--50	50--60	60--70	70--80	80--90	90--100
Gammaproteobacteria	2	0	0	0	0	0	0	0	0	124
Betaproteobacteria	2	0	0	0	0	0	0	0	0	59
Epsilonproteobacteria	6	0	0	0	0	0	0	0	0	11
Deltaproteobacteria	8	0	0	0	0	0	0	0	0	20
Alphaproteobacteria	2	0	0	0	0	0	0	0	0	89
Magnetococcus	1	0	0	0	0	0	0	0	0	0
Chrysiogenetes	1	0	0	0	0	0	0	0	0	0
Firmicutes	31	0	0	0	0	0	0	0	0	73
Tenericutes	3	0	0	0	0	0	0	0	0	16
Actinobacteria	21	0	0	0	0	0	0	0	0	59
Chlamydiae	0	0	0	0	0	0	0	0	0	8
Spirochaetes	10	0	0	0	0	0	0	0	0	4
Acidobacteria	4	0	0	0	0	0	0	0	0	1
Bacteroidetes	9	0	0	0	0	0	0	0	0	26
Fibrobacteres	1	0	0	0	0	0	0	0	0	0
Fusobacteria	1	0	0	0	0	0	0	0	0	4
Verrucomicrobia	0	0	0	0	0	0	0	0	0	4
Gemmatimonadetes	0	0	0	0	0	0	0	0	0	1
Planctomycetes	0	0	0	0	0	0	0	0	0	4
Elusimicrobia	1	0	0	0	0	0	0	0	0	1
Synergistetes	1	0	0	0	0	0	0	0	0	1
Cyanobacteria	0	0	0	0	0	0	0	0	0	16
Chlorobi	10	0	0	0	0	0	0	0	0	0
Chloroflexi	0	0	0	0	0	0	0	0	0	11
Deinococcus-Thermus	1	0	0	0	0	0	0	0	0	6
Aquificae	2	0	0	0	0	0	0	0	0	7
Thermotogae	0	0	0	0	0	0	0	0	0	11
Dictyoglomi	0	0	0	0	0	0	0	0	0	2
Nitrospirae	0	0	0	0	0	0	0	0	0	2
Thermobaculum	0	0	0	0	0	0	0	0	0	1
Deferribacteres	3	0	0	0	0	0	0	0	0	0
Euryarchaeota	7	0	0	0	0	0	0	0	0	53
Crenarchaeota	1	0	0	0	0	0	0	0	0	22
Thaumarchaeota	2	0	0	0	0	0	0	0	0	0
Nanoarchaeota	1	0	0	0	0	0	0	0	0	0
Korarchaeota	0	0	0	0	0	0	0	0	0	1
Total	131	0	0	0	0	0	0	0	0	637

(mod_M00308_1)

Phyla	Module completion ratio (%)									
	0--10	10--20	20--30	30--40	40--50	50--60	60--70	70--80	80--90	90--100
Gammaproteobacteria	32	0	0	36	0	0	58	0	0	0
Betaproteobacteria	20	0	0	18	0	0	23	0	0	0
Epsilonproteobacteria	15	0	0	2	0	0	0	0	0	0
Deltaproteobacteria	26	0	0	1	0	0	1	0	0	0
Alphaproteobacteria	36	0	0	16	0	0	39	0	0	0
Magnetococcus	1	0	0	0	0	0	0	0	0	0
Chrysiogenetes	1	0	0	0	0	0	0	0	0	0
Firmicutes	49	0	0	14	0	0	41	0	0	0
Tenericutes	18	0	0	1	0	0	0	0	0	0
Actinobacteria	36	0	0	7	0	0	37	0	0	0
Chlamydiae	8	0	0	0	0	0	0	0	0	0
Spirochaetes	11	0	0	1	0	0	2	0	0	0
Acidobacteria	1	0	0	1	0	0	3	0	0	0
Bacteroidetes	12	0	0	1	0	0	22	0	0	0
Fibrobacteres	0	0	0	1	0	0	0	0	0	0
Fusobacteria	2	0	0	0	0	0	3	0	0	0
Verrucomicrobia	2	0	0	0	0	0	2	0	0	0
Gemmatimonadetes	1	0	0	0	0	0	0	0	0	0
Planctomycetes	0	0	0	3	0	0	1	0	0	0
Elusimicrobia	1	0	0	0	0	0	1	0	0	0
Synergistetes	1	0	0	0	0	0	1	0	0	0
Cyanobacteria	2	0	0	14	0	0	0	0	0	0
Chlorobi	10	0	0	0	0	0	0	0	0	0
Chloroflexi	7	0	0	2	0	0	2	0	0	0
Deinococcus-Thermus	3	0	0	2	0	0	2	0	0	0
Aquificae	9	0	0	0	0	0	0	0	0	0
Thermotogae	5	0	0	0	0	0	6	0	0	0
Dictyoglomi	0	0	0	0	0	0	2	0	0	0
Nitrospirae	2	0	0	0	0	0	0	0	0	0
Thermobaculum	0	0	0	0	0	0	1	0	0	0
Deferribacteres	3	0	0	0	0	0	0	0	0	0
Euryarchaeota	45	0	0	3	0	0	4	0	0	8
Crenarchaeota	16	0	0	1	0	0	6	0	0	0
Thaumarchaeota	2	0	0	0	0	0	0	0	0	0
Nanoarchaeota	1	0	0	0	0	0	0	0	0	0
Korarchaeota	1	0	0	0	0	0	0	0	0	0
Total	379	0	0	124	0	0	257	0	0	8

(mod_M00309_1)

Phyla	Module completion ratio (%)									
	0--10	10--20	20--30	30--40	40--50	50--60	60--70	70--80	80--90	90--100
Gammaproteobacteria	126	0	0	0	0	0	0	0	0	0
Betaproteobacteria	61	0	0	0	0	0	0	0	0	0
Epsilonproteobacteria	17	0	0	0	0	0	0	0	0	0
Deltaproteobacteria	28	0	0	0	0	0	0	0	0	0
Alphaproteobacteria	91	0	0	0	0	0	0	0	0	0
Magnetococcus	1	0	0	0	0	0	0	0	0	0
Chrysiogenetes	1	0	0	0	0	0	0	0	0	0
Firmicutes	104	0	0	0	0	0	0	0	0	0
Tenericutes	19	0	0	0	0	0	0	0	0	0
Actinobacteria	80	0	0	0	0	0	0	0	0	0
Chlamydiae	8	0	0	0	0	0	0	0	0	0
Spirochaetes	14	0	0	0	0	0	0	0	0	0
Acidobacteria	5	0	0	0	0	0	0	0	0	0
Bacteroidetes	35	0	0	0	0	0	0	0	0	0
Fibrobacteres	1	0	0	0	0	0	0	0	0	0
Fusobacteria	5	0	0	0	0	0	0	0	0	0
Verrucomicrobia	4	0	0	0	0	0	0	0	0	0
Gemmatimonadetes	1	0	0	0	0	0	0	0	0	0
Planctomycetes	4	0	0	0	0	0	0	0	0	0
Elusimicrobia	2	0	0	0	0	0	0	0	0	0
Synergistetes	2	0	0	0	0	0	0	0	0	0
Cyanobacteria	16	0	0	0	0	0	0	0	0	0
Chlorobi	10	0	0	0	0	0	0	0	0	0
Chloroflexi	11	0	0	0	0	0	0	0	0	0
Deinococcus-Thermus	7	0	0	0	0	0	0	0	0	0
Aquificae	9	0	0	0	0	0	0	0	0	0
Thermotogae	11	0	0	0	0	0	0	0	0	0
Dictyoglomi	2	0	0	0	0	0	0	0	0	0
Nitrospirae	2	0	0	0	0	0	0	0	0	0
Thermobaculum	1	0	0	0	0	0	0	0	0	0
Deferribacteres	3	0	0	0	0	0	0	0	0	0
Euryarchaeota	46	0	0	0	0	11	0	0	0	3
Crenarchaeota	16	0	0	0	0	2	0	0	0	5
Thaumarchaeota	2	0	0	0	0	0	0	0	0	0
Nanoarchaeota	1	0	0	0	0	0	0	0	0	0
Korarchaeota	1	0	0	0	0	0	0	0	0	0
Total	747	0	0	0	0	13	0	0	0	8

(mod_M00310_1)

Phyla	Module completion ratio (%)									
	0--10	10--20	20--30	30--40	40--50	50--60	60--70	70--80	80--90	90--100
Gammaproteobacteria	126	0	0	0	0	0	0	0	0	0
Betaproteobacteria	55	0	0	0	0	0	0	4	0	2
Epsilonproteobacteria	7	0	0	0	0	0	0	0	0	10
Deltaproteobacteria	21	0	0	0	0	0	0	2	0	5
Alphaproteobacteria	90	0	0	0	0	0	0	0	0	1
Magnetococcus	1	0	0	0	0	0	0	0	0	0
Chrysiogenetes	0	0	0	0	0	1	0	0	0	0
Firmicutes	88	0	0	0	0	2	0	1	0	13
Tenericutes	19	0	0	0	0	0	0	0	0	0
Actinobacteria	74	0	0	0	0	2	0	4	0	0
Chlamydiae	8	0	0	0	0	0	0	0	0	0
Spirochaetes	13	0	0	0	0	1	0	0	0	0
Acidobacteria	5	0	0	0	0	0	0	0	0	0
Bacteroidetes	34	0	0	0	0	0	0	1	0	0
Fibrobacteres	1	0	0	0	0	0	0	0	0	0
Fusobacteria	4	0	0	0	0	0	0	0	0	1
Verrucomicrobia	4	0	0	0	0	0	0	0	0	0
Gemmatimonadetes	1	0	0	0	0	0	0	0	0	0
Planctomycetes	4	0	0	0	0	0	0	0	0	0
Elusimicrobia	1	0	0	0	0	0	0	0	0	1
Synergistetes	1	0	0	0	0	0	0	0	0	1
Cyanobacteria	16	0	0	0	0	0	0	0	0	0
Chlorobi	10	0	0	0	0	0	0	0	0	0
Chloroflexi	8	0	0	0	0	0	0	0	0	3
Deinococcus-Thermus	7	0	0	0	0	0	0	0	0	0
Aquificae	0	0	0	0	0	0	0	2	0	7
Thermotogae	0	0	0	0	0	0	0	0	0	11
Dictyoglomi	0	0	0	0	0	0	0	0	0	2
Nitrospirae	0	0	0	0	0	0	0	1	0	1
Thermobaculum	1	0	0	0	0	0	0	0	0	0
Deferribacteres	3	0	0	0	0	0	0	0	0	0
Euryarchaeota	13	0	0	0	0	0	0	6	0	41
Crenarchaeota	1	0	0	0	0	0	0	1	0	21
Thaumarchaeota	2	0	0	0	0	0	0	0	0	0
Nanoarchaeota	1	0	0	0	0	0	0	0	0	0
Korarchaeota	0	0	0	0	0	0	0	0	0	1
Total	619	0	0	0	0	6	0	22	0	121

(mod_M00311_1)

Phyla	Module completion ratio (%)									
	0--10	10--20	20--30	30--40	40--50	50--60	60--70	70--80	80--90	90--100
Gammaproteobacteria	119	0	0	0	0	7	0	0	0	0
Betaproteobacteria	56	0	0	0	0	5	0	0	0	0
Epsilonproteobacteria	0	0	0	0	0	0	0	0	0	17
Deltaproteobacteria	4	0	0	0	0	2	0	1	0	21
Alphaproteobacteria	80	0	0	0	0	10	0	1	0	0
Magnetococcus	0	0	0	0	0	1	0	0	0	0
Chrysiogenetes	0	0	0	0	0	1	0	0	0	0
Firmicutes	48	0	1	0	0	25	0	0	0	30
Tenericutes	19	0	0	0	0	0	0	0	0	0
Actinobacteria	37	0	0	0	0	40	0	0	0	3
Chlamydiae	8	0	0	0	0	0	0	0	0	0
Spirochaetes	12	0	0	0	0	0	0	0	0	2
Acidobacteria	2	0	0	0	0	1	0	0	0	2
Bacteroidetes	21	0	0	0	0	5	0	1	0	8
Fibrobacteres	1	0	0	0	0	0	0	0	0	0
Fusobacteria	3	0	0	0	0	1	0	0	0	1
Verrucomicrobia	3	0	0	0	0	1	0	0	0	0
Gemmatimonadetes	0	0	0	0	0	1	0	0	0	0
Planctomycetes	2	0	0	0	0	2	0	0	0	0
Elusimicrobia	0	0	0	0	0	0	0	0	0	2
Synergistetes	0	0	0	0	0	0	0	0	0	2
Cyanobacteria	16	0	0	0	0	0	0	0	0	0
Chlorobi	0	0	0	0	0	10	0	0	0	0
Chloroflexi	4	0	0	0	0	4	0	0	0	3
Deinococcus-Thermus	4	0	0	0	0	3	0	0	0	0
Aquificae	2	0	0	0	0	5	0	0	0	2
Thermotogae	0	0	0	0	0	0	0	0	0	11
Dictyoglomi	0	0	0	0	0	2	0	0	0	0
Nitrospirae	1	0	0	0	0	0	0	0	0	1
Thermobaculum	0	0	0	0	0	1	0	0	0	0
Deferribacteres	0	0	0	0	0	0	0	0	0	3
Euryarchaeota	4	0	0	0	0	21	0	2	0	33
Crenarchaeota	0	0	1	0	0	17	0	0	0	5
Thaumarchaeota	0	0	0	0	0	2	0	0	0	0
Nanoarchaeota	1	0	0	0	0	0	0	0	0	0
Korarchaeota	0	0	0	0	0	0	0	0	0	1
Total	447	0	2	0	0	167	0	5	0	147

(mod_M00312_1)

Phyla	Module completion ratio (%)									
	0--10	10--20	20--30	30--40	40--50	50--60	60--70	70--80	80--90	90--100
Gammaproteobacteria	126	0	0	0	0	0	0	0	0	0
Betaproteobacteria	61	0	0	0	0	0	0	0	0	0
Epsilonproteobacteria	17	0	0	0	0	0	0	0	0	0
Deltaproteobacteria	27	0	0	0	0	1	0	0	0	0
Alphaproteobacteria	91	0	0	0	0	0	0	0	0	0
Magnetococcus	1	0	0	0	0	0	0	0	0	0
Chrysiogenetes	1	0	0	0	0	0	0	0	0	0
Firmicutes	104	0	0	0	0	0	0	0	0	0
Tenericutes	19	0	0	0	0	0	0	0	0	0
Actinobacteria	80	0	0	0	0	0	0	0	0	0
Chlamydiae	8	0	0	0	0	0	0	0	0	0
Spirochaetes	14	0	0	0	0	0	0	0	0	0
Acidobacteria	3	0	0	0	0	2	0	0	0	0
Bacteroidetes	35	0	0	0	0	0	0	0	0	0
Fibrobacteres	1	0	0	0	0	0	0	0	0	0
Fusobacteria	5	0	0	0	0	0	0	0	0	0
Verrucomicrobia	4	0	0	0	0	0	0	0	0	0
Gemmatimonadetes	1	0	0	0	0	0	0	0	0	0
Planctomycetes	4	0	0	0	0	0	0	0	0	0
Elusimicrobia	2	0	0	0	0	0	0	0	0	0
Synergistetes	2	0	0	0	0	0	0	0	0	0
Cyanobacteria	16	0	0	0	0	0	0	0	0	0
Chlorobi	10	0	0	0	0	0	0	0	0	0
Chloroflexi	11	0	0	0	0	0	0	0	0	0
Deinococcus-Thermus	7	0	0	0	0	0	0	0	0	0
Aquificae	9	0	0	0	0	0	0	0	0	0
Thermotogae	11	0	0	0	0	0	0	0	0	0
Dictyoglomi	2	0	0	0	0	0	0	0	0	0
Nitrospirae	2	0	0	0	0	0	0	0	0	0
Thermobaculum	1	0	0	0	0	0	0	0	0	0
Deferribacteres	3	0	0	0	0	0	0	0	0	0
Euryarchaeota	37	0	3	0	0	1	0	14	0	5
Crenarchaeota	23	0	0	0	0	0	0	0	0	0
Thaumarchaeota	2	0	0	0	0	0	0	0	0	0
Nanoarchaeota	1	0	0	0	0	0	0	0	0	0
Korarchaeota	1	0	0	0	0	0	0	0	0	0
Total	742	0	3	0	0	4	0	14	0	5

(mod_M00313_1)

Phyla	Module completion ratio (%)									
	0--10	10--20	20--30	30--40	40--50	50--60	60--70	70--80	80--90	90--100
Gammaproteobacteria	124	0	0	0	0	0	0	0	0	2
Betaproteobacteria	50	0	0	0	0	0	0	0	0	11
Epsilonproteobacteria	15	0	0	0	0	0	0	0	0	2
Deltaproteobacteria	6	0	0	0	0	1	0	0	0	21
Alphaproteobacteria	82	0	0	0	0	0	0	0	0	9
Magnetococcus	1	0	0	0	0	0	0	0	0	0
Chrysiogenetes	0	0	0	0	0	0	0	0	0	1
Firmicutes	76	0	0	0	0	0	0	0	0	28
Tenericutes	19	0	0	0	0	0	0	0	0	0
Actinobacteria	73	0	0	0	0	1	0	0	0	6
Chlamydiae	8	0	0	0	0	0	0	0	0	0
Spirochaetes	13	0	0	0	0	0	0	0	0	1
Acidobacteria	5	0	0	0	0	0	0	0	0	0
Bacteroidetes	28	0	0	0	0	0	0	0	0	7
Fibrobacteres	0	0	0	0	0	0	0	0	0	1
Fusobacteria	5	0	0	0	0	0	0	0	0	0
Verrucomicrobia	3	0	0	0	0	0	0	0	0	1
Gemmatimonadetes	1	0	0	0	0	0	0	0	0	0
Planctomycetes	4	0	0	0	0	0	0	0	0	0
Elusimicrobia	2	0	0	0	0	0	0	0	0	0
Synergistetes	0	0	0	0	0	0	0	0	0	2
Cyanobacteria	16	0	0	0	0	0	0	0	0	0
Chlorobi	5	0	0	0	0	0	0	0	0	5
Chloroflexi	7	0	0	0	0	1	0	0	0	3
Deinococcus-Thermus	7	0	0	0	0	0	0	0	0	0
Aquificae	8	0	0	0	0	0	0	0	0	1
Thermotogae	8	0	0	0	0	0	0	0	0	3
Dictyoglomi	2	0	0	0	0	0	0	0	0	0
Nitrospirae	1	0	0	0	0	0	0	0	0	1
Thermobaculum	1	0	0	0	0	0	0	0	0	0
Deferribacteres	0	0	0	0	0	0	0	0	0	3
Euryarchaeota	22	0	0	0	0	0	0	0	0	38
Crenarchaeota	3	0	0	0	0	0	0	0	0	20
Thaumarchaeota	2	0	0	0	0	0	0	0	0	0
Nanoarchaeota	1	0	0	0	0	0	0	0	0	0
Korarchaeota	0	0	0	0	0	0	0	0	0	1
Total	598	0	0	0	0	3	0	0	0	167

(mod_M00313_2)

Phyla	Module completion ratio (%)									
	0--10	10--20	20--30	30--40	40--50	50--60	60--70	70--80	80--90	90--100
Gammaproteobacteria	114	0	0	0	0	0	0	0	0	12
Betaproteobacteria	20	0	0	0	0	0	0	0	0	41
Epsilonproteobacteria	17	0	0	0	0	0	0	0	0	0
Deltaproteobacteria	28	0	0	0	0	0	0	0	0	0
Alphaproteobacteria	59	0	0	0	0	0	0	0	0	32
Magnetococcus	1	0	0	0	0	0	0	0	0	0
Chrysiogenetes	1	0	0	0	0	0	0	0	0	0
Firmicutes	104	0	0	0	0	0	0	0	0	0
Tenericutes	19	0	0	0	0	0	0	0	0	0
Actinobacteria	67	0	0	0	0	0	0	0	0	13
Chlamydiae	8	0	0	0	0	0	0	0	0	0
Spirochaetes	14	0	0	0	0	0	0	0	0	0
Acidobacteria	5	0	0	0	0	0	0	0	0	0
Bacteroidetes	35	0	0	0	0	0	0	0	0	0
Fibrobacteres	1	0	0	0	0	0	0	0	0	0
Fusobacteria	5	0	0	0	0	0	0	0	0	0
Verrucomicrobia	3	0	0	0	0	0	0	0	0	1
Gemmatimonadetes	1	0	0	0	0	0	0	0	0	0
Planctomycetes	4	0	0	0	0	0	0	0	0	0
Elusimicrobia	2	0	0	0	0	0	0	0	0	0
Synergistetes	2	0	0	0	0	0	0	0	0	0
Cyanobacteria	16	0	0	0	0	0	0	0	0	0
Chlorobi	10	0	0	0	0	0	0	0	0	0
Chloroflexi	11	0	0	0	0	0	0	0	0	0
Deinococcus-Thermus	7	0	0	0	0	0	0	0	0	0
Aquificae	9	0	0	0	0	0	0	0	0	0
Thermotogae	11	0	0	0	0	0	0	0	0	0
Dictyoglomi	2	0	0	0	0	0	0	0	0	0
Nitrospirae	2	0	0	0	0	0	0	0	0	0
Thermobaculum	1	0	0	0	0	0	0	0	0	0
Deferribacteres	3	0	0	0	0	0	0	0	0	0
Euryarchaeota	60	0	0	0	0	0	0	0	0	0
Crenarchaeota	23	0	0	0	0	0	0	0	0	0
Thaumarchaeota	2	0	0	0	0	0	0	0	0	0
Nanoarchaeota	1	0	0	0	0	0	0	0	0	0
Korarchaeota	1	0	0	0	0	0	0	0	0	0
Total	669	0	0	0	0	0	0	0	0	99

(mod_M00314_1)

Phyla	Module completion ratio (%)									
	0--10	10--20	20--30	30--40	40--50	50--60	60--70	70--80	80--90	90--100
Gammaproteobacteria	126	0	0	0	0	0	0	0	0	0
Betaproteobacteria	61	0	0	0	0	0	0	0	0	0
Epsilonproteobacteria	17	0	0	0	0	0	0	0	0	0
Deltaproteobacteria	28	0	0	0	0	0	0	0	0	0
Alphaproteobacteria	91	0	0	0	0	0	0	0	0	0
Magnetococcus	1	0	0	0	0	0	0	0	0	0
Chrysiogenetes	1	0	0	0	0	0	0	0	0	0
Firmicutes	91	0	0	0	0	0	0	0	0	13
Tenericutes	19	0	0	0	0	0	0	0	0	0
Actinobacteria	80	0	0	0	0	0	0	0	0	0
Chlamydiae	8	0	0	0	0	0	0	0	0	0
Spirochaetes	14	0	0	0	0	0	0	0	0	0
Acidobacteria	5	0	0	0	0	0	0	0	0	0
Bacteroidetes	35	0	0	0	0	0	0	0	0	0
Fibrobacteres	1	0	0	0	0	0	0	0	0	0
Fusobacteria	5	0	0	0	0	0	0	0	0	0
Verrucomicrobia	4	0	0	0	0	0	0	0	0	0
Gemmatimonadetes	1	0	0	0	0	0	0	0	0	0
Planctomycetes	4	0	0	0	0	0	0	0	0	0
Elusimicrobia	2	0	0	0	0	0	0	0	0	0
Synergistetes	2	0	0	0	0	0	0	0	0	0
Cyanobacteria	16	0	0	0	0	0	0	0	0	0
Chlorobi	10	0	0	0	0	0	0	0	0	0
Chloroflexi	11	0	0	0	0	0	0	0	0	0
Deinococcus-Thermus	7	0	0	0	0	0	0	0	0	0
Aquificae	9	0	0	0	0	0	0	0	0	0
Thermotogae	11	0	0	0	0	0	0	0	0	0
Dictyoglomi	2	0	0	0	0	0	0	0	0	0
Nitrospirae	2	0	0	0	0	0	0	0	0	0
Thermobaculum	1	0	0	0	0	0	0	0	0	0
Deferribacteres	3	0	0	0	0	0	0	0	0	0
Euryarchaeota	60	0	0	0	0	0	0	0	0	0
Crenarchaeota	23	0	0	0	0	0	0	0	0	0
Thaumarchaeota	2	0	0	0	0	0	0	0	0	0
Nanoarchaeota	1	0	0	0	0	0	0	0	0	0
Korarchaeota	1	0	0	0	0	0	0	0	0	0
Total	755	0	0	0	0	0	0	0	0	13

(mod_M00315_1)

Phyla	Module completion ratio (%)									
	0--10	10--20	20--30	30--40	40--50	50--60	60--70	70--80	80--90	90--100
Gammaproteobacteria	126	0	0	0	0	0	0	0	0	0
Betaproteobacteria	61	0	0	0	0	0	0	0	0	0
Epsilonproteobacteria	17	0	0	0	0	0	0	0	0	0
Deltaproteobacteria	28	0	0	0	0	0	0	0	0	0
Alphaproteobacteria	91	0	0	0	0	0	0	0	0	0
Magnetococcus	1	0	0	0	0	0	0	0	0	0
Chrysiogenetes	1	0	0	0	0	0	0	0	0	0
Firmicutes	100	0	0	0	0	0	0	0	0	4
Tenericutes	19	0	0	0	0	0	0	0	0	0
Actinobacteria	80	0	0	0	0	0	0	0	0	0
Chlamydiae	8	0	0	0	0	0	0	0	0	0
Spirochaetes	14	0	0	0	0	0	0	0	0	0
Acidobacteria	5	0	0	0	0	0	0	0	0	0
Bacteroidetes	35	0	0	0	0	0	0	0	0	0
Fibrobacteres	1	0	0	0	0	0	0	0	0	0
Fusobacteria	5	0	0	0	0	0	0	0	0	0
Verrucomicrobia	4	0	0	0	0	0	0	0	0	0
Gemmatimonadetes	1	0	0	0	0	0	0	0	0	0
Planctomycetes	4	0	0	0	0	0	0	0	0	0
Elusimicrobia	2	0	0	0	0	0	0	0	0	0
Synergistetes	2	0	0	0	0	0	0	0	0	0
Cyanobacteria	16	0	0	0	0	0	0	0	0	0
Chlorobi	10	0	0	0	0	0	0	0	0	0
Chloroflexi	11	0	0	0	0	0	0	0	0	0
Deinococcus-Thermus	7	0	0	0	0	0	0	0	0	0
Aquificae	9	0	0	0	0	0	0	0	0	0
Thermotogae	11	0	0	0	0	0	0	0	0	0
Dictyoglomi	2	0	0	0	0	0	0	0	0	0
Nitrospirae	2	0	0	0	0	0	0	0	0	0
Thermobaculum	1	0	0	0	0	0	0	0	0	0
Deferribacteres	3	0	0	0	0	0	0	0	0	0
Euryarchaeota	60	0	0	0	0	0	0	0	0	0
Crenarchaeota	23	0	0	0	0	0	0	0	0	0
Thaumarchaeota	2	0	0	0	0	0	0	0	0	0
Nanoarchaeota	1	0	0	0	0	0	0	0	0	0
Korarchaeota	1	0	0	0	0	0	0	0	0	0
Total	764	0	0	0	0	0	0	0	0	4

(mod_M00316_1)

Phyla	Module completion ratio (%)									
	0--10	10--20	20--30	30--40	40--50	50--60	60--70	70--80	80--90	90--100
Gammaproteobacteria	126	0	0	0	0	0	0	0	0	0
Betaproteobacteria	61	0	0	0	0	0	0	0	0	0
Epsilonproteobacteria	17	0	0	0	0	0	0	0	0	0
Deltaproteobacteria	28	0	0	0	0	0	0	0	0	0
Alphaproteobacteria	91	0	0	0	0	0	0	0	0	0
Magnetococcus	1	0	0	0	0	0	0	0	0	0
Chrysiogenetes	1	0	0	0	0	0	0	0	0	0
Firmicutes	104	0	0	0	0	0	0	0	0	0
Tenericutes	19	0	0	0	0	0	0	0	0	0
Actinobacteria	80	0	0	0	0	0	0	0	0	0
Chlamydiae	8	0	0	0	0	0	0	0	0	0
Spirochaetes	14	0	0	0	0	0	0	0	0	0
Acidobacteria	5	0	0	0	0	0	0	0	0	0
Bacteroidetes	35	0	0	0	0	0	0	0	0	0
Fibrobacteres	1	0	0	0	0	0	0	0	0	0
Fusobacteria	5	0	0	0	0	0	0	0	0	0
Verrucomicrobia	4	0	0	0	0	0	0	0	0	0
Gemmatimonadetes	1	0	0	0	0	0	0	0	0	0
Planctomycetes	4	0	0	0	0	0	0	0	0	0
Elusimicrobia	2	0	0	0	0	0	0	0	0	0
Synergistetes	2	0	0	0	0	0	0	0	0	0
Cyanobacteria	14	0	0	0	0	0	0	0	0	2
Chlorobi	10	0	0	0	0	0	0	0	0	0
Chloroflexi	11	0	0	0	0	0	0	0	0	0
Deinococcus-Thermus	7	0	0	0	0	0	0	0	0	0
Aquificae	9	0	0	0	0	0	0	0	0	0
Thermotogae	11	0	0	0	0	0	0	0	0	0
Dictyoglomi	2	0	0	0	0	0	0	0	0	0
Nitrospirae	2	0	0	0	0	0	0	0	0	0
Thermobaculum	1	0	0	0	0	0	0	0	0	0
Deferribacteres	3	0	0	0	0	0	0	0	0	0
Euryarchaeota	60	0	0	0	0	0	0	0	0	0
Crenarchaeota	23	0	0	0	0	0	0	0	0	0
Thaumarchaeota	2	0	0	0	0	0	0	0	0	0
Nanoarchaeota	1	0	0	0	0	0	0	0	0	0
Korarchaeota	1	0	0	0	0	0	0	0	0	0
Total	766	0	0	0	0	0	0	0	0	2

(mod_M00317_1)

Phyla	Module completion ratio (%)									
	0--10	10--20	20--30	30--40	40--50	50--60	60--70	70--80	80--90	90--100
Gammaproteobacteria	93	0	0	0	0	0	0	0	0	33
Betaproteobacteria	61	0	0	0	0	0	0	0	0	0
Epsilonproteobacteria	16	0	0	0	0	0	0	0	0	1
Deltaproteobacteria	28	0	0	0	0	0	0	0	0	0
Alphaproteobacteria	74	0	0	0	0	0	0	0	0	17
Magnetococcus	1	0	0	0	0	0	0	0	0	0
Chrysiogenetes	1	0	0	0	0	0	0	0	0	0
Firmicutes	103	0	1	0	0	0	0	0	0	0
Tenericutes	19	0	0	0	0	0	0	0	0	0
Actinobacteria	80	0	0	0	0	0	0	0	0	0
Chlamydiae	8	0	0	0	0	0	0	0	0	0
Spirochaetes	14	0	0	0	0	0	0	0	0	0
Acidobacteria	5	0	0	0	0	0	0	0	0	0
Bacteroidetes	35	0	0	0	0	0	0	0	0	0
Fibrobacteres	1	0	0	0	0	0	0	0	0	0
Fusobacteria	5	0	0	0	0	0	0	0	0	0
Verrucomicrobia	4	0	0	0	0	0	0	0	0	0
Gemmatimonadetes	1	0	0	0	0	0	0	0	0	0
Planctomycetes	4	0	0	0	0	0	0	0	0	0
Elusimicrobia	2	0	0	0	0	0	0	0	0	0
Synergistetes	2	0	0	0	0	0	0	0	0	0
Cyanobacteria	16	0	0	0	0	0	0	0	0	0
Chlorobi	10	0	0	0	0	0	0	0	0	0
Chloroflexi	11	0	0	0	0	0	0	0	0	0
Deinococcus-Thermus	7	0	0	0	0	0	0	0	0	0
Aquificae	9	0	0	0	0	0	0	0	0	0
Thermotogae	11	0	0	0	0	0	0	0	0	0
Dictyoglomi	2	0	0	0	0	0	0	0	0	0
Nitrospirae	2	0	0	0	0	0	0	0	0	0
Thermobaculum	1	0	0	0	0	0	0	0	0	0
Deferribacteres	3	0	0	0	0	0	0	0	0	0
Euryarchaeota	60	0	0	0	0	0	0	0	0	0
Crenarchaeota	23	0	0	0	0	0	0	0	0	0
Thaumarchaeota	2	0	0	0	0	0	0	0	0	0
Nanoarchaeota	1	0	0	0	0	0	0	0	0	0
Korarchaeota	1	0	0	0	0	0	0	0	0	0
Total	716	0	1	0	0	0	0	0	0	51

(mod_M00318_1)

Phyla	Module completion ratio (%)									
	0--10	10--20	20--30	30--40	40--50	50--60	60--70	70--80	80--90	90--100
Gammaproteobacteria	126	0	0	0	0	0	0	0	0	0
Betaproteobacteria	61	0	0	0	0	0	0	0	0	0
Epsilonproteobacteria	17	0	0	0	0	0	0	0	0	0
Deltaproteobacteria	28	0	0	0	0	0	0	0	0	0
Alphaproteobacteria	91	0	0	0	0	0	0	0	0	0
Magnetococcus	1	0	0	0	0	0	0	0	0	0
Chrysiogenetes	1	0	0	0	0	0	0	0	0	0
Firmicutes	101	0	0	0	0	0	0	0	0	3
Tenericutes	19	0	0	0	0	0	0	0	0	0
Actinobacteria	80	0	0	0	0	0	0	0	0	0
Chlamydiae	8	0	0	0	0	0	0	0	0	0
Spirochaetes	14	0	0	0	0	0	0	0	0	0
Acidobacteria	5	0	0	0	0	0	0	0	0	0
Bacteroidetes	35	0	0	0	0	0	0	0	0	0
Fibrobacteres	1	0	0	0	0	0	0	0	0	0
Fusobacteria	5	0	0	0	0	0	0	0	0	0
Verrucomicrobia	4	0	0	0	0	0	0	0	0	0
Gemmatimonadetes	1	0	0	0	0	0	0	0	0	0
Planctomycetes	4	0	0	0	0	0	0	0	0	0
Elusimicrobia	2	0	0	0	0	0	0	0	0	0
Synergistetes	2	0	0	0	0	0	0	0	0	0
Cyanobacteria	16	0	0	0	0	0	0	0	0	0
Chlorobi	10	0	0	0	0	0	0	0	0	0
Chloroflexi	11	0	0	0	0	0	0	0	0	0
Deinococcus-Thermus	7	0	0	0	0	0	0	0	0	0
Aquificae	9	0	0	0	0	0	0	0	0	0
Thermotogae	11	0	0	0	0	0	0	0	0	0
Dictyoglomi	2	0	0	0	0	0	0	0	0	0
Nitrospirae	2	0	0	0	0	0	0	0	0	0
Thermobaculum	1	0	0	0	0	0	0	0	0	0
Deferribacteres	3	0	0	0	0	0	0	0	0	0
Euryarchaeota	60	0	0	0	0	0	0	0	0	0
Crenarchaeota	23	0	0	0	0	0	0	0	0	0
Thaumarchaeota	2	0	0	0	0	0	0	0	0	0
Nanoarchaeota	1	0	0	0	0	0	0	0	0	0
Korarchaeota	1	0	0	0	0	0	0	0	0	0
Total	765	0	0	0	0	0	0	0	0	3

(mod_M00319_1)

Phyla	Module completion ratio (%)									
	0--10	10--20	20--30	30--40	40--50	50--60	60--70	70--80	80--90	90--100
Gammaproteobacteria	124	0	0	0	0	0	0	0	0	2
Betaproteobacteria	60	0	0	0	0	0	0	0	0	1
Epsilonproteobacteria	17	0	0	0	0	0	0	0	0	0
Deltaproteobacteria	27	0	1	0	0	0	0	0	0	0
Alphaproteobacteria	85	0	0	0	0	0	0	0	0	6
Magnetococcus	1	0	0	0	0	0	0	0	0	0
Chrysiogenetes	1	0	0	0	0	0	0	0	0	0
Firmicutes	91	0	1	0	0	0	0	1	0	11
Tenericutes	16	0	0	0	0	0	0	1	0	2
Actinobacteria	75	0	0	0	0	0	0	0	0	5
Chlamydiae	6	0	2	0	0	0	0	0	0	0
Spirochaetes	11	0	1	0	0	0	0	0	0	2
Acidobacteria	5	0	0	0	0	0	0	0	0	0
Bacteroidetes	29	0	0	0	0	0	0	0	0	6
Fibrobacteres	1	0	0	0	0	0	0	0	0	0
Fusobacteria	1	0	0	0	0	0	0	0	0	4
Verrucomicrobia	3	0	0	0	0	0	0	0	0	1
Gemmatimonadetes	1	0	0	0	0	0	0	0	0	0
Planctomycetes	0	0	0	0	0	0	0	0	0	4
Elusimicrobia	2	0	0	0	0	0	0	0	0	0
Synergistetes	2	0	0	0	0	0	0	0	0	0
Cyanobacteria	16	0	0	0	0	0	0	0	0	0
Chlorobi	10	0	0	0	0	0	0	0	0	0
Chloroflexi	4	0	1	0	0	0	0	0	0	6
Deinococcus-Thermus	5	0	0	0	0	0	0	0	0	2
Aquificae	8	0	1	0	0	0	0	0	0	0
Thermotogae	11	0	0	0	0	0	0	0	0	0
Dictyoglomi	2	0	0	0	0	0	0	0	0	0
Nitrospirae	2	0	0	0	0	0	0	0	0	0
Thermobaculum	1	0	0	0	0	0	0	0	0	0
Deferribacteres	3	0	0	0	0	0	0	0	0	0
Euryarchaeota	60	0	0	0	0	0	0	0	0	0
Crenarchaeota	23	0	0	0	0	0	0	0	0	0
Thaumarchaeota	1	0	0	0	0	0	0	0	0	1
Nanoarchaeota	1	0	0	0	0	0	0	0	0	0
Korarchaeota	1	0	0	0	0	0	0	0	0	0
Total	706	0	7	0	0	0	0	2	0	53

(mod_M00320_1)

Phyla	Module completion ratio (%)									
	0--10	10--20	20--30	30--40	40--50	50--60	60--70	70--80	80--90	90--100
Gammaproteobacteria	4	0	0	0	0	0	1	0	0	121
Betaproteobacteria	1	0	0	0	0	0	3	0	0	57
Epsilonproteobacteria	0	0	0	0	0	0	0	0	0	17
Deltaproteobacteria	2	0	0	21	0	0	5	0	0	0
Alphaproteobacteria	11	0	0	0	0	0	6	0	0	74
Magnetococcus	0	0	0	0	0	0	1	0	0	0
Chrysiogenetes	0	0	0	0	0	0	1	0	0	0
Firmicutes	99	0	0	5	0	0	0	0	0	0
Tenericutes	19	0	0	0	0	0	0	0	0	0
Actinobacteria	80	0	0	0	0	0	0	0	0	0
Chlamydiae	0	0	0	0	0	0	0	0	0	8
Spirochaetes	0	0	0	14	0	0	0	0	0	0
Acidobacteria	0	0	0	5	0	0	0	0	0	0
Bacteroidetes	2	0	0	33	0	0	0	0	0	0
Fibrobacteres	0	0	0	1	0	0	0	0	0	0
Fusobacteria	1	0	0	4	0	0	0	0	0	0
Verrucomicrobia	0	0	0	4	0	0	0	0	0	0
Gemmatimonadetes	0	0	0	1	0	0	0	0	0	0
Planctomycetes	0	0	0	4	0	0	0	0	0	0
Elusimicrobia	0	0	0	2	0	0	0	0	0	0
Synergistetes	0	0	0	2	0	0	0	0	0	0
Cyanobacteria	0	0	0	16	0	0	0	0	0	0
Chlorobi	0	0	0	10	0	0	0	0	0	0
Chloroflexi	11	0	0	0	0	0	0	0	0	0
Deinococcus-Thermus	0	0	0	7	0	0	0	0	0	0
Aquificae	0	0	0	6	0	0	3	0	0	0
Thermotogae	0	0	0	11	0	0	0	0	0	0
Dictyoglomi	0	0	0	2	0	0	0	0	0	0
Nitrospirae	0	0	0	1	0	0	1	0	0	0
Thermobaculum	1	0	0	0	0	0	0	0	0	0
Deferribacteres	0	0	0	2	0	0	1	0	0	0
Euryarchaeota	59	0	0	1	0	0	0	0	0	0
Crenarchaeota	23	0	0	0	0	0	0	0	0	0
Thaumarchaeota	2	0	0	0	0	0	0	0	0	0
Nanoarchaeota	1	0	0	0	0	0	0	0	0	0
Korarchaeota	1	0	0	0	0	0	0	0	0	0
Total	317	0	0	152	0	0	22	0	0	277

(mod_M00321_1)

Phyla	Module completion ratio (%)									
	0--10	10--20	20--30	30--40	40--50	50--60	60--70	70--80	80--90	90--100
Gammaproteobacteria	126	0	0	0	0	0	0	0	0	0
Betaproteobacteria	61	0	0	0	0	0	0	0	0	0
Epsilonproteobacteria	17	0	0	0	0	0	0	0	0	0
Deltaproteobacteria	28	0	0	0	0	0	0	0	0	0
Alphaproteobacteria	91	0	0	0	0	0	0	0	0	0
Magnetococcus	1	0	0	0	0	0	0	0	0	0
Chrysiogenetes	1	0	0	0	0	0	0	0	0	0
Firmicutes	104	0	0	0	0	0	0	0	0	0
Tenericutes	19	0	0	0	0	0	0	0	0	0
Actinobacteria	80	0	0	0	0	0	0	0	0	0
Chlamydiae	8	0	0	0	0	0	0	0	0	0
Spirochaetes	14	0	0	0	0	0	0	0	0	0
Acidobacteria	5	0	0	0	0	0	0	0	0	0
Bacteroidetes	35	0	0	0	0	0	0	0	0	0
Fibrobacteres	1	0	0	0	0	0	0	0	0	0
Fusobacteria	5	0	0	0	0	0	0	0	0	0
Verrucomicrobia	4	0	0	0	0	0	0	0	0	0
Gemmatimonadetes	1	0	0	0	0	0	0	0	0	0
Planctomycetes	4	0	0	0	0	0	0	0	0	0
Elusimicrobia	2	0	0	0	0	0	0	0	0	0
Synergistetes	2	0	0	0	0	0	0	0	0	0
Cyanobacteria	6	0	0	0	0	0	0	3	0	7
Chlorobi	10	0	0	0	0	0	0	0	0	0
Chloroflexi	11	0	0	0	0	0	0	0	0	0
Deinococcus-Thermus	7	0	0	0	0	0	0	0	0	0
Aquificae	9	0	0	0	0	0	0	0	0	0
Thermotogae	11	0	0	0	0	0	0	0	0	0
Dictyoglomi	2	0	0	0	0	0	0	0	0	0
Nitrospirae	2	0	0	0	0	0	0	0	0	0
Thermobaculum	1	0	0	0	0	0	0	0	0	0
Deferribacteres	3	0	0	0	0	0	0	0	0	0
Euryarchaeota	60	0	0	0	0	0	0	0	0	0
Crenarchaeota	23	0	0	0	0	0	0	0	0	0
Thaumarchaeota	2	0	0	0	0	0	0	0	0	0
Nanoarchaeota	1	0	0	0	0	0	0	0	0	0
Korarchaeota	1	0	0	0	0	0	0	0	0	0
Total	758	0	0	0	0	0	0	3	0	7

(mod_M00322_1)

Phyla	Module completion ratio (%)									
	0--10	10--20	20--30	30--40	40--50	50--60	60--70	70--80	80--90	90--100
Gammaproteobacteria	126	0	0	0	0	0	0	0	0	0
Betaproteobacteria	61	0	0	0	0	0	0	0	0	0
Epsilonproteobacteria	17	0	0	0	0	0	0	0	0	0
Deltaproteobacteria	28	0	0	0	0	0	0	0	0	0
Alphaproteobacteria	91	0	0	0	0	0	0	0	0	0
Magnetococcus	1	0	0	0	0	0	0	0	0	0
Chrysiogenetes	1	0	0	0	0	0	0	0	0	0
Firmicutes	104	0	0	0	0	0	0	0	0	0
Tenericutes	19	0	0	0	0	0	0	0	0	0
Actinobacteria	79	0	1	0	0	0	0	0	0	0
Chlamydiae	8	0	0	0	0	0	0	0	0	0
Spirochaetes	14	0	0	0	0	0	0	0	0	0
Acidobacteria	5	0	0	0	0	0	0	0	0	0
Bacteroidetes	35	0	0	0	0	0	0	0	0	0
Fibrobacteres	1	0	0	0	0	0	0	0	0	0
Fusobacteria	5	0	0	0	0	0	0	0	0	0
Verrucomicrobia	4	0	0	0	0	0	0	0	0	0
Gemmatimonadetes	1	0	0	0	0	0	0	0	0	0
Planctomycetes	4	0	0	0	0	0	0	0	0	0
Elusimicrobia	2	0	0	0	0	0	0	0	0	0
Synergistetes	2	0	0	0	0	0	0	0	0	0
Cyanobacteria	6	0	0	0	0	0	0	0	0	10
Chlorobi	10	0	0	0	0	0	0	0	0	0
Chloroflexi	11	0	0	0	0	0	0	0	0	0
Deinococcus-Thermus	7	0	0	0	0	0	0	0	0	0
Aquificae	9	0	0	0	0	0	0	0	0	0
Thermotogae	11	0	0	0	0	0	0	0	0	0
Dictyoglomi	2	0	0	0	0	0	0	0	0	0
Nitrospirae	2	0	0	0	0	0	0	0	0	0
Thermobaculum	1	0	0	0	0	0	0	0	0	0
Deferribacteres	3	0	0	0	0	0	0	0	0	0
Euryarchaeota	60	0	0	0	0	0	0	0	0	0
Crenarchaeota	23	0	0	0	0	0	0	0	0	0
Thaumarchaeota	2	0	0	0	0	0	0	0	0	0
Nanoarchaeota	1	0	0	0	0	0	0	0	0	0
Korarchaeota	1	0	0	0	0	0	0	0	0	0
Total	757	0	1	0	0	0	0	0	0	10

(mod_M00323_1)

Phyla	Module completion ratio (%)									
	0--10	10--20	20--30	30--40	40--50	50--60	60--70	70--80	80--90	90--100
Gammaproteobacteria	126	0	0	0	0	0	0	0	0	0
Betaproteobacteria	61	0	0	0	0	0	0	0	0	0
Epsilonproteobacteria	17	0	0	0	0	0	0	0	0	0
Deltaproteobacteria	28	0	0	0	0	0	0	0	0	0
Alphaproteobacteria	91	0	0	0	0	0	0	0	0	0
Magnetococcus	1	0	0	0	0	0	0	0	0	0
Chrysiogenetes	1	0	0	0	0	0	0	0	0	0
Firmicutes	103	0	1	0	0	0	0	0	0	0
Tenericutes	19	0	0	0	0	0	0	0	0	0
Actinobacteria	80	0	0	0	0	0	0	0	0	0
Chlamydiae	8	0	0	0	0	0	0	0	0	0
Spirochaetes	14	0	0	0	0	0	0	0	0	0
Acidobacteria	5	0	0	0	0	0	0	0	0	0
Bacteroidetes	35	0	0	0	0	0	0	0	0	0
Fibrobacteres	1	0	0	0	0	0	0	0	0	0
Fusobacteria	5	0	0	0	0	0	0	0	0	0
Verrucomicrobia	4	0	0	0	0	0	0	0	0	0
Gemmatimonadetes	1	0	0	0	0	0	0	0	0	0
Planctomycetes	4	0	0	0	0	0	0	0	0	0
Elusimicrobia	2	0	0	0	0	0	0	0	0	0
Synergistetes	2	0	0	0	0	0	0	0	0	0
Cyanobacteria	6	0	0	0	0	0	0	0	0	10
Chlorobi	10	0	0	0	0	0	0	0	0	0
Chloroflexi	11	0	0	0	0	0	0	0	0	0
Deinococcus-Thermus	7	0	0	0	0	0	0	0	0	0
Aquificae	9	0	0	0	0	0	0	0	0	0
Thermotogae	11	0	0	0	0	0	0	0	0	0
Dictyoglomi	2	0	0	0	0	0	0	0	0	0
Nitrospirae	2	0	0	0	0	0	0	0	0	0
Thermobaculum	1	0	0	0	0	0	0	0	0	0
Deferribacteres	3	0	0	0	0	0	0	0	0	0
Euryarchaeota	60	0	0	0	0	0	0	0	0	0
Crenarchaeota	23	0	0	0	0	0	0	0	0	0
Thaumarchaeota	2	0	0	0	0	0	0	0	0	0
Nanoarchaeota	1	0	0	0	0	0	0	0	0	0
Korarchaeota	1	0	0	0	0	0	0	0	0	0
Total	757	0	1	0	0	0	0	0	0	10

(mod_M00324_1)

Phyla	Module completion ratio (%)									
	0--10	10--20	20--30	30--40	40--50	50--60	60--70	70--80	80--90	90--100
Gammaproteobacteria	80	0	1	0	0	0	0	0	3	42
Betaproteobacteria	43	0	0	0	0	0	0	0	1	17
Epsilonproteobacteria	15	0	2	0	0	0	0	0	0	0
Deltaproteobacteria	27	0	0	0	0	0	0	0	0	1
Alphaproteobacteria	68	0	2	0	0	1	0	0	7	13
Magnetococcus	1	0	0	0	0	0	0	0	0	0
Chrysiogenetes	1	0	0	0	0	0	0	0	0	0
Firmicutes	99	0	5	0	0	0	0	0	0	0
Tenericutes	19	0	0	0	0	0	0	0	0	0
Actinobacteria	62	0	18	0	0	0	0	0	0	0
Chlamydiae	8	0	0	0	0	0	0	0	0	0
Spirochaetes	14	0	0	0	0	0	0	0	0	0
Acidobacteria	5	0	0	0	0	0	0	0	0	0
Bacteroidetes	35	0	0	0	0	0	0	0	0	0
Fibrobacteres	1	0	0	0	0	0	0	0	0	0
Fusobacteria	5	0	0	0	0	0	0	0	0	0
Verrucomicrobia	4	0	0	0	0	0	0	0	0	0
Gemmatimonadetes	1	0	0	0	0	0	0	0	0	0
Planctomycetes	4	0	0	0	0	0	0	0	0	0
Elusimicrobia	2	0	0	0	0	0	0	0	0	0
Synergistetes	2	0	0	0	0	0	0	0	0	0
Cyanobacteria	16	0	0	0	0	0	0	0	0	0
Chlorobi	10	0	0	0	0	0	0	0	0	0
Chloroflexi	10	0	1	0	0	0	0	0	0	0
Deinococcus-Thermus	7	0	0	0	0	0	0	0	0	0
Aquificae	9	0	0	0	0	0	0	0	0	0
Thermotogae	11	0	0	0	0	0	0	0	0	0
Dictyoglomi	2	0	0	0	0	0	0	0	0	0
Nitrospirae	2	0	0	0	0	0	0	0	0	0
Thermobaculum	1	0	0	0	0	0	0	0	0	0
Deferribacteres	1	0	1	0	0	1	0	0	0	0
Euryarchaeota	54	0	6	0	0	0	0	0	0	0
Crenarchaeota	23	0	0	0	0	0	0	0	0	0
Thaumarchaeota	2	0	0	0	0	0	0	0	0	0
Nanoarchaeota	1	0	0	0	0	0	0	0	0	0
Korarchaeota	1	0	0	0	0	0	0	0	0	0
Total	646	0	36	0	0	2	0	0	11	73

(mod_M00325_1)

Phyla	Module completion ratio (%)									
	0--10	10--20	20--30	30--40	40--50	50--60	60--70	70--80	80--90	90--100
Gammaproteobacteria	25	0	0	92	0	0	4	0	0	5
Betaproteobacteria	4	0	0	45	0	0	3	0	0	9
Epsilonproteobacteria	9	0	0	7	0	0	0	0	0	1
Deltaproteobacteria	13	0	0	14	0	0	0	0	0	1
Alphaproteobacteria	9	0	0	76	0	0	1	0	0	5
Magnetococcus	0	0	0	0	0	0	1	0	0	0
Chrysiogenetes	0	0	0	1	0	0	0	0	0	0
Firmicutes	104	0	0	0	0	0	0	0	0	0
Tenericutes	19	0	0	0	0	0	0	0	0	0
Actinobacteria	80	0	0	0	0	0	0	0	0	0
Chlamydiae	8	0	0	0	0	0	0	0	0	0
Spirochaetes	12	0	0	2	0	0	0	0	0	0
Acidobacteria	2	0	0	3	0	0	0	0	0	0
Bacteroidetes	7	0	0	28	0	0	0	0	0	0
Fibrobacteres	0	0	0	1	0	0	0	0	0	0
Fusobacteria	5	0	0	0	0	0	0	0	0	0
Verrucomicrobia	2	0	0	2	0	0	0	0	0	0
Gemmatimonadetes	0	0	0	1	0	0	0	0	0	0
Planctomycetes	4	0	0	0	0	0	0	0	0	0
Elusimicrobia	1	0	0	1	0	0	0	0	0	0
Synergistetes	0	0	0	2	0	0	0	0	0	0
Cyanobacteria	15	0	0	1	0	0	0	0	0	0
Chlorobi	0	0	0	10	0	0	0	0	0	0
Chloroflexi	11	0	0	0	0	0	0	0	0	0
Deinococcus-Thermus	7	0	0	0	0	0	0	0	0	0
Aquificae	7	0	0	2	0	0	0	0	0	0
Thermotogae	11	0	0	0	0	0	0	0	0	0
Dictyoglomi	0	0	0	2	0	0	0	0	0	0
Nitrospirae	0	0	0	1	0	0	1	0	0	0
Thermobaculum	1	0	0	0	0	0	0	0	0	0
Deferribacteres	3	0	0	0	0	0	0	0	0	0
Euryarchaeota	60	0	0	0	0	0	0	0	0	0
Crenarchaeota	23	0	0	0	0	0	0	0	0	0
Thaumarchaeota	2	0	0	0	0	0	0	0	0	0
Nanoarchaeota	1	0	0	0	0	0	0	0	0	0
Korarchaeota	1	0	0	0	0	0	0	0	0	0
Total	446	0	0	291	0	0	10	0	0	21

(mod_M00326_1)

Phyla	Module completion ratio (%)									
	0--10	10--20	20--30	30--40	40--50	50--60	60--70	70--80	80--90	90--100
Gammaproteobacteria	29	0	85	0	0	0	0	2	0	10
Betaproteobacteria	5	0	56	0	0	0	0	0	0	0
Epsilonproteobacteria	10	0	7	0	0	0	0	0	0	0
Deltaproteobacteria	13	0	15	0	0	0	0	0	0	0
Alphaproteobacteria	9	0	82	0	0	0	0	0	0	0
Magnetococcus	0	0	1	0	0	0	0	0	0	0
Chrysiogenetes	0	0	1	0	0	0	0	0	0	0
Firmicutes	104	0	0	0	0	0	0	0	0	0
Tenericutes	19	0	0	0	0	0	0	0	0	0
Actinobacteria	80	0	0	0	0	0	0	0	0	0
Chlamydiae	8	0	0	0	0	0	0	0	0	0
Spirochaetes	12	0	2	0	0	0	0	0	0	0
Acidobacteria	2	0	3	0	0	0	0	0	0	0
Bacteroidetes	7	0	28	0	0	0	0	0	0	0
Fibrobacteres	0	0	1	0	0	0	0	0	0	0
Fusobacteria	5	0	0	0	0	0	0	0	0	0
Verrucomicrobia	2	0	2	0	0	0	0	0	0	0
Gemmatimonadetes	0	0	1	0	0	0	0	0	0	0
Planctomycetes	4	0	0	0	0	0	0	0	0	0
Elusimicrobia	1	0	1	0	0	0	0	0	0	0
Synergistetes	0	0	2	0	0	0	0	0	0	0
Cyanobacteria	16	0	0	0	0	0	0	0	0	0
Chlorobi	0	0	10	0	0	0	0	0	0	0
Chloroflexi	11	0	0	0	0	0	0	0	0	0
Deinococcus-Thermus	7	0	0	0	0	0	0	0	0	0
Aquificae	7	0	2	0	0	0	0	0	0	0
Thermotogae	11	0	0	0	0	0	0	0	0	0
Dictyoglomi	0	0	2	0	0	0	0	0	0	0
Nitrospirae	1	0	1	0	0	0	0	0	0	0
Thermobaculum	1	0	0	0	0	0	0	0	0	0
Deferribacteres	3	0	0	0	0	0	0	0	0	0
Euryarchaeota	60	0	0	0	0	0	0	0	0	0
Crenarchaeota	23	0	0	0	0	0	0	0	0	0
Thaumarchaeota	2	0	0	0	0	0	0	0	0	0
Nanoarchaeota	1	0	0	0	0	0	0	0	0	0
Korarchaeota	1	0	0	0	0	0	0	0	0	0
Total	454	0	302	0	0	0	0	2	0	10

(mod_M00327_1)

Phyla	Module completion ratio (%)									
	0--10	10--20	20--30	30--40	40--50	50--60	60--70	70--80	80--90	90--100
Gammaproteobacteria	126	0	0	0	0	0	0	0	0	0
Betaproteobacteria	61	0	0	0	0	0	0	0	0	0
Epsilonproteobacteria	17	0	0	0	0	0	0	0	0	0
Deltaproteobacteria	28	0	0	0	0	0	0	0	0	0
Alphaproteobacteria	90	0	0	0	0	0	0	0	0	1
Magnetococcus	1	0	0	0	0	0	0	0	0	0
Chrysiogenetes	1	0	0	0	0	0	0	0	0	0
Firmicutes	104	0	0	0	0	0	0	0	0	0
Tenericutes	19	0	0	0	0	0	0	0	0	0
Actinobacteria	80	0	0	0	0	0	0	0	0	0
Chlamydiae	8	0	0	0	0	0	0	0	0	0
Spirochaetes	14	0	0	0	0	0	0	0	0	0
Acidobacteria	5	0	0	0	0	0	0	0	0	0
Bacteroidetes	35	0	0	0	0	0	0	0	0	0
Fibrobacteres	1	0	0	0	0	0	0	0	0	0
Fusobacteria	5	0	0	0	0	0	0	0	0	0
Verrucomicrobia	4	0	0	0	0	0	0	0	0	0
Gemmatimonadetes	1	0	0	0	0	0	0	0	0	0
Planctomycetes	4	0	0	0	0	0	0	0	0	0
Elusimicrobia	2	0	0	0	0	0	0	0	0	0
Synergistetes	2	0	0	0	0	0	0	0	0	0
Cyanobacteria	16	0	0	0	0	0	0	0	0	0
Chlorobi	10	0	0	0	0	0	0	0	0	0
Chloroflexi	11	0	0	0	0	0	0	0	0	0
Deinococcus-Thermus	7	0	0	0	0	0	0	0	0	0
Aquificae	9	0	0	0	0	0	0	0	0	0
Thermotogae	11	0	0	0	0	0	0	0	0	0
Dictyoglomi	2	0	0	0	0	0	0	0	0	0
Nitrospirae	2	0	0	0	0	0	0	0	0	0
Thermobaculum	1	0	0	0	0	0	0	0	0	0
Deferribacteres	3	0	0	0	0	0	0	0	0	0
Euryarchaeota	60	0	0	0	0	0	0	0	0	0
Crenarchaeota	23	0	0	0	0	0	0	0	0	0
Thaumarchaeota	2	0	0	0	0	0	0	0	0	0
Nanoarchaeota	1	0	0	0	0	0	0	0	0	0
Korarchaeota	1	0	0	0	0	0	0	0	0	0
Total	767	0	0	0	0	0	0	0	0	1

(mod_M00328_1)

Phyla	Module completion ratio (%)									
	0--10	10--20	20--30	30--40	40--50	50--60	60--70	70--80	80--90	90--100
Gammaproteobacteria	108	0	0	5	0	0	3	0	0	10
Betaproteobacteria	56	0	0	1	0	0	0	0	0	4
Epsilonproteobacteria	17	0	0	0	0	0	0	0	0	0
Deltaproteobacteria	28	0	0	0	0	0	0	0	0	0
Alphaproteobacteria	86	0	0	5	0	0	0	0	0	0
Magnetococcus	1	0	0	0	0	0	0	0	0	0
Chrysiogenetes	0	0	0	1	0	0	0	0	0	0
Firmicutes	104	0	0	0	0	0	0	0	0	0
Tenericutes	19	0	0	0	0	0	0	0	0	0
Actinobacteria	80	0	0	0	0	0	0	0	0	0
Chlamydiae	8	0	0	0	0	0	0	0	0	0
Spirochaetes	14	0	0	0	0	0	0	0	0	0
Acidobacteria	5	0	0	0	0	0	0	0	0	0
Bacteroidetes	35	0	0	0	0	0	0	0	0	0
Fibrobacteres	1	0	0	0	0	0	0	0	0	0
Fusobacteria	5	0	0	0	0	0	0	0	0	0
Verrucomicrobia	4	0	0	0	0	0	0	0	0	0
Gemmatimonadetes	1	0	0	0	0	0	0	0	0	0
Planctomycetes	4	0	0	0	0	0	0	0	0	0
Elusimicrobia	2	0	0	0	0	0	0	0	0	0
Synergistetes	2	0	0	0	0	0	0	0	0	0
Cyanobacteria	16	0	0	0	0	0	0	0	0	0
Chlorobi	8	0	0	2	0	0	0	0	0	0
Chloroflexi	11	0	0	0	0	0	0	0	0	0
Deinococcus-Thermus	7	0	0	0	0	0	0	0	0	0
Aquificae	9	0	0	0	0	0	0	0	0	0
Thermotogae	11	0	0	0	0	0	0	0	0	0
Dictyoglomi	2	0	0	0	0	0	0	0	0	0
Nitrospirae	2	0	0	0	0	0	0	0	0	0
Thermobaculum	1	0	0	0	0	0	0	0	0	0
Deferribacteres	3	0	0	0	0	0	0	0	0	0
Euryarchaeota	60	0	0	0	0	0	0	0	0	0
Crenarchaeota	23	0	0	0	0	0	0	0	0	0
Thaumarchaeota	2	0	0	0	0	0	0	0	0	0
Nanoarchaeota	1	0	0	0	0	0	0	0	0	0
Korarchaeota	1	0	0	0	0	0	0	0	0	0
Total	737	0	0	14	0	0	3	0	0	14

(mod_M00329_1)

Phyla	Module completion ratio (%)									
	0--10	10--20	20--30	30--40	40--50	50--60	60--70	70--80	80--90	90--100
Gammaproteobacteria	126	0	0	0	0	0	0	0	0	0
Betaproteobacteria	61	0	0	0	0	0	0	0	0	0
Epsilonproteobacteria	17	0	0	0	0	0	0	0	0	0
Deltaproteobacteria	28	0	0	0	0	0	0	0	0	0
Alphaproteobacteria	87	0	0	0	0	2	0	0	0	2
Magnetococcus	1	0	0	0	0	0	0	0	0	0
Chrysiogenetes	1	0	0	0	0	0	0	0	0	0
Firmicutes	104	0	0	0	0	0	0	0	0	0
Tenericutes	19	0	0	0	0	0	0	0	0	0
Actinobacteria	80	0	0	0	0	0	0	0	0	0
Chlamydiae	8	0	0	0	0	0	0	0	0	0
Spirochaetes	14	0	0	0	0	0	0	0	0	0
Acidobacteria	5	0	0	0	0	0	0	0	0	0
Bacteroidetes	35	0	0	0	0	0	0	0	0	0
Fibrobacteres	1	0	0	0	0	0	0	0	0	0
Fusobacteria	5	0	0	0	0	0	0	0	0	0
Verrucomicrobia	4	0	0	0	0	0	0	0	0	0
Gemmatimonadetes	1	0	0	0	0	0	0	0	0	0
Planctomycetes	4	0	0	0	0	0	0	0	0	0
Elusimicrobia	2	0	0	0	0	0	0	0	0	0
Synergistetes	2	0	0	0	0	0	0	0	0	0
Cyanobacteria	16	0	0	0	0	0	0	0	0	0
Chlorobi	10	0	0	0	0	0	0	0	0	0
Chloroflexi	11	0	0	0	0	0	0	0	0	0
Deinococcus-Thermus	7	0	0	0	0	0	0	0	0	0
Aquificae	9	0	0	0	0	0	0	0	0	0
Thermotogae	11	0	0	0	0	0	0	0	0	0
Dictyoglomi	2	0	0	0	0	0	0	0	0	0
Nitrospirae	2	0	0	0	0	0	0	0	0	0
Thermobaculum	1	0	0	0	0	0	0	0	0	0
Deferribacteres	3	0	0	0	0	0	0	0	0	0
Euryarchaeota	60	0	0	0	0	0	0	0	0	0
Crenarchaeota	23	0	0	0	0	0	0	0	0	0
Thaumarchaeota	2	0	0	0	0	0	0	0	0	0
Nanoarchaeota	1	0	0	0	0	0	0	0	0	0
Korarchaeota	1	0	0	0	0	0	0	0	0	0
Total	764	0	0	0	0	2	0	0	0	2

(mod_M00330_1)

Phyla	Module completion ratio (%)									
	0--10	10--20	20--30	30--40	40--50	50--60	60--70	70--80	80--90	90--100
Gammaproteobacteria	120	0	0	1	0	0	2	0	0	3
Betaproteobacteria	61	0	0	0	0	0	0	0	0	0
Epsilonproteobacteria	17	0	0	0	0	0	0	0	0	0
Deltaproteobacteria	28	0	0	0	0	0	0	0	0	0
Alphaproteobacteria	91	0	0	0	0	0	0	0	0	0
Magnetococcus	1	0	0	0	0	0	0	0	0	0
Chrysiogenetes	1	0	0	0	0	0	0	0	0	0
Firmicutes	104	0	0	0	0	0	0	0	0	0
Tenericutes	19	0	0	0	0	0	0	0	0	0
Actinobacteria	80	0	0	0	0	0	0	0	0	0
Chlamydiae	8	0	0	0	0	0	0	0	0	0
Spirochaetes	14	0	0	0	0	0	0	0	0	0
Acidobacteria	5	0	0	0	0	0	0	0	0	0
Bacteroidetes	35	0	0	0	0	0	0	0	0	0
Fibrobacteres	1	0	0	0	0	0	0	0	0	0
Fusobacteria	5	0	0	0	0	0	0	0	0	0
Verrucomicrobia	4	0	0	0	0	0	0	0	0	0
Gemmatimonadetes	1	0	0	0	0	0	0	0	0	0
Planctomycetes	4	0	0	0	0	0	0	0	0	0
Elusimicrobia	2	0	0	0	0	0	0	0	0	0
Synergistetes	2	0	0	0	0	0	0	0	0	0
Cyanobacteria	16	0	0	0	0	0	0	0	0	0
Chlorobi	10	0	0	0	0	0	0	0	0	0
Chloroflexi	11	0	0	0	0	0	0	0	0	0
Deinococcus-Thermus	7	0	0	0	0	0	0	0	0	0
Aquificae	9	0	0	0	0	0	0	0	0	0
Thermotogae	11	0	0	0	0	0	0	0	0	0
Dictyoglomi	2	0	0	0	0	0	0	0	0	0
Nitrospirae	2	0	0	0	0	0	0	0	0	0
Thermobaculum	1	0	0	0	0	0	0	0	0	0
Deferribacteres	3	0	0	0	0	0	0	0	0	0
Euryarchaeota	60	0	0	0	0	0	0	0	0	0
Crenarchaeota	23	0	0	0	0	0	0	0	0	0
Thaumarchaeota	2	0	0	0	0	0	0	0	0	0
Nanoarchaeota	1	0	0	0	0	0	0	0	0	0
Korarchaeota	1	0	0	0	0	0	0	0	0	0
Total	762	0	0	1	0	0	2	0	0	3

(mod_M00331_1)

Phyla	Module completion ratio (%)									
	0--10	10--20	20--30	30--40	40--50	50--60	60--70	70--80	80--90	90--100
Gammaproteobacteria	42	0	0	0	0	0	1	0	4	79
Betaproteobacteria	11	3	1	7	0	0	1	0	1	37
Epsilonproteobacteria	2	1	3	7	4	0	0	0	0	0
Deltaproteobacteria	5	3	0	0	0	0	0	3	1	16
Alphaproteobacteria	62	3	0	0	1	0	0	0	4	21
Magnetococcus	0	0	0	1	0	0	0	0	0	0
Chrysiogenetes	0	0	0	0	0	0	0	0	0	1
Firmicutes	100	3	0	0	0	1	0	0	0	0
Tenericutes	19	0	0	0	0	0	0	0	0	0
Actinobacteria	80	0	0	0	0	0	0	0	0	0
Chlamydiae	0	0	8	0	0	0	0	0	0	0
Spirochaetes	11	0	0	0	0	0	0	0	0	3
Acidobacteria	0	4	0	0	0	0	1	0	0	0
Bacteroidetes	35	0	0	0	0	0	0	0	0	0
Fibrobacteres	1	0	0	0	0	0	0	0	0	0
Fusobacteria	3	1	0	1	0	0	0	0	0	0
Verrucomicrobia	2	0	0	1	0	1	0	0	0	0
Gemmatimonadetes	0	0	0	1	0	0	0	0	0	0
Planctomycetes	3	0	0	1	0	0	0	0	0	0
Elusimicrobia	2	0	0	0	0	0	0	0	0	0
Synergistetes	2	0	0	0	0	0	0	0	0	0
Cyanobacteria	16	0	0	0	0	0	0	0	0	0
Chlorobi	10	0	0	0	0	0	0	0	0	0
Chloroflexi	11	0	0	0	0	0	0	0	0	0
Deinococcus-Thermus	5	1	1	0	0	0	0	0	0	0
Aquificae	2	0	0	1	3	2	1	0	0	0
Thermotogae	10	1	0	0	0	0	0	0	0	0
Dictyoglomi	2	0	0	0	0	0	0	0	0	0
Nitrospirae	0	2	0	0	0	0	0	0	0	0
Thermobaculum	1	0	0	0	0	0	0	0	0	0
Deferribacteres	1	0	0	0	2	0	0	0	0	0
Euryarchaeota	60	0	0	0	0	0	0	0	0	0
Crenarchaeota	23	0	0	0	0	0	0	0	0	0
Thaumarchaeota	2	0	0	0	0	0	0	0	0	0
Nanoarchaeota	1	0	0	0	0	0	0	0	0	0
Korarchaeota	1	0	0	0	0	0	0	0	0	0
Total	525	22	13	20	10	4	4	3	10	157

(mod_M00332_1)

Phyla	Module completion ratio (%)									
	0--10	10--20	20--30	30--40	40--50	50--60	60--70	70--80	80--90	90--100
Gammaproteobacteria	96	0	0	0	0	1	10	7	1	11
Betaproteobacteria	43	0	0	0	0	2	9	1	3	3
Epsilonproteobacteria	17	0	0	0	0	0	0	0	0	0
Deltaproteobacteria	22	0	0	1	3	0	0	0	2	0
Alphaproteobacteria	87	0	0	0	0	4	0	0	0	0
Magnetococcus	1	0	0	0	0	0	0	0	0	0
Chrysiogenetes	1	0	0	0	0	0	0	0	0	0
Firmicutes	104	0	0	0	0	0	0	0	0	0
Tenericutes	19	0	0	0	0	0	0	0	0	0
Actinobacteria	80	0	0	0	0	0	0	0	0	0
Chlamydiae	0	0	0	0	0	1	7	0	0	0
Spirochaetes	14	0	0	0	0	0	0	0	0	0
Acidobacteria	5	0	0	0	0	0	0	0	0	0
Bacteroidetes	35	0	0	0	0	0	0	0	0	0
Fibrobacteres	1	0	0	0	0	0	0	0	0	0
Fusobacteria	5	0	0	0	0	0	0	0	0	0
Verrucomicrobia	4	0	0	0	0	0	0	0	0	0
Gemmatimonadetes	1	0	0	0	0	0	0	0	0	0
Planctomycetes	4	0	0	0	0	0	0	0	0	0
Elusimicrobia	2	0	0	0	0	0	0	0	0	0
Synergistetes	2	0	0	0	0	0	0	0	0	0
Cyanobacteria	16	0	0	0	0	0	0	0	0	0
Chlorobi	10	0	0	0	0	0	0	0	0	0
Chloroflexi	11	0	0	0	0	0	0	0	0	0
Deinococcus-Thermus	7	0	0	0	0	0	0	0	0	0
Aquificae	9	0	0	0	0	0	0	0	0	0
Thermotogae	11	0	0	0	0	0	0	0	0	0
Dictyoglomi	2	0	0	0	0	0	0	0	0	0
Nitrospirae	2	0	0	0	0	0	0	0	0	0
Thermobaculum	1	0	0	0	0	0	0	0	0	0
Deferribacteres	3	0	0	0	0	0	0	0	0	0
Euryarchaeota	60	0	0	0	0	0	0	0	0	0
Crenarchaeota	23	0	0	0	0	0	0	0	0	0
Thaumarchaeota	2	0	0	0	0	0	0	0	0	0
Nanoarchaeota	1	0	0	0	0	0	0	0	0	0
Korarchaeota	1	0	0	0	0	0	0	0	0	0
Total	702	0	0	1	3	8	26	8	6	14

(mod_M00333_1)

Phyla	Module completion ratio (%)									
	0--10	10--20	20--30	30--40	40--50	50--60	60--70	70--80	80--90	90--100
Gammaproteobacteria	100	3	0	0	0	2	2	12	3	4
Betaproteobacteria	28	0	1	1	2	3	6	9	6	5
Epsilonproteobacteria	14	0	0	1	0	1	0	1	0	0
Deltaproteobacteria	27	0	0	0	0	1	0	0	0	0
Alphaproteobacteria	22	0	4	2	1	2	31	3	9	17
Magnetococcus	1	0	0	0	0	0	0	0	0	0
Chrysiogenetes	1	0	0	0	0	0	0	0	0	0
Firmicutes	104	0	0	0	0	0	0	0	0	0
Tenericutes	19	0	0	0	0	0	0	0	0	0
Actinobacteria	80	0	0	0	0	0	0	0	0	0
Chlamydiae	8	0	0	0	0	0	0	0	0	0
Spirochaetes	14	0	0	0	0	0	0	0	0	0
Acidobacteria	2	0	0	0	2	1	0	0	0	0
Bacteroidetes	35	0	0	0	0	0	0	0	0	0
Fibrobacteres	1	0	0	0	0	0	0	0	0	0
Fusobacteria	2	0	0	0	0	2	1	0	0	0
Verrucomicrobia	4	0	0	0	0	0	0	0	0	0
Gemmatimonadetes	1	0	0	0	0	0	0	0	0	0
Planctomycetes	4	0	0	0	0	0	0	0	0	0
Elusimicrobia	2	0	0	0	0	0	0	0	0	0
Synergistetes	2	0	0	0	0	0	0	0	0	0
Cyanobacteria	16	0	0	0	0	0	0	0	0	0
Chlorobi	9	0	0	0	0	0	1	0	0	0
Chloroflexi	11	0	0	0	0	0	0	0	0	0
Deinococcus-Thermus	7	0	0	0	0	0	0	0	0	0
Aquificae	9	0	0	0	0	0	0	0	0	0
Thermotogae	11	0	0	0	0	0	0	0	0	0
Dictyoglomi	2	0	0	0	0	0	0	0	0	0
Nitrospirae	2	0	0	0	0	0	0	0	0	0
Thermobaculum	1	0	0	0	0	0	0	0	0	0
Deferribacteres	3	0	0	0	0	0	0	0	0	0
Euryarchaeota	60	0	0	0	0	0	0	0	0	0
Crenarchaeota	23	0	0	0	0	0	0	0	0	0
Thaumarchaeota	2	0	0	0	0	0	0	0	0	0
Nanoarchaeota	1	0	0	0	0	0	0	0	0	0
Korarchaeota	1	0	0	0	0	0	0	0	0	0
Total	629	3	5	4	5	12	41	25	18	26

(mod_M00334_1)

Phyla	Module completion ratio (%)									
	0--10	10--20	20--30	30--40	40--50	50--60	60--70	70--80	80--90	90--100
Gammaproteobacteria	60	7	1	2	10	20	20	2	2	2
Betaproteobacteria	30	1	0	0	1	27	2	0	0	0
Epsilonproteobacteria	15	0	0	1	1	0	0	0	0	0
Deltaproteobacteria	20	1	1	1	2	1	2	0	0	0
Alphaproteobacteria	77	2	0	1	5	6	0	0	0	0
Magnetococcus	1	0	0	0	0	0	0	0	0	0
Chrysiogenetes	1	0	0	0	0	0	0	0	0	0
Firmicutes	104	0	0	0	0	0	0	0	0	0
Tenericutes	19	0	0	0	0	0	0	0	0	0
Actinobacteria	79	1	0	0	0	0	0	0	0	0
Chlamydiae	7	1	0	0	0	0	0	0	0	0
Spirochaetes	14	0	0	0	0	0	0	0	0	0
Acidobacteria	2	0	0	0	3	0	0	0	0	0
Bacteroidetes	35	0	0	0	0	0	0	0	0	0
Fibrobacteres	1	0	0	0	0	0	0	0	0	0
Fusobacteria	5	0	0	0	0	0	0	0	0	0
Verrucomicrobia	4	0	0	0	0	0	0	0	0	0
Gemmatimonadetes	0	0	0	0	1	0	0	0	0	0
Planctomycetes	2	0	0	2	0	0	0	0	0	0
Elusimicrobia	2	0	0	0	0	0	0	0	0	0
Synergistetes	2	0	0	0	0	0	0	0	0	0
Cyanobacteria	16	0	0	0	0	0	0	0	0	0
Chlorobi	9	1	0	0	0	0	0	0	0	0
Chloroflexi	11	0	0	0	0	0	0	0	0	0
Deinococcus-Thermus	7	0	0	0	0	0	0	0	0	0
Aquificae	9	0	0	0	0	0	0	0	0	0
Thermotogae	11	0	0	0	0	0	0	0	0	0
Dictyoglomi	2	0	0	0	0	0	0	0	0	0
Nitrospirae	1	0	0	0	0	1	0	0	0	0
Thermobaculum	1	0	0	0	0	0	0	0	0	0
Deferribacteres	3	0	0	0	0	0	0	0	0	0
Euryarchaeota	60	0	0	0	0	0	0	0	0	0
Crenarchaeota	23	0	0	0	0	0	0	0	0	0
Thaumarchaeota	2	0	0	0	0	0	0	0	0	0
Nanoarchaeota	1	0	0	0	0	0	0	0	0	0
Korarchaeota	1	0	0	0	0	0	0	0	0	0
Total	637	14	2	7	23	55	24	2	2	2

(mod_M00335_1)

Phyla	Module completion ratio (%)									
	0--10	10--20	20--30	30--40	40--50	50--60	60--70	70--80	80--90	90--100
Gammaproteobacteria	1	0	0	0	0	0	6	8	83	28
Betaproteobacteria	1	0	0	0	0	0	0	2	58	0
Epsilonproteobacteria	0	0	0	0	0	0	2	15	0	0
Deltaproteobacteria	0	0	0	0	0	0	1	22	5	0
Alphaproteobacteria	1	0	0	0	0	0	1	28	58	3
Magnetococcus	0	0	0	0	0	0	0	0	1	0
Chrysiogenetes	0	0	0	0	0	0	0	0	1	0
Firmicutes	0	0	0	0	0	1	63	39	1	0
Tenericutes	0	0	0	2	6	5	6	0	0	0
Actinobacteria	0	0	0	0	0	2	17	53	8	0
Chlamydiae	0	0	0	0	0	0	8	0	0	0
Spirochaetes	0	0	0	0	0	0	4	10	0	0
Acidobacteria	0	0	0	0	0	0	0	5	0	0
Bacteroidetes	0	1	0	0	0	1	31	2	0	0
Fibrobacteres	0	0	0	0	0	0	1	0	0	0
Fusobacteria	0	0	0	0	0	0	2	3	0	0
Verrucomicrobia	0	0	0	0	0	0	4	0	0	0
Gemmatimonadetes	0	0	0	0	0	0	0	1	0	0
Planctomycetes	0	0	0	0	0	0	4	0	0	0
Elusimicrobia	0	0	0	0	0	0	2	0	0	0
Synergistetes	0	0	0	0	0	0	0	2	0	0
Cyanobacteria	0	0	0	0	0	0	16	0	0	0
Chlorobi	0	0	0	0	0	0	1	9	0	0
Chloroflexi	0	0	0	0	0	1	10	0	0	0
Deinococcus-Thermus	0	0	0	0	0	0	7	0	0	0
Aquificae	0	0	0	0	0	0	2	7	0	0
Thermotogae	0	0	0	0	0	0	1	9	1	0
Dictyoglomi	0	0	0	0	0	0	0	2	0	0
Nitrospirae	0	0	0	0	0	0	0	2	0	0
Thermobaculum	0	0	0	0	0	0	1	0	0	0
Deferribacteres	0	0	0	0	0	0	0	3	0	0
Euryarchaeota	0	0	8	52	0	0	0	0	0	0
Crenarchaeota	0	1	22	0	0	0	0	0	0	0
Thaumarchaeota	0	1	1	0	0	0	0	0	0	0
Nanoarchaeota	0	0	1	0	0	0	0	0	0	0
Korarchaeota	0	0	1	0	0	0	0	0	0	0
Total	3	3	33	54	6	10	190	222	216	31

(mod_M00336_1)

Phyla	Module completion ratio (%)									
	0--10	10--20	20--30	30--40	40--50	50--60	60--70	70--80	80--90	90--100
Gammaproteobacteria	10	0	0	0	0	2	0	90	0	24
Betaproteobacteria	1	0	0	0	0	1	0	59	0	0
Epsilonproteobacteria	0	0	0	0	0	1	0	16	0	0
Deltaproteobacteria	1	0	0	0	0	9	0	18	0	0
Alphaproteobacteria	3	0	1	0	0	21	0	65	0	1
Magnetococcus	0	0	0	0	0	0	0	1	0	0
Chrysiogenetes	0	0	0	0	0	0	0	1	0	0
Firmicutes	54	0	3	0	0	44	0	3	0	0
Tenericutes	19	0	0	0	0	0	0	0	0	0
Actinobacteria	3	0	1	0	0	7	0	69	0	0
Chlamydiae	7	0	1	0	0	0	0	0	0	0
Spirochaetes	10	0	0	0	0	4	0	0	0	0
Acidobacteria	0	0	0	0	0	1	0	4	0	0
Bacteroidetes	3	0	1	0	0	29	0	2	0	0
Fibrobacteres	1	0	0	0	0	0	0	0	0	0
Fusobacteria	3	0	0	0	0	1	0	1	0	0
Verrucomicrobia	0	0	1	0	0	3	0	0	0	0
Gemmatimonadetes	0	0	0	0	0	1	0	0	0	0
Planctomycetes	0	0	0	0	0	4	0	0	0	0
Elusimicrobia	2	0	0	0	0	0	0	0	0	0
Synergistetes	2	0	0	0	0	0	0	0	0	0
Cyanobacteria	0	0	0	0	0	16	0	0	0	0
Chlorobi	0	0	1	0	0	9	0	0	0	0
Chloroflexi	0	0	0	0	0	3	0	8	0	0
Deinococcus-Thermus	0	0	0	0	0	7	0	0	0	0
Aquificae	0	0	0	0	0	3	0	6	0	0
Thermotogae	11	0	0	0	0	0	0	0	0	0
Dictyoglomi	2	0	0	0	0	0	0	0	0	0
Nitrospirae	0	0	0	0	0	1	0	1	0	0
Thermobaculum	0	0	0	0	0	0	0	1	0	0
Deferribacteres	0	0	0	0	0	2	0	1	0	0
Euryarchaeota	34	0	2	0	0	23	0	1	0	0
Crenarchaeota	7	0	0	0	0	15	0	1	0	0
Thaumarchaeota	0	0	0	0	0	2	0	0	0	0
Nanoarchaeota	1	0	0	0	0	0	0	0	0	0
Korarchaeota	0	0	0	0	0	1	0	0	0	0
Total	174	0	11	0	0	210	0	348	0	25

(mod_M00337_1)

Phyla	Module completion ratio (%)									
	0--10	10--20	20--30	30--40	40--50	50--60	60--70	70--80	80--90	90--100
Gammaproteobacteria	126	0	0	0	0	0	0	0	0	0
Betaproteobacteria	61	0	0	0	0	0	0	0	0	0
Epsilonproteobacteria	17	0	0	0	0	0	0	0	0	0
Deltaproteobacteria	28	0	0	0	0	0	0	0	0	0
Alphaproteobacteria	91	0	0	0	0	0	0	0	0	0
Magnetococcus	1	0	0	0	0	0	0	0	0	0
Chrysiogenetes	1	0	0	0	0	0	0	0	0	0
Firmicutes	104	0	0	0	0	0	0	0	0	0
Tenericutes	19	0	0	0	0	0	0	0	0	0
Actinobacteria	80	0	0	0	0	0	0	0	0	0
Chlamydiae	8	0	0	0	0	0	0	0	0	0
Spirochaetes	14	0	0	0	0	0	0	0	0	0
Acidobacteria	5	0	0	0	0	0	0	0	0	0
Bacteroidetes	35	0	0	0	0	0	0	0	0	0
Fibrobacteres	1	0	0	0	0	0	0	0	0	0
Fusobacteria	5	0	0	0	0	0	0	0	0	0
Verrucomicrobia	4	0	0	0	0	0	0	0	0	0
Gemmatimonadetes	1	0	0	0	0	0	0	0	0	0
Planctomycetes	4	0	0	0	0	0	0	0	0	0
Elusimicrobia	2	0	0	0	0	0	0	0	0	0
Synergistetes	2	0	0	0	0	0	0	0	0	0
Cyanobacteria	16	0	0	0	0	0	0	0	0	0
Chlorobi	10	0	0	0	0	0	0	0	0	0
Chloroflexi	11	0	0	0	0	0	0	0	0	0
Deinococcus-Thermus	7	0	0	0	0	0	0	0	0	0
Aquificae	9	0	0	0	0	0	0	0	0	0
Thermotogae	11	0	0	0	0	0	0	0	0	0
Dictyoglomi	2	0	0	0	0	0	0	0	0	0
Nitrospirae	2	0	0	0	0	0	0	0	0	0
Thermobaculum	1	0	0	0	0	0	0	0	0	0
Deferribacteres	3	0	0	0	0	0	0	0	0	0
Euryarchaeota	60	0	0	0	0	0	0	0	0	0
Crenarchaeota	23	0	0	0	0	0	0	0	0	0
Thaumarchaeota	2	0	0	0	0	0	0	0	0	0
Nanoarchaeota	1	0	0	0	0	0	0	0	0	0
Korarchaeota	1	0	0	0	0	0	0	0	0	0
Total	768	0	0	0	0	0	0	0	0	0

(mod_M00338_1)

Phyla	Module completion ratio (%)									
	0--10	10--20	20--30	30--40	40--50	50--60	60--70	70--80	80--90	90--100
Gammaproteobacteria	97	0	0	0	0	18	0	0	0	11
Betaproteobacteria	55	0	0	0	0	4	0	0	0	2
Epsilonproteobacteria	17	0	0	0	0	0	0	0	0	0
Deltaproteobacteria	21	0	0	0	0	2	0	0	0	5
Alphaproteobacteria	80	0	0	0	0	4	0	0	0	7
Magnetococcus	1	0	0	0	0	0	0	0	0	0
Chrysiogenetes	1	0	0	0	0	0	0	0	0	0
Firmicutes	103	0	0	0	0	1	0	0	0	0
Tenericutes	19	0	0	0	0	0	0	0	0	0
Actinobacteria	21	0	0	0	0	37	0	0	0	22
Chlamydiae	7	0	0	0	0	1	0	0	0	0
Spirochaetes	14	0	0	0	0	0	0	0	0	0
Acidobacteria	3	0	0	0	0	2	0	0	0	0
Bacteroidetes	17	0	0	0	0	15	0	0	0	3
Fibrobacteres	1	0	0	0	0	0	0	0	0	0
Fusobacteria	5	0	0	0	0	0	0	0	0	0
Verrucomicrobia	4	0	0	0	0	0	0	0	0	0
Gemmatimonadetes	0	0	0	0	0	0	0	0	0	1
Planctomycetes	3	0	0	0	0	1	0	0	0	0
Elusimicrobia	2	0	0	0	0	0	0	0	0	0
Synergistetes	2	0	0	0	0	0	0	0	0	0
Cyanobacteria	15	0	0	0	0	0	0	0	0	1
Chlorobi	3	0	0	0	0	7	0	0	0	0
Chloroflexi	9	0	0	0	0	2	0	0	0	0
Deinococcus-Thermus	7	0	0	0	0	0	0	0	0	0
Aquificae	9	0	0	0	0	0	0	0	0	0
Thermotogae	9	0	0	0	0	2	0	0	0	0
Dictyoglomi	2	0	0	0	0	0	0	0	0	0
Nitrospirae	2	0	0	0	0	0	0	0	0	0
Thermobaculum	1	0	0	0	0	0	0	0	0	0
Deferribacteres	3	0	0	0	0	0	0	0	0	0
Euryarchaeota	48	0	0	0	0	9	0	0	0	3
Crenarchaeota	17	0	0	0	0	5	0	0	0	1
Thaumarchaeota	2	0	0	0	0	0	0	0	0	0
Nanoarchaeota	1	0	0	0	0	0	0	0	0	0
Korarchaeota	1	0	0	0	0	0	0	0	0	0
Total	602	0	0	0	0	110	0	0	0	56

(mod_M00339_1)

Phyla	Module completion ratio (%)									
	0--10	10--20	20--30	30--40	40--50	50--60	60--70	70--80	80--90	90--100
Gammaproteobacteria	121	0	0	4	0	0	0	0	0	1
Betaproteobacteria	61	0	0	0	0	0	0	0	0	0
Epsilonproteobacteria	17	0	0	0	0	0	0	0	0	0
Deltaproteobacteria	28	0	0	0	0	0	0	0	0	0
Alphaproteobacteria	91	0	0	0	0	0	0	0	0	0
Magnetococcus	1	0	0	0	0	0	0	0	0	0
Chrysiogenetes	1	0	0	0	0	0	0	0	0	0
Firmicutes	104	0	0	0	0	0	0	0	0	0
Tenericutes	19	0	0	0	0	0	0	0	0	0
Actinobacteria	80	0	0	0	0	0	0	0	0	0
Chlamydiae	8	0	0	0	0	0	0	0	0	0
Spirochaetes	14	0	0	0	0	0	0	0	0	0
Acidobacteria	5	0	0	0	0	0	0	0	0	0
Bacteroidetes	35	0	0	0	0	0	0	0	0	0
Fibrobacteres	1	0	0	0	0	0	0	0	0	0
Fusobacteria	5	0	0	0	0	0	0	0	0	0
Verrucomicrobia	4	0	0	0	0	0	0	0	0	0
Gemmatimonadetes	1	0	0	0	0	0	0	0	0	0
Planctomycetes	4	0	0	0	0	0	0	0	0	0
Elusimicrobia	2	0	0	0	0	0	0	0	0	0
Synergistetes	2	0	0	0	0	0	0	0	0	0
Cyanobacteria	16	0	0	0	0	0	0	0	0	0
Chlorobi	10	0	0	0	0	0	0	0	0	0
Chloroflexi	11	0	0	0	0	0	0	0	0	0
Deinococcus-Thermus	7	0	0	0	0	0	0	0	0	0
Aquificae	9	0	0	0	0	0	0	0	0	0
Thermotogae	11	0	0	0	0	0	0	0	0	0
Dictyoglomi	2	0	0	0	0	0	0	0	0	0
Nitrospirae	2	0	0	0	0	0	0	0	0	0
Thermobaculum	1	0	0	0	0	0	0	0	0	0
Deferribacteres	3	0	0	0	0	0	0	0	0	0
Euryarchaeota	60	0	0	0	0	0	0	0	0	0
Crenarchaeota	23	0	0	0	0	0	0	0	0	0
Thaumarchaeota	2	0	0	0	0	0	0	0	0	0
Nanoarchaeota	1	0	0	0	0	0	0	0	0	0
Korarchaeota	1	0	0	0	0	0	0	0	0	0
Total	763	0	0	4	0	0	0	0	0	1

(mod_M00340_1)

Phyla	Module completion ratio (%)									
	0--10	10--20	20--30	30--40	40--50	50--60	60--70	70--80	80--90	90--100
Gammaproteobacteria	126	0	0	0	0	0	0	0	0	0
Betaproteobacteria	61	0	0	0	0	0	0	0	0	0
Epsilonproteobacteria	17	0	0	0	0	0	0	0	0	0
Deltaproteobacteria	28	0	0	0	0	0	0	0	0	0
Alphaproteobacteria	91	0	0	0	0	0	0	0	0	0
Magnetococcus	1	0	0	0	0	0	0	0	0	0
Chrysiogenetes	1	0	0	0	0	0	0	0	0	0
Firmicutes	104	0	0	0	0	0	0	0	0	0
Tenericutes	19	0	0	0	0	0	0	0	0	0
Actinobacteria	80	0	0	0	0	0	0	0	0	0
Chlamydiae	8	0	0	0	0	0	0	0	0	0
Spirochaetes	14	0	0	0	0	0	0	0	0	0
Acidobacteria	5	0	0	0	0	0	0	0	0	0
Bacteroidetes	35	0	0	0	0	0	0	0	0	0
Fibrobacteres	1	0	0	0	0	0	0	0	0	0
Fusobacteria	5	0	0	0	0	0	0	0	0	0
Verrucomicrobia	4	0	0	0	0	0	0	0	0	0
Gemmatimonadetes	1	0	0	0	0	0	0	0	0	0
Planctomycetes	4	0	0	0	0	0	0	0	0	0
Elusimicrobia	2	0	0	0	0	0	0	0	0	0
Synergistetes	2	0	0	0	0	0	0	0	0	0
Cyanobacteria	16	0	0	0	0	0	0	0	0	0
Chlorobi	10	0	0	0	0	0	0	0	0	0
Chloroflexi	11	0	0	0	0	0	0	0	0	0
Deinococcus-Thermus	7	0	0	0	0	0	0	0	0	0
Aquificae	9	0	0	0	0	0	0	0	0	0
Thermotogae	11	0	0	0	0	0	0	0	0	0
Dictyoglomi	2	0	0	0	0	0	0	0	0	0
Nitrospirae	2	0	0	0	0	0	0	0	0	0
Thermobaculum	1	0	0	0	0	0	0	0	0	0
Deferribacteres	3	0	0	0	0	0	0	0	0	0
Euryarchaeota	60	0	0	0	0	0	0	0	0	0
Crenarchaeota	23	0	0	0	0	0	0	0	0	0
Thaumarchaeota	2	0	0	0	0	0	0	0	0	0
Nanoarchaeota	1	0	0	0	0	0	0	0	0	0
Korarchaeota	1	0	0	0	0	0	0	0	0	0
Total	768	0	0	0	0	0	0	0	0	0

(mod_M00341_1)

Phyla	Module completion ratio (%)									
	0--10	10--20	20--30	30--40	40--50	50--60	60--70	70--80	80--90	90--100
Gammaproteobacteria	126	0	0	0	0	0	0	0	0	0
Betaproteobacteria	61	0	0	0	0	0	0	0	0	0
Epsilonproteobacteria	17	0	0	0	0	0	0	0	0	0
Deltaproteobacteria	28	0	0	0	0	0	0	0	0	0
Alphaproteobacteria	91	0	0	0	0	0	0	0	0	0
Magnetococcus	1	0	0	0	0	0	0	0	0	0
Chrysiogenetes	1	0	0	0	0	0	0	0	0	0
Firmicutes	104	0	0	0	0	0	0	0	0	0
Tenericutes	19	0	0	0	0	0	0	0	0	0
Actinobacteria	80	0	0	0	0	0	0	0	0	0
Chlamydiae	8	0	0	0	0	0	0	0	0	0
Spirochaetes	14	0	0	0	0	0	0	0	0	0
Acidobacteria	5	0	0	0	0	0	0	0	0	0
Bacteroidetes	35	0	0	0	0	0	0	0	0	0
Fibrobacteres	1	0	0	0	0	0	0	0	0	0
Fusobacteria	5	0	0	0	0	0	0	0	0	0
Verrucomicrobia	4	0	0	0	0	0	0	0	0	0
Gemmatimonadetes	1	0	0	0	0	0	0	0	0	0
Planctomycetes	4	0	0	0	0	0	0	0	0	0
Elusimicrobia	2	0	0	0	0	0	0	0	0	0
Synergistetes	2	0	0	0	0	0	0	0	0	0
Cyanobacteria	16	0	0	0	0	0	0	0	0	0
Chlorobi	10	0	0	0	0	0	0	0	0	0
Chloroflexi	11	0	0	0	0	0	0	0	0	0
Deinococcus-Thermus	7	0	0	0	0	0	0	0	0	0
Aquificae	9	0	0	0	0	0	0	0	0	0
Thermotogae	11	0	0	0	0	0	0	0	0	0
Dictyoglomi	2	0	0	0	0	0	0	0	0	0
Nitrospirae	2	0	0	0	0	0	0	0	0	0
Thermobaculum	1	0	0	0	0	0	0	0	0	0
Deferribacteres	3	0	0	0	0	0	0	0	0	0
Euryarchaeota	60	0	0	0	0	0	0	0	0	0
Crenarchaeota	23	0	0	0	0	0	0	0	0	0
Thaumarchaeota	2	0	0	0	0	0	0	0	0	0
Nanoarchaeota	1	0	0	0	0	0	0	0	0	0
Korarchaeota	1	0	0	0	0	0	0	0	0	0
Total	768	0	0	0	0	0	0	0	0	0

(mod_M00342_1)

Phyla	Module completion ratio (%)									
	0--10	10--20	20--30	30--40	40--50	50--60	60--70	70--80	80--90	90--100
Gammaproteobacteria	126	0	0	0	0	0	0	0	0	0
Betaproteobacteria	61	0	0	0	0	0	0	0	0	0
Epsilonproteobacteria	17	0	0	0	0	0	0	0	0	0
Deltaproteobacteria	28	0	0	0	0	0	0	0	0	0
Alphaproteobacteria	91	0	0	0	0	0	0	0	0	0
Magnetococcus	1	0	0	0	0	0	0	0	0	0
Chrysiogenetes	1	0	0	0	0	0	0	0	0	0
Firmicutes	104	0	0	0	0	0	0	0	0	0
Tenericutes	19	0	0	0	0	0	0	0	0	0
Actinobacteria	13	0	0	0	0	15	0	2	0	50
Chlamydiae	8	0	0	0	0	0	0	0	0	0
Spirochaetes	14	0	0	0	0	0	0	0	0	0
Acidobacteria	5	0	0	0	0	0	0	0	0	0
Bacteroidetes	35	0	0	0	0	0	0	0	0	0
Fibrobacteres	1	0	0	0	0	0	0	0	0	0
Fusobacteria	5	0	0	0	0	0	0	0	0	0
Verrucomicrobia	3	0	0	0	0	0	0	0	0	1
Gemmatimonadetes	1	0	0	0	0	0	0	0	0	0
Planctomycetes	4	0	0	0	0	0	0	0	0	0
Elusimicrobia	2	0	0	0	0	0	0	0	0	0
Synergistetes	2	0	0	0	0	0	0	0	0	0
Cyanobacteria	15	0	1	0	0	0	0	0	0	0
Chlorobi	10	0	0	0	0	0	0	0	0	0
Chloroflexi	11	0	0	0	0	0	0	0	0	0
Deinococcus-Thermus	7	0	0	0	0	0	0	0	0	0
Aquificae	9	0	0	0	0	0	0	0	0	0
Thermotogae	11	0	0	0	0	0	0	0	0	0
Dictyoglomi	2	0	0	0	0	0	0	0	0	0
Nitrospirae	1	0	0	0	0	0	0	0	0	1
Thermobaculum	1	0	0	0	0	0	0	0	0	0
Deferribacteres	3	0	0	0	0	0	0	0	0	0
Euryarchaeota	0	0	0	0	0	60	0	0	0	0
Crenarchaeota	0	0	0	0	0	23	0	0	0	0
Thaumarchaeota	0	0	0	0	0	2	0	0	0	0
Nanoarchaeota	0	0	0	0	0	1	0	0	0	0
Korarchaeota	0	0	0	0	0	1	0	0	0	0
Total	611	0	1	0	0	102	0	2	0	52

(mod_M00343_1)

Phyla	Module completion ratio (%)									
	0--10	10--20	20--30	30--40	40--50	50--60	60--70	70--80	80--90	90--100
Gammaproteobacteria	126	0	0	0	0	0	0	0	0	0
Betaproteobacteria	61	0	0	0	0	0	0	0	0	0
Epsilonproteobacteria	17	0	0	0	0	0	0	0	0	0
Deltaproteobacteria	28	0	0	0	0	0	0	0	0	0
Alphaproteobacteria	91	0	0	0	0	0	0	0	0	0
Magnetococcus	1	0	0	0	0	0	0	0	0	0
Chrysiogenetes	1	0	0	0	0	0	0	0	0	0
Firmicutes	104	0	0	0	0	0	0	0	0	0
Tenericutes	19	0	0	0	0	0	0	0	0	0
Actinobacteria	27	0	0	1	0	0	52	0	0	0
Chlamydiae	8	0	0	0	0	0	0	0	0	0
Spirochaetes	14	0	0	0	0	0	0	0	0	0
Acidobacteria	5	0	0	0	0	0	0	0	0	0
Bacteroidetes	35	0	0	0	0	0	0	0	0	0
Fibrobacteres	1	0	0	0	0	0	0	0	0	0
Fusobacteria	5	0	0	0	0	0	0	0	0	0
Verrucomicrobia	3	0	0	0	0	0	1	0	0	0
Gemmatimonadetes	1	0	0	0	0	0	0	0	0	0
Planctomycetes	4	0	0	0	0	0	0	0	0	0
Elusimicrobia	2	0	0	0	0	0	0	0	0	0
Synergistetes	2	0	0	0	0	0	0	0	0	0
Cyanobacteria	16	0	0	0	0	0	0	0	0	0
Chlorobi	10	0	0	0	0	0	0	0	0	0
Chloroflexi	11	0	0	0	0	0	0	0	0	0
Deinococcus-Thermus	7	0	0	0	0	0	0	0	0	0
Aquificae	9	0	0	0	0	0	0	0	0	0
Thermotogae	11	0	0	0	0	0	0	0	0	0
Dictyoglomi	2	0	0	0	0	0	0	0	0	0
Nitrospirae	1	0	0	0	0	0	1	0	0	0
Thermobaculum	1	0	0	0	0	0	0	0	0	0
Deferribacteres	3	0	0	0	0	0	0	0	0	0
Euryarchaeota	0	0	0	0	0	0	3	0	0	57
Crenarchaeota	0	0	0	0	0	0	8	0	0	15
Thaumarchaeota	0	0	0	0	0	0	2	0	0	0
Nanoarchaeota	0	0	0	0	0	0	0	0	0	1
Korarchaeota	0	0	0	0	0	0	1	0	0	0
Total	626	0	0	1	0	0	68	0	0	73

(mod_M00344_1)

Phyla	Module completion ratio (%)									
	0--10	10--20	20--30	30--40	40--50	50--60	60--70	70--80	80--90	90--100
Gammaproteobacteria	4	0	0	22	0	0	92	0	0	8
Betaproteobacteria	2	0	0	0	0	0	43	0	0	16
Epsilonproteobacteria	0	0	0	0	0	0	17	0	0	0
Deltaproteobacteria	7	0	0	9	0	0	12	0	0	0
Alphaproteobacteria	44	0	0	10	0	0	28	0	0	9
Magnetococcus	0	0	0	1	0	0	0	0	0	0
Chrysiogenetes	0	0	0	0	0	0	1	0	0	0
Firmicutes	7	0	0	85	0	0	12	0	0	0
Tenericutes	1	0	0	18	0	0	0	0	0	0
Actinobacteria	6	0	0	49	0	0	25	0	0	0
Chlamydiae	8	0	0	0	0	0	0	0	0	0
Spirochaetes	1	0	0	13	0	0	0	0	0	0
Acidobacteria	1	0	0	4	0	0	0	0	0	0
Bacteroidetes	2	0	0	10	0	0	20	0	0	3
Fibrobacteres	0	0	0	1	0	0	0	0	0	0
Fusobacteria	1	0	0	4	0	0	0	0	0	0
Verrucomicrobia	0	0	0	3	0	0	1	0	0	0
Gemmatimonadetes	0	0	0	1	0	0	0	0	0	0
Planctomycetes	0	0	0	3	0	0	1	0	0	0
Elusimicrobia	0	0	0	2	0	0	0	0	0	0
Synergistetes	2	0	0	0	0	0	0	0	0	0
Cyanobacteria	0	0	0	3	0	0	13	0	0	0
Chlorobi	0	0	0	1	0	0	9	0	0	0
Chloroflexi	10	0	0	1	0	0	0	0	0	0
Deinococcus-Thermus	0	0	0	7	0	0	0	0	0	0
Aquificae	3	0	0	6	0	0	0	0	0	0
Thermotogae	0	0	0	11	0	0	0	0	0	0
Dictyoglomi	0	0	0	2	0	0	0	0	0	0
Nitrospirae	1	0	0	1	0	0	0	0	0	0
Thermobaculum	0	0	0	1	0	0	0	0	0	0
Deferribacteres	0	0	0	0	0	0	3	0	0	0
Euryarchaeota	37	0	0	20	0	0	3	0	0	0
Crenarchaeota	23	0	0	0	0	0	0	0	0	0
Thaumarchaeota	2	0	0	0	0	0	0	0	0	0
Nanoarchaeota	1	0	0	0	0	0	0	0	0	0
Korarchaeota	1	0	0	0	0	0	0	0	0	0
Total	164	0	0	288	0	0	280	0	0	36

(mod_M00345_1)

Phyla	Module completion ratio (%)									
	0--10	10--20	20--30	30--40	40--50	50--60	60--70	70--80	80--90	90--100
Gammaproteobacteria	2	0	0	45	0	0	77	0	0	2
Betaproteobacteria	1	0	0	28	0	0	32	0	0	0
Epsilonproteobacteria	0	0	0	10	0	0	6	0	0	1
Deltaproteobacteria	2	0	0	16	0	0	10	0	0	0
Alphaproteobacteria	46	0	0	33	0	0	12	0	0	0
Magnetococcus	0	0	0	0	0	0	1	0	0	0
Chrysiogenetes	0	0	0	0	0	0	1	0	0	0
Firmicutes	3	0	0	6	0	0	79	0	0	16
Tenericutes	1	0	0	1	0	0	17	0	0	0
Actinobacteria	4	0	0	13	0	0	59	0	0	4
Chlamydiae	8	0	0	0	0	0	0	0	0	0
Spirochaetes	4	0	0	8	0	0	2	0	0	0
Acidobacteria	2	0	0	2	0	0	1	0	0	0
Bacteroidetes	3	0	0	4	0	0	25	0	0	3
Fibrobacteres	0	0	0	0	0	0	1	0	0	0
Fusobacteria	0	0	0	1	0	0	3	0	0	1
Verrucomicrobia	0	0	0	0	0	0	4	0	0	0
Gemmatimonadetes	0	0	0	1	0	0	0	0	0	0
Planctomycetes	0	0	0	1	0	0	3	0	0	0
Elusimicrobia	0	0	0	0	0	0	2	0	0	0
Synergistetes	0	0	0	2	0	0	0	0	0	0
Cyanobacteria	0	0	0	7	0	0	9	0	0	0
Chlorobi	0	0	0	1	0	0	9	0	0	0
Chloroflexi	2	0	0	8	0	0	1	0	0	0
Deinococcus-Thermus	0	0	0	0	0	0	7	0	0	0
Aquificae	5	0	0	3	0	0	1	0	0	0
Thermotogae	0	0	0	0	0	0	11	0	0	0
Dictyoglomi	0	0	0	0	0	0	2	0	0	0
Nitrospirae	0	0	0	2	0	0	0	0	0	0
Thermobaculum	0	0	0	1	0	0	0	0	0	0
Deferribacteres	0	0	0	0	0	0	3	0	0	0
Euryarchaeota	12	0	0	40	0	0	5	0	0	3
Crenarchaeota	0	0	0	21	0	0	2	0	0	0
Thaumarchaeota	2	0	0	0	0	0	0	0	0	0
Nanoarchaeota	1	0	0	0	0	0	0	0	0	0
Korarchaeota	1	0	0	0	0	0	0	0	0	0
Total	99	0	0	254	0	0	385	0	0	30

(mod_M00346_1)

Phyla	Module completion ratio (%)									
	0--10	10--20	20--30	30--40	40--50	50--60	60--70	70--80	80--90	90--100
Gammaproteobacteria	0	1	7	11	57	22	28	0	0	0
Betaproteobacteria	1	0	2	4	24	21	4	3	2	0
Epsilonproteobacteria	0	0	2	10	5	0	0	0	0	0
Deltaproteobacteria	0	0	2	9	12	5	0	0	0	0
Alphaproteobacteria	0	1	10	27	23	17	2	3	8	0
Magnetococcus	0	0	0	1	0	0	0	0	0	0
Chrysiogenetes	0	0	0	1	0	0	0	0	0	0
Firmicutes	0	4	46	45	9	0	0	0	0	0
Tenericutes	0	3	15	1	0	0	0	0	0	0
Actinobacteria	0	0	8	23	45	4	0	0	0	0
Chlamydiae	0	0	1	7	0	0	0	0	0	0
Spirochaetes	0	0	8	5	1	0	0	0	0	0
Acidobacteria	0	0	0	1	4	0	0	0	0	0
Bacteroidetes	1	0	3	6	24	1	0	0	0	0
Fibrobacteres	0	0	0	1	0	0	0	0	0	0
Fusobacteria	0	1	2	2	0	0	0	0	0	0
Verrucomicrobia	0	0	1	1	2	0	0	0	0	0
Gemmatimonadetes	0	0	0	0	0	1	0	0	0	0
Planctomycetes	0	0	0	0	2	2	0	0	0	0
Elusimicrobia	0	0	2	0	0	0	0	0	0	0
Synergistetes	0	0	2	0	0	0	0	0	0	0
Cyanobacteria	0	0	1	4	6	5	0	0	0	0
Chlorobi	0	0	0	3	7	0	0	0	0	0
Chloroflexi	0	0	1	4	3	3	0	0	0	0
Deinococcus-Thermus	0	0	0	0	1	5	1	0	0	0
Aquificae	0	0	0	7	2	0	0	0	0	0
Thermotogae	0	0	11	0	0	0	0	0	0	0
Dictyoglomi	0	0	1	1	0	0	0	0	0	0
Nitrospirae	0	0	0	1	1	0	0	0	0	0
Thermobaculum	0	0	0	1	0	0	0	0	0	0
Deferribacteres	0	0	0	3	0	0	0	0	0	0
Euryarchaeota	0	0	9	19	22	9	1	0	0	0
Crenarchaeota	0	0	3	4	6	8	2	0	0	0
Thaumarchaeota	0	0	0	2	0	0	0	0	0	0
Nanoarchaeota	1	0	0	0	0	0	0	0	0	0
Korarchaeota	0	0	0	1	0	0	0	0	0	0
Total	3	10	137	205	256	103	38	6	10	0

(mod_M00347_1)

Phyla	Module completion ratio (%)									
	0--10	10--20	20--30	30--40	40--50	50--60	60--70	70--80	80--90	90--100
Gammaproteobacteria	121	3	2	0	0	0	0	0	0	0
Betaproteobacteria	52	1	8	0	0	0	0	0	0	0
Epsilonproteobacteria	17	0	0	0	0	0	0	0	0	0
Deltaproteobacteria	14	13	1	0	0	0	0	0	0	0
Alphaproteobacteria	79	1	11	0	0	0	0	0	0	0
Magnetococcus	1	0	0	0	0	0	0	0	0	0
Chrysiogenetes	1	0	0	0	0	0	0	0	0	0
Firmicutes	90	13	1	0	0	0	0	0	0	0
Tenericutes	19	0	0	0	0	0	0	0	0	0
Actinobacteria	66	14	0	0	0	0	0	0	0	0
Chlamydiae	8	0	0	0	0	0	0	0	0	0
Spirochaetes	13	1	0	0	0	0	0	0	0	0
Acidobacteria	5	0	0	0	0	0	0	0	0	0
Bacteroidetes	33	2	0	0	0	0	0	0	0	0
Fibrobacteres	1	0	0	0	0	0	0	0	0	0
Fusobacteria	4	1	0	0	0	0	0	0	0	0
Verrucomicrobia	3	1	0	0	0	0	0	0	0	0
Gemmatimonadetes	1	0	0	0	0	0	0	0	0	0
Planctomycetes	0	0	4	0	0	0	0	0	0	0
Elusimicrobia	2	0	0	0	0	0	0	0	0	0
Synergistetes	2	0	0	0	0	0	0	0	0	0
Cyanobacteria	16	0	0	0	0	0	0	0	0	0
Chlorobi	9	1	0	0	0	0	0	0	0	0
Chloroflexi	9	2	0	0	0	0	0	0	0	0
Deinococcus-Thermus	7	0	0	0	0	0	0	0	0	0
Aquificae	5	4	0	0	0	0	0	0	0	0
Thermotogae	11	0	0	0	0	0	0	0	0	0
Dictyoglomi	2	0	0	0	0	0	0	0	0	0
Nitrospirae	1	1	0	0	0	0	0	0	0	0
Thermobaculum	1	0	0	0	0	0	0	0	0	0
Deferribacteres	3	0	0	0	0	0	0	0	0	0
Euryarchaeota	11	2	12	0	0	3	1	0	5	26
Crenarchaeota	18	5	0	0	0	0	0	0	0	0
Thaumarchaeota	2	0	0	0	0	0	0	0	0	0
Nanoarchaeota	1	0	0	0	0	0	0	0	0	0
Korarchaeota	0	1	0	0	0	0	0	0	0	0
Total	628	66	39	0	0	3	1	0	5	26

(mod_M00348_1)

Phyla	Module completion ratio (%)									
	0--10	10--20	20--30	30--40	40--50	50--60	60--70	70--80	80--90	90--100
Gammaproteobacteria	107	0	0	0	0	0	0	0	0	19
Betaproteobacteria	29	0	1	0	0	0	0	0	0	31
Epsilonproteobacteria	17	0	0	0	0	0	0	0	0	0
Deltaproteobacteria	27	0	0	0	0	0	0	0	0	1
Alphaproteobacteria	85	0	1	0	0	0	0	1	0	4
Magnetococcus	1	0	0	0	0	0	0	0	0	0
Chrysiogenetes	1	0	0	0	0	0	0	0	0	0
Firmicutes	102	0	1	0	0	0	0	1	0	0
Tenericutes	19	0	0	0	0	0	0	0	0	0
Actinobacteria	74	0	5	0	0	1	0	0	0	0
Chlamydiae	8	0	0	0	0	0	0	0	0	0
Spirochaetes	14	0	0	0	0	0	0	0	0	0
Acidobacteria	5	0	0	0	0	0	0	0	0	0
Bacteroidetes	35	0	0	0	0	0	0	0	0	0
Fibrobacteres	1	0	0	0	0	0	0	0	0	0
Fusobacteria	5	0	0	0	0	0	0	0	0	0
Verrucomicrobia	4	0	0	0	0	0	0	0	0	0
Gemmatimonadetes	1	0	0	0	0	0	0	0	0	0
Planctomycetes	4	0	0	0	0	0	0	0	0	0
Elusimicrobia	2	0	0	0	0	0	0	0	0	0
Synergistetes	2	0	0	0	0	0	0	0	0	0
Cyanobacteria	16	0	0	0	0	0	0	0	0	0
Chlorobi	10	0	0	0	0	0	0	0	0	0
Chloroflexi	11	0	0	0	0	0	0	0	0	0
Deinococcus-Thermus	6	0	1	0	0	0	0	0	0	0
Aquificae	9	0	0	0	0	0	0	0	0	0
Thermotogae	10	0	1	0	0	0	0	0	0	0
Dictyoglomi	2	0	0	0	0	0	0	0	0	0
Nitrospirae	2	0	0	0	0	0	0	0	0	0
Thermobaculum	1	0	0	0	0	0	0	0	0	0
Deferribacteres	3	0	0	0	0	0	0	0	0	0
Euryarchaeota	60	0	0	0	0	0	0	0	0	0
Crenarchaeota	23	0	0	0	0	0	0	0	0	0
Thaumarchaeota	2	0	0	0	0	0	0	0	0	0
Nanoarchaeota	1	0	0	0	0	0	0	0	0	0
Korarchaeota	1	0	0	0	0	0	0	0	0	0
Total	700	0	10	0	0	1	0	2	0	55

(mod_M00349_1)

Phyla	Module completion ratio (%)									
	0--10	10--20	20--30	30--40	40--50	50--60	60--70	70--80	80--90	90--100
Gammaproteobacteria	64	0	5	0	0	8	0	5	0	44
Betaproteobacteria	25	0	0	0	0	0	0	3	0	33
Epsilonproteobacteria	13	0	1	0	0	0	0	1	0	2
Deltaproteobacteria	18	0	1	0	0	3	0	1	0	5
Alphaproteobacteria	46	0	0	0	0	0	0	2	0	43
Magnetococcus	0	0	0	0	0	0	0	0	0	1
Chrysiogenetes	0	0	0	0	0	0	0	0	0	1
Firmicutes	103	0	1	0	0	0	0	0	0	0
Tenericutes	19	0	0	0	0	0	0	0	0	0
Actinobacteria	78	0	2	0	0	0	0	0	0	0
Chlamydiae	8	0	0	0	0	0	0	0	0	0
Spirochaetes	9	0	1	0	0	2	0	1	0	1
Acidobacteria	5	0	0	0	0	0	0	0	0	0
Bacteroidetes	35	0	0	0	0	0	0	0	0	0
Fibrobacteres	0	0	0	0	0	1	0	0	0	0
Fusobacteria	5	0	0	0	0	0	0	0	0	0
Verrucomicrobia	4	0	0	0	0	0	0	0	0	0
Gemmatimonadetes	1	0	0	0	0	0	0	0	0	0
Planctomycetes	4	0	0	0	0	0	0	0	0	0
Elusimicrobia	2	0	0	0	0	0	0	0	0	0
Synergistetes	2	0	0	0	0	0	0	0	0	0
Cyanobacteria	16	0	0	0	0	0	0	0	0	0
Chlorobi	10	0	0	0	0	0	0	0	0	0
Chloroflexi	11	0	0	0	0	0	0	0	0	0
Deinococcus-Thermus	7	0	0	0	0	0	0	0	0	0
Aquificae	9	0	0	0	0	0	0	0	0	0
Thermotogae	11	0	0	0	0	0	0	0	0	0
Dictyoglomi	2	0	0	0	0	0	0	0	0	0
Nitrospirae	2	0	0	0	0	0	0	0	0	0
Thermobaculum	1	0	0	0	0	0	0	0	0	0
Deferribacteres	2	0	0	0	0	0	0	0	0	1
Euryarchaeota	60	0	0	0	0	0	0	0	0	0
Crenarchaeota	23	0	0	0	0	0	0	0	0	0
Thaumarchaeota	2	0	0	0	0	0	0	0	0	0
Nanoarchaeota	1	0	0	0	0	0	0	0	0	0
Korarchaeota	1	0	0	0	0	0	0	0	0	0
Total	599	0	11	0	0	14	0	13	0	131

(mod_M00350_1)

Phyla	Module completion ratio (%)									
	0--10	10--20	20--30	30--40	40--50	50--60	60--70	70--80	80--90	90--100
Gammaproteobacteria	117	0	9	0	0	0	0	0	0	0
Betaproteobacteria	60	0	1	0	0	0	0	0	0	0
Epsilonproteobacteria	16	0	1	0	0	0	0	0	0	0
Deltaproteobacteria	27	0	1	0	0	0	0	0	0	0
Alphaproteobacteria	87	0	4	0	0	0	0	0	0	0
Magnetococcus	1	0	0	0	0	0	0	0	0	0
Chrysiogenetes	1	0	0	0	0	0	0	0	0	0
Firmicutes	100	0	4	0	0	0	0	0	0	0
Tenericutes	19	0	0	0	0	0	0	0	0	0
Actinobacteria	65	0	15	0	0	0	0	0	0	0
Chlamydiae	8	0	0	0	0	0	0	0	0	0
Spirochaetes	14	0	0	0	0	0	0	0	0	0
Acidobacteria	4	0	1	0	0	0	0	0	0	0
Bacteroidetes	34	0	1	0	0	0	0	0	0	0
Fibrobacteres	1	0	0	0	0	0	0	0	0	0
Fusobacteria	4	0	1	0	0	0	0	0	0	0
Verrucomicrobia	4	0	0	0	0	0	0	0	0	0
Gemmatimonadetes	1	0	0	0	0	0	0	0	0	0
Planctomycetes	4	0	0	0	0	0	0	0	0	0
Elusimicrobia	2	0	0	0	0	0	0	0	0	0
Synergistetes	2	0	0	0	0	0	0	0	0	0
Cyanobacteria	12	0	2	0	0	2	0	0	0	0
Chlorobi	10	0	0	0	0	0	0	0	0	0
Chloroflexi	9	0	2	0	0	0	0	0	0	0
Deinococcus-Thermus	7	0	0	0	0	0	0	0	0	0
Aquificae	9	0	0	0	0	0	0	0	0	0
Thermotogae	11	0	0	0	0	0	0	0	0	0
Dictyoglomi	2	0	0	0	0	0	0	0	0	0
Nitrospirae	2	0	0	0	0	0	0	0	0	0
Thermobaculum	1	0	0	0	0	0	0	0	0	0
Deferribacteres	3	0	0	0	0	0	0	0	0	0
Euryarchaeota	60	0	0	0	0	0	0	0	0	0
Crenarchaeota	23	0	0	0	0	0	0	0	0	0
Thaumarchaeota	2	0	0	0	0	0	0	0	0	0
Nanoarchaeota	1	0	0	0	0	0	0	0	0	0
Korarchaeota	1	0	0	0	0	0	0	0	0	0
Total	724	0	42	0	0	2	0	0	0	0

(mod_M00351_1)

Phyla	Module completion ratio (%)									
	0--10	10--20	20--30	30--40	40--50	50--60	60--70	70--80	80--90	90--100
Gammaproteobacteria	126	0	0	0	0	0	0	0	0	0
Betaproteobacteria	61	0	0	0	0	0	0	0	0	0
Epsilonproteobacteria	17	0	0	0	0	0	0	0	0	0
Deltaproteobacteria	28	0	0	0	0	0	0	0	0	0
Alphaproteobacteria	91	0	0	0	0	0	0	0	0	0
Magnetococcus	1	0	0	0	0	0	0	0	0	0
Chrysiogenetes	1	0	0	0	0	0	0	0	0	0
Firmicutes	104	0	0	0	0	0	0	0	0	0
Tenericutes	19	0	0	0	0	0	0	0	0	0
Actinobacteria	80	0	0	0	0	0	0	0	0	0
Chlamydiae	8	0	0	0	0	0	0	0	0	0
Spirochaetes	14	0	0	0	0	0	0	0	0	0
Acidobacteria	5	0	0	0	0	0	0	0	0	0
Bacteroidetes	35	0	0	0	0	0	0	0	0	0
Fibrobacteres	1	0	0	0	0	0	0	0	0	0
Fusobacteria	5	0	0	0	0	0	0	0	0	0
Verrucomicrobia	4	0	0	0	0	0	0	0	0	0
Gemmatimonadetes	1	0	0	0	0	0	0	0	0	0
Planctomycetes	4	0	0	0	0	0	0	0	0	0
Elusimicrobia	2	0	0	0	0	0	0	0	0	0
Synergistetes	2	0	0	0	0	0	0	0	0	0
Cyanobacteria	16	0	0	0	0	0	0	0	0	0
Chlorobi	10	0	0	0	0	0	0	0	0	0
Chloroflexi	11	0	0	0	0	0	0	0	0	0
Deinococcus-Thermus	7	0	0	0	0	0	0	0	0	0
Aquificae	9	0	0	0	0	0	0	0	0	0
Thermotogae	11	0	0	0	0	0	0	0	0	0
Dictyoglomi	2	0	0	0	0	0	0	0	0	0
Nitrospirae	2	0	0	0	0	0	0	0	0	0
Thermobaculum	1	0	0	0	0	0	0	0	0	0
Deferribacteres	3	0	0	0	0	0	0	0	0	0
Euryarchaeota	60	0	0	0	0	0	0	0	0	0
Crenarchaeota	23	0	0	0	0	0	0	0	0	0
Thaumarchaeota	2	0	0	0	0	0	0	0	0	0
Nanoarchaeota	1	0	0	0	0	0	0	0	0	0
Korarchaeota	1	0	0	0	0	0	0	0	0	0
Total	768	0	0	0	0	0	0	0	0	0

(mod_M00352_1)

Phyla	Module completion ratio (%)									
	0--10	10--20	20--30	30--40	40--50	50--60	60--70	70--80	80--90	90--100
Gammaproteobacteria	126	0	0	0	0	0	0	0	0	0
Betaproteobacteria	61	0	0	0	0	0	0	0	0	0
Epsilonproteobacteria	17	0	0	0	0	0	0	0	0	0
Deltaproteobacteria	28	0	0	0	0	0	0	0	0	0
Alphaproteobacteria	91	0	0	0	0	0	0	0	0	0
Magnetococcus	1	0	0	0	0	0	0	0	0	0
Chrysiogenetes	1	0	0	0	0	0	0	0	0	0
Firmicutes	104	0	0	0	0	0	0	0	0	0
Tenericutes	19	0	0	0	0	0	0	0	0	0
Actinobacteria	80	0	0	0	0	0	0	0	0	0
Chlamydiae	8	0	0	0	0	0	0	0	0	0
Spirochaetes	14	0	0	0	0	0	0	0	0	0
Acidobacteria	5	0	0	0	0	0	0	0	0	0
Bacteroidetes	35	0	0	0	0	0	0	0	0	0
Fibrobacteres	1	0	0	0	0	0	0	0	0	0
Fusobacteria	5	0	0	0	0	0	0	0	0	0
Verrucomicrobia	4	0	0	0	0	0	0	0	0	0
Gemmatimonadetes	1	0	0	0	0	0	0	0	0	0
Planctomycetes	4	0	0	0	0	0	0	0	0	0
Elusimicrobia	2	0	0	0	0	0	0	0	0	0
Synergistetes	2	0	0	0	0	0	0	0	0	0
Cyanobacteria	16	0	0	0	0	0	0	0	0	0
Chlorobi	10	0	0	0	0	0	0	0	0	0
Chloroflexi	11	0	0	0	0	0	0	0	0	0
Deinococcus-Thermus	7	0	0	0	0	0	0	0	0	0
Aquificae	9	0	0	0	0	0	0	0	0	0
Thermotogae	11	0	0	0	0	0	0	0	0	0
Dictyoglomi	2	0	0	0	0	0	0	0	0	0
Nitrospirae	2	0	0	0	0	0	0	0	0	0
Thermobaculum	1	0	0	0	0	0	0	0	0	0
Deferribacteres	3	0	0	0	0	0	0	0	0	0
Euryarchaeota	60	0	0	0	0	0	0	0	0	0
Crenarchaeota	23	0	0	0	0	0	0	0	0	0
Thaumarchaeota	2	0	0	0	0	0	0	0	0	0
Nanoarchaeota	1	0	0	0	0	0	0	0	0	0
Korarchaeota	1	0	0	0	0	0	0	0	0	0
Total	768	0	0	0	0	0	0	0	0	0

(mod_M00353_1)

Phyla	Module completion ratio (%)									
	0--10	10--20	20--30	30--40	40--50	50--60	60--70	70--80	80--90	90--100
Gammaproteobacteria	126	0	0	0	0	0	0	0	0	0
Betaproteobacteria	61	0	0	0	0	0	0	0	0	0
Epsilonproteobacteria	17	0	0	0	0	0	0	0	0	0
Deltaproteobacteria	28	0	0	0	0	0	0	0	0	0
Alphaproteobacteria	91	0	0	0	0	0	0	0	0	0
Magnetococcus	1	0	0	0	0	0	0	0	0	0
Chrysiogenetes	1	0	0	0	0	0	0	0	0	0
Firmicutes	104	0	0	0	0	0	0	0	0	0
Tenericutes	19	0	0	0	0	0	0	0	0	0
Actinobacteria	80	0	0	0	0	0	0	0	0	0
Chlamydiae	8	0	0	0	0	0	0	0	0	0
Spirochaetes	14	0	0	0	0	0	0	0	0	0
Acidobacteria	5	0	0	0	0	0	0	0	0	0
Bacteroidetes	35	0	0	0	0	0	0	0	0	0
Fibrobacteres	1	0	0	0	0	0	0	0	0	0
Fusobacteria	5	0	0	0	0	0	0	0	0	0
Verrucomicrobia	4	0	0	0	0	0	0	0	0	0
Gemmatimonadetes	1	0	0	0	0	0	0	0	0	0
Planctomycetes	4	0	0	0	0	0	0	0	0	0
Elusimicrobia	2	0	0	0	0	0	0	0	0	0
Synergistetes	2	0	0	0	0	0	0	0	0	0
Cyanobacteria	16	0	0	0	0	0	0	0	0	0
Chlorobi	10	0	0	0	0	0	0	0	0	0
Chloroflexi	11	0	0	0	0	0	0	0	0	0
Deinococcus-Thermus	7	0	0	0	0	0	0	0	0	0
Aquificae	9	0	0	0	0	0	0	0	0	0
Thermotogae	11	0	0	0	0	0	0	0	0	0
Dictyoglomi	2	0	0	0	0	0	0	0	0	0
Nitrospirae	2	0	0	0	0	0	0	0	0	0
Thermobaculum	1	0	0	0	0	0	0	0	0	0
Deferribacteres	3	0	0	0	0	0	0	0	0	0
Euryarchaeota	60	0	0	0	0	0	0	0	0	0
Crenarchaeota	23	0	0	0	0	0	0	0	0	0
Thaumarchaeota	2	0	0	0	0	0	0	0	0	0
Nanoarchaeota	1	0	0	0	0	0	0	0	0	0
Korarchaeota	1	0	0	0	0	0	0	0	0	0
Total	768	0	0	0	0	0	0	0	0	0

(mod_M00354_1)

Phyla	Module completion ratio (%)									
	0--10	10--20	20--30	30--40	40--50	50--60	60--70	70--80	80--90	90--100
Gammaproteobacteria	126	0	0	0	0	0	0	0	0	0
Betaproteobacteria	61	0	0	0	0	0	0	0	0	0
Epsilonproteobacteria	17	0	0	0	0	0	0	0	0	0
Deltaproteobacteria	28	0	0	0	0	0	0	0	0	0
Alphaproteobacteria	91	0	0	0	0	0	0	0	0	0
Magnetococcus	1	0	0	0	0	0	0	0	0	0
Chrysiogenetes	1	0	0	0	0	0	0	0	0	0
Firmicutes	104	0	0	0	0	0	0	0	0	0
Tenericutes	19	0	0	0	0	0	0	0	0	0
Actinobacteria	80	0	0	0	0	0	0	0	0	0
Chlamydiae	8	0	0	0	0	0	0	0	0	0
Spirochaetes	14	0	0	0	0	0	0	0	0	0
Acidobacteria	5	0	0	0	0	0	0	0	0	0
Bacteroidetes	35	0	0	0	0	0	0	0	0	0
Fibrobacteres	1	0	0	0	0	0	0	0	0	0
Fusobacteria	5	0	0	0	0	0	0	0	0	0
Verrucomicrobia	4	0	0	0	0	0	0	0	0	0
Gemmatimonadetes	1	0	0	0	0	0	0	0	0	0
Planctomycetes	4	0	0	0	0	0	0	0	0	0
Elusimicrobia	2	0	0	0	0	0	0	0	0	0
Synergistetes	2	0	0	0	0	0	0	0	0	0
Cyanobacteria	16	0	0	0	0	0	0	0	0	0
Chlorobi	10	0	0	0	0	0	0	0	0	0
Chloroflexi	11	0	0	0	0	0	0	0	0	0
Deinococcus-Thermus	7	0	0	0	0	0	0	0	0	0
Aquificae	9	0	0	0	0	0	0	0	0	0
Thermotogae	11	0	0	0	0	0	0	0	0	0
Dictyoglomi	2	0	0	0	0	0	0	0	0	0
Nitrospirae	2	0	0	0	0	0	0	0	0	0
Thermobaculum	1	0	0	0	0	0	0	0	0	0
Deferribacteres	3	0	0	0	0	0	0	0	0	0
Euryarchaeota	60	0	0	0	0	0	0	0	0	0
Crenarchaeota	23	0	0	0	0	0	0	0	0	0
Thaumarchaeota	2	0	0	0	0	0	0	0	0	0
Nanoarchaeota	1	0	0	0	0	0	0	0	0	0
Korarchaeota	1	0	0	0	0	0	0	0	0	0
Total	768	0	0	0	0	0	0	0	0	0

(mod_M00355_1)

Phyla	Module completion ratio (%)									
	0--10	10--20	20--30	30--40	40--50	50--60	60--70	70--80	80--90	90--100
Gammaproteobacteria	126	0	0	0	0	0	0	0	0	0
Betaproteobacteria	61	0	0	0	0	0	0	0	0	0
Epsilonproteobacteria	17	0	0	0	0	0	0	0	0	0
Deltaproteobacteria	28	0	0	0	0	0	0	0	0	0
Alphaproteobacteria	91	0	0	0	0	0	0	0	0	0
Magnetococcus	1	0	0	0	0	0	0	0	0	0
Chrysiogenetes	1	0	0	0	0	0	0	0	0	0
Firmicutes	104	0	0	0	0	0	0	0	0	0
Tenericutes	19	0	0	0	0	0	0	0	0	0
Actinobacteria	80	0	0	0	0	0	0	0	0	0
Chlamydiae	8	0	0	0	0	0	0	0	0	0
Spirochaetes	14	0	0	0	0	0	0	0	0	0
Acidobacteria	5	0	0	0	0	0	0	0	0	0
Bacteroidetes	35	0	0	0	0	0	0	0	0	0
Fibrobacteres	1	0	0	0	0	0	0	0	0	0
Fusobacteria	5	0	0	0	0	0	0	0	0	0
Verrucomicrobia	4	0	0	0	0	0	0	0	0	0
Gemmatimonadetes	1	0	0	0	0	0	0	0	0	0
Planctomycetes	4	0	0	0	0	0	0	0	0	0
Elusimicrobia	2	0	0	0	0	0	0	0	0	0
Synergistetes	2	0	0	0	0	0	0	0	0	0
Cyanobacteria	16	0	0	0	0	0	0	0	0	0
Chlorobi	10	0	0	0	0	0	0	0	0	0
Chloroflexi	11	0	0	0	0	0	0	0	0	0
Deinococcus-Thermus	7	0	0	0	0	0	0	0	0	0
Aquificae	9	0	0	0	0	0	0	0	0	0
Thermotogae	11	0	0	0	0	0	0	0	0	0
Dictyoglomi	2	0	0	0	0	0	0	0	0	0
Nitrospirae	2	0	0	0	0	0	0	0	0	0
Thermobaculum	1	0	0	0	0	0	0	0	0	0
Deferribacteres	3	0	0	0	0	0	0	0	0	0
Euryarchaeota	60	0	0	0	0	0	0	0	0	0
Crenarchaeota	23	0	0	0	0	0	0	0	0	0
Thaumarchaeota	2	0	0	0	0	0	0	0	0	0
Nanoarchaeota	1	0	0	0	0	0	0	0	0	0
Korarchaeota	1	0	0	0	0	0	0	0	0	0
Total	768	0	0	0	0	0	0	0	0	0

(mod_M00356_1)

Phyla	Module completion ratio (%)									
	0--10	10--20	20--30	30--40	40--50	50--60	60--70	70--80	80--90	90--100
Gammaproteobacteria	126	0	0	0	0	0	0	0	0	0
Betaproteobacteria	61	0	0	0	0	0	0	0	0	0
Epsilonproteobacteria	17	0	0	0	0	0	0	0	0	0
Deltaproteobacteria	28	0	0	0	0	0	0	0	0	0
Alphaproteobacteria	91	0	0	0	0	0	0	0	0	0
Magnetococcus	1	0	0	0	0	0	0	0	0	0
Chrysiogenetes	1	0	0	0	0	0	0	0	0	0
Firmicutes	104	0	0	0	0	0	0	0	0	0
Tenericutes	19	0	0	0	0	0	0	0	0	0
Actinobacteria	80	0	0	0	0	0	0	0	0	0
Chlamydiae	8	0	0	0	0	0	0	0	0	0
Spirochaetes	14	0	0	0	0	0	0	0	0	0
Acidobacteria	5	0	0	0	0	0	0	0	0	0
Bacteroidetes	35	0	0	0	0	0	0	0	0	0
Fibrobacteres	1	0	0	0	0	0	0	0	0	0
Fusobacteria	5	0	0	0	0	0	0	0	0	0
Verrucomicrobia	4	0	0	0	0	0	0	0	0	0
Gemmatimonadetes	1	0	0	0	0	0	0	0	0	0
Planctomycetes	4	0	0	0	0	0	0	0	0	0
Elusimicrobia	2	0	0	0	0	0	0	0	0	0
Synergistetes	2	0	0	0	0	0	0	0	0	0
Cyanobacteria	16	0	0	0	0	0	0	0	0	0
Chlorobi	10	0	0	0	0	0	0	0	0	0
Chloroflexi	11	0	0	0	0	0	0	0	0	0
Deinococcus-Thermus	7	0	0	0	0	0	0	0	0	0
Aquificae	9	0	0	0	0	0	0	0	0	0
Thermotogae	11	0	0	0	0	0	0	0	0	0
Dictyoglomi	2	0	0	0	0	0	0	0	0	0
Nitrospirae	2	0	0	0	0	0	0	0	0	0
Thermobaculum	1	0	0	0	0	0	0	0	0	0
Deferribacteres	3	0	0	0	0	0	0	0	0	0
Euryarchaeota	28	0	0	21	0	0	5	0	0	6
Crenarchaeota	23	0	0	0	0	0	0	0	0	0
Thaumarchaeota	2	0	0	0	0	0	0	0	0	0
Nanoarchaeota	1	0	0	0	0	0	0	0	0	0
Korarchaeota	1	0	0	0	0	0	0	0	0	0
Total	736	0	0	21	0	0	5	0	0	6

(mod_M00357_1)

Phyla	Module completion ratio (%)									
	0--10	10--20	20--30	30--40	40--50	50--60	60--70	70--80	80--90	90--100
Gammaproteobacteria	22	9	0	95	0	0	0	0	0	0
Betaproteobacteria	14	8	0	39	0	0	0	0	0	0
Epsilonproteobacteria	3	0	0	14	0	0	0	0	0	0
Deltaproteobacteria	7	3	0	18	0	0	0	0	0	0
Alphaproteobacteria	37	21	0	33	0	0	0	0	0	0
Magnetococcus	1	0	0	0	0	0	0	0	0	0
Chrysiogenetes	0	0	0	1	0	0	0	0	0	0
Firmicutes	7	13	0	84	0	0	0	0	0	0
Tenericutes	0	6	0	13	0	0	0	0	0	0
Actinobacteria	8	13	0	59	0	0	0	0	0	0
Chlamydiae	7	1	0	0	0	0	0	0	0	0
Spirochaetes	3	1	0	10	0	0	0	0	0	0
Acidobacteria	1	2	0	2	0	0	0	0	0	0
Bacteroidetes	18	2	0	15	0	0	0	0	0	0
Fibrobacteres	0	0	0	1	0	0	0	0	0	0
Fusobacteria	0	0	0	5	0	0	0	0	0	0
Verrucomicrobia	0	3	0	1	0	0	0	0	0	0
Gemmatimonadetes	0	0	0	1	0	0	0	0	0	0
Planctomycetes	0	3	0	1	0	0	0	0	0	0
Elusimicrobia	0	1	0	1	0	0	0	0	0	0
Synergistetes	0	2	0	0	0	0	0	0	0	0
Cyanobacteria	3	10	0	3	0	0	0	0	0	0
Chlorobi	3	5	0	2	0	0	0	0	0	0
Chloroflexi	11	0	0	0	0	0	0	0	0	0
Deinococcus-Thermus	2	1	0	4	0	0	0	0	0	0
Aquificae	8	1	0	0	0	0	0	0	0	0
Thermotogae	0	9	0	2	0	0	0	0	0	0
Dictyoglomi	0	2	0	0	0	0	0	0	0	0
Nitrospirae	1	1	0	0	0	0	0	0	0	0
Thermobaculum	0	1	0	0	0	0	0	0	0	0
Deferribacteres	0	0	0	3	0	0	0	0	0	0
Euryarchaeota	26	7	0	11	0	9	4	0	0	3
Crenarchaeota	23	0	0	0	0	0	0	0	0	0
Thaumarchaeota	2	0	0	0	0	0	0	0	0	0
Nanoarchaeota	1	0	0	0	0	0	0	0	0	0
Korarchaeota	1	0	0	0	0	0	0	0	0	0
Total	209	125	0	418	0	9	4	0	0	3

(mod_M00358_1)

Phyla	Module completion ratio (%)									
	0--10	10--20	20--30	30--40	40--50	50--60	60--70	70--80	80--90	90--100
Gammaproteobacteria	122	0	4	0	0	0	0	0	0	0
Betaproteobacteria	55	0	6	0	0	0	0	0	0	0
Epsilonproteobacteria	17	0	0	0	0	0	0	0	0	0
Deltaproteobacteria	27	0	1	0	0	0	0	0	0	0
Alphaproteobacteria	81	0	10	0	0	0	0	0	0	0
Magnetococcus	1	0	0	0	0	0	0	0	0	0
Chrysiogenetes	1	0	0	0	0	0	0	0	0	0
Firmicutes	65	0	27	0	0	12	0	0	0	0
Tenericutes	19	0	0	0	0	0	0	0	0	0
Actinobacteria	67	0	13	0	0	0	0	0	0	0
Chlamydiae	8	0	0	0	0	0	0	0	0	0
Spirochaetes	14	0	0	0	0	0	0	0	0	0
Acidobacteria	4	0	0	0	0	1	0	0	0	0
Bacteroidetes	23	0	1	0	0	11	0	0	0	0
Fibrobacteres	1	0	0	0	0	0	0	0	0	0
Fusobacteria	5	0	0	0	0	0	0	0	0	0
Verrucomicrobia	4	0	0	0	0	0	0	0	0	0
Gemmatimonadetes	0	0	1	0	0	0	0	0	0	0
Planctomycetes	0	0	4	0	0	0	0	0	0	0
Elusimicrobia	2	0	0	0	0	0	0	0	0	0
Synergistetes	1	0	1	0	0	0	0	0	0	0
Cyanobacteria	1	0	15	0	0	0	0	0	0	0
Chlorobi	9	0	1	0	0	0	0	0	0	0
Chloroflexi	7	0	3	0	0	1	0	0	0	0
Deinococcus-Thermus	0	0	7	0	0	0	0	0	0	0
Aquificae	9	0	0	0	0	0	0	0	0	0
Thermotogae	0	0	11	0	0	0	0	0	0	0
Dictyoglomi	2	0	0	0	0	0	0	0	0	0
Nitrospirae	2	0	0	0	0	0	0	0	0	0
Thermobaculum	0	0	1	0	0	0	0	0	0	0
Deferribacteres	3	0	0	0	0	0	0	0	0	0
Euryarchaeota	30	0	13	0	0	1	0	1	0	15
Crenarchaeota	19	0	4	0	0	0	0	0	0	0
Thaumarchaeota	2	0	0	0	0	0	0	0	0	0
Nanoarchaeota	1	0	0	0	0	0	0	0	0	0
Korarchaeota	0	0	1	0	0	0	0	0	0	0
Total	602	0	124	0	0	26	0	1	0	15

(mod_M00359_1)

Phyla	Module completion ratio (%)									
	0--10	10--20	20--30	30--40	40--50	50--60	60--70	70--80	80--90	90--100
Gammaproteobacteria	0	0	0	0	0	0	1	1	9	115
Betaproteobacteria	0	0	0	0	0	0	0	0	1	60
Epsilonproteobacteria	0	0	0	0	0	0	1	1	11	4
Deltaproteobacteria	0	0	0	0	0	0	0	0	0	28
Alphaproteobacteria	0	0	0	0	1	0	0	2	88	0
Magnetococcus	0	0	0	0	0	0	0	0	1	0
Chrysiogenetes	0	0	0	0	0	0	0	0	1	0
Firmicutes	0	0	0	0	0	0	0	0	53	51
Tenericutes	0	0	0	0	0	0	0	0	0	19
Actinobacteria	0	0	0	0	0	0	0	0	8	72
Chlamydiae	0	0	0	0	0	0	0	0	6	2
Spirochaetes	0	0	0	0	0	0	0	0	1	13
Acidobacteria	0	0	0	0	0	0	0	0	0	5
Bacteroidetes	0	0	0	0	1	0	0	0	0	34
Fibrobacteres	0	0	0	0	0	0	0	0	0	1
Fusobacteria	0	0	0	0	0	0	0	0	0	5
Verrucomicrobia	0	0	0	0	0	0	0	0	0	4
Gemmatimonadetes	0	0	0	0	0	0	0	0	0	1
Planctomycetes	0	0	0	0	0	0	0	0	0	4
Elusimicrobia	0	0	0	0	0	0	0	0	1	1
Synergistetes	0	0	0	0	0	0	0	0	0	2
Cyanobacteria	0	0	0	0	0	0	0	0	5	11
Chlorobi	0	0	0	0	0	0	0	0	0	10
Chloroflexi	0	0	0	0	0	0	0	0	0	11
Deinococcus-Thermus	0	0	0	0	0	0	0	0	0	7
Aquificae	0	0	0	0	0	0	0	0	9	0
Thermotogae	0	0	0	0	0	0	0	0	5	6
Dictyoglomi	0	0	0	0	0	0	0	0	2	0
Nitrospirae	0	0	0	0	0	0	0	0	1	1
Thermobaculum	0	0	0	0	0	0	0	0	0	1
Deferribacteres	0	0	0	0	0	0	0	0	2	1
Euryarchaeota	0	0	0	0	0	0	0	0	40	20
Crenarchaeota	0	0	0	0	0	0	0	0	4	19
Thaumarchaeota	0	0	0	0	0	0	0	0	2	0
Nanoarchaeota	0	0	0	0	0	0	0	0	1	0
Korarchaeota	0	0	0	0	0	0	0	0	0	1
Total	0	0	0	0	2	0	2	4	251	509

(mod_M00360_1)

Phyla	Module completion ratio (%)									
	0--10	10--20	20--30	30--40	40--50	50--60	60--70	70--80	80--90	90--100
Gammaproteobacteria	0	0	0	0	0	0	1	0	1	124
Betaproteobacteria	0	0	0	0	0	0	0	0	1	60
Epsilonproteobacteria	0	0	0	0	0	0	0	0	2	15
Deltaproteobacteria	0	0	0	0	0	0	0	0	0	28
Alphaproteobacteria	0	0	0	0	1	0	0	0	0	90
Magnetococcus	0	0	0	0	0	0	0	0	0	1
Chrysiogenetes	0	0	0	0	0	0	0	0	0	1
Firmicutes	0	0	0	0	0	0	0	0	0	104
Tenericutes	0	0	0	0	0	0	0	0	0	19
Actinobacteria	0	0	0	0	0	0	0	0	0	80
Chlamydiae	0	0	0	0	0	0	0	0	0	8
Spirochaetes	0	0	0	0	0	0	0	0	0	14
Acidobacteria	0	0	0	0	0	0	0	0	0	5
Bacteroidetes	0	0	0	0	0	1	0	0	0	34
Fibrobacteres	0	0	0	0	0	0	0	0	0	1
Fusobacteria	0	0	0	0	0	0	0	0	0	5
Verrucomicrobia	0	0	0	0	0	0	0	0	0	4
Gemmatimonadetes	0	0	0	0	0	0	0	0	0	1
Planctomycetes	0	0	0	0	0	0	0	0	0	4
Elusimicrobia	0	0	0	0	0	0	0	0	0	2
Synergistetes	0	0	0	0	0	0	0	0	0	2
Cyanobacteria	0	0	0	0	0	0	0	0	0	16
Chlorobi	0	0	0	0	0	0	0	0	0	10
Chloroflexi	0	0	0	0	0	0	0	0	0	11
Deinococcus-Thermus	0	0	0	0	0	0	0	0	0	7
Aquificae	0	0	0	0	0	0	0	0	0	9
Thermotogae	0	0	0	0	0	0	0	0	0	11
Dictyoglomi	0	0	0	0	0	0	0	0	0	2
Nitrospirae	0	0	0	0	0	0	0	0	0	2
Thermobaculum	0	0	0	0	0	0	0	0	0	1
Deferribacteres	0	0	0	0	0	0	0	0	0	3
Euryarchaeota	0	0	0	0	0	0	0	0	0	60
Crenarchaeota	0	0	0	0	0	0	0	0	0	23
Thaumarchaeota	0	0	0	0	0	0	0	0	0	2
Nanoarchaeota	0	0	0	0	0	0	0	0	0	1
Korarchaeota	0	0	0	0	0	0	0	0	0	1
Total	0	0	0	0	1	1	1	0	4	761

(mod_M00361_1)

Phyla	Module completion ratio (%)									
	0--10	10--20	20--30	30--40	40--50	50--60	60--70	70--80	80--90	90--100
Gammaproteobacteria	6	2	0	12	0	48	58	0	0	0
Betaproteobacteria	1	2	0	5	0	25	27	0	1	0
Epsilonproteobacteria	0	0	0	2	0	9	6	0	0	0
Deltaproteobacteria	0	0	0	1	0	3	24	0	0	0
Alphaproteobacteria	9	10	0	10	0	14	48	0	0	0
Magnetococcus	0	0	0	0	0	0	1	0	0	0
Chrysiogenetes	0	0	0	0	0	0	1	0	0	0
Firmicutes	1	6	0	37	0	37	23	0	0	0
Tenericutes	11	3	0	4	0	1	0	0	0	0
Actinobacteria	0	3	0	7	0	9	60	0	1	0
Chlamydiae	0	6	0	0	0	1	1	0	0	0
Spirochaetes	1	8	0	1	0	1	3	0	0	0
Acidobacteria	0	0	0	0	0	1	4	0	0	0
Bacteroidetes	2	1	0	0	0	30	2	0	0	0
Fibrobacteres	0	0	0	0	0	0	1	0	0	0
Fusobacteria	0	0	0	2	0	3	0	0	0	0
Verrucomicrobia	0	0	0	0	0	1	1	0	2	0
Gemmatimonadetes	0	0	0	0	0	1	0	0	0	0
Planctomycetes	0	0	0	0	0	0	3	0	1	0
Elusimicrobia	1	1	0	0	0	0	0	0	0	0
Synergistetes	0	1	0	0	0	0	1	0	0	0
Cyanobacteria	0	0	0	0	0	12	4	0	0	0
Chlorobi	0	0	0	0	0	10	0	0	0	0
Chloroflexi	0	1	0	3	0	7	0	0	0	0
Deinococcus-Thermus	0	3	0	2	0	1	1	0	0	0
Aquificae	0	0	0	0	0	2	7	0	0	0
Thermotogae	0	1	0	8	0	2	0	0	0	0
Dictyoglomi	0	0	0	0	0	0	2	0	0	0
Nitrospirae	0	0	0	0	0	0	2	0	0	0
Thermobaculum	0	0	0	0	0	1	0	0	0	0
Deferribacteres	0	0	0	0	0	1	2	0	0	0
Euryarchaeota	0	2	0	16	0	29	13	0	0	0
Crenarchaeota	1	2	0	15	0	2	3	0	0	0
Thaumarchaeota	0	1	0	1	0	0	0	0	0	0
Nanoarchaeota	1	0	0	0	0	0	0	0	0	0
Korarchaeota	0	0	0	1	0	0	0	0	0	0
Total	34	53	0	127	0	251	298	0	5	0

(mod_M00362_1)

Phyla	Module completion ratio (%)									
	0--10	10--20	20--30	30--40	40--50	50--60	60--70	70--80	80--90	90--100
Gammaproteobacteria	1	4	3	0	6	25	0	28	36	23
Betaproteobacteria	1	0	2	0	1	19	0	16	20	2
Epsilonproteobacteria	0	0	0	0	1	6	0	7	3	0
Deltaproteobacteria	0	0	0	0	0	4	0	9	9	6
Alphaproteobacteria	7	2	7	0	7	16	0	38	13	1
Magnetococcus	0	0	0	0	0	0	0	0	1	0
Chrysiogenetes	0	0	0	0	0	0	0	0	1	0
Firmicutes	0	0	1	0	8	18	0	35	35	7
Tenericutes	11	3	3	0	1	1	0	0	0	0
Actinobacteria	0	0	1	0	5	5	0	18	36	15
Chlamydiae	0	6	0	0	1	1	0	0	0	0
Spirochaetes	1	8	1	0	0	0	0	4	0	0
Acidobacteria	0	0	0	0	0	0	0	2	1	2
Bacteroidetes	2	1	0	0	8	17	0	7	0	0
Fibrobacteres	0	0	0	0	0	0	0	1	0	0
Fusobacteria	0	0	0	0	0	3	0	2	0	0
Verrucomicrobia	0	0	0	0	0	1	0	2	1	0
Gemmatimonadetes	0	0	0	0	0	1	0	0	0	0
Planctomycetes	0	0	0	0	0	0	0	1	2	1
Elusimicrobia	0	1	0	0	1	0	0	0	0	0
Synergistetes	0	0	0	0	1	0	0	0	1	0
Cyanobacteria	0	0	0	0	0	1	0	10	4	1
Chlorobi	0	0	0	0	0	9	0	1	0	0
Chloroflexi	0	0	1	0	2	2	0	2	4	0
Deinococcus-Thermus	0	0	0	0	1	3	0	1	2	0
Aquificae	0	0	0	0	0	1	0	4	3	1
Thermotogae	0	0	0	0	0	1	0	8	2	0
Dictyoglomi	0	0	0	0	0	0	0	0	0	2
Nitrospirae	0	0	0	0	0	0	0	0	0	2
Thermobaculum	0	0	0	0	0	0	0	1	0	0
Deferribacteres	0	0	0	0	0	0	0	2	0	1
Euryarchaeota	0	0	5	0	5	13	0	24	9	4
Crenarchaeota	0	1	3	0	9	7	0	3	0	0
Thaumarchaeota	0	0	1	0	0	1	0	0	0	0
Nanoarchaeota	1	0	0	0	0	0	0	0	0	0
Korarchaeota	0	0	0	0	0	1	0	0	0	0
Total	24	26	28	0	57	156	0	226	183	68

(mod_M00363_1)

Phyla	Module completion ratio (%)									
	0--10	10--20	20--30	30--40	40--50	50--60	60--70	70--80	80--90	90--100
Gammaproteobacteria	125	0	0	0	0	0	0	0	0	1
Betaproteobacteria	61	0	0	0	0	0	0	0	0	0
Epsilonproteobacteria	17	0	0	0	0	0	0	0	0	0
Deltaproteobacteria	28	0	0	0	0	0	0	0	0	0
Alphaproteobacteria	91	0	0	0	0	0	0	0	0	0
Magnetococcus	1	0	0	0	0	0	0	0	0	0
Chrysiogenetes	1	0	0	0	0	0	0	0	0	0
Firmicutes	104	0	0	0	0	0	0	0	0	0
Tenericutes	19	0	0	0	0	0	0	0	0	0
Actinobacteria	80	0	0	0	0	0	0	0	0	0
Chlamydiae	8	0	0	0	0	0	0	0	0	0
Spirochaetes	14	0	0	0	0	0	0	0	0	0
Acidobacteria	5	0	0	0	0	0	0	0	0	0
Bacteroidetes	35	0	0	0	0	0	0	0	0	0
Fibrobacteres	1	0	0	0	0	0	0	0	0	0
Fusobacteria	5	0	0	0	0	0	0	0	0	0
Verrucomicrobia	4	0	0	0	0	0	0	0	0	0
Gemmatimonadetes	1	0	0	0	0	0	0	0	0	0
Planctomycetes	4	0	0	0	0	0	0	0	0	0
Elusimicrobia	2	0	0	0	0	0	0	0	0	0
Synergistetes	2	0	0	0	0	0	0	0	0	0
Cyanobacteria	16	0	0	0	0	0	0	0	0	0
Chlorobi	10	0	0	0	0	0	0	0	0	0
Chloroflexi	11	0	0	0	0	0	0	0	0	0
Deinococcus-Thermus	7	0	0	0	0	0	0	0	0	0
Aquificae	9	0	0	0	0	0	0	0	0	0
Thermotogae	11	0	0	0	0	0	0	0	0	0
Dictyoglomi	2	0	0	0	0	0	0	0	0	0
Nitrospirae	2	0	0	0	0	0	0	0	0	0
Thermobaculum	1	0	0	0	0	0	0	0	0	0
Deferribacteres	3	0	0	0	0	0	0	0	0	0
Euryarchaeota	60	0	0	0	0	0	0	0	0	0
Crenarchaeota	23	0	0	0	0	0	0	0	0	0
Thaumarchaeota	2	0	0	0	0	0	0	0	0	0
Nanoarchaeota	1	0	0	0	0	0	0	0	0	0
Korarchaeota	1	0	0	0	0	0	0	0	0	0
Total	767	0	0	0	0	0	0	0	0	1

(mod_M00364_1)

Phyla	Module completion ratio (%)									
	0--10	10--20	20--30	30--40	40--50	50--60	60--70	70--80	80--90	90--100
Gammaproteobacteria	2	0	0	88	0	0	30	0	0	6
Betaproteobacteria	1	0	0	58	0	0	2	0	0	0
Epsilonproteobacteria	0	0	0	17	0	0	0	0	0	0
Deltaproteobacteria	0	0	0	18	0	0	10	0	0	0
Alphaproteobacteria	2	0	0	61	0	0	21	0	0	7
Magnetococcus	0	0	0	1	0	0	0	0	0	0
Chrysiogenetes	0	0	0	1	0	0	0	0	0	0
Firmicutes	2	0	0	43	0	0	59	0	0	0
Tenericutes	18	0	0	0	0	0	1	0	0	0
Actinobacteria	1	0	0	21	0	0	58	0	0	0
Chlamydiae	0	0	0	7	0	0	1	0	0	0
Spirochaetes	2	0	0	12	0	0	0	0	0	0
Acidobacteria	0	0	0	5	0	0	0	0	0	0
Bacteroidetes	2	0	0	13	0	0	20	0	0	0
Fibrobacteres	0	0	0	1	0	0	0	0	0	0
Fusobacteria	0	0	0	3	0	0	2	0	0	0
Verrucomicrobia	0	0	0	4	0	0	0	0	0	0
Gemmatimonadetes	0	0	0	1	0	0	0	0	0	0
Planctomycetes	0	0	0	4	0	0	0	0	0	0
Elusimicrobia	0	0	0	2	0	0	0	0	0	0
Synergistetes	0	0	0	2	0	0	0	0	0	0
Cyanobacteria	0	0	0	5	0	0	11	0	0	0
Chlorobi	0	0	0	0	0	0	10	0	0	0
Chloroflexi	3	0	0	2	0	0	6	0	0	0
Deinococcus-Thermus	0	0	0	1	0	0	6	0	0	0
Aquificae	0	0	0	9	0	0	0	0	0	0
Thermotogae	0	0	0	11	0	0	0	0	0	0
Dictyoglomi	0	0	0	2	0	0	0	0	0	0
Nitrospirae	0	0	0	2	0	0	0	0	0	0
Thermobaculum	0	0	0	0	0	0	1	0	0	0
Deferribacteres	0	0	0	3	0	0	0	0	0	0
Euryarchaeota	0	0	0	1	0	0	59	0	0	0
Crenarchaeota	0	0	0	0	0	0	23	0	0	0
Thaumarchaeota	0	0	0	0	0	0	2	0	0	0
Nanoarchaeota	1	0	0	0	0	0	0	0	0	0
Korarchaeota	0	0	0	0	0	0	1	0	0	0
Total	34	0	0	398	0	0	323	0	0	13

(mod_M00365_1)

Phyla	Module completion ratio (%)									
	0--10	10--20	20--30	30--40	40--50	50--60	60--70	70--80	80--90	90--100
Gammaproteobacteria	90	0	0	0	0	36	0	0	0	0
Betaproteobacteria	59	0	0	0	0	2	0	0	0	0
Epsilonproteobacteria	17	0	0	0	0	0	0	0	0	0
Deltaproteobacteria	18	0	0	0	0	7	0	0	0	3
Alphaproteobacteria	65	0	0	0	0	26	0	0	0	0
Magnetococcus	1	0	0	0	0	0	0	0	0	0
Chrysiogenetes	1	0	0	0	0	0	0	0	0	0
Firmicutes	44	0	0	0	0	59	0	0	0	1
Tenericutes	18	0	0	0	0	1	0	0	0	0
Actinobacteria	2	0	0	0	0	21	0	0	0	57
Chlamydiae	7	0	0	0	0	1	0	0	0	0
Spirochaetes	6	0	0	0	0	8	0	0	0	0
Acidobacteria	5	0	0	0	0	0	0	0	0	0
Bacteroidetes	14	0	0	0	0	21	0	0	0	0
Fibrobacteres	1	0	0	0	0	0	0	0	0	0
Fusobacteria	3	0	0	0	0	2	0	0	0	0
Verrucomicrobia	4	0	0	0	0	0	0	0	0	0
Gemmatimonadetes	1	0	0	0	0	0	0	0	0	0
Planctomycetes	3	0	0	0	0	1	0	0	0	0
Elusimicrobia	2	0	0	0	0	0	0	0	0	0
Synergistetes	2	0	0	0	0	0	0	0	0	0
Cyanobacteria	4	0	0	0	0	12	0	0	0	0
Chlorobi	0	0	0	0	0	10	0	0	0	0
Chloroflexi	3	0	0	0	0	2	0	0	0	6
Deinococcus-Thermus	1	0	0	0	0	6	0	0	0	0
Aquificae	9	0	0	0	0	0	0	0	0	0
Thermotogae	8	0	0	0	0	3	0	0	0	0
Dictyoglomi	0	0	0	0	0	2	0	0	0	0
Nitrospirae	2	0	0	0	0	0	0	0	0	0
Thermobaculum	0	0	0	0	0	0	0	0	0	1
Deferribacteres	3	0	0	0	0	0	0	0	0	0
Euryarchaeota	0	0	0	0	0	1	0	0	0	59
Crenarchaeota	0	0	0	0	0	0	0	0	0	23
Thaumarchaeota	0	0	0	0	0	0	0	0	0	2
Nanoarchaeota	1	0	0	0	0	0	0	0	0	0
Korarchaeota	0	0	0	0	0	0	0	0	0	1
Total	394	0	0	0	0	221	0	0	0	153

(mod_M00366_1)

Phyla	Module completion ratio (%)									
	0--10	10--20	20--30	30--40	40--50	50--60	60--70	70--80	80--90	90--100
Gammaproteobacteria	80	0	37	0	0	9	0	0	0	0
Betaproteobacteria	59	0	2	0	0	0	0	0	0	0
Epsilonproteobacteria	17	0	0	0	0	0	0	0	0	0
Deltaproteobacteria	1	0	17	0	0	10	0	0	0	0
Alphaproteobacteria	54	0	29	0	0	8	0	0	0	0
Magnetococcus	0	0	1	0	0	0	0	0	0	0
Chrysiogenetes	0	0	1	0	0	0	0	0	0	0
Firmicutes	2	0	43	0	0	59	0	0	0	0
Tenericutes	18	0	0	0	0	1	0	0	0	0
Actinobacteria	21	0	43	0	0	16	0	0	0	0
Chlamydiae	0	0	7	0	0	1	0	0	0	0
Spirochaetes	3	0	11	0	0	0	0	0	0	0
Acidobacteria	0	0	5	0	0	0	0	0	0	0
Bacteroidetes	2	0	13	0	0	20	0	0	0	0
Fibrobacteres	0	0	1	0	0	0	0	0	0	0
Fusobacteria	0	0	3	0	0	2	0	0	0	0
Verrucomicrobia	0	0	4	0	0	0	0	0	0	0
Gemmatimonadetes	0	0	1	0	0	0	0	0	0	0
Planctomycetes	0	0	4	0	0	0	0	0	0	0
Elusimicrobia	0	0	2	0	0	0	0	0	0	0
Synergistetes	0	0	2	0	0	0	0	0	0	0
Cyanobacteria	0	0	5	0	0	11	0	0	0	0
Chlorobi	0	0	0	0	0	10	0	0	0	0
Chloroflexi	5	0	6	0	0	0	0	0	0	0
Deinococcus-Thermus	0	0	1	0	0	6	0	0	0	0
Aquificae	0	0	9	0	0	0	0	0	0	0
Thermotogae	3	0	8	0	0	0	0	0	0	0
Dictyoglomi	2	0	0	0	0	0	0	0	0	0
Nitrospirae	0	0	2	0	0	0	0	0	0	0
Thermobaculum	0	0	1	0	0	0	0	0	0	0
Deferribacteres	0	0	3	0	0	0	0	0	0	0
Euryarchaeota	0	0	60	0	0	0	0	0	0	0
Crenarchaeota	0	0	23	0	0	0	0	0	0	0
Thaumarchaeota	0	0	2	0	0	0	0	0	0	0
Nanoarchaeota	1	0	0	0	0	0	0	0	0	0
Korarchaeota	0	0	1	0	0	0	0	0	0	0
Total	268	0	347	0	0	153	0	0	0	0

(mod_M00367_1)

Phyla	Module completion ratio (%)									
	0--10	10--20	20--30	30--40	40--50	50--60	60--70	70--80	80--90	90--100
Gammaproteobacteria	90	0	0	36	0	0	0	0	0	0
Betaproteobacteria	59	0	0	2	0	0	0	0	0	0
Epsilonproteobacteria	17	0	0	0	0	0	0	0	0	0
Deltaproteobacteria	18	0	0	10	0	0	0	0	0	0
Alphaproteobacteria	65	0	0	26	0	0	0	0	0	0
Magnetococcus	1	0	0	0	0	0	0	0	0	0
Chrysiogenetes	1	0	0	0	0	0	0	0	0	0
Firmicutes	44	0	0	60	0	0	0	0	0	0
Tenericutes	18	0	0	1	0	0	0	0	0	0
Actinobacteria	22	0	0	58	0	0	0	0	0	0
Chlamydiae	7	0	0	1	0	0	0	0	0	0
Spirochaetes	7	0	0	7	0	0	0	0	0	0
Acidobacteria	5	0	0	0	0	0	0	0	0	0
Bacteroidetes	14	0	0	21	0	0	0	0	0	0
Fibrobacteres	1	0	0	0	0	0	0	0	0	0
Fusobacteria	3	0	0	2	0	0	0	0	0	0
Verrucomicrobia	4	0	0	0	0	0	0	0	0	0
Gemmatimonadetes	1	0	0	0	0	0	0	0	0	0
Planctomycetes	4	0	0	0	0	0	0	0	0	0
Elusimicrobia	2	0	0	0	0	0	0	0	0	0
Synergistetes	2	0	0	0	0	0	0	0	0	0
Cyanobacteria	4	0	0	12	0	0	0	0	0	0
Chlorobi	0	0	0	10	0	0	0	0	0	0
Chloroflexi	5	0	0	6	0	0	0	0	0	0
Deinococcus-Thermus	1	0	0	6	0	0	0	0	0	0
Aquificae	9	0	0	0	0	0	0	0	0	0
Thermotogae	11	0	0	0	0	0	0	0	0	0
Dictyoglomi	2	0	0	0	0	0	0	0	0	0
Nitrospirae	2	0	0	0	0	0	0	0	0	0
Thermobaculum	0	0	0	1	0	0	0	0	0	0
Deferribacteres	3	0	0	0	0	0	0	0	0	0
Euryarchaeota	0	0	0	60	0	0	0	0	0	0
Crenarchaeota	0	0	0	23	0	0	0	0	0	0
Thaumarchaeota	0	0	0	2	0	0	0	0	0	0
Nanoarchaeota	1	0	0	0	0	0	0	0	0	0
Korarchaeota	0	0	0	1	0	0	0	0	0	0
Total	423	0	0	345	0	0	0	0	0	0

(mod_M00368_1)

Phyla	Module completion ratio (%)									
	0--10	10--20	20--30	30--40	40--50	50--60	60--70	70--80	80--90	90--100
Gammaproteobacteria	1	0	0	125	0	0	0	0	0	0
Betaproteobacteria	1	0	0	60	0	0	0	0	0	0
Epsilonproteobacteria	0	0	0	17	0	0	0	0	0	0
Deltaproteobacteria	0	0	0	28	0	0	0	0	0	0
Alphaproteobacteria	8	0	0	83	0	0	0	0	0	0
Magnetococcus	0	0	0	1	0	0	0	0	0	0
Chrysiogenetes	0	0	0	1	0	0	0	0	0	0
Firmicutes	0	0	0	104	0	0	0	0	0	0
Tenericutes	2	0	0	17	0	0	0	0	0	0
Actinobacteria	0	0	0	80	0	0	0	0	0	0
Chlamydiae	7	0	0	1	0	0	0	0	0	0
Spirochaetes	0	0	0	14	0	0	0	0	0	0
Acidobacteria	0	0	0	5	0	0	0	0	0	0
Bacteroidetes	2	0	0	33	0	0	0	0	0	0
Fibrobacteres	0	0	0	1	0	0	0	0	0	0
Fusobacteria	0	0	0	5	0	0	0	0	0	0
Verrucomicrobia	0	0	0	4	0	0	0	0	0	0
Gemmatimonadetes	0	0	0	1	0	0	0	0	0	0
Planctomycetes	0	0	0	4	0	0	0	0	0	0
Elusimicrobia	0	0	0	2	0	0	0	0	0	0
Synergistetes	0	0	0	2	0	0	0	0	0	0
Cyanobacteria	0	0	0	16	0	0	0	0	0	0
Chlorobi	0	0	0	10	0	0	0	0	0	0
Chloroflexi	0	0	0	11	0	0	0	0	0	0
Deinococcus-Thermus	0	0	0	7	0	0	0	0	0	0
Aquificae	0	0	0	9	0	0	0	0	0	0
Thermotogae	0	0	0	11	0	0	0	0	0	0
Dictyoglomi	0	0	0	2	0	0	0	0	0	0
Nitrospirae	0	0	0	2	0	0	0	0	0	0
Thermobaculum	0	0	0	1	0	0	0	0	0	0
Deferribacteres	0	0	0	3	0	0	0	0	0	0
Euryarchaeota	1	0	0	59	0	0	0	0	0	0
Crenarchaeota	0	0	0	23	0	0	0	0	0	0
Thaumarchaeota	0	0	0	2	0	0	0	0	0	0
Nanoarchaeota	1	0	0	0	0	0	0	0	0	0
Korarchaeota	0	0	0	1	0	0	0	0	0	0
Total	23	0	0	745	0	0	0	0	0	0

(mod_M00369_1)

Phyla	Module completion ratio (%)									
	0--10	10--20	20--30	30--40	40--50	50--60	60--70	70--80	80--90	90--100
Gammaproteobacteria	126	0	0	0	0	0	0	0	0	0
Betaproteobacteria	61	0	0	0	0	0	0	0	0	0
Epsilonproteobacteria	17	0	0	0	0	0	0	0	0	0
Deltaproteobacteria	28	0	0	0	0	0	0	0	0	0
Alphaproteobacteria	91	0	0	0	0	0	0	0	0	0
Magnetococcus	1	0	0	0	0	0	0	0	0	0
Chrysiogenetes	1	0	0	0	0	0	0	0	0	0
Firmicutes	104	0	0	0	0	0	0	0	0	0
Tenericutes	19	0	0	0	0	0	0	0	0	0
Actinobacteria	80	0	0	0	0	0	0	0	0	0
Chlamydiae	8	0	0	0	0	0	0	0	0	0
Spirochaetes	14	0	0	0	0	0	0	0	0	0
Acidobacteria	5	0	0	0	0	0	0	0	0	0
Bacteroidetes	35	0	0	0	0	0	0	0	0	0
Fibrobacteres	1	0	0	0	0	0	0	0	0	0
Fusobacteria	5	0	0	0	0	0	0	0	0	0
Verrucomicrobia	4	0	0	0	0	0	0	0	0	0
Gemmatimonadetes	1	0	0	0	0	0	0	0	0	0
Planctomycetes	4	0	0	0	0	0	0	0	0	0
Elusimicrobia	2	0	0	0	0	0	0	0	0	0
Synergistetes	2	0	0	0	0	0	0	0	0	0
Cyanobacteria	16	0	0	0	0	0	0	0	0	0
Chlorobi	10	0	0	0	0	0	0	0	0	0
Chloroflexi	11	0	0	0	0	0	0	0	0	0
Deinococcus-Thermus	7	0	0	0	0	0	0	0	0	0
Aquificae	9	0	0	0	0	0	0	0	0	0
Thermotogae	11	0	0	0	0	0	0	0	0	0
Dictyoglomi	2	0	0	0	0	0	0	0	0	0
Nitrospirae	2	0	0	0	0	0	0	0	0	0
Thermobaculum	1	0	0	0	0	0	0	0	0	0
Deferribacteres	3	0	0	0	0	0	0	0	0	0
Euryarchaeota	60	0	0	0	0	0	0	0	0	0
Crenarchaeota	23	0	0	0	0	0	0	0	0	0
Thaumarchaeota	2	0	0	0	0	0	0	0	0	0
Nanoarchaeota	1	0	0	0	0	0	0	0	0	0
Korarchaeota	1	0	0	0	0	0	0	0	0	0
Total	768	0	0	0	0	0	0	0	0	0

(mod_M00370_1)

Phyla	Module completion ratio (%)									
	0--10	10--20	20--30	30--40	40--50	50--60	60--70	70--80	80--90	90--100
Gammaproteobacteria	126	0	0	0	0	0	0	0	0	0
Betaproteobacteria	61	0	0	0	0	0	0	0	0	0
Epsilonproteobacteria	17	0	0	0	0	0	0	0	0	0
Deltaproteobacteria	28	0	0	0	0	0	0	0	0	0
Alphaproteobacteria	91	0	0	0	0	0	0	0	0	0
Magnetococcus	1	0	0	0	0	0	0	0	0	0
Chrysiogenetes	1	0	0	0	0	0	0	0	0	0
Firmicutes	104	0	0	0	0	0	0	0	0	0
Tenericutes	19	0	0	0	0	0	0	0	0	0
Actinobacteria	80	0	0	0	0	0	0	0	0	0
Chlamydiae	8	0	0	0	0	0	0	0	0	0
Spirochaetes	14	0	0	0	0	0	0	0	0	0
Acidobacteria	5	0	0	0	0	0	0	0	0	0
Bacteroidetes	35	0	0	0	0	0	0	0	0	0
Fibrobacteres	1	0	0	0	0	0	0	0	0	0
Fusobacteria	5	0	0	0	0	0	0	0	0	0
Verrucomicrobia	4	0	0	0	0	0	0	0	0	0
Gemmatimonadetes	1	0	0	0	0	0	0	0	0	0
Planctomycetes	4	0	0	0	0	0	0	0	0	0
Elusimicrobia	2	0	0	0	0	0	0	0	0	0
Synergistetes	2	0	0	0	0	0	0	0	0	0
Cyanobacteria	16	0	0	0	0	0	0	0	0	0
Chlorobi	10	0	0	0	0	0	0	0	0	0
Chloroflexi	11	0	0	0	0	0	0	0	0	0
Deinococcus-Thermus	7	0	0	0	0	0	0	0	0	0
Aquificae	9	0	0	0	0	0	0	0	0	0
Thermotogae	11	0	0	0	0	0	0	0	0	0
Dictyoglomi	2	0	0	0	0	0	0	0	0	0
Nitrospirae	2	0	0	0	0	0	0	0	0	0
Thermobaculum	1	0	0	0	0	0	0	0	0	0
Deferribacteres	3	0	0	0	0	0	0	0	0	0
Euryarchaeota	60	0	0	0	0	0	0	0	0	0
Crenarchaeota	23	0	0	0	0	0	0	0	0	0
Thaumarchaeota	2	0	0	0	0	0	0	0	0	0
Nanoarchaeota	1	0	0	0	0	0	0	0	0	0
Korarchaeota	1	0	0	0	0	0	0	0	0	0
Total	768	0	0	0	0	0	0	0	0	0

(mod_M00371_1)

Phyla	Module completion ratio (%)									
	0--10	10--20	20--30	30--40	40--50	50--60	60--70	70--80	80--90	90--100
Gammaproteobacteria	126	0	0	0	0	0	0	0	0	0
Betaproteobacteria	61	0	0	0	0	0	0	0	0	0
Epsilonproteobacteria	17	0	0	0	0	0	0	0	0	0
Deltaproteobacteria	28	0	0	0	0	0	0	0	0	0
Alphaproteobacteria	91	0	0	0	0	0	0	0	0	0
Magnetococcus	1	0	0	0	0	0	0	0	0	0
Chrysiogenetes	1	0	0	0	0	0	0	0	0	0
Firmicutes	104	0	0	0	0	0	0	0	0	0
Tenericutes	19	0	0	0	0	0	0	0	0	0
Actinobacteria	80	0	0	0	0	0	0	0	0	0
Chlamydiae	8	0	0	0	0	0	0	0	0	0
Spirochaetes	14	0	0	0	0	0	0	0	0	0
Acidobacteria	5	0	0	0	0	0	0	0	0	0
Bacteroidetes	35	0	0	0	0	0	0	0	0	0
Fibrobacteres	1	0	0	0	0	0	0	0	0	0
Fusobacteria	5	0	0	0	0	0	0	0	0	0
Verrucomicrobia	4	0	0	0	0	0	0	0	0	0
Gemmatimonadetes	1	0	0	0	0	0	0	0	0	0
Planctomycetes	4	0	0	0	0	0	0	0	0	0
Elusimicrobia	2	0	0	0	0	0	0	0	0	0
Synergistetes	2	0	0	0	0	0	0	0	0	0
Cyanobacteria	16	0	0	0	0	0	0	0	0	0
Chlorobi	10	0	0	0	0	0	0	0	0	0
Chloroflexi	11	0	0	0	0	0	0	0	0	0
Deinococcus-Thermus	7	0	0	0	0	0	0	0	0	0
Aquificae	9	0	0	0	0	0	0	0	0	0
Thermotogae	11	0	0	0	0	0	0	0	0	0
Dictyoglomi	2	0	0	0	0	0	0	0	0	0
Nitrospirae	2	0	0	0	0	0	0	0	0	0
Thermobaculum	1	0	0	0	0	0	0	0	0	0
Deferribacteres	3	0	0	0	0	0	0	0	0	0
Euryarchaeota	60	0	0	0	0	0	0	0	0	0
Crenarchaeota	23	0	0	0	0	0	0	0	0	0
Thaumarchaeota	2	0	0	0	0	0	0	0	0	0
Nanoarchaeota	1	0	0	0	0	0	0	0	0	0
Korarchaeota	1	0	0	0	0	0	0	0	0	0
Total	768	0	0	0	0	0	0	0	0	0

(mod_M00372_1)

Phyla	Module completion ratio (%)									
	0--10	10--20	20--30	30--40	40--50	50--60	60--70	70--80	80--90	90--100
Gammaproteobacteria	121	5	0	0	0	0	0	0	0	0
Betaproteobacteria	61	0	0	0	0	0	0	0	0	0
Epsilonproteobacteria	17	0	0	0	0	0	0	0	0	0
Deltaproteobacteria	27	1	0	0	0	0	0	0	0	0
Alphaproteobacteria	82	9	0	0	0	0	0	0	0	0
Magnetococcus	1	0	0	0	0	0	0	0	0	0
Chrysiogenetes	1	0	0	0	0	0	0	0	0	0
Firmicutes	104	0	0	0	0	0	0	0	0	0
Tenericutes	19	0	0	0	0	0	0	0	0	0
Actinobacteria	79	1	0	0	0	0	0	0	0	0
Chlamydiae	8	0	0	0	0	0	0	0	0	0
Spirochaetes	14	0	0	0	0	0	0	0	0	0
Acidobacteria	5	0	0	0	0	0	0	0	0	0
Bacteroidetes	19	16	0	0	0	0	0	0	0	0
Fibrobacteres	1	0	0	0	0	0	0	0	0	0
Fusobacteria	5	0	0	0	0	0	0	0	0	0
Verrucomicrobia	4	0	0	0	0	0	0	0	0	0
Gemmatimonadetes	1	0	0	0	0	0	0	0	0	0
Planctomycetes	4	0	0	0	0	0	0	0	0	0
Elusimicrobia	2	0	0	0	0	0	0	0	0	0
Synergistetes	2	0	0	0	0	0	0	0	0	0
Cyanobacteria	1	15	0	0	0	0	0	0	0	0
Chlorobi	10	0	0	0	0	0	0	0	0	0
Chloroflexi	11	0	0	0	0	0	0	0	0	0
Deinococcus-Thermus	7	0	0	0	0	0	0	0	0	0
Aquificae	9	0	0	0	0	0	0	0	0	0
Thermotogae	11	0	0	0	0	0	0	0	0	0
Dictyoglomi	2	0	0	0	0	0	0	0	0	0
Nitrospirae	2	0	0	0	0	0	0	0	0	0
Thermobaculum	1	0	0	0	0	0	0	0	0	0
Deferribacteres	3	0	0	0	0	0	0	0	0	0
Euryarchaeota	59	1	0	0	0	0	0	0	0	0
Crenarchaeota	20	3	0	0	0	0	0	0	0	0
Thaumarchaeota	2	0	0	0	0	0	0	0	0	0
Nanoarchaeota	1	0	0	0	0	0	0	0	0	0
Korarchaeota	1	0	0	0	0	0	0	0	0	0
Total	717	51	0	0	0	0	0	0	0	0

(mod_M00373_1)

Phyla	Module completion ratio (%)									
	0--10	10--20	20--30	30--40	40--50	50--60	60--70	70--80	80--90	90--100
Gammaproteobacteria	84	34	7	0	1	0	0	0	0	0
Betaproteobacteria	13	21	6	18	2	0	0	1	0	0
Epsilonproteobacteria	17	0	0	0	0	0	0	0	0	0
Deltaproteobacteria	11	4	10	1	1	1	0	0	0	0
Alphaproteobacteria	24	6	9	3	21	3	2	0	16	7
Magnetococcus	0	0	0	0	1	0	0	0	0	0
Chrysiogenetes	0	0	1	0	0	0	0	0	0	0
Firmicutes	65	32	6	1	0	0	0	0	0	0
Tenericutes	19	0	0	0	0	0	0	0	0	0
Actinobacteria	30	7	14	12	13	4	0	0	0	0
Chlamydiae	7	1	0	0	0	0	0	0	0	0
Spirochaetes	10	1	0	0	0	0	3	0	0	0
Acidobacteria	1	4	0	0	0	0	0	0	0	0
Bacteroidetes	9	10	11	5	0	0	0	0	0	0
Fibrobacteres	1	0	0	0	0	0	0	0	0	0
Fusobacteria	3	1	1	0	0	0	0	0	0	0
Verrucomicrobia	1	3	0	0	0	0	0	0	0	0
Gemmatimonadetes	1	0	0	0	0	0	0	0	0	0
Planctomycetes	3	0	0	1	0	0	0	0	0	0
Elusimicrobia	2	0	0	0	0	0	0	0	0	0
Synergistetes	1	1	0	0	0	0	0	0	0	0
Cyanobacteria	15	1	0	0	0	0	0	0	0	0
Chlorobi	6	4	0	0	0	0	0	0	0	0
Chloroflexi	3	1	3	1	3	0	0	0	0	0
Deinococcus-Thermus	1	2	3	1	0	0	0	0	0	0
Aquificae	9	0	0	0	0	0	0	0	0	0
Thermotogae	9	2	0	0	0	0	0	0	0	0
Dictyoglomi	2	0	0	0	0	0	0	0	0	0
Nitrospirae	2	0	0	0	0	0	0	0	0	0
Thermobaculum	0	1	0	0	0	0	0	0	0	0
Deferribacteres	0	0	2	1	0	0	0	0	0	0
Euryarchaeota	49	11	0	0	0	0	0	0	0	0
Crenarchaeota	18	5	0	0	0	0	0	0	0	0
Thaumarchaeota	1	1	0	0	0	0	0	0	0	0
Nanoarchaeota	1	0	0	0	0	0	0	0	0	0
Korarchaeota	1	0	0	0	0	0	0	0	0	0
Total	419	153	73	44	42	8	5	1	16	7

(mod_M00373_2)

Phyla	Module completion ratio (%)									
	0--10	10--20	20--30	30--40	40--50	50--60	60--70	70--80	80--90	90--100
Gammaproteobacteria	126	0	0	0	0	0	0	0	0	0
Betaproteobacteria	61	0	0	0	0	0	0	0	0	0
Epsilonproteobacteria	17	0	0	0	0	0	0	0	0	0
Deltaproteobacteria	28	0	0	0	0	0	0	0	0	0
Alphaproteobacteria	84	0	0	0	0	0	0	0	0	7
Magnetococcus	1	0	0	0	0	0	0	0	0	0
Chrysiogenetes	1	0	0	0	0	0	0	0	0	0
Firmicutes	104	0	0	0	0	0	0	0	0	0
Tenericutes	19	0	0	0	0	0	0	0	0	0
Actinobacteria	80	0	0	0	0	0	0	0	0	0
Chlamydiae	8	0	0	0	0	0	0	0	0	0
Spirochaetes	14	0	0	0	0	0	0	0	0	0
Acidobacteria	5	0	0	0	0	0	0	0	0	0
Bacteroidetes	35	0	0	0	0	0	0	0	0	0
Fibrobacteres	1	0	0	0	0	0	0	0	0	0
Fusobacteria	5	0	0	0	0	0	0	0	0	0
Verrucomicrobia	4	0	0	0	0	0	0	0	0	0
Gemmatimonadetes	1	0	0	0	0	0	0	0	0	0
Planctomycetes	4	0	0	0	0	0	0	0	0	0
Elusimicrobia	2	0	0	0	0	0	0	0	0	0
Synergistetes	2	0	0	0	0	0	0	0	0	0
Cyanobacteria	16	0	0	0	0	0	0	0	0	0
Chlorobi	10	0	0	0	0	0	0	0	0	0
Chloroflexi	11	0	0	0	0	0	0	0	0	0
Deinococcus-Thermus	7	0	0	0	0	0	0	0	0	0
Aquificae	9	0	0	0	0	0	0	0	0	0
Thermotogae	11	0	0	0	0	0	0	0	0	0
Dictyoglomi	2	0	0	0	0	0	0	0	0	0
Nitrospirae	2	0	0	0	0	0	0	0	0	0
Thermobaculum	1	0	0	0	0	0	0	0	0	0
Deferribacteres	3	0	0	0	0	0	0	0	0	0
Euryarchaeota	60	0	0	0	0	0	0	0	0	0
Crenarchaeota	23	0	0	0	0	0	0	0	0	0
Thaumarchaeota	2	0	0	0	0	0	0	0	0	0
Nanoarchaeota	1	0	0	0	0	0	0	0	0	0
Korarchaeota	1	0	0	0	0	0	0	0	0	0
Total	761	0	0	0	0	0	0	0	0	7

(mod_M00374_1)

Phyla	Module completion ratio (%)									
	0--10	10--20	20--30	30--40	40--50	50--60	60--70	70--80	80--90	90--100
Gammaproteobacteria	2	2	5	22	22	42	31	0	0	0
Betaproteobacteria	1	0	1	7	4	38	10	0	0	0
Epsilonproteobacteria	0	1	0	10	5	1	0	0	0	0
Deltaproteobacteria	0	0	5	3	4	6	9	1	0	0
Alphaproteobacteria	1	1	1	42	38	6	2	0	0	0
Magnetococcus	0	0	0	0	1	0	0	0	0	0
Chrysiogenetes	0	0	0	1	0	0	0	0	0	0
Firmicutes	10	26	22	29	12	3	2	0	0	0
Tenericutes	19	0	0	0	0	0	0	0	0	0
Actinobacteria	1	2	4	23	20	27	3	0	0	0
Chlamydiae	0	0	0	8	0	0	0	0	0	0
Spirochaetes	8	2	1	2	1	0	0	0	0	0
Acidobacteria	0	0	0	2	0	3	0	0	0	0
Bacteroidetes	2	0	1	12	4	11	5	0	0	0
Fibrobacteres	0	0	0	1	0	0	0	0	0	0
Fusobacteria	0	2	2	1	0	0	0	0	0	0
Verrucomicrobia	0	0	0	2	0	2	0	0	0	0
Gemmatimonadetes	0	0	0	0	1	0	0	0	0	0
Planctomycetes	0	0	0	3	1	0	0	0	0	0
Elusimicrobia	0	2	0	0	0	0	0	0	0	0
Synergistetes	0	1	0	1	0	0	0	0	0	0
Cyanobacteria	1	0	3	5	2	3	2	0	0	0
Chlorobi	0	0	0	3	7	0	0	0	0	0
Chloroflexi	0	0	0	3	3	3	2	0	0	0
Deinococcus-Thermus	0	0	0	0	0	2	5	0	0	0
Aquificae	0	0	0	3	6	0	0	0	0	0
Thermotogae	0	6	3	2	0	0	0	0	0	0
Dictyoglomi	0	1	1	0	0	0	0	0	0	0
Nitrospirae	0	0	0	1	1	0	0	0	0	0
Thermobaculum	0	0	0	1	0	0	0	0	0	0
Deferribacteres	0	0	0	0	1	2	0	0	0	0
Euryarchaeota	0	1	1	12	25	10	10	1	0	0
Crenarchaeota	0	0	2	6	0	1	4	0	2	8
Thaumarchaeota	0	0	0	2	0	0	0	0	0	0
Nanoarchaeota	1	0	0	0	0	0	0	0	0	0
Korarchaeota	0	0	0	0	0	1	0	0	0	0
Total	46	47	52	207	158	161	85	2	2	8

(mod_M00375_1)

Phyla	Module completion ratio (%)									
	0--10	10--20	20--30	30--40	40--50	50--60	60--70	70--80	80--90	90--100
Gammaproteobacteria	52	58	16	0	0	0	0	0	0	0
Betaproteobacteria	11	18	11	20	1	0	0	0	0	0
Epsilonproteobacteria	15	2	0	0	0	0	0	0	0	0
Deltaproteobacteria	8	1	8	5	5	1	0	0	0	0
Alphaproteobacteria	23	12	2	3	42	9	0	0	0	0
Magnetococcus	0	0	0	0	1	0	0	0	0	0
Chrysiogenetes	0	0	0	1	0	0	0	0	0	0
Firmicutes	40	38	21	4	1	0	0	0	0	0
Tenericutes	18	1	0	0	0	0	0	0	0	0
Actinobacteria	30	7	23	10	10	0	0	0	0	0
Chlamydiae	7	0	0	1	0	0	0	0	0	0
Spirochaetes	11	3	0	0	0	0	0	0	0	0
Acidobacteria	0	1	2	2	0	0	0	0	0	0
Bacteroidetes	7	5	8	8	7	0	0	0	0	0
Fibrobacteres	1	0	0	0	0	0	0	0	0	0
Fusobacteria	4	0	0	1	0	0	0	0	0	0
Verrucomicrobia	1	1	2	0	0	0	0	0	0	0
Gemmatimonadetes	0	0	1	0	0	0	0	0	0	0
Planctomycetes	2	1	0	1	0	0	0	0	0	0
Elusimicrobia	2	0	0	0	0	0	0	0	0	0
Synergistetes	0	1	1	0	0	0	0	0	0	0
Cyanobacteria	14	2	0	0	0	0	0	0	0	0
Chlorobi	1	5	3	1	0	0	0	0	0	0
Chloroflexi	3	0	1	0	2	4	1	0	0	0
Deinococcus-Thermus	0	0	1	4	2	0	0	0	0	0
Aquificae	9	0	0	0	0	0	0	0	0	0
Thermotogae	5	4	2	0	0	0	0	0	0	0
Dictyoglomi	2	0	0	0	0	0	0	0	0	0
Nitrospirae	1	0	1	0	0	0	0	0	0	0
Thermobaculum	0	0	0	1	0	0	0	0	0	0
Deferribacteres	0	0	0	1	2	0	0	0	0	0
Euryarchaeota	34	2	21	3	0	0	0	0	0	0
Crenarchaeota	8	1	4	2	0	3	0	0	0	5
Thaumarchaeota	0	0	2	0	0	0	0	0	0	0
Nanoarchaeota	1	0	0	0	0	0	0	0	0	0
Korarchaeota	0	1	0	0	0	0	0	0	0	0
Total	310	164	130	68	73	17	1	0	0	5

(mod_M00376_1)

Phyla	Module completion ratio (%)									
	0--10	10--20	20--30	30--40	40--50	50--60	60--70	70--80	80--90	90--100
Gammaproteobacteria	2	18	95	11	0	0	0	0	0	0
Betaproteobacteria	1	3	27	10	18	1	0	1	0	0
Epsilonproteobacteria	0	10	7	0	0	0	0	0	0	0
Deltaproteobacteria	6	3	4	5	10	0	0	0	0	0
Alphaproteobacteria	1	4	29	3	4	30	20	0	0	0
Magnetococcus	0	0	0	0	0	1	0	0	0	0
Chrysiogenetes	0	0	0	0	1	0	0	0	0	0
Firmicutes	34	34	20	13	3	0	0	0	0	0
Tenericutes	19	0	0	0	0	0	0	0	0	0
Actinobacteria	7	22	8	22	10	11	0	0	0	0
Chlamydiae	0	0	7	1	0	0	0	0	0	0
Spirochaetes	9	2	3	0	0	0	0	0	0	0
Acidobacteria	0	0	2	1	2	0	0	0	0	0
Bacteroidetes	2	0	10	14	9	0	0	0	0	0
Fibrobacteres	0	1	0	0	0	0	0	0	0	0
Fusobacteria	3	1	0	1	0	0	0	0	0	0
Verrucomicrobia	0	0	1	1	2	0	0	0	0	0
Gemmatimonadetes	0	0	0	1	0	0	0	0	0	0
Planctomycetes	0	0	3	0	1	0	0	0	0	0
Elusimicrobia	1	1	0	0	0	0	0	0	0	0
Synergistetes	0	0	2	0	0	0	0	0	0	0
Cyanobacteria	1	4	11	0	0	0	0	0	0	0
Chlorobi	0	0	1	5	3	1	0	0	0	0
Chloroflexi	3	0	0	2	1	1	0	0	0	4
Deinococcus-Thermus	0	0	0	3	4	0	0	0	0	0
Aquificae	0	1	8	0	0	0	0	0	0	0
Thermotogae	7	2	2	0	0	0	0	0	0	0
Dictyoglomi	0	2	0	0	0	0	0	0	0	0
Nitrospirae	0	0	1	1	0	0	0	0	0	0
Thermobaculum	0	0	0	0	1	0	0	0	0	0
Deferribacteres	0	0	0	0	3	0	0	0	0	0
Euryarchaeota	29	7	20	4	0	0	0	0	0	0
Crenarchaeota	7	8	3	0	0	0	0	5	0	0
Thaumarchaeota	0	2	0	0	0	0	0	0	0	0
Nanoarchaeota	1	0	0	0	0	0	0	0	0	0
Korarchaeota	0	1	0	0	0	0	0	0	0	0
Total	133	126	264	98	72	45	20	6	0	4

(mod_M00376_2)

Phyla	Module completion ratio (%)									
	0--10	10--20	20--30	30--40	40--50	50--60	60--70	70--80	80--90	90--100
Gammaproteobacteria	120	0	5	0	1	0	0	0	0	0
Betaproteobacteria	27	0	25	0	8	0	0	0	0	1
Epsilonproteobacteria	16	0	1	0	0	0	0	0	0	0
Deltaproteobacteria	27	0	1	0	0	0	0	0	0	0
Alphaproteobacteria	50	0	13	0	8	13	0	0	7	0
Magnetococcus	1	0	0	0	0	0	0	0	0	0
Chrysiogenetes	0	0	1	0	0	0	0	0	0	0
Firmicutes	104	0	0	0	0	0	0	0	0	0
Tenericutes	19	0	0	0	0	0	0	0	0	0
Actinobacteria	62	0	10	0	7	1	0	0	0	0
Chlamydiae	8	0	0	0	0	0	0	0	0	0
Spirochaetes	11	0	3	0	0	0	0	0	0	0
Acidobacteria	5	0	0	0	0	0	0	0	0	0
Bacteroidetes	35	0	0	0	0	0	0	0	0	0
Fibrobacteres	1	0	0	0	0	0	0	0	0	0
Fusobacteria	5	0	0	0	0	0	0	0	0	0
Verrucomicrobia	4	0	0	0	0	0	0	0	0	0
Gemmatimonadetes	1	0	0	0	0	0	0	0	0	0
Planctomycetes	4	0	0	0	0	0	0	0	0	0
Elusimicrobia	2	0	0	0	0	0	0	0	0	0
Synergistetes	2	0	0	0	0	0	0	0	0	0
Cyanobacteria	16	0	0	0	0	0	0	0	0	0
Chlorobi	10	0	0	0	0	0	0	0	0	0
Chloroflexi	7	0	0	0	0	0	0	0	0	4
Deinococcus-Thermus	7	0	0	0	0	0	0	0	0	0
Aquificae	9	0	0	0	0	0	0	0	0	0
Thermotogae	11	0	0	0	0	0	0	0	0	0
Dictyoglomi	2	0	0	0	0	0	0	0	0	0
Nitrospirae	2	0	0	0	0	0	0	0	0	0
Thermobaculum	1	0	0	0	0	0	0	0	0	0
Deferribacteres	3	0	0	0	0	0	0	0	0	0
Euryarchaeota	60	0	0	0	0	0	0	0	0	0
Crenarchaeota	23	0	0	0	0	0	0	0	0	0
Thaumarchaeota	2	0	0	0	0	0	0	0	0	0
Nanoarchaeota	1	0	0	0	0	0	0	0	0	0
Korarchaeota	1	0	0	0	0	0	0	0	0	0
Total	659	0	59	0	24	14	0	0	7	5

(mod_M00377_1)

Phyla	Module completion ratio (%)									
	0--10	10--20	20--30	30--40	40--50	50--60	60--70	70--80	80--90	90--100
Gammaproteobacteria	0	3	18	0	22	56	0	27	0	0
Betaproteobacteria	0	2	10	0	10	35	0	4	0	0
Epsilonproteobacteria	0	3	0	0	12	2	0	0	0	0
Deltaproteobacteria	0	1	2	0	8	13	0	4	0	0
Alphaproteobacteria	0	18	15	0	23	21	0	14	0	0
Magnetococcus	0	0	1	0	0	0	0	0	0	0
Chrysiogenetes	0	0	0	0	0	1	0	0	0	0
Firmicutes	0	3	1	0	12	60	0	23	0	5
Tenericutes	0	3	3	0	10	3	0	0	0	0
Actinobacteria	0	2	6	0	23	36	0	13	0	0
Chlamydiae	0	7	1	0	0	0	0	0	0	0
Spirochaetes	0	0	4	0	7	3	0	0	0	0
Acidobacteria	0	1	1	0	2	1	0	0	0	0
Bacteroidetes	1	4	11	0	4	9	0	6	0	0
Fibrobacteres	0	0	0	0	0	1	0	0	0	0
Fusobacteria	0	0	0	0	0	2	0	3	0	0
Verrucomicrobia	0	0	0	0	1	3	0	0	0	0
Gemmatimonadetes	0	0	0	0	0	1	0	0	0	0
Planctomycetes	0	0	0	0	2	2	0	0	0	0
Elusimicrobia	0	0	0	0	2	0	0	0	0	0
Synergistetes	0	0	0	0	1	1	0	0	0	0
Cyanobacteria	1	0	8	0	5	2	0	0	0	0
Chlorobi	0	0	3	0	5	2	0	0	0	0
Chloroflexi	1	0	3	0	7	0	0	0	0	0
Deinococcus-Thermus	0	0	1	0	3	3	0	0	0	0
Aquificae	0	2	6	0	1	0	0	0	0	0
Thermotogae	0	0	0	0	0	6	0	5	0	0
Dictyoglomi	0	0	0	0	2	0	0	0	0	0
Nitrospirae	0	0	0	0	2	0	0	0	0	0
Thermobaculum	0	0	1	0	0	0	0	0	0	0
Deferribacteres	0	0	0	0	0	3	0	0	0	0
Euryarchaeota	30	19	6	0	4	1	0	0	0	0
Crenarchaeota	18	5	0	0	0	0	0	0	0	0
Thaumarchaeota	0	2	0	0	0	0	0	0	0	0
Nanoarchaeota	1	0	0	0	0	0	0	0	0	0
Korarchaeota	1	0	0	0	0	0	0	0	0	0
Total	53	75	101	0	168	267	0	99	0	5

(mod_M00378_1)

Phyla	Module completion ratio (%)									
	0--10	10--20	20--30	30--40	40--50	50--60	60--70	70--80	80--90	90--100
Gammaproteobacteria	126	0	0	0	0	0	0	0	0	0
Betaproteobacteria	61	0	0	0	0	0	0	0	0	0
Epsilonproteobacteria	17	0	0	0	0	0	0	0	0	0
Deltaproteobacteria	28	0	0	0	0	0	0	0	0	0
Alphaproteobacteria	91	0	0	0	0	0	0	0	0	0
Magnetococcus	1	0	0	0	0	0	0	0	0	0
Chrysiogenetes	1	0	0	0	0	0	0	0	0	0
Firmicutes	104	0	0	0	0	0	0	0	0	0
Tenericutes	19	0	0	0	0	0	0	0	0	0
Actinobacteria	80	0	0	0	0	0	0	0	0	0
Chlamydiae	8	0	0	0	0	0	0	0	0	0
Spirochaetes	14	0	0	0	0	0	0	0	0	0
Acidobacteria	5	0	0	0	0	0	0	0	0	0
Bacteroidetes	35	0	0	0	0	0	0	0	0	0
Fibrobacteres	1	0	0	0	0	0	0	0	0	0
Fusobacteria	5	0	0	0	0	0	0	0	0	0
Verrucomicrobia	4	0	0	0	0	0	0	0	0	0
Gemmatimonadetes	1	0	0	0	0	0	0	0	0	0
Planctomycetes	4	0	0	0	0	0	0	0	0	0
Elusimicrobia	2	0	0	0	0	0	0	0	0	0
Synergistetes	2	0	0	0	0	0	0	0	0	0
Cyanobacteria	4	0	0	0	0	0	0	0	0	12
Chlorobi	10	0	0	0	0	0	0	0	0	0
Chloroflexi	11	0	0	0	0	0	0	0	0	0
Deinococcus-Thermus	7	0	0	0	0	0	0	0	0	0
Aquificae	9	0	0	0	0	0	0	0	0	0
Thermotogae	11	0	0	0	0	0	0	0	0	0
Dictyoglomi	2	0	0	0	0	0	0	0	0	0
Nitrospirae	2	0	0	0	0	0	0	0	0	0
Thermobaculum	1	0	0	0	0	0	0	0	0	0
Deferribacteres	3	0	0	0	0	0	0	0	0	0
Euryarchaeota	31	0	0	0	0	0	0	0	0	29
Crenarchaeota	23	0	0	0	0	0	0	0	0	0
Thaumarchaeota	2	0	0	0	0	0	0	0	0	0
Nanoarchaeota	1	0	0	0	0	0	0	0	0	0
Korarchaeota	1	0	0	0	0	0	0	0	0	0
Total	727	0	0	0	0	0	0	0	0	41

(mod_M00378_2)

Phyla	Module completion ratio (%)									
	0--10	10--20	20--30	30--40	40--50	50--60	60--70	70--80	80--90	90--100
Gammaproteobacteria	125	0	0	0	0	0	0	0	0	1
Betaproteobacteria	59	0	0	0	0	0	0	0	0	2
Epsilonproteobacteria	17	0	0	0	0	0	0	0	0	0
Deltaproteobacteria	28	0	0	0	0	0	0	0	0	0
Alphaproteobacteria	86	0	0	0	0	0	0	0	0	5
Magnetococcus	1	0	0	0	0	0	0	0	0	0
Chrysiogenetes	1	0	0	0	0	0	0	0	0	0
Firmicutes	103	0	0	0	0	0	0	0	0	1
Tenericutes	19	0	0	0	0	0	0	0	0	0
Actinobacteria	33	0	0	0	0	0	0	0	0	47
Chlamydiae	8	0	0	0	0	0	0	0	0	0
Spirochaetes	14	0	0	0	0	0	0	0	0	0
Acidobacteria	5	0	0	0	0	0	0	0	0	0
Bacteroidetes	35	0	0	0	0	0	0	0	0	0
Fibrobacteres	1	0	0	0	0	0	0	0	0	0
Fusobacteria	5	0	0	0	0	0	0	0	0	0
Verrucomicrobia	4	0	0	0	0	0	0	0	0	0
Gemmatimonadetes	1	0	0	0	0	0	0	0	0	0
Planctomycetes	4	0	0	0	0	0	0	0	0	0
Elusimicrobia	2	0	0	0	0	0	0	0	0	0
Synergistetes	2	0	0	0	0	0	0	0	0	0
Cyanobacteria	16	0	0	0	0	0	0	0	0	0
Chlorobi	10	0	0	0	0	0	0	0	0	0
Chloroflexi	4	0	0	0	0	0	0	0	0	7
Deinococcus-Thermus	7	0	0	0	0	0	0	0	0	0
Aquificae	9	0	0	0	0	0	0	0	0	0
Thermotogae	11	0	0	0	0	0	0	0	0	0
Dictyoglomi	2	0	0	0	0	0	0	0	0	0
Nitrospirae	2	0	0	0	0	0	0	0	0	0
Thermobaculum	0	0	0	0	0	0	0	0	0	1
Deferribacteres	3	0	0	0	0	0	0	0	0	0
Euryarchaeota	13	0	0	0	0	0	0	0	0	47
Crenarchaeota	23	0	0	0	0	0	0	0	0	0
Thaumarchaeota	0	0	0	0	0	0	0	0	0	2
Nanoarchaeota	1	0	0	0	0	0	0	0	0	0
Korarchaeota	1	0	0	0	0	0	0	0	0	0
Total	655	0	0	0	0	0	0	0	0	113

(mod_M00378_3)

Phyla	Module completion ratio (%)									
	0--10	10--20	20--30	30--40	40--50	50--60	60--70	70--80	80--90	90--100
Gammaproteobacteria	121	0	0	4	0	0	1	0	0	0
Betaproteobacteria	59	0	0	0	0	0	2	0	0	0
Epsilonproteobacteria	17	0	0	0	0	0	0	0	0	0
Deltaproteobacteria	27	0	0	0	0	0	1	0	0	0
Alphaproteobacteria	86	0	0	0	0	0	5	0	0	0
Magnetococcus	1	0	0	0	0	0	0	0	0	0
Chrysiogenetes	1	0	0	0	0	0	0	0	0	0
Firmicutes	103	0	0	1	0	0	0	0	0	0
Tenericutes	19	0	0	0	0	0	0	0	0	0
Actinobacteria	28	0	0	4	0	0	48	0	0	0
Chlamydiae	8	0	0	0	0	0	0	0	0	0
Spirochaetes	14	0	0	0	0	0	0	0	0	0
Acidobacteria	5	0	0	0	0	0	0	0	0	0
Bacteroidetes	35	0	0	0	0	0	0	0	0	0
Fibrobacteres	1	0	0	0	0	0	0	0	0	0
Fusobacteria	5	0	0	0	0	0	0	0	0	0
Verrucomicrobia	4	0	0	0	0	0	0	0	0	0
Gemmatimonadetes	1	0	0	0	0	0	0	0	0	0
Planctomycetes	4	0	0	0	0	0	0	0	0	0
Elusimicrobia	2	0	0	0	0	0	0	0	0	0
Synergistetes	2	0	0	0	0	0	0	0	0	0
Cyanobacteria	16	0	0	0	0	0	0	0	0	0
Chlorobi	10	0	0	0	0	0	0	0	0	0
Chloroflexi	4	0	0	0	0	0	7	0	0	0
Deinococcus-Thermus	7	0	0	0	0	0	0	0	0	0
Aquificae	9	0	0	0	0	0	0	0	0	0
Thermotogae	10	0	0	1	0	0	0	0	0	0
Dictyoglomi	2	0	0	0	0	0	0	0	0	0
Nitrospirae	2	0	0	0	0	0	0	0	0	0
Thermobaculum	0	0	0	0	0	0	1	0	0	0
Deferribacteres	3	0	0	0	0	0	0	0	0	0
Euryarchaeota	12	0	0	0	0	0	39	0	0	9
Crenarchaeota	22	0	0	1	0	0	0	0	0	0
Thaumarchaeota	0	0	0	0	0	0	2	0	0	0
Nanoarchaeota	1	0	0	0	0	0	0	0	0	0
Korarchaeota	0	0	0	1	0	0	0	0	0	0
Total	641	0	0	12	0	0	106	0	0	9

(mod_M00379_1)

Phyla	Module completion ratio (%)									
	0--10	10--20	20--30	30--40	40--50	50--60	60--70	70--80	80--90	90--100
Gammaproteobacteria	126	0	0	0	0	0	0	0	0	0
Betaproteobacteria	61	0	0	0	0	0	0	0	0	0
Epsilonproteobacteria	17	0	0	0	0	0	0	0	0	0
Deltaproteobacteria	28	0	0	0	0	0	0	0	0	0
Alphaproteobacteria	91	0	0	0	0	0	0	0	0	0
Magnetococcus	1	0	0	0	0	0	0	0	0	0
Chrysiogenetes	1	0	0	0	0	0	0	0	0	0
Firmicutes	104	0	0	0	0	0	0	0	0	0
Tenericutes	19	0	0	0	0	0	0	0	0	0
Actinobacteria	80	0	0	0	0	0	0	0	0	0
Chlamydiae	8	0	0	0	0	0	0	0	0	0
Spirochaetes	14	0	0	0	0	0	0	0	0	0
Acidobacteria	5	0	0	0	0	0	0	0	0	0
Bacteroidetes	35	0	0	0	0	0	0	0	0	0
Fibrobacteres	1	0	0	0	0	0	0	0	0	0
Fusobacteria	5	0	0	0	0	0	0	0	0	0
Verrucomicrobia	4	0	0	0	0	0	0	0	0	0
Gemmatimonadetes	1	0	0	0	0	0	0	0	0	0
Planctomycetes	4	0	0	0	0	0	0	0	0	0
Elusimicrobia	2	0	0	0	0	0	0	0	0	0
Synergistetes	2	0	0	0	0	0	0	0	0	0
Cyanobacteria	16	0	0	0	0	0	0	0	0	0
Chlorobi	10	0	0	0	0	0	0	0	0	0
Chloroflexi	11	0	0	0	0	0	0	0	0	0
Deinococcus-Thermus	7	0	0	0	0	0	0	0	0	0
Aquificae	9	0	0	0	0	0	0	0	0	0
Thermotogae	11	0	0	0	0	0	0	0	0	0
Dictyoglomi	2	0	0	0	0	0	0	0	0	0
Nitrospirae	2	0	0	0	0	0	0	0	0	0
Thermobaculum	1	0	0	0	0	0	0	0	0	0
Deferribacteres	3	0	0	0	0	0	0	0	0	0
Euryarchaeota	60	0	0	0	0	0	0	0	0	0
Crenarchaeota	23	0	0	0	0	0	0	0	0	0
Thaumarchaeota	2	0	0	0	0	0	0	0	0	0
Nanoarchaeota	1	0	0	0	0	0	0	0	0	0
Korarchaeota	1	0	0	0	0	0	0	0	0	0
Total	768	0	0	0	0	0	0	0	0	0

(mod_M00380_1)

Phyla	Module completion ratio (%)									
	0--10	10--20	20--30	30--40	40--50	50--60	60--70	70--80	80--90	90--100
Gammaproteobacteria	126	0	0	0	0	0	0	0	0	0
Betaproteobacteria	61	0	0	0	0	0	0	0	0	0
Epsilonproteobacteria	17	0	0	0	0	0	0	0	0	0
Deltaproteobacteria	28	0	0	0	0	0	0	0	0	0
Alphaproteobacteria	91	0	0	0	0	0	0	0	0	0
Magnetococcus	1	0	0	0	0	0	0	0	0	0
Chrysiogenetes	1	0	0	0	0	0	0	0	0	0
Firmicutes	104	0	0	0	0	0	0	0	0	0
Tenericutes	19	0	0	0	0	0	0	0	0	0
Actinobacteria	80	0	0	0	0	0	0	0	0	0
Chlamydiae	8	0	0	0	0	0	0	0	0	0
Spirochaetes	14	0	0	0	0	0	0	0	0	0
Acidobacteria	5	0	0	0	0	0	0	0	0	0
Bacteroidetes	35	0	0	0	0	0	0	0	0	0
Fibrobacteres	1	0	0	0	0	0	0	0	0	0
Fusobacteria	5	0	0	0	0	0	0	0	0	0
Verrucomicrobia	4	0	0	0	0	0	0	0	0	0
Gemmatimonadetes	1	0	0	0	0	0	0	0	0	0
Planctomycetes	4	0	0	0	0	0	0	0	0	0
Elusimicrobia	2	0	0	0	0	0	0	0	0	0
Synergistetes	2	0	0	0	0	0	0	0	0	0
Cyanobacteria	16	0	0	0	0	0	0	0	0	0
Chlorobi	10	0	0	0	0	0	0	0	0	0
Chloroflexi	11	0	0	0	0	0	0	0	0	0
Deinococcus-Thermus	7	0	0	0	0	0	0	0	0	0
Aquificae	9	0	0	0	0	0	0	0	0	0
Thermotogae	11	0	0	0	0	0	0	0	0	0
Dictyoglomi	2	0	0	0	0	0	0	0	0	0
Nitrospirae	2	0	0	0	0	0	0	0	0	0
Thermobaculum	1	0	0	0	0	0	0	0	0	0
Deferribacteres	3	0	0	0	0	0	0	0	0	0
Euryarchaeota	60	0	0	0	0	0	0	0	0	0
Crenarchaeota	23	0	0	0	0	0	0	0	0	0
Thaumarchaeota	2	0	0	0	0	0	0	0	0	0
Nanoarchaeota	1	0	0	0	0	0	0	0	0	0
Korarchaeota	1	0	0	0	0	0	0	0	0	0
Total	768	0	0	0	0	0	0	0	0	0

(mod_M00381_1)

Phyla	Module completion ratio (%)									
	0--10	10--20	20--30	30--40	40--50	50--60	60--70	70--80	80--90	90--100
Gammaproteobacteria	126	0	0	0	0	0	0	0	0	0
Betaproteobacteria	61	0	0	0	0	0	0	0	0	0
Epsilonproteobacteria	17	0	0	0	0	0	0	0	0	0
Deltaproteobacteria	28	0	0	0	0	0	0	0	0	0
Alphaproteobacteria	91	0	0	0	0	0	0	0	0	0
Magnetococcus	1	0	0	0	0	0	0	0	0	0
Chrysiogenetes	1	0	0	0	0	0	0	0	0	0
Firmicutes	104	0	0	0	0	0	0	0	0	0
Tenericutes	19	0	0	0	0	0	0	0	0	0
Actinobacteria	80	0	0	0	0	0	0	0	0	0
Chlamydiae	8	0	0	0	0	0	0	0	0	0
Spirochaetes	14	0	0	0	0	0	0	0	0	0
Acidobacteria	5	0	0	0	0	0	0	0	0	0
Bacteroidetes	35	0	0	0	0	0	0	0	0	0
Fibrobacteres	1	0	0	0	0	0	0	0	0	0
Fusobacteria	5	0	0	0	0	0	0	0	0	0
Verrucomicrobia	4	0	0	0	0	0	0	0	0	0
Gemmatimonadetes	1	0	0	0	0	0	0	0	0	0
Planctomycetes	4	0	0	0	0	0	0	0	0	0
Elusimicrobia	2	0	0	0	0	0	0	0	0	0
Synergistetes	2	0	0	0	0	0	0	0	0	0
Cyanobacteria	16	0	0	0	0	0	0	0	0	0
Chlorobi	10	0	0	0	0	0	0	0	0	0
Chloroflexi	11	0	0	0	0	0	0	0	0	0
Deinococcus-Thermus	7	0	0	0	0	0	0	0	0	0
Aquificae	9	0	0	0	0	0	0	0	0	0
Thermotogae	11	0	0	0	0	0	0	0	0	0
Dictyoglomi	2	0	0	0	0	0	0	0	0	0
Nitrospirae	2	0	0	0	0	0	0	0	0	0
Thermobaculum	1	0	0	0	0	0	0	0	0	0
Deferribacteres	3	0	0	0	0	0	0	0	0	0
Euryarchaeota	60	0	0	0	0	0	0	0	0	0
Crenarchaeota	23	0	0	0	0	0	0	0	0	0
Thaumarchaeota	2	0	0	0	0	0	0	0	0	0
Nanoarchaeota	1	0	0	0	0	0	0	0	0	0
Korarchaeota	1	0	0	0	0	0	0	0	0	0
Total	768	0	0	0	0	0	0	0	0	0

(mod_M00382_1)

Phyla	Module completion ratio (%)									
	0--10	10--20	20--30	30--40	40--50	50--60	60--70	70--80	80--90	90--100
Gammaproteobacteria	126	0	0	0	0	0	0	0	0	0
Betaproteobacteria	61	0	0	0	0	0	0	0	0	0
Epsilonproteobacteria	17	0	0	0	0	0	0	0	0	0
Deltaproteobacteria	28	0	0	0	0	0	0	0	0	0
Alphaproteobacteria	91	0	0	0	0	0	0	0	0	0
Magnetococcus	1	0	0	0	0	0	0	0	0	0
Chrysiogenetes	1	0	0	0	0	0	0	0	0	0
Firmicutes	104	0	0	0	0	0	0	0	0	0
Tenericutes	19	0	0	0	0	0	0	0	0	0
Actinobacteria	80	0	0	0	0	0	0	0	0	0
Chlamydiae	8	0	0	0	0	0	0	0	0	0
Spirochaetes	14	0	0	0	0	0	0	0	0	0
Acidobacteria	5	0	0	0	0	0	0	0	0	0
Bacteroidetes	35	0	0	0	0	0	0	0	0	0
Fibrobacteres	1	0	0	0	0	0	0	0	0	0
Fusobacteria	5	0	0	0	0	0	0	0	0	0
Verrucomicrobia	4	0	0	0	0	0	0	0	0	0
Gemmatimonadetes	1	0	0	0	0	0	0	0	0	0
Planctomycetes	4	0	0	0	0	0	0	0	0	0
Elusimicrobia	2	0	0	0	0	0	0	0	0	0
Synergistetes	2	0	0	0	0	0	0	0	0	0
Cyanobacteria	16	0	0	0	0	0	0	0	0	0
Chlorobi	10	0	0	0	0	0	0	0	0	0
Chloroflexi	11	0	0	0	0	0	0	0	0	0
Deinococcus-Thermus	7	0	0	0	0	0	0	0	0	0
Aquificae	9	0	0	0	0	0	0	0	0	0
Thermotogae	11	0	0	0	0	0	0	0	0	0
Dictyoglomi	2	0	0	0	0	0	0	0	0	0
Nitrospirae	2	0	0	0	0	0	0	0	0	0
Thermobaculum	1	0	0	0	0	0	0	0	0	0
Deferribacteres	3	0	0	0	0	0	0	0	0	0
Euryarchaeota	60	0	0	0	0	0	0	0	0	0
Crenarchaeota	23	0	0	0	0	0	0	0	0	0
Thaumarchaeota	2	0	0	0	0	0	0	0	0	0
Nanoarchaeota	1	0	0	0	0	0	0	0	0	0
Korarchaeota	1	0	0	0	0	0	0	0	0	0
Total	768	0	0	0	0	0	0	0	0	0

(mod_M00383_1)

Phyla	Module completion ratio (%)									
	0--10	10--20	20--30	30--40	40--50	50--60	60--70	70--80	80--90	90--100
Gammaproteobacteria	126	0	0	0	0	0	0	0	0	0
Betaproteobacteria	61	0	0	0	0	0	0	0	0	0
Epsilonproteobacteria	17	0	0	0	0	0	0	0	0	0
Deltaproteobacteria	28	0	0	0	0	0	0	0	0	0
Alphaproteobacteria	91	0	0	0	0	0	0	0	0	0
Magnetococcus	1	0	0	0	0	0	0	0	0	0
Chrysiogenetes	1	0	0	0	0	0	0	0	0	0
Firmicutes	104	0	0	0	0	0	0	0	0	0
Tenericutes	19	0	0	0	0	0	0	0	0	0
Actinobacteria	80	0	0	0	0	0	0	0	0	0
Chlamydiae	8	0	0	0	0	0	0	0	0	0
Spirochaetes	14	0	0	0	0	0	0	0	0	0
Acidobacteria	5	0	0	0	0	0	0	0	0	0
Bacteroidetes	35	0	0	0	0	0	0	0	0	0
Fibrobacteres	1	0	0	0	0	0	0	0	0	0
Fusobacteria	5	0	0	0	0	0	0	0	0	0
Verrucomicrobia	4	0	0	0	0	0	0	0	0	0
Gemmatimonadetes	1	0	0	0	0	0	0	0	0	0
Planctomycetes	4	0	0	0	0	0	0	0	0	0
Elusimicrobia	2	0	0	0	0	0	0	0	0	0
Synergistetes	2	0	0	0	0	0	0	0	0	0
Cyanobacteria	16	0	0	0	0	0	0	0	0	0
Chlorobi	10	0	0	0	0	0	0	0	0	0
Chloroflexi	11	0	0	0	0	0	0	0	0	0
Deinococcus-Thermus	7	0	0	0	0	0	0	0	0	0
Aquificae	9	0	0	0	0	0	0	0	0	0
Thermotogae	11	0	0	0	0	0	0	0	0	0
Dictyoglomi	2	0	0	0	0	0	0	0	0	0
Nitrospirae	2	0	0	0	0	0	0	0	0	0
Thermobaculum	1	0	0	0	0	0	0	0	0	0
Deferribacteres	3	0	0	0	0	0	0	0	0	0
Euryarchaeota	60	0	0	0	0	0	0	0	0	0
Crenarchaeota	23	0	0	0	0	0	0	0	0	0
Thaumarchaeota	2	0	0	0	0	0	0	0	0	0
Nanoarchaeota	1	0	0	0	0	0	0	0	0	0
Korarchaeota	1	0	0	0	0	0	0	0	0	0
Total	768	0	0	0	0	0	0	0	0	0

(mod_M00384_1)

Phyla	Module completion ratio (%)									
	0--10	10--20	20--30	30--40	40--50	50--60	60--70	70--80	80--90	90--100
Gammaproteobacteria	126	0	0	0	0	0	0	0	0	0
Betaproteobacteria	61	0	0	0	0	0	0	0	0	0
Epsilonproteobacteria	17	0	0	0	0	0	0	0	0	0
Deltaproteobacteria	28	0	0	0	0	0	0	0	0	0
Alphaproteobacteria	91	0	0	0	0	0	0	0	0	0
Magnetococcus	1	0	0	0	0	0	0	0	0	0
Chrysiogenetes	1	0	0	0	0	0	0	0	0	0
Firmicutes	104	0	0	0	0	0	0	0	0	0
Tenericutes	19	0	0	0	0	0	0	0	0	0
Actinobacteria	80	0	0	0	0	0	0	0	0	0
Chlamydiae	8	0	0	0	0	0	0	0	0	0
Spirochaetes	14	0	0	0	0	0	0	0	0	0
Acidobacteria	5	0	0	0	0	0	0	0	0	0
Bacteroidetes	35	0	0	0	0	0	0	0	0	0
Fibrobacteres	1	0	0	0	0	0	0	0	0	0
Fusobacteria	5	0	0	0	0	0	0	0	0	0
Verrucomicrobia	4	0	0	0	0	0	0	0	0	0
Gemmatimonadetes	1	0	0	0	0	0	0	0	0	0
Planctomycetes	4	0	0	0	0	0	0	0	0	0
Elusimicrobia	2	0	0	0	0	0	0	0	0	0
Synergistetes	2	0	0	0	0	0	0	0	0	0
Cyanobacteria	16	0	0	0	0	0	0	0	0	0
Chlorobi	10	0	0	0	0	0	0	0	0	0
Chloroflexi	11	0	0	0	0	0	0	0	0	0
Deinococcus-Thermus	7	0	0	0	0	0	0	0	0	0
Aquificae	9	0	0	0	0	0	0	0	0	0
Thermotogae	11	0	0	0	0	0	0	0	0	0
Dictyoglomi	2	0	0	0	0	0	0	0	0	0
Nitrospirae	2	0	0	0	0	0	0	0	0	0
Thermobaculum	1	0	0	0	0	0	0	0	0	0
Deferribacteres	3	0	0	0	0	0	0	0	0	0
Euryarchaeota	60	0	0	0	0	0	0	0	0	0
Crenarchaeota	23	0	0	0	0	0	0	0	0	0
Thaumarchaeota	2	0	0	0	0	0	0	0	0	0
Nanoarchaeota	1	0	0	0	0	0	0	0	0	0
Korarchaeota	1	0	0	0	0	0	0	0	0	0
Total	768	0	0	0	0	0	0	0	0	0

(mod_M00385_1)

Phyla	Module completion ratio (%)									
	0--10	10--20	20--30	30--40	40--50	50--60	60--70	70--80	80--90	90--100
Gammaproteobacteria	126	0	0	0	0	0	0	0	0	0
Betaproteobacteria	61	0	0	0	0	0	0	0	0	0
Epsilonproteobacteria	17	0	0	0	0	0	0	0	0	0
Deltaproteobacteria	28	0	0	0	0	0	0	0	0	0
Alphaproteobacteria	91	0	0	0	0	0	0	0	0	0
Magnetococcus	1	0	0	0	0	0	0	0	0	0
Chrysiogenetes	1	0	0	0	0	0	0	0	0	0
Firmicutes	104	0	0	0	0	0	0	0	0	0
Tenericutes	19	0	0	0	0	0	0	0	0	0
Actinobacteria	80	0	0	0	0	0	0	0	0	0
Chlamydiae	8	0	0	0	0	0	0	0	0	0
Spirochaetes	14	0	0	0	0	0	0	0	0	0
Acidobacteria	5	0	0	0	0	0	0	0	0	0
Bacteroidetes	35	0	0	0	0	0	0	0	0	0
Fibrobacteres	1	0	0	0	0	0	0	0	0	0
Fusobacteria	5	0	0	0	0	0	0	0	0	0
Verrucomicrobia	4	0	0	0	0	0	0	0	0	0
Gemmatimonadetes	1	0	0	0	0	0	0	0	0	0
Planctomycetes	4	0	0	0	0	0	0	0	0	0
Elusimicrobia	2	0	0	0	0	0	0	0	0	0
Synergistetes	2	0	0	0	0	0	0	0	0	0
Cyanobacteria	16	0	0	0	0	0	0	0	0	0
Chlorobi	10	0	0	0	0	0	0	0	0	0
Chloroflexi	11	0	0	0	0	0	0	0	0	0
Deinococcus-Thermus	7	0	0	0	0	0	0	0	0	0
Aquificae	9	0	0	0	0	0	0	0	0	0
Thermotogae	11	0	0	0	0	0	0	0	0	0
Dictyoglomi	2	0	0	0	0	0	0	0	0	0
Nitrospirae	2	0	0	0	0	0	0	0	0	0
Thermobaculum	1	0	0	0	0	0	0	0	0	0
Deferribacteres	3	0	0	0	0	0	0	0	0	0
Euryarchaeota	60	0	0	0	0	0	0	0	0	0
Crenarchaeota	23	0	0	0	0	0	0	0	0	0
Thaumarchaeota	2	0	0	0	0	0	0	0	0	0
Nanoarchaeota	1	0	0	0	0	0	0	0	0	0
Korarchaeota	1	0	0	0	0	0	0	0	0	0
Total	768	0	0	0	0	0	0	0	0	0

(mod_M00386_1)

Phyla	Module completion ratio (%)									
	0--10	10--20	20--30	30--40	40--50	50--60	60--70	70--80	80--90	90--100
Gammaproteobacteria	126	0	0	0	0	0	0	0	0	0
Betaproteobacteria	61	0	0	0	0	0	0	0	0	0
Epsilonproteobacteria	17	0	0	0	0	0	0	0	0	0
Deltaproteobacteria	28	0	0	0	0	0	0	0	0	0
Alphaproteobacteria	91	0	0	0	0	0	0	0	0	0
Magnetococcus	1	0	0	0	0	0	0	0	0	0
Chrysiogenetes	1	0	0	0	0	0	0	0	0	0
Firmicutes	104	0	0	0	0	0	0	0	0	0
Tenericutes	19	0	0	0	0	0	0	0	0	0
Actinobacteria	80	0	0	0	0	0	0	0	0	0
Chlamydiae	8	0	0	0	0	0	0	0	0	0
Spirochaetes	14	0	0	0	0	0	0	0	0	0
Acidobacteria	5	0	0	0	0	0	0	0	0	0
Bacteroidetes	35	0	0	0	0	0	0	0	0	0
Fibrobacteres	1	0	0	0	0	0	0	0	0	0
Fusobacteria	5	0	0	0	0	0	0	0	0	0
Verrucomicrobia	4	0	0	0	0	0	0	0	0	0
Gemmatimonadetes	1	0	0	0	0	0	0	0	0	0
Planctomycetes	4	0	0	0	0	0	0	0	0	0
Elusimicrobia	2	0	0	0	0	0	0	0	0	0
Synergistetes	2	0	0	0	0	0	0	0	0	0
Cyanobacteria	16	0	0	0	0	0	0	0	0	0
Chlorobi	10	0	0	0	0	0	0	0	0	0
Chloroflexi	11	0	0	0	0	0	0	0	0	0
Deinococcus-Thermus	7	0	0	0	0	0	0	0	0	0
Aquificae	9	0	0	0	0	0	0	0	0	0
Thermotogae	11	0	0	0	0	0	0	0	0	0
Dictyoglomi	2	0	0	0	0	0	0	0	0	0
Nitrospirae	2	0	0	0	0	0	0	0	0	0
Thermobaculum	1	0	0	0	0	0	0	0	0	0
Deferribacteres	3	0	0	0	0	0	0	0	0	0
Euryarchaeota	60	0	0	0	0	0	0	0	0	0
Crenarchaeota	23	0	0	0	0	0	0	0	0	0
Thaumarchaeota	2	0	0	0	0	0	0	0	0	0
Nanoarchaeota	1	0	0	0	0	0	0	0	0	0
Korarchaeota	1	0	0	0	0	0	0	0	0	0
Total	768	0	0	0	0	0	0	0	0	0

(mod_M00387_1)

Phyla	Module completion ratio (%)									
	0--10	10--20	20--30	30--40	40--50	50--60	60--70	70--80	80--90	90--100
Gammaproteobacteria	126	0	0	0	0	0	0	0	0	0
Betaproteobacteria	61	0	0	0	0	0	0	0	0	0
Epsilonproteobacteria	17	0	0	0	0	0	0	0	0	0
Deltaproteobacteria	28	0	0	0	0	0	0	0	0	0
Alphaproteobacteria	91	0	0	0	0	0	0	0	0	0
Magnetococcus	1	0	0	0	0	0	0	0	0	0
Chrysiogenetes	1	0	0	0	0	0	0	0	0	0
Firmicutes	104	0	0	0	0	0	0	0	0	0
Tenericutes	19	0	0	0	0	0	0	0	0	0
Actinobacteria	80	0	0	0	0	0	0	0	0	0
Chlamydiae	8	0	0	0	0	0	0	0	0	0
Spirochaetes	14	0	0	0	0	0	0	0	0	0
Acidobacteria	5	0	0	0	0	0	0	0	0	0
Bacteroidetes	35	0	0	0	0	0	0	0	0	0
Fibrobacteres	1	0	0	0	0	0	0	0	0	0
Fusobacteria	5	0	0	0	0	0	0	0	0	0
Verrucomicrobia	4	0	0	0	0	0	0	0	0	0
Gemmatimonadetes	1	0	0	0	0	0	0	0	0	0
Planctomycetes	4	0	0	0	0	0	0	0	0	0
Elusimicrobia	2	0	0	0	0	0	0	0	0	0
Synergistetes	2	0	0	0	0	0	0	0	0	0
Cyanobacteria	16	0	0	0	0	0	0	0	0	0
Chlorobi	10	0	0	0	0	0	0	0	0	0
Chloroflexi	11	0	0	0	0	0	0	0	0	0
Deinococcus-Thermus	7	0	0	0	0	0	0	0	0	0
Aquificae	9	0	0	0	0	0	0	0	0	0
Thermotogae	11	0	0	0	0	0	0	0	0	0
Dictyoglomi	2	0	0	0	0	0	0	0	0	0
Nitrospirae	2	0	0	0	0	0	0	0	0	0
Thermobaculum	1	0	0	0	0	0	0	0	0	0
Deferribacteres	3	0	0	0	0	0	0	0	0	0
Euryarchaeota	60	0	0	0	0	0	0	0	0	0
Crenarchaeota	23	0	0	0	0	0	0	0	0	0
Thaumarchaeota	2	0	0	0	0	0	0	0	0	0
Nanoarchaeota	1	0	0	0	0	0	0	0	0	0
Korarchaeota	1	0	0	0	0	0	0	0	0	0
Total	768	0	0	0	0	0	0	0	0	0

(mod_M00388_1)

Phyla	Module completion ratio (%)									
	0--10	10--20	20--30	30--40	40--50	50--60	60--70	70--80	80--90	90--100
Gammaproteobacteria	126	0	0	0	0	0	0	0	0	0
Betaproteobacteria	61	0	0	0	0	0	0	0	0	0
Epsilonproteobacteria	17	0	0	0	0	0	0	0	0	0
Deltaproteobacteria	28	0	0	0	0	0	0	0	0	0
Alphaproteobacteria	91	0	0	0	0	0	0	0	0	0
Magnetococcus	1	0	0	0	0	0	0	0	0	0
Chrysiogenetes	1	0	0	0	0	0	0	0	0	0
Firmicutes	104	0	0	0	0	0	0	0	0	0
Tenericutes	19	0	0	0	0	0	0	0	0	0
Actinobacteria	80	0	0	0	0	0	0	0	0	0
Chlamydiae	8	0	0	0	0	0	0	0	0	0
Spirochaetes	14	0	0	0	0	0	0	0	0	0
Acidobacteria	5	0	0	0	0	0	0	0	0	0
Bacteroidetes	35	0	0	0	0	0	0	0	0	0
Fibrobacteres	1	0	0	0	0	0	0	0	0	0
Fusobacteria	5	0	0	0	0	0	0	0	0	0
Verrucomicrobia	4	0	0	0	0	0	0	0	0	0
Gemmatimonadetes	1	0	0	0	0	0	0	0	0	0
Planctomycetes	4	0	0	0	0	0	0	0	0	0
Elusimicrobia	2	0	0	0	0	0	0	0	0	0
Synergistetes	2	0	0	0	0	0	0	0	0	0
Cyanobacteria	16	0	0	0	0	0	0	0	0	0
Chlorobi	10	0	0	0	0	0	0	0	0	0
Chloroflexi	11	0	0	0	0	0	0	0	0	0
Deinococcus-Thermus	7	0	0	0	0	0	0	0	0	0
Aquificae	9	0	0	0	0	0	0	0	0	0
Thermotogae	11	0	0	0	0	0	0	0	0	0
Dictyoglomi	2	0	0	0	0	0	0	0	0	0
Nitrospirae	2	0	0	0	0	0	0	0	0	0
Thermobaculum	1	0	0	0	0	0	0	0	0	0
Deferribacteres	3	0	0	0	0	0	0	0	0	0
Euryarchaeota	60	0	0	0	0	0	0	0	0	0
Crenarchaeota	23	0	0	0	0	0	0	0	0	0
Thaumarchaeota	2	0	0	0	0	0	0	0	0	0
Nanoarchaeota	1	0	0	0	0	0	0	0	0	0
Korarchaeota	1	0	0	0	0	0	0	0	0	0
Total	768	0	0	0	0	0	0	0	0	0

(mod_M00389_1)

Phyla	Module completion ratio (%)									
	0--10	10--20	20--30	30--40	40--50	50--60	60--70	70--80	80--90	90--100
Gammaproteobacteria	126	0	0	0	0	0	0	0	0	0
Betaproteobacteria	61	0	0	0	0	0	0	0	0	0
Epsilonproteobacteria	17	0	0	0	0	0	0	0	0	0
Deltaproteobacteria	28	0	0	0	0	0	0	0	0	0
Alphaproteobacteria	91	0	0	0	0	0	0	0	0	0
Magnetococcus	1	0	0	0	0	0	0	0	0	0
Chrysiogenetes	1	0	0	0	0	0	0	0	0	0
Firmicutes	104	0	0	0	0	0	0	0	0	0
Tenericutes	19	0	0	0	0	0	0	0	0	0
Actinobacteria	80	0	0	0	0	0	0	0	0	0
Chlamydiae	8	0	0	0	0	0	0	0	0	0
Spirochaetes	14	0	0	0	0	0	0	0	0	0
Acidobacteria	5	0	0	0	0	0	0	0	0	0
Bacteroidetes	35	0	0	0	0	0	0	0	0	0
Fibrobacteres	1	0	0	0	0	0	0	0	0	0
Fusobacteria	5	0	0	0	0	0	0	0	0	0
Verrucomicrobia	4	0	0	0	0	0	0	0	0	0
Gemmatimonadetes	1	0	0	0	0	0	0	0	0	0
Planctomycetes	4	0	0	0	0	0	0	0	0	0
Elusimicrobia	2	0	0	0	0	0	0	0	0	0
Synergistetes	2	0	0	0	0	0	0	0	0	0
Cyanobacteria	16	0	0	0	0	0	0	0	0	0
Chlorobi	10	0	0	0	0	0	0	0	0	0
Chloroflexi	11	0	0	0	0	0	0	0	0	0
Deinococcus-Thermus	7	0	0	0	0	0	0	0	0	0
Aquificae	9	0	0	0	0	0	0	0	0	0
Thermotogae	11	0	0	0	0	0	0	0	0	0
Dictyoglomi	2	0	0	0	0	0	0	0	0	0
Nitrospirae	2	0	0	0	0	0	0	0	0	0
Thermobaculum	1	0	0	0	0	0	0	0	0	0
Deferribacteres	3	0	0	0	0	0	0	0	0	0
Euryarchaeota	60	0	0	0	0	0	0	0	0	0
Crenarchaeota	23	0	0	0	0	0	0	0	0	0
Thaumarchaeota	2	0	0	0	0	0	0	0	0	0
Nanoarchaeota	1	0	0	0	0	0	0	0	0	0
Korarchaeota	1	0	0	0	0	0	0	0	0	0
Total	768	0	0	0	0	0	0	0	0	0

(mod_M00390_1)

Phyla	Module completion ratio (%)									
	0--10	10--20	20--30	30--40	40--50	50--60	60--70	70--80	80--90	90--100
Gammaproteobacteria	126	0	0	0	0	0	0	0	0	0
Betaproteobacteria	61	0	0	0	0	0	0	0	0	0
Epsilonproteobacteria	17	0	0	0	0	0	0	0	0	0
Deltaproteobacteria	28	0	0	0	0	0	0	0	0	0
Alphaproteobacteria	91	0	0	0	0	0	0	0	0	0
Magnetococcus	1	0	0	0	0	0	0	0	0	0
Chrysiogenetes	1	0	0	0	0	0	0	0	0	0
Firmicutes	104	0	0	0	0	0	0	0	0	0
Tenericutes	19	0	0	0	0	0	0	0	0	0
Actinobacteria	80	0	0	0	0	0	0	0	0	0
Chlamydiae	8	0	0	0	0	0	0	0	0	0
Spirochaetes	14	0	0	0	0	0	0	0	0	0
Acidobacteria	5	0	0	0	0	0	0	0	0	0
Bacteroidetes	35	0	0	0	0	0	0	0	0	0
Fibrobacteres	1	0	0	0	0	0	0	0	0	0
Fusobacteria	5	0	0	0	0	0	0	0	0	0
Verrucomicrobia	4	0	0	0	0	0	0	0	0	0
Gemmatimonadetes	1	0	0	0	0	0	0	0	0	0
Planctomycetes	4	0	0	0	0	0	0	0	0	0
Elusimicrobia	2	0	0	0	0	0	0	0	0	0
Synergistetes	2	0	0	0	0	0	0	0	0	0
Cyanobacteria	16	0	0	0	0	0	0	0	0	0
Chlorobi	10	0	0	0	0	0	0	0	0	0
Chloroflexi	11	0	0	0	0	0	0	0	0	0
Deinococcus-Thermus	7	0	0	0	0	0	0	0	0	0
Aquificae	9	0	0	0	0	0	0	0	0	0
Thermotogae	11	0	0	0	0	0	0	0	0	0
Dictyoglomi	2	0	0	0	0	0	0	0	0	0
Nitrospirae	2	0	0	0	0	0	0	0	0	0
Thermobaculum	1	0	0	0	0	0	0	0	0	0
Deferribacteres	3	0	0	0	0	0	0	0	0	0
Euryarchaeota	28	0	0	0	0	0	1	0	0	31
Crenarchaeota	0	0	0	0	0	0	0	0	0	23
Thaumarchaeota	0	0	0	0	0	0	0	0	0	2
Nanoarchaeota	0	0	0	0	0	0	0	0	0	1
Korarchaeota	0	0	0	0	0	0	0	0	0	1
Total	709	0	0	0	0	0	1	0	0	58

(mod_M00391_1)

Phyla	Module completion ratio (%)									
	0--10	10--20	20--30	30--40	40--50	50--60	60--70	70--80	80--90	90--100
Gammaproteobacteria	126	0	0	0	0	0	0	0	0	0
Betaproteobacteria	61	0	0	0	0	0	0	0	0	0
Epsilonproteobacteria	17	0	0	0	0	0	0	0	0	0
Deltaproteobacteria	28	0	0	0	0	0	0	0	0	0
Alphaproteobacteria	91	0	0	0	0	0	0	0	0	0
Magnetococcus	1	0	0	0	0	0	0	0	0	0
Chrysiogenetes	1	0	0	0	0	0	0	0	0	0
Firmicutes	104	0	0	0	0	0	0	0	0	0
Tenericutes	19	0	0	0	0	0	0	0	0	0
Actinobacteria	80	0	0	0	0	0	0	0	0	0
Chlamydiae	8	0	0	0	0	0	0	0	0	0
Spirochaetes	14	0	0	0	0	0	0	0	0	0
Acidobacteria	5	0	0	0	0	0	0	0	0	0
Bacteroidetes	35	0	0	0	0	0	0	0	0	0
Fibrobacteres	1	0	0	0	0	0	0	0	0	0
Fusobacteria	5	0	0	0	0	0	0	0	0	0
Verrucomicrobia	4	0	0	0	0	0	0	0	0	0
Gemmatimonadetes	1	0	0	0	0	0	0	0	0	0
Planctomycetes	4	0	0	0	0	0	0	0	0	0
Elusimicrobia	2	0	0	0	0	0	0	0	0	0
Synergistetes	2	0	0	0	0	0	0	0	0	0
Cyanobacteria	16	0	0	0	0	0	0	0	0	0
Chlorobi	10	0	0	0	0	0	0	0	0	0
Chloroflexi	11	0	0	0	0	0	0	0	0	0
Deinococcus-Thermus	7	0	0	0	0	0	0	0	0	0
Aquificae	9	0	0	0	0	0	0	0	0	0
Thermotogae	11	0	0	0	0	0	0	0	0	0
Dictyoglomi	2	0	0	0	0	0	0	0	0	0
Nitrospirae	2	0	0	0	0	0	0	0	0	0
Thermobaculum	1	0	0	0	0	0	0	0	0	0
Deferribacteres	3	0	0	0	0	0	0	0	0	0
Euryarchaeota	28	0	5	0	27	0	0	0	0	0
Crenarchaeota	0	0	0	0	23	0	0	0	0	0
Thaumarchaeota	0	0	1	0	1	0	0	0	0	0
Nanoarchaeota	0	0	1	0	0	0	0	0	0	0
Korarchaeota	0	0	0	0	1	0	0	0	0	0
Total	709	0	7	0	52	0	0	0	0	0

(mod_M00392_1)

Phyla	Module completion ratio (%)									
	0--10	10--20	20--30	30--40	40--50	50--60	60--70	70--80	80--90	90--100
Gammaproteobacteria	126	0	0	0	0	0	0	0	0	0
Betaproteobacteria	61	0	0	0	0	0	0	0	0	0
Epsilonproteobacteria	17	0	0	0	0	0	0	0	0	0
Deltaproteobacteria	28	0	0	0	0	0	0	0	0	0
Alphaproteobacteria	91	0	0	0	0	0	0	0	0	0
Magnetococcus	1	0	0	0	0	0	0	0	0	0
Chrysiogenetes	1	0	0	0	0	0	0	0	0	0
Firmicutes	104	0	0	0	0	0	0	0	0	0
Tenericutes	19	0	0	0	0	0	0	0	0	0
Actinobacteria	80	0	0	0	0	0	0	0	0	0
Chlamydiae	8	0	0	0	0	0	0	0	0	0
Spirochaetes	14	0	0	0	0	0	0	0	0	0
Acidobacteria	5	0	0	0	0	0	0	0	0	0
Bacteroidetes	35	0	0	0	0	0	0	0	0	0
Fibrobacteres	1	0	0	0	0	0	0	0	0	0
Fusobacteria	5	0	0	0	0	0	0	0	0	0
Verrucomicrobia	4	0	0	0	0	0	0	0	0	0
Gemmatimonadetes	1	0	0	0	0	0	0	0	0	0
Planctomycetes	4	0	0	0	0	0	0	0	0	0
Elusimicrobia	2	0	0	0	0	0	0	0	0	0
Synergistetes	2	0	0	0	0	0	0	0	0	0
Cyanobacteria	15	0	0	1	0	0	0	0	0	0
Chlorobi	10	0	0	0	0	0	0	0	0	0
Chloroflexi	11	0	0	0	0	0	0	0	0	0
Deinococcus-Thermus	7	0	0	0	0	0	0	0	0	0
Aquificae	9	0	0	0	0	0	0	0	0	0
Thermotogae	11	0	0	0	0	0	0	0	0	0
Dictyoglomi	2	0	0	0	0	0	0	0	0	0
Nitrospirae	2	0	0	0	0	0	0	0	0	0
Thermobaculum	1	0	0	0	0	0	0	0	0	0
Deferribacteres	3	0	0	0	0	0	0	0	0	0
Euryarchaeota	59	0	0	1	0	0	0	0	0	0
Crenarchaeota	23	0	0	0	0	0	0	0	0	0
Thaumarchaeota	2	0	0	0	0	0	0	0	0	0
Nanoarchaeota	1	0	0	0	0	0	0	0	0	0
Korarchaeota	1	0	0	0	0	0	0	0	0	0
Total	766	0	0	2	0	0	0	0	0	0

(mod_M00393_1)

Phyla	Module completion ratio (%)									
	0--10	10--20	20--30	30--40	40--50	50--60	60--70	70--80	80--90	90--100
Gammaproteobacteria	126	0	0	0	0	0	0	0	0	0
Betaproteobacteria	61	0	0	0	0	0	0	0	0	0
Epsilonproteobacteria	17	0	0	0	0	0	0	0	0	0
Deltaproteobacteria	28	0	0	0	0	0	0	0	0	0
Alphaproteobacteria	91	0	0	0	0	0	0	0	0	0
Magnetococcus	1	0	0	0	0	0	0	0	0	0
Chrysiogenetes	1	0	0	0	0	0	0	0	0	0
Firmicutes	104	0	0	0	0	0	0	0	0	0
Tenericutes	19	0	0	0	0	0	0	0	0	0
Actinobacteria	80	0	0	0	0	0	0	0	0	0
Chlamydiae	8	0	0	0	0	0	0	0	0	0
Spirochaetes	14	0	0	0	0	0	0	0	0	0
Acidobacteria	5	0	0	0	0	0	0	0	0	0
Bacteroidetes	35	0	0	0	0	0	0	0	0	0
Fibrobacteres	1	0	0	0	0	0	0	0	0	0
Fusobacteria	5	0	0	0	0	0	0	0	0	0
Verrucomicrobia	4	0	0	0	0	0	0	0	0	0
Gemmatimonadetes	1	0	0	0	0	0	0	0	0	0
Planctomycetes	4	0	0	0	0	0	0	0	0	0
Elusimicrobia	2	0	0	0	0	0	0	0	0	0
Synergistetes	2	0	0	0	0	0	0	0	0	0
Cyanobacteria	16	0	0	0	0	0	0	0	0	0
Chlorobi	10	0	0	0	0	0	0	0	0	0
Chloroflexi	11	0	0	0	0	0	0	0	0	0
Deinococcus-Thermus	7	0	0	0	0	0	0	0	0	0
Aquificae	9	0	0	0	0	0	0	0	0	0
Thermotogae	11	0	0	0	0	0	0	0	0	0
Dictyoglomi	2	0	0	0	0	0	0	0	0	0
Nitrospirae	2	0	0	0	0	0	0	0	0	0
Thermobaculum	1	0	0	0	0	0	0	0	0	0
Deferribacteres	3	0	0	0	0	0	0	0	0	0
Euryarchaeota	60	0	0	0	0	0	0	0	0	0
Crenarchaeota	23	0	0	0	0	0	0	0	0	0
Thaumarchaeota	2	0	0	0	0	0	0	0	0	0
Nanoarchaeota	1	0	0	0	0	0	0	0	0	0
Korarchaeota	1	0	0	0	0	0	0	0	0	0
Total	768	0	0	0	0	0	0	0	0	0

(mod_M00394_1)

Phyla	Module completion ratio (%)									
	0--10	10--20	20--30	30--40	40--50	50--60	60--70	70--80	80--90	90--100
Gammaproteobacteria	1	0	0	0	0	0	0	20	0	105
Betaproteobacteria	1	0	0	0	0	0	0	60	0	0
Epsilonproteobacteria	0	0	0	0	0	17	0	0	0	0
Deltaproteobacteria	0	0	0	0	0	7	0	7	0	14
Alphaproteobacteria	1	0	0	0	0	12	0	78	0	0
Magnetococcus	0	0	0	0	0	0	0	0	0	1
Chrysiogenetes	0	0	0	0	0	0	0	1	0	0
Firmicutes	1	0	17	0	0	86	0	0	0	0
Tenericutes	0	0	16	0	0	3	0	0	0	0
Actinobacteria	0	0	0	0	0	12	0	68	0	0
Chlamydiae	0	0	0	0	0	8	0	0	0	0
Spirochaetes	0	0	0	0	0	10	0	4	0	0
Acidobacteria	0	0	0	0	0	5	0	0	0	0
Bacteroidetes	0	0	1	0	0	34	0	0	0	0
Fibrobacteres	0	0	0	0	0	1	0	0	0	0
Fusobacteria	0	0	0	0	0	5	0	0	0	0
Verrucomicrobia	0	0	0	0	0	4	0	0	0	0
Gemmatimonadetes	0	0	0	0	0	1	0	0	0	0
Planctomycetes	0	0	0	0	0	0	0	4	0	0
Elusimicrobia	0	0	0	0	0	2	0	0	0	0
Synergistetes	0	0	0	0	0	2	0	0	0	0
Cyanobacteria	0	0	0	0	0	0	0	16	0	0
Chlorobi	0	0	0	0	0	10	0	0	0	0
Chloroflexi	0	0	1	0	0	10	0	0	0	0
Deinococcus-Thermus	0	0	0	0	0	7	0	0	0	0
Aquificae	0	0	0	0	0	9	0	0	0	0
Thermotogae	0	0	0	0	0	11	0	0	0	0
Dictyoglomi	0	0	0	0	0	2	0	0	0	0
Nitrospirae	0	0	0	0	0	2	0	0	0	0
Thermobaculum	0	0	0	0	0	1	0	0	0	0
Deferribacteres	0	0	0	0	0	3	0	0	0	0
Euryarchaeota	0	0	60	0	0	0	0	0	0	0
Crenarchaeota	0	0	23	0	0	0	0	0	0	0
Thaumarchaeota	0	0	2	0	0	0	0	0	0	0
Nanoarchaeota	1	0	0	0	0	0	0	0	0	0
Korarchaeota	0	0	1	0	0	0	0	0	0	0
Total	5	0	121	0	0	264	0	258	0	120

(mod_M00395_1)

Phyla	Module completion ratio (%)									
	0--10	10--20	20--30	30--40	40--50	50--60	60--70	70--80	80--90	90--100
Gammaproteobacteria	126	0	0	0	0	0	0	0	0	0
Betaproteobacteria	61	0	0	0	0	0	0	0	0	0
Epsilonproteobacteria	17	0	0	0	0	0	0	0	0	0
Deltaproteobacteria	28	0	0	0	0	0	0	0	0	0
Alphaproteobacteria	91	0	0	0	0	0	0	0	0	0
Magnetococcus	1	0	0	0	0	0	0	0	0	0
Chrysiogenetes	1	0	0	0	0	0	0	0	0	0
Firmicutes	104	0	0	0	0	0	0	0	0	0
Tenericutes	19	0	0	0	0	0	0	0	0	0
Actinobacteria	80	0	0	0	0	0	0	0	0	0
Chlamydiae	8	0	0	0	0	0	0	0	0	0
Spirochaetes	14	0	0	0	0	0	0	0	0	0
Acidobacteria	5	0	0	0	0	0	0	0	0	0
Bacteroidetes	35	0	0	0	0	0	0	0	0	0
Fibrobacteres	1	0	0	0	0	0	0	0	0	0
Fusobacteria	5	0	0	0	0	0	0	0	0	0
Verrucomicrobia	4	0	0	0	0	0	0	0	0	0
Gemmatimonadetes	1	0	0	0	0	0	0	0	0	0
Planctomycetes	4	0	0	0	0	0	0	0	0	0
Elusimicrobia	2	0	0	0	0	0	0	0	0	0
Synergistetes	2	0	0	0	0	0	0	0	0	0
Cyanobacteria	16	0	0	0	0	0	0	0	0	0
Chlorobi	10	0	0	0	0	0	0	0	0	0
Chloroflexi	11	0	0	0	0	0	0	0	0	0
Deinococcus-Thermus	7	0	0	0	0	0	0	0	0	0
Aquificae	9	0	0	0	0	0	0	0	0	0
Thermotogae	11	0	0	0	0	0	0	0	0	0
Dictyoglomi	2	0	0	0	0	0	0	0	0	0
Nitrospirae	2	0	0	0	0	0	0	0	0	0
Thermobaculum	1	0	0	0	0	0	0	0	0	0
Deferribacteres	3	0	0	0	0	0	0	0	0	0
Euryarchaeota	60	0	0	0	0	0	0	0	0	0
Crenarchaeota	23	0	0	0	0	0	0	0	0	0
Thaumarchaeota	2	0	0	0	0	0	0	0	0	0
Nanoarchaeota	1	0	0	0	0	0	0	0	0	0
Korarchaeota	1	0	0	0	0	0	0	0	0	0
Total	768	0	0	0	0	0	0	0	0	0

(mod_M00396_1)

Phyla	Module completion ratio (%)									
	0--10	10--20	20--30	30--40	40--50	50--60	60--70	70--80	80--90	90--100
Gammaproteobacteria	126	0	0	0	0	0	0	0	0	0
Betaproteobacteria	61	0	0	0	0	0	0	0	0	0
Epsilonproteobacteria	17	0	0	0	0	0	0	0	0	0
Deltaproteobacteria	28	0	0	0	0	0	0	0	0	0
Alphaproteobacteria	91	0	0	0	0	0	0	0	0	0
Magnetococcus	1	0	0	0	0	0	0	0	0	0
Chrysiogenetes	1	0	0	0	0	0	0	0	0	0
Firmicutes	104	0	0	0	0	0	0	0	0	0
Tenericutes	19	0	0	0	0	0	0	0	0	0
Actinobacteria	80	0	0	0	0	0	0	0	0	0
Chlamydiae	8	0	0	0	0	0	0	0	0	0
Spirochaetes	14	0	0	0	0	0	0	0	0	0
Acidobacteria	5	0	0	0	0	0	0	0	0	0
Bacteroidetes	35	0	0	0	0	0	0	0	0	0
Fibrobacteres	1	0	0	0	0	0	0	0	0	0
Fusobacteria	5	0	0	0	0	0	0	0	0	0
Verrucomicrobia	4	0	0	0	0	0	0	0	0	0
Gemmatimonadetes	1	0	0	0	0	0	0	0	0	0
Planctomycetes	4	0	0	0	0	0	0	0	0	0
Elusimicrobia	2	0	0	0	0	0	0	0	0	0
Synergistetes	2	0	0	0	0	0	0	0	0	0
Cyanobacteria	16	0	0	0	0	0	0	0	0	0
Chlorobi	10	0	0	0	0	0	0	0	0	0
Chloroflexi	11	0	0	0	0	0	0	0	0	0
Deinococcus-Thermus	7	0	0	0	0	0	0	0	0	0
Aquificae	9	0	0	0	0	0	0	0	0	0
Thermotogae	11	0	0	0	0	0	0	0	0	0
Dictyoglomi	2	0	0	0	0	0	0	0	0	0
Nitrospirae	2	0	0	0	0	0	0	0	0	0
Thermobaculum	1	0	0	0	0	0	0	0	0	0
Deferribacteres	3	0	0	0	0	0	0	0	0	0
Euryarchaeota	60	0	0	0	0	0	0	0	0	0
Crenarchaeota	23	0	0	0	0	0	0	0	0	0
Thaumarchaeota	2	0	0	0	0	0	0	0	0	0
Nanoarchaeota	1	0	0	0	0	0	0	0	0	0
Korarchaeota	1	0	0	0	0	0	0	0	0	0
Total	768	0	0	0	0	0	0	0	0	0

(mod_M00397_1)

Phyla	Module completion ratio (%)									
	0--10	10--20	20--30	30--40	40--50	50--60	60--70	70--80	80--90	90--100
Gammaproteobacteria	126	0	0	0	0	0	0	0	0	0
Betaproteobacteria	61	0	0	0	0	0	0	0	0	0
Epsilonproteobacteria	17	0	0	0	0	0	0	0	0	0
Deltaproteobacteria	28	0	0	0	0	0	0	0	0	0
Alphaproteobacteria	91	0	0	0	0	0	0	0	0	0
Magnetococcus	1	0	0	0	0	0	0	0	0	0
Chrysiogenetes	1	0	0	0	0	0	0	0	0	0
Firmicutes	104	0	0	0	0	0	0	0	0	0
Tenericutes	19	0	0	0	0	0	0	0	0	0
Actinobacteria	80	0	0	0	0	0	0	0	0	0
Chlamydiae	8	0	0	0	0	0	0	0	0	0
Spirochaetes	14	0	0	0	0	0	0	0	0	0
Acidobacteria	5	0	0	0	0	0	0	0	0	0
Bacteroidetes	35	0	0	0	0	0	0	0	0	0
Fibrobacteres	1	0	0	0	0	0	0	0	0	0
Fusobacteria	5	0	0	0	0	0	0	0	0	0
Verrucomicrobia	4	0	0	0	0	0	0	0	0	0
Gemmatimonadetes	1	0	0	0	0	0	0	0	0	0
Planctomycetes	4	0	0	0	0	0	0	0	0	0
Elusimicrobia	2	0	0	0	0	0	0	0	0	0
Synergistetes	2	0	0	0	0	0	0	0	0	0
Cyanobacteria	16	0	0	0	0	0	0	0	0	0
Chlorobi	10	0	0	0	0	0	0	0	0	0
Chloroflexi	11	0	0	0	0	0	0	0	0	0
Deinococcus-Thermus	7	0	0	0	0	0	0	0	0	0
Aquificae	9	0	0	0	0	0	0	0	0	0
Thermotogae	11	0	0	0	0	0	0	0	0	0
Dictyoglomi	2	0	0	0	0	0	0	0	0	0
Nitrospirae	2	0	0	0	0	0	0	0	0	0
Thermobaculum	1	0	0	0	0	0	0	0	0	0
Deferribacteres	3	0	0	0	0	0	0	0	0	0
Euryarchaeota	60	0	0	0	0	0	0	0	0	0
Crenarchaeota	23	0	0	0	0	0	0	0	0	0
Thaumarchaeota	2	0	0	0	0	0	0	0	0	0
Nanoarchaeota	1	0	0	0	0	0	0	0	0	0
Korarchaeota	1	0	0	0	0	0	0	0	0	0
Total	768	0	0	0	0	0	0	0	0	0

(mod_M00398_1)

Phyla	Module completion ratio (%)									
	0--10	10--20	20--30	30--40	40--50	50--60	60--70	70--80	80--90	90--100
Gammaproteobacteria	126	0	0	0	0	0	0	0	0	0
Betaproteobacteria	61	0	0	0	0	0	0	0	0	0
Epsilonproteobacteria	17	0	0	0	0	0	0	0	0	0
Deltaproteobacteria	28	0	0	0	0	0	0	0	0	0
Alphaproteobacteria	91	0	0	0	0	0	0	0	0	0
Magnetococcus	1	0	0	0	0	0	0	0	0	0
Chrysiogenetes	1	0	0	0	0	0	0	0	0	0
Firmicutes	104	0	0	0	0	0	0	0	0	0
Tenericutes	19	0	0	0	0	0	0	0	0	0
Actinobacteria	80	0	0	0	0	0	0	0	0	0
Chlamydiae	8	0	0	0	0	0	0	0	0	0
Spirochaetes	14	0	0	0	0	0	0	0	0	0
Acidobacteria	5	0	0	0	0	0	0	0	0	0
Bacteroidetes	35	0	0	0	0	0	0	0	0	0
Fibrobacteres	1	0	0	0	0	0	0	0	0	0
Fusobacteria	5	0	0	0	0	0	0	0	0	0
Verrucomicrobia	4	0	0	0	0	0	0	0	0	0
Gemmatimonadetes	1	0	0	0	0	0	0	0	0	0
Planctomycetes	4	0	0	0	0	0	0	0	0	0
Elusimicrobia	2	0	0	0	0	0	0	0	0	0
Synergistetes	2	0	0	0	0	0	0	0	0	0
Cyanobacteria	16	0	0	0	0	0	0	0	0	0
Chlorobi	10	0	0	0	0	0	0	0	0	0
Chloroflexi	11	0	0	0	0	0	0	0	0	0
Deinococcus-Thermus	7	0	0	0	0	0	0	0	0	0
Aquificae	9	0	0	0	0	0	0	0	0	0
Thermotogae	11	0	0	0	0	0	0	0	0	0
Dictyoglomi	2	0	0	0	0	0	0	0	0	0
Nitrospirae	2	0	0	0	0	0	0	0	0	0
Thermobaculum	1	0	0	0	0	0	0	0	0	0
Deferribacteres	3	0	0	0	0	0	0	0	0	0
Euryarchaeota	60	0	0	0	0	0	0	0	0	0
Crenarchaeota	23	0	0	0	0	0	0	0	0	0
Thaumarchaeota	2	0	0	0	0	0	0	0	0	0
Nanoarchaeota	1	0	0	0	0	0	0	0	0	0
Korarchaeota	1	0	0	0	0	0	0	0	0	0
Total	768	0	0	0	0	0	0	0	0	0

(mod_M00399_1)

Phyla	Module completion ratio (%)									
	0--10	10--20	20--30	30--40	40--50	50--60	60--70	70--80	80--90	90--100
Gammaproteobacteria	126	0	0	0	0	0	0	0	0	0
Betaproteobacteria	61	0	0	0	0	0	0	0	0	0
Epsilonproteobacteria	17	0	0	0	0	0	0	0	0	0
Deltaproteobacteria	28	0	0	0	0	0	0	0	0	0
Alphaproteobacteria	91	0	0	0	0	0	0	0	0	0
Magnetococcus	1	0	0	0	0	0	0	0	0	0
Chrysiogenetes	1	0	0	0	0	0	0	0	0	0
Firmicutes	104	0	0	0	0	0	0	0	0	0
Tenericutes	19	0	0	0	0	0	0	0	0	0
Actinobacteria	80	0	0	0	0	0	0	0	0	0
Chlamydiae	8	0	0	0	0	0	0	0	0	0
Spirochaetes	14	0	0	0	0	0	0	0	0	0
Acidobacteria	5	0	0	0	0	0	0	0	0	0
Bacteroidetes	35	0	0	0	0	0	0	0	0	0
Fibrobacteres	1	0	0	0	0	0	0	0	0	0
Fusobacteria	5	0	0	0	0	0	0	0	0	0
Verrucomicrobia	4	0	0	0	0	0	0	0	0	0
Gemmatimonadetes	1	0	0	0	0	0	0	0	0	0
Planctomycetes	4	0	0	0	0	0	0	0	0	0
Elusimicrobia	2	0	0	0	0	0	0	0	0	0
Synergistetes	2	0	0	0	0	0	0	0	0	0
Cyanobacteria	16	0	0	0	0	0	0	0	0	0
Chlorobi	10	0	0	0	0	0	0	0	0	0
Chloroflexi	11	0	0	0	0	0	0	0	0	0
Deinococcus-Thermus	7	0	0	0	0	0	0	0	0	0
Aquificae	9	0	0	0	0	0	0	0	0	0
Thermotogae	11	0	0	0	0	0	0	0	0	0
Dictyoglomi	2	0	0	0	0	0	0	0	0	0
Nitrospirae	2	0	0	0	0	0	0	0	0	0
Thermobaculum	1	0	0	0	0	0	0	0	0	0
Deferribacteres	3	0	0	0	0	0	0	0	0	0
Euryarchaeota	60	0	0	0	0	0	0	0	0	0
Crenarchaeota	23	0	0	0	0	0	0	0	0	0
Thaumarchaeota	2	0	0	0	0	0	0	0	0	0
Nanoarchaeota	1	0	0	0	0	0	0	0	0	0
Korarchaeota	1	0	0	0	0	0	0	0	0	0
Total	768	0	0	0	0	0	0	0	0	0

(mod_M00400_1)

Phyla	Module completion ratio (%)									
	0--10	10--20	20--30	30--40	40--50	50--60	60--70	70--80	80--90	90--100
Gammaproteobacteria	125	0	0	1	0	0	0	0	0	0
Betaproteobacteria	60	0	0	1	0	0	0	0	0	0
Epsilonproteobacteria	17	0	0	0	0	0	0	0	0	0
Deltaproteobacteria	26	0	0	2	0	0	0	0	0	0
Alphaproteobacteria	82	0	0	9	0	0	0	0	0	0
Magnetococcus	1	0	0	0	0	0	0	0	0	0
Chrysiogenetes	1	0	0	0	0	0	0	0	0	0
Firmicutes	99	0	0	5	0	0	0	0	0	0
Tenericutes	19	0	0	0	0	0	0	0	0	0
Actinobacteria	56	0	0	24	0	0	0	0	0	0
Chlamydiae	8	0	0	0	0	0	0	0	0	0
Spirochaetes	14	0	0	0	0	0	0	0	0	0
Acidobacteria	5	0	0	0	0	0	0	0	0	0
Bacteroidetes	35	0	0	0	0	0	0	0	0	0
Fibrobacteres	1	0	0	0	0	0	0	0	0	0
Fusobacteria	5	0	0	0	0	0	0	0	0	0
Verrucomicrobia	4	0	0	0	0	0	0	0	0	0
Gemmatimonadetes	1	0	0	0	0	0	0	0	0	0
Planctomycetes	3	0	0	1	0	0	0	0	0	0
Elusimicrobia	2	0	0	0	0	0	0	0	0	0
Synergistetes	1	0	0	1	0	0	0	0	0	0
Cyanobacteria	9	0	0	7	0	0	0	0	0	0
Chlorobi	8	0	0	2	0	0	0	0	0	0
Chloroflexi	11	0	0	0	0	0	0	0	0	0
Deinococcus-Thermus	7	0	0	0	0	0	0	0	0	0
Aquificae	9	0	0	0	0	0	0	0	0	0
Thermotogae	11	0	0	0	0	0	0	0	0	0
Dictyoglomi	2	0	0	0	0	0	0	0	0	0
Nitrospirae	2	0	0	0	0	0	0	0	0	0
Thermobaculum	1	0	0	0	0	0	0	0	0	0
Deferribacteres	3	0	0	0	0	0	0	0	0	0
Euryarchaeota	0	0	0	60	0	0	0	0	0	0
Crenarchaeota	0	0	0	23	0	0	0	0	0	0
Thaumarchaeota	0	0	0	2	0	0	0	0	0	0
Nanoarchaeota	0	0	0	1	0	0	0	0	0	0
Korarchaeota	0	0	0	1	0	0	0	0	0	0
Total	628	0	0	140	0	0	0	0	0	0

(mod_M00401_1)

Phyla	Module completion ratio (%)									
	0--10	10--20	20--30	30--40	40--50	50--60	60--70	70--80	80--90	90--100
Gammaproteobacteria	126	0	0	0	0	0	0	0	0	0
Betaproteobacteria	61	0	0	0	0	0	0	0	0	0
Epsilonproteobacteria	17	0	0	0	0	0	0	0	0	0
Deltaproteobacteria	28	0	0	0	0	0	0	0	0	0
Alphaproteobacteria	91	0	0	0	0	0	0	0	0	0
Magnetococcus	1	0	0	0	0	0	0	0	0	0
Chrysiogenetes	1	0	0	0	0	0	0	0	0	0
Firmicutes	104	0	0	0	0	0	0	0	0	0
Tenericutes	19	0	0	0	0	0	0	0	0	0
Actinobacteria	80	0	0	0	0	0	0	0	0	0
Chlamydiae	8	0	0	0	0	0	0	0	0	0
Spirochaetes	14	0	0	0	0	0	0	0	0	0
Acidobacteria	5	0	0	0	0	0	0	0	0	0
Bacteroidetes	35	0	0	0	0	0	0	0	0	0
Fibrobacteres	1	0	0	0	0	0	0	0	0	0
Fusobacteria	5	0	0	0	0	0	0	0	0	0
Verrucomicrobia	4	0	0	0	0	0	0	0	0	0
Gemmatimonadetes	1	0	0	0	0	0	0	0	0	0
Planctomycetes	4	0	0	0	0	0	0	0	0	0
Elusimicrobia	2	0	0	0	0	0	0	0	0	0
Synergistetes	2	0	0	0	0	0	0	0	0	0
Cyanobacteria	16	0	0	0	0	0	0	0	0	0
Chlorobi	10	0	0	0	0	0	0	0	0	0
Chloroflexi	11	0	0	0	0	0	0	0	0	0
Deinococcus-Thermus	7	0	0	0	0	0	0	0	0	0
Aquificae	9	0	0	0	0	0	0	0	0	0
Thermotogae	11	0	0	0	0	0	0	0	0	0
Dictyoglomi	2	0	0	0	0	0	0	0	0	0
Nitrospirae	2	0	0	0	0	0	0	0	0	0
Thermobaculum	1	0	0	0	0	0	0	0	0	0
Deferribacteres	3	0	0	0	0	0	0	0	0	0
Euryarchaeota	0	0	0	60	0	0	0	0	0	0
Crenarchaeota	20	0	0	3	0	0	0	0	0	0
Thaumarchaeota	1	0	0	1	0	0	0	0	0	0
Nanoarchaeota	1	0	0	0	0	0	0	0	0	0
Korarchaeota	1	0	0	0	0	0	0	0	0	0
Total	704	0	0	64	0	0	0	0	0	0

(mod_M00402_1)

Phyla	Module completion ratio (%)									
	0--10	10--20	20--30	30--40	40--50	50--60	60--70	70--80	80--90	90--100
Gammaproteobacteria	126	0	0	0	0	0	0	0	0	0
Betaproteobacteria	61	0	0	0	0	0	0	0	0	0
Epsilonproteobacteria	17	0	0	0	0	0	0	0	0	0
Deltaproteobacteria	28	0	0	0	0	0	0	0	0	0
Alphaproteobacteria	91	0	0	0	0	0	0	0	0	0
Magnetococcus	1	0	0	0	0	0	0	0	0	0
Chrysiogenetes	1	0	0	0	0	0	0	0	0	0
Firmicutes	104	0	0	0	0	0	0	0	0	0
Tenericutes	19	0	0	0	0	0	0	0	0	0
Actinobacteria	80	0	0	0	0	0	0	0	0	0
Chlamydiae	8	0	0	0	0	0	0	0	0	0
Spirochaetes	14	0	0	0	0	0	0	0	0	0
Acidobacteria	5	0	0	0	0	0	0	0	0	0
Bacteroidetes	35	0	0	0	0	0	0	0	0	0
Fibrobacteres	1	0	0	0	0	0	0	0	0	0
Fusobacteria	5	0	0	0	0	0	0	0	0	0
Verrucomicrobia	4	0	0	0	0	0	0	0	0	0
Gemmatimonadetes	1	0	0	0	0	0	0	0	0	0
Planctomycetes	4	0	0	0	0	0	0	0	0	0
Elusimicrobia	2	0	0	0	0	0	0	0	0	0
Synergistetes	2	0	0	0	0	0	0	0	0	0
Cyanobacteria	16	0	0	0	0	0	0	0	0	0
Chlorobi	10	0	0	0	0	0	0	0	0	0
Chloroflexi	11	0	0	0	0	0	0	0	0	0
Deinococcus-Thermus	7	0	0	0	0	0	0	0	0	0
Aquificae	9	0	0	0	0	0	0	0	0	0
Thermotogae	11	0	0	0	0	0	0	0	0	0
Dictyoglomi	2	0	0	0	0	0	0	0	0	0
Nitrospirae	2	0	0	0	0	0	0	0	0	0
Thermobaculum	1	0	0	0	0	0	0	0	0	0
Deferribacteres	3	0	0	0	0	0	0	0	0	0
Euryarchaeota	60	0	0	0	0	0	0	0	0	0
Crenarchaeota	23	0	0	0	0	0	0	0	0	0
Thaumarchaeota	2	0	0	0	0	0	0	0	0	0
Nanoarchaeota	1	0	0	0	0	0	0	0	0	0
Korarchaeota	1	0	0	0	0	0	0	0	0	0
Total	768	0	0	0	0	0	0	0	0	0

(mod_M00403_1)

Phyla	Module completion ratio (%)									
	0--10	10--20	20--30	30--40	40--50	50--60	60--70	70--80	80--90	90--100
Gammaproteobacteria	125	1	0	0	0	0	0	0	0	0
Betaproteobacteria	60	1	0	0	0	0	0	0	0	0
Epsilonproteobacteria	17	0	0	0	0	0	0	0	0	0
Deltaproteobacteria	26	2	0	0	0	0	0	0	0	0
Alphaproteobacteria	82	9	0	0	0	0	0	0	0	0
Magnetococcus	1	0	0	0	0	0	0	0	0	0
Chrysiogenetes	1	0	0	0	0	0	0	0	0	0
Firmicutes	99	5	0	0	0	0	0	0	0	0
Tenericutes	19	0	0	0	0	0	0	0	0	0
Actinobacteria	56	24	0	0	0	0	0	0	0	0
Chlamydiae	8	0	0	0	0	0	0	0	0	0
Spirochaetes	14	0	0	0	0	0	0	0	0	0
Acidobacteria	5	0	0	0	0	0	0	0	0	0
Bacteroidetes	35	0	0	0	0	0	0	0	0	0
Fibrobacteres	1	0	0	0	0	0	0	0	0	0
Fusobacteria	5	0	0	0	0	0	0	0	0	0
Verrucomicrobia	4	0	0	0	0	0	0	0	0	0
Gemmatimonadetes	1	0	0	0	0	0	0	0	0	0
Planctomycetes	3	1	0	0	0	0	0	0	0	0
Elusimicrobia	2	0	0	0	0	0	0	0	0	0
Synergistetes	1	1	0	0	0	0	0	0	0	0
Cyanobacteria	9	7	0	0	0	0	0	0	0	0
Chlorobi	8	2	0	0	0	0	0	0	0	0
Chloroflexi	11	0	0	0	0	0	0	0	0	0
Deinococcus-Thermus	7	0	0	0	0	0	0	0	0	0
Aquificae	9	0	0	0	0	0	0	0	0	0
Thermotogae	11	0	0	0	0	0	0	0	0	0
Dictyoglomi	2	0	0	0	0	0	0	0	0	0
Nitrospirae	2	0	0	0	0	0	0	0	0	0
Thermobaculum	1	0	0	0	0	0	0	0	0	0
Deferribacteres	3	0	0	0	0	0	0	0	0	0
Euryarchaeota	0	60	0	0	0	0	0	0	0	0
Crenarchaeota	0	23	0	0	0	0	0	0	0	0
Thaumarchaeota	0	2	0	0	0	0	0	0	0	0
Nanoarchaeota	0	1	0	0	0	0	0	0	0	0
Korarchaeota	0	1	0	0	0	0	0	0	0	0
Total	628	140	0	0	0	0	0	0	0	0

(mod_M00404_1)

Phyla	Module completion ratio (%)									
	0--10	10--20	20--30	30--40	40--50	50--60	60--70	70--80	80--90	90--100
Gammaproteobacteria	126	0	0	0	0	0	0	0	0	0
Betaproteobacteria	61	0	0	0	0	0	0	0	0	0
Epsilonproteobacteria	17	0	0	0	0	0	0	0	0	0
Deltaproteobacteria	28	0	0	0	0	0	0	0	0	0
Alphaproteobacteria	91	0	0	0	0	0	0	0	0	0
Magnetococcus	1	0	0	0	0	0	0	0	0	0
Chrysiogenetes	1	0	0	0	0	0	0	0	0	0
Firmicutes	104	0	0	0	0	0	0	0	0	0
Tenericutes	19	0	0	0	0	0	0	0	0	0
Actinobacteria	80	0	0	0	0	0	0	0	0	0
Chlamydiae	8	0	0	0	0	0	0	0	0	0
Spirochaetes	14	0	0	0	0	0	0	0	0	0
Acidobacteria	5	0	0	0	0	0	0	0	0	0
Bacteroidetes	35	0	0	0	0	0	0	0	0	0
Fibrobacteres	1	0	0	0	0	0	0	0	0	0
Fusobacteria	5	0	0	0	0	0	0	0	0	0
Verrucomicrobia	4	0	0	0	0	0	0	0	0	0
Gemmatimonadetes	1	0	0	0	0	0	0	0	0	0
Planctomycetes	4	0	0	0	0	0	0	0	0	0
Elusimicrobia	2	0	0	0	0	0	0	0	0	0
Synergistetes	2	0	0	0	0	0	0	0	0	0
Cyanobacteria	16	0	0	0	0	0	0	0	0	0
Chlorobi	10	0	0	0	0	0	0	0	0	0
Chloroflexi	11	0	0	0	0	0	0	0	0	0
Deinococcus-Thermus	7	0	0	0	0	0	0	0	0	0
Aquificae	9	0	0	0	0	0	0	0	0	0
Thermotogae	11	0	0	0	0	0	0	0	0	0
Dictyoglomi	2	0	0	0	0	0	0	0	0	0
Nitrospirae	2	0	0	0	0	0	0	0	0	0
Thermobaculum	1	0	0	0	0	0	0	0	0	0
Deferribacteres	3	0	0	0	0	0	0	0	0	0
Euryarchaeota	60	0	0	0	0	0	0	0	0	0
Crenarchaeota	23	0	0	0	0	0	0	0	0	0
Thaumarchaeota	2	0	0	0	0	0	0	0	0	0
Nanoarchaeota	1	0	0	0	0	0	0	0	0	0
Korarchaeota	1	0	0	0	0	0	0	0	0	0
Total	768	0	0	0	0	0	0	0	0	0

(mod_M00405_1)

Phyla	Module completion ratio (%)									
	0--10	10--20	20--30	30--40	40--50	50--60	60--70	70--80	80--90	90--100
Gammaproteobacteria	126	0	0	0	0	0	0	0	0	0
Betaproteobacteria	61	0	0	0	0	0	0	0	0	0
Epsilonproteobacteria	17	0	0	0	0	0	0	0	0	0
Deltaproteobacteria	28	0	0	0	0	0	0	0	0	0
Alphaproteobacteria	91	0	0	0	0	0	0	0	0	0
Magnetococcus	1	0	0	0	0	0	0	0	0	0
Chrysiogenetes	1	0	0	0	0	0	0	0	0	0
Firmicutes	104	0	0	0	0	0	0	0	0	0
Tenericutes	19	0	0	0	0	0	0	0	0	0
Actinobacteria	80	0	0	0	0	0	0	0	0	0
Chlamydiae	8	0	0	0	0	0	0	0	0	0
Spirochaetes	14	0	0	0	0	0	0	0	0	0
Acidobacteria	5	0	0	0	0	0	0	0	0	0
Bacteroidetes	35	0	0	0	0	0	0	0	0	0
Fibrobacteres	1	0	0	0	0	0	0	0	0	0
Fusobacteria	5	0	0	0	0	0	0	0	0	0
Verrucomicrobia	4	0	0	0	0	0	0	0	0	0
Gemmatimonadetes	1	0	0	0	0	0	0	0	0	0
Planctomycetes	4	0	0	0	0	0	0	0	0	0
Elusimicrobia	2	0	0	0	0	0	0	0	0	0
Synergistetes	2	0	0	0	0	0	0	0	0	0
Cyanobacteria	16	0	0	0	0	0	0	0	0	0
Chlorobi	10	0	0	0	0	0	0	0	0	0
Chloroflexi	11	0	0	0	0	0	0	0	0	0
Deinococcus-Thermus	7	0	0	0	0	0	0	0	0	0
Aquificae	9	0	0	0	0	0	0	0	0	0
Thermotogae	11	0	0	0	0	0	0	0	0	0
Dictyoglomi	2	0	0	0	0	0	0	0	0	0
Nitrospirae	2	0	0	0	0	0	0	0	0	0
Thermobaculum	1	0	0	0	0	0	0	0	0	0
Deferribacteres	3	0	0	0	0	0	0	0	0	0
Euryarchaeota	60	0	0	0	0	0	0	0	0	0
Crenarchaeota	23	0	0	0	0	0	0	0	0	0
Thaumarchaeota	2	0	0	0	0	0	0	0	0	0
Nanoarchaeota	1	0	0	0	0	0	0	0	0	0
Korarchaeota	1	0	0	0	0	0	0	0	0	0
Total	768	0	0	0	0	0	0	0	0	0

(mod_M00406_1)

Phyla	Module completion ratio (%)									
	0--10	10--20	20--30	30--40	40--50	50--60	60--70	70--80	80--90	90--100
Gammaproteobacteria	126	0	0	0	0	0	0	0	0	0
Betaproteobacteria	61	0	0	0	0	0	0	0	0	0
Epsilonproteobacteria	17	0	0	0	0	0	0	0	0	0
Deltaproteobacteria	28	0	0	0	0	0	0	0	0	0
Alphaproteobacteria	91	0	0	0	0	0	0	0	0	0
Magnetococcus	1	0	0	0	0	0	0	0	0	0
Chrysiogenetes	1	0	0	0	0	0	0	0	0	0
Firmicutes	104	0	0	0	0	0	0	0	0	0
Tenericutes	19	0	0	0	0	0	0	0	0	0
Actinobacteria	80	0	0	0	0	0	0	0	0	0
Chlamydiae	8	0	0	0	0	0	0	0	0	0
Spirochaetes	14	0	0	0	0	0	0	0	0	0
Acidobacteria	5	0	0	0	0	0	0	0	0	0
Bacteroidetes	35	0	0	0	0	0	0	0	0	0
Fibrobacteres	1	0	0	0	0	0	0	0	0	0
Fusobacteria	5	0	0	0	0	0	0	0	0	0
Verrucomicrobia	4	0	0	0	0	0	0	0	0	0
Gemmatimonadetes	1	0	0	0	0	0	0	0	0	0
Planctomycetes	4	0	0	0	0	0	0	0	0	0
Elusimicrobia	2	0	0	0	0	0	0	0	0	0
Synergistetes	2	0	0	0	0	0	0	0	0	0
Cyanobacteria	16	0	0	0	0	0	0	0	0	0
Chlorobi	10	0	0	0	0	0	0	0	0	0
Chloroflexi	11	0	0	0	0	0	0	0	0	0
Deinococcus-Thermus	7	0	0	0	0	0	0	0	0	0
Aquificae	9	0	0	0	0	0	0	0	0	0
Thermotogae	11	0	0	0	0	0	0	0	0	0
Dictyoglomi	2	0	0	0	0	0	0	0	0	0
Nitrospirae	2	0	0	0	0	0	0	0	0	0
Thermobaculum	1	0	0	0	0	0	0	0	0	0
Deferribacteres	3	0	0	0	0	0	0	0	0	0
Euryarchaeota	60	0	0	0	0	0	0	0	0	0
Crenarchaeota	23	0	0	0	0	0	0	0	0	0
Thaumarchaeota	2	0	0	0	0	0	0	0	0	0
Nanoarchaeota	1	0	0	0	0	0	0	0	0	0
Korarchaeota	1	0	0	0	0	0	0	0	0	0
Total	768	0	0	0	0	0	0	0	0	0

(mod_M00407_1)

Phyla	Module completion ratio (%)									
	0--10	10--20	20--30	30--40	40--50	50--60	60--70	70--80	80--90	90--100
Gammaproteobacteria	126	0	0	0	0	0	0	0	0	0
Betaproteobacteria	61	0	0	0	0	0	0	0	0	0
Epsilonproteobacteria	17	0	0	0	0	0	0	0	0	0
Deltaproteobacteria	28	0	0	0	0	0	0	0	0	0
Alphaproteobacteria	91	0	0	0	0	0	0	0	0	0
Magnetococcus	1	0	0	0	0	0	0	0	0	0
Chrysiogenetes	1	0	0	0	0	0	0	0	0	0
Firmicutes	104	0	0	0	0	0	0	0	0	0
Tenericutes	19	0	0	0	0	0	0	0	0	0
Actinobacteria	80	0	0	0	0	0	0	0	0	0
Chlamydiae	8	0	0	0	0	0	0	0	0	0
Spirochaetes	14	0	0	0	0	0	0	0	0	0
Acidobacteria	5	0	0	0	0	0	0	0	0	0
Bacteroidetes	35	0	0	0	0	0	0	0	0	0
Fibrobacteres	1	0	0	0	0	0	0	0	0	0
Fusobacteria	5	0	0	0	0	0	0	0	0	0
Verrucomicrobia	4	0	0	0	0	0	0	0	0	0
Gemmatimonadetes	1	0	0	0	0	0	0	0	0	0
Planctomycetes	4	0	0	0	0	0	0	0	0	0
Elusimicrobia	2	0	0	0	0	0	0	0	0	0
Synergistetes	2	0	0	0	0	0	0	0	0	0
Cyanobacteria	16	0	0	0	0	0	0	0	0	0
Chlorobi	10	0	0	0	0	0	0	0	0	0
Chloroflexi	11	0	0	0	0	0	0	0	0	0
Deinococcus-Thermus	7	0	0	0	0	0	0	0	0	0
Aquificae	9	0	0	0	0	0	0	0	0	0
Thermotogae	11	0	0	0	0	0	0	0	0	0
Dictyoglomi	2	0	0	0	0	0	0	0	0	0
Nitrospirae	2	0	0	0	0	0	0	0	0	0
Thermobaculum	1	0	0	0	0	0	0	0	0	0
Deferribacteres	3	0	0	0	0	0	0	0	0	0
Euryarchaeota	60	0	0	0	0	0	0	0	0	0
Crenarchaeota	23	0	0	0	0	0	0	0	0	0
Thaumarchaeota	2	0	0	0	0	0	0	0	0	0
Nanoarchaeota	1	0	0	0	0	0	0	0	0	0
Korarchaeota	1	0	0	0	0	0	0	0	0	0
Total	768	0	0	0	0	0	0	0	0	0

(mod_M00408_1)

Phyla	Module completion ratio (%)									
	0--10	10--20	20--30	30--40	40--50	50--60	60--70	70--80	80--90	90--100
Gammaproteobacteria	126	0	0	0	0	0	0	0	0	0
Betaproteobacteria	61	0	0	0	0	0	0	0	0	0
Epsilonproteobacteria	17	0	0	0	0	0	0	0	0	0
Deltaproteobacteria	28	0	0	0	0	0	0	0	0	0
Alphaproteobacteria	91	0	0	0	0	0	0	0	0	0
Magnetococcus	1	0	0	0	0	0	0	0	0	0
Chrysiogenetes	1	0	0	0	0	0	0	0	0	0
Firmicutes	104	0	0	0	0	0	0	0	0	0
Tenericutes	19	0	0	0	0	0	0	0	0	0
Actinobacteria	80	0	0	0	0	0	0	0	0	0
Chlamydiae	8	0	0	0	0	0	0	0	0	0
Spirochaetes	14	0	0	0	0	0	0	0	0	0
Acidobacteria	5	0	0	0	0	0	0	0	0	0
Bacteroidetes	35	0	0	0	0	0	0	0	0	0
Fibrobacteres	1	0	0	0	0	0	0	0	0	0
Fusobacteria	5	0	0	0	0	0	0	0	0	0
Verrucomicrobia	4	0	0	0	0	0	0	0	0	0
Gemmatimonadetes	1	0	0	0	0	0	0	0	0	0
Planctomycetes	4	0	0	0	0	0	0	0	0	0
Elusimicrobia	2	0	0	0	0	0	0	0	0	0
Synergistetes	2	0	0	0	0	0	0	0	0	0
Cyanobacteria	16	0	0	0	0	0	0	0	0	0
Chlorobi	10	0	0	0	0	0	0	0	0	0
Chloroflexi	11	0	0	0	0	0	0	0	0	0
Deinococcus-Thermus	7	0	0	0	0	0	0	0	0	0
Aquificae	9	0	0	0	0	0	0	0	0	0
Thermotogae	11	0	0	0	0	0	0	0	0	0
Dictyoglomi	2	0	0	0	0	0	0	0	0	0
Nitrospirae	2	0	0	0	0	0	0	0	0	0
Thermobaculum	1	0	0	0	0	0	0	0	0	0
Deferribacteres	3	0	0	0	0	0	0	0	0	0
Euryarchaeota	60	0	0	0	0	0	0	0	0	0
Crenarchaeota	23	0	0	0	0	0	0	0	0	0
Thaumarchaeota	2	0	0	0	0	0	0	0	0	0
Nanoarchaeota	1	0	0	0	0	0	0	0	0	0
Korarchaeota	1	0	0	0	0	0	0	0	0	0
Total	768	0	0	0	0	0	0	0	0	0

(mod_M00409_1)

Phyla	Module completion ratio (%)									
	0--10	10--20	20--30	30--40	40--50	50--60	60--70	70--80	80--90	90--100
Gammaproteobacteria	126	0	0	0	0	0	0	0	0	0
Betaproteobacteria	61	0	0	0	0	0	0	0	0	0
Epsilonproteobacteria	17	0	0	0	0	0	0	0	0	0
Deltaproteobacteria	28	0	0	0	0	0	0	0	0	0
Alphaproteobacteria	91	0	0	0	0	0	0	0	0	0
Magnetococcus	1	0	0	0	0	0	0	0	0	0
Chrysiogenetes	1	0	0	0	0	0	0	0	0	0
Firmicutes	104	0	0	0	0	0	0	0	0	0
Tenericutes	19	0	0	0	0	0	0	0	0	0
Actinobacteria	80	0	0	0	0	0	0	0	0	0
Chlamydiae	8	0	0	0	0	0	0	0	0	0
Spirochaetes	14	0	0	0	0	0	0	0	0	0
Acidobacteria	5	0	0	0	0	0	0	0	0	0
Bacteroidetes	35	0	0	0	0	0	0	0	0	0
Fibrobacteres	1	0	0	0	0	0	0	0	0	0
Fusobacteria	5	0	0	0	0	0	0	0	0	0
Verrucomicrobia	4	0	0	0	0	0	0	0	0	0
Gemmatimonadetes	1	0	0	0	0	0	0	0	0	0
Planctomycetes	4	0	0	0	0	0	0	0	0	0
Elusimicrobia	2	0	0	0	0	0	0	0	0	0
Synergistetes	2	0	0	0	0	0	0	0	0	0
Cyanobacteria	16	0	0	0	0	0	0	0	0	0
Chlorobi	10	0	0	0	0	0	0	0	0	0
Chloroflexi	11	0	0	0	0	0	0	0	0	0
Deinococcus-Thermus	7	0	0	0	0	0	0	0	0	0
Aquificae	9	0	0	0	0	0	0	0	0	0
Thermotogae	11	0	0	0	0	0	0	0	0	0
Dictyoglomi	2	0	0	0	0	0	0	0	0	0
Nitrospirae	2	0	0	0	0	0	0	0	0	0
Thermobaculum	1	0	0	0	0	0	0	0	0	0
Deferribacteres	3	0	0	0	0	0	0	0	0	0
Euryarchaeota	60	0	0	0	0	0	0	0	0	0
Crenarchaeota	23	0	0	0	0	0	0	0	0	0
Thaumarchaeota	2	0	0	0	0	0	0	0	0	0
Nanoarchaeota	1	0	0	0	0	0	0	0	0	0
Korarchaeota	1	0	0	0	0	0	0	0	0	0
Total	768	0	0	0	0	0	0	0	0	0

(mod_M00410_1)

Phyla	Module completion ratio (%)									
	0--10	10--20	20--30	30--40	40--50	50--60	60--70	70--80	80--90	90--100
Gammaproteobacteria	126	0	0	0	0	0	0	0	0	0
Betaproteobacteria	61	0	0	0	0	0	0	0	0	0
Epsilonproteobacteria	17	0	0	0	0	0	0	0	0	0
Deltaproteobacteria	28	0	0	0	0	0	0	0	0	0
Alphaproteobacteria	91	0	0	0	0	0	0	0	0	0
Magnetococcus	1	0	0	0	0	0	0	0	0	0
Chrysiogenetes	1	0	0	0	0	0	0	0	0	0
Firmicutes	104	0	0	0	0	0	0	0	0	0
Tenericutes	19	0	0	0	0	0	0	0	0	0
Actinobacteria	80	0	0	0	0	0	0	0	0	0
Chlamydiae	8	0	0	0	0	0	0	0	0	0
Spirochaetes	14	0	0	0	0	0	0	0	0	0
Acidobacteria	5	0	0	0	0	0	0	0	0	0
Bacteroidetes	35	0	0	0	0	0	0	0	0	0
Fibrobacteres	1	0	0	0	0	0	0	0	0	0
Fusobacteria	5	0	0	0	0	0	0	0	0	0
Verrucomicrobia	4	0	0	0	0	0	0	0	0	0
Gemmatimonadetes	1	0	0	0	0	0	0	0	0	0
Planctomycetes	4	0	0	0	0	0	0	0	0	0
Elusimicrobia	2	0	0	0	0	0	0	0	0	0
Synergistetes	2	0	0	0	0	0	0	0	0	0
Cyanobacteria	16	0	0	0	0	0	0	0	0	0
Chlorobi	10	0	0	0	0	0	0	0	0	0
Chloroflexi	11	0	0	0	0	0	0	0	0	0
Deinococcus-Thermus	7	0	0	0	0	0	0	0	0	0
Aquificae	9	0	0	0	0	0	0	0	0	0
Thermotogae	11	0	0	0	0	0	0	0	0	0
Dictyoglomi	2	0	0	0	0	0	0	0	0	0
Nitrospirae	2	0	0	0	0	0	0	0	0	0
Thermobaculum	1	0	0	0	0	0	0	0	0	0
Deferribacteres	3	0	0	0	0	0	0	0	0	0
Euryarchaeota	60	0	0	0	0	0	0	0	0	0
Crenarchaeota	23	0	0	0	0	0	0	0	0	0
Thaumarchaeota	2	0	0	0	0	0	0	0	0	0
Nanoarchaeota	1	0	0	0	0	0	0	0	0	0
Korarchaeota	1	0	0	0	0	0	0	0	0	0
Total	768	0	0	0	0	0	0	0	0	0

(mod_M00411_1)

Phyla	Module completion ratio (%)									
	0--10	10--20	20--30	30--40	40--50	50--60	60--70	70--80	80--90	90--100
Gammaproteobacteria	126	0	0	0	0	0	0	0	0	0
Betaproteobacteria	61	0	0	0	0	0	0	0	0	0
Epsilonproteobacteria	17	0	0	0	0	0	0	0	0	0
Deltaproteobacteria	28	0	0	0	0	0	0	0	0	0
Alphaproteobacteria	91	0	0	0	0	0	0	0	0	0
Magnetococcus	1	0	0	0	0	0	0	0	0	0
Chrysiogenetes	1	0	0	0	0	0	0	0	0	0
Firmicutes	104	0	0	0	0	0	0	0	0	0
Tenericutes	19	0	0	0	0	0	0	0	0	0
Actinobacteria	80	0	0	0	0	0	0	0	0	0
Chlamydiae	8	0	0	0	0	0	0	0	0	0
Spirochaetes	14	0	0	0	0	0	0	0	0	0
Acidobacteria	5	0	0	0	0	0	0	0	0	0
Bacteroidetes	35	0	0	0	0	0	0	0	0	0
Fibrobacteres	1	0	0	0	0	0	0	0	0	0
Fusobacteria	5	0	0	0	0	0	0	0	0	0
Verrucomicrobia	4	0	0	0	0	0	0	0	0	0
Gemmatimonadetes	1	0	0	0	0	0	0	0	0	0
Planctomycetes	4	0	0	0	0	0	0	0	0	0
Elusimicrobia	2	0	0	0	0	0	0	0	0	0
Synergistetes	2	0	0	0	0	0	0	0	0	0
Cyanobacteria	16	0	0	0	0	0	0	0	0	0
Chlorobi	10	0	0	0	0	0	0	0	0	0
Chloroflexi	11	0	0	0	0	0	0	0	0	0
Deinococcus-Thermus	7	0	0	0	0	0	0	0	0	0
Aquificae	9	0	0	0	0	0	0	0	0	0
Thermotogae	11	0	0	0	0	0	0	0	0	0
Dictyoglomi	2	0	0	0	0	0	0	0	0	0
Nitrospirae	2	0	0	0	0	0	0	0	0	0
Thermobaculum	1	0	0	0	0	0	0	0	0	0
Deferribacteres	3	0	0	0	0	0	0	0	0	0
Euryarchaeota	60	0	0	0	0	0	0	0	0	0
Crenarchaeota	23	0	0	0	0	0	0	0	0	0
Thaumarchaeota	2	0	0	0	0	0	0	0	0	0
Nanoarchaeota	1	0	0	0	0	0	0	0	0	0
Korarchaeota	1	0	0	0	0	0	0	0	0	0
Total	768	0	0	0	0	0	0	0	0	0

(mod_M00411_1)

Phyla	Module completion ratio (%)									
	0--10	10--20	20--30	30--40	40--50	50--60	60--70	70--80	80--90	90--100
Gammaproteobacteria	126	0	0	0	0	0	0	0	0	0
Betaproteobacteria	61	0	0	0	0	0	0	0	0	0
Epsilonproteobacteria	17	0	0	0	0	0	0	0	0	0
Deltaproteobacteria	28	0	0	0	0	0	0	0	0	0
Alphaproteobacteria	91	0	0	0	0	0	0	0	0	0
Magnetococcus	1	0	0	0	0	0	0	0	0	0
Chrysiogenetes	1	0	0	0	0	0	0	0	0	0
Firmicutes	104	0	0	0	0	0	0	0	0	0
Tenericutes	19	0	0	0	0	0	0	0	0	0
Actinobacteria	80	0	0	0	0	0	0	0	0	0
Chlamydiae	8	0	0	0	0	0	0	0	0	0
Spirochaetes	14	0	0	0	0	0	0	0	0	0
Acidobacteria	5	0	0	0	0	0	0	0	0	0
Bacteroidetes	35	0	0	0	0	0	0	0	0	0
Fibrobacteres	1	0	0	0	0	0	0	0	0	0
Fusobacteria	5	0	0	0	0	0	0	0	0	0
Verrucomicrobia	4	0	0	0	0	0	0	0	0	0
Gemmatimonadetes	1	0	0	0	0	0	0	0	0	0
Planctomycetes	4	0	0	0	0	0	0	0	0	0
Elusimicrobia	2	0	0	0	0	0	0	0	0	0
Synergistetes	2	0	0	0	0	0	0	0	0	0
Cyanobacteria	16	0	0	0	0	0	0	0	0	0
Chlorobi	10	0	0	0	0	0	0	0	0	0
Chloroflexi	11	0	0	0	0	0	0	0	0	0
Deinococcus-Thermus	7	0	0	0	0	0	0	0	0	0
Aquificae	9	0	0	0	0	0	0	0	0	0
Thermotogae	11	0	0	0	0	0	0	0	0	0
Dictyoglomi	2	0	0	0	0	0	0	0	0	0
Nitrospirae	2	0	0	0	0	0	0	0	0	0
Thermobaculum	1	0	0	0	0	0	0	0	0	0
Deferribacteres	3	0	0	0	0	0	0	0	0	0
Euryarchaeota	60	0	0	0	0	0	0	0	0	0
Crenarchaeota	23	0	0	0	0	0	0	0	0	0
Thaumarchaeota	2	0	0	0	0	0	0	0	0	0
Nanoarchaeota	1	0	0	0	0	0	0	0	0	0
Korarchaeota	1	0	0	0	0	0	0	0	0	0
Total	768	0	0	0	0	0	0	0	0	0

(mod_M00412_1)

Phyla	Module completion ratio (%)									
	0--10	10--20	20--30	30--40	40--50	50--60	60--70	70--80	80--90	90--100
Gammaproteobacteria	126	0	0	0	0	0	0	0	0	0
Betaproteobacteria	61	0	0	0	0	0	0	0	0	0
Epsilonproteobacteria	17	0	0	0	0	0	0	0	0	0
Deltaproteobacteria	28	0	0	0	0	0	0	0	0	0
Alphaproteobacteria	91	0	0	0	0	0	0	0	0	0
Magnetococcus	1	0	0	0	0	0	0	0	0	0
Chrysiogenetes	1	0	0	0	0	0	0	0	0	0
Firmicutes	104	0	0	0	0	0	0	0	0	0
Tenericutes	19	0	0	0	0	0	0	0	0	0
Actinobacteria	80	0	0	0	0	0	0	0	0	0
Chlamydiae	8	0	0	0	0	0	0	0	0	0
Spirochaetes	14	0	0	0	0	0	0	0	0	0
Acidobacteria	5	0	0	0	0	0	0	0	0	0
Bacteroidetes	35	0	0	0	0	0	0	0	0	0
Fibrobacteres	1	0	0	0	0	0	0	0	0	0
Fusobacteria	5	0	0	0	0	0	0	0	0	0
Verrucomicrobia	4	0	0	0	0	0	0	0	0	0
Gemmatimonadetes	1	0	0	0	0	0	0	0	0	0
Planctomycetes	4	0	0	0	0	0	0	0	0	0
Elusimicrobia	2	0	0	0	0	0	0	0	0	0
Synergistetes	2	0	0	0	0	0	0	0	0	0
Cyanobacteria	16	0	0	0	0	0	0	0	0	0
Chlorobi	10	0	0	0	0	0	0	0	0	0
Chloroflexi	11	0	0	0	0	0	0	0	0	0
Deinococcus-Thermus	7	0	0	0	0	0	0	0	0	0
Aquificae	9	0	0	0	0	0	0	0	0	0
Thermotogae	11	0	0	0	0	0	0	0	0	0
Dictyoglomi	2	0	0	0	0	0	0	0	0	0
Nitrospirae	2	0	0	0	0	0	0	0	0	0
Thermobaculum	1	0	0	0	0	0	0	0	0	0
Deferribacteres	3	0	0	0	0	0	0	0	0	0
Euryarchaeota	60	0	0	0	0	0	0	0	0	0
Crenarchaeota	23	0	0	0	0	0	0	0	0	0
Thaumarchaeota	2	0	0	0	0	0	0	0	0	0
Nanoarchaeota	1	0	0	0	0	0	0	0	0	0
Korarchaeota	1	0	0	0	0	0	0	0	0	0
Total	768	0	0	0	0	0	0	0	0	0

(mod_M00413_1)

Phyla	Module completion ratio (%)									
	0--10	10--20	20--30	30--40	40--50	50--60	60--70	70--80	80--90	90--100
Gammaproteobacteria	126	0	0	0	0	0	0	0	0	0
Betaproteobacteria	59	2	0	0	0	0	0	0	0	0
Epsilonproteobacteria	17	0	0	0	0	0	0	0	0	0
Deltaproteobacteria	28	0	0	0	0	0	0	0	0	0
Alphaproteobacteria	91	0	0	0	0	0	0	0	0	0
Magnetococcus	1	0	0	0	0	0	0	0	0	0
Chrysiogenetes	1	0	0	0	0	0	0	0	0	0
Firmicutes	104	0	0	0	0	0	0	0	0	0
Tenericutes	19	0	0	0	0	0	0	0	0	0
Actinobacteria	80	0	0	0	0	0	0	0	0	0
Chlamydiae	8	0	0	0	0	0	0	0	0	0
Spirochaetes	14	0	0	0	0	0	0	0	0	0
Acidobacteria	5	0	0	0	0	0	0	0	0	0
Bacteroidetes	35	0	0	0	0	0	0	0	0	0
Fibrobacteres	1	0	0	0	0	0	0	0	0	0
Fusobacteria	5	0	0	0	0	0	0	0	0	0
Verrucomicrobia	4	0	0	0	0	0	0	0	0	0
Gemmatimonadetes	1	0	0	0	0	0	0	0	0	0
Planctomycetes	4	0	0	0	0	0	0	0	0	0
Elusimicrobia	2	0	0	0	0	0	0	0	0	0
Synergistetes	2	0	0	0	0	0	0	0	0	0
Cyanobacteria	16	0	0	0	0	0	0	0	0	0
Chlorobi	10	0	0	0	0	0	0	0	0	0
Chloroflexi	11	0	0	0	0	0	0	0	0	0
Deinococcus-Thermus	7	0	0	0	0	0	0	0	0	0
Aquificae	9	0	0	0	0	0	0	0	0	0
Thermotogae	11	0	0	0	0	0	0	0	0	0
Dictyoglomi	2	0	0	0	0	0	0	0	0	0
Nitrospirae	2	0	0	0	0	0	0	0	0	0
Thermobaculum	1	0	0	0	0	0	0	0	0	0
Deferribacteres	3	0	0	0	0	0	0	0	0	0
Euryarchaeota	1	59	0	0	0	0	0	0	0	0
Crenarchaeota	23	0	0	0	0	0	0	0	0	0
Thaumarchaeota	0	2	0	0	0	0	0	0	0	0
Nanoarchaeota	0	1	0	0	0	0	0	0	0	0
Korarchaeota	1	0	0	0	0	0	0	0	0	0
Total	704	64	0	0	0	0	0	0	0	0

(mod_M00414_1)

Phyla	Module completion ratio (%)									
	0--10	10--20	20--30	30--40	40--50	50--60	60--70	70--80	80--90	90--100
Gammaproteobacteria	126	0	0	0	0	0	0	0	0	0
Betaproteobacteria	61	0	0	0	0	0	0	0	0	0
Epsilonproteobacteria	17	0	0	0	0	0	0	0	0	0
Deltaproteobacteria	28	0	0	0	0	0	0	0	0	0
Alphaproteobacteria	91	0	0	0	0	0	0	0	0	0
Magnetococcus	1	0	0	0	0	0	0	0	0	0
Chrysiogenetes	1	0	0	0	0	0	0	0	0	0
Firmicutes	104	0	0	0	0	0	0	0	0	0
Tenericutes	19	0	0	0	0	0	0	0	0	0
Actinobacteria	80	0	0	0	0	0	0	0	0	0
Chlamydiae	8	0	0	0	0	0	0	0	0	0
Spirochaetes	14	0	0	0	0	0	0	0	0	0
Acidobacteria	5	0	0	0	0	0	0	0	0	0
Bacteroidetes	35	0	0	0	0	0	0	0	0	0
Fibrobacteres	1	0	0	0	0	0	0	0	0	0
Fusobacteria	5	0	0	0	0	0	0	0	0	0
Verrucomicrobia	4	0	0	0	0	0	0	0	0	0
Gemmatimonadetes	1	0	0	0	0	0	0	0	0	0
Planctomycetes	4	0	0	0	0	0	0	0	0	0
Elusimicrobia	2	0	0	0	0	0	0	0	0	0
Synergistetes	2	0	0	0	0	0	0	0	0	0
Cyanobacteria	16	0	0	0	0	0	0	0	0	0
Chlorobi	10	0	0	0	0	0	0	0	0	0
Chloroflexi	11	0	0	0	0	0	0	0	0	0
Deinococcus-Thermus	7	0	0	0	0	0	0	0	0	0
Aquificae	9	0	0	0	0	0	0	0	0	0
Thermotogae	11	0	0	0	0	0	0	0	0	0
Dictyoglomi	2	0	0	0	0	0	0	0	0	0
Nitrospirae	2	0	0	0	0	0	0	0	0	0
Thermobaculum	1	0	0	0	0	0	0	0	0	0
Deferribacteres	3	0	0	0	0	0	0	0	0	0
Euryarchaeota	60	0	0	0	0	0	0	0	0	0
Crenarchaeota	23	0	0	0	0	0	0	0	0	0
Thaumarchaeota	2	0	0	0	0	0	0	0	0	0
Nanoarchaeota	1	0	0	0	0	0	0	0	0	0
Korarchaeota	1	0	0	0	0	0	0	0	0	0
Total	768	0	0	0	0	0	0	0	0	0

(mod_M00414_1)

Phyla	Module completion ratio (%)									
	0--10	10--20	20--30	30--40	40--50	50--60	60--70	70--80	80--90	90--100
Gammaproteobacteria	126	0	0	0	0	0	0	0	0	0
Betaproteobacteria	61	0	0	0	0	0	0	0	0	0
Epsilonproteobacteria	17	0	0	0	0	0	0	0	0	0
Deltaproteobacteria	28	0	0	0	0	0	0	0	0	0
Alphaproteobacteria	91	0	0	0	0	0	0	0	0	0
Magnetococcus	1	0	0	0	0	0	0	0	0	0
Chrysiogenetes	1	0	0	0	0	0	0	0	0	0
Firmicutes	104	0	0	0	0	0	0	0	0	0
Tenericutes	19	0	0	0	0	0	0	0	0	0
Actinobacteria	80	0	0	0	0	0	0	0	0	0
Chlamydiae	8	0	0	0	0	0	0	0	0	0
Spirochaetes	14	0	0	0	0	0	0	0	0	0
Acidobacteria	5	0	0	0	0	0	0	0	0	0
Bacteroidetes	35	0	0	0	0	0	0	0	0	0
Fibrobacteres	1	0	0	0	0	0	0	0	0	0
Fusobacteria	5	0	0	0	0	0	0	0	0	0
Verrucomicrobia	4	0	0	0	0	0	0	0	0	0
Gemmatimonadetes	1	0	0	0	0	0	0	0	0	0
Planctomycetes	4	0	0	0	0	0	0	0	0	0
Elusimicrobia	2	0	0	0	0	0	0	0	0	0
Synergistetes	2	0	0	0	0	0	0	0	0	0
Cyanobacteria	16	0	0	0	0	0	0	0	0	0
Chlorobi	10	0	0	0	0	0	0	0	0	0
Chloroflexi	11	0	0	0	0	0	0	0	0	0
Deinococcus-Thermus	7	0	0	0	0	0	0	0	0	0
Aquificae	9	0	0	0	0	0	0	0	0	0
Thermotogae	11	0	0	0	0	0	0	0	0	0
Dictyoglomi	2	0	0	0	0	0	0	0	0	0
Nitrospirae	2	0	0	0	0	0	0	0	0	0
Thermobaculum	1	0	0	0	0	0	0	0	0	0
Deferribacteres	3	0	0	0	0	0	0	0	0	0
Euryarchaeota	60	0	0	0	0	0	0	0	0	0
Crenarchaeota	23	0	0	0	0	0	0	0	0	0
Thaumarchaeota	2	0	0	0	0	0	0	0	0	0
Nanoarchaeota	1	0	0	0	0	0	0	0	0	0
Korarchaeota	1	0	0	0	0	0	0	0	0	0
Total	768	0	0	0	0	0	0	0	0	0

(mod_M00415_1)

Phyla	Module completion ratio (%)									
	0--10	10--20	20--30	30--40	40--50	50--60	60--70	70--80	80--90	90--100
Gammaproteobacteria	126	0	0	0	0	0	0	0	0	0
Betaproteobacteria	61	0	0	0	0	0	0	0	0	0
Epsilonproteobacteria	17	0	0	0	0	0	0	0	0	0
Deltaproteobacteria	28	0	0	0	0	0	0	0	0	0
Alphaproteobacteria	91	0	0	0	0	0	0	0	0	0
Magnetococcus	1	0	0	0	0	0	0	0	0	0
Chrysiogenetes	1	0	0	0	0	0	0	0	0	0
Firmicutes	104	0	0	0	0	0	0	0	0	0
Tenericutes	19	0	0	0	0	0	0	0	0	0
Actinobacteria	80	0	0	0	0	0	0	0	0	0
Chlamydiae	8	0	0	0	0	0	0	0	0	0
Spirochaetes	14	0	0	0	0	0	0	0	0	0
Acidobacteria	5	0	0	0	0	0	0	0	0	0
Bacteroidetes	35	0	0	0	0	0	0	0	0	0
Fibrobacteres	1	0	0	0	0	0	0	0	0	0
Fusobacteria	5	0	0	0	0	0	0	0	0	0
Verrucomicrobia	4	0	0	0	0	0	0	0	0	0
Gemmatimonadetes	1	0	0	0	0	0	0	0	0	0
Planctomycetes	4	0	0	0	0	0	0	0	0	0
Elusimicrobia	2	0	0	0	0	0	0	0	0	0
Synergistetes	2	0	0	0	0	0	0	0	0	0
Cyanobacteria	16	0	0	0	0	0	0	0	0	0
Chlorobi	10	0	0	0	0	0	0	0	0	0
Chloroflexi	11	0	0	0	0	0	0	0	0	0
Deinococcus-Thermus	7	0	0	0	0	0	0	0	0	0
Aquificae	9	0	0	0	0	0	0	0	0	0
Thermotogae	11	0	0	0	0	0	0	0	0	0
Dictyoglomi	2	0	0	0	0	0	0	0	0	0
Nitrospirae	2	0	0	0	0	0	0	0	0	0
Thermobaculum	1	0	0	0	0	0	0	0	0	0
Deferribacteres	3	0	0	0	0	0	0	0	0	0
Euryarchaeota	60	0	0	0	0	0	0	0	0	0
Crenarchaeota	23	0	0	0	0	0	0	0	0	0
Thaumarchaeota	2	0	0	0	0	0	0	0	0	0
Nanoarchaeota	1	0	0	0	0	0	0	0	0	0
Korarchaeota	1	0	0	0	0	0	0	0	0	0
Total	768	0	0	0	0	0	0	0	0	0

(mod_M00416_1)

Phyla	Module completion ratio (%)									
	0--10	10--20	20--30	30--40	40--50	50--60	60--70	70--80	80--90	90--100
Gammaproteobacteria	126	0	0	0	0	0	0	0	0	0
Betaproteobacteria	60	0	1	0	0	0	0	0	0	0
Epsilonproteobacteria	17	0	0	0	0	0	0	0	0	0
Deltaproteobacteria	28	0	0	0	0	0	0	0	0	0
Alphaproteobacteria	90	0	1	0	0	0	0	0	0	0
Magnetococcus	1	0	0	0	0	0	0	0	0	0
Chrysiogenetes	1	0	0	0	0	0	0	0	0	0
Firmicutes	78	0	1	0	0	1	0	2	0	22
Tenericutes	19	0	0	0	0	0	0	0	0	0
Actinobacteria	80	0	0	0	0	0	0	0	0	0
Chlamydiae	8	0	0	0	0	0	0	0	0	0
Spirochaetes	14	0	0	0	0	0	0	0	0	0
Acidobacteria	5	0	0	0	0	0	0	0	0	0
Bacteroidetes	35	0	0	0	0	0	0	0	0	0
Fibrobacteres	1	0	0	0	0	0	0	0	0	0
Fusobacteria	5	0	0	0	0	0	0	0	0	0
Verrucomicrobia	4	0	0	0	0	0	0	0	0	0
Gemmatimonadetes	1	0	0	0	0	0	0	0	0	0
Planctomycetes	4	0	0	0	0	0	0	0	0	0
Elusimicrobia	2	0	0	0	0	0	0	0	0	0
Synergistetes	2	0	0	0	0	0	0	0	0	0
Cyanobacteria	16	0	0	0	0	0	0	0	0	0
Chlorobi	10	0	0	0	0	0	0	0	0	0
Chloroflexi	11	0	0	0	0	0	0	0	0	0
Deinococcus-Thermus	7	0	0	0	0	0	0	0	0	0
Aquificae	9	0	0	0	0	0	0	0	0	0
Thermotogae	11	0	0	0	0	0	0	0	0	0
Dictyoglomi	2	0	0	0	0	0	0	0	0	0
Nitrospirae	2	0	0	0	0	0	0	0	0	0
Thermobaculum	1	0	0	0	0	0	0	0	0	0
Deferribacteres	3	0	0	0	0	0	0	0	0	0
Euryarchaeota	60	0	0	0	0	0	0	0	0	0
Crenarchaeota	16	0	0	0	0	0	0	7	0	0
Thaumarchaeota	2	0	0	0	0	0	0	0	0	0
Nanoarchaeota	1	0	0	0	0	0	0	0	0	0
Korarchaeota	1	0	0	0	0	0	0	0	0	0
Total	733	0	3	0	0	1	0	9	0	22

(mod_M00417_1)

Phyla	Module completion ratio (%)									
	0--10	10--20	20--30	30--40	40--50	50--60	60--70	70--80	80--90	90--100
Gammaproteobacteria	55	0	0	0	0	1	0	3	0	67
Betaproteobacteria	24	0	0	0	0	0	0	1	0	36
Epsilonproteobacteria	17	0	0	0	0	0	0	0	0	0
Deltaproteobacteria	28	0	0	0	0	0	0	0	0	0
Alphaproteobacteria	51	0	0	0	0	1	0	3	0	36
Magnetococcus	1	0	0	0	0	0	0	0	0	0
Chrysiogenetes	1	0	0	0	0	0	0	0	0	0
Firmicutes	99	0	2	0	0	1	0	2	0	0
Tenericutes	19	0	0	0	0	0	0	0	0	0
Actinobacteria	80	0	0	0	0	0	0	0	0	0
Chlamydiae	6	0	0	0	0	0	0	0	0	2
Spirochaetes	14	0	0	0	0	0	0	0	0	0
Acidobacteria	4	0	1	0	0	0	0	0	0	0
Bacteroidetes	35	0	0	0	0	0	0	0	0	0
Fibrobacteres	1	0	0	0	0	0	0	0	0	0
Fusobacteria	5	0	0	0	0	0	0	0	0	0
Verrucomicrobia	3	0	1	0	0	0	0	0	0	0
Gemmatimonadetes	0	0	1	0	0	0	0	0	0	0
Planctomycetes	4	0	0	0	0	0	0	0	0	0
Elusimicrobia	2	0	0	0	0	0	0	0	0	0
Synergistetes	2	0	0	0	0	0	0	0	0	0
Cyanobacteria	16	0	0	0	0	0	0	0	0	0
Chlorobi	9	0	0	0	0	0	0	0	0	1
Chloroflexi	9	0	2	0	0	0	0	0	0	0
Deinococcus-Thermus	6	0	1	0	0	0	0	0	0	0
Aquificae	9	0	0	0	0	0	0	0	0	0
Thermotogae	11	0	0	0	0	0	0	0	0	0
Dictyoglomi	2	0	0	0	0	0	0	0	0	0
Nitrospirae	2	0	0	0	0	0	0	0	0	0
Thermobaculum	1	0	0	0	0	0	0	0	0	0
Deferribacteres	3	0	0	0	0	0	0	0	0	0
Euryarchaeota	60	0	0	0	0	0	0	0	0	0
Crenarchaeota	23	0	0	0	0	0	0	0	0	0
Thaumarchaeota	2	0	0	0	0	0	0	0	0	0
Nanoarchaeota	1	0	0	0	0	0	0	0	0	0
Korarchaeota	1	0	0	0	0	0	0	0	0	0
Total	606	0	8	0	0	3	0	9	0	142

(mod_M00418_1)

Phyla	Module completion ratio (%)									
	0--10	10--20	20--30	30--40	40--50	50--60	60--70	70--80	80--90	90--100
Gammaproteobacteria	126	0	0	0	0	0	0	0	0	0
Betaproteobacteria	60	0	0	0	0	0	0	0	0	1
Epsilonproteobacteria	17	0	0	0	0	0	0	0	0	0
Deltaproteobacteria	27	0	0	0	0	0	0	0	0	1
Alphaproteobacteria	91	0	0	0	0	0	0	0	0	0
Magnetococcus	1	0	0	0	0	0	0	0	0	0
Chrysiogenetes	1	0	0	0	0	0	0	0	0	0
Firmicutes	104	0	0	0	0	0	0	0	0	0
Tenericutes	19	0	0	0	0	0	0	0	0	0
Actinobacteria	80	0	0	0	0	0	0	0	0	0
Chlamydiae	8	0	0	0	0	0	0	0	0	0
Spirochaetes	14	0	0	0	0	0	0	0	0	0
Acidobacteria	5	0	0	0	0	0	0	0	0	0
Bacteroidetes	35	0	0	0	0	0	0	0	0	0
Fibrobacteres	1	0	0	0	0	0	0	0	0	0
Fusobacteria	5	0	0	0	0	0	0	0	0	0
Verrucomicrobia	4	0	0	0	0	0	0	0	0	0
Gemmatimonadetes	1	0	0	0	0	0	0	0	0	0
Planctomycetes	4	0	0	0	0	0	0	0	0	0
Elusimicrobia	2	0	0	0	0	0	0	0	0	0
Synergistetes	2	0	0	0	0	0	0	0	0	0
Cyanobacteria	16	0	0	0	0	0	0	0	0	0
Chlorobi	10	0	0	0	0	0	0	0	0	0
Chloroflexi	11	0	0	0	0	0	0	0	0	0
Deinococcus-Thermus	7	0	0	0	0	0	0	0	0	0
Aquificae	9	0	0	0	0	0	0	0	0	0
Thermotogae	11	0	0	0	0	0	0	0	0	0
Dictyoglomi	2	0	0	0	0	0	0	0	0	0
Nitrospirae	2	0	0	0	0	0	0	0	0	0
Thermobaculum	1	0	0	0	0	0	0	0	0	0
Deferribacteres	3	0	0	0	0	0	0	0	0	0
Euryarchaeota	60	0	0	0	0	0	0	0	0	0
Crenarchaeota	23	0	0	0	0	0	0	0	0	0
Thaumarchaeota	2	0	0	0	0	0	0	0	0	0
Nanoarchaeota	1	0	0	0	0	0	0	0	0	0
Korarchaeota	1	0	0	0	0	0	0	0	0	0
Total	766	0	0	0	0	0	0	0	0	2

(mod_M00419_1)

Phyla	Module completion ratio (%)									
	0--10	10--20	20--30	30--40	40--50	50--60	60--70	70--80	80--90	90--100
Gammaproteobacteria	126	0	0	0	0	0	0	0	0	0
Betaproteobacteria	60	0	0	0	0	0	0	0	0	1
Epsilonproteobacteria	17	0	0	0	0	0	0	0	0	0
Deltaproteobacteria	28	0	0	0	0	0	0	0	0	0
Alphaproteobacteria	91	0	0	0	0	0	0	0	0	0
Magnetococcus	1	0	0	0	0	0	0	0	0	0
Chrysiogenetes	1	0	0	0	0	0	0	0	0	0
Firmicutes	104	0	0	0	0	0	0	0	0	0
Tenericutes	19	0	0	0	0	0	0	0	0	0
Actinobacteria	80	0	0	0	0	0	0	0	0	0
Chlamydiae	8	0	0	0	0	0	0	0	0	0
Spirochaetes	14	0	0	0	0	0	0	0	0	0
Acidobacteria	5	0	0	0	0	0	0	0	0	0
Bacteroidetes	35	0	0	0	0	0	0	0	0	0
Fibrobacteres	1	0	0	0	0	0	0	0	0	0
Fusobacteria	5	0	0	0	0	0	0	0	0	0
Verrucomicrobia	4	0	0	0	0	0	0	0	0	0
Gemmatimonadetes	1	0	0	0	0	0	0	0	0	0
Planctomycetes	4	0	0	0	0	0	0	0	0	0
Elusimicrobia	2	0	0	0	0	0	0	0	0	0
Synergistetes	2	0	0	0	0	0	0	0	0	0
Cyanobacteria	16	0	0	0	0	0	0	0	0	0
Chlorobi	10	0	0	0	0	0	0	0	0	0
Chloroflexi	11	0	0	0	0	0	0	0	0	0
Deinococcus-Thermus	7	0	0	0	0	0	0	0	0	0
Aquificae	9	0	0	0	0	0	0	0	0	0
Thermotogae	11	0	0	0	0	0	0	0	0	0
Dictyoglomi	2	0	0	0	0	0	0	0	0	0
Nitrospirae	2	0	0	0	0	0	0	0	0	0
Thermobaculum	1	0	0	0	0	0	0	0	0	0
Deferribacteres	3	0	0	0	0	0	0	0	0	0
Euryarchaeota	60	0	0	0	0	0	0	0	0	0
Crenarchaeota	23	0	0	0	0	0	0	0	0	0
Thaumarchaeota	2	0	0	0	0	0	0	0	0	0
Nanoarchaeota	1	0	0	0	0	0	0	0	0	0
Korarchaeota	1	0	0	0	0	0	0	0	0	0
Total	767	0	0	0	0	0	0	0	0	1

(mod_M00423_1)

Phyla	Module completion ratio (%)									
	0--10	10--20	20--30	30--40	40--50	50--60	60--70	70--80	80--90	90--100
Gammaproteobacteria	126	0	0	0	0	0	0	0	0	0
Betaproteobacteria	60	0	0	0	0	0	1	0	0	0
Epsilonproteobacteria	15	0	0	0	0	0	0	0	0	2
Deltaproteobacteria	20	0	0	1	0	0	1	0	0	6
Alphaproteobacteria	91	0	0	0	0	0	0	0	0	0
Magnetococcus	1	0	0	0	0	0	0	0	0	0
Chrysiogenetes	1	0	0	0	0	0	0	0	0	0
Firmicutes	102	0	0	0	0	0	0	0	0	2
Tenericutes	19	0	0	0	0	0	0	0	0	0
Actinobacteria	80	0	0	0	0	0	0	0	0	0
Chlamydiae	8	0	0	0	0	0	0	0	0	0
Spirochaetes	14	0	0	0	0	0	0	0	0	0
Acidobacteria	5	0	0	0	0	0	0	0	0	0
Bacteroidetes	35	0	0	0	0	0	0	0	0	0
Fibrobacteres	1	0	0	0	0	0	0	0	0	0
Fusobacteria	5	0	0	0	0	0	0	0	0	0
Verrucomicrobia	4	0	0	0	0	0	0	0	0	0
Gemmatimonadetes	1	0	0	0	0	0	0	0	0	0
Planctomycetes	4	0	0	0	0	0	0	0	0	0
Elusimicrobia	2	0	0	0	0	0	0	0	0	0
Synergistetes	2	0	0	0	0	0	0	0	0	0
Cyanobacteria	16	0	0	0	0	0	0	0	0	0
Chlorobi	10	0	0	0	0	0	0	0	0	0
Chloroflexi	11	0	0	0	0	0	0	0	0	0
Deinococcus-Thermus	7	0	0	0	0	0	0	0	0	0
Aquificae	8	0	0	0	0	0	0	0	0	1
Thermotogae	10	0	0	0	0	0	0	0	0	1
Dictyoglomi	2	0	0	0	0	0	0	0	0	0
Nitrospirae	2	0	0	0	0	0	0	0	0	0
Thermobaculum	1	0	0	0	0	0	0	0	0	0
Deferribacteres	2	0	0	0	0	0	0	0	0	1
Euryarchaeota	36	0	0	1	0	0	2	0	0	21
Crenarchaeota	18	0	0	0	0	0	0	0	0	5
Thaumarchaeota	2	0	0	0	0	0	0	0	0	0
Nanoarchaeota	1	0	0	0	0	0	0	0	0	0
Korarchaeota	0	0	0	0	0	0	0	0	0	1
Total	722	0	0	2	0	0	4	0	0	40

(mod_M00424_1)

Phyla	Module completion ratio (%)									
	0--10	10--20	20--30	30--40	40--50	50--60	60--70	70--80	80--90	90--100
Gammaproteobacteria	126	0	0	0	0	0	0	0	0	0
Betaproteobacteria	61	0	0	0	0	0	0	0	0	0
Epsilonproteobacteria	17	0	0	0	0	0	0	0	0	0
Deltaproteobacteria	28	0	0	0	0	0	0	0	0	0
Alphaproteobacteria	91	0	0	0	0	0	0	0	0	0
Magnetococcus	1	0	0	0	0	0	0	0	0	0
Chrysiogenetes	1	0	0	0	0	0	0	0	0	0
Firmicutes	104	0	0	0	0	0	0	0	0	0
Tenericutes	19	0	0	0	0	0	0	0	0	0
Actinobacteria	80	0	0	0	0	0	0	0	0	0
Chlamydiae	8	0	0	0	0	0	0	0	0	0
Spirochaetes	14	0	0	0	0	0	0	0	0	0
Acidobacteria	5	0	0	0	0	0	0	0	0	0
Bacteroidetes	35	0	0	0	0	0	0	0	0	0
Fibrobacteres	1	0	0	0	0	0	0	0	0	0
Fusobacteria	5	0	0	0	0	0	0	0	0	0
Verrucomicrobia	4	0	0	0	0	0	0	0	0	0
Gemmatimonadetes	1	0	0	0	0	0	0	0	0	0
Planctomycetes	4	0	0	0	0	0	0	0	0	0
Elusimicrobia	2	0	0	0	0	0	0	0	0	0
Synergistetes	2	0	0	0	0	0	0	0	0	0
Cyanobacteria	16	0	0	0	0	0	0	0	0	0
Chlorobi	10	0	0	0	0	0	0	0	0	0
Chloroflexi	11	0	0	0	0	0	0	0	0	0
Deinococcus-Thermus	7	0	0	0	0	0	0	0	0	0
Aquificae	9	0	0	0	0	0	0	0	0	0
Thermotogae	11	0	0	0	0	0	0	0	0	0
Dictyoglomi	2	0	0	0	0	0	0	0	0	0
Nitrospirae	2	0	0	0	0	0	0	0	0	0
Thermobaculum	1	0	0	0	0	0	0	0	0	0
Deferribacteres	3	0	0	0	0	0	0	0	0	0
Euryarchaeota	60	0	0	0	0	0	0	0	0	0
Crenarchaeota	23	0	0	0	0	0	0	0	0	0
Thaumarchaeota	2	0	0	0	0	0	0	0	0	0
Nanoarchaeota	1	0	0	0	0	0	0	0	0	0
Korarchaeota	1	0	0	0	0	0	0	0	0	0
Total	768	0	0	0	0	0	0	0	0	0

(mod_M00424_1)

Phyla	Module completion ratio (%)									
	0--10	10--20	20--30	30--40	40--50	50--60	60--70	70--80	80--90	90--100
Gammaproteobacteria	126	0	0	0	0	0	0	0	0	0
Betaproteobacteria	61	0	0	0	0	0	0	0	0	0
Epsilonproteobacteria	17	0	0	0	0	0	0	0	0	0
Deltaproteobacteria	28	0	0	0	0	0	0	0	0	0
Alphaproteobacteria	91	0	0	0	0	0	0	0	0	0
Magnetococcus	1	0	0	0	0	0	0	0	0	0
Chrysiogenetes	1	0	0	0	0	0	0	0	0	0
Firmicutes	104	0	0	0	0	0	0	0	0	0
Tenericutes	19	0	0	0	0	0	0	0	0	0
Actinobacteria	80	0	0	0	0	0	0	0	0	0
Chlamydiae	8	0	0	0	0	0	0	0	0	0
Spirochaetes	14	0	0	0	0	0	0	0	0	0
Acidobacteria	5	0	0	0	0	0	0	0	0	0
Bacteroidetes	35	0	0	0	0	0	0	0	0	0
Fibrobacteres	1	0	0	0	0	0	0	0	0	0
Fusobacteria	5	0	0	0	0	0	0	0	0	0
Verrucomicrobia	4	0	0	0	0	0	0	0	0	0
Gemmatimonadetes	1	0	0	0	0	0	0	0	0	0
Planctomycetes	4	0	0	0	0	0	0	0	0	0
Elusimicrobia	2	0	0	0	0	0	0	0	0	0
Synergistetes	2	0	0	0	0	0	0	0	0	0
Cyanobacteria	16	0	0	0	0	0	0	0	0	0
Chlorobi	10	0	0	0	0	0	0	0	0	0
Chloroflexi	11	0	0	0	0	0	0	0	0	0
Deinococcus-Thermus	7	0	0	0	0	0	0	0	0	0
Aquificae	9	0	0	0	0	0	0	0	0	0
Thermotogae	11	0	0	0	0	0	0	0	0	0
Dictyoglomi	2	0	0	0	0	0	0	0	0	0
Nitrospirae	2	0	0	0	0	0	0	0	0	0
Thermobaculum	1	0	0	0	0	0	0	0	0	0
Deferribacteres	3	0	0	0	0	0	0	0	0	0
Euryarchaeota	60	0	0	0	0	0	0	0	0	0
Crenarchaeota	23	0	0	0	0	0	0	0	0	0
Thaumarchaeota	2	0	0	0	0	0	0	0	0	0
Nanoarchaeota	1	0	0	0	0	0	0	0	0	0
Korarchaeota	1	0	0	0	0	0	0	0	0	0
Total	768	0	0	0	0	0	0	0	0	0

(mod_M00425_1)

Phyla	Module completion ratio (%)									
	0--10	10--20	20--30	30--40	40--50	50--60	60--70	70--80	80--90	90--100
Gammaproteobacteria	126	0	0	0	0	0	0	0	0	0
Betaproteobacteria	61	0	0	0	0	0	0	0	0	0
Epsilonproteobacteria	17	0	0	0	0	0	0	0	0	0
Deltaproteobacteria	28	0	0	0	0	0	0	0	0	0
Alphaproteobacteria	91	0	0	0	0	0	0	0	0	0
Magnetococcus	1	0	0	0	0	0	0	0	0	0
Chrysiogenetes	1	0	0	0	0	0	0	0	0	0
Firmicutes	104	0	0	0	0	0	0	0	0	0
Tenericutes	19	0	0	0	0	0	0	0	0	0
Actinobacteria	80	0	0	0	0	0	0	0	0	0
Chlamydiae	8	0	0	0	0	0	0	0	0	0
Spirochaetes	14	0	0	0	0	0	0	0	0	0
Acidobacteria	5	0	0	0	0	0	0	0	0	0
Bacteroidetes	35	0	0	0	0	0	0	0	0	0
Fibrobacteres	1	0	0	0	0	0	0	0	0	0
Fusobacteria	5	0	0	0	0	0	0	0	0	0
Verrucomicrobia	4	0	0	0	0	0	0	0	0	0
Gemmatimonadetes	1	0	0	0	0	0	0	0	0	0
Planctomycetes	4	0	0	0	0	0	0	0	0	0
Elusimicrobia	2	0	0	0	0	0	0	0	0	0
Synergistetes	2	0	0	0	0	0	0	0	0	0
Cyanobacteria	15	0	1	0	0	0	0	0	0	0
Chlorobi	10	0	0	0	0	0	0	0	0	0
Chloroflexi	11	0	0	0	0	0	0	0	0	0
Deinococcus-Thermus	7	0	0	0	0	0	0	0	0	0
Aquificae	9	0	0	0	0	0	0	0	0	0
Thermotogae	11	0	0	0	0	0	0	0	0	0
Dictyoglomi	2	0	0	0	0	0	0	0	0	0
Nitrospirae	2	0	0	0	0	0	0	0	0	0
Thermobaculum	1	0	0	0	0	0	0	0	0	0
Deferribacteres	3	0	0	0	0	0	0	0	0	0
Euryarchaeota	0	0	60	0	0	0	0	0	0	0
Crenarchaeota	0	0	23	0	0	0	0	0	0	0
Thaumarchaeota	1	0	1	0	0	0	0	0	0	0
Nanoarchaeota	0	0	1	0	0	0	0	0	0	0
Korarchaeota	1	0	0	0	0	0	0	0	0	0
Total	682	0	86	0	0	0	0	0	0	0

(mod_M00426_1)

Phyla	Module completion ratio (%)									
	0--10	10--20	20--30	30--40	40--50	50--60	60--70	70--80	80--90	90--100
Gammaproteobacteria	126	0	0	0	0	0	0	0	0	0
Betaproteobacteria	61	0	0	0	0	0	0	0	0	0
Epsilonproteobacteria	17	0	0	0	0	0	0	0	0	0
Deltaproteobacteria	28	0	0	0	0	0	0	0	0	0
Alphaproteobacteria	91	0	0	0	0	0	0	0	0	0
Magnetococcus	1	0	0	0	0	0	0	0	0	0
Chrysiogenetes	1	0	0	0	0	0	0	0	0	0
Firmicutes	104	0	0	0	0	0	0	0	0	0
Tenericutes	19	0	0	0	0	0	0	0	0	0
Actinobacteria	80	0	0	0	0	0	0	0	0	0
Chlamydiae	8	0	0	0	0	0	0	0	0	0
Spirochaetes	14	0	0	0	0	0	0	0	0	0
Acidobacteria	5	0	0	0	0	0	0	0	0	0
Bacteroidetes	35	0	0	0	0	0	0	0	0	0
Fibrobacteres	1	0	0	0	0	0	0	0	0	0
Fusobacteria	5	0	0	0	0	0	0	0	0	0
Verrucomicrobia	4	0	0	0	0	0	0	0	0	0
Gemmatimonadetes	1	0	0	0	0	0	0	0	0	0
Planctomycetes	4	0	0	0	0	0	0	0	0	0
Elusimicrobia	2	0	0	0	0	0	0	0	0	0
Synergistetes	2	0	0	0	0	0	0	0	0	0
Cyanobacteria	16	0	0	0	0	0	0	0	0	0
Chlorobi	10	0	0	0	0	0	0	0	0	0
Chloroflexi	11	0	0	0	0	0	0	0	0	0
Deinococcus-Thermus	7	0	0	0	0	0	0	0	0	0
Aquificae	9	0	0	0	0	0	0	0	0	0
Thermotogae	11	0	0	0	0	0	0	0	0	0
Dictyoglomi	2	0	0	0	0	0	0	0	0	0
Nitrospirae	2	0	0	0	0	0	0	0	0	0
Thermobaculum	1	0	0	0	0	0	0	0	0	0
Deferribacteres	3	0	0	0	0	0	0	0	0	0
Euryarchaeota	60	0	0	0	0	0	0	0	0	0
Crenarchaeota	23	0	0	0	0	0	0	0	0	0
Thaumarchaeota	2	0	0	0	0	0	0	0	0	0
Nanoarchaeota	1	0	0	0	0	0	0	0	0	0
Korarchaeota	1	0	0	0	0	0	0	0	0	0
Total	768	0	0	0	0	0	0	0	0	0

(mod_M00427_1)

Phyla	Module completion ratio (%)									
	0--10	10--20	20--30	30--40	40--50	50--60	60--70	70--80	80--90	90--100
Gammaproteobacteria	126	0	0	0	0	0	0	0	0	0
Betaproteobacteria	61	0	0	0	0	0	0	0	0	0
Epsilonproteobacteria	17	0	0	0	0	0	0	0	0	0
Deltaproteobacteria	28	0	0	0	0	0	0	0	0	0
Alphaproteobacteria	91	0	0	0	0	0	0	0	0	0
Magnetococcus	1	0	0	0	0	0	0	0	0	0
Chrysiogenetes	1	0	0	0	0	0	0	0	0	0
Firmicutes	104	0	0	0	0	0	0	0	0	0
Tenericutes	19	0	0	0	0	0	0	0	0	0
Actinobacteria	80	0	0	0	0	0	0	0	0	0
Chlamydiae	8	0	0	0	0	0	0	0	0	0
Spirochaetes	14	0	0	0	0	0	0	0	0	0
Acidobacteria	5	0	0	0	0	0	0	0	0	0
Bacteroidetes	35	0	0	0	0	0	0	0	0	0
Fibrobacteres	1	0	0	0	0	0	0	0	0	0
Fusobacteria	5	0	0	0	0	0	0	0	0	0
Verrucomicrobia	4	0	0	0	0	0	0	0	0	0
Gemmatimonadetes	1	0	0	0	0	0	0	0	0	0
Planctomycetes	4	0	0	0	0	0	0	0	0	0
Elusimicrobia	2	0	0	0	0	0	0	0	0	0
Synergistetes	2	0	0	0	0	0	0	0	0	0
Cyanobacteria	16	0	0	0	0	0	0	0	0	0
Chlorobi	10	0	0	0	0	0	0	0	0	0
Chloroflexi	11	0	0	0	0	0	0	0	0	0
Deinococcus-Thermus	7	0	0	0	0	0	0	0	0	0
Aquificae	9	0	0	0	0	0	0	0	0	0
Thermotogae	11	0	0	0	0	0	0	0	0	0
Dictyoglomi	2	0	0	0	0	0	0	0	0	0
Nitrospirae	2	0	0	0	0	0	0	0	0	0
Thermobaculum	1	0	0	0	0	0	0	0	0	0
Deferribacteres	3	0	0	0	0	0	0	0	0	0
Euryarchaeota	60	0	0	0	0	0	0	0	0	0
Crenarchaeota	23	0	0	0	0	0	0	0	0	0
Thaumarchaeota	2	0	0	0	0	0	0	0	0	0
Nanoarchaeota	1	0	0	0	0	0	0	0	0	0
Korarchaeota	1	0	0	0	0	0	0	0	0	0
Total	768	0	0	0	0	0	0	0	0	0

(mod_M00428_1)

Phyla	Module completion ratio (%)									
	0--10	10--20	20--30	30--40	40--50	50--60	60--70	70--80	80--90	90--100
Gammaproteobacteria	126	0	0	0	0	0	0	0	0	0
Betaproteobacteria	61	0	0	0	0	0	0	0	0	0
Epsilonproteobacteria	17	0	0	0	0	0	0	0	0	0
Deltaproteobacteria	28	0	0	0	0	0	0	0	0	0
Alphaproteobacteria	91	0	0	0	0	0	0	0	0	0
Magnetococcus	1	0	0	0	0	0	0	0	0	0
Chrysiogenetes	1	0	0	0	0	0	0	0	0	0
Firmicutes	104	0	0	0	0	0	0	0	0	0
Tenericutes	19	0	0	0	0	0	0	0	0	0
Actinobacteria	80	0	0	0	0	0	0	0	0	0
Chlamydiae	8	0	0	0	0	0	0	0	0	0
Spirochaetes	14	0	0	0	0	0	0	0	0	0
Acidobacteria	5	0	0	0	0	0	0	0	0	0
Bacteroidetes	35	0	0	0	0	0	0	0	0	0
Fibrobacteres	1	0	0	0	0	0	0	0	0	0
Fusobacteria	5	0	0	0	0	0	0	0	0	0
Verrucomicrobia	4	0	0	0	0	0	0	0	0	0
Gemmatimonadetes	1	0	0	0	0	0	0	0	0	0
Planctomycetes	4	0	0	0	0	0	0	0	0	0
Elusimicrobia	2	0	0	0	0	0	0	0	0	0
Synergistetes	2	0	0	0	0	0	0	0	0	0
Cyanobacteria	16	0	0	0	0	0	0	0	0	0
Chlorobi	10	0	0	0	0	0	0	0	0	0
Chloroflexi	11	0	0	0	0	0	0	0	0	0
Deinococcus-Thermus	7	0	0	0	0	0	0	0	0	0
Aquificae	9	0	0	0	0	0	0	0	0	0
Thermotogae	11	0	0	0	0	0	0	0	0	0
Dictyoglomi	2	0	0	0	0	0	0	0	0	0
Nitrospirae	2	0	0	0	0	0	0	0	0	0
Thermobaculum	1	0	0	0	0	0	0	0	0	0
Deferribacteres	3	0	0	0	0	0	0	0	0	0
Euryarchaeota	60	0	0	0	0	0	0	0	0	0
Crenarchaeota	23	0	0	0	0	0	0	0	0	0
Thaumarchaeota	2	0	0	0	0	0	0	0	0	0
Nanoarchaeota	1	0	0	0	0	0	0	0	0	0
Korarchaeota	1	0	0	0	0	0	0	0	0	0
Total	768	0	0	0	0	0	0	0	0	0

(mod_M00429_1)

Phyla	Module completion ratio (%)									
	0--10	10--20	20--30	30--40	40--50	50--60	60--70	70--80	80--90	90--100
Gammaproteobacteria	10	8	0	108	0	0	0	0	0	0
Betaproteobacteria	3	15	0	43	0	0	0	0	0	0
Epsilonproteobacteria	0	5	0	12	0	0	0	0	0	0
Deltaproteobacteria	1	7	0	20	0	0	0	0	0	0
Alphaproteobacteria	40	51	0	0	0	0	0	0	0	0
Magnetococcus	0	1	0	0	0	0	0	0	0	0
Chrysiogenetes	0	0	0	1	0	0	0	0	0	0
Firmicutes	0	0	0	47	0	6	1	0	1	49
Tenericutes	13	3	0	3	0	0	0	0	0	0
Actinobacteria	3	14	0	63	0	0	0	0	0	0
Chlamydiae	7	1	0	0	0	0	0	0	0	0
Spirochaetes	5	9	0	0	0	0	0	0	0	0
Acidobacteria	0	3	0	2	0	0	0	0	0	0
Bacteroidetes	3	31	0	1	0	0	0	0	0	0
Fibrobacteres	0	0	0	1	0	0	0	0	0	0
Fusobacteria	0	4	0	1	0	0	0	0	0	0
Verrucomicrobia	0	4	0	0	0	0	0	0	0	0
Gemmatimonadetes	0	1	0	0	0	0	0	0	0	0
Planctomycetes	0	0	0	4	0	0	0	0	0	0
Elusimicrobia	0	2	0	0	0	0	0	0	0	0
Synergistetes	0	0	0	2	0	0	0	0	0	0
Cyanobacteria	3	13	0	0	0	0	0	0	0	0
Chlorobi	0	0	0	10	0	0	0	0	0	0
Chloroflexi	0	0	0	11	0	0	0	0	0	0
Deinococcus-Thermus	0	0	0	7	0	0	0	0	0	0
Aquificae	1	5	0	3	0	0	0	0	0	0
Thermotogae	0	0	0	11	0	0	0	0	0	0
Dictyoglomi	0	0	0	2	0	0	0	0	0	0
Nitrospirae	0	1	0	1	0	0	0	0	0	0
Thermobaculum	0	0	0	1	0	0	0	0	0	0
Deferribacteres	1	1	0	1	0	0	0	0	0	0
Euryarchaeota	50	10	0	0	0	0	0	0	0	0
Crenarchaeota	23	0	0	0	0	0	0	0	0	0
Thaumarchaeota	2	0	0	0	0	0	0	0	0	0
Nanoarchaeota	1	0	0	0	0	0	0	0	0	0
Korarchaeota	1	0	0	0	0	0	0	0	0	0
Total	167	189	0	355	0	6	1	0	1	49

(mod_M00430_1)

Phyla	Module completion ratio (%)									
	0--10	10--20	20--30	30--40	40--50	50--60	60--70	70--80	80--90	90--100
Gammaproteobacteria	126	0	0	0	0	0	0	0	0	0
Betaproteobacteria	61	0	0	0	0	0	0	0	0	0
Epsilonproteobacteria	17	0	0	0	0	0	0	0	0	0
Deltaproteobacteria	28	0	0	0	0	0	0	0	0	0
Alphaproteobacteria	91	0	0	0	0	0	0	0	0	0
Magnetococcus	1	0	0	0	0	0	0	0	0	0
Chrysiogenetes	1	0	0	0	0	0	0	0	0	0
Firmicutes	104	0	0	0	0	0	0	0	0	0
Tenericutes	19	0	0	0	0	0	0	0	0	0
Actinobacteria	80	0	0	0	0	0	0	0	0	0
Chlamydiae	8	0	0	0	0	0	0	0	0	0
Spirochaetes	14	0	0	0	0	0	0	0	0	0
Acidobacteria	5	0	0	0	0	0	0	0	0	0
Bacteroidetes	35	0	0	0	0	0	0	0	0	0
Fibrobacteres	1	0	0	0	0	0	0	0	0	0
Fusobacteria	5	0	0	0	0	0	0	0	0	0
Verrucomicrobia	4	0	0	0	0	0	0	0	0	0
Gemmatimonadetes	1	0	0	0	0	0	0	0	0	0
Planctomycetes	4	0	0	0	0	0	0	0	0	0
Elusimicrobia	2	0	0	0	0	0	0	0	0	0
Synergistetes	2	0	0	0	0	0	0	0	0	0
Cyanobacteria	16	0	0	0	0	0	0	0	0	0
Chlorobi	10	0	0	0	0	0	0	0	0	0
Chloroflexi	11	0	0	0	0	0	0	0	0	0
Deinococcus-Thermus	7	0	0	0	0	0	0	0	0	0
Aquificae	9	0	0	0	0	0	0	0	0	0
Thermotogae	11	0	0	0	0	0	0	0	0	0
Dictyoglomi	2	0	0	0	0	0	0	0	0	0
Nitrospirae	2	0	0	0	0	0	0	0	0	0
Thermobaculum	1	0	0	0	0	0	0	0	0	0
Deferribacteres	3	0	0	0	0	0	0	0	0	0
Euryarchaeota	60	0	0	0	0	0	0	0	0	0
Crenarchaeota	23	0	0	0	0	0	0	0	0	0
Thaumarchaeota	2	0	0	0	0	0	0	0	0	0
Nanoarchaeota	1	0	0	0	0	0	0	0	0	0
Korarchaeota	1	0	0	0	0	0	0	0	0	0
Total	768	0	0	0	0	0	0	0	0	0

(mod_M00432_1)

Phyla	Module completion ratio (%)									
	0--10	10--20	20--30	30--40	40--50	50--60	60--70	70--80	80--90	90--100
Gammaproteobacteria	10	0	0	1	0	0	0	0	0	115
Betaproteobacteria	1	0	0	0	0	0	0	0	0	60
Epsilonproteobacteria	2	0	0	0	0	0	0	0	0	15
Deltaproteobacteria	2	0	0	0	0	0	1	0	0	25
Alphaproteobacteria	25	0	0	0	0	0	1	0	0	65
Magnetococcus	0	0	0	0	0	0	0	0	0	1
Chrysiogenetes	0	0	0	0	0	0	0	0	0	1
Firmicutes	27	0	0	3	0	0	3	0	0	71
Tenericutes	19	0	0	0	0	0	0	0	0	0
Actinobacteria	5	0	0	1	0	0	2	0	0	72
Chlamydiae	8	0	0	0	0	0	0	0	0	0
Spirochaetes	9	0	0	0	0	0	0	0	0	5
Acidobacteria	0	0	0	0	0	0	1	0	0	4
Bacteroidetes	8	0	0	0	0	0	1	0	0	26
Fibrobacteres	0	0	0	0	0	0	0	0	0	1
Fusobacteria	2	0	0	0	0	0	0	0	0	3
Verrucomicrobia	0	0	0	0	0	0	0	0	0	4
Gemmatimonadetes	1	0	0	0	0	0	0	0	0	0
Planctomycetes	0	0	0	0	0	0	0	0	0	4
Elusimicrobia	0	0	0	1	0	0	0	0	0	1
Synergistetes	0	0	0	0	0	0	0	0	0	2
Cyanobacteria	1	0	0	0	0	0	0	0	0	15
Chlorobi	0	0	0	0	0	0	0	0	0	10
Chloroflexi	0	0	0	0	0	0	0	0	0	11
Deinococcus-Thermus	0	0	0	0	0	0	0	0	0	7
Aquificae	0	0	0	0	0	0	0	0	0	9
Thermotogae	3	0	0	2	0	0	0	0	0	6
Dictyoglomi	0	0	0	0	0	0	0	0	0	2
Nitrospirae	0	0	0	0	0	0	0	0	0	2
Thermobaculum	0	0	0	0	0	0	0	0	0	1
Deferribacteres	0	0	0	0	0	0	0	0	0	3
Euryarchaeota	7	0	0	2	0	0	2	0	0	49
Crenarchaeota	9	0	0	0	0	0	0	0	0	14
Thaumarchaeota	0	0	0	0	0	0	0	0	0	2
Nanoarchaeota	1	0	0	0	0	0	0	0	0	0
Korarchaeota	0	0	0	0	0	0	0	0	0	1
Total	140	0	0	10	0	0	11	0	0	607

(mod_M00433_1)

Phyla	Module completion ratio (%)									
	0--10	10--20	20--30	30--40	40--50	50--60	60--70	70--80	80--90	90--100
Gammaproteobacteria	115	0	0	11	0	0	0	0	0	0
Betaproteobacteria	51	0	0	10	0	0	0	0	0	0
Epsilonproteobacteria	15	0	0	2	0	0	0	0	0	0
Deltaproteobacteria	15	0	0	13	0	0	0	0	0	0
Alphaproteobacteria	76	0	0	15	0	0	0	0	0	0
Magnetococcus	0	0	0	1	0	0	0	0	0	0
Chrysiogenetes	0	0	0	1	0	0	0	0	0	0
Firmicutes	80	0	0	24	0	0	0	0	0	0
Tenericutes	19	0	0	0	0	0	0	0	0	0
Actinobacteria	77	0	0	3	0	0	0	0	0	0
Chlamydiae	8	0	0	0	0	0	0	0	0	0
Spirochaetes	13	0	0	1	0	0	0	0	0	0
Acidobacteria	4	0	0	1	0	0	0	0	0	0
Bacteroidetes	33	0	0	2	0	0	0	0	0	0
Fibrobacteres	1	0	0	0	0	0	0	0	0	0
Fusobacteria	4	0	0	1	0	0	0	0	0	0
Verrucomicrobia	2	0	0	2	0	0	0	0	0	0
Gemmatimonadetes	1	0	0	0	0	0	0	0	0	0
Planctomycetes	4	0	0	0	0	0	0	0	0	0
Elusimicrobia	1	0	0	1	0	0	0	0	0	0
Synergistetes	2	0	0	0	0	0	0	0	0	0
Cyanobacteria	9	0	0	7	0	0	0	0	0	0
Chlorobi	0	0	0	10	0	0	0	0	0	0
Chloroflexi	5	0	0	6	0	0	0	0	0	0
Deinococcus-Thermus	0	0	0	7	0	0	0	0	0	0
Aquificae	7	0	0	2	0	0	0	0	0	0
Thermotogae	10	0	0	1	0	0	0	0	0	0
Dictyoglomi	0	0	0	2	0	0	0	0	0	0
Nitrospirae	1	0	0	1	0	0	0	0	0	0
Thermobaculum	0	0	0	1	0	0	0	0	0	0
Deferribacteres	1	0	0	2	0	0	0	0	0	0
Euryarchaeota	50	0	0	10	0	0	0	0	0	0
Crenarchaeota	23	0	0	0	0	0	0	0	0	0
Thaumarchaeota	2	0	0	0	0	0	0	0	0	0
Nanoarchaeota	1	0	0	0	0	0	0	0	0	0
Korarchaeota	1	0	0	0	0	0	0	0	0	0
Total	631	0	0	137	0	0	0	0	0	0

(mod_M00435_1)

Phyla	Module completion ratio (%)									
	0--10	10--20	20--30	30--40	40--50	50--60	60--70	70--80	80--90	90--100
Gammaproteobacteria	99	0	0	0	0	0	1	0	0	26
Betaproteobacteria	37	0	0	4	0	0	1	0	0	19
Epsilonproteobacteria	17	0	0	0	0	0	0	0	0	0
Deltaproteobacteria	27	0	0	1	0	0	0	0	0	0
Alphaproteobacteria	76	0	0	3	0	0	1	0	0	11
Magnetococcus	1	0	0	0	0	0	0	0	0	0
Chrysiogenetes	1	0	0	0	0	0	0	0	0	0
Firmicutes	98	0	0	5	0	0	0	0	0	1
Tenericutes	19	0	0	0	0	0	0	0	0	0
Actinobacteria	68	0	0	9	0	0	3	0	0	0
Chlamydiae	8	0	0	0	0	0	0	0	0	0
Spirochaetes	14	0	0	0	0	0	0	0	0	0
Acidobacteria	5	0	0	0	0	0	0	0	0	0
Bacteroidetes	35	0	0	0	0	0	0	0	0	0
Fibrobacteres	1	0	0	0	0	0	0	0	0	0
Fusobacteria	5	0	0	0	0	0	0	0	0	0
Verrucomicrobia	4	0	0	0	0	0	0	0	0	0
Gemmatimonadetes	1	0	0	0	0	0	0	0	0	0
Planctomycetes	4	0	0	0	0	0	0	0	0	0
Elusimicrobia	2	0	0	0	0	0	0	0	0	0
Synergistetes	2	0	0	0	0	0	0	0	0	0
Cyanobacteria	16	0	0	0	0	0	0	0	0	0
Chlorobi	10	0	0	0	0	0	0	0	0	0
Chloroflexi	11	0	0	0	0	0	0	0	0	0
Deinococcus-Thermus	7	0	0	0	0	0	0	0	0	0
Aquificae	9	0	0	0	0	0	0	0	0	0
Thermotogae	11	0	0	0	0	0	0	0	0	0
Dictyoglomi	2	0	0	0	0	0	0	0	0	0
Nitrospirae	2	0	0	0	0	0	0	0	0	0
Thermobaculum	1	0	0	0	0	0	0	0	0	0
Deferribacteres	3	0	0	0	0	0	0	0	0	0
Euryarchaeota	58	0	0	2	0	0	0	0	0	0
Crenarchaeota	23	0	0	0	0	0	0	0	0	0
Thaumarchaeota	2	0	0	0	0	0	0	0	0	0
Nanoarchaeota	1	0	0	0	0	0	0	0	0	0
Korarchaeota	1	0	0	0	0	0	0	0	0	0
Total	681	0	0	24	0	0	6	0	0	57

(mod_M00436_1)

Phyla	Module completion ratio (%)									
	0--10	10--20	20--30	30--40	40--50	50--60	60--70	70--80	80--90	90--100
Gammaproteobacteria	97	0	0	1	0	0	0	0	0	28
Betaproteobacteria	32	0	0	1	0	0	2	0	0	26
Epsilonproteobacteria	17	0	0	0	0	0	0	0	0	0
Deltaproteobacteria	27	0	0	0	0	0	0	0	0	1
Alphaproteobacteria	63	0	0	1	0	0	1	0	0	26
Magnetococcus	1	0	0	0	0	0	0	0	0	0
Chrysiogenetes	1	0	0	0	0	0	0	0	0	0
Firmicutes	87	0	0	3	0	0	10	0	0	4
Tenericutes	19	0	0	0	0	0	0	0	0	0
Actinobacteria	45	0	0	4	0	0	11	0	0	20
Chlamydiae	8	0	0	0	0	0	0	0	0	0
Spirochaetes	14	0	0	0	0	0	0	0	0	0
Acidobacteria	5	0	0	0	0	0	0	0	0	0
Bacteroidetes	35	0	0	0	0	0	0	0	0	0
Fibrobacteres	1	0	0	0	0	0	0	0	0	0
Fusobacteria	4	0	0	0	0	0	1	0	0	0
Verrucomicrobia	4	0	0	0	0	0	0	0	0	0
Gemmatimonadetes	1	0	0	0	0	0	0	0	0	0
Planctomycetes	4	0	0	0	0	0	0	0	0	0
Elusimicrobia	2	0	0	0	0	0	0	0	0	0
Synergistetes	1	0	0	1	0	0	0	0	0	0
Cyanobacteria	14	0	0	0	0	0	0	0	0	2
Chlorobi	10	0	0	0	0	0	0	0	0	0
Chloroflexi	10	0	0	0	0	0	0	0	0	1
Deinococcus-Thermus	7	0	0	0	0	0	0	0	0	0
Aquificae	9	0	0	0	0	0	0	0	0	0
Thermotogae	11	0	0	0	0	0	0	0	0	0
Dictyoglomi	2	0	0	0	0	0	0	0	0	0
Nitrospirae	2	0	0	0	0	0	0	0	0	0
Thermobaculum	0	0	0	0	0	0	0	0	0	1
Deferribacteres	3	0	0	0	0	0	0	0	0	0
Euryarchaeota	60	0	0	0	0	0	0	0	0	0
Crenarchaeota	23	0	0	0	0	0	0	0	0	0
Thaumarchaeota	2	0	0	0	0	0	0	0	0	0
Nanoarchaeota	1	0	0	0	0	0	0	0	0	0
Korarchaeota	1	0	0	0	0	0	0	0	0	0
Total	623	0	0	11	0	0	25	0	0	109

(mod_M00437_1)

Phyla	Module completion ratio (%)									
	0--10	10--20	20--30	30--40	40--50	50--60	60--70	70--80	80--90	90--100
Gammaproteobacteria	126	0	0	0	0	0	0	0	0	0
Betaproteobacteria	59	0	0	0	0	0	0	0	0	2
Epsilonproteobacteria	17	0	0	0	0	0	0	0	0	0
Deltaproteobacteria	28	0	0	0	0	0	0	0	0	0
Alphaproteobacteria	91	0	0	0	0	0	0	0	0	0
Magnetococcus	1	0	0	0	0	0	0	0	0	0
Chrysiogenetes	1	0	0	0	0	0	0	0	0	0
Firmicutes	104	0	0	0	0	0	0	0	0	0
Tenericutes	19	0	0	0	0	0	0	0	0	0
Actinobacteria	80	0	0	0	0	0	0	0	0	0
Chlamydiae	8	0	0	0	0	0	0	0	0	0
Spirochaetes	14	0	0	0	0	0	0	0	0	0
Acidobacteria	5	0	0	0	0	0	0	0	0	0
Bacteroidetes	35	0	0	0	0	0	0	0	0	0
Fibrobacteres	1	0	0	0	0	0	0	0	0	0
Fusobacteria	5	0	0	0	0	0	0	0	0	0
Verrucomicrobia	4	0	0	0	0	0	0	0	0	0
Gemmatimonadetes	1	0	0	0	0	0	0	0	0	0
Planctomycetes	4	0	0	0	0	0	0	0	0	0
Elusimicrobia	2	0	0	0	0	0	0	0	0	0
Synergistetes	2	0	0	0	0	0	0	0	0	0
Cyanobacteria	16	0	0	0	0	0	0	0	0	0
Chlorobi	10	0	0	0	0	0	0	0	0	0
Chloroflexi	11	0	0	0	0	0	0	0	0	0
Deinococcus-Thermus	7	0	0	0	0	0	0	0	0	0
Aquificae	9	0	0	0	0	0	0	0	0	0
Thermotogae	11	0	0	0	0	0	0	0	0	0
Dictyoglomi	2	0	0	0	0	0	0	0	0	0
Nitrospirae	2	0	0	0	0	0	0	0	0	0
Thermobaculum	1	0	0	0	0	0	0	0	0	0
Deferribacteres	3	0	0	0	0	0	0	0	0	0
Euryarchaeota	60	0	0	0	0	0	0	0	0	0
Crenarchaeota	23	0	0	0	0	0	0	0	0	0
Thaumarchaeota	2	0	0	0	0	0	0	0	0	0
Nanoarchaeota	1	0	0	0	0	0	0	0	0	0
Korarchaeota	1	0	0	0	0	0	0	0	0	0
Total	766	0	0	0	0	0	0	0	0	2

(mod_M00438_1)

Phyla	Module completion ratio (%)									
	0--10	10--20	20--30	30--40	40--50	50--60	60--70	70--80	80--90	90--100
Gammaproteobacteria	83	0	9	0	0	0	0	31	0	3
Betaproteobacteria	21	0	12	0	0	0	0	23	0	5
Epsilonproteobacteria	16	0	0	0	0	0	0	1	0	0
Deltaproteobacteria	26	0	1	0	0	0	0	0	0	1
Alphaproteobacteria	66	0	1	0	0	0	0	17	0	7
Magnetococcus	1	0	0	0	0	0	0	0	0	0
Chrysiogenetes	1	0	0	0	0	0	0	0	0	0
Firmicutes	104	0	0	0	0	0	0	0	0	0
Tenericutes	19	0	0	0	0	0	0	0	0	0
Actinobacteria	78	0	0	0	0	1	0	1	0	0
Chlamydiae	8	0	0	0	0	0	0	0	0	0
Spirochaetes	14	0	0	0	0	0	0	0	0	0
Acidobacteria	5	0	0	0	0	0	0	0	0	0
Bacteroidetes	34	0	0	0	0	0	0	0	0	1
Fibrobacteres	1	0	0	0	0	0	0	0	0	0
Fusobacteria	5	0	0	0	0	0	0	0	0	0
Verrucomicrobia	3	0	0	0	0	0	0	1	0	0
Gemmatimonadetes	1	0	0	0	0	0	0	0	0	0
Planctomycetes	2	0	0	0	0	0	0	1	0	1
Elusimicrobia	2	0	0	0	0	0	0	0	0	0
Synergistetes	2	0	0	0	0	0	0	0	0	0
Cyanobacteria	4	0	2	0	0	2	0	0	0	8
Chlorobi	10	0	0	0	0	0	0	0	0	0
Chloroflexi	10	0	0	0	0	0	0	1	0	0
Deinococcus-Thermus	7	0	0	0	0	0	0	0	0	0
Aquificae	7	0	0	0	0	0	0	2	0	0
Thermotogae	11	0	0	0	0	0	0	0	0	0
Dictyoglomi	2	0	0	0	0	0	0	0	0	0
Nitrospirae	2	0	0	0	0	0	0	0	0	0
Thermobaculum	1	0	0	0	0	0	0	0	0	0
Deferribacteres	3	0	0	0	0	0	0	0	0	0
Euryarchaeota	60	0	0	0	0	0	0	0	0	0
Crenarchaeota	23	0	0	0	0	0	0	0	0	0
Thaumarchaeota	2	0	0	0	0	0	0	0	0	0
Nanoarchaeota	1	0	0	0	0	0	0	0	0	0
Korarchaeota	1	0	0	0	0	0	0	0	0	0
Total	636	0	25	0	0	3	0	78	0	26

(mod_M00439_1)

Phyla	Module completion ratio (%)									
	0--10	10--20	20--30	30--40	40--50	50--60	60--70	70--80	80--90	90--100
Gammaproteobacteria	68	0	0	0	3	2	0	0	9	44
Betaproteobacteria	42	0	5	0	0	0	0	0	2	12
Epsilonproteobacteria	17	0	0	0	0	0	0	0	0	0
Deltaproteobacteria	27	0	0	0	1	0	0	0	0	0
Alphaproteobacteria	60	0	1	0	10	16	0	0	4	0
Magnetococcus	1	0	0	0	0	0	0	0	0	0
Chrysiogenetes	1	0	0	0	0	0	0	0	0	0
Firmicutes	33	0	4	0	0	5	0	0	10	52
Tenericutes	2	0	1	0	0	2	0	0	7	7
Actinobacteria	78	0	0	0	0	1	0	0	1	0
Chlamydiae	1	0	0	0	0	1	0	0	1	5
Spirochaetes	5	0	0	0	0	0	0	0	1	8
Acidobacteria	5	0	0	0	0	0	0	0	0	0
Bacteroidetes	35	0	0	0	0	0	0	0	0	0
Fibrobacteres	1	0	0	0	0	0	0	0	0	0
Fusobacteria	1	0	0	0	0	0	0	0	0	4
Verrucomicrobia	0	0	1	0	1	2	0	0	0	0
Gemmatimonadetes	1	0	0	0	0	0	0	0	0	0
Planctomycetes	2	0	0	0	0	1	0	0	1	0
Elusimicrobia	2	0	0	0	0	0	0	0	0	0
Synergistetes	2	0	0	0	0	0	0	0	0	0
Cyanobacteria	15	0	0	0	1	0	0	0	0	0
Chlorobi	10	0	0	0	0	0	0	0	0	0
Chloroflexi	7	0	4	0	0	0	0	0	0	0
Deinococcus-Thermus	6	0	1	0	0	0	0	0	0	0
Aquificae	9	0	0	0	0	0	0	0	0	0
Thermotogae	10	0	1	0	0	0	0	0	0	0
Dictyoglomi	2	0	0	0	0	0	0	0	0	0
Nitrospirae	2	0	0	0	0	0	0	0	0	0
Thermobaculum	0	0	1	0	0	0	0	0	0	0
Deferribacteres	3	0	0	0	0	0	0	0	0	0
Euryarchaeota	60	0	0	0	0	0	0	0	0	0
Crenarchaeota	23	0	0	0	0	0	0	0	0	0
Thaumarchaeota	2	0	0	0	0	0	0	0	0	0
Nanoarchaeota	1	0	0	0	0	0	0	0	0	0
Korarchaeota	1	0	0	0	0	0	0	0	0	0
Total	535	0	19	0	16	30	0	0	36	132

(mod_M00440_1)

Phyla	Module completion ratio (%)									
	0--10	10--20	20--30	30--40	40--50	50--60	60--70	70--80	80--90	90--100
Gammaproteobacteria	109	0	2	0	0	1	0	0	0	14
Betaproteobacteria	60	0	1	0	0	0	0	0	0	0
Epsilonproteobacteria	13	0	0	0	0	1	0	0	1	2
Deltaproteobacteria	28	0	0	0	0	0	0	0	0	0
Alphaproteobacteria	82	0	2	0	0	0	0	0	2	5
Magnetococcus	1	0	0	0	0	0	0	0	0	0
Chrysiogenetes	1	0	0	0	0	0	0	0	0	0
Firmicutes	91	0	1	0	0	1	0	0	0	11
Tenericutes	19	0	0	0	0	0	0	0	0	0
Actinobacteria	80	0	0	0	0	0	0	0	0	0
Chlamydiae	8	0	0	0	0	0	0	0	0	0
Spirochaetes	14	0	0	0	0	0	0	0	0	0
Acidobacteria	5	0	0	0	0	0	0	0	0	0
Bacteroidetes	35	0	0	0	0	0	0	0	0	0
Fibrobacteres	1	0	0	0	0	0	0	0	0	0
Fusobacteria	3	0	1	0	0	0	0	0	0	1
Verrucomicrobia	4	0	0	0	0	0	0	0	0	0
Gemmatimonadetes	1	0	0	0	0	0	0	0	0	0
Planctomycetes	4	0	0	0	0	0	0	0	0	0
Elusimicrobia	2	0	0	0	0	0	0	0	0	0
Synergistetes	2	0	0	0	0	0	0	0	0	0
Cyanobacteria	16	0	0	0	0	0	0	0	0	0
Chlorobi	10	0	0	0	0	0	0	0	0	0
Chloroflexi	11	0	0	0	0	0	0	0	0	0
Deinococcus-Thermus	7	0	0	0	0	0	0	0	0	0
Aquificae	9	0	0	0	0	0	0	0	0	0
Thermotogae	11	0	0	0	0	0	0	0	0	0
Dictyoglomi	2	0	0	0	0	0	0	0	0	0
Nitrospirae	2	0	0	0	0	0	0	0	0	0
Thermobaculum	1	0	0	0	0	0	0	0	0	0
Deferribacteres	2	0	0	0	0	0	0	0	0	1
Euryarchaeota	60	0	0	0	0	0	0	0	0	0
Crenarchaeota	23	0	0	0	0	0	0	0	0	0
Thaumarchaeota	2	0	0	0	0	0	0	0	0	0
Nanoarchaeota	1	0	0	0	0	0	0	0	0	0
Korarchaeota	1	0	0	0	0	0	0	0	0	0
Total	721	0	7	0	0	3	0	0	3	34

(mod_M00441_1)

Phyla	Module completion ratio (%)									
	0--10	10--20	20--30	30--40	40--50	50--60	60--70	70--80	80--90	90--100
Gammaproteobacteria	83	0	0	9	0	0	34	0	0	0
Betaproteobacteria	21	0	0	12	0	0	28	0	0	0
Epsilonproteobacteria	16	0	0	0	0	0	1	0	0	0
Deltaproteobacteria	26	0	0	1	0	0	1	0	0	0
Alphaproteobacteria	66	0	0	1	0	0	24	0	0	0
Magnetococcus	1	0	0	0	0	0	0	0	0	0
Chrysiogenetes	1	0	0	0	0	0	0	0	0	0
Firmicutes	104	0	0	0	0	0	0	0	0	0
Tenericutes	19	0	0	0	0	0	0	0	0	0
Actinobacteria	78	0	0	1	0	0	1	0	0	0
Chlamydiae	8	0	0	0	0	0	0	0	0	0
Spirochaetes	14	0	0	0	0	0	0	0	0	0
Acidobacteria	5	0	0	0	0	0	0	0	0	0
Bacteroidetes	34	0	0	0	0	0	1	0	0	0
Fibrobacteres	1	0	0	0	0	0	0	0	0	0
Fusobacteria	5	0	0	0	0	0	0	0	0	0
Verrucomicrobia	3	0	0	0	0	0	1	0	0	0
Gemmatimonadetes	1	0	0	0	0	0	0	0	0	0
Planctomycetes	2	0	0	0	0	0	2	0	0	0
Elusimicrobia	2	0	0	0	0	0	0	0	0	0
Synergistetes	2	0	0	0	0	0	0	0	0	0
Cyanobacteria	4	0	0	2	0	0	7	0	0	3
Chlorobi	10	0	0	0	0	0	0	0	0	0
Chloroflexi	10	0	0	0	0	0	1	0	0	0
Deinococcus-Thermus	7	0	0	0	0	0	0	0	0	0
Aquificae	7	0	0	0	0	0	2	0	0	0
Thermotogae	11	0	0	0	0	0	0	0	0	0
Dictyoglomi	2	0	0	0	0	0	0	0	0	0
Nitrospirae	2	0	0	0	0	0	0	0	0	0
Thermobaculum	1	0	0	0	0	0	0	0	0	0
Deferribacteres	3	0	0	0	0	0	0	0	0	0
Euryarchaeota	60	0	0	0	0	0	0	0	0	0
Crenarchaeota	23	0	0	0	0	0	0	0	0	0
Thaumarchaeota	2	0	0	0	0	0	0	0	0	0
Nanoarchaeota	1	0	0	0	0	0	0	0	0	0
Korarchaeota	1	0	0	0	0	0	0	0	0	0
Total	636	0	0	26	0	0	103	0	0	3

(mod_M00442_1)

Phyla	Module completion ratio (%)									
	0--10	10--20	20--30	30--40	40--50	50--60	60--70	70--80	80--90	90--100
Gammaproteobacteria	108	0	0	1	0	0	2	0	0	15
Betaproteobacteria	60	0	0	1	0	0	0	0	0	0
Epsilonproteobacteria	17	0	0	0	0	0	0	0	0	0
Deltaproteobacteria	22	0	0	0	0	0	2	0	0	4
Alphaproteobacteria	68	0	0	1	0	0	9	0	0	13
Magnetococcus	1	0	0	0	0	0	0	0	0	0
Chrysiogenetes	1	0	0	0	0	0	0	0	0	0
Firmicutes	80	0	0	2	0	0	2	0	0	20
Tenericutes	19	0	0	0	0	0	0	0	0	0
Actinobacteria	65	0	0	1	0	0	4	0	0	10
Chlamydiae	8	0	0	0	0	0	0	0	0	0
Spirochaetes	14	0	0	0	0	0	0	0	0	0
Acidobacteria	5	0	0	0	0	0	0	0	0	0
Bacteroidetes	35	0	0	0	0	0	0	0	0	0
Fibrobacteres	1	0	0	0	0	0	0	0	0	0
Fusobacteria	1	0	0	0	0	0	0	0	0	4
Verrucomicrobia	4	0	0	0	0	0	0	0	0	0
Gemmatimonadetes	1	0	0	0	0	0	0	0	0	0
Planctomycetes	4	0	0	0	0	0	0	0	0	0
Elusimicrobia	2	0	0	0	0	0	0	0	0	0
Synergistetes	1	0	0	0	0	0	1	0	0	0
Cyanobacteria	16	0	0	0	0	0	0	0	0	0
Chlorobi	10	0	0	0	0	0	0	0	0	0
Chloroflexi	6	0	0	0	0	0	0	0	0	5
Deinococcus-Thermus	6	0	0	0	0	0	0	0	0	1
Aquificae	9	0	0	0	0	0	0	0	0	0
Thermotogae	6	0	0	0	0	0	5	0	0	0
Dictyoglomi	2	0	0	0	0	0	0	0	0	0
Nitrospirae	2	0	0	0	0	0	0	0	0	0
Thermobaculum	0	0	0	0	0	0	0	0	0	1
Deferribacteres	3	0	0	0	0	0	0	0	0	0
Euryarchaeota	60	0	0	0	0	0	0	0	0	0
Crenarchaeota	23	0	0	0	0	0	0	0	0	0
Thaumarchaeota	2	0	0	0	0	0	0	0	0	0
Nanoarchaeota	1	0	0	0	0	0	0	0	0	0
Korarchaeota	1	0	0	0	0	0	0	0	0	0
Total	664	0	0	6	0	0	25	0	0	73

Table S3. Characterization of the 205 KEGG pathway modules containing submodules based on the module completion patterns in 768 prokaryotic species

Module no.	Module type	Number of module components	Module completion pattern*	Major taxonomic pattern	Rare module**	Function	Small function category	Large function category
M00001_1	Pathway	10	A (2)	Prokaryote		Glycolysis (Embden-Meyerhof pathway), glucose => pyruvate	Central carbohydrate metabolism	Carbohydrate & lipid metabolism
M00002_1	Pathway	6	A (2)	Prokaryote		Glycolysis, core module involving three-carbon compounds	Central carbohydrate metabolism	Carbohydrate & lipid metabolism
M00003_1	Pathway	8	C	Bacteria		Gluconeogenesis, oxaloacetate => fructose-6P	Central carbohydrate metabolism	Carbohydrate & lipid metabolism
M00004_1	Pathway	7	C	Bacteria		Pentose phosphate pathway (Pentose phosphate cycle)	Central carbohydrate metabolism	Carbohydrate & lipid metabolism
M00005_1	Pathway	1	A (1)	Prokaryote		PRPP biosynthesis, ribose 5P => PRPP	Central carbohydrate metabolism	Carbohydrate & lipid metabolism
M00006_1	Pathway	2	C	Bacteria		Pentose phosphate pathway, oxidative phase, glucose 6P => ribulose 5P	Central carbohydrate metabolism	Carbohydrate & lipid metabolism
M00007_1	Pathway	4	A (1)	Prokaryote		Pentose phosphate pathway, non-oxidative phase, fructose 6P => ribose 5P	Central carbohydrate metabolism	Carbohydrate & lipid metabolism
M00008_1	Pathway	3	C	Proteobacteria/ Actinobacteria		Entner-Doudoroff pathway, glucose-6P => glyceraldehyde-3P + pyruvate	Central carbohydrate metabolism	Carbohydrate & lipid metabolism
M00009_1	Pathway	8	C	Bacteria		Citrate cycle (TCA cycle, Krebs cycle)	Central carbohydrate metabolism	Carbohydrate & lipid metabolism
M00010_1	Pathway	3	A (1)	Prokaryote		Citrate cycle, first carbon oxidation	Central carbohydrate metabolism	Carbohydrate & lipid metabolism
M00011_1	Pathway	5	C	Prokaryote		Citrate cycle, second carbon oxidation	Central carbohydrate metabolism	Carbohydrate & lipid metabolism
M00012_1	Pathway	5	C	Prokaryote		Glyoxylate cycle	Other carbohydrate metabolism	Carbohydrate & lipid metabolism
M00013_1	Pathway	5	D	Non-prokaryote		Malonate semialdehyde pathway, propanoyl-CoA => Acetyl-CoA	Other carbohydrate metabolism	Carbohydrate & lipid metabolism
M00014_1	Pathway	5	D	Non-prokaryote		Glucuronate pathway (uronate pathway)	Other carbohydrate metabolism	Carbohydrate & lipid metabolism
M00015_1	Pathway	2	A (1)	Prokaryote		Proline biosynthesis, glutamate => proline	Arginine & proline metabolism	Nucleotide & amino acid metabolism
M00016_1	Pathway	9	C	Prokaryote		Lysine biosynthesis, aspartate => lysine	Lysine metabolism	Nucleotide & amino acid metabolism
M00017_1	Pathway	7	C	Proteobacteria/Firmicutes /Actinobacteria		Methionine biosynthesis, apartate => homoserine => methionine	Cystein & methionine metabolism	Nucleotide & amino acid metabolism
M00018_1	Pathway	5	A (1)	Prokaryote		Threonine biosynthesis, apartate => homoserine => threonine	Serin & threonine metabolism	Nucleotide & amino acid metabolism
M00019_1	Pathway	7	A (2)	Gammaproteobacteria	rare	Leucine biosynthesis, pyruvate => 2-oxoisovalerate => leucine	BCAA metabolism	Nucleotide & amino acid metabolism
M00020_1	Pathway	3	C	Prokaryote		Serine biosynthesis, glycerate-3P => serine	Serin & threonine metabolism	Nucleotide & amino acid metabolism
M00021_1	Pathway	2	A (1)	Prokaryote		Cysteine biosynthesis, serine => cysteine	Cystein & methionine metabolism	Nucleotide & amino acid metabolism
M00022_1	Pathway	7	A (1)	Prokaryote		Shikimate pathway, phosphoenolpyruvate + erythrose-4P => chorismate	Aromatic amino acid metabolism	Nucleotide & amino acid metabolism
M00023_1	Pathway	3	C	Prokaryote		Tryptophan biosynthesis, chorismate => tryptophan	Aromatic amino acid metabolism	Nucleotide & amino acid metabolism
M00024_1	Pathway	3	C	Bacteria		Phenylalanine biosynthesis, chorismate => phenylalanine	Aromatic amino acid metabolism	Nucleotide & amino acid metabolism
M00025_1	Pathway	4	C	Proteobacteria/ Fusobacteria/ Gemmatimonadetes		Tyrosine biosynthesis, chorismate => tyrosine	Aromatic amino acid metabolism	Nucleotide & amino acid metabolism
M00026_1	Pathway	6	A (2)	Prokaryote		Histidine biosynthesis, PRPP => histidine	Histidine metabolism	Nucleotide & amino acid metabolism
M00027_1	Pathway	3	C	Prokaryote	rare	GABA (gamma-Aminobutyrate) shunt	Other amino acid metabolism	Nucleotide & amino acid metabolism
M00028_1	Pathway	4	C	Prokaryote		Ornithine biosynthesis, glutamate => ornithine	Arginine & proline metabolism	Nucleotide & amino acid metabolism
M00029_1	Pathway	5	D	Non-prokaryote		Urea cycle	Arginine & proline metabolism	Nucleotide & amino acid metabolism
M00030_1	Pathway	7	D	Non-prokaryote		Lysine biosynthesis, 2-oxoglutarate => 2-aminoadipate => lysine	Lysine metabolism	Nucleotide & amino acid metabolism
M00031_1	Pathway	5	C	Archaea	rare	Lysine biosynthesis, archaea, 2-aminoadipate => lysine	Lysine metabolism	Nucleotide & amino acid metabolism
M00032_1	Pathway	7	D	Non-prokaryote		Lysine degradation, lysine => saccharopine => acetoacetyl-CoA	Lysine metabolism	Nucleotide & amino acid metabolism
M00033_1	Pathway	5	C	Prokaryote	rare	Ectoine biosynthesis	Akaloid & other secondardy metabolite	Nucleotide & amino acid metabolism
M00034_1	Pathway	9	C	Gammaproteobacteria	rare	Methionine salvage pathway	Cystein & methionine metabolism	Nucleotide & amino acid metabolism
M00034_2	Pathway	10	C	Gammaproteobacteria/ Firmicutes/Cyanobacteria	rare	Methionine salvage pathway	Cystein & methionine metabolism	Nucleotide & amino acid metabolism
M00035_1	Pathway	4	C	Prokaryote		Methionine degradation	Cystein & methionine metabolism	Nucleotide & amino acid metabolism
M00036_1	Pathway	6	C	Proteobacteria	rare	Leucine degradation, leucine => acetoacetate + acetyl-CoA	BCAA metabolism	Nucleotide & amino acid metabolism
M00037_1	Pathway	4	D	Non-prokaryote		Melatonin biosynthesis, tryptophan => serotonin => melatonin	Aromatic amino acid metabolism	Nucleotide & amino acid metabolism
M00038_1	Pathway	7	B	Gammaproteobacteria	rare	Tryptophan metabolism, tryptophan => kynurenine => 2-aminomuconate	Aromatic amino acid metabolism	Nucleotide & amino acid metabolism
M00039_1	Pathway	5	D	Non-prokaryote		Lignin biosynthesis, cinnamate => lignin	Phenylpropanoid & flavonoid biosyntesis	Nucleotide & amino acid metabolism

M00040_1	Pathway	2	B	Proteobacteria	rare	Tyrosine biosynthesis, prephanate => pretyrosine => tyrosine	Aromatic amino acid metabolism	Nucleotide & amino acid metabolism
M00042_1	Pathway	4	D	Non-prokaryote		Catecholamine biosynthesis, tyrosine => dopamine => noradrenaline => adrenaline	Aromatic amino acid metabolism	Nucleotide & amino acid metabolism
M00043_1	Pathway	1	D	Non-prokaryote		Thyroid hormone biosynthesis, tyrosine => triiodothyronine/thyroxine	Aromatic amino acid metabolism	Nucleotide & amino acid metabolism
M00044_1	Pathway	5	D	Non-prokaryote		Tyrosine degradation, tyrosine => homogentisate	Aromatic amino acid metabolism	Nucleotide & amino acid metabolism
M00045_1	Pathway	4	C	Prokaryote		Histidine degradation, histidine => N-formiminoglutamate => glutamate	Histidine metabolism	Nucleotide & amino acid metabolism
M00046_1	Pathway	4	C	Proteobacteria	rare	beta-alanine biosynthesis, cytosine / uracil => beta-alanine	Other amino acid metabolism	Nucleotide & amino acid metabolism
M00047_1	Pathway	3	D	Non-prokaryote		Creatine pathway	Other amino acid metabolism	Nucleotide & amino acid metabolism
M00048_1	Pathway	9	A (2)	Prokaryote		Inosine monophosphate biosynthesis, PRPP + glutamine => IMP	Purine metabolism	Nucleotide & amino acid metabolism
M00049_1	Pathway	6	A (2)	Prokaryote		Adenine nucleotide biosynthesis, IMP => ADP/dADP,ATP/dATP	Purine metabolism	Nucleotide & amino acid metabolism
M00050_1	Pathway	6	C	Bacteria		Guanine nucleotide biosynthesis, IMP => GDP/dGDP,GTP/dGTP	Purine metabolism	Nucleotide & amino acid metabolism
M00051_1	Pathway	3	D	Non-prokaryote		Uridine monophosphate biosynthesis, glutamine (+ PRPP) => UMP	Pyrimidine metabolism	Nucleotide & amino acid metabolism
M00051_2	Pathway	4	D	Non-prokaryote		Uridine monophosphate biosynthesis, glutamine (+ PRPP) => UMP	Pyrimidine metabolism	Nucleotide & amino acid metabolism
M00051_3	Pathway	5	A (2)	Prokaryote		Uridine monophosphate biosynthesis, glutamine (+ PRPP) => UMP	Pyrimidine metabolism	Nucleotide & amino acid metabolism
M00052_1	Pathway	3	A (1)	Prokaryote		Pyrimidine ribonucleotide biosynthesis, UMP => UDP/UTP,CDP/CTP	Pyrimidine metabolism	Nucleotide & amino acid metabolism
M00053_1	Pathway	8	C	Proteobacteria/Firmicutes /Actinobacteria		Pyrimidine deoxyribonuleotide biosynthesis, CDP/CTP => dCDP/dCTP,dTDP/dTTP	Pyrimidine metabolism	Nucleotide & amino acid metabolism
M00055_1	Pathway	13	D	Non-prokaryote		N-glycan precursor biosynthesis	Glycan metabolism	Carbohydrate & lipid metabolism
M00056_1	Pathway	4	D	Non-prokaryote		O-glycan biosynthesis, mucin type core	Glycan metabolism	Carbohydrate & lipid metabolism
M00057_1	Pathway	4	D	Non-prokaryote		Glycosaminoglycan biosynthesis, linkage tetrasaccharide	Glycosaminoglycan metabolism	Carbohydrate & lipid metabolism
M00058_1	Pathway	2	D	Non-prokaryote		Glycosaminoglycan biosynthesis, chondroitin sulfate backbone	Glycosaminoglycan metabolism	Carbohydrate & lipid metabolism
M00059_1	Pathway	5	D	Non-prokaryote		Glycosaminoglycan biosynthesis, heparan sulfate backbone	Glycosaminoglycan metabolism	Carbohydrate & lipid metabolism
M00060_1	Pathway	9	C	Gammaproteobacteria	rare	Lipopolysaccharide biosynthesis, KDO2-lipid A	LPS metabolism	Carbohydrate & lipid metabolism
M00061_1	Pathway	6	C	Bacteria	rare	Uronic acid metabolism	Other carbohydrate metabolism	Carbohydrate & lipid metabolism
M00063_1	Pathway	3	C	Bacteria		CMP-KDO biosynthesis	LPS metabolism	Carbohydrate & lipid metabolism
M00064_1	Pathway	4	C	Bacteria		ADP-L-glycero-D-manno-heptose biosynthesis	LPS metabolism	Carbohydrate & lipid metabolism
M00065_1	Pathway	8	D	Non-prokaryote		GPI-anchor biosynthesis, core oligosaccharide	Glycan metabolism	Carbohydrate & lipid metabolism
M00066_1	Pathway	2	D	Non-prokaryote		Lactosylceramide biosynthesis	Lipid metabolism	Carbohydrate & lipid metabolism
M00067_1	Pathway	2	D	Non-prokaryote		Cerebroside and sulfatide biosynthesis	Lipid metabolism	Carbohydrate & lipid metabolism
M00068_1	Pathway	2	D	Non-prokaryote		Glycosphingolipid biosynthesis, globo-series, LacCer => Gb4Cer	Glycan metabolism	Carbohydrate & lipid metabolism
M00069_1	Pathway	2	D	Non-prokaryote		Glycosphingolipid biosynthesis, ganglio series, LacCer => GT3	Glycan metabolism	Carbohydrate & lipid metabolism
M00070_1	Pathway	2	D	Non-prokaryote		Glycosphingolipid biosynthesis, lacto-series, LacCer => Lc4Cer	Glycan metabolism	Carbohydrate & lipid metabolism
M00071_1	Pathway	2	D	Non-prokaryote		Glycosphingolipid biosynthesis, neolacto-series, LacCer => nLc4Cer	Glycan metabolism	Carbohydrate & lipid metabolism
M00073_1	Pathway	3	D	Non-prokaryote		N-glycan precursor trimming	Glycan metabolism	Carbohydrate & lipid metabolism
M00074_1	Pathway	6	D	Non-prokaryote		N-glycan biosynthesis, high-mannose type	Glycan metabolism	Carbohydrate & lipid metabolism
M00075_1	Pathway	2	D	Non-prokaryote		N-glycan biosynthesis, complex type	Glycan metabolism	Carbohydrate & lipid metabolism
M00075_2	Pathway	1	B	Proteobacteria/Firmicutes	rare	N-glycan biosynthesis, complex type	Glycan metabolism	Carbohydrate & lipid metabolism
M00075_3	Pathway	3	D	Non-prokaryote		N-glycan biosynthesis, complex type	Glycan metabolism	Carbohydrate & lipid metabolism
M00075_4	Pathway	3	D	Non-prokaryote		N-glycan biosynthesis, complex type	Glycan metabolism	Carbohydrate & lipid metabolism
M00076_1	Pathway	6	D	Non-prokaryote		Dermatan sulfate degradation	Glycosaminoglycan metabolism	Carbohydrate & lipid metabolism
M00077_1	Pathway	4	D	Non-prokaryote		Chondroitin sulfate degradation	Glycosaminoglycan metabolism	Carbohydrate & lipid metabolism
M00078_1	Pathway	8	D	Non-prokaryote		Heparan sulfate degradation	Glycosaminoglycan metabolism	Carbohydrate & lipid metabolism
M00079_1	Pathway	5	D	Non-prokaryote		Keratan sulfate degradation	Glycosaminoglycan metabolism	Carbohydrate & lipid metabolism
M00080_1	Pathway	12	B	Gammaproteobacteria	rare	Lipopolysaccharide biosynthesis, inner core => outer core => O-antigen	LPS metabolism	Carbohydrate & lipid metabolism
M00081_1	Pathway	3	D	Non-prokaryote		Pectin degradation	Other carbohydrate metabolism	Carbohydrate & lipid metabolism
M00082_1	Pathway	4	D	Non-prokaryote		Fatty acid biosynthesis, initiation	Fatty acid metabolism	Carbohydrate & lipid metabolism

M00083_1	Pathway	1	C	Bacteria		Fatty acid biosynthesis, elongation	Fatty acid metabolism	Carbohydrate & lipid metabolism
M00085_1	Pathway	3	D	Non-prokaryote		Fatty acid biosynthesis, elongation, mitochondria	Fatty acid metabolism	Carbohydrate & lipid metabolism
M00086_1	Pathway	1	C	Prokaryote		beta-Oxidation, acyl-CoA synthesis	Fatty acid metabolism	Carbohydrate & lipid metabolism
M00087_1	Pathway	3	C	Bacteria		beta-Oxidation	Fatty acid metabolism	Carbohydrate & lipid metabolism
M00088_1	Pathway	3	C	Prokaryote	rare	Ketone body biosynthesis, acetyl-CoA => acetoacetate/3-hydroxybutyrate/acetone	Fatty acid metabolism	Carbohydrate & lipid metabolism
M00088_2	Pathway	1	B	Prokaryote		Ketone body biosynthesis, acetyl-CoA => acetoacetate/3-hydroxybutyrate/acetone	Fatty acid metabolism	Carbohydrate & lipid metabolism
M00088_3	Pathway	1	B	Prokaryote	rare	Ketone body biosynthesis, acetyl-CoA => acetoacetate/3-hydroxybutyrate/acetone	Fatty acid metabolism	Carbohydrate & lipid metabolism
M00089_1	Pathway	4	D	Non-prokaryote		Triacylglycerol biosynthesis	Lipid metabolism	Carbohydrate & lipid metabolism
M00090_1	Pathway	3	D	Non-prokaryote		Phosphatidylcholine (PC) biosynthesis, choline => PC	Lipid metabolism	Carbohydrate & lipid metabolism
M00091_1	Pathway	2	B	Proteobacteria/Firmicutes /Actinobacteria	rare	Phosphatidylcholine (PC) biosynthesis, PE => PC	Lipid metabolism	Carbohydrate & lipid metabolism
M00092_1	Pathway	3	D	Non-prokaryote		Phosphatidylethanolamine (PE) biosynthesis, ethanolamine => PE	Lipid metabolism	Carbohydrate & lipid metabolism
M00093_1	Pathway	3	C	Bacteria		Phosphatidylethanolamine (PE) biosynthesis, PA => PS => PE	Lipid metabolism	Carbohydrate & lipid metabolism
M00094_1	Pathway	4	D	Non-prokaryote		Ceramide biosynthesis	Lipid metabolism	Carbohydrate & lipid metabolism
M00095_1	Pathway	7	C	Actinobacteria	rare	C5 isoprenoid biosynthesis, mevalonate pathway	Telpenoid backbone biosynthesis	Carbohydrate & lipid metabolism
M00096_1	Pathway	8	C	Bacteria		C5 isoprenoid biosynthesis, non-mevalonate pathway	Telpenoid backbone biosynthesis	Carbohydrate & lipid metabolism
M00097_1	Pathway	3	C	Bacteria	rare	beta-Carotene biosynthesis, GGAP => beta-carotene	Other terpenoid biosynthesis	Carbohydrate & lipid metabolism
M00097_2	Pathway	4	C	Cyanobacteria/Chlorobi	rare	beta-Carotene biosynthesis, GGAP => beta-carotene	Other terpenoid biosynthesis	Carbohydrate & lipid metabolism
M00098_1	Pathway	2	B	Betaproteobacteria/ Actinobascteria/ Cyanobacteria	rare	Acylglycerol degradation	Lipid metabolism	Carbohydrate & lipid metabolism
M00099_1	Pathway	5	D	Non-prokaryote		Sphingosine biosynthesis	Lipid metabolism	Carbohydrate & lipid metabolism
M00100_1	Pathway	2	D	Non-prokaryote		Sphingosine degradation	Lipid metabolism	Carbohydrate & lipid metabolism
M00101_1	Pathway	12	D	Non-prokaryote		Cholesterol biosynthesis, FPP => cholesterol	Sterol biosynthesis	Carbohydrate & lipid metabolism
M00102_1	Pathway	5	D	Non-prokaryote		Ergocalciferol biosynthesis	Sterol biosynthesis	Carbohydrate & lipid metabolism
M00103_1	Pathway	2	D	Non-prokaryote		Cholecalciferol biosynthesis	Sterol biosynthesis	Carbohydrate & lipid metabolism
M00104_1	Pathway	12	D	Non-prokaryote		Bile acid biosynthesis, cholesterol => cholate	Sterol biosynthesis	Carbohydrate & lipid metabolism
M00105_1	Pathway	11	D	Non-prokaryote		Bile acid biosynthesis, cholesterol => chenodeoxycholate	Sterol biosynthesis	Carbohydrate & lipid metabolism
M00106_1	Pathway	2	D	Non-prokaryote		Conjugated bile acid biosynthesis, choloyl-CoA => taurocholate/glycocholate	Sterol biosynthesis	Carbohydrate & lipid metabolism
M00107_1	Pathway	2	D	Non-prokaryote		Steroid hormone biosynthesis, cholesterol => prognenolone => progesterone	Sterol biosynthesis	Carbohydrate & lipid metabolism
M00108_1	Pathway	3	D	Non-prokaryote		C21-Steroid hormone biosynthesis, progesterone => corticosterone/aldosterone	Sterol biosynthesis	Carbohydrate & lipid metabolism
M00109_1	Pathway	4	D	Non-prokaryote		C21-Steroid hormone biosynthesis, progesterone => cortisol/cortisone	Sterol biosynthesis	Carbohydrate & lipid metabolism
M00110_1	Pathway	3	D	Non-prokaryote		C19/C18-Steroid hormone biosynthesis, pregnenolone => androstenedione => estrone	Sterol biosynthesis	Carbohydrate & lipid metabolism
M00112_1	Pathway	4	D	Non-prokaryote		Tocopherol biosynthesis	Cofactor & vitamine biosynthesis	Nucleotide & amino acid metabolism
M00113_1	Pathway	8	D	Non-prokaryote		Jasmonic acid biosynthesis	Fatty acid metabolism	Carbohydrate & lipid metabolism
M00114_1	Pathway	9	D	Non-prokaryote		Ascorbate biosynthesis, plants, glucose-6P => ascorbate	Cofactor & vitamine biosynthesis	Nucleotide & amino acid metabolism
M00115_1	Pathway	5	C	Bacteria		NAD biosynthesis, aspartate => NAD	Cofactor & vitamine biosynthesis	Nucleotide & amino acid metabolism
M00116_1	Pathway	9	C	Cyanobacteria	rare	Menaquinone biosynthesis, chorismate => menaquinone	Cofactor & vitamine biosynthesis	Nucleotide & amino acid metabolism
M00117_1	Pathway	9	C	Gammaproteobacteria	rare	Ubiquinone biosynthesis, prokaryotes, chorismate => ubiquinone	Cofactor & vitamine biosynthesis	Nucleotide & amino acid metabolism
M00118_1	Pathway	2	C	Bacteria		Glutathione biosynthesis, glutamate => glutathione	Cofactor & vitamine biosynthesis	Nucleotide & amino acid metabolism
M00119_1	Pathway	3	C	Prokaryote		Pantothenate biosynthesis, valine/L-aspartate => pantothenate	Cofactor & vitamine biosynthesis	Nucleotide & amino acid metabolism
M00119_2	Pathway	1	C	Prokaryote		Pantothenate biosynthesis, valine/L-aspartate => pantothenate	Cofactor & vitamine biosynthesis	Nucleotide & amino acid metabolism
M00119_3	Pathway	1	A (1)	Bacteria		Pantothenate biosynthesis, valine/L-aspartate => pantothenate	Cofactor & vitamine biosynthesis	Nucleotide & amino acid metabolism
M00120_1	Pathway	3	A (1)	Bacteria		Coenzyme A biosynthesis, pantothenate => CoA	Cofactor & vitamine biosynthesis	Nucleotide & amino acid metabolism

M00121_1	Pathway	6	A (2)	Prokaryote		Heme biosynthesis, glutamate => protoheme/siroheme	Cofactor & vitamine biosynthesis	Nucleotide & amino acid metabolism
M00121_2	Pathway	4	C	Bacteria		Heme biosynthesis, glutamate => protoheme/siroheme	Cofactor & vitamine biosynthesis	Nucleotide & amino acid metabolism
M00121_3	Pathway	1	B	Firmicutes		Heme biosynthesis, glutamate => protoheme/siroheme	Cofactor & vitamine biosynthesis	Nucleotide & amino acid metabolism
M00122_1	Pathway	5	C	Alphaproteobacteria	rare	Cobalamin biosynthesis, cobinamide => cobalamin	Cofactor & vitamine biosynthesis	Nucleotide & amino acid metabolism
M00122_2	Pathway	2	C	Bacteria	rare	Cobalamin biosynthesis, cobinamide => cobalamin	Cofactor & vitamine biosynthesis	Nucleotide & amino acid metabolism
M00122_3	Pathway	1	C	Prokaryote		Cobalamin biosynthesis, cobinamide => cobalamin	Cofactor & vitamine biosynthesis	Nucleotide & amino acid metabolism
M00123_1	Pathway	4	C	Prokaryote		Biotin biosynthesis, pimeloyl-CoA => biotin	Cofactor & vitamine biosynthesis	Nucleotide & amino acid metabolism
M00124_1	Pathway	6	C	Gammaproteobacteria	rare	Pyridoxal biosynthesis, erythrose-4P => pyridoxal-5P	Cofactor & vitamine biosynthesis	Nucleotide & amino acid metabolism
M00125_1	Pathway	2	A (1)	Prokaryote		Riboflavin biosynthesis, GTP => riboflavin/FMN/FAD	Cofactor & vitamine biosynthesis	Nucleotide & amino acid metabolism
M00125_2	Pathway	1	A (1)	Prokaryote		Riboflavin biosynthesis, GTP => riboflavin/FMN/FAD	Cofactor & vitamine biosynthesis	Nucleotide & amino acid metabolism
M00125_3	Pathway	3	A (1)	Bacteria		Riboflavin biosynthesis, GTP => riboflavin/FMN/FAD	Cofactor & vitamine biosynthesis	Nucleotide & amino acid metabolism
M00126_1	Pathway	5	C	Bacteria		Tetrahydrofolate biosynthesis, GTP => THF	Cofactor & vitamine biosynthesis	Nucleotide & amino acid metabolism
M00127_1	Pathway	2	C	Prokaryote		Thiamine biosynthesis, AIR => thiamine-P/thiamine-2P	Cofactor & vitamine biosynthesis	Nucleotide & amino acid metabolism
M00127_2	Pathway	1	B	Prokaryote		Thiamine biosynthesis, AIR => thiamine-P/thiamine-2P	Cofactor & vitamine biosynthesis	Nucleotide & amino acid metabolism
M00127_3	Pathway	2	C	Prokaryote		Thiamine biosynthesis, AIR => thiamine-P/thiamine-2P	Cofactor & vitamine biosynthesis	Nucleotide & amino acid metabolism
M00128_1	Pathway	7	D	Non-prokaryote		Ubiquinone biosynthesis, eukaryotes, chorismate => ubiquinone	Cofactor & vitamine biosynthesis	Nucleotide & amino acid metabolism
M00129_1	Pathway	6	D	Non-prokaryote		Ascorbate biosynthesis, animals, glucose-1P => ascorbate	Cofactor & vitamine biosynthesis	Nucleotide & amino acid metabolism
M00130_1	Pathway	4	D	Non-prokaryote		Inositol phosphate metabolism, PI=> PIP2 => Ins(1,4,5)P3 => Ins(1,3,4,5)P4	Lipid metabolism	Carbohydrate & lipid metabolism
M00131_1	Pathway	4	D	Non-prokaryote		Inositol phosphate metabolism, Ins(1,3,4,5)P4 => Ins(1,3,4)P3 => myo-inositol	Lipid metabolism	Carbohydrate & lipid metabolism
M00132_1	Pathway	3	D	Non-prokaryote		Inositol phosphate metabolism, Ins(1,3,4)P3 => phytate	Lipid metabolism	Carbohydrate & lipid metabolism
M00133_1	Pathway	2	C	Prokaryote		Polyamine biosynthesis, arginine => agmatine => putrescine => spermidine	Polyamine biosynthesis	Nucleotide & amino acid metabolism
M00133_2	Pathway	1	C	Prokaryote		Polyamine biosynthesis, arginine => agmatine => putrescine => spermidine	Polyamine biosynthesis	Nucleotide & amino acid metabolism
M00133_3	Pathway	1	C	Prokaryote		Polyamine biosynthesis, arginine => agmatine => putrescine => spermidine	Polyamine biosynthesis	Nucleotide & amino acid metabolism
M00134_1	Pathway	2	C	Prokaryote	rare	Polyamine biosynthesis, arginine => ornithine => putrescine	Polyamine biosynthesis	Nucleotide & amino acid metabolism
M00135_1	Pathway	3	C	Bacteria	rare	GABA biosynthesis, eukaryotes, putrescine => GABA	Polyamine biosynthesis	Nucleotide & amino acid metabolism
M00136_1	Pathway	4	B	Gammaproteobacteria	rare	GABA biosynthesis, prokaryotes, putrescine => GABA	Polyamine biosynthesis	Nucleotide & amino acid metabolism
M00137_1	Pathway	3	D	Non-prokaryote		Flavonoid biosynthesis, 4-coumaroyl-CoA => naringenin => apigenin	Phenylpropanoid & flavonoid biosyntesis	Nucleotide & amino acid metabolism
M00138_1	Pathway	3	D	Non-prokaryote		Flavonoid biosynthesis, naringenin => pelargonidin	Phenylpropanoid & flavonoid biosyntesis	Nucleotide & amino acid metabolism
M00139_1	Pathway	11	D	Non-prokaryote		Morphine biosynthesis, dopamine => morphine	Akaloid & other secondardy metabolite	Nucleotide & amino acid metabolism
M00140_1	Pathway	3	C	Prokaryote		C1-unit interconversion, prokaryotes	Cofactor & vitamine biosynthesis	Nucleotide & amino acid metabolism
M00141_1	Pathway	2	C	Chloroflexi	rare	C1-unit interconversion, eukaryotes	Cofactor & vitamine biosynthesis	Nucleotide & amino acid metabolism
M00141_2	Pathway	3	D	Non-prokaryote		C1-unit interconversion, eukaryotes	Cofactor & vitamine biosynthesis	Nucleotide & amino acid metabolism
M00165_1	Pathway	11	C	Cyanobacteria	rare	Reductive pentose phosphate cycle (Calvin cycle)	Carbon fixation	Energy metabolism
M00166_1	Pathway	3	C	Cyanobacteria	rare	Reductive pentose phosphate cycle, RuBP + CO2 => glyceraldehyde-3P	Carbon fixation	Energy metabolism
M00167_1	Pathway	8	C	Cyanobacteria	rare	Reductive pentose phosphate cycle, glyceraldehyde-3P => RuBP	Carbon fixation	Energy metabolism
M00168_1	Pathway	2	C	Prokaryote		CAM (Crassulacean acid metabolism), dark	Carbon fixation	Energy metabolism
M00169_1	Pathway	2	C	Bacteria		CAM (Crassulacean acid metabolism), light	Carbon fixation	Energy metabolism
M00170_1	Pathway	3	D	Non-prokaryote		C4-dicarboxylic acid cycle, phosphoenolpyruvate carboxykinase type	Carbon fixation	Energy metabolism
M00171_1	Pathway	7	D	Non-prokaryote		C4-dicarboxylic acid cycle, NAD+ -malic enzyme type	Carbon fixation	Energy metabolism
M00172_1	Pathway	4	D	Non-prokaryote		C4-dicarboxylic acid cycle, NADP+ -malic enzyme type	Carbon fixation	Energy metabolism
M00173_1	Pathway	10	C	Prokaryote	rare	Reductive citric acid cycle (Arnon-Buchanan cycle)	Carbon fixation	Energy metabolism
M00174_1	Pathway	4	C	Betaproteobacteria	rare	Methane oxidation, methylotroph, methane => CO2	Methane metabolism	Energy metabolism
M00175_1	Pathway	1	B	Prokaryote		Nitrogen fixation, N2 => ammonia	Nitrogen fixation	Energy metabolism
M00176_1	Pathway	3	C	Bacteria		Sulfur reduction, sulfate => H2S	Sulfur metabolism	Energy metabolism
M00307_1	Pathway	1	A (1)	Prokaryote		Pyruvate oxidation, pyruvate => acetyl-CoA	Central carbohydrate metabolism	Carbohydrate & lipid metabolism

M00308_1	Pathway	3	C	Euryarchaeota	rare	Semi-phosphorylative Entner-Doudoroff pathway, gluconate => glyceraldehyde-3P + pyruvate	Central carbohydrate metabolism	Carbohydrate & lipid metabolism
M00309_1	Pathway	2	B	Archaea	rare	Non-phosphorylative Entner-Doudoroff pathway, gluconate => glyceraldehyde + pyruvate	Central carbohydrate metabolism	Carbohydrate & lipid metabolism
M00338_1	Pathway	2	B	Prokaryote	rare	Cysteine biosynthesis, homocysteine + serine => cysteine	Cystein & methionine metabolism	Nucleotide & amino acid metabolism
M00344_1	Pathway	3	C	Proteobacteria/ Bacteroidetes	rare	Formaldehyde assimilation, xylulose monophosphate pathway	Methane metabolism	Energy metabolism
M00345_1	Pathway	3	C	Prokaryote	rare	Formaldehyde assimilation, ribulose monophosphate pathway	Methane metabolism	Energy metabolism
M00346_1	Pathway	9	D	Non-prokaryote		Formaldehyde assimilation, serine pathway	Methane metabolism	Energy metabolism
M00347_1	Pathway	10	B	Euryarchaeota	rare	Methanogenesis, formate => methane	Methane metabolism	Energy metabolism
M00350_1	Pathway	4	D	Non-prokaryote		Capsaicin biosynthesis, L-Phenylalanine => Capsaicin	Aromatic amino acid metabolism	Nucleotide & amino acid metabolism
M00356_1	Pathway	3	B	Euryarchaeota	rare	Methanogenesis, methanol => methane	Methane metabolism	Energy metabolism
M00357_1	Pathway	6	**C**	Euryarchaeota	rare	Methanogenesis, acetate => methane	Methane metabolism	Energy metabolism
M00358_1	Pathway	4	B	Euryarchaeota	rare	Coenzyme M biosynthesis	Methane metabolism	Energy metabolism
M00364_1	Pathway	3	C	Proteobacteria	rare	C10-C20 isoprenoid biosynthesis, bacteria	Telpenoid backbone biosynthesis	Carbohydrate & lipid metabolism
M00365_1	Pathway	2	C	Prokaryote		C10-C20 isoprenoid biosynthesis, archaea	Telpenoid backbone biosynthesis	Carbohydrate & lipid metabolism
M00366_1	Pathway	4	D	Non-prokaryote		C10-C20 isoprenoid biosynthesis, plants	Telpenoid backbone biosynthesis	Carbohydrate & lipid metabolism
M00367_1	Pathway	3	D	Non-prokaryote		C10-C20 isoprenoid biosynthesis, non-plant eukaryotes	Telpenoid backbone biosynthesis	Carbohydrate & lipid metabolism
M00368_1	Pathway	3	D	Non-prokaryote		Ethylene biosynthesis, methionine => ethylene	Cystein & methionine metabolism	Nucleotide & amino acid metabolism
M00369_1	Pathway	3	D	Non-prokaryote		Cyanogenic glycoside biosynthesis, tyrosine => dhurrin	Akaloid & other secondardy metabolite	Nucleotide & amino acid metabolism
M00370_1	Pathway	5	D	Non-prokaryote		Glucosinolate biosynthesis, tryptophan => glucobrassicin	Akaloid & other secondardy metabolite	Nucleotide & amino acid metabolism
M00371_1	Pathway	6	D	Non-prokaryote		Castasterone biosynthesis, campesterol => castasterone	Other terpenoid biosynthesis	Carbohydrate & lipid metabolism
M00372_1	Pathway	6	D	Non-prokaryote		Abscisic acid biosynthesis, beta-carotene => abscisic acid	Other terpenoid biosynthesis	Carbohydrate & lipid metabolism
M00373_1	Pathway	11	C	Alphaproteobacteria	rare	Ethylmalonyl pathway	Other carbohydrate metabolism	Carbohydrate & lipid metabolism
M00373_2	Pathway	2	B	Alphaproteobacteria	rare	Ethylmalonyl pathway	Other carbohydrate metabolism	Carbohydrate & lipid metabolism
M00374_1	Pathway	13	C	Crenarchaeota	rare	Dicarboxylate-hydroxybutyrate cycle	Carbon fixation	Energy metabolism
M00375_1	Pathway	12	C	Crenarchaeota	rare	Hydroxypropionate-hydroxybutylate cycle	Carbon fixation	Energy metabolism
M00376_1	Pathway	11	C	Chloroflexi	rare	3-Hydroxypropionate bicycle	Carbon fixation	Energy metabolism
M00376_2	Pathway	5	B	Betaproteobacteria/ Chloroflexi	rare	3-Hydroxypropionate bicycle	Carbon fixation	Energy metabolism
M00377_1	Pathway	7	C	Firmicutes	rare	Reductive acetyl-CoA pathway (Wood-Ljungdahl pathway)	Carbon fixation	Energy metabolism
M00378_1	Pathway	1	B	Cyanobacteria/ Euryarchaeota	rare	F420 biosynthesis	Methane metabolism	Energy metabolism
M00378_2	Pathway	1	B	Prokaryote		F420 biosynthesis	Methane metabolism	Energy metabolism
M00378_3	Pathway	3	B	Euryarchaeota	rare	F420 biosynthesis	Methane metabolism	Energy metabolism
M00415_1	Pathway	4	D	Non-prokaryote		Fatty acid biosynthesis, elongation, endoplasmic reticulum	Fatty acid metabolism	Carbohydrate & lipid metabolism
M00432_1	Pathway	3	A (1)	Prokaryote		Leucine biosynthesis, 2-oxoisovalerate => 2-oxoisocaproate	BCAA metabolism	Nucleotide & amino acid metabolism
M00433_1	Pathway	3	D	Non-prokaryote		Lysine biosynthesis, 2-oxoglutarate => 2-oxoadipate	Lysine metabolism	Nucleotide & amino acid metabolism

*Typical patterns for the module competion by 768 prokaryotic species are shown in Fig. 1.

**The modules completed by only less than 10% of 768 prokaryotic species are defined as " rare" modules.

Table S4. Characterization of the 263 KEGG structural complex modules containing submodules based on the module completion patterns in 768 prokaryotic species

Module no.	Module type	Number of module components	Module completion pattern*	Major taxonomic pattern	Rare module**	Function	Small function category	Large function category
M00072_1	Complex	6	D	Non-prokaryote		Oligosaccharyltransferase	Glycan metabolism	Carbohydrate & lipid metabolism
M00142_1	Complex	7	D	Non-prokaryote		NADH:ubiquinone oxidoreductase, mitochondria	ATP synthesis	Energy metabolism
M00143_1	Complex	10	D	Non-prokaryote		NADH dehydrogenase (ubiquinone) Fe-S protein/flavoprotein complex, mitochondria	ATP synthesis	Energy metabolism
M00144_1	Complex	15	C	Bacteria		NADH:quinone oxidoreductase, prokaryotes	ATP synthesis	Energy metabolism
M00144_2	Complex	13	C	Bacteria		NADH:quinone oxidoreductase, prokaryotes	ATP synthesis	Energy metabolism
M00144_3	Complex	14	C	Bacteria		NADH:quinone oxidoreductase, prokaryotes	ATP synthesis	Energy metabolism
M00145_1	Complex	11	B	Cyanobacteria	rare	NAD(P)H:quinone oxidoreductase, chloroplasts and cyanobacteria	ATP synthesis	Energy metabolism
M00146_1	Complex	14	D	Non-prokaryote		NADH dehydrogenase (ubiquinone) 1 alpha subcomplex	ATP synthesis	Energy metabolism
M00147_1	Complex	13	D	Non-prokaryote		NADH dehydrogenase (ubiquinone) 1 beta subcomplex	ATP synthesis	Energy metabolism
M00148_1	Complex	4	D	Non-prokaryote		Succinate dehydrogenase (ubiquinone)	ATP synthesis	Energy metabolism
M00149_1	Complex	4	C	Prokaryote		Succinate dehydrogenase, prokaryotes	ATP synthesis	Energy metabolism
M00150_1	Complex	4	B	Proteobacteria/ Actinobacteria	rare	Fumarate reductase, prokaryotes	ATP synthesis	Energy metabolism
M00151_1	Complex	3	C	Proteobacteria/ Magnetococcus/Aquificae		Cytochrome bc1 complex respiratory unit	ATP synthesis	Energy metabolism
M00151_2	Complex	3	B	Firmicutes	rare	Cytochrome bc1 complex respiratory unit	ATP synthesis	Energy metabolism
M00151_3	Complex	3	B	Actinobacteria	rare	Cytochrome bc1 complex respiratory unit	ATP synthesis	Energy metabolism
M00152_1	Complex	10	D	Non-prokaryote		Cytochrome bc1 complex	ATP synthesis	Energy metabolism
M00153_1	Complex	2	C	Prokaryote		Cytochrome d ubiquinol oxidase	ATP synthesis	Energy metabolism
M00154_1	Complex	15	D	Non-prokaryote		Cytochrome c oxidase	ATP synthesis	Energy metabolism
M00154_2	Complex	15	D	Non-prokaryote		Cytochrome c oxidase	ATP synthesis	Energy metabolism
M00155_1	Complex	3	C	Prokaryote		Cytochrome c oxidase, prokaryotes	ATP synthesis	Energy metabolism
M00155_2	Complex	2	C	Prokaryote	rare	Cytochrome c oxidase, prokaryotes	ATP synthesis	Energy metabolism
M00156_1	Complex	4	C	Proteobacteria		Cytochrome c oxidase, cbb3-type	ATP synthesis	Energy metabolism
M00157_1	Complex	8	A (1)	Bacteria		F-type ATPase, bacteria	ATP synthesis	Energy metabolism
M00158_1	Complex	12	D	Non-prokaryote		F-type ATPase, eukaryotes	ATP synthesis	Energy metabolism
M00159_1	Complex	8	B	Prokaryote		V-type ATPase, prokaryotes	ATP synthesis	Energy metabolism
M00160_1	Complex	13	D	Non-prokaryote		V-type ATPase, eukaryotes	ATP synthesis	Energy metabolism
M00161_1	Complex	19	B	Cyanobacteria	rare	Photosystem II	Photosynthesis	Energy metabolism
M00161_2	Complex	3	D	Non-prokaryote		Photosystem II	Photosynthesis	Energy metabolism
M00161_3	Complex	4	B	Cyanobacteria	rare	Photosystem II	Photosynthesis	Energy metabolism
M00162_1	Complex	8	B	Cyanobacteria	rare	Cytochrome b6f complex	Photosynthesis	Energy metabolism
M00163_1	Complex	14	D	Non-prokaryote		Photosystem I	Photosynthesis	Energy metabolism
M00163_2	Complex	12	B	Cyanobacteria	rare	Photosystem I	Photosynthesis	Energy metabolism
M00164_1	Complex	8	A (1)	Bacteria		ATP synthase	Photosynthesis	Energy metabolism
M00177_1	Complex	79	D	Non-prokaryote		Ribosome, eukaryotes	Ribosome	Genetic information processing
M00178_1	Complex	52	A (1)	Bacteria		Ribosome, bacteria	Ribosome	Genetic information processing
M00179_1	Complex	59	C	Archaea		Ribosome, archaea	Ribosome	Genetic information processing
M00180_1	Complex	12	D	Non-prokaryote		RNA polymerase II, eukaryotes	RNA polymerase	Genetic information processing
M00181_1	Complex	16	D	Non-prokaryote		RNA polymerase III, eukaryotes	RNA polymerase	Genetic information processing
M00182_1	Complex	12	D	Non-prokaryote		RNA polymerase I, eukaryotes	RNA polymerase	Genetic information processing
M00183_1	Complex	4	A (1)	Bacteria		RNA polymerase, bacteria	RNA polymerase	Genetic information processing
M00183_2	Complex	3	C	Proteobacteria	rare	RNA polymerase, bacteria	RNA polymerase	Genetic information processing

M00184_1	Complex	13	B	Archaea	rare	RNA polymerase, archaea	RNA polymerase	Genetic information processing
M00184_2	Complex	12	B	Archaea		RNA polymerase, archaea	RNA polymerase	Genetic information processing
M00185_1	Complex	4	C	Bacteria		Sulfate transport system	Mineral and organic ion transport system	Environmental information processing
M00186_1	Complex	3	B	Prokaryote		Tungstate transport system	Mineral and organic ion transport system	Environmental information processing
M00187_1	Complex	3	B	Archaea	rare	Putative sulfate transport system	Mineral and organic ion transport system	Environmental information processing
M00188_1	Complex	3	C	Prokaryote		Sulfonate/nitrate/taurine transport system	Mineral and organic ion transport system	Environmental information processing
M00189_1	Complex	3	C	Prokaryote		Putative molybdate transport system	Mineral and organic ion transport system	Environmental information processing
M00190_1	Complex	3	C	Prokaryote		Iron(III) transport system	Mineral and organic ion transport system	Environmental information processing
M00191_1	Complex	3	B	Prokaryote		Thiamine transport system	Mineral and organic ion transport system	Environmental information processing
M00192_1	Complex	3	B	Proteobacteria	rare	Putative thiamine transport system	Mineral and organic ion transport system	Environmental information processing
M00193_1	Complex	4	C	Prokaryote		Putative spermidine/putrescine transport system	Mineral and organic ion transport system	Environmental information processing
M00194_1	Complex	4	C	Bacteria		Maltose/maltodextrin transport system	Saccharide and polyol transport system	Environmental information processing
M00195_1	Complex	4	B	Archaea	rare	Maltooligosaccharide transport system	Saccharide and polyol transport system	Environmental information processing
M00196_1	Complex	4	B	Bacteria	rare	Multiple sugar transport system	Saccharide and polyol transport system	Environmental information processing
M00197_1	Complex	4	C	Prokaryote		Putative fructooligosaccharide transport system	Saccharide and polyol transport system	Environmental information processing
M00197_2	Complex	4	C	Bacteria	rare	Putative fructooligosaccharide transport system	Saccharide and polyol transport system	Environmental information processing
M00197_3	Complex	4	C	Archaea	rare	Putative fructooligosaccharide transport system	Saccharide and polyol transport system	Environmental information processing
M00197_4	Complex	4	C	Firmicutes	rare	Putative fructooligosaccharide transport system	Saccharide and polyol transport system	Environmental information processing
M00198_1	Complex	4	B	Proteobacteria/Firmicutes/ Actinobacteria		Putative sn-glycerol-phosphate transport system	Saccharide and polyol transport system	Environmental information processing
M00199_1	Complex	3	B	Bacteria	rare	L-Arabinose/lactose transport system	Saccharide and polyol transport system	Environmental information processing
M00200_1	Complex	4	B	Proteobacteria/ Actinobacteria	rare	Putative sorbitol/mannitol transport system	Saccharide and polyol transport system	Environmental information processing
M00201_1	Complex	4	B	Proteobacteria	rare	alpha-Glucoside transport system	Saccharide and polyol transport system	Environmental information processing
M00202_1	Complex	4	B	Proteobacteria	rare	Putative oligogalacturonide transport system	Saccharide and polyol transport system	Environmental information processing
M00203_1	Complex	4	B	Archaea	rare	Glucose/arabinose transport system	Saccharide and polyol transport system	Environmental information processing
M00204_1	Complex	4	B	Proteobacteria	rare	Trehalose/maltose transport system	Saccharide and polyol transport system	Environmental information processing
M00205_1	Complex	3	B	Firmicutes/ Actinobacteria	rare	N-Acetylglucosamine transport system	Saccharide and polyol transport system	Environmental information processing
M00206_1	Complex	4	B	Actinobacteria	rare	Cellobiose transport system	Saccharide and polyol transport system	Environmental information processing
M00207_1	Complex	4	C	Prokaryote		Putative multiple sugar transport system	Saccharide and polyol transport system	Environmental information processing
M00208_1	Complex	3	C	Prokaryote		Glycine betaine/proline transport system	Mineral and organic ion transport system	Environmental information processing
M00209_1	Complex	3	C	Prokaryote		Osmoprotectant transport system	Mineral and organic ion transport system	Environmental information processing
M00210_1	Complex	3	C	Bacteria		Putative ABC transport system	Saccharide and polyol transport system	Environmental information processing
M00211_1	Complex	2	C	Prokaryote		Putative ABC transport system	Saccharide and polyol transport system	Environmental information processing
M00212_1	Complex	3	C	Prokaryote		Ribose transport system	Saccharide and polyol transport system	Environmental information processing
M00213_1	Complex	3	B	Proteobacteria	rare	L-Arabinose transport system	Saccharide and polyol transport system	Environmental information processing
M00214_1	Complex	3	B	Bacteria	rare	Methyl-galactoside transport system	Saccharide and polyol transport system	Environmental information processing
M00215_1	Complex	3	B	Bacteria	rare	D-Xylose transport system	Saccharide and polyol transport system	Environmental information processing
M00216_1	Complex	3	B	Bacteria	rare	Multiple sugar transport system	Saccharide and polyol transport system	Environmental information processing
M00217_1	Complex	3	B	Gammaproteobacteria/ Firmicutes	rare	D-Allose transport system	Saccharide and polyol transport system	Environmental information processing
M00218_1	Complex	3	B	Proteobacteria/ Actinobacteria/ Deonococcus-Thermus	rare	Fructose transport system	Saccharide and polyol transport system	Environmental information processing
M00219_1	Complex	4	B	Bacteria	rare	AI-2 transport system	Saccharide and polyol transport system	Environmental information processing
M00220_1	Complex	4	B	Proteobacteria	rare	Rhamnose transport system	Saccharide and polyol transport system	Environmental information processing
M00221_1	Complex	3	C	Prokaryote		Putative simple sugar transport system	Saccharide and polyol transport system	Environmental information processing
M00222_1	Complex	4	A (1)	Prokaryote		Phosphate transport system	Phosphate and amino acid transport system	Environmental information processing

M00223_1	Complex	3	C	Prokaryote		Phosphonate transport system	Phosphate and amino acid transport system	Environmental information processing
M00224_1	Complex	2	B	Prokaryote	rare	Putative phosphonate transport system	Phosphate and amino acid transport system	Environmental information processing
M00225_1	Complex	4	B	Proteobacteria	rare	Lysine/arginine/ornithine transport system	Phosphate and amino acid transport system	Environmental information processing
M00226_1	Complex	4	B	Proteobacteria	rare	Histidine transport system	Phosphate and amino acid transport system	Environmental information processing
M00227_1	Complex	3	B	Proteobacteria/ Firmicutes	rare	Glutamine transport system	Phosphate and amino acid transport system	Environmental information processing
M00228_1	Complex	3	B	Bacteria		Putative glutamine transport system	Phosphate and amino acid transport system	Environmental information processing
M00229_1	Complex	4	B	Gammaproteobacteria	rare	Arginine transport system	Phosphate and amino acid transport system	Environmental information processing
M00230_1	Complex	4	B	Proteobacteria		Glutamate/aspartate transport system	Phosphate and amino acid transport system	Environmental information processing
M00231_1	Complex	4	B	Proteobacteria	rare	Octopine/nopaline transport system	Phosphate and amino acid transport system	Environmental information processing
M00232_1	Complex	4	B	Bacteria		General L-amino acid transport system	Phosphate and amino acid transport system	Environmental information processing
M00233_1	Complex	4	B	Actinobacteria	rare	Glutamate transport system	Phosphate and amino acid transport system	Environmental information processing
M00234_1	Complex	3	B	Proteobacteria	rare	Cystine transport system	Phosphate and amino acid transport system	Environmental information processing
M00235_1	Complex	4	B	Proteobacteria	rare	Arginine/ornithine transport system	Phosphate and amino acid transport system	Environmental information processing
M00236_1	Complex	3	C	Prokaryote		Putative polar amino acid transport system	Phosphate and amino acid transport system	Environmental information processing
M00237_1	Complex	5	C	Prokaryote		Branched-chain amino acid transport system	Phosphate and amino acid transport system	Environmental information processing
M00238_1	Complex	3	C	Bacteria		D-Methionine transport system	Phosphate and amino acid transport system	Environmental information processing
M00239_1	Complex	5	C	Prokaryote		Peptides/nickel transport system	Peptide and nickel transport system	Environmental information processing
M00240_1	Complex	3	A (1)	Prokaryote		Iron complex transport system	Metallic cation, iron-siderophore and vitamin B12 transport system	Environmental information processing
M00241_1	Complex	3	B	Gammaproteobacteria	rare	Vitamin B12 transport system	Metallic cation, iron-siderophore and vitamin B12 transport system	Environmental information processing
M00242_1	Complex	3	C	Prokaryote		Zinc transport system	Metallic cation, iron-siderophore and vitamin B12 transport system	Environmental information processing
M00243_1	Complex	3	B	Bacteria		Manganese/iron transport system	Metallic cation, iron-siderophore and vitamin B12 transport system	Environmental information processing
M00244_1	Complex	3	C	Prokaryote		Putative zinc/manganese transport system	Metallic cation, iron-siderophore and vitamin B12 transport system	Environmental information processing
M00245_1	Complex	4	C	Prokaryote		Cobalt transport system	Metallic cation, iron-siderophore and vitamin B12 transport system	Environmental information processing
M00246_1	Complex	4	C	Proteobacteria/ Cyanobacteria	rare	Nickel transport system	Metallic cation, iron-siderophore and vitamin B12 transport system	Environmental information processing
M00247_1	Complex	3	B	Bacteria		Putative ABC transport system	Metallic cation, iron-siderophore and vitamin B12 transport system	Environmental information processing
M00248_1	Complex	2	A (1)	Prokaryote		Putative antibiotic transport system	ABC-2 type and other transport systems	Environmental information processing
M00249_1	Complex	3	B	Proteobacteria/ Cyanobacteria/Chlorobi	rare	Capsular polysaccharide transport system	ABC-2 type and other transport systems	Environmental information processing
M00250_1	Complex	2	C	Prokaryote		Lipopolysaccharide transport system	ABC-2 type and other transport systems	Environmental information processing
M00251_1	Complex	2	B	Frmicutes	rare	Teichoic acid transport system	ABC-2 type and other transport systems	Environmental information processing
M00252_1	Complex	2	B	Prokaryote		Lipooligosaccharide transport system	ABC-2 type and other transport systems	Environmental information processing
M00253_1	Complex	2	B	Bacteria	rare	Sodium transport system	ABC-2 type and other transport systems	Environmental information processing
M00254_1	Complex	2	C	Prokaryote		ABC-2 type transport system	ABC-2 type and other transport systems	Environmental information processing
M00255_1	Complex	2	C	Prokaryote		Lipoprotein-releasing system	ABC-2 type and other transport systems	Environmental information processing
M00256_1	Complex	2	C	Bacteria		Cell division transport system	ABC-2 type and other transport systems	Environmental information processing
M00257_1	Complex	2	B	Firmicutes	rare	Hemin transport system	ABC-2 type and other transport systems	Environmental information processing
M00258_1	Complex	2	A (1)	Prokaryote		Putative ABC transport system	ABC-2 type and other transport systems	Environmental information processing
M00259_1	Complex	3	C	Proteobacteria/Chloroflexi/ Deonococcus-Thermus		Heme transport system	ABC-2 type and other transport systems	Environmental information processing
M00260_1	Complex	6	A (1)	Bacteria		DNA polymerase III complex, bacteria	DNA polymerase	Genetic information processing
M00261_1	Complex	4	D	Non-prokaryote		DNA polymerase alpha/primase complex	DNA polymerase	Genetic information processing
M00262_1	Complex	4	D	Non-prokaryote		DNA polymerase delta complex	DNA polymerase	Genetic information processing
M00263_1	Complex	4	D	Non-prokaryote		DNA polymerase epsilon complex	DNA polymerase	Genetic information processing

M00264_1	Complex	2	B	Archaea	rare	DNA polymerase II complex, archaea	DNA polymerase	Genetic information processing
M00265_1	Complex	3	B	Bacteria		PTS system, glucose-specific II component	Phosphotransferase system (PTS)	Environmental information processing
M00266_1	Complex	3	B	Bacteria	rare	PTS system, maltose and glucose-specific II component	Phosphotransferase system (PTS)	Environmental information processing
M00267_1	Complex	3	B	Bacteria	rare	PTS system, N-acetylglucosamine-specific II component	Phosphotransferase system (PTS)	Environmental information processing
M00268_1	Complex	3	B	Bacteria	rare	PTS system, arbutin-like II component	Phosphotransferase system (PTS)	Environmental information processing
M00269_1	Complex	3	B	Bacteria	rare	PTS system, sucrose-specific II component	Phosphotransferase system (PTS)	Environmental information processing
M00270_1	Complex	3	B	Bacteria	rare	PTS system, trehalose-specific II component	Phosphotransferase system (PTS)	Environmental information processing
M00271_1	Complex	3	B	Bacteria		PTS system, beta-glucosides-specific II component	Phosphotransferase system (PTS)	Environmental information processing
M00272_1	Complex	3	B	Proteobacteria/ Firmicutes	rare	PTS system, arbutin-, cellobiose-, and salicin-specific II component	Phosphotransferase system (PTS)	Environmental information processing
M00273_1	Complex	3	C	Prokaryote		PTS system, fructose-specific II component	Phosphotransferase system (PTS)	Environmental information processing
M00274_1	Complex	3	B	Bacteria		PTS system, mannitol-specific II component	Phosphotransferase system (PTS)	Environmental information processing
M00275_1	Complex	3	B	Bacteria		PTS system, cellobiose-specific II component	Phosphotransferase system (PTS)	Environmental information processing
M00276_1	Complex	4	B	Bacteria		PTS system, mannose-specific II component	Phosphotransferase system (PTS)	Environmental information processing
M00277_1	Complex	4	B	Bacteria	rare	PTS system, N-acetylgalactosamine-specific II component	Phosphotransferase system (PTS)	Environmental information processing
M00278_1	Complex	4	B	Gammaproteobacteria	rare	PTS system, sorbose-specific II component	Phosphotransferase system (PTS)	Environmental information processing
M00279_1	Complex	3	B	Prokaryote	rare	PTS system, galactitol-specific II component	Phosphotransferase system (PTS)	Environmental information processing
M00280_1	Complex	3	B	Bacteria	rare	PTS system, glucitol/sorbitol-specific II component	Phosphotransferase system (PTS)	Environmental information processing
M00281_1	Complex	3	B	Firmicutes/Fusobacteria	rare	PTS system, lactose-specific II component	Phosphotransferase system (PTS)	Environmental information processing
M00282_1	Complex	3	B	Bacteria	rare	PTS system, D-glucosamine-specific II component	Phosphotransferase system (PTS)	Environmental information processing
M00283_1	Complex	3	B	Bacteria		PTS system, ascorbate-specific II component	Phosphotransferase system (PTS)	Environmental information processing
M00284_1	Complex	6	D	Non-prokaryote		Origin recognition complex	Replication system	Genetic information processing
M00285_1	Complex	6	D	Non-prokaryote		MCM complex	Replication system	Genetic information processing
M00286_1	Complex	4	D	Non-prokaryote		GINS complex	Replication system	Genetic information processing
M00287_1	Complex	4	B	Gammaproteobacteria/ Firmicutes/ Fusobacteria	rare	PTS system, galactosamine-specific II component	Phosphotransferase system (PTS)	Environmental information processing
M00288_1	Complex	3	D	Non-prokaryote		RPA complex	Replication system	Genetic information processing
M00289_1	Complex	3	D	Non-prokaryote		RF-C complex	Replication system	Genetic information processing
M00290_1	Complex	10	D	Non-prokaryote		Holo-TFIIH complex	Repair system	Genetic information processing
M00291_1	Complex	3	D	Non-prokaryote		MRN complex	Repair system	Genetic information processing
M00292_1	Complex	3	D	Non-prokaryote		MRX complex	Repair system	Genetic information processing
M00293_1	Complex	2	D	Non-prokaryote		DNA polymerase zeta complex	DNA polymerase	Genetic information processing
M00294_1	Complex	2	D	Non-prokaryote		DNA polymerase gamma complex	DNA polymerase	Genetic information processing
M00295_1	Complex	14	D	Non-prokaryote		BRCA1-associated genome surveillance complex (BASC)	Repair system	Genetic information processing
M00296_1	Complex	5	D	Non-prokaryote		BER complex	Repair system	Genetic information processing
M00297_1	Complex	3	D	Non-prokaryote		DNA-PK complex	Repair system	Genetic information processing
M00298_1	Complex	2	B	Firmicutes	rare	Multidrug/hemolysin transport system	ABC-2 type and other transport systems	Environmental information processing
M00299_1	Complex	4	C	Prokaryote		Spermidine/putrescine transport system	Mineral and organic ion transport system	Environmental information processing
M00300_1	Complex	4	B	Proteobacteria		Putrescine transport system	Mineral and organic ion transport system	Environmental information processing
M00301_1	Complex	4	B	Alphaproteobacteria	rare	Mannopine transport system	Mineral and organic ion transport system	Environmental information processing
M00302_1	Complex	4	B	Proteobacteria/ Actinobacteria	rare	2-Aminoethylphosphonate transport system	Mineral and organic ion transport system	Environmental information processing
M00303_1	Complex	3	B	Gammaproteobacteria	rare	PTS system, N-acetylmuramic acid-specific II component	Phosphotransferase system (PTS)	Environmental information processing
M00304_1	Complex	4	B	Firmicutes	rare	PTS system, fructose-specific II component	Phosphotransferase system (PTS)	Environmental information processing
M00305_1	Complex	3	B	Gammaproteobacteria/ Firmicutes/ Fusobacteria	rare	PTS system, 2-O-A-mannosyl-D-glycerate-specific II component	Phosphotransferase system (PTS)	Environmental information processing
M00306_1	Complex	3	B	Proteobacteria/ Firmicutes	rare	PTS system, fructose-specific II-like component	Phosphotransferase system (PTS)	Environmental information processing
M00310_1	Complex	4	B	Prokaryote		Pyruvate:ferredoxin oxidoreductase	Carbohydrate metabolism	Carbohydrate & lipid metabolism

M00311_1	Complex	4	C	Prokaryote		2-oxoglutarate:ferredoxin oxidoreductase	Carbohydrate metabolism	Carbohydrate & lipid metabolism
M00312_1	Complex	4	B	Euryarchaeota	rare	2-oxoisovalerate:ferredoxin oxidoreductase	Carbohydrate metabolism	Carbohydrate & lipid metabolism
M00313_1	Complex	2	B	Prokaryote		indolepyruvate:ferredoxin oxidoreductase	Carbohydrate metabolism	Carbohydrate & lipid metabolism
M00313_2	Complex	1	B	Proteobacteria/ Actinobacteria/ Verrcomicrobia		indolepyruvate:ferredoxin oxidoreductase	Carbohydrate metabolism	Carbohydrate & lipid metabolism
M00314_1	Complex	2	B	Firmicutes	rare	Bacitracin transport system	ABC-2 type and other transport systems	Environmental information processing
M00315_1	Complex	2	B	Firmicutes	rare	Uncharacterized ABC transport system	ABC-2 type and other transport systems	Environmental information processing
M00316_1	Complex	3	B	Cyanobacteria	rare	Manganese transport system	Metallic cation, iron-siderophore and vitamin B12 transport system	Environmental information processing
M00317_1	Complex	4	B	Proteobacteria	rare	Manganese/iron transport system	Metallic cation, iron-siderophore and vitamin B12 transport system	Environmental information processing
M00318_1	Complex	3	B	Firmicutes	rare	Iron/zinc/copper transport system	Metallic cation, iron-siderophore and vitamin B12 transport system	Environmental information processing
M00319_1	Complex	4	B	Prokaryote	rare	Manganese/zinc/iron transport system	Metallic cation, iron-siderophore and vitamin B12 transport system	Environmental information processing
M00320_1	Complex	3	C	Proteobacteria/ Chlamydiae		Lipopolysaccharide export system	ABC-2 type and other transport systems	Environmental information processing
M00321_1	Complex	4	B	Cyanobacteria	rare	Bicarbonate transport system	Mineral and organic ion transport system	Environmental information processing
M00322_1	Complex	5	B	Cyanobacteria	rare	Neutral amino acid transport system	Phosphate and amino acid transport system	Environmental information processing
M00323_1	Complex	5	B	Cyanobacteria	rare	Urea transport system	Phosphate and amino acid transport system	Environmental information processing
M00324_1	Complex	5	B	Proteobacteria	rare	Dipeptide transport system	Peptide and nickel transport system	Environmental information processing
M00325_1	Complex	3	C	Proteobacteria	rare	alpha-Hemolysin/cyclolysin transport system	Bacterial secretion system	Environmental information processing
M00326_1	Complex	4	C	Gammaproteobacteria	rare	RTX toxin transport system	Bacterial secretion system	Environmental information processing
M00327_1	Complex	3	B	Alphaproteobacteria	rare	S-Layer protein transport system	Bacterial secretion system	Environmental information processing
M00328_1	Complex	3	B	Proteobacteria	rare	Hemophore/metalloprotease transport system	Bacterial secretion system	Environmental information processing
M00329_1	Complex	2	B	Alphaproteobacteria	rare	Multiple protein transport system	Bacterial secretion system	Environmental information processing
M00330_1	Complex	3	B	Gammaproteobacteria	rare	Adhesin protein transport system	Bacterial secretion system	Environmental information processing
M00331_1	Complex	11	C	Proteobacteria/ Chrysiogenetes/ Spirochaetes		Type II general secretion system	Bacterial secretion system	Environmental information processing
M00332_1	Complex	15	B	Proteobacteria	rare	Type III secretion system	Bacterial secretion system	Environmental information processing
M00333_1	Complex	12	B	Proteobacteria	rare	Type IV secretion system	Bacterial secretion system	Environmental information processing
M00334_1	Complex	9	B	Gammaproteobacteria	rare	Type VI secretion system	Bacterial secretion system	Environmental information processing
M00335_1	Complex	13	C	Proteobacteria	rare	Sec (secretion) system	Bacterial secretion system	Environmental information processing
M00336_1	Complex	4	C	Proteobacteria	rare	Twin-arginine translocation (Tat) system	Bacterial secretion system	Environmental information processing
M00337_1	Complex	16	D	Non-prokaryote		Immunoproteasome	Proteasome	Genetic information processing
M00339_1	Complex	3	B	Gammaproteobacteria	rare	RaxAB-RaxC type I secretion system	Bacterial secretion system	Environmental information processing
M00340_1	Complex	14	D	Non-prokaryote		Proteasome, 20S core particle	Proteasome	Genetic information processing
M00341_1	Complex	19	D	Non-prokaryote		Proteasome, 19S regulatory particle (PA700)	Proteasome	Genetic information processing
M00342_1	Complex	4	B	Actinobacteria/Nitrospirae Verrucomicrobia/	rare	Bacterial proteasome	Proteasome	Genetic information processing
M00343_1	Complex	3	B	Archaea	rare	Archaeal proteasome	Proteasome	Genetic information processing
M00348_1	Complex	4	B	Proteobacteria	rare	Glutathione transport system	Peptide and nickel transport system	Environmental information processing
M00349_1	Complex	4	B	Bacteria		Microcin C transport system	Peptide and nickel transport system	Environmental information processing
M00351_1	Complex	10	D	Non-prokaryote		Spliceosome, U1-snRNP	Spliceosome	Genetic information processing
M00352_1	Complex	20	D	Non-prokaryote		Spliceosome, U2-snRNP	Spliceosome	Genetic information processing
M00353_1	Complex	9	D	Non-prokaryote		Spliceosome, Prp19/CDC5L complex	Spliceosome	Genetic information processing
M00354_1	Complex	31	D	Non-prokaryote		Spliceosome, U4/U6.U5 tri-snRNP	Spliceosome	Genetic information processing
M00355_1	Complex	31	D	Non-prokaryote		Spliceosome, 35S U5-snRNP	Spliceosome	Genetic information processing
M00379_1	Complex	4	D	Non-prokaryote		SCF-MET30 complex	Ubiquitin	Genetic information processing

M00380_1	Complex	4	D	Non-prokaryote		SCF-BTRC complex	Ubiquitin	Genetic information processing
M00381_1	Complex	4	D	Non-prokaryote		SCF-SKP2 complex	Ubiquitin	Genetic information processing
M00382_1	Complex	4	D	Non-prokaryote		SCF-FBS complex	Ubiquitin	Genetic information processing
M00383_1	Complex	5	D	Non-prokaryote		ECV complex	Ubiquitin	Genetic information processing
M00384_1	Complex	3	D	Non-prokaryote		Cul3-SPOP complex	Ubiquitin	Genetic information processing
M00385_1	Complex	4	D	Non-prokaryote		Cul4-DDB1-DDB2 complex	Ubiquitin	Genetic information processing
M00386_1	Complex	4	D	Non-prokaryote		Cul4-DDB1-CSA complex	Ubiquitin	Genetic information processing
M00387_1	Complex	4	D	Non-prokaryote		SCF-FBW7 complex	Ubiquitin	Genetic information processing
M00388_1	Complex	5	D	Non-prokaryote		ECS complex	Ubiquitin	Genetic information processing
M00389_1	Complex	12	D	Non-prokaryote		APC/C complex	Ubiquitin	Genetic information processing
M00390_1	Complex	3	B	Archaea	rare	Exosome, archaea	RNA processing	Genetic information processing
M00391_1	Complex	10	D	Non-prokaryote		Exosome, eukaryotes	RNA processing	Genetic information processing
M00392_1	Complex	3	D	Non-prokaryote		Ski complex	RNA processing	Genetic information processing
M00393_1	Complex	3	D	Non-prokaryote		TRAMP complex	RNA processing	Genetic information processing
M00394_1	Complex	4	C	Proteobacteria/ Magnetococcus		RNA degradosome	RNA processing	Genetic information processing
M00395_1	Complex	5	D	Non-prokaryote		Decapping complex	RNA processing	Genetic information processing
M00396_1	Complex	7	D	Non-prokaryote		Lsm 2-8 complex	Spliceosome	Genetic information processing
M00397_1	Complex	7	D	Non-prokaryote		Lsm 1-7 complex	Spliceosome	Genetic information processing
M00398_1	Complex	7	D	Non-prokaryote		Sm core complex	Spliceosome	Genetic information processing
M00399_1	Complex	2	D	Non-prokaryote		Cap binding complex	Spliceosome	Genetic information processing
M00400_1	Complex	3	D	Non-prokaryote		p97-Ufd1-Npl4 complex	Protein processing	Genetic information processing
M00401_1	Complex	3	D	Non-prokaryote		Sec61 complex	Protein processing	Genetic information processing
M00402_1	Complex	4	D	Non-prokaryote		Translocon-associated protein (TRAP) complex	Protein processing	Genetic information processing
M00403_1	Complex	10	D	Non-prokaryote		HRD1/SEL1 ERAD complex	Protein processing	Genetic information processing
M00404_1	Complex	5	D	Non-prokaryote		COPII complex	Protein processing	Genetic information processing
M00405_1	Complex	5	D	Non-prokaryote		THC complex	RNA processing	Genetic information processing
M00406_1	Complex	8	D	Non-prokaryote		TREX complex	RNA processing	Genetic information processing
M00407_1	Complex	4	D	Non-prokaryote		SCF-CDC4 complex	Ubiquitin	Genetic information processing
M00408_1	Complex	2	D	Non-prokaryote		ESCRT-0 complex	Protein processing	Genetic information processing
M00409_1	Complex	4	D	Non-prokaryote		ESCRT-I complex	Protein processing	Genetic information processing
M00410_1	Complex	3	D	Non-prokaryote		ESCRT-II complex	Protein processing	Genetic information processing
M00411_1	Complex	4	D	Non-prokaryote		SCF-GRR1 complex	Ubiquitin	Genetic information processing
M00412_1	Complex	9	D	Non-prokaryote		ESCRT-III complex	Protein processing	Genetic information processing
M00413_1	Complex	10	D	Non-prokaryote		FA core complex	Repair system	Genetic information processing
M00414_1	Complex	4	D	Non-prokaryote		Bloom's syndrome complex	Repair system	Genetic information processing
M00416_1	Complex	4	B	Firmicutes	rare	Cytochrome aa3-600 menaquinol oxidase	ATP synthesis	Energy metabolism
M00417_1	Complex	4	B	Proteobacteria/ Chlamydiae/Chlorobi		Cytochrome o ubiquinol oxidase	ATP synthesis	Energy metabolism
M00423_1	Complex	3	B	Prokaryote	rare	Molybdate/tungstate transport system	Mineral and organic ion transport system	Environmental information processing
M00424_1	Complex	5	D	Non-prokaryote		Shelterin complex	Replication system	Genetic information processing
M00425_1	Complex	4	D	Non-prokaryote		H/ACA ribonucleoprotein complex	RNA processing	Genetic information processing
M00426_1	Complex	2	D	Non-prokaryote		Survival motor neuron (SMN) complex	RNA processing	Genetic information processing
M00427_1	Complex	33	D	Non-prokaryote		Nuclear pore complex	RNA processing	Genetic information processing
M00428_1	Complex	3	D	Non-prokaryote		eIF4F complex	RNA processing	Genetic information processing
M00429_1	Complex	6	C	Firmicutes	rare	Competence-related DNA transformation transporter	Bacterial secretion system	Environmental information processing

M00430_1	Complex	15	D	Non-prokaryote		Exon junction complex (EJC)	RNA processing	Genetic information processing
M00435_1	Complex	3	B	Proteobacteria/ Firmicutes	rare	Taurine transport system	Mineral and organic ion transport system	Environmental information processing
M00436_1	Complex	3	B	Bacteria		Sulfonate transport system	Mineral and organic ion transport system	Environmental information processing
M00437_1	Complex	3	B	Betaproteobacteria	rare	Phthalate transport system	Mineral and organic ion transport system	Environmental information processing
M00438_1	Complex	4	B	Bacteria	rare	Nitrate/nitrite transport system	Mineral and organic ion transport system	Environmental information processing
M00439_1	Complex	5	C	Bacteria		Oligopeptide transport system	Peptide and nickel transport system	Environmental information processing
M00440_1	Complex	5	B	Bacteria	rare	Nickel transport system	Peptide and nickel transport system	Environmental information processing
M00441_1	Complex	3	B	Cyanobacteria	rare	Bispecific cyanate/nitrite transport system	Mineral and organic ion transport system	Environmental information processing
M00442_1	Complex	3	B	Bacteria	rare	Putative hydroxymethylpyrimidine transport system	Mineral and organic ion transport system	Environmental information processing

*Typical patterns for the module competion by 768 prokaryotic species are shown in Fig. 1.

**The modules completed by only less than 10% of 768 prokaryotic species are defined as " rare" modules.

Table S5. Characterization of the 7 KEGG modules (4 functional sets and 3 signatures) based on the module completion patterns in 768 prokaryotic species

Module no.	Module type	Number of module components	Module completion pattern*	Major taxonomic pattern	Rare module**	Function	Large function category	Small function category
M00359_1	Functional set	21	A (2)	Prokaryote		Aminoacyl-tRNA biosynthesis, eukaryotes	Metabolism	Aminoacyl-tRNA
M00360_1	Functional set	22	A (1)	Prokaryote		Aminoacyl-tRNA biosynthesis, prokaryotes	Metabolism	Aminoacyl-tRNA
M00361_1	Functional set	6	D	Non-prokaryote		Nucleotide sugar biosynthesis, eukaryotes	Metabolism	Nucleotide sugar
M00362_1	Functional set	7	C	Prokaryote	rare	Nucleotide sugar biosynthesis, prokaryotes	Metabolism	Nucleotide sugar
M00418_1	Signature	2	B	Proteobacteria	rare	Aromatic degradation, anaerobic	Genotypic signature	Metabolic capacity
M00419_1	Signature	5	B	Betaproteobacteria	rare	Aromatic degradation, aerobic	Genotypic signature	Metabolic capacity
M00363_1	Signature	1	B	Gammaproteobacteria	rare	EHEC pathogenicity signature, Shiga toxin	Genotypic signature	Pathogenecity

*Typical patterns for the module competion by 768 prokaryotic species are shown in Fig. 1.

**The modules completed by only less than 10% of 768 prokaryotic species are defined as " rare" modules.

Table S6. Notations of Boolean algebra-like equations for all KEGG modules containing redefined ones

Module No.	Type	Boorean
M00001_1	Pathway	(K00844,K12407,K00845) (K01810,K06859,K13810) K00850 (K01623,K01624) K01803 (K00134,K00150) K00927 K01834 K01689 K00873
M00002_1	Pathway	K01803 (K00134,K00150) K00927 K01834 K01689 K00873
M00003_1	Pathway	(K01596,K01610) K01689 K01834 K00927 K00134 K01803 (K01623,K01624) (K03841,K02446,K11532,K04041)
M00004_1	Pathway	(K13937,(K00036+(K01057,K07404))) K00033 K01783 (K01807,K01808) K00615 K00616 (K01810,K13810)
M00005_1	Pathway	K00948
M00006_1	Pathway	(K13937,(K00036+(K01057,K07404))) K00033
M00007_1	Pathway	K00615 (K00616,K13810) K01783 (K01807,K01808)
M00008_1	Pathway	(K13937,(K00036+(K01057,K07404))) K01690 K01625
M00009_1	Pathway	K01647 (K01681,K01682) (K00031,K00030) (K00164+K00658+K00382) ((K01902+K01903),(K01899+K01900)) ((K00239+K00240+K00241+K00242),(K00244+K00245+K00246+K00247)) (K01676,K01679,(K01677+K01678)) (K00026,K00025,K00024)
M00010_1	Pathway	K01647 (K01681,K01682) (K00031,K00030)
M00011_1	Pathway	((K00164+K00658+K00382),(K00174+K00175+K00177+K00176)) ((K01902+K01903),(K01899+K01900)) ((K00239+K00240+K00241+K00242),(K00244+K00245+K00246+K00247),(K00234+K00235+K00236+K00237)) (K01676,K01679,(K01677+K01678)) (K00026,K00025,K00024)
M00012_1	Pathway	K01647 (K01681,K01682) K01637 K01638 (K00026,K00025,K00024)
M00013_1	Pathway	K00249 (K01692,K07511,K07514,K07515) K05605 K00140 K01578
M00014_1	Pathway	K00012 K13247 K03331 K05351 K00854
M00015_1	Pathway	((K00931+K00147),K12657) K00286
M00016_1	Pathway	(K00928,K12524,K12525,K12526) K00133 K01714 K00215 K00674 (K00821,K14267) K01439 K01778 (K01586,K12526)
M00017_1	Pathway	(K00928,K12524,K12525,K12526) K00133 (K00003,K12524,K12525) K00651 K01739 K01760 (K00548,K00549)
M00018_1	Pathway	(K00928,K12524,K12525,K12526) K00133 (K00003,K12524,K12525) (K00872,K02204,K02203) K01733
M00019_1	Pathway	(K01652+K01653+K11258) K00053 K01687 K01649 (K01702,(K01703+K01704)) K00052 K00826
M00020_1	Pathway	K00058 K00831 K01079
M00021_1	Pathway	K00640 (K01738,K12339,K13034,K10150)
M00022_1	Pathway	(K01626,K03856,K13853) (K01735,K13830,K13829) (K03785,K03786,K13832,K13830) (K00014,K13832,K13830) (K00891,K13830,K13829) (K00800,K13830) K01736
M00023_1	Pathway	((((K01657+K01658),K13503,K13501,K01656)+K00766),K13497) ((K01817+(K01656,K01609)),K13498,K13501) ((K01695+(K01696,K06001)),K01694)
M00024_1	Pathway	(K01850,K04092,K14187,K04093,K14170,K04516,K06208,K06209,K13853) (K01713,K04518,K14170) K00832
M00025_1	Pathway	(K01850,K04092,K14187,K04093,K14170,K04516,K06208,K06209,K13853) (K00210,K04517,K14187) K00832 K00817

M00026_1	Pathway	K00765 ((K01523+K01496),K11755,K14152) K01814 ((K02501+K02500),K01663) (K01693+K00817+(K04486,K05602),(K01089+K00817)) (K00013,K14152)
M00027_1	Pathway	K01580 (K13524,K07250,K00823) (K00135,K00139)
M00028_1	Pathway	(K00619,K00620,K00618,K11067,K14681,K14682) ((K00930+K00145),K12659) (K00818,K00821,K05830) (K01438,K14677,K00620)
M00029_1	Pathway	K01948 K00611 K01940 (K01755,K14681) K01476
M00030_1	Pathway	K01655 K01705 K05824 (K00825,K00838,K05825) (K00143,K14085) K00293 (K00290,K00291,K14157)
M00031_1	Pathway	(K05826+K05827) K00930 K00145 K05830 K05831
M00032_1	Pathway	((K00290,K00291)+(K00292,K00293),K14157) (K00143,K14085) K00825 K00164 K00658 K00252 (K01825,K01782,K07514,(K01692,K07515,K07511)+K00022)
M00033_1	Pathway	K00928,K12524,K12525,K12526 K00133 K00836 K06718 K06720
M00034_1	Pathway	K00789 K01611 K00797 (((K01243,K01244)+K00899),K00772) K08963 K08964 K09880 K08967 (K00815,K08969,K00832,K00838)
M00034_2	Pathway	K00789 K01611 K00797 (((K01243,K01244)+K00899),K00772) K08963 K08964 K08965 K08966 K08967 (K00815,K08969,K00832,K00838)
M00035_1	Pathway	K00789 K00558 K01251 (K01697,K10150)
M00036_1	Pathway	K00826 (((K00166+K00167),K11381)+K09699+K00382) (K00253,K00249) (K01968+K01969) (K05607,K13766) K01640
M00037_1	Pathway	K00502 K01593 K00669 K00543
M00038_1	Pathway	(K00453,K00463) (K01432,K14263,K07130) K00486 K01556 K00452 K03392 K10217
M00039_1	Pathway	K00487 K01904 K09753 K00083 (K00430,K11188,K03782)
M00040_1	Pathway	K00832 (K00220,(K15226+K15227))
M00042_1	Pathway	(K00505,K00501) (K01592,K01593) K00503 K00553
M00043_1	Pathway	K00431
M00044_1	Pathway	K03334 K00457 K00451 K01800 K01555
M00045_1	Pathway	K01745 K01712 K01468 K01479
M00046_1	Pathway	K01485 K00207 K01464 K01431
M00047_1	Pathway	K00613 K00542 K00933
M00048_1	Pathway	K00764 (K01945,K11787,K11788,K13713) (K00601,K11175,K08289,K11787) K01952 (K01933,K11787,K11788) (K01587,K11808,(K01589+K01588)) (K01923,K01587,K13713) K01756 (K00602,K01492,(K06863+K11176))
M00049_1	Pathway	K01939 K01756 K00939 (K00524,(K00525+K00526),(K10807+K10808)) K00940 K00527
M00050_1	Pathway	K00088 K01951 K00942 K00940 K00527 (K00524,(K00525+K00526),(K10807+K10808))
M00051_1	Pathway	K11540 (K00226,K00254) (K13421,(K00762+K01591))
M00051_2	Pathway	K11541 K01465 (K00226,K00254) (K13421,(K00762+K01591))
M00051_3	Pathway	(K01954,(K01955+K01956)) ((K00609+K00610),K00608) K01465 (K00226,K00254) (K13421,(K00762+K01591))
M00052_1	Pathway	(K13800,K13809,K00945,K13799) K00940 K01937

M00053_1	Pathway	(K00524,(K00525+K00526),(K10807+K10808)) K00940 K00527 K01494 K01520 (K00560,K13998) K00943 K00940
M00055_1	Pathway	K01001 (K07432+K07441) K03842 K03843 K03844 K03845 K03846 K03847 K03846 K00729 K03848 K03849 K03850
M00056_1	Pathway	K00710 (K00731,K09653) (K00727,K09662,K09663) K00739
M00057_1	Pathway	K00771 K00733 K00734 (K00735,K10157,K10158,K10812)
M00058_1	Pathway	K00746 (K13499,K00747,K03419)
M00059_1	Pathway	(K02369,K02370) (K02366,K02367) (K02368,K02370) (K02576,K02577,K02578,K02579) K01793
M00060_1	Pathway	K00677 K02535 K02536 K03269 K00748 K00912 K02527 K02517 K02560
M00061_1	Pathway	K01812 K00041 K01685 K01686 K00040 K01812
M00063_1	Pathway	K01627 K03270 K00979
M00064_1	Pathway	K03271 K03272 K03273 K03274
M00065_1	Pathway	K03857 K03434 K05283 K05284 K05285 K07542 K05286 K05287
M00066_1	Pathway	K00720 K07553
M00067_1	Pathway	K04628 K01019
M00068_1	Pathway	K01988 K00719
M00069_1	Pathway	K03370 (K03371,K03369)
M00070_1	Pathway	K03766 (K07819,K07820,K03877)
M00071_1	Pathway	K03766 (K07966,K07967,K07968,K07969)
M00073_1	Pathway	K01228 K05546 K01230
M00074_1	Pathway	K05546 K01230 K05528 (K05529+K05530) (K05529+K05531+K05532+K05533+K05534) K05535
M00075_1	Pathway	K01231 K00736
M00075_2	Pathway	K00737
M00075_3	Pathway	K00738 (K00744,K09661) K13748
M00075_4	Pathway	K00717 (K07966,K07967,K07968) (K00778,K00779)
M00076_1	Pathway	K01136 K01217 K01135 K01197 K01195
M00077_1	Pathway	K01135 K01197 K01195 K01132
M00078_1	Pathway	(K07964,K07965) K01136 K01217 K01565 K10532 K01205 K01195 K01137
M00079_1	Pathway	K01132 K12309 K01137 K12373
M00080_1	Pathway	K02527 K02841 K02843 K02850 K02848 K02849 K02844 K02840 K03278 K03279 K03280 K02847
M00081_1	Pathway	K01051 K01184 K01213
M00082_1	Pathway	K01961 K00665 K00665 K00665

M00083_1	Pathway	(K00665,(K00667+K00668),K11533,(K00647,K09458,K00648)+K00059+K02372+(K00208,K02371,K10780))
M00085_1	Pathway	(K07508,K07509) ((K00022+K07511),K07515) K07512
M00086_1	Pathway	K01897,K15013
M00087_1	Pathway	(K00232,K00249,K00255,K06445,K09479) (((K01692,K07511,K13767)+(K00022,K07516)),K01825,K01782,K07514,K07515,K10527) (K00632,K07508,K07509,K07513)
M00088_1	Pathway	K00626 K01641 K01640
M00088_2	Pathway	K00019
M00088_3	Pathway	K01574
M00089_1	Pathway	(K00629,K13506,K13507,K00630,K13508,K00631,K03621,K08591) (K00655,K13509,K13513,K13517,K13519,K13523,K14674) K01080 (K00635,K11155,K11160)
M00090_1	Pathway	(K00866,K14156) K00968 (K00994,K13644)
M00091_1	Pathway	K00551 K00570
M00092_1	Pathway	(K00894,K14156) K00967 (K00993,K13644)
M00093_1	Pathway	K00981 K00998 K01613
M00094_1	Pathway	K00654 K04708 (K04709,K04710) K04712
M00095_1	Pathway	K00626 K01641 K00021 K00869 (K00938,K13273) K01597 K01823
M00096_1	Pathway	K01662 K00099 (K00991,K12506) K00919 (K01770,K12506) K03526 K03527 K01823
M00097_1	Pathway	K02291 K10027 (K06443,K14605,K14606)
M00097_2	Pathway	K02291 K02293 K00514 (K06443,K14605,K14606)
M00098_1	Pathway	(K01046,K12298,K13534,K14073,K14074,K14075,K14076,K14452,K14674,K14675) K01054
M00099_1	Pathway	K00654 K04708 (K04709,K04710) K04712 (K01441,K12348,K12349)
M00100_1	Pathway	K04718 K01634
M00101_1	Pathway	K00801 K00511 K01852 K05917 K00222 K07750 K07748 (K09827,K13373) K09828 K01824 K00227 K00213
M00102_1	Pathway	K00559 K09829 K09830 K09831 K00223
M00103_1	Pathway	K07419 K07438
M00104_1	Pathway	K00489 K12408 K07431 K00251 K00037 K00488 K08748 K01796 K10214 K12405 K08764 K11992
M00105_1	Pathway	K00489 K12408 K00251 K00037 K00488 K08748 K01796 K10214 K12405 K08764 K11992
M00106_1	Pathway	K08748 K00659
M00107_1	Pathway	K00498 (K00070,K01822)
M00108_1	Pathway	K00513 (K00497,K07433) K07433
M00109_1	Pathway	K00512 K00513 (K00497,K07433) K00071

M00110_1	Pathway	K00512 (K00070,K01822) K07434
M00112_1	Pathway	K09833 K12502 K09834 K05928
M00113_1	Pathway	K00454 K01723 K10525 K05894 K10526 K00232 K10527 (K00632,K07513)
M00114_1	Pathway	(K01810,K06859,K13810) K01809 K01840 (K00966,K00971) K10046 K14190 K10047 K00064 (K00225,K05276)
M00115_1	Pathway	K00278 K03517 K00767 (K00969,K06210) (K01916,K01950)
M00116_1	Pathway	(K01851,K02552,K02361) K02551 K08680 K02549 K01911 K01661 K12073 K02548 K03183
M00117_1	Pathway	K03181 K03179 (K03186,K03182) K03688 K00568 K03185 K03183 K03184 K00568
M00118_1	Pathway	((K11204+K11205),K01919) K01920
M00119_1	Pathway	K00826 K00606 K00077
M00119_2	Pathway	K01579
M00119_3	Pathway	(K01918,K13799)
M00120_1	Pathway	(K00867,K03525,K09680,K01947) ((K01922+K01598),K13038) (K02318,(K00954,K02201)+K00859)
M00121_1	Pathway	(K01885,K14163) K02492 K01845 K01698 K01749 (K01719,K13542,K13543)
M00121_2	Pathway	K01599 (K00228,K02495) (K00230,K00231) K01772
M00121_3	Pathway	(K02302,(K00589,K02303,K02496,K13542,K13543)+K02304+K03794)
M00122_1	Pathway	K13786 K00798 K02232 (K02225,K02227,K08260) K02231
M00122_2	Pathway	K00768 K02226
M00122_3	Pathway	K02233
M00123_1	Pathway	K00652 K00833 K01935 K01012
M00124_1	Pathway	K03472 K03473 K00831 K00097 K03474 K00275
M00125_1	Pathway	(K01497,K14652) ((K01498+K00082),K11752)
M00125_2	Pathway	(K02858,K14652)
M00125_3	Pathway	K00794 K00793 ((K00861+K00953),K11753)
M00126_1	Pathway	K01495 (K01077,K01113) (K13939,(K13940+K00796),(K01633+K00950+K00796),(K01633+K13941)) (K11754,K01930) (K00287,K13998,K13938)
M00127_1	Pathway	K03147 (K00877,K00941,K14153)
M00127_2	Pathway	(K00878,K14154)
M00127_3	Pathway	(K00788,K14153,K14154) K00946
M00128_1	Pathway	K03181 K06125 K00591 K06126 K06127 K06134 K00591
M00129_1	Pathway	K00963 K00012 K00699 K01195,K14756 K01053 K00103
M00130_1	Pathway	(K00888,K13711) (K00889,K13712) (K01116,K05857,K05858,K05859,K05860,K05861) K00911

M00131_1	Pathway	K01106 (K01107,K15422) K01109 (K01092,K10047)
M00132_1	Pathway	K00913 K00915 K10572
M00133_1	Pathway	(K01583,K01584,K01585,K02626) K01480
M00133_2	Pathway	K01611
M00133_3	Pathway	K00797
M00134_1	Pathway	K01476 K01581
M00135_1	Pathway	K00657 K00274 (K00128,K14085,K00149)
M00136_1	Pathway	K09470 K09471 K09472 K09473
M00137_1	Pathway	K00660 K01859 K13077
M00138_1	Pathway	K00475 K00091 K05277
M00139_1	Pathway	K13382 K13383 K13384 K13385 K13386 K13391 K13392 K13393 K14976 K13394 K14975
M00140_1	Pathway	K00600 K01491 K01938
M00141_1	Pathway	K00600 K00288
M00141_2	Pathway	K00600 K13403 K13402
M00165_1	Pathway	(K01601+K01602) K00927 (K05298,K00150) (K01623,K01624) (K03841,K02446,K11532,K04041) K00615 (K01623,K01624) (K01100,K11532) K00615 (K01807,K01808) K00855
M00166_1	Pathway	(K01601+K01602) K00927 (K05298,K00150)
M00167_1	Pathway	(K01623,K01624) (K03841,K02446,K11532,K04041) K00615 (K01623,K01624) (K01100,K11532) K00615 (K01807,K01808) K00855
M00168_1	Pathway	K01595 (K00025,K00026,K00024)
M00169_1	Pathway	K00029 K01006
M00170_1	Pathway	K01595 K14454 K01610
M00171_1	Pathway	K01595 K14454 K14455 (K00025,K00026,K00024) K00028 K00814 K01006
M00172_1	Pathway	K01595 K00051 K00029 K01006
M00173_1	Pathway	((K00169+K00170+K00171+K00172),K03737) (((K01007,K01006)+K01595),(K01959+K01960),K01958) K00024 (K01676,K01679,(K01677+K01678)) ((K00239+K00240),(K00244+K00245+K00246+K00247)) (K01902+K01903) (K00174+K00175) K00031 (K01681,K01682) (K01648,(K15230+K15231),(K15232+K15233+K15234))
M00174_1	Pathway	K08684 (K00429,K03781,K03782,K00430,K11188,K00093,(K14028+K14029)) K00148 (K00122,(K00123+(K00124,K00125)+K00126+K00127),(K08348+K08349+K08350))
M00175_1	Pathway	K00531,(K02586+K02591),K02588,K00536
M00176_1	Pathway	(K13811,(K00958+K00860),(K00955+K00957),(K00956+K00957+K00860)) K00390 ((K00380+K00381),K00392)
M00307_1	Pathway	(((K00163,((K00161+K00162))+K00627+K00382),K13997),(K00169+K00170+K00171+K00172))
M00308_1	Pathway	K05308 K00874 K01625

M00309_1	Pathway	K05308 K11395
M00338_1	Pathway	(K01697,K10150) K01758
M00344_1	Pathway	K00863 K01624 K03841
M00345_1	Pathway	(((K08093,K13812)+K08094),K13831) K00850 K01624
M00346_1	Pathway	K00600 K00830 K00018 K11529 K01689 K01595 K00024 (K08692+K14067) K08691
M00347_1	Pathway	(K00123+K00125) (K00200+K00201+K00202+K00203+K11260) K00672 K01499 K00319 K00320 (K00440+K00441+K00442+K00443) (K00577+K00578+K00579+K00580+K00581+K00582+K00583+K00584) (K00399+K00400+K00401+K00402+K03421+K03422) (K03388+K03389+K03390)
M00350_1	Pathway	(K10775,K13064) K00487 K01904 K00588
M00356_1	Pathway	(K14080+K04480+K14081) (K00399+K00400+K00401+K00402+K03421+K03422) (K03388+K03389+K03390+K08264+K08265)
M00357_1	Pathway	K00925 (K00625,K13788) (K00192+K00193+K00194+K00195+K00197) (K00577+K00578+K00579+K00580+K00581+K00582+K00583+K00584) (K00399+K00400+K00401+K00402+K03421+K03422) (K03388+K03389+K03390+K08264+K08265)
M00358_1	Pathway	K08097 K05979 K05884 (K13039+K06034)
M00364_1	Pathway	K01823 K00795 (K13789,K13787)
M00365_1	Pathway	K01823 K13787
M00366_1	Pathway	K01823 K14066 K00787 K13789
M00367_1	Pathway	K01823 K00787 K00804
M00368_1	Pathway	K00789 K01762 K05933
M00369_1	Pathway	K13027 K13029 K13030
M00370_1	Pathway	(K11812,K11813) K11818 K11819 K11820 K11821
M00371_1	Pathway	(K09587,K12639) K09588 K09591 (K12637,K12638) (K09590,K12640) K12640
M00372_1	Pathway	K02294 K09838 K14594 K09840 K09841 K09842
M00373_1	Pathway	K00626 K00023 K01715 K14446 K14447 K14448 K14449 K14450 (K01965+K01966) K05606 K01847
M00373_2	Pathway	K14450 K14451
M00374_1	Pathway	((K00169+K00170+K00171+K00172),K03737) K01007 K01595 K00024 (K01676,K01679,(K01677+K01678)) ((K00239+K00240),(K00244+K00245+K00246+K00247)) (K01902+K01903) K15038 K14465 (K14466,K14467) K14534 (K15016,(K01715+(K01782,K01825,K07516))) K00626
M00375_1	Pathway	((K02160+K01961+K01962+K01963),(K01964+K15037+K15036)) (K14468,(K15017+K15039)) (K14469,(K15018+K15019+K15020)) ((K01965+K01966),K15052,(K01964+K15037+K15036)) K05606 (K01847,(K01848+K01849),K11942) K15038 K14465 (K14466,K14467) K14534 (K15016,(K01715+(K01782,K01825,K07516))) K00626
M00376_1	Pathway	((K02160+K01961+K01962+K01963),(K01964+K15037+K15036)) (K14468,(K15017+K15039)) (K14469,(K15018+K15019+K15020)) ((K01965+K01966),K15052,(K01964+K15037+K15036)) K05606 (K01847,(K01848+K01849),K11942) (K14471+K14472) ((K00239+K00240),(K00244+K00245+K00246+K00247)) (K01676,(K01677+K01678),K01679) (K14471+K14472) K08691
M00376_2	Pathway	K08691 K14449 K14470 K09709 K08691

M00377_1	Pathway	K01938 K01491 K00297 K15023 (K14138+K00190) (K00625,K13788,K15024) K00925
M00378_1	Pathway	(K11780+K11781)
M00378_2	Pathway	K14941
M00378_3	Pathway	K11212 K12234 K14940
M00415_1	Pathway	(K10247,K10205,K10248,K10249,K10244,K10203,(K10250+K15397)) K10251 K10703 K10258
M00432_1	Pathway	K01649 K01703+K01704 K00052
M00433_1	Pathway	K01655 K01705 K05824
M00072_1	Complex	K07151 K12666 K12667 K12668 K12669 K12670
M00142_1	Complex	K03878 K03879 K03880 K03881 K03882 K03883 K03884
M00143_1	Complex	K03934 K03935 K03936 K03937 K03938 K03939 K03940 K03941 K03942 K03943
M00144_1	Complex	K00330 K00331 K00331 K00332 K00333 K00334 K00335 K00336 K00337 K00338 K00339 K00340 K00341 K00342 K00343
M00144_2	Complex	K00330 K00331 K00331 K13378 K00334 K00335 K00336 K00337 K00338 K00339 K00340 K00341 K00342 K00343
M00144_3	Complex	K00330 K00331 K13380 K00334 K00335 K00336 K00337 K00338 K00339 K00340 K00341 K00342 K00343
M00145_1	Complex	K05574 K05582 K05581 K05579 K05572 K05580 K05578 K05576 K05577 K05575 K05573
M00146_1	Complex	K03945 K03946 K03947 K03948 K03949 K03950 K03951 K03952 K03953 K03954 K03955 K03956 K11352 K11353
M00147_1	Complex	K03957 K03958 K03959 K03960 K03961 K03962 K03963 K03964 K03965 K03966 K11351 K03967 K03968
M00148_1	Complex	K00236 K00237 K00234 K00235
M00149_1	Complex	K00241 K00242 K00239 K00240
M00150_1	Complex	K00244 K00245 K00246 K00247
M00151_1	Complex	K00411 K00412 K00413
M00151_2	Complex	K03886 K03887 K03888
M00151_3	Complex	K03890 K03891 K03889
M00152_1	Complex	K00411 K00412 K00413 K00414 K00415 K00416 K00417 K00418 K00419 K00420
M00153_1	Complex	K00425 K00426
M00154_1	Complex	K02257 K02262 K02256 K02261 K02263 K02264 K02265 K02266 K02267 K02268 K02269 K02272 K02258 K02259 K02260
M00154_2	Complex	K02257 K02262 K02256 K02261 K02263 K02264 K02265 K02266 K02267 K02268 K02270 K02272 K02258 K02259 K02260
M00155_1	Complex	K02275 K02274 K02276
M00155_2	Complex	K02275 K15408
M00156_1	Complex	K00404 K00405 K00407 K00406
M00157_1	Complex	K02112 K02111 K02115 K02113 K02114 K02110 K02108 K02109

M00158_1	Complex	K02133 K02132 K02136 K02137 K02134 K02135 K02128 K02126 K02127 K02125 K02138 K02140
M00159_1	Complex	K02117 K02118 K02119 K02120 K02121 K02122 K02123 K02124
M00160_1	Complex	K02145 K02147 K02148 K02149 K02150 K02151 K02152 K02153 K02154 K02146 K02144 K03662 (K02155,K03661)
M00161_1	Complex	K02703 K02706 K02705 K02704 K02707 K02708 K02713 K02711 K02709 K02710 K02712 K02714 K02718 K02724 K02723 K08902 K08903 K02716 K02717
M00161_2	Complex	K08901 K03541 K03542 K02721
M00161_3	Complex	K02720 K02719 K02722
M00162_1	Complex	K02635 K02637 K02634 K02636 K02642 K02643 K03689 K02640
M00163_1	Complex	K02689 K02690 K02691 K02692 K02693 K02694 K02697 K02699 K02696 K02698 K02700 K02701 K08905 K02695
M00163_2	Complex	K02689 K02690 K02691 K02692 K02693 K02694 K02697 K02699 K02696 K02698 K02700 K02702
M00164_1	Complex	K02112 K02111 K02115 K02113 K02114 K02110 K02108 K02109
M00177_1	Complex	K02981 K02985 K02984 K02987 K02989 K02991 K02993 K02995 K02997 K02947 K02949 K02951 K02953 K02955 K02958 K02957 K02960 K02962 K02964 K02966 K02969 K02971 K02973 K02974 K02975 K02976 K02978 K02977 K02979 K02980 K02983 K02998 K02925 K02930 K02932 K02934 K02937 K02936 K02938 K02940 K02866 K02865 K02868 K02870 K02873 K02872 K02875 K02877 K02880 K02883 K02882 K02885 K02889 K02891 K02894 K02893 K02896 K02898 K02901 K02900 K02903 K02905 K02908 K02910 K02912 K02915 K02918 K02917 K02920 K02922 K02921 K02923 K02924 K02927 K02928 K02929 K02941 K02942 K02943
M00178_1	Complex	K02945 K02967 K02982 K02986 K02988 K02990 K02992 K02994 K02996 K02946 K02948 K02950 K02952 K02954 K02956 K02959 K02961 K02963 K02965 K02968 K02863 K02886 K02906 K02926 K02931 K02933 K02935 K02939 K02864 K02867 K02871 K02874 K02876 K02878 K02879 K02881 K02884 K02887 K02888 K02890 K02892 K02895 K02899 K02902 K02904 K02907 K02909 K02911 K02913 K02914 K02916 K02919
M00179_1	Complex	K02967 K02982 K02986 K02988 K02992 K02994 K02996 K02946 K02948 K02950 K02952 K02954 K02956 K02961 K02965 K02984 K02987 K02991 K02995 K02962 K02966 K02974 K02978 K02977 K02979 K02863 K02886 K02906 K02931 K02933 K02864 K02867 K02869 K02871 K02874 K02876 K02881 K02890 K02892 K02895 K02904 K02907 K02930 K02936 K02866 K02877 K02883 K02885 K02889 K02896 K02908 K02910 K02912 K02922 K02921 K02924 K02927 K02929 K02944
M00180_1	Complex	K03006 K03010 K03011 K03008 K03012 K03015 K03017 K03013 K03014 K03016 K03009 K03007
M00181_1	Complex	K03018 K03021 K03027 K03020 K03023 K03026 K14721 K03025 K03024 K03022 K03019 K03013 K03014 K03016 K03009 K03007
M00182_1	Complex	K02999 K03002 K03027 K03020 K03000 K03004 K03005 K03013 K03014 K03016 K03009 K03007
M00183_1	Complex	K03040 K03043 K03046 K03060
M00183_2	Complex	K03040 K13797 K03060
M00184_1	Complex	K03042 K03041 K03044 K03045 K03053 K03055 K03058 K03047 K03049 K03050 K03056 K03059 K03051
M00184_2	Complex	K03042 K03041 K13798 K03053 K03055 K03058 K03047 K03049 K03050 K03056 K03059 K03051
M00185_1	Complex	K02048 K02046 K02047 K02045
M00186_1	Complex	K05772 K05773 K06857
M00187_1	Complex	K02061 K02060 K02059

M00188_1	Complex	K02051 K02050 K02049
M00189_1	Complex	K02020 K02018 K02017
M00190_1	Complex	K02012 K02011 K02010
M00191_1	Complex	K02064 K02063 K02062
M00192_1	Complex	K05777 K05778 K05779
M00193_1	Complex	K02055 K02053 K02054 K02052
M00194_1	Complex	K10108 K10109 K10110 (K10111,K10112)
M00195_1	Complex	K10113 K10114 K10115 K10116
M00196_1	Complex	K10117 K10118 K10119 K10112
M00197_1	Complex	K10108 K10109 K10110 (K10111,K10112,K10116)
M00197_2	Complex	K10117 K10118 K10119 (K10111,K10112,K10116)
M00197_3	Complex	K10113 K10114 K10115 (K10111,K10112,K10116)
M00197_4	Complex	K10120 K10121 K10122 (K10111,K10112,K10116)
M00198_1	Complex	K05813 K05814 K05815 K05816
M00199_1	Complex	K10188 K10189 K10190
M00200_1	Complex	K10227 K10228 K10229 (K10230,K10243)
M00201_1	Complex	K10232 K10233 K10234 K10235
M00202_1	Complex	K10192 K10193 K10194 K10195
M00203_1	Complex	K10196 K10197 K10198 K10199
M00204_1	Complex	K10236 K10237 K10238 K10239
M00205_1	Complex	K10200 K10201 K10202
M00206_1	Complex	K10240 K10241 K10242 K10243
M00207_1	Complex	K02027 K02025 K02026 K02023
M00208_1	Complex	K02002 K02001 K02000
M00209_1	Complex	K05845 K05846 K05847
M00210_1	Complex	K02067 K02066 K02065
M00211_1	Complex	K02069 K02068
M00212_1	Complex	K10439 K10440 K10441
M00213_1	Complex	K10537 K10538 K10539
M00214_1	Complex	K10540 K10541 K10542

M00215_1	Complex	K10543 K10544 K10545
M00216_1	Complex	K10546 K10547 K10548
M00217_1	Complex	K10549 K10550 K10551
M00218_1	Complex	K10552 K10553 K10554
M00219_1	Complex	K10555 K10556 K10557 K10558
M00220_1	Complex	K10559 K10560 K10561 K10562
M00221_1	Complex	K02058 K02057 K02056
M00222_1	Complex	K02040 K02037 K02038 K02036
M00223_1	Complex	K02044 K02042 K02041
M00224_1	Complex	K05780 K05781
M00225_1	Complex	K10013 K10015 K10016 K10017
M00226_1	Complex	K10014 K10015 K10016 K10017
M00227_1	Complex	K10036 K10037 K10038
M00228_1	Complex	K10039 K10040 K10041
M00229_1	Complex	(K09996,K09997) K09998 K09999 K10000
M00230_1	Complex	K10001 K10002 K10003 K10004
M00231_1	Complex	K10018 K10019 K10020 K10021
M00232_1	Complex	K09969 K09970 K09971 K09972
M00233_1	Complex	K10005 K10006 K10007 K10008
M00234_1	Complex	K02424 K10009 K10010
M00235_1	Complex	K10022 K10023 K10024 K10025
M00236_1	Complex	K02030 K02029 K02028
M00237_1	Complex	K01999 K01997 K01998 K01995 K01996
M00238_1	Complex	K02073 K02072 K02071
M00239_1	Complex	K02035 K02033 K02034 K02031 K02032
M00240_1	Complex	K02016 K02015 K02013
M00241_1	Complex	K06858 K06073 K06074
M00242_1	Complex	K09815 K09816 K09817
M00243_1	Complex	K09818 K09819 K09820
M00244_1	Complex	K02077 K02075 K02074

M00245_1	Complex	K02009 K02008 K02007 K02006
M00246_1	Complex	K10094 K02008 K02007 K02006
M00247_1	Complex	K01989 K05832 K05833
M00248_1	Complex	K09686 K09687
M00249_1	Complex	K09688 K10107 K09689
M00250_1	Complex	K09690 K09691
M00251_1	Complex	K09692 K09693
M00252_1	Complex	K09694 K09695
M00253_1	Complex	K09696 K09697
M00254_1	Complex	K01992 K01990
M00255_1	Complex	K09808 K09810
M00256_1	Complex	K09811 K09812
M00257_1	Complex	K09813 K09814
M00258_1	Complex	K02004 K02003
M00259_1	Complex	K02195 K02194 K02193
M00260_1	Complex	K02337 (K03763,K02342,K14159) K02338 K02340 K02341 K02343
M00261_1	Complex	K02320 K02321 K02685 K02684
M00262_1	Complex	K02327 K02328 K03504 K03505
M00263_1	Complex	K02324 K02325 K02326 K03506
M00264_1	Complex	K02322 K02323
M00265_1	Complex	K02777 K02778 K02779
M00266_1	Complex	K02777 K02790 K02791
M00267_1	Complex	K02802 K02803 K02804
M00268_1	Complex	K02777 K02749 K02750
M00269_1	Complex	K02808 K02809 K02810
M00270_1	Complex	(K02777,K02817) K02818 K02819
M00271_1	Complex	K02755 K02756 K02757
M00272_1	Complex	K02777 K02752 K02753
M00273_1	Complex	K02768 K02769 K02770
M00274_1	Complex	K02798 K02799 K02800

M00275_1	Complex	K02759 K02760 K02761
M00276_1	Complex	K02793 K02794 K02795 K02796
M00277_1	Complex	K02744 K02745 K02746 K02747
M00278_1	Complex	K02812 K02813 K02814 K02815
M00279_1	Complex	K02773 K02774 K02775
M00280_1	Complex	K02781 K02782 K02783
M00281_1	Complex	K02786 K02787 K02788
M00282_1	Complex	K02763 K02764 K02765
M00283_1	Complex	K02821 K02822 K03475
M00284_1	Complex	K02603 K02604 K02605 K02606 K02607 K02608
M00285_1	Complex	K02540 K02541 K02212 K02209 K02542 K02210
M00286_1	Complex	K10732 K10733 K10734 K10735
M00287_1	Complex	K02744 K10984 K10985 K10986
M00288_1	Complex	K07466 (K10739,K10741) K10740
M00289_1	Complex	K10754 K10755 K10756
M00290_1	Complex	K03141 K03142 K03143 K03144 K10843 K10844 K10845 K02202 K06634 K10842
M00291_1	Complex	K10865 K10866 K10867
M00292_1	Complex	K10865 K10866 K10868
M00293_1	Complex	K02350 (K03508,K13728)
M00294_1	Complex	K02332 K02333
M00295_1	Complex	K10605 K08735 K08737 K08734 K10858 K10754 K10755 K10756 K04802 K10865 K10866 K10867 K10901 K04728
M00296_1	Complex	K10803 K10798 (K02330,K03512,K10981) K10776 (K10771,K10772)
M00297_1	Complex	K10884 K10885 K06642
M00298_1	Complex	K11051 K11050
M00299_1	Complex	K11069 K11070 K11071 (K11072,K11076)
M00300_1	Complex	K11073 K11074 K11075 K11076
M00301_1	Complex	K11077 K11078 K11079 K11080
M00302_1	Complex	K11081 K11082 K11083 K11084
M00303_1	Complex	K02777 K11191 K11192
M00304_1	Complex	K11194 K11195 K11196 K02771

M00305_1	Complex	K11198 K11199 K11200
M00306_1	Complex	K11201 K11202 K11203
M00310_1	Complex	K00169 K00170 K00171 K00172
M00311_1	Complex	K00174 K00175 K00176 K00177
M00312_1	Complex	K00186 K00187 K00188 K00189
M00313_1	Complex	K00179 K00180
M00313_2	Complex	K04090
M00314_1	Complex	K11632 K11631
M00315_1	Complex	K11636 K11635
M00316_1	Complex	K11601 K11602 K11603
M00317_1	Complex	K11604 K11605 K11606 K11607
M00318_1	Complex	K11704 K11705 K11706
M00319_1	Complex	K11707 K11708 K11709 K11710
M00320_1	Complex	K07091 K11720 K06861
M00321_1	Complex	K11950 K11951 K11952 K11953
M00322_1	Complex	K11954 K11955 K11956 K11957 K11958
M00323_1	Complex	K11959 K11960 K11961 K11962 K11963
M00324_1	Complex	K12368 K12369 K12370 K12371 K12372
M00325_1	Complex	K11004 K11003 K12340
M00326_1	Complex	K12530 K12531 K12532 K12340
M00327_1	Complex	K12533 K12534 K12535
M00328_1	Complex	K12536 K12537 K12538
M00329_1	Complex	K12539 K12540
M00330_1	Complex	K12541 K12542 K12543
M00331_1	Complex	K02453 K02452 K02455 K02456 K02457 K02458 K02459 K02460 K02461 K02462 K02454
M00332_1	Complex	K03221 K04056 K04057 K04059 K03219 K04058 K03222 K03226 K03227 K03228 K03229 K03230 K03224 K03225 K03223
M00333_1	Complex	K03194 K03197 K03198 K03200 K03202 K03204 K03201 K03203 K03195 K03199 K03196 K03205
M00334_1	Complex	K11904 K11903 K11906 K11891 K11892 K11907 K11912 K11913 K11915
M00335_1	Complex	K03072 K12257 K03074 K03073 K03075 K13301 K03076 K03210 K03217 K03070 K03110 K03071 K03106
M00336_1	Complex	K03116 K03117 K03118 K03425

M00337_1	Complex	K02725 K02726 K02727 K02728 K02729 K02730 K02731 K02732 K02734 K02735 K02736 K02741 K02733 K02740 K06696 K06697
M00339_1	Complex	K13408 K13409 K13410
M00340_1	Complex	K02725 K02726 K02727 K02728 K02729 K02730 K02731 K02732 K02734 K02735 K02736 K02737 K02738 K02739
M00341_1	Complex	K03061 K03062 K03063 K03064 K03065 K03066 K03028 K03032 K03033 K03035 K03036 K03037 K03038 K03039 K03029 K03030 K03031 K06691 K10881
M00342_1	Complex	K03432 K03433 K13527 K13571
M00343_1	Complex	K03432 K03433 K03420
M00348_1	Complex	K13889 K13890 K13891 K13892
M00349_1	Complex	K13893 K13894 K13895 K13896
M00351_1	Complex	K11086 K11087 K11096 K11088 K11097 K11098 K11099 K11093 K11091 K11095
M00352_1	Complex	K11086 K11087 K11096 K11088 K11097 K11098 K11099 K11092 K11094 K12825 K12826 K12827 K12828 K12829 K12830 K12831 K12832 K12833 K12835 K12834
M00353_1	Complex	K10599 K12860 K12861 K12862 K12863 K12864 K03283 K12865 K12866
M00354_1	Complex	K11086 K11087 K11096 K11088 K11097 K11098 K11099 K12621 K12622 K12623 K12624 K12625 K12626 K12627 K12843 K12662 K09567 K12844 K12845 K12852 K12853 K12855 K12856 K12857 K12854 K12858 K12859 K13099 K11984 K12847 K12846
M00355_1	Complex	K11086 K11087 K11096 K11088 K11097 K11098 K11099 K12856 K12854 K12852 K12857 K10599 K12860 K12861 K12862 K12863 K06063 K12867 K12868 K12869 K12870 K12871 K12872 K12873 K12874 K09564 K12733 K12816 K13117 K03283 K13123
M00379_1	Complex	K03868 K03347 K03094 K10259
M00380_1	Complex	K03868 K03347 K03094 K03362
M00381_1	Complex	K03868 K03347 K03094 K03875
M00382_1	Complex	K03868 K03347 K03094 (K10099,K10100)
M00383_1	Complex	K03868 K03870 K03872 K03873 K03871
M00384_1	Complex	K03868 K03869 K10523
M00385_1	Complex	K03868 K10609 K10610 K10140
M00386_1	Complex	K03868 K10609 K10610 K10570
M00387_1	Complex	K03868 K03347 K03094 K10260
M00388_1	Complex	K10611 K10612 K03872 K03873 (K04701,K04694,K04695,K04696,K04697,K04698,K10322,K04699)
M00389_1	Complex	K03358 K03349 (K03363,K03364) K03348 K03350 K03351 K03352 K03353 (K03354,K03356) K03355 K03357 (K03359,K12456)
M00390_1	Complex	(K07573,K03679) K11600 K12589
M00391_1	Complex	K07573 (K12587,K12588) K03679 K03681 K11600 K12589 K12586 K12585 K03678 K12590
M00392_1	Complex	K12599 K12600 (K12601,K12602)
M00393_1	Complex	(K03514,K12596) K12597 K12598

M00394_1	Complex	K08300 K03732 K01689 K00962
M00395_1	Complex	(K12610,K12611,K12612) K12613 K12614 K12615 K12617
M00396_1	Complex	K12621 K12622 K12623 K12624 K12625 K12626 K12627
M00397_1	Complex	K12620 K12621 K12622 K12623 K12624 K12625 K12626
M00398_1	Complex	K11086 K11087 K11096 K11088 K11097 K11098 K11099
M00399_1	Complex	K12882 K12883
M00400_1	Complex	K13525 K14016 K14015
M00401_1	Complex	K10956 K09481 K07342
M00402_1	Complex	K13249 K13250 K13251 K04571
M00403_1	Complex	K10601 K14026 (K10088,K14008) K14027 K11519 K13989 K14025 K13525 K14015 K14016
M00404_1	Complex	K14004 K14005 K14006 K14007 K07953
M00405_1	Complex	K12878 K12879 K13174 K13175 K13176
M00406_1	Complex	K12878 K12879 K13174 K13175 K13176 K12880 K12881 K12812
M00407_1	Complex	K03868 K03347 K03094 K03361
M00408_1	Complex	K12182 K04705
M00409_1	Complex	K12183 K12184 K12185 K12186
M00410_1	Complex	K12188 K12189 K12190
M00411_1	Complex	K03868 K03347 K03094 K03360
M00412_1	Complex	K12191 K12192 K12193 K12194 K12195 K12196 K12197 K12198 K15053
M00413_1	Complex	K10888 K10889 K10890 K10892 K10893 K10894 K10606 K10896 K10898 K10993
M00414_1	Complex	K10901 K03165 K10990 K15365
M00416_1	Complex	K02827 K02826 K02828 K02829
M00417_1	Complex	K02297 K02298 K02299 K02300
M00423_1	Complex	K15495 K15496 K15497
M00424_1	Complex	K11109 K11110 K11111 K11112 K11113
M00425_1	Complex	K11128 K11129 K11130 K11131
M00426_1	Complex	K13129 K13130
M00427_1	Complex	K09291 K14295 K14296 K03345 K14297 K14298 K14004 K14299 K14300 K14301 K14302 K14303 K14304 K14305 K14306 K14307 K14308 K14309 K14310 K14311 K14312 K14313 K14314 K14315 K14316 K14317 K14318 K12172 K14319 K10577 K12160 K14320 K14321
M00428_1	Complex	K03259 K03260 K03257
M00429_1	Complex	K02237 K02238 K02240 K02243 K02244 (K02245,K02246,K02247,K02248,K02249)

M00430_1	Complex	K12876 K12877 K14323 K13025 K14324 K13114 K12875 K14325 K12881 K12812 K13171 K14285 K14326 K14327 K14328
M00435_1	Complex	K15551 K15552 K10831
M00436_1	Complex	K15553 K15554 K15555
M00437_1	Complex	K15556 K15557 K15558
M00438_1	Complex	K15576 K15577 K15578 K15579
M00439_1	Complex	K15580 K15581 K15582 K15583 K10823
M00440_1	Complex	K15584 K15585 K15586 K15587 K10824
M00441_1	Complex	K15576 K15577 K15597
M00442_1	Complex	K15598 K15599 K15600
M00363_1	Signature	K11006 K11007
M00418_1	Signature	(K07543+K07544) K07545 K07546 (K07547+K07548) (K07549+K07550)
M00419_1	Signature	K10616+K10617+K10618+K10619
M00359_1	FuncSet	K01872 K01887 K01893 K01876 K01883 K01886 K01885 K01880 K01892 K01870 K01869 K04567 K01874 (K01889+K01890) K01881 K01875 K01868 K01867 K01866 K01873
M00360_1	FuncSet	K01872 K01887 (K01893,K01876,K09759) K01883 (K01886,K01885,K09698) ((K01878+K01879),K14164,K01880) K01892 K01870 K01869 (K04567,K04566) K01874 K01889+K01890 K01881 K01875 K01868 K01867 K01866 K01873
M00361_1	FuncSet	K00963 K01784 K00972 K00012 K08678 (K00966,K00971)
M00362_1	FuncSet	K00963 K00965 K01784 (K00972,K04042,K11528) K01791 K00012 (K00966,K00971)

Table S7. Summary of metagenomic sequences of human gut microbiome

[Sanger sequence (6)]

Sample ID	CDS no.	average length of CDSs	no. of complete CDSs predicted from contigs and singlets				no. of partial CDSs predicted from contigs and singlets				no. of KO assigned CDSs	ratio of assigned KOs (%)	no. of CDSs mapped to module	module mapping ratio of KO assigned CDSs (%)
			contigs	singlets	total	ratio of complete CDSs (%)	contigs	singlets	total	ratio of partial CDS (%)				
F1-S	54,151	173.1	15,919	10,407	26,326	48.6	15,842	11,983	27,825	51.4	19,969	37.0	7,003	35.1
F1-T	65,156	173.4	14,895	11,057	25,952	39.8	18,428	20,776	39,204	60.2	26,401	40.5	9,130	34.6
F1-U	35,260	167.9	11,667	6,834	18,501	52.47	8,123	8,636	16,759	47.5	19,471	55.2	5,977	30.7
F2-V	66,461	175.3	16,876	12,164	29,040	43.7	20,921	16,500	37,421	56.3	28,991	43.6	10,160	35.0
F2-W	57,213	177.0	16,180	11,310	27,490	48.0	15,580	14,143	29,723	52.0	22,365	39.1	7,894	35.3
F2-X	57,446	176.1	13,660	11,983	25,643	44.6	14,575	17,228	31,803	55.4	21,164	36.8	7,388	34.9
F2-Y	64,942	179.3	17,313	12,417	29,730	45.8	17,698	17,514	35,212	54.2	23,512	36.2	8,140	34.6
In-A	38,778	183.1	11,726	7,977	19,703	50.8	7,380	11,695	19,075	49.2	13,700	35.3	4,742	34.6
In-B	20,063	196.0	4,732	5,099	9,831	49.0	2,541	7,691	10,232	51.0	7,669	38.2	3,013	39.3
In-D	67,740	177.8	14,133	18,088	32,221	47.6	13,566	21,953	35,519	52.4	23,907	35.3	8,264	34.6
In-E	37,652	181.4	8,477	9,814	18,291	48.6	6,271	13,090	19,361	51.4	15,313	40.7	5,860	38.3
In-M	34,330	175.5	14,473	4,396	18,869	55.0	9,175	6,286	15,461	45.0	16,157	47.1	5,461	33.8
In-R	63,356	166.0	16,871	12,705	29,576	46.7	18,701	15,079	33,780	53.3	22,498	35.5	7,558	33.6
Average	50,965	177.0	13,609	10,327	23,936	47.7	12,985	14,044	27,029	52.3	20,086	39.4	6,968	34.9

[Illumina sequence (28)]

Sample ID	CDS no.	average length of CDSs	no. of complete CDSs predicted from contigs and singlets				no. of partial CDSs predicted from contigs and singlets				no. of KO assigned CDSs	ratio of assigned KOs (%)	no. of CDSs mapped to module	module mapping ratio of KO assigned CDSs (%)
			contigs	singlets	total	ratio of complete CDSs (%)	contigs	singlets	total	ratio of partial CDS (%)				
MH_0001	28,238	204.7	9,604	0	9,604	34.0	18,634	0	18,634	66.0	11,726	41.5	4,001	34.1
MH_0005	26,938	215.9	9,002	0	9,002	33.4	17,936	0	17,936	66.6	10,916	40.5	3,603	33.0
Average	27,588	210.3	9,303	0	9,303	33.7	18,285	0	18,285	66.3	11,321	41.0	3,802	33.6

(6), (29): Reference number in the text
