# Supplementary material for: Evaluation method for the potential functionome harbored in the genome and metagenome
Source: BMC Genomics. 2012 Dec 12;13:699. doi: 10.1186/1471-2164-13-699 (PMC3541978; doi:10.1186/1471-2164-13-699)
Supplement: Additional file 2 — Figures S1–S9. Figure S1. Distribution patterns of the completion ratio of the KEGG pathway modules in 768 prokaryotic species. The completion ratio of 205 pathway modules containing submodules were evaluated in this study. Figure S2. Distribution patterns of the completion ratio of the KEGG structural complex modules in 768 prokaryotic species. The module completion ratio of 263 structural complex modules containing submodules was evaluated in this study. Figure S3. Distribution patterns of the completion ratio of the KEGG functional set and signature modules in 768 prokaryotic species. The module completion ratio of 7 functional set and signature modules was evaluated in this study. Figure S4. Distribution of KO identifiers mapped to the module for glyoxylate cycle (M00012) in other pathway modules. KO identifiers, except for K01637 and K01638 colored light green, are also shared in several other modules. Figure S5. Module completion patterns in 8 phenotypically different Bacillus-related species. (A) Pathway module. (B) Structural complex module. bsu, Bacillus subtilis ; bao, Bacillus amyloliquefaciens ; bli, Bacillus licheniformis ; bha, Bacillus halodurans ; bpf, Bacillus pseudofirmus ; oih, Oceanobacillus iheyensis ; gka, Geobacillus kaustophilus ; and gth, Geobacillus thermoglucosidasius. Green characters show rare modules, which are completed by less than 10% of 768 prokaryotic species. Figure S6. Module completion patterns in human and human gut microbiomes. (A)-1–3, Pathway module. (B)-1–3, Structural complex module. Upper histogram shows the module completion pattern in gut microbiomes from 13 healthy individuals [18]. Middle histogram shows module completion patterns in humans. Lower histogram shows module completion patterns in human gut microbiomes plus humans. Green characters show rare modules, which are completed by less than 10% of 768 prokaryotic species. Figure S7. Definition of submodules for the KEGG module with branching. The heme biosynthesis [file 1471-2164-13-699-S2.pdf]

Figure S1. Distribution patterns of the completion ratio of the KEGG pathway modules in 768 prokaryotic species. The completion ratio of 205 pathway modules containing submodules were evaluated in this study.

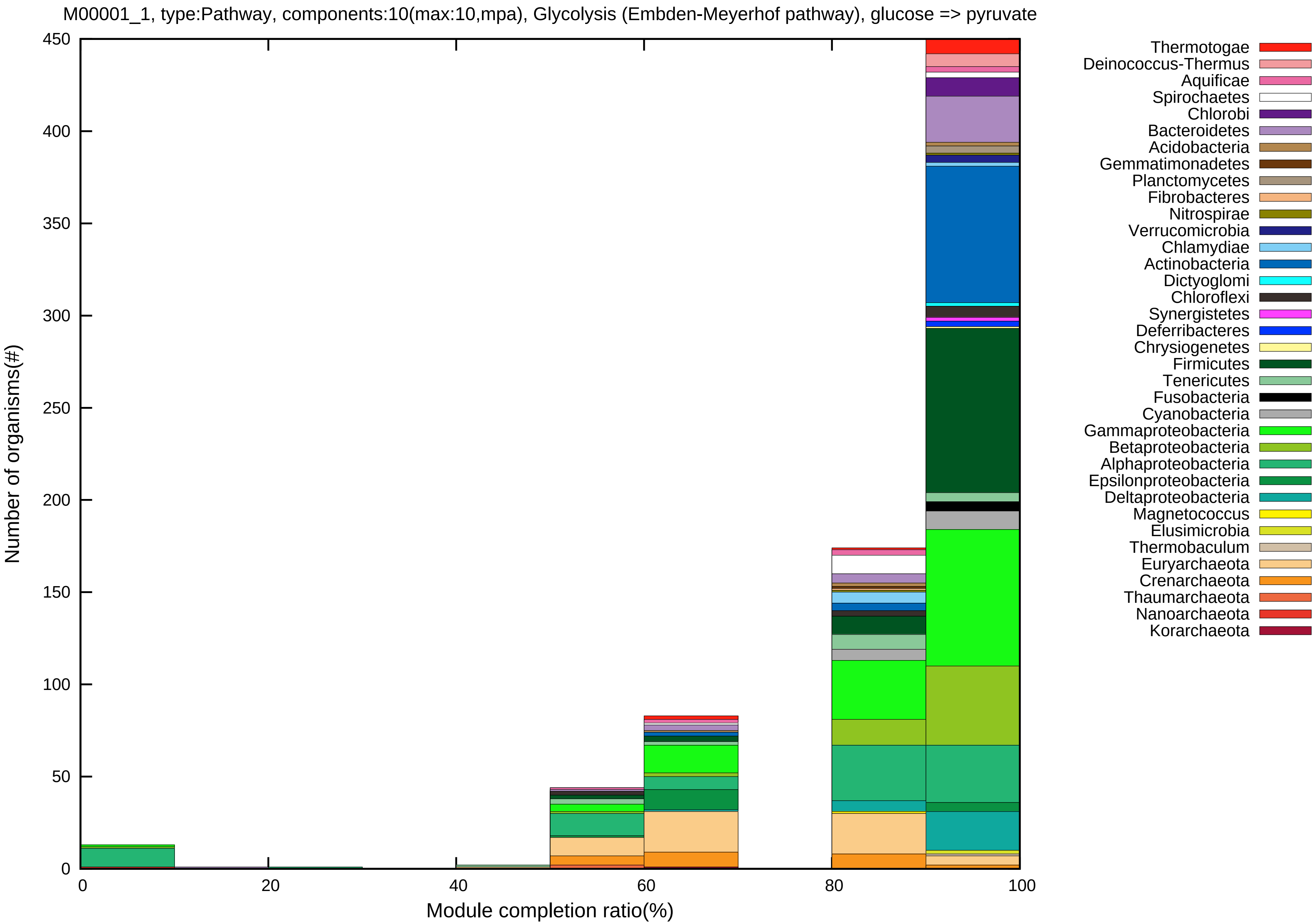

M00001_1, type:Pathway, components:10(max:10,mpa), Glycolysis (Embden-Meyerhof pathway), glucose => pyruvate
Number of organisms(#)
Module completion ratio(%)
0
50
100
150
200
250
300
350
400
450
0
20
40
60
80
100
Thermotogae
Deinococcus-Thermus
Aquificae
Spirochaetes
Chlorobi
Bacteroidetes
Acidobacteria
Gemmatimonadetes
Planctomycetes
Fibrobacteres
Nitrospirae
Verrucomicrobia
Chlamydiae
Actinobacteria
Dictyoglomi
Chloroflexi
Synergistetes
Deferribacteres
Chrysiogenetes
Firmicutes
Tenericutes
Fusobacteria
Cyanobacteria
Gammaproteobacteria
Betaproteobacteria
Alphaproteobacteria
Epsilonproteobacteria
Deltaproteobacteria
Magnetococcus
Elusimicrobia
Thermobaculum
Euryarchaeota
Crenarchaeota
Thaumarchaeota
Nanoarchaeota
Korarchaeota

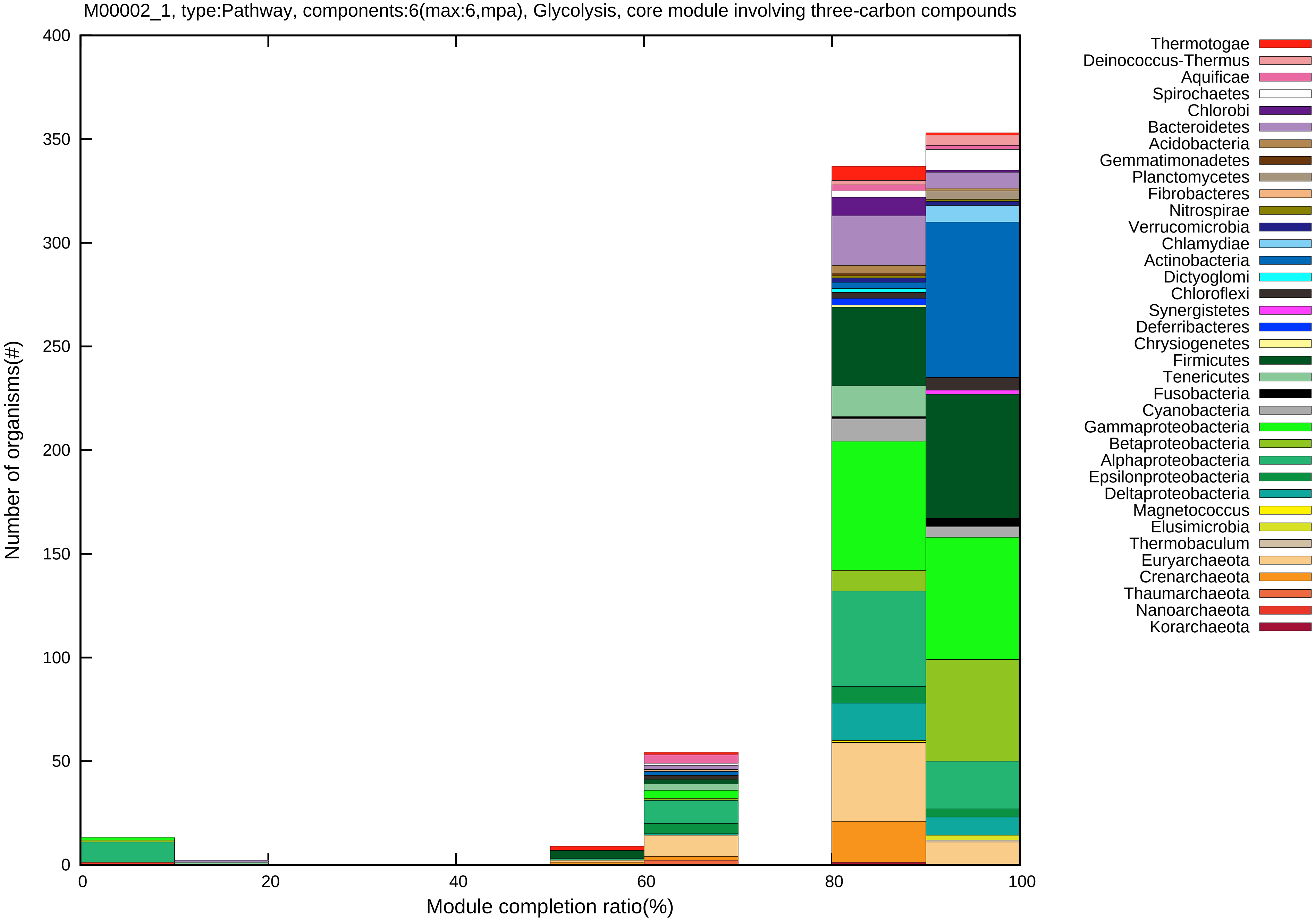

M00002_1, type:Pathway, components:6(max:6,mpa), Glycolysis, core module involving three-carbon compounds
Number of organisms(#)
Module completion ratio(%)
0
50
100
150
200
250
300
350
400
0
20
40
60
80
100
Thermotogae
Deinococcus-Thermus
Aquificae
Spirochaetes
Chlorobi
Bacteroidetes
Acidobacteria
Gemmatimonadetes
Planctomycetes
Fibrobacteres
Nitrospirae
Verrucomicrobia
Chlamydiae
Actinobacteria
Dictyoglomi
Chloroflexi
Synergistetes
Deferribacteres
Chrysiogenetes
Firmicutes
Tenericutes
Fusobacteria
Cyanobacteria
Gammaproteobacteria
Betaproteobacteria
Alphaproteobacteria
Epsilonproteobacteria
Deltaproteobacteria
Magnetococcus
Elusimicrobia
Thermobaculum
Euryarchaeota
Crenarchaeota
Thaumarchaeota
Nanoarchaeota
Korarchaeota

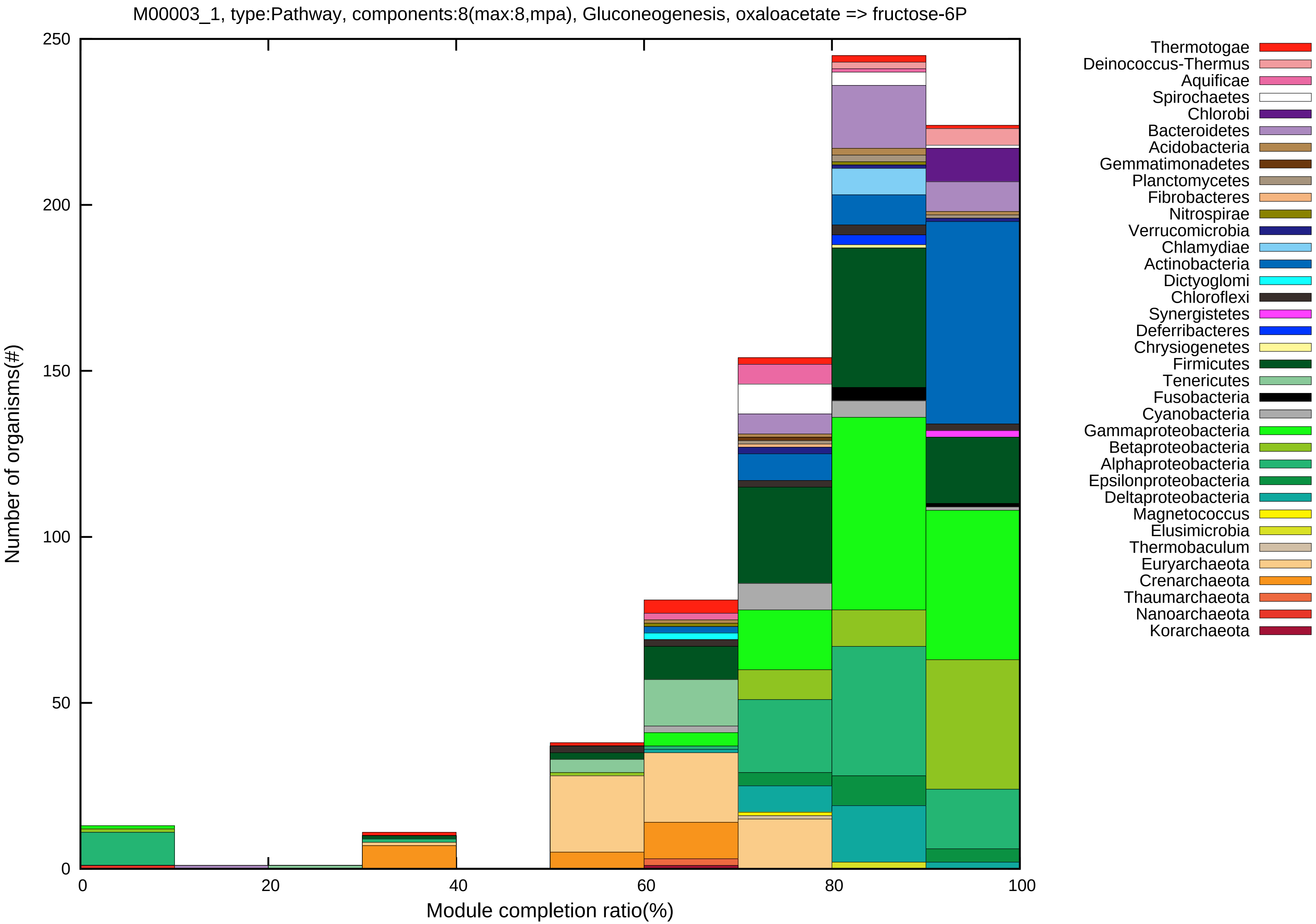

M00003_1, type:Pathway, components:8(max:8,mpa), Gluconeogenesis, oxaloacetate => fructose-6P
Number of organisms(#)
Module completion ratio(%)
0
50
100
150
200
250
0
20
40
60
80
100
Thermotogae
Deinococcus-Thermus
Aquificae
Spirochaetes
Chlorobi
Bacteroidetes
Acidobacteria
Gemmatimonadetes
Planctomycetes
Fibrobacteres
Nitrospirae
Verrucomicrobia
Chlamydiae
Actinobacteria
Dictyoglomi
Chloroflexi
Synergistetes
Deferribacteres
Chrysiogenetes
Firmicutes
Tenericutes
Fusobacteria
Cyanobacteria
Gammaproteobacteria
Betaproteobacteria
Alphaproteobacteria
Epsilonproteobacteria
Deltaproteobacteria
Magnetococcus
Elusimicrobia
Thermobaculum
Euryarchaeota
Crenarchaeota
Thaumarchaeota
Nanoarchaeota
Korarchaeota

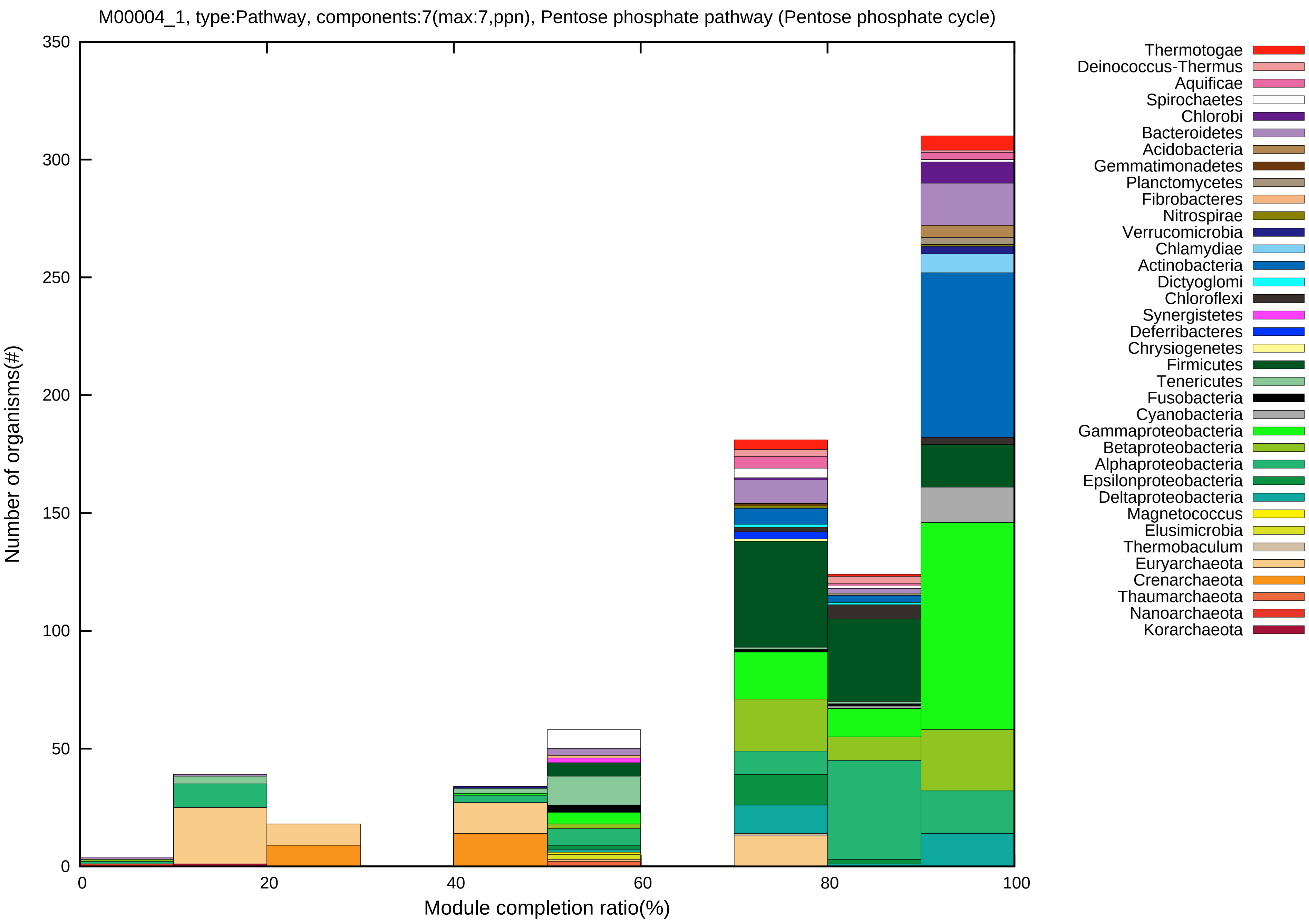
M00004_1, type:Pathway, components:7(max:7,ppn), Pentose phosphate pathway (Pentose phosphate cycle)
Number of organisms(#)
Module completion ratio(%)
0
50
100
150
200
250
300
350
0
20
40
60
80
100
Thermotogae
Deinococcus-Thermus
Aquificae
Spirochaetes
Chlorobi
Bacteroidetes
Acidobacteria
Gemmatimonadetes
Planctomycetes
Fibrobacteres
Nitrospirae
Verrucomicrobia
Chlamydiae
Actinobacteria
Dictyoglomi
Chloroflexi
Synergistetes
Deferribacteres
Chrysiogenetes
Firmicutes
Tenericutes
Fusobacteria
Cyanobacteria
Gammaproteobacteria
Betaproteobacteria
Alphaproteobacteria
Epsilonproteobacteria
Deltaproteobacteria
Magnetococcus
Elusimicrobia
Thermobaculum
Euryarchaeota
Crenarchaeota
Thaumarchaeota
Nanoarchaeota
Korarchaeota

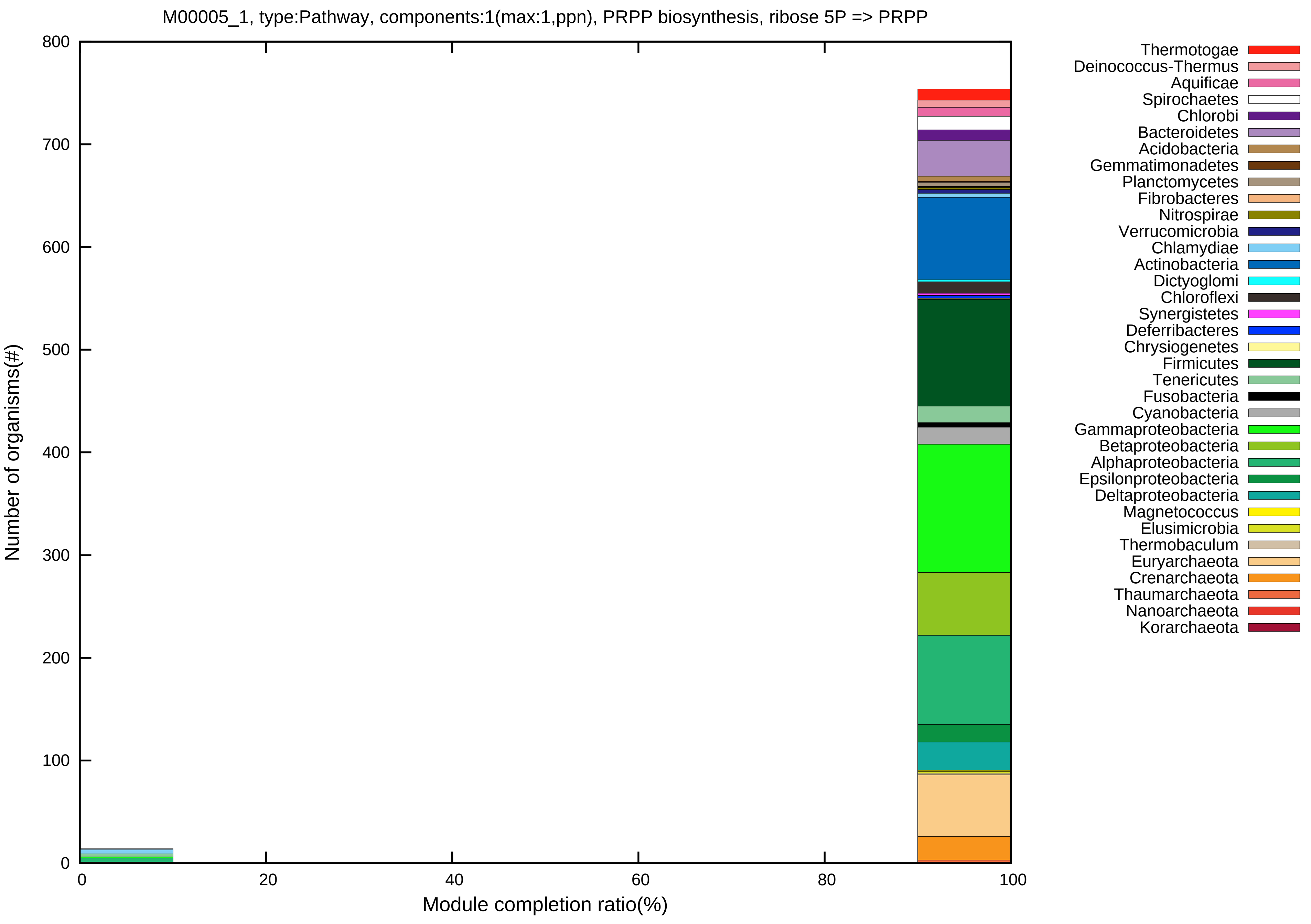
M00005_1, type:Pathway, components:1(max:1,ppn), PRPP biosynthesis, ribose 5P => PRPP
Number of organisms(#)
Module completion ratio(%)
0
100
200
300
400
500
600
700
800
0
20
40
60
80
100
Thermotogae
Deinococcus-Thermus
Aquificae
Spirochaetes
Chlorobi
Bacteroidetes
Acidobacteria
Gemmatimonadetes
Planctomycetes
Fibrobacteres
Nitrospirae
Verrucomicrobia
Chlamydiae
Actinobacteria
Dictyoglomi
Chloroflexi
Synergistetes
Deferribacteres
Chrysiogenetes
Firmicutes
Tenericutes
Fusobacteria
Cyanobacteria
Gammaproteobacteria
Betaproteobacteria
Alphaproteobacteria
Epsilonproteobacteria
Deltaproteobacteria
Magnetococcus
Elusimicrobia
Thermobaculum
Euryarchaeota
Crenarchaeota
Thaumarchaeota
Nanoarchaeota
Korarchaeota

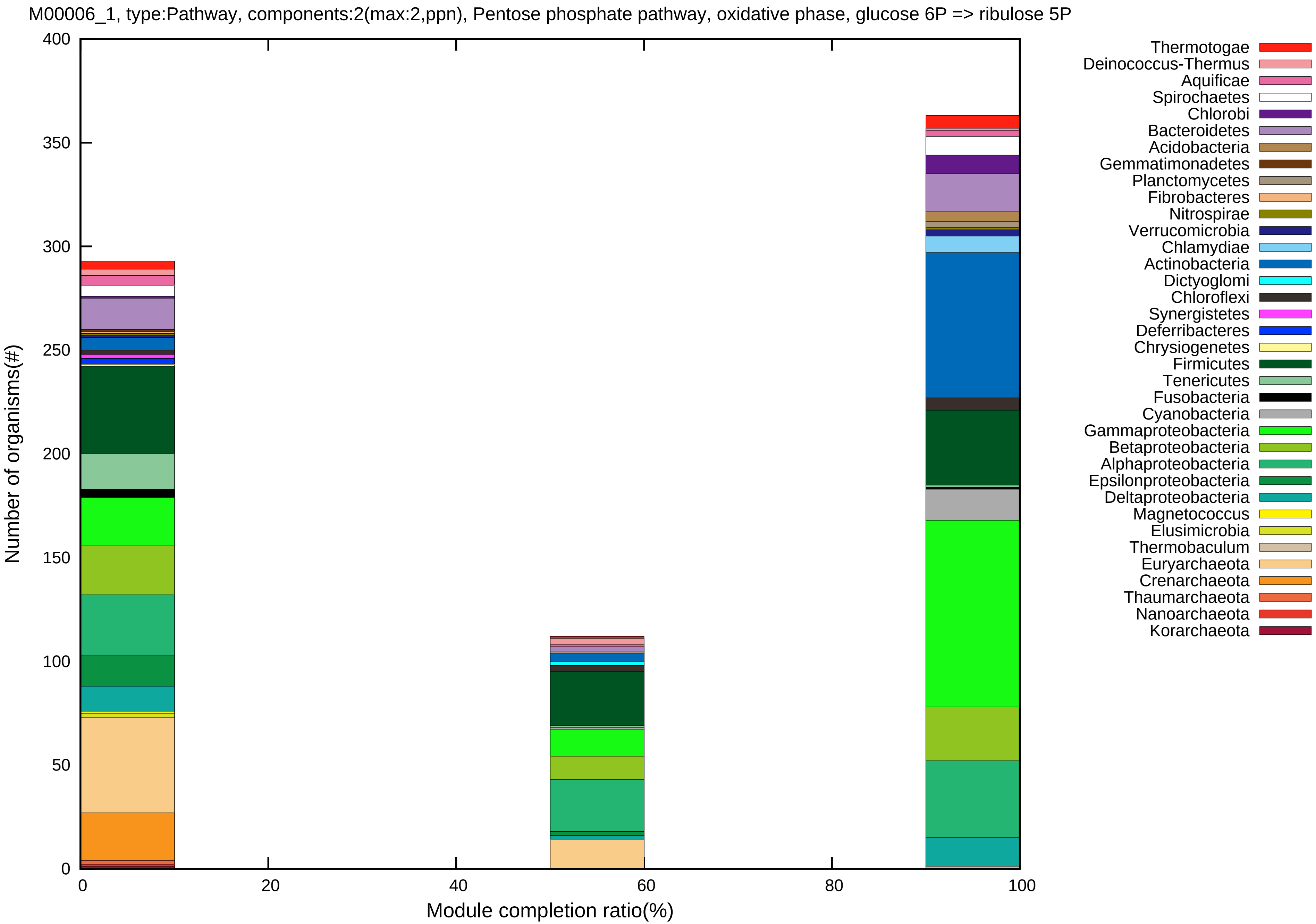

M00006_1, type:Pathway, components:2(max:2,ppn), Pentose phosphate pathway, oxidative phase, glucose 6P => ribulose 5P
Number of organisms(#)
Module completion ratio(%)
0
50
100
150
200
250
300
350
400
0
20
40
60
80
100
Thermotogae
Deinococcus-Thermus
Aquificae
Spirochaetes
Chlorobi
Bacteroidetes
Acidobacteria
Gemmatimonadetes
Planctomycetes
Fibrobacteres
Nitrospirae
Verrucomicrobia
Chlamydiae
Actinobacteria
Dictyoglomi
Chloroflexi
Synergistetes
Deferribacteres
Chrysiogenetes
Firmicutes
Tenericutes
Fusobacteria
Cyanobacteria
Gammaproteobacteria
Betaproteobacteria
Alphaproteobacteria
Epsilonproteobacteria
Deltaproteobacteria
Magnetococcus
Elusimicrobia
Thermobaculum
Euryarchaeota
Crenarchaeota
Thaumarchaeota
Nanoarchaeota
Korarchaeota

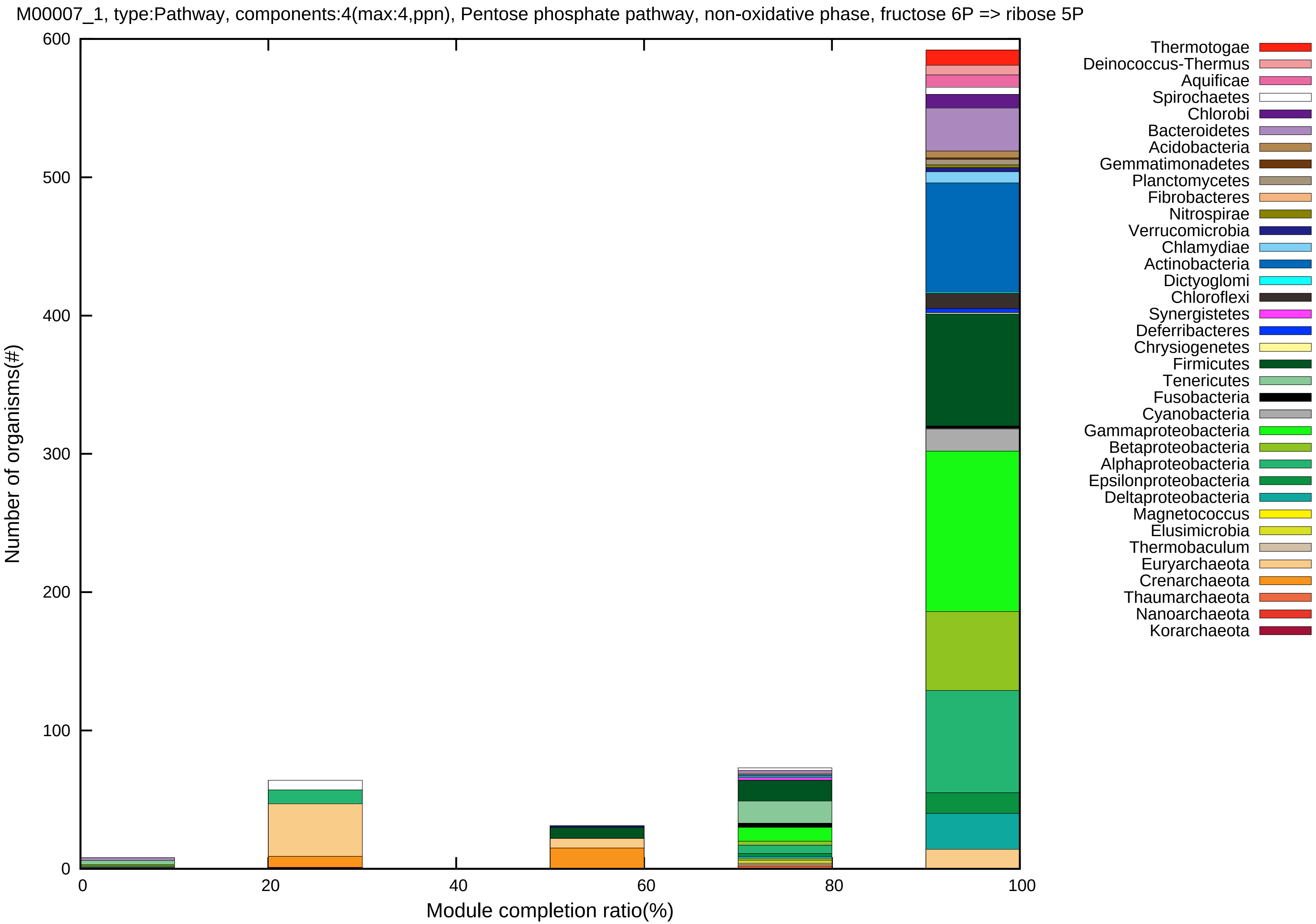

M00007_1, type:Pathway, components:4(max:4,ppn), Pentose phosphate pathway, non-oxidative phase, fructose 6P => ribose 5P
Number of organisms(#)
Module completion ratio(%)
0
100
200
300
400
500
600
0
20
40
60
80
100
Thermotogae
Deinococcus-Thermus
Aquificae
Spirochaetes
Chlorobi
Bacteroidetes
Acidobacteria
Gemmatimonadetes
Planctomycetes
Fibrobacteres
Nitrospirae
Verrucomicrobia
Chlamydiae
Actinobacteria
Dictyoglomi
Chloroflexi
Synergistetes
Deferribacteres
Chrysiogenetes
Firmicutes
Tenericutes
Fusobacteria
Cyanobacteria
Gammaproteobacteria
Betaproteobacteria
Alphaproteobacteria
Epsilonproteobacteria
Deltaproteobacteria
Magnetococcus
Elusimicrobia
Thermobaculum
Euryarchaeota
Crenarchaeota
Thaumarchaeota
Nanoarchaeota
Korarchaeota

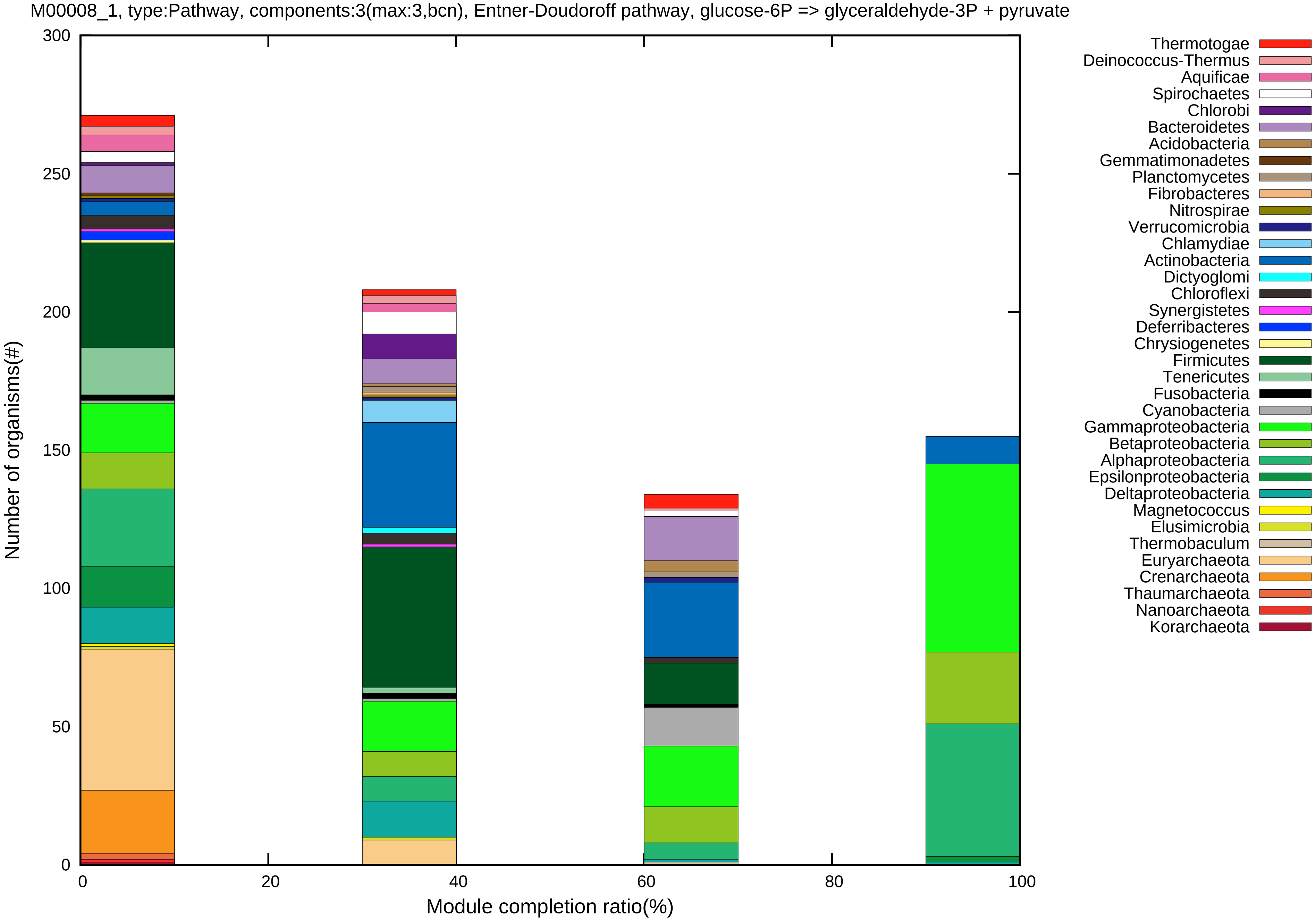
M00008_1, type:Pathway, components:3(max:3,bcn), Entner-Doudoroff pathway, glucose-6P => glyceraldehyde-3P + pyruvate
Number of organisms(#)
Module completion ratio(%)
0
50
100
150
200
250
300
0
20
40
60
80
100
Thermotogae
Deinococcus-Thermus
Aquificae
Spirochaetes
Chlorobi
Bacteroidetes
Acidobacteria
Gemmatimonadetes
Planctomycetes
Fibrobacteres
Nitrospirae
Verrucomicrobia
Chlamydiae
Actinobacteria
Dictyoglomi
Chloroflexi
Synergistetes
Deferribacteres
Chrysiogenetes
Firmicutes
Tenericutes
Fusobacteria
Cyanobacteria
Gammaproteobacteria
Betaproteobacteria
Alphaproteobacteria
Epsilonproteobacteria
Deltaproteobacteria
Magnetococcus
Elusimicrobia
Thermobaculum
Euryarchaeota
Crenarchaeota
Thaumarchaeota
Nanoarchaeota
Korarchaeota

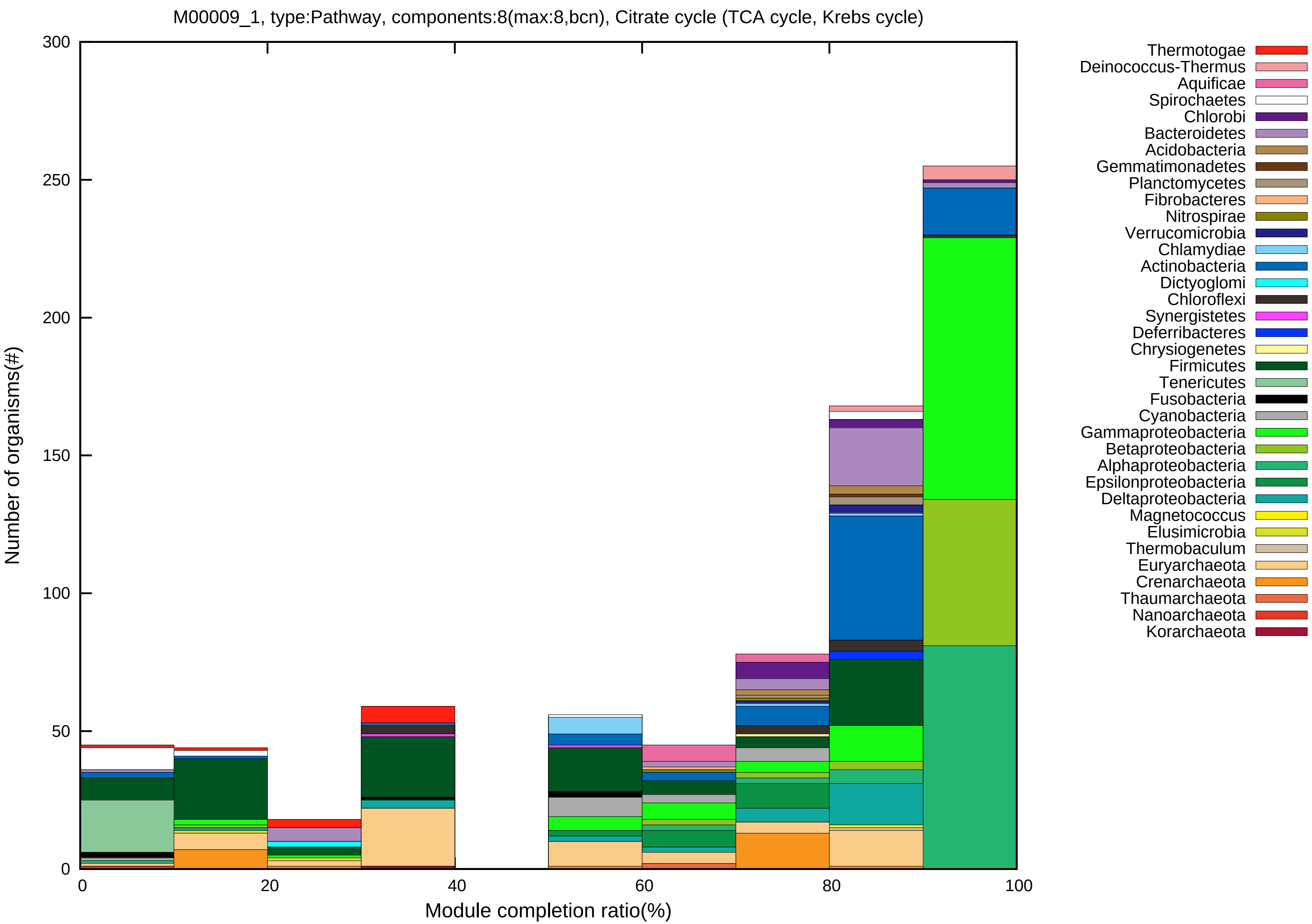
M00009_1, type:Pathway, components:8(max:8,bcn), Citrate cycle (TCA cycle, Krebs cycle)
Number of organisms(#)
Module completion ratio(%)
0
20
40
60
80
100
50
150
200
250
300
Thermotogae
Deinococcus-Thermus
Aquificae
Spirochaetes
Chlorobi
Bacteroidetes
Acidobacteria
Gemmatimonadetes
Planctomycetes
Fibrobacteres
Nitrospirae
Verrucomicrobia
Chlamydiae
Actinobacteria
Dictyoglomi
Chloroflexi
Synergistetes
Deferribacteres
Chrysiogenetes
Firmicutes
Tenericutes
Fusobacteria
Cyanobacteria
Gammaproteobacteria
Betaproteobacteria
Alphaproteobacteria
Epsilonproteobacteria
Deltaproteobacteria
Magnetococcus
Elusimicrobia
Thermobaculum
Euryarchaeota
Crenarchaeota
Thaumarchaeota
Nanoarchaeota
Korarchaeota

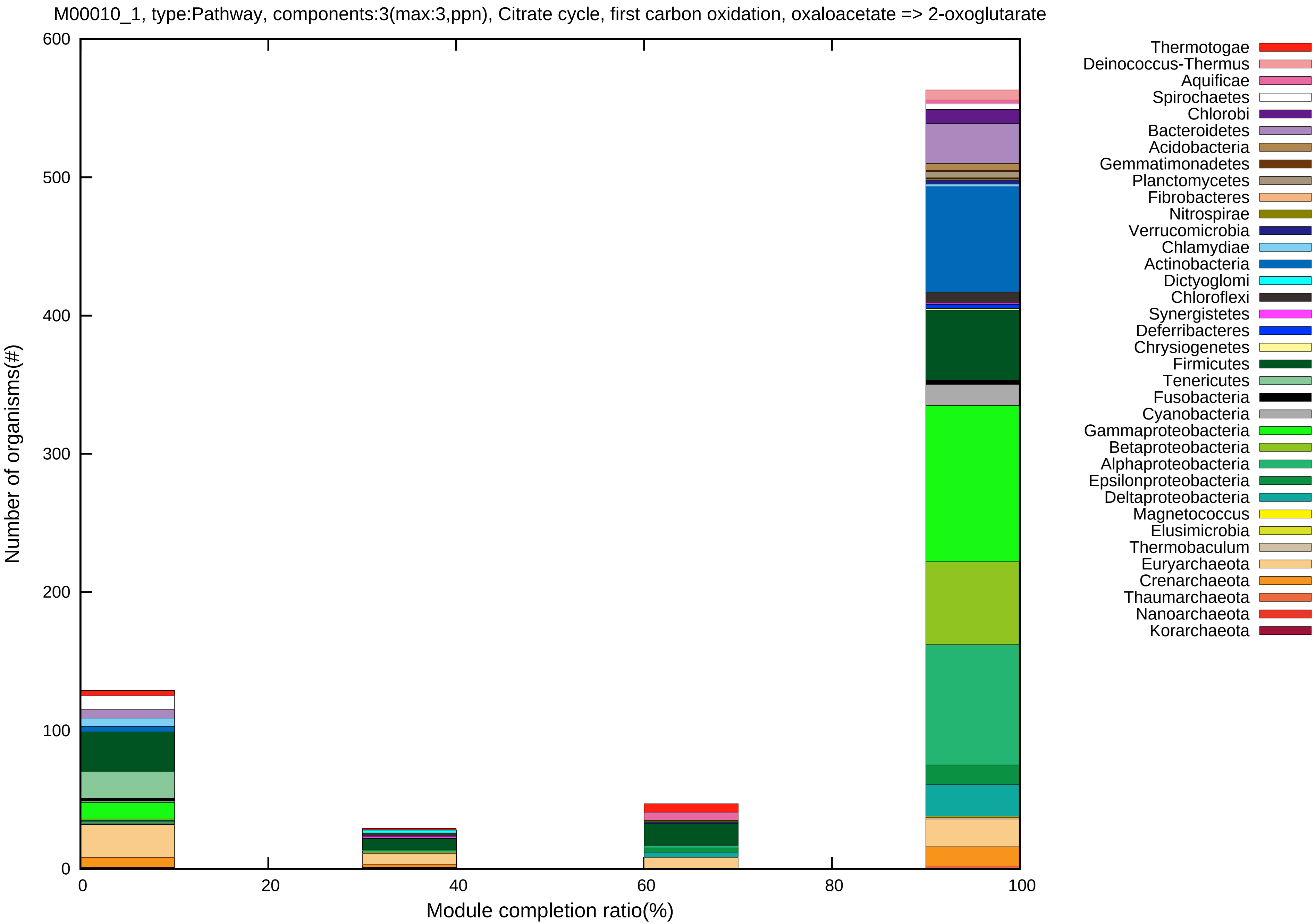
M00010_1, type:Pathway, components:3(max:3,ppn), Citrate cycle, first carbon oxidation, oxaloacetate => 2-oxoglutarate
Number of organisms(#)
Module completion ratio(%)
0
100
200
300
400
500
600
0
20
40
60
80
100
Thermotogae
Deinococcus-Thermus
Aquificae
Spirochaetes
Chlorobi
Bacteroidetes
Acidobacteria
Gemmatimonadetes
Planctomycetes
Fibrobacteres
Nitrospirae
Verrucomicrobia
Chlamydiae
Actinobacteria
Dictyoglomi
Chloroflexi
Synergistetes
Deferribacteres
Chrysiogenetes
Firmicutes
Tenericutes
Fusobacteria
Cyanobacteria
Gammaproteobacteria
Betaproteobacteria
Alphaproteobacteria
Epsilonproteobacteria
Deltaproteobacteria
Magnetococcus
Elusimicrobia
Thermobaculum
Euryarchaeota
Crenarchaeota
Thaumarchaeota
Nanoarchaeota
Korarchaeota

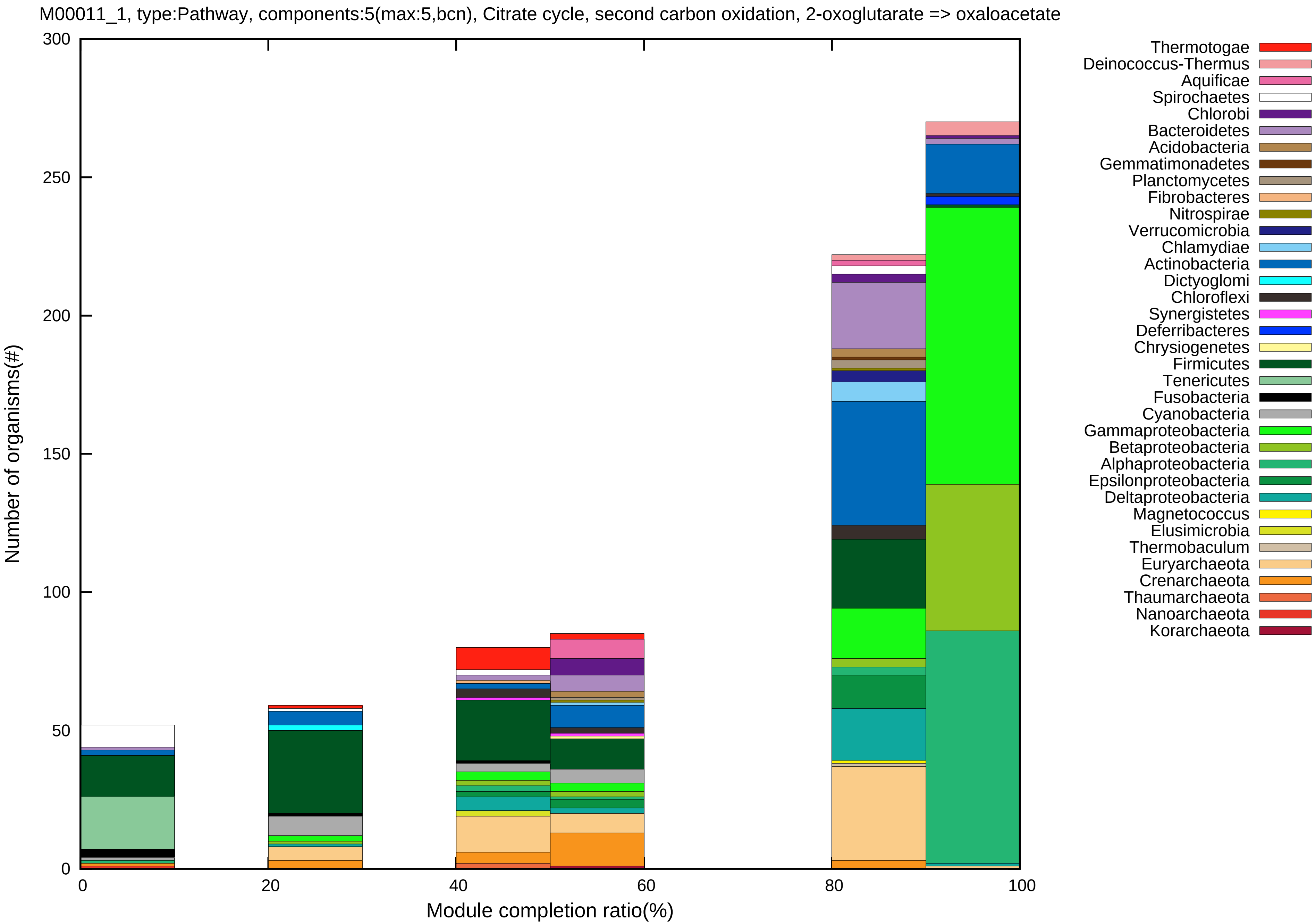
M00011_1, type:Pathway, components:5(max:5,bcn), Citrate cycle, second carbon oxidation, 2-oxoglutarate => oxaloacetate
Number of organisms(#)
Module completion ratio(%)
0
50
100
150
200
250
300
0
20
40
60
80
100
Thermotogae
Deinococcus-Thermus
Aquificae
Spirochaetes
Chlorobi
Bacteroidetes
Acidobacteria
Gemmatimonadetes
Planctomycetes
Fibrobacteres
Nitrospirae
Verrucomicrobia
Chlamydiae
Actinobacteria
Dictyoglomi
Chloroflexi
Synergistetes
Deferribacteres
Chrysiogenetes
Firmicutes
Tenericutes
Fusobacteria
Cyanobacteria
Gammaproteobacteria
Betaproteobacteria
Alphaproteobacteria
Epsilonproteobacteria
Deltaproteobacteria
Magnetococcus
Elusimicrobia
Thermobaculum
Euryarchaeota
Crenarchaeota
Thaumarchaeota
Nanoarchaeota
Korarchaeota

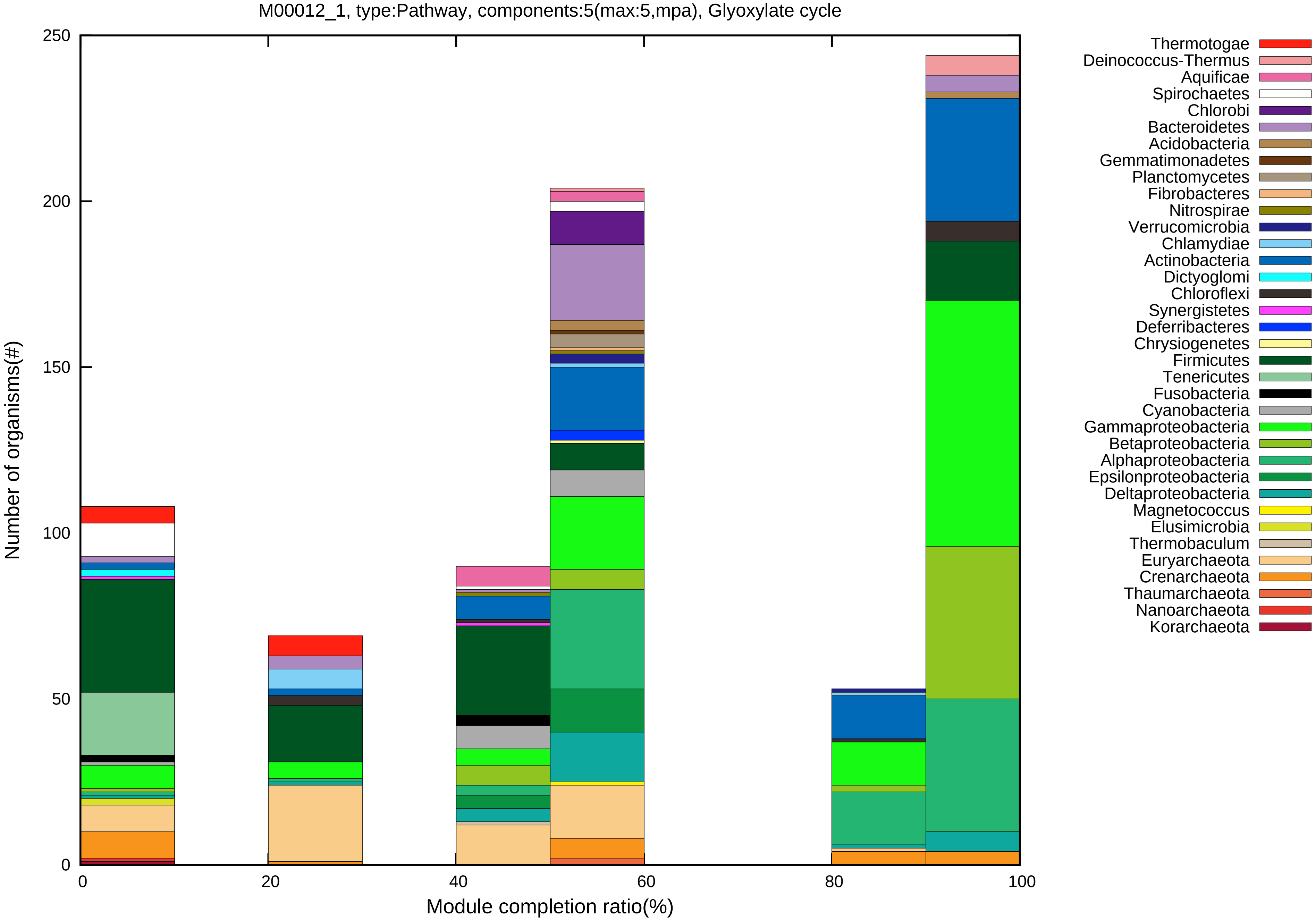

M00012_1, type:Pathway, components:5(max:5,mpa), Glyoxylate cycle
Number of organisms(#)
Module completion ratio(%)
0
50
100
150
200
250
0
20
40
60
80
100
Thermotogae
Deinococcus-Thermus
Aquificae
Spirochaetes
Chlorobi
Bacteroidetes
Acidobacteria
Gemmatimonadetes
Planctomycetes
Fibrobacteres
Nitrospirae
Verrucomicrobia
Chlamydiae
Actinobacteria
Dictyoglomi
Chloroflexi
Synergistetes
Deferribacteres
Chrysiogenetes
Firmicutes
Tenericutes
Fusobacteria
Cyanobacteria
Gammaproteobacteria
Betaproteobacteria
Alphaproteobacteria
Epsilonproteobacteria
Deltaproteobacteria
Magnetococcus
Elusimicrobia
Thermobaculum
Euryarchaeota
Crenarchaeota
Thaumarchaeota
Nanoarchaeota
Korarchaeota

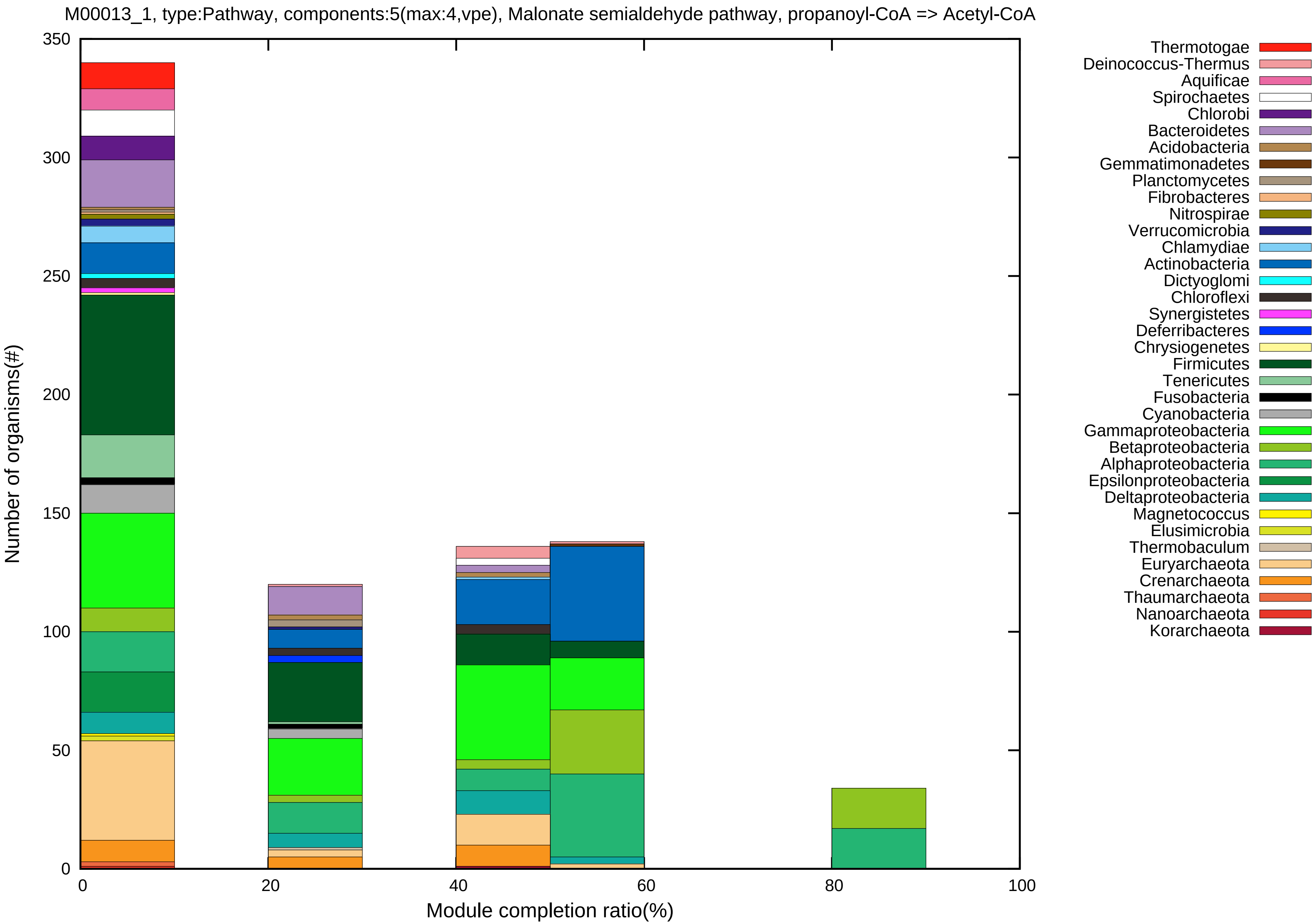

M00013_1, type:Pathway, components:5(max:4,vpe), Malonate semialdehyde pathway, propanoyl-CoA => Acetyl-CoA
Number of organisms(#)
Module completion ratio(%)
0
50
100
150
200
250
300
350
0
20
40
60
80
100
Thermotogae
Deinococcus-Thermus
Aquificae
Spirochaetes
Chlorobi
Bacteroidetes
Acidobacteria
Gemmatimonadetes
Planctomycetes
Fibrobacteres
Nitrospirae
Verrucomicrobia
Chlamydiae
Actinobacteria
Dictyoglomi
Chloroflexi
Synergistetes
Deferribacteres
Chrysiogenetes
Firmicutes
Tenericutes
Fusobacteria
Cyanobacteria
Gammaproteobacteria
Betaproteobacteria
Alphaproteobacteria
Epsilonproteobacteria
Deltaproteobacteria
Magnetococcus
Elusimicrobia
Thermobaculum
Euryarchaeota
Crenarchaeota
Thaumarchaeota
Nanoarchaeota
Korarchaeota

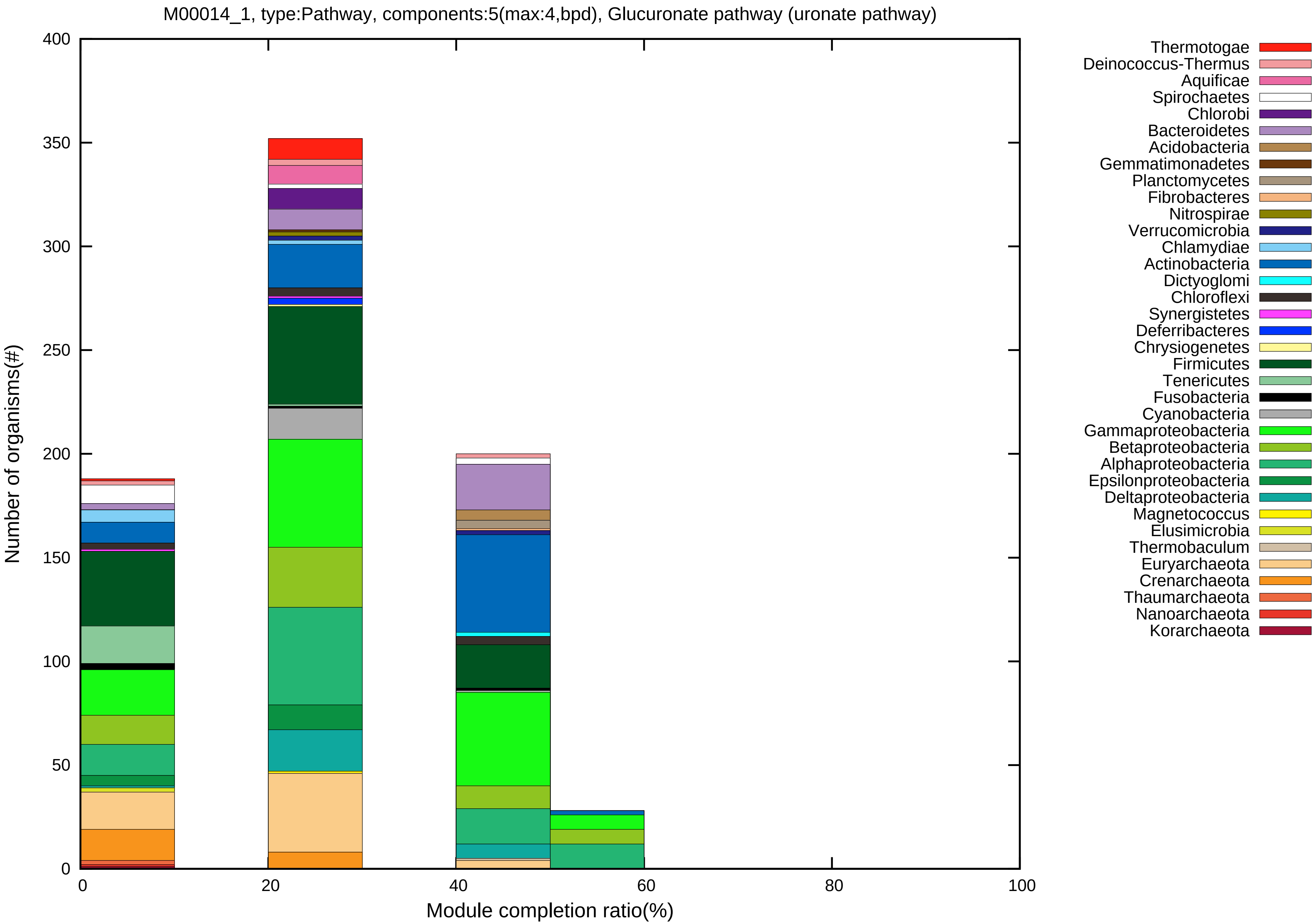

M00014_1, type:Pathway, components:5(max:4,bpd), Glucuronate pathway (uronate pathway)
Number of organisms(#)
Module completion ratio(%)
0
50
100
150
200
250
300
350
400
0
20
40
60
80
100
Thermotogae
Deinococcus-Thermus
Aquificae
Spirochaetes
Chlorobi
Bacteroidetes
Acidobacteria
Gemmatimonadetes
Planctomycetes
Fibrobacteres
Nitrospirae
Verrucomicrobia
Chlamydiae
Actinobacteria
Dictyoglomi
Chloroflexi
Synergistetes
Deferribacteres
Chrysiogenetes
Firmicutes
Tenericutes
Fusobacteria
Cyanobacteria
Gammaproteobacteria
Betaproteobacteria
Alphaproteobacteria
Epsilonproteobacteria
Deltaproteobacteria
Magnetococcus
Elusimicrobia
Thermobaculum
Euryarchaeota
Crenarchaeota
Thaumarchaeota
Nanoarchaeota
Korarchaeota

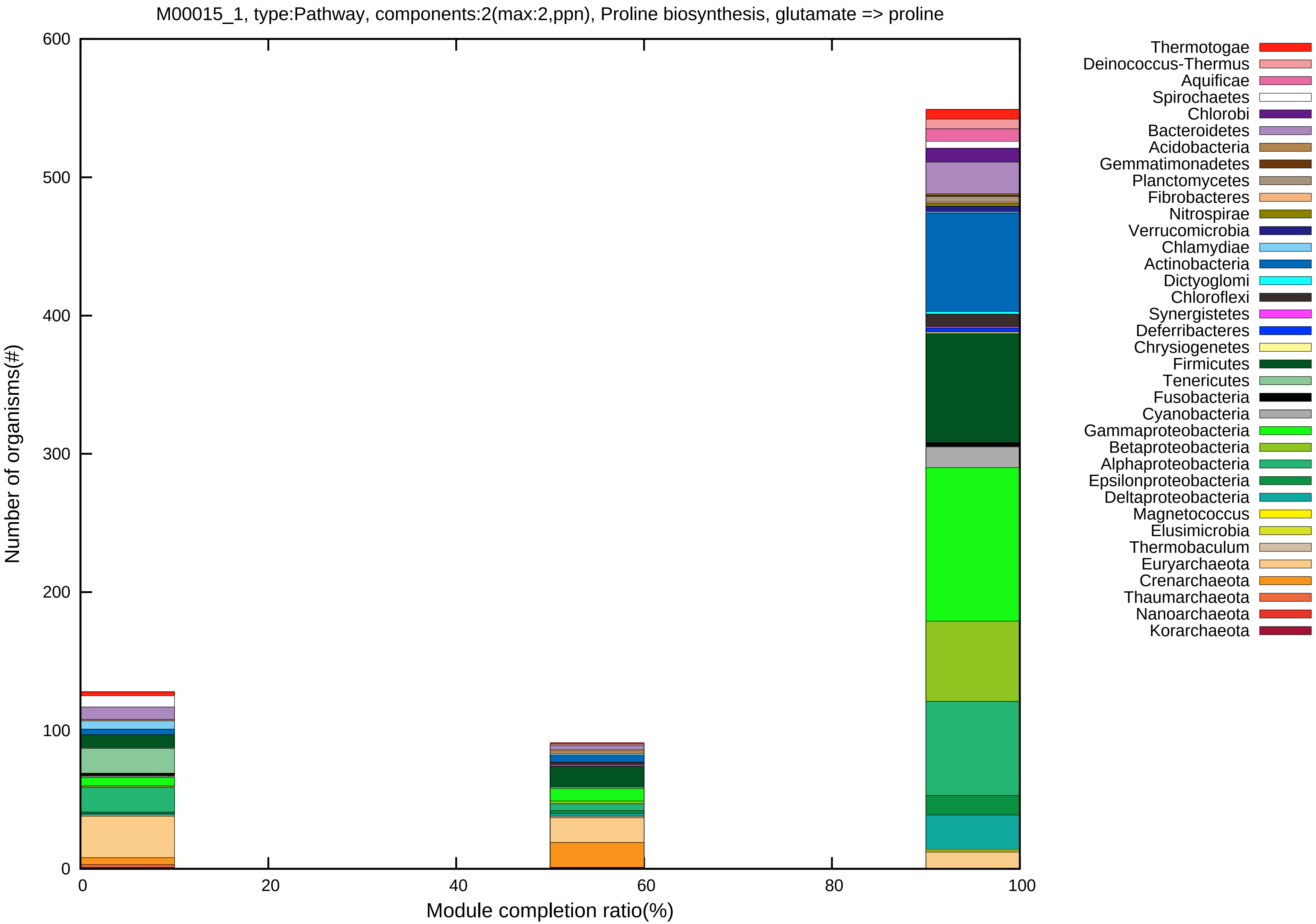

M00015_1, type:Pathway, components:2(max:2,ppn), Proline biosynthesis, glutamate => proline
Number of organisms(#)
Module completion ratio(%)
0
100
200
300
400
500
600
0
20
40
60
80
100
Thermotogae
Deinococcus-Thermus
Aquificae
Spirochaetes
Chlorobi
Bacteroidetes
Acidobacteria
Gemmatimonadetes
Planctomycetes
Fibrobacteres
Nitrospirae
Verrucomicrobia
Chlamydiae
Actinobacteria
Dictyoglomi
Chloroflexi
Synergistetes
Deferribacteres
Chrysiogenetes
Firmicutes
Tenericutes
Fusobacteria
Cyanobacteria
Gammaproteobacteria
Betaproteobacteria
Alphaproteobacteria
Epsilonproteobacteria
Deltaproteobacteria
Magnetococcus
Elusimicrobia
Thermobaculum
Euryarchaeota
Crenarchaeota
Thaumarchaeota
Nanoarchaeota
Korarchaeota

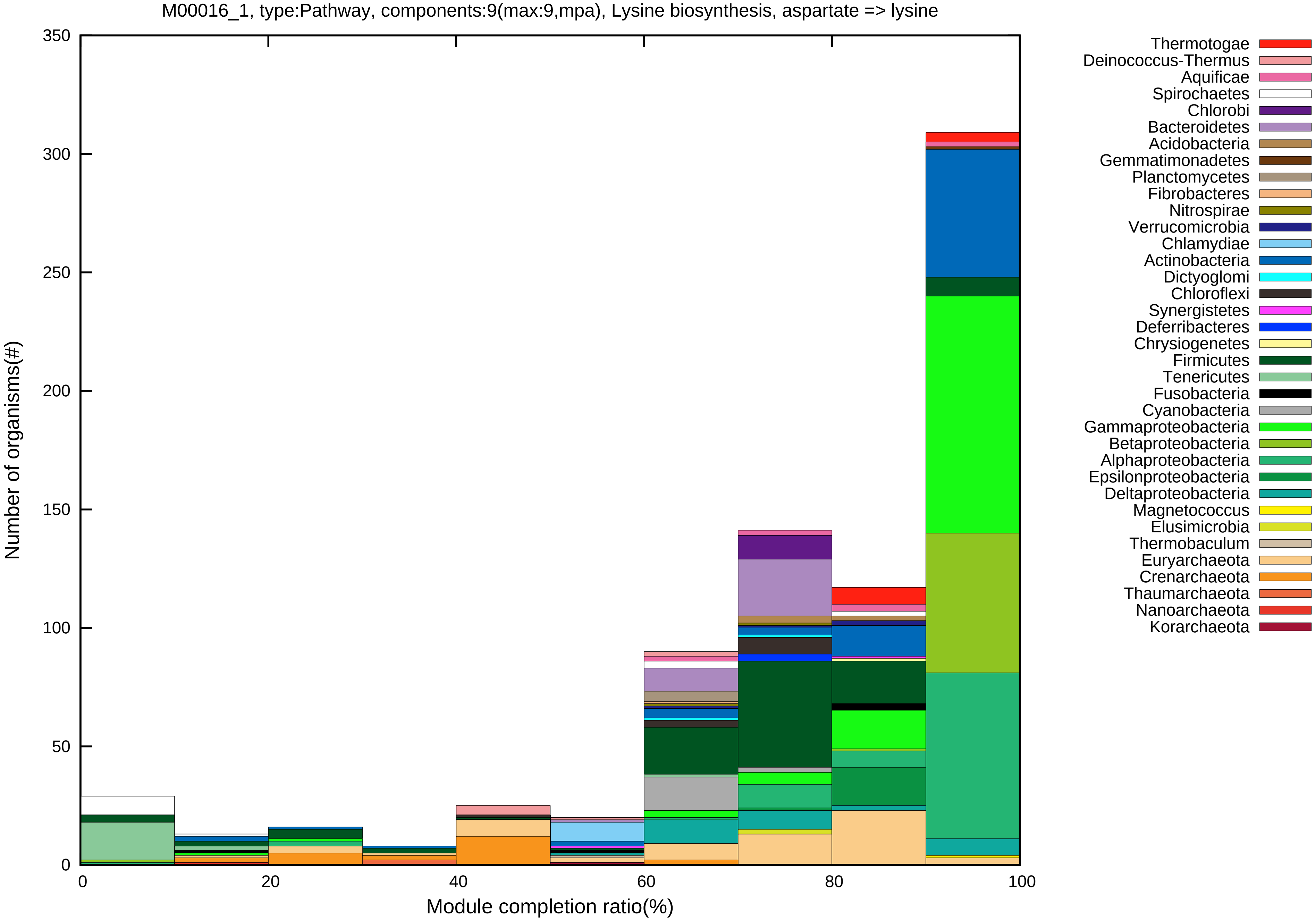

M00016_1, type:Pathway, components:9(max:9,mpa), Lysine biosynthesis, aspartate => lysine
Number of organisms(#)
Module completion ratio(%)
0
50
100
150
200
250
300
350
0
20
40
60
80
100
Thermotogae
Deinococcus-Thermus
Aquificae
Spirochaetes
Chlorobi
Bacteroidetes
Acidobacteria
Gemmatimonadetes
Planctomycetes
Fibrobacteres
Nitrospirae
Verrucomicrobia
Chlamydiae
Actinobacteria
Dictyoglomi
Chloroflexi
Synergistetes
Deferribacteres
Chrysiogenetes
Firmicutes
Tenericutes
Fusobacteria
Cyanobacteria
Gammaproteobacteria
Betaproteobacteria
Alphaproteobacteria
Epsilonproteobacteria
Deltaproteobacteria
Magnetococcus
Elusimicrobia
Thermobaculum
Euryarchaeota
Crenarchaeota
Thaumarchaeota
Nanoarchaeota
Korarchaeota

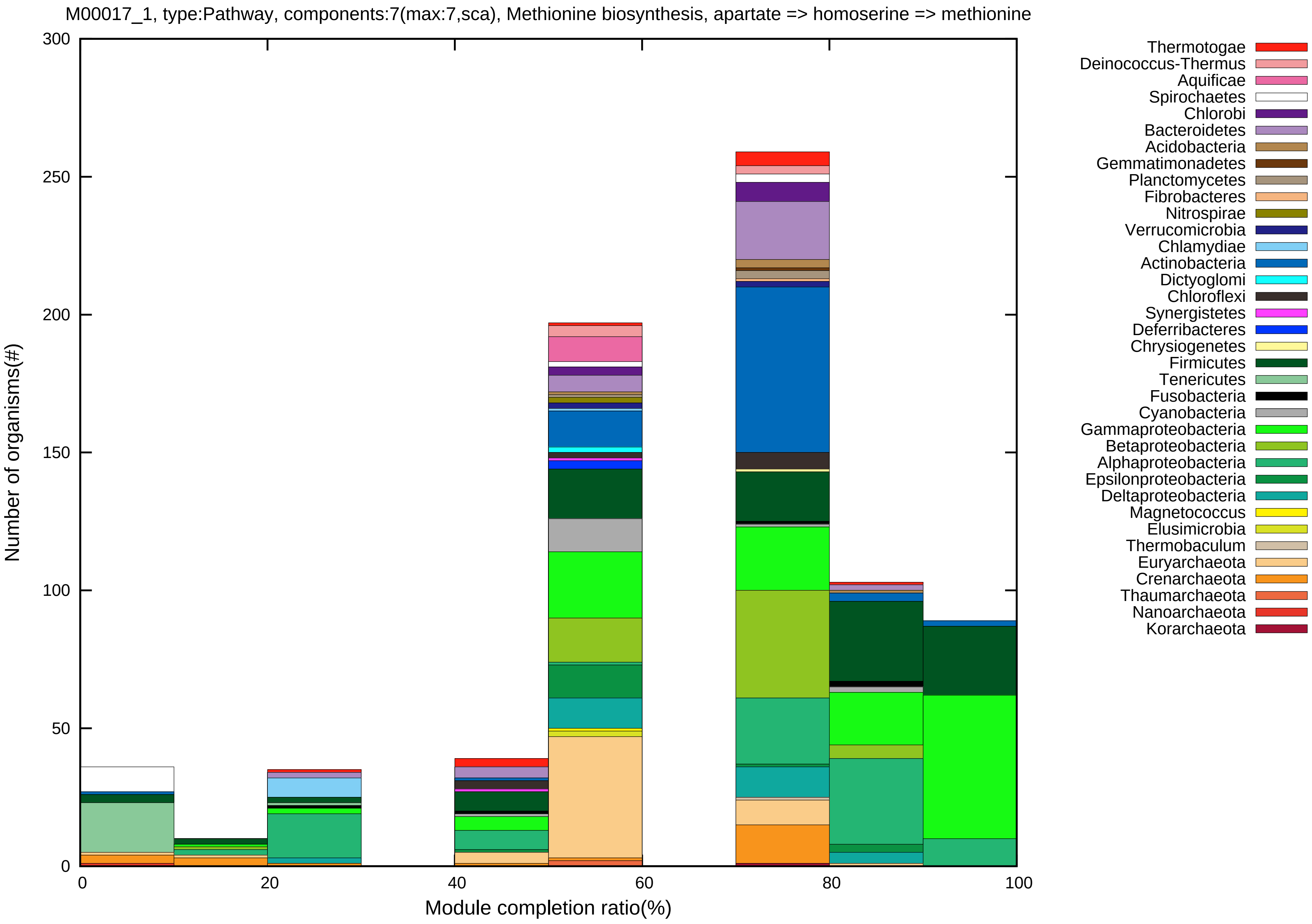
M00017_1, type:Pathway, components:7(max:7,sca), Methionine biosynthesis, apartate => homoserine => methionine
Number of organisms(#)
Module completion ratio(%)
0
50
100
150
200
250
300
0
20
40
60
80
100
Thermotogae
Deinococcus-Thermus
Aquificae
Spirochaetes
Chlorobi
Bacteroidetes
Acidobacteria
Gemmatimonadetes
Planctomycetes
Fibrobacteres
Nitrospirae
Verrucomicrobia
Chlamydiae
Actinobacteria
Dictyoglomi
Chloroflexi
Synergistetes
Deferribacteres
Chrysiogenetes
Firmicutes
Tenericutes
Fusobacteria
Cyanobacteria
Gammaproteobacteria
Betaproteobacteria
Alphaproteobacteria
Epsilonproteobacteria
Deltaproteobacteria
Magnetococcus
Elusimicrobia
Thermobaculum
Euryarchaeota
Crenarchaeota
Thaumarchaeota
Nanoarchaeota
Korarchaeota

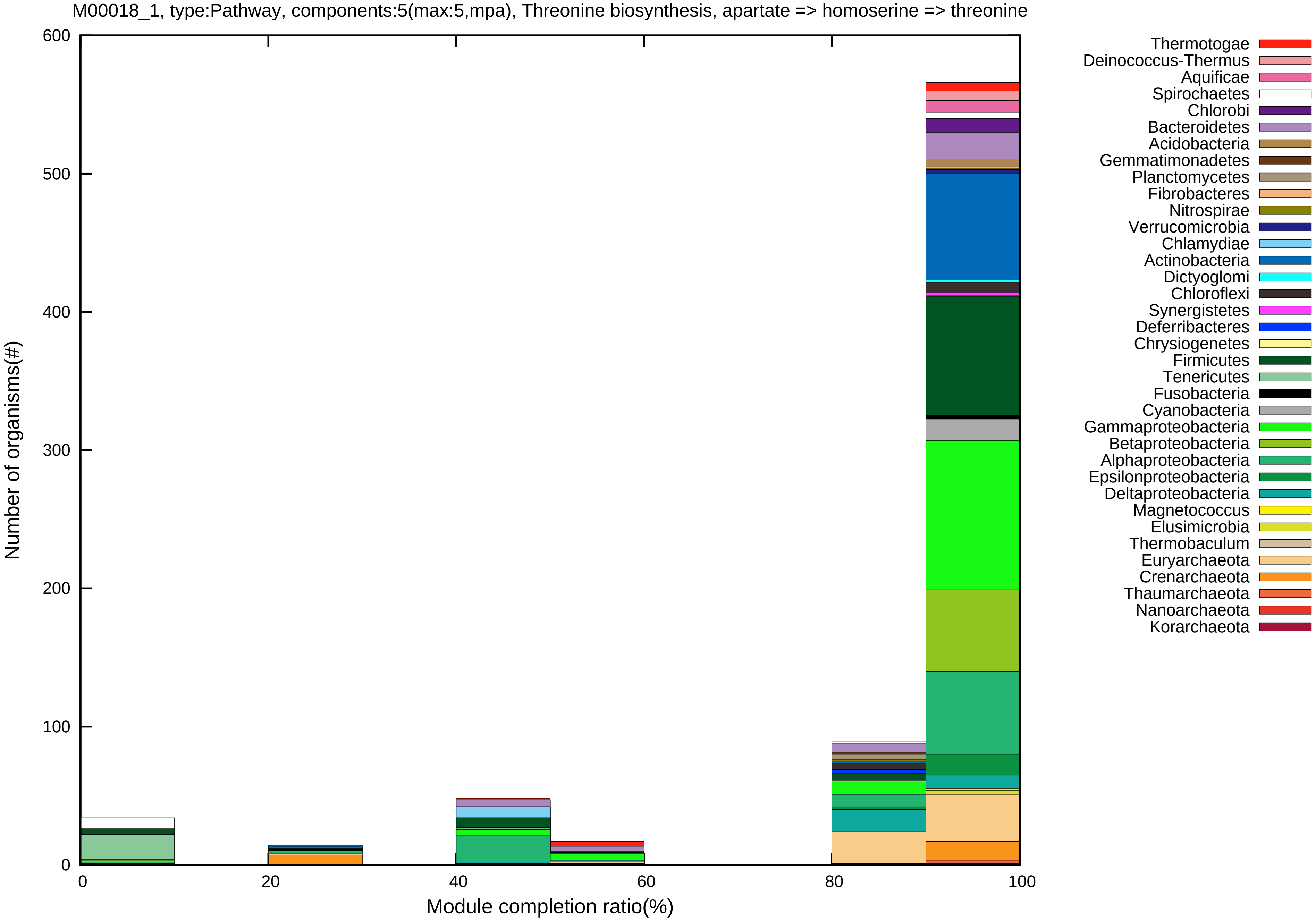
M00018_1, type:Pathway, components:5(max:5,mpa), Threonine biosynthesis, apartate => homoserine => threonine
Number of organisms(#)
Module completion ratio(%)
0
100
200
300
400
500
600
0
20
40
60
80
100
Thermotogae
Deinococcus-Thermus
Aquificae
Spirochaetes
Chlorobi
Bacteroidetes
Acidobacteria
Gemmatimonadetes
Planctomycetes
Fibrobacteres
Nitrospirae
Verrucomicrobia
Chlamydiae
Actinobacteria
Dictyoglomi
Chloroflexi
Synergistetes
Deferribacteres
Chrysiogenetes
Firmicutes
Tenericutes
Fusobacteria
Cyanobacteria
Gammaproteobacteria
Betaproteobacteria
Alphaproteobacteria
Epsilonproteobacteria
Deltaproteobacteria
Magnetococcus
Elusimicrobia
Thermobaculum
Euryarchaeota
Crenarchaeota
Thaumarchaeota
Nanoarchaeota
Korarchaeota

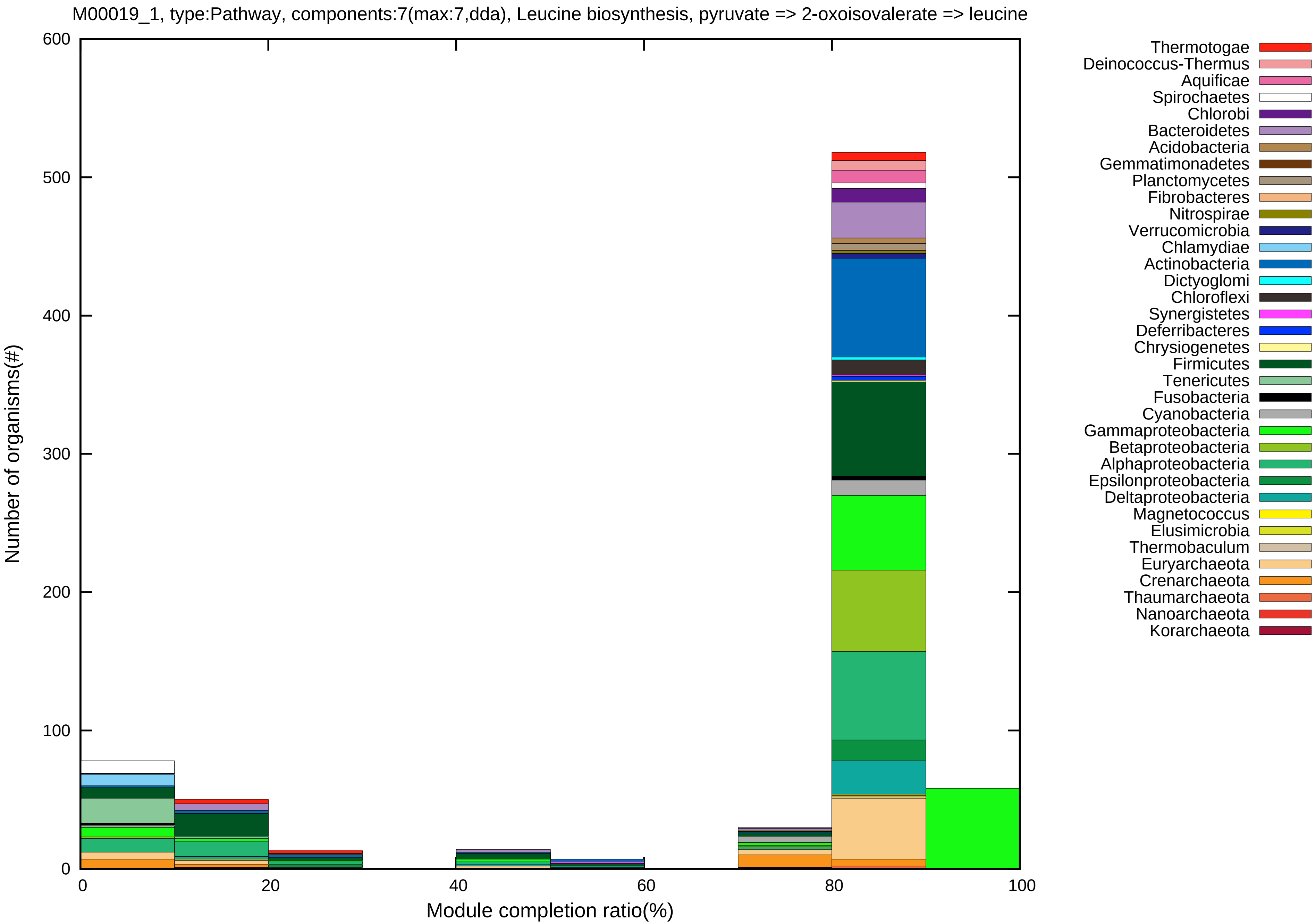
M00019_1, type:Pathway, components:7(max:7,dda), Leucine biosynthesis, pyruvate => 2-oxoisovalerate => leucine
Number of organisms(#)
Module completion ratio(%)
0
100
200
300
400
500
600
0
20
40
60
80
100
Thermotogae
Deinococcus-Thermus
Aquificae
Spirochaetes
Chlorobi
Bacteroidetes
Acidobacteria
Gemmatimonadetes
Planctomycetes
Fibrobacteres
Nitrospirae
Verrucomicrobia
Chlamydiae
Actinobacteria
Dictyoglomi
Chloroflexi
Synergistetes
Deferribacteres
Chrysiogenetes
Firmicutes
Tenericutes
Fusobacteria
Cyanobacteria
Gammaproteobacteria
Betaproteobacteria
Alphaproteobacteria
Epsilonproteobacteria
Deltaproteobacteria
Magnetococcus
Elusimicrobia
Thermobaculum
Euryarchaeota
Crenarchaeota
Thaumarchaeota
Nanoarchaeota
Korarchaeota

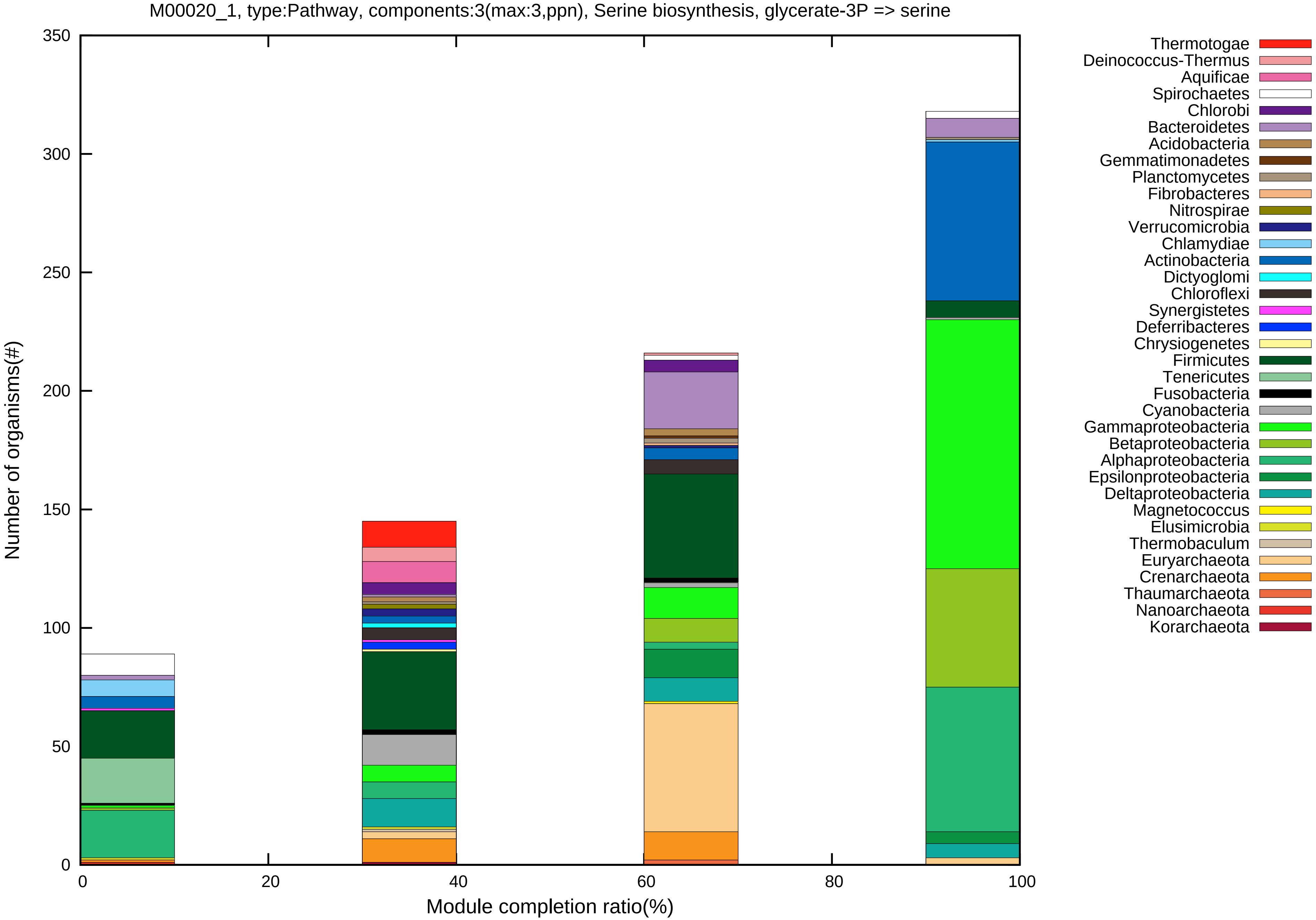

M00020_1, type:Pathway, components:3(max:3,ppn), Serine biosynthesis, glycerate-3P => serine
Number of organisms(#)
Module completion ratio(%)
0
50
100
150
200
250
300
350
0
20
40
60
80
100
Thermotogae
Deinococcus-Thermus
Aquificae
Spirochaetes
Chlorobi
Bacteroidetes
Acidobacteria
Gemmatimonadetes
Planctomycetes
Fibrobacteres
Nitrospirae
Verrucomicrobia
Chlamydiae
Actinobacteria
Dictyoglomi
Chloroflexi
Synergistetes
Deferribacteres
Chrysiogenetes
Firmicutes
Tenericutes
Fusobacteria
Cyanobacteria
Gammaproteobacteria
Betaproteobacteria
Alphaproteobacteria
Epsilonproteobacteria
Deltaproteobacteria
Magnetococcus
Elusimicrobia
Thermobaculum
Euryarchaeota
Crenarchaeota
Thaumarchaeota
Nanoarchaeota
Korarchaeota

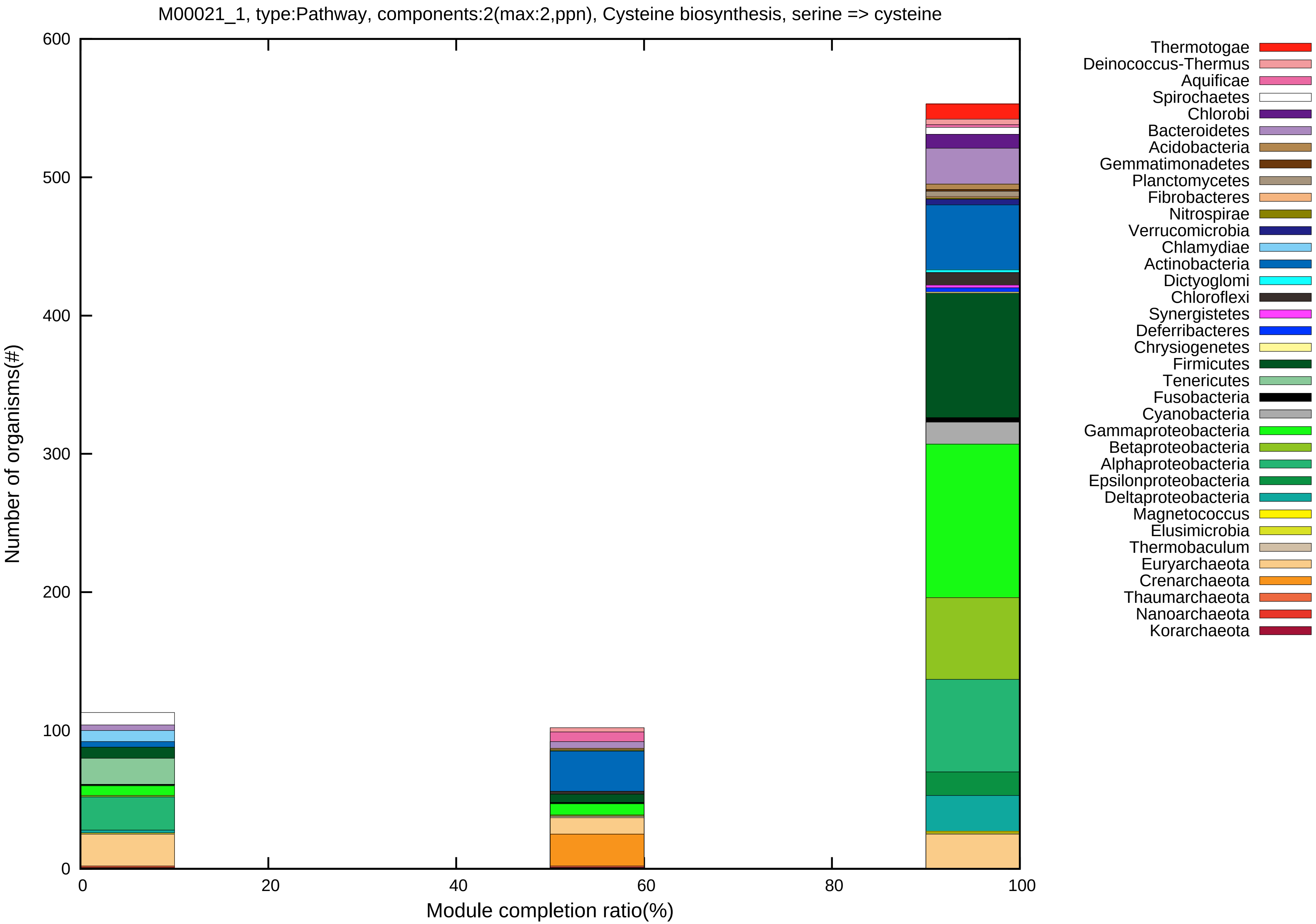
M00021_1, type:Pathway, components:2(max:2,ppn), Cysteine biosynthesis, serine => cysteine
Number of organisms(#)
Module completion ratio(%)
0
100
200
300
400
500
600
0
20
40
60
80
100
Thermotogae
Deinococcus-Thermus
Aquificae
Spirochaetes
Chlorobi
Bacteroidetes
Acidobacteria
Gemmatimonadetes
Planctomycetes
Fibrobacteres
Nitrospirae
Verrucomicrobia
Chlamydiae
Actinobacteria
Dictyoglomi
Chloroflexi
Synergistetes
Deferribacteres
Chrysiogenetes
Firmicutes
Tenericutes
Fusobacteria
Cyanobacteria
Gammaproteobacteria
Betaproteobacteria
Alphaproteobacteria
Epsilonproteobacteria
Deltaproteobacteria
Magnetococcus
Elusimicrobia
Thermobaculum
Euryarchaeota
Crenarchaeota
Thaumarchaeota
Nanoarchaeota
Korarchaeota

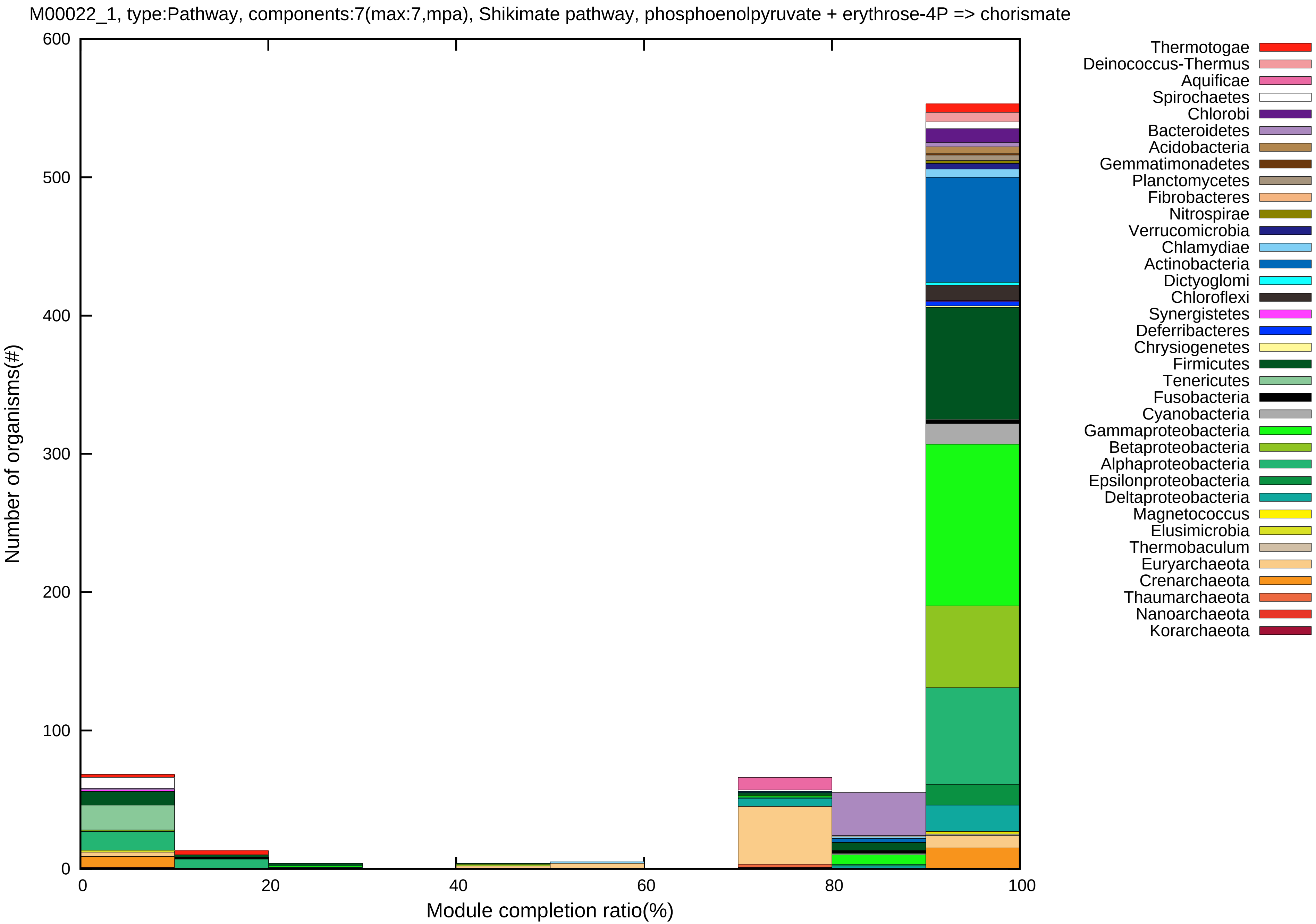

M00022_1, type:Pathway, components:7(max:7,mpa), Shikimate pathway, phosphoenolpyruvate + erythrose-4P => chorismate
Number of organisms(#)
Module completion ratio(%)
0
100
200
300
400
500
600
0
20
40
60
80
100
Thermotogae
Deinococcus-Thermus
Aquificae
Spirochaetes
Chlorobi
Bacteroidetes
Acidobacteria
Gemmatimonadetes
Planctomycetes
Fibrobacteres
Nitrospirae
Verrucomicrobia
Chlamydiae
Actinobacteria
Dictyoglomi
Chloroflexi
Synergistetes
Deferribacteres
Chrysiogenetes
Firmicutes
Tenericutes
Fusobacteria
Cyanobacteria
Gammaproteobacteria
Betaproteobacteria
Alphaproteobacteria
Epsilonproteobacteria
Deltaproteobacteria
Magnetococcus
Elusimicrobia
Thermobaculum
Euryarchaeota
Crenarchaeota
Thaumarchaeota
Nanoarchaeota
Korarchaeota

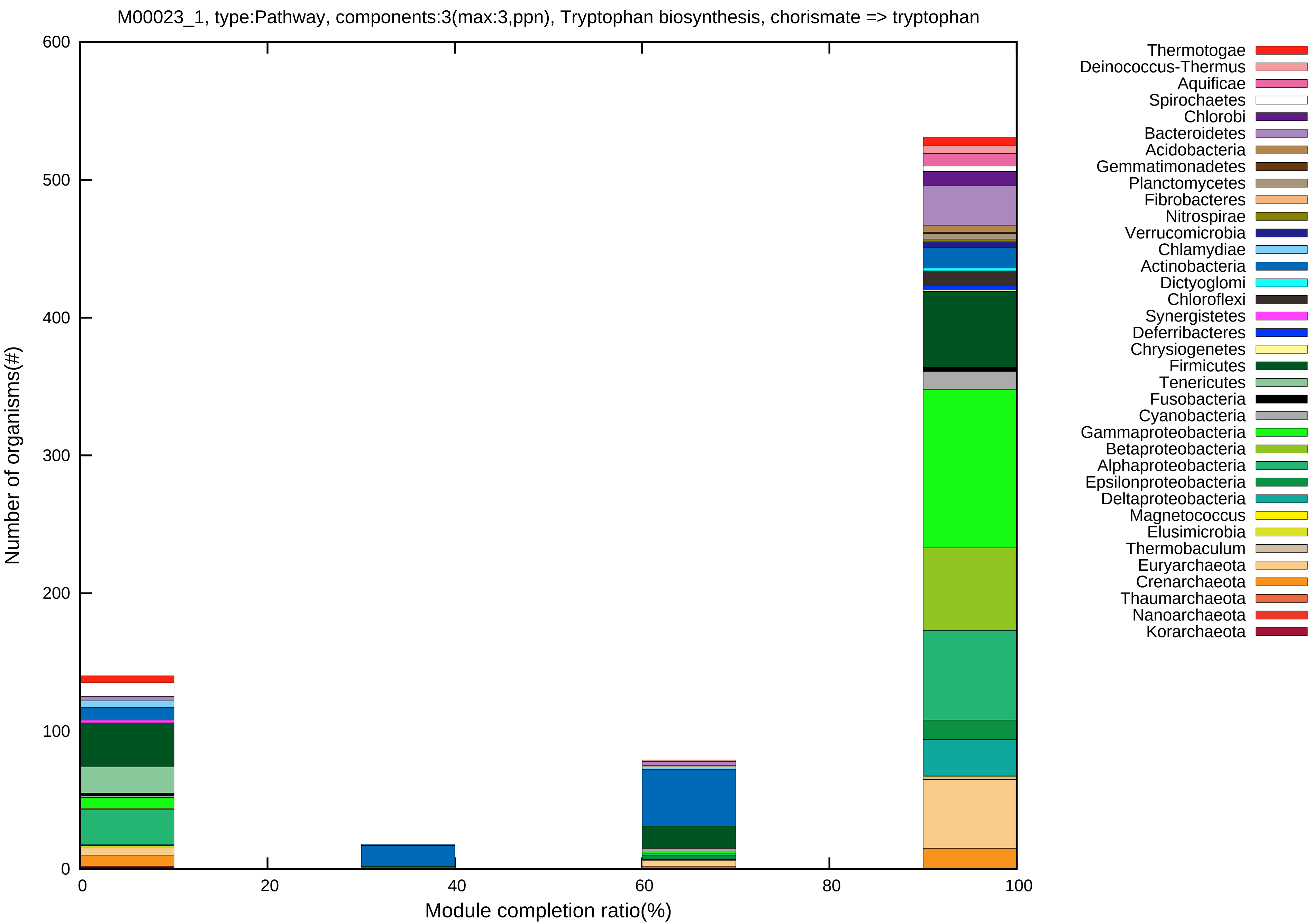

M00023_1, type:Pathway, components:3(max:3,ppn), Tryptophan biosynthesis, chorismate => tryptophan
Number of organisms(#)
Module completion ratio(%)
0
100
200
300
400
500
600
0
20
40
60
80
100
Thermotogae
Deinococcus-Thermus
Aquificae
Spirochaetes
Chlorobi
Bacteroidetes
Acidobacteria
Gemmatimonadetes
Planctomycetes
Fibrobacteres
Nitrospirae
Verrucomicrobia
Chlamydiae
Actinobacteria
Dictyoglomi
Chloroflexi
Synergistetes
Deferribacteres
Chrysiogenetes
Firmicutes
Tenericutes
Fusobacteria
Cyanobacteria
Gammaproteobacteria
Betaproteobacteria
Alphaproteobacteria
Epsilonproteobacteria
Deltaproteobacteria
Magnetococcus
Elusimicrobia
Thermobaculum
Euryarchaeota
Crenarchaeota
Thaumarchaeota
Nanoarchaeota
Korarchaeota

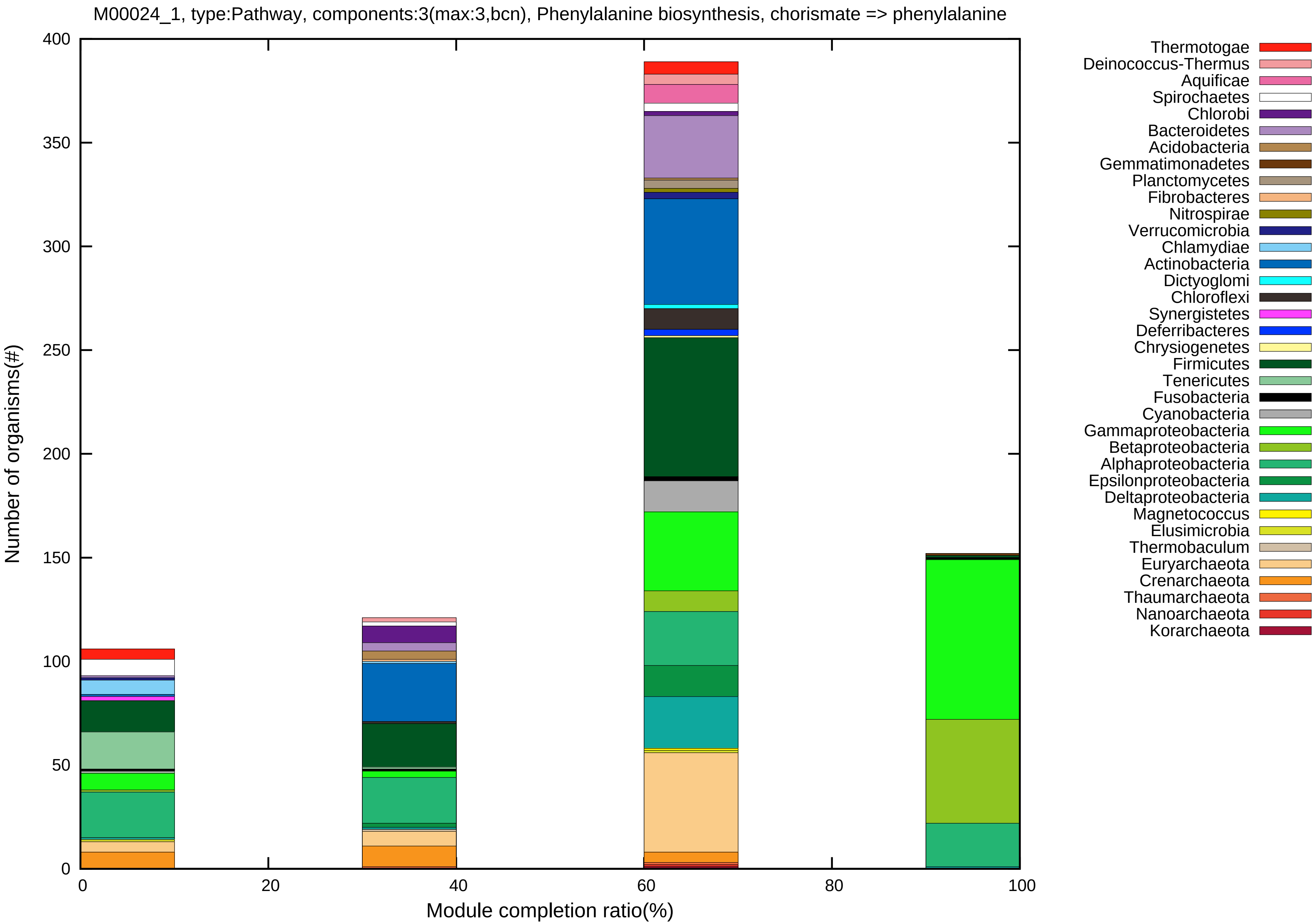
M00024_1, type:Pathway, components:3(max:3,bcn), Phenylalanine biosynthesis, chorismate => phenylalanine
Number of organisms(#)
Module completion ratio(%)
0
20
40
60
80
100
50
150
200
250
300
350
400
Thermotogae
Deinococcus-Thermus
Aquificae
Spirochaetes
Chlorobi
Bacteroidetes
Acidobacteria
Gemmatimonadetes
Planctomycetes
Fibrobacteres
Nitrospirae
Verrucomicrobia
Chlamydiae
Actinobacteria
Dictyoglomi
Chloroflexi
Synergistetes
Deferribacteres
Chrysiogenetes
Firmicutes
Tenericutes
Fusobacteria
Cyanobacteria
Gammaproteobacteria
Betaproteobacteria
Alphaproteobacteria
Epsilonproteobacteria
Deltaproteobacteria
Magnetococcus
Elusimicrobia
Thermobaculum
Euryarchaeota
Crenarchaeota
Thaumarchaeota
Nanoarchaeota
Korarchaeota

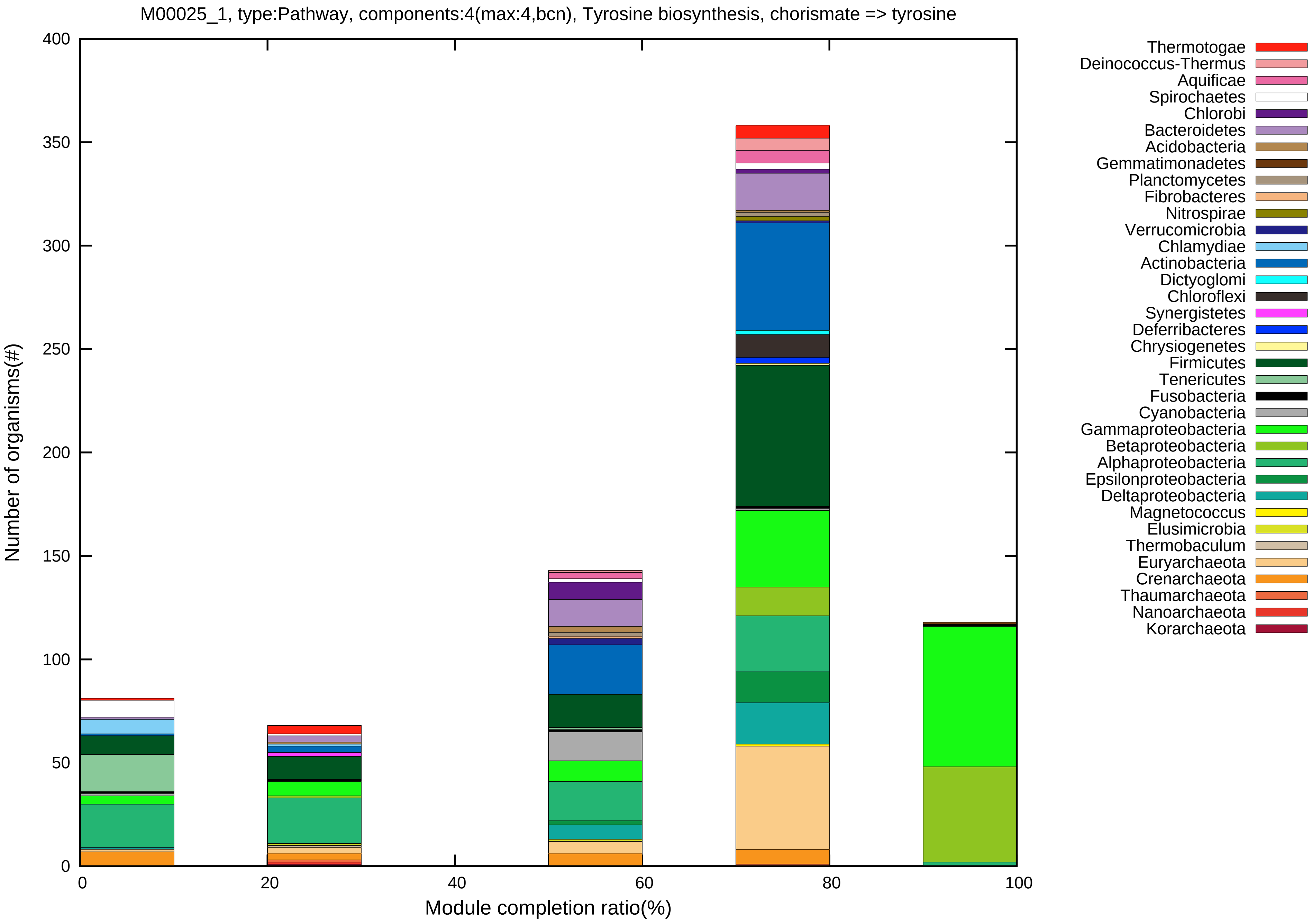
M00025_1, type:Pathway, components:4(max:4,bcn), Tyrosine biosynthesis, chorismate => tyrosine
Number of organisms(#)
Module completion ratio(%)
0
50
100
150
200
250
300
350
400
0
20
40
60
80
100
Thermotogae
Deinococcus-Thermus
Aquificae
Spirochaetes
Chlorobi
Bacteroidetes
Acidobacteria
Gemmatimonadetes
Planctomycetes
Fibrobacteres
Nitrospirae
Verrucomicrobia
Chlamydiae
Actinobacteria
Dictyoglomi
Chloroflexi
Synergistetes
Deferribacteres
Chrysiogenetes
Firmicutes
Tenericutes
Fusobacteria
Cyanobacteria
Gammaproteobacteria
Betaproteobacteria
Alphaproteobacteria
Epsilonproteobacteria
Deltaproteobacteria
Magnetococcus
Elusimicrobia
Thermobaculum
Euryarchaeota
Crenarchaeota
Thaumarchaeota
Nanoarchaeota
Korarchaeota

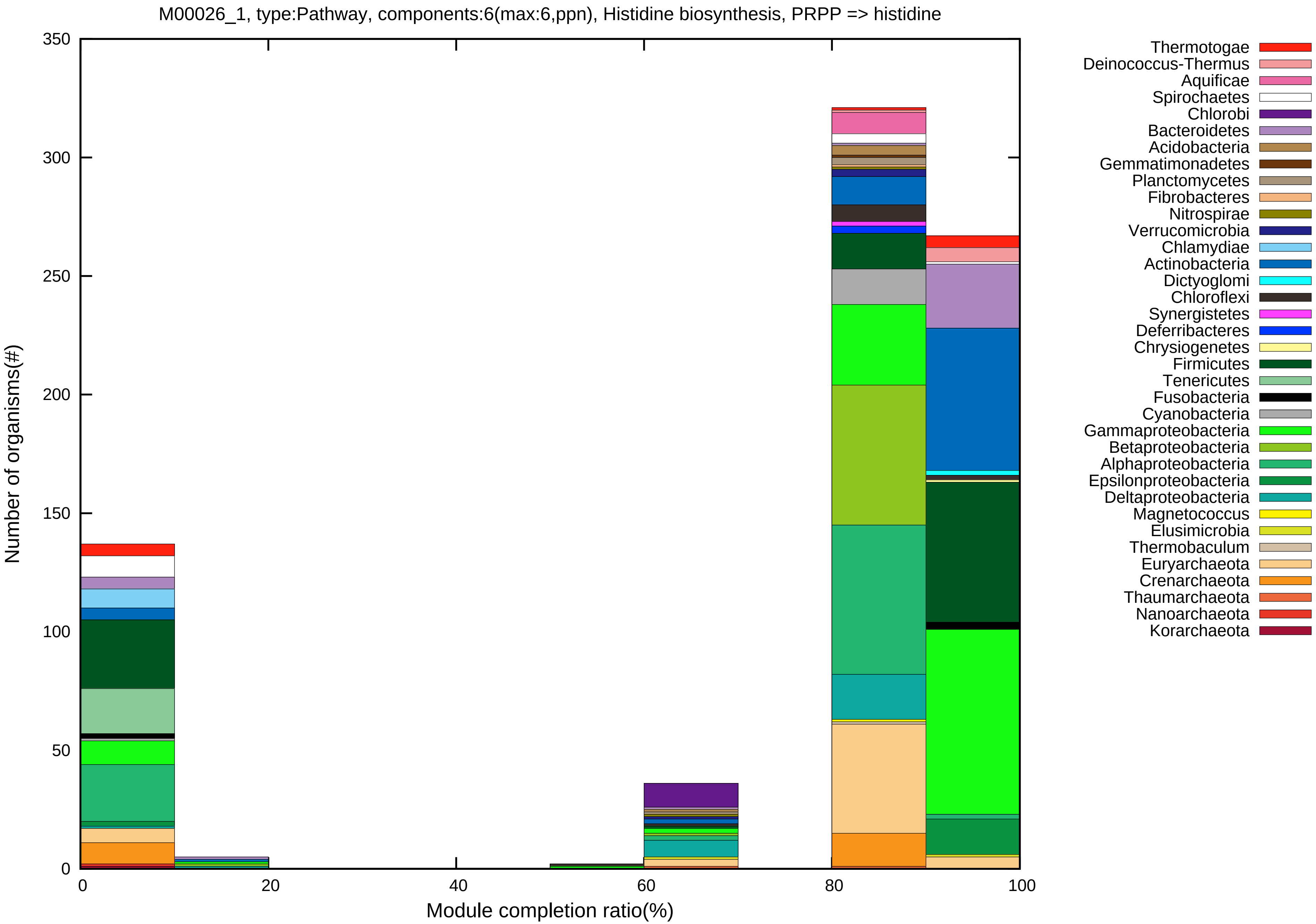

M00026_1, type:Pathway, components:6(max:6,ppn), Histidine biosynthesis, PRPP => histidine
Number of organisms(#)
Module completion ratio(%)
0
50
100
150
200
250
300
350
0
20
40
60
80
100
Thermotogae
Deinococcus-Thermus
Aquificae
Spirochaetes
Chlorobi
Bacteroidetes
Acidobacteria
Gemmatimonadetes
Planctomycetes
Fibrobacteres
Nitrospirae
Verrucomicrobia
Chlamydiae
Actinobacteria
Dictyoglomi
Chloroflexi
Synergistetes
Deferribacteres
Chrysiogenetes
Firmicutes
Tenericutes
Fusobacteria
Cyanobacteria
Gammaproteobacteria
Betaproteobacteria
Alphaproteobacteria
Epsilonproteobacteria
Deltaproteobacteria
Magnetococcus
Elusimicrobia
Thermobaculum
Euryarchaeota
Crenarchaeota
Thaumarchaeota
Nanoarchaeota
Korarchaeota

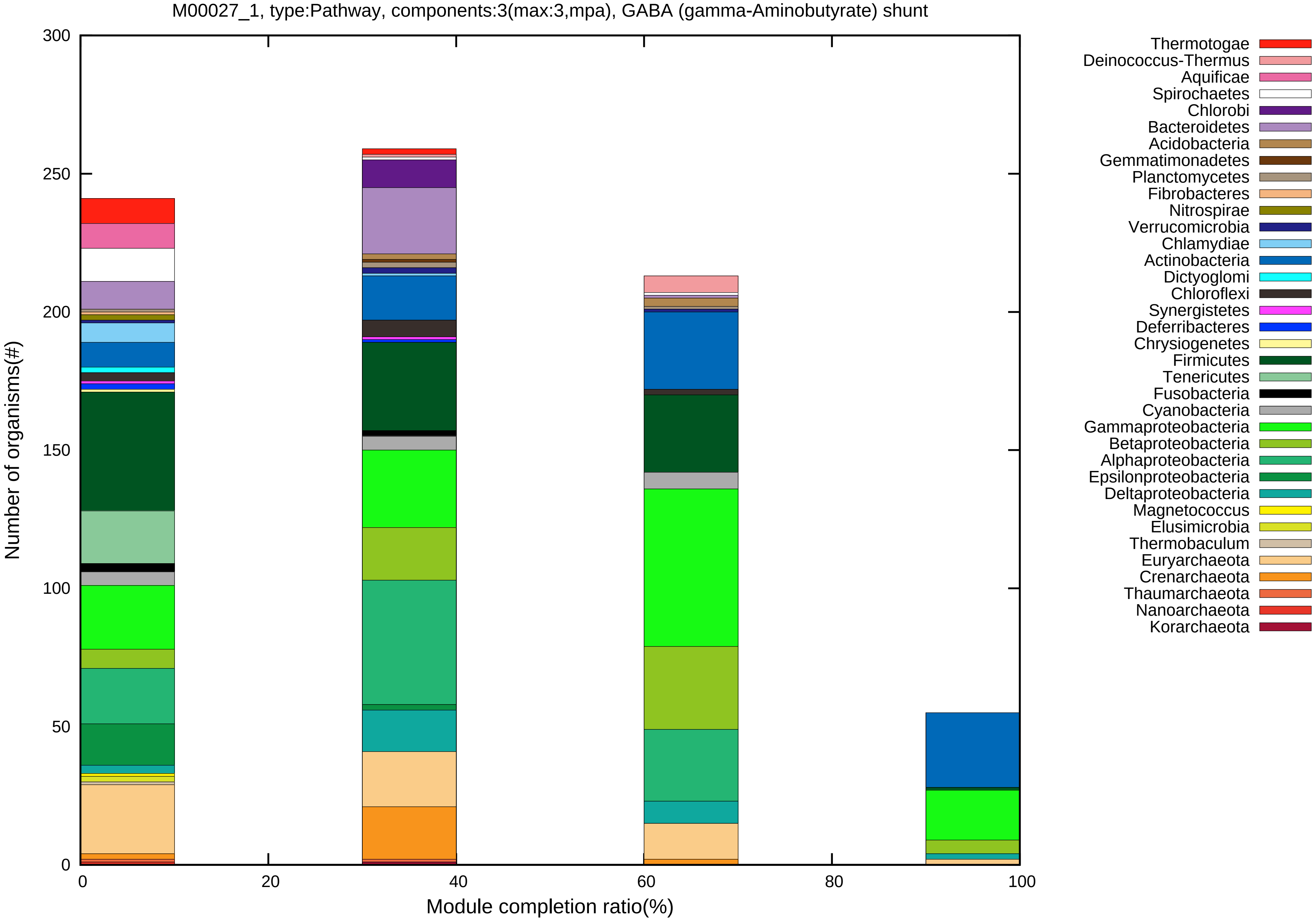

M00027_1, type:Pathway, components:3(max:3,mpa), GABA (gamma-Aminobutyrate) shunt
Number of organisms(#)
Module completion ratio(%)
0
50
100
150
200
250
300
0
20
40
60
80
100
Thermotogae
Deinococcus-Thermus
Aquificae
Spirochaetes
Chlorobi
Bacteroidetes
Acidobacteria
Gemmatimonadetes
Planctomycetes
Fibrobacteres
Nitrospirae
Verrucomicrobia
Chlamydiae
Actinobacteria
Dictyoglomi
Chloroflexi
Synergistetes
Deferribacteres
Chrysiogenetes
Firmicutes
Tenericutes
Fusobacteria
Cyanobacteria
Gammaproteobacteria
Betaproteobacteria
Alphaproteobacteria
Epsilonproteobacteria
Deltaproteobacteria
Magnetococcus
Elusimicrobia
Thermobaculum
Euryarchaeota
Crenarchaeota
Thaumarchaeota
Nanoarchaeota
Korarchaeota

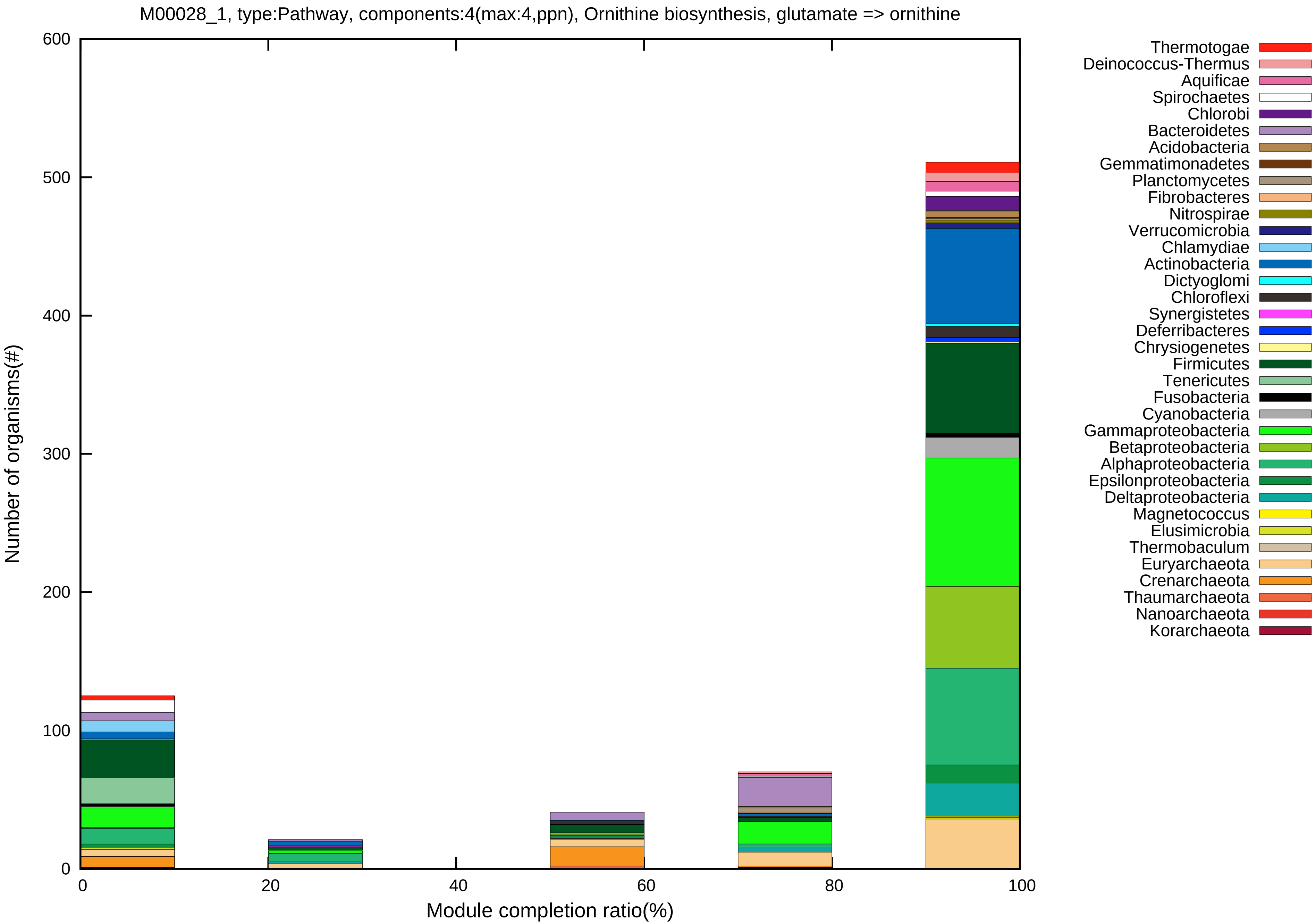

M00028_1, type:Pathway, components:4(max:4,ppn), Ornithine biosynthesis, glutamate => ornithine
Number of organisms(#)
Module completion ratio(%)
0
100
200
300
400
500
600
0
20
40
60
80
100
Thermotogae
Deinococcus-Thermus
Aquificae
Spirochaetes
Chlorobi
Bacteroidetes
Acidobacteria
Gemmatimonadetes
Planctomycetes
Fibrobacteres
Nitrospirae
Verrucomicrobia
Chlamydiae
Actinobacteria
Dictyoglomi
Chloroflexi
Synergistetes
Deferribacteres
Chrysiogenetes
Firmicutes
Tenericutes
Fusobacteria
Cyanobacteria
Gammaproteobacteria
Betaproteobacteria
Alphaproteobacteria
Epsilonproteobacteria
Deltaproteobacteria
Magnetococcus
Elusimicrobia
Thermobaculum
Euryarchaeota
Crenarchaeota
Thaumarchaeota
Nanoarchaeota
Korarchaeota

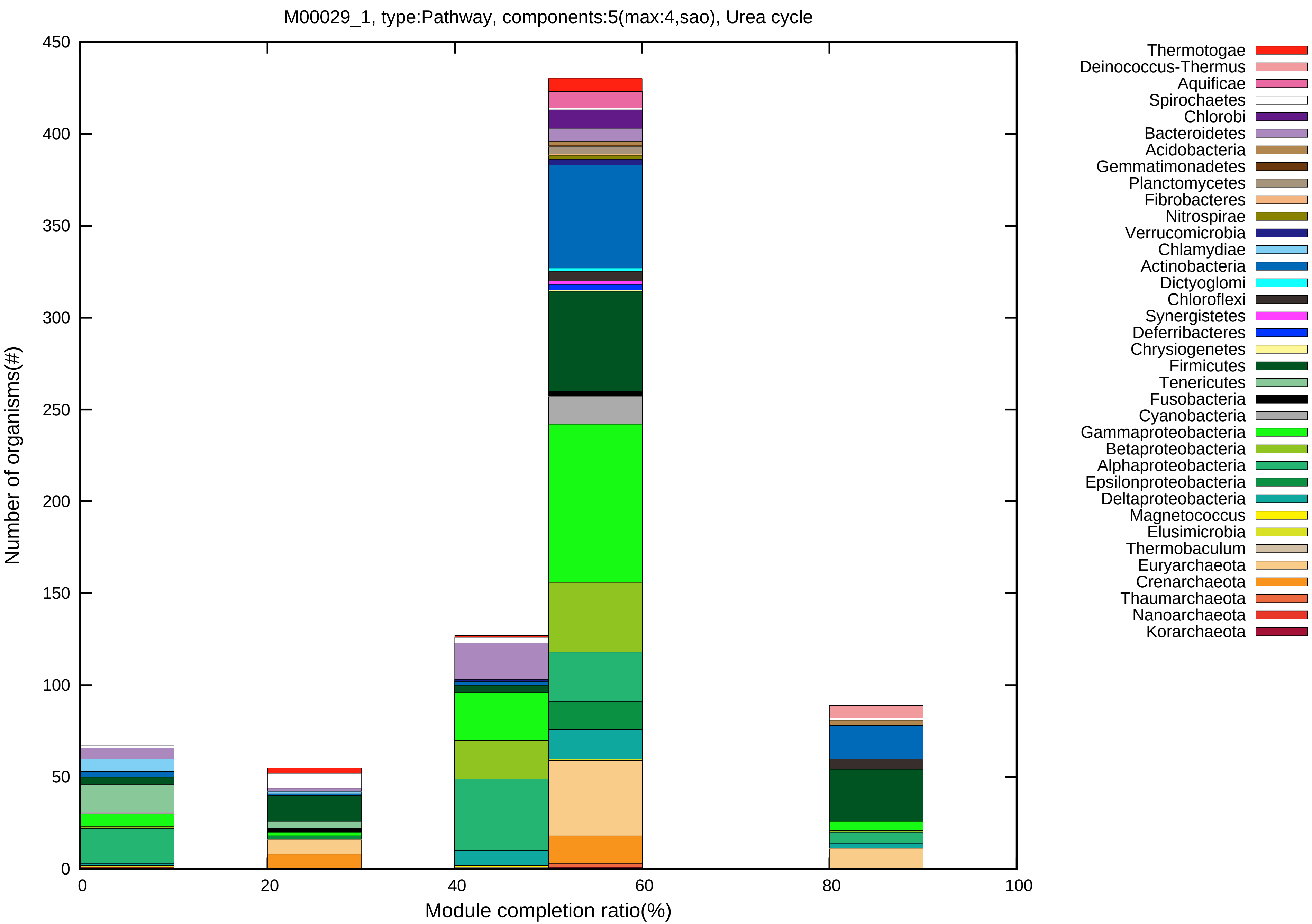
M00029_1, type:Pathway, components:5(max:4,sao), Urea cycle
Number of organisms(#)
Module completion ratio(%)
0
50
100
150
200
250
300
350
400
450
0
20
40
60
80
100
Thermotogae
Deinococcus-Thermus
Aquificae
Spirochaetes
Chlorobi
Bacteroidetes
Acidobacteria
Gemmatimonadetes
Planctomycetes
Fibrobacteres
Nitrospirae
Verrucomicrobia
Chlamydiae
Actinobacteria
Dictyoglomi
Chloroflexi
Synergistetes
Deferribacteres
Chrysiogenetes
Firmicutes
Tenericutes
Fusobacteria
Cyanobacteria
Gammaproteobacteria
Betaproteobacteria
Alphaproteobacteria
Epsilonproteobacteria
Deltaproteobacteria
Magnetococcus
Elusimicrobia
Thermobaculum
Euryarchaeota
Crenarchaeota
Thaumarchaeota
Nanoarchaeota
Korarchaeota

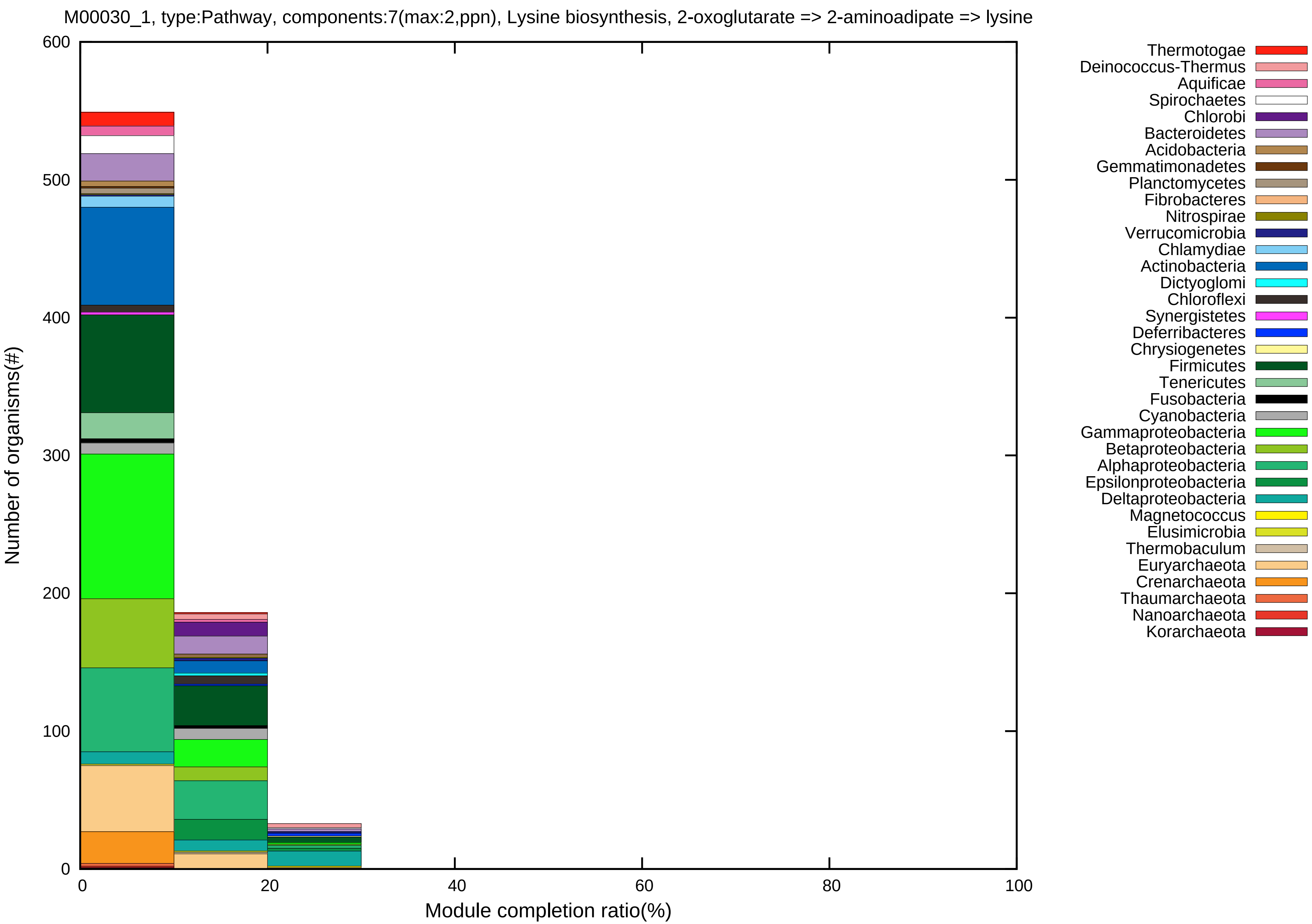
M00030_1, type:Pathway, components:7(max:2,ppn), Lysine biosynthesis, 2-oxoglutarate => 2-aminoadipate => lysine
Number of organisms(#)
Module completion ratio(%)
0
100
200
300
400
500
600
0
20
40
60
80
100
Thermotogae
Deinococcus-Thermus
Aquificae
Spirochaetes
Chlorobi
Bacteroidetes
Acidobacteria
Gemmatimonadetes
Planctomycetes
Fibrobacteres
Nitrospirae
Verrucomicrobia
Chlamydiae
Actinobacteria
Dictyoglomi
Chloroflexi
Synergistetes
Deferribacteres
Chrysiogenetes
Firmicutes
Tenericutes
Fusobacteria
Cyanobacteria
Gammaproteobacteria
Betaproteobacteria
Alphaproteobacteria
Epsilonproteobacteria
Deltaproteobacteria
Magnetococcus
Elusimicrobia
Thermobaculum
Euryarchaeota
Crenarchaeota
Thaumarchaeota
Nanoarchaeota
Korarchaeota

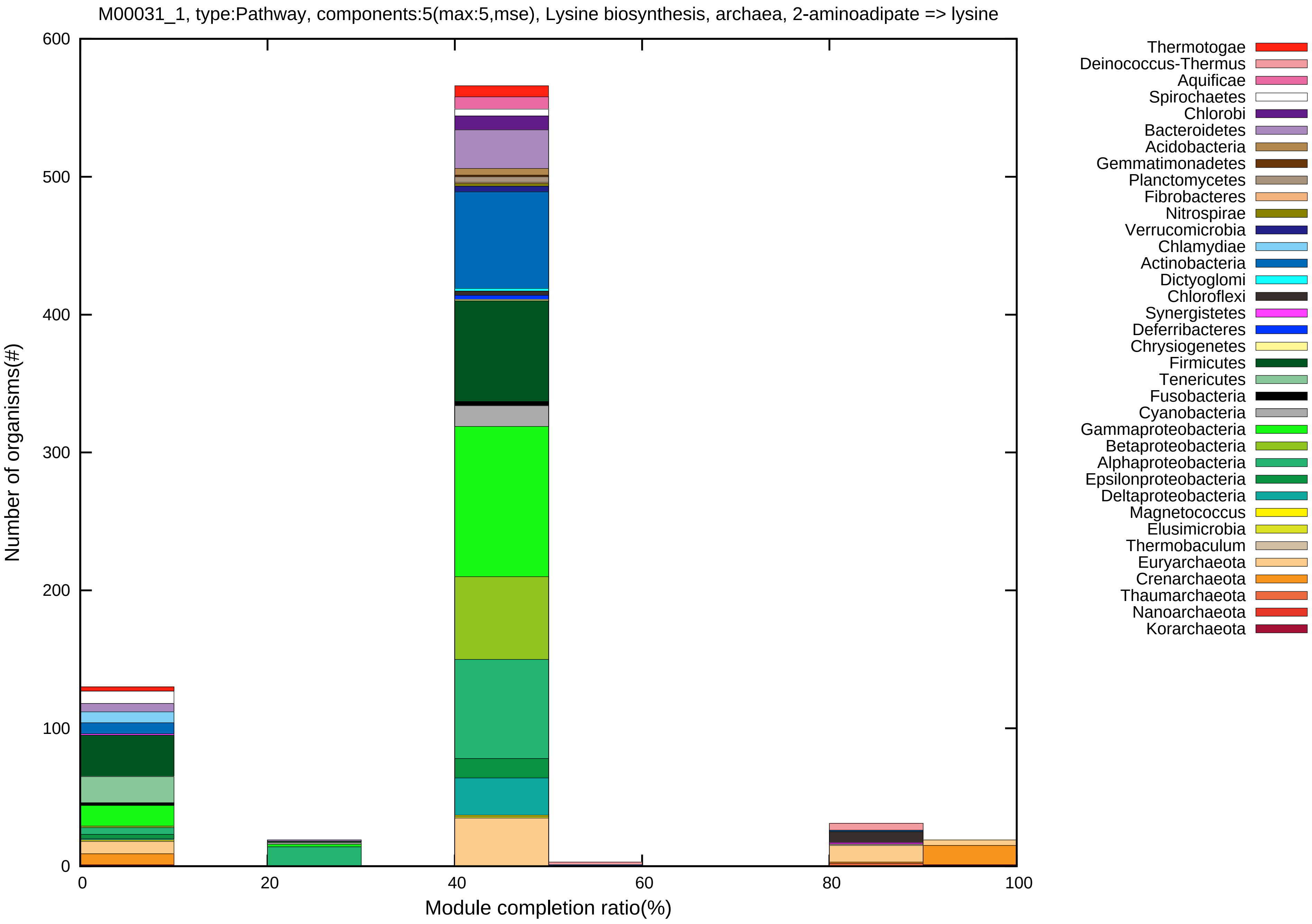

M00031_1, type:Pathway, components:5(max:5,mse), Lysine biosynthesis, archaea, 2-aminoadipate => lysine
Number of organisms(#)
Module completion ratio(%)
0
100
200
300
400
500
600
0
20
40
60
80
100
Thermotogae
Deinococcus-Thermus
Aquificae
Spirochaetes
Chlorobi
Bacteroidetes
Acidobacteria
Gemmatimonadetes
Planctomycetes
Fibrobacteres
Nitrospirae
Verrucomicrobia
Chlamydiae
Actinobacteria
Dictyoglomi
Chloroflexi
Synergistetes
Deferribacteres
Chrysiogenetes
Firmicutes
Tenericutes
Fusobacteria
Cyanobacteria
Gammaproteobacteria
Betaproteobacteria
Alphaproteobacteria
Epsilonproteobacteria
Deltaproteobacteria
Magnetococcus
Elusimicrobia
Thermobaculum
Euryarchaeota
Crenarchaeota
Thaumarchaeota
Nanoarchaeota
Korarchaeota

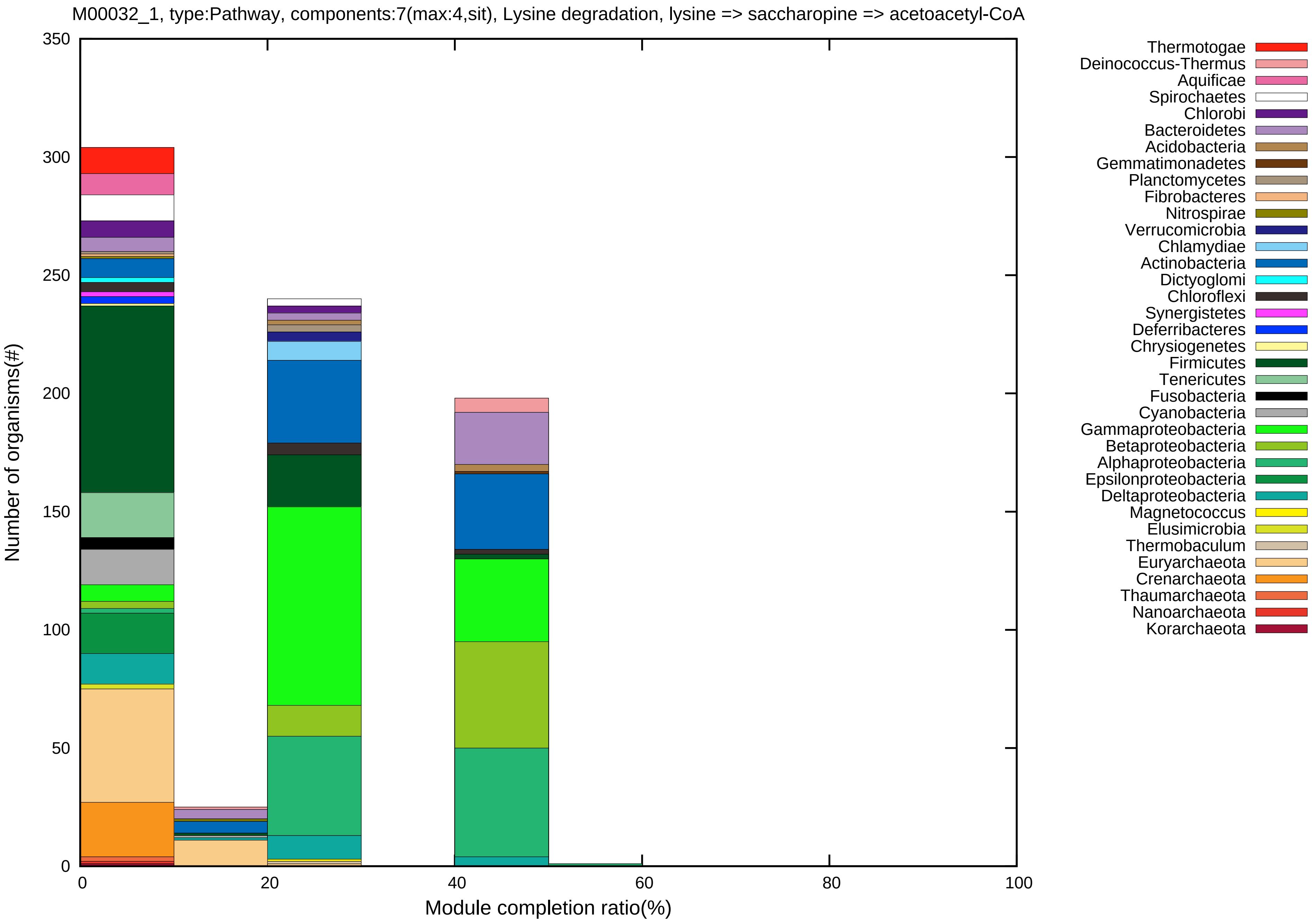

M00032_1, type:Pathway, components:7(max:4,sit), Lysine degradation, lysine => saccharopine => acetoacetyl-CoA
Number of organisms(#)
Module completion ratio(%)
0
50
100
150
200
250
300
350
0
20
40
60
80
100
Thermotogae
Deinococcus-Thermus
Aquificae
Spirochaetes
Chlorobi
Bacteroidetes
Acidobacteria
Gemmatimonadetes
Planctomycetes
Fibrobacteres
Nitrospirae
Verrucomicrobia
Chlamydiae
Actinobacteria
Dictyoglomi
Chloroflexi
Synergistetes
Deferribacteres
Chrysiogenetes
Firmicutes
Tenericutes
Fusobacteria
Cyanobacteria
Gammaproteobacteria
Betaproteobacteria
Alphaproteobacteria
Epsilonproteobacteria
Deltaproteobacteria
Magnetococcus
Elusimicrobia
Thermobaculum
Euryarchaeota
Crenarchaeota
Thaumarchaeota
Nanoarchaeota
Korarchaeota

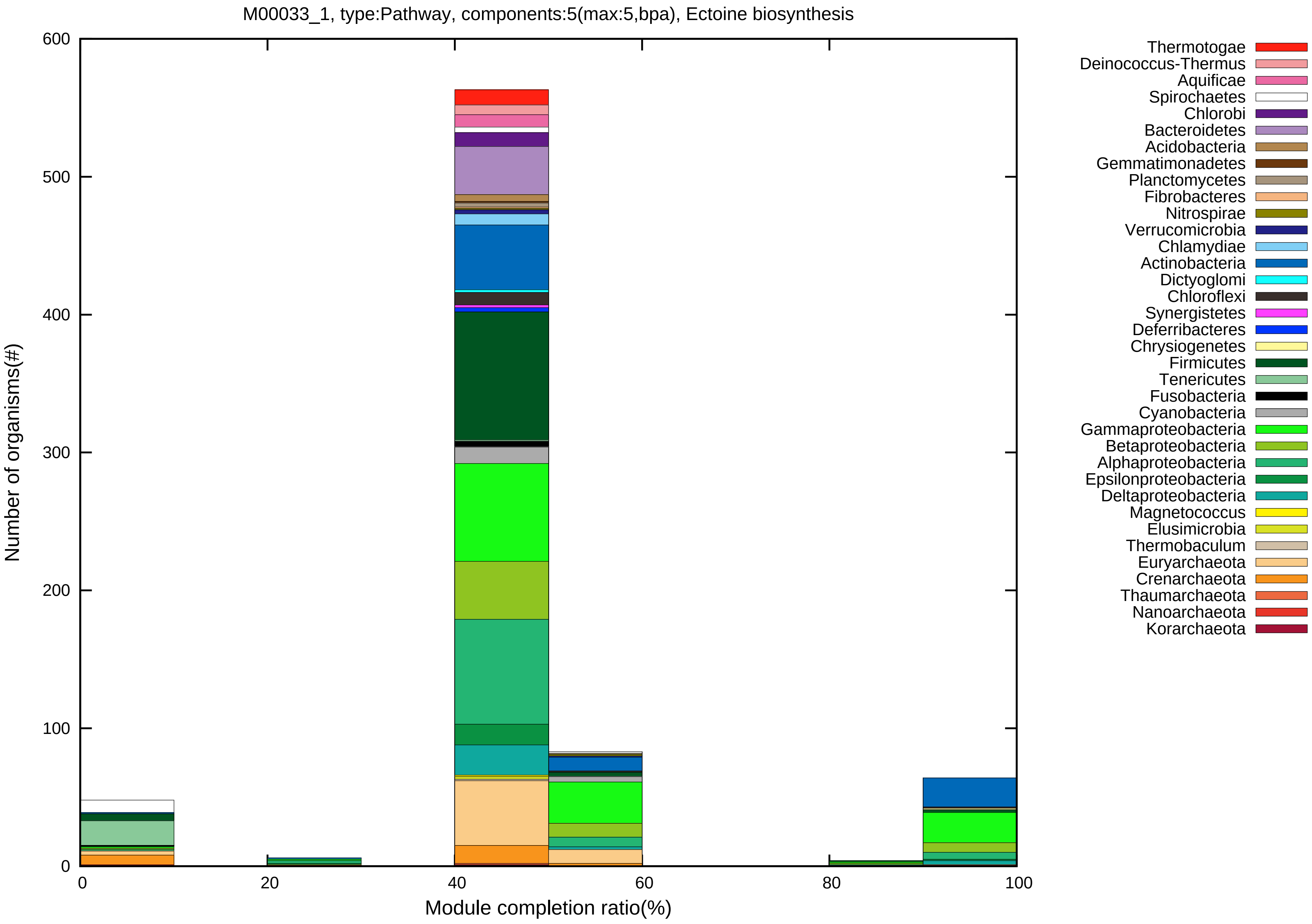
M00033_1, type:Pathway, components:5(max:5,bpa), Ectoine biosynthesis
Number of organisms(#)
Module completion ratio(%)
0
100
200
300
400
500
600
0
20
40
60
80
100
Thermotogae
Deinococcus-Thermus
Aquificae
Spirochaetes
Chlorobi
Bacteroidetes
Acidobacteria
Gemmatimonadetes
Planctomycetes
Fibrobacteres
Nitrospirae
Verrucomicrobia
Chlamydiae
Actinobacteria
Dictyoglomi
Chloroflexi
Synergistetes
Deferribacteres
Chrysiogenetes
Firmicutes
Tenericutes
Fusobacteria
Cyanobacteria
Gammaproteobacteria
Betaproteobacteria
Alphaproteobacteria
Epsilonproteobacteria
Deltaproteobacteria
Magnetococcus
Elusimicrobia
Thermobaculum
Euryarchaeota
Crenarchaeota
Thaumarchaeota
Nanoarchaeota
Korarchaeota

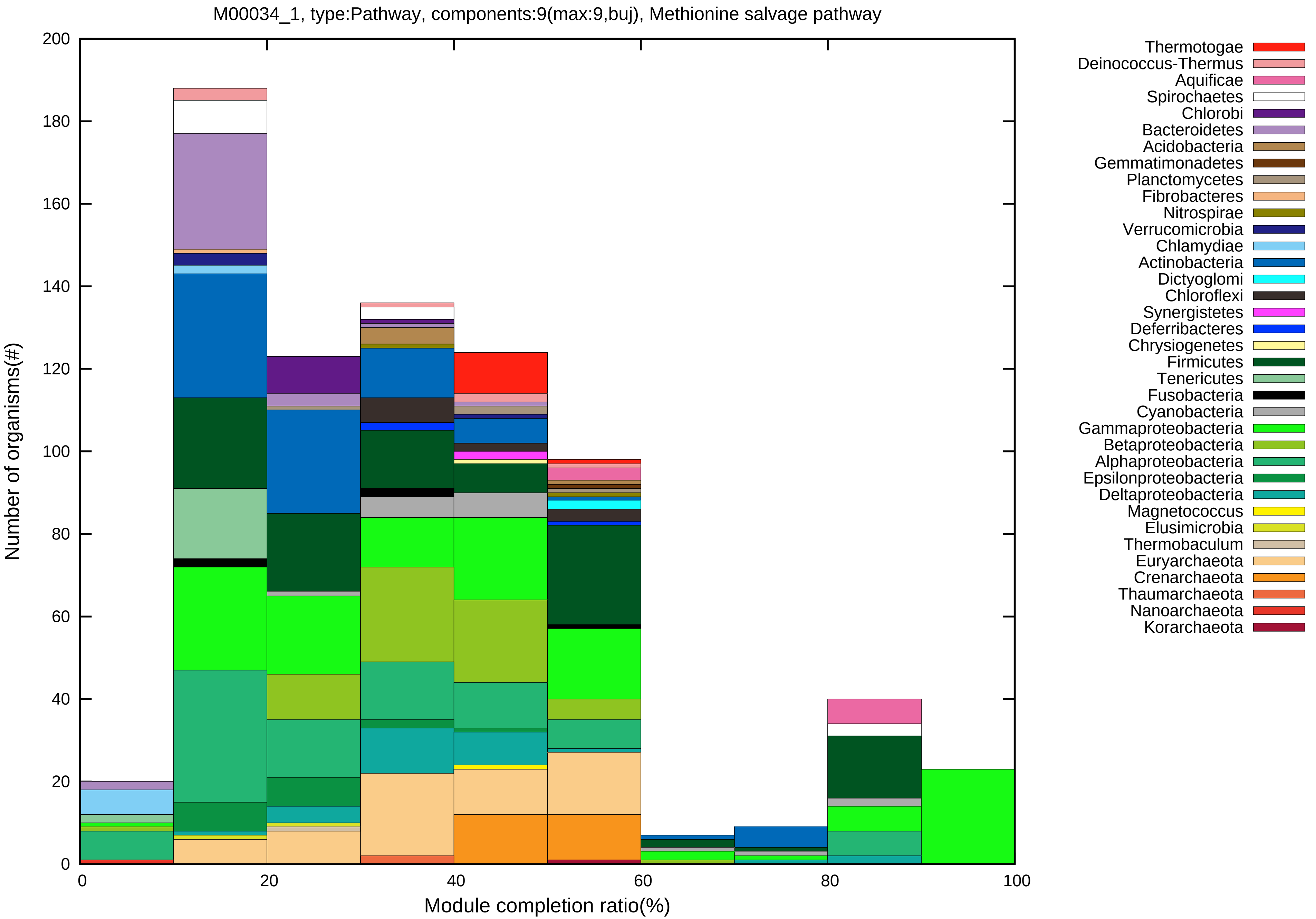
M00034_1, type:Pathway, components:9(max:9,buj), Methionine salvage pathway
Number of organisms(#)
Module completion ratio(%)
0
20
40
60
80
100
120
140
160
180
200
Thermotogae
Deinococcus-Thermus
Aquificae
Spirochaetes
Chlorobi
Bacteroidetes
Acidobacteria
Gemmatimonadetes
Planctomycetes
Fibrobacteres
Nitrospirae
Verrucomicrobia
Chlamydiae
Actinobacteria
Dictyoglomi
Chloroflexi
Synergistetes
Deferribacteres
Chrysiogenetes
Firmicutes
Tenericutes
Fusobacteria
Cyanobacteria
Gammaproteobacteria
Betaproteobacteria
Alphaproteobacteria
Epsilonproteobacteria
Deltaproteobacteria
Magnetococcus
Elusimicrobia
Thermobaculum
Euryarchaeota
Crenarchaeota
Thaumarchaeota
Nanoarchaeota
Korarchaeota

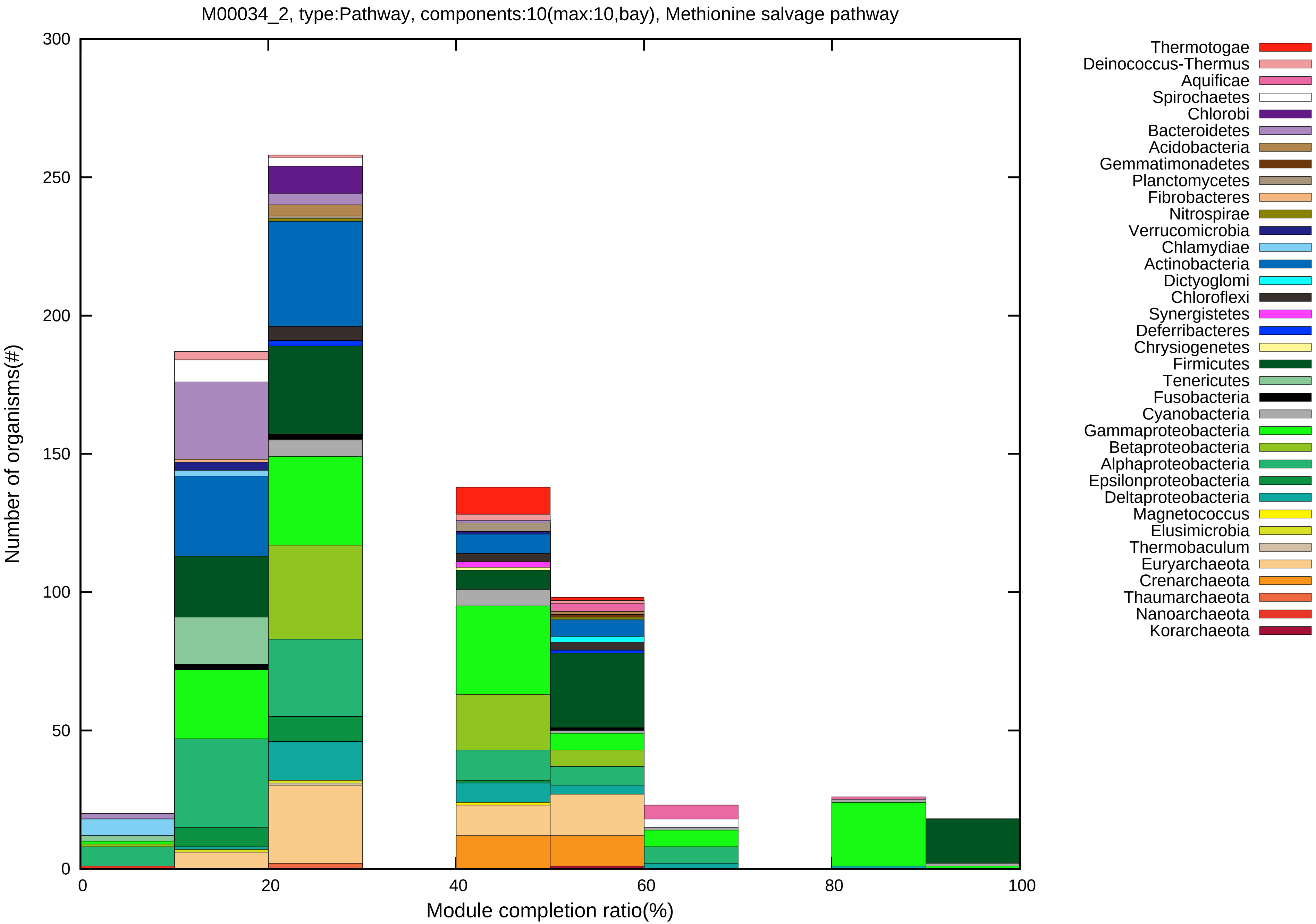

M00034_2, type:Pathway, components:10(max:10,bay), Methionine salvage pathway
Number of organisms(#)
Module completion ratio(%)
0
50
100
150
200
250
300
0
20
40
60
80
100
Thermotogae
Deinococcus-Thermus
Aquificae
Spirochaetes
Chlorobi
Bacteroidetes
Acidobacteria
Gemmatimonadetes
Planctomycetes
Fibrobacteres
Nitrospirae
Verrucomicrobia
Chlamydiae
Actinobacteria
Dictyoglomi
Chloroflexi
Synergistetes
Deferribacteres
Chrysiogenetes
Firmicutes
Tenericutes
Fusobacteria
Cyanobacteria
Gammaproteobacteria
Betaproteobacteria
Alphaproteobacteria
Epsilonproteobacteria
Deltaproteobacteria
Magnetococcus
Elusimicrobia
Thermobaculum
Euryarchaeota
Crenarchaeota
Thaumarchaeota
Nanoarchaeota
Korarchaeota

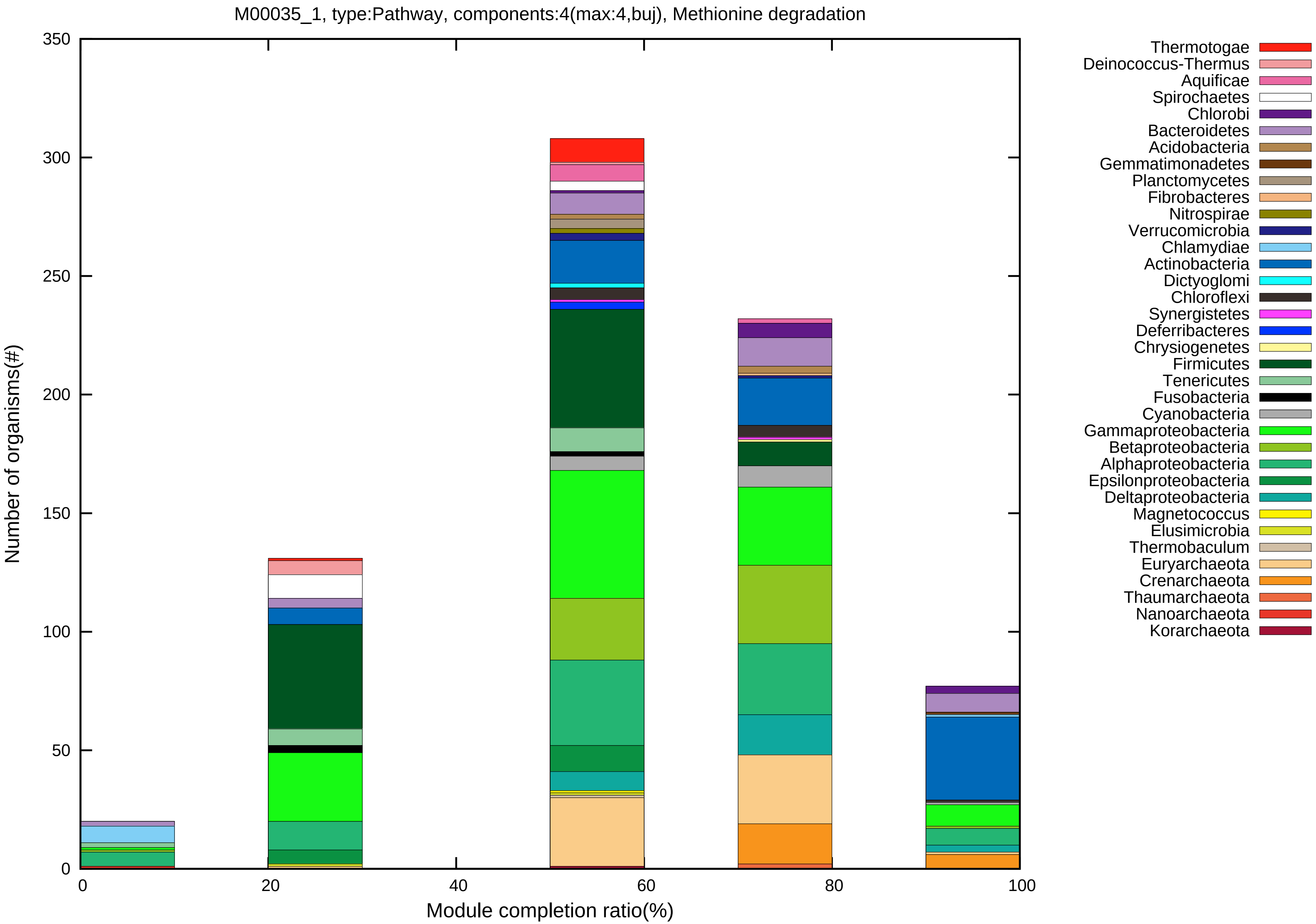

M00035_1, type:Pathway, components:4(max:4,buj), Methionine degradation
Number of organisms(#)
Module completion ratio(%)
0
50
100
150
200
250
300
350
0
20
40
60
80
100
Thermotogae
Deinococcus-Thermus
Aquificae
Spirochaetes
Chlorobi
Bacteroidetes
Acidobacteria
Gemmatimonadetes
Planctomycetes
Fibrobacteres
Nitrospirae
Verrucomicrobia
Chlamydiae
Actinobacteria
Dictyoglomi
Chloroflexi
Synergistetes
Deferribacteres
Chrysiogenetes
Firmicutes
Tenericutes
Fusobacteria
Cyanobacteria
Gammaproteobacteria
Betaproteobacteria
Alphaproteobacteria
Epsilonproteobacteria
Deltaproteobacteria
Magnetococcus
Elusimicrobia
Thermobaculum
Euryarchaeota
Crenarchaeota
Thaumarchaeota
Nanoarchaeota
Korarchaeota

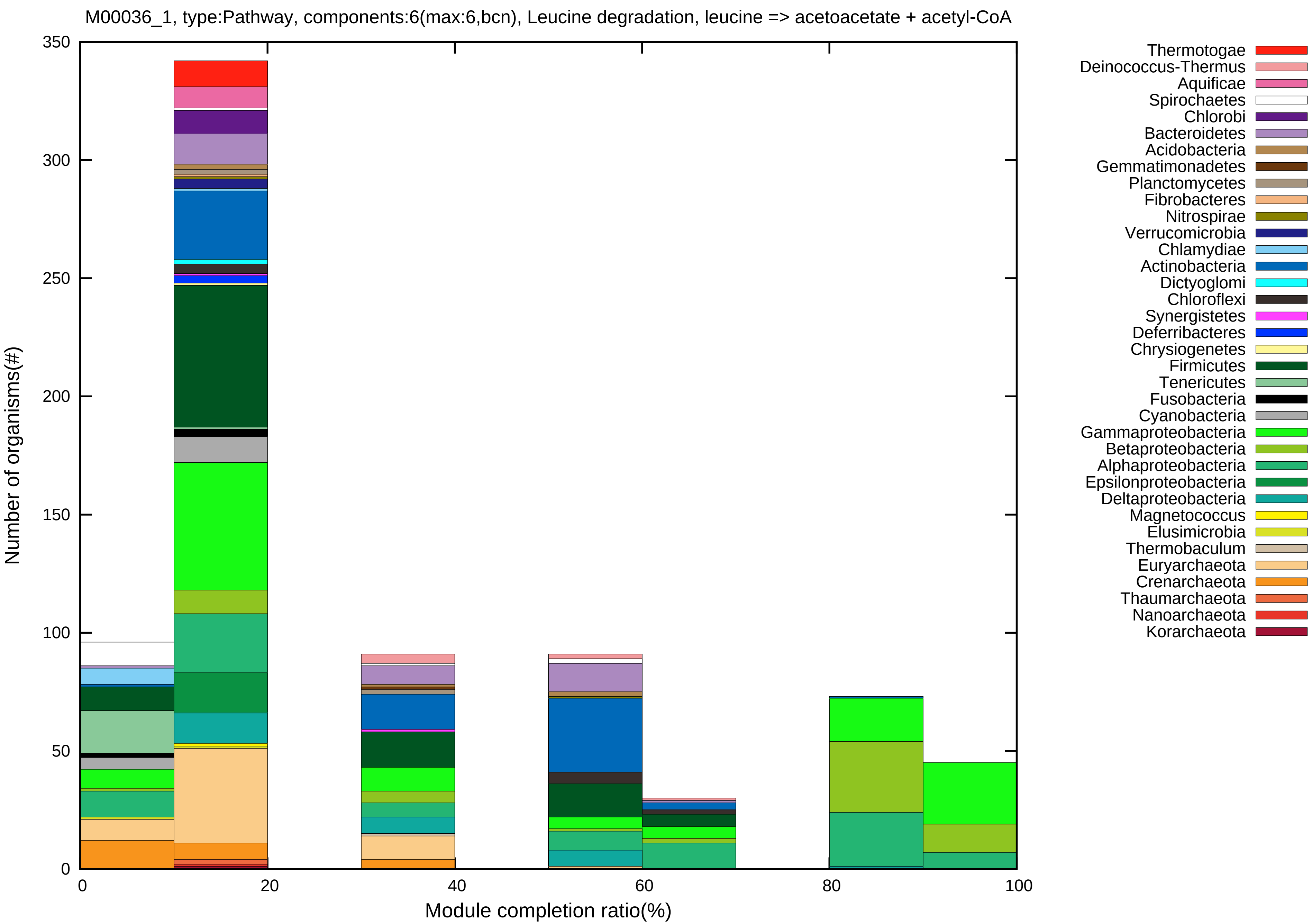
M00036_1, type:Pathway, components:6(max:6,bcn), Leucine degradation, leucine => acetoacetate + acetyl-CoA
Number of organisms(#)
Module completion ratio(%)
0
50
100
150
200
250
300
350
0
20
40
60
80
100
Thermotogae
Deinococcus-Thermus
Aquificae
Spirochaetes
Chlorobi
Bacteroidetes
Acidobacteria
Gemmatimonadetes
Planctomycetes
Fibrobacteres
Nitrospirae
Verrucomicrobia
Chlamydiae
Actinobacteria
Dictyoglomi
Chloroflexi
Synergistetes
Deferribacteres
Chrysiogenetes
Firmicutes
Tenericutes
Fusobacteria
Cyanobacteria
Gammaproteobacteria
Betaproteobacteria
Alphaproteobacteria
Epsilonproteobacteria
Deltaproteobacteria
Magnetococcus
Elusimicrobia
Thermobaculum
Euryarchaeota
Crenarchaeota
Thaumarchaeota
Nanoarchaeota
Korarchaeota

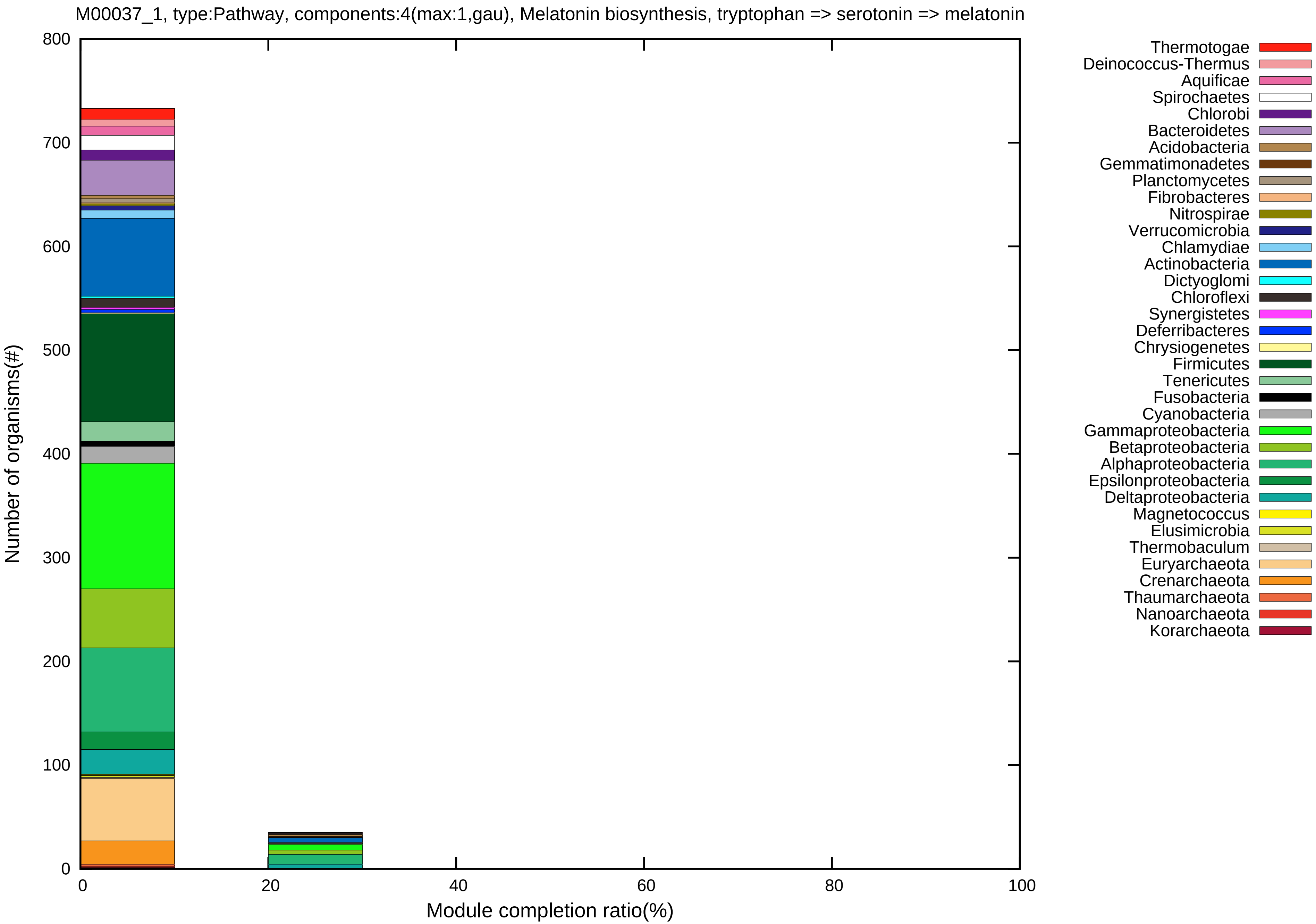

M00037_1, type:Pathway, components:4(max:1,gau), Melatonin biosynthesis, tryptophan => serotonin => melatonin
Number of organisms(#)
Module completion ratio(%)
0
100
200
300
400
500
600
700
800
0
20
40
60
80
100
Thermotogae
Deinococcus-Thermus
Aquificae
Spirochaetes
Chlorobi
Bacteroidetes
Acidobacteria
Gemmatimonadetes
Planctomycetes
Fibrobacteres
Nitrospirae
Verrucomicrobia
Chlamydiae
Actinobacteria
Dictyoglomi
Chloroflexi
Synergistetes
Deferribacteres
Chrysiogenetes
Firmicutes
Tenericutes
Fusobacteria
Cyanobacteria
Gammaproteobacteria
Betaproteobacteria
Alphaproteobacteria
Epsilonproteobacteria
Deltaproteobacteria
Magnetococcus
Elusimicrobia
Thermobaculum
Euryarchaeota
Crenarchaeota
Thaumarchaeota
Nanoarchaeota
Korarchaeota

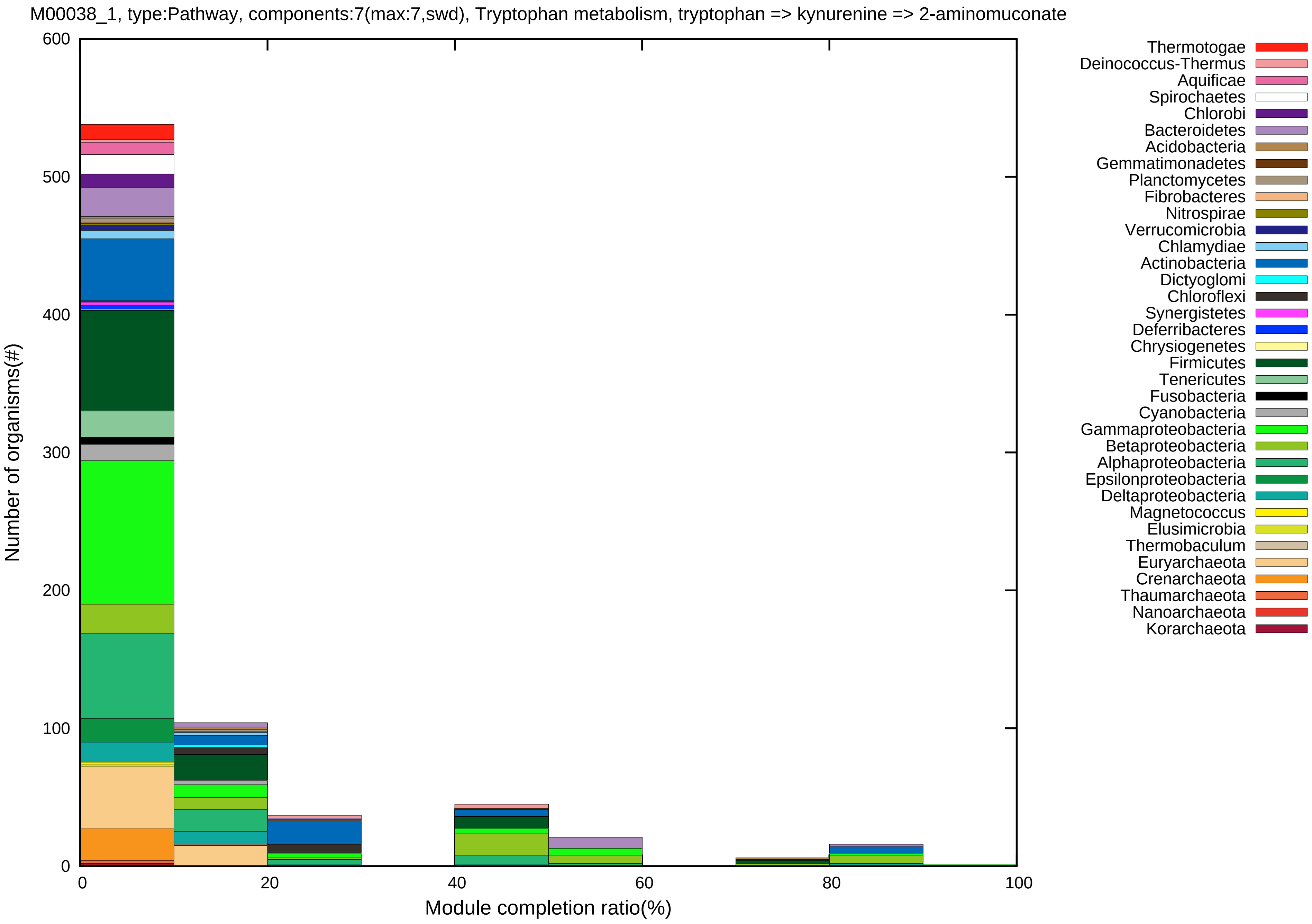
M00038_1, type:Pathway, components:7(max:7,swd), Tryptophan metabolism, tryptophan => kynurenine => 2-aminomuconate
Number of organisms(#)
Module completion ratio(%)
0
100
200
300
400
500
600
0
20
40
60
80
100
Thermotogae
Deinococcus-Thermus
Aquificae
Spirochaetes
Chlorobi
Bacteroidetes
Acidobacteria
Gemmatimonadetes
Planctomycetes
Fibrobacteres
Nitrospirae
Verrucomicrobia
Chlamydiae
Actinobacteria
Dictyoglomi
Chloroflexi
Synergistetes
Deferribacteres
Chrysiogenetes
Firmicutes
Tenericutes
Fusobacteria
Cyanobacteria
Gammaproteobacteria
Betaproteobacteria
Alphaproteobacteria
Epsilonproteobacteria
Deltaproteobacteria
Magnetococcus
Elusimicrobia
Thermobaculum
Euryarchaeota
Crenarchaeota
Thaumarchaeota
Nanoarchaeota
Korarchaeota

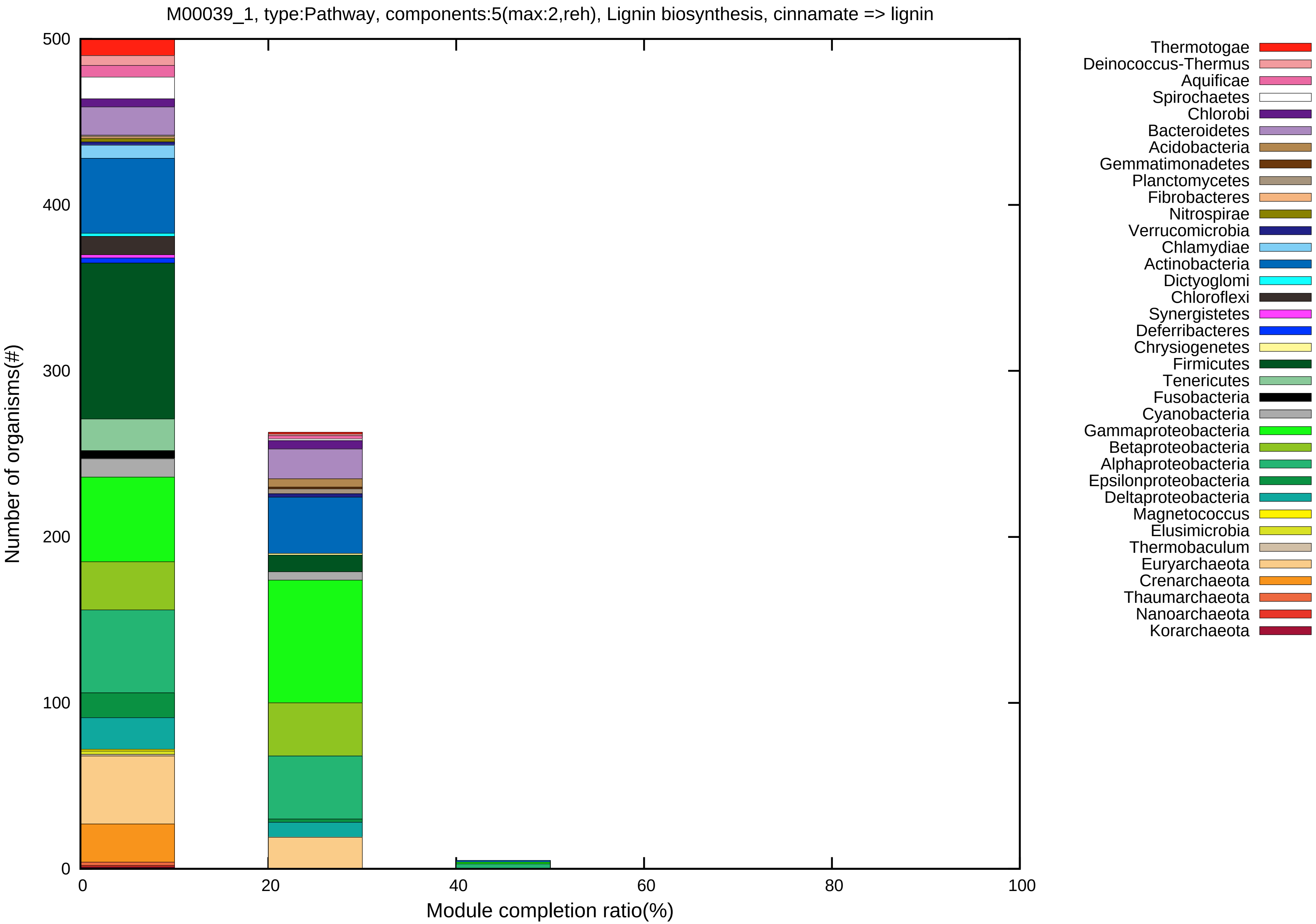

M00039_1, type:Pathway, components:5(max:2,reh), Lignin biosynthesis, cinnamate => lignin
Number of organisms(#)
Module completion ratio(%)
0
100
200
300
400
500
0
20
40
60
80
100
Thermotogae
Deinococcus-Thermus
Aquificae
Spirochaetes
Chlorobi
Bacteroidetes
Acidobacteria
Gemmatimonadetes
Planctomycetes
Fibrobacteres
Nitrospirae
Verrucomicrobia
Chlamydiae
Actinobacteria
Dictyoglomi
Chloroflexi
Synergistetes
Deferribacteres
Chrysiogenetes
Firmicutes
Tenericutes
Fusobacteria
Cyanobacteria
Gammaproteobacteria
Betaproteobacteria
Alphaproteobacteria
Epsilonproteobacteria
Deltaproteobacteria
Magnetococcus
Elusimicrobia
Thermobaculum
Euryarchaeota
Crenarchaeota
Thaumarchaeota
Nanoarchaeota
Korarchaeota

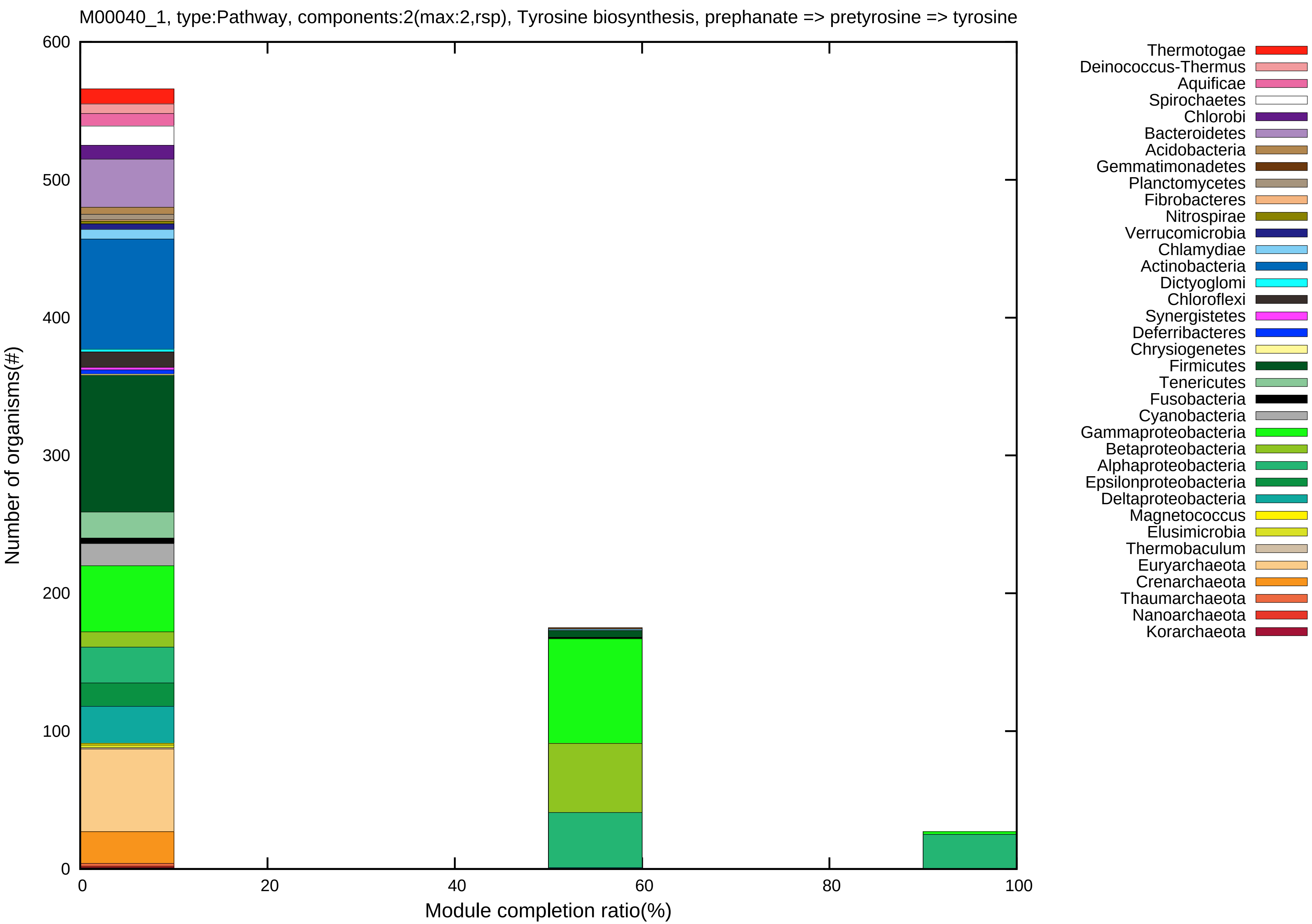

M00040_1, type:Pathway, components:2(max:2,rsp), Tyrosine biosynthesis, prephanate => pretyrosine => tyrosine
Number of organisms(#)
Module completion ratio(%)
0
100
200
300
400
500
600
0
20
40
60
80
100
Thermotogae
Deinococcus-Thermus
Aquificae
Spirochaetes
Chlorobi
Bacteroidetes
Acidobacteria
Gemmatimonadetes
Planctomycetes
Fibrobacteres
Nitrospirae
Verrucomicrobia
Chlamydiae
Actinobacteria
Dictyoglomi
Chloroflexi
Synergistetes
Deferribacteres
Chrysiogenetes
Firmicutes
Tenericutes
Fusobacteria
Cyanobacteria
Gammaproteobacteria
Betaproteobacteria
Alphaproteobacteria
Epsilonproteobacteria
Deltaproteobacteria
Magnetococcus
Elusimicrobia
Thermobaculum
Euryarchaeota
Crenarchaeota
Thaumarchaeota
Nanoarchaeota
Korarchaeota

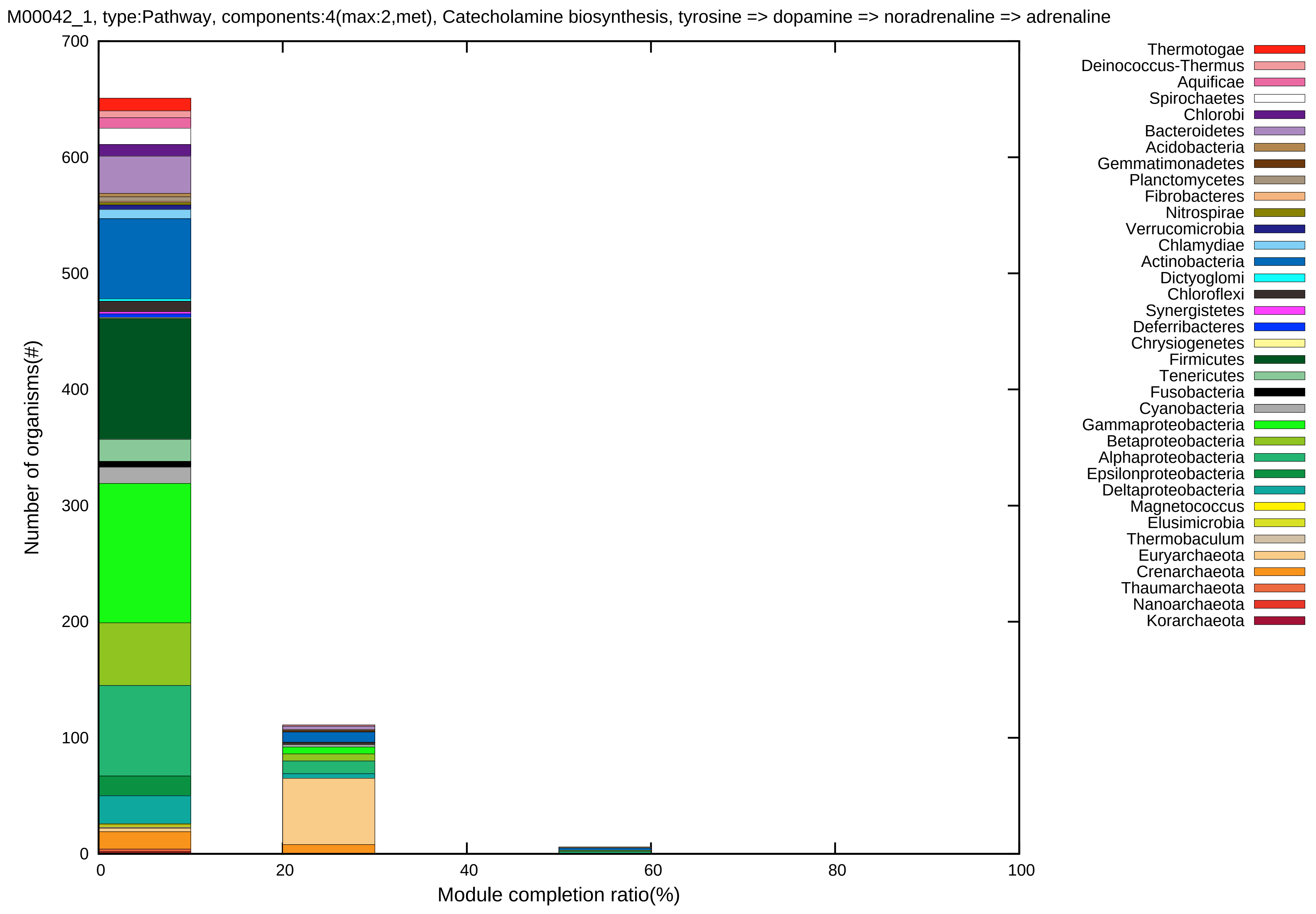
M00042_1, type:Pathway, components:4(max:2,met), Catecholamine biosynthesis, tyrosine => dopamine => noradrenaline => adrenaline
Number of organisms(#)
Module completion ratio(%)
0
100
200
300
400
500
600
700
0
20
40
60
80
100
Thermotogae
Deinococcus-Thermus
Aquificae
Spirochaetes
Chlorobi
Bacteroidetes
Acidobacteria
Gemmatimonadetes
Planctomycetes
Fibrobacteres
Nitrospirae
Verrucomicrobia
Chlamydiae
Actinobacteria
Dictyoglomi
Chloroflexi
Synergistetes
Deferribacteres
Chrysiogenetes
Firmicutes
Tenericutes
Fusobacteria
Cyanobacteria
Gammaproteobacteria
Betaproteobacteria
Alphaproteobacteria
Epsilonproteobacteria
Deltaproteobacteria
Magnetococcus
Elusimicrobia
Thermobaculum
Euryarchaeota
Crenarchaeota
Thaumarchaeota
Nanoarchaeota
Korarchaeota

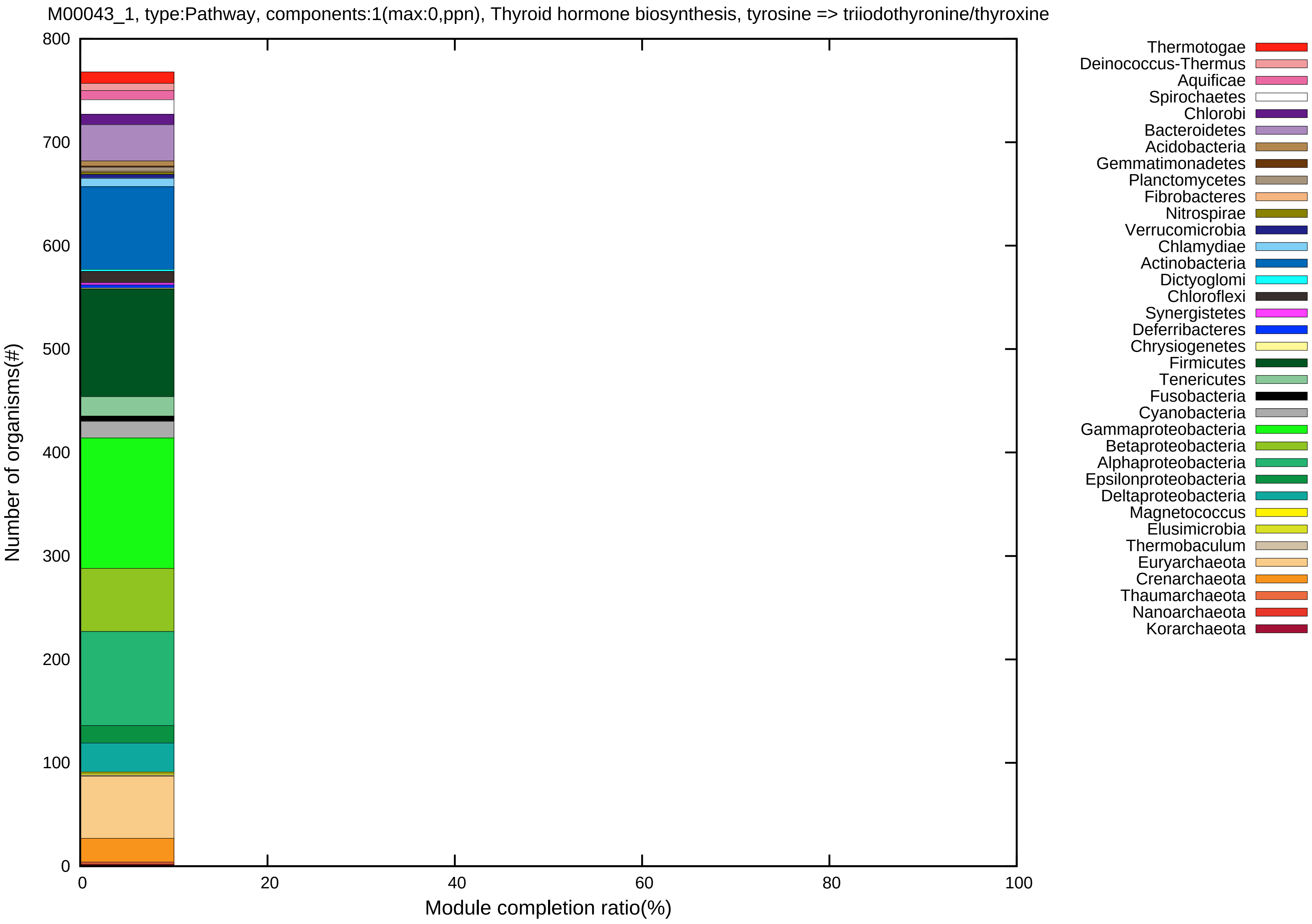

M00043_1, type:Pathway, components:1(max:0,ppn), Thyroid hormone biosynthesis, tyrosine => triiodothyronine/thyroxine
Number of organisms(#)
Module completion ratio(%)
0
100
200
300
400
500
600
700
800
0
20
40
60
80
100
Thermotogae
Deinococcus-Thermus
Aquificae
Spirochaetes
Chlorobi
Bacteroidetes
Acidobacteria
Gemmatimonadetes
Planctomycetes
Fibrobacteres
Nitrospirae
Verrucomicrobia
Chlamydiae
Actinobacteria
Dictyoglomi
Chloroflexi
Synergistetes
Deferribacteres
Chrysiogenetes
Firmicutes
Tenericutes
Fusobacteria
Cyanobacteria
Gammaproteobacteria
Betaproteobacteria
Alphaproteobacteria
Epsilonproteobacteria
Deltaproteobacteria
Magnetococcus
Elusimicrobia
Thermobaculum
Euryarchaeota
Crenarchaeota
Thaumarchaeota
Nanoarchaeota
Korarchaeota

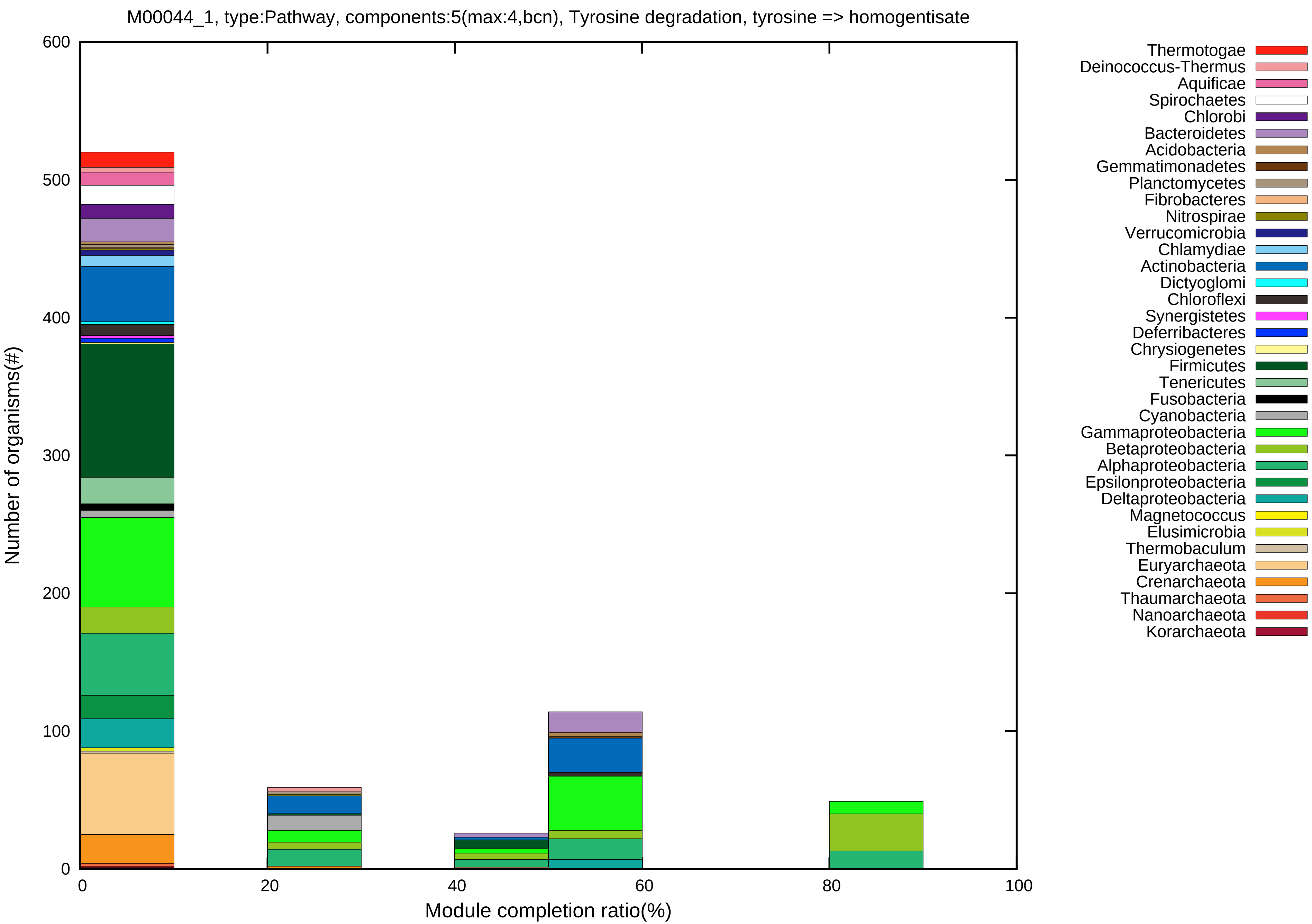
M00044_1, type:Pathway, components:5(max:4,bcn), Tyrosine degradation, tyrosine => homogentisate
Number of organisms(#)
Module completion ratio(%)
0
100
200
300
400
500
600
0
20
40
60
80
100
Thermotogae
Deinococcus-Thermus
Aquificae
Spirochaetes
Chlorobi
Bacteroidetes
Acidobacteria
Gemmatimonadetes
Planctomycetes
Fibrobacteres
Nitrospirae
Verrucomicrobia
Chlamydiae
Actinobacteria
Dictyoglomi
Chloroflexi
Synergistetes
Deferribacteres
Chrysiogenetes
Firmicutes
Tenericutes
Fusobacteria
Cyanobacteria
Gammaproteobacteria
Betaproteobacteria
Alphaproteobacteria
Epsilonproteobacteria
Deltaproteobacteria
Magnetococcus
Elusimicrobia
Thermobaculum
Euryarchaeota
Crenarchaeota
Thaumarchaeota
Nanoarchaeota
Korarchaeota

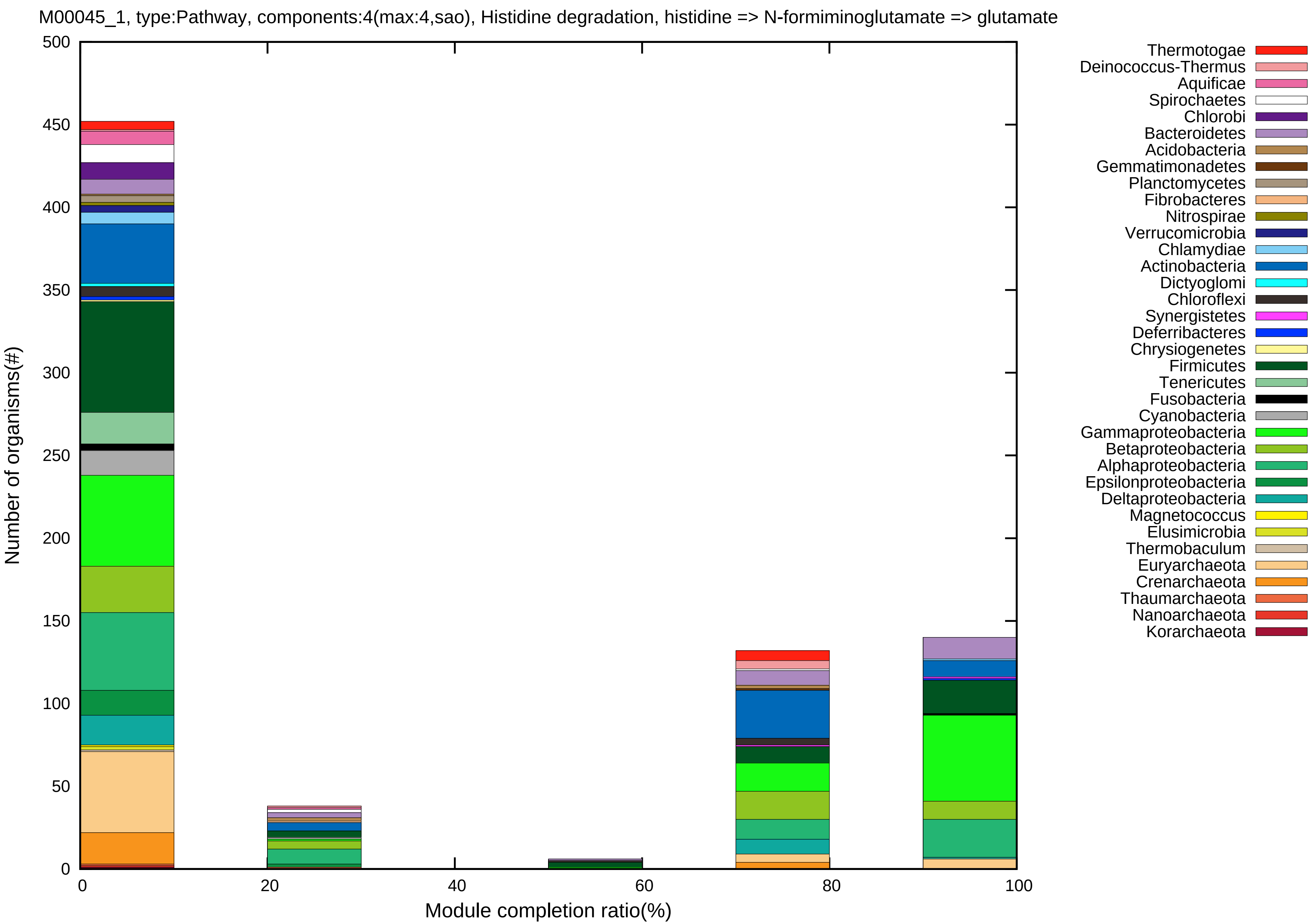

M00045_1, type:Pathway, components:4(max:4,sao), Histidine degradation, histidine => N-formiminoglutamate => glutamate
Number of organisms(#)
Module completion ratio(%)
0
50
100
150
200
250
300
350
400
450
500
0
20
40
60
80
100
Thermotogae
Deinococcus-Thermus
Aquificae
Spirochaetes
Chlorobi
Bacteroidetes
Acidobacteria
Gemmatimonadetes
Planctomycetes
Fibrobacteres
Nitrospirae
Verrucomicrobia
Chlamydiae
Actinobacteria
Dictyoglomi
Chloroflexi
Synergistetes
Deferribacteres
Chrysiogenetes
Firmicutes
Tenericutes
Fusobacteria
Cyanobacteria
Gammaproteobacteria
Betaproteobacteria
Alphaproteobacteria
Epsilonproteobacteria
Deltaproteobacteria
Magnetococcus
Elusimicrobia
Thermobaculum
Euryarchaeota
Crenarchaeota
Thaumarchaeota
Nanoarchaeota
Korarchaeota

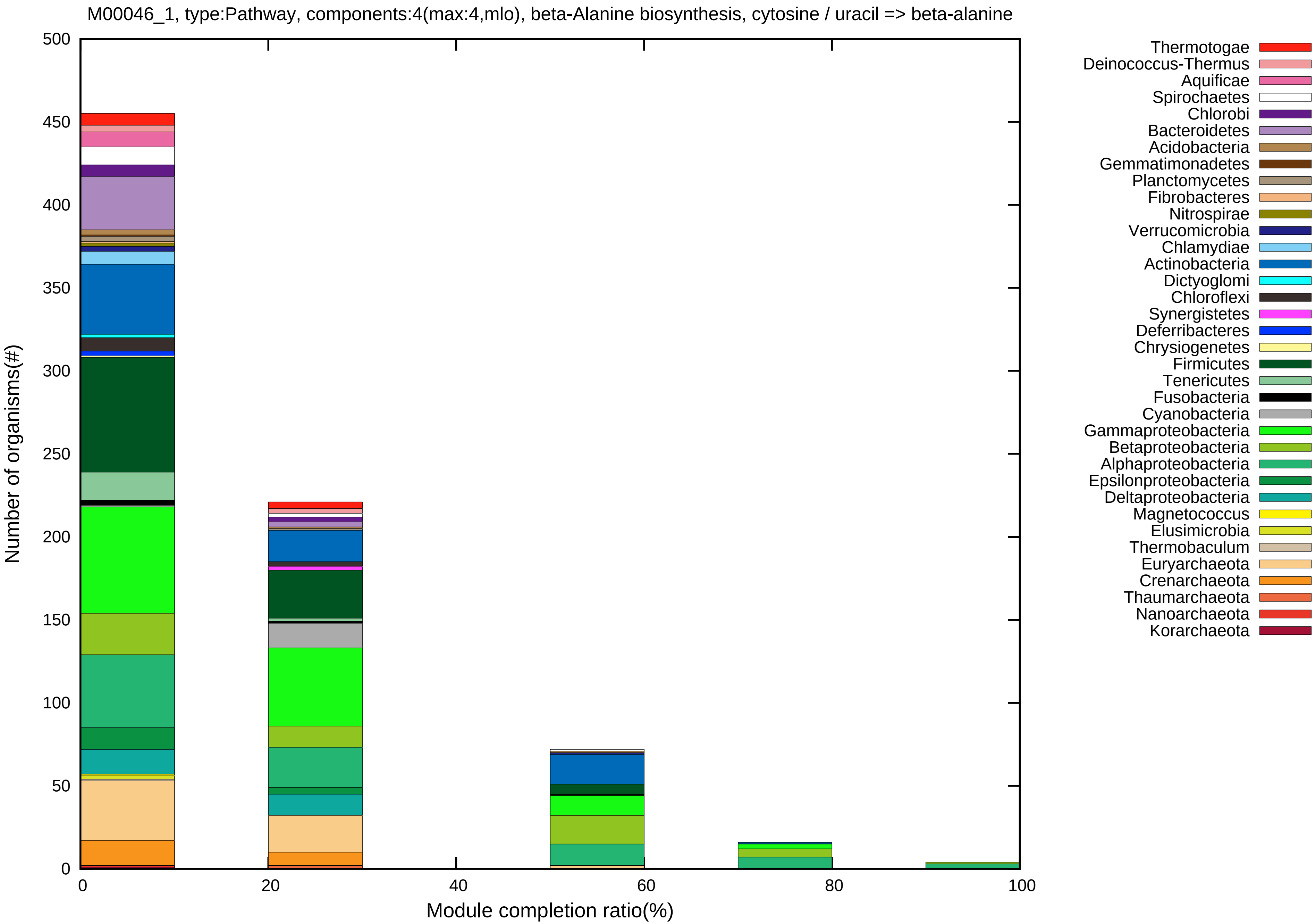

M00046_1, type:Pathway, components:4(max:4,mlo), beta-Alanine biosynthesis, cytosine / uracil => beta-alanine
Number of organisms(#)
Module completion ratio(%)
0
50
100
150
200
250
300
350
400
450
500
0
20
40
60
80
100
Thermotogae
Deinococcus-Thermus
Aquificae
Spirochaetes
Chlorobi
Bacteroidetes
Acidobacteria
Gemmatimonadetes
Planctomycetes
Fibrobacteres
Nitrospirae
Verrucomicrobia
Chlamydiae
Actinobacteria
Dictyoglomi
Chloroflexi
Synergistetes
Deferribacteres
Chrysiogenetes
Firmicutes
Tenericutes
Fusobacteria
Cyanobacteria
Gammaproteobacteria
Betaproteobacteria
Alphaproteobacteria
Epsilonproteobacteria
Deltaproteobacteria
Magnetococcus
Elusimicrobia
Thermobaculum
Euryarchaeota
Crenarchaeota
Thaumarchaeota
Nanoarchaeota
Korarchaeota

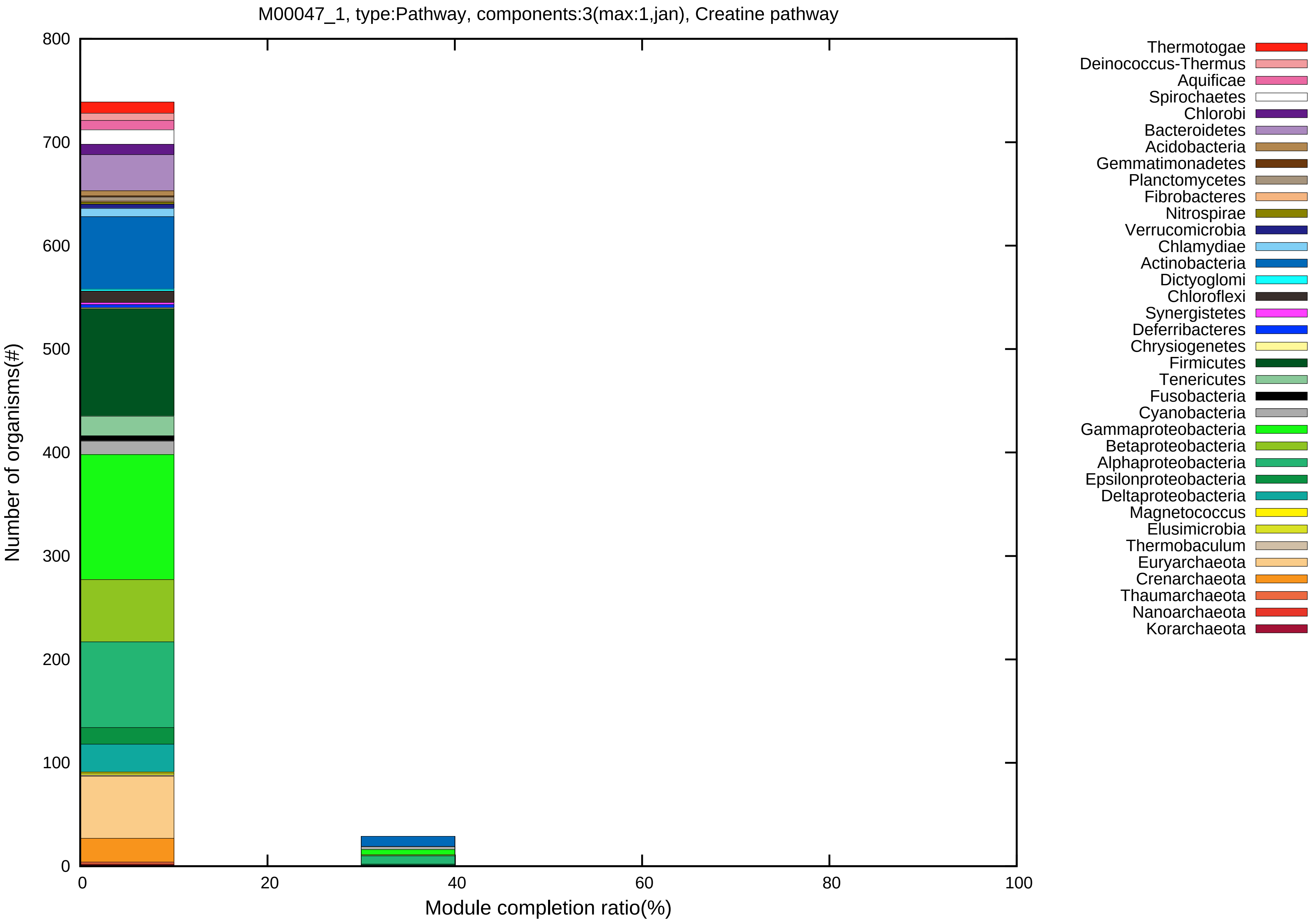

M00047_1, type:Pathway, components:3(max:1,jan), Creatine pathway
Number of organisms(#)
0
100
200
300
400
500
600
700
800
0
20
40
60
80
100
Module completion ratio(%)
Thermotogae
Deinococcus-Thermus
Aquificae
Spirochaetes
Chlorobi
Bacteroidetes
Acidobacteria
Gemmatimonadetes
Planctomycetes
Fibrobacteres
Nitrospirae
Verrucomicrobia
Chlamydiae
Actinobacteria
Dictyoglomi
Chloroflexi
Synergistetes
Deferribacteres
Chrysiogenetes
Firmicutes
Tenericutes
Fusobacteria
Cyanobacteria
Gammaproteobacteria
Betaproteobacteria
Alphaproteobacteria
Epsilonproteobacteria
Deltaproteobacteria
Magnetococcus
Elusimicrobia
Thermobaculum
Euryarchaeota
Crenarchaeota
Thaumarchaeota
Nanoarchaeota
Korarchaeota

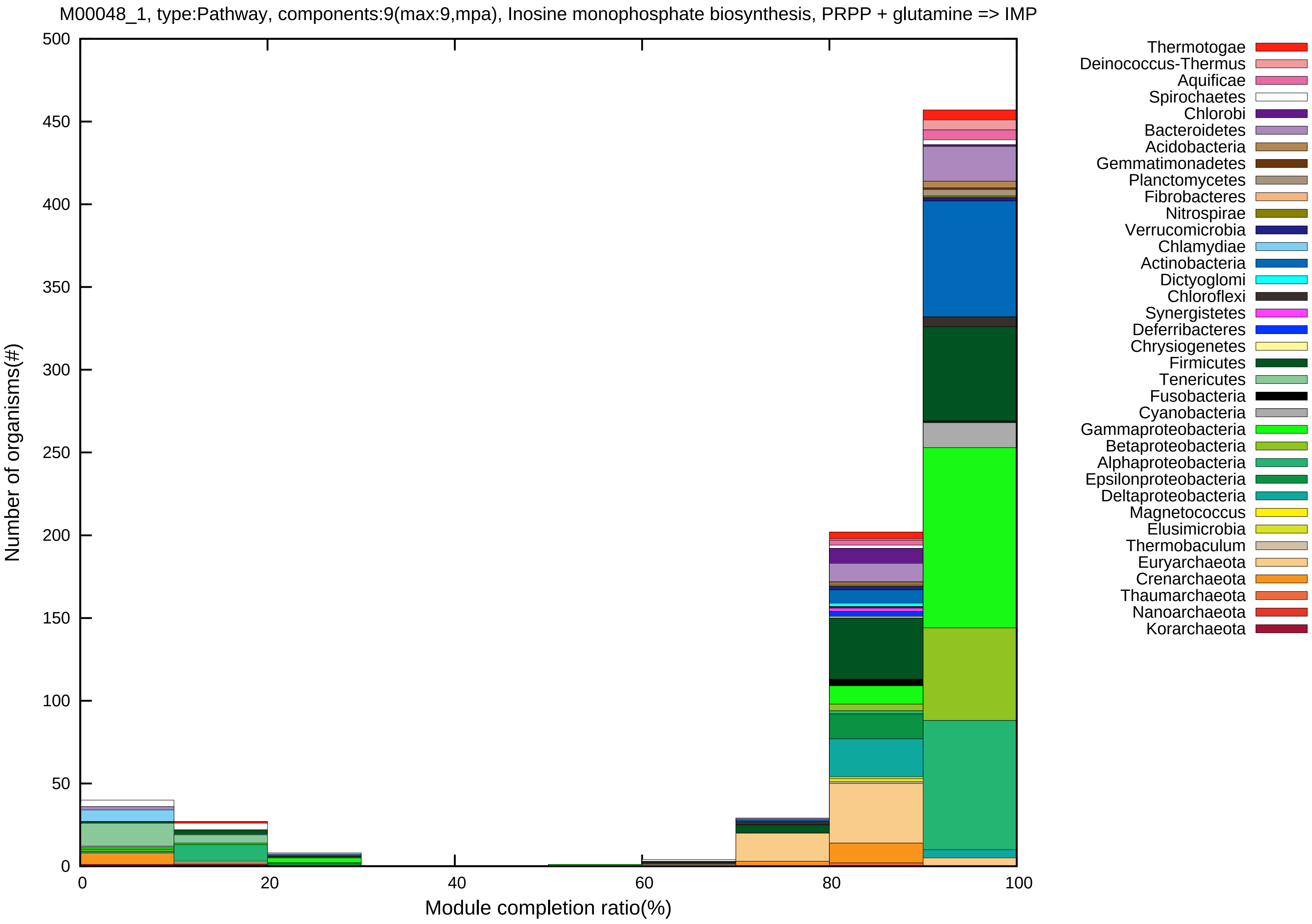
M00048_1, type:Pathway, components:9(max:9,mpa), Inosine monophosphate biosynthesis, PRPP + glutamine => IMP
Number of organisms(#)
Module completion ratio(%)
0
50
100
150
200
250
300
350
400
450
500
0
20
40
60
80
100
Thermotogae
Deinococcus-Thermus
Aquificae
Spirochaetes
Chlorobi
Bacteroidetes
Acidobacteria
Gemmatimonadetes
Planctomycetes
Fibrobacteres
Nitrospirae
Verrucomicrobia
Chlamydiae
Actinobacteria
Dictyoglomi
Chloroflexi
Synergistetes
Deferribacteres
Chrysiogenetes
Firmicutes
Tenericutes
Fusobacteria
Cyanobacteria
Gammaproteobacteria
Betaproteobacteria
Alphaproteobacteria
Epsilonproteobacteria
Deltaproteobacteria
Magnetococcus
Elusimicrobia
Thermobaculum
Euryarchaeota
Crenarchaeota
Thaumarchaeota
Nanoarchaeota
Korarchaeota

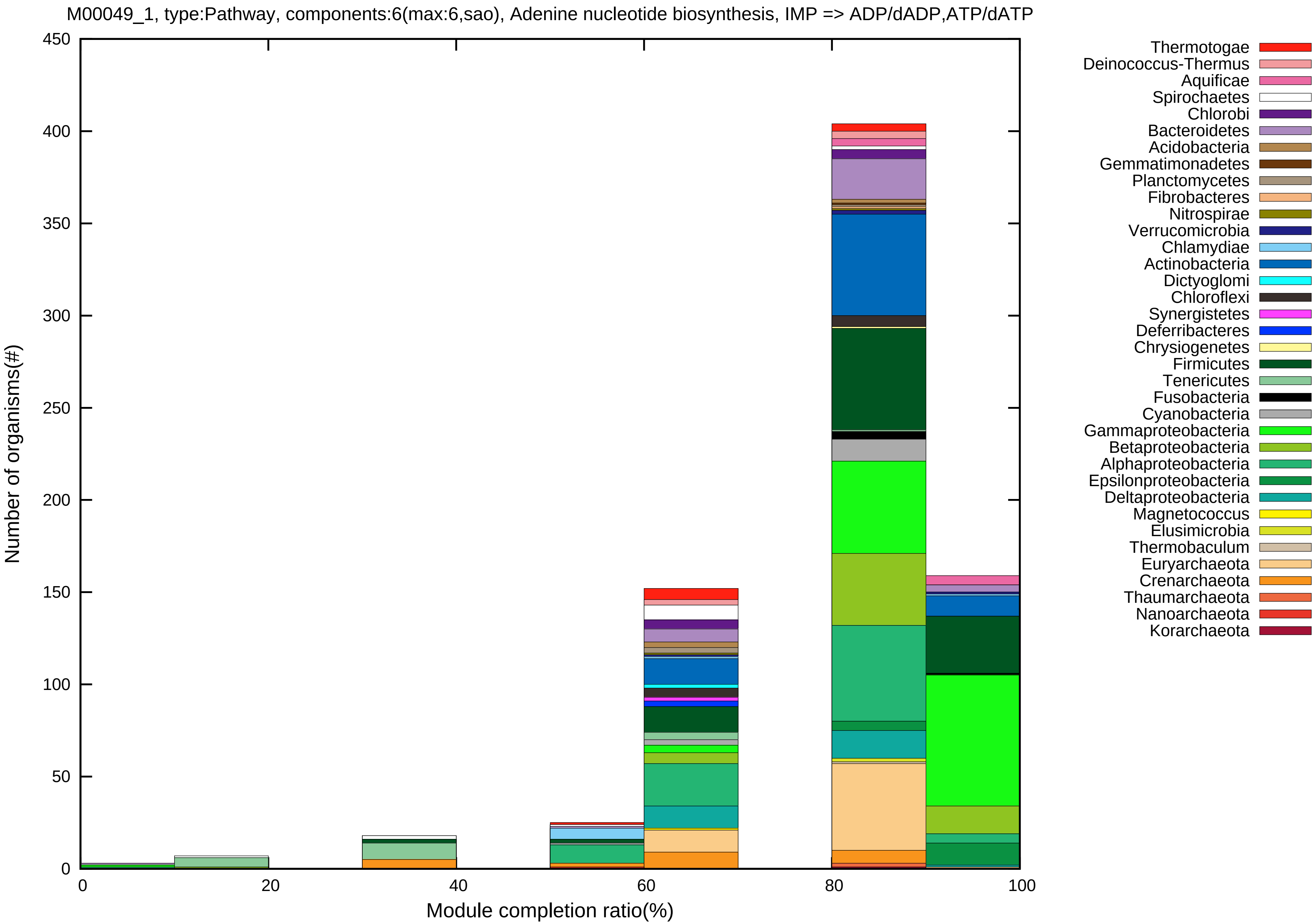
M00049_1, type:Pathway, components:6(max:6,sao), Adenine nucleotide biosynthesis, IMP => ADP/dADP,ATP/dATP
Number of organisms(#)
Module completion ratio(%)
0
50
100
150
200
250
300
350
400
450
0
20
40
60
80
100
Thermotogae
Deinococcus-Thermus
Aquificae
Spirochaetes
Chlorobi
Bacteroidetes
Acidobacteria
Gemmatimonadetes
Planctomycetes
Fibrobacteres
Nitrospirae
Verrucomicrobia
Chlamydiae
Actinobacteria
Dictyoglomi
Chloroflexi
Synergistetes
Deferribacteres
Chrysiogenetes
Firmicutes
Tenericutes
Fusobacteria
Cyanobacteria
Gammaproteobacteria
Betaproteobacteria
Alphaproteobacteria
Epsilonproteobacteria
Deltaproteobacteria
Magnetococcus
Elusimicrobia
Thermobaculum
Euryarchaeota
Crenarchaeota
Thaumarchaeota
Nanoarchaeota
Korarchaeota

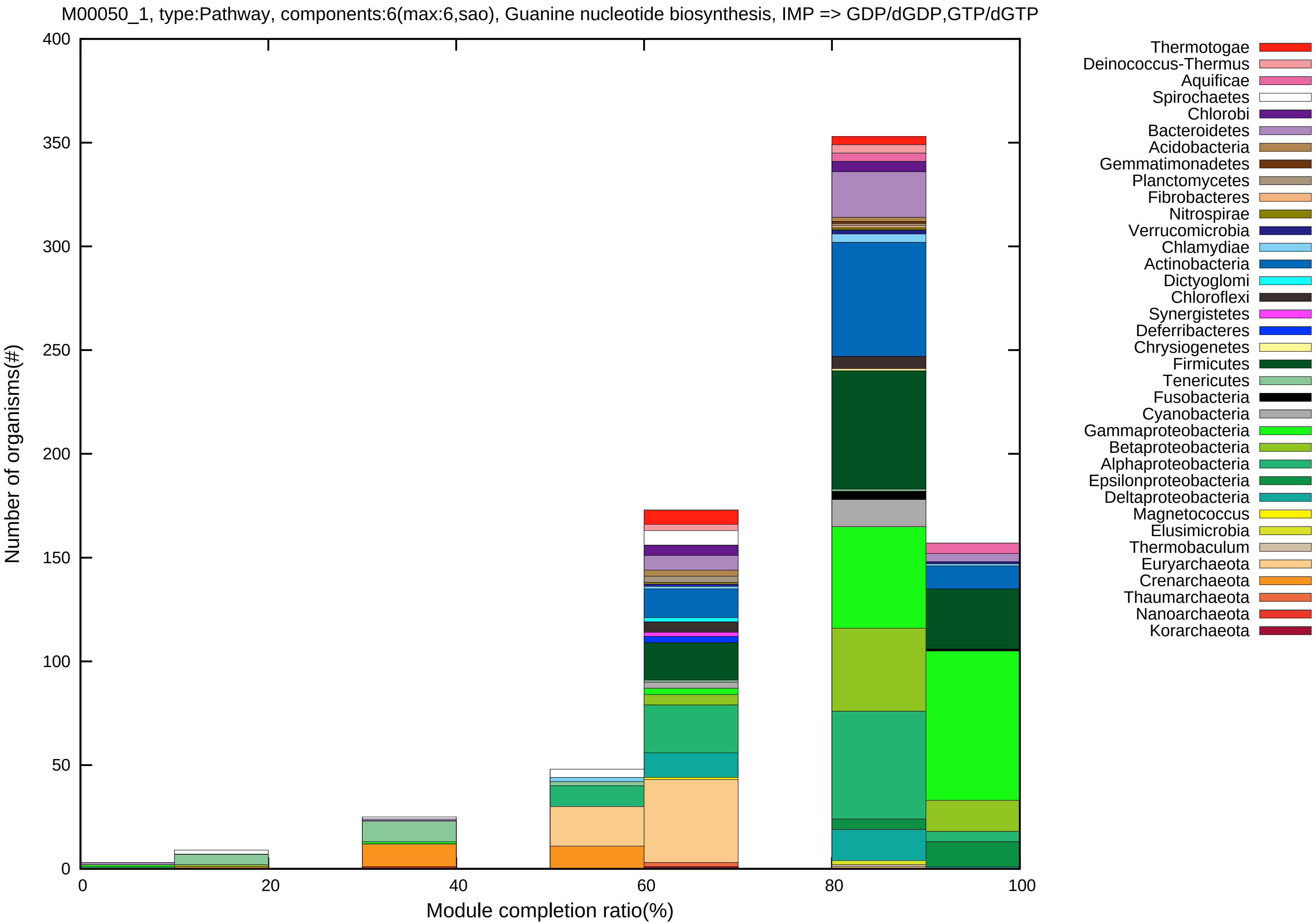
M00050_1, type:Pathway, components:6(max:6,sao), Guanine nucleotide biosynthesis, IMP => GDP/dGDP,GTP/dGTP
Number of organisms(#)
Module completion ratio(%)
0
20
40
60
80
100
50
150
200
250
300
350
400
Thermotogae
Deinococcus-Thermus
Aquificae
Spirochaetes
Chlorobi
Bacteroidetes
Acidobacteria
Gemmatimonadetes
Planctomycetes
Fibrobacteres
Nitrospirae
Verrucomicrobia
Chlamydiae
Actinobacteria
Dictyoglomi
Chloroflexi
Synergistetes
Deferribacteres
Chrysiogenetes
Firmicutes
Tenericutes
Fusobacteria
Cyanobacteria
Gammaproteobacteria
Betaproteobacteria
Alphaproteobacteria
Epsilonproteobacteria
Deltaproteobacteria
Magnetococcus
Elusimicrobia
Thermobaculum
Euryarchaeota
Crenarchaeota
Thaumarchaeota
Nanoarchaeota
Korarchaeota

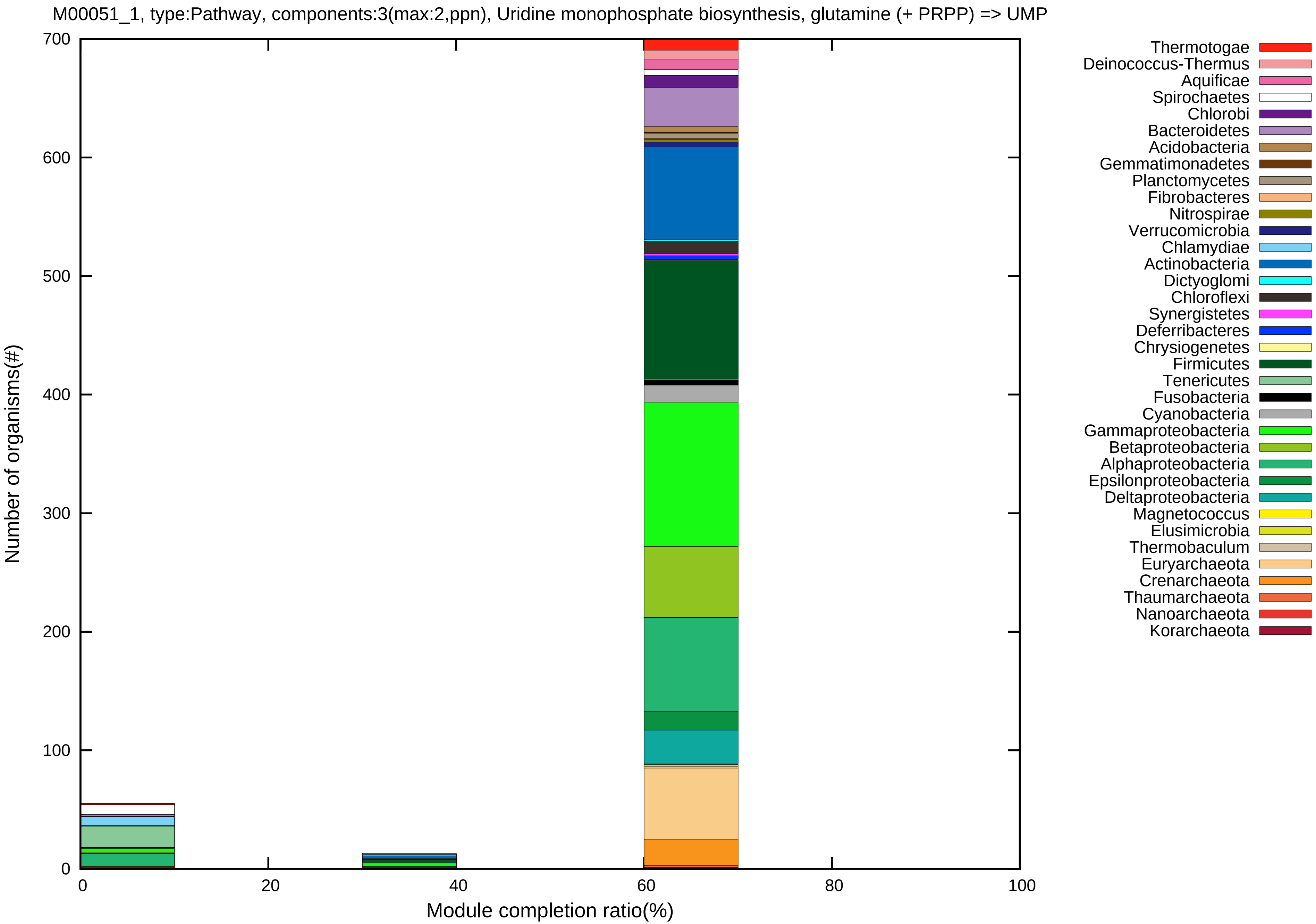
M00051_1, type:Pathway, components:3(max:2,ppn), Uridine monophosphate biosynthesis, glutamine (+ PRPP) => UMP
Number of organisms(#)
Module completion ratio(%)
0
100
200
300
400
500
600
700
0
20
40
60
80
100
Thermotogae
Deinococcus-Thermus
Aquificae
Spirochaetes
Chlorobi
Bacteroidetes
Acidobacteria
Gemmatimonadetes
Planctomycetes
Fibrobacteres
Nitrospirae
Verrucomicrobia
Chlamydiae
Actinobacteria
Dictyoglomi
Chloroflexi
Synergistetes
Deferribacteres
Chrysiogenetes
Firmicutes
Tenericutes
Fusobacteria
Cyanobacteria
Gammaproteobacteria
Betaproteobacteria
Alphaproteobacteria
Epsilonproteobacteria
Deltaproteobacteria
Magnetococcus
Elusimicrobia
Thermobaculum
Euryarchaeota
Crenarchaeota
Thaumarchaeota
Nanoarchaeota
Korarchaeota

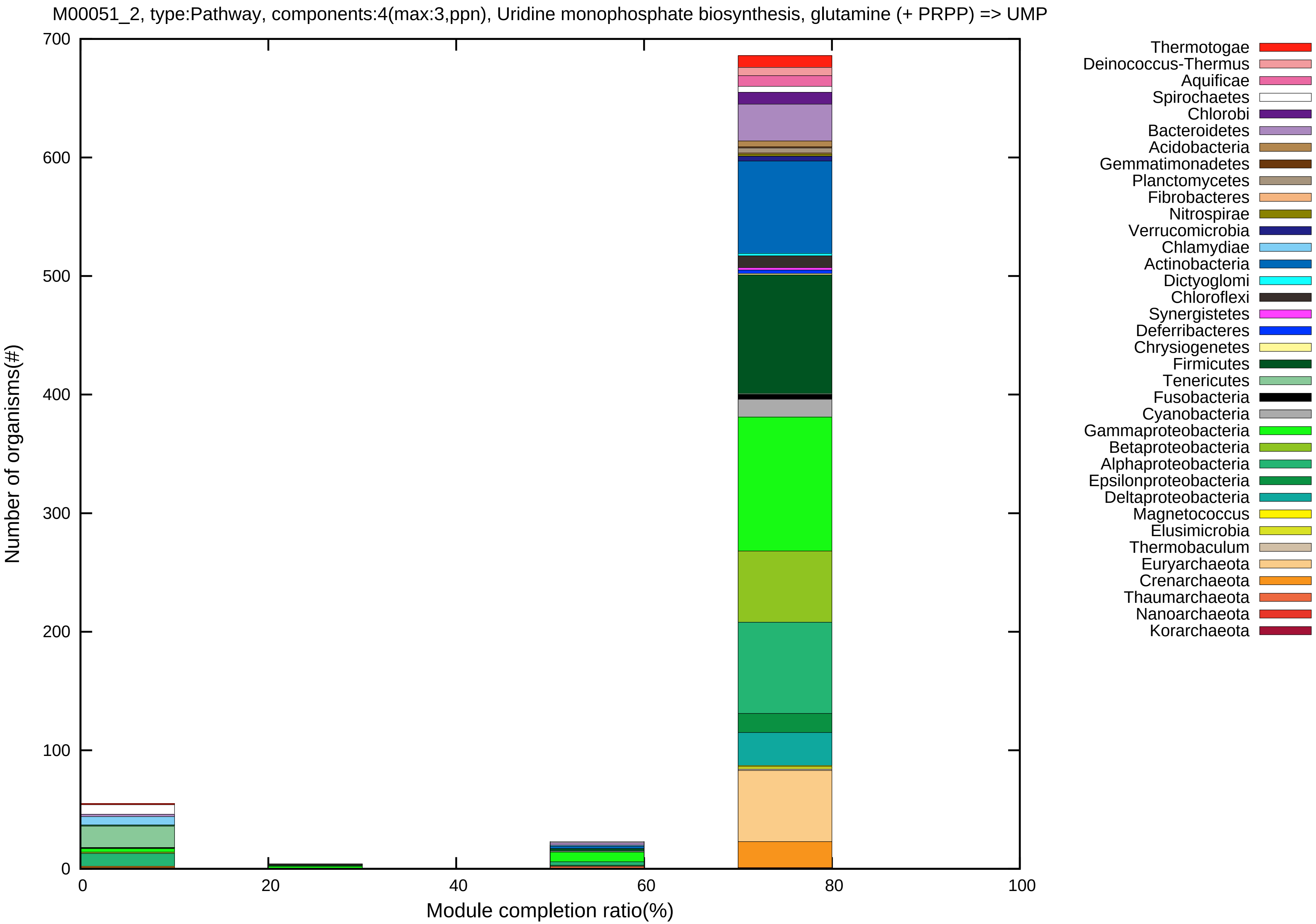

M00051_2, type:Pathway, components:4(max:3,ppn), Uridine monophosphate biosynthesis, glutamine (+ PRPP) => UMP
Number of organisms(#)
Module completion ratio(%)
0
100
200
300
400
500
600
700
0
20
40
60
80
100
Thermotogae
Deinococcus-Thermus
Aquificae
Spirochaetes
Chlorobi
Bacteroidetes
Acidobacteria
Gemmatimonadetes
Planctomycetes
Fibrobacteres
Nitrospirae
Verrucomicrobia
Chlamydiae
Actinobacteria
Dictyoglomi
Chloroflexi
Synergistetes
Deferribacteres
Chrysiogenetes
Firmicutes
Tenericutes
Fusobacteria
Cyanobacteria
Gammaproteobacteria
Betaproteobacteria
Alphaproteobacteria
Epsilonproteobacteria
Deltaproteobacteria
Magnetococcus
Elusimicrobia
Thermobaculum
Euryarchaeota
Crenarchaeota
Thaumarchaeota
Nanoarchaeota
Korarchaeota

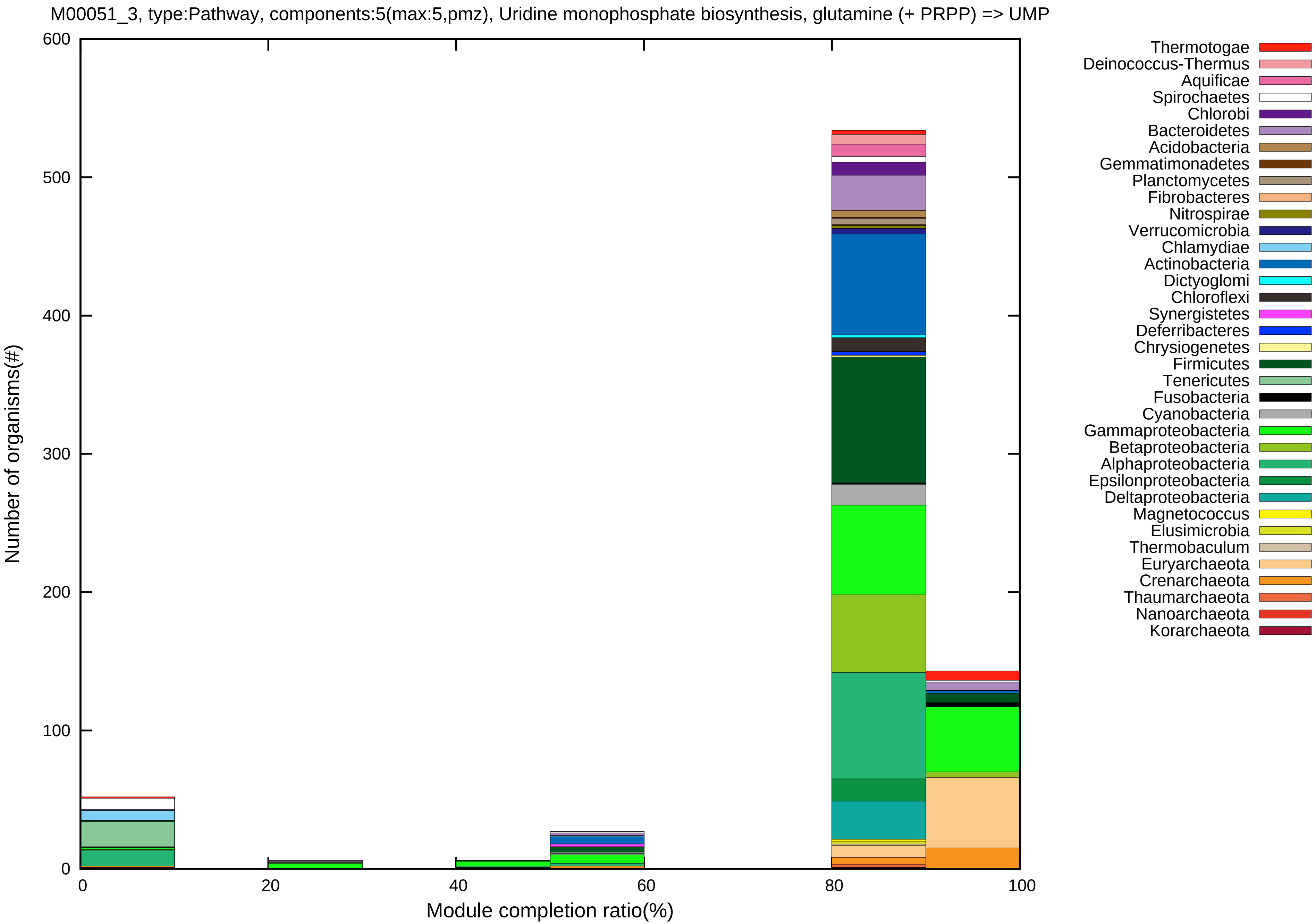
M00051_3, type:Pathway, components:5(max:5,pmz), Uridine monophosphate biosynthesis, glutamine (+ PRPP) => UMP
Number of organisms(#)
Module completion ratio(%)
0
20
40
60
80
100
100
200
300
400
500
600
Thermotogae
Deinococcus-Thermus
Aquificae
Spirochaetes
Chlorobi
Bacteroidetes
Acidobacteria
Gemmatimonadetes
Planctomycetes
Fibrobacteres
Nitrospirae
Verrucomicrobia
Chlamydiae
Actinobacteria
Dictyoglomi
Chloroflexi
Synergistetes
Deferribacteres
Chrysiogenetes
Firmicutes
Tenericutes
Fusobacteria
Cyanobacteria
Gammaproteobacteria
Betaproteobacteria
Alphaproteobacteria
Epsilonproteobacteria
Deltaproteobacteria
Magnetococcus
Elusimicrobia
Thermobaculum
Euryarchaeota
Crenarchaeota
Thaumarchaeota
Nanoarchaeota
Korarchaeota

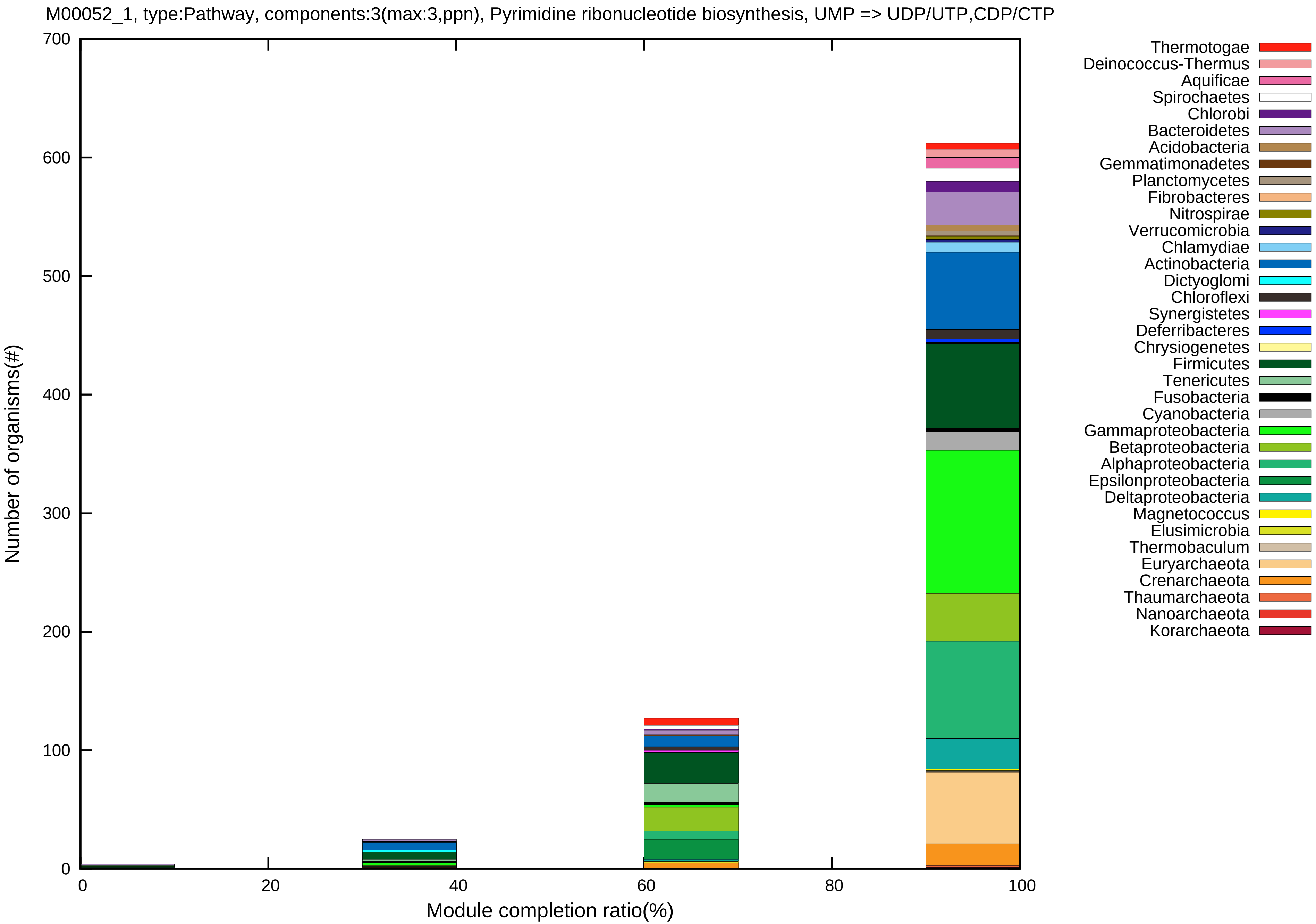

M00052_1, type:Pathway, components:3(max:3,ppn), Pyrimidine ribonucleotide biosynthesis, UMP => UDP/UTP,CDP/CTP
Number of organisms(#)
Module completion ratio(%)
0
100
200
300
400
500
600
700
0
20
40
60
80
100
Thermotogae
Deinococcus-Thermus
Aquificae
Spirochaetes
Chlorobi
Bacteroidetes
Acidobacteria
Gemmatimonadetes
Planctomycetes
Fibrobacteres
Nitrospirae
Verrucomicrobia
Chlamydiae
Actinobacteria
Dictyoglomi
Chloroflexi
Synergistetes
Deferribacteres
Chrysiogenetes
Firmicutes
Tenericutes
Fusobacteria
Cyanobacteria
Gammaproteobacteria
Betaproteobacteria
Alphaproteobacteria
Epsilonproteobacteria
Deltaproteobacteria
Magnetococcus
Elusimicrobia
Thermobaculum
Euryarchaeota
Crenarchaeota
Thaumarchaeota
Nanoarchaeota
Korarchaeota

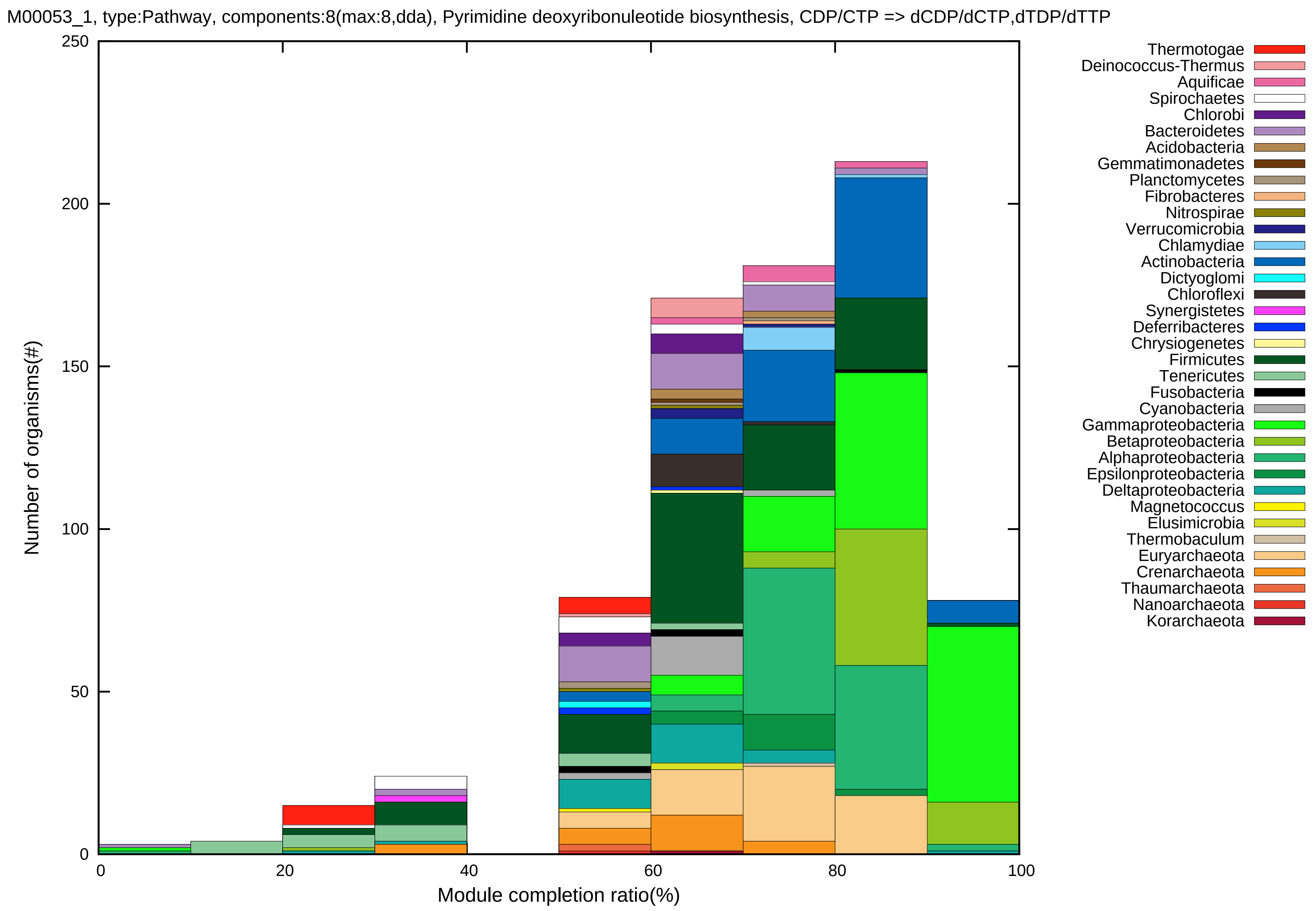
M00053_1, type:Pathway, components:8(max:8,dda), Pyrimidine deoxyribonuleotide biosynthesis, CDP/CTP => dCDP/dCTP,dTDP/dTTP
Number of organisms(#)
Module completion ratio(%)
0
50
100
150
200
250
0
20
40
60
80
100
Thermotogae
Deinococcus-Thermus
Aquificae
Spirochaetes
Chlorobi
Bacteroidetes
Acidobacteria
Gemmatimonadetes
Planctomycetes
Fibrobacteres
Nitrospirae
Verrucomicrobia
Chlamydiae
Actinobacteria
Dictyoglomi
Chloroflexi
Synergistetes
Deferribacteres
Chrysiogenetes
Firmicutes
Tenericutes
Fusobacteria
Cyanobacteria
Gammaproteobacteria
Betaproteobacteria
Alphaproteobacteria
Epsilonproteobacteria
Deltaproteobacteria
Magnetococcus
Elusimicrobia
Thermobaculum
Euryarchaeota
Crenarchaeota
Thaumarchaeota
Nanoarchaeota
Korarchaeota

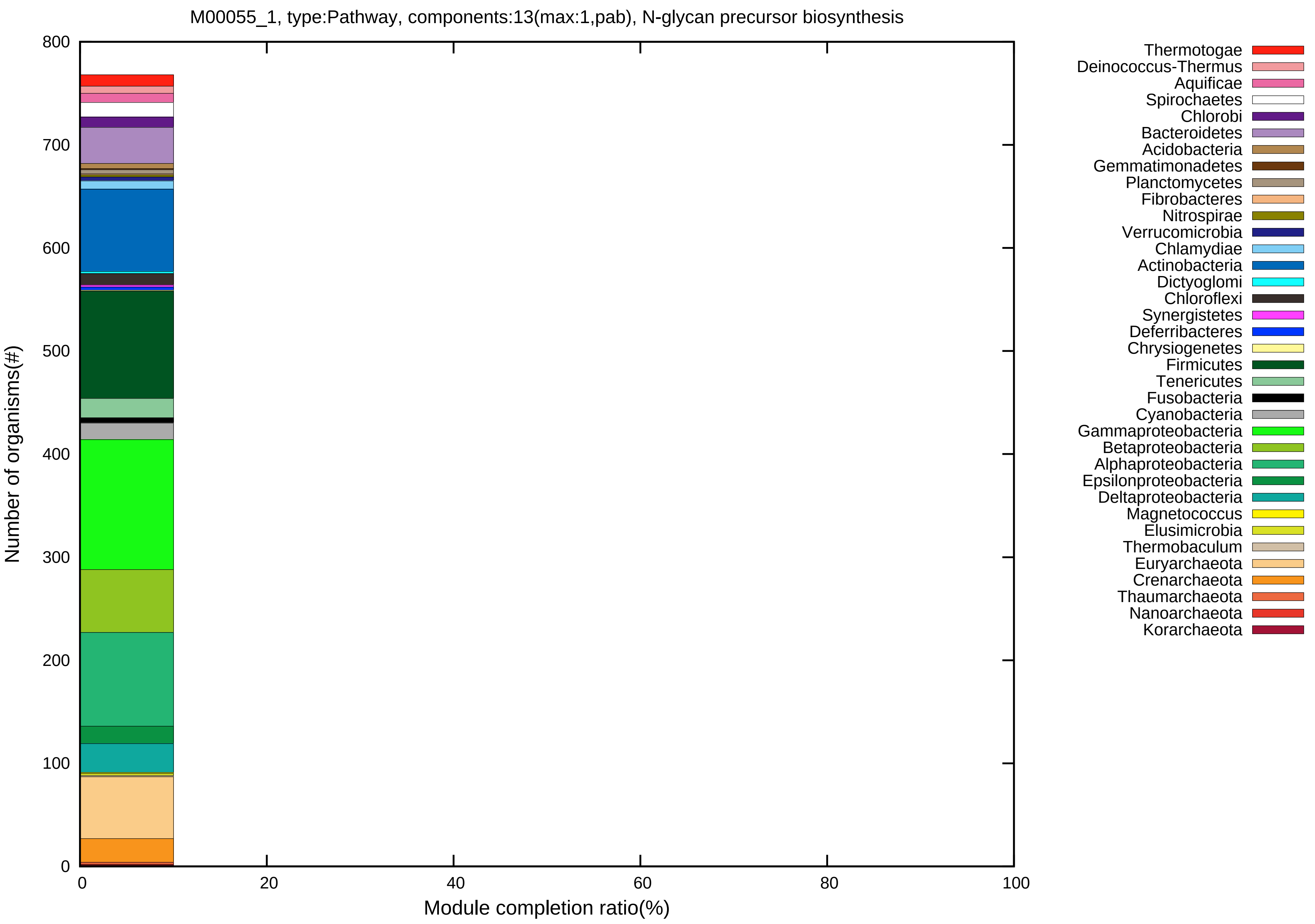

M00055_1, type:Pathway, components:13(max:1,pab), N-glycan precursor biosynthesis
Number of organisms(#)
Module completion ratio(%)
0
100
200
300
400
500
600
700
800
0
20
40
60
80
100
Thermotogae
Deinococcus-Thermus
Aquificae
Spirochaetes
Chlorobi
Bacteroidetes
Acidobacteria
Gemmatimonadetes
Planctomycetes
Fibrobacteres
Nitrospirae
Verrucomicrobia
Chlamydiae
Actinobacteria
Dictyoglomi
Chloroflexi
Synergistetes
Deferribacteres
Chrysiogenetes
Firmicutes
Tenericutes
Fusobacteria
Cyanobacteria
Gammaproteobacteria
Betaproteobacteria
Alphaproteobacteria
Epsilonproteobacteria
Deltaproteobacteria
Magnetococcus
Elusimicrobia
Thermobaculum
Euryarchaeota
Crenarchaeota
Thaumarchaeota
Nanoarchaeota
Korarchaeota

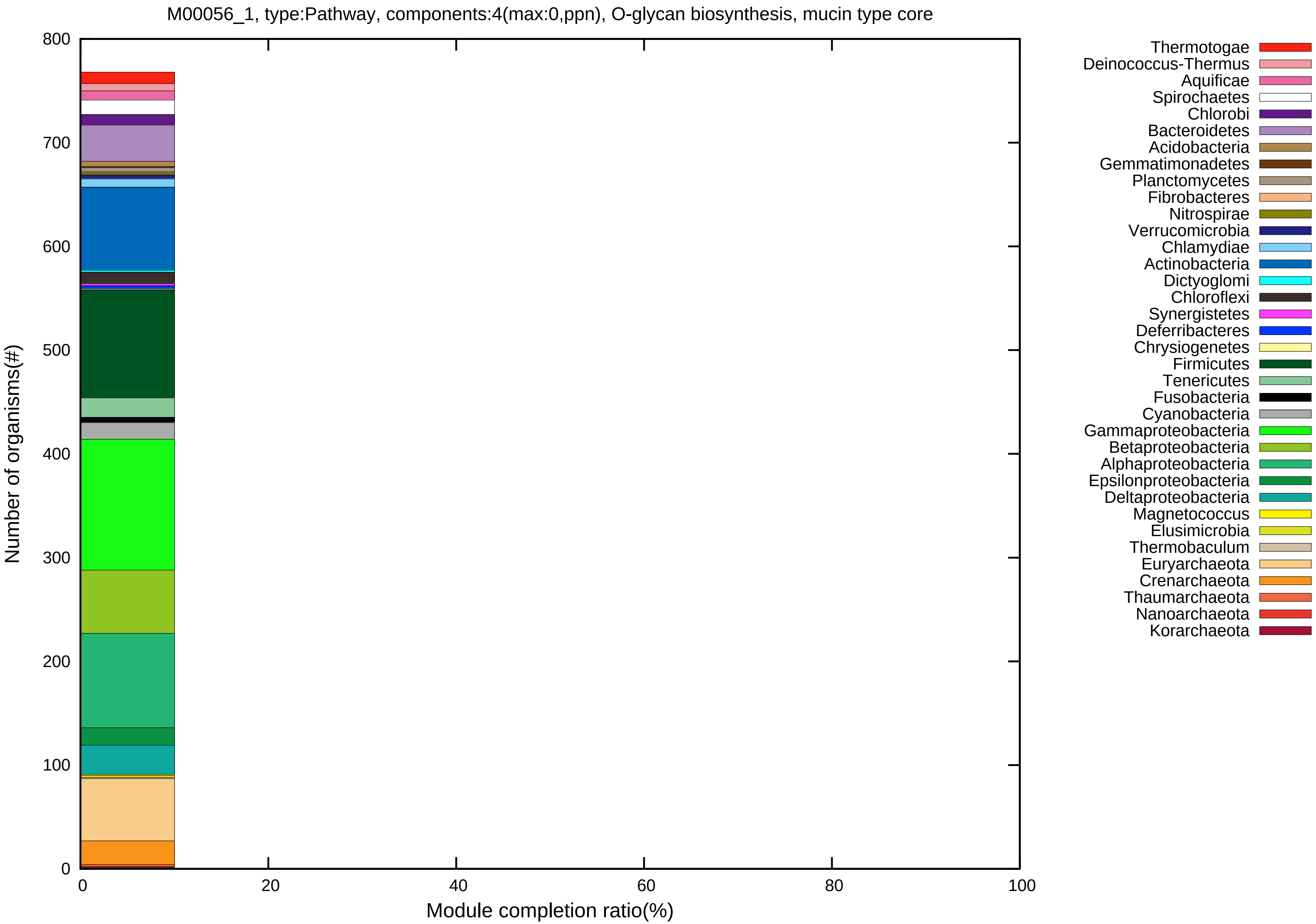
M00056_1, type:Pathway, components:4(max:0,ppn), O-glycan biosynthesis, mucin type core
Number of organisms(#)
Module completion ratio(%)
0
100
200
300
400
500
600
700
800
0
20
40
60
80
100
Thermotogae
Deinococcus-Thermus
Aquificae
Spirochaetes
Chlorobi
Bacteroidetes
Acidobacteria
Gemmatimonadetes
Planctomycetes
Fibrobacteres
Nitrospirae
Verrucomicrobia
Chlamydiae
Actinobacteria
Dictyoglomi
Chloroflexi
Synergistetes
Deferribacteres
Chrysiogenetes
Firmicutes
Tenericutes
Fusobacteria
Cyanobacteria
Gammaproteobacteria
Betaproteobacteria
Alphaproteobacteria
Epsilonproteobacteria
Deltaproteobacteria
Magnetococcus
Elusimicrobia
Thermobaculum
Euryarchaeota
Crenarchaeota
Thaumarchaeota
Nanoarchaeota
Korarchaeota

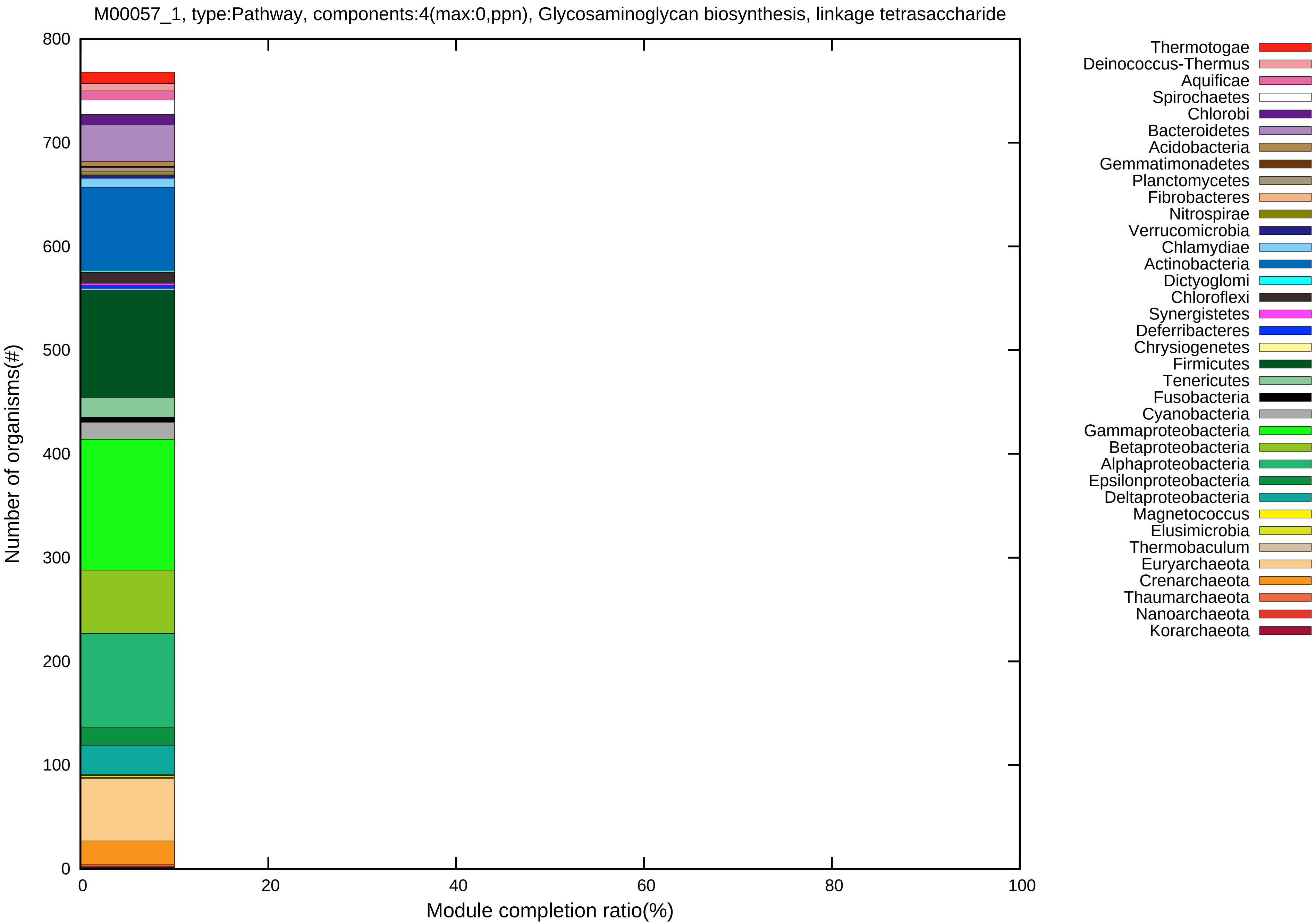

M00057_1, type:Pathway, components:4(max:0,ppn), Glycosaminoglycan biosynthesis, linkage tetrasaccharide
Number of organisms(#)
Module completion ratio(%)
0
100
200
300
400
500
600
700
800
0
20
40
60
80
100
Thermotogae
Deinococcus-Thermus
Aquificae
Spirochaetes
Chlorobi
Bacteroidetes
Acidobacteria
Gemmatimonadetes
Planctomycetes
Fibrobacteres
Nitrospirae
Verrucomicrobia
Chlamydiae
Actinobacteria
Dictyoglomi
Chloroflexi
Synergistetes
Deferribacteres
Chrysiogenetes
Firmicutes
Tenericutes
Fusobacteria
Cyanobacteria
Gammaproteobacteria
Betaproteobacteria
Alphaproteobacteria
Epsilonproteobacteria
Deltaproteobacteria
Magnetococcus
Elusimicrobia
Thermobaculum
Euryarchaeota
Crenarchaeota
Thaumarchaeota
Nanoarchaeota
Korarchaeota

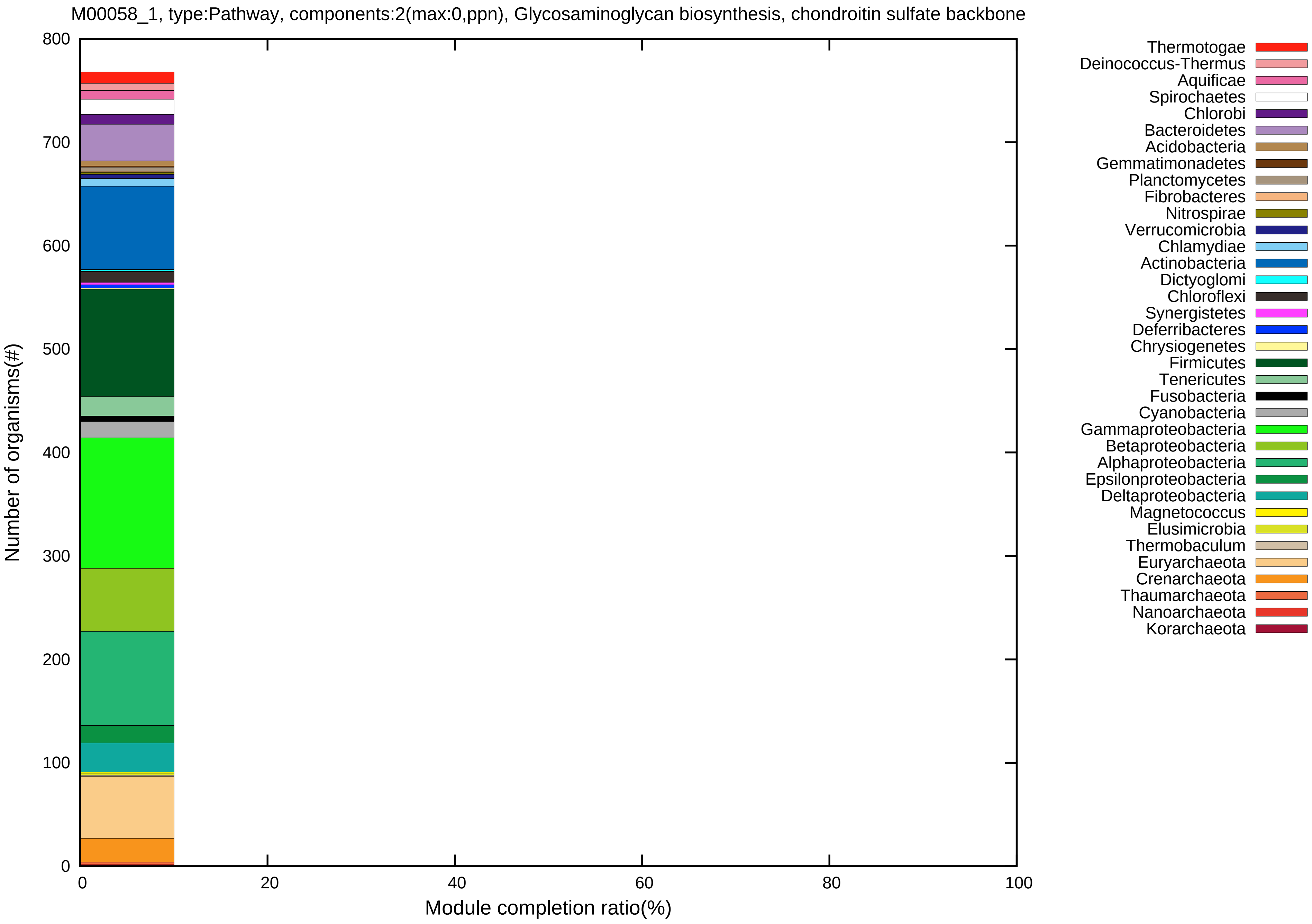

M00058_1, type:Pathway, components:2(max:0,ppn), Glycosaminoglycan biosynthesis, chondroitin sulfate backbone
Number of organisms(#)
Module completion ratio(%)
0
100
200
300
400
500
600
700
800
0
20
40
60
80
100
Thermotogae
Deinococcus-Thermus
Aquificae
Spirochaetes
Chlorobi
Bacteroidetes
Acidobacteria
Gemmatimonadetes
Planctomycetes
Fibrobacteres
Nitrospirae
Verrucomicrobia
Chlamydiae
Actinobacteria
Dictyoglomi
Chloroflexi
Synergistetes
Deferribacteres
Chrysiogenetes
Firmicutes
Tenericutes
Fusobacteria
Cyanobacteria
Gammaproteobacteria
Betaproteobacteria
Alphaproteobacteria
Epsilonproteobacteria
Deltaproteobacteria
Magnetococcus
Elusimicrobia
Thermobaculum
Euryarchaeota
Crenarchaeota
Thaumarchaeota
Nanoarchaeota
Korarchaeota

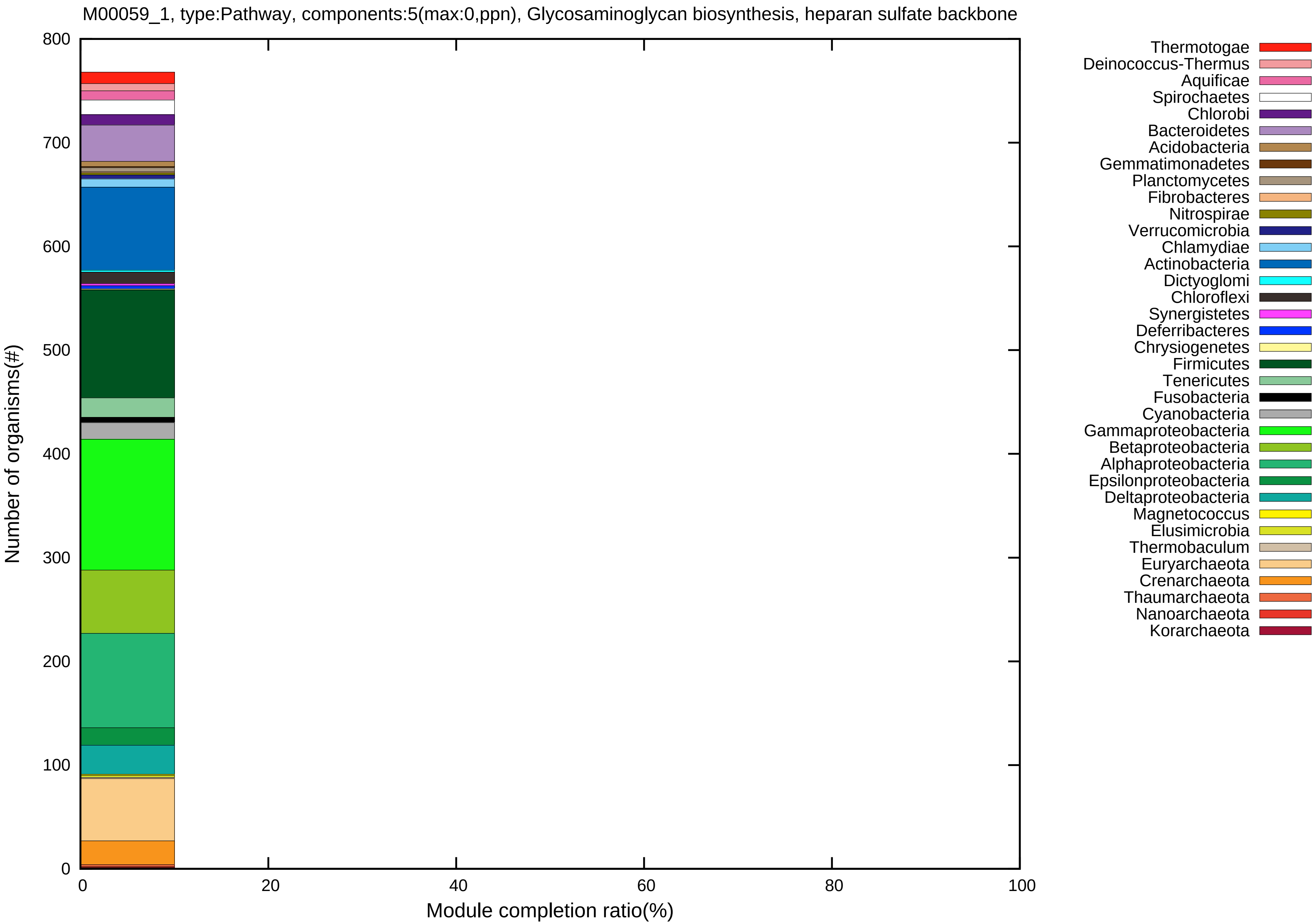
M00059_1, type:Pathway, components:5(max:0,ppn), Glycosaminoglycan biosynthesis, heparan sulfate backbone
Number of organisms(#)
Module completion ratio(%)
0
100
200
300
400
500
600
700
800
0
20
40
60
80
100
Thermotogae
Deinococcus-Thermus
Aquificae
Spirochaetes
Chlorobi
Bacteroidetes
Acidobacteria
Gemmatimonadetes
Planctomycetes
Fibrobacteres
Nitrospirae
Verrucomicrobia
Chlamydiae
Actinobacteria
Dictyoglomi
Chloroflexi
Synergistetes
Deferribacteres
Chrysiogenetes
Firmicutes
Tenericutes
Fusobacteria
Cyanobacteria
Gammaproteobacteria
Betaproteobacteria
Alphaproteobacteria
Epsilonproteobacteria
Deltaproteobacteria
Magnetococcus
Elusimicrobia
Thermobaculum
Euryarchaeota
Crenarchaeota
Thaumarchaeota
Nanoarchaeota
Korarchaeota

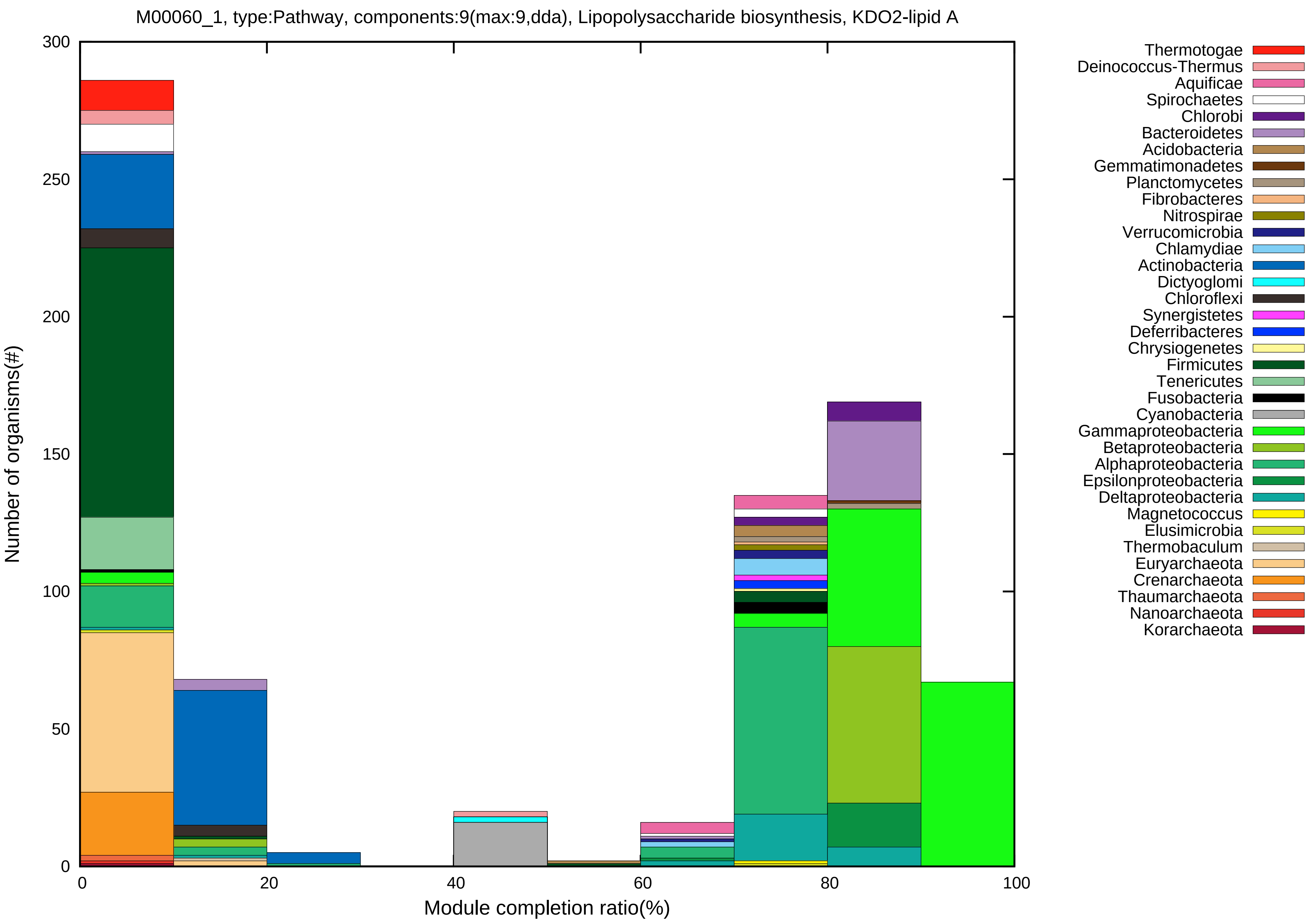
M00060_1, type:Pathway, components:9(max:9,dda), Lipopolysaccharide biosynthesis, KDO2-lipid A
Number of organisms(#)
Module completion ratio(%)
0
50
100
150
200
250
300
0
20
40
60
80
100
Thermotogae
Deinococcus-Thermus
Aquificae
Spirochaetes
Chlorobi
Bacteroidetes
Acidobacteria
Gemmatimonadetes
Planctomycetes
Fibrobacteres
Nitrospirae
Verrucomicrobia
Chlamydiae
Actinobacteria
Dictyoglomi
Chloroflexi
Synergistetes
Deferribacteres
Chrysiogenetes
Firmicutes
Tenericutes
Fusobacteria
Cyanobacteria
Gammaproteobacteria
Betaproteobacteria
Alphaproteobacteria
Epsilonproteobacteria
Deltaproteobacteria
Magnetococcus
Elusimicrobia
Thermobaculum
Euryarchaeota
Crenarchaeota
Thaumarchaeota
Nanoarchaeota
Korarchaeota

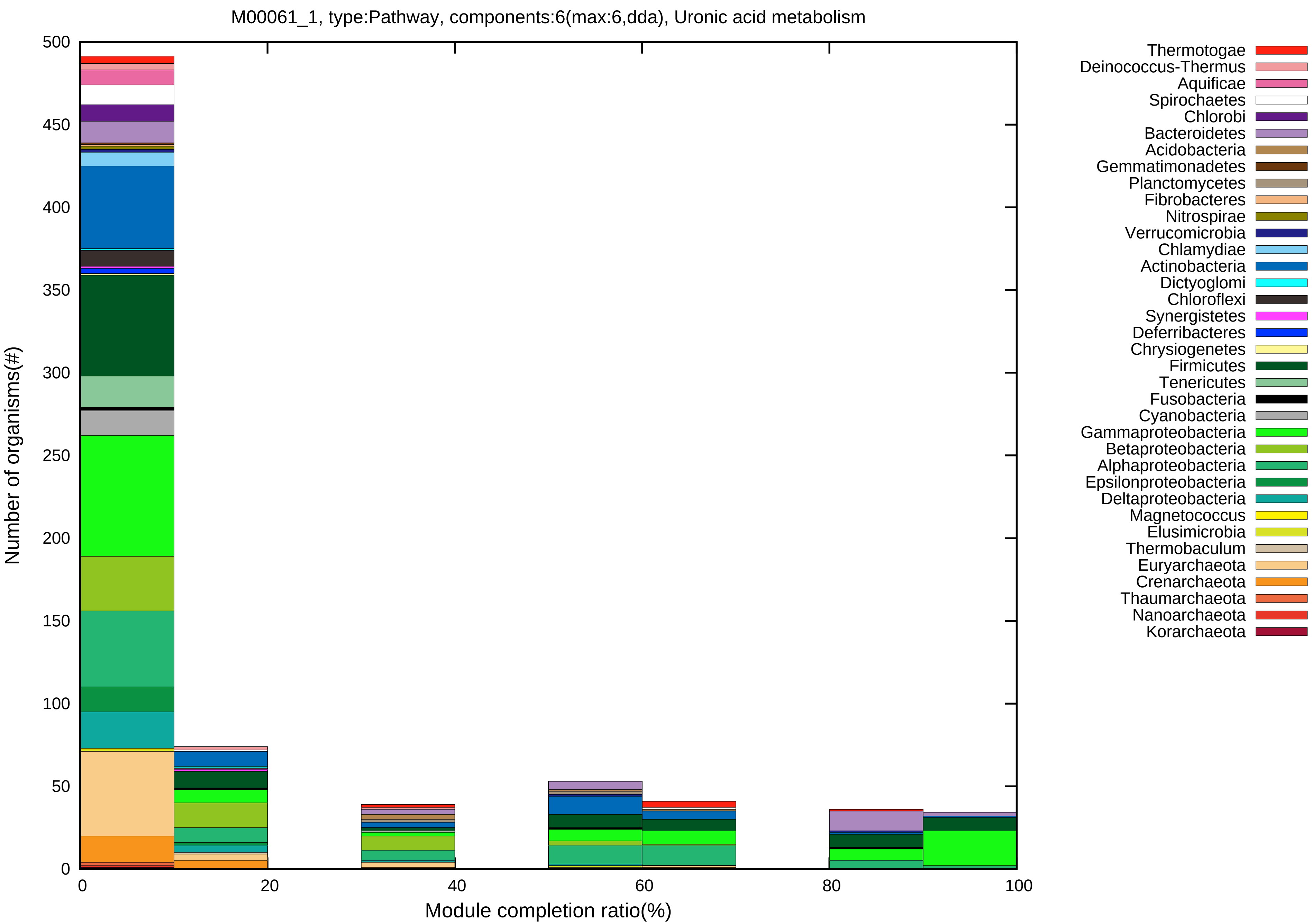

M00061_1, type:Pathway, components:6(max:6,dda), Uronic acid metabolism
Number of organisms(#)
Module completion ratio(%)
0
50
100
150
200
250
300
350
400
450
500
0
20
40
60
80
100
Thermotogae
Deinococcus-Thermus
Aquificae
Spirochaetes
Chlorobi
Bacteroidetes
Acidobacteria
Gemmatimonadetes
Planctomycetes
Fibrobacteres
Nitrospirae
Verrucomicrobia
Chlamydiae
Actinobacteria
Dictyoglomi
Chloroflexi
Synergistetes
Deferribacteres
Chrysiogenetes
Firmicutes
Tenericutes
Fusobacteria
Cyanobacteria
Gammaproteobacteria
Betaproteobacteria
Alphaproteobacteria
Epsilonproteobacteria
Deltaproteobacteria
Magnetococcus
Elusimicrobia
Thermobaculum
Euryarchaeota
Crenarchaeota
Thaumarchaeota
Nanoarchaeota
Korarchaeota

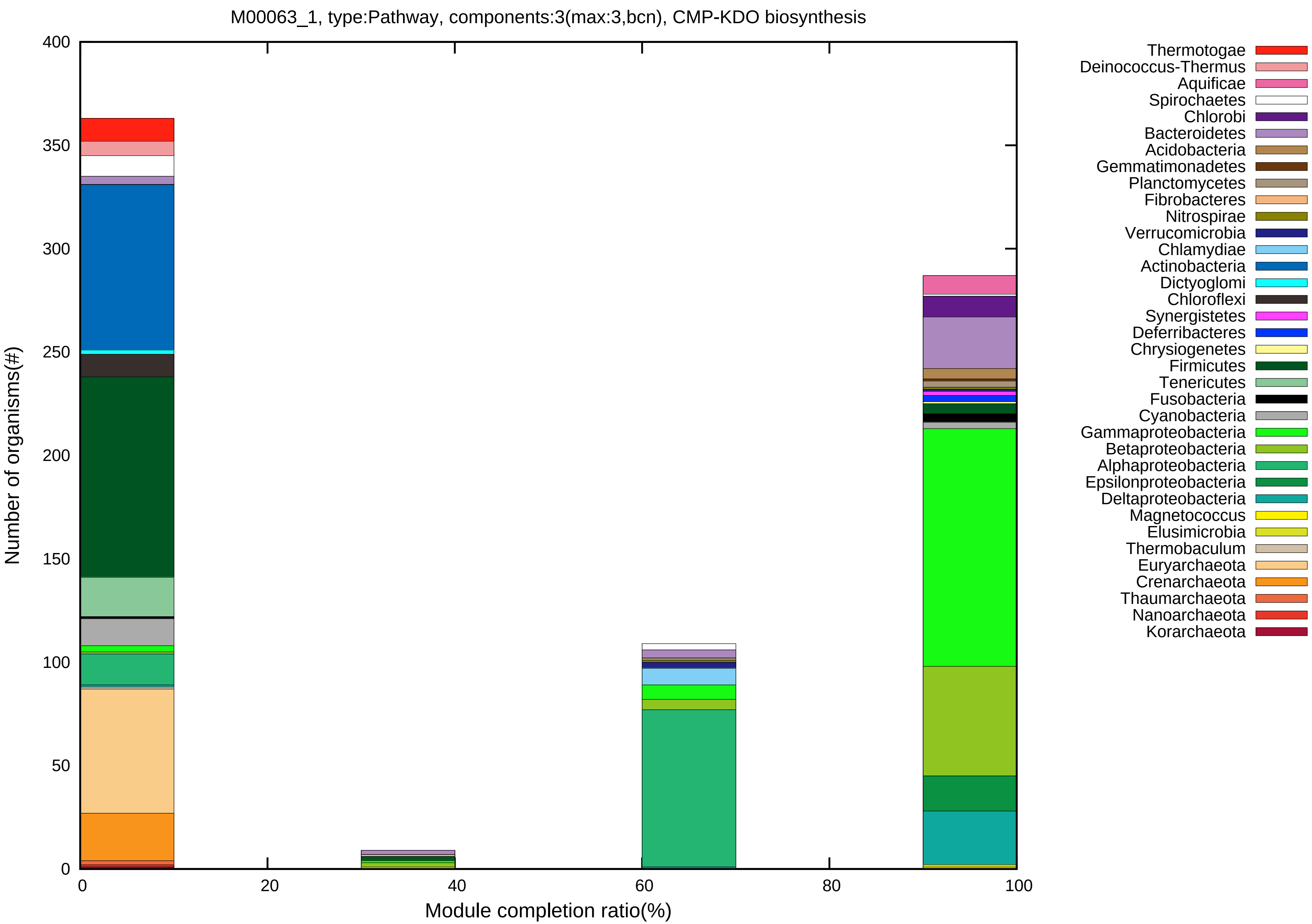
M00063_1, type:Pathway, components:3(max:3,bcn), CMP-KDO biosynthesis
Number of organisms(#)
Module completion ratio(%)
0
50
100
150
200
250
300
350
400
0
20
40
60
80
100
Thermotogae
Deinococcus-Thermus
Aquificae
Spirochaetes
Chlorobi
Bacteroidetes
Acidobacteria
Gemmatimonadetes
Planctomycetes
Fibrobacteres
Nitrospirae
Verrucomicrobia
Chlamydiae
Actinobacteria
Dictyoglomi
Chloroflexi
Synergistetes
Deferribacteres
Chrysiogenetes
Firmicutes
Tenericutes
Fusobacteria
Cyanobacteria
Gammaproteobacteria
Betaproteobacteria
Alphaproteobacteria
Epsilonproteobacteria
Deltaproteobacteria
Magnetococcus
Elusimicrobia
Thermobaculum
Euryarchaeota
Crenarchaeota
Thaumarchaeota
Nanoarchaeota
Korarchaeota

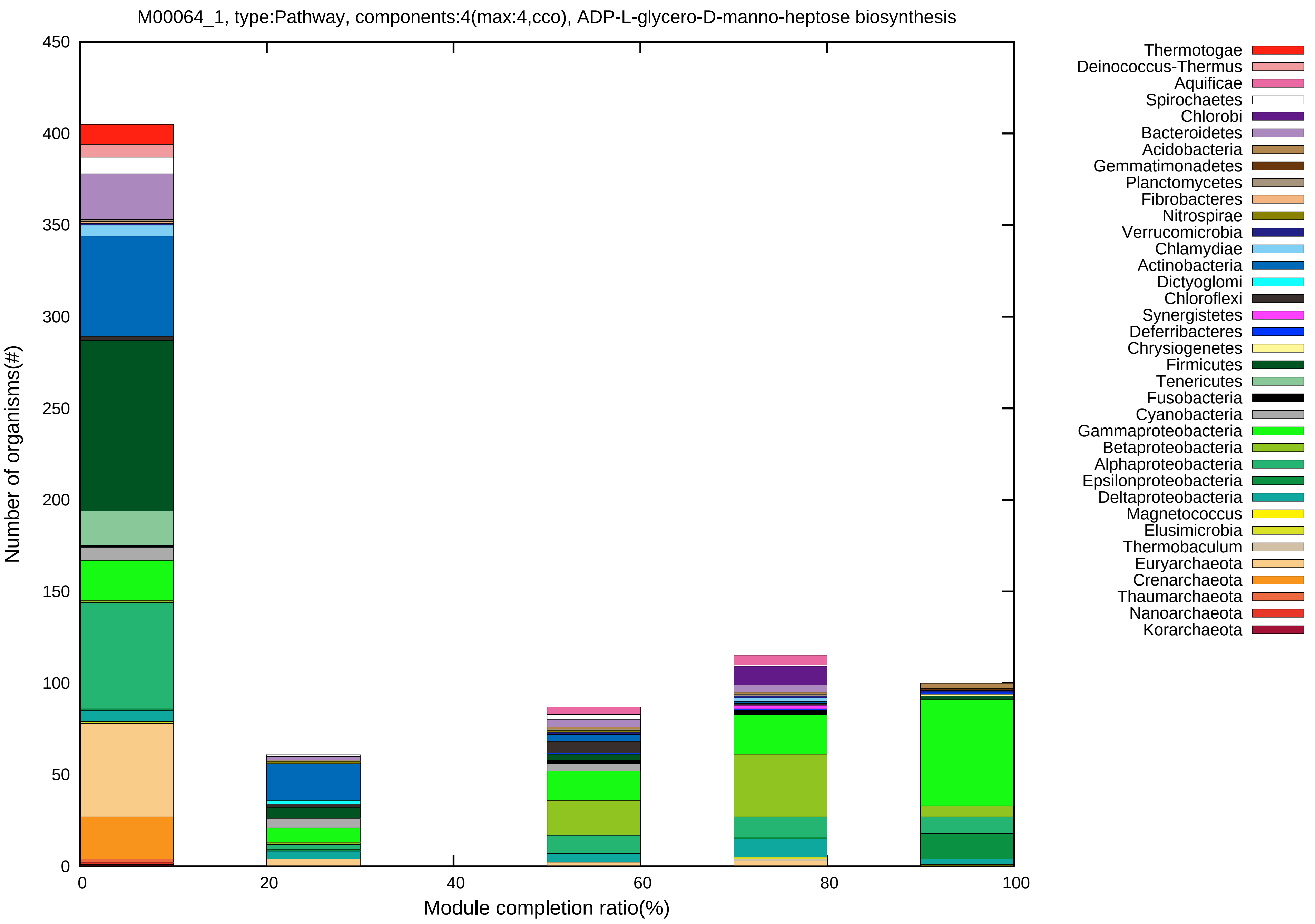

M00064_1, type:Pathway, components:4(max:4,cco), ADP-L-glycero-D-manno-heptose biosynthesis
Number of organisms(#)
Module completion ratio(%)
0
50
100
150
200
250
300
350
400
450
0
20
40
60
80
100
Thermotogae
Deinococcus-Thermus
Aquificae
Spirochaetes
Chlorobi
Bacteroidetes
Acidobacteria
Gemmatimonadetes
Planctomycetes
Fibrobacteres
Nitrospirae
Verrucomicrobia
Chlamydiae
Actinobacteria
Dictyoglomi
Chloroflexi
Synergistetes
Deferribacteres
Chrysiogenetes
Firmicutes
Tenericutes
Fusobacteria
Cyanobacteria
Gammaproteobacteria
Betaproteobacteria
Alphaproteobacteria
Epsilonproteobacteria
Deltaproteobacteria
Magnetococcus
Elusimicrobia
Thermobaculum
Euryarchaeota
Crenarchaeota
Thaumarchaeota
Nanoarchaeota
Korarchaeota

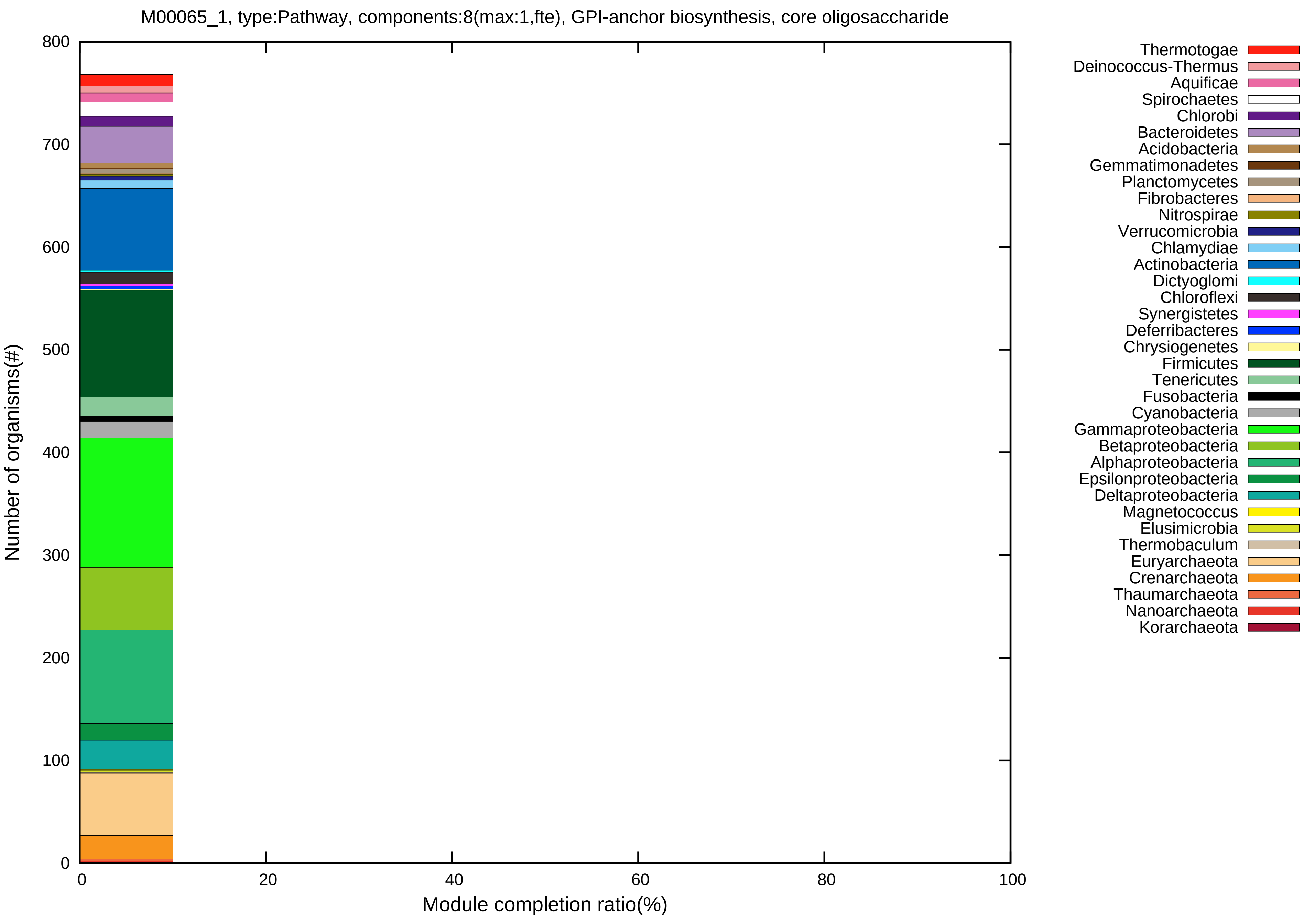
M00065_1, type:Pathway, components:8(max:1,fte), GPI-anchor biosynthesis, core oligosaccharide
Number of organisms(#)
Module completion ratio(%)
0
100
200
300
400
500
600
700
800
0
20
40
60
80
100
Thermotogae
Deinococcus-Thermus
Aquificae
Spirochaetes
Chlorobi
Bacteroidetes
Acidobacteria
Gemmatimonadetes
Planctomycetes
Fibrobacteres
Nitrospirae
Verrucomicrobia
Chlamydiae
Actinobacteria
Dictyoglomi
Chloroflexi
Synergistetes
Deferribacteres
Chrysiogenetes
Firmicutes
Tenericutes
Fusobacteria
Cyanobacteria
Gammaproteobacteria
Betaproteobacteria
Alphaproteobacteria
Epsilonproteobacteria
Deltaproteobacteria
Magnetococcus
Elusimicrobia
Thermobaculum
Euryarchaeota
Crenarchaeota
Thaumarchaeota
Nanoarchaeota
Korarchaeota

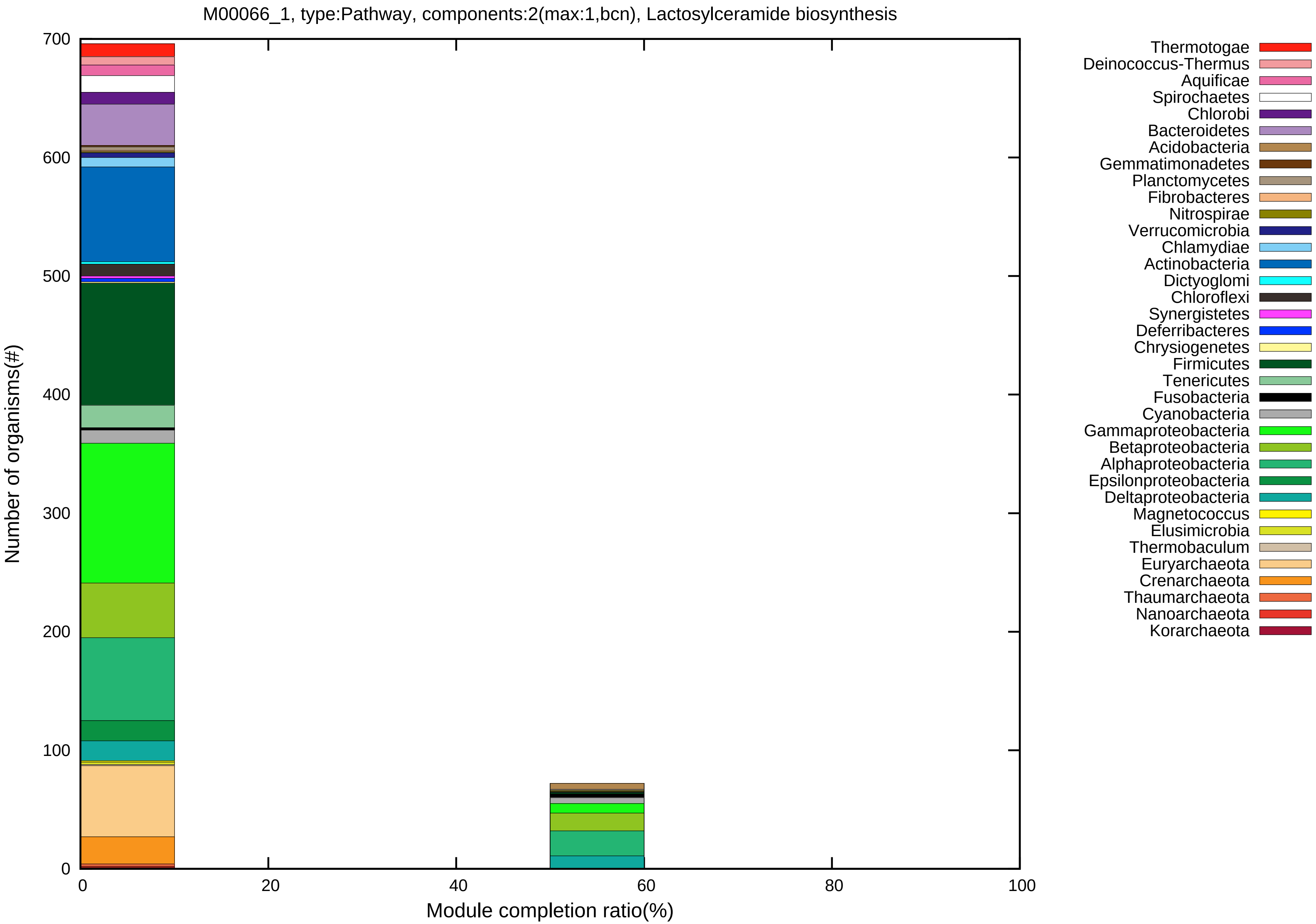
M00066_1, type:Pathway, components:2(max:1,bcn), Lactosylceramide biosynthesis
Number of organisms(#)
Module completion ratio(%)
0
100
200
300
400
500
600
700
0
20
40
60
80
100
Thermotogae
Deinococcus-Thermus
Aquificae
Spirochaetes
Chlorobi
Bacteroidetes
Acidobacteria
Gemmatimonadetes
Planctomycetes
Fibrobacteres
Nitrospirae
Verrucomicrobia
Chlamydiae
Actinobacteria
Dictyoglomi
Chloroflexi
Synergistetes
Deferribacteres
Chrysiogenetes
Firmicutes
Tenericutes
Fusobacteria
Cyanobacteria
Gammaproteobacteria
Betaproteobacteria
Alphaproteobacteria
Epsilonproteobacteria
Deltaproteobacteria
Magnetococcus
Elusimicrobia
Thermobaculum
Euryarchaeota
Crenarchaeota
Thaumarchaeota
Nanoarchaeota
Korarchaeota

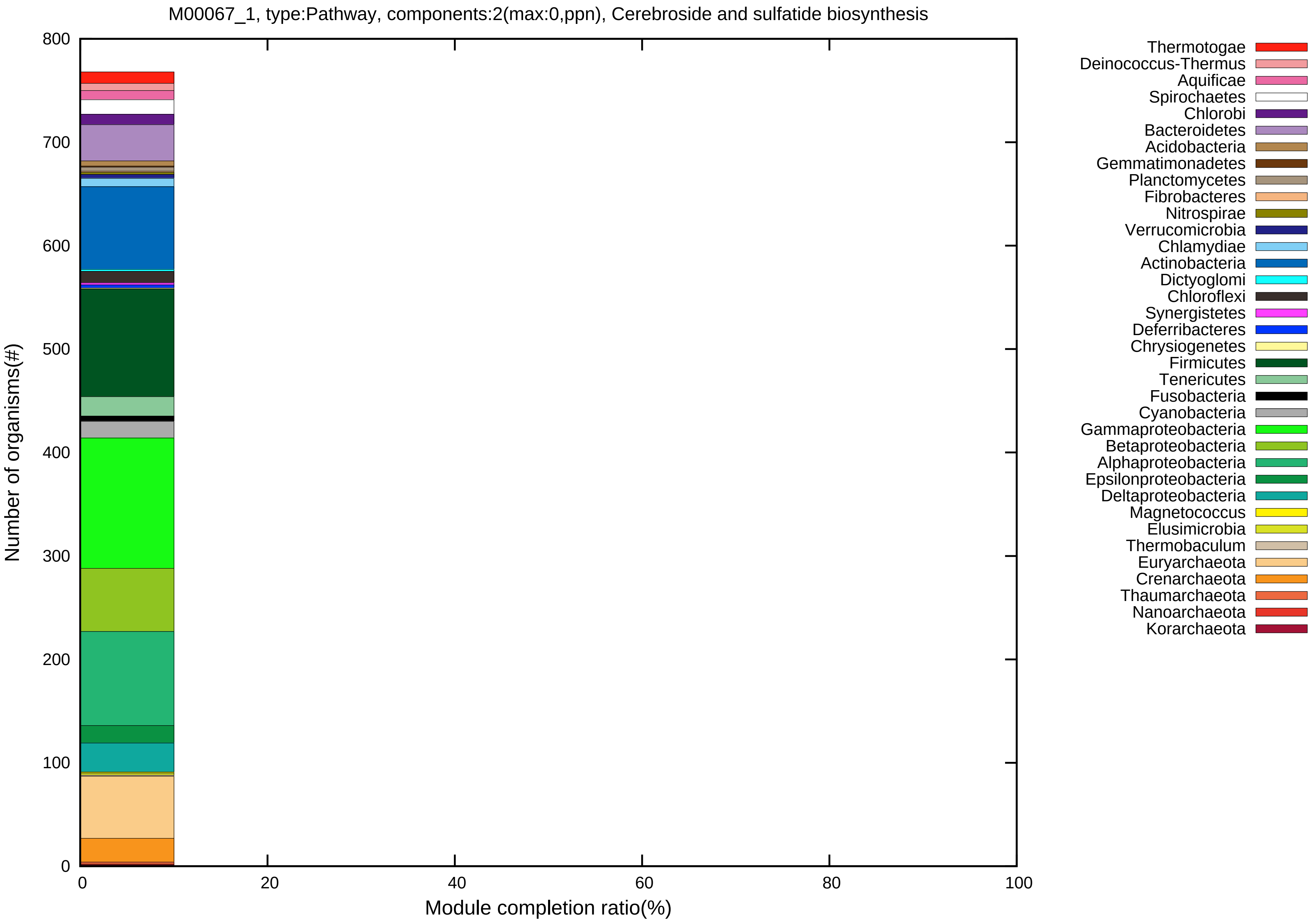

M00067_1, type:Pathway, components:2(max:0,ppn), Cerebroside and sulfatide biosynthesis
Number of organisms(#)
Module completion ratio(%)
0
100
200
300
400
500
600
700
800
0
20
40
60
80
100
Thermotogae
Deinococcus-Thermus
Aquificae
Spirochaetes
Chlorobi
Bacteroidetes
Acidobacteria
Gemmatimonadetes
Planctomycetes
Fibrobacteres
Nitrospirae
Verrucomicrobia
Chlamydiae
Actinobacteria
Dictyoglomi
Chloroflexi
Synergistetes
Deferribacteres
Chrysiogenetes
Firmicutes
Tenericutes
Fusobacteria
Cyanobacteria
Gammaproteobacteria
Betaproteobacteria
Alphaproteobacteria
Epsilonproteobacteria
Deltaproteobacteria
Magnetococcus
Elusimicrobia
Thermobaculum
Euryarchaeota
Crenarchaeota
Thaumarchaeota
Nanoarchaeota
Korarchaeota

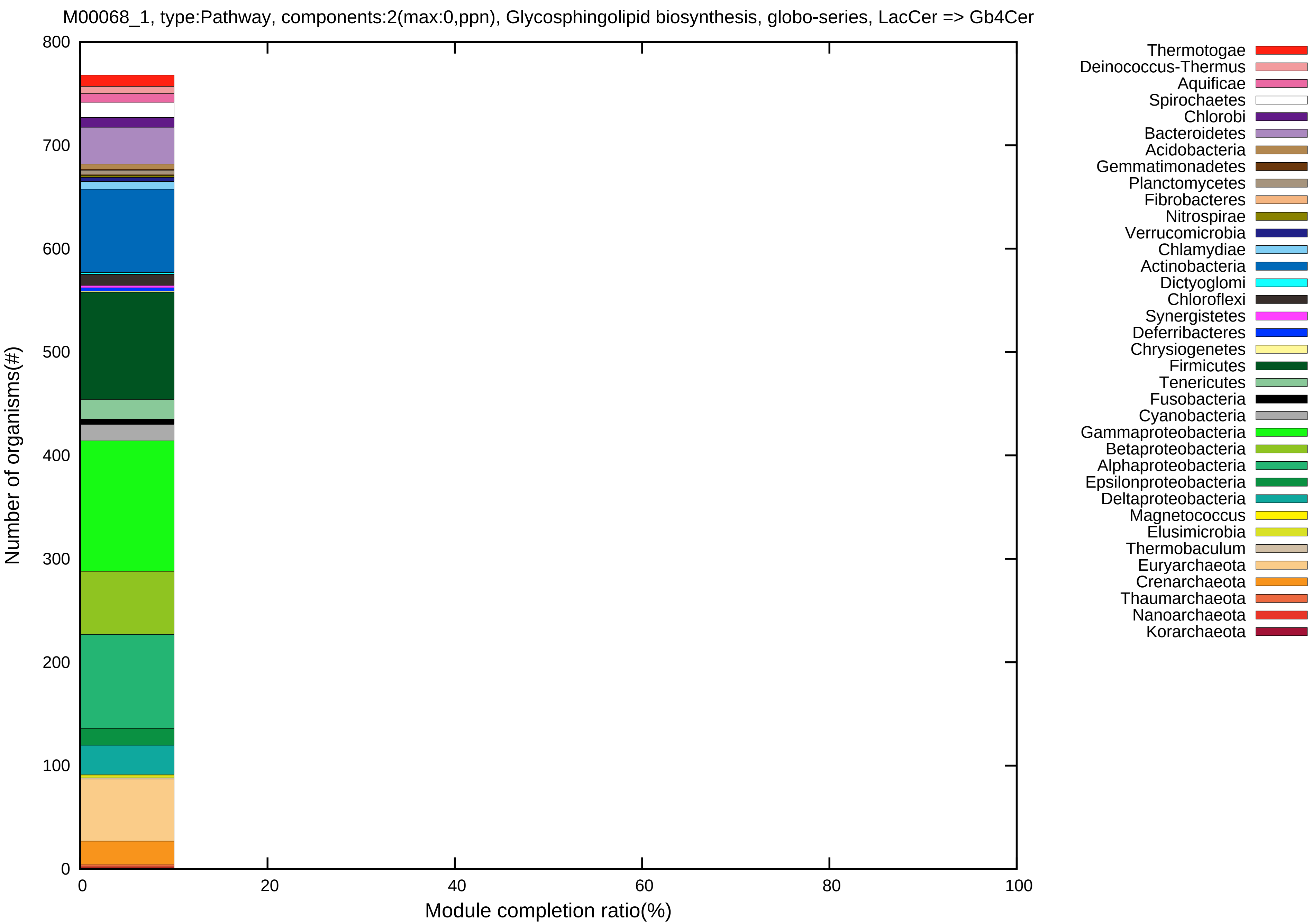
M00068_1, type:Pathway, components:2(max:0,ppn), Glycosphingolipid biosynthesis, globo-series, LacCer => Gb4Cer
Number of organisms(#)
Module completion ratio(%)
0
100
200
300
400
500
600
700
800
0
20
40
60
80
100
Thermotogae
Deinococcus-Thermus
Aquificae
Spirochaetes
Chlorobi
Bacteroidetes
Acidobacteria
Gemmatimonadetes
Planctomycetes
Fibrobacteres
Nitrospirae
Verrucomicrobia
Chlamydiae
Actinobacteria
Dictyoglomi
Chloroflexi
Synergistetes
Deferribacteres
Chrysiogenetes
Firmicutes
Tenericutes
Fusobacteria
Cyanobacteria
Gammaproteobacteria
Betaproteobacteria
Alphaproteobacteria
Epsilonproteobacteria
Deltaproteobacteria
Magnetococcus
Elusimicrobia
Thermobaculum
Euryarchaeota
Crenarchaeota
Thaumarchaeota
Nanoarchaeota
Korarchaeota

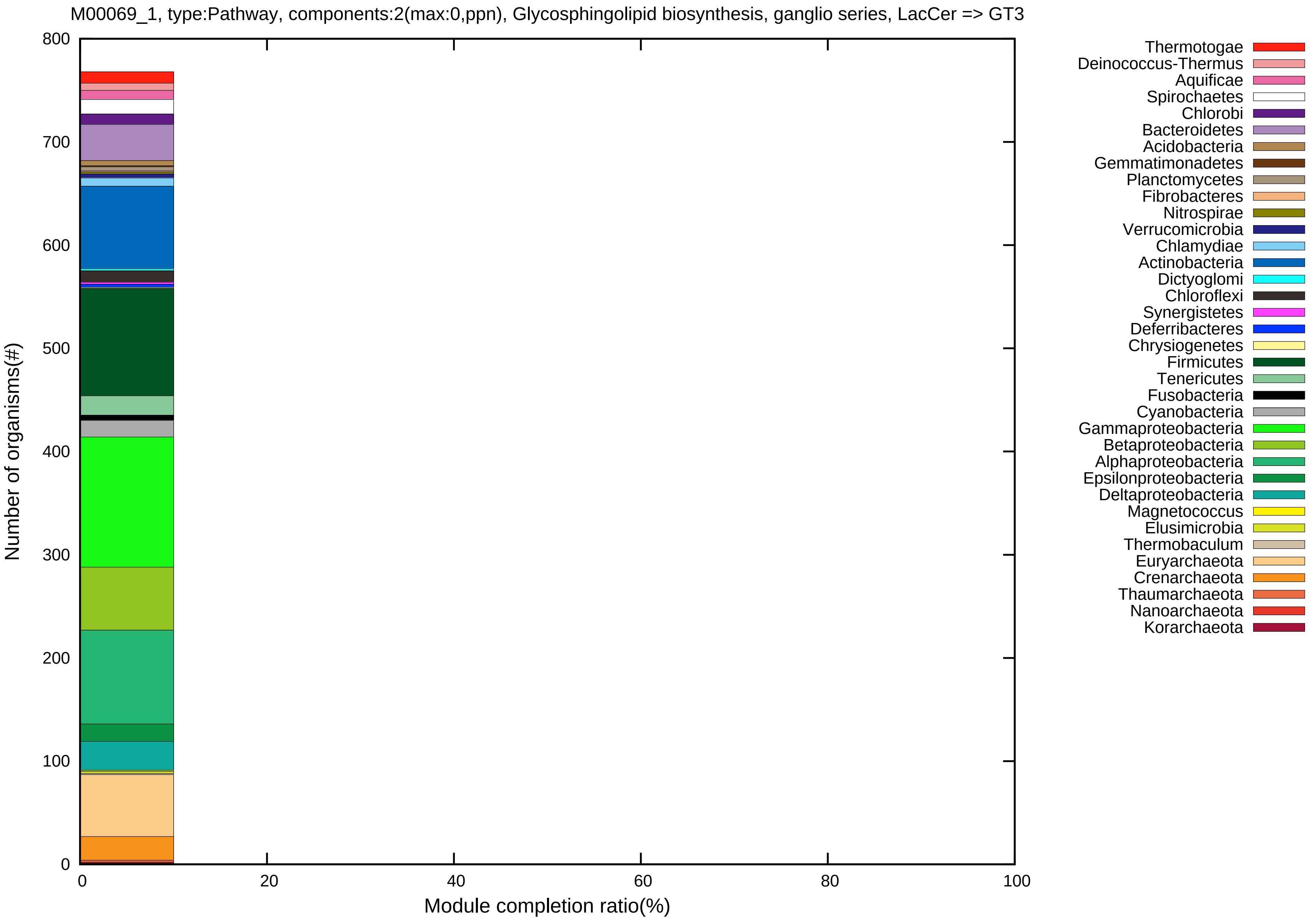

M00069_1, type:Pathway, components:2(max:0,ppn), Glycosphingolipid biosynthesis, ganglio series, LacCer => GT3
Number of organisms(#)
Module completion ratio(%)
0
100
200
300
400
500
600
700
800
0
20
40
60
80
100
Thermotogae
Deinococcus-Thermus
Aquificae
Spirochaetes
Chlorobi
Bacteroidetes
Acidobacteria
Gemmatimonadetes
Planctomycetes
Fibrobacteres
Nitrospirae
Verrucomicrobia
Chlamydiae
Actinobacteria
Dictyoglomi
Chloroflexi
Synergistetes
Deferribacteres
Chrysiogenetes
Firmicutes
Tenericutes
Fusobacteria
Cyanobacteria
Gammaproteobacteria
Betaproteobacteria
Alphaproteobacteria
Epsilonproteobacteria
Deltaproteobacteria
Magnetococcus
Elusimicrobia
Thermobaculum
Euryarchaeota
Crenarchaeota
Thaumarchaeota
Nanoarchaeota
Korarchaeota

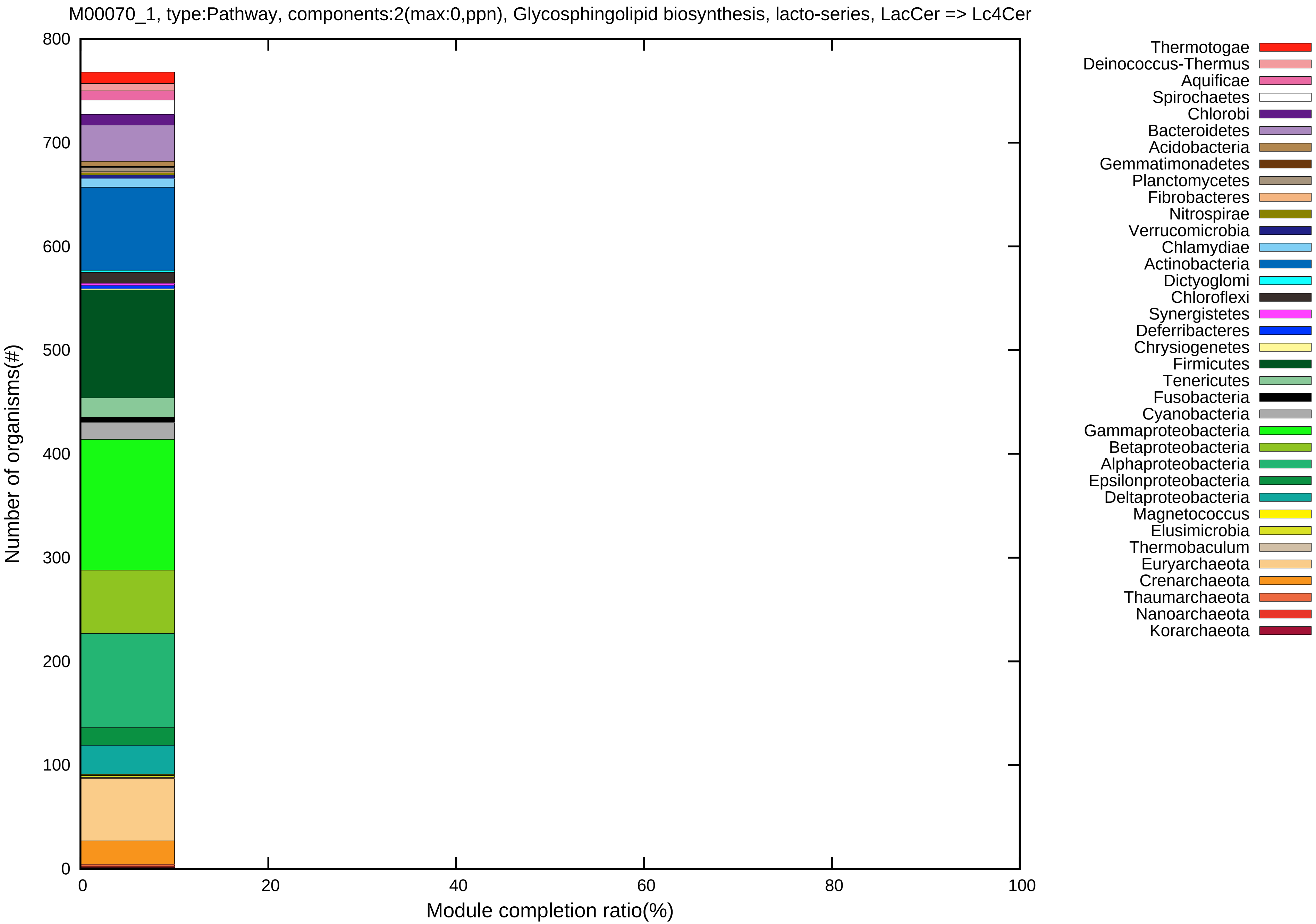
M00070_1, type:Pathway, components:2(max:0,ppn), Glycosphingolipid biosynthesis, lacto-series, LacCer => Lc4Cer
Number of organisms(#)
Module completion ratio(%)
0
100
200
300
400
500
600
700
800
0
20
40
60
80
100
Thermotogae
Deinococcus-Thermus
Aquificae
Spirochaetes
Chlorobi
Bacteroidetes
Acidobacteria
Gemmatimonadetes
Planctomycetes
Fibrobacteres
Nitrospirae
Verrucomicrobia
Chlamydiae
Actinobacteria
Dictyoglomi
Chloroflexi
Synergistetes
Deferribacteres
Chrysiogenetes
Firmicutes
Tenericutes
Fusobacteria
Cyanobacteria
Gammaproteobacteria
Betaproteobacteria
Alphaproteobacteria
Epsilonproteobacteria
Deltaproteobacteria
Magnetococcus
Elusimicrobia
Thermobaculum
Euryarchaeota
Crenarchaeota
Thaumarchaeota
Nanoarchaeota
Korarchaeota

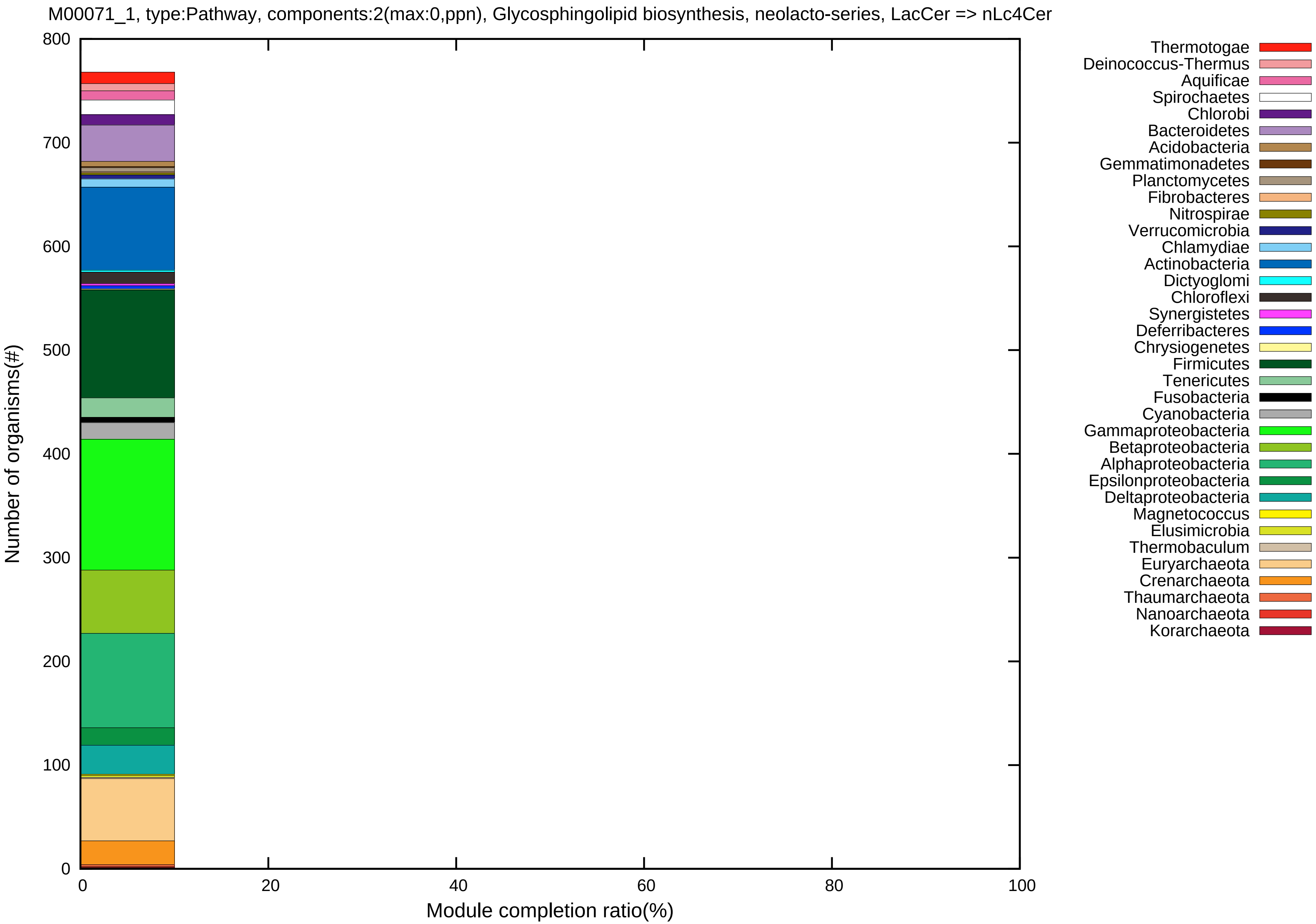

M00071_1, type:Pathway, components:2(max:0,ppn), Glycosphingolipid biosynthesis, neolacto-series, LacCer => nLc4Cer
Number of organisms(#)
0
100
200
300
400
500
600
700
800
Module completion ratio(%)
0
20
40
60
80
100
Thermotogae
Deinococcus-Thermus
Aquificae
Spirochaetes
Chlorobi
Bacteroidetes
Acidobacteria
Gemmatimonadetes
Planctomycetes
Fibrobacteres
Nitrospirae
Verrucomicrobia
Chlamydiae
Actinobacteria
Dictyoglomi
Chloroflexi
Synergistetes
Deferribacteres
Chrysiogenetes
Firmicutes
Tenericutes
Fusobacteria
Cyanobacteria
Gammaproteobacteria
Betaproteobacteria
Alphaproteobacteria
Epsilonproteobacteria
Deltaproteobacteria
Magnetococcus
Elusimicrobia
Thermobaculum
Euryarchaeota
Crenarchaeota
Thaumarchaeota
Nanoarchaeota
Korarchaeota

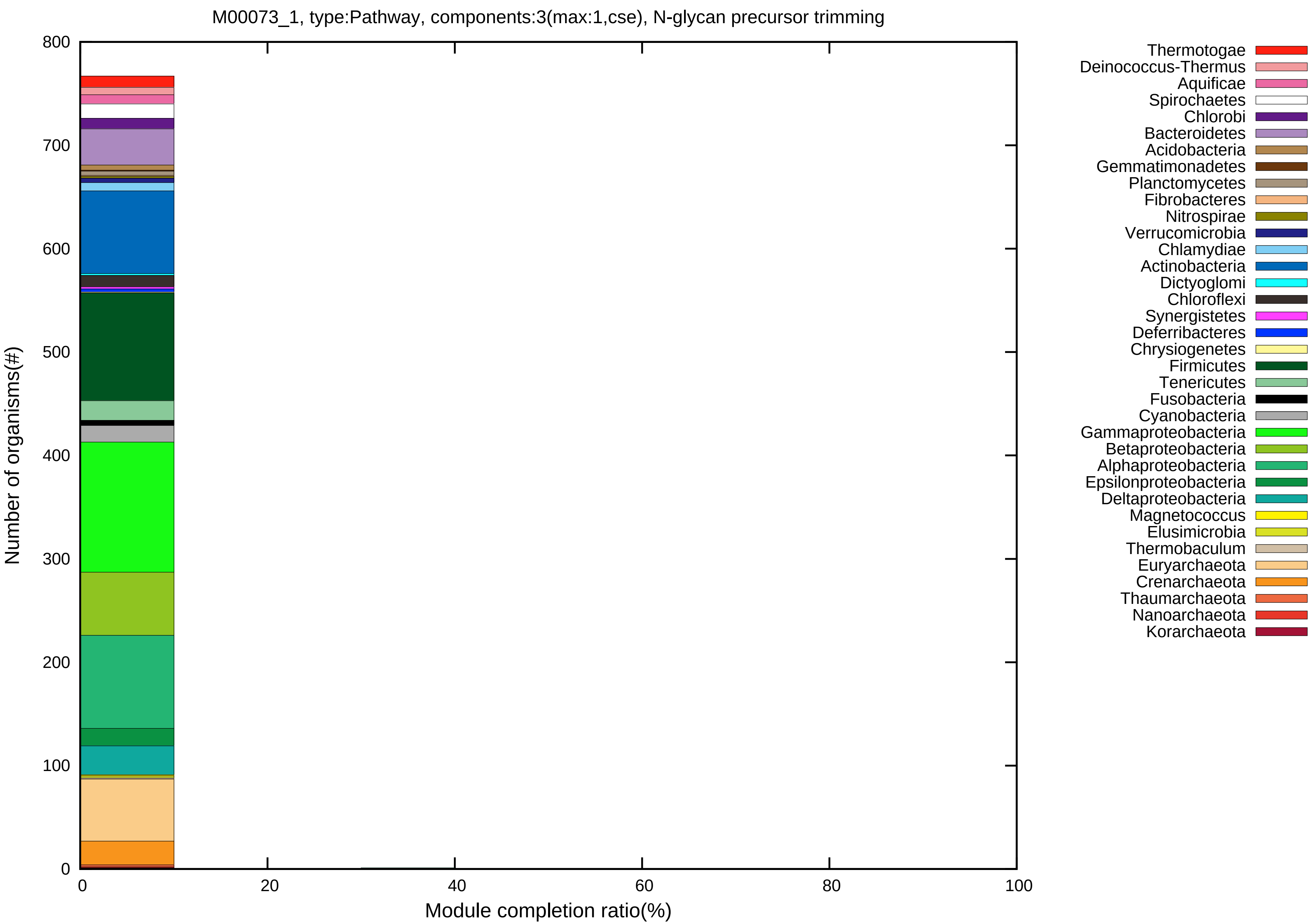

M00073_1, type:Pathway, components:3(max:1,cse), N-glycan precursor trimming
Number of organisms(#)
Module completion ratio(%)
0
100
200
300
400
500
600
700
800
0
20
40
60
80
100
Thermotogae
Deinococcus-Thermus
Aquificae
Spirochaetes
Chlorobi
Bacteroidetes
Acidobacteria
Gemmatimonadetes
Planctomycetes
Fibrobacteres
Nitrospirae
Verrucomicrobia
Chlamydiae
Actinobacteria
Dictyoglomi
Chloroflexi
Synergistetes
Deferribacteres
Chrysiogenetes
Firmicutes
Tenericutes
Fusobacteria
Cyanobacteria
Gammaproteobacteria
Betaproteobacteria
Alphaproteobacteria
Epsilonproteobacteria
Deltaproteobacteria
Magnetococcus
Elusimicrobia
Thermobaculum
Euryarchaeota
Crenarchaeota
Thaumarchaeota
Nanoarchaeota
Korarchaeota

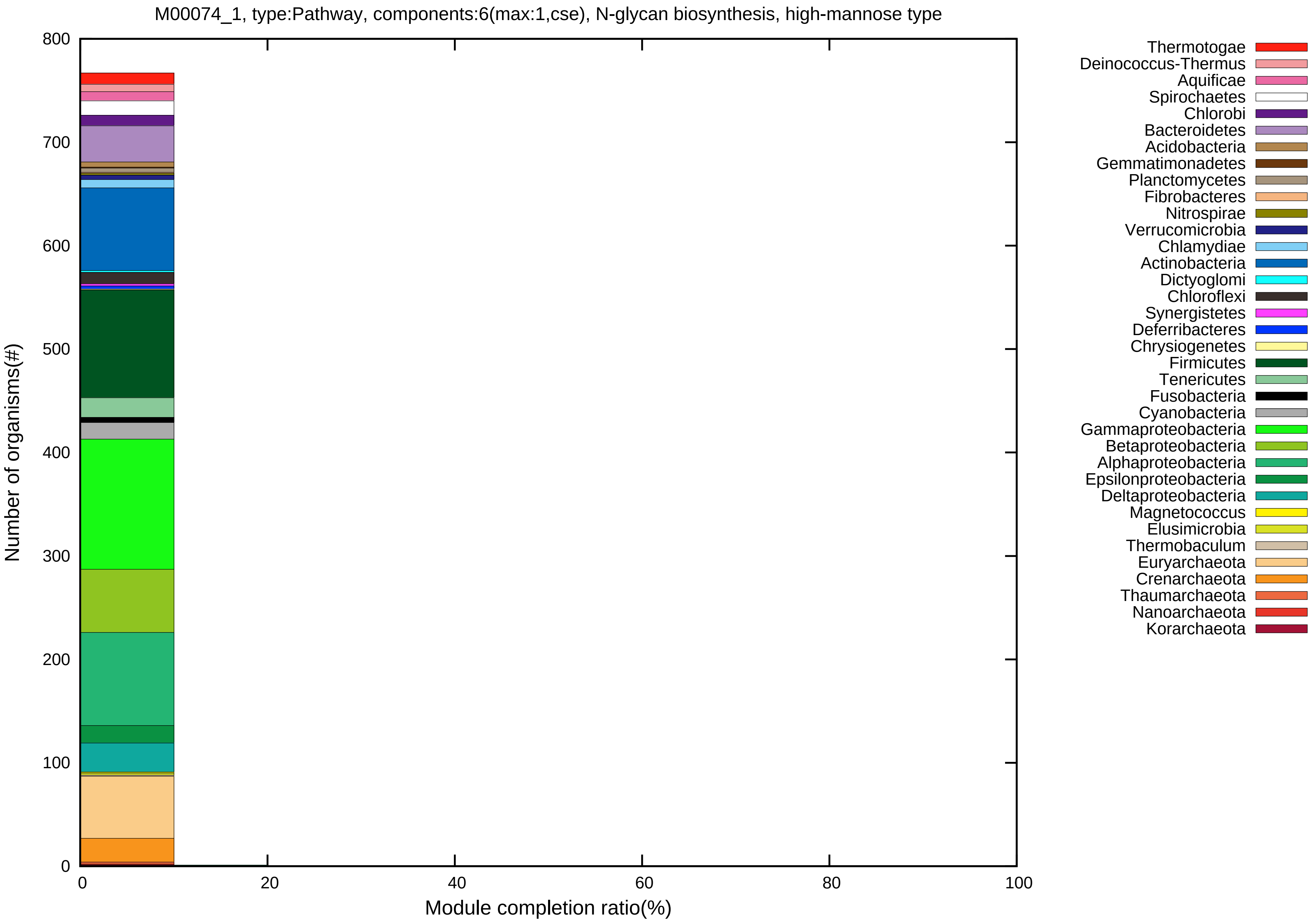
M00074_1, type:Pathway, components:6(max:1,cse), N-glycan biosynthesis, high-mannose type
Number of organisms(#)
Module completion ratio(%)
0
100
200
300
400
500
600
700
800
0
20
40
60
80
100
Thermotogae
Deinococcus-Thermus
Aquificae
Spirochaetes
Chlorobi
Bacteroidetes
Acidobacteria
Gemmatimonadetes
Planctomycetes
Fibrobacteres
Nitrospirae
Verrucomicrobia
Chlamydiae
Actinobacteria
Dictyoglomi
Chloroflexi
Synergistetes
Deferribacteres
Chrysiogenetes
Firmicutes
Tenericutes
Fusobacteria
Cyanobacteria
Gammaproteobacteria
Betaproteobacteria
Alphaproteobacteria
Epsilonproteobacteria
Deltaproteobacteria
Magnetococcus
Elusimicrobia
Thermobaculum
Euryarchaeota
Crenarchaeota
Thaumarchaeota
Nanoarchaeota
Korarchaeota

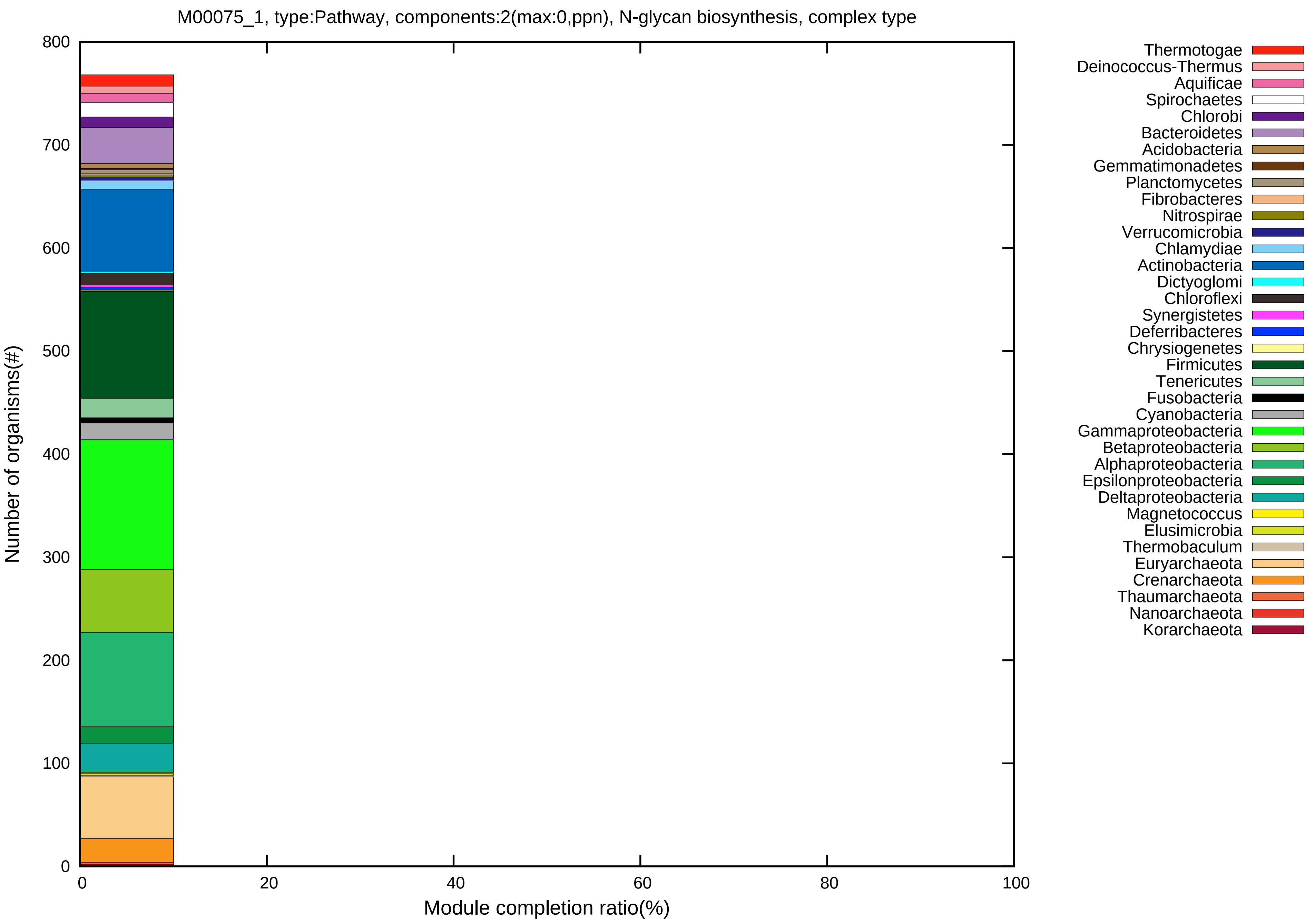
M00075_1, type:Pathway, components:2(max:0,ppn), N-glycan biosynthesis, complex type
Number of organisms(#)
Module completion ratio(%)
0
100
200
300
400
500
600
700
800
0
20
40
60
80
100
Thermotogae
Deinococcus-Thermus
Aquificae
Spirochaetes
Chlorobi
Bacteroidetes
Acidobacteria
Gemmatimonadetes
Planctomycetes
Fibrobacteres
Nitrospirae
Verrucomicrobia
Chlamydiae
Actinobacteria
Dictyoglomi
Chloroflexi
Synergistetes
Deferribacteres
Chrysiogenetes
Firmicutes
Tenericutes
Fusobacteria
Cyanobacteria
Gammaproteobacteria
Betaproteobacteria
Alphaproteobacteria
Epsilonproteobacteria
Deltaproteobacteria
Magnetococcus
Elusimicrobia
Thermobaculum
Euryarchaeota
Crenarchaeota
Thaumarchaeota
Nanoarchaeota
Korarchaeota

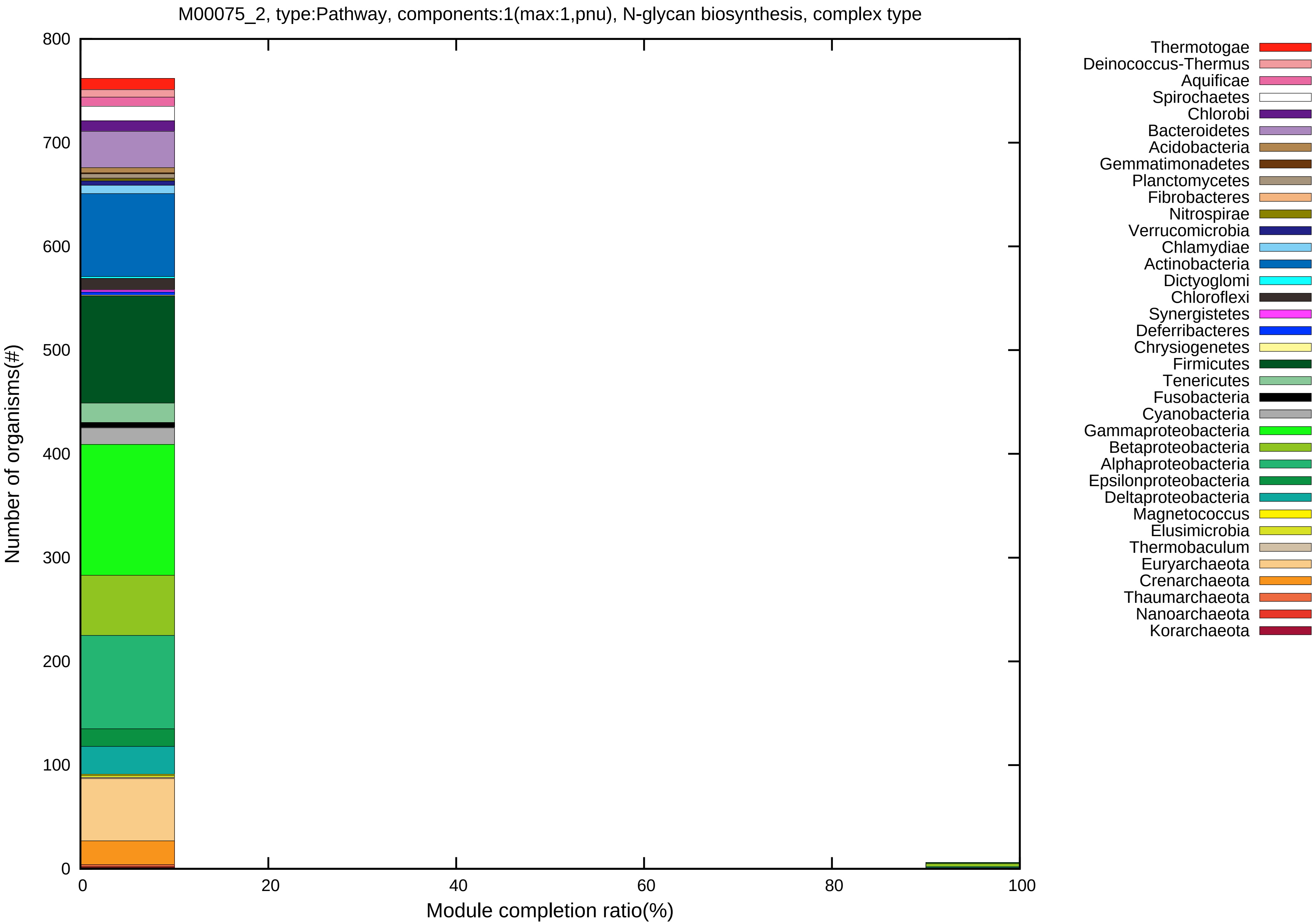

M00075_2, type:Pathway, components:1(max:1,pnu), N-glycan biosynthesis, complex type
Number of organisms(#)
Module completion ratio(%)
0
100
200
300
400
500
600
700
800
0
20
40
60
80
100
Thermotogae
Deinococcus-Thermus
Aquificae
Spirochaetes
Chlorobi
Bacteroidetes
Acidobacteria
Gemmatimonadetes
Planctomycetes
Fibrobacteres
Nitrospirae
Verrucomicrobia
Chlamydiae
Actinobacteria
Dictyoglomi
Chloroflexi
Synergistetes
Deferribacteres
Chrysiogenetes
Firmicutes
Tenericutes
Fusobacteria
Cyanobacteria
Gammaproteobacteria
Betaproteobacteria
Alphaproteobacteria
Epsilonproteobacteria
Deltaproteobacteria
Magnetococcus
Elusimicrobia
Thermobaculum
Euryarchaeota
Crenarchaeota
Thaumarchaeota
Nanoarchaeota
Korarchaeota

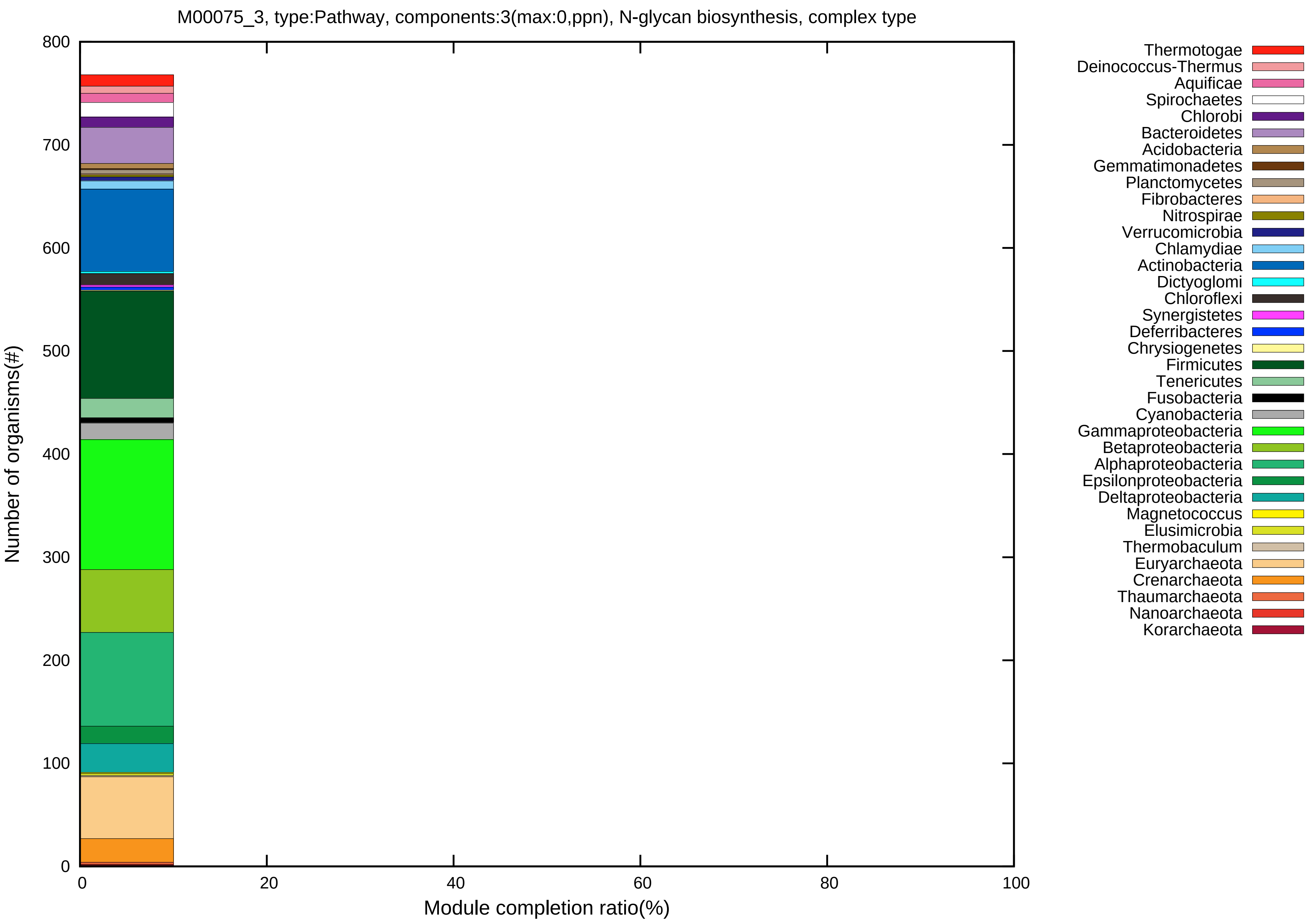
M00075_3, type:Pathway, components:3(max:0,ppn), N-glycan biosynthesis, complex type
Number of organisms(#)
Module completion ratio(%)
0
100
200
300
400
500
600
700
800
0
20
40
60
80
100
Thermotogae
Deinococcus-Thermus
Aquificae
Spirochaetes
Chlorobi
Bacteroidetes
Acidobacteria
Gemmatimonadetes
Planctomycetes
Fibrobacteres
Nitrospirae
Verrucomicrobia
Chlamydiae
Actinobacteria
Dictyoglomi
Chloroflexi
Synergistetes
Deferribacteres
Chrysiogenetes
Firmicutes
Tenericutes
Fusobacteria
Cyanobacteria
Gammaproteobacteria
Betaproteobacteria
Alphaproteobacteria
Epsilonproteobacteria
Deltaproteobacteria
Magnetococcus
Elusimicrobia
Thermobaculum
Euryarchaeota
Crenarchaeota
Thaumarchaeota
Nanoarchaeota
Korarchaeota

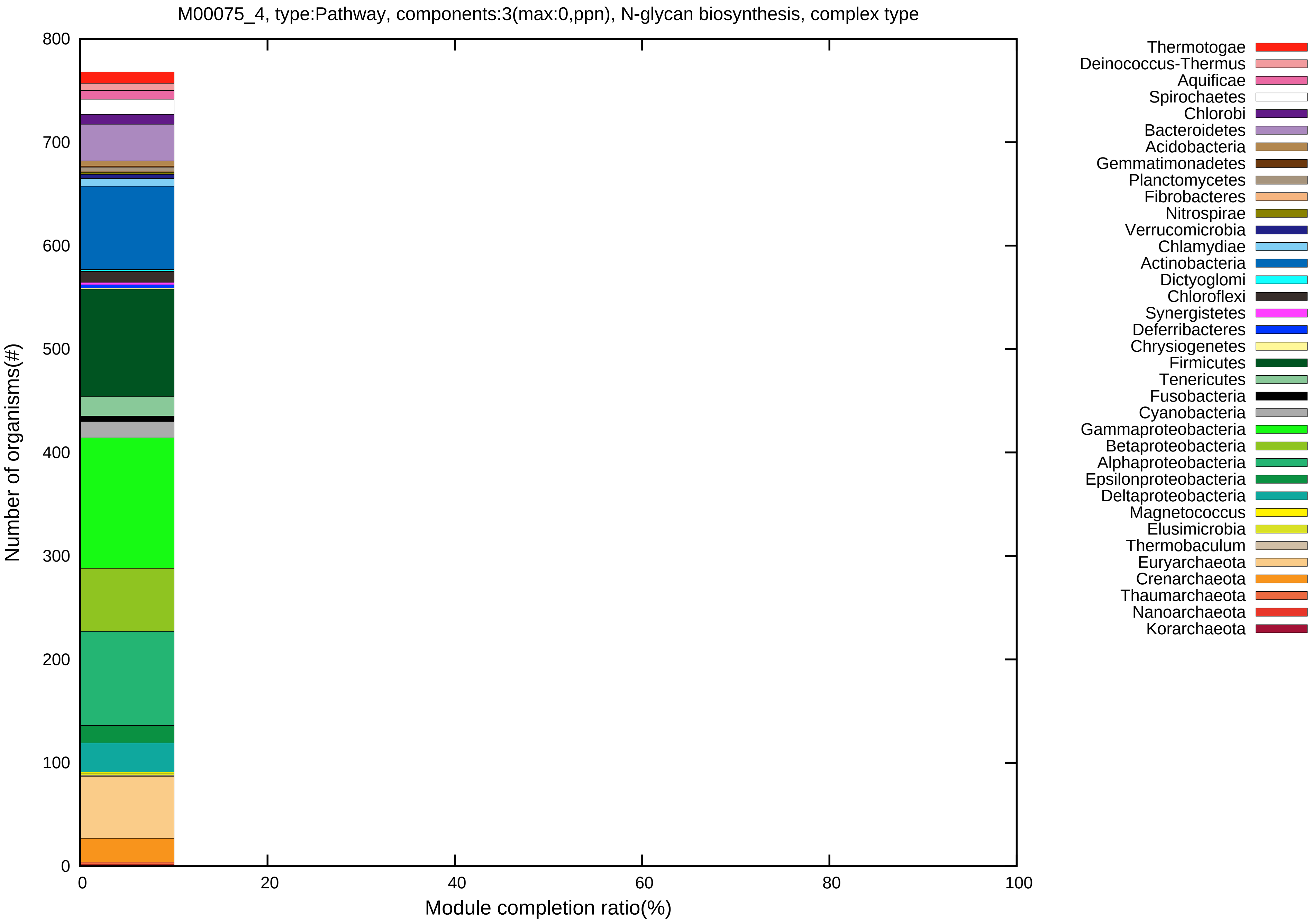

M00075_4, type:Pathway, components:3(max:0,ppn), N-glycan biosynthesis, complex type
Number of organisms(#)
Module completion ratio(%)
0
100
200
300
400
500
600
700
800
0
20
40
60
80
100
Thermotogae
Deinococcus-Thermus
Aquificae
Spirochaetes
Chlorobi
Bacteroidetes
Acidobacteria
Gemmatimonadetes
Planctomycetes
Fibrobacteres
Nitrospirae
Verrucomicrobia
Chlamydiae
Actinobacteria
Dictyoglomi
Chloroflexi
Synergistetes
Deferribacteres
Chrysiogenetes
Firmicutes
Tenericutes
Fusobacteria
Cyanobacteria
Gammaproteobacteria
Betaproteobacteria
Alphaproteobacteria
Epsilonproteobacteria
Deltaproteobacteria
Magnetococcus
Elusimicrobia
Thermobaculum
Euryarchaeota
Crenarchaeota
Thaumarchaeota
Nanoarchaeota
Korarchaeota

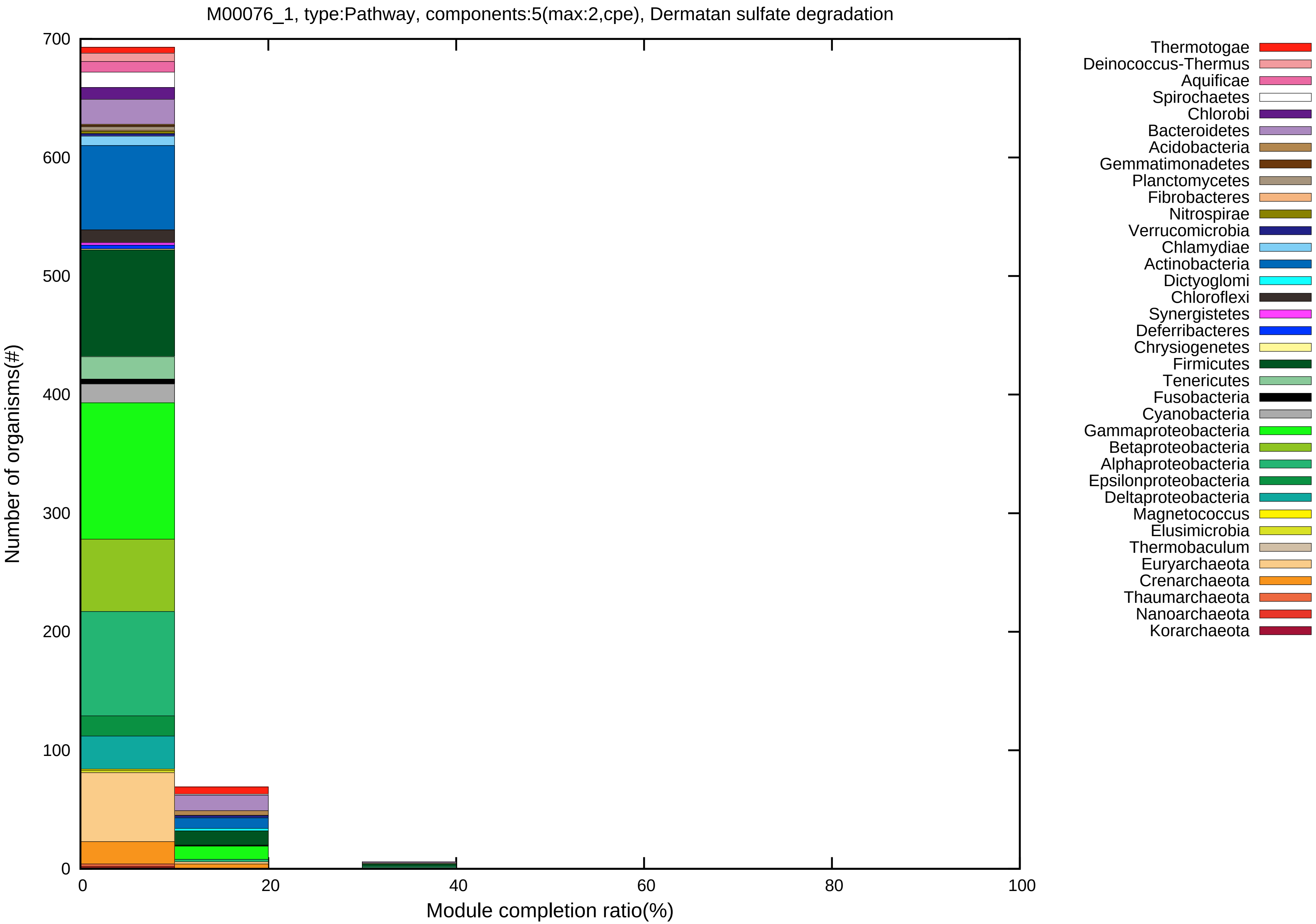
M00076_1, type:Pathway, components:5(max:2,cpe), Dermatan sulfate degradation
Number of organisms(#)
Module completion ratio(%)
0
100
200
300
400
500
600
700
0
20
40
60
80
100
Thermotogae
Deinococcus-Thermus
Aquificae
Spirochaetes
Chlorobi
Bacteroidetes
Acidobacteria
Gemmatimonadetes
Planctomycetes
Fibrobacteres
Nitrospirae
Verrucomicrobia
Chlamydiae
Actinobacteria
Dictyoglomi
Chloroflexi
Synergistetes
Deferribacteres
Chrysiogenetes
Firmicutes
Tenericutes
Fusobacteria
Cyanobacteria
Gammaproteobacteria
Betaproteobacteria
Alphaproteobacteria
Epsilonproteobacteria
Deltaproteobacteria
Magnetococcus
Elusimicrobia
Thermobaculum
Euryarchaeota
Crenarchaeota
Thaumarchaeota
Nanoarchaeota
Korarchaeota

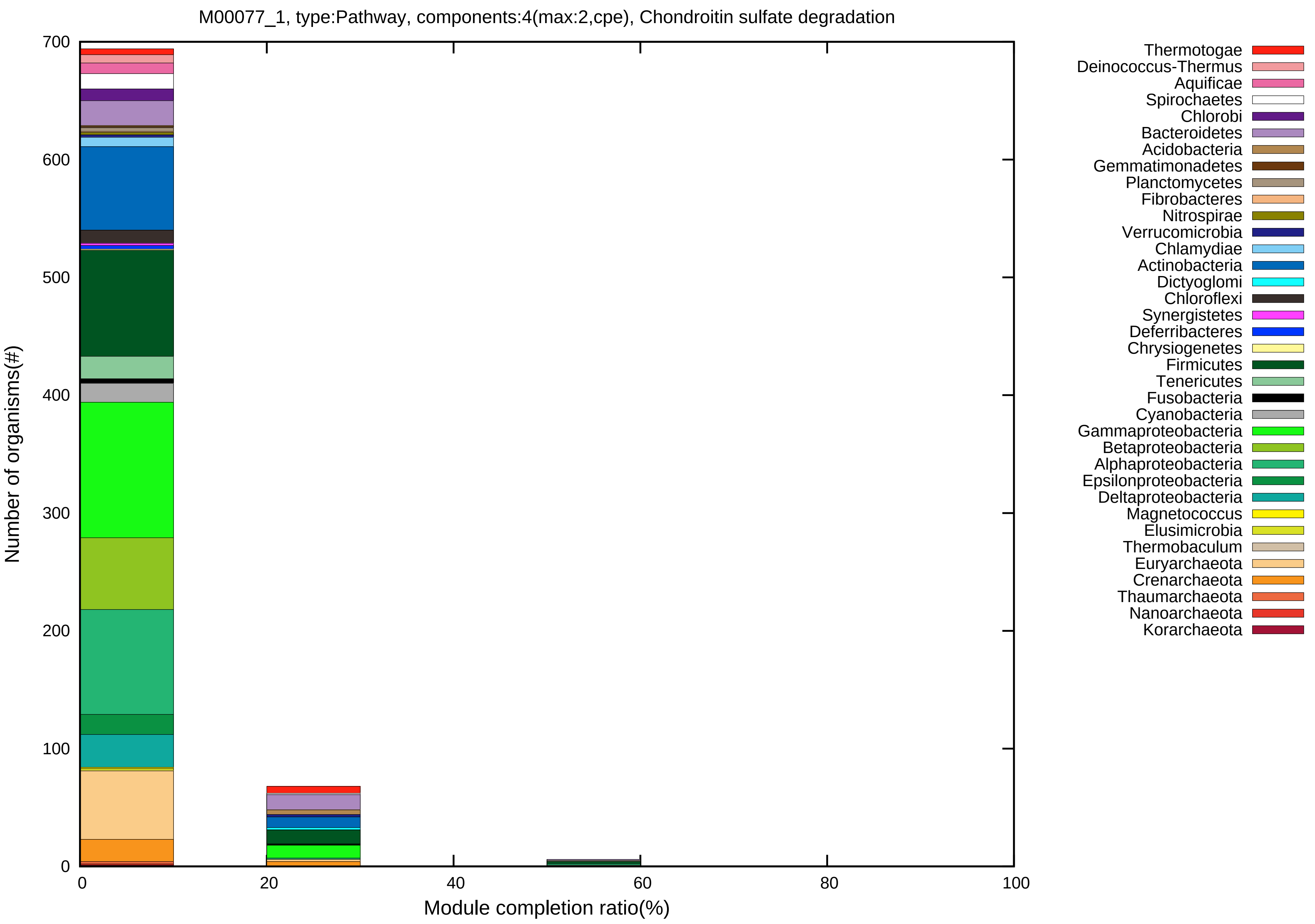

M00077_1, type:Pathway, components:4(max:2,cpe), Chondroitin sulfate degradation
Number of organisms(#)
Module completion ratio(%)
0
100
200
300
400
500
600
700
0
20
40
60
80
100
Thermotogae
Deinococcus-Thermus
Aquificae
Spirochaetes
Chlorobi
Bacteroidetes
Acidobacteria
Gemmatimonadetes
Planctomycetes
Fibrobacteres
Nitrospirae
Verrucomicrobia
Chlamydiae
Actinobacteria
Dictyoglomi
Chloroflexi
Synergistetes
Deferribacteres
Chrysiogenetes
Firmicutes
Tenericutes
Fusobacteria
Cyanobacteria
Gammaproteobacteria
Betaproteobacteria
Alphaproteobacteria
Epsilonproteobacteria
Deltaproteobacteria
Magnetococcus
Elusimicrobia
Thermobaculum
Euryarchaeota
Crenarchaeota
Thaumarchaeota
Nanoarchaeota
Korarchaeota

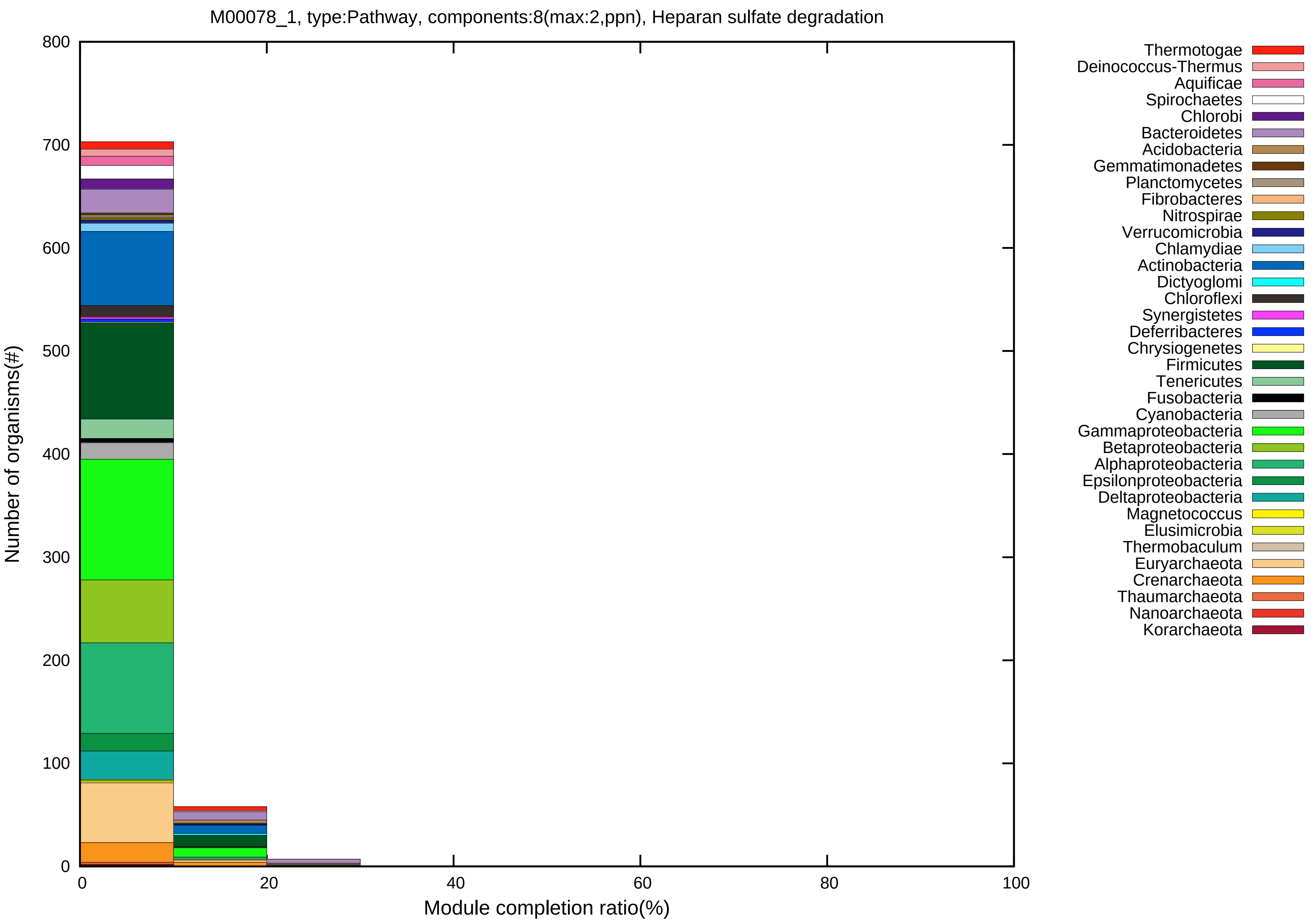

M00078_1, type:Pathway, components:8(max:2,ppn), Heparan sulfate degradation
Number of organisms(#)
Module completion ratio(%)
0
100
200
300
400
500
600
700
800
0
20
40
60
80
100
Thermotogae
Deinococcus-Thermus
Aquificae
Spirochaetes
Chlorobi
Bacteroidetes
Acidobacteria
Gemmatimonadetes
Planctomycetes
Fibrobacteres
Nitrospirae
Verrucomicrobia
Chlamydiae
Actinobacteria
Dictyoglomi
Chloroflexi
Synergistetes
Deferribacteres
Chrysiogenetes
Firmicutes
Tenericutes
Fusobacteria
Cyanobacteria
Gammaproteobacteria
Betaproteobacteria
Alphaproteobacteria
Epsilonproteobacteria
Deltaproteobacteria
Magnetococcus
Elusimicrobia
Thermobaculum
Euryarchaeota
Crenarchaeota
Thaumarchaeota
Nanoarchaeota
Korarchaeota

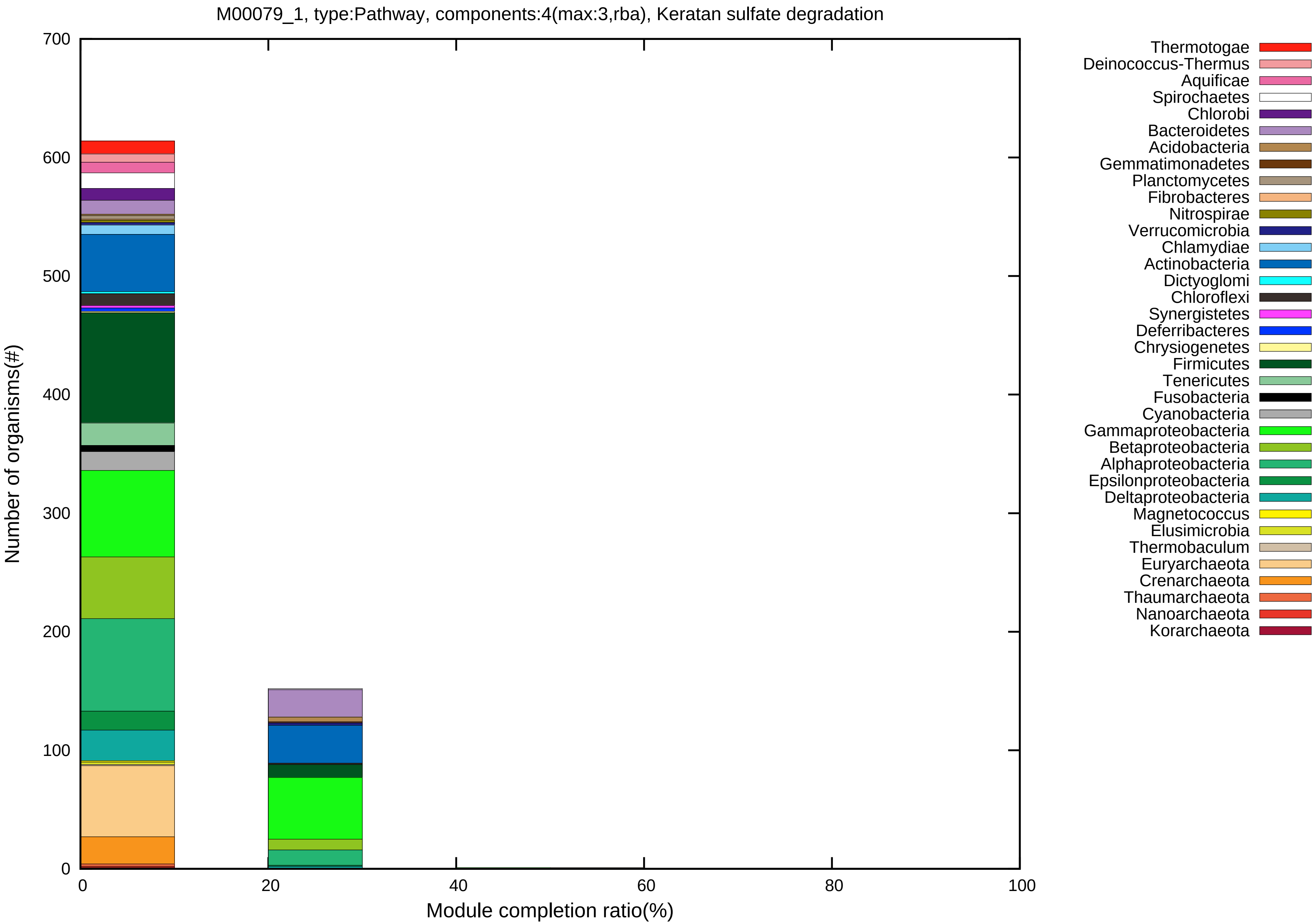

M00079_1, type:Pathway, components:4(max:3,rba), Keratan sulfate degradation
Number of organisms(#)
Module completion ratio(%)
0
100
200
300
400
500
600
700
0
20
40
60
80
100
Thermotogae
Deinococcus-Thermus
Aquificae
Spirochaetes
Chlorobi
Bacteroidetes
Acidobacteria
Gemmatimonadetes
Planctomycetes
Fibrobacteres
Nitrospirae
Verrucomicrobia
Chlamydiae
Actinobacteria
Dictyoglomi
Chloroflexi
Synergistetes
Deferribacteres
Chrysiogenetes
Firmicutes
Tenericutes
Fusobacteria
Cyanobacteria
Gammaproteobacteria
Betaproteobacteria
Alphaproteobacteria
Epsilonproteobacteria
Deltaproteobacteria
Magnetococcus
Elusimicrobia
Thermobaculum
Euryarchaeota
Crenarchaeota
Thaumarchaeota
Nanoarchaeota
Korarchaeota

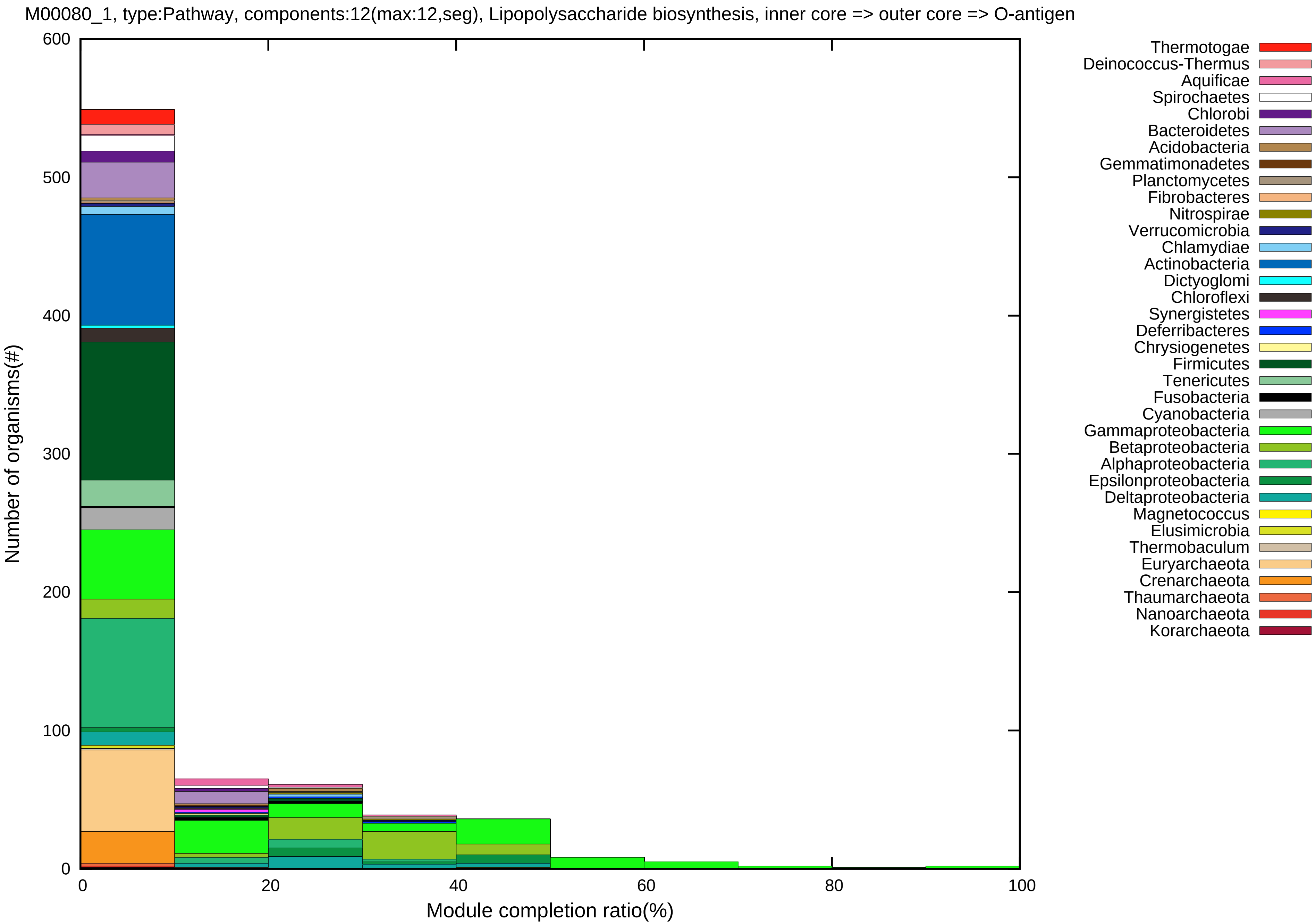

M00080_1, type:Pathway, components:12(max:12,seg), Lipopolysaccharide biosynthesis, inner core => outer core => O-antigen
Number of organisms(#)
Module completion ratio(%)
0
100
200
300
400
500
600
0
20
40
60
80
100
Thermotogae
Deinococcus-Thermus
Aquificae
Spirochaetes
Chlorobi
Bacteroidetes
Acidobacteria
Gemmatimonadetes
Planctomycetes
Fibrobacteres
Nitrospirae
Verrucomicrobia
Chlamydiae
Actinobacteria
Dictyoglomi
Chloroflexi
Synergistetes
Deferribacteres
Chrysiogenetes
Firmicutes
Tenericutes
Fusobacteria
Cyanobacteria
Gammaproteobacteria
Betaproteobacteria
Alphaproteobacteria
Epsilonproteobacteria
Deltaproteobacteria
Magnetococcus
Elusimicrobia
Thermobaculum
Euryarchaeota
Crenarchaeota
Thaumarchaeota
Nanoarchaeota
Korarchaeota

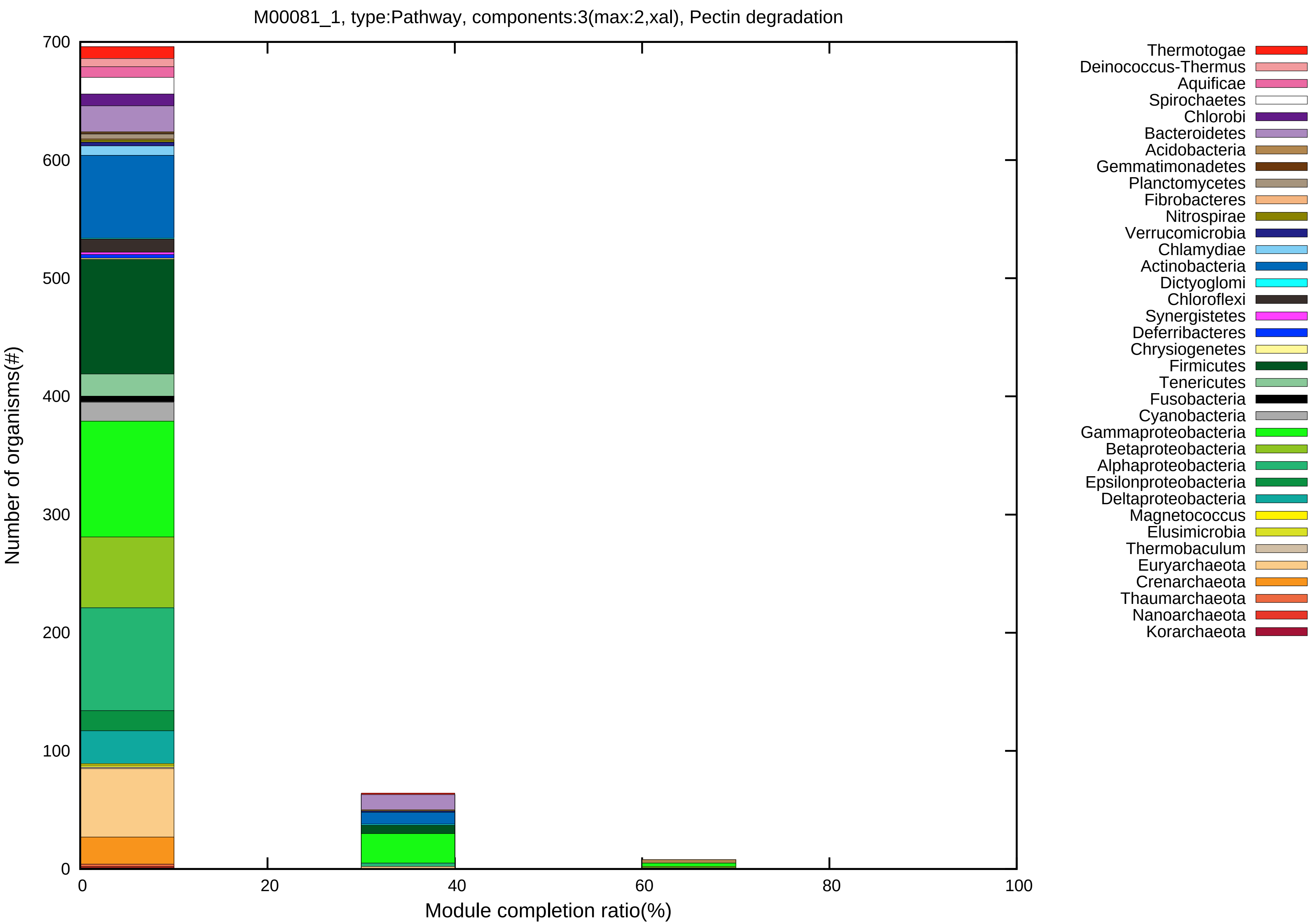

M00081_1, type:Pathway, components:3(max:2,xal), Pectin degradation
Number of organisms(#)
Module completion ratio(%)
0
100
200
300
400
500
600
700
0
20
40
60
80
100
Thermotogae
Deinococcus-Thermus
Aquificae
Spirochaetes
Chlorobi
Bacteroidetes
Acidobacteria
Gemmatimonadetes
Planctomycetes
Fibrobacteres
Nitrospirae
Verrucomicrobia
Chlamydiae
Actinobacteria
Dictyoglomi
Chloroflexi
Synergistetes
Deferribacteres
Chrysiogenetes
Firmicutes
Tenericutes
Fusobacteria
Cyanobacteria
Gammaproteobacteria
Betaproteobacteria
Alphaproteobacteria
Epsilonproteobacteria
Deltaproteobacteria
Magnetococcus
Elusimicrobia
Thermobaculum
Euryarchaeota
Crenarchaeota
Thaumarchaeota
Nanoarchaeota
Korarchaeota

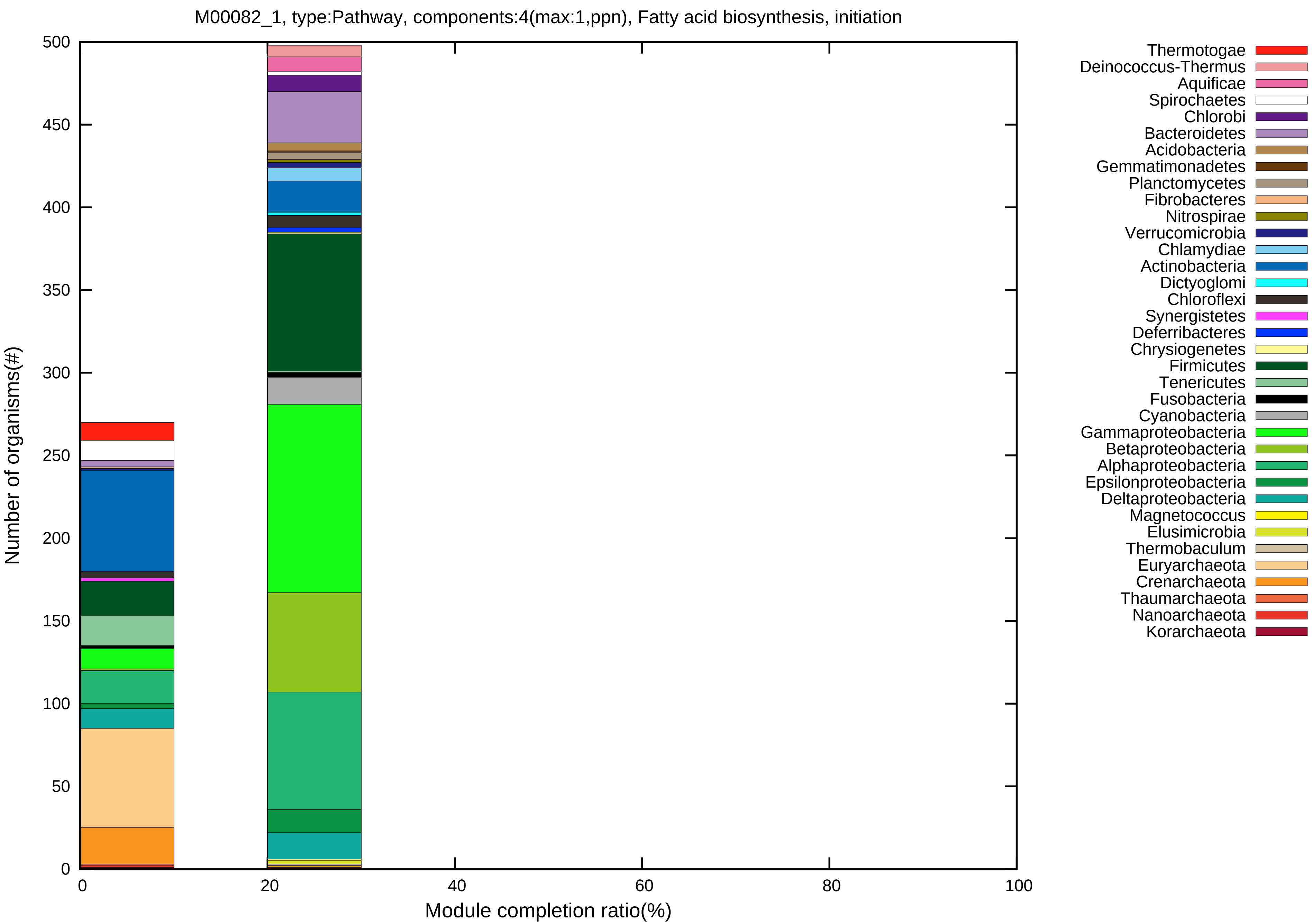
M00082_1, type:Pathway, components:4(max:1,ppn), Fatty acid biosynthesis, initiation
Number of organisms(#)
Module completion ratio(%)
0
50
100
150
200
250
300
350
400
450
500
0
20
40
60
80
100
Thermotogae
Deinococcus-Thermus
Aquificae
Spirochaetes
Chlorobi
Bacteroidetes
Acidobacteria
Gemmatimonadetes
Planctomycetes
Fibrobacteres
Nitrospirae
Verrucomicrobia
Chlamydiae
Actinobacteria
Dictyoglomi
Chloroflexi
Synergistetes
Deferribacteres
Chrysiogenetes
Firmicutes
Tenericutes
Fusobacteria
Cyanobacteria
Gammaproteobacteria
Betaproteobacteria
Alphaproteobacteria
Epsilonproteobacteria
Deltaproteobacteria
Magnetococcus
Elusimicrobia
Thermobaculum
Euryarchaeota
Crenarchaeota
Thaumarchaeota
Nanoarchaeota
Korarchaeota

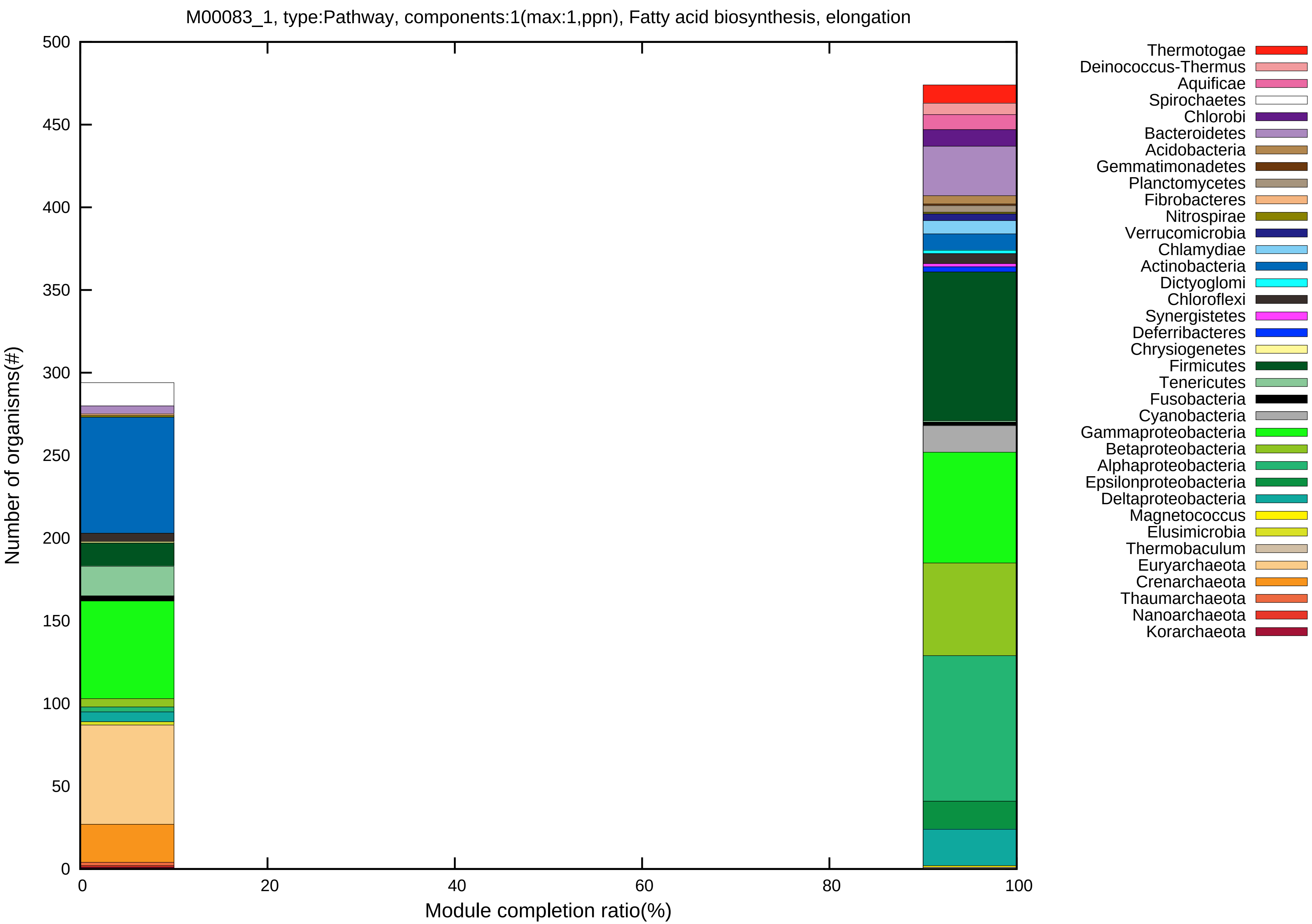

M00083_1, type:Pathway, components:1(max:1,ppn), Fatty acid biosynthesis, elongation
Number of organisms(#)
Module completion ratio(%)
0
50
100
150
200
250
300
350
400
450
500
0
20
40
60
80
100
Thermotogae
Deinococcus-Thermus
Aquificae
Spirochaetes
Chlorobi
Bacteroidetes
Acidobacteria
Gemmatimonadetes
Planctomycetes
Fibrobacteres
Nitrospirae
Verrucomicrobia
Chlamydiae
Actinobacteria
Dictyoglomi
Chloroflexi
Synergistetes
Deferribacteres
Chrysiogenetes
Firmicutes
Tenericutes
Fusobacteria
Cyanobacteria
Gammaproteobacteria
Betaproteobacteria
Alphaproteobacteria
Epsilonproteobacteria
Deltaproteobacteria
Magnetococcus
Elusimicrobia
Thermobaculum
Euryarchaeota
Crenarchaeota
Thaumarchaeota
Nanoarchaeota
Korarchaeota

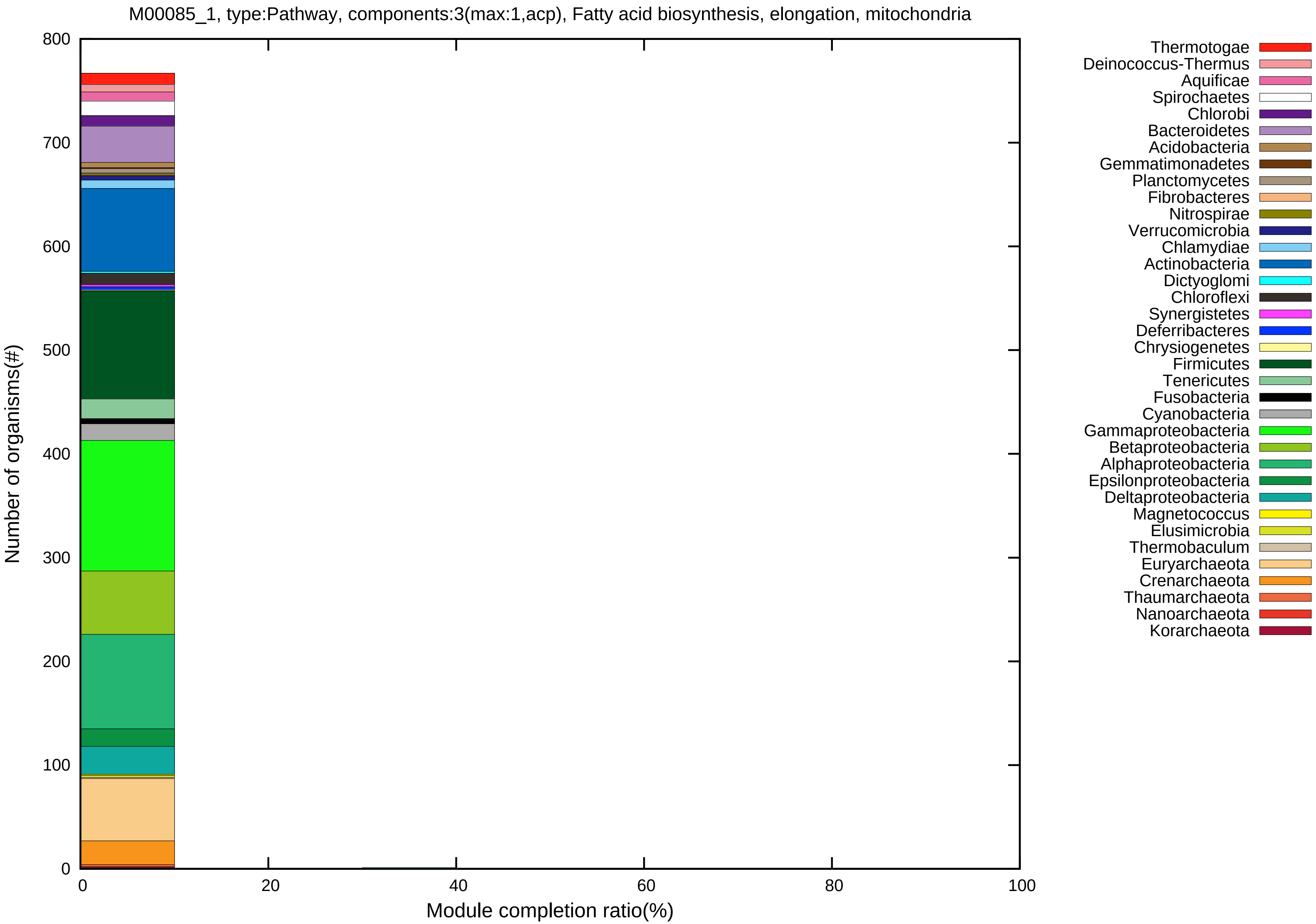

M00085_1, type:Pathway, components:3(max:1,acp), Fatty acid biosynthesis, elongation, mitochondria
Number of organisms(#)
Module completion ratio(%)
0
100
200
300
400
500
600
700
800
0
20
40
60
80
100
Thermotogae
Deinococcus-Thermus
Aquificae
Spirochaetes
Chlorobi
Bacteroidetes
Acidobacteria
Gemmatimonadetes
Planctomycetes
Fibrobacteres
Nitrospirae
Verrucomicrobia
Chlamydiae
Actinobacteria
Dictyoglomi
Chloroflexi
Synergistetes
Deferribacteres
Chrysiogenetes
Firmicutes
Tenericutes
Fusobacteria
Cyanobacteria
Gammaproteobacteria
Betaproteobacteria
Alphaproteobacteria
Epsilonproteobacteria
Deltaproteobacteria
Magnetococcus
Elusimicrobia
Thermobaculum
Euryarchaeota
Crenarchaeota
Thaumarchaeota
Nanoarchaeota
Korarchaeota

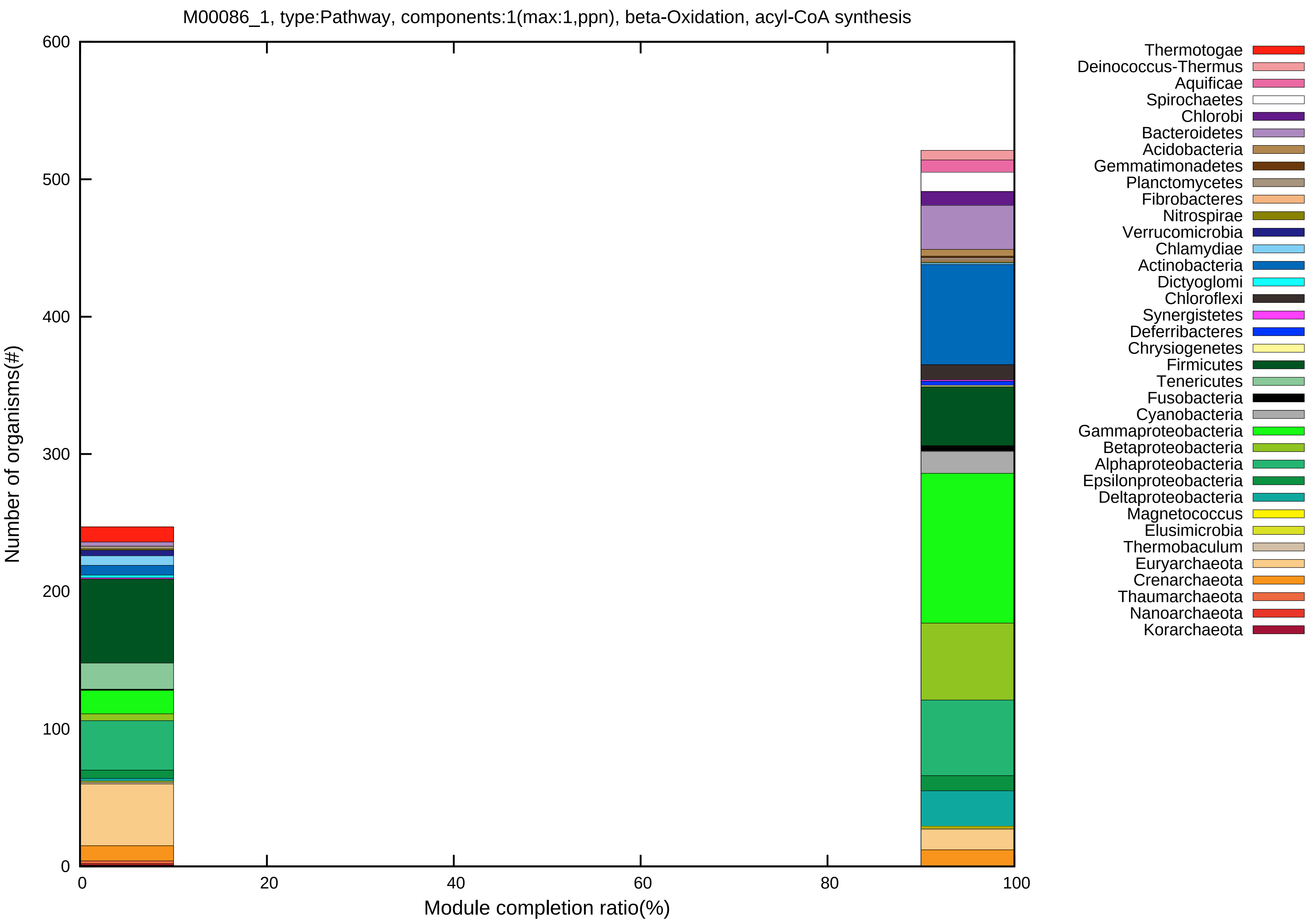

M00086_1, type:Pathway, components:1(max:1,ppn), beta-Oxidation, acyl-CoA synthesis
Number of organisms(#)
Module completion ratio(%)
0
100
200
300
400
500
600
0
20
40
60
80
100
Thermotogae
Deinococcus-Thermus
Aquificae
Spirochaetes
Chlorobi
Bacteroidetes
Acidobacteria
Gemmatimonadetes
Planctomycetes
Fibrobacteres
Nitrospirae
Verrucomicrobia
Chlamydiae
Actinobacteria
Dictyoglomi
Chloroflexi
Synergistetes
Deferribacteres
Chrysiogenetes
Firmicutes
Tenericutes
Fusobacteria
Cyanobacteria
Gammaproteobacteria
Betaproteobacteria
Alphaproteobacteria
Epsilonproteobacteria
Deltaproteobacteria
Magnetococcus
Elusimicrobia
Thermobaculum
Euryarchaeota
Crenarchaeota
Thaumarchaeota
Nanoarchaeota
Korarchaeota

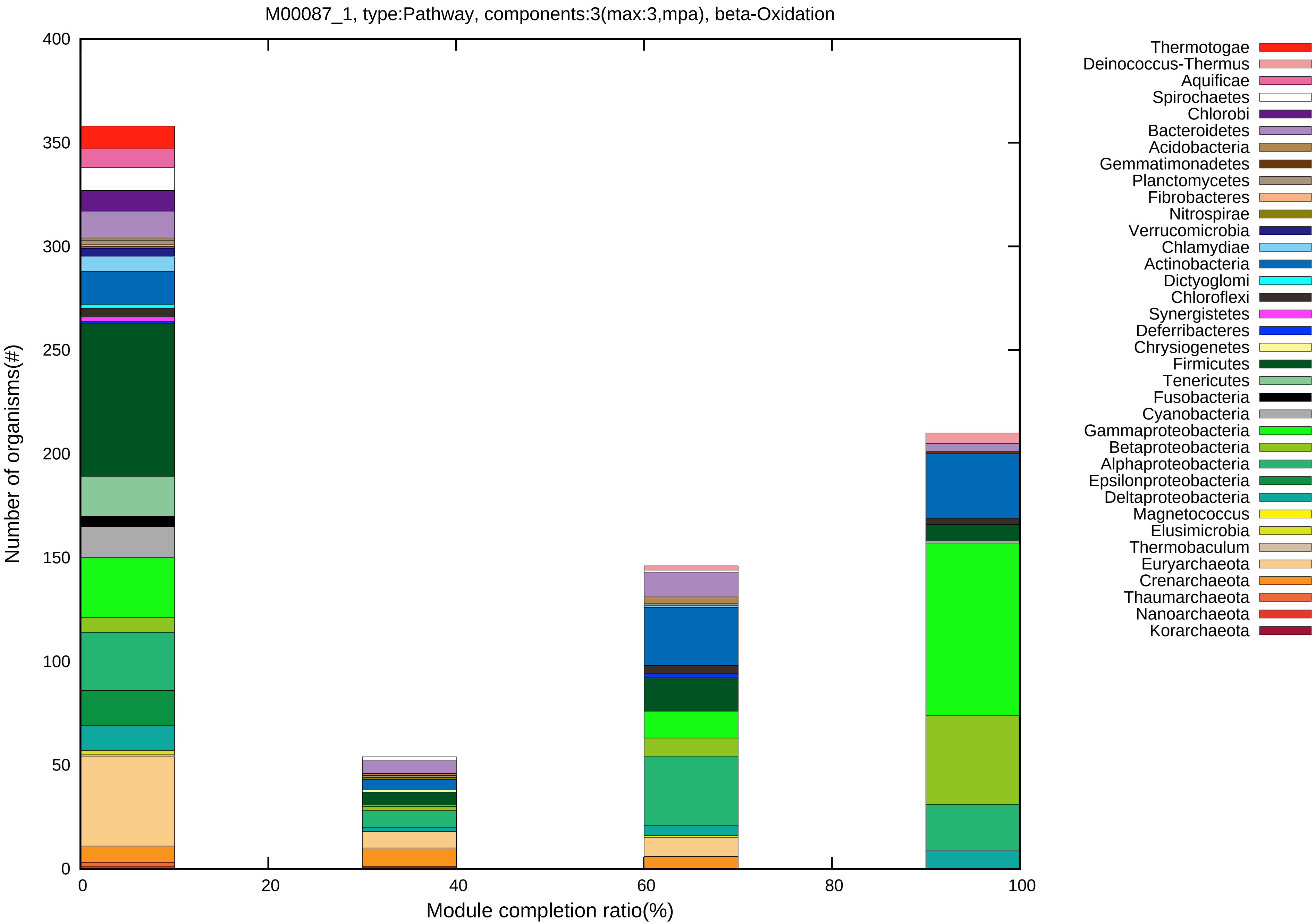

M00087_1, type:Pathway, components:3(max:3,mpa), beta-Oxidation
Number of organisms(#)
Module completion ratio(%)
0
50
100
150
200
250
300
350
400
0
20
40
60
80
100
Thermotogae
Deinococcus-Thermus
Aquificae
Spirochaetes
Chlorobi
Bacteroidetes
Acidobacteria
Gemmatimonadetes
Planctomycetes
Fibrobacteres
Nitrospirae
Verrucomicrobia
Chlamydiae
Actinobacteria
Dictyoglomi
Chloroflexi
Synergistetes
Deferribacteres
Chrysiogenetes
Firmicutes
Tenericutes
Fusobacteria
Cyanobacteria
Gammaproteobacteria
Betaproteobacteria
Alphaproteobacteria
Epsilonproteobacteria
Deltaproteobacteria
Magnetococcus
Elusimicrobia
Thermobaculum
Euryarchaeota
Crenarchaeota
Thaumarchaeota
Nanoarchaeota
Korarchaeota

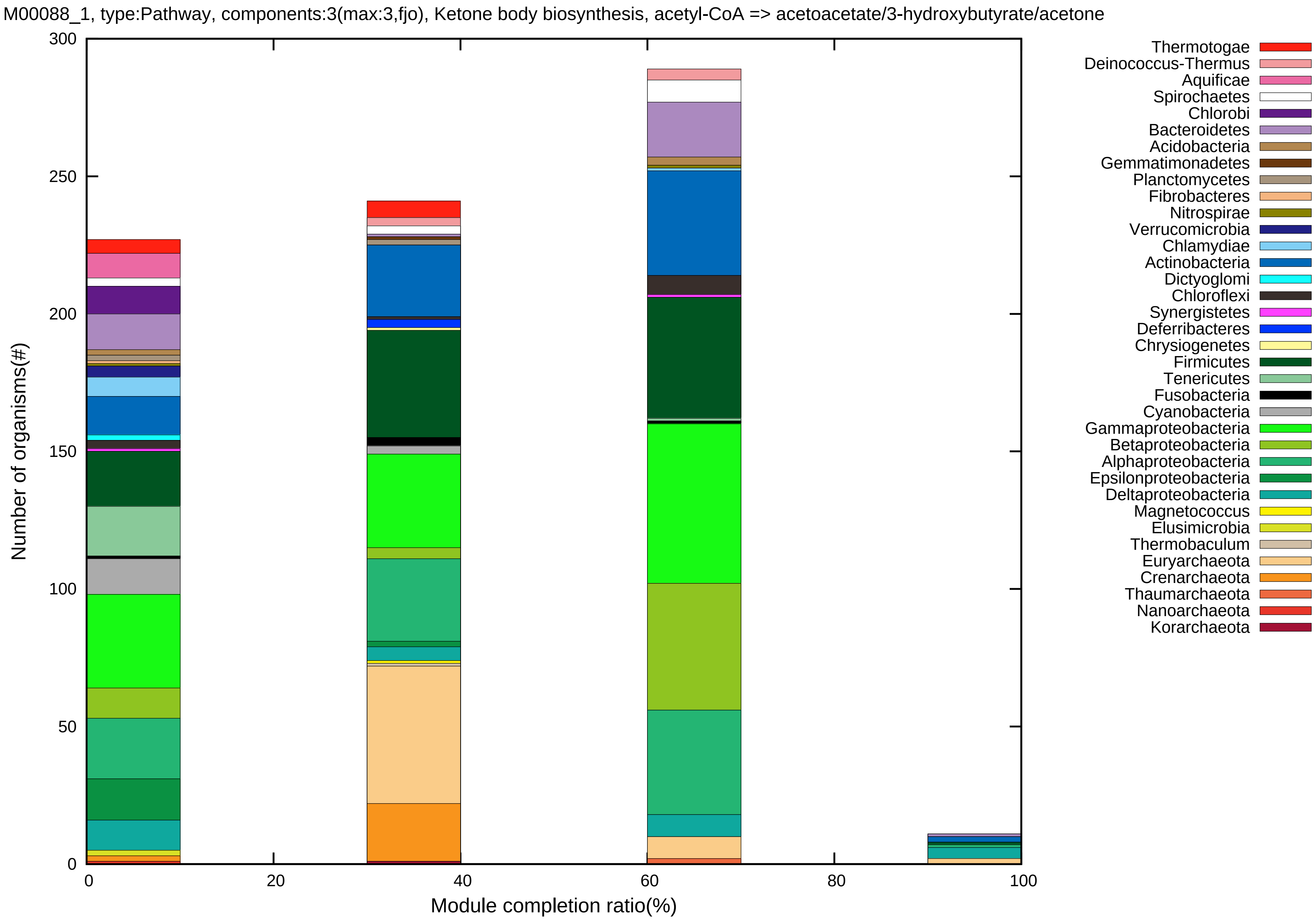

M00088_1, type:Pathway, components:3(max:3,fjo), Ketone body biosynthesis, acetyl-CoA => acetoacetate/3-hydroxybutyrate/acetone
Number of organisms(#)
0
50
100
150
200
250
300
0
20
40
60
80
100
Module completion ratio(%)
Thermotogae
Deinococcus-Thermus
Aquificae
Spirochaetes
Chlorobi
Bacteroidetes
Acidobacteria
Gemmatimonadetes
Planctomycetes
Fibrobacteres
Nitrospirae
Verrucomicrobia
Chlamydiae
Actinobacteria
Dictyoglomi
Chloroflexi
Synergistetes
Deferribacteres
Chrysiogenetes
Firmicutes
Tenericutes
Fusobacteria
Cyanobacteria
Gammaproteobacteria
Betaproteobacteria
Alphaproteobacteria
Epsilonproteobacteria
Deltaproteobacteria
Magnetococcus
Elusimicrobia
Thermobaculum
Euryarchaeota
Crenarchaeota
Thaumarchaeota
Nanoarchaeota
Korarchaeota

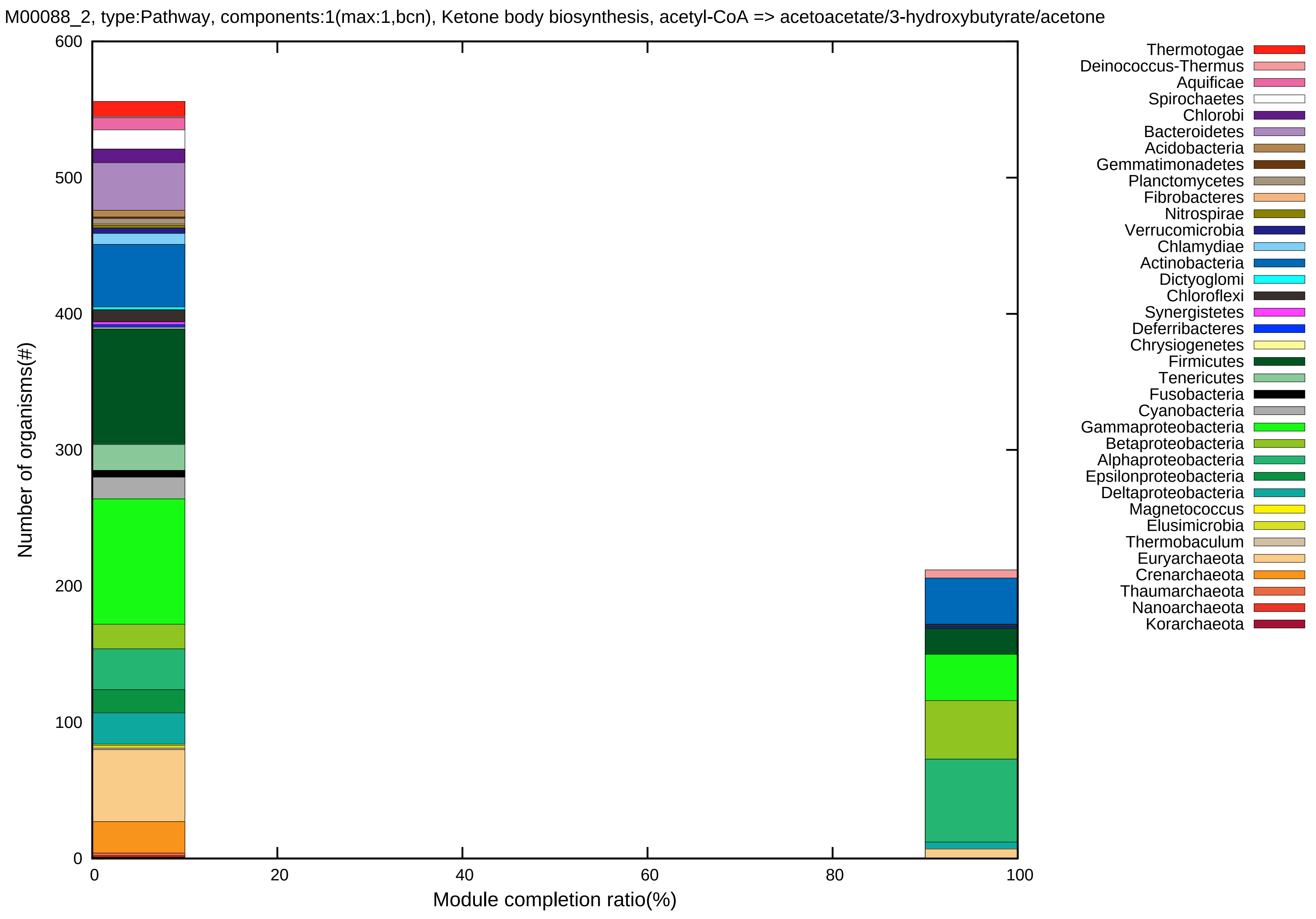

M00088_2, type:Pathway, components:1(max:1,bcn), Ketone body biosynthesis, acetyl-CoA => acetoacetate/3-hydroxybutyrate/acetone
Number of organisms(#)
Module completion ratio(%)
0
100
200
300
400
500
600
0
20
40
60
80
100
Thermotogae
Deinococcus-Thermus
Aquificae
Spirochaetes
Chlorobi
Bacteroidetes
Acidobacteria
Gemmatimonadetes
Planctomycetes
Fibrobacteres
Nitrospirae
Verrucomicrobia
Chlamydiae
Actinobacteria
Dictyoglomi
Chloroflexi
Synergistetes
Deferribacteres
Chrysiogenetes
Firmicutes
Tenericutes
Fusobacteria
Cyanobacteria
Gammaproteobacteria
Betaproteobacteria
Alphaproteobacteria
Epsilonproteobacteria
Deltaproteobacteria
Magnetococcus
Elusimicrobia
Thermobaculum
Euryarchaeota
Crenarchaeota
Thaumarchaeota
Nanoarchaeota
Korarchaeota

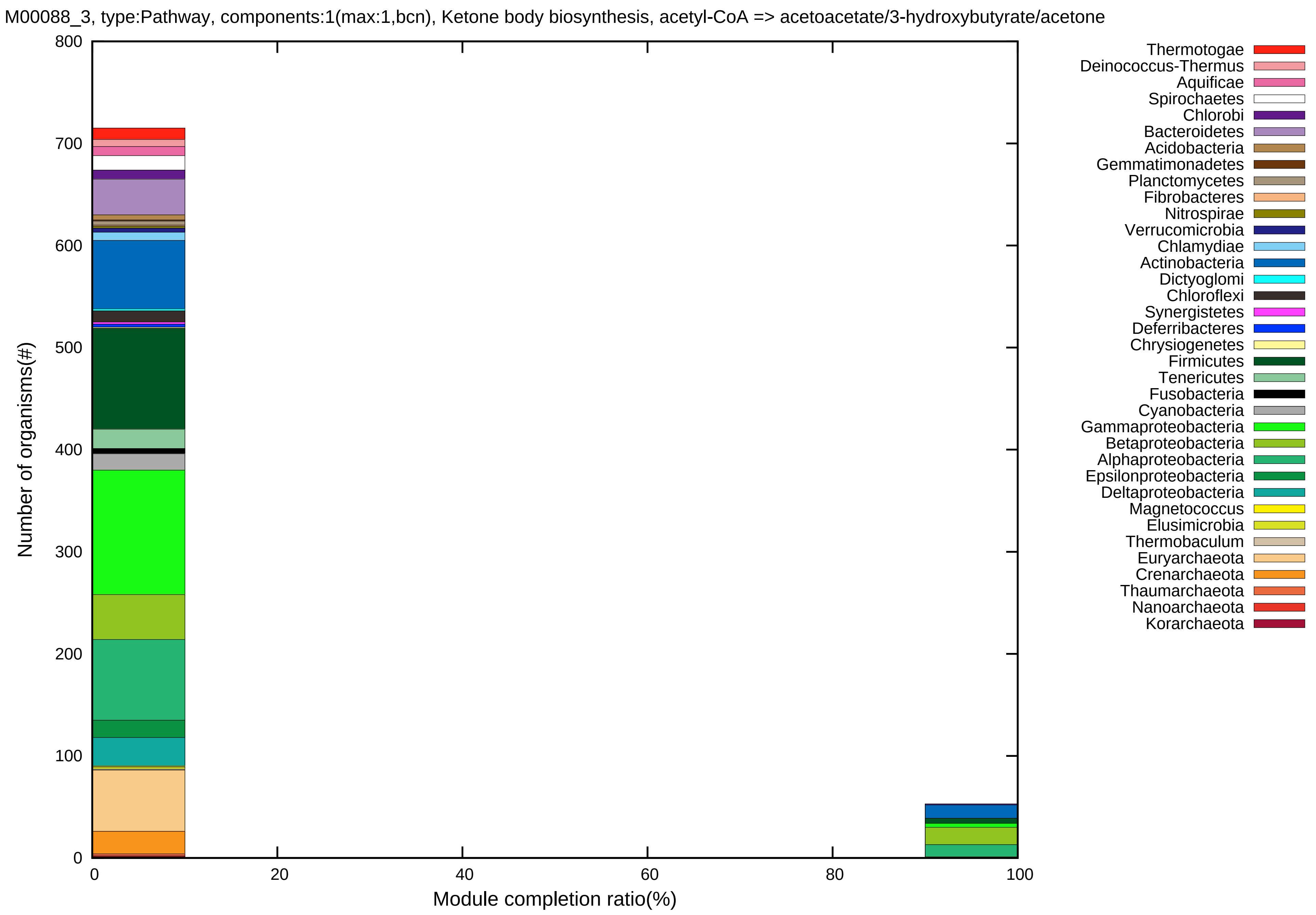

M00088_3, type:Pathway, components:1(max:1,bcn), Ketone body biosynthesis, acetyl-CoA => acetoacetate/3-hydroxybutyrate/acetone
Number of organisms(#)
Module completion ratio(%)
0
100
200
300
400
500
600
700
800
0
20
40
60
80
100
Thermotogae
Deinococcus-Thermus
Aquificae
Spirochaetes
Chlorobi
Bacteroidetes
Acidobacteria
Gemmatimonadetes
Planctomycetes
Fibrobacteres
Nitrospirae
Verrucomicrobia
Chlamydiae
Actinobacteria
Dictyoglomi
Chloroflexi
Synergistetes
Deferribacteres
Chrysiogenetes
Firmicutes
Tenericutes
Fusobacteria
Cyanobacteria
Gammaproteobacteria
Betaproteobacteria
Alphaproteobacteria
Epsilonproteobacteria
Deltaproteobacteria
Magnetococcus
Elusimicrobia
Thermobaculum
Euryarchaeota
Crenarchaeota
Thaumarchaeota
Nanoarchaeota
Korarchaeota

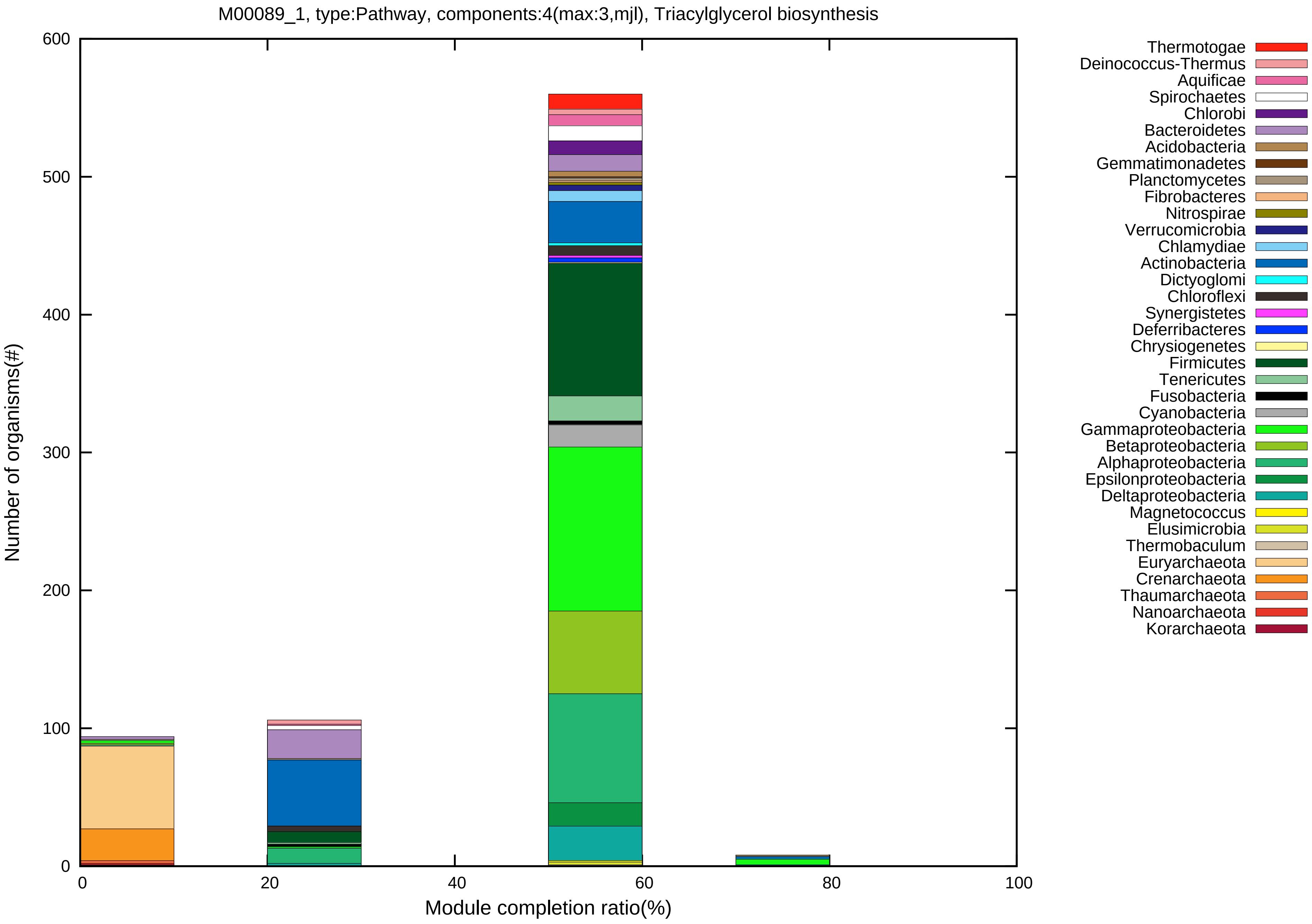

M00089_1, type:Pathway, components:4(max:3,mjl), Triacylglycerol biosynthesis
Number of organisms(#)
Module completion ratio(%)
0
100
200
300
400
500
600
0
20
40
60
80
100
Thermotogae
Deinococcus-Thermus
Aquificae
Spirochaetes
Chlorobi
Bacteroidetes
Acidobacteria
Gemmatimonadetes
Planctomycetes
Fibrobacteres
Nitrospirae
Verrucomicrobia
Chlamydiae
Actinobacteria
Dictyoglomi
Chloroflexi
Synergistetes
Deferribacteres
Chrysiogenetes
Firmicutes
Tenericutes
Fusobacteria
Cyanobacteria
Gammaproteobacteria
Betaproteobacteria
Alphaproteobacteria
Epsilonproteobacteria
Deltaproteobacteria
Magnetococcus
Elusimicrobia
Thermobaculum
Euryarchaeota
Crenarchaeota
Thaumarchaeota
Nanoarchaeota
Korarchaeota

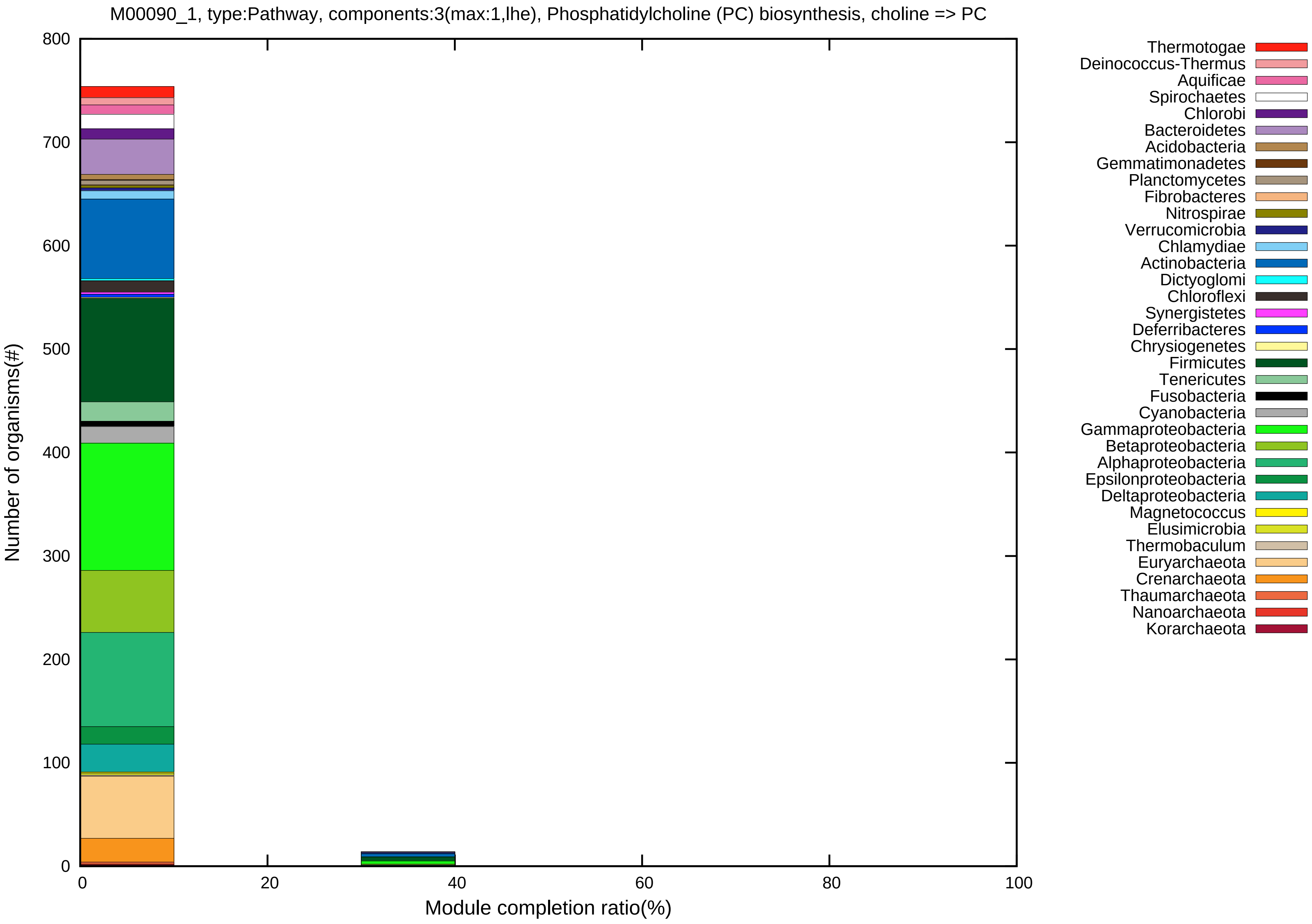
M00090_1, type:Pathway, components:3(max:1,lhe), Phosphatidylcholine (PC) biosynthesis, choline => PC
Number of organisms(#)
Module completion ratio(%)
0
100
200
300
400
500
600
700
800
0
20
40
60
80
100
Thermotogae
Deinococcus-Thermus
Aquificae
Spirochaetes
Chlorobi
Bacteroidetes
Acidobacteria
Gemmatimonadetes
Planctomycetes
Fibrobacteres
Nitrospirae
Verrucomicrobia
Chlamydiae
Actinobacteria
Dictyoglomi
Chloroflexi
Synergistetes
Deferribacteres
Chrysiogenetes
Firmicutes
Tenericutes
Fusobacteria
Cyanobacteria
Gammaproteobacteria
Betaproteobacteria
Alphaproteobacteria
Epsilonproteobacteria
Deltaproteobacteria
Magnetococcus
Elusimicrobia
Thermobaculum
Euryarchaeota
Crenarchaeota
Thaumarchaeota
Nanoarchaeota
Korarchaeota

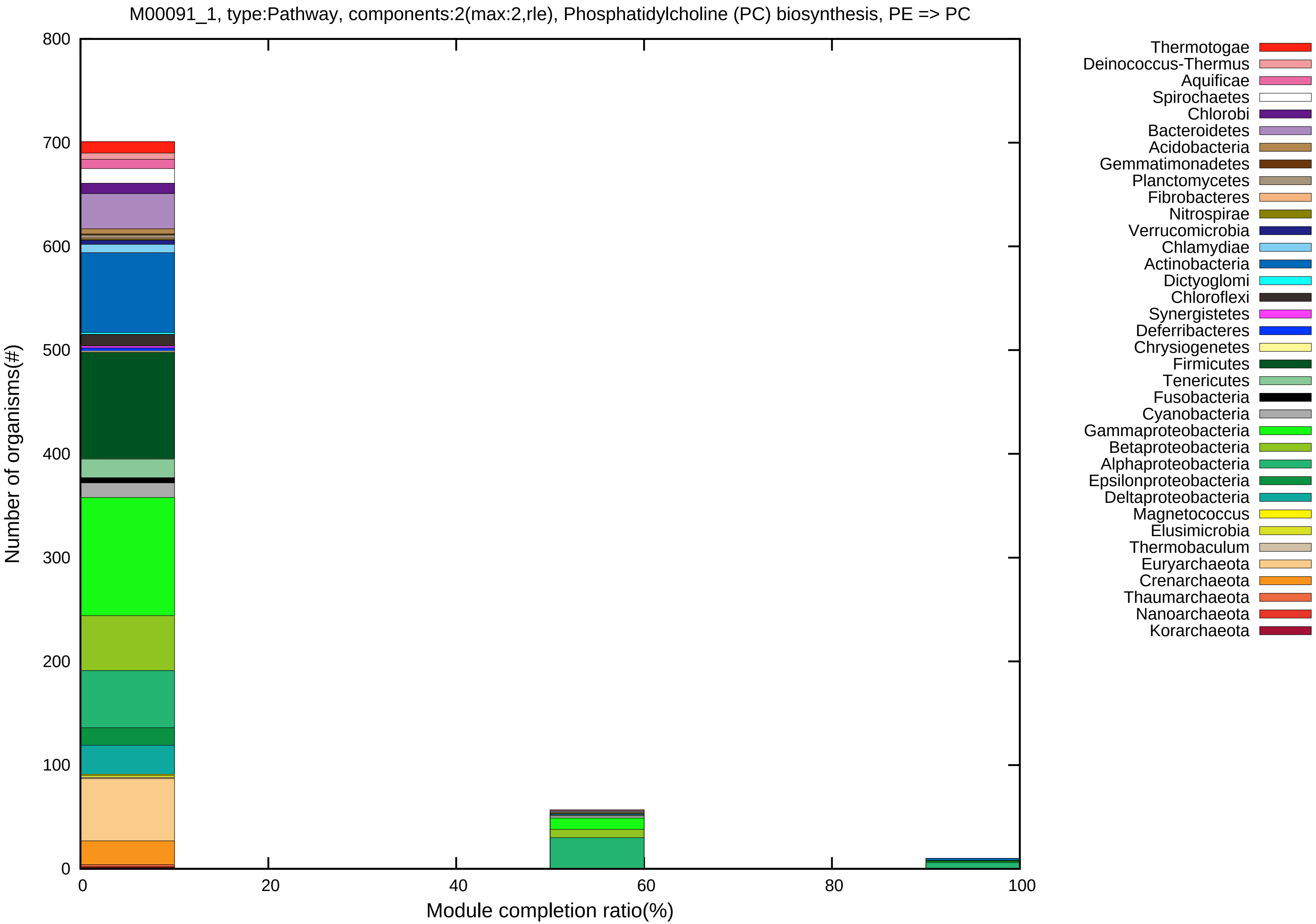

M00091_1, type:Pathway, components:2(max:2,rle), Phosphatidylcholine (PC) biosynthesis, PE => PC
Number of organisms(#)
Module completion ratio(%)
0
100
200
300
400
500
600
700
800
0
20
40
60
80
100
Thermotogae
Deinococcus-Thermus
Aquificae
Spirochaetes
Chlorobi
Bacteroidetes
Acidobacteria
Gemmatimonadetes
Planctomycetes
Fibrobacteres
Nitrospirae
Verrucomicrobia
Chlamydiae
Actinobacteria
Dictyoglomi
Chloroflexi
Synergistetes
Deferribacteres
Chrysiogenetes
Firmicutes
Tenericutes
Fusobacteria
Cyanobacteria
Gammaproteobacteria
Betaproteobacteria
Alphaproteobacteria
Epsilonproteobacteria
Deltaproteobacteria
Magnetococcus
Elusimicrobia
Thermobaculum
Euryarchaeota
Crenarchaeota
Thaumarchaeota
Nanoarchaeota
Korarchaeota

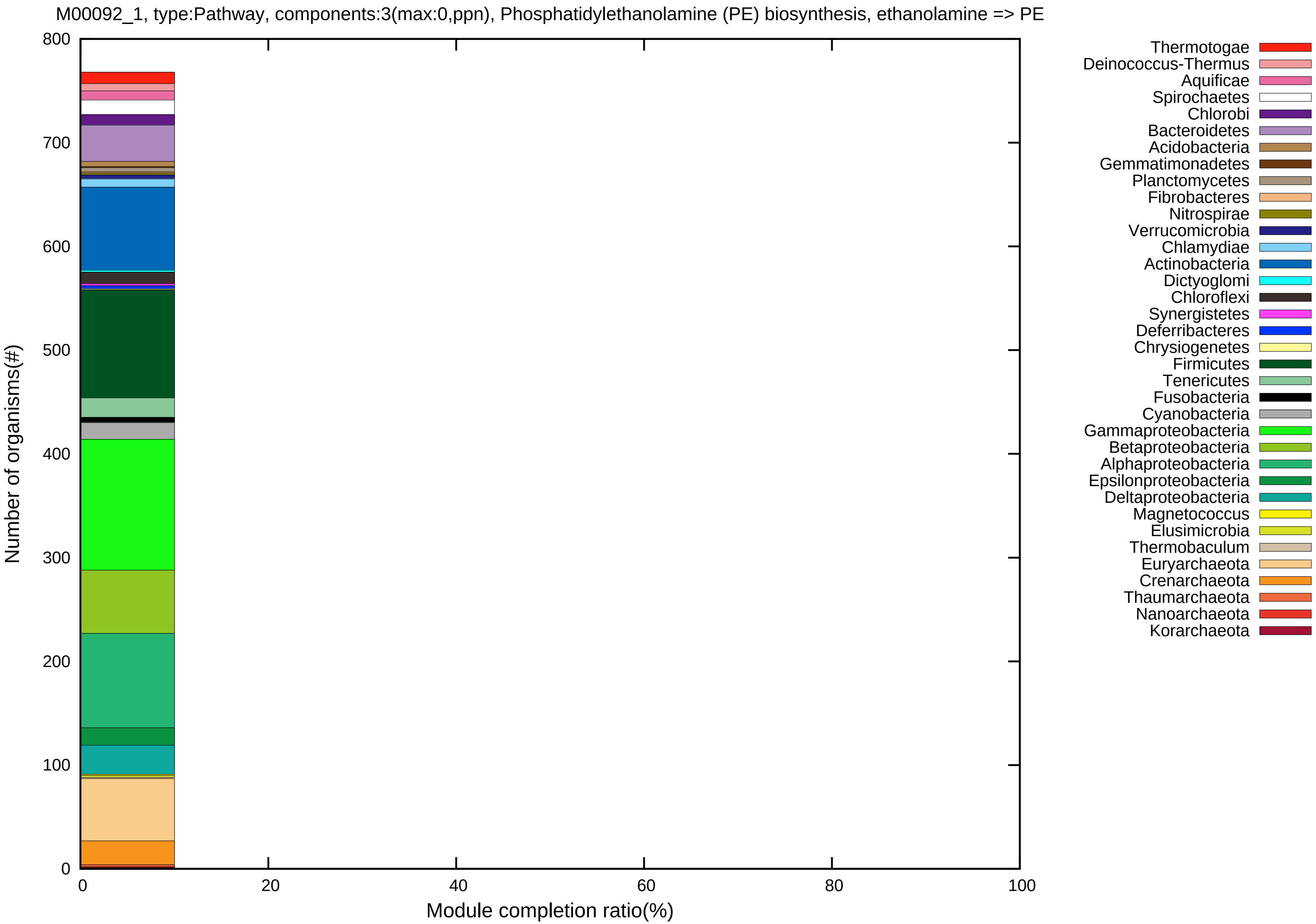
M00092_1, type:Pathway, components:3(max:0,ppn), Phosphatidylethanolamine (PE) biosynthesis, ethanolamine => PE
Number of organisms(#)
Module completion ratio(%)
0
100
200
300
400
500
600
700
800
0
20
40
60
80
100
Thermotogae
Deinococcus-Thermus
Aquificae
Spirochaetes
Chlorobi
Bacteroidetes
Acidobacteria
Gemmatimonadetes
Planctomycetes
Fibrobacteres
Nitrospirae
Verrucomicrobia
Chlamydiae
Actinobacteria
Dictyoglomi
Chloroflexi
Synergistetes
Deferribacteres
Chrysiogenetes
Firmicutes
Tenericutes
Fusobacteria
Cyanobacteria
Gammaproteobacteria
Betaproteobacteria
Alphaproteobacteria
Epsilonproteobacteria
Deltaproteobacteria
Magnetococcus
Elusimicrobia
Thermobaculum
Euryarchaeota
Crenarchaeota
Thaumarchaeota
Nanoarchaeota
Korarchaeota

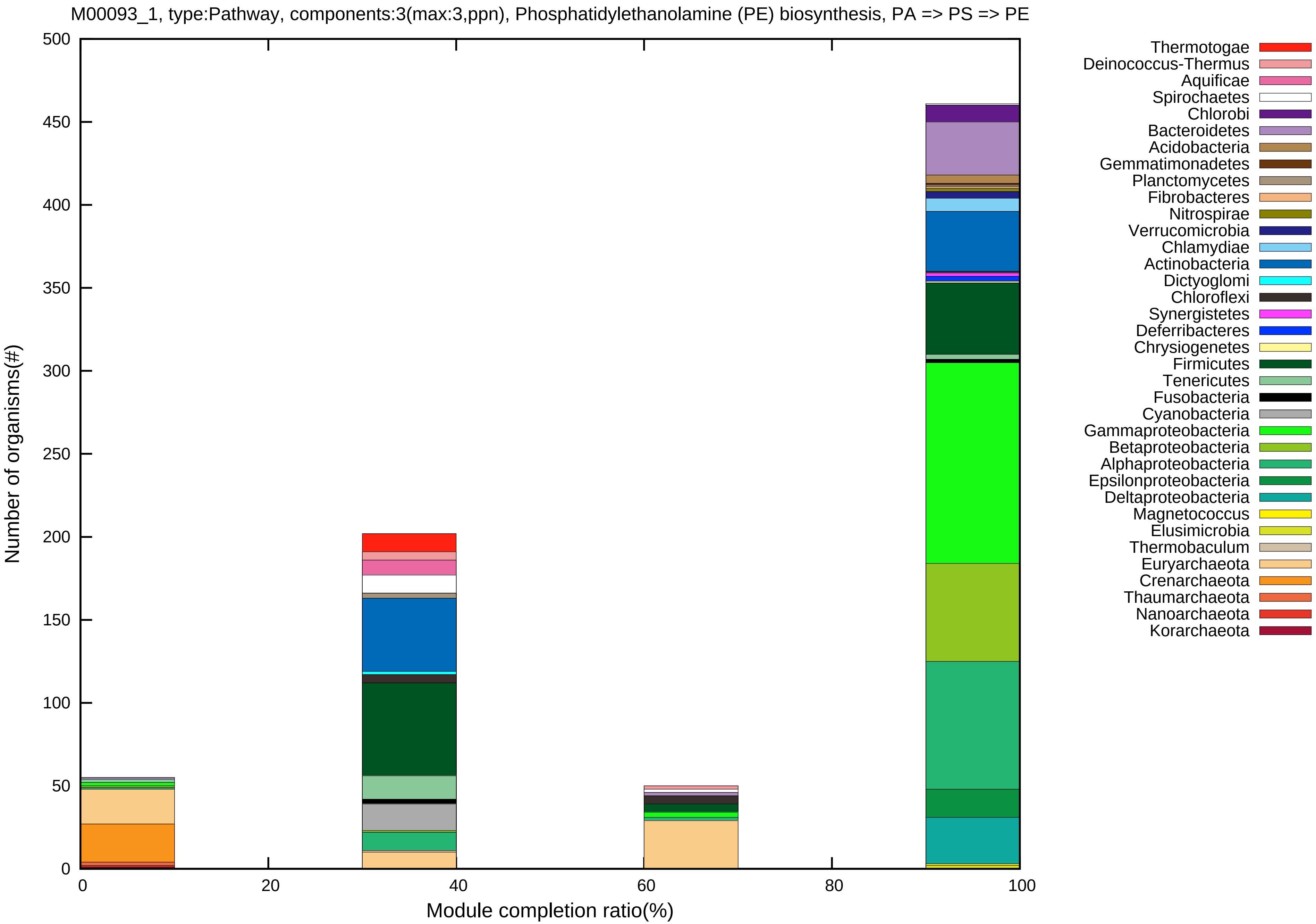

M00093_1, type:Pathway, components:3(max:3,ppn), Phosphatidylethanolamine (PE) biosynthesis, PA => PS => PE
Number of organisms(#)
Module completion ratio(%)
0
50
100
150
200
250
300
350
400
450
500
0
20
40
60
80
100
Thermotogae
Deinococcus-Thermus
Aquificae
Spirochaetes
Chlorobi
Bacteroidetes
Acidobacteria
Gemmatimonadetes
Planctomycetes
Fibrobacteres
Nitrospirae
Verrucomicrobia
Chlamydiae
Actinobacteria
Dictyoglomi
Chloroflexi
Synergistetes
Deferribacteres
Chrysiogenetes
Firmicutes
Tenericutes
Fusobacteria
Cyanobacteria
Gammaproteobacteria
Betaproteobacteria
Alphaproteobacteria
Epsilonproteobacteria
Deltaproteobacteria
Magnetococcus
Elusimicrobia
Thermobaculum
Euryarchaeota
Crenarchaeota
Thaumarchaeota
Nanoarchaeota
Korarchaeota

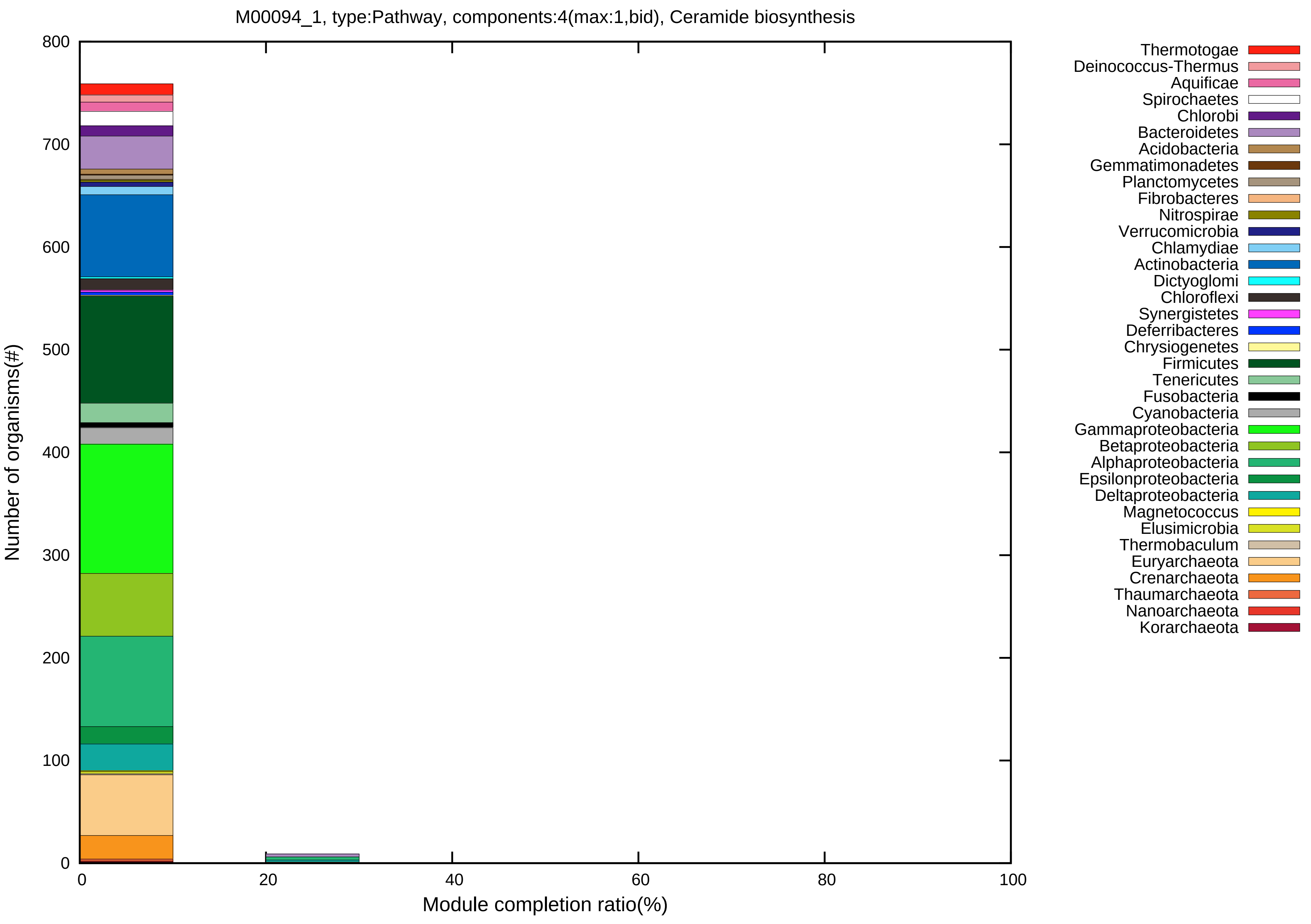
M00094_1, type:Pathway, components:4(max:1,bid), Ceramide biosynthesis
Number of organisms(#)
Module completion ratio(%)
0
100
200
300
400
500
600
700
800
0
20
40
60
80
100
Thermotogae
Deinococcus-Thermus
Aquificae
Spirochaetes
Chlorobi
Bacteroidetes
Acidobacteria
Gemmatimonadetes
Planctomycetes
Fibrobacteres
Nitrospirae
Verrucomicrobia
Chlamydiae
Actinobacteria
Dictyoglomi
Chloroflexi
Synergistetes
Deferribacteres
Chrysiogenetes
Firmicutes
Tenericutes
Fusobacteria
Cyanobacteria
Gammaproteobacteria
Betaproteobacteria
Alphaproteobacteria
Epsilonproteobacteria
Deltaproteobacteria
Magnetococcus
Elusimicrobia
Thermobaculum
Euryarchaeota
Crenarchaeota
Thaumarchaeota
Nanoarchaeota
Korarchaeota

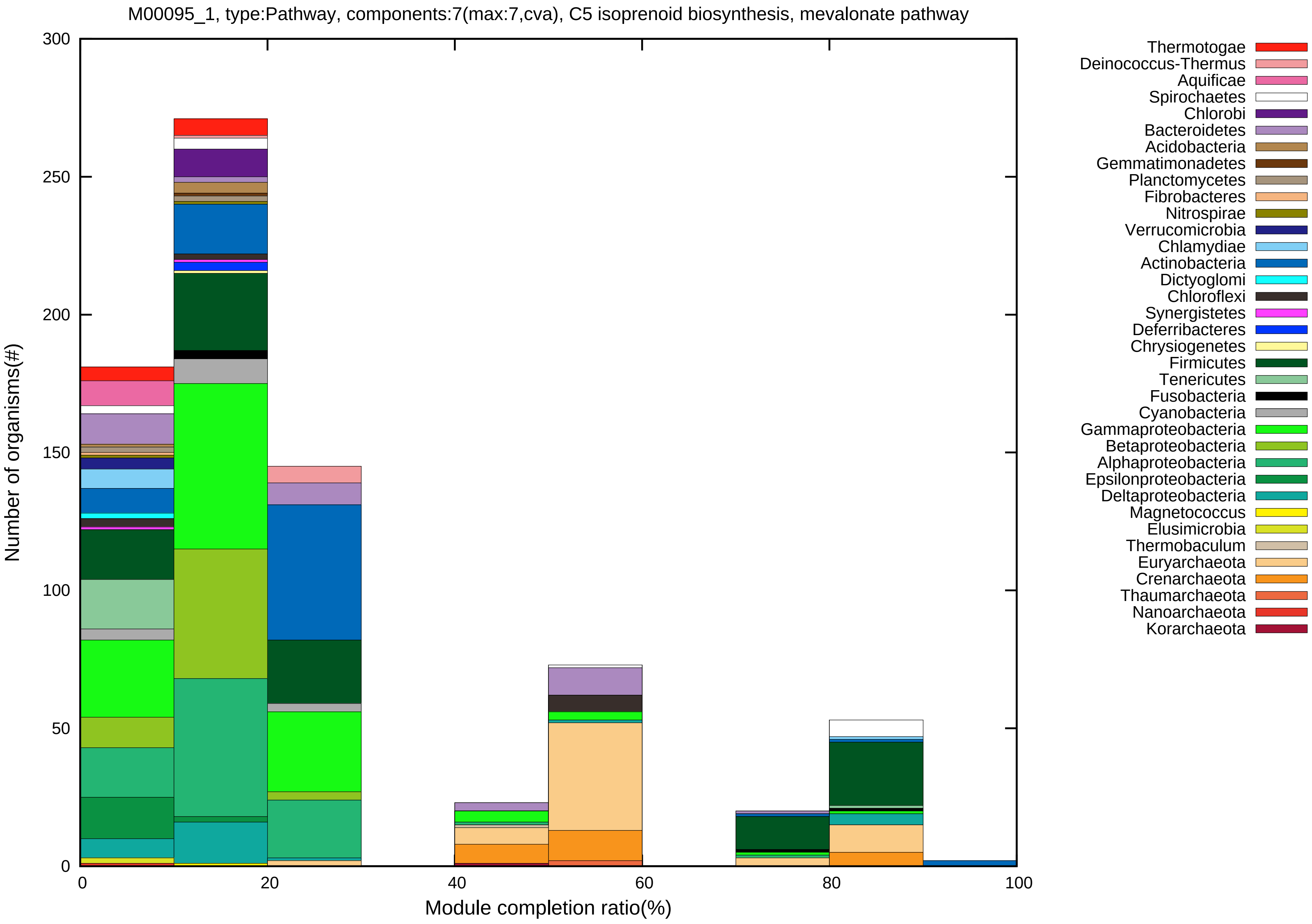

M00095_1, type:Pathway, components:7(max:7,cva), C5 isoprenoid biosynthesis, mevalonate pathway
Number of organisms(#)
Module completion ratio(%)
0
50
100
150
200
250
300
0
20
40
60
80
100
Thermotogae
Deinococcus-Thermus
Aquificae
Spirochaetes
Chlorobi
Bacteroidetes
Acidobacteria
Gemmatimonadetes
Planctomycetes
Fibrobacteres
Nitrospirae
Verrucomicrobia
Chlamydiae
Actinobacteria
Dictyoglomi
Chloroflexi
Synergistetes
Deferribacteres
Chrysiogenetes
Firmicutes
Tenericutes
Fusobacteria
Cyanobacteria
Gammaproteobacteria
Betaproteobacteria
Alphaproteobacteria
Epsilonproteobacteria
Deltaproteobacteria
Magnetococcus
Elusimicrobia
Thermobaculum
Euryarchaeota
Crenarchaeota
Thaumarchaeota
Nanoarchaeota
Korarchaeota

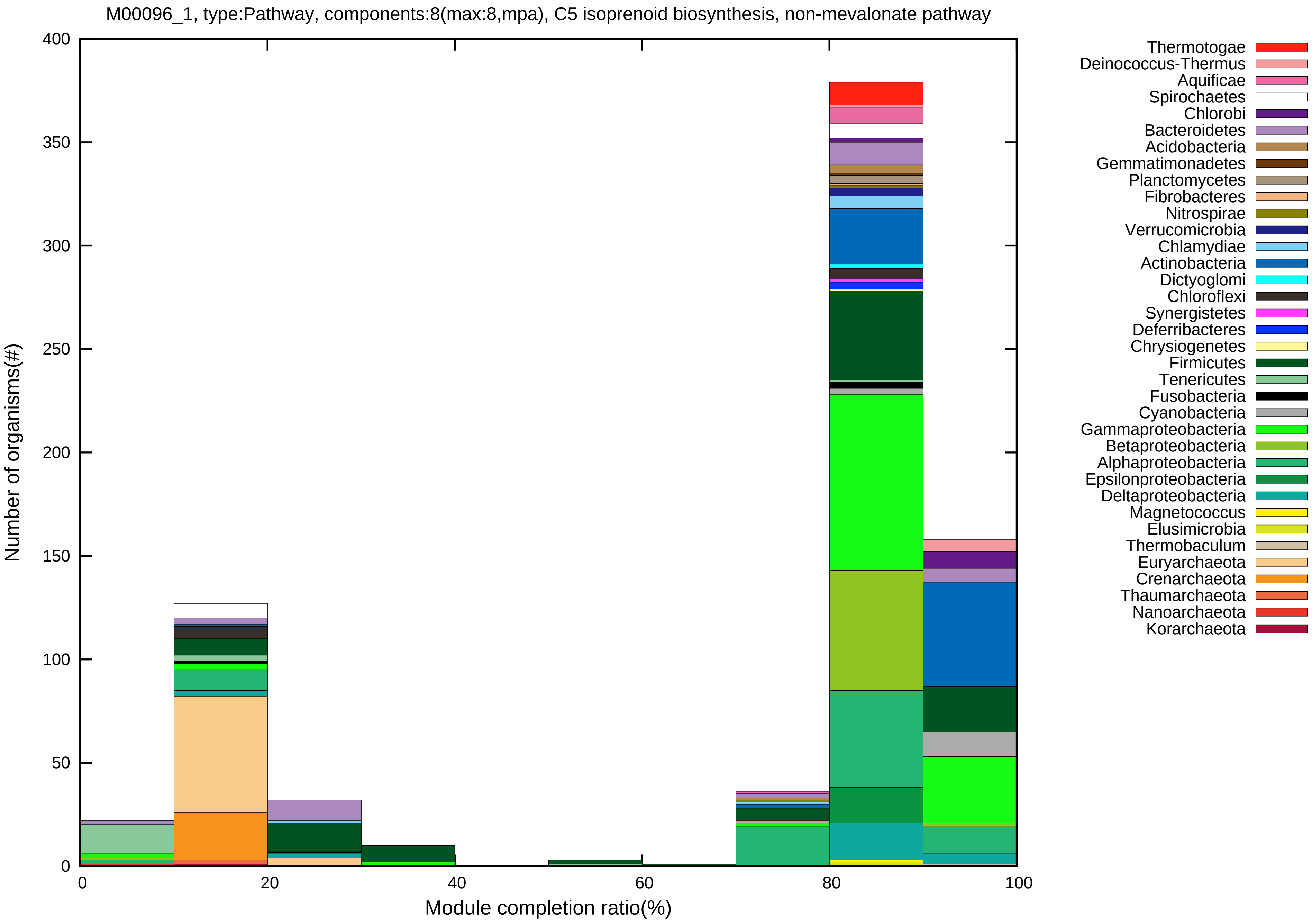
M00096_1, type:Pathway, components:8(max:8,mpa), C5 isoprenoid biosynthesis, non-mevalonate pathway
Number of organisms(#)
Module completion ratio(%)
0
50
100
150
200
250
300
350
400
0
20
40
60
80
100
Thermotogae
Deinococcus-Thermus
Aquificae
Spirochaetes
Chlorobi
Bacteroidetes
Acidobacteria
Gemmatimonadetes
Planctomycetes
Fibrobacteres
Nitrospirae
Verrucomicrobia
Chlamydiae
Actinobacteria
Dictyoglomi
Chloroflexi
Synergistetes
Deferribacteres
Chrysiogenetes
Firmicutes
Tenericutes
Fusobacteria
Cyanobacteria
Gammaproteobacteria
Betaproteobacteria
Alphaproteobacteria
Epsilonproteobacteria
Deltaproteobacteria
Magnetococcus
Elusimicrobia
Thermobaculum
Euryarchaeota
Crenarchaeota
Thaumarchaeota
Nanoarchaeota
Korarchaeota

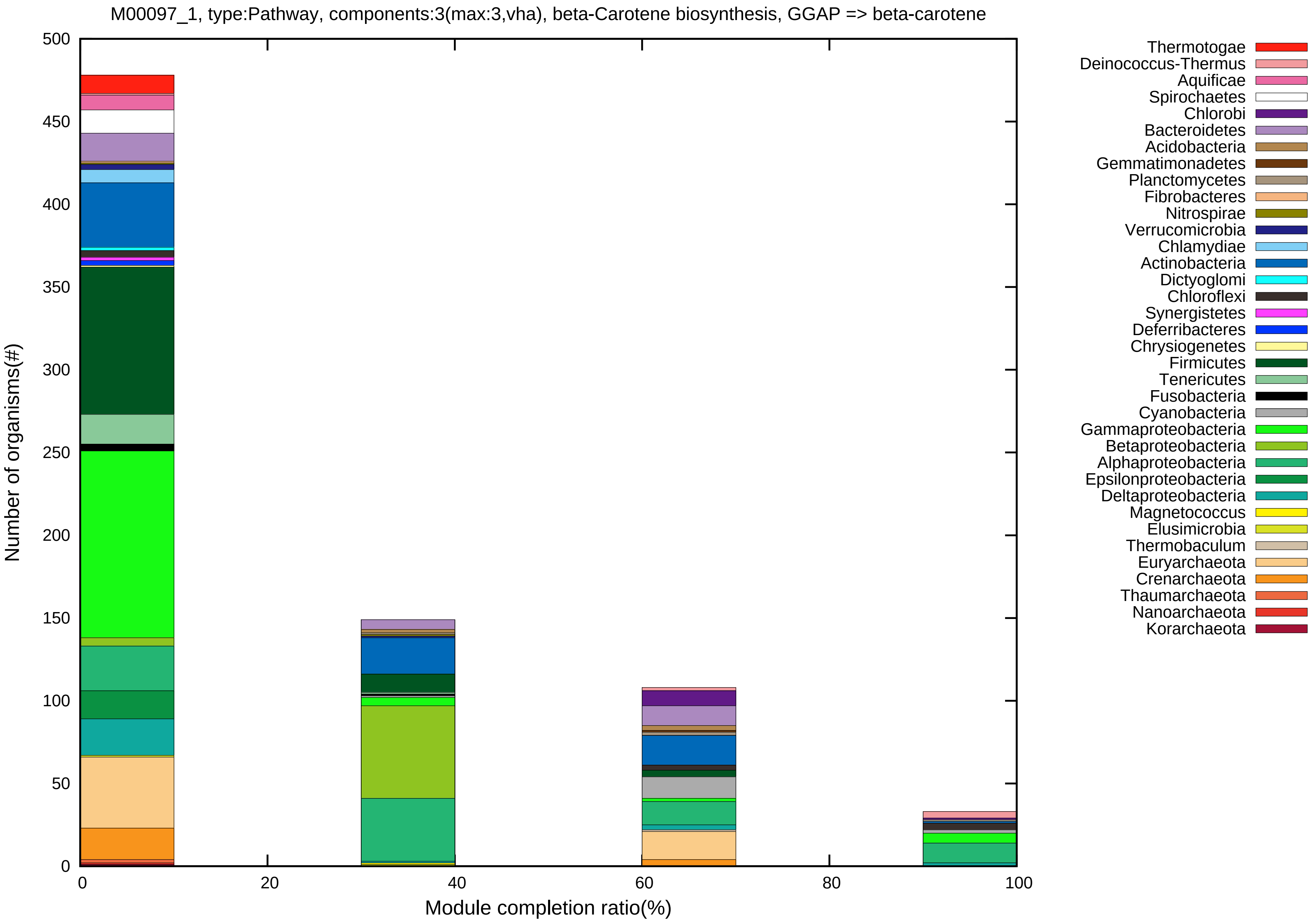
M00097_1, type:Pathway, components:3(max:3,vha), beta-Carotene biosynthesis, GGAP => beta-carotene
Number of organisms(#)
Module completion ratio(%)
0
50
100
150
200
250
300
350
400
450
500
0
20
40
60
80
100
Thermotogae
Deinococcus-Thermus
Aquificae
Spirochaetes
Chlorobi
Bacteroidetes
Acidobacteria
Gemmatimonadetes
Planctomycetes
Fibrobacteres
Nitrospirae
Verrucomicrobia
Chlamydiae
Actinobacteria
Dictyoglomi
Chloroflexi
Synergistetes
Deferribacteres
Chrysiogenetes
Firmicutes
Tenericutes
Fusobacteria
Cyanobacteria
Gammaproteobacteria
Betaproteobacteria
Alphaproteobacteria
Epsilonproteobacteria
Deltaproteobacteria
Magnetococcus
Elusimicrobia
Thermobaculum
Euryarchaeota
Crenarchaeota
Thaumarchaeota
Nanoarchaeota
Korarchaeota

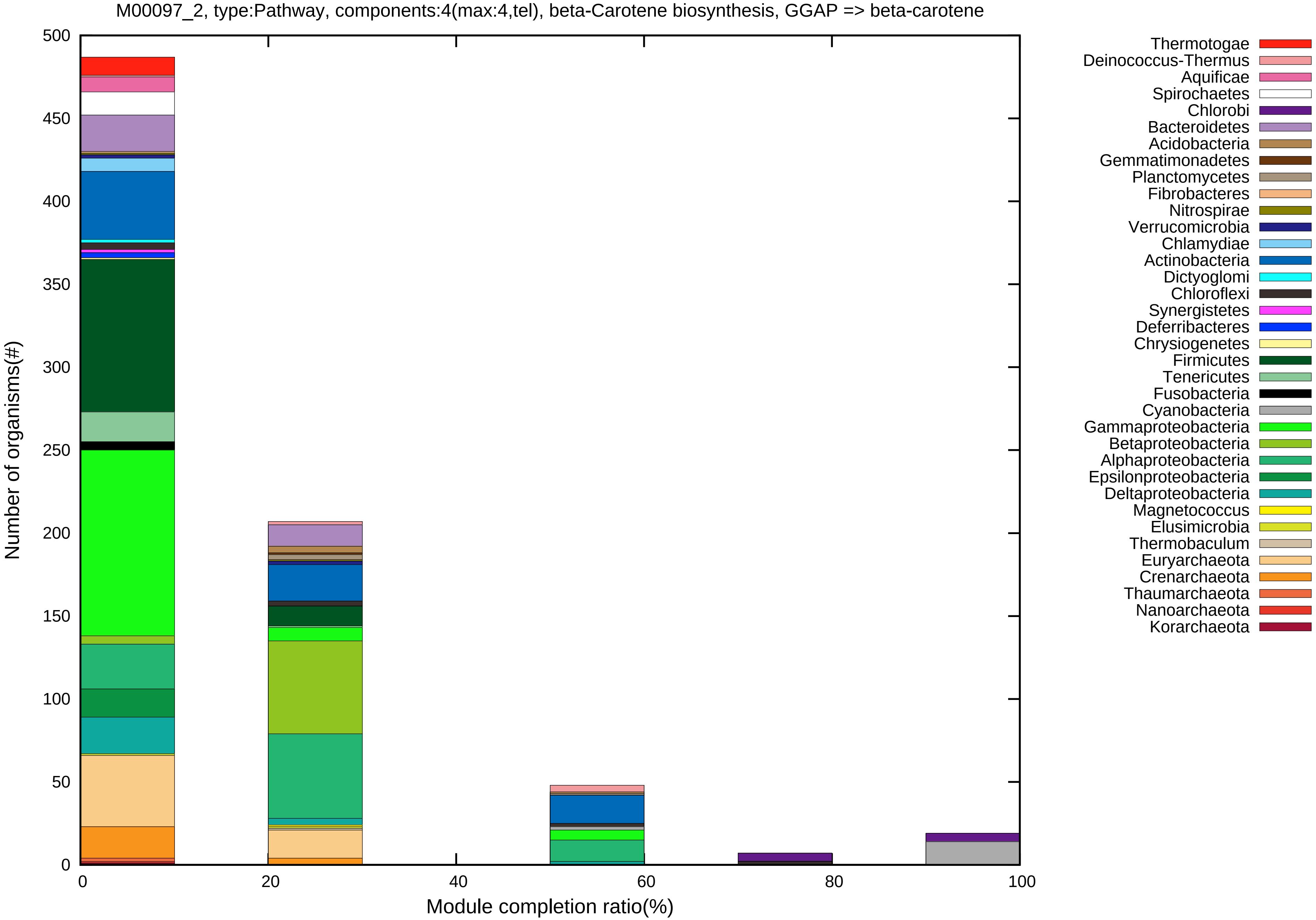
M00097_2, type:Pathway, components:4(max:4,tel), beta-Carotene biosynthesis, GGAP => beta-carotene
Number of organisms(#)
Module completion ratio(%)
0
50
100
150
200
250
300
350
400
450
500
0
20
40
60
80
100
Thermotogae
Deinococcus-Thermus
Aquificae
Spirochaetes
Chlorobi
Bacteroidetes
Acidobacteria
Gemmatimonadetes
Planctomycetes
Fibrobacteres
Nitrospirae
Verrucomicrobia
Chlamydiae
Actinobacteria
Dictyoglomi
Chloroflexi
Synergistetes
Deferribacteres
Chrysiogenetes
Firmicutes
Tenericutes
Fusobacteria
Cyanobacteria
Gammaproteobacteria
Betaproteobacteria
Alphaproteobacteria
Epsilonproteobacteria
Deltaproteobacteria
Magnetococcus
Elusimicrobia
Thermobaculum
Euryarchaeota
Crenarchaeota
Thaumarchaeota
Nanoarchaeota
Korarchaeota

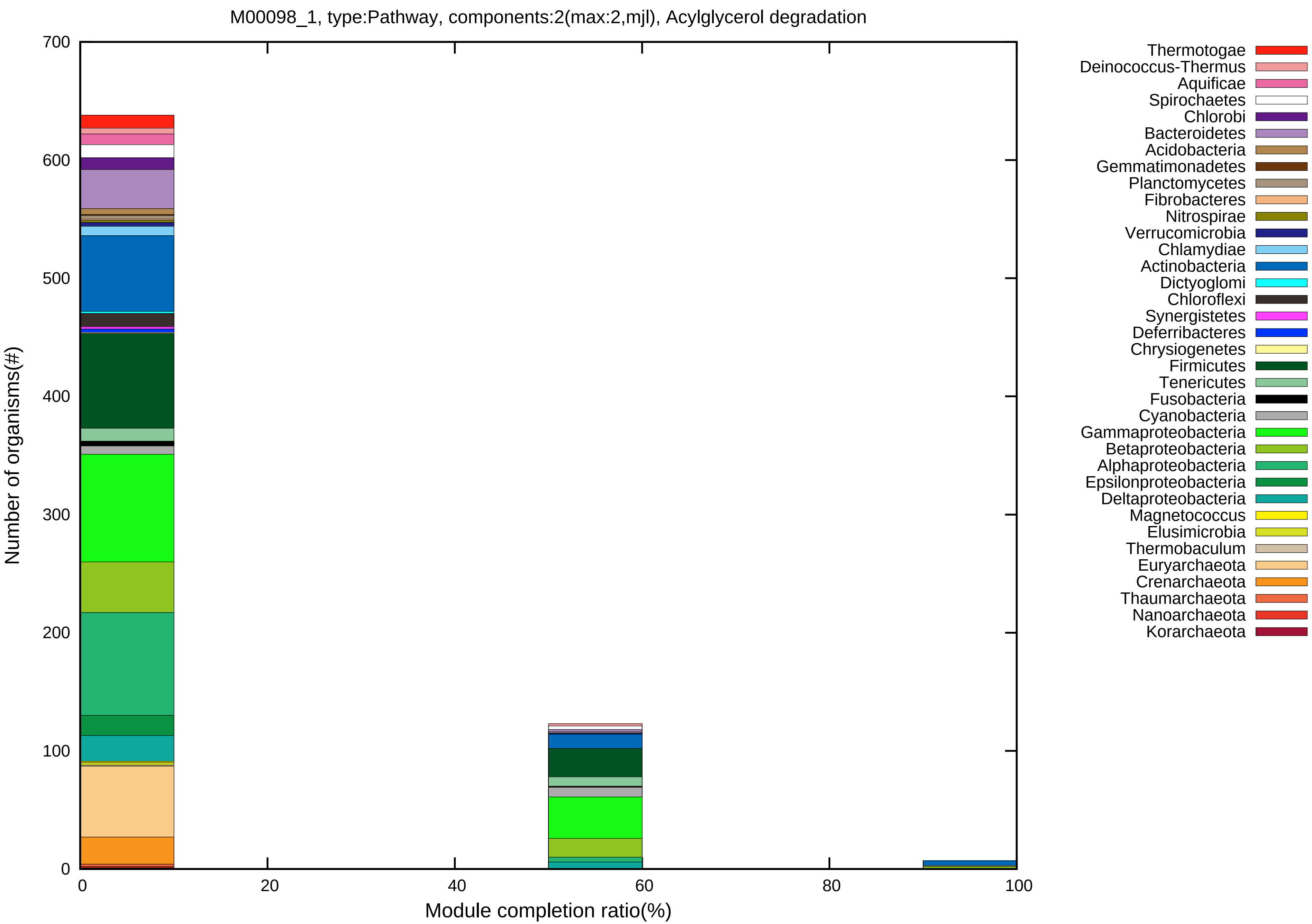

M00098_1, type:Pathway, components:2(max:2,mjl), Acylglycerol degradation
Number of organisms(#)
Module completion ratio(%)
0
100
200
300
400
500
600
700
0
20
40
60
80
100
Thermotogae
Deinococcus-Thermus
Aquificae
Spirochaetes
Chlorobi
Bacteroidetes
Acidobacteria
Gemmatimonadetes
Planctomycetes
Fibrobacteres
Nitrospirae
Verrucomicrobia
Chlamydiae
Actinobacteria
Dictyoglomi
Chloroflexi
Synergistetes
Deferribacteres
Chrysiogenetes
Firmicutes
Tenericutes
Fusobacteria
Cyanobacteria
Gammaproteobacteria
Betaproteobacteria
Alphaproteobacteria
Epsilonproteobacteria
Deltaproteobacteria
Magnetococcus
Elusimicrobia
Thermobaculum
Euryarchaeota
Crenarchaeota
Thaumarchaeota
Nanoarchaeota
Korarchaeota

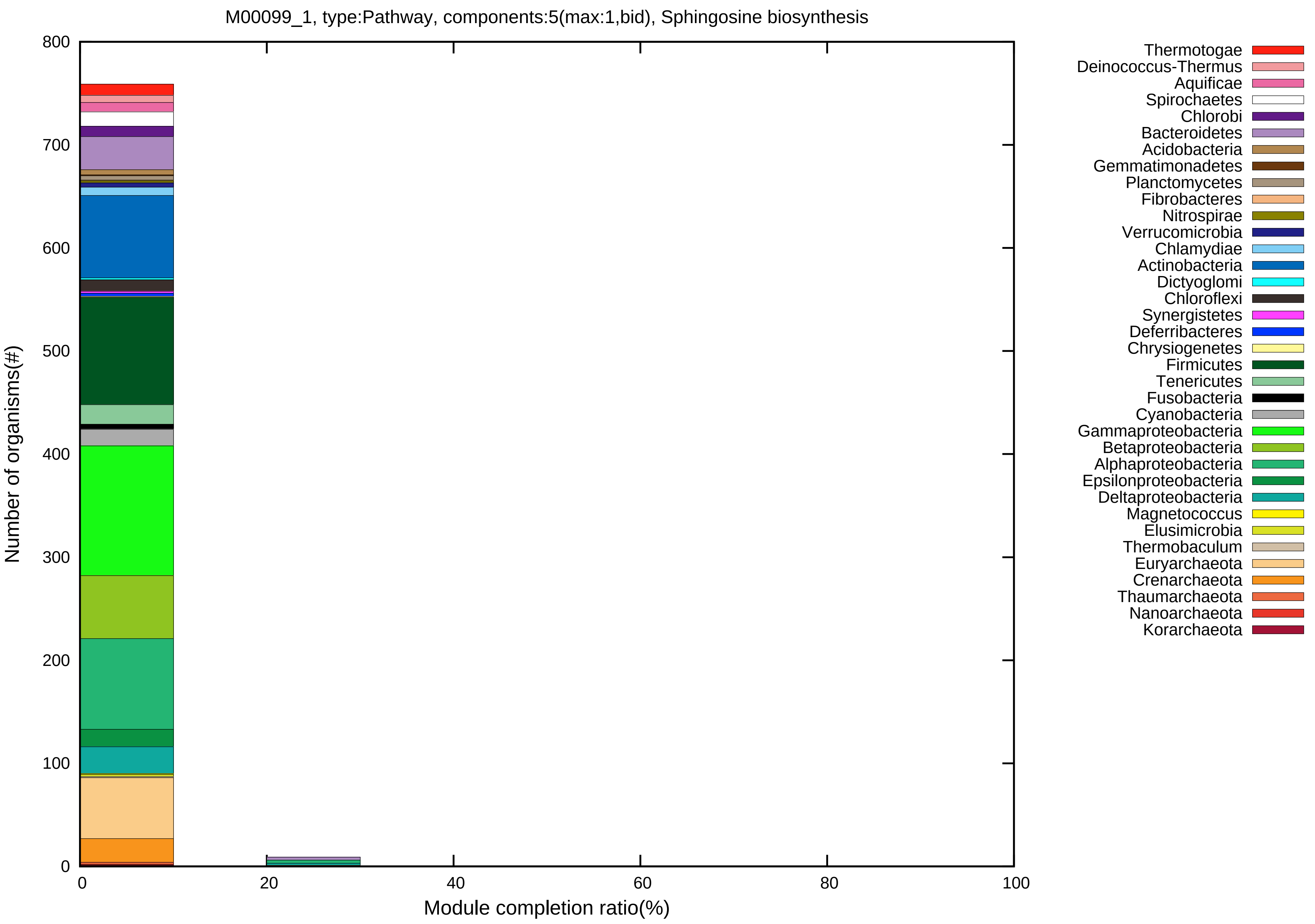

M00099_1, type:Pathway, components:5(max:1,bid), Sphingosine biosynthesis
Number of organisms(#)
Module completion ratio(%)
0
100
200
300
400
500
600
700
800
0
20
40
60
80
100
Thermotogae
Deinococcus-Thermus
Aquificae
Spirochaetes
Chlorobi
Bacteroidetes
Acidobacteria
Gemmatimonadetes
Planctomycetes
Fibrobacteres
Nitrospirae
Verrucomicrobia
Chlamydiae
Actinobacteria
Dictyoglomi
Chloroflexi
Synergistetes
Deferribacteres
Chrysiogenetes
Firmicutes
Tenericutes
Fusobacteria
Cyanobacteria
Gammaproteobacteria
Betaproteobacteria
Alphaproteobacteria
Epsilonproteobacteria
Deltaproteobacteria
Magnetococcus
Elusimicrobia
Thermobaculum
Euryarchaeota
Crenarchaeota
Thaumarchaeota
Nanoarchaeota
Korarchaeota

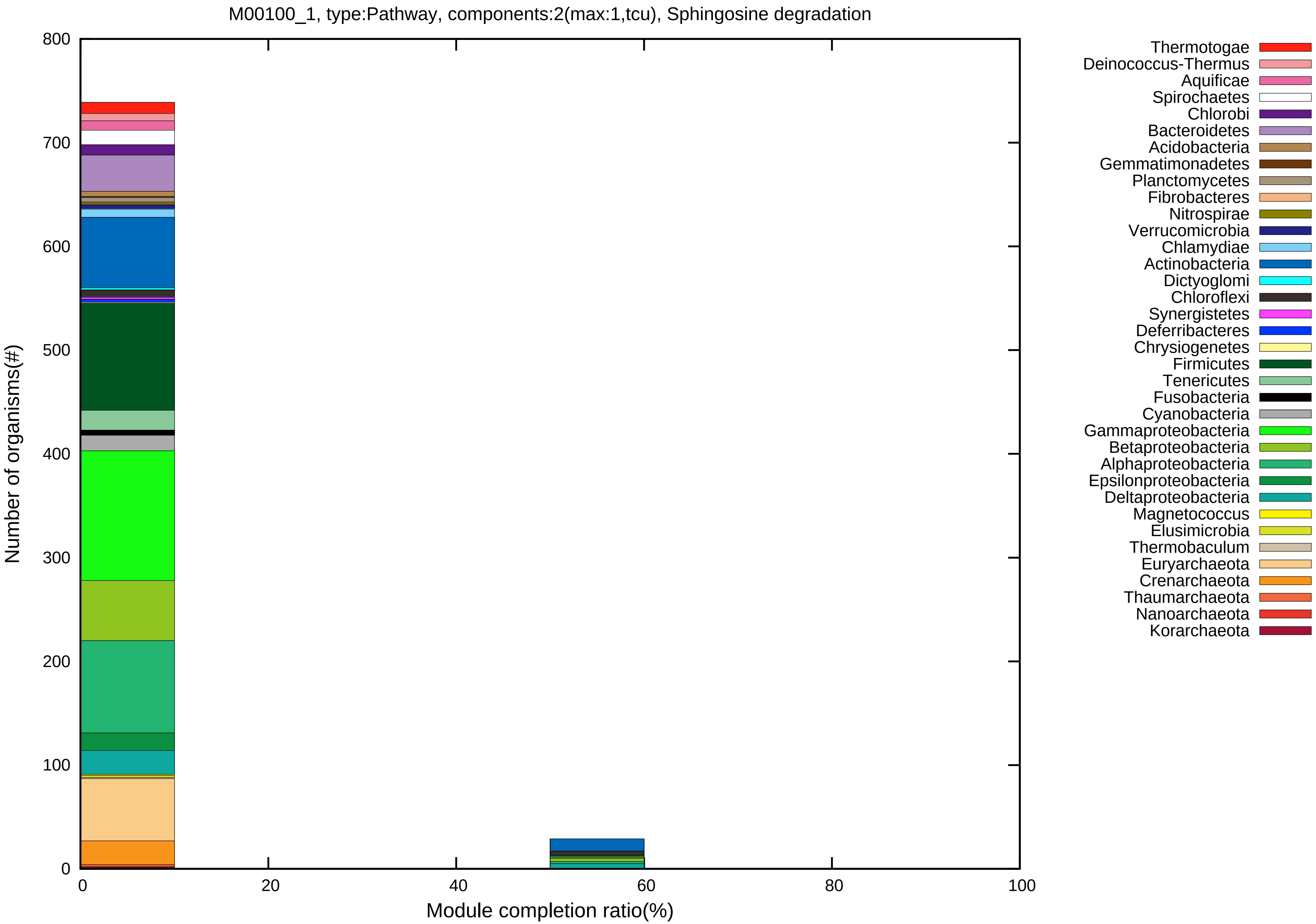

M00100_1, type:Pathway, components:2(max:1,tcu), Sphingosine degradation
Number of organisms(#)
Module completion ratio(%)
0
100
200
300
400
500
600
700
800
0
20
40
60
80
100
Thermotogae
Deinococcus-Thermus
Aquificae
Spirochaetes
Chlorobi
Bacteroidetes
Acidobacteria
Gemmatimonadetes
Planctomycetes
Fibrobacteres
Nitrospirae
Verrucomicrobia
Chlamydiae
Actinobacteria
Dictyoglomi
Chloroflexi
Synergistetes
Deferribacteres
Chrysiogenetes
Firmicutes
Tenericutes
Fusobacteria
Cyanobacteria
Gammaproteobacteria
Betaproteobacteria
Alphaproteobacteria
Epsilonproteobacteria
Deltaproteobacteria
Magnetococcus
Elusimicrobia
Thermobaculum
Euryarchaeota
Crenarchaeota
Thaumarchaeota
Nanoarchaeota
Korarchaeota

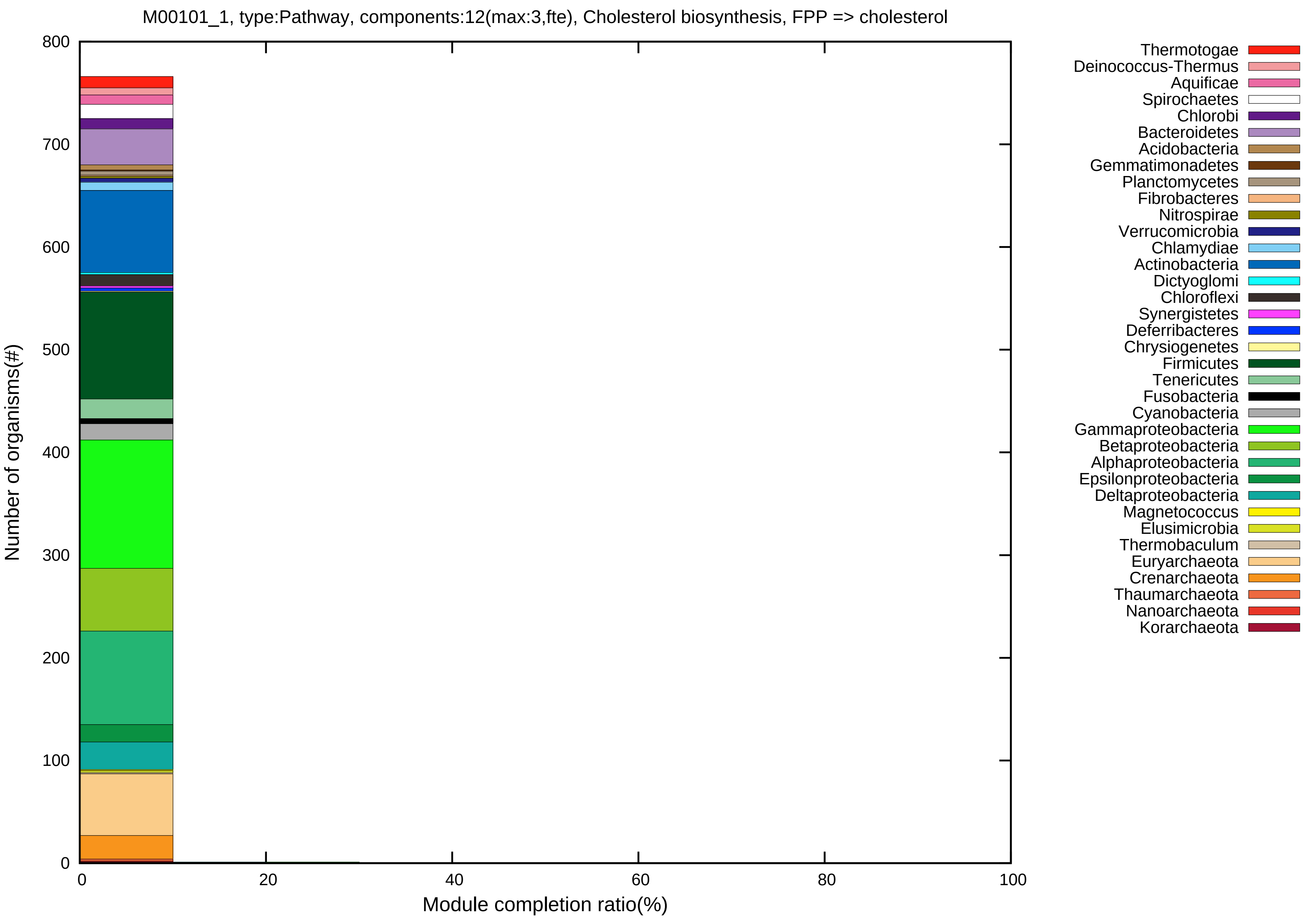

M00101_1, type:Pathway, components:12(max:3,fte), Cholesterol biosynthesis, FPP => cholesterol
Number of organisms(#)
Module completion ratio(%)
0
100
200
300
400
500
600
700
800
0
20
40
60
80
100
Thermotogae
Deinococcus-Thermus
Aquificae
Spirochaetes
Chlorobi
Bacteroidetes
Acidobacteria
Gemmatimonadetes
Planctomycetes
Fibrobacteres
Nitrospirae
Verrucomicrobia
Chlamydiae
Actinobacteria
Dictyoglomi
Chloroflexi
Synergistetes
Deferribacteres
Chrysiogenetes
Firmicutes
Tenericutes
Fusobacteria
Cyanobacteria
Gammaproteobacteria
Betaproteobacteria
Alphaproteobacteria
Epsilonproteobacteria
Deltaproteobacteria
Magnetococcus
Elusimicrobia
Thermobaculum
Euryarchaeota
Crenarchaeota
Thaumarchaeota
Nanoarchaeota
Korarchaeota

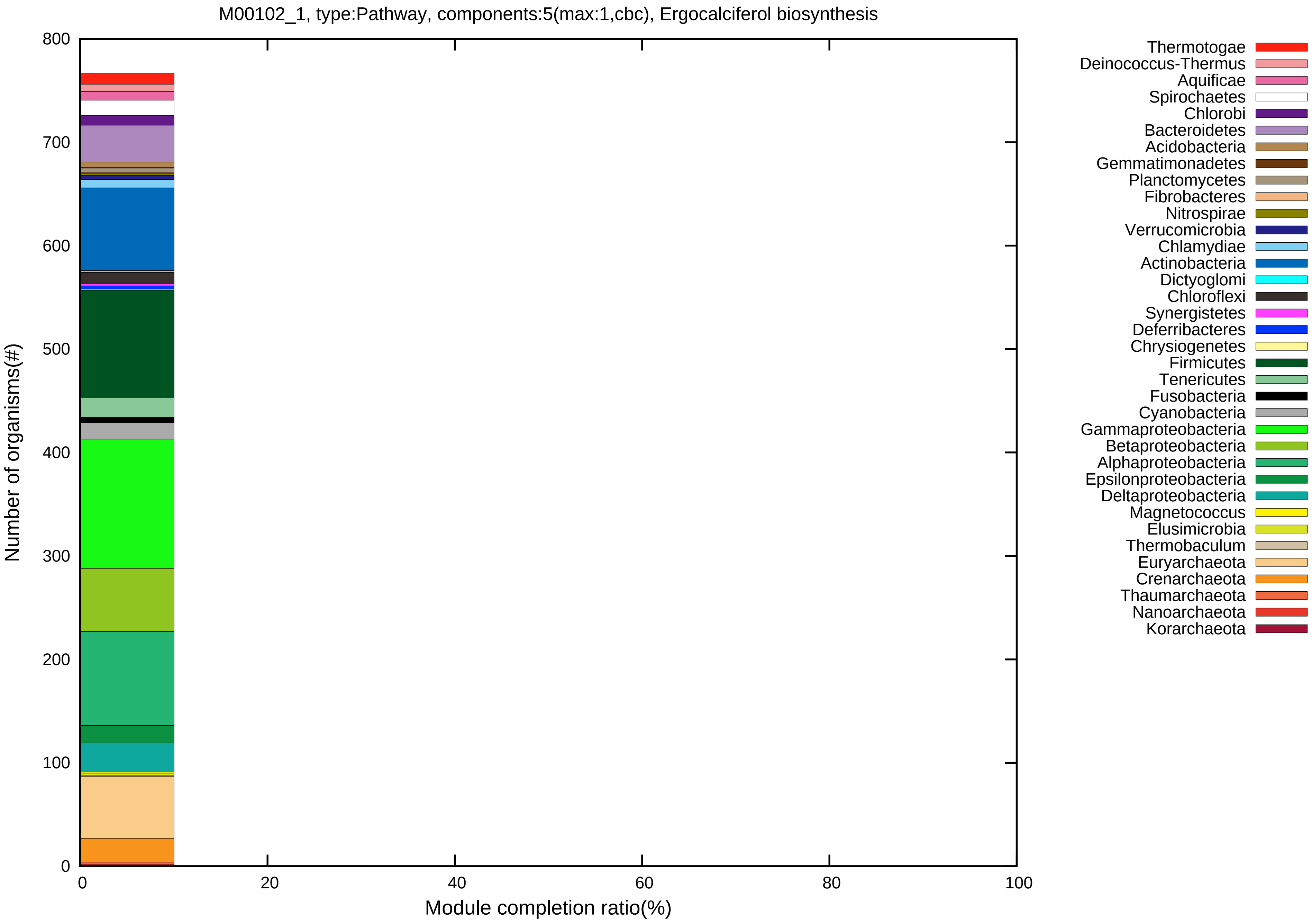

M00102_1, type:Pathway, components:5(max:1,cbc), Ergocalciferol biosynthesis
Number of organisms(#)
Module completion ratio(%)
0
100
200
300
400
500
600
700
800
0
20
40
60
80
100
Thermotogae
Deinococcus-Thermus
Aquificae
Spirochaetes
Chlorobi
Bacteroidetes
Acidobacteria
Gemmatimonadetes
Planctomycetes
Fibrobacteres
Nitrospirae
Verrucomicrobia
Chlamydiae
Actinobacteria
Dictyoglomi
Chloroflexi
Synergistetes
Deferribacteres
Chrysiogenetes
Firmicutes
Tenericutes
Fusobacteria
Cyanobacteria
Gammaproteobacteria
Betaproteobacteria
Alphaproteobacteria
Epsilonproteobacteria
Deltaproteobacteria
Magnetococcus
Elusimicrobia
Thermobaculum
Euryarchaeota
Crenarchaeota
Thaumarchaeota
Nanoarchaeota
Korarchaeota

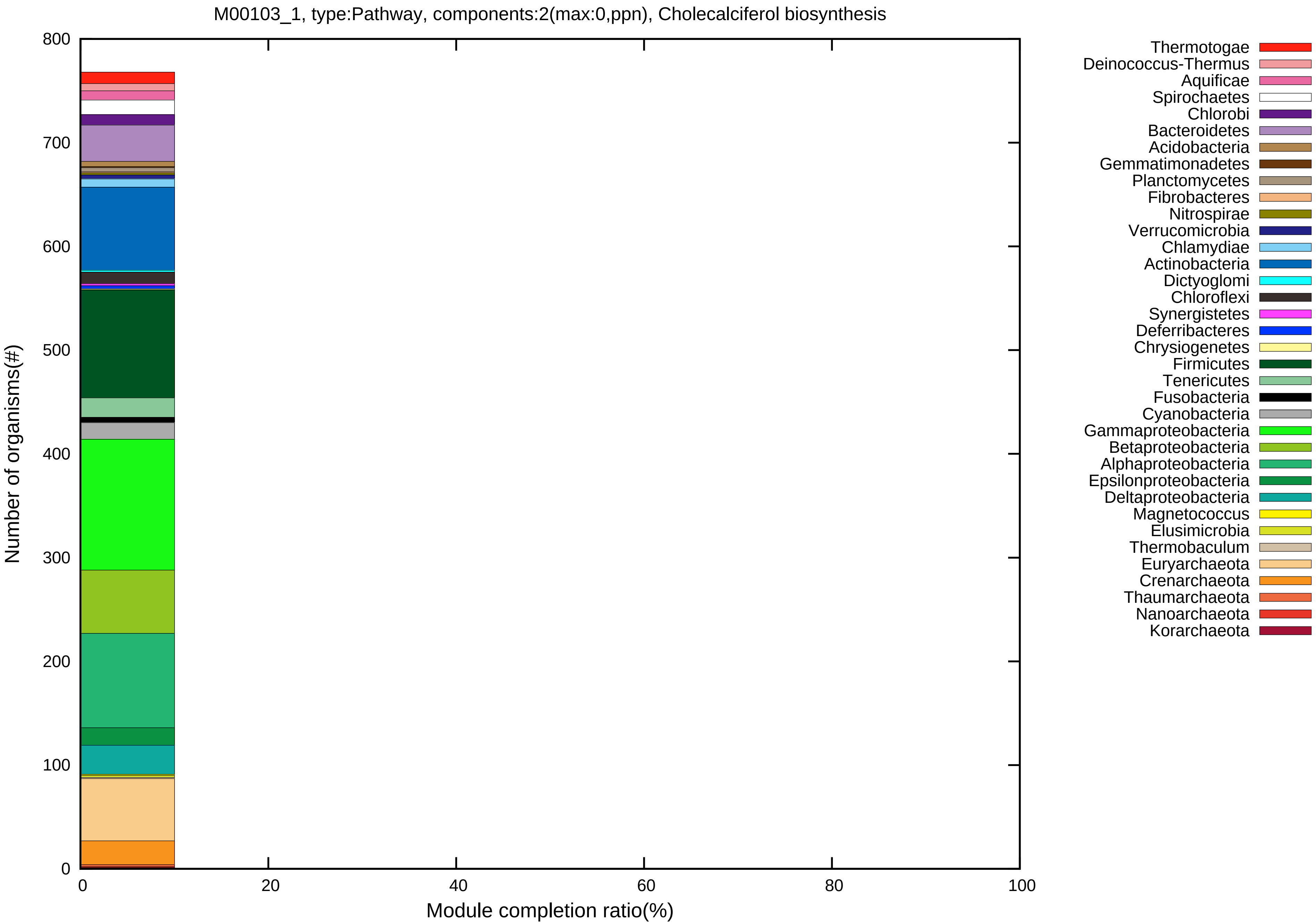

M00103_1, type:Pathway, components:2(max:0,ppn), Cholecalciferol biosynthesis
Number of organisms(#)
Module completion ratio(%)
0
100
200
300
400
500
600
700
800
0
20
40
60
80
100
Thermotogae
Deinococcus-Thermus
Aquificae
Spirochaetes
Chlorobi
Bacteroidetes
Acidobacteria
Gemmatimonadetes
Planctomycetes
Fibrobacteres
Nitrospirae
Verrucomicrobia
Chlamydiae
Actinobacteria
Dictyoglomi
Chloroflexi
Synergistetes
Deferribacteres
Chrysiogenetes
Firmicutes
Tenericutes
Fusobacteria
Cyanobacteria
Gammaproteobacteria
Betaproteobacteria
Alphaproteobacteria
Epsilonproteobacteria
Deltaproteobacteria
Magnetococcus
Elusimicrobia
Thermobaculum
Euryarchaeota
Crenarchaeota
Thaumarchaeota
Nanoarchaeota
Korarchaeota

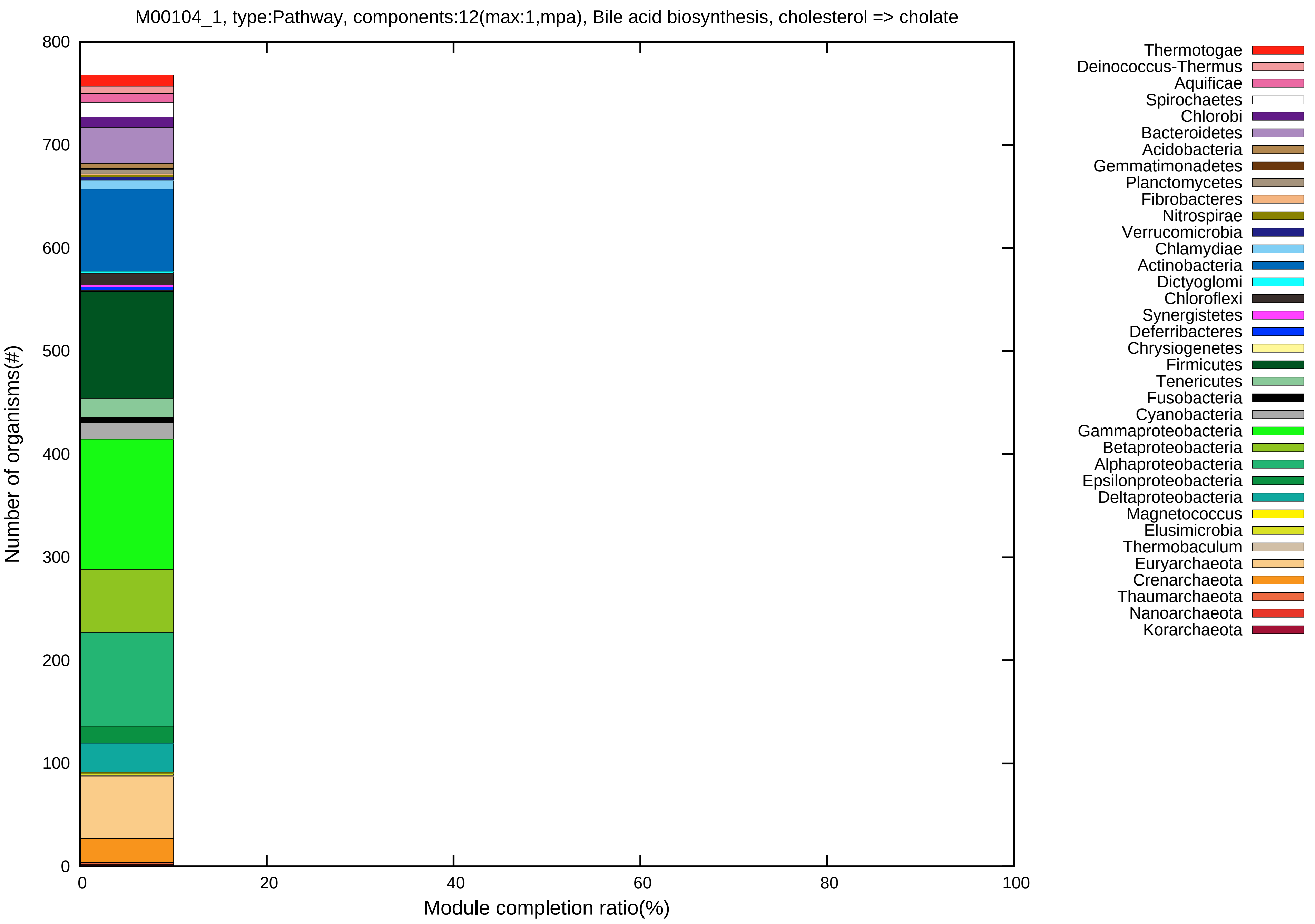

M00104_1, type:Pathway, components:12(max:1,mpa), Bile acid biosynthesis, cholesterol => cholate
Number of organisms(#)
Module completion ratio(%)
0
100
200
300
400
500
600
700
800
0
20
40
60
80
100
Thermotogae
Deinococcus-Thermus
Aquificae
Spirochaetes
Chlorobi
Bacteroidetes
Acidobacteria
Gemmatimonadetes
Planctomycetes
Fibrobacteres
Nitrospirae
Verrucomicrobia
Chlamydiae
Actinobacteria
Dictyoglomi
Chloroflexi
Synergistetes
Deferribacteres
Chrysiogenetes
Firmicutes
Tenericutes
Fusobacteria
Cyanobacteria
Gammaproteobacteria
Betaproteobacteria
Alphaproteobacteria
Epsilonproteobacteria
Deltaproteobacteria
Magnetococcus
Elusimicrobia
Thermobaculum
Euryarchaeota
Crenarchaeota
Thaumarchaeota
Nanoarchaeota
Korarchaeota

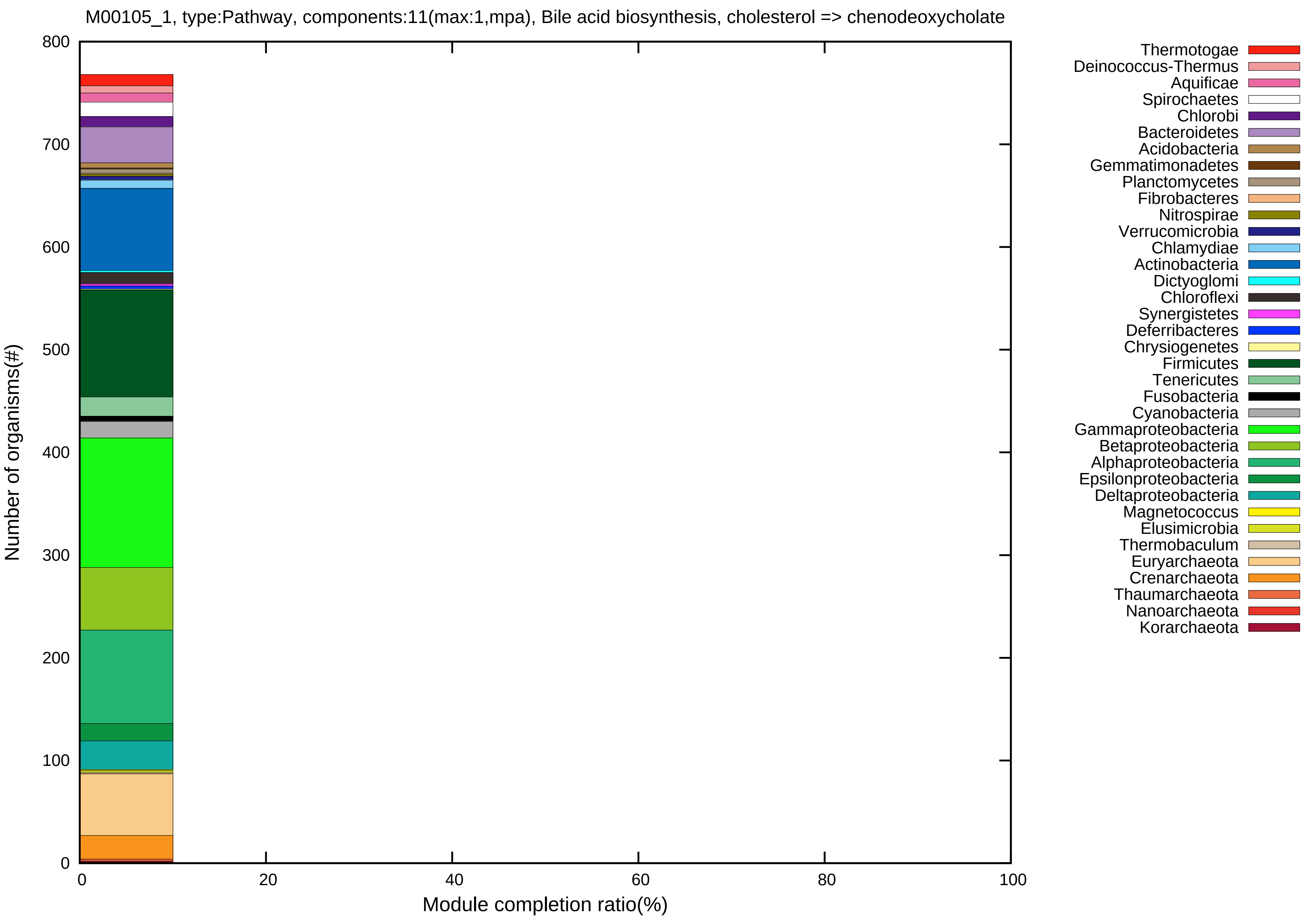
M00105_1, type:Pathway, components:11(max:1,mpa), Bile acid biosynthesis, cholesterol => chenodeoxycholate
Number of organisms(#)
Module completion ratio(%)
0
100
200
300
400
500
600
700
800
0
20
40
60
80
100
Thermotogae
Deinococcus-Thermus
Aquificae
Spirochaetes
Chlorobi
Bacteroidetes
Acidobacteria
Gemmatimonadetes
Planctomycetes
Fibrobacteres
Nitrospirae
Verrucomicrobia
Chlamydiae
Actinobacteria
Dictyoglomi
Chloroflexi
Synergistetes
Deferribacteres
Chrysiogenetes
Firmicutes
Tenericutes
Fusobacteria
Cyanobacteria
Gammaproteobacteria
Betaproteobacteria
Alphaproteobacteria
Epsilonproteobacteria
Deltaproteobacteria
Magnetococcus
Elusimicrobia
Thermobaculum
Euryarchaeota
Crenarchaeota
Thaumarchaeota
Nanoarchaeota
Korarchaeota

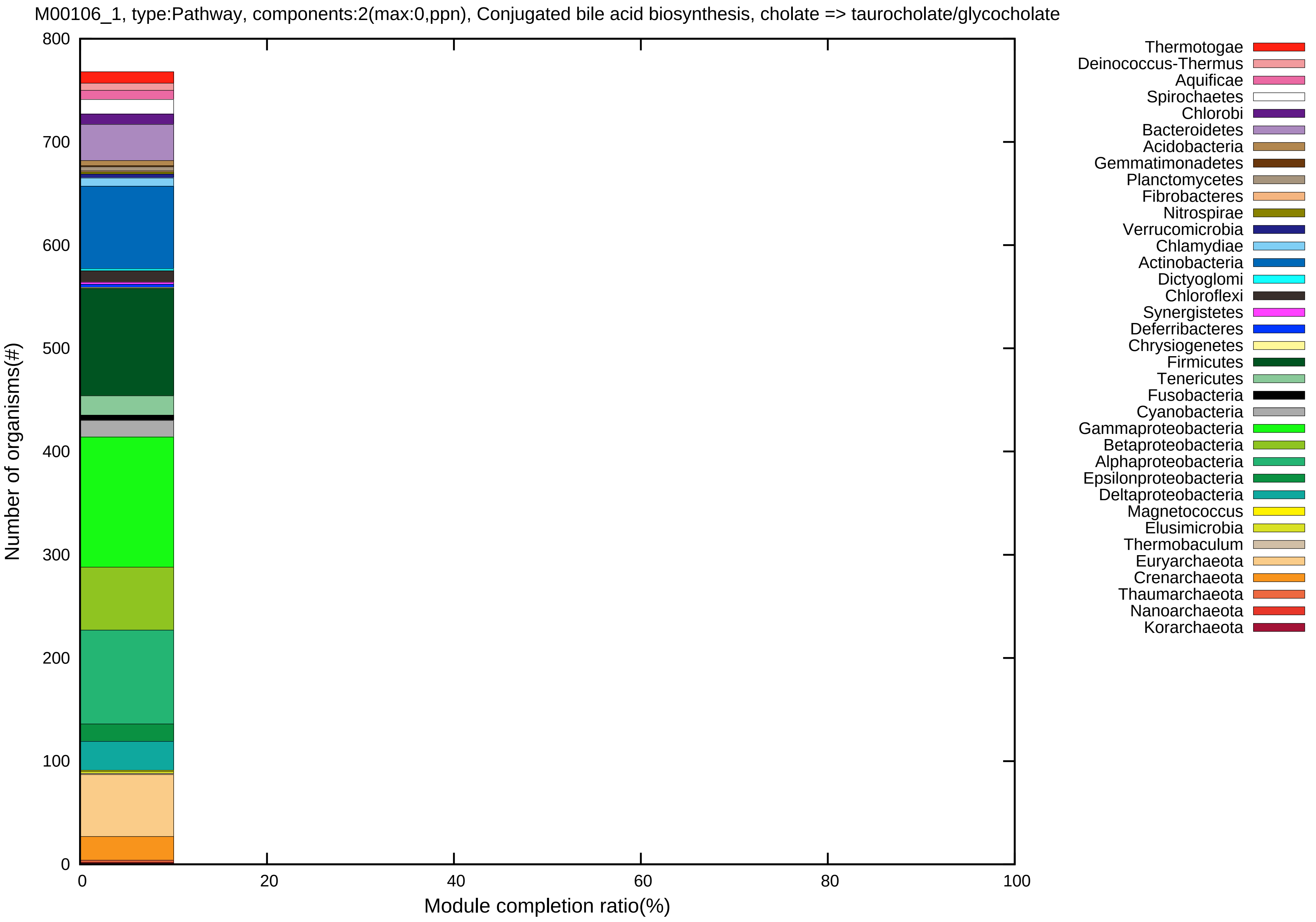

M00106_1, type:Pathway, components:2(max:0,ppn), Conjugated bile acid biosynthesis, cholate => taurocholate/glycocholate
Number of organisms(#)
Module completion ratio(%)
0
100
200
300
400
500
600
700
800
0
20
40
60
80
100
Thermotogae
Deinococcus-Thermus
Aquificae
Spirochaetes
Chlorobi
Bacteroidetes
Acidobacteria
Gemmatimonadetes
Planctomycetes
Fibrobacteres
Nitrospirae
Verrucomicrobia
Chlamydiae
Actinobacteria
Dictyoglomi
Chloroflexi
Synergistetes
Deferribacteres
Chrysiogenetes
Firmicutes
Tenericutes
Fusobacteria
Cyanobacteria
Gammaproteobacteria
Betaproteobacteria
Alphaproteobacteria
Epsilonproteobacteria
Deltaproteobacteria
Magnetococcus
Elusimicrobia
Thermobaculum
Euryarchaeota
Crenarchaeota
Thaumarchaeota
Nanoarchaeota
Korarchaeota

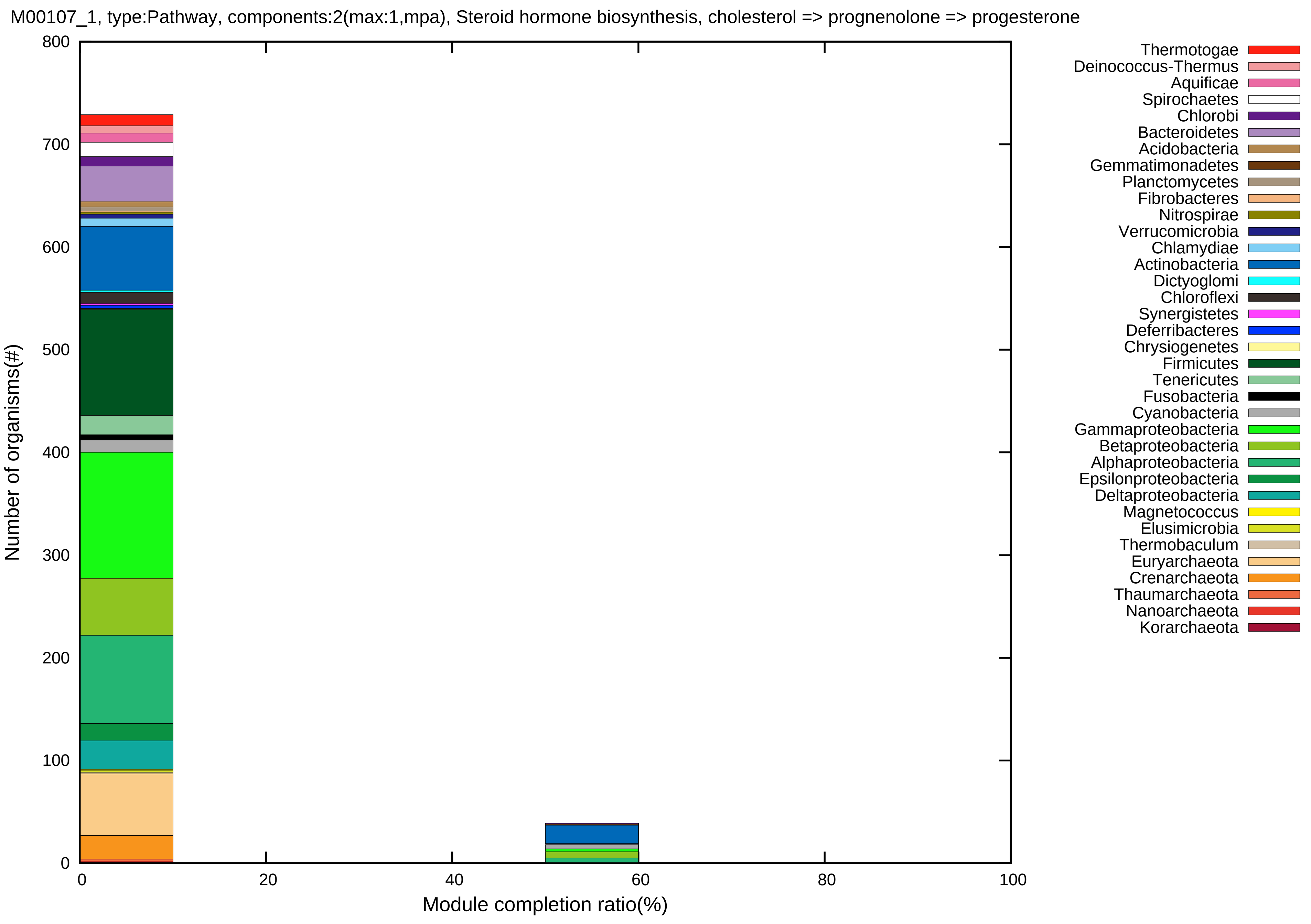

M00107_1, type:Pathway, components:2(max:1,mpa), Steroid hormone biosynthesis, cholesterol => prognenolone => progesterone
Number of organisms(#)
Module completion ratio(%)
0
100
200
300
400
500
600
700
800
0
20
40
60
80
100
Thermotogae
Deinococcus-Thermus
Aquificae
Spirochaetes
Chlorobi
Bacteroidetes
Acidobacteria
Gemmatimonadetes
Planctomycetes
Fibrobacteres
Nitrospirae
Verrucomicrobia
Chlamydiae
Actinobacteria
Dictyoglomi
Chloroflexi
Synergistetes
Deferribacteres
Chrysiogenetes
Firmicutes
Tenericutes
Fusobacteria
Cyanobacteria
Gammaproteobacteria
Betaproteobacteria
Alphaproteobacteria
Epsilonproteobacteria
Deltaproteobacteria
Magnetococcus
Elusimicrobia
Thermobaculum
Euryarchaeota
Crenarchaeota
Thaumarchaeota
Nanoarchaeota
Korarchaeota

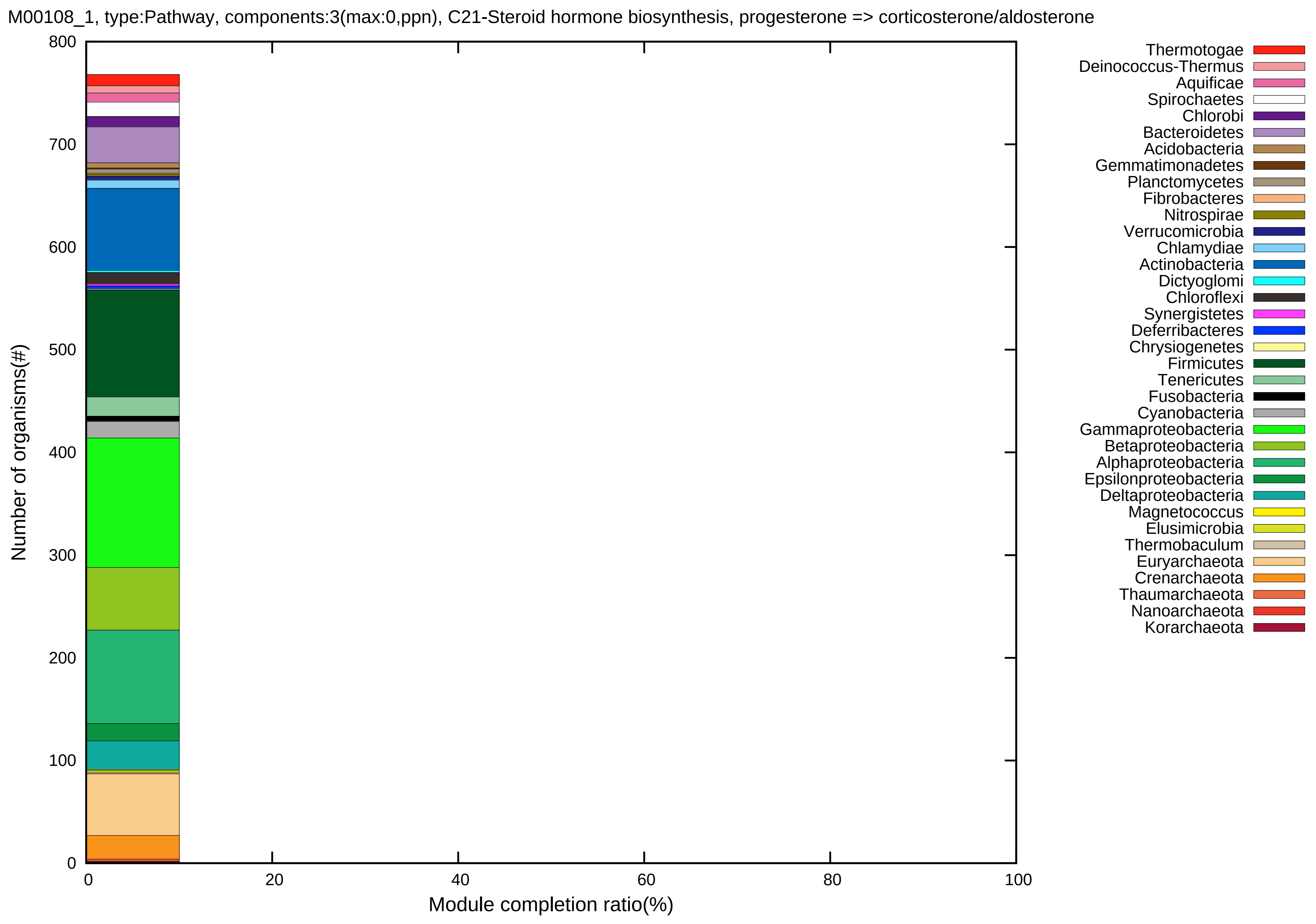
M00108_1, type:Pathway, components:3(max:0,ppn), C21-Steroid hormone biosynthesis, progesterone => corticosterone/aldosterone
Number of organisms(#)
Module completion ratio(%)
0
100
200
300
400
500
600
700
800
0
20
40
60
80
100
Thermotogae
Deinococcus-Thermus
Aquificae
Spirochaetes
Chlorobi
Bacteroidetes
Acidobacteria
Gemmatimonadetes
Planctomycetes
Fibrobacteres
Nitrospirae
Verrucomicrobia
Chlamydiae
Actinobacteria
Dictyoglomi
Chloroflexi
Synergistetes
Deferribacteres
Chrysiogenetes
Firmicutes
Tenericutes
Fusobacteria
Cyanobacteria
Gammaproteobacteria
Betaproteobacteria
Alphaproteobacteria
Epsilonproteobacteria
Deltaproteobacteria
Magnetococcus
Elusimicrobia
Thermobaculum
Euryarchaeota
Crenarchaeota
Thaumarchaeota
Nanoarchaeota
Korarchaeota

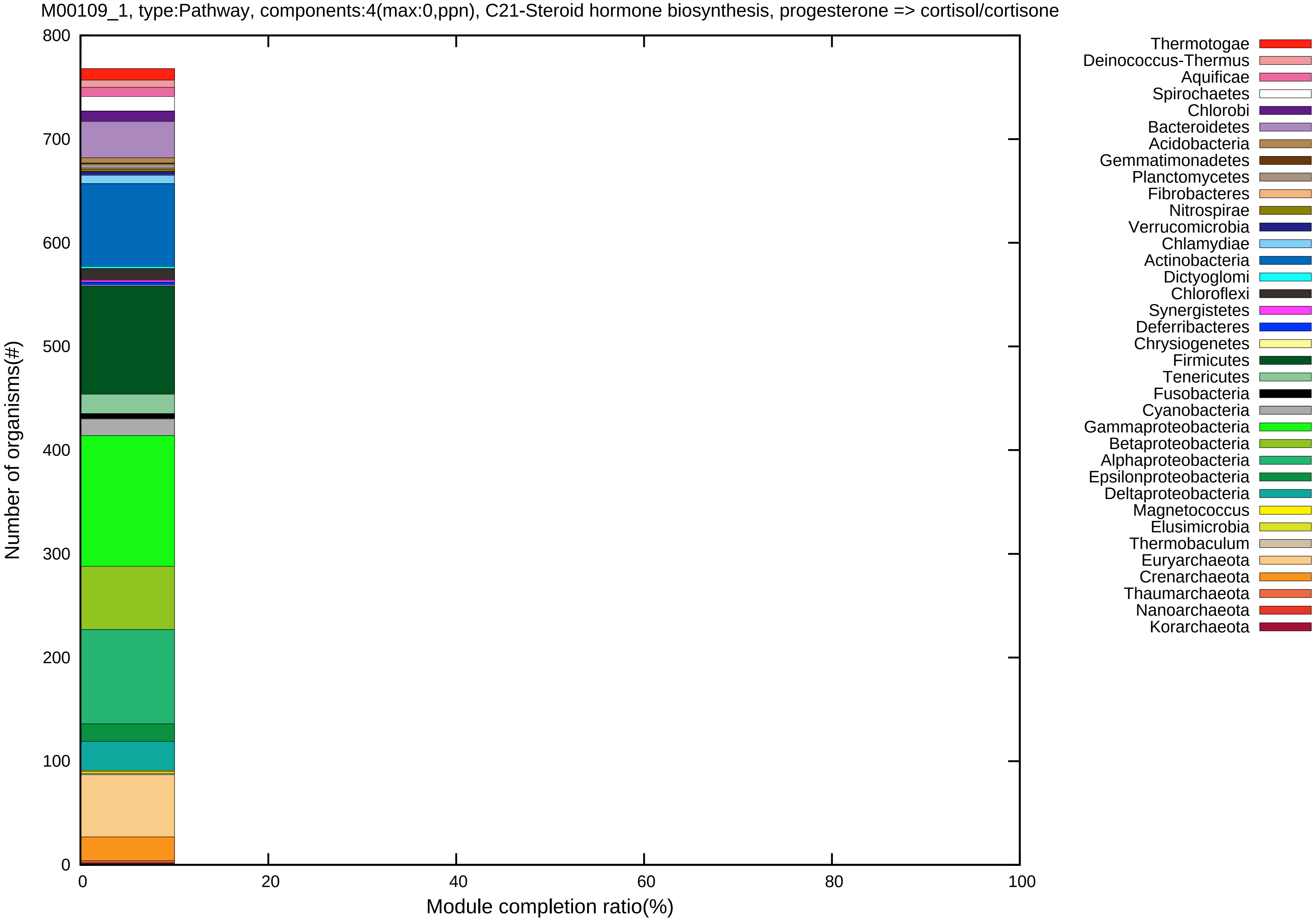

M00109_1, type:Pathway, components:4(max:0,ppn), C21-Steroid hormone biosynthesis, progesterone => cortisol/cortisone
Number of organisms(#)
Module completion ratio(%)
0
100
200
300
400
500
600
700
800
0
20
40
60
80
100
Thermotogae
Deinococcus-Thermus
Aquificae
Spirochaetes
Chlorobi
Bacteroidetes
Acidobacteria
Gemmatimonadetes
Planctomycetes
Fibrobacteres
Nitrospirae
Verrucomicrobia
Chlamydiae
Actinobacteria
Dictyoglomi
Chloroflexi
Synergistetes
Deferribacteres
Chrysiogenetes
Firmicutes
Tenericutes
Fusobacteria
Cyanobacteria
Gammaproteobacteria
Betaproteobacteria
Alphaproteobacteria
Epsilonproteobacteria
Deltaproteobacteria
Magnetococcus
Elusimicrobia
Thermobaculum
Euryarchaeota
Crenarchaeota
Thaumarchaeota
Nanoarchaeota
Korarchaeota

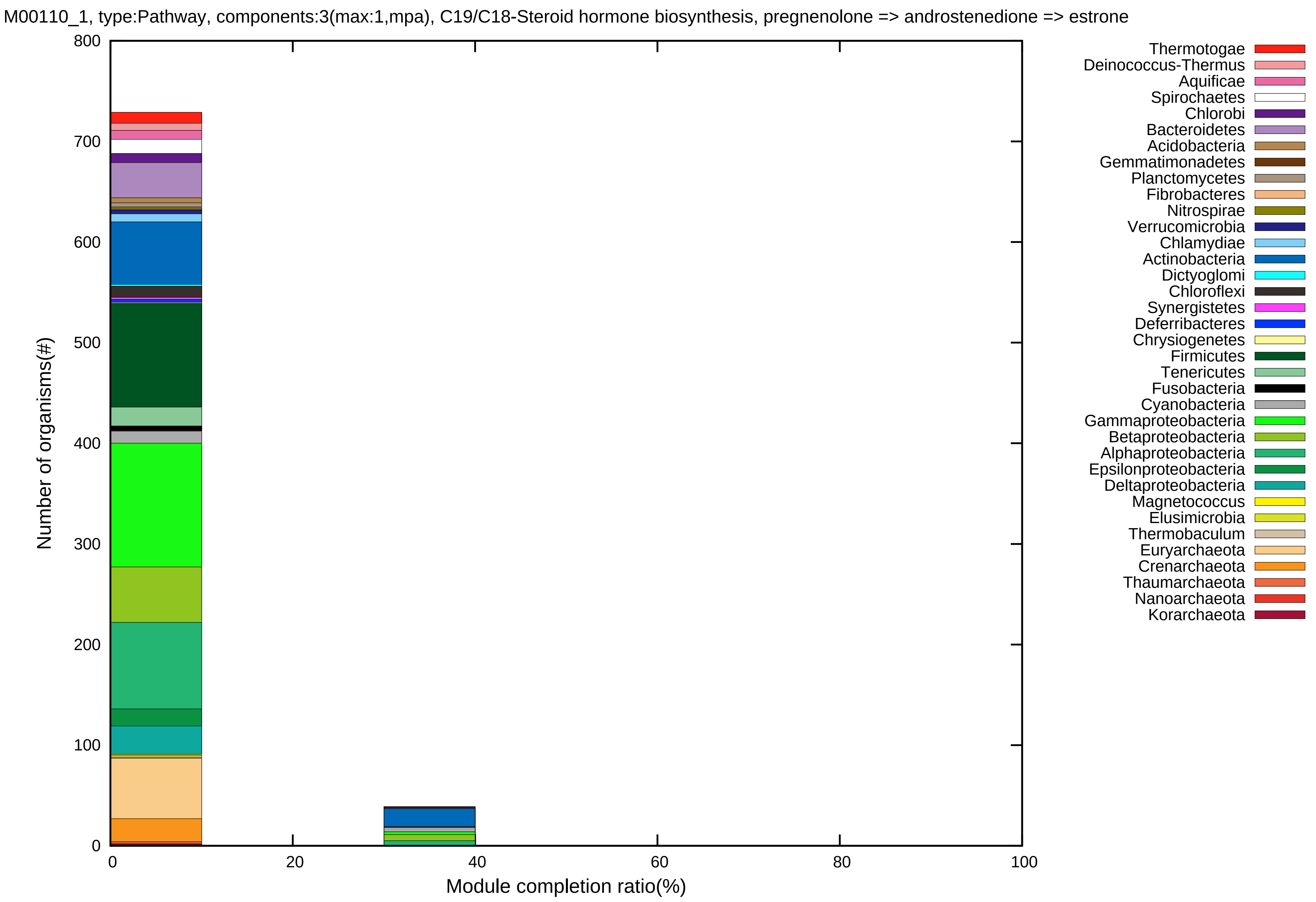

M00110_1, type:Pathway, components:3(max:1,mpa), C19/C18-Steroid hormone biosynthesis, pregnenolone => androstenedione => estrone
Number of organisms(#)
Module completion ratio(%)
0
100
200
300
400
500
600
700
800
0
20
40
60
80
100
Thermotogae
Deinococcus-Thermus
Aquificae
Spirochaetes
Chlorobi
Bacteroidetes
Acidobacteria
Gemmatimonadetes
Planctomycetes
Fibrobacteres
Nitrospirae
Verrucomicrobia
Chlamydiae
Actinobacteria
Dictyoglomi
Chloroflexi
Synergistetes
Deferribacteres
Chrysiogenetes
Firmicutes
Tenericutes
Fusobacteria
Cyanobacteria
Gammaproteobacteria
Betaproteobacteria
Alphaproteobacteria
Epsilonproteobacteria
Deltaproteobacteria
Magnetococcus
Elusimicrobia
Thermobaculum
Euryarchaeota
Crenarchaeota
Thaumarchaeota
Nanoarchaeota
Korarchaeota

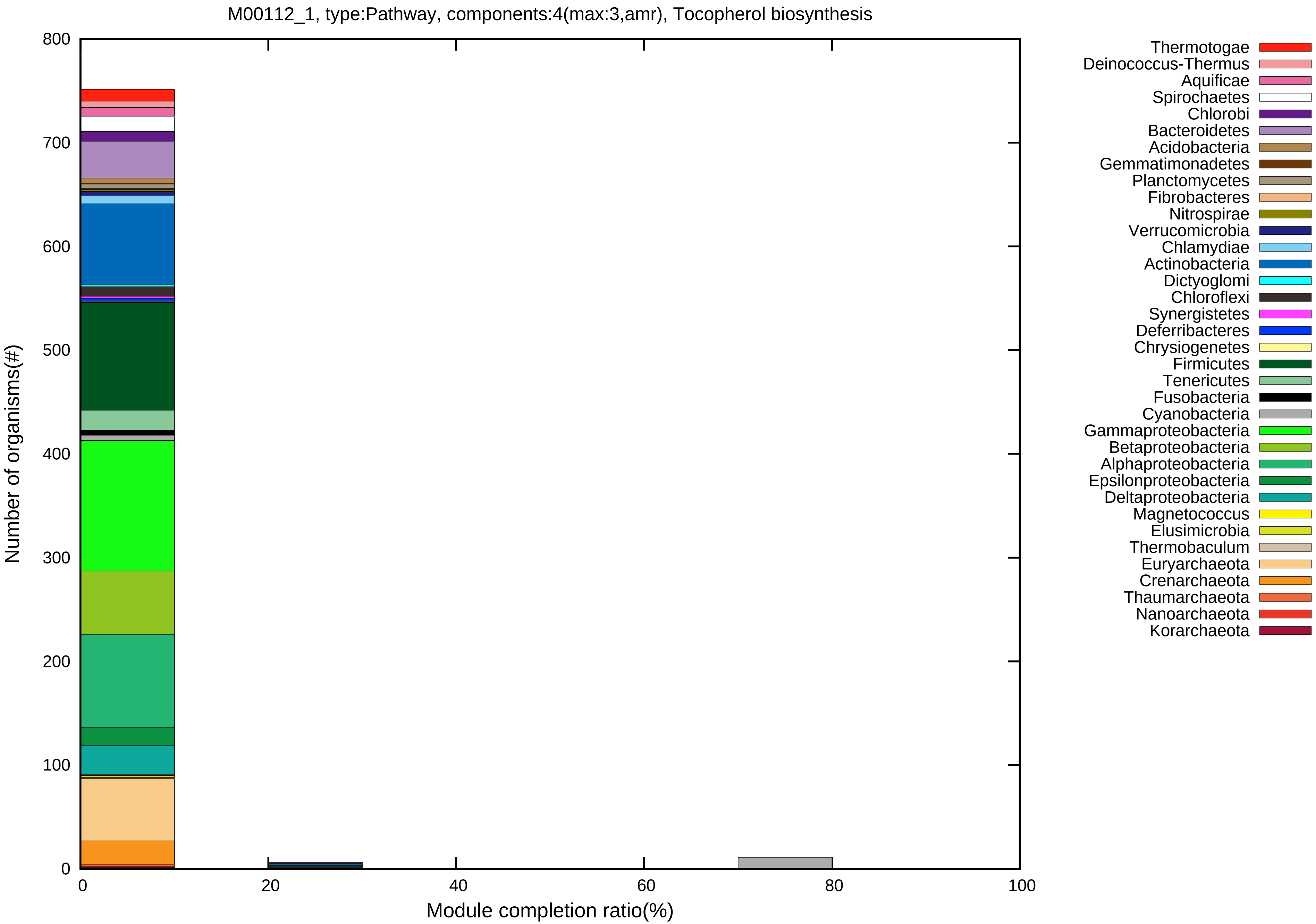

M00112_1, type:Pathway, components:4(max:3,amr), Tocopherol biosynthesis
Number of organisms(#)
Module completion ratio(%)
0
100
200
300
400
500
600
700
800
0
20
40
60
80
100
Thermotogae
Deinococcus-Thermus
Aquificae
Spirochaetes
Chlorobi
Bacteroidetes
Acidobacteria
Gemmatimonadetes
Planctomycetes
Fibrobacteres
Nitrospirae
Verrucomicrobia
Chlamydiae
Actinobacteria
Dictyoglomi
Chloroflexi
Synergistetes
Deferribacteres
Chrysiogenetes
Firmicutes
Tenericutes
Fusobacteria
Cyanobacteria
Gammaproteobacteria
Betaproteobacteria
Alphaproteobacteria
Epsilonproteobacteria
Deltaproteobacteria
Magnetococcus
Elusimicrobia
Thermobaculum
Euryarchaeota
Crenarchaeota
Thaumarchaeota
Nanoarchaeota
Korarchaeota

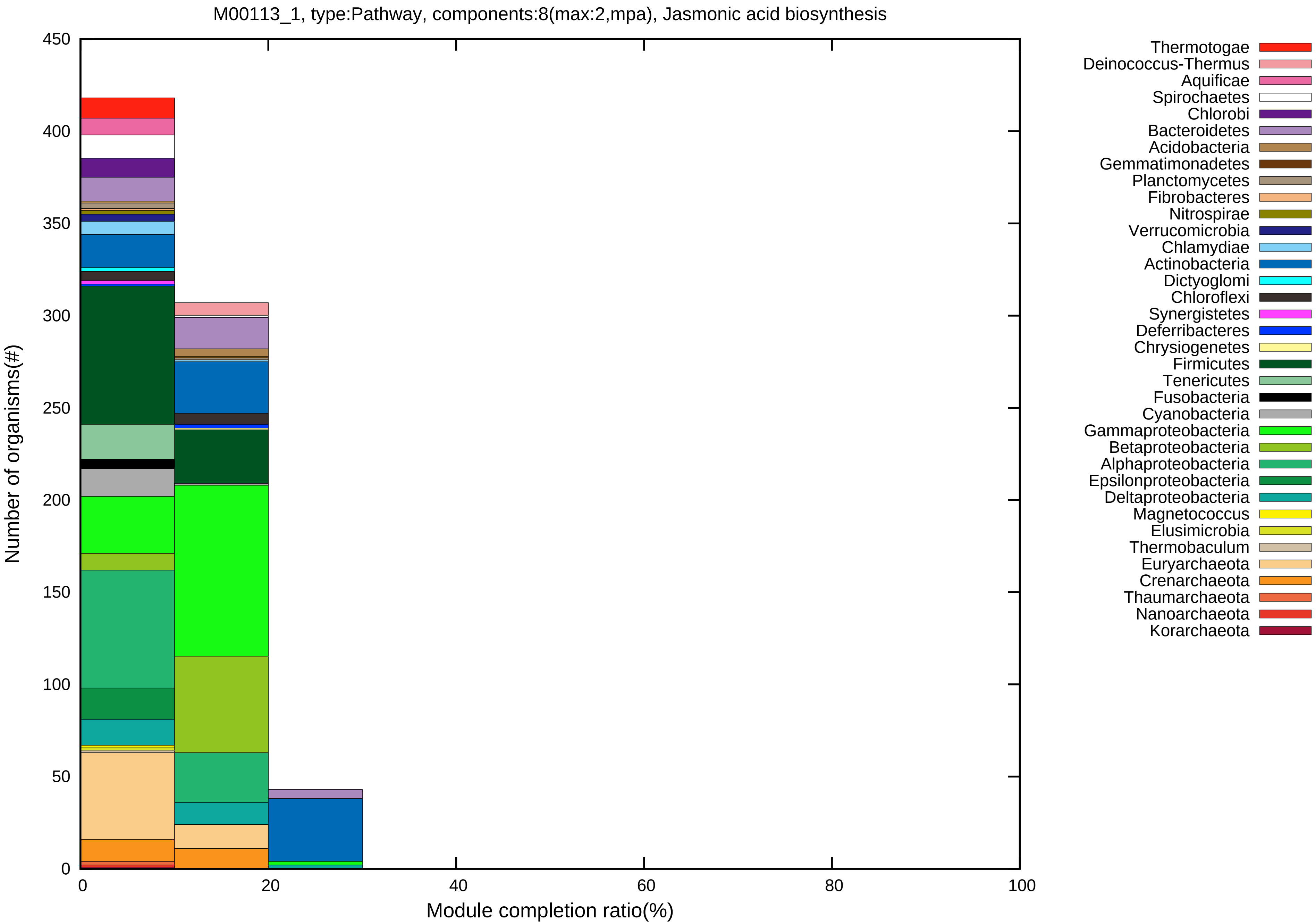

M00113_1, type:Pathway, components:8(max:2,mpa), Jasmonic acid biosynthesis
Number of organisms(#)
Module completion ratio(%)
0
50
100
150
200
250
300
350
400
450
0
20
40
60
80
100
Thermotogae
Deinococcus-Thermus
Aquificae
Spirochaetes
Chlorobi
Bacteroidetes
Acidobacteria
Gemmatimonadetes
Planctomycetes
Fibrobacteres
Nitrospirae
Verrucomicrobia
Chlamydiae
Actinobacteria
Dictyoglomi
Chloroflexi
Synergistetes
Deferribacteres
Chrysiogenetes
Firmicutes
Tenericutes
Fusobacteria
Cyanobacteria
Gammaproteobacteria
Betaproteobacteria
Alphaproteobacteria
Epsilonproteobacteria
Deltaproteobacteria
Magnetococcus
Elusimicrobia
Thermobaculum
Euryarchaeota
Crenarchaeota
Thaumarchaeota
Nanoarchaeota
Korarchaeota

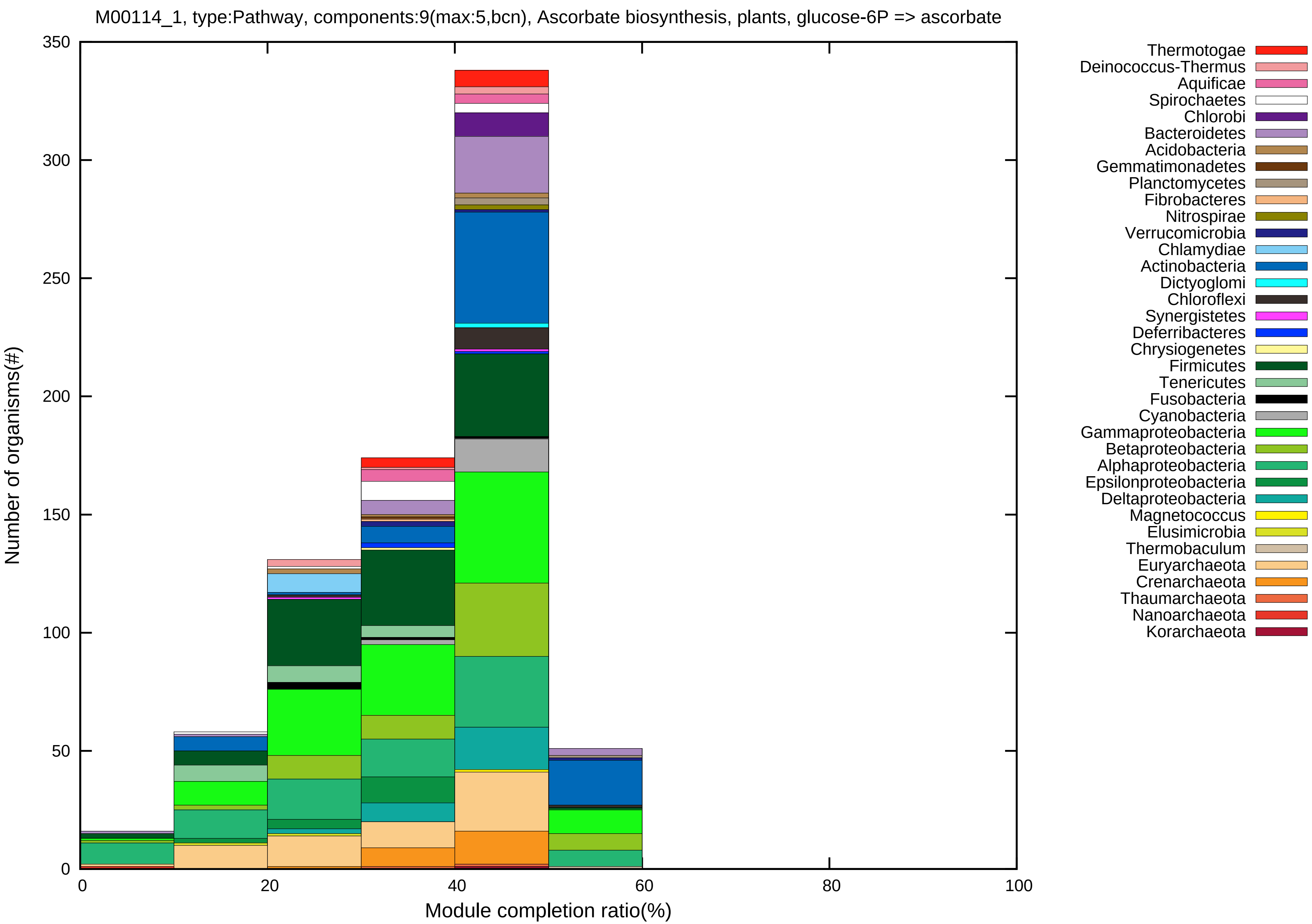

M00114_1, type:Pathway, components:9(max:5,bcn), Ascorbate biosynthesis, plants, glucose-6P => ascorbate
Number of organisms(#)
Module completion ratio(%)
0
50
100
150
200
250
300
350
0
20
40
60
80
100
Thermotogae
Deinococcus-Thermus
Aquificae
Spirochaetes
Chlorobi
Bacteroidetes
Acidobacteria
Gemmatimonadetes
Planctomycetes
Fibrobacteres
Nitrospirae
Verrucomicrobia
Chlamydiae
Actinobacteria
Dictyoglomi
Chloroflexi
Synergistetes
Deferribacteres
Chrysiogenetes
Firmicutes
Tenericutes
Fusobacteria
Cyanobacteria
Gammaproteobacteria
Betaproteobacteria
Alphaproteobacteria
Epsilonproteobacteria
Deltaproteobacteria
Magnetococcus
Elusimicrobia
Thermobaculum
Euryarchaeota
Crenarchaeota
Thaumarchaeota
Nanoarchaeota
Korarchaeota

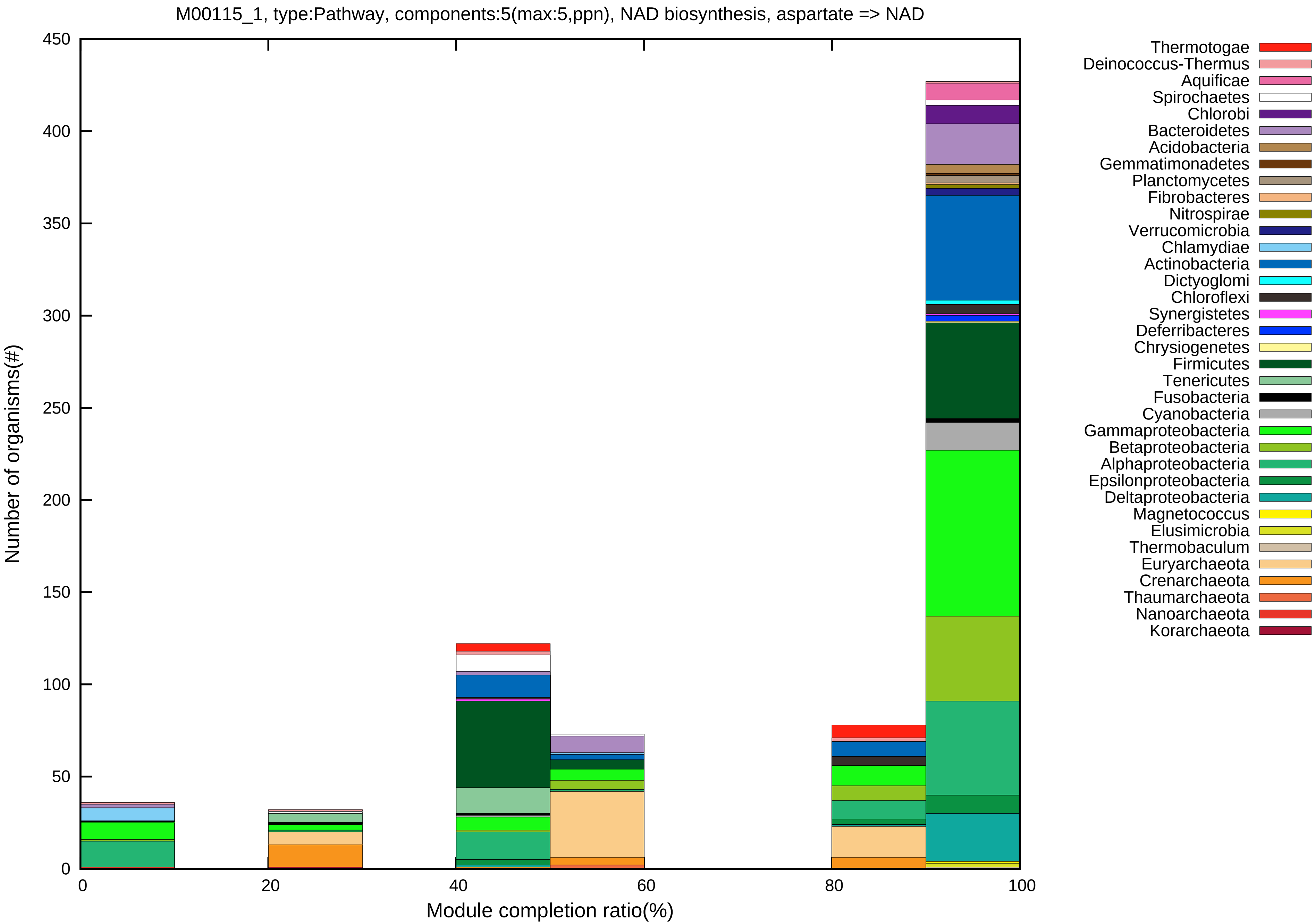
M00115_1, type:Pathway, components:5(max:5,ppn), NAD biosynthesis, aspartate => NAD
Number of organisms(#)
Module completion ratio(%)
0
50
100
150
200
250
300
350
400
450
0
20
40
60
80
100
Thermotogae
Deinococcus-Thermus
Aquificae
Spirochaetes
Chlorobi
Bacteroidetes
Acidobacteria
Gemmatimonadetes
Planctomycetes
Fibrobacteres
Nitrospirae
Verrucomicrobia
Chlamydiae
Actinobacteria
Dictyoglomi
Chloroflexi
Synergistetes
Deferribacteres
Chrysiogenetes
Firmicutes
Tenericutes
Fusobacteria
Cyanobacteria
Gammaproteobacteria
Betaproteobacteria
Alphaproteobacteria
Epsilonproteobacteria
Deltaproteobacteria
Magnetococcus
Elusimicrobia
Thermobaculum
Euryarchaeota
Crenarchaeota
Thaumarchaeota
Nanoarchaeota
Korarchaeota

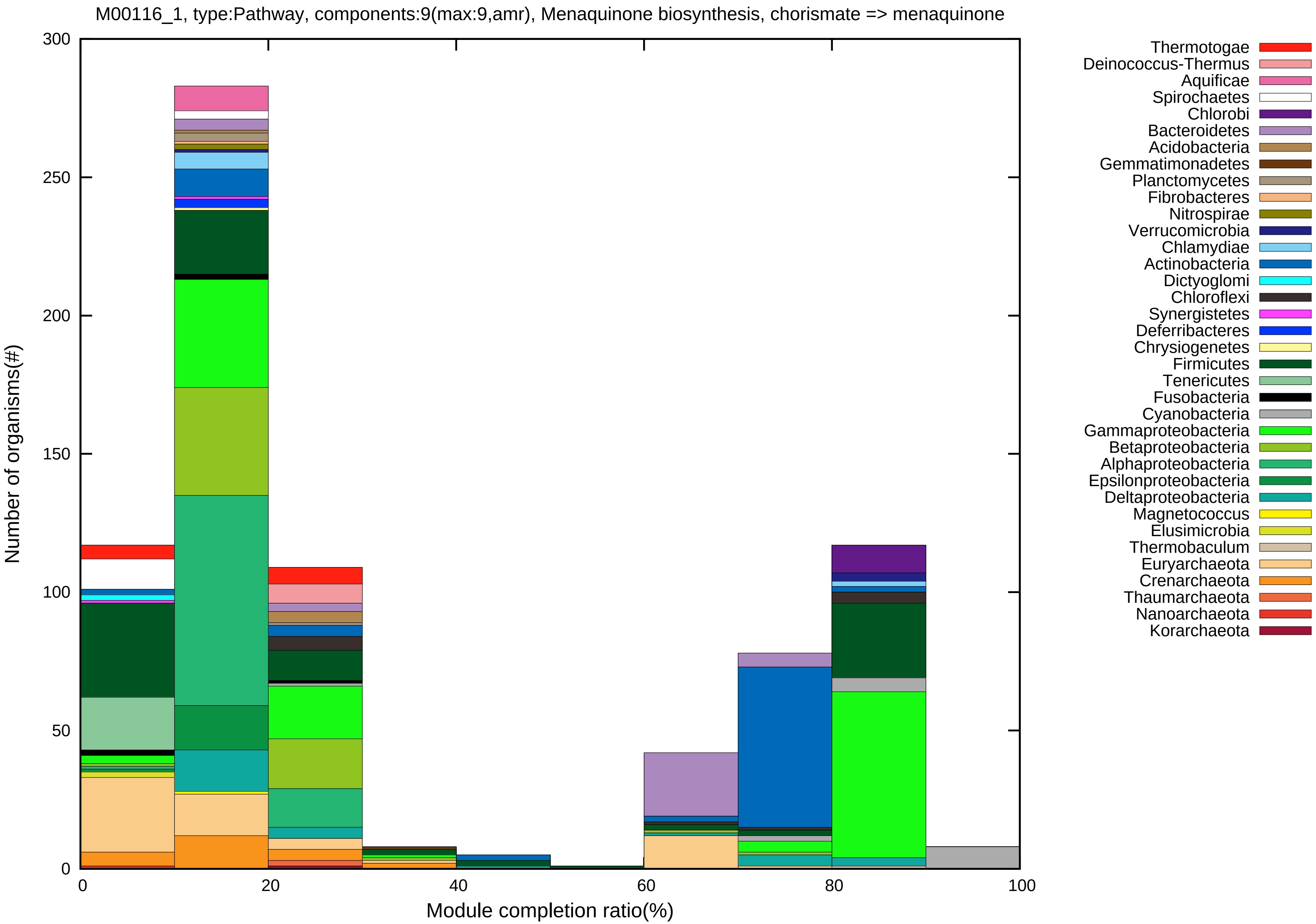

M00116_1, type:Pathway, components:9(max:9,amr), Menaquinone biosynthesis, chorismate => menaquinone
Number of organisms(#)
Module completion ratio(%)
0
50
100
150
200
250
300
0
20
40
60
80
100
Thermotogae
Deinococcus-Thermus
Aquificae
Spirochaetes
Chlorobi
Bacteroidetes
Acidobacteria
Gemmatimonadetes
Planctomycetes
Fibrobacteres
Nitrospirae
Verrucomicrobia
Chlamydiae
Actinobacteria
Dictyoglomi
Chloroflexi
Synergistetes
Deferribacteres
Chrysiogenetes
Firmicutes
Tenericutes
Fusobacteria
Cyanobacteria
Gammaproteobacteria
Betaproteobacteria
Alphaproteobacteria
Epsilonproteobacteria
Deltaproteobacteria
Magnetococcus
Elusimicrobia
Thermobaculum
Euryarchaeota
Crenarchaeota
Thaumarchaeota
Nanoarchaeota
Korarchaeota

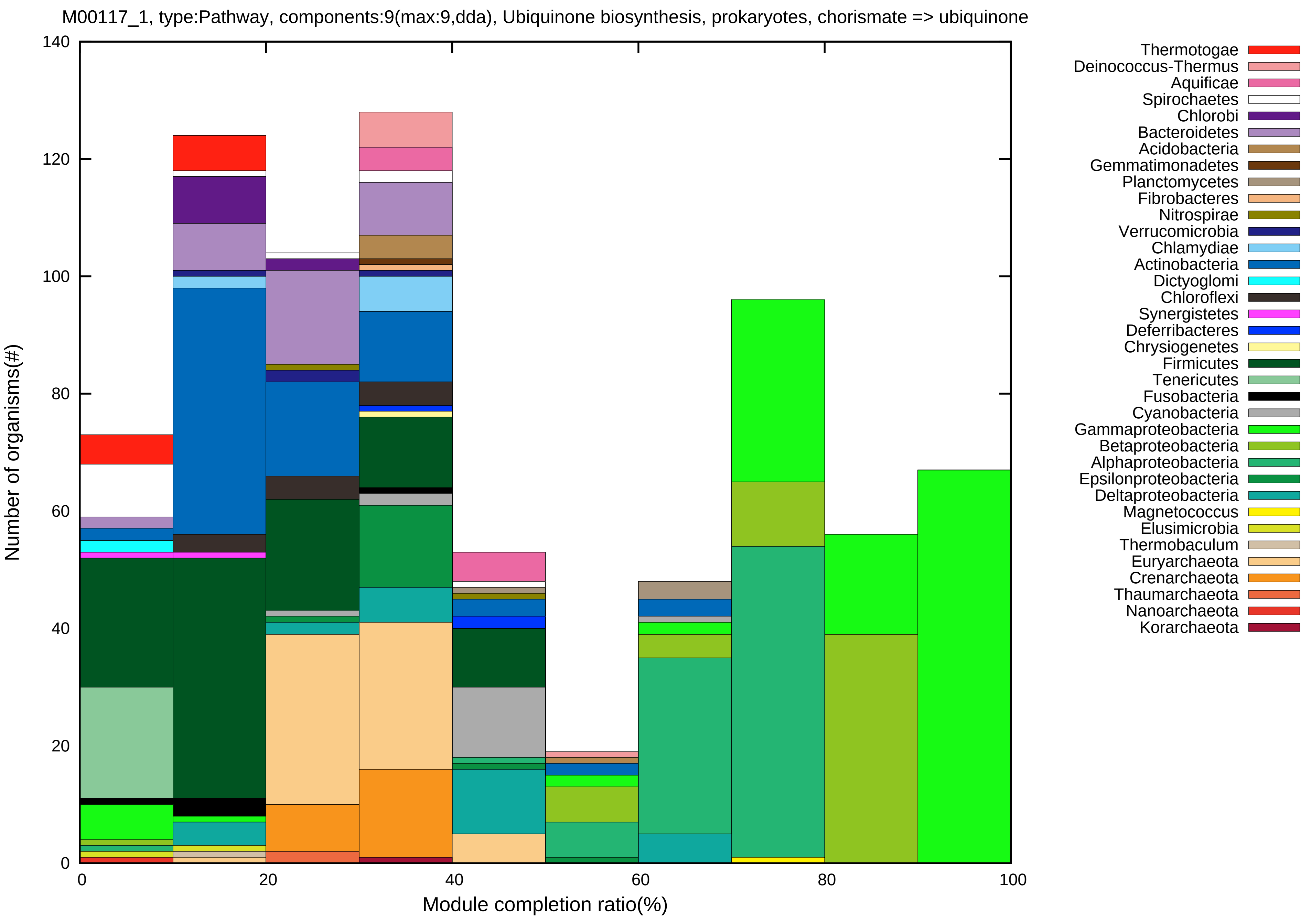
M00117_1, type:Pathway, components:9(max:9,dda), Ubiquinone biosynthesis, prokaryotes, chorismate => ubiquinone
Number of organisms(#)
Module completion ratio(%)
0
20
40
60
80
100
120
140
Thermotogae
Deinococcus-Thermus
Aquificae
Spirochaetes
Chlorobi
Bacteroidetes
Acidobacteria
Gemmatimonadetes
Planctomycetes
Fibrobacteres
Nitrospirae
Verrucomicrobia
Chlamydiae
Actinobacteria
Dictyoglomi
Chloroflexi
Synergistetes
Deferribacteres
Chrysiogenetes
Firmicutes
Tenericutes
Fusobacteria
Cyanobacteria
Gammaproteobacteria
Betaproteobacteria
Alphaproteobacteria
Epsilonproteobacteria
Deltaproteobacteria
Magnetococcus
Elusimicrobia
Thermobaculum
Euryarchaeota
Crenarchaeota
Thaumarchaeota
Nanoarchaeota
Korarchaeota

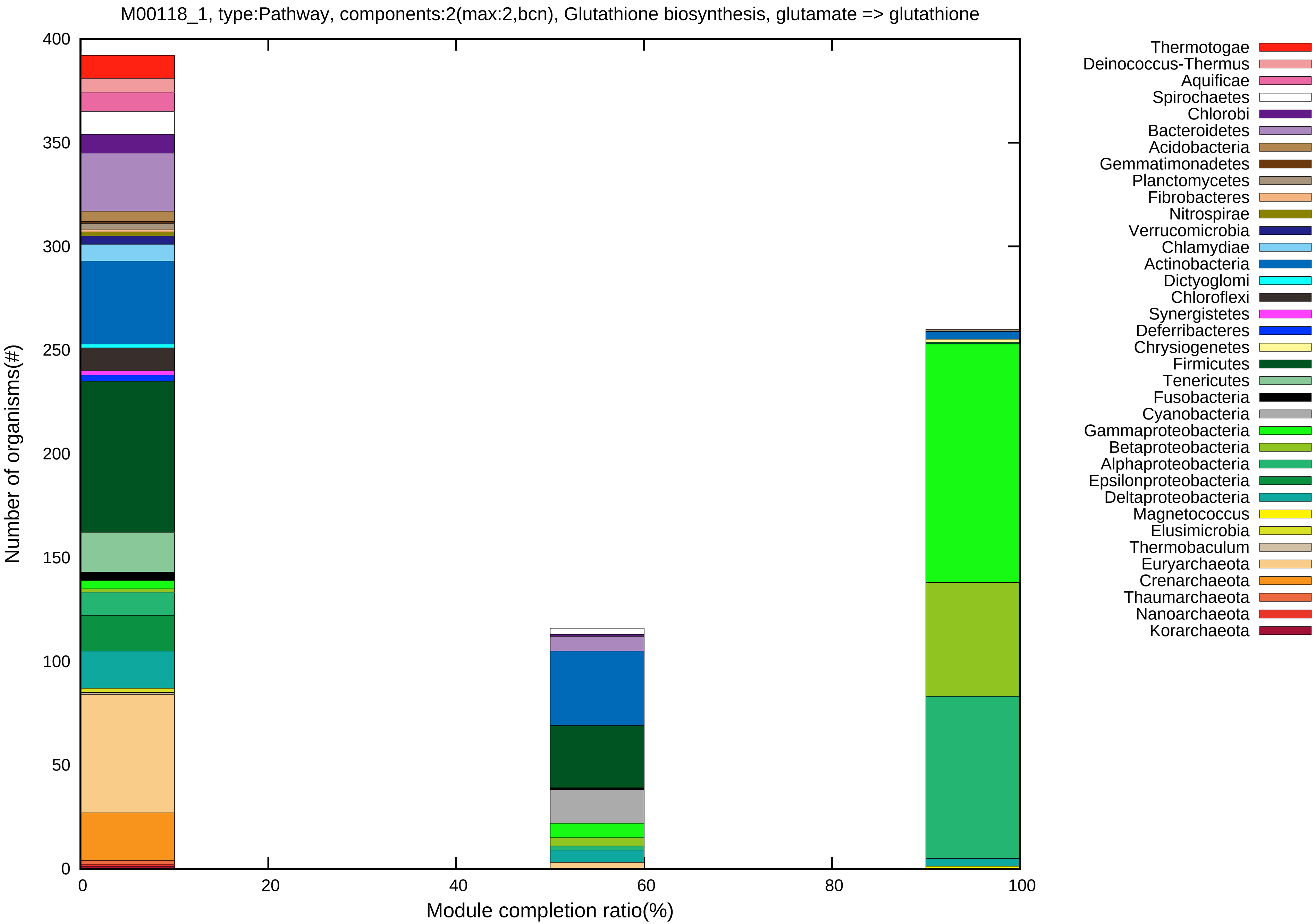

M00118_1, type:Pathway, components:2(max:2,bcn), Glutathione biosynthesis, glutamate => glutathione
Number of organisms(#)
Module completion ratio(%)
0
50
100
150
200
250
300
350
400
0
20
40
60
80
100
Thermotogae
Deinococcus-Thermus
Aquificae
Spirochaetes
Chlorobi
Bacteroidetes
Acidobacteria
Gemmatimonadetes
Planctomycetes
Fibrobacteres
Nitrospirae
Verrucomicrobia
Chlamydiae
Actinobacteria
Dictyoglomi
Chloroflexi
Synergistetes
Deferribacteres
Chrysiogenetes
Firmicutes
Tenericutes
Fusobacteria
Cyanobacteria
Gammaproteobacteria
Betaproteobacteria
Alphaproteobacteria
Epsilonproteobacteria
Deltaproteobacteria
Magnetococcus
Elusimicrobia
Thermobaculum
Euryarchaeota
Crenarchaeota
Thaumarchaeota
Nanoarchaeota
Korarchaeota

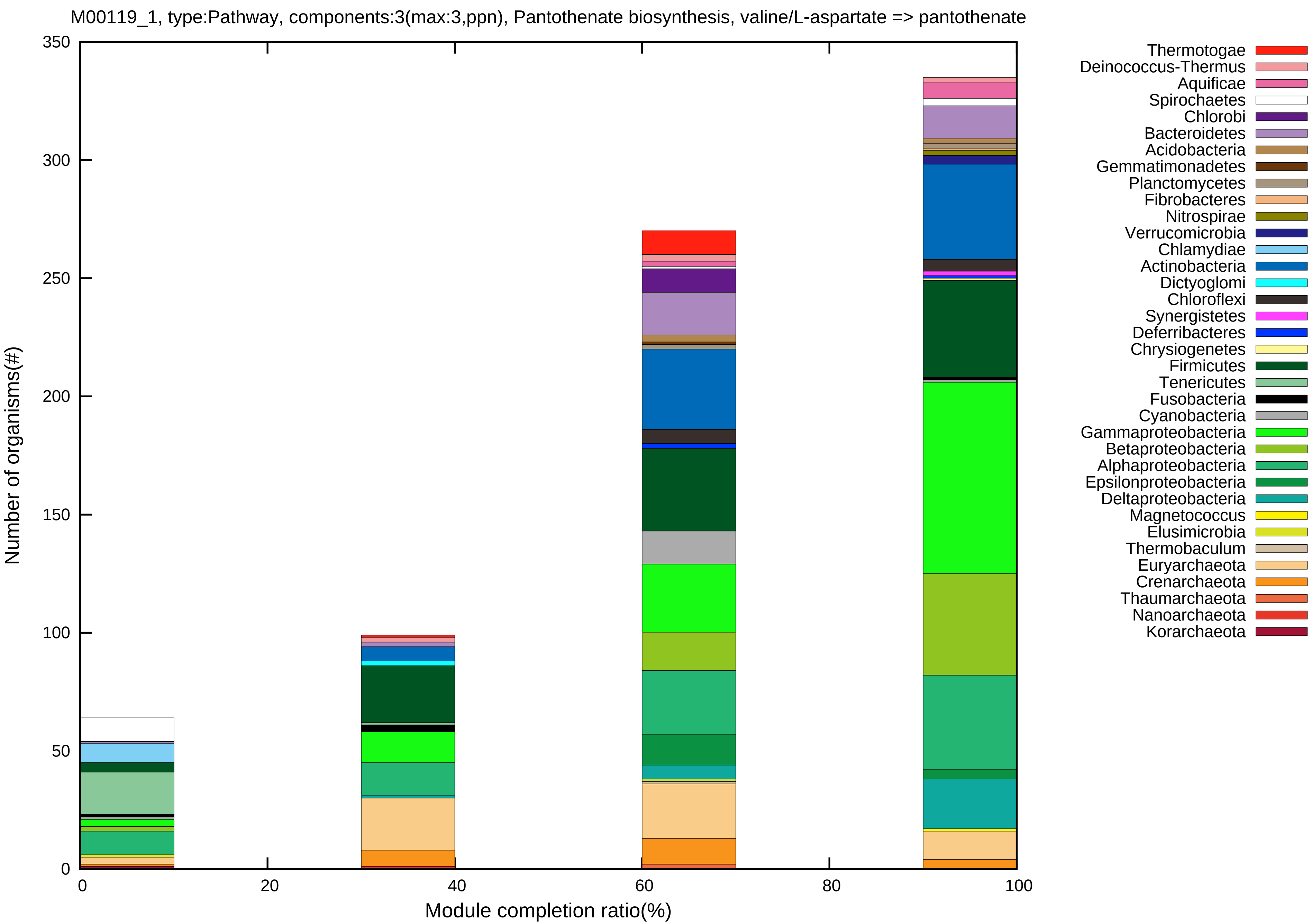

M00119_1, type:Pathway, components:3(max:3,ppn), Pantothenate biosynthesis, valine/L-aspartate => pantothenate
Number of organisms(#)
Module completion ratio(%)
0
50
100
150
200
250
300
350
0
20
40
60
80
100
Thermotogae
Deinococcus-Thermus
Aquificae
Spirochaetes
Chlorobi
Bacteroidetes
Acidobacteria
Gemmatimonadetes
Planctomycetes
Fibrobacteres
Nitrospirae
Verrucomicrobia
Chlamydiae
Actinobacteria
Dictyoglomi
Chloroflexi
Synergistetes
Deferribacteres
Chrysiogenetes
Firmicutes
Tenericutes
Fusobacteria
Cyanobacteria
Gammaproteobacteria
Betaproteobacteria
Alphaproteobacteria
Epsilonproteobacteria
Deltaproteobacteria
Magnetococcus
Elusimicrobia
Thermobaculum
Euryarchaeota
Crenarchaeota
Thaumarchaeota
Nanoarchaeota
Korarchaeota

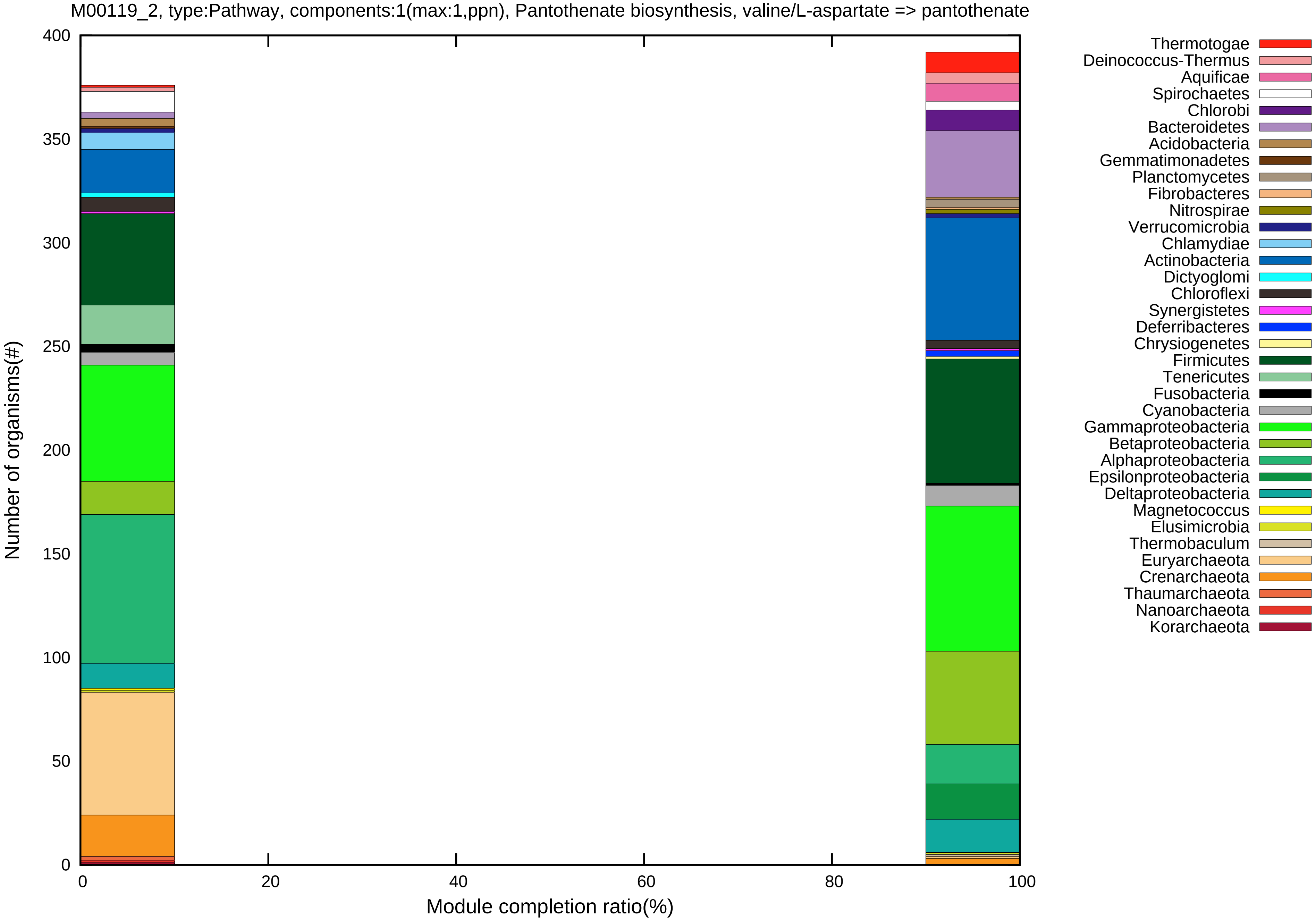

M00119_2, type:Pathway, components:1(max:1,ppn), Pantothenate biosynthesis, valine/L-aspartate => pantothenate
Number of organisms(#)
Module completion ratio(%)
0
50
100
150
200
250
300
350
400
0
20
40
60
80
100
Thermotogae
Deinococcus-Thermus
Aquificae
Spirochaetes
Chlorobi
Bacteroidetes
Acidobacteria
Gemmatimonadetes
Planctomycetes
Fibrobacteres
Nitrospirae
Verrucomicrobia
Chlamydiae
Actinobacteria
Dictyoglomi
Chloroflexi
Synergistetes
Deferribacteres
Chrysiogenetes
Firmicutes
Tenericutes
Fusobacteria
Cyanobacteria
Gammaproteobacteria
Betaproteobacteria
Alphaproteobacteria
Epsilonproteobacteria
Deltaproteobacteria
Magnetococcus
Elusimicrobia
Thermobaculum
Euryarchaeota
Crenarchaeota
Thaumarchaeota
Nanoarchaeota
Korarchaeota

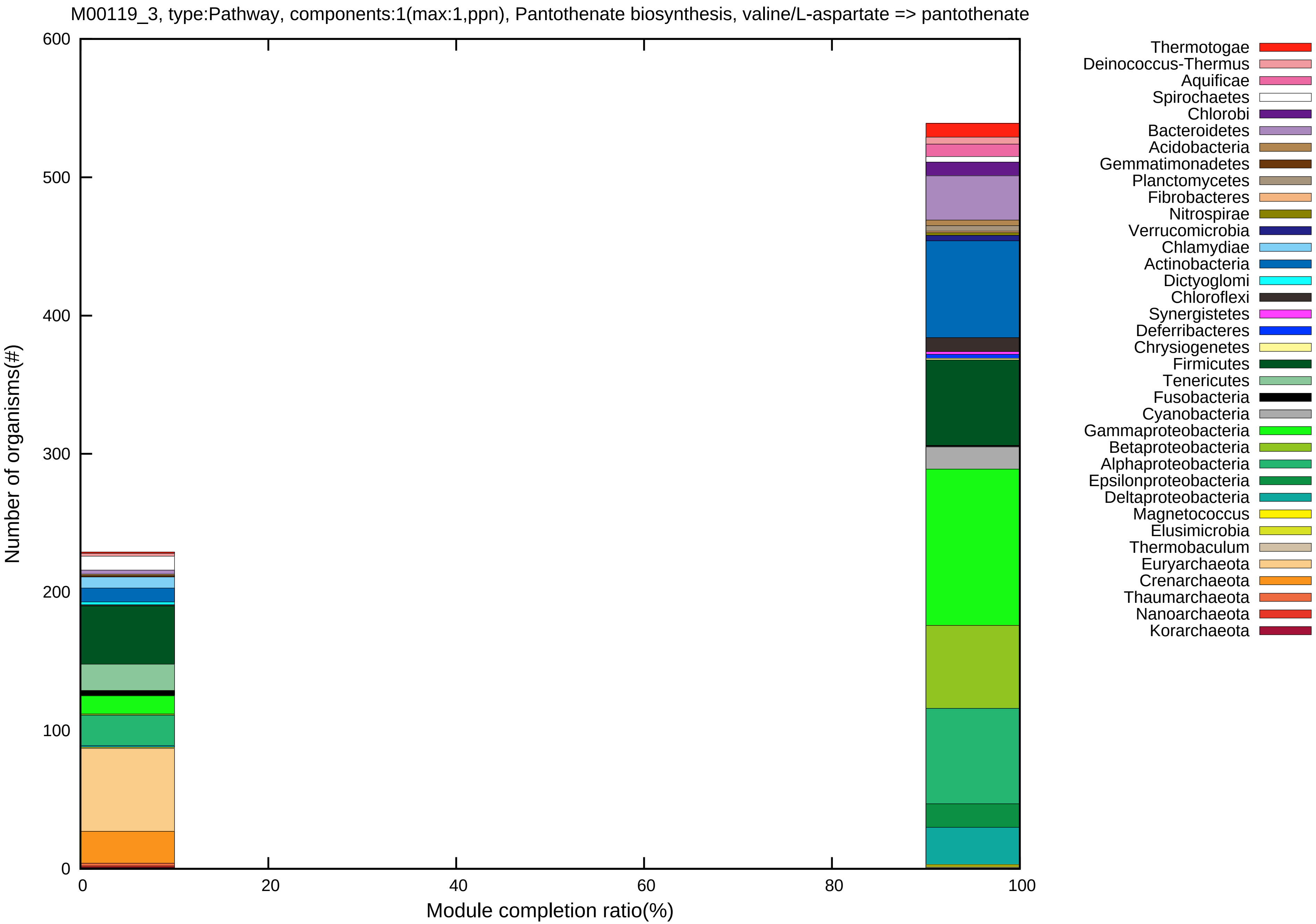
M00119_3, type:Pathway, components:1(max:1,ppn), Pantothenate biosynthesis, valine/L-aspartate => pantothenate
Number of organisms(#)
Module completion ratio(%)
0
100
200
300
400
500
600
0
20
40
60
80
100
Thermotogae
Deinococcus-Thermus
Aquificae
Spirochaetes
Chlorobi
Bacteroidetes
Acidobacteria
Gemmatimonadetes
Planctomycetes
Fibrobacteres
Nitrospirae
Verrucomicrobia
Chlamydiae
Actinobacteria
Dictyoglomi
Chloroflexi
Synergistetes
Deferribacteres
Chrysiogenetes
Firmicutes
Tenericutes
Fusobacteria
Cyanobacteria
Gammaproteobacteria
Betaproteobacteria
Alphaproteobacteria
Epsilonproteobacteria
Deltaproteobacteria
Magnetococcus
Elusimicrobia
Thermobaculum
Euryarchaeota
Crenarchaeota
Thaumarchaeota
Nanoarchaeota
Korarchaeota

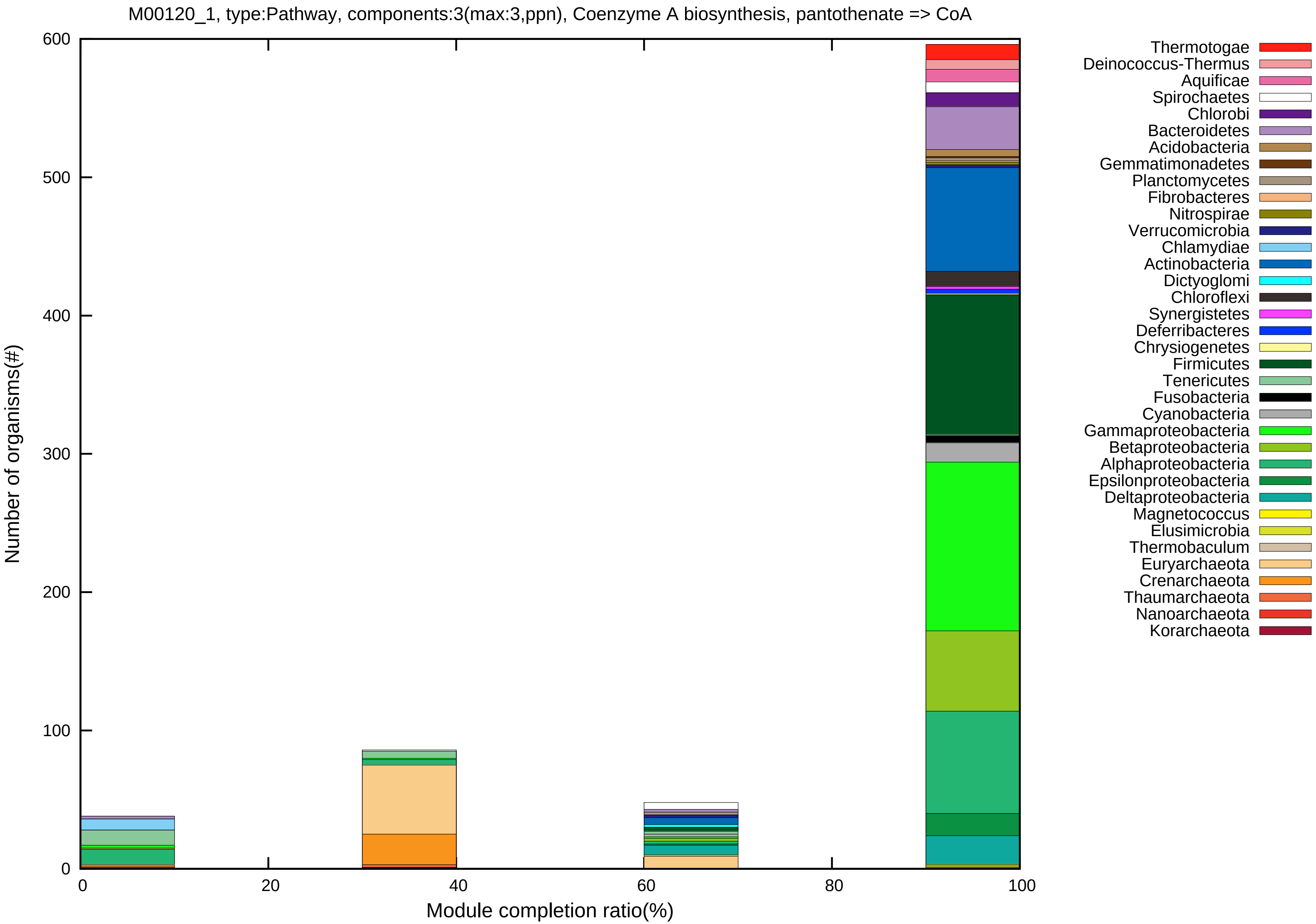

M00120_1, type:Pathway, components:3(max:3,ppn), Coenzyme A biosynthesis, pantothenate => CoA
Number of organisms(#)
Module completion ratio(%)
0
100
200
300
400
500
600
0
20
40
60
80
100
Thermotogae
Deinococcus-Thermus
Aquificae
Spirochaetes
Chlorobi
Bacteroidetes
Acidobacteria
Gemmatimonadetes
Planctomycetes
Fibrobacteres
Nitrospirae
Verrucomicrobia
Chlamydiae
Actinobacteria
Dictyoglomi
Chloroflexi
Synergistetes
Deferribacteres
Chrysiogenetes
Firmicutes
Tenericutes
Fusobacteria
Cyanobacteria
Gammaproteobacteria
Betaproteobacteria
Alphaproteobacteria
Epsilonproteobacteria
Deltaproteobacteria
Magnetococcus
Elusimicrobia
Thermobaculum
Euryarchaeota
Crenarchaeota
Thaumarchaeota
Nanoarchaeota
Korarchaeota

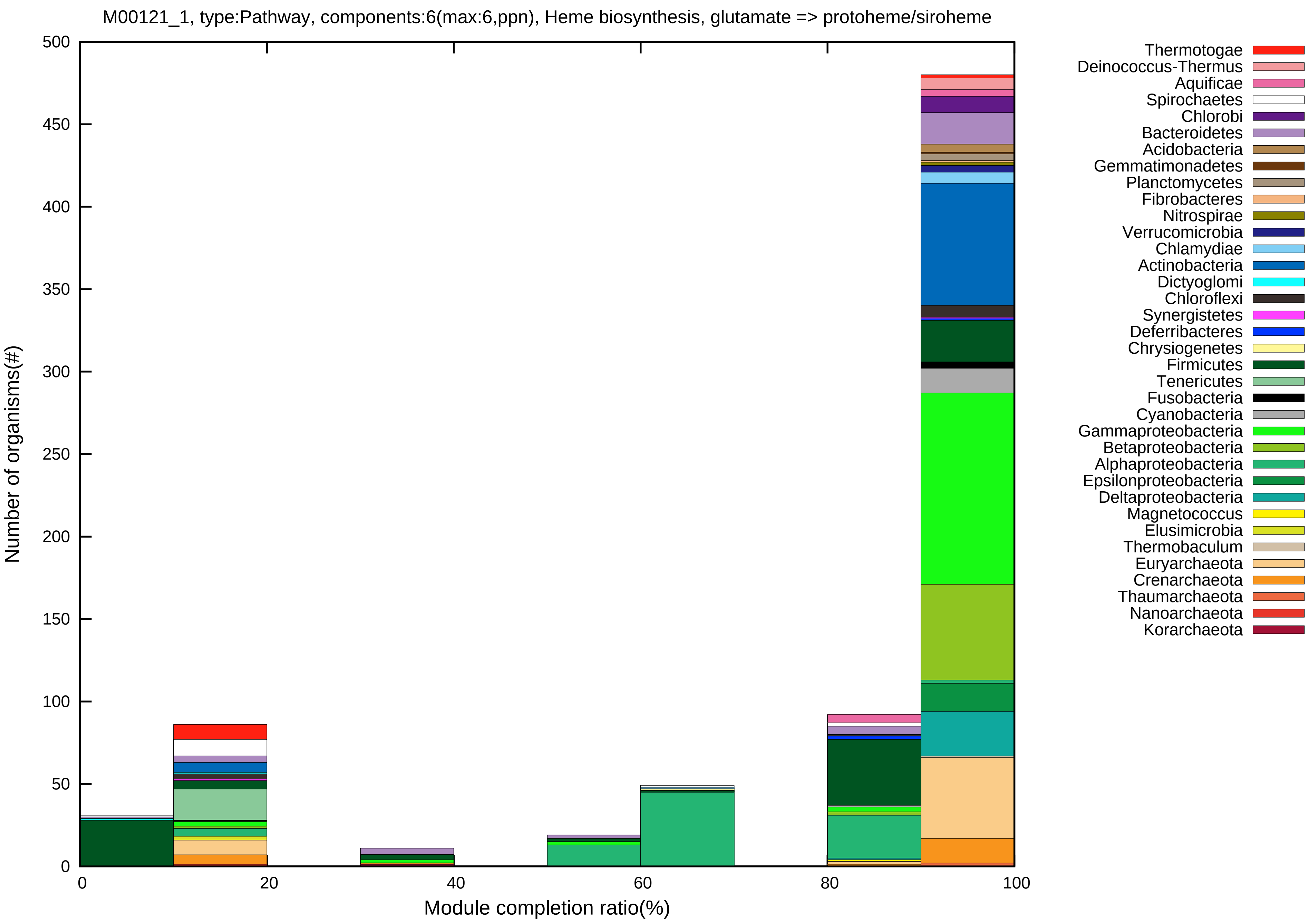
M00121_1, type:Pathway, components:6(max:6,ppn), Heme biosynthesis, glutamate => protoheme/siroheme
Number of organisms(#)
Module completion ratio(%)
0
50
100
150
200
250
300
350
400
450
500
0
20
40
60
80
100
Thermotogae
Deinococcus-Thermus
Aquificae
Spirochaetes
Chlorobi
Bacteroidetes
Acidobacteria
Gemmatimonadetes
Planctomycetes
Fibrobacteres
Nitrospirae
Verrucomicrobia
Chlamydiae
Actinobacteria
Dictyoglomi
Chloroflexi
Synergistetes
Deferribacteres
Chrysiogenetes
Firmicutes
Tenericutes
Fusobacteria
Cyanobacteria
Gammaproteobacteria
Betaproteobacteria
Alphaproteobacteria
Epsilonproteobacteria
Deltaproteobacteria
Magnetococcus
Elusimicrobia
Thermobaculum
Euryarchaeota
Crenarchaeota
Thaumarchaeota
Nanoarchaeota
Korarchaeota

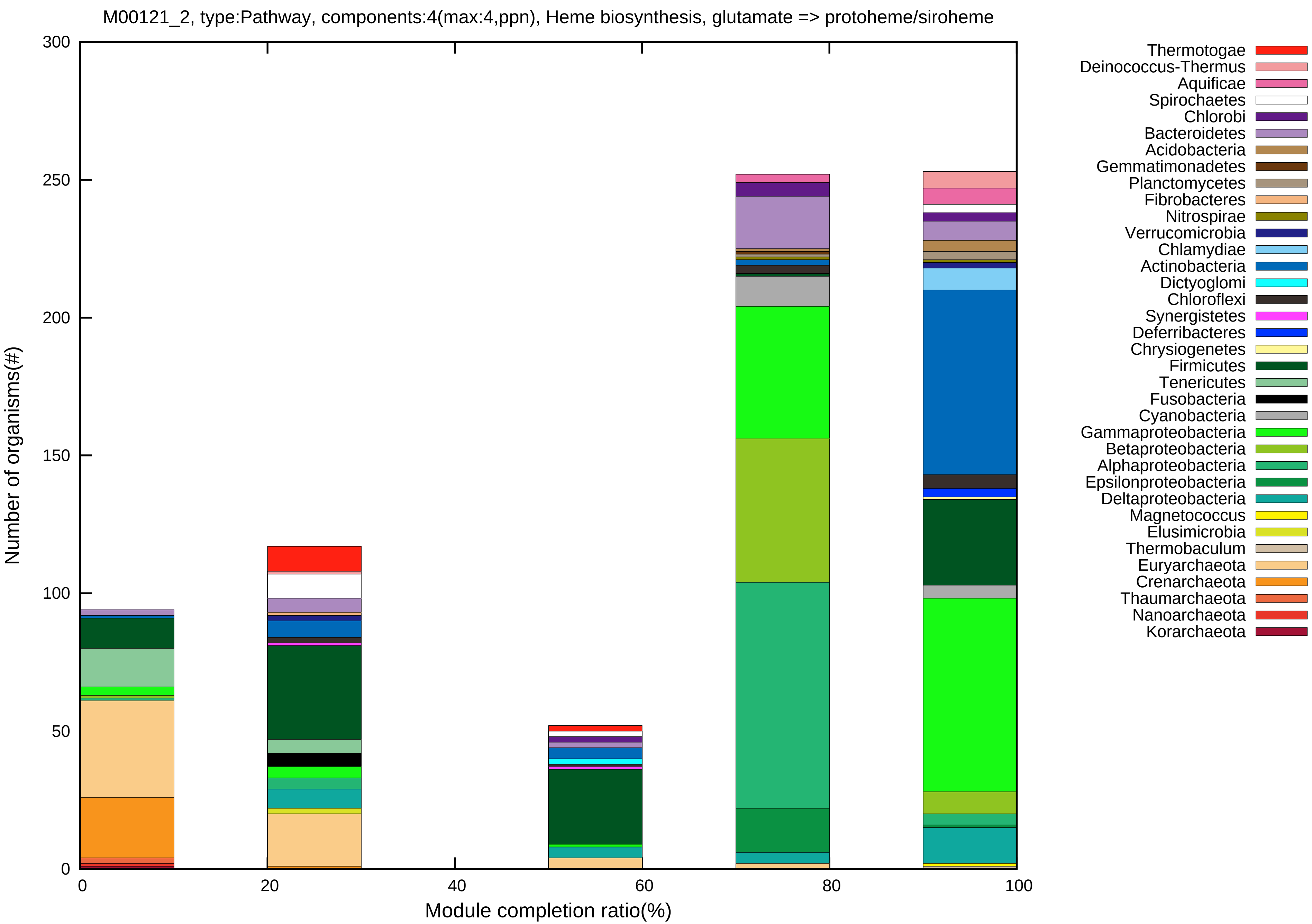
M00121_2, type:Pathway, components:4(max:4,ppn), Heme biosynthesis, glutamate => protoheme/siroheme
Number of organisms(#)
Module completion ratio(%)
0
50
100
150
200
250
300
0
20
40
60
80
100
Thermotogae
Deinococcus-Thermus
Aquificae
Spirochaetes
Chlorobi
Bacteroidetes
Acidobacteria
Gemmatimonadetes
Planctomycetes
Fibrobacteres
Nitrospirae
Verrucomicrobia
Chlamydiae
Actinobacteria
Dictyoglomi
Chloroflexi
Synergistetes
Deferribacteres
Chrysiogenetes
Firmicutes
Tenericutes
Fusobacteria
Cyanobacteria
Gammaproteobacteria
Betaproteobacteria
Alphaproteobacteria
Epsilonproteobacteria
Deltaproteobacteria
Magnetococcus
Elusimicrobia
Thermobaculum
Euryarchaeota
Crenarchaeota
Thaumarchaeota
Nanoarchaeota
Korarchaeota

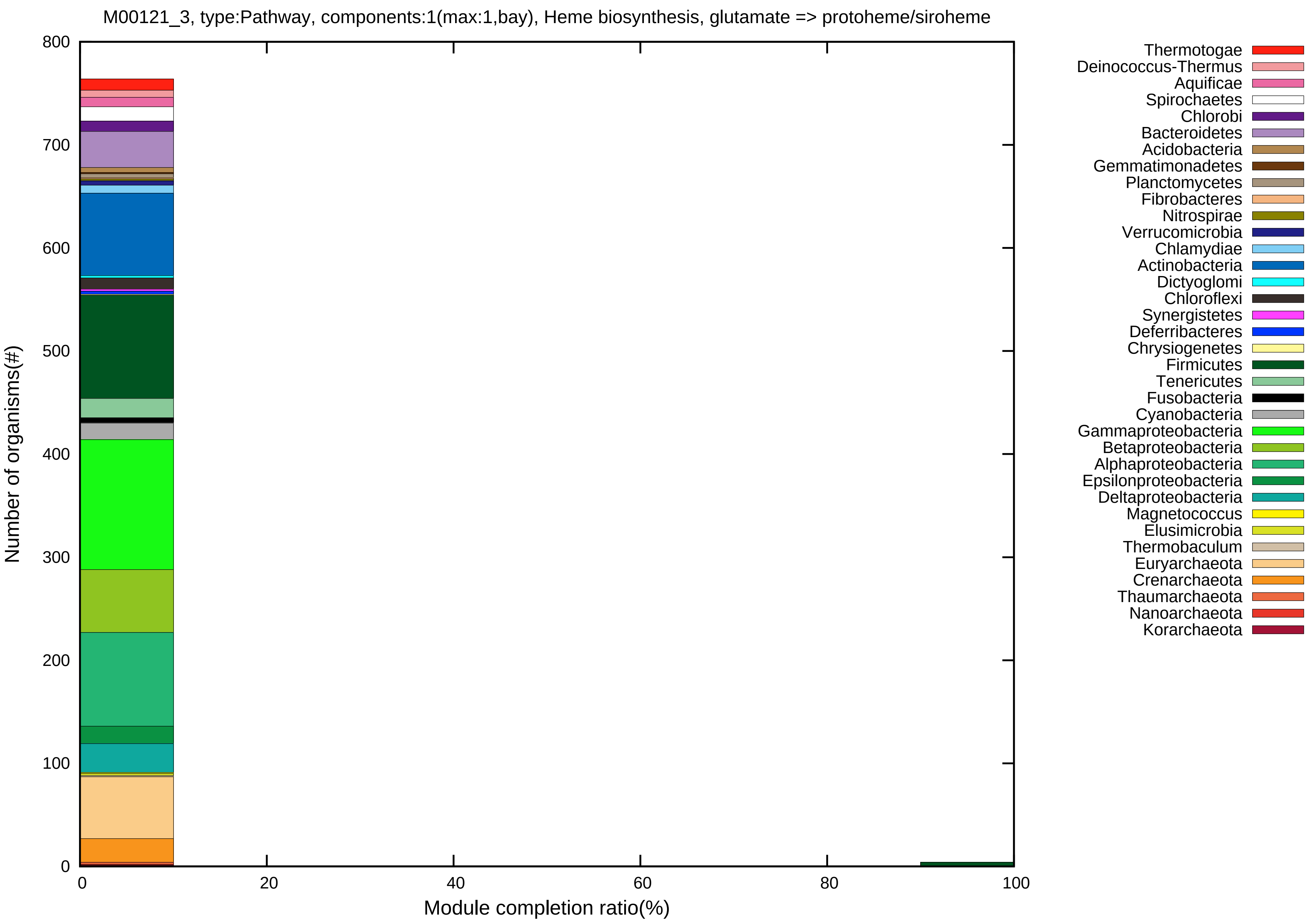
M00121_3, type:Pathway, components:1(max:1,bay), Heme biosynthesis, glutamate => protoheme/siroheme
Number of organisms(#)
Module completion ratio(%)
0
100
200
300
400
500
600
700
800
0
20
40
60
80
100
Thermotogae
Deinococcus-Thermus
Aquificae
Spirochaetes
Chlorobi
Bacteroidetes
Acidobacteria
Gemmatimonadetes
Planctomycetes
Fibrobacteres
Nitrospirae
Verrucomicrobia
Chlamydiae
Actinobacteria
Dictyoglomi
Chloroflexi
Synergistetes
Deferribacteres
Chrysiogenetes
Firmicutes
Tenericutes
Fusobacteria
Cyanobacteria
Gammaproteobacteria
Betaproteobacteria
Alphaproteobacteria
Epsilonproteobacteria
Deltaproteobacteria
Magnetococcus
Elusimicrobia
Thermobaculum
Euryarchaeota
Crenarchaeota
Thaumarchaeota
Nanoarchaeota
Korarchaeota

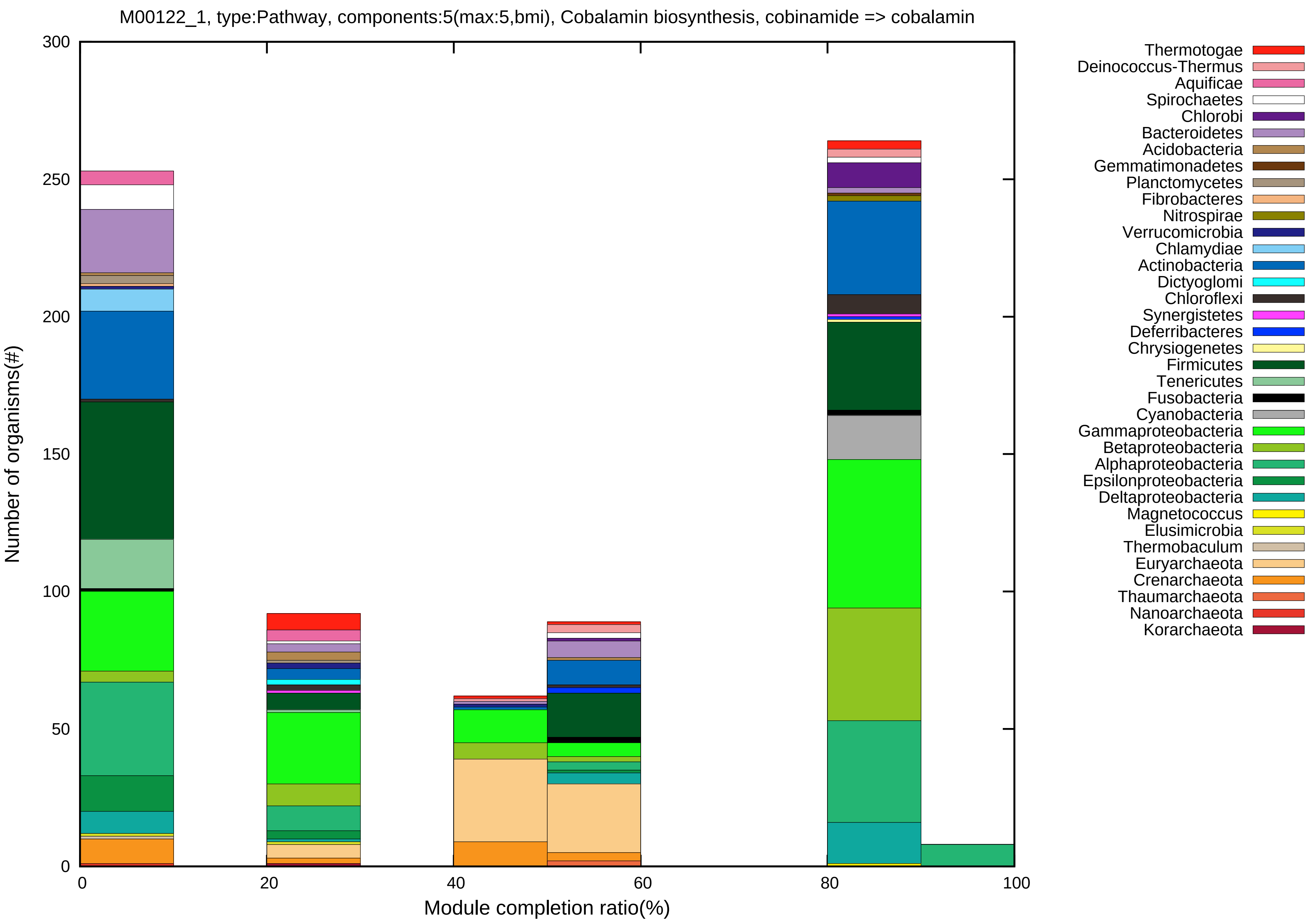

M00122_1, type:Pathway, components:5(max:5,bmi), Cobalamin biosynthesis, cobinamide => cobalamin
Number of organisms(#)
Module completion ratio(%)
0
50
100
150
200
250
300
0
20
40
60
80
100
Thermotogae
Deinococcus-Thermus
Aquificae
Spirochaetes
Chlorobi
Bacteroidetes
Acidobacteria
Gemmatimonadetes
Planctomycetes
Fibrobacteres
Nitrospirae
Verrucomicrobia
Chlamydiae
Actinobacteria
Dictyoglomi
Chloroflexi
Synergistetes
Deferribacteres
Chrysiogenetes
Firmicutes
Tenericutes
Fusobacteria
Cyanobacteria
Gammaproteobacteria
Betaproteobacteria
Alphaproteobacteria
Epsilonproteobacteria
Deltaproteobacteria
Magnetococcus
Elusimicrobia
Thermobaculum
Euryarchaeota
Crenarchaeota
Thaumarchaeota
Nanoarchaeota
Korarchaeota

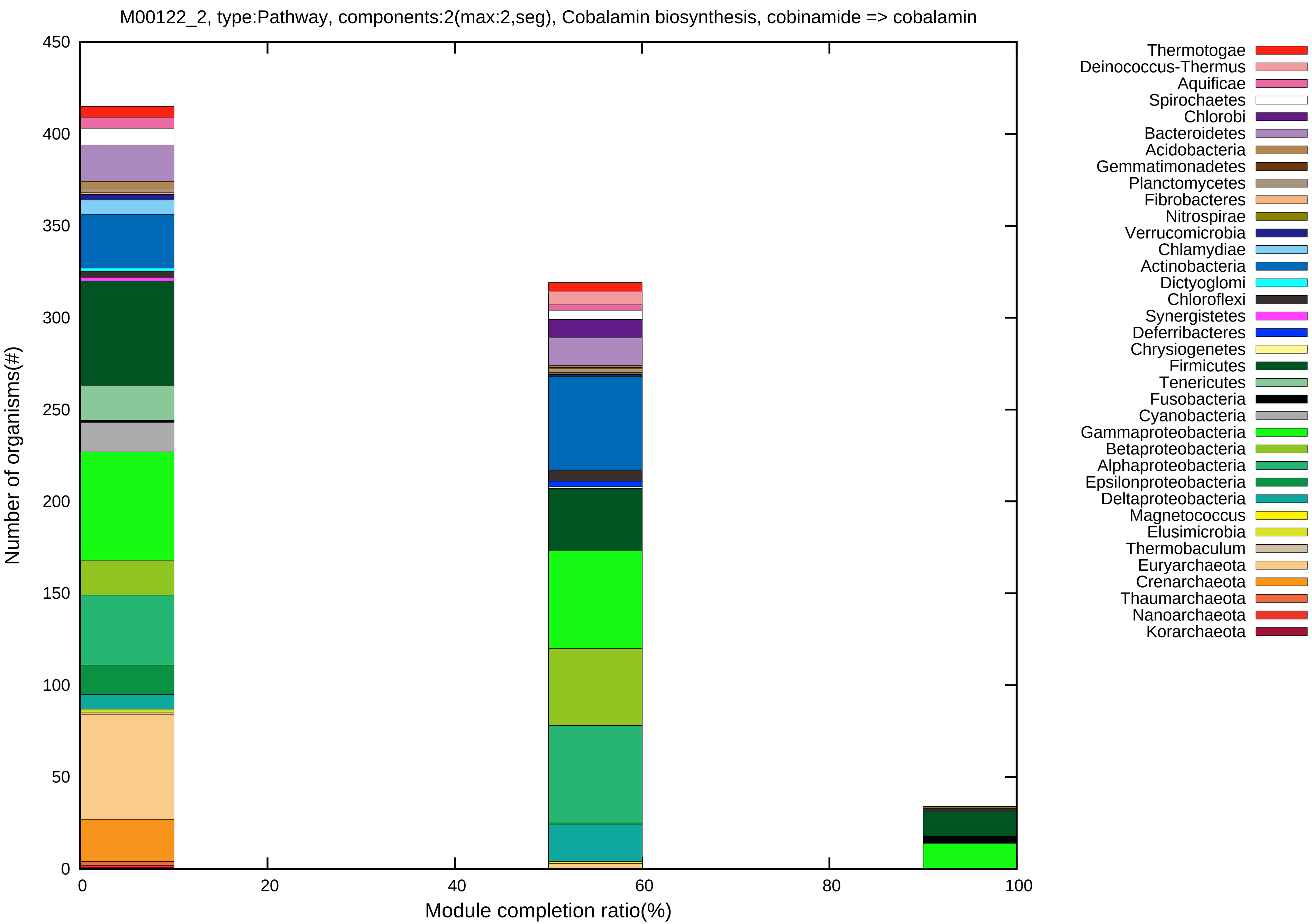

M00122_2, type:Pathway, components:2(max:2,seg), Cobalamin biosynthesis, cobinamide => cobalamin
Number of organisms(#)
Module completion ratio(%)
0
50
100
150
200
250
300
350
400
450
0
20
40
60
80
100
Thermotogae
Deinococcus-Thermus
Aquificae
Spirochaetes
Chlorobi
Bacteroidetes
Acidobacteria
Gemmatimonadetes
Planctomycetes
Fibrobacteres
Nitrospirae
Verrucomicrobia
Chlamydiae
Actinobacteria
Dictyoglomi
Chloroflexi
Synergistetes
Deferribacteres
Chrysiogenetes
Firmicutes
Tenericutes
Fusobacteria
Cyanobacteria
Gammaproteobacteria
Betaproteobacteria
Alphaproteobacteria
Epsilonproteobacteria
Deltaproteobacteria
Magnetococcus
Elusimicrobia
Thermobaculum
Euryarchaeota
Crenarchaeota
Thaumarchaeota
Nanoarchaeota
Korarchaeota

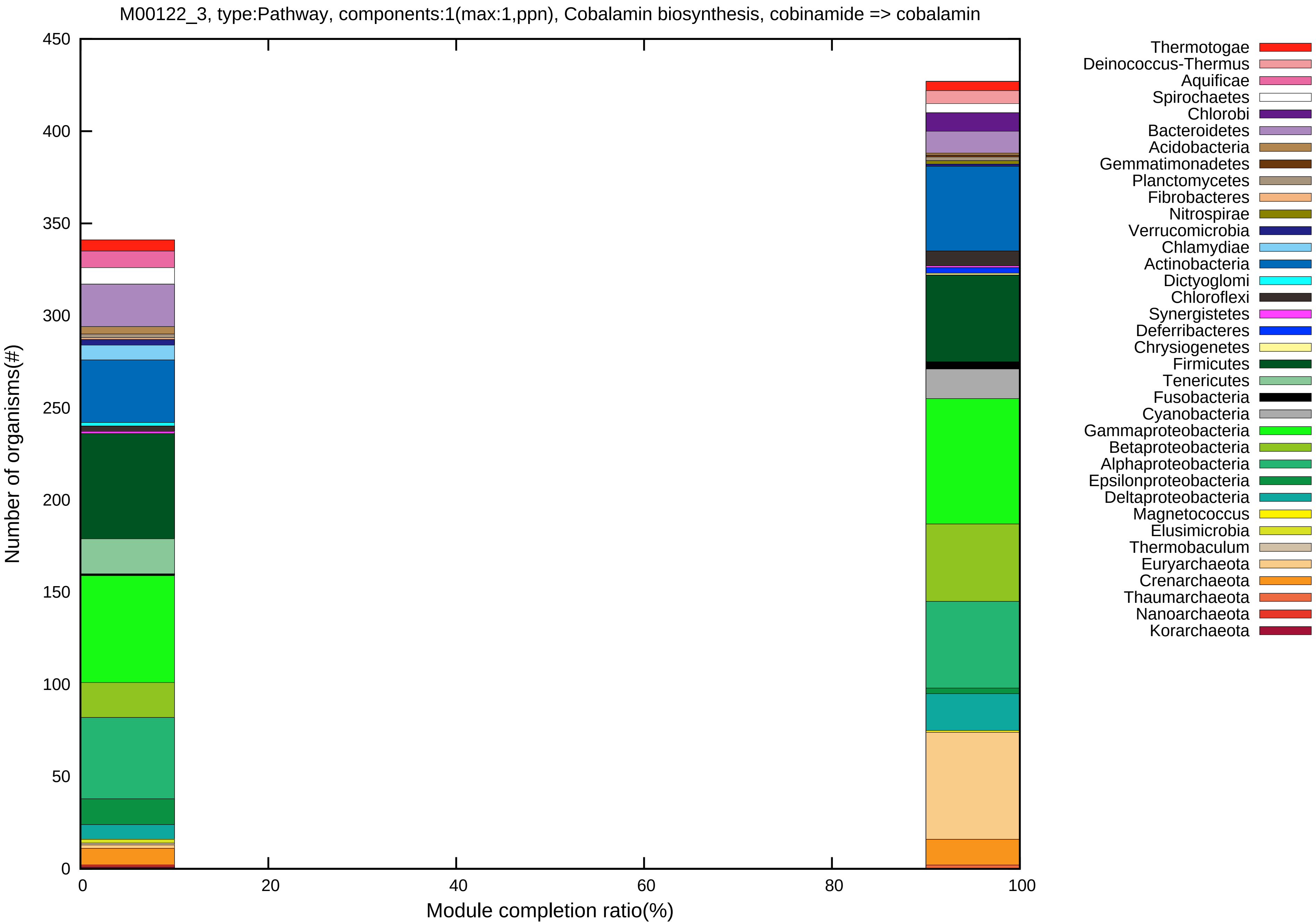
M00122_3, type:Pathway, components:1(max:1,ppn), Cobalamin biosynthesis, cobinamide => cobalamin
Number of organisms(#)
Module completion ratio(%)
0
50
100
150
200
250
300
350
400
450
0
20
40
60
80
100
Thermotogae
Deinococcus-Thermus
Aquificae
Spirochaetes
Chlorobi
Bacteroidetes
Acidobacteria
Gemmatimonadetes
Planctomycetes
Fibrobacteres
Nitrospirae
Verrucomicrobia
Chlamydiae
Actinobacteria
Dictyoglomi
Chloroflexi
Synergistetes
Deferribacteres
Chrysiogenetes
Firmicutes
Tenericutes
Fusobacteria
Cyanobacteria
Gammaproteobacteria
Betaproteobacteria
Alphaproteobacteria
Epsilonproteobacteria
Deltaproteobacteria
Magnetococcus
Elusimicrobia
Thermobaculum
Euryarchaeota
Crenarchaeota
Thaumarchaeota
Nanoarchaeota
Korarchaeota

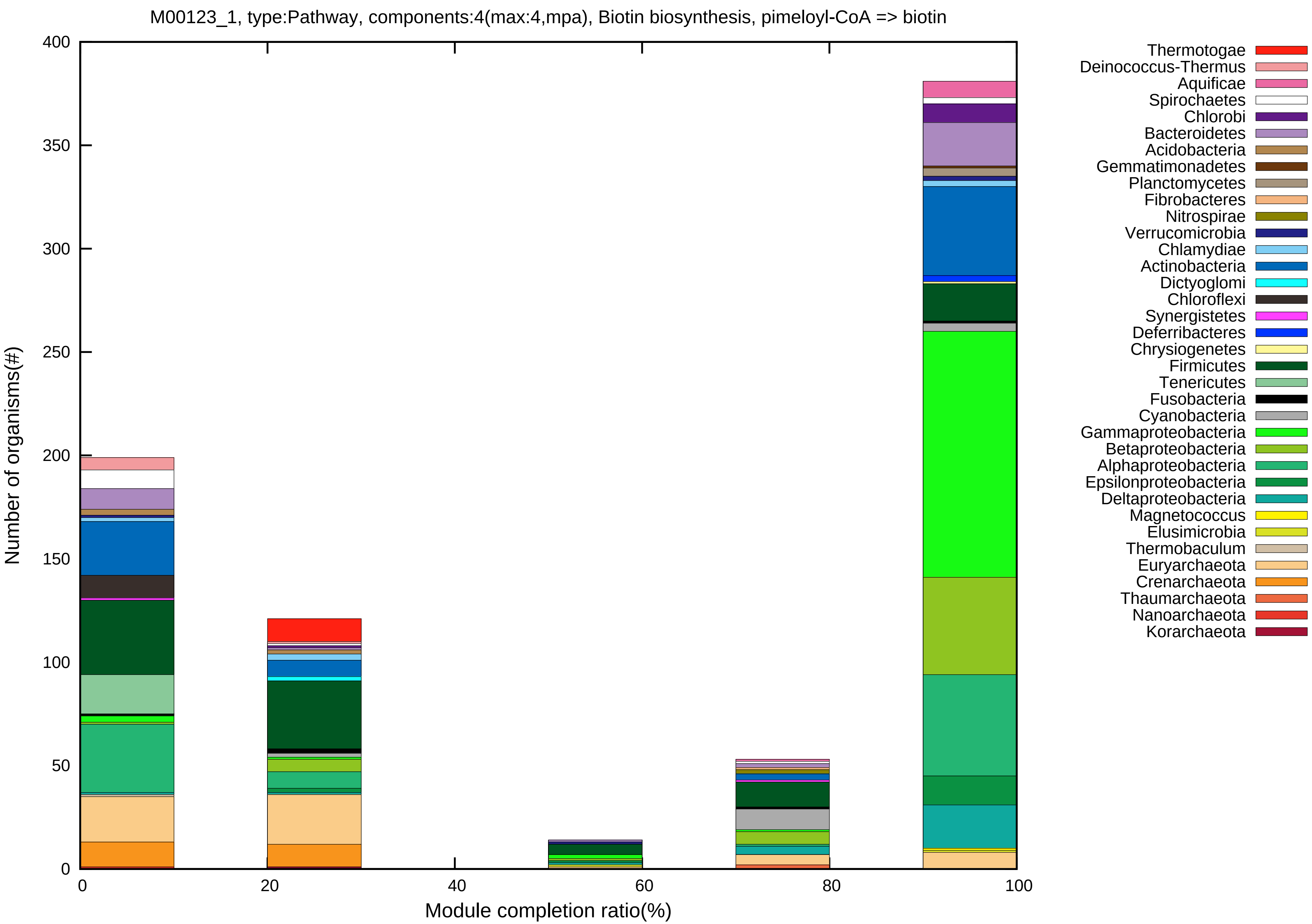

M00123_1, type:Pathway, components:4(max:4,mpa), Biotin biosynthesis, pimeloyl-CoA => biotin
Number of organisms(#)
Module completion ratio(%)
0
50
100
150
200
250
300
350
400
0
20
40
60
80
100
Thermotogae
Deinococcus-Thermus
Aquificae
Spirochaetes
Chlorobi
Bacteroidetes
Acidobacteria
Gemmatimonadetes
Planctomycetes
Fibrobacteres
Nitrospirae
Verrucomicrobia
Chlamydiae
Actinobacteria
Dictyoglomi
Chloroflexi
Synergistetes
Deferribacteres
Chrysiogenetes
Firmicutes
Tenericutes
Fusobacteria
Cyanobacteria
Gammaproteobacteria
Betaproteobacteria
Alphaproteobacteria
Epsilonproteobacteria
Deltaproteobacteria
Magnetococcus
Elusimicrobia
Thermobaculum
Euryarchaeota
Crenarchaeota
Thaumarchaeota
Nanoarchaeota
Korarchaeota

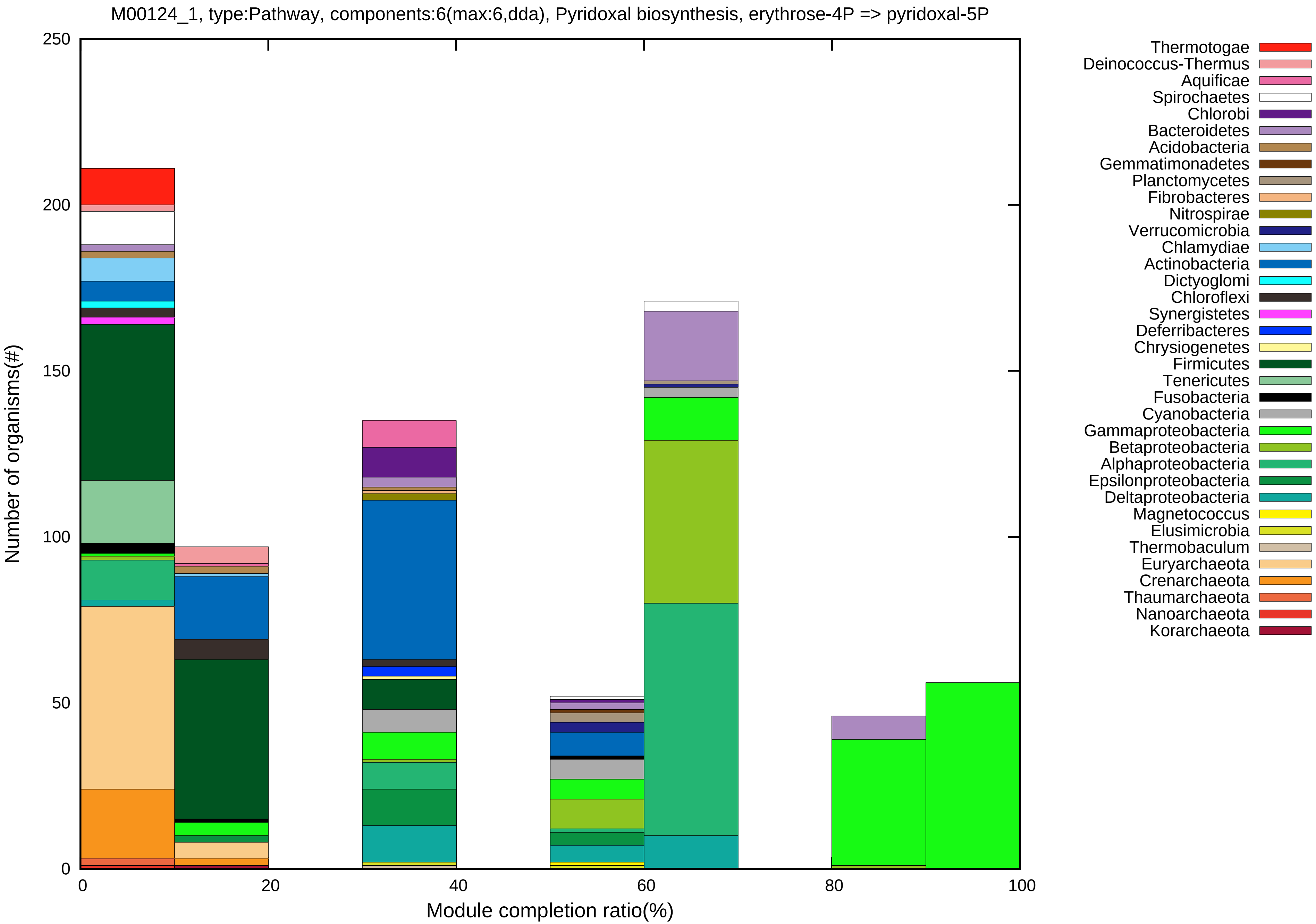

M00124_1, type:Pathway, components:6(max:6,dda), Pyridoxal biosynthesis, erythrose-4P => pyridoxal-5P
Number of organisms(#)
Module completion ratio(%)
0
50
100
150
200
250
0
20
40
60
80
100
Thermotogae
Deinococcus-Thermus
Aquificae
Spirochaetes
Chlorobi
Bacteroidetes
Acidobacteria
Gemmatimonadetes
Planctomycetes
Fibrobacteres
Nitrospirae
Verrucomicrobia
Chlamydiae
Actinobacteria
Dictyoglomi
Chloroflexi
Synergistetes
Deferribacteres
Chrysiogenetes
Firmicutes
Tenericutes
Fusobacteria
Cyanobacteria
Gammaproteobacteria
Betaproteobacteria
Alphaproteobacteria
Epsilonproteobacteria
Deltaproteobacteria
Magnetococcus
Elusimicrobia
Thermobaculum
Euryarchaeota
Crenarchaeota
Thaumarchaeota
Nanoarchaeota
Korarchaeota

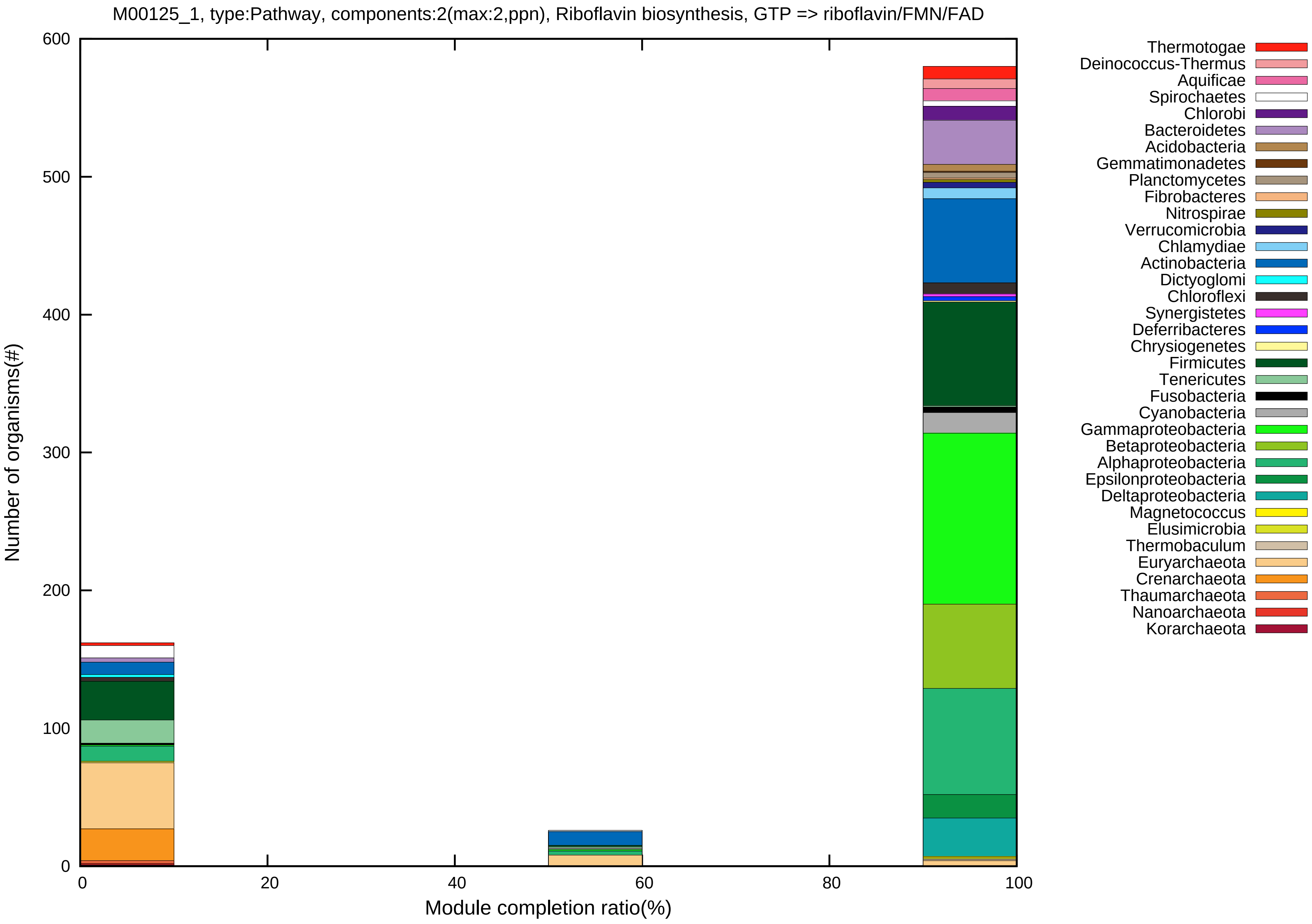

M00125_1, type:Pathway, components:2(max:2,ppn), Riboflavin biosynthesis, GTP => riboflavin/FMN/FAD
Number of organisms(#)
Module completion ratio(%)
0
100
200
300
400
500
600
0
20
40
60
80
100
Thermotogae
Deinococcus-Thermus
Aquificae
Spirochaetes
Chlorobi
Bacteroidetes
Acidobacteria
Gemmatimonadetes
Planctomycetes
Fibrobacteres
Nitrospirae
Verrucomicrobia
Chlamydiae
Actinobacteria
Dictyoglomi
Chloroflexi
Synergistetes
Deferribacteres
Chrysiogenetes
Firmicutes
Tenericutes
Fusobacteria
Cyanobacteria
Gammaproteobacteria
Betaproteobacteria
Alphaproteobacteria
Epsilonproteobacteria
Deltaproteobacteria
Magnetococcus
Elusimicrobia
Thermobaculum
Euryarchaeota
Crenarchaeota
Thaumarchaeota
Nanoarchaeota
Korarchaeota

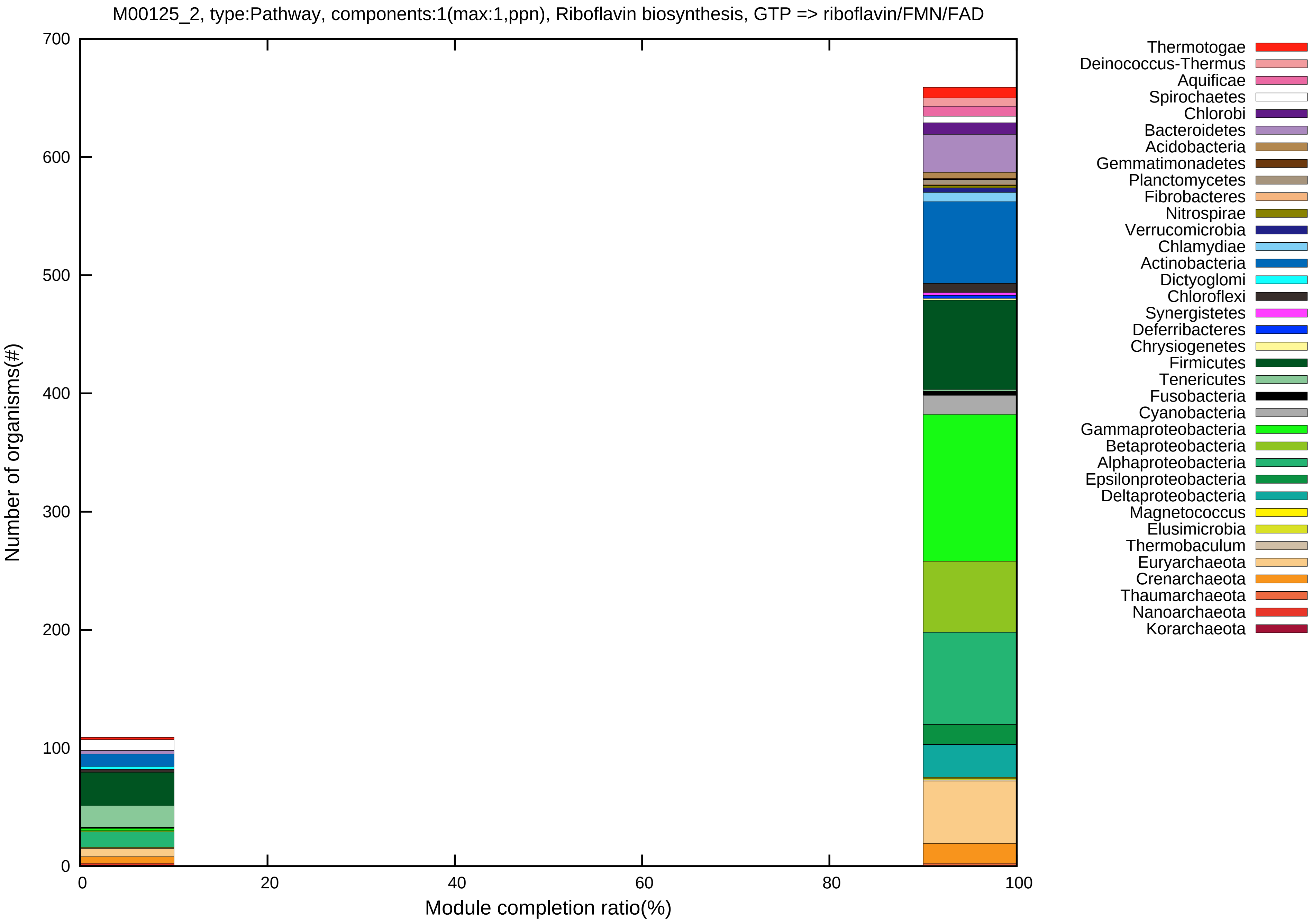
M00125_2, type:Pathway, components:1(max:1,ppn), Riboflavin biosynthesis, GTP => riboflavin/FMN/FAD
Number of organisms(#)
Module completion ratio(%)
0
100
200
300
400
500
600
700
0
20
40
60
80
100
Thermotogae
Deinococcus-Thermus
Aquificae
Spirochaetes
Chlorobi
Bacteroidetes
Acidobacteria
Gemmatimonadetes
Planctomycetes
Fibrobacteres
Nitrospirae
Verrucomicrobia
Chlamydiae
Actinobacteria
Dictyoglomi
Chloroflexi
Synergistetes
Deferribacteres
Chrysiogenetes
Firmicutes
Tenericutes
Fusobacteria
Cyanobacteria
Gammaproteobacteria
Betaproteobacteria
Alphaproteobacteria
Epsilonproteobacteria
Deltaproteobacteria
Magnetococcus
Elusimicrobia
Thermobaculum
Euryarchaeota
Crenarchaeota
Thaumarchaeota
Nanoarchaeota
Korarchaeota

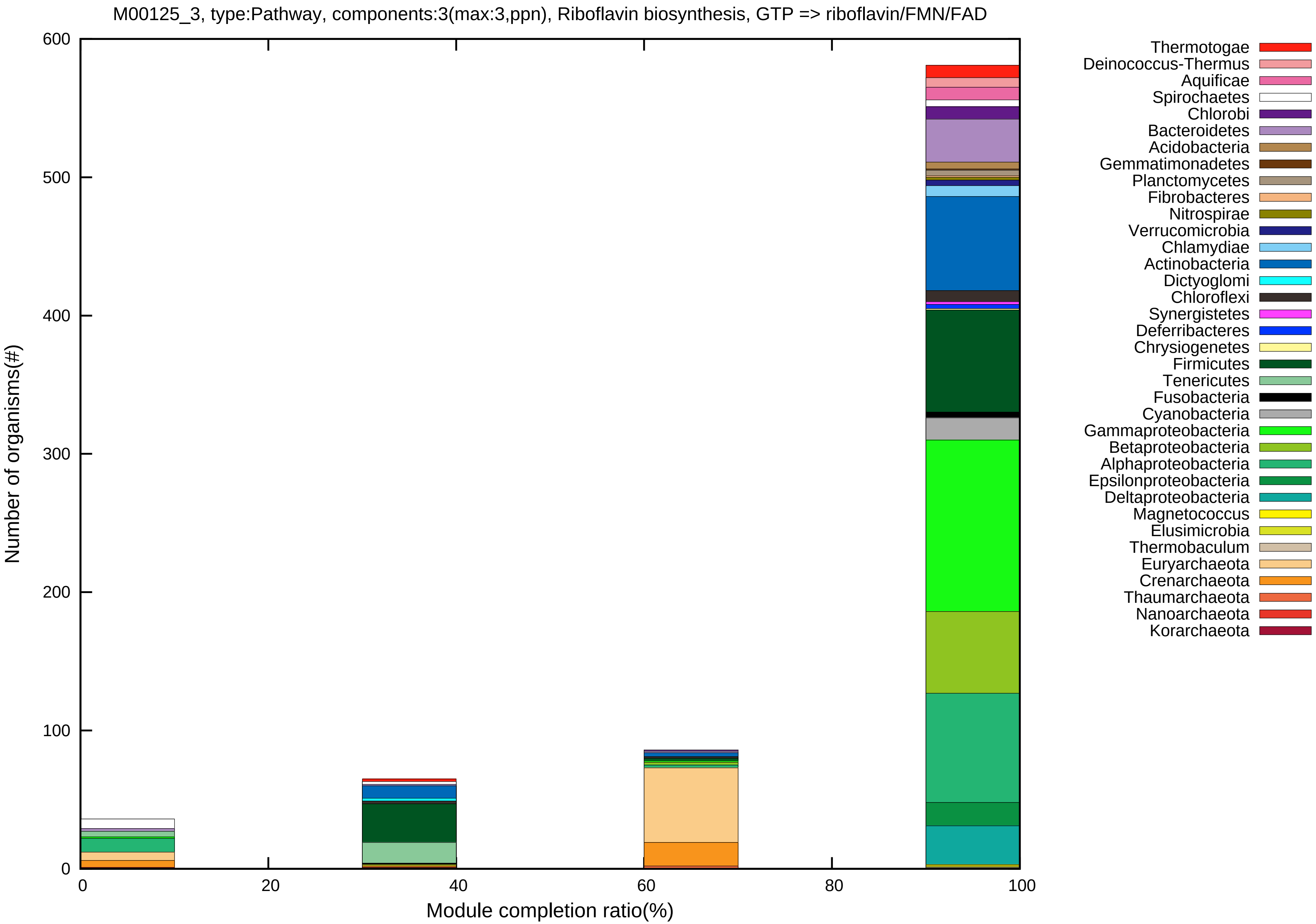
M00125_3, type:Pathway, components:3(max:3,ppn), Riboflavin biosynthesis, GTP => riboflavin/FMN/FAD
Number of organisms(#)
Module completion ratio(%)
0
100
200
300
400
500
600
0
20
40
60
80
100
Thermotogae
Deinococcus-Thermus
Aquificae
Spirochaetes
Chlorobi
Bacteroidetes
Acidobacteria
Gemmatimonadetes
Planctomycetes
Fibrobacteres
Nitrospirae
Verrucomicrobia
Chlamydiae
Actinobacteria
Dictyoglomi
Chloroflexi
Synergistetes
Deferribacteres
Chrysiogenetes
Firmicutes
Tenericutes
Fusobacteria
Cyanobacteria
Gammaproteobacteria
Betaproteobacteria
Alphaproteobacteria
Epsilonproteobacteria
Deltaproteobacteria
Magnetococcus
Elusimicrobia
Thermobaculum
Euryarchaeota
Crenarchaeota
Thaumarchaeota
Nanoarchaeota
Korarchaeota

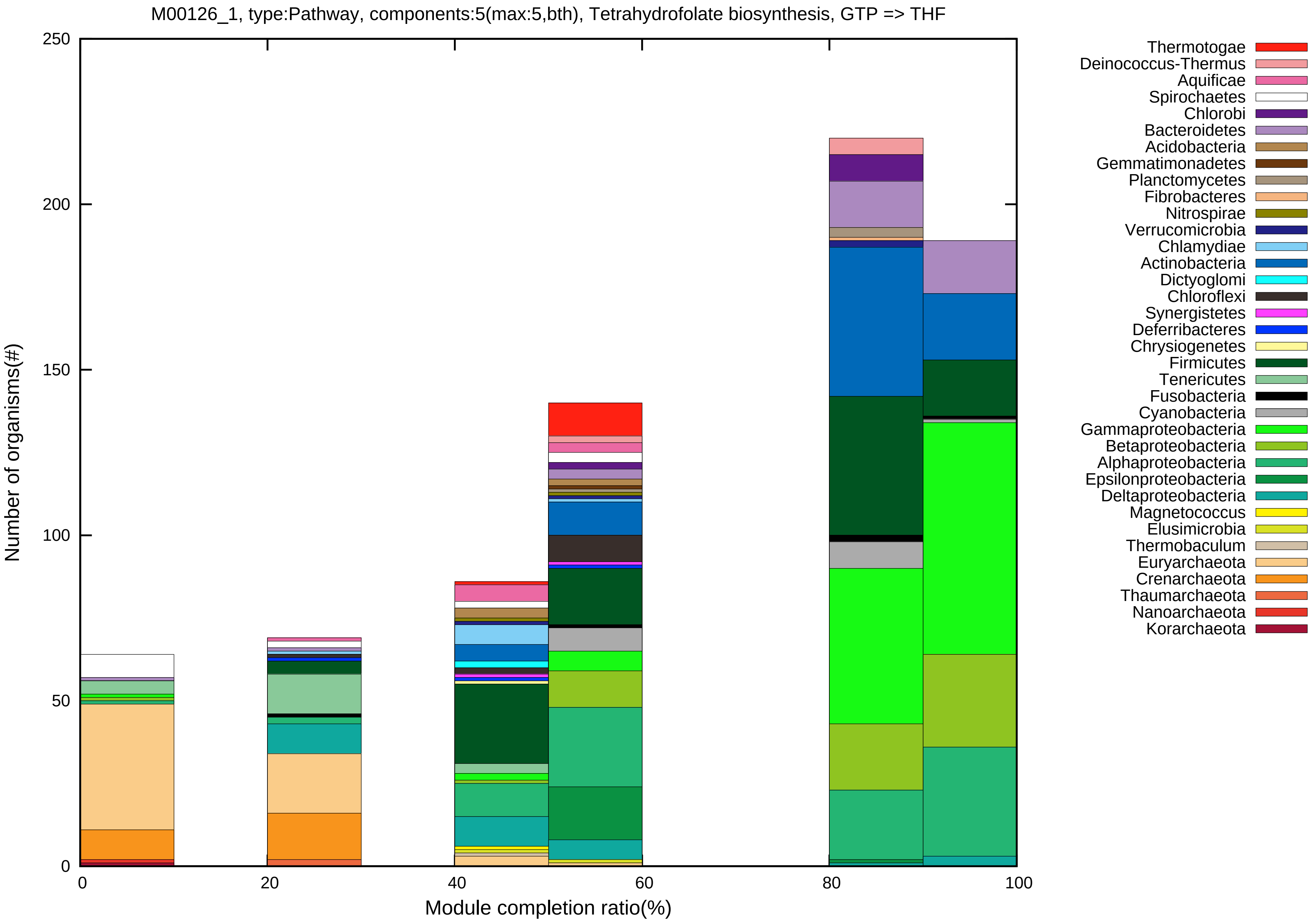
M00126_1, type:Pathway, components:5(max:5,bth), Tetrahydrofolate biosynthesis, GTP => THF
Number of organisms(#)
Module completion ratio(%)
0
50
100
150
200
250
0
20
40
60
80
100
Thermotogae
Deinococcus-Thermus
Aquificae
Spirochaetes
Chlorobi
Bacteroidetes
Acidobacteria
Gemmatimonadetes
Planctomycetes
Fibrobacteres
Nitrospirae
Verrucomicrobia
Chlamydiae
Actinobacteria
Dictyoglomi
Chloroflexi
Synergistetes
Deferribacteres
Chrysiogenetes
Firmicutes
Tenericutes
Fusobacteria
Cyanobacteria
Gammaproteobacteria
Betaproteobacteria
Alphaproteobacteria
Epsilonproteobacteria
Deltaproteobacteria
Magnetococcus
Elusimicrobia
Thermobaculum
Euryarchaeota
Crenarchaeota
Thaumarchaeota
Nanoarchaeota
Korarchaeota

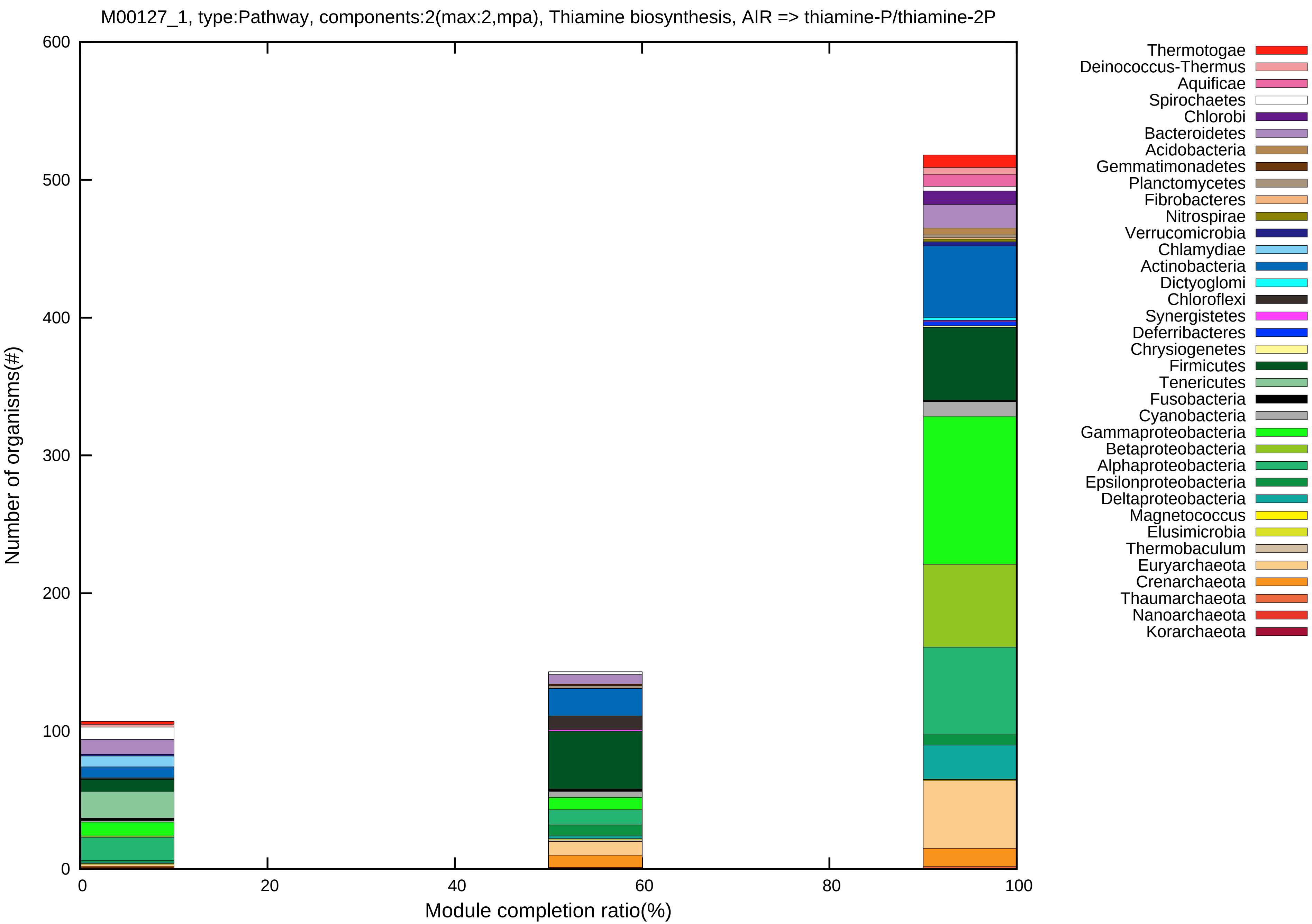

M00127_1, type:Pathway, components:2(max:2,mpa), Thiamine biosynthesis, AIR => thiamine-P/thiamine-2P
Number of organisms(#)
Module completion ratio(%)
0
100
200
300
400
500
600
0
20
40
60
80
100
Thermotogae
Deinococcus-Thermus
Aquificae
Spirochaetes
Chlorobi
Bacteroidetes
Acidobacteria
Gemmatimonadetes
Planctomycetes
Fibrobacteres
Nitrospirae
Verrucomicrobia
Chlamydiae
Actinobacteria
Dictyoglomi
Chloroflexi
Synergistetes
Deferribacteres
Chrysiogenetes
Firmicutes
Tenericutes
Fusobacteria
Cyanobacteria
Gammaproteobacteria
Betaproteobacteria
Alphaproteobacteria
Epsilonproteobacteria
Deltaproteobacteria
Magnetococcus
Elusimicrobia
Thermobaculum
Euryarchaeota
Crenarchaeota
Thaumarchaeota
Nanoarchaeota
Korarchaeota

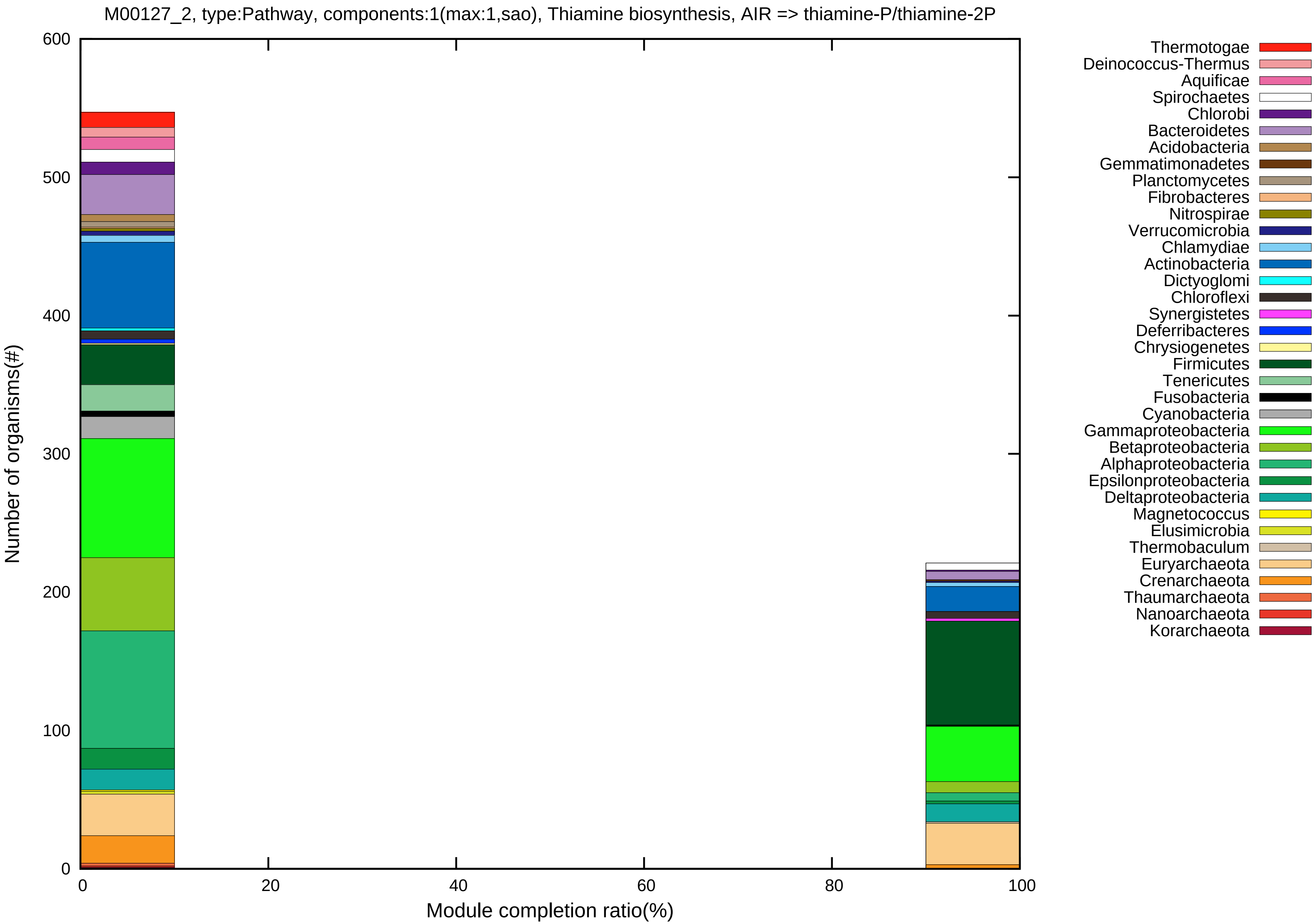

M00127_2, type:Pathway, components:1(max:1,sao), Thiamine biosynthesis, AIR => thiamine-P/thiamine-2P
Number of organisms(#)
Module completion ratio(%)
0
100
200
300
400
500
600
0
20
40
60
80
100
Thermotogae
Deinococcus-Thermus
Aquificae
Spirochaetes
Chlorobi
Bacteroidetes
Acidobacteria
Gemmatimonadetes
Planctomycetes
Fibrobacteres
Nitrospirae
Verrucomicrobia
Chlamydiae
Actinobacteria
Dictyoglomi
Chloroflexi
Synergistetes
Deferribacteres
Chrysiogenetes
Firmicutes
Tenericutes
Fusobacteria
Cyanobacteria
Gammaproteobacteria
Betaproteobacteria
Alphaproteobacteria
Epsilonproteobacteria
Deltaproteobacteria
Magnetococcus
Elusimicrobia
Thermobaculum
Euryarchaeota
Crenarchaeota
Thaumarchaeota
Nanoarchaeota
Korarchaeota

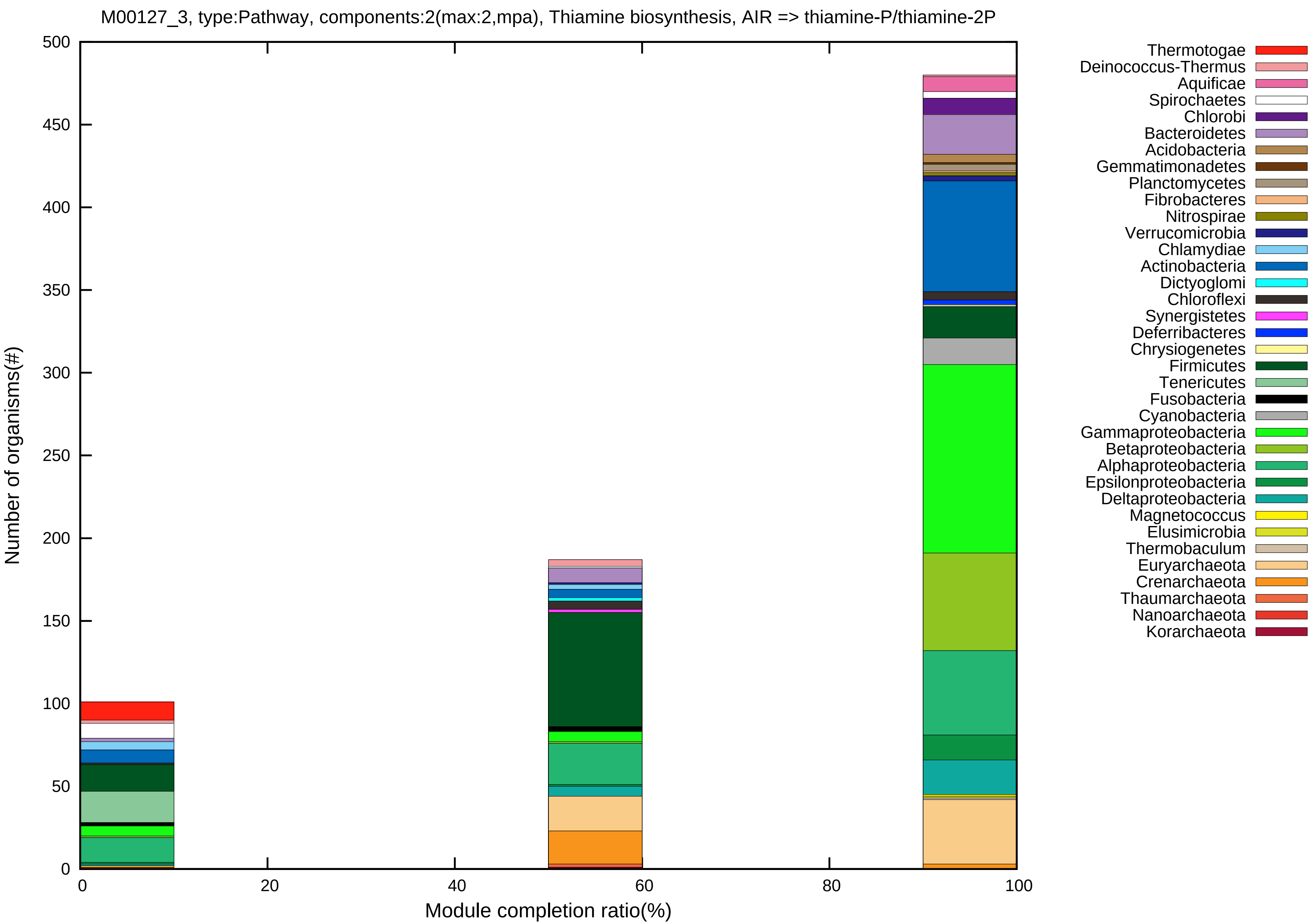

M00127_3, type:Pathway, components:2(max:2,mpa), Thiamine biosynthesis, AIR => thiamine-P/thiamine-2P
Number of organisms(#)
Module completion ratio(%)
0
50
100
150
200
250
300
350
400
450
500
0
20
40
60
80
100
Thermotogae
Deinococcus-Thermus
Aquificae
Spirochaetes
Chlorobi
Bacteroidetes
Acidobacteria
Gemmatimonadetes
Planctomycetes
Fibrobacteres
Nitrospirae
Verrucomicrobia
Chlamydiae
Actinobacteria
Dictyoglomi
Chloroflexi
Synergistetes
Deferribacteres
Chrysiogenetes
Firmicutes
Tenericutes
Fusobacteria
Cyanobacteria
Gammaproteobacteria
Betaproteobacteria
Alphaproteobacteria
Epsilonproteobacteria
Deltaproteobacteria
Magnetococcus
Elusimicrobia
Thermobaculum
Euryarchaeota
Crenarchaeota
Thaumarchaeota
Nanoarchaeota
Korarchaeota

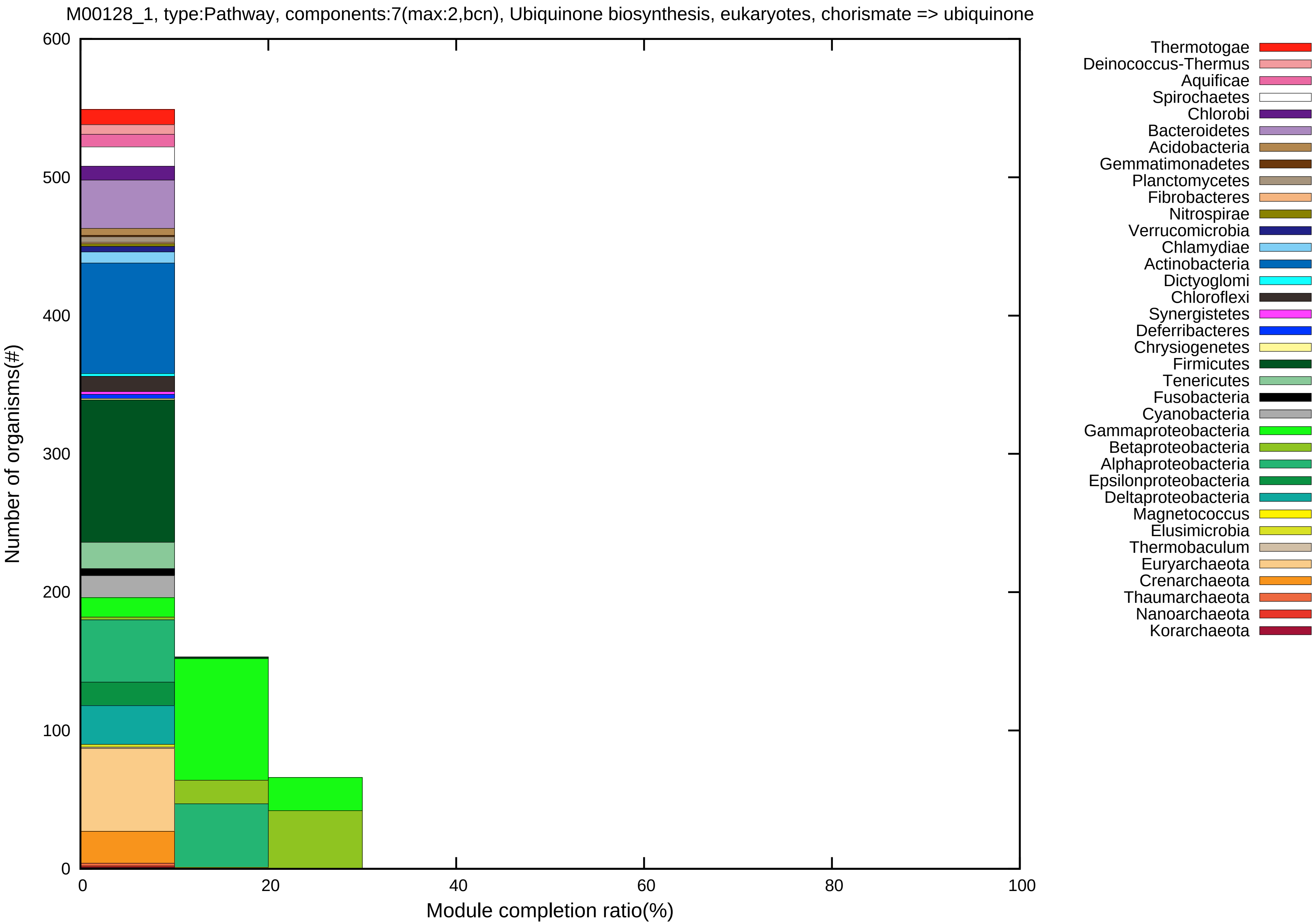

M00128_1, type:Pathway, components:7(max:2,bcn), Ubiquinone biosynthesis, eukaryotes, chorismate => ubiquinone
Number of organisms(#)
Module completion ratio(%)
0
100
200
300
400
500
600
0
20
40
60
80
100
Thermotogae
Deinococcus-Thermus
Aquificae
Spirochaetes
Chlorobi
Bacteroidetes
Acidobacteria
Gemmatimonadetes
Planctomycetes
Fibrobacteres
Nitrospirae
Verrucomicrobia
Chlamydiae
Actinobacteria
Dictyoglomi
Chloroflexi
Synergistetes
Deferribacteres
Chrysiogenetes
Firmicutes
Tenericutes
Fusobacteria
Cyanobacteria
Gammaproteobacteria
Betaproteobacteria
Alphaproteobacteria
Epsilonproteobacteria
Deltaproteobacteria
Magnetococcus
Elusimicrobia
Thermobaculum
Euryarchaeota
Crenarchaeota
Thaumarchaeota
Nanoarchaeota
Korarchaeota

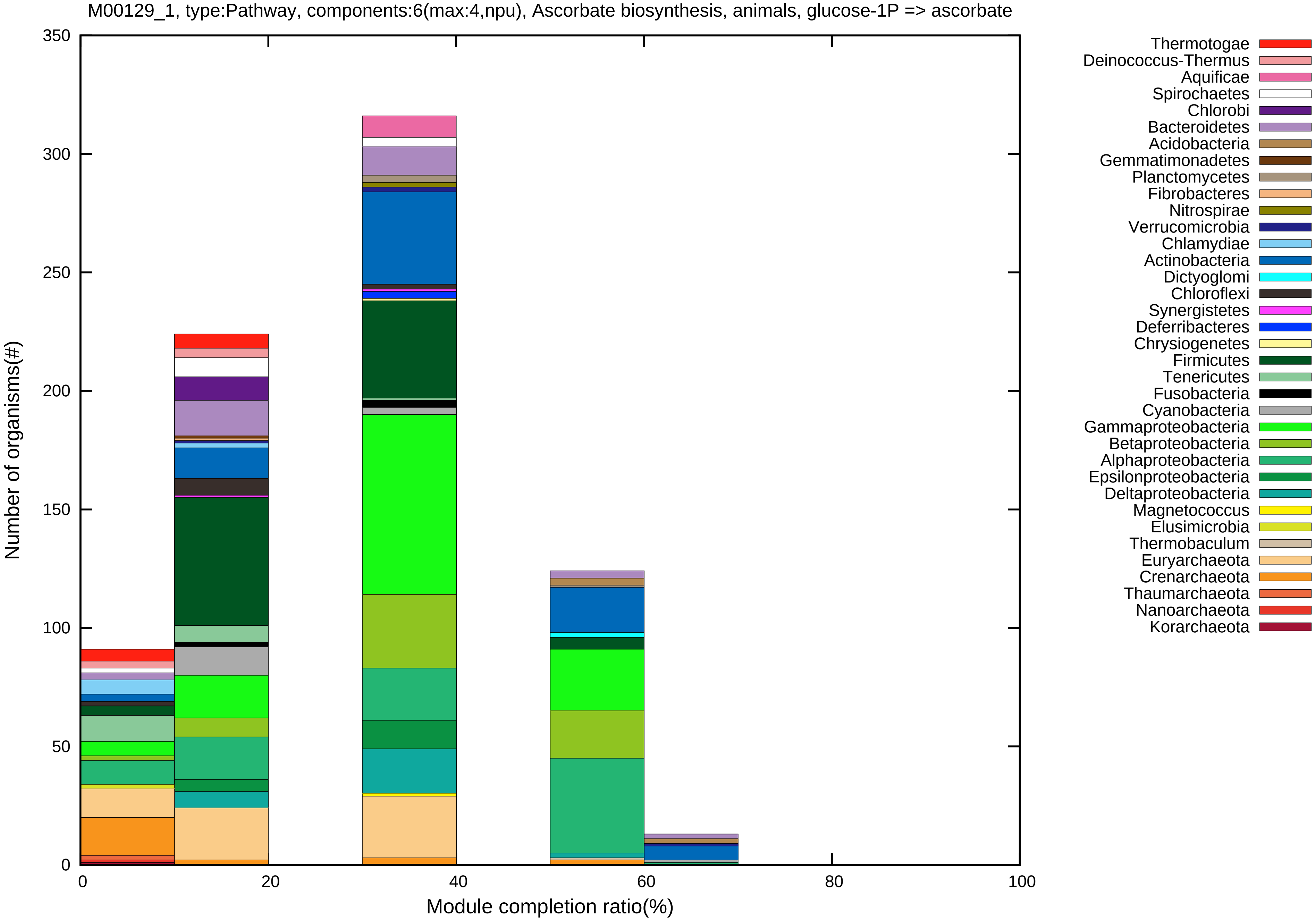

M00129_1, type:Pathway, components:6(max:4,npu), Ascorbate biosynthesis, animals, glucose-1P => ascorbate
Number of organisms(#)
Module completion ratio(%)
0
50
100
150
200
250
300
350
0
20
40
60
80
100
Thermotogae
Deinococcus-Thermus
Aquificae
Spirochaetes
Chlorobi
Bacteroidetes
Acidobacteria
Gemmatimonadetes
Planctomycetes
Fibrobacteres
Nitrospirae
Verrucomicrobia
Chlamydiae
Actinobacteria
Dictyoglomi
Chloroflexi
Synergistetes
Deferribacteres
Chrysiogenetes
Firmicutes
Tenericutes
Fusobacteria
Cyanobacteria
Gammaproteobacteria
Betaproteobacteria
Alphaproteobacteria
Epsilonproteobacteria
Deltaproteobacteria
Magnetococcus
Elusimicrobia
Thermobaculum
Euryarchaeota
Crenarchaeota
Thaumarchaeota
Nanoarchaeota
Korarchaeota

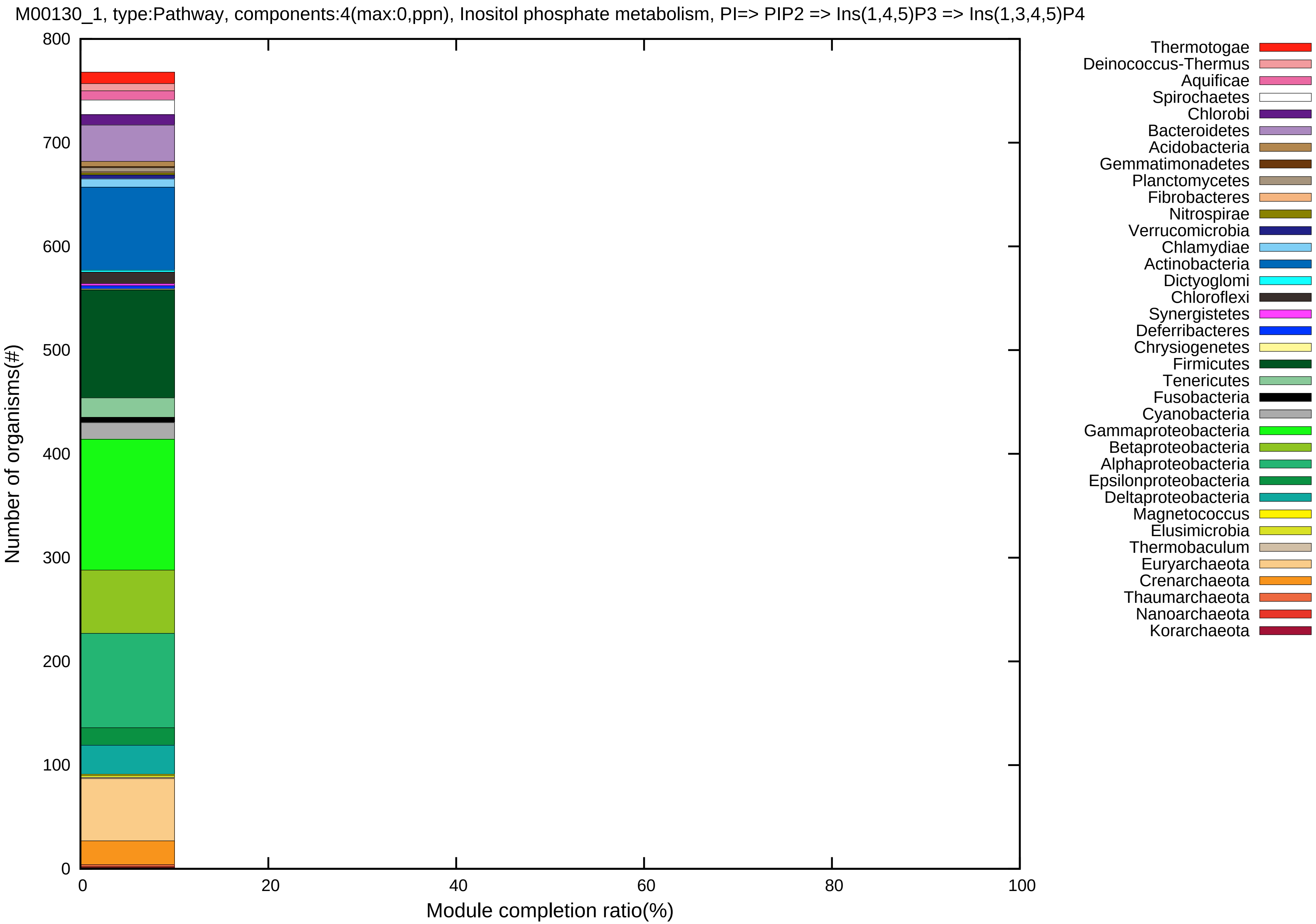
M00130_1, type:Pathway, components:4(max:0,ppn), Inositol phosphate metabolism, PI=> PIP2 => Ins(1,4,5)P3 => Ins(1,3,4,5)P4
Number of organisms(#)
Module completion ratio(%)
0
100
200
300
400
500
600
700
800
0
20
40
60
80
100
Thermotogae
Deinococcus-Thermus
Aquificae
Spirochaetes
Chlorobi
Bacteroidetes
Acidobacteria
Gemmatimonadetes
Planctomycetes
Fibrobacteres
Nitrospirae
Verrucomicrobia
Chlamydiae
Actinobacteria
Dictyoglomi
Chloroflexi
Synergistetes
Deferribacteres
Chrysiogenetes
Firmicutes
Tenericutes
Fusobacteria
Cyanobacteria
Gammaproteobacteria
Betaproteobacteria
Alphaproteobacteria
Epsilonproteobacteria
Deltaproteobacteria
Magnetococcus
Elusimicrobia
Thermobaculum
Euryarchaeota
Crenarchaeota
Thaumarchaeota
Nanoarchaeota
Korarchaeota

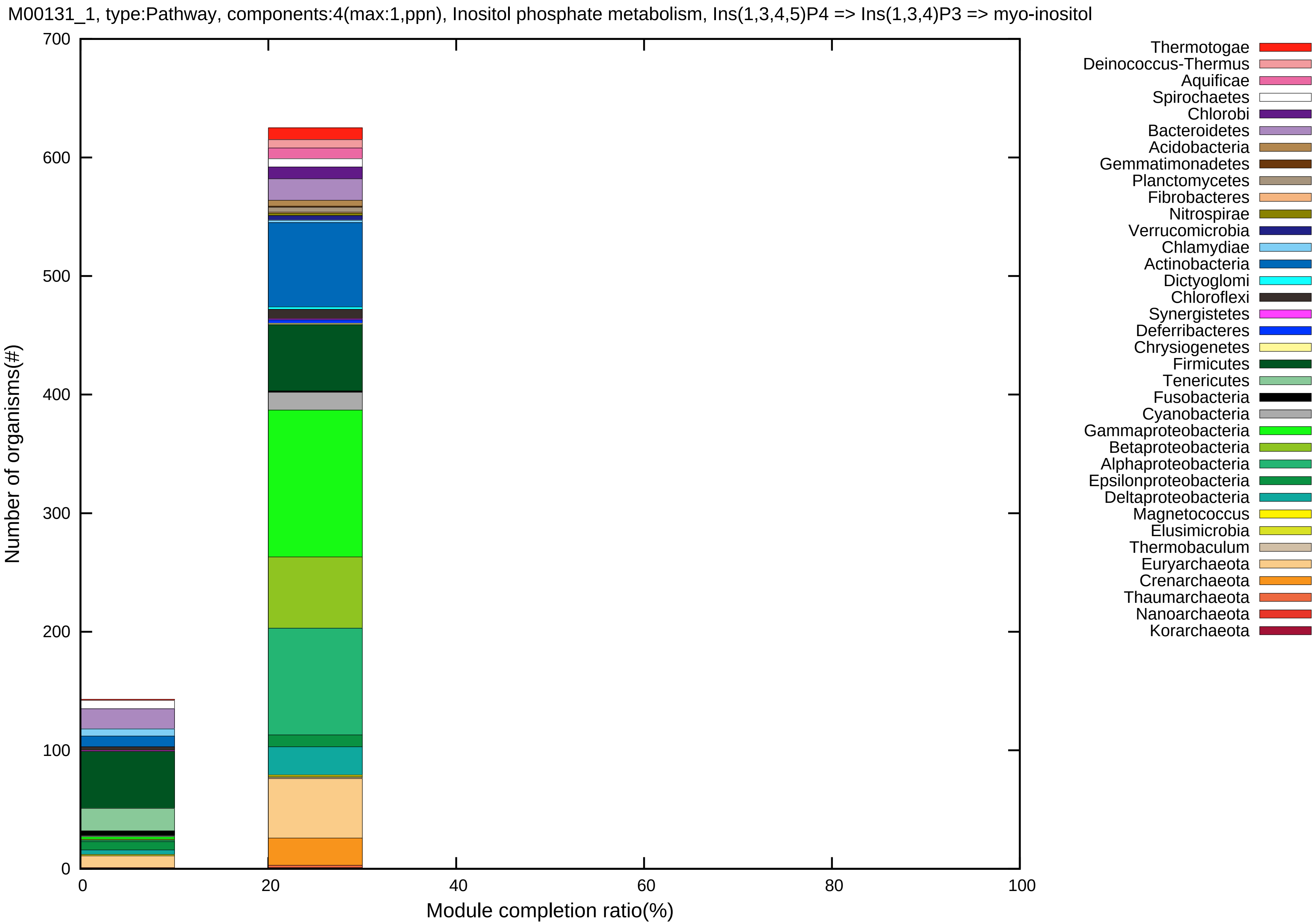
M00131_1, type:Pathway, components:4(max:1,ppn), Inositol phosphate metabolism, Ins(1,3,4,5)P4 => Ins(1,3,4)P3 => myo-inositol
Number of organisms(#)
Module completion ratio(%)
0
100
200
300
400
500
600
700
0
20
40
60
80
100
Thermotogae
Deinococcus-Thermus
Aquificae
Spirochaetes
Chlorobi
Bacteroidetes
Acidobacteria
Gemmatimonadetes
Planctomycetes
Fibrobacteres
Nitrospirae
Verrucomicrobia
Chlamydiae
Actinobacteria
Dictyoglomi
Chloroflexi
Synergistetes
Deferribacteres
Chrysiogenetes
Firmicutes
Tenericutes
Fusobacteria
Cyanobacteria
Gammaproteobacteria
Betaproteobacteria
Alphaproteobacteria
Epsilonproteobacteria
Deltaproteobacteria
Magnetococcus
Elusimicrobia
Thermobaculum
Euryarchaeota
Crenarchaeota
Thaumarchaeota
Nanoarchaeota
Korarchaeota

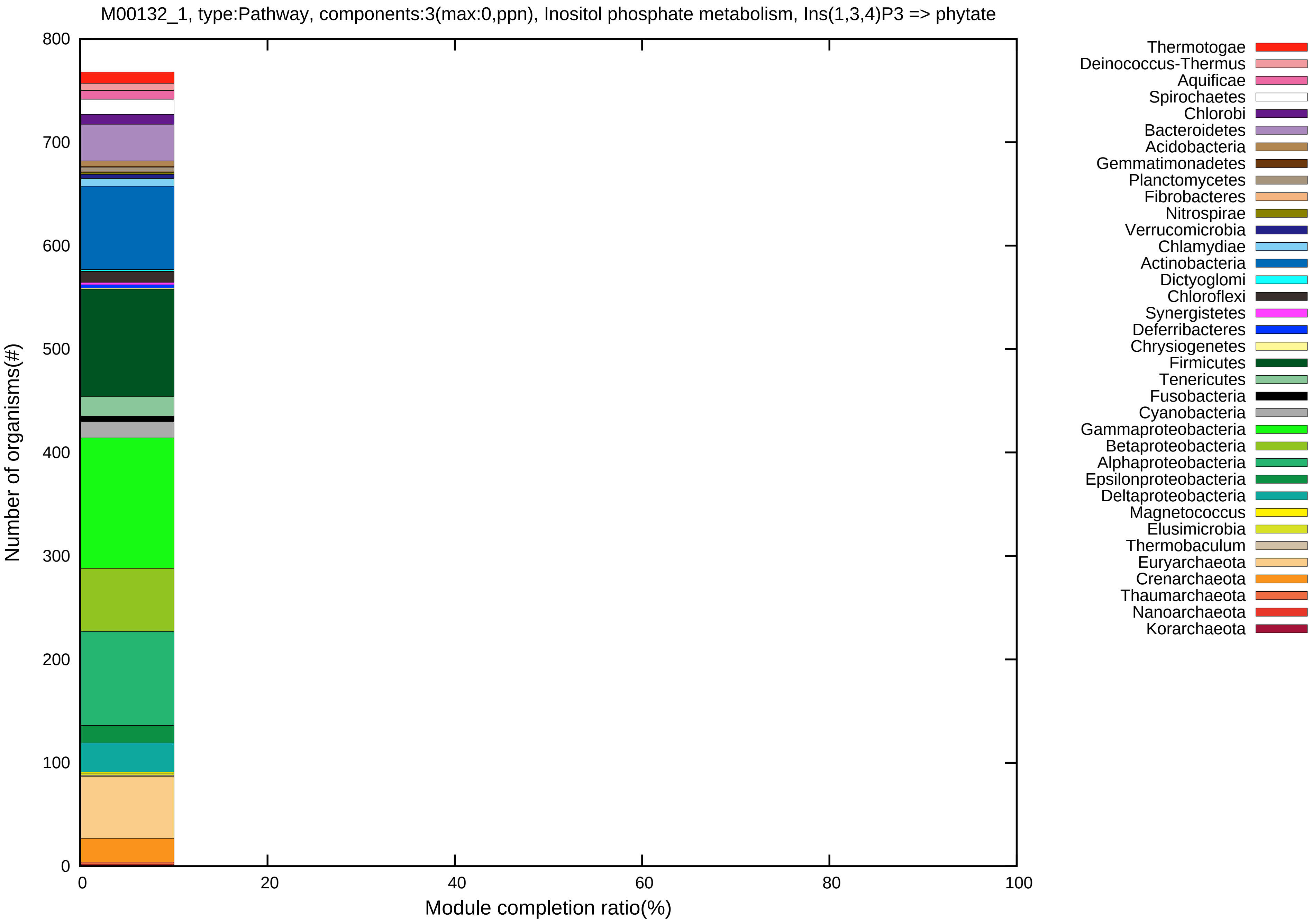
M00132_1, type:Pathway, components:3(max:0,ppn), Inositol phosphate metabolism, Ins(1,3,4)P3 => phytate
Number of organisms(#)
0
100
200
300
400
500
600
700
800
0
20
40
60
80
100
Module completion ratio(%)
Thermotogae
Deinococcus-Thermus
Aquificae
Spirochaetes
Chlorobi
Bacteroidetes
Acidobacteria
Gemmatimonadetes
Planctomycetes
Fibrobacteres
Nitrospirae
Verrucomicrobia
Chlamydiae
Actinobacteria
Dictyoglomi
Chloroflexi
Synergistetes
Deferribacteres
Chrysiogenetes
Firmicutes
Tenericutes
Fusobacteria
Cyanobacteria
Gammaproteobacteria
Betaproteobacteria
Alphaproteobacteria
Epsilonproteobacteria
Deltaproteobacteria
Magnetococcus
Elusimicrobia
Thermobaculum
Euryarchaeota
Crenarchaeota
Thaumarchaeota
Nanoarchaeota
Korarchaeota

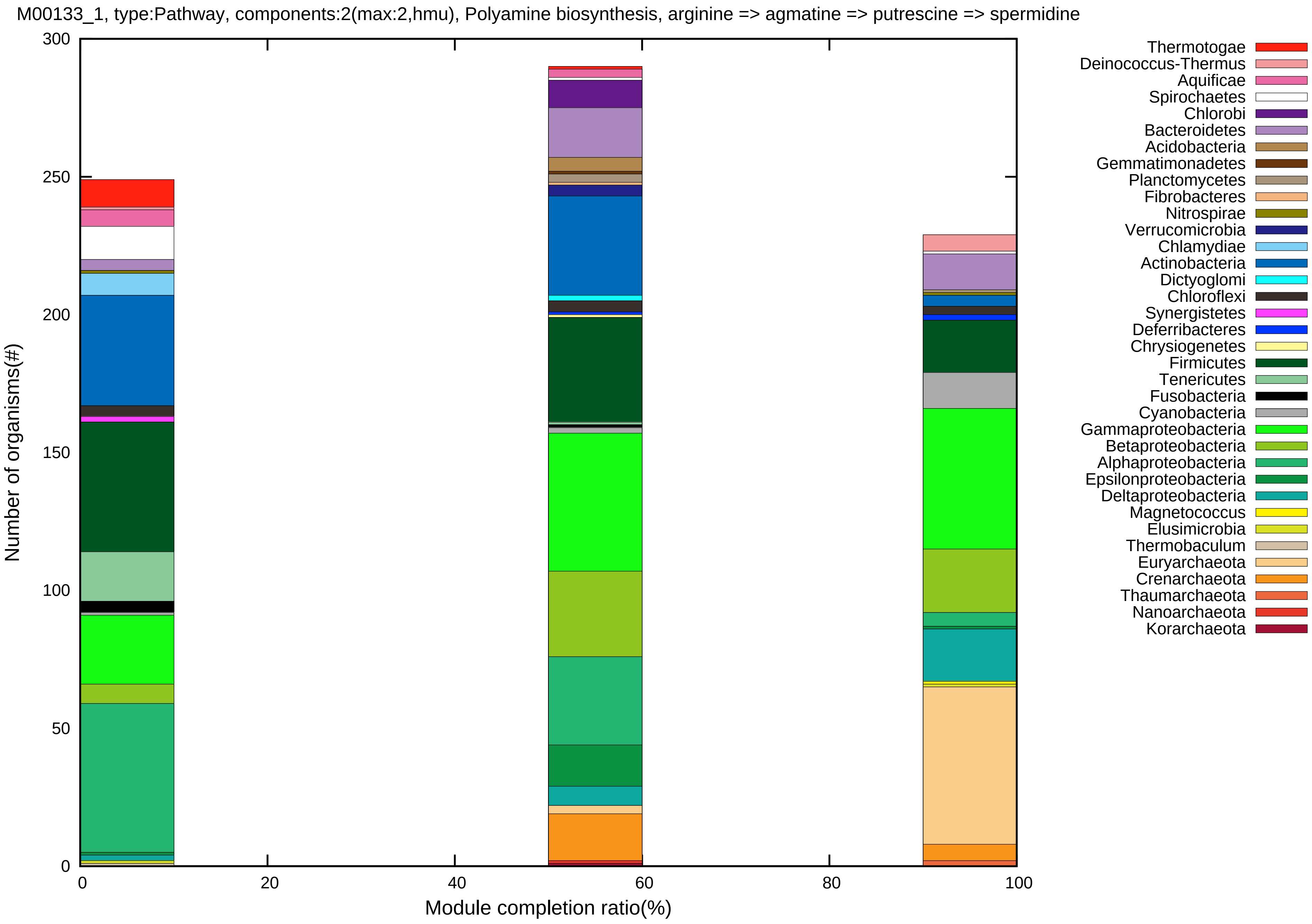

M00133_1, type:Pathway, components:2(max:2,hmu), Polyamine biosynthesis, arginine => agmatine => putrescine => spermidine
Number of organisms(#)
Module completion ratio(%)
0
50
100
150
200
250
300
0
20
40
60
80
100
Thermotogae
Deinococcus-Thermus
Aquificae
Spirochaetes
Chlorobi
Bacteroidetes
Acidobacteria
Gemmatimonadetes
Planctomycetes
Fibrobacteres
Nitrospirae
Verrucomicrobia
Chlamydiae
Actinobacteria
Dictyoglomi
Chloroflexi
Synergistetes
Deferribacteres
Chrysiogenetes
Firmicutes
Tenericutes
Fusobacteria
Cyanobacteria
Gammaproteobacteria
Betaproteobacteria
Alphaproteobacteria
Epsilonproteobacteria
Deltaproteobacteria
Magnetococcus
Elusimicrobia
Thermobaculum
Euryarchaeota
Crenarchaeota
Thaumarchaeota
Nanoarchaeota
Korarchaeota

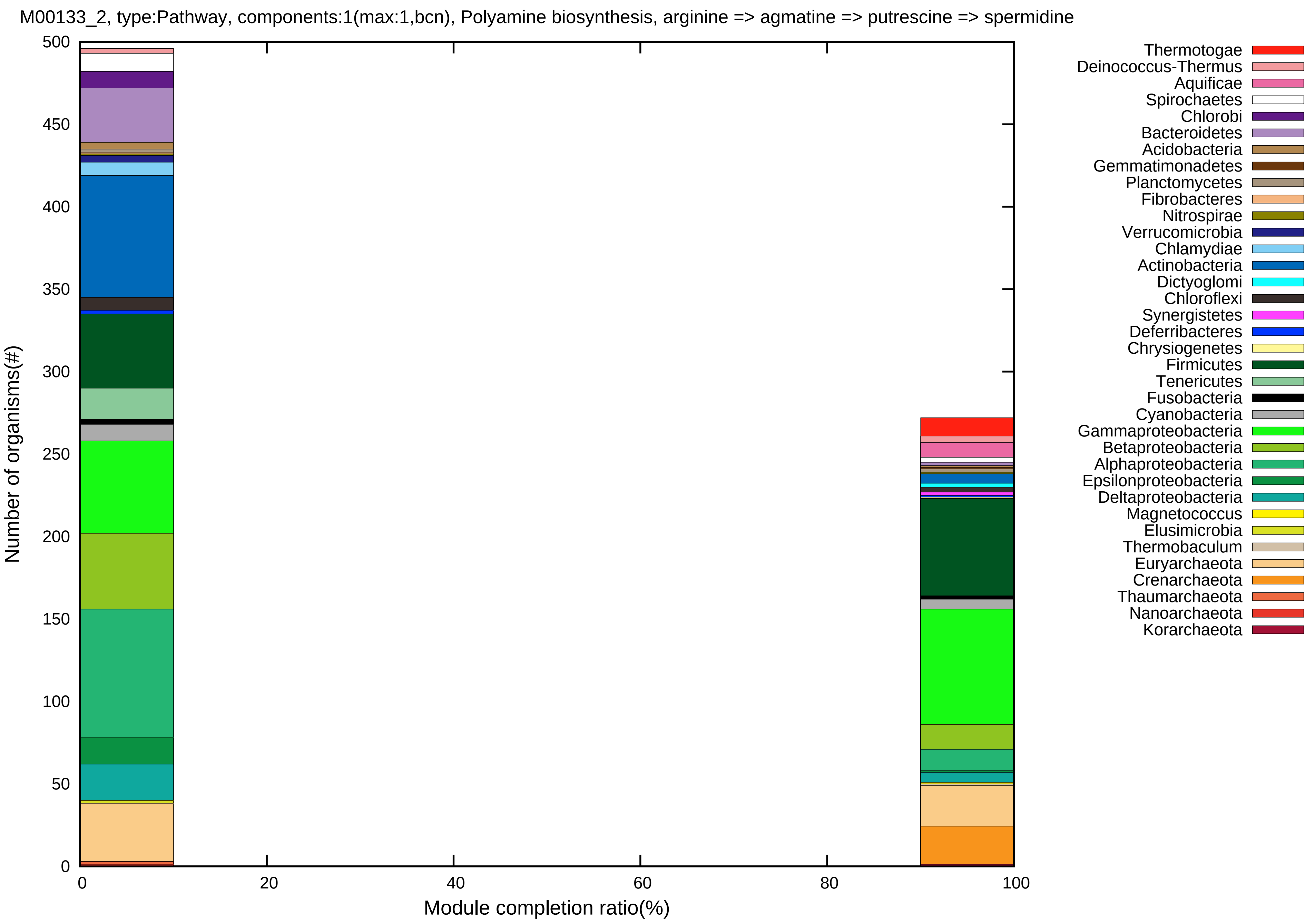
M00133_2, type:Pathway, components:1(max:1,bcn), Polyamine biosynthesis, arginine => agmatine => putrescine => spermidine
Number of organisms(#)
Module completion ratio(%)
0
50
100
150
200
250
300
350
400
450
500
0
20
40
60
80
100
Thermotogae
Deinococcus-Thermus
Aquificae
Spirochaetes
Chlorobi
Bacteroidetes
Acidobacteria
Gemmatimonadetes
Planctomycetes
Fibrobacteres
Nitrospirae
Verrucomicrobia
Chlamydiae
Actinobacteria
Dictyoglomi
Chloroflexi
Synergistetes
Deferribacteres
Chrysiogenetes
Firmicutes
Tenericutes
Fusobacteria
Cyanobacteria
Gammaproteobacteria
Betaproteobacteria
Alphaproteobacteria
Epsilonproteobacteria
Deltaproteobacteria
Magnetococcus
Elusimicrobia
Thermobaculum
Euryarchaeota
Crenarchaeota
Thaumarchaeota
Nanoarchaeota
Korarchaeota

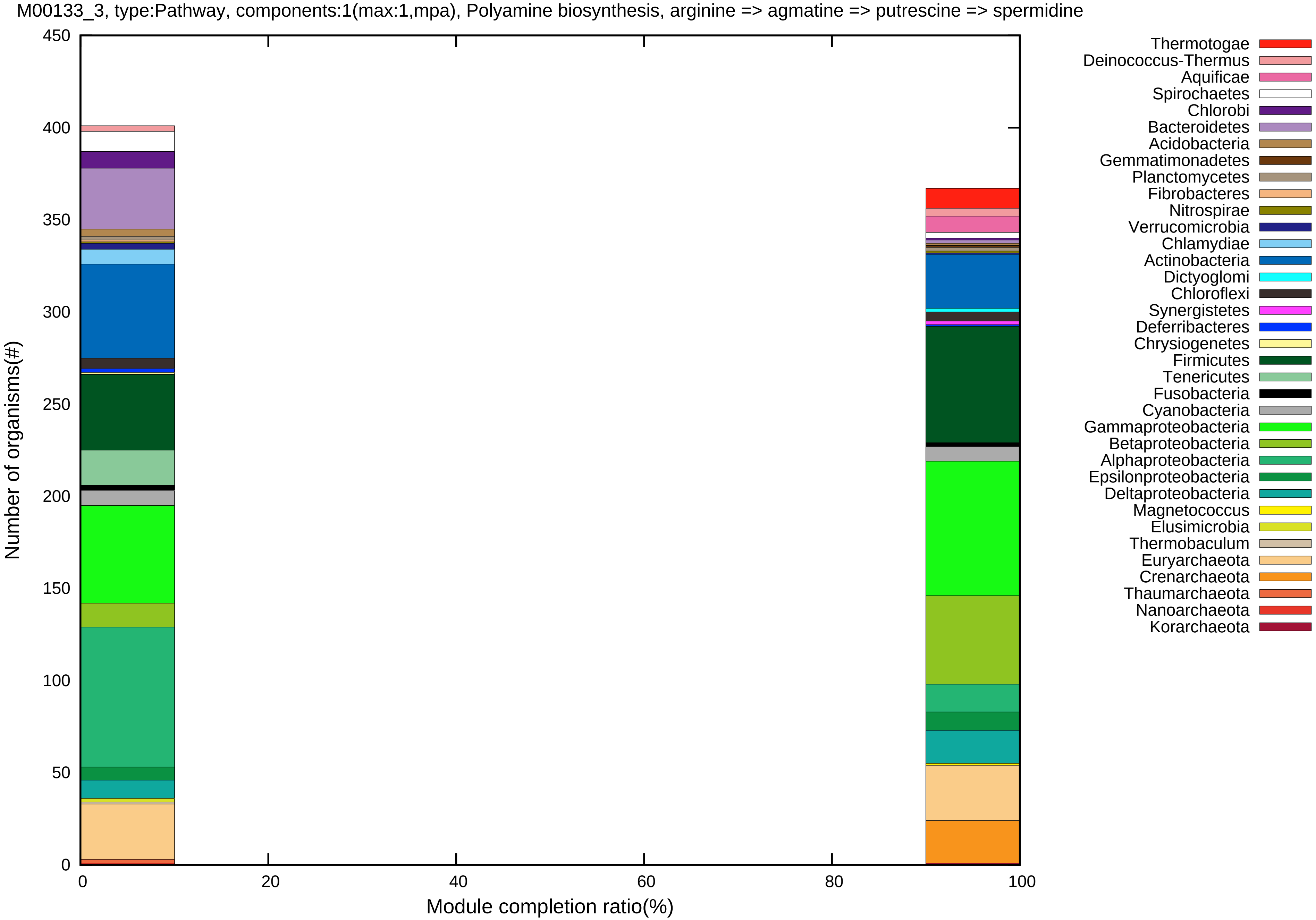

M00133_3, type:Pathway, components:1(max:1,mpa), Polyamine biosynthesis, arginine => agmatine => putrescine => spermidine
Number of organisms(#)
Module completion ratio(%)
0
50
100
150
200
250
300
350
400
450
0
20
40
60
80
100
Thermotogae
Deinococcus-Thermus
Aquificae
Spirochaetes
Chlorobi
Bacteroidetes
Acidobacteria
Gemmatimonadetes
Planctomycetes
Fibrobacteres
Nitrospirae
Verrucomicrobia
Chlamydiae
Actinobacteria
Dictyoglomi
Chloroflexi
Synergistetes
Deferribacteres
Chrysiogenetes
Firmicutes
Tenericutes
Fusobacteria
Cyanobacteria
Gammaproteobacteria
Betaproteobacteria
Alphaproteobacteria
Epsilonproteobacteria
Deltaproteobacteria
Magnetococcus
Elusimicrobia
Thermobaculum
Euryarchaeota
Crenarchaeota
Thaumarchaeota
Nanoarchaeota
Korarchaeota

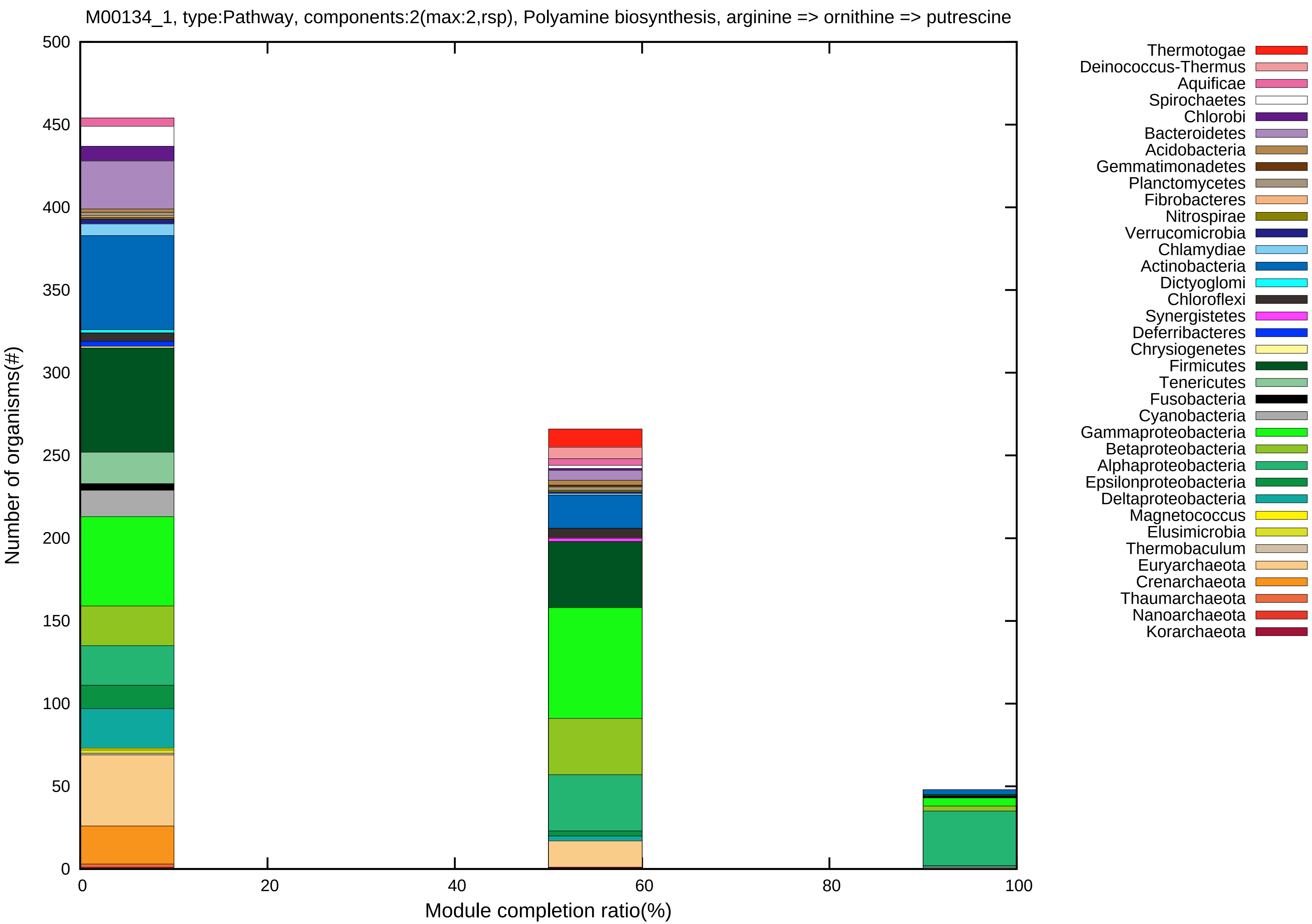

M00134_1, type:Pathway, components:2(max:2,rsp), Polyamine biosynthesis, arginine => ornithine => putrescine
Number of organisms(#)
Module completion ratio(%)
0
50
100
150
200
250
300
350
400
450
500
0
20
40
60
80
100
Thermotogae
Deinococcus-Thermus
Aquificae
Spirochaetes
Chlorobi
Bacteroidetes
Acidobacteria
Gemmatimonadetes
Planctomycetes
Fibrobacteres
Nitrospirae
Verrucomicrobia
Chlamydiae
Actinobacteria
Dictyoglomi
Chloroflexi
Synergistetes
Deferribacteres
Chrysiogenetes
Firmicutes
Tenericutes
Fusobacteria
Cyanobacteria
Gammaproteobacteria
Betaproteobacteria
Alphaproteobacteria
Epsilonproteobacteria
Deltaproteobacteria
Magnetococcus
Elusimicrobia
Thermobaculum
Euryarchaeota
Crenarchaeota
Thaumarchaeota
Nanoarchaeota
Korarchaeota

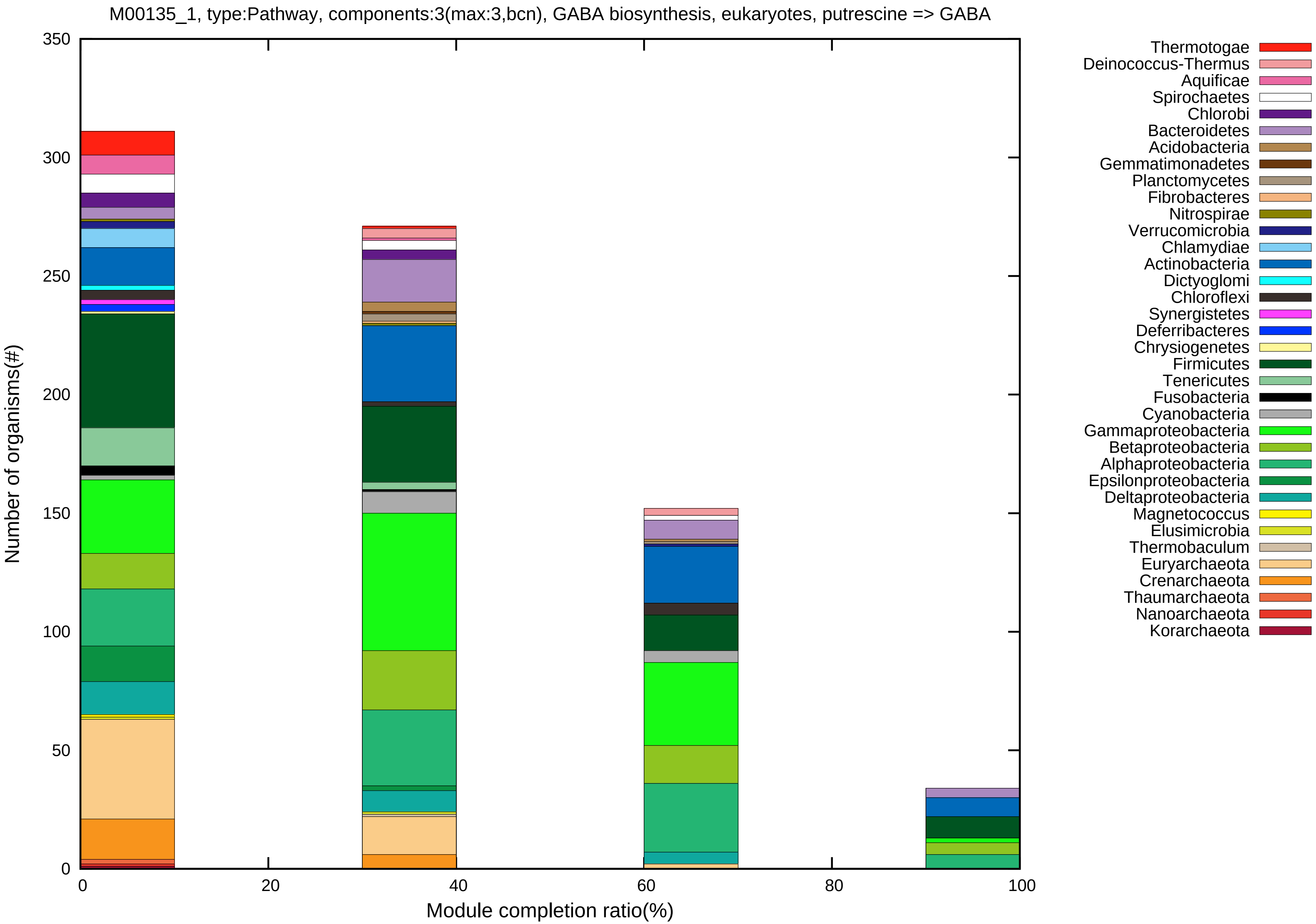

M00135_1, type:Pathway, components:3(max:3,bcn), GABA biosynthesis, eukaryotes, putrescine => GABA
Number of organisms(#)
Module completion ratio(%)
0
50
100
150
200
250
300
350
0
20
40
60
80
100
Thermotogae
Deinococcus-Thermus
Aquificae
Spirochaetes
Chlorobi
Bacteroidetes
Acidobacteria
Gemmatimonadetes
Planctomycetes
Fibrobacteres
Nitrospirae
Verrucomicrobia
Chlamydiae
Actinobacteria
Dictyoglomi
Chloroflexi
Synergistetes
Deferribacteres
Chrysiogenetes
Firmicutes
Tenericutes
Fusobacteria
Cyanobacteria
Gammaproteobacteria
Betaproteobacteria
Alphaproteobacteria
Epsilonproteobacteria
Deltaproteobacteria
Magnetococcus
Elusimicrobia
Thermobaculum
Euryarchaeota
Crenarchaeota
Thaumarchaeota
Nanoarchaeota
Korarchaeota

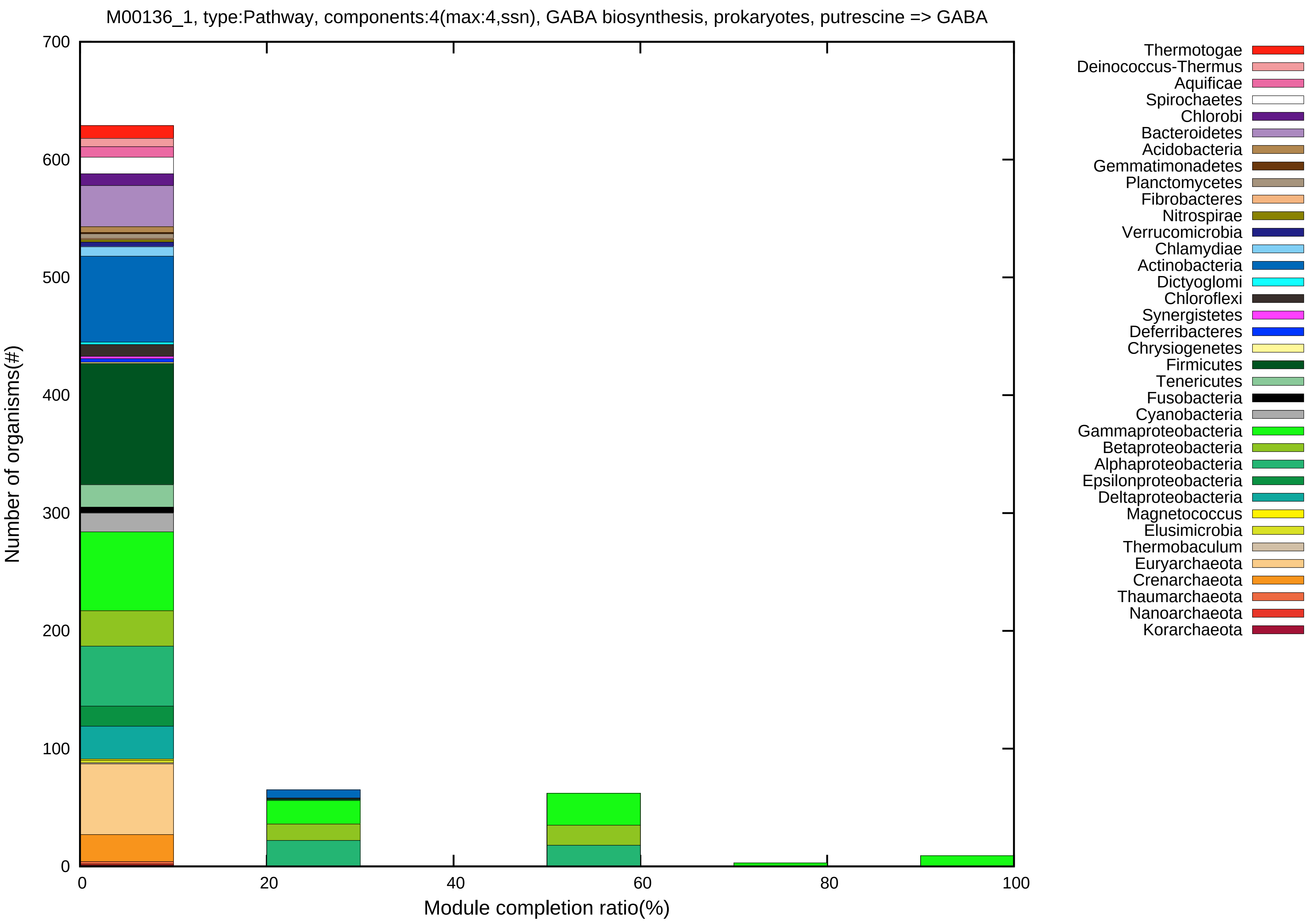

M00136_1, type:Pathway, components:4(max:4,ssn), GABA biosynthesis, prokaryotes, putrescine => GABA
Number of organisms(#)
Module completion ratio(%)
0
100
200
300
400
500
600
700
0
20
40
60
80
100
Thermotogae
Deinococcus-Thermus
Aquificae
Spirochaetes
Chlorobi
Bacteroidetes
Acidobacteria
Gemmatimonadetes
Planctomycetes
Fibrobacteres
Nitrospirae
Verrucomicrobia
Chlamydiae
Actinobacteria
Dictyoglomi
Chloroflexi
Synergistetes
Deferribacteres
Chrysiogenetes
Firmicutes
Tenericutes
Fusobacteria
Cyanobacteria
Gammaproteobacteria
Betaproteobacteria
Alphaproteobacteria
Epsilonproteobacteria
Deltaproteobacteria
Magnetococcus
Elusimicrobia
Thermobaculum
Euryarchaeota
Crenarchaeota
Thaumarchaeota
Nanoarchaeota
Korarchaeota

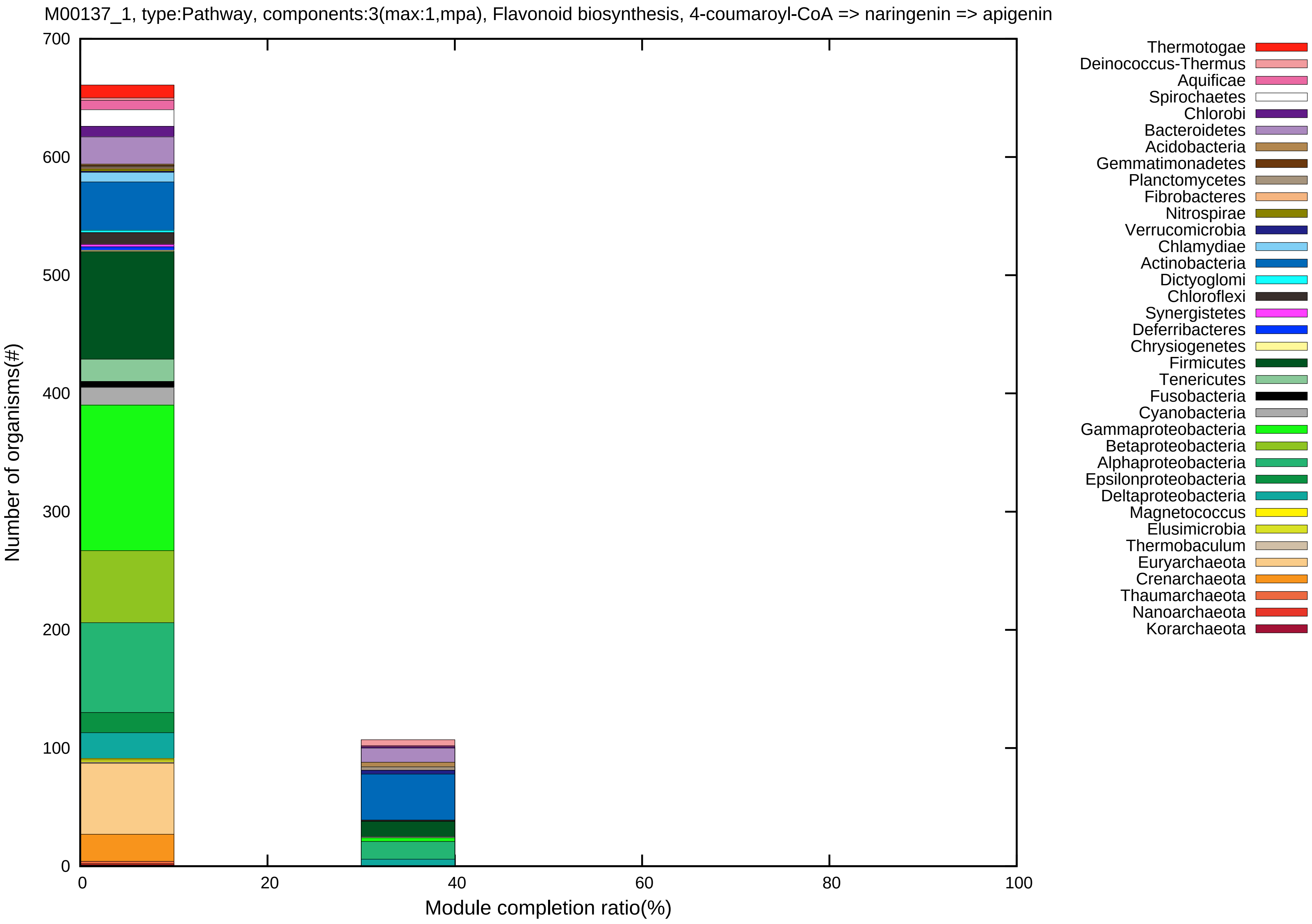

M00137_1, type:Pathway, components:3(max:1,mpa), Flavonoid biosynthesis, 4-coumaroyl-CoA => naringenin => apigenin
Number of organisms(#)
Module completion ratio(%)
0
100
200
300
400
500
600
700
0
20
40
60
80
100
Thermotogae
Deinococcus-Thermus
Aquificae
Spirochaetes
Chlorobi
Bacteroidetes
Acidobacteria
Gemmatimonadetes
Planctomycetes
Fibrobacteres
Nitrospirae
Verrucomicrobia
Chlamydiae
Actinobacteria
Dictyoglomi
Chloroflexi
Synergistetes
Deferribacteres
Chrysiogenetes
Firmicutes
Tenericutes
Fusobacteria
Cyanobacteria
Gammaproteobacteria
Betaproteobacteria
Alphaproteobacteria
Epsilonproteobacteria
Deltaproteobacteria
Magnetococcus
Elusimicrobia
Thermobaculum
Euryarchaeota
Crenarchaeota
Thaumarchaeota
Nanoarchaeota
Korarchaeota

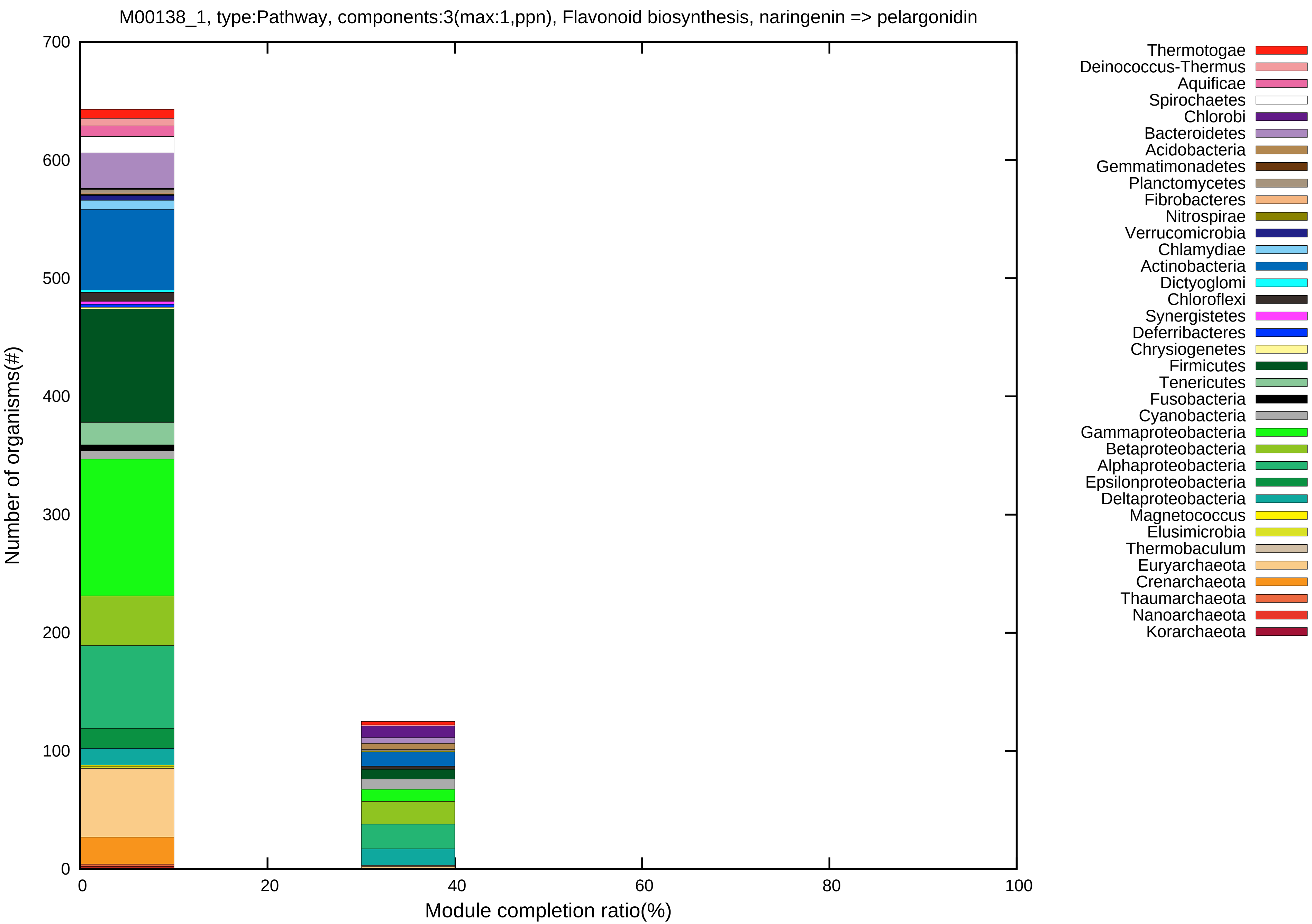

M00138_1, type:Pathway, components:3(max:1,ppn), Flavonoid biosynthesis, naringenin => pelargonidin
Number of organisms(#)
Module completion ratio(%)
0
100
200
300
400
500
600
700
0
20
40
60
80
100
Thermotogae
Deinococcus-Thermus
Aquificae
Spirochaetes
Chlorobi
Bacteroidetes
Acidobacteria
Gemmatimonadetes
Planctomycetes
Fibrobacteres
Nitrospirae
Verrucomicrobia
Chlamydiae
Actinobacteria
Dictyoglomi
Chloroflexi
Synergistetes
Deferribacteres
Chrysiogenetes
Firmicutes
Tenericutes
Fusobacteria
Cyanobacteria
Gammaproteobacteria
Betaproteobacteria
Alphaproteobacteria
Epsilonproteobacteria
Deltaproteobacteria
Magnetococcus
Elusimicrobia
Thermobaculum
Euryarchaeota
Crenarchaeota
Thaumarchaeota
Nanoarchaeota
Korarchaeota

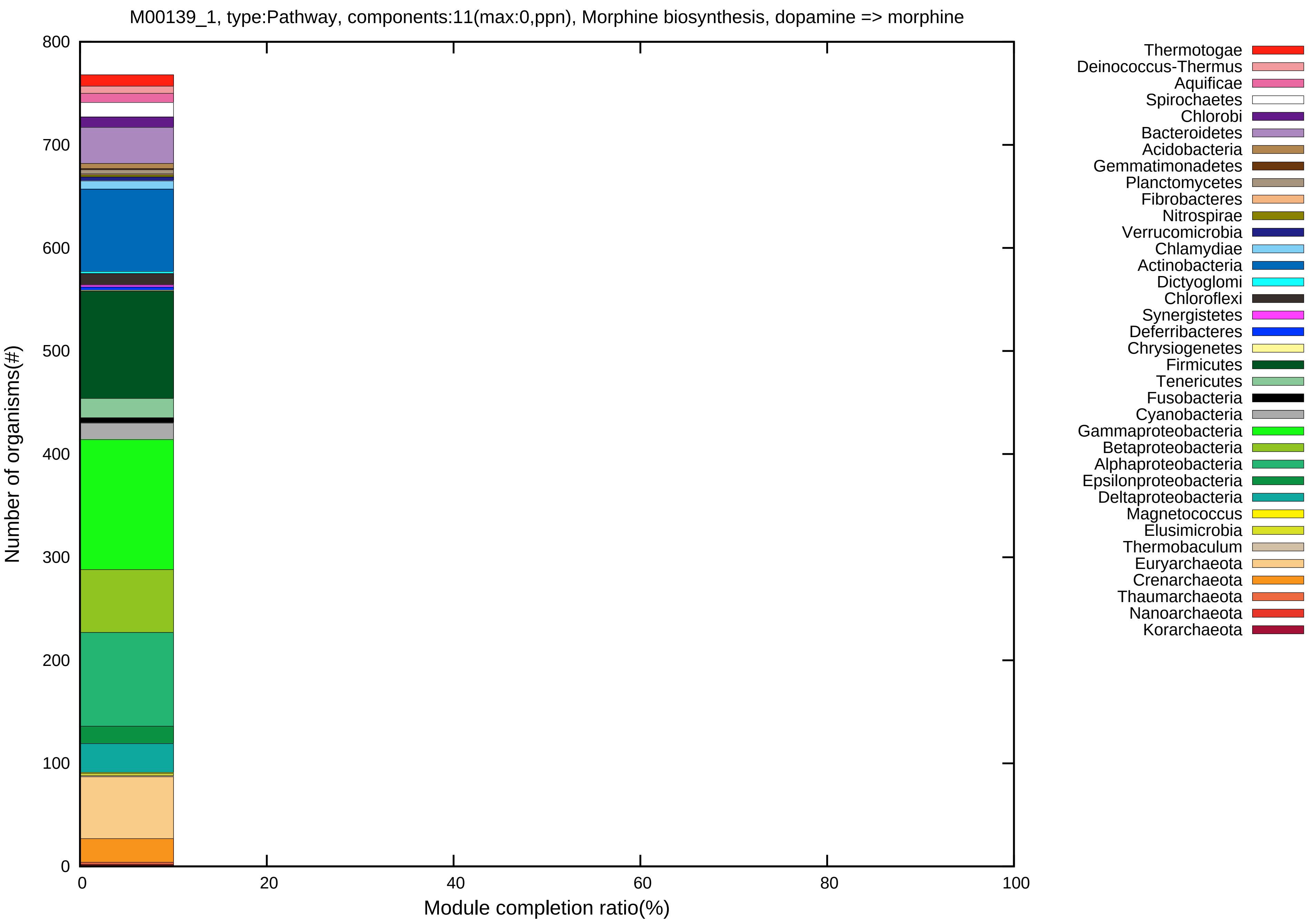

M00139_1, type:Pathway, components:11(max:0,ppn), Morphine biosynthesis, dopamine => morphine
Number of organisms(#)
Module completion ratio(%)
0
100
200
300
400
500
600
700
800
0
20
40
60
80
100
Thermotogae
Deinococcus-Thermus
Aquificae
Spirochaetes
Chlorobi
Bacteroidetes
Acidobacteria
Gemmatimonadetes
Planctomycetes
Fibrobacteres
Nitrospirae
Verrucomicrobia
Chlamydiae
Actinobacteria
Dictyoglomi
Chloroflexi
Synergistetes
Deferribacteres
Chrysiogenetes
Firmicutes
Tenericutes
Fusobacteria
Cyanobacteria
Gammaproteobacteria
Betaproteobacteria
Alphaproteobacteria
Epsilonproteobacteria
Deltaproteobacteria
Magnetococcus
Elusimicrobia
Thermobaculum
Euryarchaeota
Crenarchaeota
Thaumarchaeota
Nanoarchaeota
Korarchaeota

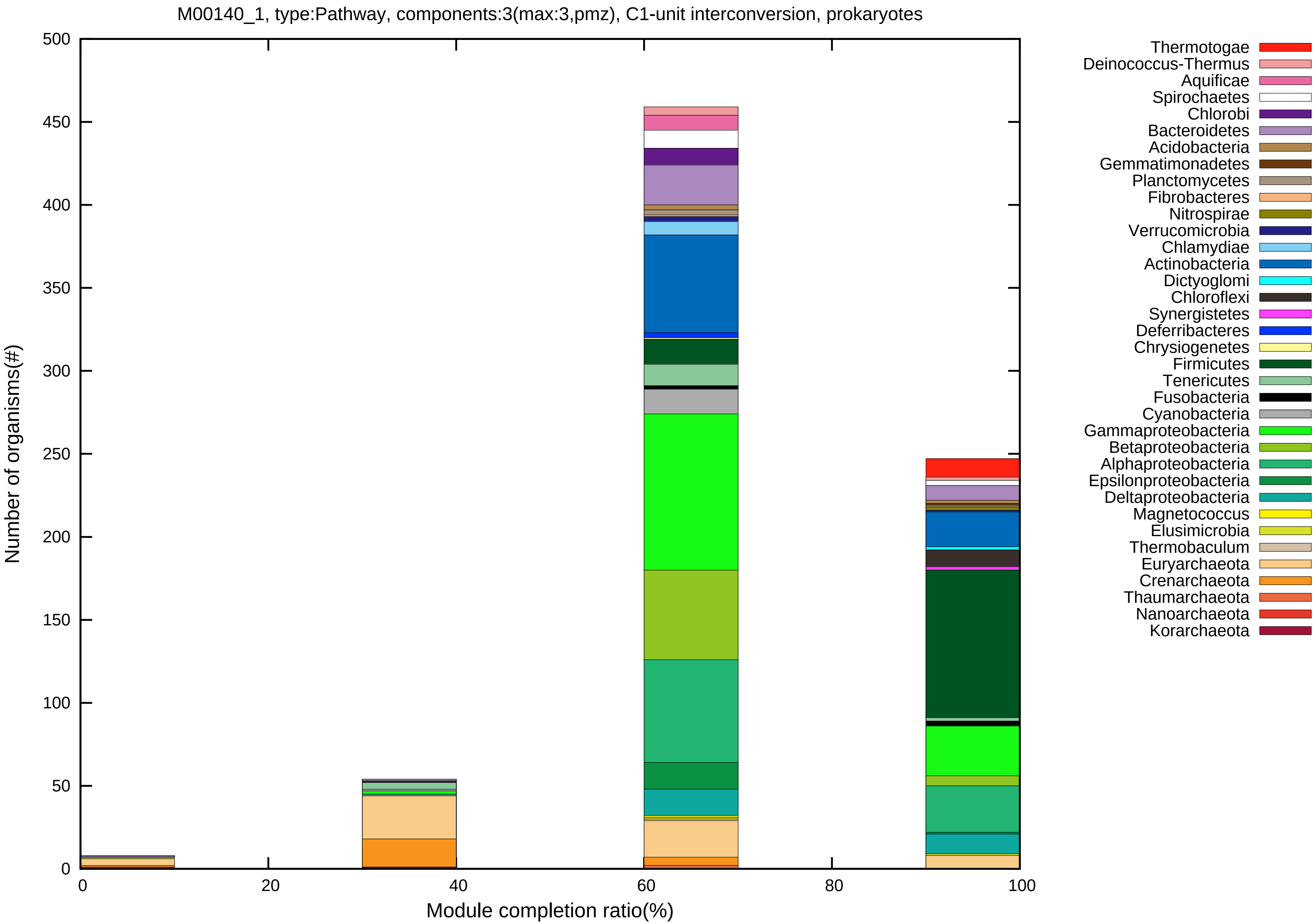
M00140_1, type:Pathway, components:3(max:3,pmz), C1-unit interconversion, prokaryotes
Number of organisms(#)
Module completion ratio(%)
0
50
100
150
200
250
300
350
400
450
500
0
20
40
60
80
100
Thermotogae
Deinococcus-Thermus
Aquificae
Spirochaetes
Chlorobi
Bacteroidetes
Acidobacteria
Gemmatimonadetes
Planctomycetes
Fibrobacteres
Nitrospirae
Verrucomicrobia
Chlamydiae
Actinobacteria
Dictyoglomi
Chloroflexi
Synergistetes
Deferribacteres
Chrysiogenetes
Firmicutes
Tenericutes
Fusobacteria
Cyanobacteria
Gammaproteobacteria
Betaproteobacteria
Alphaproteobacteria
Epsilonproteobacteria
Deltaproteobacteria
Magnetococcus
Elusimicrobia
Thermobaculum
Euryarchaeota
Crenarchaeota
Thaumarchaeota
Nanoarchaeota
Korarchaeota

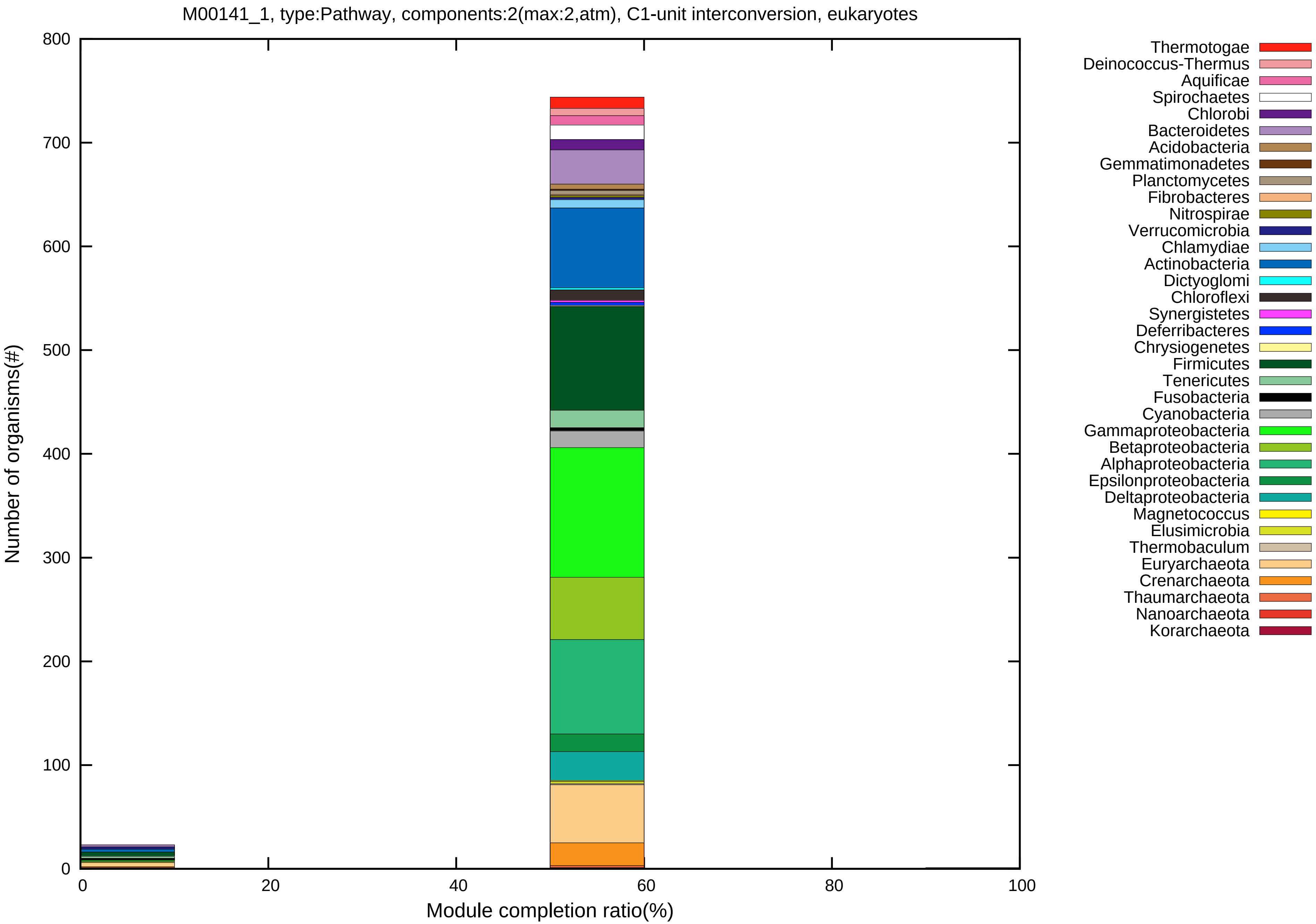
M00141_1, type:Pathway, components:2(max:2,atm), C1-unit interconversion, eukaryotes
Number of organisms(#)
Module completion ratio(%)
0
100
200
300
400
500
600
700
800
0
20
40
60
80
100
Thermotogae
Deinococcus-Thermus
Aquificae
Spirochaetes
Chlorobi
Bacteroidetes
Acidobacteria
Gemmatimonadetes
Planctomycetes
Fibrobacteres
Nitrospirae
Verrucomicrobia
Chlamydiae
Actinobacteria
Dictyoglomi
Chloroflexi
Synergistetes
Deferribacteres
Chrysiogenetes
Firmicutes
Tenericutes
Fusobacteria
Cyanobacteria
Gammaproteobacteria
Betaproteobacteria
Alphaproteobacteria
Epsilonproteobacteria
Deltaproteobacteria
Magnetococcus
Elusimicrobia
Thermobaculum
Euryarchaeota
Crenarchaeota
Thaumarchaeota
Nanoarchaeota
Korarchaeota

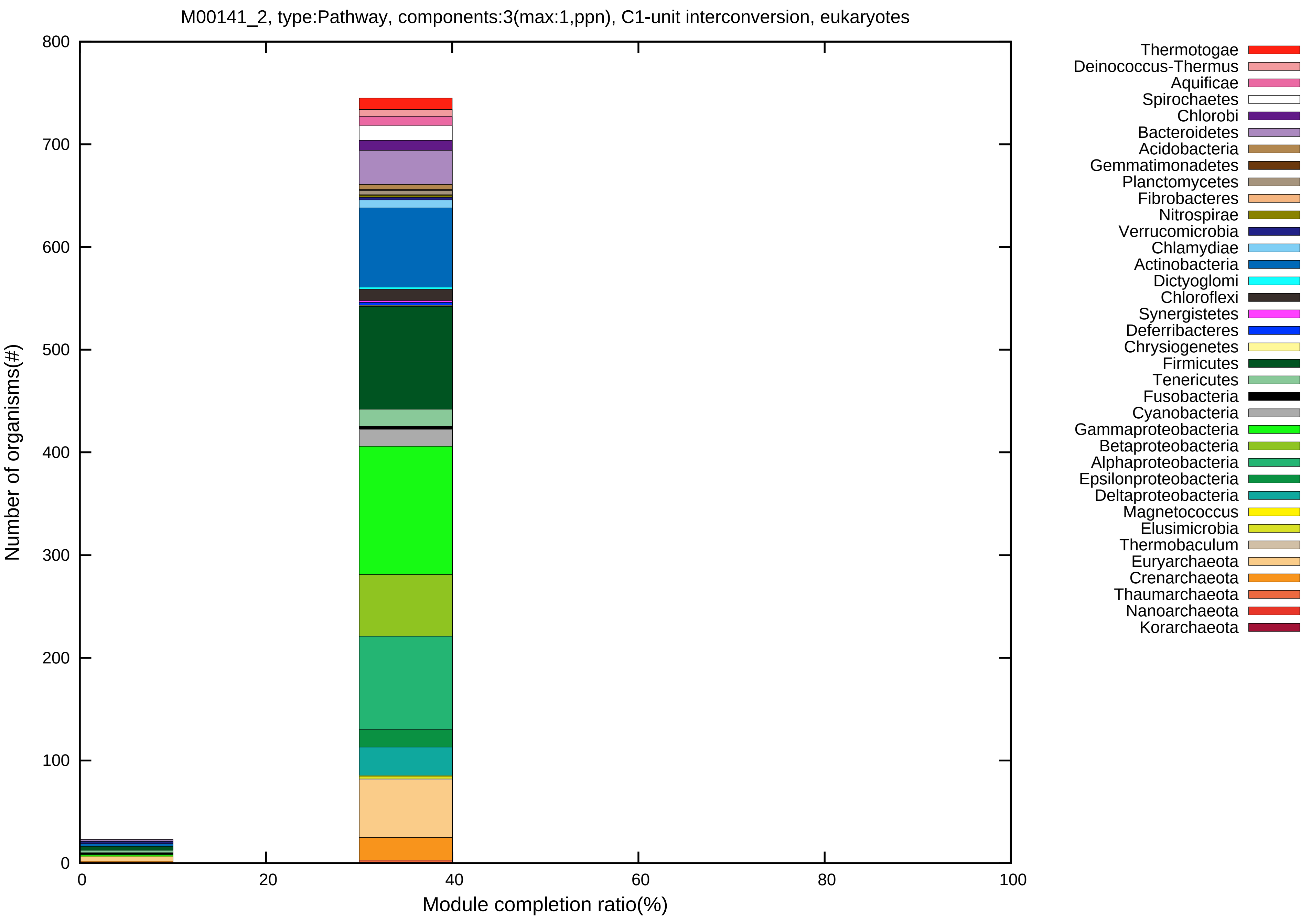
M00141_2, type:Pathway, components:3(max:1,ppn), C1-unit interconversion, eukaryotes
Number of organisms(#)
Module completion ratio(%)
0
100
200
300
400
500
600
700
800
0
20
40
60
80
100
Thermotogae
Deinococcus-Thermus
Aquificae
Spirochaetes
Chlorobi
Bacteroidetes
Acidobacteria
Gemmatimonadetes
Planctomycetes
Fibrobacteres
Nitrospirae
Verrucomicrobia
Chlamydiae
Actinobacteria
Dictyoglomi
Chloroflexi
Synergistetes
Deferribacteres
Chrysiogenetes
Firmicutes
Tenericutes
Fusobacteria
Cyanobacteria
Gammaproteobacteria
Betaproteobacteria
Alphaproteobacteria
Epsilonproteobacteria
Deltaproteobacteria
Magnetococcus
Elusimicrobia
Thermobaculum
Euryarchaeota
Crenarchaeota
Thaumarchaeota
Nanoarchaeota
Korarchaeota

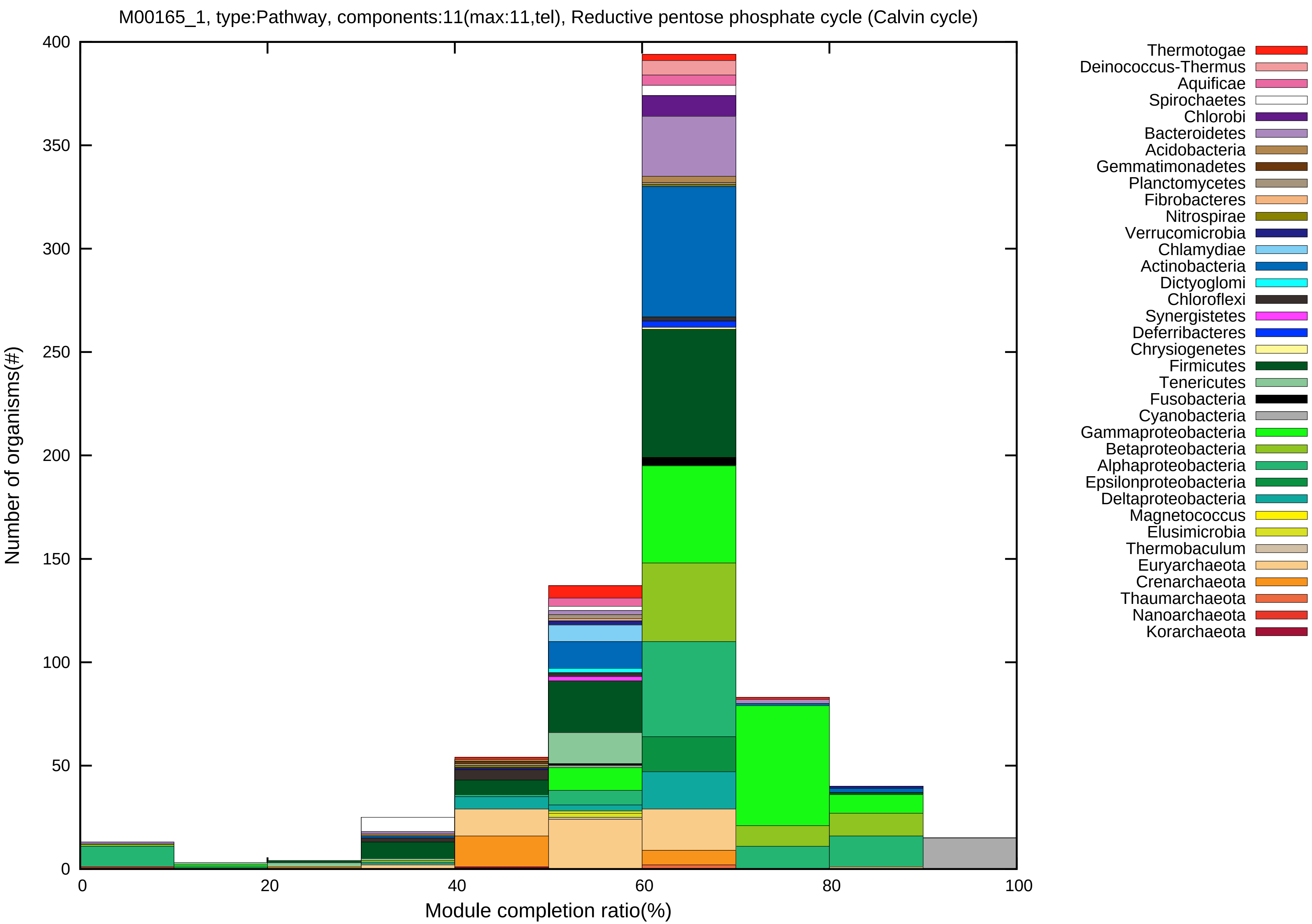

M00165_1, type:Pathway, components:11(max:11,tel), Reductive pentose phosphate cycle (Calvin cycle)
Number of organisms(#)
Module completion ratio(%)
0
50
100
150
200
250
300
350
400
0
20
40
60
80
100
Thermotogae
Deinococcus-Thermus
Aquificae
Spirochaetes
Chlorobi
Bacteroidetes
Acidobacteria
Gemmatimonadetes
Planctomycetes
Fibrobacteres
Nitrospirae
Verrucomicrobia
Chlamydiae
Actinobacteria
Dictyoglomi
Chloroflexi
Synergistetes
Deferribacteres
Chrysiogenetes
Firmicutes
Tenericutes
Fusobacteria
Cyanobacteria
Gammaproteobacteria
Betaproteobacteria
Alphaproteobacteria
Epsilonproteobacteria
Deltaproteobacteria
Magnetococcus
Elusimicrobia
Thermobaculum
Euryarchaeota
Crenarchaeota
Thaumarchaeota
Nanoarchaeota
Korarchaeota

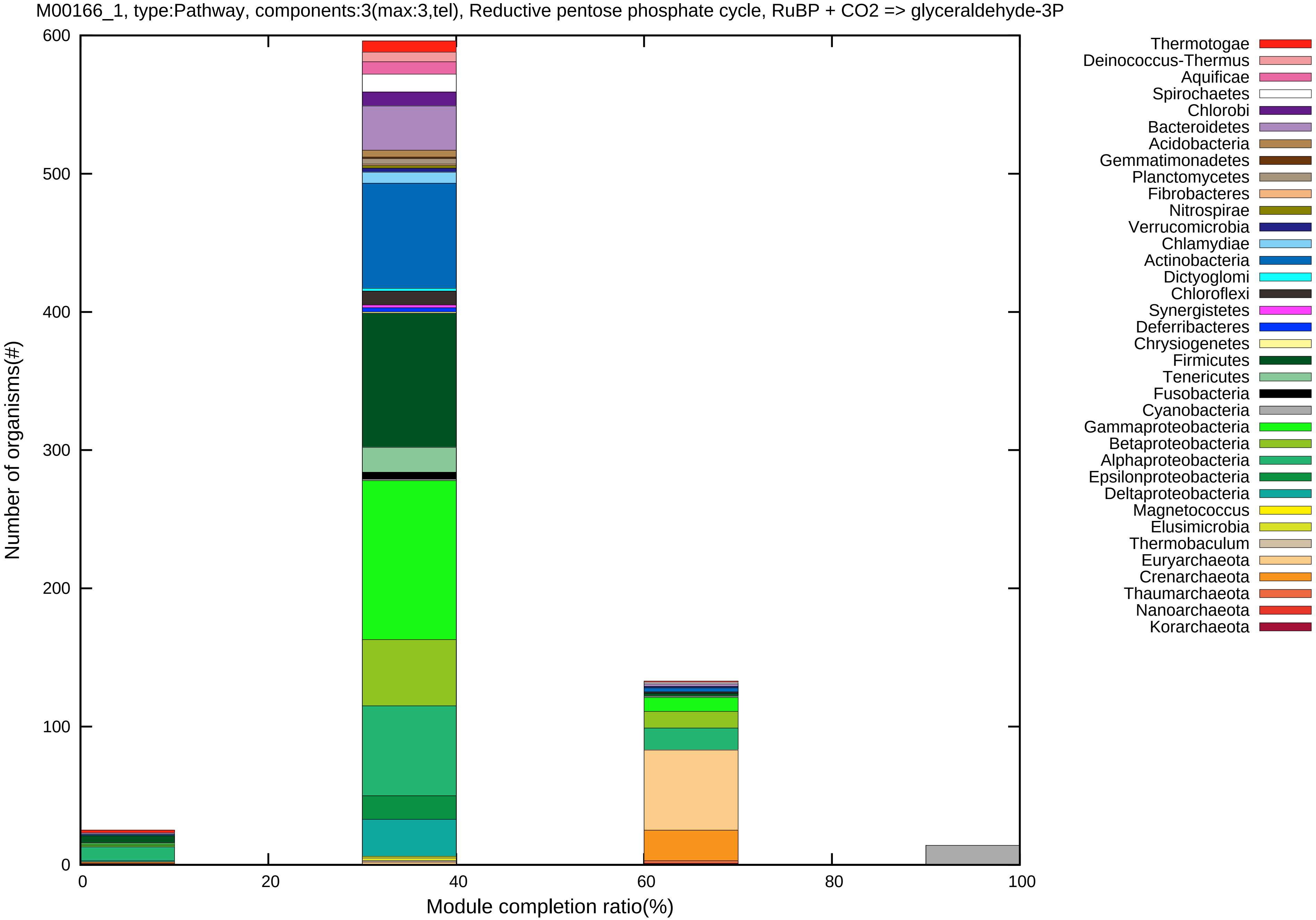

M00166_1, type:Pathway, components:3(max:3,tel), Reductive pentose phosphate cycle, RuBP + CO2 => glyceraldehyde-3P
Number of organisms(#)
Module completion ratio(%)
0
100
200
300
400
500
600
0
20
40
60
80
100
Thermotogae
Deinococcus-Thermus
Aquificae
Spirochaetes
Chlorobi
Bacteroidetes
Acidobacteria
Gemmatimonadetes
Planctomycetes
Fibrobacteres
Nitrospirae
Verrucomicrobia
Chlamydiae
Actinobacteria
Dictyoglomi
Chloroflexi
Synergistetes
Deferribacteres
Chrysiogenetes
Firmicutes
Tenericutes
Fusobacteria
Cyanobacteria
Gammaproteobacteria
Betaproteobacteria
Alphaproteobacteria
Epsilonproteobacteria
Deltaproteobacteria
Magnetococcus
Elusimicrobia
Thermobaculum
Euryarchaeota
Crenarchaeota
Thaumarchaeota
Nanoarchaeota
Korarchaeota

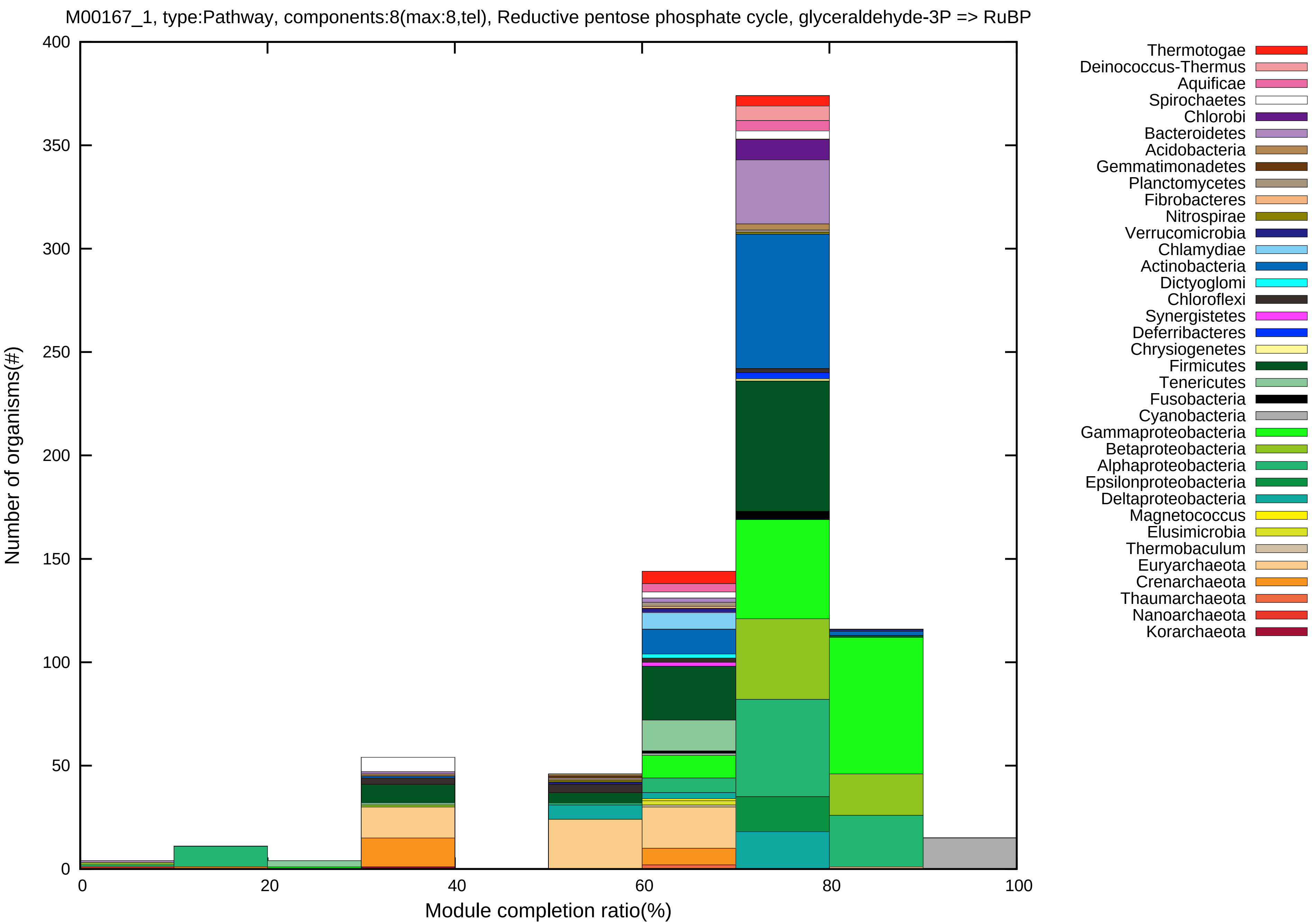
M00167_1, type:Pathway, components:8(max:8,tel), Reductive pentose phosphate cycle, glyceraldehyde-3P => RuBP
Number of organisms(#)
Module completion ratio(%)
0
50
100
150
200
250
300
350
400
0
20
40
60
80
100
Thermotogae
Deinococcus-Thermus
Aquificae
Spirochaetes
Chlorobi
Bacteroidetes
Acidobacteria
Gemmatimonadetes
Planctomycetes
Fibrobacteres
Nitrospirae
Verrucomicrobia
Chlamydiae
Actinobacteria
Dictyoglomi
Chloroflexi
Synergistetes
Deferribacteres
Chrysiogenetes
Firmicutes
Tenericutes
Fusobacteria
Cyanobacteria
Gammaproteobacteria
Betaproteobacteria
Alphaproteobacteria
Epsilonproteobacteria
Deltaproteobacteria
Magnetococcus
Elusimicrobia
Thermobaculum
Euryarchaeota
Crenarchaeota
Thaumarchaeota
Nanoarchaeota
Korarchaeota

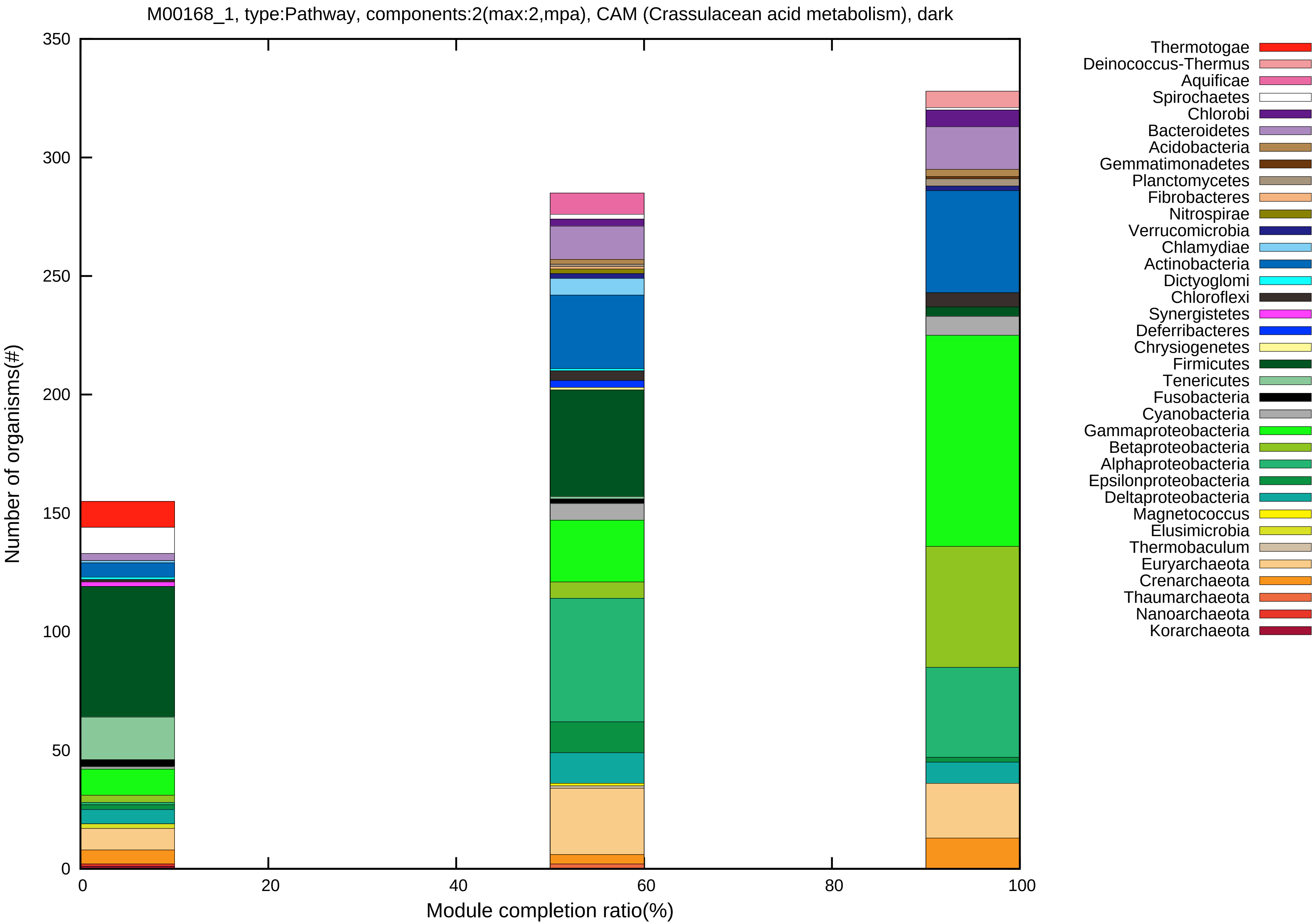

M00168_1, type:Pathway, components:2(max:2,mpa), CAM (Crassulacean acid metabolism), dark
Number of organisms(#)
Module completion ratio(%)
0
50
100
150
200
250
300
350
0
20
40
60
80
100
Thermotogae
Deinococcus-Thermus
Aquificae
Spirochaetes
Chlorobi
Bacteroidetes
Acidobacteria
Gemmatimonadetes
Planctomycetes
Fibrobacteres
Nitrospirae
Verrucomicrobia
Chlamydiae
Actinobacteria
Dictyoglomi
Chloroflexi
Synergistetes
Deferribacteres
Chrysiogenetes
Firmicutes
Tenericutes
Fusobacteria
Cyanobacteria
Gammaproteobacteria
Betaproteobacteria
Alphaproteobacteria
Epsilonproteobacteria
Deltaproteobacteria
Magnetococcus
Elusimicrobia
Thermobaculum
Euryarchaeota
Crenarchaeota
Thaumarchaeota
Nanoarchaeota
Korarchaeota

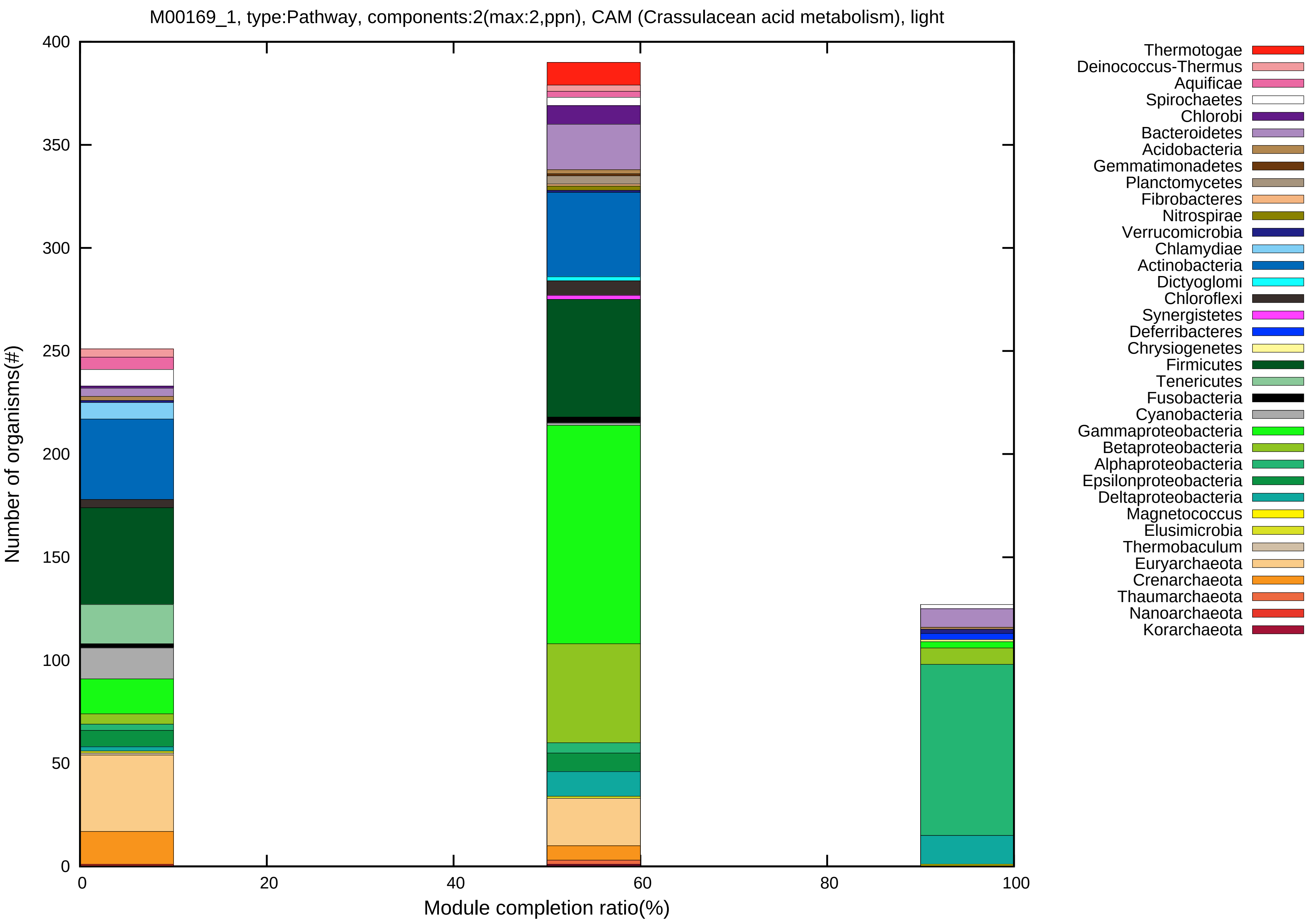
M00169_1, type:Pathway, components:2(max:2,ppn), CAM (Crassulacean acid metabolism), light
Number of organisms(#)
Module completion ratio(%)
0
50
100
150
200
250
300
350
400
0
20
40
60
80
100
Thermotogae
Deinococcus-Thermus
Aquificae
Spirochaetes
Chlorobi
Bacteroidetes
Acidobacteria
Gemmatimonadetes
Planctomycetes
Fibrobacteres
Nitrospirae
Verrucomicrobia
Chlamydiae
Actinobacteria
Dictyoglomi
Chloroflexi
Synergistetes
Deferribacteres
Chrysiogenetes
Firmicutes
Tenericutes
Fusobacteria
Cyanobacteria
Gammaproteobacteria
Betaproteobacteria
Alphaproteobacteria
Epsilonproteobacteria
Deltaproteobacteria
Magnetococcus
Elusimicrobia
Thermobaculum
Euryarchaeota
Crenarchaeota
Thaumarchaeota
Nanoarchaeota
Korarchaeota

M00170_1, type:Pathway, components:3(max:2,fjo), C4-dicarboxylic acid cycle, phosphoenolpyruvate carboxykinase type

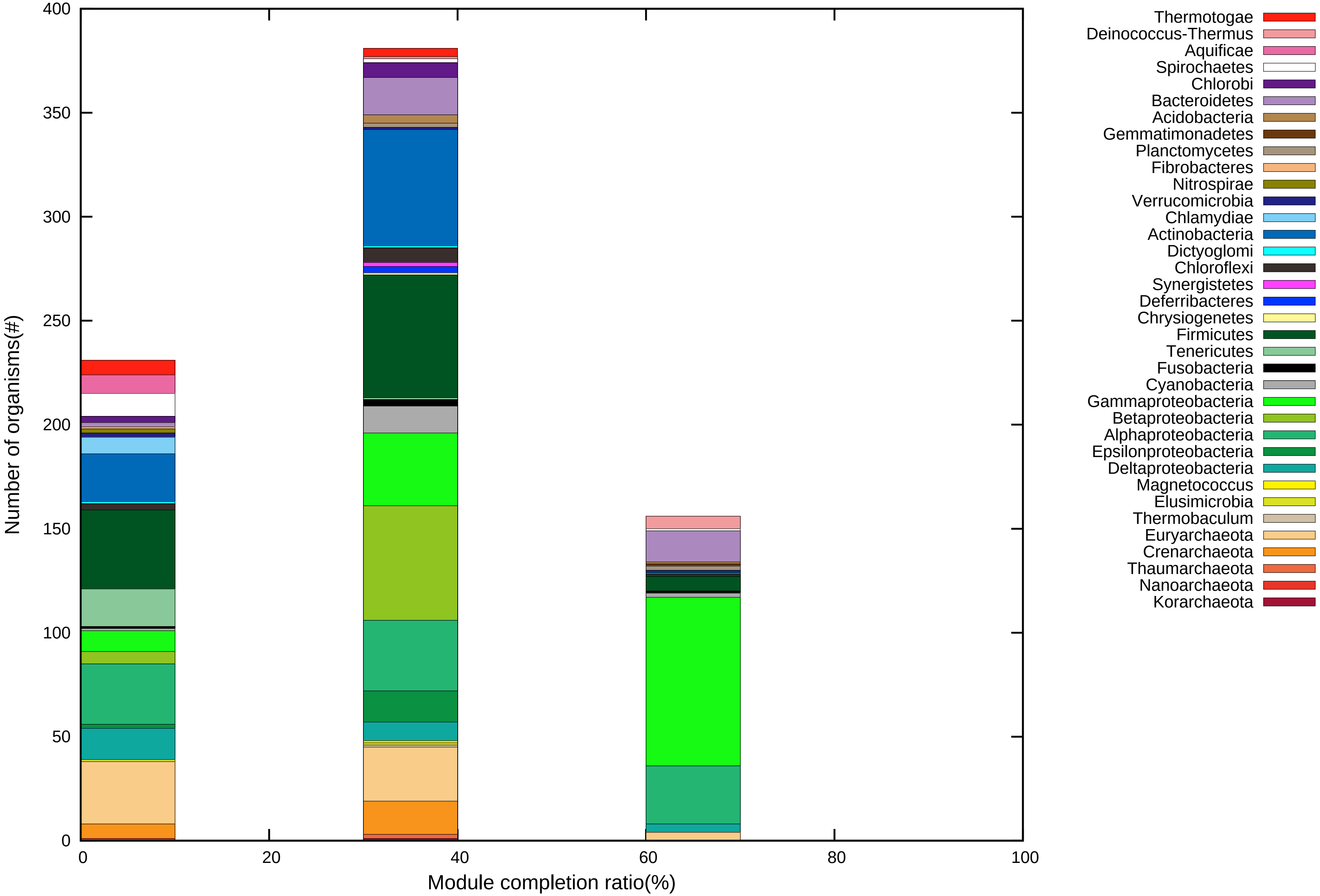

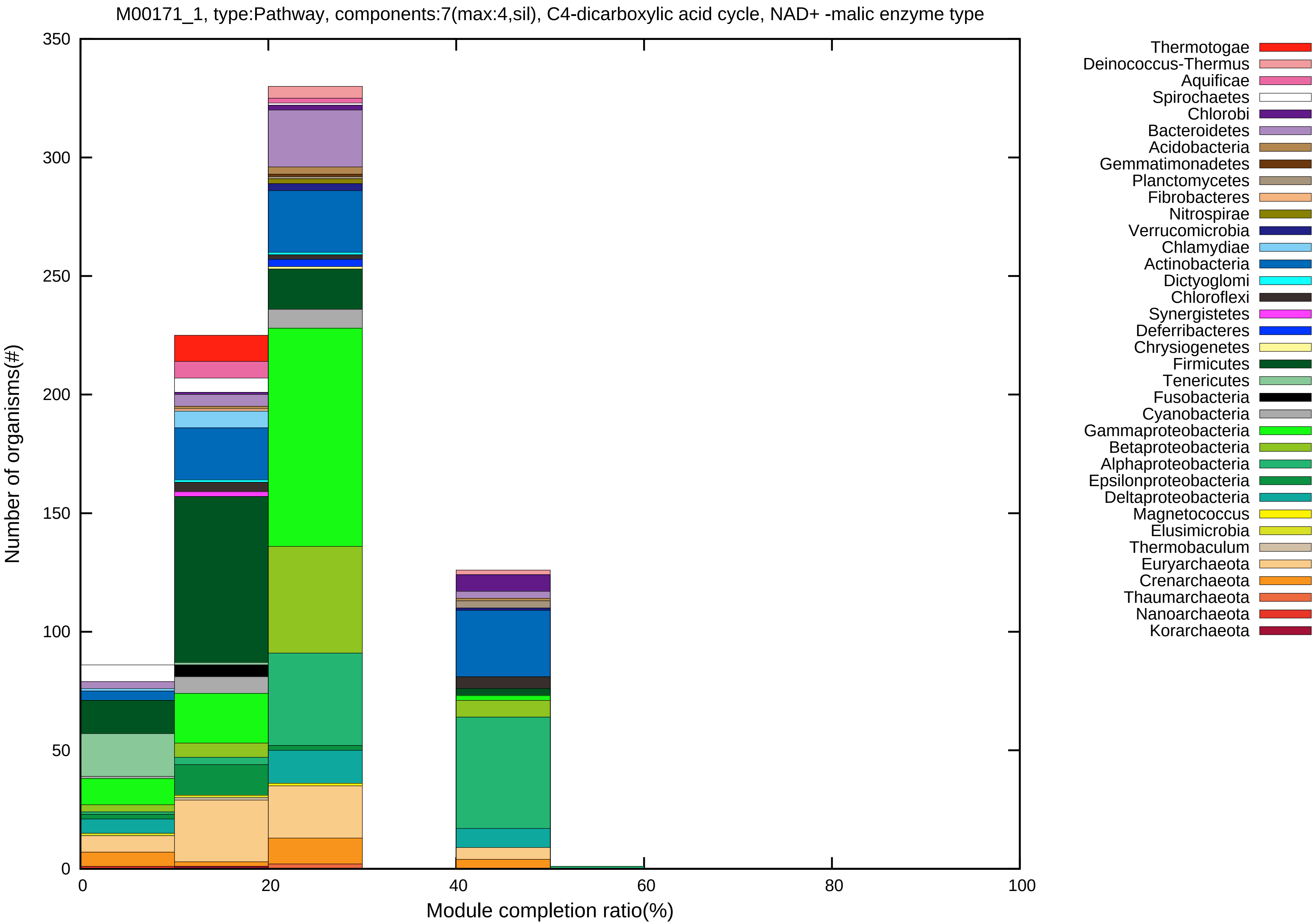

M00171_1, type:Pathway, components:7(max:4,sil), C4-dicarboxylic acid cycle, NAD+ -malic enzyme type
Number of organisms(#)
Module completion ratio(%)
0
50
100
150
200
250
300
350
0
20
40
60
80
100
Thermotogae
Deinococcus-Thermus
Aquificae
Spirochaetes
Chlorobi
Bacteroidetes
Acidobacteria
Gemmatimonadetes
Planctomycetes
Fibrobacteres
Nitrospirae
Verrucomicrobia
Chlamydiae
Actinobacteria
Dictyoglomi
Chloroflexi
Synergistetes
Deferribacteres
Chrysiogenetes
Firmicutes
Tenericutes
Fusobacteria
Cyanobacteria
Gammaproteobacteria
Betaproteobacteria
Alphaproteobacteria
Epsilonproteobacteria
Deltaproteobacteria
Magnetococcus
Elusimicrobia
Thermobaculum
Euryarchaeota
Crenarchaeota
Thaumarchaeota
Nanoarchaeota
Korarchaeota

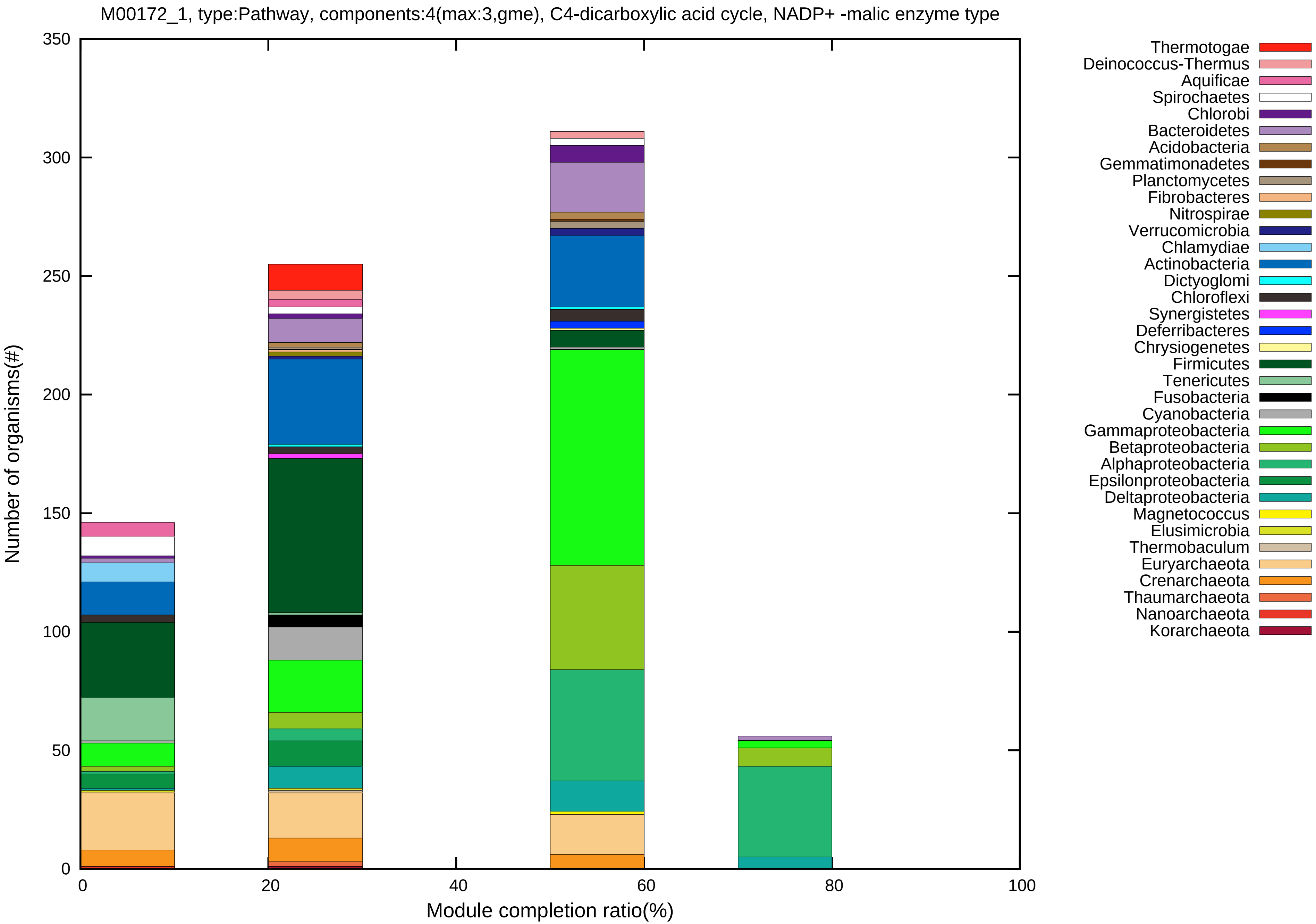

M00172_1, type:Pathway, components:4(max:3,gme), C4-dicarboxylic acid cycle, NADP+ -malic enzyme type
Number of organisms(#)
Module completion ratio(%)
0
50
100
150
200
250
300
350
0
20
40
60
80
100
Thermotogae
Deinococcus-Thermus
Aquificae
Spirochaetes
Chlorobi
Bacteroidetes
Acidobacteria
Gemmatimonadetes
Planctomycetes
Fibrobacteres
Nitrospirae
Verrucomicrobia
Chlamydiae
Actinobacteria
Dictyoglomi
Chloroflexi
Synergistetes
Deferribacteres
Chrysiogenetes
Firmicutes
Tenericutes
Fusobacteria
Cyanobacteria
Gammaproteobacteria
Betaproteobacteria
Alphaproteobacteria
Epsilonproteobacteria
Deltaproteobacteria
Magnetococcus
Elusimicrobia
Thermobaculum
Euryarchaeota
Crenarchaeota
Thaumarchaeota
Nanoarchaeota
Korarchaeota

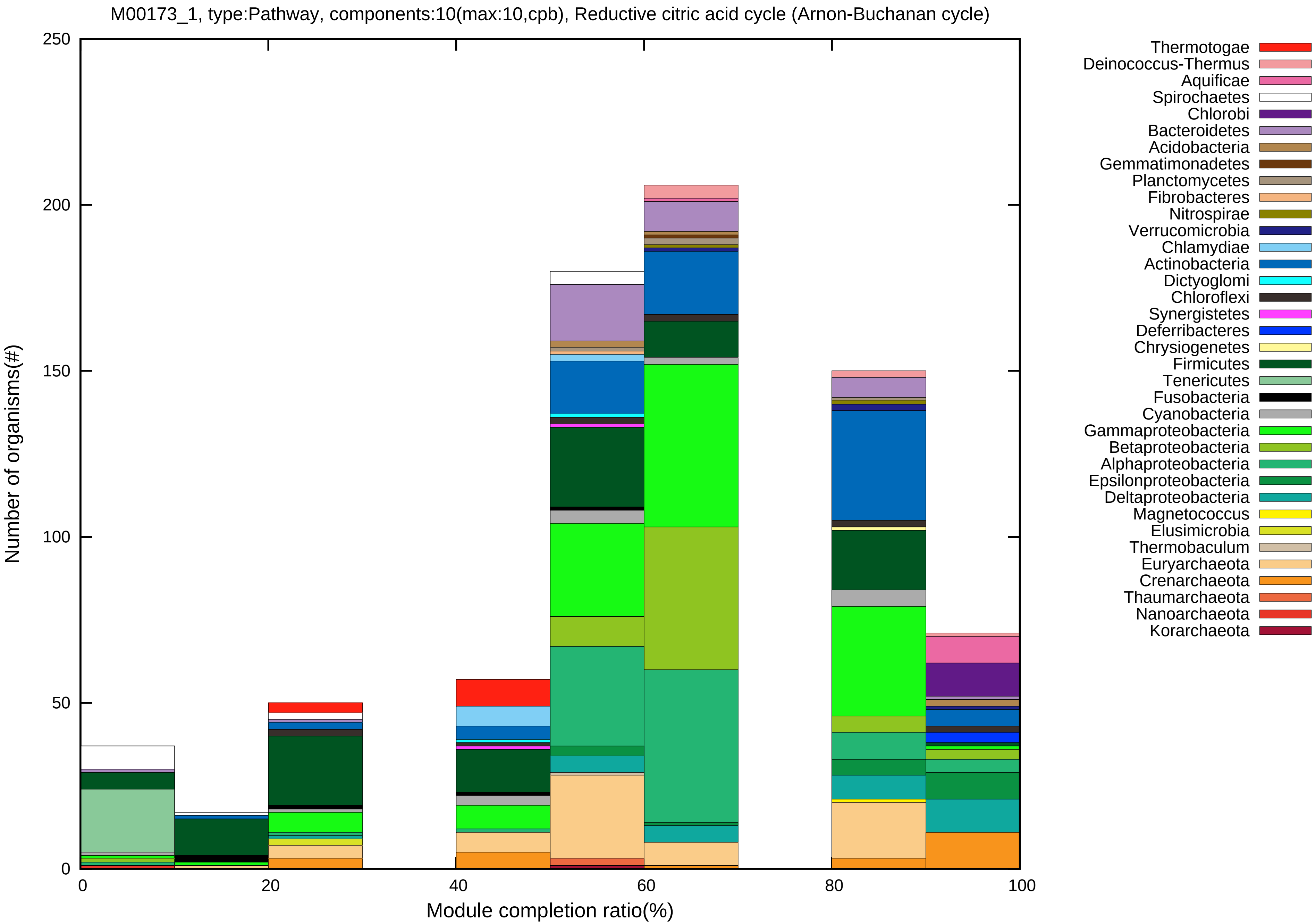

M00173_1, type:Pathway, components:10(max:10,cpb), Reductive citric acid cycle (Arnon-Buchanan cycle)
Number of organisms(#)
Module completion ratio(%)
0
50
100
150
200
250
0
20
40
60
80
100
Thermotogae
Deinococcus-Thermus
Aquificae
Spirochaetes
Chlorobi
Bacteroidetes
Acidobacteria
Gemmatimonadetes
Planctomycetes
Fibrobacteres
Nitrospirae
Verrucomicrobia
Chlamydiae
Actinobacteria
Dictyoglomi
Chloroflexi
Synergistetes
Deferribacteres
Chrysiogenetes
Firmicutes
Tenericutes
Fusobacteria
Cyanobacteria
Gammaproteobacteria
Betaproteobacteria
Alphaproteobacteria
Epsilonproteobacteria
Deltaproteobacteria
Magnetococcus
Elusimicrobia
Thermobaculum
Euryarchaeota
Crenarchaeota
Thaumarchaeota
Nanoarchaeota
Korarchaeota

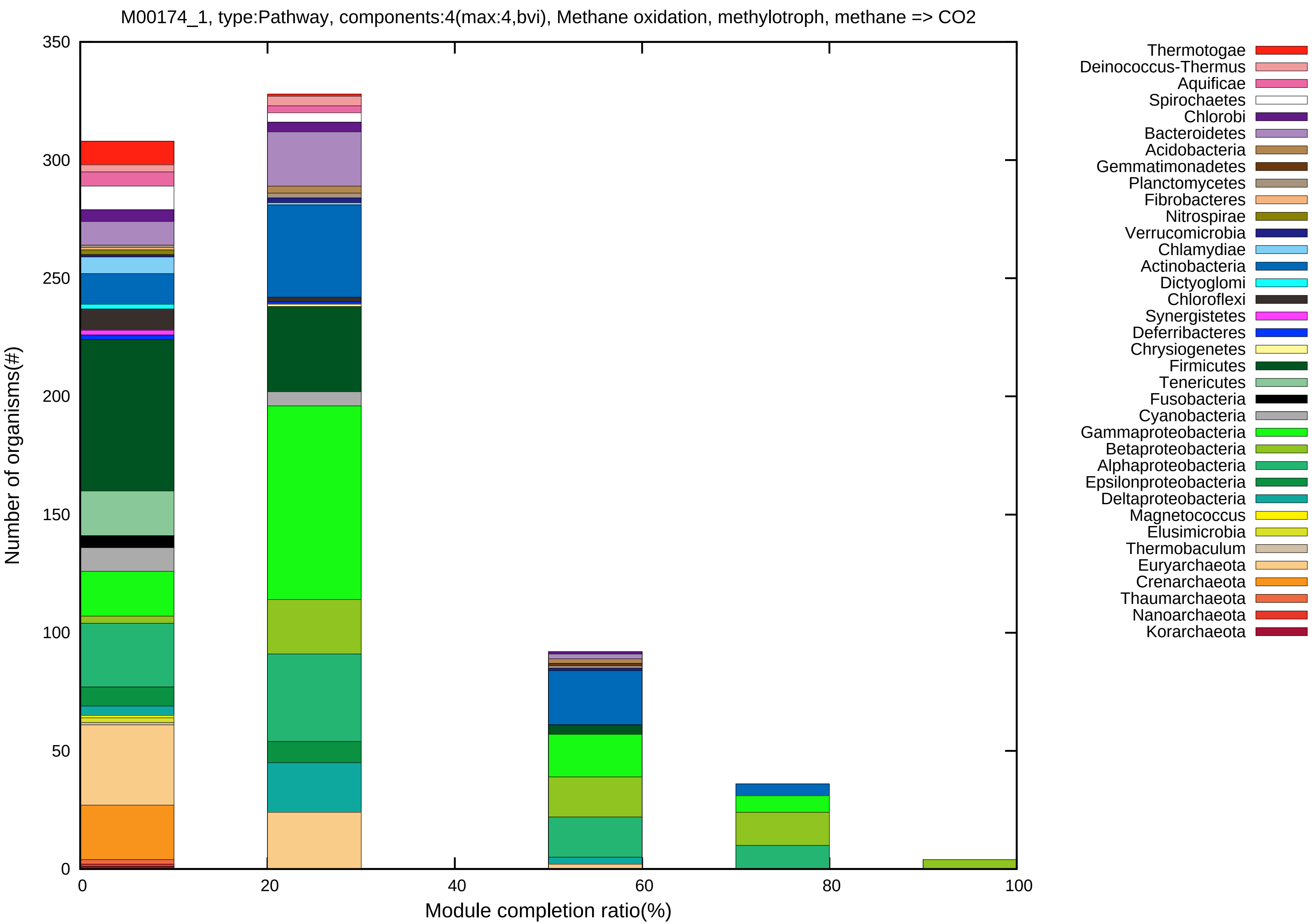
M00174_1, type:Pathway, components:4(max:4,bvi), Methane oxidation, methylotroph, methane => CO2
Number of organisms(#)
Module completion ratio(%)
0
50
100
150
200
250
300
350
0
20
40
60
80
100
Thermotogae
Deinococcus-Thermus
Aquificae
Spirochaetes
Chlorobi
Bacteroidetes
Acidobacteria
Gemmatimonadetes
Planctomycetes
Fibrobacteres
Nitrospirae
Verrucomicrobia
Chlamydiae
Actinobacteria
Dictyoglomi
Chloroflexi
Synergistetes
Deferribacteres
Chrysiogenetes
Firmicutes
Tenericutes
Fusobacteria
Cyanobacteria
Gammaproteobacteria
Betaproteobacteria
Alphaproteobacteria
Epsilonproteobacteria
Deltaproteobacteria
Magnetococcus
Elusimicrobia
Thermobaculum
Euryarchaeota
Crenarchaeota
Thaumarchaeota
Nanoarchaeota
Korarchaeota

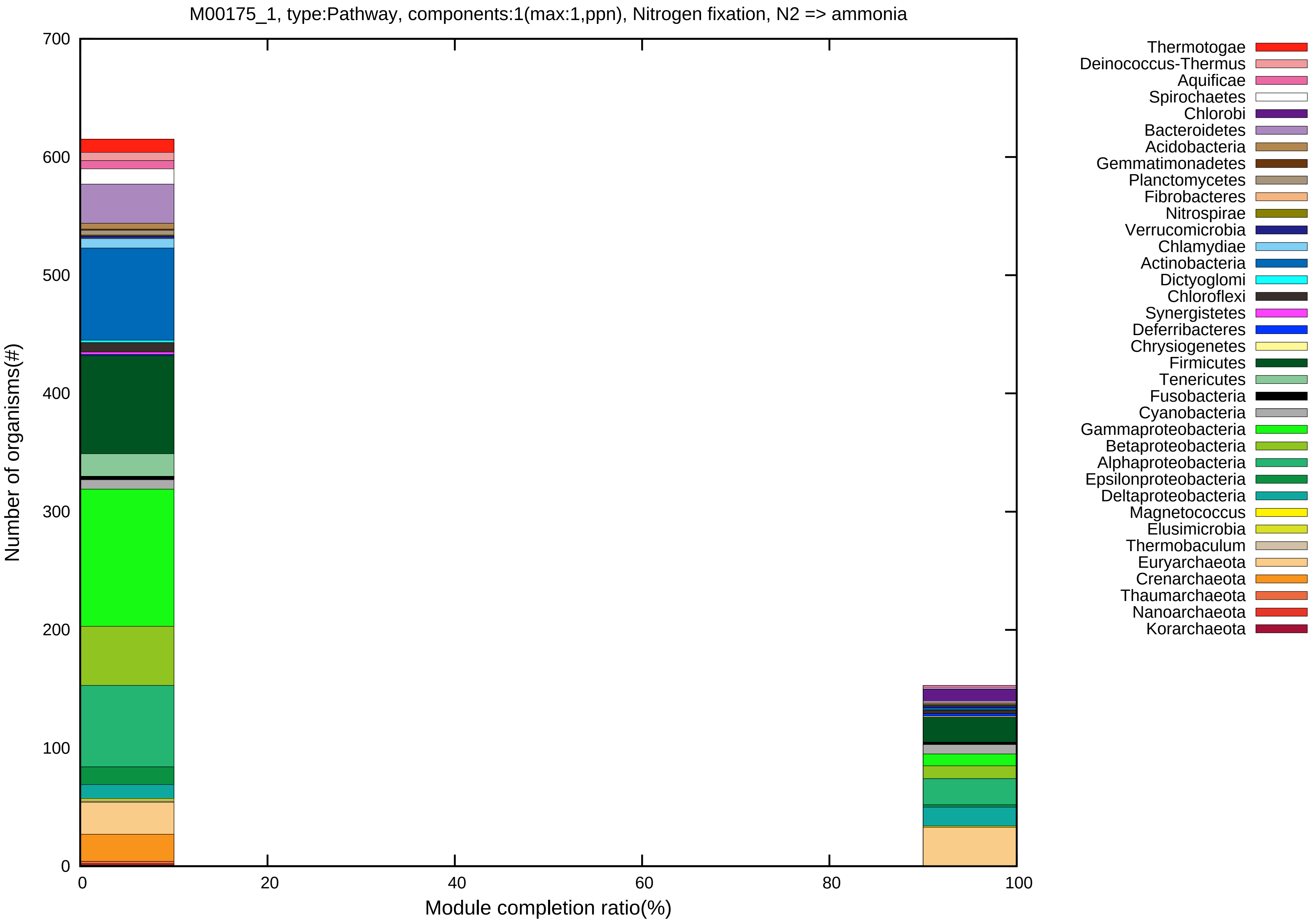

M00175_1, type:Pathway, components:1(max:1,ppn), Nitrogen fixation, N2 => ammonia
Number of organisms(#)
Module completion ratio(%)
0
100
200
300
400
500
600
700
0
20
40
60
80
100
Thermotogae
Deinococcus-Thermus
Aquificae
Spirochaetes
Chlorobi
Bacteroidetes
Acidobacteria
Gemmatimonadetes
Planctomycetes
Fibrobacteres
Nitrospirae
Verrucomicrobia
Chlamydiae
Actinobacteria
Dictyoglomi
Chloroflexi
Synergistetes
Deferribacteres
Chrysiogenetes
Firmicutes
Tenericutes
Fusobacteria
Cyanobacteria
Gammaproteobacteria
Betaproteobacteria
Alphaproteobacteria
Epsilonproteobacteria
Deltaproteobacteria
Magnetococcus
Elusimicrobia
Thermobaculum
Euryarchaeota
Crenarchaeota
Thaumarchaeota
Nanoarchaeota
Korarchaeota

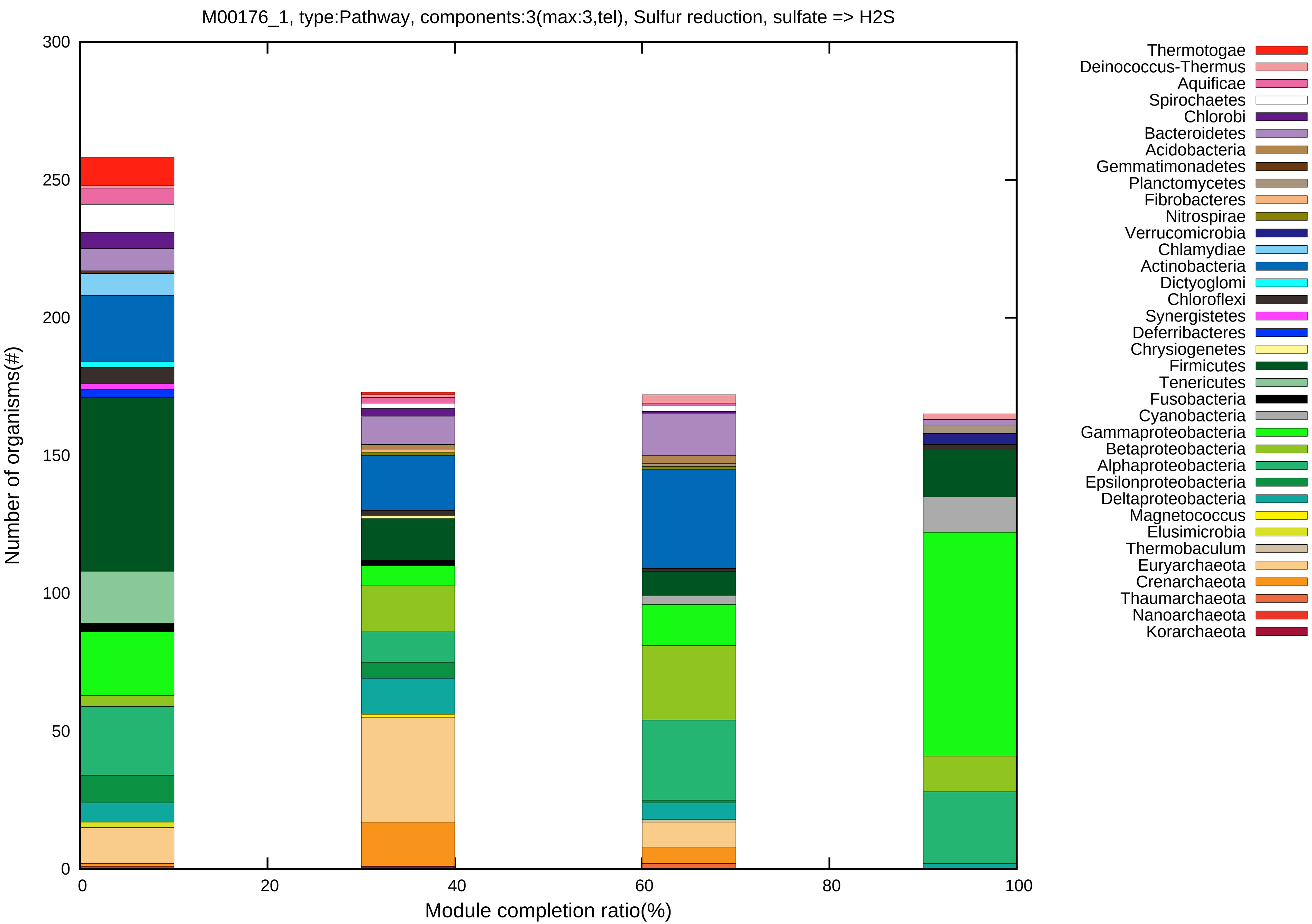
M00176_1, type:Pathway, components:3(max:3,tel), Sulfur reduction, sulfate => H2S
Number of organisms(#)
Module completion ratio(%)
0
50
100
150
200
250
300
0
20
40
60
80
100
Thermotogae
Deinococcus-Thermus
Aquificae
Spirochaetes
Chlorobi
Bacteroidetes
Acidobacteria
Gemmatimonadetes
Planctomycetes
Fibrobacteres
Nitrospirae
Verrucomicrobia
Chlamydiae
Actinobacteria
Dictyoglomi
Chloroflexi
Synergistetes
Deferribacteres
Chrysiogenetes
Firmicutes
Tenericutes
Fusobacteria
Cyanobacteria
Gammaproteobacteria
Betaproteobacteria
Alphaproteobacteria
Epsilonproteobacteria
Deltaproteobacteria
Magnetococcus
Elusimicrobia
Thermobaculum
Euryarchaeota
Crenarchaeota
Thaumarchaeota
Nanoarchaeota
Korarchaeota

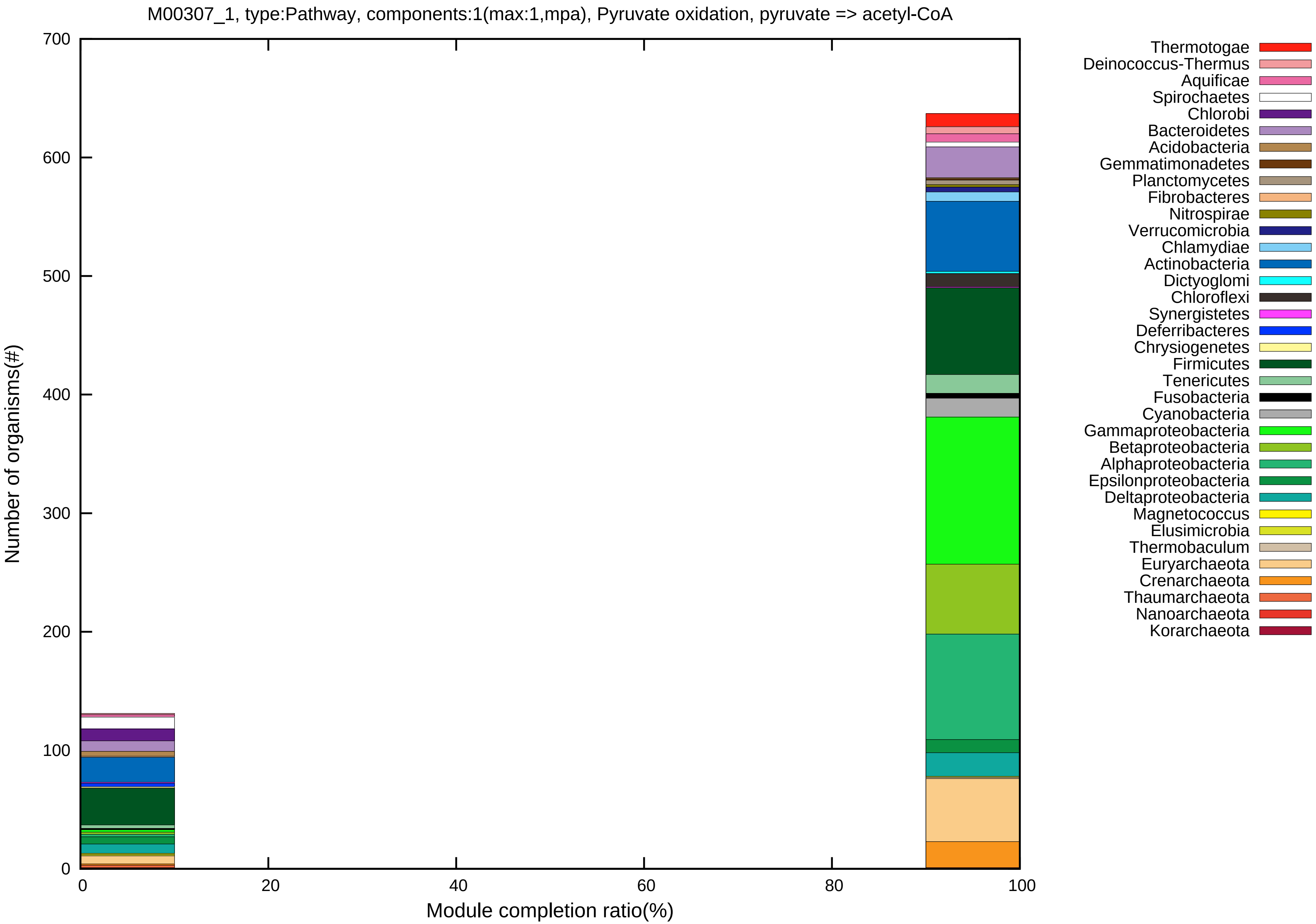

M00307_1, type:Pathway, components:1(max:1,mpa), Pyruvate oxidation, pyruvate => acetyl-CoA
Number of organisms(#)
Module completion ratio(%)
0
100
200
300
400
500
600
700
0
20
40
60
80
100
Thermotogae
Deinococcus-Thermus
Aquificae
Spirochaetes
Chlorobi
Bacteroidetes
Acidobacteria
Gemmatimonadetes
Planctomycetes
Fibrobacteres
Nitrospirae
Verrucomicrobia
Chlamydiae
Actinobacteria
Dictyoglomi
Chloroflexi
Synergistetes
Deferribacteres
Chrysiogenetes
Firmicutes
Tenericutes
Fusobacteria
Cyanobacteria
Gammaproteobacteria
Betaproteobacteria
Alphaproteobacteria
Epsilonproteobacteria
Deltaproteobacteria
Magnetococcus
Elusimicrobia
Thermobaculum
Euryarchaeota
Crenarchaeota
Thaumarchaeota
Nanoarchaeota
Korarchaeota

M00308_1, type:Pathway, components:3(max:3,hmu), Semi-phosphorylative Entner-Doudoroff pathway, gluconate => glyceraldehyde-3P + pyruvate

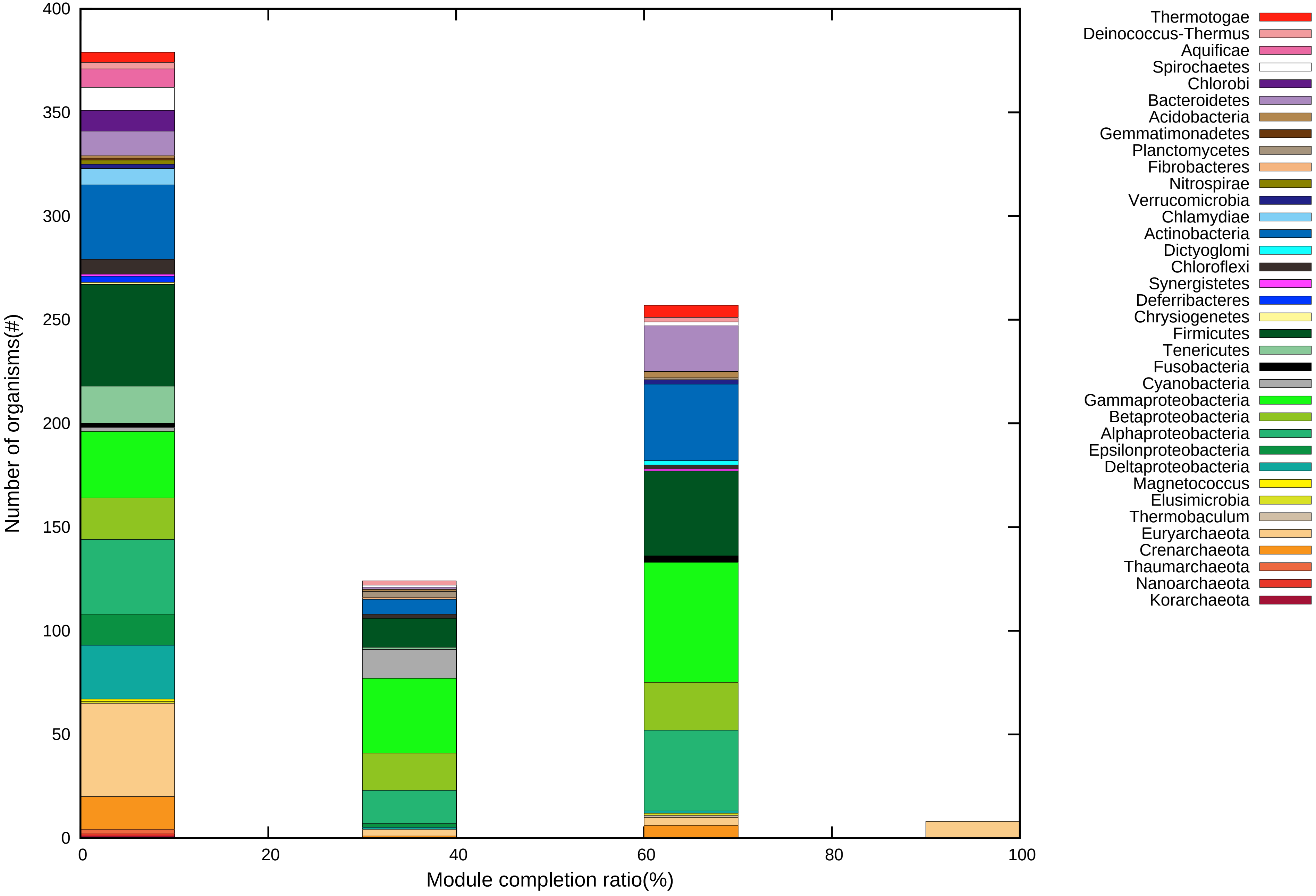

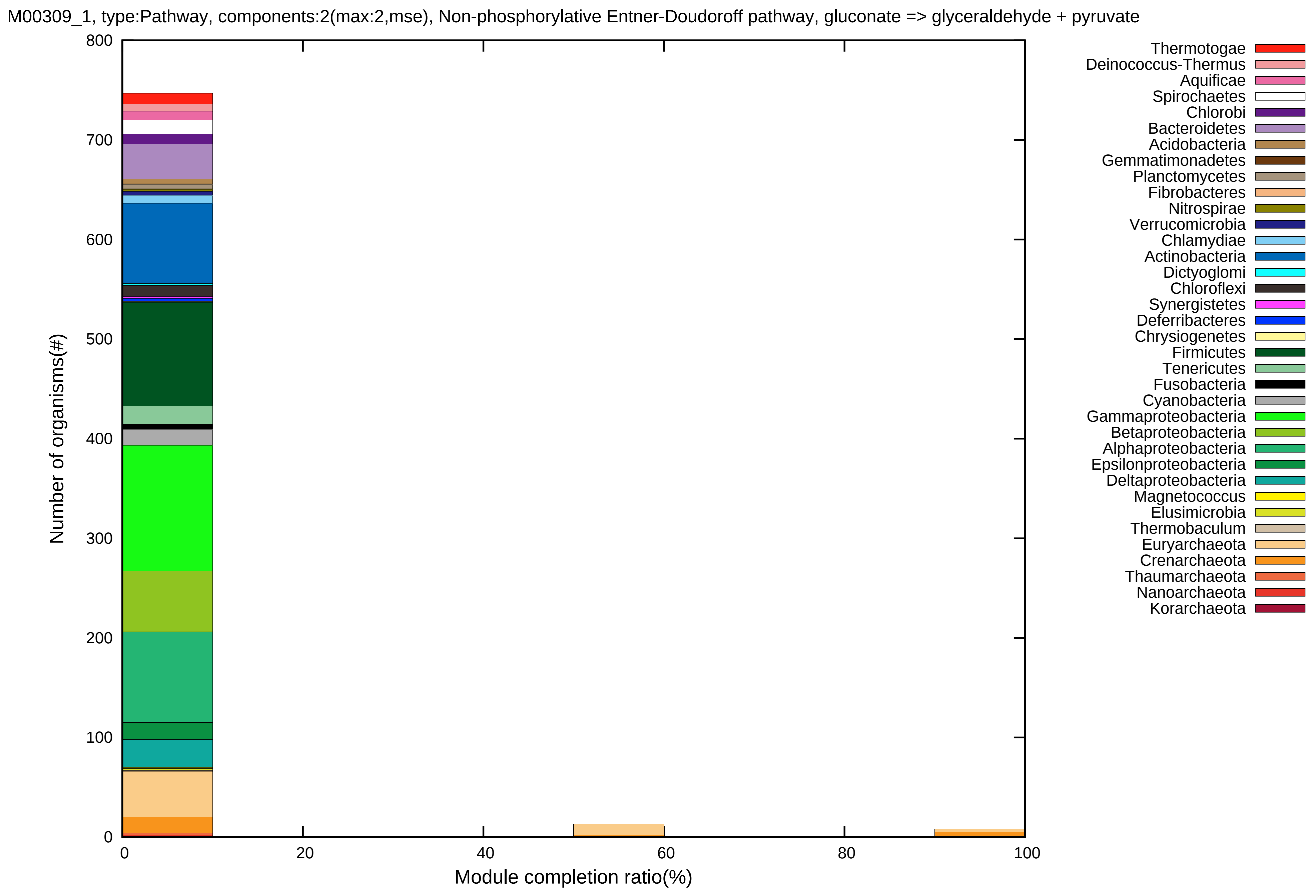

M00309_1, type:Pathway, components:2(max:2,mse), Non-phosphorylative Entner-Doudoroff pathway, gluconate => glyceraldehyde + pyruvate
Number of organisms(#)
Module completion ratio(%)
0
100
200
300
400
500
600
700
800
0
20
40
60
80
100
Thermotogae
Deinococcus-Thermus
Aquificae
Spirochaetes
Chlorobi
Bacteroidetes
Acidobacteria
Gemmatimonadetes
Planctomycetes
Fibrobacteres
Nitrospirae
Verrucomicrobia
Chlamydiae
Actinobacteria
Dictyoglomi
Chloroflexi
Synergistetes
Deferribacteres
Chrysiogenetes
Firmicutes
Tenericutes
Fusobacteria
Cyanobacteria
Gammaproteobacteria
Betaproteobacteria
Alphaproteobacteria
Epsilonproteobacteria
Deltaproteobacteria
Magnetococcus
Elusimicrobia
Thermobaculum
Euryarchaeota
Crenarchaeota
Thaumarchaeota
Nanoarchaeota
Korarchaeota

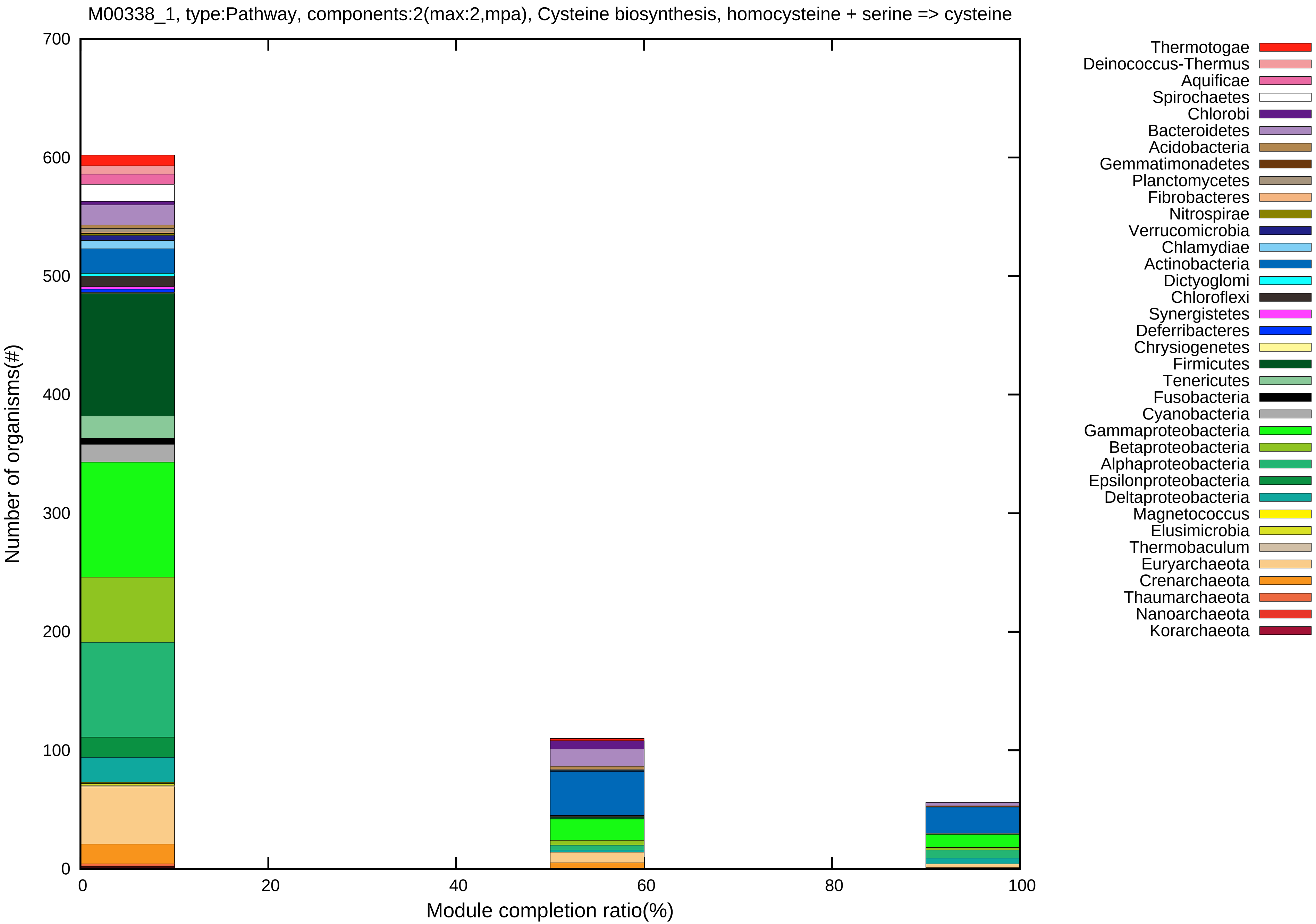
M00338_1, type:Pathway, components:2(max:2,mpa), Cysteine biosynthesis, homocysteine + serine => cysteine
Number of organisms(#)
Module completion ratio(%)
0
100
200
300
400
500
600
700
0
20
40
60
80
100
Thermotogae
Deinococcus-Thermus
Aquificae
Spirochaetes
Chlorobi
Bacteroidetes
Acidobacteria
Gemmatimonadetes
Planctomycetes
Fibrobacteres
Nitrospirae
Verrucomicrobia
Chlamydiae
Actinobacteria
Dictyoglomi
Chloroflexi
Synergistetes
Deferribacteres
Chrysiogenetes
Firmicutes
Tenericutes
Fusobacteria
Cyanobacteria
Gammaproteobacteria
Betaproteobacteria
Alphaproteobacteria
Epsilonproteobacteria
Deltaproteobacteria
Magnetococcus
Elusimicrobia
Thermobaculum
Euryarchaeota
Crenarchaeota
Thaumarchaeota
Nanoarchaeota
Korarchaeota

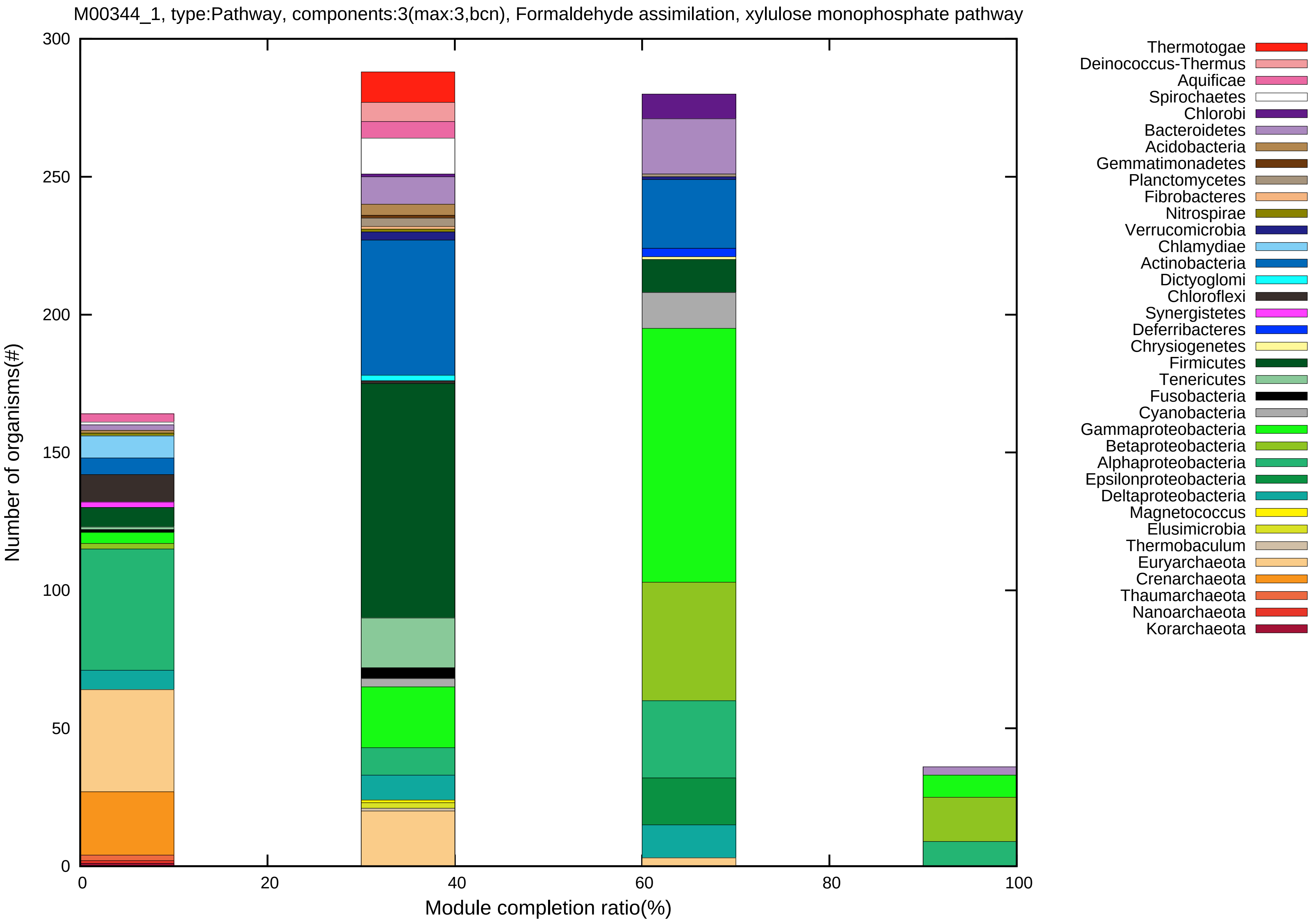

M00344_1, type:Pathway, components:3(max:3,bcn), Formaldehyde assimilation, xylulose monophosphate pathway
Number of organisms(#)
Module completion ratio(%)
0
50
100
150
200
250
300
0
20
40
60
80
100
Thermotogae
Deinococcus-Thermus
Aquificae
Spirochaetes
Chlorobi
Bacteroidetes
Acidobacteria
Gemmatimonadetes
Planctomycetes
Fibrobacteres
Nitrospirae
Verrucomicrobia
Chlamydiae
Actinobacteria
Dictyoglomi
Chloroflexi
Synergistetes
Deferribacteres
Chrysiogenetes
Firmicutes
Tenericutes
Fusobacteria
Cyanobacteria
Gammaproteobacteria
Betaproteobacteria
Alphaproteobacteria
Epsilonproteobacteria
Deltaproteobacteria
Magnetococcus
Elusimicrobia
Thermobaculum
Euryarchaeota
Crenarchaeota
Thaumarchaeota
Nanoarchaeota
Korarchaeota

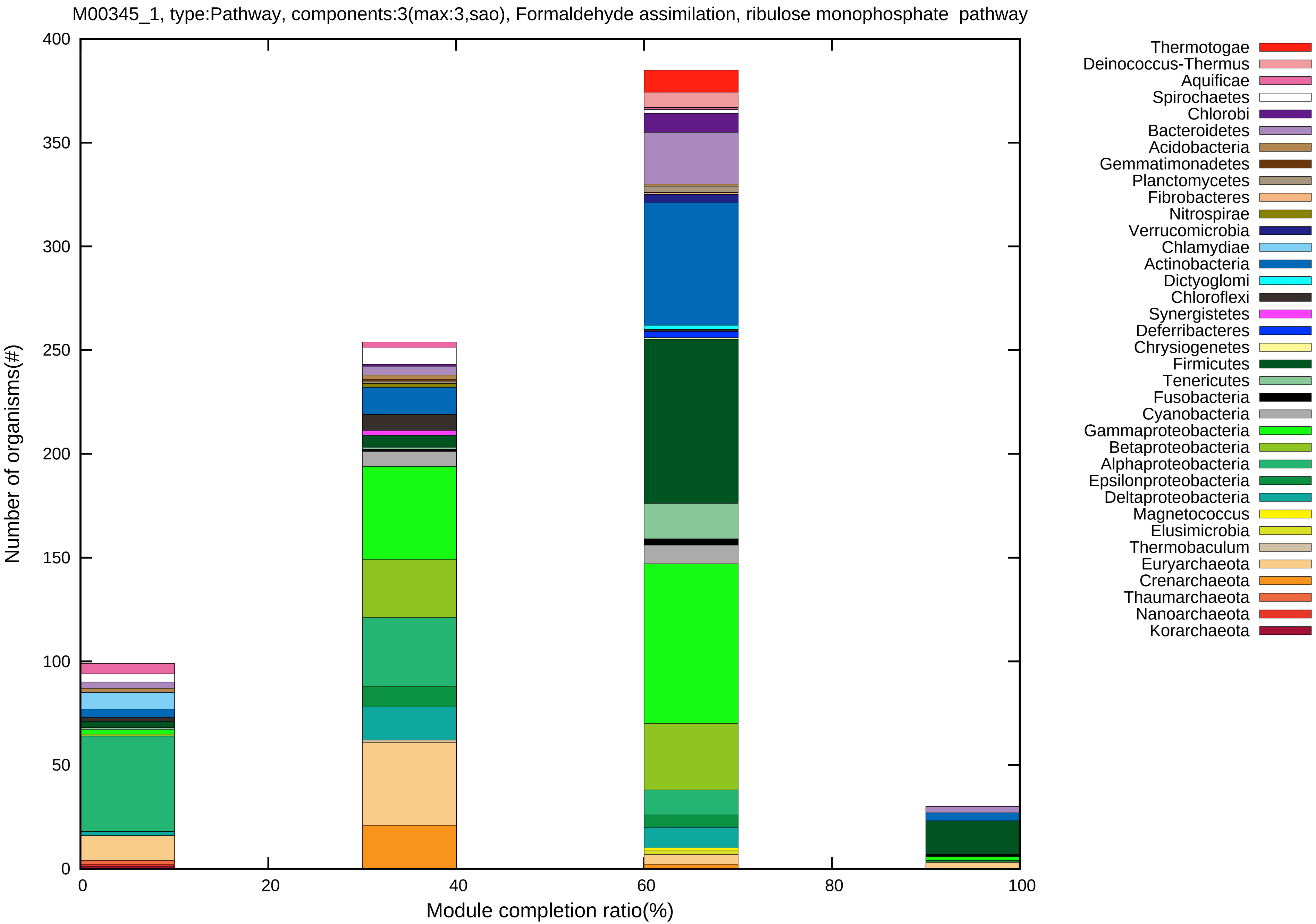

M00345_1, type:Pathway, components:3(max:3,sao), Formaldehyde assimilation, ribulose monophosphate pathway
Number of organisms(#)
Module completion ratio(%)
0
50
100
150
200
250
300
350
400
0
20
40
60
80
100
Thermotogae
Deinococcus-Thermus
Aquificae
Spirochaetes
Chlorobi
Bacteroidetes
Acidobacteria
Gemmatimonadetes
Planctomycetes
Fibrobacteres
Nitrospirae
Verrucomicrobia
Chlamydiae
Actinobacteria
Dictyoglomi
Chloroflexi
Synergistetes
Deferribacteres
Chrysiogenetes
Firmicutes
Tenericutes
Fusobacteria
Cyanobacteria
Gammaproteobacteria
Betaproteobacteria
Alphaproteobacteria
Epsilonproteobacteria
Deltaproteobacteria
Magnetococcus
Elusimicrobia
Thermobaculum
Euryarchaeota
Crenarchaeota
Thaumarchaeota
Nanoarchaeota
Korarchaeota

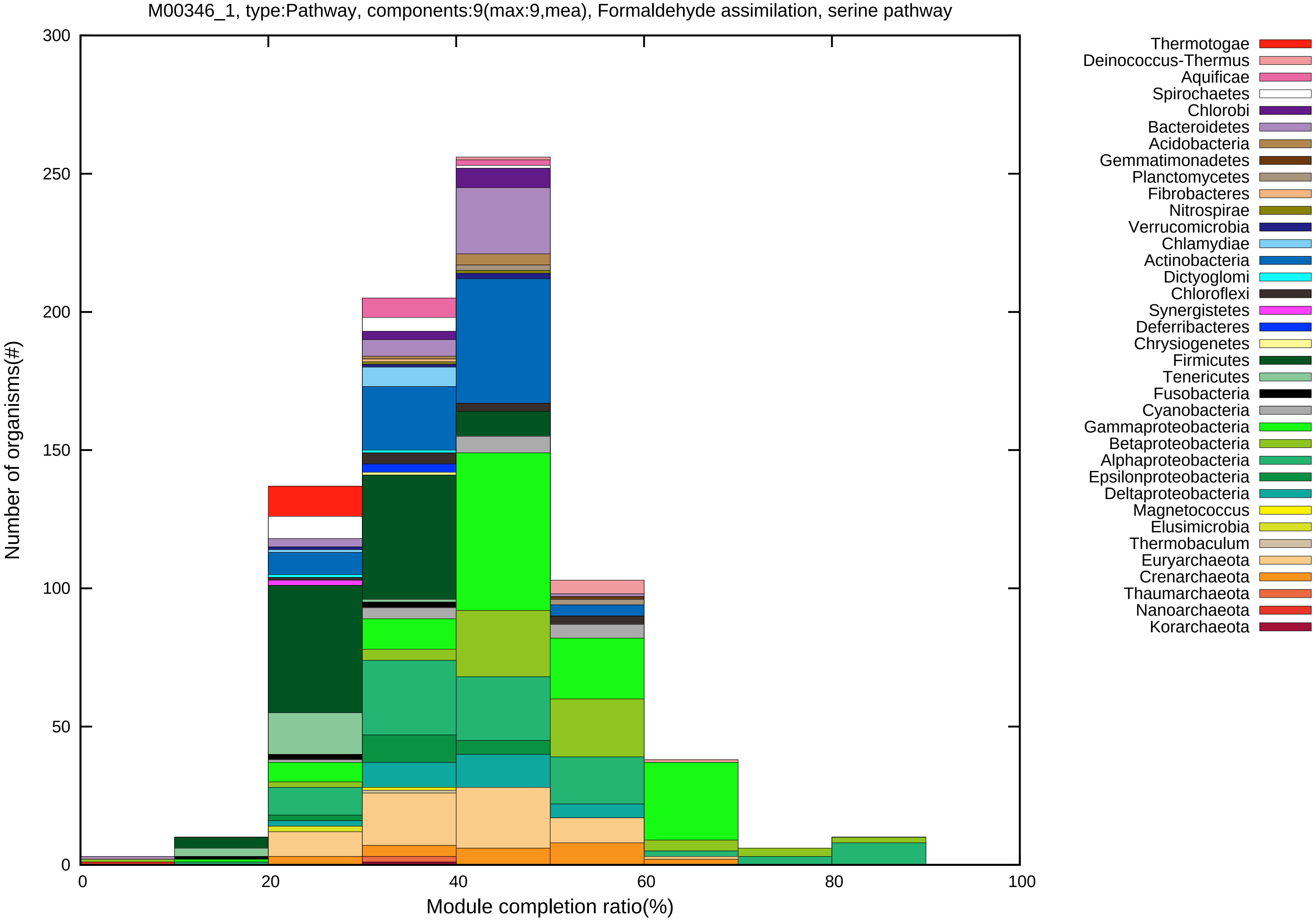

M00346_1, type:Pathway, components:9(max:9,mea), Formaldehyde assimilation, serine pathway
Number of organisms(#)
Module completion ratio(%)
0
50
100
150
200
250
300
0
20
40
60
80
100
Thermotogae
Deinococcus-Thermus
Aquificae
Spirochaetes
Chlorobi
Bacteroidetes
Acidobacteria
Gemmatimonadetes
Planctomycetes
Fibrobacteres
Nitrospirae
Verrucomicrobia
Chlamydiae
Actinobacteria
Dictyoglomi
Chloroflexi
Synergistetes
Deferribacteres
Chrysiogenetes
Firmicutes
Tenericutes
Fusobacteria
Cyanobacteria
Gammaproteobacteria
Betaproteobacteria
Alphaproteobacteria
Epsilonproteobacteria
Deltaproteobacteria
Magnetococcus
Elusimicrobia
Thermobaculum
Euryarchaeota
Crenarchaeota
Thaumarchaeota
Nanoarchaeota
Korarchaeota

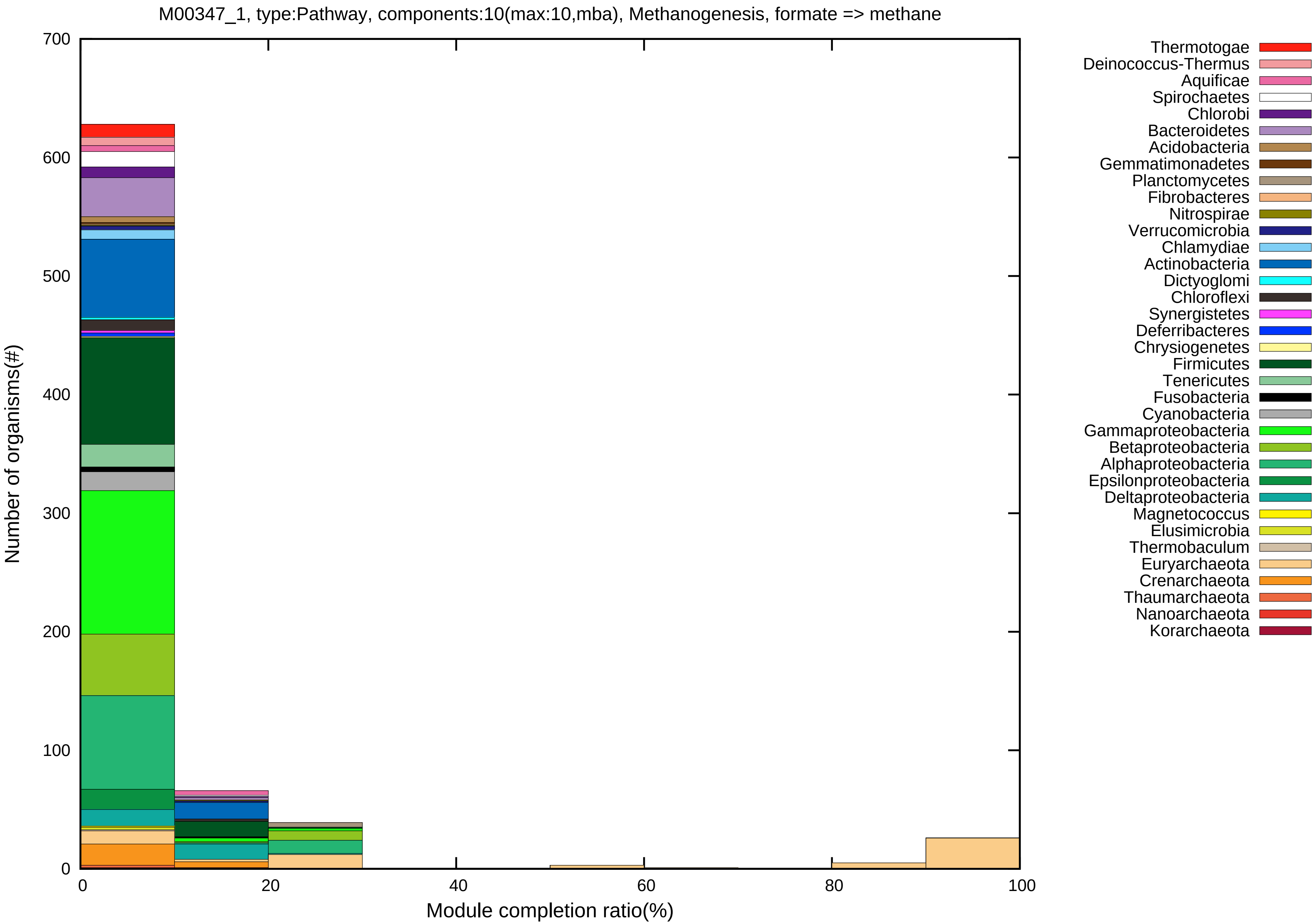

M00347_1, type:Pathway, components:10(max:10,mba), Methanogenesis, formate => methane
Number of organisms(#)
Module completion ratio(%)
0
100
200
300
400
500
600
700
0
20
40
60
80
100
Thermotogae
Deinococcus-Thermus
Aquificae
Spirochaetes
Chlorobi
Bacteroidetes
Acidobacteria
Gemmatimonadetes
Planctomycetes
Fibrobacteres
Nitrospirae
Verrucomicrobia
Chlamydiae
Actinobacteria
Dictyoglomi
Chloroflexi
Synergistetes
Deferribacteres
Chrysiogenetes
Firmicutes
Tenericutes
Fusobacteria
Cyanobacteria
Gammaproteobacteria
Betaproteobacteria
Alphaproteobacteria
Epsilonproteobacteria
Deltaproteobacteria
Magnetococcus
Elusimicrobia
Thermobaculum
Euryarchaeota
Crenarchaeota
Thaumarchaeota
Nanoarchaeota
Korarchaeota

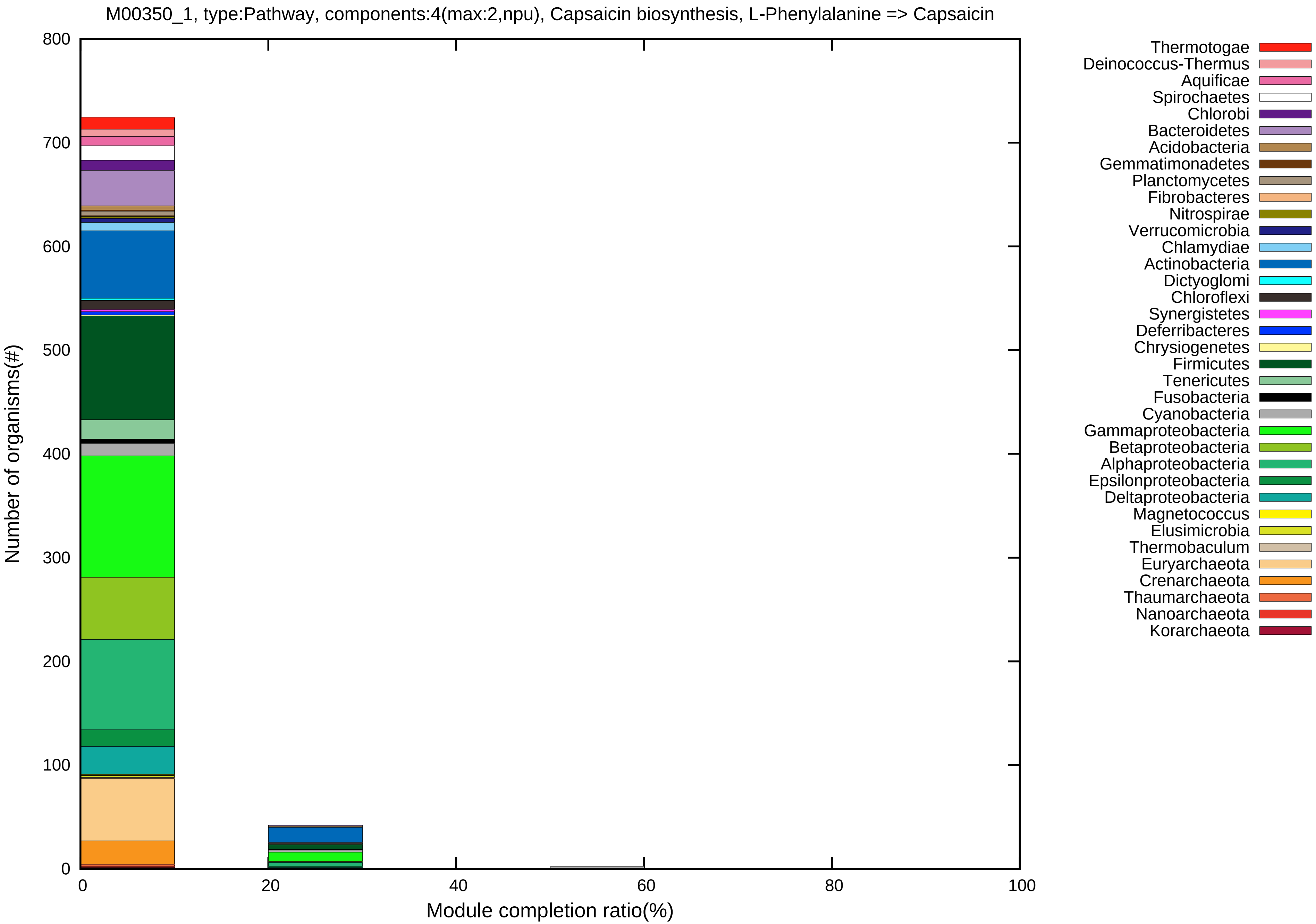
M00350_1, type:Pathway, components:4(max:2,npu), Capsaicin biosynthesis, L-Phenylalanine => Capsaicin
Number of organisms(#)
Module completion ratio(%)
0
100
200
300
400
500
600
700
800
0
20
40
60
80
100
Thermotogae
Deinococcus-Thermus
Aquificae
Spirochaetes
Chlorobi
Bacteroidetes
Acidobacteria
Gemmatimonadetes
Planctomycetes
Fibrobacteres
Nitrospirae
Verrucomicrobia
Chlamydiae
Actinobacteria
Dictyoglomi
Chloroflexi
Synergistetes
Deferribacteres
Chrysiogenetes
Firmicutes
Tenericutes
Fusobacteria
Cyanobacteria
Gammaproteobacteria
Betaproteobacteria
Alphaproteobacteria
Epsilonproteobacteria
Deltaproteobacteria
Magnetococcus
Elusimicrobia
Thermobaculum
Euryarchaeota
Crenarchaeota
Thaumarchaeota
Nanoarchaeota
Korarchaeota

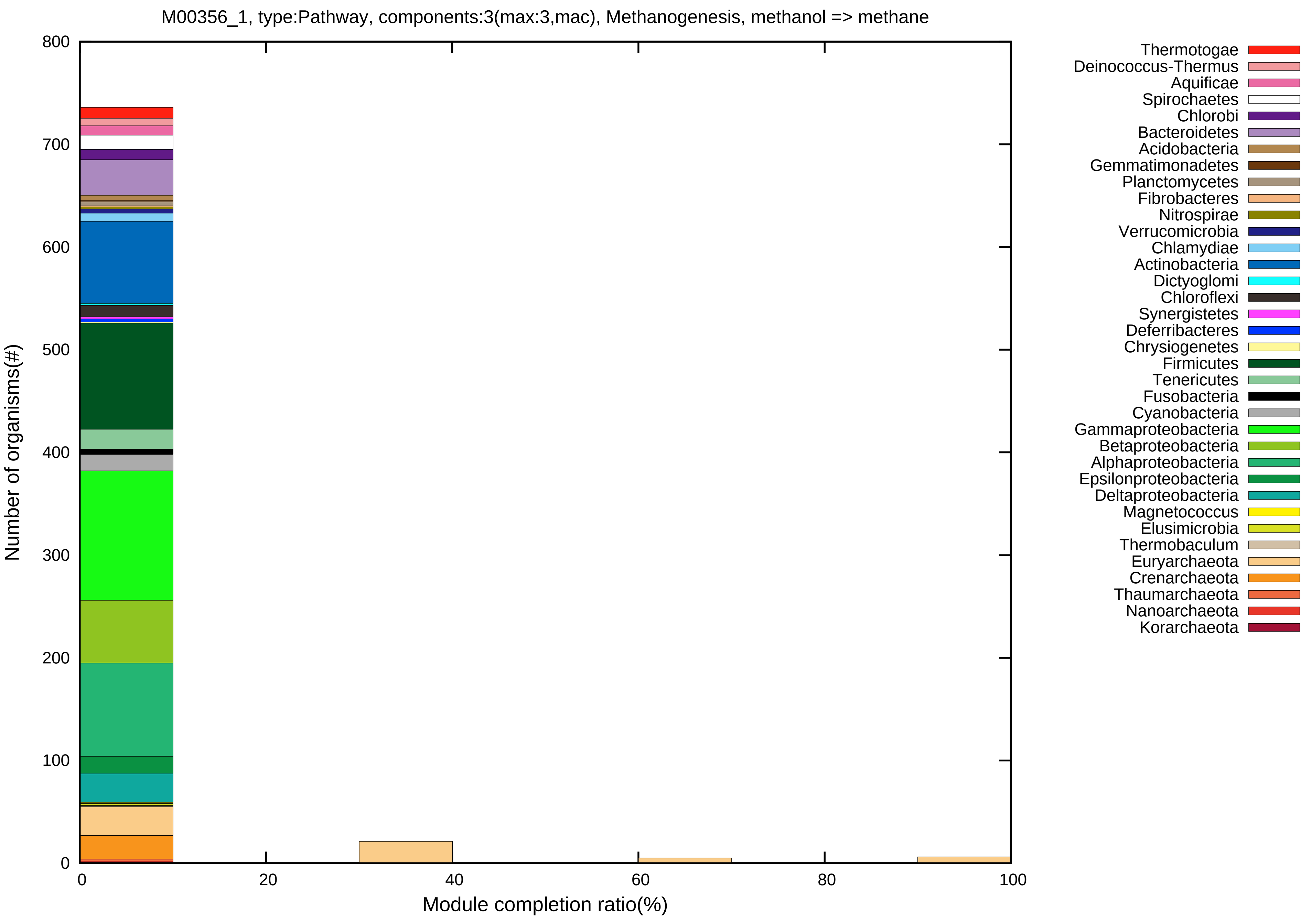
M00356_1, type:Pathway, components:3(max:3,mac), Methanogenesis, methanol => methane
Number of organisms(#)
Module completion ratio(%)
0
100
200
300
400
500
600
700
800
0
20
40
60
80
100
Thermotogae
Deinococcus-Thermus
Aquificae
Spirochaetes
Chlorobi
Bacteroidetes
Acidobacteria
Gemmatimonadetes
Planctomycetes
Fibrobacteres
Nitrospirae
Verrucomicrobia
Chlamydiae
Actinobacteria
Dictyoglomi
Chloroflexi
Synergistetes
Deferribacteres
Chrysiogenetes
Firmicutes
Tenericutes
Fusobacteria
Cyanobacteria
Gammaproteobacteria
Betaproteobacteria
Alphaproteobacteria
Epsilonproteobacteria
Deltaproteobacteria
Magnetococcus
Elusimicrobia
Thermobaculum
Euryarchaeota
Crenarchaeota
Thaumarchaeota
Nanoarchaeota
Korarchaeota

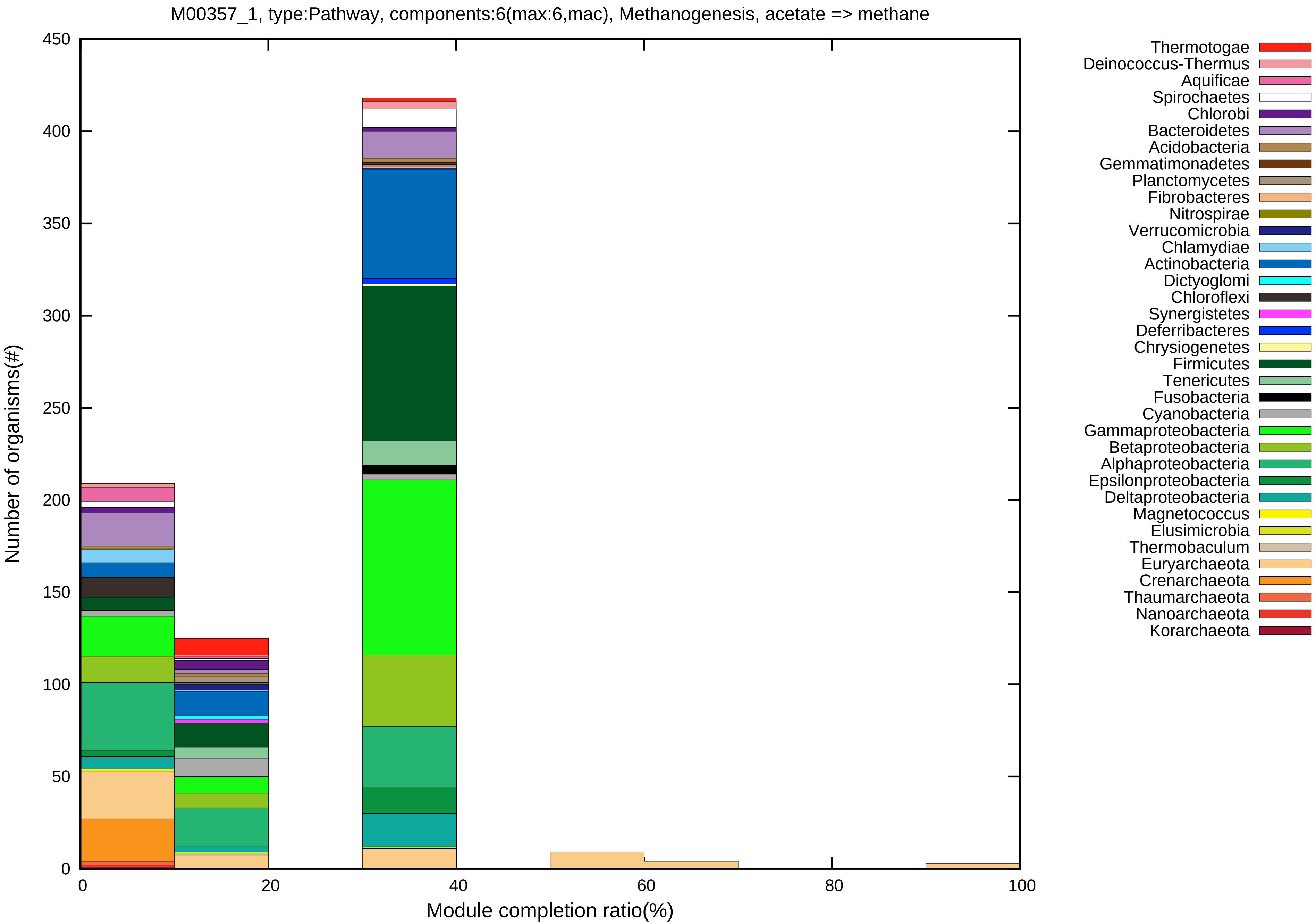
M00357_1, type:Pathway, components:6(max:6,mac), Methanogenesis, acetate => methane
Number of organisms(#)
Module completion ratio(%)
0
50
100
150
200
250
300
350
400
450
0
20
40
60
80
100
Thermotogae
Deinococcus-Thermus
Aquificae
Spirochaetes
Chlorobi
Bacteroidetes
Acidobacteria
Gemmatimonadetes
Planctomycetes
Fibrobacteres
Nitrospirae
Verrucomicrobia
Chlamydiae
Actinobacteria
Dictyoglomi
Chloroflexi
Synergistetes
Deferribacteres
Chrysiogenetes
Firmicutes
Tenericutes
Fusobacteria
Cyanobacteria
Gammaproteobacteria
Betaproteobacteria
Alphaproteobacteria
Epsilonproteobacteria
Deltaproteobacteria
Magnetococcus
Elusimicrobia
Thermobaculum
Euryarchaeota
Crenarchaeota
Thaumarchaeota
Nanoarchaeota
Korarchaeota

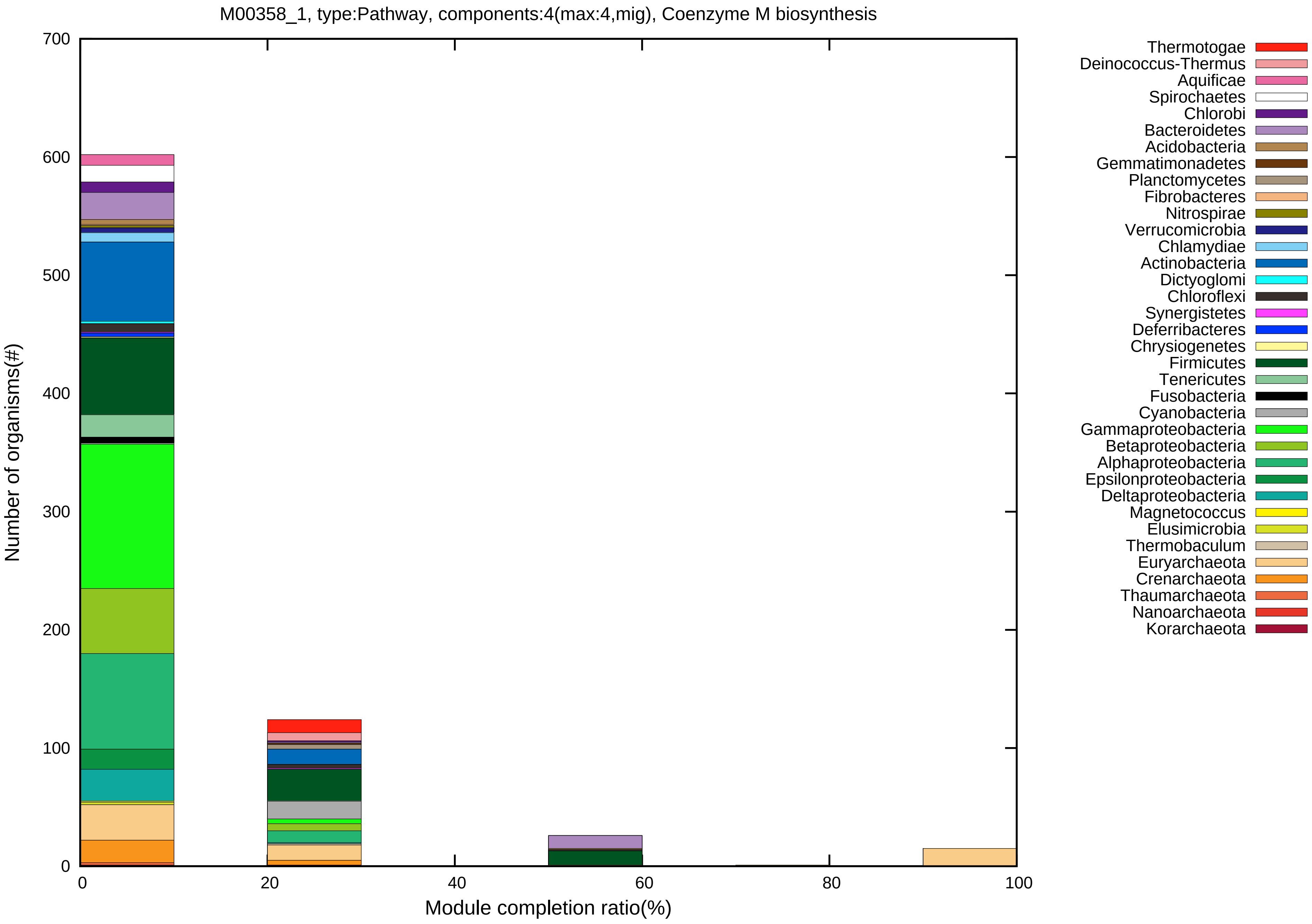
M00358_1, type:Pathway, components:4(max:4,mig), Coenzyme M biosynthesis
Number of organisms(#)
0
100
200
300
400
500
600
700
0
20
40
60
80
100
Module completion ratio(%)
Thermotogae
Deinococcus-Thermus
Aquificae
Spirochaetes
Chlorobi
Bacteroidetes
Acidobacteria
Gemmatimonadetes
Planctomycetes
Fibrobacteres
Nitrospirae
Verrucomicrobia
Chlamydiae
Actinobacteria
Dictyoglomi
Chloroflexi
Synergistetes
Deferribacteres
Chrysiogenetes
Firmicutes
Tenericutes
Fusobacteria
Cyanobacteria
Gammaproteobacteria
Betaproteobacteria
Alphaproteobacteria
Epsilonproteobacteria
Deltaproteobacteria
Magnetococcus
Elusimicrobia
Thermobaculum
Euryarchaeota
Crenarchaeota
Thaumarchaeota
Nanoarchaeota
Korarchaeota

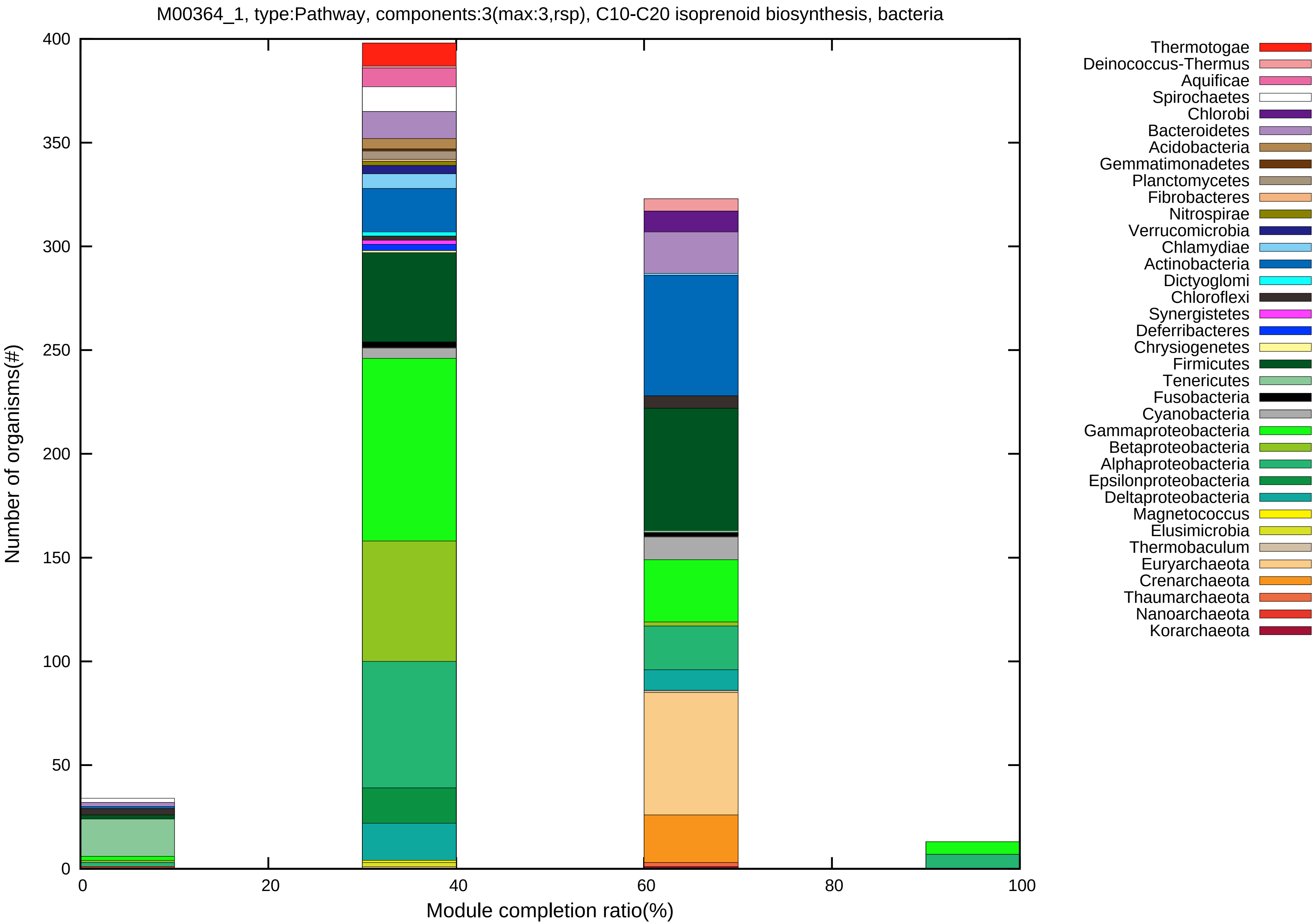

M00364_1, type:Pathway, components:3(max:3,rsp), C10-C20 isoprenoid biosynthesis, bacteria
Number of organisms(#)
Module completion ratio(%)
0
20
40
60
80
100
0
50
100
150
200
250
300
350
400
Thermotogae
Deinococcus-Thermus
Aquificae
Spirochaetes
Chlorobi
Bacteroidetes
Acidobacteria
Gemmatimonadetes
Planctomycetes
Fibrobacteres
Nitrospirae
Verrucomicrobia
Chlamydiae
Actinobacteria
Dictyoglomi
Chloroflexi
Synergistetes
Deferribacteres
Chrysiogenetes
Firmicutes
Tenericutes
Fusobacteria
Cyanobacteria
Gammaproteobacteria
Betaproteobacteria
Alphaproteobacteria
Epsilonproteobacteria
Deltaproteobacteria
Magnetococcus
Elusimicrobia
Thermobaculum
Euryarchaeota
Crenarchaeota
Thaumarchaeota
Nanoarchaeota
Korarchaeota

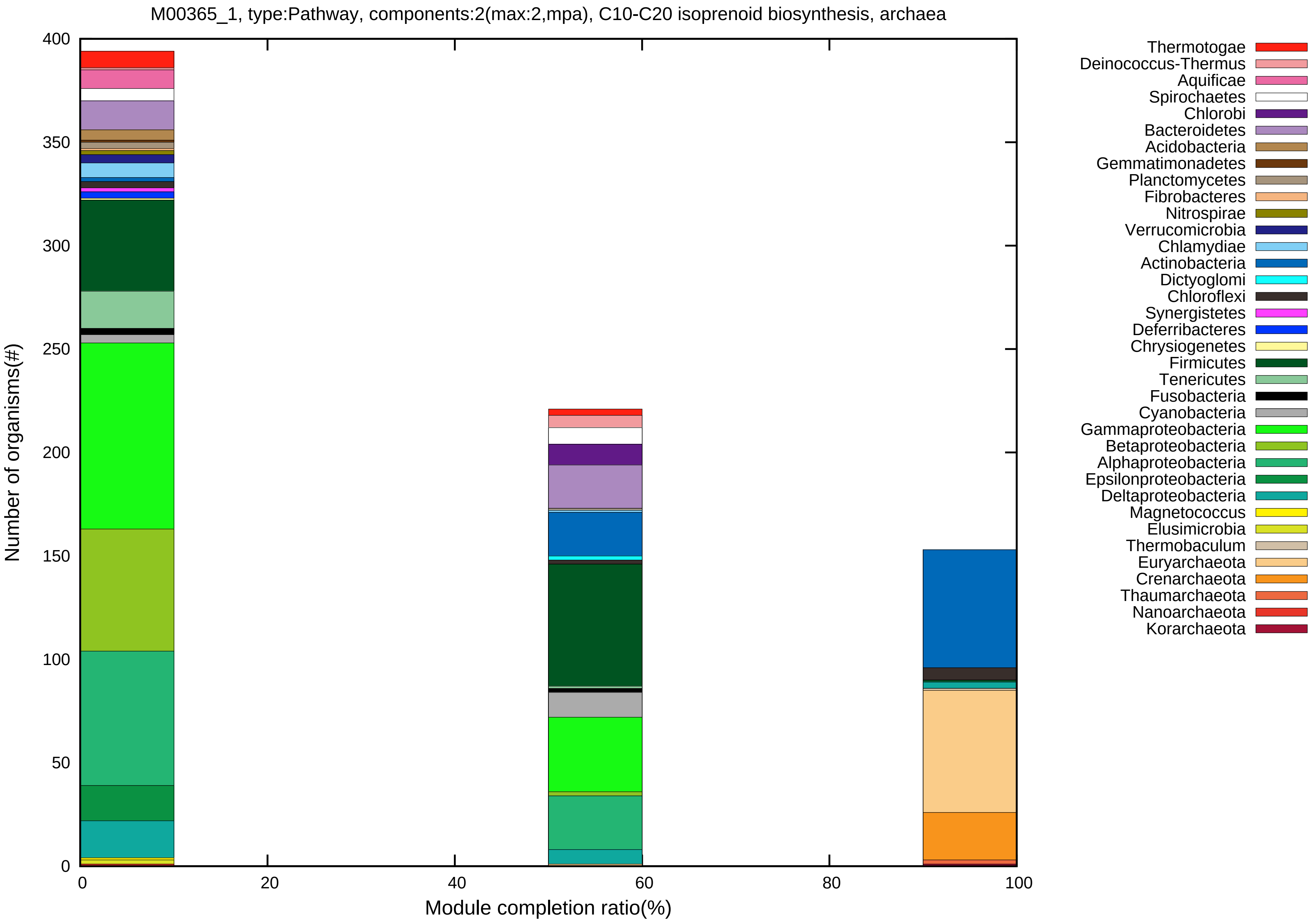

M00365_1, type:Pathway, components:2(max:2,mpa), C10-C20 isoprenoid biosynthesis, archaea
Number of organisms(#)
Module completion ratio(%)
0
50
100
150
200
250
300
350
400
0
20
40
60
80
100
Thermotogae
Deinococcus-Thermus
Aquificae
Spirochaetes
Chlorobi
Bacteroidetes
Acidobacteria
Gemmatimonadetes
Planctomycetes
Fibrobacteres
Nitrospirae
Verrucomicrobia
Chlamydiae
Actinobacteria
Dictyoglomi
Chloroflexi
Synergistetes
Deferribacteres
Chrysiogenetes
Firmicutes
Tenericutes
Fusobacteria
Cyanobacteria
Gammaproteobacteria
Betaproteobacteria
Alphaproteobacteria
Epsilonproteobacteria
Deltaproteobacteria
Magnetococcus
Elusimicrobia
Thermobaculum
Euryarchaeota
Crenarchaeota
Thaumarchaeota
Nanoarchaeota
Korarchaeota

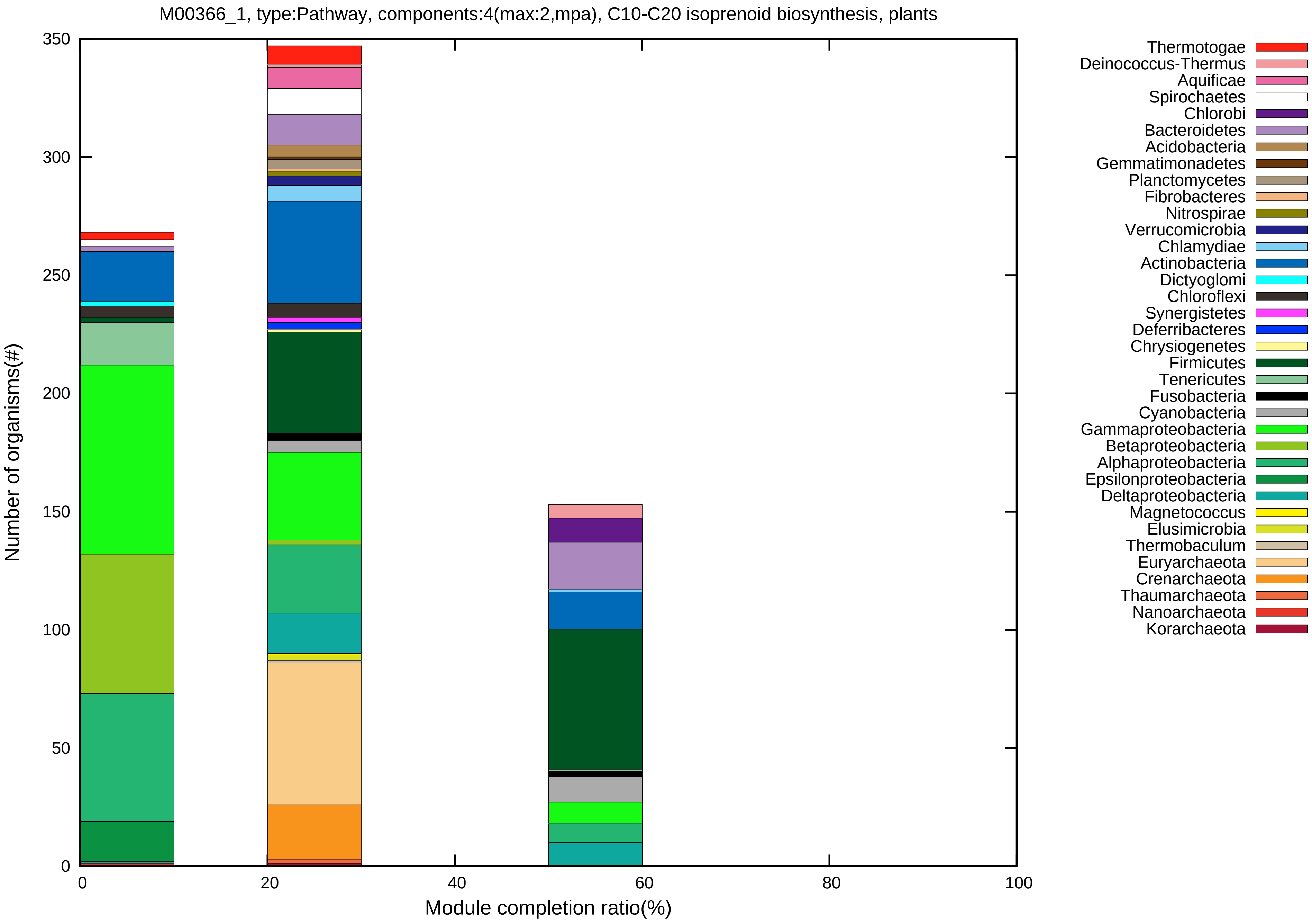

M00366_1, type:Pathway, components:4(max:2,mpa), C10-C20 isoprenoid biosynthesis, plants
Number of organisms(#)
Module completion ratio(%)
0
50
100
150
200
250
300
350
0
20
40
60
80
100
Thermotogae
Deinococcus-Thermus
Aquificae
Spirochaetes
Chlorobi
Bacteroidetes
Acidobacteria
Gemmatimonadetes
Planctomycetes
Fibrobacteres
Nitrospirae
Verrucomicrobia
Chlamydiae
Actinobacteria
Dictyoglomi
Chloroflexi
Synergistetes
Deferribacteres
Chrysiogenetes
Firmicutes
Tenericutes
Fusobacteria
Cyanobacteria
Gammaproteobacteria
Betaproteobacteria
Alphaproteobacteria
Epsilonproteobacteria
Deltaproteobacteria
Magnetococcus
Elusimicrobia
Thermobaculum
Euryarchaeota
Crenarchaeota
Thaumarchaeota
Nanoarchaeota
Korarchaeota

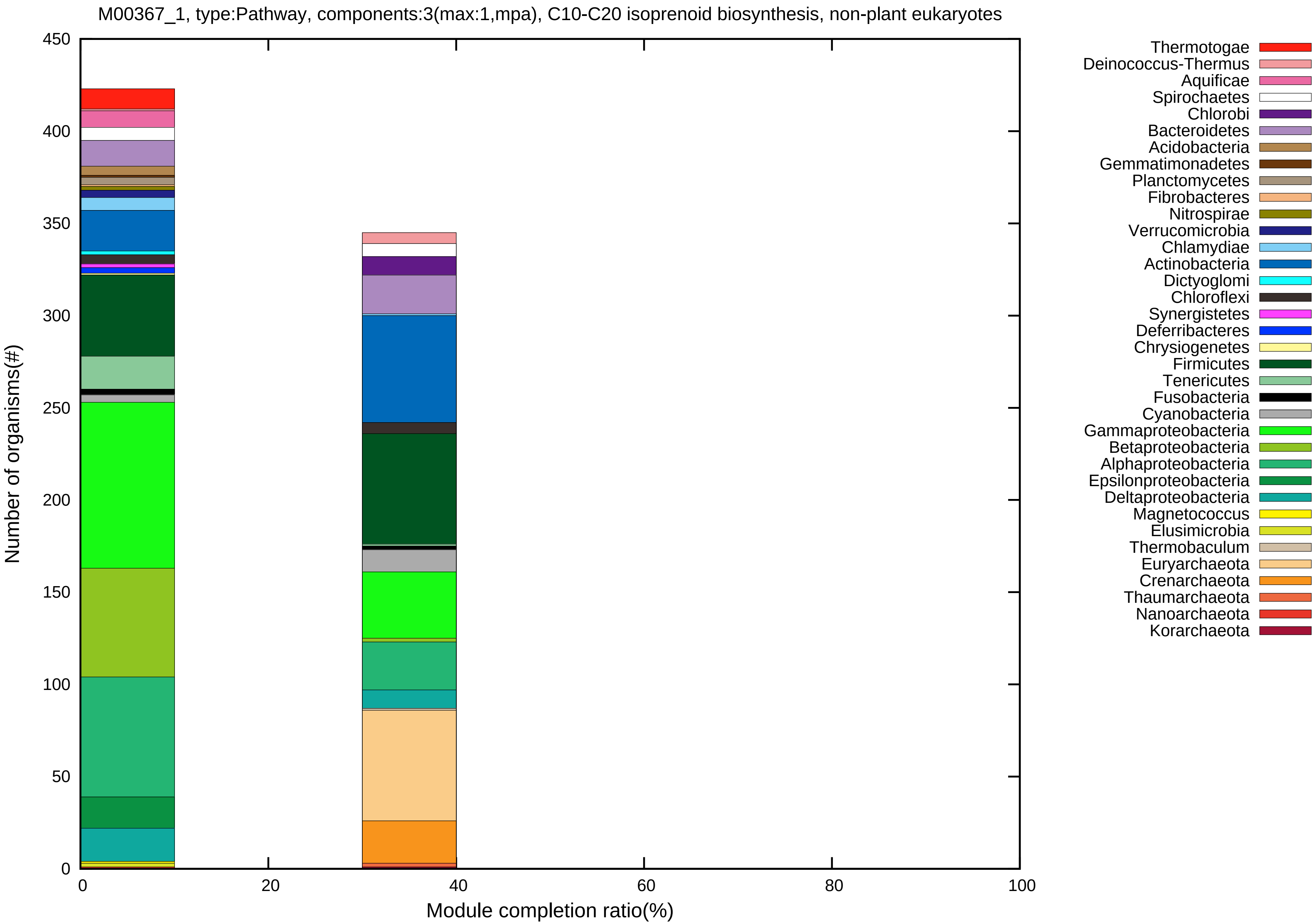

M00367_1, type:Pathway, components:3(max:1,mpa), C10-C20 isoprenoid biosynthesis, non-plant eukaryotes
Number of organisms(#)
Module completion ratio(%)
0
50
100
150
200
250
300
350
400
450
0
20
40
60
80
100
Thermotogae
Deinococcus-Thermus
Aquificae
Spirochaetes
Chlorobi
Bacteroidetes
Acidobacteria
Gemmatimonadetes
Planctomycetes
Fibrobacteres
Nitrospirae
Verrucomicrobia
Chlamydiae
Actinobacteria
Dictyoglomi
Chloroflexi
Synergistetes
Deferribacteres
Chrysiogenetes
Firmicutes
Tenericutes
Fusobacteria
Cyanobacteria
Gammaproteobacteria
Betaproteobacteria
Alphaproteobacteria
Epsilonproteobacteria
Deltaproteobacteria
Magnetococcus
Elusimicrobia
Thermobaculum
Euryarchaeota
Crenarchaeota
Thaumarchaeota
Nanoarchaeota
Korarchaeota

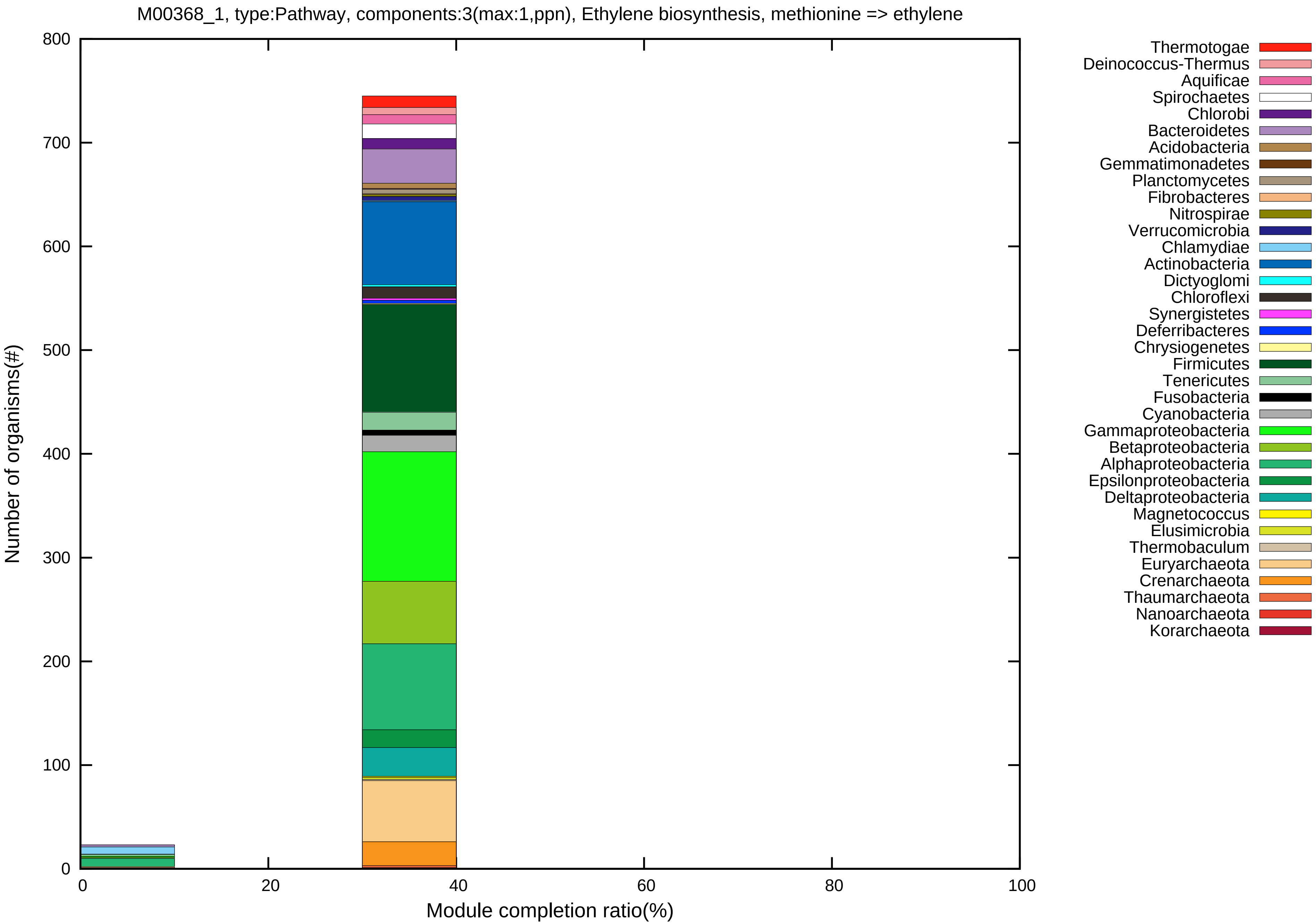
M00368_1, type:Pathway, components:3(max:1,ppn), Ethylene biosynthesis, methionine => ethylene
Number of organisms(#)
Module completion ratio(%)
0
100
200
300
400
500
600
700
800
0
20
40
60
80
100
Thermotogae
Deinococcus-Thermus
Aquificae
Spirochaetes
Chlorobi
Bacteroidetes
Acidobacteria
Gemmatimonadetes
Planctomycetes
Fibrobacteres
Nitrospirae
Verrucomicrobia
Chlamydiae
Actinobacteria
Dictyoglomi
Chloroflexi
Synergistetes
Deferribacteres
Chrysiogenetes
Firmicutes
Tenericutes
Fusobacteria
Cyanobacteria
Gammaproteobacteria
Betaproteobacteria
Alphaproteobacteria
Epsilonproteobacteria
Deltaproteobacteria
Magnetococcus
Elusimicrobia
Thermobaculum
Euryarchaeota
Crenarchaeota
Thaumarchaeota
Nanoarchaeota
Korarchaeota

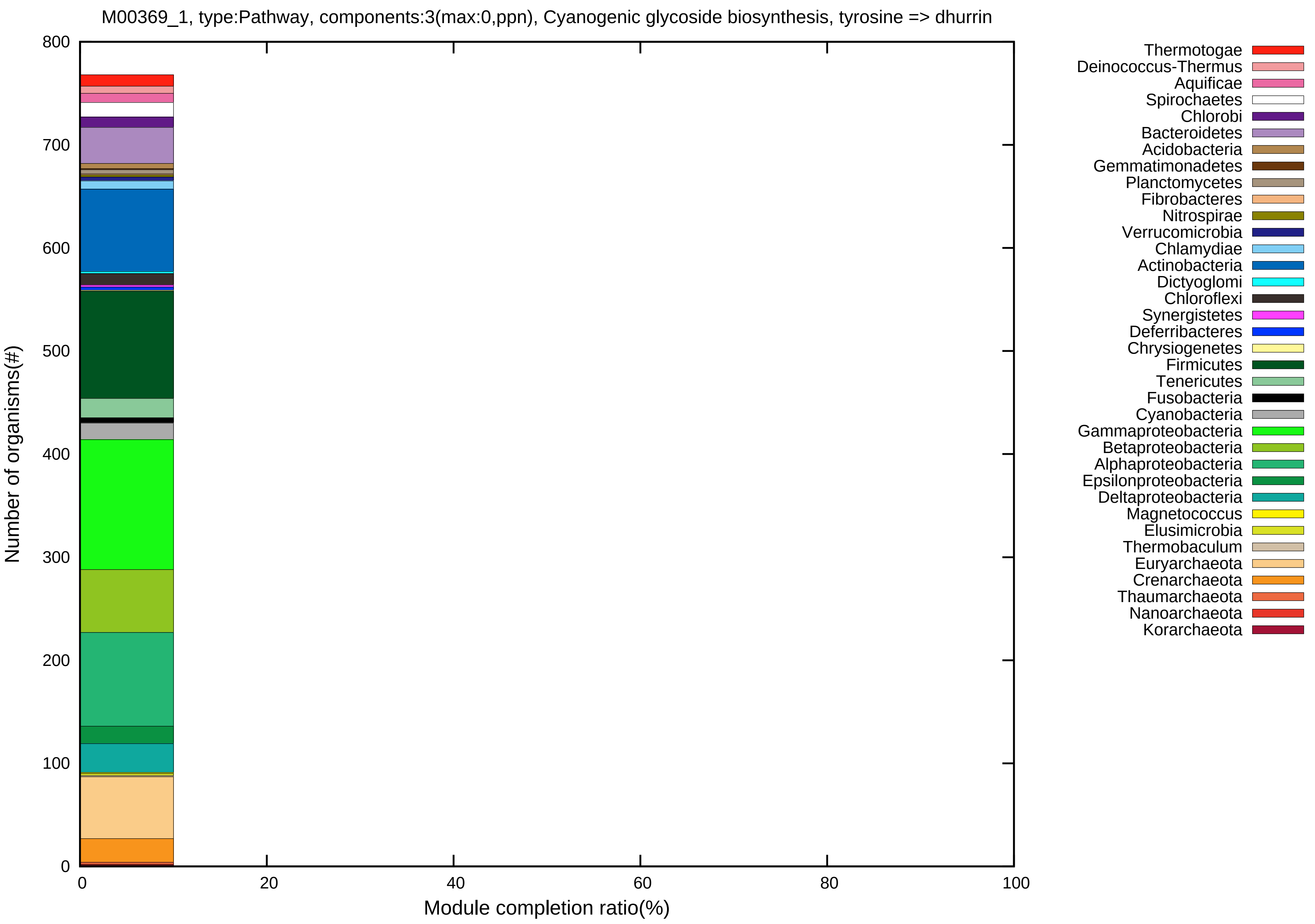

M00369_1, type:Pathway, components:3(max:0,ppn), Cyanogenic glycoside biosynthesis, tyrosine => dhurrin
Number of organisms(#)
Module completion ratio(%)
0
100
200
300
400
500
600
700
800
0
20
40
60
80
100
Thermotogae
Deinococcus-Thermus
Aquificae
Spirochaetes
Chlorobi
Bacteroidetes
Acidobacteria
Gemmatimonadetes
Planctomycetes
Fibrobacteres
Nitrospirae
Verrucomicrobia
Chlamydiae
Actinobacteria
Dictyoglomi
Chloroflexi
Synergistetes
Deferribacteres
Chrysiogenetes
Firmicutes
Tenericutes
Fusobacteria
Cyanobacteria
Gammaproteobacteria
Betaproteobacteria
Alphaproteobacteria
Epsilonproteobacteria
Deltaproteobacteria
Magnetococcus
Elusimicrobia
Thermobaculum
Euryarchaeota
Crenarchaeota
Thaumarchaeota
Nanoarchaeota
Korarchaeota

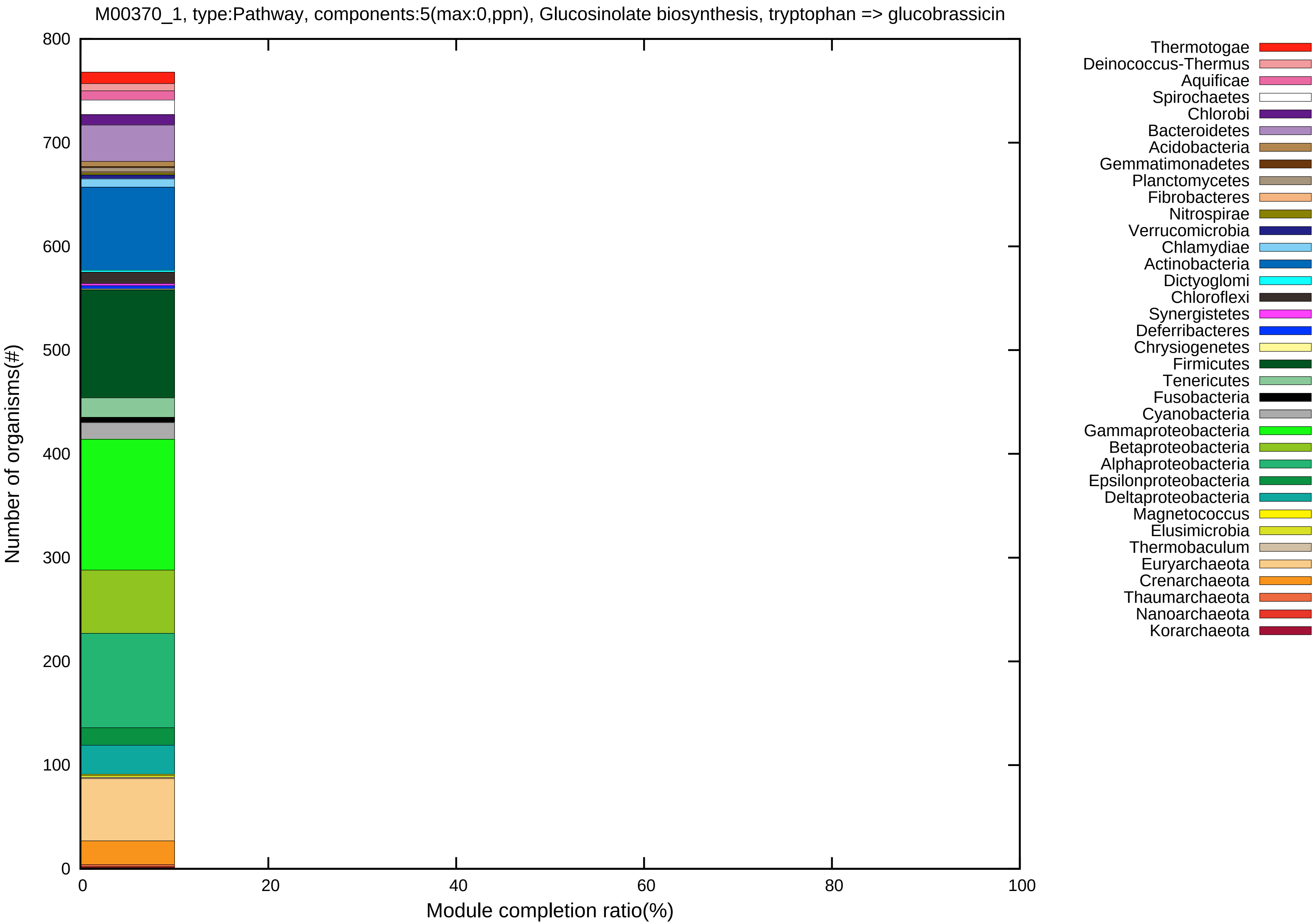

M00370_1, type:Pathway, components:5(max:0,ppn), Glucosinolate biosynthesis, tryptophan => glucobrassicin
Number of organisms(#)
Module completion ratio(%)
0
100
200
300
400
500
600
700
800
0
20
40
60
80
100
Thermotogae
Deinococcus-Thermus
Aquificae
Spirochaetes
Chlorobi
Bacteroidetes
Acidobacteria
Gemmatimonadetes
Planctomycetes
Fibrobacteres
Nitrospirae
Verrucomicrobia
Chlamydiae
Actinobacteria
Dictyoglomi
Chloroflexi
Synergistetes
Deferribacteres
Chrysiogenetes
Firmicutes
Tenericutes
Fusobacteria
Cyanobacteria
Gammaproteobacteria
Betaproteobacteria
Alphaproteobacteria
Epsilonproteobacteria
Deltaproteobacteria
Magnetococcus
Elusimicrobia
Thermobaculum
Euryarchaeota
Crenarchaeota
Thaumarchaeota
Nanoarchaeota
Korarchaeota

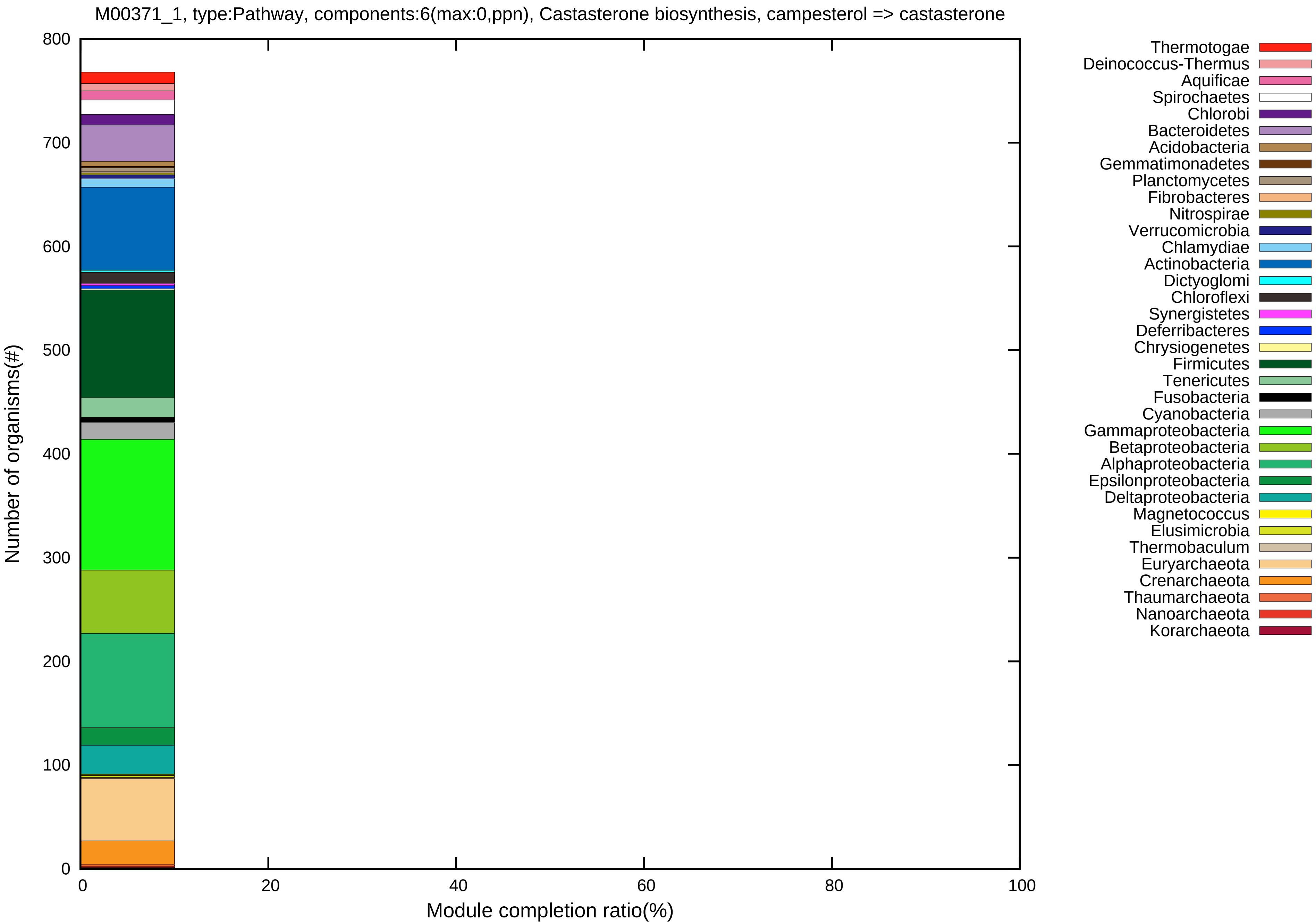

M00371_1, type:Pathway, components:6(max:0,ppn), Castasterone biosynthesis, campesterol => castasterone
Number of organisms(#)
Module completion ratio(%)
0
100
200
300
400
500
600
700
800
0
20
40
60
80
100
Thermotogae
Deinococcus-Thermus
Aquificae
Spirochaetes
Chlorobi
Bacteroidetes
Acidobacteria
Gemmatimonadetes
Planctomycetes
Fibrobacteres
Nitrospirae
Verrucomicrobia
Chlamydiae
Actinobacteria
Dictyoglomi
Chloroflexi
Synergistetes
Deferribacteres
Chrysiogenetes
Firmicutes
Tenericutes
Fusobacteria
Cyanobacteria
Gammaproteobacteria
Betaproteobacteria
Alphaproteobacteria
Epsilonproteobacteria
Deltaproteobacteria
Magnetococcus
Elusimicrobia
Thermobaculum
Euryarchaeota
Crenarchaeota
Thaumarchaeota
Nanoarchaeota
Korarchaeota

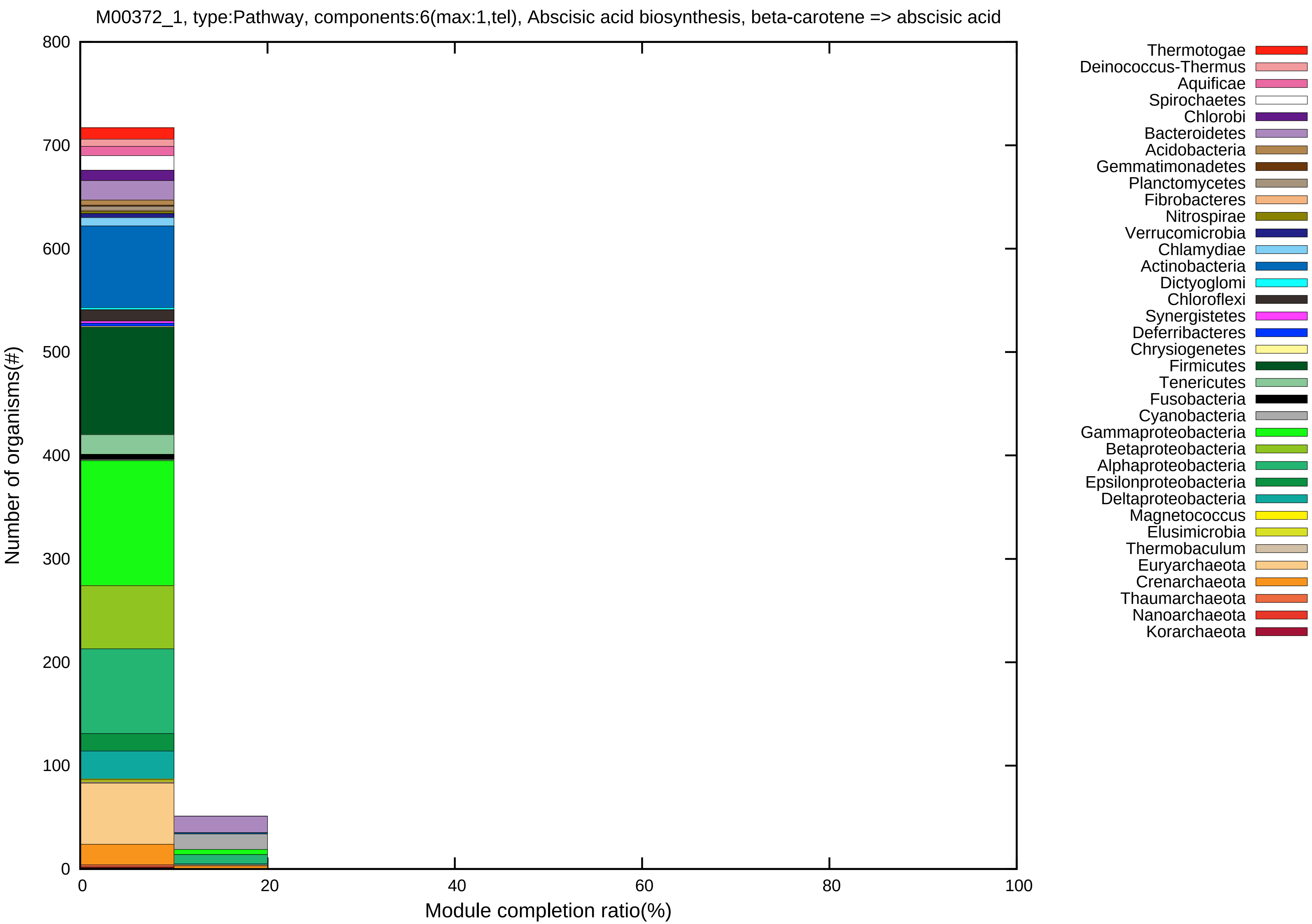

M00372_1, type:Pathway, components:6(max:1,tel), Abscisic acid biosynthesis, beta-carotene => abscisic acid
Number of organisms(#)
Module completion ratio(%)
0
100
200
300
400
500
600
700
800
0
20
40
60
80
100
Thermotogae
Deinococcus-Thermus
Aquificae
Spirochaetes
Chlorobi
Bacteroidetes
Acidobacteria
Gemmatimonadetes
Planctomycetes
Fibrobacteres
Nitrospirae
Verrucomicrobia
Chlamydiae
Actinobacteria
Dictyoglomi
Chloroflexi
Synergistetes
Deferribacteres
Chrysiogenetes
Firmicutes
Tenericutes
Fusobacteria
Cyanobacteria
Gammaproteobacteria
Betaproteobacteria
Alphaproteobacteria
Epsilonproteobacteria
Deltaproteobacteria
Magnetococcus
Elusimicrobia
Thermobaculum
Euryarchaeota
Crenarchaeota
Thaumarchaeota
Nanoarchaeota
Korarchaeota

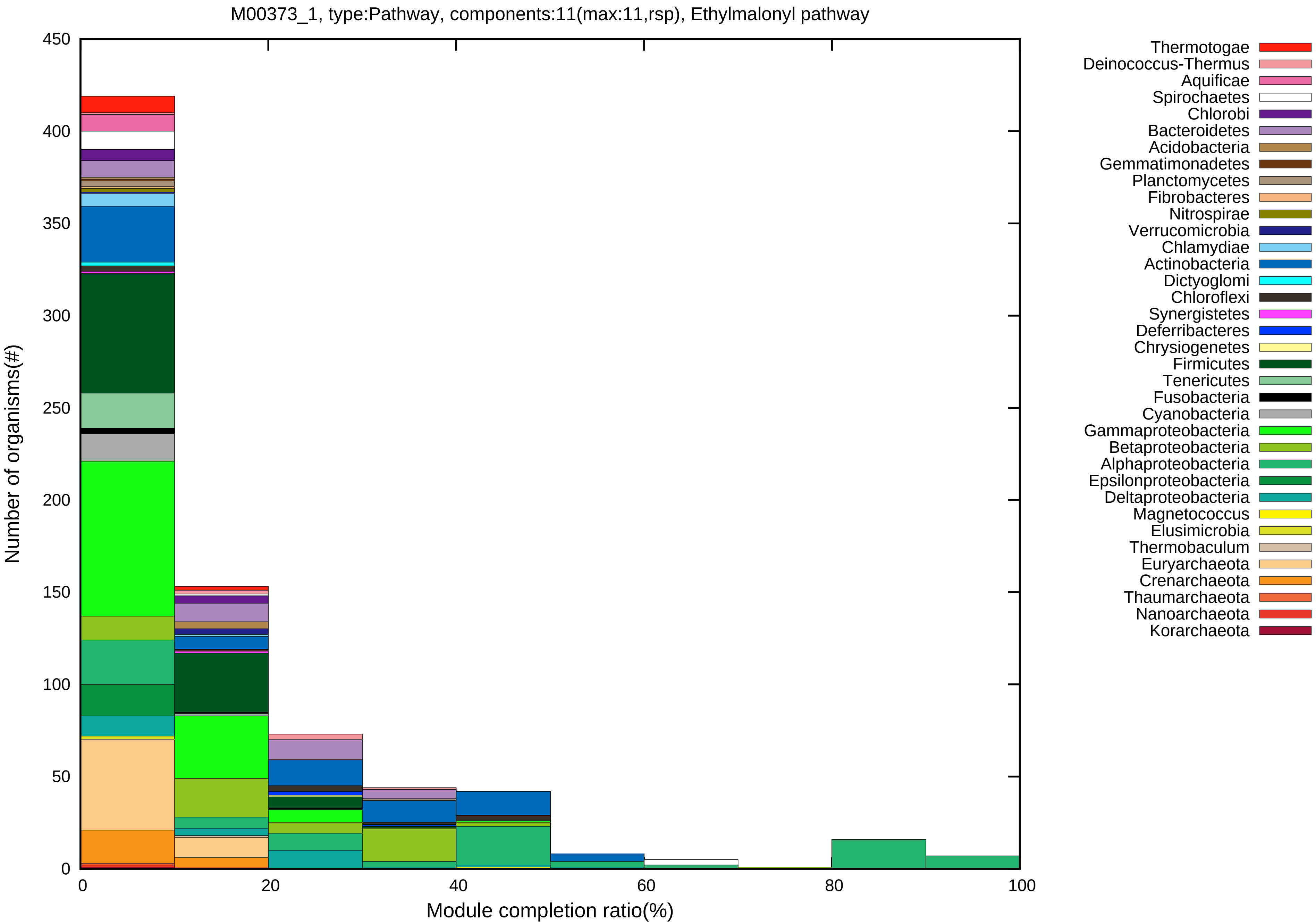

M00373_1, type:Pathway, components:11(max:11,rsp), Ethylmalonyl pathway
Number of organisms(#)
Module completion ratio(%)
0
50
100
150
200
250
300
350
400
450
0
20
40
60
80
100
Thermotogae
Deinococcus-Thermus
Aquificae
Spirochaetes
Chlorobi
Bacteroidetes
Acidobacteria
Gemmatimonadetes
Planctomycetes
Fibrobacteres
Nitrospirae
Verrucomicrobia
Chlamydiae
Actinobacteria
Dictyoglomi
Chloroflexi
Synergistetes
Deferribacteres
Chrysiogenetes
Firmicutes
Tenericutes
Fusobacteria
Cyanobacteria
Gammaproteobacteria
Betaproteobacteria
Alphaproteobacteria
Epsilonproteobacteria
Deltaproteobacteria
Magnetococcus
Elusimicrobia
Thermobaculum
Euryarchaeota
Crenarchaeota
Thaumarchaeota
Nanoarchaeota
Korarchaeota

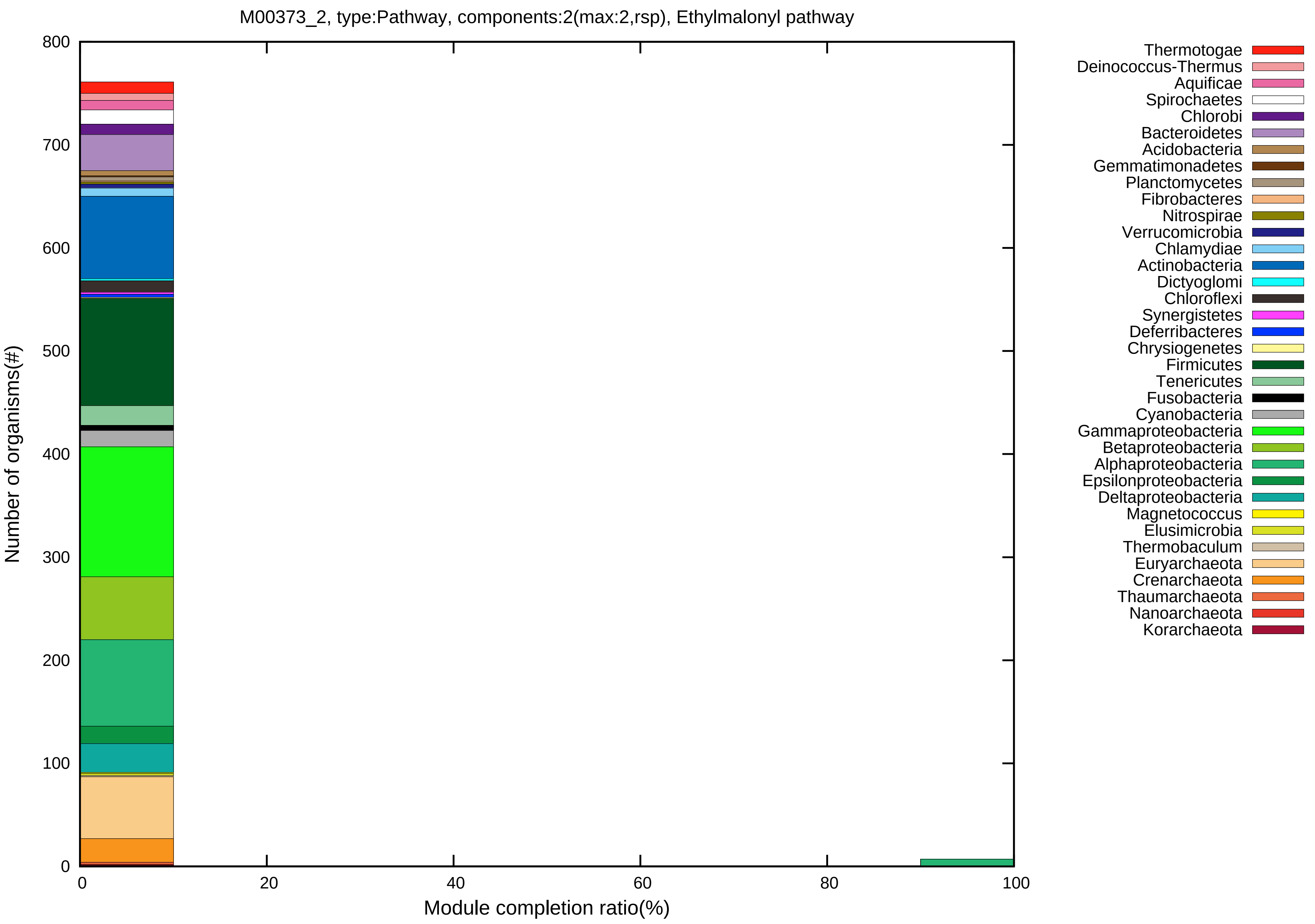

M00373_2, type:Pathway, components:2(max:2,rsp), Ethylmalonyl pathway
Number of organisms(#)
Module completion ratio(%)
0
100
200
300
400
500
600
700
800
0
20
40
60
80
100
Thermotogae
Deinococcus-Thermus
Aquificae
Spirochaetes
Chlorobi
Bacteroidetes
Acidobacteria
Gemmatimonadetes
Planctomycetes
Fibrobacteres
Nitrospirae
Verrucomicrobia
Chlamydiae
Actinobacteria
Dictyoglomi
Chloroflexi
Synergistetes
Deferribacteres
Chrysiogenetes
Firmicutes
Tenericutes
Fusobacteria
Cyanobacteria
Gammaproteobacteria
Betaproteobacteria
Alphaproteobacteria
Epsilonproteobacteria
Deltaproteobacteria
Magnetococcus
Elusimicrobia
Thermobaculum
Euryarchaeota
Crenarchaeota
Thaumarchaeota
Nanoarchaeota
Korarchaeota

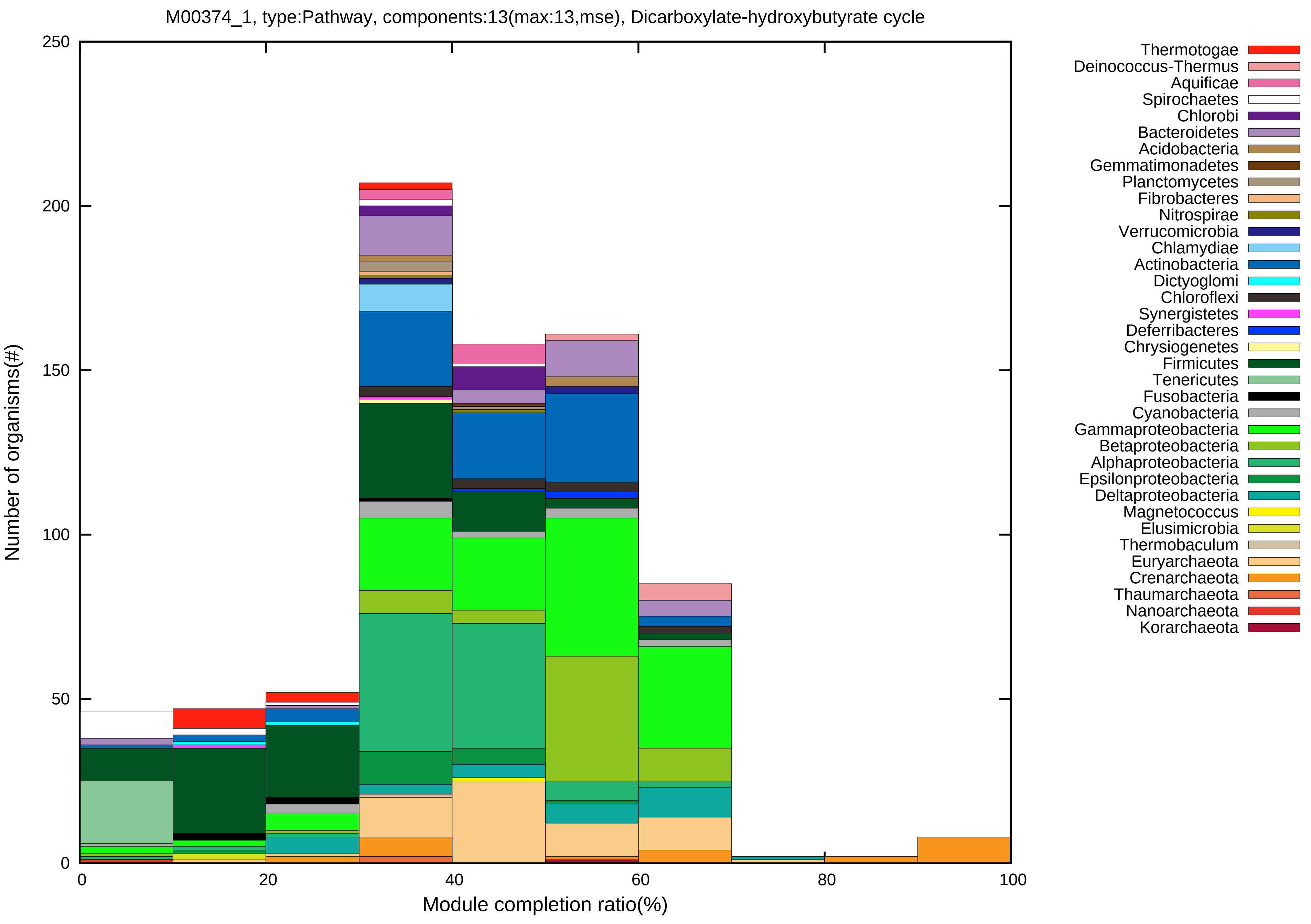

M00374_1, type:Pathway, components:13(max:13,mse), Dicarboxylate-hydroxybutyrate cycle
Number of organisms(#)
Module completion ratio(%)
0
50
100
150
200
250
0
20
40
60
80
100
Thermotogae
Deinococcus-Thermus
Aquificae
Spirochaetes
Chlorobi
Bacteroidetes
Acidobacteria
Gemmatimonadetes
Planctomycetes
Fibrobacteres
Nitrospirae
Verrucomicrobia
Chlamydiae
Actinobacteria
Dictyoglomi
Chloroflexi
Synergistetes
Deferribacteres
Chrysiogenetes
Firmicutes
Tenericutes
Fusobacteria
Cyanobacteria
Gammaproteobacteria
Betaproteobacteria
Alphaproteobacteria
Epsilonproteobacteria
Deltaproteobacteria
Magnetococcus
Elusimicrobia
Thermobaculum
Euryarchaeota
Crenarchaeota
Thaumarchaeota
Nanoarchaeota
Korarchaeota

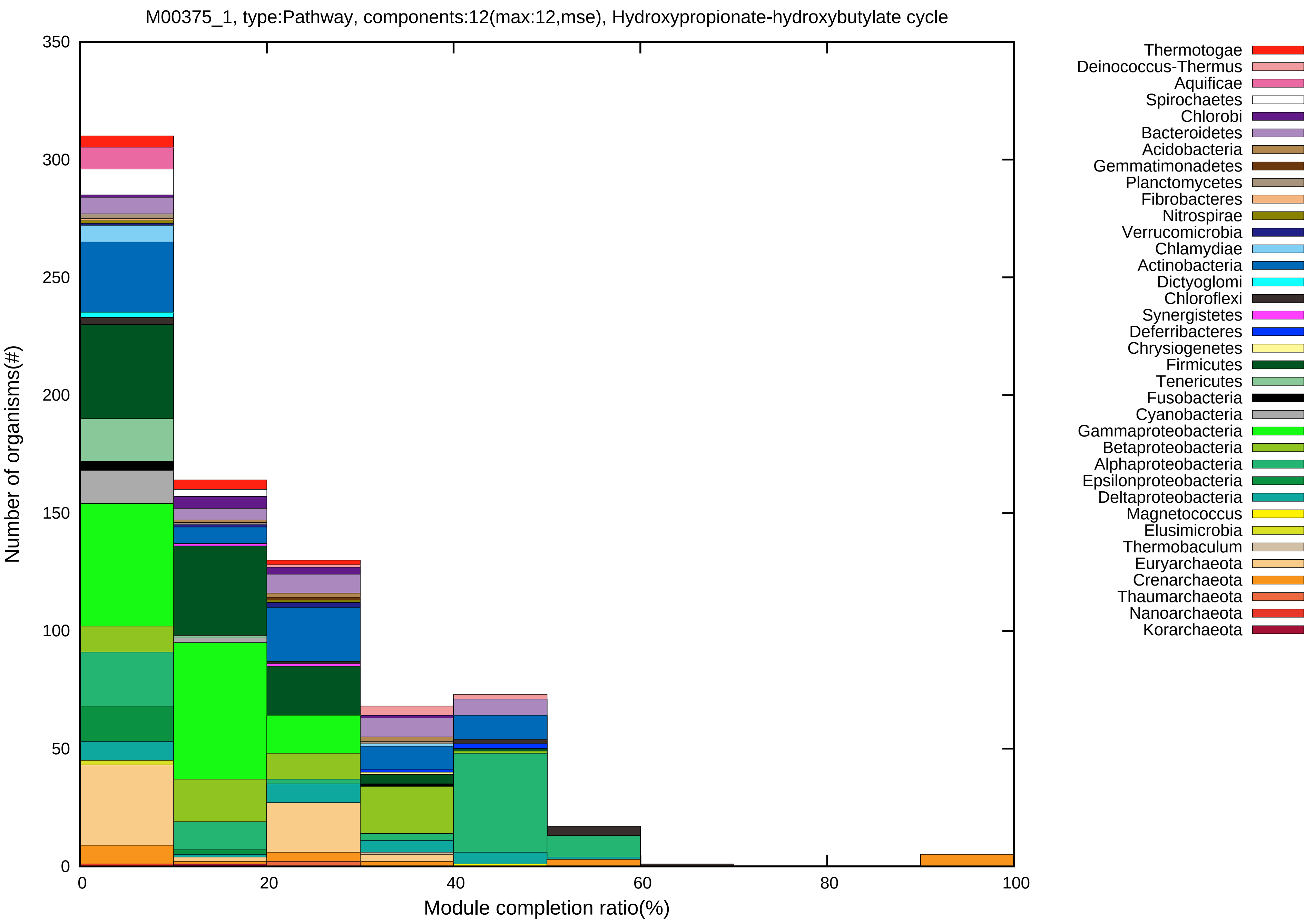
M00375_1, type:Pathway, components:12(max:12,mse), Hydroxypropionate-hydroxybutylate cycle
Number of organisms(#)
Module completion ratio(%)
0
50
100
150
200
250
300
350
0
20
40
60
80
100
Thermotogae
Deinococcus-Thermus
Aquificae
Spirochaetes
Chlorobi
Bacteroidetes
Acidobacteria
Gemmatimonadetes
Planctomycetes
Fibrobacteres
Nitrospirae
Verrucomicrobia
Chlamydiae
Actinobacteria
Dictyoglomi
Chloroflexi
Synergistetes
Deferribacteres
Chrysiogenetes
Firmicutes
Tenericutes
Fusobacteria
Cyanobacteria
Gammaproteobacteria
Betaproteobacteria
Alphaproteobacteria
Epsilonproteobacteria
Deltaproteobacteria
Magnetococcus
Elusimicrobia
Thermobaculum
Euryarchaeota
Crenarchaeota
Thaumarchaeota
Nanoarchaeota
Korarchaeota

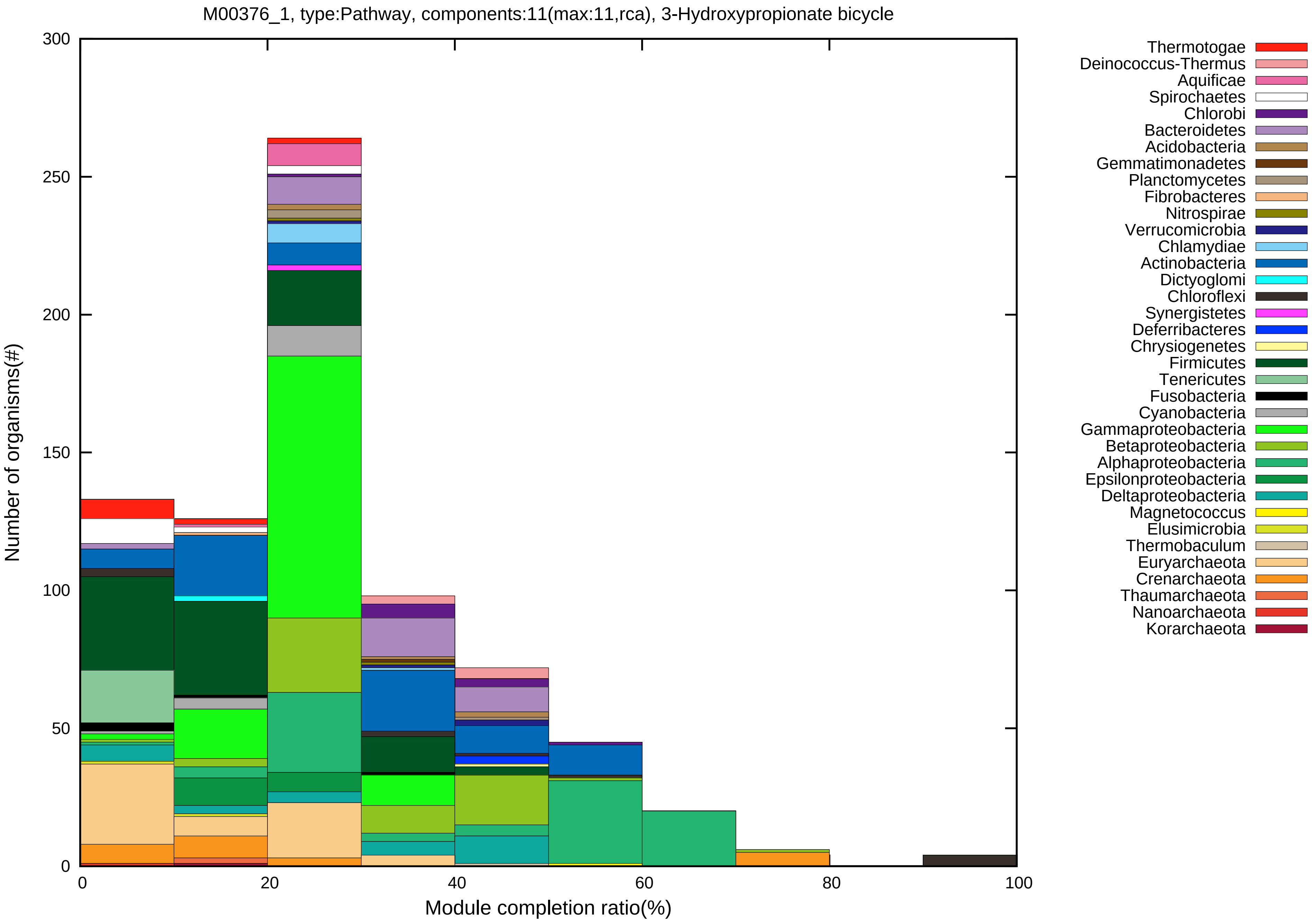
M00376_1, type:Pathway, components:11(max:11,rca), 3-Hydroxypropionate bicycle
Number of organisms(#)
Module completion ratio(%)
0
50
100
150
200
250
300
0
20
40
60
80
100
Thermotogae
Deinococcus-Thermus
Aquificae
Spirochaetes
Chlorobi
Bacteroidetes
Acidobacteria
Gemmatimonadetes
Planctomycetes
Fibrobacteres
Nitrospirae
Verrucomicrobia
Chlamydiae
Actinobacteria
Dictyoglomi
Chloroflexi
Synergistetes
Deferribacteres
Chrysiogenetes
Firmicutes
Tenericutes
Fusobacteria
Cyanobacteria
Gammaproteobacteria
Betaproteobacteria
Alphaproteobacteria
Epsilonproteobacteria
Deltaproteobacteria
Magnetococcus
Elusimicrobia
Thermobaculum
Euryarchaeota
Crenarchaeota
Thaumarchaeota
Nanoarchaeota
Korarchaeota

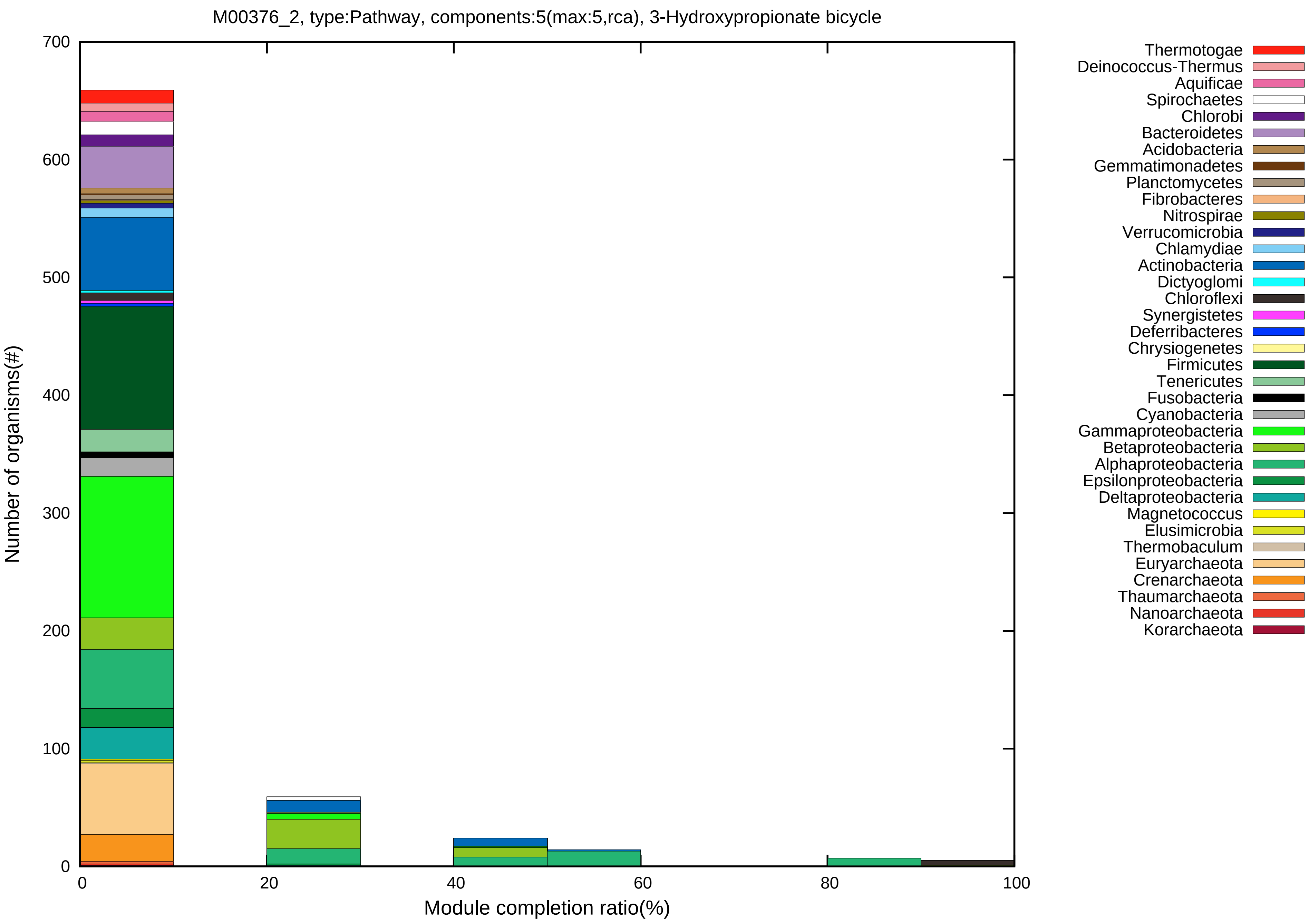

M00376_2, type:Pathway, components:5(max:5,rca), 3-Hydroxypropionate bicycle
Number of organisms(#)
Module completion ratio(%)
0
100
200
300
400
500
600
700
0
20
40
60
80
100
Thermotogae
Deinococcus-Thermus
Aquificae
Spirochaetes
Chlorobi
Bacteroidetes
Acidobacteria
Gemmatimonadetes
Planctomycetes
Fibrobacteres
Nitrospirae
Verrucomicrobia
Chlamydiae
Actinobacteria
Dictyoglomi
Chloroflexi
Synergistetes
Deferribacteres
Chrysiogenetes
Firmicutes
Tenericutes
Fusobacteria
Cyanobacteria
Gammaproteobacteria
Betaproteobacteria
Alphaproteobacteria
Epsilonproteobacteria
Deltaproteobacteria
Magnetococcus
Elusimicrobia
Thermobaculum
Euryarchaeota
Crenarchaeota
Thaumarchaeota
Nanoarchaeota
Korarchaeota

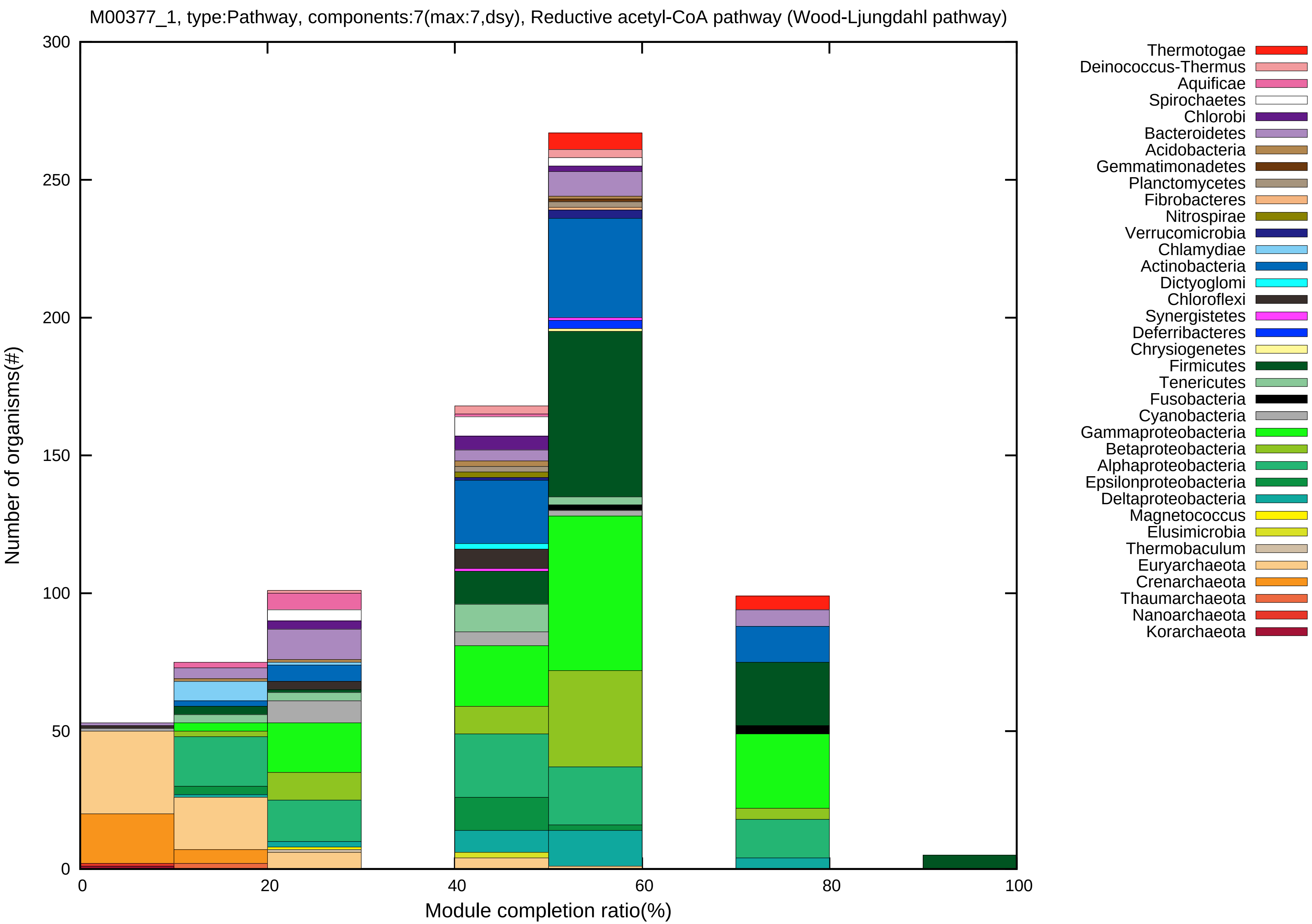

M00377_1, type:Pathway, components:7(max:7,dsy), Reductive acetyl-CoA pathway (Wood-Ljungdahl pathway)
Number of organisms(#)
Module completion ratio(%)
0
50
100
150
200
250
300
0
20
40
60
80
100
Thermotogae
Deinococcus-Thermus
Aquificae
Spirochaetes
Chlorobi
Bacteroidetes
Acidobacteria
Gemmatimonadetes
Planctomycetes
Fibrobacteres
Nitrospirae
Verrucomicrobia
Chlamydiae
Actinobacteria
Dictyoglomi
Chloroflexi
Synergistetes
Deferribacteres
Chrysiogenetes
Firmicutes
Tenericutes
Fusobacteria
Cyanobacteria
Gammaproteobacteria
Betaproteobacteria
Alphaproteobacteria
Epsilonproteobacteria
Deltaproteobacteria
Magnetococcus
Elusimicrobia
Thermobaculum
Euryarchaeota
Crenarchaeota
Thaumarchaeota
Nanoarchaeota
Korarchaeota

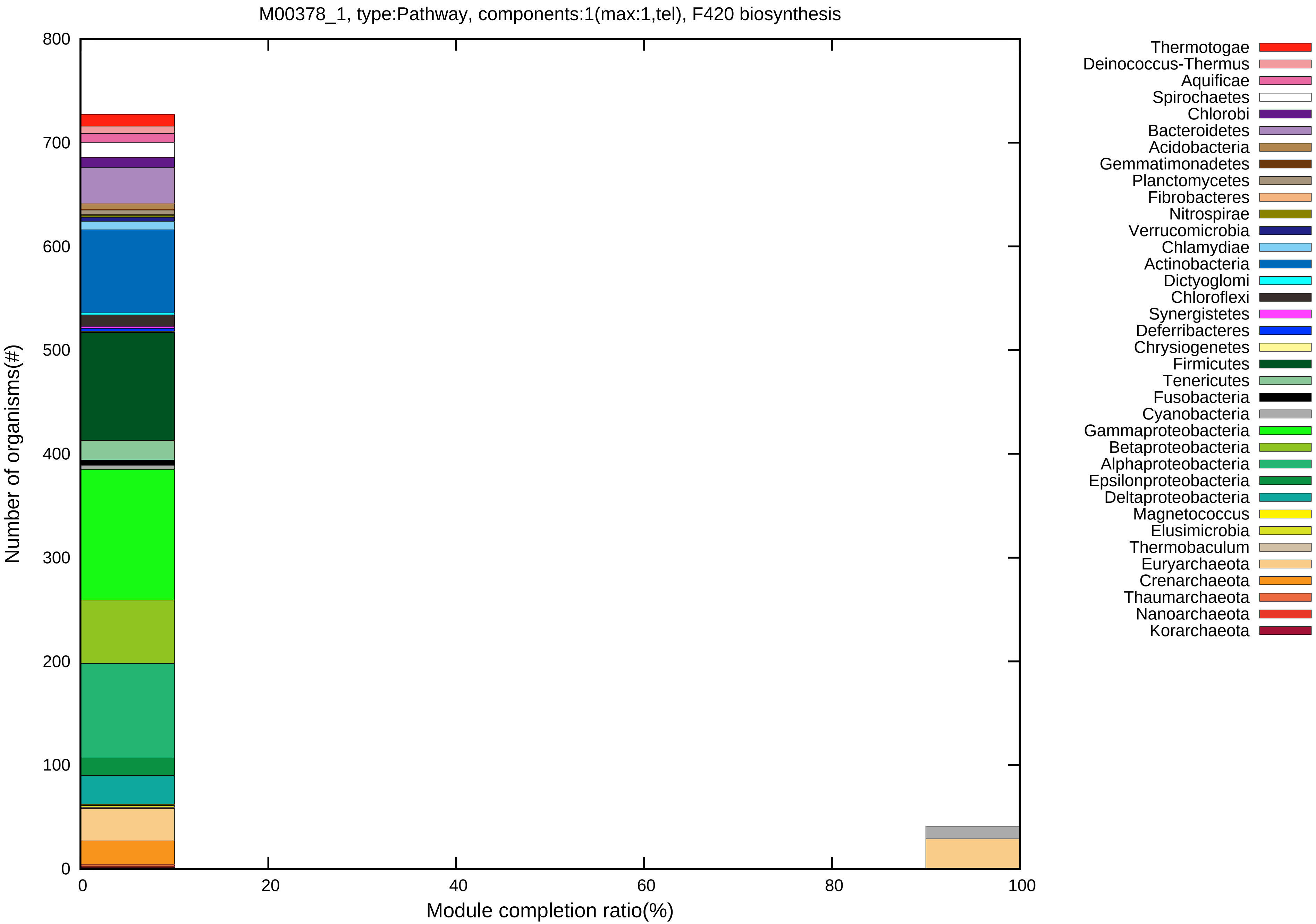

M00378_1, type:Pathway, components:1(max:1,tel), F420 biosynthesis
Number of organisms(#)
Module completion ratio(%)
0
100
200
300
400
500
600
700
800
0
20
40
60
80
100
Thermotogae
Deinococcus-Thermus
Aquificae
Spirochaetes
Chlorobi
Bacteroidetes
Acidobacteria
Gemmatimonadetes
Planctomycetes
Fibrobacteres
Nitrospirae
Verrucomicrobia
Chlamydiae
Actinobacteria
Dictyoglomi
Chloroflexi
Synergistetes
Deferribacteres
Chrysiogenetes
Firmicutes
Tenericutes
Fusobacteria
Cyanobacteria
Gammaproteobacteria
Betaproteobacteria
Alphaproteobacteria
Epsilonproteobacteria
Deltaproteobacteria
Magnetococcus
Elusimicrobia
Thermobaculum
Euryarchaeota
Crenarchaeota
Thaumarchaeota
Nanoarchaeota
Korarchaeota

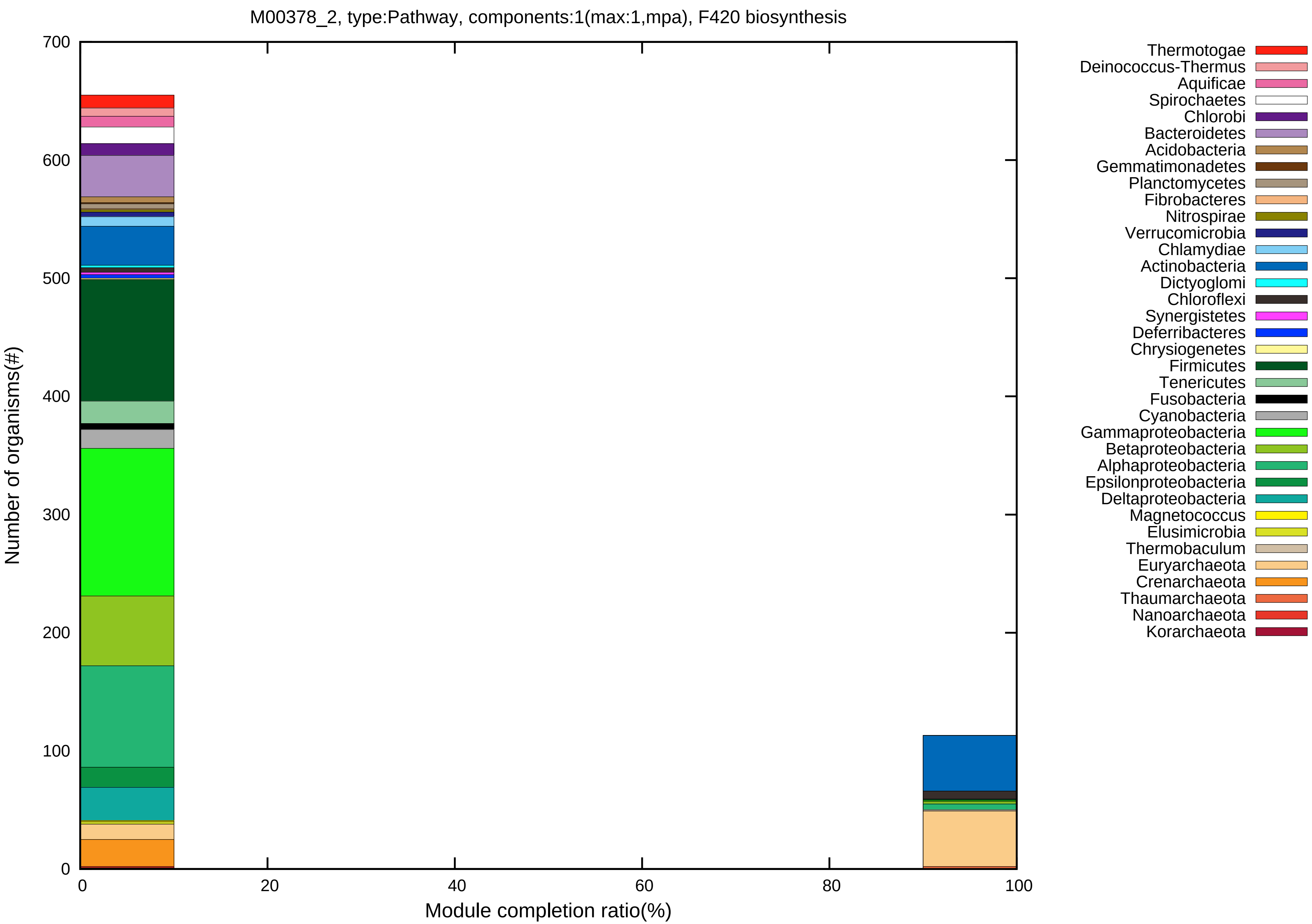
M00378_2, type:Pathway, components:1(max:1,mpa), F420 biosynthesis
Number of organisms(#)
Module completion ratio(%)
0
100
200
300
400
500
600
700
0
20
40
60
80
100
Thermotogae
Deinococcus-Thermus
Aquificae
Spirochaetes
Chlorobi
Bacteroidetes
Acidobacteria
Gemmatimonadetes
Planctomycetes
Fibrobacteres
Nitrospirae
Verrucomicrobia
Chlamydiae
Actinobacteria
Dictyoglomi
Chloroflexi
Synergistetes
Deferribacteres
Chrysiogenetes
Firmicutes
Tenericutes
Fusobacteria
Cyanobacteria
Gammaproteobacteria
Betaproteobacteria
Alphaproteobacteria
Epsilonproteobacteria
Deltaproteobacteria
Magnetococcus
Elusimicrobia
Thermobaculum
Euryarchaeota
Crenarchaeota
Thaumarchaeota
Nanoarchaeota
Korarchaeota

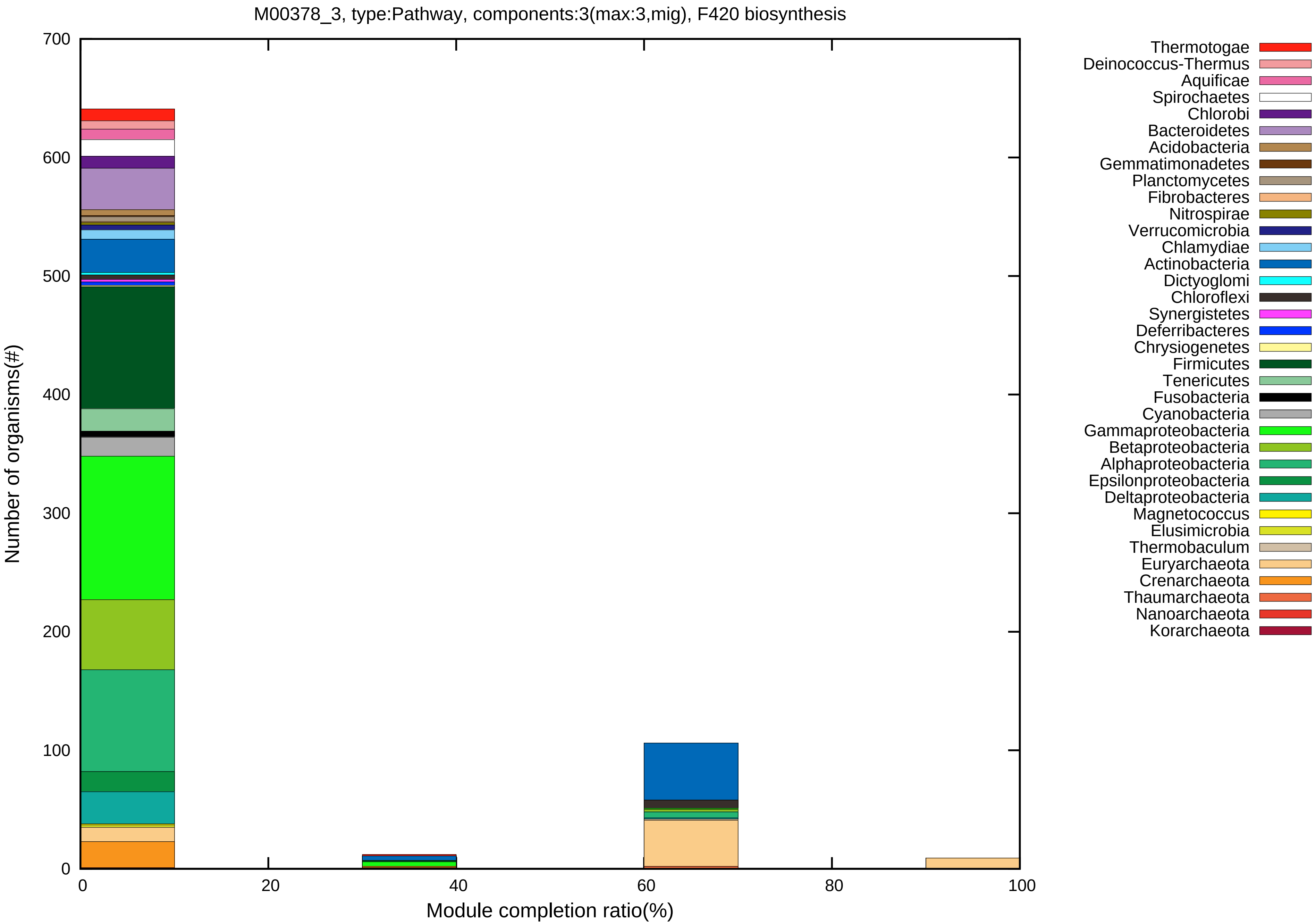
M00378_3, type:Pathway, components:3(max:3,mig), F420 biosynthesis
Number of organisms(#)
Module completion ratio(%)
0
100
200
300
400
500
600
700
0
20
40
60
80
100
Thermotogae
Deinococcus-Thermus
Aquificae
Spirochaetes
Chlorobi
Bacteroidetes
Acidobacteria
Gemmatimonadetes
Planctomycetes
Fibrobacteres
Nitrospirae
Verrucomicrobia
Chlamydiae
Actinobacteria
Dictyoglomi
Chloroflexi
Synergistetes
Deferribacteres
Chrysiogenetes
Firmicutes
Tenericutes
Fusobacteria
Cyanobacteria
Gammaproteobacteria
Betaproteobacteria
Alphaproteobacteria
Epsilonproteobacteria
Deltaproteobacteria
Magnetococcus
Elusimicrobia
Thermobaculum
Euryarchaeota
Crenarchaeota
Thaumarchaeota
Nanoarchaeota
Korarchaeota

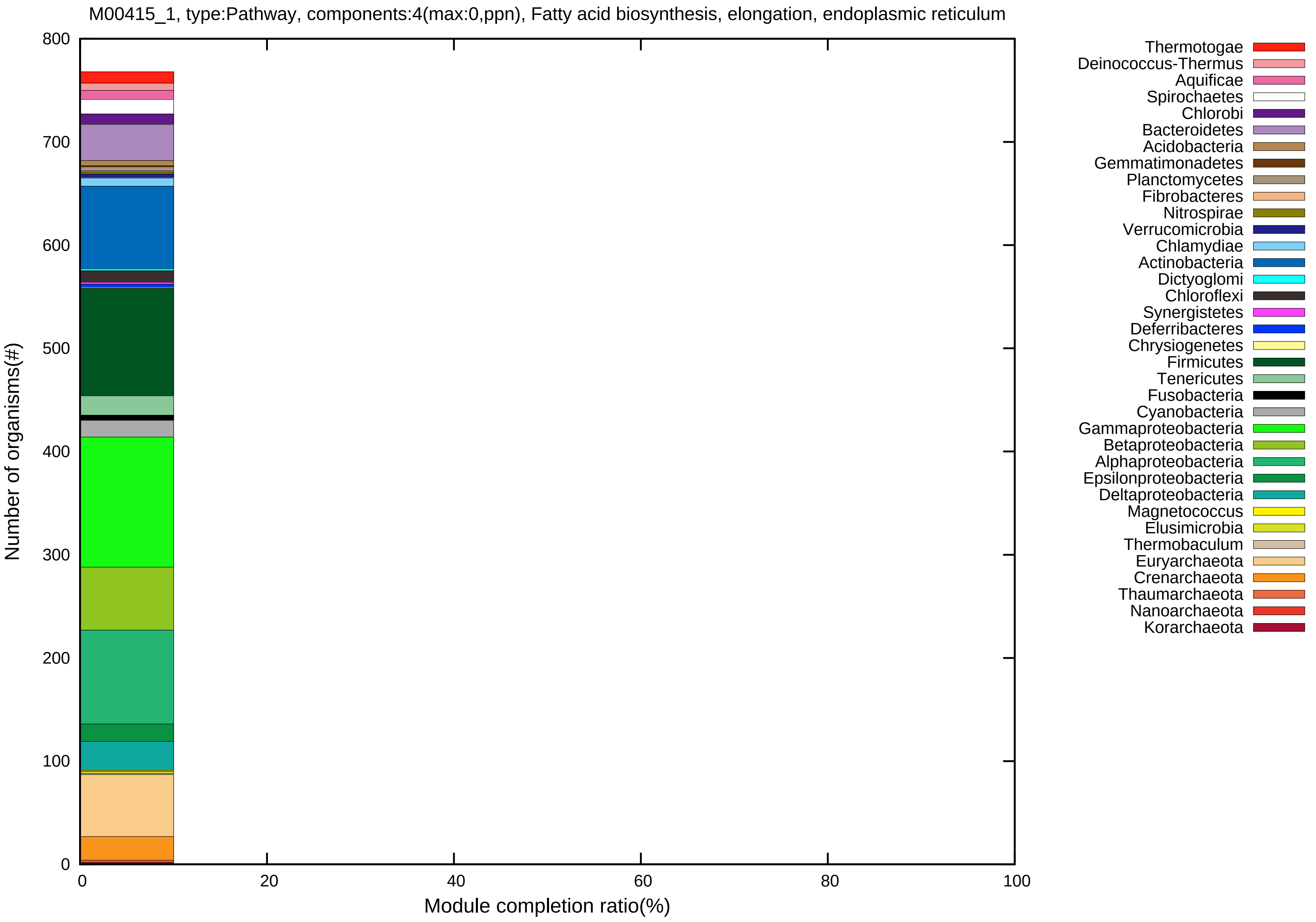

M00415_1, type:Pathway, components:4(max:0,ppn), Fatty acid biosynthesis, elongation, endoplasmic reticulum
Number of organisms(#)
Module completion ratio(%)
0
100
200
300
400
500
600
700
800
0
20
40
60
80
100
Thermotogae
Deinococcus-Thermus
Aquificae
Spirochaetes
Chlorobi
Bacteroidetes
Acidobacteria
Gemmatimonadetes
Planctomycetes
Fibrobacteres
Nitrospirae
Verrucomicrobia
Chlamydiae
Actinobacteria
Dictyoglomi
Chloroflexi
Synergistetes
Deferribacteres
Chrysiogenetes
Firmicutes
Tenericutes
Fusobacteria
Cyanobacteria
Gammaproteobacteria
Betaproteobacteria
Alphaproteobacteria
Epsilonproteobacteria
Deltaproteobacteria
Magnetococcus
Elusimicrobia
Thermobaculum
Euryarchaeota
Crenarchaeota
Thaumarchaeota
Nanoarchaeota
Korarchaeota

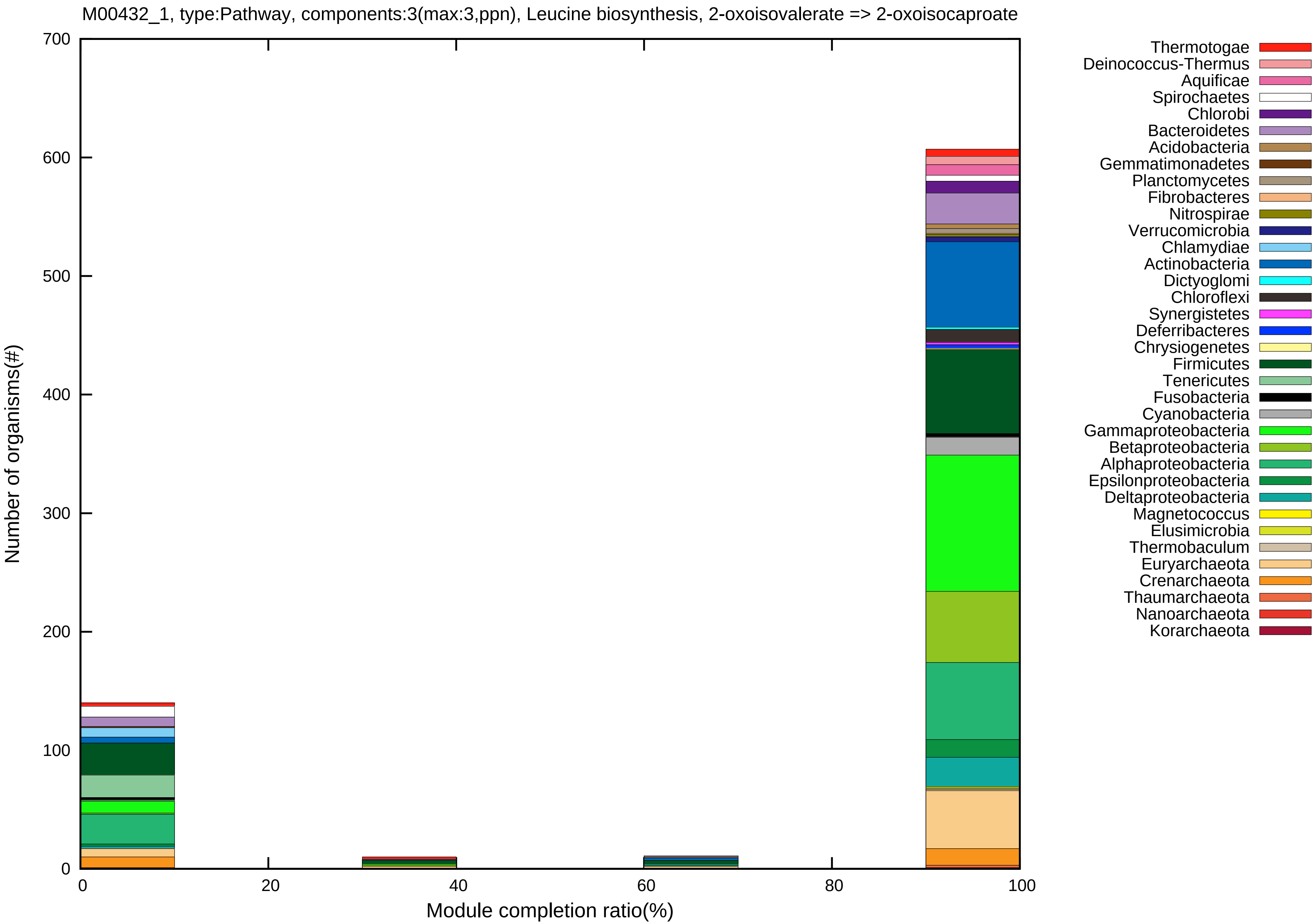

M00432_1, type:Pathway, components:3(max:3,ppn), Leucine biosynthesis, 2-oxoisovalerate => 2-oxoisocaproate
Number of organisms(#)
Module completion ratio(%)
0
100
200
300
400
500
600
700
0
20
40
60
80
100
Thermotogae
Deinococcus-Thermus
Aquificae
Spirochaetes
Chlorobi
Bacteroidetes
Acidobacteria
Gemmatimonadetes
Planctomycetes
Fibrobacteres
Nitrospirae
Verrucomicrobia
Chlamydiae
Actinobacteria
Dictyoglomi
Chloroflexi
Synergistetes
Deferribacteres
Chrysiogenetes
Firmicutes
Tenericutes
Fusobacteria
Cyanobacteria
Gammaproteobacteria
Betaproteobacteria
Alphaproteobacteria
Epsilonproteobacteria
Deltaproteobacteria
Magnetococcus
Elusimicrobia
Thermobaculum
Euryarchaeota
Crenarchaeota
Thaumarchaeota
Nanoarchaeota
Korarchaeota

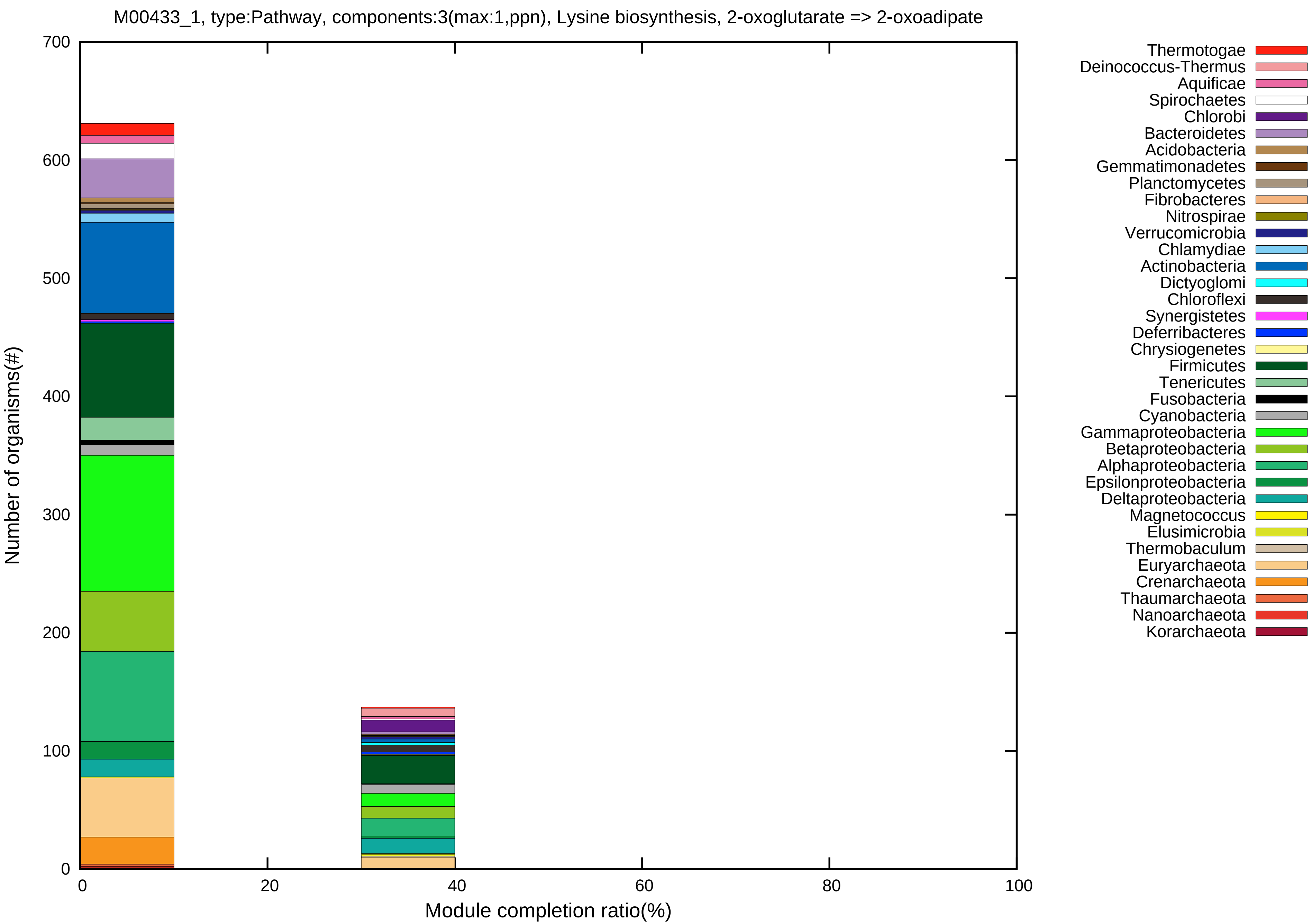

M00433_1, type:Pathway, components:3(max:1,ppn), Lysine biosynthesis, 2-oxoglutarate => 2-oxoadipate
Number of organisms(#)
Module completion ratio(%)
0
100
200
300
400
500
600
700
0
20
40
60
80
100
Thermotogae
Deinococcus-Thermus
Aquificae
Spirochaetes
Chlorobi
Bacteroidetes
Acidobacteria
Gemmatimonadetes
Planctomycetes
Fibrobacteres
Nitrospirae
Verrucomicrobia
Chlamydiae
Actinobacteria
Dictyoglomi
Chloroflexi
Synergistetes
Deferribacteres
Chrysiogenetes
Firmicutes
Tenericutes
Fusobacteria
Cyanobacteria
Gammaproteobacteria
Betaproteobacteria
Alphaproteobacteria
Epsilonproteobacteria
Deltaproteobacteria
Magnetococcus
Elusimicrobia
Thermobaculum
Euryarchaeota
Crenarchaeota
Thaumarchaeota
Nanoarchaeota
Korarchaeota

Figure S2. Distribution patterns of the completion ratio of the KEGG structural complex modules in 768 prokaryotic species. The module completion ratio of 263 structural complex modules containing submodules was evaluated in this study.

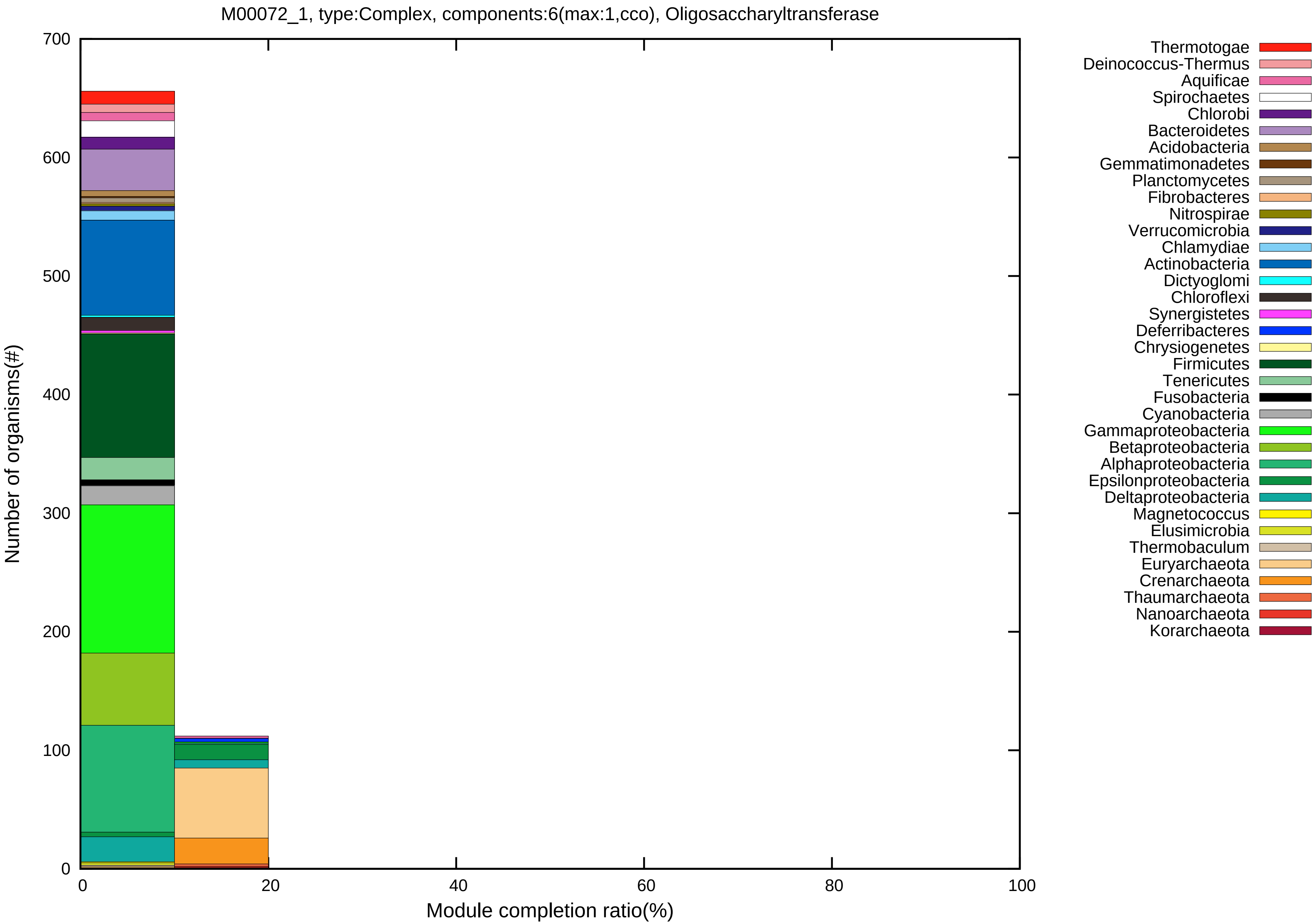
M00072_1, type:Complex, components:6(max:1,cco), Oligosaccharyltransferase
Number of organisms(#)
Module completion ratio(%)
0
100
200
300
400
500
600
700
0
20
40
60
80
100
Thermotogae
Deinococcus-Thermus
Aquificae
Spirochaetes
Chlorobi
Bacteroidetes
Acidobacteria
Gemmatimonadetes
Planctomycetes
Fibrobacteres
Nitrospirae
Verrucomicrobia
Chlamydiae
Actinobacteria
Dictyoglomi
Chloroflexi
Synergistetes
Deferribacteres
Chrysiogenetes
Firmicutes
Tenericutes
Fusobacteria
Cyanobacteria
Gammaproteobacteria
Betaproteobacteria
Alphaproteobacteria
Epsilonproteobacteria
Deltaproteobacteria
Magnetococcus
Elusimicrobia
Thermobaculum
Euryarchaeota
Crenarchaeota
Thaumarchaeota
Nanoarchaeota
Korarchaeota

M00142_1, type:Complex, components:7(max:0,ppn), NADH:ubiquinone oxidoreductase, mitochondria

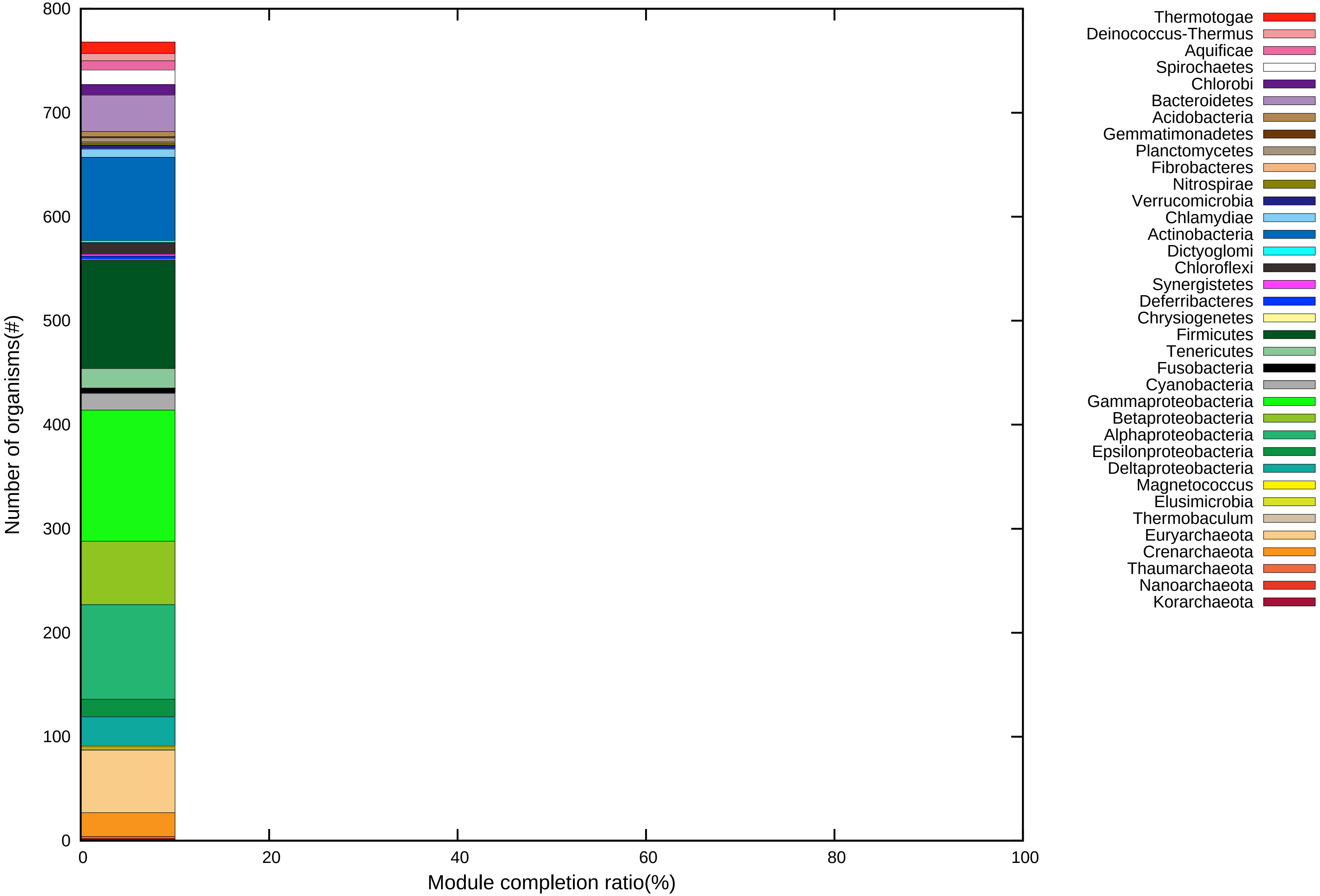

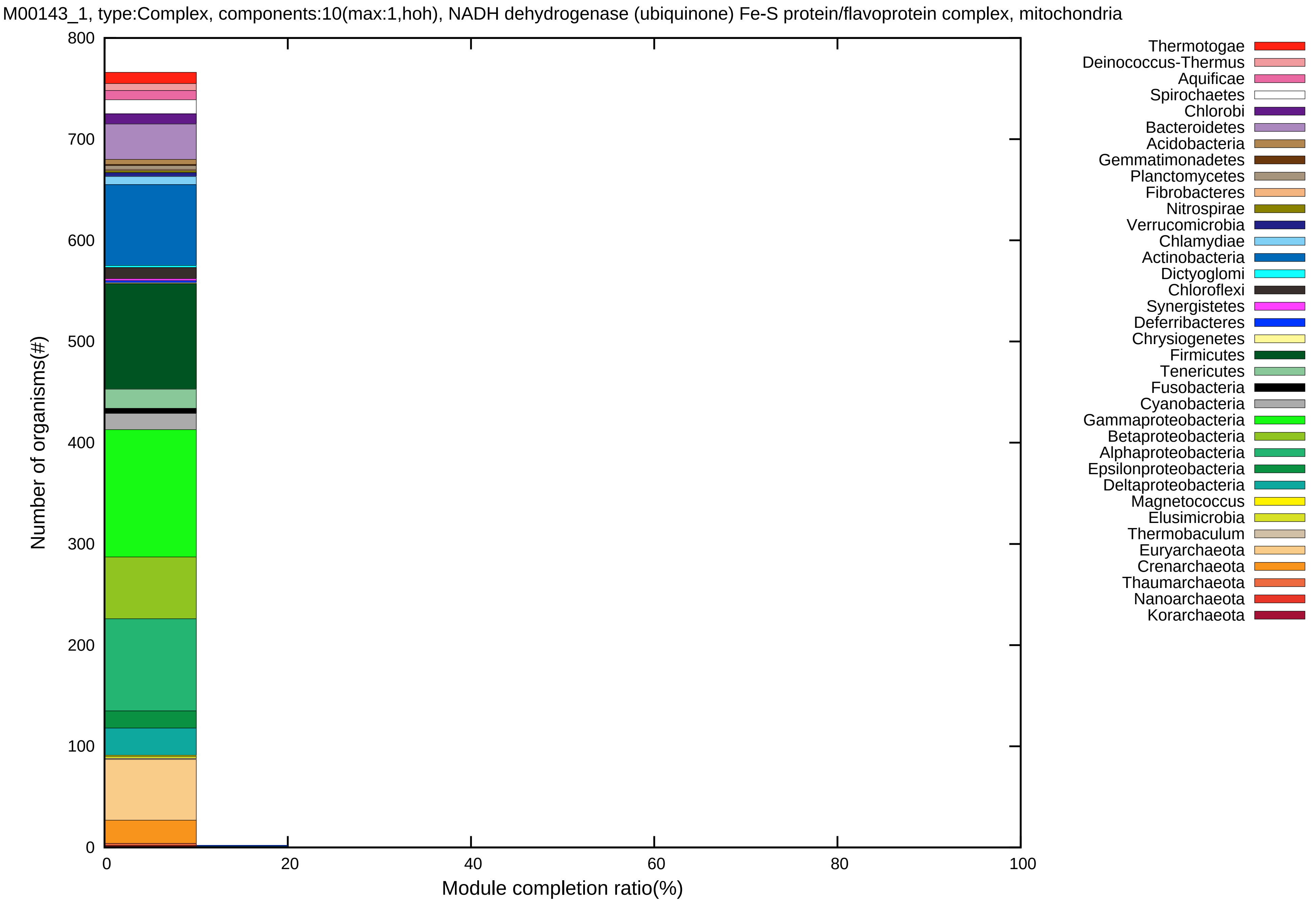
M00143_1, type:Complex, components:10(max:1,hoh), NADH dehydrogenase (ubiquinone) Fe-S protein/flavoprotein complex, mitochondria
Number of organisms(#)
Module completion ratio(%)
0
100
200
300
400
500
600
700
800
0
20
40
60
80
100
Thermotogae
Deinococcus-Thermus
Aquificae
Spirochaetes
Chlorobi
Bacteroidetes
Acidobacteria
Gemmatimonadetes
Planctomycetes
Fibrobacteres
Nitrospirae
Verrucomicrobia
Chlamydiae
Actinobacteria
Dictyoglomi
Chloroflexi
Synergistetes
Deferribacteres
Chrysiogenetes
Firmicutes
Tenericutes
Fusobacteria
Cyanobacteria
Gammaproteobacteria
Betaproteobacteria
Alphaproteobacteria
Epsilonproteobacteria
Deltaproteobacteria
Magnetococcus
Elusimicrobia
Thermobaculum
Euryarchaeota
Crenarchaeota
Thaumarchaeota
Nanoarchaeota
Korarchaeota

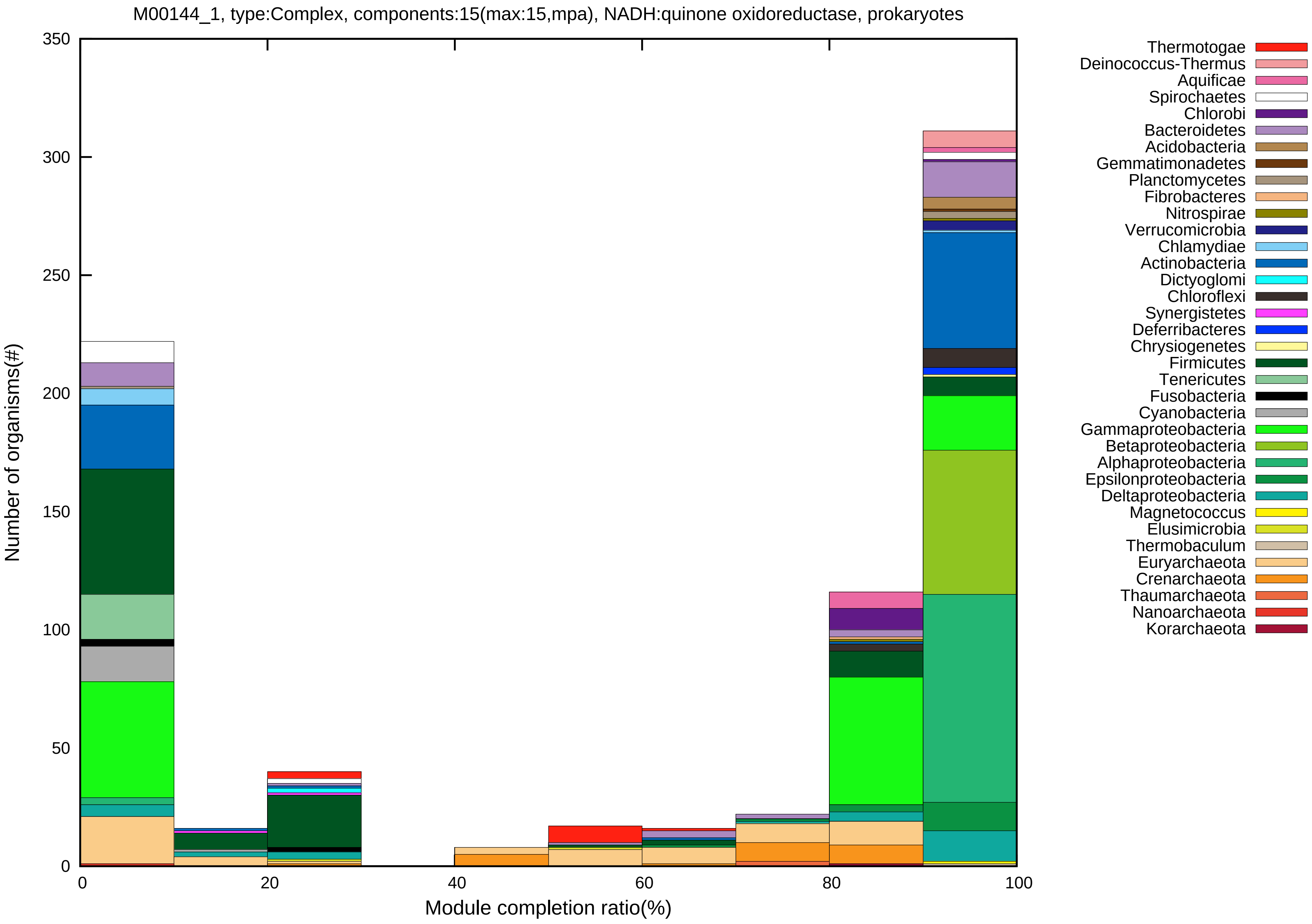
M00144_1, type:Complex, components:15(max:15,mpa), NADH:quinone oxidoreductase, prokaryotes
Number of organisms(#)
Module completion ratio(%)
0
50
100
150
200
250
300
350
0
20
40
60
80
100
Thermotogae
Deinococcus-Thermus
Aquificae
Spirochaetes
Chlorobi
Bacteroidetes
Acidobacteria
Gemmatimonadetes
Planctomycetes
Fibrobacteres
Nitrospirae
Verrucomicrobia
Chlamydiae
Actinobacteria
Dictyoglomi
Chloroflexi
Synergistetes
Deferribacteres
Chrysiogenetes
Firmicutes
Tenericutes
Fusobacteria
Cyanobacteria
Gammaproteobacteria
Betaproteobacteria
Alphaproteobacteria
Epsilonproteobacteria
Deltaproteobacteria
Magnetococcus
Elusimicrobia
Thermobaculum
Euryarchaeota
Crenarchaeota
Thaumarchaeota
Nanoarchaeota
Korarchaeota

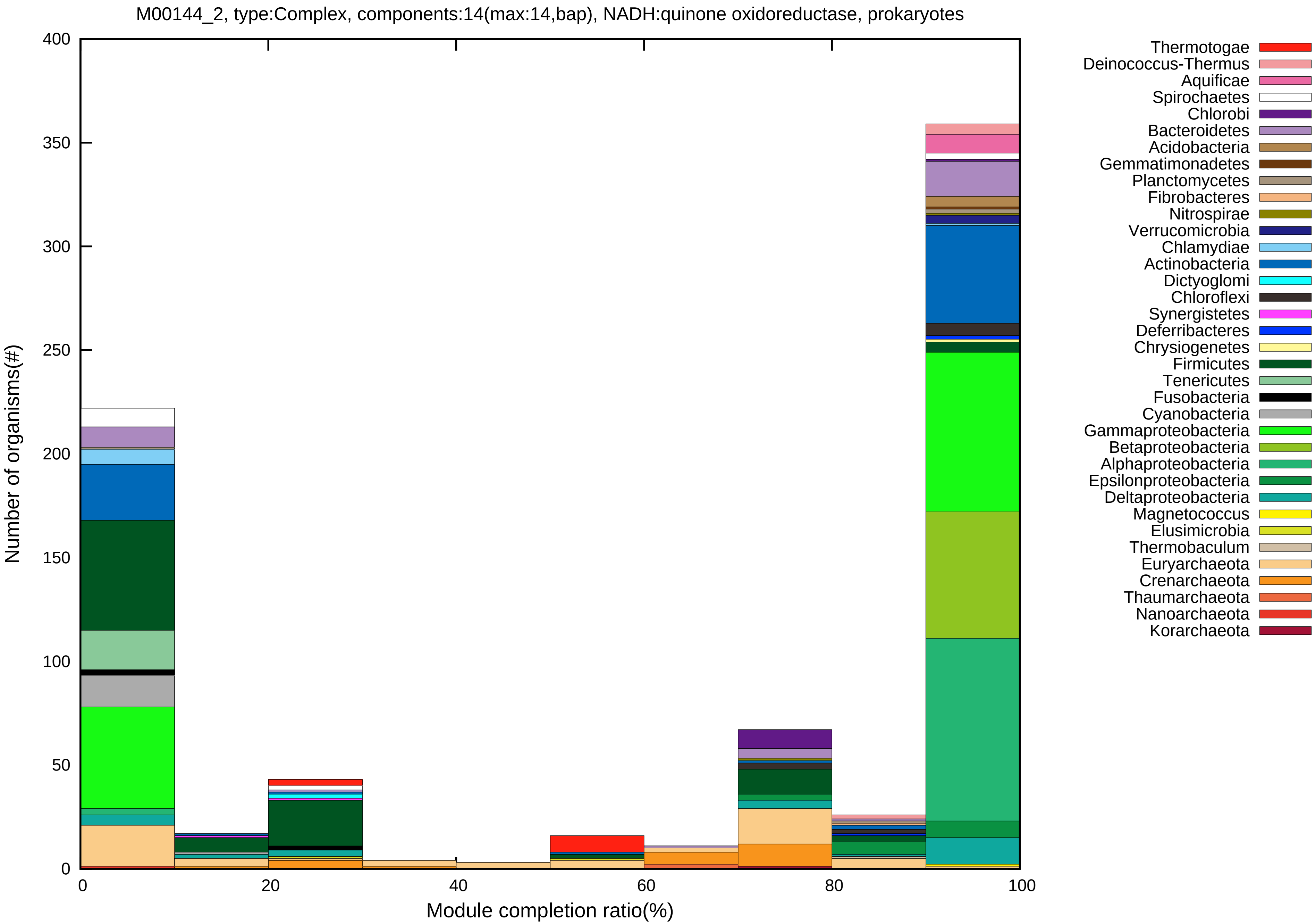

M00144_2, type:Complex, components:14(max:14,bap), NADH:quinone oxidoreductase, prokaryotes
Number of organisms(#)
Module completion ratio(%)
0
50
100
150
200
250
300
350
400
0
20
40
60
80
100
Thermotogae
Deinococcus-Thermus
Aquificae
Spirochaetes
Chlorobi
Bacteroidetes
Acidobacteria
Gemmatimonadetes
Planctomycetes
Fibrobacteres
Nitrospirae
Verrucomicrobia
Chlamydiae
Actinobacteria
Dictyoglomi
Chloroflexi
Synergistetes
Deferribacteres
Chrysiogenetes
Firmicutes
Tenericutes
Fusobacteria
Cyanobacteria
Gammaproteobacteria
Betaproteobacteria
Alphaproteobacteria
Epsilonproteobacteria
Deltaproteobacteria
Magnetococcus
Elusimicrobia
Thermobaculum
Euryarchaeota
Crenarchaeota
Thaumarchaeota
Nanoarchaeota
Korarchaeota

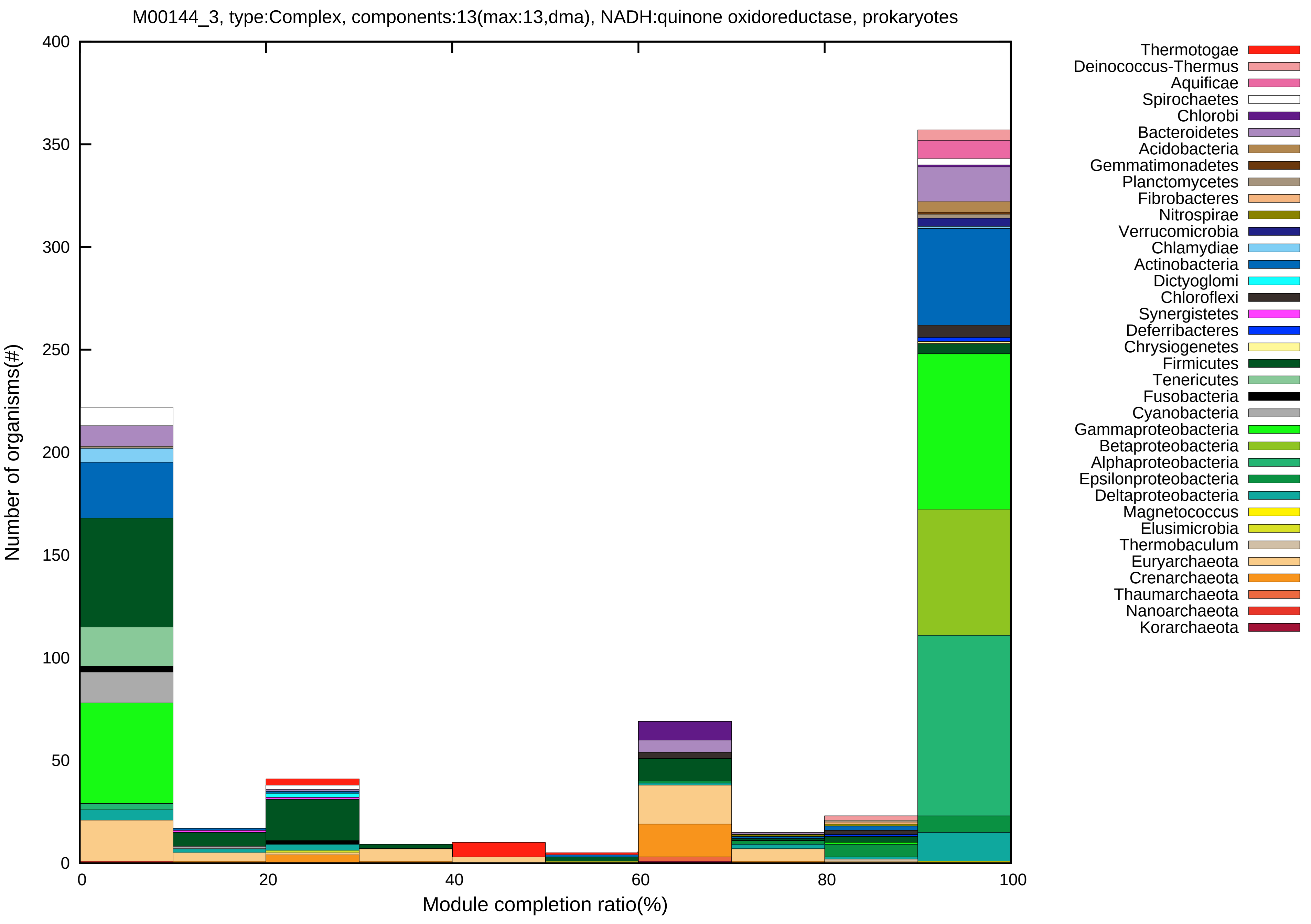

M00144_3, type:Complex, components:13(max:13,dma), NADH:quinone oxidoreductase, prokaryotes
Number of organisms(#)
Module completion ratio(%)
0
50
100
150
200
250
300
350
400
0
20
40
60
80
100
Thermotogae
Deinococcus-Thermus
Aquificae
Spirochaetes
Chlorobi
Bacteroidetes
Acidobacteria
Gemmatimonadetes
Planctomycetes
Fibrobacteres
Nitrospirae
Verrucomicrobia
Chlamydiae
Actinobacteria
Dictyoglomi
Chloroflexi
Synergistetes
Deferribacteres
Chrysiogenetes
Firmicutes
Tenericutes
Fusobacteria
Cyanobacteria
Gammaproteobacteria
Betaproteobacteria
Alphaproteobacteria
Epsilonproteobacteria
Deltaproteobacteria
Magnetococcus
Elusimicrobia
Thermobaculum
Euryarchaeota
Crenarchaeota
Thaumarchaeota
Nanoarchaeota
Korarchaeota

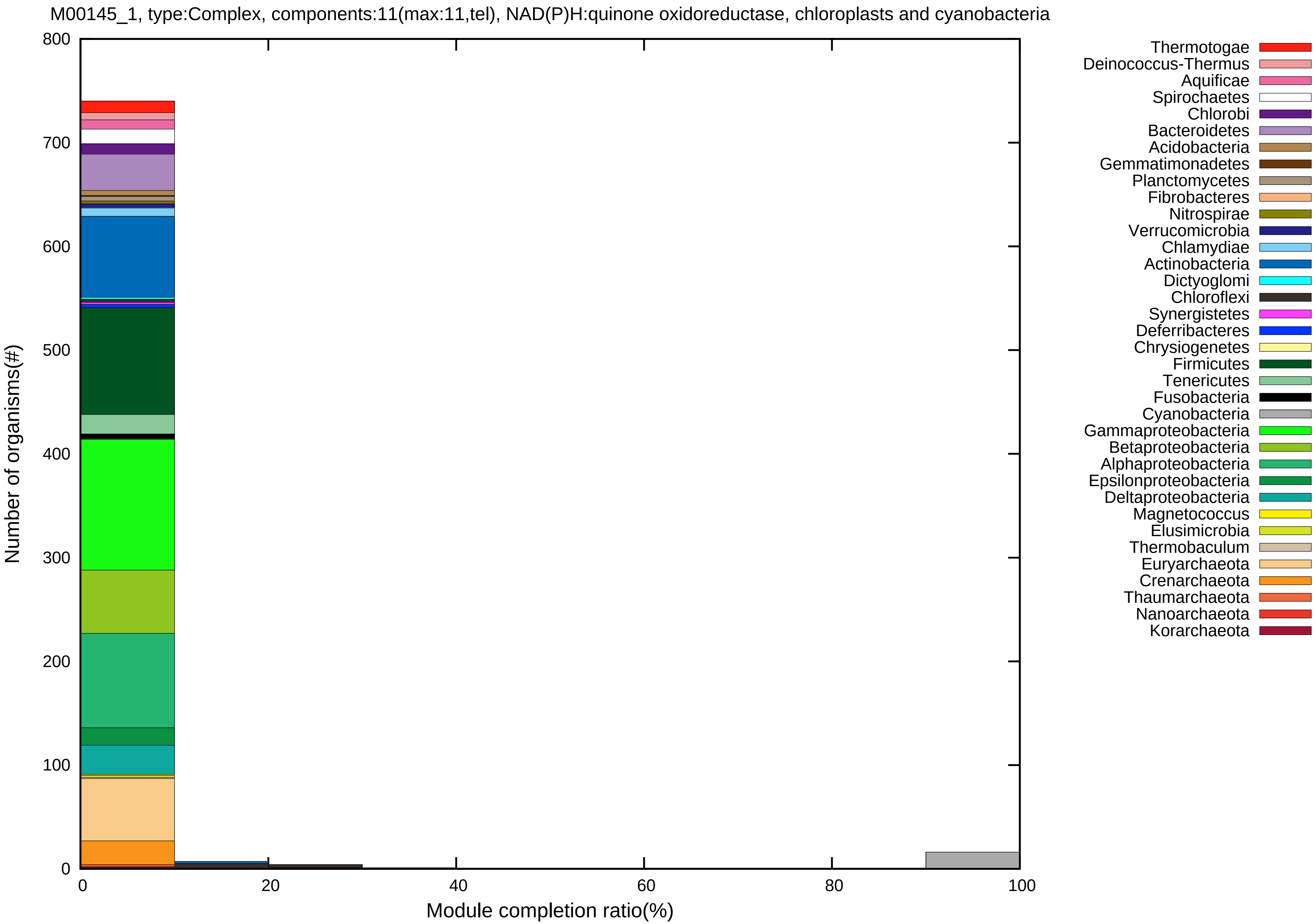
M00145_1, type:Complex, components:11(max:11,tel), NAD(P)H:quinone oxidoreductase, chloroplasts and cyanobacteria
Number of organisms(#)
Module completion ratio(%)
0
100
200
300
400
500
600
700
800
0
20
40
60
80
100
Thermotogae
Deinococcus-Thermus
Aquificae
Spirochaetes
Chlorobi
Bacteroidetes
Acidobacteria
Gemmatimonadetes
Planctomycetes
Fibrobacteres
Nitrospirae
Verrucomicrobia
Chlamydiae
Actinobacteria
Dictyoglomi
Chloroflexi
Synergistetes
Deferribacteres
Chrysiogenetes
Firmicutes
Tenericutes
Fusobacteria
Cyanobacteria
Gammaproteobacteria
Betaproteobacteria
Alphaproteobacteria
Epsilonproteobacteria
Deltaproteobacteria
Magnetococcus
Elusimicrobia
Thermobaculum
Euryarchaeota
Crenarchaeota
Thaumarchaeota
Nanoarchaeota
Korarchaeota

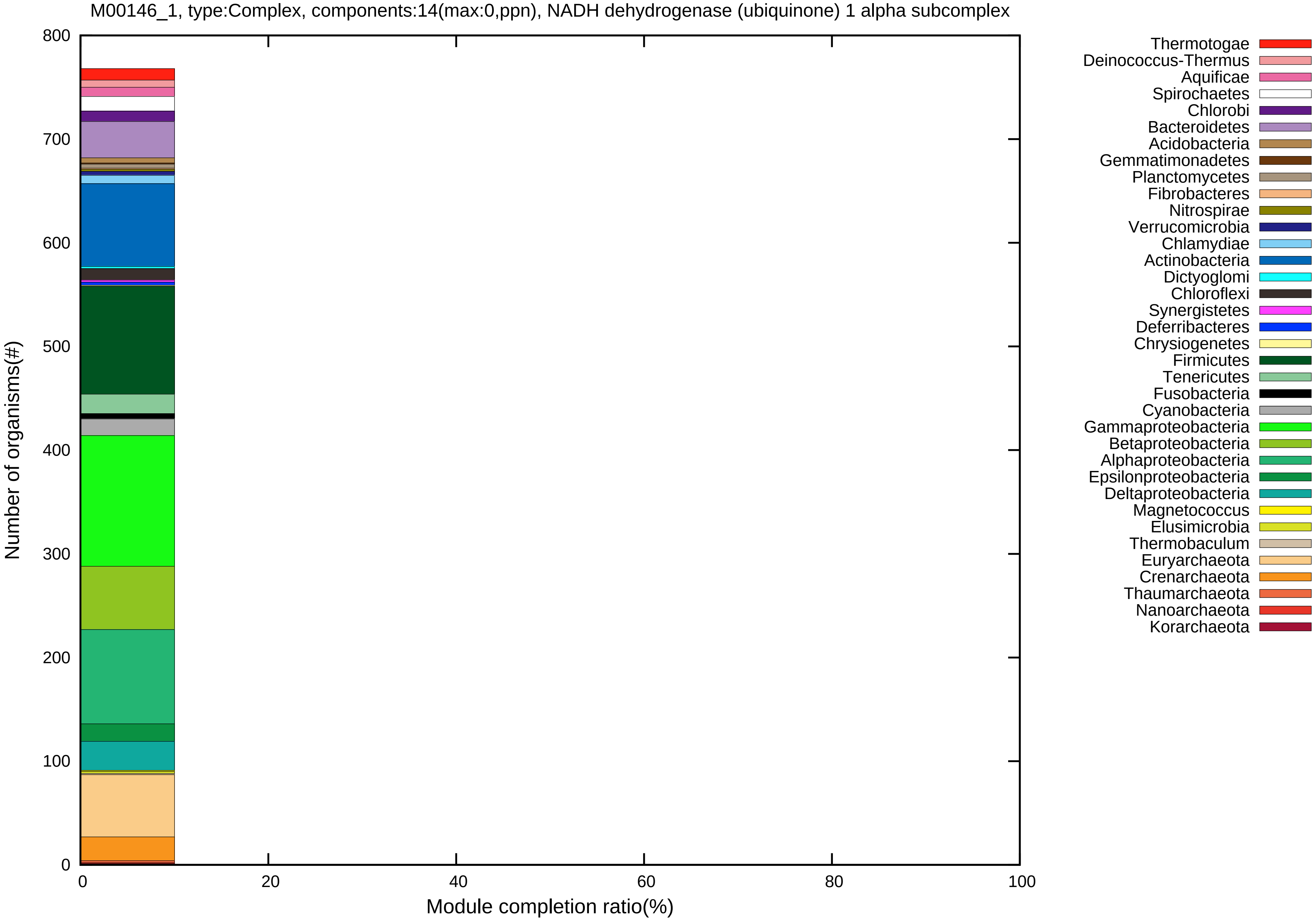

M00146_1, type:Complex, components:14(max:0,ppn), NADH dehydrogenase (ubiquinone) 1 alpha subcomplex
Number of organisms(#)
Module completion ratio(%)
0
100
200
300
400
500
600
700
800
0
20
40
60
80
100
Thermotogae
Deinococcus-Thermus
Aquificae
Spirochaetes
Chlorobi
Bacteroidetes
Acidobacteria
Gemmatimonadetes
Planctomycetes
Fibrobacteres
Nitrospirae
Verrucomicrobia
Chlamydiae
Actinobacteria
Dictyoglomi
Chloroflexi
Synergistetes
Deferribacteres
Chrysiogenetes
Firmicutes
Tenericutes
Fusobacteria
Cyanobacteria
Gammaproteobacteria
Betaproteobacteria
Alphaproteobacteria
Epsilonproteobacteria
Deltaproteobacteria
Magnetococcus
Elusimicrobia
Thermobaculum
Euryarchaeota
Crenarchaeota
Thaumarchaeota
Nanoarchaeota
Korarchaeota

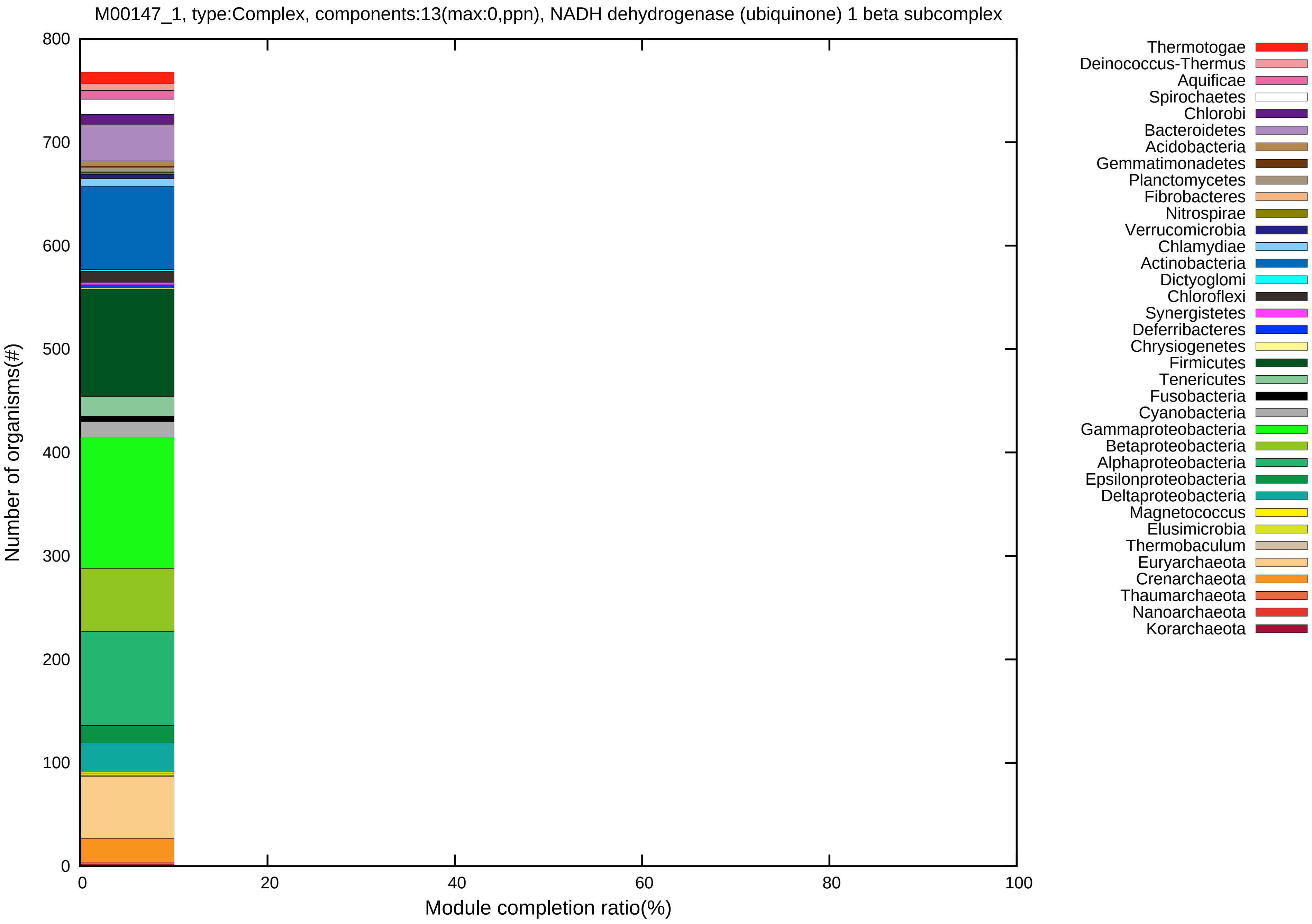

M00147_1, type:Complex, components:13(max:0,ppn), NADH dehydrogenase (ubiquinone) 1 beta subcomplex
Number of organisms(#)
Module completion ratio(%)
0
100
200
300
400
500
600
700
800
0
20
40
60
80
100
Thermotogae
Deinococcus-Thermus
Aquificae
Spirochaetes
Chlorobi
Bacteroidetes
Acidobacteria
Gemmatimonadetes
Planctomycetes
Fibrobacteres
Nitrospirae
Verrucomicrobia
Chlamydiae
Actinobacteria
Dictyoglomi
Chloroflexi
Synergistetes
Deferribacteres
Chrysiogenetes
Firmicutes
Tenericutes
Fusobacteria
Cyanobacteria
Gammaproteobacteria
Betaproteobacteria
Alphaproteobacteria
Epsilonproteobacteria
Deltaproteobacteria
Magnetococcus
Elusimicrobia
Thermobaculum
Euryarchaeota
Crenarchaeota
Thaumarchaeota
Nanoarchaeota
Korarchaeota

M00148_1, type:Complex, components:4(max:0,ppn), Succinate dehydrogenase (ubiquinone)

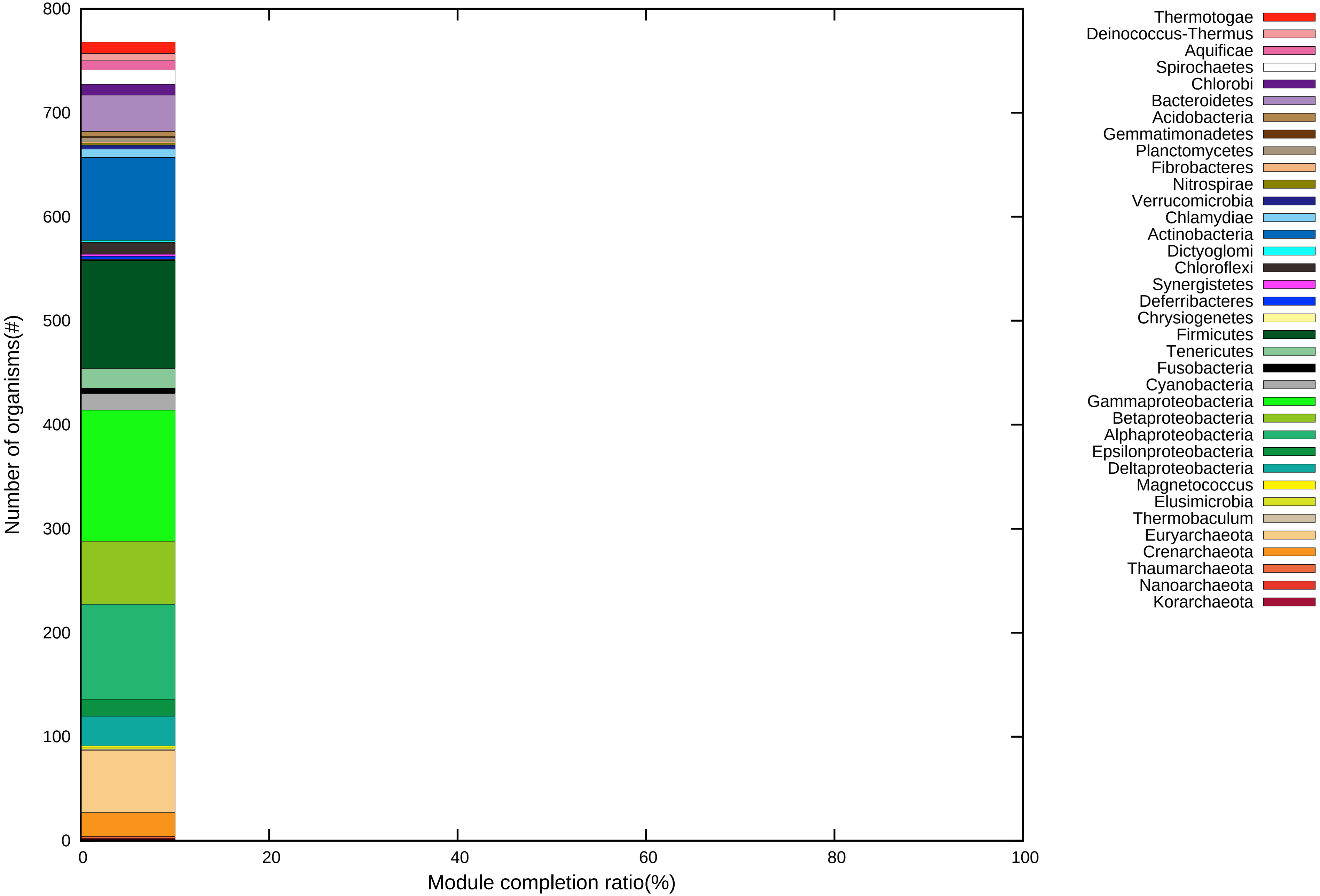

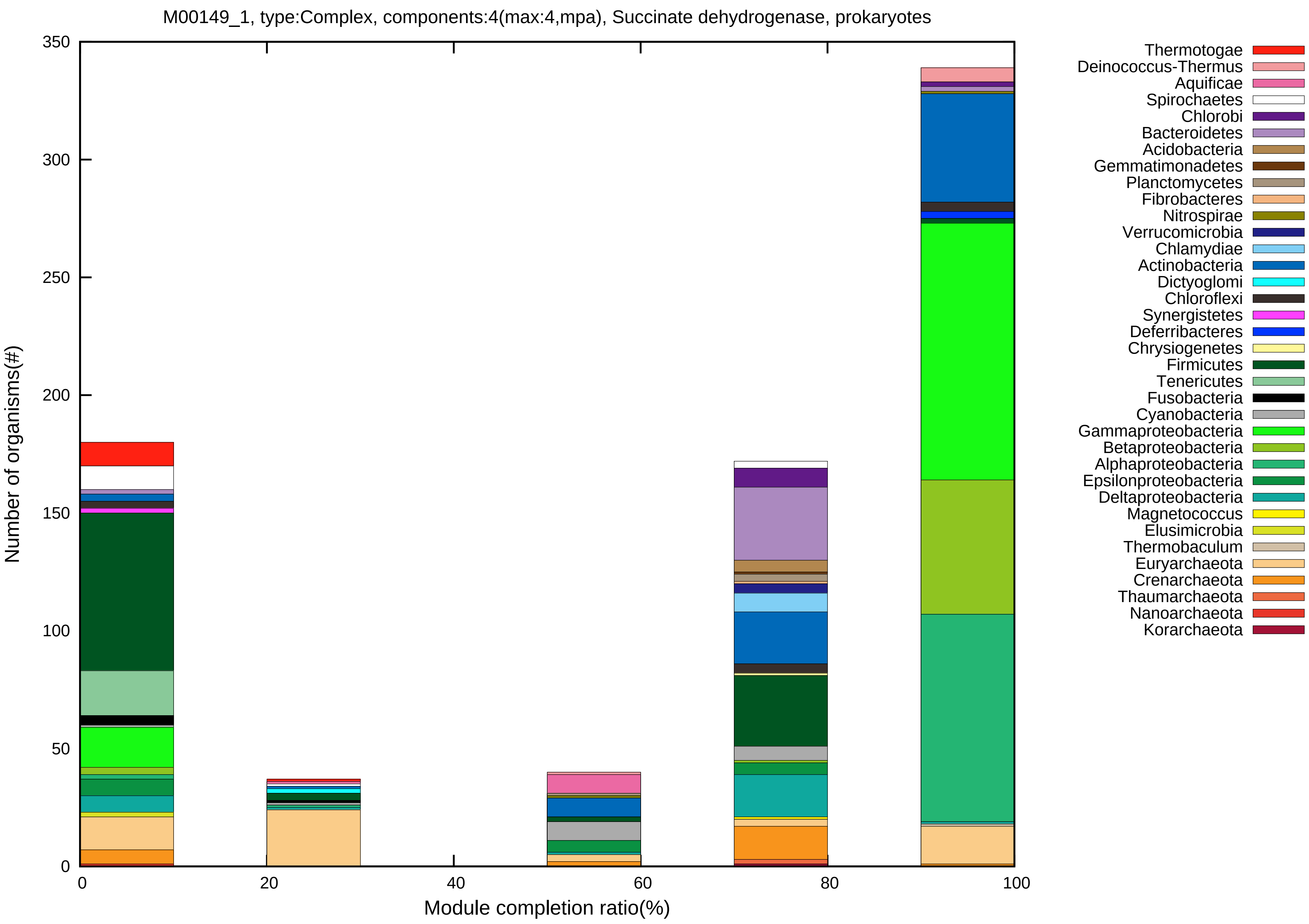

M00149_1, type:Complex, components:4(max:4,mpa), Succinate dehydrogenase, prokaryotes
Number of organisms(#)
Module completion ratio(%)
0
50
100
150
200
250
300
350
0
20
40
60
80
100
Thermotogae
Deinococcus-Thermus
Aquificae
Spirochaetes
Chlorobi
Bacteroidetes
Acidobacteria
Gemmatimonadetes
Planctomycetes
Fibrobacteres
Nitrospirae
Verrucomicrobia
Chlamydiae
Actinobacteria
Dictyoglomi
Chloroflexi
Synergistetes
Deferribacteres
Chrysiogenetes
Firmicutes
Tenericutes
Fusobacteria
Cyanobacteria
Gammaproteobacteria
Betaproteobacteria
Alphaproteobacteria
Epsilonproteobacteria
Deltaproteobacteria
Magnetococcus
Elusimicrobia
Thermobaculum
Euryarchaeota
Crenarchaeota
Thaumarchaeota
Nanoarchaeota
Korarchaeota

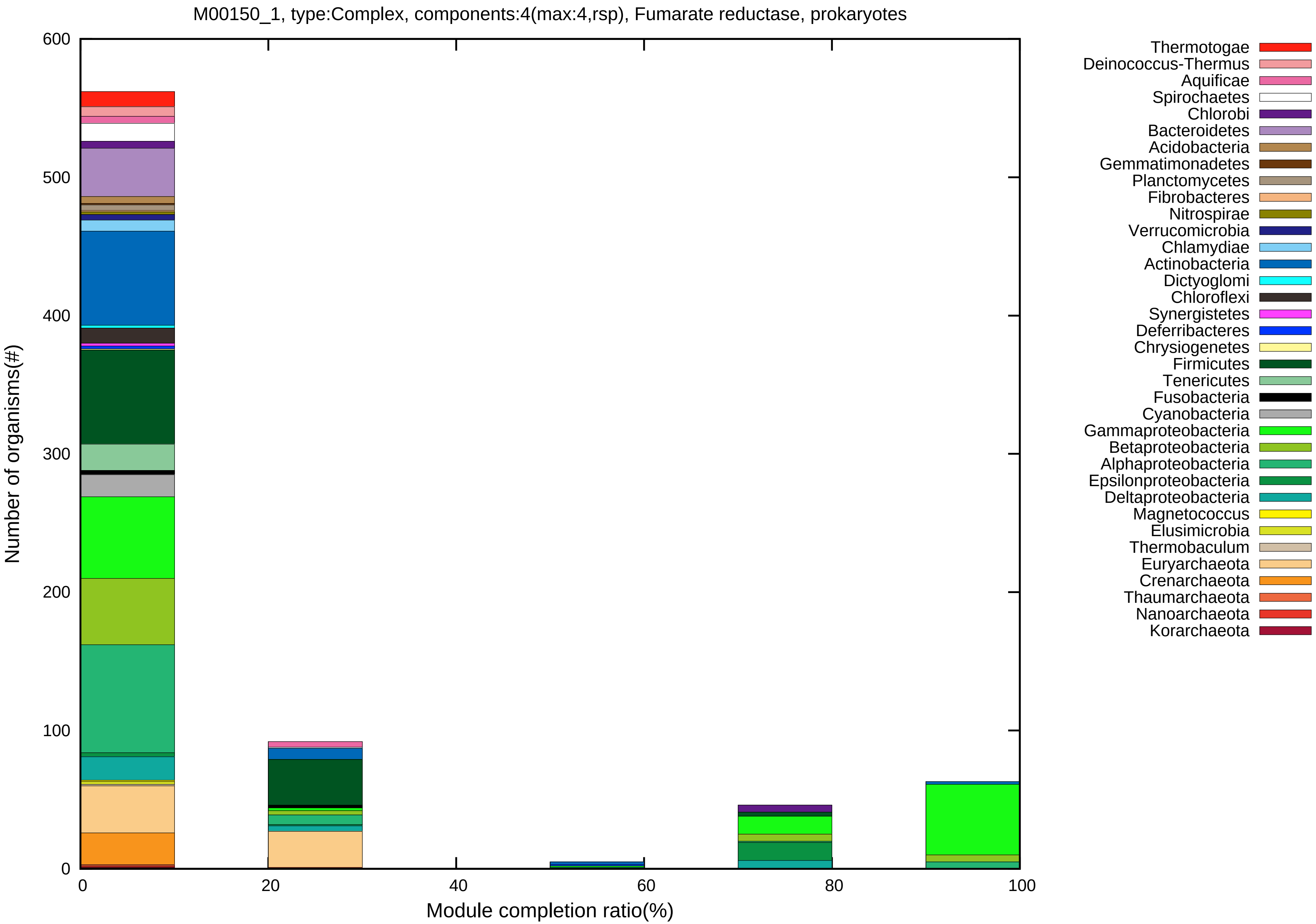

M00150_1, type:Complex, components:4(max:4,rsp), Fumarate reductase, prokaryotes
Number of organisms(#)
Module completion ratio(%)
0
100
200
300
400
500
600
0
20
40
60
80
100
Thermotogae
Deinococcus-Thermus
Aquificae
Spirochaetes
Chlorobi
Bacteroidetes
Acidobacteria
Gemmatimonadetes
Planctomycetes
Fibrobacteres
Nitrospirae
Verrucomicrobia
Chlamydiae
Actinobacteria
Dictyoglomi
Chloroflexi
Synergistetes
Deferribacteres
Chrysiogenetes
Firmicutes
Tenericutes
Fusobacteria
Cyanobacteria
Gammaproteobacteria
Betaproteobacteria
Alphaproteobacteria
Epsilonproteobacteria
Deltaproteobacteria
Magnetococcus
Elusimicrobia
Thermobaculum
Euryarchaeota
Crenarchaeota
Thaumarchaeota
Nanoarchaeota
Korarchaeota

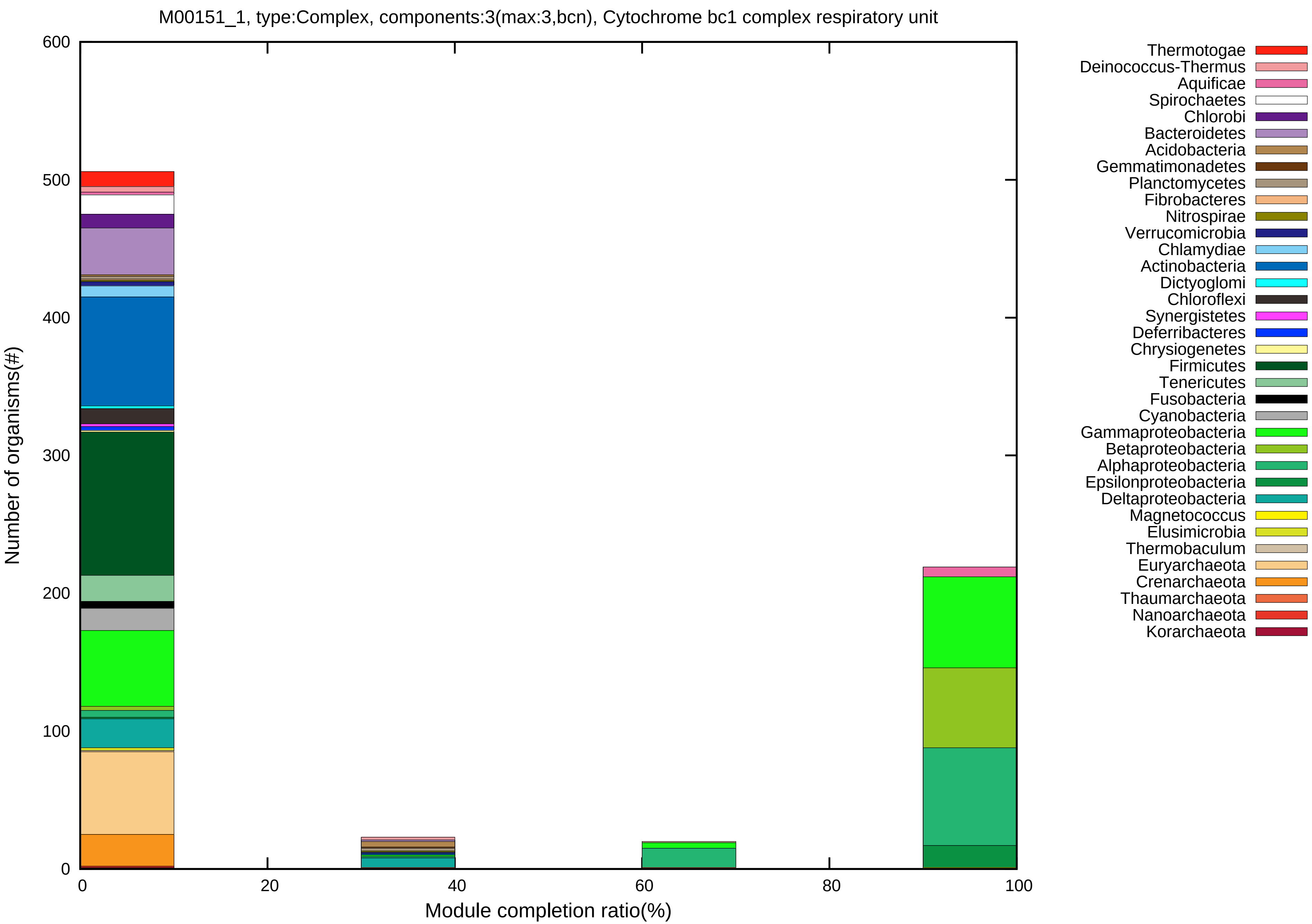
M00151_1, type:Complex, components:3(max:3,bcn), Cytochrome bc1 complex respiratory unit
Number of organisms(#)
Module completion ratio(%)
0
100
200
300
400
500
600
0
20
40
60
80
100
Thermotogae
Deinococcus-Thermus
Aquificae
Spirochaetes
Chlorobi
Bacteroidetes
Acidobacteria
Gemmatimonadetes
Planctomycetes
Fibrobacteres
Nitrospirae
Verrucomicrobia
Chlamydiae
Actinobacteria
Dictyoglomi
Chloroflexi
Synergistetes
Deferribacteres
Chrysiogenetes
Firmicutes
Tenericutes
Fusobacteria
Cyanobacteria
Gammaproteobacteria
Betaproteobacteria
Alphaproteobacteria
Epsilonproteobacteria
Deltaproteobacteria
Magnetococcus
Elusimicrobia
Thermobaculum
Euryarchaeota
Crenarchaeota
Thaumarchaeota
Nanoarchaeota
Korarchaeota

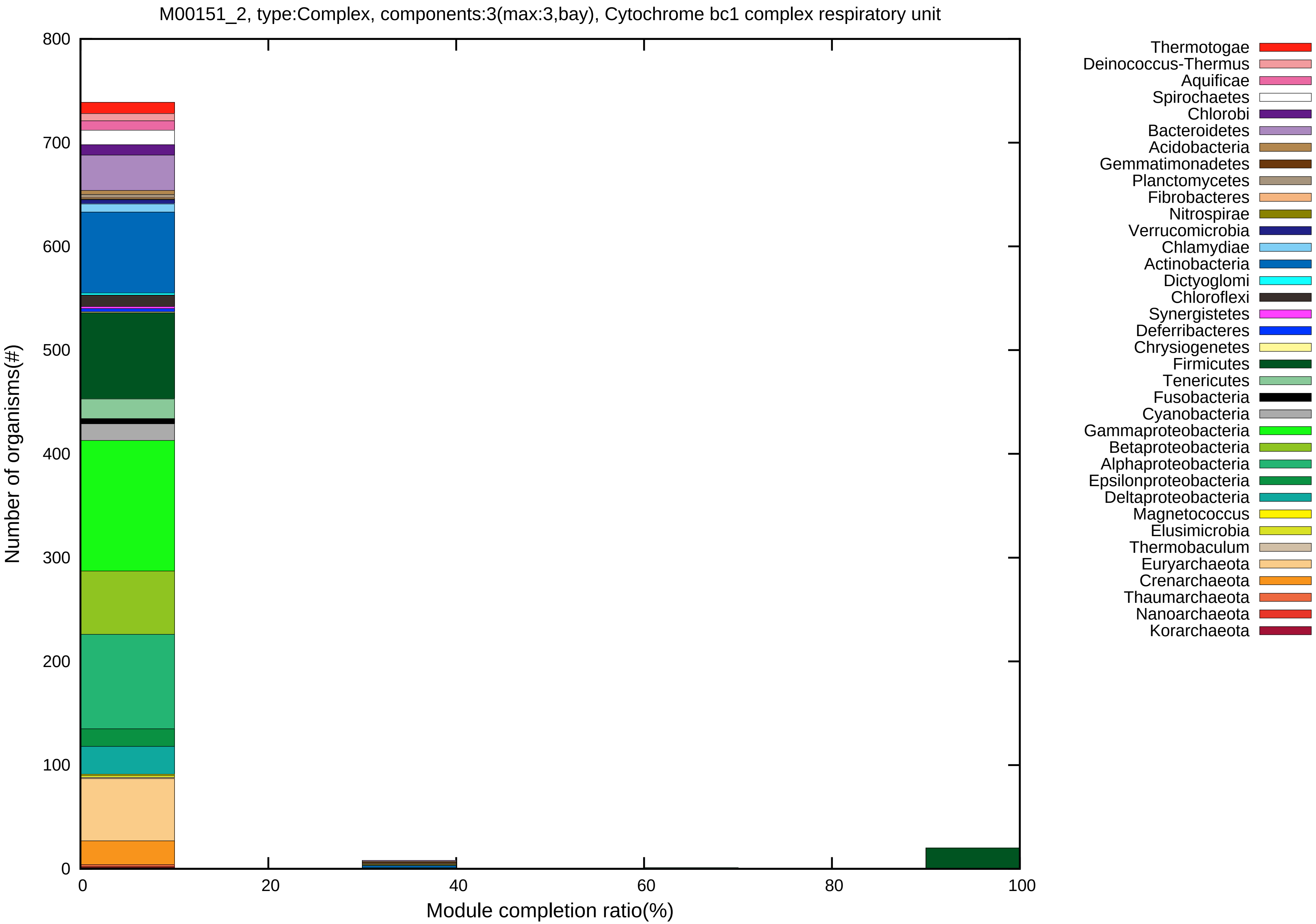

M00151_2, type:Complex, components:3(max:3,bay), Cytochrome bc1 complex respiratory unit
Number of organisms(#)
Module completion ratio(%)
0
20
40
60
80
100
100
200
300
400
500
600
700
800
Thermotogae
Deinococcus-Thermus
Aquificae
Spirochaetes
Chlorobi
Bacteroidetes
Acidobacteria
Gemmatimonadetes
Planctomycetes
Fibrobacteres
Nitrospirae
Verrucomicrobia
Chlamydiae
Actinobacteria
Dictyoglomi
Chloroflexi
Synergistetes
Deferribacteres
Chrysiogenetes
Firmicutes
Tenericutes
Fusobacteria
Cyanobacteria
Gammaproteobacteria
Betaproteobacteria
Alphaproteobacteria
Epsilonproteobacteria
Deltaproteobacteria
Magnetococcus
Elusimicrobia
Thermobaculum
Euryarchaeota
Crenarchaeota
Thaumarchaeota
Nanoarchaeota
Korarchaeota

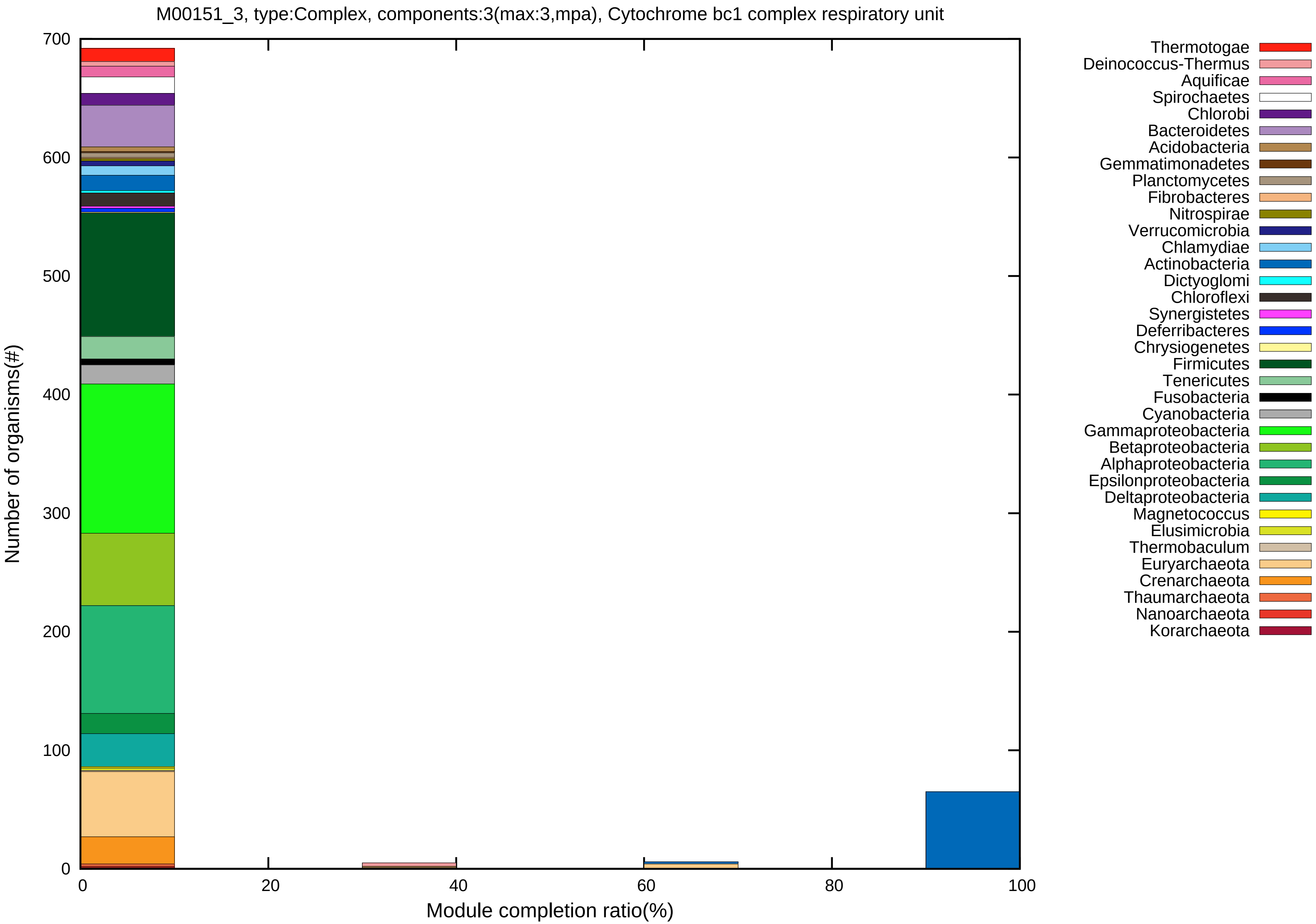
M00151_3, type:Complex, components:3(max:3,mpa), Cytochrome bc1 complex respiratory unit
Number of organisms(#)
Module completion ratio(%)
0
100
200
300
400
500
600
700
0
20
40
60
80
100
Thermotogae
Deinococcus-Thermus
Aquificae
Spirochaetes
Chlorobi
Bacteroidetes
Acidobacteria
Gemmatimonadetes
Planctomycetes
Fibrobacteres
Nitrospirae
Verrucomicrobia
Chlamydiae
Actinobacteria
Dictyoglomi
Chloroflexi
Synergistetes
Deferribacteres
Chrysiogenetes
Firmicutes
Tenericutes
Fusobacteria
Cyanobacteria
Gammaproteobacteria
Betaproteobacteria
Alphaproteobacteria
Epsilonproteobacteria
Deltaproteobacteria
Magnetococcus
Elusimicrobia
Thermobaculum
Euryarchaeota
Crenarchaeota
Thaumarchaeota
Nanoarchaeota
Korarchaeota

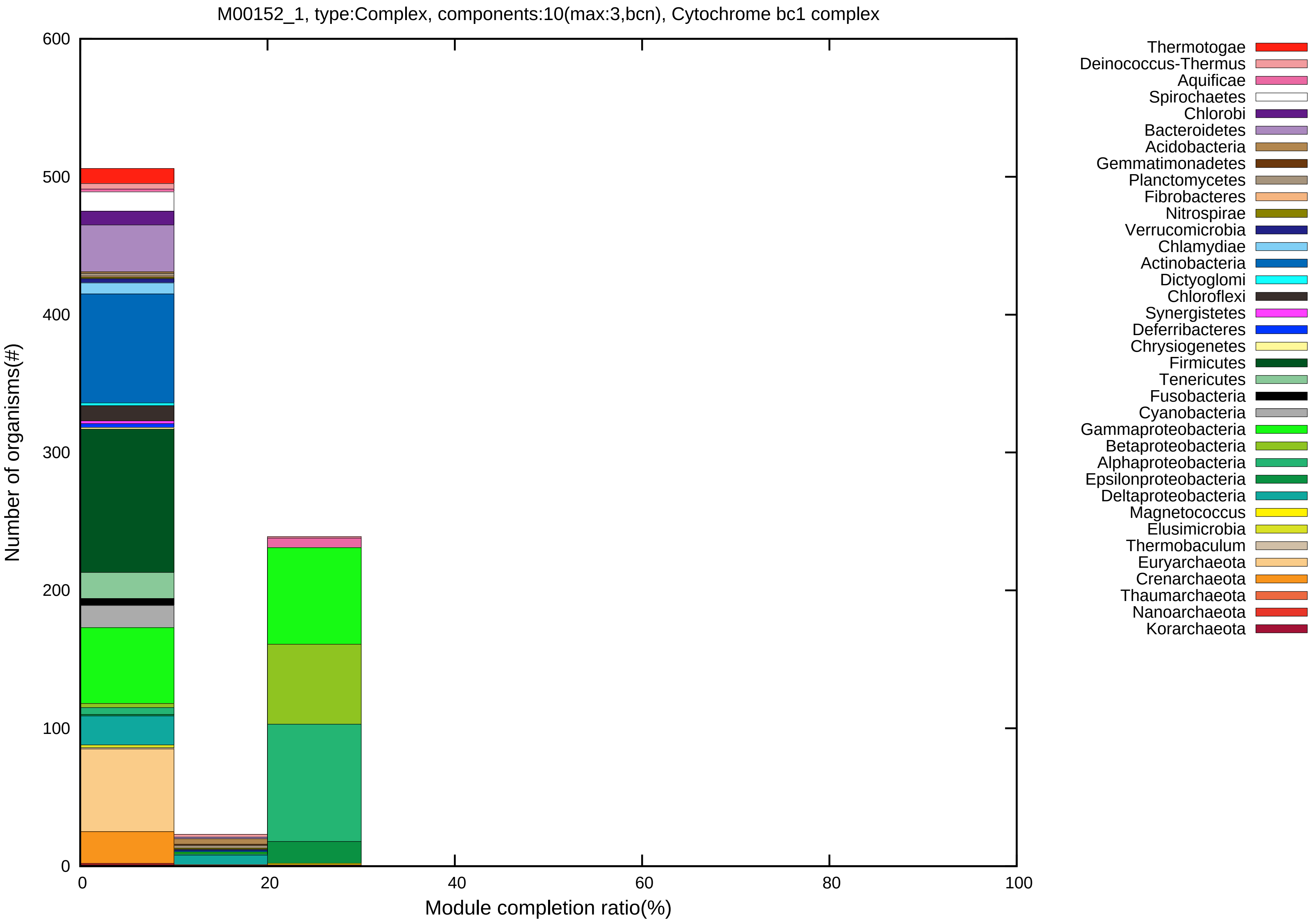
M00152_1, type:Complex, components:10(max:3,bcn), Cytochrome bc1 complex
Number of organisms(#)
0
100
200
300
400
500
600
0
20
40
60
80
100
Module completion ratio(%)
Thermotogae
Deinococcus-Thermus
Aquificae
Spirochaetes
Chlorobi
Bacteroidetes
Acidobacteria
Gemmatimonadetes
Planctomycetes
Fibrobacteres
Nitrospirae
Verrucomicrobia
Chlamydiae
Actinobacteria
Dictyoglomi
Chloroflexi
Synergistetes
Deferribacteres
Chrysiogenetes
Firmicutes
Tenericutes
Fusobacteria
Cyanobacteria
Gammaproteobacteria
Betaproteobacteria
Alphaproteobacteria
Epsilonproteobacteria
Deltaproteobacteria
Magnetococcus
Elusimicrobia
Thermobaculum
Euryarchaeota
Crenarchaeota
Thaumarchaeota
Nanoarchaeota
Korarchaeota

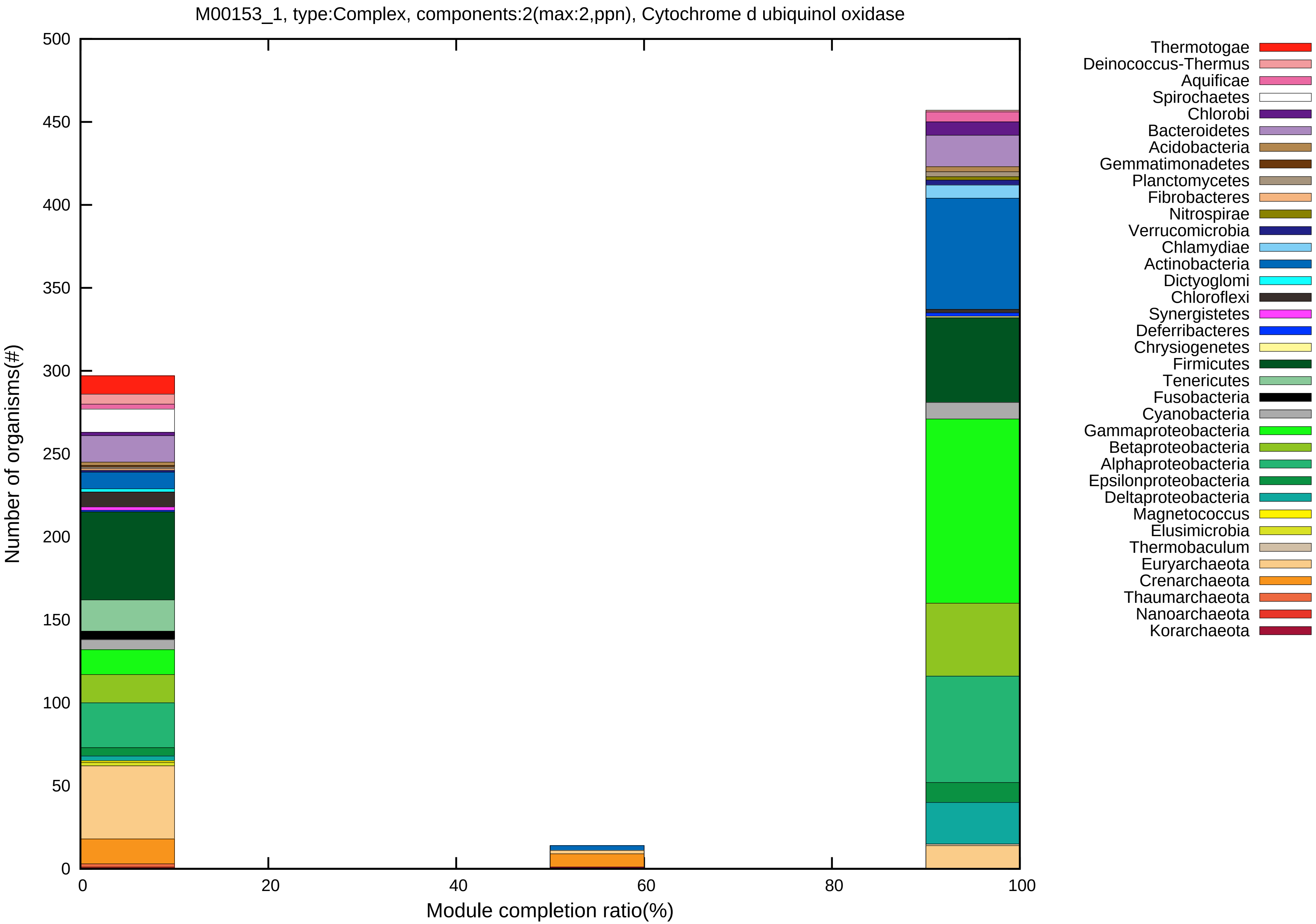

M00153_1, type:Complex, components:2(max:2,ppn), Cytochrome d ubiquinol oxidase
Number of organisms(#)
Module completion ratio(%)
0
50
100
150
200
250
300
350
400
450
500
0
20
40
60
80
100
Thermotogae
Deinococcus-Thermus
Aquificae
Spirochaetes
Chlorobi
Bacteroidetes
Acidobacteria
Gemmatimonadetes
Planctomycetes
Fibrobacteres
Nitrospirae
Verrucomicrobia
Chlamydiae
Actinobacteria
Dictyoglomi
Chloroflexi
Synergistetes
Deferribacteres
Chrysiogenetes
Firmicutes
Tenericutes
Fusobacteria
Cyanobacteria
Gammaproteobacteria
Betaproteobacteria
Alphaproteobacteria
Epsilonproteobacteria
Deltaproteobacteria
Magnetococcus
Elusimicrobia
Thermobaculum
Euryarchaeota
Crenarchaeota
Thaumarchaeota
Nanoarchaeota
Korarchaeota

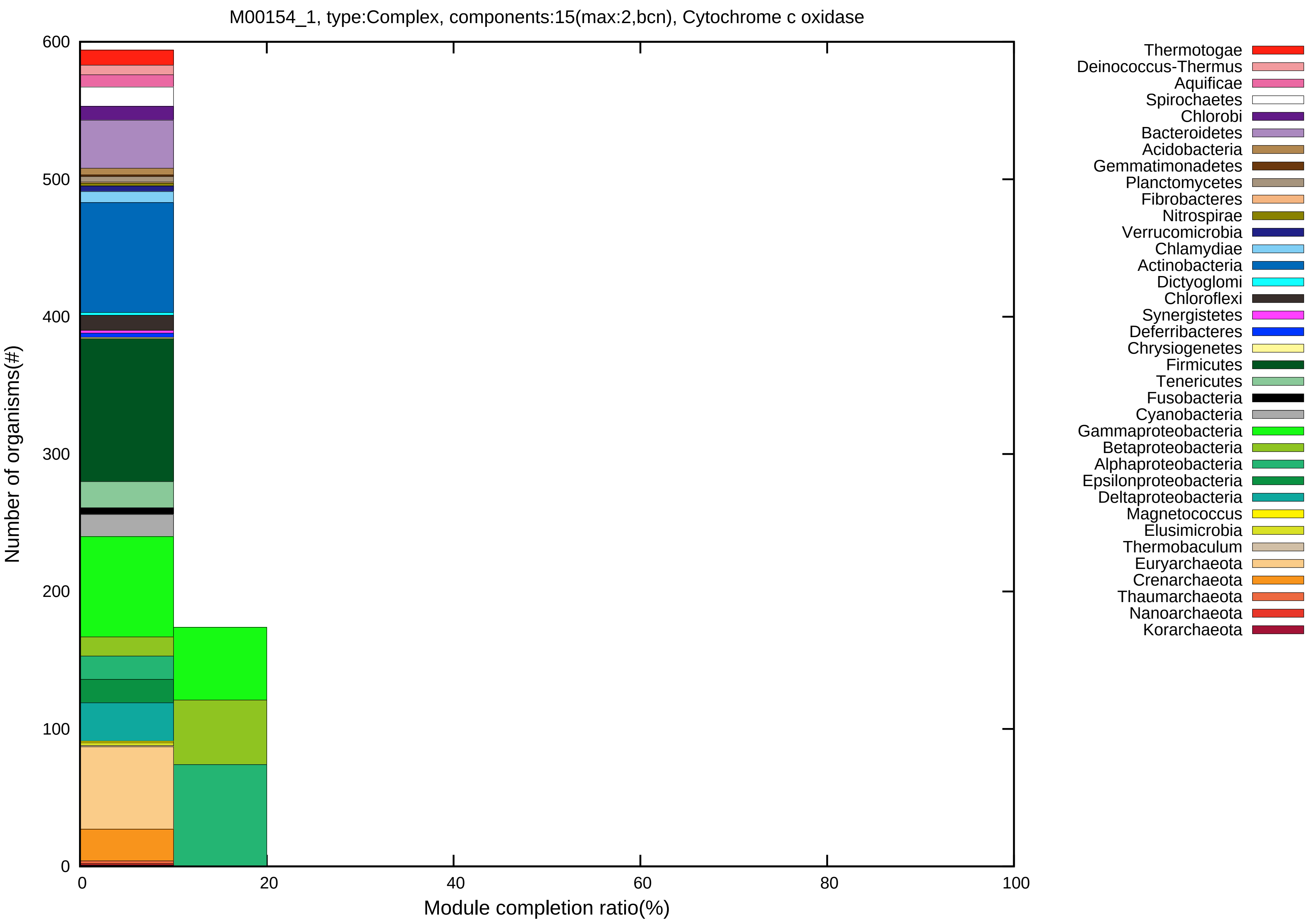

M00154_1, type:Complex, components:15(max:2,bcn), Cytochrome c oxidase
Number of organisms(#)
Module completion ratio(%)
0
100
200
300
400
500
600
0
20
40
60
80
100
Thermotogae
Deinococcus-Thermus
Aquificae
Spirochaetes
Chlorobi
Bacteroidetes
Acidobacteria
Gemmatimonadetes
Planctomycetes
Fibrobacteres
Nitrospirae
Verrucomicrobia
Chlamydiae
Actinobacteria
Dictyoglomi
Chloroflexi
Synergistetes
Deferribacteres
Chrysiogenetes
Firmicutes
Tenericutes
Fusobacteria
Cyanobacteria
Gammaproteobacteria
Betaproteobacteria
Alphaproteobacteria
Epsilonproteobacteria
Deltaproteobacteria
Magnetococcus
Elusimicrobia
Thermobaculum
Euryarchaeota
Crenarchaeota
Thaumarchaeota
Nanoarchaeota
Korarchaeota

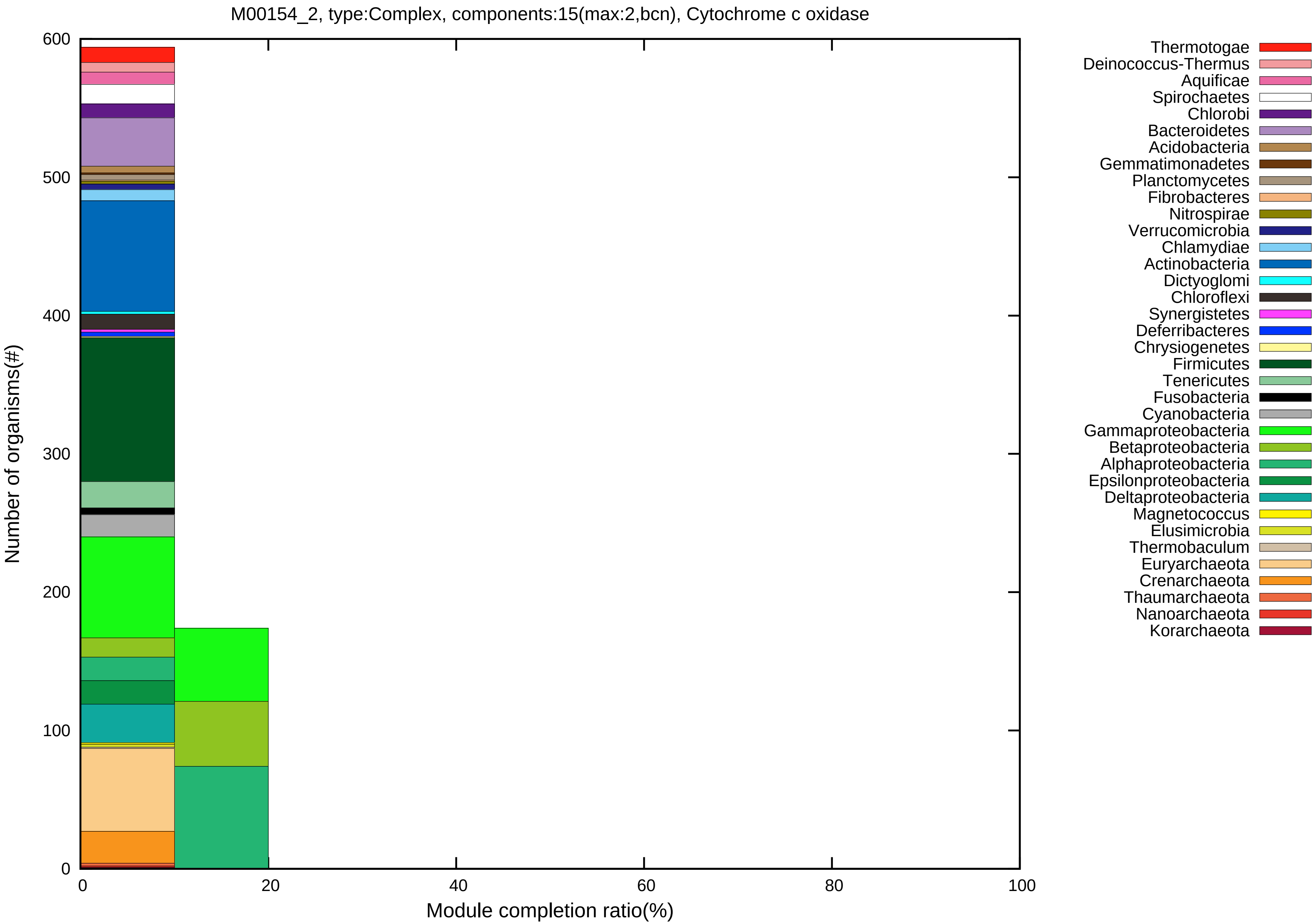

M00154_2, type:Complex, components:15(max:2,bcn), Cytochrome c oxidase
Number of organisms(#)
Module completion ratio(%)
0
100
200
300
400
500
600
0
20
40
60
80
100
Thermotogae
Deinococcus-Thermus
Aquificae
Spirochaetes
Chlorobi
Bacteroidetes
Acidobacteria
Gemmatimonadetes
Planctomycetes
Fibrobacteres
Nitrospirae
Verrucomicrobia
Chlamydiae
Actinobacteria
Dictyoglomi
Chloroflexi
Synergistetes
Deferribacteres
Chrysiogenetes
Firmicutes
Tenericutes
Fusobacteria
Cyanobacteria
Gammaproteobacteria
Betaproteobacteria
Alphaproteobacteria
Epsilonproteobacteria
Deltaproteobacteria
Magnetococcus
Elusimicrobia
Thermobaculum
Euryarchaeota
Crenarchaeota
Thaumarchaeota
Nanoarchaeota
Korarchaeota

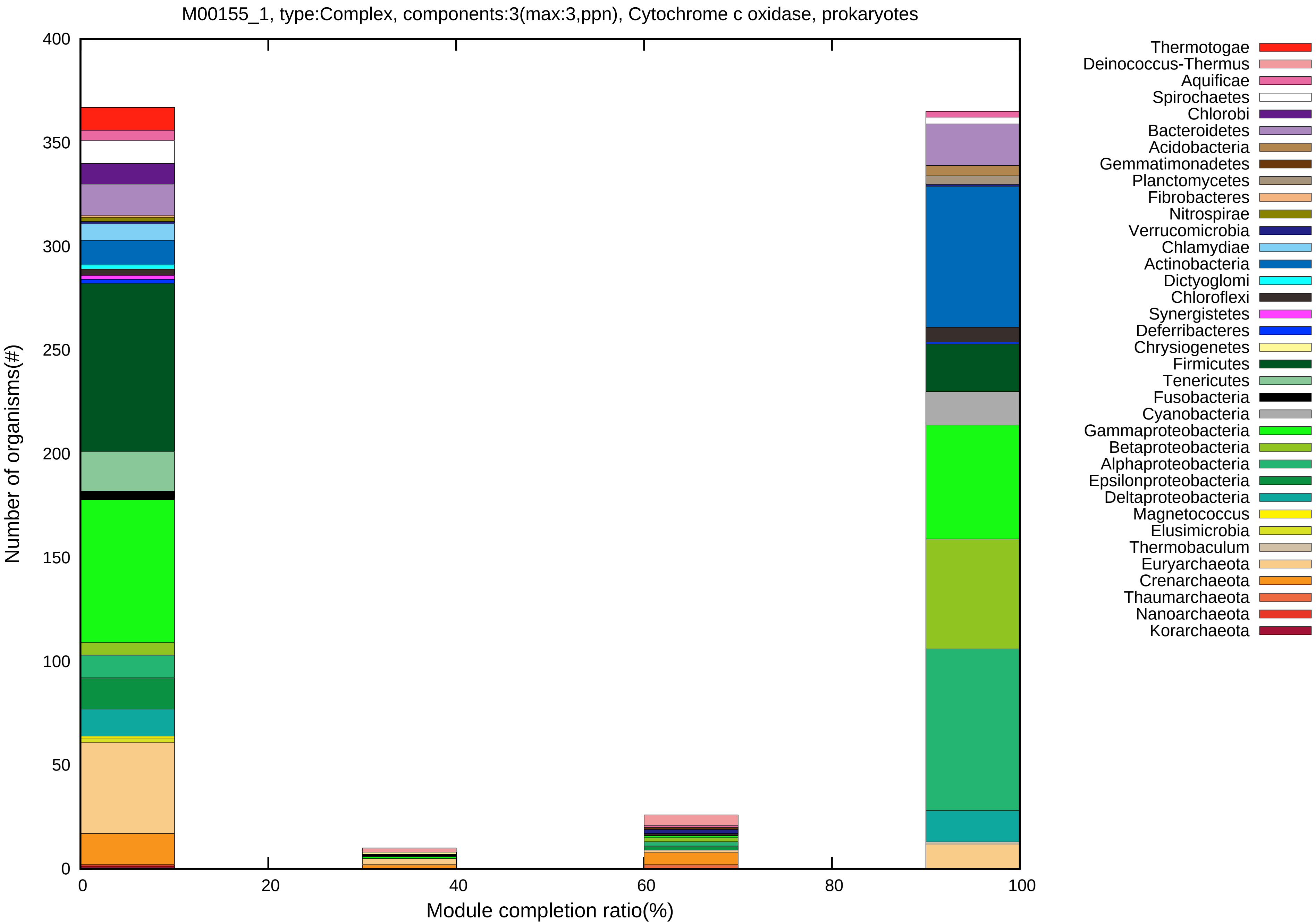
M00155_1, type:Complex, components:3(max:3,ppn), Cytochrome c oxidase, prokaryotes
Number of organisms(#)
Module completion ratio(%)
0
50
100
150
200
250
300
350
400
0
20
40
60
80
100
Thermotogae
Deinococcus-Thermus
Aquificae
Spirochaetes
Chlorobi
Bacteroidetes
Acidobacteria
Gemmatimonadetes
Planctomycetes
Fibrobacteres
Nitrospirae
Verrucomicrobia
Chlamydiae
Actinobacteria
Dictyoglomi
Chloroflexi
Synergistetes
Deferribacteres
Chrysiogenetes
Firmicutes
Tenericutes
Fusobacteria
Cyanobacteria
Gammaproteobacteria
Betaproteobacteria
Alphaproteobacteria
Epsilonproteobacteria
Deltaproteobacteria
Magnetococcus
Elusimicrobia
Thermobaculum
Euryarchaeota
Crenarchaeota
Thaumarchaeota
Nanoarchaeota
Korarchaeota

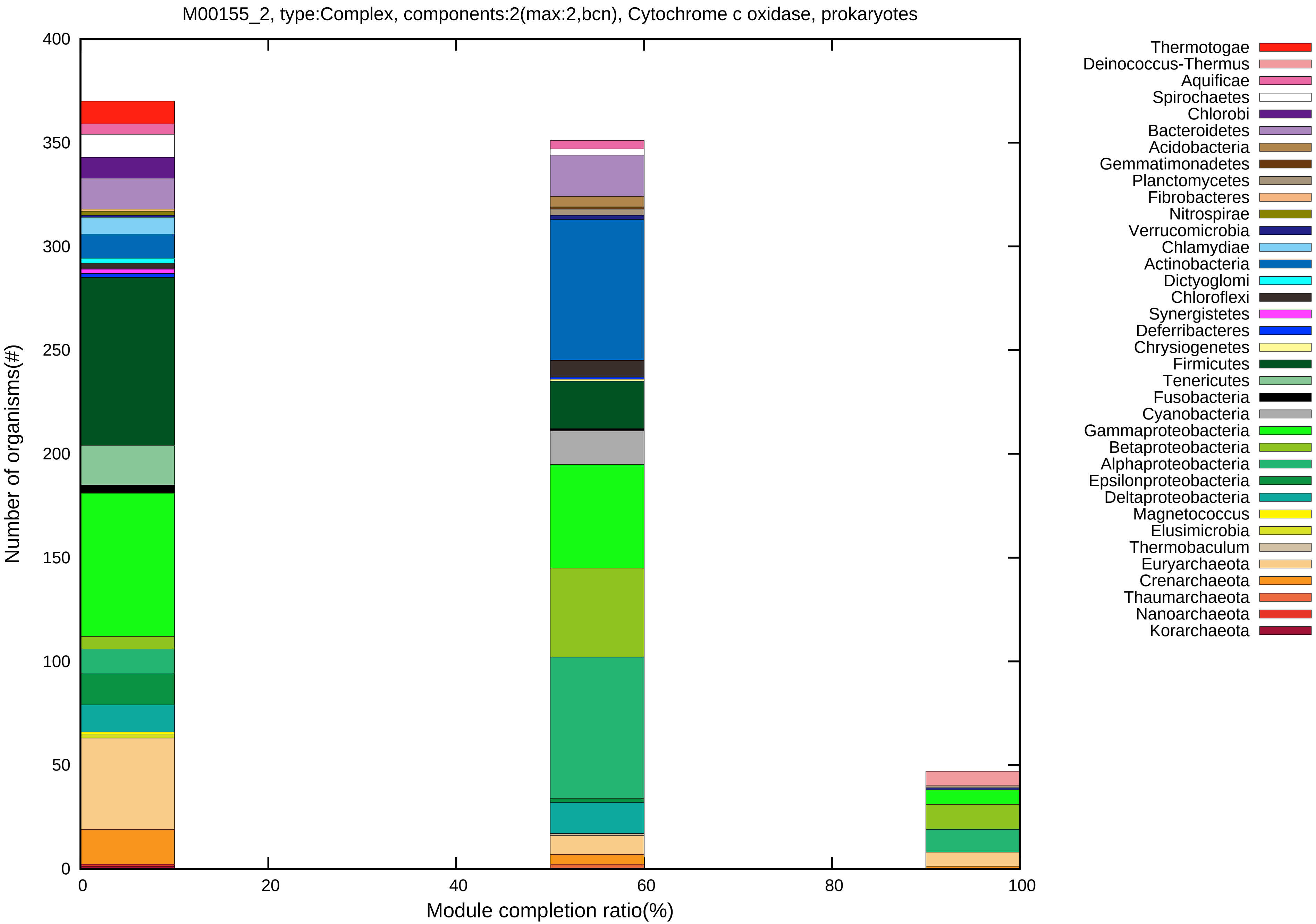
M00155_2, type:Complex, components:2(max:2,bcn), Cytochrome c oxidase, prokaryotes
Number of organisms(#)
0
50
100
150
200
250
300
350
400
0
20
40
60
80
100
Module completion ratio(%)
Thermotogae
Deinococcus-Thermus
Aquificae
Spirochaetes
Chlorobi
Bacteroidetes
Acidobacteria
Gemmatimonadetes
Planctomycetes
Fibrobacteres
Nitrospirae
Verrucomicrobia
Chlamydiae
Actinobacteria
Dictyoglomi
Chloroflexi
Synergistetes
Deferribacteres
Chrysiogenetes
Firmicutes
Tenericutes
Fusobacteria
Cyanobacteria
Gammaproteobacteria
Betaproteobacteria
Alphaproteobacteria
Epsilonproteobacteria
Deltaproteobacteria
Magnetococcus
Elusimicrobia
Thermobaculum
Euryarchaeota
Crenarchaeota
Thaumarchaeota
Nanoarchaeota
Korarchaeota

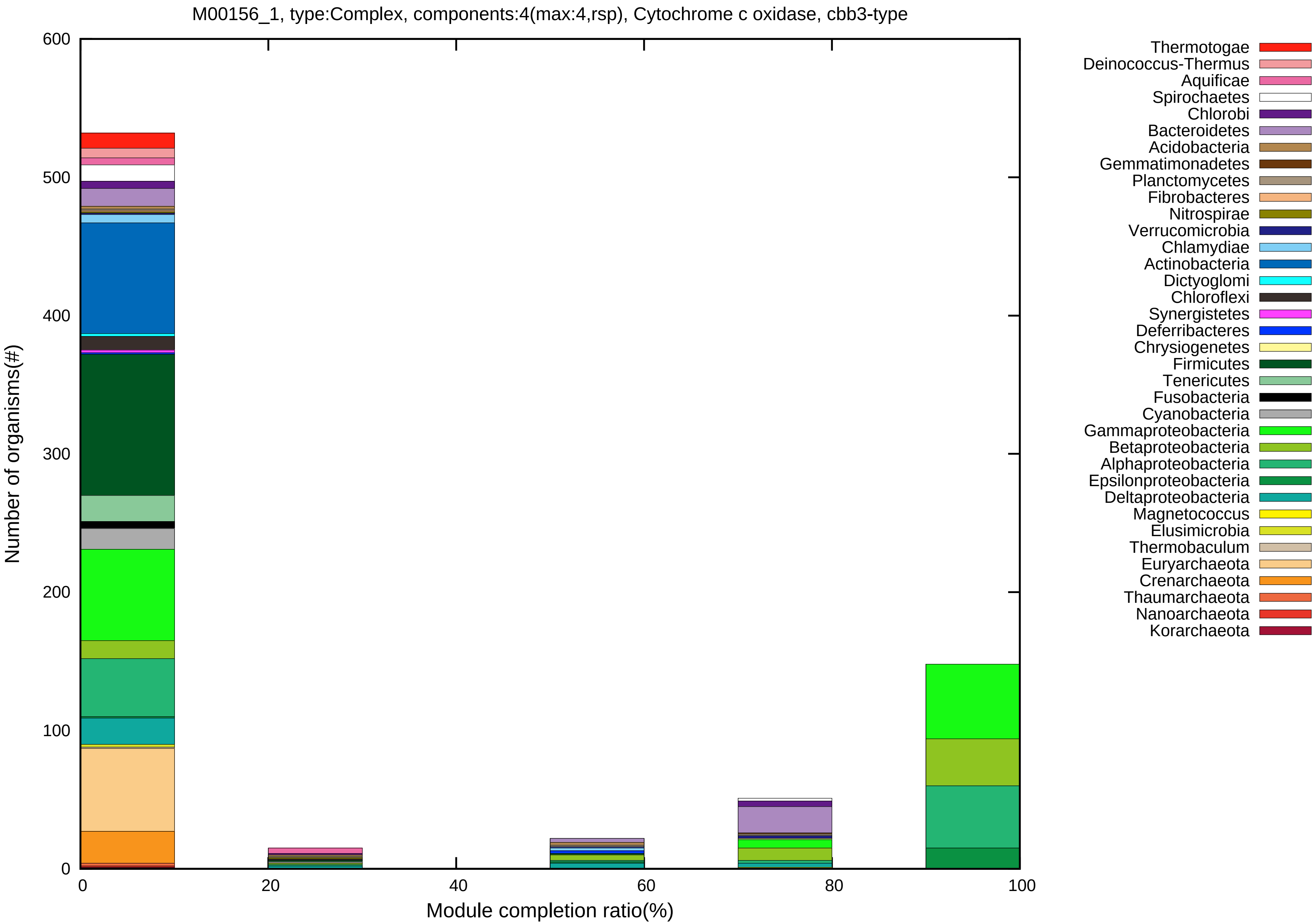

M00156_1, type:Complex, components:4(max:4,rsp), Cytochrome c oxidase, cbb3-type
Number of organisms(#)
Module completion ratio(%)
0
100
200
300
400
500
600
0
20
40
60
80
100
Thermotogae
Deinococcus-Thermus
Aquificae
Spirochaetes
Chlorobi
Bacteroidetes
Acidobacteria
Gemmatimonadetes
Planctomycetes
Fibrobacteres
Nitrospirae
Verrucomicrobia
Chlamydiae
Actinobacteria
Dictyoglomi
Chloroflexi
Synergistetes
Deferribacteres
Chrysiogenetes
Firmicutes
Tenericutes
Fusobacteria
Cyanobacteria
Gammaproteobacteria
Betaproteobacteria
Alphaproteobacteria
Epsilonproteobacteria
Deltaproteobacteria
Magnetococcus
Elusimicrobia
Thermobaculum
Euryarchaeota
Crenarchaeota
Thaumarchaeota
Nanoarchaeota
Korarchaeota

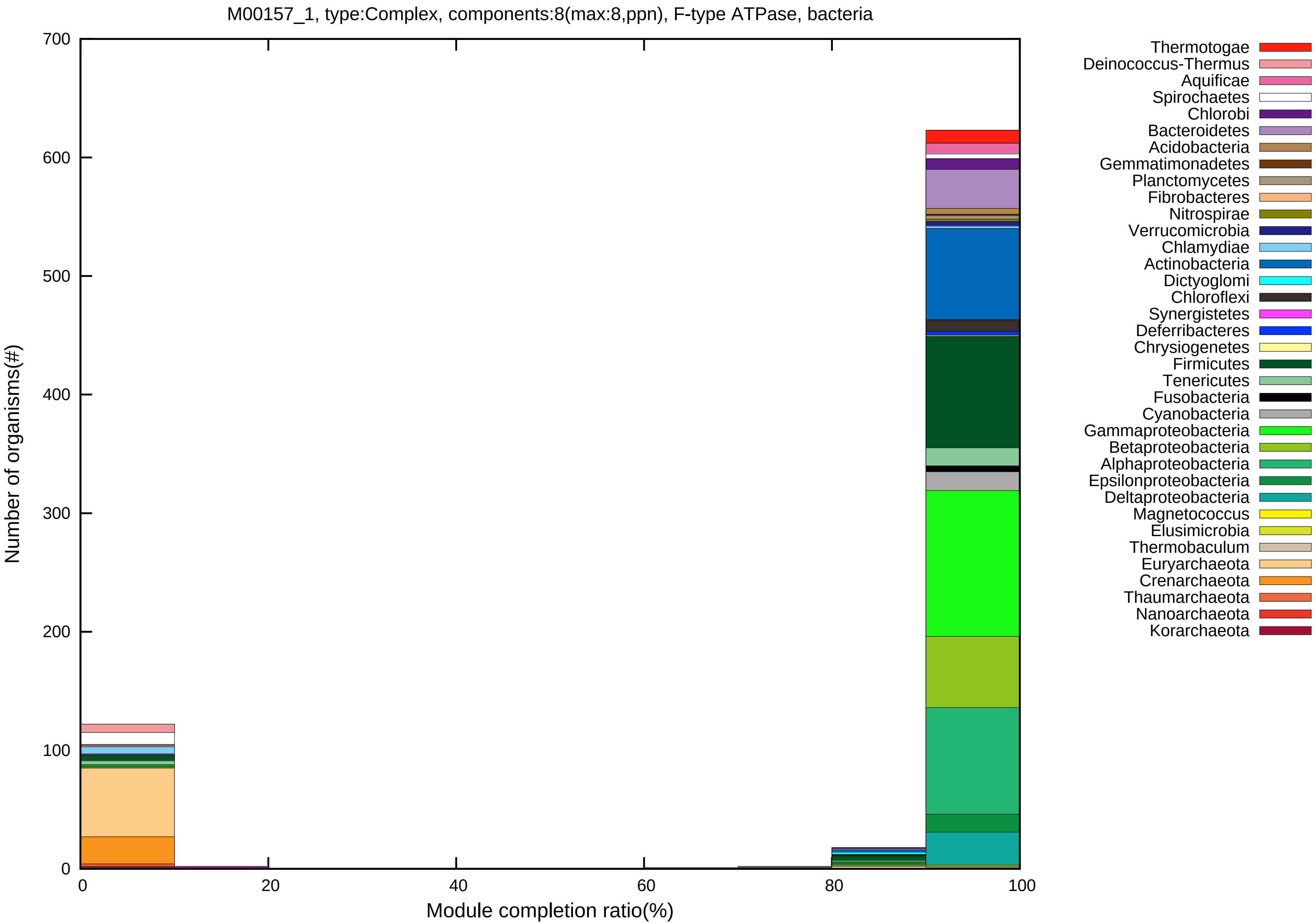

M00157_1, type:Complex, components:8(max:8,ppn), F-type ATPase, bacteria
Number of organisms(#)
Module completion ratio(%)
0
100
200
300
400
500
600
700
0
20
40
60
80
100
Thermotogae
Deinococcus-Thermus
Aquificae
Spirochaetes
Chlorobi
Bacteroidetes
Acidobacteria
Gemmatimonadetes
Planctomycetes
Fibrobacteres
Nitrospirae
Verrucomicrobia
Chlamydiae
Actinobacteria
Dictyoglomi
Chloroflexi
Synergistetes
Deferribacteres
Chrysiogenetes
Firmicutes
Tenericutes
Fusobacteria
Cyanobacteria
Gammaproteobacteria
Betaproteobacteria
Alphaproteobacteria
Epsilonproteobacteria
Deltaproteobacteria
Magnetococcus
Elusimicrobia
Thermobaculum
Euryarchaeota
Crenarchaeota
Thaumarchaeota
Nanoarchaeota
Korarchaeota

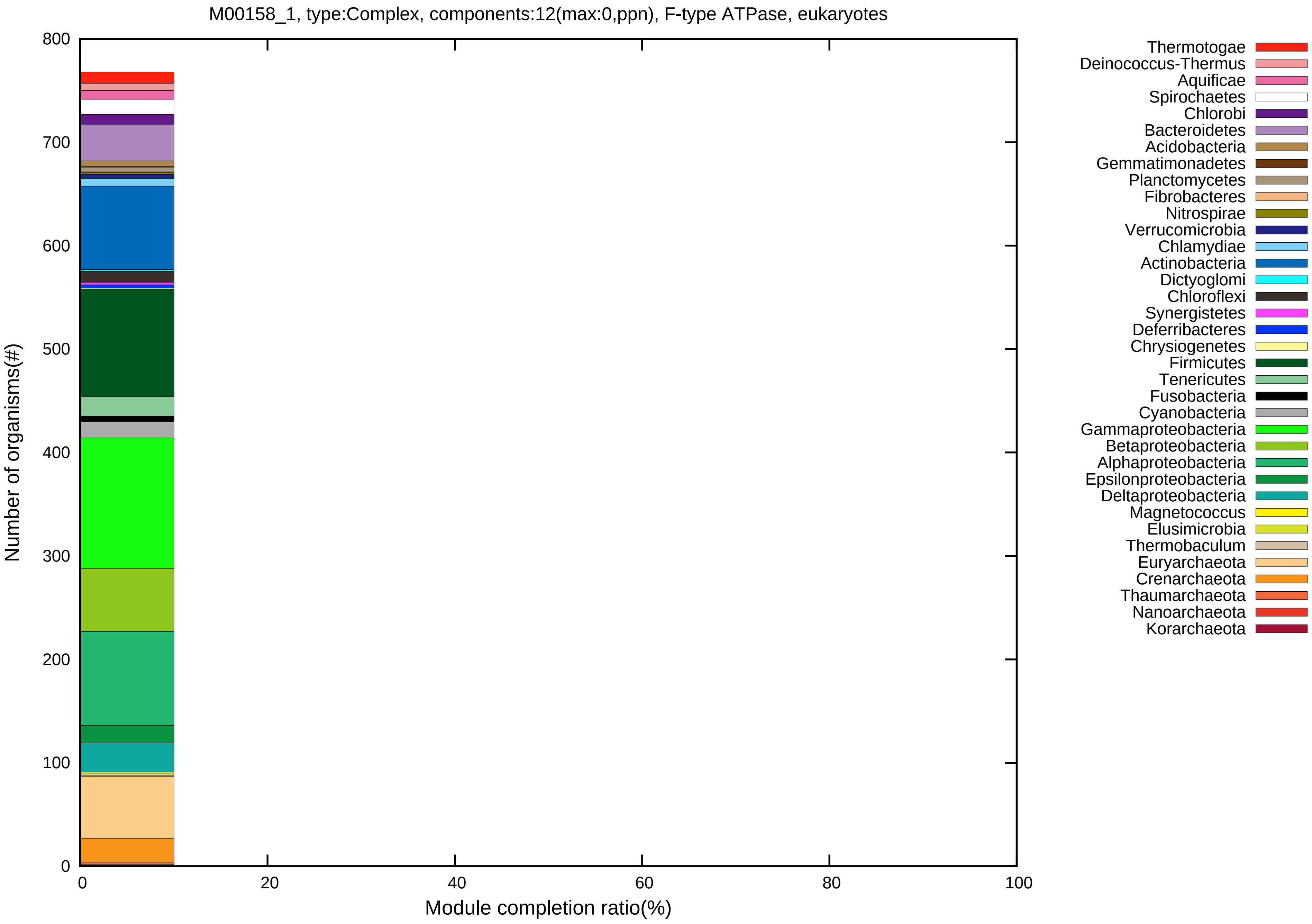

M00158_1, type:Complex, components:12(max:0,ppn), F-type ATPase, eukaryotes
Number of organisms(#)
Module completion ratio(%)
0
100
200
300
400
500
600
700
800
0
20
40
60
80
100
Thermotogae
Deinococcus-Thermus
Aquificae
Spirochaetes
Chlorobi
Bacteroidetes
Acidobacteria
Gemmatimonadetes
Planctomycetes
Fibrobacteres
Nitrospirae
Verrucomicrobia
Chlamydiae
Actinobacteria
Dictyoglomi
Chloroflexi
Synergistetes
Deferribacteres
Chrysiogenetes
Firmicutes
Tenericutes
Fusobacteria
Cyanobacteria
Gammaproteobacteria
Betaproteobacteria
Alphaproteobacteria
Epsilonproteobacteria
Deltaproteobacteria
Magnetococcus
Elusimicrobia
Thermobaculum
Euryarchaeota
Crenarchaeota
Thaumarchaeota
Nanoarchaeota
Korarchaeota

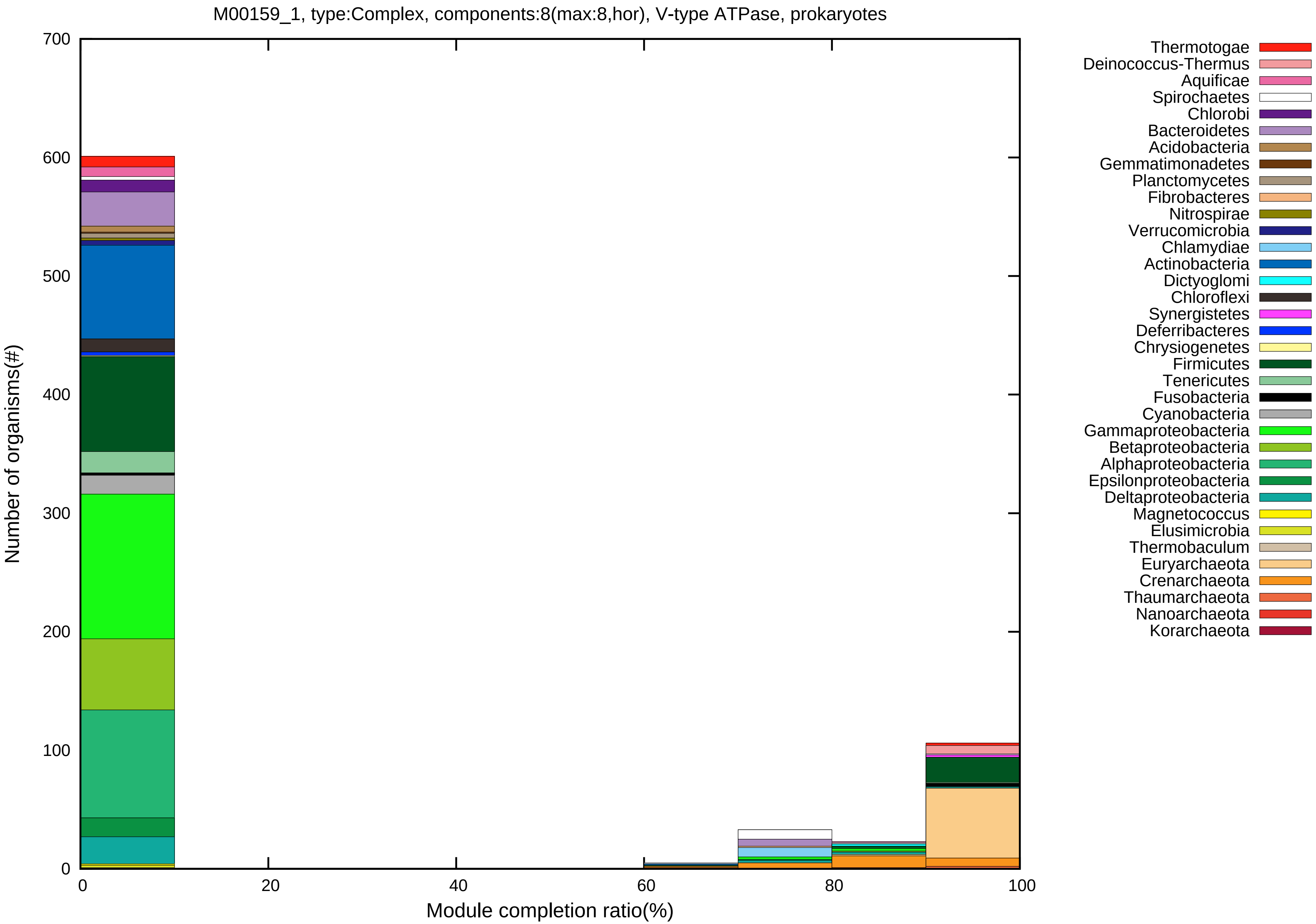
M00159_1, type:Complex, components:8(max:8,hor), V-type ATPase, prokaryotes
Number of organisms(#)
Module completion ratio(%)
0
100
200
300
400
500
600
700
0
20
40
60
80
100
Thermotogae
Deinococcus-Thermus
Aquificae
Spirochaetes
Chlorobi
Bacteroidetes
Acidobacteria
Gemmatimonadetes
Planctomycetes
Fibrobacteres
Nitrospirae
Verrucomicrobia
Chlamydiae
Actinobacteria
Dictyoglomi
Chloroflexi
Synergistetes
Deferribacteres
Chrysiogenetes
Firmicutes
Tenericutes
Fusobacteria
Cyanobacteria
Gammaproteobacteria
Betaproteobacteria
Alphaproteobacteria
Epsilonproteobacteria
Deltaproteobacteria
Magnetococcus
Elusimicrobia
Thermobaculum
Euryarchaeota
Crenarchaeota
Thaumarchaeota
Nanoarchaeota
Korarchaeota

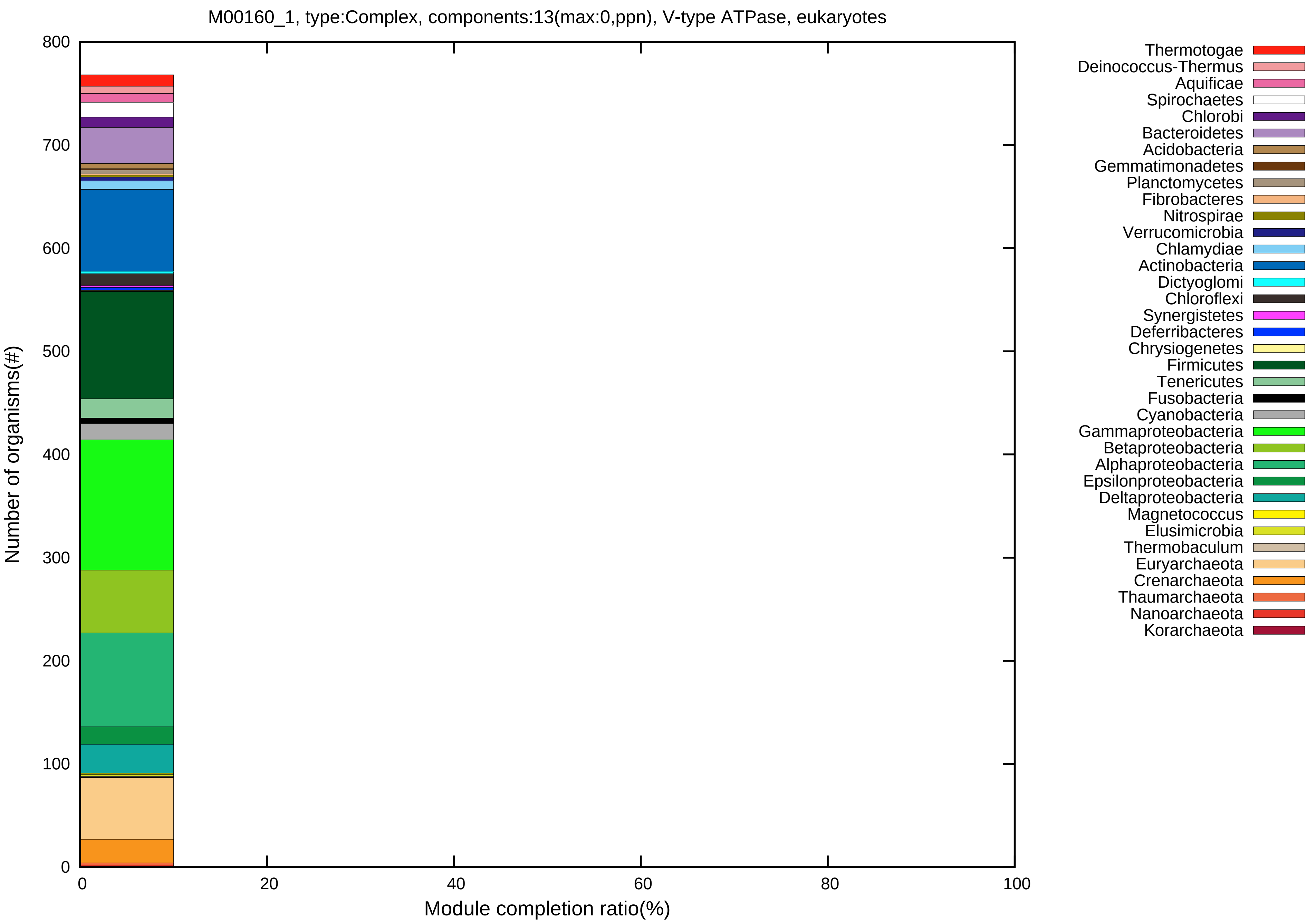
M00160_1, type:Complex, components:13(max:0,ppn), V-type ATPase, eukaryotes
Number of organisms(#)
Module completion ratio(%)
0
100
200
300
400
500
600
700
800
0
20
40
60
80
100
Thermotogae
Deinococcus-Thermus
Aquificae
Spirochaetes
Chlorobi
Bacteroidetes
Acidobacteria
Gemmatimonadetes
Planctomycetes
Fibrobacteres
Nitrospirae
Verrucomicrobia
Chlamydiae
Actinobacteria
Dictyoglomi
Chloroflexi
Synergistetes
Deferribacteres
Chrysiogenetes
Firmicutes
Tenericutes
Fusobacteria
Cyanobacteria
Gammaproteobacteria
Betaproteobacteria
Alphaproteobacteria
Epsilonproteobacteria
Deltaproteobacteria
Magnetococcus
Elusimicrobia
Thermobaculum
Euryarchaeota
Crenarchaeota
Thaumarchaeota
Nanoarchaeota
Korarchaeota

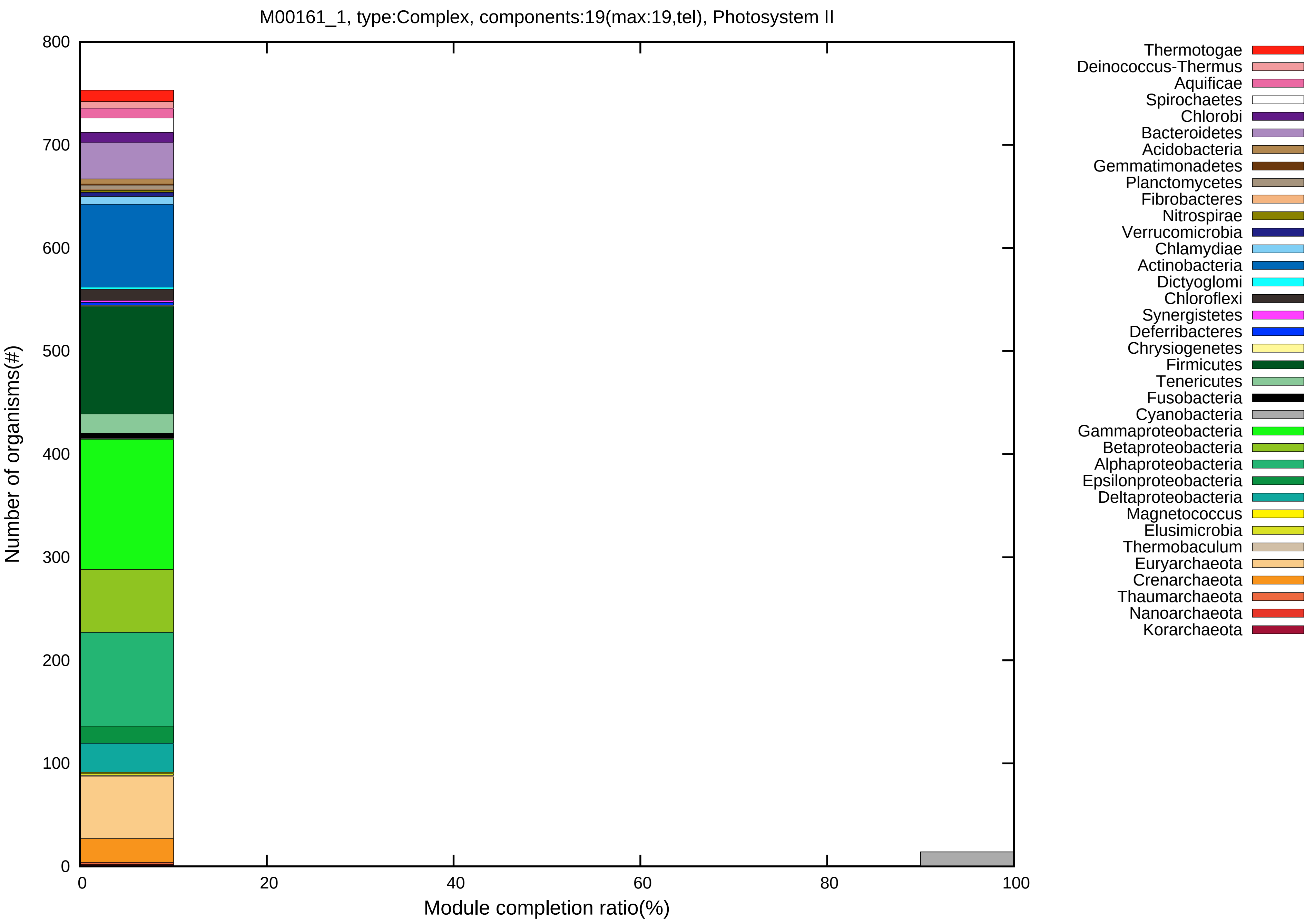
M00161_1, type:Complex, components:19(max:19,tel), Photosystem II
Number of organisms(#)
Module completion ratio(%)
0
100
200
300
400
500
600
700
800
0
20
40
60
80
100
Thermotogae
Deinococcus-Thermus
Aquificae
Spirochaetes
Chlorobi
Bacteroidetes
Acidobacteria
Gemmatimonadetes
Planctomycetes
Fibrobacteres
Nitrospirae
Verrucomicrobia
Chlamydiae
Actinobacteria
Dictyoglomi
Chloroflexi
Synergistetes
Deferribacteres
Chrysiogenetes
Firmicutes
Tenericutes
Fusobacteria
Cyanobacteria
Gammaproteobacteria
Betaproteobacteria
Alphaproteobacteria
Epsilonproteobacteria
Deltaproteobacteria
Magnetococcus
Elusimicrobia
Thermobaculum
Euryarchaeota
Crenarchaeota
Thaumarchaeota
Nanoarchaeota
Korarchaeota

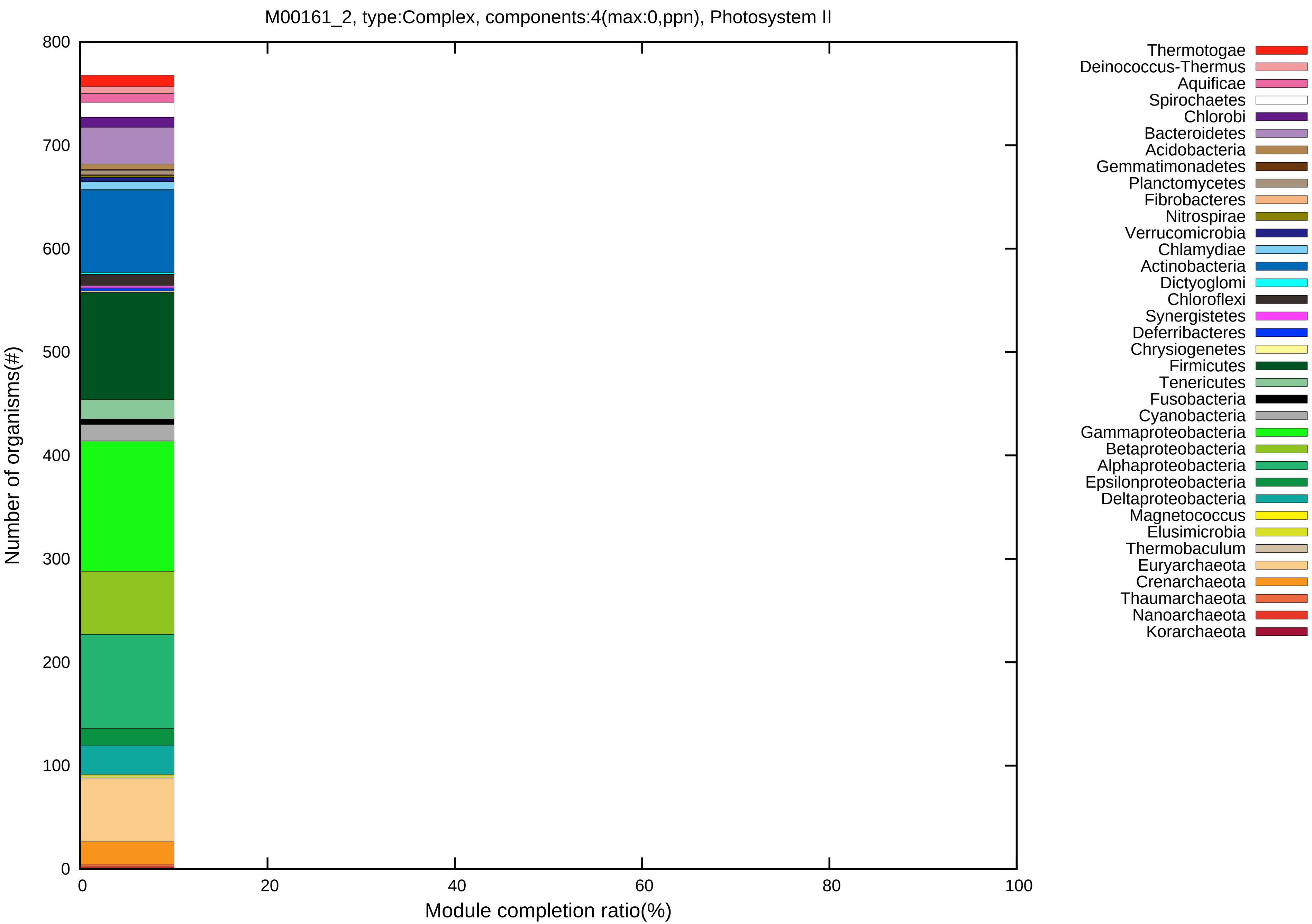

M00161_2, type:Complex, components:4(max:0,ppn), Photosystem II
Number of organisms(#)
Module completion ratio(%)
0
100
200
300
400
500
600
700
800
0
20
40
60
80
100
Thermotogae
Deinococcus-Thermus
Aquificae
Spirochaetes
Chlorobi
Bacteroidetes
Acidobacteria
Gemmatimonadetes
Planctomycetes
Fibrobacteres
Nitrospirae
Verrucomicrobia
Chlamydiae
Actinobacteria
Dictyoglomi
Chloroflexi
Synergistetes
Deferribacteres
Chrysiogenetes
Firmicutes
Tenericutes
Fusobacteria
Cyanobacteria
Gammaproteobacteria
Betaproteobacteria
Alphaproteobacteria
Epsilonproteobacteria
Deltaproteobacteria
Magnetococcus
Elusimicrobia
Thermobaculum
Euryarchaeota
Crenarchaeota
Thaumarchaeota
Nanoarchaeota
Korarchaeota

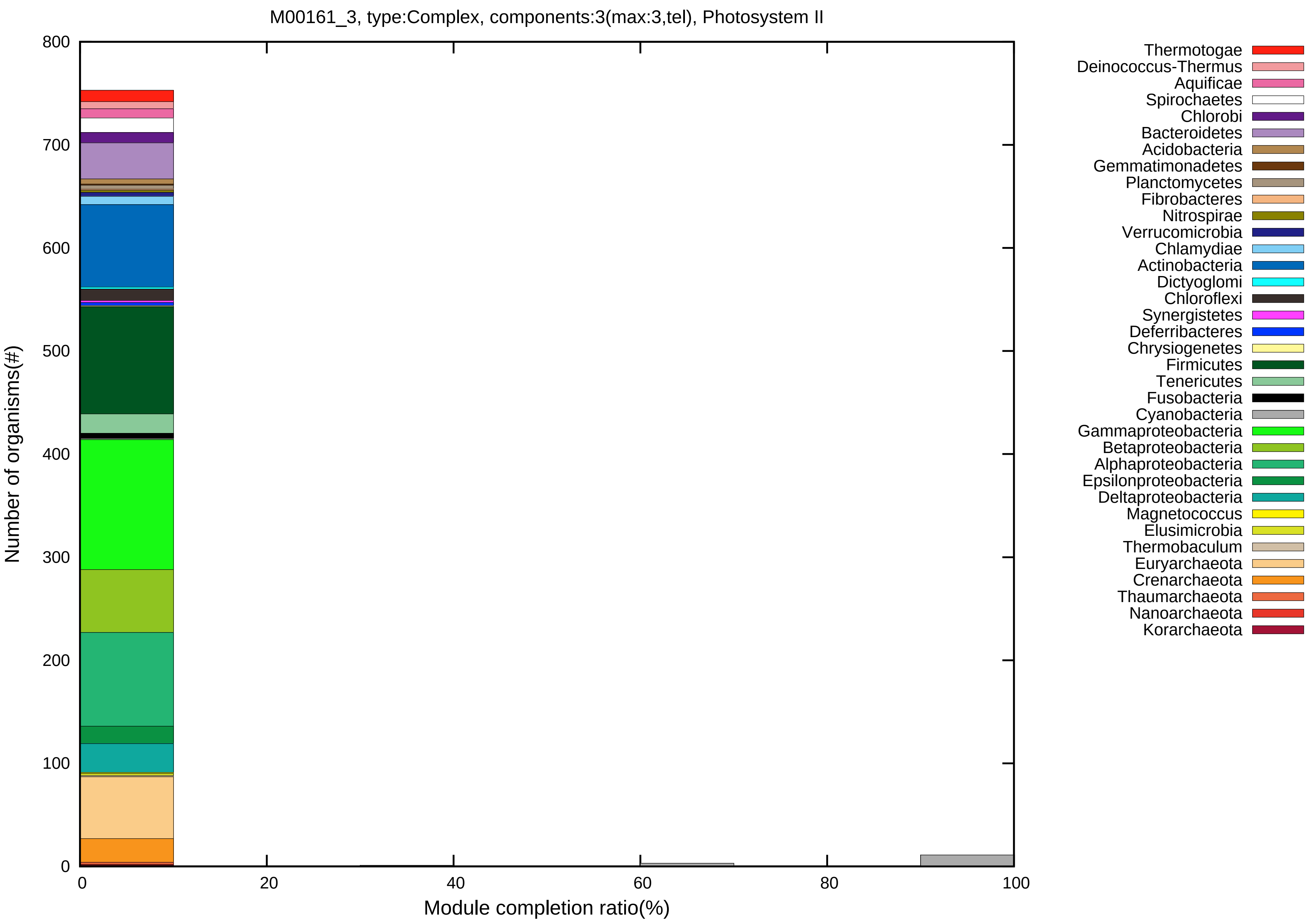
M00161_3, type:Complex, components:3(max:3,tel), Photosystem II
Number of organisms(#)
0
100
200
300
400
500
600
700
800
Module completion ratio(%)
0
20
40
60
80
100
Thermotogae
Deinococcus-Thermus
Aquificae
Spirochaetes
Chlorobi
Bacteroidetes
Acidobacteria
Gemmatimonadetes
Planctomycetes
Fibrobacteres
Nitrospirae
Verrucomicrobia
Chlamydiae
Actinobacteria
Dictyoglomi
Chloroflexi
Synergistetes
Deferribacteres
Chrysiogenetes
Firmicutes
Tenericutes
Fusobacteria
Cyanobacteria
Gammaproteobacteria
Betaproteobacteria
Alphaproteobacteria
Epsilonproteobacteria
Deltaproteobacteria
Magnetococcus
Elusimicrobia
Thermobaculum
Euryarchaeota
Crenarchaeota
Thaumarchaeota
Nanoarchaeota
Korarchaeota

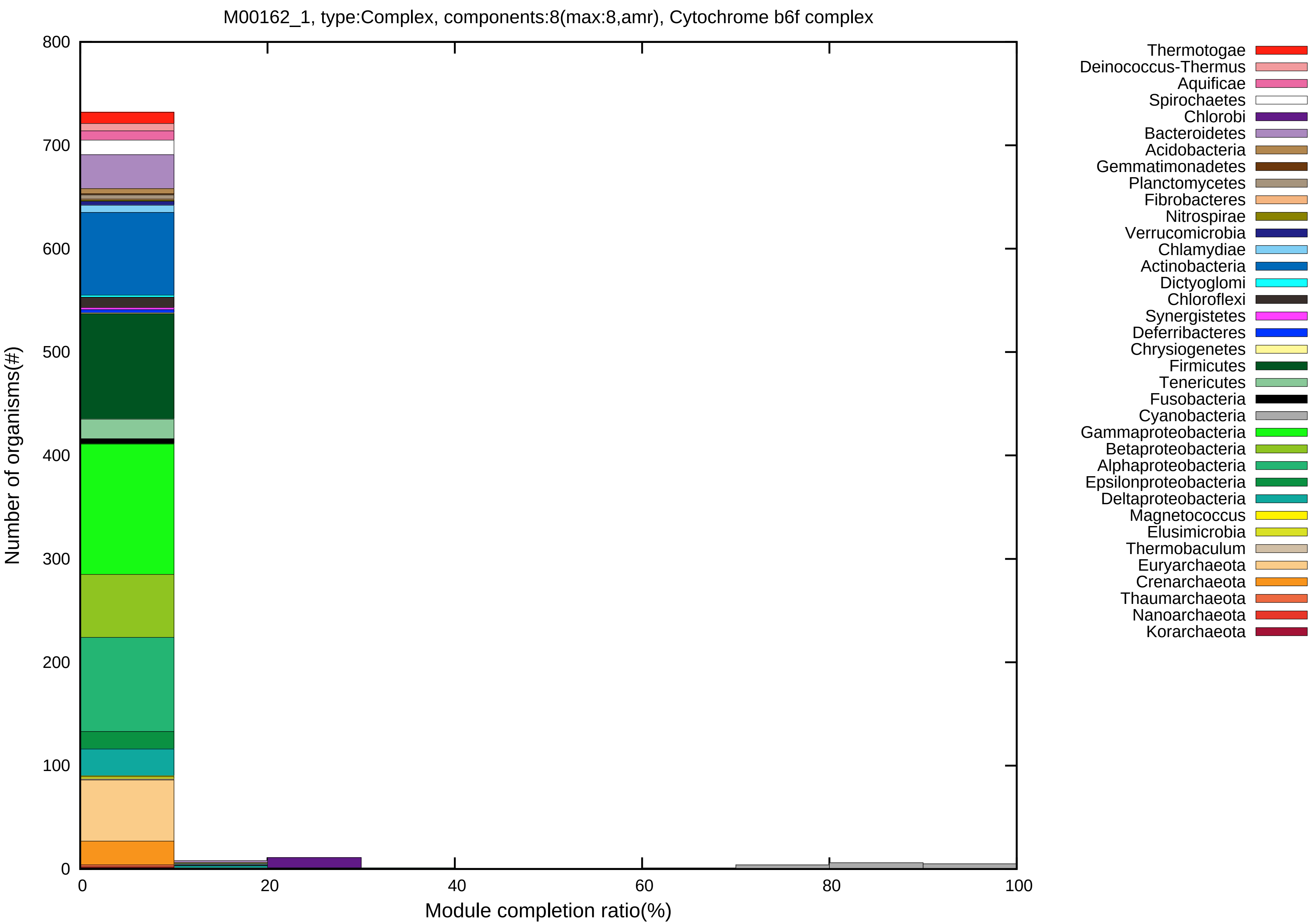

M00162_1, type:Complex, components:8(max:8,amr), Cytochrome b6f complex
Number of organisms(#)
Module completion ratio(%)
0
100
200
300
400
500
600
700
800
0
20
40
60
80
100
Thermotogae
Deinococcus-Thermus
Aquificae
Spirochaetes
Chlorobi
Bacteroidetes
Acidobacteria
Gemmatimonadetes
Planctomycetes
Fibrobacteres
Nitrospirae
Verrucomicrobia
Chlamydiae
Actinobacteria
Dictyoglomi
Chloroflexi
Synergistetes
Deferribacteres
Chrysiogenetes
Firmicutes
Tenericutes
Fusobacteria
Cyanobacteria
Gammaproteobacteria
Betaproteobacteria
Alphaproteobacteria
Epsilonproteobacteria
Deltaproteobacteria
Magnetococcus
Elusimicrobia
Thermobaculum
Euryarchaeota
Crenarchaeota
Thaumarchaeota
Nanoarchaeota
Korarchaeota

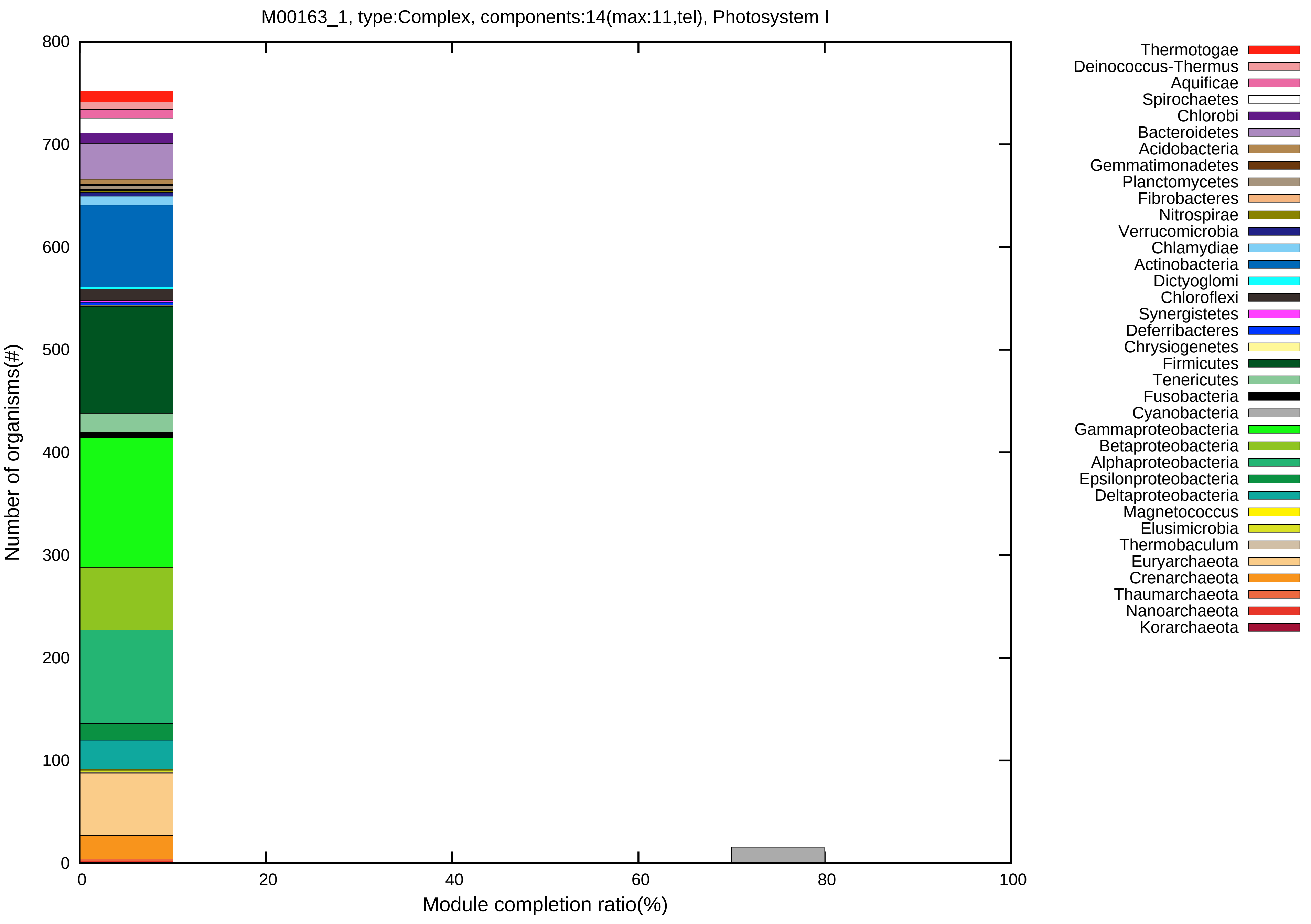

M00163_1, type:Complex, components:14(max:11,tel), Photosystem I
Number of organisms(#)
Module completion ratio(%)
0
100
200
300
400
500
600
700
800
0
20
40
60
80
100
Thermotogae
Deinococcus-Thermus
Aquificae
Spirochaetes
Chlorobi
Bacteroidetes
Acidobacteria
Gemmatimonadetes
Planctomycetes
Fibrobacteres
Nitrospirae
Verrucomicrobia
Chlamydiae
Actinobacteria
Dictyoglomi
Chloroflexi
Synergistetes
Deferribacteres
Chrysiogenetes
Firmicutes
Tenericutes
Fusobacteria
Cyanobacteria
Gammaproteobacteria
Betaproteobacteria
Alphaproteobacteria
Epsilonproteobacteria
Deltaproteobacteria
Magnetococcus
Elusimicrobia
Thermobaculum
Euryarchaeota
Crenarchaeota
Thaumarchaeota
Nanoarchaeota
Korarchaeota

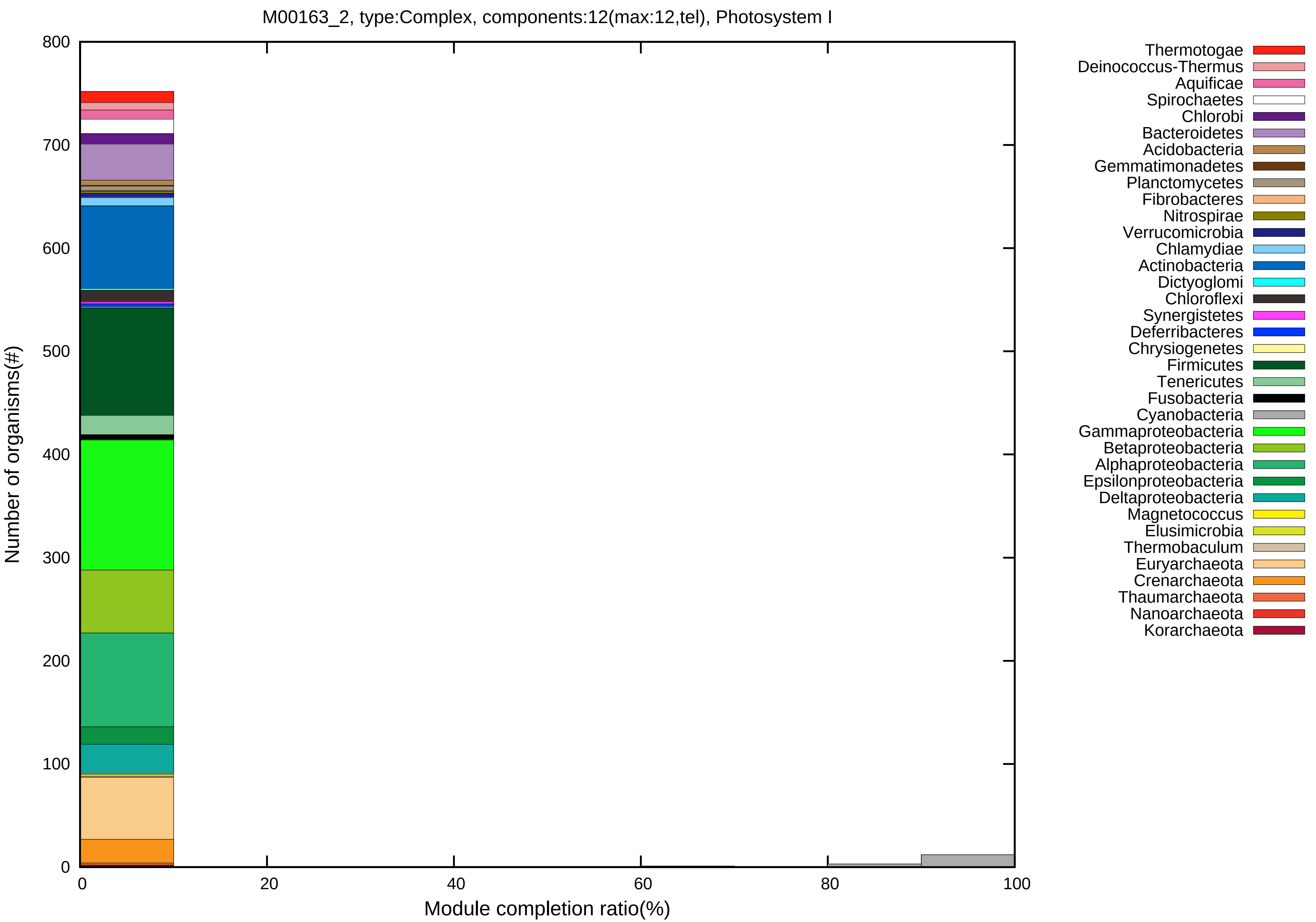
M00163_2, type:Complex, components:12(max:12,tel), Photosystem I
Number of organisms(#)
Module completion ratio(%)
0
100
200
300
400
500
600
700
800
0
20
40
60
80
100
Thermotogae
Deinococcus-Thermus
Aquificae
Spirochaetes
Chlorobi
Bacteroidetes
Acidobacteria
Gemmatimonadetes
Planctomycetes
Fibrobacteres
Nitrospirae
Verrucomicrobia
Chlamydiae
Actinobacteria
Dictyoglomi
Chloroflexi
Synergistetes
Deferribacteres
Chrysiogenetes
Firmicutes
Tenericutes
Fusobacteria
Cyanobacteria
Gammaproteobacteria
Betaproteobacteria
Alphaproteobacteria
Epsilonproteobacteria
Deltaproteobacteria
Magnetococcus
Elusimicrobia
Thermobaculum
Euryarchaeota
Crenarchaeota
Thaumarchaeota
Nanoarchaeota
Korarchaeota

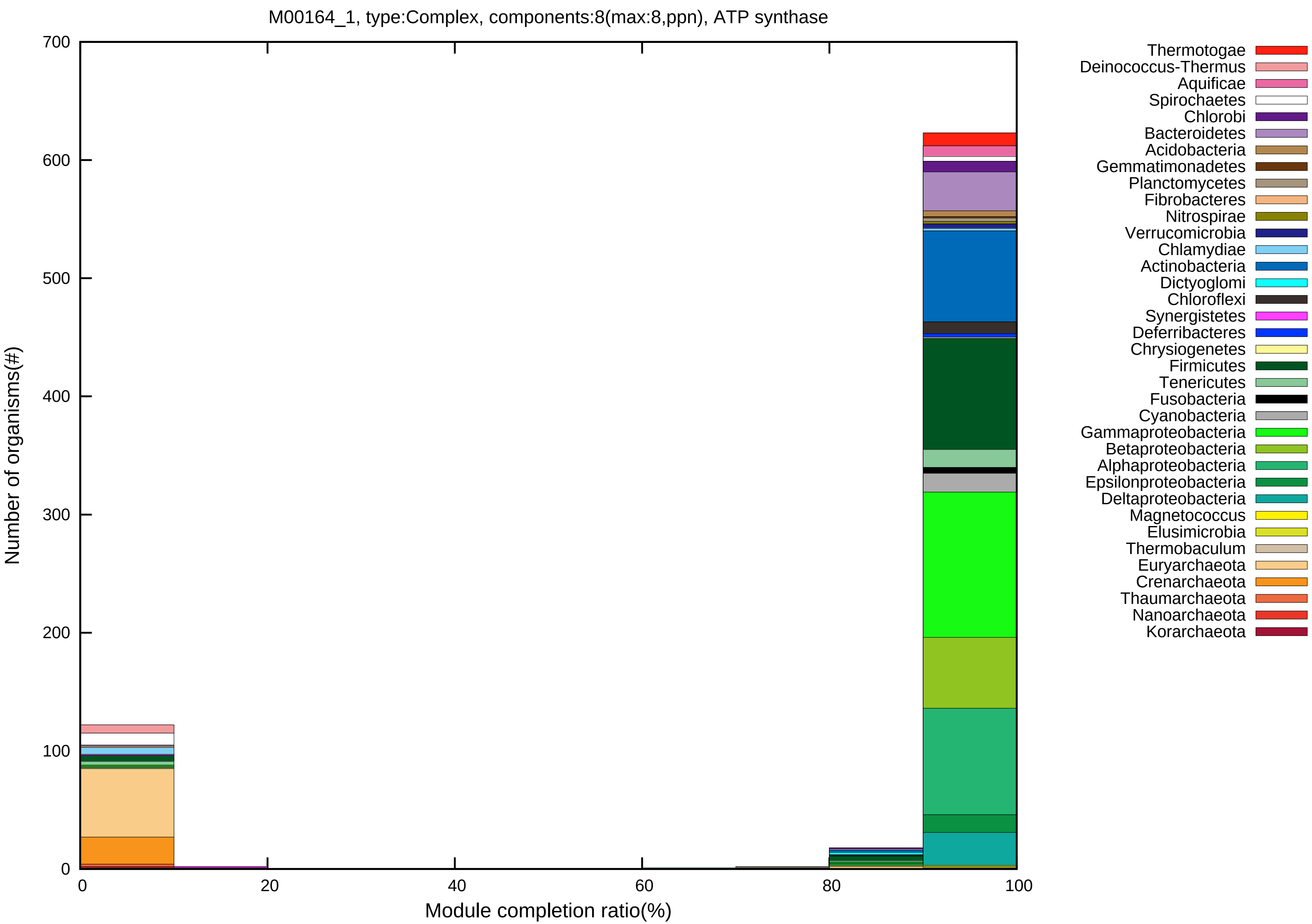

M00164_1, type:Complex, components:8(max:8,ppn), ATP synthase
Number of organisms(#)
Module completion ratio(%)
0
100
200
300
400
500
600
700
0
20
40
60
80
100
Thermotogae
Deinococcus-Thermus
Aquificae
Spirochaetes
Chlorobi
Bacteroidetes
Acidobacteria
Gemmatimonadetes
Planctomycetes
Fibrobacteres
Nitrospirae
Verrucomicrobia
Chlamydiae
Actinobacteria
Dictyoglomi
Chloroflexi
Synergistetes
Deferribacteres
Chrysiogenetes
Firmicutes
Tenericutes
Fusobacteria
Cyanobacteria
Gammaproteobacteria
Betaproteobacteria
Alphaproteobacteria
Epsilonproteobacteria
Deltaproteobacteria
Magnetococcus
Elusimicrobia
Thermobaculum
Euryarchaeota
Crenarchaeota
Thaumarchaeota
Nanoarchaeota
Korarchaeota

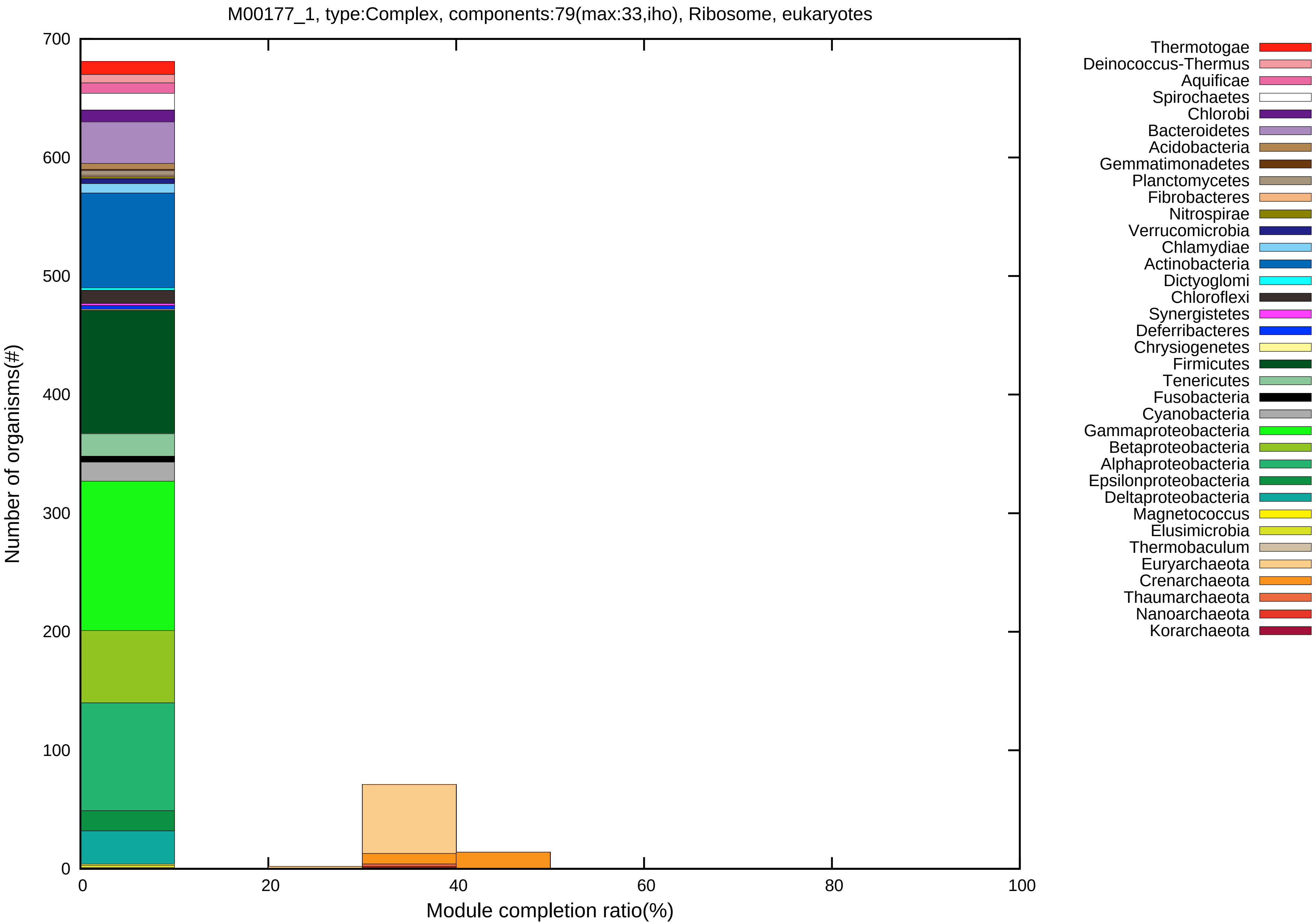

M00177_1, type:Complex, components:79(max:33,iho), Ribosome, eukaryotes
Number of organisms(#)
Module completion ratio(%)
0
100
200
300
400
500
600
700
0
20
40
60
80
100
Thermotogae
Deinococcus-Thermus
Aquificae
Spirochaetes
Chlorobi
Bacteroidetes
Acidobacteria
Gemmatimonadetes
Planctomycetes
Fibrobacteres
Nitrospirae
Verrucomicrobia
Chlamydiae
Actinobacteria
Dictyoglomi
Chloroflexi
Synergistetes
Deferribacteres
Chrysiogenetes
Firmicutes
Tenericutes
Fusobacteria
Cyanobacteria
Gammaproteobacteria
Betaproteobacteria
Alphaproteobacteria
Epsilonproteobacteria
Deltaproteobacteria
Magnetococcus
Elusimicrobia
Thermobaculum
Euryarchaeota
Crenarchaeota
Thaumarchaeota
Nanoarchaeota
Korarchaeota

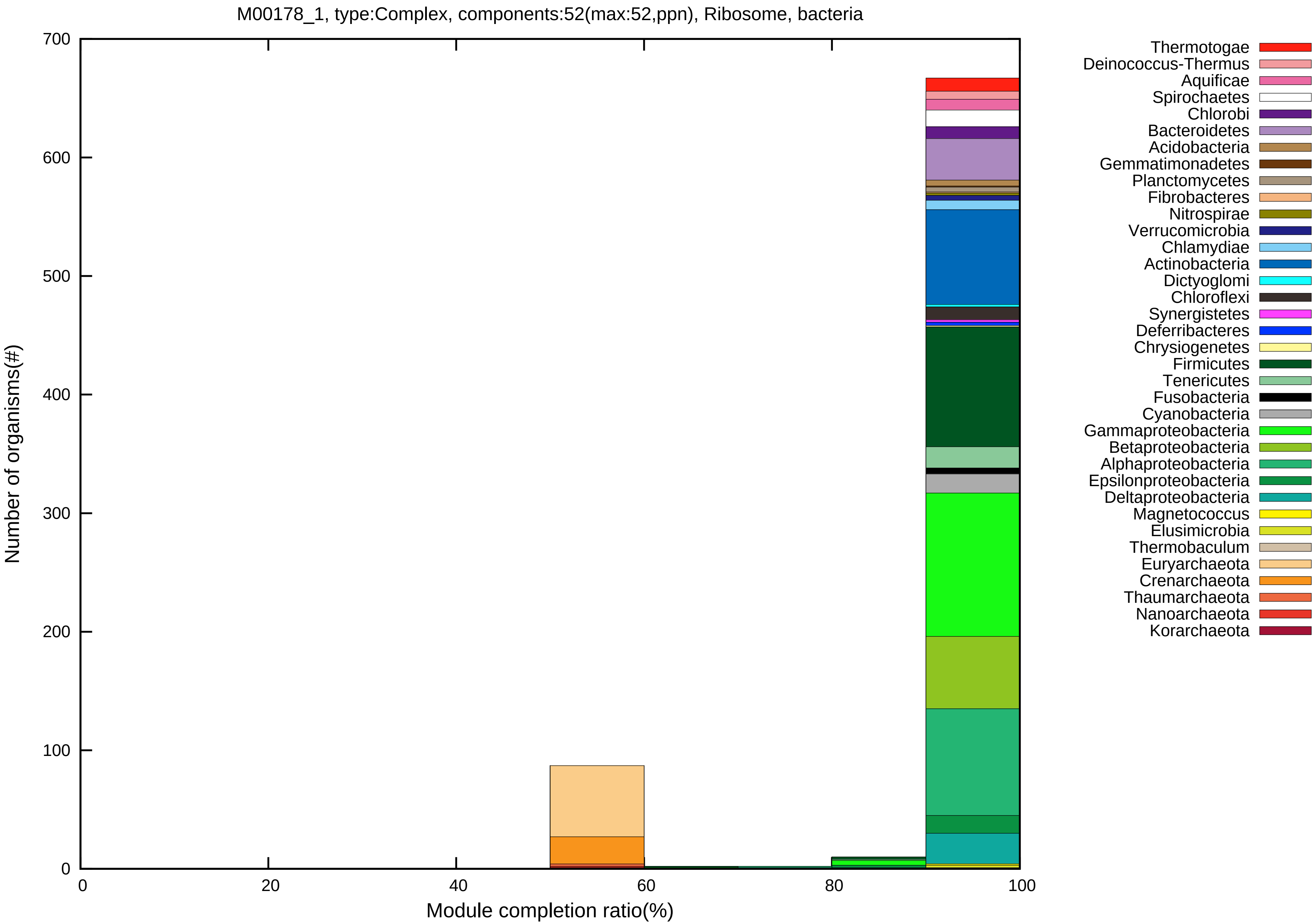

M00178_1, type:Complex, components:52(max:52,ppn), Ribosome, bacteria
Number of organisms(#)
Module completion ratio(%)
0
100
200
300
400
500
600
700
0
20
40
60
80
100
Thermotogae
Deinococcus-Thermus
Aquificae
Spirochaetes
Chlorobi
Bacteroidetes
Acidobacteria
Gemmatimonadetes
Planctomycetes
Fibrobacteres
Nitrospirae
Verrucomicrobia
Chlamydiae
Actinobacteria
Dictyoglomi
Chloroflexi
Synergistetes
Deferribacteres
Chrysiogenetes
Firmicutes
Tenericutes
Fusobacteria
Cyanobacteria
Gammaproteobacteria
Betaproteobacteria
Alphaproteobacteria
Epsilonproteobacteria
Deltaproteobacteria
Magnetococcus
Elusimicrobia
Thermobaculum
Euryarchaeota
Crenarchaeota
Thaumarchaeota
Nanoarchaeota
Korarchaeota

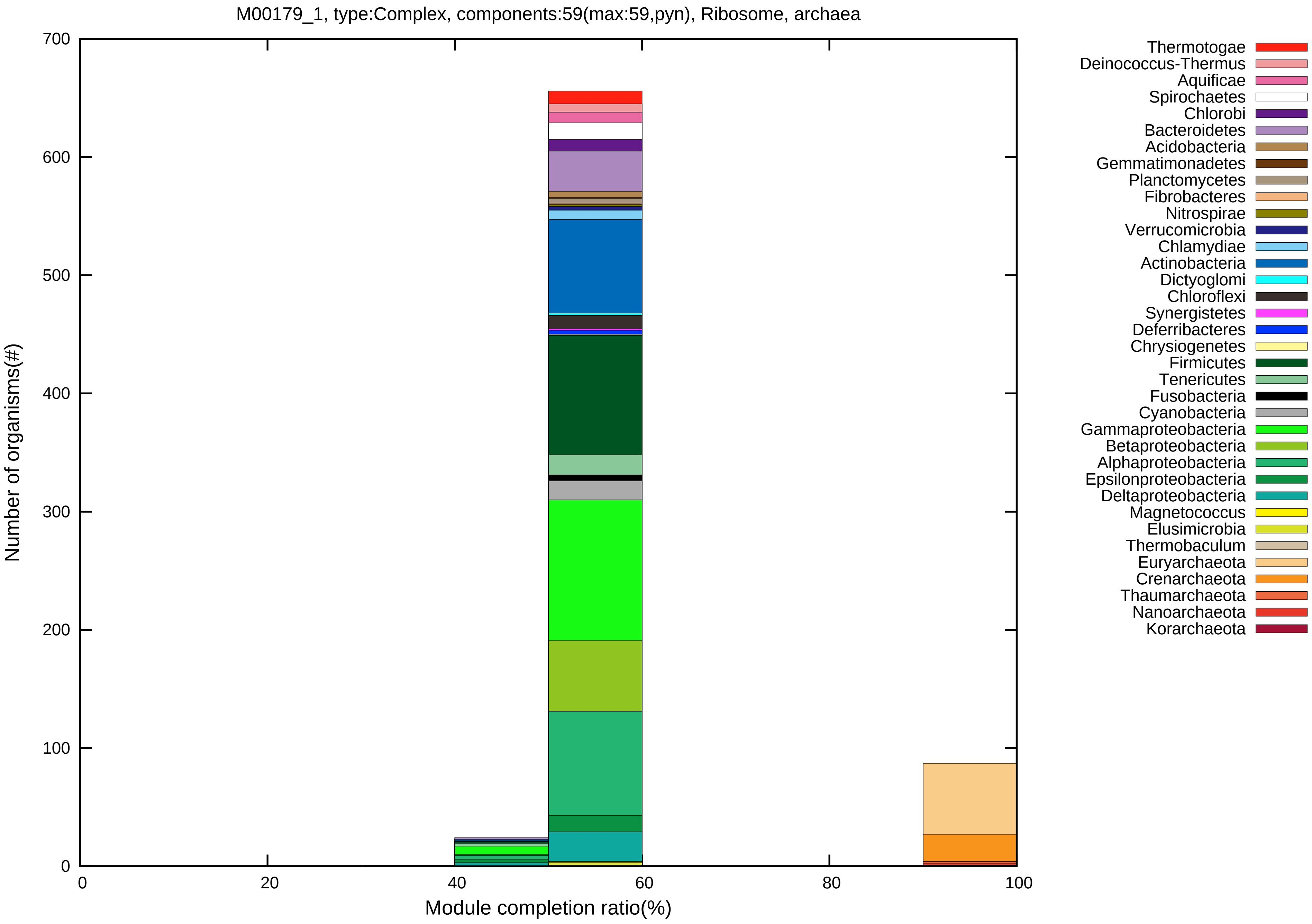

M00179_1, type:Complex, components:59(max:59,pyn), Ribosome, archaea
Number of organisms(#)
Module completion ratio(%)
0
100
200
300
400
500
600
700
0
20
40
60
80
100
Thermotogae
Deinococcus-Thermus
Aquificae
Spirochaetes
Chlorobi
Bacteroidetes
Acidobacteria
Gemmatimonadetes
Planctomycetes
Fibrobacteres
Nitrospirae
Verrucomicrobia
Chlamydiae
Actinobacteria
Dictyoglomi
Chloroflexi
Synergistetes
Deferribacteres
Chrysiogenetes
Firmicutes
Tenericutes
Fusobacteria
Cyanobacteria
Gammaproteobacteria
Betaproteobacteria
Alphaproteobacteria
Epsilonproteobacteria
Deltaproteobacteria
Magnetococcus
Elusimicrobia
Thermobaculum
Euryarchaeota
Crenarchaeota
Thaumarchaeota
Nanoarchaeota
Korarchaeota

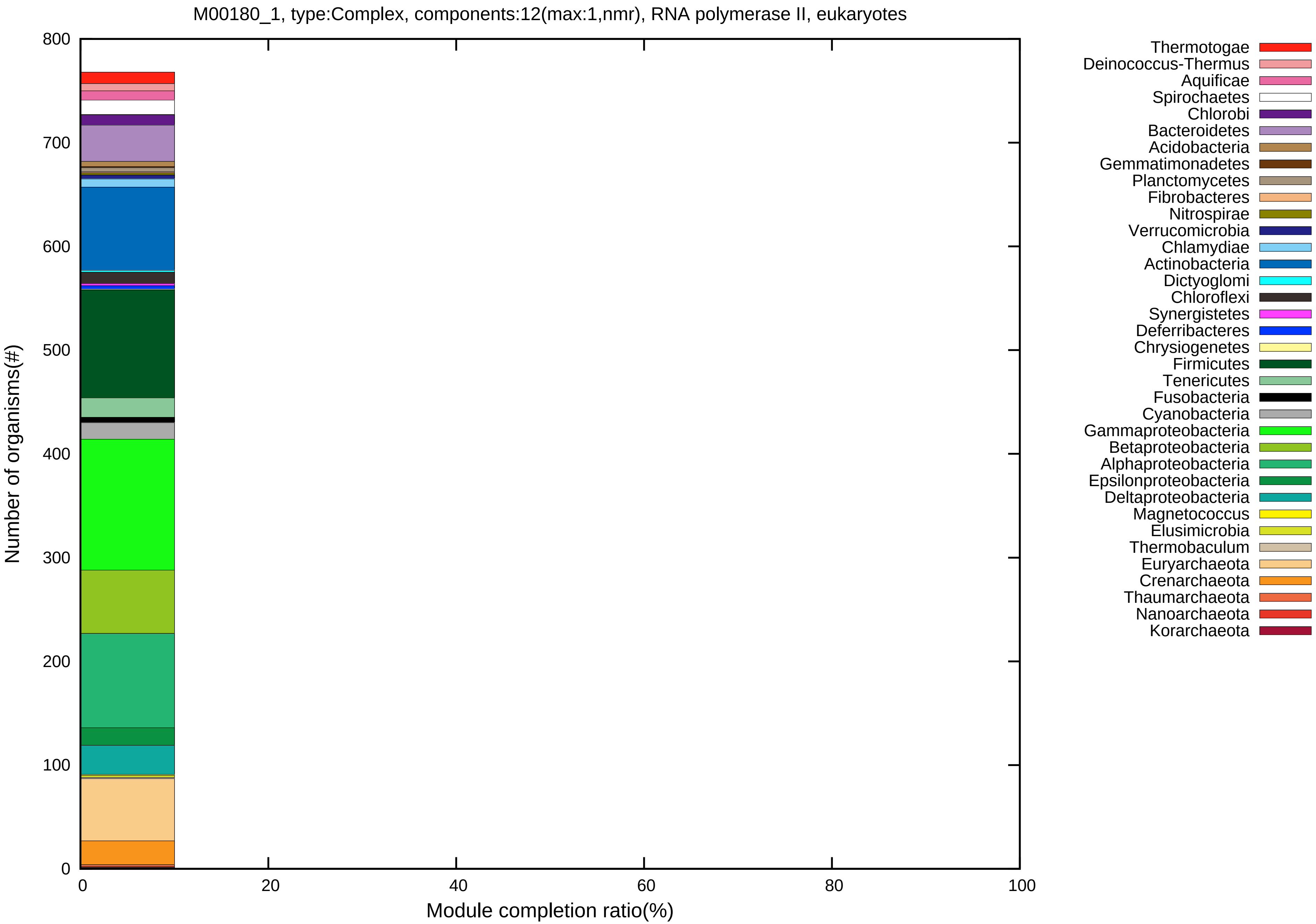

M00180_1, type:Complex, components:12(max:1,nmr), RNA polymerase II, eukaryotes
Number of organisms(#)
Module completion ratio(%)
0
100
200
300
400
500
600
700
800
0
20
40
60
80
100
Thermotogae
Deinococcus-Thermus
Aquificae
Spirochaetes
Chlorobi
Bacteroidetes
Acidobacteria
Gemmatimonadetes
Planctomycetes
Fibrobacteres
Nitrospirae
Verrucomicrobia
Chlamydiae
Actinobacteria
Dictyoglomi
Chloroflexi
Synergistetes
Deferribacteres
Chrysiogenetes
Firmicutes
Tenericutes
Fusobacteria
Cyanobacteria
Gammaproteobacteria
Betaproteobacteria
Alphaproteobacteria
Epsilonproteobacteria
Deltaproteobacteria
Magnetococcus
Elusimicrobia
Thermobaculum
Euryarchaeota
Crenarchaeota
Thaumarchaeota
Nanoarchaeota
Korarchaeota

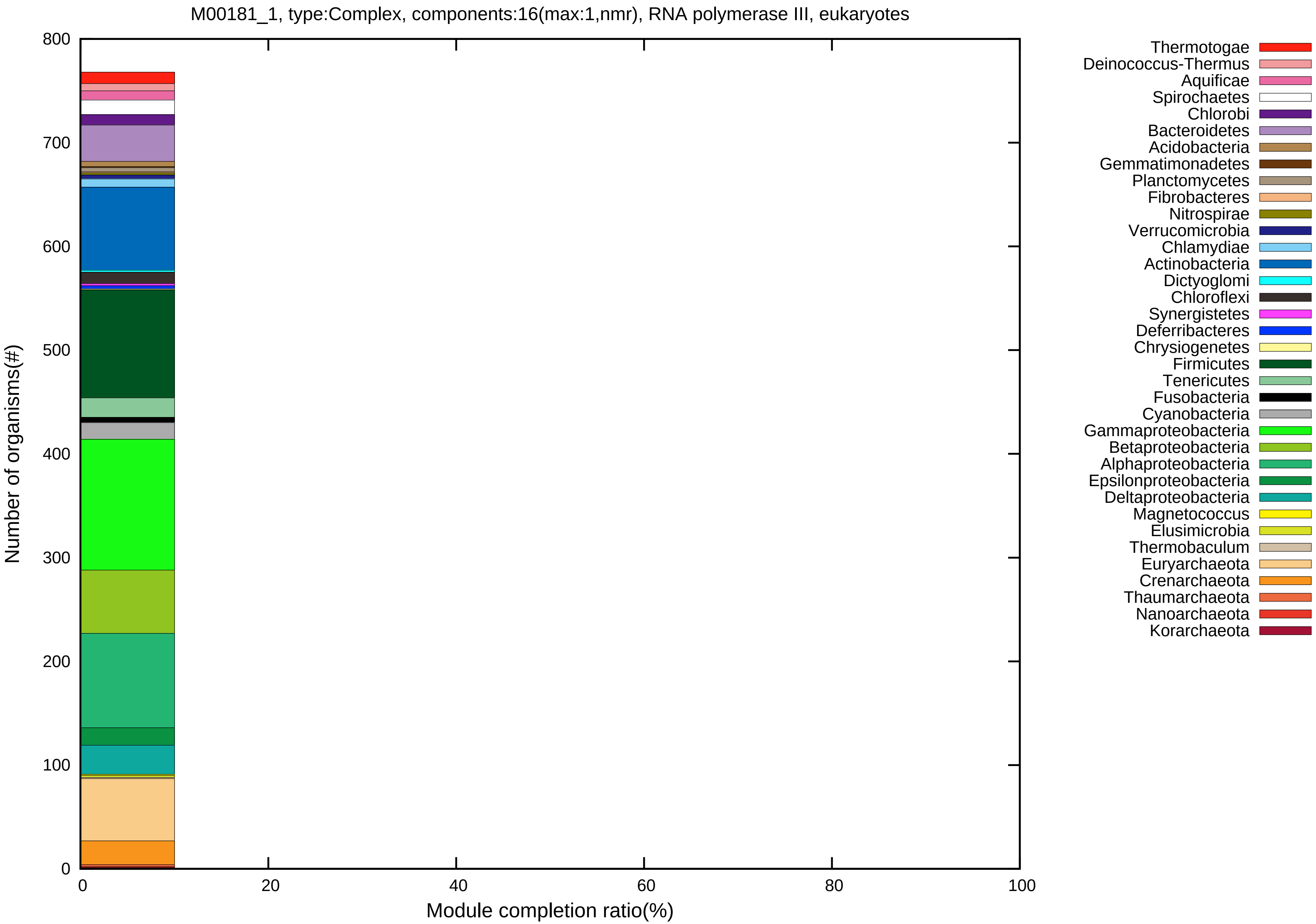

M00181_1, type:Complex, components:16(max:1,nmr), RNA polymerase III, eukaryotes
Number of organisms(#)
0
100
200
300
400
500
600
700
800
0
20
40
60
80
100
Module completion ratio(%)
Thermotogae
Deinococcus-Thermus
Aquificae
Spirochaetes
Chlorobi
Bacteroidetes
Acidobacteria
Gemmatimonadetes
Planctomycetes
Fibrobacteres
Nitrospirae
Verrucomicrobia
Chlamydiae
Actinobacteria
Dictyoglomi
Chloroflexi
Synergistetes
Deferribacteres
Chrysiogenetes
Firmicutes
Tenericutes
Fusobacteria
Cyanobacteria
Gammaproteobacteria
Betaproteobacteria
Alphaproteobacteria
Epsilonproteobacteria
Deltaproteobacteria
Magnetococcus
Elusimicrobia
Thermobaculum
Euryarchaeota
Crenarchaeota
Thaumarchaeota
Nanoarchaeota
Korarchaeota

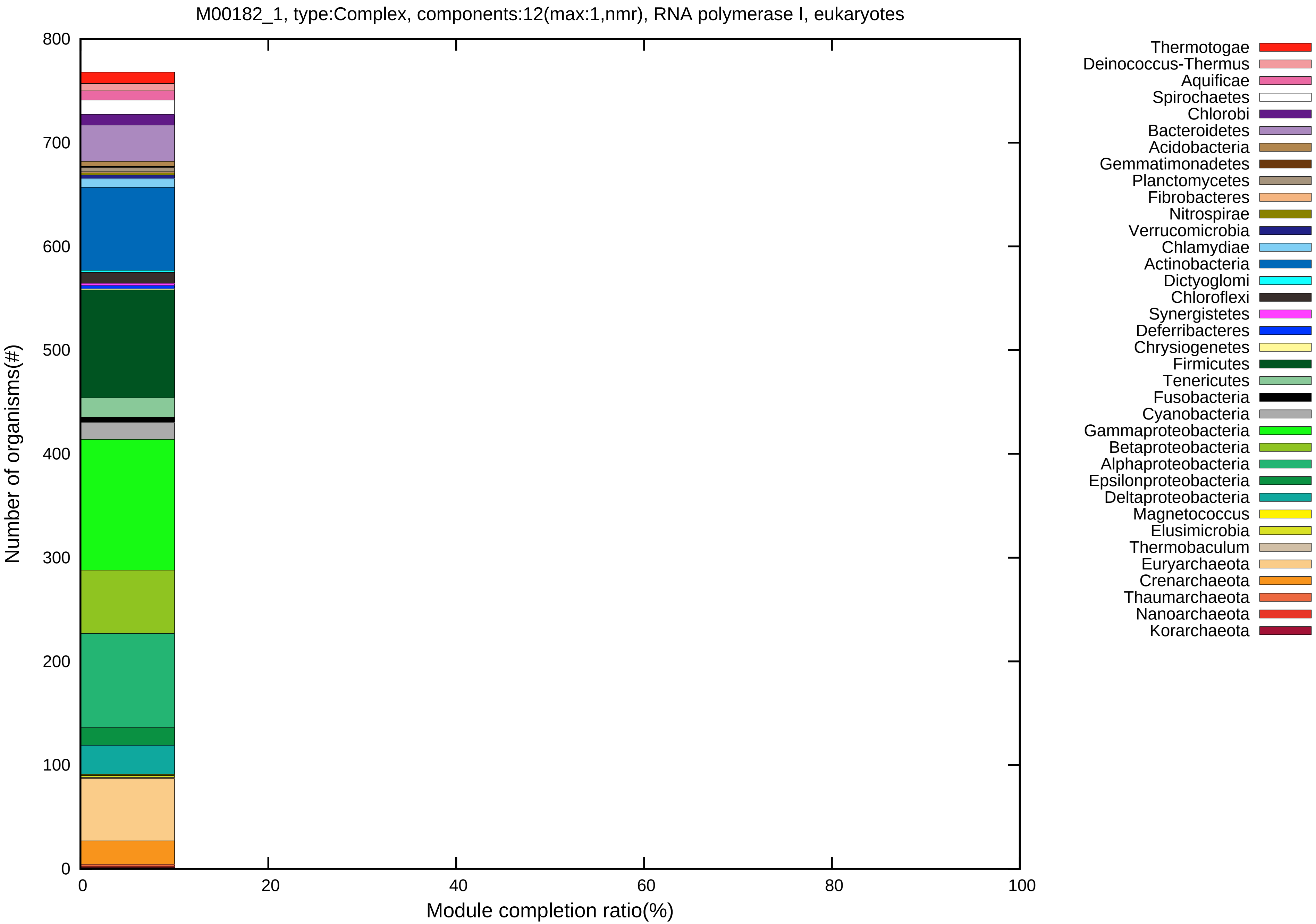

M00182_1, type:Complex, components:12(max:1,nmr), RNA polymerase I, eukaryotes
Number of organisms(#)
Module completion ratio(%)
0
100
200
300
400
500
600
700
800
0
20
40
60
80
100
Thermotogae
Deinococcus-Thermus
Aquificae
Spirochaetes
Chlorobi
Bacteroidetes
Acidobacteria
Gemmatimonadetes
Planctomycetes
Fibrobacteres
Nitrospirae
Verrucomicrobia
Chlamydiae
Actinobacteria
Dictyoglomi
Chloroflexi
Synergistetes
Deferribacteres
Chrysiogenetes
Firmicutes
Tenericutes
Fusobacteria
Cyanobacteria
Gammaproteobacteria
Betaproteobacteria
Alphaproteobacteria
Epsilonproteobacteria
Deltaproteobacteria
Magnetococcus
Elusimicrobia
Thermobaculum
Euryarchaeota
Crenarchaeota
Thaumarchaeota
Nanoarchaeota
Korarchaeota

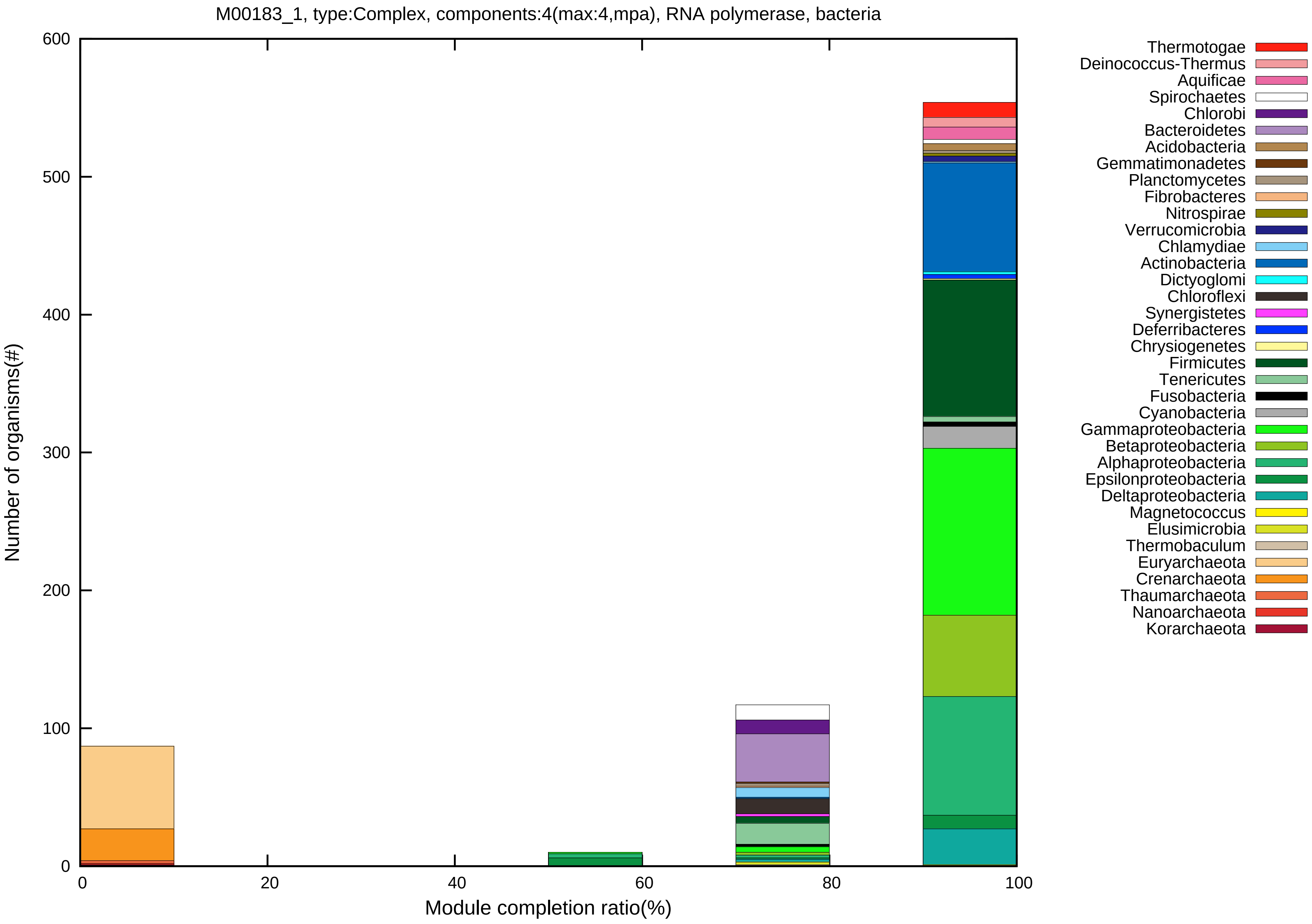

M00183_1, type:Complex, components:4(max:4,mpa), RNA polymerase, bacteria
Number of organisms(#)
Module completion ratio(%)
0
100
200
300
400
500
600
0
20
40
60
80
100
Thermotogae
Deinococcus-Thermus
Aquificae
Spirochaetes
Chlorobi
Bacteroidetes
Acidobacteria
Gemmatimonadetes
Planctomycetes
Fibrobacteres
Nitrospirae
Verrucomicrobia
Chlamydiae
Actinobacteria
Dictyoglomi
Chloroflexi
Synergistetes
Deferribacteres
Chrysiogenetes
Firmicutes
Tenericutes
Fusobacteria
Cyanobacteria
Gammaproteobacteria
Betaproteobacteria
Alphaproteobacteria
Epsilonproteobacteria
Deltaproteobacteria
Magnetococcus
Elusimicrobia
Thermobaculum
Euryarchaeota
Crenarchaeota
Thaumarchaeota
Nanoarchaeota
Korarchaeota

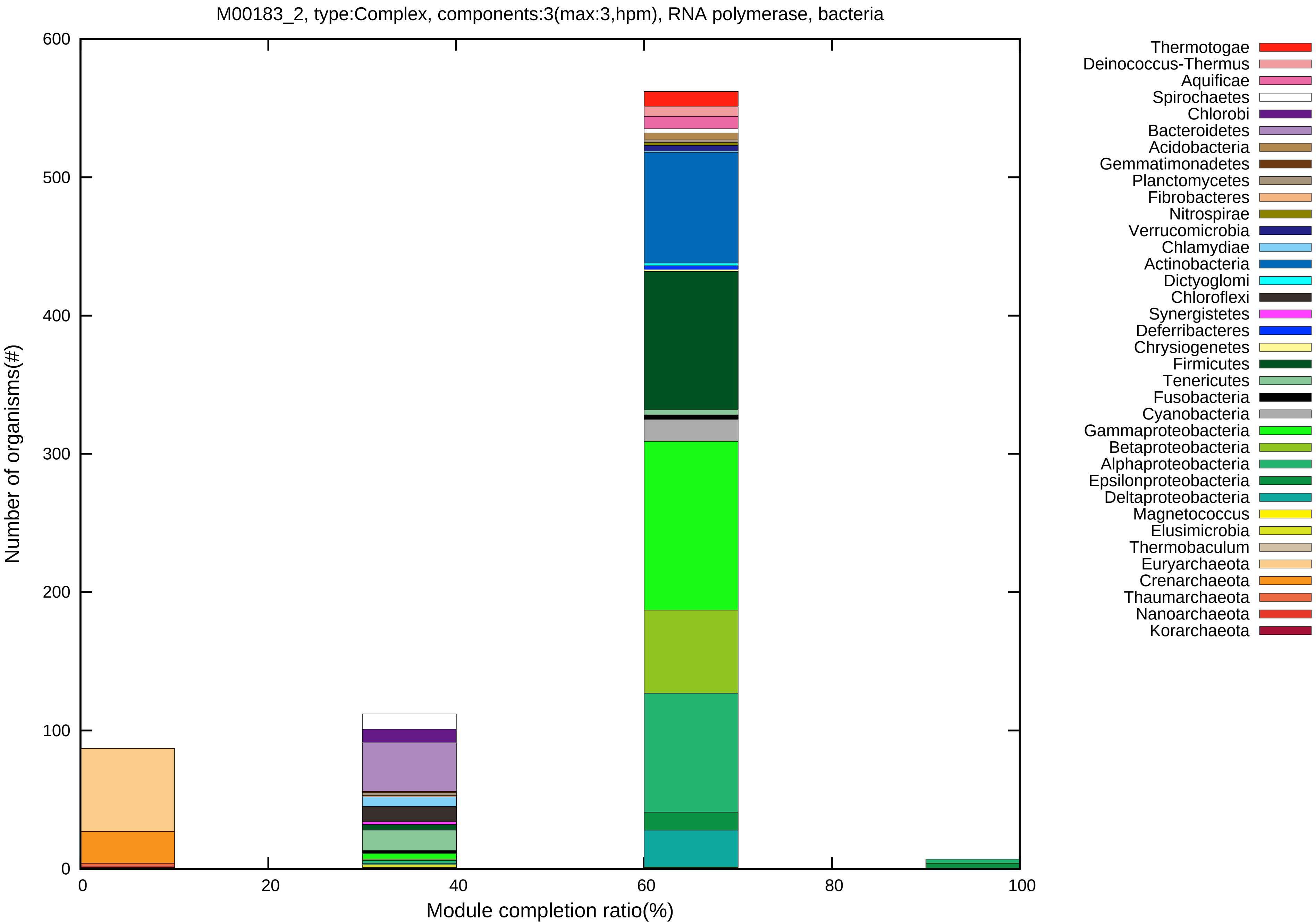

M00183_2, type:Complex, components:3(max:3,hpm), RNA polymerase, bacteria
Number of organisms(#)
Module completion ratio(%)
0
100
200
300
400
500
600
0
20
40
60
80
100
Thermotogae
Deinococcus-Thermus
Aquificae
Spirochaetes
Chlorobi
Bacteroidetes
Acidobacteria
Gemmatimonadetes
Planctomycetes
Fibrobacteres
Nitrospirae
Verrucomicrobia
Chlamydiae
Actinobacteria
Dictyoglomi
Chloroflexi
Synergistetes
Deferribacteres
Chrysiogenetes
Firmicutes
Tenericutes
Fusobacteria
Cyanobacteria
Gammaproteobacteria
Betaproteobacteria
Alphaproteobacteria
Epsilonproteobacteria
Deltaproteobacteria
Magnetococcus
Elusimicrobia
Thermobaculum
Euryarchaeota
Crenarchaeota
Thaumarchaeota
Nanoarchaeota
Korarchaeota

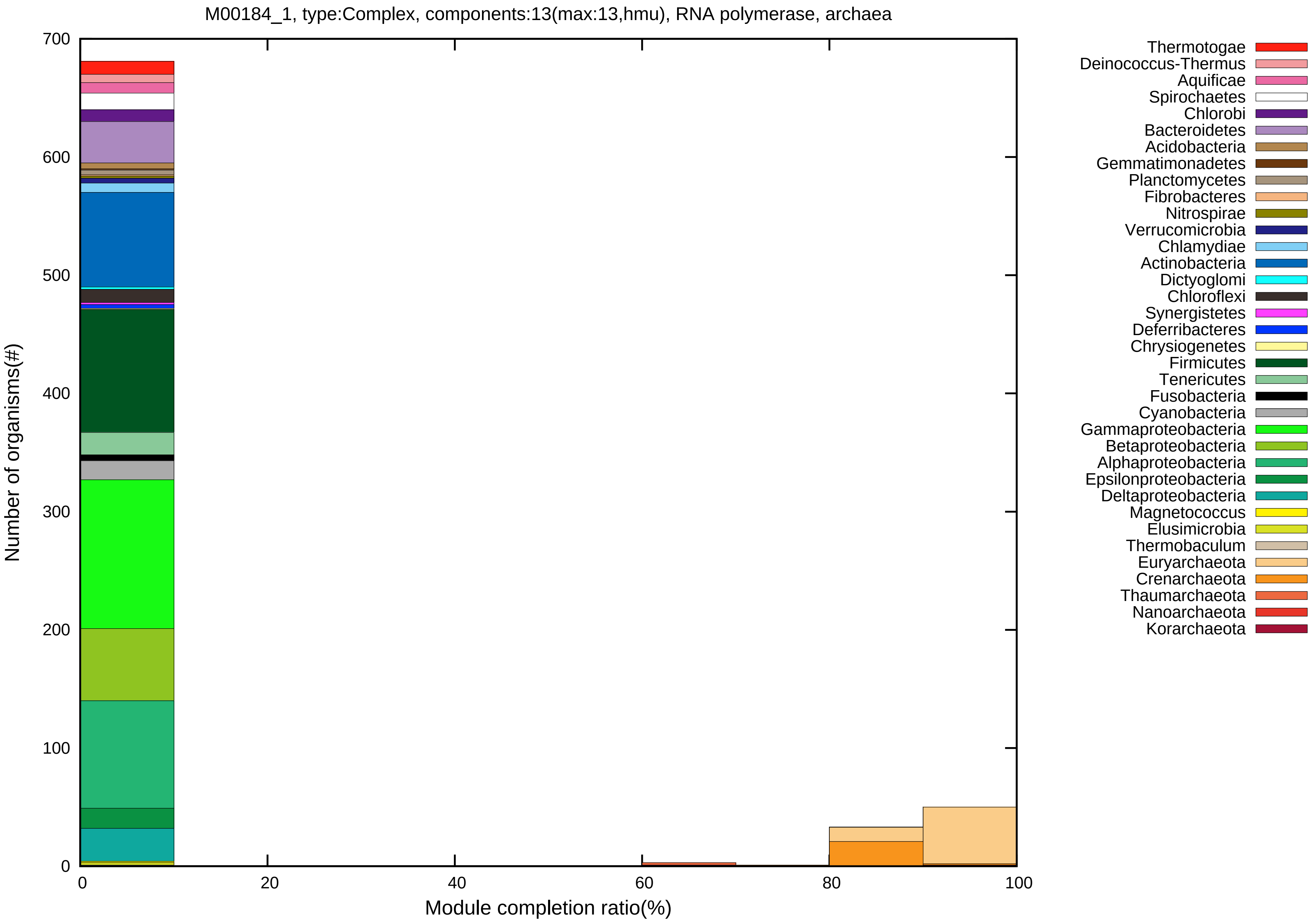

M00184_1, type:Complex, components:13(max:13,hmu), RNA polymerase, archaea
Number of organisms(#)
Module completion ratio(%)
0
100
200
300
400
500
600
700
0
20
40
60
80
100
Thermotogae
Deinococcus-Thermus
Aquificae
Spirochaetes
Chlorobi
Bacteroidetes
Acidobacteria
Gemmatimonadetes
Planctomycetes
Fibrobacteres
Nitrospirae
Verrucomicrobia
Chlamydiae
Actinobacteria
Dictyoglomi
Chloroflexi
Synergistetes
Deferribacteres
Chrysiogenetes
Firmicutes
Tenericutes
Fusobacteria
Cyanobacteria
Gammaproteobacteria
Betaproteobacteria
Alphaproteobacteria
Epsilonproteobacteria
Deltaproteobacteria
Magnetococcus
Elusimicrobia
Thermobaculum
Euryarchaeota
Crenarchaeota
Thaumarchaeota
Nanoarchaeota
Korarchaeota

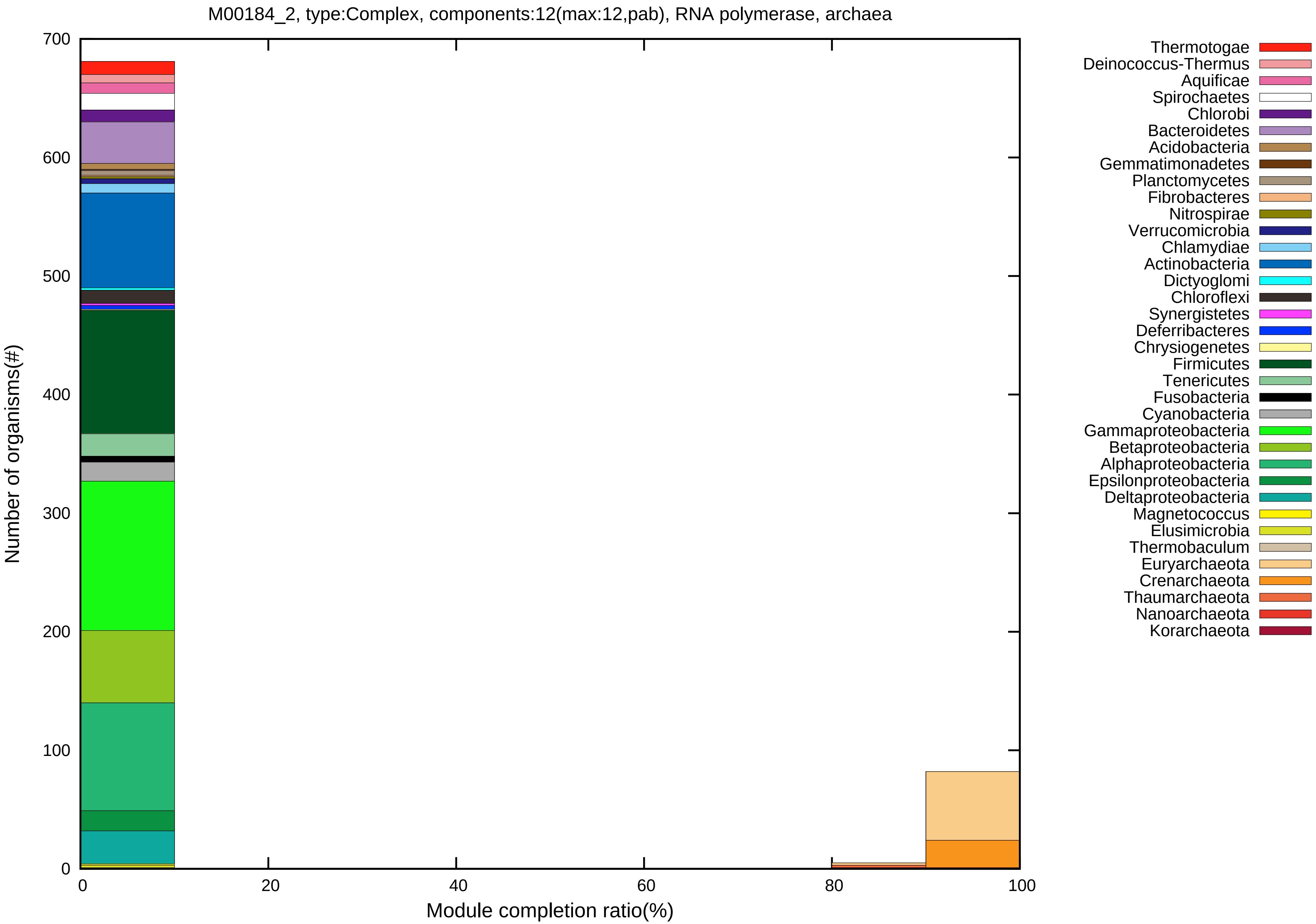

M00184_2, type:Complex, components:12(max:12,pab), RNA polymerase, archaea
Number of organisms(#)
Module completion ratio(%)
0
100
200
300
400
500
600
700
0
20
40
60
80
100
Thermotogae
Deinococcus-Thermus
Aquificae
Spirochaetes
Chlorobi
Bacteroidetes
Acidobacteria
Gemmatimonadetes
Planctomycetes
Fibrobacteres
Nitrospirae
Verrucomicrobia
Chlamydiae
Actinobacteria
Dictyoglomi
Chloroflexi
Synergistetes
Deferribacteres
Chrysiogenetes
Firmicutes
Tenericutes
Fusobacteria
Cyanobacteria
Gammaproteobacteria
Betaproteobacteria
Alphaproteobacteria
Epsilonproteobacteria
Deltaproteobacteria
Magnetococcus
Elusimicrobia
Thermobaculum
Euryarchaeota
Crenarchaeota
Thaumarchaeota
Nanoarchaeota
Korarchaeota

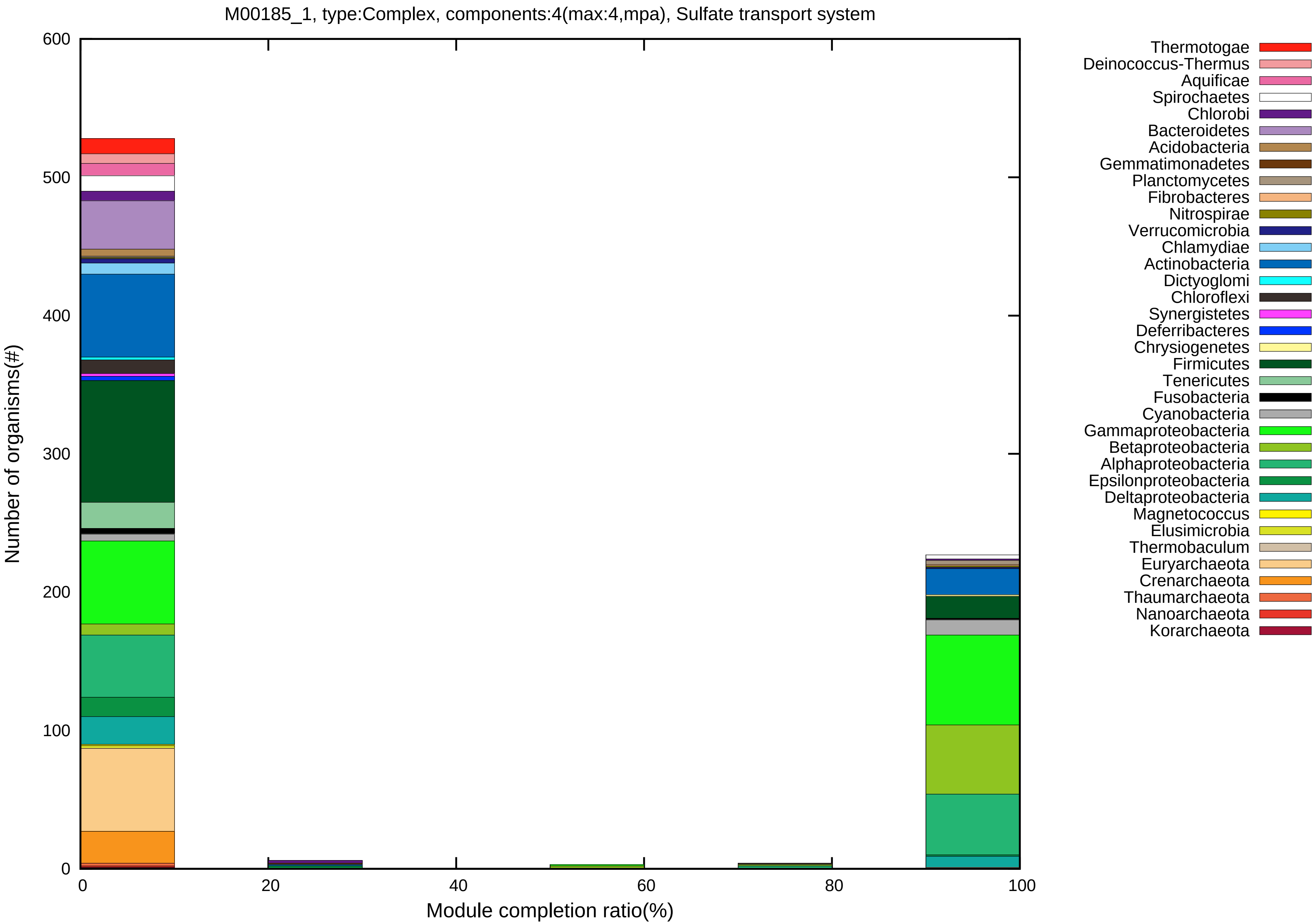

M00185_1, type:Complex, components:4(max:4,mpa), Sulfate transport system
Number of organisms(#)
Module completion ratio(%)
0
100
200
300
400
500
600
0
20
40
60
80
100
Thermotogae
Deinococcus-Thermus
Aquificae
Spirochaetes
Chlorobi
Bacteroidetes
Acidobacteria
Gemmatimonadetes
Planctomycetes
Fibrobacteres
Nitrospirae
Verrucomicrobia
Chlamydiae
Actinobacteria
Dictyoglomi
Chloroflexi
Synergistetes
Deferribacteres
Chrysiogenetes
Firmicutes
Tenericutes
Fusobacteria
Cyanobacteria
Gammaproteobacteria
Betaproteobacteria
Alphaproteobacteria
Epsilonproteobacteria
Deltaproteobacteria
Magnetococcus
Elusimicrobia
Thermobaculum
Euryarchaeota
Crenarchaeota
Thaumarchaeota
Nanoarchaeota
Korarchaeota

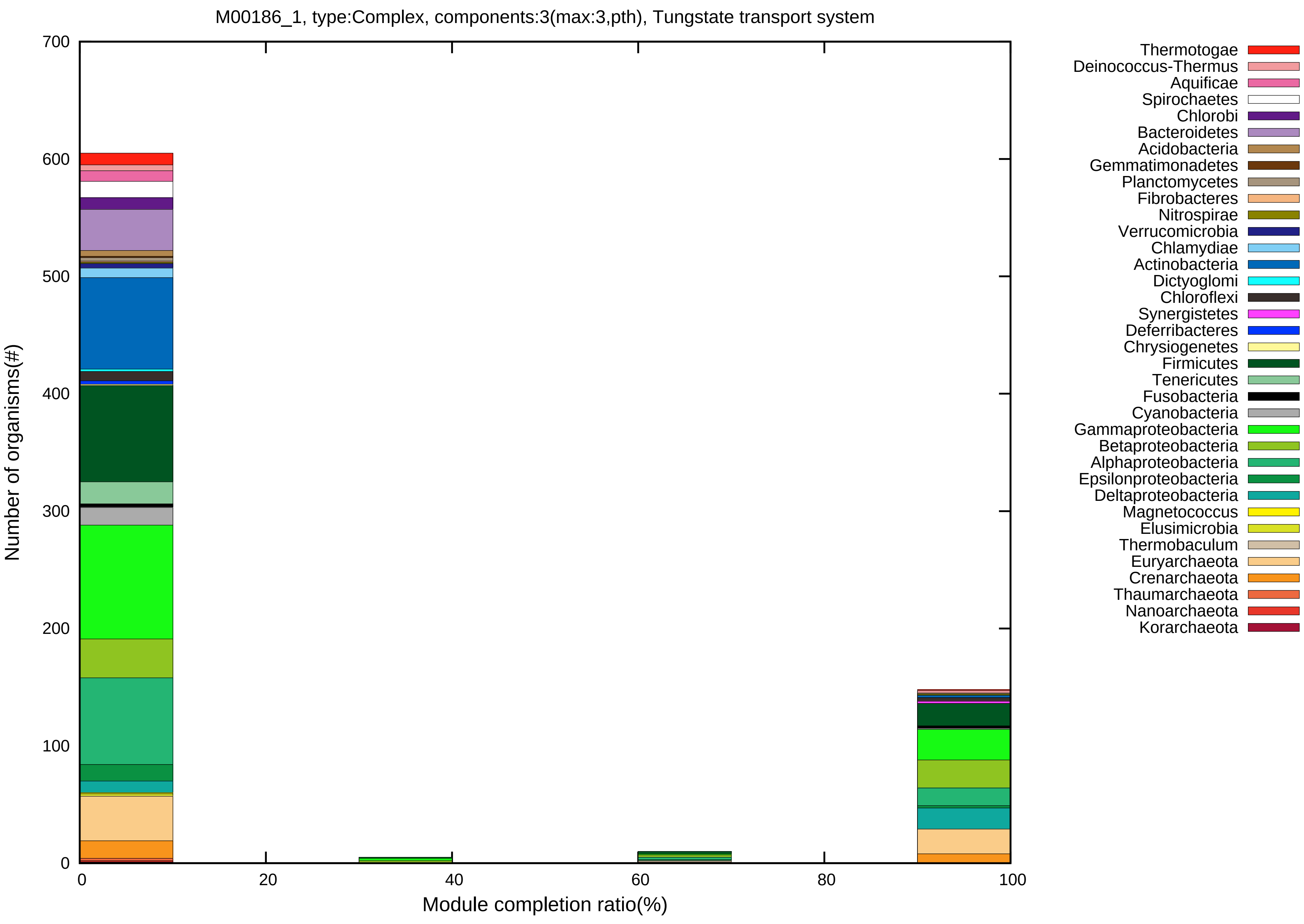

M00186_1, type:Complex, components:3(max:3,pth), Tungstate transport system
Number of organisms(#)
Module completion ratio(%)
0
100
200
300
400
500
600
700
0
20
40
60
80
100
Thermotogae
Deinococcus-Thermus
Aquificae
Spirochaetes
Chlorobi
Bacteroidetes
Acidobacteria
Gemmatimonadetes
Planctomycetes
Fibrobacteres
Nitrospirae
Verrucomicrobia
Chlamydiae
Actinobacteria
Dictyoglomi
Chloroflexi
Synergistetes
Deferribacteres
Chrysiogenetes
Firmicutes
Tenericutes
Fusobacteria
Cyanobacteria
Gammaproteobacteria
Betaproteobacteria
Alphaproteobacteria
Epsilonproteobacteria
Deltaproteobacteria
Magnetococcus
Elusimicrobia
Thermobaculum
Euryarchaeota
Crenarchaeota
Thaumarchaeota
Nanoarchaeota
Korarchaeota

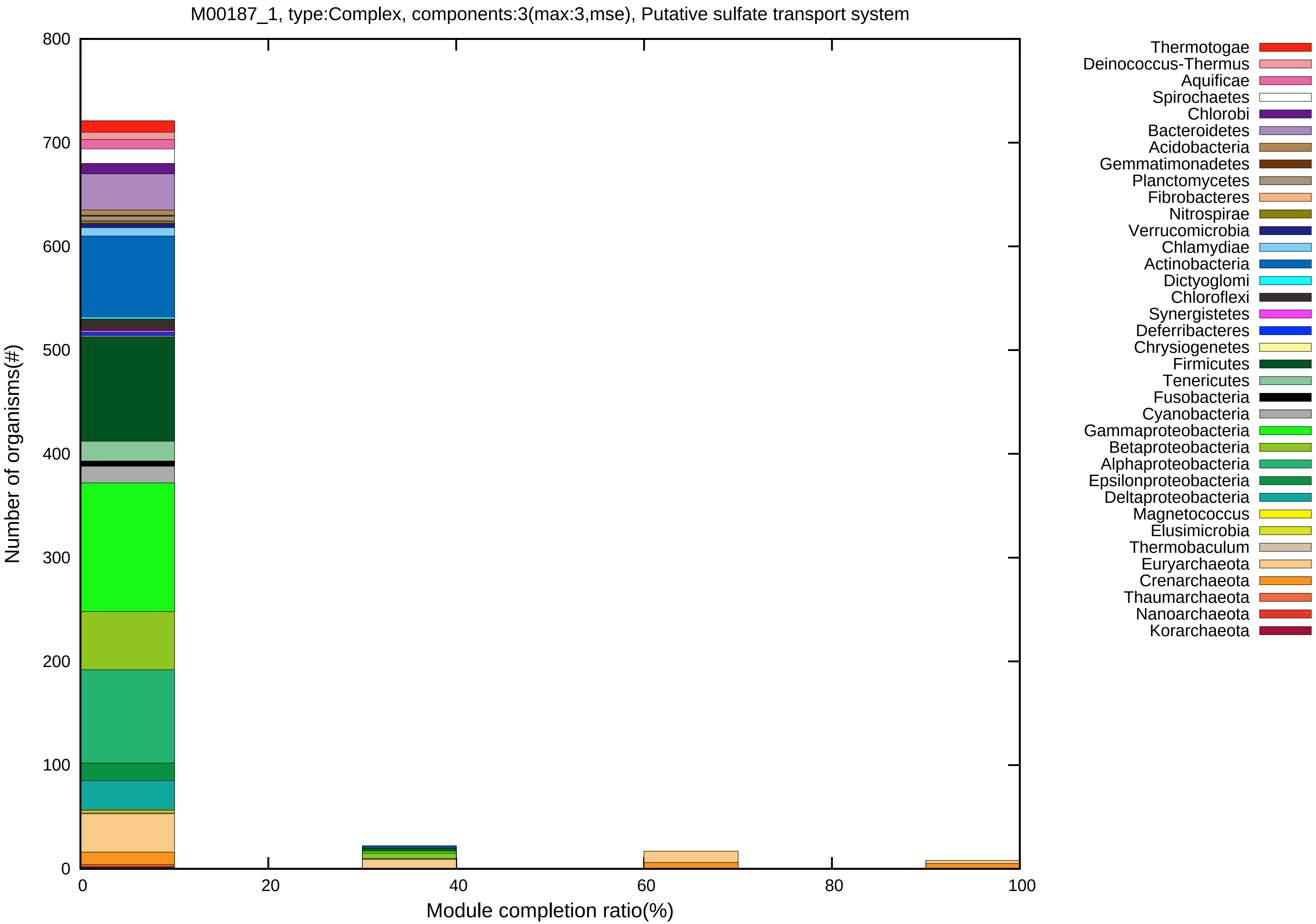

M00187_1, type:Complex, components:3(max:3,mse), Putative sulfate transport system
Number of organisms(#)
Module completion ratio(%)
0
100
200
300
400
500
600
700
800
0
20
40
60
80
100
Thermotogae
Deinococcus-Thermus
Aquificae
Spirochaetes
Chlorobi
Bacteroidetes
Acidobacteria
Gemmatimonadetes
Planctomycetes
Fibrobacteres
Nitrospirae
Verrucomicrobia
Chlamydiae
Actinobacteria
Dictyoglomi
Chloroflexi
Synergistetes
Deferribacteres
Chrysiogenetes
Firmicutes
Tenericutes
Fusobacteria
Cyanobacteria
Gammaproteobacteria
Betaproteobacteria
Alphaproteobacteria
Epsilonproteobacteria
Deltaproteobacteria
Magnetococcus
Elusimicrobia
Thermobaculum
Euryarchaeota
Crenarchaeota
Thaumarchaeota
Nanoarchaeota
Korarchaeota

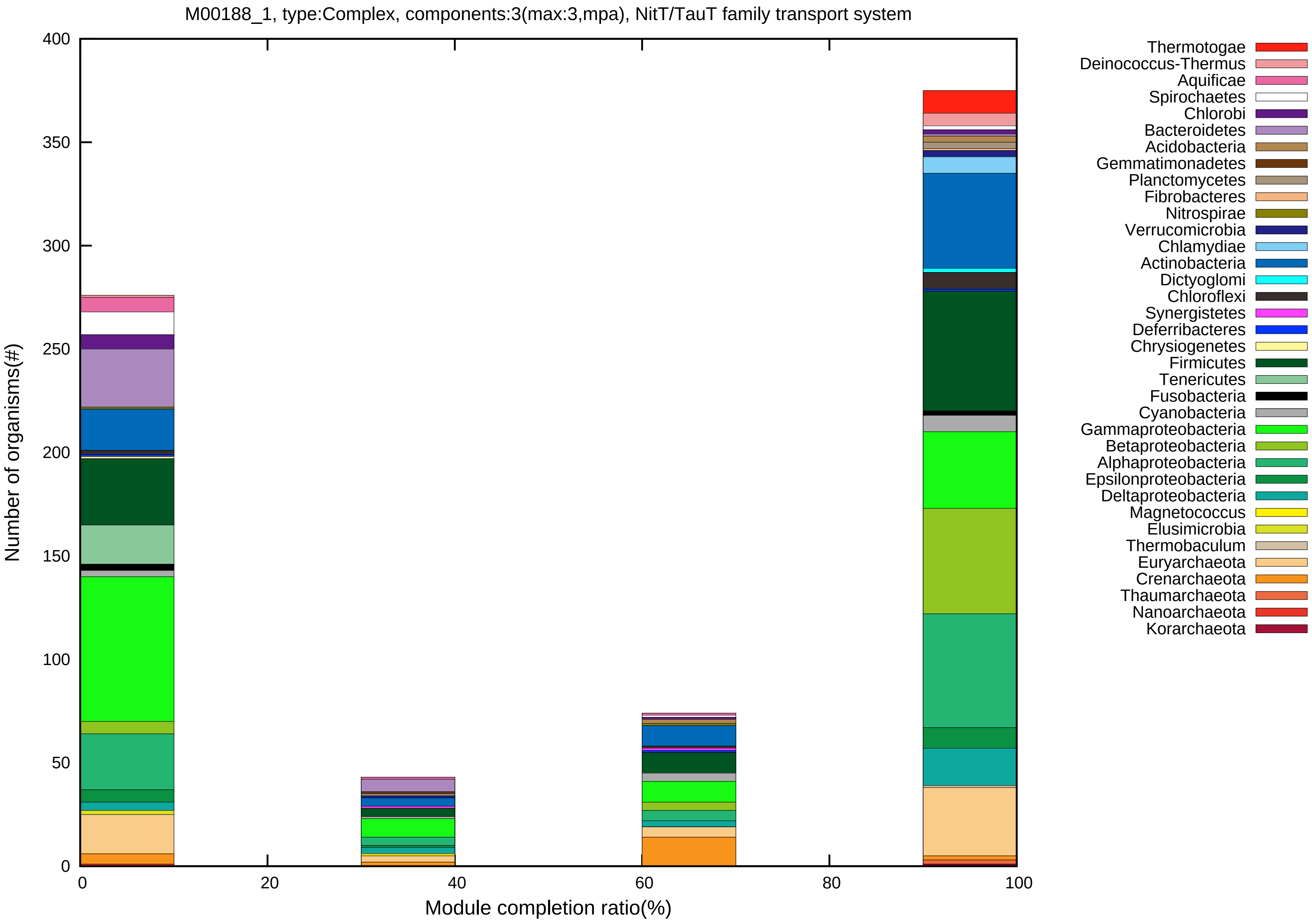
M00188_1, type:Complex, components:3(max:3,mpa), NitT/TauT family transport system
Number of organisms(#)
Module completion ratio(%)
0
50
100
150
200
250
300
350
400
0
20
40
60
80
100
Thermotogae
Deinococcus-Thermus
Aquificae
Spirochaetes
Chlorobi
Bacteroidetes
Acidobacteria
Gemmatimonadetes
Planctomycetes
Fibrobacteres
Nitrospirae
Verrucomicrobia
Chlamydiae
Actinobacteria
Dictyoglomi
Chloroflexi
Synergistetes
Deferribacteres
Chrysiogenetes
Firmicutes
Tenericutes
Fusobacteria
Cyanobacteria
Gammaproteobacteria
Betaproteobacteria
Alphaproteobacteria
Epsilonproteobacteria
Deltaproteobacteria
Magnetococcus
Elusimicrobia
Thermobaculum
Euryarchaeota
Crenarchaeota
Thaumarchaeota
Nanoarchaeota
Korarchaeota

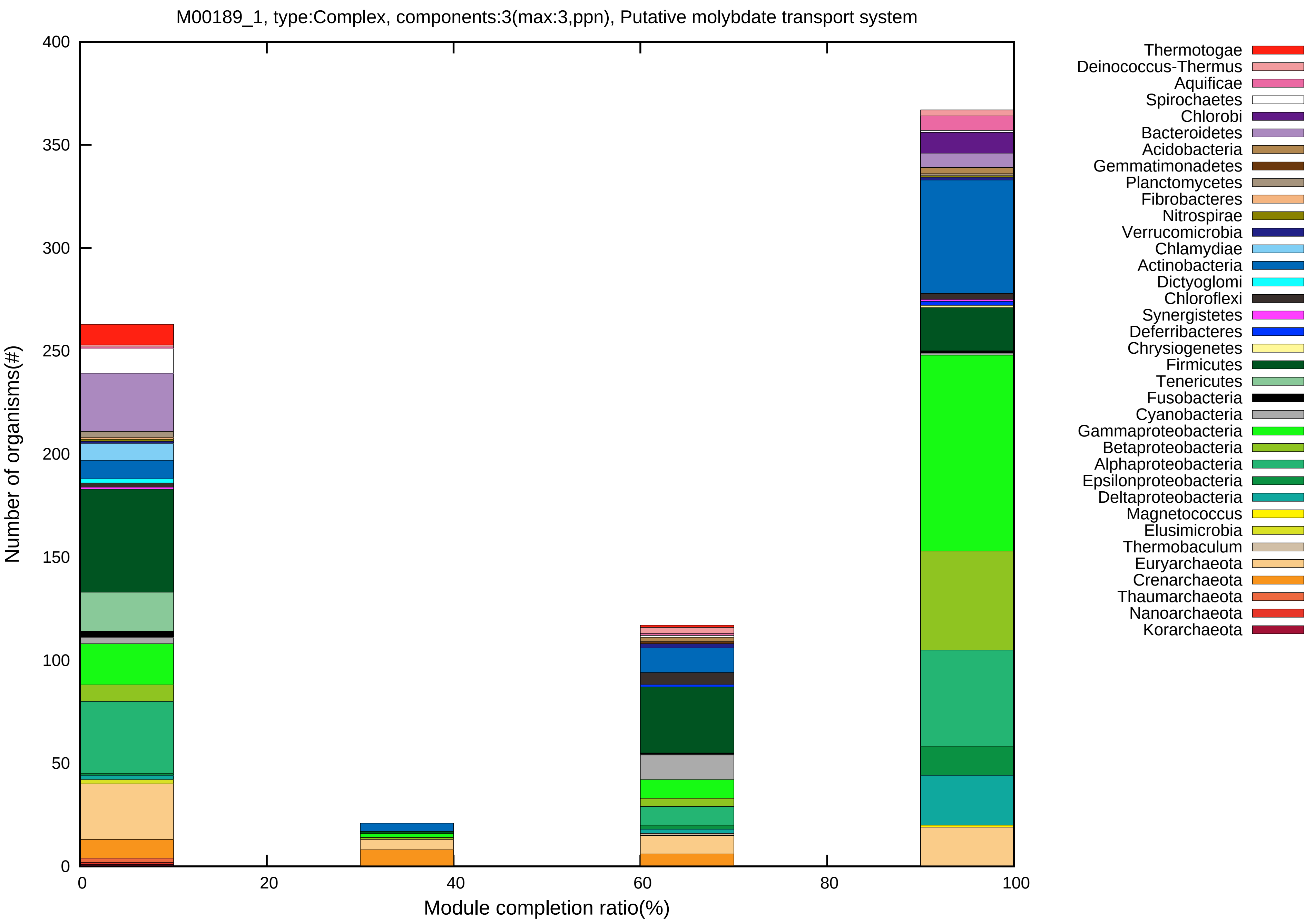

M00189_1, type:Complex, components:3(max:3,ppn), Putative molybdate transport system
Number of organisms(#)
Module completion ratio(%)
0
50
100
150
200
250
300
350
400
0
20
40
60
80
100
Thermotogae
Deinococcus-Thermus
Aquificae
Spirochaetes
Chlorobi
Bacteroidetes
Acidobacteria
Gemmatimonadetes
Planctomycetes
Fibrobacteres
Nitrospirae
Verrucomicrobia
Chlamydiae
Actinobacteria
Dictyoglomi
Chloroflexi
Synergistetes
Deferribacteres
Chrysiogenetes
Firmicutes
Tenericutes
Fusobacteria
Cyanobacteria
Gammaproteobacteria
Betaproteobacteria
Alphaproteobacteria
Epsilonproteobacteria
Deltaproteobacteria
Magnetococcus
Elusimicrobia
Thermobaculum
Euryarchaeota
Crenarchaeota
Thaumarchaeota
Nanoarchaeota
Korarchaeota

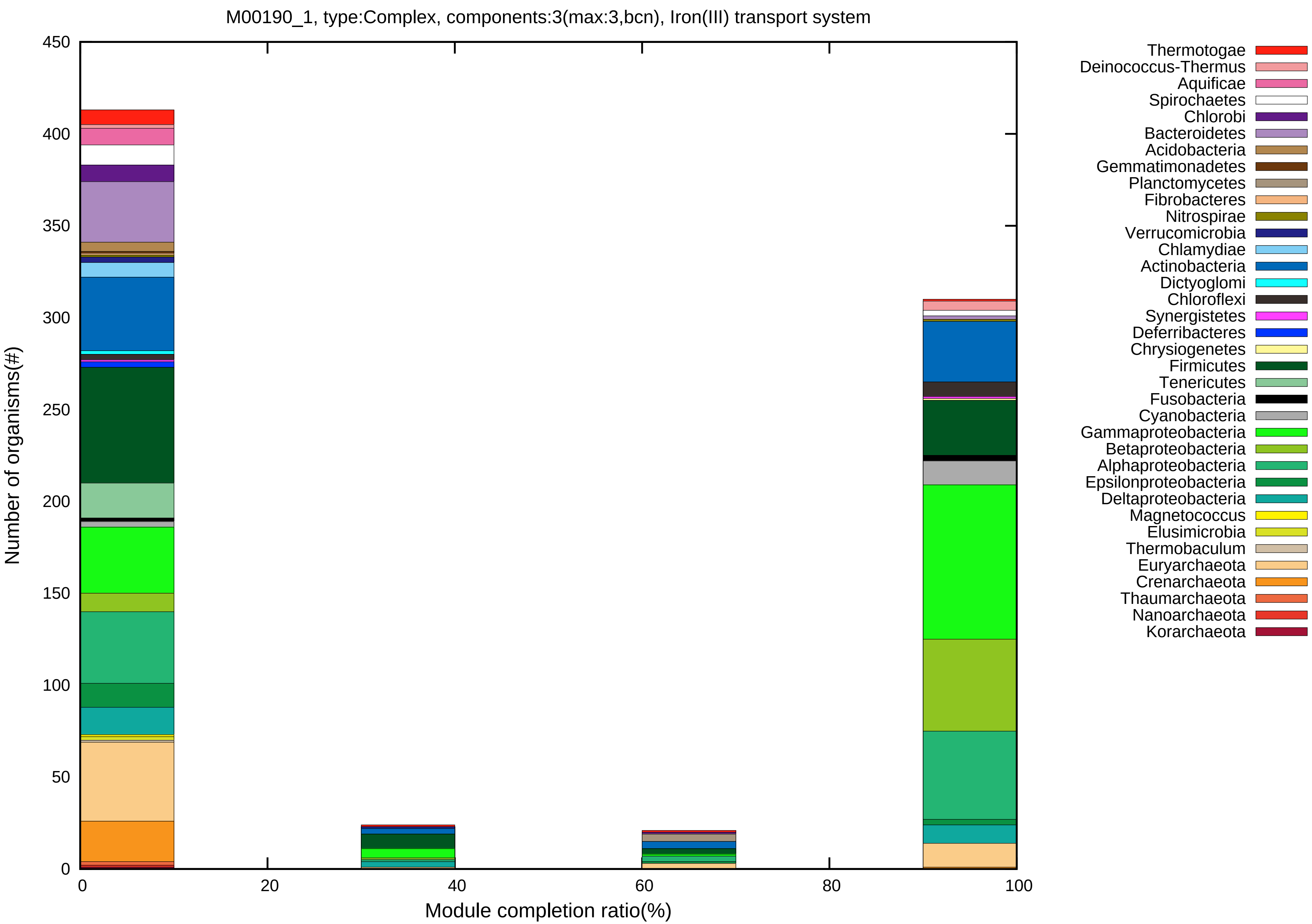
M00190_1, type:Complex, components:3(max:3,bcn), Iron(III) transport system
Number of organisms(#)
Module completion ratio(%)
0
50
100
150
200
250
300
350
400
450
0
20
40
60
80
100
Thermotogae
Deinococcus-Thermus
Aquificae
Spirochaetes
Chlorobi
Bacteroidetes
Acidobacteria
Gemmatimonadetes
Planctomycetes
Fibrobacteres
Nitrospirae
Verrucomicrobia
Chlamydiae
Actinobacteria
Dictyoglomi
Chloroflexi
Synergistetes
Deferribacteres
Chrysiogenetes
Firmicutes
Tenericutes
Fusobacteria
Cyanobacteria
Gammaproteobacteria
Betaproteobacteria
Alphaproteobacteria
Epsilonproteobacteria
Deltaproteobacteria
Magnetococcus
Elusimicrobia
Thermobaculum
Euryarchaeota
Crenarchaeota
Thaumarchaeota
Nanoarchaeota
Korarchaeota

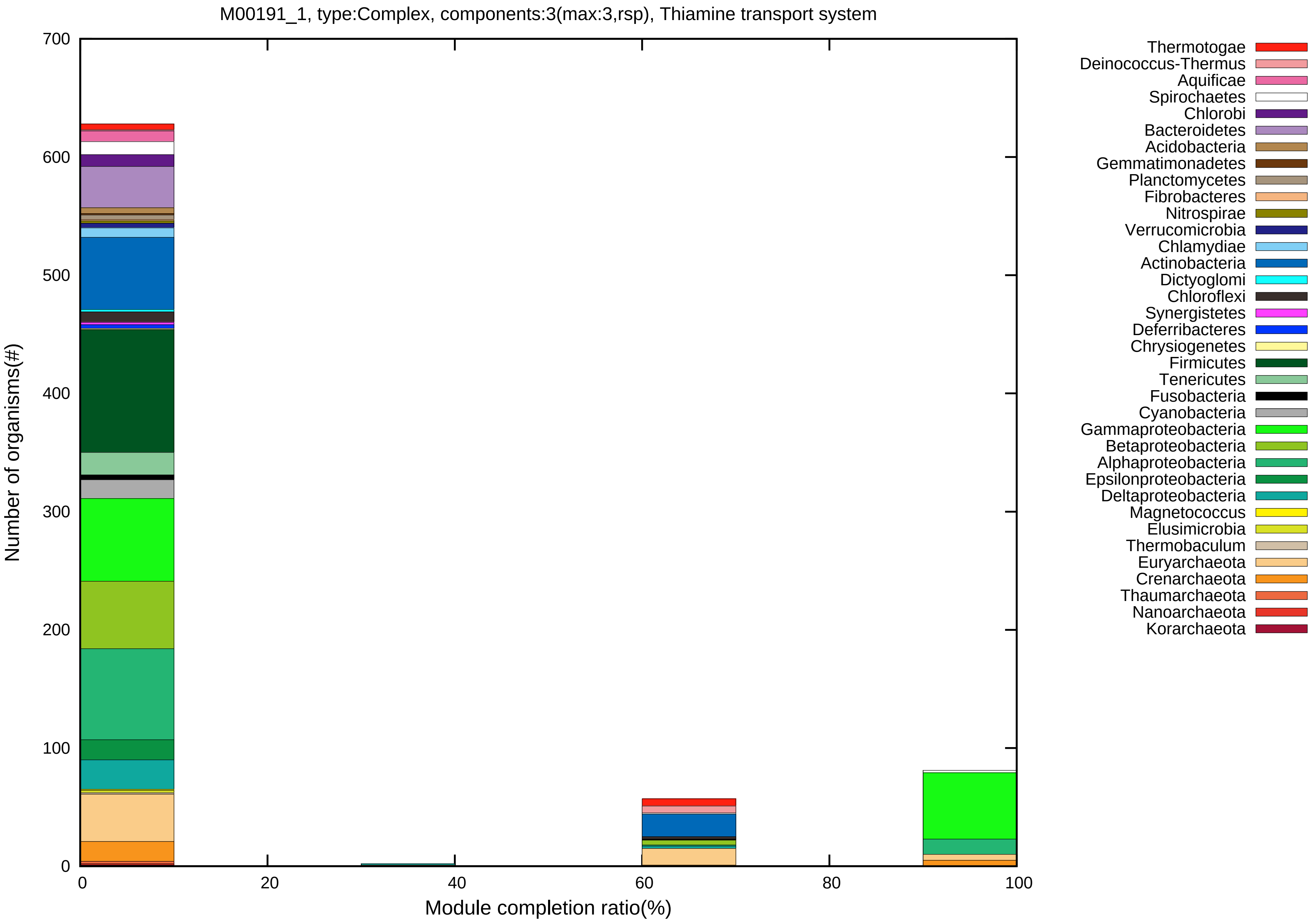

M00191_1, type:Complex, components:3(max:3,rsp), Thiamine transport system
Number of organisms(#)
Module completion ratio(%)
0
100
200
300
400
500
600
700
0
20
40
60
80
100
Thermotogae
Deinococcus-Thermus
Aquificae
Spirochaetes
Chlorobi
Bacteroidetes
Acidobacteria
Gemmatimonadetes
Planctomycetes
Fibrobacteres
Nitrospirae
Verrucomicrobia
Chlamydiae
Actinobacteria
Dictyoglomi
Chloroflexi
Synergistetes
Deferribacteres
Chrysiogenetes
Firmicutes
Tenericutes
Fusobacteria
Cyanobacteria
Gammaproteobacteria
Betaproteobacteria
Alphaproteobacteria
Epsilonproteobacteria
Deltaproteobacteria
Magnetococcus
Elusimicrobia
Thermobaculum
Euryarchaeota
Crenarchaeota
Thaumarchaeota
Nanoarchaeota
Korarchaeota

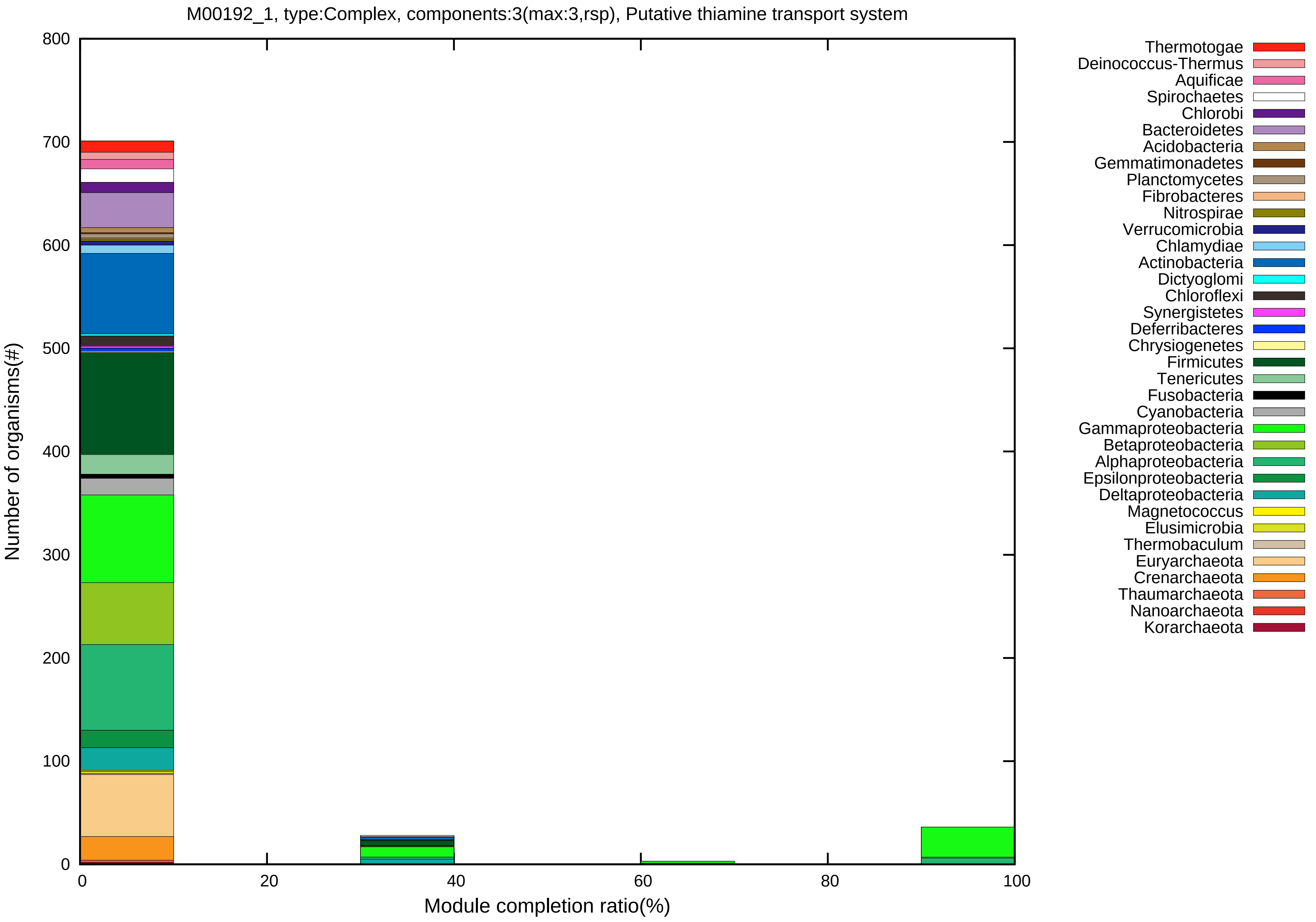

M00192_1, type:Complex, components:3(max:3,rsp), Putative thiamine transport system
Number of organisms(#)
Module completion ratio(%)
0
100
200
300
400
500
600
700
800
0
20
40
60
80
100
Thermotogae
Deinococcus-Thermus
Aquificae
Spirochaetes
Chlorobi
Bacteroidetes
Acidobacteria
Gemmatimonadetes
Planctomycetes
Fibrobacteres
Nitrospirae
Verrucomicrobia
Chlamydiae
Actinobacteria
Dictyoglomi
Chloroflexi
Synergistetes
Deferribacteres
Chrysiogenetes
Firmicutes
Tenericutes
Fusobacteria
Cyanobacteria
Gammaproteobacteria
Betaproteobacteria
Alphaproteobacteria
Epsilonproteobacteria
Deltaproteobacteria
Magnetococcus
Elusimicrobia
Thermobaculum
Euryarchaeota
Crenarchaeota
Thaumarchaeota
Nanoarchaeota
Korarchaeota

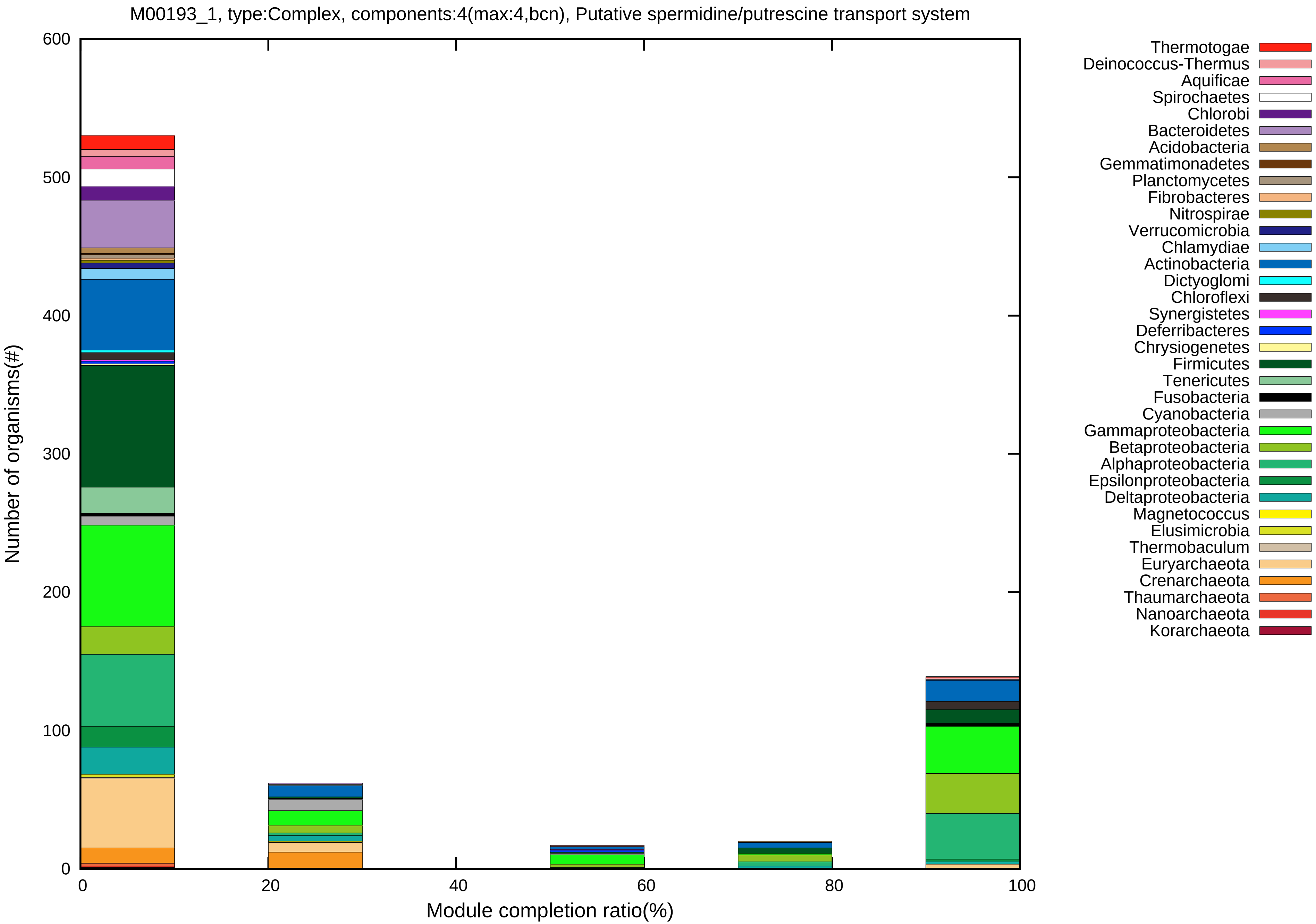

M00193_1, type:Complex, components:4(max:4,bcn), Putative spermidine/putrescine transport system
Number of organisms(#)
Module completion ratio(%)
0
100
200
300
400
500
600
0
20
40
60
80
100
Thermotogae
Deinococcus-Thermus
Aquificae
Spirochaetes
Chlorobi
Bacteroidetes
Acidobacteria
Gemmatimonadetes
Planctomycetes
Fibrobacteres
Nitrospirae
Verrucomicrobia
Chlamydiae
Actinobacteria
Dictyoglomi
Chloroflexi
Synergistetes
Deferribacteres
Chrysiogenetes
Firmicutes
Tenericutes
Fusobacteria
Cyanobacteria
Gammaproteobacteria
Betaproteobacteria
Alphaproteobacteria
Epsilonproteobacteria
Deltaproteobacteria
Magnetococcus
Elusimicrobia
Thermobaculum
Euryarchaeota
Crenarchaeota
Thaumarchaeota
Nanoarchaeota
Korarchaeota

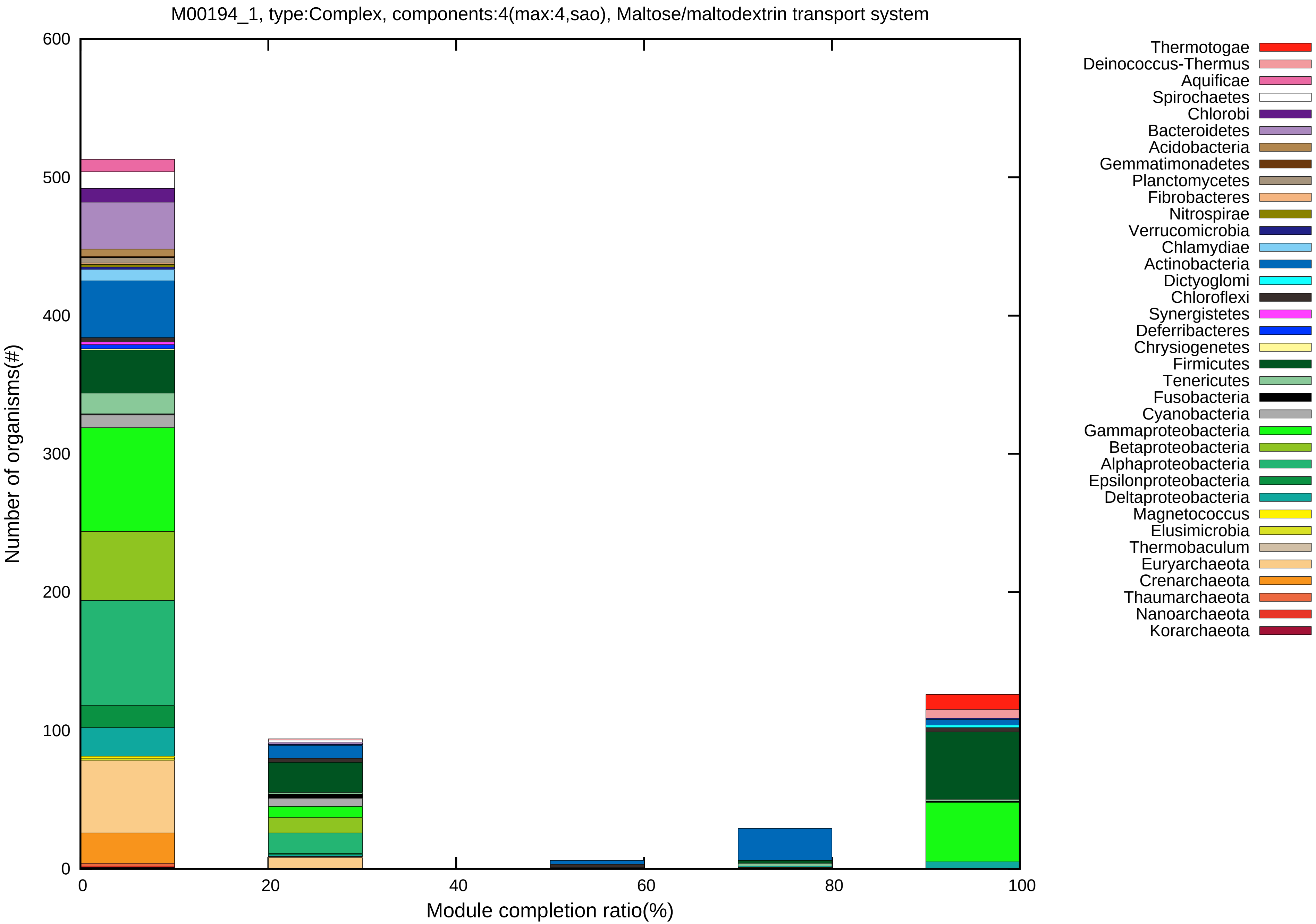
M00194_1, type:Complex, components:4(max:4,sao), Maltose/maltodextrin transport system
Number of organisms(#)
Module completion ratio(%)
0
100
200
300
400
500
600
0
20
40
60
80
100
Thermotogae
Deinococcus-Thermus
Aquificae
Spirochaetes
Chlorobi
Bacteroidetes
Acidobacteria
Gemmatimonadetes
Planctomycetes
Fibrobacteres
Nitrospirae
Verrucomicrobia
Chlamydiae
Actinobacteria
Dictyoglomi
Chloroflexi
Synergistetes
Deferribacteres
Chrysiogenetes
Firmicutes
Tenericutes
Fusobacteria
Cyanobacteria
Gammaproteobacteria
Betaproteobacteria
Alphaproteobacteria
Epsilonproteobacteria
Deltaproteobacteria
Magnetococcus
Elusimicrobia
Thermobaculum
Euryarchaeota
Crenarchaeota
Thaumarchaeota
Nanoarchaeota
Korarchaeota

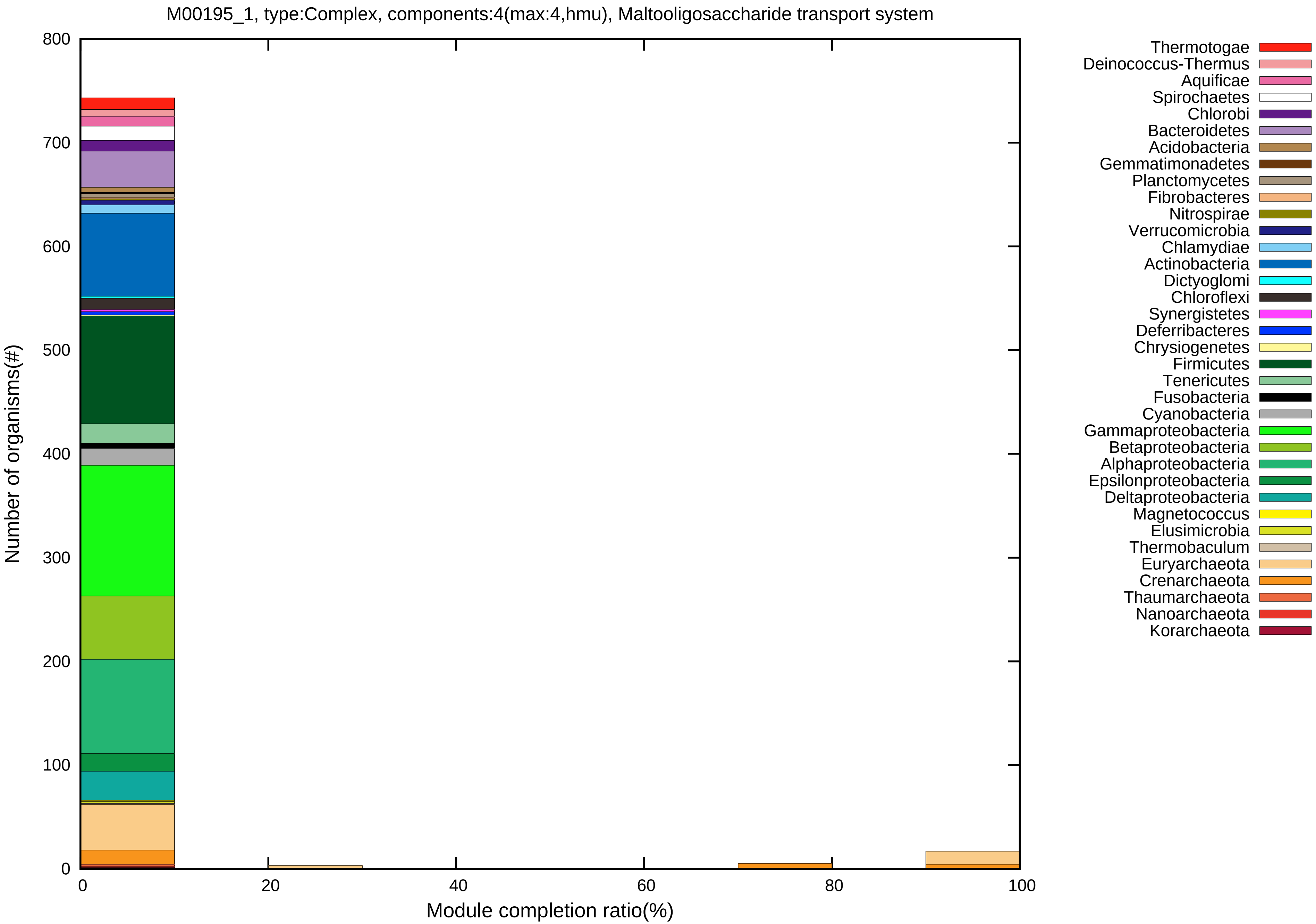

M00195_1, type:Complex, components:4(max:4,hmu), Maltooligosaccharide transport system
Number of organisms(#)
0
100
200
300
400
500
600
700
800
0
20
40
60
80
100
Module completion ratio(%)
Thermotogae
Deinococcus-Thermus
Aquificae
Spirochaetes
Chlorobi
Bacteroidetes
Acidobacteria
Gemmatimonadetes
Planctomycetes
Fibrobacteres
Nitrospirae
Verrucomicrobia
Chlamydiae
Actinobacteria
Dictyoglomi
Chloroflexi
Synergistetes
Deferribacteres
Chrysiogenetes
Firmicutes
Tenericutes
Fusobacteria
Cyanobacteria
Gammaproteobacteria
Betaproteobacteria
Alphaproteobacteria
Epsilonproteobacteria
Deltaproteobacteria
Magnetococcus
Elusimicrobia
Thermobaculum
Euryarchaeota
Crenarchaeota
Thaumarchaeota
Nanoarchaeota
Korarchaeota

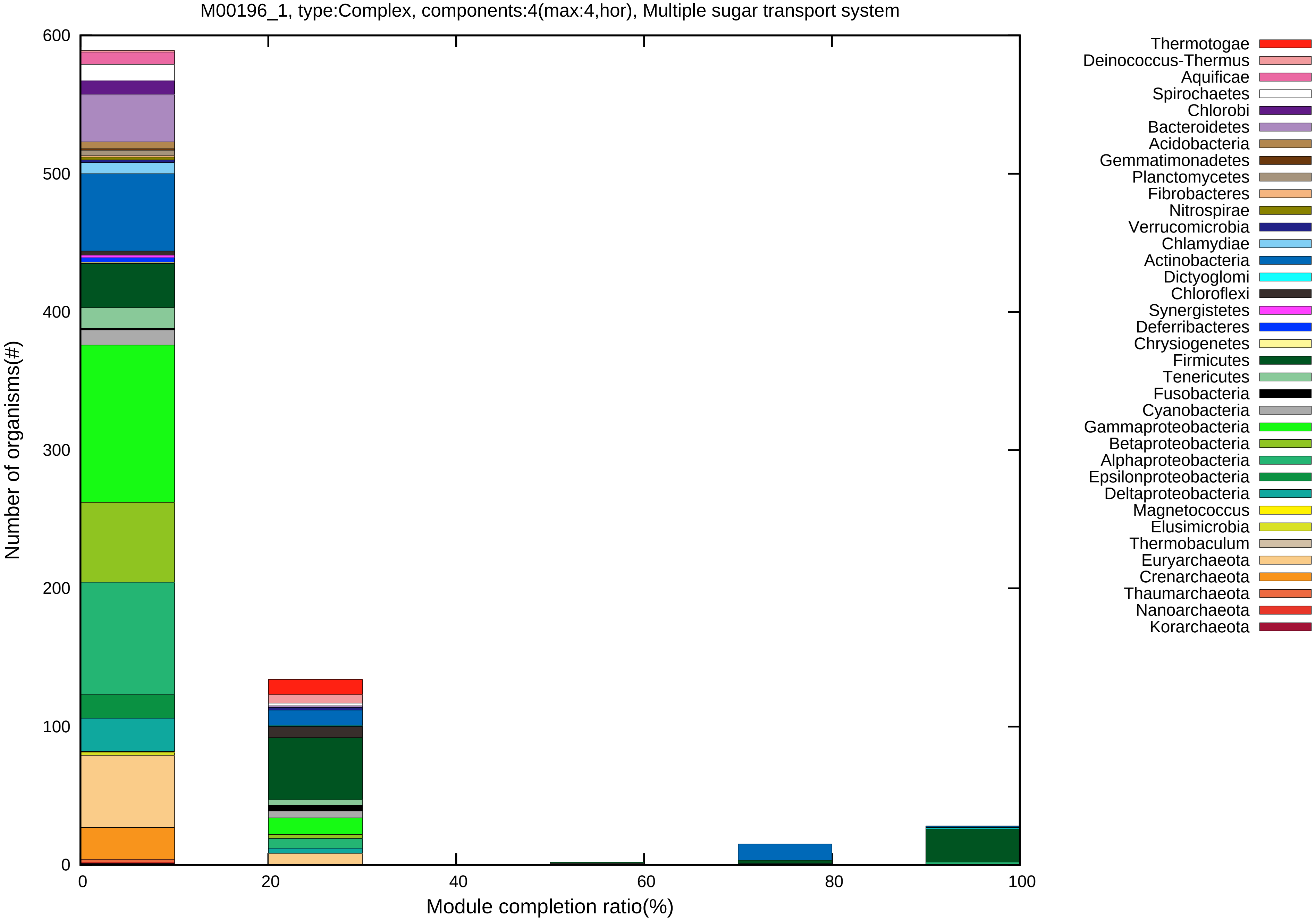
M00196_1, type:Complex, components:4(max:4,hor), Multiple sugar transport system
Number of organisms(#)
Module completion ratio(%)
0
100
200
300
400
500
600
0
20
40
60
80
100
Thermotogae
Deinococcus-Thermus
Aquificae
Spirochaetes
Chlorobi
Bacteroidetes
Acidobacteria
Gemmatimonadetes
Planctomycetes
Fibrobacteres
Nitrospirae
Verrucomicrobia
Chlamydiae
Actinobacteria
Dictyoglomi
Chloroflexi
Synergistetes
Deferribacteres
Chrysiogenetes
Firmicutes
Tenericutes
Fusobacteria
Cyanobacteria
Gammaproteobacteria
Betaproteobacteria
Alphaproteobacteria
Epsilonproteobacteria
Deltaproteobacteria
Magnetococcus
Elusimicrobia
Thermobaculum
Euryarchaeota
Crenarchaeota
Thaumarchaeota
Nanoarchaeota
Korarchaeota

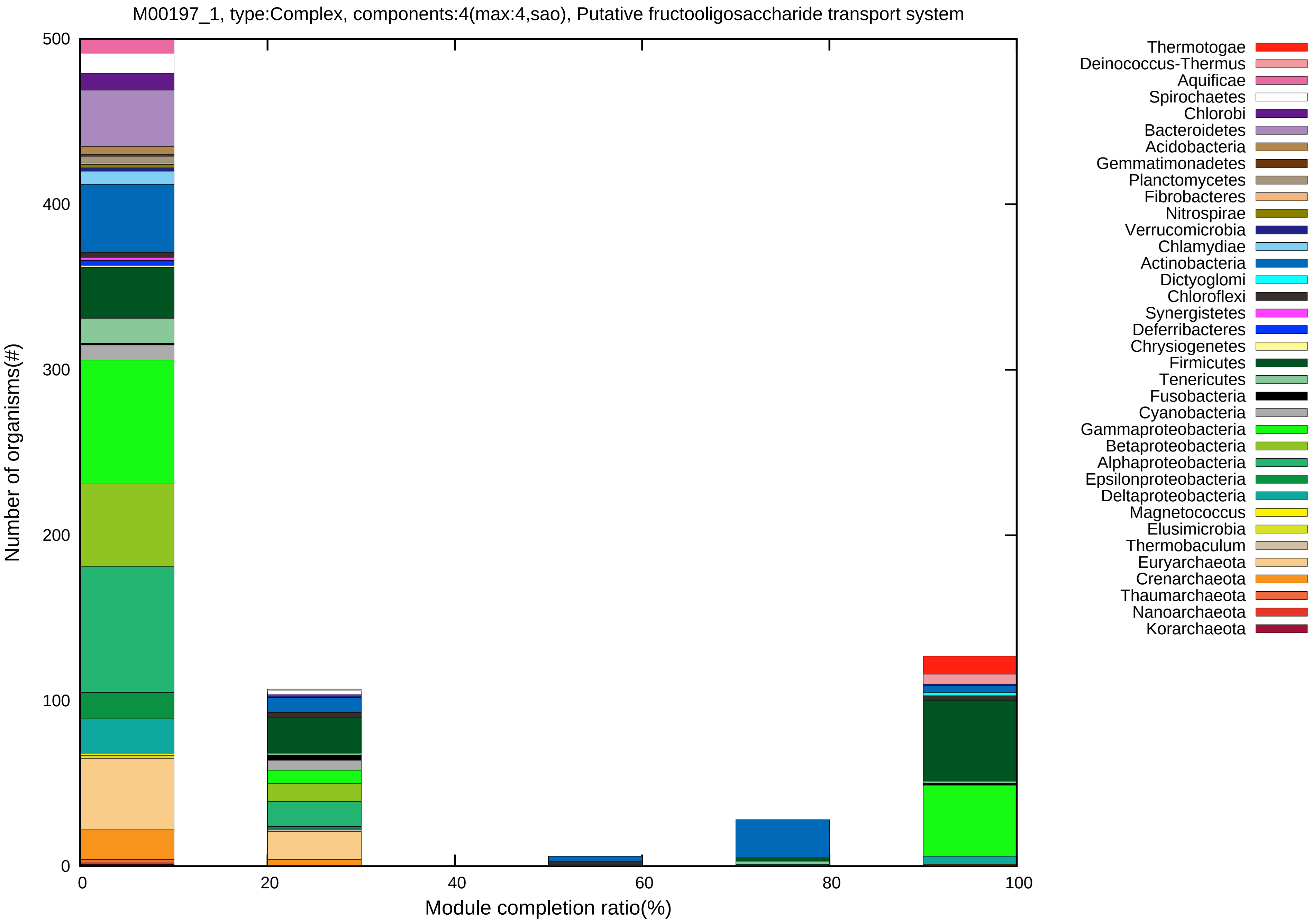

M00197_1, type:Complex, components:4(max:4,sao), Putative fructooligosaccharide transport system
Number of organisms(#)
Module completion ratio(%)
0
100
200
300
400
500
0
20
40
60
80
100
Thermotogae
Deinococcus-Thermus
Aquificae
Spirochaetes
Chlorobi
Bacteroidetes
Acidobacteria
Gemmatimonadetes
Planctomycetes
Fibrobacteres
Nitrospirae
Verrucomicrobia
Chlamydiae
Actinobacteria
Dictyoglomi
Chloroflexi
Synergistetes
Deferribacteres
Chrysiogenetes
Firmicutes
Tenericutes
Fusobacteria
Cyanobacteria
Gammaproteobacteria
Betaproteobacteria
Alphaproteobacteria
Epsilonproteobacteria
Deltaproteobacteria
Magnetococcus
Elusimicrobia
Thermobaculum
Euryarchaeota
Crenarchaeota
Thaumarchaeota
Nanoarchaeota
Korarchaeota

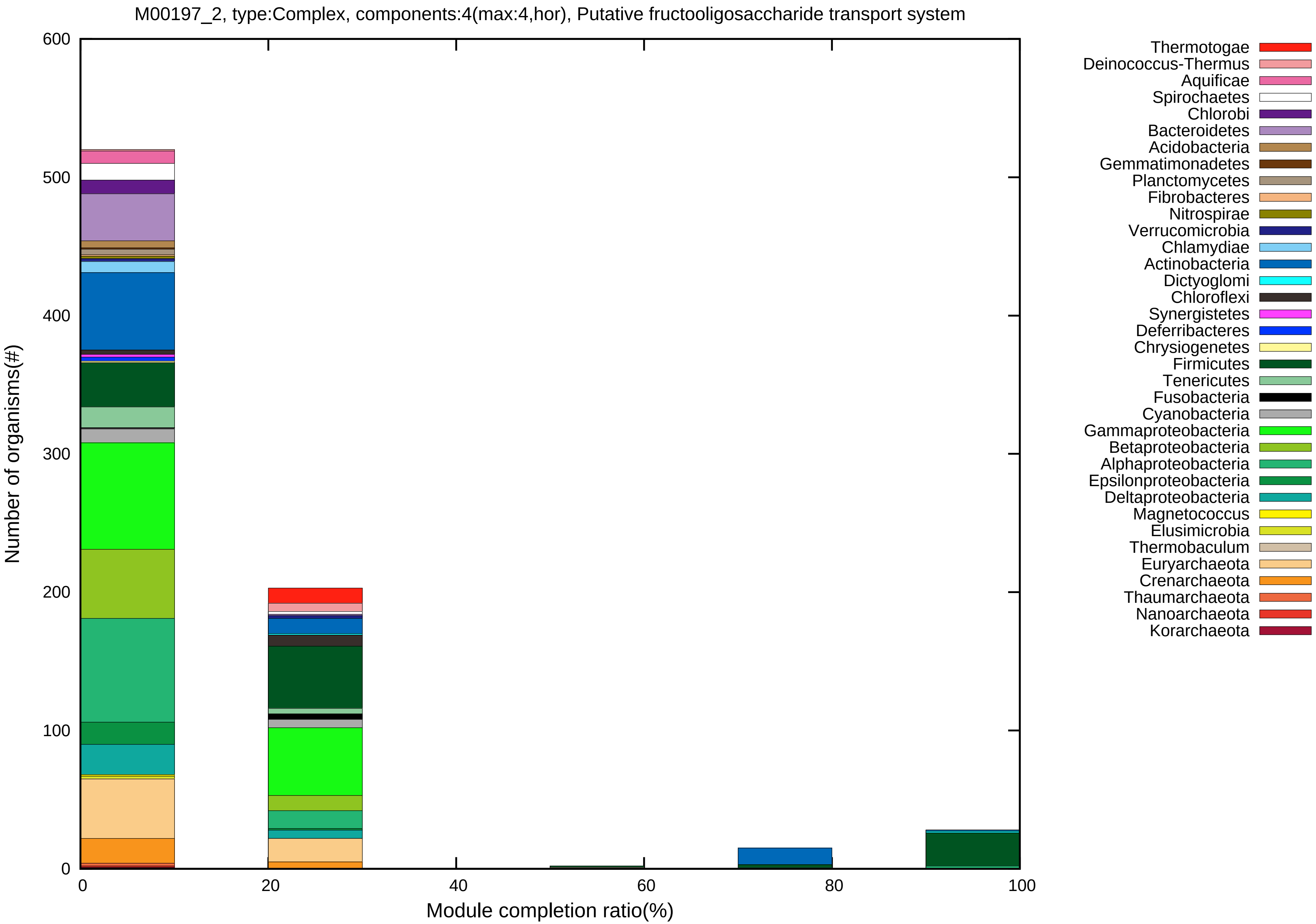
M00197_2, type:Complex, components:4(max:4,hor), Putative fructooligosaccharide transport system
Number of organisms(#)
Module completion ratio(%)
0
100
200
300
400
500
600
0
20
40
60
80
100
Thermotogae
Deinococcus-Thermus
Aquificae
Spirochaetes
Chlorobi
Bacteroidetes
Acidobacteria
Gemmatimonadetes
Planctomycetes
Fibrobacteres
Nitrospirae
Verrucomicrobia
Chlamydiae
Actinobacteria
Dictyoglomi
Chloroflexi
Synergistetes
Deferribacteres
Chrysiogenetes
Firmicutes
Tenericutes
Fusobacteria
Cyanobacteria
Gammaproteobacteria
Betaproteobacteria
Alphaproteobacteria
Epsilonproteobacteria
Deltaproteobacteria
Magnetococcus
Elusimicrobia
Thermobaculum
Euryarchaeota
Crenarchaeota
Thaumarchaeota
Nanoarchaeota
Korarchaeota

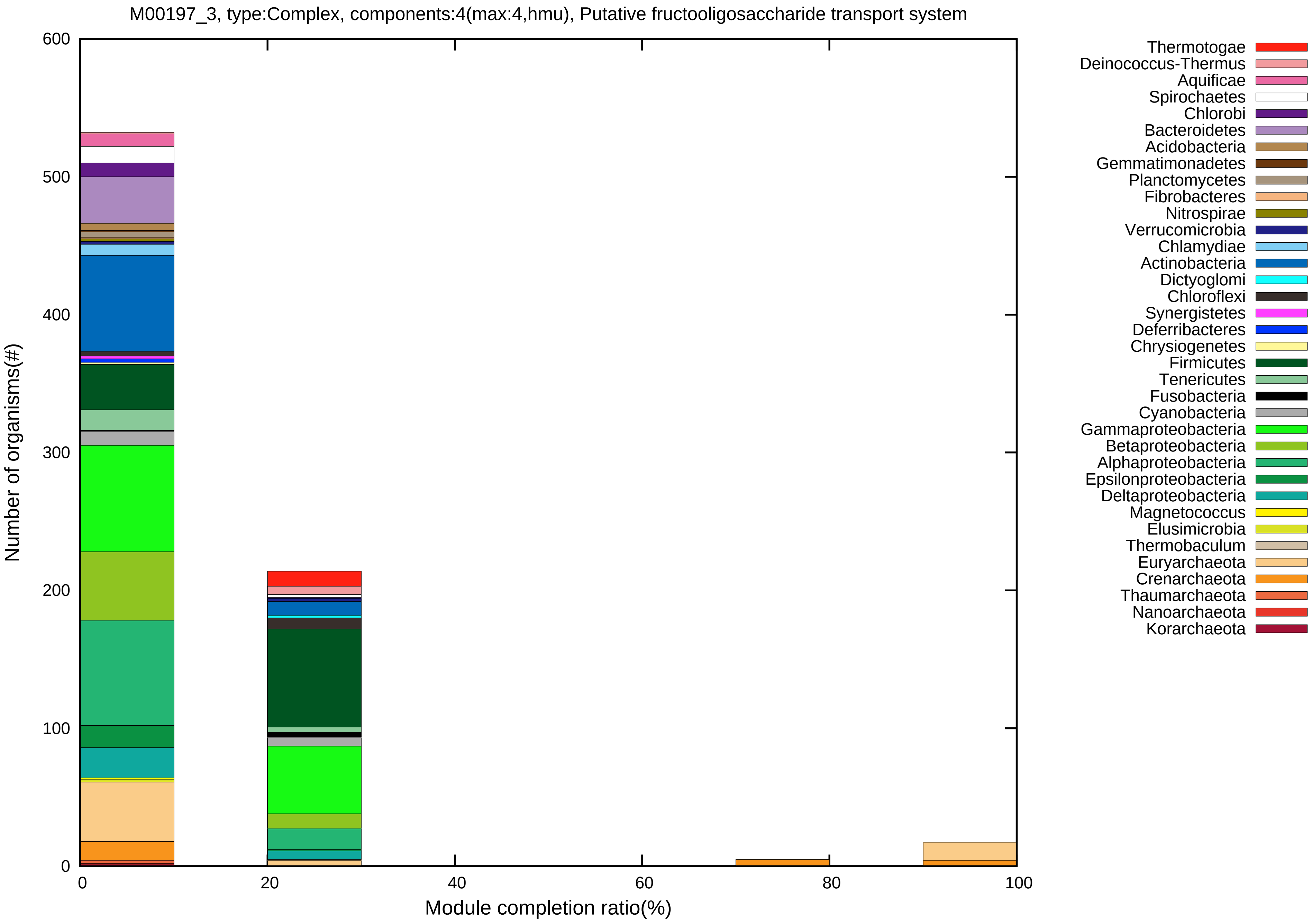

M00197_3, type:Complex, components:4(max:4,hmu), Putative fructooligosaccharide transport system
Number of organisms(#)
Module completion ratio(%)
0
100
200
300
400
500
600
0
20
40
60
80
100
Thermotogae
Deinococcus-Thermus
Aquificae
Spirochaetes
Chlorobi
Bacteroidetes
Acidobacteria
Gemmatimonadetes
Planctomycetes
Fibrobacteres
Nitrospirae
Verrucomicrobia
Chlamydiae
Actinobacteria
Dictyoglomi
Chloroflexi
Synergistetes
Deferribacteres
Chrysiogenetes
Firmicutes
Tenericutes
Fusobacteria
Cyanobacteria
Gammaproteobacteria
Betaproteobacteria
Alphaproteobacteria
Epsilonproteobacteria
Deltaproteobacteria
Magnetococcus
Elusimicrobia
Thermobaculum
Euryarchaeota
Crenarchaeota
Thaumarchaeota
Nanoarchaeota
Korarchaeota

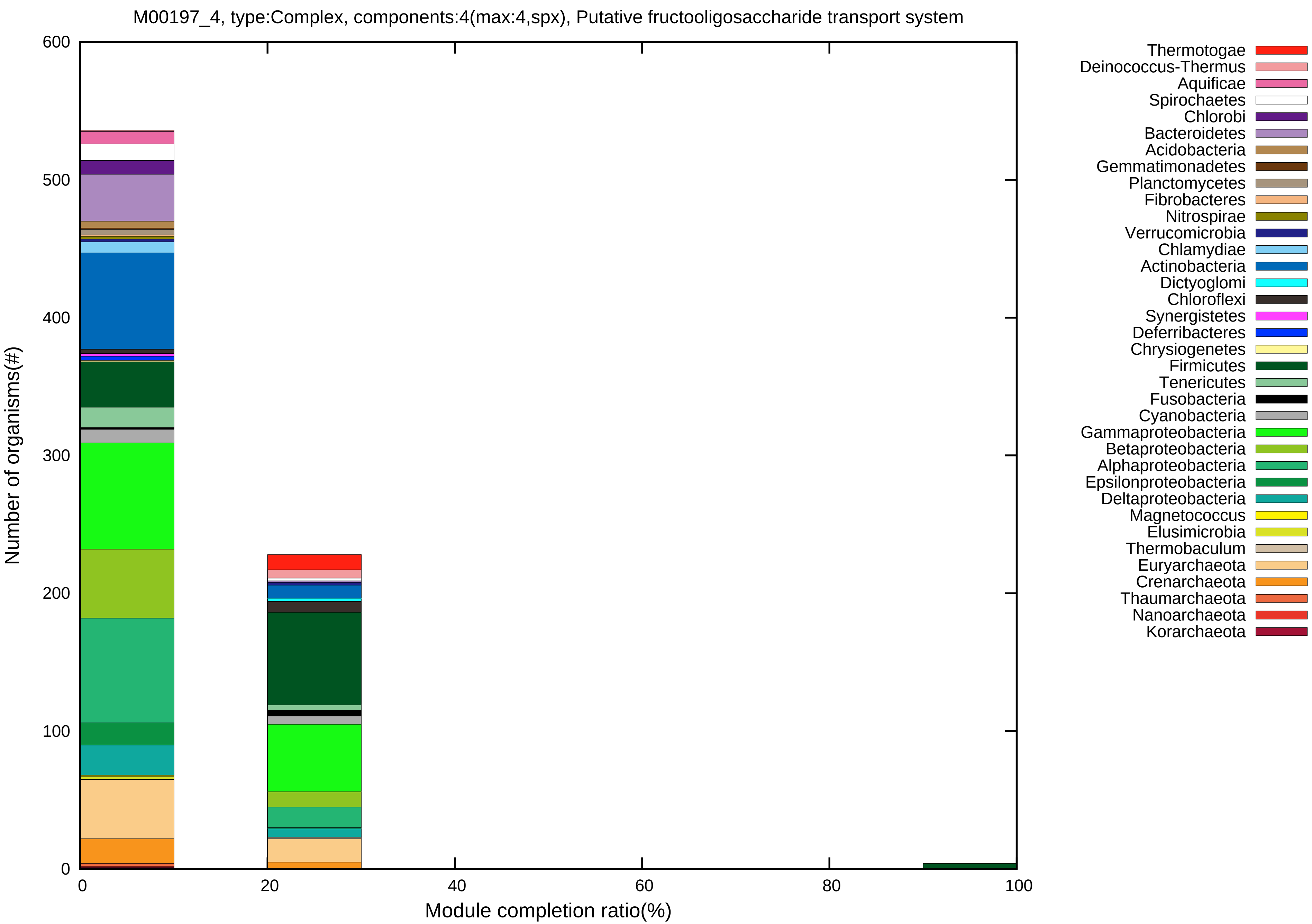

M00197_4, type:Complex, components:4(max:4,spx), Putative fructooligosaccharide transport system
Number of organisms(#)
0
100
200
300
400
500
600
0
20
40
60
80
100
Module completion ratio(%)
Thermotogae
Deinococcus-Thermus
Aquificae
Spirochaetes
Chlorobi
Bacteroidetes
Acidobacteria
Gemmatimonadetes
Planctomycetes
Fibrobacteres
Nitrospirae
Verrucomicrobia
Chlamydiae
Actinobacteria
Dictyoglomi
Chloroflexi
Synergistetes
Deferribacteres
Chrysiogenetes
Firmicutes
Tenericutes
Fusobacteria
Cyanobacteria
Gammaproteobacteria
Betaproteobacteria
Alphaproteobacteria
Epsilonproteobacteria
Deltaproteobacteria
Magnetococcus
Elusimicrobia
Thermobaculum
Euryarchaeota
Crenarchaeota
Thaumarchaeota
Nanoarchaeota
Korarchaeota

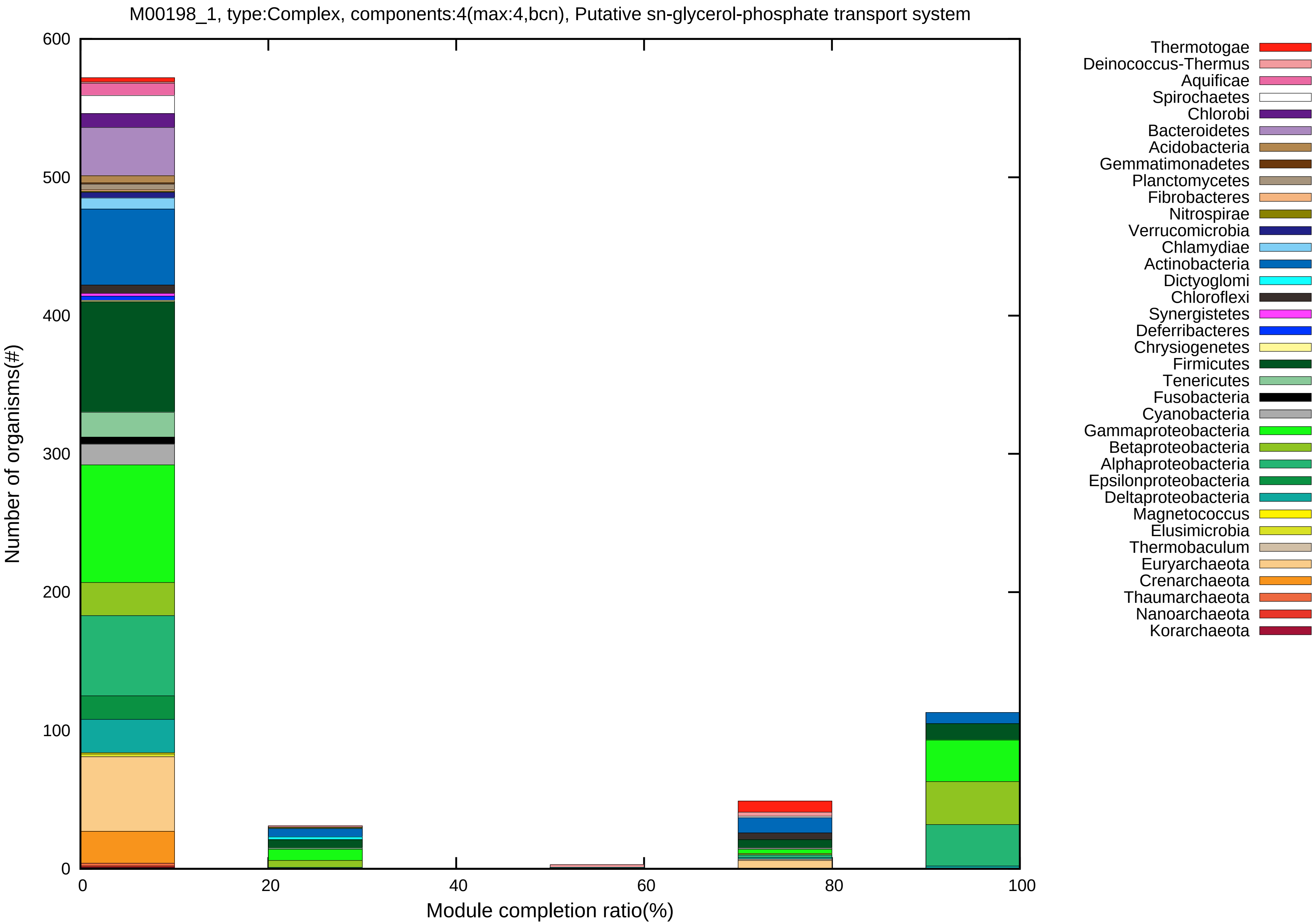

M00198_1, type:Complex, components:4(max:4,bcn), Putative sn-glycerol-phosphate transport system
Number of organisms(#)
Module completion ratio(%)
0
100
200
300
400
500
600
0
20
40
60
80
100
Thermotogae
Deinococcus-Thermus
Aquificae
Spirochaetes
Chlorobi
Bacteroidetes
Acidobacteria
Gemmatimonadetes
Planctomycetes
Fibrobacteres
Nitrospirae
Verrucomicrobia
Chlamydiae
Actinobacteria
Dictyoglomi
Chloroflexi
Synergistetes
Deferribacteres
Chrysiogenetes
Firmicutes
Tenericutes
Fusobacteria
Cyanobacteria
Gammaproteobacteria
Betaproteobacteria
Alphaproteobacteria
Epsilonproteobacteria
Deltaproteobacteria
Magnetococcus
Elusimicrobia
Thermobaculum
Euryarchaeota
Crenarchaeota
Thaumarchaeota
Nanoarchaeota
Korarchaeota

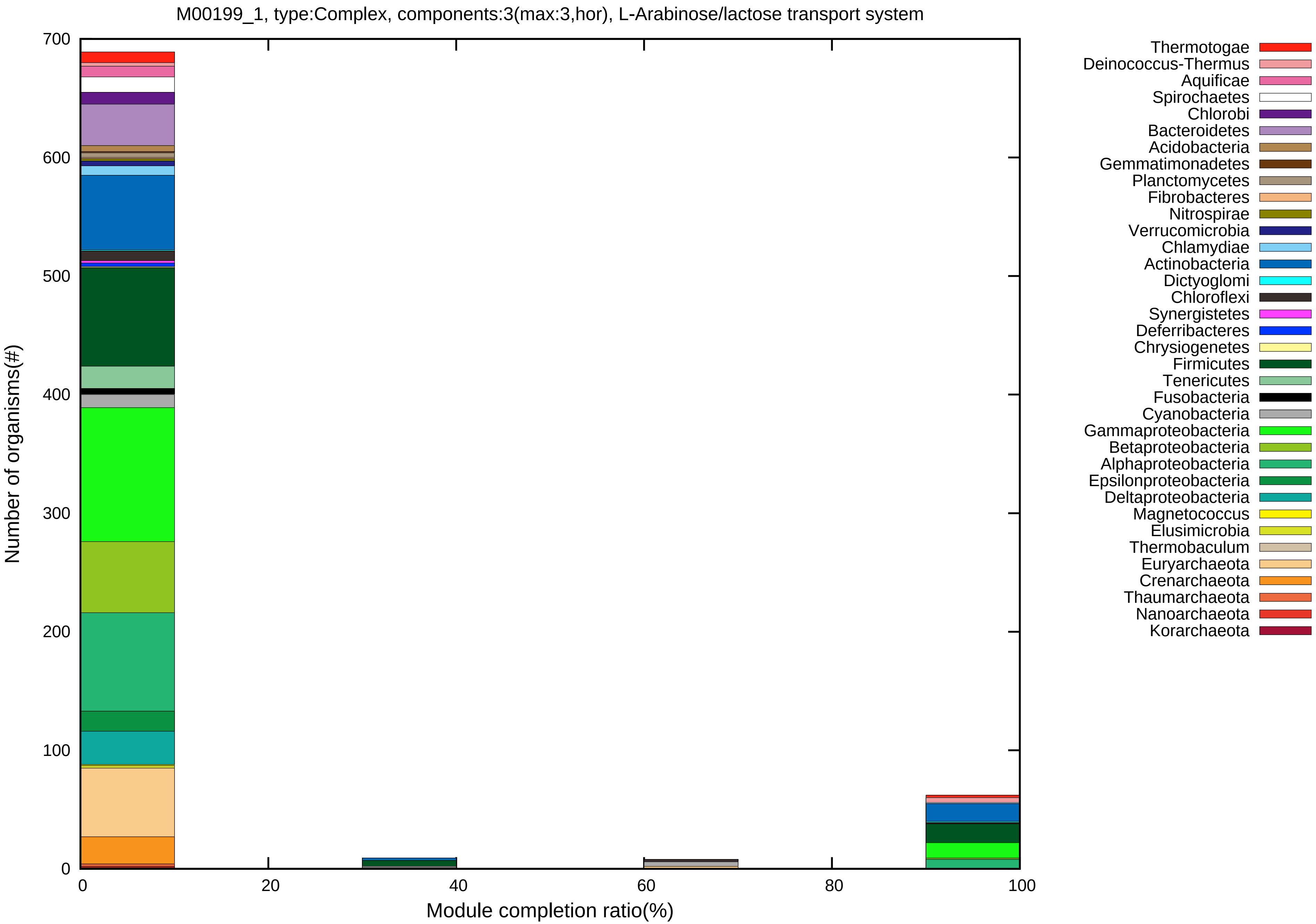
M00199_1, type:Complex, components:3(max:3,hor), L-Arabinose/lactose transport system
Number of organisms(#)
Module completion ratio(%)
0
100
200
300
400
500
600
700
0
20
40
60
80
100
Thermotogae
Deinococcus-Thermus
Aquificae
Spirochaetes
Chlorobi
Bacteroidetes
Acidobacteria
Gemmatimonadetes
Planctomycetes
Fibrobacteres
Nitrospirae
Verrucomicrobia
Chlamydiae
Actinobacteria
Dictyoglomi
Chloroflexi
Synergistetes
Deferribacteres
Chrysiogenetes
Firmicutes
Tenericutes
Fusobacteria
Cyanobacteria
Gammaproteobacteria
Betaproteobacteria
Alphaproteobacteria
Epsilonproteobacteria
Deltaproteobacteria
Magnetococcus
Elusimicrobia
Thermobaculum
Euryarchaeota
Crenarchaeota
Thaumarchaeota
Nanoarchaeota
Korarchaeota

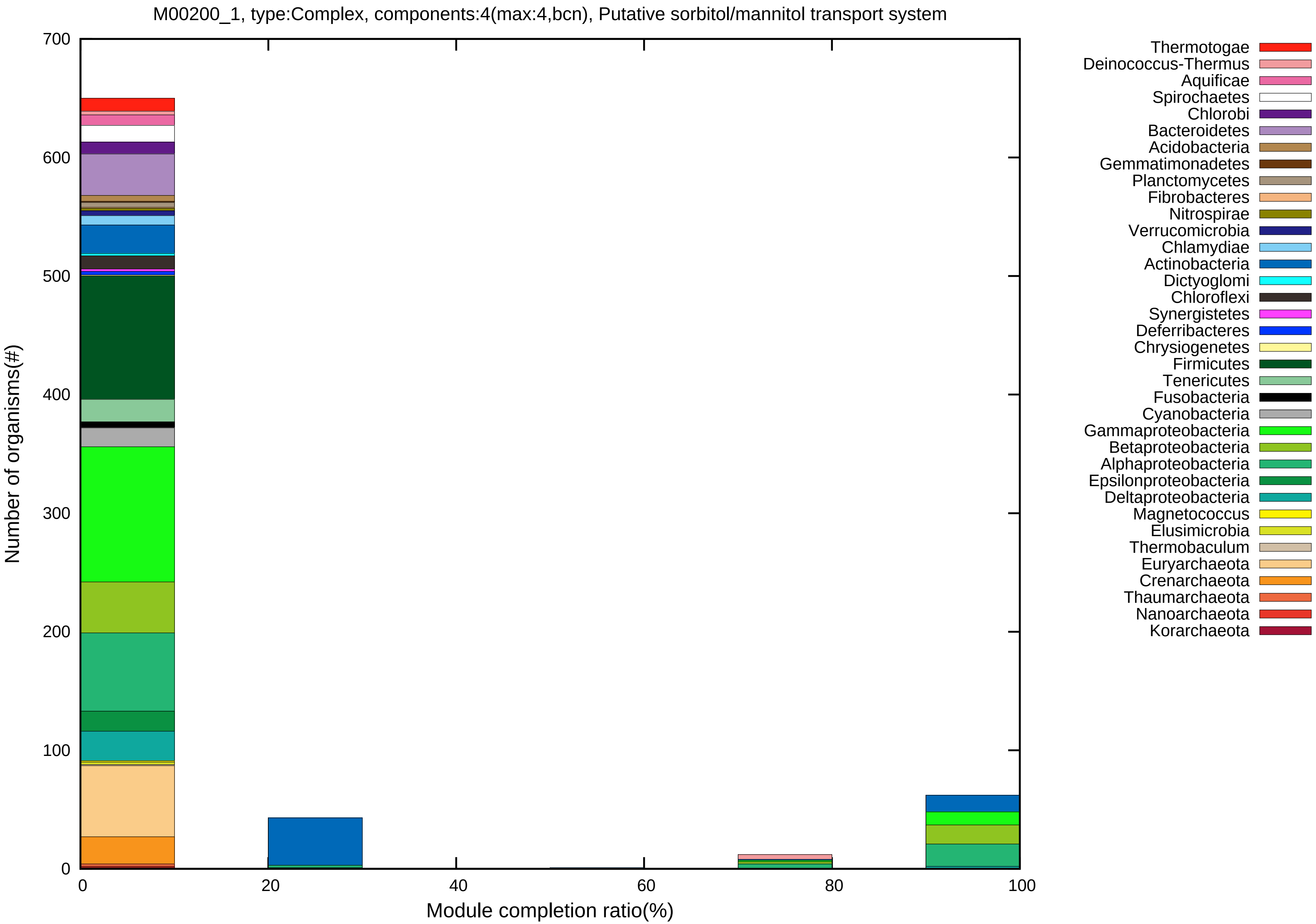

M00200_1, type:Complex, components:4(max:4,bcn), Putative sorbitol/mannitol transport system
Number of organisms(#)
Module completion ratio(%)
0
100
200
300
400
500
600
700
0
20
40
60
80
100
Thermotogae
Deinococcus-Thermus
Aquificae
Spirochaetes
Chlorobi
Bacteroidetes
Acidobacteria
Gemmatimonadetes
Planctomycetes
Fibrobacteres
Nitrospirae
Verrucomicrobia
Chlamydiae
Actinobacteria
Dictyoglomi
Chloroflexi
Synergistetes
Deferribacteres
Chrysiogenetes
Firmicutes
Tenericutes
Fusobacteria
Cyanobacteria
Gammaproteobacteria
Betaproteobacteria
Alphaproteobacteria
Epsilonproteobacteria
Deltaproteobacteria
Magnetococcus
Elusimicrobia
Thermobaculum
Euryarchaeota
Crenarchaeota
Thaumarchaeota
Nanoarchaeota
Korarchaeota

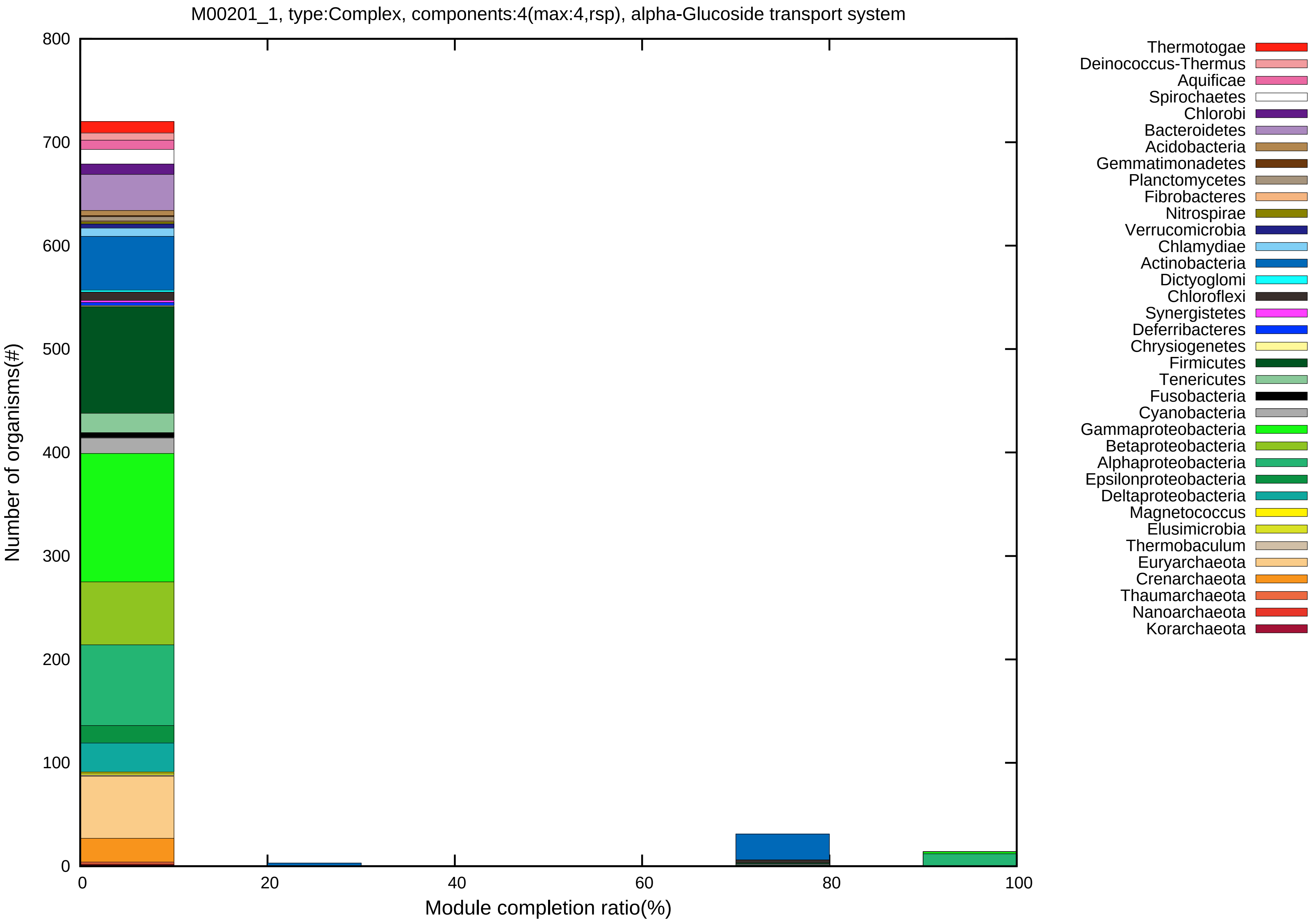

M00201_1, type:Complex, components:4(max:4,rsp), alpha-Glucoside transport system
Number of organisms(#)
Module completion ratio(%)
0
100
200
300
400
500
600
700
800
0
20
40
60
80
100
Thermotogae
Deinococcus-Thermus
Aquificae
Spirochaetes
Chlorobi
Bacteroidetes
Acidobacteria
Gemmatimonadetes
Planctomycetes
Fibrobacteres
Nitrospirae
Verrucomicrobia
Chlamydiae
Actinobacteria
Dictyoglomi
Chloroflexi
Synergistetes
Deferribacteres
Chrysiogenetes
Firmicutes
Tenericutes
Fusobacteria
Cyanobacteria
Gammaproteobacteria
Betaproteobacteria
Alphaproteobacteria
Epsilonproteobacteria
Deltaproteobacteria
Magnetococcus
Elusimicrobia
Thermobaculum
Euryarchaeota
Crenarchaeota
Thaumarchaeota
Nanoarchaeota
Korarchaeota

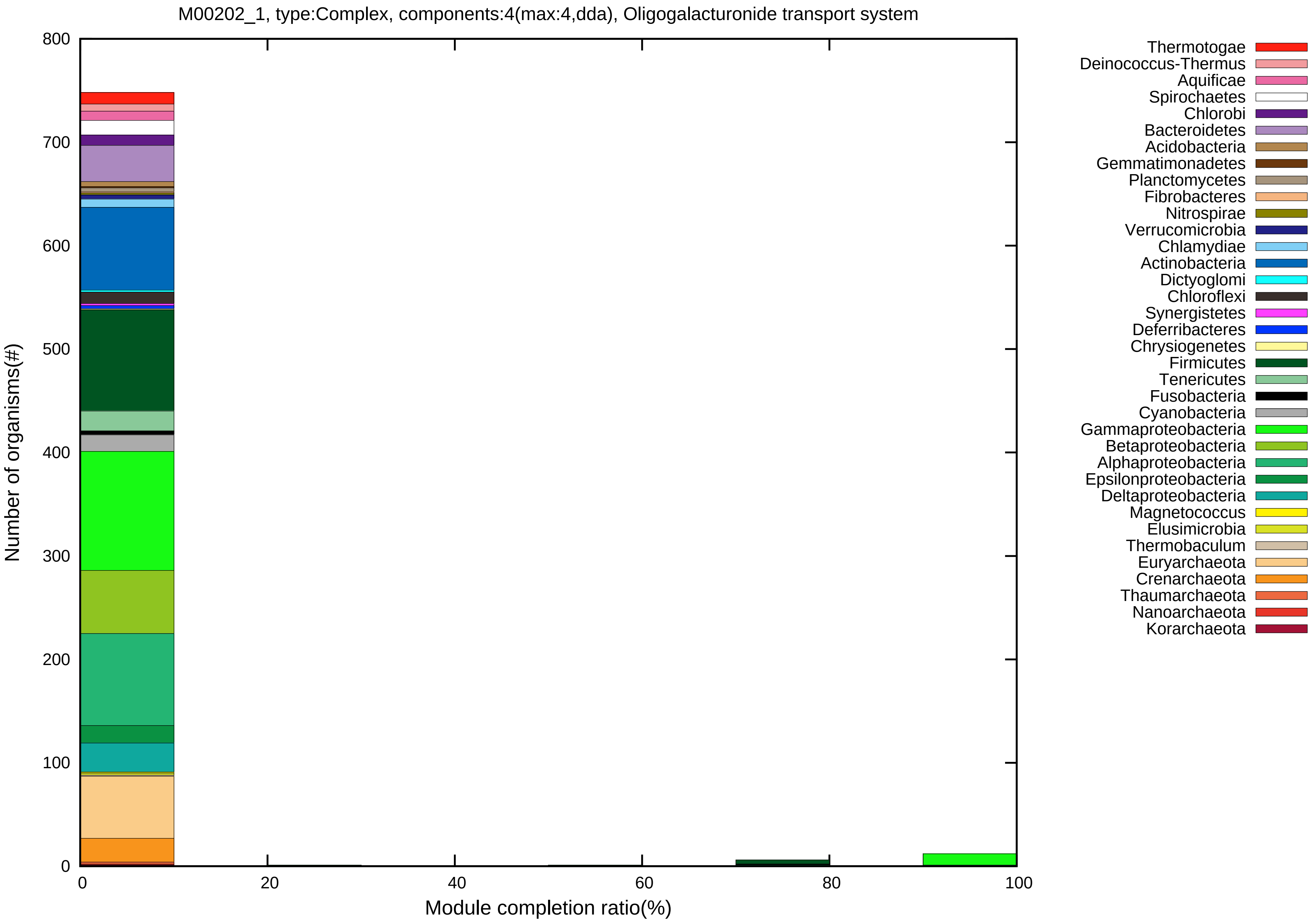

M00202_1, type:Complex, components:4(max:4,dda), Oligogalacturonide transport system
Number of organisms(#)
Module completion ratio(%)
0
100
200
300
400
500
600
700
800
0
20
40
60
80
100
Thermotogae
Deinococcus-Thermus
Aquificae
Spirochaetes
Chlorobi
Bacteroidetes
Acidobacteria
Gemmatimonadetes
Planctomycetes
Fibrobacteres
Nitrospirae
Verrucomicrobia
Chlamydiae
Actinobacteria
Dictyoglomi
Chloroflexi
Synergistetes
Deferribacteres
Chrysiogenetes
Firmicutes
Tenericutes
Fusobacteria
Cyanobacteria
Gammaproteobacteria
Betaproteobacteria
Alphaproteobacteria
Epsilonproteobacteria
Deltaproteobacteria
Magnetococcus
Elusimicrobia
Thermobaculum
Euryarchaeota
Crenarchaeota
Thaumarchaeota
Nanoarchaeota
Korarchaeota

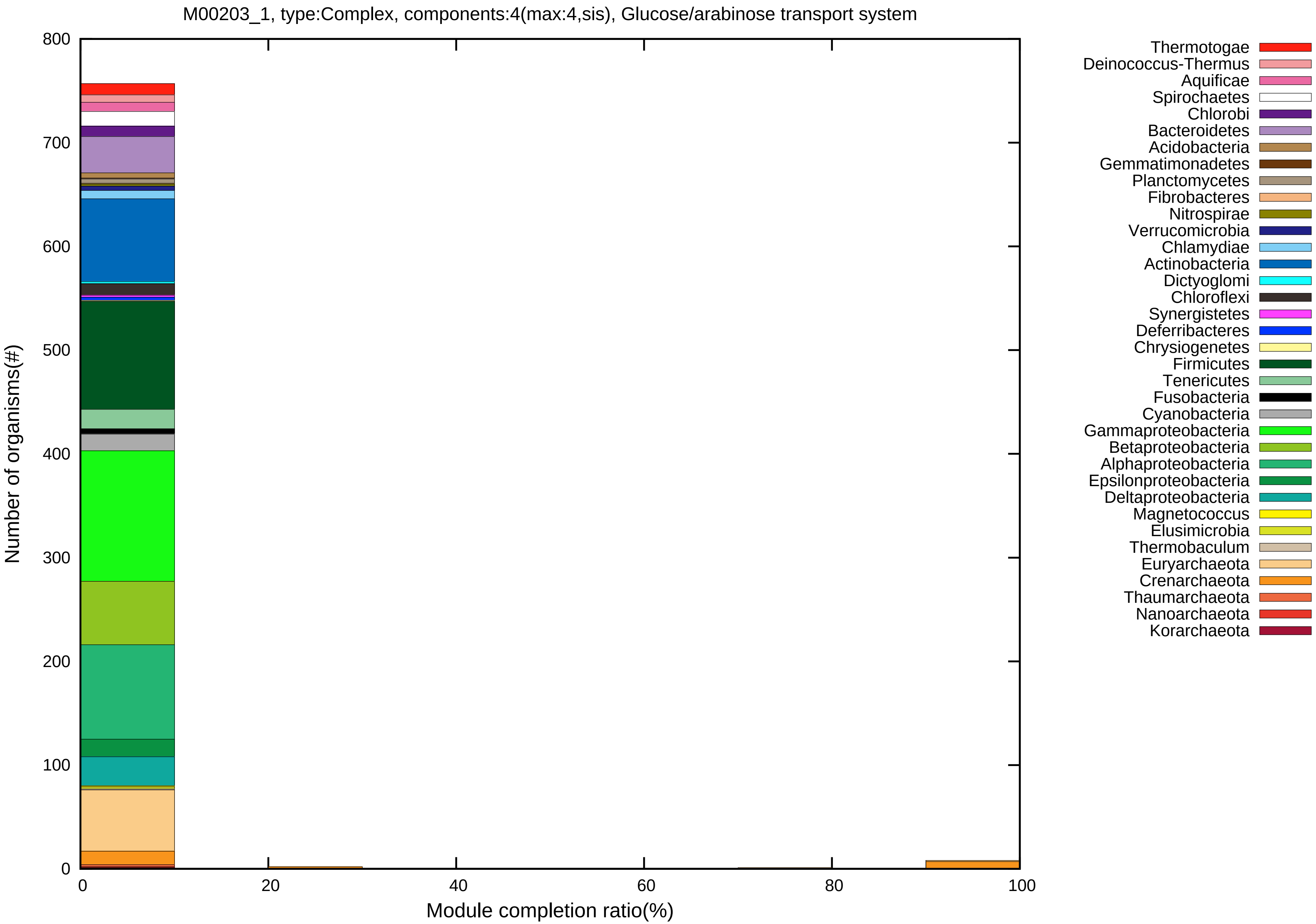
M00203_1, type:Complex, components:4(max:4,sis), Glucose/arabinose transport system
Number of organisms(#)
Module completion ratio(%)
0
100
200
300
400
500
600
700
800
0
20
40
60
80
100
Thermotogae
Deinococcus-Thermus
Aquificae
Spirochaetes
Chlorobi
Bacteroidetes
Acidobacteria
Gemmatimonadetes
Planctomycetes
Fibrobacteres
Nitrospirae
Verrucomicrobia
Chlamydiae
Actinobacteria
Dictyoglomi
Chloroflexi
Synergistetes
Deferribacteres
Chrysiogenetes
Firmicutes
Tenericutes
Fusobacteria
Cyanobacteria
Gammaproteobacteria
Betaproteobacteria
Alphaproteobacteria
Epsilonproteobacteria
Deltaproteobacteria
Magnetococcus
Elusimicrobia
Thermobaculum
Euryarchaeota
Crenarchaeota
Thaumarchaeota
Nanoarchaeota
Korarchaeota

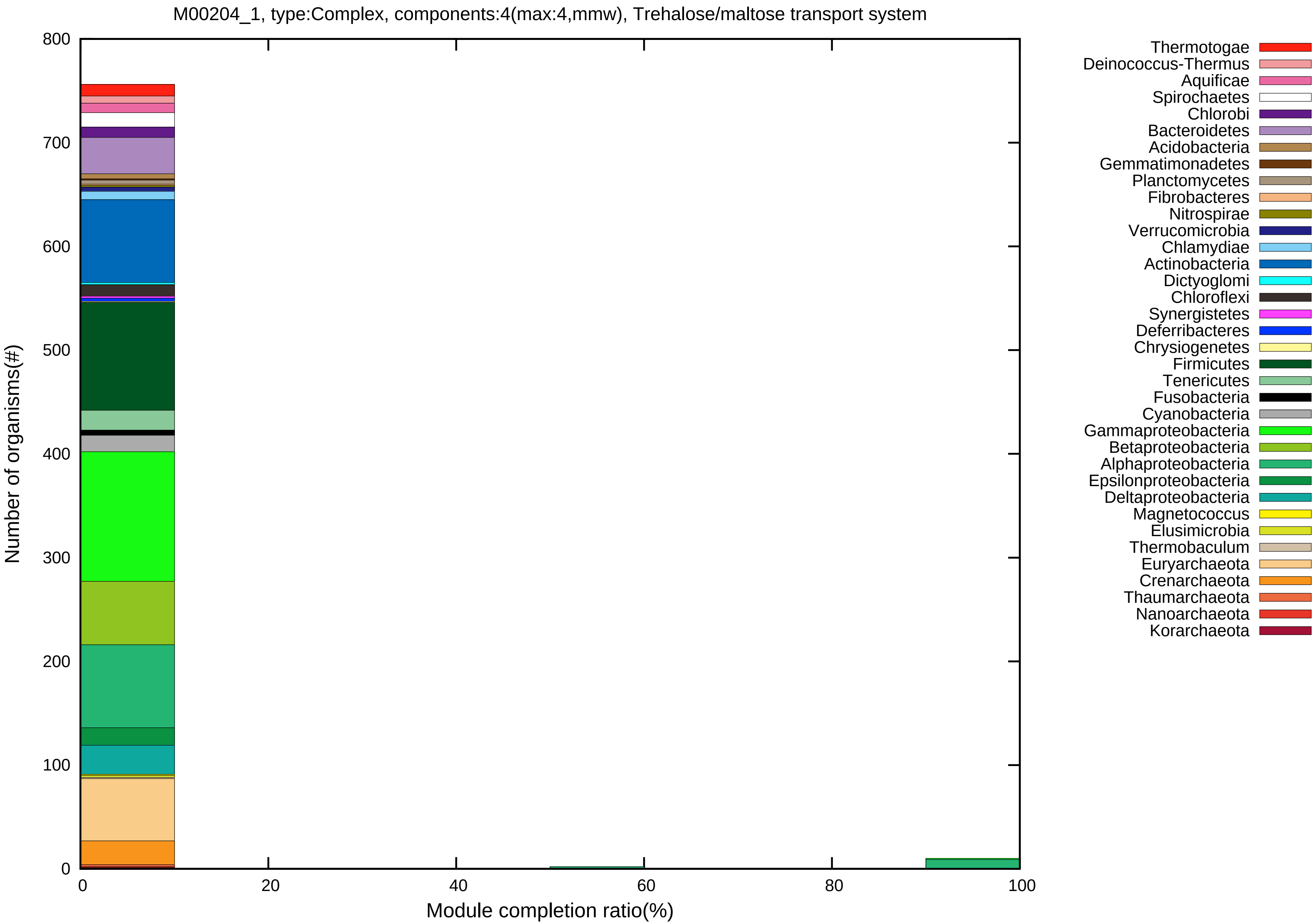
M00204_1, type:Complex, components:4(max:4,mmw), Trehalose/maltose transport system
Number of organisms(#)
Module completion ratio(%)
0
100
200
300
400
500
600
700
800
0
20
40
60
80
100
Thermotogae
Deinococcus-Thermus
Aquificae
Spirochaetes
Chlorobi
Bacteroidetes
Acidobacteria
Gemmatimonadetes
Planctomycetes
Fibrobacteres
Nitrospirae
Verrucomicrobia
Chlamydiae
Actinobacteria
Dictyoglomi
Chloroflexi
Synergistetes
Deferribacteres
Chrysiogenetes
Firmicutes
Tenericutes
Fusobacteria
Cyanobacteria
Gammaproteobacteria
Betaproteobacteria
Alphaproteobacteria
Epsilonproteobacteria
Deltaproteobacteria
Magnetococcus
Elusimicrobia
Thermobaculum
Euryarchaeota
Crenarchaeota
Thaumarchaeota
Nanoarchaeota
Korarchaeota

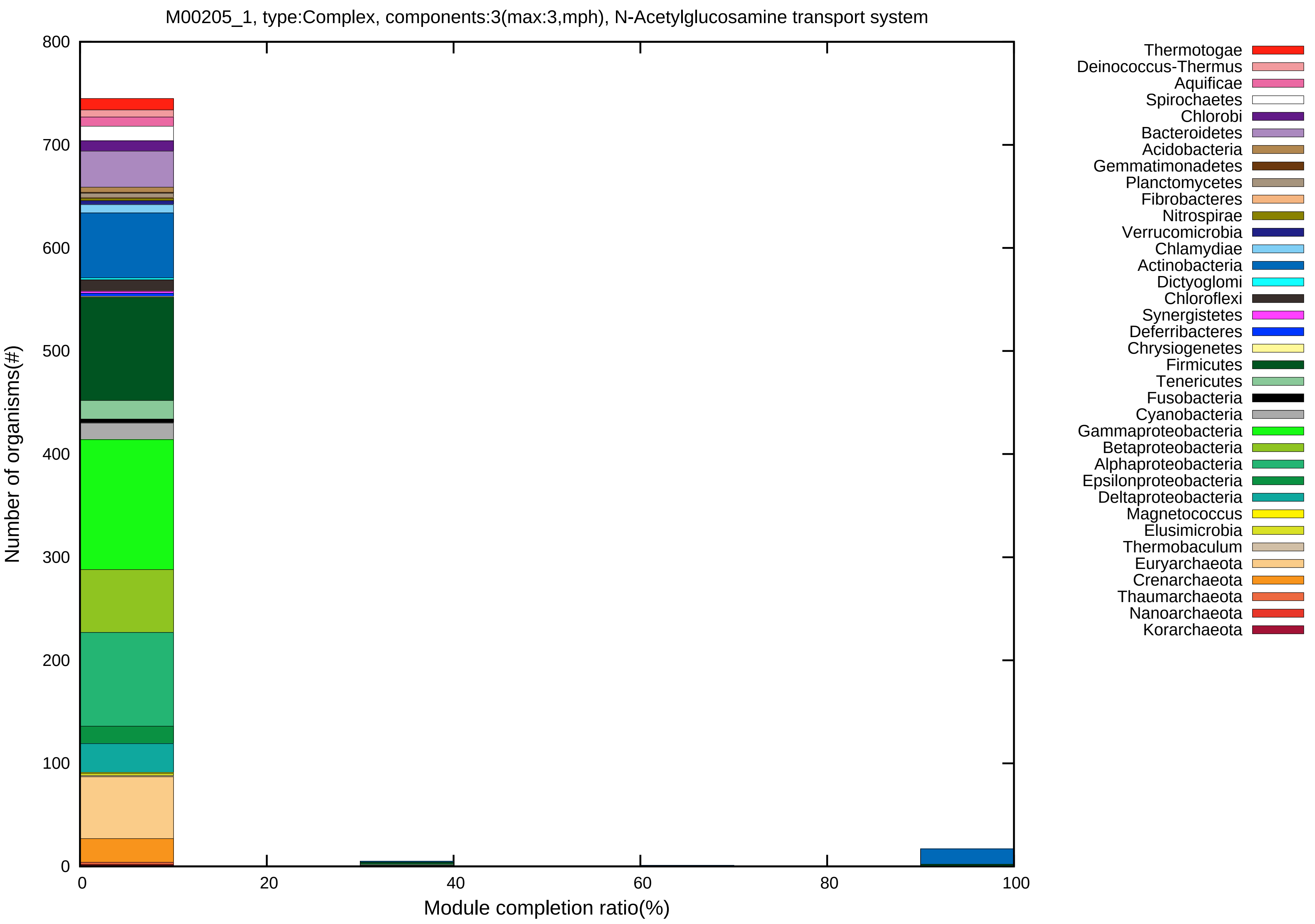

M00205_1, type:Complex, components:3(max:3,mph), N-Acetylglucosamine transport system
Number of organisms(#)
0
100
200
300
400
500
600
700
800
Module completion ratio(%)
0
20
40
60
80
100
Thermotogae
Deinococcus-Thermus
Aquificae
Spirochaetes
Chlorobi
Bacteroidetes
Acidobacteria
Gemmatimonadetes
Planctomycetes
Fibrobacteres
Nitrospirae
Verrucomicrobia
Chlamydiae
Actinobacteria
Dictyoglomi
Chloroflexi
Synergistetes
Deferribacteres
Chrysiogenetes
Firmicutes
Tenericutes
Fusobacteria
Cyanobacteria
Gammaproteobacteria
Betaproteobacteria
Alphaproteobacteria
Epsilonproteobacteria
Deltaproteobacteria
Magnetococcus
Elusimicrobia
Thermobaculum
Euryarchaeota
Crenarchaeota
Thaumarchaeota
Nanoarchaeota
Korarchaeota

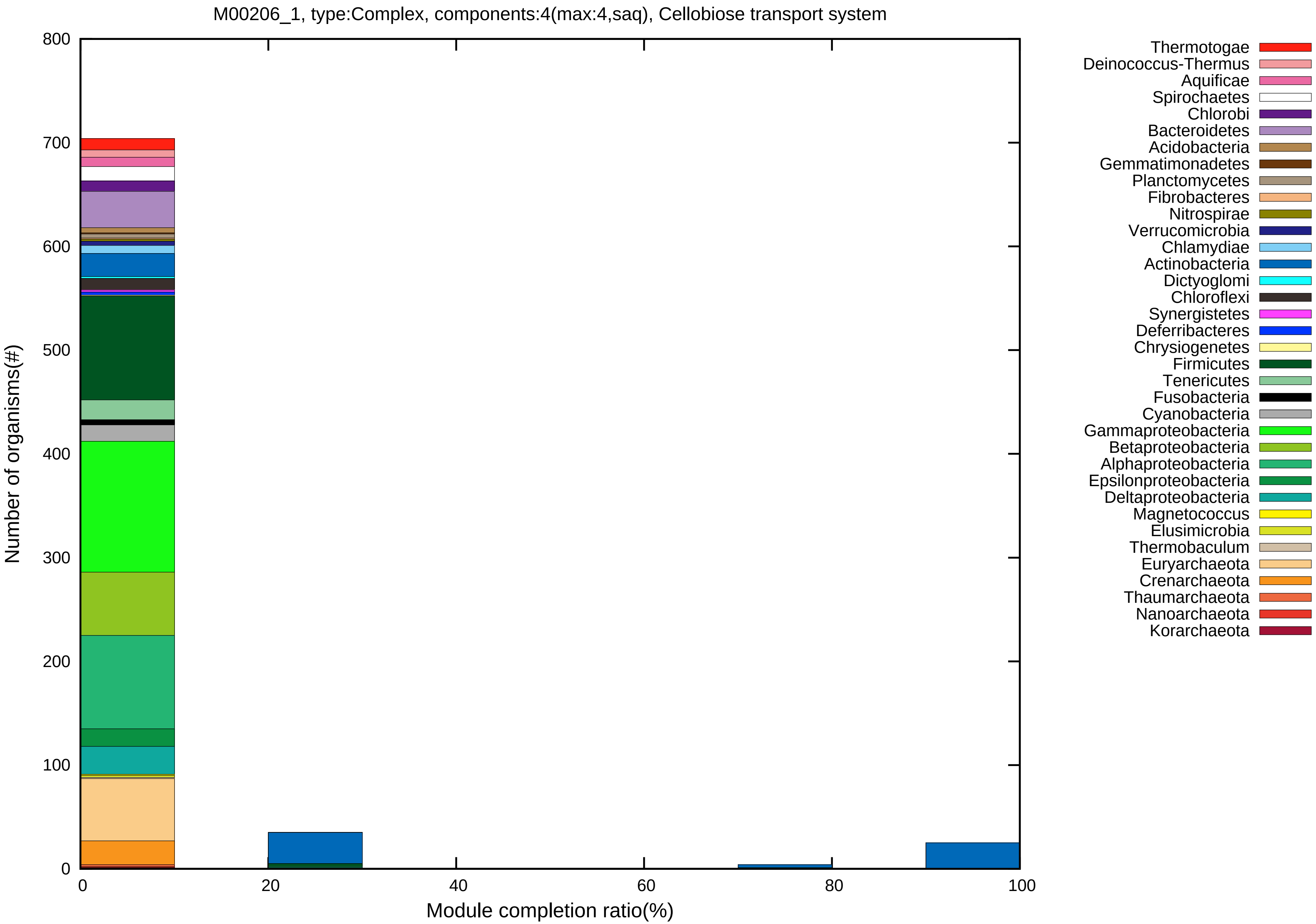

M00206_1, type:Complex, components:4(max:4,saq), Cellobiose transport system
Number of organisms(#)
Module completion ratio(%)
0
100
200
300
400
500
600
700
800
0
20
40
60
80
100
Thermotogae
Deinococcus-Thermus
Aquificae
Spirochaetes
Chlorobi
Bacteroidetes
Acidobacteria
Gemmatimonadetes
Planctomycetes
Fibrobacteres
Nitrospirae
Verrucomicrobia
Chlamydiae
Actinobacteria
Dictyoglomi
Chloroflexi
Synergistetes
Deferribacteres
Chrysiogenetes
Firmicutes
Tenericutes
Fusobacteria
Cyanobacteria
Gammaproteobacteria
Betaproteobacteria
Alphaproteobacteria
Epsilonproteobacteria
Deltaproteobacteria
Magnetococcus
Elusimicrobia
Thermobaculum
Euryarchaeota
Crenarchaeota
Thaumarchaeota
Nanoarchaeota
Korarchaeota

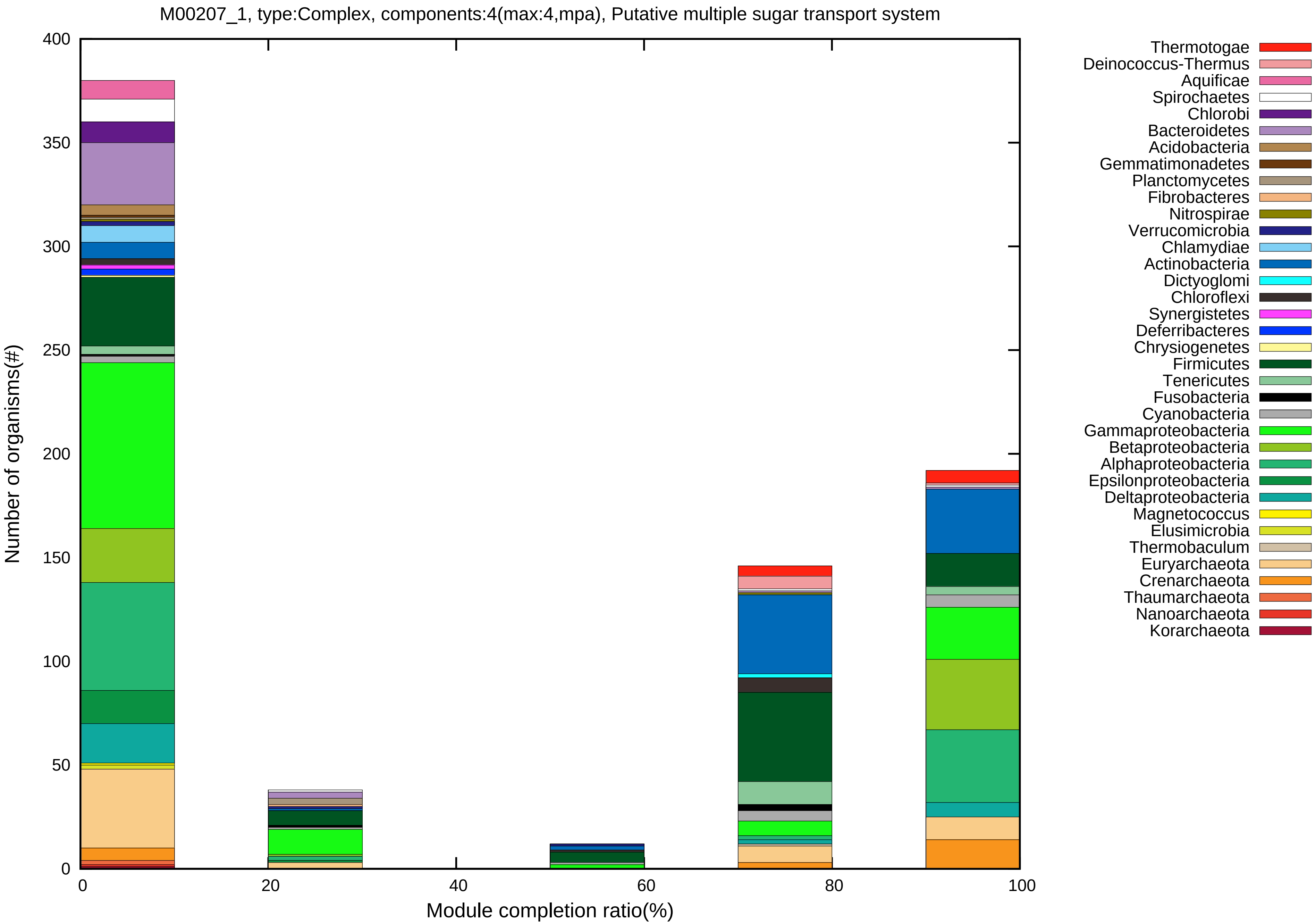

M00207_1, type:Complex, components:4(max:4,mpa), Putative multiple sugar transport system
Number of organisms(#)
Module completion ratio(%)
0
50
100
150
200
250
300
350
400
0
20
40
60
80
100
Thermotogae
Deinococcus-Thermus
Aquificae
Spirochaetes
Chlorobi
Bacteroidetes
Acidobacteria
Gemmatimonadetes
Planctomycetes
Fibrobacteres
Nitrospirae
Verrucomicrobia
Chlamydiae
Actinobacteria
Dictyoglomi
Chloroflexi
Synergistetes
Deferribacteres
Chrysiogenetes
Firmicutes
Tenericutes
Fusobacteria
Cyanobacteria
Gammaproteobacteria
Betaproteobacteria
Alphaproteobacteria
Epsilonproteobacteria
Deltaproteobacteria
Magnetococcus
Elusimicrobia
Thermobaculum
Euryarchaeota
Crenarchaeota
Thaumarchaeota
Nanoarchaeota
Korarchaeota

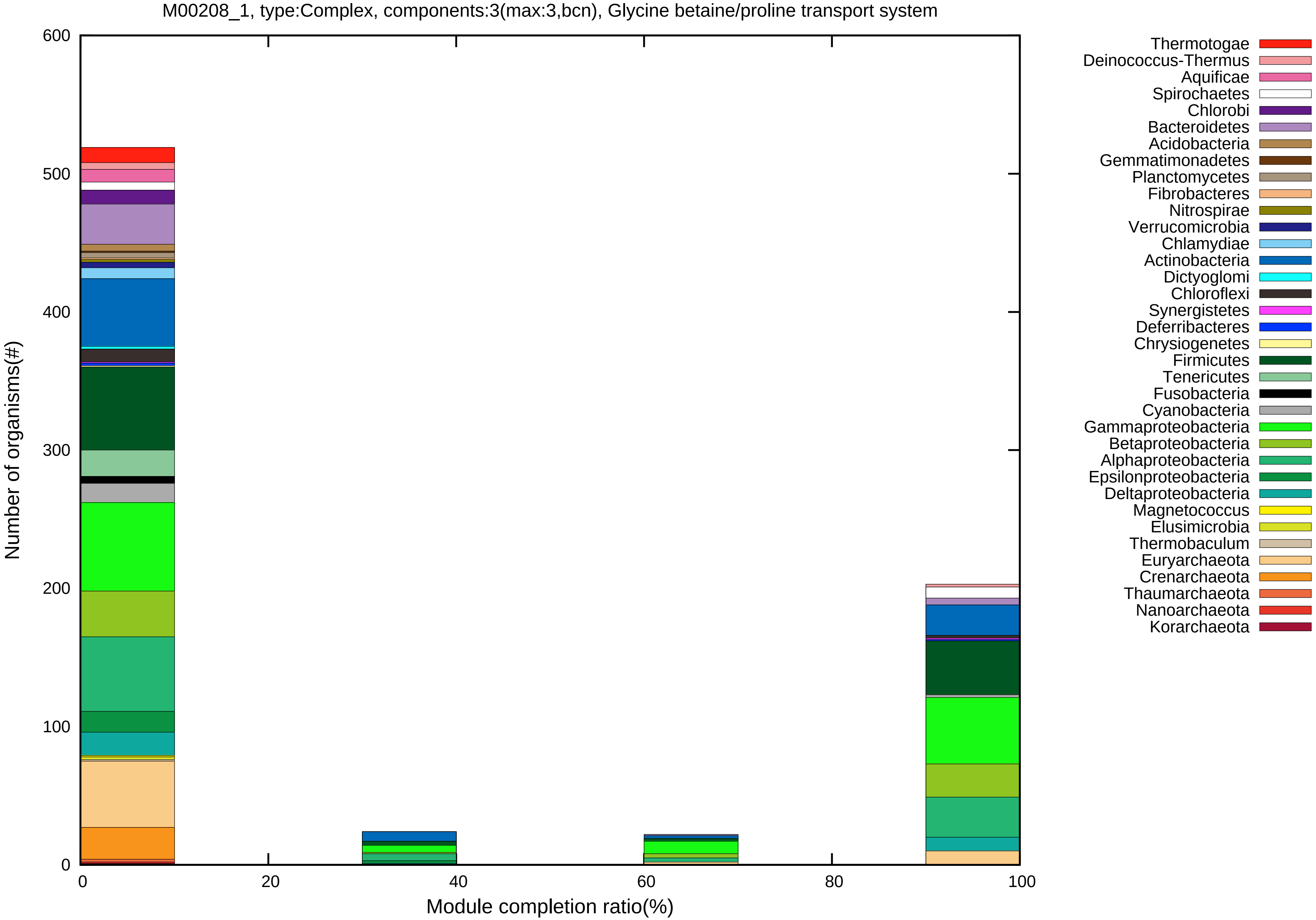
M00208_1, type:Complex, components:3(max:3,bcn), Glycine betaine/proline transport system
Number of organisms(#)
Module completion ratio(%)
0
100
200
300
400
500
600
0
20
40
60
80
100
Thermotogae
Deinococcus-Thermus
Aquificae
Spirochaetes
Chlorobi
Bacteroidetes
Acidobacteria
Gemmatimonadetes
Planctomycetes
Fibrobacteres
Nitrospirae
Verrucomicrobia
Chlamydiae
Actinobacteria
Dictyoglomi
Chloroflexi
Synergistetes
Deferribacteres
Chrysiogenetes
Firmicutes
Tenericutes
Fusobacteria
Cyanobacteria
Gammaproteobacteria
Betaproteobacteria
Alphaproteobacteria
Epsilonproteobacteria
Deltaproteobacteria
Magnetococcus
Elusimicrobia
Thermobaculum
Euryarchaeota
Crenarchaeota
Thaumarchaeota
Nanoarchaeota
Korarchaeota

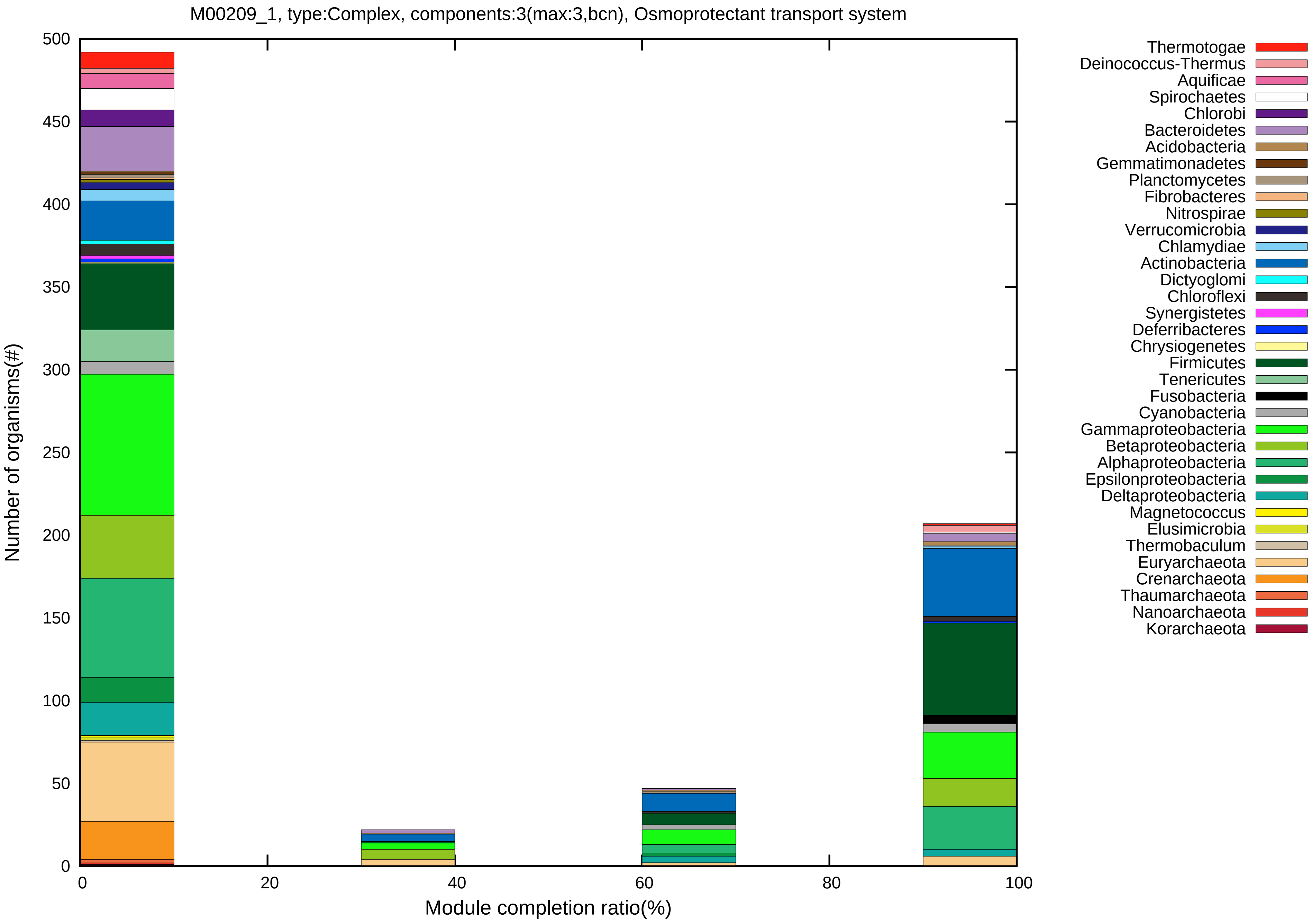

M00209_1, type:Complex, components:3(max:3,bcn), Osmoprotectant transport system
Number of organisms(#)
Module completion ratio(%)
0
50
100
150
200
250
300
350
400
450
500
0
20
40
60
80
100
Thermotogae
Deinococcus-Thermus
Aquificae
Spirochaetes
Chlorobi
Bacteroidetes
Acidobacteria
Gemmatimonadetes
Planctomycetes
Fibrobacteres
Nitrospirae
Verrucomicrobia
Chlamydiae
Actinobacteria
Dictyoglomi
Chloroflexi
Synergistetes
Deferribacteres
Chrysiogenetes
Firmicutes
Tenericutes
Fusobacteria
Cyanobacteria
Gammaproteobacteria
Betaproteobacteria
Alphaproteobacteria
Epsilonproteobacteria
Deltaproteobacteria
Magnetococcus
Elusimicrobia
Thermobaculum
Euryarchaeota
Crenarchaeota
Thaumarchaeota
Nanoarchaeota
Korarchaeota

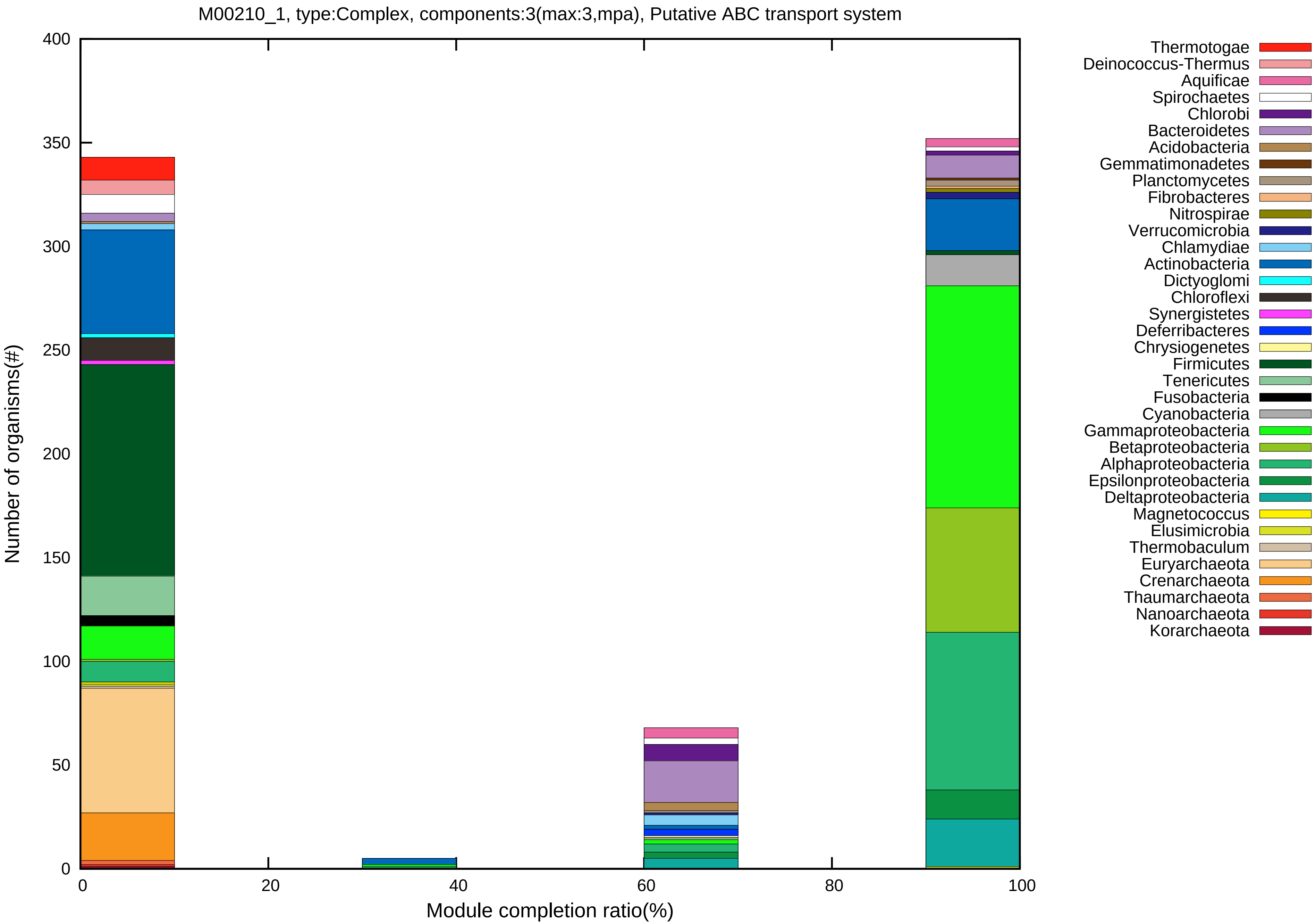

M00210_1, type:Complex, components:3(max:3,mpa), Putative ABC transport system
Number of organisms(#)
Module completion ratio(%)
0
50
100
150
200
250
300
350
400
0
20
40
60
80
100
Thermotogae
Deinococcus-Thermus
Aquificae
Spirochaetes
Chlorobi
Bacteroidetes
Acidobacteria
Gemmatimonadetes
Planctomycetes
Fibrobacteres
Nitrospirae
Verrucomicrobia
Chlamydiae
Actinobacteria
Dictyoglomi
Chloroflexi
Synergistetes
Deferribacteres
Chrysiogenetes
Firmicutes
Tenericutes
Fusobacteria
Cyanobacteria
Gammaproteobacteria
Betaproteobacteria
Alphaproteobacteria
Epsilonproteobacteria
Deltaproteobacteria
Magnetococcus
Elusimicrobia
Thermobaculum
Euryarchaeota
Crenarchaeota
Thaumarchaeota
Nanoarchaeota
Korarchaeota

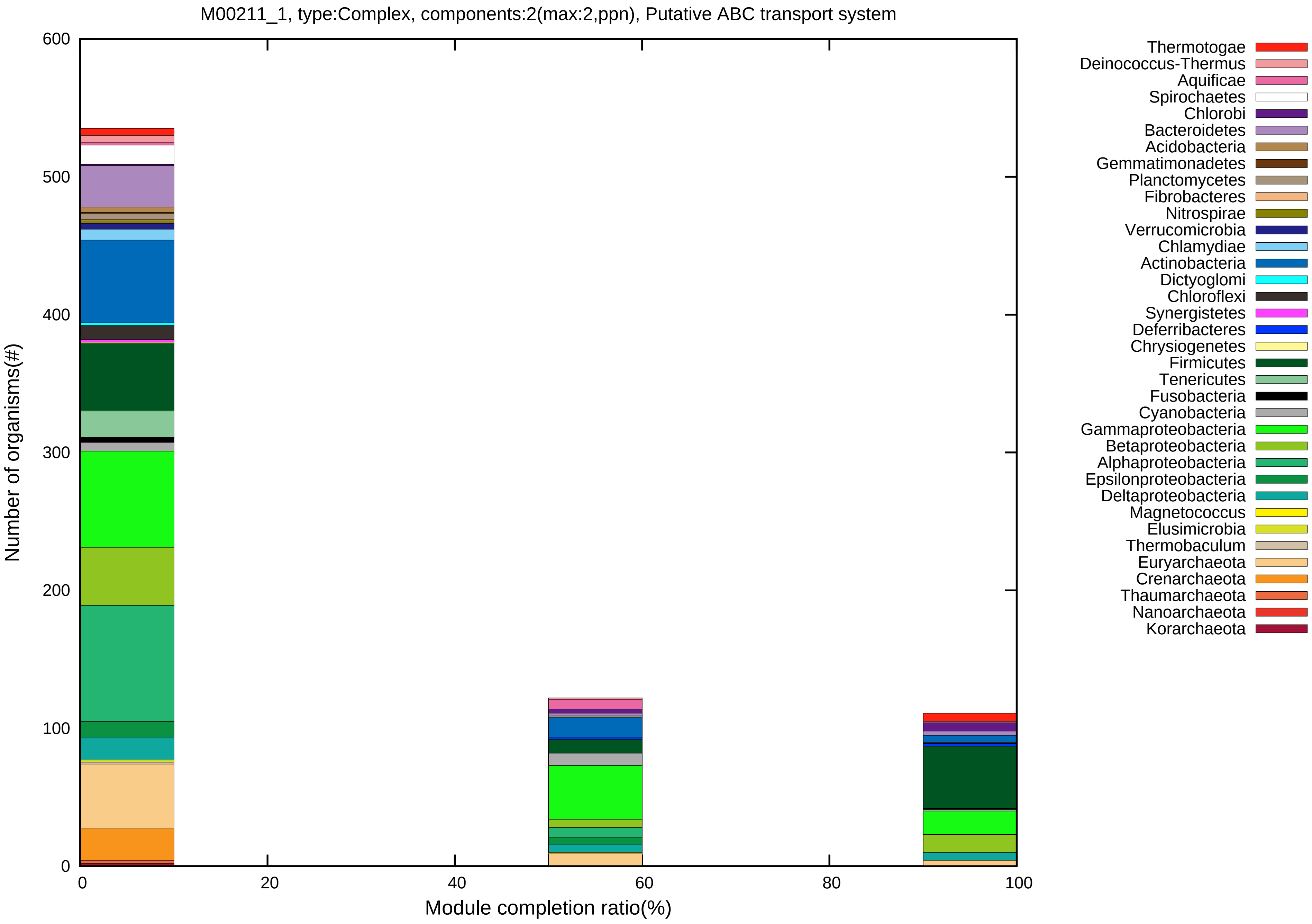
M00211_1, type:Complex, components:2(max:2,ppn), Putative ABC transport system
Number of organisms(#)
Module completion ratio(%)
0
100
200
300
400
500
600
0
20
40
60
80
100
Thermotogae
Deinococcus-Thermus
Aquificae
Spirochaetes
Chlorobi
Bacteroidetes
Acidobacteria
Gemmatimonadetes
Planctomycetes
Fibrobacteres
Nitrospirae
Verrucomicrobia
Chlamydiae
Actinobacteria
Dictyoglomi
Chloroflexi
Synergistetes
Deferribacteres
Chrysiogenetes
Firmicutes
Tenericutes
Fusobacteria
Cyanobacteria
Gammaproteobacteria
Betaproteobacteria
Alphaproteobacteria
Epsilonproteobacteria
Deltaproteobacteria
Magnetococcus
Elusimicrobia
Thermobaculum
Euryarchaeota
Crenarchaeota
Thaumarchaeota
Nanoarchaeota
Korarchaeota

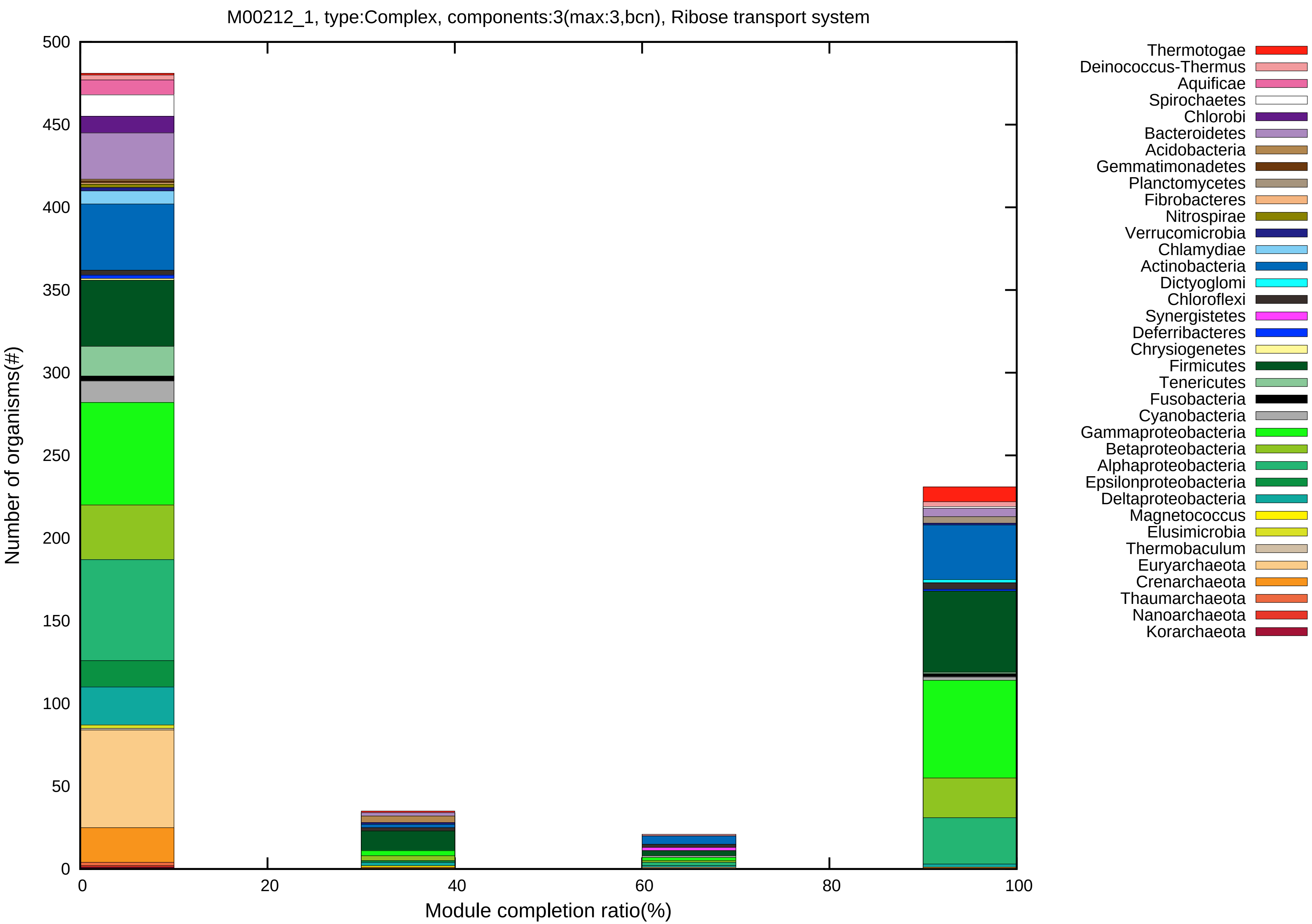
M00212_1, type:Complex, components:3(max:3,bcn), Ribose transport system
Number of organisms(#)
Module completion ratio(%)
0
50
100
150
200
250
300
350
400
450
500
0
20
40
60
80
100
Thermotogae
Deinococcus-Thermus
Aquificae
Spirochaetes
Chlorobi
Bacteroidetes
Acidobacteria
Gemmatimonadetes
Planctomycetes
Fibrobacteres
Nitrospirae
Verrucomicrobia
Chlamydiae
Actinobacteria
Dictyoglomi
Chloroflexi
Synergistetes
Deferribacteres
Chrysiogenetes
Firmicutes
Tenericutes
Fusobacteria
Cyanobacteria
Gammaproteobacteria
Betaproteobacteria
Alphaproteobacteria
Epsilonproteobacteria
Deltaproteobacteria
Magnetococcus
Elusimicrobia
Thermobaculum
Euryarchaeota
Crenarchaeota
Thaumarchaeota
Nanoarchaeota
Korarchaeota

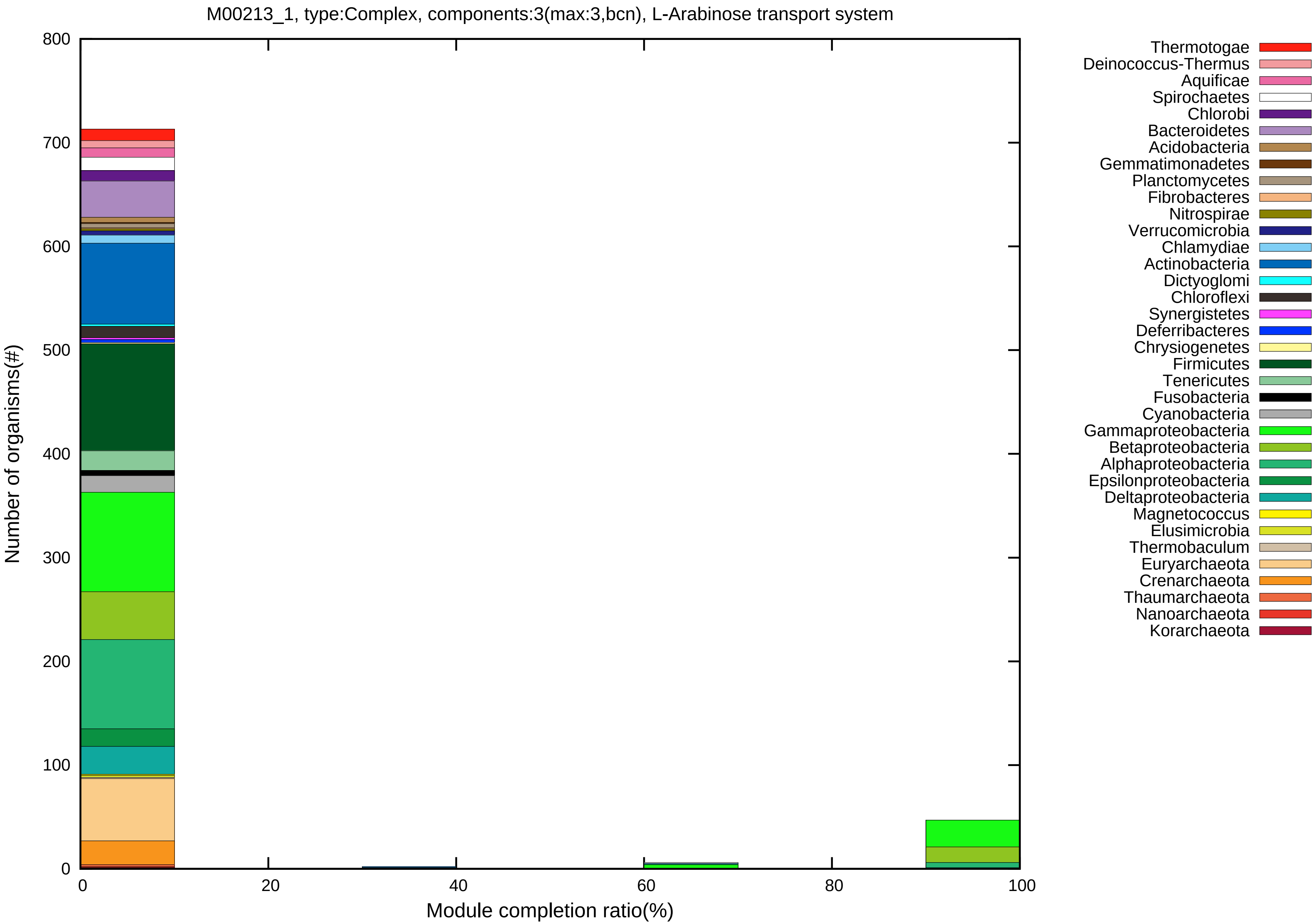

M00213_1, type:Complex, components:3(max:3,bcn), L-Arabinose transport system
Number of organisms(#)
Module completion ratio(%)
0
100
200
300
400
500
600
700
800
0
20
40
60
80
100
Thermotogae
Deinococcus-Thermus
Aquificae
Spirochaetes
Chlorobi
Bacteroidetes
Acidobacteria
Gemmatimonadetes
Planctomycetes
Fibrobacteres
Nitrospirae
Verrucomicrobia
Chlamydiae
Actinobacteria
Dictyoglomi
Chloroflexi
Synergistetes
Deferribacteres
Chrysiogenetes
Firmicutes
Tenericutes
Fusobacteria
Cyanobacteria
Gammaproteobacteria
Betaproteobacteria
Alphaproteobacteria
Epsilonproteobacteria
Deltaproteobacteria
Magnetococcus
Elusimicrobia
Thermobaculum
Euryarchaeota
Crenarchaeota
Thaumarchaeota
Nanoarchaeota
Korarchaeota

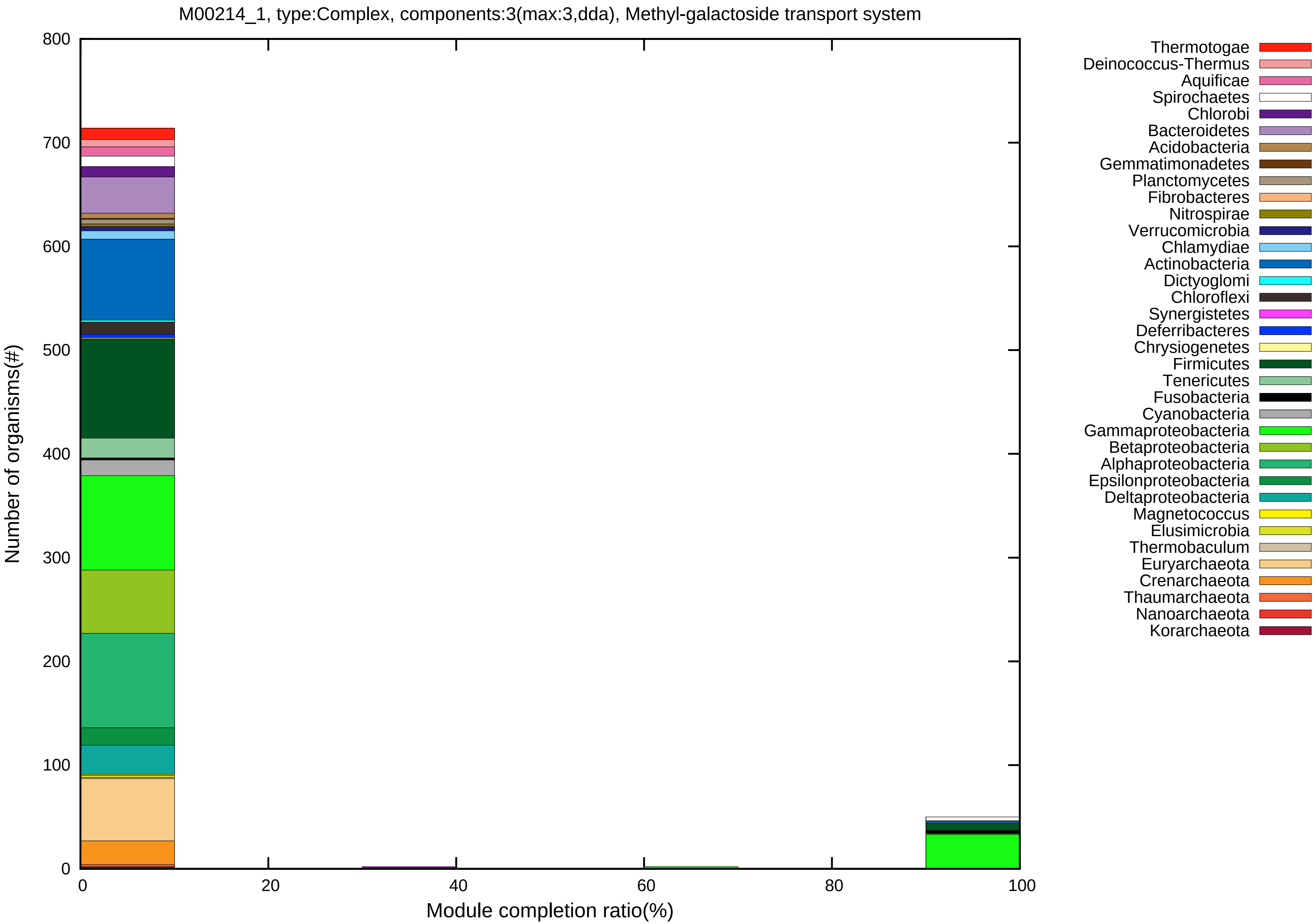

M00214_1, type:Complex, components:3(max:3,dda), Methyl-galactoside transport system
Number of organisms(#)
Module completion ratio(%)
0
100
200
300
400
500
600
700
800
0
20
40
60
80
100
Thermotogae
Deinococcus-Thermus
Aquificae
Spirochaetes
Chlorobi
Bacteroidetes
Acidobacteria
Gemmatimonadetes
Planctomycetes
Fibrobacteres
Nitrospirae
Verrucomicrobia
Chlamydiae
Actinobacteria
Dictyoglomi
Chloroflexi
Synergistetes
Deferribacteres
Chrysiogenetes
Firmicutes
Tenericutes
Fusobacteria
Cyanobacteria
Gammaproteobacteria
Betaproteobacteria
Alphaproteobacteria
Epsilonproteobacteria
Deltaproteobacteria
Magnetococcus
Elusimicrobia
Thermobaculum
Euryarchaeota
Crenarchaeota
Thaumarchaeota
Nanoarchaeota
Korarchaeota

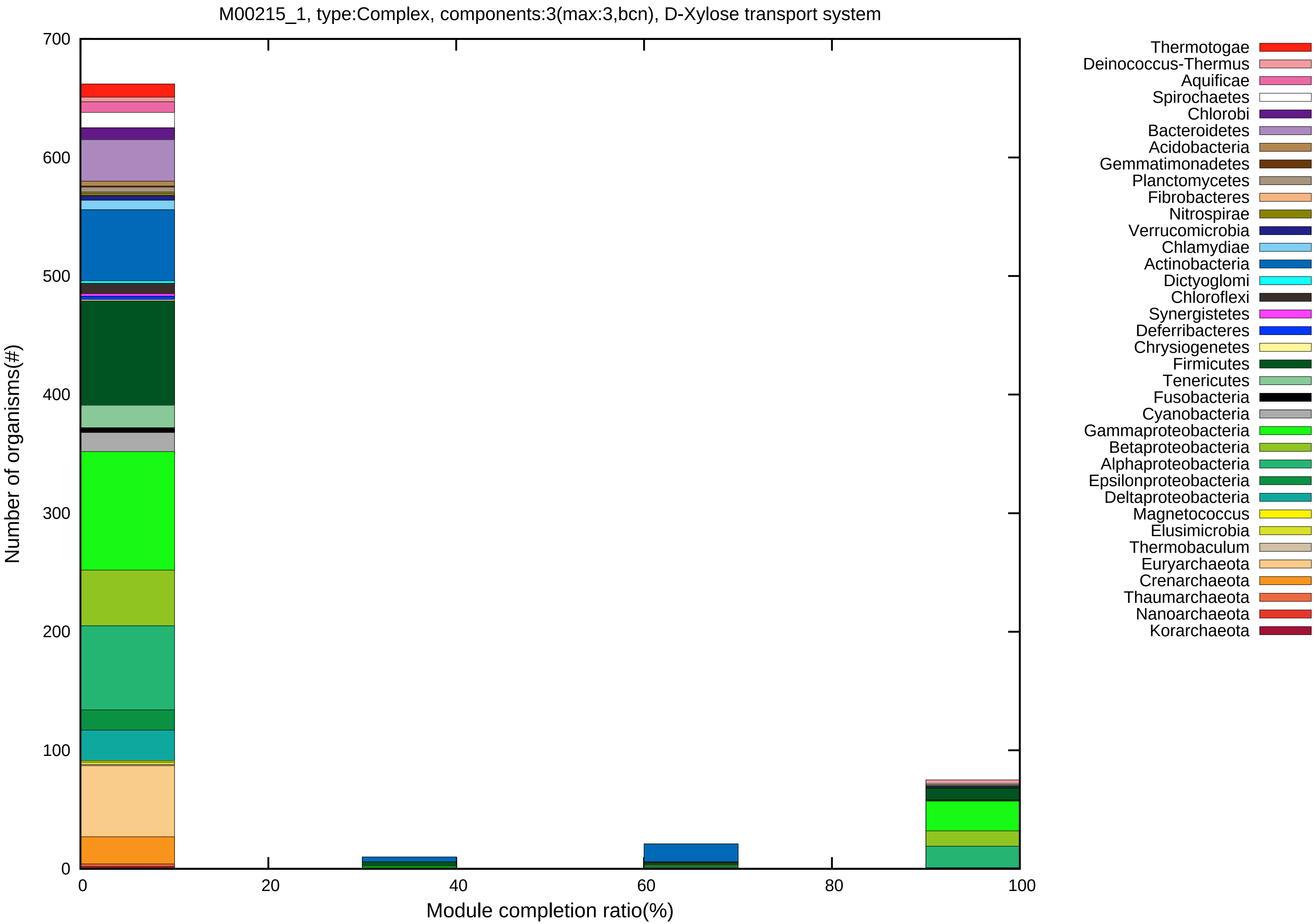
M00215_1, type:Complex, components:3(max:3,bcn), D-Xylose transport system
Number of organisms(#)
Module completion ratio(%)
0
100
200
300
400
500
600
700
0
20
40
60
80
100
Thermotogae
Deinococcus-Thermus
Aquificae
Spirochaetes
Chlorobi
Bacteroidetes
Acidobacteria
Gemmatimonadetes
Planctomycetes
Fibrobacteres
Nitrospirae
Verrucomicrobia
Chlamydiae
Actinobacteria
Dictyoglomi
Chloroflexi
Synergistetes
Deferribacteres
Chrysiogenetes
Firmicutes
Tenericutes
Fusobacteria
Cyanobacteria
Gammaproteobacteria
Betaproteobacteria
Alphaproteobacteria
Epsilonproteobacteria
Deltaproteobacteria
Magnetococcus
Elusimicrobia
Thermobaculum
Euryarchaeota
Crenarchaeota
Thaumarchaeota
Nanoarchaeota
Korarchaeota

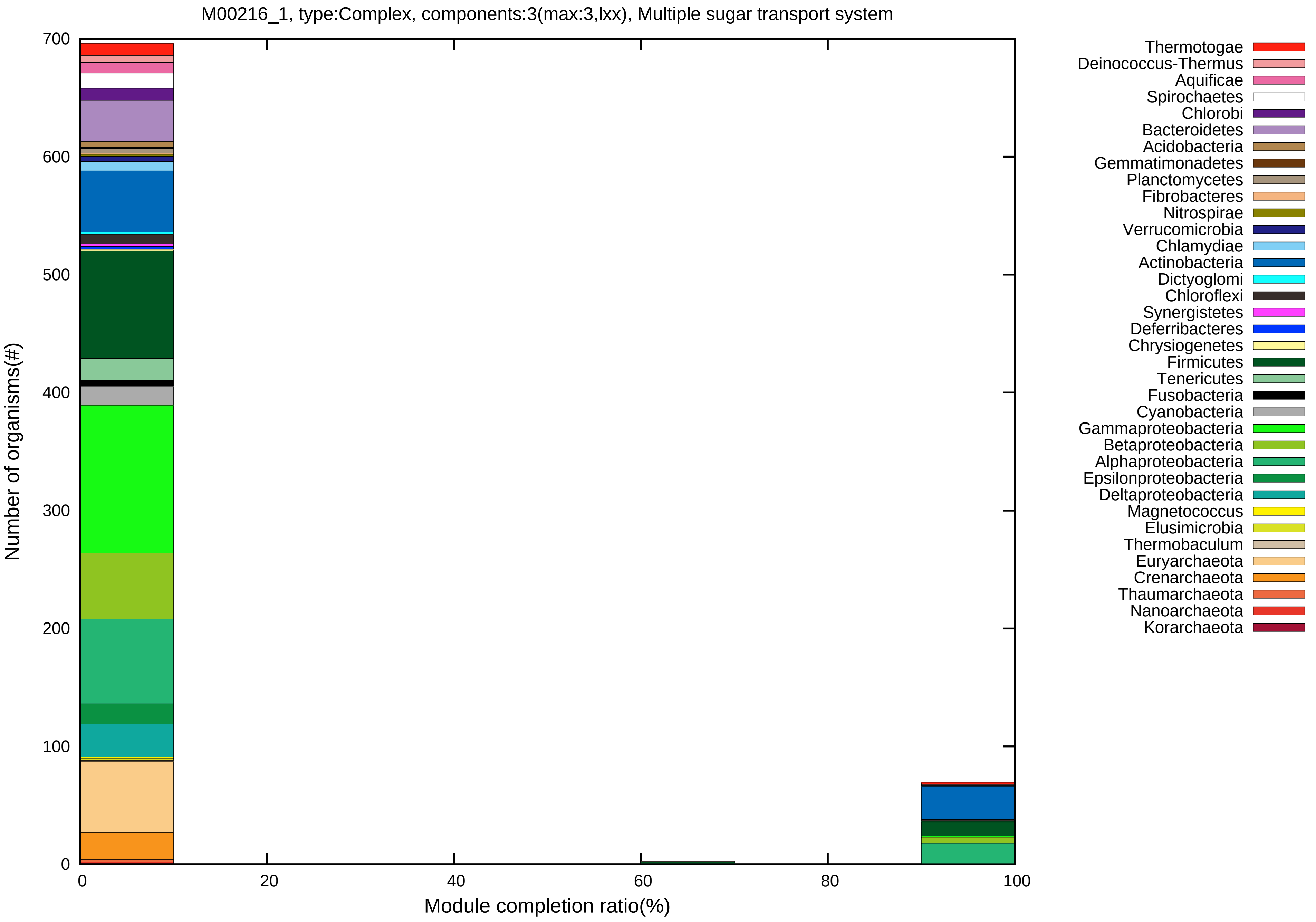

M00216_1, type:Complex, components:3(max:3,lxx), Multiple sugar transport system
Number of organisms(#)
Module completion ratio(%)
0
100
200
300
400
500
600
700
0
20
40
60
80
100
Thermotogae
Deinococcus-Thermus
Aquificae
Spirochaetes
Chlorobi
Bacteroidetes
Acidobacteria
Gemmatimonadetes
Planctomycetes
Fibrobacteres
Nitrospirae
Verrucomicrobia
Chlamydiae
Actinobacteria
Dictyoglomi
Chloroflexi
Synergistetes
Deferribacteres
Chrysiogenetes
Firmicutes
Tenericutes
Fusobacteria
Cyanobacteria
Gammaproteobacteria
Betaproteobacteria
Alphaproteobacteria
Epsilonproteobacteria
Deltaproteobacteria
Magnetococcus
Elusimicrobia
Thermobaculum
Euryarchaeota
Crenarchaeota
Thaumarchaeota
Nanoarchaeota
Korarchaeota

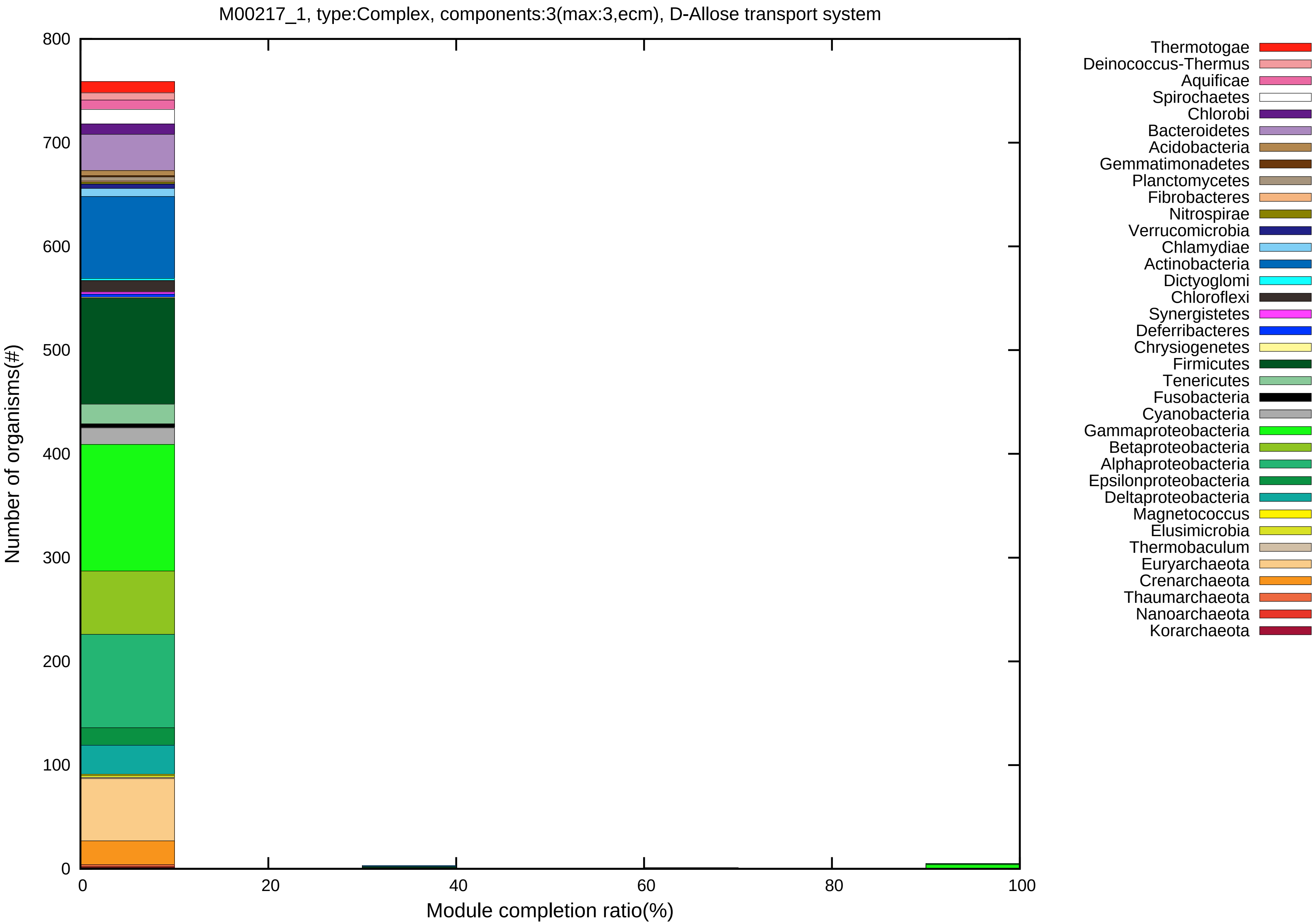

M00217_1, type:Complex, components:3(max:3,ecm), D-Allose transport system
Number of organisms(#)
Module completion ratio(%)
0
100
200
300
400
500
600
700
800
0
20
40
60
80
100
Thermotogae
Deinococcus-Thermus
Aquificae
Spirochaetes
Chlorobi
Bacteroidetes
Acidobacteria
Gemmatimonadetes
Planctomycetes
Fibrobacteres
Nitrospirae
Verrucomicrobia
Chlamydiae
Actinobacteria
Dictyoglomi
Chloroflexi
Synergistetes
Deferribacteres
Chrysiogenetes
Firmicutes
Tenericutes
Fusobacteria
Cyanobacteria
Gammaproteobacteria
Betaproteobacteria
Alphaproteobacteria
Epsilonproteobacteria
Deltaproteobacteria
Magnetococcus
Elusimicrobia
Thermobaculum
Euryarchaeota
Crenarchaeota
Thaumarchaeota
Nanoarchaeota
Korarchaeota

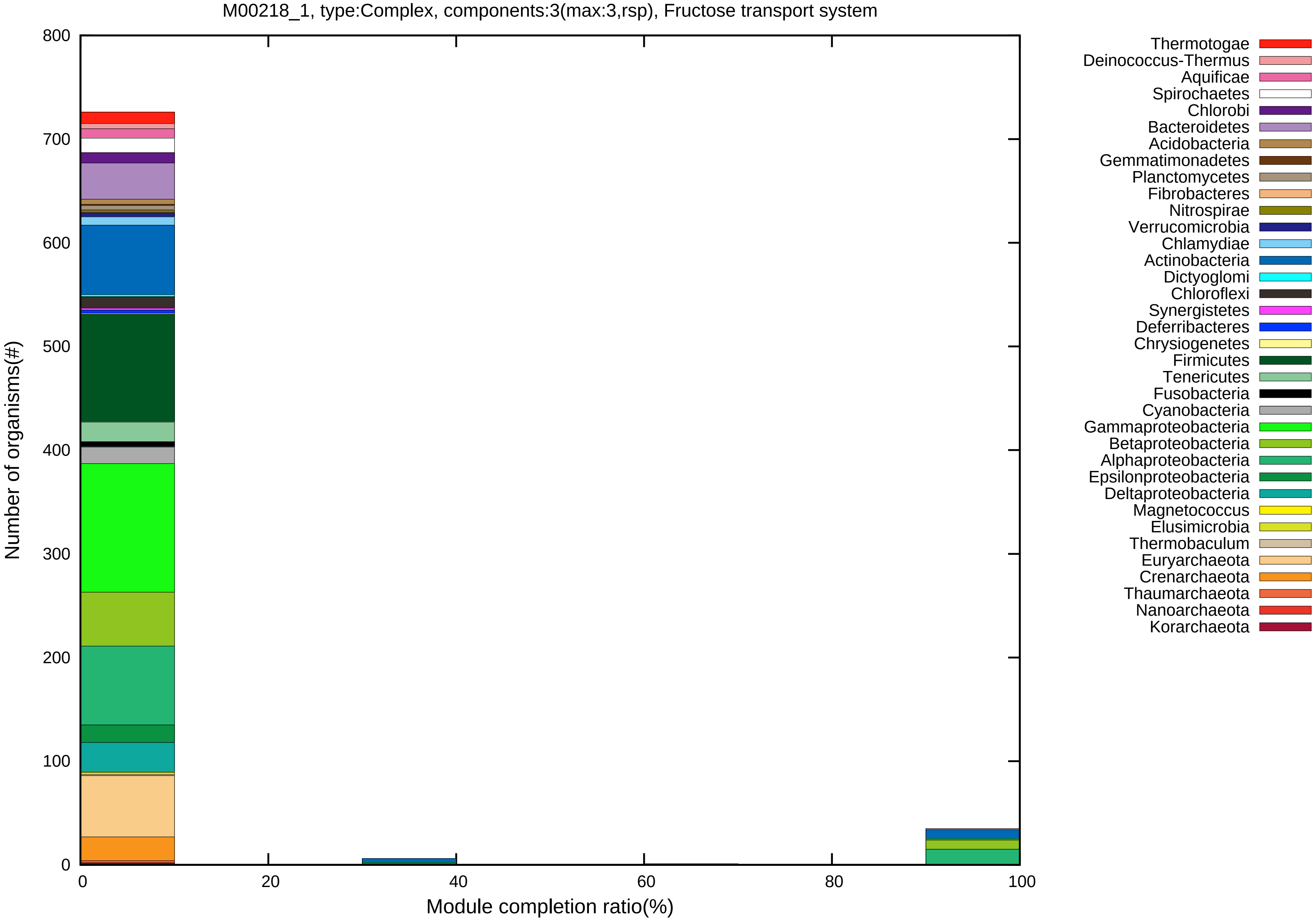

M00218_1, type:Complex, components:3(max:3,rsp), Fructose transport system
Number of organisms(#)
Module completion ratio(%)
0
100
200
300
400
500
600
700
800
0
20
40
60
80
100
Thermotogae
Deinococcus-Thermus
Aquificae
Spirochaetes
Chlorobi
Bacteroidetes
Acidobacteria
Gemmatimonadetes
Planctomycetes
Fibrobacteres
Nitrospirae
Verrucomicrobia
Chlamydiae
Actinobacteria
Dictyoglomi
Chloroflexi
Synergistetes
Deferribacteres
Chrysiogenetes
Firmicutes
Tenericutes
Fusobacteria
Cyanobacteria
Gammaproteobacteria
Betaproteobacteria
Alphaproteobacteria
Epsilonproteobacteria
Deltaproteobacteria
Magnetococcus
Elusimicrobia
Thermobaculum
Euryarchaeota
Crenarchaeota
Thaumarchaeota
Nanoarchaeota
Korarchaeota

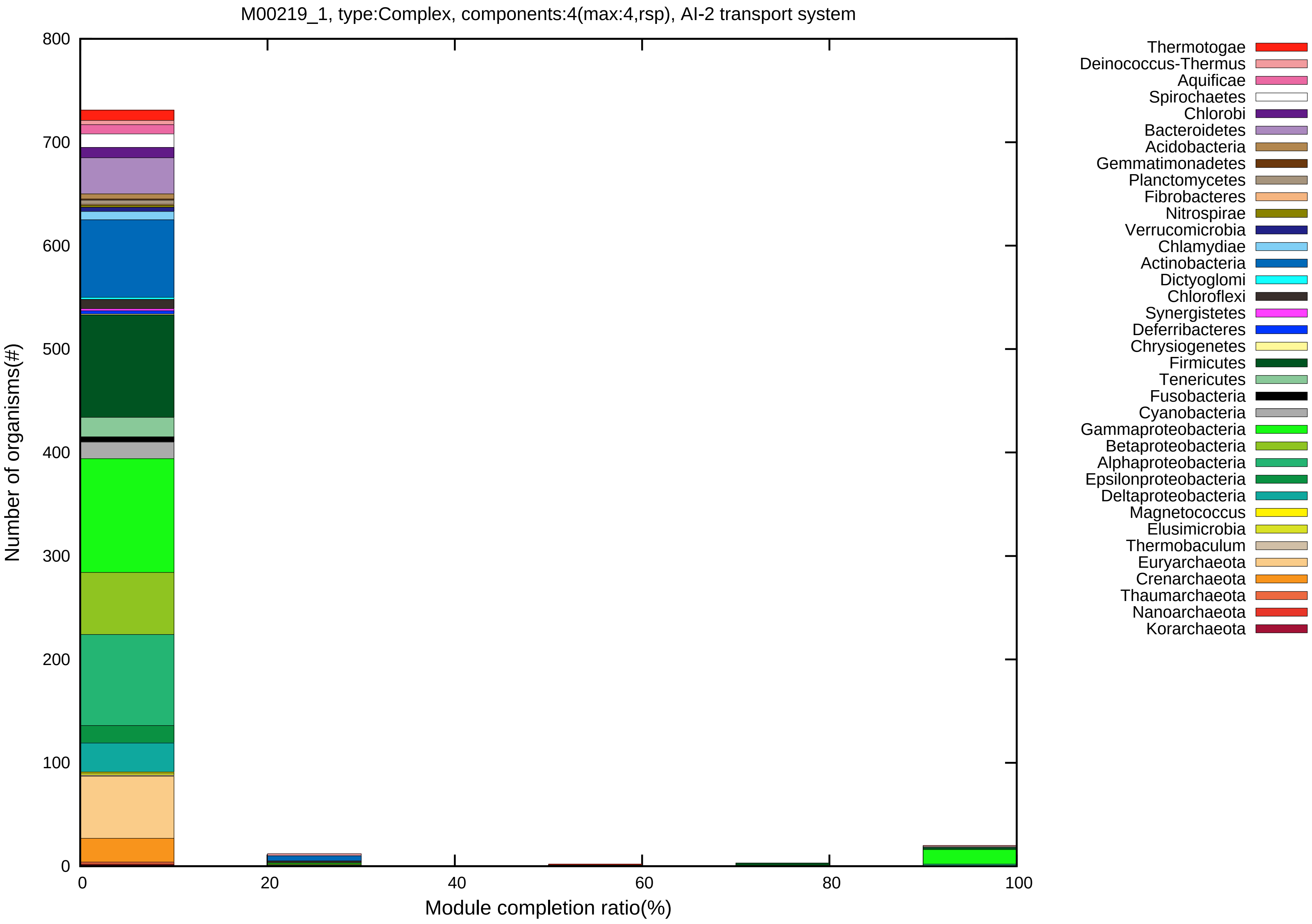
M00219_1, type:Complex, components:4(max:4,rsp), AI-2 transport system
Number of organisms(#)
0
100
200
300
400
500
600
700
800
0
20
40
60
80
100
Module completion ratio(%)
Thermotogae
Deinococcus-Thermus
Aquificae
Spirochaetes
Chlorobi
Bacteroidetes
Acidobacteria
Gemmatimonadetes
Planctomycetes
Fibrobacteres
Nitrospirae
Verrucomicrobia
Chlamydiae
Actinobacteria
Dictyoglomi
Chloroflexi
Synergistetes
Deferribacteres
Chrysiogenetes
Firmicutes
Tenericutes
Fusobacteria
Cyanobacteria
Gammaproteobacteria
Betaproteobacteria
Alphaproteobacteria
Epsilonproteobacteria
Deltaproteobacteria
Magnetococcus
Elusimicrobia
Thermobaculum
Euryarchaeota
Crenarchaeota
Thaumarchaeota
Nanoarchaeota
Korarchaeota

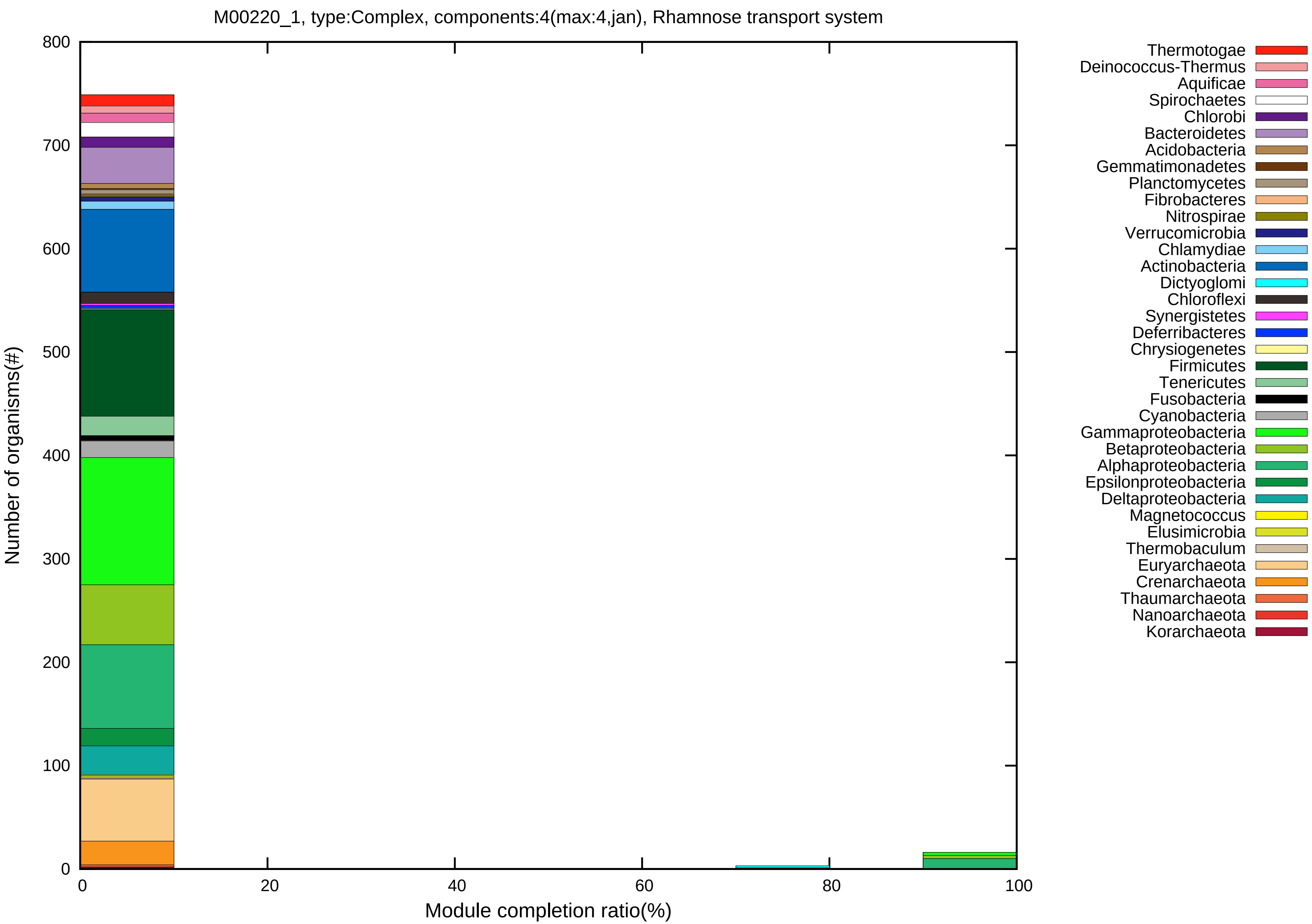

M00220_1, type:Complex, components:4(max:4,jan), Rhamnose transport system
Number of organisms(#)
Module completion ratio(%)
0
100
200
300
400
500
600
700
800
0
20
40
60
80
100
Thermotogae
Deinococcus-Thermus
Aquificae
Spirochaetes
Chlorobi
Bacteroidetes
Acidobacteria
Gemmatimonadetes
Planctomycetes
Fibrobacteres
Nitrospirae
Verrucomicrobia
Chlamydiae
Actinobacteria
Dictyoglomi
Chloroflexi
Synergistetes
Deferribacteres
Chrysiogenetes
Firmicutes
Tenericutes
Fusobacteria
Cyanobacteria
Gammaproteobacteria
Betaproteobacteria
Alphaproteobacteria
Epsilonproteobacteria
Deltaproteobacteria
Magnetococcus
Elusimicrobia
Thermobaculum
Euryarchaeota
Crenarchaeota
Thaumarchaeota
Nanoarchaeota
Korarchaeota

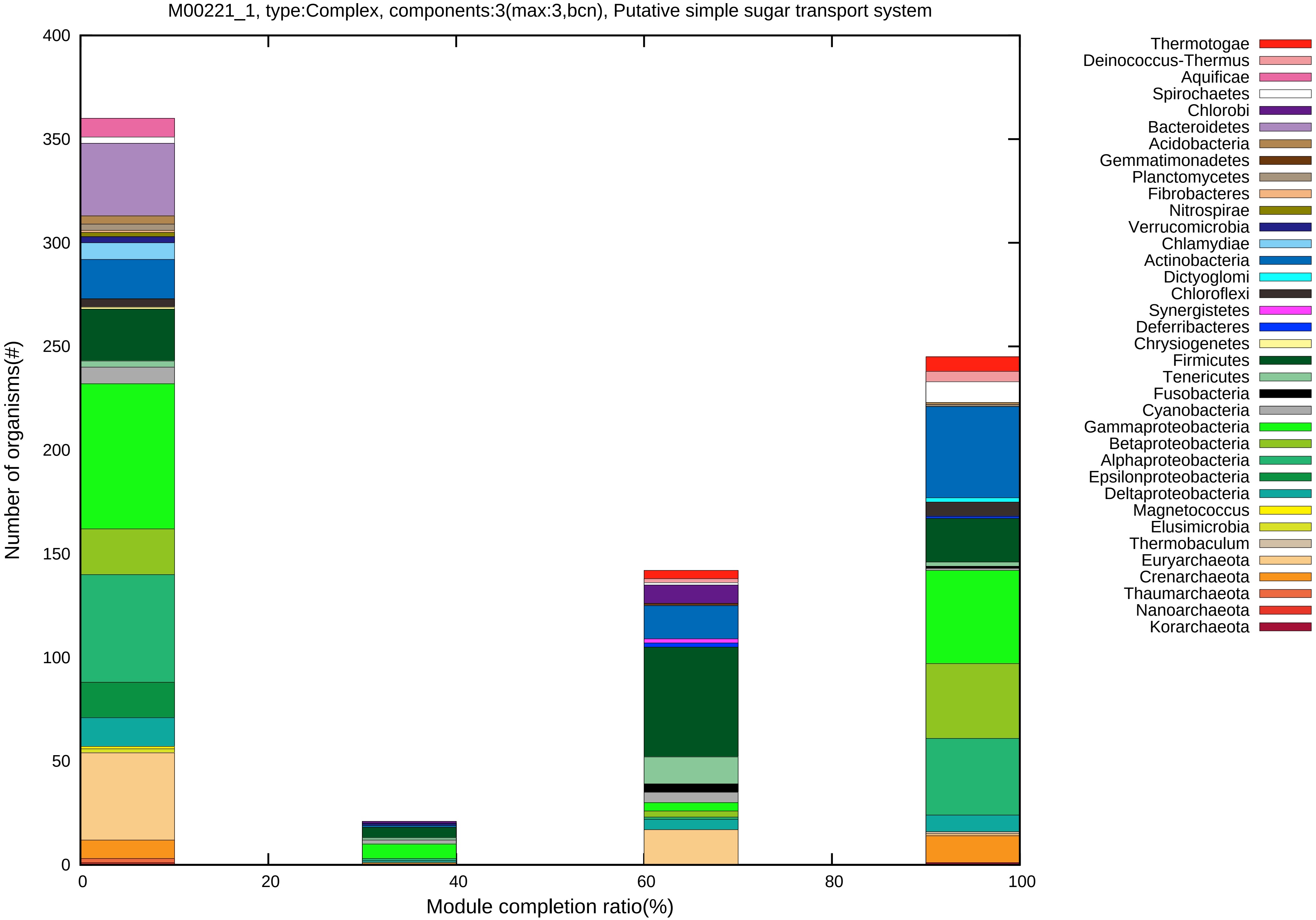

M00221_1, type:Complex, components:3(max:3,bcn), Putative simple sugar transport system
Number of organisms(#)
Module completion ratio(%)
0
50
100
150
200
250
300
350
400
0
20
40
60
80
100
Thermotogae
Deinococcus-Thermus
Aquificae
Spirochaetes
Chlorobi
Bacteroidetes
Acidobacteria
Gemmatimonadetes
Planctomycetes
Fibrobacteres
Nitrospirae
Verrucomicrobia
Chlamydiae
Actinobacteria
Dictyoglomi
Chloroflexi
Synergistetes
Deferribacteres
Chrysiogenetes
Firmicutes
Tenericutes
Fusobacteria
Cyanobacteria
Gammaproteobacteria
Betaproteobacteria
Alphaproteobacteria
Epsilonproteobacteria
Deltaproteobacteria
Magnetococcus
Elusimicrobia
Thermobaculum
Euryarchaeota
Crenarchaeota
Thaumarchaeota
Nanoarchaeota
Korarchaeota

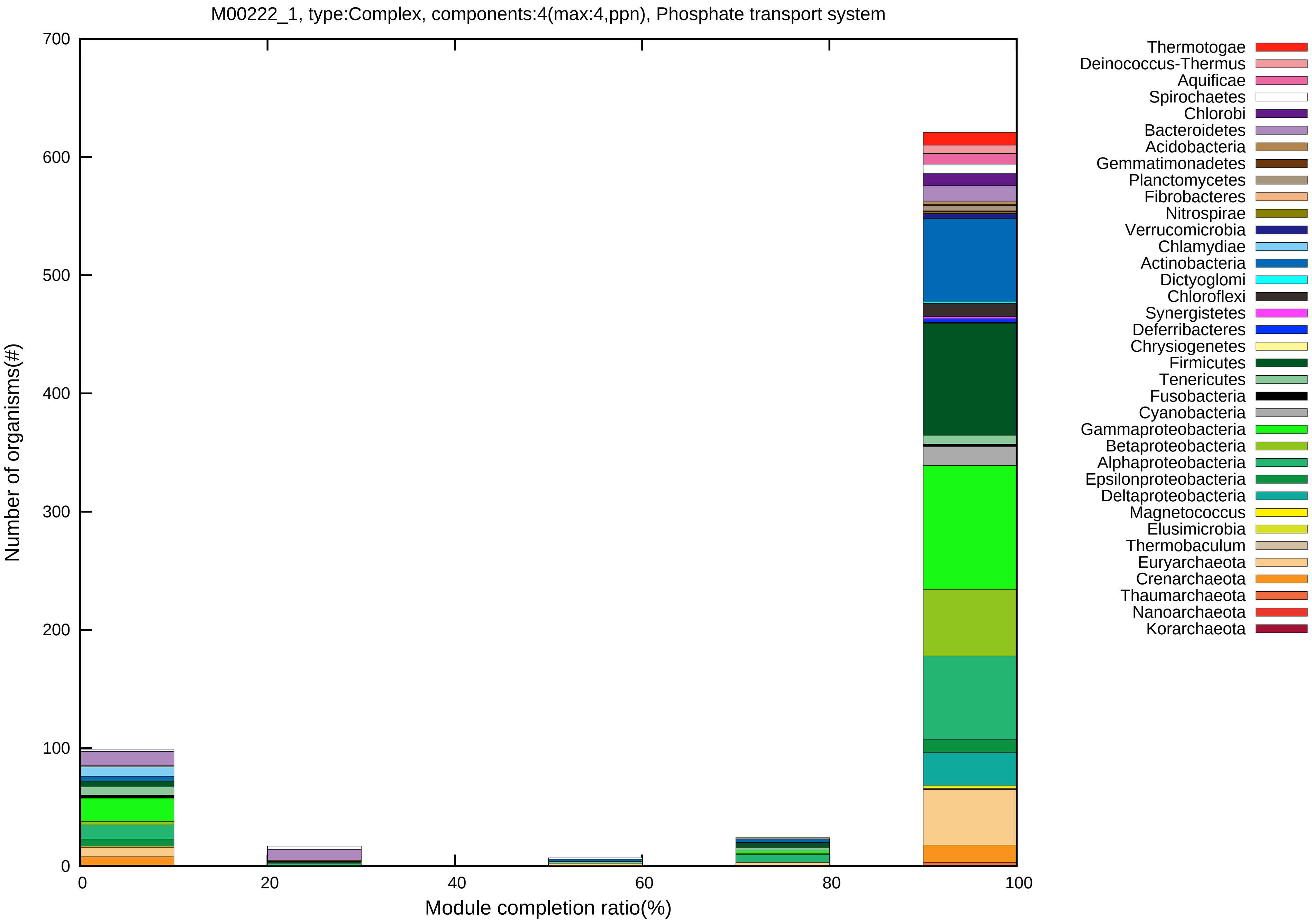

M00222_1, type:Complex, components:4(max:4,ppn), Phosphate transport system
Number of organisms(#)
700
600
500
400
300
200
100
0
0
20
40
60
80
100
Module completion ratio(%)
Thermotogae
Deinococcus-Thermus
Aquificae
Spirochaetes
Chlorobi
Bacteroidetes
Acidobacteria
Gemmatimonadetes
Planctomycetes
Fibrobacteres
Nitrospirae
Verrucomicrobia
Chlamydiae
Actinobacteria
Dictyoglomi
Chloroflexi
Synergistetes
Deferribacteres
Chrysiogenetes
Firmicutes
Tenericutes
Fusobacteria
Cyanobacteria
Gammaproteobacteria
Betaproteobacteria
Alphaproteobacteria
Epsilonproteobacteria
Deltaproteobacteria
Magnetococcus
Elusimicrobia
Thermobaculum
Euryarchaeota
Crenarchaeota
Thaumarchaeota
Nanoarchaeota
Korarchaeota

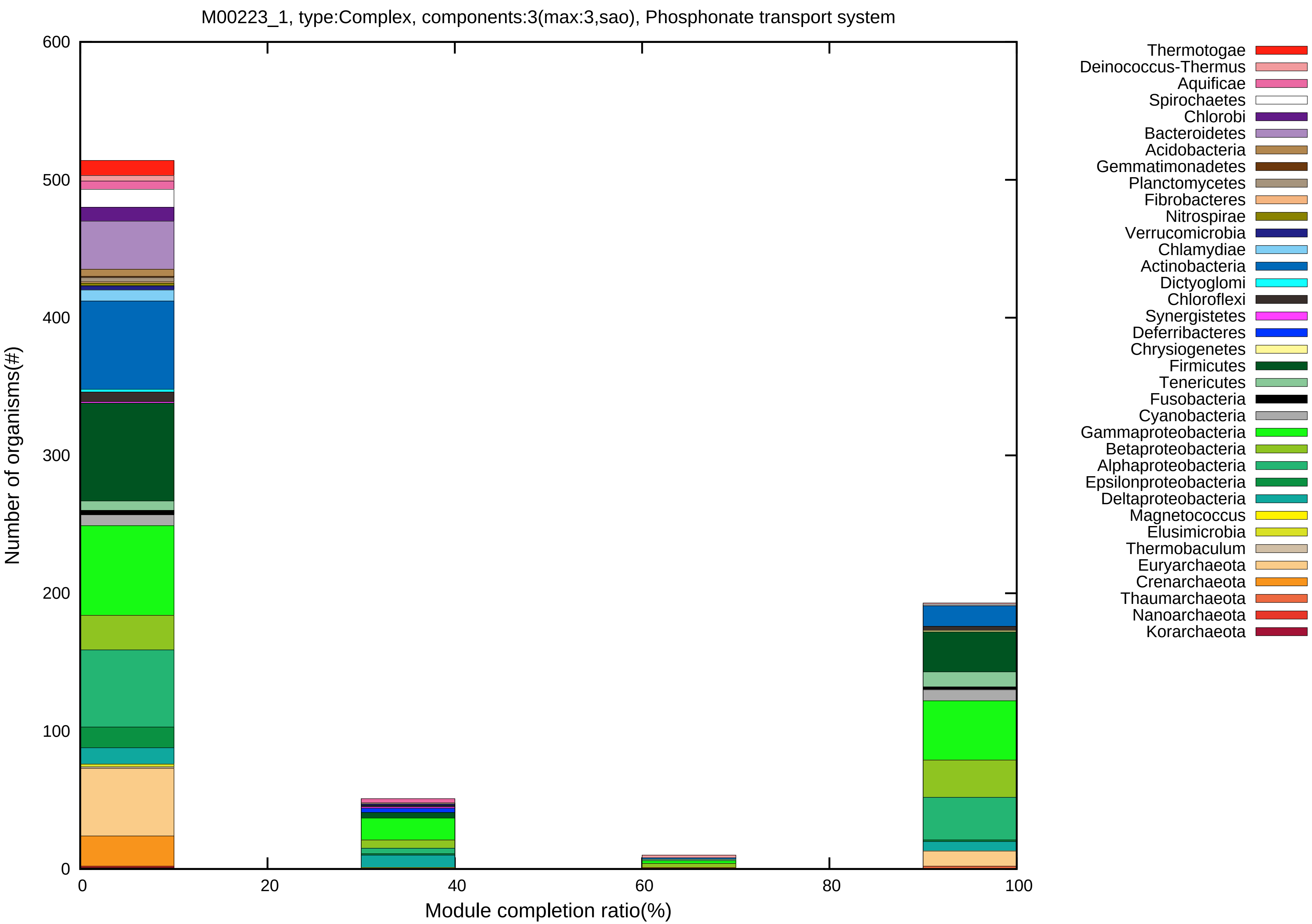

M00223_1, type:Complex, components:3(max:3,sao), Phosphonate transport system
Number of organisms(#)
Module completion ratio(%)
0
100
200
300
400
500
600
0
20
40
60
80
100
Thermotogae
Deinococcus-Thermus
Aquificae
Spirochaetes
Chlorobi
Bacteroidetes
Acidobacteria
Gemmatimonadetes
Planctomycetes
Fibrobacteres
Nitrospirae
Verrucomicrobia
Chlamydiae
Actinobacteria
Dictyoglomi
Chloroflexi
Synergistetes
Deferribacteres
Chrysiogenetes
Firmicutes
Tenericutes
Fusobacteria
Cyanobacteria
Gammaproteobacteria
Betaproteobacteria
Alphaproteobacteria
Epsilonproteobacteria
Deltaproteobacteria
Magnetococcus
Elusimicrobia
Thermobaculum
Euryarchaeota
Crenarchaeota
Thaumarchaeota
Nanoarchaeota
Korarchaeota

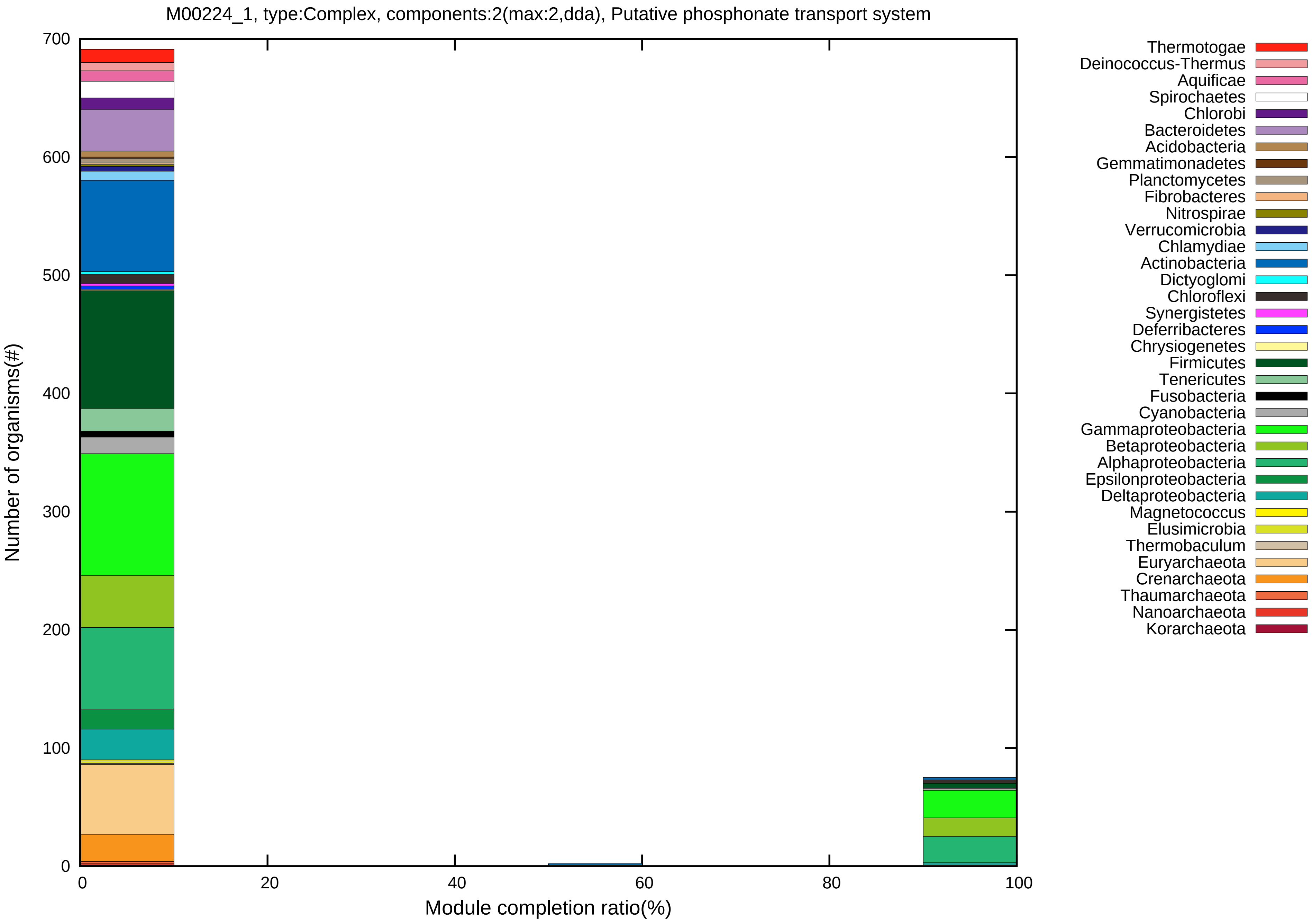
M00224_1, type:Complex, components:2(max:2,dda), Putative phosphonate transport system
Number of organisms(#)
Module completion ratio(%)
0
100
200
300
400
500
600
700
0
20
40
60
80
100
Thermotogae
Deinococcus-Thermus
Aquificae
Spirochaetes
Chlorobi
Bacteroidetes
Acidobacteria
Gemmatimonadetes
Planctomycetes
Fibrobacteres
Nitrospirae
Verrucomicrobia
Chlamydiae
Actinobacteria
Dictyoglomi
Chloroflexi
Synergistetes
Deferribacteres
Chrysiogenetes
Firmicutes
Tenericutes
Fusobacteria
Cyanobacteria
Gammaproteobacteria
Betaproteobacteria
Alphaproteobacteria
Epsilonproteobacteria
Deltaproteobacteria
Magnetococcus
Elusimicrobia
Thermobaculum
Euryarchaeota
Crenarchaeota
Thaumarchaeota
Nanoarchaeota
Korarchaeota

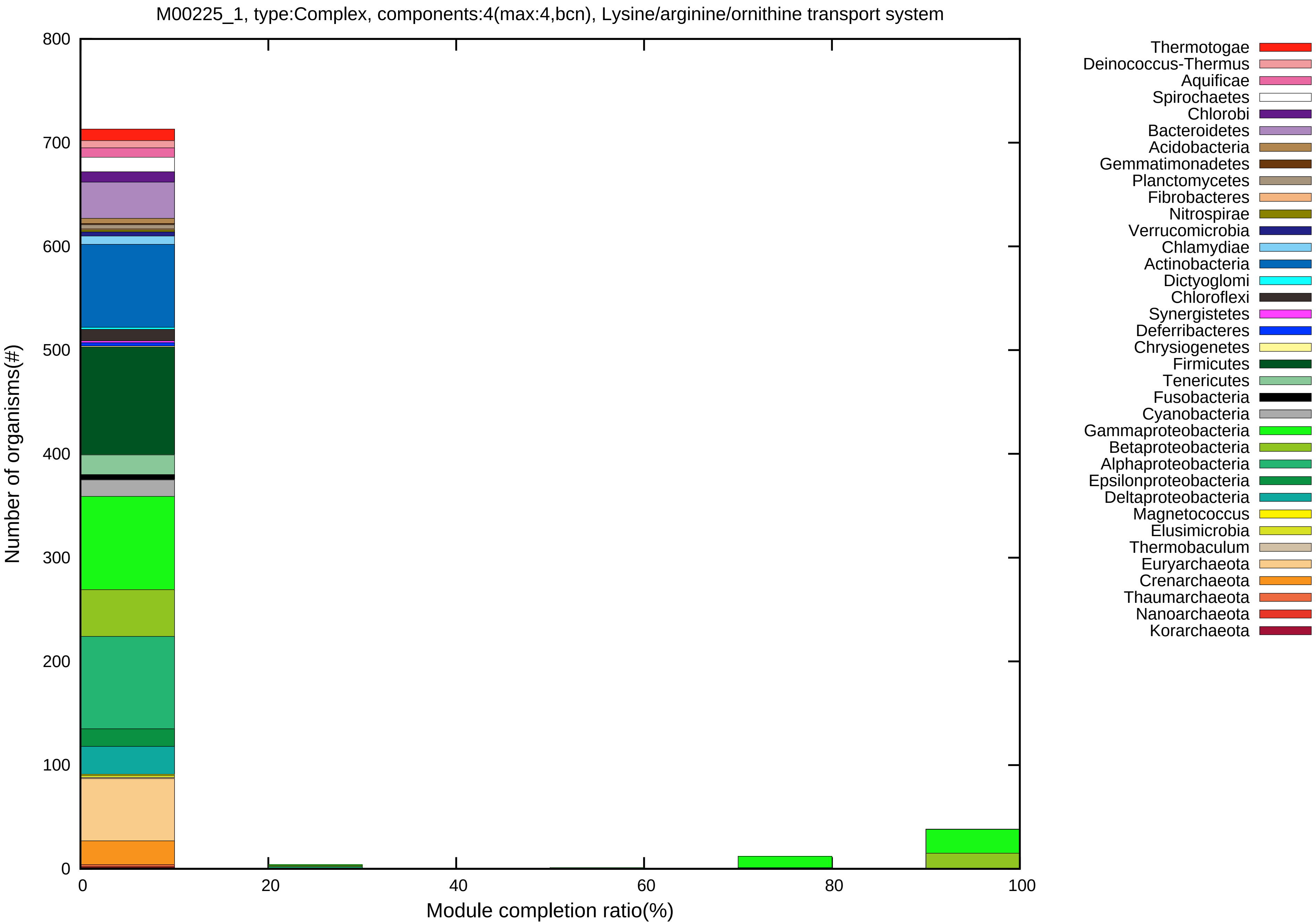

M00225_1, type:Complex, components:4(max:4,bcn), Lysine/arginine/ornithine transport system
Number of organisms(#)
Module completion ratio(%)
0
100
200
300
400
500
600
700
800
0
20
40
60
80
100
Thermotogae
Deinococcus-Thermus
Aquificae
Spirochaetes
Chlorobi
Bacteroidetes
Acidobacteria
Gemmatimonadetes
Planctomycetes
Fibrobacteres
Nitrospirae
Verrucomicrobia
Chlamydiae
Actinobacteria
Dictyoglomi
Chloroflexi
Synergistetes
Deferribacteres
Chrysiogenetes
Firmicutes
Tenericutes
Fusobacteria
Cyanobacteria
Gammaproteobacteria
Betaproteobacteria
Alphaproteobacteria
Epsilonproteobacteria
Deltaproteobacteria
Magnetococcus
Elusimicrobia
Thermobaculum
Euryarchaeota
Crenarchaeota
Thaumarchaeota
Nanoarchaeota
Korarchaeota

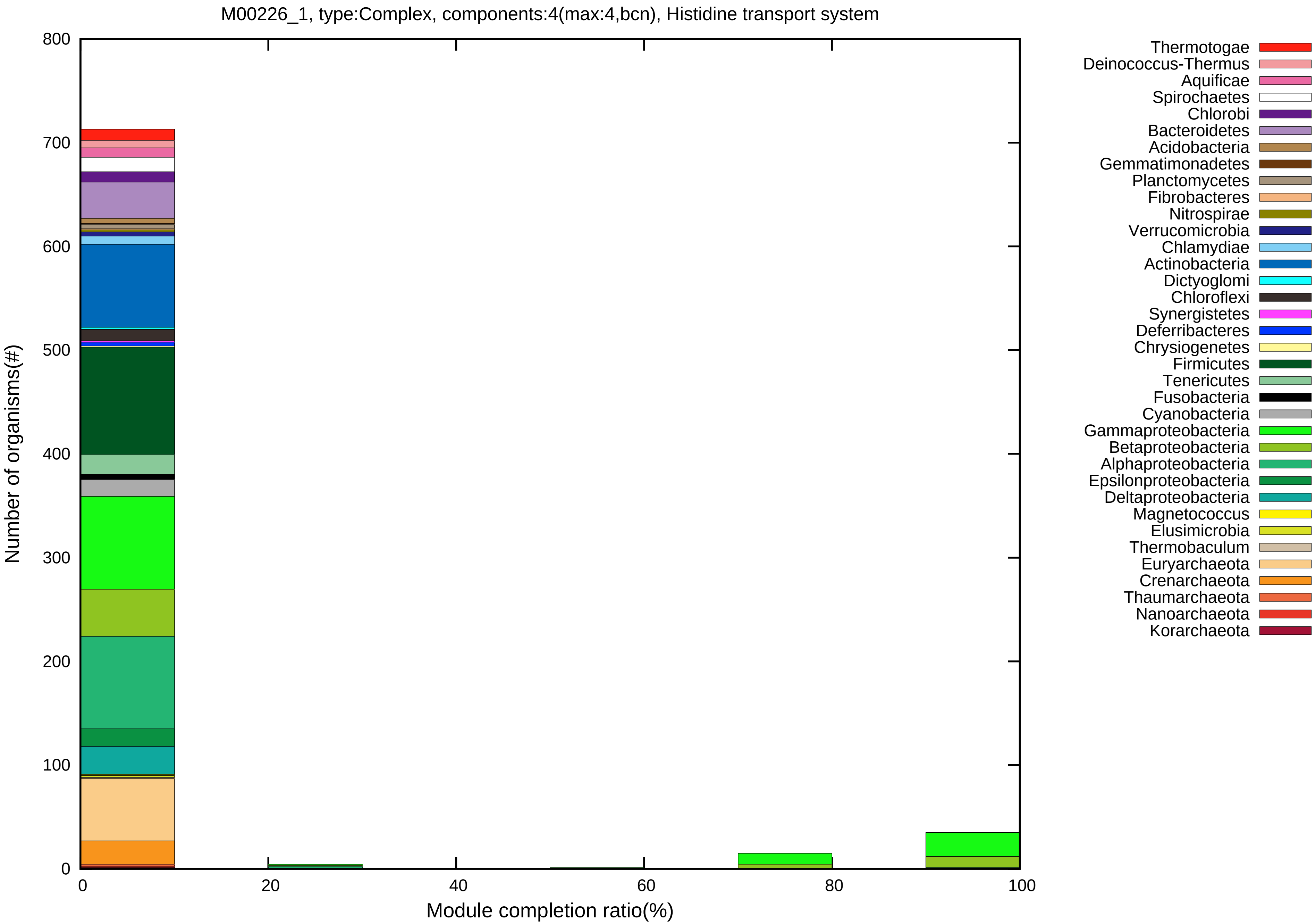

M00226_1, type:Complex, components:4(max:4,bcn), Histidine transport system
Number of organisms(#)
Module completion ratio(%)
0
100
200
300
400
500
600
700
800
0
20
40
60
80
100
Thermotogae
Deinococcus-Thermus
Aquificae
Spirochaetes
Chlorobi
Bacteroidetes
Acidobacteria
Gemmatimonadetes
Planctomycetes
Fibrobacteres
Nitrospirae
Verrucomicrobia
Chlamydiae
Actinobacteria
Dictyoglomi
Chloroflexi
Synergistetes
Deferribacteres
Chrysiogenetes
Firmicutes
Tenericutes
Fusobacteria
Cyanobacteria
Gammaproteobacteria
Betaproteobacteria
Alphaproteobacteria
Epsilonproteobacteria
Deltaproteobacteria
Magnetococcus
Elusimicrobia
Thermobaculum
Euryarchaeota
Crenarchaeota
Thaumarchaeota
Nanoarchaeota
Korarchaeota

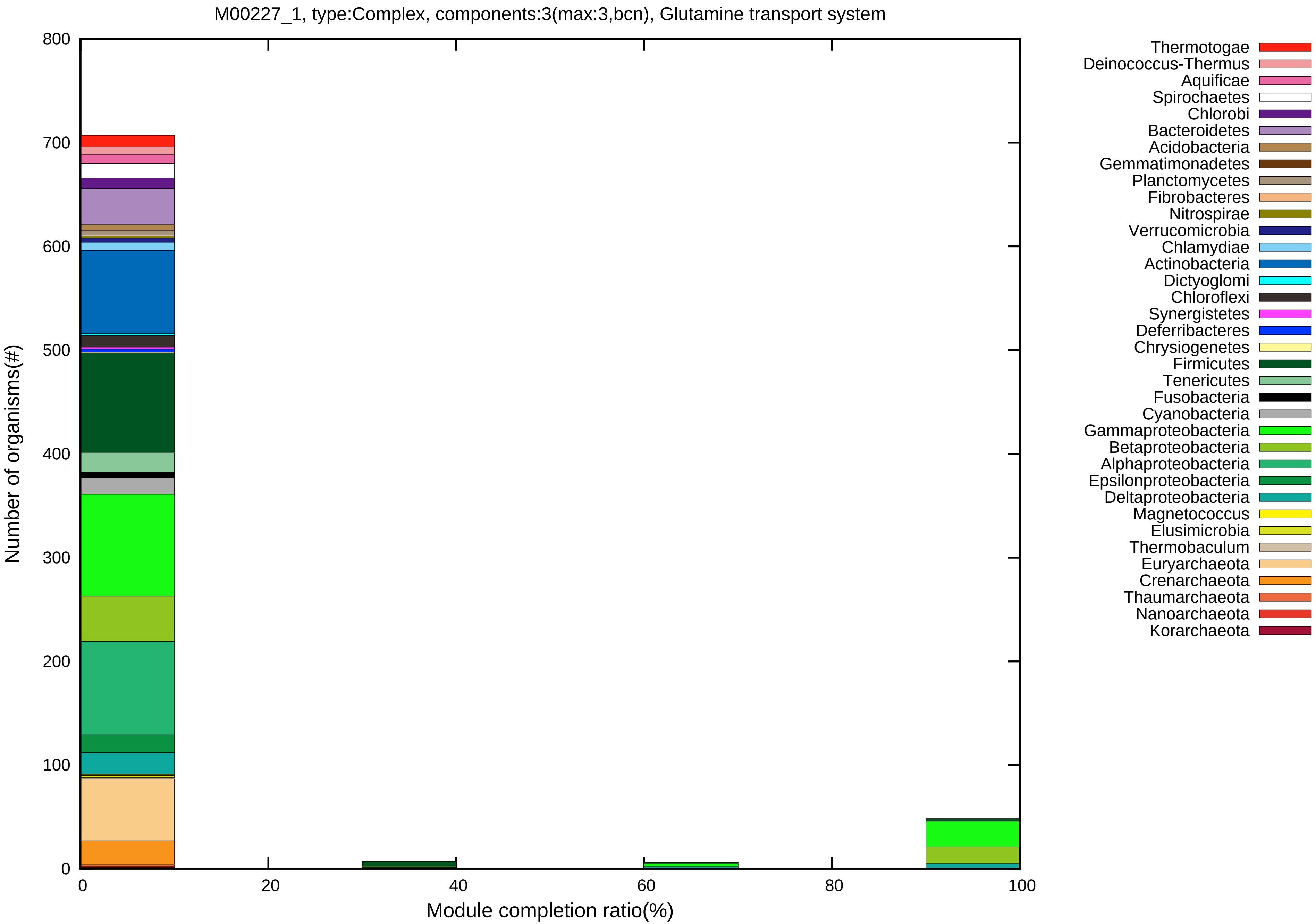
M00227_1, type:Complex, components:3(max:3,bcn), Glutamine transport system
Number of organisms(#)
Module completion ratio(%)
0
100
200
300
400
500
600
700
800
0
20
40
60
80
100
Thermotogae
Deinococcus-Thermus
Aquificae
Spirochaetes
Chlorobi
Bacteroidetes
Acidobacteria
Gemmatimonadetes
Planctomycetes
Fibrobacteres
Nitrospirae
Verrucomicrobia
Chlamydiae
Actinobacteria
Dictyoglomi
Chloroflexi
Synergistetes
Deferribacteres
Chrysiogenetes
Firmicutes
Tenericutes
Fusobacteria
Cyanobacteria
Gammaproteobacteria
Betaproteobacteria
Alphaproteobacteria
Epsilonproteobacteria
Deltaproteobacteria
Magnetococcus
Elusimicrobia
Thermobaculum
Euryarchaeota
Crenarchaeota
Thaumarchaeota
Nanoarchaeota
Korarchaeota

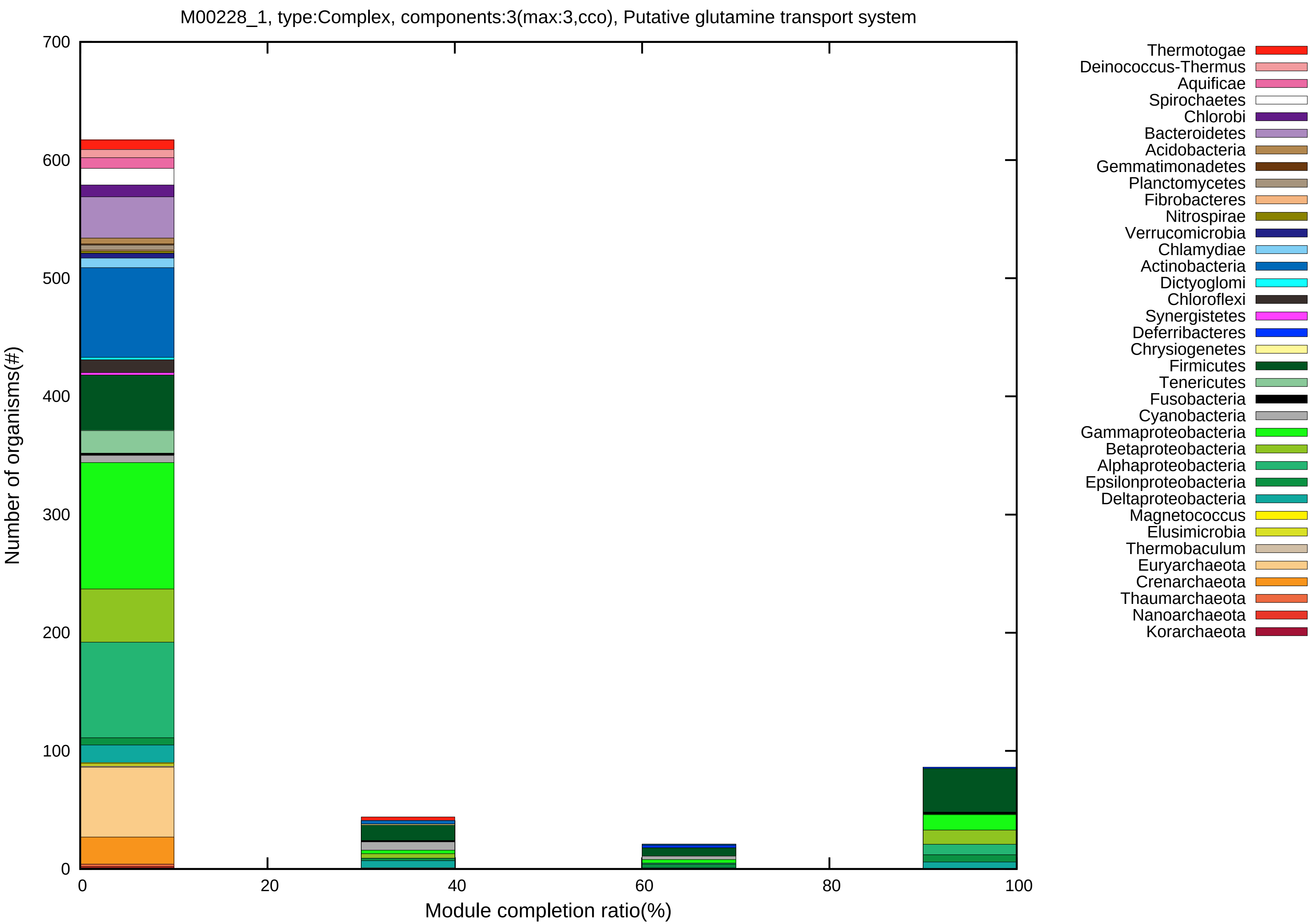
M00228_1, type:Complex, components:3(max:3,cco), Putative glutamine transport system
Number of organisms(#)
0
100
200
300
400
500
600
700
Module completion ratio(%)
0
20
40
60
80
100
Thermotogae
Deinococcus-Thermus
Aquificae
Spirochaetes
Chlorobi
Bacteroidetes
Acidobacteria
Gemmatimonadetes
Planctomycetes
Fibrobacteres
Nitrospirae
Verrucomicrobia
Chlamydiae
Actinobacteria
Dictyoglomi
Chloroflexi
Synergistetes
Deferribacteres
Chrysiogenetes
Firmicutes
Tenericutes
Fusobacteria
Cyanobacteria
Gammaproteobacteria
Betaproteobacteria
Alphaproteobacteria
Epsilonproteobacteria
Deltaproteobacteria
Magnetococcus
Elusimicrobia
Thermobaculum
Euryarchaeota
Crenarchaeota
Thaumarchaeota
Nanoarchaeota
Korarchaeota

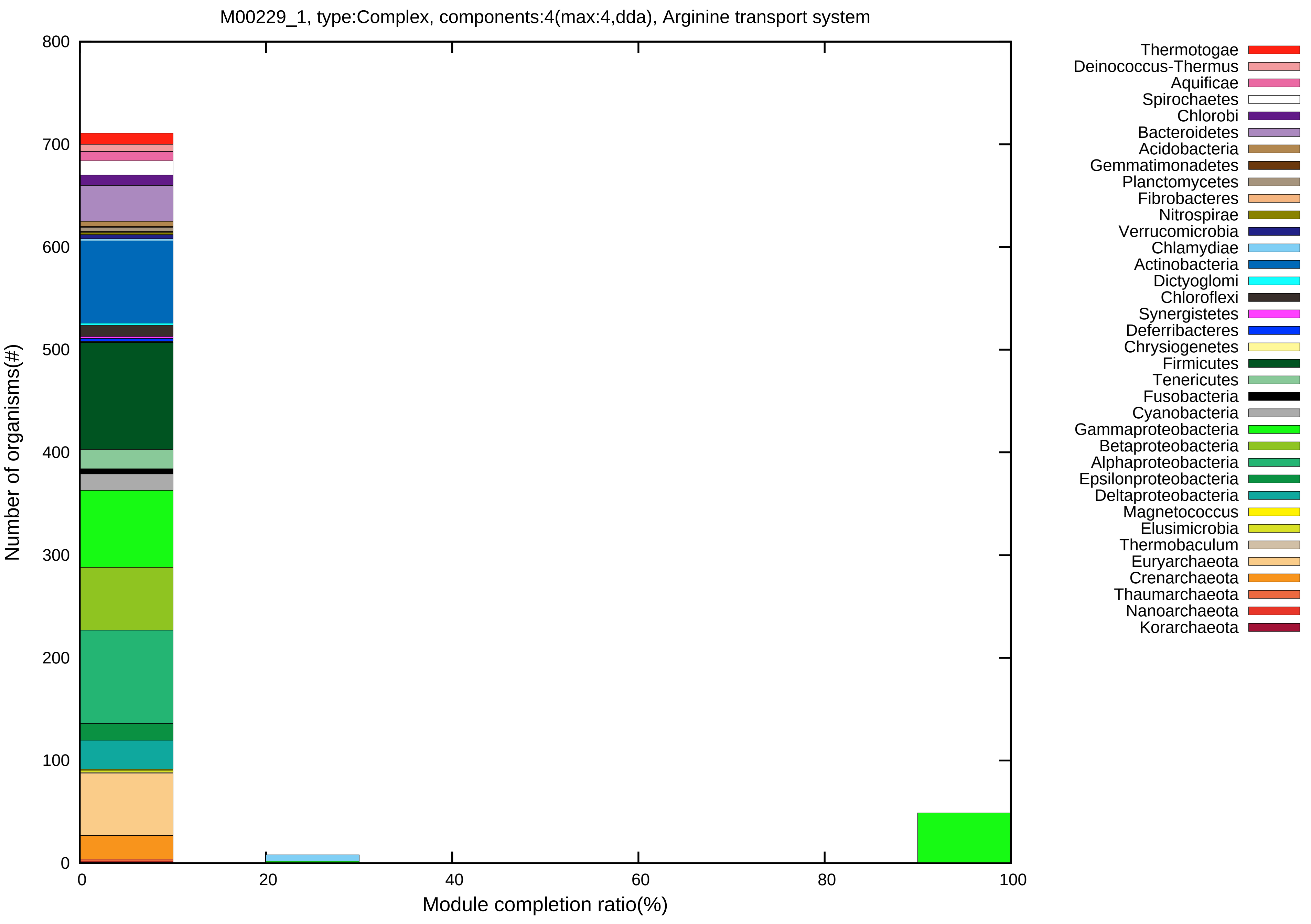
M00229_1, type:Complex, components:4(max:4,dda), Arginine transport system
Number of organisms(#)
Module completion ratio(%)
0
100
200
300
400
500
600
700
800
0
20
40
60
80
100
Thermotogae
Deinococcus-Thermus
Aquificae
Spirochaetes
Chlorobi
Bacteroidetes
Acidobacteria
Gemmatimonadetes
Planctomycetes
Fibrobacteres
Nitrospirae
Verrucomicrobia
Chlamydiae
Actinobacteria
Dictyoglomi
Chloroflexi
Synergistetes
Deferribacteres
Chrysiogenetes
Firmicutes
Tenericutes
Fusobacteria
Cyanobacteria
Gammaproteobacteria
Betaproteobacteria
Alphaproteobacteria
Epsilonproteobacteria
Deltaproteobacteria
Magnetococcus
Elusimicrobia
Thermobaculum
Euryarchaeota
Crenarchaeota
Thaumarchaeota
Nanoarchaeota
Korarchaeota

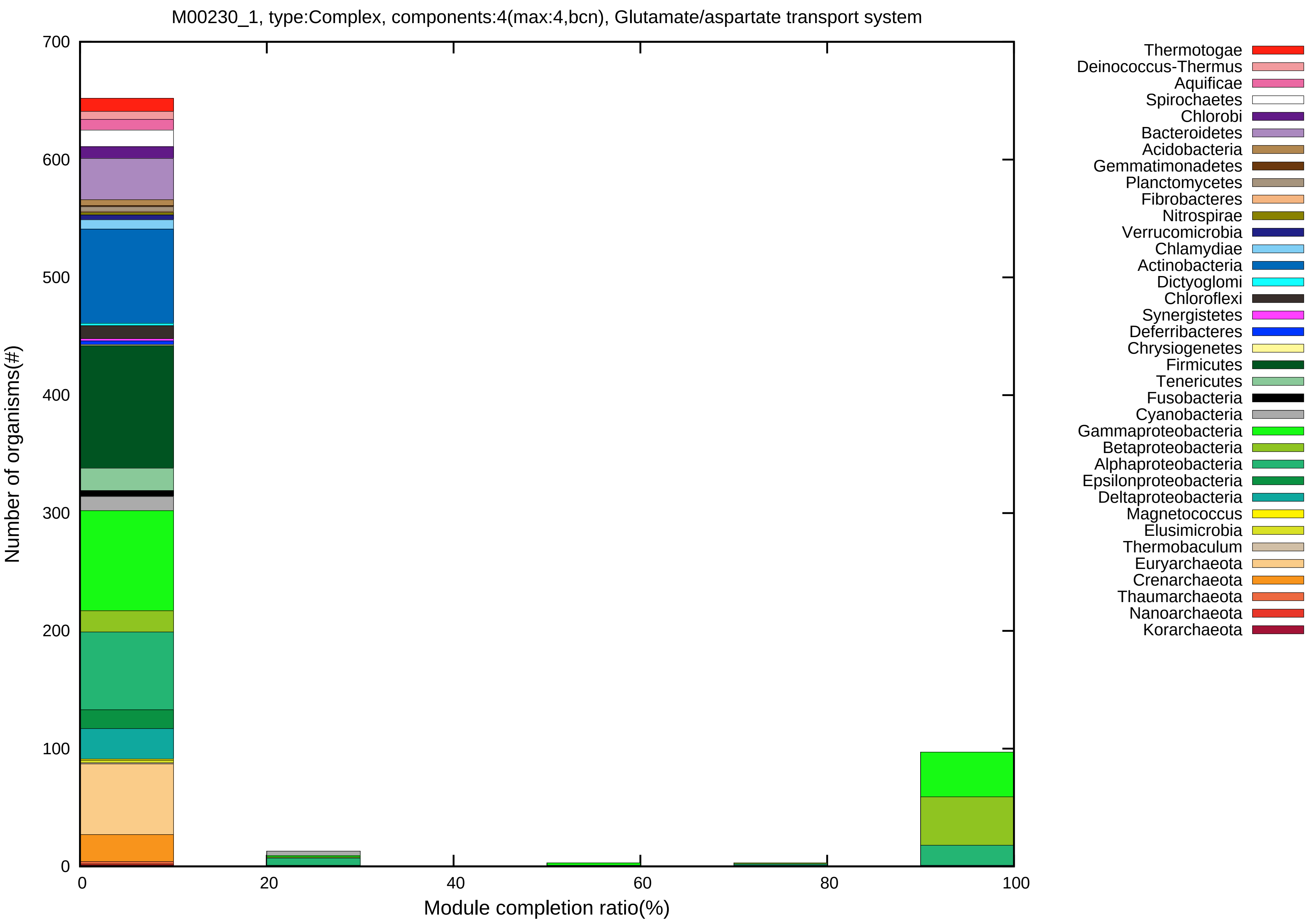
M00230_1, type:Complex, components:4(max:4,bcn), Glutamate/aspartate transport system
Number of organisms(#)
Module completion ratio(%)
0
100
200
300
400
500
600
700
0
20
40
60
80
100
Thermotogae
Deinococcus-Thermus
Aquificae
Spirochaetes
Chlorobi
Bacteroidetes
Acidobacteria
Gemmatimonadetes
Planctomycetes
Fibrobacteres
Nitrospirae
Verrucomicrobia
Chlamydiae
Actinobacteria
Dictyoglomi
Chloroflexi
Synergistetes
Deferribacteres
Chrysiogenetes
Firmicutes
Tenericutes
Fusobacteria
Cyanobacteria
Gammaproteobacteria
Betaproteobacteria
Alphaproteobacteria
Epsilonproteobacteria
Deltaproteobacteria
Magnetococcus
Elusimicrobia
Thermobaculum
Euryarchaeota
Crenarchaeota
Thaumarchaeota
Nanoarchaeota
Korarchaeota

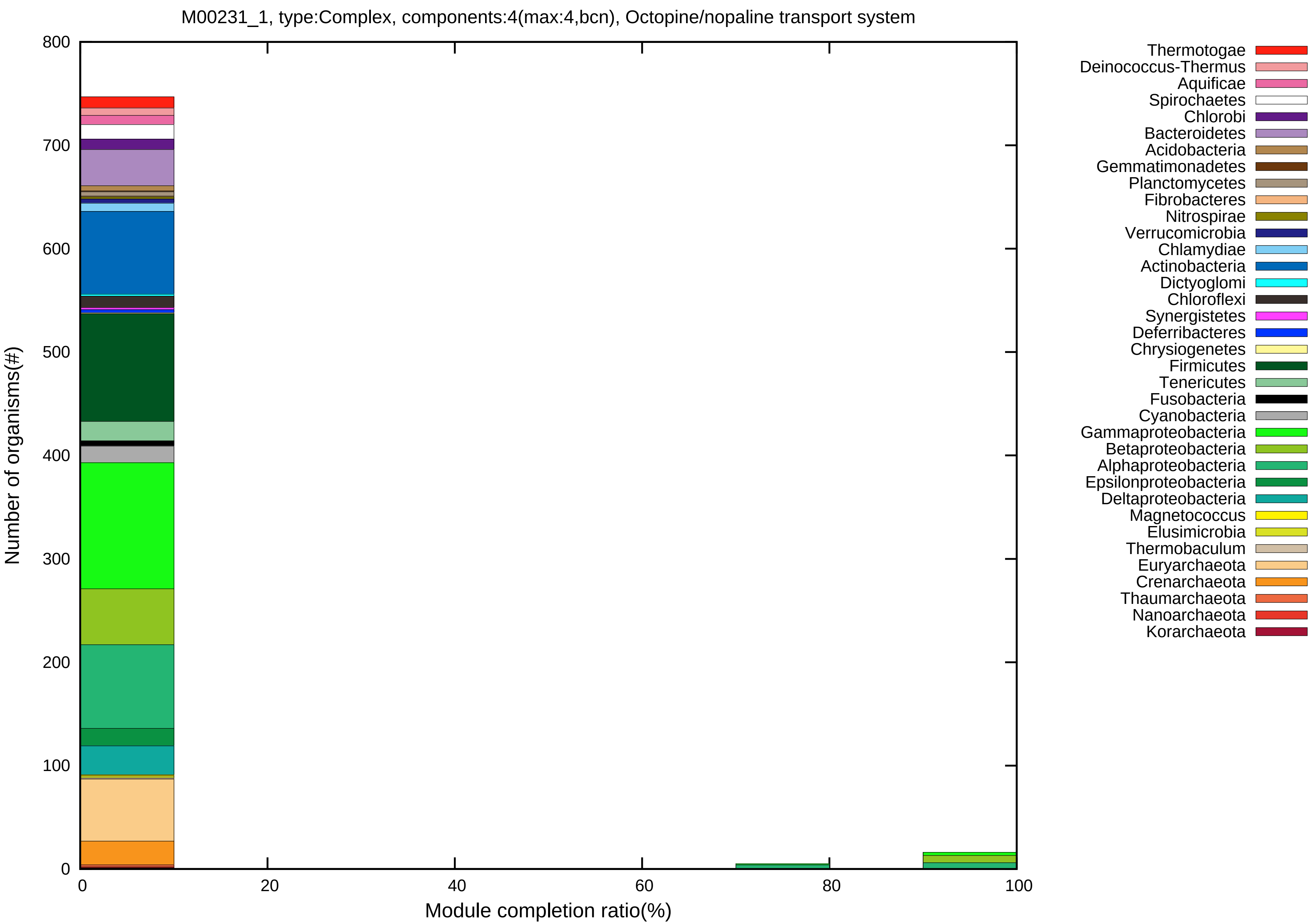

M00231_1, type:Complex, components:4(max:4,bcn), Octopine/nopaline transport system
Number of organisms(#)
Module completion ratio(%)
0
100
200
300
400
500
600
700
800
0
20
40
60
80
100
Thermotogae
Deinococcus-Thermus
Aquificae
Spirochaetes
Chlorobi
Bacteroidetes
Acidobacteria
Gemmatimonadetes
Planctomycetes
Fibrobacteres
Nitrospirae
Verrucomicrobia
Chlamydiae
Actinobacteria
Dictyoglomi
Chloroflexi
Synergistetes
Deferribacteres
Chrysiogenetes
Firmicutes
Tenericutes
Fusobacteria
Cyanobacteria
Gammaproteobacteria
Betaproteobacteria
Alphaproteobacteria
Epsilonproteobacteria
Deltaproteobacteria
Magnetococcus
Elusimicrobia
Thermobaculum
Euryarchaeota
Crenarchaeota
Thaumarchaeota
Nanoarchaeota
Korarchaeota

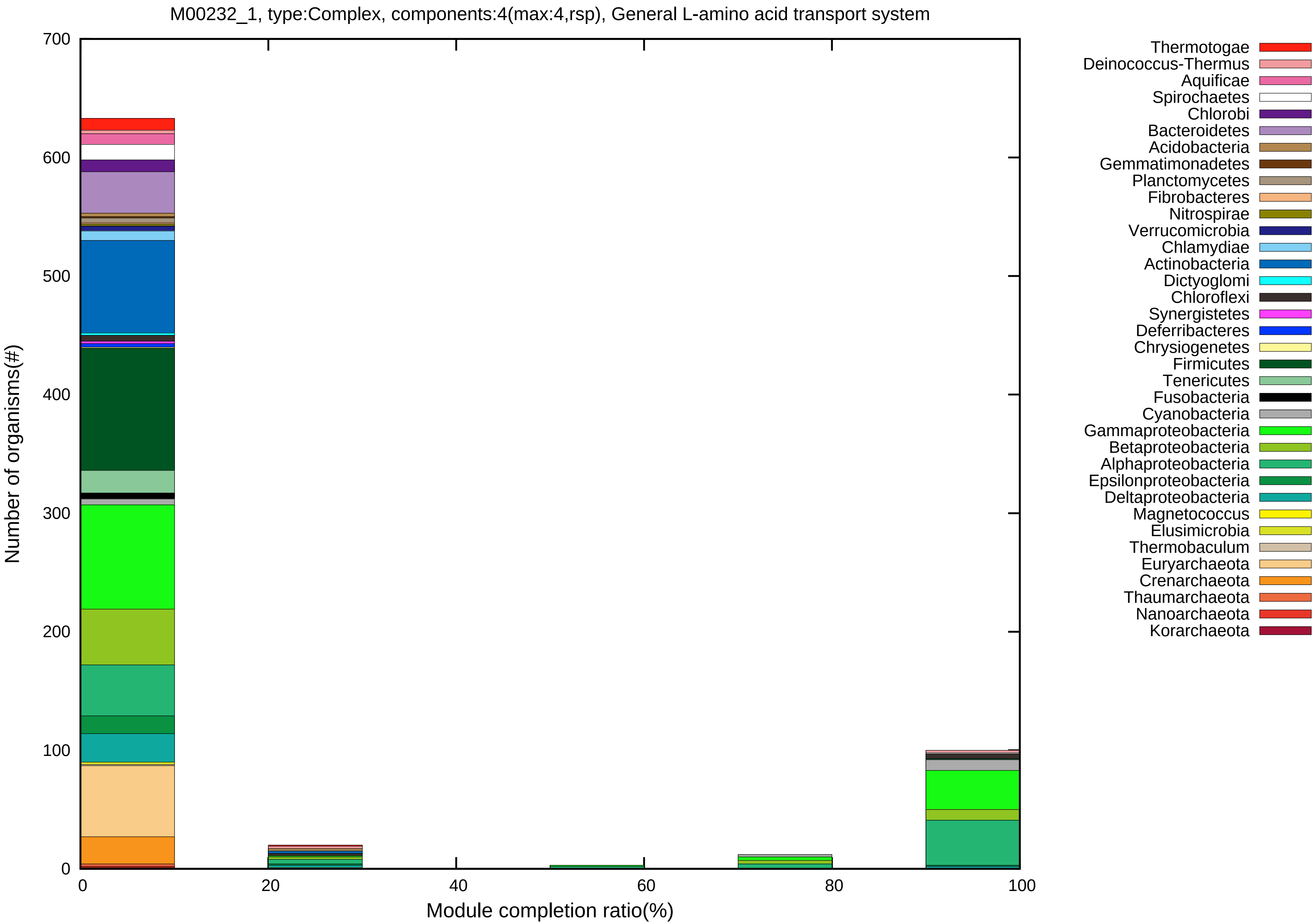

M00232_1, type:Complex, components:4(max:4,rsp), General L-amino acid transport system
Number of organisms(#)
0
100
200
300
400
500
600
700
0
20
40
60
80
100
Module completion ratio(%)
Thermotogae
Deinococcus-Thermus
Aquificae
Spirochaetes
Chlorobi
Bacteroidetes
Acidobacteria
Gemmatimonadetes
Planctomycetes
Fibrobacteres
Nitrospirae
Verrucomicrobia
Chlamydiae
Actinobacteria
Dictyoglomi
Chloroflexi
Synergistetes
Deferribacteres
Chrysiogenetes
Firmicutes
Tenericutes
Fusobacteria
Cyanobacteria
Gammaproteobacteria
Betaproteobacteria
Alphaproteobacteria
Epsilonproteobacteria
Deltaproteobacteria
Magnetococcus
Elusimicrobia
Thermobaculum
Euryarchaeota
Crenarchaeota
Thaumarchaeota
Nanoarchaeota
Korarchaeota

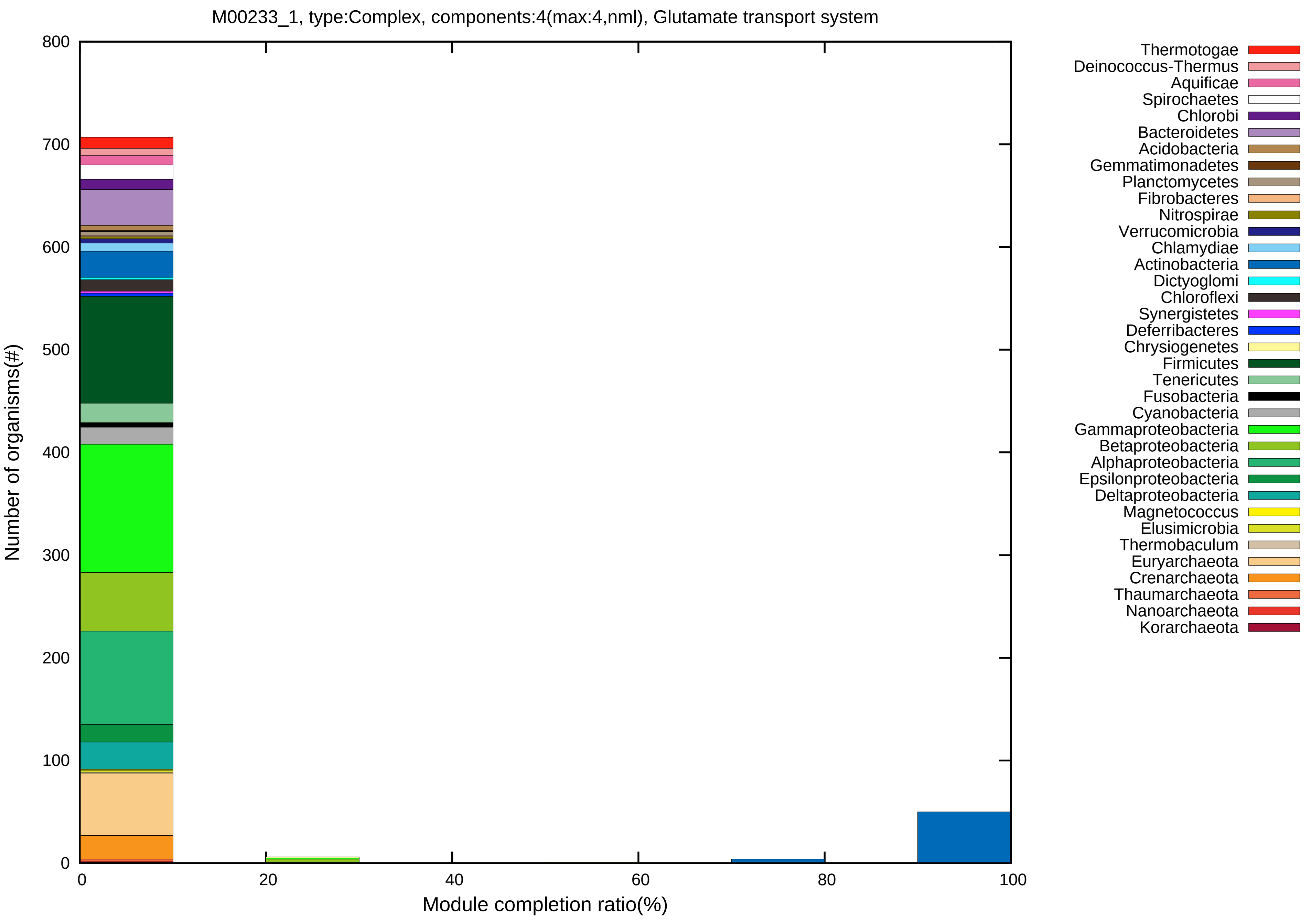

M00233_1, type:Complex, components:4(max:4,nml), Glutamate transport system
Number of organisms(#)
Module completion ratio(%)
0
100
200
300
400
500
600
700
800
0
20
40
60
80
100
Thermotogae
Deinococcus-Thermus
Aquificae
Spirochaetes
Chlorobi
Bacteroidetes
Acidobacteria
Gemmatimonadetes
Planctomycetes
Fibrobacteres
Nitrospirae
Verrucomicrobia
Chlamydiae
Actinobacteria
Dictyoglomi
Chloroflexi
Synergistetes
Deferribacteres
Chrysiogenetes
Firmicutes
Tenericutes
Fusobacteria
Cyanobacteria
Gammaproteobacteria
Betaproteobacteria
Alphaproteobacteria
Epsilonproteobacteria
Deltaproteobacteria
Magnetococcus
Elusimicrobia
Thermobaculum
Euryarchaeota
Crenarchaeota
Thaumarchaeota
Nanoarchaeota
Korarchaeota

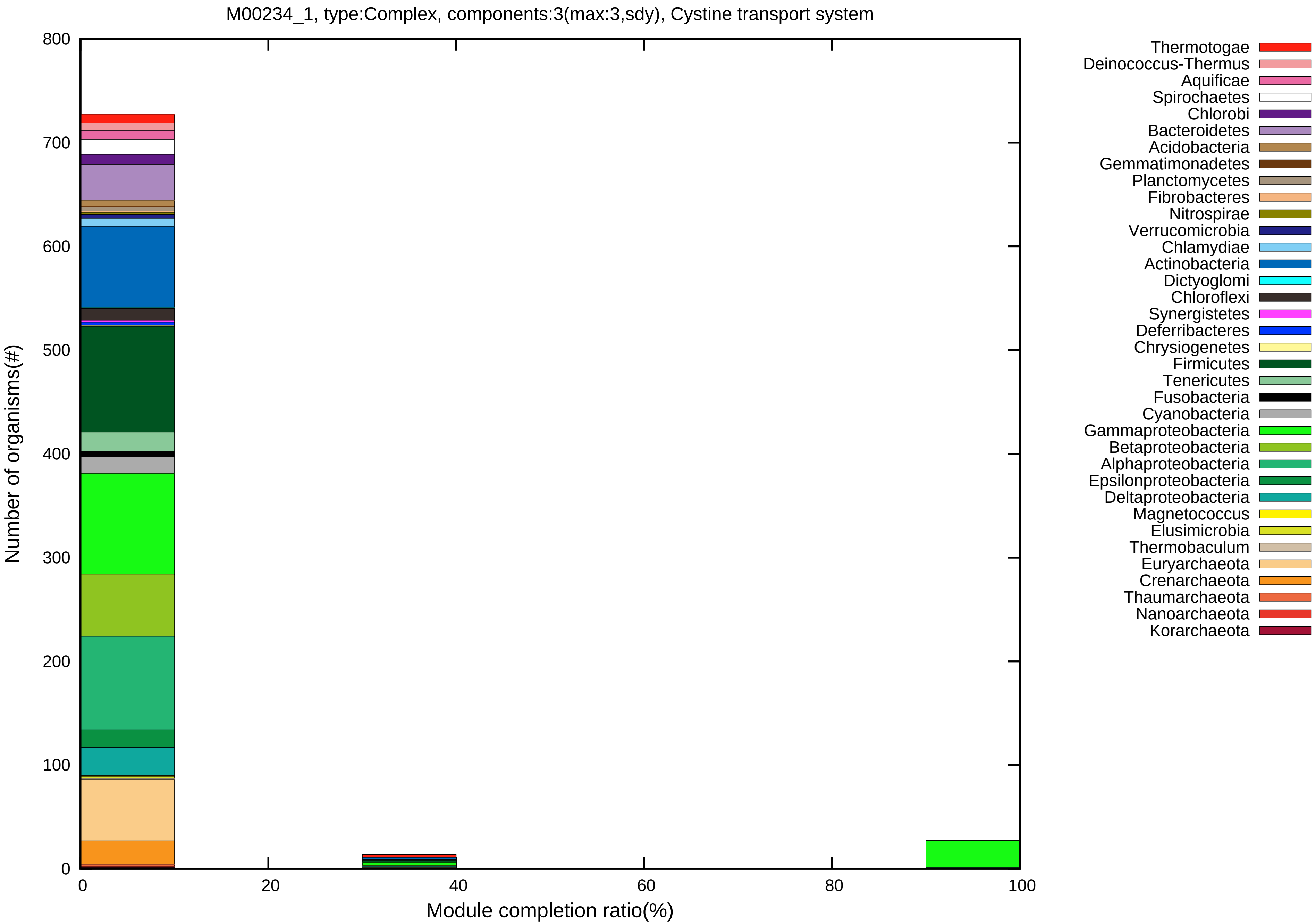

M00234_1, type:Complex, components:3(max:3,sdy), Cystine transport system
Number of organisms(#)
Module completion ratio(%)
0
100
200
300
400
500
600
700
800
0
20
40
60
80
100
Thermotogae
Deinococcus-Thermus
Aquificae
Spirochaetes
Chlorobi
Bacteroidetes
Acidobacteria
Gemmatimonadetes
Planctomycetes
Fibrobacteres
Nitrospirae
Verrucomicrobia
Chlamydiae
Actinobacteria
Dictyoglomi
Chloroflexi
Synergistetes
Deferribacteres
Chrysiogenetes
Firmicutes
Tenericutes
Fusobacteria
Cyanobacteria
Gammaproteobacteria
Betaproteobacteria
Alphaproteobacteria
Epsilonproteobacteria
Deltaproteobacteria
Magnetococcus
Elusimicrobia
Thermobaculum
Euryarchaeota
Crenarchaeota
Thaumarchaeota
Nanoarchaeota
Korarchaeota

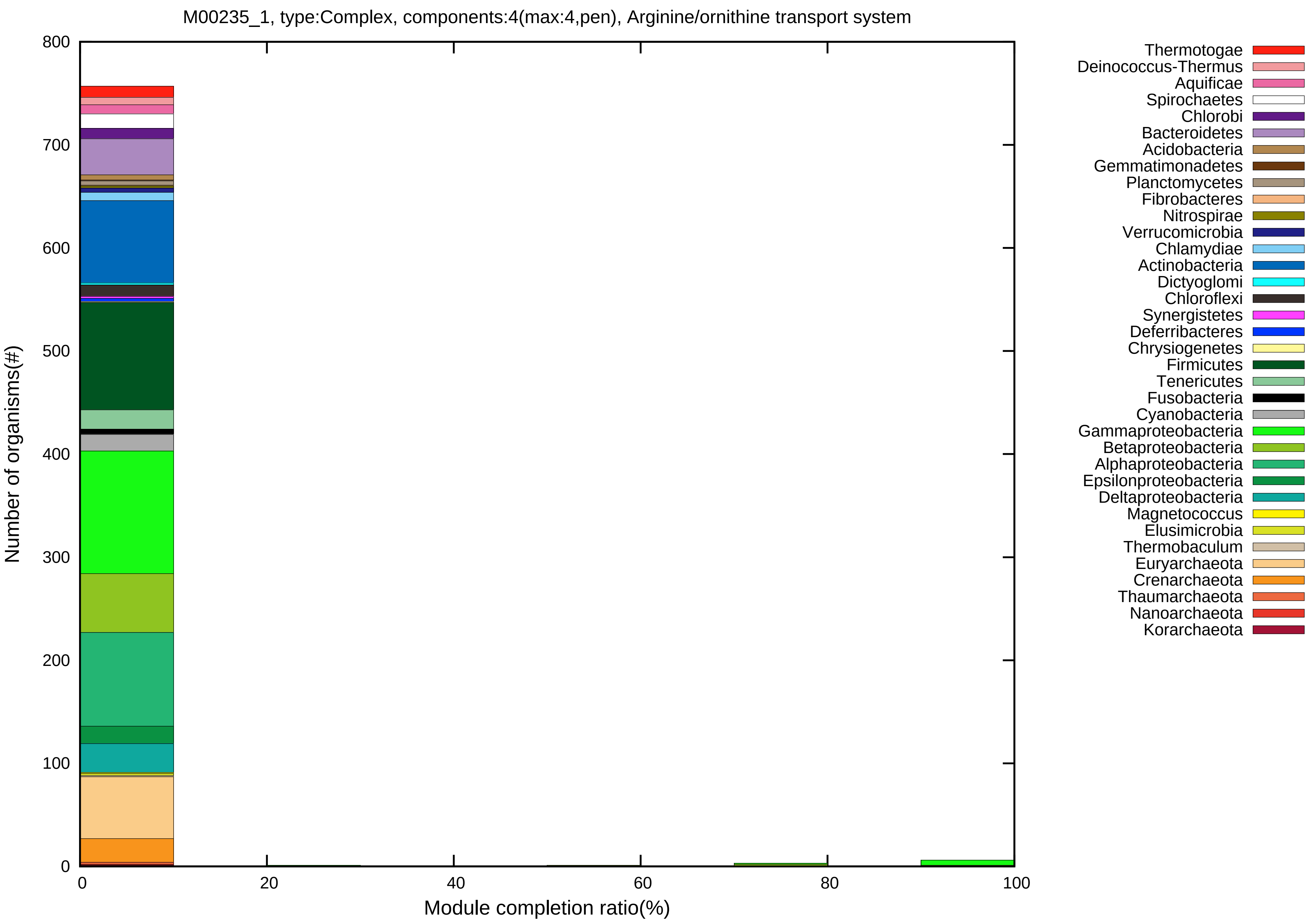
M00235_1, type:Complex, components:4(max:4,pen), Arginine/ornithine transport system
Number of organisms(#)
Module completion ratio(%)
0
100
200
300
400
500
600
700
800
0
20
40
60
80
100
Thermotogae
Deinococcus-Thermus
Aquificae
Spirochaetes
Chlorobi
Bacteroidetes
Acidobacteria
Gemmatimonadetes
Planctomycetes
Fibrobacteres
Nitrospirae
Verrucomicrobia
Chlamydiae
Actinobacteria
Dictyoglomi
Chloroflexi
Synergistetes
Deferribacteres
Chrysiogenetes
Firmicutes
Tenericutes
Fusobacteria
Cyanobacteria
Gammaproteobacteria
Betaproteobacteria
Alphaproteobacteria
Epsilonproteobacteria
Deltaproteobacteria
Magnetococcus
Elusimicrobia
Thermobaculum
Euryarchaeota
Crenarchaeota
Thaumarchaeota
Nanoarchaeota
Korarchaeota

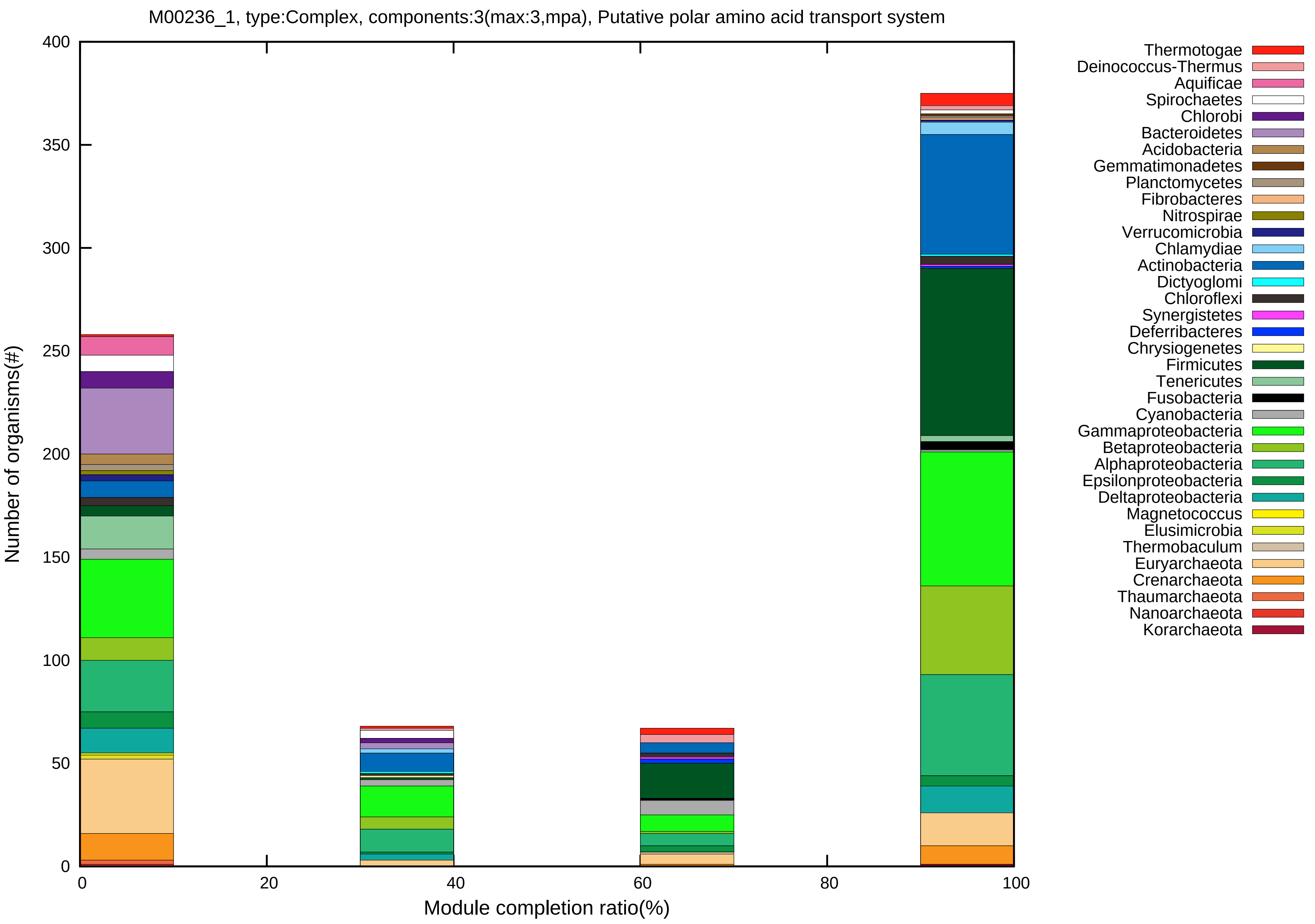

M00236_1, type:Complex, components:3(max:3,mpa), Putative polar amino acid transport system
Number of organisms(#)
Module completion ratio(%)
0
50
100
150
200
250
300
350
400
0
20
40
60
80
100
Thermotogae
Deinococcus-Thermus
Aquificae
Spirochaetes
Chlorobi
Bacteroidetes
Acidobacteria
Gemmatimonadetes
Planctomycetes
Fibrobacteres
Nitrospirae
Verrucomicrobia
Chlamydiae
Actinobacteria
Dictyoglomi
Chloroflexi
Synergistetes
Deferribacteres
Chrysiogenetes
Firmicutes
Tenericutes
Fusobacteria
Cyanobacteria
Gammaproteobacteria
Betaproteobacteria
Alphaproteobacteria
Epsilonproteobacteria
Deltaproteobacteria
Magnetococcus
Elusimicrobia
Thermobaculum
Euryarchaeota
Crenarchaeota
Thaumarchaeota
Nanoarchaeota
Korarchaeota

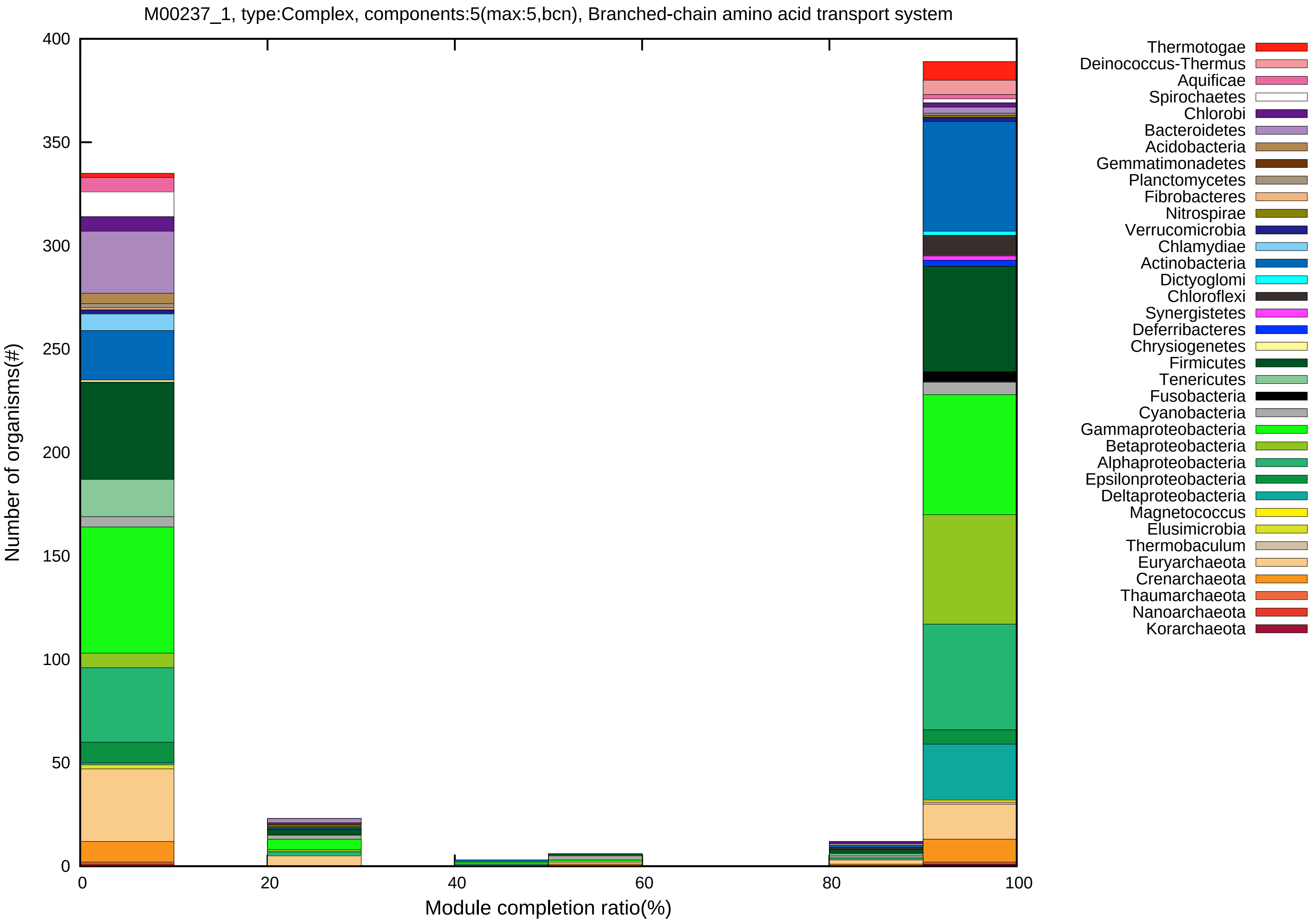

M00237_1, type:Complex, components:5(max:5,bcn), Branched-chain amino acid transport system
Number of organisms(#)
Module completion ratio(%)
0
50
100
150
200
250
300
350
400
0
20
40
60
80
100
Thermotogae
Deinococcus-Thermus
Aquificae
Spirochaetes
Chlorobi
Bacteroidetes
Acidobacteria
Gemmatimonadetes
Planctomycetes
Fibrobacteres
Nitrospirae
Verrucomicrobia
Chlamydiae
Actinobacteria
Dictyoglomi
Chloroflexi
Synergistetes
Deferribacteres
Chrysiogenetes
Firmicutes
Tenericutes
Fusobacteria
Cyanobacteria
Gammaproteobacteria
Betaproteobacteria
Alphaproteobacteria
Epsilonproteobacteria
Deltaproteobacteria
Magnetococcus
Elusimicrobia
Thermobaculum
Euryarchaeota
Crenarchaeota
Thaumarchaeota
Nanoarchaeota
Korarchaeota

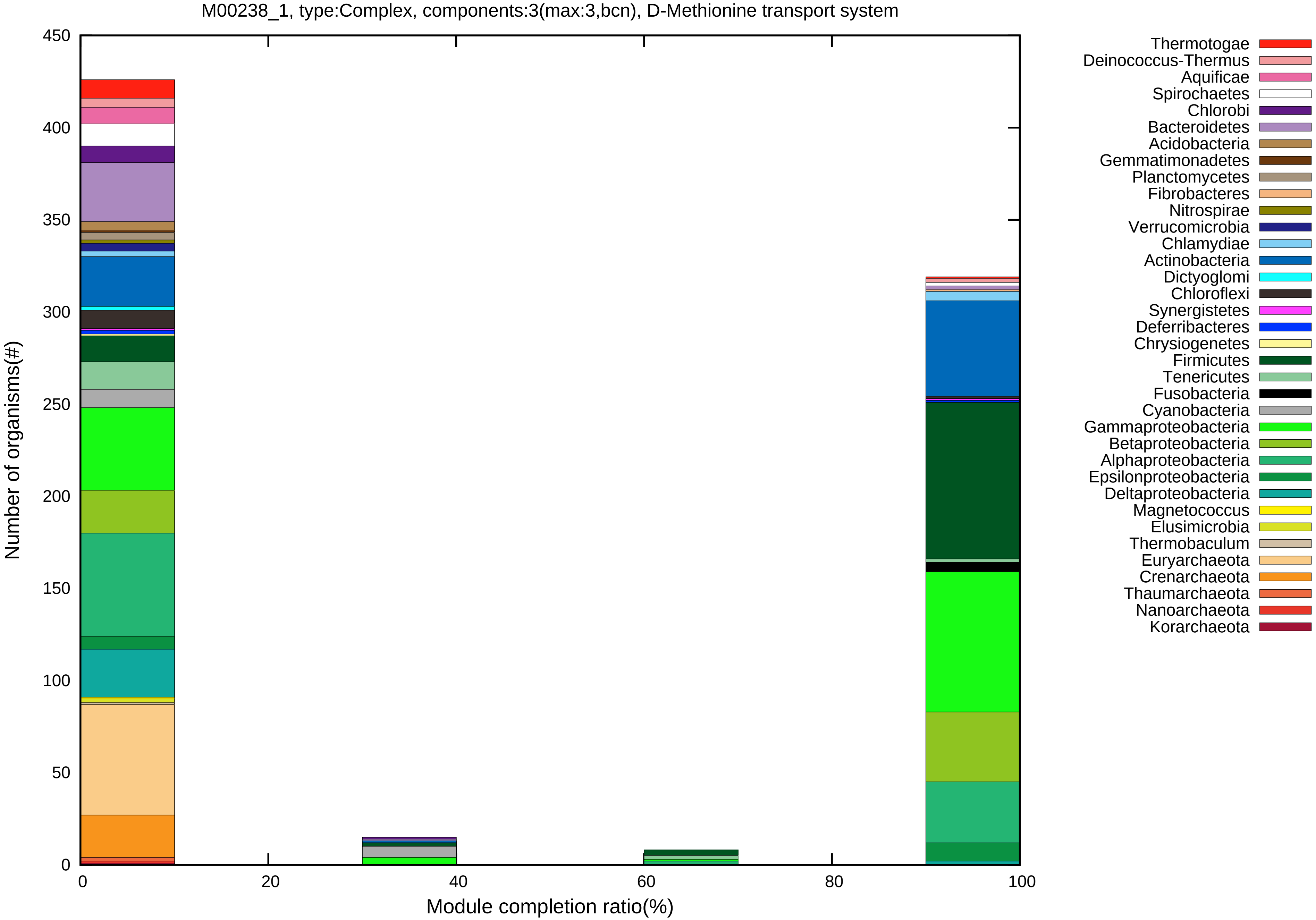

M00238_1, type:Complex, components:3(max:3,bcn), D-Methionine transport system
Number of organisms(#)
Module completion ratio(%)
0
50
100
150
200
250
300
350
400
450
0
20
40
60
80
100
Thermotogae
Deinococcus-Thermus
Aquificae
Spirochaetes
Chlorobi
Bacteroidetes
Acidobacteria
Gemmatimonadetes
Planctomycetes
Fibrobacteres
Nitrospirae
Verrucomicrobia
Chlamydiae
Actinobacteria
Dictyoglomi
Chloroflexi
Synergistetes
Deferribacteres
Chrysiogenetes
Firmicutes
Tenericutes
Fusobacteria
Cyanobacteria
Gammaproteobacteria
Betaproteobacteria
Alphaproteobacteria
Epsilonproteobacteria
Deltaproteobacteria
Magnetococcus
Elusimicrobia
Thermobaculum
Euryarchaeota
Crenarchaeota
Thaumarchaeota
Nanoarchaeota
Korarchaeota

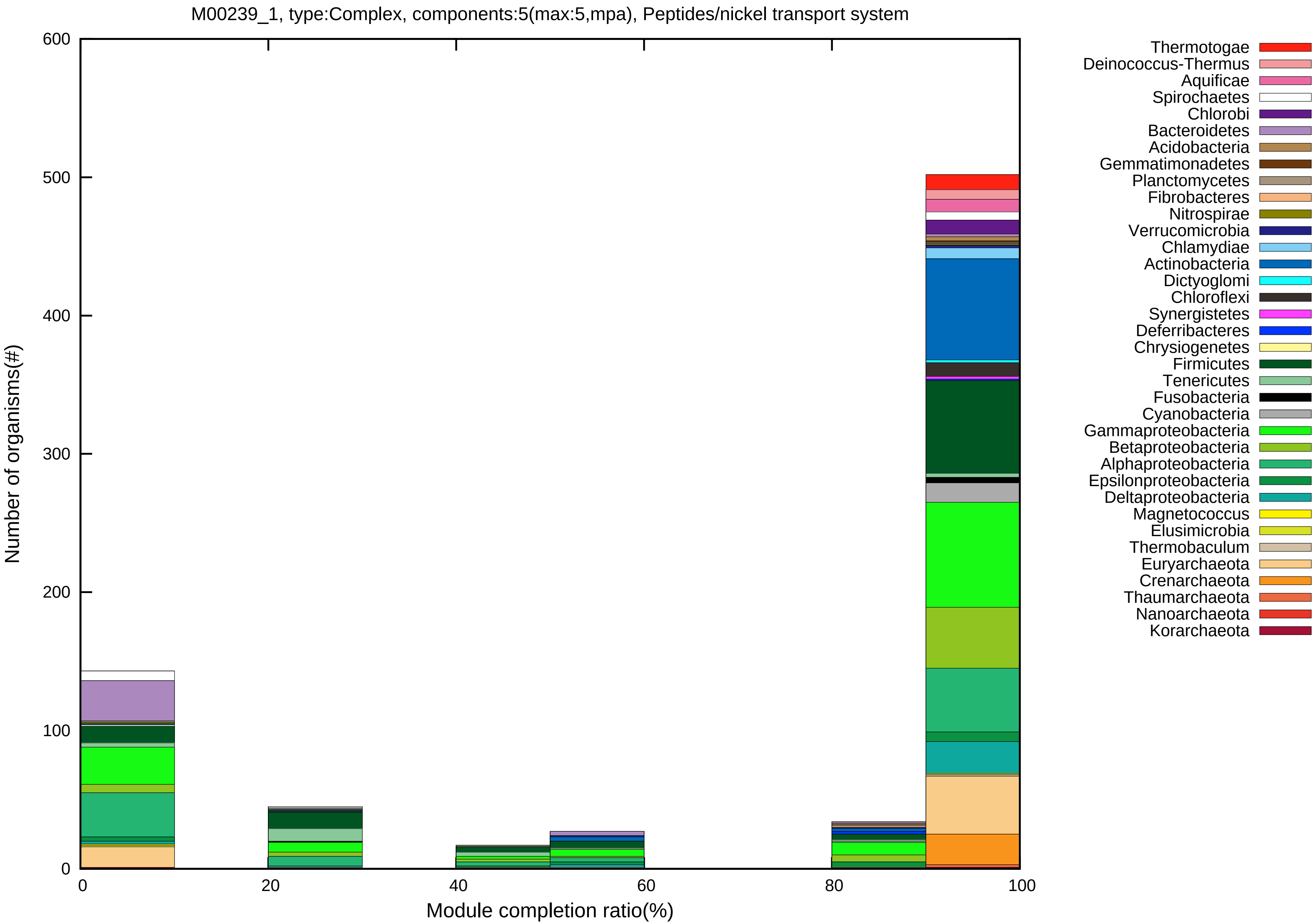

M00239_1, type:Complex, components:5(max:5,mpa), Peptides/nickel transport system
Number of organisms(#)
0
100
200
300
400
500
600
0
20
40
60
80
100
Module completion ratio(%)
Thermotogae
Deinococcus-Thermus
Aquificae
Spirochaetes
Chlorobi
Bacteroidetes
Acidobacteria
Gemmatimonadetes
Planctomycetes
Fibrobacteres
Nitrospirae
Verrucomicrobia
Chlamydiae
Actinobacteria
Dictyoglomi
Chloroflexi
Synergistetes
Deferribacteres
Chrysiogenetes
Firmicutes
Tenericutes
Fusobacteria
Cyanobacteria
Gammaproteobacteria
Betaproteobacteria
Alphaproteobacteria
Epsilonproteobacteria
Deltaproteobacteria
Magnetococcus
Elusimicrobia
Thermobaculum
Euryarchaeota
Crenarchaeota
Thaumarchaeota
Nanoarchaeota
Korarchaeota

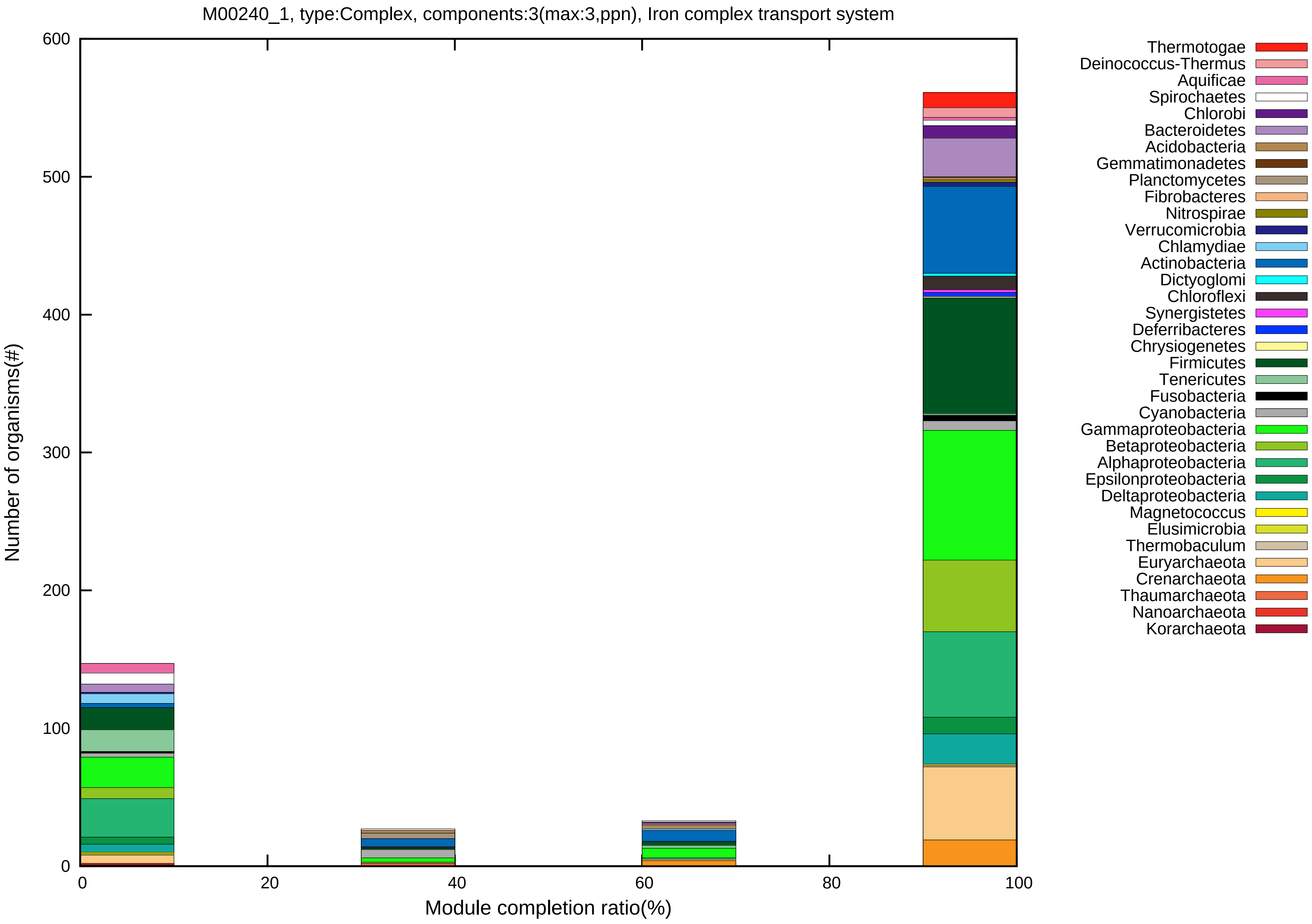
M00240_1, type:Complex, components:3(max:3,ppn), Iron complex transport system
Number of organisms(#)
Module completion ratio(%)
0
100
200
300
400
500
600
0
20
40
60
80
100
Thermotogae
Deinococcus-Thermus
Aquificae
Spirochaetes
Chlorobi
Bacteroidetes
Acidobacteria
Gemmatimonadetes
Planctomycetes
Fibrobacteres
Nitrospirae
Verrucomicrobia
Chlamydiae
Actinobacteria
Dictyoglomi
Chloroflexi
Synergistetes
Deferribacteres
Chrysiogenetes
Firmicutes
Tenericutes
Fusobacteria
Cyanobacteria
Gammaproteobacteria
Betaproteobacteria
Alphaproteobacteria
Epsilonproteobacteria
Deltaproteobacteria
Magnetococcus
Elusimicrobia
Thermobaculum
Euryarchaeota
Crenarchaeota
Thaumarchaeota
Nanoarchaeota
Korarchaeota

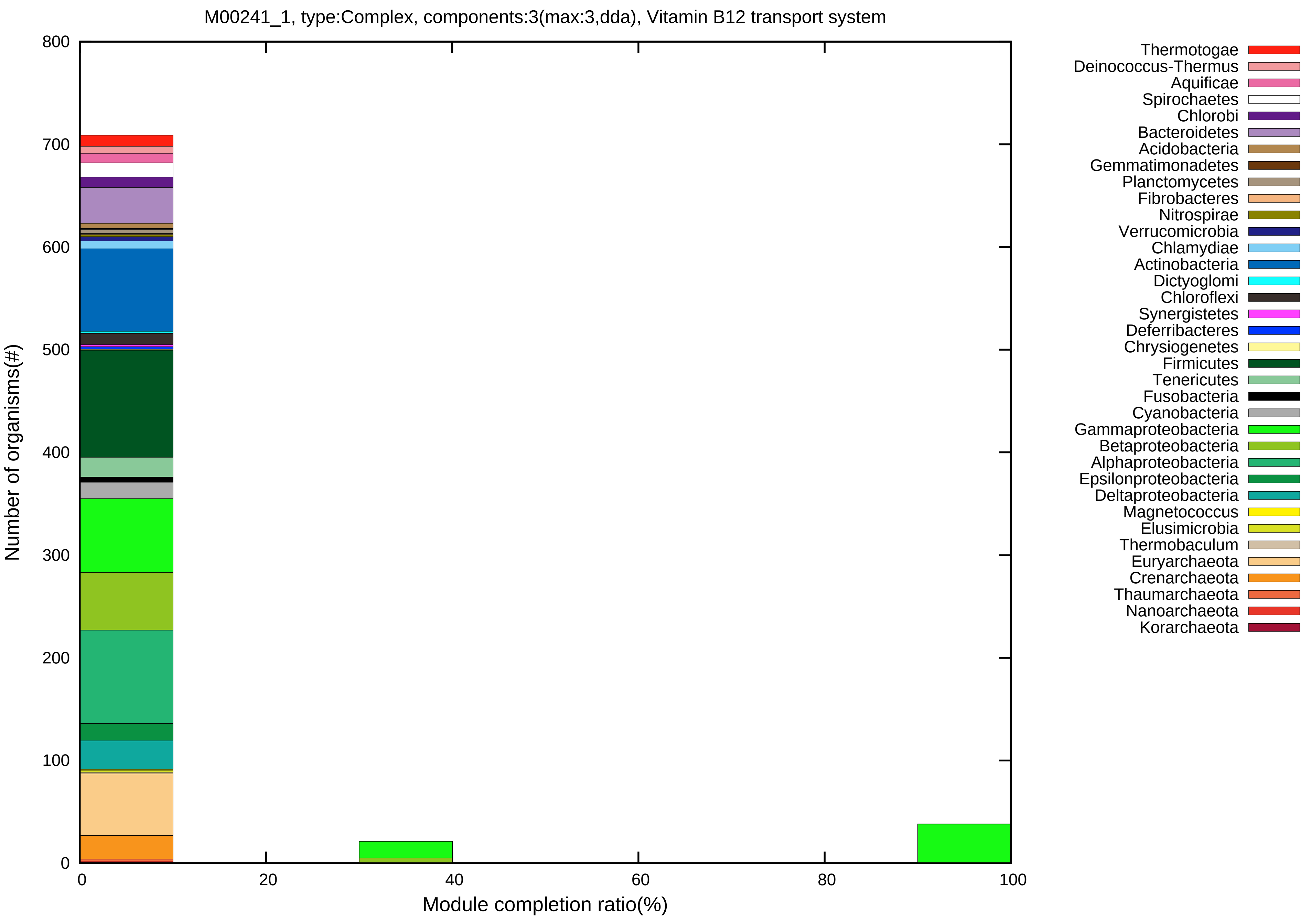
M00241_1, type:Complex, components:3(max:3,dda), Vitamin B12 transport system
Number of organisms(#)
Module completion ratio(%)
0
100
200
300
400
500
600
700
800
0
20
40
60
80
100
Thermotogae
Deinococcus-Thermus
Aquificae
Spirochaetes
Chlorobi
Bacteroidetes
Acidobacteria
Gemmatimonadetes
Planctomycetes
Fibrobacteres
Nitrospirae
Verrucomicrobia
Chlamydiae
Actinobacteria
Dictyoglomi
Chloroflexi
Synergistetes
Deferribacteres
Chrysiogenetes
Firmicutes
Tenericutes
Fusobacteria
Cyanobacteria
Gammaproteobacteria
Betaproteobacteria
Alphaproteobacteria
Epsilonproteobacteria
Deltaproteobacteria
Magnetococcus
Elusimicrobia
Thermobaculum
Euryarchaeota
Crenarchaeota
Thaumarchaeota
Nanoarchaeota
Korarchaeota

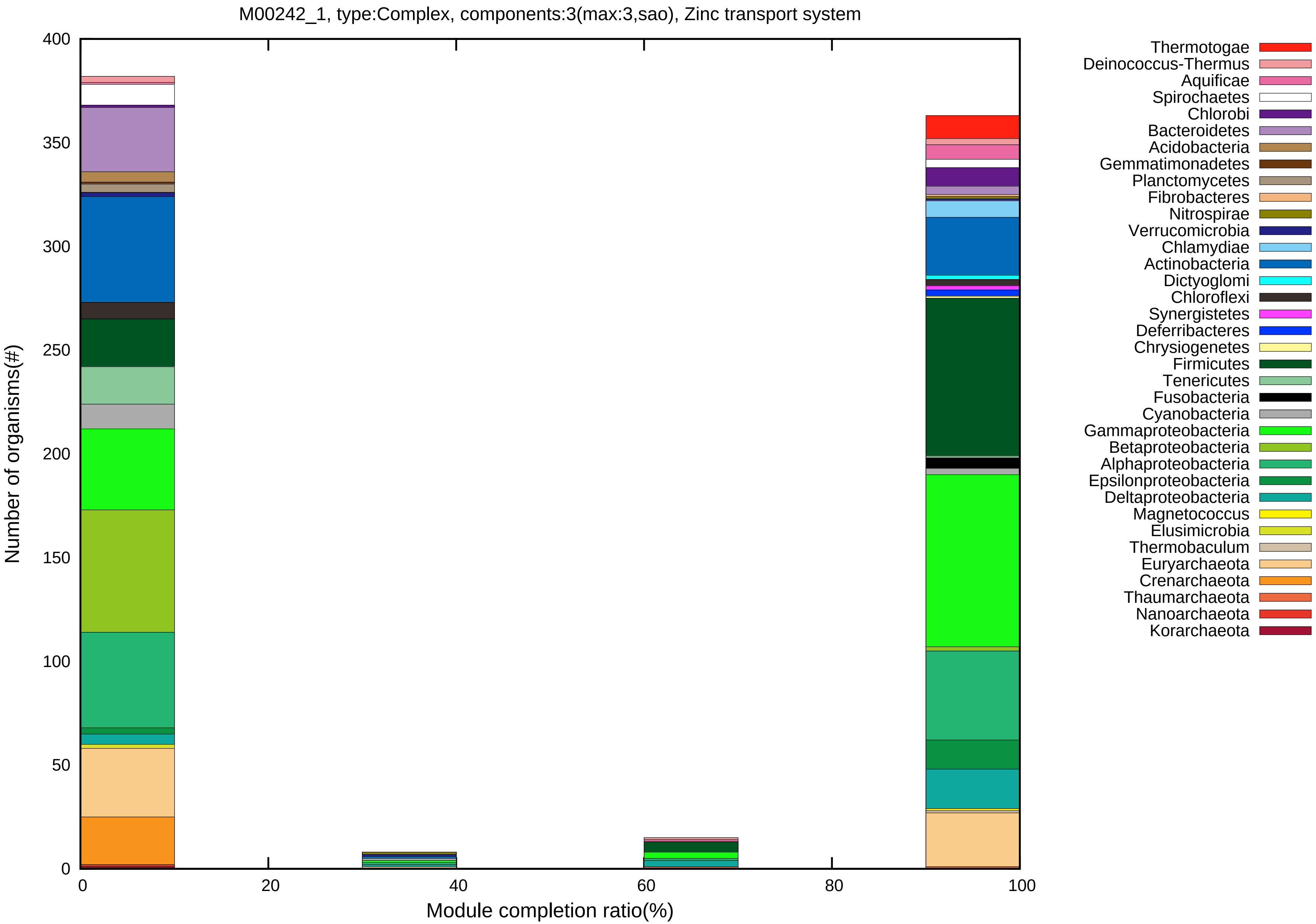

M00242_1, type:Complex, components:3(max:3,sao), Zinc transport system
Number of organisms(#)
Module completion ratio(%)
0
50
100
150
200
250
300
350
400
0
20
40
60
80
100
Thermotogae
Deinococcus-Thermus
Aquificae
Spirochaetes
Chlorobi
Bacteroidetes
Acidobacteria
Gemmatimonadetes
Planctomycetes
Fibrobacteres
Nitrospirae
Verrucomicrobia
Chlamydiae
Actinobacteria
Dictyoglomi
Chloroflexi
Synergistetes
Deferribacteres
Chrysiogenetes
Firmicutes
Tenericutes
Fusobacteria
Cyanobacteria
Gammaproteobacteria
Betaproteobacteria
Alphaproteobacteria
Epsilonproteobacteria
Deltaproteobacteria
Magnetococcus
Elusimicrobia
Thermobaculum
Euryarchaeota
Crenarchaeota
Thaumarchaeota
Nanoarchaeota
Korarchaeota

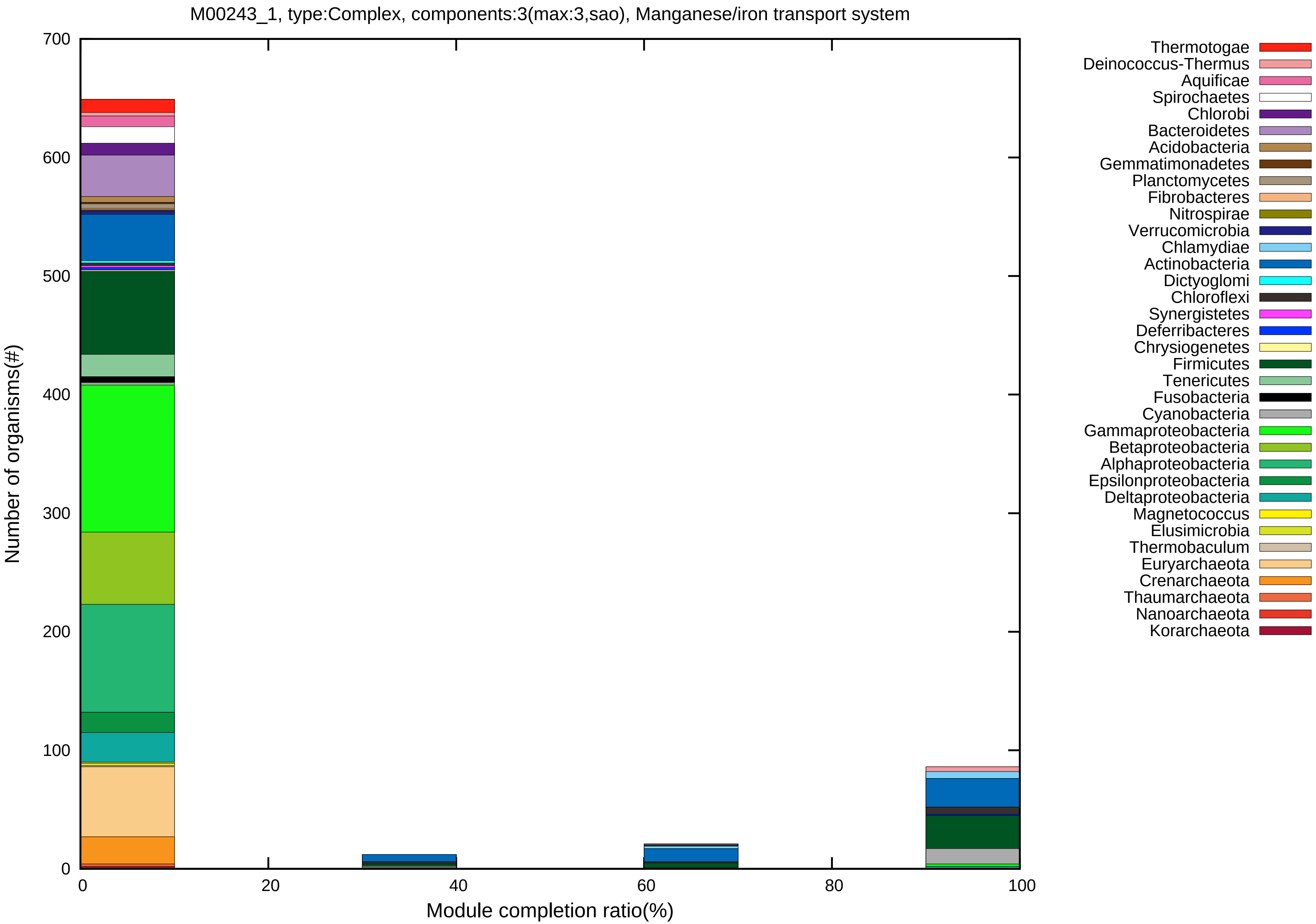

M00243_1, type:Complex, components:3(max:3,sao), Manganese/iron transport system
Number of organisms(#)
Module completion ratio(%)
0
100
200
300
400
500
600
700
0
20
40
60
80
100
Thermotogae
Deinococcus-Thermus
Aquificae
Spirochaetes
Chlorobi
Bacteroidetes
Acidobacteria
Gemmatimonadetes
Planctomycetes
Fibrobacteres
Nitrospirae
Verrucomicrobia
Chlamydiae
Actinobacteria
Dictyoglomi
Chloroflexi
Synergistetes
Deferribacteres
Chrysiogenetes
Firmicutes
Tenericutes
Fusobacteria
Cyanobacteria
Gammaproteobacteria
Betaproteobacteria
Alphaproteobacteria
Epsilonproteobacteria
Deltaproteobacteria
Magnetococcus
Elusimicrobia
Thermobaculum
Euryarchaeota
Crenarchaeota
Thaumarchaeota
Nanoarchaeota
Korarchaeota

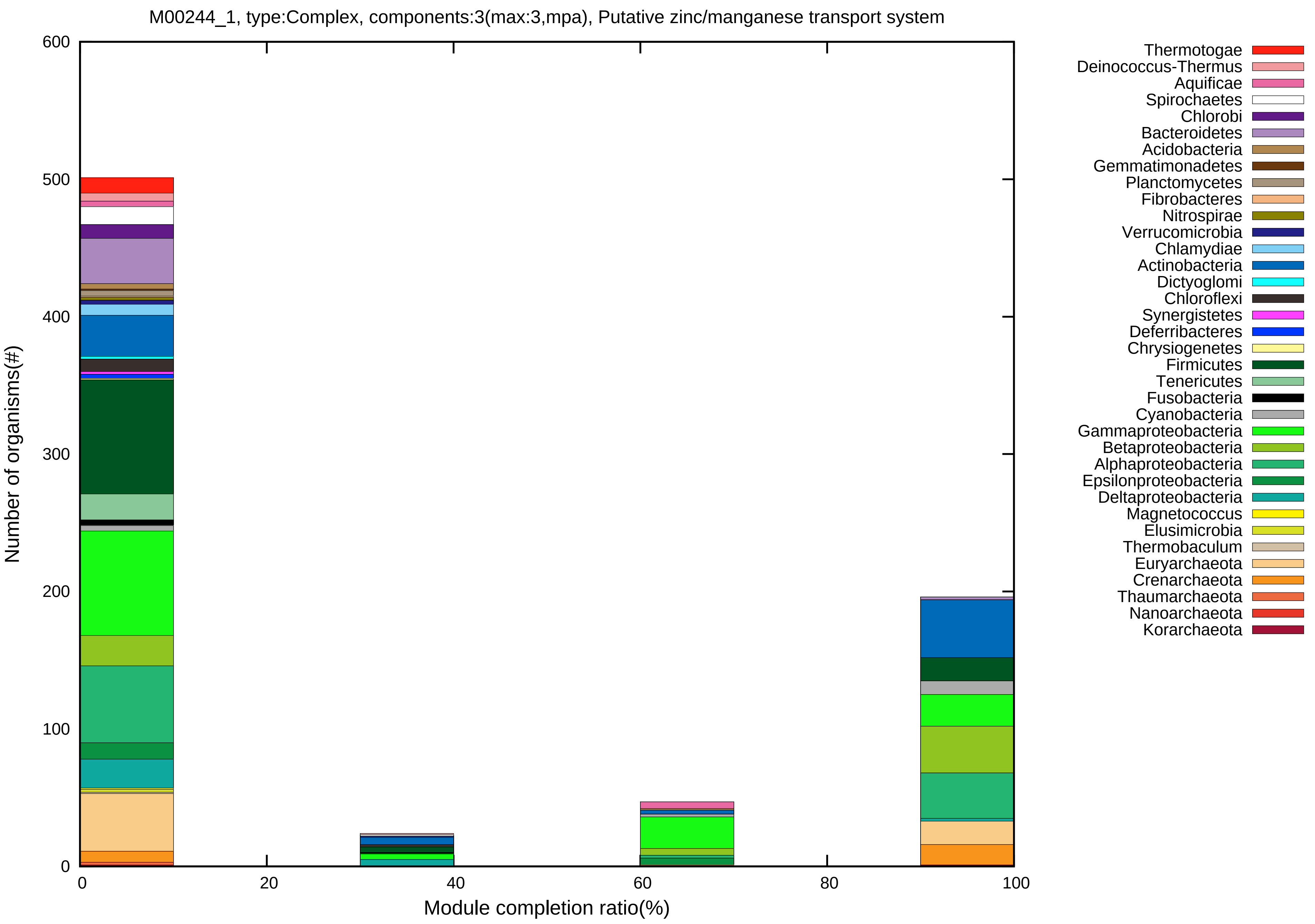

M00244_1, type:Complex, components:3(max:3,mpa), Putative zinc/manganese transport system
Number of organisms(#)
Module completion ratio(%)
0
100
200
300
400
500
600
0
20
40
60
80
100
Thermotogae
Deinococcus-Thermus
Aquificae
Spirochaetes
Chlorobi
Bacteroidetes
Acidobacteria
Gemmatimonadetes
Planctomycetes
Fibrobacteres
Nitrospirae
Verrucomicrobia
Chlamydiae
Actinobacteria
Dictyoglomi
Chloroflexi
Synergistetes
Deferribacteres
Chrysiogenetes
Firmicutes
Tenericutes
Fusobacteria
Cyanobacteria
Gammaproteobacteria
Betaproteobacteria
Alphaproteobacteria
Epsilonproteobacteria
Deltaproteobacteria
Magnetococcus
Elusimicrobia
Thermobaculum
Euryarchaeota
Crenarchaeota
Thaumarchaeota
Nanoarchaeota
Korarchaeota

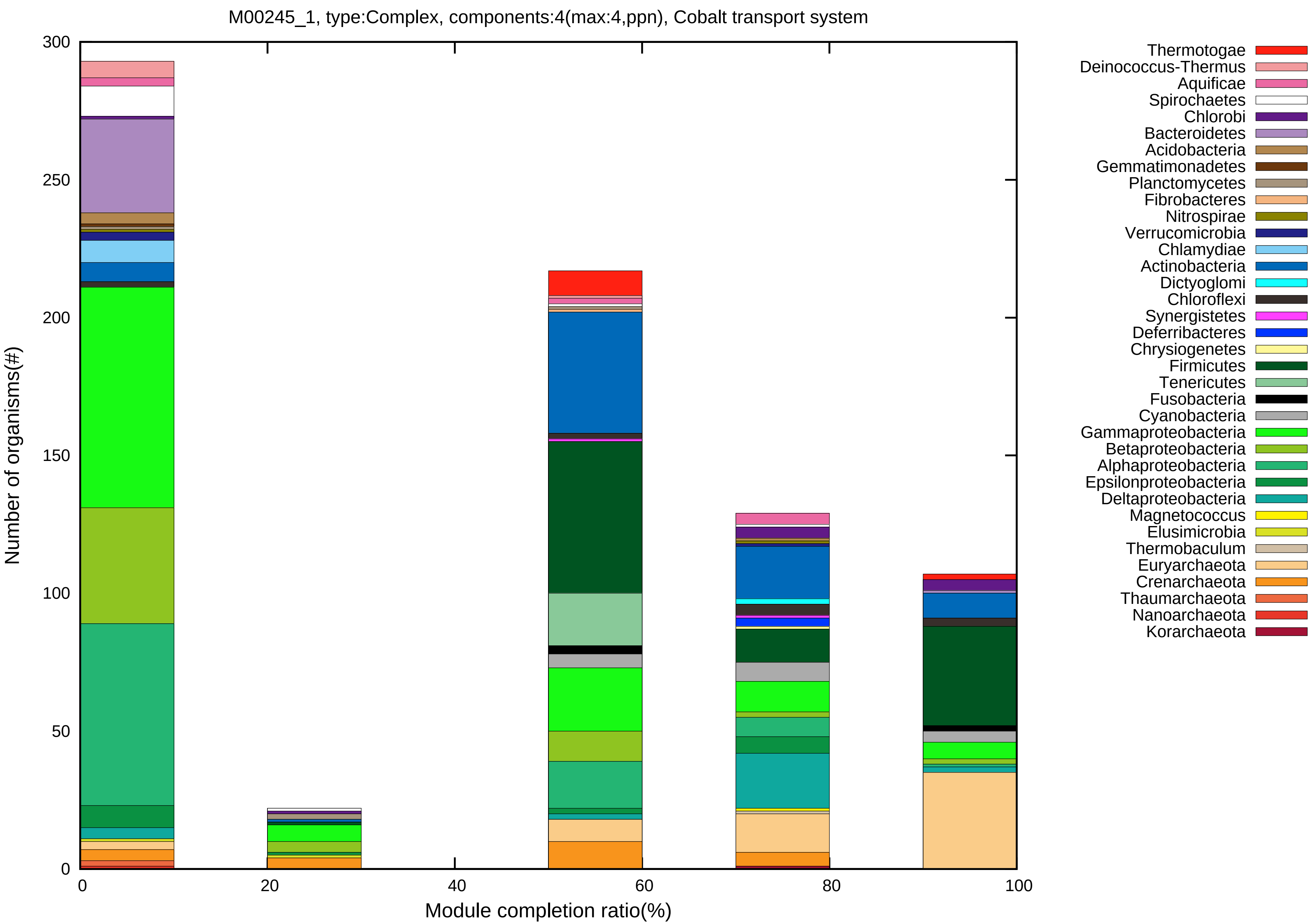

M00245_1, type:Complex, components:4(max:4,ppn), Cobalt transport system
Number of organisms(#)
Module completion ratio(%)
0
50
100
150
200
250
300
0
20
40
60
80
100
Thermotogae
Deinococcus-Thermus
Aquificae
Spirochaetes
Chlorobi
Bacteroidetes
Acidobacteria
Gemmatimonadetes
Planctomycetes
Fibrobacteres
Nitrospirae
Verrucomicrobia
Chlamydiae
Actinobacteria
Dictyoglomi
Chloroflexi
Synergistetes
Deferribacteres
Chrysiogenetes
Firmicutes
Tenericutes
Fusobacteria
Cyanobacteria
Gammaproteobacteria
Betaproteobacteria
Alphaproteobacteria
Epsilonproteobacteria
Deltaproteobacteria
Magnetococcus
Elusimicrobia
Thermobaculum
Euryarchaeota
Crenarchaeota
Thaumarchaeota
Nanoarchaeota
Korarchaeota

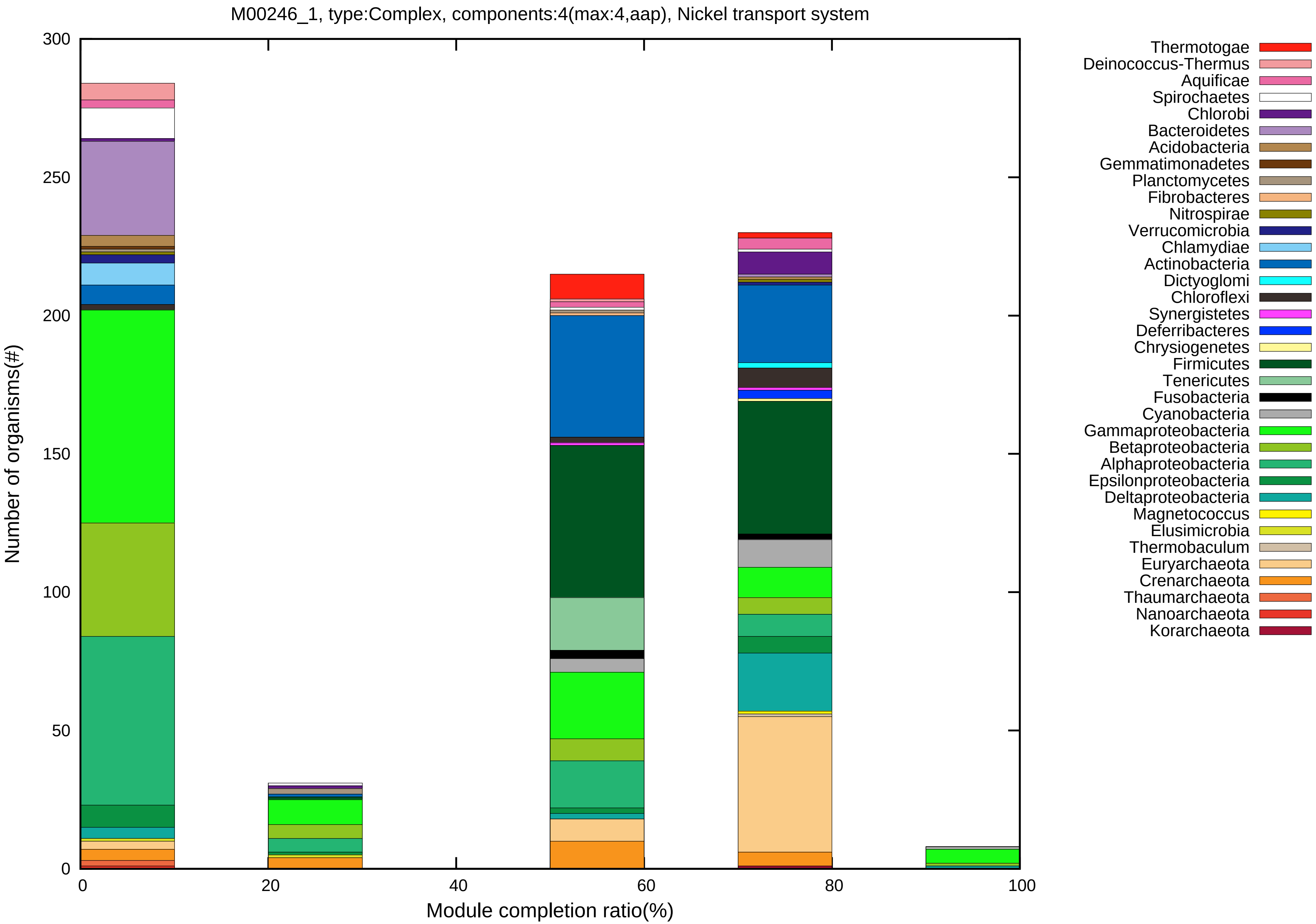

M00246_1, type:Complex, components:4(max:4,aap), Nickel transport system
Number of organisms(#)
Module completion ratio(%)
0
50
100
150
200
250
300
0
20
40
60
80
100
Thermotogae
Deinococcus-Thermus
Aquificae
Spirochaetes
Chlorobi
Bacteroidetes
Acidobacteria
Gemmatimonadetes
Planctomycetes
Fibrobacteres
Nitrospirae
Verrucomicrobia
Chlamydiae
Actinobacteria
Dictyoglomi
Chloroflexi
Synergistetes
Deferribacteres
Chrysiogenetes
Firmicutes
Tenericutes
Fusobacteria
Cyanobacteria
Gammaproteobacteria
Betaproteobacteria
Alphaproteobacteria
Epsilonproteobacteria
Deltaproteobacteria
Magnetococcus
Elusimicrobia
Thermobaculum
Euryarchaeota
Crenarchaeota
Thaumarchaeota
Nanoarchaeota
Korarchaeota

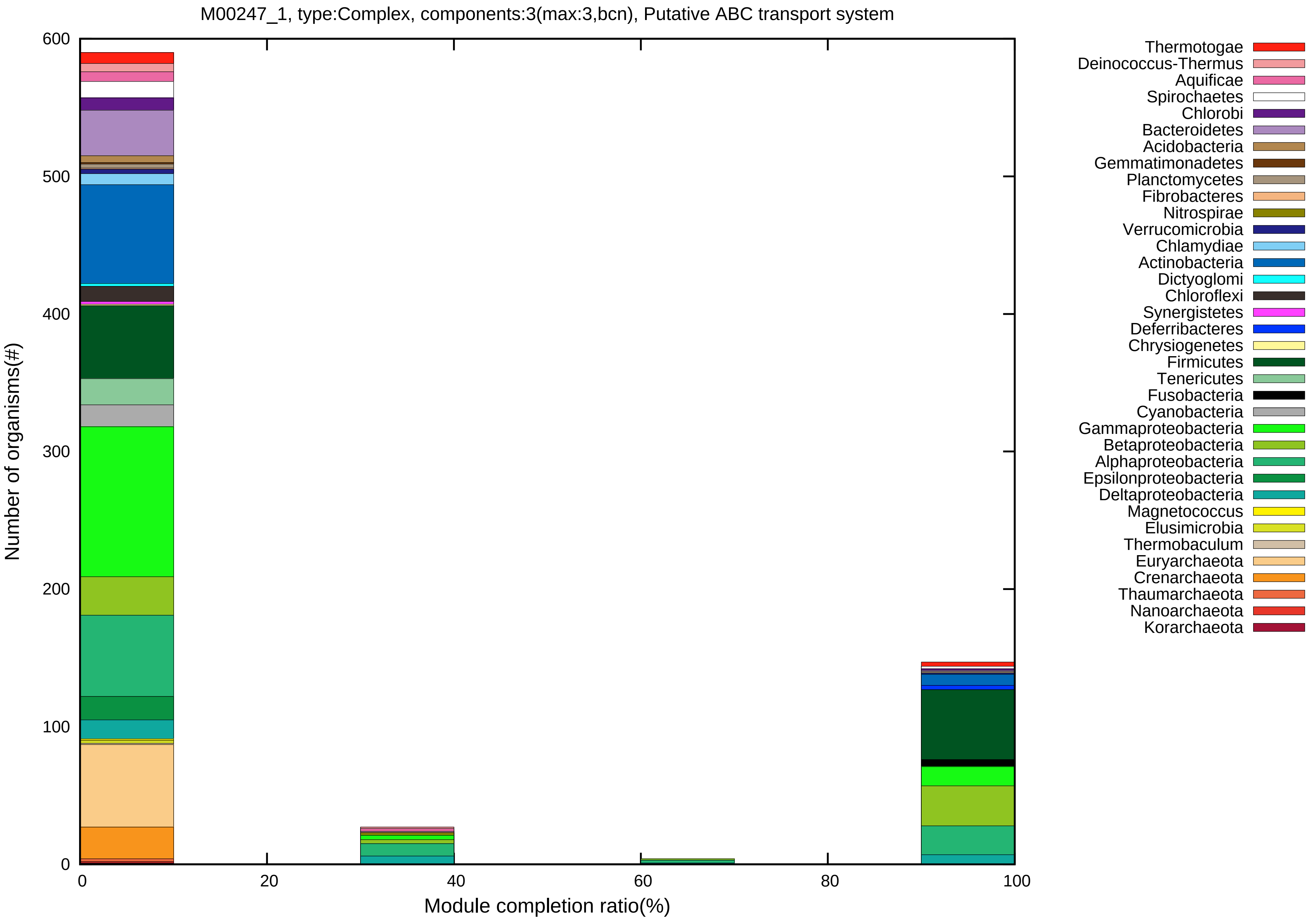

M00247_1, type:Complex, components:3(max:3,bcn), Putative ABC transport system
Number of organisms(#)
Module completion ratio(%)
0
100
200
300
400
500
600
0
20
40
60
80
100
Thermotogae
Deinococcus-Thermus
Aquificae
Spirochaetes
Chlorobi
Bacteroidetes
Acidobacteria
Gemmatimonadetes
Planctomycetes
Fibrobacteres
Nitrospirae
Verrucomicrobia
Chlamydiae
Actinobacteria
Dictyoglomi
Chloroflexi
Synergistetes
Deferribacteres
Chrysiogenetes
Firmicutes
Tenericutes
Fusobacteria
Cyanobacteria
Gammaproteobacteria
Betaproteobacteria
Alphaproteobacteria
Epsilonproteobacteria
Deltaproteobacteria
Magnetococcus
Elusimicrobia
Thermobaculum
Euryarchaeota
Crenarchaeota
Thaumarchaeota
Nanoarchaeota
Korarchaeota

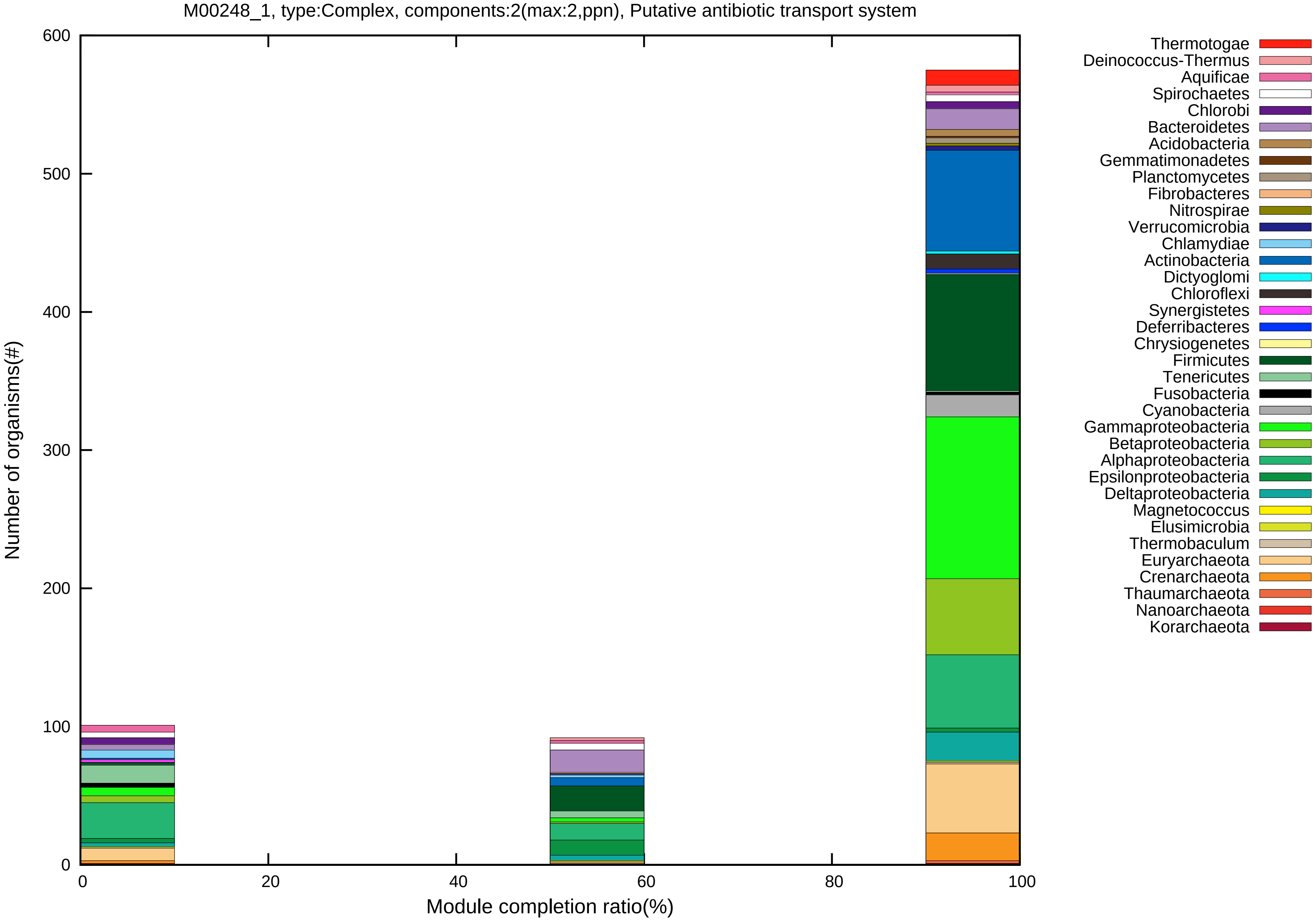

M00248_1, type:Complex, components:2(max:2,ppn), Putative antibiotic transport system
Number of organisms(#)
0
100
200
300
400
500
600
0
20
40
60
80
100
Module completion ratio(%)
Thermotogae
Deinococcus-Thermus
Aquificae
Spirochaetes
Chlorobi
Bacteroidetes
Acidobacteria
Gemmatimonadetes
Planctomycetes
Fibrobacteres
Nitrospirae
Verrucomicrobia
Chlamydiae
Actinobacteria
Dictyoglomi
Chloroflexi
Synergistetes
Deferribacteres
Chrysiogenetes
Firmicutes
Tenericutes
Fusobacteria
Cyanobacteria
Gammaproteobacteria
Betaproteobacteria
Alphaproteobacteria
Epsilonproteobacteria
Deltaproteobacteria
Magnetococcus
Elusimicrobia
Thermobaculum
Euryarchaeota
Crenarchaeota
Thaumarchaeota
Nanoarchaeota
Korarchaeota

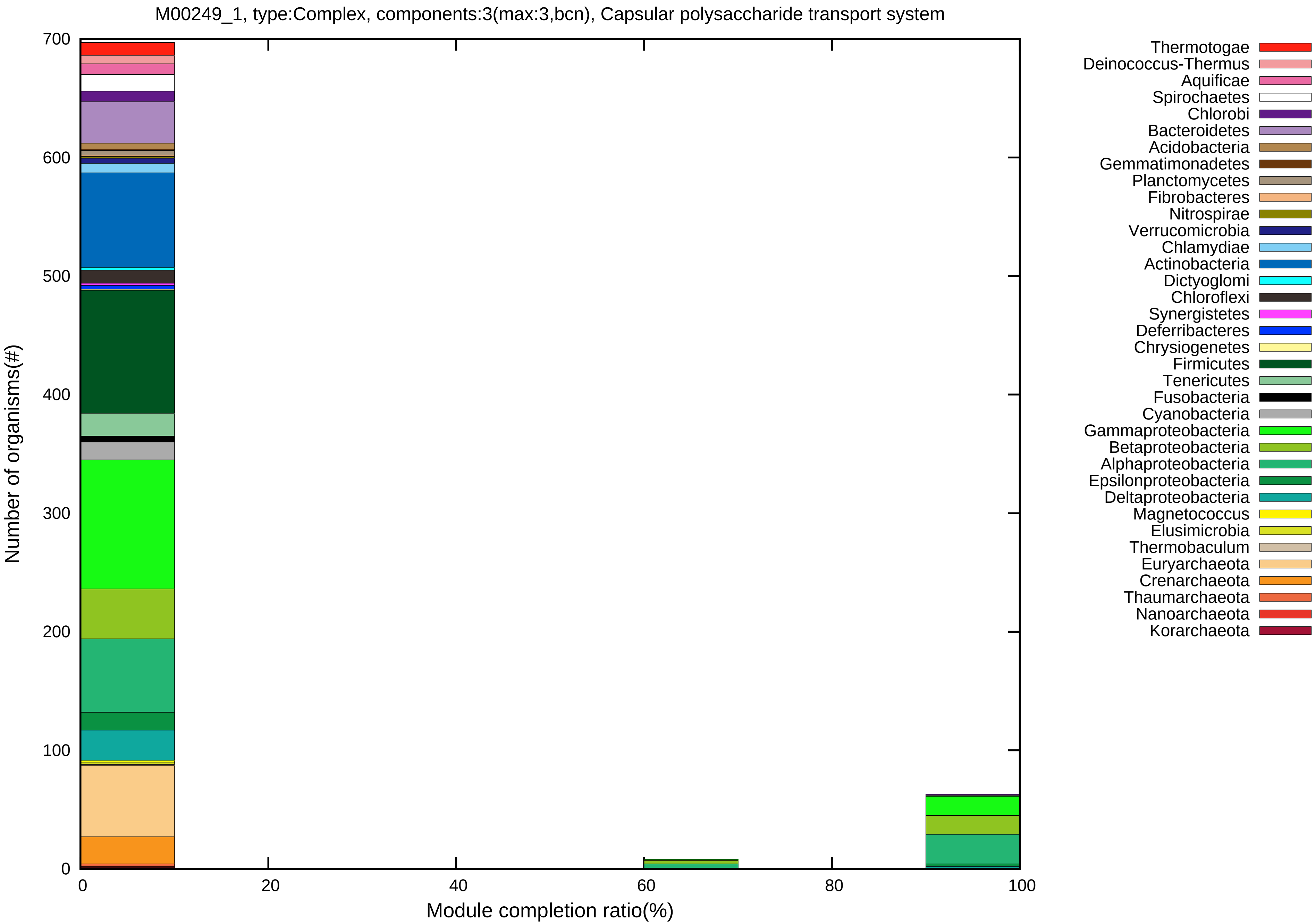

M00249_1, type:Complex, components:3(max:3,bcn), Capsular polysaccharide transport system
Number of organisms(#)
Module completion ratio(%)
0
100
200
300
400
500
600
700
0
20
40
60
80
100
Thermotogae
Deinococcus-Thermus
Aquificae
Spirochaetes
Chlorobi
Bacteroidetes
Acidobacteria
Gemmatimonadetes
Planctomycetes
Fibrobacteres
Nitrospirae
Verrucomicrobia
Chlamydiae
Actinobacteria
Dictyoglomi
Chloroflexi
Synergistetes
Deferribacteres
Chrysiogenetes
Firmicutes
Tenericutes
Fusobacteria
Cyanobacteria
Gammaproteobacteria
Betaproteobacteria
Alphaproteobacteria
Epsilonproteobacteria
Deltaproteobacteria
Magnetococcus
Elusimicrobia
Thermobaculum
Euryarchaeota
Crenarchaeota
Thaumarchaeota
Nanoarchaeota
Korarchaeota

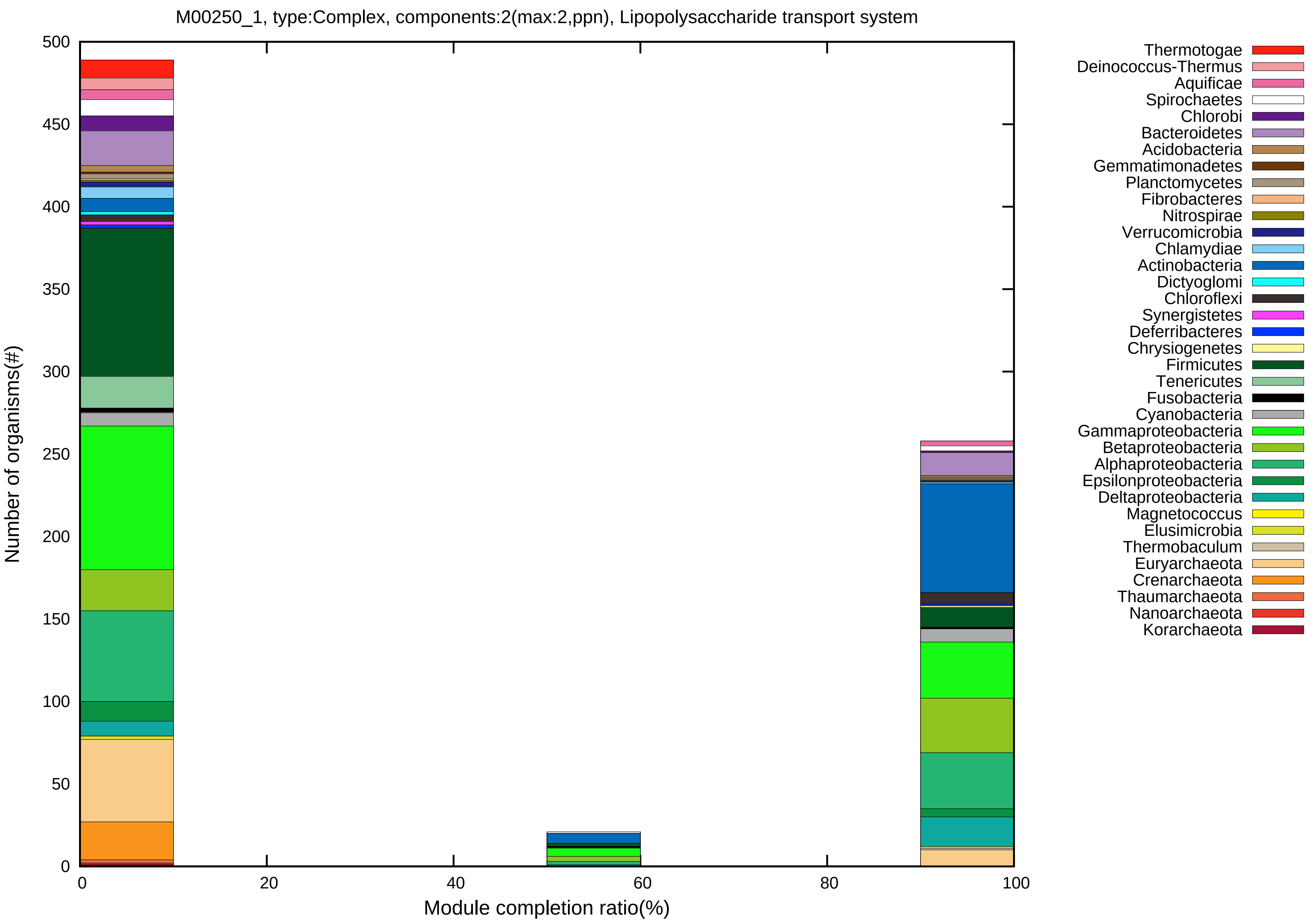

M00250_1, type:Complex, components:2(max:2,ppn), Lipopolysaccharide transport system
Number of organisms(#)
Module completion ratio(%)
0
50
100
150
200
250
300
350
400
450
500
0
20
40
60
80
100
Thermotogae
Deinococcus-Thermus
Aquificae
Spirochaetes
Chlorobi
Bacteroidetes
Acidobacteria
Gemmatimonadetes
Planctomycetes
Fibrobacteres
Nitrospirae
Verrucomicrobia
Chlamydiae
Actinobacteria
Dictyoglomi
Chloroflexi
Synergistetes
Deferribacteres
Chrysiogenetes
Firmicutes
Tenericutes
Fusobacteria
Cyanobacteria
Gammaproteobacteria
Betaproteobacteria
Alphaproteobacteria
Epsilonproteobacteria
Deltaproteobacteria
Magnetococcus
Elusimicrobia
Thermobaculum
Euryarchaeota
Crenarchaeota
Thaumarchaeota
Nanoarchaeota
Korarchaeota

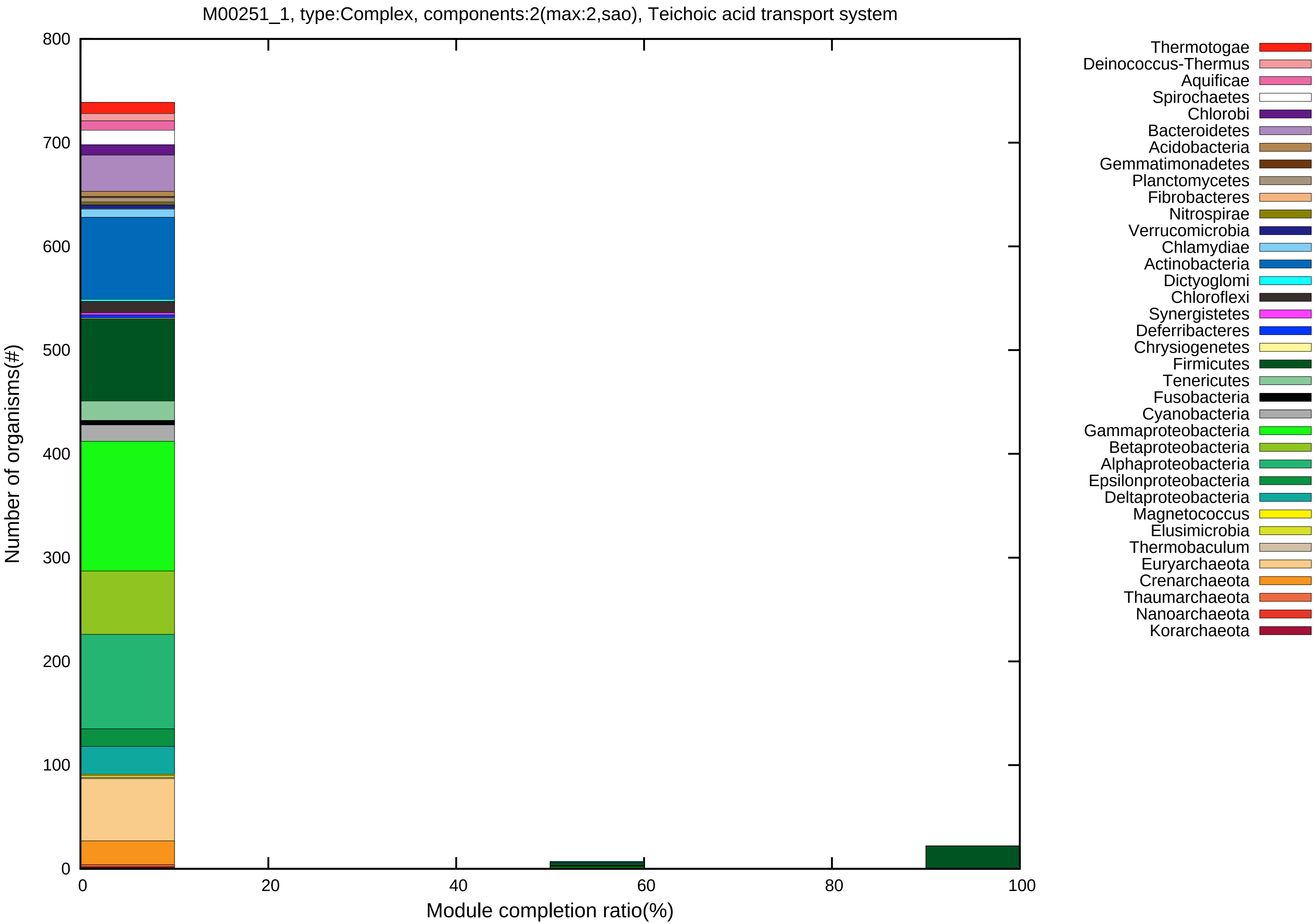
M00251_1, type:Complex, components:2(max:2,sao), Teichoic acid transport system
Number of organisms(#)
Module completion ratio(%)
0
100
200
300
400
500
600
700
800
0
20
40
60
80
100
Thermotogae
Deinococcus-Thermus
Aquificae
Spirochaetes
Chlorobi
Bacteroidetes
Acidobacteria
Gemmatimonadetes
Planctomycetes
Fibrobacteres
Nitrospirae
Verrucomicrobia
Chlamydiae
Actinobacteria
Dictyoglomi
Chloroflexi
Synergistetes
Deferribacteres
Chrysiogenetes
Firmicutes
Tenericutes
Fusobacteria
Cyanobacteria
Gammaproteobacteria
Betaproteobacteria
Alphaproteobacteria
Epsilonproteobacteria
Deltaproteobacteria
Magnetococcus
Elusimicrobia
Thermobaculum
Euryarchaeota
Crenarchaeota
Thaumarchaeota
Nanoarchaeota
Korarchaeota

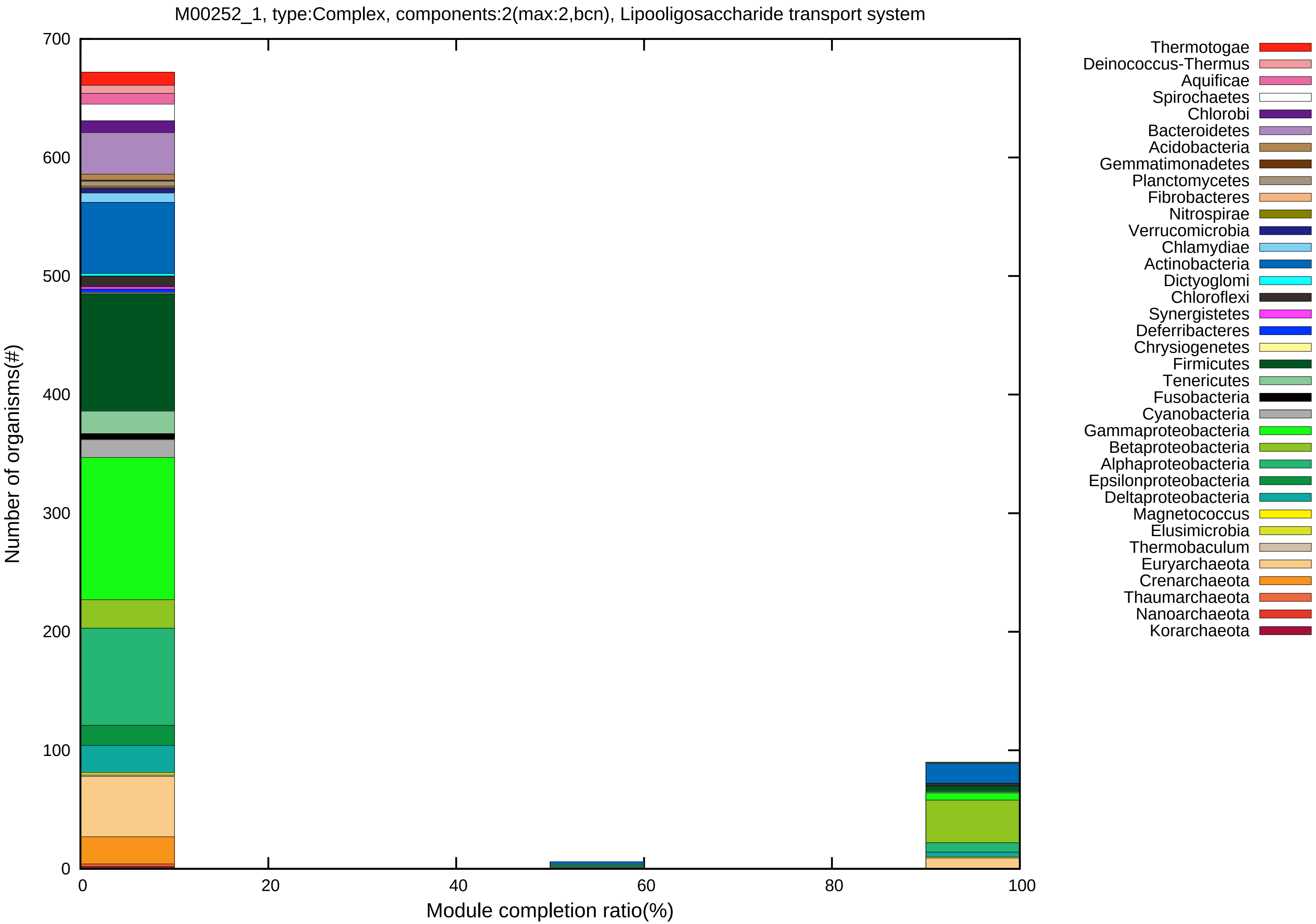

M00252_1, type:Complex, components:2(max:2,bcn), Lipooligosaccharide transport system
Number of organisms(#)
Module completion ratio(%)
0
100
200
300
400
500
600
700
0
20
40
60
80
100
Thermotogae
Deinococcus-Thermus
Aquificae
Spirochaetes
Chlorobi
Bacteroidetes
Acidobacteria
Gemmatimonadetes
Planctomycetes
Fibrobacteres
Nitrospirae
Verrucomicrobia
Chlamydiae
Actinobacteria
Dictyoglomi
Chloroflexi
Synergistetes
Deferribacteres
Chrysiogenetes
Firmicutes
Tenericutes
Fusobacteria
Cyanobacteria
Gammaproteobacteria
Betaproteobacteria
Alphaproteobacteria
Epsilonproteobacteria
Deltaproteobacteria
Magnetococcus
Elusimicrobia
Thermobaculum
Euryarchaeota
Crenarchaeota
Thaumarchaeota
Nanoarchaeota
Korarchaeota

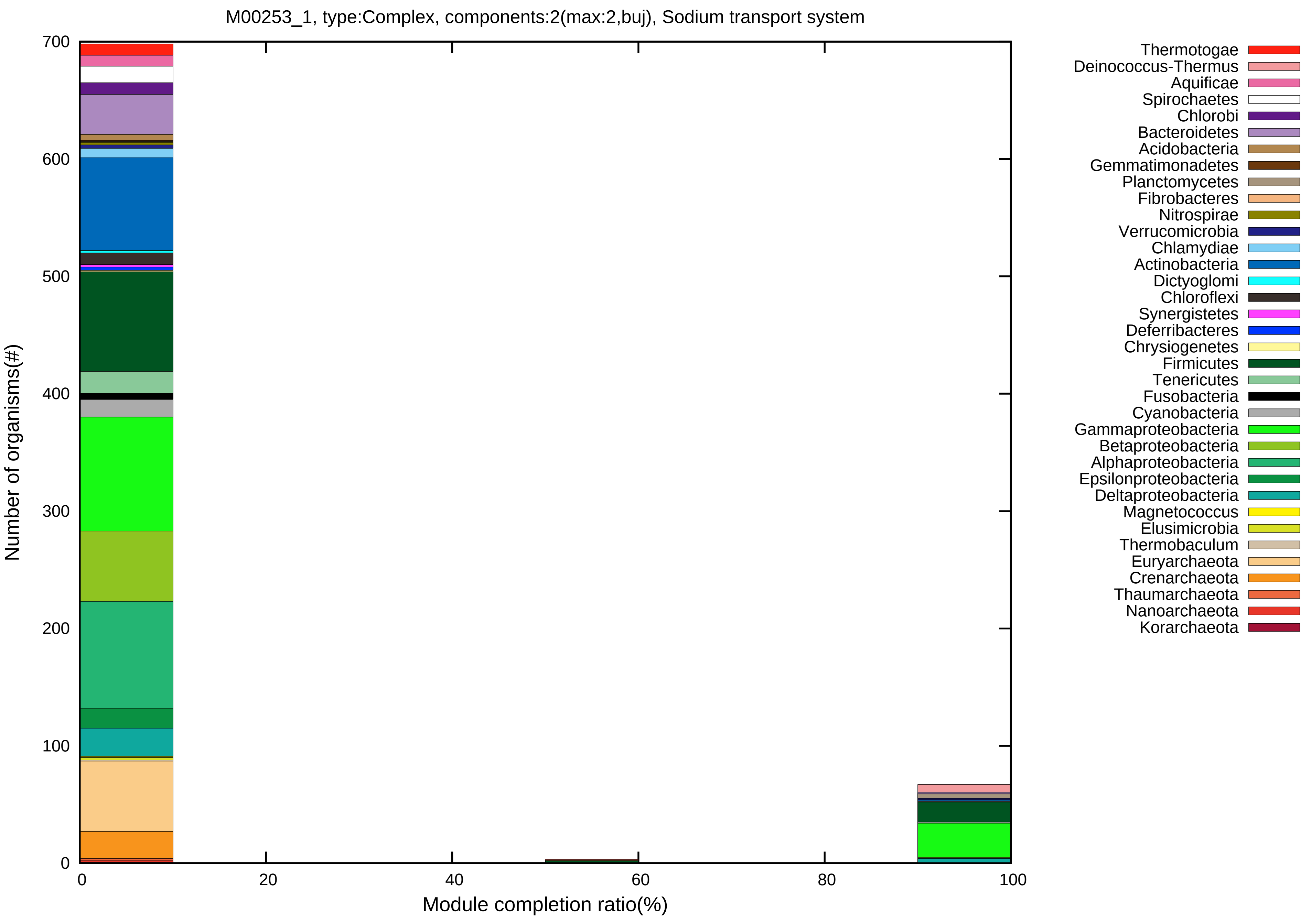
M00253_1, type:Complex, components:2(max:2,buj), Sodium transport system
Number of organisms(#)
Module completion ratio(%)
0
100
200
300
400
500
600
700
0
20
40
60
80
100
Thermotogae
Deinococcus-Thermus
Aquificae
Spirochaetes
Chlorobi
Bacteroidetes
Acidobacteria
Gemmatimonadetes
Planctomycetes
Fibrobacteres
Nitrospirae
Verrucomicrobia
Chlamydiae
Actinobacteria
Dictyoglomi
Chloroflexi
Synergistetes
Deferribacteres
Chrysiogenetes
Firmicutes
Tenericutes
Fusobacteria
Cyanobacteria
Gammaproteobacteria
Betaproteobacteria
Alphaproteobacteria
Epsilonproteobacteria
Deltaproteobacteria
Magnetococcus
Elusimicrobia
Thermobaculum
Euryarchaeota
Crenarchaeota
Thaumarchaeota
Nanoarchaeota
Korarchaeota

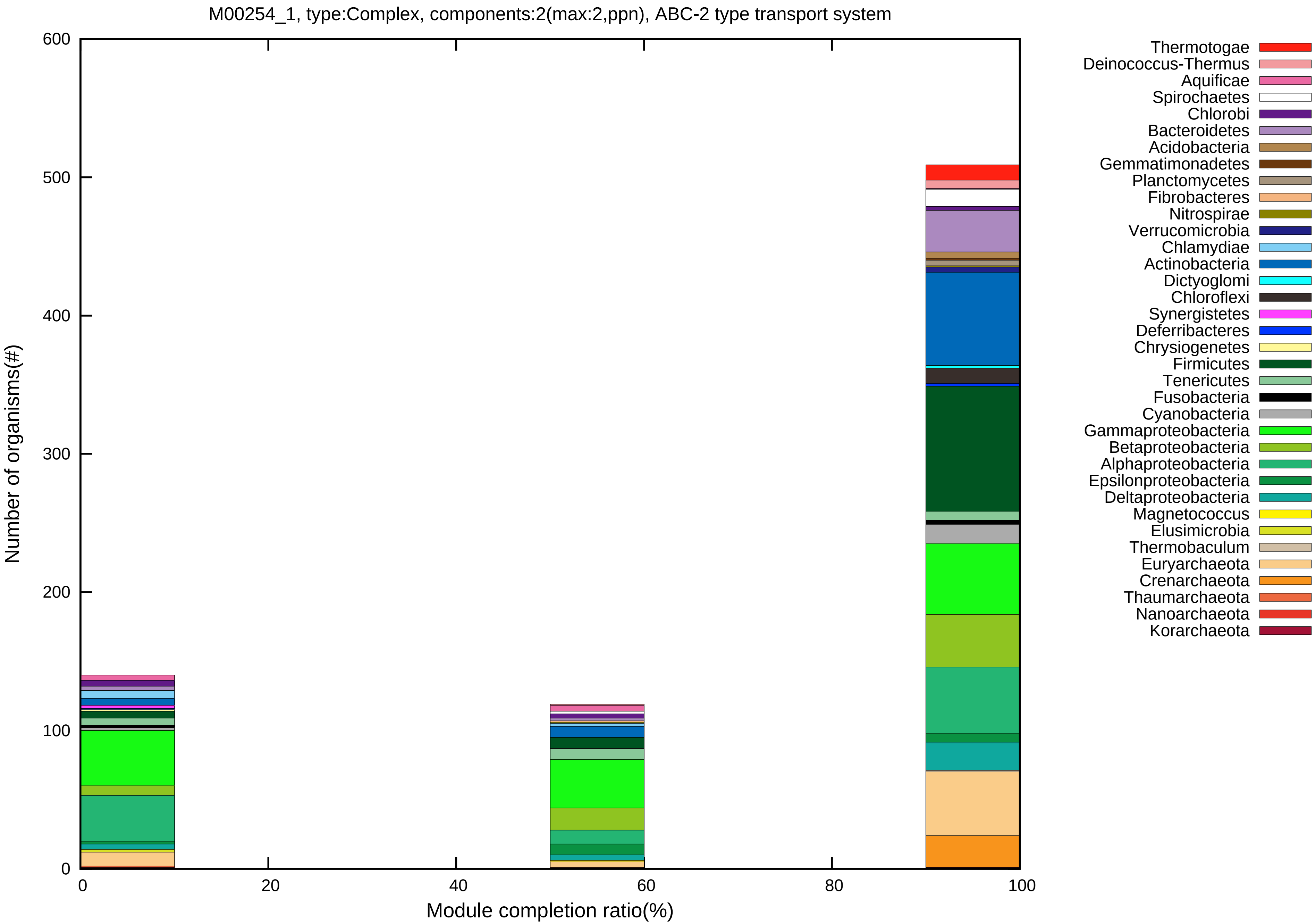

M00254_1, type:Complex, components:2(max:2,ppn), ABC-2 type transport system
Number of organisms(#)
Module completion ratio(%)
0
100
200
300
400
500
600
0
20
40
60
80
100
Thermotogae
Deinococcus-Thermus
Aquificae
Spirochaetes
Chlorobi
Bacteroidetes
Acidobacteria
Gemmatimonadetes
Planctomycetes
Fibrobacteres
Nitrospirae
Verrucomicrobia
Chlamydiae
Actinobacteria
Dictyoglomi
Chloroflexi
Synergistetes
Deferribacteres
Chrysiogenetes
Firmicutes
Tenericutes
Fusobacteria
Cyanobacteria
Gammaproteobacteria
Betaproteobacteria
Alphaproteobacteria
Epsilonproteobacteria
Deltaproteobacteria
Magnetococcus
Elusimicrobia
Thermobaculum
Euryarchaeota
Crenarchaeota
Thaumarchaeota
Nanoarchaeota
Korarchaeota

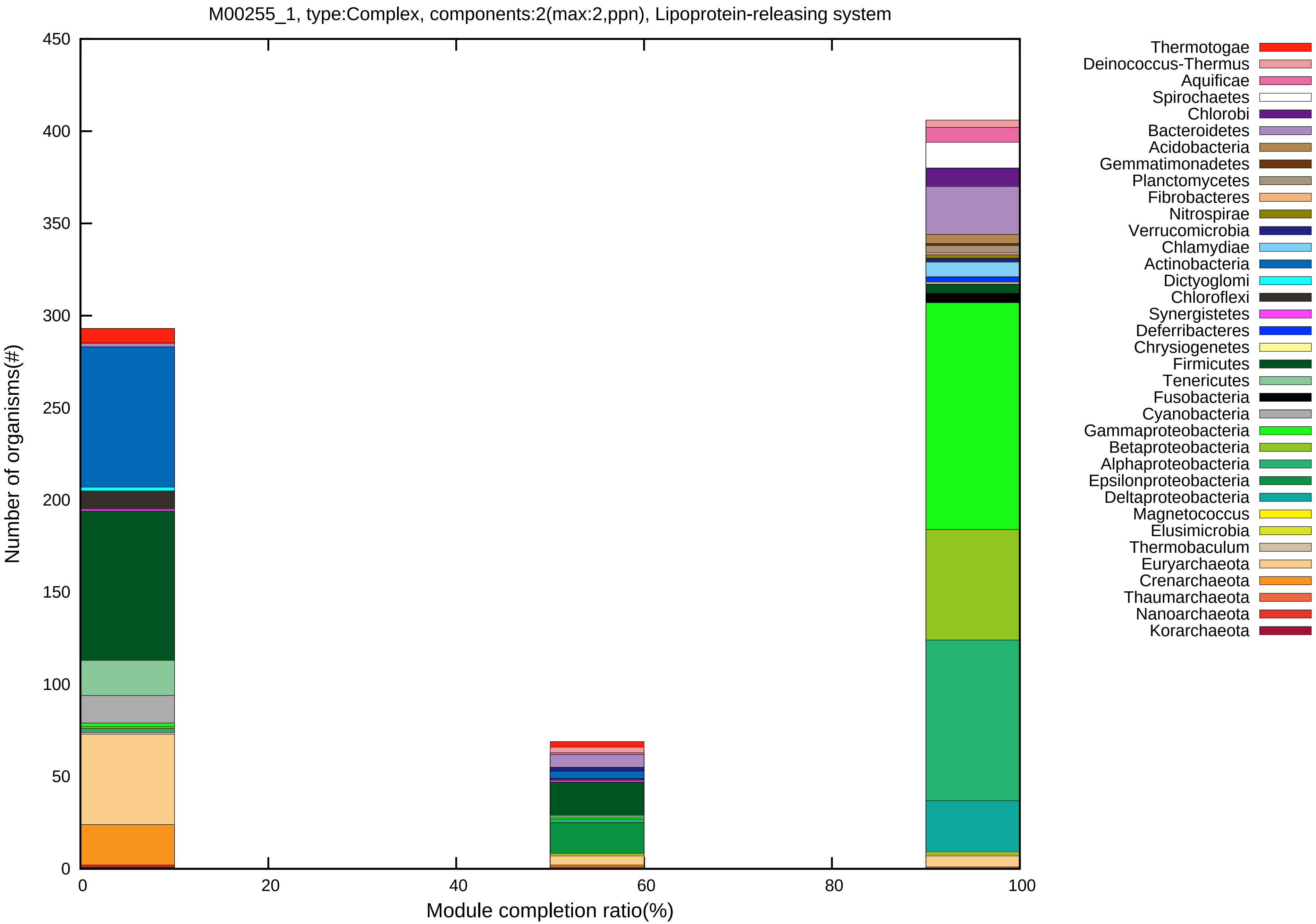

M00255_1, type:Complex, components:2(max:2,ppn), Lipoprotein-releasing system
Number of organisms(#)
Module completion ratio(%)
0
50
100
150
200
250
300
350
400
450
0
20
40
60
80
100
Thermotogae
Deinococcus-Thermus
Aquificae
Spirochaetes
Chlorobi
Bacteroidetes
Acidobacteria
Gemmatimonadetes
Planctomycetes
Fibrobacteres
Nitrospirae
Verrucomicrobia
Chlamydiae
Actinobacteria
Dictyoglomi
Chloroflexi
Synergistetes
Deferribacteres
Chrysiogenetes
Firmicutes
Tenericutes
Fusobacteria
Cyanobacteria
Gammaproteobacteria
Betaproteobacteria
Alphaproteobacteria
Epsilonproteobacteria
Deltaproteobacteria
Magnetococcus
Elusimicrobia
Thermobaculum
Euryarchaeota
Crenarchaeota
Thaumarchaeota
Nanoarchaeota
Korarchaeota

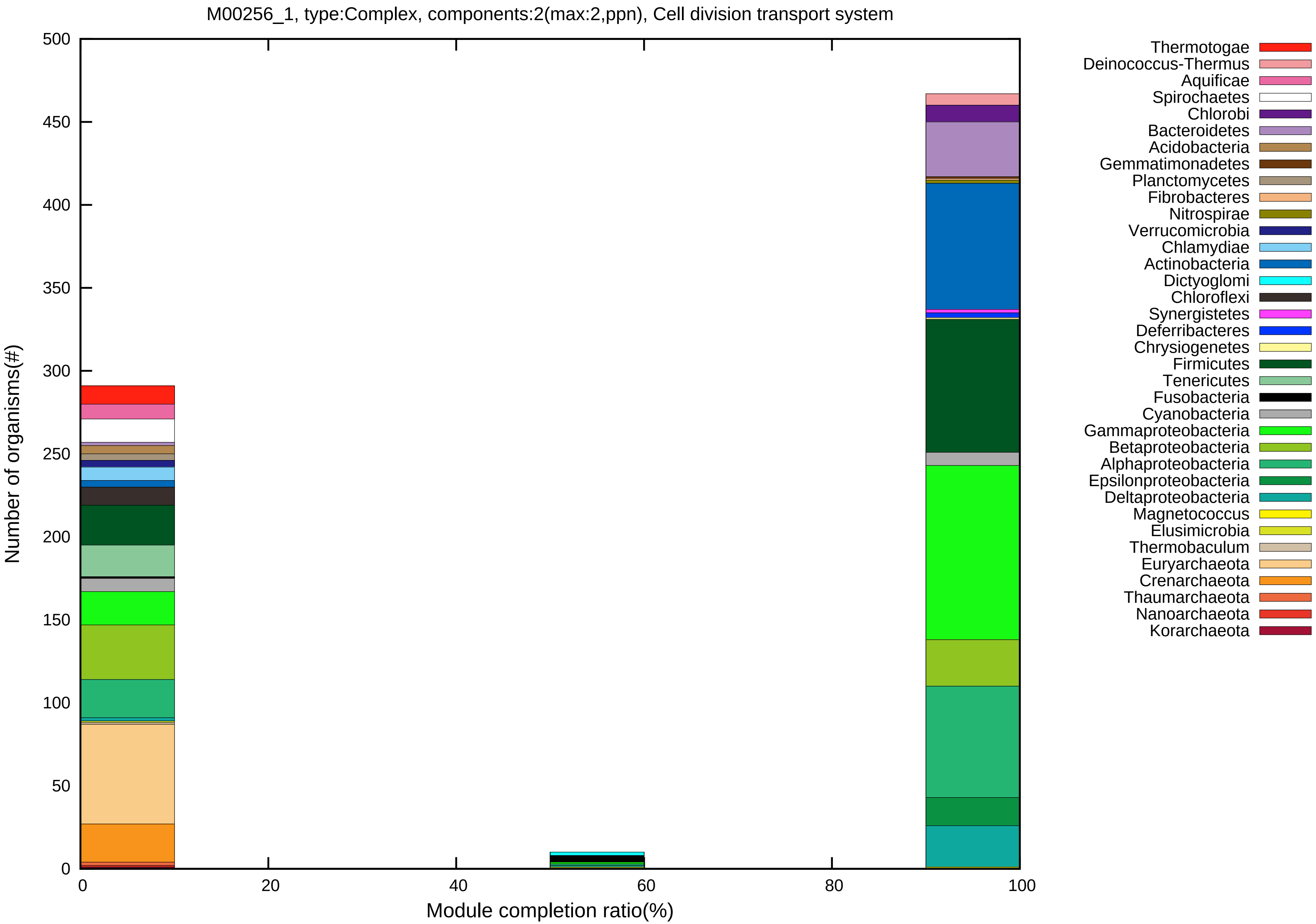

M00256_1, type:Complex, components:2(max:2,ppn), Cell division transport system
Number of organisms(#)
Module completion ratio(%)
0
50
100
150
200
250
300
350
400
450
500
0
20
40
60
80
100
Thermotogae
Deinococcus-Thermus
Aquificae
Spirochaetes
Chlorobi
Bacteroidetes
Acidobacteria
Gemmatimonadetes
Planctomycetes
Fibrobacteres
Nitrospirae
Verrucomicrobia
Chlamydiae
Actinobacteria
Dictyoglomi
Chloroflexi
Synergistetes
Deferribacteres
Chrysiogenetes
Firmicutes
Tenericutes
Fusobacteria
Cyanobacteria
Gammaproteobacteria
Betaproteobacteria
Alphaproteobacteria
Epsilonproteobacteria
Deltaproteobacteria
Magnetococcus
Elusimicrobia
Thermobaculum
Euryarchaeota
Crenarchaeota
Thaumarchaeota
Nanoarchaeota
Korarchaeota

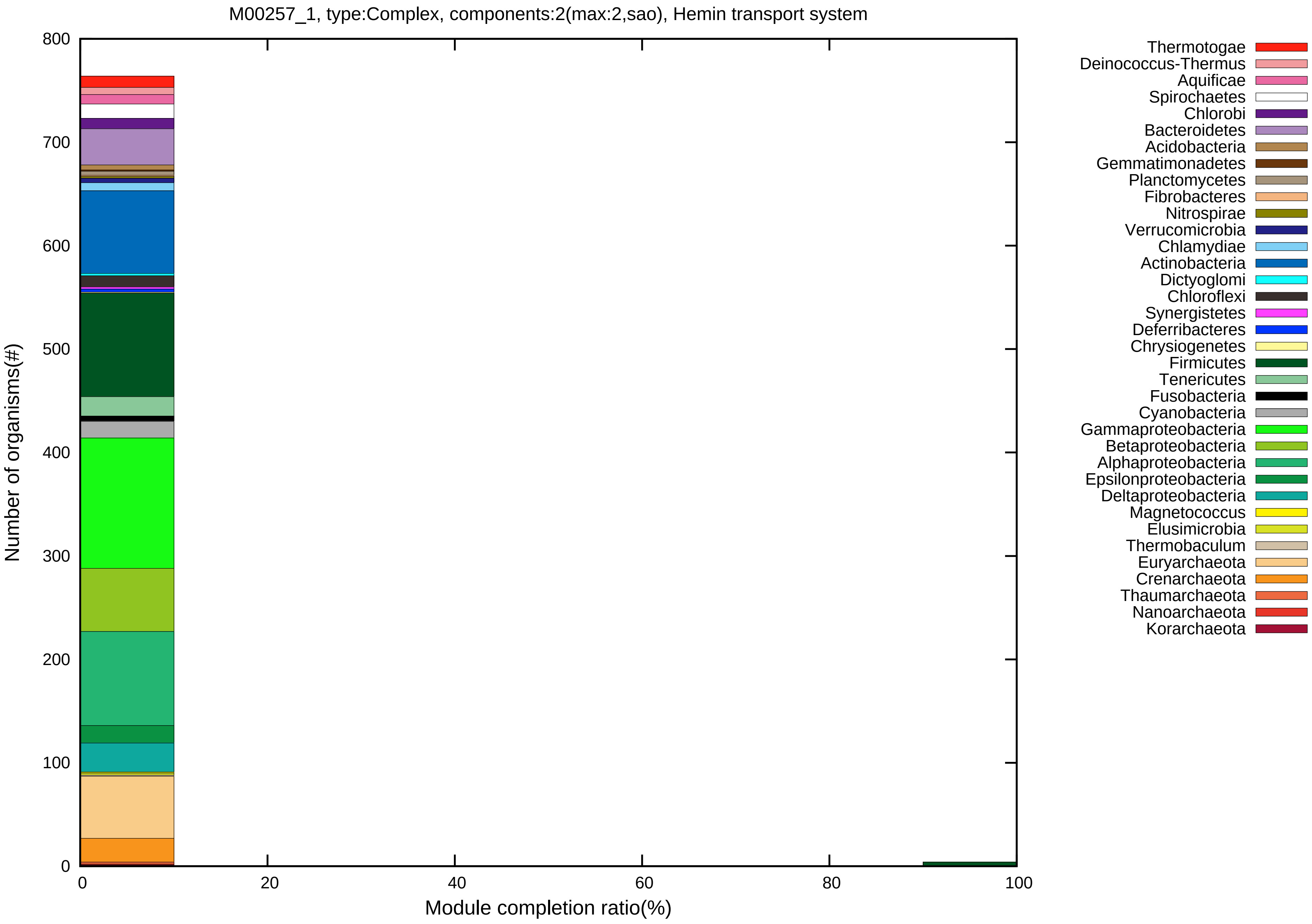

M00257_1, type:Complex, components:2(max:2,sao), Hemin transport system
Number of organisms(#)
Module completion ratio(%)
0
100
200
300
400
500
600
700
800
0
20
40
60
80
100
Thermotogae
Deinococcus-Thermus
Aquificae
Spirochaetes
Chlorobi
Bacteroidetes
Acidobacteria
Gemmatimonadetes
Planctomycetes
Fibrobacteres
Nitrospirae
Verrucomicrobia
Chlamydiae
Actinobacteria
Dictyoglomi
Chloroflexi
Synergistetes
Deferribacteres
Chrysiogenetes
Firmicutes
Tenericutes
Fusobacteria
Cyanobacteria
Gammaproteobacteria
Betaproteobacteria
Alphaproteobacteria
Epsilonproteobacteria
Deltaproteobacteria
Magnetococcus
Elusimicrobia
Thermobaculum
Euryarchaeota
Crenarchaeota
Thaumarchaeota
Nanoarchaeota
Korarchaeota

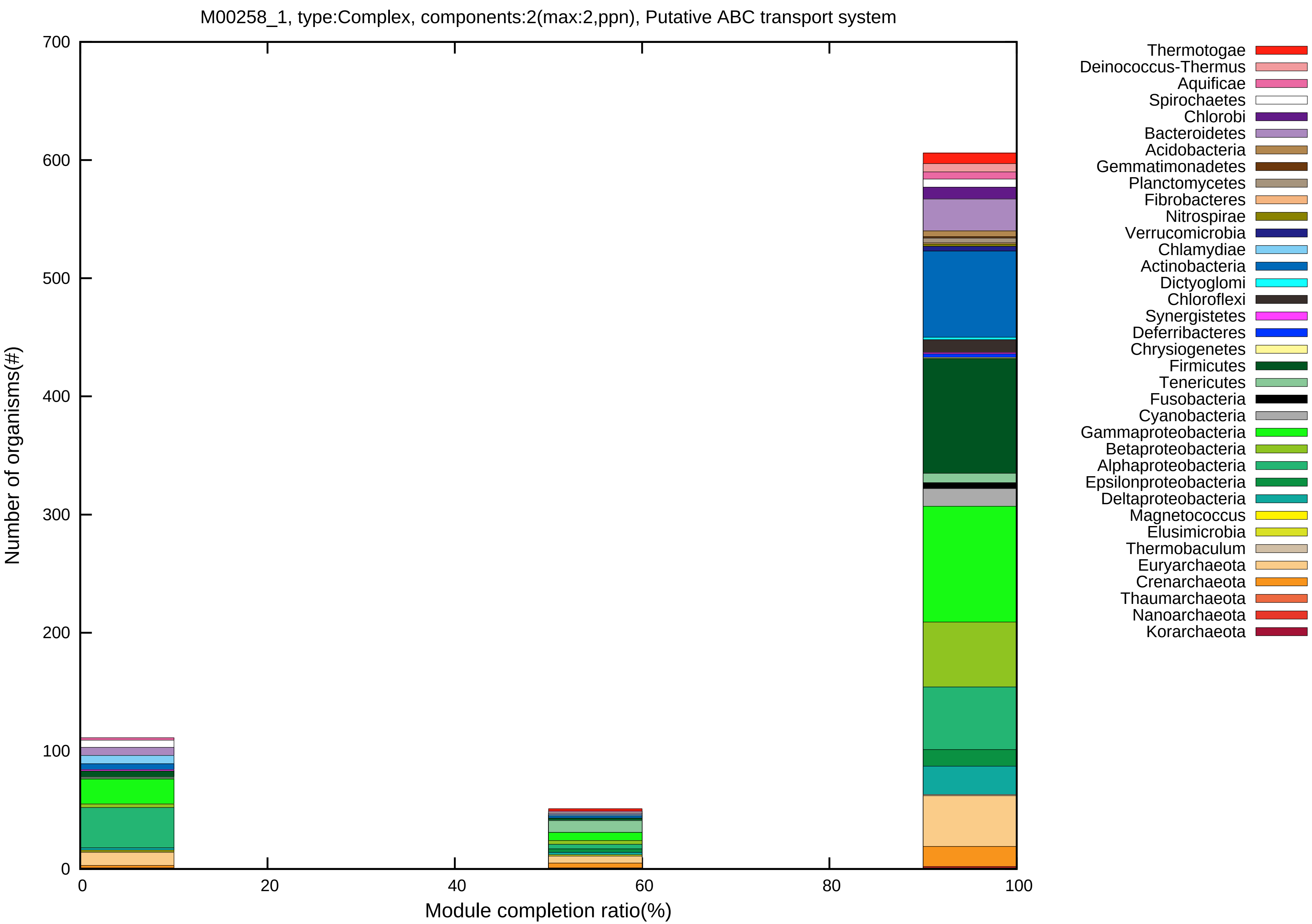

M00258_1, type:Complex, components:2(max:2,ppn), Putative ABC transport system
Number of organisms(#)
Module completion ratio(%)
0
100
200
300
400
500
600
700
0
20
40
60
80
100
Thermotogae
Deinococcus-Thermus
Aquificae
Spirochaetes
Chlorobi
Bacteroidetes
Acidobacteria
Gemmatimonadetes
Planctomycetes
Fibrobacteres
Nitrospirae
Verrucomicrobia
Chlamydiae
Actinobacteria
Dictyoglomi
Chloroflexi
Synergistetes
Deferribacteres
Chrysiogenetes
Firmicutes
Tenericutes
Fusobacteria
Cyanobacteria
Gammaproteobacteria
Betaproteobacteria
Alphaproteobacteria
Epsilonproteobacteria
Deltaproteobacteria
Magnetococcus
Elusimicrobia
Thermobaculum
Euryarchaeota
Crenarchaeota
Thaumarchaeota
Nanoarchaeota
Korarchaeota

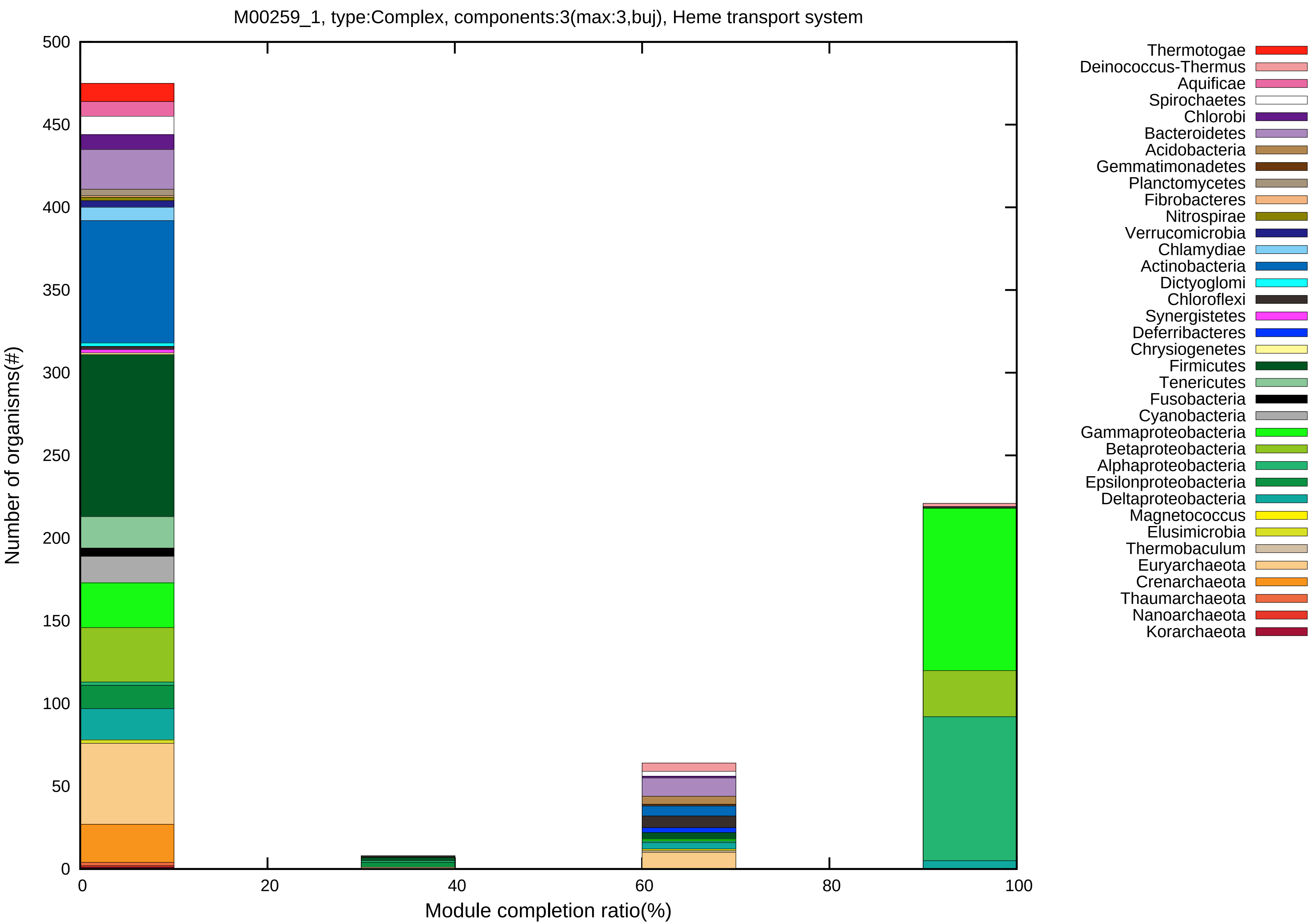

M00259_1, type:Complex, components:3(max:3,buj), Heme transport system
Number of organisms(#)
Module completion ratio(%)
0
50
100
150
200
250
300
350
400
450
500
0
20
40
60
80
100
Thermotogae
Deinococcus-Thermus
Aquificae
Spirochaetes
Chlorobi
Bacteroidetes
Acidobacteria
Gemmatimonadetes
Planctomycetes
Fibrobacteres
Nitrospirae
Verrucomicrobia
Chlamydiae
Actinobacteria
Dictyoglomi
Chloroflexi
Synergistetes
Deferribacteres
Chrysiogenetes
Firmicutes
Tenericutes
Fusobacteria
Cyanobacteria
Gammaproteobacteria
Betaproteobacteria
Alphaproteobacteria
Epsilonproteobacteria
Deltaproteobacteria
Magnetococcus
Elusimicrobia
Thermobaculum
Euryarchaeota
Crenarchaeota
Thaumarchaeota
Nanoarchaeota
Korarchaeota

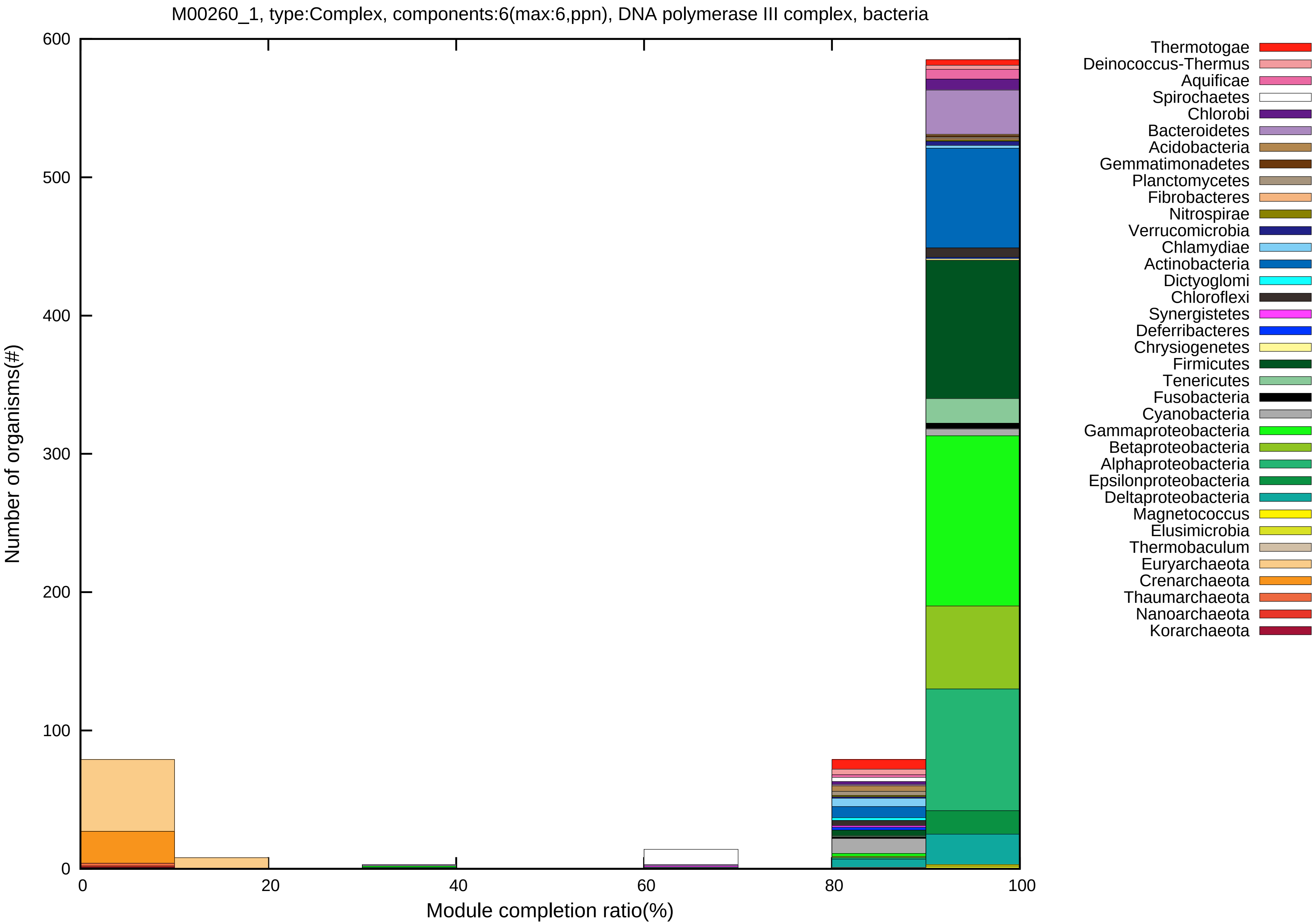

M00260_1, type:Complex, components:6(max:6,ppn), DNA polymerase III complex, bacteria
Number of organisms(#)
Module completion ratio(%)
0
100
200
300
400
500
600
0
20
40
60
80
100
Thermotogae
Deinococcus-Thermus
Aquificae
Spirochaetes
Chlorobi
Bacteroidetes
Acidobacteria
Gemmatimonadetes
Planctomycetes
Fibrobacteres
Nitrospirae
Verrucomicrobia
Chlamydiae
Actinobacteria
Dictyoglomi
Chloroflexi
Synergistetes
Deferribacteres
Chrysiogenetes
Firmicutes
Tenericutes
Fusobacteria
Cyanobacteria
Gammaproteobacteria
Betaproteobacteria
Alphaproteobacteria
Epsilonproteobacteria
Deltaproteobacteria
Magnetococcus
Elusimicrobia
Thermobaculum
Euryarchaeota
Crenarchaeota
Thaumarchaeota
Nanoarchaeota
Korarchaeota

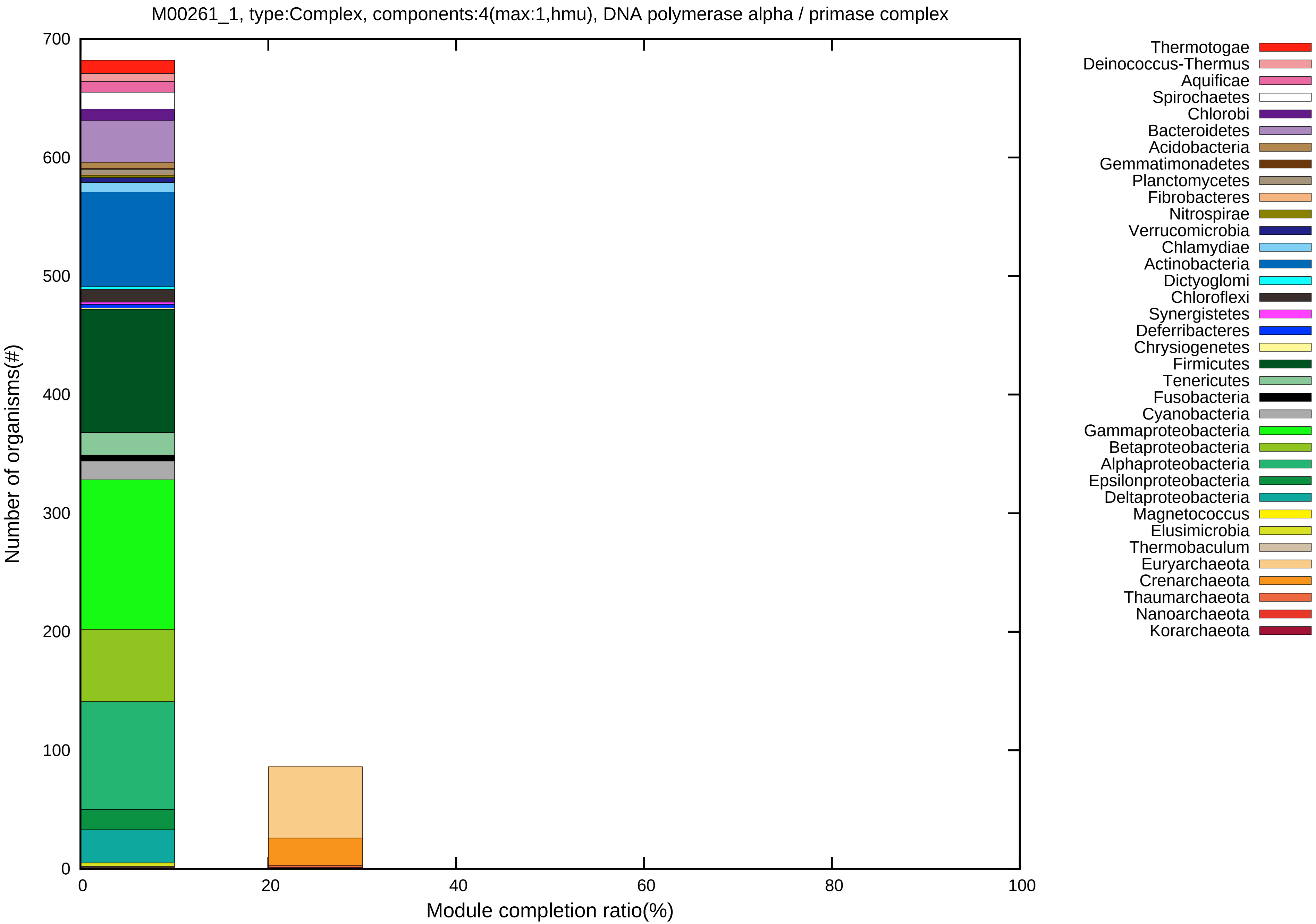

M00261_1, type:Complex, components:4(max:1,hmu), DNA polymerase alpha / primase complex
Number of organisms(#)
Module completion ratio(%)
0
100
200
300
400
500
600
700
0
20
40
60
80
100
Thermotogae
Deinococcus-Thermus
Aquificae
Spirochaetes
Chlorobi
Bacteroidetes
Acidobacteria
Gemmatimonadetes
Planctomycetes
Fibrobacteres
Nitrospirae
Verrucomicrobia
Chlamydiae
Actinobacteria
Dictyoglomi
Chloroflexi
Synergistetes
Deferribacteres
Chrysiogenetes
Firmicutes
Tenericutes
Fusobacteria
Cyanobacteria
Gammaproteobacteria
Betaproteobacteria
Alphaproteobacteria
Epsilonproteobacteria
Deltaproteobacteria
Magnetococcus
Elusimicrobia
Thermobaculum
Euryarchaeota
Crenarchaeota
Thaumarchaeota
Nanoarchaeota
Korarchaeota

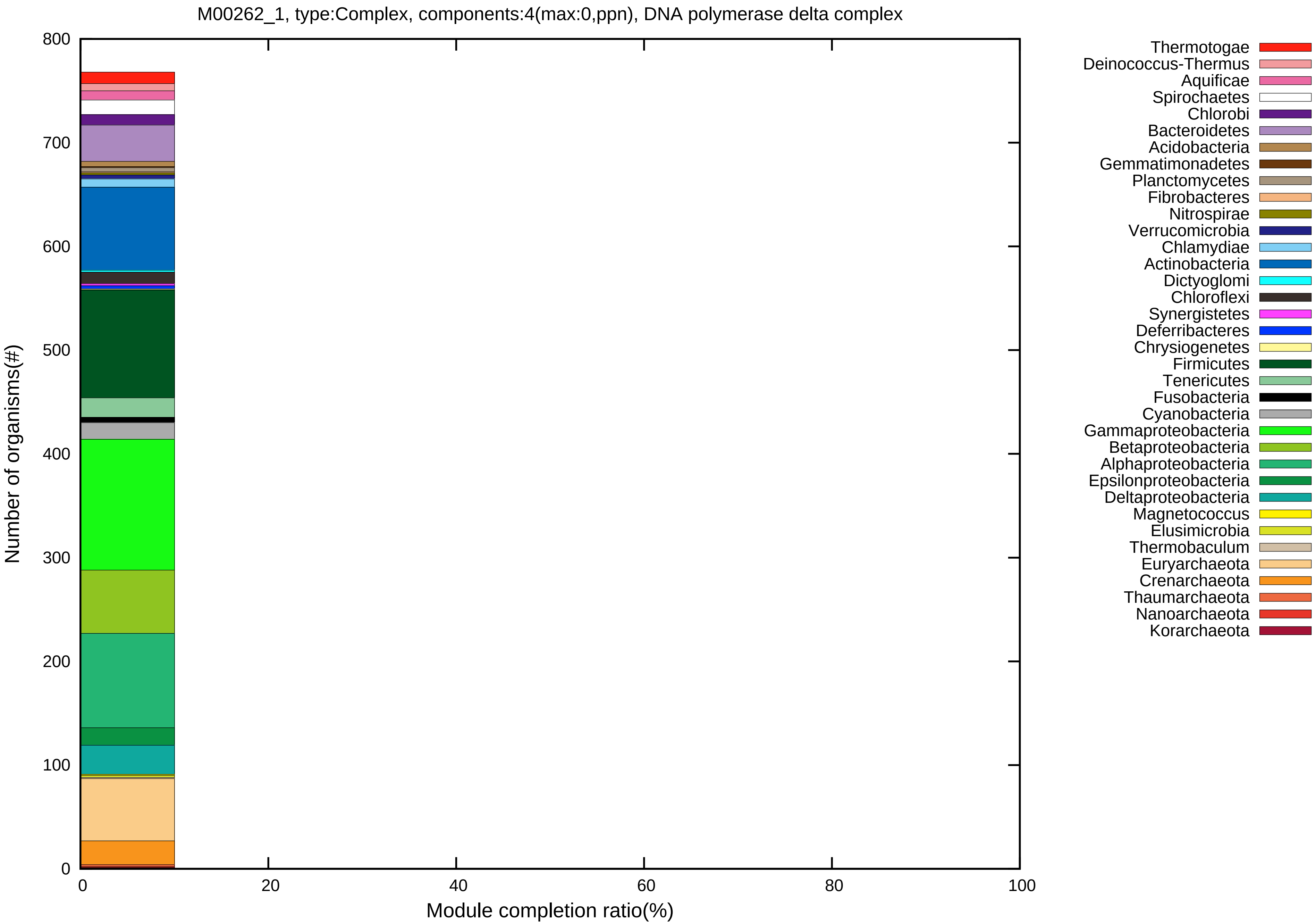
M00262_1, type:Complex, components:4(max:0,ppn), DNA polymerase delta complex
Number of organisms(#)
Module completion ratio(%)
0
20
40
60
80
100
0
100
200
300
400
500
600
700
800
Thermotogae
Deinococcus-Thermus
Aquificae
Spirochaetes
Chlorobi
Bacteroidetes
Acidobacteria
Gemmatimonadetes
Planctomycetes
Fibrobacteres
Nitrospirae
Verrucomicrobia
Chlamydiae
Actinobacteria
Dictyoglomi
Chloroflexi
Synergistetes
Deferribacteres
Chrysiogenetes
Firmicutes
Tenericutes
Fusobacteria
Cyanobacteria
Gammaproteobacteria
Betaproteobacteria
Alphaproteobacteria
Epsilonproteobacteria
Deltaproteobacteria
Magnetococcus
Elusimicrobia
Thermobaculum
Euryarchaeota
Crenarchaeota
Thaumarchaeota
Nanoarchaeota
Korarchaeota

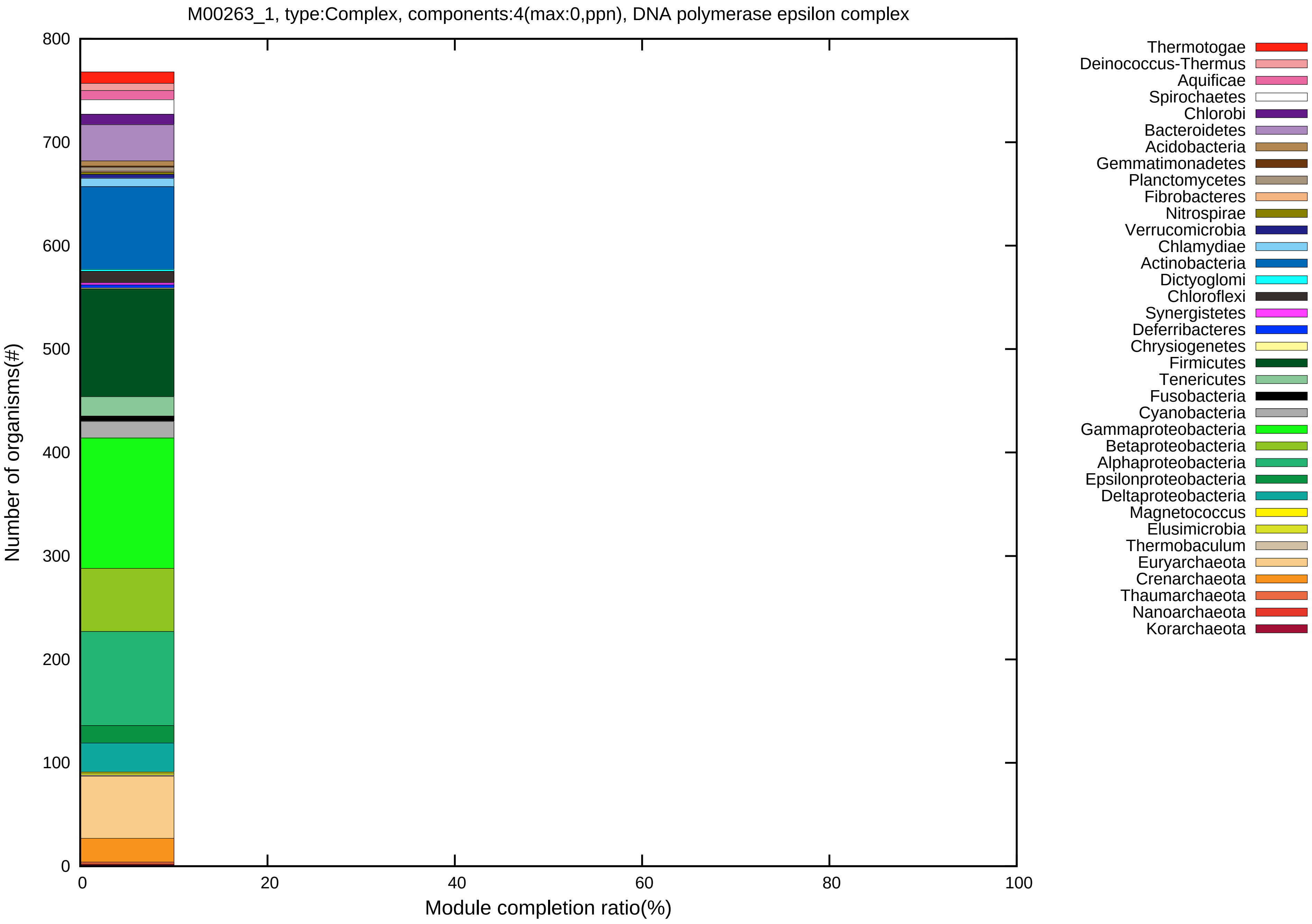

M00263_1, type:Complex, components:4(max:0,ppn), DNA polymerase epsilon complex
Number of organisms(#)
Module completion ratio(%)
0
100
200
300
400
500
600
700
800
0
20
40
60
80
100
Thermotogae
Deinococcus-Thermus
Aquificae
Spirochaetes
Chlorobi
Bacteroidetes
Acidobacteria
Gemmatimonadetes
Planctomycetes
Fibrobacteres
Nitrospirae
Verrucomicrobia
Chlamydiae
Actinobacteria
Dictyoglomi
Chloroflexi
Synergistetes
Deferribacteres
Chrysiogenetes
Firmicutes
Tenericutes
Fusobacteria
Cyanobacteria
Gammaproteobacteria
Betaproteobacteria
Alphaproteobacteria
Epsilonproteobacteria
Deltaproteobacteria
Magnetococcus
Elusimicrobia
Thermobaculum
Euryarchaeota
Crenarchaeota
Thaumarchaeota
Nanoarchaeota
Korarchaeota

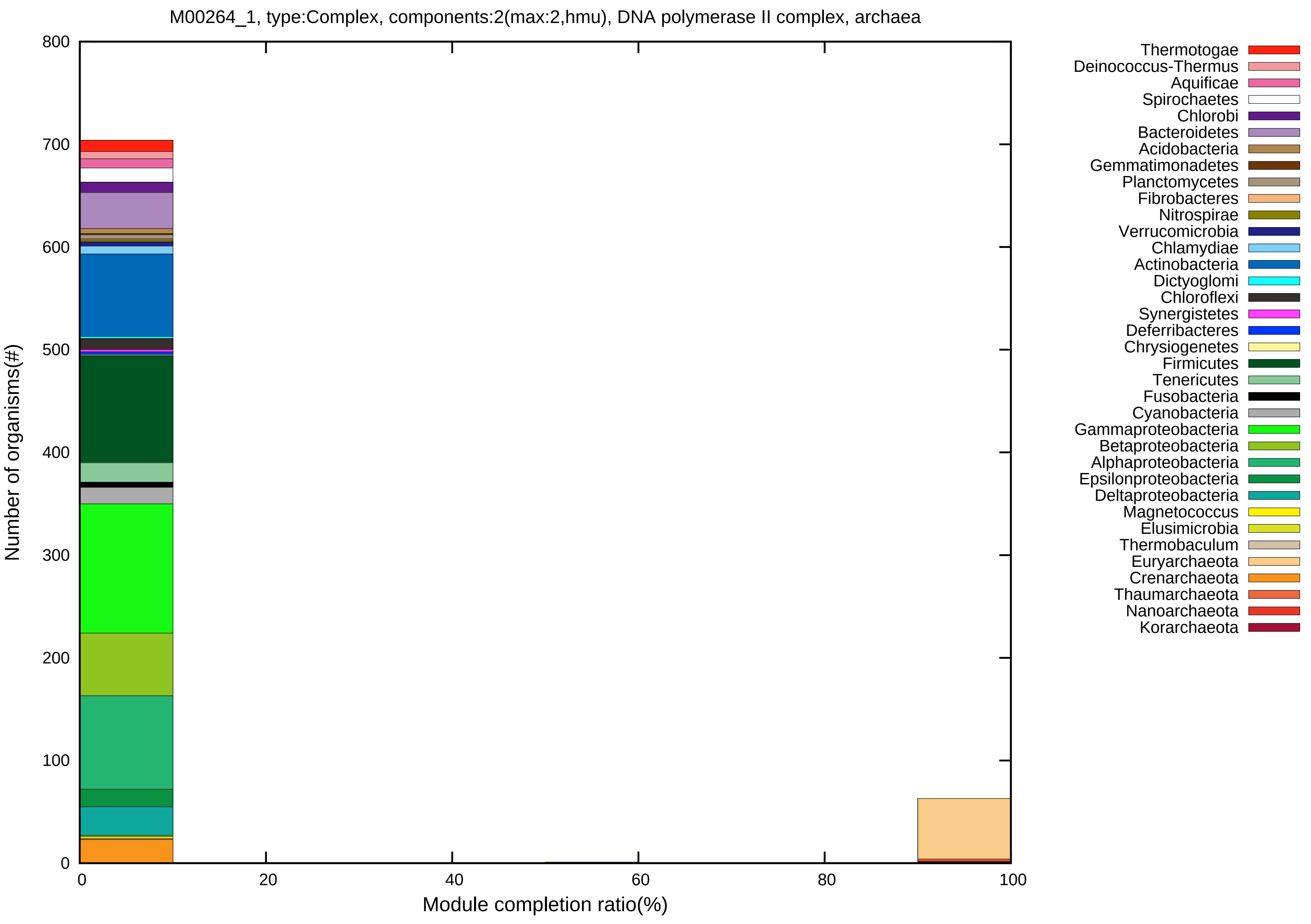

M00264_1, type:Complex, components:2(max:2,hmu), DNA polymerase II complex, archaea
Number of organisms(#)
Module completion ratio(%)
0
100
200
300
400
500
600
700
800
0
20
40
60
80
100
Thermotogae
Deinococcus-Thermus
Aquificae
Spirochaetes
Chlorobi
Bacteroidetes
Acidobacteria
Gemmatimonadetes
Planctomycetes
Fibrobacteres
Nitrospirae
Verrucomicrobia
Chlamydiae
Actinobacteria
Dictyoglomi
Chloroflexi
Synergistetes
Deferribacteres
Chrysiogenetes
Firmicutes
Tenericutes
Fusobacteria
Cyanobacteria
Gammaproteobacteria
Betaproteobacteria
Alphaproteobacteria
Epsilonproteobacteria
Deltaproteobacteria
Magnetococcus
Elusimicrobia
Thermobaculum
Euryarchaeota
Crenarchaeota
Thaumarchaeota
Nanoarchaeota
Korarchaeota

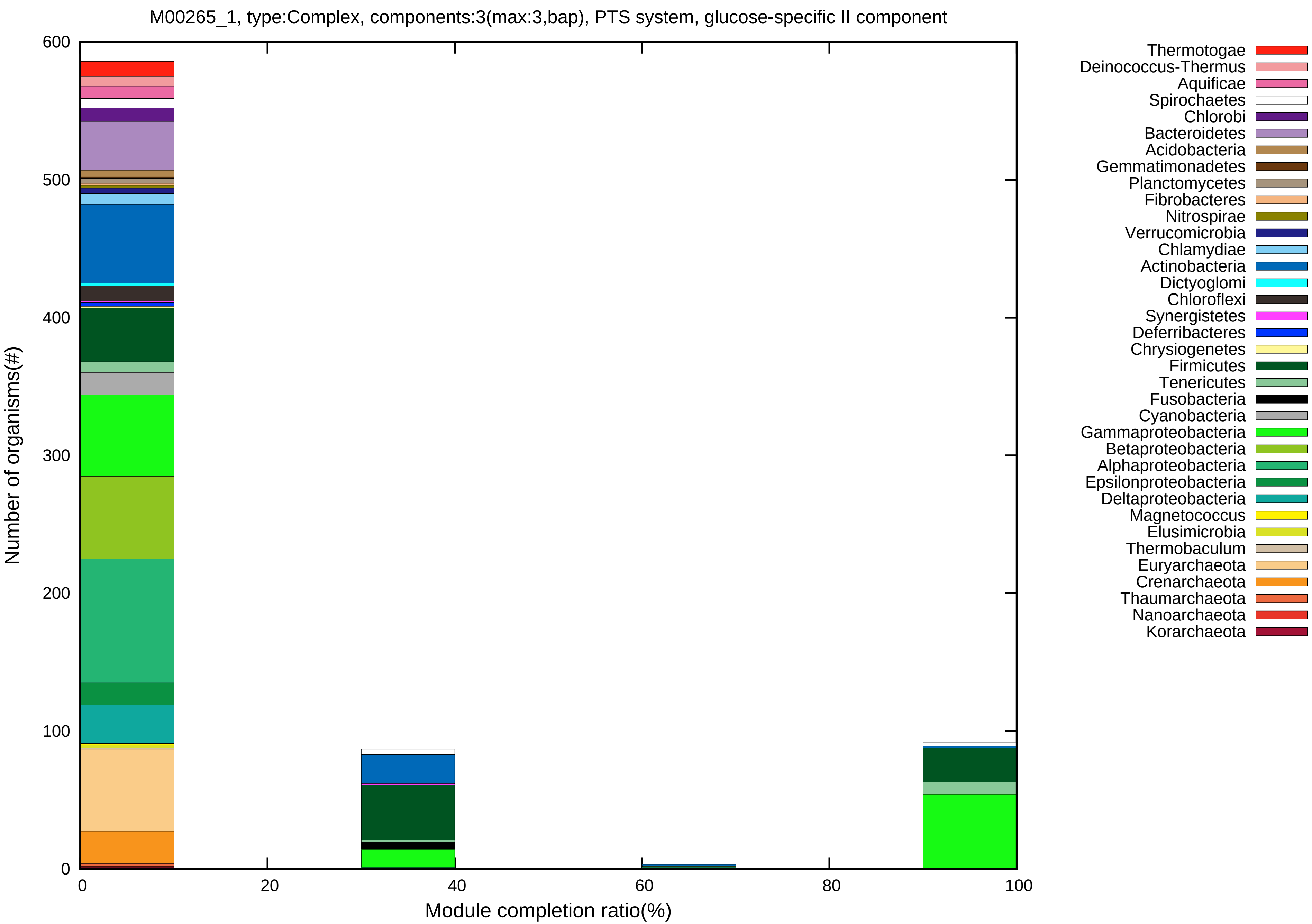

M00265_1, type:Complex, components:3(max:3,bap), PTS system, glucose-specific II component
Number of organisms(#)
Module completion ratio(%)
0
100
200
300
400
500
600
0
20
40
60
80
100
Thermotogae
Deinococcus-Thermus
Aquificae
Spirochaetes
Chlorobi
Bacteroidetes
Acidobacteria
Gemmatimonadetes
Planctomycetes
Fibrobacteres
Nitrospirae
Verrucomicrobia
Chlamydiae
Actinobacteria
Dictyoglomi
Chloroflexi
Synergistetes
Deferribacteres
Chrysiogenetes
Firmicutes
Tenericutes
Fusobacteria
Cyanobacteria
Gammaproteobacteria
Betaproteobacteria
Alphaproteobacteria
Epsilonproteobacteria
Deltaproteobacteria
Magnetococcus
Elusimicrobia
Thermobaculum
Euryarchaeota
Crenarchaeota
Thaumarchaeota
Nanoarchaeota
Korarchaeota

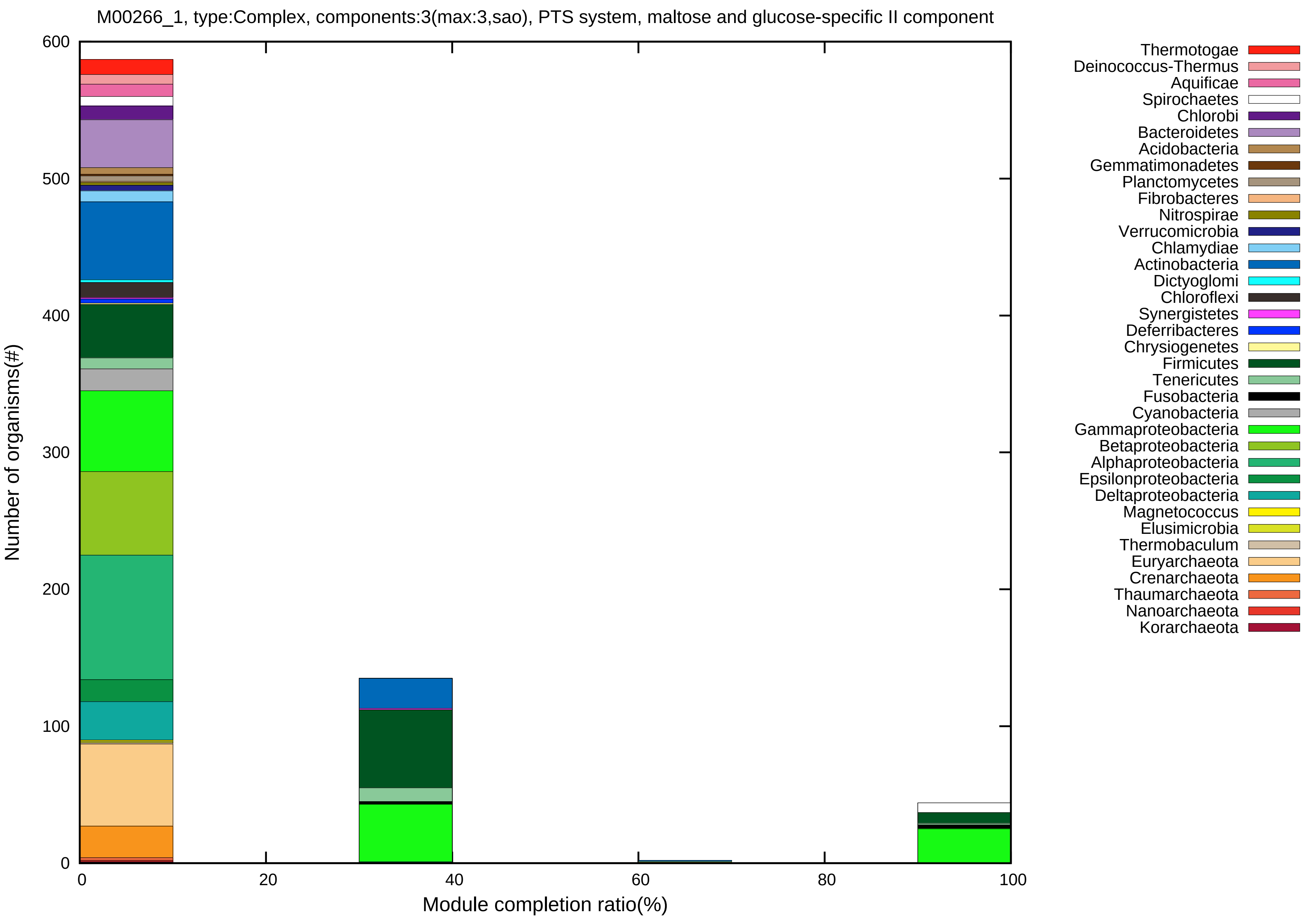
M00266_1, type:Complex, components:3(max:3,sao), PTS system, maltose and glucose-specific II component
Number of organisms(#)
Module completion ratio(%)
0
100
200
300
400
500
600
0
20
40
60
80
100
Thermotogae
Deinococcus-Thermus
Aquificae
Spirochaetes
Chlorobi
Bacteroidetes
Acidobacteria
Gemmatimonadetes
Planctomycetes
Fibrobacteres
Nitrospirae
Verrucomicrobia
Chlamydiae
Actinobacteria
Dictyoglomi
Chloroflexi
Synergistetes
Deferribacteres
Chrysiogenetes
Firmicutes
Tenericutes
Fusobacteria
Cyanobacteria
Gammaproteobacteria
Betaproteobacteria
Alphaproteobacteria
Epsilonproteobacteria
Deltaproteobacteria
Magnetococcus
Elusimicrobia
Thermobaculum
Euryarchaeota
Crenarchaeota
Thaumarchaeota
Nanoarchaeota
Korarchaeota

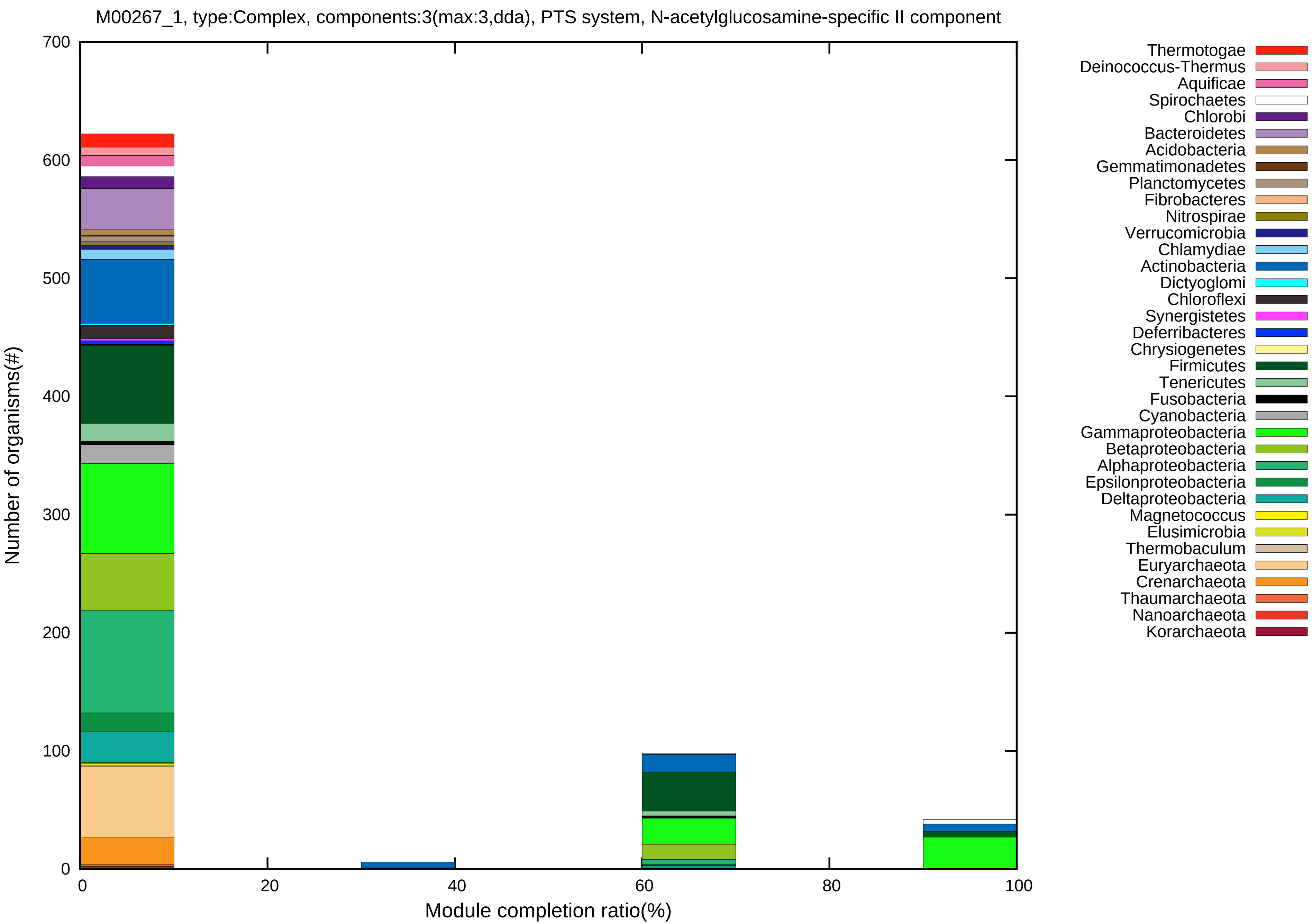
M00267_1, type:Complex, components:3(max:3,dda), PTS system, N-acetylglucosamine-specific II component
Number of organisms(#)
Module completion ratio(%)
0
100
200
300
400
500
600
700
0
20
40
60
80
100
Thermotogae
Deinococcus-Thermus
Aquificae
Spirochaetes
Chlorobi
Bacteroidetes
Acidobacteria
Gemmatimonadetes
Planctomycetes
Fibrobacteres
Nitrospirae
Verrucomicrobia
Chlamydiae
Actinobacteria
Dictyoglomi
Chloroflexi
Synergistetes
Deferribacteres
Chrysiogenetes
Firmicutes
Tenericutes
Fusobacteria
Cyanobacteria
Gammaproteobacteria
Betaproteobacteria
Alphaproteobacteria
Epsilonproteobacteria
Deltaproteobacteria
Magnetococcus
Elusimicrobia
Thermobaculum
Euryarchaeota
Crenarchaeota
Thaumarchaeota
Nanoarchaeota
Korarchaeota

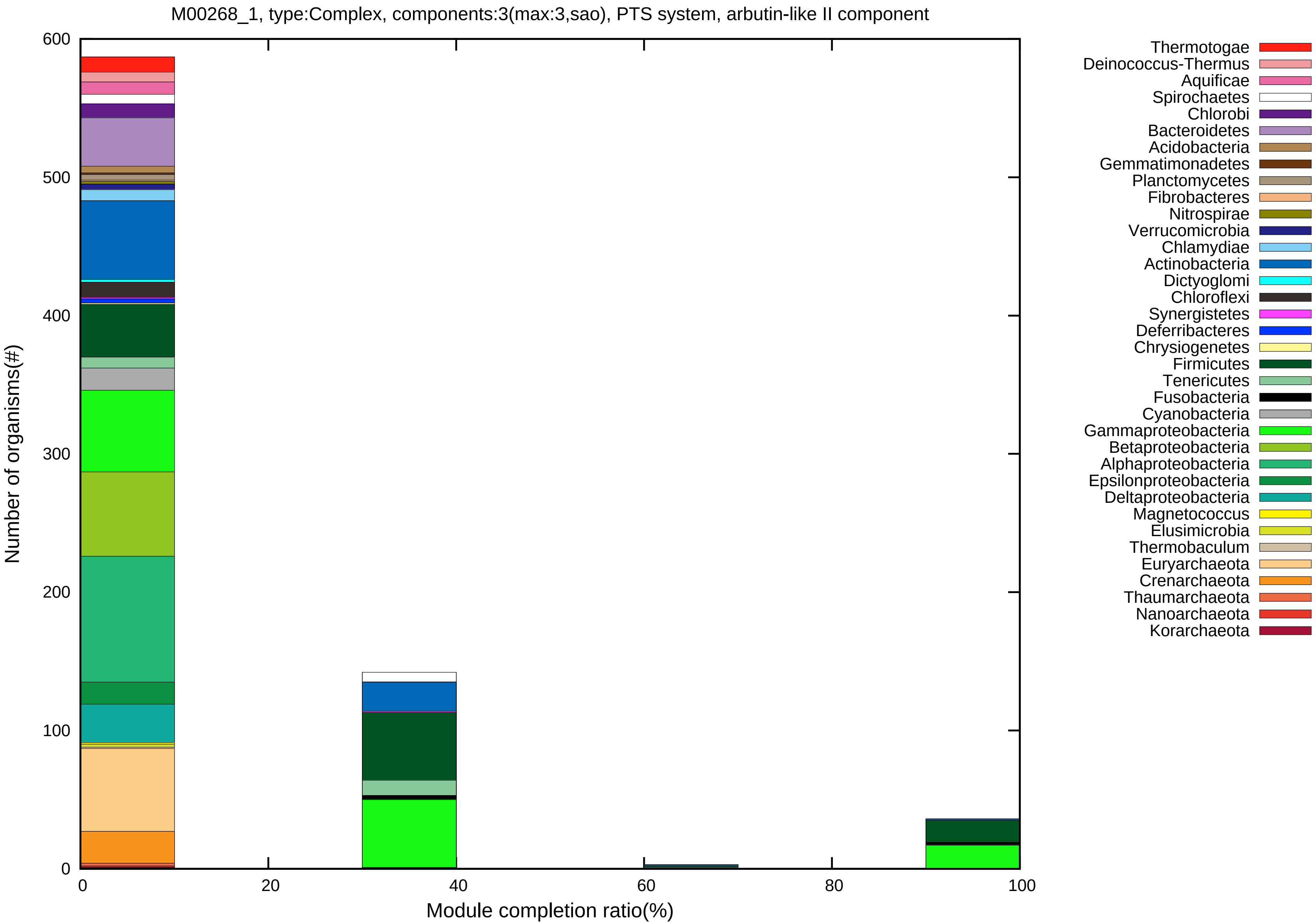

M00268_1, type:Complex, components:3(max:3,sao), PTS system, arbutin-like II component
Number of organisms(#)
Module completion ratio(%)
0
100
200
300
400
500
600
0
20
40
60
80
100
Thermotogae
Deinococcus-Thermus
Aquificae
Spirochaetes
Chlorobi
Bacteroidetes
Acidobacteria
Gemmatimonadetes
Planctomycetes
Fibrobacteres
Nitrospirae
Verrucomicrobia
Chlamydiae
Actinobacteria
Dictyoglomi
Chloroflexi
Synergistetes
Deferribacteres
Chrysiogenetes
Firmicutes
Tenericutes
Fusobacteria
Cyanobacteria
Gammaproteobacteria
Betaproteobacteria
Alphaproteobacteria
Epsilonproteobacteria
Deltaproteobacteria
Magnetococcus
Elusimicrobia
Thermobaculum
Euryarchaeota
Crenarchaeota
Thaumarchaeota
Nanoarchaeota
Korarchaeota

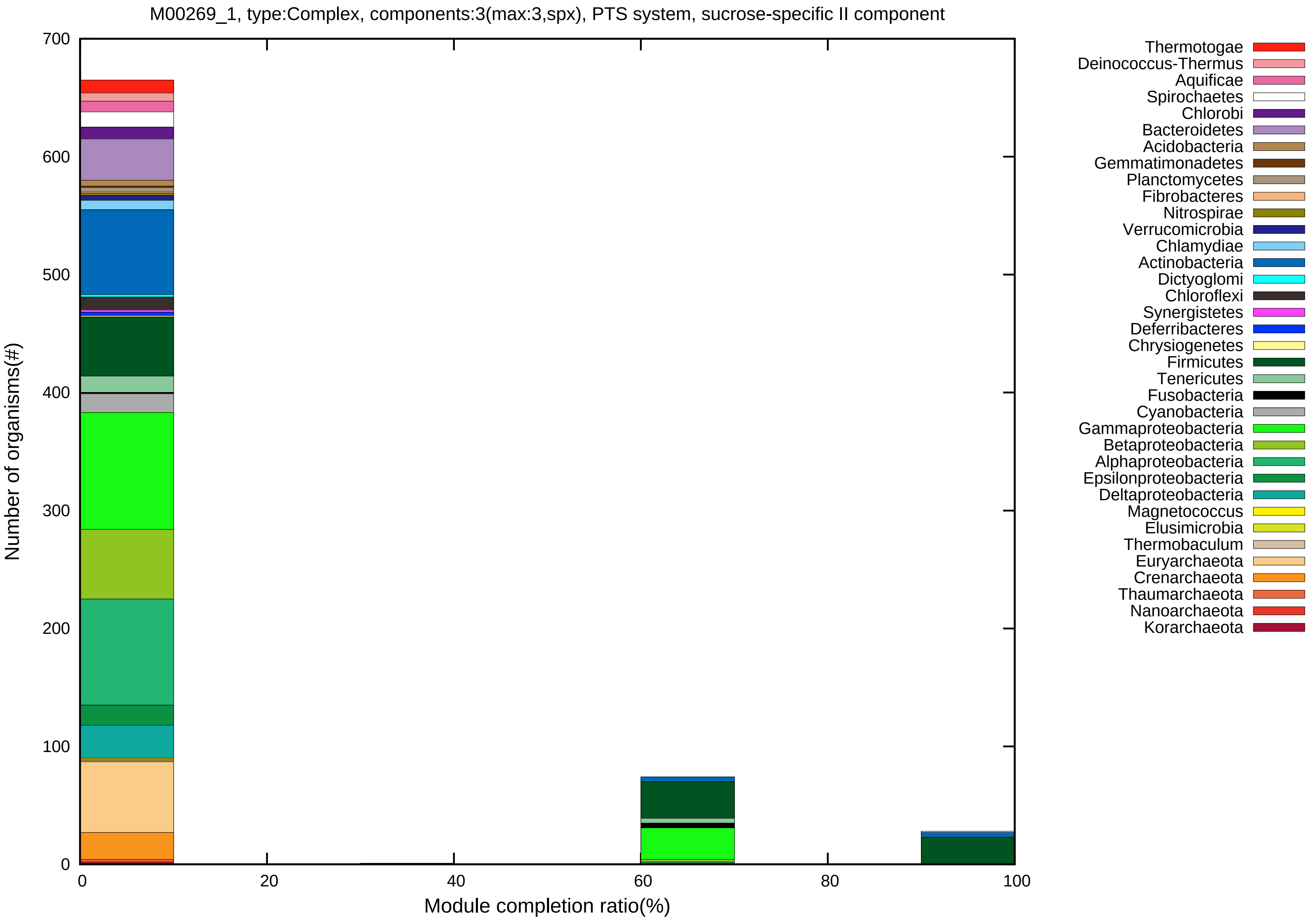

M00269_1, type:Complex, components:3(max:3,spx), PTS system, sucrose-specific II component
Number of organisms(#)
Module completion ratio(%)
0
100
200
300
400
500
600
700
0
20
40
60
80
100
Thermotogae
Deinococcus-Thermus
Aquificae
Spirochaetes
Chlorobi
Bacteroidetes
Acidobacteria
Gemmatimonadetes
Planctomycetes
Fibrobacteres
Nitrospirae
Verrucomicrobia
Chlamydiae
Actinobacteria
Dictyoglomi
Chloroflexi
Synergistetes
Deferribacteres
Chrysiogenetes
Firmicutes
Tenericutes
Fusobacteria
Cyanobacteria
Gammaproteobacteria
Betaproteobacteria
Alphaproteobacteria
Epsilonproteobacteria
Deltaproteobacteria
Magnetococcus
Elusimicrobia
Thermobaculum
Euryarchaeota
Crenarchaeota
Thaumarchaeota
Nanoarchaeota
Korarchaeota

M00270_1, type:Complex, components:3(max:3,sao), PTS system, trehalose-specific II component

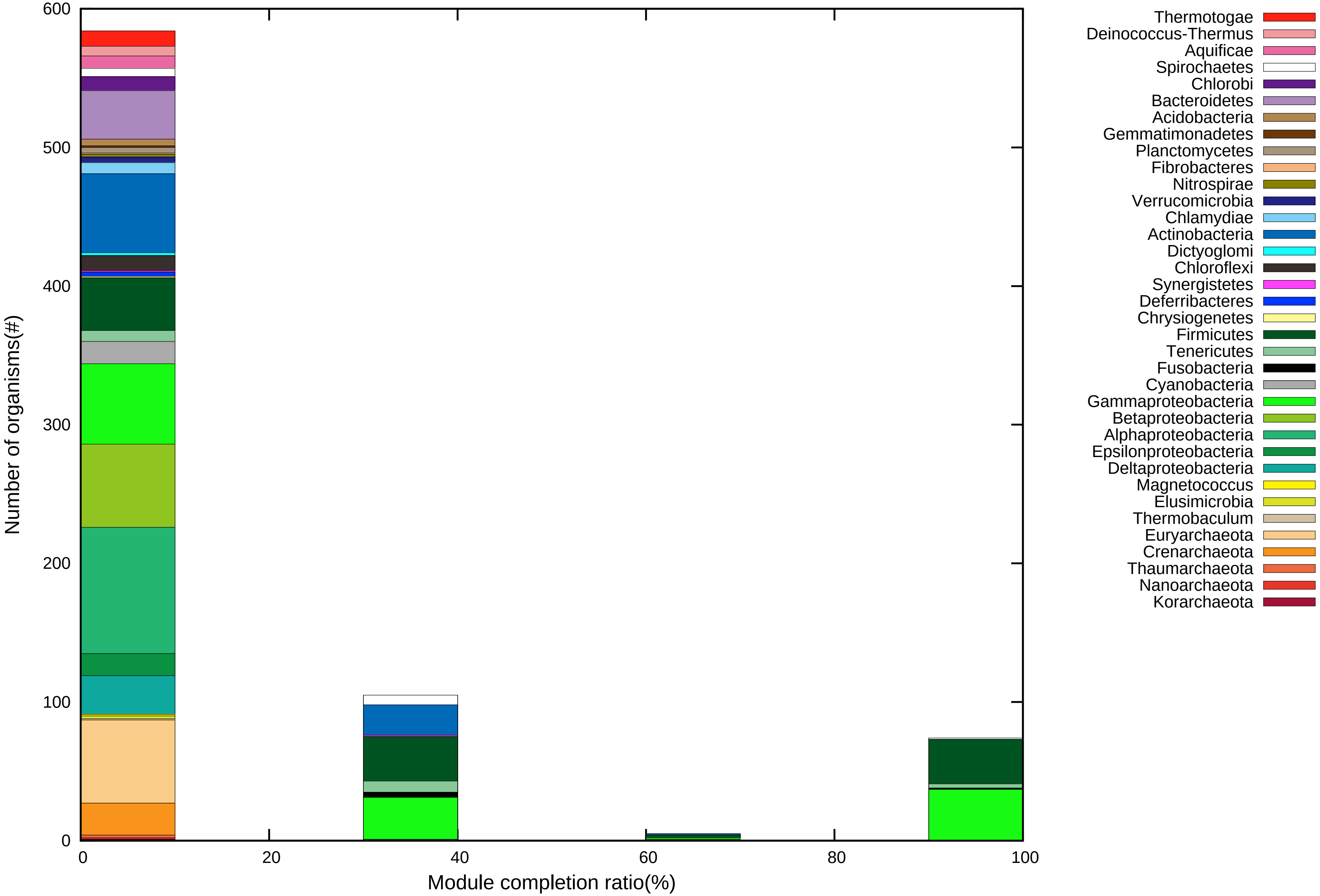

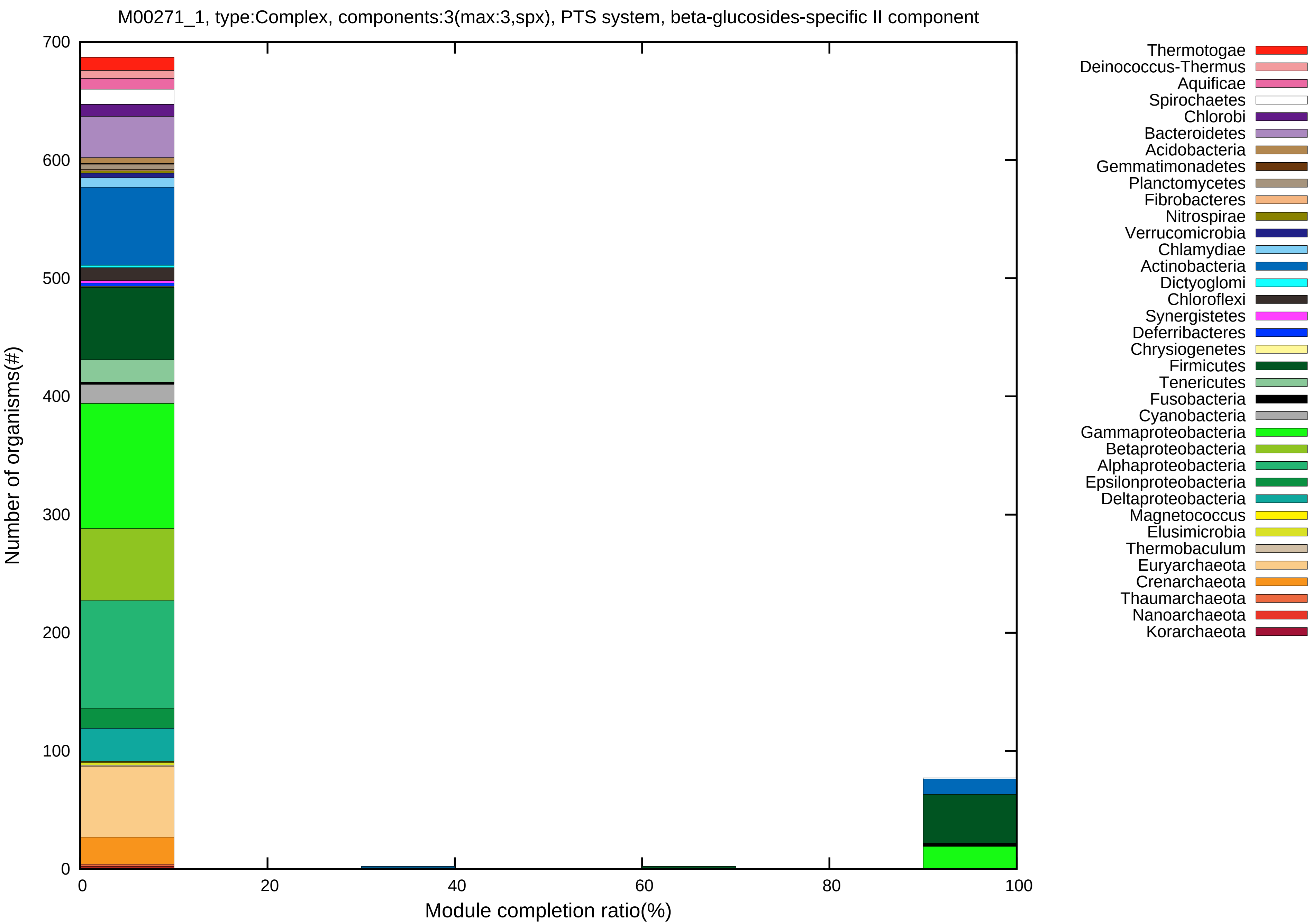

M00271_1, type:Complex, components:3(max:3,spx), PTS system, beta-glucosides-specific II component
Number of organisms(#)
Module completion ratio(%)
0
100
200
300
400
500
600
700
0
20
40
60
80
100
Thermotogae
Deinococcus-Thermus
Aquificae
Spirochaetes
Chlorobi
Bacteroidetes
Acidobacteria
Gemmatimonadetes
Planctomycetes
Fibrobacteres
Nitrospirae
Verrucomicrobia
Chlamydiae
Actinobacteria
Dictyoglomi
Chloroflexi
Synergistetes
Deferribacteres
Chrysiogenetes
Firmicutes
Tenericutes
Fusobacteria
Cyanobacteria
Gammaproteobacteria
Betaproteobacteria
Alphaproteobacteria
Epsilonproteobacteria
Deltaproteobacteria
Magnetococcus
Elusimicrobia
Thermobaculum
Euryarchaeota
Crenarchaeota
Thaumarchaeota
Nanoarchaeota
Korarchaeota

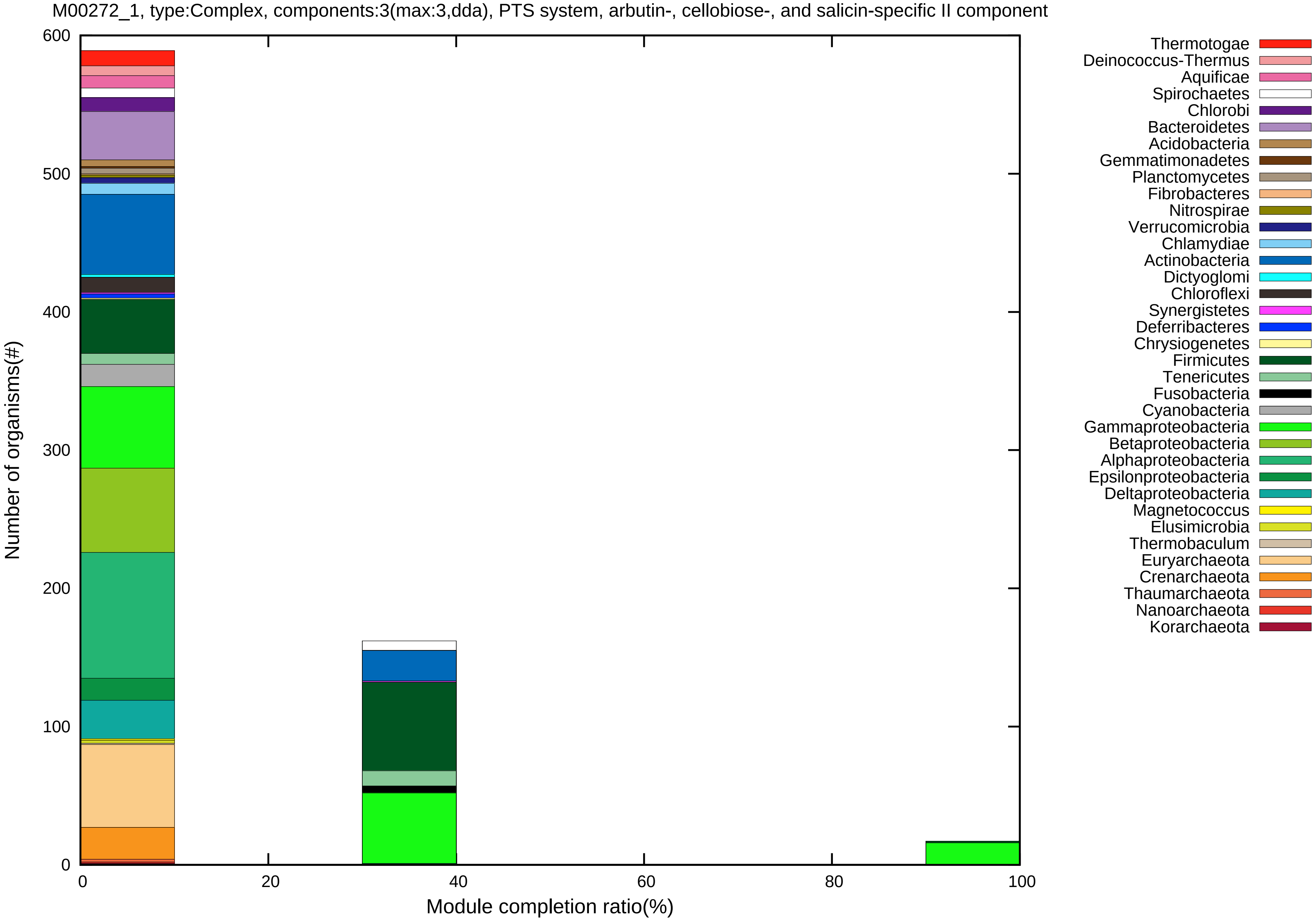

M00272_1, type:Complex, components:3(max:3,dda), PTS system, arbutin-, cellobiose-, and salicin-specific II component
Number of organisms(#)
Module completion ratio(%)
0
100
200
300
400
500
600
0
20
40
60
80
100
Thermotogae
Deinococcus-Thermus
Aquificae
Spirochaetes
Chlorobi
Bacteroidetes
Acidobacteria
Gemmatimonadetes
Planctomycetes
Fibrobacteres
Nitrospirae
Verrucomicrobia
Chlamydiae
Actinobacteria
Dictyoglomi
Chloroflexi
Synergistetes
Deferribacteres
Chrysiogenetes
Firmicutes
Tenericutes
Fusobacteria
Cyanobacteria
Gammaproteobacteria
Betaproteobacteria
Alphaproteobacteria
Epsilonproteobacteria
Deltaproteobacteria
Magnetococcus
Elusimicrobia
Thermobaculum
Euryarchaeota
Crenarchaeota
Thaumarchaeota
Nanoarchaeota
Korarchaeota

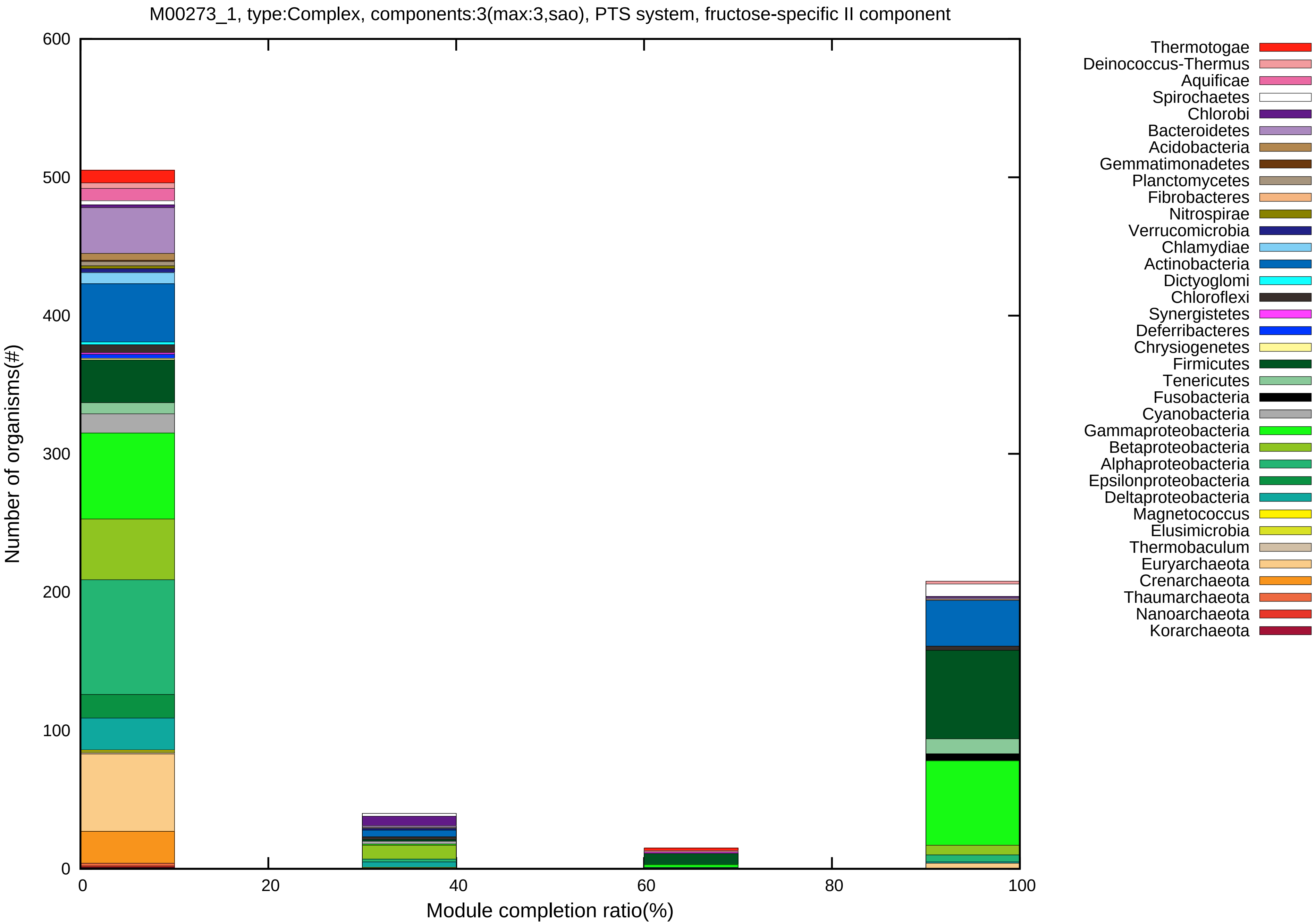

M00273_1, type:Complex, components:3(max:3,sao), PTS system, fructose-specific II component
Number of organisms(#)
Module completion ratio(%)
0
100
200
300
400
500
600
0
20
40
60
80
100
Thermotogae
Deinococcus-Thermus
Aquificae
Spirochaetes
Chlorobi
Bacteroidetes
Acidobacteria
Gemmatimonadetes
Planctomycetes
Fibrobacteres
Nitrospirae
Verrucomicrobia
Chlamydiae
Actinobacteria
Dictyoglomi
Chloroflexi
Synergistetes
Deferribacteres
Chrysiogenetes
Firmicutes
Tenericutes
Fusobacteria
Cyanobacteria
Gammaproteobacteria
Betaproteobacteria
Alphaproteobacteria
Epsilonproteobacteria
Deltaproteobacteria
Magnetococcus
Elusimicrobia
Thermobaculum
Euryarchaeota
Crenarchaeota
Thaumarchaeota
Nanoarchaeota
Korarchaeota

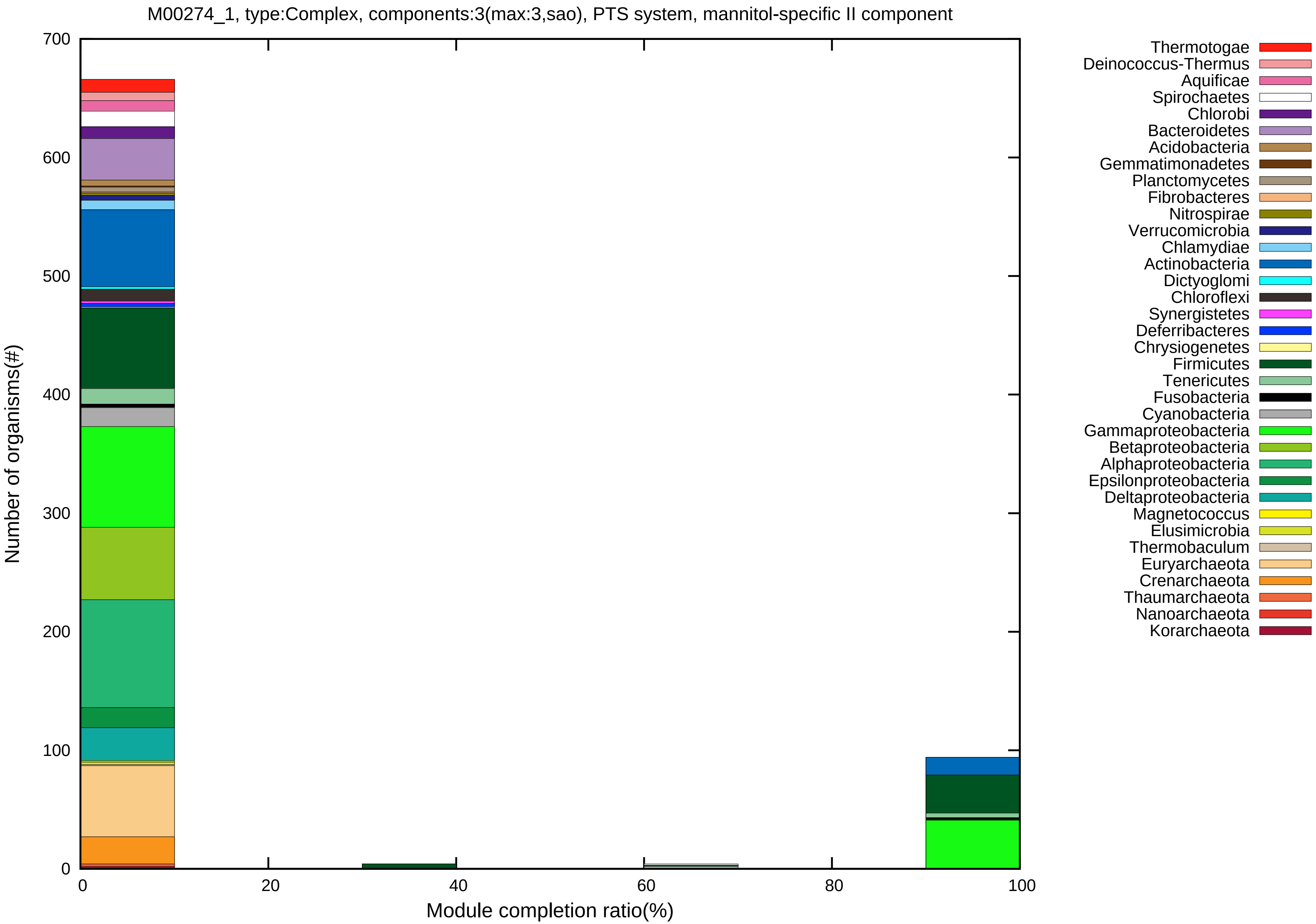
M00274_1, type:Complex, components:3(max:3,sao), PTS system, mannitol-specific II component
Number of organisms(#)
0
100
200
300
400
500
600
700
0
20
40
60
80
100
Module completion ratio(%)
Thermotogae
Deinococcus-Thermus
Aquificae
Spirochaetes
Chlorobi
Bacteroidetes
Acidobacteria
Gemmatimonadetes
Planctomycetes
Fibrobacteres
Nitrospirae
Verrucomicrobia
Chlamydiae
Actinobacteria
Dictyoglomi
Chloroflexi
Synergistetes
Deferribacteres
Chrysiogenetes
Firmicutes
Tenericutes
Fusobacteria
Cyanobacteria
Gammaproteobacteria
Betaproteobacteria
Alphaproteobacteria
Epsilonproteobacteria
Deltaproteobacteria
Magnetococcus
Elusimicrobia
Thermobaculum
Euryarchaeota
Crenarchaeota
Thaumarchaeota
Nanoarchaeota
Korarchaeota

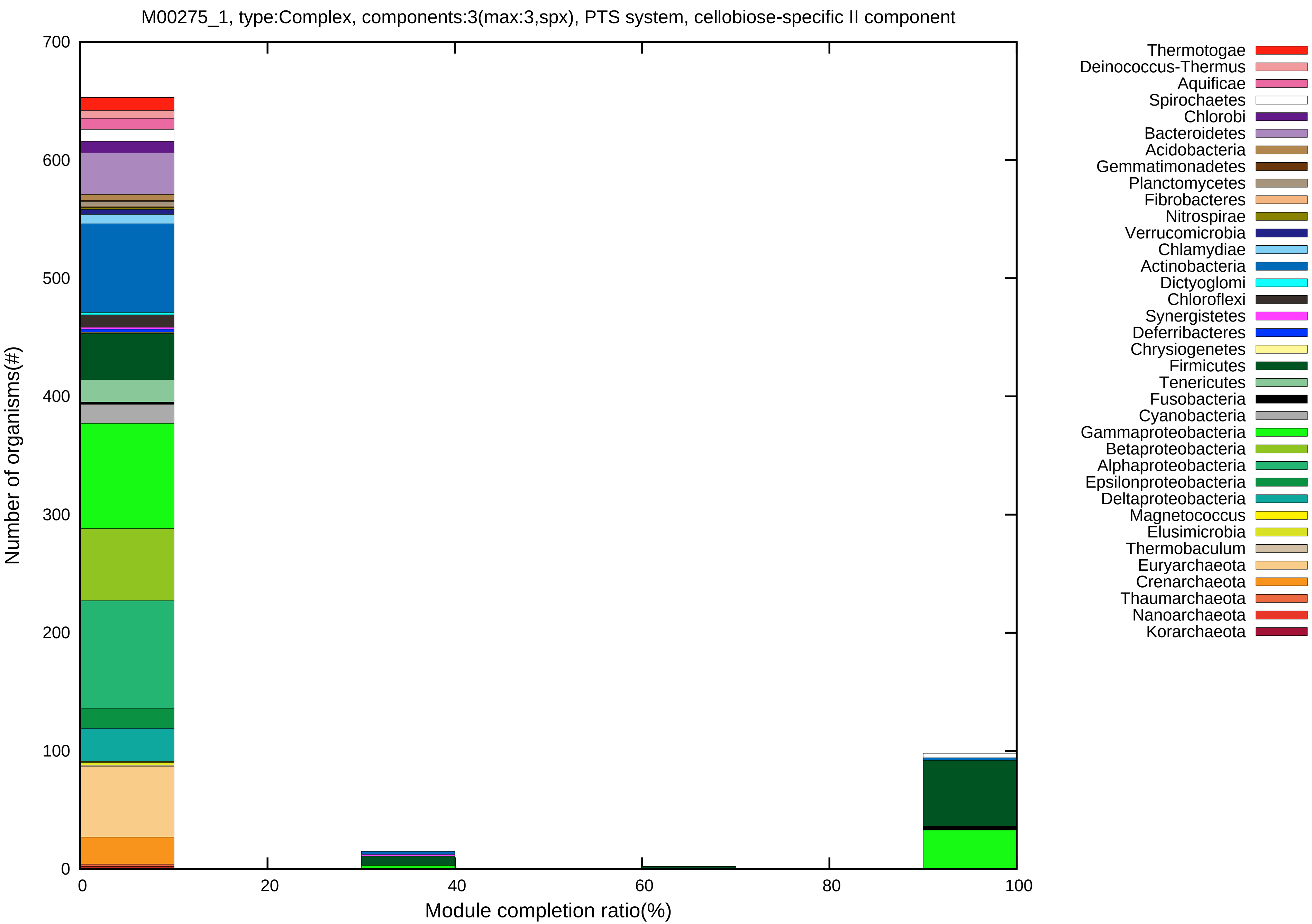

M00275_1, type:Complex, components:3(max:3,spx), PTS system, cellobiose-specific II component
Number of organisms(#)
Module completion ratio(%)
0
100
200
300
400
500
600
700
0
20
40
60
80
100
Thermotogae
Deinococcus-Thermus
Aquificae
Spirochaetes
Chlorobi
Bacteroidetes
Acidobacteria
Gemmatimonadetes
Planctomycetes
Fibrobacteres
Nitrospirae
Verrucomicrobia
Chlamydiae
Actinobacteria
Dictyoglomi
Chloroflexi
Synergistetes
Deferribacteres
Chrysiogenetes
Firmicutes
Tenericutes
Fusobacteria
Cyanobacteria
Gammaproteobacteria
Betaproteobacteria
Alphaproteobacteria
Epsilonproteobacteria
Deltaproteobacteria
Magnetococcus
Elusimicrobia
Thermobaculum
Euryarchaeota
Crenarchaeota
Thaumarchaeota
Nanoarchaeota
Korarchaeota

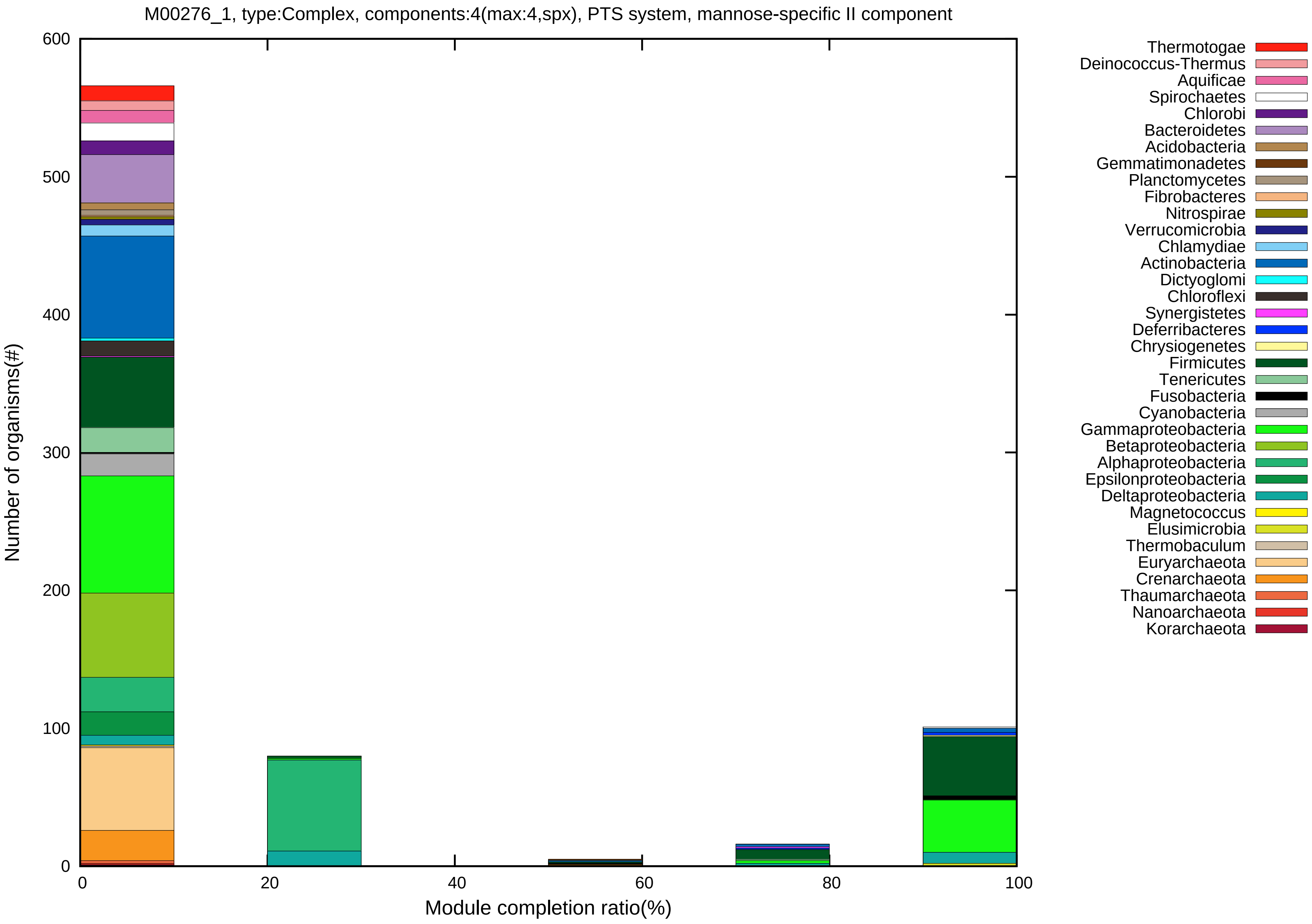

M00276_1, type:Complex, components:4(max:4,spx), PTS system, mannose-specific II component
Number of organisms(#)
Module completion ratio(%)
0
100
200
300
400
500
600
0
20
40
60
80
100
Thermotogae
Deinococcus-Thermus
Aquificae
Spirochaetes
Chlorobi
Bacteroidetes
Acidobacteria
Gemmatimonadetes
Planctomycetes
Fibrobacteres
Nitrospirae
Verrucomicrobia
Chlamydiae
Actinobacteria
Dictyoglomi
Chloroflexi
Synergistetes
Deferribacteres
Chrysiogenetes
Firmicutes
Tenericutes
Fusobacteria
Cyanobacteria
Gammaproteobacteria
Betaproteobacteria
Alphaproteobacteria
Epsilonproteobacteria
Deltaproteobacteria
Magnetococcus
Elusimicrobia
Thermobaculum
Euryarchaeota
Crenarchaeota
Thaumarchaeota
Nanoarchaeota
Korarchaeota

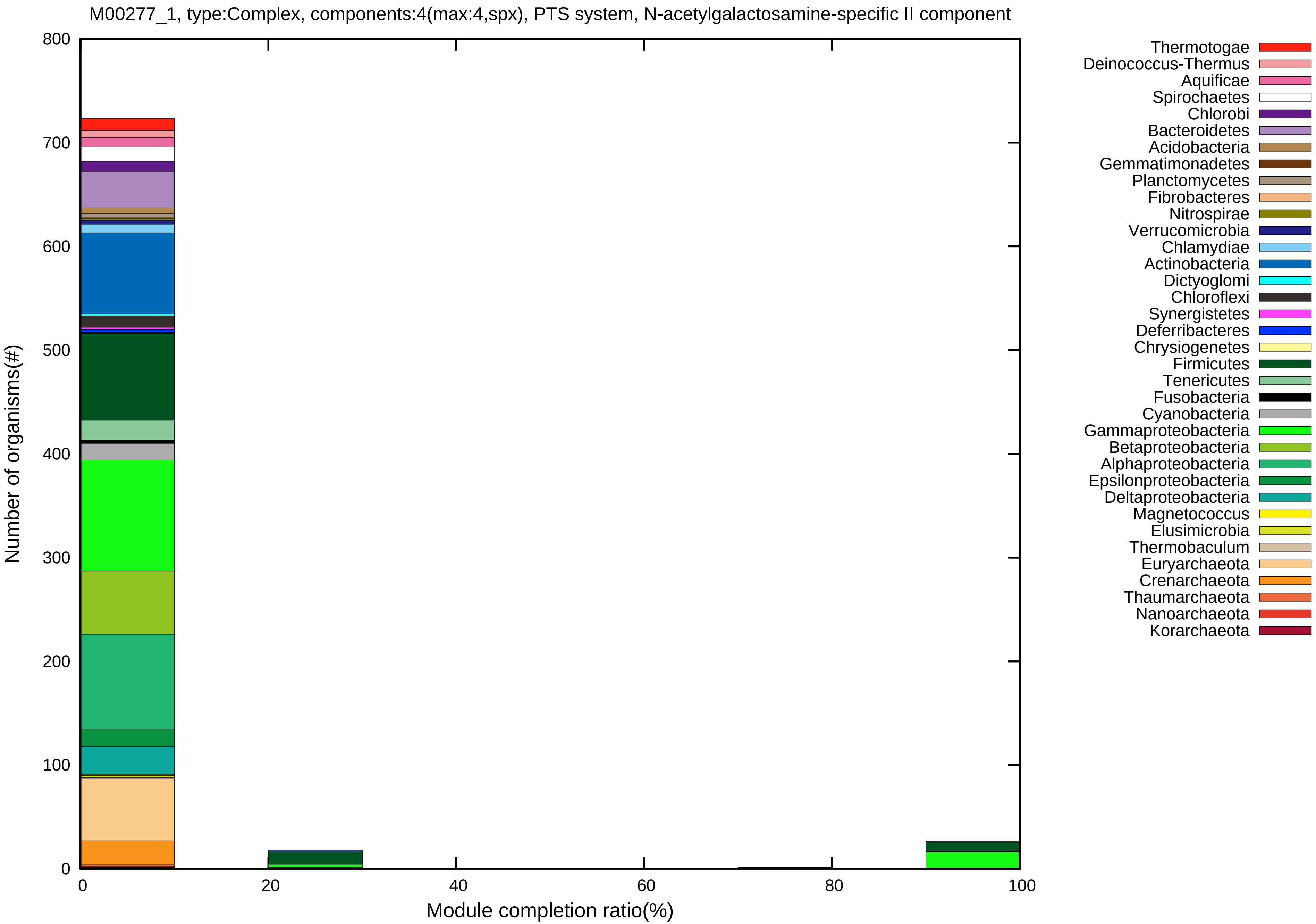

M00277_1, type:Complex, components:4(max:4,spx), PTS system, N-acetylgalactosamine-specific II component
Number of organisms(#)
0
100
200
300
400
500
600
700
800
0
20
40
60
80
100
Module completion ratio(%)
Thermotogae
Deinococcus-Thermus
Aquificae
Spirochaetes
Chlorobi
Bacteroidetes
Acidobacteria
Gemmatimonadetes
Planctomycetes
Fibrobacteres
Nitrospirae
Verrucomicrobia
Chlamydiae
Actinobacteria
Dictyoglomi
Chloroflexi
Synergistetes
Deferribacteres
Chrysiogenetes
Firmicutes
Tenericutes
Fusobacteria
Cyanobacteria
Gammaproteobacteria
Betaproteobacteria
Alphaproteobacteria
Epsilonproteobacteria
Deltaproteobacteria
Magnetococcus
Elusimicrobia
Thermobaculum
Euryarchaeota
Crenarchaeota
Thaumarchaeota
Nanoarchaeota
Korarchaeota

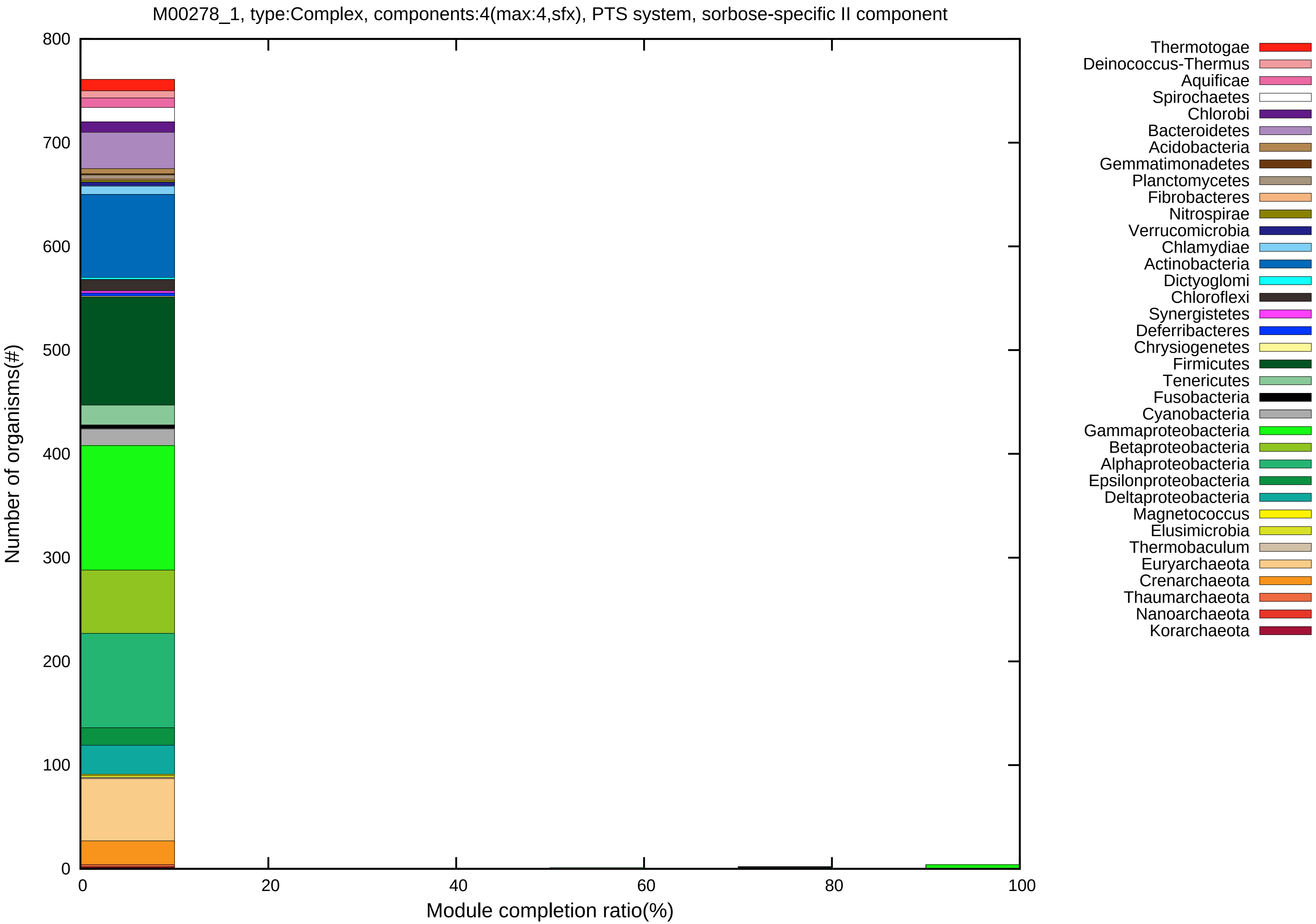

M00278_1, type:Complex, components:4(max:4,sfx), PTS system, sorbose-specific II component
Number of organisms(#)
Module completion ratio(%)
0
100
200
300
400
500
600
700
800
0
20
40
60
80
100
Thermotogae
Deinococcus-Thermus
Aquificae
Spirochaetes
Chlorobi
Bacteroidetes
Acidobacteria
Gemmatimonadetes
Planctomycetes
Fibrobacteres
Nitrospirae
Verrucomicrobia
Chlamydiae
Actinobacteria
Dictyoglomi
Chloroflexi
Synergistetes
Deferribacteres
Chrysiogenetes
Firmicutes
Tenericutes
Fusobacteria
Cyanobacteria
Gammaproteobacteria
Betaproteobacteria
Alphaproteobacteria
Epsilonproteobacteria
Deltaproteobacteria
Magnetococcus
Elusimicrobia
Thermobaculum
Euryarchaeota
Crenarchaeota
Thaumarchaeota
Nanoarchaeota
Korarchaeota

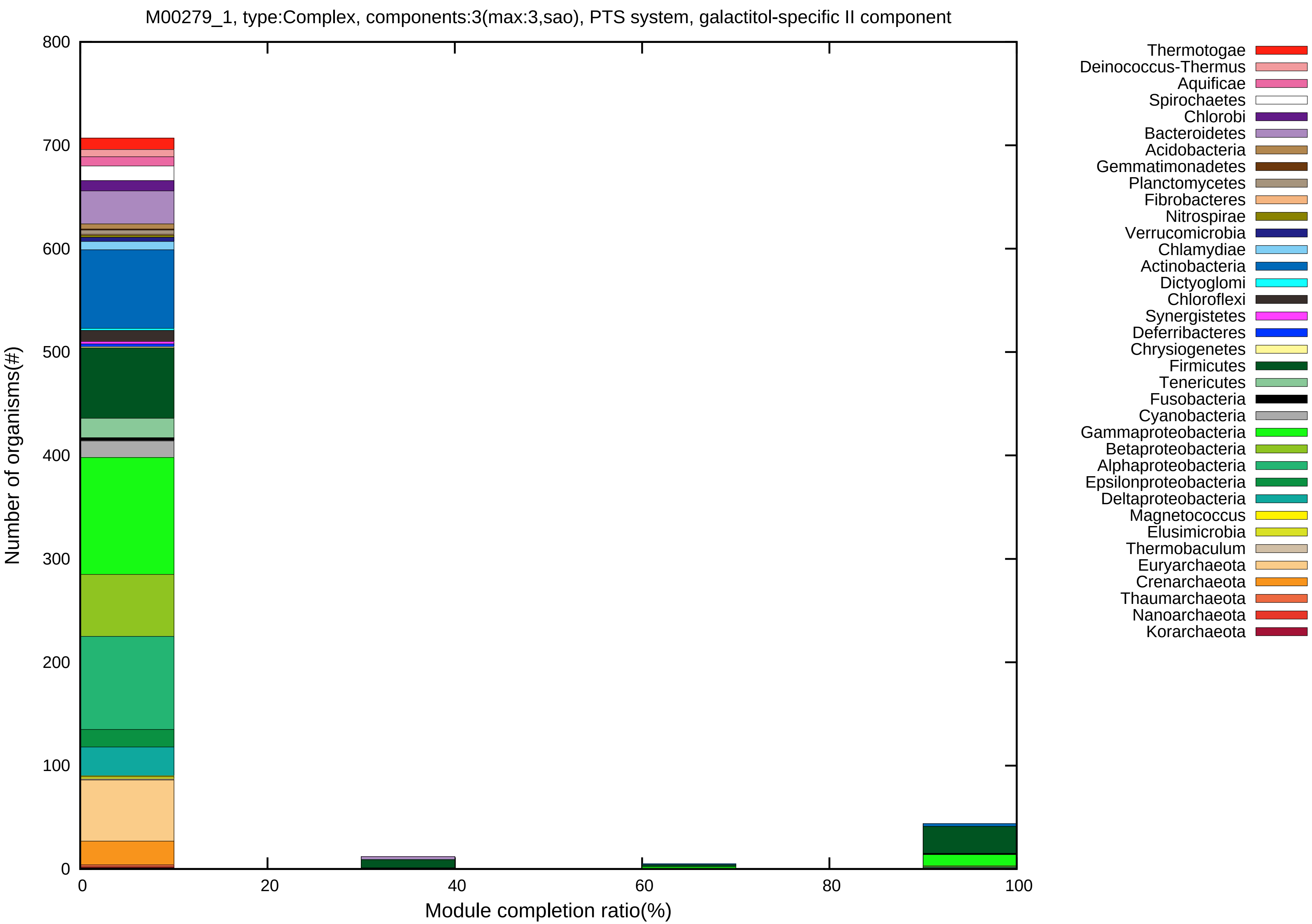
M00279_1, type:Complex, components:3(max:3,sao), PTS system, galactitol-specific II component
Number of organisms(#)
Module completion ratio(%)
0
100
200
300
400
500
600
700
800
0
20
40
60
80
100
Thermotogae
Deinococcus-Thermus
Aquificae
Spirochaetes
Chlorobi
Bacteroidetes
Acidobacteria
Gemmatimonadetes
Planctomycetes
Fibrobacteres
Nitrospirae
Verrucomicrobia
Chlamydiae
Actinobacteria
Dictyoglomi
Chloroflexi
Synergistetes
Deferribacteres
Chrysiogenetes
Firmicutes
Tenericutes
Fusobacteria
Cyanobacteria
Gammaproteobacteria
Betaproteobacteria
Alphaproteobacteria
Epsilonproteobacteria
Deltaproteobacteria
Magnetococcus
Elusimicrobia
Thermobaculum
Euryarchaeota
Crenarchaeota
Thaumarchaeota
Nanoarchaeota
Korarchaeota

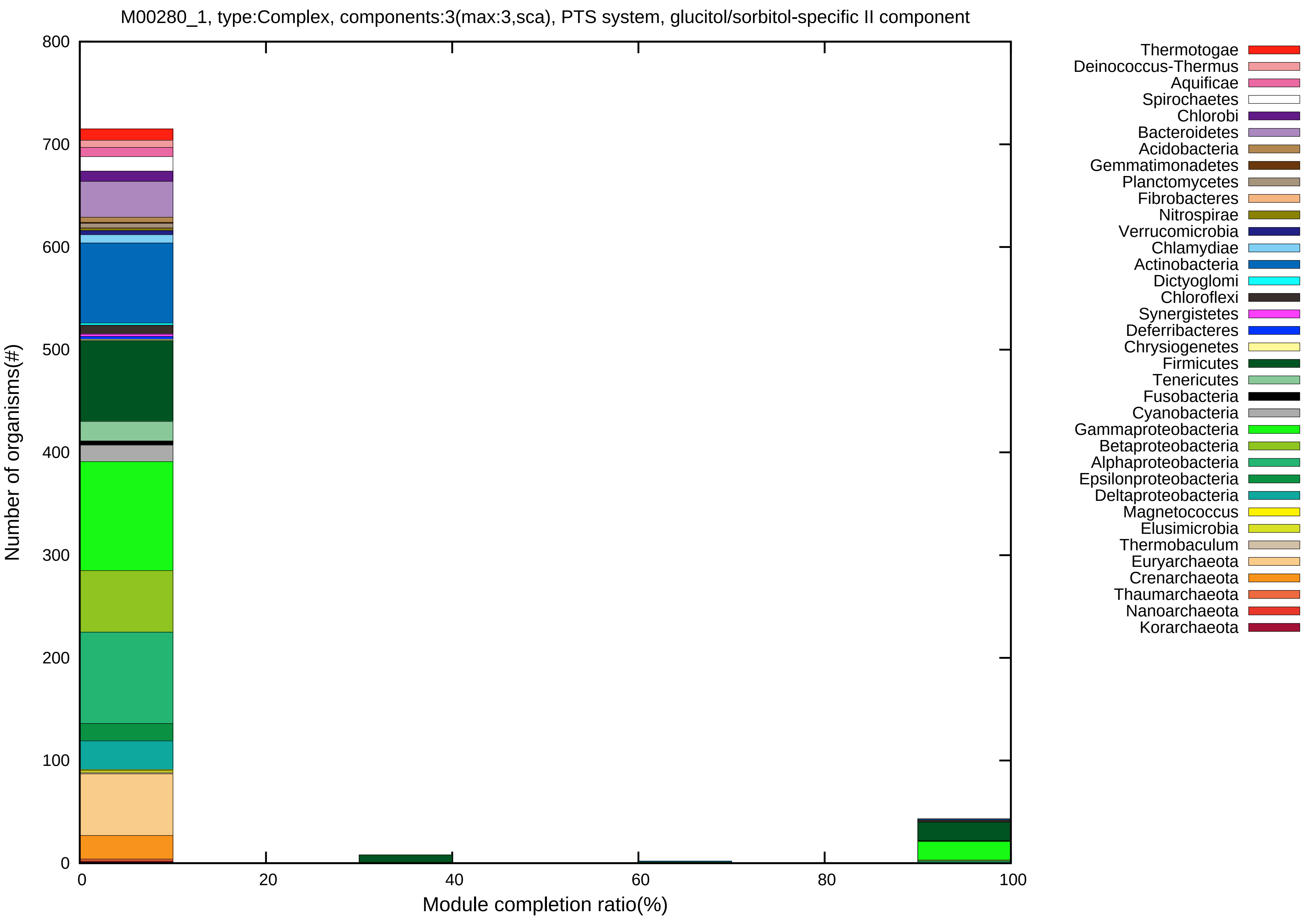
M00280_1, type:Complex, components:3(max:3,sca), PTS system, glucitol/sorbitol-specific II component
Number of organisms(#)
Module completion ratio(%)
0
100
200
300
400
500
600
700
800
0
20
40
60
80
100
Thermotogae
Deinococcus-Thermus
Aquificae
Spirochaetes
Chlorobi
Bacteroidetes
Acidobacteria
Gemmatimonadetes
Planctomycetes
Fibrobacteres
Nitrospirae
Verrucomicrobia
Chlamydiae
Actinobacteria
Dictyoglomi
Chloroflexi
Synergistetes
Deferribacteres
Chrysiogenetes
Firmicutes
Tenericutes
Fusobacteria
Cyanobacteria
Gammaproteobacteria
Betaproteobacteria
Alphaproteobacteria
Epsilonproteobacteria
Deltaproteobacteria
Magnetococcus
Elusimicrobia
Thermobaculum
Euryarchaeota
Crenarchaeota
Thaumarchaeota
Nanoarchaeota
Korarchaeota

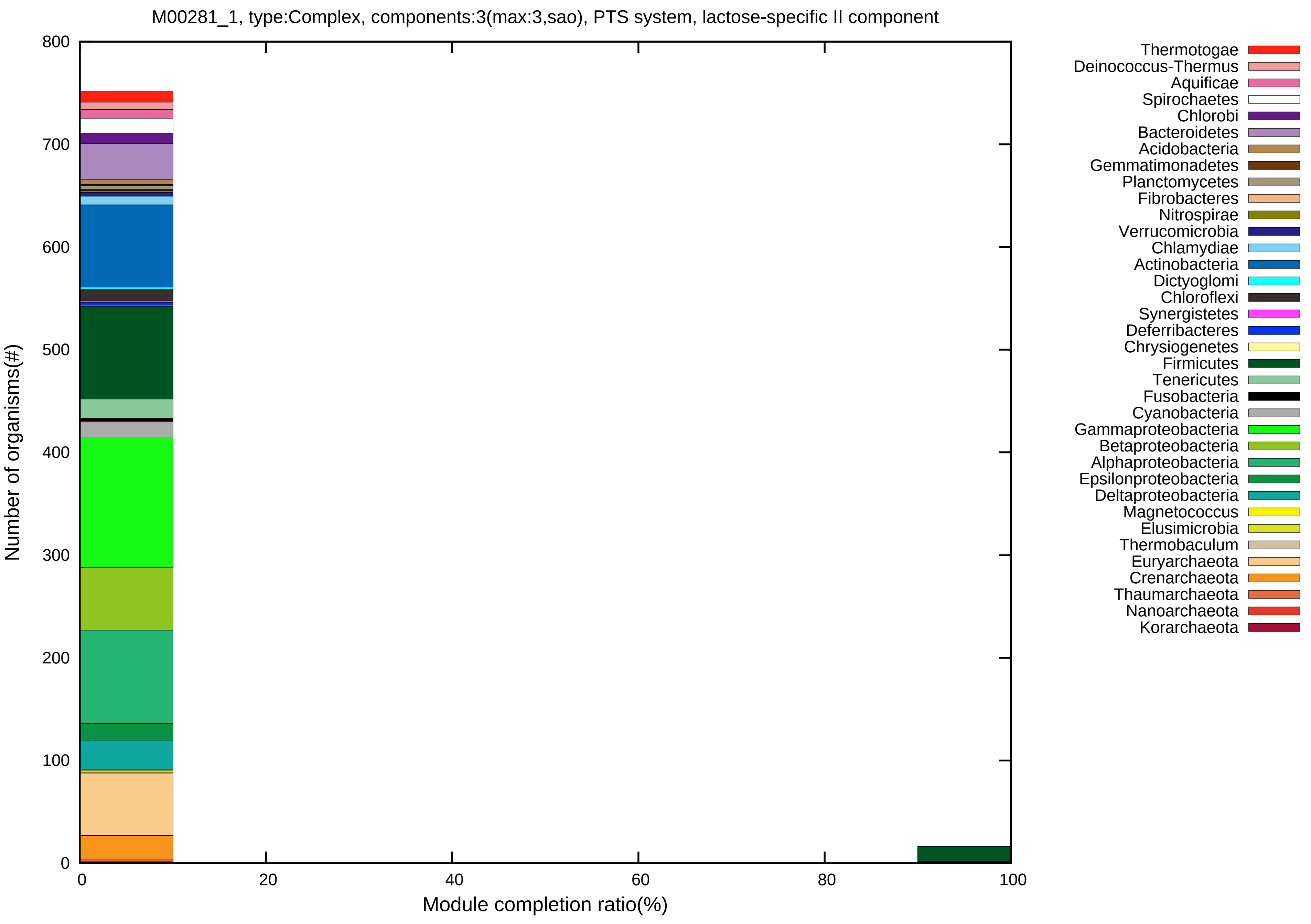

M00281_1, type:Complex, components:3(max:3,sao), PTS system, lactose-specific II component
Number of organisms(#)
Module completion ratio(%)
0
100
200
300
400
500
600
700
800
0
20
40
60
80
100
Thermotogae
Deinococcus-Thermus
Aquificae
Spirochaetes
Chlorobi
Bacteroidetes
Acidobacteria
Gemmatimonadetes
Planctomycetes
Fibrobacteres
Nitrospirae
Verrucomicrobia
Chlamydiae
Actinobacteria
Dictyoglomi
Chloroflexi
Synergistetes
Deferribacteres
Chrysiogenetes
Firmicutes
Tenericutes
Fusobacteria
Cyanobacteria
Gammaproteobacteria
Betaproteobacteria
Alphaproteobacteria
Epsilonproteobacteria
Deltaproteobacteria
Magnetococcus
Elusimicrobia
Thermobaculum
Euryarchaeota
Crenarchaeota
Thaumarchaeota
Nanoarchaeota
Korarchaeota

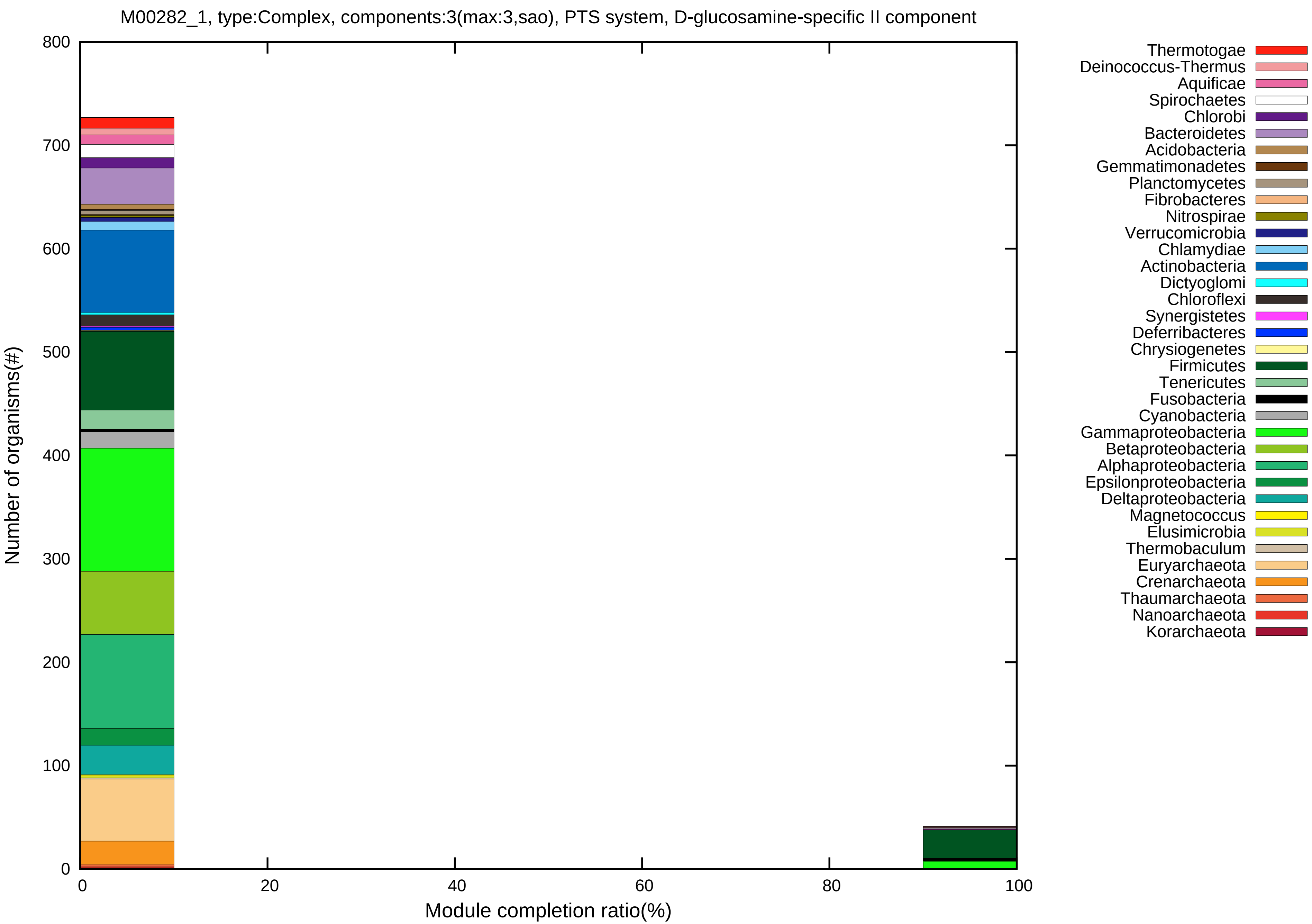

M00282_1, type:Complex, components:3(max:3,sao), PTS system, D-glucosamine-specific II component
Number of organisms(#)
Module completion ratio(%)
0
100
200
300
400
500
600
700
800
0
20
40
60
80
100
Thermotogae
Deinococcus-Thermus
Aquificae
Spirochaetes
Chlorobi
Bacteroidetes
Acidobacteria
Gemmatimonadetes
Planctomycetes
Fibrobacteres
Nitrospirae
Verrucomicrobia
Chlamydiae
Actinobacteria
Dictyoglomi
Chloroflexi
Synergistetes
Deferribacteres
Chrysiogenetes
Firmicutes
Tenericutes
Fusobacteria
Cyanobacteria
Gammaproteobacteria
Betaproteobacteria
Alphaproteobacteria
Epsilonproteobacteria
Deltaproteobacteria
Magnetococcus
Elusimicrobia
Thermobaculum
Euryarchaeota
Crenarchaeota
Thaumarchaeota
Nanoarchaeota
Korarchaeota

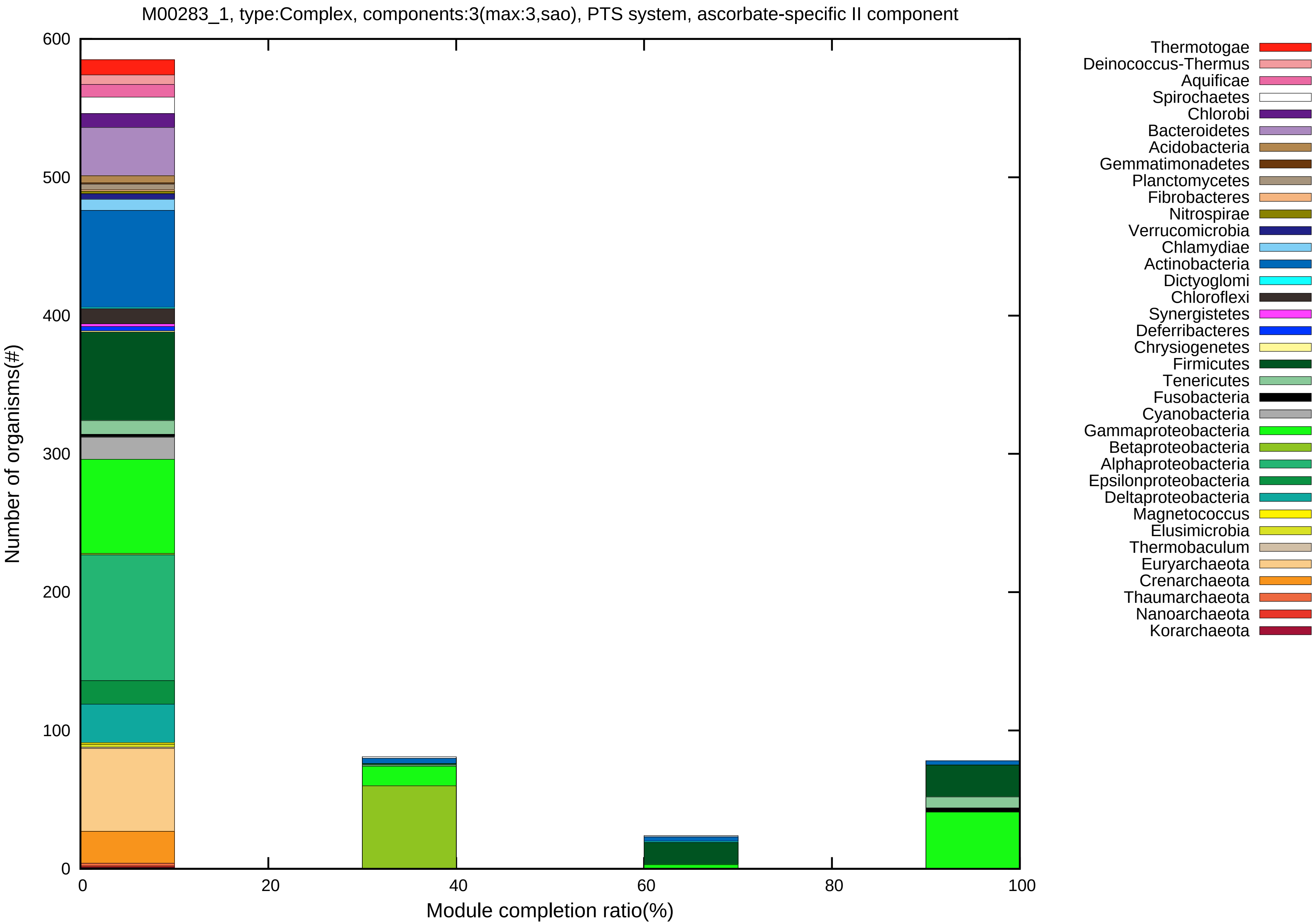

M00283_1, type:Complex, components:3(max:3,sao), PTS system, ascorbate-specific II component
Number of organisms(#)
Module completion ratio(%)
0
100
200
300
400
500
600
0
20
40
60
80
100
Thermotogae
Deinococcus-Thermus
Aquificae
Spirochaetes
Chlorobi
Bacteroidetes
Acidobacteria
Gemmatimonadetes
Planctomycetes
Fibrobacteres
Nitrospirae
Verrucomicrobia
Chlamydiae
Actinobacteria
Dictyoglomi
Chloroflexi
Synergistetes
Deferribacteres
Chrysiogenetes
Firmicutes
Tenericutes
Fusobacteria
Cyanobacteria
Gammaproteobacteria
Betaproteobacteria
Alphaproteobacteria
Epsilonproteobacteria
Deltaproteobacteria
Magnetococcus
Elusimicrobia
Thermobaculum
Euryarchaeota
Crenarchaeota
Thaumarchaeota
Nanoarchaeota
Korarchaeota

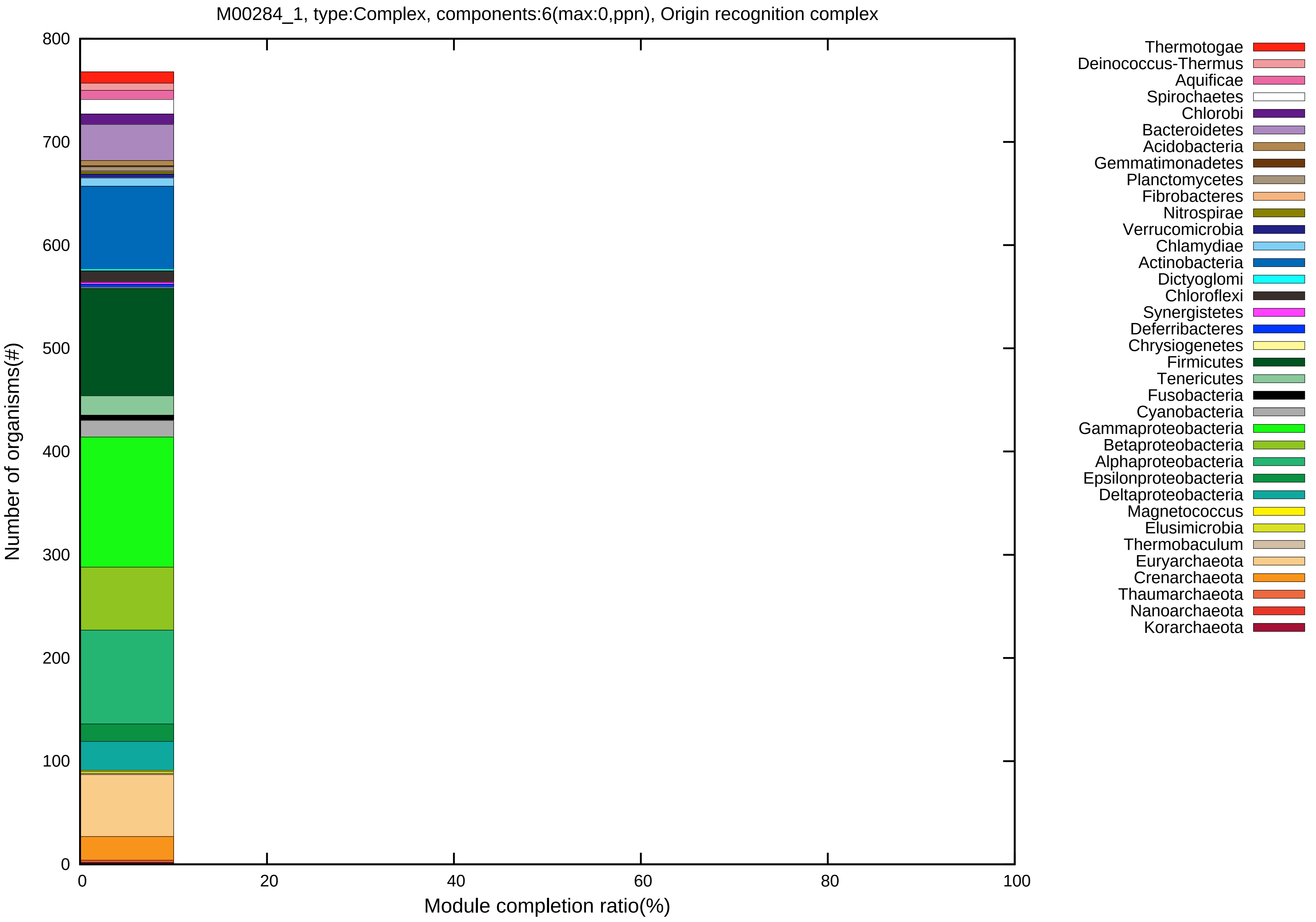
M00284_1, type:Complex, components:6(max:0,ppn), Origin recognition complex
Number of organisms(#)
Module completion ratio(%)
0
100
200
300
400
500
600
700
800
0
20
40
60
80
100
Thermotogae
Deinococcus-Thermus
Aquificae
Spirochaetes
Chlorobi
Bacteroidetes
Acidobacteria
Gemmatimonadetes
Planctomycetes
Fibrobacteres
Nitrospirae
Verrucomicrobia
Chlamydiae
Actinobacteria
Dictyoglomi
Chloroflexi
Synergistetes
Deferribacteres
Chrysiogenetes
Firmicutes
Tenericutes
Fusobacteria
Cyanobacteria
Gammaproteobacteria
Betaproteobacteria
Alphaproteobacteria
Epsilonproteobacteria
Deltaproteobacteria
Magnetococcus
Elusimicrobia
Thermobaculum
Euryarchaeota
Crenarchaeota
Thaumarchaeota
Nanoarchaeota
Korarchaeota

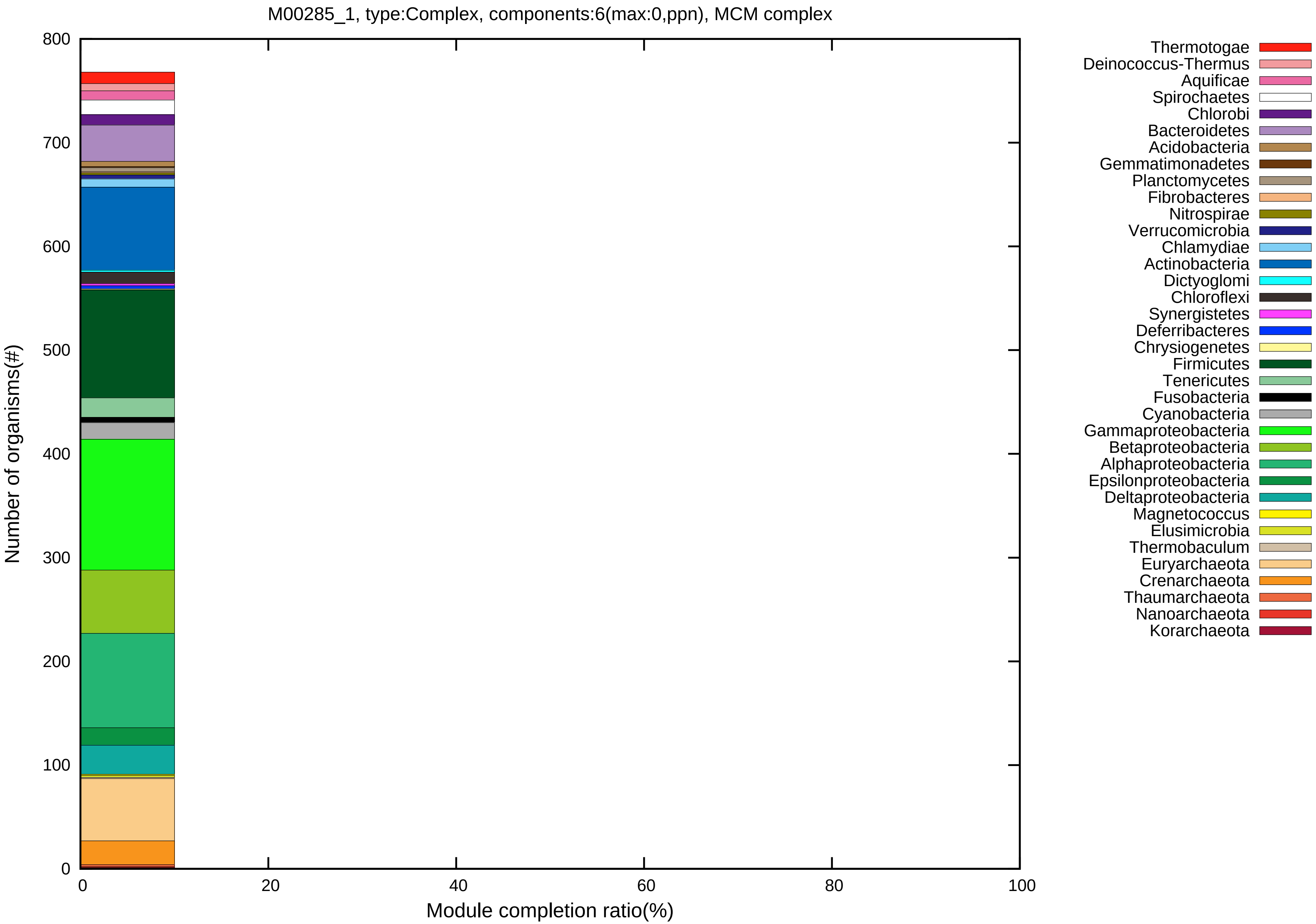

M00285_1, type:Complex, components:6(max:0,ppn), MCM complex
Number of organisms(#)
Module completion ratio(%)
0
100
200
300
400
500
600
700
800
0
20
40
60
80
100
Thermotogae
Deinococcus-Thermus
Aquificae
Spirochaetes
Chlorobi
Bacteroidetes
Acidobacteria
Gemmatimonadetes
Planctomycetes
Fibrobacteres
Nitrospirae
Verrucomicrobia
Chlamydiae
Actinobacteria
Dictyoglomi
Chloroflexi
Synergistetes
Deferribacteres
Chrysiogenetes
Firmicutes
Tenericutes
Fusobacteria
Cyanobacteria
Gammaproteobacteria
Betaproteobacteria
Alphaproteobacteria
Epsilonproteobacteria
Deltaproteobacteria
Magnetococcus
Elusimicrobia
Thermobaculum
Euryarchaeota
Crenarchaeota
Thaumarchaeota
Nanoarchaeota
Korarchaeota

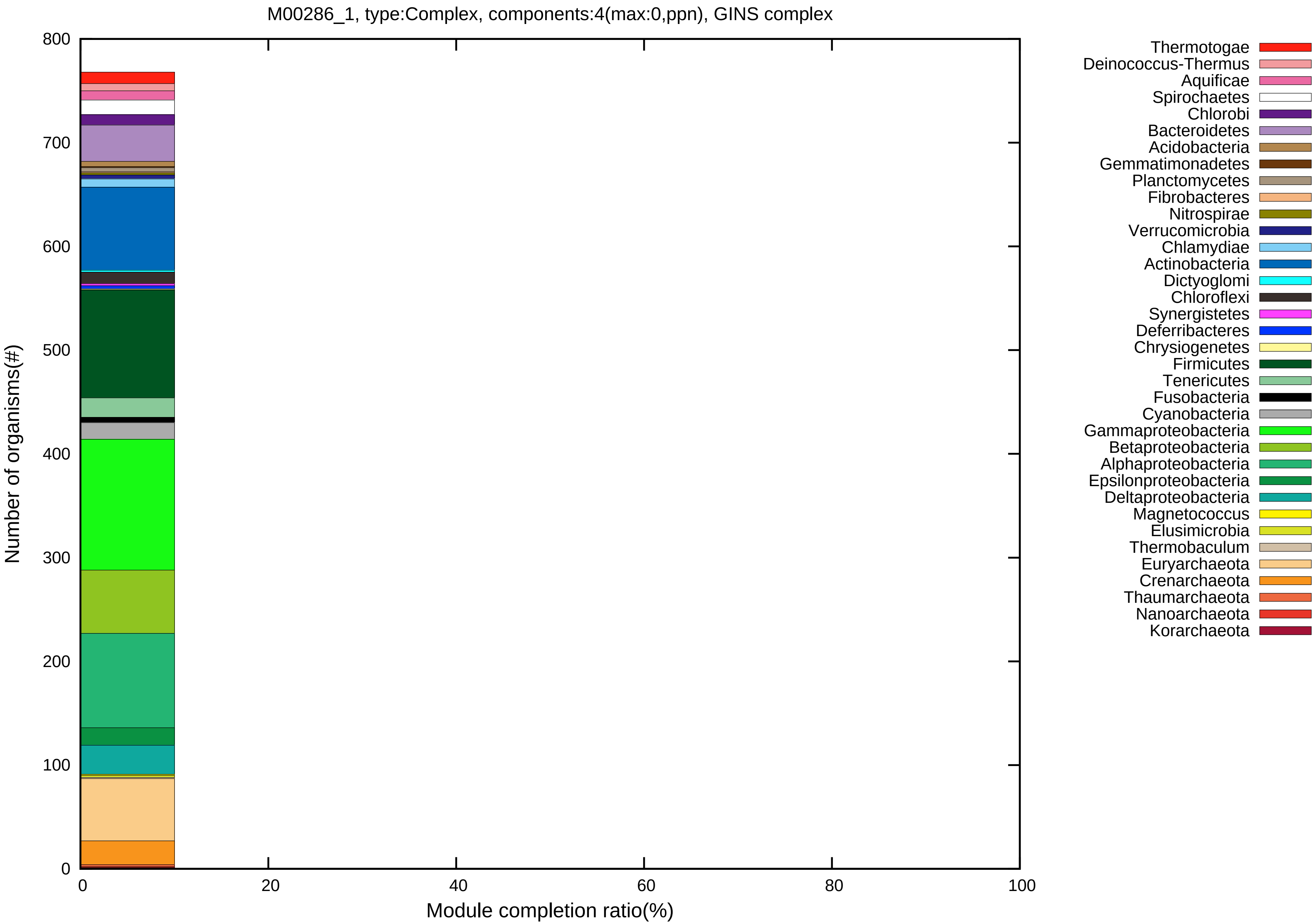
M00286_1, type:Complex, components:4(max:0,ppn), GINS complex
Number of organisms(#)
Module completion ratio(%)
0
100
200
300
400
500
600
700
800
0
20
40
60
80
100
Thermotogae
Deinococcus-Thermus
Aquificae
Spirochaetes
Chlorobi
Bacteroidetes
Acidobacteria
Gemmatimonadetes
Planctomycetes
Fibrobacteres
Nitrospirae
Verrucomicrobia
Chlamydiae
Actinobacteria
Dictyoglomi
Chloroflexi
Synergistetes
Deferribacteres
Chrysiogenetes
Firmicutes
Tenericutes
Fusobacteria
Cyanobacteria
Gammaproteobacteria
Betaproteobacteria
Alphaproteobacteria
Epsilonproteobacteria
Deltaproteobacteria
Magnetococcus
Elusimicrobia
Thermobaculum
Euryarchaeota
Crenarchaeota
Thaumarchaeota
Nanoarchaeota
Korarchaeota

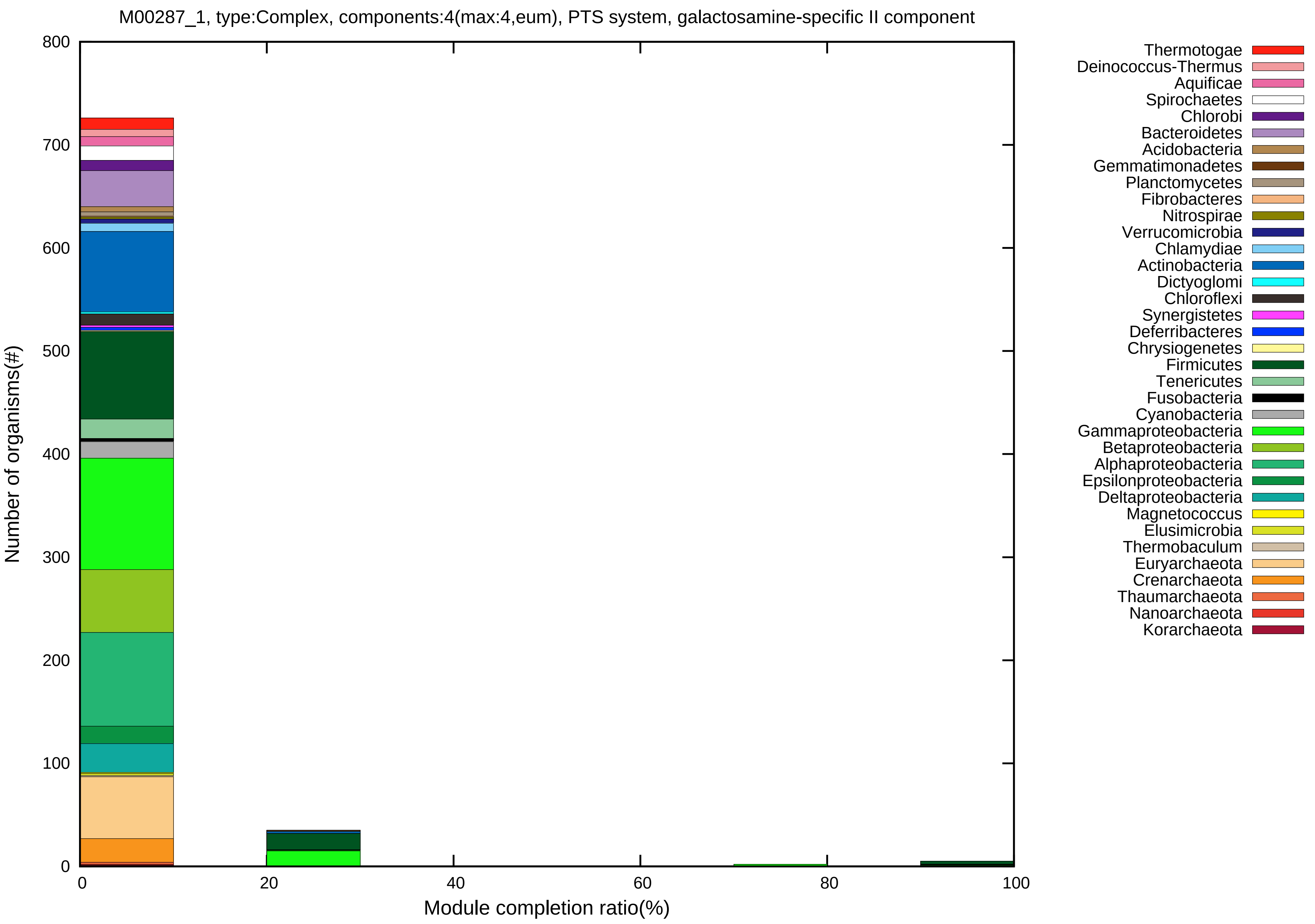

M00287_1, type:Complex, components:4(max:4,eum), PTS system, galactosamine-specific II component
Number of organisms(#)
Module completion ratio(%)
0
100
200
300
400
500
600
700
800
0
20
40
60
80
100
Thermotogae
Deinococcus-Thermus
Aquificae
Spirochaetes
Chlorobi
Bacteroidetes
Acidobacteria
Gemmatimonadetes
Planctomycetes
Fibrobacteres
Nitrospirae
Verrucomicrobia
Chlamydiae
Actinobacteria
Dictyoglomi
Chloroflexi
Synergistetes
Deferribacteres
Chrysiogenetes
Firmicutes
Tenericutes
Fusobacteria
Cyanobacteria
Gammaproteobacteria
Betaproteobacteria
Alphaproteobacteria
Epsilonproteobacteria
Deltaproteobacteria
Magnetococcus
Elusimicrobia
Thermobaculum
Euryarchaeota
Crenarchaeota
Thaumarchaeota
Nanoarchaeota
Korarchaeota

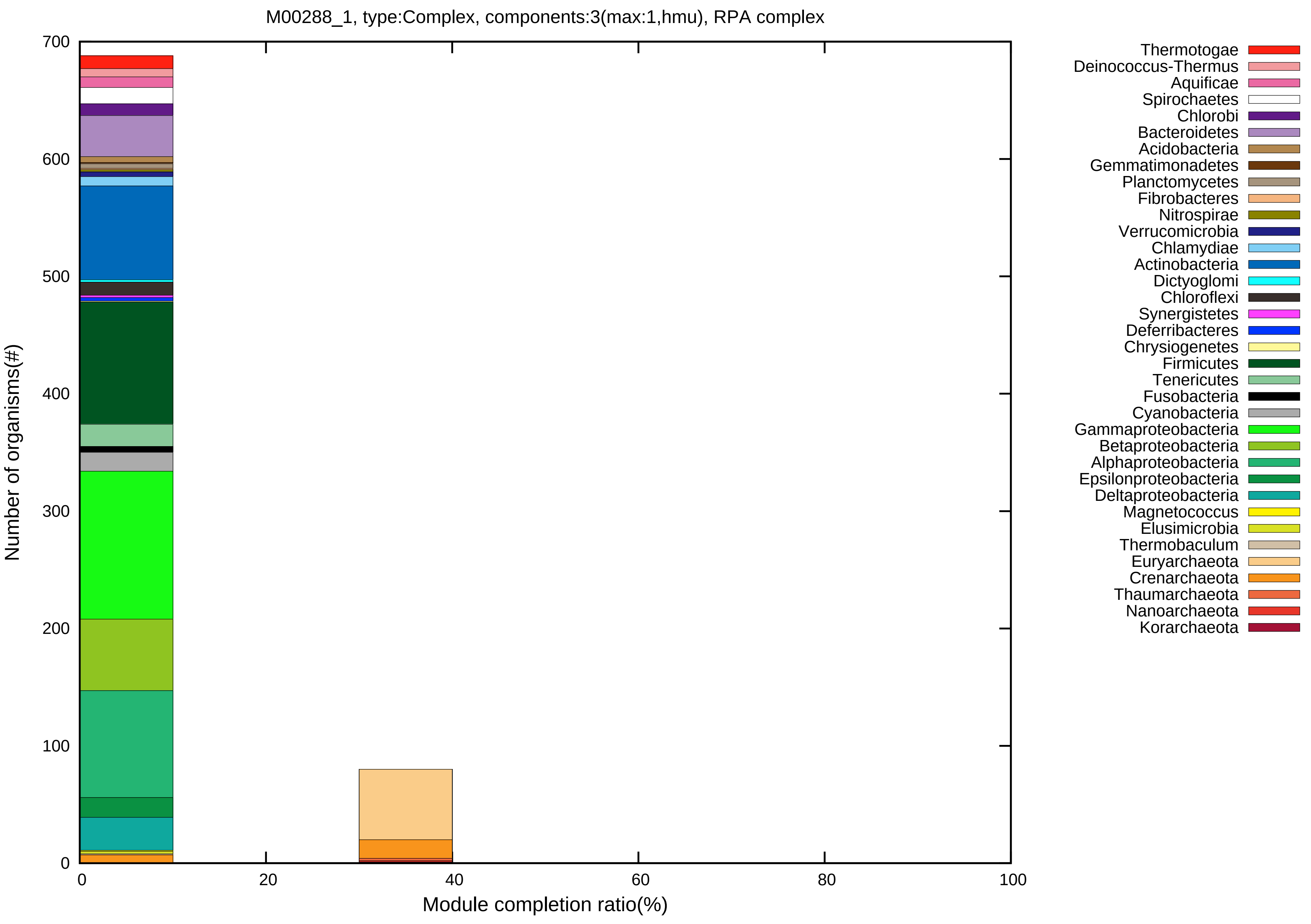
M00288_1, type:Complex, components:3(max:1,hmu), RPA complex
Number of organisms(#)
Module completion ratio(%)
0
100
200
300
400
500
600
700
0
20
40
60
80
100
Thermotogae
Deinococcus-Thermus
Aquificae
Spirochaetes
Chlorobi
Bacteroidetes
Acidobacteria
Gemmatimonadetes
Planctomycetes
Fibrobacteres
Nitrospirae
Verrucomicrobia
Chlamydiae
Actinobacteria
Dictyoglomi
Chloroflexi
Synergistetes
Deferribacteres
Chrysiogenetes
Firmicutes
Tenericutes
Fusobacteria
Cyanobacteria
Gammaproteobacteria
Betaproteobacteria
Alphaproteobacteria
Epsilonproteobacteria
Deltaproteobacteria
Magnetococcus
Elusimicrobia
Thermobaculum
Euryarchaeota
Crenarchaeota
Thaumarchaeota
Nanoarchaeota
Korarchaeota

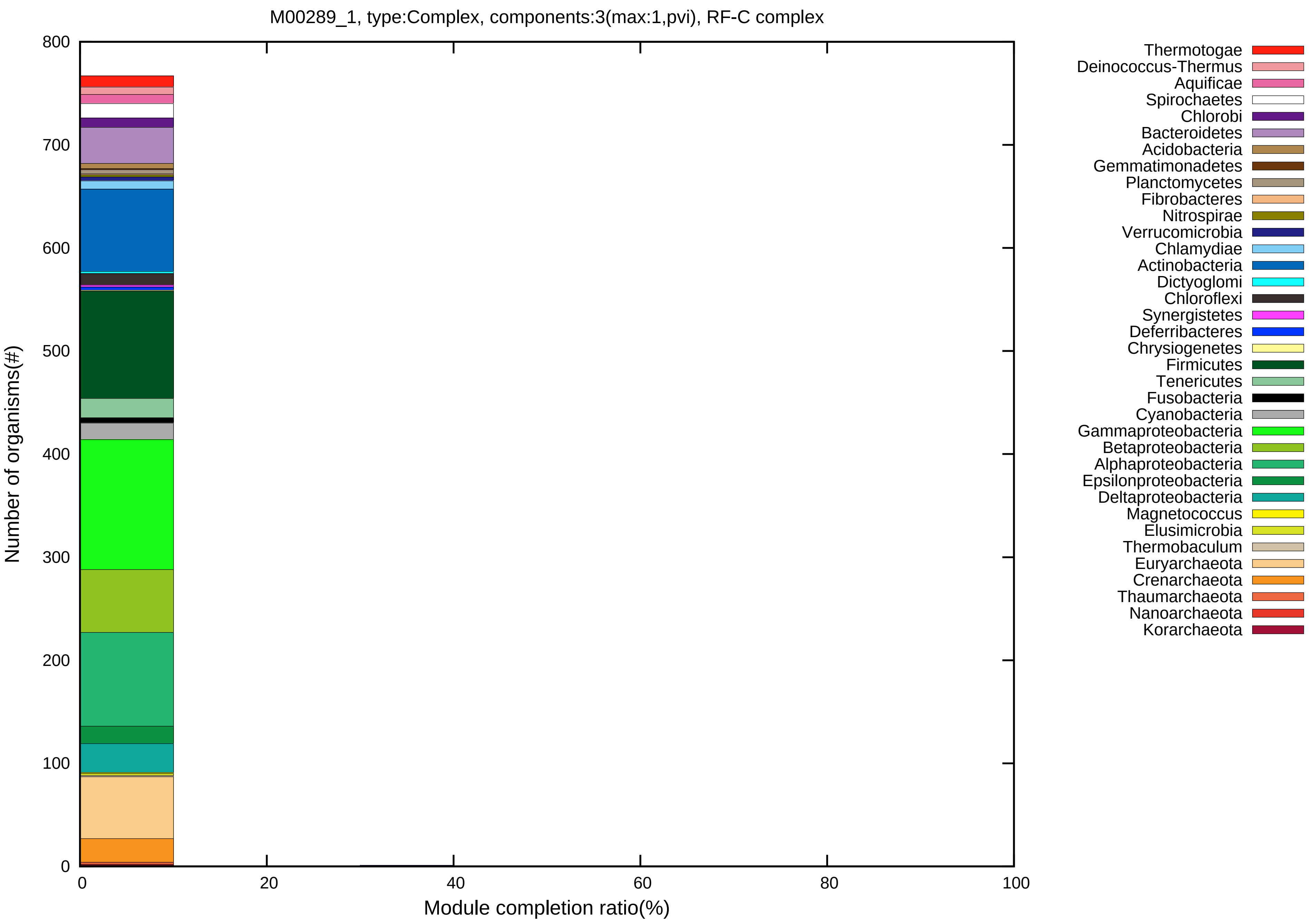

M00289_1, type:Complex, components:3(max:1,pvi), RF-C complex
Number of organisms(#)
0
100
200
300
400
500
600
700
800
Module completion ratio(%)
0
20
40
60
80
100
Thermotogae
Deinococcus-Thermus
Aquificae
Spirochaetes
Chlorobi
Bacteroidetes
Acidobacteria
Gemmatimonadetes
Planctomycetes
Fibrobacteres
Nitrospirae
Verrucomicrobia
Chlamydiae
Actinobacteria
Dictyoglomi
Chloroflexi
Synergistetes
Deferribacteres
Chrysiogenetes
Firmicutes
Tenericutes
Fusobacteria
Cyanobacteria
Gammaproteobacteria
Betaproteobacteria
Alphaproteobacteria
Epsilonproteobacteria
Deltaproteobacteria
Magnetococcus
Elusimicrobia
Thermobaculum
Euryarchaeota
Crenarchaeota
Thaumarchaeota
Nanoarchaeota
Korarchaeota

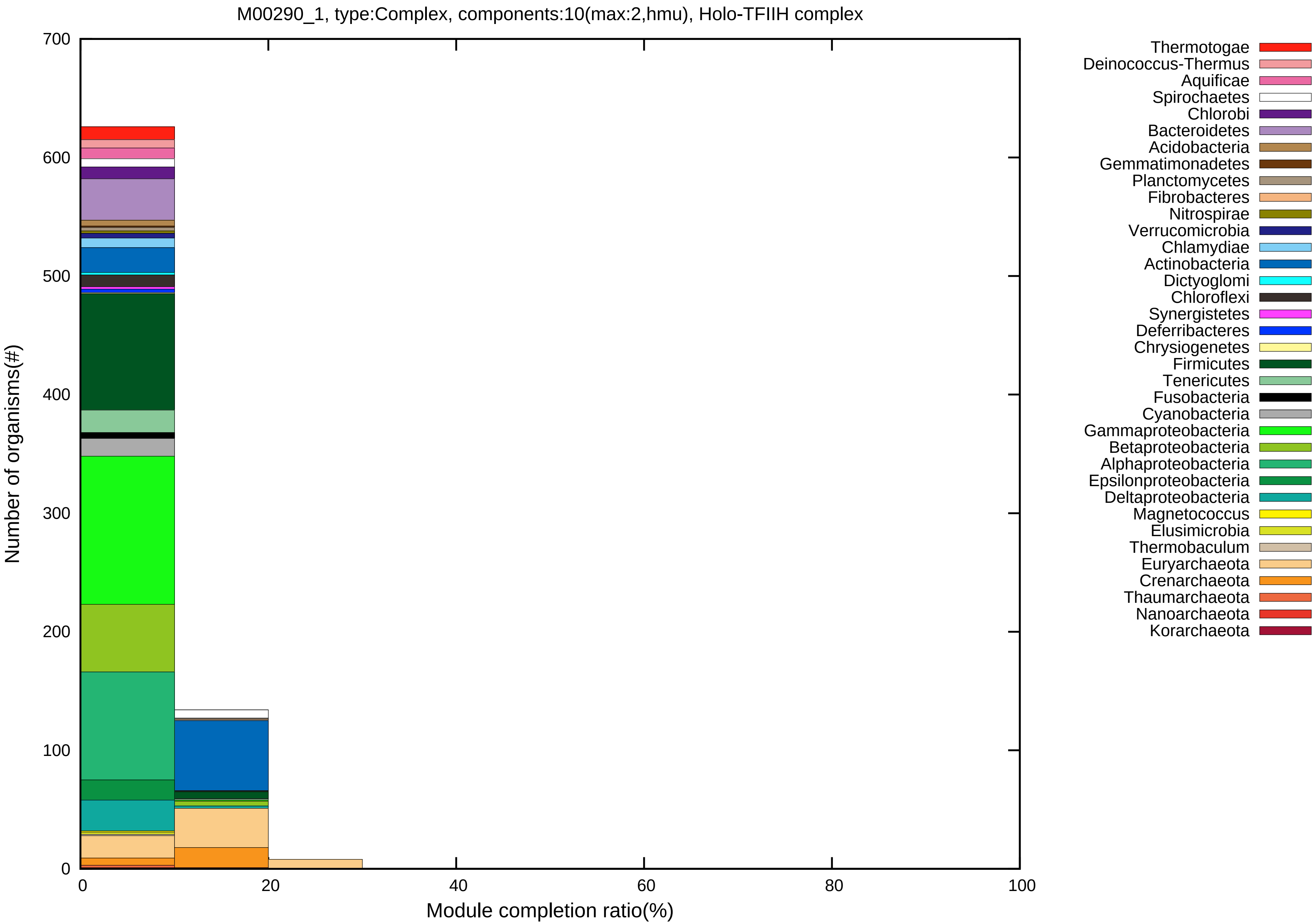
M00290_1, type:Complex, components:10(max:2,hmu), Holo-TFIIH complex
Number of organisms(#)
Module completion ratio(%)
0
100
200
300
400
500
600
700
0
20
40
60
80
100
Thermotogae
Deinococcus-Thermus
Aquificae
Spirochaetes
Chlorobi
Bacteroidetes
Acidobacteria
Gemmatimonadetes
Planctomycetes
Fibrobacteres
Nitrospirae
Verrucomicrobia
Chlamydiae
Actinobacteria
Dictyoglomi
Chloroflexi
Synergistetes
Deferribacteres
Chrysiogenetes
Firmicutes
Tenericutes
Fusobacteria
Cyanobacteria
Gammaproteobacteria
Betaproteobacteria
Alphaproteobacteria
Epsilonproteobacteria
Deltaproteobacteria
Magnetococcus
Elusimicrobia
Thermobaculum
Euryarchaeota
Crenarchaeota
Thaumarchaeota
Nanoarchaeota
Korarchaeota

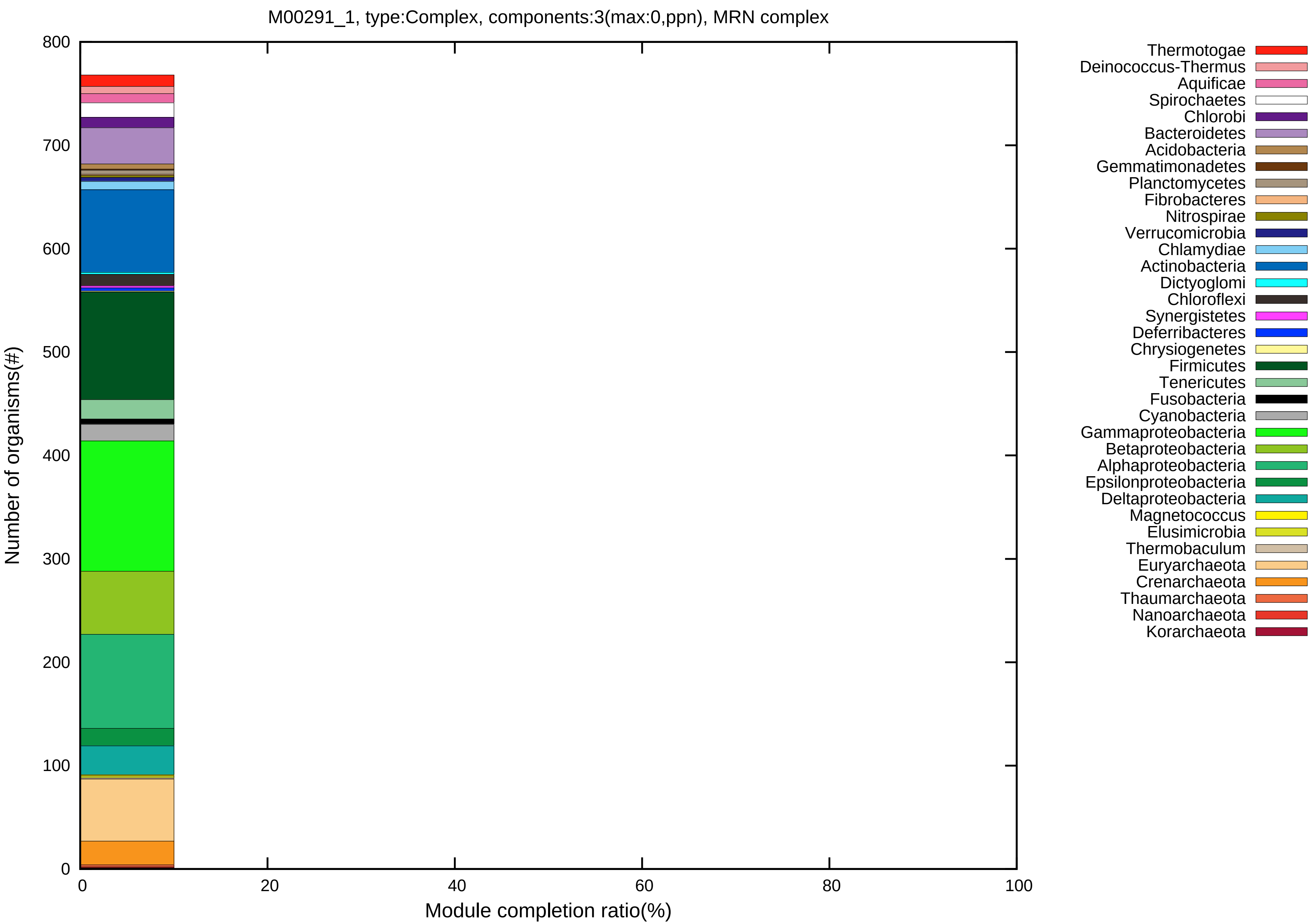

M00291_1, type:Complex, components:3(max:0,ppn), MRN complex
Number of organisms(#)
Module completion ratio(%)
0
100
200
300
400
500
600
700
800
0
20
40
60
80
100
Thermotogae
Deinococcus-Thermus
Aquificae
Spirochaetes
Chlorobi
Bacteroidetes
Acidobacteria
Gemmatimonadetes
Planctomycetes
Fibrobacteres
Nitrospirae
Verrucomicrobia
Chlamydiae
Actinobacteria
Dictyoglomi
Chloroflexi
Synergistetes
Deferribacteres
Chrysiogenetes
Firmicutes
Tenericutes
Fusobacteria
Cyanobacteria
Gammaproteobacteria
Betaproteobacteria
Alphaproteobacteria
Epsilonproteobacteria
Deltaproteobacteria
Magnetococcus
Elusimicrobia
Thermobaculum
Euryarchaeota
Crenarchaeota
Thaumarchaeota
Nanoarchaeota
Korarchaeota

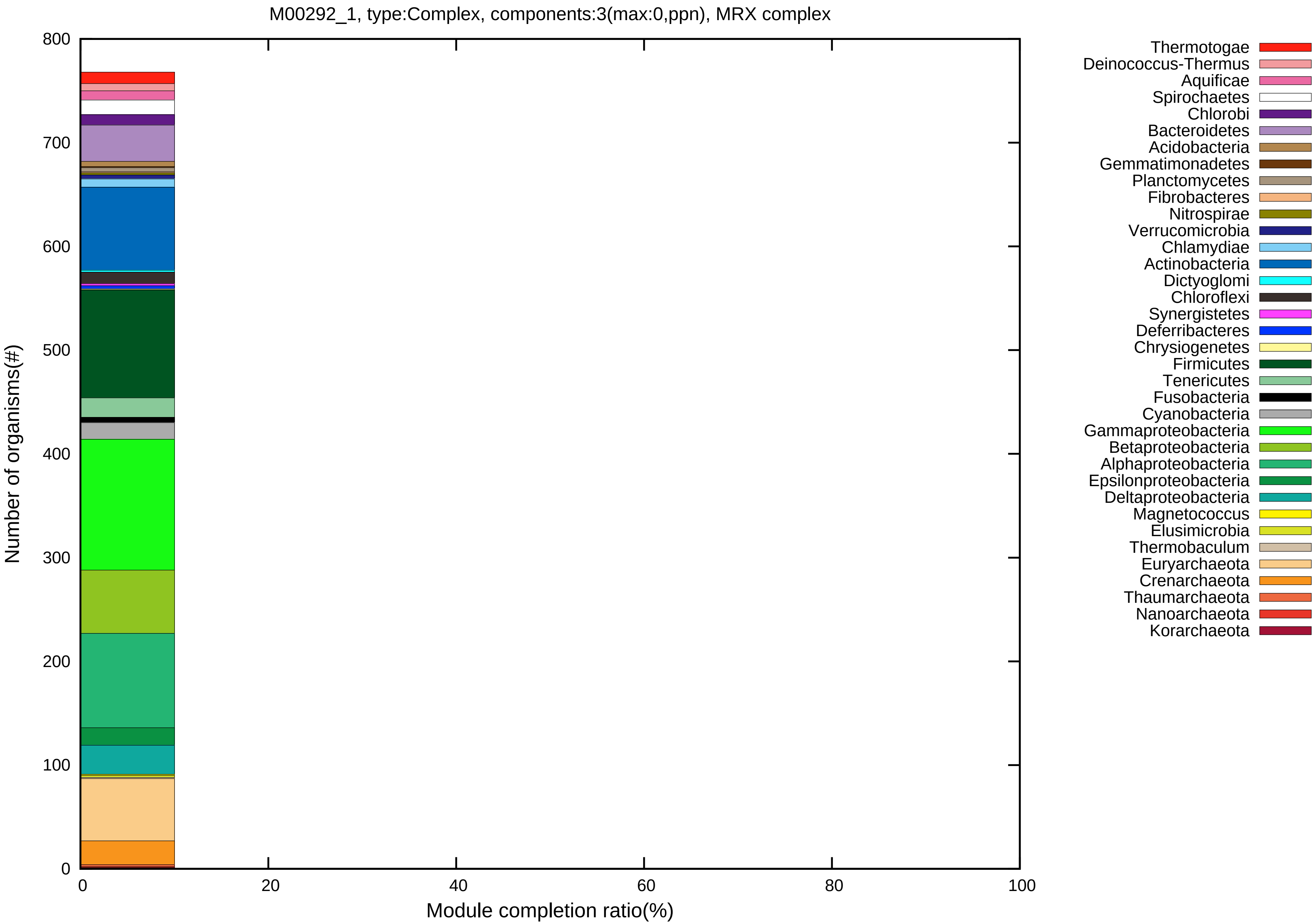

M00292_1, type:Complex, components:3(max:0,ppn), MRX complex
Number of organisms(#)
Module completion ratio(%)
0
100
200
300
400
500
600
700
800
0
20
40
60
80
100
Thermotogae
Deinococcus-Thermus
Aquificae
Spirochaetes
Chlorobi
Bacteroidetes
Acidobacteria
Gemmatimonadetes
Planctomycetes
Fibrobacteres
Nitrospirae
Verrucomicrobia
Chlamydiae
Actinobacteria
Dictyoglomi
Chloroflexi
Synergistetes
Deferribacteres
Chrysiogenetes
Firmicutes
Tenericutes
Fusobacteria
Cyanobacteria
Gammaproteobacteria
Betaproteobacteria
Alphaproteobacteria
Epsilonproteobacteria
Deltaproteobacteria
Magnetococcus
Elusimicrobia
Thermobaculum
Euryarchaeota
Crenarchaeota
Thaumarchaeota
Nanoarchaeota
Korarchaeota

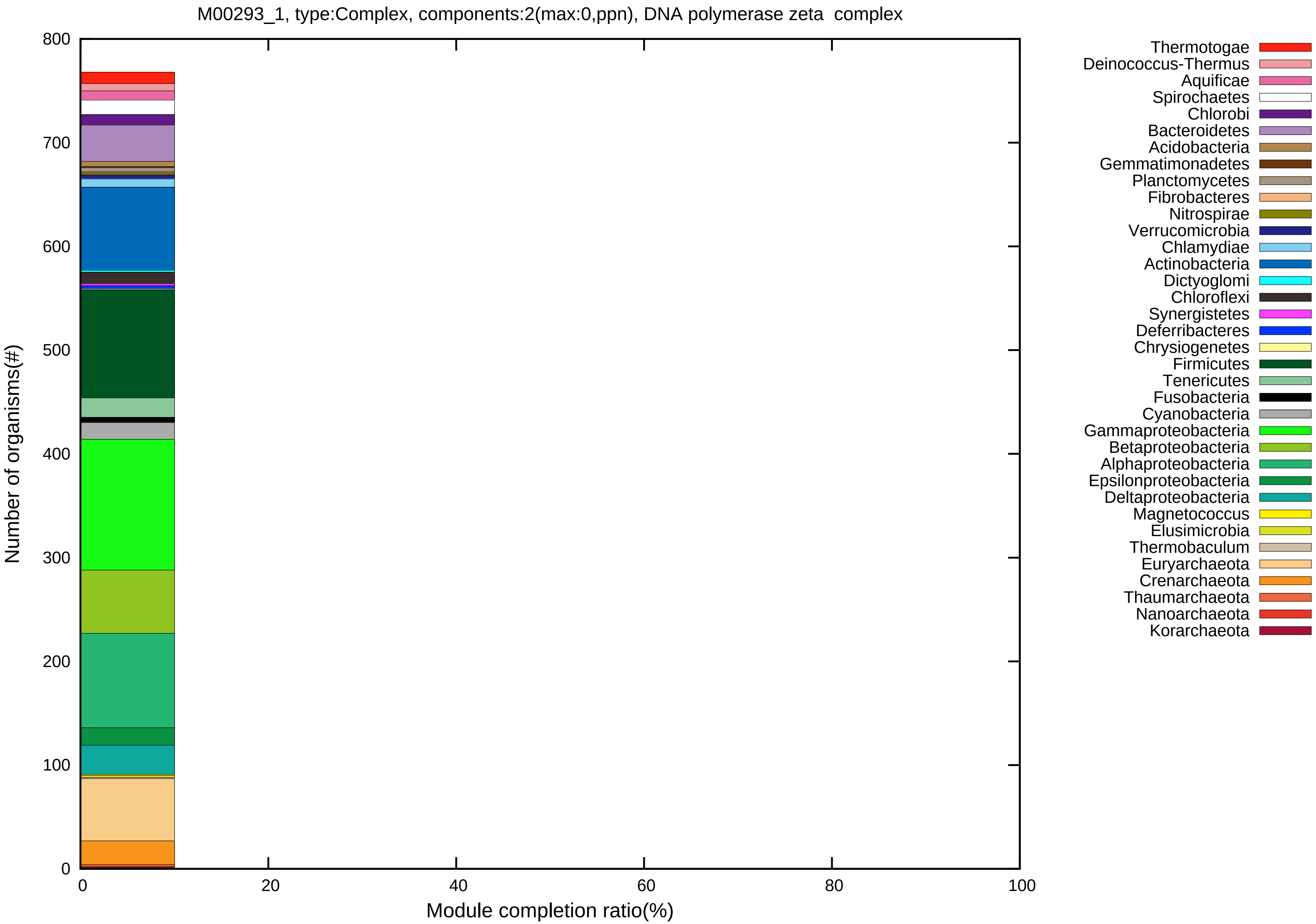

M00293_1, type:Complex, components:2(max:0,ppn), DNA polymerase zeta complex
Number of organisms(#)
Module completion ratio(%)
0
100
200
300
400
500
600
700
800
0
20
40
60
80
100
Thermotogae
Deinococcus-Thermus
Aquificae
Spirochaetes
Chlorobi
Bacteroidetes
Acidobacteria
Gemmatimonadetes
Planctomycetes
Fibrobacteres
Nitrospirae
Verrucomicrobia
Chlamydiae
Actinobacteria
Dictyoglomi
Chloroflexi
Synergistetes
Deferribacteres
Chrysiogenetes
Firmicutes
Tenericutes
Fusobacteria
Cyanobacteria
Gammaproteobacteria
Betaproteobacteria
Alphaproteobacteria
Epsilonproteobacteria
Deltaproteobacteria
Magnetococcus
Elusimicrobia
Thermobaculum
Euryarchaeota
Crenarchaeota
Thaumarchaeota
Nanoarchaeota
Korarchaeota

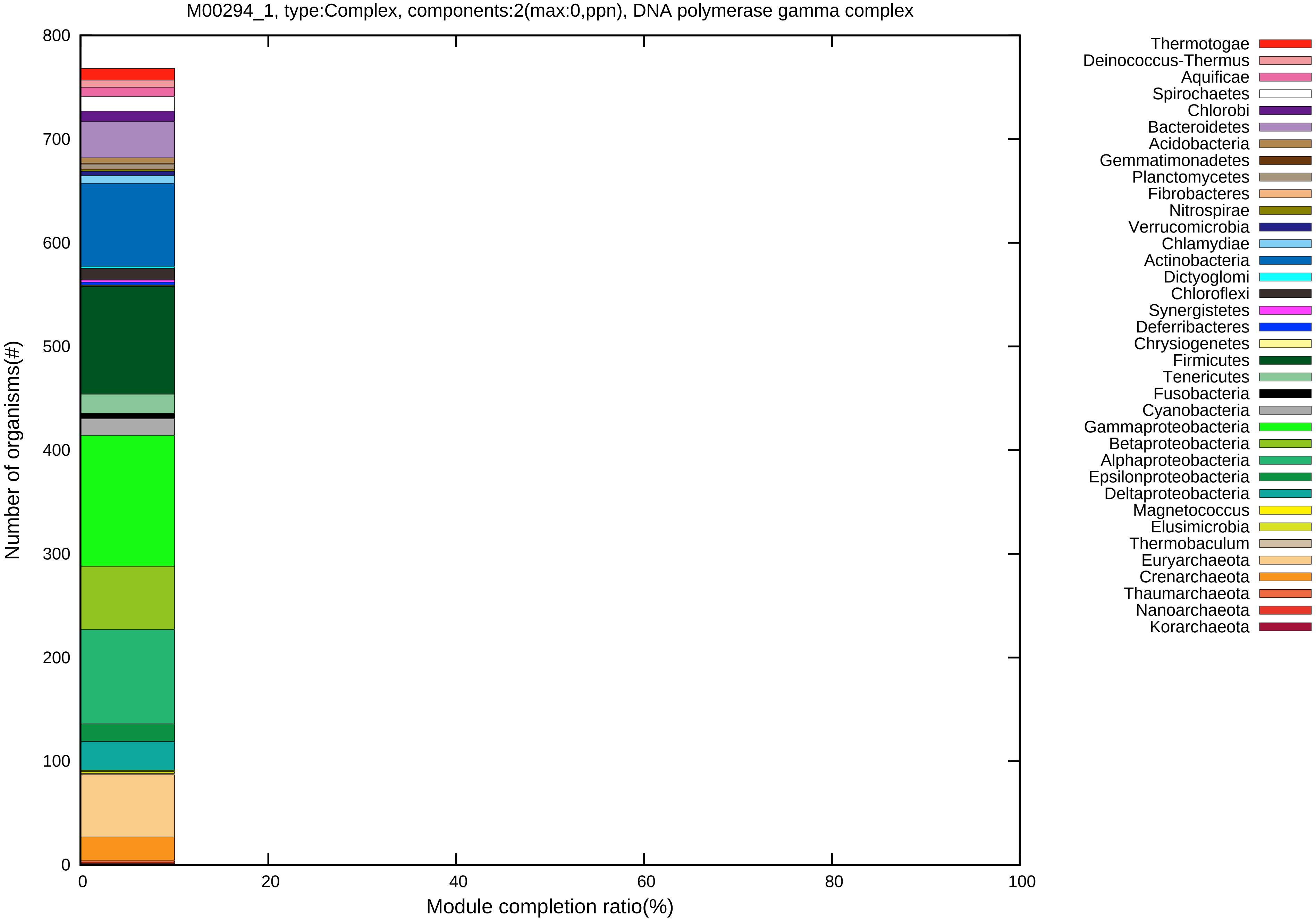

M00294_1, type:Complex, components:2(max:0,ppn), DNA polymerase gamma complex
Number of organisms(#)
0
100
200
300
400
500
600
700
800
0
20
40
60
80
100
Module completion ratio(%)
Thermotogae
Deinococcus-Thermus
Aquificae
Spirochaetes
Chlorobi
Bacteroidetes
Acidobacteria
Gemmatimonadetes
Planctomycetes
Fibrobacteres
Nitrospirae
Verrucomicrobia
Chlamydiae
Actinobacteria
Dictyoglomi
Chloroflexi
Synergistetes
Deferribacteres
Chrysiogenetes
Firmicutes
Tenericutes
Fusobacteria
Cyanobacteria
Gammaproteobacteria
Betaproteobacteria
Alphaproteobacteria
Epsilonproteobacteria
Deltaproteobacteria
Magnetococcus
Elusimicrobia
Thermobaculum
Euryarchaeota
Crenarchaeota
Thaumarchaeota
Nanoarchaeota
Korarchaeota

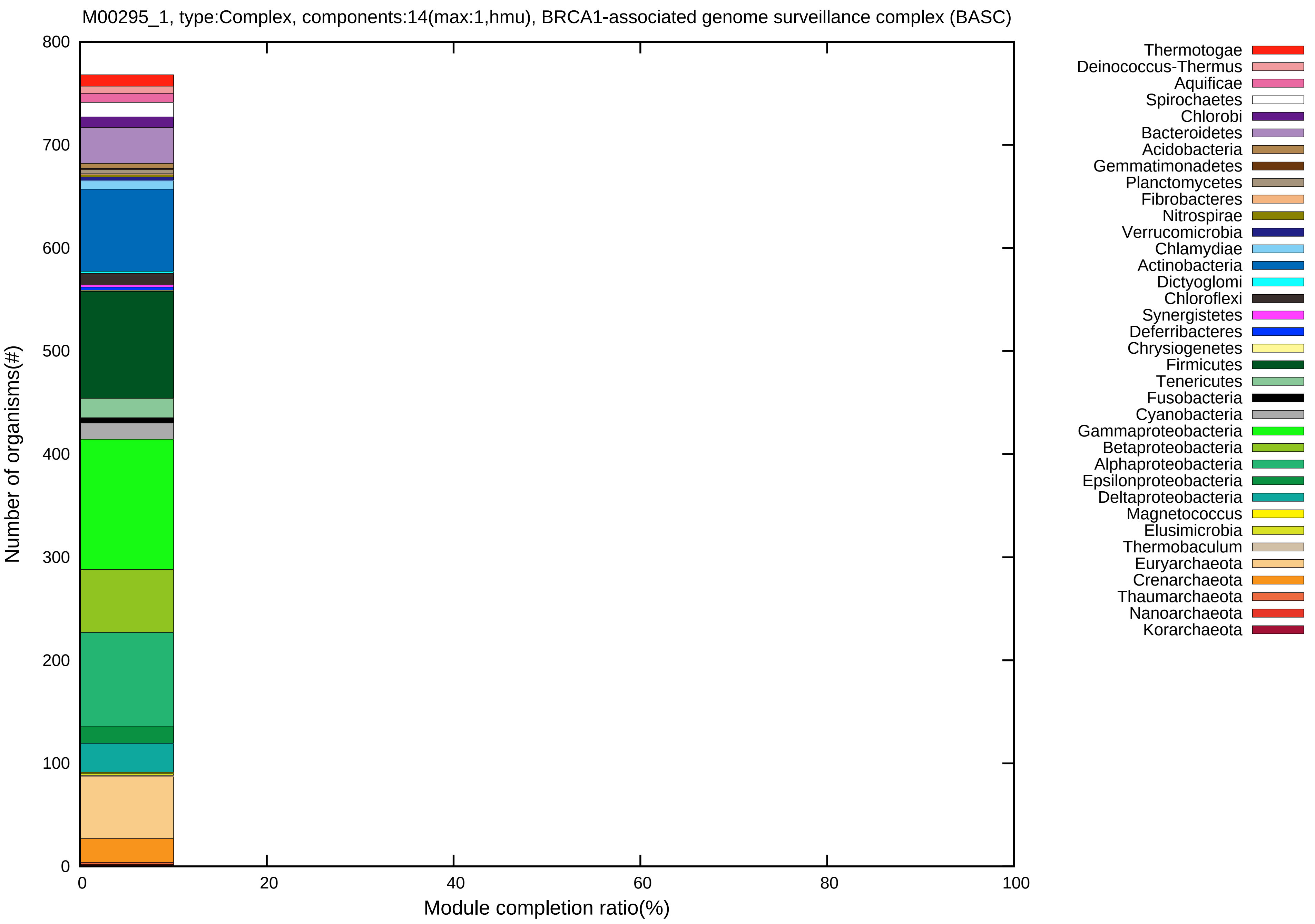

M00295_1, type:Complex, components:14(max:1,hmu), BRCA1-associated genome surveillance complex (BASC)
Number of organisms(#)
Module completion ratio(%)
0
100
200
300
400
500
600
700
800
0
20
40
60
80
100
Thermotogae
Deinococcus-Thermus
Aquificae
Spirochaetes
Chlorobi
Bacteroidetes
Acidobacteria
Gemmatimonadetes
Planctomycetes
Fibrobacteres
Nitrospirae
Verrucomicrobia
Chlamydiae
Actinobacteria
Dictyoglomi
Chloroflexi
Synergistetes
Deferribacteres
Chrysiogenetes
Firmicutes
Tenericutes
Fusobacteria
Cyanobacteria
Gammaproteobacteria
Betaproteobacteria
Alphaproteobacteria
Epsilonproteobacteria
Deltaproteobacteria
Magnetococcus
Elusimicrobia
Thermobaculum
Euryarchaeota
Crenarchaeota
Thaumarchaeota
Nanoarchaeota
Korarchaeota

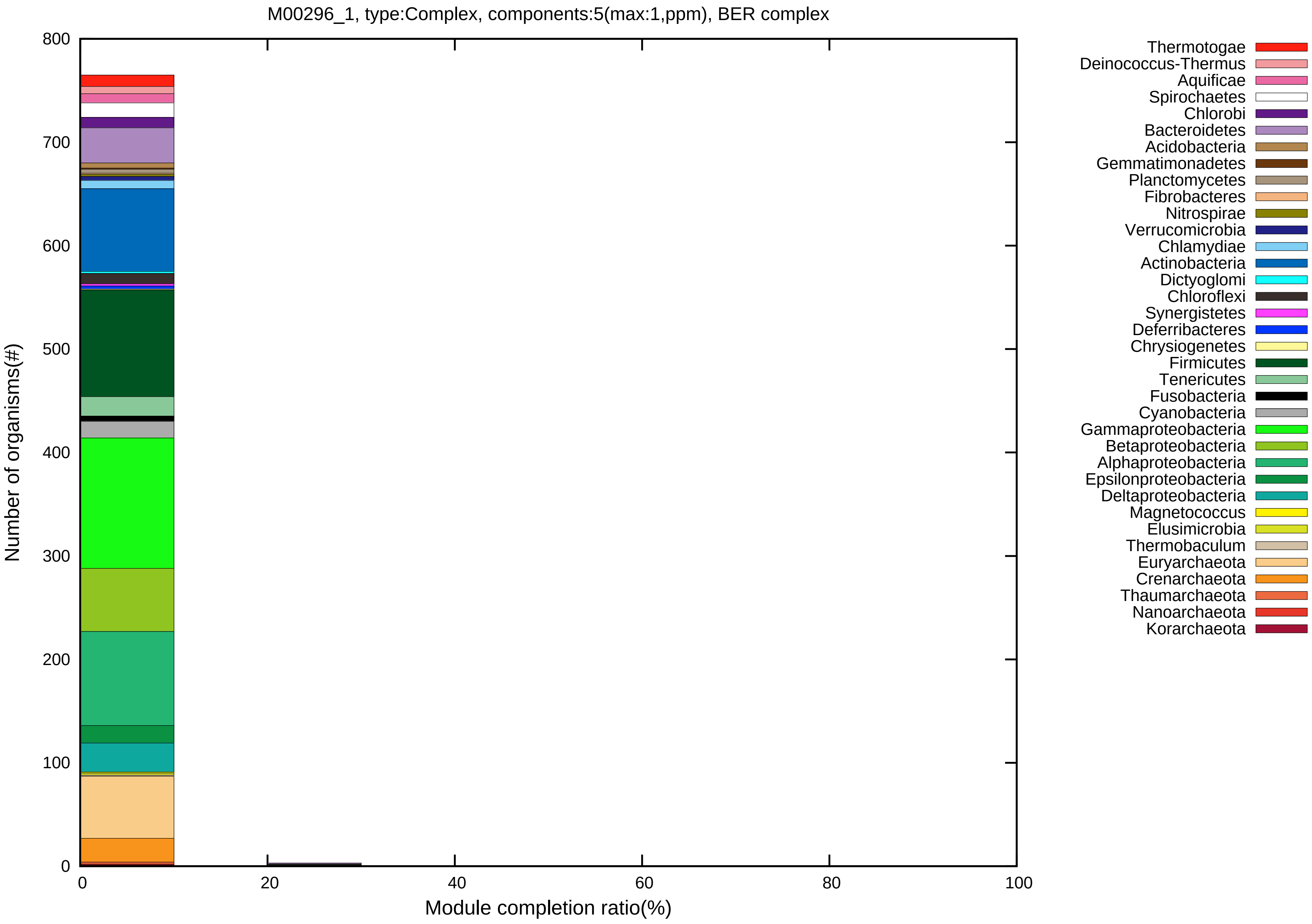

M00296_1, type:Complex, components:5(max:1,ppm), BER complex
Number of organisms(#)
Module completion ratio(%)
0
100
200
300
400
500
600
700
800
0
20
40
60
80
100
Thermotogae
Deinococcus-Thermus
Aquificae
Spirochaetes
Chlorobi
Bacteroidetes
Acidobacteria
Gemmatimonadetes
Planctomycetes
Fibrobacteres
Nitrospirae
Verrucomicrobia
Chlamydiae
Actinobacteria
Dictyoglomi
Chloroflexi
Synergistetes
Deferribacteres
Chrysiogenetes
Firmicutes
Tenericutes
Fusobacteria
Cyanobacteria
Gammaproteobacteria
Betaproteobacteria
Alphaproteobacteria
Epsilonproteobacteria
Deltaproteobacteria
Magnetococcus
Elusimicrobia
Thermobaculum
Euryarchaeota
Crenarchaeota
Thaumarchaeota
Nanoarchaeota
Korarchaeota

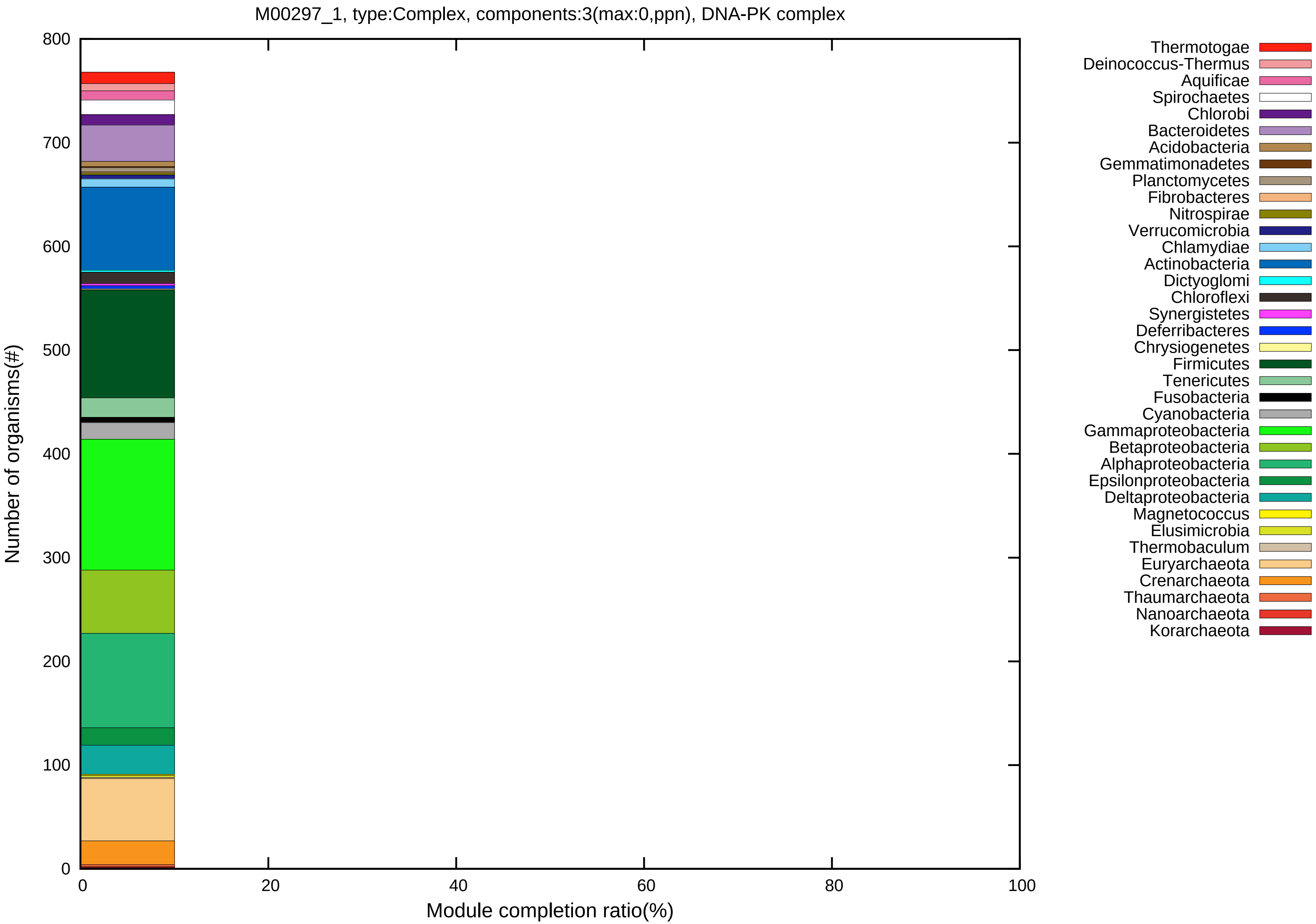

M00297_1, type:Complex, components:3(max:0,ppn), DNA-PK complex
Number of organisms(#)
0
100
200
300
400
500
600
700
800
0
20
40
60
80
100
Module completion ratio(%)
Thermotogae
Deinococcus-Thermus
Aquificae
Spirochaetes
Chlorobi
Bacteroidetes
Acidobacteria
Gemmatimonadetes
Planctomycetes
Fibrobacteres
Nitrospirae
Verrucomicrobia
Chlamydiae
Actinobacteria
Dictyoglomi
Chloroflexi
Synergistetes
Deferribacteres
Chrysiogenetes
Firmicutes
Tenericutes
Fusobacteria
Cyanobacteria
Gammaproteobacteria
Betaproteobacteria
Alphaproteobacteria
Epsilonproteobacteria
Deltaproteobacteria
Magnetococcus
Elusimicrobia
Thermobaculum
Euryarchaeota
Crenarchaeota
Thaumarchaeota
Nanoarchaeota
Korarchaeota

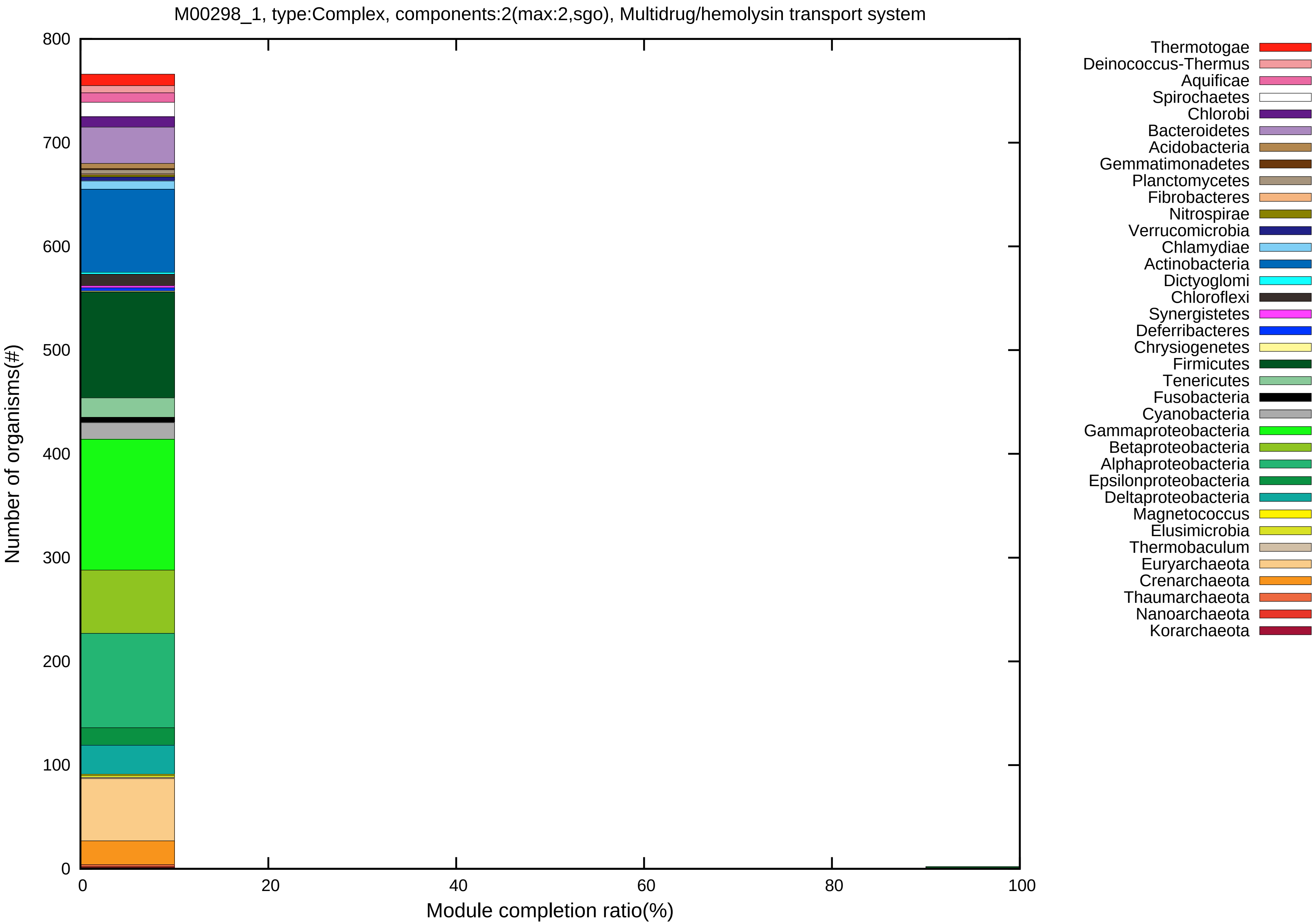
M00298_1, type:Complex, components:2(max:2,sgo), Multidrug/hemolysin transport system
Number of organisms(#)
Module completion ratio(%)
0
100
200
300
400
500
600
700
800
0
20
40
60
80
100
Thermotogae
Deinococcus-Thermus
Aquificae
Spirochaetes
Chlorobi
Bacteroidetes
Acidobacteria
Gemmatimonadetes
Planctomycetes
Fibrobacteres
Nitrospirae
Verrucomicrobia
Chlamydiae
Actinobacteria
Dictyoglomi
Chloroflexi
Synergistetes
Deferribacteres
Chrysiogenetes
Firmicutes
Tenericutes
Fusobacteria
Cyanobacteria
Gammaproteobacteria
Betaproteobacteria
Alphaproteobacteria
Epsilonproteobacteria
Deltaproteobacteria
Magnetococcus
Elusimicrobia
Thermobaculum
Euryarchaeota
Crenarchaeota
Thaumarchaeota
Nanoarchaeota
Korarchaeota

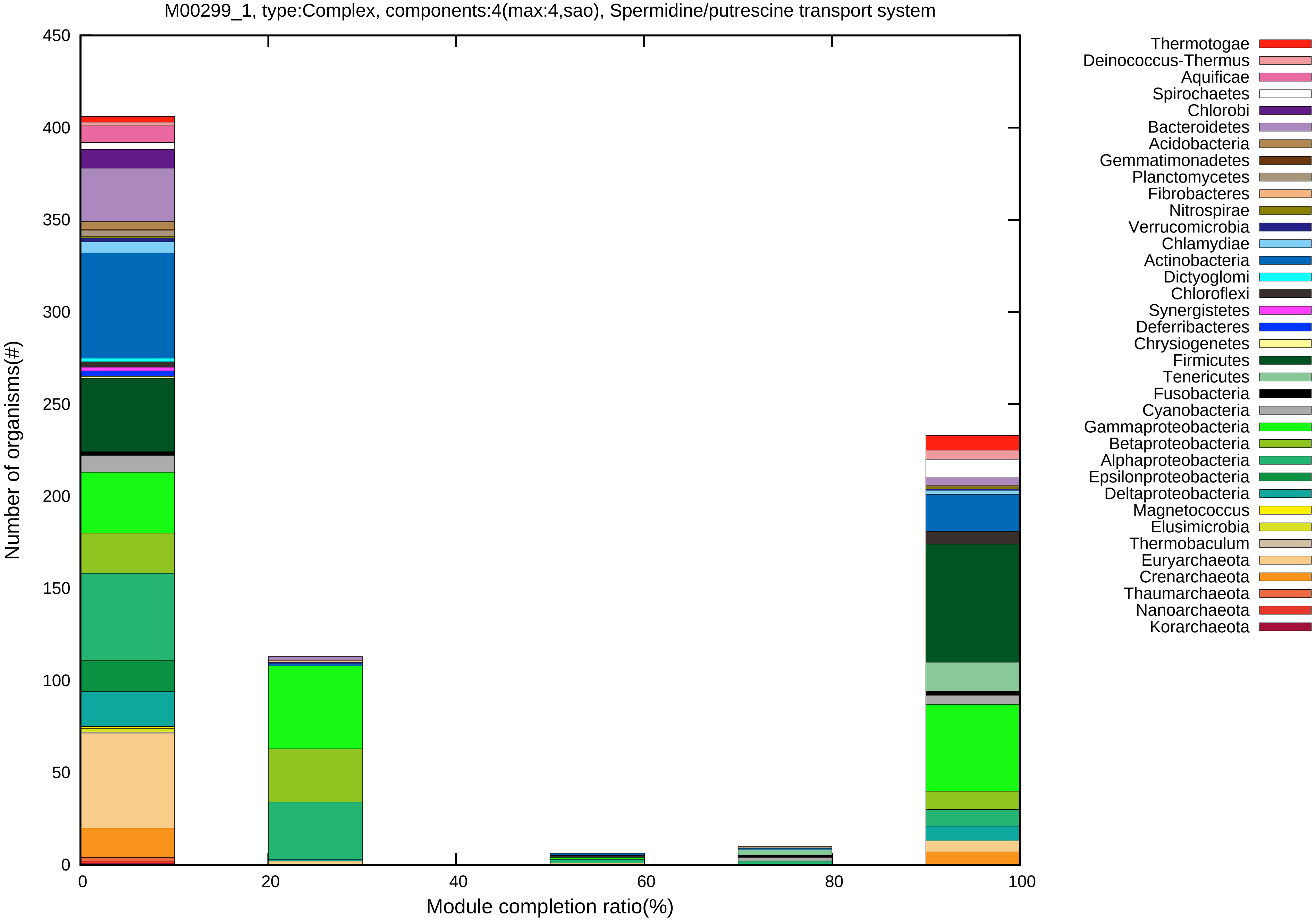
M00299_1, type:Complex, components:4(max:4,sao), Spermidine/putrescine transport system
Number of organisms(#)
Module completion ratio(%)
0
50
100
150
200
250
300
350
400
450
0
20
40
60
80
100
Thermotogae
Deinococcus-Thermus
Aquificae
Spirochaetes
Chlorobi
Bacteroidetes
Acidobacteria
Gemmatimonadetes
Planctomycetes
Fibrobacteres
Nitrospirae
Verrucomicrobia
Chlamydiae
Actinobacteria
Dictyoglomi
Chloroflexi
Synergistetes
Deferribacteres
Chrysiogenetes
Firmicutes
Tenericutes
Fusobacteria
Cyanobacteria
Gammaproteobacteria
Betaproteobacteria
Alphaproteobacteria
Epsilonproteobacteria
Deltaproteobacteria
Magnetococcus
Elusimicrobia
Thermobaculum
Euryarchaeota
Crenarchaeota
Thaumarchaeota
Nanoarchaeota
Korarchaeota

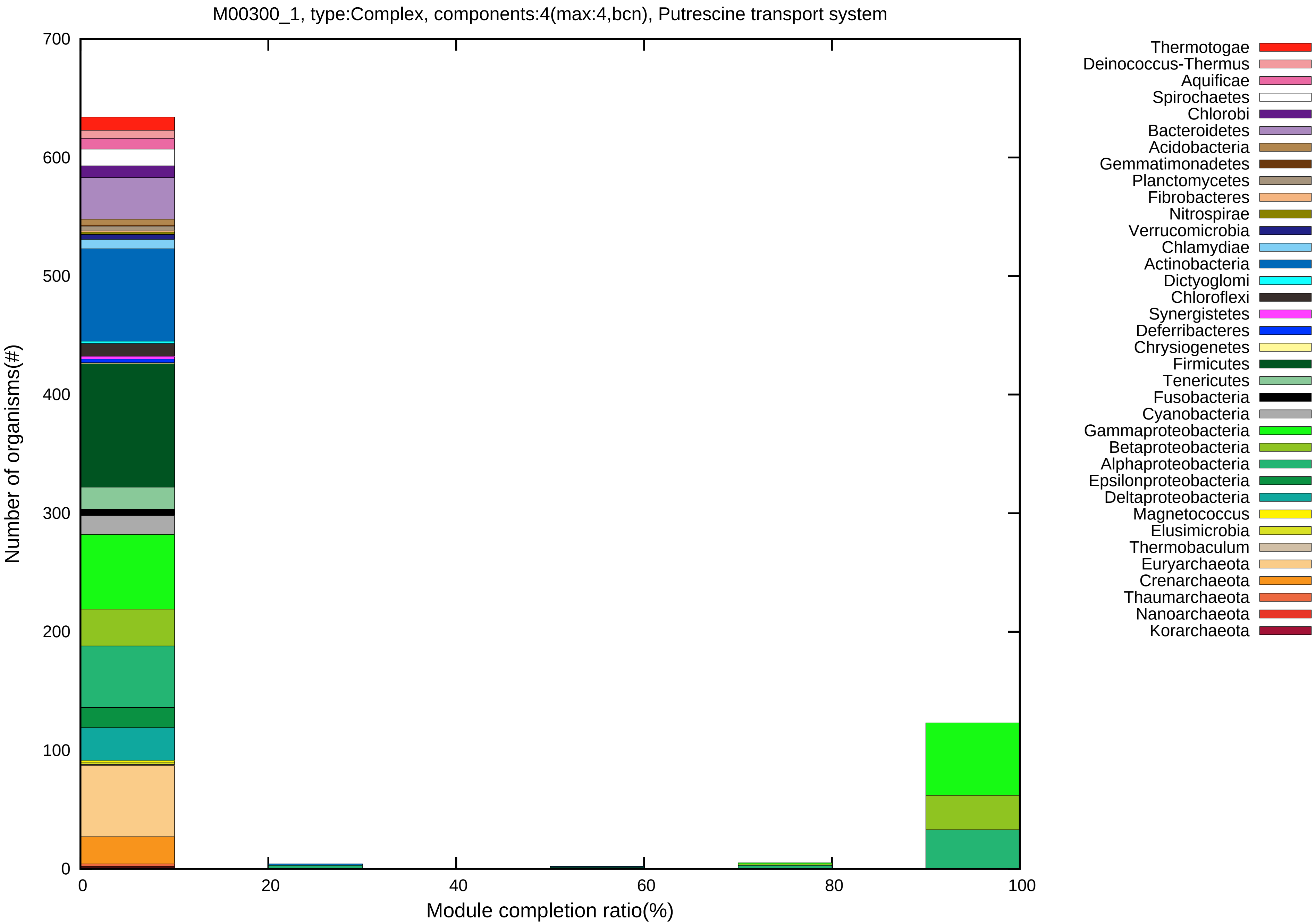

M00300_1, type:Complex, components:4(max:4,bcn), Putrescine transport system
Number of organisms(#)
Module completion ratio(%)
0
100
200
300
400
500
600
700
0
20
40
60
80
100
Thermotogae
Deinococcus-Thermus
Aquificae
Spirochaetes
Chlorobi
Bacteroidetes
Acidobacteria
Gemmatimonadetes
Planctomycetes
Fibrobacteres
Nitrospirae
Verrucomicrobia
Chlamydiae
Actinobacteria
Dictyoglomi
Chloroflexi
Synergistetes
Deferribacteres
Chrysiogenetes
Firmicutes
Tenericutes
Fusobacteria
Cyanobacteria
Gammaproteobacteria
Betaproteobacteria
Alphaproteobacteria
Epsilonproteobacteria
Deltaproteobacteria
Magnetococcus
Elusimicrobia
Thermobaculum
Euryarchaeota
Crenarchaeota
Thaumarchaeota
Nanoarchaeota
Korarchaeota

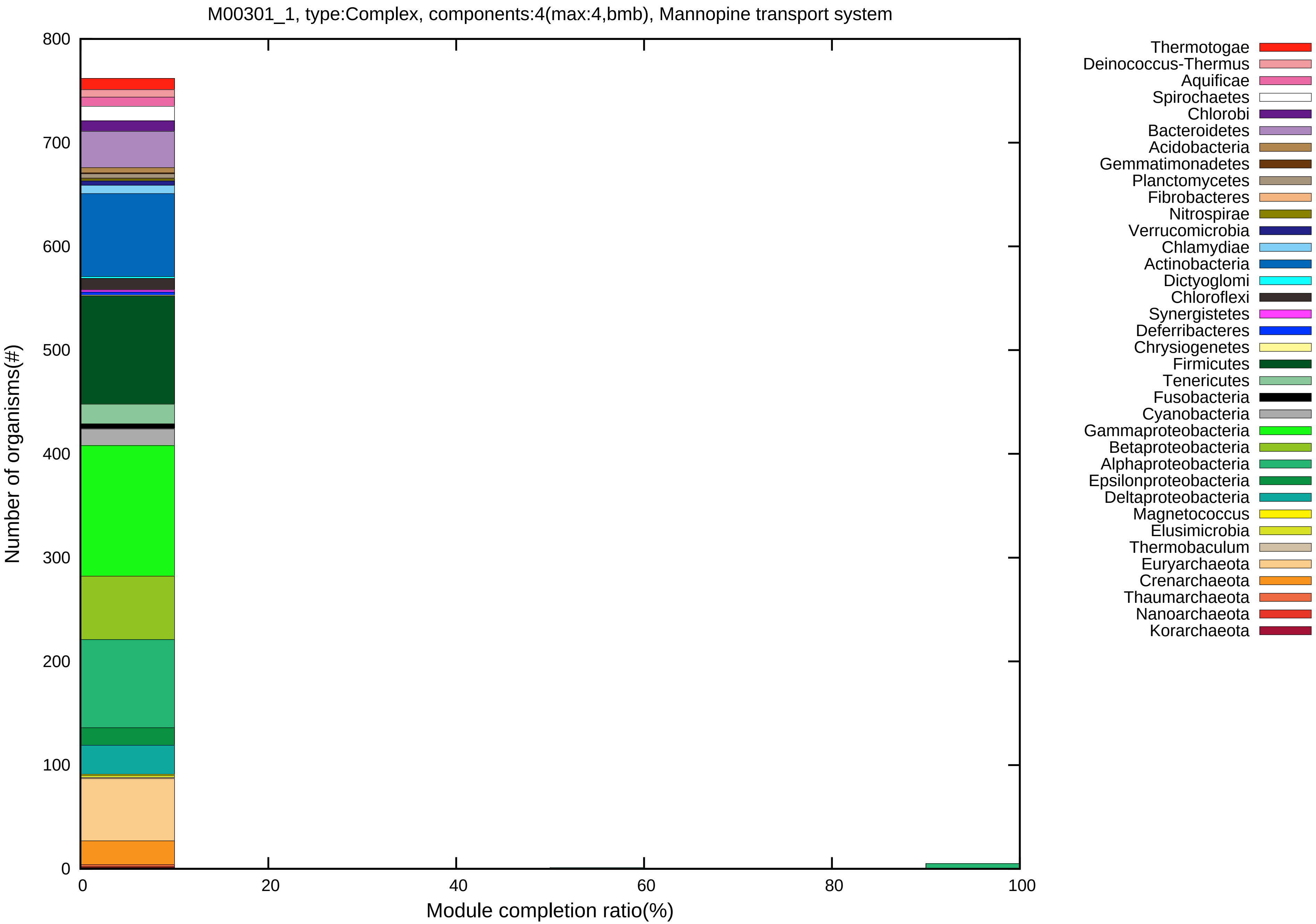

M00301_1, type:Complex, components:4(max:4,bmb), Mannopine transport system
Number of organisms(#)
Module completion ratio(%)
0
100
200
300
400
500
600
700
800
0
20
40
60
80
100
Thermotogae
Deinococcus-Thermus
Aquificae
Spirochaetes
Chlorobi
Bacteroidetes
Acidobacteria
Gemmatimonadetes
Planctomycetes
Fibrobacteres
Nitrospirae
Verrucomicrobia
Chlamydiae
Actinobacteria
Dictyoglomi
Chloroflexi
Synergistetes
Deferribacteres
Chrysiogenetes
Firmicutes
Tenericutes
Fusobacteria
Cyanobacteria
Gammaproteobacteria
Betaproteobacteria
Alphaproteobacteria
Epsilonproteobacteria
Deltaproteobacteria
Magnetococcus
Elusimicrobia
Thermobaculum
Euryarchaeota
Crenarchaeota
Thaumarchaeota
Nanoarchaeota
Korarchaeota

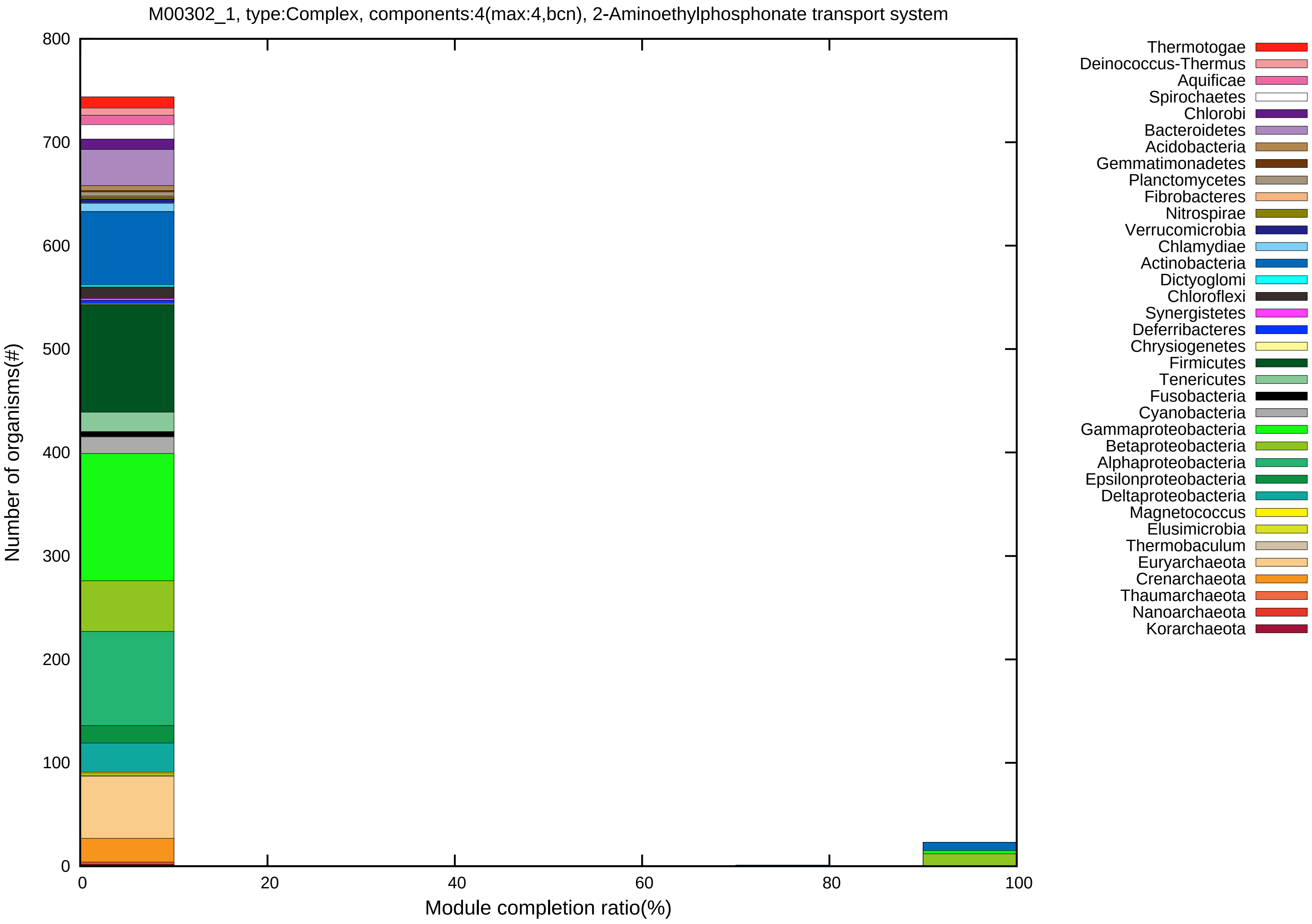

M00302_1, type:Complex, components:4(max:4,bcn), 2-Aminoethylphosphonate transport system
Number of organisms(#)
Module completion ratio(%)
0
100
200
300
400
500
600
700
800
0
20
40
60
80
100
Thermotogae
Deinococcus-Thermus
Aquificae
Spirochaetes
Chlorobi
Bacteroidetes
Acidobacteria
Gemmatimonadetes
Planctomycetes
Fibrobacteres
Nitrospirae
Verrucomicrobia
Chlamydiae
Actinobacteria
Dictyoglomi
Chloroflexi
Synergistetes
Deferribacteres
Chrysiogenetes
Firmicutes
Tenericutes
Fusobacteria
Cyanobacteria
Gammaproteobacteria
Betaproteobacteria
Alphaproteobacteria
Epsilonproteobacteria
Deltaproteobacteria
Magnetococcus
Elusimicrobia
Thermobaculum
Euryarchaeota
Crenarchaeota
Thaumarchaeota
Nanoarchaeota
Korarchaeota

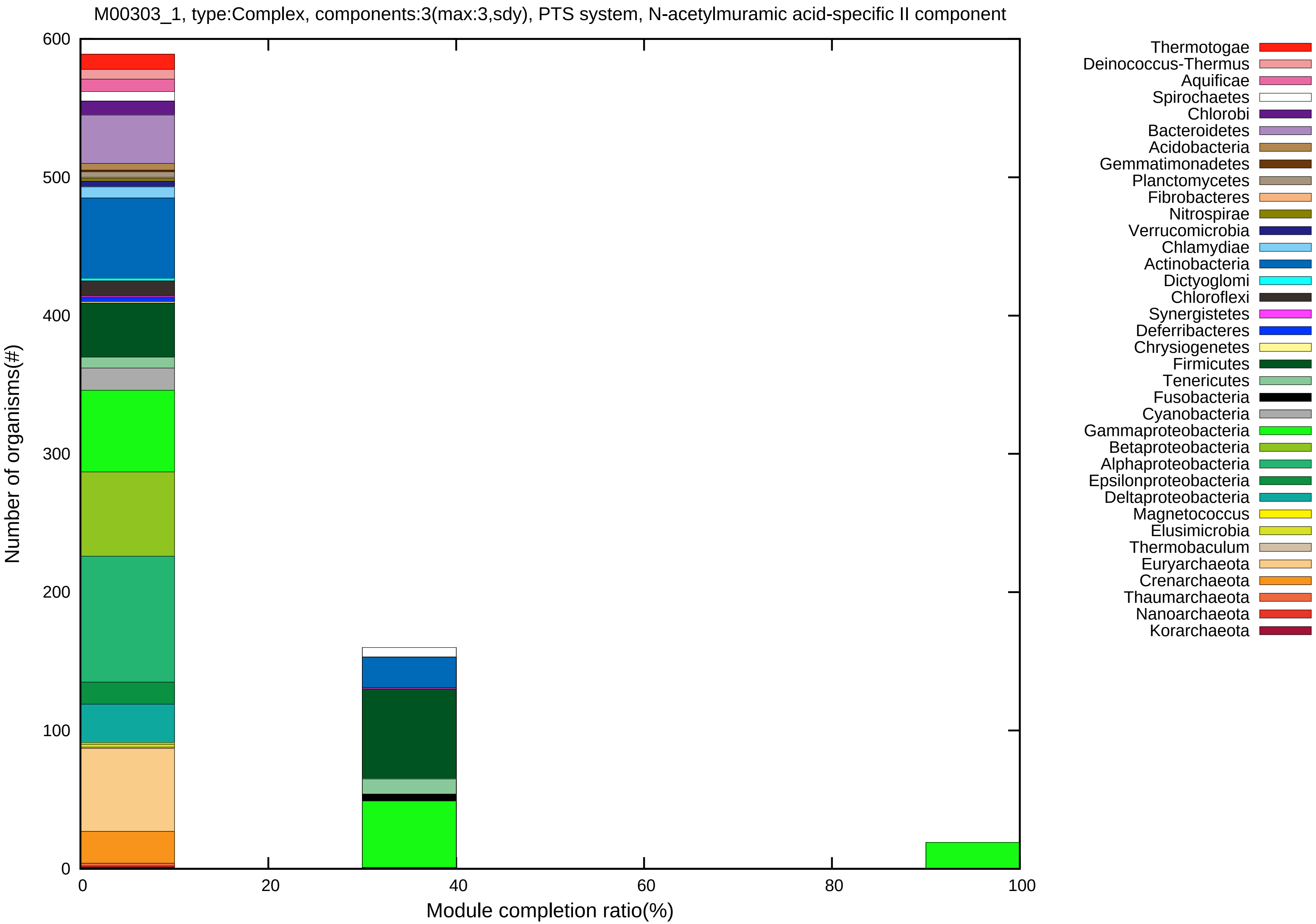
M00303_1, type:Complex, components:3(max:3,sdy), PTS system, N-acetylmuramic acid-specific II component
Number of organisms(#)
Module completion ratio(%)
0
100
200
300
400
500
600
0
20
40
60
80
100
Thermotogae
Deinococcus-Thermus
Aquificae
Spirochaetes
Chlorobi
Bacteroidetes
Acidobacteria
Gemmatimonadetes
Planctomycetes
Fibrobacteres
Nitrospirae
Verrucomicrobia
Chlamydiae
Actinobacteria
Dictyoglomi
Chloroflexi
Synergistetes
Deferribacteres
Chrysiogenetes
Firmicutes
Tenericutes
Fusobacteria
Cyanobacteria
Gammaproteobacteria
Betaproteobacteria
Alphaproteobacteria
Epsilonproteobacteria
Deltaproteobacteria
Magnetococcus
Elusimicrobia
Thermobaculum
Euryarchaeota
Crenarchaeota
Thaumarchaeota
Nanoarchaeota
Korarchaeota

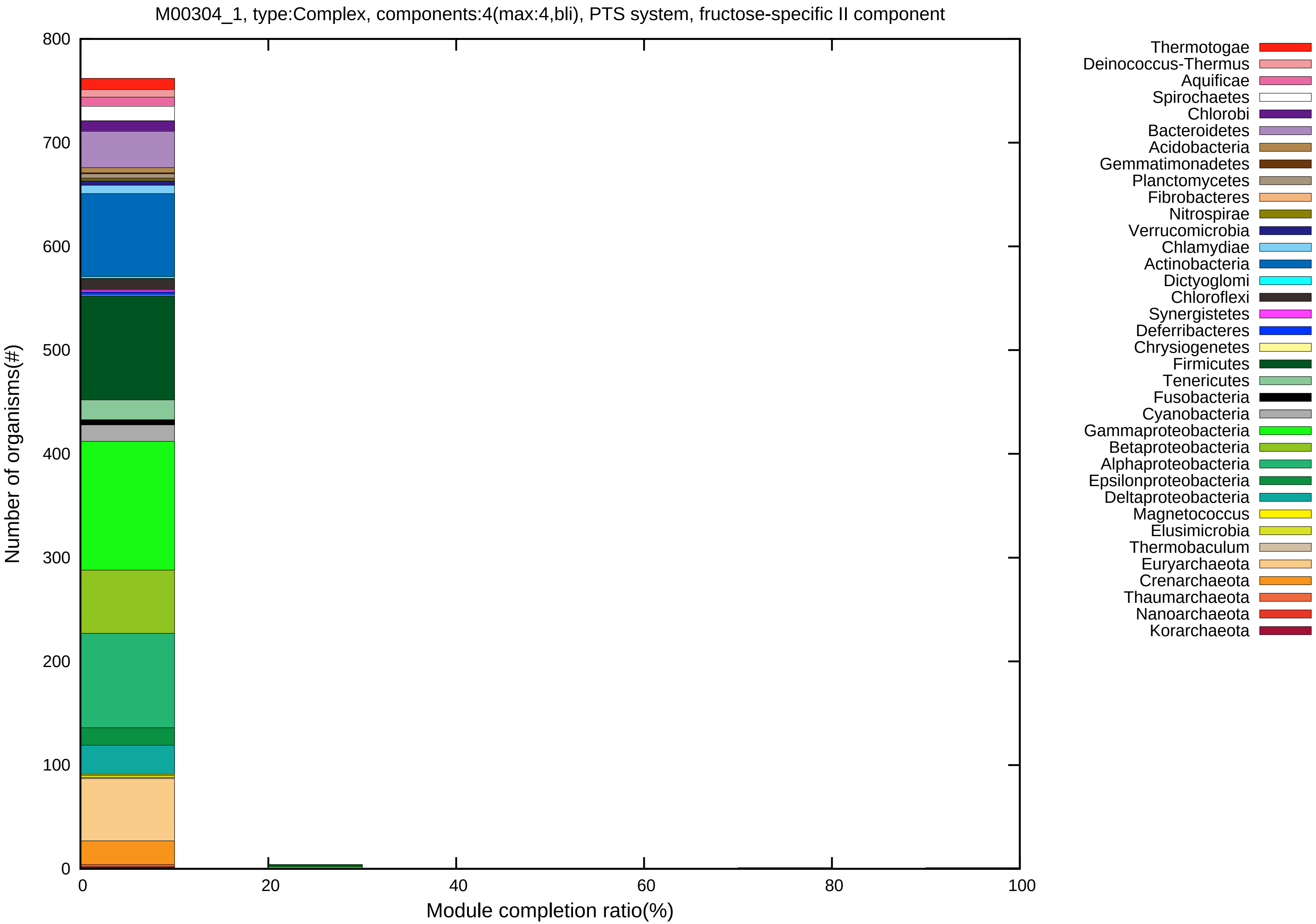
M00304_1, type:Complex, components:4(max:4,bli), PTS system, fructose-specific II component
Number of organisms(#)
Module completion ratio(%)
0
100
200
300
400
500
600
700
800
0
20
40
60
80
100
Thermotogae
Deinococcus-Thermus
Aquificae
Spirochaetes
Chlorobi
Bacteroidetes
Acidobacteria
Gemmatimonadetes
Planctomycetes
Fibrobacteres
Nitrospirae
Verrucomicrobia
Chlamydiae
Actinobacteria
Dictyoglomi
Chloroflexi
Synergistetes
Deferribacteres
Chrysiogenetes
Firmicutes
Tenericutes
Fusobacteria
Cyanobacteria
Gammaproteobacteria
Betaproteobacteria
Alphaproteobacteria
Epsilonproteobacteria
Deltaproteobacteria
Magnetococcus
Elusimicrobia
Thermobaculum
Euryarchaeota
Crenarchaeota
Thaumarchaeota
Nanoarchaeota
Korarchaeota

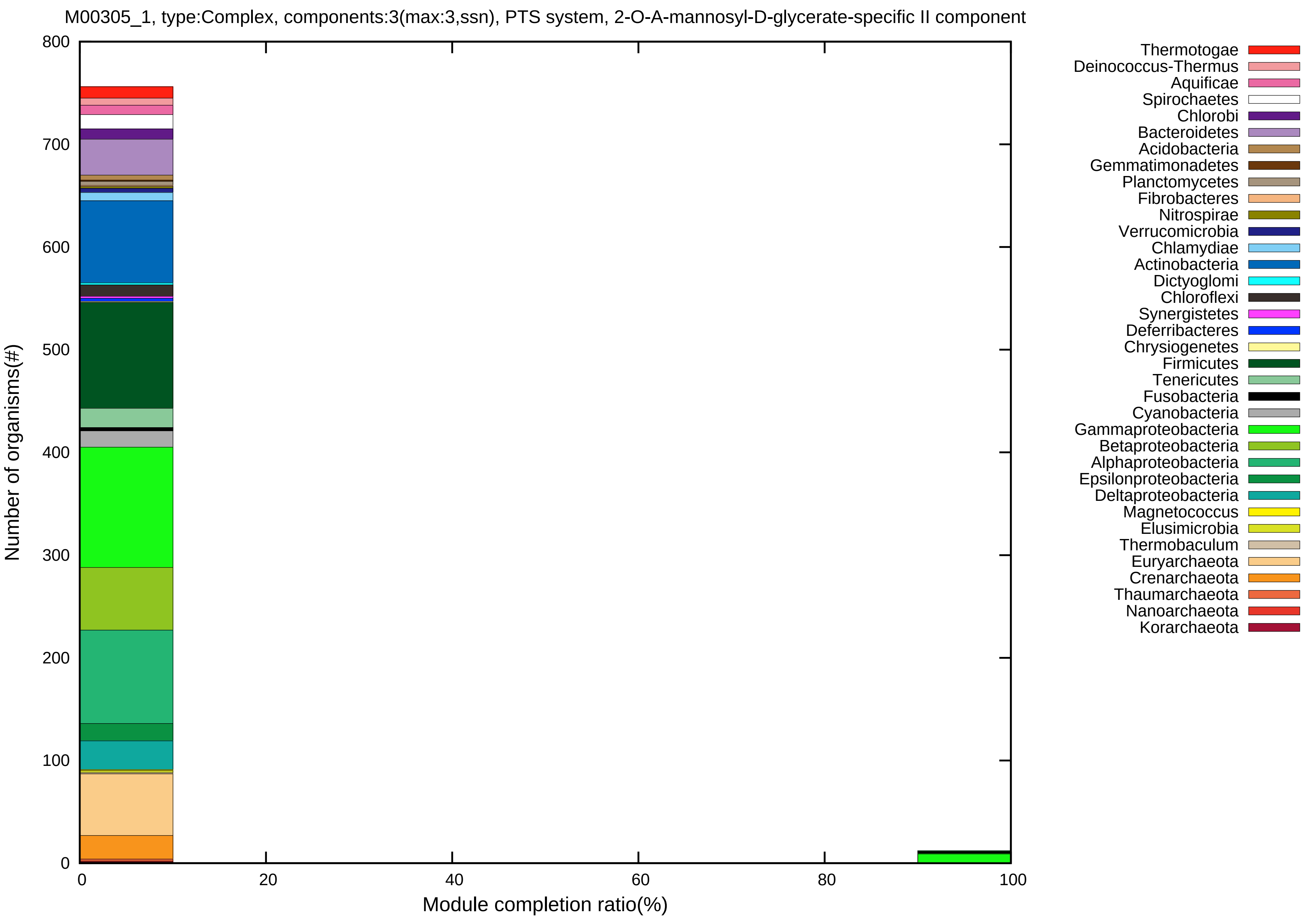
M00305_1, type:Complex, components:3(max:3,ssn), PTS system, 2-O-A-mannosyl-D-glycerate-specific II component
Number of organisms(#)
Module completion ratio(%)
0
100
200
300
400
500
600
700
800
0
20
40
60
80
100
Thermotogae
Deinococcus-Thermus
Aquificae
Spirochaetes
Chlorobi
Bacteroidetes
Acidobacteria
Gemmatimonadetes
Planctomycetes
Fibrobacteres
Nitrospirae
Verrucomicrobia
Chlamydiae
Actinobacteria
Dictyoglomi
Chloroflexi
Synergistetes
Deferribacteres
Chrysiogenetes
Firmicutes
Tenericutes
Fusobacteria
Cyanobacteria
Gammaproteobacteria
Betaproteobacteria
Alphaproteobacteria
Epsilonproteobacteria
Deltaproteobacteria
Magnetococcus
Elusimicrobia
Thermobaculum
Euryarchaeota
Crenarchaeota
Thaumarchaeota
Nanoarchaeota
Korarchaeota

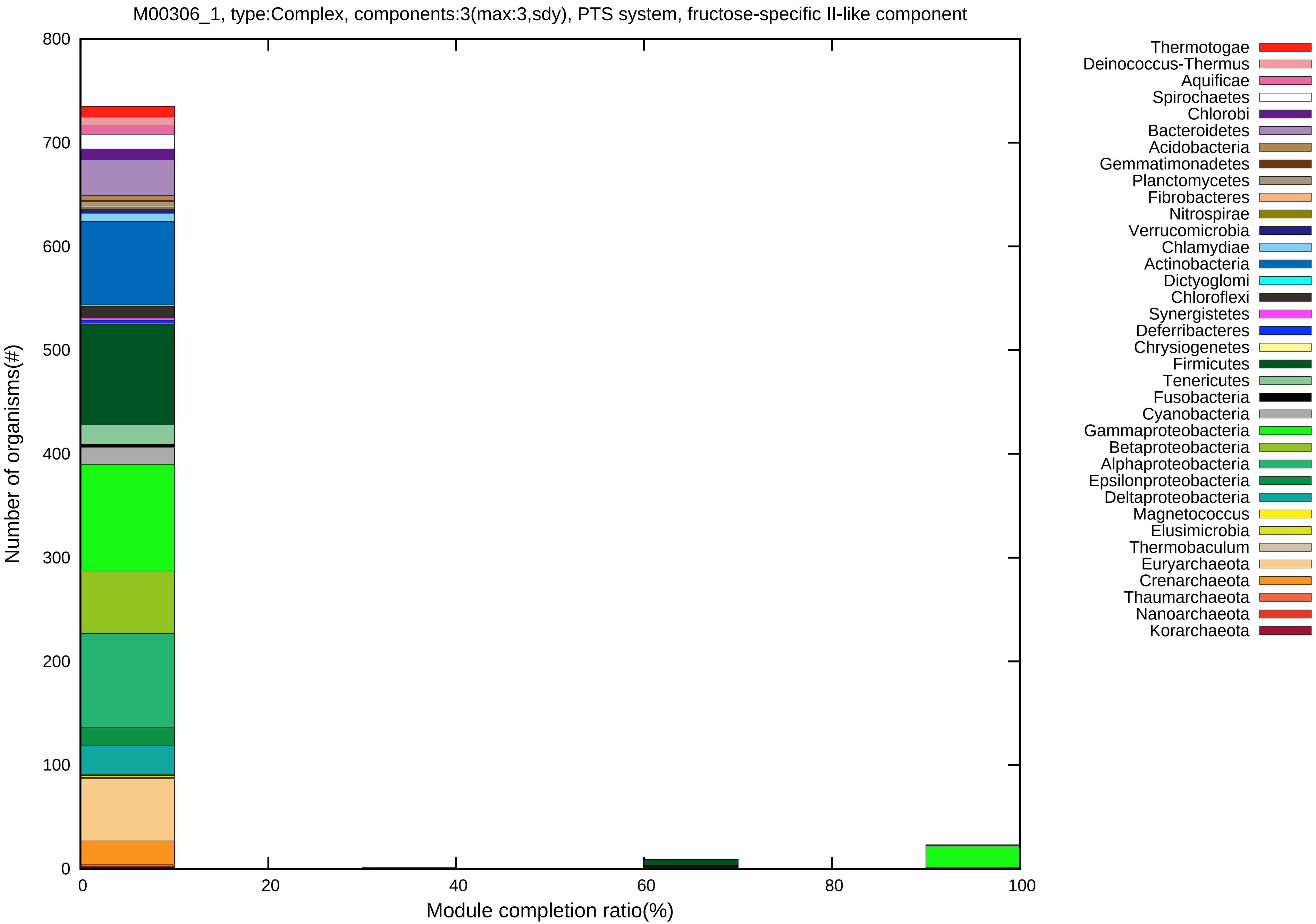
M00306_1, type:Complex, components:3(max:3,sdy), PTS system, fructose-specific II-like component
Number of organisms(#)
Module completion ratio(%)
0
100
200
300
400
500
600
700
800
0
20
40
60
80
100
Thermotogae
Deinococcus-Thermus
Aquificae
Spirochaetes
Chlorobi
Bacteroidetes
Acidobacteria
Gemmatimonadetes
Planctomycetes
Fibrobacteres
Nitrospirae
Verrucomicrobia
Chlamydiae
Actinobacteria
Dictyoglomi
Chloroflexi
Synergistetes
Deferribacteres
Chrysiogenetes
Firmicutes
Tenericutes
Fusobacteria
Cyanobacteria
Gammaproteobacteria
Betaproteobacteria
Alphaproteobacteria
Epsilonproteobacteria
Deltaproteobacteria
Magnetococcus
Elusimicrobia
Thermobaculum
Euryarchaeota
Crenarchaeota
Thaumarchaeota
Nanoarchaeota
Korarchaeota

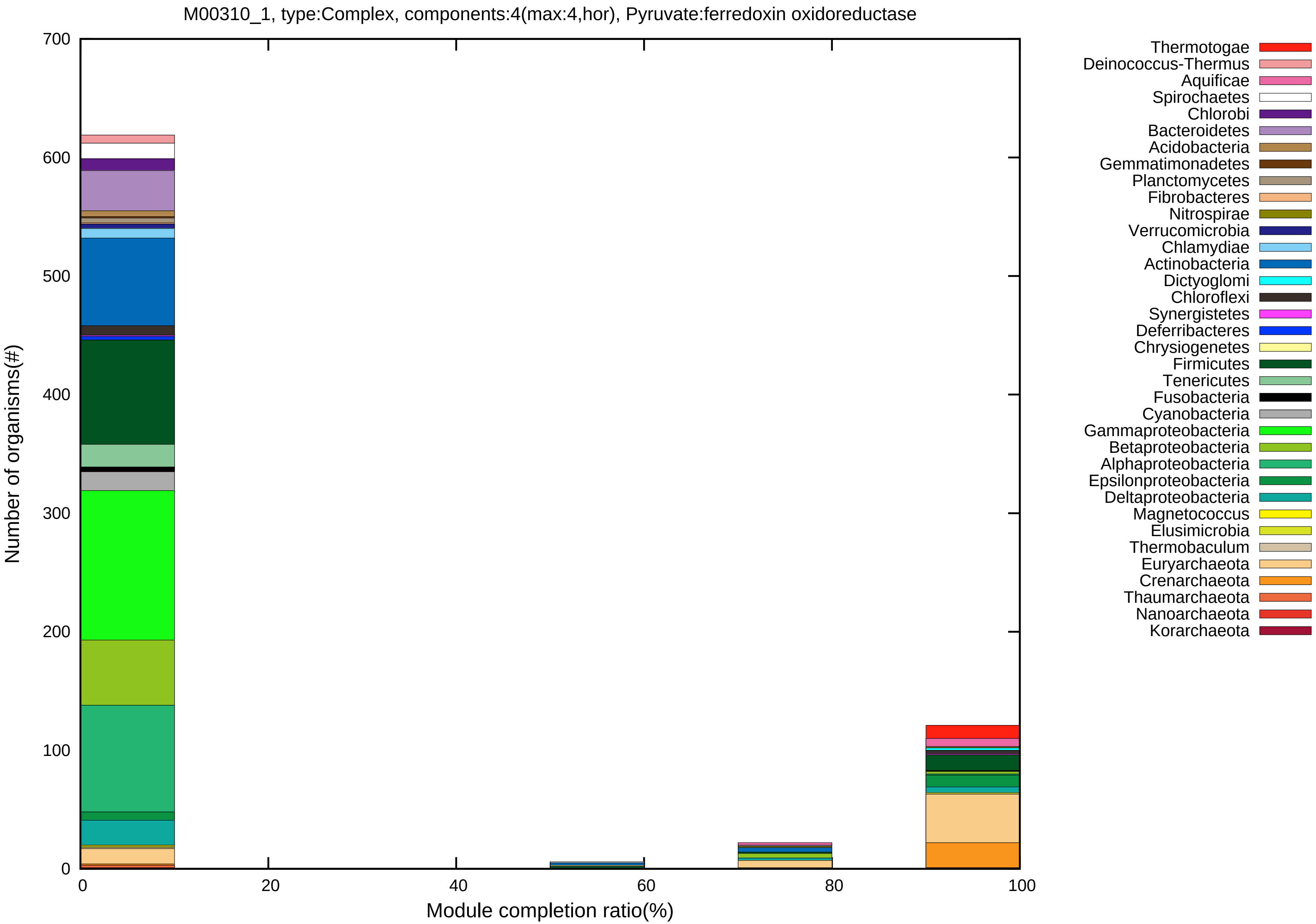

M00310_1, type:Complex, components:4(max:4,hor), Pyruvate:ferredoxin oxidoreductase
Number of organisms(#)
Module completion ratio(%)
0
100
200
300
400
500
600
700
0
20
40
60
80
100
Thermotogae
Deinococcus-Thermus
Aquificae
Spirochaetes
Chlorobi
Bacteroidetes
Acidobacteria
Gemmatimonadetes
Planctomycetes
Fibrobacteres
Nitrospirae
Verrucomicrobia
Chlamydiae
Actinobacteria
Dictyoglomi
Chloroflexi
Synergistetes
Deferribacteres
Chrysiogenetes
Firmicutes
Tenericutes
Fusobacteria
Cyanobacteria
Gammaproteobacteria
Betaproteobacteria
Alphaproteobacteria
Epsilonproteobacteria
Deltaproteobacteria
Magnetococcus
Elusimicrobia
Thermobaculum
Euryarchaeota
Crenarchaeota
Thaumarchaeota
Nanoarchaeota
Korarchaeota

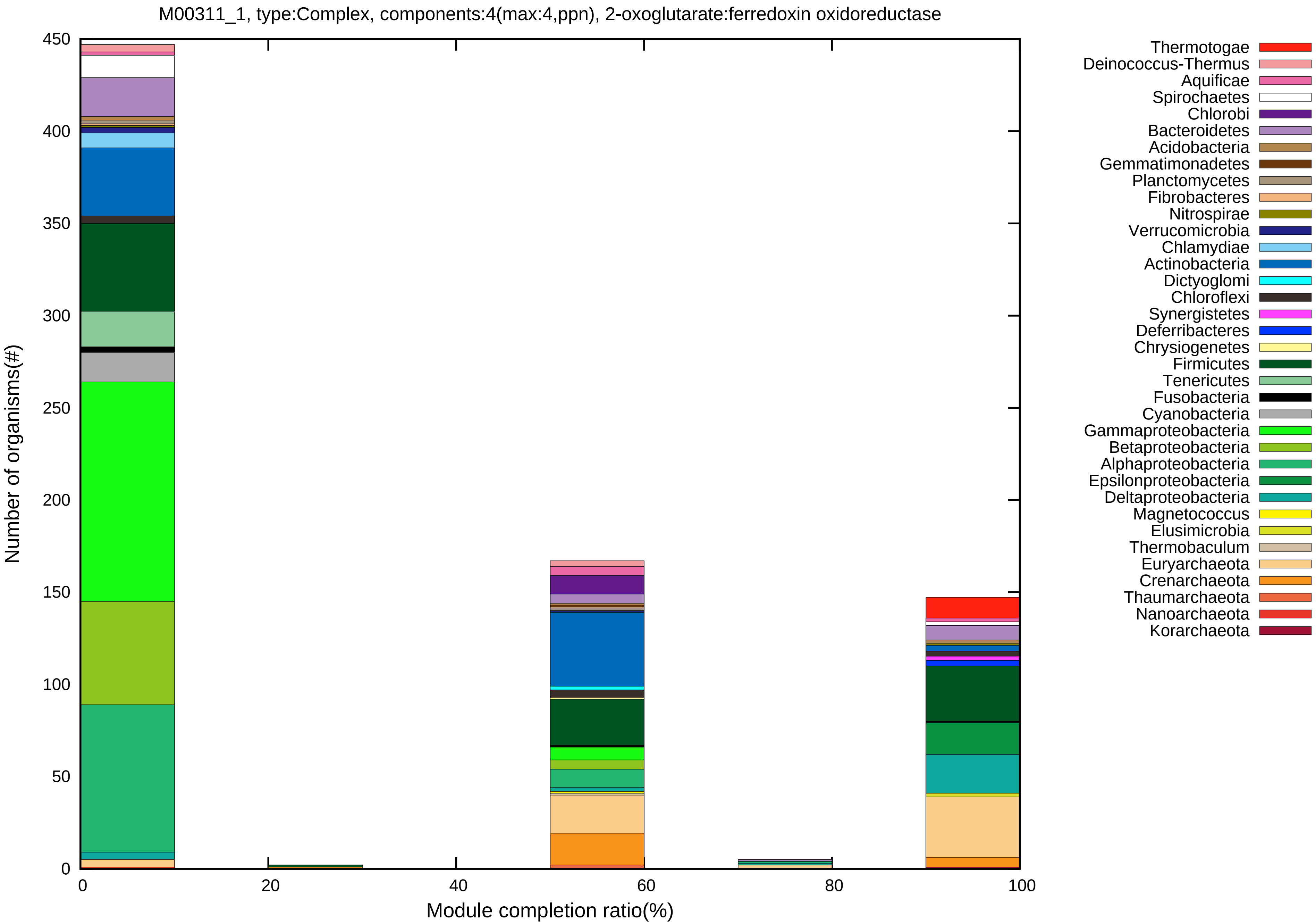
M00311_1, type:Complex, components:4(max:4,ppn), 2-oxoglutarate:ferredoxin oxidoreductase
Number of organisms(#)
Module completion ratio(%)
0
50
100
150
200
250
300
350
400
450
0
20
40
60
80
100
Thermotogae
Deinococcus-Thermus
Aquificae
Spirochaetes
Chlorobi
Bacteroidetes
Acidobacteria
Gemmatimonadetes
Planctomycetes
Fibrobacteres
Nitrospirae
Verrucomicrobia
Chlamydiae
Actinobacteria
Dictyoglomi
Chloroflexi
Synergistetes
Deferribacteres
Chrysiogenetes
Firmicutes
Tenericutes
Fusobacteria
Cyanobacteria
Gammaproteobacteria
Betaproteobacteria
Alphaproteobacteria
Epsilonproteobacteria
Deltaproteobacteria
Magnetococcus
Elusimicrobia
Thermobaculum
Euryarchaeota
Crenarchaeota
Thaumarchaeota
Nanoarchaeota
Korarchaeota

M00312_1, type:Complex, components:4(max:4,pab), 2-oxoisovalerate:ferredoxin oxidoreductase

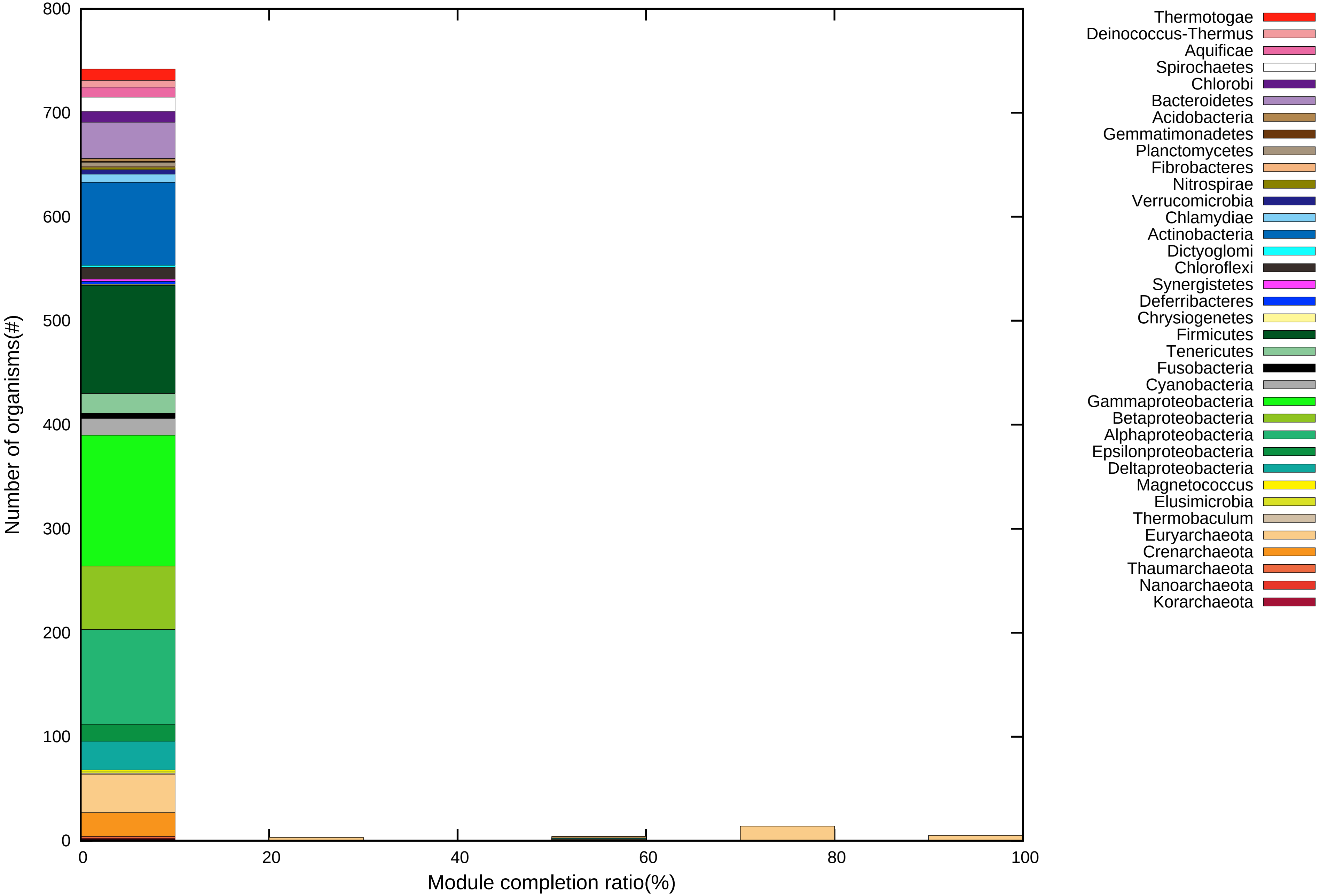

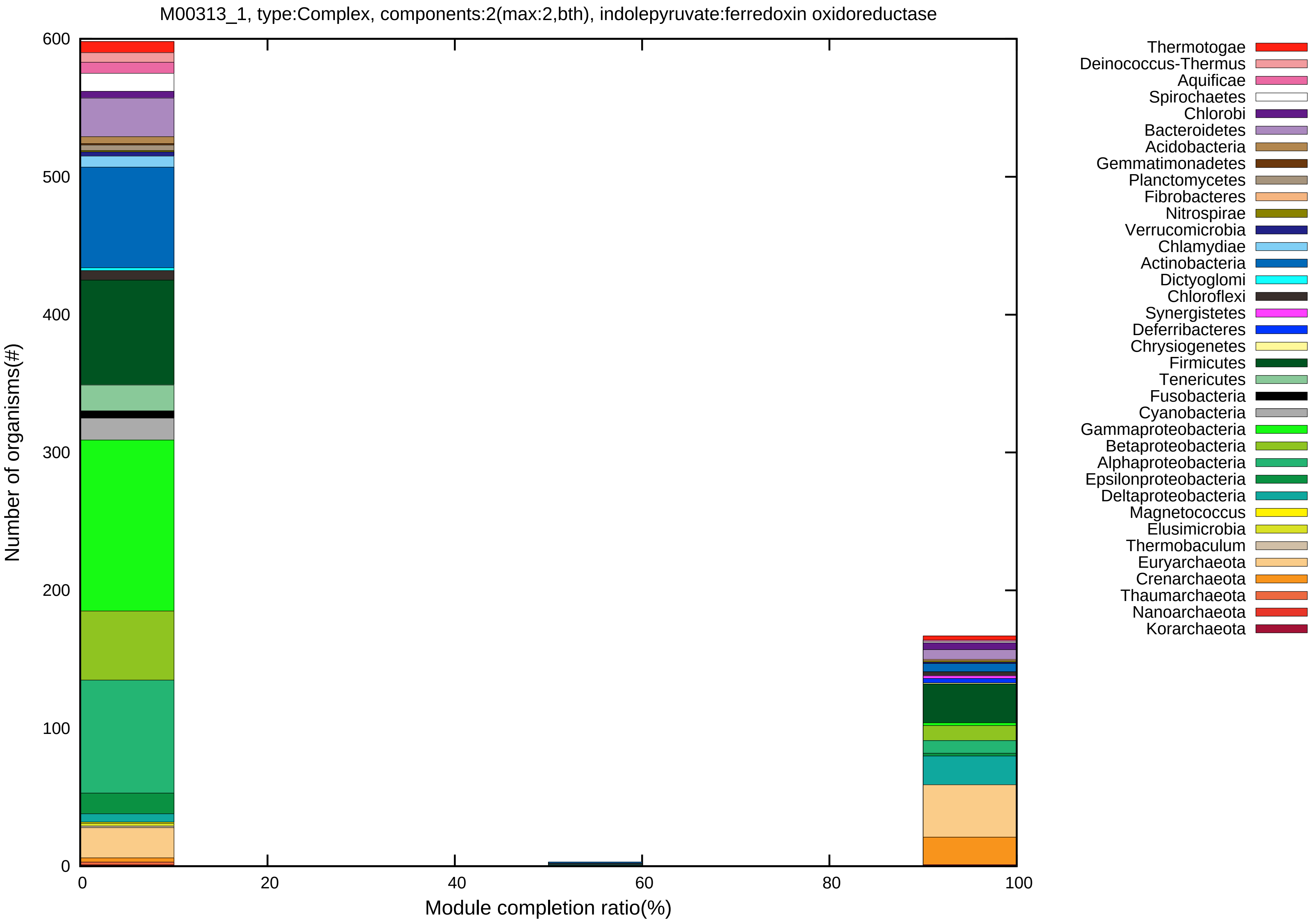
M00313_1, type:Complex, components:2(max:2,bth), indolepyruvate:ferredoxin oxidoreductase
Number of organisms(#)
Module completion ratio(%)
0
100
200
300
400
500
600
0
20
40
60
80
100
Thermotogae
Deinococcus-Thermus
Aquificae
Spirochaetes
Chlorobi
Bacteroidetes
Acidobacteria
Gemmatimonadetes
Planctomycetes
Fibrobacteres
Nitrospirae
Verrucomicrobia
Chlamydiae
Actinobacteria
Dictyoglomi
Chloroflexi
Synergistetes
Deferribacteres
Chrysiogenetes
Firmicutes
Tenericutes
Fusobacteria
Cyanobacteria
Gammaproteobacteria
Betaproteobacteria
Alphaproteobacteria
Epsilonproteobacteria
Deltaproteobacteria
Magnetococcus
Elusimicrobia
Thermobaculum
Euryarchaeota
Crenarchaeota
Thaumarchaeota
Nanoarchaeota
Korarchaeota

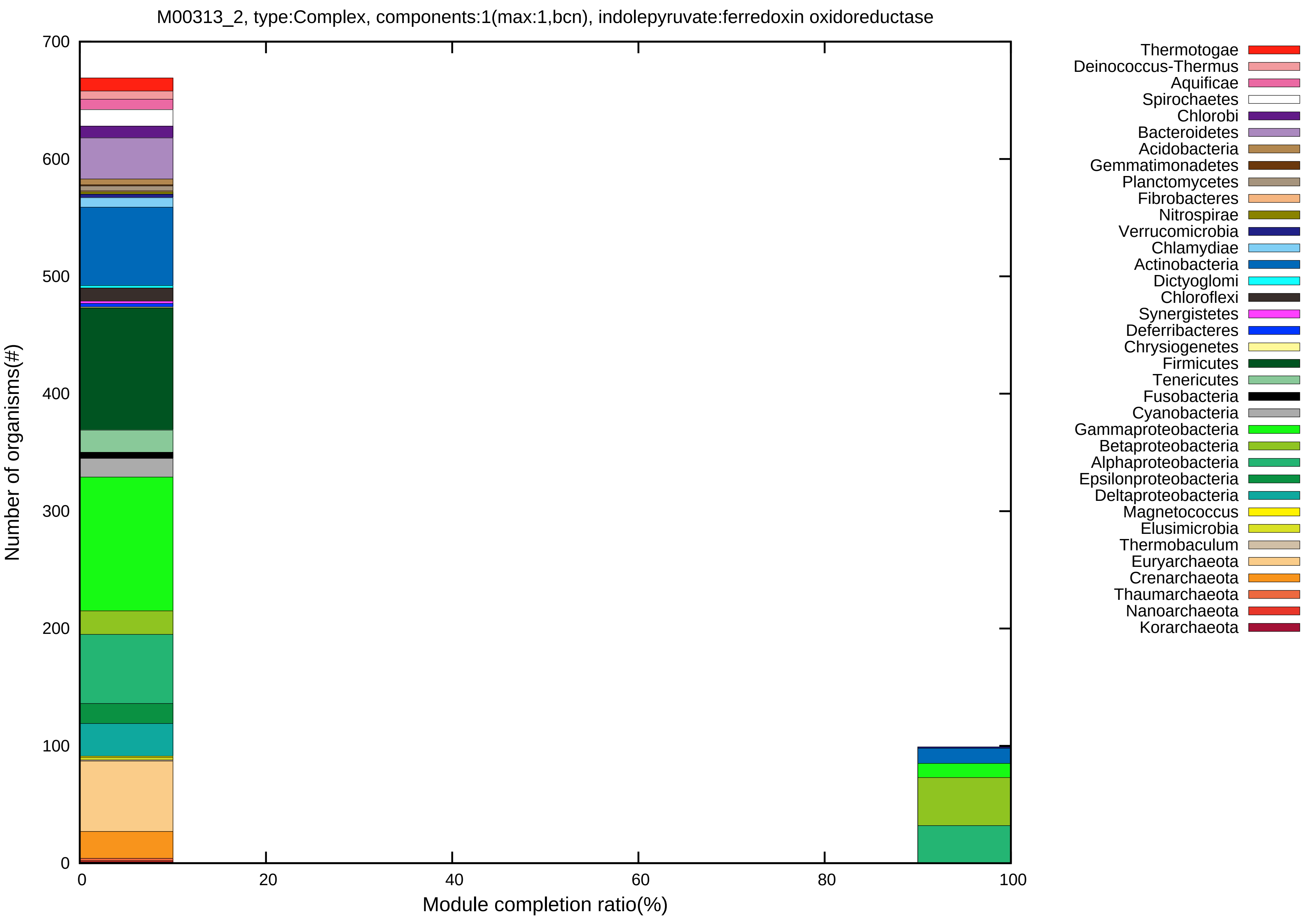

M00313_2, type:Complex, components:1(max:1,bcn), indolepyruvate:ferredoxin oxidoreductase
Number of organisms(#)
Module completion ratio(%)
0
100
200
300
400
500
600
700
0
20
40
60
80
100
Thermotogae
Deinococcus-Thermus
Aquificae
Spirochaetes
Chlorobi
Bacteroidetes
Acidobacteria
Gemmatimonadetes
Planctomycetes
Fibrobacteres
Nitrospirae
Verrucomicrobia
Chlamydiae
Actinobacteria
Dictyoglomi
Chloroflexi
Synergistetes
Deferribacteres
Chrysiogenetes
Firmicutes
Tenericutes
Fusobacteria
Cyanobacteria
Gammaproteobacteria
Betaproteobacteria
Alphaproteobacteria
Epsilonproteobacteria
Deltaproteobacteria
Magnetococcus
Elusimicrobia
Thermobaculum
Euryarchaeota
Crenarchaeota
Thaumarchaeota
Nanoarchaeota
Korarchaeota

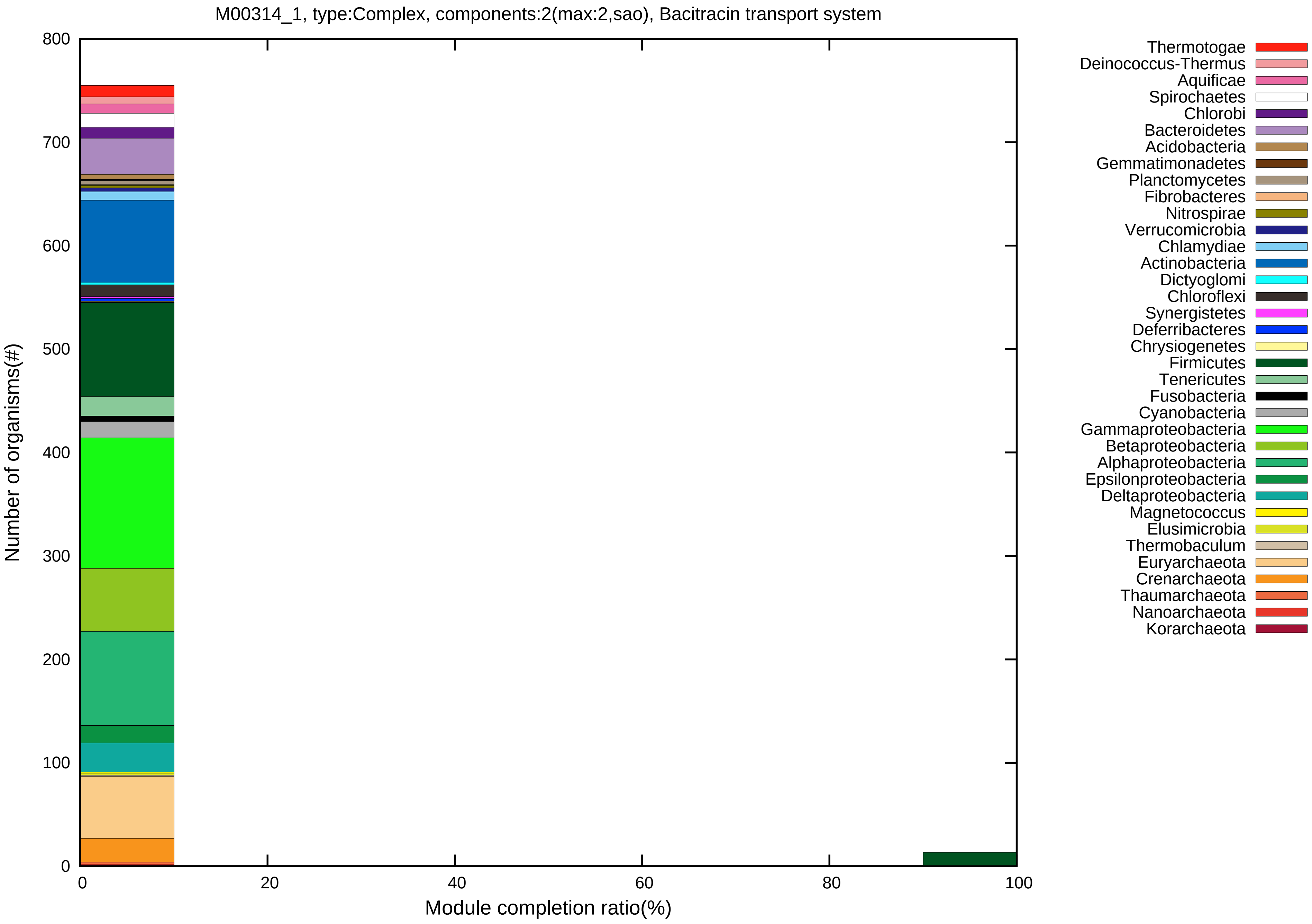
M00314_1, type:Complex, components:2(max:2,sao), Bacitracin transport system
Number of organisms(#)
Module completion ratio(%)
0
100
200
300
400
500
600
700
800
0
20
40
60
80
100
Thermotogae
Deinococcus-Thermus
Aquificae
Spirochaetes
Chlorobi
Bacteroidetes
Acidobacteria
Gemmatimonadetes
Planctomycetes
Fibrobacteres
Nitrospirae
Verrucomicrobia
Chlamydiae
Actinobacteria
Dictyoglomi
Chloroflexi
Synergistetes
Deferribacteres
Chrysiogenetes
Firmicutes
Tenericutes
Fusobacteria
Cyanobacteria
Gammaproteobacteria
Betaproteobacteria
Alphaproteobacteria
Epsilonproteobacteria
Deltaproteobacteria
Magnetococcus
Elusimicrobia
Thermobaculum
Euryarchaeota
Crenarchaeota
Thaumarchaeota
Nanoarchaeota
Korarchaeota

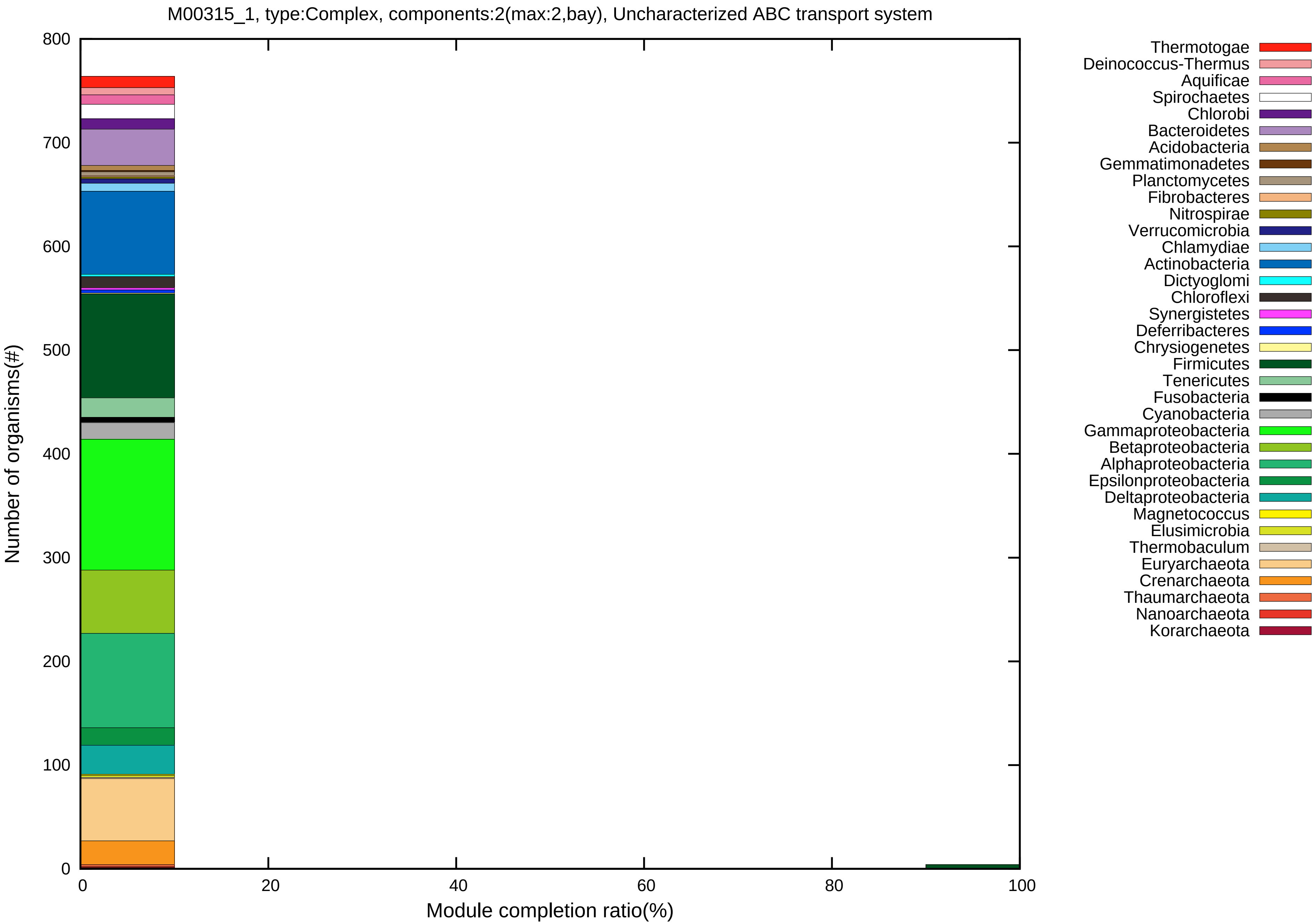
M00315_1, type:Complex, components:2(max:2,bay), Uncharacterized ABC transport system
Number of organisms(#)
Module completion ratio(%)
0
100
200
300
400
500
600
700
800
0
20
40
60
80
100
Thermotogae
Deinococcus-Thermus
Aquificae
Spirochaetes
Chlorobi
Bacteroidetes
Acidobacteria
Gemmatimonadetes
Planctomycetes
Fibrobacteres
Nitrospirae
Verrucomicrobia
Chlamydiae
Actinobacteria
Dictyoglomi
Chloroflexi
Synergistetes
Deferribacteres
Chrysiogenetes
Firmicutes
Tenericutes
Fusobacteria
Cyanobacteria
Gammaproteobacteria
Betaproteobacteria
Alphaproteobacteria
Epsilonproteobacteria
Deltaproteobacteria
Magnetococcus
Elusimicrobia
Thermobaculum
Euryarchaeota
Crenarchaeota
Thaumarchaeota
Nanoarchaeota
Korarchaeota

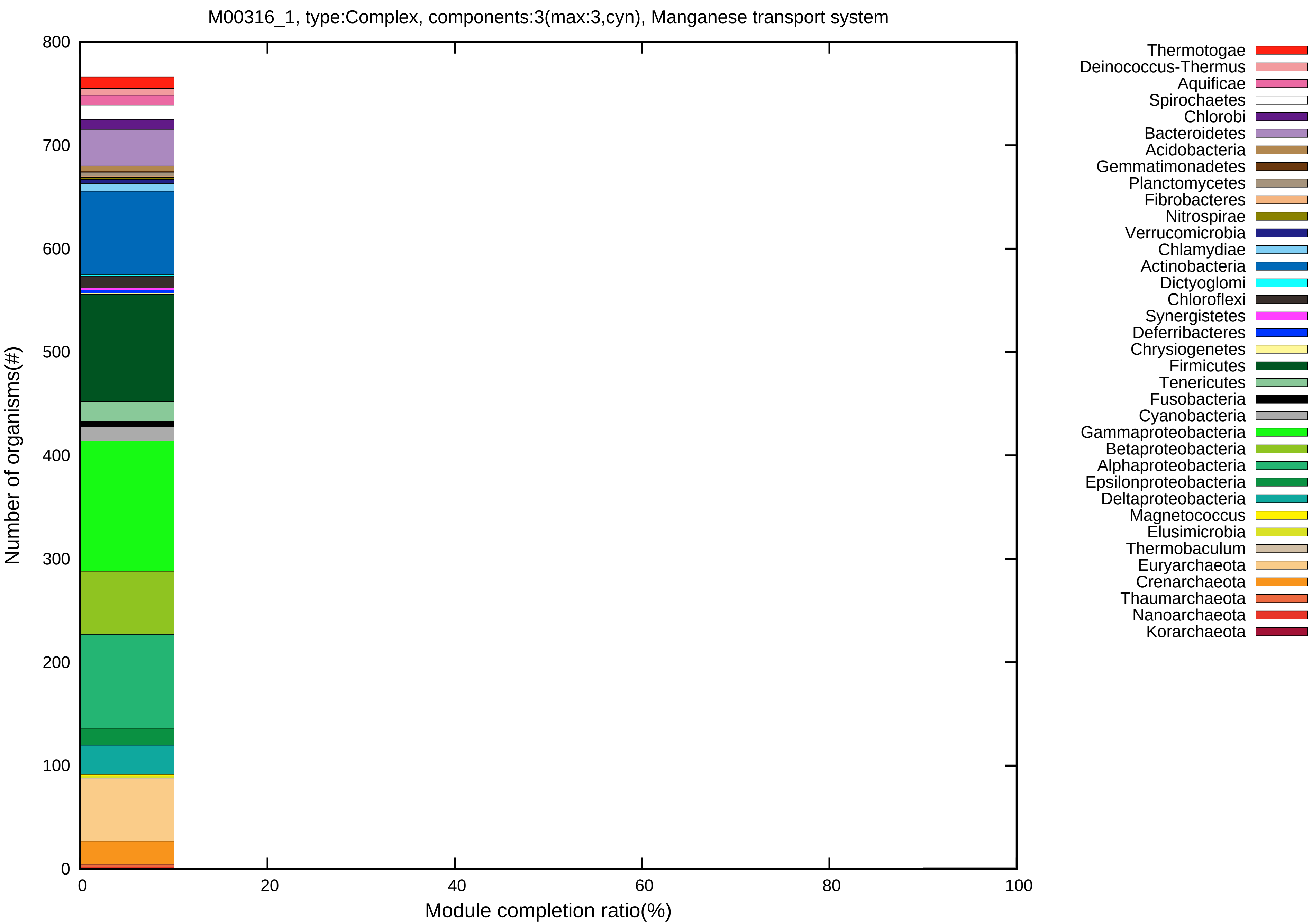

M00316_1, type:Complex, components:3(max:3,cyn), Manganese transport system
Number of organisms(#)
Module completion ratio(%)
0
100
200
300
400
500
600
700
800
0
20
40
60
80
100
Thermotogae
Deinococcus-Thermus
Aquificae
Spirochaetes
Chlorobi
Bacteroidetes
Acidobacteria
Gemmatimonadetes
Planctomycetes
Fibrobacteres
Nitrospirae
Verrucomicrobia
Chlamydiae
Actinobacteria
Dictyoglomi
Chloroflexi
Synergistetes
Deferribacteres
Chrysiogenetes
Firmicutes
Tenericutes
Fusobacteria
Cyanobacteria
Gammaproteobacteria
Betaproteobacteria
Alphaproteobacteria
Epsilonproteobacteria
Deltaproteobacteria
Magnetococcus
Elusimicrobia
Thermobaculum
Euryarchaeota
Crenarchaeota
Thaumarchaeota
Nanoarchaeota
Korarchaeota

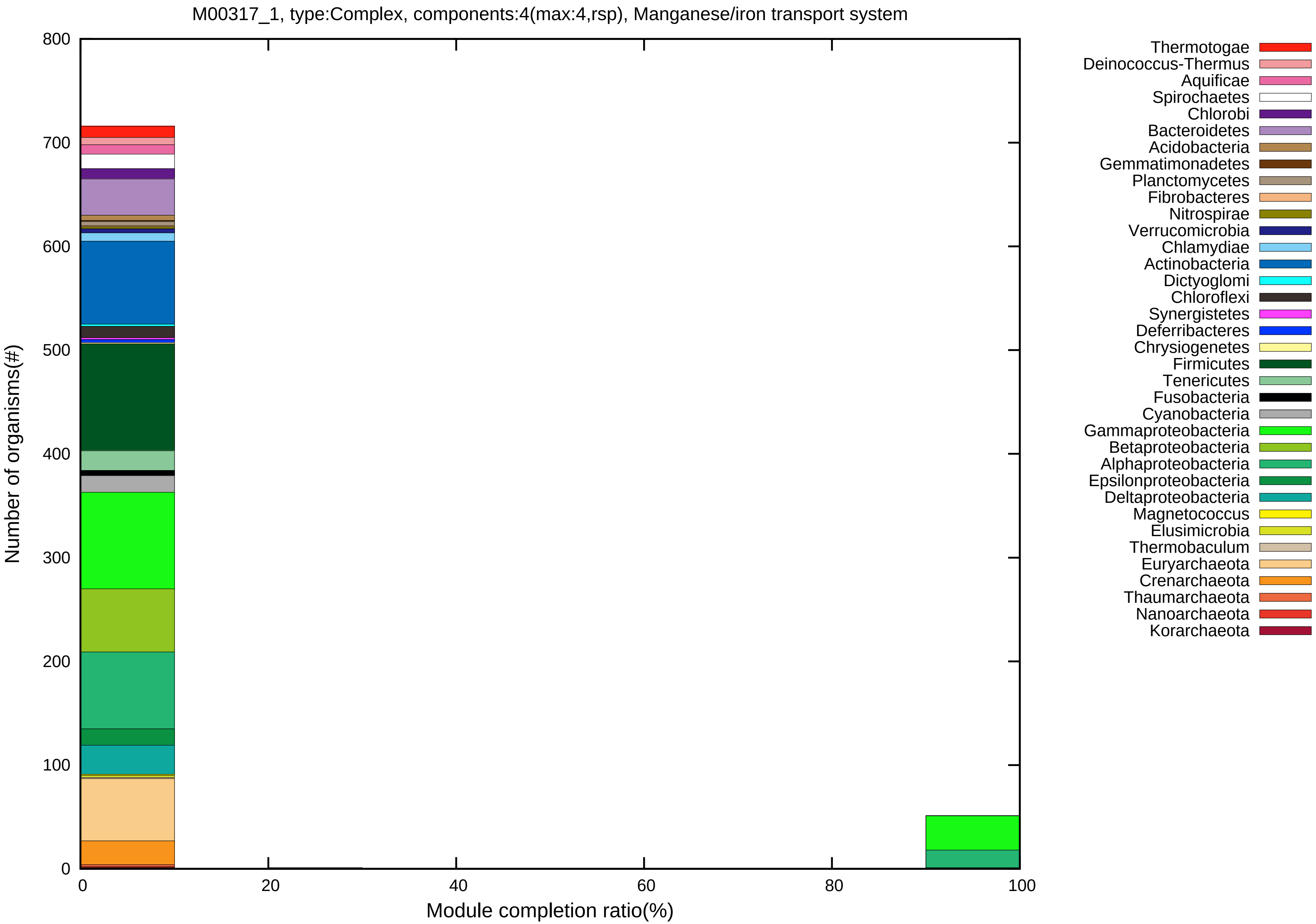
M00317_1, type:Complex, components:4(max:4,rsp), Manganese/iron transport system
Number of organisms(#)
Module completion ratio(%)
0
100
200
300
400
500
600
700
800
0
20
40
60
80
100
Thermotogae
Deinococcus-Thermus
Aquificae
Spirochaetes
Chlorobi
Bacteroidetes
Acidobacteria
Gemmatimonadetes
Planctomycetes
Fibrobacteres
Nitrospirae
Verrucomicrobia
Chlamydiae
Actinobacteria
Dictyoglomi
Chloroflexi
Synergistetes
Deferribacteres
Chrysiogenetes
Firmicutes
Tenericutes
Fusobacteria
Cyanobacteria
Gammaproteobacteria
Betaproteobacteria
Alphaproteobacteria
Epsilonproteobacteria
Deltaproteobacteria
Magnetococcus
Elusimicrobia
Thermobaculum
Euryarchaeota
Crenarchaeota
Thaumarchaeota
Nanoarchaeota
Korarchaeota

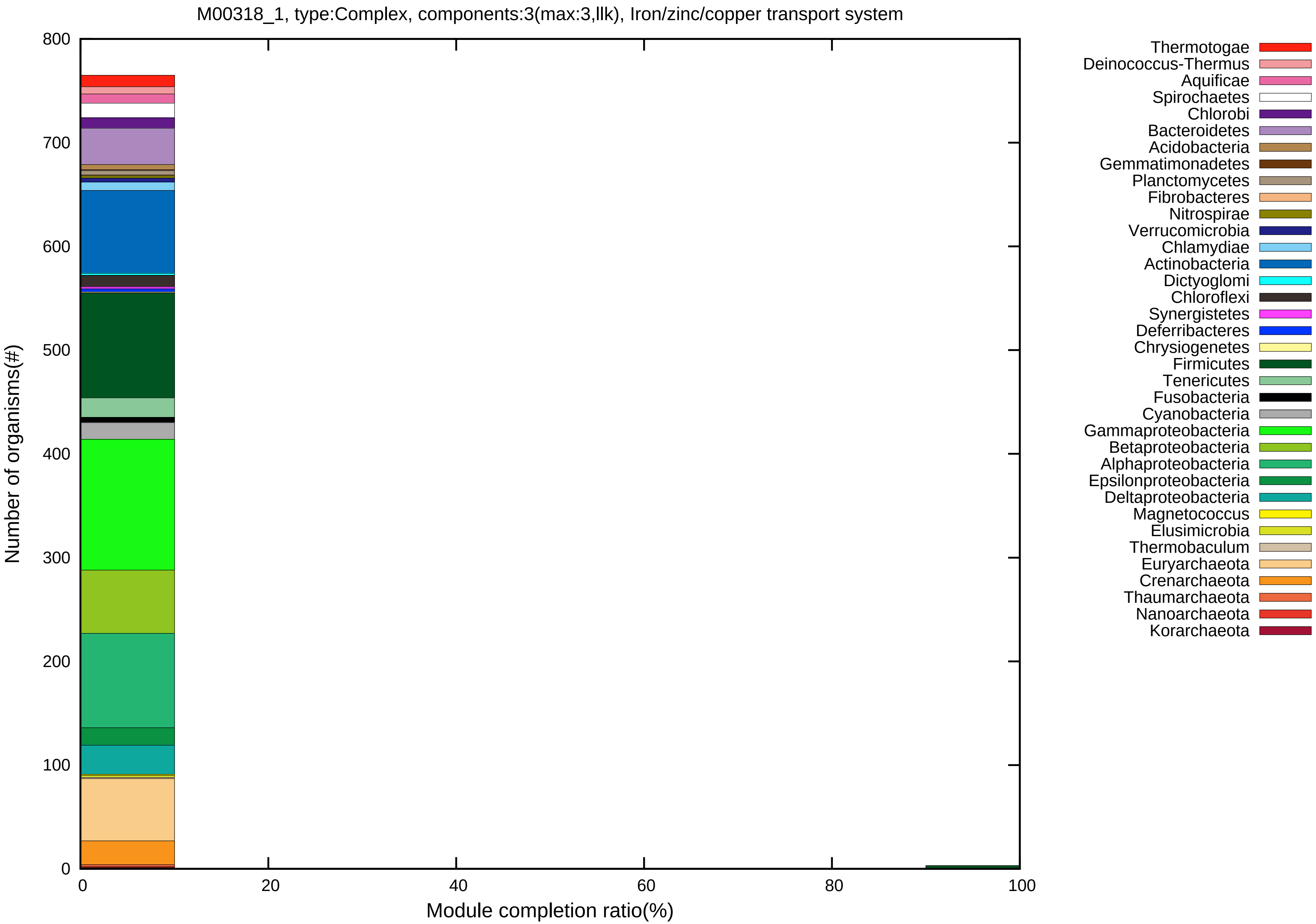
M00318_1, type:Complex, components:3(max:3,llk), Iron/zinc/copper transport system
Number of organisms(#)
Module completion ratio(%)
0
100
200
300
400
500
600
700
800
0
20
40
60
80
100
Thermotogae
Deinococcus-Thermus
Aquificae
Spirochaetes
Chlorobi
Bacteroidetes
Acidobacteria
Gemmatimonadetes
Planctomycetes
Fibrobacteres
Nitrospirae
Verrucomicrobia
Chlamydiae
Actinobacteria
Dictyoglomi
Chloroflexi
Synergistetes
Deferribacteres
Chrysiogenetes
Firmicutes
Tenericutes
Fusobacteria
Cyanobacteria
Gammaproteobacteria
Betaproteobacteria
Alphaproteobacteria
Epsilonproteobacteria
Deltaproteobacteria
Magnetococcus
Elusimicrobia
Thermobaculum
Euryarchaeota
Crenarchaeota
Thaumarchaeota
Nanoarchaeota
Korarchaeota

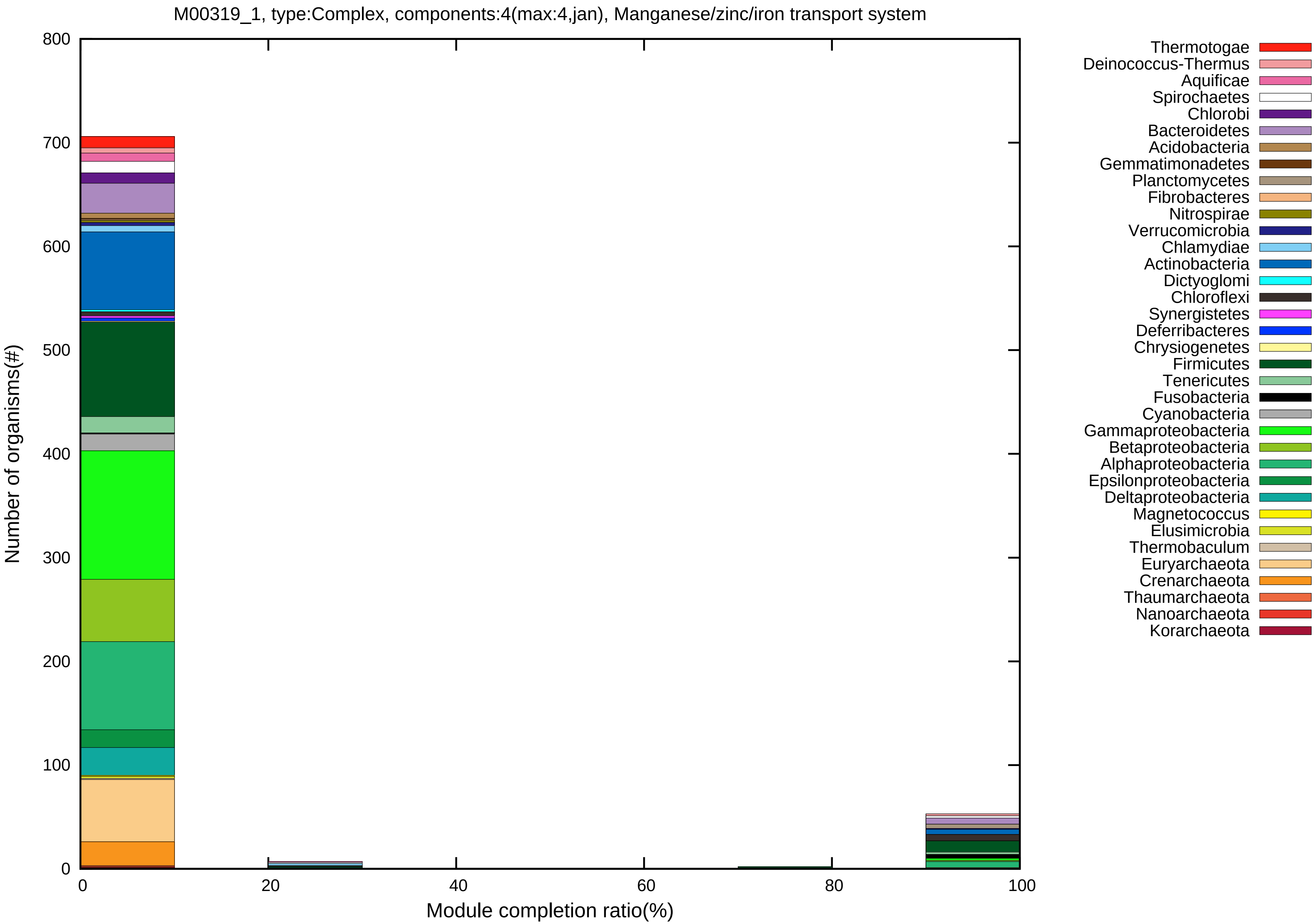

M00319_1, type:Complex, components:4(max:4,jan), Manganese/zinc/iron transport system
Number of organisms(#)
Module completion ratio(%)
0
100
200
300
400
500
600
700
800
0
20
40
60
80
100
Thermotogae
Deinococcus-Thermus
Aquificae
Spirochaetes
Chlorobi
Bacteroidetes
Acidobacteria
Gemmatimonadetes
Planctomycetes
Fibrobacteres
Nitrospirae
Verrucomicrobia
Chlamydiae
Actinobacteria
Dictyoglomi
Chloroflexi
Synergistetes
Deferribacteres
Chrysiogenetes
Firmicutes
Tenericutes
Fusobacteria
Cyanobacteria
Gammaproteobacteria
Betaproteobacteria
Alphaproteobacteria
Epsilonproteobacteria
Deltaproteobacteria
Magnetococcus
Elusimicrobia
Thermobaculum
Euryarchaeota
Crenarchaeota
Thaumarchaeota
Nanoarchaeota
Korarchaeota

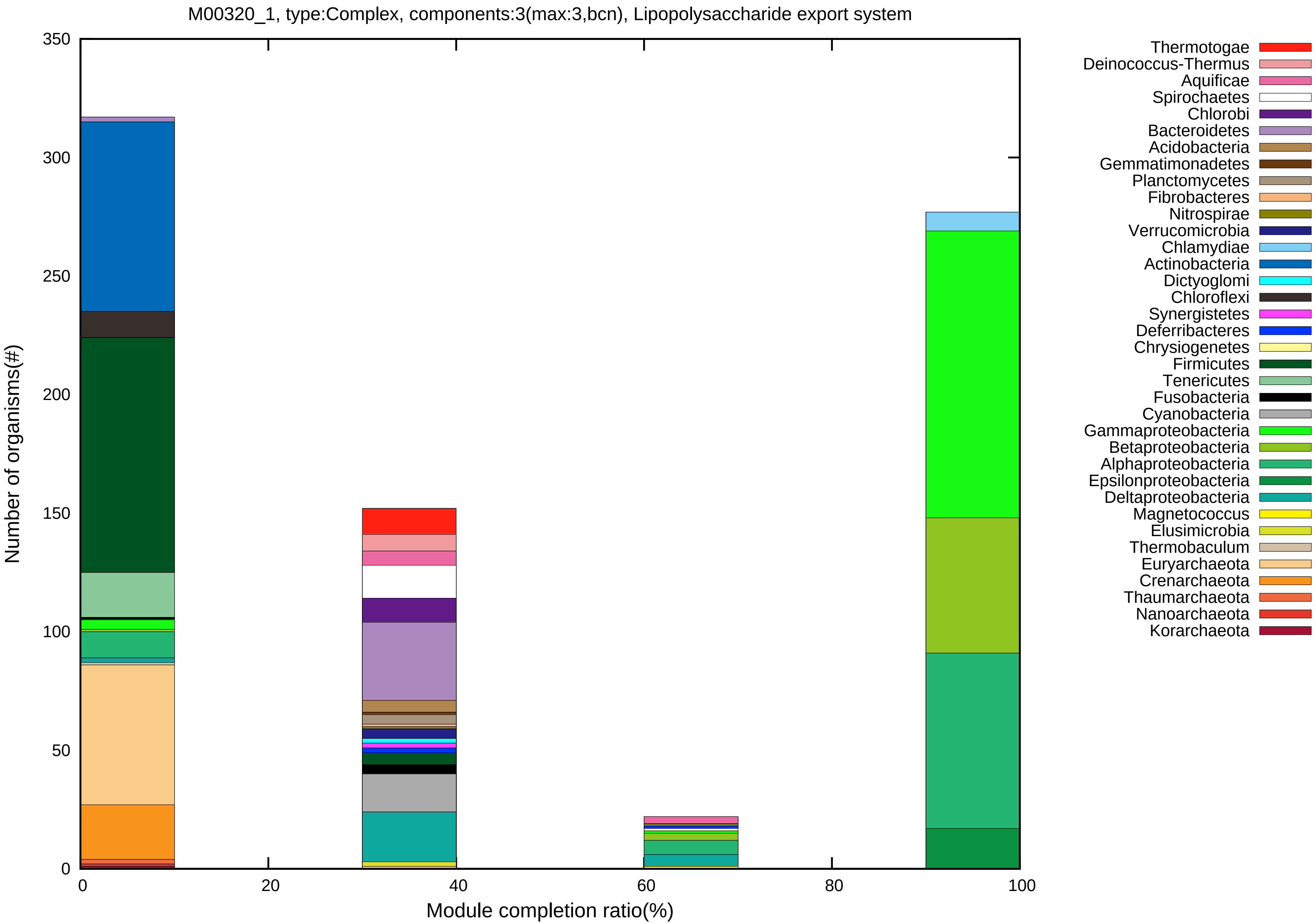

M00320_1, type:Complex, components:3(max:3,bcn), Lipopolysaccharide export system
Number of organisms(#)
Module completion ratio(%)
0
50
100
150
200
250
300
350
0
20
40
60
80
100
Thermotogae
Deinococcus-Thermus
Aquificae
Spirochaetes
Chlorobi
Bacteroidetes
Acidobacteria
Gemmatimonadetes
Planctomycetes
Fibrobacteres
Nitrospirae
Verrucomicrobia
Chlamydiae
Actinobacteria
Dictyoglomi
Chloroflexi
Synergistetes
Deferribacteres
Chrysiogenetes
Firmicutes
Tenericutes
Fusobacteria
Cyanobacteria
Gammaproteobacteria
Betaproteobacteria
Alphaproteobacteria
Epsilonproteobacteria
Deltaproteobacteria
Magnetococcus
Elusimicrobia
Thermobaculum
Euryarchaeota
Crenarchaeota
Thaumarchaeota
Nanoarchaeota
Korarchaeota

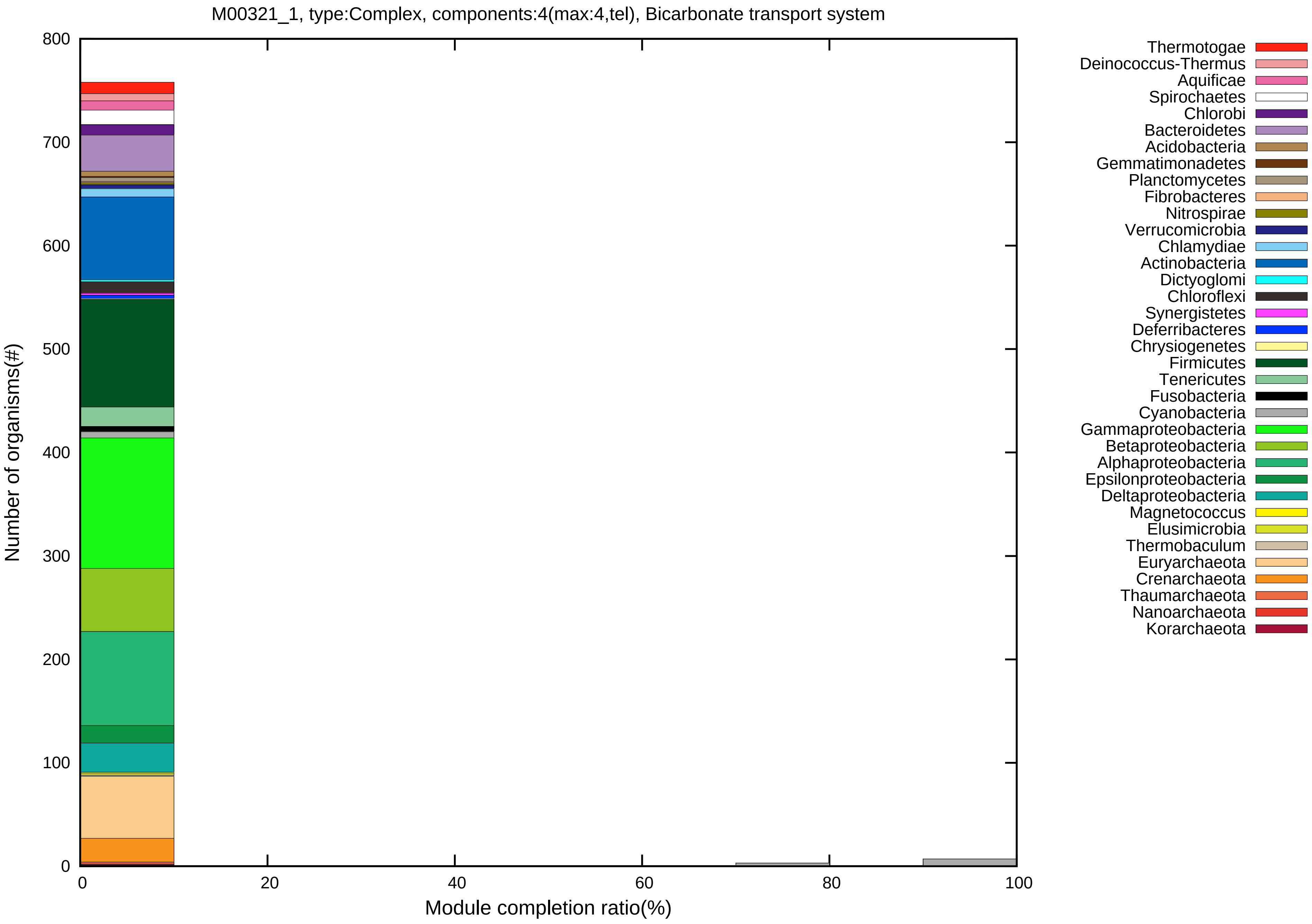

M00321_1, type:Complex, components:4(max:4,tel), Bicarbonate transport system
Number of organisms(#)
Module completion ratio(%)
0
100
200
300
400
500
600
700
800
0
20
40
60
80
100
Thermotogae
Deinococcus-Thermus
Aquificae
Spirochaetes
Chlorobi
Bacteroidetes
Acidobacteria
Gemmatimonadetes
Planctomycetes
Fibrobacteres
Nitrospirae
Verrucomicrobia
Chlamydiae
Actinobacteria
Dictyoglomi
Chloroflexi
Synergistetes
Deferribacteres
Chrysiogenetes
Firmicutes
Tenericutes
Fusobacteria
Cyanobacteria
Gammaproteobacteria
Betaproteobacteria
Alphaproteobacteria
Epsilonproteobacteria
Deltaproteobacteria
Magnetococcus
Elusimicrobia
Thermobaculum
Euryarchaeota
Crenarchaeota
Thaumarchaeota
Nanoarchaeota
Korarchaeota

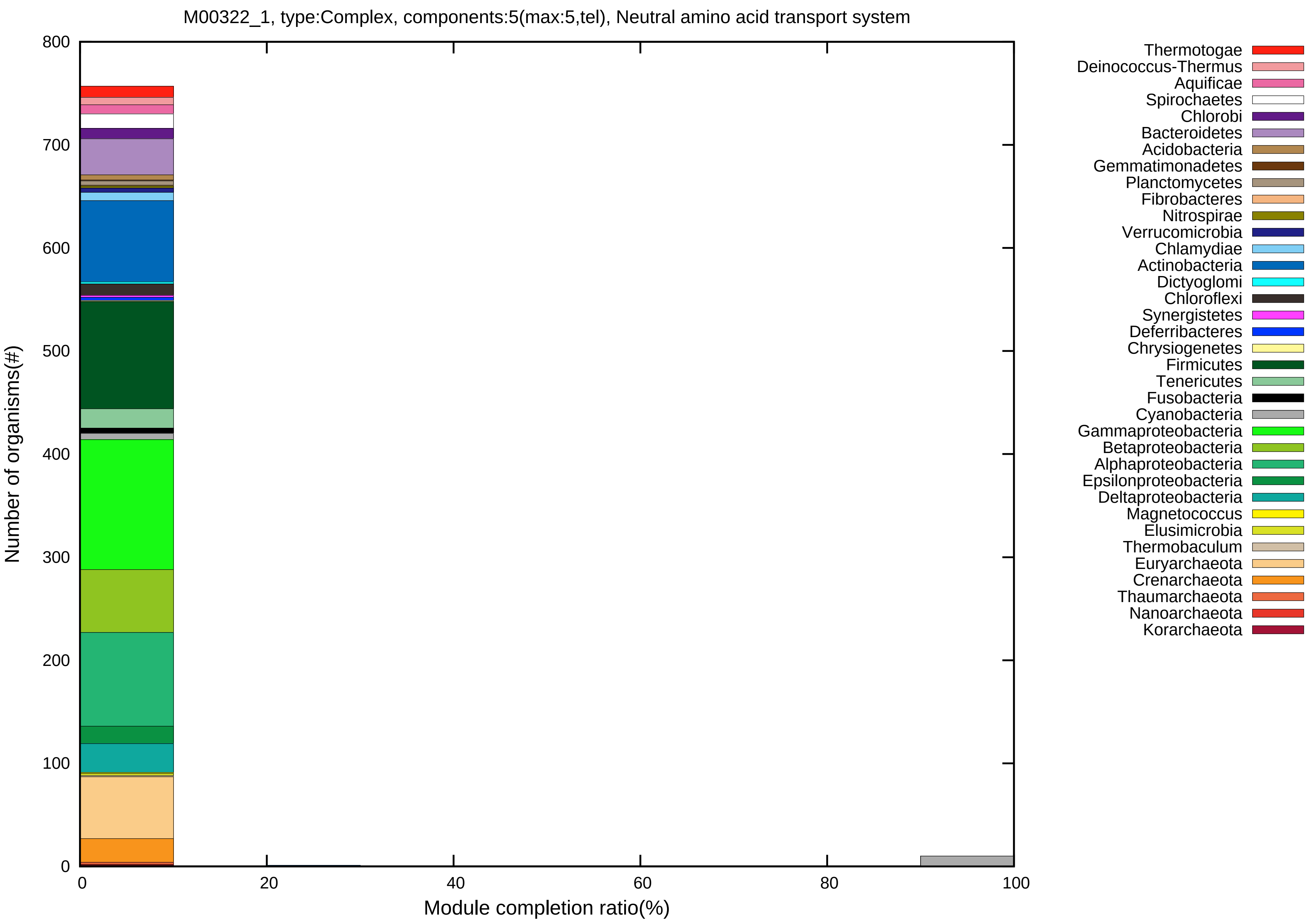

M00322_1, type:Complex, components:5(max:5,tel), Neutral amino acid transport system
Number of organisms(#)
Module completion ratio(%)
0
100
200
300
400
500
600
700
800
0
20
40
60
80
100
Thermotogae
Deinococcus-Thermus
Aquificae
Spirochaetes
Chlorobi
Bacteroidetes
Acidobacteria
Gemmatimonadetes
Planctomycetes
Fibrobacteres
Nitrospirae
Verrucomicrobia
Chlamydiae
Actinobacteria
Dictyoglomi
Chloroflexi
Synergistetes
Deferribacteres
Chrysiogenetes
Firmicutes
Tenericutes
Fusobacteria
Cyanobacteria
Gammaproteobacteria
Betaproteobacteria
Alphaproteobacteria
Epsilonproteobacteria
Deltaproteobacteria
Magnetococcus
Elusimicrobia
Thermobaculum
Euryarchaeota
Crenarchaeota
Thaumarchaeota
Nanoarchaeota
Korarchaeota

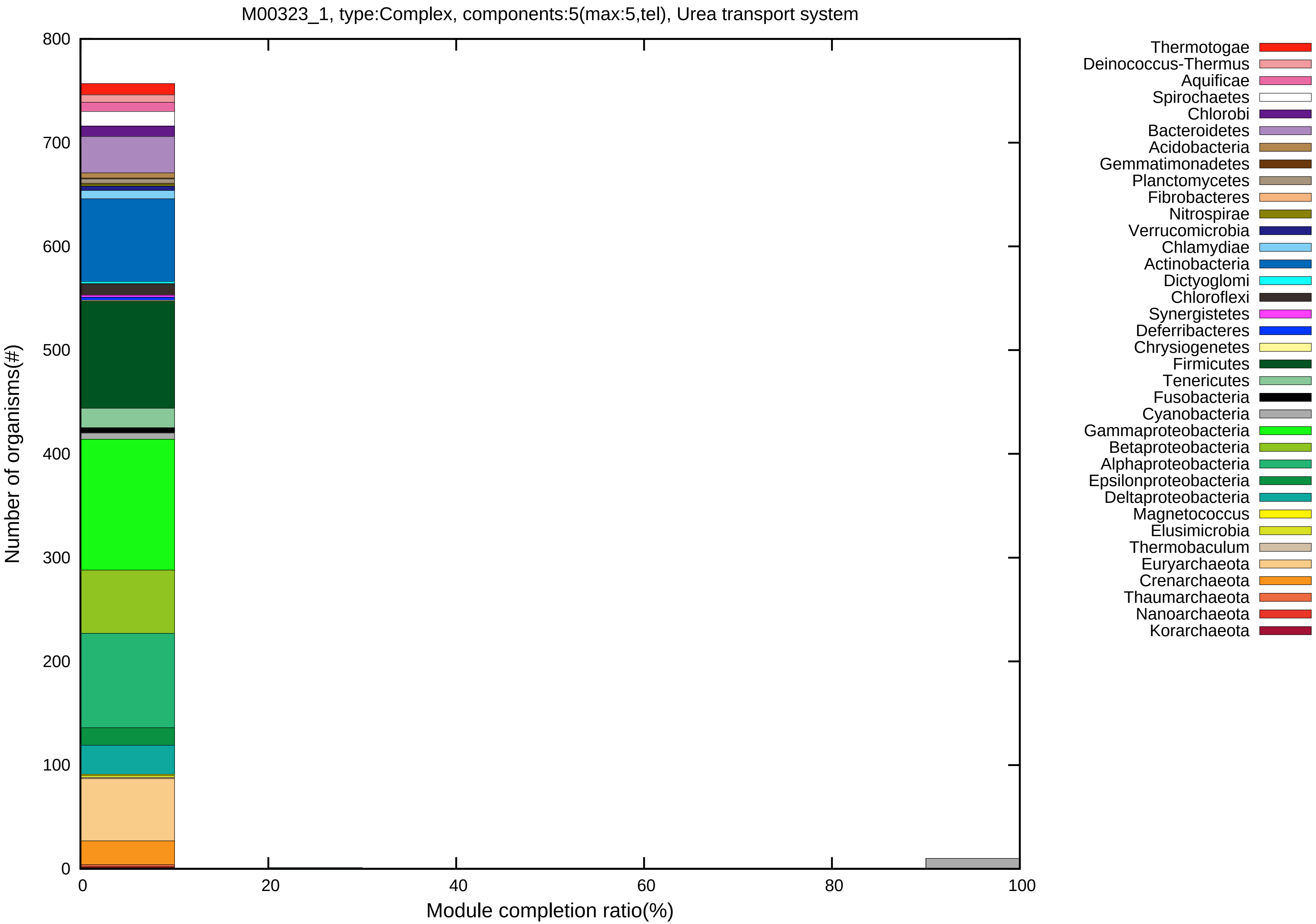

M00323_1, type:Complex, components:5(max:5,tel), Urea transport system
Number of organisms(#)
Module completion ratio(%)
0
100
200
300
400
500
600
700
800
0
20
40
60
80
100
Thermotogae
Deinococcus-Thermus
Aquificae
Spirochaetes
Chlorobi
Bacteroidetes
Acidobacteria
Gemmatimonadetes
Planctomycetes
Fibrobacteres
Nitrospirae
Verrucomicrobia
Chlamydiae
Actinobacteria
Dictyoglomi
Chloroflexi
Synergistetes
Deferribacteres
Chrysiogenetes
Firmicutes
Tenericutes
Fusobacteria
Cyanobacteria
Gammaproteobacteria
Betaproteobacteria
Alphaproteobacteria
Epsilonproteobacteria
Deltaproteobacteria
Magnetococcus
Elusimicrobia
Thermobaculum
Euryarchaeota
Crenarchaeota
Thaumarchaeota
Nanoarchaeota
Korarchaeota

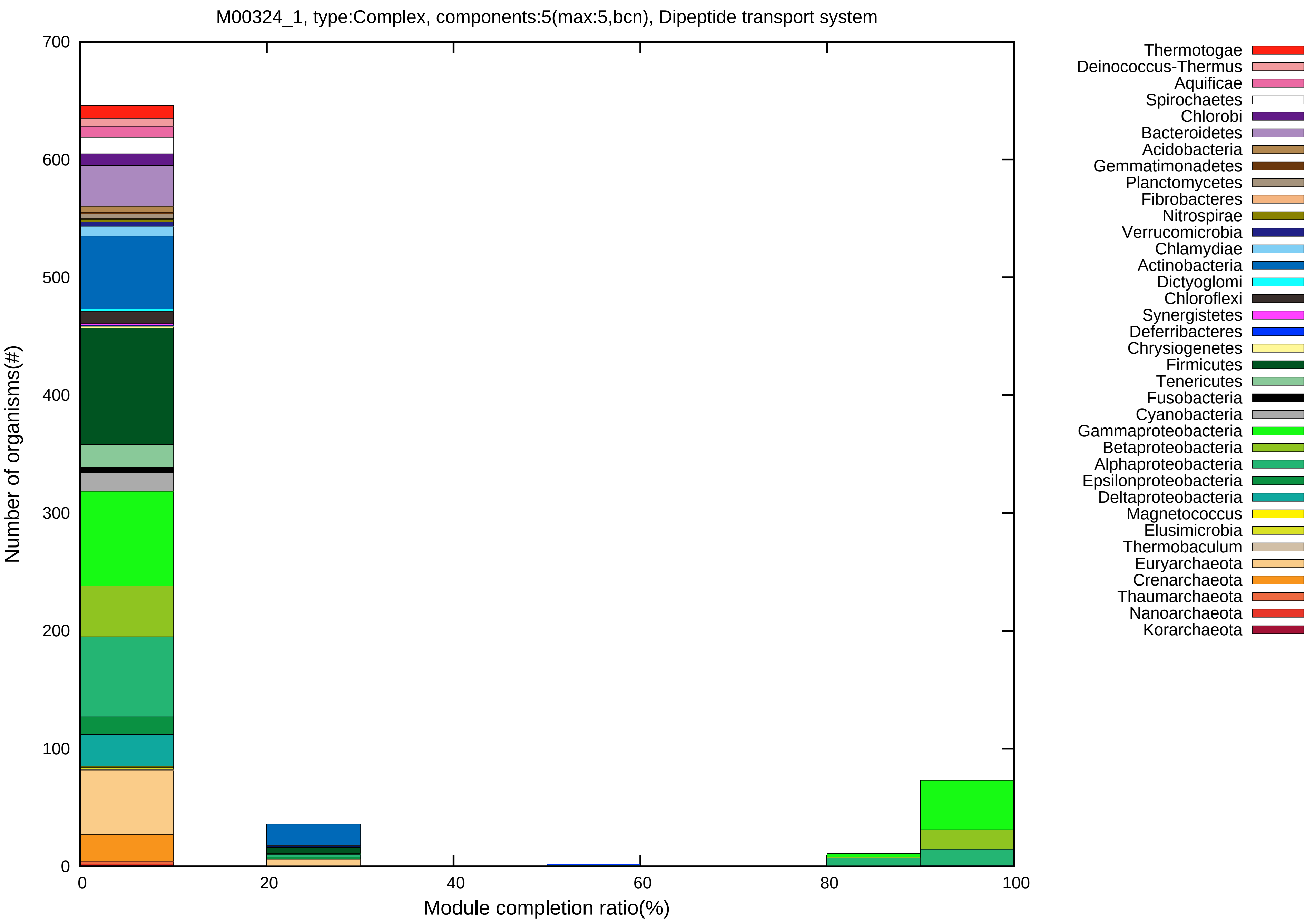

M00324_1, type:Complex, components:5(max:5,bcn), Dipeptide transport system
Number of organisms(#)
Module completion ratio(%)
0
100
200
300
400
500
600
700
0
20
40
60
80
100
Thermotogae
Deinococcus-Thermus
Aquificae
Spirochaetes
Chlorobi
Bacteroidetes
Acidobacteria
Gemmatimonadetes
Planctomycetes
Fibrobacteres
Nitrospirae
Verrucomicrobia
Chlamydiae
Actinobacteria
Dictyoglomi
Chloroflexi
Synergistetes
Deferribacteres
Chrysiogenetes
Firmicutes
Tenericutes
Fusobacteria
Cyanobacteria
Gammaproteobacteria
Betaproteobacteria
Alphaproteobacteria
Epsilonproteobacteria
Deltaproteobacteria
Magnetococcus
Elusimicrobia
Thermobaculum
Euryarchaeota
Crenarchaeota
Thaumarchaeota
Nanoarchaeota
Korarchaeota

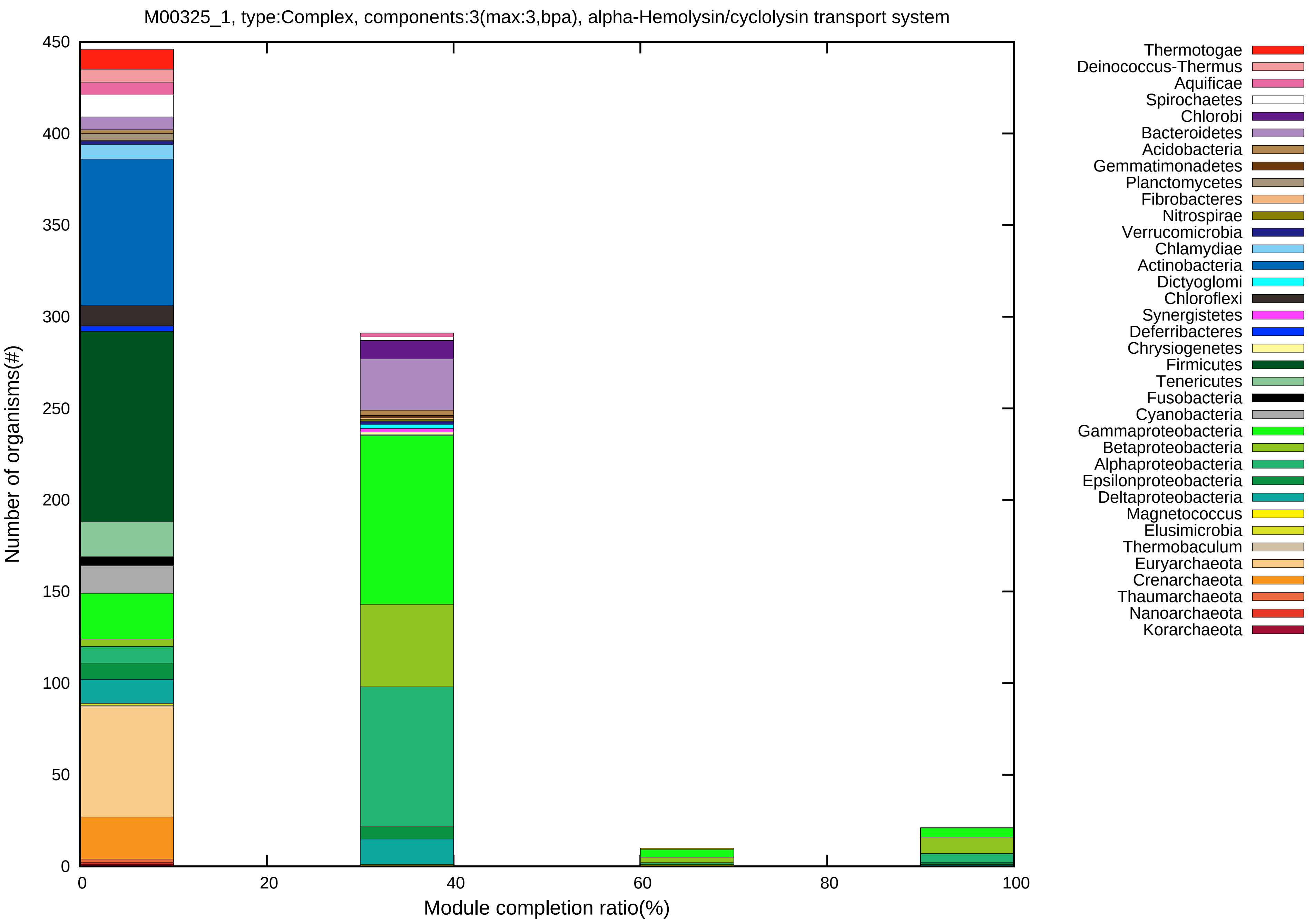
M00325_1, type:Complex, components:3(max:3,bpa), alpha-Hemolysin/cyclolysin transport system
Number of organisms(#)
Module completion ratio(%)
0
50
100
150
200
250
300
350
400
450
0
20
40
60
80
100
Thermotogae
Deinococcus-Thermus
Aquificae
Spirochaetes
Chlorobi
Bacteroidetes
Acidobacteria
Gemmatimonadetes
Planctomycetes
Fibrobacteres
Nitrospirae
Verrucomicrobia
Chlamydiae
Actinobacteria
Dictyoglomi
Chloroflexi
Synergistetes
Deferribacteres
Chrysiogenetes
Firmicutes
Tenericutes
Fusobacteria
Cyanobacteria
Gammaproteobacteria
Betaproteobacteria
Alphaproteobacteria
Epsilonproteobacteria
Deltaproteobacteria
Magnetococcus
Elusimicrobia
Thermobaculum
Euryarchaeota
Crenarchaeota
Thaumarchaeota
Nanoarchaeota
Korarchaeota

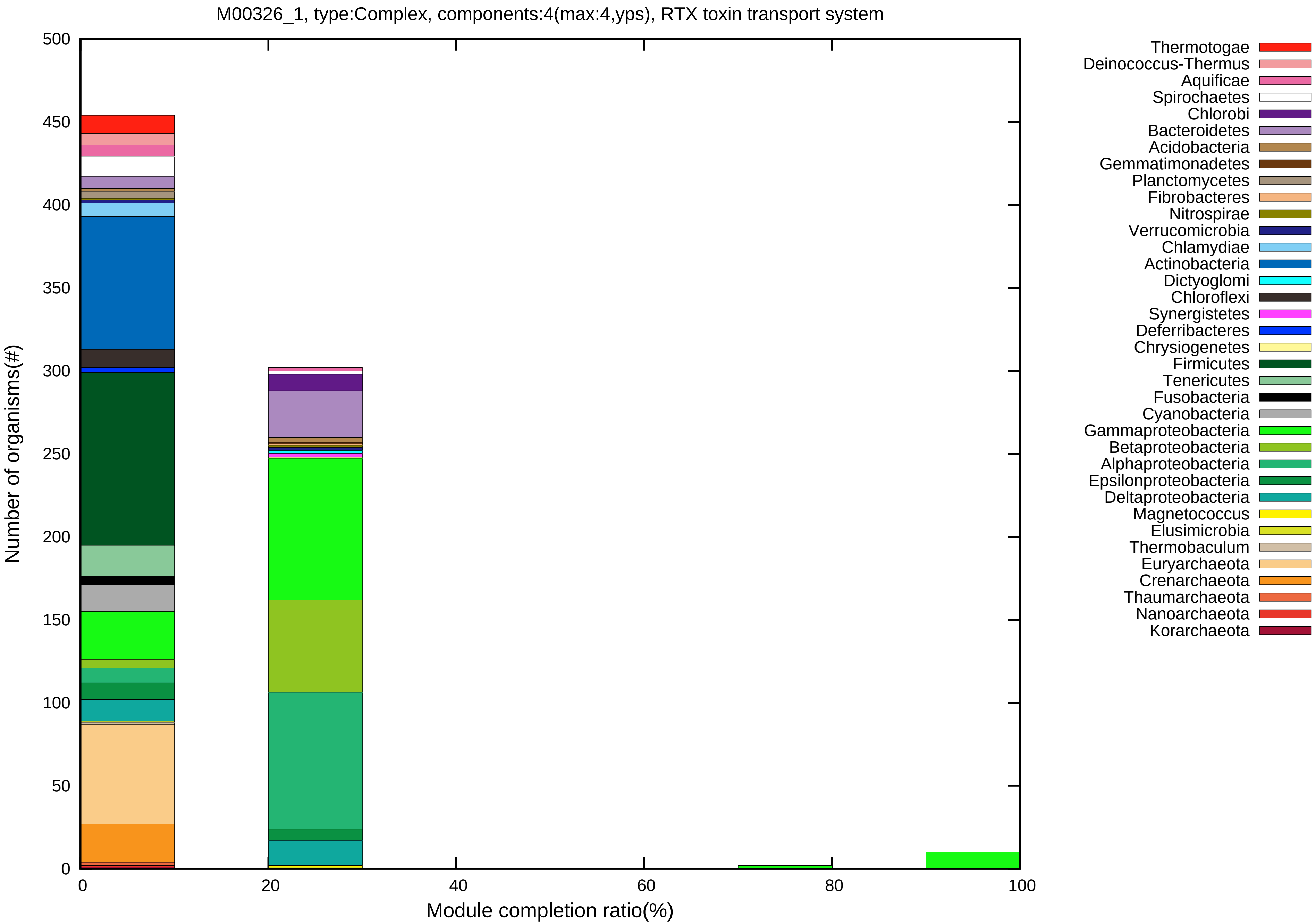

M00326_1, type:Complex, components:4(max:4,yps), RTX toxin transport system
Number of organisms(#)
Module completion ratio(%)
0
50
100
150
200
250
300
350
400
450
500
0
20
40
60
80
100
Thermotogae
Deinococcus-Thermus
Aquificae
Spirochaetes
Chlorobi
Bacteroidetes
Acidobacteria
Gemmatimonadetes
Planctomycetes
Fibrobacteres
Nitrospirae
Verrucomicrobia
Chlamydiae
Actinobacteria
Dictyoglomi
Chloroflexi
Synergistetes
Deferribacteres
Chrysiogenetes
Firmicutes
Tenericutes
Fusobacteria
Cyanobacteria
Gammaproteobacteria
Betaproteobacteria
Alphaproteobacteria
Epsilonproteobacteria
Deltaproteobacteria
Magnetococcus
Elusimicrobia
Thermobaculum
Euryarchaeota
Crenarchaeota
Thaumarchaeota
Nanoarchaeota
Korarchaeota

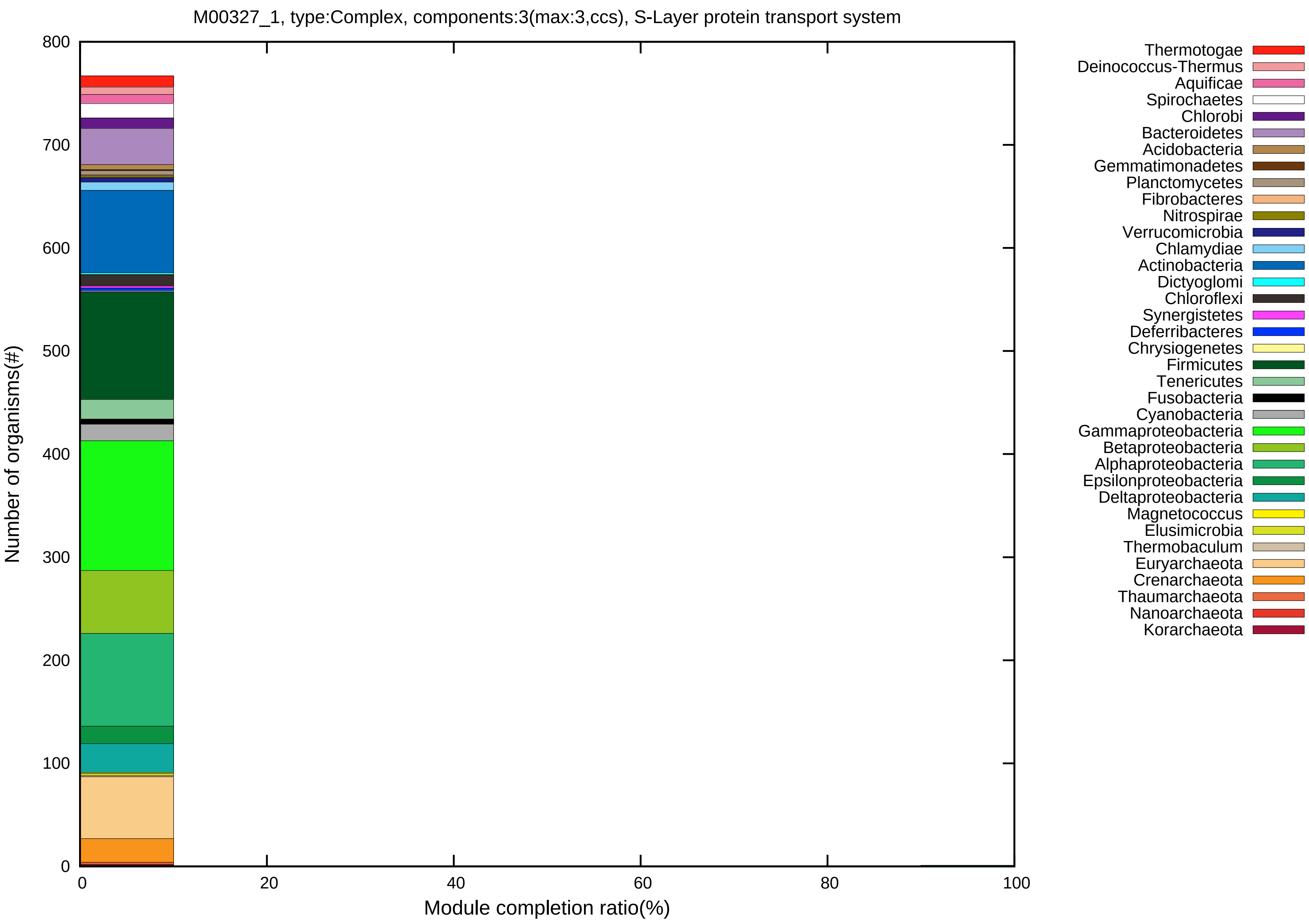
M00327_1, type:Complex, components:3(max:3,ccs), S-Layer protein transport system
Number of organisms(#)
Module completion ratio(%)
0
100
200
300
400
500
600
700
800
0
20
40
60
80
100
Thermotogae
Deinococcus-Thermus
Aquificae
Spirochaetes
Chlorobi
Bacteroidetes
Acidobacteria
Gemmatimonadetes
Planctomycetes
Fibrobacteres
Nitrospirae
Verrucomicrobia
Chlamydiae
Actinobacteria
Dictyoglomi
Chloroflexi
Synergistetes
Deferribacteres
Chrysiogenetes
Firmicutes
Tenericutes
Fusobacteria
Cyanobacteria
Gammaproteobacteria
Betaproteobacteria
Alphaproteobacteria
Epsilonproteobacteria
Deltaproteobacteria
Magnetococcus
Elusimicrobia
Thermobaculum
Euryarchaeota
Crenarchaeota
Thaumarchaeota
Nanoarchaeota
Korarchaeota

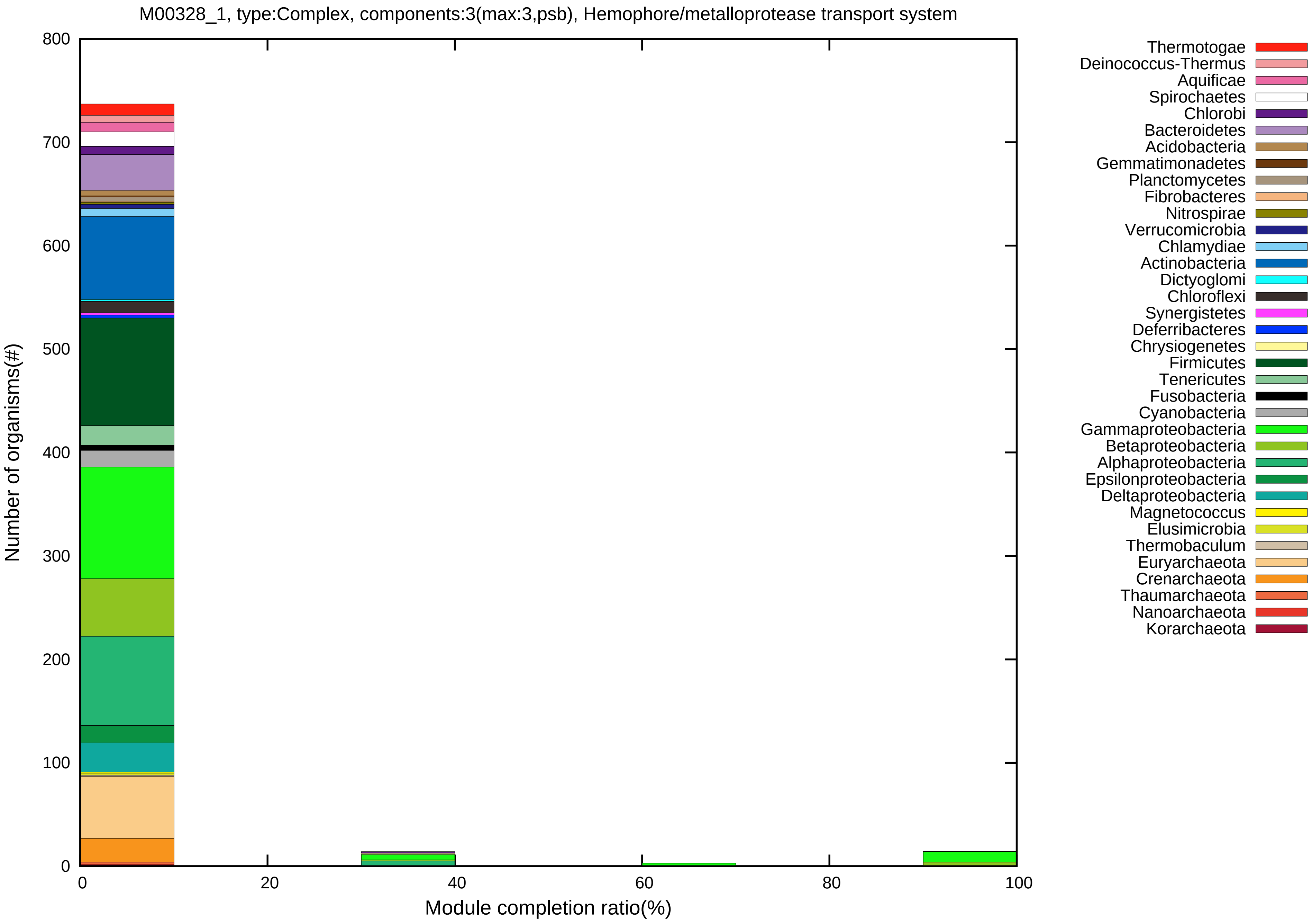
M00328_1, type:Complex, components:3(max:3,psb), Hemophore/metalloprotease transport system
Number of organisms(#)
Module completion ratio(%)
0
100
200
300
400
500
600
700
800
0
20
40
60
80
100
Thermotogae
Deinococcus-Thermus
Aquificae
Spirochaetes
Chlorobi
Bacteroidetes
Acidobacteria
Gemmatimonadetes
Planctomycetes
Fibrobacteres
Nitrospirae
Verrucomicrobia
Chlamydiae
Actinobacteria
Dictyoglomi
Chloroflexi
Synergistetes
Deferribacteres
Chrysiogenetes
Firmicutes
Tenericutes
Fusobacteria
Cyanobacteria
Gammaproteobacteria
Betaproteobacteria
Alphaproteobacteria
Epsilonproteobacteria
Deltaproteobacteria
Magnetococcus
Elusimicrobia
Thermobaculum
Euryarchaeota
Crenarchaeota
Thaumarchaeota
Nanoarchaeota
Korarchaeota

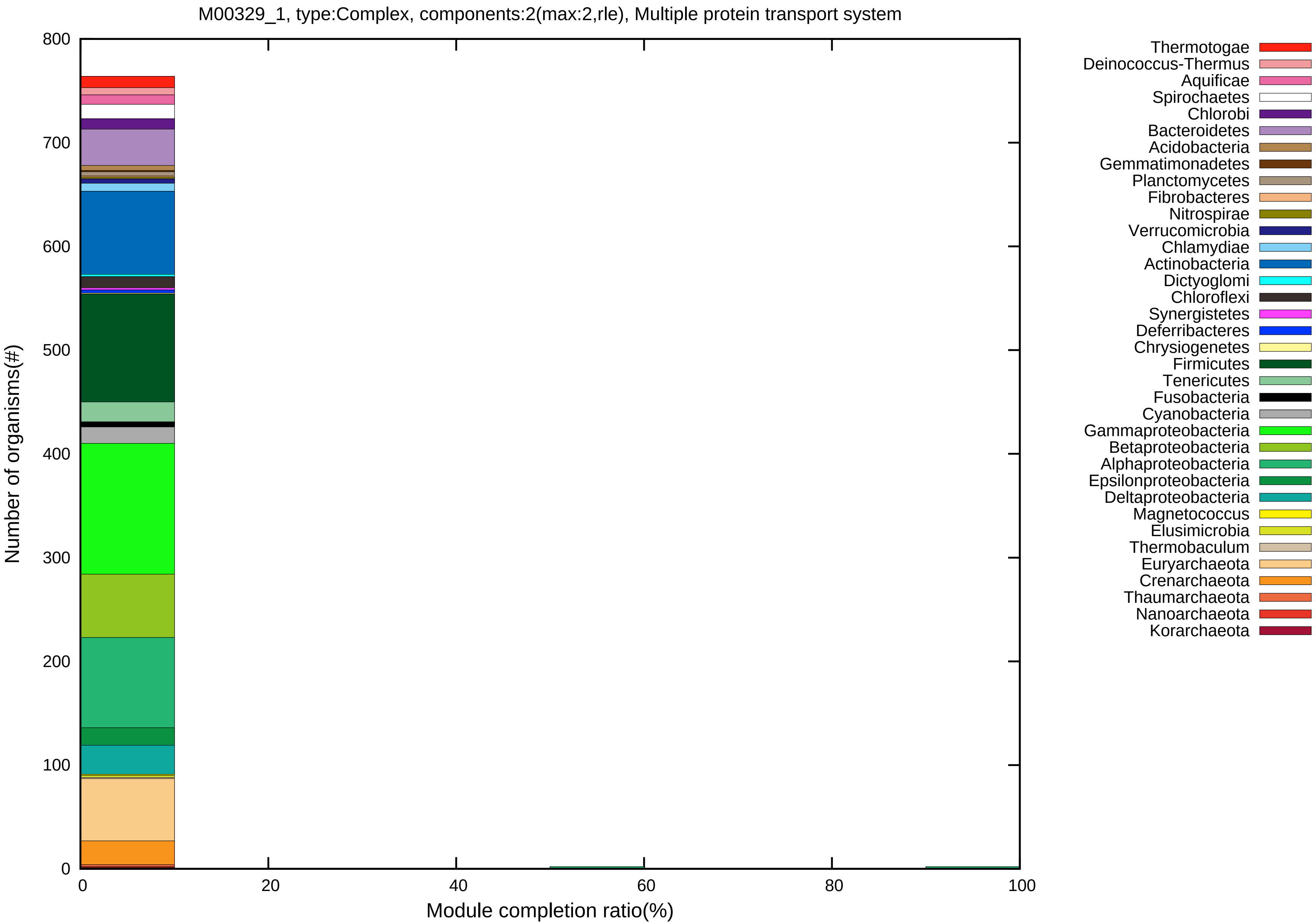

M00329_1, type:Complex, components:2(max:2,rle), Multiple protein transport system
Number of organisms(#)
Module completion ratio(%)
0
100
200
300
400
500
600
700
800
0
20
40
60
80
100
Thermotogae
Deinococcus-Thermus
Aquificae
Spirochaetes
Chlorobi
Bacteroidetes
Acidobacteria
Gemmatimonadetes
Planctomycetes
Fibrobacteres
Nitrospirae
Verrucomicrobia
Chlamydiae
Actinobacteria
Dictyoglomi
Chloroflexi
Synergistetes
Deferribacteres
Chrysiogenetes
Firmicutes
Tenericutes
Fusobacteria
Cyanobacteria
Gammaproteobacteria
Betaproteobacteria
Alphaproteobacteria
Epsilonproteobacteria
Deltaproteobacteria
Magnetococcus
Elusimicrobia
Thermobaculum
Euryarchaeota
Crenarchaeota
Thaumarchaeota
Nanoarchaeota
Korarchaeota

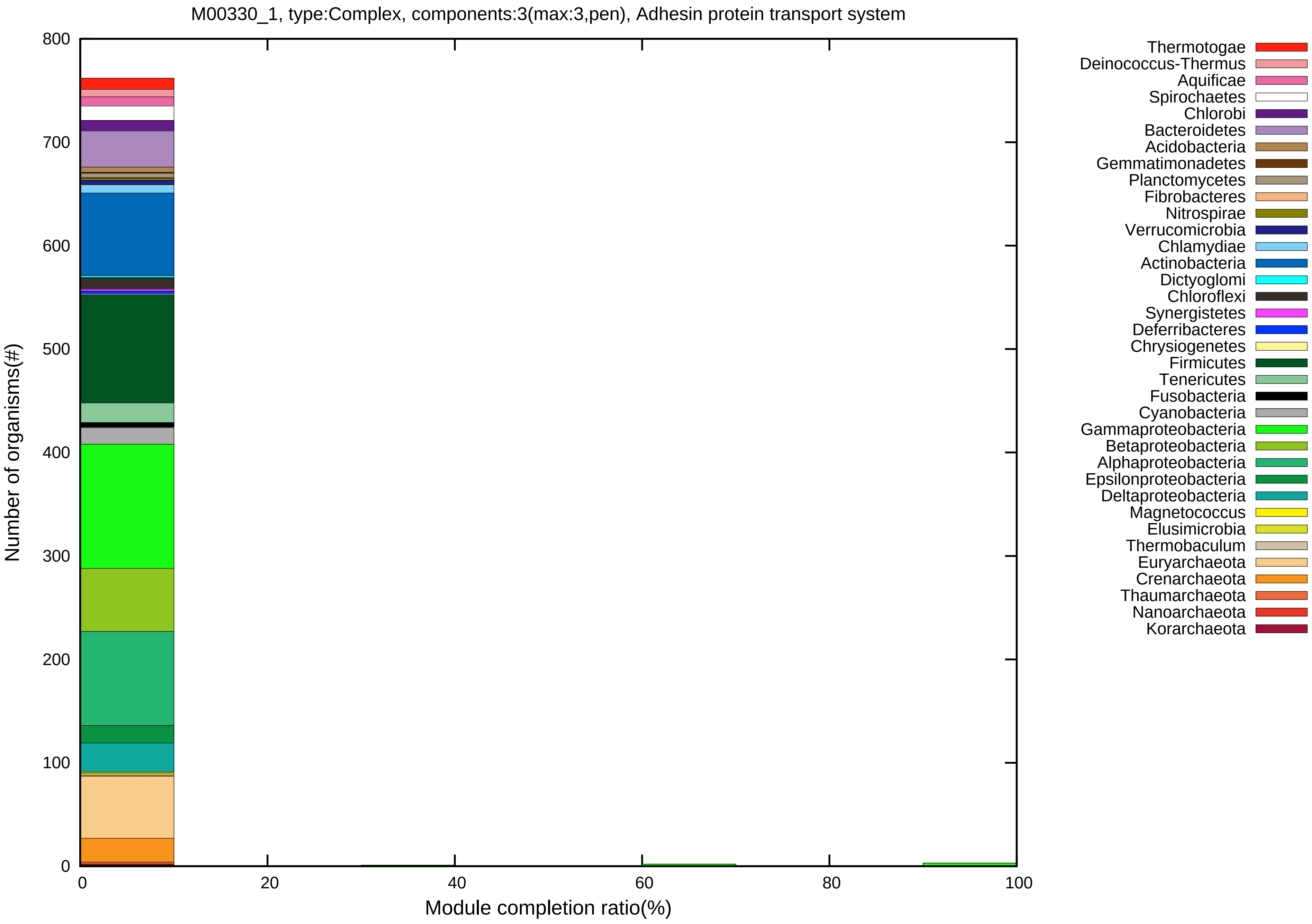
M00330_1, type:Complex, components:3(max:3,pen), Adhesin protein transport system
Number of organisms(#)
Module completion ratio(%)
0
100
200
300
400
500
600
700
800
0
20
40
60
80
100
Thermotogae
Deinococcus-Thermus
Aquificae
Spirochaetes
Chlorobi
Bacteroidetes
Acidobacteria
Gemmatimonadetes
Planctomycetes
Fibrobacteres
Nitrospirae
Verrucomicrobia
Chlamydiae
Actinobacteria
Dictyoglomi
Chloroflexi
Synergistetes
Deferribacteres
Chrysiogenetes
Firmicutes
Tenericutes
Fusobacteria
Cyanobacteria
Gammaproteobacteria
Betaproteobacteria
Alphaproteobacteria
Epsilonproteobacteria
Deltaproteobacteria
Magnetococcus
Elusimicrobia
Thermobaculum
Euryarchaeota
Crenarchaeota
Thaumarchaeota
Nanoarchaeota
Korarchaeota

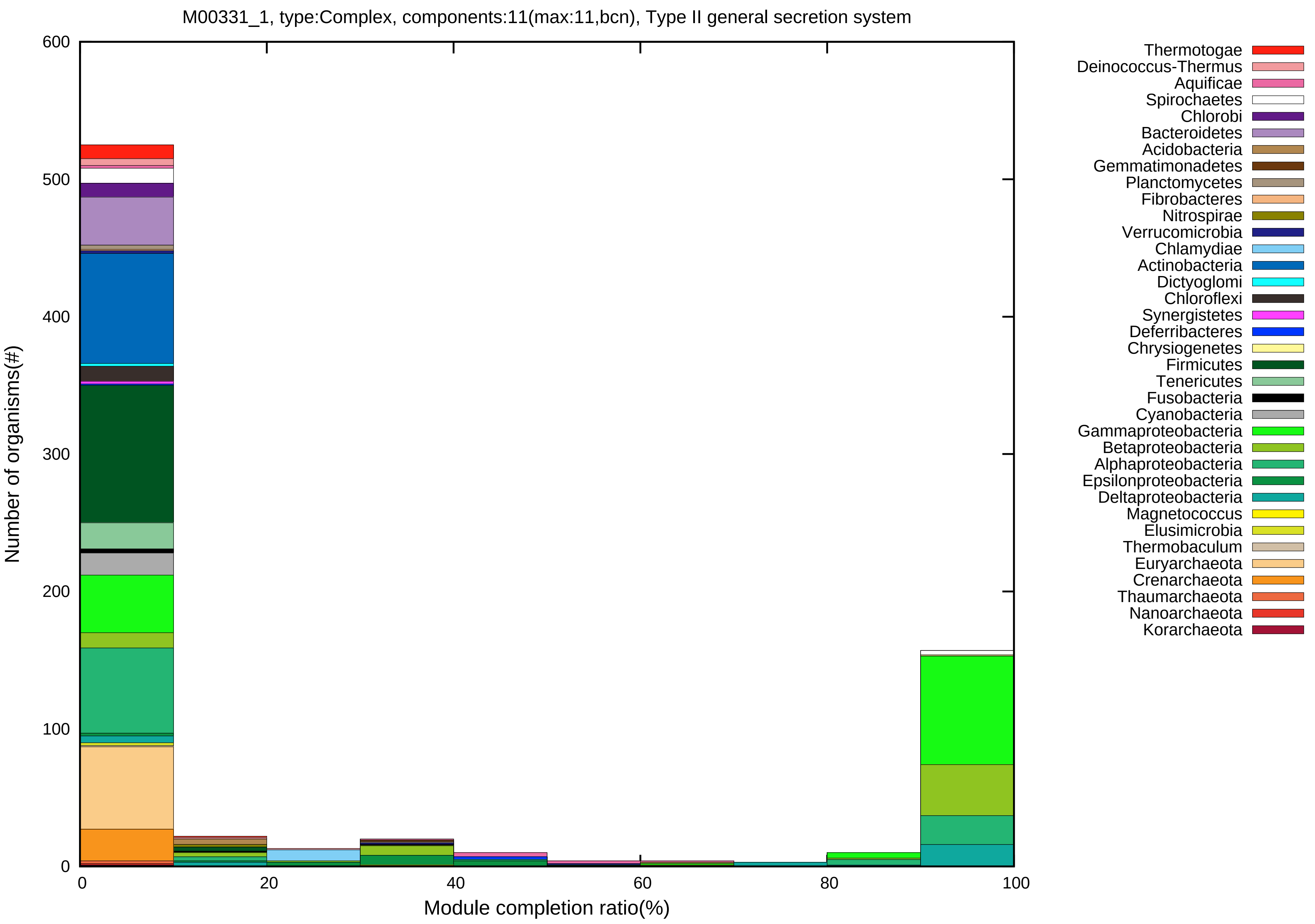
M00331_1, type:Complex, components:11(max:11,bcn), Type II general secretion system
Number of organisms(#)
Module completion ratio(%)
0
100
200
300
400
500
600
0
20
40
60
80
100
Thermotogae
Deinococcus-Thermus
Aquificae
Spirochaetes
Chlorobi
Bacteroidetes
Acidobacteria
Gemmatimonadetes
Planctomycetes
Fibrobacteres
Nitrospirae
Verrucomicrobia
Chlamydiae
Actinobacteria
Dictyoglomi
Chloroflexi
Synergistetes
Deferribacteres
Chrysiogenetes
Firmicutes
Tenericutes
Fusobacteria
Cyanobacteria
Gammaproteobacteria
Betaproteobacteria
Alphaproteobacteria
Epsilonproteobacteria
Deltaproteobacteria
Magnetococcus
Elusimicrobia
Thermobaculum
Euryarchaeota
Crenarchaeota
Thaumarchaeota
Nanoarchaeota
Korarchaeota

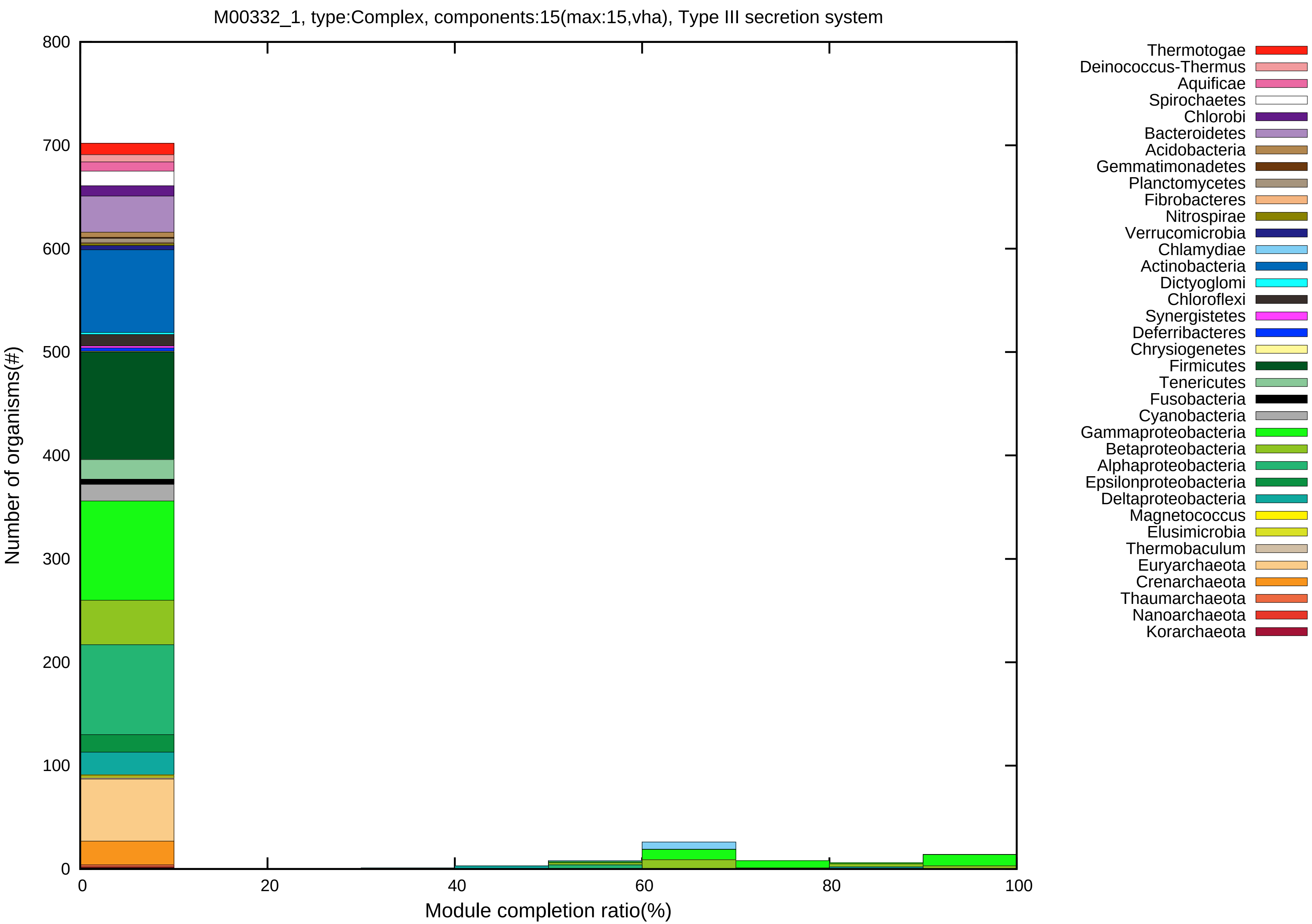
M00332_1, type:Complex, components:15(max:15,vha), Type III secretion system
Number of organisms(#)
Module completion ratio(%)
0
20
40
60
80
100
0
100
200
300
400
500
600
700
800
Thermotogae
Deinococcus-Thermus
Aquificae
Spirochaetes
Chlorobi
Bacteroidetes
Acidobacteria
Gemmatimonadetes
Planctomycetes
Fibrobacteres
Nitrospirae
Verrucomicrobia
Chlamydiae
Actinobacteria
Dictyoglomi
Chloroflexi
Synergistetes
Deferribacteres
Chrysiogenetes
Firmicutes
Tenericutes
Fusobacteria
Cyanobacteria
Gammaproteobacteria
Betaproteobacteria
Alphaproteobacteria
Epsilonproteobacteria
Deltaproteobacteria
Magnetococcus
Elusimicrobia
Thermobaculum
Euryarchaeota
Crenarchaeota
Thaumarchaeota
Nanoarchaeota
Korarchaeota

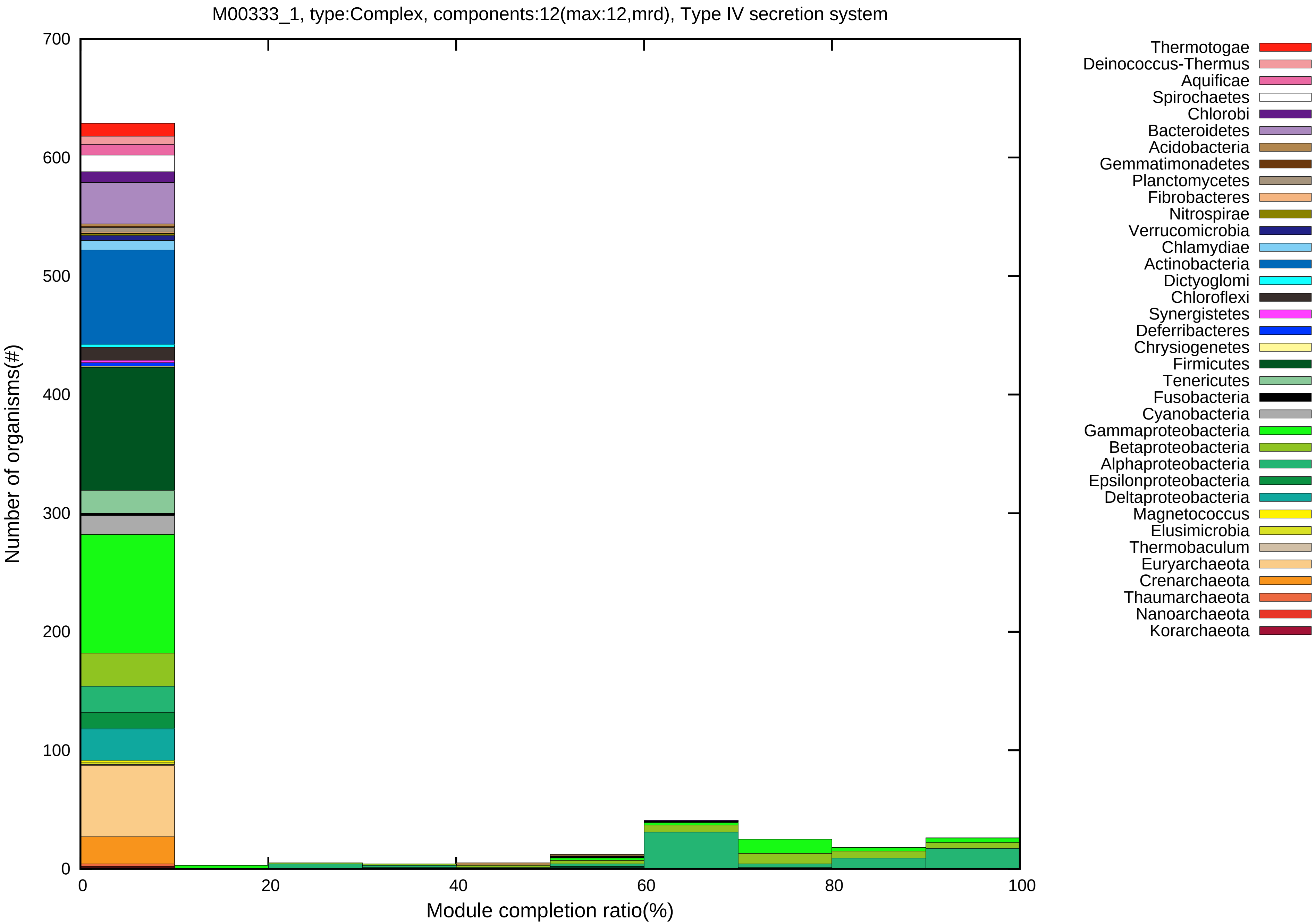

M00333_1, type:Complex, components:12(max:12,mrd), Type IV secretion system
Number of organisms(#)
Module completion ratio(%)
0
100
200
300
400
500
600
700
0
20
40
60
80
100
Thermotogae
Deinococcus-Thermus
Aquificae
Spirochaetes
Chlorobi
Bacteroidetes
Acidobacteria
Gemmatimonadetes
Planctomycetes
Fibrobacteres
Nitrospirae
Verrucomicrobia
Chlamydiae
Actinobacteria
Dictyoglomi
Chloroflexi
Synergistetes
Deferribacteres
Chrysiogenetes
Firmicutes
Tenericutes
Fusobacteria
Cyanobacteria
Gammaproteobacteria
Betaproteobacteria
Alphaproteobacteria
Epsilonproteobacteria
Deltaproteobacteria
Magnetococcus
Elusimicrobia
Thermobaculum
Euryarchaeota
Crenarchaeota
Thaumarchaeota
Nanoarchaeota
Korarchaeota

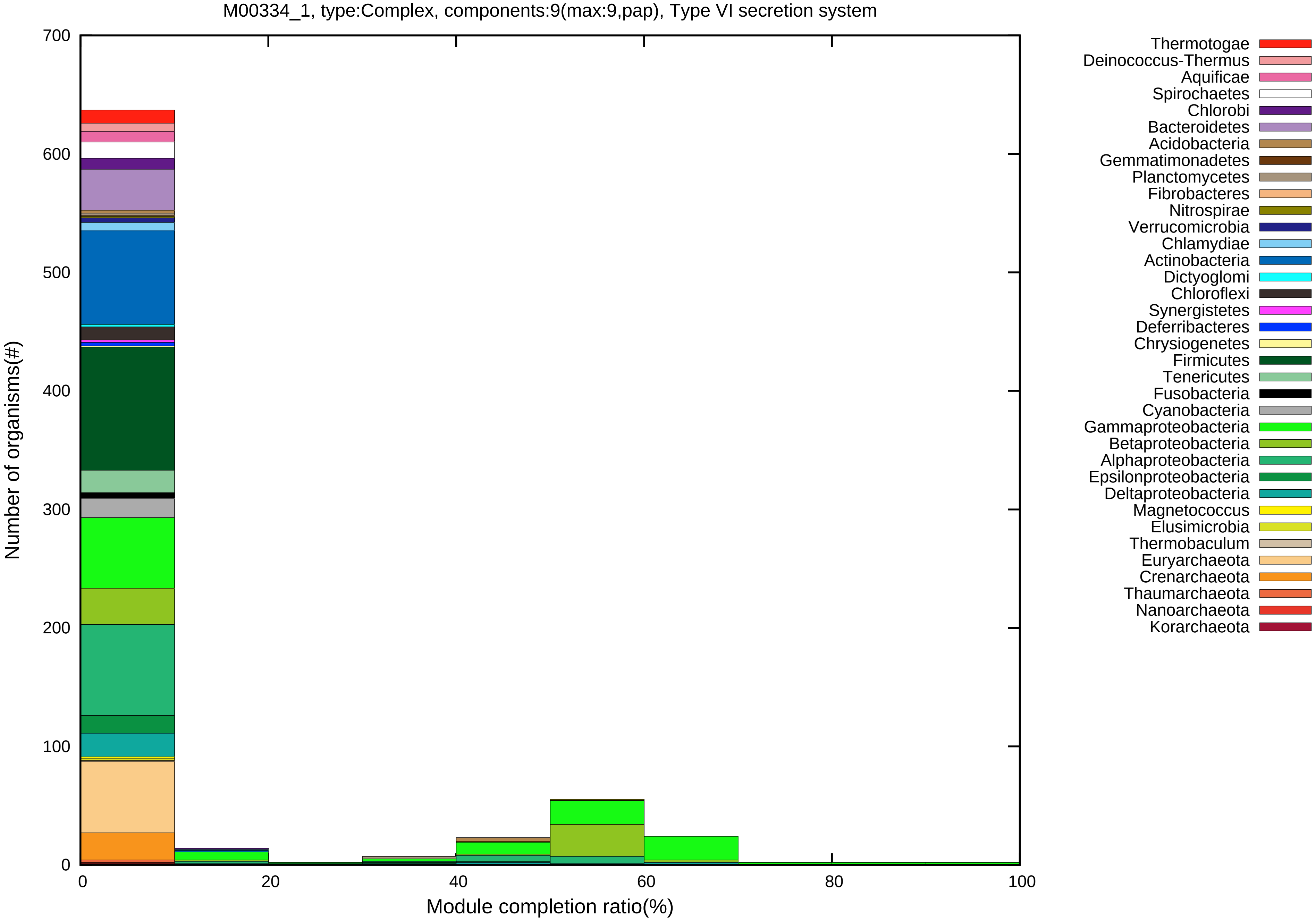

M00334_1, type:Complex, components:9(max:9,pap), Type VI secretion system
Number of organisms(#)
Module completion ratio(%)
0
100
200
300
400
500
600
700
0
20
40
60
80
100
Thermotogae
Deinococcus-Thermus
Aquificae
Spirochaetes
Chlorobi
Bacteroidetes
Acidobacteria
Gemmatimonadetes
Planctomycetes
Fibrobacteres
Nitrospirae
Verrucomicrobia
Chlamydiae
Actinobacteria
Dictyoglomi
Chloroflexi
Synergistetes
Deferribacteres
Chrysiogenetes
Firmicutes
Tenericutes
Fusobacteria
Cyanobacteria
Gammaproteobacteria
Betaproteobacteria
Alphaproteobacteria
Epsilonproteobacteria
Deltaproteobacteria
Magnetococcus
Elusimicrobia
Thermobaculum
Euryarchaeota
Crenarchaeota
Thaumarchaeota
Nanoarchaeota
Korarchaeota

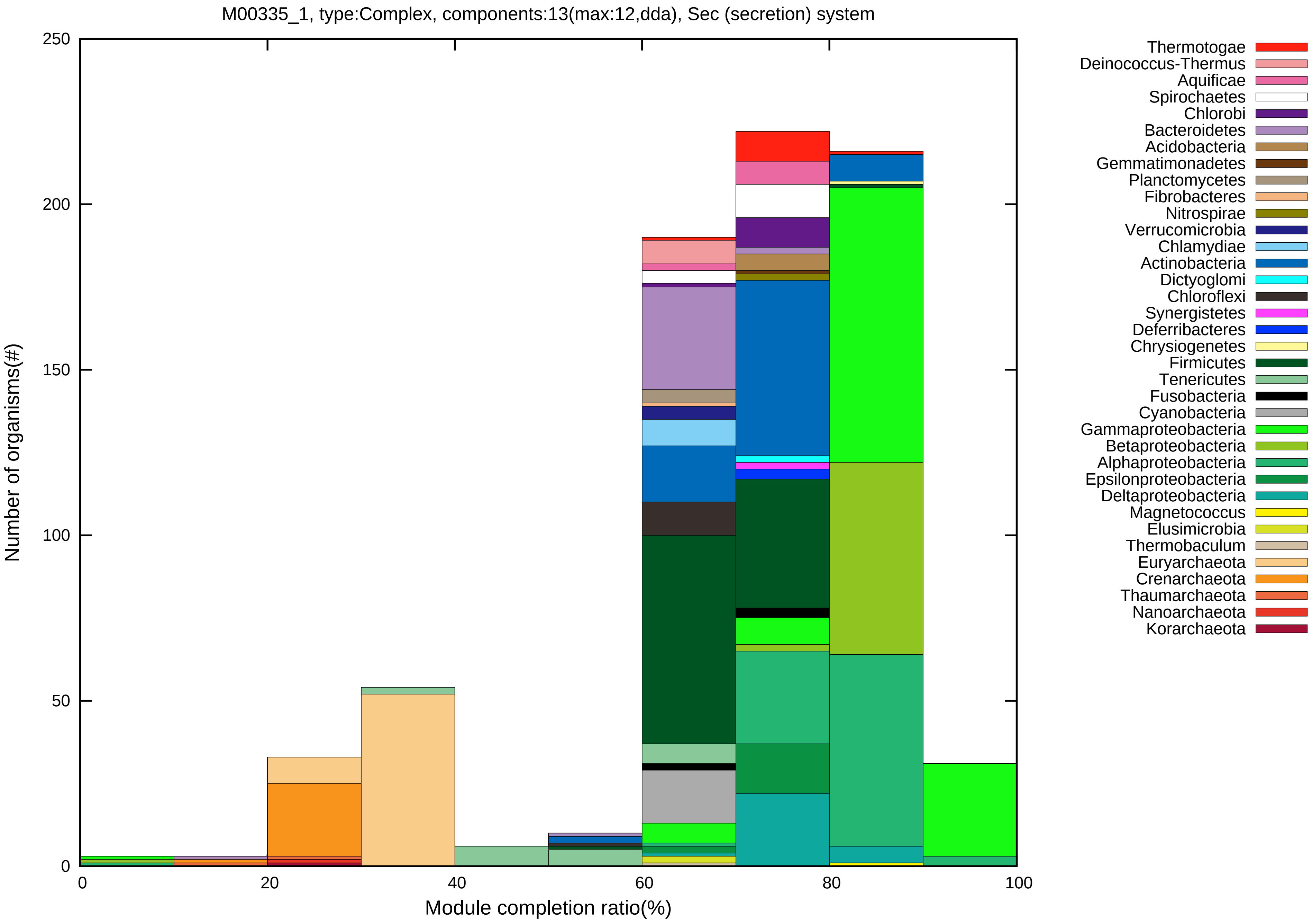

M00335_1, type:Complex, components:13(max:12,dda), Sec (secretion) system
Number of organisms(#)
Module completion ratio(%)
0
50
100
150
200
250
0
20
40
60
80
100
Thermotogae
Deinococcus-Thermus
Aquificae
Spirochaetes
Chlorobi
Bacteroidetes
Acidobacteria
Gemmatimonadetes
Planctomycetes
Fibrobacteres
Nitrospirae
Verrucomicrobia
Chlamydiae
Actinobacteria
Dictyoglomi
Chloroflexi
Synergistetes
Deferribacteres
Chrysiogenetes
Firmicutes
Tenericutes
Fusobacteria
Cyanobacteria
Gammaproteobacteria
Betaproteobacteria
Alphaproteobacteria
Epsilonproteobacteria
Deltaproteobacteria
Magnetococcus
Elusimicrobia
Thermobaculum
Euryarchaeota
Crenarchaeota
Thaumarchaeota
Nanoarchaeota
Korarchaeota

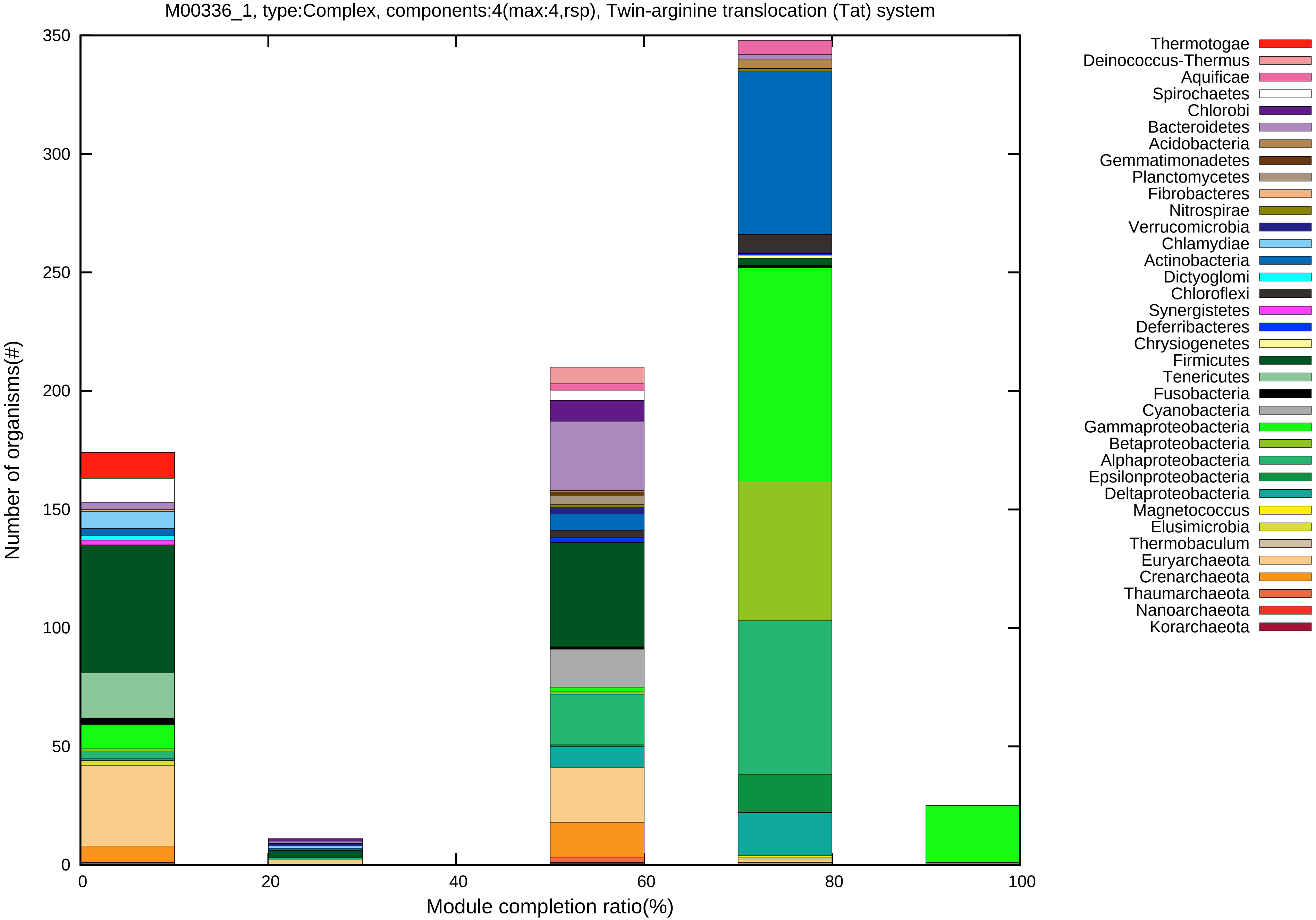
M00336_1, type:Complex, components:4(max:4,rsp), Twin-arginine translocation (Tat) system
Number of organisms(#)
Module completion ratio(%)
0
50
100
150
200
250
300
350
0
20
40
60
80
100
Thermotogae
Deinococcus-Thermus
Aquificae
Spirochaetes
Chlorobi
Bacteroidetes
Acidobacteria
Gemmatimonadetes
Planctomycetes
Fibrobacteres
Nitrospirae
Verrucomicrobia
Chlamydiae
Actinobacteria
Dictyoglomi
Chloroflexi
Synergistetes
Deferribacteres
Chrysiogenetes
Firmicutes
Tenericutes
Fusobacteria
Cyanobacteria
Gammaproteobacteria
Betaproteobacteria
Alphaproteobacteria
Epsilonproteobacteria
Deltaproteobacteria
Magnetococcus
Elusimicrobia
Thermobaculum
Euryarchaeota
Crenarchaeota
Thaumarchaeota
Nanoarchaeota
Korarchaeota

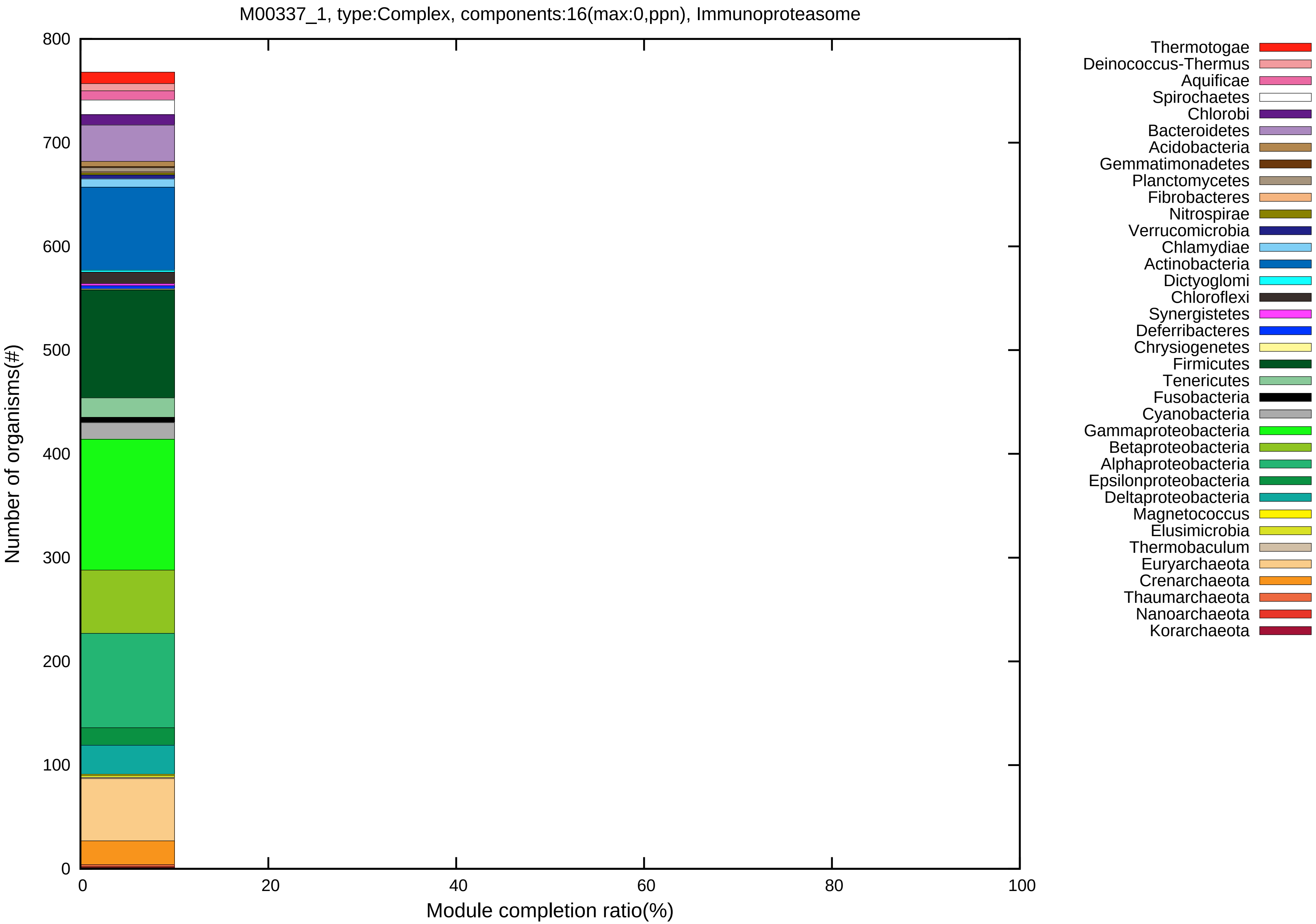
M00337_1, type:Complex, components:16(max:0,ppn), Immunoproteasome
Number of organisms(#)
Module completion ratio(%)
0
100
200
300
400
500
600
700
800
0
20
40
60
80
100
Thermotogae
Deinococcus-Thermus
Aquificae
Spirochaetes
Chlorobi
Bacteroidetes
Acidobacteria
Gemmatimonadetes
Planctomycetes
Fibrobacteres
Nitrospirae
Verrucomicrobia
Chlamydiae
Actinobacteria
Dictyoglomi
Chloroflexi
Synergistetes
Deferribacteres
Chrysiogenetes
Firmicutes
Tenericutes
Fusobacteria
Cyanobacteria
Gammaproteobacteria
Betaproteobacteria
Alphaproteobacteria
Epsilonproteobacteria
Deltaproteobacteria
Magnetococcus
Elusimicrobia
Thermobaculum
Euryarchaeota
Crenarchaeota
Thaumarchaeota
Nanoarchaeota
Korarchaeota

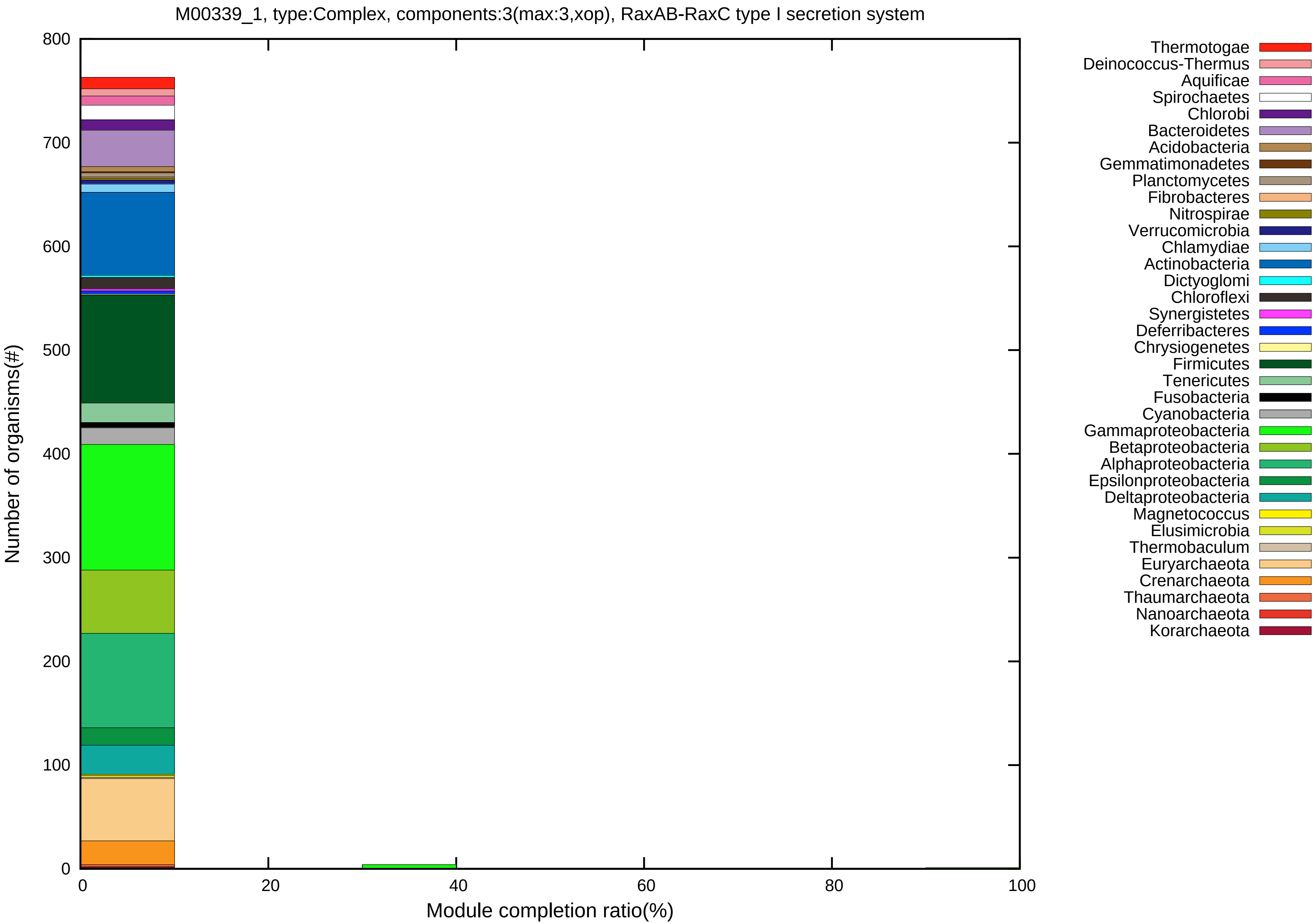

M00339_1, type:Complex, components:3(max:3,xop), RaxAB-RaxC type I secretion system
Number of organisms(#)
Module completion ratio(%)
0
100
200
300
400
500
600
700
800
0
20
40
60
80
100
Thermotogae
Deinococcus-Thermus
Aquificae
Spirochaetes
Chlorobi
Bacteroidetes
Acidobacteria
Gemmatimonadetes
Planctomycetes
Fibrobacteres
Nitrospirae
Verrucomicrobia
Chlamydiae
Actinobacteria
Dictyoglomi
Chloroflexi
Synergistetes
Deferribacteres
Chrysiogenetes
Firmicutes
Tenericutes
Fusobacteria
Cyanobacteria
Gammaproteobacteria
Betaproteobacteria
Alphaproteobacteria
Epsilonproteobacteria
Deltaproteobacteria
Magnetococcus
Elusimicrobia
Thermobaculum
Euryarchaeota
Crenarchaeota
Thaumarchaeota
Nanoarchaeota
Korarchaeota

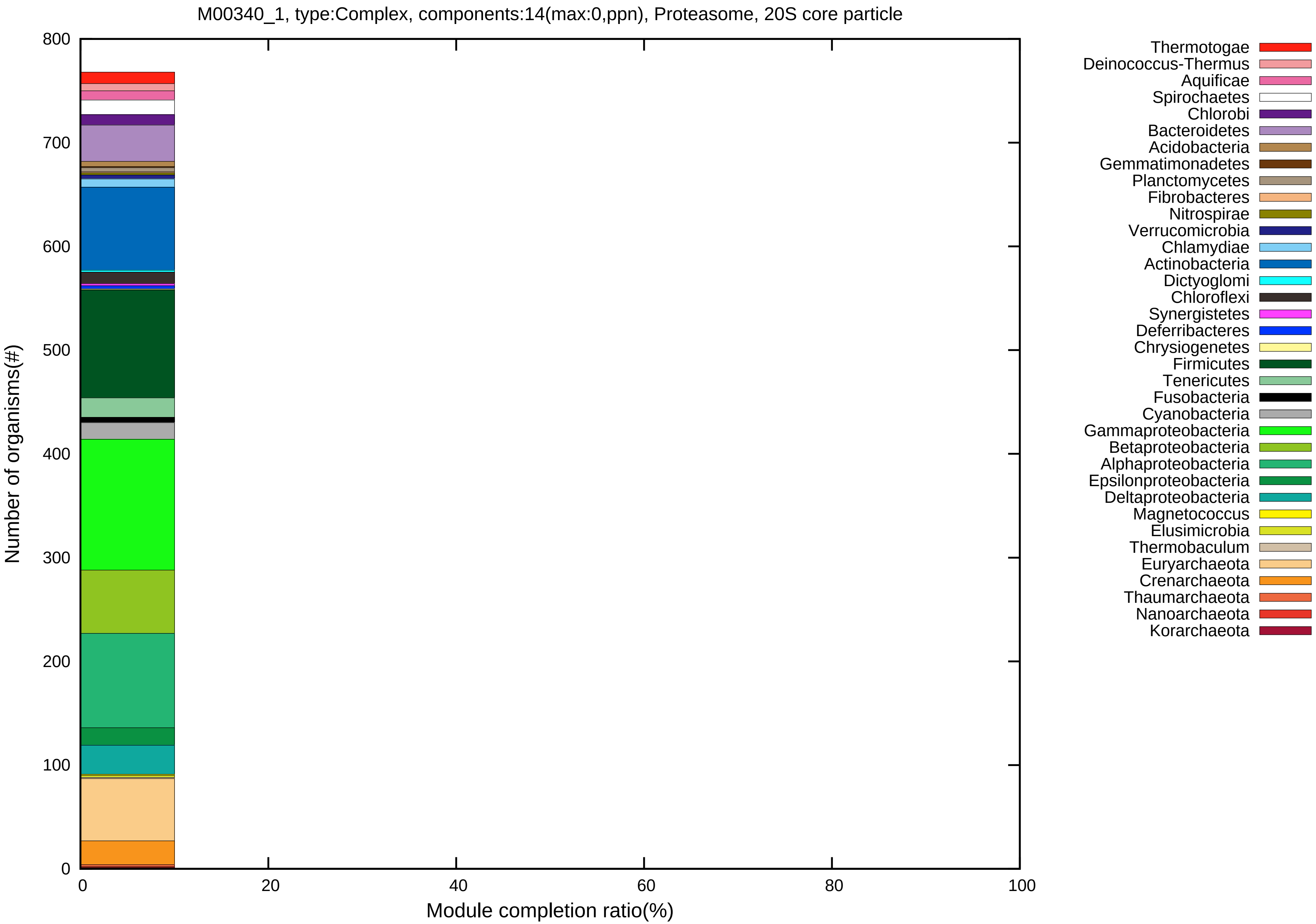

M00340_1, type:Complex, components:14(max:0,ppn), Proteasome, 20S core particle
Number of organisms(#)
Module completion ratio(%)
0
100
200
300
400
500
600
700
800
0
20
40
60
80
100
Thermotogae
Deinococcus-Thermus
Aquificae
Spirochaetes
Chlorobi
Bacteroidetes
Acidobacteria
Gemmatimonadetes
Planctomycetes
Fibrobacteres
Nitrospirae
Verrucomicrobia
Chlamydiae
Actinobacteria
Dictyoglomi
Chloroflexi
Synergistetes
Deferribacteres
Chrysiogenetes
Firmicutes
Tenericutes
Fusobacteria
Cyanobacteria
Gammaproteobacteria
Betaproteobacteria
Alphaproteobacteria
Epsilonproteobacteria
Deltaproteobacteria
Magnetococcus
Elusimicrobia
Thermobaculum
Euryarchaeota
Crenarchaeota
Thaumarchaeota
Nanoarchaeota
Korarchaeota

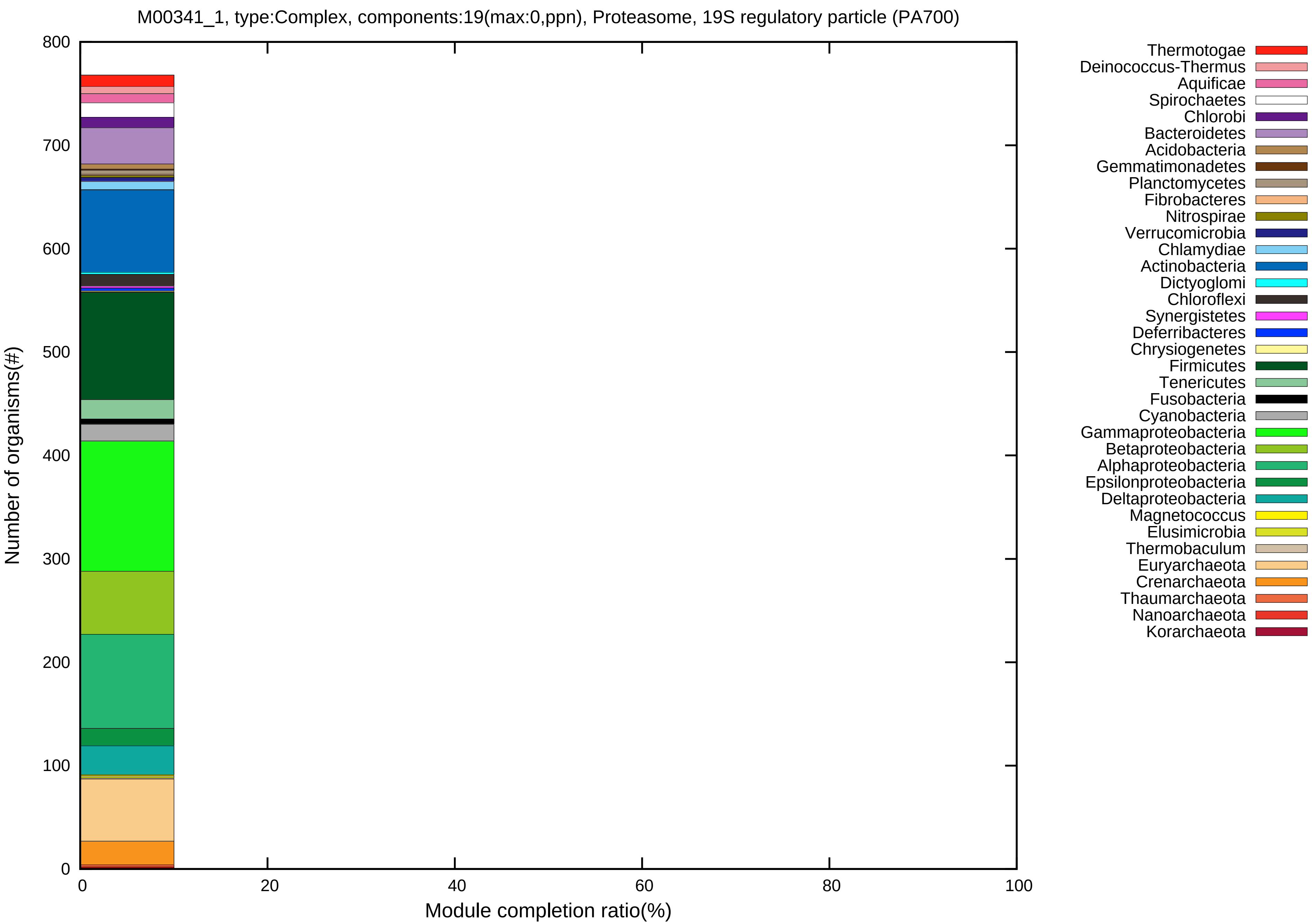

M00341_1, type:Complex, components:19(max:0,ppn), Proteasome, 19S regulatory particle (PA700)
Number of organisms(#)
Module completion ratio(%)
0
100
200
300
400
500
600
700
800
0
20
40
60
80
100
Thermotogae
Deinococcus-Thermus
Aquificae
Spirochaetes
Chlorobi
Bacteroidetes
Acidobacteria
Gemmatimonadetes
Planctomycetes
Fibrobacteres
Nitrospirae
Verrucomicrobia
Chlamydiae
Actinobacteria
Dictyoglomi
Chloroflexi
Synergistetes
Deferribacteres
Chrysiogenetes
Firmicutes
Tenericutes
Fusobacteria
Cyanobacteria
Gammaproteobacteria
Betaproteobacteria
Alphaproteobacteria
Epsilonproteobacteria
Deltaproteobacteria
Magnetococcus
Elusimicrobia
Thermobaculum
Euryarchaeota
Crenarchaeota
Thaumarchaeota
Nanoarchaeota
Korarchaeota

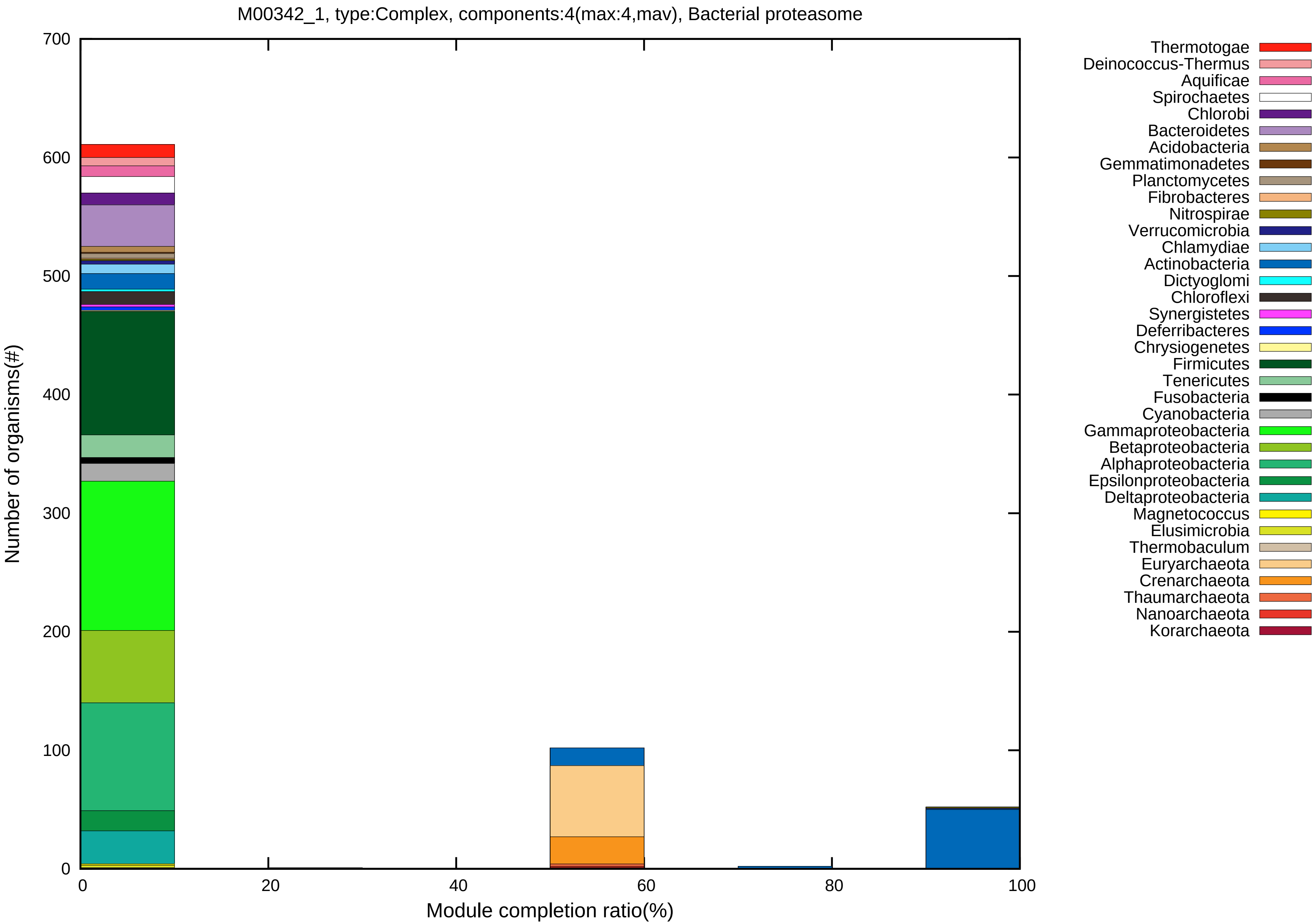

M00342_1, type:Complex, components:4(max:4,mav), Bacterial proteasome
Number of organisms(#)
Module completion ratio(%)
0
100
200
300
400
500
600
700
0
20
40
60
80
100
Thermotogae
Deinococcus-Thermus
Aquificae
Spirochaetes
Chlorobi
Bacteroidetes
Acidobacteria
Gemmatimonadetes
Planctomycetes
Fibrobacteres
Nitrospirae
Verrucomicrobia
Chlamydiae
Actinobacteria
Dictyoglomi
Chloroflexi
Synergistetes
Deferribacteres
Chrysiogenetes
Firmicutes
Tenericutes
Fusobacteria
Cyanobacteria
Gammaproteobacteria
Betaproteobacteria
Alphaproteobacteria
Epsilonproteobacteria
Deltaproteobacteria
Magnetococcus
Elusimicrobia
Thermobaculum
Euryarchaeota
Crenarchaeota
Thaumarchaeota
Nanoarchaeota
Korarchaeota

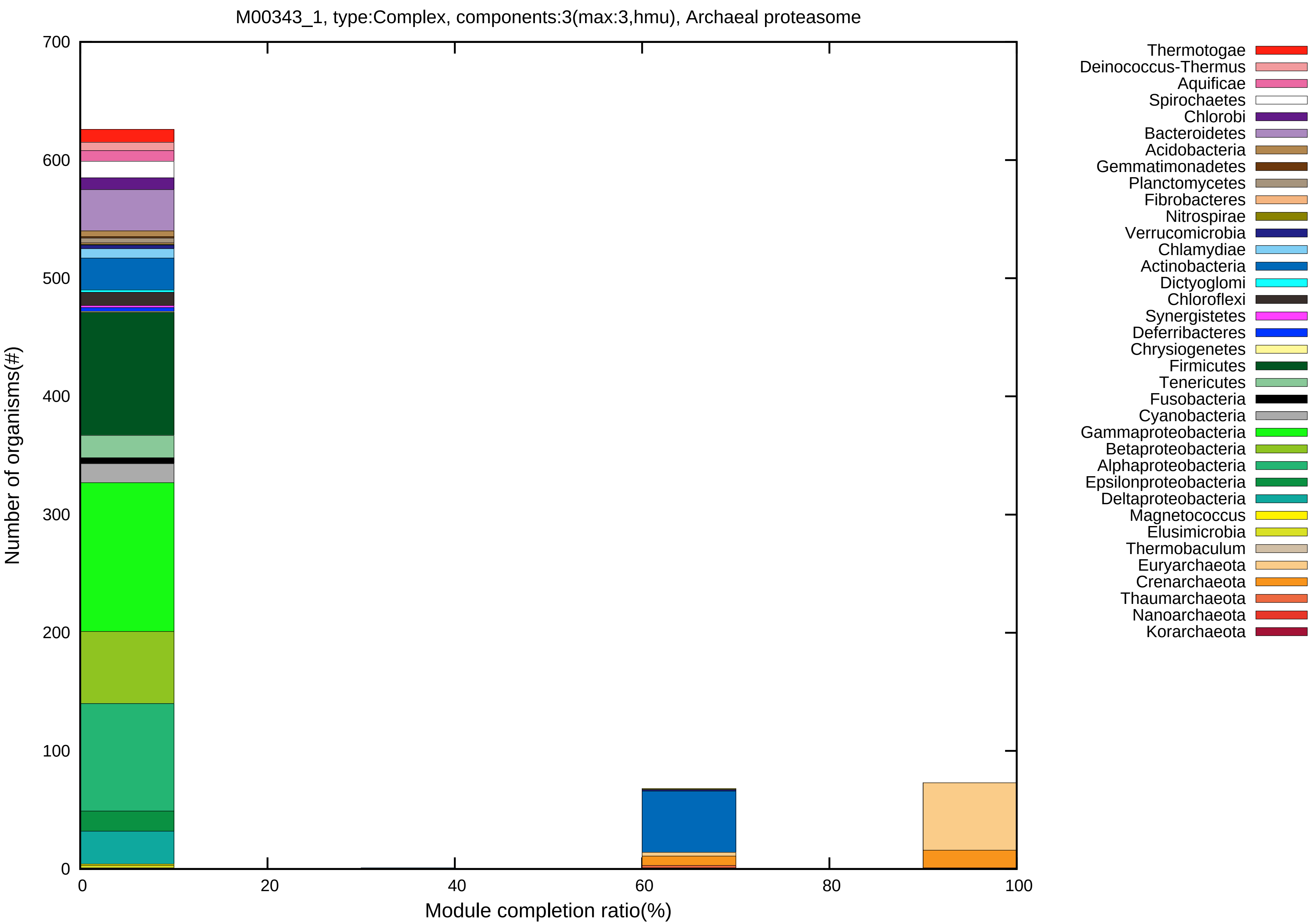
M00343_1, type:Complex, components:3(max:3,hmu), Archaeal proteasome
Number of organisms(#)
Module completion ratio(%)
0
100
200
300
400
500
600
700
0
20
40
60
80
100
Thermotogae
Deinococcus-Thermus
Aquificae
Spirochaetes
Chlorobi
Bacteroidetes
Acidobacteria
Gemmatimonadetes
Planctomycetes
Fibrobacteres
Nitrospirae
Verrucomicrobia
Chlamydiae
Actinobacteria
Dictyoglomi
Chloroflexi
Synergistetes
Deferribacteres
Chrysiogenetes
Firmicutes
Tenericutes
Fusobacteria
Cyanobacteria
Gammaproteobacteria
Betaproteobacteria
Alphaproteobacteria
Epsilonproteobacteria
Deltaproteobacteria
Magnetococcus
Elusimicrobia
Thermobaculum
Euryarchaeota
Crenarchaeota
Thaumarchaeota
Nanoarchaeota
Korarchaeota

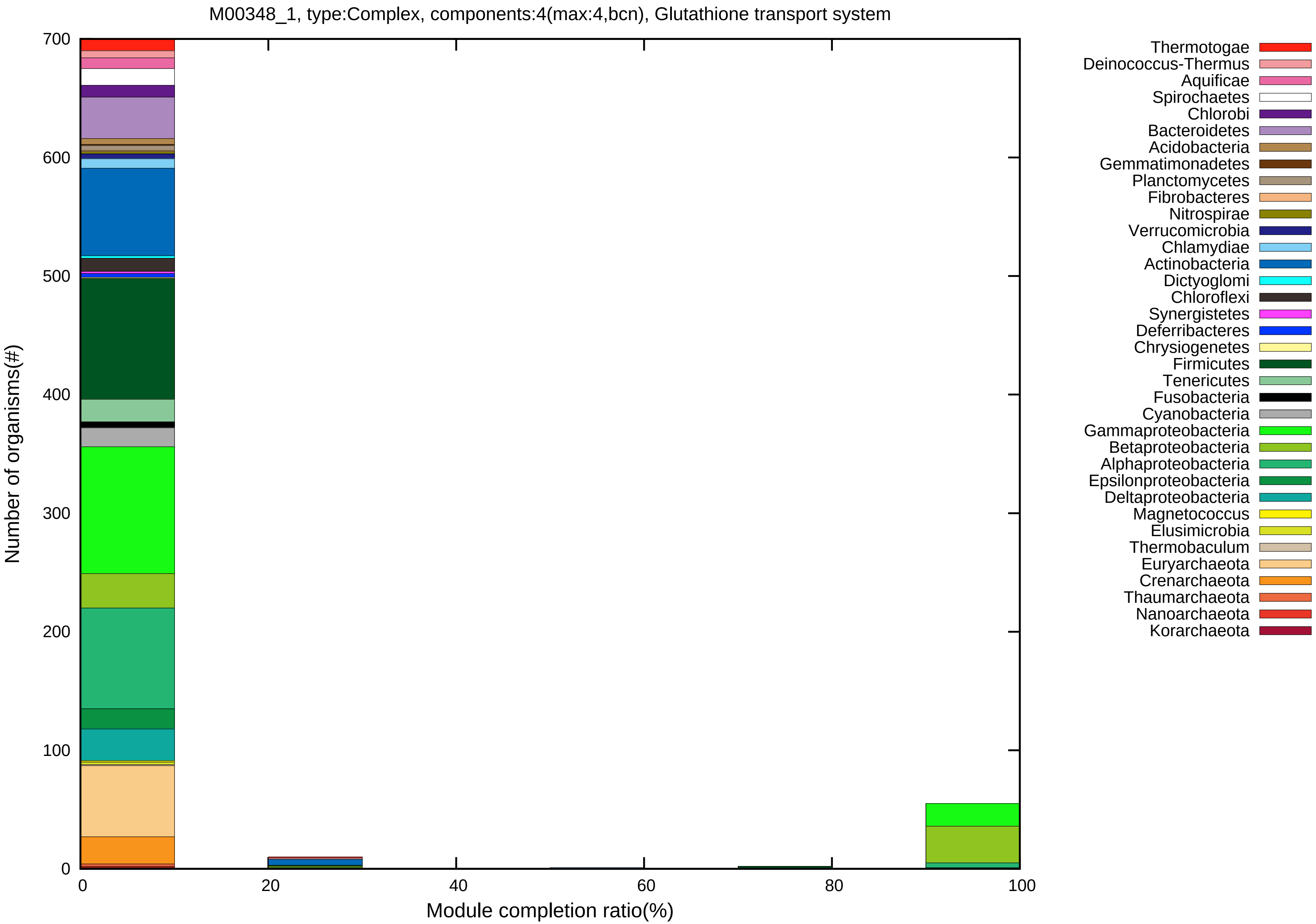

M00348_1, type:Complex, components:4(max:4,bcn), Glutathione transport system
Number of organisms(#)
Module completion ratio(%)
0
100
200
300
400
500
600
700
0
20
40
60
80
100
Thermotogae
Deinococcus-Thermus
Aquificae
Spirochaetes
Chlorobi
Bacteroidetes
Acidobacteria
Gemmatimonadetes
Planctomycetes
Fibrobacteres
Nitrospirae
Verrucomicrobia
Chlamydiae
Actinobacteria
Dictyoglomi
Chloroflexi
Synergistetes
Deferribacteres
Chrysiogenetes
Firmicutes
Tenericutes
Fusobacteria
Cyanobacteria
Gammaproteobacteria
Betaproteobacteria
Alphaproteobacteria
Epsilonproteobacteria
Deltaproteobacteria
Magnetococcus
Elusimicrobia
Thermobaculum
Euryarchaeota
Crenarchaeota
Thaumarchaeota
Nanoarchaeota
Korarchaeota

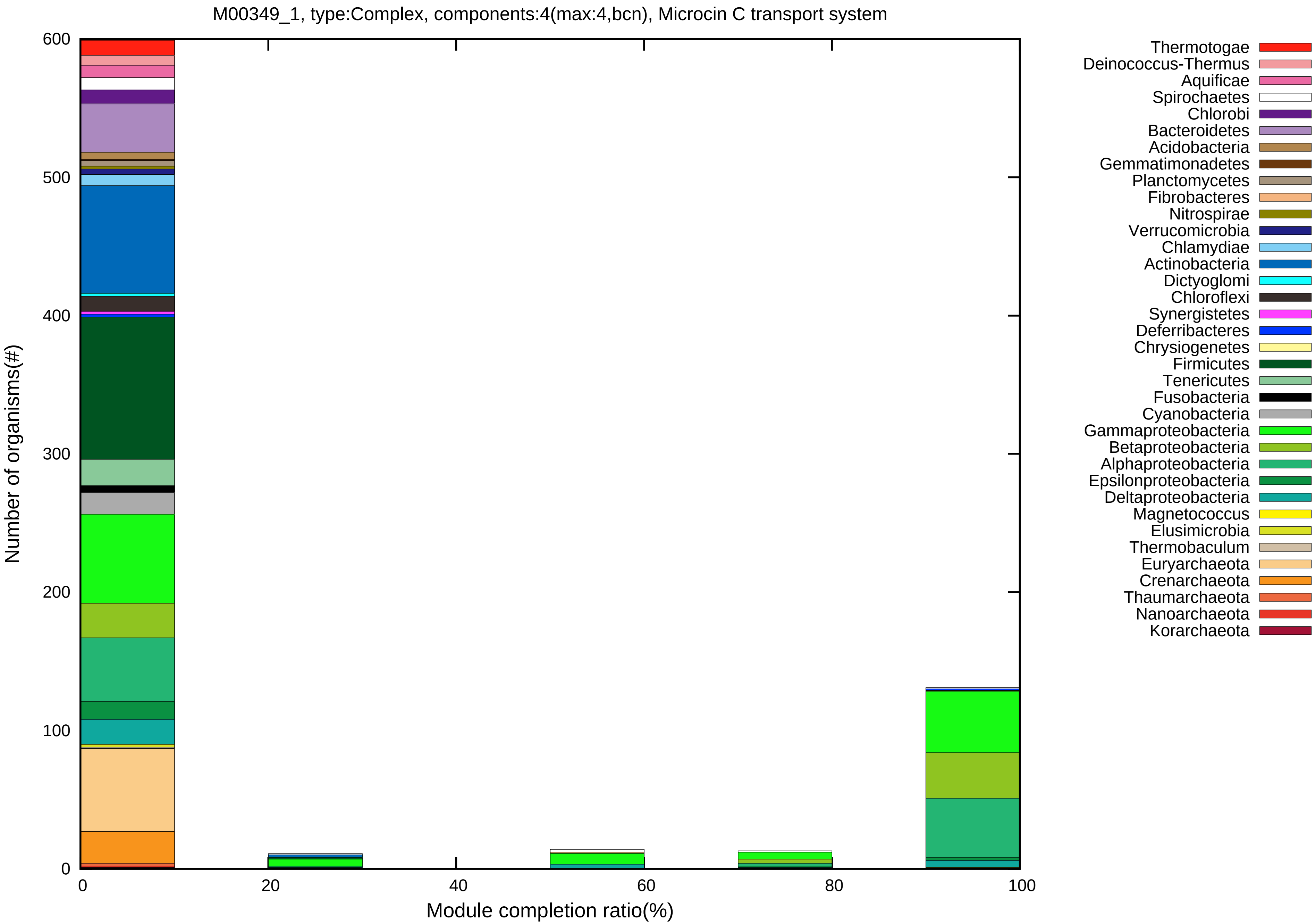

M00349_1, type:Complex, components:4(max:4,bcn), Microcin C transport system
Number of organisms(#)
0
100
200
300
400
500
600
0
20
40
60
80
100
Module completion ratio(%)
Thermotogae
Deinococcus-Thermus
Aquificae
Spirochaetes
Chlorobi
Bacteroidetes
Acidobacteria
Gemmatimonadetes
Planctomycetes
Fibrobacteres
Nitrospirae
Verrucomicrobia
Chlamydiae
Actinobacteria
Dictyoglomi
Chloroflexi
Synergistetes
Deferribacteres
Chrysiogenetes
Firmicutes
Tenericutes
Fusobacteria
Cyanobacteria
Gammaproteobacteria
Betaproteobacteria
Alphaproteobacteria
Epsilonproteobacteria
Deltaproteobacteria
Magnetococcus
Elusimicrobia
Thermobaculum
Euryarchaeota
Crenarchaeota
Thaumarchaeota
Nanoarchaeota
Korarchaeota

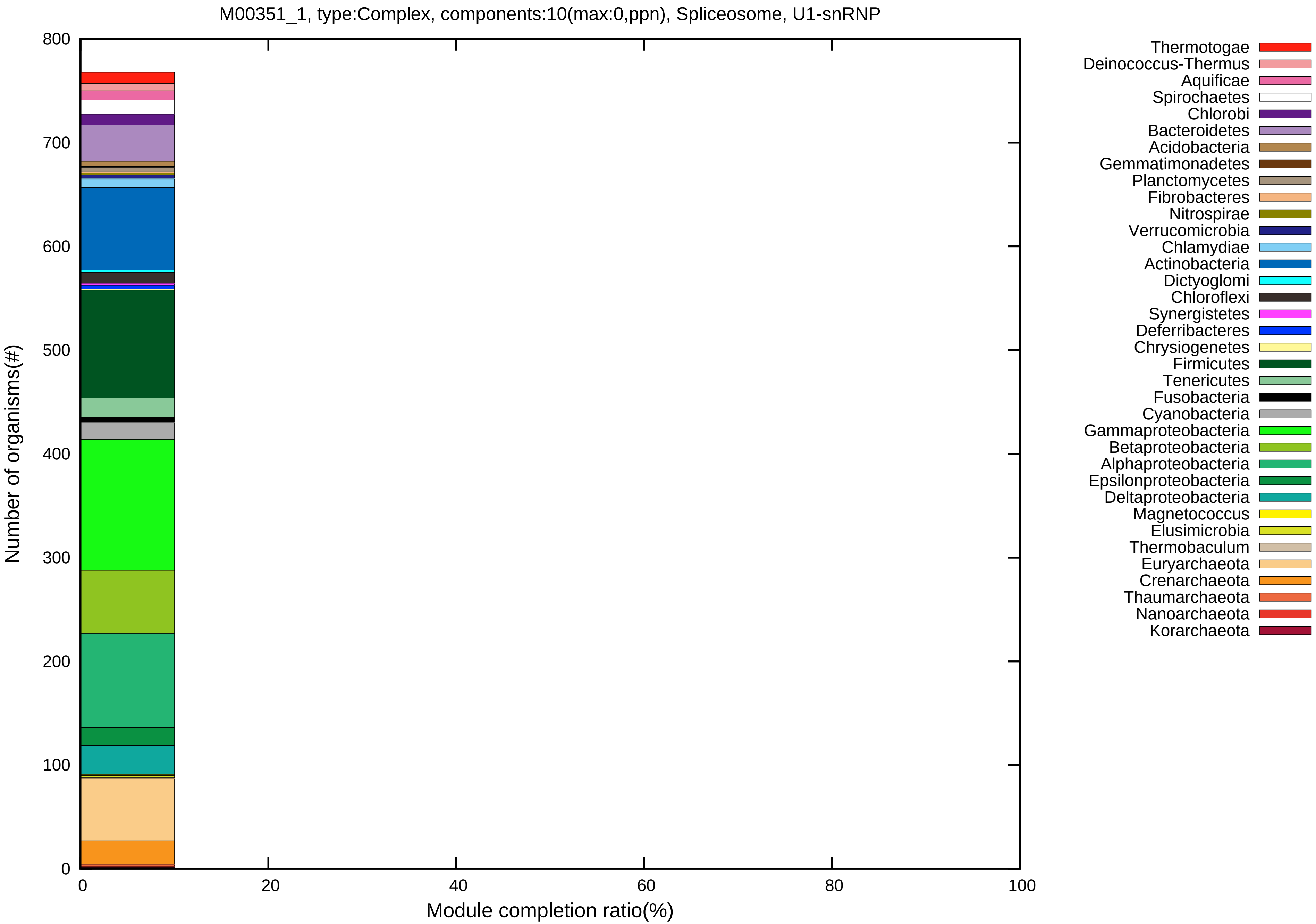
M00351_1, type:Complex, components:10(max:0,ppn), Spliceosome, U1-snRNP
Number of organisms(#)
Module completion ratio(%)
0
100
200
300
400
500
600
700
800
0
20
40
60
80
100
Thermotogae
Deinococcus-Thermus
Aquificae
Spirochaetes
Chlorobi
Bacteroidetes
Acidobacteria
Gemmatimonadetes
Planctomycetes
Fibrobacteres
Nitrospirae
Verrucomicrobia
Chlamydiae
Actinobacteria
Dictyoglomi
Chloroflexi
Synergistetes
Deferribacteres
Chrysiogenetes
Firmicutes
Tenericutes
Fusobacteria
Cyanobacteria
Gammaproteobacteria
Betaproteobacteria
Alphaproteobacteria
Epsilonproteobacteria
Deltaproteobacteria
Magnetococcus
Elusimicrobia
Thermobaculum
Euryarchaeota
Crenarchaeota
Thaumarchaeota
Nanoarchaeota
Korarchaeota

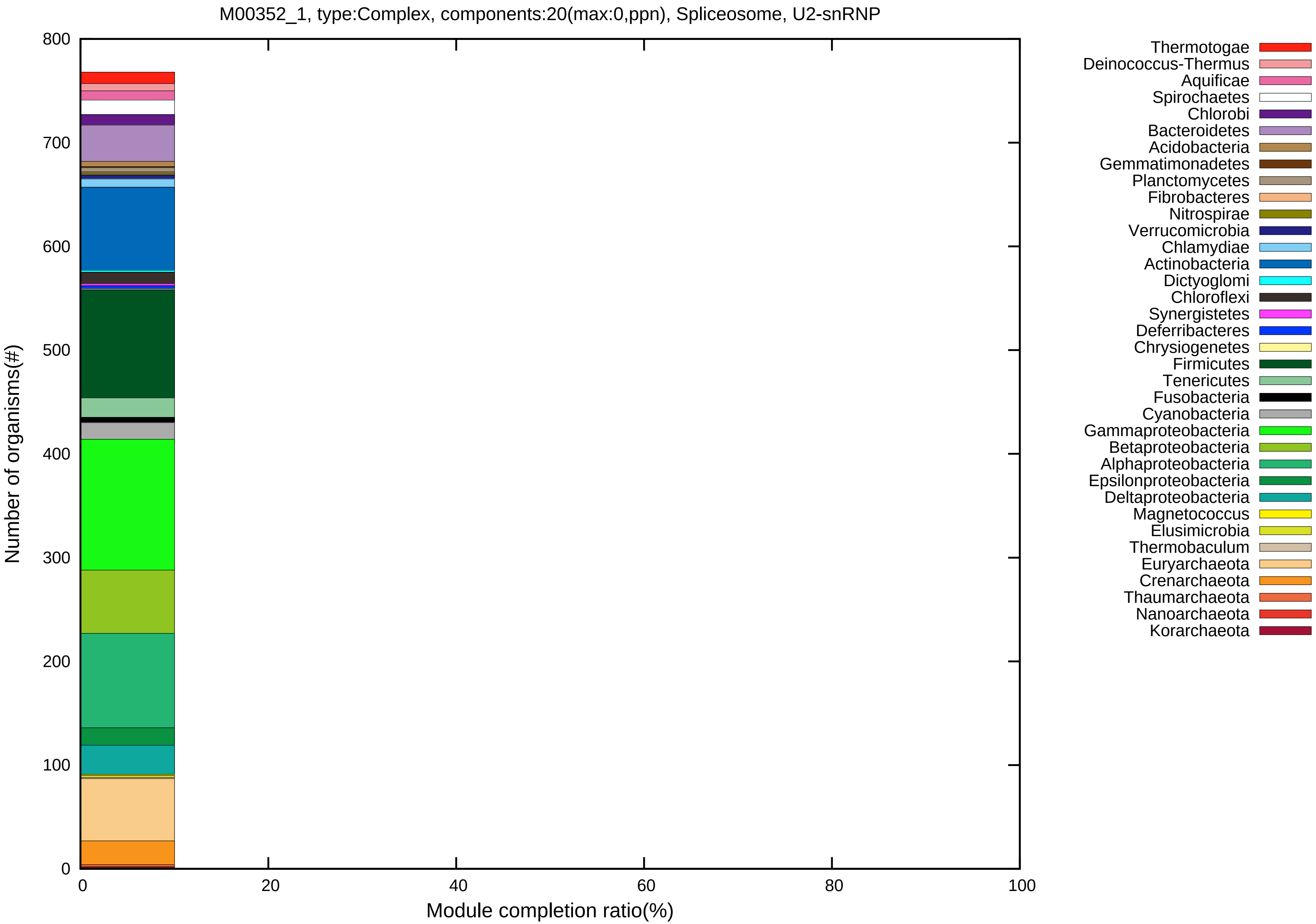
M00352_1, type:Complex, components:20(max:0,ppn), Spliceosome, U2-snRNP
Number of organisms(#)
Module completion ratio(%)
0
100
200
300
400
500
600
700
800
0
20
40
60
80
100
Thermotogae
Deinococcus-Thermus
Aquificae
Spirochaetes
Chlorobi
Bacteroidetes
Acidobacteria
Gemmatimonadetes
Planctomycetes
Fibrobacteres
Nitrospirae
Verrucomicrobia
Chlamydiae
Actinobacteria
Dictyoglomi
Chloroflexi
Synergistetes
Deferribacteres
Chrysiogenetes
Firmicutes
Tenericutes
Fusobacteria
Cyanobacteria
Gammaproteobacteria
Betaproteobacteria
Alphaproteobacteria
Epsilonproteobacteria
Deltaproteobacteria
Magnetococcus
Elusimicrobia
Thermobaculum
Euryarchaeota
Crenarchaeota
Thaumarchaeota
Nanoarchaeota
Korarchaeota

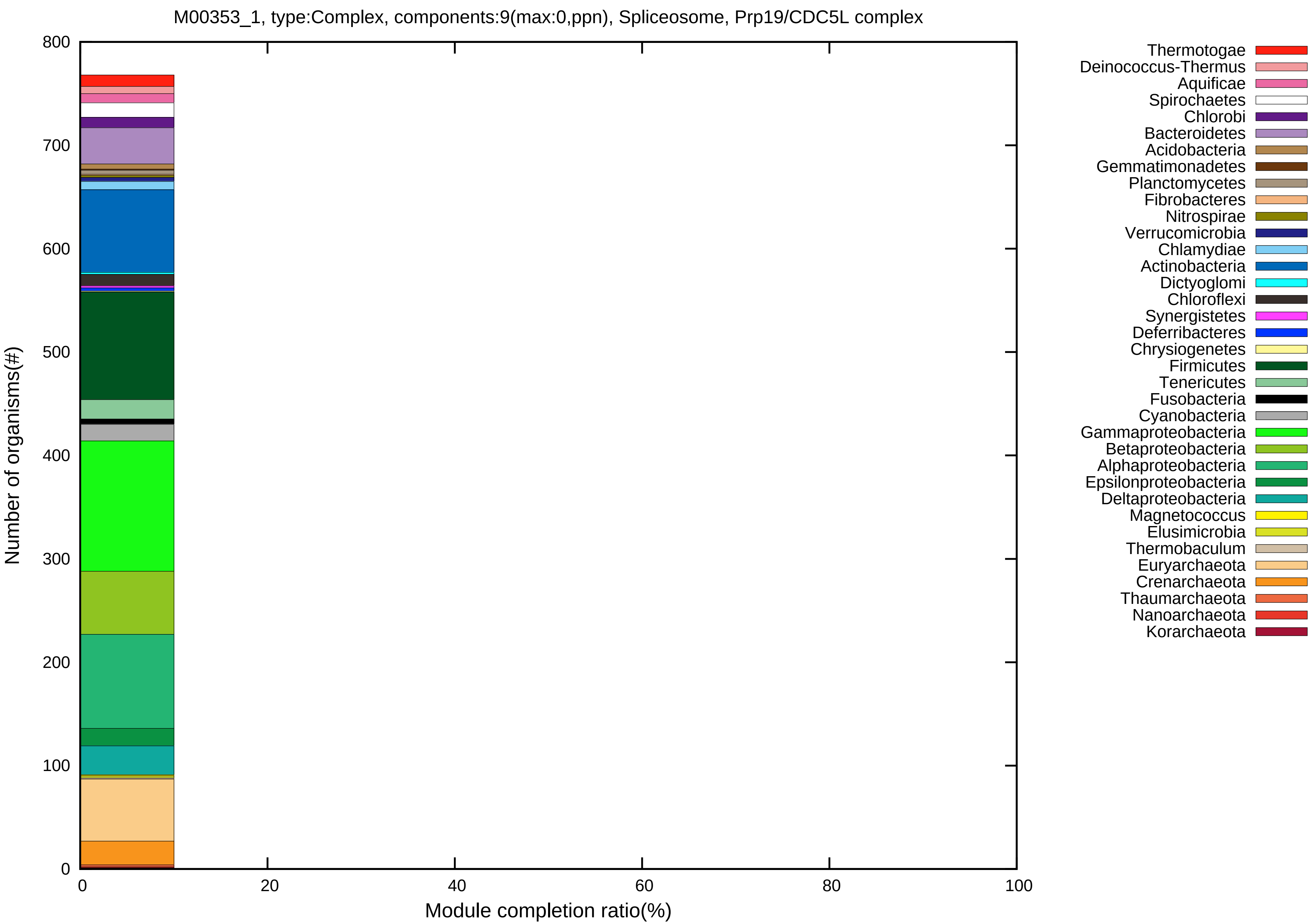

M00353_1, type:Complex, components:9(max:0,ppn), Spliceosome, Prp19/CDC5L complex
Number of organisms(#)
Module completion ratio(%)
0
100
200
300
400
500
600
700
800
0
20
40
60
80
100
Thermotogae
Deinococcus-Thermus
Aquificae
Spirochaetes
Chlorobi
Bacteroidetes
Acidobacteria
Gemmatimonadetes
Planctomycetes
Fibrobacteres
Nitrospirae
Verrucomicrobia
Chlamydiae
Actinobacteria
Dictyoglomi
Chloroflexi
Synergistetes
Deferribacteres
Chrysiogenetes
Firmicutes
Tenericutes
Fusobacteria
Cyanobacteria
Gammaproteobacteria
Betaproteobacteria
Alphaproteobacteria
Epsilonproteobacteria
Deltaproteobacteria
Magnetococcus
Elusimicrobia
Thermobaculum
Euryarchaeota
Crenarchaeota
Thaumarchaeota
Nanoarchaeota
Korarchaeota

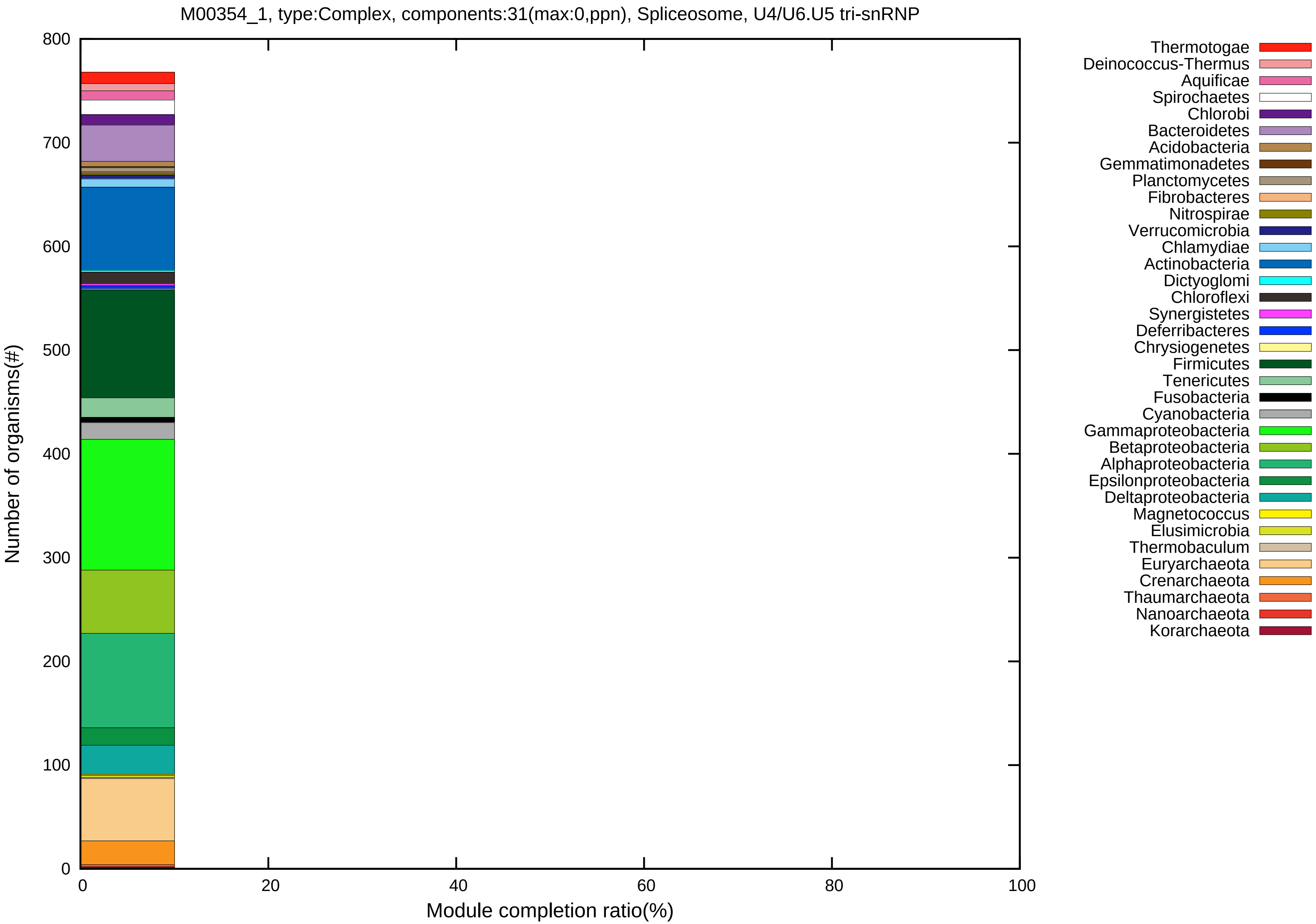

M00354_1, type:Complex, components:31(max:0,ppn), Spliceosome, U4/U6.U5 tri-snRNP
Number of organisms(#)
0
100
200
300
400
500
600
700
800
Module completion ratio(%)
0
20
40
60
80
100
Thermotogae
Deinococcus-Thermus
Aquificae
Spirochaetes
Chlorobi
Bacteroidetes
Acidobacteria
Gemmatimonadetes
Planctomycetes
Fibrobacteres
Nitrospirae
Verrucomicrobia
Chlamydiae
Actinobacteria
Dictyoglomi
Chloroflexi
Synergistetes
Deferribacteres
Chrysiogenetes
Firmicutes
Tenericutes
Fusobacteria
Cyanobacteria
Gammaproteobacteria
Betaproteobacteria
Alphaproteobacteria
Epsilonproteobacteria
Deltaproteobacteria
Magnetococcus
Elusimicrobia
Thermobaculum
Euryarchaeota
Crenarchaeota
Thaumarchaeota
Nanoarchaeota
Korarchaeota

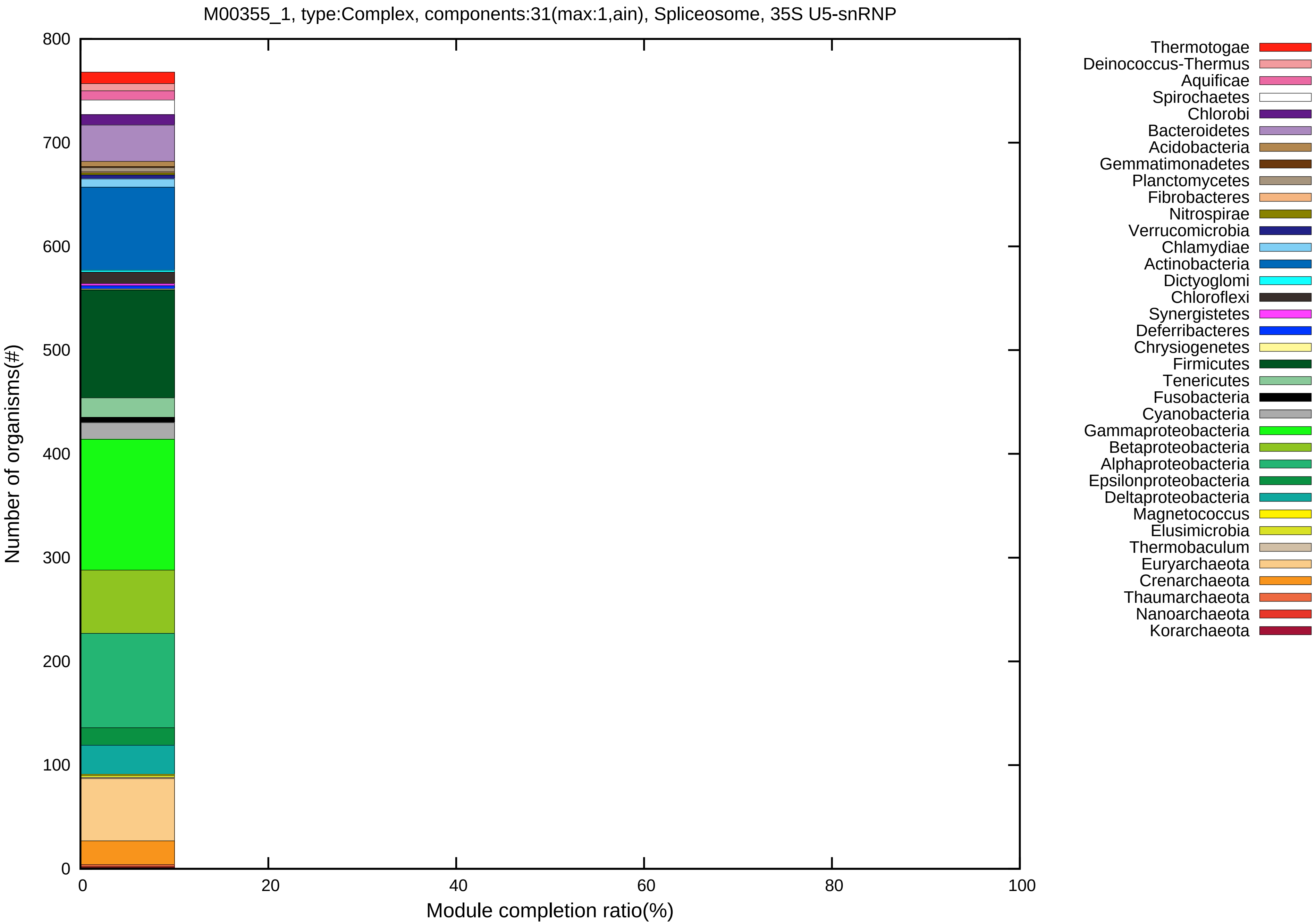
M00355_1, type:Complex, components:31(max:1,ain), Spliceosome, 35S U5-snRNP
Number of organisms(#)
Module completion ratio(%)
0
100
200
300
400
500
600
700
800
0
20
40
60
80
100
Thermotogae
Deinococcus-Thermus
Aquificae
Spirochaetes
Chlorobi
Bacteroidetes
Acidobacteria
Gemmatimonadetes
Planctomycetes
Fibrobacteres
Nitrospirae
Verrucomicrobia
Chlamydiae
Actinobacteria
Dictyoglomi
Chloroflexi
Synergistetes
Deferribacteres
Chrysiogenetes
Firmicutes
Tenericutes
Fusobacteria
Cyanobacteria
Gammaproteobacteria
Betaproteobacteria
Alphaproteobacteria
Epsilonproteobacteria
Deltaproteobacteria
Magnetococcus
Elusimicrobia
Thermobaculum
Euryarchaeota
Crenarchaeota
Thaumarchaeota
Nanoarchaeota
Korarchaeota

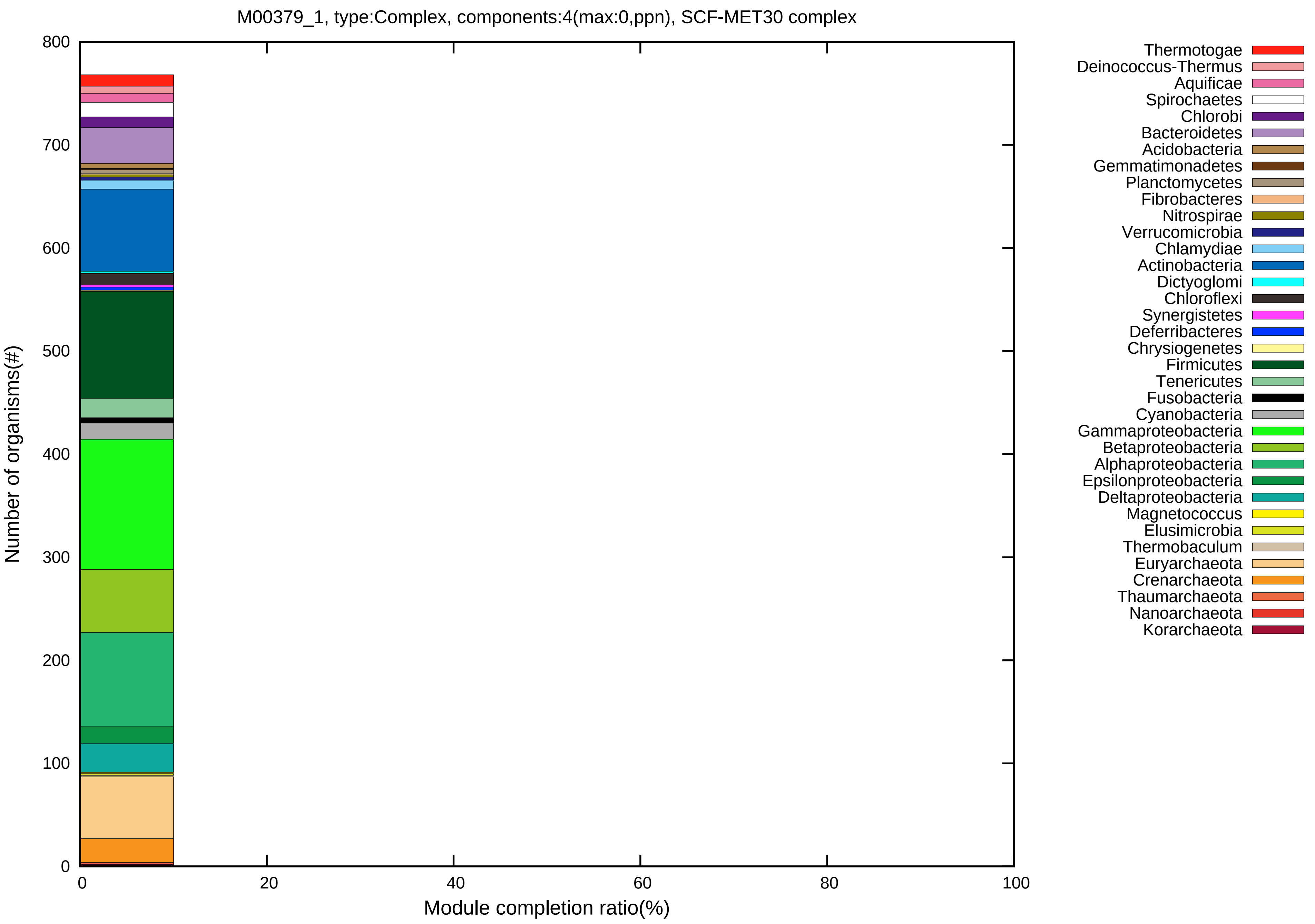

M00379_1, type:Complex, components:4(max:0,ppn), SCF-MET30 complex
Number of organisms(#)
Module completion ratio(%)
0
100
200
300
400
500
600
700
800
0
20
40
60
80
100
Thermotogae
Deinococcus-Thermus
Aquificae
Spirochaetes
Chlorobi
Bacteroidetes
Acidobacteria
Gemmatimonadetes
Planctomycetes
Fibrobacteres
Nitrospirae
Verrucomicrobia
Chlamydiae
Actinobacteria
Dictyoglomi
Chloroflexi
Synergistetes
Deferribacteres
Chrysiogenetes
Firmicutes
Tenericutes
Fusobacteria
Cyanobacteria
Gammaproteobacteria
Betaproteobacteria
Alphaproteobacteria
Epsilonproteobacteria
Deltaproteobacteria
Magnetococcus
Elusimicrobia
Thermobaculum
Euryarchaeota
Crenarchaeota
Thaumarchaeota
Nanoarchaeota
Korarchaeota

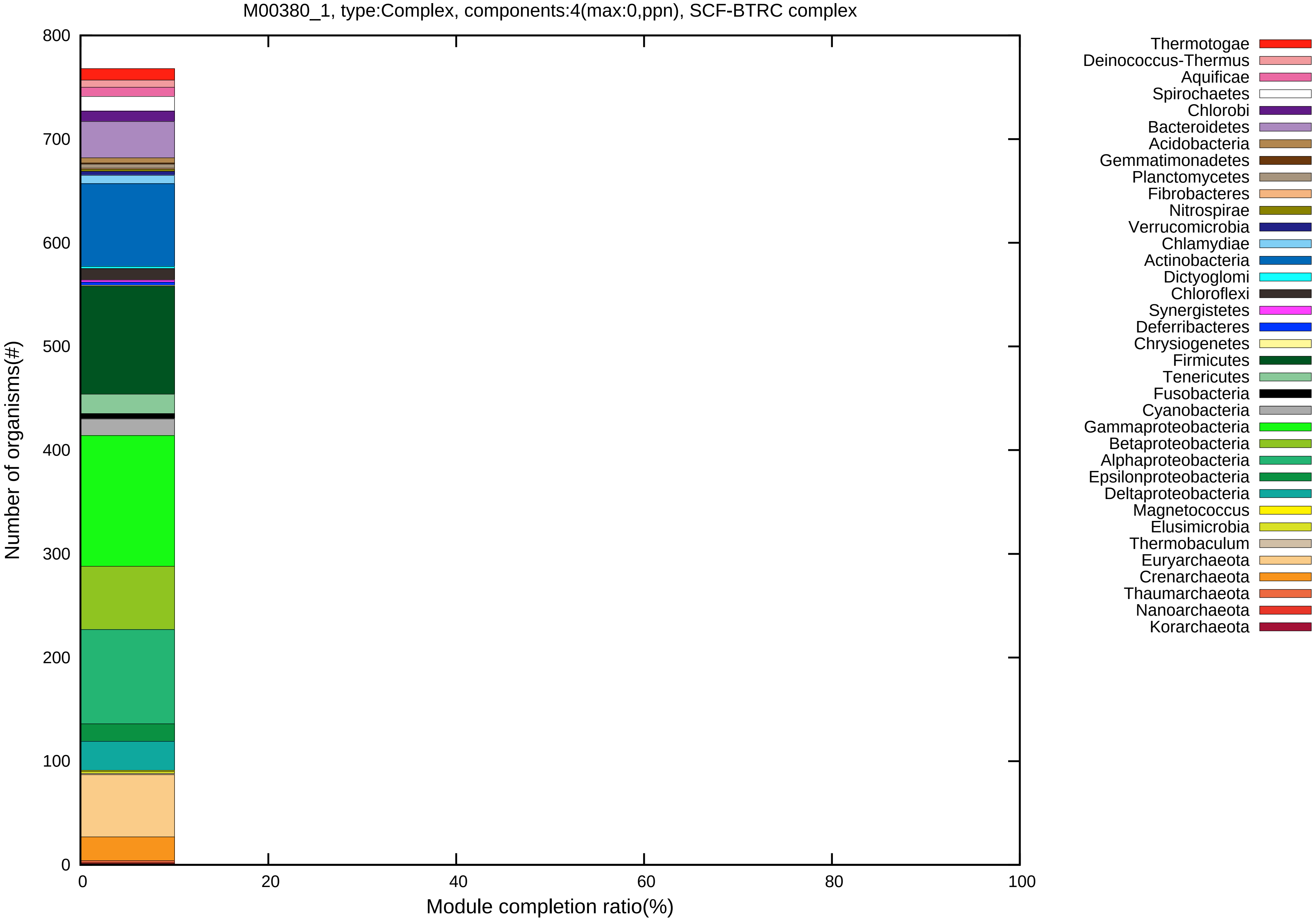
M00380_1, type:Complex, components:4(max:0,ppn), SCF-BTRC complex
Number of organisms(#)
0
100
200
300
400
500
600
700
800
0
20
40
60
80
100
Module completion ratio(%)
Thermotogae
Deinococcus-Thermus
Aquificae
Spirochaetes
Chlorobi
Bacteroidetes
Acidobacteria
Gemmatimonadetes
Planctomycetes
Fibrobacteres
Nitrospirae
Verrucomicrobia
Chlamydiae
Actinobacteria
Dictyoglomi
Chloroflexi
Synergistetes
Deferribacteres
Chrysiogenetes
Firmicutes
Tenericutes
Fusobacteria
Cyanobacteria
Gammaproteobacteria
Betaproteobacteria
Alphaproteobacteria
Epsilonproteobacteria
Deltaproteobacteria
Magnetococcus
Elusimicrobia
Thermobaculum
Euryarchaeota
Crenarchaeota
Thaumarchaeota
Nanoarchaeota
Korarchaeota

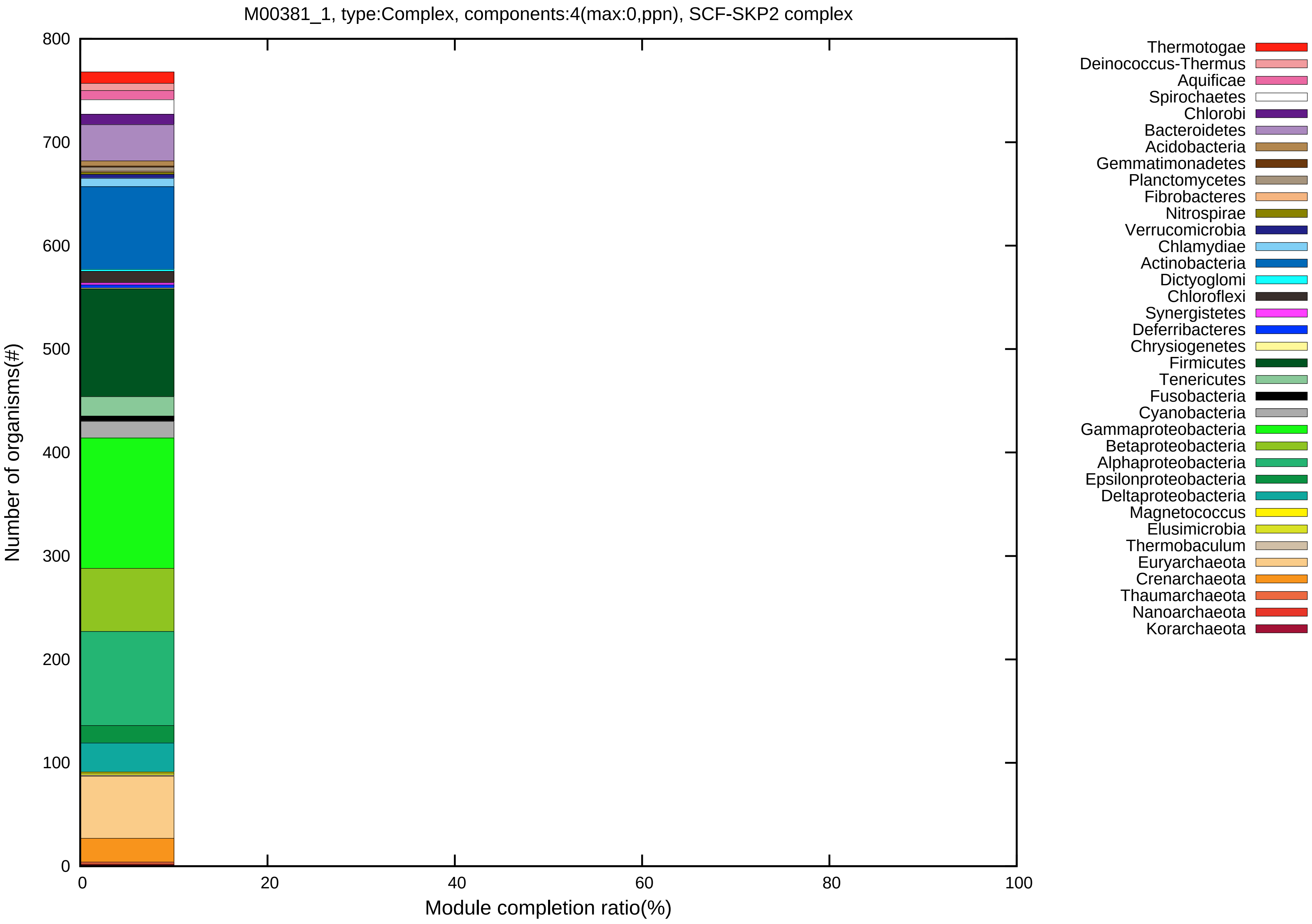

M00381_1, type:Complex, components:4(max:0,ppn), SCF-SKP2 complex
Number of organisms(#)
Module completion ratio(%)
0
100
200
300
400
500
600
700
800
0
20
40
60
80
100
Thermotogae
Deinococcus-Thermus
Aquificae
Spirochaetes
Chlorobi
Bacteroidetes
Acidobacteria
Gemmatimonadetes
Planctomycetes
Fibrobacteres
Nitrospirae
Verrucomicrobia
Chlamydiae
Actinobacteria
Dictyoglomi
Chloroflexi
Synergistetes
Deferribacteres
Chrysiogenetes
Firmicutes
Tenericutes
Fusobacteria
Cyanobacteria
Gammaproteobacteria
Betaproteobacteria
Alphaproteobacteria
Epsilonproteobacteria
Deltaproteobacteria
Magnetococcus
Elusimicrobia
Thermobaculum
Euryarchaeota
Crenarchaeota
Thaumarchaeota
Nanoarchaeota
Korarchaeota

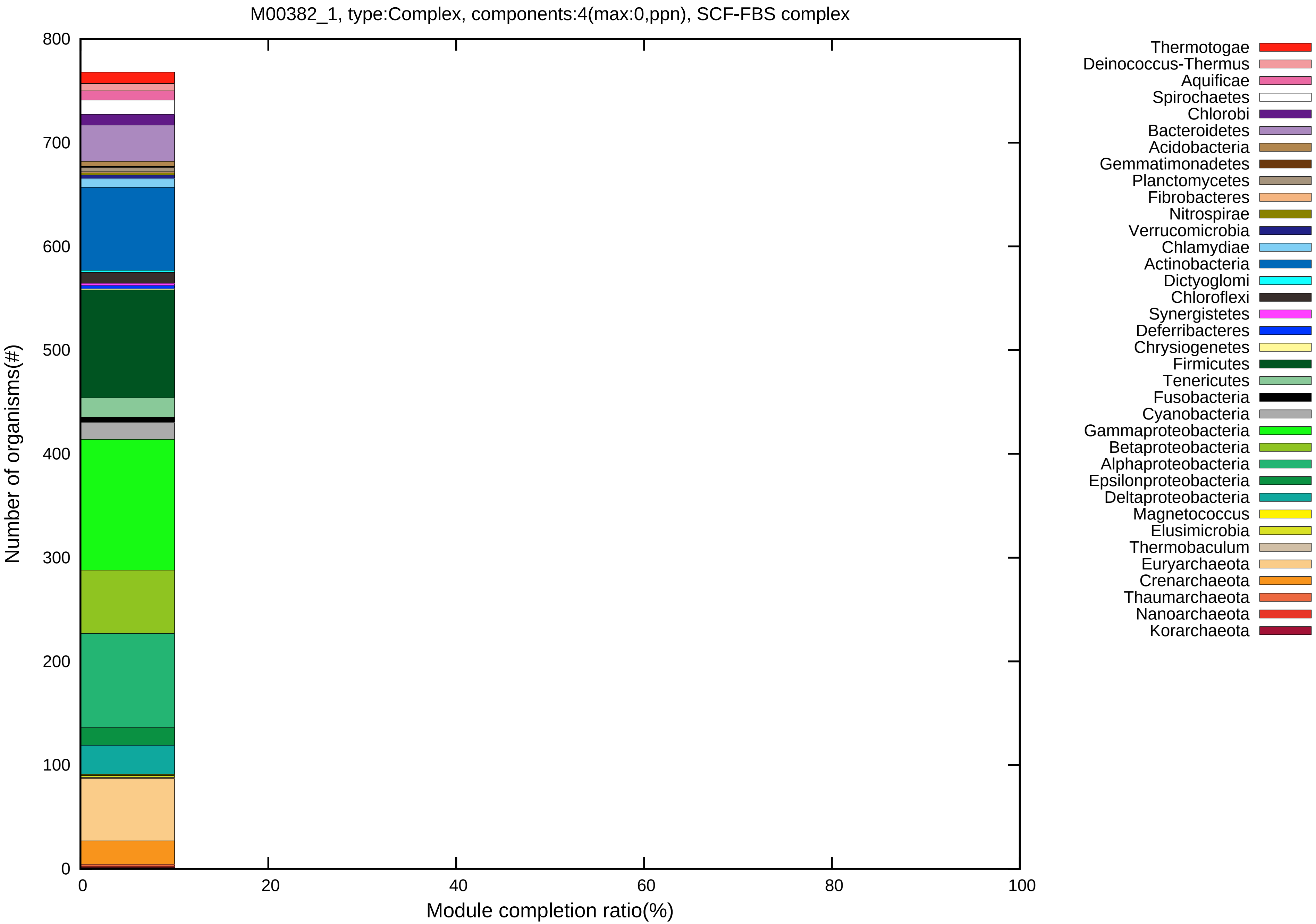

M00382_1, type:Complex, components:4(max:0,ppn), SCF-FBS complex
Number of organisms(#)
Module completion ratio(%)
0
100
200
300
400
500
600
700
800
0
20
40
60
80
100
Thermotogae
Deinococcus-Thermus
Aquificae
Spirochaetes
Chlorobi
Bacteroidetes
Acidobacteria
Gemmatimonadetes
Planctomycetes
Fibrobacteres
Nitrospirae
Verrucomicrobia
Chlamydiae
Actinobacteria
Dictyoglomi
Chloroflexi
Synergistetes
Deferribacteres
Chrysiogenetes
Firmicutes
Tenericutes
Fusobacteria
Cyanobacteria
Gammaproteobacteria
Betaproteobacteria
Alphaproteobacteria
Epsilonproteobacteria
Deltaproteobacteria
Magnetococcus
Elusimicrobia
Thermobaculum
Euryarchaeota
Crenarchaeota
Thaumarchaeota
Nanoarchaeota
Korarchaeota

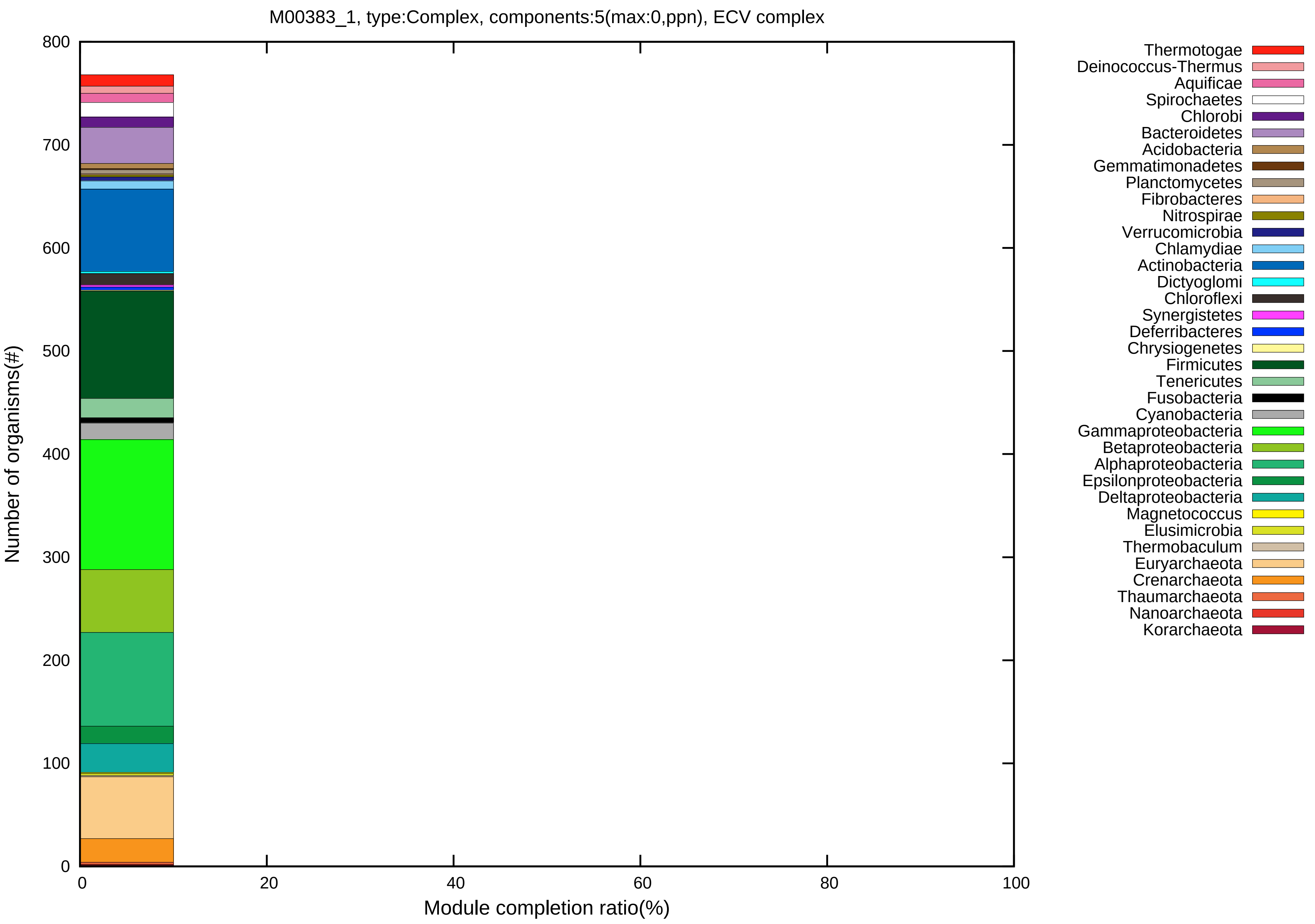

M00383_1, type:Complex, components:5(max:0,ppn), ECV complex
Number of organisms(#)
Module completion ratio(%)
0
100
200
300
400
500
600
700
800
0
20
40
60
80
100
Thermotogae
Deinococcus-Thermus
Aquificae
Spirochaetes
Chlorobi
Bacteroidetes
Acidobacteria
Gemmatimonadetes
Planctomycetes
Fibrobacteres
Nitrospirae
Verrucomicrobia
Chlamydiae
Actinobacteria
Dictyoglomi
Chloroflexi
Synergistetes
Deferribacteres
Chrysiogenetes
Firmicutes
Tenericutes
Fusobacteria
Cyanobacteria
Gammaproteobacteria
Betaproteobacteria
Alphaproteobacteria
Epsilonproteobacteria
Deltaproteobacteria
Magnetococcus
Elusimicrobia
Thermobaculum
Euryarchaeota
Crenarchaeota
Thaumarchaeota
Nanoarchaeota
Korarchaeota

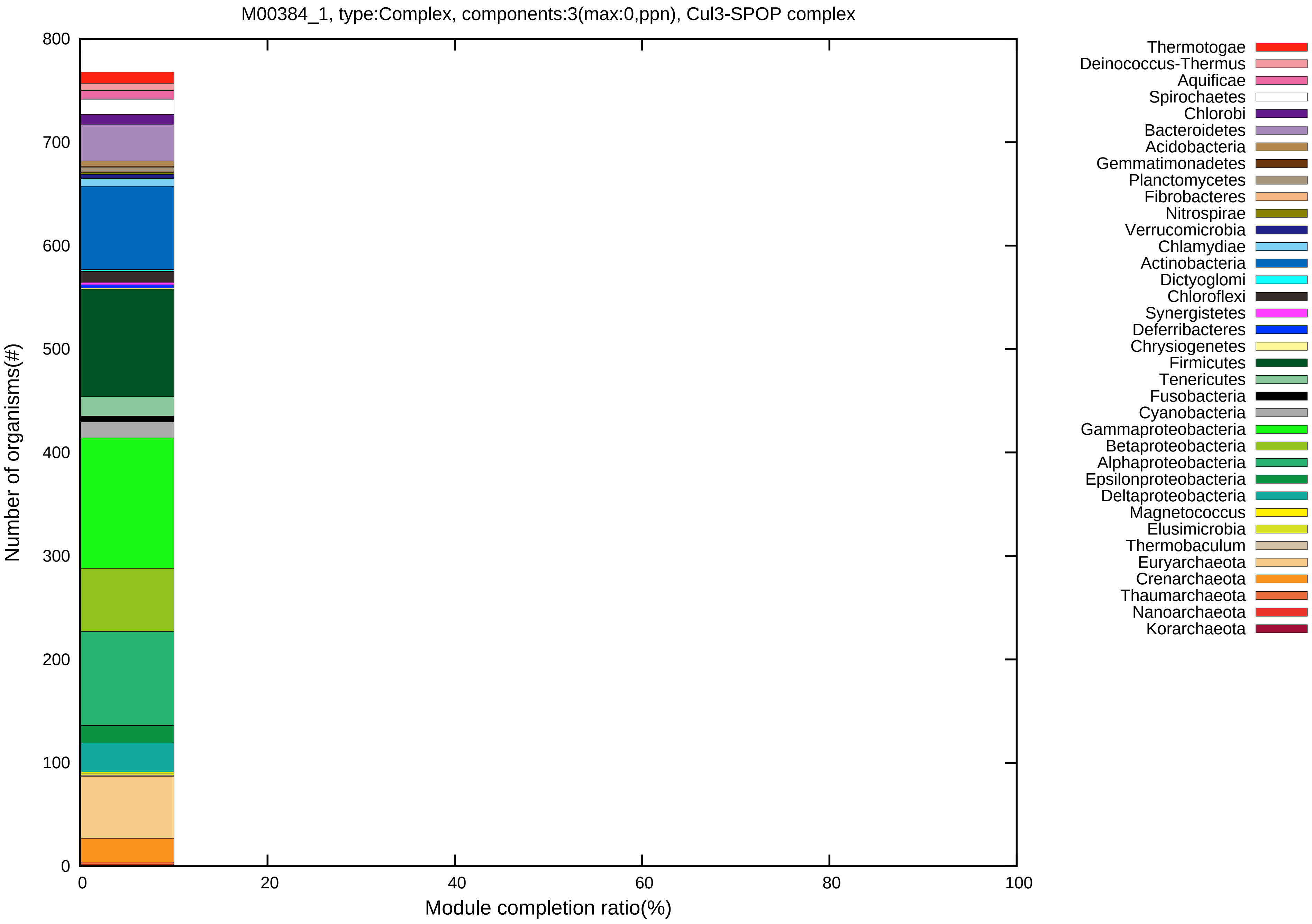

M00384_1, type:Complex, components:3(max:0,ppn), Cul3-SPOP complex
Number of organisms(#)
Module completion ratio(%)
0
100
200
300
400
500
600
700
800
0
20
40
60
80
100
Thermotogae
Deinococcus-Thermus
Aquificae
Spirochaetes
Chlorobi
Bacteroidetes
Acidobacteria
Gemmatimonadetes
Planctomycetes
Fibrobacteres
Nitrospirae
Verrucomicrobia
Chlamydiae
Actinobacteria
Dictyoglomi
Chloroflexi
Synergistetes
Deferribacteres
Chrysiogenetes
Firmicutes
Tenericutes
Fusobacteria
Cyanobacteria
Gammaproteobacteria
Betaproteobacteria
Alphaproteobacteria
Epsilonproteobacteria
Deltaproteobacteria
Magnetococcus
Elusimicrobia
Thermobaculum
Euryarchaeota
Crenarchaeota
Thaumarchaeota
Nanoarchaeota
Korarchaeota

M00385_1, type:Complex, components:4(max:0,ppn), Cul4-DDB1-DDB2 complex

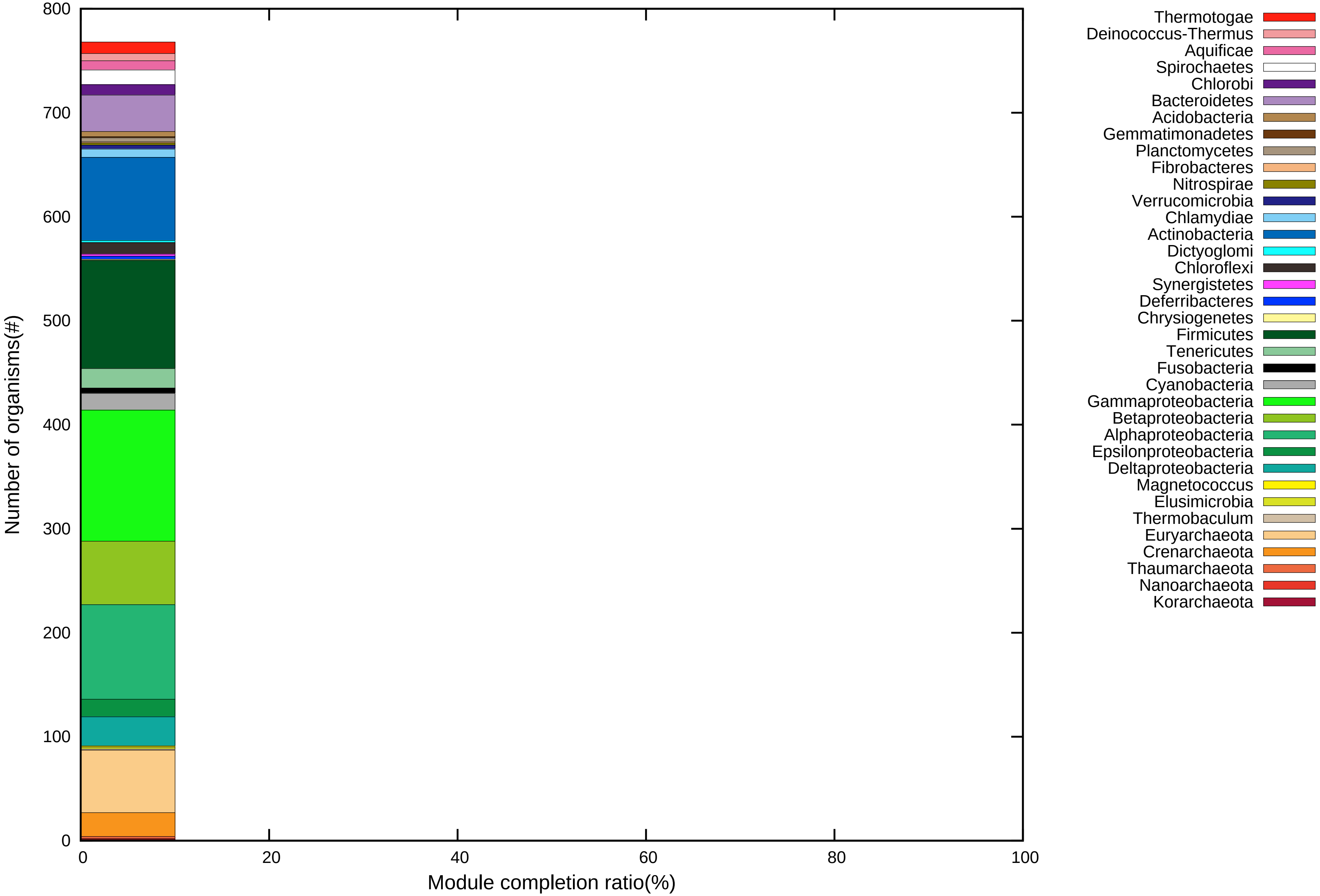

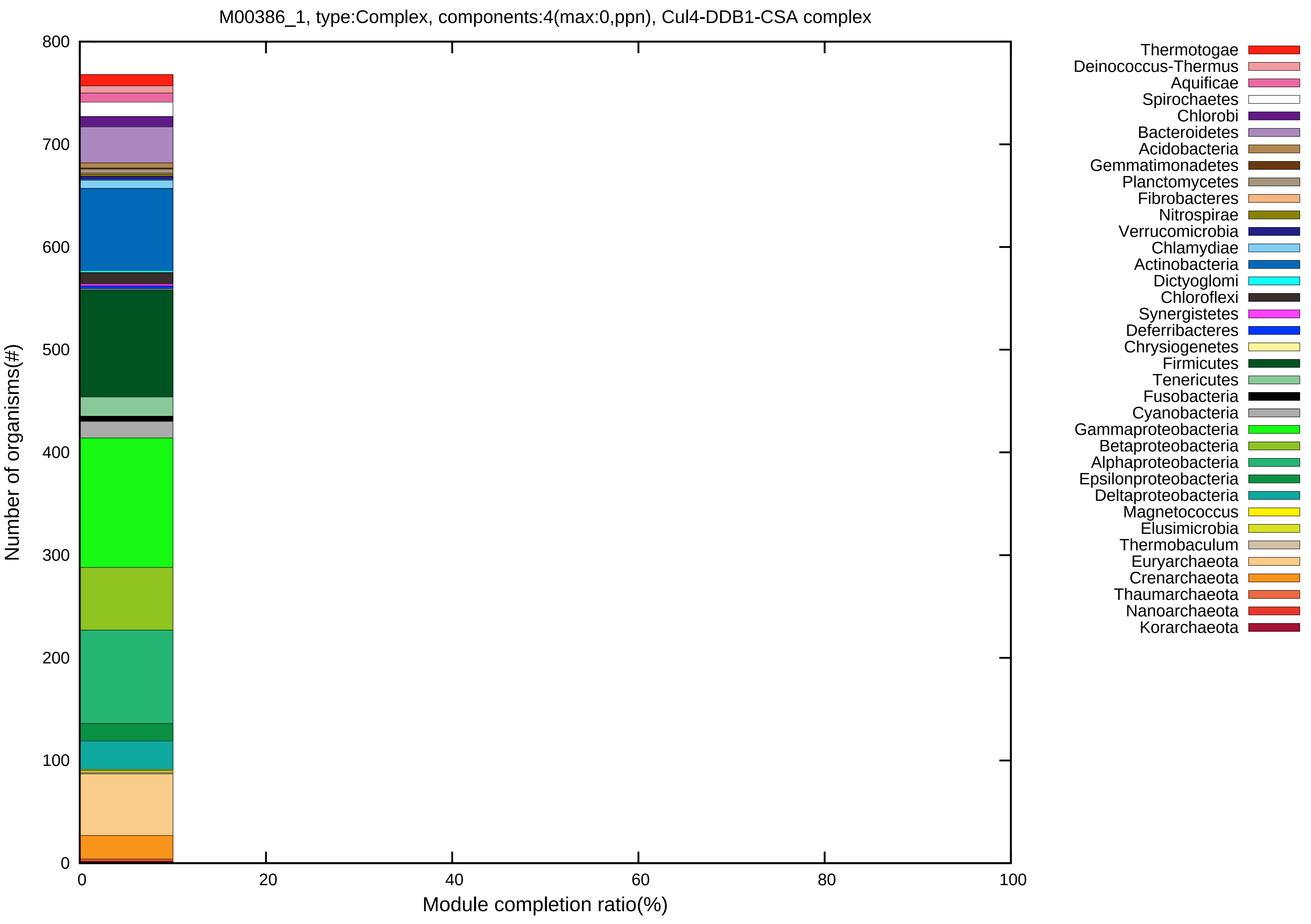

M00386_1, type:Complex, components:4(max:0,ppn), Cul4-DDB1-CSA complex
Number of organisms(#)
Module completion ratio(%)
0
100
200
300
400
500
600
700
800
0
20
40
60
80
100
Thermotogae
Deinococcus-Thermus
Aquificae
Spirochaetes
Chlorobi
Bacteroidetes
Acidobacteria
Gemmatimonadetes
Planctomycetes
Fibrobacteres
Nitrospirae
Verrucomicrobia
Chlamydiae
Actinobacteria
Dictyoglomi
Chloroflexi
Synergistetes
Deferribacteres
Chrysiogenetes
Firmicutes
Tenericutes
Fusobacteria
Cyanobacteria
Gammaproteobacteria
Betaproteobacteria
Alphaproteobacteria
Epsilonproteobacteria
Deltaproteobacteria
Magnetococcus
Elusimicrobia
Thermobaculum
Euryarchaeota
Crenarchaeota
Thaumarchaeota
Nanoarchaeota
Korarchaeota

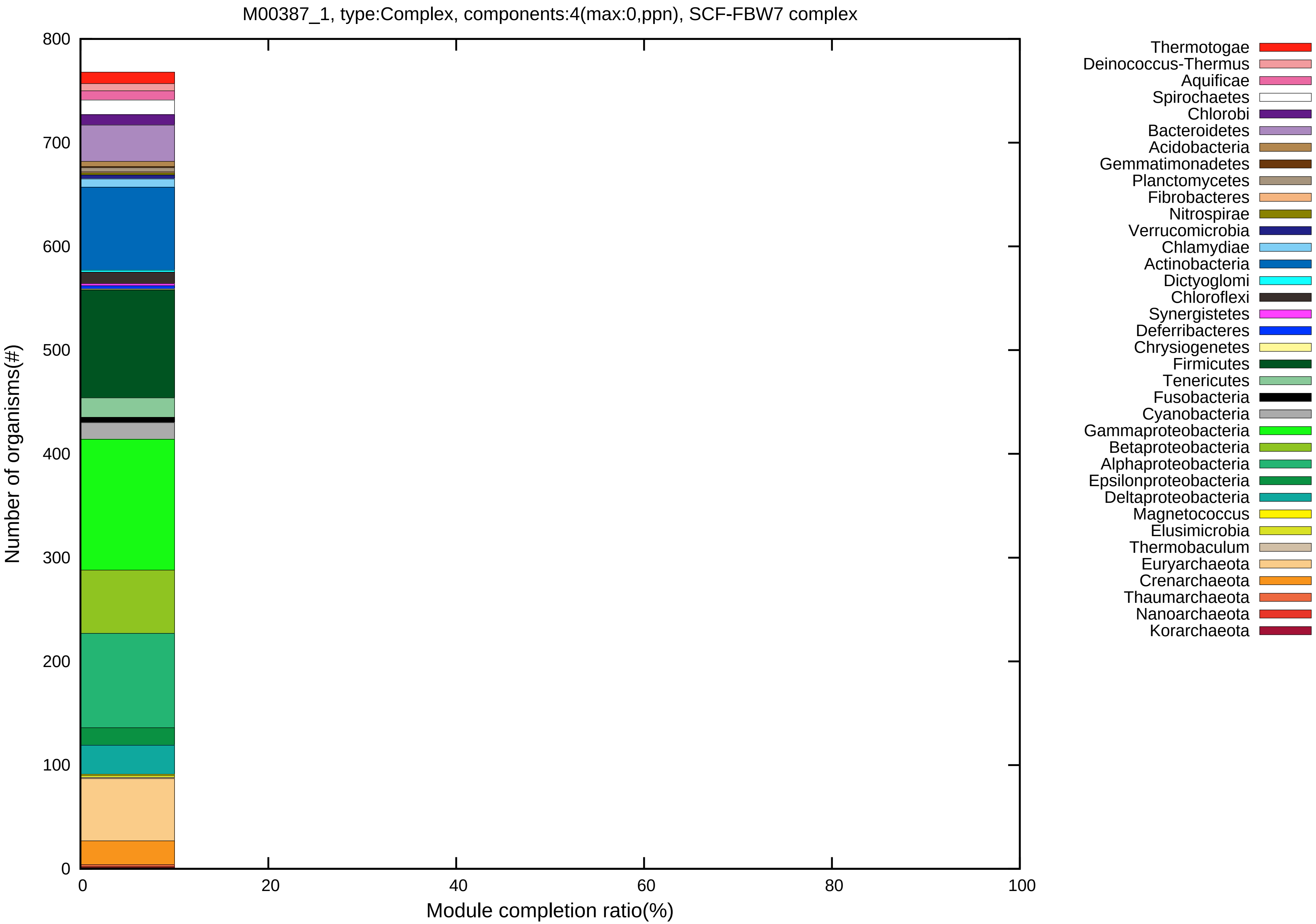

M00387_1, type:Complex, components:4(max:0,ppn), SCF-FBW7 complex
Number of organisms(#)
Module completion ratio(%)
0
100
200
300
400
500
600
700
800
0
20
40
60
80
100
Thermotogae
Deinococcus-Thermus
Aquificae
Spirochaetes
Chlorobi
Bacteroidetes
Acidobacteria
Gemmatimonadetes
Planctomycetes
Fibrobacteres
Nitrospirae
Verrucomicrobia
Chlamydiae
Actinobacteria
Dictyoglomi
Chloroflexi
Synergistetes
Deferribacteres
Chrysiogenetes
Firmicutes
Tenericutes
Fusobacteria
Cyanobacteria
Gammaproteobacteria
Betaproteobacteria
Alphaproteobacteria
Epsilonproteobacteria
Deltaproteobacteria
Magnetococcus
Elusimicrobia
Thermobaculum
Euryarchaeota
Crenarchaeota
Thaumarchaeota
Nanoarchaeota
Korarchaeota

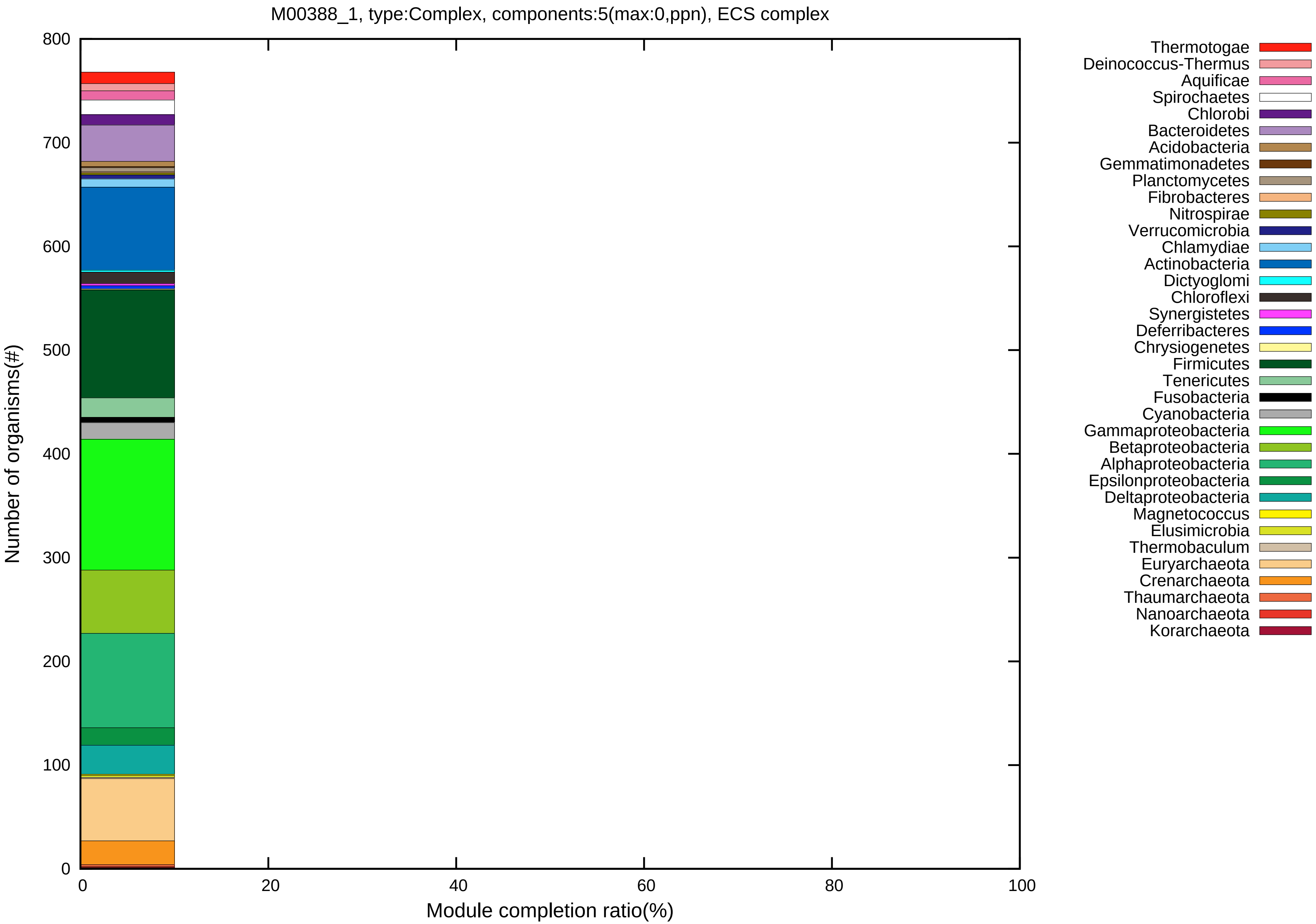

M00388_1, type:Complex, components:5(max:0,ppn), ECS complex
Number of organisms(#)
Module completion ratio(%)
0
100
200
300
400
500
600
700
800
0
20
40
60
80
100
Thermotogae
Deinococcus-Thermus
Aquificae
Spirochaetes
Chlorobi
Bacteroidetes
Acidobacteria
Gemmatimonadetes
Planctomycetes
Fibrobacteres
Nitrospirae
Verrucomicrobia
Chlamydiae
Actinobacteria
Dictyoglomi
Chloroflexi
Synergistetes
Deferribacteres
Chrysiogenetes
Firmicutes
Tenericutes
Fusobacteria
Cyanobacteria
Gammaproteobacteria
Betaproteobacteria
Alphaproteobacteria
Epsilonproteobacteria
Deltaproteobacteria
Magnetococcus
Elusimicrobia
Thermobaculum
Euryarchaeota
Crenarchaeota
Thaumarchaeota
Nanoarchaeota
Korarchaeota

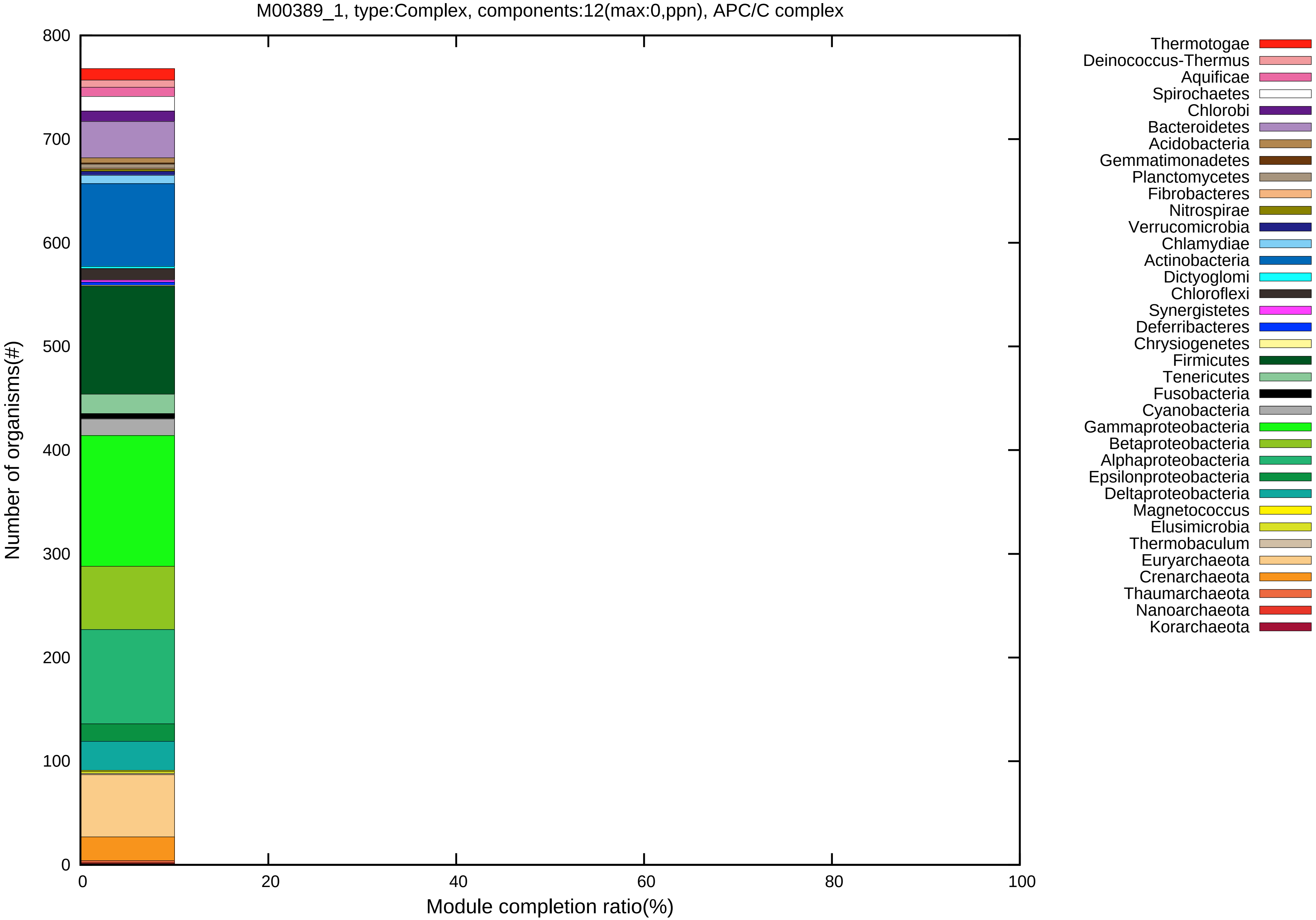
M00389_1, type:Complex, components:12(max:0,ppn), APC/C complex
Number of organisms(#)
Module completion ratio(%)
0
100
200
300
400
500
600
700
800
0
20
40
60
80
100
Thermotogae
Deinococcus-Thermus
Aquificae
Spirochaetes
Chlorobi
Bacteroidetes
Acidobacteria
Gemmatimonadetes
Planctomycetes
Fibrobacteres
Nitrospirae
Verrucomicrobia
Chlamydiae
Actinobacteria
Dictyoglomi
Chloroflexi
Synergistetes
Deferribacteres
Chrysiogenetes
Firmicutes
Tenericutes
Fusobacteria
Cyanobacteria
Gammaproteobacteria
Betaproteobacteria
Alphaproteobacteria
Epsilonproteobacteria
Deltaproteobacteria
Magnetococcus
Elusimicrobia
Thermobaculum
Euryarchaeota
Crenarchaeota
Thaumarchaeota
Nanoarchaeota
Korarchaeota

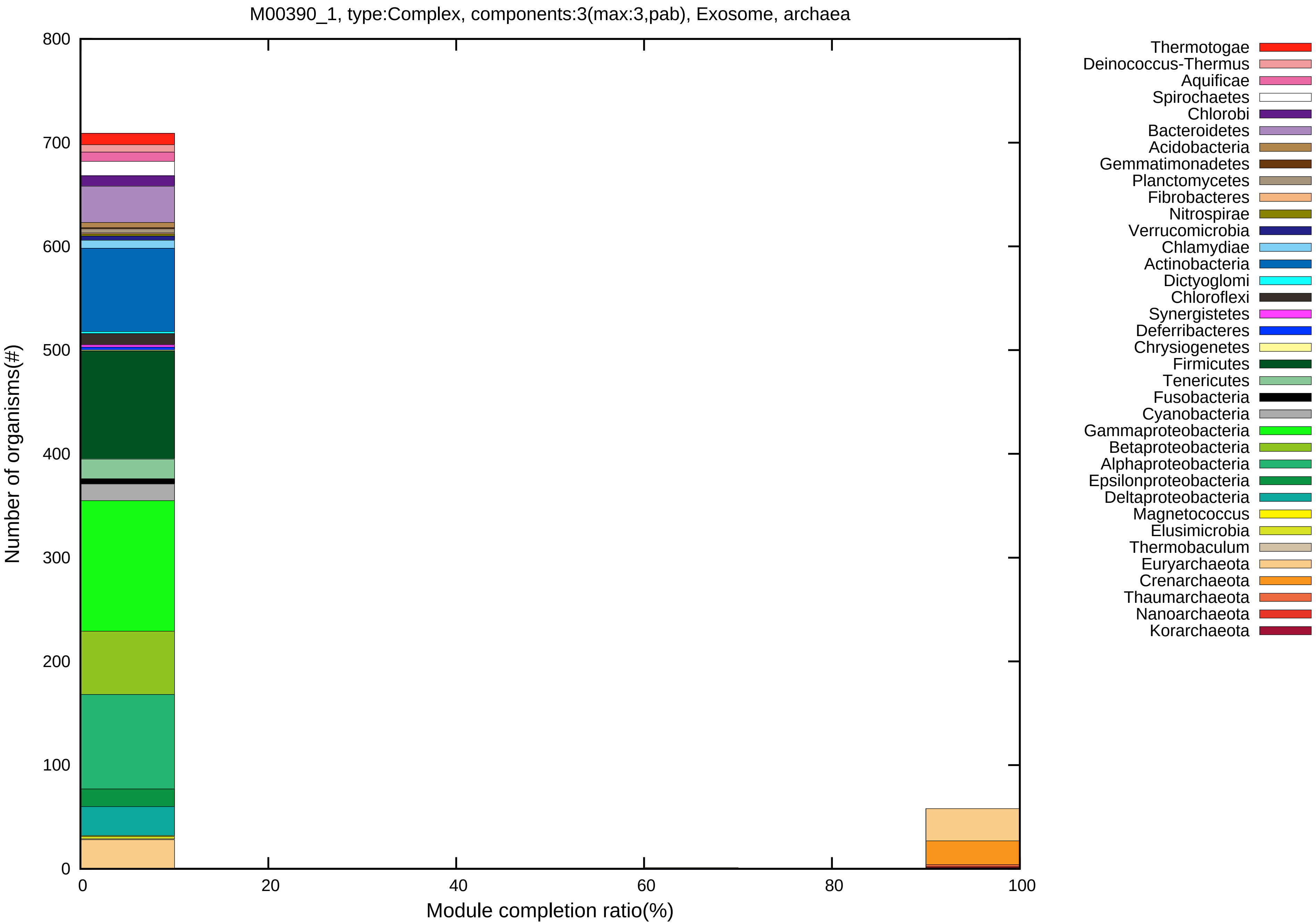
M00390_1, type:Complex, components:3(max:3,pab), Exosome, archaea
Number of organisms(#)
Module completion ratio(%)
0
100
200
300
400
500
600
700
800
0
20
40
60
80
100
Thermotogae
Deinococcus-Thermus
Aquificae
Spirochaetes
Chlorobi
Bacteroidetes
Acidobacteria
Gemmatimonadetes
Planctomycetes
Fibrobacteres
Nitrospirae
Verrucomicrobia
Chlamydiae
Actinobacteria
Dictyoglomi
Chloroflexi
Synergistetes
Deferribacteres
Chrysiogenetes
Firmicutes
Tenericutes
Fusobacteria
Cyanobacteria
Gammaproteobacteria
Betaproteobacteria
Alphaproteobacteria
Epsilonproteobacteria
Deltaproteobacteria
Magnetococcus
Elusimicrobia
Thermobaculum
Euryarchaeota
Crenarchaeota
Thaumarchaeota
Nanoarchaeota
Korarchaeota

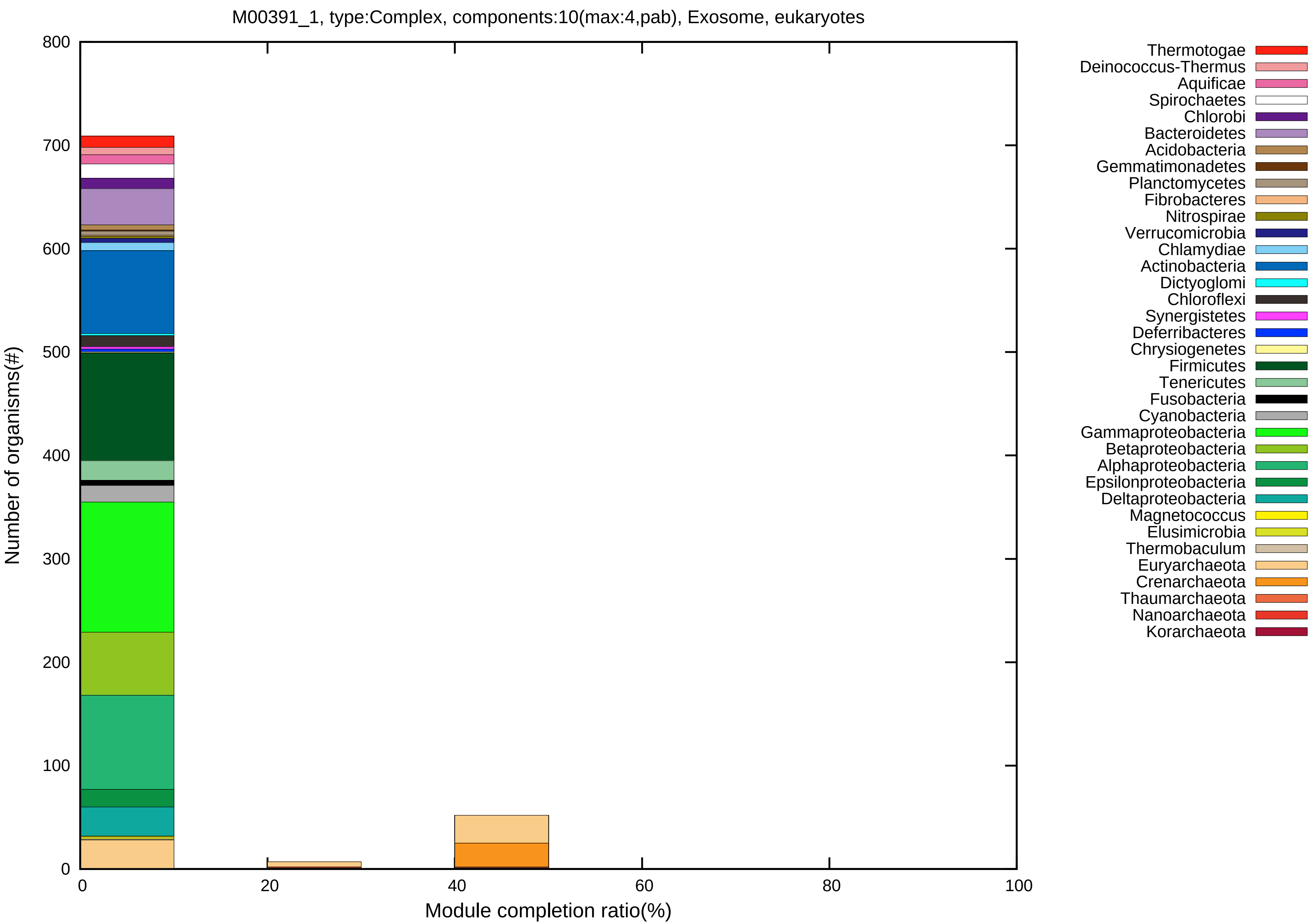

M00391_1, type:Complex, components:10(max:4,pab), Exosome, eukaryotes
Number of organisms(#)
Module completion ratio(%)
0
100
200
300
400
500
600
700
800
0
20
40
60
80
100
Thermotogae
Deinococcus-Thermus
Aquificae
Spirochaetes
Chlorobi
Bacteroidetes
Acidobacteria
Gemmatimonadetes
Planctomycetes
Fibrobacteres
Nitrospirae
Verrucomicrobia
Chlamydiae
Actinobacteria
Dictyoglomi
Chloroflexi
Synergistetes
Deferribacteres
Chrysiogenetes
Firmicutes
Tenericutes
Fusobacteria
Cyanobacteria
Gammaproteobacteria
Betaproteobacteria
Alphaproteobacteria
Epsilonproteobacteria
Deltaproteobacteria
Magnetococcus
Elusimicrobia
Thermobaculum
Euryarchaeota
Crenarchaeota
Thaumarchaeota
Nanoarchaeota
Korarchaeota

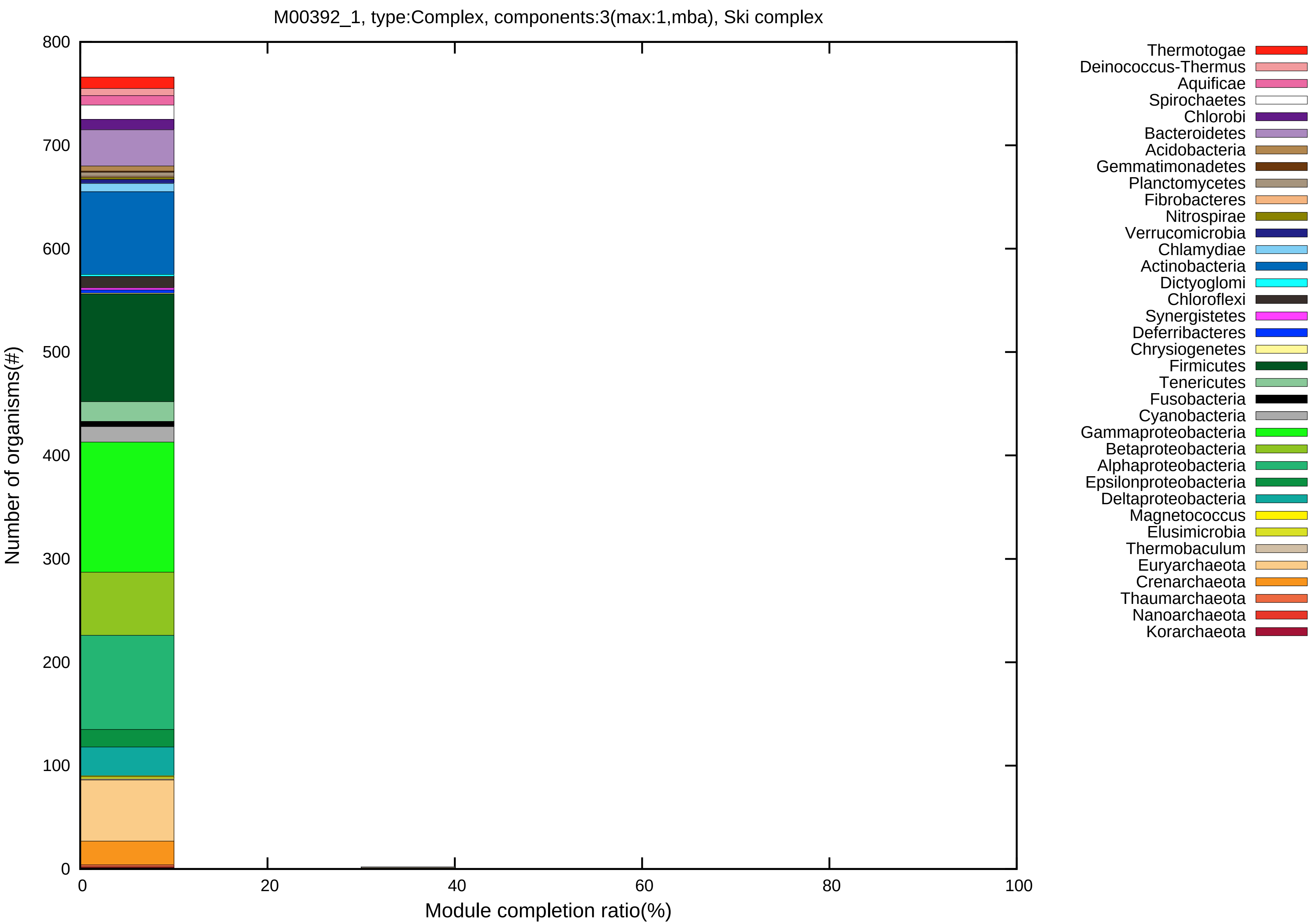

M00392_1, type:Complex, components:3(max:1,mba), Ski complex
Number of organisms(#)
Module completion ratio(%)
0
20
40
60
80
100
0
100
200
300
400
500
600
700
800
Thermotogae
Deinococcus-Thermus
Aquificae
Spirochaetes
Chlorobi
Bacteroidetes
Acidobacteria
Gemmatimonadetes
Planctomycetes
Fibrobacteres
Nitrospirae
Verrucomicrobia
Chlamydiae
Actinobacteria
Dictyoglomi
Chloroflexi
Synergistetes
Deferribacteres
Chrysiogenetes
Firmicutes
Tenericutes
Fusobacteria
Cyanobacteria
Gammaproteobacteria
Betaproteobacteria
Alphaproteobacteria
Epsilonproteobacteria
Deltaproteobacteria
Magnetococcus
Elusimicrobia
Thermobaculum
Euryarchaeota
Crenarchaeota
Thaumarchaeota
Nanoarchaeota
Korarchaeota

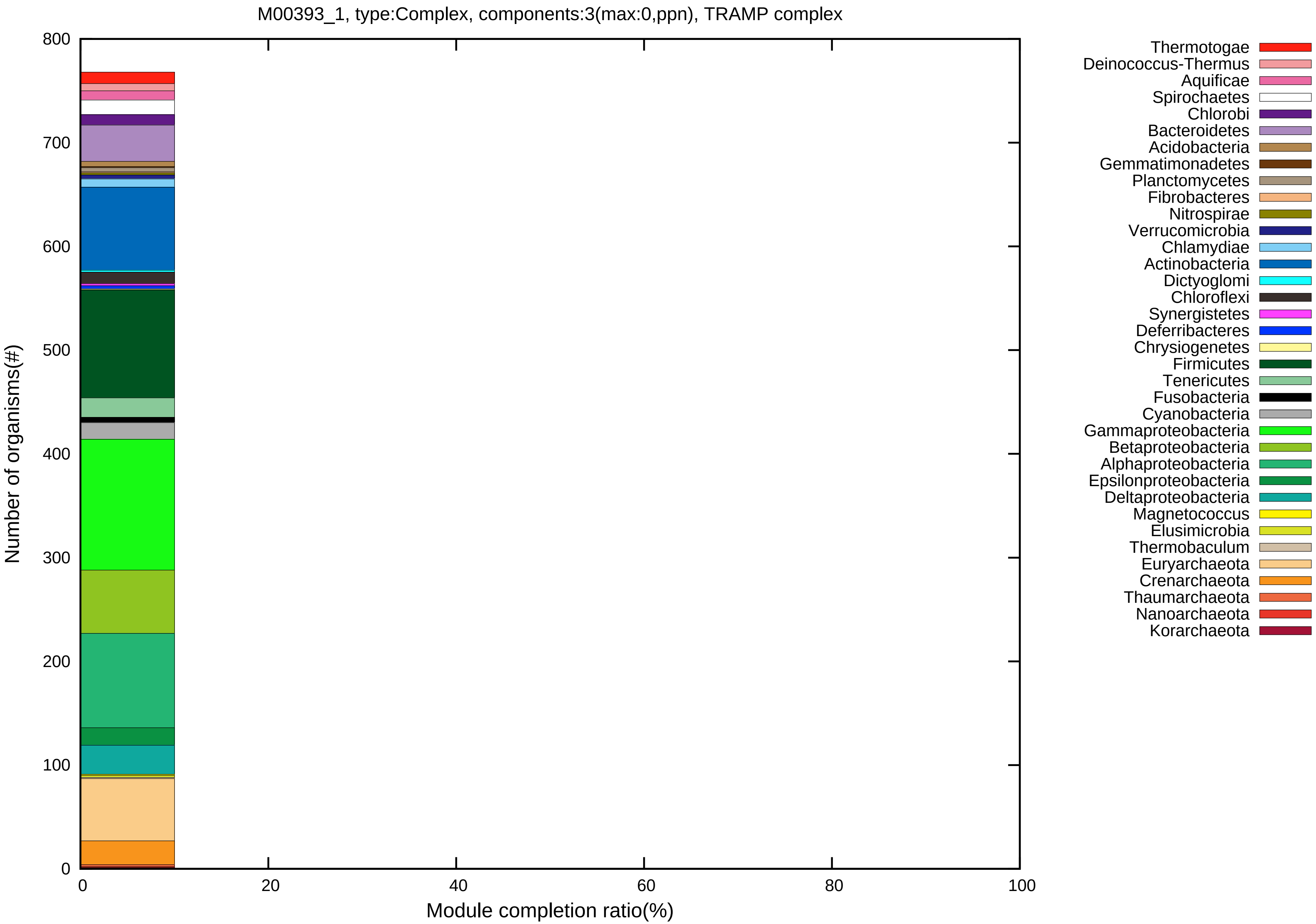

M00393_1, type:Complex, components:3(max:0,ppn), TRAMP complex
Number of organisms(#)
Module completion ratio(%)
0
100
200
300
400
500
600
700
800
0
20
40
60
80
100
Thermotogae
Deinococcus-Thermus
Aquificae
Spirochaetes
Chlorobi
Bacteroidetes
Acidobacteria
Gemmatimonadetes
Planctomycetes
Fibrobacteres
Nitrospirae
Verrucomicrobia
Chlamydiae
Actinobacteria
Dictyoglomi
Chloroflexi
Synergistetes
Deferribacteres
Chrysiogenetes
Firmicutes
Tenericutes
Fusobacteria
Cyanobacteria
Gammaproteobacteria
Betaproteobacteria
Alphaproteobacteria
Epsilonproteobacteria
Deltaproteobacteria
Magnetococcus
Elusimicrobia
Thermobaculum
Euryarchaeota
Crenarchaeota
Thaumarchaeota
Nanoarchaeota
Korarchaeota

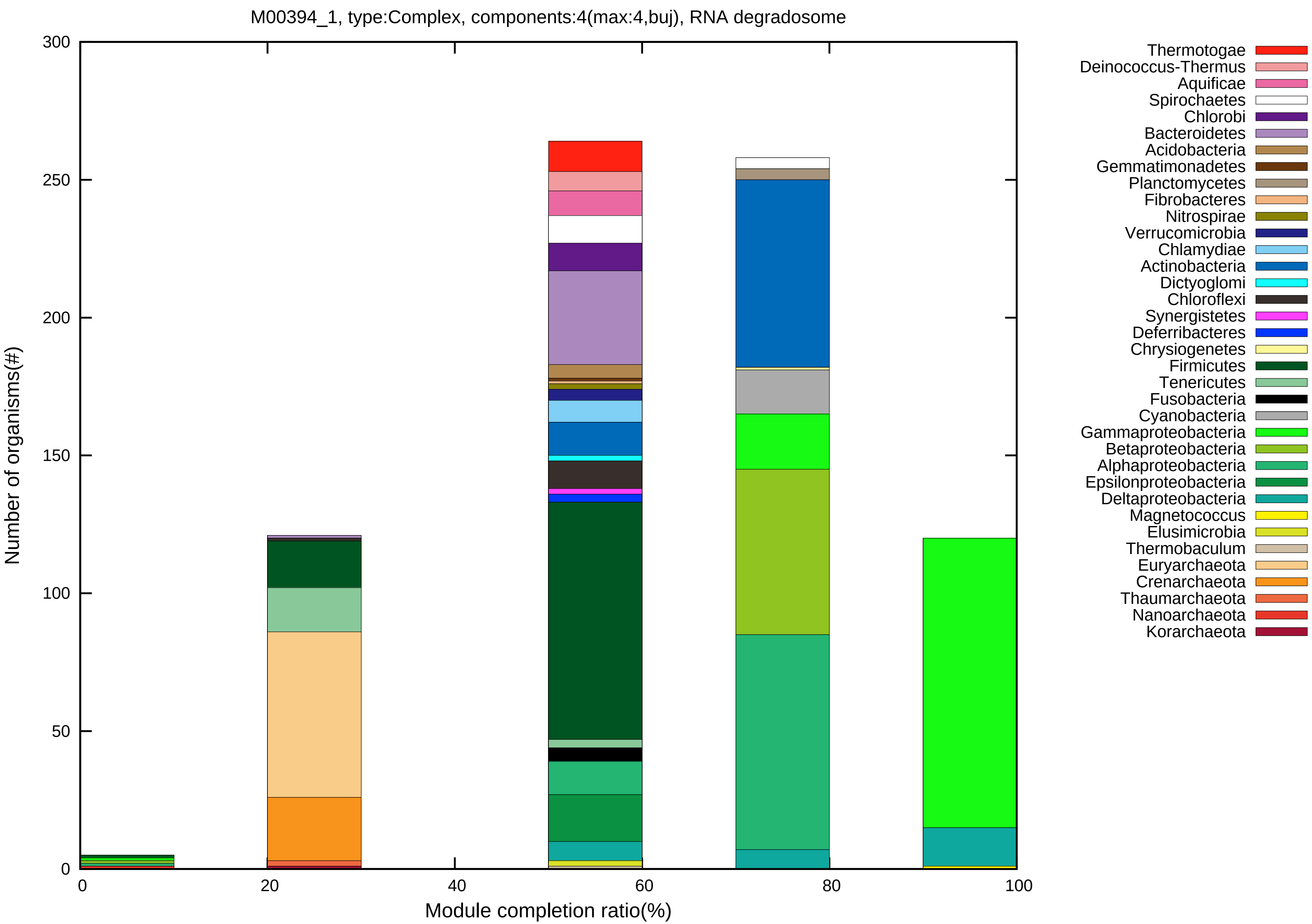
M00394_1, type:Complex, components:4(max:4,buj), RNA degradosome
Number of organisms(#)
0
50
100
150
200
250
300
0
20
40
60
80
100
Module completion ratio(%)
Thermotogae
Deinococcus-Thermus
Aquificae
Spirochaetes
Chlorobi
Bacteroidetes
Acidobacteria
Gemmatimonadetes
Planctomycetes
Fibrobacteres
Nitrospirae
Verrucomicrobia
Chlamydiae
Actinobacteria
Dictyoglomi
Chloroflexi
Synergistetes
Deferribacteres
Chrysiogenetes
Firmicutes
Tenericutes
Fusobacteria
Cyanobacteria
Gammaproteobacteria
Betaproteobacteria
Alphaproteobacteria
Epsilonproteobacteria
Deltaproteobacteria
Magnetococcus
Elusimicrobia
Thermobaculum
Euryarchaeota
Crenarchaeota
Thaumarchaeota
Nanoarchaeota
Korarchaeota

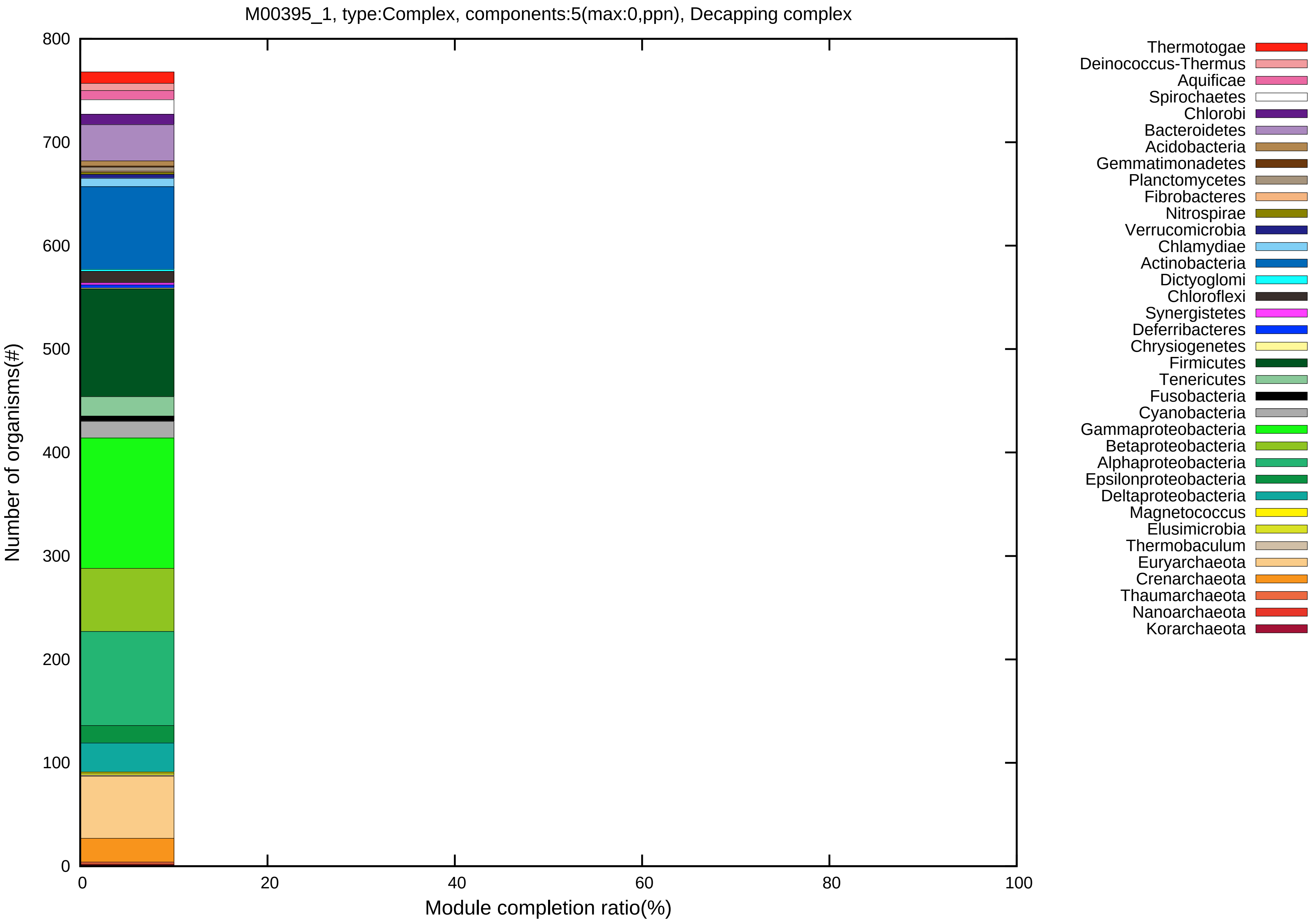
M00395_1, type:Complex, components:5(max:0,ppn), Decapping complex
Number of organisms(#)
Module completion ratio(%)
0
100
200
300
400
500
600
700
800
0
20
40
60
80
100
Thermotogae
Deinococcus-Thermus
Aquificae
Spirochaetes
Chlorobi
Bacteroidetes
Acidobacteria
Gemmatimonadetes
Planctomycetes
Fibrobacteres
Nitrospirae
Verrucomicrobia
Chlamydiae
Actinobacteria
Dictyoglomi
Chloroflexi
Synergistetes
Deferribacteres
Chrysiogenetes
Firmicutes
Tenericutes
Fusobacteria
Cyanobacteria
Gammaproteobacteria
Betaproteobacteria
Alphaproteobacteria
Epsilonproteobacteria
Deltaproteobacteria
Magnetococcus
Elusimicrobia
Thermobaculum
Euryarchaeota
Crenarchaeota
Thaumarchaeota
Nanoarchaeota
Korarchaeota

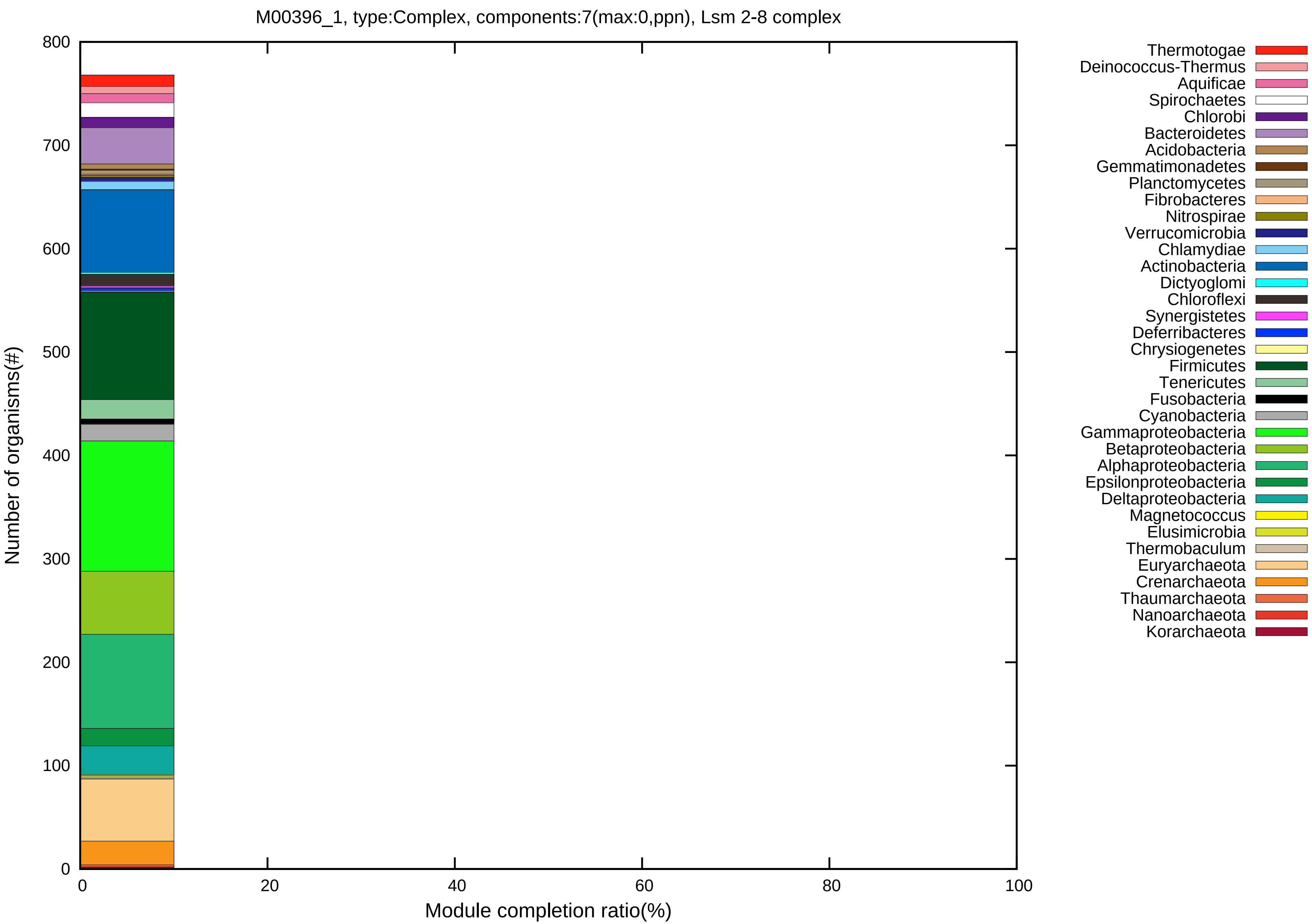

M00396_1, type:Complex, components:7(max:0,ppn), Lsm 2-8 complex
Number of organisms(#)
Module completion ratio(%)
0
100
200
300
400
500
600
700
800
0
20
40
60
80
100
Thermotogae
Deinococcus-Thermus
Aquificae
Spirochaetes
Chlorobi
Bacteroidetes
Acidobacteria
Gemmatimonadetes
Planctomycetes
Fibrobacteres
Nitrospirae
Verrucomicrobia
Chlamydiae
Actinobacteria
Dictyoglomi
Chloroflexi
Synergistetes
Deferribacteres
Chrysiogenetes
Firmicutes
Tenericutes
Fusobacteria
Cyanobacteria
Gammaproteobacteria
Betaproteobacteria
Alphaproteobacteria
Epsilonproteobacteria
Deltaproteobacteria
Magnetococcus
Elusimicrobia
Thermobaculum
Euryarchaeota
Crenarchaeota
Thaumarchaeota
Nanoarchaeota
Korarchaeota

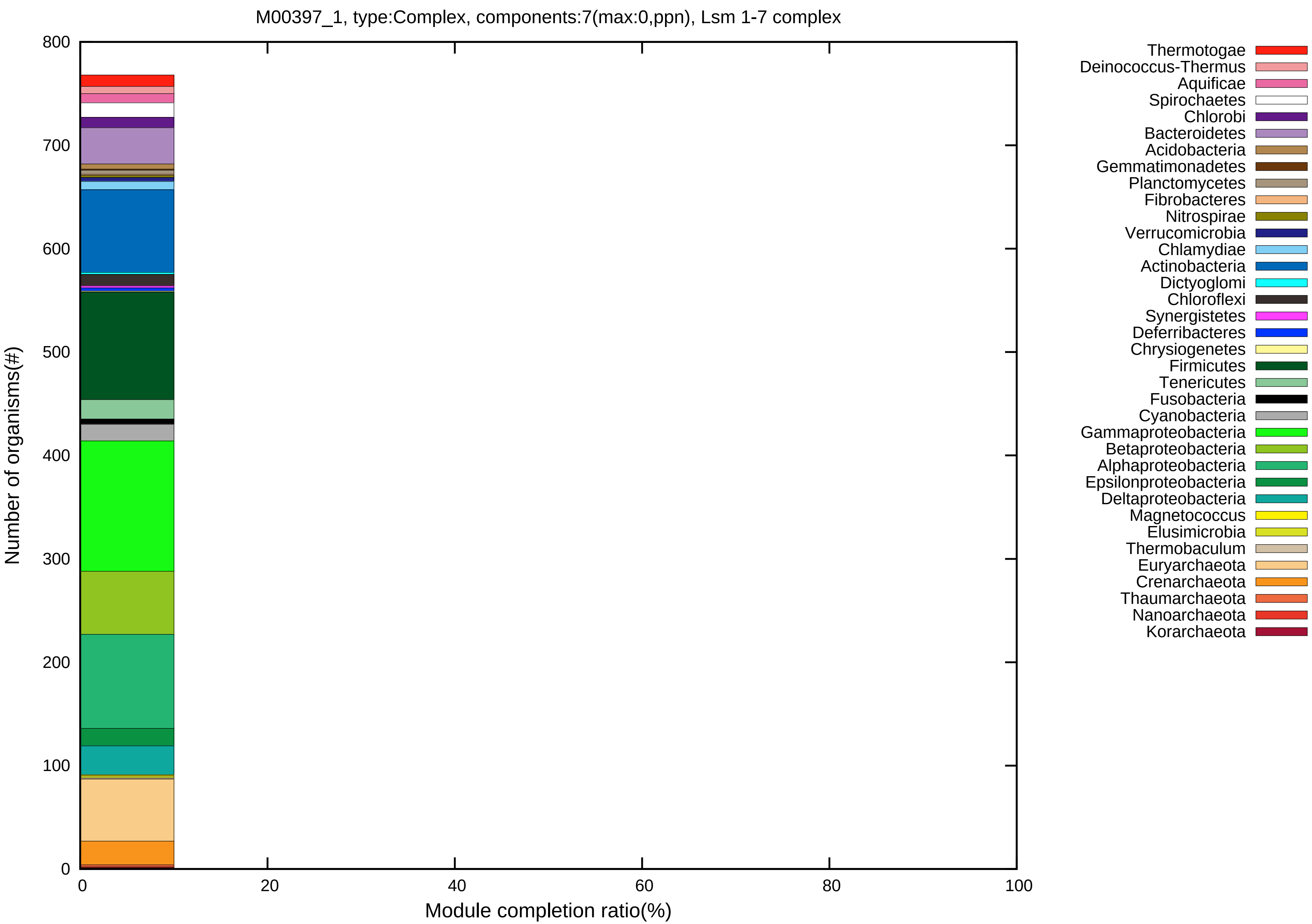
M00397_1, type:Complex, components:7(max:0,ppn), Lsm 1-7 complex
Number of organisms(#)
0
100
200
300
400
500
600
700
800
0
20
40
60
80
100
Module completion ratio(%)
Thermotogae
Deinococcus-Thermus
Aquificae
Spirochaetes
Chlorobi
Bacteroidetes
Acidobacteria
Gemmatimonadetes
Planctomycetes
Fibrobacteres
Nitrospirae
Verrucomicrobia
Chlamydiae
Actinobacteria
Dictyoglomi
Chloroflexi
Synergistetes
Deferribacteres
Chrysiogenetes
Firmicutes
Tenericutes
Fusobacteria
Cyanobacteria
Gammaproteobacteria
Betaproteobacteria
Alphaproteobacteria
Epsilonproteobacteria
Deltaproteobacteria
Magnetococcus
Elusimicrobia
Thermobaculum
Euryarchaeota
Crenarchaeota
Thaumarchaeota
Nanoarchaeota
Korarchaeota

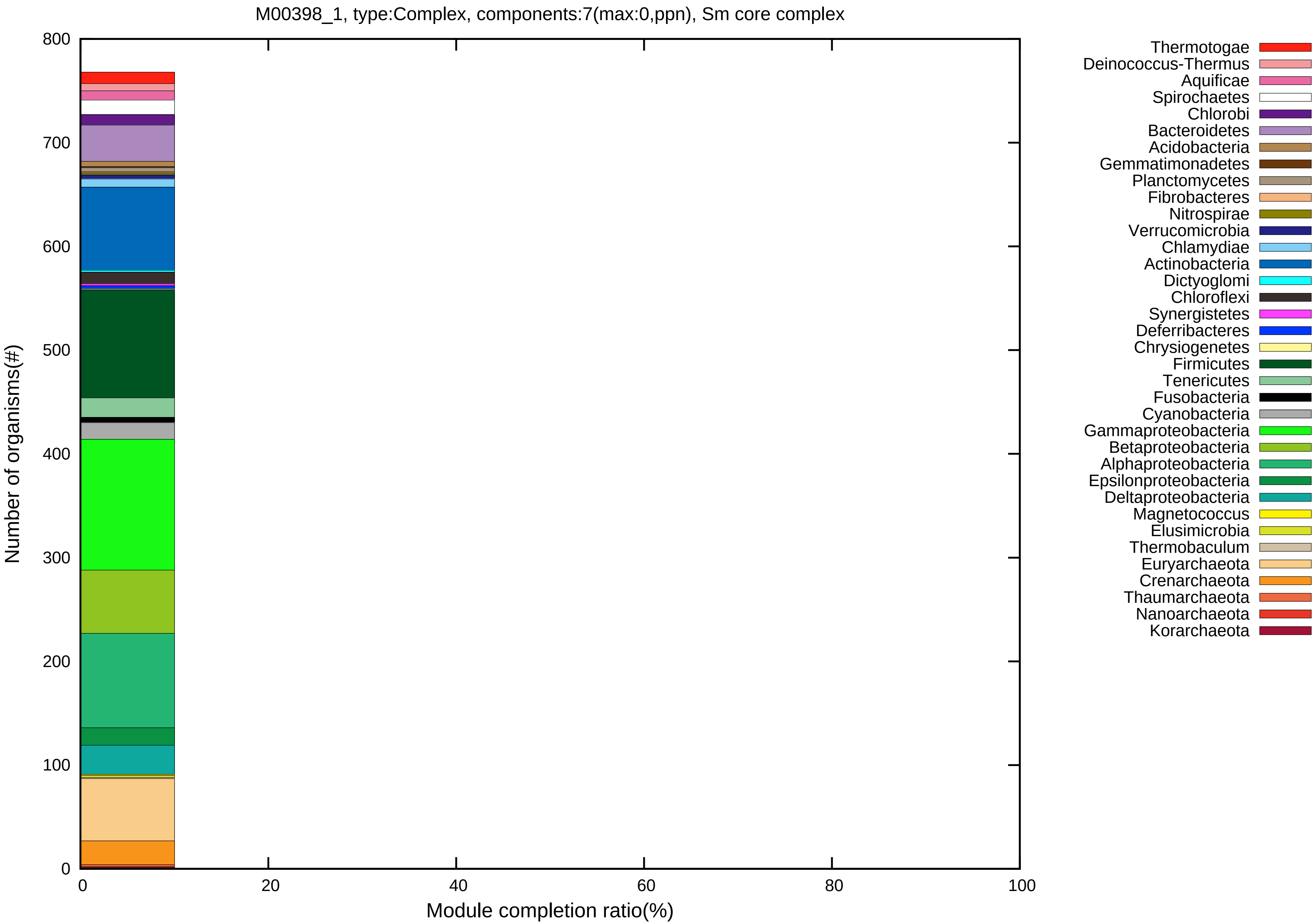
M00398_1, type:Complex, components:7(max:0,ppn), Sm core complex
Number of organisms(#)
Module completion ratio(%)
0
100
200
300
400
500
600
700
800
0
20
40
60
80
100
Thermotogae
Deinococcus-Thermus
Aquificae
Spirochaetes
Chlorobi
Bacteroidetes
Acidobacteria
Gemmatimonadetes
Planctomycetes
Fibrobacteres
Nitrospirae
Verrucomicrobia
Chlamydiae
Actinobacteria
Dictyoglomi
Chloroflexi
Synergistetes
Deferribacteres
Chrysiogenetes
Firmicutes
Tenericutes
Fusobacteria
Cyanobacteria
Gammaproteobacteria
Betaproteobacteria
Alphaproteobacteria
Epsilonproteobacteria
Deltaproteobacteria
Magnetococcus
Elusimicrobia
Thermobaculum
Euryarchaeota
Crenarchaeota
Thaumarchaeota
Nanoarchaeota
Korarchaeota

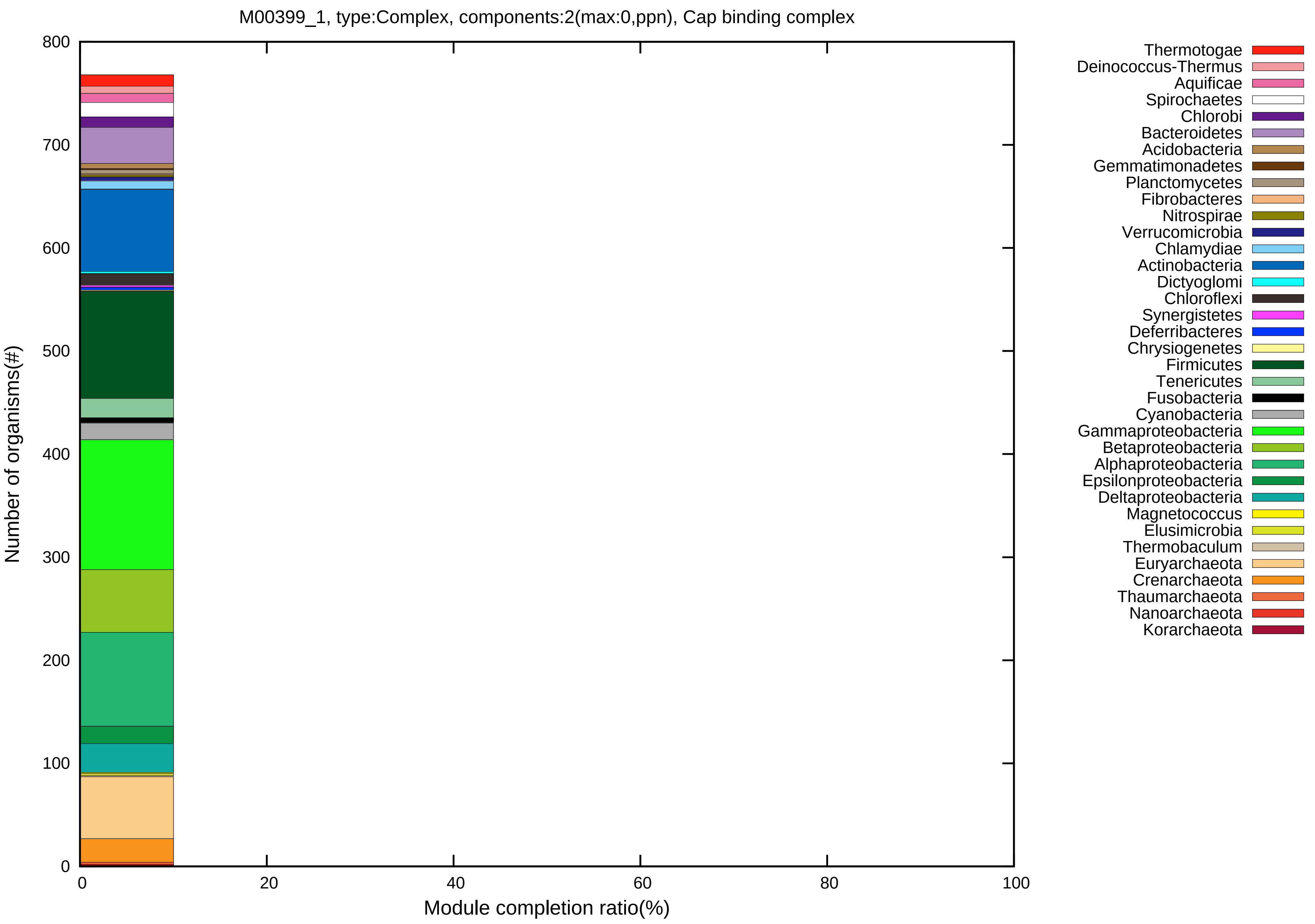
M00399_1, type:Complex, components:2(max:0,ppn), Cap binding complex
Number of organisms(#)
Module completion ratio(%)
0
100
200
300
400
500
600
700
800
0
20
40
60
80
100
Thermotogae
Deinococcus-Thermus
Aquificae
Spirochaetes
Chlorobi
Bacteroidetes
Acidobacteria
Gemmatimonadetes
Planctomycetes
Fibrobacteres
Nitrospirae
Verrucomicrobia
Chlamydiae
Actinobacteria
Dictyoglomi
Chloroflexi
Synergistetes
Deferribacteres
Chrysiogenetes
Firmicutes
Tenericutes
Fusobacteria
Cyanobacteria
Gammaproteobacteria
Betaproteobacteria
Alphaproteobacteria
Epsilonproteobacteria
Deltaproteobacteria
Magnetococcus
Elusimicrobia
Thermobaculum
Euryarchaeota
Crenarchaeota
Thaumarchaeota
Nanoarchaeota
Korarchaeota

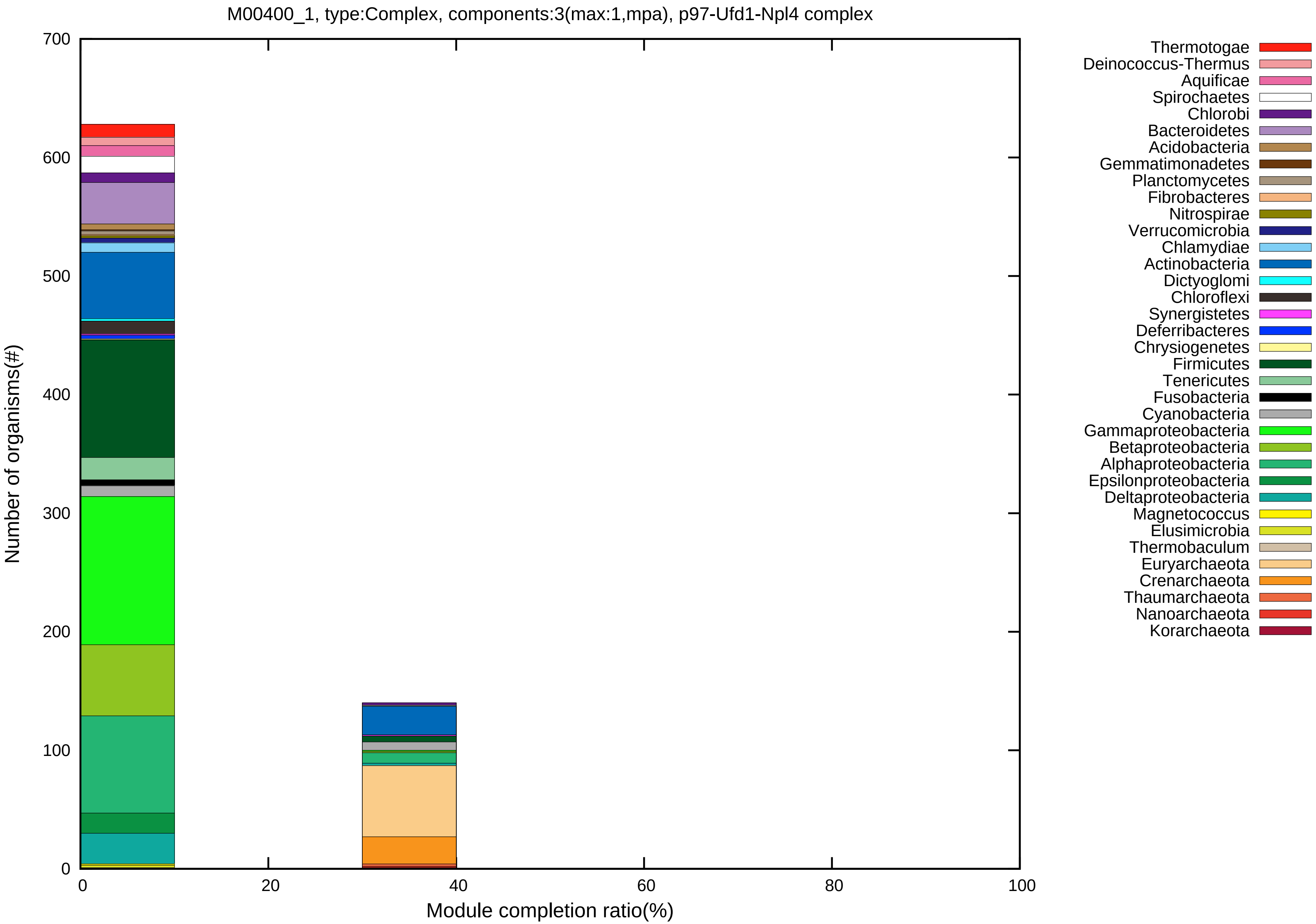
M00400_1, type:Complex, components:3(max:1,mpa), p97-Ufd1-Npl4 complex
Number of organisms(#)
Module completion ratio(%)
0
100
200
300
400
500
600
700
0
20
40
60
80
100
Thermotogae
Deinococcus-Thermus
Aquificae
Spirochaetes
Chlorobi
Bacteroidetes
Acidobacteria
Gemmatimonadetes
Planctomycetes
Fibrobacteres
Nitrospirae
Verrucomicrobia
Chlamydiae
Actinobacteria
Dictyoglomi
Chloroflexi
Synergistetes
Deferribacteres
Chrysiogenetes
Firmicutes
Tenericutes
Fusobacteria
Cyanobacteria
Gammaproteobacteria
Betaproteobacteria
Alphaproteobacteria
Epsilonproteobacteria
Deltaproteobacteria
Magnetococcus
Elusimicrobia
Thermobaculum
Euryarchaeota
Crenarchaeota
Thaumarchaeota
Nanoarchaeota
Korarchaeota

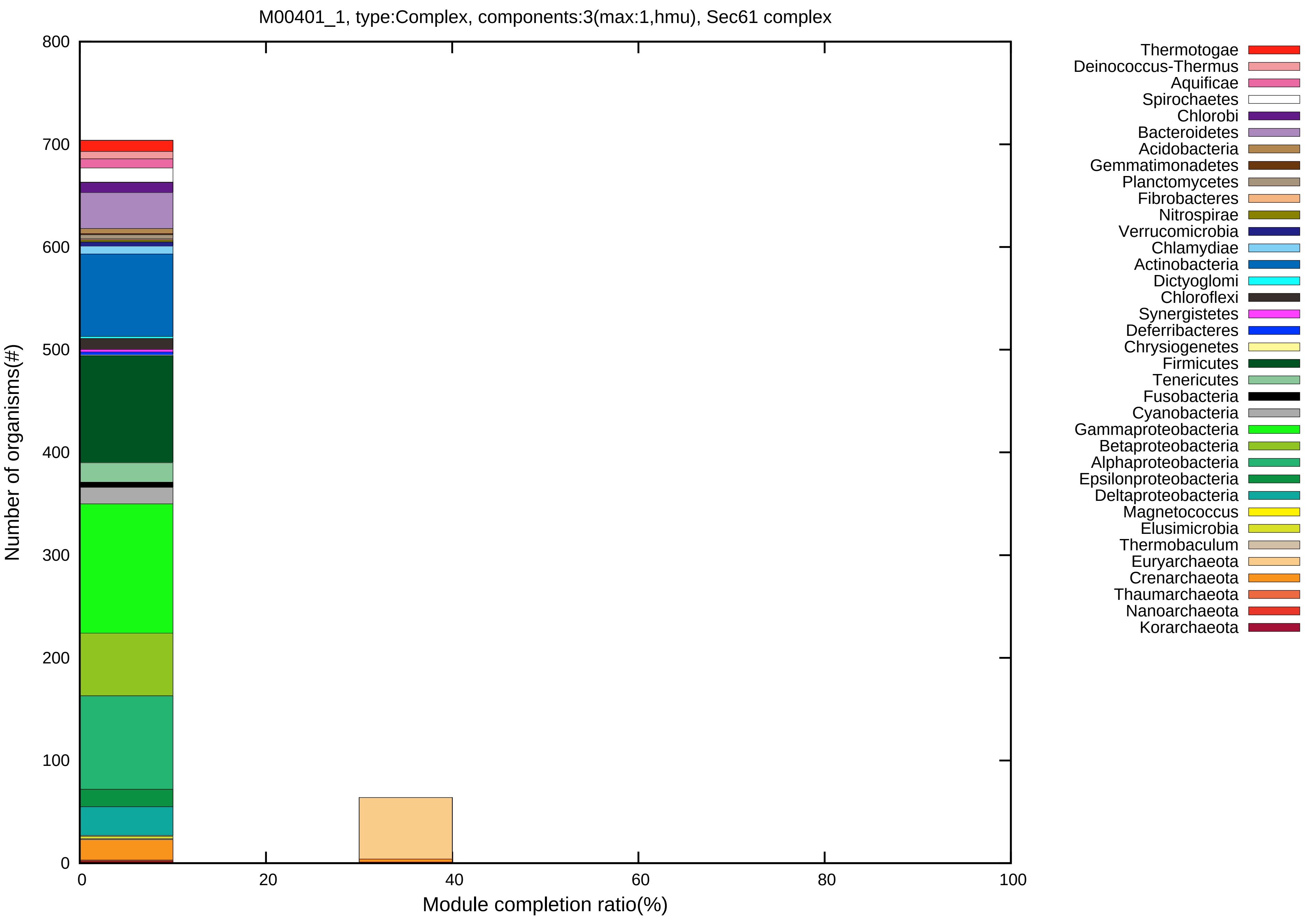

M00401_1, type:Complex, components:3(max:1,hmu), Sec61 complex
Number of organisms(#)
Module completion ratio(%)
0
100
200
300
400
500
600
700
800
0
20
40
60
80
100
Thermotogae
Deinococcus-Thermus
Aquificae
Spirochaetes
Chlorobi
Bacteroidetes
Acidobacteria
Gemmatimonadetes
Planctomycetes
Fibrobacteres
Nitrospirae
Verrucomicrobia
Chlamydiae
Actinobacteria
Dictyoglomi
Chloroflexi
Synergistetes
Deferribacteres
Chrysiogenetes
Firmicutes
Tenericutes
Fusobacteria
Cyanobacteria
Gammaproteobacteria
Betaproteobacteria
Alphaproteobacteria
Epsilonproteobacteria
Deltaproteobacteria
Magnetococcus
Elusimicrobia
Thermobaculum
Euryarchaeota
Crenarchaeota
Thaumarchaeota
Nanoarchaeota
Korarchaeota

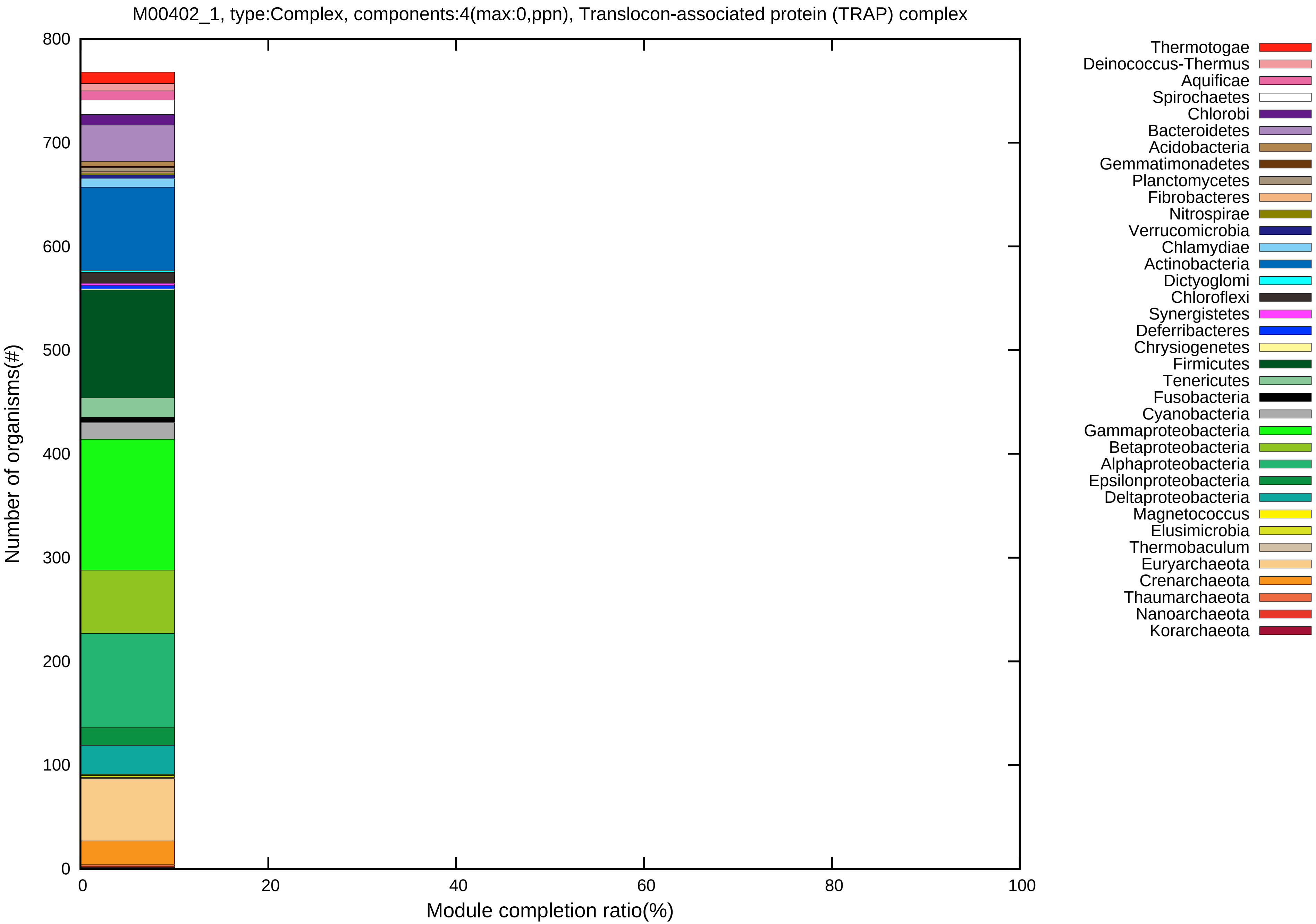

M00402_1, type:Complex, components:4(max:0,ppn), Translocon-associated protein (TRAP) complex
Number of organisms(#)
Module completion ratio(%)
0
100
200
300
400
500
600
700
800
0
20
40
60
80
100
Thermotogae
Deinococcus-Thermus
Aquificae
Spirochaetes
Chlorobi
Bacteroidetes
Acidobacteria
Gemmatimonadetes
Planctomycetes
Fibrobacteres
Nitrospirae
Verrucomicrobia
Chlamydiae
Actinobacteria
Dictyoglomi
Chloroflexi
Synergistetes
Deferribacteres
Chrysiogenetes
Firmicutes
Tenericutes
Fusobacteria
Cyanobacteria
Gammaproteobacteria
Betaproteobacteria
Alphaproteobacteria
Epsilonproteobacteria
Deltaproteobacteria
Magnetococcus
Elusimicrobia
Thermobaculum
Euryarchaeota
Crenarchaeota
Thaumarchaeota
Nanoarchaeota
Korarchaeota

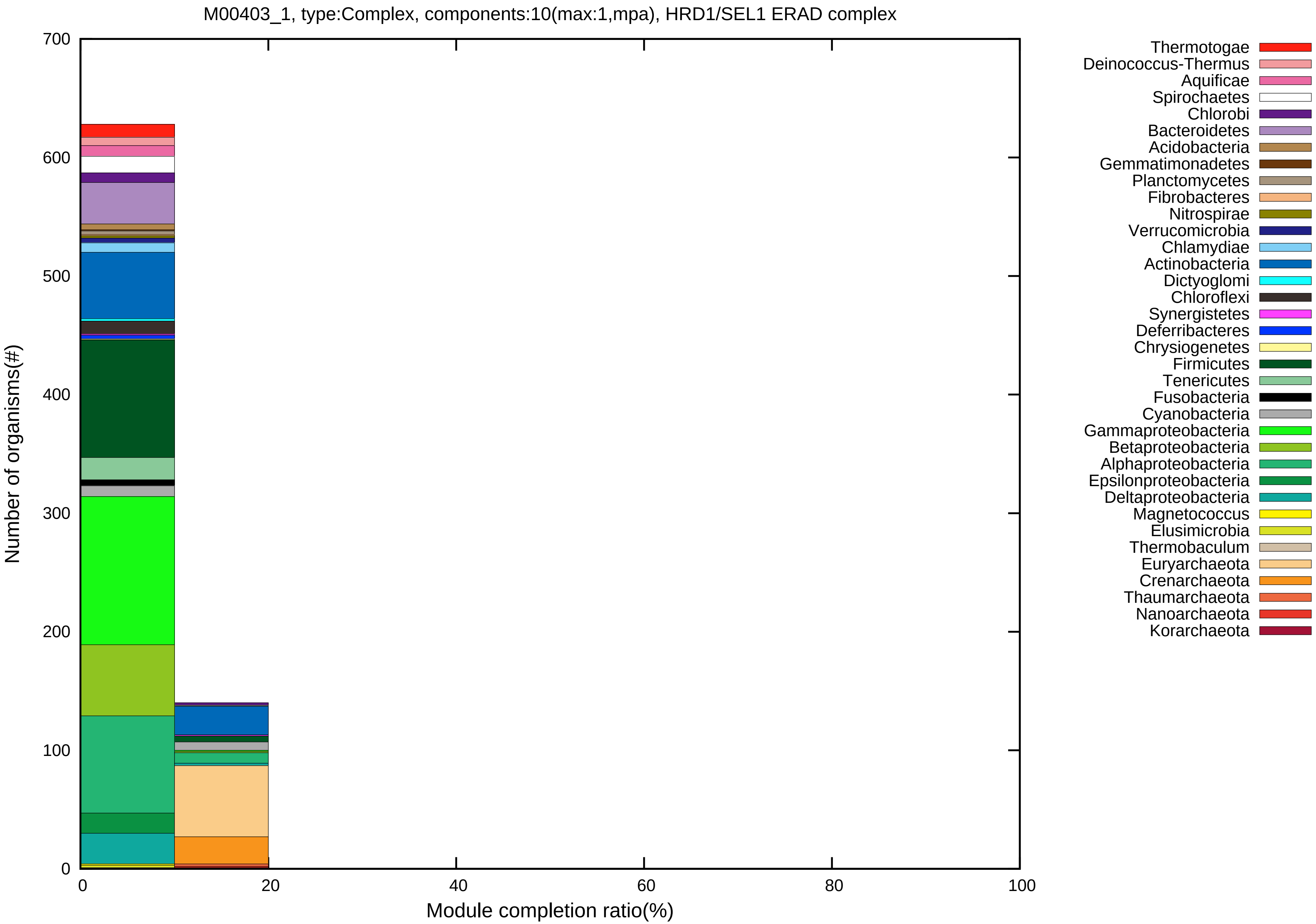
M00403_1, type:Complex, components:10(max:1,mpa), HRD1/SEL1 ERAD complex
Number of organisms(#)
Module completion ratio(%)
0
100
200
300
400
500
600
700
0
20
40
60
80
100
Thermotogae
Deinococcus-Thermus
Aquificae
Spirochaetes
Chlorobi
Bacteroidetes
Acidobacteria
Gemmatimonadetes
Planctomycetes
Fibrobacteres
Nitrospirae
Verrucomicrobia
Chlamydiae
Actinobacteria
Dictyoglomi
Chloroflexi
Synergistetes
Deferribacteres
Chrysiogenetes
Firmicutes
Tenericutes
Fusobacteria
Cyanobacteria
Gammaproteobacteria
Betaproteobacteria
Alphaproteobacteria
Epsilonproteobacteria
Deltaproteobacteria
Magnetococcus
Elusimicrobia
Thermobaculum
Euryarchaeota
Crenarchaeota
Thaumarchaeota
Nanoarchaeota
Korarchaeota

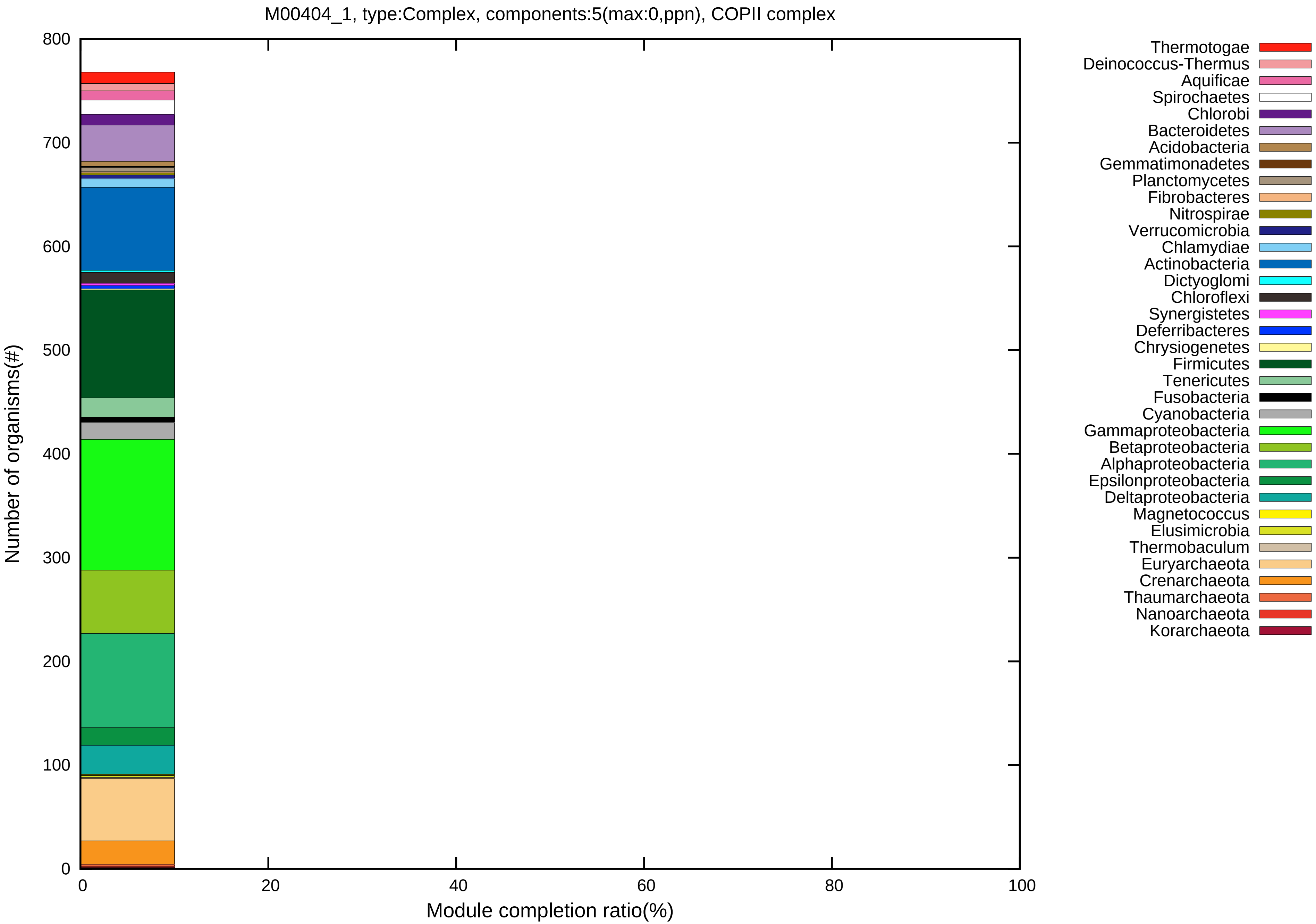

M00404_1, type:Complex, components:5(max:0,ppn), COPII complex
Number of organisms(#)
Module completion ratio(%)
0
100
200
300
400
500
600
700
800
0
20
40
60
80
100
Thermotogae
Deinococcus-Thermus
Aquificae
Spirochaetes
Chlorobi
Bacteroidetes
Acidobacteria
Gemmatimonadetes
Planctomycetes
Fibrobacteres
Nitrospirae
Verrucomicrobia
Chlamydiae
Actinobacteria
Dictyoglomi
Chloroflexi
Synergistetes
Deferribacteres
Chrysiogenetes
Firmicutes
Tenericutes
Fusobacteria
Cyanobacteria
Gammaproteobacteria
Betaproteobacteria
Alphaproteobacteria
Epsilonproteobacteria
Deltaproteobacteria
Magnetococcus
Elusimicrobia
Thermobaculum
Euryarchaeota
Crenarchaeota
Thaumarchaeota
Nanoarchaeota
Korarchaeota

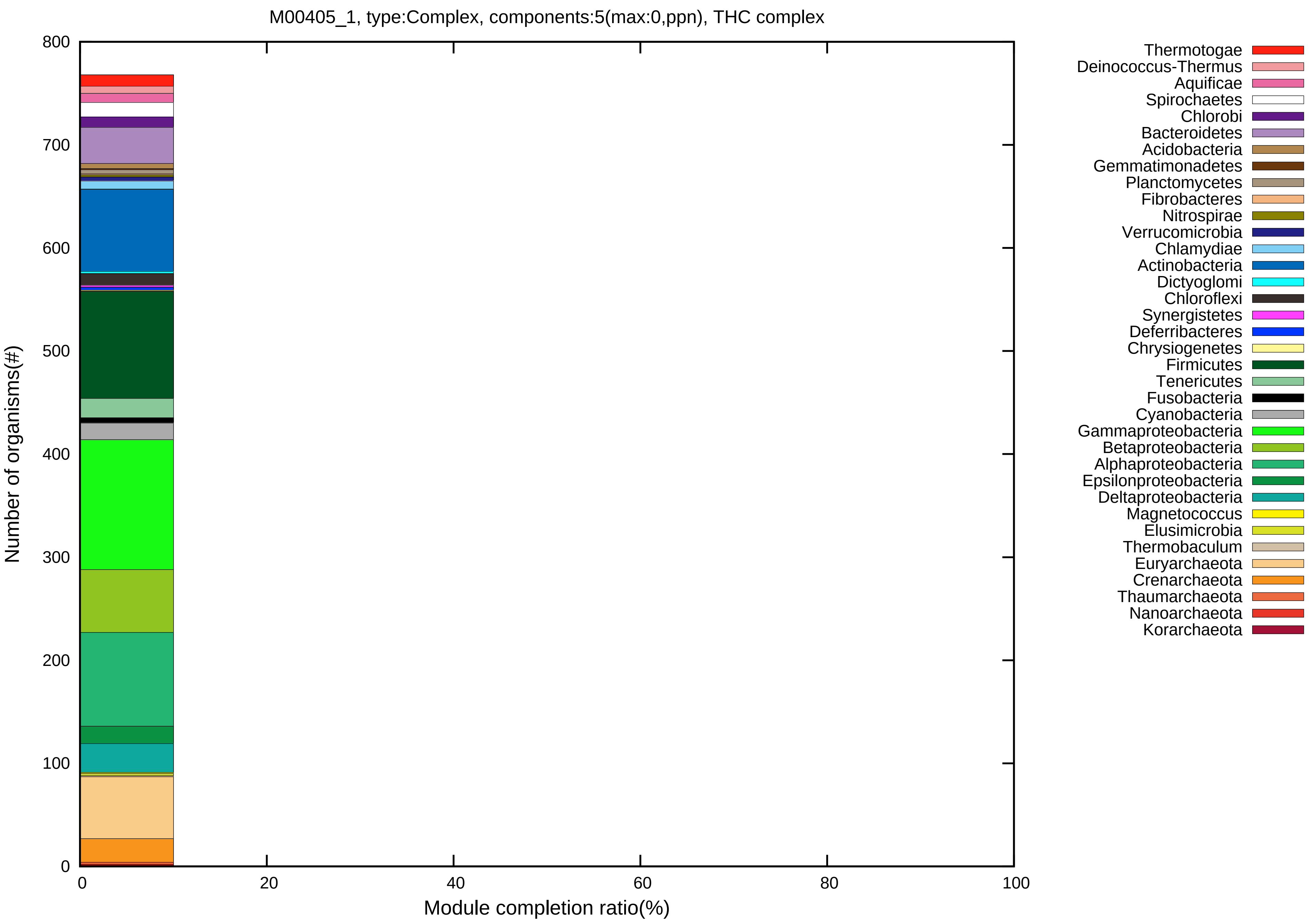
M00405_1, type:Complex, components:5(max:0,ppn), THC complex
Number of organisms(#)
Module completion ratio(%)
0
100
200
300
400
500
600
700
800
0
20
40
60
80
100
Thermotogae
Deinococcus-Thermus
Aquificae
Spirochaetes
Chlorobi
Bacteroidetes
Acidobacteria
Gemmatimonadetes
Planctomycetes
Fibrobacteres
Nitrospirae
Verrucomicrobia
Chlamydiae
Actinobacteria
Dictyoglomi
Chloroflexi
Synergistetes
Deferribacteres
Chrysiogenetes
Firmicutes
Tenericutes
Fusobacteria
Cyanobacteria
Gammaproteobacteria
Betaproteobacteria
Alphaproteobacteria
Epsilonproteobacteria
Deltaproteobacteria
Magnetococcus
Elusimicrobia
Thermobaculum
Euryarchaeota
Crenarchaeota
Thaumarchaeota
Nanoarchaeota
Korarchaeota

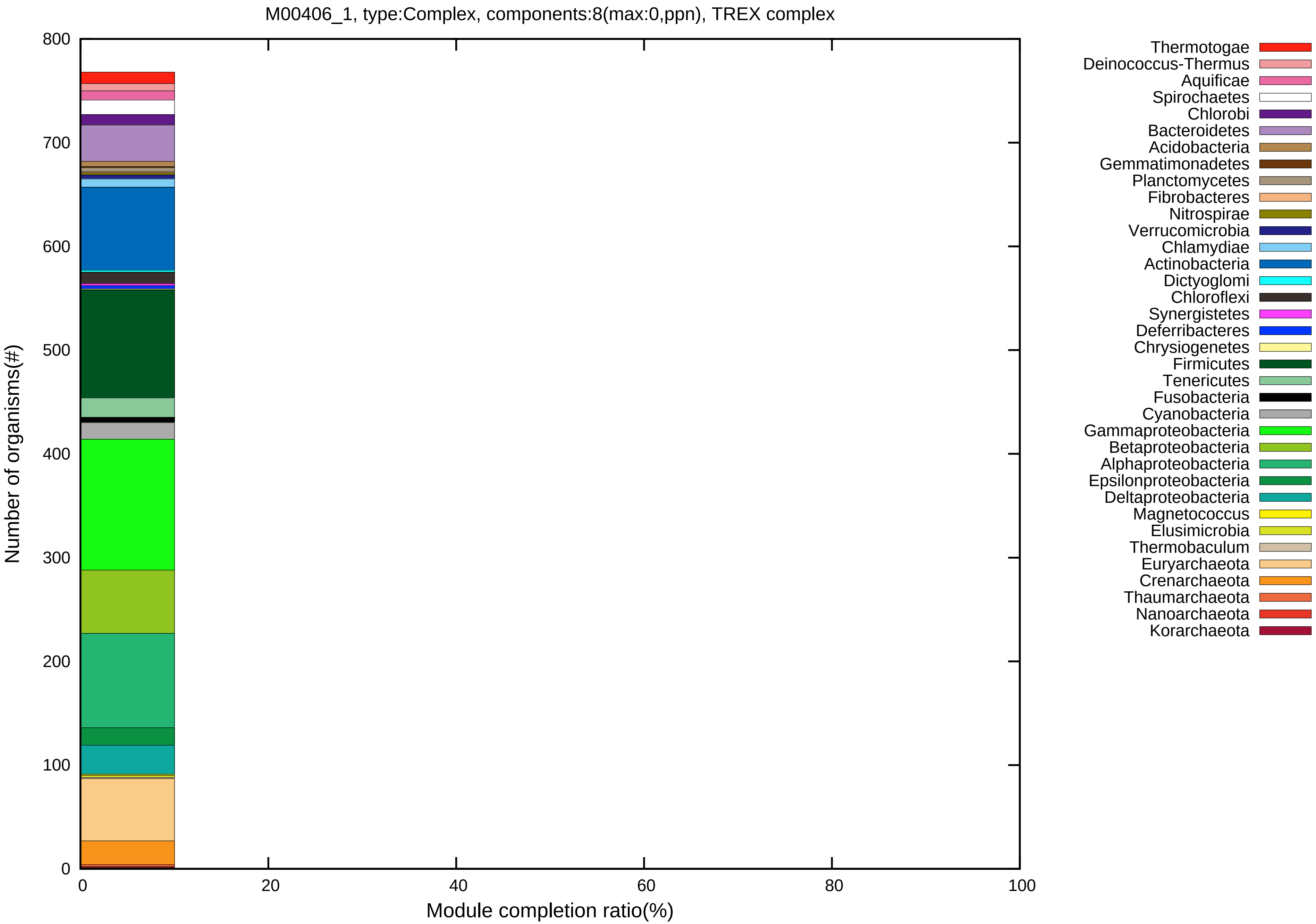
M00406_1, type:Complex, components:8(max:0,ppn), TREX complex
Number of organisms(#)
0
100
200
300
400
500
600
700
800
0
20
40
60
80
100
Module completion ratio(%)
Thermotogae
Deinococcus-Thermus
Aquificae
Spirochaetes
Chlorobi
Bacteroidetes
Acidobacteria
Gemmatimonadetes
Planctomycetes
Fibrobacteres
Nitrospirae
Verrucomicrobia
Chlamydiae
Actinobacteria
Dictyoglomi
Chloroflexi
Synergistetes
Deferribacteres
Chrysiogenetes
Firmicutes
Tenericutes
Fusobacteria
Cyanobacteria
Gammaproteobacteria
Betaproteobacteria
Alphaproteobacteria
Epsilonproteobacteria
Deltaproteobacteria
Magnetococcus
Elusimicrobia
Thermobaculum
Euryarchaeota
Crenarchaeota
Thaumarchaeota
Nanoarchaeota
Korarchaeota

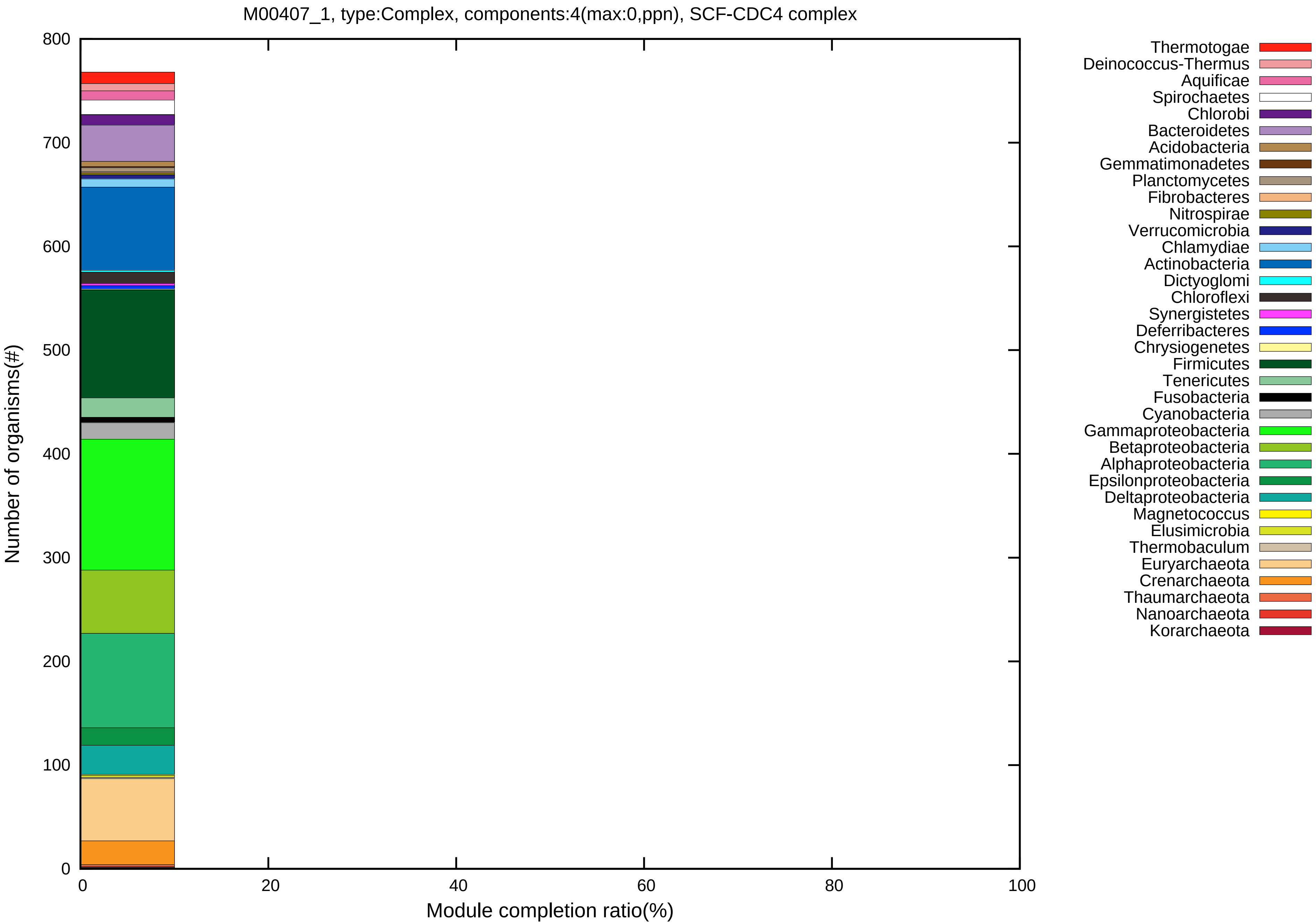
M00407_1, type:Complex, components:4(max:0,ppn), SCF-CDC4 complex
Number of organisms(#)
Module completion ratio(%)
0
100
200
300
400
500
600
700
800
0
20
40
60
80
100
Thermotogae
Deinococcus-Thermus
Aquificae
Spirochaetes
Chlorobi
Bacteroidetes
Acidobacteria
Gemmatimonadetes
Planctomycetes
Fibrobacteres
Nitrospirae
Verrucomicrobia
Chlamydiae
Actinobacteria
Dictyoglomi
Chloroflexi
Synergistetes
Deferribacteres
Chrysiogenetes
Firmicutes
Tenericutes
Fusobacteria
Cyanobacteria
Gammaproteobacteria
Betaproteobacteria
Alphaproteobacteria
Epsilonproteobacteria
Deltaproteobacteria
Magnetococcus
Elusimicrobia
Thermobaculum
Euryarchaeota
Crenarchaeota
Thaumarchaeota
Nanoarchaeota
Korarchaeota

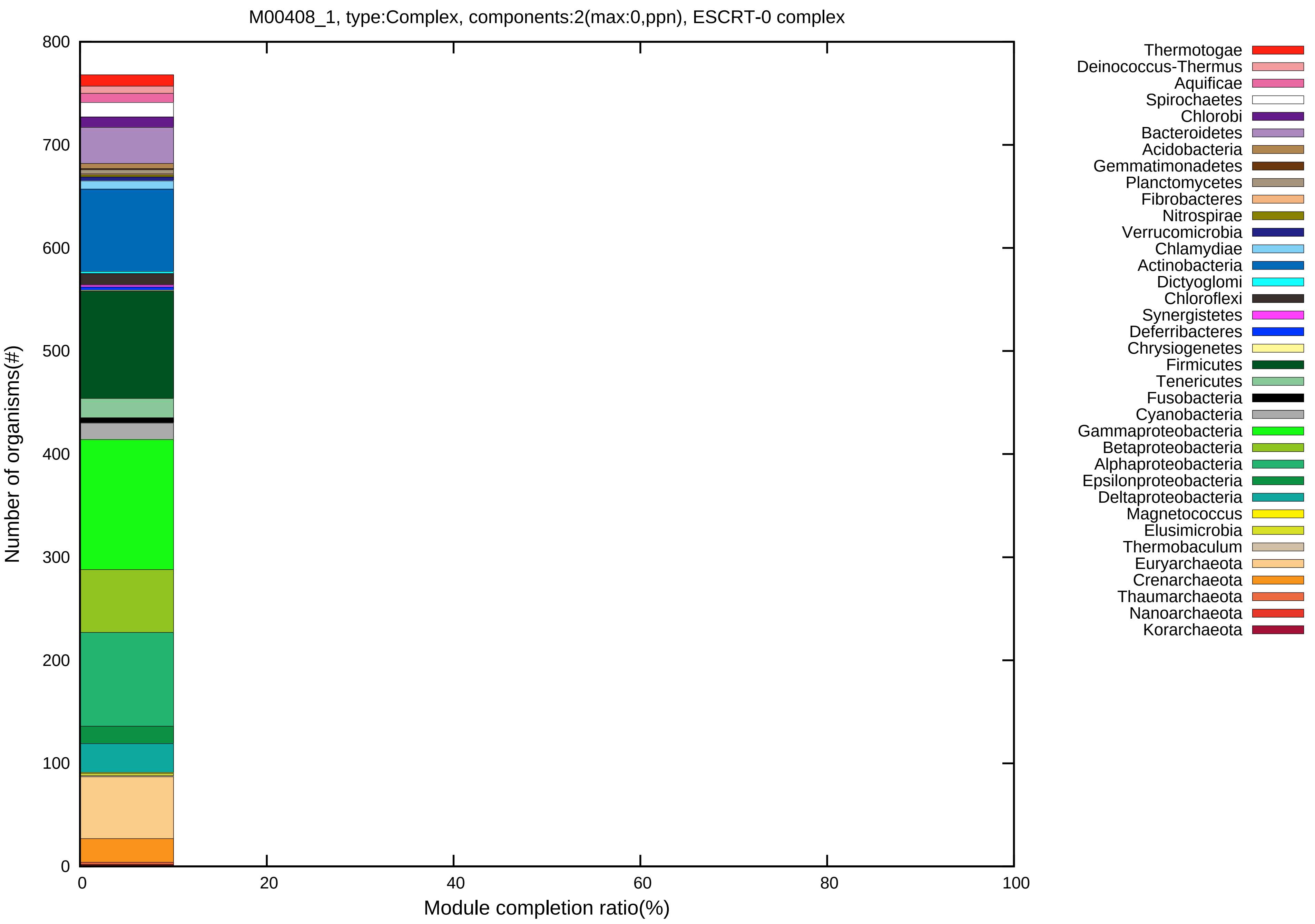

M00408_1, type:Complex, components:2(max:0,ppn), ESCRT-0 complex
Number of organisms(#)
Module completion ratio(%)
0
100
200
300
400
500
600
700
800
0
20
40
60
80
100
Thermotogae
Deinococcus-Thermus
Aquificae
Spirochaetes
Chlorobi
Bacteroidetes
Acidobacteria
Gemmatimonadetes
Planctomycetes
Fibrobacteres
Nitrospirae
Verrucomicrobia
Chlamydiae
Actinobacteria
Dictyoglomi
Chloroflexi
Synergistetes
Deferribacteres
Chrysiogenetes
Firmicutes
Tenericutes
Fusobacteria
Cyanobacteria
Gammaproteobacteria
Betaproteobacteria
Alphaproteobacteria
Epsilonproteobacteria
Deltaproteobacteria
Magnetococcus
Elusimicrobia
Thermobaculum
Euryarchaeota
Crenarchaeota
Thaumarchaeota
Nanoarchaeota
Korarchaeota

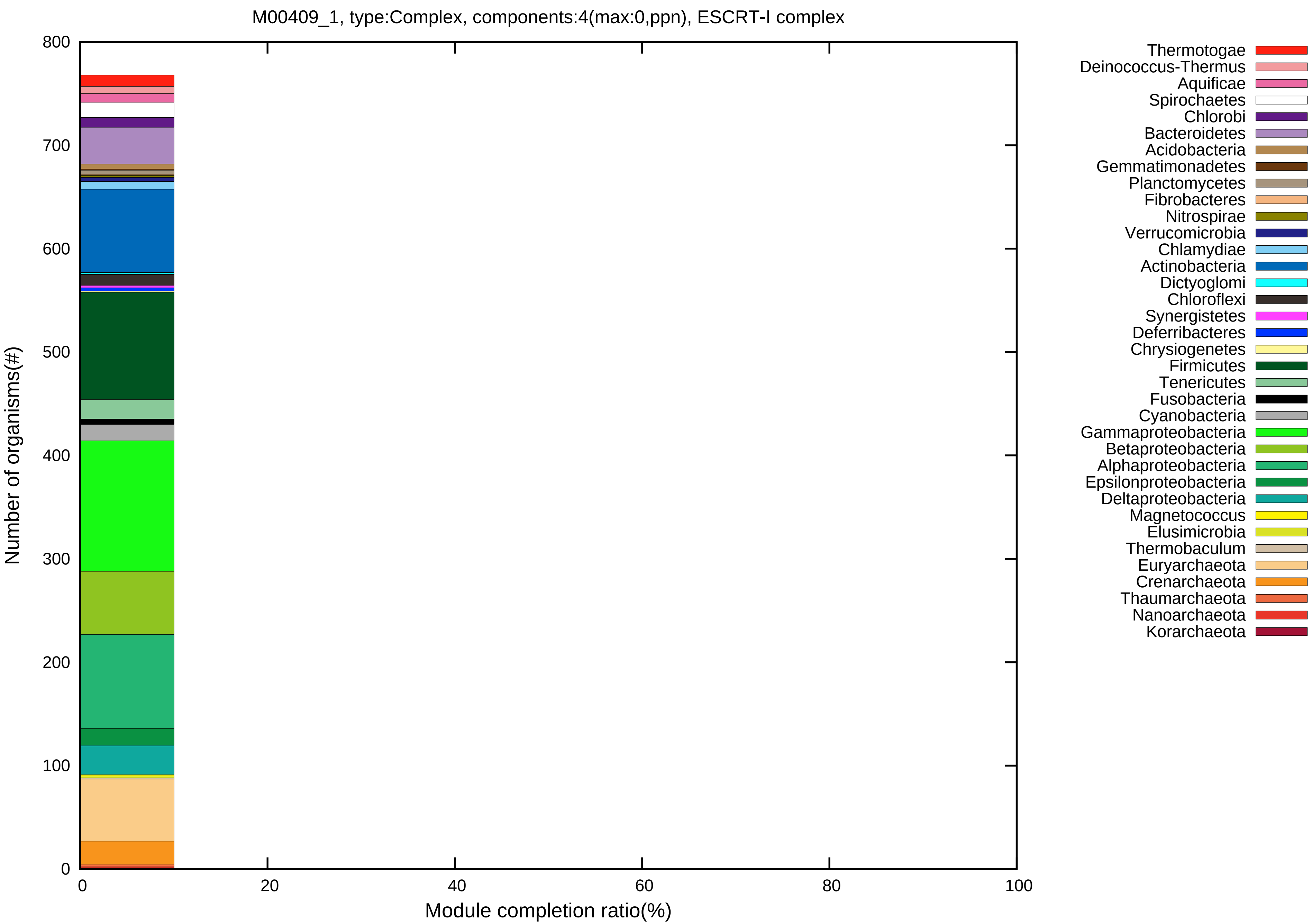
M00409_1, type:Complex, components:4(max:0,ppn), ESCRT-I complex
Number of organisms(#)
Module completion ratio(%)
0
100
200
300
400
500
600
700
800
0
20
40
60
80
100
Thermotogae
Deinococcus-Thermus
Aquificae
Spirochaetes
Chlorobi
Bacteroidetes
Acidobacteria
Gemmatimonadetes
Planctomycetes
Fibrobacteres
Nitrospirae
Verrucomicrobia
Chlamydiae
Actinobacteria
Dictyoglomi
Chloroflexi
Synergistetes
Deferribacteres
Chrysiogenetes
Firmicutes
Tenericutes
Fusobacteria
Cyanobacteria
Gammaproteobacteria
Betaproteobacteria
Alphaproteobacteria
Epsilonproteobacteria
Deltaproteobacteria
Magnetococcus
Elusimicrobia
Thermobaculum
Euryarchaeota
Crenarchaeota
Thaumarchaeota
Nanoarchaeota
Korarchaeota

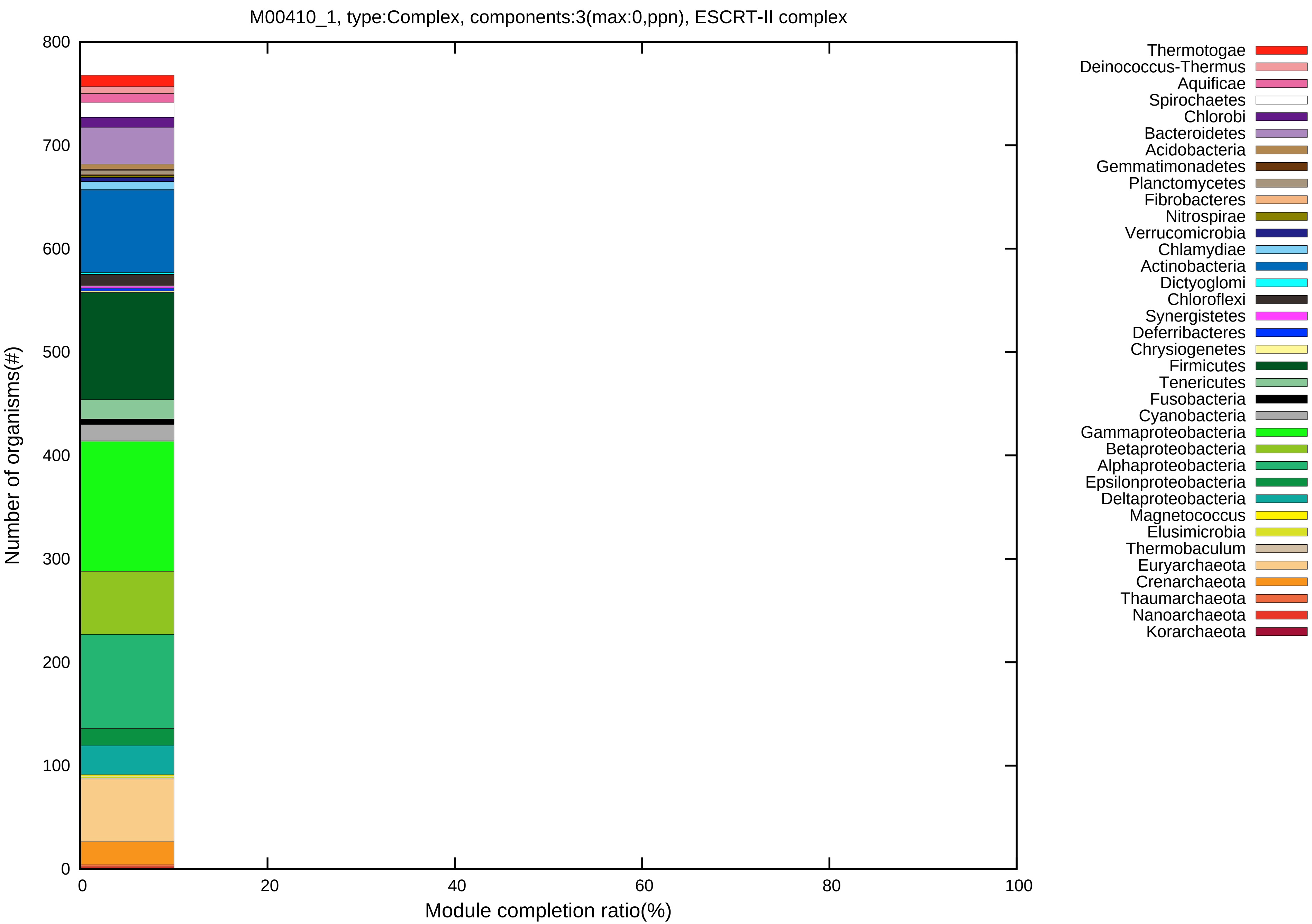
M00410_1, type:Complex, components:3(max:0,ppn), ESCRT-II complex
Number of organisms(#)
Module completion ratio(%)
0
100
200
300
400
500
600
700
800
0
20
40
60
80
100
Thermotogae
Deinococcus-Thermus
Aquificae
Spirochaetes
Chlorobi
Bacteroidetes
Acidobacteria
Gemmatimonadetes
Planctomycetes
Fibrobacteres
Nitrospirae
Verrucomicrobia
Chlamydiae
Actinobacteria
Dictyoglomi
Chloroflexi
Synergistetes
Deferribacteres
Chrysiogenetes
Firmicutes
Tenericutes
Fusobacteria
Cyanobacteria
Gammaproteobacteria
Betaproteobacteria
Alphaproteobacteria
Epsilonproteobacteria
Deltaproteobacteria
Magnetococcus
Elusimicrobia
Thermobaculum
Euryarchaeota
Crenarchaeota
Thaumarchaeota
Nanoarchaeota
Korarchaeota

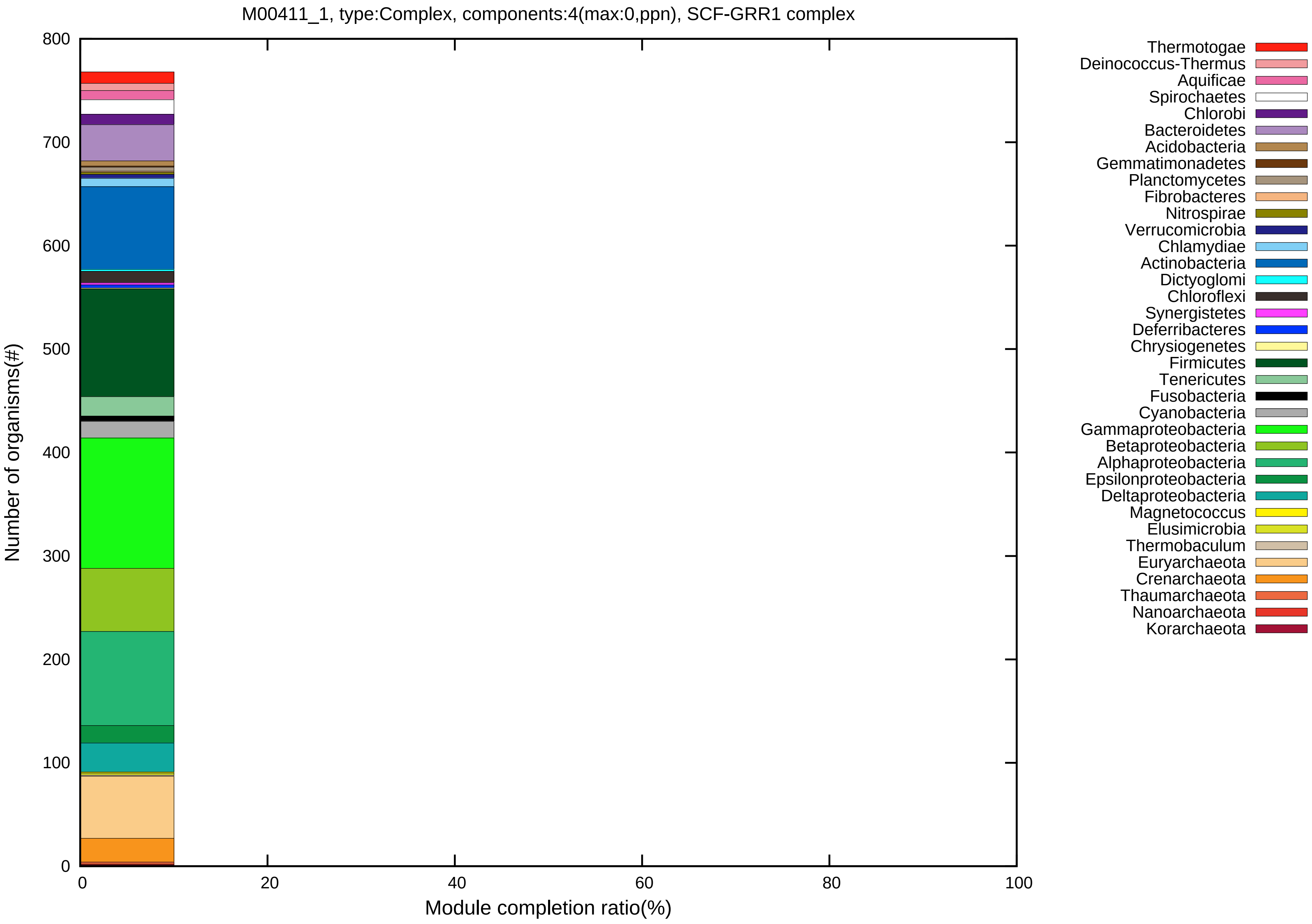

M00411_1, type:Complex, components:4(max:0,ppn), SCF-GRR1 complex
Number of organisms(#)
Module completion ratio(%)
0
100
200
300
400
500
600
700
800
0
20
40
60
80
100
Thermotogae
Deinococcus-Thermus
Aquificae
Spirochaetes
Chlorobi
Bacteroidetes
Acidobacteria
Gemmatimonadetes
Planctomycetes
Fibrobacteres
Nitrospirae
Verrucomicrobia
Chlamydiae
Actinobacteria
Dictyoglomi
Chloroflexi
Synergistetes
Deferribacteres
Chrysiogenetes
Firmicutes
Tenericutes
Fusobacteria
Cyanobacteria
Gammaproteobacteria
Betaproteobacteria
Alphaproteobacteria
Epsilonproteobacteria
Deltaproteobacteria
Magnetococcus
Elusimicrobia
Thermobaculum
Euryarchaeota
Crenarchaeota
Thaumarchaeota
Nanoarchaeota
Korarchaeota

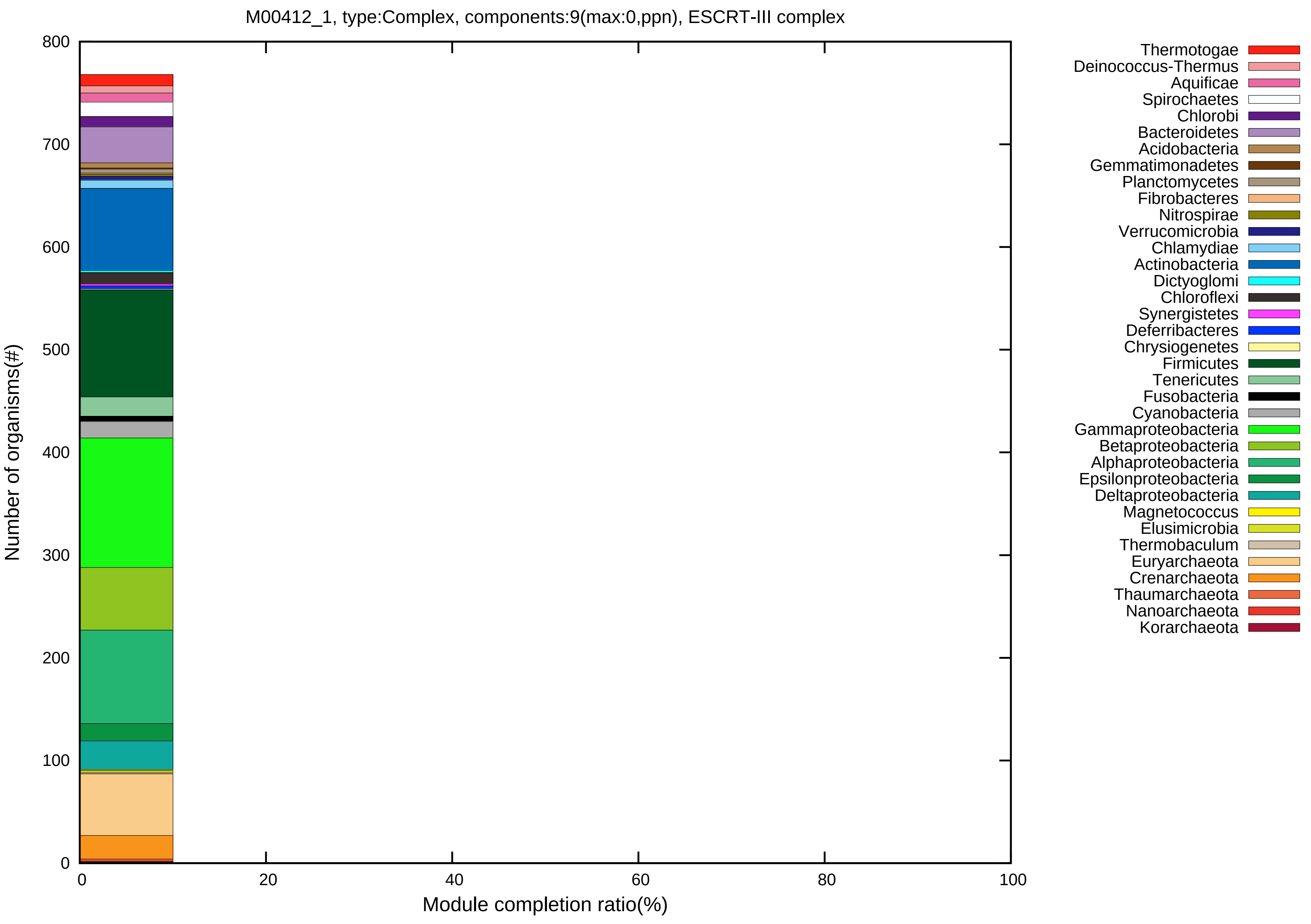

M00412_1, type:Complex, components:9(max:0,ppn), ESCRT-III complex
Number of organisms(#)
Module completion ratio(%)
0
100
200
300
400
500
600
700
800
0
20
40
60
80
100
Thermotogae
Deinococcus-Thermus
Aquificae
Spirochaetes
Chlorobi
Bacteroidetes
Acidobacteria
Gemmatimonadetes
Planctomycetes
Fibrobacteres
Nitrospirae
Verrucomicrobia
Chlamydiae
Actinobacteria
Dictyoglomi
Chloroflexi
Synergistetes
Deferribacteres
Chrysiogenetes
Firmicutes
Tenericutes
Fusobacteria
Cyanobacteria
Gammaproteobacteria
Betaproteobacteria
Alphaproteobacteria
Epsilonproteobacteria
Deltaproteobacteria
Magnetococcus
Elusimicrobia
Thermobaculum
Euryarchaeota
Crenarchaeota
Thaumarchaeota
Nanoarchaeota
Korarchaeota

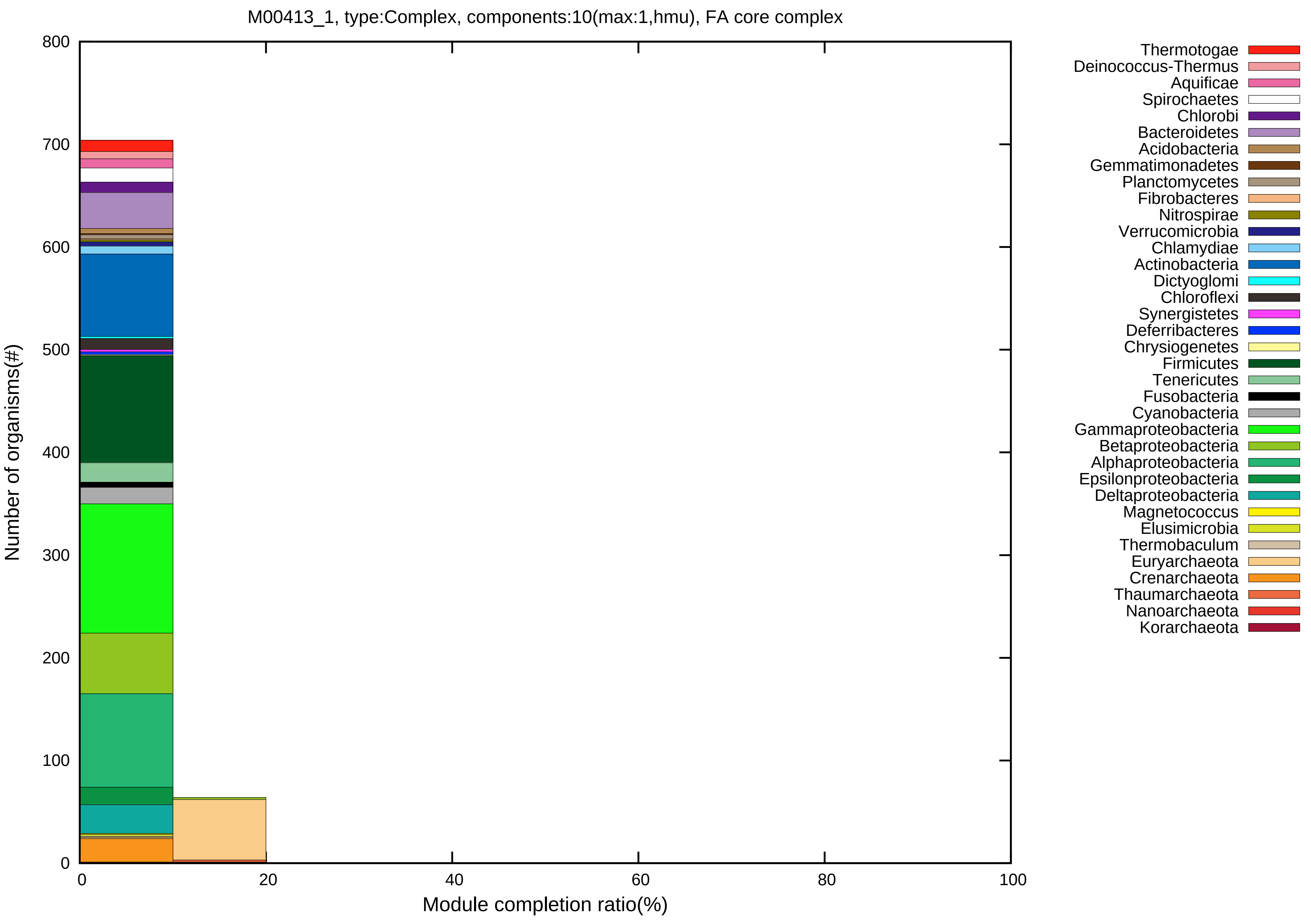

M00413_1, type:Complex, components:10(max:1,hmu), FA core complex
Number of organisms(#)
Module completion ratio(%)
0
100
200
300
400
500
600
700
800
0
20
40
60
80
100
Thermotogae
Deinococcus-Thermus
Aquificae
Spirochaetes
Chlorobi
Bacteroidetes
Acidobacteria
Gemmatimonadetes
Planctomycetes
Fibrobacteres
Nitrospirae
Verrucomicrobia
Chlamydiae
Actinobacteria
Dictyoglomi
Chloroflexi
Synergistetes
Deferribacteres
Chrysiogenetes
Firmicutes
Tenericutes
Fusobacteria
Cyanobacteria
Gammaproteobacteria
Betaproteobacteria
Alphaproteobacteria
Epsilonproteobacteria
Deltaproteobacteria
Magnetococcus
Elusimicrobia
Thermobaculum
Euryarchaeota
Crenarchaeota
Thaumarchaeota
Nanoarchaeota
Korarchaeota

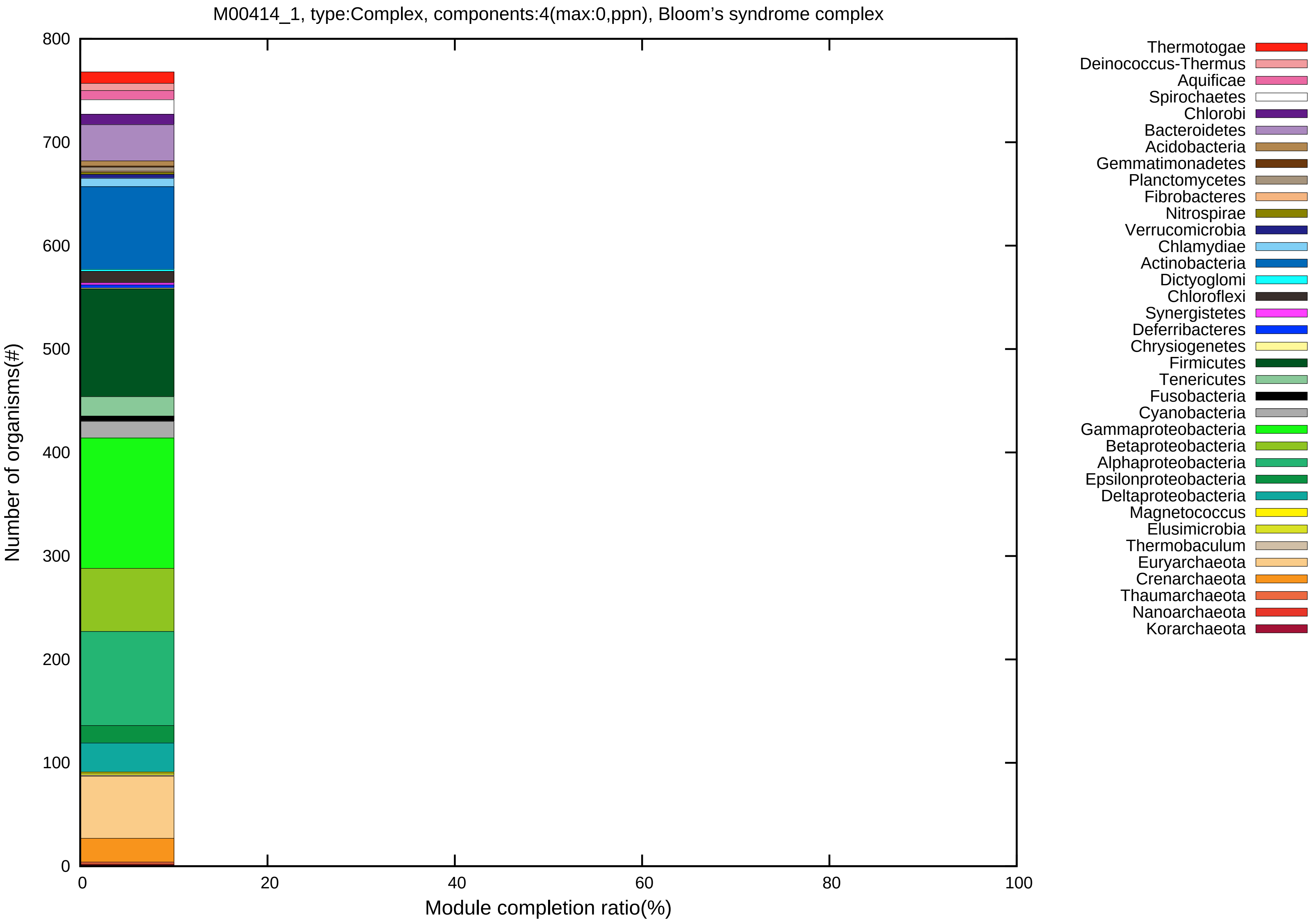
M00414_1, type:Complex, components:4(max:0,ppn), Bloom's syndrome complex
Number of organisms(#)
Module completion ratio(%)
0
100
200
300
400
500
600
700
800
0
20
40
60
80
100
Thermotogae
Deinococcus-Thermus
Aquificae
Spirochaetes
Chlorobi
Bacteroidetes
Acidobacteria
Gemmatimonadetes
Planctomycetes
Fibrobacteres
Nitrospirae
Verrucomicrobia
Chlamydiae
Actinobacteria
Dictyoglomi
Chloroflexi
Synergistetes
Deferribacteres
Chrysiogenetes
Firmicutes
Tenericutes
Fusobacteria
Cyanobacteria
Gammaproteobacteria
Betaproteobacteria
Alphaproteobacteria
Epsilonproteobacteria
Deltaproteobacteria
Magnetococcus
Elusimicrobia
Thermobaculum
Euryarchaeota
Crenarchaeota
Thaumarchaeota
Nanoarchaeota
Korarchaeota

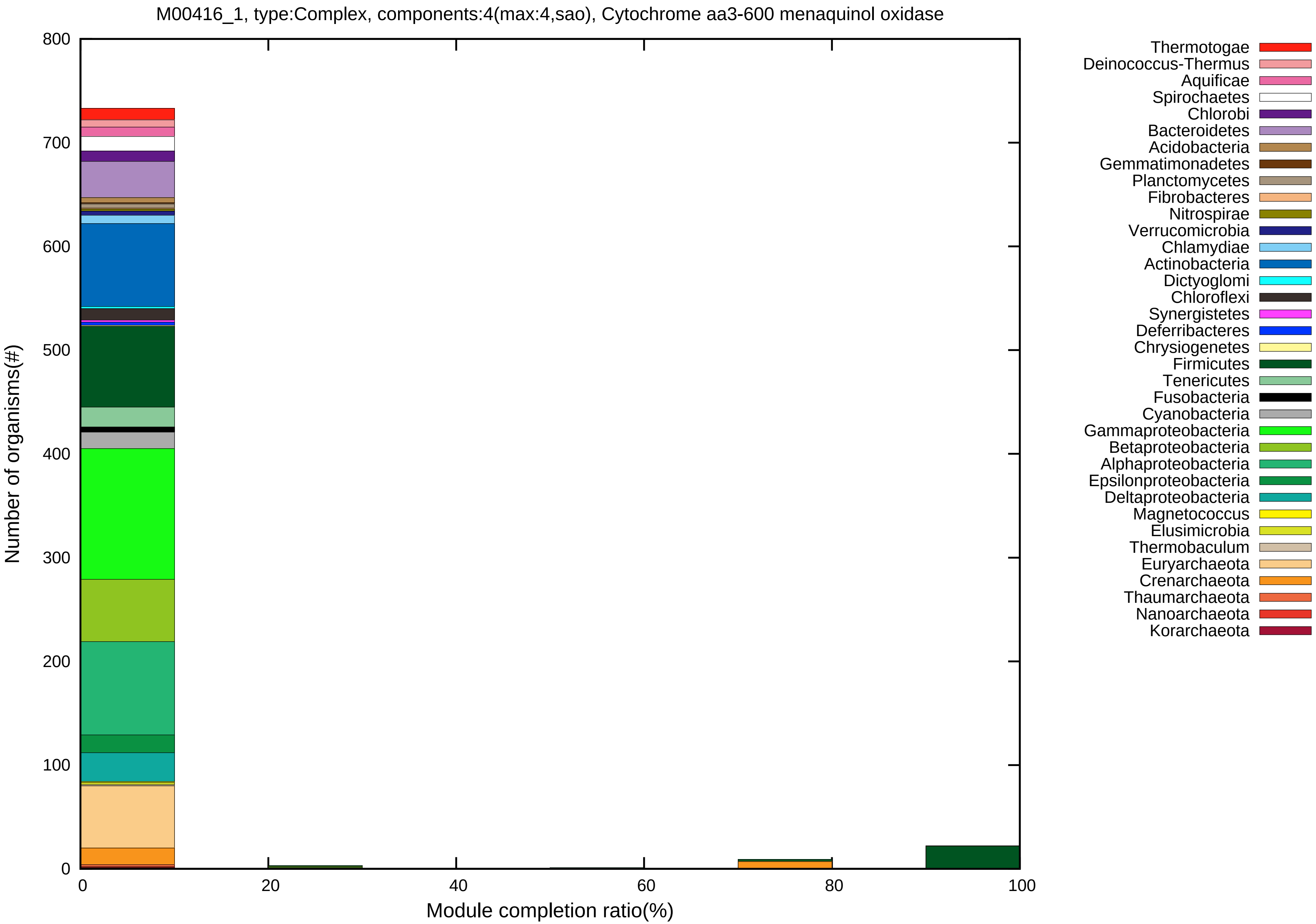
M00416_1, type:Complex, components:4(max:4,sao), Cytochrome aa3-600 menaquinol oxidase
Number of organisms(#)
Module completion ratio(%)
0
100
200
300
400
500
600
700
800
0
20
40
60
80
100
Thermotogae
Deinococcus-Thermus
Aquificae
Spirochaetes
Chlorobi
Bacteroidetes
Acidobacteria
Gemmatimonadetes
Planctomycetes
Fibrobacteres
Nitrospirae
Verrucomicrobia
Chlamydiae
Actinobacteria
Dictyoglomi
Chloroflexi
Synergistetes
Deferribacteres
Chrysiogenetes
Firmicutes
Tenericutes
Fusobacteria
Cyanobacteria
Gammaproteobacteria
Betaproteobacteria
Alphaproteobacteria
Epsilonproteobacteria
Deltaproteobacteria
Magnetococcus
Elusimicrobia
Thermobaculum
Euryarchaeota
Crenarchaeota
Thaumarchaeota
Nanoarchaeota
Korarchaeota

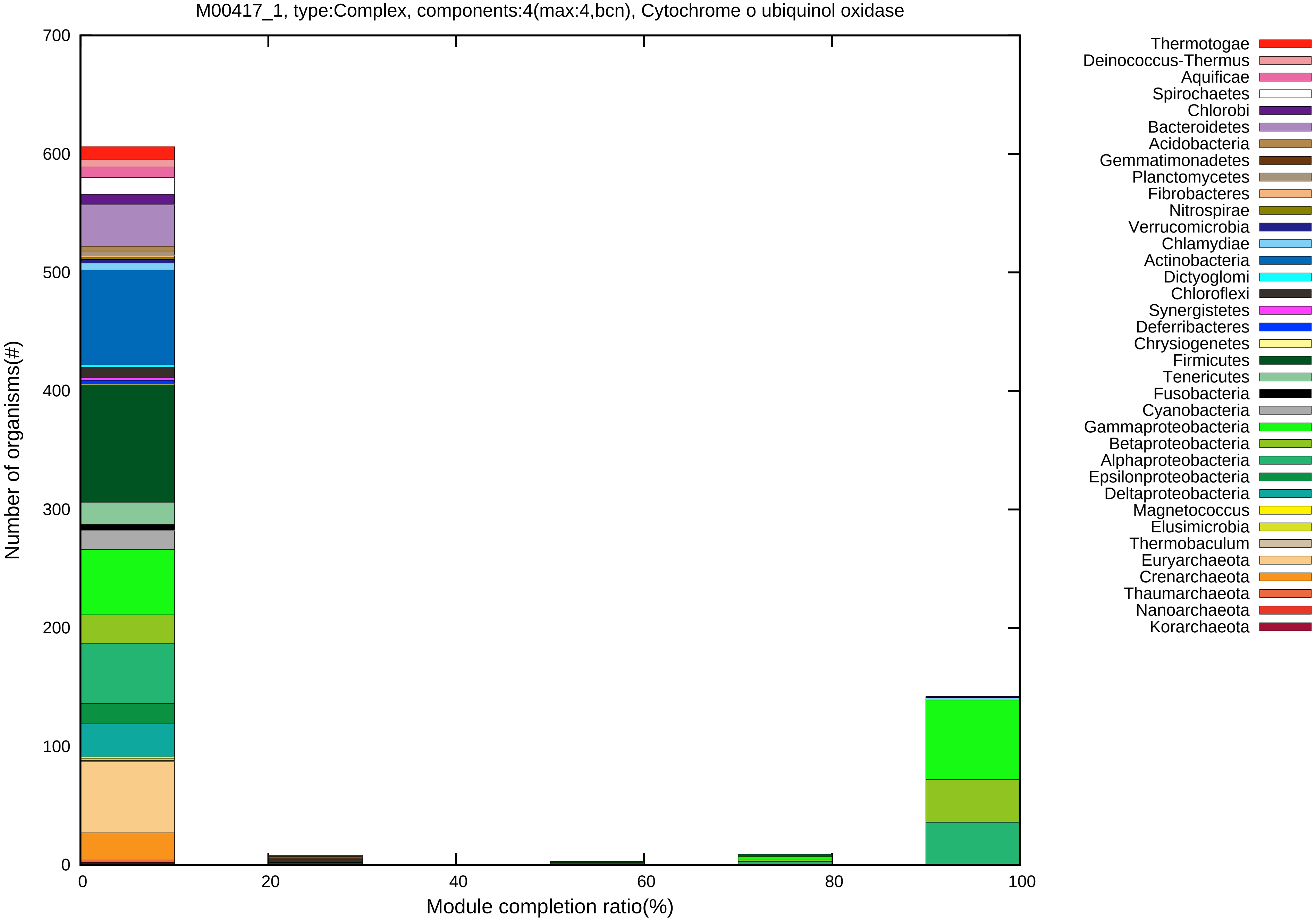
M00417_1, type:Complex, components:4(max:4,bcn), Cytochrome o ubiquinol oxidase
Number of organisms(#)
Module completion ratio(%)
0
100
200
300
400
500
600
700
20
40
60
80
Thermotogae
Deinococcus-Thermus
Aquificae
Spirochaetes
Chlorobi
Bacteroidetes
Acidobacteria
Gemmatimonadetes
Planctomycetes
Fibrobacteres
Nitrospirae
Verrucomicrobia
Chlamydiae
Actinobacteria
Dictyoglomi
Chloroflexi
Synergistetes
Deferribacteres
Chrysiogenetes
Firmicutes
Tenericutes
Fusobacteria
Cyanobacteria
Gammaproteobacteria
Betaproteobacteria
Alphaproteobacteria
Epsilonproteobacteria
Deltaproteobacteria
Magnetococcus
Elusimicrobia
Thermobaculum
Euryarchaeota
Crenarchaeota
Thaumarchaeota
Nanoarchaeota
Korarchaeota

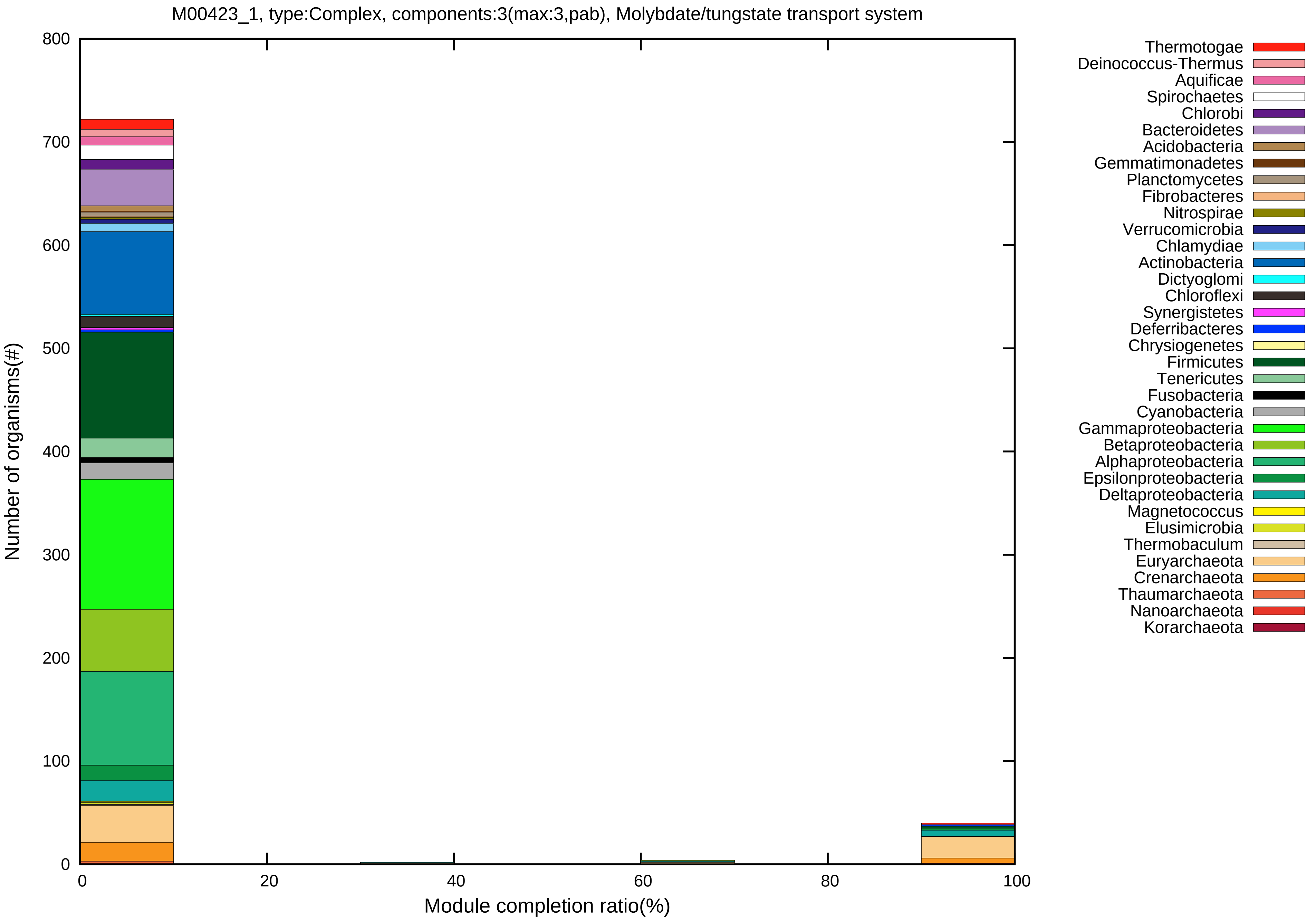

M00423_1, type:Complex, components:3(max:3,pab), Molybdate/tungstate transport system
Number of organisms(#)
Module completion ratio(%)
0
100
200
300
400
500
600
700
800
0
20
40
60
80
100
Thermotogae
Deinococcus-Thermus
Aquificae
Spirochaetes
Chlorobi
Bacteroidetes
Acidobacteria
Gemmatimonadetes
Planctomycetes
Fibrobacteres
Nitrospirae
Verrucomicrobia
Chlamydiae
Actinobacteria
Dictyoglomi
Chloroflexi
Synergistetes
Deferribacteres
Chrysiogenetes
Firmicutes
Tenericutes
Fusobacteria
Cyanobacteria
Gammaproteobacteria
Betaproteobacteria
Alphaproteobacteria
Epsilonproteobacteria
Deltaproteobacteria
Magnetococcus
Elusimicrobia
Thermobaculum
Euryarchaeota
Crenarchaeota
Thaumarchaeota
Nanoarchaeota
Korarchaeota

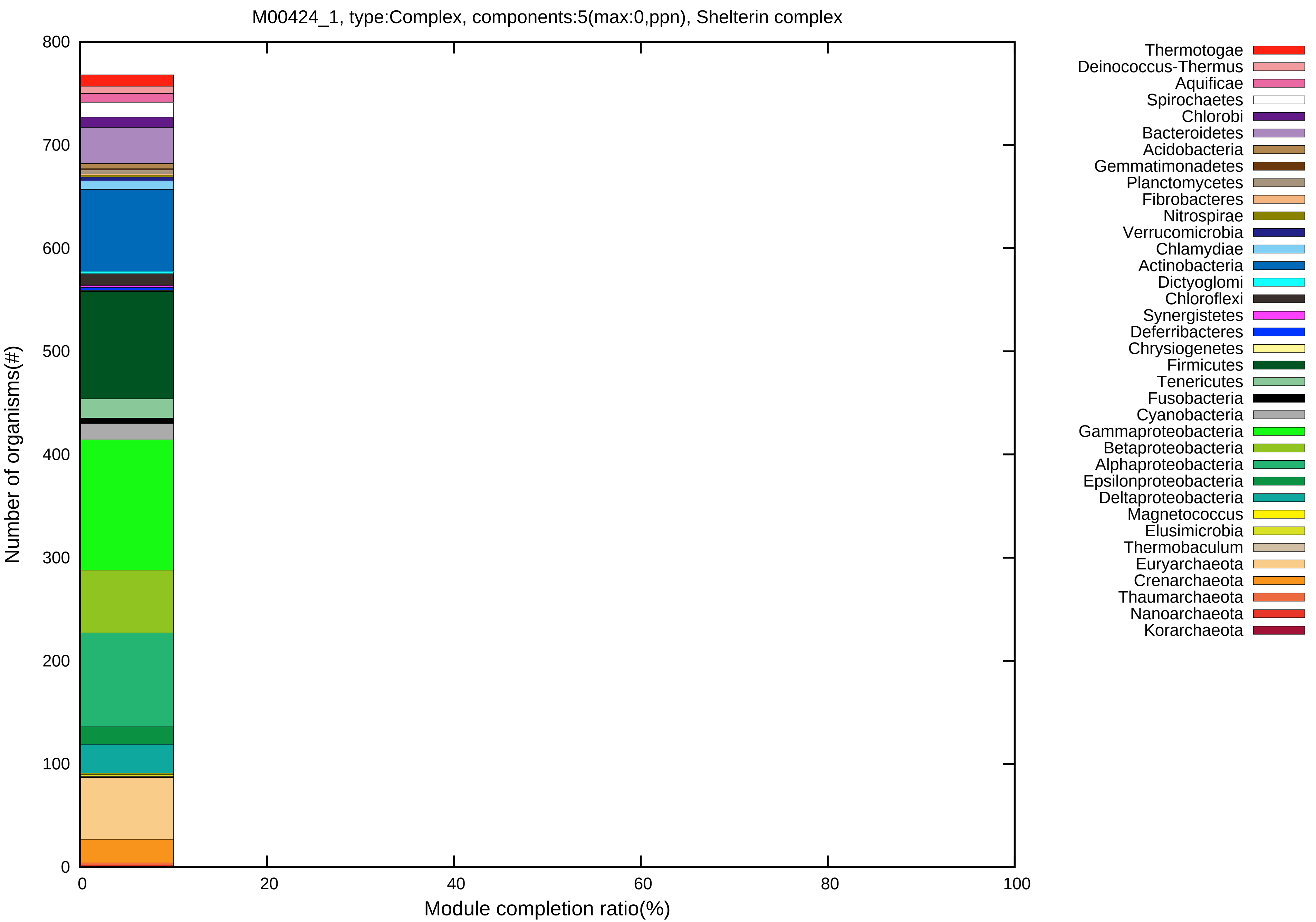

M00424_1, type:Complex, components:5(max:0,ppn), Shelterin complex
Number of organisms(#)
Module completion ratio(%)
0
100
200
300
400
500
600
700
800
0
20
40
60
80
100
Thermotogae
Deinococcus-Thermus
Aquificae
Spirochaetes
Chlorobi
Bacteroidetes
Acidobacteria
Gemmatimonadetes
Planctomycetes
Fibrobacteres
Nitrospirae
Verrucomicrobia
Chlamydiae
Actinobacteria
Dictyoglomi
Chloroflexi
Synergistetes
Deferribacteres
Chrysiogenetes
Firmicutes
Tenericutes
Fusobacteria
Cyanobacteria
Gammaproteobacteria
Betaproteobacteria
Alphaproteobacteria
Epsilonproteobacteria
Deltaproteobacteria
Magnetococcus
Elusimicrobia
Thermobaculum
Euryarchaeota
Crenarchaeota
Thaumarchaeota
Nanoarchaeota
Korarchaeota

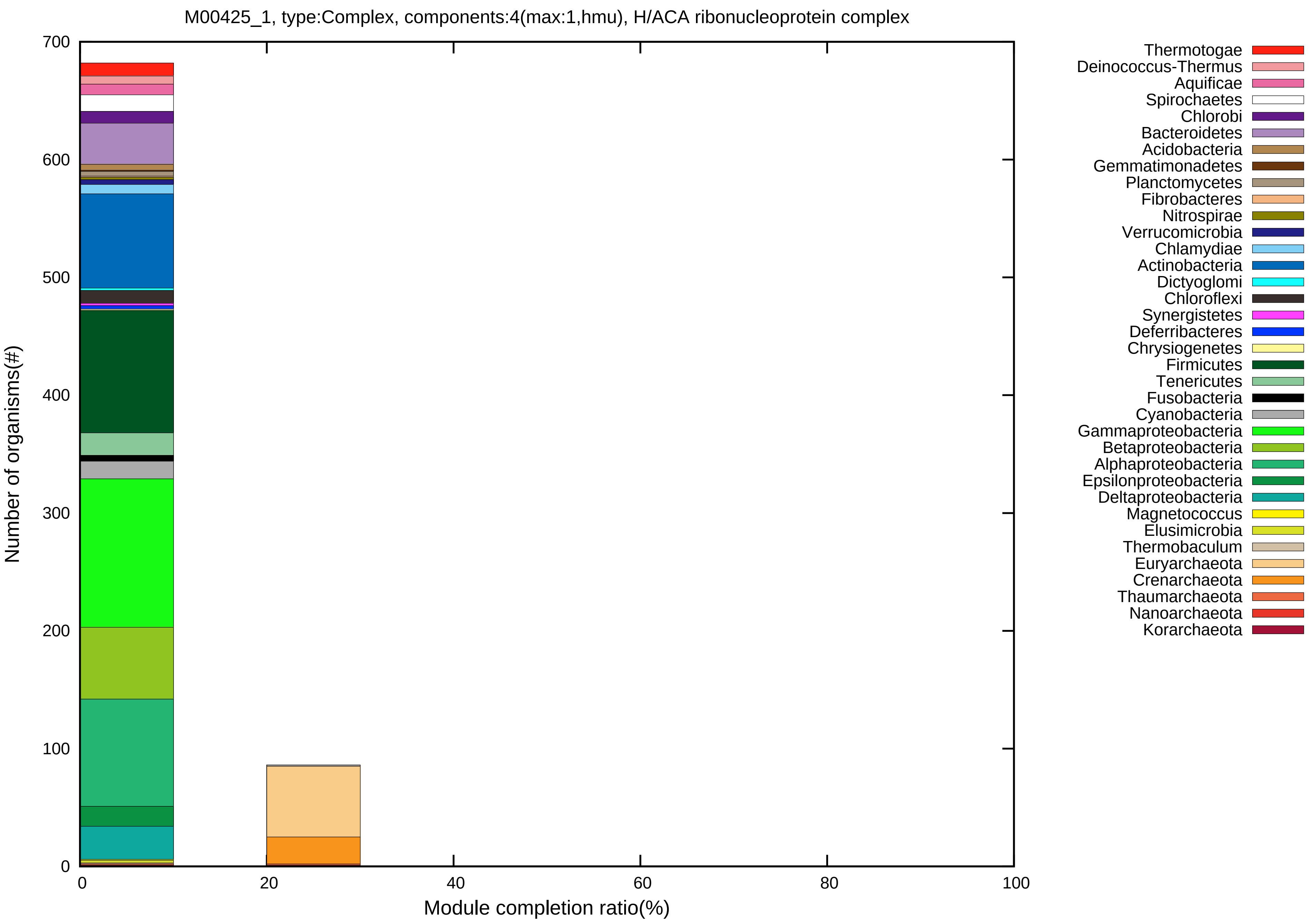
M00425_1, type:Complex, components:4(max:1,hmu), H/ACA ribonucleoprotein complex
Number of organisms(#)
Module completion ratio(%)
0
100
200
300
400
500
600
700
0
20
40
60
80
100
Thermotogae
Deinococcus-Thermus
Aquificae
Spirochaetes
Chlorobi
Bacteroidetes
Acidobacteria
Gemmatimonadetes
Planctomycetes
Fibrobacteres
Nitrospirae
Verrucomicrobia
Chlamydiae
Actinobacteria
Dictyoglomi
Chloroflexi
Synergistetes
Deferribacteres
Chrysiogenetes
Firmicutes
Tenericutes
Fusobacteria
Cyanobacteria
Gammaproteobacteria
Betaproteobacteria
Alphaproteobacteria
Epsilonproteobacteria
Deltaproteobacteria
Magnetococcus
Elusimicrobia
Thermobaculum
Euryarchaeota
Crenarchaeota
Thaumarchaeota
Nanoarchaeota
Korarchaeota

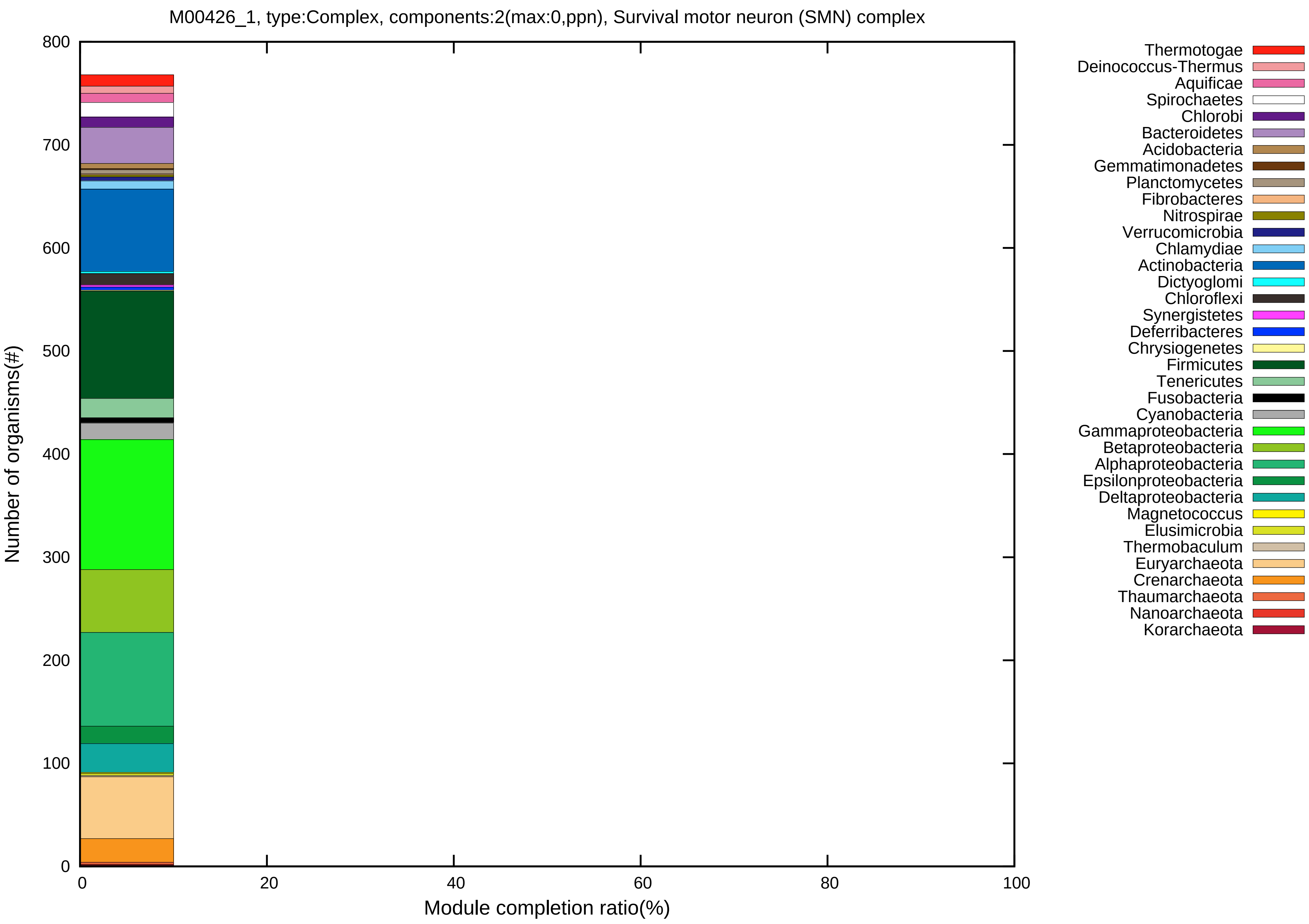
M00426_1, type:Complex, components:2(max:0,ppn), Survival motor neuron (SMN) complex
Number of organisms(#)
0
100
200
300
400
500
600
700
800
0
20
40
60
80
100
Module completion ratio(%)
Thermotogae
Deinococcus-Thermus
Aquificae
Spirochaetes
Chlorobi
Bacteroidetes
Acidobacteria
Gemmatimonadetes
Planctomycetes
Fibrobacteres
Nitrospirae
Verrucomicrobia
Chlamydiae
Actinobacteria
Dictyoglomi
Chloroflexi
Synergistetes
Deferribacteres
Chrysiogenetes
Firmicutes
Tenericutes
Fusobacteria
Cyanobacteria
Gammaproteobacteria
Betaproteobacteria
Alphaproteobacteria
Epsilonproteobacteria
Deltaproteobacteria
Magnetococcus
Elusimicrobia
Thermobaculum
Euryarchaeota
Crenarchaeota
Thaumarchaeota
Nanoarchaeota
Korarchaeota

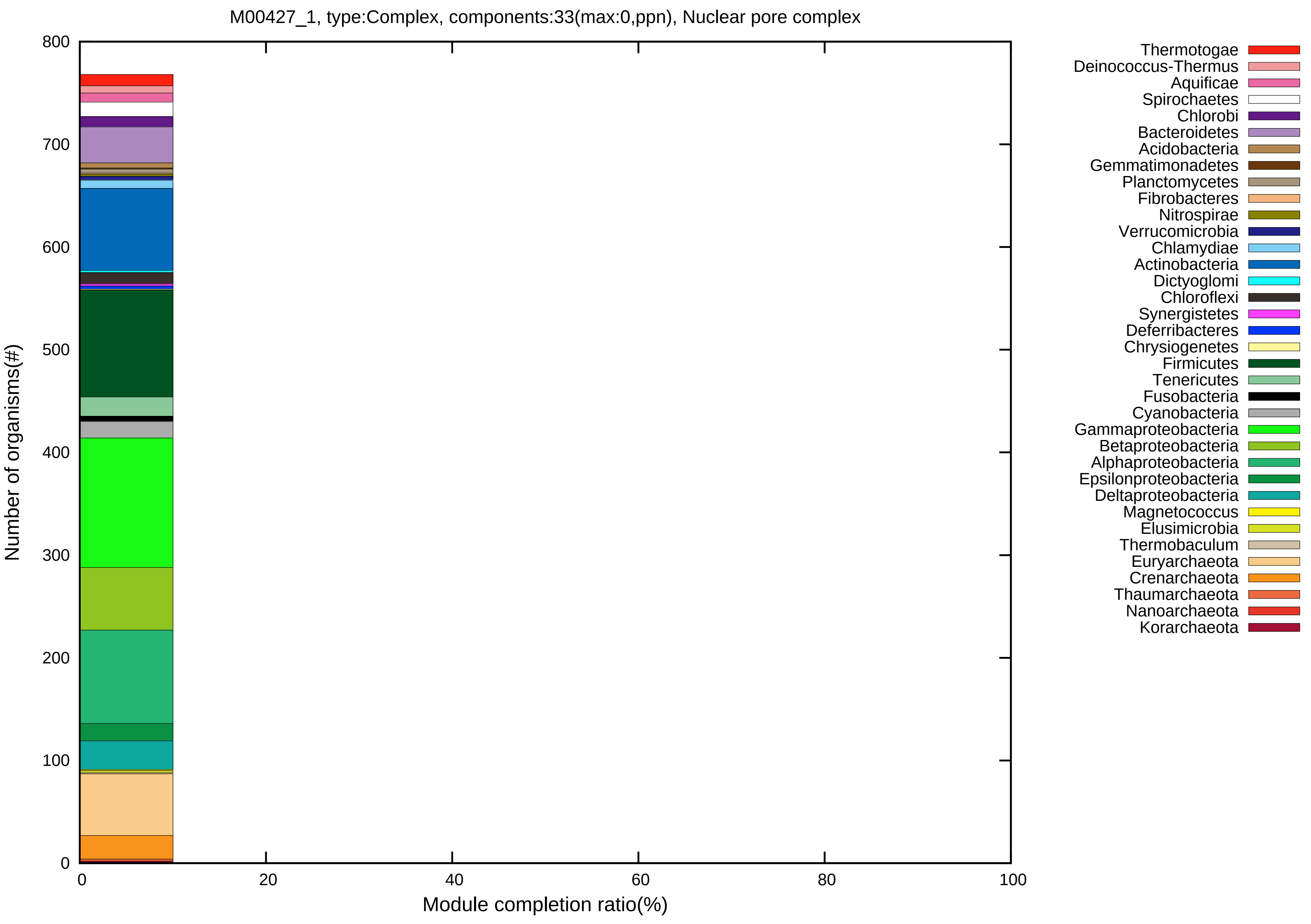

M00427_1, type:Complex, components:33(max:0,ppn), Nuclear pore complex
Number of organisms(#)
0
100
200
300
400
500
600
700
800
0
20
40
60
80
100
Module completion ratio(%)
Thermotogae
Deinococcus-Thermus
Aquificae
Spirochaetes
Chlorobi
Bacteroidetes
Acidobacteria
Gemmatimonadetes
Planctomycetes
Fibrobacteres
Nitrospirae
Verrucomicrobia
Chlamydiae
Actinobacteria
Dictyoglomi
Chloroflexi
Synergistetes
Deferribacteres
Chrysiogenetes
Firmicutes
Tenericutes
Fusobacteria
Cyanobacteria
Gammaproteobacteria
Betaproteobacteria
Alphaproteobacteria
Epsilonproteobacteria
Deltaproteobacteria
Magnetococcus
Elusimicrobia
Thermobaculum
Euryarchaeota
Crenarchaeota
Thaumarchaeota
Nanoarchaeota
Korarchaeota

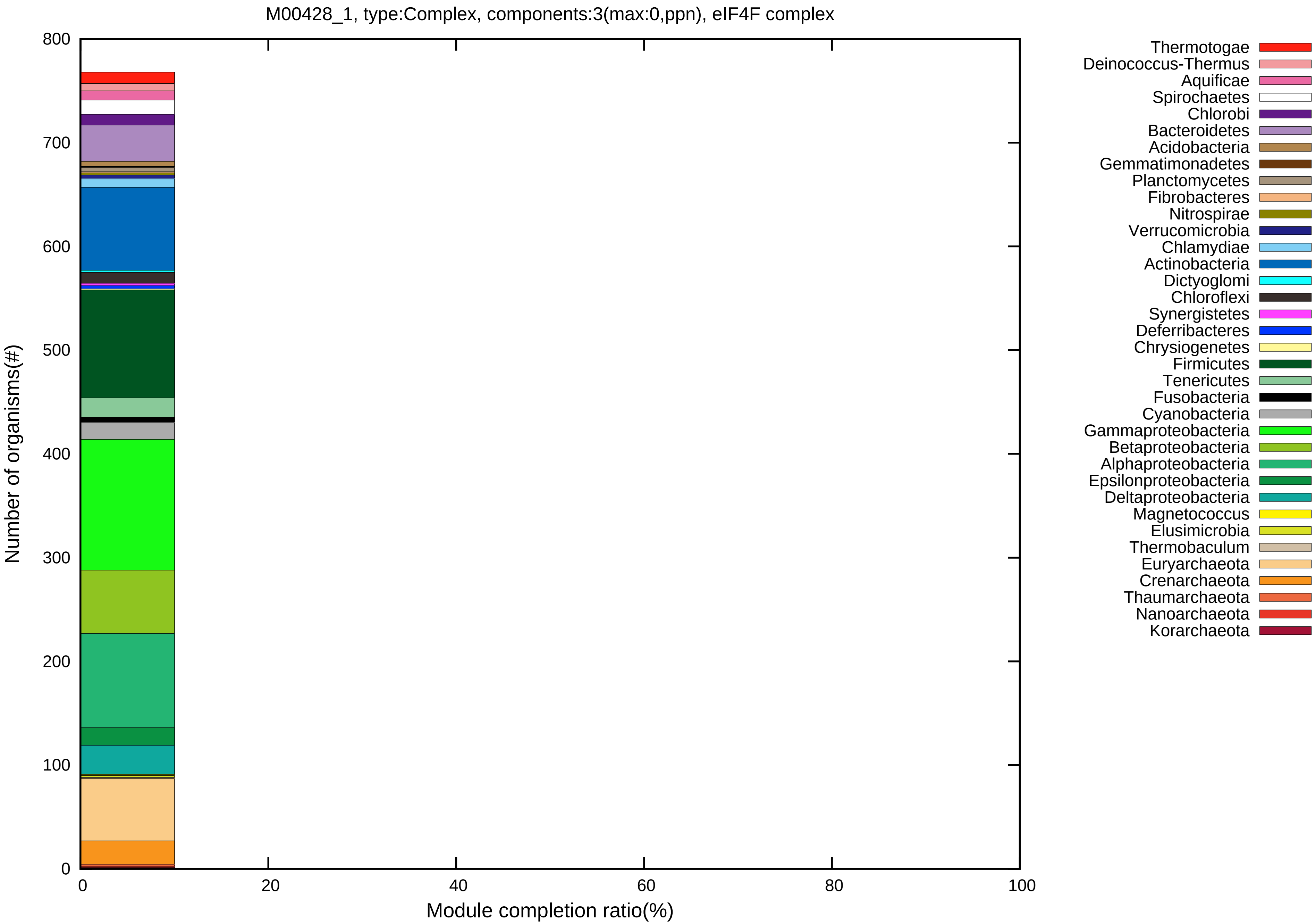
M00428_1, type:Complex, components:3(max:0,ppn), eIF4F complex
Number of organisms(#)
Module completion ratio(%)
0
100
200
300
400
500
600
700
800
0
20
40
60
80
100
Thermotogae
Deinococcus-Thermus
Aquificae
Spirochaetes
Chlorobi
Bacteroidetes
Acidobacteria
Gemmatimonadetes
Planctomycetes
Fibrobacteres
Nitrospirae
Verrucomicrobia
Chlamydiae
Actinobacteria
Dictyoglomi
Chloroflexi
Synergistetes
Deferribacteres
Chrysiogenetes
Firmicutes
Tenericutes
Fusobacteria
Cyanobacteria
Gammaproteobacteria
Betaproteobacteria
Alphaproteobacteria
Epsilonproteobacteria
Deltaproteobacteria
Magnetococcus
Elusimicrobia
Thermobaculum
Euryarchaeota
Crenarchaeota
Thaumarchaeota
Nanoarchaeota
Korarchaeota

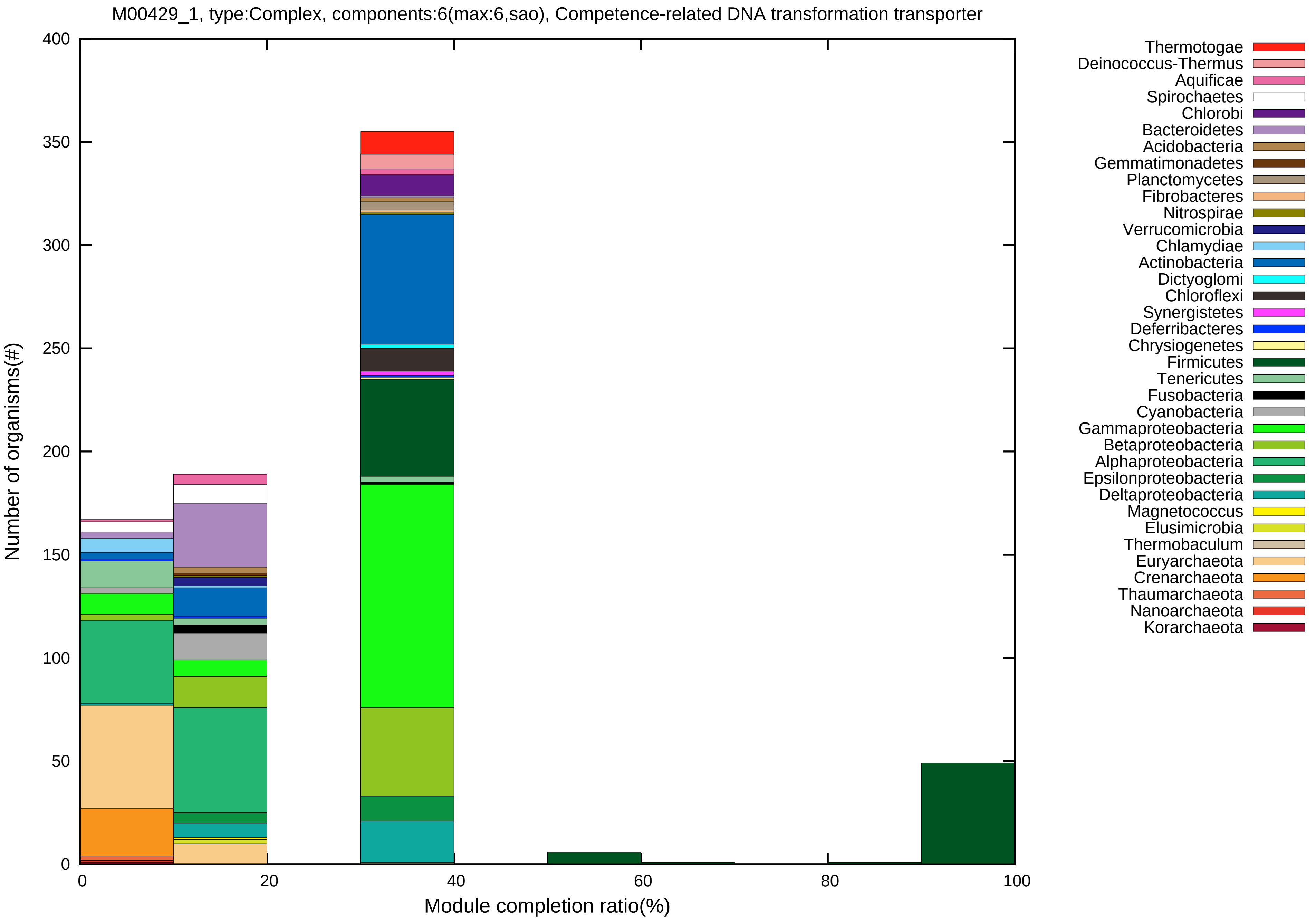
M00429_1, type:Complex, components:6(max:6,sao), Competence-related DNA transformation transporter
Number of organisms(#)
Module completion ratio(%)
0
50
100
150
200
250
300
350
400
0
20
40
60
80
100
Thermotogae
Deinococcus-Thermus
Aquificae
Spirochaetes
Chlorobi
Bacteroidetes
Acidobacteria
Gemmatimonadetes
Planctomycetes
Fibrobacteres
Nitrospirae
Verrucomicrobia
Chlamydiae
Actinobacteria
Dictyoglomi
Chloroflexi
Synergistetes
Deferribacteres
Chrysiogenetes
Firmicutes
Tenericutes
Fusobacteria
Cyanobacteria
Gammaproteobacteria
Betaproteobacteria
Alphaproteobacteria
Epsilonproteobacteria
Deltaproteobacteria
Magnetococcus
Elusimicrobia
Thermobaculum
Euryarchaeota
Crenarchaeota
Thaumarchaeota
Nanoarchaeota
Korarchaeota

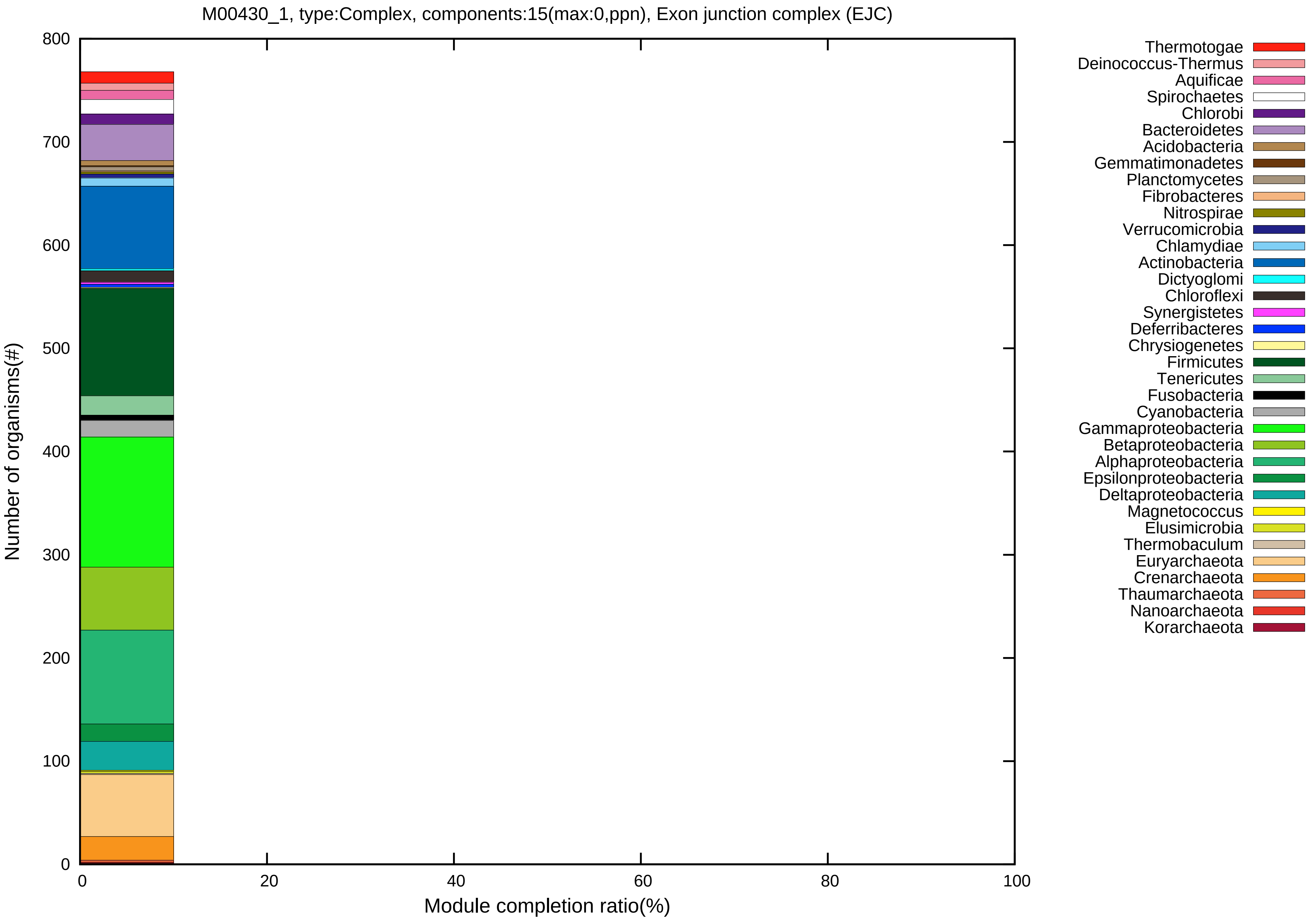
M00430_1, type:Complex, components:15(max:0,ppn), Exon junction complex (EJC)
Number of organisms(#)
Module completion ratio(%)
0
100
200
300
400
500
600
700
800
0
20
40
60
80
100
Thermotogae
Deinococcus-Thermus
Aquificae
Spirochaetes
Chlorobi
Bacteroidetes
Acidobacteria
Gemmatimonadetes
Planctomycetes
Fibrobacteres
Nitrospirae
Verrucomicrobia
Chlamydiae
Actinobacteria
Dictyoglomi
Chloroflexi
Synergistetes
Deferribacteres
Chrysiogenetes
Firmicutes
Tenericutes
Fusobacteria
Cyanobacteria
Gammaproteobacteria
Betaproteobacteria
Alphaproteobacteria
Epsilonproteobacteria
Deltaproteobacteria
Magnetococcus
Elusimicrobia
Thermobaculum
Euryarchaeota
Crenarchaeota
Thaumarchaeota
Nanoarchaeota
Korarchaeota

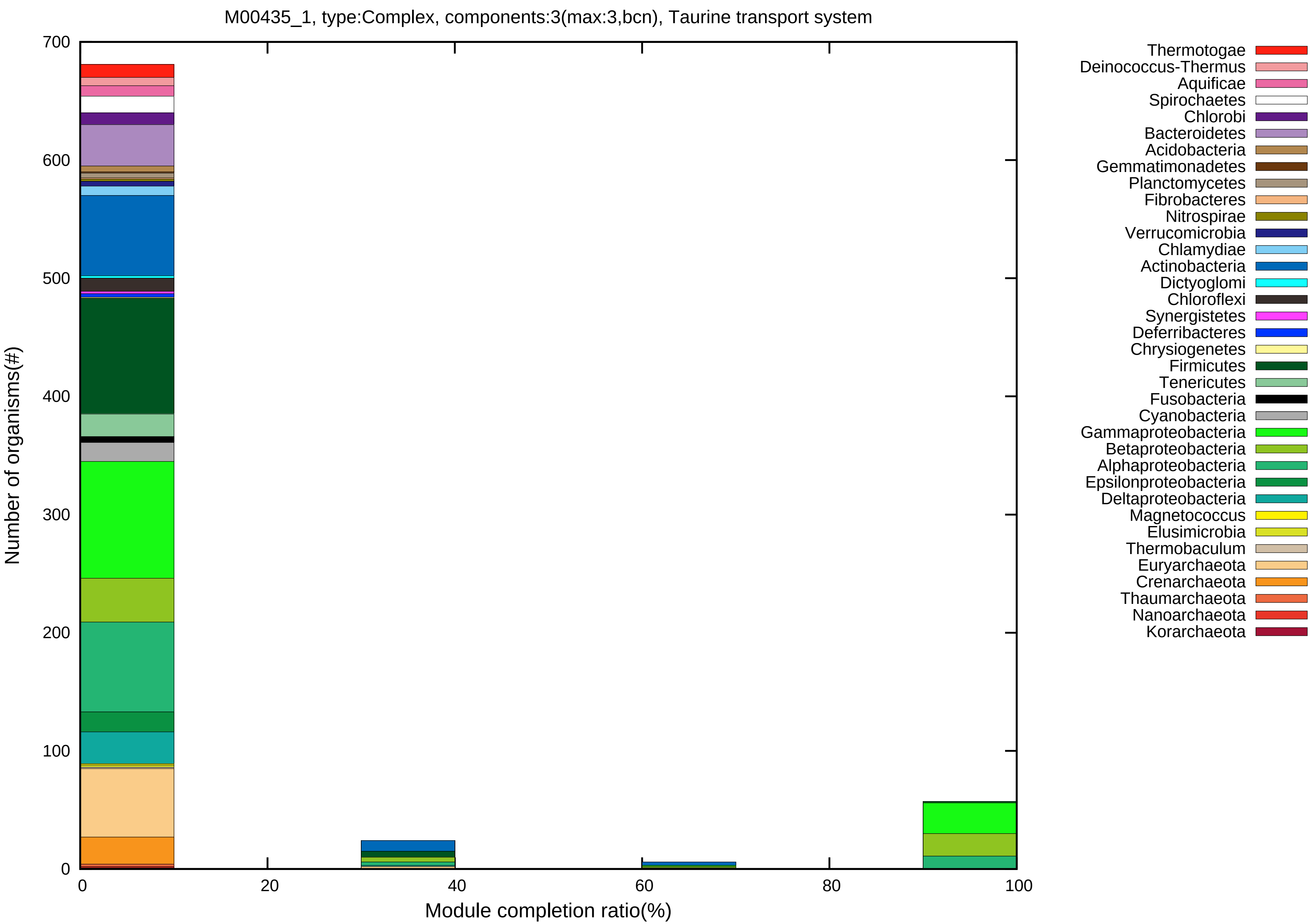
M00435_1, type:Complex, components:3(max:3,bcn), Taurine transport system
Number of organisms(#)
Module completion ratio(%)
0
100
200
300
400
500
600
700
0
20
40
60
80
100
Thermotogae
Deinococcus-Thermus
Aquificae
Spirochaetes
Chlorobi
Bacteroidetes
Acidobacteria
Gemmatimonadetes
Planctomycetes
Fibrobacteres
Nitrospirae
Verrucomicrobia
Chlamydiae
Actinobacteria
Dictyoglomi
Chloroflexi
Synergistetes
Deferribacteres
Chrysiogenetes
Firmicutes
Tenericutes
Fusobacteria
Cyanobacteria
Gammaproteobacteria
Betaproteobacteria
Alphaproteobacteria
Epsilonproteobacteria
Deltaproteobacteria
Magnetococcus
Elusimicrobia
Thermobaculum
Euryarchaeota
Crenarchaeota
Thaumarchaeota
Nanoarchaeota
Korarchaeota

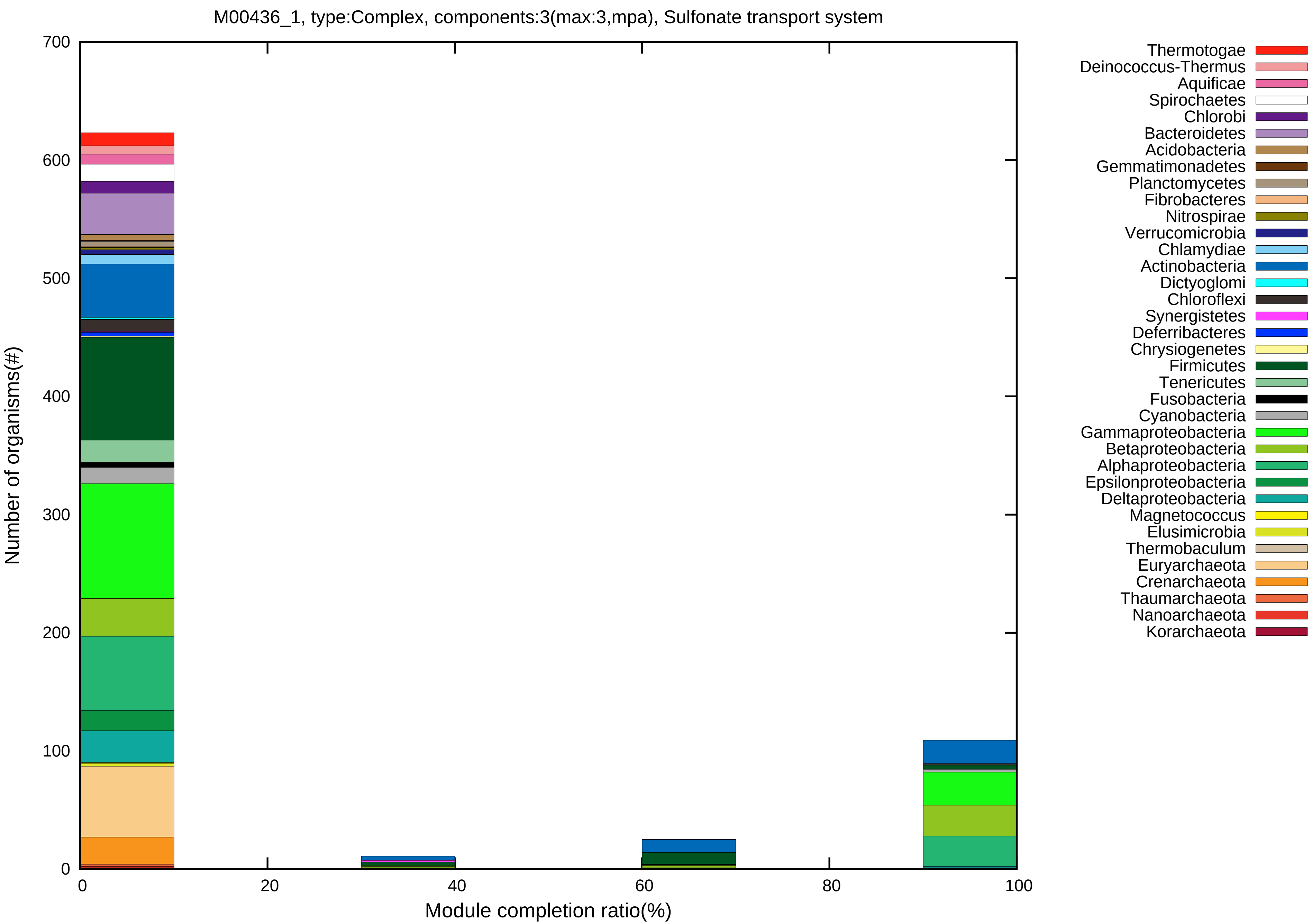

M00436_1, type:Complex, components:3(max:3,mpa), Sulfonate transport system
Number of organisms(#)
Module completion ratio(%)
0
100
200
300
400
500
600
700
0
20
40
60
80
100
Thermotogae
Deinococcus-Thermus
Aquificae
Spirochaetes
Chlorobi
Bacteroidetes
Acidobacteria
Gemmatimonadetes
Planctomycetes
Fibrobacteres
Nitrospirae
Verrucomicrobia
Chlamydiae
Actinobacteria
Dictyoglomi
Chloroflexi
Synergistetes
Deferribacteres
Chrysiogenetes
Firmicutes
Tenericutes
Fusobacteria
Cyanobacteria
Gammaproteobacteria
Betaproteobacteria
Alphaproteobacteria
Epsilonproteobacteria
Deltaproteobacteria
Magnetococcus
Elusimicrobia
Thermobaculum
Euryarchaeota
Crenarchaeota
Thaumarchaeota
Nanoarchaeota
Korarchaeota

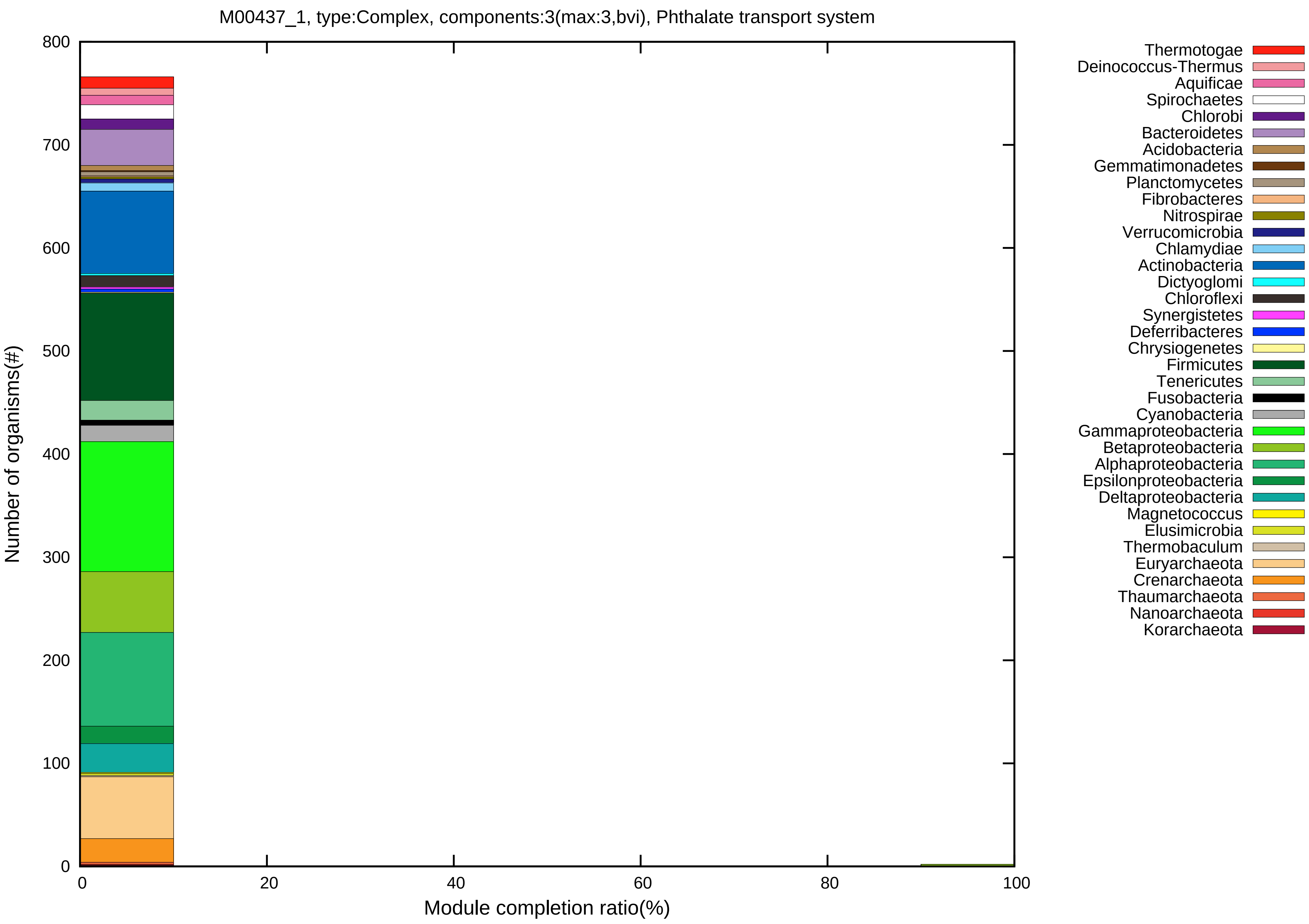
M00437_1, type:Complex, components:3(max:3,bvi), Phthalate transport system
Number of organisms(#)
Module completion ratio(%)
0
100
200
300
400
500
600
700
800
0
20
40
60
80
100
Thermotogae
Deinococcus-Thermus
Aquificae
Spirochaetes
Chlorobi
Bacteroidetes
Acidobacteria
Gemmatimonadetes
Planctomycetes
Fibrobacteres
Nitrospirae
Verrucomicrobia
Chlamydiae
Actinobacteria
Dictyoglomi
Chloroflexi
Synergistetes
Deferribacteres
Chrysiogenetes
Firmicutes
Tenericutes
Fusobacteria
Cyanobacteria
Gammaproteobacteria
Betaproteobacteria
Alphaproteobacteria
Epsilonproteobacteria
Deltaproteobacteria
Magnetococcus
Elusimicrobia
Thermobaculum
Euryarchaeota
Crenarchaeota
Thaumarchaeota
Nanoarchaeota
Korarchaeota

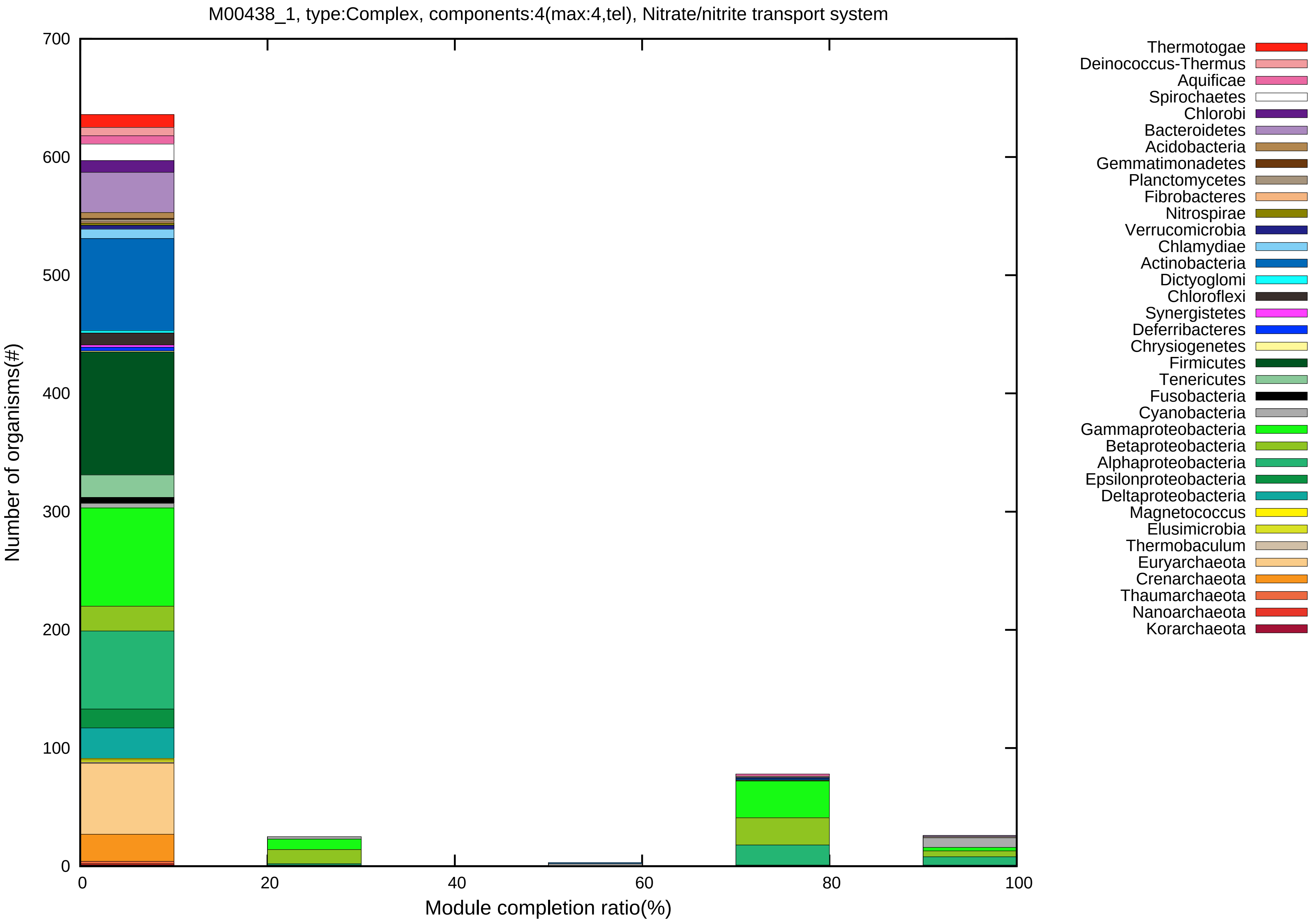
M00438_1, type:Complex, components:4(max:4,tel), Nitrate/nitrite transport system
Number of organisms(#)
Module completion ratio(%)
0
100
200
300
400
500
600
700
0
20
40
60
80
100
Thermotogae
Deinococcus-Thermus
Aquificae
Spirochaetes
Chlorobi
Bacteroidetes
Acidobacteria
Gemmatimonadetes
Planctomycetes
Fibrobacteres
Nitrospirae
Verrucomicrobia
Chlamydiae
Actinobacteria
Dictyoglomi
Chloroflexi
Synergistetes
Deferribacteres
Chrysiogenetes
Firmicutes
Tenericutes
Fusobacteria
Cyanobacteria
Gammaproteobacteria
Betaproteobacteria
Alphaproteobacteria
Epsilonproteobacteria
Deltaproteobacteria
Magnetococcus
Elusimicrobia
Thermobaculum
Euryarchaeota
Crenarchaeota
Thaumarchaeota
Nanoarchaeota
Korarchaeota

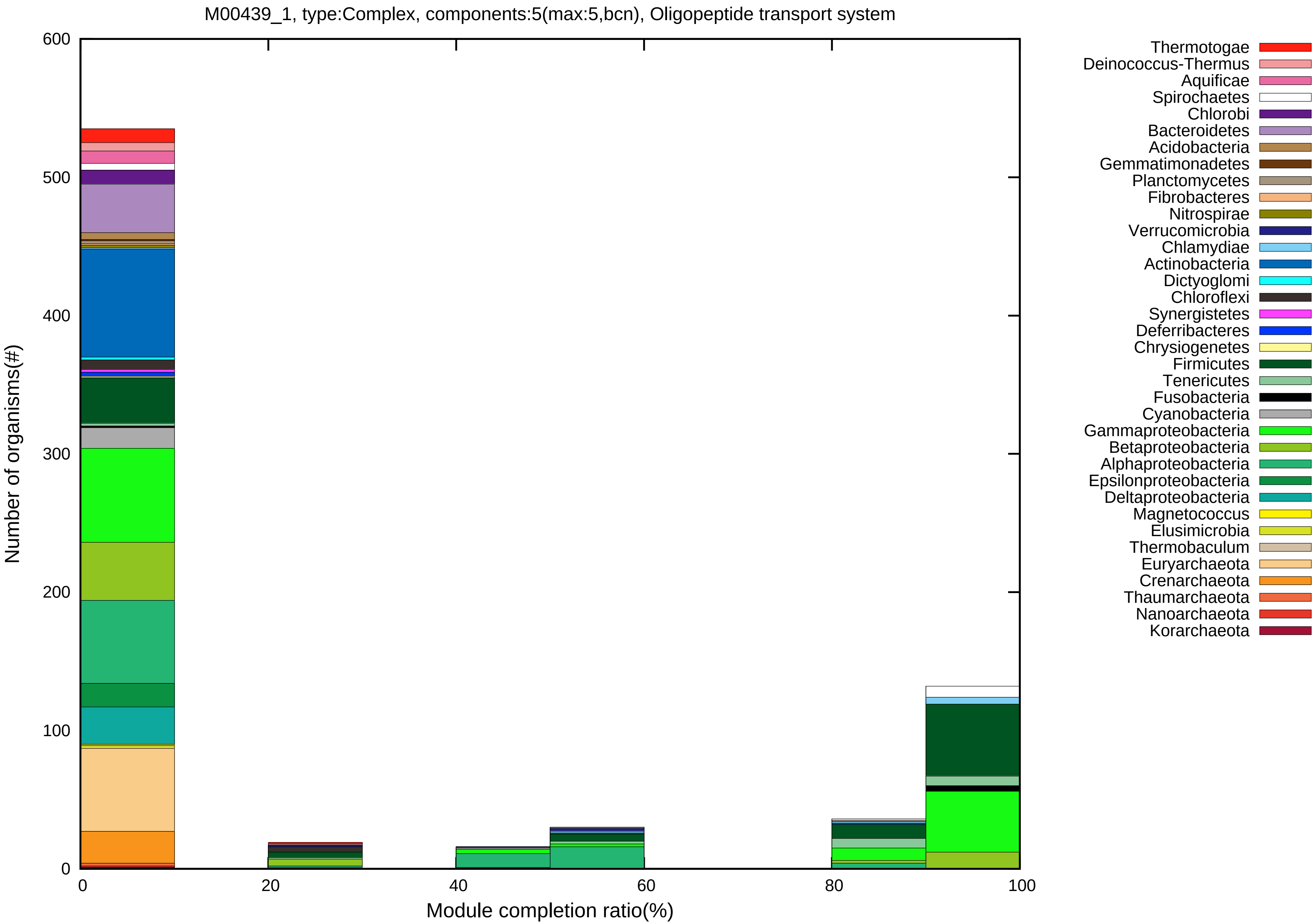

M00439_1, type:Complex, components:5(max:5,bcn), Oligopeptide transport system
Number of organisms(#)
Module completion ratio(%)
0
100
200
300
400
500
600
0
20
40
60
80
100
Thermotogae
Deinococcus-Thermus
Aquificae
Spirochaetes
Chlorobi
Bacteroidetes
Acidobacteria
Gemmatimonadetes
Planctomycetes
Fibrobacteres
Nitrospirae
Verrucomicrobia
Chlamydiae
Actinobacteria
Dictyoglomi
Chloroflexi
Synergistetes
Deferribacteres
Chrysiogenetes
Firmicutes
Tenericutes
Fusobacteria
Cyanobacteria
Gammaproteobacteria
Betaproteobacteria
Alphaproteobacteria
Epsilonproteobacteria
Deltaproteobacteria
Magnetococcus
Elusimicrobia
Thermobaculum
Euryarchaeota
Crenarchaeota
Thaumarchaeota
Nanoarchaeota
Korarchaeota

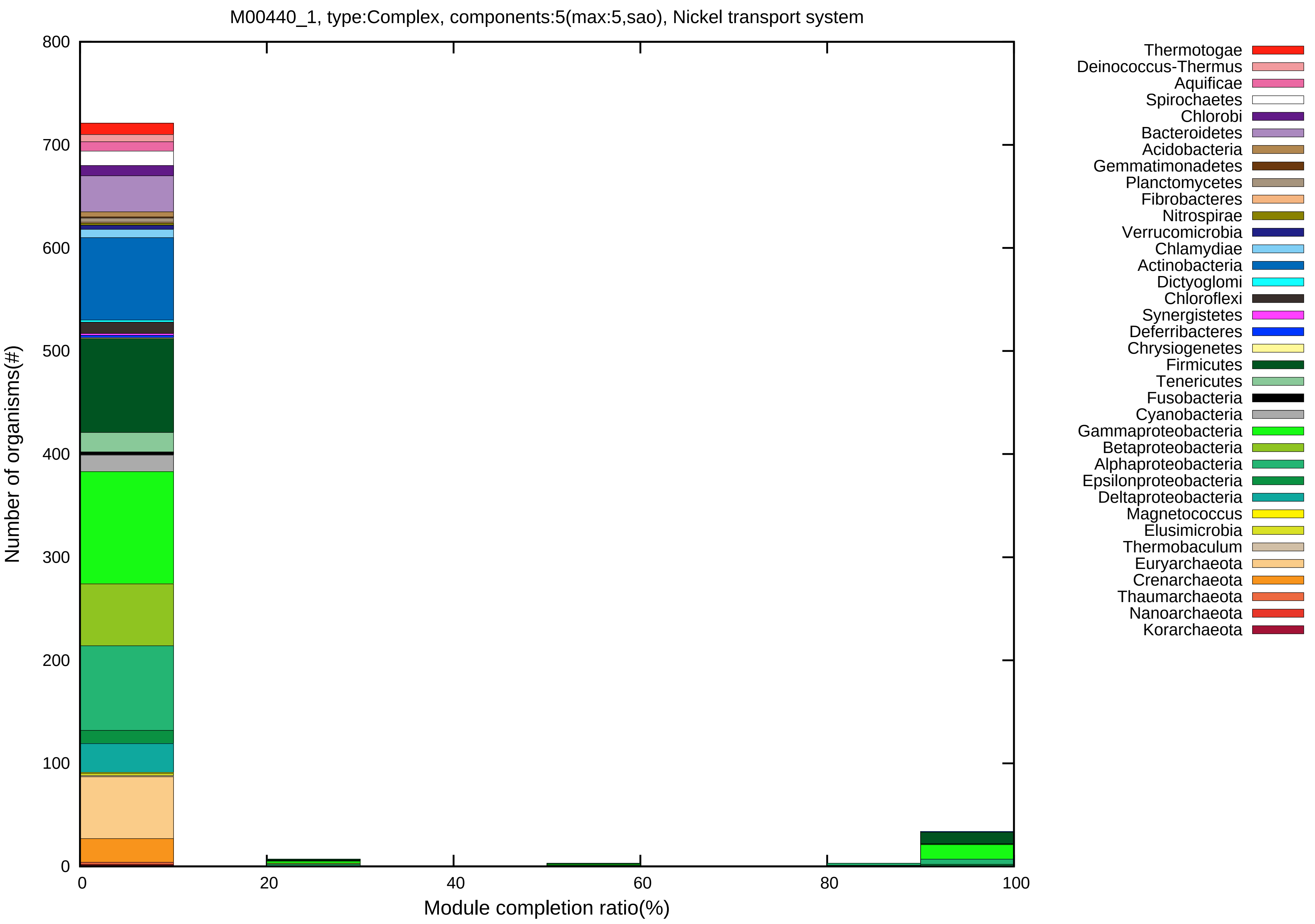
M00440_1, type:Complex, components:5(max:5,sao), Nickel transport system
Number of organisms(#)
Module completion ratio(%)
0
100
200
300
400
500
600
700
800
0
20
40
60
80
100
Thermotogae
Deinococcus-Thermus
Aquificae
Spirochaetes
Chlorobi
Bacteroidetes
Acidobacteria
Gemmatimonadetes
Planctomycetes
Fibrobacteres
Nitrospirae
Verrucomicrobia
Chlamydiae
Actinobacteria
Dictyoglomi
Chloroflexi
Synergistetes
Deferribacteres
Chrysiogenetes
Firmicutes
Tenericutes
Fusobacteria
Cyanobacteria
Gammaproteobacteria
Betaproteobacteria
Alphaproteobacteria
Epsilonproteobacteria
Deltaproteobacteria
Magnetococcus
Elusimicrobia
Thermobaculum
Euryarchaeota
Crenarchaeota
Thaumarchaeota
Nanoarchaeota
Korarchaeota

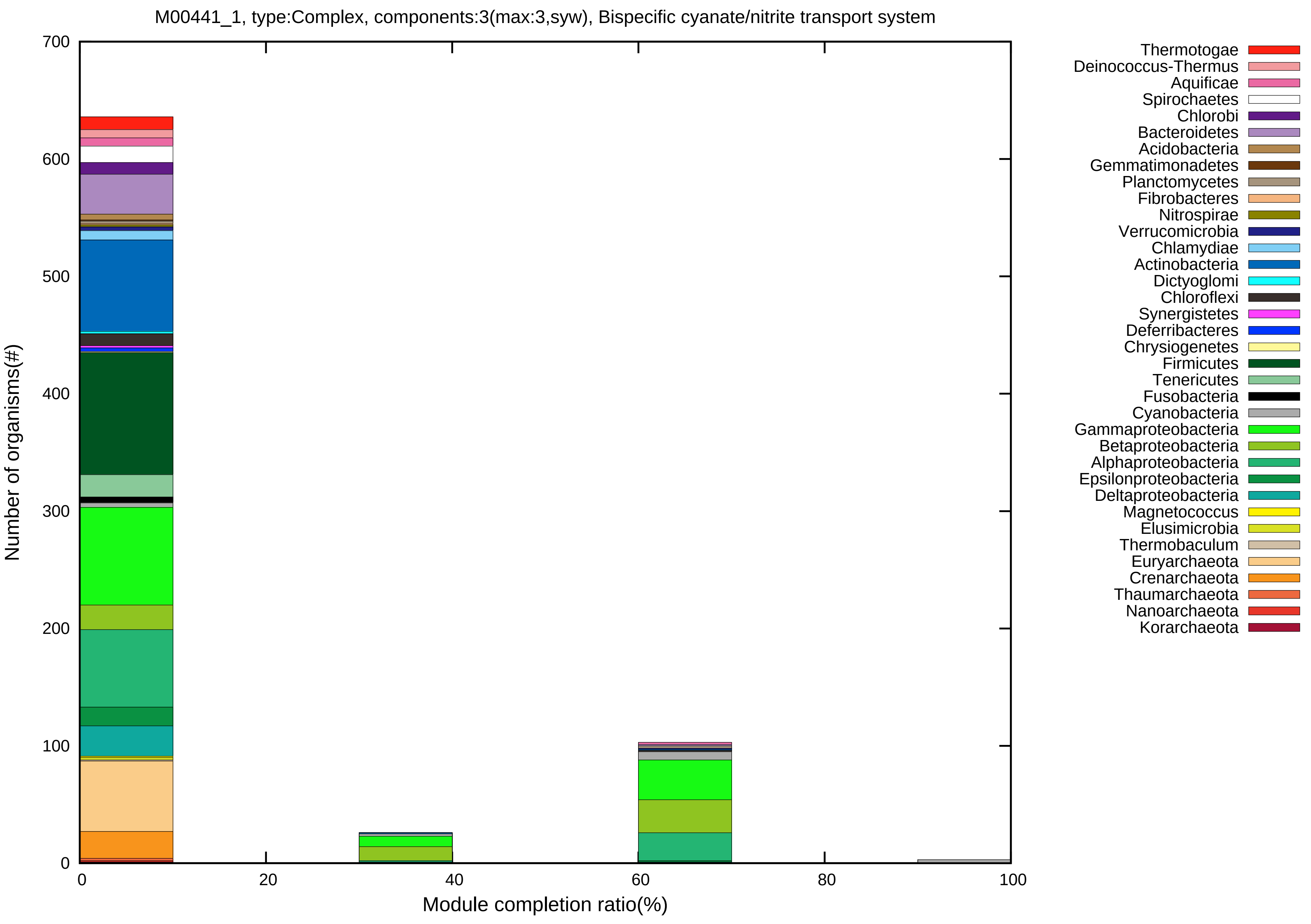

M00441_1, type:Complex, components:3(max:3,syw), Bispecific cyanate/nitrite transport system
Number of organisms(#)
Module completion ratio(%)
0
100
200
300
400
500
600
700
0
20
40
60
80
100
Thermotogae
Deinococcus-Thermus
Aquificae
Spirochaetes
Chlorobi
Bacteroidetes
Acidobacteria
Gemmatimonadetes
Planctomycetes
Fibrobacteres
Nitrospirae
Verrucomicrobia
Chlamydiae
Actinobacteria
Dictyoglomi
Chloroflexi
Synergistetes
Deferribacteres
Chrysiogenetes
Firmicutes
Tenericutes
Fusobacteria
Cyanobacteria
Gammaproteobacteria
Betaproteobacteria
Alphaproteobacteria
Epsilonproteobacteria
Deltaproteobacteria
Magnetococcus
Elusimicrobia
Thermobaculum
Euryarchaeota
Crenarchaeota
Thaumarchaeota
Nanoarchaeota
Korarchaeota

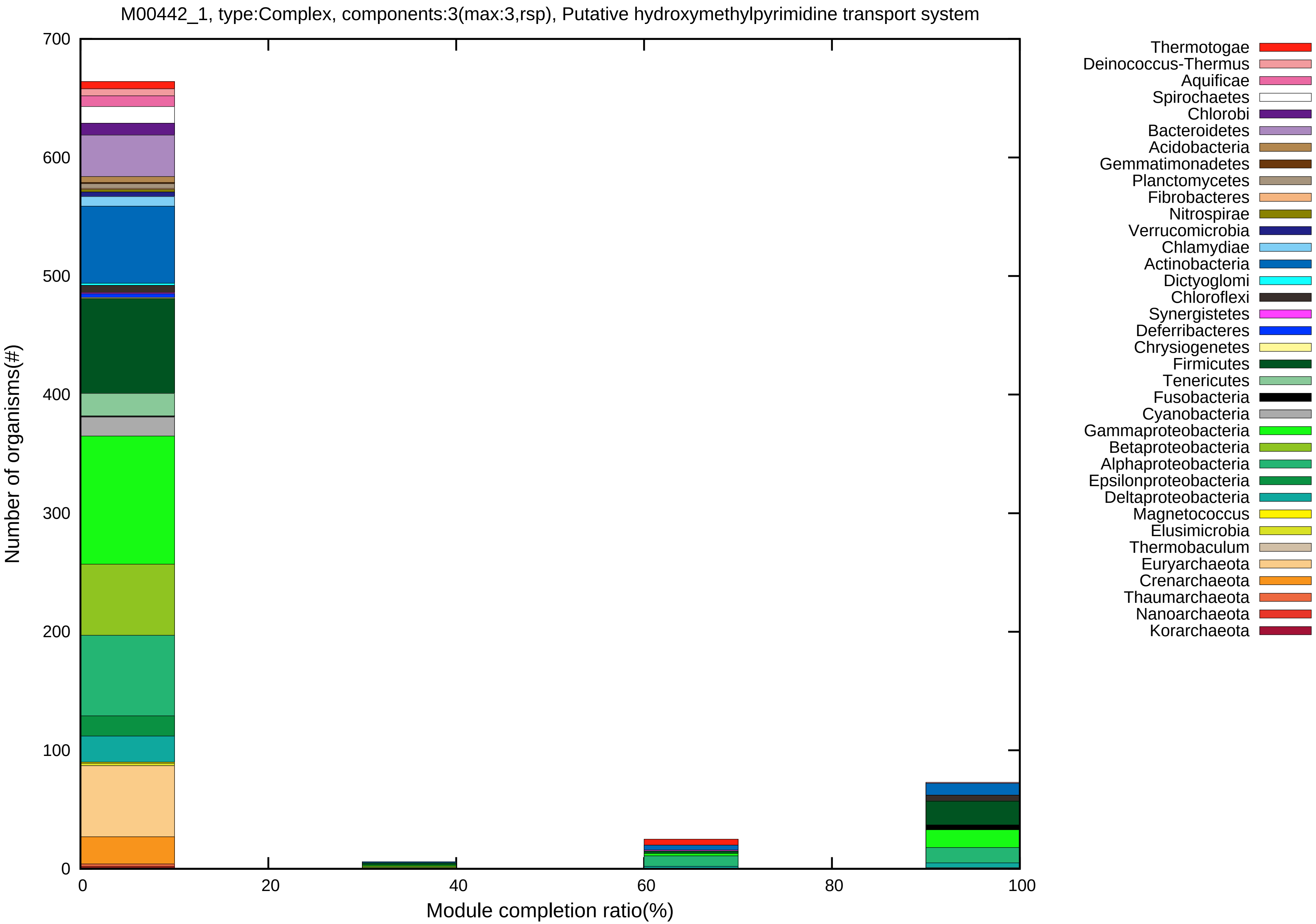

M00442_1, type:Complex, components:3(max:3,rsp), Putative hydroxymethylpyrimidine transport system
Number of organisms(#)
0
100
200
300
400
500
600
700
0
20
40
60
80
100
Module completion ratio(%)
Thermotogae
Deinococcus-Thermus
Aquificae
Spirochaetes
Chlorobi
Bacteroidetes
Acidobacteria
Gemmatimonadetes
Planctomycetes
Fibrobacteres
Nitrospirae
Verrucomicrobia
Chlamydiae
Actinobacteria
Dictyoglomi
Chloroflexi
Synergistetes
Deferribacteres
Chrysiogenetes
Firmicutes
Tenericutes
Fusobacteria
Cyanobacteria
Gammaproteobacteria
Betaproteobacteria
Alphaproteobacteria
Epsilonproteobacteria
Deltaproteobacteria
Magnetococcus
Elusimicrobia
Thermobaculum
Euryarchaeota
Crenarchaeota
Thaumarchaeota
Nanoarchaeota
Korarchaeota

Figure S3. Distribution patterns of the completion ratio of the KEGG functional set and signature modules in 768 prokaryotic species. The module completion ratio of 7 functional set and signatutre modules was evaluated in this study.

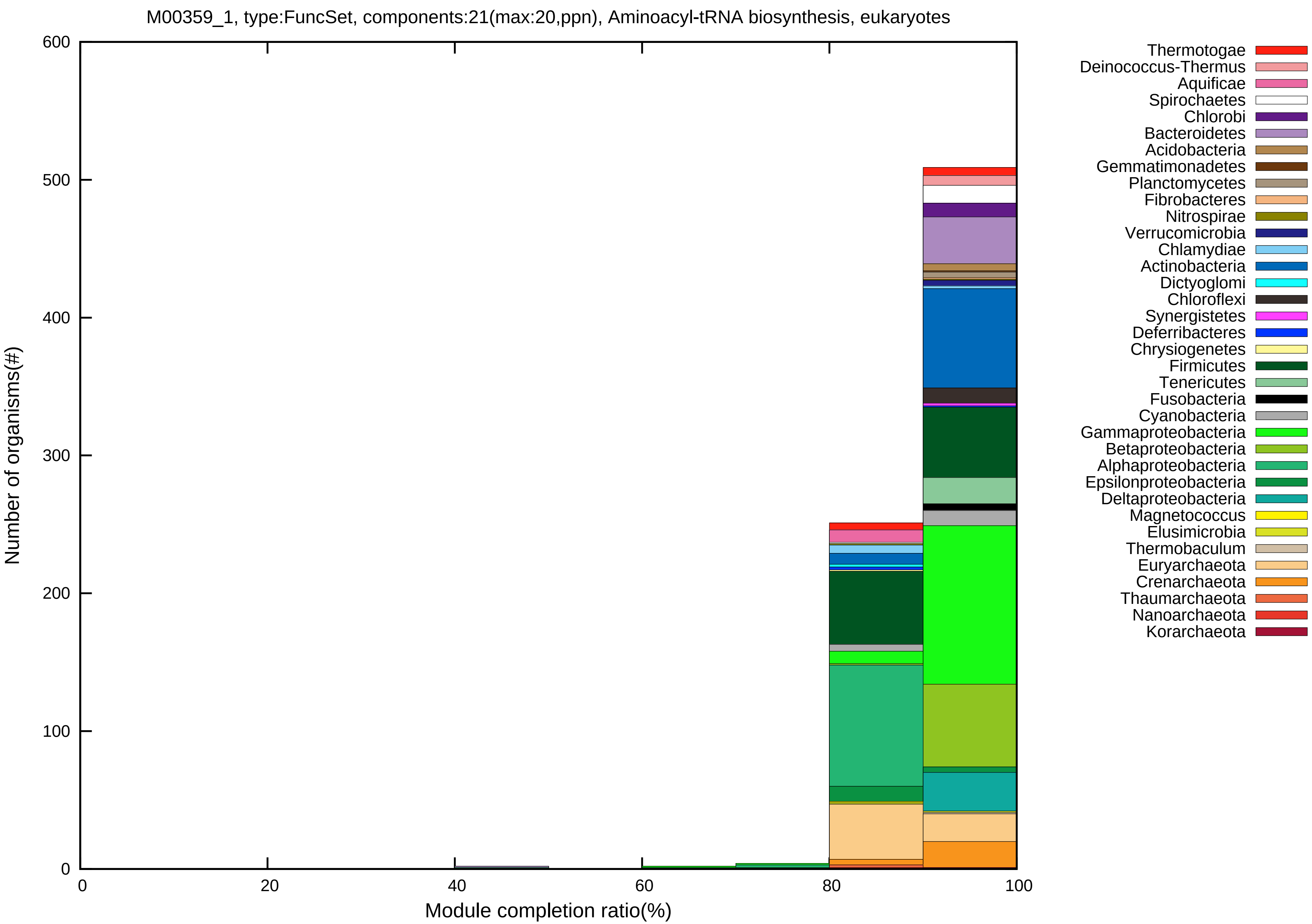
M00359_1, type:FuncSet, components:21(max:20,ppn), Aminoacyl-tRNA biosynthesis, eukaryotes
Number of organisms(#)
Module completion ratio(%)
0
100
200
300
400
500
600
0
20
40
60
80
100
Thermotogae
Deinococcus-Thermus
Aquificae
Spirochaetes
Chlorobi
Bacteroidetes
Acidobacteria
Gemmatimonadetes
Planctomycetes
Fibrobacteres
Nitrospirae
Verrucomicrobia
Chlamydiae
Actinobacteria
Dictyoglomi
Chloroflexi
Synergistetes
Deferribacteres
Chrysiogenetes
Firmicutes
Tenericutes
Fusobacteria
Cyanobacteria
Gammaproteobacteria
Betaproteobacteria
Alphaproteobacteria
Epsilonproteobacteria
Deltaproteobacteria
Magnetococcus
Elusimicrobia
Thermobaculum
Euryarchaeota
Crenarchaeota
Thaumarchaeota
Nanoarchaeota
Korarchaeota

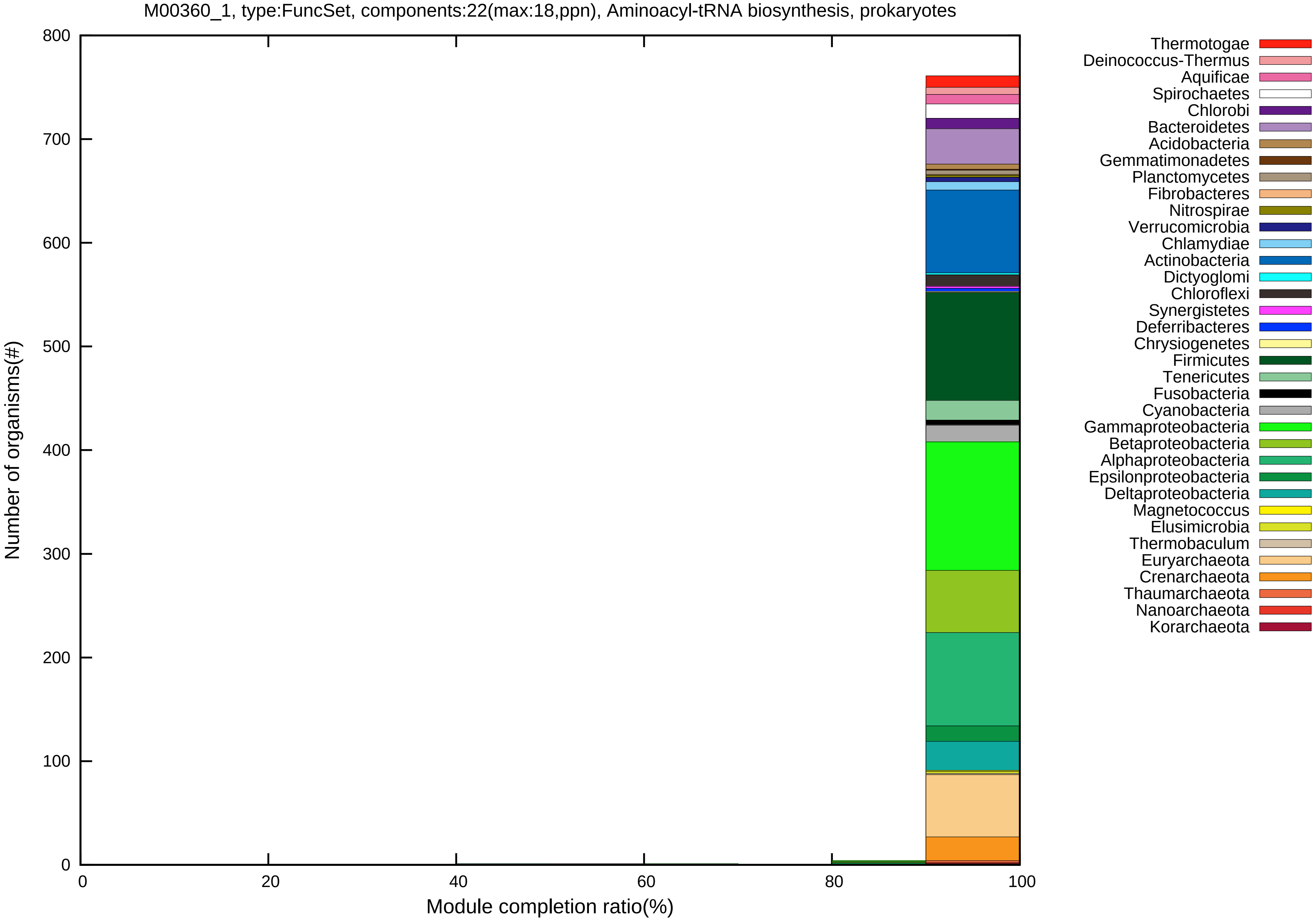

M00360_1, type:FuncSet, components:22(max:18,ppn), Aminoacyl-tRNA biosynthesis, prokaryotes
Number of organisms(#)
Module completion ratio(%)
0
100
200
300
400
500
600
700
800
0
20
40
60
80
100
Thermotogae
Deinococcus-Thermus
Aquificae
Spirochaetes
Chlorobi
Bacteroidetes
Acidobacteria
Gemmatimonadetes
Planctomycetes
Fibrobacteres
Nitrospirae
Verrucomicrobia
Chlamydiae
Actinobacteria
Dictyoglomi
Chloroflexi
Synergistetes
Deferribacteres
Chrysiogenetes
Firmicutes
Tenericutes
Fusobacteria
Cyanobacteria
Gammaproteobacteria
Betaproteobacteria
Alphaproteobacteria
Epsilonproteobacteria
Deltaproteobacteria
Magnetococcus
Elusimicrobia
Thermobaculum
Euryarchaeota
Crenarchaeota
Thaumarchaeota
Nanoarchaeota
Korarchaeota

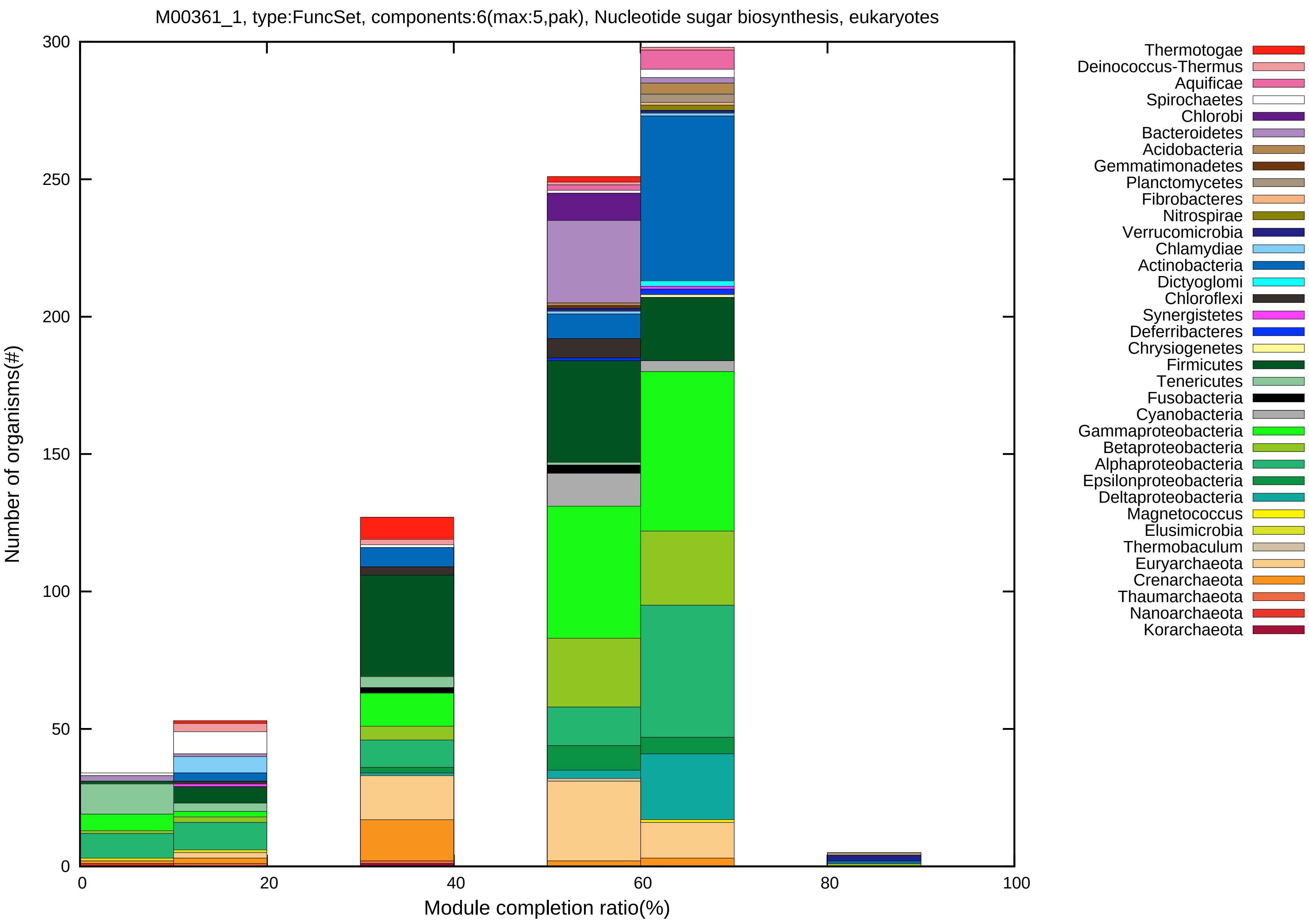

M00361_1, type:FuncSet, components:6(max:5,pak), Nucleotide sugar biosynthesis, eukaryotes
Number of organisms(#)
Module completion ratio(%)
0
50
100
150
200
250
300
0
20
40
60
80
100
Thermotogae
Deinococcus-Thermus
Aquificae
Spirochaetes
Chlorobi
Bacteroidetes
Acidobacteria
Gemmatimonadetes
Planctomycetes
Fibrobacteres
Nitrospirae
Verrucomicrobia
Chlamydiae
Actinobacteria
Dictyoglomi
Chloroflexi
Synergistetes
Deferribacteres
Chrysiogenetes
Firmicutes
Tenericutes
Fusobacteria
Cyanobacteria
Gammaproteobacteria
Betaproteobacteria
Alphaproteobacteria
Epsilonproteobacteria
Deltaproteobacteria
Magnetococcus
Elusimicrobia
Thermobaculum
Euryarchaeota
Crenarchaeota
Thaumarchaeota
Nanoarchaeota
Korarchaeota

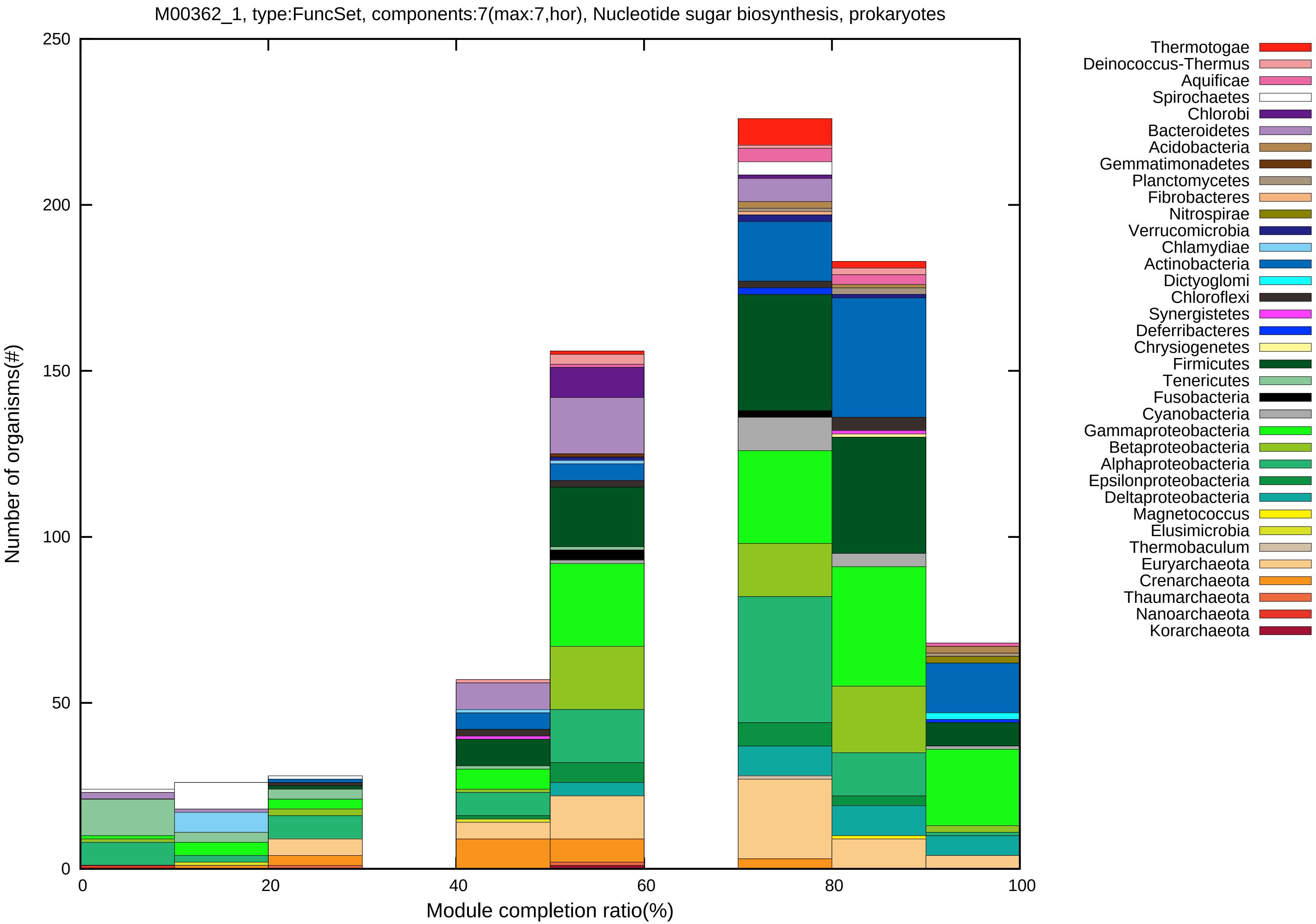
M00362_1, type:FuncSet, components:7(max:7,hor), Nucleotide sugar biosynthesis, prokaryotes
Number of organisms(#)
Module completion ratio(%)
0
50
100
150
200
250
0
20
40
60
80
100
Thermotogae
Deinococcus-Thermus
Aquificae
Spirochaetes
Chlorobi
Bacteroidetes
Acidobacteria
Gemmatimonadetes
Planctomycetes
Fibrobacteres
Nitrospirae
Verrucomicrobia
Chlamydiae
Actinobacteria
Dictyoglomi
Chloroflexi
Synergistetes
Deferribacteres
Chrysiogenetes
Firmicutes
Tenericutes
Fusobacteria
Cyanobacteria
Gammaproteobacteria
Betaproteobacteria
Alphaproteobacteria
Epsilonproteobacteria
Deltaproteobacteria
Magnetococcus
Elusimicrobia
Thermobaculum
Euryarchaeota
Crenarchaeota
Thaumarchaeota
Nanoarchaeota
Korarchaeota

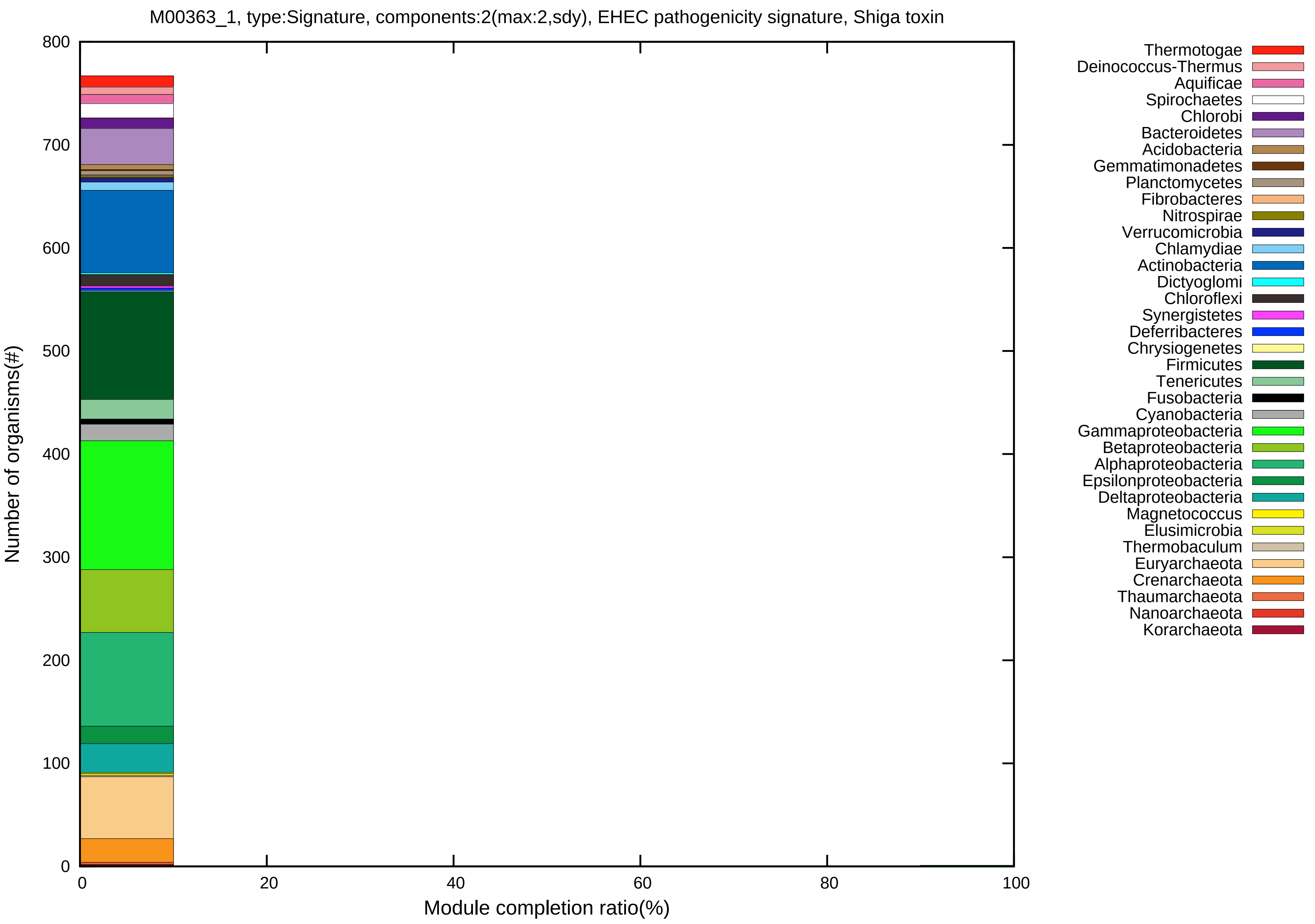

M00363_1, type:Signature, components:2(max:2,sdy), EHEC pathogenicity signature, Shiga toxin
Number of organisms(#)
0
100
200
300
400
500
600
700
800
Module completion ratio(%)
0
20
40
60
80
100
Thermotogae
Deinococcus-Thermus
Aquificae
Spirochaetes
Chlorobi
Bacteroidetes
Acidobacteria
Gemmatimonadetes
Planctomycetes
Fibrobacteres
Nitrospirae
Verrucomicrobia
Chlamydiae
Actinobacteria
Dictyoglomi
Chloroflexi
Synergistetes
Deferribacteres
Chrysiogenetes
Firmicutes
Tenericutes
Fusobacteria
Cyanobacteria
Gammaproteobacteria
Betaproteobacteria
Alphaproteobacteria
Epsilonproteobacteria
Deltaproteobacteria
Magnetococcus
Elusimicrobia
Thermobaculum
Euryarchaeota
Crenarchaeota
Thaumarchaeota
Nanoarchaeota
Korarchaeota

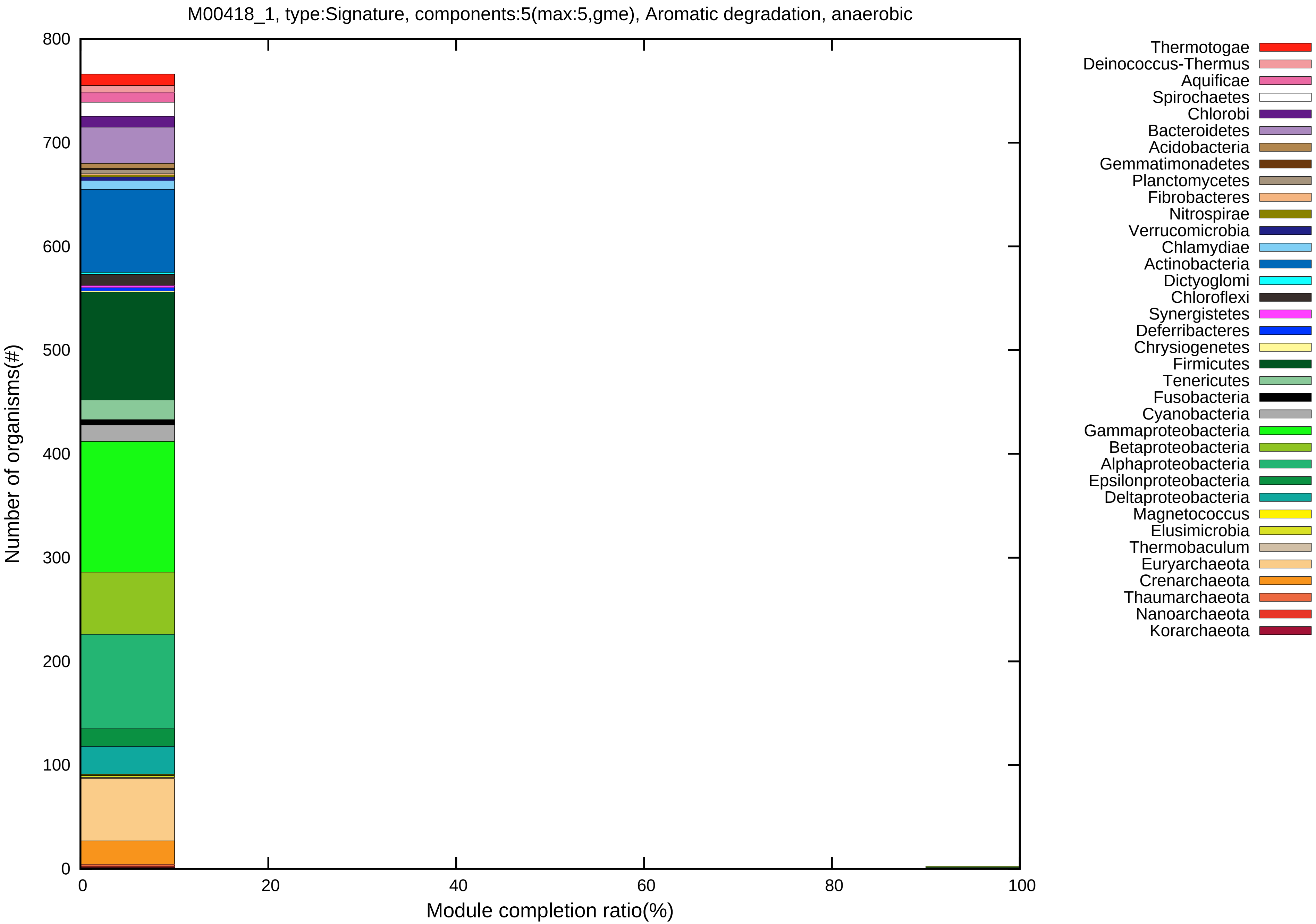

M00418_1, type:Signature, components:5(max:5,gme), Aromatic degradation, anaerobic
Number of organisms(#)
Module completion ratio(%)
0
100
200
300
400
500
600
700
800
0
20
40
60
80
100
Thermotogae
Deinococcus-Thermus
Aquificae
Spirochaetes
Chlorobi
Bacteroidetes
Acidobacteria
Gemmatimonadetes
Planctomycetes
Fibrobacteres
Nitrospirae
Verrucomicrobia
Chlamydiae
Actinobacteria
Dictyoglomi
Chloroflexi
Synergistetes
Deferribacteres
Chrysiogenetes
Firmicutes
Tenericutes
Fusobacteria
Cyanobacteria
Gammaproteobacteria
Betaproteobacteria
Alphaproteobacteria
Epsilonproteobacteria
Deltaproteobacteria
Magnetococcus
Elusimicrobia
Thermobaculum
Euryarchaeota
Crenarchaeota
Thaumarchaeota
Nanoarchaeota
Korarchaeota

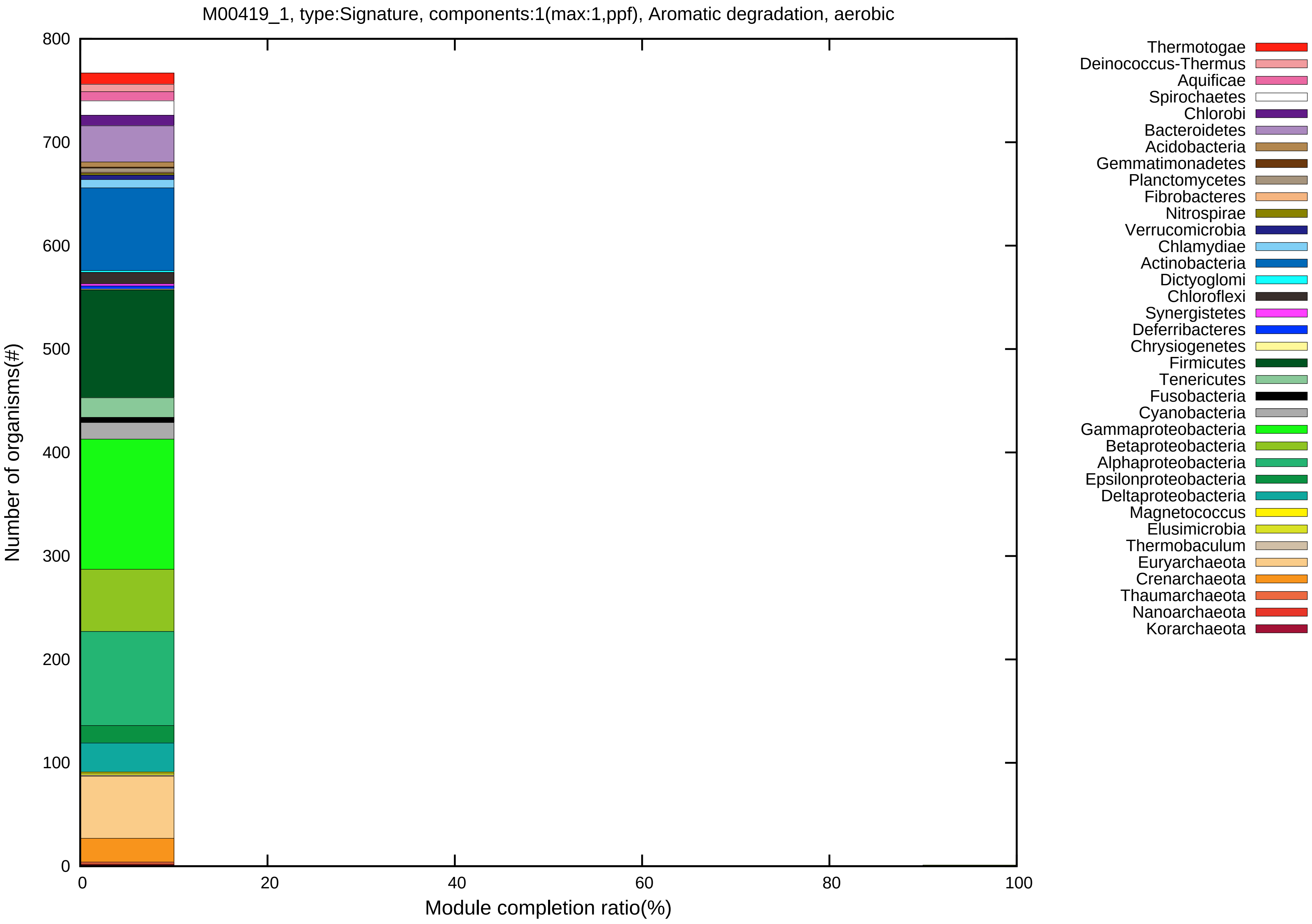

M00419_1, type:Signature, components:1(max:1,ppf), Aromatic degradation, aerobic
Number of organisms(#)
Module completion ratio(%)
0
100
200
300
400
500
600
700
800
0
20
40
60
80
100
Thermotogae
Deinococcus-Thermus
Aquificae
Spirochaetes
Chlorobi
Bacteroidetes
Acidobacteria
Gemmatimonadetes
Planctomycetes
Fibrobacteres
Nitrospirae
Verrucomicrobia
Chlamydiae
Actinobacteria
Dictyoglomi
Chloroflexi
Synergistetes
Deferribacteres
Chrysiogenetes
Firmicutes
Tenericutes
Fusobacteria
Cyanobacteria
Gammaproteobacteria
Betaproteobacteria
Alphaproteobacteria
Epsilonproteobacteria
Deltaproteobacteria
Magnetococcus
Elusimicrobia
Thermobaculum
Euryarchaeota
Crenarchaeota
Thaumarchaeota
Nanoarchaeota
Korarchaeota

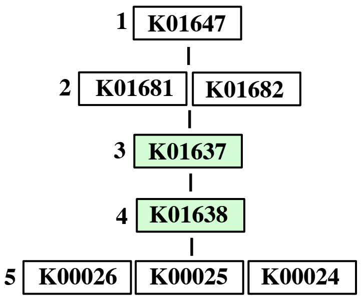

KEGG **Glyoxylate cycle**

[Module menu | Organism menu | Module entry | KO/Compound list]

Entry	M00012
Name	Glyoxylate cycle
Definition	K01647 (K01681,K01682) K01637 K01638 (K00026,K00025,K00024)
	Ortholog table / Taxonomy
Type	Pathway module

KEGG **ORTHOLOGY: K01647** Help

Entry	K01647 KO
Name	CS, gltA
Definition	citrate synthase [EC:2.3.3.1]
Pathway	ko00020 Citrate cycle (TCA cycle)<br>ko00630 Glyoxylate and dicarboxylate metabolism
Module	M00009 Citrate cycle (TCA cycle, Krebs cycle)<br>M00010 Citrate cycle, first carbon oxidation, oxaloacetate => 2-oxoglutarate<br>M00012 Glyoxylate cycle
Entry	K01681 KO
Name	ACO, acnA
Definition	aconitate hydratase 1 [EC:4.2.1.3]
Pathway	ko00020 Citrate cycle (TCA cycle)<br>ko00630 Glyoxylate and dicarboxylate metabolism<br>ko00720 Carbon fixation pathways in prokaryotes
Module	M00009 Citrate cycle (TCA cycle, Krebs cycle)<br>M00010 Citrate cycle, first carbon oxidation, oxaloacetate => 2-oxoglutarate<br>M00012 Glyoxylate cycle<br>M00173 Reductive citric acid cycle (Arnon-Buchanan cycle)
Entry	K01682 KO
Name	acnB
Definition	aconitate hydratase 2 / 2-methylisocitrate dehydratase [EC:4.2.1.3 4.2.1.99]
Pathway	ko00020 Citrate cycle (TCA cycle)<br>ko00630 Glyoxylate and dicarboxylate metabolism<br>ko00640 Propanoate metabolism<br>ko00720 Carbon fixation pathways in prokaryotes
Module	M00009 Citrate cycle (TCA cycle, Krebs cycle)<br>M00010 Citrate cycle, first carbon oxidation, oxaloacetate => 2-oxoglutarate<br>M00012 Glyoxylate cycle<br>M00173 Reductive citric acid cycle (Arnon-Buchanan cycle)

KEGG **ORTHOLOGY: K00026** Help

Entry	K00026 KO
Name	MDH2
Definition	malate dehydrogenase [EC:1.1.1.37]
Pathway	ko00020 Citrate cycle (TCA cycle)<br>ko00620 Pyruvate metabolism<br>ko00630 Glyoxylate and dicarboxylate metabolism<br>ko00710 Carbon fixation in photosynthetic organisms
Module	M00009 Citrate cycle (TCA cycle, Krebs cycle)<br>M00011 Citrate cycle, second carbon oxidation, 2-oxoglutarate => oxaloacetate<br>M00012 Glyoxylate cycle<br>M00168 CAM (Crassulacean acid metabolism), dark<br>M00171 C4-dicarboxylic acid cycle, NAD+ -malic enzyme type
Entry	K00025 KO
Name	MDH1
Definition	malate dehydrogenase [EC:1.1.1.37]
Pathway	ko00020 Citrate cycle (TCA cycle)<br>ko00620 Pyruvate metabolism<br>ko00630 Glyoxylate and dicarboxylate metabolism<br>ko00710 Carbon fixation in photosynthetic organisms<br>ko04964 Proximal tubule bicarbonate reclamation
Module	M00009 Citrate cycle (TCA cycle, Krebs cycle)<br>M00011 Citrate cycle, second carbon oxidation, 2-oxoglutarate => oxaloacetate<br>M00012 Glyoxylate cycle<br>M00168 CAM (Crassulacean acid metabolism), dark<br>M00171 C4-dicarboxylic acid cycle, NAD+ -malic enzyme type
Entry	K00024 KO
Name	mdh
Definition	malate dehydrogenase [EC:1.1.1.37]
Pathway	ko00020 Citrate cycle (TCA cycle)<br>ko00620 Pyruvate metabolism<br>ko00630 Glyoxylate and dicarboxylate metabolism<br>ko00680 Methane metabolism<br>ko00710 Carbon fixation in photosynthetic organisms<br>ko00720 Carbon fixation pathways in prokaryotes
Module	M00009 Citrate cycle (TCA cycle, Krebs cycle)<br>M00011 Citrate cycle, second carbon oxidation, 2-oxoglutarate => oxaloacetate<br>M00012 Glyoxylate cycle<br>M00168 CAM (Crassulacean acid metabolism), dark<br>M00171 C4-dicarboxylic acid cycle, NAD+ -malic enzyme type<br>M00173 Reductive citric acid cycle (Arnon-Buchanan cycle)<br>M00346 Formaldehyde assimilation, serine pathway<br>M00374 Dicarboxylate-hydroxybutyrate cycle

Figure S4. Distribution of KO identifiers mapped to the module for glyoxylate cycle (M00012) in other pathway modules. KO identifiers, except for K01637 and K01638 colored light green, are also shared in several other modules.

Figure S5. Module completion patterns in 8 phenotypically different *Bacillus*-related species. (A) Pathway module. (B) Structural complex module. bsu, *Bacillus subtilis* ; bao, *Bacillus amyloliquefaciens* ; bli, *Bacillus licheniformis* ; bha, *Bacillus halodurans* ; bpf, *Bacillus pseudofirmus* ; oih, *Oceanobacillus iheyensis* ; gka, *Geobacillus kaustophilus* ; and gth, *Geobacillus thermoglucosidasius*. Green characters show rare modules, which are completed by less than 10% of 768 prokaryotic species.

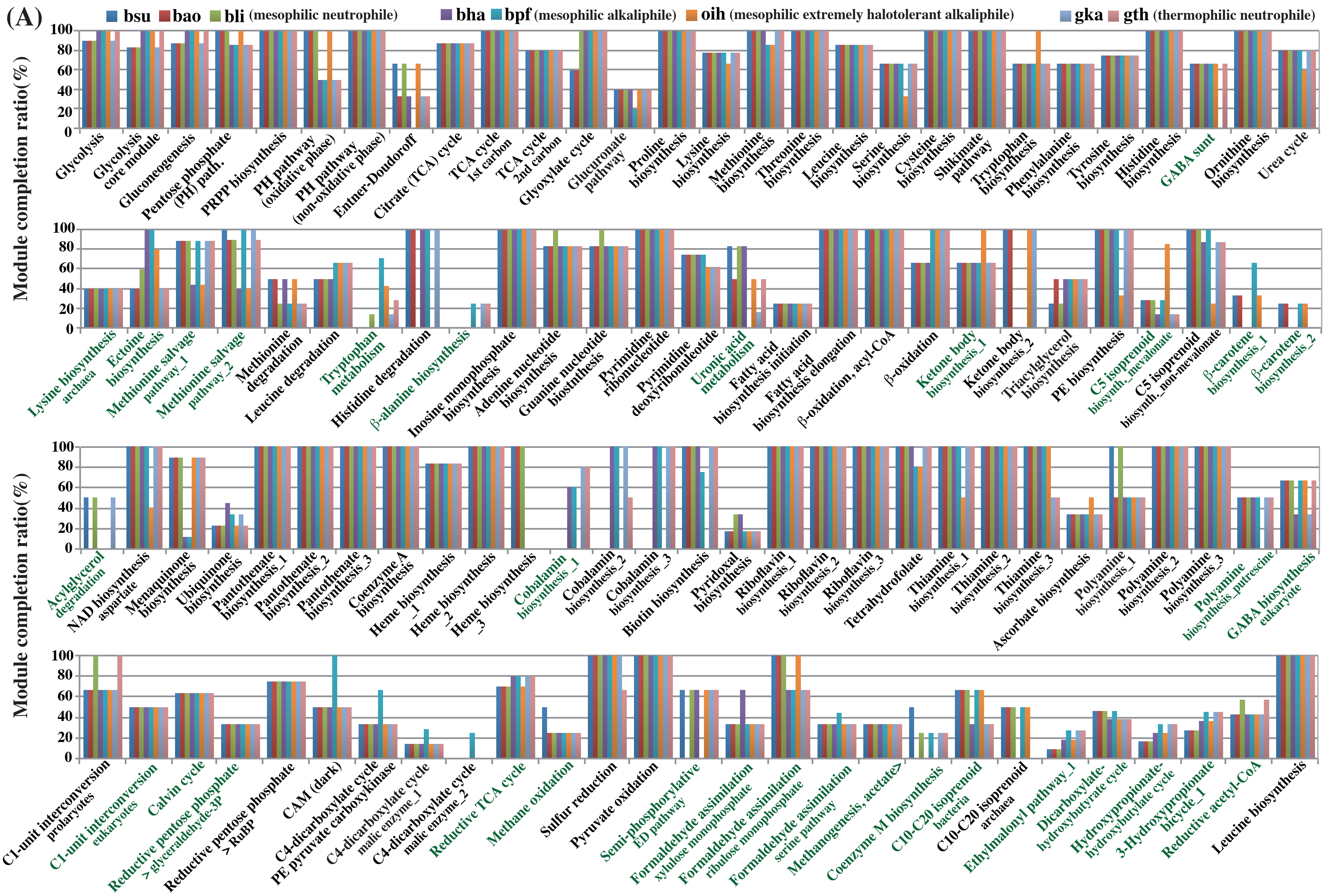

(A)
bsu bao bli (mesophilic neutrophile)
bha bpf (mesophilic alkaliphile)
oih (mesophilic extremely halotolerant alkaliphile)
gka gth (thermophilic neutrophile)
Module completion ratio(%)
Glycolysis
Glycolysis core module
Gluconeogenesis
Pentose phosphate (PH) path.
PRPP biosynthesis
PH pathway (oxidative phase)
PH pathway (non-oxidative phase)
Entner-Doudoroff
Citrate (TCA) cycle
TCA cycle 1st carbon
TCA cycle 2nd carbon
Glyoxylate cycle
Glucuronate pathway
Proline biosynthesis
Lysine biosynthesis
Methionine biosynthesis
Threonine biosynthesis
Leucine biosynthesis
Serine biosynthesis
Cysteine biosynthesis
Shikimate pathway
Tryptophan biosynthesis
Phenylalanine biosynthesis
Tyrosine biosynthesis
Histidine biosynthesis
GABA sunt
Ornithine biosynthesis
Urea cycle
Lysine biosynthesis archaea
Ectoine biosynthesis
Methionine salvage pathway_1
Methionine salvage pathway_2
Methionine degradation
Leucine degradation
Tryptophan metabolism
Histidine degradation
β-alanine biosynthesis
Inosine monophosphate biosynthesis
Adenine nucleotide biosynthesis
Guanine nucleotide biostnthesis
Pyrimidine ribonucleotide
Pyrimidine deoxyribonuleotide
Uronic acid metabolism
Fatty acid biosynthesis initiation
Fatty acid biosynthesis elongation
β-oxidation, acyl-CoA
β-oxidation
Ketone body biosynthesis_1
Ketone body biosynthesis_2
Triacylglycerol biosynthesis
PE biosynthesis
C5 isoprenoid biosynth._mevalonate
C5 isoprenoid biosynth._non-mevalonate
β-carotene biosynthesis_1
β-carotene biosynthesis_2
Acylglycerol degradation
NAD biosynthesis aspartate
Menaquinone biosynthesis
Ubiquinone biosynthesis
Pantothenate biosynthesis_1
Pantothenate biosynthesis_2
Pantothenate biosynthesis_3
Coenzyme A biosynthesis
Heme biosynthesis _1
Heme biosynthesis _2
Heme biosynthesis _3
Cobalamin biosynthesis _1
Cobalamin biosynthesis_2
Cobalamin biosynthesis_3
Biotin biosynthesis
Pyridoxal biosynthesis
Riboflavin biosynthesis_1
Riboflavin biosynthesis_2
Riboflavin biosynthesis_3
Tetrahydrofolate
Thiamine biosynthesis_1
Thiamine biosynthesis_2
Thiamine biosynthesis_3
Ascorbate biosynthesis
Polyamine biosynthesis_1
Polyamine biosynthesis_2
Polyamine biosynthesis_3
Polyamine biosynthesis_putrescine
GABA biosynthesis eukaryote
C1-unit interconversion prolaryotes
C1-unit interconversion eukaryotes
Calvin cycle
Reductive pentose phosphate > glyceraldehyde-3P
Reductive pentose phosphate > RuBP
CAM (dark)
C4-dicarboxylate cycle PE pyruvate carboxykinase
C4-dicarboxylate cycle malic enzyme_1
C4-dicarboxylate cycle malic enzyme_2
Reductive TCA cycle
Methane oxidation
Sulfur reduction
Pyruvate oxidation
Semi-phosphorylative ED pathway
Formaldehyde assimilation xylulose monophosphate
Formaldehyde assimilation ribulose monophosphate
Formaldehyde assimilation serine pathway
Methanogenesis, acetate>
Coenzyme M biosynthesis
C10-C20 isoprenoid bacteria
C10-C20 isoprenoid archaea
Ethylmalonyl pathway_1
Dicarboxylate-hydroxybutyrate cycle
Hydroxypropionate-hydroxybutylate cycle
3-Hydroxypropionate bicycle_1
Reductive acetyl-CoA
Leucine biosynthesis

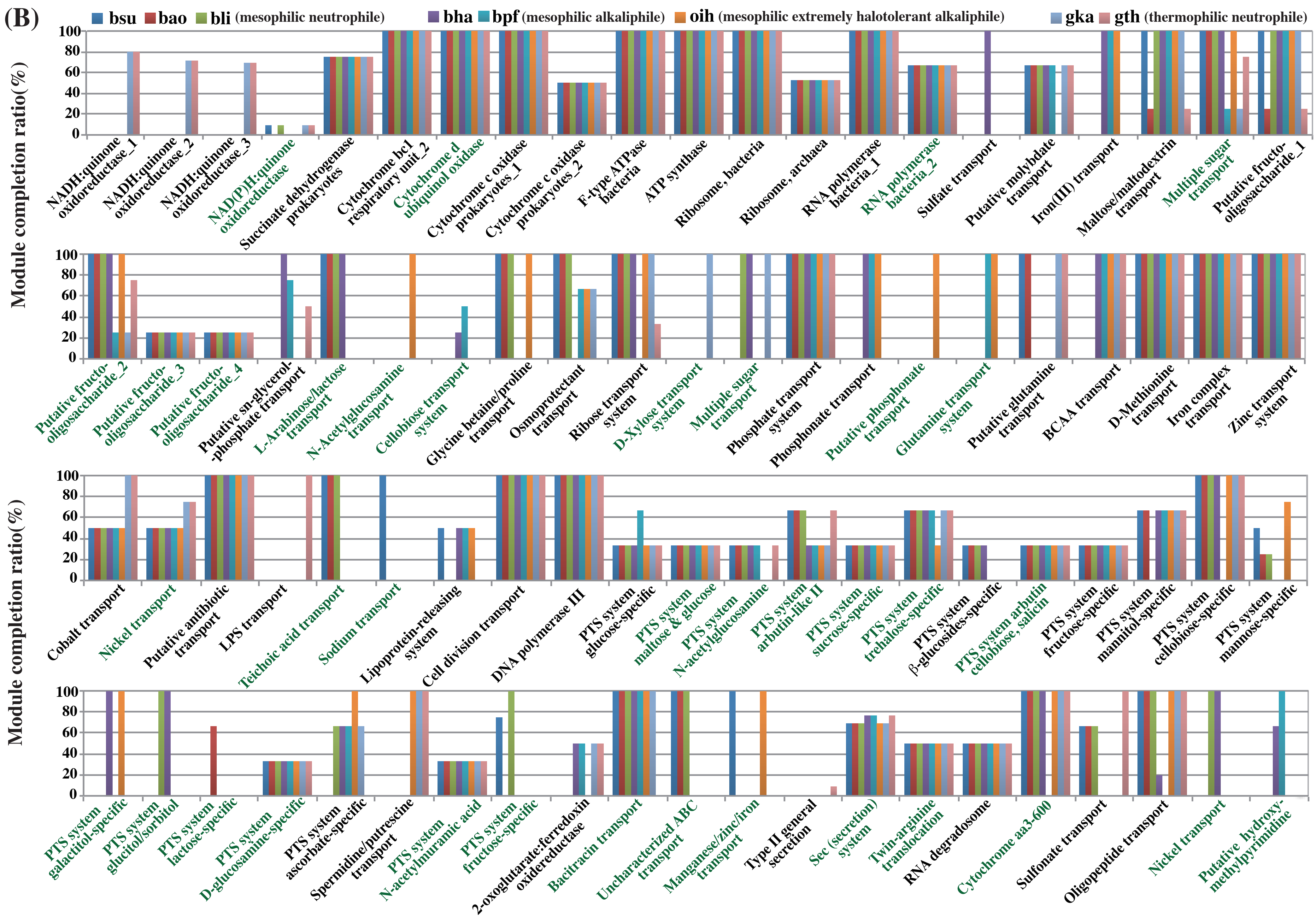
(B)
bsu bao bli (mesophilic neutrophile)
bha bpf (mesophilic alkaliphile)
oih (mesophilic extremely halotolerant alkaliphile)
gka gth (thermophilic neutrophile)
Module completion ratio(%)
NADH:quinone oxidoreductase_1
NADH:quinone oxidoreductase_2
NADH:quinone oxidoreductase_3
NAD(P)H:quinone oxidoreductase
Succinate dehydrogenase prokaryotes
Cytochrome bc1 respiratory unit_2
Cytochrome d ubiquinol oxidase
Cytochrome c oxidase prokaryotes_1
Cytochrome c oxidase prokaryotes_2
F-type ATPase bacteria
ATP synthase
Ribosome, bacteria
Ribosome, archaea
RNA polymerase bacteria_1
RNA polymerase bacteria_2
Sulfate transport
Putative molybdate transport
Iron(III) transport
Maltose/maltodextrin transport
Multiple sugar transport
Putative fructo-oligosaccharide_1
Putative fructo-oligosaccharide_2
Putative fructo-oligosaccharide_3
Putative fructo-oligosaccharide_4
Putative sn-glycerol-phosphate transport
L-Arabinose/lactose transport
N-Acetylglucosamine transport
Cellobiose transport system
Glycine betaine/proline transport
Osmoprotectant transport
Ribose transport system
D-Xylose transport system
Multiple sugar transport
Phosphate transport system
Phosphonate transport
Putative phosphonate transport
Glutamine transport system
Putative glutamine transport
BCAA transport
D-Methionine transport
Iron complex transport
Zinc transport system
Cobalt transport
Nickel transport
Putative antibiotic transport
LPS transport
Teichoic acid transport
Sodium transport
Lipoprotein-releasing system
Cell division transport
DNA polymerase III
PTS system glucose-specific
PTS system maltose & glucose
PTS system N-acetylglucosamine
PTS system arbutin-like II
PTS system sucrose-specific
PTS system trehalose-specific
PTS system β-glucosides-specific
PTS system arbutin cellobiose, salicin
PTS system fructose-specific
PTS system mannitol-specific
PTS system cellobiose-specific
PTS system mannose-specific
PTS system galactitol-specific
PTS system glucitol/sorbitol
PTS system lactose-specific
PTS system D-glucosamine-specific
PTS system ascorbate-specific
Spermidine/putrescine transport
PTS system N-acetylmuramic acid
PTS system fructose-specific
2-oxoglutarate:ferredoxin oxidereductase
Bacitracin transport
Uncharacterized ABC transport
Manganese/zinc/iron transport
Type II general secretion
Sec (secretion) system
Twin-arginine translocation
RNA degradosome
Cytochrome aa3-600
Sulfonate transport
Oligopeptide transport
Nickel transport
Putative hydroxy-methylpyrimidine

Figure S6. Module completion patterns in human and human gut microbiomes. (A)-1–3, Pathway module. (B)-1–3, Structural complex module. Upper histogram shows the module completion pattern in gut microbiomes from 13 healthy individuals [17]. Middle histogram shows module completion patterns in humans. Lower histogram shows module completion patterns in human gut microbiomes plus humans. Green characters show rare modules, which are completed by less than 10% of 768 prokaryotic species.

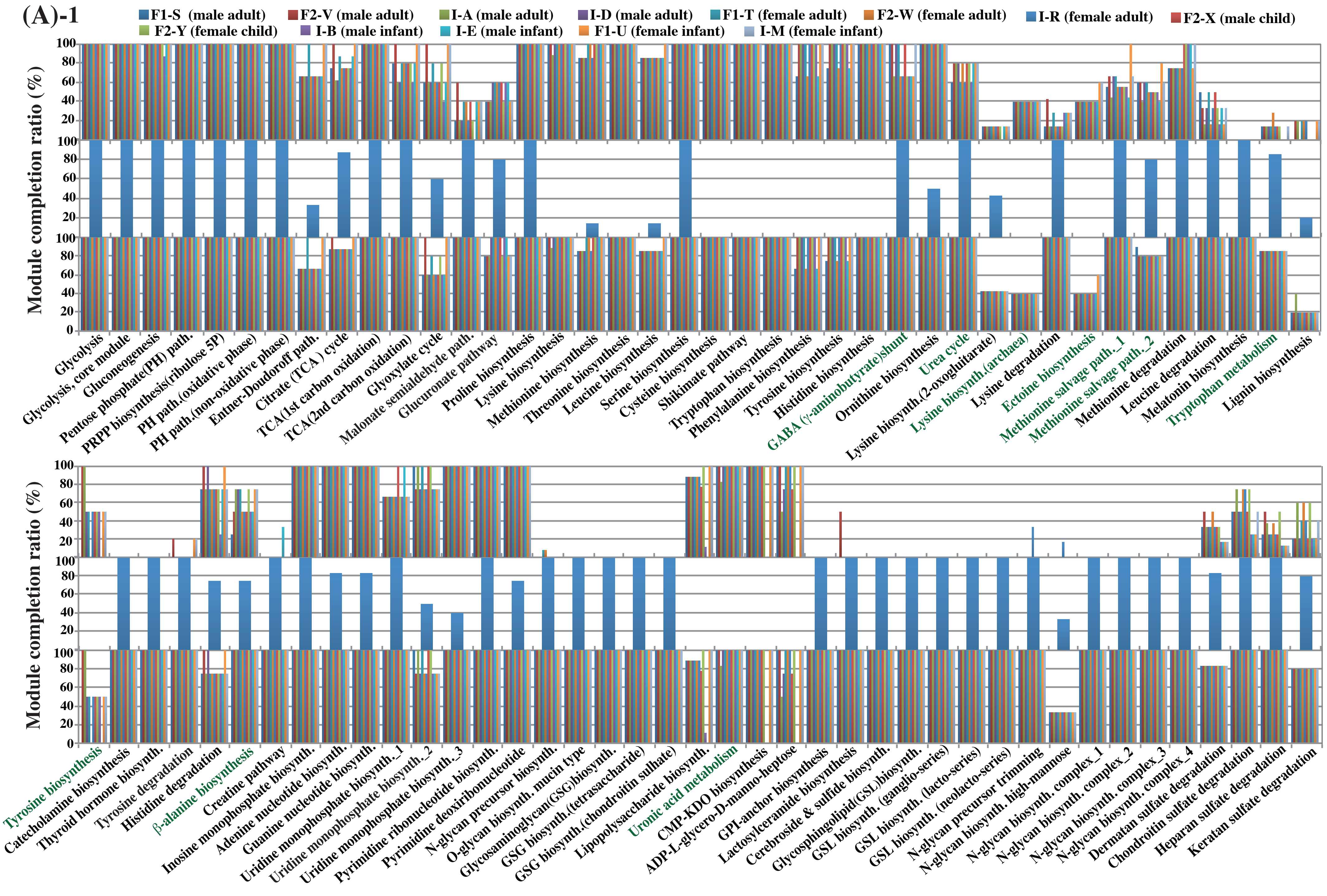

(A)-1
F1-S (male adult)
F2-V (male adult)
I-A (male adult)
I-D (male adult)
F1-T (female adult)
F2-W (female adult)
I-R (female adult)
F2-X (male child)
F2-Y (female child)
I-B (male infant)
I-E (male infant)
F1-U (female infant)
I-M (female infant)
Module completion ratio (%)
Glycolysis
Glycolysis, core module
Gluconeogenesis
Pentose phosphate(PH) path.
PRPP biosynthesis(ribulose 5P)
PH path.(oxidative phase)
PH path.(non-oxidative phase)
Entner-Doudoroff path.
Citrate (TCA) cycle
TCA(1st carbon oxidation)
TCA(2nd carbon oxidation)
Glyoxylate cycle
Malonate semialdehyde path.
Glucuronate pathway
Proline biosynthesis
Lysine biosynthesis
Methionine biosynthesis
Threonine biosynthesis
Leucine biosynthesis
Serine biosynthesis
Cysteine biosynthesis
Shikimate pathway
Tryptophan biosynthesis
Phenylalanine biosynthesis
Tyrosine biosynthesis
Histidine biosynthesis
GABA (γ-aminobutyrate)shunt
Ornithine biosynthesis
Urea cycle
Lysine biosynth.(2-oxoglutarate)
Lysine biosynth.(archaea)
Lysine degradation
Ectoine biosynthesis
Methionine salvage path._1
Methionine salvage path._2
Methionine degradation
Leucine degradation
Melatonin biosynthesis
Tryptophan metabolism
Lignin biosynthesis
Module completion ratio (%)
Tyrosine biosynthesis
Catecholamine biosynthesis
Thyroid hormone biosynth.
Tyrosine degradation
Histidine degradation
β-alanine biosynthesis
Creatine pathway
Inosine monophosphate biosynth.
Adenine nucleotide biosynth.
Guanine nucleotide biosynth.
Uridine monophosphate biosynth._1
Uridine monophosphate biosynth._2
Uridine monophosphate biosynth._3
Pyrimidine ribonucleotide biosynth.
Pyrimidine deoxiribonucleotide
N-glycan precursor biosynth.
O-glycan biosynth. mucin type
Glycosaminoglycan(GSG)biosynth.
GSG biosynth.(tetrasaccharide)
GSG biosynth.(chondroitin sulfate)
Lipopolysaccharide biosynth.
Uronic acid metabolism
CMP-KDO biosynthesis
ADP-L-glycero-D-manno-heptose
GPI-anchor biosynthesis
Lactosylceramide biosynthesis
Cerebroside & sulfide biosynth.
Glycosphingolipid(GSL)biosynth.
GSL biosynth. (ganglio-series)
GSL biosynth. (lacto-series)
GSL biosynth. (neolacto-series)
N-glycan precursor trimming
N-glycan biosynth. high-mannose
N-glycan biosynth. complex_1
N-glycan biosynth. complex_2
N-glycan biosynth. complex_3
N-glycan biosynth. complex_4
Dermatan sulfate degradation
Chondroitin sulfate degradation
Heparan sulfate degradation
Keratan sulfate degradation

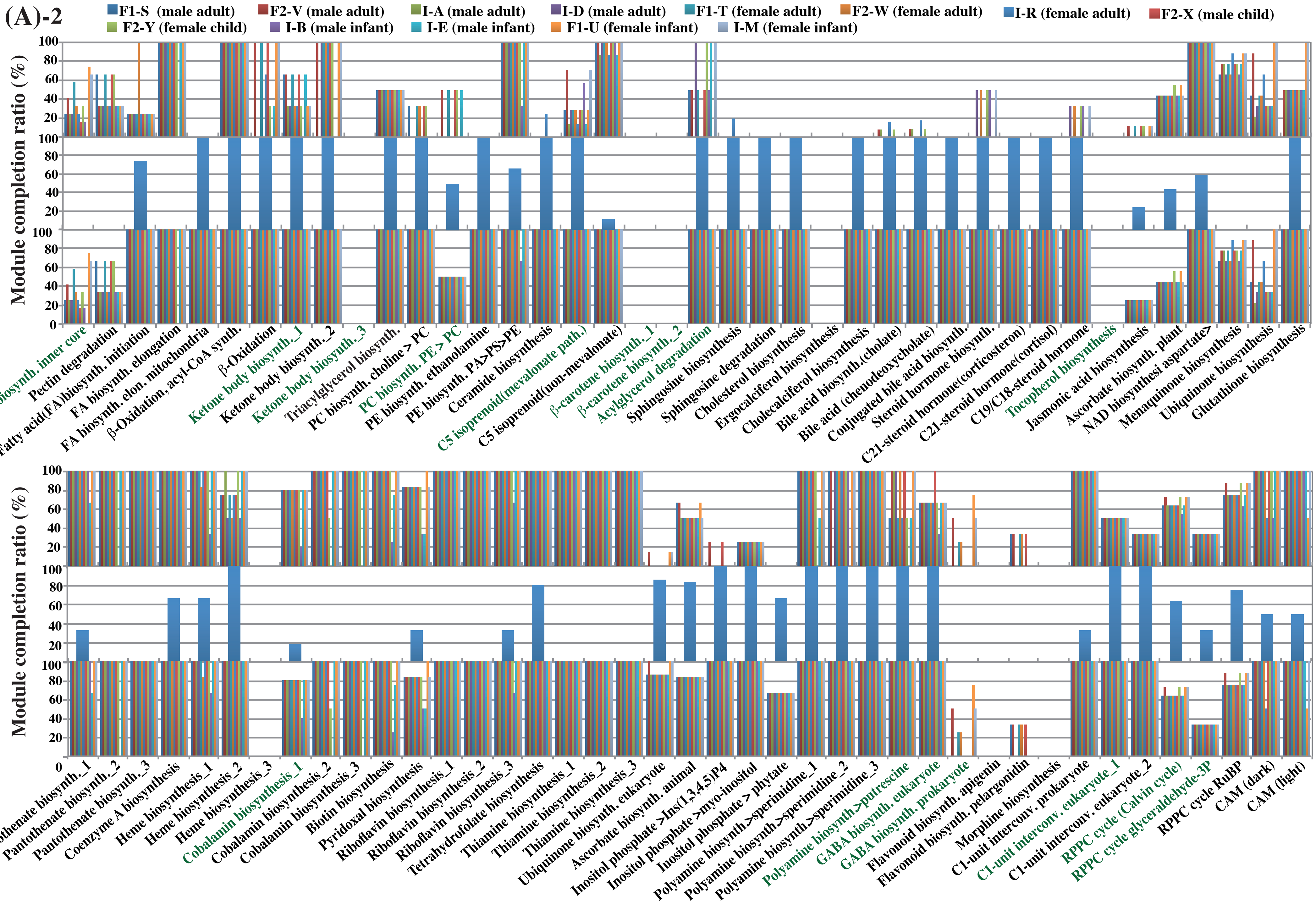

(A)-2
F1-S (male adult)
F2-V (male adult)
I-A (male adult)
I-D (male adult)
F1-T (female adult)
F2-W (female adult)
I-R (female adult)
F2-X (male child)
F2-Y (female child)
I-B (male infant)
I-E (male infant)
F1-U (female infant)
I-M (female infant)
Module completion ratio (%)
LPS biosynth. inner core
Pectin degradation
Fatty acid(FA)biosynth. initiation
FA biosynth. elongation
FA biosynth. elon. mitochondria
β-Oxidation, acyl-CoA synth.
β-Oxidation
Ketone body biosynth._1
Ketone body biosynth._2
Ketone body biosynth._3
Triacylglycerol biosynth.
PC biosynth. choline > PC
PC biosynth. PE > PC
PE biosynth. ethanolamine
PE biosynth. PA>PS>PE
Ceramide biosynthesis
C5 isoprenoid(mevalonate path.)
C5 isoprenoid(non-mevalonate)
β-carotene biosynth._1
β-carotene biosynth._2
Acylglycerol degradation
Sphingosine biosynthesis
Sphingosine degradation
Cholesterol biosynthesis
Ergocalciferol biosynthesis
Cholecalciferol biosynthesis
Bile acid biosynth.(cholate)
Bile acid (chenodeoxycholate)
Conjugated bile acid biosynth.
Steroid hormone biosynth.
C21-steroid hormone(corticosteron)
C21-steroid hormone(cortisol)
C19/C18-steroid hormone
Tocopherol biosynthesis
Jasmonic acid biosynthesis
Ascorbate biosynth. plant
NAD biosynthesi aspartate>
Menaquinone biosynthesis
Ubiquinone biosynthesis
Glutathione biosynthesis
Pantothenate biosynth._1
Pantothenate biosynth._2
Pantothenate biosynth._3
Coenzyme A biosynthesis
Heme biosynthesis_1
Heme biosynthesis_2
Heme biosynthesis_3
Cobalamin biosynthesis_1
Cobalamin biosynthesis_2
Cobalamin biosynthesis_3
Biotin biosynthesis
Pyridoxal biosynthesis
Riboflavin biosynthesis_1
Riboflavin biosynthesis_2
Riboflavin biosynthesis_3
Tetrahydrofolate biosynthesis
Thiamine biosynthesis_1
Thiamine biosynthesis_2
Thiamine biosynthesis_3
Ubiquinone biosynth. eukaryote
Ascorbate biosynth. animal
Inositol phosphate >Ins(1,3,4,5)P4
Inositol phosphate >myo-inositol
Inositol phosphate > phytate
Polyamine biosynth.>sperimidine_1
Polyamine biosynth.>sperimidine_2
Polyamine biosynth.>sperimidine_3
Polyamine biosynth.>putrescine
GABA biosynth. eukaryote
GABA biosynth. prokaryote
Flavonoid biosynth. apigenin
Flavonoid biosynth. pelargonidin
Morphine biosynthesis
C1-unit intercony. prokaryote
C1-unit intercony. eukaryote_1
C1-unit intercony. eukaryote_2
RPPC cycle (Calvin cycle)
RPPC cycle glyceraldehyde-3P
RPPC cycle RuBP
CAM (dark)
CAM (light)

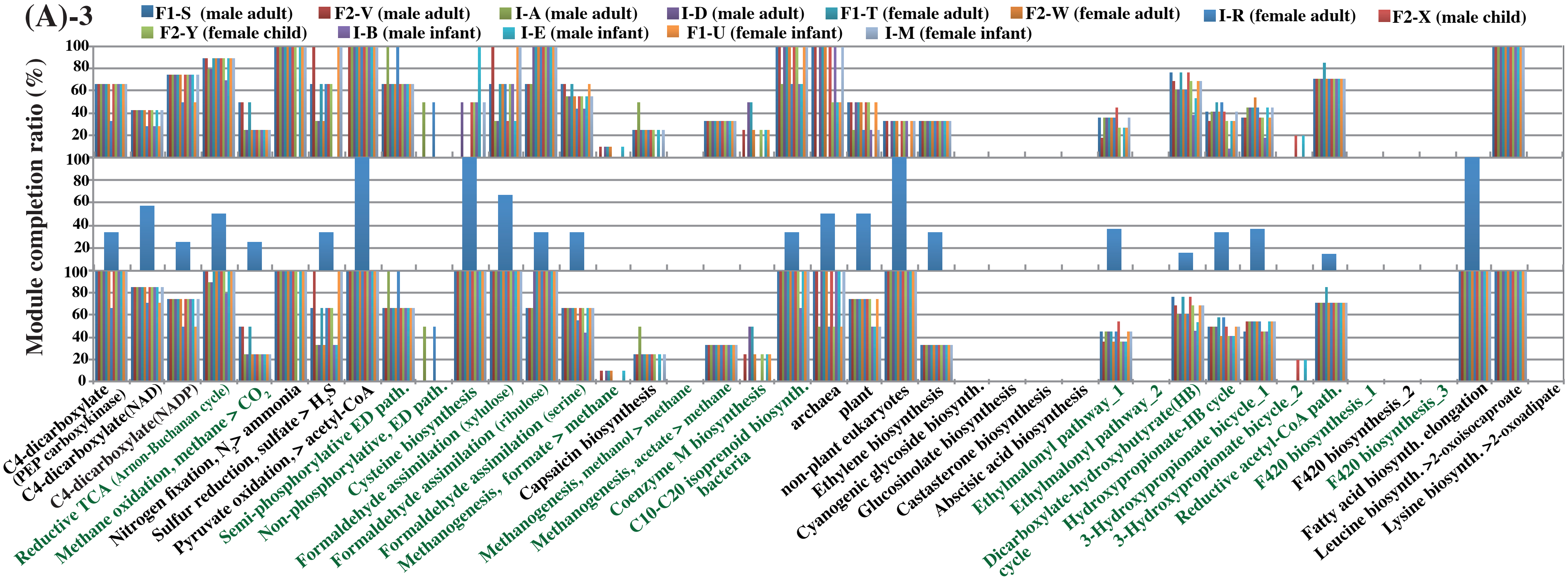

(A)-3
F1-S (male adult)
F2-V (male adult)
I-A (male adult)
I-D (male adult)
F1-T (female adult)
F2-W (female adult)
I-R (female adult)
F2-X (male child)
F2-Y (female child)
I-B (male infant)
I-E (male infant)
F1-U (female infant)
I-M (female infant)
Module completion ratio (%)
C4-dicarboxylate (PEP carboxykinase)
C4-dicarboxylate(NAD)
C4-dicarboxylate(NADP)
Reductive TCA (Arnon-Buchanan cycle)
Methane oxidation, methane > CO_2
Nitrogen fixation, N_2 > ammonia
Sulfur reduction, sulfate > H_2S
Pyruvate oxidation, > acetyl-CoA
Semi-phosphorylative ED path.
Non-phosphorylative, ED path.
Cysteine biosynthesis
Formaldehyde assimilation (xylulose)
Formaldehyde assimilation (ribulose)
Formaldehyde assimilation (serine)
Methanogenesis, formate > methane
Capsaicin biosynthesis
Methanogenesis, methanol > methane
Methanogenesis, acetate > methane
Coenzyme M biosynthesis
C10-C20 isoprenoid biosynth. bacteria
archaea
plant
non-plant eukaryotes
Ethylene biosynthesis
Cyanogenic glycoside biosynth.
Glucosinolate biosynthesis
Castasterone biosynthesis
Abscisic acid biosynthesis
Ethylmalonyl pathway_1
Ethylmalonyl pathway_2
Dicarboxylate-hydroxybutyrate(HB) cycle
Hydroxypropionate-HB cycle
3-Hydroxypropionate bicycle_1
3-Hydroxypropionate bicycle_2
Reductive acetyl-CoA path.
F420 biosynthesis_1
F420 biosynthesis_2
F420 biosynthesis_3
Fatty acid biosynth. elongation
Leucine biosynth. >2-oxoisocaproate
Lysine biosynth. >2-oxoadipate

(B)-1

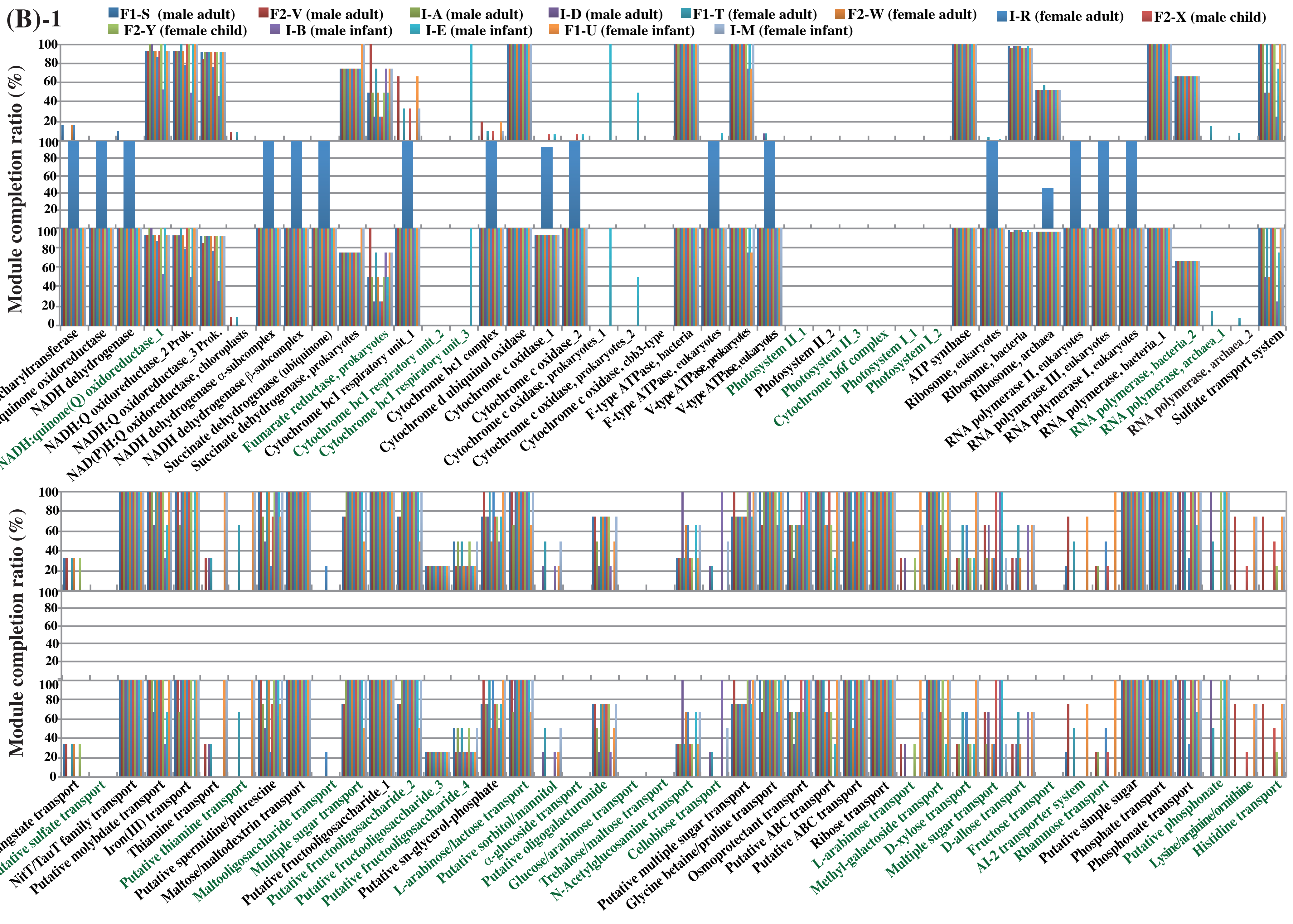
F1-S (male adult)
F2-V (male adult)
I-A (male adult)
I-D (male adult)
F1-T (female adult)
F2-W (female adult)
I-R (female adult)
F2-X (male child)
F2-Y (female child)
I-B (male infant)
I-E (male infant)
F1-U (female infant)
I-M (female infant)
Module completion ratio (%)
100
80
60
40
20
0
Oligosaccharyltransferase
NADH:ubiquinone oxidoreductase
NADH dehydrogenase
NADH:quinone(Q) oxidoreductase_1
NADH:Q oxidoreductase_2 Prok.
NADH:Q oxidoreductase_3 Prok.
NAD(P)H:Q oxidoreductase, chloroplasts
NADH dehydrogenase α-subcomplex
NADH dehydrogenase β-subcomplex
Succinate dehydrogenase (ubiquinone)
Succinate dehydrogenase, prokaryotes
Fumarate reductase, prokaryotes
Cytochrome bc1 respiratory unit_1
Cytochrome bc1 respiratory unit_2
Cytochrome bc1 respiratory unit_3
Cytochrome bc1 complex
Cytochrome d ubiquinol oxidase
Cytochrome c oxidase_1
Cytochrome c oxidase_2
Cytochrome c oxidase, prokaryotes_1
Cytochrome c oxidase, prokaryotes_2
Cytochrome c oxidase, cbb3-type
F-type ATPase, bacteria
F-type ATPase, eukaryotes
V-type ATPase, prokaryotes
V-type ATPase, eukaryotes
Photosystem II_1
Photosystem II_2
Photosystem II_3
Cytochrome b6f complex
Photosystem I_1
Photosystem I_2
ATP synthase
Ribosome, eukaryotes
Ribosome, bacteria
Ribosome, archaea
RNA polymerase II, eukaryotes
RNA polymerase III, eukaryotes
RNA polymerase I, eukaryotes
RNA polymerase, bacteria_1
RNA polymerase, bacteria_2
RNA polymerase, archaea_1
RNA polymerase, archaea_2
Sulfate transport system
Tungstate transport
Putative sulfate transport
NitT/TauT family transport
Putative molybdate transport
Iron(III) transport
Thiamine transport
Putative thiamine transport
Putative spermidine/putrescine
Maltose/maltodextrin transport
Maltooligosaccharide transport
Multiple sugar transport
Putative fructooligosaccharide_1
Putative fructooligosaccharide_2
Putative fructooligosaccharide_3
Putative fructooligosaccharide_4
Putative sn-glycerol-phosphate
L-arabinose/lactose transport
Putative sorbitol/mannitol
α-glucoside transport
Putative oligogalacturonide
Glucose/arabinose transport
Trehalose/maltose transport
N-Acetylglucosamine transport
Cellobiose transport
Putative multiple sugar transport
Glycine betaine/proline transport
Osmoprotectant transport
Putative ABC transport
Putative ABC transport
Ribose transport
L-arabinose transport
Methyl-galactoside transport
D-xylose transport
Multiple sugar transport
D-allose transport
Fructose transport
AI-2 transporter system
Rhamnose transport
Putative simple sugar
Phosphate transport
Phosphonate transport
Putative phosphonate
Lysine/arginine/ornithine
Histidine transport

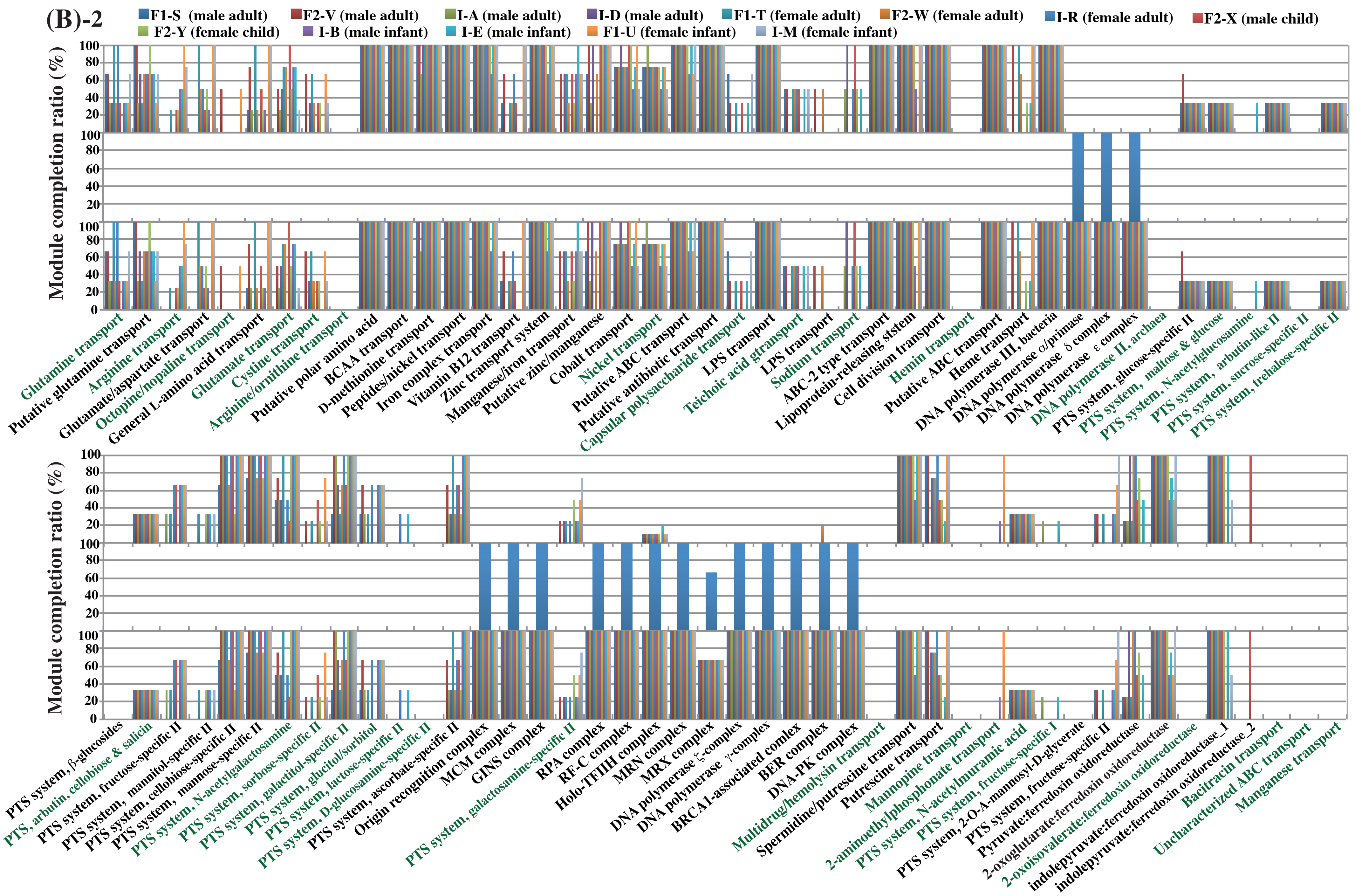

(B)-2
F1-S (male adult)
F2-V (male adult)
I-A (male adult)
I-D (male adult)
F1-T (female adult)
F2-W (female adult)
I-R (female adult)
F2-X (male child)
F2-Y (female child)
I-B (male infant)
I-E (male infant)
F1-U (female infant)
I-M (female infant)
Module completion ratio (%)
100
80
60
40
20
0
Glutamine transport
Putative glutamine transport
Arginine transport
Glutamate/aspartate transport
Octopine/nopaline transport
General L-amino acid transport
Glutamate transport
Cystine transport
Arginine/ornithine transport
Putative polar amino acid
BCAA transport
D-methionine transport
Peptides/nickel transport
Iron complex transport
Vitamin B12 transport
Zinc transport system
Manganese/iron transport
Putative zinc/manganese
Cobalt transport
Nickel transport
Putative ABC transport
Putative antibiotic transport
Capsular polysaccharide transport
LPS transport
Teichoic acid gtransport
LPS transport
Sodium transport
ABC-2 type transport
Lipoprotein-releasing ststem
Cell division transport
Hemin transport
Putative ABC transport
Heme transport
DNA polymerase III, bacteria
DNA polymerase α/primase
DNA polymerase δ complex
DNA polymerase ε complex
DNA polymerase II, archaea
PTS system, glucose-specific II
PTS system, maltose & glucose
PTS system, N-acetylglucosamine
PTS system, arbutin-like II
PTS system, sucrose-specific II
PTS system, trehalose-specific II
PTS system, β-glucosides
PTS, arbutin, cellobiose & salicin
PTS system, fructose-specific II
PTS system, mannitol-specific II
PTS system, cellobiose-specific II
PTS system, mannose-specific II
PTS system, N-acetylgalactosamine
PTS system, sorbose-specific II
PTS system, galactitol-specific II
PTS system, glucitol/sorbitol
PTS system, lactose-specific II
PTS system, D-glucosamine-specific II
PTS system, ascorbate-specific II
Origin recognition complex
MCM complex
GINS complex
PTS system, galactosamine-specific II
RPA complex
RF-C complex
Holo-TFIIH complex
MRN complex
MRX complex
DNA polymerase ζ-complex
DNA polymerase γ-complex
BRCA1-associated comlex
BER complex
DNA-PK complex
Multidrug/hemolysin transport
Spermidine/putrescine transport
Putrescine transport
Mannopine transport
2-aminoethylphosphonate transport
PTS system, N-acetylmuramic acid
PTS system, fructose-specific I
PTS system, 2-O-A-mannosyl-D-glycerate
PTS system, fructose-specific II
Pyruvate:ferredoxin oxidoreductase
2-oxoglutarate:ferredoxin oxidoreductase
2-oxoisovalerate:ferredoxin oxidoreductase
indolepyruvate:ferredoxin oxidoreductase_1
indolepyruvate:ferredoxin oxidoreductase_2
Bacitracin transport
Uncharacterized ABC transport
Manganese transport

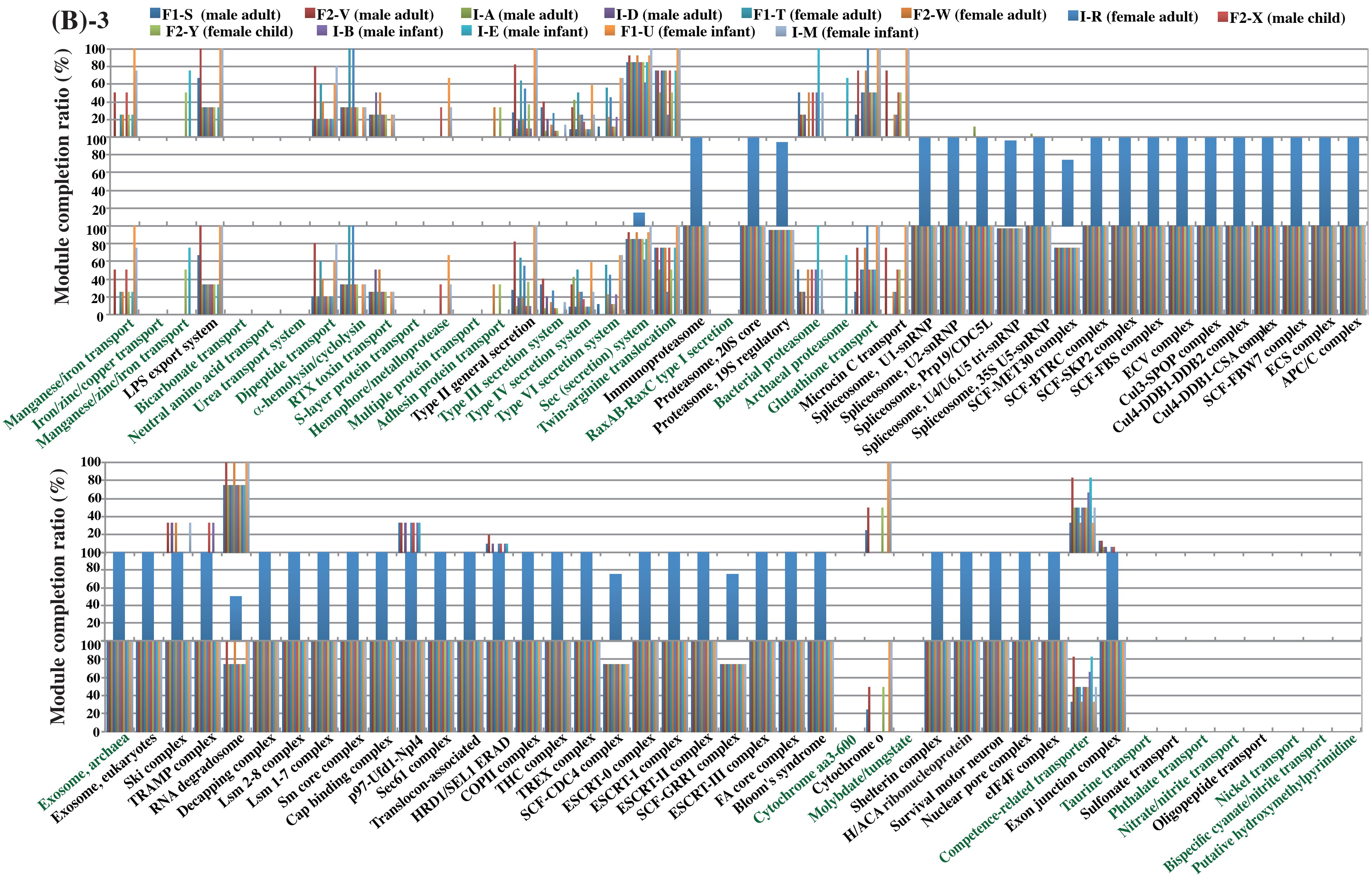
(B)-3
F1-S (male adult)
F2-V (male adult)
I-A (male adult)
I-D (male adult)
F1-T (female adult)
F2-W (female adult)
I-R (female adult)
F2-X (male child)
F2-Y (female child)
I-B (male infant)
I-E (male infant)
F1-U (female infant)
I-M (female infant)
Module completion ratio (%)
Manganese/iron transport
Iron/zinc/copper transport
Manganese/zinc/iron transport
LPS export system
Bicarbonate transport
Neutral amino acid transport
Urea transport system
Dipeptide transport
α-hemolysin/cyclolysin
RTX toxin transport
S-layer protein transport
Hemophore/metalloprotease
Multiple protein transport
Adhesin protein transport
Type II general secretion
Type III secretion system
Type IV secretion system
Type VI secretion system
Sec (secretion) system
Twin-arginine translocation
Immunoproteasome
RaxAB-RaxC type I secretion
Proteasome, 20S core
Proteasome, 19S regulatory
Bacterial proteasome
Archaeal proteasome
Glutathione transport
Microcin C transport
Spliceosome, U1-snRNP
Spliceosome, U2-snRNP
Spliceosome, Prp19/CDC5L
Spliceosome, U4/U6.U5 tri-snRNP
Spliceosome, 35S U5-snRNP
SCF-MET30 complex
SCF-BTRC complex
SCF-SKP2 complex
SCF-FBS complex
ECV complex
Cul3-SPOP complex
Cul4-DDB1-DDB2 complex
Cul4-DDB1-CSA complex
SCF-FBW7 complex
ECS complex
APC/C complex
Module completion ratio (%)
Exosome, archaea
Exosome, eukaryotes
Ski complex
TRAMP complex
RNA degradosome
Decapping complex
Lsm 2-8 complex
Lsm 1-7 complex
Sm core complex
Cap binding complex
p97-Ufd1-Npl4
Sec61 complex
Translocon-associated
HRD1/SEL1 ERAD
COPII complex
THC complex
TREX complex
SCF-CDC4 complex
ESCRT-0 complex
ESCRT-I complex
ESCRT-II complex
SCF-GRR1 complex
ESCRT-III complex
FA core complex
Bloom's syndrome
Cytochrome aa3-600
Cytochrome o
Molybdate/tungstate
Shelterin complex
H/ACA ribonucleoprotein
Survival motor neuron
Nuclear pore complex
eIF4F complex
Competence-related transporter
Exon junction complex
Taurine transport
Sulfonate transport
Phthalate transport
Nitrate/nitrite transport
Oligopeptide transport
Nickel transport
Bispecific cyanate/nitrite transport
Putative hydroxymethylpyrimidine

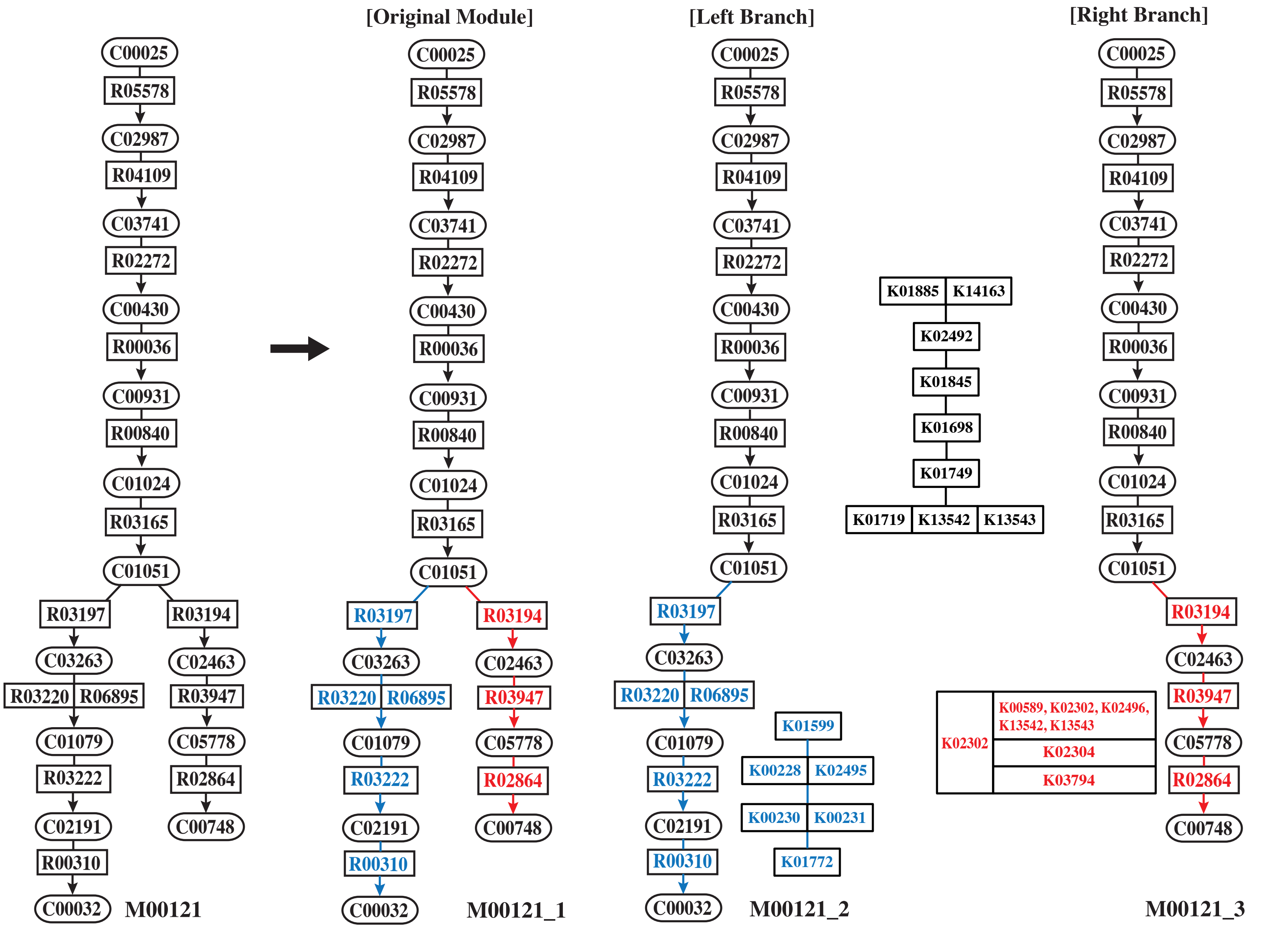

Figure S7. Definition of submodules for the KEGG module with branching
The heme biosynthesis pathway (glutamate => protoheme => siroheme) module (M00212) has branching at the intermediate compound uroporphyrinogen III (C01051), where this module was divided into 2 parts. Submodules are defined as M00121_1 (original), M00121_2 (left-side branching), and M00121_3 (right-side branching). Ovals with C numbers, rectangles with R numbers, and K numbers represent metabolites, enzymatic reactions, and KO, respectively. KO is used for mapping functional annotation of genes to the modules. Black K numbers indicate KO common to all 3 newly redefined submodules (M00121_1, M00121_2, and M00121_3), and blue and red K numbers correspond to reactions specific to M00121_2 and M00121_3, respectively.

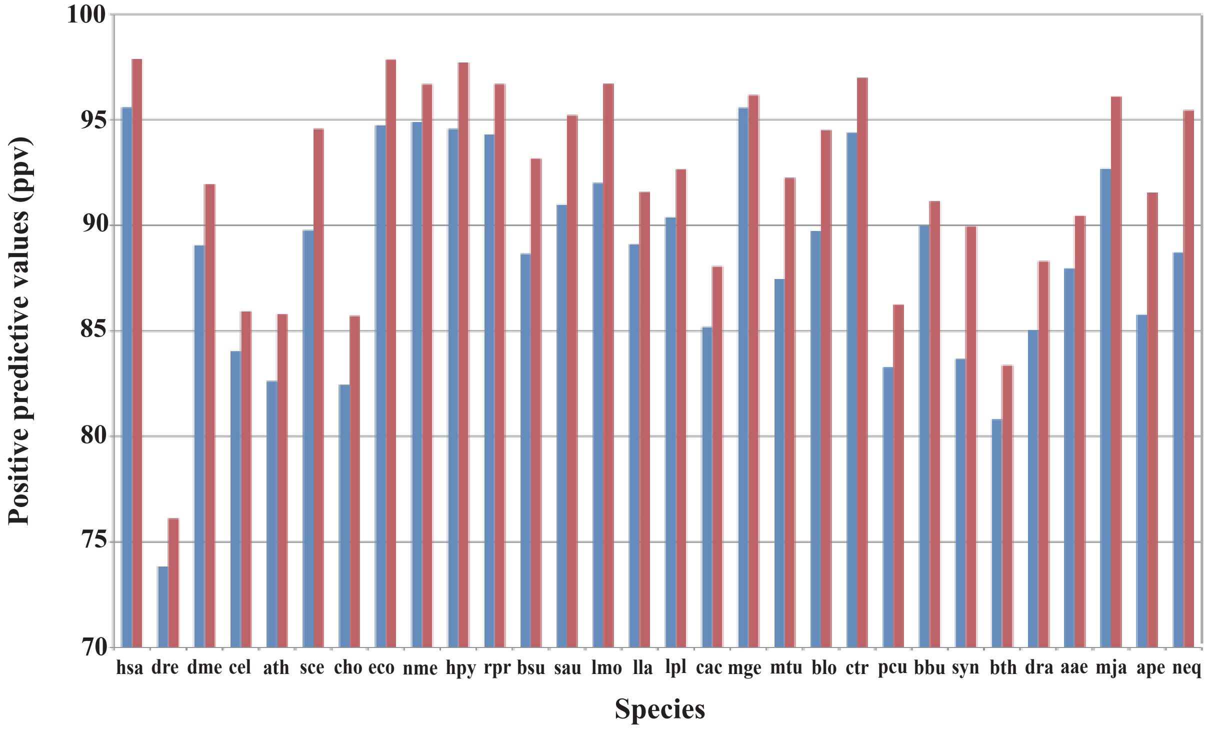

Figure S8. Positive predictive values (ppv) of the KO reassignment tests by KAAS.

We performed KO reassingment tests for 30 species (7 eukaryotes, 20 bacteria, 3 archaea) by original (old) and improved (new) KAAS and found that new KAAS showed two - five percent improvements compared with the old KAAS. Three letter codes in X axis indicate species. abbreviations as follows: hsa: *Homo sapiens*, dre: *Danio rerio*, dme: *Drosophila melanogaster*, cel: *Caenorhabditis elegans*, ath: *Arabidopsis thaliana*, sce: *Saccharomyces cerevisiae,* cho: *Cryptosporidium hominis*, eco: *Escherichia coli*, nme: *Neisseria meningitidis*, hpy:*Helicobacter pylori*, rpr: *Ricketsia prowazekii*, bsu:*Bacillus subtilis*, sau: *Staphylococcus aureus*, lmo: *Listeria monocytogenes*, lla: *Lactococcus lactis*, lpl: *Lactobacillus plantarum*, cau: *Chloroflexus aurantiacus*, mge: *Mycoplasma genitalium*, mtu: *Mycobacterium tuberculosis*, blo: *Bifidobacterium longum*, ctr:*Chlamydia trachomatis*, pcu:*Protochlamydia amoebophila*, bbu:*Borrelia burgdorferi*, syn: *Synechocystis* sp., bth: *Bacteroides thetaiotaomicron*, dra: *Deinococcus radiodurans*, aae:*Aquifex aeolicus*, mja: *Methanocaldococcus jannaschii*, ape:*Aeropyrum pernix*, neq:*Nanoarchaeum equitans.*Blue bar: old KAAS, Red bar: new KAAS

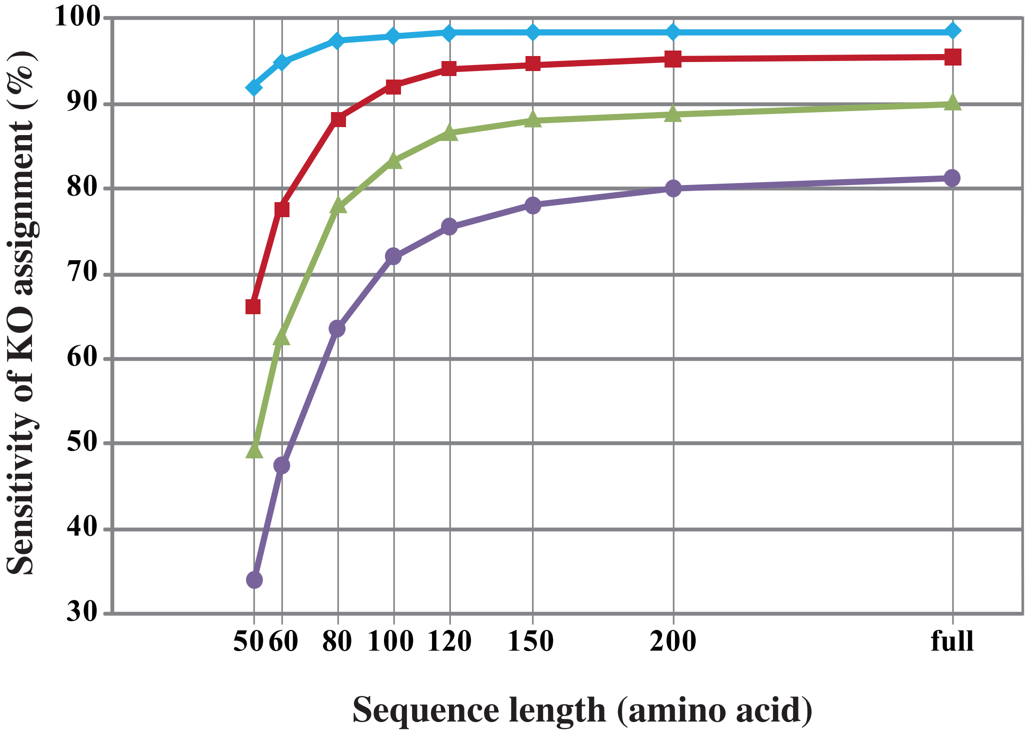

Figure S9. Effect of database dependency on accuracy of the KO assignment.

Escherichia coli isolated from Norwegian infant (Draft genome sequenced by 454 GS FLX Titanium). Blue diamonds show the results using the data set without proteins from the genera *Escherichia, Salmonella, Shigella,* and *Yersinia* (1,239 species). Similarly, redsquares, green triangles, and purple dots show the results without proteins from the order *Enterobacteriales* (1,200 species), class *Gammaproteobacteria* (1,040 species), and phylum *Proteobacteria* (755 species), respectively. KO identifiers specific to the genera *Escherichia, Salmonella, Shigella*, and *Yersinia* (16 KO identifiers), order *Enterobacteriales* (90), class *Gammaproteobacteria* (203), or phylum *Proteobacteria* (370) were removed in advance from the protein data set. Here, the accuracy is defined by the sensitivity TP/(TP+FN), where TP and FN are the numbers of true positives and false negatives, respectively. We also used truncated proteins to confirm effect of amino acid (a.a.) sequence lengths on the accuarcy of KO assignments. The 4,410 proteins from *E. coli* isolate were randomly fragmented into 50, 60, 80, 100, 120, 150, and 200 a.a. in ength, and each length of a.a. sequences was used for verification of the accuracy of KO assignment.
